# Mining of novel secondary metabolite biosynthetic gene clusters from acid mine drainage

**DOI:** 10.1038/s41597-022-01866-6

**Published:** 2022-12-09

**Authors:** Ling Wang, Wan Liu, Jieliang Liang, Linna Zhao, Qiang Li, Chenfen Zhou, Hui Cen, Qingbei Weng, Guoqing Zhang

**Affiliations:** 1 School of Life Sciences, Guizhou Normal University, Guiyang, Guizhou 550025 China; 2 National Genomics Data Center, CAS Key Laboratory of Computational Biology, Bio-Med Big Data Center, Shanghai Institute of Nutrition and Health, University of Chinese Academy of Sciences, Chinese Academy of Sciences, Shanghai, 200031 China; 3 School of Life Sciences, South China Normal University, Guangzhou, Guangdong 510631 China; 4 Suzhou BiomeMatch Therapeutics Co., Ltd. No.351, Guoshoujing Road, Shanghai, 201203 China; 5 Qiannan Normal University for Nationalities, Duyun, Guizhou 558000 China

**Keywords:** Water microbiology, Metagenomics, Water microbiology

## Abstract

Acid mine drainage (AMD) is usually acidic (pH < 4) and contains high concentrations of dissolved metals and metalloids, making AMD a typical representative of extreme environments. Recent studies have shown that microbes play a key role in AMD bioremediation, and secondary metabolite biosynthetic gene clusters (smBGCs) from AMD microbes are important resources for the synthesis of antibacterial and anticancer drugs. Here, 179 samples from 13 mineral types were used to analyze the putative novel microorganisms and secondary metabolites in AMD environments. Among 7,007 qualified metagenome-assembled genomes (MAGs) mined from these datasets, 6,340 MAGs could not be assigned to any GTDB species representative. Overall, 11,856 smBGCs in eight categories were obtained from 7,007 qualified MAGs, and 10,899 smBGCs were identified as putative novel smBGCs. We anticipate that these datasets will accelerate research in the field of AMD bioremediation, aid in the discovery of novel secondary metabolites, and facilitate investigation into gene functions, metabolic pathways, and CNPS cycles in AMD.

## Abstract (summary)

**Table:** 

Measurement(s)	Metagenome
Technology Type(s)	Next Generation Sequencing
Sample Characteristic - Environment	acid mine drainage
Sample Characteristic - Location	Guizhou Province • Marsberg • Municipality of Canaa dos Carajas • North Wales • Town of Vershire • State of Washington • Guangdong Province • Germany • County (Sweden) • Commonwealth of Pennsylvania • Province of Ontario • Hunan Province • Jiangxi Province • Guangxi Zhuang Autonomous Region • Anhui Province • Fujian Province

## Background & Summary

Acid mine drainage (AMD) is a type of acidic (pH < 4) and metal-enriched water that results from the accelerated oxidative dissolution of exposed minerals, principally sulfides, and is associated with mining^1,2^. The strong acidity and heavy metal toxicity of AMD has caused severe pollution to surrounding water systems and soils^2–4^, making AMD one of the most serious environmental problems arising during the mining of mineral resources^5,6^. Metabolically-active acidophilic microorganisms have been observed in AMD^7,8^, including microbes primarily from the Bacteria (such as Proteobacteria, Nitrospirae, Actinobacteria, Firmicutes, and Acidobacteria) and Archaea domains^9^.

Microbes in AMD play a key role in the bioremediation of AMD environments^10,11^. For example, *Acidithiobacillus*^12^, one of the most common genera in AMD, includes microbes with chemolithotrophic metabolisms that are able to oxidize Fe^2+^ and sulfur compounds (such as *Acidithiobacillus ferrooxidans*, *Acidithiobacillus ferridurans*, and *Acidithiobacillus ferrivorans*)^9,13,14^, or oxidize sulfur compounds alone (such as *Acidithiobacillus caldus*, *Acidithiobacillus thiooxidans*, and *Acidithiobacillus albertensis*)^15–17^. Sulfate-reducing bacteria (SRB), a group of diverse anaerobic microorganisms that are ubiquitous in natural habitats, have been utilized in AMD remediation^11^.

Secondary metabolite biosynthetic gene clusters (smBGCs) found in AMD microbes are important resources for the synthesis of antibacterial and anticancer drugs^18,19^. A previous study reported that microbes including *Penicillium* sp., *Penicillium rubrum*, *Penicillium solitum*, *Penicillium clavigerum*, *Chaetomium funicola*, and *Pithomyces* sp. were separated and cultivated from water and sediment samples in a pit lake formed by the former Berkeley copper mine, among which worthwhile secondary metabolites were found^20^. For example, berkelic acid, a secondary metabolite of *Penicillium sp*., had anti-OVCAR-3 activity in *NCI-DTP60*; berkeleydione, the terpenoid secondary metabolite of *Penicillium rubrum*, showed selective activity against non-small cell lung cancer NCI-H460 in *NCI-DTP 60*; and CHCl_3_ extracted from *Penicillium solitum* strongly inhibited MMP-3 and caspase-1. In addition, cyclodipeptide synthases (CDPSs) that were capable of synthesizing cyclodipeptide, a precursor of 2,5-diketopiperazines, were found to be produced by 23 metagenome-assembled genomes (MAGs) (LMSG_G000006317.1–LMSG_G000006339.1) in *Diplorickettsiaceae* in this study^21–23^. Therefore, mining smBGCs from AMD may reveal valuable secondary metabolites^18^.

**Fig. 1:**
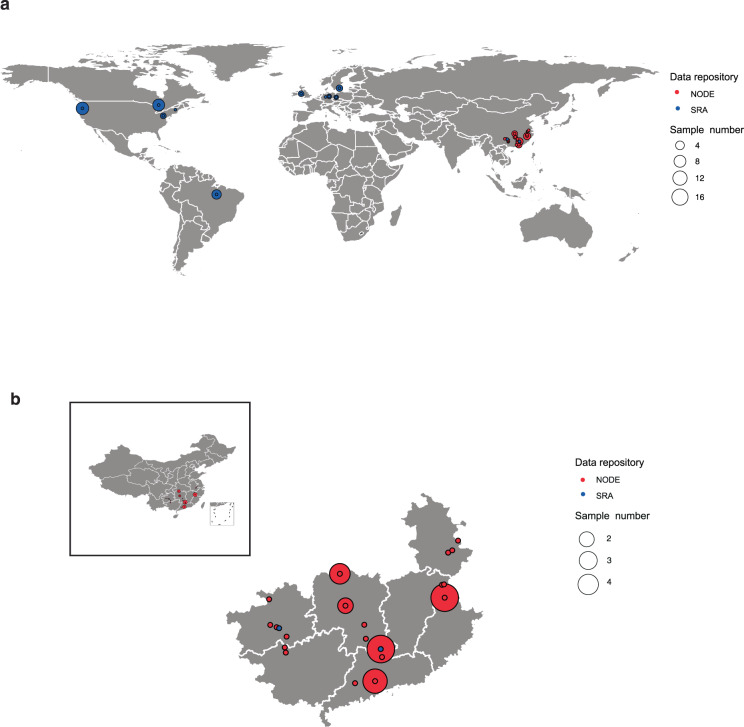
Geographic distribution of sampling sites in this study. (**a**) Geographic distribution of sampling sites for all samples (the latitude and longitude of SRS1810936 was retrieved according to the geographic location of this sample). (**b**) Geographic distribution of sampling sites for the acid mine drainage (AMD) metagenomic datasets for China.

**Fig. 2:**
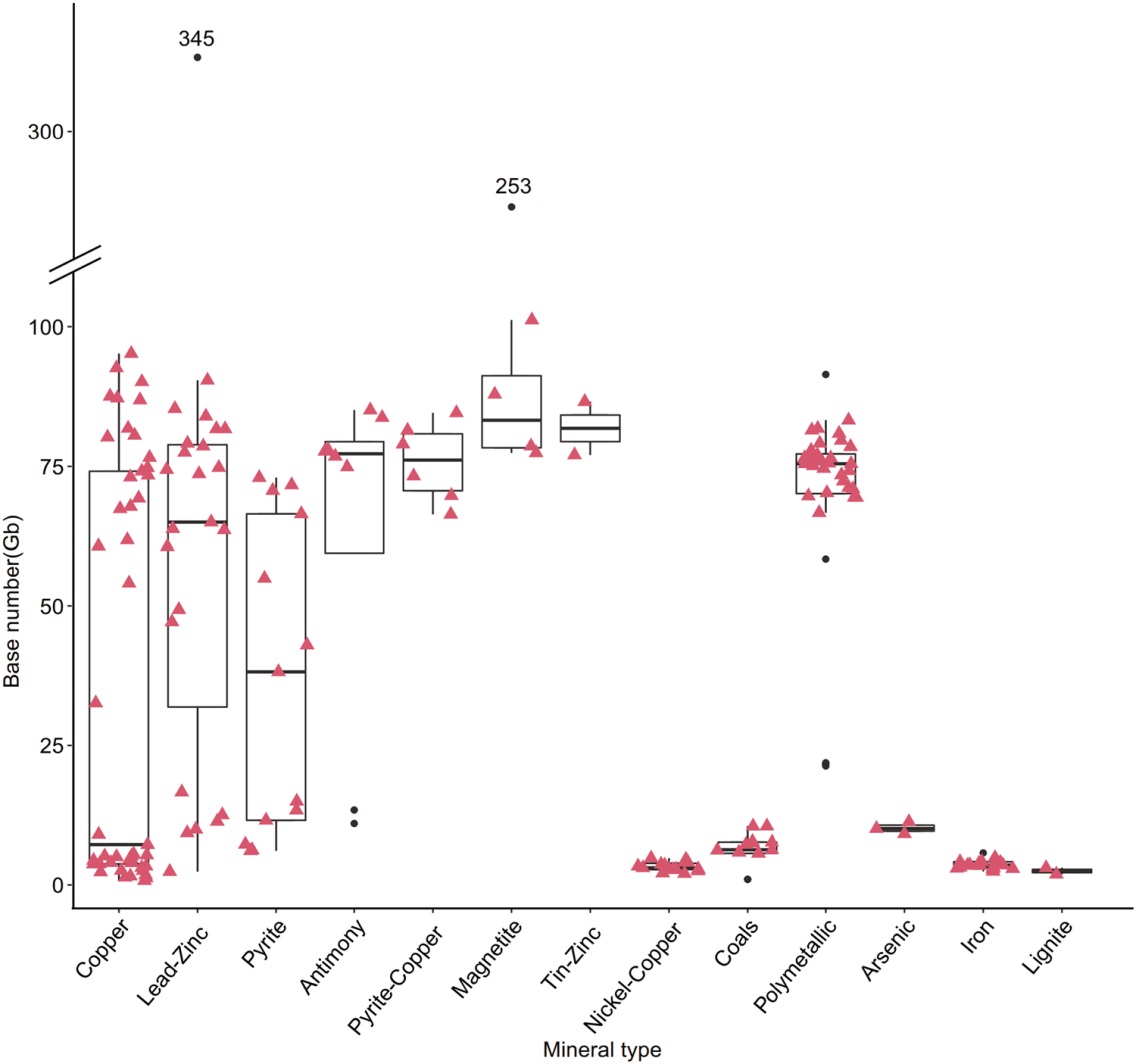
Base number distributions of samples from 13 types of minerals. The median base number of samples was similar among lead-zinc mines, antimony mines, pyrite-copper mines, magnetite mines, tin-zinc mines, and polymetallic mines. The upper and lower whiskers extend from the hinge within 1.5 x the inter-quantile range to the highest and lowest values, respectively. The outlier points (black) are the ones outside that range.

**Fig. 3:**
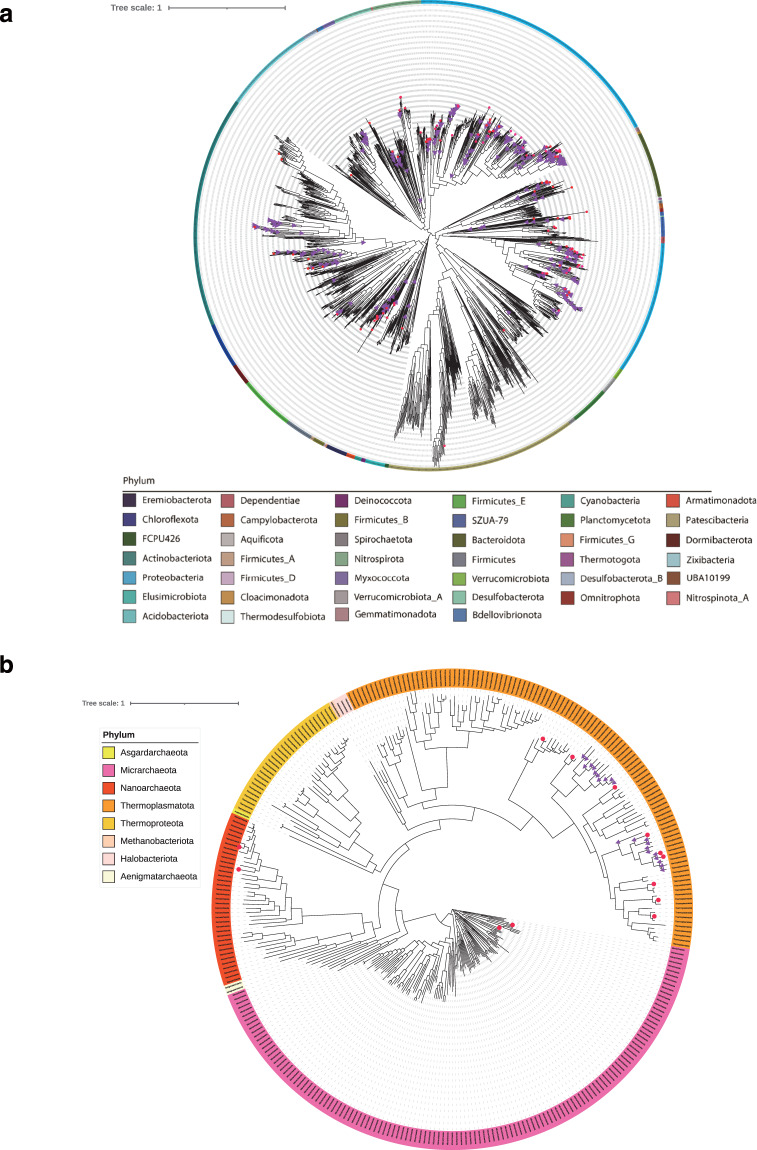
Maximum-likelihood phylogenetic trees of bacterial and archaeal MAGs at the phylum level. Major lineages are assigned arbitrary colours and named. Lineages with GTDB representative species assignment are highlighted with red dots, while lineages with existed genera assignment (genus with NCBI taxonomy ID) are marked with purple triangles. (**a**) Maximum-likelihood phylogenetic trees of bacterial MAGs were inferred from a concatenated alignment of 120 bacterial single-copy marker genes. The tree includes 8 named archaeal phyla. (**b**) Maximum-likelihood phylogenetic trees of archaeal MAGs inferred from a concatenated alignment of 122 archaeal single-copy marker genes. The tree includes 40 named bacterial phyla.

**Fig. 4:**
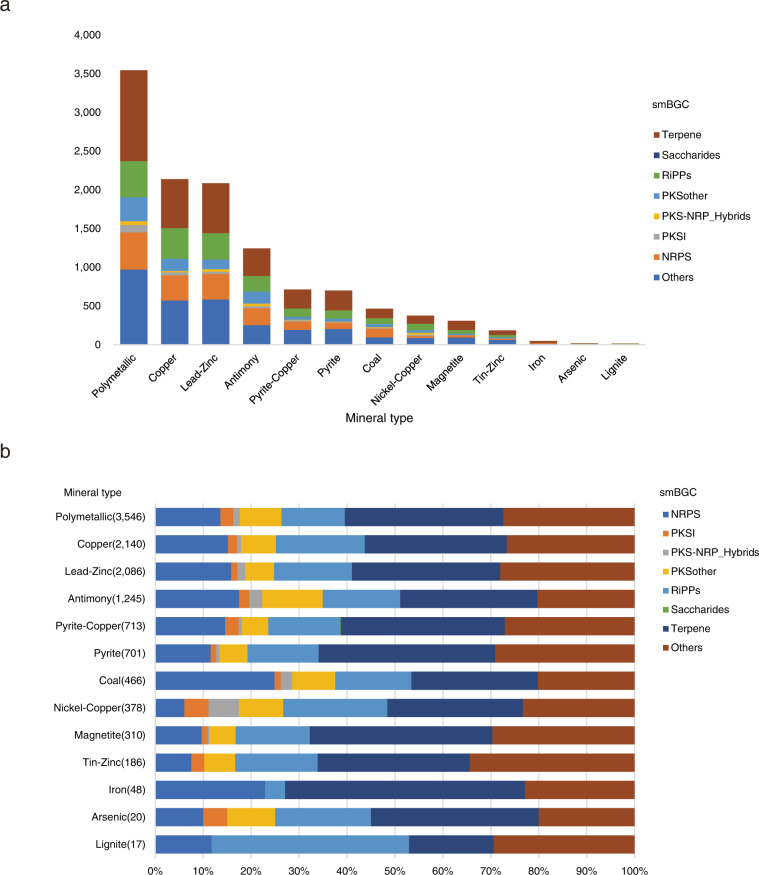
Secondary metabolite biosynthetic gene cluster (smBGC) distributions in 13 types of minerals. (**a**) The number of smBGCs in different types of minerals. (**b**) Relative frequency of smBGC types across 13 types of minerals.

In this study, data were collected and the GTDB species representative assignment for the binned MAGs and putative novel smBGCs of 111 samples from nine mineral types were analyzed. The same method was used to reanalyze public metagenomic datasets consisting of 68 samples of eight mineral types from seven countries. In total, this study obtained the analysis results of metagenomic datasets covering 179 samples of 13 projects across 13 mineral types from seven countries (Table 1, Supplementary Table 1, Figs. 1a,2). A total of 7,007 MAGs mined from the datasets exceeded the medium-quality level of the MIMAG standard^24^, including 981 MAGs determined to be high quality (Table 2, Supplementary Table 2). Further taxonomic analysis by GTDB-Tk showed that 1,394 MAGs were classified into 150 existed genera, while 5,613 MAGs were not assigned to existed genera; total of 667 MAGs could be assigned to 154 GTDB species representatives, while 6,340 MAGs were not assigned (Fig. 3, Supplementary Table 2). Overall, 11,856 smBGCs in eight categories were obtained from 7,007 MAGs (Table 3, Supplementary Table 3), and 10,899 smBGCs were identified as putative novel smBGCs for discovering novel secondary metabolites by querying each smBGC sequence against the NCBI nucleotide sequence collection (Supplementary Table 3). The analysis of the number of smBGCs in all mineral types showed that the greatest number of smBGCs was found in polymetallic mines, and the second largest number was found in copper mines. The descending order of smBGC abundance in the remaining mineral types was as follows: lead-zinc mines, antimony mines, pyrite-copper mines, pyrite mines, coal mines, nickel-copper mines, magnetite mines, tin-zinc mines, iron mines, arsenic mines, and lignite mines (Fig. 4a).

**Table 1:** Data information for each mineral type.

Mineral type	Sample number	Base number (Gb)	Base number per sample (Gb)	Country	Data source
Antimony	8	500.66	62.58	China	NODE: OEP001841
Arsenic	3	30.60	10.20	China	NODE: OEP001841
Coal	13	78.31	6.02	China and USA	SRA: SRP218093, SRP226684
Copper	39	1,660.60	42.58	Brazil, China, Germany, United Kingdom, and USA	NODE: OEP001841; SRA: SRP093762, SRP149873, SRP201756, SRP288126
Iron	17	62.81	3.69	USA	SRA: SRP009106
Lead-Zinc	24	1,638.82	68.28	China	NODE: OEP001841; SRA: ERP002170
Lignite	1	5.00	5.00	Germany	SRA: SRP093591
Magnetite	5	598.44	119.69	China	NODE: OEP001841
Nickel-Copper	15	49.15	3.28	Canada	SRA: SRP102076
Polymetallic	33	2,495.94	75.62	China and Sweden	NODE: OEP001841; SRA: SRP132763
Pyrite	13	477.59	36.74	China and Germany	NODE: OEP001841; SRA: SRP096619
Pyrite-Copper	6	454.49	75.75	China	NODE: OEP001841
Tin-Zinc	2	163.63	81.81	China	NODE: OEP001841

Giga base is a unit of length for DNA molecules, consisting of one billion nucleotides; abbreviated Gb, or Gbp for giga base pair (http://en.wikipedia.org/wiki/Base_pair).

**Table 2:** Quality control standards and metagenome-assembled genome (MAG) numbers in each quality level.

Quality level	Completeness	Contamination	Quality score	Number
High quality	≥90%	≤5%	>50	981
Medium quality	50%~90%	≤5%	>50	6,026

High-quality MAG requires the presence of the 23S, 16S, and 5S rRNA genes and at least 18 tRNAs.

**Table 3:** Numbers and percentages of smBGCs in eight categories classified by BIG-SCAPE.

Type of smBGCs	Number of smBGCs	Percentage of smBGCs
Terpene	3,751	31.64%
RiPPs	1,864	15.72%
NRPS	1,738	14.66%
PKSother	936	7.89%
PKS I	250	2.11%
PKS-NRP_Hybrids	181	1.53%
Saccharides	1	0.01%
Others	3,135	26.44%

## Methods

The workflow of data processing is depicted in Supplementary Fig. 1.

### Date source

AMD metagenomic datasets of 179 samples from 13 mineral types obtained from seven countries were used to analyze GTDB species representative assignment for the binned MAGs and putative novel smBGCs (Table 1, Supplementary Table 1, Figs. 1a,2), including 68 public and 111 private samples. The datasets of 68 publicly available samples were downloaded from the SRA database (up to November 17, 2020) using the following search strategies: (((((Mine AMD) OR acid mine drainage) OR mine tailings) OR acidic stream) AND WGS [Strategy]) AND METAGENOMIC [Source] and (mine drainage metagenome [Organism]) AND WGS [Strategy] AND METAGENOMIC [Source], and the Illumina sequence data were kept. A total of 111 private samples across nine mineral types were collected and sequenced in this study. Among them, 87 samples across four mineral types newly collected in this study came from the same mineral types as the datasets downloaded from the SRA database, and 24 samples were obtained from five new mineral types. A total of 122 samples from 10 mineral types constituted the AMD metagenomic datasets for China (Table 1, Fig. 1b).

### Quality control of raw data and metagenomic assembly

Trimmomatic is a flexible and efficient preprocessing tool used for reads processing of Illumina next-generation sequencing data, primarily for the filtering of adapter and low-quality sequences^25^. Quality control of the raw data for 179 samples in this study was performed using Trimmomatic (version 0.39) with Phred quality score cutoff of 20 and a minimum read length of 50 to remove the low-quality sequences. MetaSPAdes performs better in assembly compared to the other assembly tools, but it is time-consuming and requires very high memory^26,27^. MEGAHIT and metaSPAdes are both widely used tools for metagenome assembly^28–30^. Although metaSPAdes can provide high-quality assemblies across diverse data sets, MEGAHIT can provide acceptable assemblies with low memory usage and computational time^31^. Therefore, by a comprehensive consideration of the large volume of AMD samples analyzed and the affordable computational resources, we chose MEGAHIT^28,29^ as the software for metagenome assembly. The analysis of metagenome assembly was performed by MEGAHIT (version 1.2.9) in meta-sensitive mode to generate assembled contigs.

### Metagenomic binning

Compared to original binning software, automated methods with multiple binning methods, such as MAGO, MetaWRAP or DAS Tool, combine the strengths of a flexible set of established binning algorithms to generate more or better bins^32–34^. MetaWRAP is a widely used tool for the metagenome binning of both environmental^35–41^ and host-associated^42–44^ samples, and it can obtain the largest number of high-quality draft genomes in tested datasets with relatively less computational requirements^33,45^. Additionally, MAGO used DAS Tool for bin refinement, and MetaWRAP outperformed DAS Tool for datasets of varied complexity^33^. Therefore, we selected MetaWRAP for metagenomic binning in this study. For each assembly, contigs were binned using the binning module (parameter: –maxbin2 –concoct –metabat2), consolidated into a de-replicated bin set using the bin_refinement module (parameter: -c 50 -x 5), and the quality of bins was further improved by using the reassemble_bins module within MetaWRAP (version 1.3.2). A total of 8,035 binned MAGs were obtained from 179 samples by MetaWRAP taking 1224 hours of wall time using an HPC with multiple 2.10 GHz Intel Xeon E7-4380 CPUs and 2 TB of RAM.

The completeness and contamination of all MAGs were estimated using CheckM (version 1.1.2) with a lineage-specific workflow^46,47^. Based on these results, we selected 7,007 MAGs that were estimated to be at least 50% complete, with less than 5% contamination and that had a quality score of >50^36^. As additional indicators of completeness, we identified tRNA genes using tRNAscan-SE (version 2.0.9)^48^ and rRNA genes using Infernal (version 1.1.2)^49^ with models from the Rfam database^50^. Based on these results, we found that 981 of the 7,007 MAGs were classified as high quality based on the MIMAG standard (≥90% completeness, ≤5% contamination, ≥18/20 tRNA genes and the presence of 5S, 16S and 23S rRNA genes), with the remaining classified as medium quality (Table 2, Supplementary Table 2).

### Taxonomic assignment for bacterial and archaeal genomes

GTDB-Tk is a computationally efficient tool providing objective taxonomic assignment for bacterial and archaeal genomes based on the Genome Taxonomy Database (GTDB, http://gtdb.ecogenomic.org), and it is widely used for the classification of draft genomes directly from environmental- and human-associated samples^51^. Taxonomic analysis of each MAG was initially assigned using GTDB-Tk (version 1.4.0) based on the GTDB taxonomy R05-RS95^52^, and forty-eight phyla (eight archaeal phyla and 40 bacterial phyla) were obtained. GTDB-Tk analysis of 7,007 MAGs required 23 hours of wall time using an HPC with multiple 2.10 GHz Intel Xeon E7-4380 CPUs and 2 TB of RAM.

Based on the results of the GTDB-Tk analysis, a total of 1,707 MAGs were assigned to archaeal phyla, while 5,300 MAGs were assigned to bacterial phyla; 6,026 medium-quality MAGs were assigned to seven archaeal phyla and 38 bacterial phyla, while 981 high-quality MAGs were classified to four archaeal phyla and 31 bacterial phyla (Supplementary Table 2). In the genus level analysis, a total of 1,394 MAGs were classified into 150 extant genera, while 5,613 MAGs were not assigned. A total of 667 MAGs were assigned to GTDB representative genomes of 154 species, while 6,340 MAGs were not assigned to any GTDB species representative, data that would provide a large number of microbial resources for further research in the field of AMD bioremediation. *A. ferrooxidans*, *A. ferrivorans*, and *A. thiooxidan* have been demonstrated to be functional in AMD recovery^9,14,16^. In this study, *A. ferrooxidans* was found in copper mines, and *A. ferrivorans* and *A. thiooxidan* were found in polymetallic mines (Supplementary Table 2).

### Constructing a phylogeny of nonredundant MAGs

dRep can reduce the computational time for pairwise genome comparisons by sequentially applying a fast, inaccurate estimation of genome distance and a slow, accurate measure of average nucleotide identity, thereby achieving a 28 fold increase in speed with perfect recall and precision compared to previously developed algorithms^53^. All of the produced 7,007 qualified bin sets were aggregated and de-replicated at 95% average nucleotide identity (ANI) using dRep (version 3.2.0, parameters: -comp 50 -con 5 -sa 0.95 –pa 0.9), resulting in a total of 1,992 species-level qualified MAGs^54^. These 1,992 de-replicated MAGs were further refined using a maximum-likelihood phylogeny inferred from a concatenation of 120 bacterial or 122 archaeal marker genes produced by GTDB-Tk^51^. Bacterial and archaeal approximate maximum likelihood trees were built using FastTree (version 2.1.10) with WAG + GAMMA models^47,55–57^, and visualized by iTOL^58^.

A striking feature of these trees is the large number of major lineages without assignment of a GTDB species representative (Fig. 3)^51^. There were 24 phyla in Bacteria without assignment of a GTDB species representative, and very limited MAGs were assigned to GTDB species representatives of Bacteria in the 16 phyla of Proteobacteria, Actinobacteriota, Nitrospirota, Firmicutes_E, Firmicutes, SZUA-79, Bacteroidota, Campylobacterota, Desulfobacterota, Spirochaetota, Firmicutes_B, Patescibacteria, Acidobacteriota, Aquificota, Bdellovibrionota, and Deinococcota (Fig. 3a). No MAGs were assigned to GTDB species representatives of Archaea in the phyla of Halobacteriota, Methanobacteriota, Thermoproteota, Asgardarchaeota, and Aenigmatarchaeota, and very limited MAGs were assigned to GTDB species representatives of Archaea in the phyla of Nanoarchaeota, Micrarchaeota, and Thermoplasmatota (Fig. 3b).

### Mining of secondary metabolite biosynthetic gene clusters

Antibiotics & Secondary Metabolite Analysis Shell (antiSMASH, https://antismash.secondarymetabolites.org) is a tool that enables rapid identification, annotation, and analysis of smBGCs in genomes^59^. Since its first release in 2011, it has been the most widely used bioinformatics software for predicting smBGCs and the standard tool for smBGCs mining^60^. A total of 11,856 putative smBGCs were mined from 7,007 qualified MAGs across 13 mineral types using antiSMASH (version 5.1.2) called as follows: –cf-create-clusters –cb-general –cb-knownclusters –cb-subclusters –asf –pfam2go –smcog-trees –genefinding-tool prodigal, and in addition ignoring contigs with lengths shorter than 5 kb. antiSMASH analysis of 7,007 MAGs required 24 hours wall time using an HPC with multiple 2.60 GHz Intel (R) Xeon (R) Gold 6126 CPUs and 196 GB of RAM.

Using a threshold of 75% identity over 80% of the query length, 10,899 (91.93%) of 11,856 putative smBGCs were identified as putative novel smBGCs querying against the NCBI nucleotide sequence collection (downloaded 27 Jan 2021) by the command ‘blastn’ within the NCBI BLAST+ package (version 2.11)^61^ with an E-value cutoff of 1 × 10^−1^. Although many modular clusters were fragmented, we identified over 154 smBGC regions >50 kb in length and more than 1,834 > 30 kb. These smBGCs were further classified into eight categories using BIG-SCAPE with default parameters^62^. Among these eight smBGC categories, terpene had the largest number and made up the highest percentage of smBGCs at 3,751 smBGCs and 31.64%, respectively (Table 3, Supplementary Table 3).

## Data Records

The rawdata from the 111 private samples was deposited in NODE (https://www.biosino.org/node/project/detail/OEP001841)^63^, GSA (CRA006735)^64^, and NCBI SRA (PRJNA666025)^65^. A total of 7,007 MAGs with completeness ≥50%, contamination ≤5%, and had a quality score of >50 (the medium-quality level of the MIMAG standard) were obtained from 13 mineral types by metagenomic assembly and binning^47^. A total of 981 (14.00%) MAGs were assigned as high quality according to the MIMAG standard^24^. All 7,007 MAGs from the current study have been deposited in eLMSG (an eLibrary of Microbial Systematics and Genomics, https://www.biosino.org/elmsg/index) under accession numbers LMSG_G000004334.1–LMSG_G000011340.1^66^, NODE (https://www.biosino.org/node/analysis/detail/OEZ008530)^67^, and GenBank (PRJNA834572)^68^.

All 11,856 putative smBGCs from 7,007 MAGs of 13 mineral types were deposited in NODE (https://www.biosino.org/node/analysis/detail/OEZ008529)^69^ and GenBank (KFVK00000000)^70^. The classes of secondary metabolites synthesized by each smBGC across 13 mineral types were assigned (Fig. 4b). Non-ribosomal peptide synthetase (NRPS), post-translationally modified peptides (RiPPs), and terpene were found in all mineral types. The 13 mineral types in this study had relatively low numbers of smBGCs in the remaining smBGC categories, including type I polyketide synthesase (PKS I), PKSother, and PKS-NRP_hybrids. Saccharides are only found in pyrite-copper mines.

## Technical Validation

In order to ensure that the datasets from the SRA database only contained AMD metagenomic data, the metadata of these datasets from the SRA database and the scientific literature were manually curated. To select metagenomic datasets, only datasets for which the library strategy was WGS and the library source was METAGENOMIC were chosen. Because the pH values of AMD were usually 2–4^1^, datasets such as SRS1650501-SRS1650503, SRS872561, SRS962537, SRS963313, SRS963552, SRS963574, SRS963594, SRS963611, and SRS963627, whose pH values were greater than 4, were removed to further filter the AMD metagenomic datasets. For datasets that did not provide pH values, metadata in the SRA database and in the scientific literature were reviewed to preserve only AMD metagenomic datasets^71–75^.

The latitude and longitude of SRS1810936 was retrieved according to the geographic location of this sample. The mineral types of SRS5255199, SRS5255198, SRS5255197, and SRS2947527 were obtained through manual review of the metadata in the SRA database and scientific literature^76^.

The smBGCs number and type varied even within the same dRep cluster (Supplementary Table 4). Therefore, we used the 7,007 MAGs before de-replication for the smBGCs prediction. A total of 6,026 from 7,007 MAG belonged to medium quality according to the MIMAG standard^24^. Using the draft genome for the smBGCs mining by using antiSMASH would cause the number of detected gene clusters to be artificially high, and some contigs with gene cluster fragments might be left undetected^77^. In order to obtain better smBGCs, we ignored contigs with lengths shorter than 5 kb to increase the chance of the smBGCs we mined to have roles in secondary metabolite synthesis^78^. Although many modular clusters were fragmented, we identified over 154 BGC regions >50 kb in length and more than 1,834 > 30 kb.

**Fig. 5:**
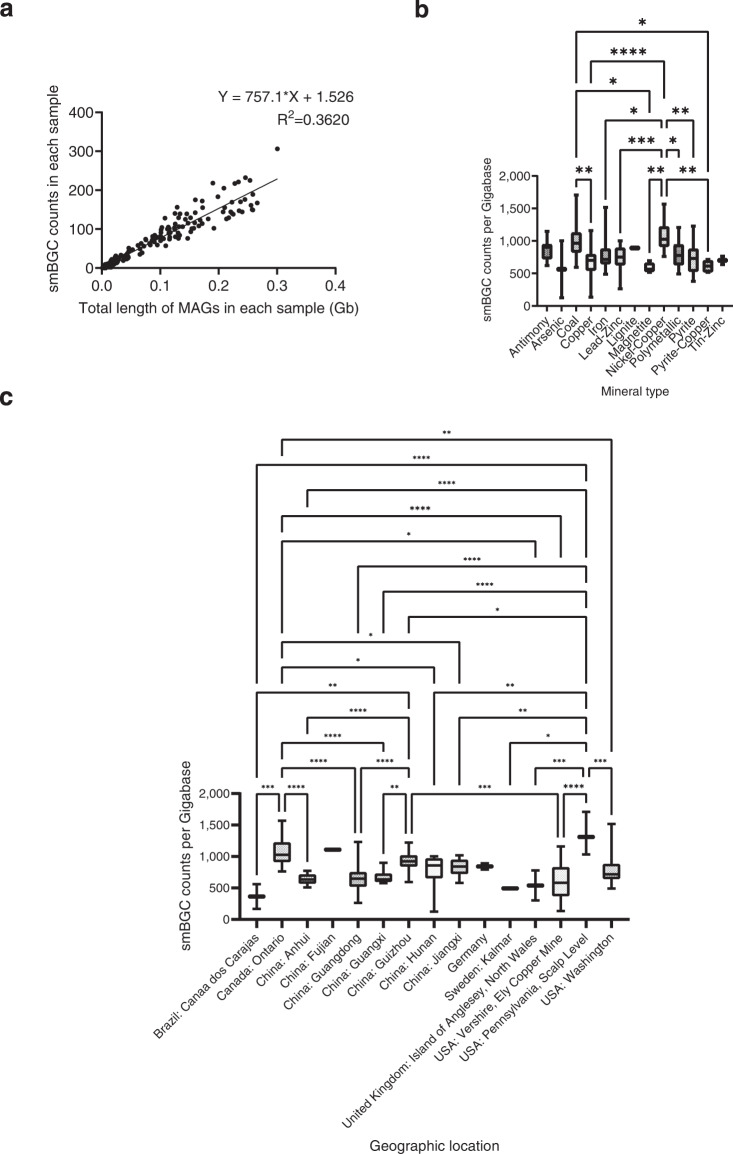
The diversity of secondary metabolite biosynthetic gene clusters (smBGCs) in different mineral types and geographic locations. (**a**) Correlation between the total number of smBGCs in each sample and the total length of quality MAGs in each sample. (**b**) smBGC counts per Gigabase (the total number of smBGCs in each sample divided by the total length of quality MAGs in each sample) plotted according to mineral type. (**c**) smBGC counts per Gigabase (the total number of smBGCs in each sample divided by the total length of quality MAGs in each sample) plotted according to geographic location. Data were analyzed using one-way ANOVA followed by Turkey’s test (^*^P < 0.05, ^**^P < 0.01, ^***^P < 0.001, and ^****^P < 0.0001).

We used linear regression to examine the sample size associated with the diversity of secondary metabolite biosynthetic gene clusters by GraphPad Prism (version 9.3.1). The total number of smBGCs in each sample showed a moderate positive correlation (R^2^ = 0.3620) with the total length of quality MAGs in each sample (Fig. 5a), demonstrating that the number of smBGCs may also be affected by other factors.

The box plots of smBGC counts per Gigabase in different geographic locations or mineral types were generated using GraphPad Prism (version 9.3.1). One-way ANOVA followed by Turkey’s test was used to analyze the differences among groups (P < 0.05) by GraphPad Prism (version 9.3.1). Notably, the smBGCs were most abundant in Canada: Ontario and USA: Pennsylvania, Scalp Level by the analysis of geographic location, while Coal mine and Nickel-Copper mine had relatively greater abundances of smBGCs according to the analysis of mineral type (Fig. 5b,c).

## Usage Notes

The datasets analyzed in this study were the largest AMD metagenomic datasets considered to date. Among the 68 samples from the SRA database, only 11 (16%) of the samples were from AMD metagenomic datasets from China. Through the collection and sequencing of 111 AMD samples in this study, the metagenomic data of AMD in southeastern China were obtained. This complemented the publicly available datasets in order to provide a better overview of the putative novel microorganisms and secondary metabolite resources in the AMD environment. These datasets can be further employed in research on AMD bioremediation, the mining of novel secondary metabolites for drug synthesis, and for the analysis of gene functions, metabolic pathways, and CNPS cycles in AMD.

## Supplementary information

Supplementary Figure 1

Supplementary Table 1

Supplementary Table 2

Supplementary Table 3

Supplementary Table 4

## Data Availability

The version and parameters of all of the bioinformatics tools used in this work are described in the Methods section.

## References

[1] Nancucheo I (2017). Recent Developments for Remediating Acidic Mine Waters Using Sulfidogenic Bacteria. Biomed Res. Int..

[2] Grimalt JO, Ferrer M, Macpherson E (1999). The mine tailing accident in Aznalcollar. Sci. Total Environ..

[3] Glukhova LB (2018). Isolation, Characterization, and Metal Response of Novel, Acid-Tolerant Penicillium spp. from Extremely Metal-Rich Waters at a Mining Site in Transbaikal (Siberia, Russia). Microb. Ecol..

[4] Schmidt U (2003). Enhancing phytoextraction: the effect of chemical soil manipulation on mobility, plant accumulation, and leaching of heavy metals. J. Environ. Qual..

[5] Johnson DB, Hallberg KB (2003). The microbiology of acidic mine waters. Res. Microbiol..

[6] Denef VJ, Mueller RS, Banfield JF (2010). AMD biofilms: using model communities to study microbial evolution and ecological complexity in nature. ISME J..

[7] Kuang JL (2013). Contemporary environmental variation determines microbial diversity patterns in acid mine drainage. ISME J..

[8] Fahy A (2015). 16S rRNA and As-Related Functional Diversity: Contrasting Fingerprints in Arsenic-Rich Sediments from an Acid Mine Drainage. Microb. Ecol..

[9] Mendez-Garcia C (2015). Microbial diversity and metabolic networks in acid mine drainage habitats. Front. Microbiol..

[10] Abinandan S, Subashchandrabose SR, Venkateswarlu K, Megharaj M (2018). Microalgae-bacteria biofilms: a sustainable synergistic approach in remediation of acid mine drainage. Appl. Microbiol. Biotechnol..

[11] Qian Z, Tianwei H, Mackey HR, van Loosdrecht MCM, Guanghao C (2019). Recent advances in dissimilatory sulfate reduction: From metabolic study to application. Water Res..

[12] Williams KP, Kelly DP (2013). Proposal for a new class within the phylum Proteobacteria, Acidithiobacillia classis nov., with the type order Acidithiobacillales, and emended description of the class Gammaproteobacteria. Int. J. Syst. Evol. Microbiol..

[13] Hedrich S, Johnson DB (2013). Acidithiobacillus ferridurans sp. nov., an acidophilic iron-, sulfur- and hydrogen-metabolizing chemolithotrophic gammaproteobacterium. Int. J. Syst. Evol. Microbiol..

[14] Hallberg KB, Gonzalez-Toril E, Johnson DB (2010). Acidithiobacillus ferrivorans, sp. nov.; facultatively anaerobic, psychrotolerant iron-, and sulfur-oxidizing acidophiles isolated from metal mine-impacted environments. Extremophiles.

[15] Chen L (2012). Acidithiobacillus caldus sulfur oxidation model based on transcriptome analysis between the wild type and sulfur oxygenase reductase defective mutant. PLoS One.

[16] Gupta A, Saha A, Sar P (2021). Thermoplasmata and Nitrososphaeria as dominant archaeal members in acid mine drainage sediment of Malanjkhand Copper Project, India. Arch. Microbiol..

[17] Yang L (2019). Acidithiobacillus thiooxidans and its potential application. Appl. Microbiol. Biotechnol..

[18] Stierle AA, Stierle DB (2014). Bioactive secondary metabolites from acid mine waste extremophiles. Nat. Prod. Commun..

[19] Keller NP (2019). Fungal secondary metabolism: regulation, function and drug discovery. Nat. Rev. Microbiol..

[20] Stierle DB, Stierle AA, Hobbs JD, Stokken J, Clardy J (2004). Berkeleydione and berkeleytrione, new bioactive metabolites from an acid mine organism. Org. Lett..

[21] Moutiez M, Belin P, Gondry M (2017). Aminoacyl-tRNA-Utilizing Enzymes in Natural Product Biosynthesis. Chem. Rev..

[22] Gondry M (2018). A Comprehensive Overview of the Cyclodipeptide Synthase Family Enriched with the Characterization of 32 New Enzymes. Front. Microbiol..

[23] Borthwick AD (2012). 2,5-Diketopiperazines: synthesis, reactions, medicinal chemistry, and bioactive natural products. Chem. Rev..

[24] Bowers RM (2017). Minimum information about a single amplified genome (MISAG) and a metagenome-assembled genome (MIMAG) of bacteria and archaea. Nat. Biotechnol..

[25] Bolger AM, Lohse M, Usadel B (2014). Trimmomatic: a flexible trimmer for Illumina sequence data. Bioinformatics.

[26] Forouzan E, Shariati P, Mousavi Maleki MS, Karkhane AA, Yakhchali B (2018). Practical evaluation of 11 de novo assemblers in metagenome assembly. J. Microbiol. Methods.

[27] Pasolli E (2019). Extensive Unexplored Human Microbiome Diversity Revealed by Over 150,000 Genomes from Metagenomes Spanning Age, Geography, and Lifestyle. Cell.

[28] Li D (2016). MEGAHIT v1.0: A fast and scalable metagenome assembler driven by advanced methodologies and community practices. Methods.

[29] Li D, Liu CM, Luo R, Sadakane K, Lam TW (2015). MEGAHIT: an ultra-fast single-node solution for large and complex metagenomics assembly via succinct de Bruijn graph. Bioinformatics.

[30] Nurk S, Meleshko D, Korobeynikov A, Pevzner P (2017). A. metaSPAdes: a new versatile metagenomic assembler. Genome Res..

[31] Fritz A (2019). CAMISIM: simulating metagenomes and microbial communities. Microbiome.

[32] Sieber CMK (2018). Recovery of genomes from metagenomes via a dereplication, aggregation and scoring strategy. Nat. Microbiol..

[33] Uritskiy GV, DiRuggiero J, Taylor J (2018). MetaWRAP-a flexible pipeline for genome-resolved metagenomic data analysis. Microbiome.

[34] Murovec B, Deutsch L, Stres B (2020). Computational Framework for High-Quality Production and Large-Scale Evolutionary Analysis of Metagenome Assembled Genomes. Mol. Biol. Evol..

[35] Dong X (2020). Thermogenic hydrocarbon biodegradation by diverse depth-stratified microbial populations at a Scotian Basin cold seep. Nat. Commun..

[36] Xu B (2022). A holistic genome dataset of bacteria, archaea and viruses of the Pearl River estuary. Sci. Data.

[37] Zhou L, Huang S, Gong J, Xu P, Huang X (2022). 500 metagenome-assembled microbial genomes from 30 subtropical estuaries in South China. Sci. Data.

[38] Zhang H (2022). Metagenome sequencing and 768 microbial genomes from cold seep in South China Sea. Sci. Data.

[39] Lee S (2021). Methane-derived carbon flows into host-virus networks at different trophic levels in soil. Proc. Natl. Acad. Sci. U S A.

[40] Bay SK (2021). Trace gas oxidizers are widespread and active members of soil microbial communities. Nat. Microbiol..

[41] Li J (2022). Intracellular silicification by early-branching magnetotactic bacteria. Sci. Adv..

[42] Yang H (2022). ABO genotype alters the gut microbiota by regulating GalNAc levels in pigs. Nature.

[43] von Schwartzenberg RJ (2021). Caloric restriction disrupts the microbiota and colonization resistance. Nature.

[44] Saheb Kashaf S, Almeida A, Segre JA, Finn RD (2021). Recovering prokaryotic genomes from host-associated, short-read shotgun metagenomic sequencing data. Nat. Protoc..

[45] Yang C (2021). A review of computational tools for generating metagenome-assembled genomes from metagenomic sequencing data. Comput. Struct. Biotechnol. J..

[46] Parks DH, Imelfort M, Skennerton CT, Hugenholtz P, Tyson GW (2015). CheckM: assessing the quality of microbial genomes recovered from isolates, single cells, and metagenomes. Genome Res..

[47] Nayfach S (2021). Publisher Correction: A genomic catalog of Earth’s microbiomes. Nat. Biotechnol..

[48] Chan PP, Lin BY, Mak AJ, Lowe TM (2021). tRNAscan-SE 2.0: improved detection and functional classification of transfer RNA genes. Nucleic Acids Res..

[49] Nawrocki EP, Eddy SR (2013). Infernal 1.1: 100-fold faster RNA homology searches. Bioinformatics.

[50] Kalvari I (2018). Rfam 13.0: shifting to a genome-centric resource for non-coding RNA families. Nucleic Acids Res..

[51] Chaumeil PA, Mussig AJ, Hugenholtz P, Parks DH (2019). GTDB-Tk: a toolkit to classify genomes with the Genome Taxonomy Database. Bioinformatics.

[52] Parks DH (2022). GTDB: an ongoing census of bacterial and archaeal diversity through a phylogenetically consistent, rank normalized and complete genome-based taxonomy. Nucleic Acids Res..

[53] Olm MR, Brown CT, Brooks B, Banfield JF (2017). dRep: a tool for fast and accurate genomic comparisons that enables improved genome recovery from metagenomes through de-replication. ISME J..

[54] Nayfach S (2021). Author Correction: A genomic catalog of Earth’s microbiomes. Nat. Biotechnol..

[55] Price MN, Dehal PS, Arkin AP (2010). FastTree 2—approximately maximum-likelihood trees for large alignments. PLoS One.

[56] Price MN, Dehal PS, Arkin AP (2009). FastTree: computing large minimum evolution trees with profiles instead of a distance matrix. Mol. Biol. Evol..

[57] Liu K, Linder CR, Warnow T (2011). RAxML and FastTree: comparing two methods for large-scale maximum likelihood phylogeny estimation. PLoS One.

[58] Letunic I, Bork P (2021). Interactive Tree Of Life (iTOL) v5: an online tool for phylogenetic tree display and annotation. Nucleic Acids Res..

[59] Blin K (2019). antiSMASH 5.0: updates to the secondary metabolite genome mining pipeline. Nucleic Acids Res..

[60] Blin K (2021). antiSMASH 6.0: improving cluster detection and comparison capabilities. Nucleic Acids Res..

[61] Camacho C (2009). BLAST+: architecture and applications. BMC Bioinformatics.

[62] Navarro-Munoz JC (2020). A computational framework to explore large-scale biosynthetic diversity. Nat. Chem. Biol..

[63] data*NODE The National Omics Data Encyclopedia* https://www.biosino.org/node/project/detail/OEP001841 (2021).

[64] (2022). dataGSA Genome Sequence Archive.

[65] (2022). NCBI Sequence Read Archive.

[66] data*eLMSG an eLibrary of Microbial Systematics and Genomics* https://www.biosino.org/elmsg/amdDetail (2022).

[67] data*NODE The National Omics Data Encyclopedia* https://www.biosino.org/node/analysis/detail/OEZ008530 (2022).

[68] dataZhang Q, Wang L, Liu W (2022). GenBank.

[69] data*NODE The National Omics Data Encyclopedia* https://www.biosino.org/node/analysis/detail/OEZ008529 (2022).

[70] dataZhang Q, Wang L, Liu W (2022). GenBank.

[71] Giddings LA (2020). Characterization of an acid rock drainage microbiome and transcriptome at the Ely Copper Mine Superfund site. PLoS One.

[72] Chen LX (2013). Shifts in microbial community composition and function in the acidification of a lead/zinc mine tailings. Environ. Microbiol..

[73] Krause S, Bremges A, Munch PC, McHardy AC, Gescher J (2017). Characterisation of a stable laboratory co-culture of acidophilic nanoorganisms. Sci. Rep..

[74] Muhling M (2016). Reconstruction of the Metabolic Potential of Acidophilic Sideroxydans Strains from the Metagenome of an Microaerophilic Enrichment Culture of Acidophilic Iron-Oxidizing Bacteria from a Pilot Plant for the Treatment of Acid Mine Drainage Reveals Metabolic Versatility and Adaptation to Life at Low pH. Front. Microbiol..

[75] Arif S, Nacke H, Hoppert M (2021). Metagenome-Assembled Genome Sequences of a Biofilm Derived from Marsberg Copper Mine. Microbiol. Resour. Announc..

[76] Liljeqvist, M. *et al*. Metagenomic analysis reveals adaptations to a cold-adapted lifestyle in a low-temperature acid mine drainage stream. *FEMS Microbiol. Ecol*. **91** (2015). 10.1093/femsec/fiv011 25764459

[77] Blin K (2013). antiSMASH 2.0—a versatile platform for genome mining of secondary metabolite producers. Nucleic Acids Res..

[78] Wei B (2021). An atlas of bacterial secondary metabolite biosynthesis gene clusters. Environ. Microbiol..

