# Supplementary material for: Mining of novel secondary metabolite biosynthetic gene clusters from acid mine drainage
Source: Sci Data. 2022 Dec 9;9:760. doi: 10.1038/s41597-022-01866-6 (PMC9734747; doi:10.1038/s41597-022-01866-6)
Supplement: Supplementary file 1 — Supplementary Figure 1 [file 41597_2022_1866_MOESM1_ESM.pdf]

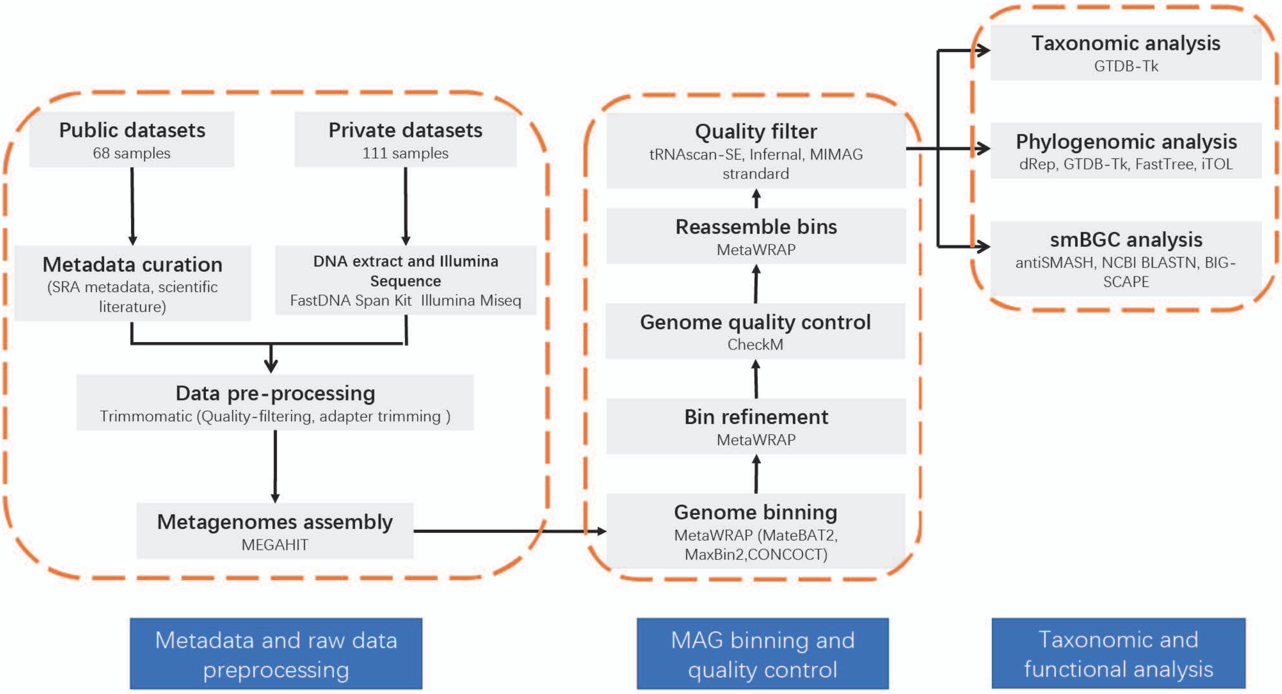

Supplimentary Figure 1. Schematic overview of sampling and metagenomic analysis performed in this study. Each rectangle symbolizes processes containing descriptions (in bold), methods or tools used in the corresponding analysis.
