# Supplementary material for: Mining of novel secondary metabolite biosynthetic gene clusters from acid mine drainage
Source: Sci Data. 2022 Dec 9;9:760. doi: 10.1038/s41597-022-01866-6 (PMC9734747; doi:10.1038/s41597-022-01866-6)
Supplement: Supplementary file 2 — Supplementary Table 1 [file 41597_2022_1866_MOESM2_ESM.pdf]

Supplementary Table 1. AMD metagenomic datasets of 179 samples from 13 mineral types of seven countries

Project	Run ID	Sample	Run ID	Sequence data (base pair)	File size (Gigabyte)	Sampling date	Release date	Geographic location	Mineral type	Environmental package	Coordinates	DOI
SRP226684	SRR10333320	SRS5562336	SRR10333320	5666609585	13.60	2018-11	2019/10/24	China: Guizhou, Fuquan	Coal	soil	26.3237 N 107.1424 E	NA
SRP226684	SRR10333321	SRS5562335	SRR10333321	7664765302	18.40	2018-11	2019/10/24	China: Guizhou, Fuquan	Coal	soil	26.3236 N 107.1424 E	NA
SRP226684	SRR10333322	SRS5562334	SRR10333322	5853436660	14.00	2018-11	2019/10/24	China: Guizhou, Fuquan	Coal	soil	26.3235 N 107.1424 E	NA
SRP226684	SRR10333323	SRS5562333	SRR10333323	6308675872	15.20	2018-11	2019/10/24	China: Guizhou, Fuquan	Coal	soil	26.3234 N 107.1424 E	NA
SRP226684	SRR10333324	SRS5562332	SRR10333324	7769566990	18.60	2018-11	2019/10/24	China: Guizhou, Fuquan	Coal	soil	26.3233 N 107.1424 E	NA
SRP226684	SRR10333325	SRS5562331	SRR10333325	10539387093	26.00	2018-11	2019/10/24	China: Guizhou, Fuquan	Coal	soil	26.3232 N 107.1424 E	NA
SRP226684	SRR10333326	SRS5562330	SRR10333326	6226290030	15.00	2018-11	2019/10/24	China: Guizhou, Fuquan	Coal	soil	26.3231 N 107.1424 E	NA
SRP226684	SRR10333327	SRS5562329	SRR10333327	10521990232	26.00	2018-11	2019/10/24	China: Guizhou, Fuquan	Coal	soil	26.3230 N 107.1424 E	NA
SRP226684	SRR10333328	SRS5562328	SRR10333328	7264920079	17.40	2018-11	2019/10/24	China: Guizhou, Fuquan	Coal	soil	26.3229 N 107.1424 E	NA
SRP226684	SRR10333329	SRS5562327	SRR10333329	7480612039	18.00	2018-11	2019/10/24	China: Guizhou, Fuquan	Coal	soil	26.3228 N 107.1424 E	NA
SRP288126	SRR12886061	SRS7564330	SRR12886061	4395775938	9.80	2018-11	2020/10/23	Germany: Kilianstollen, Marsberg	Copper	biofilm	51.4529 N 8.8597 E	10.1128/MRA.01253-20
SRP093762	SRR5161788	SRS1910798	SRR5161788	3376284098	0.50	2014-07	2017/1/15	Brazil: Canaa dos Carajas	Copper	water	6.44321 S 50.03583 W	NA
SRP093762	SRR5161789	SRS1910799	SRR5161789	2334854614	0.21	2015-06	2017/1/15	Brazil: Canaa dos Carajas	Copper	sediment	6.44321 S 50.03583 W	NA
SRP093762	SRR5161792	SRS1910802	SRR5161792	1583447487	3.50	2016-04	2017/1/15	Brazil: Canaa dos Carajas	Copper	sediment	6.44321 S 50.03583 W	NA
SRP093762	SRR5161794	SRS1910798	SRR5161794	3752109491	1.45	2014-07	2017/1/15	Brazil: Canaa dos Carajas	Copper	water	6.44321 S 50.03583 W	NA
SRP093762	SRR5161795	SRS1910798	SRR5161795	3780596855	1.55	2014-07	2017/1/15	Brazil: Canaa dos Carajas	Copper	water	6.44321 S 50.03583 W	NA
SRP093762	SRR5161798	SRS1910798	SRR5161798	2611146026	0.93	2014-07	2017/1/15	Brazil: Canaa dos Carajas	Copper	water	6.44321 S 50.03583 W	NA
SRP093762	SRR5161800	SRS1910802	SRR5161800	775922328	1.67	2016-04	2017/1/15	Brazil: Canaa dos Carajas	Copper	water	6.44321 S 50.03583 W	NA
SRP093762	SRR5161805	SRS1910798	SRR5161805	5496384926	1.63	2014-07	2017/1/15	Brazil: Canaa dos Carajas	Copper	water	6.44321 S 50.03583 W	NA
SRP093762	SRR5161807	SRS1910802	SRR5161807	1398428431	3.10	2016-04	2017/1/15	Brazil: Canaa dos Carajas	Copper	water	6.44321 S 50.03583 W	NA
SRP149873	SRR7268657	SRS3384136	SRR7268657	9054098057	20.80	2014-10	2018/6/6	United Kingdom: Island of Anglesey, North Wales	Copper	sediment	53.38711 N 4.34961 W	NA
SRP149873	SRR7303207	SRS3413320	SRR7303207	1257392964	3.00	2014-10	2018/6/13	United Kingdom: Island of Anglesey, North Wales	Copper	sediment	53.38711 N 4.34961 W	NA
SRP149873	SRR7303208	SRS3413319	SRR7303208	1781791079	4.70	2014-10	2018/6/13	United Kingdom: Island of Anglesey, North Wales	Copper	sediment	53.38711 N 4.34961 W	NA
SRP201756	SRR9320187	SRS4989160	SRR9320187	5170312161	12.40	2017-07-28	2019/7/1	USA: Vershire, Ely Copper Mine	Copper	sediment	43.91924 N 72.28636 W	10.1371/journal.pone.0237599
SRP201756	SRR9320188	SRS4989159	SRR9320188	5366031524	13.00	2017-07-28	2019/7/1	USA: Vershire, Ely Copper Mine	Copper	sediment	43.91924 N 72.28635 W	10.1371/journal.pone.0237599
SRP201756	SRR9320191	SRS4989156	SRR9320191	3122081707	7.40	2018-01-14	2019/7/1	USA: Vershire, Ely Copper Mine	Copper	sediment	43.91924 N 72.28638 W	10.1371/journal.pone.0237599
SRP201756	SRR9320192	SRS4989155	SRR9320192	4717421819	11.20	2017-07-28	2019/7/1	USA: Vershire, Ely Copper Mine	Copper	sediment	43.91924 N 72.28637 W	10.1371/journal.pone.0237599
SRP201756	SRR9320193	SRS4989154	SRR9320193	3978397897	9.60	2018-01-14	2019/7/1	USA: Vershire, Ely Copper Mine	Copper	sediment	43.91924 N 72.2864 W	10.1371/journal.pone.0237599
SRP201756	SRR9320194	SRS4989153	SRR9320194	2587829123	6.20	2018-01-14	2019/7/1	USA: Vershire, Ely Copper Mine	Copper	sediment	43.91924 N 72.28639 W	10.1371/journal.pone.0237599
SRP201756	SRR9320195	SRS4989152	SRR9320195	7232754188	17.60	2017-07-28	2019/7/1	USA: Vershire, Ely Copper Mine	Copper	water	43.91924 N 72.28642 W	10.1371/journal.pone.0237599
SRP201756	SRR9320196	SRS4989151	SRR9320196	4553183032	11.00	2017-07-28	2019/7/1	USA: Vershire, Ely Copper Mine	Copper	water	43.91924 N 72.28641 W	10.1371/journal.pone.0237599
SRP201756	SRR9320197	SRS4989150	SRR9320197	5188992799	12.60	2017-07-28	2019/7/1	USA: Vershire, Ely Copper Mine	Copper	water	43.91924 N 72.28644 W	10.1371/journal.pone.0237599
SRP201756	SRR9320198	SRS4989149	SRR9320198	4006642393	9.60	2017-07-28	2019/7/1	USA: Vershire, Ely Copper Mine	Copper	water	43.91924 N 72.28643 W	10.1371/journal.pone.0237599
SRP201756	SRR9320199	SRS4989148	SRR9320199	5052414648	12.20	2017-07-28	2019/7/1	USA: Vershire, Ely Copper Mine	Copper	water	43.91924 N 72.28645 W	10.1371/journal.pone.0237599
SRP009106	SRR359000	SRS269165	SRR359000	3622359800	1.06	missing	2012/2/29	USA: Washington, Richmond Mine C75	Iron	biofilm	44.67733 N 122.52219 W	10.1126/science.1218389
SRP009106	SRR359001	SRS269166	SRR359001	4032263400	1.66	missing	2012/2/29	USA: Washington, Richmond Mine C75	Iron	biofilm	44.67733 N 122.52219 W	10.1126/science.1218389
SRP009106	SRR359002	SRS269167	SRR359002	4162020200	1.17	missing	2012/2/29	USA: Washington, Richmond Mine C75	Iron	biofilm	44.67733 N 122.52219 W	10.1126/science.1218389
SRP009106	SRR359019	SRS269168	SRR359019	3172798000	1.14	missing	2012/2/29	USA: Washington, Richmond Mine C75	Iron	biofilm	44.67733 N 122.52219 W	10.1126/science.1218389
SRP009106	SRR359020	SRS269169	SRR359020	4889973000	1.14	missing	2012/2/29	USA: Washington, Richmond Mine C75	Iron	biofilm	44.67733 N 122.52219 W	10.1126/science.1218389
SRP009106	SRR359021	SRS269170	SRR359021	3899000600	1.49	missing	2012/2/29	USA: Washington, Richmond Mine C75	Iron	biofilm	44.67733 N 122.52219 W	10.1126/science.1218389
SRP009106	SRR359022	SRS269171	SRR359022	2875970200	1.72	missing	2012/2/29	USA: Washington, Richmond Mine C75	Iron	biofilm	44.67733 N 122.52219 W	10.1126/science.1218389
SRP009106	SRR359023	SRS269172	SRR359023	2436500600	1.17	missing	2012/2/29	USA: Washington, Richmond Mine C75	Iron	biofilm	44.67733 N 122.52219 W	10.1126/science.1218389
SRP009106	SRR359024	SRS269173	SRR359024	2992426200	1.23	missing	2012/2/29	USA: Washington, Richmond Mine C10	Iron	biofilm	44.67733 N 122.52219 W	10.1126/science.1218389
SRP009106	SRR359025	SRS269174	SRR359025	3567349200	1.40	missing	2012/2/29	USA: Washington, Richmond Mine C10	Iron	biofilm	44.67733 N 122.52219 W	10.1126/science.1218389
SRP009106	SRR359026	SRS269175	SRR359026	4241209400	0.51	missing	2012/2/29	USA: Washington, Richmond Mine C10	Iron	biofilm	44.67733 N 122.52219 W	10.1126/science.1218389
SRP009106	SRR359027	SRS269176	SRR359027	2926889000	1.19	missing	2012/2/29	USA: Washington, Richmond Mine AB20	Iron	biofilm	44.67733 N 122.52219 W	10.1126/science.1218389
SRP009106	SRR359028	SRS269177	SRR359028	3495710600	10.80	missing	2012/2/29	USA: Washington, Richmond Mine AB20	Iron	biofilm	44.67733 N 122.52219 W	10.1126/science.1218389
SRP009106	SRR359029	SRS269178	SRR359029	3121150600	1.32	missing	2012/2/29	USA: Washington, Richmond Mine AB20	Iron	biofilm	44.67733 N 122.52219 W	10.1126/science.1218389
SRP009106	SRR359030	SRS269179	SRR359030	5743950400	0.51	missing	2012/2/29	USA: Washington, Richmond Mine AB20	Iron	biofilm	44.67733 N 122.52219 W	10.1126/science.1218389
SRP009106	SRR359031	SRS269180	SRR359031	4157008000	0.11	missing	2012/2/29	USA: Washington, Richmond Mine ABmuck	Iron	biofilm	44.67733 N 122.52219 W	10.1126/science.1218389
SRP009106	SRR359032	SRS269181	SRR359032	3469019400	10.60	missing	2012/2/29	USA: Washington, Richmond Mine ABmuck	Iron	biofilm	44.67733 N 122.52219 W	10.1126/science.1218389
ERP002170	ERR225990	ERS211215	ERR225990	2394384480	7.60	missing	2010/7/1	China: Guangdong, Fankou	Lead-Zinc	sediment	25.04931 N 113.65947 E	10.1111/1462-2920.12114
SRP093591	SRR5040535	SRS1810936	SRR5040535	1925182336	1.43	missing	2016/11/26	Germany: Lusatia, lignite mining district	Lignite	water	51.26940 N 14.61787 E	10.3389/fmicb.2016.02082
SRP093591	SRR5040536	SRS1810936	SRR5040536	3077902790	1.62	missing	2016/11/26	Germany: Lusatia, lignite mining district	Lignite	water	51.26940 N 14.61787 E	10.3389/fmicb.2016.02082
SRP132763	SRR6714249	SRS2947527	SRR6714249	21587468568	52.00	2014	2018/2/13	Sweden: Kalmar	Polymetallic	water	56.65897 N 16.36564 E	NA
SRP132763	SRR6714250	SRS2947527	SRR6714250	21894042962	54.00	2014	2018/2/13	Sweden: Kalmar	Polymetallic	water	56.65897 N 16.36564 E	NA
SRP132763	SRR6714251	SRS2947527	SRR6714251	21321786786	52.00	2014	2018/2/13	Sweden: Kalmar	Polymetallic	water	56.65897 N 16.36564 E	NA
SRP132763	SRR6714252	SRS2947527	SRR6714252	21763237400	54.00	2014	2018/2/13	Sweden: Kalmar	Polymetallic	water	56.65897 N 16.36564 E	NA
SRP218093	SRR9951156	SRS5255199	SRR9951156	1004455687	2.50	2014-06	2020/1/4	USA: Pennsylvania, Scalp Level	Coal	biofilm	40.2498 N 78.8489 W	NA
SRP218093	SRR9951157	SRS5255198	SRR9951157	1010671074	2.40	2014-06	2020/1/4	USA: Pennsylvania, Scalp Level	Coal	biofilm	40.2498 N 78.8489 W	NA
SRP218093	SRR9951158	SRS5255197	SRR9951158	998439874	2.40	2014-06	2020/1/4	USA: Pennsylvania, Scalp Level	Coal	biofilm	40.2498 N 78.8489 W	NA
SRP102076	SRR5352522	SRS2053959	SRR5352522	2811260318	8.00	2014-09	2017/3/17	Canada: Ontario	Nickel-Copper	water	46.65 N 81.32 W	NA
SRP102076	SRR5352523	SRS2053960	SRR5352523	2817628592	8.00	2014-09	2017/3/17	Canada: Ontario	Nickel-Copper	water	46.65 N 81.32 W	NA
SRP102076	SRR5352524	SRS2053962	SRR5352524	2139628022	6.20	2014-09	2017/3/17	Canada: Ontario	Nickel-Copper	water	46.65 N 81.32 W	NA
SRP102076	SRR5352525	SRS2053961	SRR5352525	3714439034	10.60	2014-11	2017/3/17	Canada: Ontario	Nickel-Copper	water	46.65 N 81.32 W	NA
SRP102076	SRR5352526	SRS2053963	SRR5352526	3070835056	8.80	2014-11	2017/3/17	Canada: Ontario	Nickel-Copper	water	46.65 N 81.32 W	NA
SRP102076	SRR5352527	SRS2053971	SRR5352527	4797264866	13.60	2014-11	2017/3/17	Canada: Ontario	Nickel-Copper	water	46.65 N 81.32 W	NA
SRP102076	SRR5352528	SRS2053964	SRR5352528	4152275312	11.80	2014-09	2017/3/17	Canada: Ontario	Nickel-Copper	water	46.65 N 81.32 W	NA
SRP102076	SRR5352529	SRS2053966	SRR5352529	4043559238	11.40	2014-09	2017/3/17	Canada: Ontario	Nickel-Copper	water	46.65 N 81.32 W	NA
SRP102076	SRR5352530	SRS2053965	SRR5352530	2730198384	7.80	2014-09	2017/3/17	Canada: Ontario	Nickel-Copper	water	46.65 N 81.32 W	NA
SRP102076	SRR5352531	SRS2053967	SRR5352531	4583474536	13.00	2014-11	2017/3/17	Canada: Ontario	Nickel-Copper	water	46.65 N 81.32 W	NA
SRP102076	SRR5352532	SRS2053968	SRR5352532	2052498304	5.80	2014-09	2017/3/17	Canada: Ontario	Nickel-Copper	water	46.65 N 81.32 W	NA
SRP102076	SRR5352533	SRS2053969	SRR5352533	3534494448	8.50	2014-11	2017/4/19	Canada: Ontario	Nickel-Copper	water	46.65 N 81.32 W	NA
SRP102076	SRR5352534	SRS2053970	SRR5352534	3361108094	9.60	2014-09	2017/3/17	Canada: Ontario	Nickel-Copper	water	46.65 N 81.32 W	NA
SRP102076	SRR5352535	SRS2053972	SRR5352535	2518256596	7.20	2014-11	2017/3/17	Canada: Ontario	Nickel-Copper	water	46.65 N 81.32 W	NA
SRP102076	SRR5352536	SRS2053973	SRR5352536	2818879174	8.00	2014-09	2017/3/17	Canada: Ontario	Nickel-Copper	water	46.65 N 81.32 W	NA
SRP096619	SRR5169067	SRS1916008	SRR5169067	11589706854	54.00	2014-08-04	2017/1/17	Germany: Drei Kronen und Ehrt (Harz Mountains)	Pyrite	biofilm	51.76413 N 10.82539 E	10.1038/s41598-017-03315-6
SRP096619	SRR5169068	SRS1916009	SRR5169068	14984776446	70.00	2014-08-04	2017/1/17	Germany: Drei Kronen und Ehrt (Harz Mountains)	Pyrite	biofilm	51.76413 N 10.82539 E	10.1038/s41598-017-03315-6
OEP001841	OER128081	OES073711	OER128081	60670392600	70.00	2017/8/1	NA	China: Hunan, Shuikoushan	Lead-Zinc	sediment	26.53399 N 112.60751 E	NA
OEP001841	OER128082	OES073742	OER128082	74130584400	86.00	2017/9/1	NA	China: Jiangxi, Yongping	Copper	sediment	28.19144 N 117.76680 E	NA
OEP001841	OER128083	OES073735	OER128083	71668482000	82.00	2017/9/1	NA	China: Guangdong, Yunfu	Pyrite	sediment	22.96413 N 112.01483 E	NA
OEP001841	OER128084	OES073734	OER128084	66505791600	76.00	2017/9/1	NA	China: Guangdong, Yunfu	Pyrite	sediment	22.96480 N 112.01442 E	NA
OEP001841	OER128085	OES073745	OER128085	63860720400	74.00	2017/9/1	NA	China: Jiangxi, Yinshan	Lead-Zinc	sediment	28.97790 N 117.60154 E	NA
OEP001841	OER128086	OES073738	OER128086	54934868400	64.00	2017/9/1	NA	China: Guangdong, Yunfu	Pyrite	sediment	22.96400 N 112.01499 E	NA
OEP001841	OER128087	OES073737	OER128087	70684928400	82.00	2017/9/1	NA	China: Guangdong, Yunfu	Pyrite	sediment	22.96513 N 112.01455 E	NA
OEP001841	OER128088	OES073736	OER128088	72976766400	84.00	2017/9/1	NA	China: Guangdong, Yunfu	Pyrite	sediment	22.96442 N 112.01401 E	NA
OEP001841	OER128089	OES073748	OER128089	73661193000	84.00	2017/9/1	NA	China: Jiangxi, Yinshan	Lead-Zinc	sediment	28.97724 N 117.60100 E	NA
OEP001841	OER128090	OES073744	OER128090	63652179600	74.00	2017/9/1	NA	China: Jiangxi, Yinshan	Lead-Zinc	sediment	28.97777 N 117.60132 E	NA
OEP001841	OER128091	OES073681	OER128091	54064090800	62.00	2017/9/1	NA	China: Jiangxi, Dexing	Copper	sediment	29.00254 N 117.72751 E	NA
OEP001841	OER128092	OES073741	OER128092	92616323400	106.00	2017/9/1	NA	China: Jiangxi, Yongping	Copper	sediment	28.19167 N 117.76679 E	NA
OEP001841	OER128093	OES073679	OER128093	87542679600	100.00	2017/9/1	NA	China: Jiangxi, Dexing	Copper	sediment	29.00251 N 117.72754 E	NA
OEP001841	OER128094	OES073680	OER128094	87225340800	100.00	2017/9/1	NA	China: Jiangxi, Dexing	Copper	sediment	29.00205 N 117.72761 E	NA
OEP001841	OER128095	OES073712	OER128095	81719136000	94.00	2017/8/1	NA	China: Hunan, Shuikoushan	Lead-Zinc	sediment	26.53451 N 112.60740 E	NA
OEP001841	OER128096	OES073687	OER128096	90399309600	104.00	2017/8/1	NA	China: Hunan, Huangshaping	Lead-Zinc	sediment	25.67774 N 112.70612 E	NA
OEP001841	OER128097	OES073688	OER128097	83981041800	96.00	2017/8/1	NA	China: Hunan, Huangshaping	Lead-Zinc	sediment	25.67864 N 112.70689 E	NA
OEP001841	OER128098	OES073743	OER128098	95180438400	56.00	2017/9/1	NA	China: Jiangxi, Yongping	Copper	sediment	28.19104 N 117.76723 E	NA
OEP001841	OER128099	OES073713	OER128099	81708682800	94.00	2017/8/1	NA	China: Hunan, Shuikoushan	Lead-Zinc	sediment	26.53373 N 112.60794 E	NA
OEP001841	OER128100	OES073739	OER128100	74760999600	86.00	2017/9/1	NA	China: Jiangxi, Yongping	Copper	sediment	28.19111N 117.766944 E	NA
OEP001841	OER128101	OES073746	OER128101	47126827800	54.00	2017/9/1	NA	China: Jiangxi, Yinshan	Lead-Zinc	sediment	28.97713 N 117.60179 E	NA
OEP001841	OER128102	OES073747	OER128102	65038125600	74.00	2017/9/1	NA	China: Jiangxi, Yinshan	Lead-Zinc	sediment	28.97701 N 117.60166 E	NA
OEP001841	OER128103	OES073740	OER128103	61859157000	72.00	2017/9/1	NA	China: Jiangxi, Yongping	Copper	sediment	28.19121 N 117.76714 E	NA
OEP001841	OER128104	OES073710	OER128104	85336296600	98.00	2017/8/1	NA	China: Hunan, Shuikoushan	Lead-Zinc	sediment	26.53394 N 112.60750 E	NA
OEP001841	OER128105	OES073690	OER128105	75400021200	86.00	2017/8/1	NA	China: Guizhou, Jiuan	Polymetallic	sediment	26.52342 N 106.57084 E	NA
OEP001841	OER128106	OES073692	OER128106	75407721600	86.00	2017/8/1	NA	China: Guizhou, Jiuan	Polymetallic	sediment	26.52340 N 106.57101 E	NA
OEP001841	OER128107	OES073689	OER128107	78452504400	90.00	2017/8/1	NA	China: Guizhou, Jiuan	Polymetallic	sediment	26.52341 N 106.57044 E	NA
OEP001841	OER128108	OES073701	OER128108	73386692400	84.00	2017/8/1	NA	China: Guangxi, Longshenao	Polymetallic	sediment	24.83398 N 107.56856 E	NA
OEP001841	OER128109	OES073703	OER128109	70201506000	80.00	2017/8/1	NA	China: Guangxi, Longshenao	Polymetallic	sediment	24.83311 N 107.56905 E	NA
OEP001841	OER128110	OES073702	OER128110	91395280200	104.00	2017/8/1	NA	China: Guangxi, Longshenao	Polymetallic	sediment	24.83428 N 107.56987 E	NA
OEP001841	OER128111	OES073695	OER128111	81423097200	94.00	2017/8/1	NA	China: Guizhou, Longli	Polymetallic	sediment	26.38489 N 106.97926 E	NA
OEP001841	OER128112	OES073704	OER128112	81722317800	94.00	2017/8/1	NA	China: Guangxi, Longshenao	Polymetallic	sediment	24.83497 N 107.56874 E	NA
OEP001841	OER128113	OES073696	OER128113	58381980600	68.00	2017/8/1	NA	China: Guizhou, Longli	Polymetallic	sediment	26.38501 N 106.97967 E	NA
OEP001841	OER128114	OES073749	OER128114	74155674000	86.00	2017/8/1	NA	China: Guizhou, Zhijin	Polymetallic	sediment	26.59952 N 105.72847 E	NA
OEP001841	OER128115	OES073694	OER128115	69357787200	80.00	2017/8/1	NA	China: Guizhou, Jiuan	Polymetallic	sediment	26.52490 N 106.57054 E	NA
OEP001841	OER128116	OES073750	OER128116	71141686200	82.00	2017/8/1	NA	China: Guizhou, Zhijin	Polymetallic	sediment	26.60078 N 105.72879 E	NA
OEP001841	OER128117	OES073752	OER128117	74546034600	86.00	2017/8/1	NA	China: Guizhou, Zhijin	Polymetallic	sediment	26.60100 N 105.72895 E	NA
OEP001841	OER128118	OES073751	OER128118	69619090200	80.00	2017/8/1	NA	China: Guizhou, Zhijin	Polymetallic	sediment	26.60056 N 105.72823 E	NA
OEP001841	OER128119	OES073753	OER128119	72289009800	84.00	2017/8/1	NA	China: Guizhou, Zhijin	Polymetallic	sediment	26.59978 N 105.72803 E	NA
OEP001841	OER128120	OES073665	OER128120	74898471600	86.00	2017/8/1	NA	China: Guizhou, Banpo	Antimony	sediment	25.80975 N 107.61861 E	NA
OEP001841	OER128121	OES073754	OER128121	66626176200	76.00	2017/8/1	NA	China: Guizhou, Zhijin	Polymetallic	sediment	26.60048 N 105.72877 E	NA
OEP001841	OER128122	OES073666	OER128122	77977669200	90.00	2017/8/1	NA	China: Guizhou, Banpo	Antimony	sediment	25.80952 N 107.61943 E	NA
OEP001841	OER128123	OES073667	OER128123	83741757600	96.00	2017/8/1	NA	China: Guizhou, Banpo	Antimony	sediment	25.80953 N 107.61812 E	NA
OEP001841	OER128124	OES073668	OER128124	85082971800	98.00	2017/8/1	NA	China: Guizhou, Banpo	Antimony	sediment	25.80943 N 107.61872 E	NA
OEP001841	OER128125	OES073669	OER128125	76806594600	88.00	2017/8/1	NA	China: Guizhou, Banpo	Antimony	sediment	25.80991 N 107.61917 E	NA
OEP001841	OER128126	OES073670	OER128126	77701457400	90.00	2017/8/1	NA	China: Guizhou, Banpo	Antimony	sediment	25.80977 N 107.61804 E	NA
OEP001841	OER128127	OES073697	OER128127	75921951000	88.00	2017/8/1	NA	China: Guizhou, Longli	Polymetallic	sediment	26.38476 N 106.97943 E	NA
OEP001841	OER128128	OES073698	OER128128	76949973600	88.00	2017/8/1	NA	China: Guizhou, Longli	Polymetallic	sediment	26.38403 N 106.97936 E	NA
OEP001841	OER128129	OES073699	OER128129	80824381200	92.00	2017/8/1	NA	China: Guizhou, Longli	Polymetallic	sediment	26.38529 N 106.97886 E	NA
OEP001841	OER128130	OES073729	OER128130	69408460800	80.00	2017/8/1	NA	China: Guangxi, Wuyi	Polymetallic	sediment	25.14804 N 107.50085 E	NA
OEP001841	OER128131	OES073731	OER128131	77787606600	90.00	2017/8/1	NA	China: Guangxi, Wuyi	Polymetallic	sediment	25.14854 N 107.50044 E	NA
OEP001841	OER128132	OES073730	OER128132	75907885200	88.00	2017/8/1	NA	China: Guangxi, Wuyi	Polymetallic	sediment	25.14815 N 107.50097 E	NA
OEP001841	OER128133	OES073733	OER128133	79586565000	92.00	2017/8/1	NA	China: Guangxi, Wuyi	Polymetallic	sediment	25.14787 N 107.50102 E	NA
OEP001841	OER128134	OES073732	OER128134	75509835000	86.00	2017/8/1	NA	China: Guangxi, Wuyi	Polymetallic	sediment	25.14896 N 107.50062 E	NA
OEP001841	OER128135	OES073677	OER128135	77042609400	88.00	2017/8/1	NA	China: Guangxi, Dashan	Tin-Zinc	sediment	25.14957 N 107.50345 E	NA
OEP001841	OER128136	OES073715	OER128136	86881948800	100.00	2017/10/1	NA	China: Anhui, Tongling	Copper	sediment	30.94045 N 117.99034 E	NA
OEP001841	OER128137	OES073678	OER128137	86586682200	100.00	2017/8/1	NA	China: Guangxi, Dashan	Tin-Zinc	sediment	25.14945 N 107.50348 E	NA
OEP001841	OER128138	OES073716	OER128138	67430559600	78.00	2017/10/1	NA	China: Anhui, Tongling	Copper	sediment	30.94079 N 117.98923 E	NA
OEP001841	OER128139	OES073700	OER128139	70906320000	82.00	2017/8/1	NA	China: Guangxi, Longshenao	Polymetallic	sediment	24.83368 N 107.56887 E	NA
OEP001841	OER128140	OES073725	OER128140	75031002000	86.00	2017/10/1	NA	China: Anhui, Wuhu	Polymetallic	sediment	31.08277 N 118.25650 E	NA
OEP001841	OER128141	OES073727	OER128141	76413946800	88.00	2017/10/1	NA	China: Anhui, Wuhu	Polymetallic	sediment	31.08301 N 118.25624 E	NA
OEP001841	OER128142	OES073717	OER128142	69321118200	80.00	2017/10/1	NA	China: Anhui, Tongling	Copper	sediment	30.93995 N 117.98902 E	NA
OEP001841	OER128143	OES073718	OER128143	80555413200	92.00	2017/10/1	NA	China: Anhui, Tongling	Copper	sediment	30.94054 N 117.98997 E	NA
OEP001841	OER128144	OES073728	OER128144	76720532400	88.00	2017/10/1	NA	China: Anhui, Wuhu	Polymetallic	sediment	31.08291 N 118.25676 E	NA
OEP001841	OER128145	OES073707	OER128145	77416177800	88.00	2017/10/1	NA	China: Anhui, Maanshan	Magnetite	sediment	31.67776 N 118.62794 E	NA
OEP001841	OER128146	OES073714	OER128146	73075816200	84.00	2017/10/1	NA	China: Anhui, Tongling	Copper	sediment	30.94038 N 117.98977 E	NA
OEP001841	OER128147	OES073682	OER128147	74783776200	86.00	2017/9/1	NA	China: Guangdong, Fankou	Lead-Zinc	sediment	25.04902 N 113.66334 E	NA
OEP001841	OER128148	OES073719	OER128148	76584707400	88.00	2017/10/1	NA	China: Anhui, Tongling	Copper	sediment	30.94537 N 117.99243 E	NA
OEP001841	OER128149	OES073683	OER128149	77506624200	88.00	2017/9/1	NA	China: Guangdong, Fankou	Lead-Zinc	sediment	25.04902 N 113.66401 E	NA
OEP001841	OER128150	OES073671	OER128150	73268585400	84.00	2017/9/1	NA	China: Guangdong, Daobaoshan	Pyrite-Copper	sediment	24.55382 N 113.745228 E	NA
OEP001841	OER128151	OES073684	OER128151	74417749200	86.00	2017/9/1	NA	China: Guangdong, Fankou	Lead-Zinc	sediment	25.04911 N 113.66402 E	NA
OEP001841	OER128152	OES073673	OER128152	81442501800	94.00	2017/9/1	NA	China: Guangdong, Daobaoshan	Pyrite-Copper	sediment	24.55954 N 113.745074 E	NA
OEP001841	OER128153	OES073685	OER128153	79126610400	90.00	2017/9/1	NA	China: Guangdong, Fankou	Lead-Zinc	sediment	25.04903 N 113.66357 E	NA
OEP001841	OER128154	OES073672	OER128154	78982314000	90.00	2017/9/1	NA	China: Guangdong, Daobaoshan	Pyrite-Copper	sediment	24.55645 N 113.745245 E	NA
OEP001841	OER128155	OES073674	OER128155	66426703800	76.00	2017/9/1	NA	China: Guangdong, Daobaoshan	Pyrite-Copper	sediment	24.55204 N 113.74549 E	NA
OEP001841	OER128156	OES073675	OER128156	84587011200	98.00	2017/9/1	NA	China: Guangdong, Daobaoshan	Pyrite-Copper	sediment	24.55190 N 113.74509 E	NA
OEP001841	OER128157	OES073691	OER128157	76477618800	88.00	2017/8/1	NA	China: Guizhou, Jiuan	Polymetallic	sediment	26.52349 N 106.57095 E	NA
OEP001841	OER128158	OES073686	OER128158	78620863200	90.00	2017/9/1	NA	China: Guangdong, Fankou	Lead-Zinc	sediment	25.04978 N 113.66339 E	NA
OEP001841	OER128159	OES073693	OER128159	79073918400	90.00	2017/8/1	NA	China: Guizhou, Jiuan	Polymetallic	sediment	26.52360 N 106.57112 E	NA
OEP001841	OER128160	OES073705	OER128160	2.53305E+11	100.00	2017/10/1	NA	China: Anhui, Maanshan	Magnetite	sediment	31.67721 N 118.62743 E	NA
OEP001841	OER128161	OES073755	OER128161	73419621000	84.00	2017/9/1	NA	China: Fujian, Zijin	Copper	sediment	26.27256 N 118.26912 E	NA
OEP001841	OER128162	OES073724	OER128162	83204371800	96.00	2017/10/1	NA	China: Anhui, Wuhu	Polymetallic	sediment	31.08256 N 118.25620 E	NA
OEP001841	OER128163	OES073706	OER128163	87891264000	100.00	2017/10/1	NA	China: Anhui, Maanshan	Magnetite	sediment	31.67797 N 118.62755 E	NA
OEP001841	OER128164	OES073709	OER128164	1.01192E+11	116.00	2017/10/1	NA	China: Anhui, Maanshan	Magnetite	sediment	31.67800 N 118.62801 E	NA
OEP001841	OER128165	OES073708	OER128165	78630691200	90.00	2017/10/1	NA	China: Anhui, Maanshan	Magnetite	sediment	31.67721 N 118.627434 E	NA
OEP001841	OER128166	OES073726	OER128166	76146548400	88.00	2017/10/1	NA	China: Anhui, Wuhu	Polymetallic	sediment	31.08204 N 118.25691 E	NA
OEP001841	OER128167	OES073720	OER128167	81807285000	94.00	2017/10/1	NA	China: Anhui, Tongling	Copper	sediment	30.94547 N 117.99276 E	NA
OEP001841	OER128168	OES073676	OER128168	69784088400	80.00	2017/9/1	NA	China: Guangdong, Daobaoshan	Pyrite-Copper	sediment	24.55236 N 113.74557 E	NA
OEP001841	OER128169	OES073721	OER128169	80229447000	92.00	2017/10/1	NA	China: Anhui, Tongling	Copper	sediment	30.94578 N 117.99301 E	NA
OEP001841	OER128170	OES073723	OER128170	67837720200	78.00	2017/10/1	NA	China: Anhui, Tongling	Copper	sediment	30.94545 N 117.99378 E	NA
OEP001841	OER128171	OES073722	OER128171	90138793200	104.00	2017/10/1	NA	China: Anhui, Tongling	Copper	sediment	30.94595 N 117.99244 E	NA
OEP001841	OER128065	OES073653	OER128065	13428528000	15.20	2015/12/1	NA	China: Hunan	Antimony	sediment	27.70000 N 111.40000 E	NA
OEP001841	OER128066	OES073654	OER128066	11020313400	12.60	2015/12/1	NA	China: Hunan	Antimony	sediment	27.70000 N 111.40000 E	NA
OEP001841	OER128067	OES073659	OER128067	16640274988	17.40	2014/10/1	NA	China: Guangdong, Fankou	Lead-Zinc	water	25.05000 N 113.67000 E	NA
OEP001841	OER128068	OES073658	OER128068	3.4559E+11	213.00	2014/10/1	NA	China: Guangdong, Fankou	Lead-Zinc	water	25.68000 N 112.71000 E	NA
OEP001841	OER128069	OES073650	OER128069	11396402534	12.90	2014/12/1	NA	China: Guangdong, Fankou	Lead-Zinc	sediment	25.05000 N 113.67000 E	NA
OEP001841	OER128070	OES073649	OER128070	12553252424	14.30	2014/12/1	NA	China: Guangdong, Fankou	Lead-Zinc	sediment	25.05000 N 113.67000 E	NA
OEP001841	OER128071	OES073652	OER128071	9983676654	11.30	2014/12/1	NA	China: Guangdong, Fankou	Lead-Zinc	sediment	25.05000 N 113.67000 E	NA
OEP001841	OER128072	OES073651	OER128072	9336681674	10.70	2014/12/1	NA	China: Guangdong, Fankou	Lead-Zinc	sediment	25.05000 N 113.67000 E	NA
OEP001841	OER128073	OES073663	OER128073	49317697800	56.00	2015/10/1	NA	China: Guangdong, Fankou	Lead-Zinc	sediment	28.19000 N 117.77000 E	NA
OEP001841	OER128074	OES073661	OER128074	38182686000	44.00	2015/10/1	NA	China: Guangdong, Daobaoshan	Pyrite	sediment	28.19000 N 117.77000 E	NA

OEP001841	OER128075	OES073662	OER128075	32601061200	38.00	2015/10/1	NA	China: Guangxi, Dachang	Copper	sediment	28.19000 N 117.77000 E	NA
OEP001841	OER128076	OES073660	OER128076	60764010600	70.00	2015/10/1	NA	China: Jiangxi, ChengMenShan	Copper	sediment	28.19000 N 117.77000 E	NA
OEP001841	OER128077	OES073655	OER128077	10105962600	11.40	missing	NA	China: Hunan	Arsenic	sediment	29.65000 N 111.04000 E	NA
OEP001841	OER128078	OES073657	OER128078	9182316000	10.40	missing	NA	China: Hunan	Arsenic	sediment	29.65000 N 111.04000 E	NA
OEP001841	OER128079	OES073656	OER128079	11308829400	12.80	missing	NA	China: Hunan	Arsenic	sediment	29.65000 N 111.04000 E	NA
OEP001841	OER128080	OES073664	OER128080	42981525000	50.00	2015/10/1	NA	China: Guangdong, Yunfu	Pyrite	sediment	28.19000 N 117.77000 E	NA
OEP001841	OER131351	OES074477	OER131351	6264789166	13.00	2016/1/1	NA	China: Guangdong, Guangzhou	Pyrite	water	23.09000 N 113.30000 E	NA
OEP001841	OER131352	OES074478	OER131352	7279541829	15.20	2016/6/1	NA	China: Guangdong, Guangzhou	Pyrite	water	23.09000 N 113.30000 E	NA
OEP001841	OER131353	OES074479	OER131353	6130226880	12.80	2016/7/1	NA	China: Guangdong, Guangzhou	Pyrite	water	23.09000 N 113.30000 E	NA
OEP001841	OER131354	OES074480	OER131354	13409545050	14.00	2016/8/1	NA	China: Guangdong, Guangzhou	Pyrite	water	23.09000 N 113.30000 E	NA
