# Supplementary material for: Mining of novel secondary metabolite biosynthetic gene clusters from acid mine drainage
Source: Sci Data. 2022 Dec 9;9:760. doi: 10.1038/s41597-022-01866-6 (PMC9734747; doi:10.1038/s41597-022-01866-6)
Supplement: Supplementary file 3 — Supplementary Table 2 [file 41597_2022_1866_MOESM3_ESM.pdf]

Supplementary Table 2. A total of 7,007 metagenome-assembled genomes (MAGs) were mined from the datasets

Genome ID	Retained after dRep	dRep cluster ID	Mineral type	Completeness(%)	Contamination(%)	Quality score	Number of 5srRNA	Number of 16srRNA	Number of 23srRNA	Number of tRNA	Quality tier	N50(base pair)	Genome length (base pair)	GC content (%)	Contig number	GTDB taxonomy
LMSG_G000004663.1	yes	906_0	Copper	60.93	0.86	56.62	2	0	1	15	Medium quality	131471	2526541	59.70	45	d__Bacteria;p__Acidobacteriota;c__Acidobacteriae;o__Acidobacteriales;f__Acidobacteriaceae;g__Granulicella_A;s__
LMSG_G000004661.1	yes	1445_0	Copper	95.30	1.00	90.29	0	1	0	19	Medium quality	40703	2734532	68.80	117	d__Bacteria;p__Proteobacteria;c__Alphaproteobacteria;o__Caulobacterales;f__Caulobacteraceae;g__Palsa-881;s__
LMSG_G000004334.1	yes	633_1	Copper	97.94	0.00	97.94	1	1	1	20	High quality	22216	2722178	66.90	205	d__Bacteria;p__Eremiobacterota;c__Eremiobacteria;o__UBP12;f__UBA5184;g__;s__
LMSG_G000004662.1	yes	508_0	Copper	72.94	0.00	72.94	1	1	4	20	Medium quality	54342	763742	30.60	32	d__Bacteria;p__Patescibacteria;c__;o__;f__;g__;s__
LMSG_G000004335.1	yes	450_0	Copper	98.01	0.99	93.06	2	5	4	20	High quality	86238	4844210	62.50	88	d__Bacteria;p__Chloroflexota;c__Ktedonobacteria;o__Ktedonobacterales;f__;g__;s__
LMSG_G000004336.1	yes	418_0	Copper	100.00	0.00	100.00	1	1	1	19	High quality	165454	2891598	53.00	28	d__Bacteria;p__FCPU426;c__Palsa-1180;o__Palsa-1180;f__Palsa-1180;g__Bog-1183;s__
LMSG_G000004664.1	yes	924_0	Copper	97.22	2.56	84.40	0	0	0	20	Medium quality	47192	6231052	56.80	274	d__Bacteria;p__Acidobacteriota;c__Acidobacteriae;o__Acidobacteriales;f__Koribacteraceae;g__Sulfotelmatobacter;s__
LMSG_G000004665.1	yes	638_0	Copper	96.48	0.00	96.48	1	0	0	19	Medium quality	214092	2637078	63.40	27	d__Bacteria;p__Eremiobacterota;c__Eremiobacteria;o__UBP12;f__UBA5184;g__PALSA-1484;s__
LMSG_G000004666.1	yes	1573_1	Copper	88.57	1.72	79.95	1	1	1	19	Medium quality	29719	2896215	59.50	125	d__Bacteria;p__Acidobacteriota;c__Acidobacteriae;o__Acidobacteriales;f__Acidobacteriaceae;g__;s__
LMSG_G000004667.1	yes	907_0	Copper	70.25	0.86	65.94	1	1	1	14	Medium quality	24819	2716522	62.20	178	d__Bacteria;p__Acidobacteriota;c__Acidobacteriae;o__Acidobacteriales;f__Acidobacteriaceae;g__Terracidiphilus;s__
LMSG_G000004668.1	yes	1730_1	Copper	86.51	0.00	86.51	1	1	1	16	Medium quality	35305	842049	39.20	51	d__Bacteria;p__Dependentiae;c__Babeliae;o__Babeliales;f__Babeliaceae;g__BOG-1370;s__
LMSG_G000004669.1	yes	1046_0	Copper	97.28	1.74	88.56	2	0	0	20	Medium quality	31836	10897844	68.90	556	d__Bacteria;p__Planctomycetota;c__Planctomycetes;o__Isosphaerales;f__Isosphaeraceae;g__;s__
LMSG_G000004670.1	yes	1401_0	Copper	93.62	0.92	89.04	1	0	0	16	Medium quality	29960	5297675	65.10	240	d__Bacteria;p__Acidobacteriota;c__Acidobacteriae;o__Bryobacterales;f__Bryobacteraceae;g__;s__
LMSG_G000004343.1	yes	1121_0	Copper	94.16	3.93	74.54	1	1	1	18	High quality	70284	3342692	68.90	83	d__Bacteria;p__Proteobacteria;c__Gammaproteobacteria;o__Steroidobacterales;f__Steroidobacteraceae;g__;s__
LMSG_G000004671.1	yes	1045_1	Copper	96.12	0.00	96.12	0	0	0	18	Medium quality	19041	6395287	65.60	494	d__Bacteria;p__Planctomycetota;c__Planctomycetes;o__Isosphaerales;f__Isosphaeraceae;g__;s__
LMSG_G000004337.1	yes	302_0	Copper	99.14	2.14	88.46	1	6	8	20	High quality	38914	3208434	52.30	195	d__Bacteria;p__Actinobacteriota;c__Acidimicrobiia;o__Acidimicrobiales;f__;g__;s__
LMSG_G000004672.1	yes	2_0	Copper	73.52	0.07	73.19	0	0	0	19	Medium quality	14164	982780	44.30	88	d__Bacteria;p__Dependentiae;c__Babeliae;o__Babeliales;f__GWF2-32-72;g__;s__
LMSG_G000004673.1	yes	1090_0	Copper	80.66	4.70	57.19	1	0	0	16	Medium quality	24762	3719349	66.80	198	d__Bacteria;p__Proteobacteria;c__Gammaproteobacteria;o__Steroidobacterales;f__Steroidobacteraceae;g__;s__
LMSG_G000004674.1	yes	1026_0	Copper	90.63	2.81	76.59	0	1	1	20	Medium quality	42119	3245199	67.60	105	d__Bacteria;p__Elusimicrobiota;c__Elusimicrobia;o__UBA1565;f__UBA9628;g__PALSA-1176;s__
LMSG_G000004675.1	yes	1764_0	Copper	63.50	0.00	63.50	1	1	1	20	Medium quality	548072	551879	47.10	5	d__Bacteria;p__Patescibacteria;c__Paceibacteria;o__UBA9983;f__UBA2163;g__GWA1-54-10;s__
LMSG_G000004676.1	yes	395_0	Copper	74.75	0.00	74.75	1	2	2	20	Medium quality	83068	1495103	36.00	52	d__Bacteria;p__Patescibacteria;c__Microgenomatia;o__Levybacterales;f__UBA12049;g__;s__
LMSG_G000004677.1	yes	396_0	Copper	72.27	0.00	72.27	1	1	2	20	Medium quality	164227	1374959	36.30	22	d__Bacteria;p__Patescibacteria;c__Microgenomatia;o__Levybacterales;f__UBA12049;g__;s__
LMSG_G000004678.1	yes	641_0	Copper	98.23	2.31	86.68	0	1	1	18	Medium quality	29469	4614150	58.20	245	d__Bacteria;p__Chloroflexota;c__Ktedonobacteria;o__Ktedonobacterales;f__;g__;s__
LMSG_G000004345.1	yes	507_0	Copper	95.37	1.03	90.23	1	4	2	20	High quality	55585	3670980	53.70	118	d__Bacteria;p__Armatimonadota;c__Chthonomonadetes;o__Chthonomonadales;f__Chthonomonadaceae;g__;s__
LMSG_G000004679.1	yes	1605_0	Copper	85.64	0.23	84.52	1	2	5	19	Medium quality	50424	1250482	42.50	81	d__Bacteria;p__Verrucomicrobiota_A;c__Chlamydiia;o__Parachlamydiales;f__Rhabdochlamydiaceae;g__PALSA-1444;s__
LMSG_G000004680.1	yes	3_1	Copper	95.37	0.00	95.37	1	0	1	18	Medium quality	83970	4549639	50.20	114	d__Bacteria;p__Armatimonadota;c__Chthonomonadetes;o__Chthonomonadales;f__;g__;s__
LMSG_G000004681.1	yes	1133_0	Copper	97.83	4.01	77.77	0	1	0	20	Medium quality	145512	3143885	62.20	47	d__Bacteria;p__Eremiobacterota;c__Eremiobacteria;o__UBP12;f__UBA5184;g__PALSA-1484;s__
LMSG_G000004682.1	yes	1097_0	Copper	80.26	0.87	75.92	1	0	1	18	Medium quality	44156	1912718	41.00	85	d__Bacteria;p__Proteobacteria;c__Alphaproteobacteria;o__UBA9655;f__UBA9655;g__UBA9655;s__
LMSG_G000004344.1	yes	1020_1	Copper	92.13	3.75	73.41	1	1	1	19	High quality	66286	2407815	62.60	107	d__Bacteria;p__Elusimicrobiota;c__Elusimicrobia;o__UBA1565;f__UBA9628;g__;s__
LMSG_G000006206.1	yes	1603_0	Copper	99.80	1.60	91.80	3	1	0	19	Medium quality	87847	2859231	51.10	41	d__Bacteria;p__Spirochaetota;c__Spirochaetia;o__Spirochaetales;f__;g__;s__
LMSG_G000004683.1	yes	1298_3	Copper	73.10	0.86	68.79	1	0	1	16	Medium quality	6669	3192245	70.50	603	d__Bacteria;p__Proteobacteria;c__Gammaproteobacteria;o__Burkholderiales;f__Burkholderiaceae;g__Thiomonas;s__
LMSG_G000004346.1	yes	313_0	Copper	93.05	0.93	88.43	1	2	2	20	High quality	472191	2871635	49.10	37	d__Bacteria;p__Armatimonadota;c__Fimbriimonadia;o__Fimbriimonadales;f__Fimbriimonadaceae;g__;s__
LMSG_G000004684.1	yes	494_0	Copper	95.04	0.24	93.87	1	0	1	19	Medium quality	226901	2484933	57.00	48	d__Bacteria;p__Cyanobacteria;c__Cyanobacteriia;o__Neosynechococcales;f__Neosynechococcaceae;g__;s__
LMSG_G000004685.1	yes	1051_0	Copper	94.18	0.00	94.18	2	0	0	20	Medium quality	34180	6177492	63.80	316	d__Bacteria;p__Planctomycetota;c__Planctomycetes;o__Isosphaerales;f__Isosphaeraceae;g__;s__
LMSG_G000006323.1	yes	1588_1	Copper	98.25	1.36	91.47	1	0	0	18	Medium quality	122366	3933066	33.90	70	d__Bacteria;p__Proteobacteria;c__Gammaproteobacteria;o__Diplorickettsiales;f__Diplorickettsiaceae;g__;s__
LMSG_G000004686.1	yes	1622_1	Copper	88.68	0.68	85.31	1	1	0	18	Medium quality	55478	1858587	40.20	80	d__Bacteria;p__Verrucomicrobiota_A;c__Chlamydiia;o__Parachlamydiales;f__SM23-39;g__;s__
LMSG_G000004687.1	yes	779_0	Copper	82.47	1.14	76.78	0	1	0	17	Medium quality	8169	2021343	62.00	319	d__Bacteria;p__Actinobacteriota;c__Acidimicrobiia;o__Acidimicrobiales;f__Bog-793;g__;s__
LMSG_G000004688.1	yes	1608_0	Copper	81.18	0.00	81.18	0	0	0	18	Medium quality	18184	2006107	44.90	171	d__Bacteria;p__Proteobacteria;c__Gammaproteobacteria;o__DSM-16500;f__DSM-16500;g__;s__
LMSG_G000004338.1	yes	315_1	Copper	96.20	0.99	91.25	2	1	1	19	High quality	23438	3237855	46.50	214	d__Bacteria;p__Chloroflexota;c__Ktedonobacteria;o__Ktedonobacterales;f__;g__;s__
LMSG_G000004693.1	yes	1823_0	Copper	62.40	0.00	62.40	0	2	1	16	Medium quality	8372	480076	42.50	71	d__Bacteria;p__Patescibacteria;c__Paceibacteria;o__UBA9983;f__UBA2163;g__C7867-001;s__
LMSG_G000004689.1	yes	1406_0	Copper	97.86	0.00	97.86	1	0	0	19	Medium quality	181494	4061573	62.00	45	d__Bacteria;p__Acidobacteriota;c__Acidobacteriae;o__Acidobacteriales;f__SCQP01;g__;s__
LMSG_G000004347.1	yes	1030_1	Copper	93.63	0.28	92.21	1	1	1	19	High quality	14658	3155094	69.70	305	d__Bacteria;p__Acidobacteriota;c__Acidobacteriae;o__Acidobacteriales;f__SCQP01;g__SCQP01;s__
LMSG_G000004348.1	yes	1655_0	Copper	97.32	0.89	92.86	1	1	3	19	High quality	301334	2752682	50.60	34	d__Bacteria;p__Bdellovibrionota;c__Bdellovibrionia;o__Bdellovibrionales;f__UBA1609;g__RBG-16-40-8;s__
LMSG_G000004690.1	yes	1520_2	Copper	87.82	4.78	63.91	1	0	1	19	Medium quality	21978	6577221	63.10	410	d__Bacteria;p__Acidobacteriota;c__Acidobacteriae;o__Bryobacterales;f__Bryobacteraceae;g__;s__
LMSG_G000004691.1	yes	363_1	Copper	68.42	0.00	68.42	1	0	1	17	Medium quality	31599	736962	36.40	43	d__Bacteria;p__Patescibacteria;c__Saccharimonadia;o__Saccharimonadales;f__UBA4665;g__;s__
LMSG_G000004692.1	yes	650_0	Copper	72.85	0.50	70.38	0	0	0	15	Medium quality	4032	3680457	71.00	1028	d__Bacteria;p__Firmicutes_E;c__Thermaerobacteria;o__;f__;g__;s__
LMSG_G000004700.1	yes	1837_1	Copper	94.09	2.27	82.73	1	0	2	20	Medium quality	263714	5887807	41.80	66	d__Bacteria;p__Planctomycetota;c__Planctomycetes;o__Gemmatales;f__Gemmataceae;g__;s__
LMSG_G000004694.1	yes	1811_0	Copper	65.73	0.00	65.73	0	1	0	18	Medium quality	100265	757486	58.50	18	d__Bacteria;p__Patescibacteria;c__Paceibacteria;o__UBA6257;f__2-01-FULL-56-20;g__;s__
LMSG_G000004695.1	yes	1626_0	Copper	73.21	1.35	66.46	0	1	0	16	Medium quality	8112	1402659	40.70	219	d__Bacteria;p__Verrucomicrobiota_A;c__Chlamydiia;o__Parachlamydiales;f__SM23-39;g__;s__
LMSG_G000004696.1	yes	1173_0	Copper	60.34	0.00	60.34	3	0	2	12	Medium quality	4973	1158656	41.30	264	d__Bacteria;p__Proteobacteria;c__Gammaproteobacteria;o__UBA6186;f__UBA6186;g__;s__
LMSG_G000004697.1	yes	327_0	Copper	67.59	0.46	65.28	1	0	1	18	Medium quality	72095	822790	42.60	27	d__Bacteria;p__Patescibacteria;c__Saccharimonadia;o__Saccharimonadales;f__UBA4665;g__UBA5150;s__
LMSG_G000004349.1	yes	326_1	Copper	95.72	2.28	84.33	1	5	8	20	High quality	105215	2995819	40.20	76	d__Bacteria;p__Cyanobacteria;c__Vampirovibrionia;o__Obscuribacterales;f__Obscuribacteraceae;g__PSRHO1;s__
LMSG_G000004698.1	yes	1493_0	Copper	94.74	0.73	91.11	0	0	0	18	Medium quality	12612	2681642	66.60	300	d__Bacteria;p__Proteobacteria;c__Alphaproteobacteria;o__Acetobacterales;f__Acetobacteraceae;g__;s__
LMSG_G000004699.1	yes	1021_0	Copper	53.83	0.00	53.83	0	0	0	9	Medium quality	3264	1323575	62.30	442	d__Bacteria;p__Elusimicrobiota;c__Elusimicrobia;o__UBA1565;f__UBA9628;g__02-02-FULL-57-9;s__
LMSG_G000004707.1	yes	1194_0	Copper	79.20	2.14	68.52	1	0	1	19	Medium quality	11181	2242223	57.20	268	d__Bacteria;p__Actinobacteriota;c__Acidimicrobiia;o__Acidimicrobiales;f__;g__;s__
LMSG_G000004701.1	yes	1380_0	Copper	63.44	1.29	56.99	1	0	0	18	Medium quality	15133	3588406	71.70	267	d__Bacteria;p__Myxococcota;c__Myxococcia;o__Myxococcales;f__;g__;s__
LMSG_G000004702.1	yes	875_0	Copper	75.25	3.44	58.05	0	0	0	13	Medium quality	2941	4007909	63.40	1548	d__Bacteria;p__Myxococcota;c__Polyangia;o__Polyangiales;f__Polyangiaceae;g__;s__
LMSG_G000004703.1	yes	1452_0	Copper	67.91	2.53	55.25	0	0	0	17	Medium quality	4632	3481889	66.70	860	d__Bacteria;p__Proteobacteria;c__Alphaproteobacteria;o__Rhizobiales;f__Beijerinckiaceae;g__;s__
LMSG_G000004704.1	yes	1768_0	Copper	56.38	0.00	56.38	1	2	1	20	Medium quality	243740	720282	58.00	9	d__Bacteria;p__Patescibacteria;c__Paceibacteria;o__UBA9983;f__UBA2163;g__C7867-001;s__
LMSG_G000004705.1	yes	1681_0	Copper	84.15	0.46	81.84	0	1	0	19	Medium quality	6887	1975561	55.40	380	d__Bacteria;p__Dormibacterota;c__Dormibacteria;o__UBA8260;f__;g__;s__
LMSG_G000004706.1	yes	1628_0	Copper	63.86	0.00	63.86	1	1	1	19	Medium quality	37019	1149767	34.30	51	d__Bacteria;p__Patescibacteria;c__Microgenomatia;o__Daviesbacterales;f__UBA10151;g__;s__
LMSG_G000004708.1	yes	1627_0	Copper	83.27	0.68	79.90	0	1	0	19	Medium quality	30493	1369703	45.50	82	d__Bacteria;p__Verrucomicrobiota_A;c__Chlamydiia;o__Parachlamydiales;f__SM23-39;g__;s__
LMSG_G000004709.1	yes	683_1	Copper	88.46	2.99	73.51	0	0	0	17	Medium quality	6062	2182308	41.80	476	d__Bacteria;p__Actinobacteriota;c__Acidimicrobiia;o__Acidimicrobiales;f__;g__;s__
LMSG_G000004710.1	yes	1119_2	Copper	81.32	4.39	59.36	1	1	1	17	Medium quality	73905	2894269	68.70	68	d__Bacteria;p__Proteobacteria;c__Gammaproteobacteria;o__Steroidobacterales;f__Steroidobacteraceae;g__;s__
LMSG_G000006308.1	yes	1149_2	Copper	87.46	3.51	69.90	0	0	0	19	Medium quality	10529	3191370	55.10	405	d__Bacteria;p__Verrucomicrobiota;c__Verrucomicrobiae;o__Pedosphaerales;f__Pedosphaeraceae;g__UBA11358;s__
LMSG_G000004711.1	yes	1360_0	Copper	94.56	1.12	88.95	0	0	0	18	Medium quality	13031	2362914	70.50	252	d__Bacteria;p__FCPU426;c__;o__;f__;g__;s__
LMSG_G000004712.1	yes	1535_1	Copper	93.63	2.57	80.77	0	1	1	20	Medium quality	52571	5325920	62.00	216	d__Bacteria;p__Proteobacteria;c__Alphaproteobacteria;o__Rhizobiales;f__Xanthobacteraceae;g__Bradyrhizobium;s__

LMSG_G000004350.1	yes	250_0	Copper	95.70	4.95	70.95	3	3	2	19	High quality	59487	5914989	51.20	199	d__Bacteria;p__Chloroflexota;c__Ktedonobacteria;o__Ktedonobacterales;f__Ktedonobacteraceae;g__;s__
LMSG_G000004352.1	yes	1050_0	Copper	93.79	2.43	81.64	3	1	2	20	High quality	29313	10883609	62.70	622	d__Bacteria;p__Planctomycetota;c__Planctomycetes;o__Isosphaerales;f__Isosphaeraceae;g__Singulisphaera;s__
LMSG_G000004713.1	yes	1656_0	Copper	77.81	0.00	77.81	0	0	0	17	Medium quality	6953	1874354	49.60	328	d__Bacteria;p__Bdellovibrionota;c__Bdellovibrionia;o__Bdellovibrionales;f__UBA1609;g__RBG-16-40-8;s__
LMSG_G000004714.1	yes	1048_1	Copper	87.78	0.00	87.78	1	0	1	19	Medium quality	6797	6487860	65.60	1426	d__Bacteria;p__Planctomycetota;c__Planctomycetes;o__Isosphaerales;f__Isosphaeraceae;g__;s__
LMSG_G000004715.1	yes	1604_0	Copper	92.73	0.80	88.73	1	0	2	16	Medium quality	14082	2638369	48.70	259	d__Bacteria;p__Spirochaetota;c__Spirochaetia;o__Spirochaetales;f__;g__;s__
LMSG_G000004716.1	yes	1003_0	Copper	89.93	1.75	81.16	1	1	1	15	Medium quality	10768	4723326	62.10	555	d__Bacteria;p__Acidobacteriota;c__Acidobacteriae;o__Bryobacterales;f__Bryobacteraceae;g__BOG-234;s__
LMSG_G000004717.1	yes	1812_0	Copper	58.71	0.00	58.71	1	1	2	20	Medium quality	314167	859852	53.60	8	d__Bacteria;p__Patescibacteria;c__Paceibacteria;o__UBA6257;f__2-01-FULL-56-20;g__;s__
LMSG_G000004718.1	yes	1478_0	Copper	97.26	4.01	77.21	0	0	0	19	Medium quality	71684	3370386	67.80	67	d__Bacteria;p__Proteobacteria;c__Alphaproteobacteria;o__Acetobacterales;f__Acetobacteraceae;g__BOG-908;s__
LMSG_G000004351.1	yes	1570_1	Copper	98.86	0.00	98.86	1	2	2	20	High quality	69484	4055293	55.20	88	d__Bacteria;p__Planctomycetota;c__Phycisphaerae;o__UBA1161;f__;g__;s__
LMSG_G000004719.1	yes	839_0	Copper	89.72	0.71	86.16	1	0	0	20	Medium quality	35811	3310474	71.00	133	d__Bacteria;p__Actinobacteriota;c__Thermoleophilia;o__Solirubrobacterales;f__Solirubrobacteraceae;g__Palsa-465;s__
LMSG_G000004339.1	yes	806_2	Copper	96.58	1.28	90.17	1	1	1	19	High quality	61205	2026927	67.80	57	d__Bacteria;p__Actinobacteriota;c__Acidimicrobiia;o__Acidimicrobiales;f__RAAP-2;g__RAAP-2;s__
LMSG_G000004727.1	yes	851_0	Pyrite	83.52	2.22	72.45	1	0	0	20	Medium quality	13510	2468952	73.00	230	d__Bacteria;p__Actinobacteriota;c__Actinomycetia;o__Actinomycetales;f__Cellulomonadaceae;g__Cellulomonas;s__
LMSG_G000004720.1	yes	1137_0	Pyrite	81.00	1.37	74.17	2	1	0	6	Medium quality	7707	1596996	43.20	283	d__Bacteria;p__Firmicutes;c__Bacilli;o__Lactobacillales;f__Carnobacteriaceae;g__Alkalibacterium;s__Alkalibacterium thalassium
LMSG_G000004721.1	yes	1613_0	Pyrite	66.39	1.27	60.05	0	1	0	14	Medium quality	2520	1016140	51.10	449	d__Bacteria;p__Verrucomicrobiota_A;c__Chlamydiia;o__Parachlamydiales;f__Rhabdochlamydiaceae;g__;s__
LMSG_G000004722.1	yes	538_0	Pyrite	81.09	1.43	73.93	0	0	0	13	Medium quality	9205	2586948	50.60	395	d__Bacteria;p__Bacteroidota;c__Bacteroidia;o__Bacteroidales;f__VadinHA17;g__SR-FBR-E99;s__
LMSG_G000004723.1	yes	1488_0	Pyrite	91.49	1.99	81.54	0	0	0	17	Medium quality	25143	2600107	65.10	200	d__Bacteria;p__Proteobacteria;c__Alphaproteobacteria;o__Acetobacterales;f__Acetobacteraceae;g__Acidocella;s__
LMSG_G000004724.1	yes	1185_1	Pyrite	93.33	0.00	93.33	0	0	0	17	Medium quality	70645	2409510	61.40	57	d__Bacteria;p__Proteobacteria;c__Gammaproteobacteria;o__Pseudomonadales;f__Moraxellaceae;g__;s__
LMSG_G000004725.1	yes	336_0	Pyrite	68.73	0.86	64.42	1	0	0	16	Medium quality	11641	606257	48.90	81	d__Bacteria;p__Patescibacteria;c__Saccharimonadia;o__Saccharimonadales;f__Saccharimonadaceae;g__UBA1547;s__
LMSG_G000004726.1	yes	416_0	Pyrite	85.07	0.04	84.90	2	4	1	15	Medium quality	10538	3390576	47.30	474	d__Bacteria;p__Bacteroidota;c__Bacteroidia;o__Bacteroidales;f__Prolixibacteraceae;g__UBA1413;s__
LMSG_G000004728.1	yes	904_0	Pyrite	72.48	0.86	68.17	0	0	0	17	Medium quality	34330	2427586	61.30	117	d__Bacteria;p__Acidobacteriota;c__Acidobacteriae;o__Acidobacteriales;f__Acidobacteriaceae;g__Granulicella_A;s__
LMSG_G000004729.1	yes	1393_0	Pyrite	59.72	1.65	51.48	0	0	0	7	Medium quality	1753	1515473	64.10	887	d__Bacteria;p__Desulfobacterota;c__Desulfomonilia_A;o__UBA1062;f__UBA1062;g__UBA1062;s__
LMSG_G000004730.1	yes	1162_0	Pyrite	76.06	2.86	61.76	1	0	0	16	Medium quality	8396	2472831	66.40	348	d__Bacteria;p__Proteobacteria;c__Gammaproteobacteria;o__Steroidobacterales;f__Steroidobacteraceae;g__;s__
LMSG_G000004731.1	yes	332_0	Pyrite	64.52	0.43	62.39	1	2	5	19	Medium quality	59837	920325	46.10	47	d__Bacteria;p__Patescibacteria;c__Saccharimonadia;o__Saccharimonadales;f__Saccharimonadaceae;g__UBA1547;s__
LMSG_G000006207.1	yes	1351_0	Pyrite	95.55	2.81	81.52	2	1	0	19	Medium quality	11878	2787609	69.60	353	d__Bacteria;p__Acidobacteriota;c__Holophagae;o__Holophagales;f__Holophagaceae;g__;s__
LMSG_G000004732.1	yes	321_0	Pyrite	84.06	2.07	73.71	2	2	2	19	Medium quality	10562	3100107	37.00	391	d__Bacteria;p__Firmicutes;c__Bacilli;o__Bacillales;f__Bacillaceae_G;g__;s__
LMSG_G000004733.1	yes	337_0	Pyrite	65.02	0.66	61.70	1	1	0	19	Medium quality	158270	794205	49.40	7	d__Bacteria;p__Patescibacteria;c__Saccharimonadia;o__Saccharimonadales;f__Saccharimonadaceae;g__UBA1547;s__
LMSG_G000004739.1	yes	905_0	Pyrite	51.70	0.00	51.70	2	1	2	17	Medium quality	78185	2166794	59.70	145	d__Bacteria;p__Acidobacteriota;c__Acidobacteriae;o__Acidobacteriales;f__Acidobacteriaceae;g__Granulicella_A;s__
LMSG_G000004734.1	yes	1403_0	Pyrite	78.55	1.71	69.99	0	0	0	15	Medium quality	5566	2996592	65.70	600	d__Bacteria;p__Proteobacteria;c__Gammaproteobacteria;o__Burkholderiales;f__Burkholderiaceae;g__Bordetella_B;s__
LMSG_G000004735.1	yes	311_1	Pyrite	95.88	0.93	91.26	0	2	1	18	Medium quality	44990	4140905	51.80	168	d__Bacteria;p__Armatimonadota;c__Chthonomonadetes;o__Chthonomonadales;f__Chthonomonadaceae;g__;s__
LMSG_G000004736.1	yes	265_0	Pyrite	84.11	0.90	79.62	1	1	1	17	Medium quality	4868	1358513	27.90	363	d__Bacteria;p__Campylobacterota;c__Campylobacteria;o__Campylobacterales;f__Arcobacteraceae;g__Aliarcobacter;s__Aliarcobacter cryaerophilus_A
LMSG_G000004737.1	yes	1339_2	Pyrite	66.47	0.11	65.93	0	0	0	15	Medium quality	6984	1628441	57.00	319	d__Bacteria;p__Proteobacteria;c__Gammaproteobacteria;o__Burkholderiales;f__Ferrovaceae;g__Ferrovum;s__
LMSG_G000004738.1	yes	342_0	Pyrite	65.36	0.00	65.36	1	3	23	18	Medium quality	32494	907565	48.70	72	d__Bacteria;p__Patescibacteria;c__Saccharimonadia;o__Saccharimonadales;f__UBA4665;g__GCA-2746885;s__
LMSG_G000004353.1	yes	1335_1	Pyrite	99.39	0.00	99.39	2	2	2	19	High quality	149135	2101261	53.20	90	d__Bacteria;p__Proteobacteria;c__Gammaproteobacteria;o__Burkholderiales;f__Ferrovaceae;g__Ferrovum;s__
LMSG_G000004740.1	yes	352_0	Pyrite	100.00	1.08	94.63	1	0	1	19	Medium quality	37273	2962911	40.70	177	d__Bacteria;p__Bacteroidota;c__Bacteroidia;o__Bacteroidales;f__Paludibacteraceae;g__Paludibacter_A;s__
LMSG_G000004741.1	yes	1430_1	Pyrite	99.33	0.75	95.60	0	0	0	19	Medium quality	51643	3082760	59.70	103	d__Bacteria;p__Proteobacteria;c__Alphaproteobacteria;o__Acetobacterales;f__Acetobacteraceae;g__BOG-908;s__
LMSG_G000004742.1	yes	1262_0	Pyrite	91.95	1.97	82.13	0	0	0	17	Medium quality	30592	2901613	66.90	164	d__Bacteria;p__Proteobacteria;c__Gammaproteobacteria;o__Xanthomonadales;f__Rhodanobacteraceae;g__Rhodanobacter;s__
LMSG_G000006260.1	yes	921_0	Copper	92.88	2.59	79.95	1	1	1	17	Medium quality	183867	3239607	59.60	30	d__Bacteria;p__Acidobacteriota;c__Acidobacteriae;o__Acidobacteriales;f__Acidobacteriaceae;g__;s__
LMSG_G000004743.1	yes	1080_0	Copper	70.82	3.74	52.11	0	1	0	12	Medium quality	35282	2273048	68.40	98	d__Bacteria;p__Proteobacteria;c__Gammaproteobacteria;o__Steroidobacterales;f__Steroidobacteraceae;g__;s__
LMSG_G000004354.1	yes	600_1	Copper	98.29	1.28	91.88	2	18	29	20	High quality	44169	3203346	53.40	307	d__Bacteria;p__Actinobacteriota;c__Acidimicrobiia;o__Acidimicrobiales;f__Acidimicrobiaceae;g__;s__
LMSG_G000004340.1	yes	790_2	Copper	93.03	1.71	84.49	1	1	1	20	High quality	74045	1979381	70.70	49	d__Bacteria;p__Actinobacteriota;c__Acidimicrobiia;o__Acidimicrobiales;f__RAAP-2;g__RAAP-2;s__
LMSG_G000004744.1	yes	1590_0	Copper	89.43	1.17	83.59	3	2	1	19	Medium quality	28155	2087504	41.00	118	d__Bacteria;p__Proteobacteria;c__Gammaproteobacteria;o__Legionellales;f__Legionellaceae;g__2-12-FULL-35-11;s__
LMSG_G000006208.1	yes	1332_1	Copper	96.80	0.47	94.44	2	1	0	19	Medium quality	89189	2157756	64.30	44	d__Bacteria;p__Proteobacteria;c__Gammaproteobacteria;o__Burkholderiales;f__Ferrovaceae;g__;s__
LMSG_G000004745.1	yes	1049_1	Copper	99.61	1.74	90.89	0	0	0	20	Medium quality	47840	8476534	65.20	278	d__Bacteria;p__Planctomycetota;c__Planctomycetes;o__Isosphaerales;f__Isosphaeraceae;g__;s__
LMSG_G000004746.1	yes	918_0	Copper	65.82	0.91	61.28	1	1	1	16	Medium quality	60003	2165677	60.20	112	d__Bacteria;p__Acidobacteriota;c__Acidobacteriae;o__Acidobacteriales;f__Acidobacteriaceae;g__;s__
LMSG_G000004751.1	yes	910_1	Copper	98.08	0.86	93.77	1	0	1	19	Medium quality	80480	3711475	61.10	82	d__Bacteria;p__Acidobacteriota;c__Acidobacteriae;o__Acidobacteriales;f__Acidobacteriaceae;g__Terracidiphilus;s__
LMSG_G000004747.1	yes	314_0	Copper	62.08	0.10	61.57	0	0	0	19	Medium quality	13947	674648	52.80	66	d__Bacteria;p__Patescibacteria;c__Saccharimonadia;o__UBA4664;f__UBA4664;g__;s__
LMSG_G000004748.1	yes	1511_2	Copper	88.47	2.65	75.21	0	0	0	17	Medium quality	17572	3295113	69.90	262	d__Bacteria;p__Proteobacteria;c__Alphaproteobacteria;o__Acetobacterales;f__Acetobacteraceae;g__;s__
LMSG_G000004749.1	yes	1624_1	Copper	88.34	0.68	84.97	1	0	1	17	Medium quality	96803	1589857	43.70	57	d__Bacteria;p__Verrucomicrobiota_A;c__Chlamydiia;o__Parachlamydiales;f__SM23-39;g__PALSA-1448;s__
LMSG_G000004750.1	yes	1618_0	Copper	77.72	2.70	64.21	0	1	0	16	Medium quality	6049	1549435	53.50	296	d__Bacteria;p__Verrucomicrobiota_A;c__Chlamydiia;o__Parachlamydiales;f__SM23-39;g__;s__
LMSG_G000004752.1	yes	1339_1	Copper	71.03	1.72	62.41	0	1	0	17	Medium quality	119480	1864816	56.70	100	d__Bacteria;p__Proteobacteria;c__Gammaproteobacteria;o__Burkholderiales;f__Ferrovaceae;g__;s__
LMSG_G000004753.1	yes	1361_0	Copper	59.98	0.16	59.16	0	0	1	12	Medium quality	2144	1645585	67.20	811	d__Bacteria;p__FCPU426;c__;o__;f__;g__;s__
LMSG_G000004754.1	yes	797_0	Copper	77.58	0.00	77.58	1	1	1	18	Medium quality	247643	2047389	65.80	111	d__Bacteria;p__Actinobacteriota;c__Acidimicrobiia;o__Acidimicrobiales;f__RAAP-2;g__RAAP-2;s__
LMSG_G000004755.1	yes	1813_0	Copper	56.73	0.00	56.73	0	1	0	17	Medium quality	22076	665599	58.30	40	d__Bacteria;p__Patescibacteria;c__Paceibacteria;o__UBA6257;f__2-01-FULL-56-20;g__;s__
LMSG_G000004756.1	yes	1842_0	Copper	66.29	1.75	57.54	0	0	0	11	Medium quality	2427	1540400	48.00	686	d__Bacteria;p__Bdellovibrionota;c__FAC87;o__UBA2466;f__UBA2466;g__;s__
LMSG_G000004759.1	yes	634_0	Copper	85.97	1.22	79.88	1	0	1	19	Medium quality	41771	2814782	62.20	132	d__Bacteria;p__Proteobacteria;c__Gammaproteobacteria;o__Acidiferrobacterales;f__Sulfurifustaceae;g__Sulfuricaulis;s__
LMSG_G000004757.1	yes	914_1	Copper	90.51	1.72	81.89	0	2	0	17	Medium quality	29757	4427244	62.60	221	d__Bacteria;p__Acidobacteriota;c__Acidobacteriae;o__Acidobacteriales;f__Acidobacteriaceae;g__Terracidiphilus;s__
LMSG_G000004758.1	yes	134_2	Copper	77.57	0.00	77.57	1	1	1	18	Medium quality	21693	850967	47.50	50	d__Archaea;p__Micrarchaeota;c__Micrarchaeia;o__Micrarchaeales;f__;g__;s__
LMSG_G000004760.1	yes	1594_0	Copper	73.62	1.26	67.33	1	0	0	13	Medium quality	6234	1127886	46.50	201	d__Bacteria;p__Proteobacteria;c__Gammaproteobacteria;o__Legionellales;f__Legionellaceae;g__;s__
LMSG_G000004761.1	yes	1481_1	Copper	57.06	0.00	57.06	0	0	0	10	Medium quality	4709	1360527	62.00	352	d__Bacteria;p__Proteobacteria;c__Alphaproteobacteria;o__Acetobacterales;f__Acetobacteraceae;g__Acidocella;s__
LMSG_G000004762.1	yes	982_1	Lead-Zinc	96.85	0.62	93.75	0	0	0	18	Medium quality	28408	2276555	57.50	150	d__Bacteria;p__Proteobacteria;c__Gammaproteobacteria;o__Acidithiobacillales;f__Acidithiobacillaceae;g__Acidithiobacillus;s__
LMSG_G000004776.1	yes	61_1	Lead-Zinc	66.66	0.00	66.66	2	0	1	19	Medium quality	34101	691318	50.00	39	d__Archaea;p__Micrarchaeota;c__Micrarchaeia;o__Micrarchaeales;f__Micrarchaeaceae;g__;s__
LMSG_G000004777.1	yes	98_0	Lead-Zinc	60.74	0.00	60.74	0	0	2	18	Medium quality	36609	550073	46.60	26	d__Archaea;p__Micrarchaeota;c__Micrarchaeia;o__Micrarchaeales;f__Micrarchaeaceae;g__Micrarchaeum;s__
LMSG_G000004778.1	yes	14_0	Lead-Zinc	79.85	3.88	60.44	0	1	1	18	Medium quality	24350	883078	36.30	95	d__Archaea;p__Nanoarchaeota;c__Nanoarchaeia;o__Parvarchaeales;f__Parvarchaeaceae;g__Parvarchaeum;s__

LMSG_G000004780.1	yes	959_1	Lead-Zinc	86.45	1.82	77.36	1	1	1	17	Medium quality	46073	2496764	59.60	281	d__Bacteria;p__Nitrospirota;c__Leptospirillia;o__Leptospirillales;f__Leptospirillaceae;g__UBA4572;s__
LMSG_G000004779.1	yes	456_2	Lead-Zinc	92.56	0.43	90.43	1	0	1	20	Medium quality	70848	2487556	56.30	90	d__Bacteria;p__Actinobacteriota;c__Acidimicrobiia;o__Acidimicrobiales;f__;g__;s__
LMSG_G000004781.1	yes	1746_1	Lead-Zinc	79.08	3.47	61.72	0	0	0	17	Medium quality	7184	2126250	30.00	394	d__Bacteria;p__SZUA-79;c__SZUA-79;o__Acidulodesulfobacterales;f__SZUA-79;g__Acididesulfobacter;s__
LMSG_G000004356.1	yes	1735_1	Lead-Zinc	93.72	2.20	82.74	1	2	2	18	High quality	71484	1909771	37.60	124	d__Bacteria;p__SZUA-79;c__SZUA-79;o__Acidulodesulfobacterales;f__SZUA-79;g__Acidulodesulfobacterium;s__
LMSG_G000004355.1	yes	1109_1	Lead-Zinc	95.21	1.65	86.96	1	5	9	20	High quality	20796	5004620	55.10	511	d__Bacteria;p__Firmicutes_E;c__Sulfobacillia;o__Sulfobacillales;f__;g__;s__
LMSG_G000006261.1	yes	1539_1	Lead-Zinc	96.64	0.61	93.60	1	1	1	17	Medium quality	37631	2110747	63.40	83	d__Bacteria;p__Proteobacteria;c__Gammaproteobacteria;o__Acidiferrobacterales;f__Acidiferrobacteraceae;g__;s__
LMSG_G000004763.1	yes	1474_1	Lead-Zinc	98.25	0.03	98.10	0	0	0	19	Medium quality	36617	3357222	68.30	140	d__Bacteria;p__Proteobacteria;c__Alphaproteobacteria;o__Acetobacterales;f__Acetobacteraceae;g__Acidiphilium;s__Acidiphilium multivorum
LMSG_G000004764.1	yes	1108_1	Lead-Zinc	91.98	0.42	89.86	0	1	1	17	Medium quality	73417	3294582	58.80	117	d__Bacteria;p__Firmicutes_E;c__Sulfobacillia;o__Sulfobacillales;f__Sulfobacillaceae;g__Sulfobacillus_B;s__
LMSG_G000004765.1	yes	1500_1	Lead-Zinc	81.95	0.54	79.24	0	0	0	16	Medium quality	5420	3107626	65.70	683	d__Bacteria;p__Proteobacteria;c__Alphaproteobacteria;o__Acetobacterales;f__Acetobacteraceae;g__G45-3;s__
LMSG_G000004766.1	yes	1106_1	Lead-Zinc	95.00	1.37	88.17	0	0	1	16	Medium quality	36733	3513388	59.10	233	d__Bacteria;p__Firmicutes_E;c__Sulfobacillia;o__Sulfobacillales;f__Sulfobacillaceae;g__Sulfobacillus_B;s__
LMSG_G000004767.1	yes	694_1	Lead-Zinc	98.29	1.45	91.03	1	0	0	20	Medium quality	91127	2542162	56.40	80	d__Bacteria;p__Actinobacteriota;c__Acidimicrobiia;o__Acidimicrobiales;f__Acidimicrobiaceae;g__Ferrithrix;s__
LMSG_G000004768.1	yes	1107_1	Lead-Zinc	89.84	1.33	83.18	0	1	1	20	Medium quality	93910	3023417	60.40	94	d__Bacteria;p__Firmicutes_E;c__Sulfobacillia;o__Sulfobacillales;f__Sulfobacillaceae;g__Sulfobacillus_B;s__
LMSG_G000004771.1	yes	1028_1	Lead-Zinc	94.44	0.00	94.44	0	0	0	18	Medium quality	40042	3933731	59.90	175	d__Bacteria;p__Acidobacteriota;c__Acidobacteriae;o__Acidobacteriales;f__Acidobacteriaceae;g__Acidobacterium;s__
LMSG_G000004769.1	yes	884_1	Lead-Zinc	97.24	0.00	97.24	0	0	0	18	Medium quality	27276	2475892	57.30	118	d__Bacteria;p__Acidobacteriota;c__Acidobacteriae;o__Acidobacteriales;f__Acidobacteriaceae;g__;s__
LMSG_G000004770.1	yes	1550_0	Lead-Zinc	76.52	0.65	73.27	0	0	0	14	Medium quality	22303	2081606	66.60	171	d__Bacteria;p__Proteobacteria;c__Gammaproteobacteria;o__Acidiferrobacterales;f__Acidiferrobacteraceae;g__Acidiferrobacter;s__
LMSG_G000004772.1	yes	838_0	Lead-Zinc	98.11	1.07	92.77	0	1	0	19	Medium quality	58357	3166674	69.60	113	d__Bacteria;p__Actinobacteriota;c__Thermoleophilia;o__Solirubrobacterales;f__Solirubrobacteraceae;g__Palsa-465;s__
LMSG_G000006262.1	yes	1538_1	Lead-Zinc	93.90	0.61	90.86	1	1	1	17	Medium quality	94144	2042697	64.00	33	d__Bacteria;p__Proteobacteria;c__Gammaproteobacteria;o__Acidiferrobacterales;f__Acidiferrobacteraceae;g__;s__
LMSG_G000004773.1	yes	1466_2	Lead-Zinc	86.10	1.11	80.56	0	0	0	18	Medium quality	16754	2769508	55.90	388	d__Bacteria;p__Proteobacteria;c__Alphaproteobacteria;o__Acetobacterales;f__Acetobacteraceae;g__Acidiphilium;s__
LMSG_G000004341.1	yes	1540_1	Lead-Zinc	95.73	1.22	89.64	1	1	1	19	High quality	39077	3158443	59.10	199	d__Bacteria;p__Proteobacteria;c__Gammaproteobacteria;o__Acidiferrobacterales;f__Acidiferrobacteraceae;g__Acidiferrobacter;s__
LMSG_G000004774.1	yes	389_0	Lead-Zinc	66.20	0.93	61.58	1	1	1	19	Medium quality	61287	795439	38.40	21	d__Bacteria;p__Patescibacteria;c__Saccharimonadia;o__Saccharimonadales;f__UBA4665;g__;s__
LMSG_G000004775.1	yes	1088_1	Lead-Zinc	68.96	0.00	68.96	0	1	0	15	Medium quality	42670	3069601	67.30	126	d__Bacteria;p__Proteobacteria;c__Gammaproteobacteria;o__Steroidobacterales;f__Steroidobacteraceae;g__;s__
LMSG_G000004782.1	yes	32_1	Antimony	94.63	2.11	84.09	0	0	0	15	Medium quality	60507	3340972	33.60	136	d__Bacteria;p__Bacteroidota;c__Bacteroidia;o__Flavobacteriales;f__Flavobacteriaceae;g__Lutibacter;s__
LMSG_G000004783.1	yes	225_0	Antimony	74.64	2.83	60.49	1	0	2	11	Medium quality	5070	1229696	38.40	299	d__Bacteria;p__Firmicutes;c__Bacilli;o__Erysipelotrichales;f__Erysipelotrichaceae;g__UBA6182;s__
LMSG_G000004784.1	yes	493_1	Antimony	96.89	0.41	94.86	1	0	1	18	Medium quality	41422	1600065	43.10	60	d__Bacteria;p__Aquificota;c__Aquificae;o__Aquificales;f__Aquificaceae;g__UBA11096;s__UBA11096 sp003534055
LMSG_G000004785.1	yes	1615_0	Antimony	92.56	1.02	87.48	0	0	0	16	Medium quality	13773	1489495	32.60	188	d__Bacteria;p__Aquificota;c__Aquificae;o__Hydrogenothermales;f__Hydrogenothermaceae;g__Sulfurihydrogenibium;s__
LMSG_G000004786.1	yes	255_0	Antimony	73.81	2.59	60.88	1	8	4	17	Medium quality	16723	1990110	42.80	298	d__Bacteria;p__Proteobacteria;c__Gammaproteobacteria;o__Pseudomonadales;f__Moraxellaceae;g__Acinetobacter;s__
LMSG_G000004787.1	yes	262_1	Antimony	72.51	0.00	72.51	5	3	1	18	Medium quality	38539	4119657	47.00	170	d__Bacteria;p__Proteobacteria;c__Gammaproteobacteria;o__Enterobacterales;f__Shewanellaceae;g__Shewanella;s__
LMSG_G000004788.1	yes	1239_0	Antimony	98.96	0.95	94.20	0	0	0	19	Medium quality	94831	2451482	52.10	42	d__Bacteria;p__Proteobacteria;c__Gammaproteobacteria;o__Burkholderiales;f__Gallionellaceae;g__Gallionella;s__
LMSG_G000004789.1	yes	390_0	Antimony	82.85	2.56	70.03	2	0	0	18	Medium quality	12837	2688170	53.30	353	d__Bacteria;p__Cyanobacteria;c__Vampirovibrionia;o__Vampirovibrionales;f__Vampirovibrionaceae;g__Vampirovibrio;s__
LMSG_G000004790.1	yes	307_1	Antimony	95.64	0.11	95.07	0	0	0	16	Medium quality	86166	1983031	46.10	85	d__Bacteria;p__Proteobacteria;c__Gammaproteobacteria;o__Burkholderiales;f__Burkholderiaceae;g__Polynucleobacter;s__Polynucleobacter sp000342265
LMSG_G000004791.1	yes	271_1	Antimony	75.90	0.82	71.82	1	0	0	17	Medium quality	19431	1585822	40.30	136	d__Bacteria;p__Campylobacterota;c__Campylobacteria;o__Campylobacterales;f__Sulfurimonadaceae;g__Sulfurimonas;s__Sulfurimonas sp002452425
LMSG_G000004792.1	yes	270_1	Antimony	89.72	2.04	79.52	2	0	0	16	Medium quality	21797	1859691	38.00	122	d__Bacteria;p__Campylobacterota;c__Campylobacteria;o__Campylobacterales;f__Sulfurimonadaceae;g__Sulfurimonas;s__
LMSG_G000004357.1	yes	1643_1	Copper	98.34	2.46	86.05	1	1	1	18	High quality	77924	3790736	51.70	83	d__Bacteria;p__Bacteroidota;c__Kryptonia;o__Kryptoniales;f__;g__;s__
LMSG_G000004793.1	yes	183_1	Copper	81.54	0.00	81.54	1	1	1	19	Medium quality	26687	1235926	40.30	81	d__Archaea;p__Thermoplasmatota;c__Thermoplasmata;o__Thermoplasmatales;f__Thermoplasmataceae;g__Cuniculiplasma;s__
LMSG_G000004342.1	yes	557_0	Copper	90.13	3.20	74.13	1	1	1	19	High quality	14527	1367722	69.80	143	d__Archaea;p__Thermoplasmatota;c__Thermoplasmata;o__UBA184;f__UBA184;g__;s__
LMSG_G000004794.1	yes	997_1	Copper	93.80	2.56	80.98	0	0	1	20	Medium quality	59205	3033534	60.00	78	d__Bacteria;p__Acidobacteriota;c__Acidobacteriae;o__UBA7540;f__;g__;s__
LMSG_G000004795.1	yes	398_1	Copper	77.42	0.00	77.42	1	1	2	20	Medium quality	287171	1292208	28.60	65	d__Bacteria;p__Patescibacteria;c__;o__;f__;g__;s__
LMSG_G000004358.1	yes	595_1	Copper	97.24	1.94	87.54	1	1	1	19	High quality	179066	2917841	59.80	100	d__Archaea;p__Thermoproteota;c__Nitrososphaeria;o__Nitrososphaerales;f__UBA183;g__UBA183;s__
LMSG_G000004796.1	yes	86_1	Copper	71.80	0.47	69.47	0	1	1	16	Medium quality	17026	693842	49.10	90	d__Archaea;p__Micrarchaeota;c__Micrarchaeia;o__Micrarchaeales;f__Micrarchaeaceae;g__;s__
LMSG_G000004359.1	yes	984_1	Copper	95.52	3.54	77.84	2	1	5	20	High quality	105977	2627137	46.60	63	d__Bacteria;p__SZUA-79;c__;o__;f__;g__;s__
LMSG_G000004797.1	yes	1561_0	Copper	56.95	0.21	55.90	0	0	0	13	Medium quality	6417	2238389	63.30	381	d__Bacteria;p__Desulfobacterota_B;c__Binatia;o__Binatales;f__Binataceae;g__;s__
LMSG_G000006209.1	yes	1685_0	Copper	98.67	1.88	89.29	1	2	0	12	Medium quality	114962	3990175	36.00	117	d__Bacteria;p__Firmicutes;c__Bacilli;o__Bacillales;f__Planococcaceae;g__Ureibacillus;s__Ureibacillus sp003977595
LMSG_G000004360.1	yes	876_1	Copper	92.30	0.43	90.17	1	1	2	20	High quality	80541	2266263	64.30	47	d__Bacteria;p__Actinobacteriota;c__Acidimicrobiia;o__Acidimicrobiales;f__;g__;s__
LMSG_G000004800.1	yes	188_1	Copper	86.65	0.00	86.65	1	2	0	19	Medium quality	70706	1386794	40.70	51	d__Archaea;p__Thermoplasmatota;c__Thermoplasmata;o__Thermoplasmatales;f__Thermoplasmataceae;g__Cuniculiplasma;s__
LMSG_G000004361.1	yes	542_1	Copper	96.66	1.72	88.05	1	2	2	19	High quality	47655	1900308	69.60	77	d__Archaea;p__Thermoplasmatota;c__Thermoplasmata;o__UBA184;f__UBA184;g__;s__
LMSG_G000004798.1	yes	16_1	Copper	79.75	0.93	75.08	1	1	1	19	Medium quality	80607	707260	43.80	29	d__Archaea;p__Nanoarchaeota;c__Nanoarchaeia;o__Parvarchaeales;f__Parvarchaeaceae;g__;s__
LMSG_G000004799.1	yes	128_1	Copper	84.73	0.93	80.06	1	1	1	20	Medium quality	43725	1080066	51.00	38	d__Archaea;p__Micrarchaeota;c__Micrarchaeia;o__Micrarchaeales;f__;g__;s__
LMSG_G000004801.1	yes	1548_1	Copper	88.41	1.78	79.52	1	1	1	18	Medium quality	63121	2314254	64.30	189	d__Bacteria;p__Proteobacteria;c__Gammaproteobacteria;o__Acidiferrobacterales;f__Acidiferrobacteraceae;g__Acidiferrobacter;s__
LMSG_G000004802.1	yes	742_1	Copper	92.26	2.56	79.44	0	1	0	16	Medium quality	44583	2519274	69.30	105	d__Bacteria;p__Actinobacteriota;c__Acidimicrobiia;o__Acidimicrobiales;f__Bog-793;g__Palsa-601;s__
LMSG_G000004803.1	yes	1087_0	Copper	79.37	3.57	61.52	1	1	1	14	Medium quality	16963	3310082	68.10	298	d__Bacteria;p__Proteobacteria;c__Gammaproteobacteria;o__Steroidobacterales;f__Steroidobacteraceae;g__;s__
LMSG_G000004804.1	yes	1559_1	Copper	85.71	1.26	79.41	1	1	1	13	Medium quality	21967	2749482	66.00	165	d__Bacteria;p__Desulfobacterota_B;c__Binatia;o__Binatales;f__Binataceae;g__;s__
LMSG_G000004805.1	yes	29_1	Copper	75.15	0.00	75.15	1	0	1	18	Medium quality	52073	909913	52.70	29	d__Archaea;p__Micrarchaeota;c__Micrarchaeia;o__Micrarchaeales;f__;g__;s__
LMSG_G000004806.1	yes	994_1	Copper	93.75	3.06	78.44	0	2	1	20	Medium quality	48421	5661231	57.90	217	d__Bacteria;p__Acidobacteriota;c__Acidobacteriae;o__UBA7540;f__UBA7540;g__RH2-MAG17b;s__
LMSG_G000004362.1	yes	1640_1	Copper	98.75	4.10	78.26	1	1	1	19	High quality	102459	5219449	49.40	90	d__Bacteria;p__Bacteroidota;c__Kryptonia;o__Kryptoniales;f__;g__;s__
LMSG_G000004807.1	yes	1728_1	Copper	57.52	0.81	53.46	1	1	1	20	Medium quality	29018	1248096	38.60	92	d__Bacteria;p__Dependentiae;c__Babeliae;o__Babeliales;f__Vermiphilaceae;g__;s__
LMSG_G000004810.1	yes	1125_2	Copper	95.37	0.00	95.37	0	1	0	18	Medium quality	26338	2384515	65.00	134	d__Bacteria;p__Eremiobacterota;c__Eremiobacteria;o__UBP12;f__UBA5184;g__;s__
LMSG_G000004808.1	yes	56_1	Copper	80.84	0.00	80.84	1	1	2	20	Medium quality	135378	806151	45.40	20	d__Archaea;p__Micrarchaeota;c__Micrarchaeia;o__Micrarchaeales;f__Micrarchaeaceae;g__Micrarchaeum;s__
LMSG_G000004809.1	yes	996_1	Copper	92.28	1.71	83.74	2	0	1	18	Medium quality	20161	3358641	61.00	298	d__Bacteria;p__Acidobacteriota;c__Acidobacteriae;o__UBA7540;f__UBA7540;g__;s__
LMSG_G000004811.1	yes	1475_1	Lead-Zinc	82.62	1.49	75.16	0	0	0	19	Medium quality	29678	2647438	68.10	140	d__Bacteria;p__Proteobacteria;c__Alphaproteobacteria;o__Acetobacterales;f__Acetobacteraceae;g__Acidiphilium;s__
LMSG_G000004812.1	yes	244_1	Lead-Zinc	80.00	2.73	66.37	1	1	1	17	Medium quality	26277	2124923	51.10	118	d__Bacteria;p__Chloroflexota;c__Anaerolineae;o__Anaerolineales;f__Anaerolineaceae;g__Bellilinea;s__
LMSG_G000006263.1	yes	1284_0	Lead-Zinc	93.87	1.70	85.35	4	1	1	17	Medium quality	54244	3778465	63.40	124	d__Bacteria;p__Proteobacteria;c__Gammaproteobacteria;o__Burkholderiales;f__Burkholderiaceae;g__Acidovorax_D;s__Acidovorax_D temperans

LMSG_G000004813.1	yes	1271_0	Lead-Zinc	81.55	1.83	72.39	0	0	0	13	Medium quality	19453	2275345	64.40	142	d__Bacteria;p__Proteobacteria;c__Gammaproteobacteria;o__Burkholderiales;f__Thiobacillaceae;g__;s__
LMSG_G000004814.1	yes	1329_0	Lead-Zinc	57.69	0.70	54.20	0	0	0	12	Medium quality	43646	1488513	61.80	52	d__Bacteria;p__Proteobacteria;c__Gammaproteobacteria;o__Burkholderiales;f__Ferrovaceae;g__;s__
LMSG_G000004816.1	yes	1319_0	Lead-Zinc	77.58	3.45	60.34	2	0	0	16	Medium quality	23672	2462815	65.00	132	d__Bacteria;p__Proteobacteria;c__Gammaproteobacteria;o__Burkholderiales;f__Rhodocyclaceae;g__UBA2250;s__
LMSG_G000004363.1	yes	790_1	Lead-Zinc	98.29	3.85	79.06	1	1	2	20	High quality	136201	2064185	70.40	30	d__Bacteria;p__Actinobacteriota;c__Acidimicrobiia;o__Acidimicrobiales;f__RAAP-2;g__RAAP-2;s__
LMSG_G000004364.1	yes	1758_0	Lead-Zinc	92.13	0.94	87.42	1	1	1	18	High quality	62989	1867413	33.00	97	d__Bacteria;p__Firmicutes;c__Bacilli;o__Erysipelotrichales;f__Erysipelotrichaceae;g__UBA2212;s__
LMSG_G000004815.1	yes	465_0	Lead-Zinc	60.73	0.33	59.10	0	1	0	13	Medium quality	6676	2389191	40.60	467	d__Bacteria;p__Firmicutes_B;c__Desulfitobacteriia;o__Desulfitobacteriales;f__Desulfitobacteriaceae;g__;s__
LMSG_G000004365.1	yes	877_1	Lead-Zinc	99.47	1.58	91.58	1	1	2	20	High quality	55930	2994781	71.90	110	d__Bacteria;p__Actinobacteriota;c__Actinomycetia;o__Actinomycetales;f__;g__;s__
LMSG_G000004817.1	yes	1222_1	Lead-Zinc	76.24	1.62	68.15	0	1	0	13	Medium quality	25339	2481517	57.80	227	d__Bacteria;p__Proteobacteria;c__Gammaproteobacteria;o__Burkholderiales;f__;g__;s__
LMSG_G000004818.1	yes	1100_0	Lead-Zinc	89.28	2.21	78.25	2	0	0	19	Medium quality	30011	3427665	46.10	175	d__Bacteria;p__Firmicutes_B;c__Thermincolia;o__Carboxydocellales;f__;g__;s__
LMSG_G000004819.1	yes	1315_0	Lead-Zinc	91.49	0.92	86.87	2	0	0	15	Medium quality	81886	3349031	63.00	99	d__Bacteria;p__Proteobacteria;c__Gammaproteobacteria;o__Burkholderiales;f__Rhodocyclaceae;g__Azonexus;s__Azonexus agitata
LMSG_G000004820.1	yes	1101_0	Lead-Zinc	90.11	0.63	86.95	0	7	0	11	Medium quality	31789	1578418	51.30	101	d__Bacteria;p__Firmicutes_B;c__Desulfotomaculia;o__Desulfotomaculales;f__Desulfotomaculaceae;g__Desulfotomaculum;s__Desulfotomaculum profundi
LMSG_G000004821.1	yes	1298_2	Lead-Zinc	89.94	0.80	85.94	1	0	1	15	Medium quality	48749	3533339	69.80	106	d__Bacteria;p__Proteobacteria;c__Gammaproteobacteria;o__Burkholderiales;f__Burkholderiaceae;g__Thiomonas;s__
LMSG_G000004366.1	yes	1504_1	Lead-Zinc	96.35	0.25	95.11	1	1	1	19	High quality	35544	3473114	70.00	146	d__Bacteria;p__Proteobacteria;c__Alphaproteobacteria;o__Acetobacterales;f__Acetobacteraceae;g__;s__
LMSG_G000006210.1	yes	1533_1	Lead-Zinc	98.88	0.89	94.44	1	1	0	19	Medium quality	96305	2526393	58.40	44	d__Bacteria;p__Proteobacteria;c__Gammaproteobacteria;o__Burkholderiales;f__Gallionellaceae;g__Gallionella;s__
LMSG_G000004822.1	yes	1391_0	Lead-Zinc	73.68	3.51	56.14	1	0	0	18	Medium quality	24632	3738001	58.90	215	d__Bacteria;p__Desulfobacterota;c__Desulfuromonadia;o__Geobacterales;f__Geobacteraceae;g__;s__
LMSG_G000004823.1	yes	1773_0	Lead-Zinc	75.28	0.00	75.28	1	1	11	19	Medium quality	89634	605869	56.30	36	d__Bacteria;p__Patescibacteria;c__Paceibacteria;o__UBA9983;f__UBA2163;g__C7867-001;s__
LMSG_G000004824.1	yes	1270_1	Lead-Zinc	91.99	3.06	76.70	0	1	0	18	Medium quality	30698	2656219	65.30	122	d__Bacteria;p__Proteobacteria;c__Gammaproteobacteria;o__Burkholderiales;f__Thiobacillaceae;g__;s__
LMSG_G000004825.1	yes	256_0	Lead-Zinc	95.96	0.19	95.03	5	0	2	18	Medium quality	18808	2933118	38.50	225	d__Bacteria;p__Proteobacteria;c__Gammaproteobacteria;o__Pseudomonadales;f__Moraxellaceae;g__Acinetobacter;s__Acinetobacter junii
LMSG_G000004826.1	yes	1083_0	Lead-Zinc	68.96	0.00	68.96	0	1	0	13	Medium quality	43751	2805161	67.70	110	d__Bacteria;p__Proteobacteria;c__Gammaproteobacteria;o__Steroidobacterales;f__Steroidobacteraceae;g__;s__
LMSG_G000004827.1	yes	1300_0	Lead-Zinc	73.44	2.59	60.51	0	1	0	14	Medium quality	43900	2998819	69.70	95	d__Bacteria;p__Proteobacteria;c__Gammaproteobacteria;o__Burkholderiales;f__Burkholderiaceae;g__Thiomonas;s__
LMSG_G000004828.1	yes	227_0	Lead-Zinc	73.28	0.17	72.45	1	0	0	8	Medium quality	4482	2762572	47.20	729	d__Bacteria;p__Firmicutes_A;c__Clostridia;o__Acetivibrionales;f__Acetivibrionaceae;g__;s__
LMSG_G000004833.1	yes	1193_1	Lead-Zinc	93.51	0.56	90.74	1	0	0	19	Medium quality	52208	2893620	50.30	95	d__Bacteria;p__Proteobacteria;c__Gammaproteobacteria;o__Pseudomonadales;f__Moraxellaceae;g__;s__
LMSG_G000004829.1	yes	942_0	Lead-Zinc	92.21	2.07	81.86	1	0	2	18	Medium quality	21293	2043071	40.30	140	d__Bacteria;p__Nitrospirota;c__Thermodesulfovibrionia;o__Thermodesulfovibrionales;f__UBA9935;g__UBA665;s__UBA665 sp002299835
LMSG_G000004830.1	yes	1399_0	Lead-Zinc	72.52	2.57	59.67	0	0	0	14	Medium quality	3920	2752144	62.50	776	d__Bacteria;p__Proteobacteria;c__Alphaproteobacteria;o__Rhodospirillales;f__Magnetospirillaceae;g__;s__
LMSG_G000004831.1	yes	1352_1	Lead-Zinc	94.75	2.68	81.36	1	0	4	16	Medium quality	28113	2696800	58.10	223	d__Bacteria;p__Desulfobacterota;c__Desulfobulbia;o__Desulfobulbales;f__Desulfurivibrionaceae;g__UBA2262;s__
LMSG_G000004832.1	yes	1706_1	Lead-Zinc	80.63	1.74	71.91	0	0	0	14	Medium quality	8349	1256316	41.30	208	d__Bacteria;p__Proteobacteria;c__Gammaproteobacteria;o__UBA6186;f__UBA6186;g__;s__
LMSG_G000006264.1	yes	1387_0	Lead-Zinc	97.41	2.26	86.12	1	1	1	17	Medium quality	46133	3455375	55.50	182	d__Bacteria;p__Desulfobacterota;c__Desulfuromonadia;o__Geobacterales;f__Pseudopelobacteraceae;g__Trichlorobacter;s__Trichlorobacter lovleyi
LMSG_G000004834.1	yes	1220_0	Lead-Zinc	85.54	3.55	67.77	0	0	0	14	Medium quality	53976	2428321	57.00	69	d__Bacteria;p__Proteobacteria;c__Gammaproteobacteria;o__Burkholderiales;f__;g__;s__
LMSG_G000004367.1	yes	1060_1	Lead-Zinc	98.64	0.68	95.27	1	2	1	20	High quality	109921	1652002	42.10	24	d__Bacteria;p__Verrucomicrobiota;c__Verrucomicrobiae;o__Chthoniobacterales;f__UBA6821;g__;s__
LMSG_G000004835.1	yes	1221_1	Lead-Zinc	62.61	0.00	62.61	0	0	0	13	Medium quality	139159	1693764	58.30	18	d__Bacteria;p__Proteobacteria;c__Gammaproteobacteria;o__Burkholderiales;f__Gallionellaceae;g__;s__
LMSG_G000004836.1	yes	841_0	Lead-Zinc	73.94	2.92	59.36	1	0	1	19	Medium quality	5381	1568086	65.90	335	d__Bacteria;p__Actinobacteriota;c__Coriobacteriia;o__OPB41;f__UBA2279;g__UBA6100;s__
LMSG_G000004368.1	yes	923_1	Lead-Zinc	95.90	0.86	91.59	1	1	1	20	High quality	37673	4468736	60.00	296	d__Bacteria;p__Acidobacteriota;c__Acidobacteriae;o__Acidobacteriales;f__Acidobacteriaceae;g__;s__
LMSG_G000004369.1	yes	667_1	Lead-Zinc	95.83	1.85	86.58	1	1	1	19	High quality	27866	2609762	74.80	146	d__Bacteria;p__Chloroflexota;c__Ellin6529;o__CSP1-4;f__CSP1-4;g__;s__
LMSG_G000004837.1	yes	1593_0	Lead-Zinc	87.79	0.78	83.92	2	0	1	15	Medium quality	22831	2039600	37.20	115	d__Bacteria;p__Proteobacteria;c__Gammaproteobacteria;o__Legionellales;f__Legionellaceae;g__;s__
LMSG_G000004838.1	yes	138_1	Lead-Zinc	83.72	0.93	79.05	0	2	1	20	Medium quality	25029	814605	42.80	54	d__Archaea;p__Micrarchaeota;c__Micrarchaeia;o__Micrarchaeales;f__Micrarchaeaceae;g__Micrarchaeum;s__
LMSG_G000004839.1	yes	1230_1	Lead-Zinc	71.40	2.20	60.43	1	0	0	16	Medium quality	9619	2733176	58.10	390	d__Bacteria;p__Proteobacteria;c__Gammaproteobacteria;o__Burkholderiales;f__Gallionellaceae;g__Sideroxydans;s__
LMSG_G000004840.1	yes	1435_1	Lead-Zinc	96.81	1.01	91.76	0	1	0	18	Medium quality	26829	3458517	69.10	201	d__Bacteria;p__Proteobacteria;c__Alphaproteobacteria;o__Rhodobacterales;f__Rhodobacteraceae;g__Allgaiera;s__
LMSG_G000004370.1	yes	1342_1	Lead-Zinc	97.74	0.65	94.52	3	1	1	18	High quality	32564	3659376	55.90	169	d__Bacteria;p__Desulfobacterota;c__Desulfuromonadia;o__Geobacterales;f__Pseudopelobacteraceae;g__Pseudopelobacter;s__
LMSG_G000006211.1	yes	276_1	Lead-Zinc	98.27	0.27	96.91	2	2	0	19	Medium quality	47664	3175021	37.10	121	d__Bacteria;p__Bacteroidota;c__Bacteroidia;o__Chitinophagales;f__Chitinophagaceae;g__Hydrotalea;s__Hydrotalea flava
LMSG_G000004841.1	yes	1075_0	Lead-Zinc	71.41	0.99	66.47	0	0	0	10	Medium quality	9258	1794640	47.40	288	d__Bacteria;p__Firmicutes_D;c__Dethiobacteria;o__Dethiobacterales;f__Dethiobacteraceae;g__Dethiobacter;s__
LMSG_G000006265.1	yes	466_0	Lead-Zinc	97.70	0.30	96.22	3	4	1	17	Medium quality	57693	4765855	41.70	181	d__Bacteria;p__Firmicutes_B;c__Desulfitobacteriia;o__Desulfitobacteriales;f__Desulfitobacteriaceae;g__Desulfosporosinus;s__
LMSG_G000004842.1	yes	1056_0	Lead-Zinc	66.82	0.00	66.82	0	1	0	12	Medium quality	5285	1882041	42.00	427	d__Bacteria;p__Bdellovibrionota;c__Bdellovibrionia;o__Bdellovibrionales;f__Bdellovibrionaceae;g__GaO074139;s__
LMSG_G000004844.1	yes	492_0	Lead-Zinc	67.72	0.87	63.36	0	1	0	14	Medium quality	5581	2018403	54.80	417	d__Bacteria;p__Firmicutes;c__Alicyclobacillia;o__Alicyclobacillales;f__Alicyclobacillaceae;g__;s__
LMSG_G000004843.1	yes	663_0	Lead-Zinc	69.66	0.95	64.92	1	0	0	16	Medium quality	3219	1845159	70.90	626	d__Bacteria;p__Actinobacteriota;c__Thermoleophilia;o__Gaiellales;f__Gaiellaceae;g__Palsa-739;s__
LMSG_G000004845.1	yes	834_0	Lead-Zinc	63.44	0.85	59.17	0	0	0	12	Medium quality	4125	2408001	69.10	678	d__Bacteria;p__Actinobacteriota;c__Thermoleophilia;o__Solirubrobacterales;f__Solirubrobacteraceae;g__Palsa-465;s__
LMSG_G000004371.1	yes	212_1	Lead-Zinc	98.40	0.81	94.37	1	1	6	20	High quality	33174	1759665	41.00	135	d__Archaea;p__Thermoplasmatota;c__Thermoplasmata;o__Thermoplasmatales;f__Thermoplasmataceae;g__;s__
LMSG_G000004846.1	yes	610_2	Lead-Zinc	68.76	0.68	65.39	1	0	1	17	Medium quality	13731	1899213	53.00	224	d__Bacteria;p__Actinobacteriota;c__Acidimicrobiia;o__Acidimicrobiales;f__Acidimicrobiaceae;g__SCQM01;s__
LMSG_G000004847.1	yes	1460_0	Lead-Zinc	79.25	0.69	75.83	0	1	0	13	Medium quality	6941	3068337	63.60	525	d__Bacteria;p__Proteobacteria;c__Alphaproteobacteria;o__Rhizobiales;f__Hyphomicrobiaceae;g__Hyphomicrobium;s__
LMSG_G000004848.1	yes	1086_1	Lead-Zinc	53.91	0.00	53.91	1	0	1	16	Medium quality	13655	2939041	66.90	252	d__Bacteria;p__Proteobacteria;c__Gammaproteobacteria;o__Steroidobacterales;f__Steroidobacteraceae;g__;s__
LMSG_G000004852.1	yes	1676_0	Pyrite	77.25	0.00	77.25	1	1	1	19	Medium quality	98580	798712	40.20	12	d__Archaea;p__Micrarchaeota;c__Micrarchaeia;o__UBA8480;f__;g__;s__
LMSG_G000004849.1	yes	657_0	Pyrite	79.27	0.33	77.62	1	0	1	10	Medium quality	8078	2295036	67.70	348	d__Bacteria;p__Firmicutes_E;c__Sulfobacillia;o__;f__;g__;s__
LMSG_G000004850.1	yes	1378_1	Pyrite	85.16	0.00	85.16	1	1	1	17	Medium quality	32576	3527231	71.90	153	d__Bacteria;p__Myxococcota;c__Myxococcia;o__Myxococcales;f__;g__;s__
LMSG_G000004851.1	yes	824_1	Pyrite	87.81	1.28	81.40	1	1	1	15	Medium quality	13684	1983200	64.20	184	d__Bacteria;p__Actinobacteriota;c__Acidimicrobiia;o__Acidimicrobiales;f__;g__;s__
LMSG_G000004856.1	yes	99_1	Pyrite	75.70	0.93	71.03	1	1	0	19	Medium quality	63152	839692	42.70	35	d__Archaea;p__Micrarchaeota;c__Micrarchaeia;o__Micrarchaeales;f__;g__;s__
LMSG_G000004853.1	yes	1734_1	Pyrite	88.65	0.00	88.65	0	1	0	18	Medium quality	25850	1609031	38.30	124	d__Bacteria;p__SZUA-79;c__SZUA-79;o__Acidulodesulfobacterales;f__SZUA-79;g__Acidulodesulfobacterium;s__
LMSG_G000004854.1	yes	1596_0	Pyrite	54.34	0.00	54.34	1	0	1	9	Medium quality	2643	478868	55.70	198	d__Archaea;p__Micrarchaeota;c__Micrarchaeia;o__Micrarchaeales;f__;g__;s__
LMSG_G000004855.1	yes	378_0	Pyrite	59.29	1.03	54.15	0	2	0	16	Medium quality	4602	749212	46.10	222	d__Bacteria;p__Patescibacteria;c__Saccharimonadia;o__Saccharimonadales;f__UBA4665;g__;s__
LMSG_G000004857.1	yes	356_1	Pyrite	56.46	0.00	56.46	0	0	0	10	Medium quality	2629	1694152	63.00	740	d__Bacteria;p__Dormibacterota;c__Dormibacteria;o__Dormibacterales;f__;g__;s__
LMSG_G000006317.1	yes	35_0	Pyrite	71.15	2.86	56.84	1	0	0	18	Medium quality	3780	2613817	38.40	749	d__Bacteria;p__Proteobacteria;c__Gammaproteobacteria;o__Legionellales;f__Legionellaceae;g__Legionella;s__
LMSG_G000004858.1	yes	597_0	Pyrite	69.09	0.00	69.09	1	0	0	18	Medium quality	85781	906843	58.50	24	d__Archaea;p__Thermoproteota;c__Nitrososphaeria;o__Nitrososphaerales;f__UBA183;g__UBA183;s__
LMSG_G000004859.1	yes	26_0	Pyrite	73.70	0.00	73.70	1	2	2	19	Medium quality	50753	621811	48.00	16	d__Archaea;p__Nanoarchaeota;c__Nanoarchaeia;o__Parvarchaeales;f__Parvarchaeaceae;g__;s__
LMSG_G000004860.1	yes	795_0	Pyrite	72.06	0.00	72.06	0	0	0	16	Medium quality	20328	1234285	68.40	84	d__Bacteria;p__Actinobacteriota;c__Acidimicrobiia;o__Acidimicrobiales;f__RAAP-2;g__RAAP-2;s__
LMSG_G000004861.1	yes	107_0	Pyrite	76.47	0.31	74.92	1	1	1	19	Medium quality	24239	624445	54.40	37	d__Archaea;p__Micrarchaeota;c__Micrarchaeia;o__Micrarchaeales;f__;g__;s__
LMSG_G000004862.1	yes	397_0	Pyrite	73.82	0.00	73.82	1	1	1	20	Medium quality	90648	985045	30.20	27	d__Bacteria;p__Patescibacteria;c__Dojkabacteria;o__;f__;g__;s__

LMSG_G000004863.1	yes	560_0	Pyrite	86.35	3.33	69.69	1	1	2	17	Medium quality	19400	1786757	72.10	147	d__Archaea;p__Thermoplasmatota;c__Thermoplasmata;o__UBA184;f__UBA184;g__;s__
LMSG_G000004372.1	yes	811_0	Pyrite	94.68	1.45	87.42	1	1	1	20	High quality	34827	1862048	71.90	77	d__Bacteria;p__Actinobacteriota;c__Acidimicrobiia;o__Acidimicrobiales;f__RAAP-2;g__;s__
LMSG_G000004864.1	yes	1749_0	Pyrite	59.89	0.00	59.89	0	0	0	15	Medium quality	90908	703804	42.00	16	d__Bacteria;p__SZUA-79;c__SZUA-79;o__Acidulodesulfobacterales;f__SZUA-79;g__;s__
LMSG_G000004865.1	yes	1644_1	Pyrite	98.34	2.09	87.87	0	0	0	19	Medium quality	112209	4194735	51.30	91	d__Bacteria;p__Bacteroidota;c__Kryptonia;o__;f__;g__;s__
LMSG_G000004373.1	yes	403_0	Pyrite	95.38	0.95	90.62	1	1	2	20	High quality	32618	3050203	42.50	160	d__Bacteria;p__Bacteroidota;c__Bacteroidia;o__AKYH767;f__Palsa-948;g__;s__
LMSG_G000004866.1	yes	1126_0	Pyrite	83.35	1.85	74.10	0	1	0	12	Medium quality	5909	2356079	62.50	509	d__Bacteria;p__Eremiobacterota;c__Eremiobacteria;o__UBP12;f__UBA5184;g__PALSA-1504;s__
LMSG_G000004867.1	yes	598_0	Pyrite	71.84	0.97	66.99	0	0	1	14	Medium quality	62823	881893	62.50	32	d__Archaea;p__Thermoproteota;c__Nitrososphaeria;o__Nitrososphaerales;f__UBA183;g__UBA183;s__
LMSG_G000004868.1	yes	1407_0	Pyrite	83.17	0.93	78.50	1	0	1	19	Medium quality	41216	1328665	55.20	53	d__Archaea;p__Micrarchaeota;c__Micrarchaeia;o__Micrarchaeales;f__Micrarchaeaceae;g__;s__
LMSG_G000004869.1	yes	771_0	Pyrite	70.49	2.14	59.81	0	0	0	13	Medium quality	6640	1746292	73.20	329	d__Bacteria;p__Actinobacteriota;c__Acidimicrobiia;o__Acidimicrobiales;f__Bog-793;g__;s__
LMSG_G000004873.1	yes	582_1	Pyrite	84.38	0.00	84.38	0	4	0	18	Medium quality	126598	1693565	63.50	159	d__Archaea;p__Thermoproteota;c__Nitrososphaeria;o__UBA164;f__;g__;s__
LMSG_G000004870.1	yes	21_1	Pyrite	80.99	0.93	76.32	1	1	1	19	Medium quality	541655	1047588	40.70	13	d__Archaea;p__Nanoarchaeota;c__Nanoarchaeia;o__Parvarchaeales;f__Parvarchaeaceae;g__;s__
LMSG_G000004374.1	yes	573_0	Pyrite	93.96	0.29	92.53	1	1	1	20	High quality	117360	2144631	61.40	47	d__Bacteria;p__Proteobacteria;c__Gammaproteobacteria;o__UBA2770;f__UBA2770;g__;s__
LMSG_G000004871.1	yes	749_1	Pyrite	95.72	1.28	89.31	1	0	1	20	Medium quality	51907	3196423	71.80	111	d__Bacteria;p__Actinobacteriota;c__Acidimicrobiia;o__Acidimicrobiales;f__Palsa-688;g__;s__
LMSG_G000004872.1	yes	589_0	Pyrite	72.33	1.94	62.63	1	0	0	17	Medium quality	256351	1466673	61.60	15	d__Archaea;p__Thermoproteota;c__Nitrososphaeria;o__Nitrososphaerales;f__UBA183;g__UBA183;s__
LMSG_G000004874.1	yes	1454_0	Pyrite	91.94	2.27	80.61	0	0	0	17	Medium quality	14086	4115658	63.50	393	d__Bacteria;p__Proteobacteria;c__Alphaproteobacteria;o__Rhizobiales;f__Xanthobacteraceae;g__BOG-931;s__
LMSG_G000004375.1	yes	1471_0	Pyrite	91.65	2.00	81.65	1	1	1	19	High quality	9507	3614496	64.90	497	d__Bacteria;p__Proteobacteria;c__Alphaproteobacteria;o__ATCC43930;f__Stellaceae;g__;s__
LMSG_G000004875.1	yes	985_0	Pyrite	86.50	0.00	86.50	0	1	0	17	Medium quality	195769	2371220	51.60	62	d__Bacteria;p__Desulfobacterota;c__;o__;f__;g__;s__
LMSG_G000004876.1	yes	150_2	Pyrite	55.67	0.80	51.67	0	0	0	11	Medium quality	6950	1349880	52.30	234	d__Archaea;p__Thermoplasmatota;c__Thermoplasmata;o__UBA10834;f__;g__;s__
LMSG_G000004376.1	yes	318_1	Pyrite	94.44	0.19	93.52	1	2	1	20	High quality	86766	3674596	54.30	87	d__Bacteria;p__Armatimonadota;c__Chthonomonadetes;o__Chthonomonadales;f__;g__;s__
LMSG_G000004877.1	yes	1831_1	Pyrite	60.08	0.00	60.08	2	2	3	20	Medium quality	27392	616891	47.50	39	d__Bacteria;p__Patescibacteria;c__Paceibacteria;o__UBA6257;f__Colwellbacteraceae;g__;s__
LMSG_G000004878.1	yes	137_1	Pyrite	81.77	0.93	77.10	1	0	1	18	Medium quality	98245	801949	51.30	30	d__Archaea;p__Micrarchaeota;c__Micrarchaeia;o__Micrarchaeales;f__Micrarchaeaceae;g__;s__
LMSG_G000004879.1	yes	109_0	Pyrite	80.21	0.47	77.88	1	2	4	20	Medium quality	40143	855448	52.80	40	d__Archaea;p__Micrarchaeota;c__Micrarchaeia;o__Micrarchaeales;f__;g__;s__
LMSG_G000004882.1	yes	104_0	Pyrite	69.25	0.93	64.58	1	1	1	17	Medium quality	11376	645458	49.50	63	d__Archaea;p__Micrarchaeota;c__Micrarchaeia;o__Micrarchaeales;f__Micrarchaeaceae;g__;s__
LMSG_G000004880.1	yes	1598_0	Pyrite	54.81	0.75	51.08	0	0	0	11	Medium quality	2304	3297321	61.00	1557	d__Bacteria;p__Spirochaetota;c__Spirochaetia;o__Spirochaetales;f__ARS1246;g__;s__
LMSG_G000004881.1	yes	1031_0	Pyrite	78.53	0.96	73.76	0	0	0	17	Medium quality	7090	2468219	69.90	405	d__Bacteria;p__Acidobacteriota;c__Acidobacteriae;o__Acidobacteriales;f__SCQP01;g__SCQP01;s__
LMSG_G000004883.1	yes	187_1	Pyrite	89.47	0.81	85.44	0	1	2	19	Medium quality	12128	1534232	43.30	169	d__Archaea;p__Thermoplasmatota;c__Thermoplasmata;o__Thermoplasmatales;f__Thermoplasmataceae;g__B-DKE;s__
LMSG_G000004884.1	yes	12_1	Pyrite	74.75	0.97	69.90	1	1	1	15	Medium quality	20781	568862	33.20	45	d__Archaea;p__Nanoarchaeota;c__Nanoarchaeia;o__Parvarchaeales;f__Parvarchaeaceae;g__Parvarchaeum;s__
LMSG_G000004888.1	yes	1530_1	Pyrite	99.60	0.02	99.51	7	0	1	18	Medium quality	175831	5387568	57.40	61	d__Bacteria;p__Proteobacteria;c__Gammaproteobacteria;o__Enterobacterales;f__Enterobacteriaceae;g__Klebsiella;s__Klebsiella pneumoniae
LMSG_G000004885.1	yes	545_0	Pyrite	72.15	0.80	68.15	0	0	0	14	Medium quality	4080	1621918	64.80	450	d__Archaea;p__Thermoplasmatota;c__Thermoplasmata;o__UBA184;f__UBA184;g__;s__
LMSG_G000004886.1	yes	763_2	Pyrite	76.92	3.85	57.69	1	1	0	18	Medium quality	18328	2615831	69.80	253	d__Bacteria;p__Actinobacteriota;c__Acidimicrobiia;o__Acidimicrobiales;f__RAAP-2;g__;s__
LMSG_G000004887.1	yes	518_1	Pyrite	96.56	0.00	96.56	1	0	1	19	Medium quality	121804	2142691	49.20	25	d__Bacteria;p__Elusimicrobiota;c__Elusimicrobia;o__UBA1565;f__;g__;s__
LMSG_G000004889.1	yes	1190_0	Pyrite	68.10	1.94	58.41	1	0	0	13	Medium quality	4991	1293743	70.30	296	d__Bacteria;p__Proteobacteria;c__Gammaproteobacteria;o__UBA2770;f__UBA2770;g__;s__
LMSG_G000004890.1	yes	93_1	Pyrite	75.70	0.00	75.70	1	1	2	17	Medium quality	20721	684187	46.80	44	d__Archaea;p__Micrarchaeota;c__Micrarchaeia;o__Micrarchaeales;f__Micrarchaeaceae;g__UBA581;s__
LMSG_G000004891.1	yes	1546_1	Pyrite	96.34	2.23	85.22	1	0	1	18	Medium quality	67588	2447967	64.30	109	d__Bacteria;p__Proteobacteria;c__Gammaproteobacteria;o__Acidiferrobacterales;f__Acidiferrobacteraceae;g__Acidiferrobacter;s__
LMSG_G000006212.1	yes	152_1	Pyrite	99.00	0.80	95.00	1	2	0	20	Medium quality	72733	1901280	49.80	51	d__Archaea;p__Thermoplasmatota;c__Thermoplasmata;o__;f__;g__;s__
LMSG_G000004892.1	yes	1117_1	Pyrite	95.19	1.70	86.69	0	0	0	16	Medium quality	37466	2433488	60.50	94	d__Bacteria;p__Proteobacteria;c__Gammaproteobacteria;o__SLND01;f__;g__;s__
LMSG_G000004377.1	yes	623_1	Pyrite	90.27	1.85	81.02	1	1	4	18	High quality	37549	2079498	69.00	195	d__Bacteria;p__Dormibacterota;c__Dormibacteria;o__UBA8260;f__Bog-877;g__;s__
LMSG_G000004893.1	yes	690_1	Pyrite	87.74	0.85	83.47	0	1	0	16	Medium quality	13710	2428066	48.30	249	d__Bacteria;p__Actinobacteriota;c__Acidimicrobiia;o__Acidimicrobiales;f__Acidimicrobiaceae;g__Ferrithrix;s__
LMSG_G000004894.1	yes	515_1	Pyrite	93.83	0.00	93.83	0	1	0	19	Medium quality	50370	2030671	49.60	111	d__Bacteria;p__Elusimicrobiota;c__Elusimicrobia;o__UBA1565;f__;g__;s__
LMSG_G000006213.1	yes	157_1	Pyrite	90.73	0.00	90.73	1	1	0	15	Medium quality	16414	1519096	51.50	133	d__Archaea;p__Thermoplasmatota;c__Thermoplasmata;o__;f__;g__;s__
LMSG_G000006214.1	yes	604_1	Pyrite	95.72	2.99	80.77	1	1	0	20	Medium quality	41478	3232720	56.80	146	d__Bacteria;p__Actinobacteriota;c__Acidimicrobiia;o__Acidimicrobiales;f__Acidimicrobiaceae;g__Ferrimicrobium;s__
LMSG_G000004895.1	yes	1682_0	Pyrite	68.97	1.11	63.41	0	2	0	15	Medium quality	2795	1482236	51.40	604	d__Bacteria;p__Dormibacterota;c__Dormibacteria;o__UBA8260;f__;g__;s__
LMSG_G000004896.1	yes	782_1	Pyrite	97.43	0.85	93.16	2	0	0	20	Medium quality	69526	2215235	61.30	44	d__Bacteria;p__Actinobacteriota;c__Acidimicrobiia;o__Acidimicrobiales;f__Acidimicrobiaceae;g__Ferrimicrobium;s__
LMSG_G000004897.1	yes	935_0	Pyrite	56.19	0.00	56.19	2	1	1	2	Medium quality	3912	1200911	42.40	346	d__Bacteria;p__Firmicutes;c__Bacilli;o__Bacillales;f__Anoxybacillaceae;g__Anoxybacillus;s__Anoxybacillus thermocopriae
LMSG_G000004898.1	yes	1737_1	Pyrite	60.87	0.10	60.40	0	1	0	6	Medium quality	10985	1080152	36.80	179	d__Bacteria;p__SZUA-79;c__SZUA-79;o__Acidulodesulfobacterales;f__SZUA-79;g__Acidulodesulfobacterium;s__
LMSG_G000004899.1	yes	1281_0	Pyrite	99.10	0.58	96.18	1	0	0	19	Medium quality	192491	4583909	65.20	40	d__Bacteria;p__Proteobacteria;c__Gammaproteobacteria;o__Burkholderiales;f__Burkholderiaceae;g__Aquabacterium;s__
LMSG_G000004378.1	yes	1384_0	Pyrite	100.00	0.85	95.77	3	4	1	20	High quality	45363	2017639	65.10	65	d__Bacteria;p__Deinococcota;c__Deinococci;o__Deinococcales;f__Thermaceae;g__Thermus;s__Thermus scotoductus
LMSG_G000004379.1	yes	974_1	Pyrite	96.85	0.00	96.85	2	2	1	18	High quality	60941	2001233	60.10	69	d__Bacteria;p__Proteobacteria;c__Gammaproteobacteria;o__Acidithiobacillales;f__Acidithiobacillaceae;g__Acidithiobacillus_A;s__
LMSG_G000004380.1	yes	566_1	Pyrite	98.00	1.60	90.00	2	2	2	20	High quality	41057	1512273	70.10	128	d__Archaea;p__Thermoplasmatota;c__Thermoplasmata;o__UBA184;f__UBA184;g__;s__
LMSG_G000004900.1	yes	1076_1	Pyrite	78.04	0.72	74.42	1	0	1	12	Medium quality	86869	2103620	66.00	43	d__Bacteria;p__Proteobacteria;c__Gammaproteobacteria;o__Steroidobacterales;f__Steroidobacteraceae;g__;s__
LMSG_G000004901.1	yes	1292_0	Pyrite	99.94	1.38	93.06	0	0	0	18	Medium quality	533695	4740941	65.80	29	d__Bacteria;p__Proteobacteria;c__Gammaproteobacteria;o__Burkholderiales;f__Burkholderiaceae;g__Ralstonia;s__Ralstonia mannitolilytica
LMSG_G000004902.1	yes	1264_0	Pyrite	58.12	0.32	56.54	0	0	0	6	Medium quality	4605	1609441	71.80	368	d__Bacteria;p__Proteobacteria;c__Gammaproteobacteria;o__Xanthomonadales;f__Xanthomonadaceae;g__;s__
LMSG_G000004903.1	yes	763_1	Pyrite	87.94	3.94	68.24	0	1	0	17	Medium quality	13341	2547930	70.80	297	d__Bacteria;p__Actinobacteriota;c__Acidimicrobiia;o__Acidimicrobiales;f__RAAP-2;g__;s__
LMSG_G000004381.1	yes	605_1	Pyrite	95.72	2.14	85.04	2	1	2	20	High quality	65011	2880619	57.30	82	d__Bacteria;p__Actinobacteriota;c__Acidimicrobiia;o__Acidimicrobiales;f__Acidimicrobiaceae;g__Ferrimicrobium;s__
LMSG_G000004904.1	yes	1189_2	Pyrite	74.32	0.75	70.55	0	0	0	14	Medium quality	67630	1352961	67.00	118	d__Bacteria;p__Proteobacteria;c__Gammaproteobacteria;o__;f__;g__;s__
LMSG_G000004905.1	yes	689_1	Pyrite	100.00	0.85	95.73	1	0	1	18	Medium quality	30207	2462589	48.40	126	d__Bacteria;p__Actinobacteriota;c__Acidimicrobiia;o__Acidimicrobiales;f__Acidimicrobiaceae;g__Ferrithrix;s__
LMSG_G000004906.1	yes	1299_2	Pyrite	94.92	1.34	88.25	0	0	0	19	Medium quality	43611	3730064	70.20	131	d__Bacteria;p__Proteobacteria;c__Gammaproteobacteria;o__Burkholderiales;f__Burkholderiaceae;g__Thiomonas;s__
LMSG_G000004382.1	yes	258_0	Pyrite	99.17	0.55	96.44	2	4	4	19	High quality	184435	3143974	42.70	55	d__Bacteria;p__Proteobacteria;c__Gammaproteobacteria;o__Pseudomonadales;f__Moraxellaceae;g__Acinetobacter;s__Acinetobacter schindleri
LMSG_G000004907.1	yes	1337_1	Pyrite	94.39	0.24	93.21	3	0	0	19	Medium quality	109194	2097706	56.50	53	d__Bacteria;p__Proteobacteria;c__Gammaproteobacteria;o__Burkholderiales;f__Ferrovaceae;g__Ferrovum;s__
LMSG_G000004908.1	yes	219_1	Pyrite	96.77	0.81	92.74	1	0	2	19	Medium quality	94213	1521142	44.00	32	d__Archaea;p__Thermoplasmatota;c__Thermoplasmata;o__Thermoplasmatales;f__GCA-001856825;g__GCA-001856825;s__
LMSG_G000004909.1	yes	1745_0	Pyrite	87.06	2.06	76.77	1	0	1	18	Medium quality	45074	1780101	38.10	168	d__Bacteria;p__SZUA-79;c__SZUA-79;o__Acidulodesulfobacterales;f__SZUA-79;g__Acidulodesulfobacterium;s__
LMSG_G000004910.1	yes	37_1	Pyrite	75.93	0.00	75.93	1	1	0	15	Medium quality	9888	800996	28.60	128	d__Archaea;p__Micrarchaeota;c__Micrarchaeia;o__Micrarchaeales;f__;g__;s__
LMSG_G000004383.1	yes	729_1	Pyrite	97.15	2.99	82.20	2	2	2	20	High quality	40807	3491467	70.10	133	d__Bacteria;p__Actinobacteriota;c__Acidimicrobiia;o__Acidimicrobiales;f__UBA8190;g__;s__
LMSG_G000004911.1	yes	38_1	Pyrite	78.27	0.00	78.27	1	1	1	18	Medium quality	42622	872209	35.10	51	d__Archaea;p__Micrarchaeota;c__Micrarchaeia;o__Micrarchaeales;f__;g__;s__
LMSG_G000004912.1	yes	429_1	Pyrite	97.72	0.00	97.72	0	1	0	19	Medium quality	16392	4015412	52.40	446	d__Bacteria;p__Planctomycetota;c__Phycisphaerae;o__UBA1161;f__;g__;s__
LMSG_G000006315.1	yes	750_1	Pyrite	89.28	3.85	70.05	0	1	0	17	Medium quality	12809	2950888	72.40	353	d__Bacteria;p__Actinobacteriota;c__Acidimicrobiia;o__Acidimicrobiales;f__Palsa-688;g__;s__

LMSG_G000006215.1	yes	186_1	Pyrite	95.92	0.00	95.92	1	2	0	20	Medium quality	28768	1716782	42.10	126	d__Archaea;p__Thermoplasmatota;c__Thermoplasmata;o__Thermoplasmatales;f__Thermoplasmataceae;g__;s__
LMSG_G000004913.1	yes	882_0	Pyrite	95.07	1.94	85.36	2	0	0	20	Medium quality	212371	3573737	70.20	22	d__Bacteria;p__Actinobacteriota;c__Actinomycetia;o__Propionibacteriales;f__Nocardioidaceae;g__Nocardioides;s__Nocardioides allogilvus
LMSG_G000004914.1	yes	1017_1	Pyrite	94.55	0.00	94.55	0	2	0	19	Medium quality	37129	3099170	68.10	126	d__Bacteria;p__Firmicutes_E;c__DTU015;o__;f__;g__;s__
LMSG_G000004915.1	yes	827_0	Pyrite	74.84	1.28	68.43	0	1	0	18	Medium quality	8578	2071656	53.70	280	d__Bacteria;p__Actinobacteriota;c__Acidimicrobiia;o__Acidimicrobiales;f__Acidimicrobiaceae;g__SCQM01;s__
LMSG_G000004916.1	yes	198_1	Pyrite	84.83	0.66	81.53	0	2	0	16	Medium quality	11340	1198787	47.30	163	d__Archaea;p__Thermoplasmatota;c__Thermoplasmata;o__Thermoplasmatales;f__Thermoplasmataceae;g__UBA509;s__
LMSG_G000004384.1	yes	1152_0	Pyrite	90.59	3.03	75.43	1	1	1	18	High quality	31593	3752265	67.40	389	d__Bacteria;p__Firmicutes_E;c__Sulfobacillia;o__Sulfobacillales;f__;g__;s__
LMSG_G000006216.1	yes	953_1	Pyrite	94.48	1.97	84.64	1	1	0	19	Medium quality	71837	2261068	49.80	72	d__Bacteria;p__Nitrospirota;c__Leptospirillia;o__Leptospirillales;f__Leptospirillaceae;g__Leptospirillum;s__Leptospirillum ferrooxidans
LMSG_G000004917.1	yes	1738_0	Pyrite	72.41	2.59	59.48	0	1	0	16	Medium quality	36510	1458815	37.40	121	d__Bacteria;p__SZUA-79;c__SZUA-79;o__Acidulodesulfobacterales;f__SZUA-79;g__Acidulodesulfobacterium;s__
LMSG_G000004918.1	yes	726_2	Lead-Zinc	86.13	2.14	75.45	0	0	0	18	Medium quality	16859	2823818	71.20	190	d__Bacteria;p__Actinobacteriota;c__Acidimicrobiia;o__Acidimicrobiales;f__UBA8190;g__;s__
LMSG_G000004385.1	yes	229_1	Lead-Zinc	100.00	0.00	100.00	2	3	4	20	High quality	25233	876776	29.60	64	d__Bacteria;p__Firmicutes;c__Bacilli;o__Mycoplasmatales;f__Metamycoplasmataceae;g__Mycoplasmopsis;s__Mycoplasmopsis bovis
LMSG_G000004919.1	yes	1305_1	Lead-Zinc	97.82	0.45	95.55	0	1	0	19	Medium quality	39277	3123104	66.30	121	d__Bacteria;p__Proteobacteria;c__Gammaproteobacteria;o__Burkholderiales;f__Burkholderiaceae;g__Thiomonas;s__Thiomonas delicata
LMSG_G000004920.1	yes	1544_1	Lead-Zinc	83.25	1.93	73.60	0	0	0	12	Medium quality	12332	1974907	66.80	194	d__Bacteria;p__Proteobacteria;c__Gammaproteobacteria;o__Acidiferrobacterales;f__Acidiferrobacteraceae;g__Acidiferrobacter;s__
LMSG_G000004921.1	yes	894_1	Lead-Zinc	97.09	0.00	97.09	0	0	0	19	Medium quality	33517	3508312	56.00	171	d__Bacteria;p__Acidobacteriota;c__Acidobacteriae;o__Acidobacteriales;f__Acidobacteriaceae;g__;s__
LMSG_G000004386.1	yes	1082_1	Lead-Zinc	94.92	3.00	79.92	1	1	1	18	High quality	91145	3406862	66.40	62	d__Bacteria;p__Proteobacteria;c__Gammaproteobacteria;o__Steroidobacterales;f__Steroidobacteraceae;g__;s__
LMSG_G000004922.1	yes	521_0	Lead-Zinc	70.21	1.98	60.31	1	0	2	15	Medium quality	4025	3885656	66.10	1361	d__Bacteria;p__Chloroflexota;c__Ktedonobacteria;o__Ktedonobacterales;f__;g__;s__
LMSG_G000006266.1	yes	1077_1	Lead-Zinc	93.65	3.48	76.25	1	1	1	16	Medium quality	66310	3270354	66.70	107	d__Bacteria;p__Proteobacteria;c__Gammaproteobacteria;o__Steroidobacterales;f__Steroidobacteraceae;g__;s__
LMSG_G000004923.1	yes	1404_1	Lead-Zinc	99.35	0.27	98.00	1	0	0	18	Medium quality	203617	6473091	66.20	57	d__Bacteria;p__Proteobacteria;c__Gammaproteobacteria;o__Pseudomonadales;f__Pseudomonadaceae;g__Pseudomonas;s__Pseudomonas aeruginosa
LMSG_G000004924.1	yes	1684_0	Lead-Zinc	64.71	0.00	64.71	1	0	0	17	Medium quality	13673	723161	29.90	87	d__Archaea;p__Nanoarchaeota;c__Nanoarchaeia;o__Pacearchaeales;f__GW2011-AR1;g__JWKY01;s__
LMSG_G000004925.1	yes	45_0	Lead-Zinc	62.48	1.25	56.25	0	0	0	16	Medium quality	4707	657268	41.20	153	d__Archaea;p__Micrarchaeota;c__Micrarchaeia;o__Micrarchaeales;f__;g__;s__
LMSG_G000004926.1	yes	1367_0	Lead-Zinc	88.26	2.14	77.58	1	0	1	19	Medium quality	9899	2804040	67.40	408	d__Bacteria;p__Acidobacteriota;c__Aminicenantia;o__UBA2199;f__UBA2199;g__UBA2199;s__
LMSG_G000004387.1	yes	707_0	Lead-Zinc	98.93	0.00	98.93	1	6	3	20	High quality	56975	1861431	44.00	96	d__Archaea;p__Methanobacteriota;c__Methanobacteria;o__Methanobacteriales;f__Methanobacteriaceae;g__;s__
LMSG_G000006267.1	yes	1269_2	Lead-Zinc	97.53	0.21	96.51	1	1	1	17	Medium quality	42655	2745484	66.90	121	d__Bacteria;p__Proteobacteria;c__Gammaproteobacteria;o__Burkholderiales;f__Burkholderiaceae;g__GJ-E10;s__
LMSG_G000004927.1	yes	1696_0	Lead-Zinc	84.96	1.11	79.41	0	0	0	14	Medium quality	3925	1438512	38.50	470	d__Bacteria;p__Firmicutes;c__Bacilli;o__Acholeplasmatales;f__Acholeplasmataceae;g__UBA2284;s__
LMSG_G000004928.1	yes	1542_0	Lead-Zinc	76.37	1.72	67.75	0	1	0	14	Medium quality	44475	2007217	63.20	72	d__Bacteria;p__Proteobacteria;c__Gammaproteobacteria;o__Acidiferrobacterales;f__Acidiferrobacteraceae;g__Acidiferrobacter;s__
LMSG_G000006316.1	yes	1713_0	Lead-Zinc	91.10	1.65	82.86	0	0	0	15	Medium quality	7925	2170255	46.10	431	d__Bacteria;p__Cloacimonadota;c__Cloacimonadia;o__Cloacimonadales;f__Cloacimonadaceae;g__;s__
LMSG_G000004929.1	yes	1841_0	Lead-Zinc	71.52	3.59	53.55	0	0	0	16	Medium quality	2625	1355156	53.80	608	d__Archaea;p__Halobacteriota;c__Methanomicrobia;o__Methanomicrobiales;f__Methanoregulaceae;g__UBA9949;s__
LMSG_G000004930.1	yes	533_0	Lead-Zinc	88.86	0.00	88.86	1	2	0	15	Medium quality	17636	2344075	56.20	251	d__Bacteria;p__Cloacimonadota;c__Cloacimonadia;o__Cloacimonadales;f__Cloacimonadaceae;g__;s__
LMSG_G000004931.1	yes	1551_0	Lead-Zinc	82.92	1.83	73.78	1	1	1	14	Medium quality	72558	1954261	63.50	42	d__Bacteria;p__Proteobacteria;c__Gammaproteobacteria;o__Acidiferrobacterales;f__Acidiferrobacteraceae;g__Acidiferrobacter;s__
LMSG_G000004932.1	yes	908_0	Copper	75.05	1.72	66.43	1	1	1	10	Medium quality	33142	2917158	60.70	126	d__Bacteria;p__Acidobacteriota;c__Acidobacteriae;o__Acidobacteriales;f__Acidobacteriaceae;g__Terracidiphilus;s__
LMSG_G000004933.1	yes	209_1	Copper	88.39	1.88	78.99	1	1	1	17	Medium quality	18207	1435132	44.80	122	d__Archaea;p__Thermoplasmatota;c__Thermoplasmata;o__Thermoplasmatales;f__Thermoplasmataceae;g__UBA582;s__
LMSG_G000004934.1	yes	915_0	Copper	70.88	0.27	69.56	0	0	0	13	Medium quality	13322	2133115	61.60	288	d__Bacteria;p__Acidobacteriota;c__Acidobacteriae;o__Acidobacteriales;f__Acidobacteriaceae;g__;s__
LMSG_G000004935.1	yes	648_0	Copper	72.41	0.99	67.46	1	2	1	15	Medium quality	4444	3020931	70.20	754	d__Bacteria;p__Firmicutes_E;c__Thermaerobacteria;o__;f__;g__;s__
LMSG_G000004936.1	yes	1047_0	Copper	81.64	2.62	68.56	0	0	0	18	Medium quality	5193	7277913	67.70	1677	d__Bacteria;p__Planctomycetota;c__Planctomycetes;o__Isosphaerales;f__Isosphaeraceae;g__;s__
LMSG_G000004937.1	yes	1804_0	Copper	61.18	0.00	61.18	1	1	2	19	Medium quality	342864	681297	58.40	23	d__Bacteria;p__Patescibacteria;c__Paceibacteria;o__UBA6257;f__2-01-FULL-56-20;g__;s__
LMSG_G000004938.1	yes	143_0	Copper	76.01	1.40	69.01	1	2	0	17	Medium quality	15385	717292	46.40	64	d__Archaea;p__Micrarchaeota;c__Micrarchaeia;o__Micrarchaeales;f__;g__;s__
LMSG_G000004388.1	yes	810_1	Copper	97.43	2.53	84.81	1	1	1	20	High quality	77167	1795377	71.00	43	d__Bacteria;p__Actinobacteriota;c__Acidimicrobiia;o__Acidimicrobiales;f__RAAP-2;g__;s__
LMSG_G000004939.1	yes	1531_0	Copper	98.76	0.75	95.00	1	0	1	18	Medium quality	498909	4841998	51.80	20	d__Bacteria;p__Proteobacteria;c__Gammaproteobacteria;o__Enterobacterales;f__Enterobacteriaceae;g__Citrobacter;s__Citrobacter freundii
LMSG_G000004389.1	yes	357_1	Copper	96.58	1.28	90.17	1	1	2	20	High quality	108543	3134593	64.80	158	d__Bacteria;p__Actinobacteriota;c__Acidimicrobiia;o__Acidimicrobiales;f__;g__;s__
LMSG_G000004940.1	yes	1720_0	Copper	80.51	0.00	80.51	2	2	2	20	Medium quality	44835	972769	38.50	40	d__Bacteria;p__Dependentiae;c__Babeliae;o__Babeliales;f__Babeliaceae;g__BOG-1370;s__
LMSG_G000004941.1	yes	123_0	Copper	78.19	0.58	75.32	1	0	1	18	Medium quality	19713	825732	53.00	54	d__Archaea;p__Micrarchaeota;c__Micrarchaeia;o__Micrarchaeales;f__;g__;s__
LMSG_G000004390.1	yes	437_1	Copper	97.72	0.00	97.72	1	1	2	20	High quality	39787	3412679	56.40	184	d__Bacteria;p__Planctomycetota;c__Phycisphaerae;o__;f__;g__;s__
LMSG_G000004942.1	yes	58_0	Copper	81.93	0.00	81.93	1	1	0	20	Medium quality	211083	734681	45.00	7	d__Archaea;p__Micrarchaeota;c__Micrarchaeia;o__Micrarchaeales;f__Micrarchaeaceae;g__;s__
LMSG_G000004943.1	yes	24_1	Copper	79.53	0.97	74.68	1	1	1	18	Medium quality	114288	695668	41.30	32	d__Archaea;p__Nanoarchaeota;c__Nanoarchaeia;o__Parvarchaeales;f__Parvarchaeaceae;g__;s__
LMSG_G000006217.1	yes	190_1	Copper	97.13	0.25	95.89	1	2	0	19	Medium quality	95290	1551666	45.10	31	d__Archaea;p__Thermoplasmatota;c__Thermoplasmata;o__Thermoplasmatales;f__Thermoplasmataceae;g__UBA509;s__
LMSG_G000004944.1	yes	792_1	Copper	70.34	0.00	70.34	0	1	0	15	Medium quality	10099	1956350	66.00	218	d__Bacteria;p__Actinobacteriota;c__Acidimicrobiia;o__Acidimicrobiales;f__RAAP-2;g__RAAP-2;s__
LMSG_G000004945.1	yes	594_1	Copper	98.05	2.91	83.49	1	0	0	19	Medium quality	69583	2574255	60.30	82	d__Archaea;p__Thermoproteota;c__Nitrososphaeria;o__Nitrososphaerales;f__UBA183;g__UBA183;s__
LMSG_G000004946.1	yes	95_1	Copper	79.40	0.93	74.73	1	2	2	19	Medium quality	76064	892466	48.00	26	d__Archaea;p__Micrarchaeota;c__Micrarchaeia;o__Micrarchaeales;f__Micrarchaeaceae;g__Micrarchaeum;s__
LMSG_G000004391.1	yes	770_1	Copper	96.58	2.14	85.90	1	1	1	20	High quality	48491	2794863	72.90	99	d__Bacteria;p__Actinobacteriota;c__Acidimicrobiia;o__Acidimicrobiales;f__Bog-793;g__;s__
LMSG_G000004949.1	yes	1562_0	Copper	85.71	2.57	72.85	0	1	0	19	Medium quality	31700	3524704	63.30	150	d__Bacteria;p__Desulfobacterota_B;c__Binatia;o__Binatales;f__Binataceae;g__;s__
LMSG_G000004947.1	yes	1006_0	Copper	56.30	0.00	56.30	0	0	0	14	Medium quality	5924	1799114	61.20	331	d__Bacteria;p__Acidobacteriota;c__Acidobacteriae;o__Acidobacteriales;f__SCQP01;g__;s__
LMSG_G000004392.1	yes	998_1	Copper	95.45	1.71	86.91	2	2	2	20	High quality	114143	3390814	59.70	52	d__Bacteria;p__Acidobacteriota;c__Acidobacteriae;o__UBA7540;f__UBA7540;g__;s__
LMSG_G000004948.1	yes	67_0	Copper	59.81	0.00	59.81	0	0	0	16	Medium quality	48841	678297	50.00	19	d__Archaea;p__Micrarchaeota;c__Micrarchaeia;o__Micrarchaeales;f__Micrarchaeaceae;g__;s__
LMSG_G000006268.1	yes	220_1	Copper	91.93	2.42	79.84	1	1	1	17	Medium quality	19099	1575415	44.80	127	d__Archaea;p__Thermoplasmatota;c__Thermoplasmata;o__Thermoplasmatales;f__GCA-001856825;g__GCA-001856825;s__
LMSG_G000004950.1	yes	1029_0	Copper	93.37	1.54	85.68	0	0	0	18	Medium quality	54040	3115918	59.80	90	d__Bacteria;p__Acidobacteriota;c__Acidobacteriae;o__Acidobacteriales;f__Acidobacteriaceae;g__Acidobacterium;s__
LMSG_G000004951.1	yes	132_1	Copper	84.57	0.00	84.57	1	1	2	20	Medium quality	142579	894386	42.50	14	d__Archaea;p__Micrarchaeota;c__Micrarchaeia;o__Micrarchaeales;f__;g__;s__
LMSG_G000004953.1	yes	625_0	Copper	58.42	0.88	54.04	1	0	1	12	Medium quality	10137	1258313	69.70	152	d__Bacteria;p__Dormibacterota;c__Dormibacteria;o__UBA8260;f__Bog-877;g__;s__
LMSG_G000004393.1	yes	1654_1	Copper	95.47	0.89	91.01	1	1	1	20	High quality	174256	2646869	47.40	42	d__Bacteria;p__Bdellovibrionota;c__Bdellovibrionia;o__Bdellovibrionales;f__UBA1609;g__RBG-16-40-8;s__
LMSG_G000004952.1	yes	565_0	Copper	77.46	0.00	77.46	1	2	2	18	Medium quality	150810	1506728	70.20	25	d__Archaea;p__Thermoplasmatota;c__Thermoplasmata;o__UBA184;f__UBA184;g__;s__
LMSG_G000004954.1	yes	1502_1	Copper	99.00	0.25	97.76	0	1	0	19	Medium quality	30405	3263755	67.50	197	d__Bacteria;p__Proteobacteria;c__Alphaproteobacteria;o__Acetobacterales;f__Acetobacteraceae;g__G45-3;s__
LMSG_G000004955.1	yes	869_1	Copper	93.85	1.59	85.90	0	1	1	19	Medium quality	20763	4736931	71.40	360	d__Bacteria;p__Actinobacteriota;c__Actinomycetia;o__Streptosporangiales;f__Streptosporangiaceae;g__Palsa-506;s__
LMSG_G000004957.1	yes	1089_2	Copper	64.50	2.61	51.44	1	0	1	11	Medium quality	17782	1904039	67.20	158	d__Bacteria;p__Proteobacteria;c__Gammaproteobacteria;o__Steroidobacterales;f__Steroidobacteraceae;g__;s__
LMSG_G000004956.1	yes	965_1	Copper	89.24	1.82	80.15	1	0	1	18	Medium quality	59267	2354036	59.60	114	d__Bacteria;p__Nitrospirota;c__Leptospirillia;o__Leptospirillales;f__Leptospirillaceae;g__UBA4572;s__UBA4572 sp002387725
LMSG_G000004958.1	yes	1086_2	Copper	72.26	1.21	66.23	1	1	1	12	Medium quality	54627	1879221	69.10	52	d__Bacteria;p__Proteobacteria;c__Gammaproteobacteria;o__Steroidobacterales;f__Steroidobacteraceae;g__;s__

LMSG_G000004959.1	yes	865_0	Copper	95.05	0.00	95.05	0	0	0	19	Medium quality	20736	2962316	71.60	190	d__Bacteria;p__Actinobacteriota;c__Actinomycetia;o__Mycobacteriales;f__Mycobacteriaceae;g__Tomitella;s__
LMSG_G000004960.1	yes	1017_2	Copper	67.04	0.43	64.88	1	0	1	14	Medium quality	2247	1848533	69.30	904	d__Bacteria;p__Firmicutes_E;c__DTU015;o__;f__;g__;s__
LMSG_G000004961.1	yes	860_0	Copper	94.37	1.97	84.52	1	0	1	17	Medium quality	50492	3823562	67.10	150	d__Bacteria;p__Actinobacteriota;c__Actinomycetia;o__Mycobacteriales;f__Pseudonocardiaceae;g__Saccharomonospora;s__
LMSG_G000004962.1	yes	878_0	Copper	83.60	2.28	72.20	0	0	0	17	Medium quality	6413	2536038	71.80	494	d__Bacteria;p__Actinobacteriota;c__Actinomycetia;o__RP-AC37;f__;g__;s__
LMSG_G000004965.1	yes	263_0	Copper	89.92	1.51	82.37	2	0	0	18	Medium quality	60967	2205557	32.10	53	d__Bacteria;p__Bacteroidota;c__Bacteroidia;o__Flavobacteriales;f__Flavobacteriaceae;g__;s__
LMSG_G000006218.1	yes	1174_0	Copper	92.74	0.99	87.81	1	1	0	18	Medium quality	29395	2153931	51.70	115	d__Bacteria;p__Proteobacteria;c__Gammaproteobacteria;o__Burkholderiales;f__Burkholderiaceae;g__Paenalcaligenes;s__
LMSG_G000004963.1	yes	267_0	Copper	84.54	4.34	62.84	1	2	1	17	Medium quality	15498	2587928	41.90	273	d__Bacteria;p__Bacteroidota;c__Bacteroidia;o__Flavobacteriales;f__Flavobacteriaceae;g__;s__
LMSG_G000004394.1	yes	866_0	Copper	96.61	1.34	89.93	4	3	1	20	High quality	35112	5886810	72.90	228	d__Bacteria;p__Actinobacteriota;c__Actinomycetia;o__Streptosporangiales;f__Streptosporangiaceae;g__Spirillospora;s__Spirillospora hallensis
LMSG_G000004964.1	yes	1649_0	Copper	67.43	1.30	60.92	2	0	1	6	Medium quality	2423	1477138	49.20	680	d__Bacteria;p__Proteobacteria;c__Gammaproteobacteria;o__Burkholderiales;f__Burkholderiaceae;g__Paracandidimonas;s__
LMSG_G000004973.1	yes	861_0	Copper	93.61	0.10	93.12	3	0	1	18	Medium quality	46140	3759813	67.40	143	d__Bacteria;p__Actinobacteriota;c__Actinomycetia;o__Mycobacteriales;f__Pseudonocardiaceae;g__Saccharomonospora;s__Saccharomonospora viridis
LMSG_G000004966.1	yes	1356_0	Copper	81.10	3.42	64.03	0	0	0	15	Medium quality	5963	1991221	59.90	386	d__Bacteria;p__Proteobacteria;c__Gammaproteobacteria;o__Pseudomonadales;f__Halomonadaceae;g__Halomonas;s__
LMSG_G000004967.1	yes	656_0	Copper	93.56	0.99	88.61	3	0	1	19	Medium quality	68277	3321241	65.00	129	d__Bacteria;p__Firmicutes_E;c__Sulfobacillia;o__;f__;g__;s__
LMSG_G000004395.1	yes	268_0	Copper	92.08	1.69	83.62	1	1	1	20	High quality	94521	2782013	33.70	93	d__Bacteria;p__Bacteroidota;c__Bacteroidia;o__Flavobacteriales;f__Flavobacteriaceae;g__RR4-38;s__
LMSG_G000004968.1	yes	803_0	Copper	84.61	3.42	67.52	1	0	1	20	Medium quality	35591	2177991	66.00	224	d__Bacteria;p__Actinobacteriota;c__Acidimicrobiia;o__Acidimicrobiales;f__RAAP-2;g__RAAP-2;s__
LMSG_G000004969.1	yes	890_0	Copper	85.70	1.92	76.09	1	0	0	15	Medium quality	30797	4377476	56.80	235	d__Bacteria;p__Acidobacteriota;c__Acidobacteriae;o__Acidobacteriales;f__Acidobacteriaceae;g__;s__
LMSG_G000004970.1	yes	944_0	Copper	54.31	0.00	54.31	0	0	0	7	Medium quality	3163	1597248	37.60	536	d__Bacteria;p__Firmicutes;c__Bacilli;o__Bacillales;f__Amphibacillaceae;g__;s__
LMSG_G000004971.1	yes	1012_0	Copper	65.80	1.97	55.95	0	0	0	16	Medium quality	4173	2210825	61.40	642	d__Bacteria;p__Actinobacteriota;c__Actinomycetia;o__Mycobacteriales;f__Micromonosporaceae;g__Stackebrandtia;s__
LMSG_G000004972.1	yes	846_0	Copper	95.18	1.16	89.39	2	0	0	20	Medium quality	91887	4583465	68.90	102	d__Bacteria;p__Actinobacteriota;c__Actinomycetia;o__Streptosporangiales;f__Streptosporangiaceae;g__;s__
LMSG_G000004977.1	yes	1653_0	Copper	60.03	0.27	58.69	1	3	2	12	Medium quality	4981	1560665	39.60	372	d__Bacteria;p__Bacteroidota;c__Bacteroidia;o__Sphingobacteriales;f__Sphingobacteriaceae;g__;s__
LMSG_G000004974.1	yes	852_0	Copper	53.44	0.00	53.44	1	0	1	14	Medium quality	7355	2166446	73.60	329	d__Bacteria;p__Actinobacteriota;c__Actinomycetia;o__Actinomycetales;f__Actinomycetaceae;g__Oceanitalea;s__
LMSG_G000004975.1	yes	1350_2	Copper	99.12	0.00	99.12	2	0	0	18	Medium quality	114491	2955240	67.30	43	d__Bacteria;p__Acidobacteriota;c__Holophagae;o__Holophagales;f__Holophagaceae;g__;s__
LMSG_G000004976.1	yes	1004_0	Copper	90.99	2.40	79.01	0	1	0	16	Medium quality	10446	2813988	58.40	395	d__Bacteria;p__Firmicutes;c__Bacilli;o__Thermoactinomycetales;f__Thermoactinomycetaceae;g__;s__
LMSG_G000004978.1	yes	1013_0	Copper	87.28	0.72	83.67	1	1	1	17	Medium quality	18975	2243158	59.00	181	d__Bacteria;p__Actinobacteriota;c__Actinomycetia;o__Actinomycetales;f__Demequinaceae;g__;s__
LMSG_G000004979.1	yes	847_0	Copper	98.46	0.55	95.73	0	0	0	20	Medium quality	53449	3805890	69.00	138	d__Bacteria;p__Actinobacteriota;c__Actinomycetia;o__Mycobacteriales;f__Micromonosporaceae;g__Glycomyces;s__
LMSG_G000004396.1	yes	175_1	Copper	96.05	0.31	94.50	1	1	2	20	High quality	85191	1501832	48.60	38	d__Archaea;p__Thermoplasmatota;c__Thermoplasmata;o__Thermoplasmatales;f__Thermoplasmataceae;g__UBA582;s__
LMSG_G000004980.1	yes	577_1	Copper	91.34	1.72	82.73	0	2	1	20	Medium quality	25582	2283643	62.10	278	d__Archaea;p__Thermoplasmatota;c__Thermoplasmata;o__UBA184;f__UBA184;g__;s__
LMSG_G000004981.1	yes	1253_0	Copper	78.32	1.30	71.84	0	1	0	15	Medium quality	4083	2270169	61.90	607	d__Bacteria;p__Proteobacteria;c__Gammaproteobacteria;o__Pseudomonadales;f__Alcanivoracaceae;g__Alcanivorax_A;s__
LMSG_G000004982.1	yes	1128_3	Copper	89.86	1.85	80.61	1	4	5	18	Medium quality	98347	2253666	63.40	177	d__Bacteria;p__Eremiobacterota;c__Eremiobacteria;o__UBP12;f__UBA5184;g__;s__
LMSG_G000004986.1	yes	1634_0	Copper	93.41	0.00	93.41	2	0	1	18	Medium quality	38726	2754176	37.90	131	d__Bacteria;p__Bacteroidota;c__Bacteroidia;o__Sphingobacteriales;f__Sphingobacteriaceae;g__;s__
LMSG_G000004983.1	yes	855_0	Copper	86.37	1.81	77.31	1	0	1	19	Medium quality	10717	2530106	68.60	298	d__Bacteria;p__Actinobacteriota;c__Actinomycetia;o__Actinomycetales;f__Microbacteriaceae;g__Microbacterium;s__
LMSG_G000004397.1	yes	1750_0	Copper	98.46	1.15	92.70	3	1	1	19	High quality	53901	3263623	52.80	86	d__Bacteria;p__Firmicutes;c__Bacilli;o__Thermoactinomycetales;f__Thermoactinomycetaceae;g__Melghirimyces;s__Melghirimyces thermohalophilus
LMSG_G000004984.1	yes	1346_0	Copper	78.75	3.51	61.21	0	0	0	15	Medium quality	5663	2677025	71.70	567	d__Bacteria;p__Actinobacteriota;c__Actinomycetia;o__Actinomycetales;f__Dermabacteraceae;g__Brachybacterium;s__
LMSG_G000004398.1	yes	291_0	Copper	92.02	0.90	87.54	1	1	1	18	High quality	14159	1859415	43.90	196	d__Bacteria;p__Proteobacteria;c__Gammaproteobacteria;o__Burkholderiales;f__Burkholderiaceae;g__;s__
LMSG_G000004985.1	yes	858_0	Copper	94.57	2.81	80.52	0	3	0	19	Medium quality	22403	3454942	70.30	221	d__Bacteria;p__Actinobacteriota;c__Actinomycetia;o__Actinomycetales;f__Beutenbergiaceae;g__Ruania;s__Ruania albidiflava
LMSG_G000004987.1	yes	610_1	Copper	91.76	1.28	85.35	1	0	0	16	Medium quality	60477	2459395	52.30	119	d__Bacteria;p__Actinobacteriota;c__Acidimicrobiia;o__Acidimicrobiales;f__Acidimicrobiaceae;g__SCQM01;s__
LMSG_G000004988.1	yes	1595_0	Copper	90.99	2.20	80.02	1	0	0	11	Medium quality	15831	2538177	56.60	310	d__Bacteria;p__Proteobacteria;c__Gammaproteobacteria;o__Pseudomonadales;f__Cellvibrionaceae;g__Marinimicrobium;s__
LMSG_G000004399.1	yes	1161_0	Copper	99.50	2.24	88.31	1	1	1	20	High quality	119309	5043607	64.00	84	d__Bacteria;p__Proteobacteria;c__Alphaproteobacteria;o__Rhodospirillales;f__Magnetospirillaceae;g__Telmatospirillum;s__
LMSG_G000004400.1	yes	850_0	Copper	100.00	1.05	94.77	1	1	1	20	High quality	48504	3434716	66.60	132	d__Bacteria;p__Actinobacteriota;c__Actinomycetia;o__Actinomycetales;f__Brevibacteriaceae;g__Brevibacterium;s__
LMSG_G000004989.1	yes	1810_0	Copper	77.90	0.61	74.84	1	1	1	20	Medium quality	61863	876261	55.20	60	d__Bacteria;p__Patescibacteria;c__Paceibacteria;o__UBA6257;f__2-01-FULL-56-20;g__;s__
LMSG_G000004990.1	yes	658_0	Copper	88.94	0.99	83.99	1	0	0	17	Medium quality	61955	2724776	68.40	84	d__Bacteria;p__Firmicutes_E;c__Sulfobacillia;o__;f__;g__;s__
LMSG_G000006269.1	yes	236_0	Copper	93.45	0.93	88.83	3	2	2	15	Medium quality	21128	2050525	49.50	154	d__Bacteria;p__Proteobacteria;c__Gammaproteobacteria;o__Pseudomonadales;f__Alcanivoracaceae;g__;s__
LMSG_G000006324.1	yes	290_1	Copper	74.71	1.31	68.14	0	1	0	9	Medium quality	3167	2001041	34.70	713	d__Bacteria;p__Proteobacteria;c__Gammaproteobacteria;o__UBA6186;f__UBA6186;g__;s__
LMSG_G000004401.1	yes	497_0	Copper	97.30	0.00	97.30	4	1	1	19	High quality	65561	3377211	50.90	146	d__Bacteria;p__Firmicutes;c__Bacilli;o__Thermoactinomycetales;f__Novibacillaceae;g__Novibacillus;s__Novibacillus thermophilus
LMSG_G000004991.1	yes	862_0	Copper	95.42	2.47	83.06	0	0	0	20	Medium quality	55664	4556911	65.60	144	d__Bacteria;p__Actinobacteriota;c__Actinomycetia;o__Streptosporangiales;f__Streptosporangiaceae;g__Nocardiopsis;s__
LMSG_G000004992.1	yes	189_1	Copper	69.71	1.08	64.34	1	1	0	12	Medium quality	36709	1216983	42.80	60	d__Archaea;p__Thermoplasmatota;c__Thermoplasmata;o__Thermoplasmatales;f__Thermoplasmataceae;g__UBA509;s__
LMSG_G000006219.1	yes	654_0	Copper	92.37	0.99	87.42	2	2	0	19	Medium quality	36787	3475542	67.30	129	d__Bacteria;p__Firmicutes_E;c__Sulfobacillia;o__;f__;g__;s__
LMSG_G000004993.1	yes	867_0	Copper	98.80	1.47	91.45	1	0	0	17	Medium quality	40972	4807983	68.90	215	d__Bacteria;p__Actinobacteriota;c__Actinomycetia;o__Jiangellales;f__Jiangellaceae;g__Haloactinopolyspora;s__
LMSG_G000004994.1	yes	18_0	Copper	91.39	2.66	78.10	0	1	0	17	Medium quality	10841	2207054	56.50	276	d__Bacteria;p__Proteobacteria;c__Gammaproteobacteria;o__Burkholderiales;f__Burkholderiaceae;g__Castellaniella;s__
LMSG_G000004995.1	yes	1195_0	Copper	76.89	1.46	69.60	2	1	0	15	Medium quality	194946	3688728	66.40	33	d__Bacteria;p__Proteobacteria;c__Gammaproteobacteria;o__Xanthomonadales;f__Xanthomonadaceae;g__Stenotrophomonas;s__Stenotrophomonas maltophilia
LMSG_G000004996.1	yes	1111_0	Copper	82.03	0.00	82.03	0	1	1	11	Medium quality	32777	1956575	55.40	86	d__Bacteria;p__Firmicutes_E;c__Sulfobacillia;o__Sulfobacillales;f__Sulfobacillaceae;g__Sulfobacillus_B;s__
LMSG_G000004997.1	yes	57_0	Copper	78.03	0.93	73.36	1	1	2	20	Medium quality	40969	879343	44.70	53	d__Archaea;p__Micrarchaeota;c__Micrarchaeia;o__Micrarchaeales;f__Micrarchaeaceae;g__;s__
LMSG_G000004402.1	yes	1132_1	Copper	99.07	0.00	99.07	1	1	1	20	High quality	232591	2336138	62.80	38	d__Bacteria;p__Eremiobacterota;c__Eremiobacteria;o__UBP12;f__UBA5184;g__;s__
LMSG_G000004998.1	yes	1258_0	Copper	77.15	3.51	59.58	1	0	0	16	Medium quality	30874	2054871	70.50	106	d__Bacteria;p__Proteobacteria;c__Gammaproteobacteria;o__Xanthomonadales;f__Rhodanobacteraceae;g__Metallibacterium;s__
LMSG_G000004403.1	yes	471_1	Copper	93.33	2.50	80.83	2	2	1	19	High quality	26393	3739436	57.00	243	d__Bacteria;p__Firmicutes;c__Alicyclobacillia;o__Alicyclobacillales;f__Acidibacillaceae;g__;s__
LMSG_G000004999.1	yes	735_1	Copper	100.00	1.28	93.59	0	1	0	18	Medium quality	52002	3047003	73.10	97	d__Bacteria;p__Actinobacteriota;c__Acidimicrobiia;o__Acidimicrobiales;f__RAAP-2;g__;s__
LMSG_G000005000.1	yes	1196_0	Copper	89.62	1.84	80.42	0	0	0	17	Medium quality	5684	3071594	66.00	643	d__Bacteria;p__Proteobacteria;c__Alphaproteobacteria;o__Ferrovibrionales;f__Ferrovibrionaceae;g__;s__
LMSG_G000005001.1	yes	655_1	Copper	94.55	1.32	87.95	1	0	1	17	Medium quality	14501	3039007	68.90	315	d__Bacteria;p__Firmicutes_E;c__Sulfobacillia;o__;f__;g__;s__
LMSG_G000005002.1	yes	1131_2	Copper	75.39	0.46	73.08	1	0	1	14	Medium quality	7435	1777187	62.90	328	d__Bacteria;p__Eremiobacterota;c__Eremiobacteria;o__UBP12;f__UBA5184;g__PALSA-1484;s__
LMSG_G000004404.1	yes	168_1	Copper	97.06	0.00	97.06	1	1	1	20	High quality	45534	1671794	35.70	90	d__Archaea;p__Thermoplasmatota;c__Thermoplasmata;o__UBA184;f__;g__;s__
LMSG_G000005003.1	yes	1299_1	Copper	94.71	2.92	80.11	1	0	0	16	Medium quality	12448	3547349	70.10	426	d__Bacteria;p__Proteobacteria;c__Gammaproteobacteria;o__Burkholderiales;f__Burkholderiaceae;g__Thiomonas;s__
LMSG_G000005004.1	yes	1736_1	Lead-Zinc	81.60	0.86	77.29	1	1	0	18	Medium quality	34660	1899216	35.20	94	d__Bacteria;p__SZUA-79;c__SZUA-79;o__Acidulodesulfobacterales;f__SZUA-79;g__Acidulodesulfobacterium;s__

LMSG_G000005005.1	yes	478_2	Lead-Zinc	80.03	1.97	70.19	2	2	1	17	Medium quality	7488	2376367	47.30	376	d__Bacteria;p__Firmicutes;c__Alicyclobacillia;o__Alicyclobacillales;f__Acidibacillaceae;g__;s__
LMSG_G000006220.1	yes	653_1	Lead-Zinc	95.04	0.20	94.05	2	1	0	20	Medium quality	87679	3124015	67.80	65	d__Bacteria;p__Firmicutes_E;c__Sulfobacillia;o__;f__;g__;s__
LMSG_G000005006.1	yes	1308_0	Lead-Zinc	98.63	0.86	94.34	0	1	0	19	Medium quality	49505	2979686	66.80	90	d__Bacteria;p__Proteobacteria;c__Gammaproteobacteria;o__Burkholderiales;f__Burkholderiaceae;g__Thiomonas;s__
LMSG_G000005007.1	yes	1095_1	Lead-Zinc	89.24	2.15	78.49	2	13	19	19	Medium quality	16127	2169953	37.50	306	d__Bacteria;p__Proteobacteria;c__Alphaproteobacteria;o__Paracaedibacterales;f__UBA11393;g__;s__
LMSG_G000005008.1	yes	171_0	Lead-Zinc	71.83	1.61	63.77	1	0	2	17	Medium quality	20547	1974294	40.00	238	d__Archaea;p__Thermoplasmatota;c__Thermoplasmata;o__Thermoplasmatales;f__Thermoplasmataceae;g__Cuniculiplasma;s__
LMSG_G000005009.1	yes	1502_2	Lead-Zinc	63.46	1.37	56.62	0	0	0	17	Medium quality	54838	2030704	66.80	72	d__Bacteria;p__Proteobacteria;c__Alphaproteobacteria;o__Acetobacterales;f__Acetobacteraceae;g__G45-3;s__
LMSG_G000006270.1	yes	933_1	Lead-Zinc	99.07	0.45	96.85	1	1	1	13	Medium quality	35463	3780657	46.50	223	d__Bacteria;p__Firmicutes;c__Bacilli;o__Bacillales;f__Bacillaceae;g__Bacillus;s__Bacillus velezensis
LMSG_G000005010.1	yes	10_1	Lead-Zinc	84.70	1.94	75.00	1	2	2	18	Medium quality	130125	915611	36.60	33	d__Archaea;p__Nanoarchaeota;c__Nanoarchaeia;o__Parvarchaeales;f__Parvarchaeaceae;g__Parvarchaeum;s__
LMSG_G000005011.1	yes	1833_0	Lead-Zinc	67.23	0.00	67.23	1	1	1	19	Medium quality	40727	477555	38.50	19	d__Bacteria;p__Patescibacteria;c__Paceibacteria;o__UBA6257;f__UBA9933;g__UM-FILTER-39-64;s__
LMSG_G000005012.1	yes	1229_1	Lead-Zinc	79.96	2.26	68.66	0	0	0	14	Medium quality	18990	2196595	60.70	173	d__Bacteria;p__Proteobacteria;c__Gammaproteobacteria;o__Burkholderiales;f__Gallionellaceae;g__Sideroxydans;s__
LMSG_G000005013.1	yes	34_1	Lead-Zinc	68.55	2.19	57.58	0	0	0	14	Medium quality	6518	2244184	38.30	403	d__Bacteria;p__Proteobacteria;c__Gammaproteobacteria;o__Legionellales;f__Legionellaceae;g__Legionella;s__
LMSG_G000005014.1	yes	1397_1	Lead-Zinc	99.37	0.50	96.89	0	0	0	19	Medium quality	46587	3147597	65.20	108	d__Bacteria;p__Proteobacteria;c__Alphaproteobacteria;o__Rhodospirillales;f__Magnetospirillaceae;g__;s__
LMSG_G000005015.1	yes	1400_1	Lead-Zinc	95.07	1.81	86.02	0	0	0	18	Medium quality	13860	6173026	66.80	605	d__Bacteria;p__Acidobacteriota;c__Acidobacteriae;o__Bryobacterales;f__Bryobacteraceae;g__UBA690;s__
LMSG_G000005016.1	yes	1219_0	Lead-Zinc	66.64	2.52	54.07	0	0	0	15	Medium quality	31824	1790710	56.70	84	d__Bacteria;p__Proteobacteria;c__Gammaproteobacteria;o__Burkholderiales;f__Nitrosomonadaceae;g__;s__
LMSG_G000005017.1	yes	584_0	Lead-Zinc	92.61	1.16	86.81	0	1	0	16	Medium quality	249715	3416340	63.80	28	d__Bacteria;p__Proteobacteria;c__Alphaproteobacteria;o__Rhizobiales;f__Methylopilaceae;g__Ga0077545;s__Ga0077545 sp003347645
LMSG_G000005023.1	yes	1390_0	Lead-Zinc	90.43	1.29	83.98	0	1	0	18	Medium quality	37324	3148470	56.60	149	d__Bacteria;p__Desulfobacterota;c__Desulfuromonadia;o__Geobacterales;f__Geobacteraceae;g__H2GEO;s__
LMSG_G000005018.1	yes	1084_1	Lead-Zinc	79.82	1.83	70.68	1	0	1	16	Medium quality	48644	3533487	65.70	126	d__Bacteria;p__Proteobacteria;c__Gammaproteobacteria;o__Steroidobacterales;f__Steroidobacteraceae;g__;s__
LMSG_G000005019.1	yes	1398_1	Lead-Zinc	99.50	0.54	96.79	0	0	0	19	Medium quality	76480	3599687	67.00	93	d__Bacteria;p__Proteobacteria;c__Alphaproteobacteria;o__Rhodospirillales;f__Magnetospirillaceae;g__;s__
LMSG_G000005020.1	yes	1085_0	Lead-Zinc	83.30	4.53	60.68	1	0	1	18	Medium quality	67678	4218173	64.60	143	d__Bacteria;p__Proteobacteria;c__Gammaproteobacteria;o__Steroidobacterales;f__Steroidobacteraceae;g__;s__
LMSG_G000005021.1	yes	1286_1	Lead-Zinc	86.14	1.15	80.40	1	0	0	19	Medium quality	22266	2922505	66.80	216	d__Bacteria;p__Proteobacteria;c__Gammaproteobacteria;o__Burkholderiales;f__Burkholderiaceae;g__Hylemonella;s__
LMSG_G000005022.1	yes	1248_1	Lead-Zinc	92.71	3.04	77.51	1	0	1	16	Medium quality	19524	2398783	62.70	182	d__Bacteria;p__Proteobacteria;c__Gammaproteobacteria;o__Burkholderiales;f__Thiobacillaceae;g__Sulfuritortus;s__
LMSG_G000005024.1	yes	1389_1	Lead-Zinc	74.56	1.75	65.79	0	0	0	16	Medium quality	18503	3720993	63.00	281	d__Bacteria;p__Desulfobacterota;c__Desulfuromonadia;o__Geobacterales;f__Geobacteraceae;g__Geobacter;s__
LMSG_G000005027.1	yes	537_0	Lead-Zinc	78.85	3.67	60.49	0	0	0	14	Medium quality	5226	3897156	43.00	863	d__Bacteria;p__Bacteroidota;c__Bacteroidia;o__Bacteroidales;f__Prolixibacteraceae;g__UBA6024;s__
LMSG_G000005025.1	yes	532_0	Lead-Zinc	79.37	1.96	69.59	1	1	0	16	Medium quality	7336	2072046	58.90	378	d__Bacteria;p__Proteobacteria;c__Alphaproteobacteria;o__Micavibrionales;f__UBA2020;g__PALSA-926;s__
LMSG_G000005026.1	yes	1717_0	Lead-Zinc	93.96	0.57	91.09	1	0	1	18	Medium quality	35894	1792667	45.20	113	d__Bacteria;p__Proteobacteria;c__Gammaproteobacteria;o__Francisellales;f__;g__;s__
LMSG_G000005028.1	yes	1069_0	Lead-Zinc	80.83	0.28	79.45	1	2	1	19	Medium quality	38785	1640126	60.60	75	d__Bacteria;p__Actinobacteriota;c__Coriobacteriia;o__OPB41;f__UBA912;g__UBA912;s__
LMSG_G000005030.1	yes	1737_2	Lead-Zinc	87.78	0.05	87.55	1	1	1	18	Medium quality	30918	1663138	36.20	182	d__Bacteria;p__SZUA-79;c__SZUA-79;o__Acidulodesulfobacterales;f__SZUA-79;g__Acidulodesulfobacterium;s__
LMSG_G000005029.1	yes	1296_1	Lead-Zinc	77.25	3.91	57.70	1	0	0	15	Medium quality	62825	3384450	70.90	89	d__Bacteria;p__Proteobacteria;c__Gammaproteobacteria;o__Burkholderiales;f__Burkholderiaceae;g__Thiomonas;s__
LMSG_G000006311.1	yes	1592_1	Lead-Zinc	91.81	1.32	85.24	2	3	1	16	Medium quality	15693	2204188	40.60	252	d__Bacteria;p__Proteobacteria;c__Gammaproteobacteria;o__Legionellales;f__Legionellaceae;g__DDPF01;s__
LMSG_G000005031.1	yes	1128_2	Lead-Zinc	81.46	0.93	76.84	2	4	16	16	Medium quality	17499	2243549	63.60	339	d__Bacteria;p__Eremiobacterota;c__Eremiobacteria;o__UBP12;f__UBA5184;g__;s__
LMSG_G000005032.1	yes	1273_1	Lead-Zinc	82.47	1.97	72.62	0	1	0	17	Medium quality	18721	2542668	65.40	168	d__Bacteria;p__Proteobacteria;c__Gammaproteobacteria;o__Burkholderiales;f__Thiobacillaceae;g__Thiobacillus;s__
LMSG_G000004405.1	yes	1099_1	Lead-Zinc	96.50	2.25	85.24	3	1	2	18	High quality	29718	3378053	49.90	188	d__Bacteria;p__Firmicutes_B;c__Thermincolia;o__Carboxydocellales;f__;g__;s__
LMSG_G000004406.1	yes	1752_1	Lead-Zinc	97.59	0.00	97.59	1	1	1	18	High quality	30512	2269291	52.50	121	d__Bacteria;p__Spirochaetota;c__Spirochaetia;o__Treponematales;f__UBA8932;g__UBA8932;s__UBA8932 sp002427685
LMSG_G000005033.1	yes	199_0	Lead-Zinc	89.70	1.61	81.64	2	1	1	19	Medium quality	17134	2004940	45.30	168	d__Archaea;p__Thermoplasmatota;c__Thermoplasmata;o__Thermoplasmatales;f__Thermoplasmataceae;g__UBA509;s__
LMSG_G000004407.1	yes	161_1	Lead-Zinc	95.21	0.21	94.16	1	1	1	19	High quality	52128	1398667	37.40	91	d__Archaea;p__Thermoplasmatota;c__Thermoplasmata;o__Thermoplasmatales;f__Thermoplasmataceae;g__Ferroplasma;s__Ferroplasma acidiphilum
LMSG_G000004408.1	yes	755_0	Lead-Zinc	97.43	2.14	86.75	1	1	1	20	High quality	77854	3788608	70.60	97	d__Bacteria;p__Actinobacteriota;c__Acidimicrobiia;o__Acidimicrobiales;f__Bog-793;g__;s__
LMSG_G000005034.1	yes	952_1	Lead-Zinc	93.57	1.82	84.48	0	0	0	19	Medium quality	32776	2341931	51.40	124	d__Bacteria;p__Nitrospirota;c__Leptospirillia;o__Leptospirillales;f__Leptospirillaceae;g__Leptospirillum;s__
LMSG_G000005035.1	yes	609_1	Lead-Zinc	99.14	1.28	92.73	0	0	0	19	Medium quality	68133	2575277	53.60	82	d__Bacteria;p__Actinobacteriota;c__Acidimicrobiia;o__Acidimicrobiales;f__Acidimicrobiaceae;g__SCQM01;s__
LMSG_G000005036.1	yes	41_1	Lead-Zinc	83.80	0.00	83.80	1	1	1	19	Medium quality	224929	881913	35.30	24	d__Archaea;p__Micrarchaeota;c__Micrarchaeia;o__Micrarchaeales;f__Micrarchaeaceae;g__Mancarchaeum;s__
LMSG_G000005037.1	yes	1466_1	Lead-Zinc	96.26	0.20	95.28	0	0	0	19	Medium quality	26461	2810302	56.10	363	d__Bacteria;p__Proteobacteria;c__Alphaproteobacteria;o__Acetobacterales;f__Acetobacteraceae;g__Acidiphilium;s__
LMSG_G000005038.1	yes	1199_1	Copper	90.24	1.22	84.15	1	0	1	18	Medium quality	73942	2796205	61.70	63	d__Bacteria;p__Proteobacteria;c__Gammaproteobacteria;o__Acidiferrobacterales;f__Sulfurifustaceae;g__;s__
LMSG_G000005039.1	yes	998_2	Copper	54.31	0.00	54.31	0	1	1	15	Medium quality	7445	1950239	60.10	355	d__Bacteria;p__Acidobacteriota;c__Acidobacteriae;o__UBA7540;f__UBA7540;g__;s__
LMSG_G000004409.1	yes	580_1	Copper	92.28	2.80	78.27	1	6	5	20	High quality	2102991	2117443	51.90	21	d__Archaea;p__Thermoproteota;c__Thermoproteia;o__Marsarchaeales;f__Marsarchaeaceae;g__ECH-B-1;s__
LMSG_G000004410.1	yes	1007_0	Copper	97.41	4.55	74.69	1	1	2	19	High quality	98955	3752824	62.00	71	d__Bacteria;p__Acidobacteriota;c__Acidobacteriae;o__Acidobacteriales;f__SCQP01;g__;s__
LMSG_G000005040.1	yes	108_0	Copper	76.32	0.00	76.32	1	2	2	20	Medium quality	33536	737774	55.00	29	d__Archaea;p__Micrarchaeota;c__Micrarchaeia;o__Micrarchaeales;f__;g__;s__
LMSG_G000005041.1	yes	427_2	Copper	86.93	1.14	81.25	0	0	0	19	Medium quality	17482	3650256	61.70	369	d__Bacteria;p__Planctomycetota;c__Phycisphaerae;o__UBA1161;f__;g__;s__
LMSG_G000005042.1	yes	594_2	Copper	66.99	2.91	52.43	0	0	0	11	Medium quality	95166	1421974	62.60	31	d__Archaea;p__Thermoproteota;c__Nitrososphaeria;o__Nitrososphaerales;f__UBA183;g__UBA183;s__
LMSG_G000006271.1	yes	741_1	Copper	99.57	2.99	84.62	1	1	1	14	Medium quality	23145	2538220	69.00	161	d__Bacteria;p__Actinobacteriota;c__Acidimicrobiia;o__Acidimicrobiales;f__Bog-793;g__Palsa-601;s__
LMSG_G000005043.1	yes	1732_0	Copper	60.43	0.00	60.43	1	0	1	14	Medium quality	3481	885544	28.60	348	d__Archaea;p__Micrarchaeota;c__Micrarchaeia;o__Micrarchaeales;f__;g__;s__
LMSG_G000005044.1	yes	116_0	Copper	65.26	0.00	65.26	1	0	1	18	Medium quality	37276	542088	54.50	27	d__Archaea;p__Micrarchaeota;c__Micrarchaeia;o__Micrarchaeales;f__;g__;s__
LMSG_G000005045.1	yes	19_1	Copper	74.68	0.93	70.01	1	1	1	17	Medium quality	40697	766757	48.00	38	d__Archaea;p__Nanoarchaeota;c__Nanoarchaeia;o__Parvarchaeales;f__Parvarchaeaceae;g__;s__
LMSG_G000005046.1	yes	1843_0	Copper	60.67	0.97	55.82	0	1	1	14	Medium quality	6007	1299943	50.30	244	d__Archaea;p__Thermoproteota;c__Nitrososphaeria;o__UBA164;f__;g__;s__
LMSG_G000006221.1	yes	554_1	Copper	92.66	1.60	84.66	1	2	0	18	Medium quality	85669	2095504	69.80	44	d__Archaea;p__Thermoplasmatota;c__Thermoplasmata;o__UBA184;f__UBA184;g__;s__
LMSG_G000005047.1	yes	1446_1	Copper	94.48	0.00	94.48	0	0	0	18	Medium quality	21645	3713947	67.60	259	d__Bacteria;p__Proteobacteria;c__Alphaproteobacteria;o__Acetobacterales;f__Acetobacteraceae;g__;s__
LMSG_G000005048.1	yes	222_0	Copper	50.98	0.00	50.98	0	1	0	13	Medium quality	15867	581548	44.40	46	d__Archaea;p__Thermoplasmatota;c__Thermoplasmata;o__Thermoplasmatales;f__GCA-001856825;g__GCA-001856825;s__
LMSG_G000005049.1	yes	1744_0	Copper	67.67	0.00	67.67	0	0	0	8	Medium quality	16810	1095076	36.70	105	d__Bacteria;p__SZUA-79;c__SZUA-79;o__Acidulodesulfobacterales;f__SZUA-79;g__Acidulodesulfobacterium;s__
LMSG_G000005050.1	yes	4_0	Copper	79.75	1.87	70.41	1	1	1	19	Medium quality	28710	944182	41.30	57	d__Archaea;p__Nanoarchaeota;c__Nanoarchaeia;o__Parvarchaeales;f__Parvarchaeaceae;g__;s__
LMSG_G000005055.1	yes	632_2	Copper	85.47	0.56	82.70	1	0	1	17	Medium quality	13340	2002869	70.60	251	d__Bacteria;p__Dormibacterota;c__Dormibacteria;o__UBA8260;f__Bog-877;g__;s__
LMSG_G000005051.1	yes	1566_1	Copper	91.38	0.00	91.38	0	0	0	19	Medium quality	120486	3089805	66.10	167	d__Bacteria;p__Desulfobacterota_B;c__Binatia;o__Binatales;f__Binataceae;g__;s__
LMSG_G000005052.1	yes	91_0	Copper	64.48	0.93	59.81	1	0	0	15	Medium quality	10021	574005	48.50	70	d__Archaea;p__Micrarchaeota;c__Micrarchaeia;o__Micrarchaeales;f__;g__;s__
LMSG_G000005053.1	yes	1733_0	Copper	89.32	0.00	89.32	0	1	0	15	Medium quality	60196	1683308	36.00	103	d__Bacteria;p__SZUA-79;c__SZUA-79;o__Acidulodesulfobacterales;f__SZUA-79;g__;s__
LMSG_G000005054.1	yes	1703_1	Copper	99.62	0.00	99.62	1	0	1	20	Medium quality	251693	2802694	37.40	30	d__Bacteria;p__Firmicutes;c__Bacilli;o__Lactobacillales;f__Enterococcaceae;g__Enterococcus;s__Enterococcus faecalis
LMSG_G000004411.1	yes	780_0	Copper	92.30	1.28	85.89	2	1	2	19	High quality	10617	3740383	57.80	595	d__Bacteria;p__Actinobacteriota;c__Acidimicrobiia;o__Acidimicrobiales;f__Acidimicrobiaceae;g__Acidithrix;s__

LMSG_G000005056.1	yes	547_2	Copper	66.27	0.80	62.27	1	0	0	16	Medium quality	15107	1117817	67.20	117	d__Archaea;p__Thermoplasmatota;c__Thermoplasmata;o__UBA184;f__UBA184;g__;s__
LMSG_G000005057.1	yes	1034_0	Copper	81.05	1.98	71.15	0	1	0	15	Medium quality	3611	2367024	58.40	780	d__Bacteria;p__Firmicutes_E;c__Sulfobacillia;o__;f__;g__;s__
LMSG_G000005058.1	yes	106_0	Copper	74.45	0.00	74.45	1	2	1	19	Medium quality	95617	673381	53.60	11	d__Archaea;p__Micrarchaeota;c__Micrarchaeia;o__Micrarchaeales;f__;g__;s__
LMSG_G000005059.1	yes	207_1	Copper	86.87	0.16	86.07	1	2	4	17	Medium quality	5197	1835561	44.90	526	d__Archaea;p__Thermoplasmatota;c__Thermoplasmata;o__Thermoplasmatales;f__Thermoplasmataceae;g__B-DKE;s__
LMSG_G000005060.1	yes	1679_1	Copper	68.48	0.00	68.48	1	1	2	17	Medium quality	5837	687982	47.70	167	d__Bacteria;p__Patescibacteria;c__Paceibacteria;o__;f__;g__;s__
LMSG_G000005061.1	yes	1353_0	Lead-Zinc	58.34	0.00	58.34	0	0	0	10	Medium quality	2818	2028383	58.10	831	d__Bacteria;p__Desulfobacterota;c__Desulfobulbia;o__Desulfobulbales;f__Desulfurivibrionaceae;g__UBA2262;s__
LMSG_G000005062.1	yes	1511_3	Lead-Zinc	96.26	3.98	76.36	0	0	0	19	Medium quality	121409	3544607	70.10	53	d__Bacteria;p__Proteobacteria;c__Alphaproteobacteria;o__Acetobacterales;f__Acetobacteraceae;g__;s__
LMSG_G000006222.1	yes	695_1	Lead-Zinc	98.29	1.28	91.88	1	1	0	20	Medium quality	310381	2747161	48.80	30	d__Bacteria;p__Actinobacteriota;c__Acidimicrobiia;o__Acidimicrobiales;f__Acidimicrobiaceae;g__Aciditrix;s__
LMSG_G000005063.1	yes	1242_1	Lead-Zinc	87.86	2.17	77.00	0	0	0	14	Medium quality	19988	2622730	64.60	189	d__Bacteria;p__Proteobacteria;c__Gammaproteobacteria;o__Burkholderiales;f__Sulfuricellaceae;g__Sulfurirhabdus;s__
LMSG_G000005064.1	yes	832_0	Lead-Zinc	97.43	2.56	84.61	0	0	0	19	Medium quality	34844	3791412	72.20	171	d__Bacteria;p__Actinobacteriota;c__Thermoleophilia;o__Solirubrobacterales;f__Solirubrobacteraceae;g__Palsa-465;s__
LMSG_G000005065.1	yes	821_1	Lead-Zinc	98.29	2.14	87.61	0	1	0	20	Medium quality	25018	2772185	64.80	188	d__Bacteria;p__Actinobacteriota;c__Acidimicrobiia;o__Acidimicrobiales;f__;g__;s__
LMSG_G000005066.1	yes	1180_1	Lead-Zinc	89.70	2.25	78.47	1	1	1	15	Medium quality	23744	2369719	62.90	162	d__Bacteria;p__Elusimicrobiota;c__Elusimicrobia;o__Elusimicrobiales;f__UBA9959;g__UBA9959;s__
LMSG_G000006313.1	yes	1226_1	Lead-Zinc	95.85	0.00	95.85	2	1	0	19	Medium quality	162248	2263949	57.70	20	d__Bacteria;p__Proteobacteria;c__Gammaproteobacteria;o__Burkholderiales;f__Sulfuriferulaceae;g__UBA2487;s__
LMSG_G000005067.1	yes	1227_1	Lead-Zinc	73.27	0.00	73.27	0	1	0	14	Medium quality	8578	1933276	53.50	315	d__Bacteria;p__Proteobacteria;c__Gammaproteobacteria;o__Burkholderiales;f__Sulfuriferulaceae;g__UBA2487;s__
LMSG_G000005068.1	yes	1612_1	Lead-Zinc	87.50	0.00	87.50	1	0	0	19	Medium quality	18679	1364545	42.70	97	d__Bacteria;p__Verrucomicrobiota_A;c__Chlamydiia;o__Parachlamydiales;f__SM23-39;g__;s__
LMSG_G000005070.1	yes	481_0	Lead-Zinc	72.41	0.00	72.41	1	0	1	8	Medium quality	7071	2198021	48.80	383	d__Bacteria;p__Firmicutes_B;c__Desulfitobacteriia;o__Desulfitobacteriales;f__;g__;s__
LMSG_G000005069.1	yes	433_1	Lead-Zinc	94.31	0.00	94.31	1	0	1	20	Medium quality	26591	3720005	53.50	320	d__Bacteria;p__Planctomycetota;c__Phycisphaerae;o__UBA1161;f__;g__;s__
LMSG_G000004412.1	yes	754_1	Lead-Zinc	96.50	0.43	94.37	1	1	1	20	High quality	20302	3110239	69.10	231	d__Bacteria;p__Actinobacteriota;c__Acidimicrobiia;o__Acidimicrobiales;f__Bog-793;g__;s__
LMSG_G000006309.1	yes	385_1	Lead-Zinc	59.45	0.99	54.50	0	1	0	17	Medium quality	14520	599157	45.50	50	d__Bacteria;p__Patescibacteria;c__Saccharimonadia;o__Saccharimonadales;f__UBA4665;g__;s__
LMSG_G000005071.1	yes	925_0	Lead-Zinc	92.67	3.88	73.28	1	0	1	17	Medium quality	114642	3732792	63.10	71	d__Bacteria;p__Acidobacteriota;c__Acidobacteriae;o__Acidobacteriales;f__Acidobacteriaceae;g__Terracidiphilus;s__
LMSG_G000005076.1	yes	1154_0	Lead-Zinc	64.69	2.23	53.56	0	0	0	11	Medium quality	3482	2036134	64.80	696	d__Bacteria;p__Firmicutes_E;c__Symbiobacteriia;o__;f__;g__;s__
LMSG_G000005072.1	yes	1505_1	Lead-Zinc	86.04	3.23	69.88	0	0	0	19	Medium quality	15995	3790396	70.90	326	d__Bacteria;p__Proteobacteria;c__Alphaproteobacteria;o__Acetobacterales;f__Acetobacteraceae;g__Palsa-883;s__
LMSG_G000005073.1	yes	36_1	Lead-Zinc	81.93	0.00	81.93	1	1	0	20	Medium quality	60297	879219	37.60	45	d__Archaea;p__Micrarchaeota;c__Micrarchaeia;o__Micrarchaeales;f__;g__;s__
LMSG_G000005074.1	yes	902_1	Lead-Zinc	95.54	0.05	95.28	1	0	0	16	Medium quality	27673	3973239	61.50	218	d__Bacteria;p__Acidobacteriota;c__Acidobacteriae;o__Acidobacteriales;f__Acidobacteriaceae;g__Granulicella_A;s__
LMSG_G000005075.1	yes	1297_2	Lead-Zinc	68.43	1.72	59.81	0	0	0	14	Medium quality	14221	3412480	70.60	355	d__Bacteria;p__Proteobacteria;c__Gammaproteobacteria;o__Burkholderiales;f__Burkholderiaceae;g__Thiomonas;s__
LMSG_G000005077.1	yes	1216_1	Lead-Zinc	88.70	1.05	83.44	1	0	0	18	Medium quality	61576	2827355	55.90	73	d__Bacteria;p__Proteobacteria;c__Gammaproteobacteria;o__Burkholderiales;f__Gallionellaceae;g__;s__
LMSG_G000004413.1	yes	202_1	Lead-Zinc	95.11	2.11	84.57	1	2	1	20	High quality	59824	1401865	48.70	62	d__Archaea;p__Thermoplasmatota;c__Thermoplasmata;o__Thermoplasmatales;f__Thermoplasmataceae;g__UBA582;s__
LMSG_G000004414.1	yes	1420_0	Lead-Zinc	98.35	4.12	77.73	1	1	1	20	High quality	98683	3820155	64.60	71	d__Bacteria;p__Proteobacteria;c__Alphaproteobacteria;o__Sphingomonadales;f__Sphingomonadaceae;g__Blastomonas;s__Blastomonas fulva
LMSG_G000004415.1	yes	547_1	Lead-Zinc	98.66	1.60	90.66	2	1	3	20	High quality	317802	1525741	67.20	48	d__Archaea;p__Thermoplasmatota;c__Thermoplasmata;o__UBA184;f__UBA184;g__;s__
LMSG_G000005078.1	yes	10_2	Lead-Zinc	69.01	1.94	59.31	0	1	0	18	Medium quality	49006	697142	36.30	34	d__Archaea;p__Nanoarchaeota;c__Nanoarchaeia;o__Parvarchaeales;f__Parvarchaeaceae;g__Parvarchaeum;s__
LMSG_G000004416.1	yes	257_1	Lead-Zinc	99.63	0.96	94.84	1	1	1	19	High quality	227073	3890078	39.00	32	d__Bacteria;p__Proteobacteria;c__Gammaproteobacteria;o__Pseudomonadales;f__Moraxellaceae;g__Acinetobacter;s__Acinetobacter baumannii
LMSG_G000005079.1	yes	40_1	Lead-Zinc	83.17	0.00	83.17	1	1	1	19	Medium quality	47805	880308	29.30	66	d__Archaea;p__Micrarchaeota;c__Micrarchaeia;o__Micrarchaeales;f__Micrarchaeaceae;g__Mancarchaeum;s__
LMSG_G000004417.1	yes	211_1	Lead-Zinc	93.20	0.97	88.35	1	2	2	20	High quality	110206	1479130	47.50	78	d__Archaea;p__Thermoproteota;c__Nitrososphaeria;o__UBA164;f__;g__;s__
LMSG_G000005081.1	yes	1620_0	Lead-Zinc	75.15	0.81	71.09	1	0	0	14	Medium quality	4163	1077769	34.20	288	d__Archaea;p__Thermoplasmatota;c__Thermoplasmata;o__Thermoplasmatales;f__Thermoplasmataceae;g__Picrophilus;s__
LMSG_G000005080.1	yes	210_0	Lead-Zinc	58.87	0.81	54.84	0	0	0	8	Medium quality	7070	725748	45.20	105	d__Archaea;p__Thermoplasmatota;c__Thermoplasmata;o__Thermoplasmatales;f__Thermoplasmataceae;g__UBA582;s__
LMSG_G000005082.1	yes	1600_0	Copper	86.79	2.14	76.11	1	1	0	16	Medium quality	6108	2830423	59.90	617	d__Bacteria;p__Actinobacteriota;c__Acidimicrobiia;o__Acidimicrobiales;f__;g__;s__
LMSG_G000005083.1	yes	1553_1	Copper	66.54	0.42	64.44	1	1	0	12	Medium quality	2929	1944725	66.00	694	d__Bacteria;p__Desulfobacterota_B;c__Binatia;o__Binatales;f__Binataceae;g__;s__
LMSG_G000005084.1	yes	544_1	Copper	95.06	0.80	91.06	1	0	0	14	Medium quality	24673	1690876	68.60	111	d__Archaea;p__Thermoplasmatota;c__Thermoplasmata;o__UBA184;f__UBA184;g__;s__
LMSG_G000005085.1	yes	1175_1	Copper	94.96	0.37	93.09	0	1	1	19	Medium quality	26139	1991036	64.60	164	d__Bacteria;p__Elusimicrobiota;c__Elusimicrobia;o__UBA1565;f__;g__;s__
LMSG_G000005086.1	yes	73_1	Copper	83.33	2.80	69.32	1	1	3	15	Medium quality	19330	918266	52.30	61	d__Archaea;p__Micrarchaeota;c__Micrarchaeia;o__Micrarchaeales;f__Micrarchaeaceae;g__;s__
LMSG_G000004418.1	yes	1147_1	Copper	97.52	1.02	92.40	1	1	4	19	High quality	45581	3246805	65.50	125	d__Bacteria;p__Firmicutes_E;c__Sulfobacillia;o__Sulfobacillales;f__;g__;s__
LMSG_G000005087.1	yes	1255_1	Copper	97.01	2.45	84.75	2	0	0	18	Medium quality	86927	2996119	66.40	65	d__Bacteria;p__Proteobacteria;c__Gammaproteobacteria;o__Xanthomonadales;f__Rhodanobacteraceae;g__Metallibacterium;s__
LMSG_G000005088.1	yes	74_1	Copper	83.17	0.93	78.50	1	2	1	19	Medium quality	167183	1006788	49.70	14	d__Archaea;p__Micrarchaeota;c__Micrarchaeia;o__Micrarchaeales;f__Micrarchaeaceae;g__;s__
LMSG_G000005089.1	yes	926_1	Copper	79.26	1.78	70.37	0	0	0	16	Medium quality	4558	2894219	64.10	753	d__Bacteria;p__Acidobacteriota;c__Acidobacteriae;o__Acidobacteriales;f__Acidobacteriaceae;g__PALSA-350;s__
LMSG_G000004419.1	yes	181_2	Copper	96.32	1.61	88.26	1	2	2	20	High quality	43263	1354446	48.40	47	d__Archaea;p__Thermoplasmatota;c__Thermoplasmata;o__Thermoplasmatales;f__Thermoplasmataceae;g__UBA582;s__
LMSG_G000006223.1	yes	154_0	Copper	97.00	2.40	85.00	1	2	0	20	Medium quality	60083	1855157	50.00	63	d__Archaea;p__Thermoplasmatota;c__Thermoplasmata;o__;f__;g__;s__
LMSG_G000004422.1	yes	1005_1	Copper	96.15	0.00	96.15	1	1	2	20	High quality	31156	2950756	62.00	156	d__Bacteria;p__Acidobacteriota;c__Acidobacteriae;o__Acidobacteriales;f__SCQP01;g__;s__
LMSG_G000004420.1	yes	1496_0	Copper	98.58	0.40	96.57	1	1	1	20	High quality	238546	7330699	62.70	52	d__Bacteria;p__Proteobacteria;c__Alphaproteobacteria;o__Rhizobiales;f__Anderseniellaceae;g__SCPD01;s__SCPD01 sp003574785
LMSG_G000004421.1	yes	558_1	Copper	96.26	2.40	84.26	1	2	2	19	High quality	117073	1973901	71.80	34	d__Archaea;p__Thermoplasmatota;c__Thermoplasmata;o__UBA184;f__UBA184;g__;s__
LMSG_G000005090.1	yes	588_1	Copper	81.71	0.00	81.71	2	0	1	17	Medium quality	69745	1221534	61.70	96	d__Archaea;p__Thermoproteota;c__Nitrososphaeria;o__Nitrososphaerales;f__UBA183;g__UBA183;s__
LMSG_G000005107.1	yes	443_1	Lead-Zinc	96.69	2.18	85.80	2	0	4	19	Medium quality	35997	4068598	57.60	260	d__Bacteria;p__Chloroflexota;c__Dehalococcoidia;o__FW602-bin16;f__;g__;s__
LMSG_G000005095.1	yes	1267_0	Lead-Zinc	83.45	2.40	71.46	1	1	1	14	Medium quality	8125	2979693	62.40	443	d__Bacteria;p__Proteobacteria;c__Gammaproteobacteria;o__Xanthomonadales;f__Rhodanobacteraceae;g__Dokdonella;s__
LMSG_G000005091.1	yes	614_0	Lead-Zinc	64.35	0.00	64.35	0	0	0	14	Medium quality	4534	1854953	54.30	443	d__Bacteria;p__Actinobacteriota;c__Acidimicrobiia;o__Acidimicrobiales;f__Acidimicrobiaceae;g__SCQM01;s__
LMSG_G000005092.1	yes	447_0	Lead-Zinc	53.50	0.00	53.50	0	1	1	7	Medium quality	26802	1991934	65.70	97	d__Bacteria;p__Chloroflexota;c__UBA6077;o__UBA6077;f__UBA6077;g__;s__
LMSG_G000005093.1	yes	664_0	Lead-Zinc	84.39	3.23	68.27	1	1	1	19	Medium quality	32048	8613950	67.50	421	d__Bacteria;p__Myxococcota;c__Polyangia;o__Polyangiales;f__Polyangiaceae;g__;s__
LMSG_G000005094.1	yes	1287_1	Lead-Zinc	95.65	0.96	90.84	1	0	0	18	Medium quality	49376	2275689	66.60	87	d__Bacteria;p__Proteobacteria;c__Gammaproteobacteria;o__Burkholderiales;f__Burkholderiaceae;g__Serpentinomonas;s__Serpentinomonas mccroryi
LMSG_G000005096.1	yes	1418_1	Lead-Zinc	97.44	1.67	89.10	0	0	0	19	Medium quality	65026	3642160	63.60	93	d__Bacteria;p__Proteobacteria;c__Alphaproteobacteria;o__Sphingomonadales;f__Sphingomonadaceae;g__Sphingobium;s__
LMSG_G000005097.1	yes	296_1	Lead-Zinc	71.28	1.98	61.38	1	1	4	20	Medium quality	610567	941080	45.70	14	d__Bacteria;p__Patescibacteria;c__ABY1;o__BM507;f__UBA12465;g__UBA12465;s__
LMSG_G000005098.1	yes	1763_0	Lead-Zinc	77.87	1.02	72.78	1	1	2	20	Medium quality	105082	637663	56.60	30	d__Bacteria;p__Patescibacteria;c__Paceibacteria;o__UBA9983;f__UBA2163;g__C7867-001;s__
LMSG_G000005099.1	yes	483_0	Lead-Zinc	70.25	0.99	65.30	3	2	0	20	Medium quality	10095	3341178	62.90	377	d__Bacteria;p__Firmicutes_E;c__Symbiobacteriia;o__Symbiobacteriales;f__Symbiobacteriaceae;g__;s__
LMSG_G000005100.1	yes	1243_1	Lead-Zinc	91.25	2.65	78.00	1	0	0	17	Medium quality	33494	2325563	58.30	118	d__Bacteria;p__Proteobacteria;c__Gammaproteobacteria;o__Burkholderiales;f__;g__;s__
LMSG_G000005101.1	yes	333_0	Lead-Zinc	65.59	2.15	54.84	1	1	1	18	Medium quality	14105	596305	45.60	56	d__Bacteria;p__Patescibacteria;c__Saccharimonadia;o__Saccharimonadales;f__Saccharimonadaceae;g__UBA1547;s__
LMSG_G000006224.1	yes	476_1	Lead-Zinc	97.02	3.15	81.29	2	2	0	17	Medium quality	35334	3403975	52.70	153	d__Bacteria;p__Firmicutes_B;c__Desulfitobacteriia;o__Desulfitobacteriales;f__;g__;s__
LMSG_G000005102.1	yes	1072_0	Lead-Zinc	79.63	2.52	67.03	0	0	0	16	Medium quality	6500	2897565	65.10	603	d__Bacteria;p__Desulfobacterota;c__MBNT15;o__MBNT15;f__MBNT15;g__UBA2219;s__

LMSG_G000005103.1	yes	1244_0	Lead-Zinc	92.73	2.69	79.31	0	0	0	18	Medium quality	90333	2406017	59.30	48	d__Bacteria;p__Proteobacteria;c__Gammaproteobacteria;o__Burkholderiales;f__;g__;s__
LMSG_G000005104.1	yes	1333_0	Lead-Zinc	83.07	2.59	70.14	0	0	0	17	Medium quality	14327	2202961	62.60	231	d__Bacteria;p__Proteobacteria;c__Gammaproteobacteria;o__Burkholderiales;f__Ferrovaceae;g__;s__
LMSG_G000004423.1	yes	932_3	Lead-Zinc	97.27	4.07	76.92	1	2	2	19	High quality	52393	3986119	53.30	117	d__Bacteria;p__Nitrospirota;c__Thermodesulfovibrionia;o__Thermodesulfovibrionales;f__UBA6898;g__Sulfobium;s__
LMSG_G000005105.1	yes	1819_0	Lead-Zinc	65.16	2.81	51.12	2	1	5	16	Medium quality	34375	1010375	49.00	74	d__Bacteria;p__Patescibacteria;c__ABY1;o__SG8-24;f__2-12-FULL-60-25;g__SZUA-46;s__
LMSG_G000004424.1	yes	1366_0	Lead-Zinc	96.12	1.29	89.67	1	1	1	19	High quality	45603	3815461	65.90	128	d__Bacteria;p__Desulfobacterota;c__Desulfarculia;o__Desulfarculales;f__Desulfarculaceae;g__;s__
LMSG_G000005106.1	yes	480_1	Lead-Zinc	83.77	1.15	78.04	1	5	0	10	Medium quality	41241	2641766	50.70	164	d__Bacteria;p__Firmicutes_B;c__Desulfitobacteriia;o__Desulfitobacteriales;f__Desulfitobacteriaceae;g__;s__
LMSG_G000005113.1	yes	1213_0	Lead-Zinc	54.22	0.00	54.22	2	1	1	13	Medium quality	36159	1259366	59.60	49	d__Bacteria;p__Actinobacteriota;c__Thermoleophilia;o__BMS3ABIN01;f__BMS3ABIN01;g__;s__
LMSG_G000005108.1	yes	1248_2	Lead-Zinc	68.31	2.37	56.47	0	0	0	11	Medium quality	12518	1866687	63.40	200	d__Bacteria;p__Proteobacteria;c__Gammaproteobacteria;o__Burkholderiales;f__Thiobacillaceae;g__Sulfuritortus;s__
LMSG_G000005109.1	yes	134_1	Lead-Zinc	83.64	0.07	83.29	1	1	2	19	Medium quality	211151	1219208	46.80	31	d__Archaea;p__Micrarchaeota;c__Micrarchaeia;o__Micrarchaeales;f__;g__;s__
LMSG_G000005110.1	yes	1794_0	Lead-Zinc	66.77	0.00	66.77	1	1	5	18	Medium quality	8421	561164	38.90	117	d__Bacteria;p__Patescibacteria;c__Paceibacteria;o__UBA9983;f__UBA9973;g__UBA9973;s__
LMSG_G000004425.1	yes	676_0	Lead-Zinc	93.67	2.31	82.10	1	1	1	20	High quality	22898	4231728	71.70	317	d__Bacteria;p__Chloroflexota;c__Ellin6529;o__CSP1-4;f__CSP1-4;g__;s__
LMSG_G000005111.1	yes	1218_3	Lead-Zinc	84.08	4.47	61.74	0	0	0	11	Medium quality	23212	2164675	57.70	133	d__Bacteria;p__Proteobacteria;c__Gammaproteobacteria;o__Burkholderiales;f__Nitrosomonadaceae;g__;s__
LMSG_G000005112.1	yes	704_0	Lead-Zinc	93.62	1.96	83.82	1	0	0	18	Medium quality	42825	2877279	67.40	110	d__Bacteria;p__Firmicutes_E;c__DTU015;o__;f__;g__;s__
LMSG_G000005118.1	yes	1389_2	Lead-Zinc	80.70	3.51	63.16	1	1	0	15	Medium quality	70855	3432683	62.70	85	d__Bacteria;p__Desulfobacterota;c__Desulfuromonadia;o__Geobacterales;f__Geobacteraceae;g__Geobacter;s__
LMSG_G000004426.1	yes	1376_0	Lead-Zinc	99.13	4.84	74.94	1	1	1	20	High quality	68452	4096604	72.30	89	d__Bacteria;p__Myxococcota;c__Myxococcia;o__Myxococcales;f__Anaeromyxobacteraceae;g__Anaeromyxobacter;s__
LMSG_G000005114.1	yes	1355_0	Lead-Zinc	83.33	1.68	74.96	2	1	0	16	Medium quality	40998	2188165	62.00	75	d__Bacteria;p__Desulfobacterota;c__Desulfobulbia;o__Desulfobulbales;f__Desulfurivibrionaceae;g__;s__
LMSG_G000005115.1	yes	941_0	Lead-Zinc	79.12	1.11	73.57	0	1	0	13	Medium quality	5492	1639594	43.20	388	d__Bacteria;p__Nitrospirota;c__Thermodesulfovibrionia;o__Thermodesulfovibrionales;f__UBA9935;g__GWB2-47-37;s__
LMSG_G000004427.1	yes	669_1	Lead-Zinc	91.66	1.13	86.01	1	1	1	20	High quality	49925	2996054	72.00	111	d__Bacteria;p__Chloroflexota;c__Ellin6529;o__CSP1-4;f__CSP1-4;g__;s__
LMSG_G000005116.1	yes	415_0	Lead-Zinc	53.37	0.00	53.37	0	3	0	16	Medium quality	8042	549556	42.60	83	d__Bacteria;p__Patescibacteria;c__Microgenomatia;o__GWA2-44-7;f__UBA8517;g__UBA8517;s__
LMSG_G000004428.1	yes	1197_0	Lead-Zinc	94.11	2.94	79.41	1	1	1	19	High quality	159738	2780311	56.40	28	d__Bacteria;p__UBA10199;c__UBA10199;o__2-02-FULL-44-16;f__;g__;s__
LMSG_G000005117.1	yes	1825_1	Lead-Zinc	74.99	0.00	74.99	1	0	3	19	Medium quality	50641	700423	39.50	49	d__Bacteria;p__Patescibacteria;c__Paceibacteria;o__UBA6257;f__UBA9933;g__;s__
LMSG_G000004429.1	yes	1010_0	Lead-Zinc	91.19	0.90	86.68	1	1	1	18	High quality	22216	2784934	69.90	161	d__Bacteria;p__Actinobacteriota;c__Actinomycetia;o__Propionibacteriales;f__Propionibacteriaceae;g__Fen-668;s__
LMSG_G000005119.1	yes	1184_0	Lead-Zinc	88.20	4.49	65.73	0	1	0	16	Medium quality	54651	2991502	61.50	95	d__Bacteria;p__Elusimicrobiota;c__Elusimicrobia;o__Elusimicrobiales;f__UBA9959;g__UBA9959;s__
LMSG_G000005120.1	yes	1071_1	Lead-Zinc	96.63	1.59	88.70	0	2	5	19	Medium quality	66726	2751748	65.40	152	d__Bacteria;p__Desulfobacterota;c__MBNT15;o__MBNT15;f__MBNT15;g__CG2-30-66-27;s__CG2-30-66-27 sp002279275
LMSG_G000005125.1	yes	1182_0	Lead-Zinc	52.54	0.00	52.54	0	0	0	7	Medium quality	35040	1365082	67.60	107	d__Bacteria;p__Elusimicrobiota;c__Elusimicrobia;o__Elusimicrobiales;f__UBA9959;g__UBA9959;s__
LMSG_G000005121.1	yes	1179_0	Lead-Zinc	85.01	2.25	73.78	0	1	0	19	Medium quality	57914	3528360	59.40	88	d__Bacteria;p__Elusimicrobiota;c__Elusimicrobia;o__Elusimicrobiales;f__UBA9959;g__UBA9959;s__
LMSG_G000006225.1	yes	937_0	Lead-Zinc	98.63	4.07	78.28	1	1	0	17	Medium quality	27402	3353499	51.40	180	d__Bacteria;p__Nitrospirota;c__Thermodesulfovibrionia;o__Thermodesulfovibrionales;f__UBA9935;g__;s__
LMSG_G000005122.1	yes	1770_1	Lead-Zinc	75.28	0.00	75.28	1	2	2	20	Medium quality	50761	747362	58.80	19	d__Bacteria;p__Patescibacteria;c__Paceibacteria;o__UBA9983;f__UBA2163;g__C7867-001;s__
LMSG_G000005123.1	yes	259_1	Lead-Zinc	97.01	0.00	97.01	2	0	1	18	Medium quality	34570	1929026	46.20	100	d__Bacteria;p__Proteobacteria;c__Gammaproteobacteria;o__Thiomicrospirales;f__Thiomicrospiraceae;g__GCA-002282575;s__GCA-002282575 sp002281095
LMSG_G000005124.1	yes	1102_0	Lead-Zinc	90.22	1.06	84.91	2	0	0	19	Medium quality	28071	2601210	47.20	135	d__Bacteria;p__Firmicutes_B;c__Thermincolia;o__Thermincolales;f__UBA2595;g__GW-Firmicutes-8;s__
LMSG_G000005126.1	yes	1800_1	Lead-Zinc	75.09	0.00	75.09	0	1	0	19	Medium quality	23227	712871	45.80	55	d__Bacteria;p__Patescibacteria;c__Paceibacteria;o__UBA6257;f__GWB1-50-10;g__GWA2-45-9;s__
LMSG_G000005127.1	yes	1274_0	Lead-Zinc	64.97	2.74	51.29	0	1	0	15	Medium quality	24183	1984099	65.70	125	d__Bacteria;p__Proteobacteria;c__Gammaproteobacteria;o__Burkholderiales;f__Thiobacillaceae;g__Thiobacillus;s__
LMSG_G000005128.1	yes	651_1	Lead-Zinc	93.06	0.09	92.61	0	1	0	15	Medium quality	45833	3102761	67.60	134	d__Bacteria;p__Firmicutes_E;c__Sulfobacillia;o__;f__;g__;s__
LMSG_G000005129.1	yes	1118_1	Lead-Zinc	81.44	3.99	61.49	1	1	1	13	Medium quality	4146	2582797	69.50	722	d__Bacteria;p__Proteobacteria;c__Gammaproteobacteria;o__Steroidobacterales;f__Steroidobacteraceae;g__;s__
LMSG_G000005130.1	yes	1417_1	Lead-Zinc	93.62	1.47	86.25	0	0	0	17	Medium quality	93724	3394664	62.10	68	d__Bacteria;p__Proteobacteria;c__Alphaproteobacteria;o__Sphingomonadales;f__Sphingomonadaceae;g__Sphingobium;s__
LMSG_G000006226.1	yes	1241_1	Lead-Zinc	92.33	0.95	87.60	2	1	0	18	Medium quality	32599	2547346	64.00	116	d__Bacteria;p__Proteobacteria;c__Gammaproteobacteria;o__Burkholderiales;f__Sulfuricellaceae;g__UBA2239;s__
LMSG_G000005131.1	yes	1767_0	Lead-Zinc	80.30	0.00	80.30	1	1	3	20	Medium quality	103929	879991	55.80	57	d__Bacteria;p__Patescibacteria;c__Paceibacteria;o__UBA9983;f__UBA2163;g__C7867-001;s__
LMSG_G000005132.1	yes	1128_1	Lead-Zinc	94.49	2.14	83.80	1	0	0	16	Medium quality	8971	2226288	63.30	336	d__Bacteria;p__Eremiobacterota;c__Eremiobacteria;o__UBP12;f__UBA5184;g__;s__
LMSG_G000005133.1	yes	842_0	Lead-Zinc	85.97	3.19	70.00	0	0	0	16	Medium quality	15937	2274485	69.70	288	d__Bacteria;p__Actinobacteriota;c__Coriobacteriia;o__OPB41;f__PALSA-660;g__;s__
LMSG_G000005134.1	yes	1694_0	Lead-Zinc	74.35	2.03	64.22	0	0	0	16	Medium quality	3942	1394697	50.10	442	d__Bacteria;p__Verrucomicrobiota_A;c__Chlamydiia;o__FEN-1388;f__FEN-1388;g__;s__
LMSG_G000005139.1	yes	1285_0	Lead-Zinc	78.31	2.83	64.17	0	0	0	13	Medium quality	5795	2726384	67.80	530	d__Bacteria;p__Proteobacteria;c__Gammaproteobacteria;o__Burkholderiales;f__Burkholderiaceae;g__Diaphorobacter;s__Diaphorobacter nitroreducens
LMSG_G000005135.1	yes	1818_1	Lead-Zinc	82.02	3.68	63.64	1	3	2	19	Medium quality	150758	1457742	53.90	36	d__Bacteria;p__Patescibacteria;c__ABY1;o__SG8-24;f__2-12-FULL-60-25;g__SZUA-46;s__
LMSG_G000005136.1	yes	1725_0	Lead-Zinc	71.28	0.20	70.29	0	1	1	16	Medium quality	19739	1027813	47.60	72	d__Bacteria;p__Patescibacteria;c__Doudnabacteria;o__UBA920;f__UBA920;g__PALSA-1336;s__
LMSG_G000005137.1	yes	1023_0	Lead-Zinc	90.63	2.62	77.53	0	1	0	19	Medium quality	38532	2805838	69.00	123	d__Bacteria;p__Elusimicrobiota;c__Elusimicrobia;o__UBA1565;f__UBA9628;g__GWA2-66-18;s__
LMSG_G000005138.1	yes	1053_0	Lead-Zinc	95.21	0.60	92.23	0	0	0	19	Medium quality	16747	3840067	35.20	379	d__Bacteria;p__Bacteroidota;c__Ignavibacteria;o__SJA-28;f__B-1AR;g__UBA2330;s__
LMSG_G000005144.1	yes	1419_0	Lead-Zinc	66.76	2.85	52.52	0	0	0	16	Medium quality	3847	2545767	65.80	746	d__Bacteria;p__Proteobacteria;c__Alphaproteobacteria;o__Sphingomonadales;f__Sphingomonadaceae;g__Sphingobium;s__Sphingobium sp001650725
LMSG_G000005140.1	yes	1727_0	Lead-Zinc	86.50	3.30	69.98	1	1	1	20	Medium quality	14085	3652262	66.40	315	d__Bacteria;p__Actinobacteriota;c__Thermoleophilia;o__RBG-16-64-13;f__RBG-16-64-13;g__;s__
LMSG_G000005141.1	yes	1787_1	Lead-Zinc	71.09	0.00	71.09	0	1	0	19	Medium quality	24820	577965	39.40	38	d__Bacteria;p__Patescibacteria;c__Paceibacteria;o__UBA6257;f__UBA9933;g__;s__
LMSG_G000005142.1	yes	242_0	Lead-Zinc	61.93	0.00	61.93	1	1	1	19	Medium quality	67732	1099942	49.00	20	d__Bacteria;p__Patescibacteria;c__Paceibacteria;o__UBA9983;f__UBA6899;g__UBA6899;s__
LMSG_G000005143.1	yes	1074_1	Lead-Zinc	75.11	0.00	75.11	0	0	0	10	Medium quality	6503	1569615	49.10	297	d__Bacteria;p__Firmicutes_D;c__Dethiobacteria;o__Dethiobacterales;f__Dethiobacteraceae;g__Dethiobacter;s__
LMSG_G000005145.1	yes	7_0	Polymetallic	78.39	1.78	69.50	0	1	0	16	Medium quality	8448	1083008	35.50	148	d__Archaea;p__Nanoarchaeota;c__Nanoarchaeia;o__Parvarchaeales;f__Parvarchaeaceae;g__Parvarchaeum;s__
LMSG_G000005146.1	yes	1747_1	Polymetallic	79.41	1.68	71.01	2	0	0	19	Medium quality	44961	2167099	31.80	96	d__Bacteria;p__SZUA-79;c__SZUA-79;o__Acidulodesulfobacterales;f__SZUA-79;g__Acididesulfobacter;s__
LMSG_G000005147.1	yes	898_0	Polymetallic	63.77	0.00	63.77	0	1	0	14	Medium quality	19202	2261186	58.30	152	d__Bacteria;p__Acidobacteriota;c__Acidobacteriae;o__Acidobacteriales;f__Acidobacteriaceae;g__;s__
LMSG_G000004430.1	yes	815_1	Polymetallic	98.29	1.28	91.88	1	1	2	18	High quality	49494	2091692	45.50	63	d__Bacteria;p__Actinobacteriota;c__Acidimicrobiia;o__Acidimicrobiales;f__Bog-793;g__;s__
LMSG_G000005148.1	yes	726_1	Polymetallic	93.16	2.23	82.01	0	0	0	20	Medium quality	29122	3457206	71.10	220	d__Bacteria;p__Actinobacteriota;c__Acidimicrobiia;o__Acidimicrobiales;f__UBA8190;g__;s__
LMSG_G000005149.1	yes	717_1	Polymetallic	95.72	2.14	85.04	1	0	1	20	Medium quality	38562	4044525	65.80	284	d__Bacteria;p__Actinobacteriota;c__Acidimicrobiia;o__Acidimicrobiales;f__Bog-793;g__Palsa-601;s__
LMSG_G000004431.1	yes	624_1	Polymetallic	95.37	1.23	89.20	1	1	2	20	High quality	21493	3118436	67.00	351	d__Bacteria;p__Dormibacterota;c__Dormibacteria;o__UBA8260;f__Bog-877;g__;s__
LMSG_G000005150.1	yes	373_1	Polymetallic	72.92	0.00	72.92	1	1	1	18	Medium quality	140497	714053	33.50	9	d__Bacteria;p__Patescibacteria;c__Saccharimonadia;o__Saccharimonadales;f__SZUA-47;g__;s__
LMSG_G000005151.1	yes	896_0	Polymetallic	71.83	1.72	63.21	1	1	1	14	Medium quality	6285	3191724	63.00	634	d__Bacteria;p__Acidobacteriota;c__Acidobacteriae;o__Acidobacteriales;f__Acidobacteriaceae;g__Palsa-343;s__
LMSG_G000005152.1	yes	602_1	Polymetallic	93.90	0.30	92.38	0	1	0	18	Medium quality	75503	1909380	60.70	41	d__Bacteria;p__Proteobacteria;c__Gammaproteobacteria;o__Acidiferrobacterales;f__Acidiferrobacteraceae;g__;s__
LMSG_G000005153.1	yes	367_1	Polymetallic	80.95	1.91	71.39	1	1	1	19	Medium quality	17908	649988	35.80	56	d__Bacteria;p__Patescibacteria;c__Saccharimonadia;o__Saccharimonadales;f__UBA4665;g__;s__
LMSG_G000005154.1	yes	25_1	Polymetallic	77.99	0.97	73.14	1	1	2	19	Medium quality	63899	740721	42.30	19	d__Archaea;p__Nanoarchaeota;c__Nanoarchaeia;o__Parvarchaeales;f__Parvarchaeaceae;g__;s__
LMSG_G000005155.1	yes	653_2	Polymetallic	86.62	0.99	81.67	0	1	0	11	Medium quality	7222	2594544	68.70	488	d__Bacteria;p__Firmicutes_E;c__Sulfobacillia;o__;f__;g__;s__
LMSG_G000005156.1	yes	611_1	Polymetallic	98.29	1.28	91.88	0	0	0	20	Medium quality	24825	3261274	54.60	224	d__Bacteria;p__Actinobacteriota;c__Acidimicrobiia;o__Acidimicrobiales;f__Acidimicrobiaceae;g__SCQM01;s__
LMSG_G000005157.1	yes	1748_2	Polymetallic	71.72	1.12	66.11	1	0	0	16	Medium quality	7112	1457740	32.20	262	d__Bacteria;p__SZUA-79;c__SZUA-79;o__Acidulodesulfobacterales;f__SZUA-79;g__Acididesulfobacter;s__
LMSG_G000005158.1	yes	44_1	Polymetallic	71.96	0.93	67.29	1	0	1	16	Medium quality	23998	793902	42.30	48	d__Archaea;p__Micrarchaeota;c__Micrarchaeia;o__Micrarchaeales;f__;g__;s__

LMSG_G000005159.1	yes	964_1	Polymetallic	82.32	0.91	77.78	1	2	0	18	Medium quality	53318	1962172	59.50	61	d__Bacteria;p__Nitrospirota;c__Leptospirillia;o__Leptospirillales;f__Leptospirillaceae;g__UBA4572;s__
LMSG_G000005160.1	yes	90_1	Polymetallic	68.84	0.47	66.51	0	1	0	12	Medium quality	14446	480775	54.90	49	d__Archaea;p__Micrarchaeota;c__Micrarchaeia;o__Micrarchaeales;f__Micrarchaeaceae;g__Micrarchaeum;s__
LMSG_G000005161.1	yes	375_1	Polymetallic	74.60	1.12	68.99	1	1	1	20	Medium quality	45185	820830	35.40	48	d__Bacteria;p__Patescibacteria;c__Saccharimonadia;o__Saccharimonadales;f__UBA4665;g__;s__
LMSG_G000005162.1	yes	592_1	Polymetallic	87.95	0.00	87.95	1	1	1	17	Medium quality	12648	1348052	60.70	153	d__Archaea;p__Thermoproteota;c__Nitrososphaeria;o__Nitrososphaerales;f__UBA183;g__UBA183;s__
LMSG_G000005163.1	yes	769_0	Polymetallic	57.54	0.00	57.54	0	0	0	7	Medium quality	3897	1639255	73.90	468	d__Bacteria;p__Actinobacteriota;c__Acidimicrobiia;o__Acidimicrobiales;f__Bog-793;g__;s__
LMSG_G000005164.1	yes	837_1	Polymetallic	72.60	0.43	70.47	1	0	0	20	Medium quality	7035	2369871	68.70	414	d__Bacteria;p__Actinobacteriota;c__Thermoleophilia;o__Solirubrobacterales;f__Solirubrobacteraceae;g__Palsa-465;s__
LMSG_G000005165.1	yes	101_1	Polymetallic	85.98	2.34	74.30	1	2	2	20	Medium quality	32161	964967	48.10	47	d__Archaea;p__Micrarchaeota;c__Micrarchaeia;o__Micrarchaeales;f__;g__;s__
LMSG_G000005166.1	yes	825_1	Polymetallic	98.00	0.85	93.73	1	0	1	20	Medium quality	17218	2805425	60.60	253	d__Bacteria;p__Actinobacteriota;c__Acidimicrobiia;o__Acidimicrobiales;f__RAAP-2;g__;s__
LMSG_G000005167.1	yes	184_1	Polymetallic	88.70	0.81	84.67	2	2	2	18	Medium quality	79003	1482575	42.40	35	d__Archaea;p__Thermoplasmatota;c__Thermoplasmata;o__Thermoplasmatales;f__Thermoplasmataceae;g__Cuniculiplasma;s__
LMSG_G000005168.1	yes	616_1	Polymetallic	99.14	0.85	94.87	1	0	1	19	Medium quality	49445	2577277	48.20	128	d__Bacteria;p__Actinobacteriota;c__Acidimicrobiia;o__Acidimicrobiales;f__Acidimicrobiaceae;g__SCQM01;s__
LMSG_G000005169.1	yes	59_1	Polymetallic	81.46	0.00	81.46	1	2	1	20	Medium quality	71517	734680	46.30	31	d__Archaea;p__Micrarchaeota;c__Micrarchaeia;o__Micrarchaeales;f__Micrarchaeaceae;g__;s__
LMSG_G000005170.1	yes	111_1	Polymetallic	88.31	0.93	83.64	1	1	1	19	Medium quality	40380	971996	52.40	31	d__Archaea;p__Micrarchaeota;c__Micrarchaeia;o__Micrarchaeales;f__;g__;s__
LMSG_G000005171.1	yes	360_1	Polymetallic	66.84	0.56	64.04	0	0	0	15	Medium quality	22516	636091	40.00	50	d__Bacteria;p__Patescibacteria;c__Saccharimonadia;o__Saccharimonadales;f__UBA4665;g__UBA5150;s__
LMSG_G000004432.1	yes	737_1	Polymetallic	100.00	0.85	95.73	1	1	1	19	High quality	52804	2742008	71.00	76	d__Bacteria;p__Actinobacteriota;c__Acidimicrobiia;o__Acidimicrobiales;f__RAAP-2;g__;s__
LMSG_G000004433.1	yes	702_1	Polymetallic	98.29	1.28	91.88	1	1	1	20	High quality	51081	3557757	70.30	134	d__Bacteria;p__Actinobacteriota;c__Acidimicrobiia;o__Acidimicrobiales;f__Palsa-688;g__;s__
LMSG_G000005172.1	yes	148_0	Polymetallic	50.03	0.00	50.03	0	0	0	16	Medium quality	9161	617714	44.70	87	d__Archaea;p__Micrarchaeota;c__Micrarchaeia;o__Micrarchaeales;f__;g__;s__
LMSG_G000005173.1	yes	456_1	Polymetallic	91.45	0.43	89.32	0	0	0	20	Medium quality	67552	2522758	56.20	84	d__Bacteria;p__Actinobacteriota;c__Acidimicrobiia;o__Acidimicrobiales;f__;g__;s__
LMSG_G000005174.1	yes	354_1	Polymetallic	77.87	0.00	77.87	1	0	2	16	Medium quality	13886	619544	31.10	76	d__Bacteria;p__Patescibacteria;c__Saccharimonadia;o__Saccharimonadales;f__UBA4665;g__;s__
LMSG_G000004434.1	yes	574_1	Polymetallic	93.67	0.00	93.67	1	2	1	19	High quality	73723	2006326	63.10	54	d__Bacteria;p__Proteobacteria;c__Gammaproteobacteria;o__UBA2770;f__UBA2770;g__;s__
LMSG_G000004435.1	yes	516_2	Polymetallic	96.56	0.00	96.56	1	1	15	20	High quality	204072	2197472	49.50	102	d__Bacteria;p__Elusimicrobiota;c__Elusimicrobia;o__UBA1565;f__;g__;s__
LMSG_G000005175.1	yes	221_1	Polymetallic	86.69	1.61	78.63	1	2	1	18	Medium quality	18609	1298680	44.50	98	d__Archaea;p__Thermoplasmatota;c__Thermoplasmata;o__Thermoplasmatales;f__GCA-001856825;g__GCA-001856825;s__GCA-001856825 sp001856825
LMSG_G000005176.1	yes	126_1	Polymetallic	74.29	1.87	64.95	1	1	2	19	Medium quality	92972	742721	42.20	13	d__Archaea;p__Micrarchaeota;c__Micrarchaeia;o__Micrarchaeales;f__Micrarchaeaceae;g__;s__
LMSG_G000005177.1	yes	765_0	Polymetallic	84.61	3.63	66.46	0	1	0	17	Medium quality	45111	2682825	69.60	119	d__Bacteria;p__Actinobacteriota;c__Acidimicrobiia;o__Acidimicrobiales;f__RAAP-2;g__;s__
LMSG_G000005178.1	yes	1302_1	Polymetallic	78.77	0.86	74.46	1	0	0	13	Medium quality	11380	2438240	70.50	251	d__Bacteria;p__Proteobacteria;c__Gammaproteobacteria;o__Burkholderiales;f__Burkholderiaceae;g__Thiomonas;s__
LMSG_G000005179.1	yes	1290_0	Polymetallic	71.70	3.85	52.47	0	0	0	10	Medium quality	2973	2546690	72.90	882	d__Bacteria;p__Proteobacteria;c__Gammaproteobacteria;o__Burkholderiales;f__Burkholderiaceae;g__Rubrivivax;s__
LMSG_G000005180.1	yes	254_1	Polymetallic	67.86	0.00	67.86	3	5	9	14	Medium quality	6196	4508478	51.50	955	d__Bacteria;p__Chloroflexota;c__Ktedonobacteria;o__Ktedonobacterales;f__Ktedonobacteraceae;g__;s__
LMSG_G000005181.1	yes	963_1	Polymetallic	85.01	0.91	80.47	0	0	0	18	Medium quality	28579	1777033	57.90	91	d__Bacteria;p__Nitrospirota;c__Leptospirillia;o__Leptospirillales;f__Leptospirillaceae;g__UBA4572;s__
LMSG_G000005182.1	yes	1330_0	Polymetallic	64.65	0.77	60.79	2	1	1	15	Medium quality	5600	1414740	55.90	272	d__Bacteria;p__Proteobacteria;c__Gammaproteobacteria;o__Burkholderiales;f__Ferrovaceae;g__;s__
LMSG_G000005183.1	yes	696_1	Polymetallic	95.72	1.28	89.31	1	0	0	20	Medium quality	138717	2724198	49.50	65	d__Bacteria;p__Actinobacteriota;c__Acidimicrobiia;o__Acidimicrobiales;f__Acidimicrobiaceae;g__Acidithrix;s__
LMSG_G000005184.1	yes	1526_1	Polymetallic	86.81	1.66	78.52	0	1	0	17	Medium quality	73296	3859918	63.40	88	d__Bacteria;p__Proteobacteria;c__Alphaproteobacteria;o__Acetobacterales;f__Acetobacteraceae;g__Rhodopila;s__
LMSG_G000005185.1	yes	1510_1	Polymetallic	83.90	2.74	70.22	0	0	0	15	Medium quality	7911	3337721	68.10	514	d__Bacteria;p__Proteobacteria;c__Alphaproteobacteria;o__Acetobacterales;f__Acetobacteraceae;g__BOG-908;s__
LMSG_G000005186.1	yes	793_1	Polymetallic	96.58	1.28	90.17	1	0	1	19	Medium quality	31192	1873237	67.10	124	d__Bacteria;p__Actinobacteriota;c__Acidimicrobiia;o__Acidimicrobiales;f__RAAP-2;g__RAAP-2;s__
LMSG_G000005187.1	yes	980_2	Polymetallic	57.89	0.00	57.89	1	1	0	16	Medium quality	14711	2171994	57.00	302	d__Bacteria;p__Proteobacteria;c__Gammaproteobacteria;o__Acidithiobacillales;f__Acidithiobacillaceae;g__Acidithiobacillus;s__Acidithiobacillus ferrivorans
LMSG_G000005188.1	yes	1331_1	Polymetallic	96.76	0.50	94.26	0	0	0	18	Medium quality	99581	2066311	60.50	40	d__Bacteria;p__Proteobacteria;c__Gammaproteobacteria;o__Burkholderiales;f__Ferrovaceae;g__;s__
LMSG_G000005189.1	yes	1472_0	Polymetallic	97.79	1.00	92.82	1	0	1	18	Medium quality	180140	5333635	66.20	49	d__Bacteria;p__Proteobacteria;c__Alphaproteobacteria;o__Reyranellales;f__Reyranellaceae;g__SYSU-D60007;s__SYSU-D60007 sp003574685
LMSG_G000005190.1	yes	309_1	Polymetallic	86.72	0.93	82.10	0	2	1	17	Medium quality	9761	4444056	44.80	678	d__Bacteria;p__Armatimonadota;c__Chthonomonadetes;o__Chthonomonadales;f__Chthonomonadaceae;g__;s__
LMSG_G000005192.1	yes	1081_0	Polymetallic	50.15	0.00	50.15	0	0	0	14	Medium quality	30494	3266922	67.40	223	d__Bacteria;p__Proteobacteria;c__Gammaproteobacteria;o__Steroidobacterales;f__Steroidobacteraceae;g__;s__
LMSG_G000005191.1	yes	954_0	Polymetallic	76.21	0.91	71.67	1	0	1	13	Medium quality	24347	1513964	50.30	109	d__Bacteria;p__Nitrospirota;c__Leptospirillia;o__Leptospirillales;f__Leptospirillaceae;g__Leptospirillum;s__
LMSG_G000005193.1	yes	323_1	Polymetallic	97.53	0.85	93.26	1	0	1	19	Medium quality	27493	2951917	51.10	215	d__Bacteria;p__Proteobacteria;c__Gammaproteobacteria;o__Pseudomonadales;f__Moraxellaceae;g__;s__
LMSG_G000004436.1	yes	1569_1	Polymetallic	96.21	0.00	96.21	1	1	2	20	High quality	61794	3780289	57.00	111	d__Bacteria;p__Planctomycetota;c__Phycisphaerae;o__UBA1161;f__;g__;s__
LMSG_G000004437.1	yes	1054_1	Polymetallic	98.47	0.87	94.13	1	12	21	19	High quality	176000	3667409	59.10	180	d__Bacteria;p__Proteobacteria;c__Alphaproteobacteria;o__UBA1301;f__UBA1301;g__UBA6038;s__
LMSG_G000005194.1	yes	350_1	Polymetallic	84.80	0.68	81.42	0	0	0	14	Medium quality	3762	2030181	39.90	586	d__Bacteria;p__Bacteroidota;c__Bacteroidia;o__Bacteroidales;f__Paludibacteraceae;g__Paludibacter_A;s__
LMSG_G000005195.1	yes	848_0	Polymetallic	61.58	0.00	61.58	0	0	0	11	Medium quality	4482	1760643	66.90	430	d__Bacteria;p__Actinobacteriota;c__Actinomycetia;o__Actinomycetales;f__Microbacteriaceae;g__Schumannella;s__
LMSG_G000005196.1	yes	1528_1	Polymetallic	74.04	4.15	53.32	0	0	0	13	Medium quality	14838	3450866	64.80	304	d__Bacteria;p__Proteobacteria;c__Alphaproteobacteria;o__Acetobacterales;f__Acetobacteraceae;g__Rhodopila;s__
LMSG_G000004438.1	yes	177_1	Polymetallic	92.96	0.81	88.93	1	3	2	20	High quality	38111	1688279	38.00	148	d__Archaea;p__Thermoplasmatota;c__Thermoplasmata;o__Thermoplasmatales;f__Thermoplasmataceae;g__GCA-000496135;s__
LMSG_G000005197.1	yes	1035_0	Polymetallic	82.37	1.15	76.63	1	0	1	15	Medium quality	6210	2493605	62.00	560	d__Bacteria;p__Firmicutes_E;c__Sulfobacillia;o__;f__;g__;s__
LMSG_G000005198.1	yes	303_0	Polymetallic	65.43	0.00	65.43	1	2	3	19	Medium quality	51573	1355136	47.80	65	d__Bacteria;p__Patescibacteria;c__Saccharimonadia;o__Saccharimonadales;f__UBA4665;g__GCA-2746885;s__
LMSG_G000005199.1	yes	1485_1	Polymetallic	100.00	0.50	97.52	0	0	0	19	Medium quality	43095	2824003	63.70	109	d__Bacteria;p__Proteobacteria;c__Alphaproteobacteria;o__Acetobacterales;f__Acetobacteraceae;g__Acidocella;s__
LMSG_G000006272.1	yes	922_1	Polymetallic	97.84	3.45	80.60	1	1	1	17	Medium quality	180133	3294190	62.80	29	d__Bacteria;p__Acidobacteriota;c__Acidobacteriae;o__Acidobacteriales;f__Acidobacteriaceae;g__;s__
LMSG_G000005200.1	yes	807_1	Polymetallic	85.23	1.28	78.82	1	0	1	18	Medium quality	13846	1913139	72.40	211	d__Bacteria;p__Actinobacteriota;c__Acidimicrobiia;o__Acidimicrobiales;f__RAAP-2;g__RAAP-2;s__
LMSG_G000005201.1	yes	819_1	Polymetallic	97.43	2.14	86.75	0	1	0	19	Medium quality	61297	2342878	69.20	68	d__Bacteria;p__Actinobacteriota;c__Acidimicrobiia;o__Acidimicrobiales;f__;g__;s__
LMSG_G000005202.1	yes	364_1	Polymetallic	70.67	1.12	65.06	0	1	2	19	Medium quality	29789	764937	33.10	68	d__Bacteria;p__Patescibacteria;c__Saccharimonadia;o__Saccharimonadales;f__UBA4665;g__UBA4729;s__
LMSG_G000005203.1	yes	767_1	Polymetallic	97.24	1.50	89.77	0	1	0	20	Medium quality	11568	3211369	72.60	626	d__Bacteria;p__Actinobacteriota;c__Acidimicrobiia;o__Acidimicrobiales;f__Bog-793;g__;s__
LMSG_G000006227.1	yes	472_2	Polymetallic	93.97	3.72	75.39	1	2	0	20	Medium quality	24241	4120117	54.90	256	d__Bacteria;p__Firmicutes;c__Alicyclobacillia;o__Alicyclobacillales;f__Acidibacillaceae;g__;s__
LMSG_G000005204.1	yes	487_0	Polymetallic	87.27	3.40	70.28	1	0	0	13	Medium quality	15809	1904633	54.20	174	d__Bacteria;p__Firmicutes;c__Alicyclobacillia;o__Alicyclobacillales;f__Acidibacillaceae;g__;s__
LMSG_G000005205.1	yes	796_1	Polymetallic	97.60	0.85	93.33	0	1	0	18	Medium quality	27613	1984987	66.40	113	d__Bacteria;p__Actinobacteriota;c__Acidimicrobiia;o__Acidimicrobiales;f__RAAP-2;g__RAAP-2;s__
LMSG_G000005206.1	yes	900_1	Polymetallic	96.98	0.00	96.98	0	1	0	17	Medium quality	54654	4143136	61.70	133	d__Bacteria;p__Acidobacteriota;c__Acidobacteriae;o__Acidobacteriales;f__Acidobacteriaceae;g__Granulicella_A;s__
LMSG_G000005207.1	yes	136_1	Polymetallic	82.71	0.47	80.38	1	1	2	20	Medium quality	54989	926472	33.40	38	d__Archaea;p__Micrarchaeota;c__Micrarchaeia;o__Micrarchaeales;f__Micrarchaeaceae;g__;s__
LMSG_G000004439.1	yes	723_1	Polymetallic	94.87	1.99	84.90	1	1	1	19	High quality	22857	3788990	64.50	282	d__Bacteria;p__Actinobacteriota;c__Acidimicrobiia;o__Acidimicrobiales;f__Palsa-688;g__;s__
LMSG_G000004440.1	yes	840_0	Polymetallic	97.22	1.99	87.25	1	1	1	20	High quality	14484	3401606	70.00	332	d__Bacteria;p__Actinobacteriota;c__Thermoleophilia;o__Solirubrobacterales;f__Solirubrobacteraceae;g__Palsa-465;s__
LMSG_G000005208.1	yes	431_0	Polymetallic	96.59	0.07	96.26	0	2	1	20	Medium quality	69318	4067093	54.00	203	d__Bacteria;p__Planctomycetota;c__Phycisphaerae;o__UBA1161;f__;g__;s__
LMSG_G000004441.1	yes	1130_1	Polymetallic	96.81	0.93	92.19	1	2	1	20	High quality	56235	2546966	61.70	57	d__Bacteria;p__Eremiobacterota;c__Eremiobacteria;o__UBP12;f__UBA5184;g__;s__
LMSG_G000004442.1	yes	775_1	Polymetallic	98.86	1.28	92.45	1	1	1	18	High quality	60380	2771283	70.80	74	d__Bacteria;p__Actinobacteriota;c__Acidimicrobiia;o__Acidimicrobiales;f__Bog-793;g__;s__
LMSG_G000005209.1	yes	1002_0	Polymetallic	88.60	0.43	86.47	1	1	1	18	Medium quality	17125	2624903	63.40	201	d__Bacteria;p__Acidobacteriota;c__Acidobacteriae;o__Acidobacteriales;f__Acidobacteriaceae;g__Palsa-288;s__
LMSG_G000005210.1	yes	891_1	Polymetallic	95.08	1.04	89.86	0	1	0	18	Medium quality	20598	4419305	56.70	295	d__Bacteria;p__Acidobacteriota;c__Acidobacteriae;o__Acidobacteriales;f__Acidobacteriaceae;g__;s__

LMSG_G000005214.1	yes	888_0	Polymetallic	90.09	2.56	77.27	0	0	0	16	Medium quality	8217	2316337	59.00	374	d__Bacteria;p__Acidobacteriota;c__Acidobacteriae;o__Acidobacteriales;f__Acidobacteriaceae;g__;s__
LMSG_G000005211.1	yes	1402_1	Polymetallic	96.26	0.03	96.11	4	0	1	19	Medium quality	45163	6711194	68.00	241	d__Bacteria;p__Proteobacteria;c__Gammaproteobacteria;o__Burkholderiales;f__Burkholderiaceae;g__Achromobacter;s__Achromobacter sp001299435
LMSG_G000005212.1	yes	914_2	Polymetallic	71.95	3.45	54.71	1	1	1	14	Medium quality	11450	3562241	62.90	371	d__Bacteria;p__Acidobacteriota;c__Acidobacteriae;o__Acidobacteriales;f__Acidobacteriaceae;g__Terracidiphilus;s__
LMSG_G000005213.1	yes	486_2	Polymetallic	62.88	0.03	62.76	2	0	0	12	Medium quality	2158	1675532	45.90	843	d__Bacteria;p__Firmicutes;c__Alicyclobacillia;o__Alicyclobacillales;f__Acidibacillaceae;g__;s__
LMSG_G000004446.1	yes	517_1	Polymetallic	96.56	0.00	96.56	1	2	1	20	High quality	24612	2202822	50.50	141	d__Bacteria;p__Elusimicrobiota;c__Elusimicrobia;o__UBA1565;f__;g__;s__
LMSG_G000004443.1	yes	808_2	Polymetallic	92.45	3.85	73.22	1	1	1	20	High quality	62974	2099342	72.20	60	d__Bacteria;p__Actinobacteriota;c__Acidimicrobiia;o__Acidimicrobiales;f__RAAP-2;g__RAAP-2;s__
LMSG_G000004444.1	yes	472_1	Polymetallic	96.66	2.50	84.16	2	3	1	20	High quality	59485	3893559	55.10	152	d__Bacteria;p__Firmicutes;c__Alicyclobacillia;o__Alicyclobacillales;f__Acidibacillaceae;g__;s__
LMSG_G000005215.1	yes	1303_1	Polymetallic	99.54	0.10	99.04	2	0	1	18	Medium quality	42259	3313992	69.00	135	d__Bacteria;p__Proteobacteria;c__Gammaproteobacteria;o__Burkholderiales;f__Burkholderiaceae;g__Thiomonas;s__
LMSG_G000005216.1	yes	1187_1	Polymetallic	97.46	0.19	96.54	1	0	1	19	Medium quality	53204	2524046	61.70	82	d__Bacteria;p__Proteobacteria;c__Gammaproteobacteria;o__Pseudomonadales;f__Moraxellaceae;g__;s__
LMSG_G000004445.1	yes	1055_1	Polymetallic	97.82	0.30	96.34	1	2	3	20	High quality	195047	3644609	59.90	83	d__Bacteria;p__Proteobacteria;c__Alphaproteobacteria;o__UBA1301;f__UBA1301;g__UBA6038;s__
LMSG_G000005217.1	yes	1293_1	Polymetallic	97.42	0.21	96.35	0	0	0	13	Medium quality	46899	2947641	65.70	95	d__Bacteria;p__Proteobacteria;c__Gammaproteobacteria;o__Burkholderiales;f__Burkholderiaceae;g__Thiomonas;s__
LMSG_G000006228.1	yes	1295_1	Polymetallic	98.06	0.99	93.11	2	1	0	20	Medium quality	55019	3202328	69.50	102	d__Bacteria;p__Proteobacteria;c__Gammaproteobacteria;o__Burkholderiales;f__Burkholderiaceae;g__Thiomonas;s__
LMSG_G000004447.1	yes	478_1	Polymetallic	97.30	1.03	92.18	4	3	2	20	High quality	155058	3213504	46.20	43	d__Bacteria;p__Firmicutes;c__Alicyclobacillia;o__Alicyclobacillales;f__Acidibacillaceae;g__;s__
LMSG_G000005218.1	yes	214_1	Polymetallic	71.23	0.93	66.61	0	0	0	18	Medium quality	2784	1061084	38.70	431	d__Bacteria;p__Armatimonadota;c__Fimbriimonadia;o__Fimbriimonadales;f__Fimbriimonadaceae;g__;s__
LMSG_G000005219.1	yes	920_1	Polymetallic	82.09	0.20	81.08	1	1	1	15	Medium quality	63075	2706652	59.70	136	d__Bacteria;p__Acidobacteriota;c__Acidobacteriae;o__Acidobacteriales;f__Acidobacteriaceae;g__;s__
LMSG_G000005220.1	yes	1408_1	Polymetallic	90.04	3.10	74.57	0	1	0	17	Medium quality	13271	3701855	69.50	359	d__Bacteria;p__Proteobacteria;c__Alphaproteobacteria;o__Caulobacterales;f__Caulobacteraceae;g__;s__
LMSG_G000004448.1	yes	1444_1	Polymetallic	97.56	1.30	91.07	1	1	1	19	High quality	107019	3021290	66.60	51	d__Bacteria;p__Proteobacteria;c__Alphaproteobacteria;o__Caulobacterales;f__Caulobacteraceae;g__Palsa-881;s__
LMSG_G000006273.1	yes	753_1	Polymetallic	99.14	0.43	97.01	1	1	1	17	Medium quality	89607	2043673	63.90	41	d__Bacteria;p__Actinobacteriota;c__Acidimicrobiia;o__Acidimicrobiales;f__RAAP-2;g__RAAP-2;s__
LMSG_G000004449.1	yes	1617_1	Polymetallic	93.03	2.25	81.77	1	1	1	19	High quality	15983	2154924	49.00	208	d__Bacteria;p__Verrucomicrobiota_A;c__Chlamydiia;o__Parachlamydiales;f__SM23-39;g__;s__
LMSG_G000005221.1	yes	1508_0	Polymetallic	78.97	4.03	58.83	0	0	0	14	Medium quality	11647	3301093	70.40	326	d__Bacteria;p__Proteobacteria;c__Alphaproteobacteria;o__Acetobacterales;f__Acetobacteraceae;g__Palsa-883;s__
LMSG_G000005222.1	yes	768_1	Polymetallic	98.29	1.38	91.41	0	1	0	19	Medium quality	43332	3632925	74.10	161	d__Bacteria;p__Actinobacteriota;c__Acidimicrobiia;o__Acidimicrobiales;f__Bog-793;g__;s__
LMSG_G000005223.1	yes	807_2	Polymetallic	95.67	2.14	84.99	1	0	0	20	Medium quality	33586	2056731	71.80	109	d__Bacteria;p__Actinobacteriota;c__Acidimicrobiia;o__Acidimicrobiales;f__RAAP-2;g__RAAP-2;s__
LMSG_G000005226.1	yes	1482_1	Polymetallic	95.27	1.00	90.30	0	0	0	19	Medium quality	111276	2583030	64.70	30	d__Bacteria;p__Proteobacteria;c__Alphaproteobacteria;o__Acetobacterales;f__Acetobacteraceae;g__Acidocella;s__
LMSG_G000005224.1	yes	1507_1	Polymetallic	62.06	0.00	62.06	0	0	0	15	Medium quality	9288	2886241	69.70	440	d__Bacteria;p__Proteobacteria;c__Alphaproteobacteria;o__Acetobacterales;f__Acetobacteraceae;g__Palsa-883;s__
LMSG_G000005225.1	yes	1514_1	Polymetallic	63.94	1.72	55.32	0	0	0	17	Medium quality	18526	3602582	70.70	250	d__Bacteria;p__Proteobacteria;c__Alphaproteobacteria;o__Acetobacterales;f__Acetobacteraceae;g__;s__
LMSG_G000005227.1	yes	798_1	Polymetallic	79.20	1.28	72.79	0	0	0	16	Medium quality	36841	1550630	68.10	75	d__Bacteria;p__Actinobacteriota;c__Acidimicrobiia;o__Acidimicrobiales;f__RAAP-2;g__RAAP-2;s__
LMSG_G000005228.1	yes	1040_0	Polymetallic	69.58	3.07	54.24	0	0	0	17	Medium quality	2666	1784078	63.70	773	d__Bacteria;p__Verrucomicrobiota;c__Verrucomicrobiae;o__Methylacidiphilales;f__Methylacidiphilaceae;g__LP2A;s__
LMSG_G000005229.1	yes	1096_1	Polymetallic	93.54	1.08	88.17	0	0	0	18	Medium quality	22792	1366340	41.80	82	d__Bacteria;p__Proteobacteria;c__Alphaproteobacteria;o__Paracaedibacterales;f__;g__;s__
LMSG_G000005230.1	yes	1294_1	Polymetallic	87.36	1.24	81.16	0	0	0	17	Medium quality	10758	2578837	65.30	343	d__Bacteria;p__Proteobacteria;c__Gammaproteobacteria;o__Burkholderiales;f__Burkholderiaceae;g__Thiomonas;s__
LMSG_G000005231.1	yes	1621_0	Polymetallic	79.08	1.01	74.02	1	0	1	15	Medium quality	3382	1819550	52.00	621	d__Bacteria;p__Verrucomicrobiota_A;c__Chlamydiia;o__;f__;g__;s__
LMSG_G000006325.1	yes	1052_1	Polymetallic	97.67	0.29	96.22	1	0	0	19	Medium quality	24758	2119985	41.80	152	d__Bacteria;p__Proteobacteria;c__Gammaproteobacteria;o__Diplorickettsiales;f__Diplorickettsiaceae;g__;s__
LMSG_G000005232.1	yes	1044_1	Polymetallic	96.63	0.32	95.05	1	0	1	19	Medium quality	22506	5652326	64.00	366	d__Bacteria;p__Planctomycetota;c__Planctomycetes;o__Isosphaerales;f__Isosphaeraceae;g__;s__
LMSG_G000004450.1	yes	703_1	Polymetallic	96.77	2.59	83.81	1	1	1	20	High quality	22814	3634759	71.20	367	d__Bacteria;p__Actinobacteriota;c__Acidimicrobiia;o__Acidimicrobiales;f__Palsa-688;g__;s__
LMSG_G000005233.1	yes	528_1	Polymetallic	92.04	1.81	83.01	1	0	1	20	Medium quality	15215	5627625	59.90	506	d__Bacteria;p__Planctomycetota;c__Planctomycetes;o__Gemmatales;f__Gemmataceae;g__;s__
LMSG_G000005234.1	yes	1484_1	Polymetallic	75.65	1.49	68.19	0	0	0	16	Medium quality	27417	2136199	65.30	108	d__Bacteria;p__Proteobacteria;c__Alphaproteobacteria;o__Acetobacterales;f__Acetobacteraceae;g__Acidocella;s__
LMSG_G000004451.1	yes	721_1	Polymetallic	93.16	2.14	82.48	1	1	1	19	High quality	63304	4193584	69.80	113	d__Bacteria;p__Actinobacteriota;c__Acidimicrobiia;o__Acidimicrobiales;f__Palsa-688;g__;s__
LMSG_G000006274.1	yes	228_0	Polymetallic	98.18	4.69	74.76	2	4	1	15	Medium quality	194128	4840829	37.80	51	d__Bacteria;p__Firmicutes;c__Bacilli;o__Bacillales;f__Bacillaceae_L;g__Bacillus_Q;s__Bacillus_Q onubensis
LMSG_G000005235.1	yes	666_0	Polymetallic	98.68	3.08	83.28	2	0	0	20	Medium quality	49024	8582222	67.30	252	d__Bacteria;p__Actinobacteriota;c__Actinomycetia;o__Mycobacteriales;f__Mycobacteriaceae;g__Nocardia;s__Nocardia sp003123685
LMSG_G000004452.1	yes	530_1	Polymetallic	93.42	2.39	81.47	1	1	1	20	High quality	98066	2261300	58.00	47	d__Bacteria;p__Proteobacteria;c__Alphaproteobacteria;o__Micavibrionales;f__UBA2020;g__PALSA-926;s__
LMSG_G000005236.1	yes	1296_2	Polymetallic	61.20	1.72	52.58	1	0	0	14	Medium quality	7813	2443857	70.50	413	d__Bacteria;p__Proteobacteria;c__Gammaproteobacteria;o__Burkholderiales;f__Burkholderiaceae;g__Thiomonas;s__
LMSG_G000005238.1	yes	1516_0	Polymetallic	65.51	1.72	56.89	1	1	1	13	Medium quality	12764	3469846	69.40	323	d__Bacteria;p__Proteobacteria;c__Alphaproteobacteria;o__Acetobacterales;f__Acetobacteraceae;g__;s__
LMSG_G000005237.1	yes	1477_1	Polymetallic	88.29	1.28	81.91	0	0	0	18	Medium quality	14640	2645314	60.60	263	d__Bacteria;p__Proteobacteria;c__Alphaproteobacteria;o__Acetobacterales;f__Acetobacteraceae;g__Acidiphilium;s__
LMSG_G000005239.1	yes	1492_1	Polymetallic	94.76	1.66	86.47	0	0	0	19	Medium quality	46966	2524252	66.50	89	d__Bacteria;p__Proteobacteria;c__Alphaproteobacteria;o__Acetobacterales;f__Acetobacteraceae;g__;s__
LMSG_G000005240.1	yes	813_1	Polymetallic	97.43	2.61	84.38	0	1	0	19	Medium quality	33271	1793769	63.80	147	d__Bacteria;p__Actinobacteriota;c__Acidimicrobiia;o__Acidimicrobiales;f__RAAP-2;g__RAAP-2;s__
LMSG_G000005242.1	yes	800_1	Polymetallic	88.03	1.85	78.78	0	1	0	18	Medium quality	15783	2016208	67.70	172	d__Bacteria;p__Actinobacteriota;c__Acidimicrobiia;o__Acidimicrobiales;f__RAAP-2;g__RAAP-2;s__
LMSG_G000005241.1	yes	913_1	Polymetallic	75.02	3.88	55.63	0	0	0	14	Medium quality	45782	2618989	63.70	112	d__Bacteria;p__Acidobacteriota;c__Acidobacteriae;o__Acidobacteriales;f__Acidobacteriaceae;g__Terracidiphilus;s__
LMSG_G000005243.1	yes	434_1	Polymetallic	88.63	0.10	88.12	0	1	2	20	Medium quality	32188	3487455	54.70	264	d__Bacteria;p__Planctomycetota;c__Phycisphaerae;o__UBA1161;f__;g__;s__
LMSG_G000005244.1	yes	722_1	Polymetallic	90.59	2.14	79.91	0	1	0	19	Medium quality	51654	4372899	69.90	144	d__Bacteria;p__Actinobacteriota;c__Acidimicrobiia;o__Acidimicrobiales;f__Palsa-688;g__;s__
LMSG_G000005245.1	yes	1490_1	Polymetallic	95.52	0.75	91.79	0	0	0	19	Medium quality	53043	2932280	63.70	90	d__Bacteria;p__Proteobacteria;c__Alphaproteobacteria;o__Acetobacterales;f__Acetobacteraceae;g__Acidocella;s__
LMSG_G000004453.1	yes	1149_1	Polymetallic	94.25	0.68	90.88	1	1	1	20	High quality	23685	3613731	55.10	269	d__Bacteria;p__Verrucomicrobiota;c__Verrucomicrobiae;o__Pedosphaerales;f__Pedosphaeraceae;g__UBA11358;s__
LMSG_G000005247.1	yes	805_1	Polymetallic	73.44	0.86	69.13	0	1	0	18	Medium quality	16806	1734694	67.20	138	d__Bacteria;p__Actinobacteriota;c__Acidimicrobiia;o__Acidimicrobiales;f__RAAP-2;g__RAAP-2;s__
LMSG_G000005246.1	yes	1131_1	Polymetallic	66.38	2.04	56.20	0	0	0	11	Medium quality	3833	1422055	63.30	463	d__Bacteria;p__Eremiobacterota;c__Eremiobacteria;o__UBP12;f__UBA5184;g__;s__
LMSG_G000005248.1	yes	1513_1	Polymetallic	98.00	0.91	93.44	0	0	0	19	Medium quality	64524	3781321	70.10	84	d__Bacteria;p__Proteobacteria;c__Alphaproteobacteria;o__Acetobacterales;f__Acetobacteraceae;g__;s__
LMSG_G000004454.1	yes	701_1	Polymetallic	98.29	1.28	91.88	1	1	1	19	High quality	64201	3666052	68.20	156	d__Bacteria;p__Actinobacteriota;c__Acidimicrobiia;o__Acidimicrobiales;f__Palsa-688;g__;s__
LMSG_G000005249.1	yes	1591_0	Polymetallic	79.31	3.45	62.07	0	1	0	16	Medium quality	6988	2225655	40.70	416	d__Bacteria;p__Proteobacteria;c__Gammaproteobacteria;o__Legionellales;f__Legionellaceae;g__;s__
LMSG_G000005250.1	yes	873_1	Polymetallic	89.58	3.92	69.98	2	0	1	20	Medium quality	9271	6164531	71.30	830	d__Bacteria;p__Actinobacteriota;c__Actinomycetia;o__Streptosporangiales;f__Streptosporangiaceae;g__;s__
LMSG_G000005251.1	yes	960_1	Polymetallic	82.28	0.00	82.28	1	1	1	17	Medium quality	60706	2162585	59.10	115	d__Bacteria;p__Nitrospirota;c__Leptospirillia;o__Leptospirillales;f__Leptospirillaceae;g__UBA4572;s__
LMSG_G000005252.1	yes	1489_1	Polymetallic	76.28	2.09	65.84	0	0	0	17	Medium quality	6727	2541325	62.10	533	d__Bacteria;p__Proteobacteria;c__Alphaproteobacteria;o__Acetobacterales;f__Acetobacteraceae;g__Acidocella;s__
LMSG_G000005253.1	yes	1091_0	Polymetallic	85.44	4.45	63.19	0	1	0	17	Medium quality	45431	4173790	65.90	192	d__Bacteria;p__Proteobacteria;c__Gammaproteobacteria;o__Steroidobacterales;f__Steroidobacteraceae;g__;s__
LMSG_G000005254.1	yes	1509_1	Polymetallic	83.20	0.17	82.38	0	0	0	17	Medium quality	26868	2550463	71.80	132	d__Bacteria;p__Proteobacteria;c__Alphaproteobacteria;o__Acetobacterales;f__Acetobacteraceae;g__;s__
LMSG_G000005255.1	yes	304_0	Polymetallic	73.98	2.10	63.48	1	16	20	15	Medium quality	4328	1876157	50.40	553	d__Bacteria;p__Bdellovibrionota;c__FAC87;o__UBA2466;f__UBA2466;g__;s__
LMSG_G000005258.1	yes	1587_1	Polymetallic	85.98	0.78	82.11	0	0	0	11	Medium quality	21796	1329194	41.50	84	d__Bacteria;p__Proteobacteria;c__Gammaproteobacteria;o__DSM-16500;f__DSM-16500;g__;s__
LMSG_G000005256.1	yes	1511_1	Polymetallic	88.41	1.74	79.71	0	1	0	18	Medium quality	17192	2871953	70.20	233	d__Bacteria;p__Proteobacteria;c__Alphaproteobacteria;o__Acetobacterales;f__Acetobacteraceae;g__;s__
LMSG_G000005257.1	yes	442_0	Polymetallic	68.59	1.14	62.91	0	0	0	17	Medium quality	2503	3491071	50.80	1548	d__Bacteria;p__Planctomycetota;c__Planctomycetes;o__Gemmatales;f__Gemmataceae;g__;s__
LMSG_G000005259.1	yes	1037_1	Polymetallic	93.37	1.16	87.56	0	0	0	19	Medium quality	13039	6260000	57.60	671	d__Bacteria;p__Planctomycetota;c__Planctomycetes;o__Isosphaerales;f__Isosphaeraceae;g__;s__

LMSG_G000005266.1	yes	1359_2	Antimony	91.55	2.78	77.63	1	0	1	16	Medium quality	10690	2980291	55.00	357	d__Bacteria;p__Nitrospirota;c__UBA9217;o__UBA9217;f__UBA9217;g__;s__
LMSG_G000005260.1	yes	1307_0	Antimony	74.50	0.86	70.21	0	0	0	14	Medium quality	35501	2200046	64.60	93	d__Bacteria;p__Proteobacteria;c__Gammaproteobacteria;o__Burkholderiales;f__Burkholderiaceae;g__Thiomonas;s__
LMSG_G000005261.1	yes	1061_1	Antimony	87.93	0.00	87.93	1	0	1	18	Medium quality	19068	1648864	35.70	127	d__Bacteria;p__Thermodesulfobiota;c__Thermodesulfobiia;o__Thermodesulfobiales;f__Thermodesulfobiaceae;g__Thermodesulfobium;s__
LMSG_G000005262.1	yes	1652_0	Antimony	58.02	0.34	56.30	1	0	0	10	Medium quality	6071	2050407	43.30	440	d__Bacteria;p__Bacteroidota;c__Bacteroidia;o__Chitinophagales;f__Chitinophagaceae;g__Flavihumibacter;s__Flavihumibacter sp000814325
LMSG_G000005263.1	yes	677_0	Antimony	88.53	1.83	79.36	1	1	1	19	Medium quality	41476	3741723	72.20	142	d__Bacteria;p__Chloroflexota;c__Ellin6529;o__CSP1-4;f__CSP1-4;g__;s__
LMSG_G000005264.1	yes	1309_1	Antimony	92.67	2.18	81.75	0	0	0	16	Medium quality	27394	3151450	68.60	167	d__Bacteria;p__Proteobacteria;c__Gammaproteobacteria;o__Burkholderiales;f__Palsa-1005;g__VBCG01;s__
LMSG_G000005265.1	yes	883_0	Antimony	93.87	1.49	86.44	0	1	0	18	Medium quality	37815	2200347	71.50	87	d__Bacteria;p__Actinobacteriota;c__Actinomycetia;o__Actinomycetales;f__Dermatophilaceae;g__Tetrasphaera;s__
LMSG_G000005267.1	yes	1503_1	Antimony	91.61	1.24	85.40	1	0	1	16	Medium quality	28723	3074138	70.50	158	d__Bacteria;p__Proteobacteria;c__Alphaproteobacteria;o__Acetobacterales;f__Acetobacteraceae;g__BOG-930;s__
LMSG_G000005268.1	yes	1125_1	Antimony	98.14	0.93	93.52	1	0	1	18	Medium quality	21209	2343841	64.70	165	d__Bacteria;p__Eremiobacterota;c__Eremiobacteria;o__UBP12;f__UBA5184;g__;s__
LMSG_G000005269.1	yes	845_0	Antimony	75.00	0.00	75.00	0	0	0	17	Medium quality	10121	1882030	68.20	251	d__Bacteria;p__Actinobacteriota;c__Actinomycetia;o__Nanopelagicales;f__UBA5976;g__;s__
LMSG_G000005270.1	yes	1436_0	Antimony	78.36	3.81	59.30	0	0	0	16	Medium quality	4951	2587115	69.20	607	d__Bacteria;p__Proteobacteria;c__Alphaproteobacteria;o__Rhodobacterales;f__Rhodobacteraceae;g__QY30;s__
LMSG_G000005271.1	yes	881_1	Antimony	76.58	1.72	67.96	1	1	1	17	Medium quality	12045	3005417	73.40	337	d__Bacteria;p__Actinobacteriota;c__Actinomycetia;o__Nanopelagicales;f__FW305-bin1;g__;s__
LMSG_G000005272.1	yes	317_1	Antimony	82.97	0.10	82.49	1	1	5	19	Medium quality	12766	1739642	42.90	186	d__Bacteria;p__Omnitrophota;c__Koll11;o__UBA10015;f__GCA-002753745;g__;s__
LMSG_G000005273.1	yes	804_1	Antimony	87.96	4.70	64.46	0	0	0	19	Medium quality	45791	1852389	69.00	66	d__Bacteria;p__Actinobacteriota;c__Acidimicrobiia;o__Acidimicrobiales;f__RAAP-2;g__RAAP-2;s__
LMSG_G000005274.1	yes	1666_1	Antimony	97.18	3.15	81.45	1	0	1	19	Medium quality	29233	3308862	33.10	216	d__Bacteria;p__Bacteroidota;c__Ignavibacteria;o__Ignavibacteriales;f__Ignavibacteriaceae;g__FEN-1297;s__
LMSG_G000005281.1	yes	1237_0	Antimony	70.48	1.46	63.17	1	0	0	16	Medium quality	33375	1685448	56.50	64	d__Bacteria;p__Proteobacteria;c__Gammaproteobacteria;o__Burkholderiales;f__Gallionellaceae;g__Gallionella;s__
LMSG_G000005275.1	yes	512_1	Antimony	90.70	1.81	81.66	1	0	0	17	Medium quality	13352	4080855	62.50	580	d__Bacteria;p__Spirochaetota;c__Spirochaetia;o__Treponematales;f__UBA8932;g__;s__
LMSG_G000005276.1	yes	1317_0	Antimony	60.35	1.37	53.49	1	0	0	11	Medium quality	5455	1669807	66.50	359	d__Bacteria;p__Proteobacteria;c__Gammaproteobacteria;o__Burkholderiales;f__Rhodocyclaceae;g__;s__
LMSG_G000005277.1	yes	345_0	Antimony	66.99	0.00	66.99	1	0	1	19	Medium quality	40093	845429	45.00	32	d__Bacteria;p__Patescibacteria;c__Saccharimonadia;o__Saccharimonadales;f__UBA4665;g__;s__
LMSG_G000005278.1	yes	881_2	Antimony	54.19	0.00	54.19	0	0	0	13	Medium quality	4915	2421527	73.60	568	d__Bacteria;p__Actinobacteriota;c__Actinomycetia;o__Nanopelagicales;f__FW305-bin1;g__;s__
LMSG_G000005279.1	yes	1347_0	Antimony	72.30	1.08	66.93	1	1	1	18	Medium quality	5414	3722477	65.40	787	d__Bacteria;p__Planctomycetota;c__Phycisphaerae;o__Phycisphaerales;f__Phycisphaeraceae;g__;s__
LMSG_G000005280.1	yes	1234_0	Antimony	94.89	3.91	75.35	2	0	0	18	Medium quality	38020	2956635	53.50	108	d__Bacteria;p__Proteobacteria;c__Gammaproteobacteria;o__Burkholderiales;f__Gallionellaceae;g__39-52-133;s__
LMSG_G000005285.1	yes	1018_1	Antimony	84.64	2.59	71.69	1	1	1	16	Medium quality	5247	4851808	67.20	1081	d__Bacteria;p__Verrucomicrobiota;c__Verrucomicrobiae;o__Pedosphaerales;f__UBA11320;g__;s__
LMSG_G000005282.1	yes	346_0	Antimony	76.44	2.97	61.58	0	0	0	17	Medium quality	8049	4635237	71.40	657	d__Bacteria;p__Myxococcota;c__Polyangia;o__Palsa-1104;f__;g__;s__
LMSG_G000005283.1	yes	500_0	Antimony	72.57	1.72	63.95	0	1	0	16	Medium quality	4901	1644377	63.40	402	d__Bacteria;p__Proteobacteria;c__Gammaproteobacteria;o__UBA5335;f__UBA5335;g__Macondimonas;s__Macondimonas sp002862435
LMSG_G000005284.1	yes	1038_0	Antimony	54.54	0.00	54.54	0	0	0	12	Medium quality	6453	3532213	72.20	610	d__Bacteria;p__Myxococcota;c__Polyangia;o__Polyangiales;f__;g__;s__
LMSG_G000004455.1	yes	1164_1	Antimony	100.00	2.61	86.95	1	2	1	20	High quality	85893	5057525	64.60	99	d__Bacteria;p__Acidobacteriota;c__Thermoanaerobaculia;o__Gp7-AA8;f__Gp7-AA8;g__;s__
LMSG_G000004456.1	yes	1024_0	Antimony	94.38	2.31	82.82	1	1	1	19	High quality	12322	3074240	71.10	380	d__Bacteria;p__Elusimicrobiota;c__Elusimicrobia;o__UBA1565;f__UBA9628;g__GWA2-66-18;s__
LMSG_G000006229.1	yes	678_1	Antimony	92.95	3.70	74.44	1	1	0	17	Medium quality	20652	2773499	73.90	213	d__Bacteria;p__Chloroflexota;c__Ellin6529;o__CSP1-4;f__CSP1-4;g__;s__
LMSG_G000005286.1	yes	1698_0	Antimony	56.05	0.00	56.05	1	0	0	12	Medium quality	2284	983374	39.30	468	d__Archaea;p__Nanoarchaeota;c__Nanoarchaeia;o__Woesearchaeales;f__BM511;g__;s__
LMSG_G000005287.1	yes	1373_2	Antimony	78.77	2.22	67.68	1	1	0	15	Medium quality	3918	2782511	74.00	806	d__Bacteria;p__Myxococcota;c__Myxococcia;o__Myxococcales;f__Anaeromyxobacteraceae;g__Anaeromyxobacter;s__
LMSG_G000005294.1	yes	1771_0	Antimony	61.09	0.66	57.78	0	0	1	15	Medium quality	14269	494342	60.40	52	d__Bacteria;p__Patescibacteria;c__Paceibacteria;o__UBA9983;f__UBA2163;g__C7867-001;s__
LMSG_G000005288.1	yes	1795_0	Antimony	57.11	0.00	57.11	0	2	1	13	Medium quality	23491	439383	50.80	37	d__Bacteria;p__Patescibacteria;c__Paceibacteria;o__UBA9983;f__UBA11359;g__;s__
LMSG_G000005289.1	yes	1372_1	Antimony	95.16	2.80	81.19	2	0	0	19	Medium quality	9728	3986797	73.10	577	d__Bacteria;p__Myxococcota;c__Myxococcia;o__Myxococcales;f__Anaeromyxobacteraceae;g__Anaeromyxobacter;s__
LMSG_G000005290.1	yes	1129_2	Antimony	74.56	1.85	65.31	1	0	0	16	Medium quality	18531	1765619	62.00	214	d__Bacteria;p__Eremiobacterota;c__Eremiobacteria;o__UBP12;f__UBA5184;g__PALSA-1484;s__
LMSG_G000005291.1	yes	1222_2	Antimony	96.99	1.49	89.55	0	1	0	19	Medium quality	21509	3092935	57.30	193	d__Bacteria;p__Proteobacteria;c__Gammaproteobacteria;o__Burkholderiales;f__;g__;s__
LMSG_G000005292.1	yes	1482_2	Antimony	98.61	1.83	89.44	0	0	0	19	Medium quality	32767	2949305	65.10	137	d__Bacteria;p__Proteobacteria;c__Alphaproteobacteria;o__Acetobacterales;f__Acetobacteraceae;g__Acidocella;s__
LMSG_G000005293.1	yes	1442_0	Antimony	64.96	2.59	52.01	0	0	0	9	Medium quality	8243	1818441	67.90	248	d__Bacteria;p__Proteobacteria;c__Alphaproteobacteria;o__Sphingomonadales;f__Sphingomonadaceae;g__Novosphingobium;s__
LMSG_G000006318.1	yes	1585_1	Antimony	73.41	1.36	66.63	1	1	0	16	Medium quality	84693	1718175	41.10	65	d__Bacteria;p__Proteobacteria;c__Gammaproteobacteria;o__UBA1113;f__UBA1113;g__;s__
LMSG_G000005296.1	yes	1524_0	Antimony	64.68	2.88	50.30	0	1	0	15	Medium quality	16299	2590001	64.30	207	d__Bacteria;p__Verrucomicrobiota;c__Verrucomicrobiae;o__Opitutales;f__Opitutaceae;g__;s__
LMSG_G000005295.1	yes	1772_0	Antimony	61.61	1.79	52.68	0	1	1	16	Medium quality	8261	533438	58.30	75	d__Bacteria;p__Patescibacteria;c__Paceibacteria;o__UBA9983;f__UBA2163;g__C7867-001;s__
LMSG_G000005297.1	yes	1159_1	Antimony	97.51	1.49	90.05	0	0	0	19	Medium quality	31025	3467747	66.50	169	d__Bacteria;p__Proteobacteria;c__Alphaproteobacteria;o__Rhodospirillales;f__Magnetospirillaceae;g__Telmatospirillum;s__
LMSG_G000005298.1	yes	1057_0	Antimony	84.54	2.87	70.18	1	1	2	18	Medium quality	14642	2892450	45.30	401	d__Bacteria;p__Bacteroidota;c__Bacteroidia;o__Bacteroidales;f__Bacteroidaceae;g__Prevotella;s__Prevotella copri
LMSG_G000005299.1	yes	260_1	Antimony	81.64	2.99	66.68	1	0	8	19	Medium quality	59374	836021	50.60	58	d__Bacteria;p__Patescibacteria;c__Saccharimonadia;o__Saccharimonadales;f__Saccharimonadaceae;g__;s__
LMSG_G000005300.1	yes	388_0	Antimony	60.59	0.46	58.28	2	5	12	18	Medium quality	34764	622312	44.40	46	d__Bacteria;p__Patescibacteria;c__Saccharimonadia;o__Saccharimonadales;f__UBA4665;g__;s__
LMSG_G000005301.1	yes	618_1	Antimony	70.51	2.26	59.22	0	0	0	15	Medium quality	7073	2397358	50.50	461	d__Bacteria;p__Desulfobacterota;c__Desulfobacteria;o__Desulfobacterales;f__Desulfatirhabdiaceae;g__RAAP-1;s__
LMSG_G000006275.1	yes	1089_1	Antimony	92.08	4.61	69.02	1	1	1	17	Medium quality	39217	3716492	66.80	177	d__Bacteria;p__Proteobacteria;c__Gammaproteobacteria;o__Steroidobacterales;f__Steroidobacteraceae;g__;s__
LMSG_G000005302.1	yes	539_0	Antimony	77.49	2.50	65.02	1	0	0	12	Medium quality	3000	1640935	36.90	635	d__Bacteria;p__Proteobacteria;c__Gammaproteobacteria;o__Enterobacterales;f__Succinivibrionaceae;g__Succinivibrio;s__Succinivibrio sp000431835
LMSG_G000005303.1	yes	1374_1	Antimony	88.65	0.68	85.25	1	1	1	15	Medium quality	5583	2827384	75.30	630	d__Bacteria;p__Myxococcota;c__Myxococcia;o__Myxococcales;f__Anaeromyxobacteraceae;g__Anaeromyxobacter;s__
LMSG_G000004457.1	yes	252_0	Antimony	94.57	1.07	89.23	2	1	1	19	High quality	10111	2412310	40.50	330	d__Bacteria;p__Firmicutes;c__Bacilli;o__Lactobacillales;f__Streptococcaceae;g__Lactococcus;s__
LMSG_G000005304.1	yes	1386_0	Antimony	59.64	1.75	50.87	0	0	0	16	Medium quality	20806	1756565	59.40	138	d__Bacteria;p__Desulfobacterota;c__Desulfuromonadia;o__Geobacterales;f__Pseudopelobacteraceae;g__Trichlorobacter;s__
LMSG_G000005305.1	yes	1808_0	Antimony	54.25	0.00	54.25	1	1	2	19	Medium quality	95291	844000	59.00	42	d__Bacteria;p__Patescibacteria;c__Paceibacteria;o__UBA6257;f__2-01-FULL-56-20;g__;s__
LMSG_G000005306.1	yes	1762_1	Antimony	79.59	2.74	65.88	0	1	1	20	Medium quality	33812	679954	54.40	50	d__Bacteria;p__Patescibacteria;c__Paceibacteria;o__UBA9983;f__UBA2163;g__C7867-001;s__
LMSG_G000005307.1	yes	1803_1	Antimony	70.50	0.47	68.15	0	0	2	17	Medium quality	16648	560520	57.10	70	d__Bacteria;p__Patescibacteria;c__Paceibacteria;o__UBA6257;f__2-01-FULL-56-20;g__;s__
LMSG_G000005308.1	yes	475_1	Antimony	98.97	2.85	84.73	6	0	0	17	Medium quality	179005	3304014	46.10	25	d__Bacteria;p__Firmicutes_B;c__Desulfitobacteriia;o__Desulfitobacteriales;f__;g__;s__
LMSG_G000005309.1	yes	1163_0	Antimony	76.36	4.05	56.09	0	0	0	19	Medium quality	8903	3316320	58.00	447	d__Bacteria;p__Verrucomicrobiota;c__Verrucomicrobiae;o__Pedosphaerales;f__Pedosphaeraceae;g__UBA11358;s__
LMSG_G000004458.1	yes	1022_1	Antimony	95.50	1.50	88.01	1	1	1	19	High quality	22528	3334959	67.50	230	d__Bacteria;p__Elusimicrobiota;c__Elusimicrobia;o__UBA1565;f__UBA9628;g__GWA2-66-18;s__
LMSG_G000005313.1	yes	1000_1	Antimony	90.51	0.86	86.20	0	1	0	19	Medium quality	65812	3332687	60.50	85	d__Bacteria;p__Acidobacteriota;c__Acidobacteriae;o__Acidobacteriales;f__Acidobacteriaceae;g__;s__
LMSG_G000005310.1	yes	1236_1	Antimony	90.79	1.31	84.26	0	0	0	15	Medium quality	45407	2079576	57.10	64	d__Bacteria;p__Proteobacteria;c__Gammaproteobacteria;o__Burkholderiales;f__Gallionellaceae;g__Gallionella;s__
LMSG_G000005311.1	yes	880_1	Antimony	92.90	3.05	77.63	1	0	0	16	Medium quality	16083	2922413	70.00	240	d__Bacteria;p__Actinobacteriota;c__Actinomycetia;o__Propionibacteriales;f__Propionibacteriaceae;g__;s__
LMSG_G000006230.1	yes	469_0	Antimony	91.31	1.71	82.77	1	2	0	20	Medium quality	23568	3710889	66.90	240	d__Bacteria;p__Cyanobacteria;c__Sericytochromatia;o__S15B-MN24;f__;g__;s__
LMSG_G000005312.1	yes	856_0	Antimony	50.99	0.00	50.99	0	0	0	12	Medium quality	11465	1589912	72.00	184	d__Bacteria;p__Actinobacteriota;c__Actinomycetia;o__Actinomycetales;f__Cellulomonadaceae;g__Cellulomonas;s__
LMSG_G000005314.1	yes	1298_1	Antimony	63.43	0.00	63.43	1	0	0	15	Medium quality	16672	3115593	70.40	244	d__Bacteria;p__Proteobacteria;c__Gammaproteobacteria;o__Burkholderiales;f__Burkholderiaceae;g__Thiomonas;s__
LMSG_G000005315.1	yes	473_1	Antimony	93.20	1.17	87.34	0	0	0	18	Medium quality	19902	2810802	51.50	206	d__Bacteria;p__Proteobacteria;c__Gammaproteobacteria;o__Pseudomonadales;f__Moraxellaceae;g__;s__

LMSG_G000005316.1	yes	912_0	Antimony	73.70	1.15	67.96	0	1	0	14	Medium quality	39321	2171912	63.80	74	d__Bacteria;p__Acidobacteriota;c__Acidobacteriae;o__Acidobacteriales;f__Acidobacteriaceae;g__Terracidiphilus;s__
LMSG_G000004459.1	yes	292_1	Antimony	91.27	0.78	87.40	2	3	7	19	High quality	92173	1840772	38.40	72	d__Bacteria;p__Proteobacteria;c__Gammaproteobacteria;o__UBA9339;f__UBA9339;g__UBA9339;s__
LMSG_G000005322.1	yes	1314_1	Antimony	87.98	0.92	83.37	0	0	0	15	Medium quality	9207	3124861	61.60	407	d__Bacteria;p__Proteobacteria;c__Gammaproteobacteria;o__Burkholderiales;f__Rhodocyclaceae;g__Azonexus;s__
LMSG_G000006276.1	yes	231_0	Antimony	96.53	1.61	88.47	5	6	4	17	Medium quality	85048	6021709	29.30	277	d__Bacteria;p__Firmicutes_A;c__Clostridia;o__Clostridiales;f__Clostridiaceae;g__Clostridium;s__
LMSG_G000005317.1	yes	1392_0	Antimony	62.45	1.75	53.68	0	0	0	15	Medium quality	16546	3400343	58.00	301	d__Bacteria;p__Desulfobacterota;c__Desulfuromonadia;o__Geobacterales;f__Geobacteraceae;g__Geotalea;s__
LMSG_G000005318.1	yes	938_1	Antimony	90.33	2.03	80.20	1	0	0	18	Medium quality	28253	2570184	37.80	129	d__Bacteria;p__Verrucomicrobiota;c__Lentisphaeria;o__Victivallales;f__GWF2-38-69;g__GWF2-38-69;s__
LMSG_G000005319.1	yes	1160_1	Antimony	100.00	1.24	93.79	0	0	0	19	Medium quality	85070	4336287	64.40	89	d__Bacteria;p__Proteobacteria;c__Alphaproteobacteria;o__Rhodospirillales;f__Magnetospirillaceae;g__Telmatospirillum;s__
LMSG_G000005320.1	yes	1584_1	Antimony	98.25	0.19	97.29	0	0	0	19	Medium quality	124848	1941735	41.70	23	d__Bacteria;p__Proteobacteria;c__Gammaproteobacteria;o__UBA1113;f__UBA1113;g__;s__
LMSG_G000005321.1	yes	237_1	Antimony	79.36	0.00	79.36	0	1	0	18	Medium quality	8371	747123	33.40	112	d__Archaea;p__Micrarchaeota;c__Micrarchaeia;o__UBA8480;f__UBA97;g__;s__
LMSG_G000005323.1	yes	1325_0	Antimony	66.59	1.22	60.50	0	0	0	14	Medium quality	8452	2344488	56.80	318	d__Bacteria;p__Proteobacteria;c__Gammaproteobacteria;o__Acidiferrobacterales;f__Sulfurifustaceae;g__;s__
LMSG_G000004460.1	yes	402_1	Antimony	96.57	1.43	89.43	1	1	1	20	High quality	61493	3449121	45.10	114	d__Bacteria;p__Bacteroidota;c__Bacteroidia;o__AKYH767;f__Palsa-948;g__;s__
LMSG_G000005324.1	yes	340_0	Antimony	66.99	0.50	64.52	1	1	2	20	Medium quality	87282	752299	37.60	22	d__Bacteria;p__Patescibacteria;c__Saccharimonadia;o__Saccharimonadales;f__2-12-FULL-41-12;g__;s__
LMSG_G000005325.1	yes	366_0	Antimony	60.34	0.00	60.34	0	0	0	17	Medium quality	114745	722657	32.80	24	d__Bacteria;p__Patescibacteria;c__Saccharimonadia;o__Saccharimonadales;f__UBA4665;g__UBA6224;s__
LMSG_G000005326.1	yes	325_0	Antimony	86.75	1.74	78.03	0	0	0	15	Medium quality	15612	1950569	37.80	180	d__Bacteria;p__Proteobacteria;c__Gammaproteobacteria;o__DSM-16500;f__DSM-16500;g__Aquicella;s__
LMSG_G000006277.1	yes	619_0	Antimony	90.96	3.55	73.22	1	1	1	17	Medium quality	23794	3225898	51.90	197	d__Bacteria;p__Desulfobacterota;c__Desulfobacteria;o__Desulfobacterales;f__Desulfatirhabdiaceae;g__RAAP-1;s__
LMSG_G000005327.1	yes	528_2	Antimony	95.45	1.14	89.77	1	0	2	20	Medium quality	26985	6167801	59.90	345	d__Bacteria;p__Planctomycetota;c__Planctomycetes;o__Gemmatales;f__Gemmataceae;g__;s__
LMSG_G000004461.1	yes	1369_1	Antimony	97.43	0.00	97.43	1	1	1	20	High quality	197075	2832467	30.20	59	d__Bacteria;p__Bacteroidota;c__Kapabacteria;o__Kapabacteriales;f__UBA2268;g__GWF2-33-9;s__
LMSG_G000005328.1	yes	1082_2	Antimony	81.86	3.74	63.15	1	1	0	15	Medium quality	30744	2811644	67.80	166	d__Bacteria;p__Proteobacteria;c__Gammaproteobacteria;o__Steroidobacterales;f__Steroidobacteraceae;g__;s__
LMSG_G000005331.1	yes	1785_0	Antimony	63.85	2.25	52.62	1	2	1	14	Medium quality	41374	644164	43.00	33	d__Bacteria;p__Patescibacteria;c__Paceibacteria;o__UBA6257;f__UBA9933;g__WO2-47-17b;s__
LMSG_G000005329.1	yes	1809_0	Antimony	54.25	0.00	54.25	1	0	2	18	Medium quality	67257	734791	57.80	27	d__Bacteria;p__Patescibacteria;c__Paceibacteria;o__UBA6257;f__2-01-FULL-56-20;g__;s__
LMSG_G000005330.1	yes	1122_0	Antimony	81.50	2.39	69.58	1	1	1	14	Medium quality	25171	3062322	66.90	142	d__Bacteria;p__Proteobacteria;c__Gammaproteobacteria;o__Steroidobacterales;f__Steroidobacteraceae;g__;s__
LMSG_G000005332.1	yes	1704_1	Antimony	85.01	0.00	85.01	4	4	8	20	Medium quality	108520	946695	36.00	56	d__Bacteria;p__Patescibacteria;c__Paceibacteria_A;o__Moranbacterales;f__UBA2206;g__UBA9630;s__
LMSG_G000005333.1	yes	299_1	Antimony	97.06	0.93	92.44	0	1	0	19	Medium quality	71756	2647897	56.40	57	d__Bacteria;p__Eremiobacterota;c__Eremiobacteria;o__UBP12;f__UBA5184;g__PALSA-1484;s__
LMSG_G000005334.1	yes	282_1	Antimony	76.04	1.72	67.42	0	0	0	16	Medium quality	4710	2918704	47.10	686	d__Bacteria;p__Bacteroidota;c__Bacteroidia;o__Chitinophagales;f__Chitinophagaceae;g__Palsa-955;s__
LMSG_G000005335.1	yes	295_0	Antimony	77.36	1.12	71.75	1	1	1	20	Medium quality	126366	937979	26.80	16	d__Bacteria;p__Patescibacteria;c__ABY1;o__SCPX01;f__;g__;s__
LMSG_G000005336.1	yes	505_0	Antimony	99.83	2.14	89.12	0	0	0	19	Medium quality	104830	3759079	53.00	65	d__Bacteria;p__Desulfobacterota;c__Syntrophobacteria;o__Syntrophobacterales;f__Syntrophobacteraceae;g__SbD1;s__
LMSG_G000005337.1	yes	1027_1	Antimony	68.77	0.89	64.31	0	0	0	14	Medium quality	2962	2155922	53.90	850	d__Bacteria;p__Bdellovibrionota;c__Bdellovibrionia_A;o__UBA1018;f__UBA1018;g__;s__
LMSG_G000005341.1	yes	789_1	Antimony	92.61	2.14	81.93	0	0	0	18	Medium quality	31599	2819043	60.70	228	d__Bacteria;p__Actinobacteriota;c__Acidimicrobiia;o__Acidimicrobiales;f__RAAP-2;g__RAAP-2;s__
LMSG_G000004462.1	yes	1015_1	Antimony	96.62	4.73	72.98	1	1	2	19	High quality	44500	5162468	65.20	174	d__Bacteria;p__Verrucomicrobiota;c__Verrucomicrobiae;o__Pedosphaerales;f__Pedosphaeraceae;g__;s__
LMSG_G000005338.1	yes	1388_1	Antimony	97.38	0.00	97.38	1	0	1	19	Medium quality	108280	3645842	61.70	58	d__Bacteria;p__Desulfobacterota;c__Desulfuromonadia;o__Geobacterales;f__Geobacteraceae;g__;s__
LMSG_G000005339.1	yes	1263_0	Antimony	90.05	2.82	75.96	0	1	0	18	Medium quality	44194	3463844	64.80	151	d__Bacteria;p__Proteobacteria;c__Gammaproteobacteria;o__Xanthomonadales;f__Rhodanobacteraceae;g__Rhodanobacter;s__
LMSG_G000005340.1	yes	1523_1	Antimony	66.36	0.00	66.36	1	1	1	15	Medium quality	9182	2537087	63.30	374	d__Bacteria;p__Proteobacteria;c__Alphaproteobacteria;o__UBA1301;f__UBA1301;g__UBA6038;s__
LMSG_G000006231.1	yes	1341_1	Antimony	100.00	1.04	94.82	2	1	0	19	Medium quality	265872	3869685	51.20	45	d__Bacteria;p__Desulfobacterota;c__Desulfuromonadia;o__Geobacterales;f__Pseudopelobacteraceae;g__Pseudopelobacter;s__
LMSG_G000005342.1	yes	1011_0	Antimony	63.47	1.57	55.62	0	0	0	13	Medium quality	5342	1756435	69.20	377	d__Bacteria;p__Actinobacteriota;c__Actinomycetia;o__Propionibacteriales;f__Propionibacteriaceae;g__Fen-668;s__
LMSG_G000006314.1	yes	1650_1	Antimony	74.42	2.14	63.71	1	0	1	14	Medium quality	3781	2639415	46.60	769	d__Bacteria;p__Bacteroidota;c__Bacteroidia;o__Bacteroidales;f__;g__;s__
LMSG_G000005343.1	yes	1463_2	Antimony	88.06	3.03	72.94	0	0	0	17	Medium quality	14792	3082685	64.60	255	d__Bacteria;p__Proteobacteria;c__Alphaproteobacteria;o__Rhizobiales;f__Beijerinckiaceae;g__Rhodoblastus;s__
LMSG_G000005344.1	yes	1527_1	Antimony	84.61	1.99	74.66	0	0	0	17	Medium quality	13342	4589128	63.00	413	d__Bacteria;p__Proteobacteria;c__Alphaproteobacteria;o__Acetobacterales;f__Acetobacteraceae;g__Rhodopila;s__
LMSG_G000004463.1	yes	498_1	Antimony	96.55	0.00	96.55	1	1	1	19	High quality	31913	6165801	62.10	335	d__Bacteria;p__Planctomycetota;c__Planctomycetes;o__Planctomycetales;f__UBA10511;g__;s__
LMSG_G000005345.1	yes	1554_1	Antimony	89.96	0.89	85.50	1	1	1	14	Medium quality	24390	3502369	65.30	205	d__Bacteria;p__Desulfobacterota_B;c__Binatia;o__Binatales;f__Binataceae;g__;s__
LMSG_G000005346.1	yes	1589_1	Antimony	85.73	1.24	79.55	0	0	0	15	Medium quality	8085	2775246	51.60	473	d__Bacteria;p__Desulfobacterota;c__Desulfobacteria;o__Desulfobacterales;f__SURF-3;g__SURF-3;s__
LMSG_G000005347.1	yes	931_1	Antimony	92.27	2.00	82.27	0	2	0	18	Medium quality	57405	3098661	57.30	79	d__Bacteria;p__Nitrospirota;c__Thermodesulfovibrionia;o__Thermodesulfovibrionales;f__UBA6898;g__Sulfobium;s__
LMSG_G000005348.1	yes	413_0	Antimony	64.85	1.58	56.98	1	2	2	20	Medium quality	42621	927902	35.10	44	d__Bacteria;p__Patescibacteria;c__Microgenomatia;o__Levybacterales;f__UBA12049;g__;s__
LMSG_G000004464.1	yes	1395_2	Antimony	97.43	1.71	88.89	1	1	2	20	High quality	40103	3635064	66.90	146	d__Bacteria;p__Acidobacteriota;c__UBA4820;o__UBA4820;f__;g__;s__
LMSG_G000005349.1	yes	467_0	Antimony	80.53	1.31	73.99	1	2	1	16	Medium quality	11427	2242309	43.10	280	d__Bacteria;p__Firmicutes_B;c__Desulfitobacteriia;o__Desulfitobacteriales;f__Desulfitobacteriaceae;g__Desulfosporosinus;s__
LMSG_G000005350.1	yes	451_0	Antimony	89.05	0.75	85.32	0	0	0	19	Medium quality	35757	2803084	70.00	109	d__Bacteria;p__Proteobacteria;c__Alphaproteobacteria;o__Acetobacterales;f__Acetobacteraceae;g__BOG-930;s__
LMSG_G000004465.1	yes	438_1	Antimony	95.35	1.20	89.34	1	1	2	19	High quality	10683	3130779	56.60	398	d__Bacteria;p__Planctomycetota;c__Phycisphaerae;o__UBA1161;f__;g__;s__
LMSG_G000005352.1	yes	863_0	Antimony	78.27	2.83	64.15	0	0	0	12	Medium quality	10512	3643023	69.60	446	d__Bacteria;p__Actinobacteriota;c__Actinomycetia;o__Mycobacteriales;f__Mycobacteriaceae;g__Mycobacterium;s__Mycobacterium europaeum
LMSG_G000005351.1	yes	1781_1	Antimony	75.99	0.00	75.99	1	1	1	17	Medium quality	17487	705350	36.30	59	d__Bacteria;p__Patescibacteria;c__Paceibacteria;o__UBA6257;f__UBA9933;g__;s__
LMSG_G000005357.1	yes	864_0	Antimony	87.16	3.58	69.25	0	0	0	12	Medium quality	25788	4343232	69.80	264	d__Bacteria;p__Actinobacteriota;c__Actinomycetia;o__Mycobacteriales;f__Mycobacteriaceae;g__Mycobacterium;s__
LMSG_G000005353.1	yes	1487_1	Antimony	81.33	1.00	76.36	0	0	0	17	Medium quality	18887	2320536	65.90	151	d__Bacteria;p__Proteobacteria;c__Alphaproteobacteria;o__Acetobacterales;f__Acetobacteraceae;g__Acidocella;s__
LMSG_G000005354.1	yes	1491_0	Antimony	84.48	0.86	80.17	0	0	0	18	Medium quality	57463	2295682	65.60	59	d__Bacteria;p__Proteobacteria;c__Alphaproteobacteria;o__Acetobacterales;f__Acetobacteraceae;g__Acidocella;s__
LMSG_G000005355.1	yes	849_0	Antimony	88.09	2.01	78.06	0	1	0	20	Medium quality	16437	2151787	69.40	215	d__Bacteria;p__Actinobacteriota;c__Actinomycetia;o__Actinomycetales;f__Microbacteriaceae;g__Schumannella;s__
LMSG_G000005356.1	yes	1742_1	Antimony	90.32	2.59	77.39	2	0	5	18	Medium quality	19201	1548409	38.90	147	d__Bacteria;p__SZUA-79;c__SZUA-79;o__Acidulodesulfobacterales;f__SZUA-79;g__Acidulodesulfobacterium;s__
LMSG_G000004466.1	yes	853_1	Antimony	94.86	0.67	91.50	1	1	1	20	High quality	36913	1928704	69.30	80	d__Bacteria;p__Actinobacteriota;c__Actinomycetia;o__Actinomycetales;f__Microbacteriaceae;g__Gryllotalpicola;s__
LMSG_G000005358.1	yes	808_3	Antimony	88.46	1.85	79.21	1	1	1	20	Medium quality	36558	1891657	72.50	80	d__Bacteria;p__Actinobacteriota;c__Acidimicrobiia;o__Acidimicrobiales;f__RAAP-2;g__RAAP-2;s__
LMSG_G000005359.1	yes	511_1	Antimony	81.60	1.41	74.56	1	1	1	17	Medium quality	10861	3377236	66.50	401	d__Bacteria;p__Spirochaetota;c__Spirochaetia;o__Treponematales;f__UBA8932;g__UBA1306;s__
LMSG_G000005363.1	yes	1448_0	Antimony	62.06	0.00	62.06	0	0	0	8	Medium quality	11930	1869982	63.00	207	d__Bacteria;p__Proteobacteria;c__Alphaproteobacteria;o__Parvibaculales;f__Parvibaculaceae;g__;s__
LMSG_G000005360.1	yes	1816_0	Antimony	64.15	0.00	64.15	0	1	1	18	Medium quality	32525	664960	59.40	27	d__Bacteria;p__Patescibacteria;c__Paceibacteria;o__UBA6257;f__2-01-FULL-56-20;g__;s__
LMSG_G000005361.1	yes	1114_1	Antimony	70.68	0.00	70.68	0	0	0	16	Medium quality	22660	1913476	66.50	118	d__Bacteria;p__Proteobacteria;c__Gammaproteobacteria;o__Xanthomonadales;f__Rhodanobacteraceae;g__66-474;s__
LMSG_G000005362.1	yes	1532_1	Antimony	84.00	4.13	63.37	1	0	0	16	Medium quality	26748	1972368	57.00	97	d__Bacteria;p__Proteobacteria;c__Gammaproteobacteria;o__Burkholderiales;f__Gallionellaceae;g__Gallionella;s__
LMSG_G000005364.1	yes	1556_0	Antimony	85.71	1.68	77.31	1	2	1	17	Medium quality	48430	3172706	63.30	101	d__Bacteria;p__Desulfobacterota_B;c__Binatia;o__Binatales;f__Binataceae;g__;s__
LMSG_G000004467.1	yes	1014_1	Antimony	96.62	3.38	79.73	1	1	1	18	High quality	69427	4557931	64.00	99	d__Bacteria;p__Verrucomicrobiota;c__Verrucomicrobiae;o__Pedosphaerales;f__AV2;g__;s__
LMSG_G000004468.1	yes	1668_1	Antimony	90.92	0.56	88.13	4	1	3	19	High quality	27249	3387546	34.90	227	d__Bacteria;p__Bacteroidota;c__Ignavibacteria;o__Ignavibacteriales;f__Ignavibacteriaceae;g__FEN-1297;s__

LMSG_G000004469.1	yes	499_0	Antimony	100.00	3.42	82.91	1	1	2	20	High quality	112883	4334944	58.00	71	d__Bacteria;p__Acidobacteriota;c__Acidobacteriae;o__Acidobacteriales;f__Koribacteraceae;g__TOLSYN;s__
LMSG_G000005365.1	yes	893_0	Antimony	71.47	1.72	62.85	0	1	0	18	Medium quality	15994	3747461	56.80	334	d__Bacteria;p__Acidobacteriota;c__Acidobacteriae;o__Acidobacteriales;f__Acidobacteriaceae;g__;s__
LMSG_G000005366.1	yes	1201_1	Antimony	97.56	1.29	91.13	1	0	1	18	Medium quality	38903	2322265	63.00	124	d__Bacteria;p__Proteobacteria;c__Gammaproteobacteria;o__Acidiferrobacterales;f__Sulfurifustaceae;g__;s__
LMSG_G000004470.1	yes	1625_1	Antimony	93.24	0.68	89.87	1	1	1	19	High quality	44362	2006309	44.70	108	d__Bacteria;p__Verrucomicrobiota_A;c__Chlamydiia;o__Parachlamydiales;f__SM23-39;g__PALSA-1448;s__
LMSG_G000005367.1	yes	979_1	Antimony	96.11	1.86	86.80	0	0	0	17	Medium quality	13308	2352286	59.40	242	d__Bacteria;p__Proteobacteria;c__Gammaproteobacteria;o__Acidithiobacillales;f__Acidithiobacillaceae;g__Acidithiobacillus;s__
LMSG_G000005368.1	yes	1437_1	Antimony	97.12	1.36	90.31	0	0	0	18	Medium quality	107911	3320884	63.00	48	d__Bacteria;p__Proteobacteria;c__Alphaproteobacteria;o__Rhodobacterales;f__Rhodobacteraceae;g__Allgaiera;s__
LMSG_G000005371.1	yes	1586_1	Antimony	95.67	0.00	95.67	0	0	0	18	Medium quality	75965	2200671	41.10	97	d__Bacteria;p__Proteobacteria;c__Gammaproteobacteria;o__UBA1113;f__UBA1113;g__;s__
LMSG_G000005369.1	yes	1705_0	Antimony	59.57	1.54	51.87	0	1	0	19	Medium quality	14343	869840	39.70	83	d__Bacteria;p__Patescibacteria;c__Paceibacteria_A;o__Moranbacterales;f__UBA2206;g__UBA9630;s__
LMSG_G000005370.1	yes	1272_0	Antimony	66.37	1.72	57.75	0	0	0	10	Medium quality	8679	1867600	64.90	518	d__Bacteria;p__Proteobacteria;c__Gammaproteobacteria;o__Burkholderiales;f__Thiobacillaceae;g__;s__
LMSG_G000005372.1	yes	1783_0	Antimony	82.60	0.00	82.60	1	1	1	20	Medium quality	14906	833754	36.70	85	d__Bacteria;p__Patescibacteria;c__GCA-002792135;o__;f__;g__;s__
LMSG_G000006232.1	yes	809_1	Antimony	96.39	3.03	81.23	1	1	0	20	Medium quality	68300	1932936	71.10	52	d__Bacteria;p__Actinobacteriota;c__Acidimicrobiia;o__Acidimicrobiales;f__RAAP-2;g__RAAP-2;s__
LMSG_G000005373.1	yes	662_0	Antimony	91.29	2.31	79.72	2	0	1	18	Medium quality	19531	3343924	54.40	331	d__Bacteria;p__Desulfobacterota;c__Desulfobulbia;o__Desulfobulbales;f__Desulfocapsaceae;g__;s__
LMSG_G000005374.1	yes	535_1	Antimony	100.00	0.16	99.18	0	0	0	19	Medium quality	122889	3267075	36.50	42	d__Bacteria;p__Bacteroidota;c__Bacteroidia;o__Bacteroidales;f__Paludibacteraceae;g__Paludibacter;s__
LMSG_G000005375.1	yes	1435_2	Antimony	92.56	1.50	85.06	0	1	0	18	Medium quality	14135	3351235	68.50	326	d__Bacteria;p__Proteobacteria;c__Alphaproteobacteria;o__Rhodobacterales;f__Rhodobacteraceae;g__Allgaiera;s__
LMSG_G000004471.1	yes	1375_1	Antimony	90.96	0.65	87.74	1	1	1	18	High quality	6882	4172601	74.10	787	d__Bacteria;p__Myxococcota;c__Myxococcia;o__Myxococcales;f__Anaeromyxobacteraceae;g__Anaeromyxobacter;s__
LMSG_G000005376.1	yes	445_1	Antimony	77.77	0.93	73.15	1	1	1	20	Medium quality	83903	1058385	48.70	18	d__Bacteria;p__Patescibacteria;c__Gracilibacteria;o__;f__;g__;s__
LMSG_G000005377.1	yes	274_1	Antimony	69.90	0.66	66.62	0	1	0	10	Medium quality	3965	1991386	45.60	555	d__Bacteria;p__Bacteroidota;c__Bacteroidia;o__Chitinophagales;f__Chitinophagaceae;g__;s__
LMSG_G000005378.1	yes	1760_1	Antimony	69.09	0.00	69.09	1	0	1	16	Medium quality	10196	508007	46.90	60	d__Bacteria;p__Patescibacteria;c__Paceibacteria;o__UBA9983;f__UBA2163;g__C7867-001;s__
LMSG_G000005379.1	yes	1599_0	Antimony	94.47	2.63	81.30	1	0	0	17	Medium quality	30083	6579832	40.70	326	d__Bacteria;p__Bacteroidota;c__Bacteroidia;o__Bacteroidales;f__UBA4181;g__;s__
LMSG_G000005380.1	yes	301_1	Antimony	72.36	0.00	72.36	1	1	4	20	Medium quality	600444	1099786	41.60	9	d__Bacteria;p__Patescibacteria;c__Microgenomatia;o__UBA1406;f__GWC2-37-13;g__;s__
LMSG_G000005382.1	yes	1008_1	Antimony	71.33	0.10	70.82	0	0	0	19	Medium quality	9958	649257	60.50	85	d__Bacteria;p__Patescibacteria;c__Paceibacteria;o__UBA9983;f__UBA2163;g__UBA10103;s__
LMSG_G000005381.1	yes	1552_1	Antimony	82.49	0.42	80.39	0	1	0	15	Medium quality	11972	2466829	63.20	288	d__Bacteria;p__Desulfobacterota_B;c__Binatia;o__Binatales;f__Binataceae;g__;s__
LMSG_G000004472.1	yes	294_1	Antimony	93.63	4.91	69.09	1	1	1	19	High quality	295752	3304746	53.20	68	d__Bacteria;p__Chloroflexota;c__Anaerolineae;o__Anaerolineales;f__Anaerolineaceae;g__UBA6107;s__
LMSG_G000005387.1	yes	312_1	Antimony	95.37	0.93	90.75	0	1	1	18	Medium quality	17346	4139788	52.20	330	d__Bacteria;p__Armatimonadota;c__Chthonomonadetes;o__Chthonomonadales;f__Chthonomonadaceae;g__;s__
LMSG_G000005383.1	yes	1782_1	Antimony	72.42	3.93	52.76	1	3	3	20	Medium quality	94006	1106163	37.20	42	d__Bacteria;p__Patescibacteria;c__Paceibacteria_A;o__;f__;g__;s__
LMSG_G000005384.1	yes	329_1	Antimony	65.97	1.72	57.35	1	0	0	17	Medium quality	9830	607958	46.50	68	d__Bacteria;p__Patescibacteria;c__Saccharimonadia;o__Saccharimonadales;f__Saccharimonadaceae;g__UBA1547;s__
LMSG_G000005385.1	yes	331_1	Antimony	67.93	2.30	56.44	0	1	0	18	Medium quality	29822	704083	44.20	38	d__Bacteria;p__Patescibacteria;c__Saccharimonadia;o__Saccharimonadales;f__Saccharimonadaceae;g__UBA1547;s__
LMSG_G000005386.1	yes	1206_1	Antimony	86.21	1.03	81.09	1	0	0	14	Medium quality	11583	3270269	71.40	389	d__Bacteria;p__Acidobacteriota;c__Vicinamibacteria;o__Vicinamibacterales;f__Fen-181;g__FEN-299;s__
LMSG_G000005388.1	yes	1344_1	Antimony	87.41	2.15	76.66	0	0	0	17	Medium quality	27601	3433789	56.30	179	d__Bacteria;p__Desulfobacterota;c__Desulfuromonadia;o__Geobacterales;f__Pseudopelobacteraceae;g__Pseudopelobacter;s__
LMSG_G000005389.1	yes	1186_1	Antimony	95.79	0.46	93.48	0	0	0	18	Medium quality	34178	2539079	62.00	103	d__Bacteria;p__Proteobacteria;c__Gammaproteobacteria;o__Pseudomonadales;f__Moraxellaceae;g__;s__
LMSG_G000005390.1	yes	1453_0	Antimony	64.46	2.02	54.34	0	1	0	15	Medium quality	7734	3379886	67.20	620	d__Bacteria;p__Proteobacteria;c__Alphaproteobacteria;o__Rhizobiales;f__Beijerinckiaceae;g__;s__
LMSG_G000005391.1	yes	1729_1	Antimony	58.13	1.02	53.05	1	2	1	20	Medium quality	112296	1251918	37.30	38	d__Bacteria;p__Dependentiae;c__Babeliae;o__Babeliales;f__Vermiphilaceae;g__;s__
LMSG_G000005392.1	yes	1204_2	Antimony	71.93	0.19	70.99	2	0	1	11	Medium quality	6417	1717628	71.40	335	d__Bacteria;p__Acidobacteriota;c__Thermoanaerobaculia;o__Thermoanaerobaculales;f__Thermoanaerobaculaceae;g__RBG-13-68-16;s__
LMSG_G000005393.1	yes	1520_1	Antimony	88.28	0.92	83.70	0	0	0	18	Medium quality	12587	5181075	63.40	523	d__Bacteria;p__Acidobacteriota;c__Acidobacteriae;o__Bryobacterales;f__Bryobacteraceae;g__;s__
LMSG_G000005394.1	yes	1686_0	Antimony	53.67	0.72	50.10	0	0	0	8	Medium quality	2179	1060597	52.60	523	d__Bacteria;p__Verrucomicrobiota;c__Verrucomicrobiae;o__Chthoniobacterales;f__UBA6821;g__;s__
LMSG_G000005395.1	yes	1766_1	Antimony	75.28	3.06	60.00	0	0	1	20	Medium quality	57018	570521	55.40	43	d__Bacteria;p__Patescibacteria;c__Paceibacteria;o__UBA9983;f__UBA2163;g__C7867-001;s__
LMSG_G000005396.1	yes	857_0	Antimony	73.41	1.16	67.63	1	0	0	20	Medium quality	23911	3583497	71.10	277	d__Bacteria;p__Actinobacteriota;c__Actinomycetia;o__Actinomycetales;f__Cellulomonadaceae;g__Cellulomonas;s__
LMSG_G000005397.1	yes	1443_0	Antimony	50.86	0.00	50.86	0	0	0	4	Medium quality	2922	1604353	65.70	579	d__Bacteria;p__Proteobacteria;c__Alphaproteobacteria;o__Acetobacterales;f__Acetobacteraceae;g__;s__
LMSG_G000005401.1	yes	1345_1	Antimony	93.41	2.90	78.90	2	0	1	15	Medium quality	92043	4080526	55.00	189	d__Bacteria;p__Desulfobacterota;c__Desulfuromonadia;o__Geobacterales;f__Pseudopelobacteraceae;g__Pseudopelobacter;s__
LMSG_G000005398.1	yes	1383_1	Antimony	93.02	0.99	88.06	0	1	0	15	Medium quality	11177	2223399	63.70	275	d__Bacteria;p__Desulfobacterota;c__Desulfobulbia;o__Desulfobulbales;f__;g__;s__
LMSG_G000005399.1	yes	1041_1	Antimony	90.66	2.61	77.64	1	0	0	19	Medium quality	7928	6143438	68.80	982	d__Bacteria;p__Planctomycetota;c__Planctomycetes;o__Isosphaerales;f__Isosphaeraceae;g__;s__
LMSG_G000005400.1	yes	502_1	Antimony	99.83	1.18	93.96	1	0	0	19	Medium quality	37111	4285811	55.80	209	d__Bacteria;p__Desulfobacterota;c__Syntrophobacteria;o__Syntrophobacterales;f__Syntrophobacteraceae;g__SbD1;s__
LMSG_G000005402.1	yes	1042_1	Antimony	69.38	1.16	63.57	1	0	0	20	Medium quality	4469	4906020	62.30	1356	d__Bacteria;p__Planctomycetota;c__Planctomycetes;o__Isosphaerales;f__Isosphaeraceae;g__;s__
LMSG_G000005403.1	yes	1784_1	Antimony	70.41	0.56	67.61	0	1	0	15	Medium quality	10337	631836	51.40	85	d__Bacteria;p__Patescibacteria;c__Paceibacteria;o__UBA6257;f__UBA9933;g__;s__
LMSG_G000006233.1	yes	1043_1	Antimony	98.23	0.49	95.77	2	1	0	19	Medium quality	12391	4918199	64.90	547	d__Bacteria;p__Planctomycetota;c__Planctomycetes;o__Isosphaerales;f__Isosphaeraceae;g__;s__
LMSG_G000005404.1	yes	1349_1	Antimony	80.01	0.44	77.82	1	1	1	16	Medium quality	15734	2871556	70.10	486	d__Bacteria;p__Acidobacteriota;c__Holophagae;o__Holophagales;f__Holophagaceae;g__;s__
LMSG_G000005405.1	yes	1260_1	Antimony	82.99	3.07	67.64	0	0	0	17	Medium quality	9330	2477554	67.80	295	d__Bacteria;p__Proteobacteria;c__Gammaproteobacteria;o__Xanthomonadales;f__Rhodanobacteraceae;g__Rhodanobacter;s__
LMSG_G000005406.1	yes	230_1	Antimony	90.23	0.46	87.92	6	0	2	18	Medium quality	19262	3508290	29.90	281	d__Bacteria;p__Firmicutes_A;c__Clostridia;o__Clostridiales;f__Clostridiaceae;g__;s__
LMSG_G000005407.1	yes	1301_1	Antimony	91.28	0.00	91.28	0	1	0	16	Medium quality	43639	2703081	68.70	134	d__Bacteria;p__Proteobacteria;c__Gammaproteobacteria;o__Burkholderiales;f__Burkholderiaceae;g__Thiomonas;s__
LMSG_G000006234.1	yes	1385_1	Antimony	90.52	3.33	73.86	2	2	0	16	Medium quality	16415	2393410	61.00	210	d__Bacteria;p__Desulfobacterota;c__Desulfuromonadia;o__Geobacterales;f__Pseudopelobacteraceae;g__Trichlorobacter;s__
LMSG_G000005408.1	yes	330_1	Antimony	58.34	0.43	56.21	1	1	1	17	Medium quality	25680	712202	47.00	45	d__Bacteria;p__Patescibacteria;c__Saccharimonadia;o__Saccharimonadales;f__Saccharimonadaceae;g__UBA1547;s__
LMSG_G000005409.1	yes	410_1	Antimony	62.87	0.00	62.87	0	1	1	20	Medium quality	603255	992261	42.00	3	d__Bacteria;p__Patescibacteria;c__Microgenomatia;o__Levybacterales;f__UBA12049;g__PJMG01;s__
LMSG_G000005410.1	yes	674_2	Antimony	70.11	1.85	60.86	1	0	1	17	Medium quality	5184	2399521	72.30	546	d__Bacteria;p__Chloroflexota;c__Ellin6529;o__CSP1-4;f__CSP1-4;g__;s__
LMSG_G000004473.1	yes	316_1	Antimony	97.19	0.99	92.24	1	1	1	19	High quality	30508	3237706	45.80	185	d__Bacteria;p__Chloroflexota;c__Ktedonobacteria;o__Ktedonobacterales;f__;g__;s__
LMSG_G000005411.1	yes	799_1	Polymetallic	93.92	2.14	83.24	1	0	1	20	Medium quality	60831	2242477	63.40	88	d__Bacteria;p__Actinobacteriota;c__Acidimicrobiia;o__Acidimicrobiales;f__RAAP-2;g__RAAP-2;s__
LMSG_G000005412.1	yes	897_0	Polymetallic	81.41	4.31	59.86	0	0	0	14	Medium quality	33183	3402180	62.20	154	d__Bacteria;p__Acidobacteriota;c__Acidobacteriae;o__Acidobacteriales;f__Acidobacteriaceae;g__;s__
LMSG_G000005413.1	yes	1440_0	Polymetallic	76.73	2.49	64.26	0	1	0	15	Medium quality	4322	2473144	60.10	641	d__Bacteria;p__Proteobacteria;c__Alphaproteobacteria;o__Rhizobiales;f__Beijerinckiaceae;g__Methylovirgula;s__
LMSG_G000005414.1	yes	155_1	Polymetallic	74.54	0.44	72.33	1	1	0	16	Medium quality	3215	1672718	54.00	619	d__Archaea;p__Thermoplasmatota;c__Thermoplasmata;o__UBA10834;f__;g__;s__
LMSG_G000005415.1	yes	1571_1	Polymetallic	70.00	0.00	70.00	1	1	1	16	Medium quality	7839	2784672	56.00	391	d__Bacteria;p__Planctomycetota;c__Phycisphaerae;o__UBA1161;f__;g__;s__
LMSG_G000004474.1	yes	631_1	Polymetallic	94.90	0.46	92.59	1	1	1	20	High quality	15031	2345339	70.70	246	d__Bacteria;p__Dormibacterota;c__Dormibacteria;o__UBA8260;f__Bog-877;g__;s__
LMSG_G000005416.1	yes	1297_1	Polymetallic	74.62	0.00	74.62	1	0	1	11	Medium quality	10999	3036078	70.20	342	d__Bacteria;p__Proteobacteria;c__Gammaproteobacteria;o__Burkholderiales;f__Burkholderiaceae;g__Thiomonas;s__
LMSG_G000005417.1	yes	446_1	Polymetallic	86.44	0.10	85.93	4	1	3	18	Medium quality	10824	2997138	57.40	451	d__Bacteria;p__Planctomycetota;c__Phycisphaerae;o__UBA1161;f__;g__;s__
LMSG_G000005418.1	yes	85_1	Polymetallic	85.51	0.00	85.51	1	1	1	19	Medium quality	197561	907420	46.40	14	d__Archaea;p__Micrarchaeota;c__Micrarchaeia;o__Micrarchaeales;f__Micrarchaeaceae;g__;s__
LMSG_G000005419.1	yes	531_1	Polymetallic	88.31	2.62	75.20	1	1	1	17	Medium quality	10455	2306415	57.70	302	d__Bacteria;p__Proteobacteria;c__Alphaproteobacteria;o__Micavibrionales;f__UBA2020;g__PALSA-926;s__
LMSG_G000005420.1	yes	1483_1	Polymetallic	94.52	0.50	92.04	0	0	0	19	Medium quality	24831	2364007	64.00	136	d__Bacteria;p__Proteobacteria;c__Alphaproteobacteria;o__Acetobacterales;f__Acetobacteraceae;g__Acidocella;s__
LMSG_G000005421.1	yes	1328_1	Polymetallic	78.70	2.08	68.28	0	0	0	13	Medium quality	7880	1768595	59.00	303	d__Bacteria;p__Proteobacteria;c__Gammaproteobacteria;o__Burkholderiales;f__Ferrovaceae;g__;s__
LMSG_G000004475.1	yes	603_1	Polymetallic	98.29	1.28	91.88	1	1	1	19	High quality	39778	2625729	54.90	160	d__Bacteria;p__Actinobacteriota;c__Acidimicrobiia;o__Acidimicrobiales;f__Acidimicrobiaceae;g__;s__

LMSG_G000005422.1	yes	814_1	Polymetallic	96.58	1.28	90.17	0	1	0	19	Medium quality	34004	1787398	64.60	91	d__Bacteria;p__Actinobacteriota;c__Acidimicrobiia;o__Acidimicrobiales;f__RAAP-2;g__RAAP-2;s__
LMSG_G000005423.1	yes	1327_1	Polymetallic	91.65	1.61	83.62	0	0	0	15	Medium quality	28712	2052294	59.40	110	d__Bacteria;p__Proteobacteria;c__Gammaproteobacteria;o__Burkholderiales;f__Ferrovaceae;g__;s__
LMSG_G000005424.1	yes	1066_1	Polymetallic	98.85	0.63	95.72	0	1	0	20	Medium quality	81028	2905210	60.90	74	d__Bacteria;p__Proteobacteria;c__Alphaproteobacteria;o__Rhizobiales;f__Beijerinckiaceae;g__Rhodoblastus;s__
LMSG_G000005425.1	yes	310_1	Polymetallic	89.98	0.93	85.36	1	2	2	17	Medium quality	13249	4163858	52.10	455	d__Bacteria;p__Armatimonadota;c__Chthonomonadetes;o__Chthonomonadales;f__Chthonomonadaceae;g__;s__
LMSG_G000006235.1	yes	1337_3	Polymetallic	90.28	0.59	87.32	2	1	0	18	Medium quality	38270	1838445	56.60	89	d__Bacteria;p__Proteobacteria;c__Gammaproteobacteria;o__Burkholderiales;f__Ferrovaceae;g__Ferrovum;s__
LMSG_G000004476.1	yes	788_1	Polymetallic	96.58	1.28	90.17	1	1	1	20	High quality	63622	2224791	61.10	94	d__Bacteria;p__Actinobacteriota;c__Acidimicrobiia;o__Acidimicrobiales;f__RAAP-2;g__RAAP-2;s__
LMSG_G000004477.1	yes	546_1	Polymetallic	97.46	1.60	89.46	1	1	1	20	High quality	55017	1643646	67.20	113	d__Archaea;p__Thermoplasmatota;c__Thermoplasmata;o__UBA184;f__UBA184;g__;s__
LMSG_G000005426.1	yes	474_1	Polymetallic	59.92	0.84	55.73	0	0	0	9	Medium quality	6538	1456987	45.20	293	d__Bacteria;p__Firmicutes;c__Alicyclobacillia;o__Alicyclobacillales;f__Acidibacillaceae;g__;s__
LMSG_G000006278.1	yes	1119_1	Polymetallic	92.75	2.94	78.08	1	1	1	16	Medium quality	8968	2999126	69.10	468	d__Bacteria;p__Proteobacteria;c__Gammaproteobacteria;o__Steroidobacterales;f__Steroidobacteraceae;g__;s__
LMSG_G000004478.1	yes	486_1	Polymetallic	93.87	0.77	90.03	2	2	1	20	High quality	93669	2426571	45.30	70	d__Bacteria;p__Firmicutes;c__Alicyclobacillia;o__Alicyclobacillales;f__Acidibacillaceae;g__;s__
LMSG_G000005427.1	yes	919_1	Polymetallic	89.87	1.68	81.47	0	1	0	16	Medium quality	22802	2969447	60.20	183	d__Bacteria;p__Acidobacteriota;c__Acidobacteriae;o__Acidobacteriales;f__Acidobacteriaceae;g__;s__
LMSG_G000005428.1	yes	1192_1	Polymetallic	90.18	0.23	89.03	0	1	0	19	Medium quality	38412	2825680	52.10	173	d__Bacteria;p__Proteobacteria;c__Gammaproteobacteria;o__Pseudomonadales;f__Moraxellaceae;g__;s__
LMSG_G000005429.1	yes	917_1	Polymetallic	89.20	1.13	83.55	0	1	0	17	Medium quality	21177	3042990	62.90	409	d__Bacteria;p__Acidobacteriota;c__Acidobacteriae;o__Acidobacteriales;f__Acidobacteriaceae;g__;s__
LMSG_G000006236.1	yes	601_1	Polymetallic	96.58	1.28	90.17	1	1	0	19	Medium quality	17329	2475176	52.10	208	d__Bacteria;p__Actinobacteriota;c__Acidimicrobiia;o__Acidimicrobiales;f__Acidimicrobiaceae;g__;s__
LMSG_G000005430.1	yes	1098_1	Polymetallic	86.17	1.30	79.68	0	0	0	14	Medium quality	4091	1489585	42.70	451	d__Bacteria;p__Proteobacteria;c__Alphaproteobacteria;o__Paracaedibacterales;f__UBA11393;g__Finniella;s__
LMSG_G000004479.1	yes	916_1	Polymetallic	93.04	0.00	93.04	1	1	1	18	High quality	22717	3281003	61.10	220	d__Bacteria;p__Acidobacteriota;c__Acidobacteriae;o__Acidobacteriales;f__Acidobacteriaceae;g__;s__
LMSG_G000005431.1	yes	1462_1	Polymetallic	96.55	2.19	85.58	0	0	0	19	Medium quality	30953	3521870	64.30	153	d__Bacteria;p__Proteobacteria;c__Alphaproteobacteria;o__Rhizobiales;f__Beijerinckiaceae;g__Rhodoblastus;s__
LMSG_G000005432.1	yes	1340_0	Polymetallic	57.75	0.00	57.75	1	0	1	9	Medium quality	4057	1143897	58.90	320	d__Bacteria;p__Proteobacteria;c__Gammaproteobacteria;o__Burkholderiales;f__Ferrovaceae;g__Ferrovum;s__
LMSG_G000005433.1	yes	1304_0	Polymetallic	62.44	1.81	53.38	0	0	0	11	Medium quality	8747	1915364	69.70	259	d__Bacteria;p__Proteobacteria;c__Gammaproteobacteria;o__Burkholderiales;f__Burkholderiaceae;g__Thiomonas;s__
LMSG_G000004482.1	yes	1382_1	Polymetallic	98.06	0.65	94.84	1	1	1	19	High quality	172213	6012558	54.70	59	d__Bacteria;p__Desulfobacterota;c__Desulfobacteria;o__Desulfobacterales;f__Desulfosarcinaceae;g__Desulfatitalea;s__
LMSG_G000005434.1	yes	1721_1	Polymetallic	64.49	1.10	58.99	0	0	0	18	Medium quality	9560	688621	45.40	107	d__Bacteria;p__Patescibacteria;c__ABY1;o__Magasanikbacterales;f__GWA2-37-8;g__2-02-FULL-45-10;s__
LMSG_G000004480.1	yes	693_0	Polymetallic	100.00	1.71	91.46	2	2	1	18	High quality	23737	2902560	65.60	189	d__Bacteria;p__Actinobacteriota;c__Acidimicrobiia;o__Acidimicrobiales;f__Acidimicrobiaceae;g__Acidithrix;s__
LMSG_G000004481.1	yes	725_1	Polymetallic	98.13	1.85	88.88	1	1	1	20	High quality	51966	3755288	64.20	138	d__Bacteria;p__Actinobacteriota;c__Acidimicrobiia;o__Acidimicrobiales;f__Palsa-688;g__;s__
LMSG_G000005435.1	yes	1775_0	Polymetallic	58.94	0.00	58.94	1	2	2	20	Medium quality	539823	652612	43.90	18	d__Bacteria;p__Patescibacteria;c__Paceibacteria;o__UBA9983;f__UBA11359_D;g__;s__
LMSG_G000005436.1	yes	414_0	Polymetallic	64.48	0.99	59.53	1	2	1	18	Medium quality	12219	904944	34.20	121	d__Bacteria;p__Patescibacteria;c__Microgenomatia;o__Levybacterales;f__UBA12049;g__PPGL01;s__
LMSG_G000005437.1	yes	1512_1	Polymetallic	95.68	2.62	82.59	0	0	0	19	Medium quality	66533	4288513	69.40	100	d__Bacteria;p__Proteobacteria;c__Alphaproteobacteria;o__Acetobacterales;f__Acetobacteraceae;g__;s__
LMSG_G000005438.1	yes	1793_1	Polymetallic	81.16	0.00	81.16	1	1	3	18	Medium quality	327172	870478	46.60	26	d__Bacteria;p__Patescibacteria;c__Paceibacteria;o__UBA9983;f__UBA9973;g__;s__
LMSG_G000005439.1	yes	1480_1	Polymetallic	98.00	0.50	95.52	0	0	0	19	Medium quality	43739	2534118	57.70	121	d__Bacteria;p__Proteobacteria;c__Alphaproteobacteria;o__Acetobacterales;f__Acetobacteraceae;g__Acidocella;s__
LMSG_G000005440.1	yes	1689_0	Polymetallic	67.21	0.36	65.42	1	1	1	18	Medium quality	86757	713132	33.50	16	d__Archaea;p__Nanoarchaeota;c__Nanoarchaeia;o__Pacearchaeales;f__GW2011-AR1;g__RBG-13-33-26;s__
LMSG_G000004483.1	yes	232_1	Polymetallic	91.81	1.55	84.09	1	1	1	19	High quality	129517	3953239	51.70	135	d__Bacteria;p__Chloroflexota;c__Anaerolineae;o__Anaerolineales;f__EnvOPS12;g__Fen-1038;s__
LMSG_G000005441.1	yes	1664_0	Polymetallic	58.61	1.12	53.03	0	0	0	11	Medium quality	10818	1982157	32.00	274	d__Bacteria;p__Bacteroidota;c__Ignavibacteria;o__Ignavibacteriales;f__Ignavibacteriaceae;g__FEN-1297;s__
LMSG_G000005443.1	yes	1377_0	Polymetallic	65.53	1.56	57.71	1	0	1	14	Medium quality	5344	2539297	71.60	514	d__Bacteria;p__Myxococcota;c__Myxococcia;o__Myxococcales;f__Anaeromyxobacteraceae;g__;s__
LMSG_G000004484.1	yes	436_1	Polymetallic	97.72	0.00	97.72	1	1	1	20	High quality	74756	3671726	55.10	161	d__Bacteria;p__Planctomycetota;c__Phycisphaerae;o__UBA1161;f__;g__;s__
LMSG_G000005442.1	yes	1394_1	Polymetallic	94.87	2.71	81.34	0	1	1	20	Medium quality	22469	3224532	65.50	223	d__Bacteria;p__Acidobacteriota;c__UBA4820;o__UBA4820;f__;g__;s__
LMSG_G000005447.1	yes	1660_1	Polymetallic	96.08	1.17	90.22	0	0	0	18	Medium quality	52858	4430361	40.30	147	d__Bacteria;p__Bacteroidota;c__Ignavibacteria;o__Ignavibacteriales;f__Ignavibacteriaceae;g__FEN-1297;s__
LMSG_G000005444.1	yes	1204_1	Polymetallic	82.05	4.22	60.96	0	1	0	16	Medium quality	6388	3299101	71.50	643	d__Bacteria;p__Acidobacteriota;c__Thermoanaerobaculia;o__Thermoanaerobaculales;f__Thermoanaerobaculaceae;g__RBG-13-68-16;s__
LMSG_G000005445.1	yes	1049_2	Polymetallic	72.61	1.81	63.55	1	1	1	18	Medium quality	3099	6021770	64.90	2229	d__Bacteria;p__Planctomycetota;c__Planctomycetes;o__Isosphaerales;f__Isosphaeraceae;g__;s__
LMSG_G000005446.1	yes	1240_1	Polymetallic	91.63	3.03	76.49	1	0	0	19	Medium quality	32202	3564519	63.80	204	d__Bacteria;p__Proteobacteria;c__Gammaproteobacteria;o__Burkholderiales;f__Sulfuricellaceae;g__;s__
LMSG_G000005448.1	yes	423_0	Polymetallic	65.48	0.00	65.48	1	1	7	18	Medium quality	19907	837052	41.60	70	d__Bacteria;p__Patescibacteria;c__Microgenomatia;o__Curtissbacterales;f__GWA2-41-24;g__;s__
LMSG_G000005449.1	yes	1396_1	Polymetallic	94.85	0.75	91.12	0	1	0	15	Medium quality	22871	3835391	68.90	241	d__Bacteria;p__Proteobacteria;c__Alphaproteobacteria;o__Rhodospirillales;f__Magnetospirillaceae;g__;s__
LMSG_G000004485.1	yes	835_0	Polymetallic	93.23	1.97	83.41	1	1	1	19	High quality	12066	3452387	66.30	398	d__Bacteria;p__Actinobacteriota;c__Thermoleophilia;o__Solirubrobacterales;f__Solirubrobacteraceae;g__Palsa-465;s__
LMSG_G000005450.1	yes	1623_0	Polymetallic	79.31	1.35	72.56	1	0	1	17	Medium quality	6040	1321839	47.00	242	d__Bacteria;p__Verrucomicrobiota_A;c__Chlamydiia;o__Parachlamydiales;f__SM23-39;g__;s__
LMSG_G000005453.1	yes	808_4	Polymetallic	87.38	1.28	80.97	1	1	2	18	Medium quality	28751	2059961	73.10	187	d__Bacteria;p__Actinobacteriota;c__Acidimicrobiia;o__Acidimicrobiales;f__RAAP-2;g__RAAP-2;s__
LMSG_G000005451.1	yes	1674_1	Polymetallic	71.99	3.35	55.24	0	1	0	14	Medium quality	8426	2528013	36.30	406	d__Bacteria;p__Bacteroidota;c__Ignavibacteria;o__Ignavibacteriales;f__Melioribacteraceae;g__Fen-1258;s__
LMSG_G000005452.1	yes	1821_1	Polymetallic	71.91	1.12	66.30	1	1	1	20	Medium quality	38671	567617	55.10	20	d__Bacteria;p__Patescibacteria;c__Paceibacteria;o__UBA6257;f__GWB1-50-10;g__;s__
LMSG_G000006319.1	yes	1683_1	Polymetallic	64.71	0.93	60.04	0	0	0	18	Medium quality	17359	582626	34.30	65	d__Archaea;p__Nanoarchaeota;c__Nanoarchaeia;o__Pacearchaeales;f__GW2011-AR1;g__GW2011-AR1;s__
LMSG_G000006279.1	yes	1373_1	Polymetallic	94.87	1.64	86.70	1	1	1	16	Medium quality	14946	3450843	74.80	319	d__Bacteria;p__Myxococcota;c__Myxococcia;o__Myxococcales;f__Anaeromyxobacteraceae;g__Anaeromyxobacter;s__
LMSG_G000004486.1	yes	1748_3	Polymetallic	91.57	1.12	85.96	1	1	1	18	High quality	52116	1987406	32.50	77	d__Bacteria;p__SZUA-79;c__SZUA-79;o__Acidulodesulfobacterales;f__SZUA-79;g__Acididesulfobacter;s__
LMSG_G000005462.1	yes	1269_1	Polymetallic	84.27	1.52	76.66	1	1	0	17	Medium quality	30280	2318766	66.50	108	d__Bacteria;p__Proteobacteria;c__Gammaproteobacteria;o__Burkholderiales;f__Burkholderiaceae;g__GJ-E10;s__
LMSG_G000005456.1	yes	422_1	Polymetallic	66.55	0.00	66.55	1	0	20	20	Medium quality	67588	939449	49.70	67	d__Bacteria;p__Patescibacteria;c__Doudnabacteria;o__UBA920;f__UBA920;g__PALSA-1336;s__
LMSG_G000005454.1	yes	463_1	Polymetallic	67.24	1.72	58.62	0	1	0	14	Medium quality	23848	4137235	60.20	386	d__Bacteria;p__Desulfobacterota;c__Syntrophia;o__Syntrophales;f__UBA5619;g__UBA5619;s__
LMSG_G000005455.1	yes	409_1	Polymetallic	72.22	0.00	72.22	1	1	8	19	Medium quality	877503	1313170	50.00	35	d__Bacteria;p__Patescibacteria;c__ABY1;o__UBA10025;f__UBA10025;g__;s__
LMSG_G000006280.1	yes	1564_1	Polymetallic	94.59	1.75	85.87	1	1	1	16	Medium quality	8098	2996822	41.60	451	d__Bacteria;p__Bacteroidota;c__Ignavibacteria;o__Ignavibacteriales;f__Melioribacteraceae;g__;s__
LMSG_G000005457.1	yes	1779_1	Polymetallic	74.15	0.00	74.15	1	2	1	18	Medium quality	230391	734660	41.60	27	d__Bacteria;p__Patescibacteria;c__Paceibacteria;o__UBA6257;f__UBA9933;g__;s__
LMSG_G000004487.1	yes	1068_1	Polymetallic	98.83	0.00	98.83	1	2	10	20	High quality	95916	3918278	59.00	145	d__Bacteria;p__Zixibacteria;c__MSB-5A5;o__;f__;g__;s__
LMSG_G000005458.1	yes	1113_1	Polymetallic	84.03	1.03	78.91	1	0	1	15	Medium quality	6582	2102745	56.40	404	d__Bacteria;p__Nitrospinota_A;c__UBA7883;o__;f__;g__;s__
LMSG_G000005459.1	yes	1701_1	Polymetallic	64.52	0.00	64.52	4	1	10	19	Medium quality	115701	1357368	49.00	39	d__Bacteria;p__Patescibacteria;c__Microgenomatia;o__UBA10105;f__UBA927;g__UBA927;s__
LMSG_G000006237.1	yes	536_1	Polymetallic	91.57	2.15	80.82	1	1	0	19	Medium quality	13916	3637844	43.30	426	d__Bacteria;p__Bacteroidota;c__Bacteroidia;o__Bacteroidales;f__Prolixibacteraceae;g__;s__
LMSG_G000005460.1	yes	1364_0	Polymetallic	62.47	0.81	58.42	0	0	0	11	Medium quality	13972	2109196	59.60	266	d__Bacteria;p__Desulfobacterota;c__Desulfobaccia;o__Desulfobaccales;f__0-14-0-80-60-11;g__SURF-52;s__
LMSG_G000006238.1	yes	1565_1	Polymetallic	100.00	0.61	96.96	1	2	0	19	Medium quality	66415	2848672	67.30	96	d__Bacteria;p__Proteobacteria;c__Gammaproteobacteria;o__Burkholderiales;f__Thiobacillaceae;g__UBA3361;s__
LMSG_G000005461.1	yes	110_1	Polymetallic	70.46	0.09	70.01	1	1	3	19	Medium quality	11445	1093895	46.60	124	d__Bacteria;p__Patescibacteria;c__Microgenomatia;o__Levybacterales;f__UBA12049;g__;s__
LMSG_G000005463.1	yes	1455_0	Polymetallic	88.38	1.45	81.13	0	0	0	15	Medium quality	6422	3432165	64.00	683	d__Bacteria;p__Desulfobacterota;c__Desulfobacteria;o__Desulfobacterales;f__Desulfatibacillaceae;g__;s__
LMSG_G000005464.1	yes	524_0	Polymetallic	83.68	1.36	76.87	0	0	0	15	Medium quality	6760	1733362	55.40	298	d__Bacteria;p__Nitrospirota;c__;o__;f__;g__;s__
LMSG_G000005465.1	yes	1354_1	Polymetallic	85.23	2.53	72.59	1	0	0	17	Medium quality	5211	2133940	62.90	493	d__Bacteria;p__Desulfobacterota;c__Desulfobulbia;o__Desulfobulbales;f__Desulfurivibrionaceae;g__;s__
LMSG_G000005469.1	yes	930_1	Polymetallic	94.12	2.00	84.12	0	2	1	18	Medium quality	32147	2962139	55.50	125	d__Bacteria;p__Nitrospirota;c__Thermodesulfovibrionia;o__Thermodesulfovibrionales;f__UBA6898;g__Sulfobium;s__
LMSG_G000005466.1	yes	1411_1	Polymetallic	89.44	2.67	76.11	1	0	0	17	Medium quality	8248	3583707	61.30	528	d__Bacteria;p__Chloroflexota;c__Anaerolineae;o__Thermoflexales;f__J036;g__;s__

LMSG_G000005467.1	yes	957_1	Polymetallic	88.97	1.82	79.88	1	0	1	16	Medium quality	43135	2635055	59.70	224	d__Bacteria;p__Nitrospirota;c__Leptospirillia;o__Leptospirillales;f__Leptospirillaceae;g__UBA4572;s__
LMSG_G000006281.1	yes	1657_1	Polymetallic	95.43	1.68	87.06	1	1	1	15	Medium quality	20258	3146429	40.30	285	d__Bacteria;p__Bacteroidota;c__Ignavibacteria;o__Ignavibacteriales;f__Melioribacteraceae;g__Fen-1258;s__
LMSG_G000005468.1	yes	1183_1	Polymetallic	89.51	1.45	82.24	0	0	0	18	Medium quality	46103	2805542	63.60	112	d__Bacteria;p__Elusimicrobiota;c__Elusimicrobia;o__Elusimicrobiales;f__UBA9959;g__UBA9959;s__
LMSG_G000005470.1	yes	1424_0	Polymetallic	97.88	0.41	95.84	0	0	0	18	Medium quality	128232	3306712	64.30	44	d__Bacteria;p__Proteobacteria;c__Alphaproteobacteria;o__Sphingomonadales;f__Sphingomonadaceae;g__Novosphingobium;s__Novosphingobium sp003171715
LMSG_G000005472.1	yes	349_0	Polymetallic	65.80	0.99	60.85	0	0	0	7	Medium quality	2395	1604915	68.80	763	d__Bacteria;p__Firmicutes_E;c__Sulfobacillia;o__;f__;g__;s__
LMSG_G000005471.1	yes	1268_1	Polymetallic	83.71	1.23	77.54	1	0	0	18	Medium quality	55116	2111089	65.60	67	d__Bacteria;p__Proteobacteria;c__Gammaproteobacteria;o__Burkholderiales;f__Burkholderiaceae;g__GJ-E10;s__
LMSG_G000006239.1	yes	1381_1	Polymetallic	97.71	2.10	87.23	1	1	0	18	Medium quality	17417	5690767	59.70	522	d__Bacteria;p__Desulfobacterota;c__Desulfobacteria;o__Desulfobacterales;f__Desulfosarcinaceae;g__Desulfatitalea;s__
LMSG_G000004488.1	yes	1663_1	Polymetallic	99.44	0.84	95.26	1	1	1	18	High quality	154490	3958188	32.10	58	d__Bacteria;p__Bacteroidota;c__Ignavibacteria;o__Ignavibacteriales;f__Ignavibacteriaceae;g__FEN-1297;s__
LMSG_G000004489.1	yes	182_1	Polymetallic	96.32	0.00	96.32	1	1	2	19	High quality	122957	1366973	41.50	40	d__Archaea;p__Thermoplasmatota;c__Thermoplasmata;o__Thermoplasmatales;f__Thermoplasmataceae;g__Cuniculiplasma;s__
LMSG_G000005473.1	yes	1802_1	Polymetallic	80.14	0.00	80.14	0	0	0	20	Medium quality	19916	694128	38.00	72	d__Bacteria;p__Patescibacteria;c__Paceibacteria;o__UBA6257;f__GWB1-50-10;g__;s__
LMSG_G000005474.1	yes	1365_1	Polymetallic	78.02	2.03	67.89	1	1	2	20	Medium quality	9868	3528068	66.60	455	d__Bacteria;p__Desulfobacterota;c__Desulfarculia;o__Desulfarculales;f__Desulfarculaceae;g__;s__
LMSG_G000005475.1	yes	444_1	Polymetallic	73.12	3.56	55.35	1	1	2	13	Medium quality	2636	3637929	53.40	1545	d__Bacteria;p__Chloroflexota;c__Dehalococcoidia;o__FW602-bin16;f__;g__;s__
LMSG_G000005476.1	yes	1323_1	Polymetallic	76.82	2.28	65.44	1	0	1	17	Medium quality	3718	1881045	58.50	553	d__Bacteria;p__Proteobacteria;c__Gammaproteobacteria;o__Burkholderiales;f__RBG-16-58-11;g__RBG-16-58-11;s__
LMSG_G000006240.1	yes	843_1	Polymetallic	92.21	0.00	92.21	1	1	0	20	Medium quality	69697	1947976	65.30	62	d__Bacteria;p__Actinobacteriota;c__Actinomycetia;o__Nanopelagicales;f__UBA5976;g__;s__
LMSG_G000005477.1	yes	1820_1	Polymetallic	56.43	0.00	56.43	1	1	2	17	Medium quality	32164	693940	46.10	42	d__Bacteria;p__Patescibacteria;c__ABY1;o__BM507;f__UBA917;g__;s__
LMSG_G000005478.1	yes	1700_1	Polymetallic	80.30	1.25	74.04	1	2	7	20	Medium quality	146391	931674	45.30	114	d__Bacteria;p__Patescibacteria;c__Paceibacteria;o__UBA9983;f__UBA1539;g__UBA1550;s__
LMSG_G000005479.1	yes	981_1	Polymetallic	84.29	1.86	74.98	2	2	0	18	Medium quality	11720	1914948	57.90	200	d__Bacteria;p__Proteobacteria;c__Gammaproteobacteria;o__Acidithiobacillales;f__Acidithiobacillaceae;g__Acidithiobacillus;s__
LMSG_G000005480.1	yes	1707_0	Polymetallic	55.95	0.85	51.68	1	1	1	17	Medium quality	15145	564977	60.80	44	d__Bacteria;p__Patescibacteria;c__Paceibacteria;o__UBA9983;f__UBA2163;g__;s__
LMSG_G000005481.1	yes	1134_1	Polymetallic	89.50	2.55	76.75	0	2	0	19	Medium quality	13065	3651021	52.80	362	d__Bacteria;p__Bacteroidota;c__Kapabacteria;o__Palsa-1295;f__Palsa-1295;g__PALSA-1295;s__
LMSG_G000005482.1	yes	1463_1	Polymetallic	97.33	1.94	87.62	0	0	0	19	Medium quality	68865	3576184	64.30	86	d__Bacteria;p__Proteobacteria;c__Alphaproteobacteria;o__Rhizobiales;f__Beijerinckiaceae;g__Rhodoblastus;s__
LMSG_G000005483.1	yes	1583_1	Polymetallic	64.62	0.00	64.62	0	0	0	18	Medium quality	10258	820936	46.00	109	d__Bacteria;p__Patescibacteria;c__UBA1384;o__;f__;g__;s__
LMSG_G000005484.1	yes	1753_1	Polymetallic	79.98	1.12	74.37	1	2	2	20	Medium quality	105963	816083	49.20	10	d__Bacteria;p__Patescibacteria;c__Paceibacteria;o__UBA6257;f__GWB1-50-10;g__;s__
LMSG_G000005485.1	yes	678_2	Polymetallic	66.37	0.00	66.37	1	0	1	13	Medium quality	12619	1882849	73.50	194	d__Bacteria;p__Chloroflexota;c__Ellin6529;o__CSP1-4;f__CSP1-4;g__;s__
LMSG_G000004490.1	yes	909_1	Polymetallic	94.12	1.72	85.50	1	1	1	18	High quality	39035	4098938	59.50	172	d__Bacteria;p__Acidobacteriota;c__Acidobacteriae;o__Acidobacteriales;f__Acidobacteriaceae;g__Terracidiphilus;s__
LMSG_G000005486.1	yes	1796_1	Polymetallic	74.29	1.12	68.68	0	1	1	20	Medium quality	50649	548759	42.50	22	d__Bacteria;p__Patescibacteria;c__Paceibacteria;o__UBA6257;f__GWB1-50-10;g__;s__
LMSG_G000005487.1	yes	1059_1	Polymetallic	58.44	0.00	58.44	1	0	0	17	Medium quality	5957	516019	37.20	109	d__Archaea;p__Nanoarchaeota;c__Nanoarchaeia;o__Pacearchaeales;f__UBA73;g__UBA73;s__
LMSG_G000004491.1	yes	1519_1	Polymetallic	93.91	0.87	89.57	1	1	2	19	High quality	33983	5522967	63.40	269	d__Bacteria;p__Acidobacteriota;c__Acidobacteriae;o__Bryobacterales;f__Bryobacteraceae;g__;s__
LMSG_G000005488.1	yes	1674_2	Polymetallic	72.75	4.31	51.20	2	0	2	17	Medium quality	7504	3589826	36.50	643	d__Bacteria;p__Bacteroidota;c__Ignavibacteria;o__Ignavibacteriales;f__Melioribacteraceae;g__Fen-1258;s__
LMSG_G000005491.1	yes	949_1	Polymetallic	94.18	1.82	85.09	0	2	0	19	Medium quality	64096	3025127	57.10	87	d__Bacteria;p__Nitrospirota;c__Thermodesulfovibrionia;o__Thermodesulfovibrionales;f__JdFR-88;g__;s__
LMSG_G000005489.1	yes	22_1	Polymetallic	81.69	0.93	77.02	1	4	1	19	Medium quality	167891	861953	39.80	22	d__Archaea;p__Nanoarchaeota;c__Nanoarchaeia;o__Parvarchaeales;f__Parvarchaeaceae;g__;s__
LMSG_G000005490.1	yes	1218_2	Polymetallic	94.60	1.18	88.68	1	0	1	17	Medium quality	35535	2079637	58.00	91	d__Bacteria;p__Proteobacteria;c__Gammaproteobacteria;o__Burkholderiales;f__Nitrosomonadaceae;g__;s__
LMSG_G000004492.1	yes	520_1	Polymetallic	93.89	2.09	83.44	1	3	2	19	High quality	12585	3989485	65.40	442	d__Bacteria;p__Chloroflexota;c__Ktedonobacteria;o__Ktedonobacterales;f__;g__;s__
LMSG_G000005492.1	yes	932_1	Polymetallic	79.79	2.11	69.26	1	1	2	12	Medium quality	14062	2744318	53.60	255	d__Bacteria;p__Nitrospirota;c__Thermodesulfovibrionia;o__Thermodesulfovibrionales;f__UBA6898;g__Sulfobium;s__
LMSG_G000004493.1	yes	929_1	Polymetallic	90.42	1.82	81.33	1	2	1	18	High quality	86011	3370673	57.30	66	d__Bacteria;p__Nitrospirota;c__Thermodesulfovibrionia;o__Thermodesulfovibrionales;f__UBA6898;g__Sulfobium;s__
LMSG_G000005493.1	yes	1761_1	Polymetallic	69.80	0.00	69.80	1	2	2	19	Medium quality	19159	674331	61.10	51	d__Bacteria;p__Patescibacteria;c__Paceibacteria;o__UBA9983;f__UBA2163;g__C7867-001;s__
LMSG_G000005494.1	yes	297_0	Polymetallic	66.13	0.00	66.13	0	0	0	16	Medium quality	8040	872772	51.00	167	d__Bacteria;p__Patescibacteria;c__ABY1;o__BM507;f__GWE2-39-37;g__;s__
LMSG_G000005495.1	yes	1178_1	Polymetallic	72.42	2.25	61.19	1	1	0	14	Medium quality	7086	2849914	59.50	515	d__Bacteria;p__Elusimicrobiota;c__Elusimicrobia;o__Elusimicrobiales;f__UBA9959;g__UBA9959;s__
LMSG_G000005496.1	yes	1302_2	Polymetallic	70.30	1.72	61.72	0	0	0	15	Medium quality	10329	2031250	70.20	214	d__Bacteria;p__Proteobacteria;c__Gammaproteobacteria;o__Burkholderiales;f__Burkholderiaceae;g__Thiomonas;s__
LMSG_G000005497.1	yes	1251_1	Polymetallic	82.04	3.33	65.38	2	2	0	17	Medium quality	12023	3551781	64.40	354	d__Bacteria;p__Proteobacteria;c__Gammaproteobacteria;o__Burkholderiales;f__SG8-39;g__2-12-FULL-64-23;s__
LMSG_G000005498.1	yes	948_0	Polymetallic	74.54	2.27	63.18	2	0	2	18	Medium quality	96547	2631856	52.50	58	d__Bacteria;p__Nitrospirota;c__Thermodesulfovibrionia;o__Thermodesulfovibrionales;f__JdFR-88;g__;s__
LMSG_G000005499.1	yes	1835_1	Polymetallic	65.84	1.98	55.94	0	1	2	17	Medium quality	21894	661880	39.10	59	d__Bacteria;p__Patescibacteria;c__Doudnabacteria;o__UBA920;f__;g__;s__
LMSG_G000005500.1	yes	1662_1	Polymetallic	98.60	0.56	95.81	0	1	0	18	Medium quality	29011	3071048	37.50	170	d__Bacteria;p__Bacteroidota;c__Ignavibacteria;o__Ignavibacteriales;f__Ignavibacteriaceae;g__;s__
LMSG_G000004494.1	yes	247_1	Polymetallic	91.97	0.00	91.97	1	1	1	20	High quality	157874	1146225	55.50	21	d__Archaea;p__Micrarchaeota;c__Micrarchaeia;o__UBA10214;f__UBA10161;g__;s__
LMSG_G000005501.1	yes	1722_1	Polymetallic	73.06	0.00	73.06	1	1	0	19	Medium quality	41667	988802	33.10	32	d__Bacteria;p__Patescibacteria;c__Microgenomatia;o__UBA1406;f__GWC2-37-13;g__GWC2-37-13;s__
LMSG_G000005502.1	yes	1606_0	Polymetallic	73.69	1.49	66.26	0	1	0	17	Medium quality	5850	1362796	38.50	266	d__Bacteria;p__Verrucomicrobiota_A;c__Chlamydiia;o__Parachlamydiales;f__Rhabdochlamydiaceae;g__;s__
LMSG_G000005503.1	yes	1836_0	Polymetallic	60.39	1.19	54.45	1	1	2	18	Medium quality	26002	555027	38.20	50	d__Bacteria;p__Patescibacteria;c__Doudnabacteria;o__UBA920;f__;g__;s__
LMSG_G000005504.1	yes	794_1	Polymetallic	97.43	2.14	86.75	1	0	0	20	Medium quality	27739	1937627	68.80	124	d__Bacteria;p__Actinobacteriota;c__Acidimicrobiia;o__Acidimicrobiales;f__RAAP-2;g__RAAP-2;s__
LMSG_G000005505.1	yes	1726_0	Polymetallic	62.92	0.00	62.92	0	0	1	14	Medium quality	2701	1977973	48.30	809	d__Bacteria;p__Chloroflexota;c__Dehalococcoidia;o__GIF9;f__;g__;s__
LMSG_G000005506.1	yes	1363_1	Polymetallic	96.77	2.60	83.76	1	0	1	20	Medium quality	24776	4110973	58.20	222	d__Bacteria;p__Desulfobacterota;c__Desulfobaccia;o__Desulfobaccales;f__0-14-0-80-60-11;g__SURF-52;s__
LMSG_G000005507.1	yes	245_0	Polymetallic	59.53	1.82	50.44	0	1	0	13	Medium quality	9820	1769357	50.50	242	d__Bacteria;p__Chloroflexota;c__Anaerolineae;o__Anaerolineales;f__Anaerolineaceae;g__UBA6107;s__
LMSG_G000005508.1	yes	298_1	Polymetallic	76.23	2.33	64.56	1	1	2	16	Medium quality	4842	2796442	46.90	738	d__Bacteria;p__Bacteroidota;c__UBA10030;o__UBA10030;f__UBA8401;g__UBA8401;s__
LMSG_G000005510.1	yes	248_1	Polymetallic	83.17	1.40	76.17	1	0	1	20	Medium quality	35964	1300279	58.60	86	d__Archaea;p__Micrarchaeota;c__Micrarchaeia;o__UBA10214;f__UBA10161;g__;s__
LMSG_G000005509.1	yes	1723_1	Polymetallic	72.21	2.25	60.98	1	3	3	20	Medium quality	29800	830809	42.90	55	d__Bacteria;p__Patescibacteria;c__Paceibacteria;o__UBA6257;f__2-01-FULL-56-20;g__;s__
LMSG_G000004495.1	yes	947_1	Polymetallic	99.09	1.82	90.00	1	2	3	19	High quality	101759	2979829	54.40	56	d__Bacteria;p__Nitrospirota;c__Thermodesulfovibrionia;o__Thermodesulfovibrionales;f__JdFR-88;g__;s__
LMSG_G000004496.1	yes	680_1	Polymetallic	94.90	0.93	90.28	1	1	1	20	High quality	31004	2738765	71.90	176	d__Bacteria;p__Chloroflexota;c__Ellin6529;o__CSP1-4;f__CSP1-4;g__;s__
LMSG_G000005515.1	yes	1578_1	Polymetallic	91.20	3.76	72.39	0	1	0	19	Medium quality	59091	3243331	67.70	127	d__Bacteria;p__Gemmatimonadota;c__Gemmatimonadetes;o__Gemmatimonadales;f__Gemmatimonadaceae;g__Fen-1231;s__
LMSG_G000006241.1	yes	1658_1	Polymetallic	94.97	1.17	89.13	1	1	0	18	Medium quality	19218	2594254	29.20	176	d__Bacteria;p__Bacteroidota;c__Ignavibacteria;o__Ignavibacteriales;f__Melioribacteraceae;g__XYB12-FULL-38-5;s__
LMSG_G000005511.1	yes	1670_0	Polymetallic	77.68	3.74	58.97	0	0	0	10	Medium quality	16764	3064111	40.20	306	d__Bacteria;p__Bacteroidota;c__Ignavibacteria;o__Ignavibacteriales;f__Melioribacteraceae;g__;s__
LMSG_G000005512.1	yes	1434_1	Polymetallic	57.91	0.00	57.91	0	0	0	12	Medium quality	11531	2172460	69.00	237	d__Bacteria;p__Proteobacteria;c__Alphaproteobacteria;o__Rhodobacterales;f__Rhodobacteraceae;g__Allgaiera;s__
LMSG_G000005513.1	yes	280_1	Polymetallic	60.83	0.59	57.91	1	1	1	19	Medium quality	24145	852269	37.30	53	d__Bacteria;p__Patescibacteria;c__Microgenomatia;o__Levybacterales;f__UBA12049;g__;s__
LMSG_G000005514.1	yes	1661_1	Polymetallic	90.50	0.56	87.71	0	1	0	18	Medium quality	79294	3833254	36.90	125	d__Bacteria;p__Bacteroidota;c__Ignavibacteria;o__Ignavibacteriales;f__Ignavibacteriaceae;g__FEN-1297;s__
LMSG_G000005518.1	yes	1702_1	Polymetallic	67.45	0.47	65.10	0	0	0	17	Medium quality	7455	579285	50.30	102	d__Bacteria;p__Patescibacteria;c__Paceibacteria;o__UBA9983;f__UBA11359_A;g__;s__
LMSG_G000004497.1	yes	1358_1	Polymetallic	95.00	0.00	95.00	1	1	1	19	High quality	39001	3678741	59.30	134	d__Bacteria;p__Nitrospirota;c__UBA9217;o__UBA9217;f__;g__;s__
LMSG_G000005516.1	yes	526_1	Polymetallic	95.24	0.00	95.24	0	0	0	19	Medium quality	45366	3600461	35.10	130	d__Bacteria;p__Bacteroidota;c__Kapabacteria;o__Kapabacteriales;f__XYC2-FULL-35-21;g__XYC2-FULL-35-21;s__
LMSG_G000005517.1	yes	1777_0	Polymetallic	66.24	3.25	50.01	0	0	0	17	Medium quality	16990	647383	34.60	135	d__Bacteria;p__Patescibacteria;c__Paceibacteria;o__UBA9983;f__UBA9973;g__UBA9973;s__

LMSG_G000004498.1	yes	1665_1	Polymetallic	94.97	2.23	83.80	1	1	1	19	High quality	162326	4709277	36.30	61	d__Bacteria;p__Bacteroidota;c__Ignavibacteria;o__Ignavibacteriales;f__Ignavibacteriaceae;g__FEN-1297;s__
LMSG_G000005519.1	yes	668_1	Polymetallic	91.43	3.70	72.92	0	0	0	19	Medium quality	32904	3446657	72.30	193	d__Bacteria;p__Chloroflexota;c__Ellin6529;o__CSP1-4;f__CSP1-4;g__;s__
LMSG_G000005520.1	yes	1828_1	Polymetallic	71.84	0.66	68.53	0	1	1	19	Medium quality	12032	603486	42.30	69	d__Bacteria;p__Patescibacteria;c__Paceibacteria;o__UBA6257;f__GWB1-50-10;g__;s__
LMSG_G000004499.1	yes	1601_0	Polymetallic	98.01	2.63	84.84	1	1	1	19	High quality	44305	4716215	49.30	209	d__Bacteria;p__Desulfobacterota;c__Desulfobacteria;o__Desulfobacterales;f__Desulfobacteraceae;g__Desulfobacula;s__
LMSG_G000005521.1	yes	417_0	Polymetallic	56.60	0.00	56.60	1	1	1	17	Medium quality	17179	798727	41.90	61	d__Bacteria;p__Patescibacteria;c__Microgenomatia;o__GWA2-44-7;f__UBA8517;g__UBA5941;s__
LMSG_G000005522.1	yes	504_0	Polymetallic	99.19	1.77	90.32	2	0	1	19	Medium quality	32183	5501895	56.20	260	d__Bacteria;p__Desulfobacterota;c__Syntrophobacteria;o__Syntrophobacterales;f__Syntrophobacteraceae;g__SbD1;s__
LMSG_G000004500.1	yes	945_1	Polymetallic	98.18	0.00	98.18	1	2	1	19	High quality	91848	2856903	53.10	54	d__Bacteria;p__Nitrospirota;c__Thermodesulfovibrionia;o__Thermodesulfovibrionales;f__JdFR-88;g__;s__
LMSG_G000005523.1	yes	1659_0	Polymetallic	74.15	0.00	74.15	0	1	0	11	Medium quality	8904	3279184	35.00	432	d__Bacteria;p__Bacteroidota;c__Ignavibacteria;o__Ignavibacteriales;f__Melioribacteraceae;g__XYB12-FULL-38-5;s__
LMSG_G000004501.1	yes	173_1	Polymetallic	96.46	0.00	96.46	1	1	2	19	High quality	51671	1505636	47.80	57	d__Archaea;p__Thermoplasmatota;c__Thermoplasmata;o__Thermoplasmatales;f__Thermoplasmataceae;g__UBA582;s__
LMSG_G000005524.1	yes	328_1	Polymetallic	77.27	0.00	77.27	0	1	0	20	Medium quality	143751	911353	39.30	25	d__Bacteria;p__Patescibacteria;c__Microgenomatia;o__Shapirobacterales;f__UBA12405;g__;s__
LMSG_G000004502.1	yes	213_1	Polymetallic	90.61	1.53	82.96	1	1	2	20	High quality	14138	1327449	41.10	151	d__Archaea;p__Thermoproteota;c__Nitrososphaeria;o__Nitrososphaerales;f__Nitrosopumilaceae;g__Nitrosotenuis;s__
LMSG_G000005525.1	yes	1675_0	Polymetallic	71.04	4.19	50.10	0	0	0	12	Medium quality	5891	2714897	38.10	530	d__Bacteria;p__Bacteroidota;c__Ignavibacteria;o__Ignavibacteriales;f__Melioribacteraceae;g__XYB12-FULL-38-5;s__
LMSG_G000006320.1	yes	1786_1	Polymetallic	68.61	0.00	68.61	1	1	3	20	Medium quality	55995	676565	45.50	31	d__Bacteria;p__Patescibacteria;c__Paceibacteria;o__Portnoybacterales;f__;g__;s__
LMSG_G000005526.1	yes	1582_1	Polymetallic	85.04	0.00	85.04	1	1	7	20	Medium quality	367392	1670748	43.30	89	d__Archaea;p__Nanoarchaeota;c__Nanoarchaeia;o__Woesearchaeales;f__UBA9642;g__UBA9642;s__
LMSG_G000006282.1	yes	934_1	Polymetallic	95.45	2.45	83.18	1	1	2	16	Medium quality	30792	3072217	55.60	146	d__Bacteria;p__Nitrospirota;c__Thermodesulfovibrionia;o__Thermodesulfovibrionales;f__UBA6898;g__PALSA-1316;s__
LMSG_G000004503.1	yes	275_1	Polymetallic	99.75	0.03	99.59	2	1	1	20	High quality	51364	3099848	37.10	98	d__Bacteria;p__Bacteroidota;c__Bacteroidia;o__Chitinophagales;f__Chitinophagaceae;g__Hydrotalea;s__
LMSG_G000005527.1	yes	1797_1	Polymetallic	74.05	1.12	68.44	0	1	1	18	Medium quality	22512	756786	51.20	43	d__Bacteria;p__Patescibacteria;c__Paceibacteria;o__UBA6257;f__GWB1-50-10;g__;s__
LMSG_G000004504.1	yes	1648_1	Polymetallic	98.88	1.64	90.69	1	1	1	18	High quality	96584	3536541	53.10	62	d__Bacteria;p__Bacteroidota;c__Kryptonia;o__Kryptoniales;f__;g__;s__
LMSG_G000005528.1	yes	347_0	Polymetallic	85.14	1.32	78.54	1	0	0	17	Medium quality	13099	2794329	67.00	254	d__Bacteria;p__Chloroflexota;c__Dehalococcoidia;o__UBA2991;f__UBA2991;g__;s__
LMSG_G000005529.1	yes	1464_1	Polymetallic	97.97	3.38	81.08	1	0	1	18	Medium quality	42560	4541965	56.30	161	d__Bacteria;p__Verrucomicrobiota;c__Verrucomicrobiae;o__Pedosphaerales;f__Pedosphaeraceae;g__UBA11358;s__
LMSG_G000005530.1	yes	1671_0	Polymetallic	78.44	1.72	69.82	0	1	0	14	Medium quality	6241	2895847	37.70	517	d__Bacteria;p__Bacteroidota;c__Ignavibacteria;o__Ignavibacteriales;f__Ignavibacteriaceae;g__FEN-1297;s__
LMSG_G000005531.1	yes	1829_0	Polymetallic	55.69	0.41	53.66	1	1	2	20	Medium quality	37214	924137	45.60	41	d__Bacteria;p__Dependentiae;c__Babeliae;o__Babeliales;f__UBA12409;g__;s__
LMSG_G000005532.1	yes	1798_1	Polymetallic	75.65	1.19	69.71	1	0	1	19	Medium quality	14433	633374	51.80	65	d__Bacteria;p__Patescibacteria;c__Paceibacteria;o__UBA6257;f__GWB1-50-10;g__;s__
LMSG_G000004505.1	yes	1062_1	Polymetallic	98.36	2.46	86.07	1	2	1	20	High quality	274589	3313950	60.90	20	d__Bacteria;p__Bacteroidota;c__UBA10030;o__UBA10030;f__UBA10030;g__;s__
LMSG_G000005533.1	yes	501_0	Polymetallic	72.27	0.99	67.32	1	1	1	20	Medium quality	34140	1402056	62.60	67	d__Bacteria;p__Patescibacteria;c__ABY1;o__SG8-24;f__SG8-24;g__;s__
LMSG_G000005536.1	yes	1343_1	Polymetallic	78.44	0.65	75.22	0	1	0	16	Medium quality	8949	2637031	60.30	464	d__Bacteria;p__Desulfobacterota;c__Desulfuromonadia;o__Geobacterales;f__Pseudopelobacteraceae;g__Pseudopelobacter;s__
LMSG_G000005534.1	yes	578_0	Polymetallic	90.88	4.44	68.66	0	0	0	16	Medium quality	21401	3653429	66.70	289	d__Bacteria;p__Acidobacteriota;c__Vicinamibacteria;o__Vicinamibacterales;f__SCN-69-37;g__SCUN01;s__
LMSG_G000005535.1	yes	253_1	Polymetallic	98.70	1.54	91.02	1	0	0	20	Medium quality	23170	2230701	57.30	182	d__Bacteria;p__Firmicutes_B;c__Desulfotomaculia;o__Ammonifexales;f__Desulforudaceae;g__SURF-60;s__SURF-60 sp003604975
LMSG_G000006283.1	yes	1311_1	Polymetallic	92.75	4.34	71.07	1	1	1	17	Medium quality	10149	3640959	70.00	506	d__Bacteria;p__Proteobacteria;c__Gammaproteobacteria;o__Burkholderiales;f__SG8-39;g__;s__
LMSG_G000005537.1	yes	1801_1	Polymetallic	78.92	0.00	78.92	1	2	1	20	Medium quality	16063	753643	42.60	68	d__Bacteria;p__Patescibacteria;c__Paceibacteria;o__UBA6257;f__GWB1-50-10;g__;s__
LMSG_G000005538.1	yes	510_1	Polymetallic	93.45	0.00	93.45	0	1	3	19	Medium quality	42681	1769459	51.20	69	d__Archaea;p__Micrarchaeota;c__Micrarchaeia;o__B9-G16;f__;g__;s__
LMSG_G000005539.1	yes	675_0	Polymetallic	52.58	0.00	52.58	0	0	0	13	Medium quality	7714	1453467	68.20	279	d__Bacteria;p__Chloroflexota;c__Ellin6529;o__CSP1-4;f__CSP1-4;g__Palsa-1032;s__
LMSG_G000005540.1	yes	844_1	Polymetallic	91.68	1.43	84.54	0	1	0	20	Medium quality	50020	2076238	69.40	73	d__Bacteria;p__Actinobacteriota;c__Actinomycetia;o__Nanopelagicales;f__UBA5976;g__;s__
LMSG_G000004506.1	yes	1070_1	Polymetallic	95.79	0.84	91.59	1	1	1	18	High quality	21466	2761707	63.70	208	d__Bacteria;p__Desulfobacterota;c__MBNT15;o__MBNT15;f__MBNT15;g__;s__
LMSG_G000005542.1	yes	1322_1	Polymetallic	98.18	2.24	86.99	0	0	0	19	Medium quality	82596	3365327	56.80	58	d__Bacteria;p__Proteobacteria;c__Gammaproteobacteria;o__Burkholderiales;f__RBG-16-58-11;g__RBG-16-58-11;s__
LMSG_G000004507.1	yes	943_0	Polymetallic	91.81	1.21	85.75	1	1	1	20	High quality	16073	2661820	41.80	299	d__Bacteria;p__Nitrospirota;c__Thermodesulfovibrionia;o__Thermodesulfovibrionales;f__UBA1546;g__;s__
LMSG_G000005541.1	yes	1778_0	Polymetallic	60.92	1.90	51.43	1	0	1	20	Medium quality	25131	737047	55.80	49	d__Bacteria;p__Patescibacteria;c__Paceibacteria;o__UBA9983;f__UBA2163;g__;s__
LMSG_G000005543.1	yes	672_1	Polymetallic	92.08	1.85	82.83	0	1	0	20	Medium quality	18465	2870835	72.60	242	d__Bacteria;p__Chloroflexota;c__Ellin6529;o__CSP1-4;f__CSP1-4;g__;s__
LMSG_G000004508.1	yes	1461_1	Polymetallic	99.58	1.41	92.53	1	1	1	19	High quality	74272	3893170	64.50	109	d__Bacteria;p__Proteobacteria;c__Alphaproteobacteria;o__Rhizobiales;f__Beijerinckiaceae;g__;s__
LMSG_G000005544.1	yes	1792_1	Polymetallic	75.42	0.00	75.42	1	1	2	18	Medium quality	16097	501444	46.90	40	d__Bacteria;p__Patescibacteria;c__Paceibacteria;o__UBA9983;f__UBA9973;g__;s__
LMSG_G000006242.1	yes	791_1	Polymetallic	92.53	3.09	77.10	1	1	0	18	Medium quality	67009	2175517	65.90	157	d__Bacteria;p__Actinobacteriota;c__Acidimicrobiia;o__Acidimicrobiales;f__RAAP-2;g__RAAP-2;s__
LMSG_G000005545.1	yes	1822_1	Polymetallic	73.78	0.00	73.78	1	1	1	16	Medium quality	100036	653352	50.30	50	d__Bacteria;p__Patescibacteria;c__Paceibacteria;o__UBA6257;f__GWB1-50-10;g__;s__
LMSG_G000005549.1	yes	1780_1	Polymetallic	71.68	1.72	63.06	1	0	2	18	Medium quality	61643	647826	36.40	40	d__Bacteria;p__Patescibacteria;c__Paceibacteria;o__UBA6257;f__UBA9933;g__;s__
LMSG_G000005546.1	yes	1450_0	Polymetallic	72.19	3.01	57.16	0	1	0	12	Medium quality	12548	2928951	64.20	315	d__Bacteria;p__Proteobacteria;c__Alphaproteobacteria;o__Rhizobiales;f__Xanthobacteraceae;g__Pseudolabrys;s__
LMSG_G000005547.1	yes	1755_0	Polymetallic	76.53	0.47	74.20	1	1	2	17	Medium quality	4819	1136558	52.20	294	d__Archaea;p__Micrarchaeota;c__Micrarchaeia;o__UBA10214;f__;g__;s__
LMSG_G000005548.1	yes	1350_1	Polymetallic	87.30	1.75	78.53	1	1	0	18	Medium quality	11376	2739592	68.20	303	d__Bacteria;p__Acidobacteriota;c__Holophagae;o__Holophagales;f__Holophagaceae;g__;s__
LMSG_G000005550.1	yes	1433_1	Polymetallic	89.40	1.31	82.84	1	1	1	17	Medium quality	25402	3268132	66.70	184	d__Bacteria;p__Proteobacteria;c__Alphaproteobacteria;o__Rhodobacterales;f__Rhodobacteraceae;g__;s__
LMSG_G000005551.1	yes	1805_1	Polymetallic	78.92	1.12	73.31	1	2	2	19	Medium quality	55000	758909	51.00	20	d__Bacteria;p__Patescibacteria;c__Paceibacteria;o__UBA6257;f__GWB1-50-10;g__;s__
LMSG_G000005552.1	yes	509_1	Polymetallic	91.04	2.34	79.36	2	0	0	17	Medium quality	6687	1163031	57.00	226	d__Archaea;p__Micrarchaeota;c__Micrarchaeia;o__UBA10214;f__UBA10214;g__;s__
LMSG_G000005553.1	yes	1579_1	Polymetallic	82.84	3.30	66.36	1	1	0	16	Medium quality	8835	3705036	68.50	500	d__Bacteria;p__Gemmatimonadota;c__Gemmatimonadetes;o__Gemmatimonadales;f__Gemmatimonadaceae;g__SCN-70-22;s__
LMSG_G000005554.1	yes	1789_0	Polymetallic	62.35	1.12	56.74	1	2	2	19	Medium quality	43273	913281	41.90	31	d__Bacteria;p__Patescibacteria;c__Microgenomatia;o__Curtissbacterales;f__GWA2-41-24;g__;s__
LMSG_G000005555.1	yes	989_0	Polymetallic	87.58	4.09	67.14	1	0	1	15	Medium quality	13391	2389575	70.30	242	d__Bacteria;p__Acidobacteriota;c__UBA4820;o__UBA4820;f__;g__;s__
LMSG_G000005556.1	yes	1827_1	Polymetallic	73.40	0.75	69.66	0	1	1	19	Medium quality	34834	662327	39.40	58	d__Bacteria;p__Patescibacteria;c__Paceibacteria;o__UBA6257;f__UBA9933;g__;s__
LMSG_G000005557.1	yes	1073_1	Polymetallic	81.97	2.15	71.22	0	0	0	18	Medium quality	9761	1212002	44.30	169	d__Bacteria;p__Omnitrophota;c__Koll11;o__GIF10;f__UBA6249;g__;s__
LMSG_G000005558.1	yes	631_2	Tin-Zinc	97.68	0.00	97.68	2	0	2	20	Medium quality	32253	2690499	70.40	175	d__Bacteria;p__Dormibacterota;c__Dormibacteria;o__UBA8260;f__Bog-877;g__;s__
LMSG_G000005559.1	yes	172_0	Tin-Zinc	71.32	1.80	62.33	1	1	1	16	Medium quality	44926	1126855	39.40	54	d__Archaea;p__Thermoplasmatota;c__Thermoplasmata;o__Thermoplasmatales;f__Thermoplasmataceae;g__Cuniculiplasma;s__
LMSG_G000004509.1	yes	181_1	Tin-Zinc	92.29	3.23	76.17	1	1	1	18	High quality	11370	1621149	47.80	202	d__Archaea;p__Thermoplasmatota;c__Thermoplasmata;o__Thermoplasmatales;f__Thermoplasmataceae;g__UBA582;s__
LMSG_G000005560.1	yes	1145_1	Tin-Zinc	99.00	1.03	93.83	0	3	0	18	Medium quality	38091	4174332	53.60	231	d__Bacteria;p__Firmicutes_E;c__Sulfobacillia;o__Sulfobacillales;f__Sulfobacillaceae;g__Sulfobacillus_C;s__
LMSG_G000005561.1	yes	691_1	Tin-Zinc	93.16	2.14	82.48	0	1	0	18	Medium quality	36580	2460730	64.10	103	d__Bacteria;p__Actinobacteriota;c__Acidimicrobiia;o__Acidimicrobiales;f__Acidimicrobiaceae;g__Acidithrix;s__
LMSG_G000005562.1	yes	361_0	Tin-Zinc	72.58	1.43	65.43	1	0	0	13	Medium quality	3376	708829	44.60	264	d__Bacteria;p__Patescibacteria;c__Saccharimonadia;o__Saccharimonadales;f__UBA4665;g__UBA4729;s__
LMSG_G000005563.1	yes	617_1	Tin-Zinc	80.82	1.62	72.71	0	7	23	18	Medium quality	2940	2180697	48.90	903	d__Bacteria;p__Actinobacteriota;c__Acidimicrobiia;o__Acidimicrobiales;f__RAAP-2;g__;s__
LMSG_G000005564.1	yes	1441_0	Tin-Zinc	85.23	3.04	70.02	1	0	0	16	Medium quality	5661	2707660	59.90	579	d__Bacteria;p__Proteobacteria;c__Alphaproteobacteria;o__Rhizobiales;f__Beijerinckiaceae;g__Methylovirgula;s__
LMSG_G000005565.1	yes	344_1	Tin-Zinc	79.37	1.19	73.43	0	0	0	15	Medium quality	19247	701952	49.60	65	d__Bacteria;p__Patescibacteria;c__Saccharimonadia;o__Saccharimonadales;f__UBA4665;g__UBA6224;s__
LMSG_G000005566.1	yes	972_1	Tin-Zinc	88.40	3.64	70.22	2	2	0	17	Medium quality	10903	2297077	56.20	292	d__Bacteria;p__Nitrospirota;c__Leptospirillia;o__Leptospirillales;f__Leptospirillaceae;g__Leptospirillum_A;s__
LMSG_G000005567.1	yes	1540_2	Tin-Zinc	84.55	3.70	66.06	1	1	1	16	Medium quality	24372	2114564	60.50	146	d__Bacteria;p__Proteobacteria;c__Gammaproteobacteria;o__Acidiferrobacterales;f__Acidiferrobacteraceae;g__Acidiferrobacter;s__
LMSG_G000005568.1	yes	1151_1	Tin-Zinc	93.72	1.44	86.52	0	0	0	18	Medium quality	20157	3810511	68.40	308	d__Bacteria;p__Firmicutes_E;c__Sulfobacillia;o__Sulfobacillales;f__;g__;s__

LMSG_G000005569.1	yes	1427_1	Tin-Zinc	95.33	2.08	84.95	1	0	1	18	Medium quality	40907	2280073	66.80	107	d__Bacteria;p__Proteobacteria;c__Alphaproteobacteria;o__Sphingomonadales;f__Sphingomonadaceae;g__Qipengyuania;s__
LMSG_G000005570.1	yes	522_0	Tin-Zinc	64.67	1.23	58.53	0	1	0	13	Medium quality	2766	3111313	65.40	1151	d__Bacteria;p__Chloroflexota;c__Ktedonobacteria;o__Ktedonobacterales;f__;g__;s__
LMSG_G000005571.1	yes	1115_1	Tin-Zinc	93.86	2.59	80.93	0	0	0	15	Medium quality	19791	2440444	55.50	172	d__Bacteria;p__Proteobacteria;c__Gammaproteobacteria;o__Halothiobacillales;f__Halothiobacillaceae;g__Halothiobacillus;s__
LMSG_G000005572.1	yes	880_2	Tin-Zinc	52.58	0.00	52.58	0	0	0	16	Medium quality	6864	2426699	70.40	384	d__Bacteria;p__Actinobacteriota;c__Actinomycetia;o__Propionibacteriales;f__Propionibacteriaceae;g__;s__
LMSG_G000005573.1	yes	176_1	Tin-Zinc	65.14	0.81	61.11	1	0	1	17	Medium quality	20121	1315068	41.20	113	d__Archaea;p__Thermoplasmatota;c__Thermoplasmata;o__Thermoplasmatales;f__Thermoplasmataceae;g__GCA-000496135;s__
LMSG_G000005574.1	yes	1223_1	Tin-Zinc	74.65	2.95	59.92	0	0	0	11	Medium quality	7413	1745111	53.40	261	d__Bacteria;p__Proteobacteria;c__Gammaproteobacteria;o__Burkholderiales;f__Sulfuriferulaceae;g__Sulfuriferula_A;s__
LMSG_G000005575.1	yes	868_0	Tin-Zinc	62.06	1.76	53.28	0	0	0	13	Medium quality	8699	1786356	71.50	271	d__Bacteria;p__Actinobacteriota;c__Actinomycetia;o__Actinomycetales;f__Angustibacteraceae;g__;s__
LMSG_G000004510.1	yes	1_1	Tin-Zinc	97.43	0.66	94.13	1	1	1	18	High quality	17948	2065710	37.10	220	d__Bacteria;p__Actinobacteriota;c__Acidimicrobiia;o__Acidimicrobiales;f__;g__;s__
LMSG_G000005576.1	yes	464_1	Tin-Zinc	92.65	1.97	82.81	2	0	1	20	Medium quality	11095	2518578	52.80	326	d__Bacteria;p__Firmicutes;c__Alicyclobacillia;o__Alicyclobacillales;f__Acidibacillaceae;g__Acidibacillus;s__Acidibacillus ferrooxidans
LMSG_G000005580.1	yes	879_1	Tin-Zinc	70.76	2.81	56.71	0	0	0	16	Medium quality	5287	2297564	71.50	528	d__Bacteria;p__Actinobacteriota;c__Actinomycetia;o__Propionibacteriales;f__Propionibacteriaceae;g__;s__
LMSG_G000005577.1	yes	27_0	Tin-Zinc	80.01	1.94	70.31	1	1	2	19	Medium quality	86417	804953	44.00	22	d__Archaea;p__Nanoarchaeota;c__Nanoarchaeia;o__Parvarchaeales;f__Parvarchaeaceae;g__;s__
LMSG_G000005578.1	yes	1431_1	Tin-Zinc	69.53	2.92	54.95	0	0	0	15	Medium quality	2696	1399549	66.40	582	d__Bacteria;p__Proteobacteria;c__Alphaproteobacteria;o__Sphingomonadales;f__Sphingomonadaceae;g__Sphingomonas_A;s__
LMSG_G000005579.1	yes	1709_1	Tin-Zinc	76.96	1.17	71.10	1	0	1	18	Medium quality	20796	990935	43.70	68	d__Bacteria;p__Patescibacteria;c__CPR2;o__UBA1875;f__;g__;s__
LMSG_G000004511.1	yes	576_1	Tin-Zinc	96.80	1.60	88.80	1	2	2	19	High quality	46978	2095924	64.30	101	d__Archaea;p__Thermoplasmatota;c__Thermoplasmata;o__UBA184;f__UBA184;g__;s__
LMSG_G000005581.1	yes	1545_1	Tin-Zinc	69.66	0.00	69.66	1	1	1	12	Medium quality	39563	1880043	63.60	101	d__Bacteria;p__Proteobacteria;c__Gammaproteobacteria;o__Acidiferrobacterales;f__Acidiferrobacteraceae;g__Acidiferrobacter;s__
LMSG_G000004512.1	yes	823_1	Tin-Zinc	90.59	1.14	84.90	1	1	1	19	High quality	20378	2591577	67.90	188	d__Bacteria;p__Actinobacteriota;c__Acidimicrobiia;o__Acidimicrobiales;f__QHCF01;g__;s__
LMSG_G000005582.1	yes	491_0	Tin-Zinc	87.67	2.76	73.90	1	0	1	18	Medium quality	15657	3144765	51.20	366	d__Bacteria;p__Firmicutes;c__Alicyclobacillia;o__Alicyclobacillales;f__Alicyclobacillaceae;g__QXHL01;s__
LMSG_G000005583.1	yes	1695_0	Tin-Zinc	61.04	1.23	54.92	0	0	0	16	Medium quality	5609	554200	39.50	126	d__Bacteria;p__Patescibacteria;c__Paceibacteria;o__UBA6257;f__UBA9933;g__;s__
LMSG_G000005585.1	yes	1224_0	Tin-Zinc	96.89	3.41	79.83	1	0	1	17	Medium quality	44314	2548499	53.80	96	d__Bacteria;p__Proteobacteria;c__Gammaproteobacteria;o__Burkholderiales;f__Sulfuriferulaceae;g__Sulfuriferula_A;s__
LMSG_G000005584.1	yes	626_0	Tin-Zinc	73.45	0.93	68.83	1	2	0	20	Medium quality	31429	2870271	67.60	177	d__Bacteria;p__Dormibacterota;c__Dormibacteria;o__UBA8260;f__Bog-877;g__;s__
LMSG_G000006284.1	yes	1368_1	Tin-Zinc	93.18	0.00	93.18	1	2	10	17	Medium quality	66852	1960228	46.30	95	d__Bacteria;p__Actinobacteriota;c__UBA9087;o__UBA10029;f__UBA10029;g__CG2-30-50-142;s__
LMSG_G000005586.1	yes	744_1	Tin-Zinc	95.64	2.99	80.69	1	0	1	20	Medium quality	31850	2630712	70.30	140	d__Bacteria;p__Actinobacteriota;c__Acidimicrobiia;o__Acidimicrobiales;f__Bog-793;g__Palsa-601;s__
LMSG_G000005587.1	yes	1326_0	Tin-Zinc	58.02	0.00	58.02	0	1	0	10	Medium quality	3866	1098802	57.90	310	d__Bacteria;p__Proteobacteria;c__Gammaproteobacteria;o__Burkholderiales;f__Ferrovaceae;g__Ferrovum;s__
LMSG_G000005588.1	yes	84_1	Polymetallic	79.25	0.93	74.58	0	1	1	16	Medium quality	15229	937019	50.00	79	d__Archaea;p__Micrarchaeota;c__Micrarchaeia;o__Micrarchaeales;f__;g__;s__
LMSG_G000004513.1	yes	195_1	Polymetallic	98.07	0.81	94.04	1	1	1	20	High quality	52434	1454141	43.70	40	d__Archaea;p__Thermoplasmatota;c__Thermoplasmata;o__Thermoplasmatales;f__Thermoplasmataceae;g__UBA447;s__
LMSG_G000005589.1	yes	146_1	Polymetallic	82.39	0.00	82.39	1	2	2	19	Medium quality	28328	958334	40.70	87	d__Archaea;p__Micrarchaeota;c__Micrarchaeia;o__Micrarchaeales;f__;g__;s__
LMSG_G000005590.1	yes	749_2	Polymetallic	68.80	1.28	62.39	0	0	0	14	Medium quality	7141	2177724	71.70	336	d__Bacteria;p__Actinobacteriota;c__Acidimicrobiia;o__Acidimicrobiales;f__Palsa-688;g__;s__
LMSG_G000005591.1	yes	859_0	Polymetallic	54.12	0.00	54.12	0	0	0	16	Medium quality	3231	2563798	68.40	865	d__Bacteria;p__Actinobacteriota;c__Actinomycetia;o__Actinomycetales;f__Beutenbergiaceae;g__Haloactinobacterium;s__
LMSG_G000005592.1	yes	777_0	Polymetallic	67.54	2.14	56.86	0	0	0	12	Medium quality	3223	2384262	71.40	787	d__Bacteria;p__Actinobacteriota;c__Acidimicrobiia;o__Acidimicrobiales;f__Bog-793;g__;s__
LMSG_G000005593.1	yes	887_0	Polymetallic	59.97	1.35	53.24	0	0	0	13	Medium quality	3536	1348515	60.60	421	d__Bacteria;p__Acidobacteriota;c__Acidobacteriae;o__Acidobacteriales;f__Acidobacteriaceae;g__;s__
LMSG_G000005594.1	yes	405_1	Polymetallic	75.70	0.00	75.70	1	1	1	20	Medium quality	183340	931325	26.50	17	d__Bacteria;p__Patescibacteria;c__;o__;f__;g__;s__
LMSG_G000005595.1	yes	180_0	Copper	80.05	2.42	67.96	1	2	1	19	Medium quality	30268	1637630	39.00	108	d__Archaea;p__Thermoplasmatota;c__Thermoplasmata;o__Thermoplasmatales;f__Thermoplasmataceae;g__Cuniculiplasma;s__
LMSG_G000005596.1	yes	319_0	Copper	82.57	2.01	72.50	1	3	1	18	Medium quality	4516	3171557	65.20	829	d__Bacteria;p__Firmicutes_E;c__Sulfobacillia;o__;f__;g__;s__
LMSG_G000005597.1	yes	1139_1	Copper	98.00	1.03	92.83	0	1	0	14	Medium quality	39410	3568599	49.60	136	d__Bacteria;p__Firmicutes_E;c__Sulfobacillia;o__Sulfobacillales;f__Sulfobacillaceae;g__Sulfobacillus;s__
LMSG_G000005598.1	yes	1016_1	Copper	95.33	0.00	95.33	0	1	0	20	Medium quality	116801	2285986	66.80	35	d__Bacteria;p__Deinococcota;c__Deinococci;o__Deinococcales;f__;g__;s__
LMSG_G000005599.1	yes	752_0	Copper	70.04	2.75	56.27	0	0	0	16	Medium quality	6811	2298340	73.30	380	d__Bacteria;p__Actinobacteriota;c__Acidimicrobiia;o__Acidimicrobiales;f__Palsa-688;g__;s__
LMSG_G000004514.1	yes	158_1	Copper	92.23	0.81	88.17	1	1	1	19	High quality	53248	1438152	39.00	44	d__Archaea;p__Thermoplasmatota;c__Thermoplasmata;o__Thermoplasmatales;f__Thermoplasmataceae;g__Ferroplasma;s__
LMSG_G000006285.1	yes	1166_1	Copper	94.55	0.00	94.55	1	1	1	14	Medium quality	21085	3438311	59.60	326	d__Bacteria;p__Firmicutes_E;c__Sulfobacillia;o__Sulfobacillales;f__;g__;s__
LMSG_G000006286.1	yes	1148_1	Copper	96.53	0.00	96.53	1	3	3	17	Medium quality	70395	2986476	66.10	97	d__Bacteria;p__Firmicutes_E;c__Sulfobacillia;o__Sulfobacillales;f__;g__;s__
LMSG_G000004515.1	yes	979_2	Copper	97.47	0.00	97.47	1	1	1	18	High quality	35232	2104468	59.50	80	d__Bacteria;p__Proteobacteria;c__Gammaproteobacteria;o__Acidithiobacillales;f__Acidithiobacillaceae;g__Acidithiobacillus;s__Acidithiobacillus ferrooxidans
LMSG_G000005600.1	yes	1153_0	Copper	94.47	0.66	91.17	0	1	1	14	Medium quality	22797	3524083	68.40	355	d__Bacteria;p__Firmicutes_E;c__Sulfobacillia;o__Sulfobacillales;f__;g__;s__
LMSG_G000005601.1	yes	956_1	Copper	55.35	0.00	55.35	0	0	0	15	Medium quality	28762	1328224	56.90	86	d__Bacteria;p__Nitrospirota;c__Leptospirillia;o__Leptospirillales;f__Leptospirillaceae;g__UBA4572;s__
LMSG_G000005602.1	yes	206_1	Copper	81.85	3.23	65.73	1	2	2	17	Medium quality	19483	2583911	39.00	238	d__Archaea;p__Thermoplasmatota;c__Thermoplasmata;o__Thermoplasmatales;f__Thermoplasmataceae;g__Cuniculiplasma;s__
LMSG_G000005603.1	yes	1548_2	Copper	73.00	4.40	51.00	1	0	0	12	Medium quality	7669	2168858	65.90	668	d__Bacteria;p__Proteobacteria;c__Gammaproteobacteria;o__Acidiferrobacterales;f__Acidiferrobacteraceae;g__Acidiferrobacter;s__
LMSG_G000005604.1	yes	1078_1	Copper	89.08	4.28	67.67	1	1	0	18	Medium quality	39274	3197399	66.70	139	d__Bacteria;p__Proteobacteria;c__Gammaproteobacteria;o__Steroidobacterales;f__Steroidobacteraceae;g__;s__
LMSG_G000004516.1	yes	817_1	Copper	94.87	1.71	86.33	1	1	1	20	High quality	22817	3092035	60.60	208	d__Bacteria;p__Actinobacteriota;c__Acidimicrobiia;o__Acidimicrobiales;f__;g__;s__
LMSG_G000005605.1	yes	606_1	Copper	97.43	1.28	91.02	1	0	0	19	Medium quality	44864	2670835	58.60	135	d__Bacteria;p__Actinobacteriota;c__Acidimicrobiia;o__Acidimicrobiales;f__Acidimicrobiaceae;g__Ferrimicrobium;s__
LMSG_G000005606.1	yes	1143_1	Copper	94.00	0.33	92.34	0	2	0	19	Medium quality	20371	4381871	55.00	317	d__Bacteria;p__Firmicutes_E;c__Sulfobacillia;o__Sulfobacillales;f__Sulfobacillaceae;g__Sulfobacillus_B;s__
LMSG_G000005607.1	yes	159_1	Copper	78.52	1.63	70.39	1	0	1	11	Medium quality	6372	1486803	38.30	278	d__Archaea;p__Thermoplasmatota;c__Thermoplasmata;o__Thermoplasmatales;f__Thermoplasmataceae;g__Ferroplasma;s__
LMSG_G000004517.1	yes	167_1	Copper	96.34	1.63	88.21	2	2	2	18	High quality	58700	1513674	33.70	42	d__Archaea;p__Thermoplasmatota;c__Thermoplasmata;o__Thermoplasmatales;f__Thermoplasmataceae;g__Acidiplasma;s__
LMSG_G000005608.1	yes	644_1	Copper	92.57	0.09	92.12	0	0	0	19	Medium quality	20909	3813228	70.20	288	d__Bacteria;p__Firmicutes_E;c__Thermaerobacteria;o__;f__;g__;s__
LMSG_G000005609.1	yes	174_1	Copper	88.12	0.81	84.09	1	0	2	18	Medium quality	10363	1390310	48.30	166	d__Archaea;p__Thermoplasmatota;c__Thermoplasmata;o__Thermoplasmatales;f__Thermoplasmataceae;g__UBA582;s__
LMSG_G000006287.1	yes	762_1	Copper	98.29	3.50	80.77	1	1	1	16	Medium quality	21229	2894425	70.90	201	d__Bacteria;p__Actinobacteriota;c__Acidimicrobiia;o__Acidimicrobiales;f__RAAP-2;g__;s__
LMSG_G000005610.1	yes	1741_1	Copper	74.57	1.10	69.08	0	0	0	11	Medium quality	16466	1309060	37.90	122	d__Bacteria;p__SZUA-79;c__SZUA-79;o__Acidulodesulfobacterales;f__SZUA-79;g__Acidulodesulfobacterium;s__
LMSG_G000004518.1	yes	1735_2	Copper	91.81	0.05	91.58	1	1	1	19	High quality	70513	1908000	37.40	217	d__Bacteria;p__SZUA-79;c__SZUA-79;o__Acidulodesulfobacterales;f__SZUA-79;g__Acidulodesulfobacterium;s__Acidulodesulfobacterium ferriphilum
LMSG_G000005611.1	yes	125_1	Copper	77.10	0.00	77.10	1	1	1	20	Medium quality	529549	844591	44.30	6	d__Archaea;p__Micrarchaeota;c__Micrarchaeia;o__Micrarchaeales;f__;g__;s__
LMSG_G000005612.1	yes	727_0	Copper	84.84	0.85	80.57	0	1	0	17	Medium quality	16516	3011047	75.60	253	d__Bacteria;p__Actinobacteriota;c__Acidimicrobiia;o__Acidimicrobiales;f__UBA8190;g__;s__
LMSG_G000005613.1	yes	774_1	Copper	85.28	1.28	78.87	1	1	1	18	Medium quality	18337	2390366	72.60	153	d__Bacteria;p__Actinobacteriota;c__Acidimicrobiia;o__Acidimicrobiales;f__Bog-793;g__;s__
LMSG_G000005614.1	yes	541_1	Copper	87.86	1.60	79.86	1	1	1	19	Medium quality	47539	1815158	68.70	52	d__Archaea;p__Thermoplasmatota;c__Thermoplasmata;o__UBA184;f__UBA184;g__;s__
LMSG_G000004519.1	yes	720_1	Copper	98.14	0.93	93.52	1	1	1	20	High quality	52955	3129320	73.90	102	d__Bacteria;p__Dormibacterota;c__Dormibacteria;o__UBA8260;f__Bog-877;g__;s__
LMSG_G000005615.1	yes	802_1	Copper	94.87	2.23	83.72	0	0	0	20	Medium quality	17387	2228652	68.90	190	d__Bacteria;p__Actinobacteriota;c__Acidimicrobiia;o__Acidimicrobiales;f__RAAP-2;g__RAAP-2;s__
LMSG_G000004520.1	yes	435_1	Copper	97.72	0.00	97.72	1	2	2	20	High quality	44463	4276998	54.60	205	d__Bacteria;p__Planctomycetota;c__Phycisphaerae;o__UBA1161;f__;g__;s__

LMSG_G000005616.1	yes	46_0	Copper	69.90	0.93	65.23	0	0	0	14	Medium quality	5611	751647	42.40	161	d__Archaea;p__Micrarchaeota;c__Micrarchaeia;o__Micrarchaeales;f__Micrarchaeaceae;g__;s__
LMSG_G000005617.1	yes	645_0	Copper	63.06	1.32	56.46	1	0	0	13	Medium quality	9577	3331333	69.90	389	d__Bacteria;p__Firmicutes_E;c__Thermaerobacteria;o__;f__;g__;s__
LMSG_G000005618.1	yes	68_1	Copper	83.17	0.00	83.17	1	1	1	19	Medium quality	26839	1053150	56.80	50	d__Archaea;p__Micrarchaeota;c__Micrarchaeia;o__Micrarchaeales;f__Micrarchaeaceae;g__;s__
LMSG_G000005619.1	yes	642_0	Copper	60.34	1.72	51.72	0	0	0	11	Medium quality	5064	2761520	75.10	659	d__Bacteria;p__Firmicutes_E;c__Thermaerobacteria;o__;f__;g__;s__
LMSG_G000004521.1	yes	205_1	Copper	95.52	4.84	71.33	1	2	2	20	High quality	65390	1351411	44.30	44	d__Archaea;p__Thermoplasmatota;c__Thermoplasmata;o__Thermoplasmatales;f__Thermoplasmataceae;g__UBA582;s__
LMSG_G000005620.1	yes	740_1	Copper	77.93	0.86	73.62	0	0	0	17	Medium quality	40670	2871689	72.00	124	d__Bacteria;p__Actinobacteriota;c__Acidimicrobiia;o__Acidimicrobiales;f__RAAP-2;g__;s__
LMSG_G000006288.1	yes	215_1	Copper	90.48	2.42	78.39	1	1	2	17	Medium quality	9818	1456783	43.30	187	d__Archaea;p__Thermoplasmatota;c__Thermoplasmata;o__Thermoplasmatales;f__GCA-001856825;g__GCA-001856825;s__
LMSG_G000004522.1	yes	453_1	Copper	90.59	2.14	79.91	1	3	1	19	High quality	35271	2240047	50.50	119	d__Bacteria;p__Actinobacteriota;c__Acidimicrobiia;o__Acidimicrobiales;f__;g__;s__
LMSG_G000005621.1	yes	1168_0	Copper	96.50	0.67	93.17	0	0	0	18	Medium quality	54934	3862503	61.10	134	d__Bacteria;p__Firmicutes_E;c__Sulfobacillia;o__Sulfobacillales;f__Sulfobacillaceae;g__;s__
LMSG_G000006289.1	yes	743_1	Copper	95.72	1.28	89.31	1	1	1	17	Medium quality	88965	3210727	71.90	65	d__Bacteria;p__Actinobacteriota;c__Acidimicrobiia;o__Acidimicrobiales;f__Bog-793;g__Palsa-601;s__
LMSG_G000005622.1	yes	647_0	Copper	71.78	4.29	50.33	0	0	0	15	Medium quality	15794	4197594	72.60	343	d__Bacteria;p__Firmicutes_E;c__Thermaerobacteria;o__;f__;g__;s__
LMSG_G000005623.1	yes	372_0	Copper	63.88	0.00	63.88	0	0	0	14	Medium quality	29365	619494	44.00	35	d__Bacteria;p__Patescibacteria;c__Saccharimonadia;o__Saccharimonadales;f__UBA4665;g__;s__
LMSG_G000005624.1	yes	761_0	Copper	76.36	3.42	59.27	1	1	1	12	Medium quality	4023	3322043	69.20	932	d__Bacteria;p__Actinobacteriota;c__Acidimicrobiia;o__Acidimicrobiales;f__Bog-793;g__;s__
LMSG_G000005625.1	yes	81_0	Copper	78.50	0.93	73.83	1	2	1	18	Medium quality	54975	600410	52.40	16	d__Archaea;p__Micrarchaeota;c__Micrarchaeia;o__Micrarchaeales;f__Micrarchaeaceae;g__;s__
LMSG_G000005626.1	yes	630_1	Copper	87.50	0.93	82.88	2	2	5	19	Medium quality	54542	2409854	69.90	212	d__Bacteria;p__Dormibacterota;c__Dormibacteria;o__UBA8260;f__Bog-877;g__;s__
LMSG_G000006243.1	yes	320_0	Copper	95.04	0.33	93.39	2	1	0	18	Medium quality	44320	3179973	71.30	117	d__Bacteria;p__Firmicutes_E;c__Sulfobacillia;o__;f__;g__;s__
LMSG_G000006244.1	yes	591_1	Copper	98.54	0.97	93.69	1	1	0	18	Medium quality	261934	1851588	62.60	44	d__Archaea;p__Thermoproteota;c__Nitrososphaeria;o__Nitrososphaerales;f__UBA183;g__UBA183;s__
LMSG_G000004523.1	yes	224_0	Copper	93.14	1.77	84.27	1	2	1	19	High quality	86953	1474403	46.70	40	d__Archaea;p__Thermoplasmatota;c__Thermoplasmata;o__Thermoplasmatales;f__GCA-001856825;g__GCA-001856825;s__
LMSG_G000005627.1	yes	135_0	Copper	83.33	1.87	73.99	1	1	0	18	Medium quality	30454	923268	46.40	49	d__Archaea;p__Micrarchaeota;c__Micrarchaeia;o__Micrarchaeales;f__;g__;s__
LMSG_G000004524.1	yes	151_0	Copper	99.13	1.60	91.13	1	2	1	20	High quality	77450	1875023	55.40	36	d__Archaea;p__Thermoplasmatota;c__Thermoplasmata;o__;f__;g__;s__
LMSG_G000005628.1	yes	636_1	Polymetallic	93.98	0.00	93.98	0	0	0	19	Medium quality	19896	2542409	73.40	183	d__Bacteria;p__Dormibacterota;c__Dormibacteria;o__UBA8260;f__UBA8260;g__;s__
LMSG_G000005629.1	yes	660_1	Polymetallic	84.81	0.00	84.81	1	1	0	17	Medium quality	66061	1602765	64.30	52	d__Bacteria;p__Firmicutes_E;c__Sulfobacillia;o__;f__;g__;s__
LMSG_G000005630.1	yes	751_0	Polymetallic	85.62	2.71	72.09	0	0	0	17	Medium quality	7560	2736057	72.70	472	d__Bacteria;p__Actinobacteriota;c__Acidimicrobiia;o__Acidimicrobiales;f__Palsa-688;g__;s__
LMSG_G000005631.1	yes	437_2	Polymetallic	60.18	0.38	58.29	0	0	0	13	Medium quality	6788	2048419	56.50	363	d__Bacteria;p__Planctomycetota;c__Phycisphaerae;o__;f__;g__;s__
LMSG_G000005632.1	yes	1336_1	Polymetallic	95.33	0.06	95.06	0	2	0	19	Medium quality	75873	2102996	57.90	59	d__Bacteria;p__Proteobacteria;c__Gammaproteobacteria;o__Burkholderiales;f__Ferrovaceae;g__Ferrovum;s__
LMSG_G000005633.1	yes	659_1	Polymetallic	89.27	0.99	84.32	1	0	0	14	Medium quality	52431	1524778	69.00	60	d__Bacteria;p__Firmicutes_E;c__Sulfobacillia;o__;f__;g__;s__
LMSG_G000005634.1	yes	140_0	Polymetallic	70.93	1.87	61.59	1	0	0	16	Medium quality	7686	527617	44.10	81	d__Archaea;p__Micrarchaeota;c__Micrarchaeia;o__Micrarchaeales;f__Micrarchaeaceae;g__Micrarchaeum;s__
LMSG_G000004525.1	yes	585_1	Polymetallic	97.57	0.97	92.72	1	1	1	20	High quality	94991	1912390	64.20	43	d__Archaea;p__Thermoproteota;c__Nitrososphaeria;o__Nitrososphaerales;f__UBA183;g__BOG-1369;s__
LMSG_G000005635.1	yes	808_1	Polymetallic	79.91	0.43	77.78	1	1	1	18	Medium quality	17152	1661863	72.00	153	d__Bacteria;p__Actinobacteriota;c__Acidimicrobiia;o__Acidimicrobiales;f__RAAP-2;g__RAAP-2;s__
LMSG_G000005636.1	yes	156_1	Polymetallic	85.40	1.60	77.40	2	1	0	18	Medium quality	15662	1691898	52.20	155	d__Archaea;p__Thermoplasmatota;c__Thermoplasmata;o__;f__;g__;s__
LMSG_G000005638.1	yes	607_1	Polymetallic	95.72	1.28	89.31	2	0	0	18	Medium quality	40457	2506063	55.50	122	d__Bacteria;p__Actinobacteriota;c__Acidimicrobiia;o__Acidimicrobiales;f__Acidimicrobiaceae;g__Ferrimicrobium;s__Ferrimicrobium acidiphilum
LMSG_G000005637.1	yes	837_2	Polymetallic	84.68	1.14	78.99	0	0	0	19	Medium quality	8066	2261900	68.70	348	d__Bacteria;p__Actinobacteriota;c__Thermoleophilia;o__Solirubrobacterales;f__Solirubrobacteraceae;g__Palsa-465;s__
LMSG_G000005639.1	yes	670_1	Polymetallic	81.48	2.93	66.82	0	0	0	14	Medium quality	7681	2771284	72.40	420	d__Bacteria;p__Chloroflexota;c__Ellin6529;o__CSP1-4;f__CSP1-4;g__;s__
LMSG_G000005640.1	yes	87_0	Polymetallic	80.99	4.21	59.97	1	1	1	18	Medium quality	13373	819842	48.70	75	d__Archaea;p__Micrarchaeota;c__Micrarchaeia;o__Micrarchaeales;f__;g__;s__
LMSG_G000005641.1	yes	661_1	Polymetallic	90.75	0.99	85.80	0	1	0	16	Medium quality	77062	1521314	66.80	36	d__Bacteria;p__Firmicutes_E;c__Sulfobacillia;o__;f__;g__;s__
LMSG_G000005642.1	yes	889_1	Polymetallic	82.54	0.85	78.27	0	0	0	15	Medium quality	15713	3471217	57.50	291	d__Bacteria;p__Acidobacteriota;c__Acidobacteriae;o__Acidobacteriales;f__Acidobacteriaceae;g__;s__
LMSG_G000004526.1	yes	776_1	Polymetallic	93.63	1.57	85.80	1	1	1	18	High quality	27631	2753676	72.70	139	d__Bacteria;p__Actinobacteriota;c__Acidimicrobiia;o__Acidimicrobiales;f__Bog-793;g__;s__
LMSG_G000005643.1	yes	743_2	Polymetallic	66.14	0.85	61.87	0	0	0	16	Medium quality	13782	2489442	72.70	210	d__Bacteria;p__Actinobacteriota;c__Acidimicrobiia;o__Acidimicrobiales;f__Bog-793;g__Palsa-601;s__
LMSG_G000005644.1	yes	1103_0	Polymetallic	77.94	1.02	72.86	4	0	0	16	Medium quality	13448	2317499	43.60	217	d__Bacteria;p__Firmicutes;c__Bacilli;o__Bacillales;f__Bacillaceae_A;g__Bacillus_Y;s__Bacillus_Y subterraneus_A
LMSG_G000005645.1	yes	160_1	Polymetallic	87.07	4.07	66.75	1	2	1	15	Medium quality	34255	1632439	36.60	158	d__Archaea;p__Thermoplasmatota;c__Thermoplasmata;o__Thermoplasmatales;f__Thermoplasmataceae;g__Ferroplasma;s__
LMSG_G000006245.1	yes	287_1	Polymetallic	96.78	0.95	92.05	2	1	0	18	Medium quality	147411	1877882	40.50	46	d__Bacteria;p__Proteobacteria;c__Gammaproteobacteria;o__Burkholderiales;f__Ferrovaceae;g__PN-J185;s__
LMSG_G000005646.1	yes	1188_0	Polymetallic	66.91	1.03	61.79	2	1	0	18	Medium quality	7608	1863995	50.30	302	d__Bacteria;p__Firmicutes;c__Alicyclobacillia;o__Alicyclobacillales;f__Acidibacillaceae;g__;s__
LMSG_G000005647.1	yes	490_0	Polymetallic	93.13	0.15	92.41	2	0	0	16	Medium quality	17758	3589343	52.00	350	d__Bacteria;p__Firmicutes;c__Alicyclobacillia;o__Alicyclobacillales;f__Alicyclobacillaceae;g__Alicyclobacillus_B;s__
LMSG_G000005648.1	yes	1288_0	Polymetallic	88.09	1.44	80.87	0	0	0	16	Medium quality	11070	4469851	61.40	504	d__Bacteria;p__Proteobacteria;c__Gammaproteobacteria;o__Burkholderiales;f__Burkholderiaceae;g__Noviherbaspirillum;s__
LMSG_G000005649.1	yes	1275_0	Polymetallic	77.58	3.45	60.34	2	1	2	18	Medium quality	13634	3303329	64.50	370	d__Bacteria;p__Proteobacteria;c__Gammaproteobacteria;o__Burkholderiales;f__Thiobacillaceae;g__Thiobacillus;s__
LMSG_G000005650.1	yes	489_1	Polymetallic	94.22	1.65	85.99	0	1	0	20	Medium quality	12465	4509173	54.90	551	d__Bacteria;p__Firmicutes;c__Alicyclobacillia;o__Alicyclobacillales;f__Alicyclobacillaceae;g__;s__
LMSG_G000004527.1	yes	488_0	Polymetallic	94.35	1.54	86.66	5	2	2	20	High quality	45198	3042116	45.60	126	d__Bacteria;p__Firmicutes;c__Alicyclobacillia;o__Alicyclobacillales;f__Acidibacillaceae;g__Acidibacillus_A;s__
LMSG_G000005651.1	yes	1157_0	Polymetallic	70.79	0.00	70.79	1	1	7	20	Medium quality	451231	836751	58.00	19	d__Bacteria;p__Patescibacteria;c__Paceibacteria_A;o__Moranbacterales;f__UBA1568;g__;s__
LMSG_G000006290.1	yes	1165_1	Polymetallic	94.00	1.55	86.26	1	1	2	17	Medium quality	17519	3822095	57.90	495	d__Bacteria;p__Firmicutes_E;c__Sulfobacillia;o__Sulfobacillales;f__Sulfobacillaceae;g__Sulfobacillus;s__
LMSG_G000004528.1	yes	484_0	Polymetallic	95.34	1.54	87.65	3	1	1	20	High quality	73604	2618410	44.20	127	d__Bacteria;p__Firmicutes;c__Alicyclobacillia;o__Alicyclobacillales;f__Acidibacillaceae;g__;s__
LMSG_G000004529.1	yes	1269_3	Polymetallic	98.14	0.00	98.14	2	1	2	19	High quality	44188	2867142	66.50	93	d__Bacteria;p__Proteobacteria;c__Gammaproteobacteria;o__Burkholderiales;f__Burkholderiaceae;g__GJ-E10;s__GJ-E10 sp000828975
LMSG_G000005652.1	yes	1138_1	Polymetallic	96.00	0.33	94.34	0	1	0	18	Medium quality	27583	4782366	49.70	266	d__Bacteria;p__Firmicutes_E;c__Sulfobacillia;o__Sulfobacillales;f__Sulfobacillaceae;g__Sulfobacillus;s__
LMSG_G000005653.1	yes	1105_1	Polymetallic	97.00	0.33	95.34	0	1	0	17	Medium quality	40629	4432142	57.20	169	d__Bacteria;p__Firmicutes_E;c__Sulfobacillia;o__Sulfobacillales;f__Sulfobacillaceae;g__Sulfobacillus_B;s__
LMSG_G000005654.1	yes	1276_0	Polymetallic	88.40	2.10	77.89	1	1	0	15	Medium quality	39254	2560720	66.20	100	d__Bacteria;p__Proteobacteria;c__Gammaproteobacteria;o__Burkholderiales;f__Thiobacillaceae;g__Thiobacillus;s__
LMSG_G000005655.1	yes	1405_0	Polymetallic	99.29	1.94	89.61	0	1	0	19	Medium quality	56162	4517259	59.10	119	d__Bacteria;p__Proteobacteria;c__Gammaproteobacteria;o__Tenderiales;f__Tenderiaceae;g__;s__
LMSG_G000005656.1	yes	1265_0	Polymetallic	81.30	4.79	57.36	0	0	0	14	Medium quality	10879	2368250	69.30	275	d__Bacteria;p__Proteobacteria;c__Gammaproteobacteria;o__Xanthomonadales;f__Xanthomonadaceae;g__Thermomonas;s__
LMSG_G000005657.1	yes	1549_1	Polymetallic	76.93	1.52	69.31	1	1	0	11	Medium quality	16445	2020186	64.90	151	d__Bacteria;p__Proteobacteria;c__Gammaproteobacteria;o__Acidiferrobacterales;f__Acidiferrobacteraceae;g__Acidiferrobacter;s__Acidiferrobacter sp003184265
LMSG_G000005658.1	yes	485_0	Polymetallic	89.48	0.77	85.64	2	1	0	19	Medium quality	19386	2323869	46.00	175	d__Bacteria;p__Firmicutes;c__Alicyclobacillia;o__Alicyclobacillales;f__Acidibacillaceae;g__;s__
LMSG_G000005659.1	yes	86_3	Polymetallic	81.30	0.00	81.30	1	2	2	19	Medium quality	32235	756738	48.60	27	d__Archaea;p__Micrarchaeota;c__Micrarchaeia;o__Micrarchaeales;f__Micrarchaeaceae;g__;s__
LMSG_G000006291.1	yes	179_1	Polymetallic	90.41	1.61	82.35	1	2	2	17	Medium quality	167600	1560134	39.70	26	d__Archaea;p__Thermoplasmatota;c__Thermoplasmata;o__Thermoplasmatales;f__Thermoplasmataceae;g__Cuniculiplasma;s__
LMSG_G000005660.1	yes	1501_2	Polymetallic	96.89	0.75	93.16	0	0	0	18	Medium quality	21330	2882546	66.30	181	d__Bacteria;p__Proteobacteria;c__Alphaproteobacteria;o__Acetobacterales;f__Acetobacteraceae;g__G45-3;s__G45-3 sp003352165
LMSG_G000005661.1	yes	728_1	Polymetallic	97.43	2.99	82.48	1	0	1	19	Medium quality	15226	3500708	73.00	330	d__Bacteria;p__Actinobacteriota;c__Acidimicrobiia;o__Acidimicrobiales;f__UBA8190;g__;s__
LMSG_G000005662.1	yes	1549_2	Polymetallic	86.68	2.44	74.49	0	1	0	14	Medium quality	15301	2233029	64.10	204	d__Bacteria;p__Proteobacteria;c__Gammaproteobacteria;o__Acidiferrobacterales;f__Acidiferrobacteraceae;g__Acidiferrobacter;s__Acidiferrobacter thiooxydans
LMSG_G000004530.1	yes	1104_1	Polymetallic	93.06	1.05	87.81	1	1	1	18	High quality	41279	3639504	56.70	282	d__Bacteria;p__Firmicutes_E;c__Sulfobacillia;o__Sulfobacillales;f__;g__;s__

LMSG_G000005663.1	yes	200_0	Polymetallic	77.41	0.81	73.38	2	2	2	16	Medium quality	58068	1105483	45.90	34	d__Archaea;p__Thermoplasmatota;c__Thermoplasmata;o__Thermoplasmatales;f__Thermoplasmataceae;g__UBA509;s__
LMSG_G000005664.1	yes	969_0	Polymetallic	72.80	3.51	55.26	1	0	0	17	Medium quality	20653	2064887	55.80	146	d__Bacteria;p__Nitrospirota;c__Leptospirillia;o__Leptospirillales;f__Leptospirillaceae;g__Leptospirillum_A;s__
LMSG_G000005665.1	yes	1112_0	Polymetallic	85.53	0.33	83.87	0	2	0	17	Medium quality	22260	3085709	57.90	262	d__Bacteria;p__Firmicutes_E;c__Sulfobacillia;o__Sulfobacillales;f__Sulfobacillaceae;g__Sulfobacillus_B;s__
LMSG_G000005666.1	yes	1541_0	Polymetallic	83.66	2.13	72.99	1	0	1	18	Medium quality	12367	1861932	67.20	190	d__Bacteria;p__Proteobacteria;c__Gammaproteobacteria;o__Acidiferrobacterales;f__Acidiferrobacteraceae;g__;s__
LMSG_G000005667.1	yes	1150_1	Polymetallic	95.70	0.66	92.40	0	1	1	19	Medium quality	98391	3022303	68.70	137	d__Bacteria;p__Firmicutes_E;c__Sulfobacillia;o__Sulfobacillales;f__;g__;s__
LMSG_G000005668.1	yes	1144_0	Polymetallic	95.00	0.03	94.83	0	4	2	19	Medium quality	61849	2883708	54.00	107	d__Bacteria;p__Firmicutes_E;c__Sulfobacillia;o__Sulfobacillales;f__Sulfobacillaceae;g__Sulfobacillus_C;s__
LMSG_G000005669.1	yes	8_0	Magnetite	69.66	1.94	59.96	1	1	1	18	Medium quality	39177	623700	36.20	62	d__Archaea;p__Nanoarchaeota;c__Nanoarchaeia;o__Parvarchaeales;f__Parvarchaeaceae;g__Parvarchaeum;s__
LMSG_G000005670.1	yes	1688_1	Magnetite	72.19	0.00	72.19	1	1	3	19	Medium quality	254772	812988	37.30	11	d__Archaea;p__Nanoarchaeota;c__Nanoarchaeia;o__Pacearchaeales;f__GW2011-AR1;g__GW2011-AR1;s__
LMSG_G000005671.1	yes	1814_0	Magnetite	64.15	0.99	59.20	0	2	0	20	Medium quality	46414	851670	56.90	71	d__Bacteria;p__Patescibacteria;c__Paceibacteria;o__UBA6257;f__2-01-FULL-56-20;g__;s__
LMSG_G000005672.1	yes	112_1	Magnetite	81.93	0.93	77.26	1	1	1	19	Medium quality	47298	1026476	44.00	29	d__Archaea;p__Micrarchaeota;c__Micrarchaeia;o__Micrarchaeales;f__;g__;s__
LMSG_G000006310.1	yes	692_2	Magnetite	97.43	1.28	91.02	1	1	0	18	Medium quality	43156	2620397	64.10	88	d__Bacteria;p__Actinobacteriota;c__Acidimicrobiia;o__Acidimicrobiales;f__Acidimicrobiaceae;g__Acidithrix;s__
LMSG_G000005673.1	yes	885_1	Magnetite	94.30	1.21	88.25	0	1	1	18	Medium quality	13285	2414292	59.10	320	d__Bacteria;p__Acidobacteriota;c__Acidobacteriae;o__Acidobacteriales;f__Acidobacteriaceae;g__;s__
LMSG_G000005674.1	yes	1009_0	Magnetite	99.13	0.34	97.43	2	0	0	19	Medium quality	468098	4303472	57.00	23	d__Bacteria;p__Proteobacteria;c__Gammaproteobacteria;o__Pseudomonadales;f__Cellvibrionaceae;g__Microbulbifer;s__
LMSG_G000006292.1	yes	760_1	Magnetite	91.88	2.21	80.81	1	1	1	16	Medium quality	34927	2575268	71.20	107	d__Bacteria;p__Actinobacteriota;c__Acidimicrobiia;o__Acidimicrobiales;f__RAAP-2;g__;s__
LMSG_G000005675.1	yes	144_1	Magnetite	75.70	2.80	61.69	1	1	0	20	Medium quality	31650	685687	31.00	31	d__Archaea;p__Micrarchaeota;c__Micrarchaeia;o__Micrarchaeales;f__;g__;s__
LMSG_G000005676.1	yes	699_1	Magnetite	94.87	4.27	73.51	1	0	1	18	Medium quality	12952	3680377	68.40	367	d__Bacteria;p__Actinobacteriota;c__Acidimicrobiia;o__Acidimicrobiales;f__Palsa-688;g__;s__
LMSG_G000005677.1	yes	368_1	Magnetite	79.40	2.04	69.19	1	0	1	19	Medium quality	98034	670637	33.90	16	d__Bacteria;p__Patescibacteria;c__Saccharimonadia;o__Saccharimonadales;f__UBA4665;g__;s__
LMSG_G000005678.1	yes	47_0	Magnetite	54.04	0.00	54.04	1	1	0	18	Medium quality	12131	916733	38.10	106	d__Archaea;p__Nanoarchaeota;c__Nanoarchaeia;o__Parvarchaeales;f__Parvarchaeaceae;g__Parvarchaeum;s__
LMSG_G000005679.1	yes	599_1	Magnetite	98.29	0.43	96.16	0	0	0	20	Medium quality	107038	2682491	47.40	74	d__Bacteria;p__Actinobacteriota;c__Acidimicrobiia;o__Acidimicrobiales;f__;g__;s__
LMSG_G000004531.1	yes	192_0	Magnetite	93.90	4.84	69.71	1	2	2	20	High quality	42082	1762965	46.00	87	d__Archaea;p__Thermoplasmatota;c__Thermoplasmata;o__Thermoplasmatales;f__Thermoplasmataceae;g__UBA509;s__
LMSG_G000005680.1	yes	725_2	Magnetite	86.67	0.43	84.54	1	0	1	16	Medium quality	11563	2664058	64.20	293	d__Bacteria;p__Actinobacteriota;c__Acidimicrobiia;o__Acidimicrobiales;f__Palsa-688;g__;s__
LMSG_G000005682.1	yes	1208_1	Magnetite	81.86	4.01	61.82	1	0	1	18	Medium quality	6819	1702639	63.00	305	d__Bacteria;p__Proteobacteria;c__Gammaproteobacteria;o__UBA2770;f__UBA2770;g__;s__
LMSG_G000005681.1	yes	28_0	Magnetite	72.90	3.27	56.55	1	1	2	18	Medium quality	15739	535742	40.90	45	d__Archaea;p__Nanoarchaeota;c__Nanoarchaeia;o__Parvarchaeales;f__Parvarchaeaceae;g__;s__
LMSG_G000004532.1	yes	759_1	Magnetite	97.43	1.28	91.02	1	1	1	19	High quality	54179	2334772	70.90	67	d__Bacteria;p__Actinobacteriota;c__Acidimicrobiia;o__Acidimicrobiales;f__RAAP-2;g__;s__
LMSG_G000006293.1	yes	1521_1	Magnetite	98.27	0.86	93.96	1	1	1	16	Medium quality	70988	3783274	61.50	85	d__Bacteria;p__Acidobacteriota;c__Acidobacteriae;o__Acidobacteriales;f__Acidobacteriaceae;g__Terracidiphilus;s__
LMSG_G000006312.1	yes	102_0	Magnetite	81.61	2.80	67.60	1	2	1	19	Medium quality	45062	1143089	49.20	37	d__Archaea;p__Micrarchaeota;c__Micrarchaeia;o__Micrarchaeales;f__;g__;s__
LMSG_G000005683.1	yes	374_1	Magnetite	77.25	0.00	77.25	1	2	2	17	Medium quality	93633	563357	31.90	27	d__Bacteria;p__Patescibacteria;c__Saccharimonadia;o__Saccharimonadales;f__SZUA-47;g__;s__
LMSG_G000005684.1	yes	646_1	Magnetite	89.10	2.31	77.55	1	1	1	14	Medium quality	18268	4386000	68.80	384	d__Bacteria;p__Firmicutes_E;c__Thermaerobacteria;o__;f__;g__;s__
LMSG_G000005685.1	yes	88_1	Magnetite	71.96	0.00	71.96	0	0	0	15	Medium quality	21455	485078	54.40	26	d__Archaea;p__Micrarchaeota;c__Micrarchaeia;o__Micrarchaeales;f__Micrarchaeaceae;g__Micrarchaeum;s__
LMSG_G000005688.1	yes	1515_0	Magnetite	91.40	2.82	77.31	0	0	0	18	Medium quality	18090	4212228	70.90	330	d__Bacteria;p__Proteobacteria;c__Alphaproteobacteria;o__Acetobacterales;f__Acetobacteraceae;g__;s__
LMSG_G000004533.1	yes	685_1	Magnetite	99.14	1.28	92.73	1	4	11	20	High quality	65657	2407114	40.50	98	d__Bacteria;p__Actinobacteriota;c__Acidimicrobiia;o__Acidimicrobiales;f__;g__;s__
LMSG_G000005686.1	yes	122_0	Magnetite	78.19	0.93	73.52	1	0	0	19	Medium quality	23924	802025	47.00	44	d__Archaea;p__Micrarchaeota;c__Micrarchaeia;o__Micrarchaeales;f__;g__;s__
LMSG_G000005687.1	yes	370_1	Magnetite	72.27	0.00	72.27	1	1	1	18	Medium quality	500670	936265	40.50	6	d__Bacteria;p__Patescibacteria;c__Saccharimonadia;o__Saccharimonadales;f__UBA4665;g__;s__
LMSG_G000005689.1	yes	556_1	Magnetite	89.10	1.66	80.80	1	1	2	19	Medium quality	8061	1464493	71.10	252	d__Archaea;p__Thermoplasmatota;c__Thermoplasmata;o__UBA184;f__UBA184;g__;s__
LMSG_G000004534.1	yes	503_1	Magnetite	99.83	1.47	92.46	1	3	6	20	High quality	76636	5185567	54.30	164	d__Bacteria;p__Desulfobacterota;c__Syntrophobacteria;o__Syntrophobacterales;f__Syntrophobacteraceae;g__SbD1;s__
LMSG_G000004535.1	yes	165_1	Copper	97.69	2.44	85.50	1	2	2	20	High quality	29762	1537180	34.20	83	d__Archaea;p__Thermoplasmatota;c__Thermoplasmata;o__Thermoplasmatales;f__Thermoplasmataceae;g__Acidiplasma;s__Acidiplasma aeolicum
LMSG_G000005690.1	yes	1141_1	Copper	92.50	0.33	90.84	0	2	3	18	Medium quality	26694	4687609	51.90	391	d__Bacteria;p__Firmicutes_E;c__Sulfobacillia;o__Sulfobacillales;f__Sulfobacillaceae;g__Sulfobacillus_C;s__
LMSG_G000005691.1	yes	376_1	Copper	69.03	0.00	69.03	0	1	0	20	Medium quality	10060	811934	35.70	113	d__Bacteria;p__Patescibacteria;c__Saccharimonadia;o__Saccharimonadales;f__UBA4665;g__;s__
LMSG_G000005692.1	yes	407_1	Copper	75.70	0.00	75.70	1	4	2	20	Medium quality	88495	1017116	27.10	115	d__Bacteria;p__Patescibacteria;c__Dojkabacteria;o__;f__;g__;s__
LMSG_G000005693.1	yes	353_1	Copper	60.00	0.00	60.00	0	0	0	13	Medium quality	24440	536438	29.30	48	d__Bacteria;p__Patescibacteria;c__Saccharimonadia;o__Saccharimonadales;f__SZUA-47;g__;s__
LMSG_G000005694.1	yes	454_2	Copper	78.20	0.85	73.93	0	0	1	14	Medium quality	23896	1535102	48.30	115	d__Bacteria;p__Actinobacteriota;c__Acidimicrobiia;o__Acidimicrobiales;f__;g__;s__
LMSG_G000005695.1	yes	968_1	Copper	80.75	0.91	76.21	0	0	0	17	Medium quality	55618	2211621	55.90	82	d__Bacteria;p__Nitrospirota;c__Leptospirillia;o__Leptospirillales;f__Leptospirillaceae;g__Leptospirillum_A;s__
LMSG_G000004536.1	yes	163_1	Copper	98.73	0.00	98.73	1	2	1	20	High quality	65600	2109100	30.00	76	d__Archaea;p__Thermoplasmatota;c__Thermoplasmata;o__Thermoplasmatales;f__Thermoplasmataceae;g__Ferroplasma;s__
LMSG_G000005696.1	yes	1151_2	Copper	95.70	0.53	93.06	0	4	6	20	Medium quality	24421	3702057	67.40	290	d__Bacteria;p__Firmicutes_E;c__Sulfobacillia;o__Sulfobacillales;f__;g__;s__
LMSG_G000005697.1	yes	197_0	Copper	68.99	2.42	56.90	1	1	0	10	Medium quality	14032	1261229	42.10	142	d__Archaea;p__Thermoplasmatota;c__Thermoplasmata;o__Thermoplasmatales;f__Thermoplasmataceae;g__UBA447;s__
LMSG_G000006246.1	yes	973_1	Copper	95.06	0.62	91.98	2	2	0	18	Medium quality	76252	2220448	58.50	58	d__Bacteria;p__Proteobacteria;c__Gammaproteobacteria;o__Acidithiobacillales;f__Acidithiobacillaceae;g__Acidithiobacillus_A;s__
LMSG_G000005698.1	yes	185_1	Copper	80.24	1.61	72.18	1	1	1	19	Medium quality	21068	1947197	41.40	160	d__Archaea;p__Thermoplasmatota;c__Thermoplasmata;o__Thermoplasmatales;f__Thermoplasmataceae;g__UBA582;s__UBA582 sp002502705
LMSG_G000006294.1	yes	1167_1	Copper	95.44	1.32	88.84	1	2	1	16	Medium quality	23988	3933944	59.90	297	d__Bacteria;p__Firmicutes_E;c__Sulfobacillia;o__Sulfobacillales;f__;g__;s__
LMSG_G000005699.1	yes	94_1	Copper	79.43	0.23	78.27	1	1	0	20	Medium quality	88705	1018947	45.10	22	d__Archaea;p__Micrarchaeota;c__Micrarchaeia;o__Micrarchaeales;f__Micrarchaeaceae;g__Micrarchaeum;s__
LMSG_G000005700.1	yes	977_1	Lead-Zinc	93.78	0.16	93.01	1	0	0	14	Medium quality	59674	2308713	62.30	67	d__Bacteria;p__Proteobacteria;c__Gammaproteobacteria;o__Acidithiobacillales;f__Acidithiobacillaceae;g__RIZI01;s__RIZI01 sp003721225
LMSG_G000004537.1	yes	673_1	Lead-Zinc	91.66	1.85	82.41	1	1	1	19	High quality	31841	2767323	71.30	134	d__Bacteria;p__Chloroflexota;c__Ellin6529;o__CSP1-4;f__CSP1-4;g__;s__
LMSG_G000005701.1	yes	103_1	Lead-Zinc	82.86	0.93	78.19	1	2	0	18	Medium quality	23050	1019705	51.50	62	d__Archaea;p__Micrarchaeota;c__Micrarchaeia;o__Micrarchaeales;f__;g__;s__
LMSG_G000005702.1	yes	66_1	Lead-Zinc	84.91	0.00	84.91	1	1	0	19	Medium quality	29393	1067776	52.10	56	d__Archaea;p__Micrarchaeota;c__Micrarchaeia;o__Micrarchaeales;f__Micrarchaeaceae;g__;s__
LMSG_G000005703.1	yes	548_0	Lead-Zinc	70.93	2.40	58.93	1	0	1	18	Medium quality	4387	1062280	68.00	279	d__Archaea;p__Thermoplasmatota;c__Thermoplasmata;o__UBA184;f__UBA184;g__;s__
LMSG_G000005704.1	yes	23_1	Lead-Zinc	81.23	0.93	76.56	2	1	1	18	Medium quality	106883	867999	39.00	30	d__Archaea;p__Nanoarchaeota;c__Nanoarchaeia;o__Parvarchaeales;f__Parvarchaeaceae;g__;s__
LMSG_G000005705.1	yes	1563_0	Lead-Zinc	79.41	0.84	75.21	0	0	0	18	Medium quality	9186	2993784	62.40	482	d__Bacteria;p__Desulfobacterota_B;c__Binatia;o__Binatales;f__Binataceae;g__;s__
LMSG_G000004538.1	yes	1522_0	Lead-Zinc	99.12	1.71	90.58	1	1	1	18	High quality	128116	5007706	62.60	85	d__Bacteria;p__Acidobacteriota;c__Acidobacteriae;o__Acidobacteriales;f__Acidobacteriaceae;g__Palsa-343;s__
LMSG_G000005706.1	yes	39_1	Lead-Zinc	76.09	0.00	76.09	1	0	2	18	Medium quality	10690	839456	34.80	105	d__Archaea;p__Micrarchaeota;c__Micrarchaeia;o__Micrarchaeales;f__;g__;s__
LMSG_G000005707.1	yes	218_1	Lead-Zinc	78.22	0.81	74.19	1	1	2	18	Medium quality	95844	1211191	43.80	38	d__Archaea;p__Thermoplasmatota;c__Thermoplasmata;o__Thermoplasmatales;f__GCA-001856825;g__GCA-001856825;s__
LMSG_G000005708.1	yes	428_1	Lead-Zinc	97.72	0.00	97.72	0	4	4	20	Medium quality	53035	3001235	58.90	198	d__Bacteria;p__Planctomycetota;c__Phycisphaerae;o__UBA1161;f__;g__;s__
LMSG_G000005710.1	yes	1316_1	Lead-Zinc	93.20	0.20	92.20	1	0	0	17	Medium quality	42082	3179945	66.00	111	d__Bacteria;p__Proteobacteria;c__Gammaproteobacteria;o__Burkholderiales;f__Rhodocyclaceae;g__Sterolibacterium;s__
LMSG_G000005709.1	yes	1250_2	Lead-Zinc	70.88	2.34	59.19	1	0	0	17	Medium quality	19116	2528431	59.60	277	d__Bacteria;p__Proteobacteria;c__Gammaproteobacteria;o__Burkholderiales;f__Sulfuricellaceae;g__Sulfuricella;s__
LMSG_G000004539.1	yes	1235_1	Lead-Zinc	97.61	1.51	90.08	1	2	1	18	High quality	163787	3175253	54.90	38	d__Bacteria;p__Proteobacteria;c__Gammaproteobacteria;o__Burkholderiales;f__Gallionellaceae;g__Gallionella;s__
LMSG_G000005714.1	yes	516_1	Lead-Zinc	96.56	0.00	96.56	1	0	2	18	Medium quality	36999	2472329	49.30	223	d__Bacteria;p__Elusimicrobiota;c__Elusimicrobia;o__UBA1565;f__;g__;s__
LMSG_G000005711.1	yes	1497_0	Lead-Zinc	99.56	0.87	95.20	0	0	0	20	Medium quality	345253	5202522	65.50	22	d__Bacteria;p__Proteobacteria;c__Alphaproteobacteria;o__Dongiales;f__Dongiaceae;g__SYSU-D60009;s__

LMSG_G000005712.1	yes	1039_0	Lead-Zinc	75.36	1.71	66.84	1	0	0	17	Medium quality	3840	2824221	64.80	863	d__Bacteria;p__Actinobacteriota;c__Actinomycetia;o__Actinomycetales;f__Microbacteriaceae;g__Leucobacter;s__
LMSG_G000005713.1	yes	142_1	Lead-Zinc	68.28	0.93	63.61	1	0	0	19	Medium quality	18367	911735	46.10	117	d__Archaea;p__Micrarchaeota;c__Micrarchaeia;o__Micrarchaeales;f__;g__;s__
LMSG_G000005715.1	yes	89_0	Lead-Zinc	65.69	0.00	65.69	1	2	1	17	Medium quality	19529	556821	53.80	44	d__Archaea;p__Micrarchaeota;c__Micrarchaeia;o__Micrarchaeales;f__Micrarchaeaceae;g__Micrarchaeum;s__
LMSG_G000005716.1	yes	1156_1	Lead-Zinc	95.51	0.00	95.51	0	0	0	19	Medium quality	76396	2638559	40.80	123	d__Bacteria;p__Eremiobacterota;c__UBP9;o__;f__;g__;s__
LMSG_G000004540.1	yes	871_1	Lead-Zinc	99.46	2.58	86.54	1	1	1	20	High quality	50375	5622546	69.10	227	d__Bacteria;p__Actinobacteriota;c__Actinomycetia;o__Streptosporangiales;f__Streptosporangiaceae;g__Palsa-506;s__
LMSG_G000005717.1	yes	115_1	Lead-Zinc	79.75	0.00	79.75	1	1	1	20	Medium quality	772409	797405	56.30	23	d__Archaea;p__Micrarchaeota;c__Micrarchaeia;o__Micrarchaeales;f__;g__;s__
LMSG_G000005718.1	yes	1250_1	Lead-Zinc	92.65	4.66	69.35	1	0	1	18	Medium quality	20288	2930679	59.40	211	d__Bacteria;p__Proteobacteria;c__Gammaproteobacteria;o__Burkholderiales;f__Sulfuricellaceae;g__Sulfuricella;s__
LMSG_G000004541.1	yes	831_0	Lead-Zinc	97.43	0.43	95.30	1	1	1	18	High quality	91361	3192524	67.60	100	d__Bacteria;p__Actinobacteriota;c__Thermoleophilia;o__Solirubrobacterales;f__Solirubrobacteraceae;g__;s__
LMSG_G000005719.1	yes	119_1	Lead-Zinc	85.66	0.00	85.66	1	1	1	17	Medium quality	12481	1011104	49.40	100	d__Archaea;p__Micrarchaeota;c__Micrarchaeia;o__Micrarchaeales;f__;g__;s__
LMSG_G000005720.1	yes	15_1	Lead-Zinc	78.51	0.93	73.84	1	1	1	19	Medium quality	61702	676718	43.00	42	d__Archaea;p__Nanoarchaeota;c__Nanoarchaeia;o__Parvarchaeales;f__Parvarchaeaceae;g__;s__
LMSG_G000004542.1	yes	939_1	Lead-Zinc	97.27	0.00	97.27	1	2	2	19	High quality	48162	2868214	45.60	116	d__Bacteria;p__Nitrospirota;c__Thermodesulfovibrionia;o__Thermodesulfovibrionales;f__UBA9935;g__GWB2-47-37;s__
LMSG_G000005722.1	yes	70_1	Lead-Zinc	73.75	0.14	73.04	1	0	1	18	Medium quality	23624	872091	46.10	92	d__Archaea;p__Micrarchaeota;c__Micrarchaeia;o__Micrarchaeales;f__Micrarchaeaceae;g__;s__
LMSG_G000005721.1	yes	730_1	Lead-Zinc	89.27	2.99	74.32	0	1	0	12	Medium quality	19063	2370451	70.80	156	d__Bacteria;p__Actinobacteriota;c__Acidimicrobiia;o__Acidimicrobiales;f__RAAP-2;g__Palsa-461;s__
LMSG_G000005723.1	yes	1506_2	Lead-Zinc	62.15	0.25	60.91	0	0	0	15	Medium quality	5539	2238125	71.00	450	d__Bacteria;p__Proteobacteria;c__Alphaproteobacteria;o__Acetobacterales;f__Acetobacteraceae;g__Palsa-883;s__
LMSG_G000004543.1	yes	927_1	Lead-Zinc	95.85	0.86	91.54	1	1	1	19	High quality	86747	3351060	62.00	77	d__Bacteria;p__Acidobacteriota;c__Acidobacteriae;o__Acidobacteriales;f__Acidobacteriaceae;g__Terracidiphilus;s__
LMSG_G000004544.1	yes	684_1	Lead-Zinc	94.87	1.28	88.46	1	1	1	20	High quality	19488	2543346	37.80	207	d__Bacteria;p__Actinobacteriota;c__Acidimicrobiia;o__Acidimicrobiales;f__;g__;s__
LMSG_G000005724.1	yes	79_1	Lead-Zinc	82.39	1.87	73.05	2	1	2	19	Medium quality	54689	847708	47.50	21	d__Archaea;p__Micrarchaeota;c__Micrarchaeia;o__Micrarchaeales;f__Micrarchaeaceae;g__;s__
LMSG_G000005725.1	yes	78_1	Lead-Zinc	74.83	0.93	70.16	1	1	0	17	Medium quality	14559	645893	47.60	62	d__Archaea;p__Micrarchaeota;c__Micrarchaeia;o__Micrarchaeales;f__Micrarchaeaceae;g__;s__
LMSG_G000005726.1	yes	961_0	Lead-Zinc	91.70	0.91	87.16	0	0	0	17	Medium quality	41526	2529539	59.70	102	d__Bacteria;p__Nitrospirota;c__Leptospirillia;o__Leptospirillales;f__Leptospirillaceae;g__UBA4572;s__
LMSG_G000004545.1	yes	1558_1	Lead-Zinc	96.63	1.68	88.23	1	1	1	19	High quality	176009	4034597	61.50	97	d__Bacteria;p__Desulfobacterota_B;c__Binatia;o__Binatales;f__Binataceae;g__;s__
LMSG_G000004546.1	yes	901_1	Lead-Zinc	95.90	0.86	91.59	1	1	1	19	High quality	67562	3818508	60.80	88	d__Bacteria;p__Acidobacteriota;c__Acidobacteriae;o__Acidobacteriales;f__Acidobacteriaceae;g__Granulicella_A;s__
LMSG_G000005727.1	yes	1036_1	Lead-Zinc	98.38	2.33	86.76	0	0	0	20	Medium quality	74198	6761234	57.10	160	d__Bacteria;p__Planctomycetota;c__Planctomycetes;o__Isosphaerales;f__Isosphaeraceae;g__;s__
LMSG_G000005728.1	yes	712_0	Lead-Zinc	87.26	1.32	80.64	1	1	0	17	Medium quality	8990	4258979	72.80	645	d__Bacteria;p__Firmicutes_E;c__Thermaerobacteria;o__;f__;g__;s__
LMSG_G000005729.1	yes	612_1	Lead-Zinc	84.85	2.23	73.70	0	0	0	18	Medium quality	9962	1897832	54.30	251	d__Bacteria;p__Actinobacteriota;c__Acidimicrobiia;o__Acidimicrobiales;f__Acidimicrobiaceae;g__SCQM01;s__
LMSG_G000005730.1	yes	748_0	Lead-Zinc	68.14	0.85	63.87	0	0	0	13	Medium quality	6645	2102573	69.00	370	d__Bacteria;p__Actinobacteriota;c__Acidimicrobiia;o__Acidimicrobiales;f__Bog-793;g__Palsa-601;s__
LMSG_G000005731.1	yes	371_1	Lead-Zinc	68.97	0.99	64.02	1	1	1	18	Medium quality	42408	888715	43.90	31	d__Bacteria;p__Patescibacteria;c__Saccharimonadia;o__Saccharimonadales;f__UBA4665;g__;s__
LMSG_G000004547.1	yes	1555_1	Lead-Zinc	97.47	0.84	93.27	1	1	1	18	High quality	37860	3551012	63.60	154	d__Bacteria;p__Desulfobacterota_B;c__Binatia;o__Binatales;f__Binataceae;g__;s__
LMSG_G000004548.1	yes	439_1	Lead-Zinc	96.21	0.00	96.21	1	1	2	20	High quality	38254	3717805	57.20	167	d__Bacteria;p__Planctomycetota;c__Phycisphaerae;o__UBA1161;f__;g__;s__
LMSG_G000005732.1	yes	404_1	Lead-Zinc	77.42	0.63	74.29	1	1	4	20	Medium quality	16479	1262348	28.90	233	d__Bacteria;p__Patescibacteria;c__;o__;f__;g__;s__
LMSG_G000005733.1	yes	756_1	Lead-Zinc	88.52	4.78	64.63	1	0	1	19	Medium quality	9009	2775015	68.70	417	d__Bacteria;p__Actinobacteriota;c__Acidimicrobiia;o__Acidimicrobiales;f__RAAP-2;g__;s__
LMSG_G000005734.1	yes	649_1	Lead-Zinc	84.45	1.52	76.86	2	1	1	16	Medium quality	17343	3860347	75.00	326	d__Bacteria;p__Firmicutes_E;c__Thermaerobacteria;o__;f__;g__;s__
LMSG_G000005735.1	yes	590_1	Lead-Zinc	60.19	0.00	60.19	0	0	0	13	Medium quality	186541	761939	60.80	54	d__Archaea;p__Thermoproteota;c__Nitrososphaeria;o__Nitrososphaerales;f__UBA183;g__UBA183;s__
LMSG_G000004549.1	yes	348_1	Lead-Zinc	100.00	1.28	93.59	1	1	1	20	High quality	80428	2605620	66.70	55	d__Bacteria;p__Actinobacteriota;c__Acidimicrobiia;o__Acidimicrobiales;f__;g__;s__
LMSG_G000005736.1	yes	632_1	Lead-Zinc	87.68	1.85	78.43	1	1	1	15	Medium quality	34680	2151741	70.70	99	d__Bacteria;p__Dormibacterota;c__Dormibacteria;o__UBA8260;f__Bog-877;g__;s__
LMSG_G000004550.1	yes	621_1	Lead-Zinc	95.37	1.11	89.82	1	1	1	20	High quality	32021	2819998	66.90	206	d__Bacteria;p__Dormibacterota;c__Dormibacteria;o__UBA8260;f__Bog-877;g__;s__
LMSG_G000004551.1	yes	745_1	Lead-Zinc	97.86	1.28	91.45	1	1	1	20	High quality	26185	2618986	69.40	124	d__Bacteria;p__Actinobacteriota;c__Acidimicrobiia;o__Acidimicrobiales;f__Bog-793;g__Palsa-601;s__
LMSG_G000005737.1	yes	630_2	Lead-Zinc	92.12	0.93	87.50	1	0	1	17	Medium quality	44298	2009644	70.10	188	d__Bacteria;p__Dormibacterota;c__Dormibacteria;o__UBA8260;f__Bog-877;g__;s__
LMSG_G000005738.1	yes	145_1	Lead-Zinc	79.43	1.87	70.09	1	1	0	19	Medium quality	55530	936775	31.10	74	d__Archaea;p__Micrarchaeota;c__Micrarchaeia;o__Micrarchaeales;f__;g__;s__
LMSG_G000005739.1	yes	587_0	Lead-Zinc	84.87	1.94	75.17	1	0	0	17	Medium quality	38265	1940709	59.60	104	d__Archaea;p__Thermoproteota;c__Nitrososphaeria;o__Nitrososphaerales;f__UBA183;g__BOG-1369;s__
LMSG_G000005740.1	yes	892_1	Lead-Zinc	86.36	0.00	86.36	0	0	0	16	Medium quality	12746	4172943	58.00	473	d__Bacteria;p__Acidobacteriota;c__Acidobacteriae;o__Acidobacteriales;f__Acidobacteriaceae;g__;s__
LMSG_G000005742.1	yes	902_2	Lead-Zinc	94.39	2.63	81.22	0	1	0	17	Medium quality	22286	3653742	61.10	233	d__Bacteria;p__Acidobacteriota;c__Acidobacteriae;o__Acidobacteriales;f__Acidobacteriaceae;g__Granulicella_A;s__
LMSG_G000005741.1	yes	118_1	Lead-Zinc	80.37	1.25	74.14	1	2	2	19	Medium quality	19582	954134	48.50	62	d__Archaea;p__Micrarchaeota;c__Micrarchaeia;o__Micrarchaeales;f__;g__;s__
LMSG_G000004552.1	yes	724_1	Lead-Zinc	94.87	1.28	88.46	1	2	1	18	High quality	78006	3225790	64.50	190	d__Bacteria;p__Actinobacteriota;c__Acidimicrobiia;o__Acidimicrobiales;f__Palsa-688;g__;s__
LMSG_G000005743.1	yes	20_0	Lead-Zinc	79.36	1.87	70.02	1	2	1	18	Medium quality	36868	845560	45.50	45	d__Archaea;p__Nanoarchaeota;c__Nanoarchaeia;o__Parvarchaeales;f__Parvarchaeaceae;g__;s__
LMSG_G000004553.1	yes	211_2	Lead-Zinc	93.68	2.91	79.12	1	3	1	20	High quality	37844	1915564	48.00	188	d__Archaea;p__Thermoproteota;c__Nitrososphaeria;o__UBA164;f__;g__;s__
LMSG_G000005752.1	yes	454_1	Lead-Zinc	91.45	0.43	89.32	0	0	0	16	Medium quality	43969	1898677	50.00	91	d__Bacteria;p__Actinobacteriota;c__Acidimicrobiia;o__Acidimicrobiales;f__;g__;s__
LMSG_G000005744.1	yes	131_1	Lead-Zinc	83.80	0.93	79.13	1	1	2	19	Medium quality	65319	1049737	48.20	26	d__Archaea;p__Micrarchaeota;c__Micrarchaeia;o__Micrarchaeales;f__;g__;s__
LMSG_G000006247.1	yes	572_1	Lead-Zinc	93.67	1.72	85.05	1	1	0	19	Medium quality	70326	2027673	62.20	52	d__Bacteria;p__Proteobacteria;c__Gammaproteobacteria;o__UBA2770;f__UBA2770;g__;s__
LMSG_G000005745.1	yes	1560_1	Lead-Zinc	96.63	3.36	79.83	0	0	0	18	Medium quality	29169	4051484	63.10	219	d__Bacteria;p__Desulfobacterota_B;c__Binatia;o__Binatales;f__Binataceae;g__;s__
LMSG_G000005746.1	yes	129_1	Lead-Zinc	79.04	0.00	79.04	1	1	1	20	Medium quality	29585	855211	43.20	45	d__Archaea;p__Micrarchaeota;c__Micrarchaeia;o__Micrarchaeales;f__;g__;s__
LMSG_G000005747.1	yes	1693_1	Lead-Zinc	73.24	0.66	69.93	1	1	1	18	Medium quality	497156	646542	45.70	10	d__Bacteria;p__Patescibacteria;c__Paceibacteria;o__UBA6257;f__Colwellbacteraceae;g__;s__
LMSG_G000004554.1	yes	216_1	Lead-Zinc	95.69	0.81	91.66	1	1	2	20	High quality	76975	1406828	46.30	43	d__Archaea;p__Thermoplasmatota;c__Thermoplasmata;o__Thermoplasmatales;f__GCA-001856825;g__GCA-001856825;s__
LMSG_G000005748.1	yes	13_1	Lead-Zinc	82.44	1.94	72.74	0	2	0	18	Medium quality	25387	946057	35.50	82	d__Archaea;p__Nanoarchaeota;c__Nanoarchaeia;o__Parvarchaeales;f__Parvarchaeaceae;g__Parvarchaeum;s__
LMSG_G000005749.1	yes	495_0	Lead-Zinc	87.49	0.33	85.84	0	0	0	17	Medium quality	8277	2868830	62.50	442	d__Bacteria;p__Firmicutes_E;c__Thermaerobacteria;o__;f__;g__;s__
LMSG_G000005750.1	yes	746_1	Lead-Zinc	93.20	2.99	78.25	0	1	0	17	Medium quality	35934	2855765	72.00	139	d__Bacteria;p__Actinobacteriota;c__Acidimicrobiia;o__Acidimicrobiales;f__RAAP-2;g__;s__
LMSG_G000005751.1	yes	1171_1	Lead-Zinc	100.00	0.19	99.05	2	0	0	19	Medium quality	207743	2667968	53.40	24	d__Bacteria;p__Proteobacteria;c__Gammaproteobacteria;o__Halothiobacillales;f__Halothiobacillaceae;g__Halothiobacillus;s__
LMSG_G000004555.1	yes	764_0	Lead-Zinc	95.72	3.85	76.49	1	1	1	18	High quality	44725	2511992	71.30	131	d__Bacteria;p__Actinobacteriota;c__Acidimicrobiia;o__Acidimicrobiales;f__RAAP-2;g__;s__
LMSG_G000005753.1	yes	818_1	Lead-Zinc	86.75	0.85	82.48	0	1	0	20	Medium quality	18088	2710204	63.70	344	d__Bacteria;p__Actinobacteriota;c__Acidimicrobiia;o__Acidimicrobiales;f__;g__;s__
LMSG_G000004556.1	yes	1639_1	Lead-Zinc	97.65	4.10	77.16	1	1	1	18	High quality	26323	3527373	47.00	197	d__Bacteria;p__Bacteroidota;c__Kryptonia;o__Kryptoniales;f__;g__;s__
LMSG_G000004557.1	yes	1673_1	Lead-Zinc	99.44	1.12	93.86	1	1	2	20	High quality	23849	3186087	36.30	200	d__Bacteria;p__Bacteroidota;c__Ignavibacteria;o__Ignavibacteriales;f__Melioribacteraceae;g__Fen-1258;s__
LMSG_G000005754.1	yes	1568_1	Lead-Zinc	91.11	3.36	74.31	0	0	0	18	Medium quality	26698	3230056	60.90	208	d__Bacteria;p__Desulfobacterota_B;c__Binatia;o__Binatales;f__Binataceae;g__;s__
LMSG_G000005755.1	yes	1788_0	Lead-Zinc	70.66	1.72	62.04	1	1	1	19	Medium quality	11067	524889	40.20	56	d__Bacteria;p__Patescibacteria;c__Paceibacteria;o__UBA6257;f__UBA9933;g__;s__
LMSG_G000004558.1	yes	1547_1	Lead-Zinc	96.63	0.91	92.06	1	1	1	19	High quality	23349	2346567	62.90	154	d__Bacteria;p__Proteobacteria;c__Gammaproteobacteria;o__Acidiferrobacterales;f__Acidiferrobacteraceae;g__Acidiferrobacter;s__
LMSG_G000005756.1	yes	482_1	Lead-Zinc	91.83	0.49	89.38	2	0	0	18	Medium quality	43514	4126479	63.40	152	d__Bacteria;p__Firmicutes_E;c__DTU015;o__;f__;g__;s__
LMSG_G000005757.1	yes	430_1	Lead-Zinc	89.43	0.00	89.43	0	0	1	14	Medium quality	10903	2819098	54.00	395	d__Bacteria;p__Planctomycetota;c__Phycisphaerae;o__UBA1161;f__;g__;s__
LMSG_G000005758.1	yes	736_1	Lead-Zinc	95.63	1.28	89.22	0	1	0	19	Medium quality	26376	2421734	71.10	175	d__Bacteria;p__Actinobacteriota;c__Acidimicrobiia;o__Acidimicrobiales;f__RAAP-2;g__;s__
LMSG_G000005759.1	yes	1724_1	Lead-Zinc	66.70	0.00	66.70	1	1	2	19	Medium quality	21267	1015481	43.20	92	d__Bacteria;p__Patescibacteria;c__Doudnabacteria;o__UBA920;f__UBA920;g__PALSA-1336;s__
LMSG_G000005760.1	yes	681_1	Lead-Zinc	70.68	1.72	62.06	1	0	1	16	Medium quality	7254	2228739	71.80	409	d__Bacteria;p__Chloroflexota;c__Ellin6529;o__CSP1-4;f__CSP1-4;g__;s__
LMSG_G000005761.1	yes	65_1	Lead-Zinc	84.57	0.93	79.90	1	1	2	19	Medium quality	53702	1135287	48.20	28	d__Archaea;p__Micrarchaeota;c__Micrarchaeia;o__Micrarchaeales;f__Micrarchaeaceae;g__;s__
LMSG_G000004559.1	yes	820_1	Lead-Zinc	97.86	0.85	93.59	1	1	1	19	High quality	33943	3174920	68.10	185	d__Bacteria;p__Actinobacteriota;c__Acidimicrobiia;o__Acidimicrobiales;f__;g__;s__

LMSG_G000005762.1	yes	412_0	Lead-Zinc	63.03	0.00	63.03	1	1	2	19	Medium quality	420460	824957	36.60	26	d__Bacteria;p__Patescibacteria;c__Microgenomatia;o__Levybacterales;f__UBA12049;g__;s__
LMSG_G000005763.1	yes	1432_0	Lead-Zinc	82.07	3.42	64.96	0	0	0	19	Medium quality	4341	2137827	66.30	564	d__Bacteria;p__Proteobacteria;c__Alphaproteobacteria;o__Sphingomonadales;f__Sphingomonadaceae;g__Sphingomonas_A;s__
LMSG_G000005764.1	yes	105_1	Lead-Zinc	80.52	0.00	80.52	1	2	1	19	Medium quality	103903	772054	53.40	29	d__Archaea;p__Micrarchaeota;c__Micrarchaeia;o__Micrarchaeales;f__;g__;s__
LMSG_G000004560.1	yes	563_1	Lead-Zinc	95.46	1.60	87.46	1	2	1	18	High quality	42066	1785038	72.50	79	d__Archaea;p__Thermoplasmatota;c__Thermoplasmata;o__UBA184;f__UBA184;g__;s__
LMSG_G000005765.1	yes	1669_1	Lead-Zinc	92.43	1.12	86.85	0	1	1	20	Medium quality	16122	4103219	35.40	362	d__Bacteria;p__Bacteroidota;c__Ignavibacteria;o__Ignavibacteriales;f__Ignavibacteriaceae;g__FEN-1297;s__
LMSG_G000005766.1	yes	1610_1	Lead-Zinc	69.44	0.93	64.82	0	1	0	17	Medium quality	61051	1094706	40.40	49	d__Bacteria;p__Patescibacteria;c__Microgenomatia;o__Daviesbacterales;f__UBA10151;g__UBA10151;s__
LMSG_G000005767.1	yes	1757_1	Lead-Zinc	73.80	1.12	68.19	1	1	1	19	Medium quality	82965	957850	35.40	59	d__Bacteria;p__Patescibacteria;c__Paceibacteria;o__UBA9983;f__UBA9973;g__UBA8515;s__
LMSG_G000005768.1	yes	62_0	Lead-Zinc	70.71	0.93	66.04	1	1	1	19	Medium quality	51515	631513	48.40	22	d__Archaea;p__Micrarchaeota;c__Micrarchaeia;o__Micrarchaeales;f__;g__;s__
LMSG_G000005769.1	yes	1228_2	Lead-Zinc	95.85	0.00	95.85	1	0	0	18	Medium quality	125124	2394157	50.70	27	d__Bacteria;p__Proteobacteria;c__Gammaproteobacteria;o__Burkholderiales;f__Sulfuriferulaceae;g__UBA2487;s__
LMSG_G000005771.1	yes	757_0	Lead-Zinc	70.08	1.28	63.67	1	1	1	19	Medium quality	32927	2775715	67.30	228	d__Bacteria;p__Actinobacteriota;c__Acidimicrobiia;o__Acidimicrobiales;f__RAAP-2;g__;s__
LMSG_G000005770.1	yes	958_1	Lead-Zinc	86.36	0.91	81.82	2	1	2	13	Medium quality	44679	1938037	60.00	116	d__Bacteria;p__Nitrospirota;c__Leptospirillia;o__Leptospirillales;f__Leptospirillaceae;g__UBA4572;s__
LMSG_G000005772.1	yes	1832_2	Lead-Zinc	56.27	0.11	55.72	1	1	3	13	Medium quality	4035	504039	51.50	158	d__Bacteria;p__Patescibacteria;c__Paceibacteria;o__UBA6257;f__Colwellbacteraceae;g__;s__
LMSG_G000004561.1	yes	1567_1	Lead-Zinc	93.85	1.68	85.45	1	1	1	19	High quality	182312	3904131	62.70	46	d__Bacteria;p__Desulfobacterota_B;c__Binatia;o__Binatales;f__Binataceae;g__;s__
LMSG_G000005773.1	yes	33_1	Lead-Zinc	83.02	0.00	83.02	1	2	0	20	Medium quality	30624	905357	29.30	86	d__Archaea;p__Micrarchaeota;c__Micrarchaeia;o__Micrarchaeales;f__Micrarchaeaceae;g__;s__
LMSG_G000005774.1	yes	1212_1	Lead-Zinc	90.37	0.86	86.06	0	0	0	19	Medium quality	34934	1836928	62.30	80	d__Bacteria;p__Actinobacteriota;c__Thermoleophilia;o__BMS3ABIN01;f__BMS3ABIN01;g__;s__
LMSG_G000006248.1	yes	217_1	Lead-Zinc	95.16	1.61	87.10	1	1	0	19	Medium quality	203410	1554518	44.30	32	d__Archaea;p__Thermoplasmatota;c__Thermoplasmata;o__Thermoplasmatales;f__GCA-001856825;g__GCA-001856825;s__
LMSG_G000005775.1	yes	427_1	Lead-Zinc	91.81	1.14	86.13	0	1	1	19	Medium quality	9623	3948346	61.60	554	d__Bacteria;p__Planctomycetota;c__Phycisphaerae;o__UBA1161;f__;g__;s__
LMSG_G000005777.1	yes	1189_1	Lead-Zinc	88.21	0.95	83.49	1	3	2	14	Medium quality	57761	1761633	66.60	136	d__Bacteria;p__Proteobacteria;c__Gammaproteobacteria;o__UBA2770;f__UBA2770;g__;s__
LMSG_G000005776.1	yes	960_2	Lead-Zinc	77.89	0.00	77.89	3	1	3	13	Medium quality	30454	1836441	58.70	192	d__Bacteria;p__Nitrospirota;c__Leptospirillia;o__Leptospirillales;f__Leptospirillaceae;g__UBA4572;s__
LMSG_G000004562.1	yes	540_1	Lead-Zinc	90.66	3.12	75.09	1	2	1	18	High quality	12896	1995461	69.80	225	d__Archaea;p__Thermoplasmatota;c__Thermoplasmata;o__UBA184;f__UBA184;g__;s__
LMSG_G000004563.1	yes	936_1	Lead-Zinc	94.54	2.25	83.28	1	1	1	19	High quality	58683	3145527	49.70	147	d__Bacteria;p__Nitrospirota;c__Thermodesulfovibrionia;o__Thermodesulfovibrionales;f__UBA9935;g__;s__
LMSG_G000005778.1	yes	593_1	Lead-Zinc	83.81	0.97	78.96	0	1	1	14	Medium quality	27661	1492666	60.20	84	d__Archaea;p__Thermoproteota;c__Nitrososphaeria;o__Nitrososphaerales;f__UBA183;g__UBA183;s__
LMSG_G000005779.1	yes	700_0	Lead-Zinc	58.39	0.16	57.62	1	0	0	20	Medium quality	5752	4395457	67.50	1265	d__Bacteria;p__Actinobacteriota;c__Acidimicrobiia;o__Acidimicrobiales;f__Palsa-688;g__;s__
LMSG_G000005780.1	yes	1218_1	Lead-Zinc	96.16	1.18	90.24	0	1	0	19	Medium quality	36629	2361851	57.00	108	d__Bacteria;p__Proteobacteria;c__Gammaproteobacteria;o__Burkholderiales;f__Nitrosomonadaceae;g__;s__
LMSG_G000004564.1	yes	452_1	Lead-Zinc	94.87	1.28	88.46	1	1	1	20	High quality	75973	2556997	52.00	89	d__Bacteria;p__Actinobacteriota;c__Acidimicrobiia;o__Acidimicrobiales;f__;g__;s__
LMSG_G000004565.1	yes	580_2	Lead-Zinc	93.22	1.87	83.88	1	4	14	20	High quality	185760	2340020	51.60	200	d__Archaea;p__Thermoproteota;c__Thermoproteia;o__Marsarchaeales;f__Marsarchaeaceae;g__ECH-B-1;s__
LMSG_G000004566.1	yes	988_0	Lead-Zinc	95.93	4.88	71.54	2	1	2	20	High quality	77684	2610401	40.00	47	d__Bacteria;p__SZUA-79;c__SZUA-79;o__;f__;g__;s__
LMSG_G000005781.1	yes	986_0	Lead-Zinc	66.74	0.00	66.74	0	0	0	12	Medium quality	3267	1825635	54.20	648	d__Bacteria;p__SZUA-79;c__;o__;f__;g__;s__
LMSG_G000005782.1	yes	75_0	Pyrite-Copper	79.12	0.93	74.45	1	1	1	18	Medium quality	32972	882950	49.90	37	d__Archaea;p__Micrarchaeota;c__Micrarchaeia;o__Micrarchaeales;f__Micrarchaeaceae;g__;s__
LMSG_G000005783.1	yes	806_1	Pyrite-Copper	91.21	1.28	84.80	0	1	0	17	Medium quality	53596	1809222	66.80	75	d__Bacteria;p__Actinobacteriota;c__Acidimicrobiia;o__Acidimicrobiales;f__RAAP-2;g__RAAP-2;s__
LMSG_G000005784.1	yes	124_0	Pyrite-Copper	51.90	0.00	51.90	1	0	1	18	Medium quality	7443	646405	43.70	113	d__Archaea;p__Micrarchaeota;c__Micrarchaeia;o__Micrarchaeales;f__;g__;s__
LMSG_G000006295.1	yes	1641_0	Pyrite-Copper	91.00	2.46	78.71	1	1	1	17	Medium quality	9947	4281570	49.50	536	d__Bacteria;p__Bacteroidota;c__Kryptonia;o__Kryptoniales;f__;g__;s__
LMSG_G000005785.1	yes	687_0	Pyrite-Copper	97.43	1.28	91.02	0	0	0	18	Medium quality	16082	2815507	45.60	225	d__Bacteria;p__Actinobacteriota;c__Acidimicrobiia;o__Acidimicrobiales;f__;g__;s__
LMSG_G000004567.1	yes	1200_1	Pyrite-Copper	93.29	1.83	84.15	1	1	1	18	High quality	150883	2711492	60.90	40	d__Bacteria;p__Proteobacteria;c__Gammaproteobacteria;o__Acidiferrobacterales;f__Sulfurifustaceae;g__;s__
LMSG_G000005786.1	yes	385_2	Pyrite-Copper	60.18	0.93	55.56	0	0	0	13	Medium quality	20398	535067	45.80	36	d__Bacteria;p__Patescibacteria;c__Saccharimonadia;o__Saccharimonadales;f__UBA4665;g__;s__
LMSG_G000005787.1	yes	1739_1	Pyrite-Copper	87.60	3.42	70.51	0	1	0	12	Medium quality	47265	1822330	39.90	101	d__Bacteria;p__SZUA-79;c__SZUA-79;o__Acidulodesulfobacterales;f__SZUA-79;g__Acidulodesulfobacterium;s__
LMSG_G000005788.1	yes	1740_1	Pyrite-Copper	77.15	3.45	59.91	1	0	1	18	Medium quality	93724	1746116	35.90	63	d__Bacteria;p__SZUA-79;c__SZUA-79;o__Acidulodesulfobacterales;f__SZUA-79;g__Acidulodesulfobacterium;s__
LMSG_G000005789.1	yes	362_1	Pyrite-Copper	71.79	0.00	71.79	1	0	0	18	Medium quality	124207	755624	37.50	29	d__Bacteria;p__Patescibacteria;c__Saccharimonadia;o__Saccharimonadales;f__UBA4665;g__UBA6224;s__
LMSG_G000005790.1	yes	946_1	Pyrite-Copper	99.09	1.36	92.28	0	2	1	19	Medium quality	45295	2784064	53.20	83	d__Bacteria;p__Nitrospirota;c__Thermodesulfovibrionia;o__Thermodesulfovibrionales;f__JdFR-88;g__;s__
LMSG_G000005791.1	yes	411_1	Pyrite-Copper	60.28	0.00	60.28	1	3	5	19	Medium quality	26214	1005645	40.50	67	d__Bacteria;p__Patescibacteria;c__Microgenomatia;o__Levybacterales;f__UBA12049;g__;s__
LMSG_G000005792.1	yes	928_0	Pyrite-Copper	82.11	3.59	64.16	1	0	1	13	Medium quality	12832	2606941	63.90	277	d__Bacteria;p__Acidobacteriota;c__Acidobacteriae;o__Acidobacteriales;f__Acidobacteriaceae;g__Terracidiphilus;s__
LMSG_G000005793.1	yes	1631_1	Pyrite-Copper	59.14	1.63	51.01	2	2	8	20	Medium quality	55653	1206175	42.80	56	d__Bacteria;p__Dependentiae;c__Babeliae;o__Babeliales;f__Vermiphilaceae;g__;s__
LMSG_G000004568.1	yes	552_1	Pyrite-Copper	94.15	0.80	90.15	1	1	1	18	High quality	23868	1658640	70.00	181	d__Archaea;p__Thermoplasmatota;c__Thermoplasmata;o__UBA184;f__UBA184;g__;s__
LMSG_G000005794.1	yes	671_0	Pyrite-Copper	66.37	0.00	66.37	1	0	0	18	Medium quality	20229	2362009	73.40	143	d__Bacteria;p__Chloroflexota;c__Ellin6529;o__CSP1-4;f__CSP1-4;g__;s__
LMSG_G000005795.1	yes	971_1	Pyrite-Copper	91.51	2.73	77.88	0	0	0	17	Medium quality	109425	2201566	53.60	41	d__Bacteria;p__Nitrospirota;c__Leptospirillia;o__Leptospirillales;f__Leptospirillaceae;g__Leptospirillum_A;s__
LMSG_G000005796.1	yes	52_1	Pyrite-Copper	84.57	0.93	79.90	2	1	0	20	Medium quality	100288	851801	46.10	63	d__Archaea;p__Micrarchaeota;c__Micrarchaeia;o__Micrarchaeales;f__;g__;s__
LMSG_G000005797.1	yes	570_0	Pyrite-Copper	89.46	4.80	65.46	1	1	0	16	Medium quality	49627	2056368	68.00	101	d__Archaea;p__Thermoplasmatota;c__Thermoplasmata;o__UBA184;f__UBA184;g__;s__
LMSG_G000005798.1	yes	523_1	Pyrite-Copper	96.36	1.89	86.90	0	0	0	19	Medium quality	59744	2256990	55.50	79	d__Bacteria;p__Nitrospirota;c__;o__;f__;g__;s__
LMSG_G000004569.1	yes	441_1	Pyrite-Copper	97.72	0.00	97.72	1	2	2	20	High quality	51878	3752706	55.70	133	d__Bacteria;p__Planctomycetota;c__Phycisphaerae;o__UBA1161;f__;g__;s__
LMSG_G000005799.1	yes	679_0	Pyrite-Copper	56.89	0.00	56.89	0	1	0	18	Medium quality	54501	2570010	74.20	81	d__Bacteria;p__Chloroflexota;c__Ellin6529;o__CSP1-4;f__CSP1-4;g__;s__
LMSG_G000005803.1	yes	76_1	Pyrite-Copper	77.10	0.00	77.10	0	1	1	20	Medium quality	22640	666047	49.50	40	d__Archaea;p__Micrarchaeota;c__Micrarchaeia;o__Micrarchaeales;f__;g__;s__
LMSG_G000005800.1	yes	810_2	Pyrite-Copper	66.20	0.00	66.20	1	1	0	17	Medium quality	28468	1409968	71.50	74	d__Bacteria;p__Actinobacteriota;c__Acidimicrobiia;o__Acidimicrobiales;f__RAAP-2;g__RAAP-2;s__
LMSG_G000005801.1	yes	836_1	Pyrite-Copper	82.82	1.42	75.70	0	1	0	17	Medium quality	6702	2658068	69.00	508	d__Bacteria;p__Actinobacteriota;c__Thermoleophilia;o__Solirubrobacterales;f__Solirubrobacteraceae;g__Palsa-465;s__
LMSG_G000005802.1	yes	96_1	Pyrite-Copper	57.16	0.00	57.16	1	1	1	14	Medium quality	44813	549526	46.90	38	d__Archaea;p__Micrarchaeota;c__Micrarchaeia;o__Micrarchaeales;f__Micrarchaeaceae;g__Micrarchaeum;s__
LMSG_G000005804.1	yes	613_1	Pyrite-Copper	99.14	1.62	91.03	2	0	0	19	Medium quality	43221	2349840	52.50	86	d__Bacteria;p__Actinobacteriota;c__Acidimicrobiia;o__Acidimicrobiales;f__Acidimicrobiaceae;g__SCQM01;s__
LMSG_G000005805.1	yes	53_1	Pyrite-Copper	77.72	1.87	68.38	1	2	0	19	Medium quality	26339	724276	44.00	40	d__Archaea;p__Micrarchaeota;c__Micrarchaeia;o__Micrarchaeales;f__;g__;s__
LMSG_G000005806.1	yes	203_1	Pyrite-Copper	87.86	0.00	87.86	1	2	1	18	Medium quality	21353	1258793	44.20	92	d__Archaea;p__Thermoplasmatota;c__Thermoplasmata;o__Thermoplasmatales;f__Thermoplasmataceae;g__UBA582;s__
LMSG_G000005807.1	yes	406_1	Pyrite-Copper	75.70	0.00	75.70	1	1	3	20	Medium quality	195516	891833	26.40	42	d__Bacteria;p__Patescibacteria;c__Dojkabacteria;o__;f__;g__;s__
LMSG_G000005808.1	yes	911_1	Pyrite-Copper	93.96	1.65	85.70	1	0	0	18	Medium quality	71903	3829181	60.80	111	d__Bacteria;p__Acidobacteriota;c__Acidobacteriae;o__Acidobacteriales;f__Acidobacteriaceae;g__Terracidiphilus;s__
LMSG_G000006296.1	yes	1537_1	Pyrite-Copper	93.59	0.91	89.02	1	1	1	17	Medium quality	118341	2195833	63.90	45	d__Bacteria;p__Proteobacteria;c__Gammaproteobacteria;o__Acidiferrobacterales;f__Acidiferrobacteraceae;g__;s__
LMSG_G000005809.1	yes	1177_0	Pyrite-Copper	67.17	2.04	56.97	1	0	1	15	Medium quality	3766	1564236	64.80	505	d__Bacteria;p__Elusimicrobiota;c__Elusimicrobia;o__UBA1565;f__;g__;s__
LMSG_G000005810.1	yes	1534_1	Pyrite-Copper	83.77	1.85	74.51	0	0	0	18	Medium quality	19201	2534777	64.70	187	d__Bacteria;p__Proteobacteria;c__Gammaproteobacteria;o__Xanthomonadales;f__Rhodanobacteraceae;g__Metallibacterium;s__
LMSG_G000005811.1	yes	1712_1	Pyrite-Copper	69.80	0.00	69.80	1	2	1	17	Medium quality	36021	906396	44.10	50	d__Bacteria;p__Patescibacteria;c__Paceibacteria_A;o__Moranbacterales;f__UBA2206;g__;s__
LMSG_G000005813.1	yes	1498_0	Pyrite-Copper	99.56	2.62	86.46	0	0	0	19	Medium quality	289849	5089456	65.00	39	d__Bacteria;p__Proteobacteria;c__Alphaproteobacteria;o__Dongiales;f__Dongiaceae;g__SYSU-D60014;s__SYSU-D60014 sp003576685
LMSG_G000005812.1	yes	1247_1	Pyrite-Copper	94.93	0.00	94.93	0	1	0	17	Medium quality	38261	2195277	60.90	83	d__Bacteria;p__Proteobacteria;c__Gammaproteobacteria;o__Burkholderiales;f__Sulfuriferulaceae;g__UBA2487;s__
LMSG_G000004570.1	yes	322_1	Pyrite-Copper	94.44	1.04	89.22	1	1	1	20	High quality	10860	2807838	36.40	382	d__Bacteria;p__Cyanobacteria;c__Vampirovibrionia;o__Obscuribacterales;f__;g__;s__
LMSG_G000005814.1	yes	147_1	Pyrite-Copper	86.60	0.00	86.60	1	1	2	20	Medium quality	28144	968903	41.30	48	d__Archaea;p__Micrarchaeota;c__Micrarchaeia;o__Micrarchaeales;f__;g__;s__
LMSG_G000005816.1	yes	139_1	Pyrite-Copper	82.24	0.93	77.57	1	0	2	20	Medium quality	28234	801951	43.10	44	d__Archaea;p__Micrarchaeota;c__Micrarchaeia;o__Micrarchaeales;f__Micrarchaeaceae;g__Micrarchaeum;s__
LMSG_G000004571.1	yes	686_1	Pyrite-Copper	98.29	1.28	91.88	2	1	2	18	High quality	37447	2764817	40.80	116	d__Bacteria;p__Actinobacteriota;c__Acidimicrobiia;o__Acidimicrobiales;f__;g__;s__

LMSG_G000005815.1	yes	386_0	Pyrite-Copper	72.35	2.25	61.12	1	1	1	16	Medium quality	48888	722514	43.30	22	d__Bacteria;p__Patescibacteria;c__Saccharimonadia;o__Saccharimonadales;f__UBA4665;g__;s__
LMSG_G000004572.1	yes	816_1	Pyrite-Copper	91.45	1.28	85.04	1	1	1	18	High quality	46337	1966087	45.40	106	d__Bacteria;p__Actinobacteriota;c__Acidimicrobiia;o__Acidimicrobiales;f__Bog-793;g__;s__
LMSG_G000005817.1	yes	48_1	Pyrite-Copper	81.77	0.93	77.10	1	2	1	18	Medium quality	22445	854217	51.00	45	d__Archaea;p__Micrarchaeota;c__Micrarchaeia;o__Micrarchaeales;f__Micrarchaeaceae;g__;s__
LMSG_G000005818.1	yes	1199_2	Pyrite-Copper	85.97	0.61	82.93	1	0	0	15	Medium quality	46070	2270013	61.80	86	d__Bacteria;p__Proteobacteria;c__Gammaproteobacteria;o__Acidiferrobacterales;f__Sulfurifustaceae;g__;s__
LMSG_G000005819.1	yes	1362_0	Pyrite-Copper	81.73	1.51	74.20	0	0	0	16	Medium quality	3870	2700183	67.70	849	d__Bacteria;p__FCPU426;c__;o__;f__;g__;s__
LMSG_G000005820.1	yes	1129_1	Pyrite-Copper	84.10	0.00	84.10	0	1	0	15	Medium quality	51442	1998804	62.20	184	d__Bacteria;p__Eremiobacterota;c__Eremiobacteria;o__UBP12;f__UBA5184;g__PALSA-1484;s__
LMSG_G000004574.1	yes	1379_1	Pyrite-Copper	94.62	0.65	91.40	1	1	1	18	High quality	44286	4531287	71.80	162	d__Bacteria;p__Myxococcota;c__Myxococcia;o__Myxococcales;f__;g__;s__
LMSG_G000004573.1	yes	571_1	Pyrite-Copper	93.67	0.00	93.67	2	1	2	20	High quality	54069	2039734	61.20	63	d__Bacteria;p__Proteobacteria;c__Gammaproteobacteria;o__UBA2770;f__UBA2770;g__;s__
LMSG_G000005821.1	yes	1765_1	Pyrite-Copper	75.13	0.00	75.13	1	1	3	20	Medium quality	91117	545413	57.70	52	d__Bacteria;p__Patescibacteria;c__Paceibacteria;o__UBA9983;f__UBA2163;g__C7867-001;s__
LMSG_G000005822.1	yes	1577_0	Pyrite-Copper	68.58	1.71	60.04	0	0	0	12	Medium quality	3727	1866596	57.80	560	d__Bacteria;p__Actinobacteriota;c__Acidimicrobiia;o__Acidimicrobiales;f__Acidimicrobiaceae;g__Acidithrix;s__
LMSG_G000005823.1	yes	950_1	Pyrite-Copper	92.02	0.91	87.48	0	2	0	18	Medium quality	37155	2229343	57.30	96	d__Bacteria;p__Nitrospirota;c__Thermodesulfovibrionia;o__Thermodesulfovibrionales;f__JdFR-88;g__;s__
LMSG_G000004575.1	yes	569_1	Pyrite-Copper	93.86	0.84	89.65	1	1	2	19	High quality	20165	2563100	69.30	195	d__Archaea;p__Thermoplasmatota;c__Thermoplasmata;o__UBA184;f__UBA184;g__;s__
LMSG_G000005824.1	yes	1146_1	Pyrite-Copper	98.00	0.33	96.34	0	2	0	19	Medium quality	85779	4014047	52.50	110	d__Bacteria;p__Firmicutes_E;c__Sulfobacillia;o__Sulfobacillales;f__Sulfobacillaceae;g__Sulfobacillus_C;s__
LMSG_G000005825.1	yes	951_0	Pyrite-Copper	85.89	1.82	76.80	0	1	0	15	Medium quality	6794	1925472	58.10	327	d__Bacteria;p__Nitrospirota;c__Thermodesulfovibrionia;o__Thermodesulfovibrionales;f__JdFR-88;g__;s__
LMSG_G000005827.1	yes	1776_0	Pyrite-Copper	68.28	1.12	62.67	1	2	5	19	Medium quality	36097	644922	45.70	39	d__Bacteria;p__Patescibacteria;c__Paceibacteria;o__UBA9983;f__UBA11359_D;g__;s__
LMSG_G000006297.1	yes	783_1	Pyrite-Copper	95.72	1.71	87.18	1	1	1	17	Medium quality	20903	2353669	73.20	190	d__Bacteria;p__Actinobacteriota;c__UBA4738;o__UBA4738;f__UBA4738;g__;s__
LMSG_G000005826.1	yes	1769_0	Pyrite-Copper	78.73	0.00	78.73	1	2	1	16	Medium quality	17590	592729	60.30	58	d__Bacteria;p__Patescibacteria;c__Paceibacteria;o__UBA9983;f__UBA2163;g__C7867-001;s__
LMSG_G000004576.1	yes	1636_1	Pyrite-Copper	97.09	0.00	97.09	1	3	1	19	High quality	106080	4183141	46.30	106	d__Bacteria;p__Desulfobacterota;c__Desulfomonilia;o__Desulfomonilales;f__Desulfomonilaceae;g__;s__
LMSG_G000004577.1	yes	496_1	Pyrite-Copper	93.67	1.79	84.72	1	1	1	20	High quality	14179	5385613	63.20	570	d__Bacteria;p__Planctomycetota;c__Planctomycetes;o__Pirellulales;f__UBA11386;g__;s__
LMSG_G000005828.1	yes	1543_0	Pyrite-Copper	70.73	2.47	58.37	0	1	0	17	Medium quality	44488	1964765	63.00	58	d__Bacteria;p__Proteobacteria;c__Gammaproteobacteria;o__Acidiferrobacterales;f__Acidiferrobacteraceae;g__Acidiferrobacter;s__
LMSG_G000005829.1	yes	1597_0	Pyrite-Copper	94.30	2.29	82.84	1	0	1	15	Medium quality	14412	2486370	46.60	226	d__Bacteria;p__Actinobacteriota;c__Acidimicrobiia;o__Acidimicrobiales;f__Acidimicrobiaceae;g__Acidithrix;s__
LMSG_G000005830.1	yes	781_1	Pyrite-Copper	95.15	2.14	84.47	0	0	0	16	Medium quality	13731	1796656	66.40	155	d__Bacteria;p__Actinobacteriota;c__Acidimicrobiia;o__Acidimicrobiales;f__Acidimicrobiaceae;g__Ferrimicrobium;s__
LMSG_G000005831.1	yes	60_1	Pyrite-Copper	77.10	0.93	72.43	1	1	2	20	Medium quality	22942	712224	51.20	67	d__Archaea;p__Micrarchaeota;c__Micrarchaeia;o__Micrarchaeales;f__Micrarchaeaceae;g__;s__
LMSG_G000005832.1	yes	1135_0	Pyrite-Copper	72.07	1.25	65.82	0	0	0	14	Medium quality	3232	2563999	54.10	919	d__Bacteria;p__Bacteroidota;c__Kapabacteria;o__Palsa-1295;f__Palsa-1295;g__PALSA-1295;s__
LMSG_G000005833.1	yes	1240_2	Pyrite-Copper	80.37	2.92	65.76	1	0	0	13	Medium quality	13254	2663386	65.10	251	d__Bacteria;p__Proteobacteria;c__Gammaproteobacteria;o__Burkholderiales;f__Sulfuricellaceae;g__;s__
LMSG_G000005834.1	yes	688_1	Pyrite-Copper	98.29	0.43	96.16	1	0	0	20	Medium quality	38925	2462993	48.10	118	d__Bacteria;p__Actinobacteriota;c__Acidimicrobiia;o__Acidimicrobiales;f__Acidimicrobiaceae;g__Ferrithrix;s__
LMSG_G000005835.1	yes	992_1	Pyrite-Copper	86.85	2.56	74.03	0	1	1	13	Medium quality	8189	3092018	57.50	435	d__Bacteria;p__Acidobacteriota;c__Acidobacteriae;o__UBA7540;f__UBA7540;g__SCRD01;s__
LMSG_G000006249.1	yes	1202_0	Pyrite-Copper	90.24	2.48	77.85	2	1	0	19	Medium quality	80448	3272660	62.20	140	d__Bacteria;p__Proteobacteria;c__Gammaproteobacteria;o__Acidiferrobacterales;f__Sulfurifustaceae;g__;s__
LMSG_G000005836.1	yes	523_2	Pyrite-Copper	81.51	2.83	67.34	1	0	0	17	Medium quality	20425	1845318	56.00	232	d__Bacteria;p__Nitrospirota;c__;o__;f__;g__;s__
LMSG_G000005837.1	yes	380_1	Pyrite-Copper	63.79	0.00	63.79	0	1	0	15	Medium quality	26452	563947	46.20	30	d__Bacteria;p__Patescibacteria;c__Saccharimonadia;o__Saccharimonadales;f__UBA4665;g__;s__
LMSG_G000005838.1	yes	400_1	Pyrite-Copper	66.50	0.99	61.55	1	0	1	16	Medium quality	12706	1042596	39.40	115	d__Bacteria;p__Patescibacteria;c__Microgenomatia;o__Levybacterales;f__UBA12049;g__;s__
LMSG_G000005839.1	yes	1637_1	Pyrite-Copper	73.00	1.43	65.86	1	1	1	15	Medium quality	5688	2793925	45.80	539	d__Bacteria;p__Desulfobacterota;c__Desulfomonilia;o__Desulfomonilales;f__Desulfomonilaceae;g__;s__
LMSG_G000005840.1	yes	1716_0	Pyrite-Copper	68.15	0.66	64.85	1	1	5	17	Medium quality	7923	818903	46.70	132	d__Bacteria;p__Patescibacteria;c__ABY1;o__Magasanikbacterales;f__GWA2-37-8;g__2-02-FULL-45-10;s__
LMSG_G000005841.1	yes	1383_2	Pyrite-Copper	89.78	2.48	77.38	1	1	1	17	Medium quality	22853	2325080	64.50	278	d__Bacteria;p__Desulfobacterota;c__Desulfobulbia;o__Desulfobulbales;f__Desulfurivibrionaceae;g__;s__
LMSG_G000005842.1	yes	1711_0	Pyrite-Copper	82.02	0.00	82.02	0	0	1	18	Medium quality	168775	721060	39.80	34	d__Bacteria;p__Patescibacteria;c__CPR2;o__UBA1875;f__;g__;s__
LMSG_G000005843.1	yes	1790_0	Pyrite-Copper	74.25	0.52	71.64	1	2	6	20	Medium quality	112769	1160259	41.50	43	d__Bacteria;p__Patescibacteria;c__ABY1;o__BM507;f__GWE2-39-37;g__;s__
LMSG_G000005844.1	yes	525_0	Pyrite-Copper	65.45	0.00	65.45	0	0	0	16	Medium quality	13754	1724415	54.50	271	d__Bacteria;p__Nitrospirota;c__;o__;f__;g__;s__
LMSG_G000005845.1	yes	204_0	Pyrite-Copper	95.06	3.68	76.64	0	2	5	20	Medium quality	14308	2004288	60.90	211	d__Archaea;p__Thermoplasmatota;c__Thermoplasmata;o__UBA10834;f__UBA10834;g__;s__
LMSG_G000005846.1	yes	305_0	Pyrite-Copper	77.00	3.25	60.75	2	14	23	20	Medium quality	6404	2029408	50.80	240	d__Bacteria;p__Desulfobacterota;c__Dissulfuribacteria;o__Dissulfuribacterales;f__UBA5754;g__;s__
LMSG_G000005848.1	yes	513_1	Pyrite-Copper	99.09	2.27	87.73	0	1	1	19	Medium quality	145470	2343851	44.50	83	d__Bacteria;p__Nitrospirota;c__Thermodesulfovibrionia;o__;f__;g__;s__
LMSG_G000005847.1	yes	278_0	Pyrite-Copper	65.39	2.25	54.16	0	2	1	17	Medium quality	25820	930958	43.10	111	d__Bacteria;p__Patescibacteria;c__Microgenomatia;o__UBA1406;f__GWC2-37-13;g__;s__
LMSG_G000005849.1	yes	191_1	Pyrite-Copper	95.92	0.00	95.92	1	0	1	17	Medium quality	29652	1451990	45.60	108	d__Archaea;p__Thermoplasmatota;c__Thermoplasmata;o__Thermoplasmatales;f__Thermoplasmataceae;g__UBA509;s__
LMSG_G000005850.1	yes	1697_0	Pyrite-Copper	67.52	0.86	63.21	1	2	4	20	Medium quality	180947	620432	41.70	22	d__Bacteria;p__Patescibacteria;c__Paceibacteria;o__UBA6257;f__HR35;g__;s__
LMSG_G000005851.1	yes	784_0	Pyrite-Copper	70.79	2.09	60.34	1	1	1	19	Medium quality	43956	1239426	63.70	81	d__Bacteria;p__Patescibacteria;c__ABY1;o__SG8-24;f__GWF2-40-263;g__;s__
LMSG_G000005852.1	yes	1215_0	Pyrite-Copper	62.57	2.42	50.47	0	0	0	11	Medium quality	2308	1626211	53.70	766	d__Bacteria;p__Chloroflexota;c__Dehalococcoidia;o__Dehalococcoidales;f__UBA3254;g__UBA3254;s__
LMSG_G000006250.1	yes	945_2	Pyrite-Copper	90.49	4.78	66.61	1	5	0	17	Medium quality	15245	2808853	53.40	458	d__Bacteria;p__Nitrospirota;c__Thermodesulfovibrionia;o__Thermodesulfovibrionales;f__JdFR-88;g__;s__
LMSG_G000005853.1	yes	381_0	Pyrite-Copper	58.95	0.10	58.44	2	1	1	15	Medium quality	38058	815279	43.80	63	d__Bacteria;p__Patescibacteria;c__Saccharimonadia;o__Saccharimonadales;f__UBA4665;g__;s__
LMSG_G000005854.1	yes	1395_1	Pyrite-Copper	95.72	3.21	79.70	1	0	1	19	Medium quality	62288	3490204	66.40	234	d__Bacteria;p__Acidobacteriota;c__UBA4820;o__UBA4820;f__;g__;s__
LMSG_G000005860.1	yes	1719_1	Pyrite-Copper	70.05	1.12	64.44	1	1	3	17	Medium quality	213300	902275	38.80	29	d__Bacteria;p__Patescibacteria;c__Microgenomatia;o__UBA1406;f__GWC2-37-13;g__;s__
LMSG_G000005855.1	yes	1210_0	Pyrite-Copper	90.94	4.31	69.39	0	1	0	18	Medium quality	81669	1843366	58.50	93	d__Bacteria;p__Actinobacteriota;c__Thermoleophilia;o__BMS3ABIN01;f__BMS3ABIN01;g__;s__
LMSG_G000005856.1	yes	392_0	Pyrite-Copper	57.92	1.10	52.42	1	0	1	18	Medium quality	66873	646312	48.70	23	d__Bacteria;p__Patescibacteria;c__Microgenomatia;o__;f__;g__;s__
LMSG_G000005857.1	yes	113_0	Pyrite-Copper	85.51	0.93	80.84	1	1	1	20	Medium quality	24042	971447	48.00	80	d__Archaea;p__Micrarchaeota;c__Micrarchaeia;o__Micrarchaeales;f__;g__;s__
LMSG_G000005858.1	yes	987_0	Pyrite-Copper	92.43	2.99	77.50	0	0	0	19	Medium quality	72981	2124062	55.30	118	d__Bacteria;p__SZUA-79;c__;o__;f__;g__;s__
LMSG_G000005859.1	yes	999_0	Pyrite-Copper	59.37	0.97	54.51	1	1	1	11	Medium quality	31336	2885035	57.60	251	d__Bacteria;p__Acidobacteriota;c__Acidobacteriae;o__UBA7540;f__UBA7540;g__SCRD01;s__
LMSG_G000005862.1	yes	470_0	Pyrite-Copper	76.49	4.37	54.65	1	0	1	17	Medium quality	6829	2970405	66.80	643	d__Bacteria;p__Cyanobacteria;c__Sericytochromatia;o__S15B-MN24;f__;g__;s__
LMSG_G000005861.1	yes	394_0	Pyrite-Copper	55.12	0.71	51.56	2	1	4	18	Medium quality	55468	925058	51.30	59	d__Bacteria;p__Patescibacteria;c__Microgenomatia;o__GWA2-44-7;f__Amesbacteraceae;g__;s__
LMSG_G000004578.1	yes	233_0	Pyrite-Copper	99.09	4.19	78.14	1	1	5	20	High quality	106186	4269406	53.30	239	d__Bacteria;p__Chloroflexota;c__Anaerolineae;o__Anaerolineales;f__EnvOPS12;g__Fen-1038;s__
LMSG_G000005863.1	yes	281_0	Pyrite-Copper	64.60	0.56	61.80	1	1	1	19	Medium quality	27173	1061923	41.00	75	d__Bacteria;p__Patescibacteria;c__Microgenomatia;o__UBA1406;f__GWC2-37-13;g__GWC2-37-13;s__
LMSG_G000006321.1	yes	1690_0	Pyrite-Copper	71.26	0.93	66.59	1	1	0	18	Medium quality	142796	588343	35.50	10	d__Archaea;p__Nanoarchaeota;c__Nanoarchaeia;o__Pacearchaeales;f__GW2011-AR1;g__ASMP01;s__
LMSG_G000005864.1	yes	1806_0	Pyrite-Copper	75.28	0.00	75.28	0	3	3	18	Medium quality	201998	745876	39.10	14	d__Bacteria;p__Patescibacteria;c__Paceibacteria;o__UBA6257;f__UBA9933;g__;s__
LMSG_G000004579.1	yes	1645_1	Pyrite-Copper	97.52	2.48	85.14	1	1	1	19	High quality	70495	3952491	52.10	150	d__Bacteria;p__Bacteroidota;c__Kryptonia;o__;f__;g__;s__
LMSG_G000005865.1	yes	1710_0	Pyrite-Copper	67.97	1.12	62.36	1	1	1	18	Medium quality	31189	857189	43.30	92	d__Bacteria;p__Patescibacteria;c__CPR2;o__UBA1875;f__;g__;s__
LMSG_G000005866.1	yes	83_0	Pyrite-Copper	81.77	3.40	64.79	1	1	3	18	Medium quality	64149	771859	47.60	73	d__Archaea;p__Micrarchaeota;c__Micrarchaeia;o__Micrarchaeales;f__Micrarchaeaceae;g__;s__
LMSG_G000005867.1	yes	1691_0	Pyrite-Copper	60.53	1.11	54.97	1	1	3	19	Medium quality	24605	555065	46.60	56	d__Bacteria;p__Patescibacteria;c__Paceibacteria;o__GWC2-36-17_A;f__UBA10092;g__UBA10092;s__
LMSG_G000005871.1	yes	387_1	Pyrite-Copper	68.97	0.50	66.50	1	1	1	19	Medium quality	66604	698408	41.30	24	d__Bacteria;p__Patescibacteria;c__Saccharimonadia;o__Saccharimonadales;f__UBA4665;g__;s__
LMSG_G000005868.1	yes	428_2	Pyrite-Copper	78.92	0.43	76.79	0	1	1	18	Medium quality	5504	1948017	59.50	463	d__Bacteria;p__Planctomycetota;c__Phycisphaerae;o__UBA1161;f__;g__;s__
LMSG_G000005869.1	yes	1211_0	Pyrite-Copper	90.51	2.23	79.38	0	3	0	17	Medium quality	57078	1820301	57.50	101	d__Bacteria;p__Actinobacteriota;c__Thermoleophilia;o__BMS3ABIN01;f__BMS3ABIN01;g__;s__
LMSG_G000005870.1	yes	279_0	Pyrite-Copper	59.90	0.00	59.90	1	1	1	20	Medium quality	101793	902291	41.70	37	d__Bacteria;p__Patescibacteria;c__Microgenomatia;o__Levybacterales;f__UBA12049;g__;s__
LMSG_G000005874.1	yes	130_0	Pyrite-Copper	80.52	0.07	80.17	0	1	2	18	Medium quality	51725	828710	44.30	33	d__Archaea;p__Micrarchaeota;c__Micrarchaeia;o__Micrarchaeales;f__;g__;s__
LMSG_G000005872.1	yes	1839_0	Pyrite-Copper	60.64	0.00	60.64	1	1	5	19	Medium quality	27975	1015689	45.50	67	d__Bacteria;p__Patescibacteria;c__Microgenomatia;o__Daviesbacterales;f__UBA10151;g__;s__
LMSG_G000005873.1	yes	1667_1	Pyrite-Copper	77.36	3.67	59.03	0	1	2	16	Medium quality	4517	4203295	29.70	1192	d__Archaea;p__Asgardarchaeota;c__Heimdallarchaeia;o__LC-3;f__LC-3;g__;s__
LMSG_G000005875.1	yes	420_0	Pyrite-Copper	54.36	0.50	51.89	1	1	2	17	Medium quality	571692	891649	49.50	19	d__Bacteria;p__Patescibacteria;c__Microgenomatia;o__UBA1400;f__UBA12108;g__;s__
LMSG_G000005876.1	yes	940_1	Pyrite-Copper	97.22	1.82	88.13	1	0	2	19	Medium quality	25858	2533272	43.00	156	d__Bacteria;p__Nitrospirota;c__Thermodesulfovibrionia;o__Thermodesulfovibrionales;f__UBA9935;g__GWB2-47-37;s__
LMSG_G000005877.1	yes	477_0	Pyrite-Copper	89.26	3.40	72.26	1	1	1	13	Medium quality	10756	3832050	51.30	490	d__Bacteria;p__Firmicutes_B;c__Desulfitobacteriia;o__Desulfitobacteriales;f__;g__;s__

LMSG_G000006251.1	yes	1348_1	Pyrite-Copper	91.63	2.19	80.67	2	1	0	20	Medium quality	8022	2891146	62.00	495	d__Bacteria;p__Acidobacteriota;c__Holophagae;o__Holophagales;f__Holophagaceae;g__;s__
LMSG_G000004580.1	yes	708_1	Pyrite-Copper	93.16	2.14	82.48	1	1	1	19	High quality	36941	2838849	72.50	126	d__Bacteria;p__Actinobacteriota;c__Acidimicrobiia;o__Acidimicrobiales;f__;g__;s__
LMSG_G000005878.1	yes	448_0	Pyrite-Copper	80.19	3.17	64.35	1	0	1	14	Medium quality	6875	3318382	61.40	619	d__Bacteria;p__Chloroflexota;c__UBA6077;o__UBA6077;f__UBA6077;g__;s__
LMSG_G000005879.1	yes	9_1	Pyrite-Copper	84.70	0.97	79.85	3	1	3	19	Medium quality	47796	956327	39.30	33	d__Archaea;p__Nanoarchaeota;c__Nanoarchaeia;o__Parvarchaeales;f__Parvarchaeaceae;g__Parvarchaeum;s__
LMSG_G000005880.1	yes	461_1	Pyrite-Copper	69.24	0.49	66.79	1	0	0	14	Medium quality	4928	2004804	62.10	469	d__Bacteria;p__Firmicutes_G;c__DTU065;o__;f__;g__;s__
LMSG_G000006252.1	yes	705_0	Pyrite-Copper	94.11	0.98	89.21	2	1	0	19	Medium quality	95007	3643527	66.20	62	d__Bacteria;p__Firmicutes_E;c__DTU015;o__;f__;g__;s__
LMSG_G000005881.1	yes	1807_0	Pyrite-Copper	73.05	2.95	58.33	0	0	2	15	Medium quality	9059	877072	52.60	158	d__Bacteria;p__Patescibacteria;c__ABY1;o__BM507;f__GWE2-39-37;g__;s__
LMSG_G000005882.1	yes	1672_1	Pyrite-Copper	94.78	1.12	89.20	2	0	0	16	Medium quality	12186	3122500	39.90	395	d__Bacteria;p__Bacteroidota;c__Ignavibacteria;o__Ignavibacteriales;f__Melioribacteraceae;g__;s__
LMSG_G000006298.1	yes	408_1	Pyrite-Copper	91.94	0.42	89.83	1	1	1	16	Medium quality	15399	2091225	59.10	187	d__Bacteria;p__Deinococcota;c__Deinococci;o__Deinococcales;f__Thermaceae;g__;s__
LMSG_G000005883.1	yes	792_2	Pyrite-Copper	66.89	1.03	61.77	0	0	0	14	Medium quality	7758	2130721	65.50	497	d__Bacteria;p__Actinobacteriota;c__Acidimicrobiia;o__Acidimicrobiales;f__RAAP-2;g__RAAP-2;s__
LMSG_G000005884.1	yes	652_1	Pyrite-Copper	74.82	0.00	74.82	0	0	0	9	Medium quality	7132	2480285	66.20	396	d__Bacteria;p__Firmicutes_E;c__Sulfobacillia;o__;f__;g__;s__
LMSG_G000005885.1	yes	86_2	Pyrite-Copper	77.57	0.93	72.90	1	1	1	18	Medium quality	24814	728908	48.90	46	d__Archaea;p__Micrarchaeota;c__Micrarchaeia;o__Micrarchaeales;f__Micrarchaeaceae;g__;s__
LMSG_G000005886.1	yes	223_0	Pyrite-Copper	81.04	3.23	64.92	1	1	0	14	Medium quality	35030	1134732	46.60	51	d__Archaea;p__Thermoplasmatota;c__Thermoplasmata;o__Thermoplasmatales;f__GCA-001856825;g__GCA-001856825;s__
LMSG_G000004581.1	yes	1638_1	Pyrite-Copper	98.88	3.28	82.49	1	1	1	19	High quality	102908	4431254	51.30	80	d__Bacteria;p__Bacteroidota;c__Kryptonia;o__;f__;g__;s__
LMSG_G000005887.1	yes	1228_1	Pyrite-Copper	93.48	0.00	93.48	0	1	0	17	Medium quality	28163	2593547	51.00	171	d__Bacteria;p__Proteobacteria;c__Gammaproteobacteria;o__Burkholderiales;f__Sulfuriferulaceae;g__UBA2487;s__
LMSG_G000005890.1	yes	1181_0	Pyrite-Copper	84.95	1.12	79.34	0	1	0	18	Medium quality	71922	2085589	64.30	45	d__Bacteria;p__Elusimicrobiota;c__Elusimicrobia;o__Elusimicrobiales;f__UBA9959;g__UBA9959;s__
LMSG_G000005888.1	yes	1838_1	Pyrite-Copper	64.35	0.19	63.43	2	3	3	20	Medium quality	942606	1051733	42.70	26	d__Bacteria;p__Patescibacteria;c__Microgenomatia;o__Daviesbacterales;f__UBA10151;g__;s__
LMSG_G000005889.1	yes	1677_1	Pyrite-Copper	80.25	0.97	75.40	0	2	2	19	Medium quality	32156	1404115	45.50	72	d__Archaea;p__Thermoproteota;c__Nitrososphaeria;o__UBA164;f__UBA164;g__UBA160;s__
LMSG_G000005891.1	yes	1826_1	Pyrite-Copper	74.12	0.00	74.12	1	0	1	17	Medium quality	47036	615923	39.00	44	d__Bacteria;p__Patescibacteria;c__Paceibacteria;o__UBA6257;f__UBA9933;g__;s__
LMSG_G000005892.1	yes	80_1	Pyrite-Copper	83.64	1.87	74.30	1	1	1	18	Medium quality	188237	774863	51.30	24	d__Archaea;p__Micrarchaeota;c__Micrarchaeia;o__Micrarchaeales;f__Micrarchaeaceae;g__;s__
LMSG_G000005893.1	yes	1714_1	Pyrite-Copper	79.58	0.00	79.58	1	1	1	20	Medium quality	696638	704347	36.90	15	d__Bacteria;p__Patescibacteria;c__Paceibacteria;o__UBA6257;f__02-FULL-45-10c;g__;s__
LMSG_G000005894.1	yes	462_1	Pyrite-Copper	90.42	4.16	69.63	0	1	0	15	Medium quality	7919	3929428	65.60	596	d__Bacteria;p__Chloroflexota;c__FW602-bin22;o__FW602-bin22;f__;g__;s__
LMSG_G000004582.1	yes	201_1	Pyrite-Copper	97.53	0.00	97.53	1	2	2	20	High quality	74654	1656499	49.20	35	d__Archaea;p__Thermoplasmatota;c__Thermoplasmata;o__Thermoplasmatales;f__Thermoplasmataceae;g__UBA582;s__
LMSG_G000005895.1	yes	692_1	Pyrite-Copper	98.29	1.28	91.88	0	0	0	20	Medium quality	41869	3047483	64.30	131	d__Bacteria;p__Actinobacteriota;c__Acidimicrobiia;o__Acidimicrobiales;f__Acidimicrobiaceae;g__Acidithrix;s__
LMSG_G000004583.1	yes	1136_1	Lead-Zinc	93.48	0.00	93.48	1	1	2	19	High quality	101939	2037297	31.20	104	d__Bacteria;p__Omnitrophota;c__UBA8468;o__UBA8468;f__B48-G9;g__;s__
LMSG_G000004584.1	yes	801_1	Lead-Zinc	97.43	1.28	91.02	1	1	1	20	High quality	36375	2193268	69.90	133	d__Bacteria;p__Actinobacteriota;c__Acidimicrobiia;o__Acidimicrobiales;f__RAAP-2;g__RAAP-2;s__
LMSG_G000004585.1	yes	1155_1	Lead-Zinc	90.90	0.78	86.99	1	1	1	20	High quality	11862	2413181	40.80	298	d__Bacteria;p__Eremiobacterota;c__UBP9;o__;f__;g__;s__
LMSG_G000005896.1	yes	1501_1	Lead-Zinc	91.34	0.75	87.61	0	0	0	18	Medium quality	25939	2939907	65.10	168	d__Bacteria;p__Proteobacteria;c__Alphaproteobacteria;o__Acetobacterales;f__Acetobacteraceae;g__G45-3;s__
LMSG_G000004586.1	yes	698_1	Lead-Zinc	92.30	1.71	83.76	1	1	1	19	High quality	43537	3820355	66.30	144	d__Bacteria;p__Actinobacteriota;c__Acidimicrobiia;o__Acidimicrobiales;f__Palsa-688;g__;s__
LMSG_G000005897.1	yes	1715_0	Lead-Zinc	57.92	0.10	57.41	0	0	0	17	Medium quality	5271	418922	38.40	91	d__Bacteria;p__Patescibacteria;c__Paceibacteria;o__UBA6257;f__02-FULL-45-10c;g__;s__
LMSG_G000005898.1	yes	455_0	Lead-Zinc	72.08	2.99	57.13	1	1	1	18	Medium quality	4125	2022658	53.00	621	d__Bacteria;p__Actinobacteriota;c__Acidimicrobiia;o__Acidimicrobiales;f__;g__;s__
LMSG_G000004587.1	yes	1207_1	Lead-Zinc	94.23	3.42	77.14	1	1	1	19	High quality	80607	3893661	63.00	79	d__Bacteria;p__Acidobacteriota;c__Acidobacteriae;o__UBA7541;f__;g__;s__
LMSG_G000005899.1	yes	468_1	Lead-Zinc	92.51	2.50	80.00	2	0	2	18	Medium quality	24463	4127674	60.50	247	d__Bacteria;p__Firmicutes_B;c__Desulfotomaculia;o__;f__;g__;s__
LMSG_G000004588.1	yes	421_0	Lead-Zinc	98.87	1.12	93.26	1	1	2	20	High quality	233616	2022204	37.80	33	d__Bacteria;p__FCPU426;c__Palsa-1180;o__Palsa-1180;f__;g__;s__
LMSG_G000005900.1	yes	43_1	Lead-Zinc	86.60	0.93	81.93	1	2	1	20	Medium quality	51332	904233	39.40	54	d__Archaea;p__Micrarchaeota;c__Micrarchaeia;o__Micrarchaeales;f__;g__;s__
LMSG_G000005902.1	yes	1687_1	Lead-Zinc	68.92	0.93	64.25	1	1	3	19	Medium quality	73547	742141	38.90	18	d__Archaea;p__Nanoarchaeota;c__Nanoarchaeia;o__Pacearchaeales;f__GW2011-AR1;g__GW2011-AR1;s__
LMSG_G000005901.1	yes	995_1	Lead-Zinc	81.70	2.56	68.88	1	1	1	18	Medium quality	4904	2505041	59.00	610	d__Bacteria;p__Acidobacteriota;c__Acidobacteriae;o__UBA7540;f__UBA7540;g__;s__
LMSG_G000005903.1	yes	234_1	Lead-Zinc	73.76	0.00	73.76	1	1	3	18	Medium quality	39074	927956	43.70	56	d__Bacteria;p__Patescibacteria;c__Saccharimonadia;o__Saccharimonadales;f__UBA4665;g__UBA5150;s__
LMSG_G000005904.1	yes	869_2	Lead-Zinc	62.93	1.72	54.31	1	1	1	8	Medium quality	8335	3578193	72.10	592	d__Bacteria;p__Actinobacteriota;c__Actinomycetia;o__Streptosporangiales;f__Streptosporangiaceae;g__Palsa-506;s__
LMSG_G000005905.1	yes	1142_0	Lead-Zinc	72.29	1.33	65.63	1	0	1	17	Medium quality	3050	3878891	51.60	1418	d__Bacteria;p__Firmicutes_E;c__Sulfobacillia;o__Sulfobacillales;f__Sulfobacillaceae;g__Sulfobacillus_C;s__
LMSG_G000005906.1	yes	1499_1	Lead-Zinc	98.50	0.25	97.26	0	0	0	19	Medium quality	54001	3153467	66.50	93	d__Bacteria;p__Proteobacteria;c__Alphaproteobacteria;o__Acetobacterales;f__Acetobacteraceae;g__G45-3;s__
LMSG_G000005907.1	yes	120_1	Lead-Zinc	80.99	0.07	80.64	1	1	1	17	Medium quality	35998	949327	46.90	35	d__Archaea;p__Micrarchaeota;c__Micrarchaeia;o__Micrarchaeales;f__;g__;s__
LMSG_G000005908.1	yes	114_1	Lead-Zinc	78.81	0.00	78.81	1	1	1	20	Medium quality	147204	829067	46.50	9	d__Archaea;p__Micrarchaeota;c__Micrarchaeia;o__Micrarchaeales;f__Micrarchaeaceae;g__;s__
LMSG_G000005909.1	yes	384_1	Lead-Zinc	63.58	0.00	63.58	1	1	1	19	Medium quality	77546	852984	42.90	27	d__Bacteria;p__Patescibacteria;c__Saccharimonadia;o__Saccharimonadales;f__UBA4665;g__;s__
LMSG_G000004589.1	yes	1176_1	Lead-Zinc	95.43	0.00	95.43	1	1	2	20	High quality	59508	2256865	63.40	94	d__Bacteria;p__Elusimicrobiota;c__Elusimicrobia;o__UBA1565;f__;g__;s__
LMSG_G000005910.1	yes	426_1	Lead-Zinc	94.91	0.46	92.60	1	0	0	19	Medium quality	44524	2168554	39.10	88	d__Bacteria;p__Thermotogota;c__Thermotogae;o__Mesoaciditogales;f__Mesoaciditogaceae;g__ZAV-03;s__
LMSG_G000005911.1	yes	739_1	Lead-Zinc	93.16	2.14	82.48	0	1	0	20	Medium quality	25956	2870649	72.80	168	d__Bacteria;p__Actinobacteriota;c__Acidimicrobiia;o__Acidimicrobiales;f__RAAP-2;g__;s__
LMSG_G000005912.1	yes	440_1	Polymetallic	92.04	1.14	86.36	0	2	1	20	Medium quality	37690	3973915	56.90	191	d__Bacteria;p__Planctomycetota;c__Phycisphaerae;o__Phycisphaerales;f__;g__;s__
LMSG_G000005913.1	yes	49_1	Polymetallic	71.49	0.93	66.82	1	0	1	20	Medium quality	17240	778087	49.50	61	d__Archaea;p__Micrarchaeota;c__Micrarchaeia;o__Micrarchaeales;f__;g__;s__
LMSG_G000005914.1	yes	527_0	Polymetallic	75.72	1.10	70.23	0	0	0	17	Medium quality	8729	1092758	37.60	177	d__Bacteria;p__Proteobacteria;c__Alphaproteobacteria;o__;f__;g__;s__
LMSG_G000005915.1	yes	613_2	Polymetallic	86.81	0.43	84.68	1	0	1	17	Medium quality	20796	2214583	52.80	144	d__Bacteria;p__Actinobacteriota;c__Acidimicrobiia;o__Acidimicrobiales;f__Acidimicrobiaceae;g__SCQM01;s__
LMSG_G000005916.1	yes	1680_1	Polymetallic	88.42	1.03	83.28	0	1	1	20	Medium quality	96124	2168436	57.00	50	d__Bacteria;p__Dormibacterota;c__Dormibacteria;o__UBA8260;f__;g__;s__
LMSG_G000005917.1	yes	1065_1	Polymetallic	83.99	0.00	83.99	1	2	1	18	Medium quality	13155	4658269	59.70	570	d__Bacteria;p__Planctomycetota;c__Phycisphaerae;o__UBA1161;f__;g__;s__
LMSG_G000005918.1	yes	1467_0	Polymetallic	89.43	1.74	80.73	0	1	0	19	Medium quality	12912	2781350	56.70	268	d__Bacteria;p__Proteobacteria;c__Alphaproteobacteria;o__Acetobacterales;f__Acetobacteraceae;g__Acidiphilium;s__
LMSG_G000005919.1	yes	399_0	Polymetallic	64.30	2.25	53.07	0	0	0	16	Medium quality	33931	696227	29.70	43	d__Bacteria;p__Patescibacteria;c__Dojkabacteria;o__;f__;g__;s__
LMSG_G000005920.1	yes	324_1	Polymetallic	82.47	0.47	80.10	0	2	0	19	Medium quality	13199	2771638	32.90	334	d__Bacteria;p__Cyanobacteria;c__Vampirovibrionia;o__Obscuribacterales;f__;g__;s__
LMSG_G000005923.1	yes	369_0	Polymetallic	58.62	0.00	58.62	0	2	0	12	Medium quality	35790	530004	34.70	19	d__Bacteria;p__Patescibacteria;c__Saccharimonadia;o__Saccharimonadales;f__UBA4665;g__;s__
LMSG_G000005921.1	yes	341_0	Polymetallic	55.37	0.00	55.37	0	0	0	13	Medium quality	31623	614153	43.70	37	d__Bacteria;p__Patescibacteria;c__Saccharimonadia;o__Saccharimonadales;f__UBA4665;g__BOG-1338;s__
LMSG_G000005922.1	yes	685_2	Polymetallic	84.63	1.28	78.22	0	0	0	19	Medium quality	17482	2212101	40.10	299	d__Bacteria;p__Actinobacteriota;c__Acidimicrobiia;o__Acidimicrobiales;f__;g__;s__
LMSG_G000005924.1	yes	419_0	Polymetallic	75.84	0.00	75.84	1	4	1	20	Medium quality	28762	840024	31.20	168	d__Bacteria;p__Proteobacteria;c__Alphaproteobacteria;o__Rickettsiales;f__AB1-6;g__;s__
LMSG_G000005925.1	yes	432_0	Polymetallic	61.48	1.75	52.71	0	0	0	13	Medium quality	11973	3263457	54.30	326	d__Bacteria;p__Planctomycetota;c__Phycisphaerae;o__UBA1161;f__;g__;s__
LMSG_G000005926.1	yes	5_0	Polymetallic	71.65	2.80	57.64	1	2	0	16	Medium quality	15313	650092	41.80	58	d__Archaea;p__Nanoarchaeota;c__Nanoarchaeia;o__Parvarchaeales;f__Parvarchaeaceae;g__;s__
LMSG_G000005928.1	yes	71_0	Copper	50.31	0.00	50.31	1	0	0	14	Medium quality	10631	515231	48.70	57	d__Archaea;p__Micrarchaeota;c__Micrarchaeia;o__Micrarchaeales;f__Micrarchaeaceae;g__;s__
LMSG_G000005927.1	yes	1619_0	Copper	91.89	1.35	85.14	1	0	0	20	Medium quality	34706	1323692	41.50	57	d__Bacteria;p__Verrucomicrobiota_A;c__Chlamydiia;o__Parachlamydiales;f__SM23-39;g__;s__
LMSG_G000005929.1	yes	1602_1	Copper	90.12	0.90	85.62	0	1	0	20	Medium quality	63662	1740622	43.10	56	d__Bacteria;p__Verrucomicrobiota_A;c__Chlamydiia;o__2-12-FULL-49-11;f__2-12-FULL-49-11;g__;s__
LMSG_G000006253.1	yes	324_2	Copper	95.72	0.57	92.88	2	1	0	20	Medium quality	69817	3177311	33.80	81	d__Bacteria;p__Cyanobacteria;c__Vampirovibrionia;o__Obscuribacterales;f__;g__;s__
LMSG_G000005930.1	yes	365_1	Magnetite	73.44	0.00	73.44	0	0	0	19	Medium quality	63141	743207	31.10	15	d__Bacteria;p__Patescibacteria;c__Saccharimonadia;o__Saccharimonadales;f__UBA4665;g__;s__
LMSG_G000005931.1	yes	1282_0	Magnetite	92.18	0.83	88.04	0	0	0	18	Medium quality	57261	3445102	63.50	110	d__Bacteria;p__Proteobacteria;c__Gammaproteobacteria;o__Burkholderiales;f__Rhodocyclaceae;g__;s__
LMSG_G000005932.1	yes	506_1	Magnetite	96.58	0.85	92.31	0	0	0	20	Medium quality	125856	1925096	50.00	25	d__Bacteria;p__Actinobacteriota;c__Acidimicrobiia;o__Acidimicrobiales;f__Bog-793;g__;s__
LMSG_G000005934.1	yes	401_0	Magnetite	64.01	1.12	58.40	0	0	1	17	Medium quality	16362	809043	36.90	108	d__Bacteria;p__Patescibacteria;c__Microgenomatia;o__Levybacterales;f__UBA12049;g__;s__
LMSG_G000005933.1	yes	3_2	Magnetite	88.20	0.00	88.20	2	0	3	18	Medium quality	43610	4481300	49.80	239	d__Bacteria;p__Armatimonadota;c__Chthonomonadetes;o__Chthonomonadales;f__;g__;s__
LMSG_G000005935.1	yes	1830_0	Magnetite	69.47	0.00	69.47	1	1	1	18	Medium quality	195535	752629	45.70	24	d__Bacteria;p__Patescibacteria;c__Doudnabacteria;o__UBA920;f__;g__;s__

LMSG_G000005936.1	yes	441_2	Magnetite	84.62	4.55	61.90	1	1	1	16	Medium quality	28283	3538779	55.40	203	d__Bacteria;p__Planctomycetota;c__Phycisphaerae;o__UBA1161;f__;g__;s__
LMSG_G000005937.1	yes	620_1	Magnetite	86.93	0.31	85.39	1	1	2	20	Medium quality	33498	2138202	58.80	123	d__Bacteria;p__Dormibacterota;c__Dormibacteria;o__UBA8260;f__;g__;s__
LMSG_G000005938.1	yes	82_0	Magnetite	54.42	0.00	54.42	1	0	1	16	Medium quality	11180	477258	53.10	50	d__Archaea;p__Micrarchaeota;c__Micrarchaeia;o__Micrarchaeales;f__Micrarchaeaceae;g__;s__
LMSG_G000005939.1	yes	383_0	Magnetite	67.98	0.17	67.16	1	0	1	18	Medium quality	69889	831205	43.80	20	d__Bacteria;p__Patescibacteria;c__Saccharimonadia;o__Saccharimonadales;f__UBA4665;g__;s__
LMSG_G000005940.1	yes	382_1	Magnetite	56.03	0.00	56.03	1	0	1	17	Medium quality	55444	714981	45.40	20	d__Bacteria;p__Patescibacteria;c__Saccharimonadia;o__Saccharimonadales;f__UBA4665;g__;s__
LMSG_G000005941.1	yes	31_0	Magnetite	54.75	0.93	50.08	1	0	1	19	Medium quality	23082	562127	51.20	39	d__Archaea;p__Micrarchaeota;c__Micrarchaeia;o__Micrarchaeales;f__;g__;s__
LMSG_G000005942.1	yes	153_0	Magnetite	70.67	3.60	52.67	1	1	0	17	Medium quality	7289	2054761	49.50	350	d__Archaea;p__Thermoplasmatota;c__Thermoplasmata;o__Methanomassiliicoccales;f__;g__;s__
LMSG_G000005943.1	yes	100_1	Magnetite	57.39	0.00	57.39	0	2	0	15	Medium quality	68687	646837	47.00	30	d__Archaea;p__Micrarchaeota;c__Micrarchaeia;o__Micrarchaeales;f__;g__;s__
LMSG_G000005944.1	yes	1746_3	Polymetallic	70.55	1.12	64.94	1	1	0	13	Medium quality	10359	2044170	30.20	243	d__Bacteria;p__SZUA-79;c__SZUA-79;o__Acidulodesulfobacterales;f__SZUA-79;g__Acididesulfobacter;s__
LMSG_G000006254.1	yes	976_1	Polymetallic	92.50	0.62	89.40	2	1	0	18	Medium quality	35359	2287149	53.60	93	d__Bacteria;p__Proteobacteria;c__Gammaproteobacteria;o__Acidithiobacillales;f__Acidithiobacillaceae;g__Acidithiobacillus;s__Acidithiobacillus thiooxidans
LMSG_G000004590.1	yes	706_0	Copper	99.14	2.99	84.19	1	1	2	20	High quality	121338	6208352	70.10	92	d__Bacteria;p__Actinobacteriota;c__Acidimicrobiia;o__Acidimicrobiales;f__Ilumatobacteraceae;g__Desertimonas;s__Desertimonas flava
LMSG_G000005945.1	yes	1607_1	Copper	89.16	1.13	83.50	1	0	1	18	Medium quality	20270	1172878	44.30	139	d__Bacteria;p__Verrucomicrobiota_A;c__Chlamydiia;o__Parachlamydiales;f__Rhabdochlamydiaceae;g__;s__
LMSG_G000004591.1	yes	164_1	Copper	98.46	0.00	98.46	1	2	2	20	High quality	45561	2156471	32.70	118	d__Archaea;p__Thermoplasmatota;c__Thermoplasmata;o__Thermoplasmatales;f__Thermoplasmataceae;g__Ferroplasma;s__
LMSG_G000005946.1	yes	1140_1	Copper	98.50	0.00	98.50	0	1	1	19	Medium quality	120164	4360124	52.00	232	d__Bacteria;p__Firmicutes_E;c__Sulfobacillia;o__Sulfobacillales;f__Sulfobacillaceae;g__Sulfobacillus;s__Sulfobacillus benefaciens_A
LMSG_G000005947.1	yes	379_0	Magnetite	57.09	0.99	52.14	0	0	0	16	Medium quality	39259	519784	46.90	24	d__Bacteria;p__Patescibacteria;c__Saccharimonadia;o__Saccharimonadales;f__UBA4665;g__;s__
LMSG_G000005949.1	yes	63_0	Pyrite-Copper	69.93	0.00	69.93	1	1	1	19	Medium quality	93194	698791	52.30	12	d__Archaea;p__Micrarchaeota;c__Micrarchaeia;o__Micrarchaeales;f__;g__;s__
LMSG_G000005948.1	yes	975_0	Pyrite-Copper	68.79	0.00	68.79	1	0	0	17	Medium quality	36632	1370373	58.50	61	d__Bacteria;p__Proteobacteria;c__Gammaproteobacteria;o__Acidithiobacillales;f__Acidithiobacillaceae;g__Acidithiobacillus_A;s__
LMSG_G000005950.1	yes	990_0	Pyrite-Copper	57.38	0.90	52.87	1	0	1	15	Medium quality	6623	1997727	60.40	424	d__Bacteria;p__Acidobacteriota;c__UBA4820;o__UBA4820;f__;g__;s__
LMSG_G000005951.1	yes	117_0	Pyrite-Copper	63.16	0.00	63.16	1	1	1	17	Medium quality	42696	757522	45.70	25	d__Archaea;p__Micrarchaeota;c__Micrarchaeia;o__Micrarchaeales;f__;g__;s__
LMSG_G000005955.1	yes	519_0	Pyrite-Copper	70.90	0.00	70.90	0	0	0	15	Medium quality	72497	1575734	48.70	38	d__Bacteria;p__Elusimicrobiota;c__Elusimicrobia;o__UBA1565;f__;g__;s__
LMSG_G000005952.1	yes	635_0	Pyrite-Copper	65.49	1.25	59.26	1	0	1	18	Medium quality	5698	1053297	62.20	287	d__Archaea;p__Nanoarchaeota;c__Nanoarchaeia;o__Woesearchaeales;f__UBA525;g__UBA153;s__
LMSG_G000005953.1	yes	932_2	Pyrite-Copper	79.09	1.97	69.25	0	0	2	17	Medium quality	18247	3517929	52.70	395	d__Bacteria;p__Nitrospirota;c__Thermodesulfovibrionia;o__Thermodesulfovibrionales;f__UBA6898;g__Sulfobium;s__
LMSG_G000005954.1	yes	1370_0	Pyrite-Copper	82.53	0.60	79.55	0	0	0	17	Medium quality	9239	2976604	36.80	478	d__Bacteria;p__Bacteroidota;c__Kapabacteria;o__Kapabacteriales;f__XYC2-FULL-35-21;g__;s__
LMSG_G000005956.1	yes	1678_0	Pyrite-Copper	75.15	0.65	71.89	0	0	0	18	Medium quality	6428	1914360	44.70	429	d__Archaea;p__Halobacteriota;c__Methanosarcinia;o__Methanosarcinales;f__Methanoperedenaceae;g__Methanoperedens;s__
LMSG_G000005957.1	yes	249_0	Pyrite-Copper	59.00	0.00	59.00	1	0	0	20	Medium quality	7059	1123017	55.70	267	d__Archaea;p__Micrarchaeota;c__Micrarchaeia;o__UBA8480;f__UBA8480;g__;s__
LMSG_G000004592.1	yes	1357_1	Pyrite-Copper	93.60	2.00	83.60	2	1	2	18	High quality	102973	3360546	51.80	125	d__Bacteria;p__Nitrospirota;c__Thermodesulfovibrionia;o__Thermodesulfovibrionales;f__UBA9159;g__;s__
LMSG_G000005958.1	yes	393_0	Pyrite-Copper	62.87	0.00	62.87	1	5	4	18	Medium quality	343001	1087921	45.70	29	d__Bacteria;p__Patescibacteria;c__Microgenomatia;o__Shapirobacterales;f__;g__;s__
LMSG_G000005959.1	yes	1791_0	Pyrite-Copper	60.45	0.56	57.65	0	1	5	18	Medium quality	19827	829988	42.60	91	d__Bacteria;p__Patescibacteria;c__Microgenomatia;o__UBA1406;f__GWC2-37-13;g__GWC2-37-13;s__
LMSG_G000005960.1	yes	391_0	Pyrite-Copper	59.57	0.50	57.10	0	0	0	16	Medium quality	91323	1024189	45.20	43	d__Bacteria;p__Patescibacteria;c__Microgenomatia;o__GWA2-44-7;f__Amesbacteraceae;g__XYB1-FULL-44-23;s__
LMSG_G000005964.1	yes	121_0	Pyrite-Copper	66.97	0.00	66.97	1	1	0	20	Medium quality	48516	858584	46.10	30	d__Archaea;p__Micrarchaeota;c__Micrarchaeia;o__Micrarchaeales;f__;g__;s__
LMSG_G000005961.1	yes	1718_0	Pyrite-Copper	75.92	1.42	68.81	1	1	1	3	Medium quality	6529	1267079	30.90	341	d__Bacteria;p__Proteobacteria;c__Gammaproteobacteria;o__Burkholderiales;f__UBA11063;g__;s__
LMSG_G000005962.1	yes	1756_0	Pyrite-Copper	89.60	1.14	83.91	0	0	0	19	Medium quality	82519	1829519	39.50	35	d__Bacteria;p__Omnitrophota;c__Koll11;o__4484-171;f__4484-171;g__;s__
LMSG_G000005963.1	yes	1651_0	Pyrite-Copper	67.39	2.38	55.49	0	0	0	9	Medium quality	4261	2655524	47.40	734	d__Bacteria;p__Bacteroidota;c__Bacteroidia;o__Bacteroidales;f__UBA12481;g__;s__
LMSG_G000004593.1	yes	1214_0	Pyrite-Copper	94.39	2.16	83.62	1	1	2	20	High quality	65122	2235300	55.70	65	d__Bacteria;p__Actinobacteriota;c__Thermoleophilia;o__BMS3ABIN01;f__BMS3ABIN01;g__;s__
LMSG_G000005965.1	yes	479_0	Pyrite-Copper	66.43	3.08	51.05	3	1	1	18	Medium quality	50745	2375195	47.00	192	d__Bacteria;p__Firmicutes;c__Alicyclobacillia;o__Alicyclobacillales;f__Acidibacillaceae;g__;s__
LMSG_G000005966.1	yes	1632_0	Pyrite-Copper	91.85	1.09	86.39	1	0	0	15	Medium quality	33525	3478665	46.60	161	d__Bacteria;p__Bacteroidota;c__UBA10030;o__UBA10030;f__;g__;s__
LMSG_G000004594.1	yes	178_1	Pyrite	90.54	1.61	82.48	1	1	1	20	High quality	24787	1712743	38.40	116	d__Archaea;p__Thermoplasmatota;c__Thermoplasmata;o__Thermoplasmatales;f__Thermoplasmataceae;g__GCA-000496135;s__GCA-000496135 sp000496135
LMSG_G000004595.1	yes	166_1	Pyrite	97.56	1.63	89.43	1	2	2	20	High quality	22730	1843423	32.60	122	d__Archaea;p__Thermoplasmatota;c__Thermoplasmata;o__Thermoplasmatales;f__Thermoplasmataceae;g__Acidiplasma;s__
LMSG_G000005967.1	yes	978_1	Pyrite	87.15	1.55	79.39	0	0	0	17	Medium quality	14121	1967872	60.00	198	d__Bacteria;p__Proteobacteria;c__Gammaproteobacteria;o__Acidithiobacillales;f__Acidithiobacillaceae;g__Acidithiobacillus;s__
LMSG_G000005968.1	yes	96_2	Pyrite	80.66	0.00	80.66	1	1	1	17	Medium quality	41875	954768	45.20	207	d__Archaea;p__Micrarchaeota;c__Micrarchaeia;o__Micrarchaeales;f__Micrarchaeaceae;g__Micrarchaeum;s__
LMSG_G000005969.1	yes	967_1	Pyrite	93.08	1.82	83.99	2	0	0	19	Medium quality	67981	3057333	55.60	125	d__Bacteria;p__Nitrospirota;c__Leptospirillia;o__Leptospirillales;f__Leptospirillaceae;g__Leptospirillum_A;s__
LMSG_G000005970.1	yes	965_2	Pyrite	80.52	0.00	80.52	1	1	1	18	Medium quality	46856	2302896	60.00	103	d__Bacteria;p__Nitrospirota;c__Leptospirillia;o__Leptospirillales;f__Leptospirillaceae;g__UBA4572;s__
LMSG_G000005971.1	yes	6_1	Pyrite	83.73	1.94	74.03	1	1	1	19	Medium quality	88571	965136	40.40	22	d__Archaea;p__Nanoarchaeota;c__Nanoarchaeia;o__Parvarchaeales;f__Parvarchaeaceae;g__Parvarchaeum;s__
LMSG_G000005972.1	yes	1748_1	Pyrite	91.81	0.00	91.81	0	1	0	19	Medium quality	58412	2462103	32.70	93	d__Bacteria;p__SZUA-79;c__SZUA-79;o__Acidulodesulfobacterales;f__SZUA-79;g__Acididesulfobacter;s__Acididesulfobacter guangdongensis
LMSG_G000005973.1	yes	1746_2	Pyrite	88.23	1.68	79.83	0	0	0	19	Medium quality	30084	2568719	30.00	146	d__Bacteria;p__SZUA-79;c__SZUA-79;o__Acidulodesulfobacterales;f__SZUA-79;g__Acididesulfobacter;s__Acididesulfobacter diazotrophicus
LMSG_G000005974.1	yes	1291_0	Coal	79.58	4.79	55.65	0	0	0	15	Medium quality	2765	3655730	67.70	2364	d__Bacteria;p__Proteobacteria;c__Gammaproteobacteria;o__Burkholderiales;f__Burkholderiaceae;g__Massilia;s__
LMSG_G000005975.1	yes	1451_0	Coal	94.16	2.80	80.19	1	0	1	16	Medium quality	18759	4097919	59.40	459	d__Bacteria;p__Proteobacteria;c__Alphaproteobacteria;o__Rhizobiales;f__Xanthobacteraceae;g__Pseudolabrys;s__
LMSG_G000005976.1	yes	1447_0	Coal	61.46	1.99	51.51	0	1	0	11	Medium quality	4147	3562752	66.90	978	d__Bacteria;p__Proteobacteria;c__Alphaproteobacteria;o__Acetobacterales;f__Acetobacteraceae;g__Palsa-883;s__
LMSG_G000005977.1	yes	716_0	Coal	53.50	0.20	52.51	0	1	0	14	Medium quality	3389	2080928	66.30	641	d__Bacteria;p__Gemmatimonadota;c__Gemmatimonadetes;o__Gemmatimonadales;f__GWC2-71-9;g__;s__
LMSG_G000004596.1	yes	715_1	Coal	99.14	1.03	94.02	1	1	1	19	High quality	117954	4368427	69.90	65	d__Bacteria;p__Acidobacteriota;c__Thermoanaerobaculia;o__UBA5066;f__Gp7-AA6;g__;s__
LMSG_G000005978.1	yes	1094_0	Coal	89.55	1.39	82.61	1	0	1	16	Medium quality	23619	2984702	69.20	199	d__Bacteria;p__Proteobacteria;c__Gammaproteobacteria;o__Steroidobacterales;f__Steroidobacteraceae;g__13-2-20CM-66-19;s__
LMSG_G000005979.1	yes	1574_0	Coal	73.70	2.49	61.24	0	0	0	15	Medium quality	6505	3731325	55.10	852	d__Bacteria;p__Acidobacteriota;c__Acidobacteriae;o__Acidobacteriales;f__Gp1-AA117;g__Gp1-AA17;s__
LMSG_G000005980.1	yes	640_0	Coal	69.14	2.16	58.34	0	0	0	14	Medium quality	2413	2069270	61.80	932	d__Bacteria;p__Eremiobacterota;c__Eremiobacteria;o__UBP12;f__UBA5184;g__PALSA-1484;s__
LMSG_G000004597.1	yes	1127_0	Coal	97.68	0.00	97.68	1	1	1	19	High quality	173861	2410398	63.40	20	d__Bacteria;p__Eremiobacterota;c__Eremiobacteria;o__UBP12;f__UBA5184;g__PALSA-1484;s__
LMSG_G000005981.1	yes	240_0	Coal	61.37	0.97	56.52	1	0	1	16	Medium quality	3061	913653	37.70	314	d__Archaea;p__Thermoproteota;c__Nitrososphaeria;o__Nitrososphaerales;f__Nitrosopumilaceae;g__Nitrosotalea;s__
LMSG_G000005982.1	yes	1536_0	Coal	95.18	2.82	81.08	0	1	0	18	Medium quality	57754	5595619	61.60	160	d__Bacteria;p__Proteobacteria;c__Alphaproteobacteria;o__Rhizobiales;f__Xanthobacteraceae;g__Bradyrhizobium;s__
LMSG_G000005983.1	yes	246_0	Coal	62.10	1.10	56.60	1	1	1	13	Medium quality	3702	3749129	51.60	1113	d__Bacteria;p__Chloroflexota;c__Ktedonobacteria;o__Ktedonobacterales;f__Ktedonobacteraceae;g__;s__
LMSG_G000005984.1	yes	1259_1	Coal	95.05	2.17	84.21	0	0	0	17	Medium quality	21201	3464704	64.60	271	d__Bacteria;p__Proteobacteria;c__Gammaproteobacteria;o__Xanthomonadales;f__Rhodanobacteraceae;g__Rhodanobacter;s__
LMSG_G000004598.1	yes	1580_0	Coal	96.70	3.30	80.22	1	1	1	20	High quality	216455	3705256	61.70	37	d__Bacteria;p__Gemmatimonadota;c__Gemmatimonadetes;o__Gemmatimonadales;f__Gemmatimonadaceae;g__Fen-1247;s__
LMSG_G000004599.1	yes	293_1	Coal	93.80	0.93	89.18	1	1	2	20	High quality	57631	3664773	57.40	98	d__Bacteria;p__Acidobacteriota;c__Acidobacteriae;o__UBA7541;f__UBA7541;g__;s__
LMSG_G000005985.1	yes	1310_0	Coal	90.21	2.29	78.79	0	0	0	17	Medium quality	10049	2955704	68.30	368	d__Bacteria;p__Proteobacteria;c__Gammaproteobacteria;o__Burkholderiales;f__Palsa-1005;g__VBCG01;s__
LMSG_G000005986.1	yes	1032_0	Coal	53.50	0.00	53.50	0	0	0	8	Medium quality	3758	3162210	62.60	987	d__Bacteria;p__Acidobacteriota;c__Acidobacteriae;o__Bryobacterales;f__Bryobacteraceae;g__;s__
LMSG_G000005987.1	yes	241_0	Coal	85.61	0.65	82.38	1	0	0	18	Medium quality	6247	1298841	36.60	252	d__Archaea;p__Thermoproteota;c__Nitrososphaeria;o__Nitrososphaerales;f__Nitrosopumilaceae;g__Nitrosotalea;s__

LMSG_G000005988.1	yes	1575_0	Coal	98.42	2.85	84.18	2	0	2	19	Medium quality	33731	4985350	55.50	356	d__Bacteria;p__Acidobacteriota;c__Acidobacteriae;o__Acidobacteriales;f__Gp1-AA117;g__Gp1-AA17;s__
LMSG_G000004600.1	yes	1266_0	Coal	98.39	0.59	95.44	1	1	2	20	High quality	178429	2831150	64.10	22	d__Bacteria;p__Proteobacteria;c__Gammaproteobacteria;o__Xanthomonadales;f__Xanthomonadaceae;g__Lysobacter_B;s__
LMSG_G000006299.1	yes	643_0	Coal	92.95	1.65	84.70	1	4	2	17	Medium quality	11709	3911201	61.80	559	d__Bacteria;p__Chloroflexota;c__Ktedonobacteria;o__Ktedonobacterales;f__;g__;s__
LMSG_G000005989.1	yes	1581_0	Coal	85.16	2.26	73.85	1	1	1	19	Medium quality	6584	3848477	67.10	733	d__Bacteria;p__Gemmatimonadota;c__Gemmatimonadetes;o__Gemmatimonadales;f__Gemmatimonadaceae;g__;s__
LMSG_G000005994.1	yes	1092_0	Coal	79.67	3.58	61.79	1	1	1	11	Medium quality	16544	3160799	69.10	288	d__Bacteria;p__Proteobacteria;c__Gammaproteobacteria;o__Steroidobacterales;f__Steroidobacteraceae;g__;s__
LMSG_G000005990.1	yes	772_0	Coal	97.43	1.28	91.02	0	1	0	19	Medium quality	23519	2866669	73.30	170	d__Bacteria;p__Actinobacteriota;c__Acidimicrobiia;o__Acidimicrobiales;f__Bog-793;g__;s__
LMSG_G000005991.1	yes	1504_2	Coal	75.66	1.74	66.96	0	1	0	15	Medium quality	10959	2660173	70.30	299	d__Bacteria;p__Proteobacteria;c__Alphaproteobacteria;o__Acetobacterales;f__Acetobacteraceae;g__BOG-930;s__
LMSG_G000005992.1	yes	637_1	Coal	94.90	0.93	90.28	0	0	0	18	Medium quality	10863	2440384	63.90	291	d__Bacteria;p__Eremiobacterota;c__Eremiobacteria;o__UBP12;f__UBA5184;g__PALSA-1484;s__
LMSG_G000006300.1	yes	559_1	Coal	93.06	1.60	85.06	1	1	2	17	Medium quality	53925	1977784	72.00	87	d__Archaea;p__Thermoplasmatota;c__Thermoplasmata;o__UBA184;f__UBA184;g__;s__
LMSG_G000005993.1	yes	734_0	Coal	85.29	2.74	71.62	0	1	0	16	Medium quality	15039	2893172	72.90	218	d__Bacteria;p__Actinobacteriota;c__Acidimicrobiia;o__Acidimicrobiales;f__RAAP-2;g__PALSA-457;s__
LMSG_G000004601.1	yes	1116_1	Coal	96.46	2.04	86.27	1	1	1	19	High quality	62558	2840449	57.90	99	d__Bacteria;p__Proteobacteria;c__Gammaproteobacteria;o__SLND01;f__;g__;s__
LMSG_G000005995.1	yes	424_0	Coal	63.97	0.72	60.40	1	2	0	12	Medium quality	2721	777467	54.30	333	d__Bacteria;p__Patescibacteria;c__Saccharimonadia;o__UBA4664;f__SCTI01;g__;s__
LMSG_G000005996.1	yes	899_0	Coal	91.61	1.72	82.99	0	1	0	15	Medium quality	27320	3747753	63.10	194	d__Bacteria;p__Acidobacteriota;c__Acidobacteriae;o__Acidobacteriales;f__Acidobacteriaceae;g__Palsa-343;s__
LMSG_G000004602.1	yes	719_1	Coal	98.29	1.66	89.99	1	1	1	20	High quality	71258	3840096	69.80	106	d__Bacteria;p__Actinobacteriota;c__Acidimicrobiia;o__Acidimicrobiales;f__;g__;s__
LMSG_G000005997.1	yes	1495_1	Coal	99.56	2.83	85.43	0	1	0	18	Medium quality	42331	4966495	68.90	215	d__Bacteria;p__Proteobacteria;c__Alphaproteobacteria;o__ATCC43930;f__Stellaceae;g__;s__
LMSG_G000005998.1	yes	731_1	Coal	95.72	2.14	85.04	0	1	0	19	Medium quality	57256	2473916	71.00	68	d__Bacteria;p__Actinobacteriota;c__Acidimicrobiia;o__Acidimicrobiales;f__RAAP-2;g__Palsa-461;s__
LMSG_G000005999.1	yes	786_0	Coal	62.09	2.31	50.56	0	0	0	12	Medium quality	3718	1065247	63.00	334	d__Bacteria;p__Actinobacteriota;c__Acidimicrobiia;o__Acidimicrobiales;f__RAAP-2;g__RAAP-2;s__
LMSG_G000006000.1	yes	1449_0	Coal	58.62	0.86	54.31	0	0	0	11	Medium quality	4172	1702118	64.70	460	d__Bacteria;p__Proteobacteria;c__Alphaproteobacteria;o__ATCC43930;f__Stellaceae;g__;s__
LMSG_G000006001.1	yes	343_0	Coal	75.15	0.00	75.15	1	6	0	18	Medium quality	34667	970313	53.20	65	d__Bacteria;p__Patescibacteria;c__Saccharimonadia;o__Saccharimonadales;f__UBA4665;g__;s__
LMSG_G000006002.1	yes	1124_0	Coal	97.22	1.03	92.08	1	0	1	18	Medium quality	19614	2123972	64.50	169	d__Bacteria;p__Eremiobacterota;c__Eremiobacteria;o__UBP12;f__UBA5184;g__PALSA-1504;s__
LMSG_G000004603.1	yes	833_0	Coal	94.08	0.28	92.66	1	1	1	19	High quality	26183	4312148	71.10	231	d__Bacteria;p__Actinobacteriota;c__Thermoleophilia;o__Solirubrobacterales;f__Solirubrobacteraceae;g__Palsa-465;s__
LMSG_G000004606.1	yes	1647_1	Coal	96.30	1.82	87.21	1	1	4	18	High quality	156480	1999789	56.80	42	d__Bacteria;p__Nitrospirota;c__Leptospirillia;o__Leptospirillales;f__Leptospirillaceae;g__Leptospirillum_A;s__
LMSG_G000004604.1	yes	561_0	Coal	98.00	3.20	82.00	1	2	2	19	High quality	124707	2039419	71.90	61	d__Archaea;p__Thermoplasmatota;c__Thermoplasmata;o__UBA184;f__UBA184;g__;s__
LMSG_G000004605.1	yes	1033_2	Coal	90.11	0.28	88.69	1	1	1	19	High quality	34327	3766525	67.70	263	d__Bacteria;p__Acidobacteriota;c__Acidobacteriae;o__Acidobacteriales;f__SCQP01;g__;s__
LMSG_G000006003.1	yes	1093_1	Coal	83.33	3.70	64.86	0	1	0	12	Medium quality	52238	2793577	68.30	91	d__Bacteria;p__Proteobacteria;c__Gammaproteobacteria;o__Steroidobacterales;f__Steroidobacteraceae;g__FEN-1191;s__
LMSG_G000004607.1	yes	829_0	Coal	94.87	2.22	83.76	1	2	1	20	High quality	81119	3325602	69.50	65	d__Bacteria;p__Actinobacteriota;c__Thermoleophilia;o__Solirubrobacterales;f__Solirubrobacteraceae;g__;s__
LMSG_G000006004.1	yes	55_0	Coal	81.77	1.71	73.21	1	1	1	20	Medium quality	24312	806683	43.60	50	d__Archaea;p__Micrarchaeota;c__Micrarchaeia;o__Micrarchaeales;f__;g__;s__
LMSG_G000006005.1	yes	778_1	Coal	60.89	1.14	55.20	1	0	0	14	Medium quality	3144	1460766	60.60	543	d__Bacteria;p__Actinobacteriota;c__Acidimicrobiia;o__Acidimicrobiales;f__Bog-793;g__;s__
LMSG_G000004610.1	yes	238_2	Coal	97.97	0.00	97.97	1	2	3	19	High quality	33823	1737070	38.50	87	d__Archaea;p__Thermoproteota;c__Nitrososphaeria;o__Nitrososphaerales;f__Nitrosopumilaceae;g__Nitrosotalea;s__
LMSG_G000006006.1	yes	586_0	Coal	64.36	1.05	59.14	0	0	0	13	Medium quality	1876	989646	63.30	542	d__Archaea;p__Thermoproteota;c__Nitrososphaeria;o__Nitrososphaerales;f__UBA183;g__BOG-1369;s__
LMSG_G000004608.1	yes	629_1	Coal	94.90	0.00	94.90	1	1	1	20	High quality	22216	2257500	71.00	170	d__Bacteria;p__Dormibacterota;c__Dormibacteria;o__UBA8260;f__Bog-877;g__;s__
LMSG_G000004609.1	yes	1063_1	Coal	98.85	1.15	93.11	1	1	1	19	High quality	78891	4243963	62.00	152	d__Bacteria;p__Spirochaetota;c__Spirochaetia;o__Spirochaetales;f__;g__;s__
LMSG_G000006007.1	yes	1312_0	Coal	53.22	0.47	50.86	1	0	1	10	Medium quality	6889	1636436	69.50	264	d__Bacteria;p__Proteobacteria;c__Gammaproteobacteria;o__Burkholderiales;f__SG8-39;g__SG8-39;s__
LMSG_G000006008.1	yes	1130_2	Coal	83.52	1.85	74.27	0	0	0	18	Medium quality	7766	2072243	62.50	375	d__Bacteria;p__Eremiobacterota;c__Eremiobacteria;o__UBP12;f__UBA5184;g__;s__
LMSG_G000004611.1	yes	830_0	Coal	94.01	3.19	78.06	1	2	1	20	High quality	34347	2231799	67.70	259	d__Bacteria;p__Actinobacteriota;c__Thermoleophilia;o__Solirubrobacterales;f__Solirubrobacteraceae;g__Palsa-744;s__
LMSG_G000006009.1	yes	1254_1	Coal	79.31	1.72	70.69	2	0	0	17	Medium quality	13785	2211153	69.30	257	d__Bacteria;p__Proteobacteria;c__Gammaproteobacteria;o__Xanthomonadales;f__Rhodanobacteraceae;g__;s__
LMSG_G000006010.1	yes	639_0	Coal	98.88	0.00	98.88	1	0	0	19	Medium quality	137094	3137786	65.80	35	d__Bacteria;p__Eremiobacterota;c__Eremiobacteria;o__UBP12;f__UBA5184;g__PALSA-1484;s__
LMSG_G000006011.1	yes	1646_0	Coal	71.72	3.17	55.86	1	0	1	16	Medium quality	2564	2968878	52.90	1269	d__Bacteria;p__Bacteroidota;c__Kryptonia;o__Kryptoniales;f__;g__;s__
LMSG_G000006012.1	yes	886_1	Coal	80.79	1.03	75.62	1	1	1	15	Medium quality	5227	1936477	59.90	435	d__Bacteria;p__Acidobacteriota;c__Acidobacteriae;o__Acidobacteriales;f__Acidobacteriaceae;g__;s__
LMSG_G000006013.1	yes	766_0	Coal	88.03	1.28	81.62	1	1	1	17	Medium quality	36899	2554698	71.70	108	d__Bacteria;p__Actinobacteriota;c__Acidimicrobiia;o__Acidimicrobiales;f__RAAP-2;g__;s__
LMSG_G000004612.1	yes	208_1	Coal	95.43	0.00	95.43	1	2	1	19	High quality	26203	1274570	46.50	101	d__Archaea;p__Thermoplasmatota;c__Thermoplasmata;o__Thermoplasmatales;f__Thermoplasmataceae;g__B-DKE;s__
LMSG_G000006014.1	yes	773_0	Coal	84.74	1.71	76.20	0	1	0	15	Medium quality	9724	2968825	72.30	458	d__Bacteria;p__Actinobacteriota;c__Acidimicrobiia;o__Acidimicrobiales;f__Bog-793;g__;s__
LMSG_G000006019.1	yes	895_0	Coal	88.30	0.90	83.79	0	1	0	19	Medium quality	25313	4005296	56.70	381	d__Bacteria;p__Acidobacteriota;c__Acidobacteriae;o__Acidobacteriales;f__Acidobacteriaceae;g__;s__
LMSG_G000006015.1	yes	714_0	Coal	78.95	0.83	74.79	1	0	1	18	Medium quality	26516	2616188	74.20	149	d__Bacteria;p__Dormibacterota;c__Dormibacteria;o__UBA8260;f__Bog-877;g__;s__
LMSG_G000006016.1	yes	758_1	Coal	95.72	2.56	82.90	0	0	0	18	Medium quality	32442	2983902	73.00	138	d__Bacteria;p__Actinobacteriota;c__Acidimicrobiia;o__Acidimicrobiales;f__RAAP-2;g__;s__
LMSG_G000006017.1	yes	826_0	Coal	83.38	3.85	64.15	0	0	0	17	Medium quality	77093	3360644	69.70	84	d__Bacteria;p__Actinobacteriota;c__Acidimicrobiia;o__Acidimicrobiales;f__RAAP-2;g__Bog-473;s__
LMSG_G000004613.1	yes	713_1	Coal	99.07	0.00	99.07	1	1	1	18	High quality	19404	2807766	74.20	233	d__Bacteria;p__Dormibacterota;c__Dormibacteria;o__UBA8260;f__Bog-877;g__;s__
LMSG_G000006018.1	yes	732_1	Coal	93.55	3.59	75.61	0	0	0	17	Medium quality	18009	2912428	69.50	190	d__Bacteria;p__Actinobacteriota;c__Acidimicrobiia;o__Acidimicrobiales;f__RAAP-2;g__Palsa-461;s__
LMSG_G000004617.1	yes	555_1	Coal	93.86	2.46	81.56	1	2	1	20	High quality	84971	2166167	69.80	47	d__Archaea;p__Thermoplasmatota;c__Thermoplasmata;o__UBA184;f__UBA184;g__;s__
LMSG_G000004614.1	yes	150_1	Coal	99.13	1.60	91.13	1	1	2	20	High quality	104627	1955938	52.70	35	d__Archaea;p__Thermoplasmatota;c__Thermoplasmata;o__;f__;g__;s__
LMSG_G000004615.1	yes	568_1	Coal	96.62	2.40	84.62	1	2	2	20	High quality	86956	2959275	68.90	87	d__Archaea;p__Thermoplasmatota;c__Thermoplasmata;o__UBA184;f__UBA184;g__;s__
LMSG_G000006301.1	yes	872_1	Coal	90.73	1.34	84.05	1	1	1	16	Medium quality	17605	3383009	70.30	252	d__Bacteria;p__Actinobacteriota;c__Actinomycetia;o__Streptosporangiales;f__Streptosporangiaceae;g__UBA9676;s__
LMSG_G000004616.1	yes	564_1	Coal	91.42	1.60	83.42	1	1	1	19	High quality	31537	1685673	71.50	74	d__Archaea;p__Thermoplasmatota;c__Thermoplasmata;o__UBA184;f__UBA184;g__;s__
LMSG_G000006020.1	yes	730_2	Coal	94.87	1.14	89.18	0	0	0	18	Medium quality	28616	2321996	70.80	147	d__Bacteria;p__Actinobacteriota;c__Acidimicrobiia;o__Acidimicrobiales;f__RAAP-2;g__Palsa-461;s__
LMSG_G000004618.1	yes	709_0	Coal	98.29	1.28	91.88	1	1	1	19	High quality	32274	3056693	67.10	130	d__Bacteria;p__Actinobacteriota;c__Acidimicrobiia;o__Acidimicrobiales;f__RAAP-2;g__;s__
LMSG_G000004619.1	yes	575_1	Coal	95.40	0.94	90.70	1	2	2	19	High quality	71035	2012996	59.50	90	d__Bacteria;p__Proteobacteria;c__Gammaproteobacteria;o__UBA2770;f__UBA2770;g__;s__
LMSG_G000004620.1	yes	1033_1	Coal	95.29	0.17	94.44	1	1	2	20	High quality	37385	3799637	68.00	177	d__Bacteria;p__Acidobacteriota;c__Acidobacteriae;o__Acidobacteriales;f__SCQP01;g__;s__
LMSG_G000004621.1	yes	870_1	Coal	97.05	4.01	77.00	1	1	1	20	High quality	95641	5826126	67.00	154	d__Bacteria;p__Actinobacteriota;c__Actinomycetia;o__Streptosporangiales;f__Streptosporangiaceae;g__Palsa-506;s__
LMSG_G000006021.1	yes	141_1	Coal	75.23	0.00	75.23	1	1	1	19	Medium quality	62366	860186	45.30	19	d__Archaea;p__Micrarchaeota;c__Micrarchaeia;o__Micrarchaeales;f__;g__;s__
LMSG_G000006022.1	yes	235_0	Coal	64.10	0.85	59.83	1	1	2	19	Medium quality	20444	928885	47.00	69	d__Bacteria;p__Patescibacteria;c__Saccharimonadia;o__Saccharimonadales;f__UBA4665;g__UBA5150;s__
LMSG_G000006023.1	yes	562_1	Coal	88.00	0.80	84.00	1	2	1	19	Medium quality	84214	1849545	72.10	76	d__Archaea;p__Thermoplasmatota;c__Thermoplasmata;o__UBA184;f__UBA184;g__;s__
LMSG_G000006024.1	yes	553_0	Coal	74.68	3.59	56.74	0	0	1	16	Medium quality	8930	1561507	69.40	241	d__Archaea;p__Thermoplasmatota;c__Thermoplasmata;o__UBA184;f__UBA184;g__;s__
LMSG_G000006025.1	yes	828_1	Coal	82.99	0.65	79.74	0	0	0	18	Medium quality	7488	2575958	70.80	472	d__Bacteria;p__Actinobacteriota;c__Thermoleophilia;o__Solirubrobacterales;f__Solirubrobacteraceae;g__;s__
LMSG_G000006026.1	yes	1506_1	Coal	81.18	1.62	73.08	0	1	0	18	Medium quality	6302	3180365	70.90	657	d__Bacteria;p__Proteobacteria;c__Alphaproteobacteria;o__Acetobacterales;f__Acetobacteraceae;g__Palsa-883;s__
LMSG_G000006027.1	yes	1117_2	Coal	93.17	1.18	87.29	0	0	0	16	Medium quality	21099	2325063	60.90	171	d__Bacteria;p__Proteobacteria;c__Gammaproteobacteria;o__SLND01;f__;g__;s__
LMSG_G000006030.1	yes	359_1	Coal	58.78	0.00	58.78	0	1	0	18	Medium quality	9768	681117	40.30	101	d__Bacteria;p__Patescibacteria;c__Saccharimonadia;o__Saccharimonadales;f__UBA4665;g__;s__
LMSG_G000006028.1	yes	711_1	Coal	87.12	1.49	79.70	2	1	0	19	Medium quality	15781	4152529	72.40	361	d__Bacteria;p__Firmicutes_E;c__Thermaerobacteria;o__;f__;g__;s__
LMSG_G000006029.1	yes	718_0	Coal	52.02	0.00	52.02	0	0	0	9	Medium quality	3292	1590840	66.00	513	d__Bacteria;p__Actinobacteriota;c__Acidimicrobiia;o__Acidimicrobiales;f__Bog-793;g__Palsa-601;s__
LMSG_G000004622.1	yes	627_1	Coal	95.83	0.00	95.83	1	1	1	20	High quality	26139	3428616	64.30	345	d__Bacteria;p__Dormibacterota;c__Dormibacteria;o__UBA8260;f__Bog-877;g__;s__

LMSG_G000006031.1	yes	1630_0	Coal	83.65	2.91	69.09	1	1	1	18	Medium quality	7313	2703930	46.10	500	d__Archaea;p__Thermoproteota;c__Nitrososphaeria;o__Nitrososphaerales;f__;g__;s__
LMSG_G000006032.1	yes	733_1	Coal	95.72	2.99	80.77	1	0	1	18	Medium quality	36154	2688883	73.80	131	d__Bacteria;p__Actinobacteriota;c__Acidimicrobiia;o__Acidimicrobiales;f__RAAP-2;g__Bog-756;s__
LMSG_G000006033.1	yes	1064_0	Coal	95.21	1.15	89.47	0	0	0	18	Medium quality	17721	3636046	64.10	299	d__Bacteria;p__Spirochaetota;c__Spirochaetia;o__Spirochaetales;f__;g__;s__
LMSG_G000006034.1	yes	970_1	Coal	92.38	1.82	83.29	1	0	0	18	Medium quality	73230	2038030	56.60	69	d__Bacteria;p__Nitrospirota;c__Leptospirillia;o__Leptospirillales;f__Leptospirillaceae;g__UBA4572;s__
LMSG_G000006035.1	yes	149_0	Coal	77.43	0.00	77.43	1	1	0	16	Medium quality	22484	758308	51.10	58	d__Archaea;p__Micrarchaeota;c__Micrarchaeia;o__Micrarchaeales;f__;g__;s__
LMSG_G000006036.1	yes	874_0	Coal	96.52	4.49	74.07	1	0	0	19	Medium quality	19375	6222640	70.80	488	d__Bacteria;p__Actinobacteriota;c__Actinomycetia;o__Streptosporangiales;f__Streptosporangiaceae;g__;s__
LMSG_G000006302.1	yes	549_1	Coal	95.46	0.80	91.46	1	2	1	17	Medium quality	22389	1831151	71.00	123	d__Archaea;p__Thermoplasmatota;c__Thermoplasmata;o__UBA184;f__UBA184;g__;s__
LMSG_G000006037.1	yes	254_2	Coal	75.23	3.08	59.83	2	1	2	18	Medium quality	2333	4678030	51.90	2169	d__Bacteria;p__Chloroflexota;c__Ktedonobacteria;o__Ktedonobacterales;f__Ktedonobacteraceae;g__;s__
LMSG_G000006038.1	yes	543_0	Coal	59.32	1.82	50.22	1	1	3	12	Medium quality	2033	1156502	69.20	598	d__Archaea;p__Thermoplasmatota;c__Thermoplasmata;o__UBA184;f__UBA184;g__;s__
LMSG_G000006039.1	yes	871_2	Coal	99.46	2.14	88.77	0	1	0	20	Medium quality	44137	5857151	69.30	280	d__Bacteria;p__Actinobacteriota;c__Actinomycetia;o__Streptosporangiales;f__Streptosporangiaceae;g__Palsa-506;s__
LMSG_G000006040.1	yes	892_2	Coal	79.71	1.72	71.09	0	1	0	18	Medium quality	8005	3201828	58.60	485	d__Bacteria;p__Acidobacteriota;c__Acidobacteriae;o__Acidobacteriales;f__Acidobacteriaceae;g__;s__
LMSG_G000006041.1	yes	133_1	Coal	75.07	1.40	68.07	1	0	0	18	Medium quality	15999	679924	52.60	68	d__Archaea;p__Micrarchaeota;c__Micrarchaeia;o__Micrarchaeales;f__;g__;s__
LMSG_G000004624.1	yes	1465_0	Copper	93.09	2.52	80.49	2	1	2	18	High quality	27469	4109985	59.50	249	d__Bacteria;p__Desulfobacterota_B;c__Binatia;o__Binatales;f__Binataceae;g__;s__
LMSG_G000004623.1	yes	682_0	Copper	95.83	0.93	91.21	1	1	1	20	High quality	121725	3133587	70.60	45	d__Bacteria;p__Chloroflexota;c__Ellin6529;o__CSP1-4;f__CSP1-4;g__C-114;s__
LMSG_G000006042.1	yes	458_0	Copper	89.76	1.50	82.29	2	7	13	19	Medium quality	5522	3710294	58.80	900	d__Bacteria;p__Deinococcota;c__Deinococci;o__Deinococcales;f__Thermaceae;g__Meiothermus;s__
LMSG_G000006043.1	yes	1412_0	Copper	60.94	0.28	59.57	0	0	0	11	Medium quality	1892	2362240	65.80	1331	d__Bacteria;p__Chloroflexota;c__Ktedonobacteria;o__Ktedonobacterales;f__;g__;s__
LMSG_G000006044.1	yes	710_0	Copper	100.00	1.42	92.88	0	1	0	20	Medium quality	32133	4148038	67.20	249	d__Bacteria;p__Actinobacteriota;c__Acidimicrobiia;o__Acidimicrobiales;f__RAAP-2;g__Bog-756;s__
LMSG_G000006045.1	yes	459_0	Copper	68.50	1.38	61.62	0	1	0	16	Medium quality	22324	2948850	57.10	216	d__Bacteria;p__Chloroflexota;c__Chloroflexia;o__54-19;f__;g__;s__
LMSG_G000004625.1	yes	1371_0	Copper	96.22	1.55	88.49	1	1	1	19	High quality	31028	4643943	41.70	229	d__Bacteria;p__Bacteroidota;c__Bacteroidia;o__Cytophagales;f__Cyclobacteriaceae;g__ELB16-189;s__
LMSG_G000006046.1	yes	1116_2	Copper	83.63	2.40	71.63	1	1	1	18	Medium quality	6761	2058818	58.90	386	d__Bacteria;p__Proteobacteria;c__Gammaproteobacteria;o__SLND01;f__;g__;s__
LMSG_G000006047.1	yes	1751_0	Copper	73.14	1.98	63.24	2	0	1	17	Medium quality	3810	2626290	65.10	750	d__Bacteria;p__Chloroflexota;c__Dehalococcoidia;o__UBA2991;f__UBA2991;g__;s__
LMSG_G000006048.1	yes	1313_0	Copper	91.45	3.93	71.81	0	1	0	19	Medium quality	7387	3978371	66.60	706	d__Bacteria;p__Proteobacteria;c__Gammaproteobacteria;o__Burkholderiales;f__SG8-39;g__SG8-39;s__
LMSG_G000004626.1	yes	1494_0	Copper	90.20	2.15	79.48	4	2	1	19	High quality	9759	4366045	54.90	571	d__Bacteria;p__Chloroflexota;c__Ktedonobacteria;o__Ktedonobacterales;f__Ktedonobacteraceae;g__CF-113;s__
LMSG_G000006049.1	yes	1557_0	Copper	70.68	0.00	70.68	0	0	0	15	Medium quality	15238	2347352	65.90	225	d__Bacteria;p__Desulfobacterota_B;c__Binatia;o__Binatales;f__Binataceae;g__;s__
LMSG_G000006050.1	yes	196_1	Iron	93.23	0.81	89.20	1	0	2	17	Medium quality	18359	1765303	42.40	151	d__Archaea;p__Thermoplasmatota;c__Thermoplasmata;o__Thermoplasmatales;f__Thermoplasmataceae;g__UBA447;s__UBA447 sp002503205
LMSG_G000006051.1	yes	198_2	Iron	99.59	0.00	99.59	0	1	2	20	Medium quality	14744	1612684	46.50	169	d__Archaea;p__Thermoplasmatota;c__Thermoplasmata;o__Thermoplasmatales;f__Thermoplasmataceae;g__UBA509;s__UBA509 sp002498845
LMSG_G000006052.1	yes	92_1	Iron	72.42	0.93	67.75	1	1	1	18	Medium quality	4440	792398	47.20	222	d__Archaea;p__Micrarchaeota;c__Micrarchaeia;o__Micrarchaeales;f__Micrarchaeaceae;g__UBA581;s__UBA581 sp002501775
LMSG_G000006053.1	yes	162_1	Iron	95.72	0.81	91.66	1	0	3	17	Medium quality	13121	1828989	36.30	201	d__Archaea;p__Thermoplasmatota;c__Thermoplasmata;o__Thermoplasmatales;f__Thermoplasmataceae;g__Ferroplasma;s__Ferroplasma sp002505185
LMSG_G000004627.1	yes	966_2	Iron	93.22	1.82	84.13	1	1	1	19	High quality	56806	2638478	54.30	129	d__Bacteria;p__Nitrospirota;c__Leptospirillia;o__Leptospirillales;f__Leptospirillaceae;g__Leptospirillum_A;s__Leptospirillum_A rubarum
LMSG_G000006054.1	yes	11_1	Iron	82.52	0.97	77.67	1	1	1	18	Medium quality	19356	849356	33.50	59	d__Archaea;p__Nanoarchaeota;c__Nanoarchaeia;o__Parvarchaeales;f__Parvarchaeaceae;g__Parvarchaeum;s__Parvarchaeum sp002502045
LMSG_G000004628.1	yes	169_1	Pyrite	96.32	0.00	96.32	1	1	2	20	High quality	1137964	1770134	38.00	10	d__Archaea;p__Thermoplasmatota;c__Thermoplasmata;o__Thermoplasmatales;f__Thermoplasmataceae;g__Cuniculiplasma;s__
LMSG_G000004629.1	yes	193_1	Pyrite	98.61	0.00	98.61	1	1	2	20	High quality	110500	1861560	44.40	44	d__Archaea;p__Thermoplasmatota;c__Thermoplasmata;o__Thermoplasmatales;f__Thermoplasmataceae;g__B-DKE;s__B-DKE sp002204705
LMSG_G000006055.1	yes	1115_2	Nickel-Copper	97.70	0.57	94.83	0	1	0	18	Medium quality	44220	2305885	55.40	90	d__Bacteria;p__Proteobacteria;c__Gammaproteobacteria;o__Halothiobacillales;f__Halothiobacillaceae;g__Halothiobacillus;s__Halothiobacillus sp002282715
LMSG_G000006056.1	yes	1261_0	Nickel-Copper	97.24	1.61	89.17	0	0	0	18	Medium quality	114307	3817445	68.40	64	d__Bacteria;p__Proteobacteria;c__Gammaproteobacteria;o__Xanthomonadales;f__Rhodanobacteraceae;g__Dyella_A;s__Dyella_A sp002279065
LMSG_G000004630.1	yes	1306_1	Nickel-Copper	98.06	1.72	89.48	1	1	1	20	High quality	91551	3310213	64.00	74	d__Bacteria;p__Proteobacteria;c__Gammaproteobacteria;o__Burkholderiales;f__Burkholderiaceae;g__Thiomonas;s__Thiomonas arsenitoxydans
LMSG_G000006057.1	yes	1458_1	Nickel-Copper	78.29	2.00	68.27	0	1	0	16	Medium quality	7193	5031015	66.00	937	d__Bacteria;p__Proteobacteria;c__Alphaproteobacteria;o__Rhizobiales;f__Xanthobacteraceae;g__Aquabacter;s__Aquabacter sp002257205
LMSG_G000006058.1	yes	1280_1	Nickel-Copper	93.39	2.71	79.82	0	0	0	18	Medium quality	31220	3681219	62.70	217	d__Bacteria;p__Proteobacteria;c__Gammaproteobacteria;o__Burkholderiales;f__Burkholderiaceae;g__Polaromonas;s__Polaromonas sp002256925
LMSG_G000006059.1	yes	1233_1	Nickel-Copper	99.52	1.46	92.24	1	0	1	19	Medium quality	257601	3285938	52.50	27	d__Bacteria;p__Proteobacteria;c__Gammaproteobacteria;o__Burkholderiales;f__Gallionellaceae;g__39-52-133;s__39-52-133 sp002278935
LMSG_G000006255.1	yes	1456_1	Nickel-Copper	97.69	0.66	94.42	1	1	0	19	Medium quality	42003	6228027	66.20	255	d__Bacteria;p__Proteobacteria;c__Alphaproteobacteria;o__Reyranellales;f__Reyranellaceae;g__Reyranella;s__Reyranella sp002281855
LMSG_G000006060.1	yes	284_1	Nickel-Copper	90.33	0.90	85.82	0	0	0	17	Medium quality	5482	2754001	36.20	693	d__Bacteria;p__Bacteroidota;c__Bacteroidia;o__Chitinophagales;f__Chitinophagaceae;g__Sediminibacterium;s__Sediminibacterium sp002281875
LMSG_G000006061.1	yes	306_1	Nickel-Copper	96.33	0.83	92.21	1	0	0	19	Medium quality	317867	2604360	55.10	70	d__Bacteria;p__Proteobacteria;c__Gammaproteobacteria;o__Burkholderiales;f__Burkholderiaceae;g__Limnohabitans;s__Limnohabitans sp002282445
LMSG_G000006063.1	yes	1279_1	Nickel-Copper	73.67	1.56	65.89	1	0	0	17	Medium quality	19139	3616403	64.00	306	d__Bacteria;p__Proteobacteria;c__Gammaproteobacteria;o__Burkholderiales;f__Burkholderiaceae;g__Polaromonas;s__Polaromonas sp002278925
LMSG_G000006062.1	yes	283_1	Nickel-Copper	67.50	2.87	53.14	0	0	0	7	Medium quality	1892	1795049	40.30	995	d__Bacteria;p__Bacteroidota;c__Bacteroidia;o__Chitinophagales;f__Chitinophagaceae;g__Sediminibacterium;s__Sediminibacterium sp002256765
LMSG_G000006064.1	yes	1413_1	Nickel-Copper	97.41	0.72	93.79	0	1	0	19	Medium quality	53732	2314405	56.50	65	d__Bacteria;p__Proteobacteria;c__Alphaproteobacteria;o__Sphingomonadales;f__Sphingomonadaceae;g__Sphingorhabdus_B;s__Sphingorhabdus_B sp002282385
LMSG_G000006065.1	yes	269_1	Nickel-Copper	99.18	1.02	94.10	0	1	0	18	Medium quality	62092	2190700	40.10	69	d__Bacteria;p__Campylobacterota;c__Campylobacteria;o__Campylobacterales;f__Sulfurimonadaceae;g__Sulfuricurvum;s__Sulfuricurvum sp002282015
LMSG_G000004631.1	yes	1283_1	Nickel-Copper	98.28	4.42	76.19	1	1	1	19	High quality	74045	4530417	63.40	159	d__Bacteria;p__Proteobacteria;c__Gammaproteobacteria;o__Burkholderiales;f__Burkholderiaceae;g__Acidovorax_B;s__Acidovorax_B defluvii
LMSG_G000004632.1	yes	425_1	Nickel-Copper	96.77	0.00	96.77	1	2	2	20	High quality	18010	2092735	39.20	197	d__Bacteria;p__Proteobacteria;c__Alphaproteobacteria;o__16-39-46;f__16-39-46;g__16-39-46;s__16-39-46 sp002257235
LMSG_G000006066.1	yes	1731_1	Nickel-Copper	98.15	0.82	94.06	0	0	0	19	Medium quality	77408	1974727	41.70	37	d__Bacteria;p__Campylobacterota;c__Campylobacteria;o__Campylobacterales;f__Sulfurovaceae;g__Sulfurovum;s__Sulfurovum sp002281755
LMSG_G000006067.1	yes	534_1	Nickel-Copper	99.51	0.00	99.51	1	0	1	19	Medium quality	92389	4064609	39.40	95	d__Bacteria;p__Bacteroidota;c__Bacteroidia;o__Sphingobacteriales;f__Sphingobacteriaceae;g__Daejeonella;s__Daejeonella sp002257025
LMSG_G000006068.1	yes	308_1	Nickel-Copper	81.94	0.62	78.85	0	1	0	14	Medium quality	29618	1683207	45.50	143	d__Bacteria;p__Proteobacteria;c__Gammaproteobacteria;o__Burkholderiales;f__Burkholderiaceae;g__Polynucleobacter;s__
LMSG_G000006069.1	yes	1414_0	Nickel-Copper	87.39	0.22	86.28	0	0	0	16	Medium quality	14222	2033018	55.50	212	d__Bacteria;p__Proteobacteria;c__Alphaproteobacteria;o__Sphingomonadales;f__Sphingomonadaceae;g__Sphingorhabdus_B;s__Sphingorhabdus_B sp002278855
LMSG_G000004633.1	yes	284_2	Nickel-Copper	99.01	0.00	99.01	1	1	1	19	High quality	184619	3209456	36.10	35	d__Bacteria;p__Bacteroidota;c__Bacteroidia;o__Chitinophagales;f__Chitinophagaceae;g__Sediminibacterium;s__Sediminibacterium sp002281575
LMSG_G000006070.1	yes	1172_0	Nickel-Copper	96.40	0.57	93.53	1	0	1	18	Medium quality	29978	2392696	57.00	133	d__Bacteria;p__Proteobacteria;c__Gammaproteobacteria;o__Halothiobacillales;f__Halothiobacillaceae;g__28-57-27;s__28-57-27 sp002281735
LMSG_G000006071.1	yes	1320_0	Nickel-Copper	83.03	0.35	81.31	1	0	1	13	Medium quality	4534	1860745	43.90	481	d__Bacteria;p__Proteobacteria;c__Gammaproteobacteria;o__Burkholderiales;f__Methylophilaceae;g__Methylophilus;s__Methylophilus sp002281475
LMSG_G000004634.1	yes	1058_0	Nickel-Copper	98.21	0.89	93.75	1	1	1	19	High quality	133555	4161229	41.00	62	d__Bacteria;p__Bdellovibrionota;c__Bdellovibrionia;o__Bdellovibrionales;f__Bdellovibrionaceae;g__UBA2316;s__UBA2316 sp002256935
LMSG_G000006072.1	yes	1249_0	Nickel-Copper	84.91	1.38	78.03	1	0	0	14	Medium quality	23175	3148611	61.30	242	d__Bacteria;p__Proteobacteria;c__Gammaproteobacteria;o__Burkholderiales;f__Thiobacillaceae;g__PFJX01;s__PFJX01 sp002281555
LMSG_G000006078.1	yes	334_0	Nickel-Copper	61.25	0.85	56.98	0	0	0	19	Medium quality	62607	760352	49.60	22	d__Bacteria;p__Patescibacteria;c__Saccharimonadia;o__Saccharimonadales;f__Saccharimonadaceae;g__;s__
LMSG_G000006073.1	yes	1421_0	Nickel-Copper	65.51	0.00	65.51	0	0	0	13	Medium quality	19006	2439177	70.10	262	d__Bacteria;p__Proteobacteria;c__Alphaproteobacteria;o__Caulobacterales;f__Caulobacteraceae;g__PMMR1;s__

LMSG_G000006074.1	yes	1611_0	Nickel-Copper	72.85	1.30	66.36	0	0	1	14	Medium quality	2530	1040358	34.60	446	d__Bacteria;p__Proteobacteria;c__Alphaproteobacteria;o__Rickettsiales;f__UBA6177;g__;s__
LMSG_G000006075.1	yes	335_0	Nickel-Copper	76.36	0.00	76.36	1	2	2	16	Medium quality	43888	665130	48.30	35	d__Bacteria;p__Patescibacteria;c__Saccharimonadia;o__Saccharimonadales;f__Saccharimonadaceae;g__TM7x;s__
LMSG_G000006076.1	yes	1425_0	Nickel-Copper	98.83	0.80	94.85	0	0	0	18	Medium quality	92348	3519503	59.30	53	d__Bacteria;p__Proteobacteria;c__Alphaproteobacteria;o__Sphingomonadales;f__Sphingomonadaceae;g__Novosphingobium;s__Novosphingobium sp002280675
LMSG_G000006077.1	yes	1205_1	Nickel-Copper	87.70	1.14	82.02	1	0	1	16	Medium quality	23029	2860545	68.60	160	d__Bacteria;p__Proteobacteria;c__Alphaproteobacteria;o__Caulobacterales;f__Caulobacteraceae;g__Brevundimonas;s__Brevundimonas subvibrioides_C
LMSG_G000004635.1	yes	1422_1	Nickel-Copper	99.56	1.32	92.97	1	1	1	19	High quality	442471	3047244	64.80	16	d__Bacteria;p__Proteobacteria;c__Alphaproteobacteria;o__Sphingomonadales;f__Sphingomonadaceae;g__Sandarakinorhabdus;s__Sandarakinorhabdus sp002280855
LMSG_G000004636.1	yes	266_0	Nickel-Copper	98.86	1.08	93.46	2	5	6	20	High quality	60764	4233403	33.80	157	d__Bacteria;p__Bacteroidota;c__Bacteroidia;o__Flavobacteriales;f__Flavobacteriaceae;g__Flavobacterium;s__Flavobacterium sp002280815
LMSG_G000006079.1	yes	1409_1	Nickel-Copper	96.57	1.54	88.89	0	0	0	19	Medium quality	55057	3770434	66.70	104	d__Bacteria;p__Proteobacteria;c__Alphaproteobacteria;o__Caulobacterales;f__Caulobacteraceae;g__Caulobacter;s__Caulobacter sp002280875
LMSG_G000006080.1	yes	1458_2	Nickel-Copper	84.71	1.03	79.55	0	0	0	15	Medium quality	41419	3615970	67.10	184	d__Bacteria;p__Proteobacteria;c__Alphaproteobacteria;o__Rhizobiales;f__Xanthobacteraceae;g__Aquabacter;s__Aquabacter sp002304505
LMSG_G000004637.1	yes	583_1	Nickel-Copper	98.79	1.35	92.04	1	7	6	20	High quality	194301	4680684	61.60	136	d__Bacteria;p__Proteobacteria;c__Alphaproteobacteria;o__Rhizobiales;f__Hyphomicrobiaceae;g__Hyphomicrobium;s__Hyphomicrobium sp002279935
LMSG_G000004638.1	yes	1410_0	Nickel-Copper	92.67	1.62	84.56	1	1	1	19	High quality	97567	3724249	64.50	134	d__Bacteria;p__Proteobacteria;c__Alphaproteobacteria;o__Caulobacterales;f__Hyphomonadaceae;g__UBA7672;s__UBA7672 sp002280725
LMSG_G000006081.1	yes	854_0	Nickel-Copper	80.80	1.52	73.23	0	1	0	19	Medium quality	24145	2213646	70.30	155	d__Bacteria;p__Actinobacteriota;c__Actinomycetia;o__Actinomycetales;f__Microbacteriaceae;g__Yonghaparkia;s__Yonghaparkia sp002280615
LMSG_G000006082.1	yes	1438_0	Nickel-Copper	95.02	1.21	88.97	0	1	0	17	Medium quality	31358	3325407	66.60	150	d__Bacteria;p__Proteobacteria;c__Alphaproteobacteria;o__Rhodobacterales;f__Rhodobacteraceae;g__NCEA01;s__NCEA01 sp002280515
LMSG_G000004639.1	yes	1415_1	Nickel-Copper	96.65	2.05	86.40	1	1	1	19	High quality	47407	2981052	62.30	118	d__Bacteria;p__Proteobacteria;c__Alphaproteobacteria;o__Sphingomonadales;f__Sphingomonadaceae;g__Sphingomonas;s__Sphingomonas sp002280555
LMSG_G000006083.1	yes	1429_0	Nickel-Copper	97.77	1.19	91.80	0	0	0	19	Medium quality	187971	3039334	64.20	33	d__Bacteria;p__Proteobacteria;c__Alphaproteobacteria;o__Sphingomonadales;f__Sphingomonadaceae;g__Erythrobacter_B;s__
LMSG_G000006084.1	yes	1416_0	Nickel-Copper	82.43	0.85	78.19	0	1	0	16	Medium quality	23721	2626186	66.20	151	d__Bacteria;p__Proteobacteria;c__Alphaproteobacteria;o__Sphingomonadales;f__Sphingomonadaceae;g__Sphingomonas;s__Sphingomonas sp002280565
LMSG_G000006085.1	yes	1609_0	Nickel-Copper	81.86	1.50	74.35	1	0	1	14	Medium quality	3830	3446933	45.10	1011	d__Bacteria;p__Bacteroidota;c__Bacteroidia;o__Cytophagales;f__Cyclobacteriaceae;g__Algoriphagus;s__
LMSG_G000006086.1	yes	339_0	Nickel-Copper	60.82	0.00	60.82	1	1	3	19	Medium quality	30850	639119	48.90	29	d__Bacteria;p__Patescibacteria;c__Saccharimonadia;o__Saccharimonadales;f__Saccharimonadaceae;g__UBA1547;s__
LMSG_G000004640.1	yes	1428_1	Nickel-Copper	98.86	0.84	94.67	1	1	1	19	High quality	174995	2797377	67.90	40	d__Bacteria;p__Proteobacteria;c__Alphaproteobacteria;o__Sphingomonadales;f__Sphingomonadaceae;g__Croceibacterium;s__Croceibacterium sp002279825
LMSG_G000006087.1	yes	285_0	Nickel-Copper	60.58	0.00	60.58	0	0	0	7	Medium quality	2232	2178363	47.10	927	d__Bacteria;p__Bacteroidota;c__Bacteroidia;o__Chitinophagales;f__Chitinophagaceae;g__;s__
LMSG_G000006088.1	yes	1277_1	Nickel-Copper	87.68	1.98	77.77	1	1	1	17	Medium quality	26346	3049927	61.60	170	d__Bacteria;p__Proteobacteria;c__Gammaproteobacteria;o__Burkholderiales;f__Thiobacillaceae;g__Thiobacillus;s__Thiobacillus sp002279795
LMSG_G000006089.1	yes	1459_0	Nickel-Copper	85.18	0.65	81.93	1	1	0	17	Medium quality	15997	3845800	68.60	324	d__Bacteria;p__Proteobacteria;c__Alphaproteobacteria;o__Rhizobiales;f__Beijerinckiaceae;g__Bosea;s__Bosea sp002279705
LMSG_G000006090.1	yes	1278_0	Nickel-Copper	95.73	0.63	92.58	1	0	0	18	Medium quality	268955	2667528	64.50	38	d__Bacteria;p__Proteobacteria;c__Gammaproteobacteria;o__Burkholderiales;f__Thiobacillaceae;g__Thiobacillus;s__Thiobacillus sp002281775
LMSG_G000006091.1	yes	243_0	Nickel-Copper	55.92	0.00	55.92	0	2	2	13	Medium quality	2103	1167661	53.70	542	d__Bacteria;p__Proteobacteria;c__Alphaproteobacteria;o__Rickettsiales;f__UBA3002;g__UBA6189;s__
LMSG_G000006092.1	yes	1426_0	Nickel-Copper	91.58	3.83	72.46	0	1	0	19	Medium quality	17656	3196012	64.00	239	d__Bacteria;p__Proteobacteria;c__Alphaproteobacteria;o__Sphingomonadales;f__Sphingomonadaceae;g__Novosphingobium;s__
LMSG_G000006093.1	yes	1439_0	Nickel-Copper	95.47	0.93	90.80	0	1	0	18	Medium quality	33237	3122161	65.70	156	d__Bacteria;p__Proteobacteria;c__Alphaproteobacteria;o__Rhodobacterales;f__Rhodobacteraceae;g__Tabrizicola;s__Tabrizicola sp002280315
LMSG_G000006094.1	yes	1423_0	Nickel-Copper	93.55	0.72	89.96	1	0	1	19	Medium quality	57520	3665915	62.40	107	d__Bacteria;p__Proteobacteria;c__Alphaproteobacteria;o__Sphingomonadales;f__Sphingomonadaceae;g__Novosphingobium;s__Novosphingobium sp002256985
LMSG_G000006095.1	yes	1457_1	Nickel-Copper	95.08	0.70	91.60	0	0	0	18	Medium quality	48491	4156137	67.70	193	d__Bacteria;p__Proteobacteria;c__Alphaproteobacteria;o__Rhizobiales;f__Xanthobacteraceae;g__Aquabacter;s__Aquabacter sp002279855
LMSG_G000006096.1	yes	581_0	Nickel-Copper	94.73	1.16	88.93	0	0	0	14	Medium quality	18549	3023774	50.80	315	d__Bacteria;p__Proteobacteria;c__Gammaproteobacteria;o__Enterobacterales;f__Alteromonadaceae;g__Alishewanella;s__Alishewanella agri
LMSG_G000006097.1	yes	579_0	Nickel-Copper	71.57	2.92	56.96	0	1	0	17	Medium quality	4349	2978214	62.40	837	d__Bacteria;p__Proteobacteria;c__Alphaproteobacteria;o__Caulobacterales;f__Hyphomonadaceae;g__Hyphomonas;s__Hyphomonas sp002282565
LMSG_G000006098.1	yes	1473_1	Nickel-Copper	93.52	0.41	91.45	0	0	0	19	Medium quality	19783	3807592	63.70	301	d__Bacteria;p__Proteobacteria;c__Alphaproteobacteria;o__Acetobacterales;f__Acetobacteraceae;g__Acidiphilium;s__Acidiphilium rubrum
LMSG_G000006100.1	yes	1475_2	Nickel-Copper	96.01	0.50	93.53	0	0	0	19	Medium quality	85976	3275356	66.90	77	d__Bacteria;p__Proteobacteria;c__Alphaproteobacteria;o__Acetobacterales;f__Acetobacteraceae;g__Acidiphilium;s__Acidiphilium sp002255515
LMSG_G000006099.1	yes	1470_0	Nickel-Copper	81.27	1.59	73.32	0	0	0	19	Medium quality	19747	2611334	56.90	187	d__Bacteria;p__Proteobacteria;c__Alphaproteobacteria;o__Acetobacterales;f__Acetobacteraceae;g__Acidocella;s__Acidocella sp002281005
LMSG_G000006101.1	yes	1479_0	Nickel-Copper	99.25	0.05	99.03	1	0	0	20	Medium quality	58214	3111504	59.80	139	d__Bacteria;p__Proteobacteria;c__Alphaproteobacteria;o__Acetobacterales;f__Acetobacteraceae;g__20-60-12;s__20-60-12 sp002255495
LMSG_G000006102.1	yes	1217_0	Nickel-Copper	57.47	1.34	50.79	0	0	0	15	Medium quality	2016	1584279	56.80	813	d__Bacteria;p__Proteobacteria;c__Gammaproteobacteria;o__Burkholderiales;f__;g__;s__
LMSG_G000006103.1	yes	1529_0	Nickel-Copper	75.67	0.17	74.85	0	1	0	17	Medium quality	6957	4737339	64.90	853	d__Bacteria;p__Proteobacteria;c__Alphaproteobacteria;o__Acetobacterales;f__Acetobacteraceae;g__;s__
LMSG_G000006256.1	yes	1170_1	Nickel-Copper	97.55	0.29	96.12	1	1	0	17	Medium quality	55020	2338259	53.50	133	d__Bacteria;p__Proteobacteria;c__Gammaproteobacteria;o__Halothiobacillales;f__Halothiobacillaceae;g__Halothiobacillus;s__Halothiobacillus sp002281295
LMSG_G000004641.1	yes	1203_1	Nickel-Copper	90.25	2.56	77.43	1	3	9	19	High quality	15771	3221493	70.40	356	d__Bacteria;p__Acidobacteriota;c__Thermoanaerobaculia;o__Thermoanaerobaculales;f__Thermoanaerobaculaceae;g__RBG-13-68-16;s__RBG-13-68-16 sp002279285
LMSG_G000006104.1	yes	1476_1	Nickel-Copper	100.00	0.00	100.00	0	0	0	19	Medium quality	99483	2967385	60.10	62	d__Bacteria;p__Proteobacteria;c__Alphaproteobacteria;o__Acetobacterales;f__Acetobacteraceae;g__Acidiphilium;s__Acidiphilium sp002279355
LMSG_G000006303.1	yes	288_1	Nickel-Copper	97.23	0.98	92.36	1	2	1	17	Medium quality	117204	1759129	43.40	36	d__Bacteria;p__Proteobacteria;c__Gammaproteobacteria;o__Burkholderiales;f__Ferrovaceae;g__PN-J185;s__PN-J185 sp001431705
LMSG_G000006105.1	yes	1486_0	Nickel-Copper	67.76	2.20	56.74	0	0	0	12	Medium quality	2795	1836513	64.20	739	d__Bacteria;p__Proteobacteria;c__Alphaproteobacteria;o__Acetobacterales;f__Acetobacteraceae;g__Acidocella;s__
LMSG_G000006322.1	yes	1774_0	Nickel-Copper	53.81	0.00	53.81	1	2	1	17	Medium quality	19237	637727	58.30	47	d__Bacteria;p__Patescibacteria;c__Paceibacteria;o__UBA9983;f__UBA2163;g__C7867-001;s__
LMSG_G000006106.1	yes	785_1	Nickel-Copper	85.39	1.28	78.98	1	0	1	20	Medium quality	7651	1750743	62.50	308	d__Bacteria;p__Actinobacteriota;c__Acidimicrobiia;o__Acidimicrobiales;f__RAAP-2;g__RAAP-2;s__RAAP-2 sp002255315
LMSG_G000004642.1	yes	1468_1	Nickel-Copper	90.39	0.08	89.99	1	1	1	18	High quality	7250	2462457	57.60	409	d__Bacteria;p__Proteobacteria;c__Alphaproteobacteria;o__Acetobacterales;f__Acetobacteraceae;g__Acidocella;s__Acidocella sp002255575
LMSG_G000006107.1	yes	289_0	Nickel-Copper	77.01	0.00	77.01	1	5	4	20	Medium quality	83174	1045489	53.50	56	d__Bacteria;p__Patescibacteria;c__Paceibacteria;o__UBA9983;f__UBA2163;g__C7867-001;s__C7867-001 sp002255585
LMSG_G000006108.1	yes	529_0	Nickel-Copper	70.73	1.15	64.99	0	0	0	17	Medium quality	2549	6145561	63.40	2654	d__Bacteria;p__Planctomycetota;c__Planctomycetes;o__Pirellulales;f__PALSA-1355;g__;s__
LMSG_G000004643.1	yes	1245_0	Nickel-Copper	99.64	2.69	86.22	1	1	1	20	High quality	75443	3157378	64.20	81	d__Bacteria;p__Proteobacteria;c__Gammaproteobacteria;o__21-64-14;f__21-64-14;g__21-64-14;s__21-64-14 sp002255365
LMSG_G000006109.1	yes	807_3	Nickel-Copper	86.04	2.14	75.36	0	1	0	17	Medium quality	14222	1887594	72.70	193	d__Bacteria;p__Actinobacteriota;c__Acidimicrobiia;o__Acidimicrobiales;f__RAAP-2;g__RAAP-2;s__RAAP-2 sp002255565
LMSG_G000006110.1	yes	822_0	Copper	94.01	2.14	83.33	0	1	0	19	Medium quality	15201	2484174	64.00	234	d__Bacteria;p__Actinobacteriota;c__Acidimicrobiia;o__Acidimicrobiales;f__;g__;s__
LMSG_G000006111.1	yes	1079_0	Copper	89.81	4.76	65.99	2	1	2	14	Medium quality	42176	3029657	66.70	127	d__Bacteria;p__Proteobacteria;c__Gammaproteobacteria;o__Steroidobacterales;f__Steroidobacteraceae;g__;s__
LMSG_G000006112.1	yes	194_0	Copper	88.26	1.61	80.20	1	1	2	18	Medium quality	65102	1708166	45.20	62	d__Archaea;p__Thermoplasmatota;c__Thermoplasmata;o__Thermoplasmatales;f__Thermoplasmataceae;g__UBA582;s__
LMSG_G000006113.1	yes	962_0	Copper	69.29	0.00	69.29	0	1	0	9	Medium quality	12379	1381613	60.90	158	d__Bacteria;p__Nitrospirota;c__Leptospirillia;o__Leptospirillales;f__Leptospirillaceae;g__UBA4572;s__
LMSG_G000006114.1	yes	1109_2	Copper	93.56	1.65	85.31	1	0	0	19	Medium quality	17160	4928171	55.00	440	d__Bacteria;p__Firmicutes_E;c__Sulfobacillia;o__Sulfobacillales;f__;g__;s__
LMSG_G000004644.1	yes	1209_0	Copper	93.67	0.57	90.80	1	1	1	20	High quality	48970	2479498	58.60	105	d__Bacteria;p__Proteobacteria;c__Gammaproteobacteria;o__UBA2770;f__UBA2770;g__;s__
LMSG_G000006115.1	yes	377_0	Copper	61.96	0.00	61.96	1	1	1	18	Medium quality	18192	834540	36.50	67	d__Bacteria;p__Patescibacteria;c__Saccharimonadia;o__Saccharimonadales;f__UBA4665;g__;s__
LMSG_G000006116.1	yes	884_2	Copper	92.78	2.29	81.31	1	0	1	17	Medium quality	23810	2090761	57.00	204	d__Bacteria;p__Acidobacteriota;c__Acidobacteriae;o__Acidobacteriales;f__Acidobacteriaceae;g__;s__
LMSG_G000006117.1	yes	457_0	Copper	56.26	0.24	55.08	0	0	0	11	Medium quality	2230	1425369	55.90	670	d__Bacteria;p__Actinobacteriota;c__Acidimicrobiia;o__Acidimicrobiales;f__;g__;s__

LMSG_G000006118.1	yes	608_0	Copper	85.32	4.02	65.24	0	0	0	19	Medium quality	7428	2281168	55.50	373	d__Bacteria;p__Actinobacteriota;c__Acidimicrobiia;o__Acidimicrobiales;f__Acidimicrobiaceae;g__Ferrimicrobium;s__
LMSG_G000006119.1	yes	775_2	Copper	94.43	2.14	83.75	0	1	0	20	Medium quality	12362	2387358	70.10	243	d__Bacteria;p__Actinobacteriota;c__Acidimicrobiia;o__Acidimicrobiales;f__Bog-793;g__;s__
LMSG_G000004645.1	yes	903_0	Copper	95.04	0.00	95.04	1	1	1	19	High quality	34044	4195489	60.70	208	d__Bacteria;p__Acidobacteriota;c__Acidobacteriae;o__Acidobacteriales;f__Acidobacteriaceae;g__Granulicella_A;s__
LMSG_G000006120.1	yes	787_0	Copper	68.09	1.47	60.74	0	0	0	18	Medium quality	2211	1529663	57.80	762	d__Bacteria;p__Actinobacteriota;c__Acidimicrobiia;o__Acidimicrobiales;f__RAAP-2;g__RAAP-2;s__
LMSG_G000006257.1	yes	1614_0	Copper	91.83	0.00	91.83	2	2	0	19	Medium quality	23989	1604557	54.00	117	d__Bacteria;p__Verrucomicrobiota_A;c__Chlamydiia;o__Parachlamydiales;f__Rhabdochlamydiaceae;g__;s__
LMSG_G000006121.1	yes	127_1	Copper	83.64	0.00	83.64	1	1	0	19	Medium quality	78915	878559	41.50	51	d__Archaea;p__Micrarchaeota;c__Micrarchaeia;o__Micrarchaeales;f__Micrarchaeaceae;g__;s__
LMSG_G000004647.1	yes	628_1	Copper	96.29	0.00	96.29	1	1	1	20	High quality	9113	2421045	68.10	383	d__Bacteria;p__Dormibacterota;c__Dormibacteria;o__UBA8260;f__Bog-877;g__;s__
LMSG_G000006122.1	yes	64_0	Copper	68.14	0.00	68.14	2	0	2	15	Medium quality	9831	572621	53.20	78	d__Archaea;p__Micrarchaeota;c__Micrarchaeia;o__Micrarchaeales;f__;g__;s__
LMSG_G000006123.1	yes	1110_0	Copper	65.69	2.31	54.14	1	0	1	14	Medium quality	3991	3984605	57.10	1261	d__Bacteria;p__Firmicutes_E;c__Sulfobacillia;o__Sulfobacillales;f__;g__;s__
LMSG_G000006124.1	yes	1078_2	Copper	63.94	0.00	63.94	0	0	0	14	Medium quality	59963	1963193	67.40	218	d__Bacteria;p__Proteobacteria;c__Gammaproteobacteria;o__Steroidobacterales;f__Steroidobacteraceae;g__;s__
LMSG_G000006125.1	yes	1517_0	Copper	89.14	1.99	79.19	0	1	0	14	Medium quality	12012	2932169	68.30	353	d__Bacteria;p__Proteobacteria;c__Alphaproteobacteria;o__Acetobacterales;f__Acetobacteraceae;g__;s__
LMSG_G000006126.1	yes	622_0	Copper	60.97	0.00	60.97	0	0	0	13	Medium quality	2644	1686240	66.40	691	d__Bacteria;p__Dormibacterota;c__Dormibacteria;o__UBA8260;f__Bog-877;g__;s__
LMSG_G000006127.1	yes	54_0	Copper	74.45	0.93	69.78	1	1	2	20	Medium quality	89734	795159	45.80	29	d__Archaea;p__Micrarchaeota;c__Micrarchaeia;o__Micrarchaeales;f__;g__;s__
LMSG_G000004646.1	yes	567_0	Copper	97.60	1.60	89.60	1	1	1	20	High quality	65768	1832064	68.50	201	d__Archaea;p__Thermoplasmatota;c__Thermoplasmata;o__UBA184;f__UBA184;g__;s__
LMSG_G000006128.1	yes	738_0	Copper	64.65	0.00	64.65	1	0	0	11	Medium quality	16358	1955085	70.60	165	d__Bacteria;p__Actinobacteriota;c__Acidimicrobiia;o__Acidimicrobiales;f__RAAP-2;g__;s__
LMSG_G000006132.1	yes	615_0	Copper	59.31	1.42	52.19	0	0	0	18	Medium quality	4294	1922299	51.80	487	d__Bacteria;p__Actinobacteriota;c__Acidimicrobiia;o__Acidimicrobiales;f__Acidimicrobiaceae;g__SCQM01;s__
LMSG_G000006304.1	yes	1256_1	Copper	96.28	4.04	76.07	1	1	1	16	Medium quality	42266	3174724	65.80	531	d__Bacteria;p__Proteobacteria;c__Gammaproteobacteria;o__Xanthomonadales;f__Rhodanobacteraceae;g__Metallibacterium;s__Metallibacterium scheffleri
LMSG_G000006129.1	yes	355_0	Copper	75.77	2.77	61.92	1	1	10	20	Medium quality	26315	745198	32.60	97	d__Bacteria;p__Patescibacteria;c__Saccharimonadia;o__Saccharimonadales;f__UBA4665;g__;s__
LMSG_G000006130.1	yes	747_0	Copper	58.77	0.00	58.77	1	0	0	13	Medium quality	17320	1719308	69.20	266	d__Bacteria;p__Actinobacteriota;c__Acidimicrobiia;o__Acidimicrobiales;f__RAAP-2;g__;s__
LMSG_G000006131.1	yes	1191_0	Copper	68.41	0.85	64.14	0	0	0	13	Medium quality	3049	1503462	45.60	606	d__Bacteria;p__Actinobacteriota;c__Acidimicrobiia;o__Acidimicrobiales;f__Bog-793;g__;s__
LMSG_G000006133.1	yes	17_1	Copper	81.31	0.97	76.46	1	1	1	18	Medium quality	18629	776632	34.40	75	d__Archaea;p__Nanoarchaeota;c__Nanoarchaeia;o__Parvarchaeales;f__Parvarchaeaceae;g__Parvarchaeum;s__Parvarchaeum sp002503215
LMSG_G000006134.1	yes	97_0	Copper	67.94	0.93	63.27	0	0	1	14	Medium quality	7684	741244	43.40	145	d__Archaea;p__Micrarchaeota;c__Micrarchaeia;o__Micrarchaeales;f__Micrarchaeaceae;g__Micrarchaeum;s__
LMSG_G000006135.1	yes	42_1	Copper	82.39	0.00	82.39	1	1	1	19	Medium quality	51868	921238	39.50	25	d__Archaea;p__Micrarchaeota;c__Micrarchaeia;o__Micrarchaeales;f__Micrarchaeaceae;g__Mancarchaeum;s__Mancarchaeum acidiphilum
LMSG_G000004648.1	yes	605_2	Copper	96.58	2.14	85.90	1	1	2	20	High quality	114114	3079955	57.10	84	d__Bacteria;p__Actinobacteriota;c__Acidimicrobiia;o__Acidimicrobiales;f__Acidimicrobiaceae;g__Ferrimicrobium;s__
LMSG_G000004649.1	yes	1359_1	Copper	95.04	1.82	85.95	2	5	8	20	High quality	11482	3200045	55.00	427	d__Bacteria;p__Nitrospirota;c__UBA9217;o__UBA9217;f__UBA9217;g__;s__
LMSG_G000006136.1	yes	460_1	Copper	68.62	2.24	57.42	0	0	0	6	Medium quality	2600	2884473	44.00	1189	d__Bacteria;p__Bacteroidota;c__Bacteroidia;o__Bacteroidales;f__VadinHA17;g__LD21;s__
LMSG_G000006137.1	yes	1334_0	Copper	55.83	0.00	55.83	0	2	4	11	Medium quality	8114	1129102	55.80	172	d__Bacteria;p__Proteobacteria;c__Gammaproteobacteria;o__Burkholderiales;f__Ferrovaceae;g__;s__
LMSG_G000004650.1	yes	1123_1	Copper	92.25	0.46	89.94	2	1	2	19	High quality	15738	2241403	64.60	161	d__Bacteria;p__Eremiobacterota;c__Eremiobacteria;o__UBP12;f__UBA5184;g__PALSA-1504;s__
LMSG_G000006138.1	yes	449_1	Copper	89.43	3.14	73.74	0	0	0	18	Medium quality	8758	4276074	59.50	663	d__Bacteria;p__Chloroflexota;c__Anaerolineae;o__UBA4142;f__UBA4142;g__UBA4142;s__
LMSG_G000006139.1	yes	1252_0	Copper	60.99	1.50	53.51	1	0	1	15	Medium quality	8406	2005193	63.90	258	d__Bacteria;p__Proteobacteria;c__Gammaproteobacteria;o__Burkholderiales;f__SG8-39;g__2-12-FULL-64-23;s__
LMSG_G000006141.1	yes	991_0	Copper	64.90	0.98	60.01	0	0	0	10	Medium quality	2918	1736841	61.70	675	d__Bacteria;p__Acidobacteriota;c__Acidobacteriae;o__UBA7541;f__UBA7541;g__;s__
LMSG_G000006140.1	yes	239_1	Copper	85.11	0.00	85.11	2	6	1	15	Medium quality	2807	2026503	38.50	760	d__Archaea;p__Thermoproteota;c__Nitrososphaeria;o__Nitrososphaerales;f__Nitrosopumilaceae;g__Nitrosotalea;s__
LMSG_G000006143.1	yes	674_1	Copper	73.11	0.93	68.49	0	0	0	15	Medium quality	2739	2483348	71.80	921	d__Bacteria;p__Chloroflexota;c__Ellin6529;o__CSP1-4;f__CSP1-4;g__;s__
LMSG_G000006142.1	yes	983_1	Copper	86.62	0.84	82.42	1	2	1	14	Medium quality	7628	2455421	46.00	405	d__Bacteria;p__SZUA-79;c__;o__;f__;g__;s__
LMSG_G000004651.1	yes	665_1	Copper	95.83	2.06	85.55	1	2	1	19	High quality	28031	3826390	68.20	208	d__Bacteria;p__Chloroflexota;c__Ellin6529;o__CSP1-4;f__CSP1-4;g__Fen-1039;s__
LMSG_G000006144.1	yes	1198_1	Copper	95.97	2.44	83.78	0	2	0	20	Medium quality	39014	3154826	59.60	184	d__Bacteria;p__Proteobacteria;c__Gammaproteobacteria;o__Acidiferrobacterales;f__Sulfurifustaceae;g__;s__
LMSG_G000006145.1	yes	1120_1	Copper	83.42	2.75	69.69	0	0	0	15	Medium quality	9195	2471442	65.40	360	d__Bacteria;p__Proteobacteria;c__Gammaproteobacteria;o__Steroidobacterales;f__Steroidobacteraceae;g__;s__
LMSG_G000006146.1	yes	1572_1	Copper	73.31	0.10	72.81	0	1	0	18	Medium quality	9634	3055755	53.70	416	d__Bacteria;p__Acidobacteriota;c__Acidobacteriae;o__Acidobacteriales;f__Acidobacteriaceae;g__;s__
LMSG_G000006147.1	yes	993_0	Copper	75.97	0.85	71.70	0	0	0	17	Medium quality	4891	3502280	58.70	790	d__Bacteria;p__Acidobacteriota;c__Acidobacteriae;o__UBA7540;f__UBA7540;g__RH2-MAG17b;s__
LMSG_G000006152.1	yes	1025_0	Copper	77.56	0.37	75.69	0	0	0	18	Medium quality	3288	2583695	67.80	903	d__Bacteria;p__Elusimicrobiota;c__Elusimicrobia;o__UBA1565;f__UBA9628;g__GWA2-66-18;s__
LMSG_G000006148.1	yes	1067_0	Copper	74.05	1.09	68.63	0	0	0	17	Medium quality	5229	2335882	60.70	509	d__Bacteria;p__Proteobacteria;c__Alphaproteobacteria;o__Rhizobiales;f__Beijerinckiaceae;g__;s__
LMSG_G000006149.1	yes	238_1	Copper	73.10	0.00	73.10	1	0	1	14	Medium quality	3187	1312307	39.00	443	d__Archaea;p__Thermoproteota;c__Nitrososphaeria;o__Nitrososphaerales;f__Nitrosopumilaceae;g__Nitrosotalea;s__
LMSG_G000006150.1	yes	1225_0	Copper	68.14	3.62	50.07	3	2	2	12	Medium quality	8609	1593898	56.50	270	d__Bacteria;p__Proteobacteria;c__Gammaproteobacteria;o__Burkholderiales;f__Sulfuriferulaceae;g__Sulfuriferula_A;s__
LMSG_G000006151.1	yes	1518_0	Copper	82.69	1.43	75.54	2	0	1	16	Medium quality	5750	3848712	69.60	819	d__Bacteria;p__Proteobacteria;c__Alphaproteobacteria;o__Acetobacterales;f__Acetobacteraceae;g__PALSA-911;s__
LMSG_G000004652.1	yes	251_0	Copper	90.26	2.97	75.41	3	1	3	20	High quality	8641	4959374	51.40	723	d__Bacteria;p__Chloroflexota;c__Ktedonobacteria;o__Ktedonobacterales;f__Ktedonobacteraceae;g__;s__
LMSG_G000006153.1	yes	1238_0	Copper	64.57	2.19	53.62	0	0	0	11	Medium quality	14960	1745329	57.70	144	d__Bacteria;p__Proteobacteria;c__Gammaproteobacteria;o__Burkholderiales;f__Gallionellaceae;g__Gallionella;s__
LMSG_G000006154.1	yes	1001_0	Copper	60.00	0.17	59.14	0	0	0	15	Medium quality	5894	2580133	59.60	541	d__Bacteria;p__Acidobacteriota;c__Acidobacteriae;o__Acidobacteriales;f__Acidobacteriaceae;g__;s__
LMSG_G000006155.1	yes	1576_1	Copper	95.39	0.86	91.10	1	0	0	19	Medium quality	29681	2758368	64.10	143	d__Bacteria;p__Proteobacteria;c__Gammaproteobacteria;o__Burkholderiales;f__Burkholderiaceae;g__Thiomonas;s__
LMSG_G000006306.1	yes	1469_1	Copper	98.00	0.03	97.84	1	1	1	17	Medium quality	61237	2981505	57.20	104	d__Bacteria;p__Proteobacteria;c__Alphaproteobacteria;o__Acetobacterales;f__Acetobacteraceae;g__Acidocella;s__
LMSG_G000006156.1	yes	1231_0	Copper	61.12	0.32	59.54	0	1	0	10	Medium quality	7752	1642370	56.10	277	d__Bacteria;p__Proteobacteria;c__Gammaproteobacteria;o__Burkholderiales;f__Gallionellaceae;g__Sideroxydans;s__
LMSG_G000006305.1	yes	1328_2	Copper	97.01	1.52	89.43	1	1	1	17	Medium quality	37319	2228210	59.30	127	d__Bacteria;p__Proteobacteria;c__Gammaproteobacteria;o__Burkholderiales;f__Ferrovaceae;g__;s__
LMSG_G000004653.1	yes	812_1	Copper	94.87	0.85	90.60	1	2	1	20	High quality	12465	1987653	63.50	196	d__Bacteria;p__Actinobacteriota;c__Acidimicrobiia;o__Acidimicrobiales;f__RAAP-2;g__RAAP-2;s__
LMSG_G000006157.1	yes	69_0	Copper	55.37	0.93	50.70	0	0	0	15	Medium quality	4907	504645	52.10	126	d__Archaea;p__Micrarchaeota;c__Micrarchaeia;o__Micrarchaeales;f__Micrarchaeaceae;g__UBA12276;s__
LMSG_G000006158.1	yes	300_0	Copper	68.64	1.12	63.03	1	4	13	18	Medium quality	13845	628665	54.40	72	d__Bacteria;p__Patescibacteria;c__Paceibacteria;o__UBA6257;f__GWB1-50-10;g__;s__
LMSG_G000006159.1	yes	1815_1	Copper	59.62	0.00	59.62	1	5	10	20	Medium quality	16039	736702	50.30	67	d__Bacteria;p__Patescibacteria;c__Paceibacteria;o__UBA6257;f__2-01-FULL-56-20;g__;s__
LMSG_G000006160.1	yes	1759_1	Copper	64.44	0.00	64.44	3	1	4	17	Medium quality	9327	616831	48.70	82	d__Bacteria;p__Patescibacteria;c__Paceibacteria;o__UBA9983;f__UBA2163;g__C7867-001;s__
LMSG_G000004654.1	yes	697_1	Copper	94.87	1.28	88.46	1	1	8	20	High quality	24956	2907037	49.50	180	d__Bacteria;p__Actinobacteriota;c__Acidimicrobiia;o__Acidimicrobiales;f__Acidimicrobiaceae;g__Acidithrix;s__
LMSG_G000006162.1	yes	550_1	Copper	59.47	0.00	59.47	1	0	1	15	Medium quality	3441	1665327	68.50	464	d__Archaea;p__Thermoplasmatota;c__Thermoplasmata;o__UBA184;f__UBA184;g__;s__
LMSG_G000004655.1	yes	551_1	Copper	97.82	1.60	89.82	1	1	1	20	High quality	26461	2236588	68.20	133	d__Archaea;p__Thermoplasmatota;c__Thermoplasmata;o__UBA184;f__UBA184;g__;s__
LMSG_G000006161.1	yes	514_1	Copper	79.61	2.27	68.29	1	3	2	16	Medium quality	8592	2019928	46.90	255	d__Archaea;p__Thermoproteota;c__Nitrososphaeria;o__Nitrososphaerales;f__UBA141;g__;s__
LMSG_G000006163.1	yes	30_1	Copper	85.20	0.00	85.20	1	1	2	20	Medium quality	102678	975511	47.40	15	d__Archaea;p__Micrarchaeota;c__Micrarchaeia;o__Micrarchaeales;f__Micrarchaeaceae;g__;s__
LMSG_G000006164.1	yes	51_1	Copper	72.11	0.93	67.44	1	2	0	15	Medium quality	6952	603184	48.30	117	d__Archaea;p__Micrarchaeota;c__Micrarchaeia;o__Micrarchaeales;f__;g__;s__
LMSG_G000006165.1	yes	72_1	Copper	84.57	0.00	84.57	1	1	1	19	Medium quality	26756	869511	48.60	62	d__Archaea;p__Micrarchaeota;c__Micrarchaeia;o__Micrarchaeales;f__Micrarchaeaceae;g__;s__
LMSG_G000004656.1	yes	1257_1	Coal	94.20	1.37	87.38	1	3	1	19	High quality	15533	2595156	68.20	211	d__Bacteria;p__Proteobacteria;c__Gammaproteobacteria;o__Xanthomonadales;f__Rhodanobacteraceae;g__Metallibacterium;s__
LMSG_G000006166.1	yes	1337_2	Coal	89.78	1.52	82.16	1	1	0	16	Medium quality	39821	1805625	56.10	96	d__Bacteria;p__Proteobacteria;c__Gammaproteobacteria;o__Burkholderiales;f__Ferrovaceae;g__Ferrovum;s__

LMSG_G000006167.1	yes	980_1	Coal	93.57	0.00	93.57	1	0	1	15	Medium quality	15989	2143493	57.80	174	d__Bacteria;p__Proteobacteria;c__Gammaproteobacteria;o__Acidithiobacillales;f__Acidithiobacillaceae;g__Acidithiobacillus;s__Acidithiobacillus ferrooxidans_A
LMSG_G000004657.1	yes	1616_0	Coal	90.03	0.68	86.66	1	7	1	19	High quality	15430	1684958	41.10	159	d__Bacteria;p__Verrucomicrobiota_A;c__Chlamydiia;o__Parachlamydiales;f__Rhabdochlamydiaceae;g__;s__
LMSG_G000006168.1	yes	1338_0	Coal	82.81	1.21	76.76	0	0	0	16	Medium quality	8903	1977144	54.70	360	d__Bacteria;p__Proteobacteria;c__Gammaproteobacteria;o__Burkholderiales;f__Ferrovaceae;g__Ferrovum;s__Ferrovum myxofaciens
LMSG_G000004658.1	yes	1525_0	Lignite	99.31	2.05	89.04	1	1	1	20	High quality	187473	4477752	65.00	43	d__Bacteria;p__Verrucomicrobiota;c__Verrucomicrobiae;o__Opitutales;f__Opitutaceae;g__;s__
LMSG_G000006169.1	yes	1232_0	Lignite	56.19	0.95	51.43	1	0	0	18	Medium quality	78792	3028986	54.70	80	d__Bacteria;p__Proteobacteria;c__Gammaproteobacteria;o__Burkholderiales;f__Gallionellaceae;g__SiderOxydans;s__
LMSG_G000006170.1	yes	1321_0	Lignite	96.04	0.92	91.47	2	0	0	17	Medium quality	10598	2759013	43.70	383	d__Bacteria;p__Proteobacteria;c__Gammaproteobacteria;o__Burkholderiales;f__Methylophilaceae;g__Methylotenera;s__
LMSG_G000006171.1	yes	273_0	Lignite	62.60	0.52	60.01	0	1	0	13	Medium quality	2102	2291870	36.20	1189	d__Bacteria;p__Bacteroidota;c__Bacteroidia;o__Chitinophagales;f__Chitinophagaceae;g__Hydrotalea;s__
LMSG_G000006172.1	yes	1230_2	Lignite	96.35	1.59	88.42	0	1	0	16	Medium quality	40031	3186516	57.60	127	d__Bacteria;p__Proteobacteria;c__Gammaproteobacteria;o__Burkholderiales;f__Gallionellaceae;g__Sideroxydans;s__
LMSG_G000006173.1	yes	1159_2	Lignite	95.30	1.54	87.61	1	0	1	18	Medium quality	7389	3346868	66.20	720	d__Bacteria;p__Proteobacteria;c__Alphaproteobacteria;o__Rhodospirillales;f__Magnetospirillaceae;g__Telmatospirillum;s__
LMSG_G000006180.1	yes	226_0	Copper	69.18	3.24	52.99	0	0	0	13	Medium quality	2452	3336582	51.50	1462	d__Bacteria;p__Proteobacteria;c__Gammaproteobacteria;o__Methylococcales;f__Methylomonadaceae;g__Methylomonas;s__
LMSG_G000006174.1	yes	1834_0	Copper	75.02	0.00	75.02	1	2	1	19	Medium quality	49278	765938	47.80	21	d__Bacteria;p__Patescibacteria;c__Paceibacteria;o__UBA6257;f__2-01-FULL-56-20;g__;s__
LMSG_G000006175.1	yes	1633_0	Copper	67.67	0.93	63.00	0	1	3	18	Medium quality	8878	701568	46.10	178	d__Archaea;p__Aenigmatarchaeota;c__Aenigmatarchaeia;o__Aenigmatarchaeales;f__Aenigmatarchaeaceae;g__;s__
LMSG_G000006258.1	yes	596_0	Copper	97.24	0.97	92.39	1	1	0	19	Medium quality	45437	1575568	62.50	61	d__Archaea;p__Thermoproteota;c__Nitrososphaeria;o__Nitrososphaerales;f__UBA183;g__UBA183;s__
LMSG_G000004659.1	yes	1158_0	Copper	98.01	0.22	96.91	1	1	1	20	High quality	147073	2794070	72.30	38	d__Bacteria;p__Chloroflexota;c__Ellin6529;o__P2-11E;f__P2-11E;g__CF-39;s__
LMSG_G000006176.1	yes	77_0	Copper	75.77	0.00	75.77	1	1	1	18	Medium quality	12012	683177	52.30	72	d__Archaea;p__Micrarchaeota;c__Micrarchaeia;o__Micrarchaeales;f__Micrarchaeaceae;g__;s__
LMSG_G000006177.1	yes	358_0	Copper	55.25	0.00	55.25	0	0	0	13	Medium quality	2589	2172248	71.70	833	d__Bacteria;p__Chloroflexota;c__UBA6077;o__UBA6077;f__CF-72;g__;s__
LMSG_G000006178.1	yes	1324_0	Copper	86.66	2.04	76.44	0	1	0	15	Medium quality	6342	2540990	57.10	483	d__Bacteria;p__Proteobacteria;c__Gammaproteobacteria;o__Burkholderiales;f__Methylophilaceae;g__MM2;s__
LMSG_G000006179.1	yes	1629_0	Copper	59.23	1.33	52.57	0	0	0	18	Medium quality	4299	986500	44.50	342	d__Archaea;p__Halobacteriota;c__Methanosarcinia;o__Methanosarcinales;f__Methanoperedenaceae;g__Methanoperedens;s__
LMSG_G000006181.1	yes	1318_0	Copper	88.31	4.32	66.72	1	0	1	13	Medium quality	9358	3793968	66.60	535	d__Bacteria;p__Proteobacteria;c__Gammaproteobacteria;o__Burkholderiales;f__Rhodocyclaceae;g__;s__
LMSG_G000006182.1	yes	261_0	Copper	60.33	0.00	60.33	2	0	3	19	Medium quality	42143	611093	55.00	65	d__Bacteria;p__Patescibacteria;c__Saccharimonadia;o__Saccharimonadales;f__Saccharimonadaceae;g__AMD01;s__AMD01 sp004100265
LMSG_G000006183.1	yes	1824_0	Copper	60.36	0.18	59.46	0	1	0	18	Medium quality	19046	547969	52.00	84	d__Bacteria;p__Patescibacteria;c__Paceibacteria;o__UBA6257;f__Brennerbacteraceae;g__UBA1567;s__
LMSG_G000006184.1	yes	1635_0	Copper	70.71	0.00	70.71	1	5	0	18	Medium quality	28833	536975	37.60	82	d__Archaea;p__Aenigmatarchaeota;c__Aenigmatarchaeia;o__CG10238-14;f__SCSR01;g__;s__
LMSG_G000006185.1	yes	1799_0	Copper	76.67	1.19	70.73	1	0	3	18	Medium quality	31862	747272	50.10	84	d__Bacteria;p__Patescibacteria;c__Paceibacteria;o__UBA6257;f__GWB1-50-10;g__;s__
LMSG_G000006186.1	yes	338_0	Copper	63.67	0.85	59.40	1	1	1	20	Medium quality	496818	791056	45.90	3	d__Bacteria;p__Patescibacteria;c__Saccharimonadia;o__Saccharimonadales;f__Saccharimonadaceae;g__UBA1547;s__UBA1547 sp004100325
LMSG_G000006187.1	yes	264_0	Copper	67.87	0.88	63.49	0	1	0	15	Medium quality	3006	2428469	54.10	834	d__Bacteria;p__Nitrospirota;c__Nitrospiria;o__Nitrospirales;f__Nitrospiraceae;g__Nitrospira;s__
LMSG_G000006188.1	yes	1642_0	Copper	94.67	3.31	78.14	0	0	0	17	Medium quality	5178	2401131	54.50	601	d__Bacteria;p__Desulfobacterota;c__GWC2-55-46;o__GWC2-55-46;f__SCQA01;g__;s__
LMSG_G000006189.1	yes	1817_0	Copper	66.62	0.10	66.11	1	1	1	18	Medium quality	8789	405291	50.70	60	d__Bacteria;p__Patescibacteria;c__Paceibacteria;o__UBA6257;f__Colwellbacteraceae;g__;s__
LMSG_G000006190.1	yes	1289_0	Copper	97.87	0.78	93.99	1	0	0	19	Medium quality	51199	4014293	57.80	129	d__Bacteria;p__Proteobacteria;c__Gammaproteobacteria;o__Burkholderiales;f__Burkholderiaceae;g__Herminiimonas;s__
LMSG_G000006191.1	yes	1019_0	Copper	95.97	0.53	93.30	1	0	0	18	Medium quality	21281	2733047	54.50	216	d__Bacteria;p__Proteobacteria;c__Gammaproteobacteria;o__Burkholderiales;f__Burkholderiaceae;g__Limnobacter;s__
LMSG_G000006192.1	yes	1754_0	Copper	75.48	2.25	64.25	1	1	1	16	Medium quality	9393	743463	49.20	101	d__Bacteria;p__Patescibacteria;c__Paceibacteria;o__UBA6257;f__GWB1-50-10;g__;s__
LMSG_G000006193.1	yes	1246_0	Copper	88.33	2.29	76.88	1	1	1	18	Medium quality	12334	3339197	65.90	386	d__Bacteria;p__Proteobacteria;c__Gammaproteobacteria;o__Burkholderiales;f__SG8-41;g__SG8-41;s__
LMSG_G000006194.1	yes	1840_0	Copper	62.47	0.00	62.47	0	0	2	15	Medium quality	10220	805603	34.20	109	d__Bacteria;p__Patescibacteria;c__Microgenomatia;o__Daviesbacterales;f__UBA10151;g__;s__
LMSG_G000006307.1	yes	272_1	Copper	98.02	0.49	95.56	1	1	1	16	Medium quality	98934	2773140	32.30	54	d__Bacteria;p__Bacteroidota;c__Bacteroidia;o__Chitinophagales;f__Chitinophagaceae;g__Hydrotalea;s__
LMSG_G000006195.1	yes	1692_0	Copper	71.49	0.00	71.49	1	1	0	16	Medium quality	21399	628064	31.90	49	d__Archaea;p__Nanoarchaeota;c__Nanoarchaeia;o__Pacearchaeales;f__GW2011-AR1;g__RBG-13-33-26;s__
LMSG_G000006196.1	yes	1699_0	Copper	77.99	0.50	75.48	0	0	0	13	Medium quality	5210	1756797	33.40	401	d__Bacteria;p__Bacteroidota;c__Bacteroidia;o__AKYH767;f__B-17BO;g__;s__
LMSG_G000004660.1	yes	170_0	Polymetallic	96.32	0.00	96.32	1	1	1	20	High quality	244859	1878148	39.50	19	d__Archaea;p__Thermoplasmatota;c__Thermoplasmata;o__Thermoplasmatales;f__Thermoplasmataceae;g__Cuniculiplasma;s__
LMSG_G000006197.1	yes	955_1	Polymetallic	99.37	0.00	99.37	2	0	0	18	Medium quality	56515	2348308	62.40	58	d__Bacteria;p__Proteobacteria;c__Gammaproteobacteria;o__Acidithiobacillales;f__Acidithiobacillaceae;g__Acidithiobacillus_A;s__Acidithiobacillus_A caldus
LMSG_G000006198.1	yes	1832_1	Arsenic	82.02	0.31	80.46	3	2	4	20	Medium quality	38187	597184	51.10	47	d__Bacteria;p__Patescibacteria;c__Paceibacteria;o__UBA6257;f__Colwellbacteraceae;g__;s__
LMSG_G000006199.1	yes	1708_0	Arsenic	56.73	0.99	51.78	0	0	0	17	Medium quality	8509	518175	41.20	83	d__Bacteria;p__Patescibacteria;c__UBA1384;o__;f__;g__;s__
LMSG_G000006200.1	yes	351_0	Arsenic	96.50	0.81	92.47	1	0	1	19	Medium quality	14033	2513055	40.30	257	d__Bacteria;p__Bacteroidota;c__Bacteroidia;o__Bacteroidales;f__Paludibacteraceae;g__Paludibacter_A;s__
LMSG_G000006201.1	yes	50_1	Arsenic	86.60	2.80	72.59	1	1	1	20	Medium quality	62505	975251	50.90	36	d__Archaea;p__Micrarchaeota;c__Micrarchaeia;o__Micrarchaeales;f__;g__;s__
LMSG_G000006202.1	yes	277_0	Arsenic	88.99	3.30	72.49	1	0	0	15	Medium quality	12886	3728102	36.80	527	d__Bacteria;p__Bacteroidota;c__Bacteroidia;o__Chitinophagales;f__Chitinophagaceae;g__Palsa-955;s__
LMSG_G000006203.1	yes	1743_1	Arsenic	83.54	0.86	79.23	0	0	0	18	Medium quality	13201	1727502	39.50	243	d__Bacteria;p__SZUA-79;c__SZUA-79;o__Acidulodesulfobacterales;f__SZUA-79;g__Acidulodesulfobacterium;s__
LMSG_G000006259.1	yes	286_1	Arsenic	92.89	0.47	90.53	2	1	0	15	Medium quality	84015	1646328	40.20	28	d__Bacteria;p__Proteobacteria;c__Gammaproteobacteria;o__Burkholderiales;f__Ferrovaceae;g__PN-J185;s__PN-J185 sp001581925
LMSG_G000006204.1	yes	1169_0	Arsenic	83.33	0.57	80.46	1	1	1	18	Medium quality	124527	1659607	40.70	58	d__Bacteria;p__Proteobacteria;c__Gammaproteobacteria;o__UBA6186;f__UBA6186;g__;s__
LMSG_G000006205.1	yes	966_1	Pyrite	70.35	0.00	70.35	1	0	0	18	Medium quality	64803	2037604	54.80	140	d__Bacteria;p__Nitrospirota;c__Leptospirillia;o__Leptospirillales;f__Leptospirillaceae;g__Leptospirillum_A;s__Leptospirillum_A ferriphilum
LMSG_G000006340.1	no	1667_1	Pyrite-Copper	64.95	1.87	55.61	0	0	1	15	Medium quality	6809	3245957	30.00	568	d__Archaea;p__Asgardarchaeota;c__Heimdallarchaeia;o__LC-3;f__LC-3;g__;s__
LMSG_G000006341.1	no	510_1	Polymetallic	83.64	0.07	83.29	0	0	0	18	Medium quality	11525	1479787	51.40	180	d__Archaea;p__Micrarchaeota;c__Micrarchaeia;o__B9-G16;f__;g__;s__
LMSG_G000006342.1	no	510_1	Polymetallic	92.52	0.00	92.52	0	1	2	18	Medium quality	42207	1607791	51.40	73	d__Archaea;p__Micrarchaeota;c__Micrarchaeia;o__B9-G16;f__;g__;s__
LMSG_G000006343.1	no	100_1	Copper	52.02	0.38	50.11	0	1	0	13	Medium quality	8969	521030	48.50	77	d__Archaea;p__Micrarchaeota;c__Micrarchaeia;o__Micrarchaeales;f__;g__;s__
LMSG_G000006344.1	no	100_1	Magnetite	50.85	0.00	50.85	0	1	0	13	Medium quality	39436	529408	47.50	19	d__Archaea;p__Micrarchaeota;c__Micrarchaeia;o__Micrarchaeales;f__;g__;s__
LMSG_G000006345.1	no	101_1	Polymetallic	65.88	0.00	65.88	1	0	1	14	Medium quality	29444	702802	48.90	32	d__Archaea;p__Micrarchaeota;c__Micrarchaeia;o__Micrarchaeales;f__;g__;s__
LMSG_G000006346.1	no	101_1	Polymetallic	80.84	1.87	71.50	1	1	2	17	Medium quality	22985	886410	48.20	51	d__Archaea;p__Micrarchaeota;c__Micrarchaeia;o__Micrarchaeales;f__;g__;s__
LMSG_G000006347.1	no	101_1	Polymetallic	74.76	3.74	56.07	1	0	2	17	Medium quality	25018	788616	48.30	45	d__Archaea;p__Micrarchaeota;c__Micrarchaeia;o__Micrarchaeales;f__;g__;s__
LMSG_G000006348.1	no	103_1	Lead-Zinc	79.75	0.93	75.08	1	1	0	17	Medium quality	13727	798282	52.00	70	d__Archaea;p__Micrarchaeota;c__Micrarchaeia;o__Micrarchaeales;f__;g__;s__
LMSG_G000006349.1	no	103_1	Lead-Zinc	72.58	1.87	63.24	1	1	0	17	Medium quality	15938	757752	52.00	63	d__Archaea;p__Micrarchaeota;c__Micrarchaeia;o__Micrarchaeales;f__;g__;s__
LMSG_G000006350.1	no	103_1	Lead-Zinc	78.66	2.23	67.52	1	0	0	19	Medium quality	12377	927522	51.70	121	d__Archaea;p__Micrarchaeota;c__Micrarchaeia;o__Micrarchaeales;f__;g__;s__
LMSG_G000006351.1	no	103_1	Pyrite-Copper	81.61	0.93	76.94	1	1	0	18	Medium quality	15210	926786	51.90	96	d__Archaea;p__Micrarchaeota;c__Micrarchaeia;o__Micrarchaeales;f__;g__;s__
LMSG_G000006352.1	no	103_1	Lead-Zinc	80.06	1.87	70.72	1	1	0	19	Medium quality	15669	919285	51.70	75	d__Archaea;p__Micrarchaeota;c__Micrarchaeia;o__Micrarchaeales;f__;g__;s__
LMSG_G000006353.1	no	103_1	Copper	81.15	1.87	71.81	1	1	1	19	Medium quality	37502	1019975	51.80	42	d__Archaea;p__Micrarchaeota;c__Micrarchaeia;o__Micrarchaeales;f__;g__;s__
LMSG_G000006354.1	no	103_1	Copper	81.32	1.87	71.98	1	0	1	16	Medium quality	15646	939641	52.30	79	d__Archaea;p__Micrarchaeota;c__Micrarchaeia;o__Micrarchaeales;f__;g__;s__
LMSG_G000006355.1	no	103_1	Copper	78.34	1.87	69.00	1	1	1	17	Medium quality	43960	816487	51.70	35	d__Archaea;p__Micrarchaeota;c__Micrarchaeia;o__Micrarchaeales;f__;g__;s__
LMSG_G000006356.1	no	103_1	Magnetite	74.76	1.87	65.42	0	2	1	14	Medium quality	15271	697960	52.50	57	d__Archaea;p__Micrarchaeota;c__Micrarchaeia;o__Micrarchaeales;f__;g__;s__
LMSG_G000006357.1	no	105_1	Copper	59.65	0.00	59.65	1	1	1	16	Medium quality	123673	481658	53.20	9	d__Archaea;p__Micrarchaeota;c__Micrarchaeia;o__Micrarchaeales;f__;g__;s__
LMSG_G000006358.1	no	105_1	Copper	62.30	0.00	62.30	1	0	1	17	Medium quality	57601	520026	53.20	16	d__Archaea;p__Micrarchaeota;c__Micrarchaeia;o__Micrarchaeales;f__;g__;s__
LMSG_G000006359.1	no	105_1	Copper	64.64	0.00	64.64	1	2	1	15	Medium quality	50194	557113	53.40	13	d__Archaea;p__Micrarchaeota;c__Micrarchaeia;o__Micrarchaeales;f__;g__;s__

LMSG_G000006360.1	no	105_1	Copper	78.66	0.00	78.66	1	1	1	18	Medium quality	72544	715642	53.10	22	d__Archaea;p__Micrarchaeota;c__Micrarchaeia;o__Micrarchaeales;f__;g__;s__
LMSG_G000006361.1	no	105_1	Copper	79.59	0.00	79.59	1	1	1	19	Medium quality	121800	777230	53.30	11	d__Archaea;p__Micrarchaeota;c__Micrarchaeia;o__Micrarchaeales;f__;g__;s__
LMSG_G000006362.1	no	105_1	Polymetallic	78.19	0.93	73.52	1	2	0	19	Medium quality	22577	679851	53.80	41	d__Archaea;p__Micrarchaeota;c__Micrarchaeia;o__Micrarchaeales;f__;g__;s__
LMSG_G000006363.1	no	105_1	Lead-Zinc	79.59	0.93	74.92	1	2	2	19	Medium quality	45088	754236	53.50	28	d__Archaea;p__Micrarchaeota;c__Micrarchaeia;o__Micrarchaeales;f__;g__;s__
LMSG_G000006364.1	no	105_1	Lead-Zinc	69.31	0.00	69.31	1	1	1	19	Medium quality	43365	608160	53.80	22	d__Archaea;p__Micrarchaeota;c__Micrarchaeia;o__Micrarchaeales;f__;g__;s__
LMSG_G000006365.1	no	105_1	Lead-Zinc	80.52	0.00	80.52	1	1	1	19	Medium quality	74716	783928	53.40	22	d__Archaea;p__Micrarchaeota;c__Micrarchaeia;o__Micrarchaeales;f__;g__;s__
LMSG_G000006366.1	no	105_1	Lead-Zinc	80.52	0.93	75.85	1	1	1	19	Medium quality	48078	725880	53.70	22	d__Archaea;p__Micrarchaeota;c__Micrarchaeia;o__Micrarchaeales;f__;g__;s__
LMSG_G000006367.1	no	105_1	Magnetite	55.68	0.00	55.68	0	1	1	18	Medium quality	20359	502808	53.50	30	d__Archaea;p__Micrarchaeota;c__Micrarchaeia;o__Micrarchaeales;f__;g__;s__
LMSG_G000006368.1	no	111_1	Pyrite	75.55	0.07	75.20	0	0	0	15	Medium quality	7854	597746	53.20	105	d__Archaea;p__Micrarchaeota;c__Micrarchaeia;o__Micrarchaeales;f__;g__;s__
LMSG_G000006369.1	no	111_1	Polymetallic	87.38	0.93	82.71	1	1	1	19	Medium quality	45294	975126	52.30	31	d__Archaea;p__Micrarchaeota;c__Micrarchaeia;o__Micrarchaeales;f__;g__;s__
LMSG_G000006370.1	no	111_1	Polymetallic	71.02	1.87	61.68	0	1	0	17	Medium quality	63382	668306	51.80	20	d__Archaea;p__Micrarchaeota;c__Micrarchaeia;o__Micrarchaeales;f__;g__;s__
LMSG_G000006371.1	no	111_1	Polymetallic	78.50	1.87	69.16	1	1	1	18	Medium quality	37285	802529	52.20	30	d__Archaea;p__Micrarchaeota;c__Micrarchaeia;o__Micrarchaeales;f__;g__;s__
LMSG_G000006372.1	no	111_1	Polymetallic	83.64	0.93	78.97	1	1	1	17	Medium quality	38546	925856	52.40	30	d__Archaea;p__Micrarchaeota;c__Micrarchaeia;o__Micrarchaeales;f__;g__;s__
LMSG_G000006373.1	no	111_1	Copper	78.03	0.93	73.36	1	0	0	13	Medium quality	11059	713389	53.20	85	d__Archaea;p__Micrarchaeota;c__Micrarchaeia;o__Micrarchaeales;f__;g__;s__
LMSG_G000006374.1	no	111_1	Copper	84.57	0.93	79.90	1	0	1	18	Medium quality	32994	900076	52.50	34	d__Archaea;p__Micrarchaeota;c__Micrarchaeia;o__Micrarchaeales;f__;g__;s__
LMSG_G000006375.1	no	111_1	Copper	64.79	0.00	64.79	1	0	1	12	Medium quality	6528	588720	53.50	98	d__Archaea;p__Micrarchaeota;c__Micrarchaeia;o__Micrarchaeales;f__;g__;s__
LMSG_G000006376.1	no	111_1	Lead-Zinc	71.49	0.93	66.82	1	1	0	15	Medium quality	18120	717699	52.40	46	d__Archaea;p__Micrarchaeota;c__Micrarchaeia;o__Micrarchaeales;f__;g__;s__
LMSG_G000006377.1	no	112_1	Lead-Zinc	67.36	2.80	53.35	1	1	1	17	Medium quality	29100	878544	44.30	38	d__Archaea;p__Micrarchaeota;c__Micrarchaeia;o__Micrarchaeales;f__;g__;s__
LMSG_G000006378.1	no	112_1	Lead-Zinc	75.85	0.93	71.18	0	1	1	17	Medium quality	41209	965796	44.30	37	d__Archaea;p__Micrarchaeota;c__Micrarchaeia;o__Micrarchaeales;f__;g__;s__
LMSG_G000006379.1	no	112_1	Lead-Zinc	79.59	1.87	70.25	1	1	1	18	Medium quality	42347	959085	43.90	31	d__Archaea;p__Micrarchaeota;c__Micrarchaeia;o__Micrarchaeales;f__;g__;s__
LMSG_G000006380.1	no	112_1	Lead-Zinc	80.06	2.80	66.05	1	2	1	18	Medium quality	33730	1028070	44.10	41	d__Archaea;p__Micrarchaeota;c__Micrarchaeia;o__Micrarchaeales;f__;g__;s__
LMSG_G000006381.1	no	112_1	Magnetite	61.99	0.00	61.99	0	1	0	17	Medium quality	18230	640934	44.70	45	d__Archaea;p__Micrarchaeota;c__Micrarchaeia;o__Micrarchaeales;f__;g__;s__
LMSG_G000006382.1	no	112_1	Magnetite	78.19	3.27	61.84	1	1	1	19	Medium quality	50035	996745	43.90	31	d__Archaea;p__Micrarchaeota;c__Micrarchaeia;o__Micrarchaeales;f__;g__;s__
LMSG_G000006383.1	no	115_1	Lead-Zinc	57.71	0.00	57.71	0	2	1	16	Medium quality	173611	562546	57.00	10	d__Archaea;p__Micrarchaeota;c__Micrarchaeia;o__Micrarchaeales;f__;g__;s__
LMSG_G000006384.1	no	115_1	Pyrite-Copper	79.75	0.00	79.75	1	2	1	20	Medium quality	610078	776880	56.40	3	d__Archaea;p__Micrarchaeota;c__Micrarchaeia;o__Micrarchaeales;f__;g__;s__
LMSG_G000006385.1	no	118_1	Lead-Zinc	83.17	1.87	73.83	0	2	1	19	Medium quality	25365	990523	48.30	52	d__Archaea;p__Micrarchaeota;c__Micrarchaeia;o__Micrarchaeales;f__;g__;s__
LMSG_G000006386.1	no	119_1	Copper	86.29	1.87	76.95	1	2	2	18	Medium quality	43528	1056786	49.20	34	d__Archaea;p__Micrarchaeota;c__Micrarchaeia;o__Micrarchaeales;f__;g__;s__
LMSG_G000006387.1	no	119_1	Lead-Zinc	75.07	1.87	65.73	1	1	0	16	Medium quality	24713	885384	49.40	47	d__Archaea;p__Micrarchaeota;c__Micrarchaeia;o__Micrarchaeales;f__;g__;s__
LMSG_G000006388.1	no	119_1	Magnetite	84.73	2.13	74.07	1	0	0	19	Medium quality	29748	1028905	49.20	53	d__Archaea;p__Micrarchaeota;c__Micrarchaeia;o__Micrarchaeales;f__;g__;s__
LMSG_G000006389.1	no	120_1	Lead-Zinc	81.97	3.74	63.28	1	1	1	20	Medium quality	18936	937658	47.00	77	d__Archaea;p__Micrarchaeota;c__Micrarchaeia;o__Micrarchaeales;f__;g__;s__
LMSG_G000006390.1	no	120_1	Lead-Zinc	84.66	0.93	79.99	1	1	1	20	Medium quality	21234	862255	47.10	51	d__Archaea;p__Micrarchaeota;c__Micrarchaeia;o__Micrarchaeales;f__;g__;s__
LMSG_G000006391.1	no	120_1	Lead-Zinc	54.51	0.00	54.51	1	0	0	11	Medium quality	25033	549821	47.90	29	d__Archaea;p__Micrarchaeota;c__Micrarchaeia;o__Micrarchaeales;f__;g__;s__
LMSG_G000006392.1	no	120_1	Tin-Zinc	79.28	3.74	60.59	1	0	1	20	Medium quality	72295	886717	47.10	40	d__Archaea;p__Micrarchaeota;c__Micrarchaeia;o__Micrarchaeales;f__;g__;s__
LMSG_G000006393.1	no	120_1	Tin-Zinc	76.56	3.74	57.87	1	1	1	19	Medium quality	36267	886083	47.10	45	d__Archaea;p__Micrarchaeota;c__Micrarchaeia;o__Micrarchaeales;f__;g__;s__
LMSG_G000006394.1	no	125_1	Lead-Zinc	74.76	0.07	74.41	1	1	0	20	Medium quality	24676	789351	44.50	60	d__Archaea;p__Micrarchaeota;c__Micrarchaeia;o__Micrarchaeales;f__;g__;s__
LMSG_G000006395.1	no	128_1	Copper	82.86	1.87	73.52	1	1	2	19	Medium quality	44228	1033938	51.40	38	d__Archaea;p__Micrarchaeota;c__Micrarchaeia;o__Micrarchaeales;f__;g__;s__
LMSG_G000006396.1	no	128_1	Copper	79.12	1.87	69.78	1	0	1	20	Medium quality	30929	1077454	51.10	51	d__Archaea;p__Micrarchaeota;c__Micrarchaeia;o__Micrarchaeales;f__;g__;s__
LMSG_G000006397.1	no	128_1	Copper	79.12	0.93	74.45	1	0	1	19	Medium quality	36850	968370	51.40	42	d__Archaea;p__Micrarchaeota;c__Micrarchaeia;o__Micrarchaeales;f__;g__;s__
LMSG_G000006398.1	no	128_1	Copper	77.25	0.93	72.58	1	1	1	20	Medium quality	47063	993170	51.40	30	d__Archaea;p__Micrarchaeota;c__Micrarchaeia;o__Micrarchaeales;f__;g__;s__
LMSG_G000006399.1	no	128_1	Lead-Zinc	51.86	0.00	51.86	1	0	1	14	Medium quality	6930	575918	51.80	88	d__Archaea;p__Micrarchaeota;c__Micrarchaeia;o__Micrarchaeales;f__;g__;s__
LMSG_G000006400.1	no	129_1	Copper	79.28	2.80	65.27	1	2	1	19	Medium quality	23403	829981	43.10	50	d__Archaea;p__Micrarchaeota;c__Micrarchaeia;o__Micrarchaeales;f__;g__;s__
LMSG_G000006401.1	no	129_1	Copper	68.30	1.40	61.30	1	0	2	15	Medium quality	9627	666268	43.70	90	d__Archaea;p__Micrarchaeota;c__Micrarchaeia;o__Micrarchaeales;f__;g__;s__
LMSG_G000006402.1	no	129_1	Copper	79.98	0.93	75.31	1	1	1	20	Medium quality	69148	877681	43.20	39	d__Archaea;p__Micrarchaeota;c__Micrarchaeia;o__Micrarchaeales;f__;g__;s__
LMSG_G000006403.1	no	129_1	Magnetite	79.98	3.74	61.29	1	0	1	19	Medium quality	29753	843711	43.20	38	d__Archaea;p__Micrarchaeota;c__Micrarchaeia;o__Micrarchaeales;f__;g__;s__
LMSG_G000006404.1	no	129_1	Lead-Zinc	75.32	1.94	65.62	2	1	1	20	Medium quality	41248	903230	43.30	53	d__Archaea;p__Micrarchaeota;c__Micrarchaeia;o__Micrarchaeales;f__;g__;s__
LMSG_G000006405.1	no	129_1	Lead-Zinc	79.98	0.93	75.31	1	1	1	20	Medium quality	36081	979935	42.90	46	d__Archaea;p__Micrarchaeota;c__Micrarchaeia;o__Micrarchaeales;f__;g__;s__
LMSG_G000006406.1	no	129_1	Lead-Zinc	77.18	0.00	77.18	1	0	2	20	Medium quality	40917	818152	43.30	49	d__Archaea;p__Micrarchaeota;c__Micrarchaeia;o__Micrarchaeales;f__;g__;s__
LMSG_G000006407.1	no	129_1	Pyrite-Copper	80.33	3.29	63.90	0	1	1	19	Medium quality	17369	861788	43.60	83	d__Archaea;p__Micrarchaeota;c__Micrarchaeia;o__Micrarchaeales;f__;g__;s__
LMSG_G000006408.1	no	129_1	Lead-Zinc	81.85	0.93	77.18	1	0	1	20	Medium quality	43710	868795	43.20	72	d__Archaea;p__Micrarchaeota;c__Micrarchaeia;o__Micrarchaeales;f__;g__;s__
LMSG_G000006409.1	no	131_1	Lead-Zinc	82.79	1.44	75.61	1	1	2	18	Medium quality	37986	1059159	48.20	43	d__Archaea;p__Micrarchaeota;c__Micrarchaeia;o__Micrarchaeales;f__;g__;s__
LMSG_G000006410.1	no	131_1	Lead-Zinc	80.99	0.93	76.32	1	0	2	18	Medium quality	46937	1008731	48.20	36	d__Archaea;p__Micrarchaeota;c__Micrarchaeia;o__Micrarchaeales;f__;g__;s__
LMSG_G000006411.1	no	131_1	Lead-Zinc	72.51	2.34	60.83	0	2	1	18	Medium quality	8575	867114	48.50	113	d__Archaea;p__Micrarchaeota;c__Micrarchaeia;o__Micrarchaeales;f__;g__;s__
LMSG_G000006412.1	no	132_1	Magnetite	85.04	0.93	80.37	1	1	2	20	Medium quality	64887	917762	42.50	27	d__Archaea;p__Micrarchaeota;c__Micrarchaeia;o__Micrarchaeales;f__;g__;s__
LMSG_G000006413.1	no	132_1	Lead-Zinc	85.98	1.87	76.64	1	1	0	20	Medium quality	70661	915643	42.40	28	d__Archaea;p__Micrarchaeota;c__Micrarchaeia;o__Micrarchaeales;f__;g__;s__
LMSG_G000006414.1	no	132_1	Lead-Zinc	86.91	2.80	72.90	1	1	2	19	Medium quality	50614	1051805	42.50	70	d__Archaea;p__Micrarchaeota;c__Micrarchaeia;o__Micrarchaeales;f__;g__;s__
LMSG_G000006415.1	no	132_1	Lead-Zinc	63.40	0.47	61.07	1	1	0	15	Medium quality	12296	695542	42.70	71	d__Archaea;p__Micrarchaeota;c__Micrarchaeia;o__Micrarchaeales;f__;g__;s__
LMSG_G000006416.1	no	132_1	Magnetite	60.82	0.00	60.82	1	1	2	18	Medium quality	64189	685003	41.70	38	d__Archaea;p__Micrarchaeota;c__Micrarchaeia;o__Micrarchaeales;f__;g__;s__
LMSG_G000006417.1	no	133_1	Coal	81.89	2.80	67.88	1	2	0	20	Medium quality	16859	829595	52.50	69	d__Archaea;p__Micrarchaeota;c__Micrarchaeia;o__Micrarchaeales;f__;g__;s__
LMSG_G000006418.1	no	134_1	Lead-Zinc	53.36	0.00	53.36	1	1	0	13	Medium quality	5969	532846	47.40	102	d__Archaea;p__Micrarchaeota;c__Micrarchaeia;o__Micrarchaeales;f__;g__;s__
LMSG_G000006419.1	no	134_1	Lead-Zinc	80.84	0.90	76.35	1	1	1	18	Medium quality	31493	858147	47.20	41	d__Archaea;p__Micrarchaeota;c__Micrarchaeia;o__Micrarchaeales;f__;g__;s__
LMSG_G000006420.1	no	134_2	Copper	82.24	2.80	68.23	1	1	1	17	Medium quality	56052	1032585	46.80	48	d__Archaea;p__Micrarchaeota;c__Micrarchaeia;o__Micrarchaeales;f__;g__;s__
LMSG_G000006421.1	no	141_1	Coal	76.63	2.88	62.26	1	0	0	18	Medium quality	9286	769484	45.40	95	d__Archaea;p__Micrarchaeota;c__Micrarchaeia;o__Micrarchaeales;f__;g__;s__
LMSG_G000006422.1	no	141_1	Coal	73.67	1.40	66.67	1	1	1	18	Medium quality	12798	818557	45.80	101	d__Archaea;p__Micrarchaeota;c__Micrarchaeia;o__Micrarchaeales;f__;g__;s__
LMSG_G000006423.1	no	141_1	Lead-Zinc	78.03	1.87	68.69	1	1	2	19	Medium quality	36259	873292	45.40	44	d__Archaea;p__Micrarchaeota;c__Micrarchaeia;o__Micrarchaeales;f__;g__;s__
LMSG_G000006424.1	no	141_1	Lead-Zinc	76.47	0.93	71.80	1	1	1	20	Medium quality	20713	854881	45.60	60	d__Archaea;p__Micrarchaeota;c__Micrarchaeia;o__Micrarchaeales;f__;g__;s__
LMSG_G000006425.1	no	141_1	Lead-Zinc	78.81	4.21	57.79	1	1	1	19	Medium quality	13753	866619	45.80	100	d__Archaea;p__Micrarchaeota;c__Micrarchaeia;o__Micrarchaeales;f__;g__;s__
LMSG_G000006426.1	no	141_1	Lead-Zinc	71.02	2.80	57.01	1	0	0	18	Medium quality	7670	742007	45.60	116	d__Archaea;p__Micrarchaeota;c__Micrarchaeia;o__Micrarchaeales;f__;g__;s__
LMSG_G000006427.1	no	142_1	Magnetite	62.61	0.00	62.61	2	2	1	18	Medium quality	25970	819022	46.20	51	d__Archaea;p__Micrarchaeota;c__Micrarchaeia;o__Micrarchaeales;f__;g__;s__
LMSG_G000006428.1	no	142_1	Lead-Zinc	55.91	0.47	53.58	1	0	1	19	Medium quality	11227	826304	46.30	116	d__Archaea;p__Micrarchaeota;c__Micrarchaeia;o__Micrarchaeales;f__;g__;s__
LMSG_G000006429.1	no	144_1	Copper	63.17	2.18	52.27	1	0	0	14	Medium quality	4336	624795	31.00	162	d__Archaea;p__Micrarchaeota;c__Micrarchaeia;o__Micrarchaeales;f__;g__;s__
LMSG_G000006430.1	no	145_1	Lead-Zinc	75.70	4.05	55.46	1	1	1	19	Medium quality	51865	947984	30.90	30	d__Archaea;p__Micrarchaeota;c__Micrarchaeia;o__Micrarchaeales;f__;g__;s__
LMSG_G000006431.1	no	145_1	Magnetite	63.02	1.87	53.68	0	0	1	18	Medium quality	8496	735917	30.70	99	d__Archaea;p__Micrarchaeota;c__Micrarchaeia;o__Micrarchaeales;f__;g__;s__
LMSG_G000006432.1	no	146_1	Polymetallic	70.48	2.80	56.47	1	1	0	17	Medium quality	17304	902008	40.60	75	d__Archaea;p__Micrarchaeota;c__Micrarchaeia;o__Micrarchaeales;f__;g__;s__
LMSG_G000006433.1	no	146_1	Polymetallic	72.11	1.87	62.77	1	1	0	18	Medium quality	25637	814620	40.70	43	d__Archaea;p__Micrarchaeota;c__Micrarchaeia;o__Micrarchaeales;f__;g__;s__
LMSG_G000006434.1	no	147_1	Polymetallic	58.09	0.00	58.09	0	0	0	14	Medium quality	23429	642122	41.50	36	d__Archaea;p__Micrarchaeota;c__Micrarchaeia;o__Micrarchaeales;f__;g__;s__
LMSG_G000006435.1	no	147_1	Polymetallic	81.93	2.34	70.25	1	1	0	19	Medium quality	21418	854148	41.30	55	d__Archaea;p__Micrarchaeota;c__Micrarchaeia;o__Micrarchaeales;f__;g__;s__
LMSG_G000006436.1	no	147_1	Polymetallic	68.38	1.87	59.04	1	0	1	19	Medium quality	5576	758169	41.40	147	d__Archaea;p__Micrarchaeota;c__Micrarchaeia;o__Micrarchaeales;f__;g__;s__

LMSG_G000006437.1	no	147_1	Polymetallic	59.11	0.00	59.11	1	1	1	18	Medium quality	6210	681112	41.20	136	d__Archaea;p__Micrarchaeota;c__Micrarchaeia;o__Micrarchaeales;f__;g__;s__
LMSG_G000006438.1	no	147_1	Polymetallic	83.80	0.00	83.80	1	1	1	19	Medium quality	31774	908044	41.30	41	d__Archaea;p__Micrarchaeota;c__Micrarchaeia;o__Micrarchaeales;f__;g__;s__
LMSG_G000006439.1	no	147_1	Polymetallic	79.59	2.44	67.37	1	0	0	19	Medium quality	26221	952430	41.10	60	d__Archaea;p__Micrarchaeota;c__Micrarchaeia;o__Micrarchaeales;f__;g__;s__
LMSG_G000006440.1	no	147_1	Pyrite-Copper	76.71	0.00	76.71	1	1	1	17	Medium quality	27301	882540	41.30	49	d__Archaea;p__Micrarchaeota;c__Micrarchaeia;o__Micrarchaeales;f__;g__;s__
LMSG_G000006441.1	no	147_1	Pyrite-Copper	78.66	0.93	73.99	1	1	1	18	Medium quality	20239	910736	41.50	65	d__Archaea;p__Micrarchaeota;c__Micrarchaeia;o__Micrarchaeales;f__;g__;s__
LMSG_G000006442.1	no	147_1	Polymetallic	76.32	0.47	73.99	0	1	3	19	Medium quality	20274	874575	41.30	61	d__Archaea;p__Micrarchaeota;c__Micrarchaeia;o__Micrarchaeales;f__;g__;s__
LMSG_G000006443.1	no	147_1	Polymetallic	63.16	1.94	53.46	1	0	1	17	Medium quality	29146	796328	40.80	40	d__Archaea;p__Micrarchaeota;c__Micrarchaeia;o__Micrarchaeales;f__;g__;s__
LMSG_G000006444.1	no	147_1	Pyrite-Copper	69.27	0.00	69.27	0	0	1	15	Medium quality	19953	693357	41.30	55	d__Archaea;p__Micrarchaeota;c__Micrarchaeia;o__Micrarchaeales;f__;g__;s__
LMSG_G000006445.1	no	147_1	Polymetallic	57.71	0.00	57.71	0	0	0	14	Medium quality	24727	700925	41.10	38	d__Archaea;p__Micrarchaeota;c__Micrarchaeia;o__Micrarchaeales;f__;g__;s__
LMSG_G000006446.1	no	147_1	Pyrite	78.03	2.80	64.02	1	1	1	19	Medium quality	19155	945726	41.30	85	d__Archaea;p__Micrarchaeota;c__Micrarchaeia;o__Micrarchaeales;f__;g__;s__
LMSG_G000006447.1	no	29_1	Copper	73.75	0.00	73.75	1	1	1	16	Medium quality	46591	844397	52.70	28	d__Archaea;p__Micrarchaeota;c__Micrarchaeia;o__Micrarchaeales;f__;g__;s__
LMSG_G000006448.1	no	36_1	Lead-Zinc	81.85	1.40	74.85	1	1	0	19	Medium quality	21990	970029	37.40	82	d__Archaea;p__Micrarchaeota;c__Micrarchaeia;o__Micrarchaeales;f__;g__;s__
LMSG_G000006449.1	no	36_1	Pyrite	81.93	0.00	81.93	1	1	0	19	Medium quality	44016	866706	37.40	38	d__Archaea;p__Micrarchaeota;c__Micrarchaeia;o__Micrarchaeales;f__;g__;s__
LMSG_G000006450.1	no	36_1	Pyrite	78.19	0.93	73.52	1	1	0	18	Medium quality	12465	809029	37.50	91	d__Archaea;p__Micrarchaeota;c__Micrarchaeia;o__Micrarchaeales;f__;g__;s__
LMSG_G000006451.1	no	36_1	Pyrite	74.22	0.00	74.22	1	0	0	18	Medium quality	7870	773909	37.50	117	d__Archaea;p__Micrarchaeota;c__Micrarchaeia;o__Micrarchaeales;f__;g__;s__
LMSG_G000006452.1	no	36_1	Pyrite	78.19	0.00	78.19	1	1	0	19	Medium quality	28625	858241	37.50	46	d__Archaea;p__Micrarchaeota;c__Micrarchaeia;o__Micrarchaeales;f__;g__;s__
LMSG_G000006453.1	no	36_1	Lead-Zinc	76.32	0.00	76.32	1	1	0	19	Medium quality	26408	737322	37.70	48	d__Archaea;p__Micrarchaeota;c__Micrarchaeia;o__Micrarchaeales;f__;g__;s__
LMSG_G000006454.1	no	36_1	Polymetallic	79.12	0.00	79.12	1	1	0	19	Medium quality	24679	838950	37.50	52	d__Archaea;p__Micrarchaeota;c__Micrarchaeia;o__Micrarchaeales;f__;g__;s__
LMSG_G000006455.1	no	36_1	Copper	64.48	0.00	64.48	1	1	0	13	Medium quality	5428	527809	36.60	111	d__Archaea;p__Micrarchaeota;c__Micrarchaeia;o__Micrarchaeales;f__;g__;s__
LMSG_G000006456.1	no	36_1	Copper	79.12	0.00	79.12	1	1	0	19	Medium quality	15133	839803	37.40	82	d__Archaea;p__Micrarchaeota;c__Micrarchaeia;o__Micrarchaeales;f__;g__;s__
LMSG_G000006457.1	no	36_1	Copper	78.19	0.07	77.84	0	0	0	20	Medium quality	43568	859908	37.40	67	d__Archaea;p__Micrarchaeota;c__Micrarchaeia;o__Micrarchaeales;f__;g__;s__
LMSG_G000006458.1	no	36_1	Copper	63.94	1.87	54.60	1	1	0	16	Medium quality	17969	680185	38.10	48	d__Archaea;p__Micrarchaeota;c__Micrarchaeia;o__Micrarchaeales;f__;g__;s__
LMSG_G000006459.1	no	36_1	Copper	79.12	0.93	74.45	1	1	0	19	Medium quality	20662	827394	37.40	60	d__Archaea;p__Micrarchaeota;c__Micrarchaeia;o__Micrarchaeales;f__;g__;s__
LMSG_G000006460.1	no	36_1	Lead-Zinc	76.32	0.93	71.65	0	1	0	17	Medium quality	16012	776855	37.70	66	d__Archaea;p__Micrarchaeota;c__Micrarchaeia;o__Micrarchaeales;f__;g__;s__
LMSG_G000006461.1	no	36_1	Pyrite-Copper	78.19	0.00	78.19	1	1	0	19	Medium quality	23862	942710	37.60	80	d__Archaea;p__Micrarchaeota;c__Micrarchaeia;o__Micrarchaeales;f__;g__;s__
LMSG_G000006462.1	no	36_1	Pyrite-Copper	80.37	0.00	80.37	1	1	0	19	Medium quality	48577	839731	37.70	48	d__Archaea;p__Micrarchaeota;c__Micrarchaeia;o__Micrarchaeales;f__;g__;s__
LMSG_G000006463.1	no	37_1	Pyrite	68.93	1.87	59.59	1	1	0	16	Medium quality	3614	688764	28.30	237	d__Archaea;p__Micrarchaeota;c__Micrarchaeia;o__Micrarchaeales;f__;g__;s__
LMSG_G000006464.1	no	37_1	Pyrite	79.90	0.93	75.23	2	1	2	18	Medium quality	46845	828799	28.70	30	d__Archaea;p__Micrarchaeota;c__Micrarchaeia;o__Micrarchaeales;f__;g__;s__
LMSG_G000006465.1	no	37_1	Lead-Zinc	60.71	0.00	60.71	1	1	0	16	Medium quality	3293	644173	28.90	234	d__Archaea;p__Micrarchaeota;c__Micrarchaeia;o__Micrarchaeales;f__;g__;s__
LMSG_G000006466.1	no	37_1	Polymetallic	69.15	0.93	64.48	0	1	0	16	Medium quality	6550	662106	28.20	133	d__Archaea;p__Micrarchaeota;c__Micrarchaeia;o__Micrarchaeales;f__;g__;s__
LMSG_G000006467.1	no	37_1	Magnetite	76.16	0.93	71.49	1	1	1	17	Medium quality	20987	740228	28.30	52	d__Archaea;p__Micrarchaeota;c__Micrarchaeia;o__Micrarchaeales;f__;g__;s__
LMSG_G000006468.1	no	37_1	Lead-Zinc	77.10	0.31	75.55	0	1	1	17	Medium quality	18689	778385	28.60	93	d__Archaea;p__Micrarchaeota;c__Micrarchaeia;o__Micrarchaeales;f__;g__;s__
LMSG_G000006469.1	no	37_1	Lead-Zinc	65.65	0.00	65.65	1	1	2	17	Medium quality	81521	700873	28.40	59	d__Archaea;p__Micrarchaeota;c__Micrarchaeia;o__Micrarchaeales;f__;g__;s__
LMSG_G000006470.1	no	37_1	Magnetite	75.78	1.17	69.94	1	1	0	18	Medium quality	21453	718849	28.10	50	d__Archaea;p__Micrarchaeota;c__Micrarchaeia;o__Micrarchaeales;f__;g__;s__
LMSG_G000006471.1	no	37_1	Magnetite	71.57	0.93	66.90	1	0	1	18	Medium quality	15894	775037	28.30	110	d__Archaea;p__Micrarchaeota;c__Micrarchaeia;o__Micrarchaeales;f__;g__;s__
LMSG_G000006472.1	no	38_1	Lead-Zinc	66.74	0.93	62.07	1	2	1	17	Medium quality	6103	773786	35.20	159	d__Archaea;p__Micrarchaeota;c__Micrarchaeia;o__Micrarchaeales;f__;g__;s__
LMSG_G000006473.1	no	38_1	Pyrite	75.25	0.00	75.25	0	1	1	15	Medium quality	8426	801084	35.20	140	d__Archaea;p__Micrarchaeota;c__Micrarchaeia;o__Micrarchaeales;f__;g__;s__
LMSG_G000006474.1	no	38_1	Pyrite	78.27	0.00	78.27	0	1	1	17	Medium quality	24410	838369	35.10	41	d__Archaea;p__Micrarchaeota;c__Micrarchaeia;o__Micrarchaeales;f__;g__;s__
LMSG_G000006475.1	no	38_1	Pyrite	76.40	0.00	76.40	0	0	2	17	Medium quality	11854	803253	35.30	103	d__Archaea;p__Micrarchaeota;c__Micrarchaeia;o__Micrarchaeales;f__;g__;s__
LMSG_G000006476.1	no	38_1	Pyrite	51.98	0.00	51.98	1	0	0	15	Medium quality	6155	591745	35.40	132	d__Archaea;p__Micrarchaeota;c__Micrarchaeia;o__Micrarchaeales;f__;g__;s__
LMSG_G000006477.1	no	38_1	Lead-Zinc	75.46	0.00	75.46	1	0	2	19	Medium quality	43929	854840	35.10	56	d__Archaea;p__Micrarchaeota;c__Micrarchaeia;o__Micrarchaeales;f__;g__;s__
LMSG_G000006478.1	no	38_1	Lead-Zinc	79.20	0.93	74.53	1	1	1	19	Medium quality	40045	1127796	35.40	89	d__Archaea;p__Micrarchaeota;c__Micrarchaeia;o__Micrarchaeales;f__;g__;s__
LMSG_G000006479.1	no	38_1	Copper	77.26	0.93	72.59	1	2	0	18	Medium quality	15518	837986	35.20	109	d__Archaea;p__Micrarchaeota;c__Micrarchaeia;o__Micrarchaeales;f__;g__;s__
LMSG_G000006480.1	no	38_1	Lead-Zinc	76.40	0.00	76.40	1	1	1	19	Medium quality	23487	843265	35.20	51	d__Archaea;p__Micrarchaeota;c__Micrarchaeia;o__Micrarchaeales;f__;g__;s__
LMSG_G000006481.1	no	38_1	Pyrite-Copper	70.17	0.00	70.17	1	1	0	18	Medium quality	6491	775356	35.60	161	d__Archaea;p__Micrarchaeota;c__Micrarchaeia;o__Micrarchaeales;f__;g__;s__
LMSG_G000006482.1	no	39_1	Lead-Zinc	70.32	0.00	70.32	1	0	1	17	Medium quality	16969	780380	35.10	99	d__Archaea;p__Micrarchaeota;c__Micrarchaeia;o__Micrarchaeales;f__;g__;s__
LMSG_G000006483.1	no	39_1	Polymetallic	74.49	3.34	57.78	1	1	2	18	Medium quality	9670	798086	35.20	157	d__Archaea;p__Micrarchaeota;c__Micrarchaeia;o__Micrarchaeales;f__;g__;s__
LMSG_G000006484.1	no	43_1	Polymetallic	79.12	2.80	65.11	1	0	2	18	Medium quality	52531	793583	39.40	32	d__Archaea;p__Micrarchaeota;c__Micrarchaeia;o__Micrarchaeales;f__;g__;s__
LMSG_G000006485.1	no	43_1	Polymetallic	86.60	0.93	81.93	1	1	0	18	Medium quality	33914	854896	39.10	33	d__Archaea;p__Micrarchaeota;c__Micrarchaeia;o__Micrarchaeales;f__;g__;s__
LMSG_G000006486.1	no	43_1	Polymetallic	77.72	0.93	73.05	1	1	0	19	Medium quality	35145	781142	39.20	34	d__Archaea;p__Micrarchaeota;c__Micrarchaeia;o__Micrarchaeales;f__;g__;s__
LMSG_G000006487.1	no	43_1	Polymetallic	79.59	0.93	74.92	1	1	1	17	Medium quality	32171	785484	39.40	36	d__Archaea;p__Micrarchaeota;c__Micrarchaeia;o__Micrarchaeales;f__;g__;s__
LMSG_G000006488.1	no	43_1	Polymetallic	66.04	1.08	60.65	0	1	1	19	Medium quality	25096	987277	40.10	109	d__Archaea;p__Micrarchaeota;c__Micrarchaeia;o__Micrarchaeales;f__;g__;s__
LMSG_G000006489.1	no	43_1	Lead-Zinc	87.53	2.80	73.52	1	2	0	20	Medium quality	43092	931784	39.50	36	d__Archaea;p__Micrarchaeota;c__Micrarchaeia;o__Micrarchaeales;f__;g__;s__
LMSG_G000006490.1	no	43_1	Lead-Zinc	82.08	1.87	72.74	1	1	0	18	Medium quality	11818	791236	39.30	93	d__Archaea;p__Micrarchaeota;c__Micrarchaeia;o__Micrarchaeales;f__;g__;s__
LMSG_G000006491.1	no	43_1	Lead-Zinc	78.44	2.95	63.71	2	1	0	18	Medium quality	5968	1318130	38.40	259	d__Archaea;p__Micrarchaeota;c__Micrarchaeia;o__Micrarchaeales;f__;g__;s__
LMSG_G000006492.1	no	44_1	Polymetallic	76.63	1.87	67.29	1	1	1	18	Medium quality	20971	877558	42.00	58	d__Archaea;p__Micrarchaeota;c__Micrarchaeia;o__Micrarchaeales;f__;g__;s__
LMSG_G000006493.1	no	44_1	Polymetallic	71.02	0.93	66.35	1	1	1	16	Medium quality	22611	853427	42.10	52	d__Archaea;p__Micrarchaeota;c__Micrarchaeia;o__Micrarchaeales;f__;g__;s__
LMSG_G000006494.1	no	44_1	Polymetallic	75.70	2.80	61.69	1	1	1	18	Medium quality	20065	968377	41.90	65	d__Archaea;p__Micrarchaeota;c__Micrarchaeia;o__Micrarchaeales;f__;g__;s__
LMSG_G000006495.1	no	44_1	Polymetallic	76.16	3.74	57.47	1	1	1	18	Medium quality	22518	890950	42.10	56	d__Archaea;p__Micrarchaeota;c__Micrarchaeia;o__Micrarchaeales;f__;g__;s__
LMSG_G000006496.1	no	49_1	Polymetallic	58.48	0.00	58.48	0	0	0	17	Medium quality	29533	553624	49.20	27	d__Archaea;p__Micrarchaeota;c__Micrarchaeia;o__Micrarchaeales;f__;g__;s__
LMSG_G000006497.1	no	50_1	Polymetallic	79.12	1.40	72.12	0	0	0	17	Medium quality	18303	673075	51.70	50	d__Archaea;p__Micrarchaeota;c__Micrarchaeia;o__Micrarchaeales;f__;g__;s__
LMSG_G000006498.1	no	50_1	Polymetallic	53.34	0.00	53.34	1	2	0	17	Medium quality	46830	527174	50.90	17	d__Archaea;p__Micrarchaeota;c__Micrarchaeia;o__Micrarchaeales;f__;g__;s__
LMSG_G000006499.1	no	50_1	Polymetallic	75.62	0.93	70.95	1	2	1	20	Medium quality	63088	816130	51.00	21	d__Archaea;p__Micrarchaeota;c__Micrarchaeia;o__Micrarchaeales;f__;g__;s__
LMSG_G000006500.1	no	50_1	Polymetallic	58.33	0.00	58.33	1	0	0	17	Medium quality	36667	596780	51.40	30	d__Archaea;p__Micrarchaeota;c__Micrarchaeia;o__Micrarchaeales;f__;g__;s__
LMSG_G000006501.1	no	51_1	Copper	60.82	1.40	53.82	0	2	1	14	Medium quality	6812	580899	48.20	106	d__Archaea;p__Micrarchaeota;c__Micrarchaeia;o__Micrarchaeales;f__;g__;s__
LMSG_G000006502.1	no	52_1	Copper	72.58	0.00	72.58	0	0	0	17	Medium quality	27624	638654	46.40	33	d__Archaea;p__Micrarchaeota;c__Micrarchaeia;o__Micrarchaeales;f__;g__;s__
LMSG_G000006503.1	no	52_1	Polymetallic	66.90	0.93	62.23	1	0	1	18	Medium quality	60316	623936	46.40	19	d__Archaea;p__Micrarchaeota;c__Micrarchaeia;o__Micrarchaeales;f__;g__;s__
LMSG_G000006504.1	no	52_1	Polymetallic	65.26	0.93	60.59	0	1	1	18	Medium quality	39125	729099	46.40	21	d__Archaea;p__Micrarchaeota;c__Micrarchaeia;o__Micrarchaeales;f__;g__;s__
LMSG_G000006505.1	no	52_1	Polymetallic	72.41	0.97	67.56	1	1	1	19	Medium quality	51253	743546	46.20	27	d__Archaea;p__Micrarchaeota;c__Micrarchaeia;o__Micrarchaeales;f__;g__;s__
LMSG_G000006506.1	no	52_1	Pyrite-Copper	83.64	0.93	78.97	1	2	0	20	Medium quality	30291	813514	45.90	37	d__Archaea;p__Micrarchaeota;c__Micrarchaeia;o__Micrarchaeales;f__;g__;s__
LMSG_G000006507.1	no	52_1	Pyrite-Copper	83.64	0.93	78.97	1	2	0	20	Medium quality	85105	818714	46.10	28	d__Archaea;p__Micrarchaeota;c__Micrarchaeia;o__Micrarchaeales;f__;g__;s__
LMSG_G000006508.1	no	52_1	Pyrite-Copper	61.30	0.00	61.30	0	0	0	18	Medium quality	21797	626539	46.80	76	d__Archaea;p__Micrarchaeota;c__Micrarchaeia;o__Micrarchaeales;f__;g__;s__
LMSG_G000006509.1	no	52_1	Pyrite-Copper	80.84	2.80	66.83	1	0	1	19	Medium quality	34990	778001	45.60	35	d__Archaea;p__Micrarchaeota;c__Micrarchaeia;o__Micrarchaeales;f__;g__;s__
LMSG_G000006510.1	no	53_1	Pyrite-Copper	61.52	0.00	61.52	0	2	0	18	Medium quality	51539	552905	44.20	27	d__Archaea;p__Micrarchaeota;c__Micrarchaeia;o__Micrarchaeales;f__;g__;s__
LMSG_G000006511.1	no	53_1	Pyrite-Copper	58.48	0.00	58.48	0	2	0	16	Medium quality	12243	592311	44.30	78	d__Archaea;p__Micrarchaeota;c__Micrarchaeia;o__Micrarchaeales;f__;g__;s__
LMSG_G000006512.1	no	53_1	Pyrite	69.70	2.41	57.66	1	0	0	20	Medium quality	33759	732247	44.20	47	d__Archaea;p__Micrarchaeota;c__Micrarchaeia;o__Micrarchaeales;f__;g__;s__
LMSG_G000006513.1	no	53_1	Pyrite	72.98	3.74	54.29	1	0	0	18	Medium quality	6964	675211	44.20	116	d__Archaea;p__Micrarchaeota;c__Micrarchaeia;o__Micrarchaeales;f__;g__;s__

LMSG_G000006514.1	no	53_1	Pyrite	54.04	0.00	54.04	1	0	0	16	Medium quality	7284	596337	44.30	96	d__Archaea;p__Micrarchaeota;c__Micrarchaeia;o__Micrarchaeales;f__;g__;s__
LMSG_G000006515.1	no	53_1	Copper	52.41	0.00	52.41	1	0	1	16	Medium quality	9484	569605	44.20	89	d__Archaea;p__Micrarchaeota;c__Micrarchaeia;o__Micrarchaeales;f__;g__;s__
LMSG_G000006516.1	no	53_1	Polymetallic	60.74	0.93	56.07	1	0	1	13	Medium quality	14639	617096	44.10	79	d__Archaea;p__Micrarchaeota;c__Micrarchaeia;o__Micrarchaeales;f__;g__;s__
LMSG_G000006517.1	no	53_1	Copper	55.48	0.93	50.81	1	0	0	14	Medium quality	10824	517005	44.20	62	d__Archaea;p__Micrarchaeota;c__Micrarchaeia;o__Micrarchaeales;f__;g__;s__
LMSG_G000006518.1	no	53_1	Polymetallic	68.93	1.87	59.59	1	0	1	18	Medium quality	11570	792181	43.40	107	d__Archaea;p__Micrarchaeota;c__Micrarchaeia;o__Micrarchaeales;f__;g__;s__
LMSG_G000006519.1	no	53_1	Copper	68.69	0.93	64.02	1	0	0	20	Medium quality	15979	786116	44.00	64	d__Archaea;p__Micrarchaeota;c__Micrarchaeia;o__Micrarchaeales;f__;g__;s__
LMSG_G000006520.1	no	53_1	Lead-Zinc	63.18	1.87	53.84	1	0	0	17	Medium quality	22943	696671	44.20	72	d__Archaea;p__Micrarchaeota;c__Micrarchaeia;o__Micrarchaeales;f__;g__;s__
LMSG_G000006521.1	no	61_1	Tin-Zinc	55.76	0.00	55.76	1	0	1	16	Medium quality	29072	571453	50.40	41	d__Archaea;p__Micrarchaeota;c__Micrarchaeia;o__Micrarchaeales;f__;g__;s__
LMSG_G000006522.1	no	76_1	Copper	70.32	0.00	70.32	1	1	4	18	Medium quality	38922	669128	50.10	67	d__Archaea;p__Micrarchaeota;c__Micrarchaeia;o__Micrarchaeales;f__;g__;s__
LMSG_G000006523.1	no	76_1	Pyrite	76.63	0.00	76.63	1	2	0	20	Medium quality	22643	643335	49.40	41	d__Archaea;p__Micrarchaeota;c__Micrarchaeia;o__Micrarchaeales;f__;g__;s__
LMSG_G000006524.1	no	76_1	Copper	71.96	2.57	59.11	2	5	3	17	Medium quality	9418	890873	49.00	165	d__Archaea;p__Micrarchaeota;c__Micrarchaeia;o__Micrarchaeales;f__;g__;s__
LMSG_G000006525.1	no	76_1	Copper	64.97	0.00	64.97	0	1	1	12	Medium quality	13841	493627	49.80	45	d__Archaea;p__Micrarchaeota;c__Micrarchaeia;o__Micrarchaeales;f__;g__;s__
LMSG_G000006526.1	no	76_1	Polymetallic	73.05	0.93	68.38	0	1	1	17	Medium quality	18804	599137	49.40	80	d__Archaea;p__Micrarchaeota;c__Micrarchaeia;o__Micrarchaeales;f__;g__;s__
LMSG_G000006527.1	no	76_1	Polymetallic	75.70	0.00	75.70	1	1	1	20	Medium quality	29843	721062	49.40	38	d__Archaea;p__Micrarchaeota;c__Micrarchaeia;o__Micrarchaeales;f__;g__;s__
LMSG_G000006528.1	no	76_1	Polymetallic	64.48	0.00	64.48	1	1	0	17	Medium quality	15654	549690	49.20	46	d__Archaea;p__Micrarchaeota;c__Micrarchaeia;o__Micrarchaeales;f__;g__;s__
LMSG_G000006529.1	no	76_1	Copper	75.23	0.00	75.23	1	1	2	20	Medium quality	29287	684570	49.60	34	d__Archaea;p__Micrarchaeota;c__Micrarchaeia;o__Micrarchaeales;f__;g__;s__
LMSG_G000006530.1	no	76_1	Magnetite	65.80	2.80	51.79	1	1	1	17	Medium quality	37104	655797	50.50	43	d__Archaea;p__Micrarchaeota;c__Micrarchaeia;o__Micrarchaeales;f__;g__;s__
LMSG_G000006531.1	no	76_1	Lead-Zinc	67.36	0.00	67.36	0	0	1	16	Medium quality	22418	585401	49.60	36	d__Archaea;p__Micrarchaeota;c__Micrarchaeia;o__Micrarchaeales;f__;g__;s__
LMSG_G000006532.1	no	76_1	Pyrite-Copper	64.01	0.00	64.01	0	1	1	17	Medium quality	37257	542831	49.20	23	d__Archaea;p__Micrarchaeota;c__Micrarchaeia;o__Micrarchaeales;f__;g__;s__
LMSG_G000006533.1	no	76_1	Pyrite-Copper	75.70	0.93	71.03	1	1	1	20	Medium quality	24204	665982	49.30	37	d__Archaea;p__Micrarchaeota;c__Micrarchaeia;o__Micrarchaeales;f__;g__;s__
LMSG_G000006534.1	no	76_1	Pyrite-Copper	75.23	0.38	73.32	2	1	1	17	Medium quality	72070	691677	49.70	59	d__Archaea;p__Micrarchaeota;c__Micrarchaeia;o__Micrarchaeales;f__;g__;s__
LMSG_G000006535.1	no	76_1	Lead-Zinc	70.46	0.00	70.46	1	2	4	18	Medium quality	24388	747707	49.40	72	d__Archaea;p__Micrarchaeota;c__Micrarchaeia;o__Micrarchaeales;f__;g__;s__
LMSG_G000006536.1	no	76_1	Magnetite	50.70	0.00	50.70	1	0	0	15	Medium quality	30331	487806	48.70	26	d__Archaea;p__Micrarchaeota;c__Micrarchaeia;o__Micrarchaeales;f__;g__;s__
LMSG_G000006537.1	no	84_1	Polymetallic	72.92	2.80	58.91	0	0	1	16	Medium quality	5018	932499	50.60	261	d__Archaea;p__Micrarchaeota;c__Micrarchaeia;o__Micrarchaeales;f__;g__;s__
LMSG_G000006538.1	no	84_1	Polymetallic	69.94	0.93	65.27	0	0	1	13	Medium quality	18419	724316	50.40	53	d__Archaea;p__Micrarchaeota;c__Micrarchaeia;o__Micrarchaeales;f__;g__;s__
LMSG_G000006539.1	no	84_1	Polymetallic	69.54	0.93	64.87	0	1	1	13	Medium quality	41776	832020	50.20	36	d__Archaea;p__Micrarchaeota;c__Micrarchaeia;o__Micrarchaeales;f__;g__;s__
LMSG_G000006540.1	no	86_1	Magnetite	51.94	0.00	51.94	1	1	0	13	Medium quality	9166	539685	49.50	94	d__Archaea;p__Micrarchaeota;c__Micrarchaeia;o__Micrarchaeales;f__;g__;s__
LMSG_G000006541.1	no	99_1	Copper	67.28	0.00	67.28	1	1	1	16	Medium quality	29842	657766	42.70	34	d__Archaea;p__Micrarchaeota;c__Micrarchaeia;o__Micrarchaeales;f__;g__;s__
LMSG_G000006542.1	no	99_1	Lead-Zinc	70.56	2.88	56.19	1	1	1	19	Medium quality	11611	910241	42.80	99	d__Archaea;p__Micrarchaeota;c__Micrarchaeia;o__Micrarchaeales;f__;g__;s__
LMSG_G000006543.1	no	114_1	Lead-Zinc	69.47	0.00	69.47	1	1	1	17	Medium quality	20848	522756	47.30	38	d__Archaea;p__Micrarchaeota;c__Micrarchaeia;o__Micrarchaeales;f__Micrarchaeaceae;g__;s__
LMSG_G000006544.1	no	114_1	Lead-Zinc	74.71	0.00	74.71	1	1	1	19	Medium quality	96310	732471	46.80	15	d__Archaea;p__Micrarchaeota;c__Micrarchaeia;o__Micrarchaeales;f__Micrarchaeaceae;g__;s__
LMSG_G000006545.1	no	126_1	Polymetallic	65.96	0.93	61.29	1	0	2	17	Medium quality	97015	586651	42.70	9	d__Archaea;p__Micrarchaeota;c__Micrarchaeia;o__Micrarchaeales;f__Micrarchaeaceae;g__;s__
LMSG_G000006546.1	no	126_1	Polymetallic	70.56	2.34	58.88	1	1	2	20	Medium quality	93014	666496	42.30	18	d__Archaea;p__Micrarchaeota;c__Micrarchaeia;o__Micrarchaeales;f__Micrarchaeaceae;g__;s__
LMSG_G000006547.1	no	127_1	Polymetallic	72.42	0.00	72.42	0	1	0	19	Medium quality	24476	683428	41.30	40	d__Archaea;p__Micrarchaeota;c__Micrarchaeia;o__Micrarchaeales;f__Micrarchaeaceae;g__;s__
LMSG_G000006548.1	no	127_1	Polymetallic	72.42	0.00	72.42	1	1	1	19	Medium quality	25808	689076	41.40	39	d__Archaea;p__Micrarchaeota;c__Micrarchaeia;o__Micrarchaeales;f__Micrarchaeaceae;g__;s__
LMSG_G000006549.1	no	127_1	Polymetallic	82.71	0.00	82.71	1	1	0	20	Medium quality	30605	660188	41.40	32	d__Archaea;p__Micrarchaeota;c__Micrarchaeia;o__Micrarchaeales;f__Micrarchaeaceae;g__;s__
LMSG_G000006550.1	no	127_1	Polymetallic	80.84	0.00	80.84	0	1	0	18	Medium quality	30271	707574	41.20	38	d__Archaea;p__Micrarchaeota;c__Micrarchaeia;o__Micrarchaeales;f__Micrarchaeaceae;g__;s__
LMSG_G000006551.1	no	127_1	Polymetallic	82.71	0.00	82.71	1	2	0	19	Medium quality	29241	855631	41.20	47	d__Archaea;p__Micrarchaeota;c__Micrarchaeia;o__Micrarchaeales;f__Micrarchaeaceae;g__;s__
LMSG_G000006552.1	no	127_1	Polymetallic	82.71	0.00	82.71	1	1	0	19	Medium quality	34664	773188	41.30	41	d__Archaea;p__Micrarchaeota;c__Micrarchaeia;o__Micrarchaeales;f__Micrarchaeaceae;g__;s__
LMSG_G000006553.1	no	127_1	Magnetite	79.90	0.93	75.23	0	0	0	19	Medium quality	26190	652984	41.30	34	d__Archaea;p__Micrarchaeota;c__Micrarchaeia;o__Micrarchaeales;f__Micrarchaeaceae;g__;s__
LMSG_G000006554.1	no	127_1	Lead-Zinc	84.57	4.67	61.21	1	1	1	18	Medium quality	32977	854439	40.70	41	d__Archaea;p__Micrarchaeota;c__Micrarchaeia;o__Micrarchaeales;f__Micrarchaeaceae;g__;s__
LMSG_G000006555.1	no	127_1	Lead-Zinc	79.90	0.93	75.23	1	1	0	18	Medium quality	52077	677383	41.30	40	d__Archaea;p__Micrarchaeota;c__Micrarchaeia;o__Micrarchaeales;f__Micrarchaeaceae;g__;s__
LMSG_G000006556.1	no	127_1	Lead-Zinc	81.77	2.80	67.76	1	0	0	19	Medium quality	50794	843884	41.10	47	d__Archaea;p__Micrarchaeota;c__Micrarchaeia;o__Micrarchaeales;f__Micrarchaeaceae;g__;s__
LMSG_G000006557.1	no	127_1	Lead-Zinc	81.30	0.93	76.63	1	0	1	18	Medium quality	27054	734623	40.90	37	d__Archaea;p__Micrarchaeota;c__Micrarchaeia;o__Micrarchaeales;f__Micrarchaeaceae;g__;s__
LMSG_G000006558.1	no	127_1	Pyrite-Copper	78.03	0.93	73.36	1	1	0	19	Medium quality	25807	722731	41.30	51	d__Archaea;p__Micrarchaeota;c__Micrarchaeia;o__Micrarchaeales;f__Micrarchaeaceae;g__;s__
LMSG_G000006559.1	no	127_1	Lead-Zinc	83.64	1.87	74.30	1	1	1	20	Medium quality	31483	786991	40.80	42	d__Archaea;p__Micrarchaeota;c__Micrarchaeia;o__Micrarchaeales;f__Micrarchaeaceae;g__;s__
LMSG_G000006560.1	no	127_1	Magnetite	81.77	0.93	77.10	1	1	1	19	Medium quality	41142	713570	41.30	39	d__Archaea;p__Micrarchaeota;c__Micrarchaeia;o__Micrarchaeales;f__Micrarchaeaceae;g__;s__
LMSG_G000006561.1	no	127_1	Magnetite	80.84	4.67	57.48	1	2	0	19	Medium quality	33123	813366	41.00	59	d__Archaea;p__Micrarchaeota;c__Micrarchaeia;o__Micrarchaeales;f__Micrarchaeaceae;g__;s__
LMSG_G000006562.1	no	127_1	Coal	76.50	0.00	76.50	1	0	1	18	Medium quality	8042	689286	41.10	115	d__Archaea;p__Micrarchaeota;c__Micrarchaeia;o__Micrarchaeales;f__Micrarchaeaceae;g__;s__
LMSG_G000006563.1	no	136_1	Polymetallic	64.40	0.47	62.07	1	0	1	19	Medium quality	54628	800995	33.40	30	d__Archaea;p__Micrarchaeota;c__Micrarchaeia;o__Micrarchaeales;f__Micrarchaeaceae;g__;s__
LMSG_G000006564.1	no	136_1	Magnetite	55.14	0.47	52.81	1	0	1	19	Medium quality	32473	744470	33.70	40	d__Archaea;p__Micrarchaeota;c__Micrarchaeia;o__Micrarchaeales;f__Micrarchaeaceae;g__;s__
LMSG_G000006565.1	no	136_1	Magnetite	65.34	1.25	59.11	0	0	0	18	Medium quality	31228	686344	33.10	37	d__Archaea;p__Micrarchaeota;c__Micrarchaeia;o__Micrarchaeales;f__Micrarchaeaceae;g__;s__
LMSG_G000006566.1	no	136_1	Magnetite	71.88	2.34	60.20	0	0	1	17	Medium quality	38563	699451	33.70	35	d__Archaea;p__Micrarchaeota;c__Micrarchaeia;o__Micrarchaeales;f__Micrarchaeaceae;g__;s__
LMSG_G000006567.1	no	136_1	Magnetite	78.03	1.71	69.47	1	1	1	20	Medium quality	21079	914362	33.50	82	d__Archaea;p__Micrarchaeota;c__Micrarchaeia;o__Micrarchaeales;f__Micrarchaeaceae;g__;s__
LMSG_G000006568.1	no	136_1	Magnetite	63.31	0.93	58.64	1	0	0	16	Medium quality	7162	757552	33.40	142	d__Archaea;p__Micrarchaeota;c__Micrarchaeia;o__Micrarchaeales;f__Micrarchaeaceae;g__;s__
LMSG_G000006569.1	no	136_1	Polymetallic	66.37	0.47	64.04	1	1	1	19	Medium quality	8075	728130	33.40	128	d__Archaea;p__Micrarchaeota;c__Micrarchaeia;o__Micrarchaeales;f__Micrarchaeaceae;g__;s__
LMSG_G000006570.1	no	136_1	Polymetallic	57.64	0.47	55.31	0	0	0	13	Medium quality	2644	667590	33.70	304	d__Archaea;p__Micrarchaeota;c__Micrarchaeia;o__Micrarchaeales;f__Micrarchaeaceae;g__;s__
LMSG_G000006571.1	no	136_1	Polymetallic	78.19	0.31	76.64	1	1	1	19	Medium quality	25680	887355	33.40	55	d__Archaea;p__Micrarchaeota;c__Micrarchaeia;o__Micrarchaeales;f__Micrarchaeaceae;g__;s__
LMSG_G000006572.1	no	136_1	Polymetallic	64.95	0.00	64.95	0	0	0	18	Medium quality	29336	827141	31.80	157	d__Archaea;p__Micrarchaeota;c__Micrarchaeia;o__Micrarchaeales;f__Micrarchaeaceae;g__;s__
LMSG_G000006573.1	no	136_1	Polymetallic	57.32	0.93	52.65	0	0	0	19	Medium quality	7399	604905	33.60	140	d__Archaea;p__Micrarchaeota;c__Micrarchaeia;o__Micrarchaeales;f__Micrarchaeaceae;g__;s__
LMSG_G000006574.1	no	137_1	Lead-Zinc	61.68	1.01	56.65	0	0	2	18	Medium quality	36340	619465	52.10	48	d__Archaea;p__Micrarchaeota;c__Micrarchaeia;o__Micrarchaeales;f__Micrarchaeaceae;g__;s__
LMSG_G000006575.1	no	137_1	Copper	63.55	1.56	55.77	1	0	2	13	Medium quality	8281	611609	52.90	110	d__Archaea;p__Micrarchaeota;c__Micrarchaeia;o__Micrarchaeales;f__Micrarchaeaceae;g__;s__
LMSG_G000006576.1	no	137_1	Lead-Zinc	50.46	0.00	50.46	0	0	0	11	Medium quality	17606	397486	54.00	30	d__Archaea;p__Micrarchaeota;c__Micrarchaeia;o__Micrarchaeales;f__Micrarchaeaceae;g__;s__
LMSG_G000006577.1	no	137_1	Pyrite	76.09	1.87	66.75	1	2	3	18	Medium quality	21276	764649	51.20	51	d__Archaea;p__Micrarchaeota;c__Micrarchaeia;o__Micrarchaeales;f__Micrarchaeaceae;g__;s__
LMSG_G000006578.1	no	137_1	Pyrite	81.77	1.87	72.43	1	1	1	18	Medium quality	21827	845552	50.40	62	d__Archaea;p__Micrarchaeota;c__Micrarchaeia;o__Micrarchaeales;f__Micrarchaeaceae;g__;s__
LMSG_G000006579.1	no	137_1	Pyrite	81.77	0.93	77.10	1	1	3	19	Medium quality	26374	847713	50.60	55	d__Archaea;p__Micrarchaeota;c__Micrarchaeia;o__Micrarchaeales;f__Micrarchaeaceae;g__;s__
LMSG_G000006580.1	no	137_1	Pyrite	79.59	0.93	74.92	2	2	4	18	Medium quality	23855	754010	51.10	47	d__Archaea;p__Micrarchaeota;c__Micrarchaeia;o__Micrarchaeales;f__Micrarchaeaceae;g__;s__
LMSG_G000006581.1	no	137_1	Lead-Zinc	81.77	0.93	77.10	1	1	1	18	Medium quality	36337	734681	51.90	32	d__Archaea;p__Micrarchaeota;c__Micrarchaeia;o__Micrarchaeales;f__Micrarchaeaceae;g__;s__
LMSG_G000006582.1	no	137_1	Lead-Zinc	72.51	0.93	67.84	1	1	0	16	Medium quality	4222	628482	52.80	193	d__Archaea;p__Micrarchaeota;c__Micrarchaeia;o__Micrarchaeales;f__Micrarchaeaceae;g__;s__
LMSG_G000006583.1	no	137_1	Lead-Zinc	75.46	0.93	70.79	0	0	5	14	Medium quality	45102	658489	52.10	36	d__Archaea;p__Micrarchaeota;c__Micrarchaeia;o__Micrarchaeales;f__Micrarchaeaceae;g__;s__
LMSG_G000006584.1	no	137_1	Lead-Zinc	59.59	0.93	54.92	1	1	0	17	Medium quality	33137	538419	51.50	26	d__Archaea;p__Micrarchaeota;c__Micrarchaeia;o__Micrarchaeales;f__Micrarchaeaceae;g__;s__
LMSG_G000006585.1	no	137_1	Polymetallic	81.77	0.93	77.10	1	1	1	18	Medium quality	26746	778579	51.30	47	d__Archaea;p__Micrarchaeota;c__Micrarchaeia;o__Micrarchaeales;f__Micrarchaeaceae;g__;s__
LMSG_G000006586.1	no	137_1	Polymetallic	80.60	2.80	66.59	1	0	1	18	Medium quality	32301	783155	51.10	35	d__Archaea;p__Micrarchaeota;c__Micrarchaeia;o__Micrarchaeales;f__Micrarchaeaceae;g__;s__
LMSG_G000006587.1	no	137_1	Polymetallic	78.97	0.93	74.30	1	0	1	17	Medium quality	33176	744545	51.30	29	d__Archaea;p__Micrarchaeota;c__Micrarchaeia;o__Micrarchaeales;f__Micrarchaeaceae;g__;s__
LMSG_G000006588.1	no	137_1	Polymetallic	81.77	1.87	72.43	1	1	1	16	Medium quality	33003	752967	51.30	33	d__Archaea;p__Micrarchaeota;c__Micrarchaeia;o__Micrarchaeales;f__Micrarchaeaceae;g__;s__
LMSG_G000006589.1	no	137_1	Copper	77.10	2.80	63.09	1	1	1	18	Medium quality	31369	694575	51.00	36	d__Archaea;p__Micrarchaeota;c__Micrarchaeia;o__Micrarchaeales;f__Micrarchaeaceae;g__;s__
LMSG_G000006590.1	no	137_1	Polymetallic	80.52	1.87	71.18	1	1	2	18	Medium quality	29630	767260	51.30	42	d__Archaea;p__Micrarchaeota;c__Micrarchaeia;o__Micrarchaeales;f__Micrarchaeaceae;g__;s__

LMSG_G000006591.1	no	137_1	Magnetite	58.64	0.93	53.97	1	0	1	16	Medium quality	36529	526929	51.80	20	d__Archaea;p__Micrarchaeota;c__Micrarchaeia;o__Micrarchaeales;f__Micrarchaeaceae;g__;s__
LMSG_G000006592.1	no	137_1	Magnetite	64.48	0.93	59.81	0	0	1	15	Medium quality	18604	552326	52.80	32	d__Archaea;p__Micrarchaeota;c__Micrarchaeia;o__Micrarchaeales;f__Micrarchaeaceae;g__;s__
LMSG_G000006593.1	no	137_1	Pyrite	79.90	1.87	70.56	1	1	1	16	Medium quality	21855	760618	51.30	41	d__Archaea;p__Micrarchaeota;c__Micrarchaeia;o__Micrarchaeales;f__Micrarchaeaceae;g__;s__
LMSG_G000006594.1	no	30_1	Copper	70.48	0.00	70.48	1	1	1	18	Medium quality	8061	809378	47.90	128	d__Archaea;p__Micrarchaeota;c__Micrarchaeia;o__Micrarchaeales;f__Micrarchaeaceae;g__;s__
LMSG_G000006595.1	no	30_1	Copper	84.26	0.00	84.26	1	1	1	19	Medium quality	24912	929604	47.60	46	d__Archaea;p__Micrarchaeota;c__Micrarchaeia;o__Micrarchaeales;f__Micrarchaeaceae;g__;s__
LMSG_G000006596.1	no	30_1	Copper	85.20	0.00	85.20	1	1	1	20	Medium quality	65127	953706	47.50	27	d__Archaea;p__Micrarchaeota;c__Micrarchaeia;o__Micrarchaeales;f__Micrarchaeaceae;g__;s__
LMSG_G000006597.1	no	30_1	Copper	85.20	0.00	85.20	1	1	2	20	Medium quality	56099	961808	47.50	24	d__Archaea;p__Micrarchaeota;c__Micrarchaeia;o__Micrarchaeales;f__Micrarchaeaceae;g__;s__
LMSG_G000006598.1	no	33_1	Copper	77.18	0.00	77.18	1	2	1	20	Medium quality	29005	723350	29.30	36	d__Archaea;p__Micrarchaeota;c__Micrarchaeia;o__Micrarchaeales;f__Micrarchaeaceae;g__;s__
LMSG_G000006599.1	no	33_1	Lead-Zinc	75.07	0.93	70.40	0	1	1	19	Medium quality	45328	974771	29.80	89	d__Archaea;p__Micrarchaeota;c__Micrarchaeia;o__Micrarchaeales;f__Micrarchaeaceae;g__;s__
LMSG_G000006600.1	no	33_1	Lead-Zinc	72.97	0.00	72.97	1	0	1	19	Medium quality	6523	890374	29.60	188	d__Archaea;p__Micrarchaeota;c__Micrarchaeia;o__Micrarchaeales;f__Micrarchaeaceae;g__;s__
LMSG_G000006601.1	no	33_1	Lead-Zinc	79.28	0.00	79.28	1	2	1	20	Medium quality	20066	878232	29.40	65	d__Archaea;p__Micrarchaeota;c__Micrarchaeia;o__Micrarchaeales;f__Micrarchaeaceae;g__;s__
LMSG_G000006602.1	no	36_1	Lead-Zinc	75.07	0.00	75.07	1	0	0	17	Medium quality	12326	960988	37.20	154	d__Archaea;p__Micrarchaeota;c__Micrarchaeia;o__Micrarchaeales;f__Micrarchaeaceae;g__;s__
LMSG_G000006603.1	no	36_1	Lead-Zinc	54.51	0.00	54.51	1	1	0	17	Medium quality	9421	536372	38.80	92	d__Archaea;p__Micrarchaeota;c__Micrarchaeia;o__Micrarchaeales;f__Micrarchaeaceae;g__;s__
LMSG_G000006604.1	no	36_1	Polymetallic	56.85	0.93	52.18	0	1	0	15	Medium quality	22636	502369	38.30	34	d__Archaea;p__Micrarchaeota;c__Micrarchaeia;o__Micrarchaeales;f__Micrarchaeaceae;g__;s__
LMSG_G000006605.1	no	36_1	Lead-Zinc	76.32	0.00	76.32	1	1	0	17	Medium quality	21716	739492	37.70	45	d__Archaea;p__Micrarchaeota;c__Micrarchaeia;o__Micrarchaeales;f__Micrarchaeaceae;g__;s__
LMSG_G000006606.1	no	36_1	Lead-Zinc	78.19	0.00	78.19	1	1	1	17	Medium quality	9910	843057	37.70	112	d__Archaea;p__Micrarchaeota;c__Micrarchaeia;o__Micrarchaeales;f__Micrarchaeaceae;g__;s__
LMSG_G000006607.1	no	36_1	Pyrite-Copper	77.25	0.93	72.58	1	1	0	19	Medium quality	19862	818833	37.90	104	d__Archaea;p__Micrarchaeota;c__Micrarchaeia;o__Micrarchaeales;f__Micrarchaeaceae;g__;s__
LMSG_G000006608.1	no	36_1	Lead-Zinc	53.89	0.00	53.89	0	2	0	18	Medium quality	19220	567093	38.70	62	d__Archaea;p__Micrarchaeota;c__Micrarchaeia;o__Micrarchaeales;f__Micrarchaeaceae;g__;s__
LMSG_G000006609.1	no	36_1	Arsenic	76.01	0.93	71.34	1	1	1	19	Medium quality	16340	757838	37.80	54	d__Archaea;p__Micrarchaeota;c__Micrarchaeia;o__Micrarchaeales;f__Micrarchaeaceae;g__;s__
LMSG_G000006610.1	no	48_1	Copper	80.06	0.93	75.39	1	1	0	19	Medium quality	28325	913960	50.50	44	d__Archaea;p__Micrarchaeota;c__Micrarchaeia;o__Micrarchaeales;f__Micrarchaeaceae;g__;s__
LMSG_G000006611.1	no	48_1	Lead-Zinc	72.07	3.74	53.38	1	1	1	17	Medium quality	12048	682015	51.10	72	d__Archaea;p__Micrarchaeota;c__Micrarchaeia;o__Micrarchaeales;f__Micrarchaeaceae;g__;s__
LMSG_G000006612.1	no	48_1	Pyrite-Copper	79.43	2.18	68.53	1	0	1	17	Medium quality	22026	911487	50.70	54	d__Archaea;p__Micrarchaeota;c__Micrarchaeia;o__Micrarchaeales;f__Micrarchaeaceae;g__;s__
LMSG_G000006613.1	no	48_1	Pyrite-Copper	71.49	1.87	62.15	1	1	1	12	Medium quality	12747	715974	51.20	86	d__Archaea;p__Micrarchaeota;c__Micrarchaeia;o__Micrarchaeales;f__Micrarchaeaceae;g__;s__
LMSG_G000006614.1	no	48_1	Magnetite	84.11	2.80	70.10	1	2	1	19	Medium quality	41233	1016185	50.00	41	d__Archaea;p__Micrarchaeota;c__Micrarchaeia;o__Micrarchaeales;f__Micrarchaeaceae;g__;s__
LMSG_G000006615.1	no	48_1	Pyrite-Copper	61.41	0.93	56.74	1	0	1	18	Medium quality	13778	727326	51.20	71	d__Archaea;p__Micrarchaeota;c__Micrarchaeia;o__Micrarchaeales;f__Micrarchaeaceae;g__;s__
LMSG_G000006616.1	no	56_1	Polymetallic	75.00	0.00	75.00	1	0	0	20	Medium quality	26005	635288	46.00	39	d__Archaea;p__Micrarchaeota;c__Micrarchaeia;o__Micrarchaeales;f__Micrarchaeaceae;g__;s__
LMSG_G000006617.1	no	56_1	Polymetallic	71.49	0.00	71.49	0	0	0	19	Medium quality	26758	575390	46.10	35	d__Archaea;p__Micrarchaeota;c__Micrarchaeia;o__Micrarchaeales;f__Micrarchaeaceae;g__;s__
LMSG_G000006618.1	no	56_1	Coal	79.90	0.61	76.85	1	1	1	20	Medium quality	49764	768229	45.60	44	d__Archaea;p__Micrarchaeota;c__Micrarchaeia;o__Micrarchaeales;f__Micrarchaeaceae;g__;s__
LMSG_G000006619.1	no	59_1	Polymetallic	81.46	0.00	81.46	1	2	1	20	Medium quality	40534	751643	46.30	41	d__Archaea;p__Micrarchaeota;c__Micrarchaeia;o__Micrarchaeales;f__Micrarchaeaceae;g__;s__
LMSG_G000006620.1	no	59_1	Polymetallic	79.43	0.00	79.43	1	1	1	18	Medium quality	42162	679116	46.10	27	d__Archaea;p__Micrarchaeota;c__Micrarchaeia;o__Micrarchaeales;f__Micrarchaeaceae;g__;s__
LMSG_G000006621.1	no	59_1	Polymetallic	80.52	0.00	80.52	1	1	1	20	Medium quality	38582	677881	46.40	32	d__Archaea;p__Micrarchaeota;c__Micrarchaeia;o__Micrarchaeales;f__Micrarchaeaceae;g__;s__
LMSG_G000006622.1	no	59_1	Magnetite	79.59	1.87	70.25	1	1	1	20	Medium quality	38586	708703	46.20	31	d__Archaea;p__Micrarchaeota;c__Micrarchaeia;o__Micrarchaeales;f__Micrarchaeaceae;g__;s__
LMSG_G000006623.1	no	59_1	Magnetite	79.59	0.00	79.59	1	1	1	20	Medium quality	38677	788790	46.00	70	d__Archaea;p__Micrarchaeota;c__Micrarchaeia;o__Micrarchaeales;f__Micrarchaeaceae;g__;s__
LMSG_G000006624.1	no	59_1	Magnetite	82.39	4.67	59.03	1	2	1	20	Medium quality	21727	800977	46.50	98	d__Archaea;p__Micrarchaeota;c__Micrarchaeia;o__Micrarchaeales;f__Micrarchaeaceae;g__;s__
LMSG_G000006625.1	no	59_1	Magnetite	76.71	0.93	72.04	1	0	2	20	Medium quality	32052	741327	45.90	68	d__Archaea;p__Micrarchaeota;c__Micrarchaeia;o__Micrarchaeales;f__Micrarchaeaceae;g__;s__
LMSG_G000006626.1	no	59_1	Lead-Zinc	81.46	1.71	72.90	1	1	2	19	Medium quality	195520	774979	45.80	37	d__Archaea;p__Micrarchaeota;c__Micrarchaeia;o__Micrarchaeales;f__Micrarchaeaceae;g__;s__
LMSG_G000006627.1	no	59_1	Pyrite	74.92	0.00	74.92	1	2	1	18	Medium quality	16469	590212	46.90	48	d__Archaea;p__Micrarchaeota;c__Micrarchaeia;o__Micrarchaeales;f__Micrarchaeaceae;g__;s__
LMSG_G000006628.1	no	59_1	Copper	71.65	0.00	71.65	1	2	2	20	Medium quality	31071	703490	46.10	61	d__Archaea;p__Micrarchaeota;c__Micrarchaeia;o__Micrarchaeales;f__Micrarchaeaceae;g__;s__
LMSG_G000006629.1	no	59_1	Lead-Zinc	71.57	0.93	66.90	1	1	2	20	Medium quality	41954	616557	46.60	64	d__Archaea;p__Micrarchaeota;c__Micrarchaeia;o__Micrarchaeales;f__Micrarchaeaceae;g__;s__
LMSG_G000006630.1	no	59_1	Lead-Zinc	72.11	0.00	72.11	1	1	1	19	Medium quality	23812	759525	45.60	99	d__Archaea;p__Micrarchaeota;c__Micrarchaeia;o__Micrarchaeales;f__Micrarchaeaceae;g__;s__
LMSG_G000006631.1	no	59_1	Lead-Zinc	76.94	0.93	72.27	0	1	2	20	Medium quality	66360	773123	45.80	56	d__Archaea;p__Micrarchaeota;c__Micrarchaeia;o__Micrarchaeales;f__Micrarchaeaceae;g__;s__
LMSG_G000006632.1	no	59_1	Lead-Zinc	74.92	0.93	70.25	1	1	1	17	Medium quality	50234	617151	46.20	46	d__Archaea;p__Micrarchaeota;c__Micrarchaeia;o__Micrarchaeales;f__Micrarchaeaceae;g__;s__
LMSG_G000006633.1	no	59_1	Pyrite-Copper	82.39	0.93	77.72	1	1	3	20	Medium quality	53987	774623	45.80	44	d__Archaea;p__Micrarchaeota;c__Micrarchaeia;o__Micrarchaeales;f__Micrarchaeaceae;g__;s__
LMSG_G000006634.1	no	59_1	Lead-Zinc	68.38	0.93	63.71	0	1	1	19	Medium quality	42247	608039	46.00	50	d__Archaea;p__Micrarchaeota;c__Micrarchaeia;o__Micrarchaeales;f__Micrarchaeaceae;g__;s__
LMSG_G000006635.1	no	59_1	Pyrite-Copper	73.86	0.93	69.19	1	1	1	20	Medium quality	21407	640468	46.10	42	d__Archaea;p__Micrarchaeota;c__Micrarchaeia;o__Micrarchaeales;f__Micrarchaeaceae;g__;s__
LMSG_G000006636.1	no	59_1	Copper	77.65	0.93	72.98	1	2	1	20	Medium quality	6563	711773	46.30	160	d__Archaea;p__Micrarchaeota;c__Micrarchaeia;o__Micrarchaeales;f__Micrarchaeaceae;g__;s__
LMSG_G000006637.1	no	60_1	Pyrite-Copper	68.69	0.00	68.69	1	1	2	18	Medium quality	25874	609581	51.10	48	d__Archaea;p__Micrarchaeota;c__Micrarchaeia;o__Micrarchaeales;f__Micrarchaeaceae;g__;s__
LMSG_G000006638.1	no	60_1	Lead-Zinc	78.50	1.87	69.16	1	1	0	20	Medium quality	24631	805391	50.90	45	d__Archaea;p__Micrarchaeota;c__Micrarchaeia;o__Micrarchaeales;f__Micrarchaeaceae;g__;s__
LMSG_G000006639.1	no	61_1	Tin-Zinc	69.87	0.93	65.20	1	0	1	18	Medium quality	24800	637627	50.10	44	d__Archaea;p__Micrarchaeota;c__Micrarchaeia;o__Micrarchaeales;f__Micrarchaeaceae;g__;s__
LMSG_G000006640.1	no	61_1	Polymetallic	71.80	2.34	60.12	1	0	1	17	Medium quality	15049	881491	49.40	74	d__Archaea;p__Micrarchaeota;c__Micrarchaeia;o__Micrarchaeales;f__Micrarchaeaceae;g__;s__
LMSG_G000006641.1	no	65_1	Lead-Zinc	71.18	0.00	71.18	0	1	0	18	Medium quality	61944	934656	48.50	18	d__Archaea;p__Micrarchaeota;c__Micrarchaeia;o__Micrarchaeales;f__Micrarchaeaceae;g__;s__
LMSG_G000006642.1	no	66_1	Copper	85.66	2.80	71.65	1	1	1	19	Medium quality	65264	1121381	52.10	37	d__Archaea;p__Micrarchaeota;c__Micrarchaeia;o__Micrarchaeales;f__Micrarchaeaceae;g__;s__
LMSG_G000006643.1	no	66_1	Polymetallic	54.59	0.00	54.59	1	1	1	13	Medium quality	32860	704076	52.70	31	d__Archaea;p__Micrarchaeota;c__Micrarchaeia;o__Micrarchaeales;f__Micrarchaeaceae;g__;s__
LMSG_G000006644.1	no	66_1	Lead-Zinc	66.43	0.93	61.76	1	0	1	19	Medium quality	28846	891387	52.60	43	d__Archaea;p__Micrarchaeota;c__Micrarchaeia;o__Micrarchaeales;f__Micrarchaeaceae;g__;s__
LMSG_G000006645.1	no	66_1	Lead-Zinc	82.43	0.00	82.43	1	1	0	19	Medium quality	19737	981748	52.40	65	d__Archaea;p__Micrarchaeota;c__Micrarchaeia;o__Micrarchaeales;f__Micrarchaeaceae;g__;s__
LMSG_G000006646.1	no	66_1	Lead-Zinc	78.84	4.67	55.48	1	1	1	19	Medium quality	24326	1021345	52.00	62	d__Archaea;p__Micrarchaeota;c__Micrarchaeia;o__Micrarchaeales;f__Micrarchaeaceae;g__;s__
LMSG_G000006647.1	no	66_1	Pyrite-Copper	81.93	0.14	81.22	1	1	0	16	Medium quality	15381	946314	52.90	111	d__Archaea;p__Micrarchaeota;c__Micrarchaeia;o__Micrarchaeales;f__Micrarchaeaceae;g__;s__
LMSG_G000006648.1	no	66_1	Lead-Zinc	61.52	0.00	61.52	1	1	1	19	Medium quality	31675	784525	52.20	34	d__Archaea;p__Micrarchaeota;c__Micrarchaeia;o__Micrarchaeales;f__Micrarchaeaceae;g__;s__
LMSG_G000006649.1	no	66_1	Magnetite	51.09	0.00	51.09	0	2	0	13	Medium quality	49736	557881	53.20	15	d__Archaea;p__Micrarchaeota;c__Micrarchaeia;o__Micrarchaeales;f__Micrarchaeaceae;g__;s__
LMSG_G000006650.1	no	66_1	Magnetite	52.75	0.00	52.75	1	2	2	15	Medium quality	53569	672034	51.60	23	d__Archaea;p__Micrarchaeota;c__Micrarchaeia;o__Micrarchaeales;f__Micrarchaeaceae;g__;s__
LMSG_G000006651.1	no	66_1	Magnetite	58.65	1.44	51.47	1	1	1	19	Medium quality	22945	931755	51.60	69	d__Archaea;p__Micrarchaeota;c__Micrarchaeia;o__Micrarchaeales;f__Micrarchaeaceae;g__;s__
LMSG_G000006652.1	no	68_1	Copper	82.24	0.00	82.24	1	1	1	20	Medium quality	30382	1120571	56.60	55	d__Archaea;p__Micrarchaeota;c__Micrarchaeia;o__Micrarchaeales;f__Micrarchaeaceae;g__;s__
LMSG_G000006653.1	no	68_1	Copper	78.50	0.00	78.50	1	1	1	19	Medium quality	77184	1101147	56.70	27	d__Archaea;p__Micrarchaeota;c__Micrarchaeia;o__Micrarchaeales;f__Micrarchaeaceae;g__;s__
LMSG_G000006654.1	no	68_1	Polymetallic	83.17	0.00	83.17	1	0	1	19	Medium quality	15192	1036946	56.90	80	d__Archaea;p__Micrarchaeota;c__Micrarchaeia;o__Micrarchaeales;f__Micrarchaeaceae;g__;s__
LMSG_G000006655.1	no	68_1	Polymetallic	60.63	0.93	55.96	0	1	1	13	Medium quality	5680	615113	57.80	118	d__Archaea;p__Micrarchaeota;c__Micrarchaeia;o__Micrarchaeales;f__Micrarchaeaceae;g__;s__
LMSG_G000006656.1	no	68_1	Polymetallic	81.30	0.00	81.30	1	1	1	19	Medium quality	22159	983705	57.20	58	d__Archaea;p__Micrarchaeota;c__Micrarchaeia;o__Micrarchaeales;f__Micrarchaeaceae;g__;s__
LMSG_G000006657.1	no	68_1	Lead-Zinc	83.17	0.93	78.50	1	1	1	19	Medium quality	22047	1069358	56.60	61	d__Archaea;p__Micrarchaeota;c__Micrarchaeia;o__Micrarchaeales;f__Micrarchaeaceae;g__;s__
LMSG_G000006658.1	no	68_1	Lead-Zinc	72.89	0.00	72.89	1	1	2	19	Medium quality	20785	961341	57.20	52	d__Archaea;p__Micrarchaeota;c__Micrarchaeia;o__Micrarchaeales;f__Micrarchaeaceae;g__;s__
LMSG_G000006659.1	no	68_1	Lead-Zinc	79.29	0.07	78.94	1	1	1	18	Medium quality	20868	1047591	56.80	66	d__Archaea;p__Micrarchaeota;c__Micrarchaeia;o__Micrarchaeales;f__Micrarchaeaceae;g__;s__
LMSG_G000006660.1	no	68_1	Pyrite-Copper	70.71	1.01	65.68	0	0	2	13	Medium quality	6262	771546	57.70	141	d__Archaea;p__Micrarchaeota;c__Micrarchaeia;o__Micrarchaeales;f__Micrarchaeaceae;g__;s__
LMSG_G000006661.1	no	68_1	Lead-Zinc	79.12	0.93	74.45	1	0	0	17	Medium quality	13666	1033854	57.10	93	d__Archaea;p__Micrarchaeota;c__Micrarchaeia;o__Micrarchaeales;f__Micrarchaeaceae;g__;s__
LMSG_G000006662.1	no	68_1	Magnetite	71.89	0.00	71.89	1	1	1	19	Medium quality	20754	916783	57.10	56	d__Archaea;p__Micrarchaeota;c__Micrarchaeia;o__Micrarchaeales;f__Micrarchaeaceae;g__;s__
LMSG_G000006663.1	no	68_1	Magnetite	52.95	0.00	52.95	1	0	0	13	Medium quality	17097	637571	56.80	48	d__Archaea;p__Micrarchaeota;c__Micrarchaeia;o__Micrarchaeales;f__Micrarchaeaceae;g__;s__
LMSG_G000006664.1	no	68_1	Magnetite	64.95	1.87	55.61	2	0	1	16	Medium quality	10585	776262	57.20	83	d__Archaea;p__Micrarchaeota;c__Micrarchaeia;o__Micrarchaeales;f__Micrarchaeaceae;g__;s__
LMSG_G000006665.1	no	68_1	Copper	76.63	0.00	76.63	1	1	0	17	Medium quality	18510	930520	56.80	65	d__Archaea;p__Micrarchaeota;c__Micrarchaeia;o__Micrarchaeales;f__Micrarchaeaceae;g__;s__
LMSG_G000006666.1	no	70_1	Lead-Zinc	70.89	0.00	70.89	1	0	1	18	Medium quality	17146	673384	46.60	61	d__Archaea;p__Micrarchaeota;c__Micrarchaeia;o__Micrarchaeales;f__Micrarchaeaceae;g__;s__
LMSG_G000006667.1	no	72_1	Copper	82.63	0.00	82.63	1	3	1	16	Medium quality	7522	714615	49.10	114	d__Archaea;p__Micrarchaeota;c__Micrarchaeia;o__Micrarchaeales;f__Micrarchaeaceae;g__;s__

LMSG_G000006668.1	no	72_1	Copper	82.71	0.00	82.71	1	1	1	18	Medium quality	22735	850723	48.80	62	d__Archaea;p__Micrarchaeota;c__Micrarchaeia;o__Micrarchaeales;f__Micrarchaeaceae;g__;s__
LMSG_G000006669.1	no	72_1	Copper	84.57	0.00	84.57	1	3	1	19	Medium quality	23266	863824	48.60	65	d__Archaea;p__Micrarchaeota;c__Micrarchaeia;o__Micrarchaeales;f__Micrarchaeaceae;g__;s__
LMSG_G000006670.1	no	73_1	Polymetallic	63.62	0.93	58.95	1	1	1	16	Medium quality	8039	603104	52.80	84	d__Archaea;p__Micrarchaeota;c__Micrarchaeia;o__Micrarchaeales;f__Micrarchaeaceae;g__;s__
LMSG_G000006671.1	no	73_1	Magnetite	73.05	1.87	63.71	1	2	0	17	Medium quality	23789	831987	52.20	48	d__Archaea;p__Micrarchaeota;c__Micrarchaeia;o__Micrarchaeales;f__Micrarchaeaceae;g__;s__
LMSG_G000006672.1	no	74_1	Magnetite	78.97	2.80	64.96	1	2	1	18	Medium quality	54847	997307	49.50	25	d__Archaea;p__Micrarchaeota;c__Micrarchaeia;o__Micrarchaeales;f__Micrarchaeaceae;g__;s__
LMSG_G000006673.1	no	76_1	Lead-Zinc	63.55	1.40	56.55	0	2	0	17	Medium quality	17060	472921	49.50	62	d__Archaea;p__Micrarchaeota;c__Micrarchaeia;o__Micrarchaeales;f__Micrarchaeaceae;g__;s__
LMSG_G000006674.1	no	78_1	Lead-Zinc	71.26	4.10	50.78	1	2	2	17	Medium quality	7262	737373	47.60	115	d__Archaea;p__Micrarchaeota;c__Micrarchaeia;o__Micrarchaeales;f__Micrarchaeaceae;g__;s__
LMSG_G000006675.1	no	79_1	Lead-Zinc	57.16	0.00	57.16	0	1	1	19	Medium quality	77601	613425	48.40	9	d__Archaea;p__Micrarchaeota;c__Micrarchaeia;o__Micrarchaeales;f__Micrarchaeaceae;g__;s__
LMSG_G000006676.1	no	79_1	Lead-Zinc	73.98	2.80	59.97	0	1	2	16	Medium quality	36360	626607	48.70	21	d__Archaea;p__Micrarchaeota;c__Micrarchaeia;o__Micrarchaeales;f__Micrarchaeaceae;g__;s__
LMSG_G000006677.1	no	79_1	Lead-Zinc	54.36	0.00	54.36	0	1	2	18	Medium quality	55170	516905	48.80	15	d__Archaea;p__Micrarchaeota;c__Micrarchaeia;o__Micrarchaeales;f__Micrarchaeaceae;g__;s__
LMSG_G000006678.1	no	79_1	Pyrite-Copper	54.36	0.00	54.36	0	1	1	18	Medium quality	169507	532400	48.80	13	d__Archaea;p__Micrarchaeota;c__Micrarchaeia;o__Micrarchaeales;f__Micrarchaeaceae;g__;s__
LMSG_G000006679.1	no	80_1	Lead-Zinc	62.14	0.00	62.14	1	1	2	14	Medium quality	227826	385348	51.70	3	d__Archaea;p__Micrarchaeota;c__Micrarchaeia;o__Micrarchaeales;f__Micrarchaeaceae;g__;s__
LMSG_G000006680.1	no	80_1	Lead-Zinc	79.90	4.19	58.97	1	1	2	18	Medium quality	42898	757510	51.50	57	d__Archaea;p__Micrarchaeota;c__Micrarchaeia;o__Micrarchaeales;f__Micrarchaeaceae;g__;s__
LMSG_G000006681.1	no	80_1	Copper	70.56	0.00	70.56	1	2	1	15	Medium quality	113064	543484	52.00	9	d__Archaea;p__Micrarchaeota;c__Micrarchaeia;o__Micrarchaeales;f__Micrarchaeaceae;g__;s__
LMSG_G000006682.1	no	80_1	Copper	70.09	0.00	70.09	1	1	1	15	Medium quality	69542	525670	52.20	13	d__Archaea;p__Micrarchaeota;c__Micrarchaeia;o__Micrarchaeales;f__Micrarchaeaceae;g__;s__
LMSG_G000006683.1	no	80_1	Copper	52.80	0.00	52.80	0	2	1	15	Medium quality	67292	416299	52.20	11	d__Archaea;p__Micrarchaeota;c__Micrarchaeia;o__Micrarchaeales;f__Micrarchaeaceae;g__;s__
LMSG_G000006684.1	no	85_1	Lead-Zinc	81.46	0.93	76.79	1	1	1	19	Medium quality	470891	909096	46.60	2	d__Archaea;p__Micrarchaeota;c__Micrarchaeia;o__Micrarchaeales;f__Micrarchaeaceae;g__;s__
LMSG_G000006685.1	no	85_1	Copper	84.57	2.34	72.89	0	1	1	19	Medium quality	505381	902982	46.70	28	d__Archaea;p__Micrarchaeota;c__Micrarchaeia;o__Micrarchaeales;f__Micrarchaeaceae;g__;s__
LMSG_G000006686.1	no	85_1	Copper	84.57	0.00	84.57	1	3	2	19	Medium quality	222988	955919	46.20	14	d__Archaea;p__Micrarchaeota;c__Micrarchaeia;o__Micrarchaeales;f__Micrarchaeaceae;g__;s__
LMSG_G000006687.1	no	85_1	Copper	75.01	0.31	73.46	1	0	0	14	Medium quality	7331	559763	48.10	97	d__Archaea;p__Micrarchaeota;c__Micrarchaeia;o__Micrarchaeales;f__Micrarchaeaceae;g__;s__
LMSG_G000006688.1	no	85_1	Copper	84.57	0.00	84.57	1	2	1	19	Medium quality	603026	899512	46.40	5	d__Archaea;p__Micrarchaeota;c__Micrarchaeia;o__Micrarchaeales;f__Micrarchaeaceae;g__;s__
LMSG_G000006689.1	no	85_1	Lead-Zinc	73.52	0.93	68.85	1	1	1	19	Medium quality	92062	831477	46.70	14	d__Archaea;p__Micrarchaeota;c__Micrarchaeia;o__Micrarchaeales;f__Micrarchaeaceae;g__;s__
LMSG_G000006690.1	no	85_1	Lead-Zinc	57.71	0.00	57.71	2	1	0	14	Medium quality	7440	501166	47.60	91	d__Archaea;p__Micrarchaeota;c__Micrarchaeia;o__Micrarchaeales;f__Micrarchaeaceae;g__;s__
LMSG_G000006691.1	no	85_1	Lead-Zinc	80.52	0.00	80.52	1	1	1	19	Medium quality	41049	708612	47.40	25	d__Archaea;p__Micrarchaeota;c__Micrarchaeia;o__Micrarchaeales;f__Micrarchaeaceae;g__;s__
LMSG_G000006692.1	no	85_1	Pyrite-Copper	84.26	0.93	79.59	1	1	1	18	Medium quality	45121	874495	47.00	37	d__Archaea;p__Micrarchaeota;c__Micrarchaeia;o__Micrarchaeales;f__Micrarchaeaceae;g__;s__
LMSG_G000006693.1	no	85_1	Lead-Zinc	79.59	0.93	74.92	1	1	1	19	Medium quality	174798	718094	46.90	8	d__Archaea;p__Micrarchaeota;c__Micrarchaeia;o__Micrarchaeales;f__Micrarchaeaceae;g__;s__
LMSG_G000006694.1	no	86_1	Copper	59.34	0.00	59.34	0	0	1	17	Medium quality	27526	611070	49.60	45	d__Archaea;p__Micrarchaeota;c__Micrarchaeia;o__Micrarchaeales;f__Micrarchaeaceae;g__;s__
LMSG_G000006695.1	no	86_1	Copper	69.39	0.93	64.72	0	0	0	15	Medium quality	13837	623522	49.40	59	d__Archaea;p__Micrarchaeota;c__Micrarchaeia;o__Micrarchaeales;f__Micrarchaeaceae;g__;s__
LMSG_G000006696.1	no	86_1	Copper	61.91	2.34	50.23	1	0	0	16	Medium quality	15513	547859	49.50	52	d__Archaea;p__Micrarchaeota;c__Micrarchaeia;o__Micrarchaeales;f__Micrarchaeaceae;g__;s__
LMSG_G000006697.1	no	86_1	Pyrite-Copper	61.37	0.00	61.37	1	0	1	15	Medium quality	15722	633996	49.40	66	d__Archaea;p__Micrarchaeota;c__Micrarchaeia;o__Micrarchaeales;f__Micrarchaeaceae;g__;s__
LMSG_G000006698.1	no	86_1	Pyrite-Copper	64.49	0.00	64.49	1	2	2	17	Medium quality	15162	638158	49.40	61	d__Archaea;p__Micrarchaeota;c__Micrarchaeia;o__Micrarchaeales;f__Micrarchaeaceae;g__;s__
LMSG_G000006699.1	no	86_1	Pyrite-Copper	63.00	0.00	63.00	0	0	1	16	Medium quality	15763	581821	49.50	54	d__Archaea;p__Micrarchaeota;c__Micrarchaeia;o__Micrarchaeales;f__Micrarchaeaceae;g__;s__
LMSG_G000006700.1	no	86_2	Lead-Zinc	80.37	1.87	71.03	1	2	1	18	Medium quality	18750	772308	48.70	56	d__Archaea;p__Micrarchaeota;c__Micrarchaeia;o__Micrarchaeales;f__Micrarchaeaceae;g__;s__
LMSG_G000006701.1	no	86_2	Lead-Zinc	72.27	1.87	62.93	1	1	1	19	Medium quality	29027	656022	48.90	56	d__Archaea;p__Micrarchaeota;c__Micrarchaeia;o__Micrarchaeales;f__Micrarchaeaceae;g__;s__
LMSG_G000006702.1	no	86_2	Lead-Zinc	75.70	0.93	71.03	1	2	1	16	Medium quality	15435	714949	48.90	51	d__Archaea;p__Micrarchaeota;c__Micrarchaeia;o__Micrarchaeales;f__Micrarchaeaceae;g__;s__
LMSG_G000006703.1	no	86_2	Lead-Zinc	76.49	0.93	71.82	1	2	1	16	Medium quality	13028	665268	48.90	59	d__Archaea;p__Micrarchaeota;c__Micrarchaeia;o__Micrarchaeales;f__Micrarchaeaceae;g__;s__
LMSG_G000006704.1	no	86_3	Lead-Zinc	69.85	1.87	60.51	0	1	1	16	Medium quality	10852	697856	48.70	83	d__Archaea;p__Micrarchaeota;c__Micrarchaeia;o__Micrarchaeales;f__Micrarchaeaceae;g__;s__
LMSG_G000006705.1	no	86_3	Copper	71.33	0.00	71.33	1	1	2	17	Medium quality	46515	650785	48.90	21	d__Archaea;p__Micrarchaeota;c__Micrarchaeia;o__Micrarchaeales;f__Micrarchaeaceae;g__;s__
LMSG_G000006706.1	no	86_3	Copper	63.92	0.00	63.92	1	2	1	18	Medium quality	16120	596502	48.80	64	d__Archaea;p__Micrarchaeota;c__Micrarchaeia;o__Micrarchaeales;f__Micrarchaeaceae;g__;s__
LMSG_G000006707.1	no	86_3	Copper	59.21	1.40	52.21	2	0	1	15	Medium quality	10872	760825	48.70	107	d__Archaea;p__Micrarchaeota;c__Micrarchaeia;o__Micrarchaeales;f__Micrarchaeaceae;g__;s__
LMSG_G000006708.1	no	86_3	Copper	81.30	0.93	76.63	1	2	2	19	Medium quality	35014	824494	48.30	36	d__Archaea;p__Micrarchaeota;c__Micrarchaeia;o__Micrarchaeales;f__Micrarchaeaceae;g__;s__
LMSG_G000006709.1	no	86_3	Copper	82.24	0.93	77.57	1	2	1	19	Medium quality	130248	859890	48.20	25	d__Archaea;p__Micrarchaeota;c__Micrarchaeia;o__Micrarchaeales;f__Micrarchaeaceae;g__;s__
LMSG_G000006710.1	no	86_3	Polymetallic	50.66	0.00	50.66	1	1	1	17	Medium quality	62823	575668	48.60	41	d__Archaea;p__Micrarchaeota;c__Micrarchaeia;o__Micrarchaeales;f__Micrarchaeaceae;g__;s__
LMSG_G000006711.1	no	86_3	Copper	57.71	0.93	53.04	1	0	2	17	Medium quality	20472	584079	48.50	32	d__Archaea;p__Micrarchaeota;c__Micrarchaeia;o__Micrarchaeales;f__Micrarchaeaceae;g__;s__
LMSG_G000006712.1	no	86_3	Copper	59.96	0.93	55.29	1	1	0	17	Medium quality	30246	609114	48.40	27	d__Archaea;p__Micrarchaeota;c__Micrarchaeia;o__Micrarchaeales;f__Micrarchaeaceae;g__;s__
LMSG_G000006713.1	no	86_3	Pyrite	84.11	0.93	79.44	1	1	1	19	Medium quality	297592	893027	47.90	3	d__Archaea;p__Micrarchaeota;c__Micrarchaeia;o__Micrarchaeales;f__Micrarchaeaceae;g__;s__
LMSG_G000006714.1	no	86_3	Pyrite	84.11	0.93	79.44	1	2	1	19	Medium quality	896442	898142	47.90	3	d__Archaea;p__Micrarchaeota;c__Micrarchaeia;o__Micrarchaeales;f__Micrarchaeaceae;g__;s__
LMSG_G000006715.1	no	86_3	Pyrite	84.11	0.93	79.44	1	1	1	19	Medium quality	223354	882115	48.20	6	d__Archaea;p__Micrarchaeota;c__Micrarchaeia;o__Micrarchaeales;f__Micrarchaeaceae;g__;s__
LMSG_G000006716.1	no	86_3	Pyrite	84.11	0.93	79.44	1	1	1	19	Medium quality	116857	897584	47.90	9	d__Archaea;p__Micrarchaeota;c__Micrarchaeia;o__Micrarchaeales;f__Micrarchaeaceae;g__;s__
LMSG_G000006717.1	no	40_1	Lead-Zinc	80.37	0.07	80.02	1	1	1	19	Medium quality	10960	1007736	29.20	143	d__Archaea;p__Micrarchaeota;c__Micrarchaeia;o__Micrarchaeales;f__Micrarchaeaceae;g__Mancarchaeum;s__
LMSG_G000006718.1	no	40_1	Lead-Zinc	74.61	1.01	69.58	2	1	2	18	Medium quality	4083	1353697	29.70	388	d__Archaea;p__Micrarchaeota;c__Micrarchaeia;o__Micrarchaeales;f__Micrarchaeaceae;g__Mancarchaeum;s__
LMSG_G000006719.1	no	40_1	Lead-Zinc	82.24	0.00	82.24	1	1	1	19	Medium quality	84380	789875	29.50	41	d__Archaea;p__Micrarchaeota;c__Micrarchaeia;o__Micrarchaeales;f__Micrarchaeaceae;g__Mancarchaeum;s__
LMSG_G000006720.1	no	40_1	Lead-Zinc	72.74	0.00	72.74	1	1	1	18	Medium quality	4822	903326	29.00	253	d__Archaea;p__Micrarchaeota;c__Micrarchaeia;o__Micrarchaeales;f__Micrarchaeaceae;g__Mancarchaeum;s__
LMSG_G000006721.1	no	40_1	Polymetallic	80.06	0.00	80.06	1	1	1	18	Medium quality	12198	1055979	30.00	189	d__Archaea;p__Micrarchaeota;c__Micrarchaeia;o__Micrarchaeales;f__Micrarchaeaceae;g__Mancarchaeum;s__
LMSG_G000006722.1	no	40_1	Tin-Zinc	71.02	0.00	71.02	1	1	1	18	Medium quality	11378	831091	29.70	157	d__Archaea;p__Micrarchaeota;c__Micrarchaeia;o__Micrarchaeales;f__Micrarchaeaceae;g__Mancarchaeum;s__
LMSG_G000006723.1	no	40_1	Copper	82.42	0.00	82.42	1	2	1	19	Medium quality	36927	1067064	29.70	120	d__Archaea;p__Micrarchaeota;c__Micrarchaeia;o__Micrarchaeales;f__Micrarchaeaceae;g__Mancarchaeum;s__
LMSG_G000006724.1	no	40_1	Copper	77.10	0.93	72.43	0	1	1	17	Medium quality	8536	697768	29.70	125	d__Archaea;p__Micrarchaeota;c__Micrarchaeia;o__Micrarchaeales;f__Micrarchaeaceae;g__Mancarchaeum;s__
LMSG_G000006725.1	no	40_1	Copper	83.17	0.47	80.84	1	1	1	19	Medium quality	11550	1030747	29.20	156	d__Archaea;p__Micrarchaeota;c__Micrarchaeia;o__Micrarchaeales;f__Micrarchaeaceae;g__Mancarchaeum;s__
LMSG_G000006726.1	no	40_1	Copper	68.22	0.00	68.22	0	1	1	19	Medium quality	28260	793475	29.20	59	d__Archaea;p__Micrarchaeota;c__Micrarchaeia;o__Micrarchaeales;f__Micrarchaeaceae;g__Mancarchaeum;s__
LMSG_G000006727.1	no	40_1	Copper	82.24	0.00	82.24	1	1	1	20	Medium quality	52330	760774	29.30	40	d__Archaea;p__Micrarchaeota;c__Micrarchaeia;o__Micrarchaeales;f__Micrarchaeaceae;g__Mancarchaeum;s__
LMSG_G000006728.1	no	40_1	Polymetallic	82.24	0.00	82.24	1	1	1	20	Medium quality	48522	841782	29.20	33	d__Archaea;p__Micrarchaeota;c__Micrarchaeia;o__Micrarchaeales;f__Micrarchaeaceae;g__Mancarchaeum;s__
LMSG_G000006729.1	no	40_1	Polymetallic	83.17	0.93	78.50	1	1	1	20	Medium quality	24237	874556	29.00	64	d__Archaea;p__Micrarchaeota;c__Micrarchaeia;o__Micrarchaeales;f__Micrarchaeaceae;g__Mancarchaeum;s__
LMSG_G000006730.1	no	40_1	Copper	55.91	0.00	55.91	1	1	1	16	Medium quality	21944	785370	29.20	84	d__Archaea;p__Micrarchaeota;c__Micrarchaeia;o__Micrarchaeales;f__Micrarchaeaceae;g__Mancarchaeum;s__
LMSG_G000006731.1	no	40_1	Copper	62.61	0.00	62.61	0	1	1	19	Medium quality	26881	627005	29.40	45	d__Archaea;p__Micrarchaeota;c__Micrarchaeia;o__Micrarchaeales;f__Micrarchaeaceae;g__Mancarchaeum;s__
LMSG_G000006732.1	no	40_1	Copper	74.14	0.00	74.14	0	1	1	19	Medium quality	14872	996486	28.90	113	d__Archaea;p__Micrarchaeota;c__Micrarchaeia;o__Micrarchaeales;f__Micrarchaeaceae;g__Mancarchaeum;s__
LMSG_G000006733.1	no	40_1	Copper	61.48	0.00	61.48	0	1	0	17	Medium quality	7596	960313	28.50	168	d__Archaea;p__Micrarchaeota;c__Micrarchaeia;o__Micrarchaeales;f__Micrarchaeaceae;g__Mancarchaeum;s__
LMSG_G000006734.1	no	41_1	Lead-Zinc	79.59	0.00	79.59	1	2	2	17	Medium quality	26857	810854	35.90	59	d__Archaea;p__Micrarchaeota;c__Micrarchaeia;o__Micrarchaeales;f__Micrarchaeaceae;g__Mancarchaeum;s__
LMSG_G000006735.1	no	42_1	Copper	79.59	0.00	79.59	1	1	1	18	Medium quality	197197	806617	39.20	38	d__Archaea;p__Micrarchaeota;c__Micrarchaeia;o__Micrarchaeales;f__Micrarchaeaceae;g__Mancarchaeum;s__Mancarchaeum acidiphilum
LMSG_G000006736.1	no	138_1	Copper	76.09	0.93	71.42	1	1	1	20	Medium quality	27579	706218	43.00	46	d__Archaea;p__Micrarchaeota;c__Micrarchaeia;o__Micrarchaeales;f__Micrarchaeaceae;g__Micrarchaeum;s__
LMSG_G000006737.1	no	138_1	Lead-Zinc	80.92	1.87	71.58	1	1	1	19	Medium quality	49378	787242	43.30	52	d__Archaea;p__Micrarchaeota;c__Micrarchaeia;o__Micrarchaeales;f__Micrarchaeaceae;g__Micrarchaeum;s__
LMSG_G000006738.1	no	138_1	Lead-Zinc	79.12	0.93	74.45	1	1	1	19	Medium quality	43454	705988	43.20	28	d__Archaea;p__Micrarchaeota;c__Micrarchaeia;o__Micrarchaeales;f__Micrarchaeaceae;g__Micrarchaeum;s__
LMSG_G000006739.1	no	138_1	Lead-Zinc	78.12	0.93	73.45	0	1	2	19	Medium quality	20158	739870	43.40	65	d__Archaea;p__Micrarchaeota;c__Micrarchaeia;o__Micrarchaeales;f__Micrarchaeaceae;g__Micrarchaeum;s__
LMSG_G000006740.1	no	138_1	Polymetallic	66.04	0.00	66.04	1	1	1	19	Medium quality	33054	613325	43.20	28	d__Archaea;p__Micrarchaeota;c__Micrarchaeia;o__Micrarchaeales;f__Micrarchaeaceae;g__Micrarchaeum;s__
LMSG_G000006741.1	no	138_1	Polymetallic	73.44	0.93	68.77	1	0	3	19	Medium quality	38106	740335	42.90	39	d__Archaea;p__Micrarchaeota;c__Micrarchaeia;o__Micrarchaeales;f__Micrarchaeaceae;g__Micrarchaeum;s__
LMSG_G000006742.1	no	138_1	Polymetallic	72.51	1.87	63.17	1	1	2	18	Medium quality	39800	669083	43.00	34	d__Archaea;p__Micrarchaeota;c__Micrarchaeia;o__Micrarchaeales;f__Micrarchaeaceae;g__Micrarchaeum;s__
LMSG_G000006743.1	no	138_1	Polymetallic	69.31	0.93	64.64	1	1	2	16	Medium quality	25921	588442	43.20	34	d__Archaea;p__Micrarchaeota;c__Micrarchaeia;o__Micrarchaeales;f__Micrarchaeaceae;g__Micrarchaeum;s__

LMSG_G000006744.1	no	138_1	Tin-Zinc	82.79	2.80	68.78	1	1	1	19	Medium quality	94689	838597	42.60	28	d__Archaea;p__Micrarchaeota;c__Micrarchaeia;o__Micrarchaeales;f__Micrarchaeaceae;g__Micrarchaeum;s__
LMSG_G000006745.1	no	138_1	Tin-Zinc	81.85	2.80	67.84	1	0	1	19	Medium quality	80248	820926	42.60	34	d__Archaea;p__Micrarchaeota;c__Micrarchaeia;o__Micrarchaeales;f__Micrarchaeaceae;g__Micrarchaeum;s__
LMSG_G000006746.1	no	138_1	Polymetallic	73.44	3.97	53.59	1	1	2	18	Medium quality	35111	691929	42.70	32	d__Archaea;p__Micrarchaeota;c__Micrarchaeia;o__Micrarchaeales;f__Micrarchaeaceae;g__Micrarchaeum;s__
LMSG_G000006747.1	no	138_1	Copper	71.24	0.93	66.57	1	0	0	19	Medium quality	17016	620558	43.10	48	d__Archaea;p__Micrarchaeota;c__Micrarchaeia;o__Micrarchaeales;f__Micrarchaeaceae;g__Micrarchaeum;s__
LMSG_G000006748.1	no	138_1	Copper	82.79	1.87	73.45	1	2	2	20	Medium quality	40907	850060	42.70	37	d__Archaea;p__Micrarchaeota;c__Micrarchaeia;o__Micrarchaeales;f__Micrarchaeaceae;g__Micrarchaeum;s__
LMSG_G000006749.1	no	138_1	Lead-Zinc	80.92	3.74	62.23	0	2	1	20	Medium quality	38593	822797	42.80	29	d__Archaea;p__Micrarchaeota;c__Micrarchaeia;o__Micrarchaeales;f__Micrarchaeaceae;g__Micrarchaeum;s__
LMSG_G000006750.1	no	138_1	Lead-Zinc	79.05	0.93	74.38	1	1	1	20	Medium quality	34099	765899	42.80	34	d__Archaea;p__Micrarchaeota;c__Micrarchaeia;o__Micrarchaeales;f__Micrarchaeaceae;g__Micrarchaeum;s__
LMSG_G000006751.1	no	138_1	Pyrite-Copper	71.65	0.00	71.65	0	1	1	16	Medium quality	135595	548435	43.40	25	d__Archaea;p__Micrarchaeota;c__Micrarchaeia;o__Micrarchaeales;f__Micrarchaeaceae;g__Micrarchaeum;s__
LMSG_G000006752.1	no	138_1	Lead-Zinc	63.23	0.93	58.56	0	1	1	16	Medium quality	34384	468121	43.60	19	d__Archaea;p__Micrarchaeota;c__Micrarchaeia;o__Micrarchaeales;f__Micrarchaeaceae;g__Micrarchaeum;s__
LMSG_G000006753.1	no	138_1	Arsenic	82.79	1.87	73.45	1	1	1	20	Medium quality	41158	850149	42.70	34	d__Archaea;p__Micrarchaeota;c__Micrarchaeia;o__Micrarchaeales;f__Micrarchaeaceae;g__Micrarchaeum;s__
LMSG_G000006754.1	no	139_1	Pyrite	72.81	0.00	72.81	0	1	1	20	Medium quality	7340	803799	43.50	157	d__Archaea;p__Micrarchaeota;c__Micrarchaeia;o__Micrarchaeales;f__Micrarchaeaceae;g__Micrarchaeum;s__
LMSG_G000006755.1	no	139_1	Pyrite-Copper	75.70	0.00	75.70	2	1	1	20	Medium quality	77909	695310	43.10	44	d__Archaea;p__Micrarchaeota;c__Micrarchaeia;o__Micrarchaeales;f__Micrarchaeaceae;g__Micrarchaeum;s__
LMSG_G000006756.1	no	56_1	Copper	78.97	0.00	78.97	1	1	0	20	Medium quality	90752	728307	45.70	30	d__Archaea;p__Micrarchaeota;c__Micrarchaeia;o__Micrarchaeales;f__Micrarchaeaceae;g__Micrarchaeum;s__
LMSG_G000006757.1	no	56_1	Polymetallic	61.91	0.00	61.91	0	1	1	19	Medium quality	18858	482811	46.50	32	d__Archaea;p__Micrarchaeota;c__Micrarchaeia;o__Micrarchaeales;f__Micrarchaeaceae;g__Micrarchaeum;s__
LMSG_G000006758.1	no	56_1	Polymetallic	70.56	0.00	70.56	0	0	1	18	Medium quality	25222	576078	46.00	33	d__Archaea;p__Micrarchaeota;c__Micrarchaeia;o__Micrarchaeales;f__Micrarchaeaceae;g__Micrarchaeum;s__
LMSG_G000006759.1	no	56_1	Tin-Zinc	79.90	0.00	79.90	1	1	1	20	Medium quality	59377	727838	45.90	30	d__Archaea;p__Micrarchaeota;c__Micrarchaeia;o__Micrarchaeales;f__Micrarchaeaceae;g__Micrarchaeum;s__
LMSG_G000006760.1	no	56_1	Tin-Zinc	80.84	0.00	80.84	1	0	1	20	Medium quality	45979	729975	45.90	32	d__Archaea;p__Micrarchaeota;c__Micrarchaeia;o__Micrarchaeales;f__Micrarchaeaceae;g__Micrarchaeum;s__
LMSG_G000006761.1	no	56_1	Polymetallic	67.99	0.00	67.99	0	0	0	20	Medium quality	26713	590103	46.10	40	d__Archaea;p__Micrarchaeota;c__Micrarchaeia;o__Micrarchaeales;f__Micrarchaeaceae;g__Micrarchaeum;s__
LMSG_G000006762.1	no	56_1	Polymetallic	79.20	0.00	79.20	1	1	1	19	Medium quality	21609	755849	45.70	51	d__Archaea;p__Micrarchaeota;c__Micrarchaeia;o__Micrarchaeales;f__Micrarchaeaceae;g__Micrarchaeum;s__
LMSG_G000006763.1	no	56_1	Coal	80.37	0.00	80.37	1	1	2	20	Medium quality	37507	733938	46.00	40	d__Archaea;p__Micrarchaeota;c__Micrarchaeia;o__Micrarchaeales;f__Micrarchaeaceae;g__Micrarchaeum;s__
LMSG_G000006764.1	no	56_1	Coal	80.14	0.00	80.14	1	1	1	20	Medium quality	17936	783199	45.70	60	d__Archaea;p__Micrarchaeota;c__Micrarchaeia;o__Micrarchaeales;f__Micrarchaeaceae;g__Micrarchaeum;s__
LMSG_G000006765.1	no	59_1	Polymetallic	55.55	0.00	55.55	1	0	1	12	Medium quality	1919	456412	46.70	252	d__Archaea;p__Micrarchaeota;c__Micrarchaeia;o__Micrarchaeales;f__Micrarchaeaceae;g__Micrarchaeum;s__
LMSG_G000006766.1	no	59_1	Magnetite	73.98	0.93	69.31	1	1	1	20	Medium quality	28083	727662	46.20	78	d__Archaea;p__Micrarchaeota;c__Micrarchaeia;o__Micrarchaeales;f__Micrarchaeaceae;g__Micrarchaeum;s__
LMSG_G000006767.1	no	88_1	Lead-Zinc	66.35	0.00	66.35	2	1	0	17	Medium quality	16174	509446	53.70	62	d__Archaea;p__Micrarchaeota;c__Micrarchaeia;o__Micrarchaeales;f__Micrarchaeaceae;g__Micrarchaeum;s__
LMSG_G000006768.1	no	88_1	Lead-Zinc	71.65	0.47	69.32	0	1	0	15	Medium quality	13986	587290	53.90	54	d__Archaea;p__Micrarchaeota;c__Micrarchaeia;o__Micrarchaeales;f__Micrarchaeaceae;g__Micrarchaeum;s__
LMSG_G000006769.1	no	88_1	Pyrite-Copper	77.57	4.67	54.21	1	1	1	19	Medium quality	47394	709841	53.20	47	d__Archaea;p__Micrarchaeota;c__Micrarchaeia;o__Micrarchaeales;f__Micrarchaeaceae;g__Micrarchaeum;s__
LMSG_G000006770.1	no	88_1	Lead-Zinc	70.79	1.40	63.79	1	0	0	17	Medium quality	7112	548322	53.90	106	d__Archaea;p__Micrarchaeota;c__Micrarchaeia;o__Micrarchaeales;f__Micrarchaeaceae;g__Micrarchaeum;s__
LMSG_G000006771.1	no	88_1	Copper	69.39	0.93	64.72	1	2	0	17	Medium quality	25882	584290	53.90	51	d__Archaea;p__Micrarchaeota;c__Micrarchaeia;o__Micrarchaeales;f__Micrarchaeaceae;g__Micrarchaeum;s__
LMSG_G000006772.1	no	88_1	Copper	68.76	0.93	64.09	1	0	1	19	Medium quality	22982	637651	53.20	40	d__Archaea;p__Micrarchaeota;c__Micrarchaeia;o__Micrarchaeales;f__Micrarchaeaceae;g__Micrarchaeum;s__
LMSG_G000006773.1	no	88_1	Copper	60.51	0.00	60.51	1	1	0	13	Medium quality	8456	415804	54.70	66	d__Archaea;p__Micrarchaeota;c__Micrarchaeia;o__Micrarchaeales;f__Micrarchaeaceae;g__Micrarchaeum;s__
LMSG_G000006774.1	no	88_1	Magnetite	74.29	1.87	64.95	1	1	0	16	Medium quality	22326	603483	54.10	37	d__Archaea;p__Micrarchaeota;c__Micrarchaeia;o__Micrarchaeales;f__Micrarchaeaceae;g__Micrarchaeum;s__
LMSG_G000006775.1	no	88_1	Magnetite	60.20	0.00	60.20	0	1	1	15	Medium quality	18791	446724	54.10	38	d__Archaea;p__Micrarchaeota;c__Micrarchaeia;o__Micrarchaeales;f__Micrarchaeaceae;g__Micrarchaeum;s__
LMSG_G000006776.1	no	88_1	Copper	73.83	0.47	71.50	0	2	1	19	Medium quality	18003	552037	53.40	44	d__Archaea;p__Micrarchaeota;c__Micrarchaeia;o__Micrarchaeales;f__Micrarchaeaceae;g__Micrarchaeum;s__
LMSG_G000006777.1	no	90_1	Polymetallic	70.56	1.94	60.86	0	1	1	14	Medium quality	8203	655050	54.90	114	d__Archaea;p__Micrarchaeota;c__Micrarchaeia;o__Micrarchaeales;f__Micrarchaeaceae;g__Micrarchaeum;s__
LMSG_G000006778.1	no	90_1	Polymetallic	67.91	0.93	63.24	1	0	0	17	Medium quality	11322	627626	54.60	69	d__Archaea;p__Micrarchaeota;c__Micrarchaeia;o__Micrarchaeales;f__Micrarchaeaceae;g__Micrarchaeum;s__
LMSG_G000006779.1	no	90_1	Polymetallic	66.90	0.93	62.23	1	0	1	17	Medium quality	15864	662668	54.00	59	d__Archaea;p__Micrarchaeota;c__Micrarchaeia;o__Micrarchaeales;f__Micrarchaeaceae;g__Micrarchaeum;s__
LMSG_G000006780.1	no	94_1	Polymetallic	63.51	0.00	63.51	0	2	0	15	Medium quality	78381	545000	45.30	14	d__Archaea;p__Micrarchaeota;c__Micrarchaeia;o__Micrarchaeales;f__Micrarchaeaceae;g__Micrarchaeum;s__
LMSG_G000006781.1	no	95_1	Magnetite	77.96	3.27	61.61	1	0	0	18	Medium quality	34426	896666	48.00	53	d__Archaea;p__Micrarchaeota;c__Micrarchaeia;o__Micrarchaeales;f__Micrarchaeaceae;g__Micrarchaeum;s__
LMSG_G000006782.1	no	96_1	Pyrite-Copper	63.23	1.40	56.23	0	0	1	17	Medium quality	17944	844090	46.30	68	d__Archaea;p__Micrarchaeota;c__Micrarchaeia;o__Micrarchaeales;f__Micrarchaeaceae;g__Micrarchaeum;s__
LMSG_G000006783.1	no	96_2	Lead-Zinc	76.31	0.93	71.64	1	0	0	18	Medium quality	20938	824340	46.30	67	d__Archaea;p__Micrarchaeota;c__Micrarchaeia;o__Micrarchaeales;f__Micrarchaeaceae;g__Micrarchaeum;s__
LMSG_G000006784.1	no	96_2	Lead-Zinc	71.26	0.00	71.26	1	2	1	18	Medium quality	28357	756754	46.10	38	d__Archaea;p__Micrarchaeota;c__Micrarchaeia;o__Micrarchaeales;f__Micrarchaeaceae;g__Micrarchaeum;s__
LMSG_G000006785.1	no	96_2	Lead-Zinc	75.23	0.00	75.23	1	1	1	19	Medium quality	24967	837348	46.00	50	d__Archaea;p__Micrarchaeota;c__Micrarchaeia;o__Micrarchaeales;f__Micrarchaeaceae;g__Micrarchaeum;s__
LMSG_G000006786.1	no	96_2	Lead-Zinc	71.47	0.93	66.80	1	2	1	18	Medium quality	18155	778529	46.30	67	d__Archaea;p__Micrarchaeota;c__Micrarchaeia;o__Micrarchaeales;f__Micrarchaeaceae;g__Micrarchaeum;s__
LMSG_G000006787.1	no	96_2	Lead-Zinc	79.05	2.34	67.37	1	1	3	20	Medium quality	6617	1961386	46.90	615	d__Archaea;p__Micrarchaeota;c__Micrarchaeia;o__Micrarchaeales;f__Micrarchaeaceae;g__Micrarchaeum;s__
LMSG_G000006788.1	no	96_2	Copper	63.64	0.00	63.64	2	0	1	19	Medium quality	24207	575660	46.50	30	d__Archaea;p__Micrarchaeota;c__Micrarchaeia;o__Micrarchaeales;f__Micrarchaeaceae;g__Micrarchaeum;s__
LMSG_G000006789.1	no	96_2	Polymetallic	78.97	0.00	78.97	1	0	0	20	Medium quality	20401	841043	46.40	71	d__Archaea;p__Micrarchaeota;c__Micrarchaeia;o__Micrarchaeales;f__Micrarchaeaceae;g__Micrarchaeum;s__
LMSG_G000006790.1	no	96_2	Copper	72.52	2.80	58.51	1	0	2	17	Medium quality	9816	1102722	45.40	222	d__Archaea;p__Micrarchaeota;c__Micrarchaeia;o__Micrarchaeales;f__Micrarchaeaceae;g__Micrarchaeum;s__
LMSG_G000006791.1	no	96_2	Copper	81.77	0.93	77.10	1	1	1	20	Medium quality	110673	929330	46.10	31	d__Archaea;p__Micrarchaeota;c__Micrarchaeia;o__Micrarchaeales;f__Micrarchaeaceae;g__Micrarchaeum;s__
LMSG_G000006792.1	no	96_2	Polymetallic	81.77	1.87	72.43	1	0	1	20	Medium quality	39341	1067705	45.70	115	d__Archaea;p__Micrarchaeota;c__Micrarchaeia;o__Micrarchaeales;f__Micrarchaeaceae;g__Micrarchaeum;s__
LMSG_G000006793.1	no	96_2	Polymetallic	61.68	0.93	57.01	1	1	0	18	Medium quality	35630	734399	46.20	29	d__Archaea;p__Micrarchaeota;c__Micrarchaeia;o__Micrarchaeales;f__Micrarchaeaceae;g__Micrarchaeum;s__
LMSG_G000006794.1	no	96_2	Pyrite	74.85	1.32	68.27	1	0	2	19	Medium quality	3757	823870	46.60	265	d__Archaea;p__Micrarchaeota;c__Micrarchaeia;o__Micrarchaeales;f__Micrarchaeaceae;g__Micrarchaeum;s__
LMSG_G000006795.1	no	96_2	Pyrite	74.29	0.00	74.29	1	1	3	18	Medium quality	167313	790797	46.00	31	d__Archaea;p__Micrarchaeota;c__Micrarchaeia;o__Micrarchaeales;f__Micrarchaeaceae;g__Micrarchaeum;s__
LMSG_G000006796.1	no	96_2	Tin-Zinc	77.53	1.17	71.69	1	0	1	19	Medium quality	42635	884681	46.10	49	d__Archaea;p__Micrarchaeota;c__Micrarchaeia;o__Micrarchaeales;f__Micrarchaeaceae;g__Micrarchaeum;s__Micrarchaeum acidiphilum
LMSG_G000006797.1	no	96_2	Copper	62.86	0.93	58.19	1	2	1	18	Medium quality	34827	598139	46.10	54	d__Archaea;p__Micrarchaeota;c__Micrarchaeia;o__Micrarchaeales;f__Micrarchaeaceae;g__Micrarchaeum;s__Micrarchaeum acidiphilum
LMSG_G000006798.1	no	96_2	Pyrite	81.77	0.93	77.10	1	1	1	19	Medium quality	264617	967744	45.70	13	d__Archaea;p__Micrarchaeota;c__Micrarchaeia;o__Micrarchaeales;f__Micrarchaeaceae;g__Micrarchaeum;s__Micrarchaeum acidiphilum
LMSG_G000006799.1	no	96_2	Pyrite	81.77	0.93	77.10	1	1	1	19	Medium quality	264732	982019	45.80	13	d__Archaea;p__Micrarchaeota;c__Micrarchaeia;o__Micrarchaeales;f__Micrarchaeaceae;g__Micrarchaeum;s__Micrarchaeum acidiphilum
LMSG_G000006800.1	no	96_2	Copper	78.03	0.50	75.52	0	0	2	18	Medium quality	10752	714097	46.90	83	d__Archaea;p__Micrarchaeota;c__Micrarchaeia;o__Micrarchaeales;f__Micrarchaeaceae;g__Micrarchaeum;s__Micrarchaeum acidiphilum
LMSG_G000006801.1	no	96_2	Copper	66.56	0.00	66.56	1	0	2	17	Medium quality	2453	712252	46.30	224	d__Archaea;p__Micrarchaeota;c__Micrarchaeia;o__Micrarchaeales;f__Micrarchaeaceae;g__Micrarchaeum;s__Micrarchaeum acidiphilum
LMSG_G000006802.1	no	93_1	Pyrite	56.69	0.00	56.69	0	2	1	15	Medium quality	13884	500756	48.10	60	d__Archaea;p__Micrarchaeota;c__Micrarchaeia;o__Micrarchaeales;f__Micrarchaeaceae;g__UBA581;s__
LMSG_G000006803.1	no	93_1	Pyrite	58.87	0.93	54.20	0	1	2	13	Medium quality	17110	491342	48.70	45	d__Archaea;p__Micrarchaeota;c__Micrarchaeia;o__Micrarchaeales;f__Micrarchaeaceae;g__UBA581;s__
LMSG_G000006804.1	no	93_1	Lead-Zinc	58.64	0.00	58.64	0	1	0	7	Medium quality	16069	261417	47.40	22	d__Archaea;p__Micrarchaeota;c__Micrarchaeia;o__Micrarchaeales;f__Micrarchaeaceae;g__UBA581;s__
LMSG_G000006805.1	no	93_1	Lead-Zinc	60.98	0.00	60.98	1	0	1	14	Medium quality	58634	386154	46.40	15	d__Archaea;p__Micrarchaeota;c__Micrarchaeia;o__Micrarchaeales;f__Micrarchaeaceae;g__UBA581;s__
LMSG_G000006806.1	no	93_1	Pyrite-Copper	80.37	1.87	71.03	1	1	1	17	Medium quality	19671	746896	47.60	62	d__Archaea;p__Micrarchaeota;c__Micrarchaeia;o__Micrarchaeales;f__Micrarchaeaceae;g__UBA581;s__
LMSG_G000006807.1	no	93_1	Lead-Zinc	58.17	0.93	53.50	2	1	1	13	Medium quality	13936	394192	46.80	36	d__Archaea;p__Micrarchaeota;c__Micrarchaeia;o__Micrarchaeales;f__Micrarchaeaceae;g__UBA581;s__
LMSG_G000006808.1	no	92_1	Pyrite-Copper	76.32	4.67	52.96	1	0	0	16	Medium quality	10220	782830	48.20	94	d__Archaea;p__Micrarchaeota;c__Micrarchaeia;o__Micrarchaeales;f__Micrarchaeaceae;g__UBA581;s__UBA581 sp002501775
LMSG_G000006809.1	no	247_1	Polymetallic	91.04	0.31	89.49	1	2	3	20	High quality	148973	1142747	55.60	45	d__Archaea;p__Micrarchaeota;c__Micrarchaeia;o__UBA10214;f__UBA10161;g__;s__
LMSG_G000006810.1	no	247_1	Polymetallic	88.63	0.00	88.63	1	1	0	20	Medium quality	38302	1132465	55.60	53	d__Archaea;p__Micrarchaeota;c__Micrarchaeia;o__UBA10214;f__UBA10161;g__;s__
LMSG_G000006811.1	no	247_1	Polymetallic	87.30	0.00	87.30	1	1	1	20	Medium quality	60254	1057033	55.50	45	d__Archaea;p__Micrarchaeota;c__Micrarchaeia;o__UBA10214;f__UBA10161;g__;s__
LMSG_G000006812.1	no	247_1	Polymetallic	90.10	0.00	90.10	1	1	0	19	Medium quality	108311	1100348	55.60	27	d__Archaea;p__Micrarchaeota;c__Micrarchaeia;o__UBA10214;f__UBA10161;g__;s__
LMSG_G000006813.1	no	248_1	Polymetallic	88.04	2.80	74.03	1	1	0	20	Medium quality	26331	1282943	58.50	102	d__Archaea;p__Micrarchaeota;c__Micrarchaeia;o__UBA10214;f__UBA10161;g__;s__
LMSG_G000006814.1	no	248_1	Polymetallic	75.93	1.87	66.59	1	2	1	18	Medium quality	6094	1046317	58.80	246	d__Archaea;p__Micrarchaeota;c__Micrarchaeia;o__UBA10214;f__UBA10161;g__1-14-0-10-45-29;s__
LMSG_G000006815.1	no	509_1	Polymetallic	63.50	0.93	58.83	1	0	3	15	Medium quality	2433	874617	57.10	402	d__Archaea;p__Micrarchaeota;c__Micrarchaeia;o__UBA10214;f__UBA10214;g__;s__
LMSG_G000006816.1	no	237_1	Antimony	71.25	0.00	71.25	1	0	0	16	Medium quality	8542	666218	33.20	98	d__Archaea;p__Micrarchaeota;c__Micrarchaeia;o__UBA8480;f__UBA97;g__;s__

LMSG_G000006817.1	no	237_1	Antimony	64.29	0.00	64.29	1	0	0	15	Medium quality	4367	619690	33.20	166	d__Archaea;p__Micrarchaeota;c__Micrarchaeia;o__UBA8480;f__UBA97;g__;s__
LMSG_G000006337.1	no	1683_1	Polymetallic	61.13	2.01	51.07	0	0	1	13	Medium quality	15741	631879	34.30	86	d__Archaea;p__Nanoarchaeota;c__Nanoarchaeia;o__Pacearchaeales;f__GW2011-AR1;g__GW2011-AR1;s__
LMSG_G000006818.1	no	1683_1	Lead-Zinc	53.91	0.47	51.58	1	0	0	14	Medium quality	2451	461223	35.20	202	d__Archaea;p__Nanoarchaeota;c__Nanoarchaeia;o__Pacearchaeales;f__GW2011-AR1;g__GW2011-AR1;s__
LMSG_G000006819.1	no	1687_1	Lead-Zinc	68.78	0.93	64.11	1	1	1	19	Medium quality	26130	752188	38.90	44	d__Archaea;p__Nanoarchaeota;c__Nanoarchaeia;o__Pacearchaeales;f__GW2011-AR1;g__GW2011-AR1;s__
LMSG_G000006820.1	no	1687_1	Pyrite-Copper	62.14	0.47	59.81	1	1	4	19	Medium quality	29424	702261	39.40	50	d__Archaea;p__Nanoarchaeota;c__Nanoarchaeia;o__Pacearchaeales;f__GW2011-AR1;g__GW2011-AR1;s__
LMSG_G000006821.1	no	1687_1	Pyrite-Copper	54.61	0.00	54.61	1	1	0	18	Medium quality	9605	684921	38.80	109	d__Archaea;p__Nanoarchaeota;c__Nanoarchaeia;o__Pacearchaeales;f__GW2011-AR1;g__GW2011-AR1;s__
LMSG_G000006822.1	no	1688_1	Pyrite-Copper	58.57	0.00	58.57	1	1	4	16	Medium quality	7314	668075	37.30	124	d__Archaea;p__Nanoarchaeota;c__Nanoarchaeia;o__Pacearchaeales;f__GW2011-AR1;g__GW2011-AR1;s__
LMSG_G000006823.1	no	1059_1	Antimony	51.44	0.00	51.44	1	0	0	15	Medium quality	4580	490167	37.50	128	d__Archaea;p__Nanoarchaeota;c__Nanoarchaeia;o__Pacearchaeales;f__UBA73;g__ARS50;s__
LMSG_G000006824.1	no	1059_1	Polymetallic	54.98	0.00	54.98	1	0	0	17	Medium quality	7523	514556	37.10	94	d__Archaea;p__Nanoarchaeota;c__Nanoarchaeia;o__Pacearchaeales;f__UBA73;g__UBA73;s__
LMSG_G000006825.1	no	15_1	Magnetite	78.04	0.93	73.37	1	1	2	16	Medium quality	31572	624680	43.00	42	d__Archaea;p__Nanoarchaeota;c__Nanoarchaeia;o__Parvarchaeales;f__Parvarchaeaceae;g__;s__
LMSG_G000006826.1	no	15_1	Magnetite	74.30	2.02	64.18	1	1	1	16	Medium quality	17168	716617	42.90	65	d__Archaea;p__Nanoarchaeota;c__Nanoarchaeia;o__Parvarchaeales;f__Parvarchaeaceae;g__;s__
LMSG_G000006827.1	no	15_1	Lead-Zinc	54.57	0.00	54.57	0	0	0	14	Medium quality	5914	394202	43.80	89	d__Archaea;p__Nanoarchaeota;c__Nanoarchaeia;o__Parvarchaeales;f__Parvarchaeaceae;g__;s__
LMSG_G000006828.1	no	15_1	Polymetallic	76.17	2.80	62.16	1	1	2	17	Medium quality	35358	723239	42.90	36	d__Archaea;p__Nanoarchaeota;c__Nanoarchaeia;o__Parvarchaeales;f__Parvarchaeaceae;g__;s__
LMSG_G000006829.1	no	15_1	Polymetallic	60.20	0.93	55.53	0	0	0	16	Medium quality	40938	495919	43.40	48	d__Archaea;p__Nanoarchaeota;c__Nanoarchaeia;o__Parvarchaeales;f__Parvarchaeaceae;g__;s__
LMSG_G000006830.1	no	15_1	Polymetallic	74.30	0.93	69.63	1	1	2	16	Medium quality	33541	644140	43.30	32	d__Archaea;p__Nanoarchaeota;c__Nanoarchaeia;o__Parvarchaeales;f__Parvarchaeaceae;g__;s__
LMSG_G000006831.1	no	15_1	Polymetallic	78.35	2.80	64.34	1	2	1	17	Medium quality	30146	710146	43.20	31	d__Archaea;p__Nanoarchaeota;c__Nanoarchaeia;o__Parvarchaeales;f__Parvarchaeaceae;g__;s__
LMSG_G000006832.1	no	15_1	Polymetallic	76.01	0.93	71.34	1	1	1	18	Medium quality	36228	703937	43.20	40	d__Archaea;p__Nanoarchaeota;c__Nanoarchaeia;o__Parvarchaeales;f__Parvarchaeaceae;g__;s__
LMSG_G000006833.1	no	15_1	Copper	65.00	2.80	50.99	2	0	7	18	Medium quality	4627	701731	43.70	224	d__Archaea;p__Nanoarchaeota;c__Nanoarchaeia;o__Parvarchaeales;f__Parvarchaeaceae;g__;s__
LMSG_G000006834.1	no	15_1	Copper	77.10	1.87	67.76	1	3	1	18	Medium quality	45180	653513	43.10	50	d__Archaea;p__Nanoarchaeota;c__Nanoarchaeia;o__Parvarchaeales;f__Parvarchaeaceae;g__;s__
LMSG_G000006835.1	no	15_1	Copper	72.43	0.93	67.76	1	0	1	15	Medium quality	22012	623256	43.00	45	d__Archaea;p__Nanoarchaeota;c__Nanoarchaeia;o__Parvarchaeales;f__Parvarchaeaceae;g__;s__
LMSG_G000006836.1	no	15_1	Lead-Zinc	78.51	0.93	73.84	1	1	1	17	Medium quality	43179	681931	43.00	37	d__Archaea;p__Nanoarchaeota;c__Nanoarchaeia;o__Parvarchaeales;f__Parvarchaeaceae;g__;s__
LMSG_G000006837.1	no	15_1	Lead-Zinc	78.04	0.93	73.37	1	1	1	17	Medium quality	41860	683142	43.20	35	d__Archaea;p__Nanoarchaeota;c__Nanoarchaeia;o__Parvarchaeales;f__Parvarchaeaceae;g__;s__
LMSG_G000006838.1	no	15_1	Lead-Zinc	77.10	0.93	72.43	1	1	2	17	Medium quality	28361	600067	43.40	37	d__Archaea;p__Nanoarchaeota;c__Nanoarchaeia;o__Parvarchaeales;f__Parvarchaeaceae;g__;s__
LMSG_G000006839.1	no	15_1	Lead-Zinc	77.57	0.93	72.90	1	3	2	18	Medium quality	22533	668511	43.20	43	d__Archaea;p__Nanoarchaeota;c__Nanoarchaeia;o__Parvarchaeales;f__Parvarchaeaceae;g__;s__
LMSG_G000006840.1	no	15_1	Copper	64.64	0.00	64.64	1	2	0	17	Medium quality	20832	530343	42.90	41	d__Archaea;p__Nanoarchaeota;c__Nanoarchaeia;o__Parvarchaeales;f__Parvarchaeaceae;g__;s__
LMSG_G000006841.1	no	15_1	Lead-Zinc	67.83	2.80	53.82	1	1	0	14	Medium quality	14836	543274	43.40	48	d__Archaea;p__Nanoarchaeota;c__Nanoarchaeia;o__Parvarchaeales;f__Parvarchaeaceae;g__;s__
LMSG_G000006842.1	no	16_1	Copper	78.82	0.93	74.15	1	1	1	19	Medium quality	111911	707706	43.90	24	d__Archaea;p__Nanoarchaeota;c__Nanoarchaeia;o__Parvarchaeales;f__Parvarchaeaceae;g__;s__
LMSG_G000006843.1	no	16_1	Copper	72.25	0.97	67.40	1	1	2	19	Medium quality	205887	760471	43.70	45	d__Archaea;p__Nanoarchaeota;c__Nanoarchaeia;o__Parvarchaeales;f__Parvarchaeaceae;g__;s__
LMSG_G000006844.1	no	16_1	Copper	75.70	0.93	71.03	1	1	1	19	Medium quality	135005	679008	44.00	18	d__Archaea;p__Nanoarchaeota;c__Nanoarchaeia;o__Parvarchaeales;f__Parvarchaeaceae;g__;s__
LMSG_G000006845.1	no	16_1	Polymetallic	73.21	0.93	68.54	1	1	2	16	Medium quality	92889	657012	43.80	40	d__Archaea;p__Nanoarchaeota;c__Nanoarchaeia;o__Parvarchaeales;f__Parvarchaeaceae;g__;s__
LMSG_G000006846.1	no	16_1	Polymetallic	70.09	0.93	65.42	1	2	2	16	Medium quality	49160	600745	44.20	49	d__Archaea;p__Nanoarchaeota;c__Nanoarchaeia;o__Parvarchaeales;f__Parvarchaeaceae;g__;s__
LMSG_G000006847.1	no	16_1	Polymetallic	75.15	0.93	70.48	1	2	2	18	Medium quality	26943	646745	43.60	32	d__Archaea;p__Nanoarchaeota;c__Nanoarchaeia;o__Parvarchaeales;f__Parvarchaeaceae;g__;s__
LMSG_G000006848.1	no	16_1	Polymetallic	57.71	0.00	57.71	1	0	1	16	Medium quality	21821	482360	45.00	31	d__Archaea;p__Nanoarchaeota;c__Nanoarchaeia;o__Parvarchaeales;f__Parvarchaeaceae;g__;s__
LMSG_G000006849.1	no	16_1	Magnetite	79.28	1.25	73.05	1	2	1	19	Medium quality	44930	653766	44.00	32	d__Archaea;p__Nanoarchaeota;c__Nanoarchaeia;o__Parvarchaeales;f__Parvarchaeaceae;g__;s__
LMSG_G000006850.1	no	16_1	Magnetite	52.88	0.00	52.88	1	0	1	12	Medium quality	19889	383688	45.00	23	d__Archaea;p__Nanoarchaeota;c__Nanoarchaeia;o__Parvarchaeales;f__Parvarchaeaceae;g__;s__
LMSG_G000006851.1	no	16_1	Pyrite-Copper	57.01	0.00	57.01	1	0	2	12	Medium quality	19920	331830	45.70	25	d__Archaea;p__Nanoarchaeota;c__Nanoarchaeia;o__Parvarchaeales;f__Parvarchaeaceae;g__;s__
LMSG_G000006852.1	no	16_1	Arsenic	67.52	1.87	58.18	1	1	2	18	Medium quality	30727	604873	44.00	32	d__Archaea;p__Nanoarchaeota;c__Nanoarchaeia;o__Parvarchaeales;f__Parvarchaeaceae;g__;s__
LMSG_G000006853.1	no	19_1	Copper	56.93	0.00	56.93	1	1	1	14	Medium quality	43405	526836	48.70	29	d__Archaea;p__Nanoarchaeota;c__Nanoarchaeia;o__Parvarchaeales;f__Parvarchaeaceae;g__;s__
LMSG_G000006854.1	no	21_1	Pyrite-Copper	76.32	0.93	71.65	1	1	1	19	Medium quality	42824	950298	40.80	34	d__Archaea;p__Nanoarchaeota;c__Nanoarchaeia;o__Parvarchaeales;f__Parvarchaeaceae;g__;s__
LMSG_G000006855.1	no	22_1	Copper	81.69	0.93	77.02	1	2	1	19	Medium quality	76212	808606	39.90	37	d__Archaea;p__Nanoarchaeota;c__Nanoarchaeia;o__Parvarchaeales;f__Parvarchaeaceae;g__;s__
LMSG_G000006856.1	no	22_1	Copper	80.76	0.93	76.09	1	2	1	19	Medium quality	71341	845889	39.80	30	d__Archaea;p__Nanoarchaeota;c__Nanoarchaeia;o__Parvarchaeales;f__Parvarchaeaceae;g__;s__
LMSG_G000006857.1	no	22_1	Copper	79.82	0.93	75.15	1	1	2	19	Medium quality	28829	870002	39.90	52	d__Archaea;p__Nanoarchaeota;c__Nanoarchaeia;o__Parvarchaeales;f__Parvarchaeaceae;g__;s__
LMSG_G000006858.1	no	22_1	Copper	81.69	0.93	77.02	1	1	1	19	Medium quality	36423	820985	39.90	42	d__Archaea;p__Nanoarchaeota;c__Nanoarchaeia;o__Parvarchaeales;f__Parvarchaeaceae;g__;s__
LMSG_G000006859.1	no	22_1	Copper	81.69	0.93	77.02	1	1	1	19	Medium quality	75653	841518	40.00	36	d__Archaea;p__Nanoarchaeota;c__Nanoarchaeia;o__Parvarchaeales;f__Parvarchaeaceae;g__;s__
LMSG_G000006860.1	no	22_1	Polymetallic	74.09	0.00	74.09	0	1	1	19	Medium quality	9020	785771	39.80	161	d__Archaea;p__Nanoarchaeota;c__Nanoarchaeia;o__Parvarchaeales;f__Parvarchaeaceae;g__;s__
LMSG_G000006861.1	no	22_1	Polymetallic	56.69	0.93	52.02	1	2	2	18	Medium quality	5116	737611	39.80	169	d__Archaea;p__Nanoarchaeota;c__Nanoarchaeia;o__Parvarchaeales;f__Parvarchaeaceae;g__;s__
LMSG_G000006862.1	no	22_1	Polymetallic	71.88	0.00	71.88	0	1	1	17	Medium quality	8194	851322	40.00	148	d__Archaea;p__Nanoarchaeota;c__Nanoarchaeia;o__Parvarchaeales;f__Parvarchaeaceae;g__;s__
LMSG_G000006863.1	no	22_1	Polymetallic	78.89	0.93	74.22	0	2	4	19	Medium quality	132716	843181	39.90	13	d__Archaea;p__Nanoarchaeota;c__Nanoarchaeia;o__Parvarchaeales;f__Parvarchaeaceae;g__;s__
LMSG_G000006864.1	no	22_1	Polymetallic	80.76	3.74	62.07	1	3	2	19	Medium quality	35334	967395	39.80	51	d__Archaea;p__Nanoarchaeota;c__Nanoarchaeia;o__Parvarchaeales;f__Parvarchaeaceae;g__;s__
LMSG_G000006865.1	no	22_1	Polymetallic	78.89	1.25	72.66	1	2	1	19	Medium quality	39418	857884	39.90	42	d__Archaea;p__Nanoarchaeota;c__Nanoarchaeia;o__Parvarchaeales;f__Parvarchaeaceae;g__;s__
LMSG_G000006866.1	no	22_1	Magnetite	81.69	0.93	77.02	1	2	2	19	Medium quality	41172	826917	39.90	38	d__Archaea;p__Nanoarchaeota;c__Nanoarchaeia;o__Parvarchaeales;f__Parvarchaeaceae;g__;s__
LMSG_G000006867.1	no	22_1	Pyrite-Copper	80.76	0.93	76.09	1	1	1	18	Medium quality	55448	854127	40.00	31	d__Archaea;p__Nanoarchaeota;c__Nanoarchaeia;o__Parvarchaeales;f__Parvarchaeaceae;g__;s__
LMSG_G000006868.1	no	22_1	Magnetite	66.74	1.87	57.40	1	1	1	17	Medium quality	39006	652384	39.70	32	d__Archaea;p__Nanoarchaeota;c__Nanoarchaeia;o__Parvarchaeales;f__Parvarchaeaceae;g__;s__
LMSG_G000006869.1	no	22_1	Magnetite	54.12	0.00	54.12	1	0	0	13	Medium quality	43981	501915	39.30	23	d__Archaea;p__Nanoarchaeota;c__Nanoarchaeia;o__Parvarchaeales;f__Parvarchaeaceae;g__;s__
LMSG_G000006870.1	no	22_1	Magnetite	71.41	1.87	62.07	1	1	1	17	Medium quality	34589	677888	39.60	42	d__Archaea;p__Nanoarchaeota;c__Nanoarchaeia;o__Parvarchaeales;f__Parvarchaeaceae;g__;s__
LMSG_G000006871.1	no	22_1	Pyrite-Copper	63.47	0.93	58.80	0	1	1	16	Medium quality	35475	635621	40.20	27	d__Archaea;p__Nanoarchaeota;c__Nanoarchaeia;o__Parvarchaeales;f__Parvarchaeaceae;g__;s__
LMSG_G000006872.1	no	23_1	Lead-Zinc	80.29	0.93	75.62	1	1	2	18	Medium quality	196962	889466	39.10	32	d__Archaea;p__Nanoarchaeota;c__Nanoarchaeia;o__Parvarchaeales;f__Parvarchaeaceae;g__;s__
LMSG_G000006873.1	no	23_1	Lead-Zinc	80.29	0.93	75.62	1	1	1	18	Medium quality	263495	894515	39.10	41	d__Archaea;p__Nanoarchaeota;c__Nanoarchaeia;o__Parvarchaeales;f__Parvarchaeaceae;g__;s__
LMSG_G000006874.1	no	23_1	Lead-Zinc	79.36	3.74	60.67	1	1	2	18	Medium quality	51321	968433	39.00	54	d__Archaea;p__Nanoarchaeota;c__Nanoarchaeia;o__Parvarchaeales;f__Parvarchaeaceae;g__;s__
LMSG_G000006875.1	no	23_1	Pyrite-Copper	79.28	1.25	73.05	1	1	1	18	Medium quality	20471	818432	38.70	74	d__Archaea;p__Nanoarchaeota;c__Nanoarchaeia;o__Parvarchaeales;f__Parvarchaeaceae;g__;s__
LMSG_G000006876.1	no	23_1	Lead-Zinc	80.29	1.87	70.95	1	1	1	18	Medium quality	264213	876705	39.10	39	d__Archaea;p__Nanoarchaeota;c__Nanoarchaeia;o__Parvarchaeales;f__Parvarchaeaceae;g__;s__
LMSG_G000006877.1	no	24_1	Lead-Zinc	75.64	0.97	70.79	1	1	1	17	Medium quality	69780	615600	41.60	49	d__Archaea;p__Nanoarchaeota;c__Nanoarchaeia;o__Parvarchaeales;f__Parvarchaeaceae;g__;s__
LMSG_G000006878.1	no	24_1	Lead-Zinc	60.98	0.93	56.31	0	1	1	16	Medium quality	16471	478988	41.70	44	d__Archaea;p__Nanoarchaeota;c__Nanoarchaeia;o__Parvarchaeales;f__Parvarchaeaceae;g__;s__
LMSG_G000006879.1	no	24_1	Lead-Zinc	70.95	0.97	66.10	1	1	1	16	Medium quality	21442	544239	41.70	45	d__Archaea;p__Nanoarchaeota;c__Nanoarchaeia;o__Parvarchaeales;f__Parvarchaeaceae;g__;s__
LMSG_G000006880.1	no	24_1	Lead-Zinc	60.92	0.97	56.07	1	1	1	13	Medium quality	9856	401088	41.10	53	d__Archaea;p__Nanoarchaeota;c__Nanoarchaeia;o__Parvarchaeales;f__Parvarchaeaceae;g__;s__
LMSG_G000006881.1	no	24_1	Pyrite	76.61	1.05	71.39	1	1	1	18	Medium quality	26165	595087	41.70	45	d__Archaea;p__Nanoarchaeota;c__Nanoarchaeia;o__Parvarchaeales;f__Parvarchaeaceae;g__;s__
LMSG_G000006882.1	no	25_1	Copper	75.07	0.93	70.40	1	2	2	19	Medium quality	37832	655790	42.30	26	d__Archaea;p__Nanoarchaeota;c__Nanoarchaeia;o__Parvarchaeales;f__Parvarchaeaceae;g__;s__
LMSG_G000006883.1	no	25_1	Polymetallic	77.99	2.91	63.43	1	2	2	19	Medium quality	71481	727015	42.40	19	d__Archaea;p__Nanoarchaeota;c__Nanoarchaeia;o__Parvarchaeales;f__Parvarchaeaceae;g__;s__
LMSG_G000006884.1	no	25_1	Polymetallic	74.11	1.46	66.83	1	1	1	19	Medium quality	47310	702058	42.20	39	d__Archaea;p__Nanoarchaeota;c__Nanoarchaeia;o__Parvarchaeales;f__Parvarchaeaceae;g__;s__
LMSG_G000006885.1	no	25_1	Polymetallic	72.16	0.00	72.16	1	1	2	18	Medium quality	38848	701591	42.30	23	d__Archaea;p__Nanoarchaeota;c__Nanoarchaeia;o__Parvarchaeales;f__Parvarchaeaceae;g__;s__
LMSG_G000006886.1	no	25_1	Polymetallic	77.80	2.80	63.79	1	2	2	19	Medium quality	48003	722577	42.40	19	d__Archaea;p__Nanoarchaeota;c__Nanoarchaeia;o__Parvarchaeales;f__Parvarchaeaceae;g__;s__
LMSG_G000006887.1	no	25_1	Polymetallic	73.71	0.97	68.86	1	2	1	19	Medium quality	33412	626031	42.40	27	d__Archaea;p__Nanoarchaeota;c__Nanoarchaeia;o__Parvarchaeales;f__Parvarchaeaceae;g__;s__
LMSG_G000006888.1	no	25_1	Magnetite	66.59	2.91	52.03	1	2	2	19	Medium quality	41453	663560	42.00	43	d__Archaea;p__Nanoarchaeota;c__Nanoarchaeia;o__Parvarchaeales;f__Parvarchaeaceae;g__;s__
LMSG_G000006889.1	no	25_1	Magnetite	71.41	2.80	57.40	2	2	1	18	Medium quality	25866	641464	42.10	55	d__Archaea;p__Nanoarchaeota;c__Nanoarchaeia;o__Parvarchaeales;f__Parvarchaeaceae;g__;s__
LMSG_G000006890.1	no	6_1	Polymetallic	52.22	0.07	51.87	1	0	0	6	Medium quality	1598	351847	41.80	221	d__Archaea;p__Nanoarchaeota;c__Nanoarchaeia;o__Parvarchaeales;f__Parvarchaeaceae;g__;s__
LMSG_G000006891.1	no	10_1	Lead-Zinc	84.70	2.91	70.14	1	2	1	18	Medium quality	134274	931402	36.70	34	d__Archaea;p__Nanoarchaeota;c__Nanoarchaeia;o__Parvarchaeales;f__Parvarchaeaceae;g__Parvarchaeum;s__
LMSG_G000006892.1	no	10_2	Lead-Zinc	60.08	1.87	50.74	0	0	0	16	Medium quality	15549	575212	36.50	63	d__Archaea;p__Nanoarchaeota;c__Nanoarchaeia;o__Parvarchaeales;f__Parvarchaeaceae;g__Parvarchaeum;s__

LMSG_G000006893.1	no	10_2	Copper	73.05	3.24	56.87	1	2	1	18	Medium quality	22772	854404	36.30	51	d__Archaea;p__Nanoarchaeota;c__Nanoarchaeia;o__Parvarchaeales;f__Parvarchaeaceae;g__Parvarchaeum;s__
LMSG_G000006894.1	no	10_2	Pyrite	73.83	2.80	59.82	1	0	1	19	Medium quality	4669	863935	36.70	236	d__Archaea;p__Nanoarchaeota;c__Nanoarchaeia;o__Parvarchaeales;f__Parvarchaeaceae;g__Parvarchaeum;s__
LMSG_G000006895.1	no	12_1	Lead-Zinc	75.00	2.75	61.25	1	1	1	19	Medium quality	6493	729886	32.90	154	d__Archaea;p__Nanoarchaeota;c__Nanoarchaeia;o__Parvarchaeales;f__Parvarchaeaceae;g__Parvarchaeum;s__
LMSG_G000006896.1	no	12_1	Lead-Zinc	79.85	4.85	55.58	0	1	2	18	Medium quality	58768	758759	32.60	46	d__Archaea;p__Nanoarchaeota;c__Nanoarchaeia;o__Parvarchaeales;f__Parvarchaeaceae;g__Parvarchaeum;s__
LMSG_G000006897.1	no	12_1	Copper	80.82	4.85	56.55	0	1	1	17	Medium quality	21913	773165	32.80	68	d__Archaea;p__Nanoarchaeota;c__Nanoarchaeia;o__Parvarchaeales;f__Parvarchaeaceae;g__Parvarchaeum;s__
LMSG_G000006898.1	no	12_1	Copper	81.79	3.88	62.38	1	2	2	17	Medium quality	86291	784122	32.70	40	d__Archaea;p__Nanoarchaeota;c__Nanoarchaeia;o__Parvarchaeales;f__Parvarchaeaceae;g__Parvarchaeum;s__
LMSG_G000006899.1	no	12_1	Copper	81.54	3.74	62.85	1	1	1	16	Medium quality	43458	747325	32.70	34	d__Archaea;p__Nanoarchaeota;c__Nanoarchaeia;o__Parvarchaeales;f__Parvarchaeaceae;g__Parvarchaeum;s__
LMSG_G000006900.1	no	12_1	Copper	80.60	4.67	57.24	0	1	2	17	Medium quality	55712	742717	32.80	50	d__Archaea;p__Nanoarchaeota;c__Nanoarchaeia;o__Parvarchaeales;f__Parvarchaeaceae;g__Parvarchaeum;s__
LMSG_G000006901.1	no	12_1	Copper	70.87	2.91	56.31	1	2	1	16	Medium quality	29300	731648	32.70	45	d__Archaea;p__Nanoarchaeota;c__Nanoarchaeia;o__Parvarchaeales;f__Parvarchaeaceae;g__Parvarchaeum;s__
LMSG_G000006902.1	no	12_1	Pyrite	68.38	1.87	59.04	1	1	0	17	Medium quality	6864	624016	33.10	115	d__Archaea;p__Nanoarchaeota;c__Nanoarchaeia;o__Parvarchaeales;f__Parvarchaeaceae;g__Parvarchaeum;s__
LMSG_G000006903.1	no	13_1	Polymetallic	82.76	4.85	58.49	2	1	1	19	Medium quality	26529	781919	35.30	47	d__Archaea;p__Nanoarchaeota;c__Nanoarchaeia;o__Parvarchaeales;f__Parvarchaeaceae;g__Parvarchaeum;s__
LMSG_G000006904.1	no	13_1	Polymetallic	79.85	1.94	70.15	1	1	0	18	Medium quality	25780	714254	35.30	50	d__Archaea;p__Nanoarchaeota;c__Nanoarchaeia;o__Parvarchaeales;f__Parvarchaeaceae;g__Parvarchaeum;s__
LMSG_G000006905.1	no	13_1	Polymetallic	70.48	0.93	65.81	1	0	0	18	Medium quality	30706	528226	35.50	31	d__Archaea;p__Nanoarchaeota;c__Nanoarchaeia;o__Parvarchaeales;f__Parvarchaeaceae;g__Parvarchaeum;s__
LMSG_G000006906.1	no	13_1	Copper	64.72	0.97	59.87	0	0	0	15	Medium quality	23662	780813	35.30	93	d__Archaea;p__Nanoarchaeota;c__Nanoarchaeia;o__Parvarchaeales;f__Parvarchaeaceae;g__Parvarchaeum;s__
LMSG_G000006907.1	no	13_1	Lead-Zinc	82.44	4.37	60.60	1	1	1	19	Medium quality	53705	860667	35.50	78	d__Archaea;p__Nanoarchaeota;c__Nanoarchaeia;o__Parvarchaeales;f__Parvarchaeaceae;g__Parvarchaeum;s__
LMSG_G000006908.1	no	13_1	Lead-Zinc	81.79	1.94	72.09	1	2	0	19	Medium quality	17806	893583	35.00	82	d__Archaea;p__Nanoarchaeota;c__Nanoarchaeia;o__Parvarchaeales;f__Parvarchaeaceae;g__Parvarchaeum;s__
LMSG_G000006909.1	no	6_1	Lead-Zinc	57.60	0.93	52.93	1	0	0	13	Medium quality	4203	425899	41.40	112	d__Archaea;p__Nanoarchaeota;c__Nanoarchaeia;o__Parvarchaeales;f__Parvarchaeaceae;g__Parvarchaeum;s__
LMSG_G000006910.1	no	6_1	Tin-Zinc	64.32	2.66	51.01	0	0	1	16	Medium quality	28877	654785	40.80	61	d__Archaea;p__Nanoarchaeota;c__Nanoarchaeia;o__Parvarchaeales;f__Parvarchaeaceae;g__Parvarchaeum;s__
LMSG_G000006911.1	no	6_1	Copper	77.58	4.85	53.31	1	1	2	18	Medium quality	58702	914703	40.30	60	d__Archaea;p__Nanoarchaeota;c__Nanoarchaeia;o__Parvarchaeales;f__Parvarchaeaceae;g__Parvarchaeum;s__
LMSG_G000006912.1	no	6_1	Copper	83.73	2.91	69.17	1	2	2	17	Medium quality	146888	863438	40.50	22	d__Archaea;p__Nanoarchaeota;c__Nanoarchaeia;o__Parvarchaeales;f__Parvarchaeaceae;g__Parvarchaeum;s__
LMSG_G000006913.1	no	6_1	Copper	82.28	1.94	72.58	2	3	2	18	Medium quality	78278	881403	40.30	60	d__Archaea;p__Nanoarchaeota;c__Nanoarchaeia;o__Parvarchaeales;f__Parvarchaeaceae;g__Parvarchaeum;s__
LMSG_G000006914.1	no	6_1	Copper	77.91	0.97	73.06	1	1	1	18	Medium quality	61847	826637	40.30	29	d__Archaea;p__Nanoarchaeota;c__Nanoarchaeia;o__Parvarchaeales;f__Parvarchaeaceae;g__Parvarchaeum;s__
LMSG_G000006915.1	no	6_1	Polymetallic	62.38	2.18	51.48	0	0	0	12	Medium quality	8225	490995	41.20	70	d__Archaea;p__Nanoarchaeota;c__Nanoarchaeia;o__Parvarchaeales;f__Parvarchaeaceae;g__Parvarchaeum;s__
LMSG_G000006916.1	no	6_1	Magnetite	66.90	2.80	52.89	0	2	0	17	Medium quality	14437	738590	40.60	84	d__Archaea;p__Nanoarchaeota;c__Nanoarchaeia;o__Parvarchaeales;f__Parvarchaeaceae;g__Parvarchaeum;s__
LMSG_G000006917.1	no	6_1	Copper	79.85	2.91	65.29	1	2	1	18	Medium quality	47052	779038	40.30	28	d__Archaea;p__Nanoarchaeota;c__Nanoarchaeia;o__Parvarchaeales;f__Parvarchaeaceae;g__Parvarchaeum;s__
LMSG_G000006918.1	no	6_1	Copper	54.12	0.00	54.12	0	1	1	11	Medium quality	16308	332631	41.80	32	d__Archaea;p__Nanoarchaeota;c__Nanoarchaeia;o__Parvarchaeales;f__Parvarchaeaceae;g__Parvarchaeum;s__
LMSG_G000006919.1	no	6_1	Polymetallic	68.45	0.93	63.78	0	1	1	17	Medium quality	18655	682217	40.70	66	d__Archaea;p__Nanoarchaeota;c__Nanoarchaeia;o__Parvarchaeales;f__Parvarchaeaceae;g__Parvarchaeum;s__
LMSG_G000006920.1	no	6_1	Copper	57.08	0.93	52.41	1	0	1	14	Medium quality	53399	579597	39.60	21	d__Archaea;p__Nanoarchaeota;c__Nanoarchaeia;o__Parvarchaeales;f__Parvarchaeaceae;g__Parvarchaeum;s__
LMSG_G000006921.1	no	6_1	Copper	50.97	0.00	50.97	1	1	0	16	Medium quality	31007	586750	39.50	32	d__Archaea;p__Nanoarchaeota;c__Nanoarchaeia;o__Parvarchaeales;f__Parvarchaeaceae;g__Parvarchaeum;s__
LMSG_G000006922.1	no	6_1	Copper	76.94	1.94	67.24	1	1	0	18	Medium quality	33330	722921	40.70	31	d__Archaea;p__Nanoarchaeota;c__Nanoarchaeia;o__Parvarchaeales;f__Parvarchaeaceae;g__Parvarchaeum;s__
LMSG_G000006923.1	no	6_1	Arsenic	65.42	1.87	56.08	1	1	0	16	Medium quality	19433	653081	40.90	66	d__Archaea;p__Nanoarchaeota;c__Nanoarchaeia;o__Parvarchaeales;f__Parvarchaeaceae;g__Parvarchaeum;s__
LMSG_G000006924.1	no	9_1	Copper	84.63	1.94	74.93	1	1	1	19	Medium quality	81739	784553	39.20	22	d__Archaea;p__Nanoarchaeota;c__Nanoarchaeia;o__Parvarchaeales;f__Parvarchaeaceae;g__Parvarchaeum;s__
LMSG_G000006925.1	no	9_1	Copper	84.63	1.94	74.93	1	0	0	19	Medium quality	28248	813562	39.10	53	d__Archaea;p__Nanoarchaeota;c__Nanoarchaeia;o__Parvarchaeales;f__Parvarchaeaceae;g__Parvarchaeum;s__
LMSG_G000006926.1	no	9_1	Copper	80.71	0.97	75.86	1	1	1	16	Medium quality	24978	593167	40.00	46	d__Archaea;p__Nanoarchaeota;c__Nanoarchaeia;o__Parvarchaeales;f__Parvarchaeaceae;g__Parvarchaeum;s__
LMSG_G000006927.1	no	9_1	Copper	79.83	3.04	64.65	0	1	1	19	Medium quality	17897	741416	39.30	64	d__Archaea;p__Nanoarchaeota;c__Nanoarchaeia;o__Parvarchaeales;f__Parvarchaeaceae;g__Parvarchaeum;s__
LMSG_G000006928.1	no	9_1	Copper	51.94	0.00	51.94	0	1	1	17	Medium quality	54481	579115	38.90	25	d__Archaea;p__Nanoarchaeota;c__Nanoarchaeia;o__Parvarchaeales;f__Parvarchaeaceae;g__Parvarchaeum;s__
LMSG_G000006929.1	no	9_1	Polymetallic	84.63	4.85	60.36	0	1	1	19	Medium quality	53732	777556	39.40	28	d__Archaea;p__Nanoarchaeota;c__Nanoarchaeia;o__Parvarchaeales;f__Parvarchaeaceae;g__Parvarchaeum;s__
LMSG_G000006930.1	no	9_1	Polymetallic	80.53	0.93	75.86	0	1	1	17	Medium quality	61622	718939	39.40	19	d__Archaea;p__Nanoarchaeota;c__Nanoarchaeia;o__Parvarchaeales;f__Parvarchaeaceae;g__Parvarchaeum;s__
LMSG_G000006931.1	no	9_1	Lead-Zinc	77.91	0.97	73.06	1	0	1	19	Medium quality	76819	695151	39.60	41	d__Archaea;p__Nanoarchaeota;c__Nanoarchaeia;o__Parvarchaeales;f__Parvarchaeaceae;g__Parvarchaeum;s__
LMSG_G000006932.1	no	9_1	Lead-Zinc	66.18	1.29	59.71	0	0	0	19	Medium quality	7153	618943	39.40	100	d__Archaea;p__Nanoarchaeota;c__Nanoarchaeia;o__Parvarchaeales;f__Parvarchaeaceae;g__Parvarchaeum;s__
LMSG_G000006933.1	no	9_1	Lead-Zinc	84.63	1.12	79.03	1	1	1	19	Medium quality	107654	778598	39.40	54	d__Archaea;p__Nanoarchaeota;c__Nanoarchaeia;o__Parvarchaeales;f__Parvarchaeaceae;g__Parvarchaeum;s__
LMSG_G000006934.1	no	9_1	Lead-Zinc	64.71	0.93	60.04	0	1	0	16	Medium quality	35355	545714	39.10	23	d__Archaea;p__Nanoarchaeota;c__Nanoarchaeia;o__Parvarchaeales;f__Parvarchaeaceae;g__Parvarchaeum;s__
LMSG_G000006935.1	no	9_1	Lead-Zinc	62.37	0.00	62.37	1	0	2	16	Medium quality	54067	493563	39.90	14	d__Archaea;p__Nanoarchaeota;c__Nanoarchaeia;o__Parvarchaeales;f__Parvarchaeaceae;g__Parvarchaeum;s__
LMSG_G000006936.1	no	11_1	Polymetallic	71.76	2.91	57.20	1	0	0	19	Medium quality	21140	674292	33.40	51	d__Archaea;p__Nanoarchaeota;c__Nanoarchaeia;o__Parvarchaeales;f__Parvarchaeaceae;g__Parvarchaeum;s__Parvarchaeum sp002502045
LMSG_G000006937.1	no	11_1	Copper	65.29	1.94	55.59	1	1	1	19	Medium quality	23253	744729	32.90	64	d__Archaea;p__Nanoarchaeota;c__Nanoarchaeia;o__Parvarchaeales;f__Parvarchaeaceae;g__Parvarchaeum;s__Parvarchaeum sp002502045
LMSG_G000006938.1	no	11_1	Copper	73.05	3.88	53.64	0	1	1	17	Medium quality	27824	812725	33.20	99	d__Archaea;p__Nanoarchaeota;c__Nanoarchaeia;o__Parvarchaeales;f__Parvarchaeaceae;g__Parvarchaeum;s__Parvarchaeum sp002502045
LMSG_G000006939.1	no	11_1	Copper	71.92	3.20	55.93	1	1	1	18	Medium quality	8404	805610	33.90	132	d__Archaea;p__Nanoarchaeota;c__Nanoarchaeia;o__Parvarchaeales;f__Parvarchaeaceae;g__Parvarchaeum;s__Parvarchaeum sp002502045
LMSG_G000006940.1	no	17_1	Lead-Zinc	82.76	1.94	73.06	1	1	2	20	Medium quality	18232	1942179	33.90	331	d__Archaea;p__Nanoarchaeota;c__Nanoarchaeia;o__Parvarchaeales;f__Parvarchaeaceae;g__Parvarchaeum;s__Parvarchaeum sp002503215
LMSG_G000006941.1	no	17_1	Lead-Zinc	83.25	1.94	73.55	1	2	1	19	Medium quality	63607	817258	34.50	24	d__Archaea;p__Nanoarchaeota;c__Nanoarchaeia;o__Parvarchaeales;f__Parvarchaeaceae;g__Parvarchaeum;s__Parvarchaeum sp002503215
LMSG_G000006942.1	no	17_1	Polymetallic	83.25	3.06	67.95	1	1	1	20	Medium quality	73563	1399609	35.00	211	d__Archaea;p__Nanoarchaeota;c__Nanoarchaeia;o__Parvarchaeales;f__Parvarchaeaceae;g__Parvarchaeum;s__Parvarchaeum sp002503215
LMSG_G000006943.1	no	17_1	Iron	81.79	1.94	72.09	1	0	1	18	Medium quality	8728	853388	34.20	126	d__Archaea;p__Nanoarchaeota;c__Nanoarchaeia;o__Parvarchaeales;f__Parvarchaeaceae;g__Parvarchaeum;s__Parvarchaeum sp002503215
LMSG_G000006944.1	no	1582_1	Polymetallic	84.57	0.00	84.57	1	1	4	20	Medium quality	60256	1536134	43.40	101	d__Archaea;p__Nanoarchaeota;c__Nanoarchaeia;o__Woesearchaeales;f__UBA9642;g__UBA9642;s__
LMSG_G000006945.1	no	1582_1	Polymetallic	85.04	0.00	85.04	1	3	4	20	Medium quality	207813	1474148	43.40	34	d__Archaea;p__Nanoarchaeota;c__Nanoarchaeia;o__Woesearchaeales;f__UBA9642;g__UBA9642;s__
LMSG_G000006946.1	no	1582_1	Polymetallic	82.24	0.00	82.24	1	0	0	20	Medium quality	271828	1413255	43.40	14	d__Archaea;p__Nanoarchaeota;c__Nanoarchaeia;o__Woesearchaeales;f__UBA9642;g__UBA9642;s__
LMSG_G000006947.1	no	1582_1	Polymetallic	85.04	0.00	85.04	1	1	0	20	Medium quality	159119	1410353	43.40	17	d__Archaea;p__Nanoarchaeota;c__Nanoarchaeia;o__Woesearchaeales;f__UBA9642;g__UBA9642;s__
LMSG_G000006948.1	no	150_1	Coal	99.13	1.60	91.13	1	1	1	19	High quality	48989	1922548	52.80	61	d__Archaea;p__Thermoplasmatota;c__Thermoplasmata;o__;f__;g__;s__
LMSG_G000006949.1	no	150_1	Coal	99.13	1.60	91.13	1	2	2	20	High quality	76626	1931116	52.80	43	d__Archaea;p__Thermoplasmatota;c__Thermoplasmata;o__;f__;g__;s__
LMSG_G000006950.1	no	150_1	Copper	95.93	2.40	83.93	1	1	2	19	High quality	32509	2068173	52.50	95	d__Archaea;p__Thermoplasmatota;c__Thermoplasmata;o__;f__;g__;s__
LMSG_G000006951.1	no	152_1	Lead-Zinc	99.13	2.50	86.62	1	1	1	20	High quality	57640	2175995	49.80	99	d__Archaea;p__Thermoplasmatota;c__Thermoplasmata;o__;f__;g__;s__
LMSG_G000006952.1	no	152_1	Lead-Zinc	97.15	2.40	85.15	1	1	1	20	High quality	34606	1882688	50.00	93	d__Archaea;p__Thermoplasmatota;c__Thermoplasmata;o__;f__;g__;s__
LMSG_G000006953.1	no	152_1	Pyrite	88.80	3.52	71.20	1	0	0	15	Medium quality	7115	1516234	50.10	263	d__Archaea;p__Thermoplasmatota;c__Thermoplasmata;o__;f__;g__;s__
LMSG_G000006954.1	no	152_1	Lead-Zinc	66.40	2.40	54.40	1	0	0	16	Medium quality	4372	1382846	49.80	372	d__Archaea;p__Thermoplasmatota;c__Thermoplasmata;o__;f__;g__;s__
LMSG_G000006955.1	no	152_1	Lead-Zinc	67.10	1.60	59.10	1	1	0	14	Medium quality	10517	1216394	49.40	127	d__Archaea;p__Thermoplasmatota;c__Thermoplasmata;o__;f__;g__;s__
LMSG_G000006956.1	no	152_1	Copper	73.40	0.80	69.40	1	2	1	17	Medium quality	41161	1195254	50.70	42	d__Archaea;p__Thermoplasmatota;c__Thermoplasmata;o__;f__;g__;s__
LMSG_G000006957.1	no	152_1	Lead-Zinc	87.41	0.96	82.61	1	1	0	19	Medium quality	5369	1646138	49.90	422	d__Archaea;p__Thermoplasmatota;c__Thermoplasmata;o__;f__;g__;s__
LMSG_G000006958.1	no	152_1	Lead-Zinc	68.00	1.60	60.00	1	1	0	19	Medium quality	40380	1396105	50.30	107	d__Archaea;p__Thermoplasmatota;c__Thermoplasmata;o__;f__;g__;s__
LMSG_G000006959.1	no	152_1	Lead-Zinc	86.73	3.07	71.40	1	0	0	17	Medium quality	4243	1788340	50.20	502	d__Archaea;p__Thermoplasmatota;c__Thermoplasmata;o__;f__;g__;s__
LMSG_G000006960.1	no	152_1	Pyrite-Copper	87.00	2.40	75.00	1	1	0	20	Medium quality	151829	1827591	50.00	62	d__Archaea;p__Thermoplasmatota;c__Thermoplasmata;o__;f__;g__;s__
LMSG_G000006961.1	no	152_1	Pyrite-Copper	83.00	1.60	75.00	1	1	0	18	Medium quality	57838	1420744	50.70	46	d__Archaea;p__Thermoplasmatota;c__Thermoplasmata;o__;f__;g__;s__
LMSG_G000006962.1	no	152_1	Lead-Zinc	93.37	2.58	80.45	1	0	1	20	Medium quality	40516	1879181	49.90	131	d__Archaea;p__Thermoplasmatota;c__Thermoplasmata;o__;f__;g__;s__
LMSG_G000006963.1	no	152_1	Copper	72.45	0.80	68.45	0	2	0	16	Medium quality	30060	1370767	49.90	75	d__Archaea;p__Thermoplasmatota;c__Thermoplasmata;o__;f__;g__;s__
LMSG_G000006964.1	no	152_1	Pyrite-Copper	86.20	4.00	66.20	1	2	0	18	Medium quality	39092	1586765	50.60	78	d__Archaea;p__Thermoplasmatota;c__Thermoplasmata;o__;f__;g__;s__
LMSG_G000006965.1	no	152_1	Pyrite	98.33	1.60	90.33	1	1	0	19	Medium quality	79647	1866965	49.90	56	d__Archaea;p__Thermoplasmatota;c__Thermoplasmata;o__;f__;g__;s__

LMSG_G000006966.1	no	152_1	Polymetallic	98.20	1.60	90.20	1	1	0	20	Medium quality	36238	1904158	49.90	93	d__Archaea;p__Thermoplasmatota;c__Thermoplasmata;o__;f__;g__;s__
LMSG_G000006967.1	no	152_1	Pyrite-Copper	98.20	2.40	86.20	1	2	0	19	Medium quality	46259	1857686	50.00	68	d__Archaea;p__Thermoplasmatota;c__Thermoplasmata;o__;f__;g__;s__
LMSG_G000006968.1	no	152_1	Pyrite-Copper	98.20	2.99	83.23	1	1	0	20	Medium quality	117863	1870685	49.90	68	d__Archaea;p__Thermoplasmatota;c__Thermoplasmata;o__;f__;g__;s__
LMSG_G000006969.1	no	152_1	Copper	90.20	2.40	78.20	1	1	0	17	Medium quality	49027	1524547	50.60	60	d__Archaea;p__Thermoplasmatota;c__Thermoplasmata;o__;f__;g__;s__
LMSG_G000006970.1	no	155_1	Polymetallic	73.00	0.92	68.39	0	1	0	16	Medium quality	3771	1437728	54.00	463	d__Archaea;p__Thermoplasmatota;c__Thermoplasmata;o__;f__;g__;s__
LMSG_G000006971.1	no	156_1	Magnetite	64.75	1.60	56.75	1	0	0	12	Medium quality	15653	926745	52.60	78	d__Archaea;p__Thermoplasmatota;c__Thermoplasmata;o__;f__;g__;s__
LMSG_G000006972.1	no	157_1	Magnetite	85.40	0.80	81.40	3	1	0	19	Medium quality	17572	1463121	51.40	111	d__Archaea;p__Thermoplasmatota;c__Thermoplasmata;o__;f__;g__;s__
LMSG_G000006973.1	no	157_1	Magnetite	84.93	3.07	69.60	1	1	1	18	Medium quality	14938	1464610	51.40	135	d__Archaea;p__Thermoplasmatota;c__Thermoplasmata;o__;f__;g__;s__
LMSG_G000006974.1	no	157_1	Magnetite	65.88	1.40	58.88	0	0	1	15	Medium quality	14408	1162804	51.10	119	d__Archaea;p__Thermoplasmatota;c__Thermoplasmata;o__;f__;g__;s__
LMSG_G000006975.1	no	157_1	Copper	95.00	1.33	88.34	1	1	2	18	High quality	34580	1776831	51.30	79	d__Archaea;p__Thermoplasmatota;c__Thermoplasmata;o__;f__;g__;s__
LMSG_G000006976.1	no	157_1	Copper	95.62	1.60	87.62	1	2	2	19	High quality	17857	1797416	51.20	225	d__Archaea;p__Thermoplasmatota;c__Thermoplasmata;o__;f__;g__;s__
LMSG_G000006977.1	no	157_1	Copper	97.40	3.20	81.40	1	1	2	19	High quality	31269	2030271	51.00	120	d__Archaea;p__Thermoplasmatota;c__Thermoplasmata;o__;f__;g__;s__
LMSG_G000006978.1	no	157_1	Copper	91.80	1.60	83.80	1	1	1	19	High quality	51162	1643846	51.50	64	d__Archaea;p__Thermoplasmatota;c__Thermoplasmata;o__;f__;g__;s__
LMSG_G000006979.1	no	157_1	Copper	95.80	1.60	87.80	1	1	1	19	High quality	36525	1771071	51.20	76	d__Archaea;p__Thermoplasmatota;c__Thermoplasmata;o__;f__;g__;s__
LMSG_G000006980.1	no	157_1	Copper	98.20	3.20	82.20	1	0	1	18	Medium quality	59925	1897059	51.10	81	d__Archaea;p__Thermoplasmatota;c__Thermoplasmata;o__;f__;g__;s__
LMSG_G000006981.1	no	157_1	Pyrite-Copper	58.72	0.00	58.72	0	0	2	11	Medium quality	5216	1047865	51.50	220	d__Archaea;p__Thermoplasmatota;c__Thermoplasmata;o__;f__;g__;s__
LMSG_G000006982.1	no	157_1	Pyrite-Copper	82.44	4.00	62.44	2	1	1	19	Medium quality	23517	1765880	50.80	134	d__Archaea;p__Thermoplasmatota;c__Thermoplasmata;o__;f__;g__;s__
LMSG_G000006983.1	no	157_1	Copper	78.40	2.13	67.74	0	0	0	16	Medium quality	14290	1752324	50.50	268	d__Archaea;p__Thermoplasmatota;c__Thermoplasmata;o__;f__;g__;s__
LMSG_G000006984.1	no	215_1	Copper	95.16	4.84	70.97	1	2	1	20	High quality	39875	1708341	43.30	92	d__Archaea;p__Thermoplasmatota;c__Thermoplasmata;o__Thermoplasmatales;f__GCA-001856825;g__GCA-001856825;s__
LMSG_G000006985.1	no	215_1	Copper	84.54	1.61	76.48	1	2	0	17	Medium quality	24956	1252972	43.40	84	d__Archaea;p__Thermoplasmatota;c__Thermoplasmata;o__Thermoplasmatales;f__GCA-001856825;g__GCA-001856825;s__
LMSG_G000006986.1	no	215_1	Copper	81.45	1.61	73.39	1	1	1	18	Medium quality	74772	1799126	42.90	117	d__Archaea;p__Thermoplasmatota;c__Thermoplasmata;o__Thermoplasmatales;f__GCA-001856825;g__GCA-001856825;s__
LMSG_G000006987.1	no	215_1	Copper	72.77	0.81	68.74	1	0	0	12	Medium quality	6499	1209069	42.70	215	d__Archaea;p__Thermoplasmatota;c__Thermoplasmata;o__Thermoplasmatales;f__GCA-001856825;g__GCA-001856825;s__
LMSG_G000006988.1	no	215_1	Lead-Zinc	64.91	0.81	60.88	1	1	2	17	Medium quality	91278	1124953	42.90	35	d__Archaea;p__Thermoplasmatota;c__Thermoplasmata;o__Thermoplasmatales;f__GCA-001856825;g__GCA-001856825;s__
LMSG_G000006989.1	no	215_1	Lead-Zinc	56.85	0.81	52.82	1	2	1	15	Medium quality	62308	859996	43.70	23	d__Archaea;p__Thermoplasmatota;c__Thermoplasmata;o__Thermoplasmatales;f__GCA-001856825;g__GCA-001856825;s__
LMSG_G000006990.1	no	215_1	Pyrite-Copper	65.32	0.81	61.29	1	1	2	20	Medium quality	64461	1104512	43.40	24	d__Archaea;p__Thermoplasmatota;c__Thermoplasmata;o__Thermoplasmatales;f__GCA-001856825;g__GCA-001856825;s__
LMSG_G000006991.1	no	215_1	Pyrite	86.02	2.99	71.08	1	1	1	20	Medium quality	24665	1508499	43.30	105	d__Archaea;p__Thermoplasmatota;c__Thermoplasmata;o__Thermoplasmatales;f__GCA-001856825;g__GCA-001856825;s__
LMSG_G000006992.1	no	216_1	Pyrite	94.35	0.81	90.32	1	2	1	19	High quality	37706	1381981	46.40	65	d__Archaea;p__Thermoplasmatota;c__Thermoplasmata;o__Thermoplasmatales;f__GCA-001856825;g__GCA-001856825;s__
LMSG_G000006993.1	no	216_1	Lead-Zinc	91.12	0.81	87.09	2	1	2	18	High quality	55724	1265610	46.30	44	d__Archaea;p__Thermoplasmatota;c__Thermoplasmata;o__Thermoplasmatales;f__GCA-001856825;g__GCA-001856825;s__
LMSG_G000006994.1	no	216_1	Pyrite-Copper	69.35	2.42	57.26	1	1	0	17	Medium quality	59116	934136	46.60	30	d__Archaea;p__Thermoplasmatota;c__Thermoplasmata;o__Thermoplasmatales;f__GCA-001856825;g__GCA-001856825;s__
LMSG_G000006995.1	no	217_1	Lead-Zinc	77.41	0.81	73.38	1	1	1	19	Medium quality	229325	1392929	44.20	20	d__Archaea;p__Thermoplasmatota;c__Thermoplasmata;o__Thermoplasmatales;f__GCA-001856825;g__GCA-001856825;s__
LMSG_G000006996.1	no	217_1	Pyrite-Copper	77.41	1.61	69.35	0	2	2	19	Medium quality	43293	1554579	44.30	68	d__Archaea;p__Thermoplasmatota;c__Thermoplasmata;o__Thermoplasmatales;f__GCA-001856825;g__GCA-001856825;s__
LMSG_G000006997.1	no	218_1	Lead-Zinc	73.18	4.30	51.68	2	0	1	16	Medium quality	6702	1448867	44.10	242	d__Archaea;p__Thermoplasmatota;c__Thermoplasmata;o__Thermoplasmatales;f__GCA-001856825;g__GCA-001856825;s__
LMSG_G000006998.1	no	219_1	Pyrite	94.35	1.19	88.41	1	1	1	19	High quality	42514	1427811	44.20	51	d__Archaea;p__Thermoplasmatota;c__Thermoplasmata;o__Thermoplasmatales;f__GCA-001856825;g__GCA-001856825;s__
LMSG_G000006999.1	no	219_1	Pyrite	96.77	0.87	92.43	2	1	2	20	High quality	93583	1463259	44.10	47	d__Archaea;p__Thermoplasmatota;c__Thermoplasmata;o__Thermoplasmatales;f__GCA-001856825;g__GCA-001856825;s__
LMSG_G000007000.1	no	219_1	Copper	91.93	2.42	79.84	1	1	1	18	High quality	15454	1423654	44.10	141	d__Archaea;p__Thermoplasmatota;c__Thermoplasmata;o__Thermoplasmatales;f__GCA-001856825;g__GCA-001856825;s__
LMSG_G000007001.1	no	219_1	Pyrite	95.40	1.85	86.14	1	1	2	19	High quality	45929	1448502	44.10	87	d__Archaea;p__Thermoplasmatota;c__Thermoplasmata;o__Thermoplasmatales;f__GCA-001856825;g__GCA-001856825;s__
LMSG_G000007002.1	no	219_1	Pyrite	64.74	0.81	60.71	1	0	1	9	Medium quality	2544	816871	44.70	360	d__Archaea;p__Thermoplasmatota;c__Thermoplasmata;o__Thermoplasmatales;f__GCA-001856825;g__GCA-001856825;s__
LMSG_G000007003.1	no	219_1	Copper	69.50	3.63	51.36	1	1	2	14	Medium quality	10146	1053546	44.00	122	d__Archaea;p__Thermoplasmatota;c__Thermoplasmata;o__Thermoplasmatales;f__GCA-001856825;g__GCA-001856825;s__
LMSG_G000007004.1	no	219_1	Pyrite	95.96	4.03	75.80	1	0	2	19	Medium quality	55409	1534901	44.00	38	d__Archaea;p__Thermoplasmatota;c__Thermoplasmata;o__Thermoplasmatales;f__GCA-001856825;g__GCA-001856825;s__
LMSG_G000007005.1	no	219_1	Copper	67.51	1.61	59.45	1	0	0	13	Medium quality	6843	1255843	43.80	258	d__Archaea;p__Thermoplasmatota;c__Thermoplasmata;o__Thermoplasmatales;f__GCA-001856825;g__GCA-001856825;s__
LMSG_G000007006.1	no	219_1	Copper	61.69	0.00	61.69	1	1	1	13	Medium quality	17079	721049	44.50	58	d__Archaea;p__Thermoplasmatota;c__Thermoplasmata;o__Thermoplasmatales;f__GCA-001856825;g__GCA-001856825;s__
LMSG_G000007007.1	no	219_1	Magnetite	78.22	3.23	62.10	1	0	0	11	Medium quality	13530	1097210	44.10	111	d__Archaea;p__Thermoplasmatota;c__Thermoplasmata;o__Thermoplasmatales;f__GCA-001856825;g__GCA-001856825;s__
LMSG_G000007008.1	no	219_1	Lead-Zinc	81.85	1.61	73.79	1	0	1	16	Medium quality	43539	1535603	43.90	69	d__Archaea;p__Thermoplasmatota;c__Thermoplasmata;o__Thermoplasmatales;f__GCA-001856825;g__GCA-001856825;s__
LMSG_G000007009.1	no	219_1	Lead-Zinc	90.32	3.23	74.20	1	0	1	18	Medium quality	51706	1734263	44.10	70	d__Archaea;p__Thermoplasmatota;c__Thermoplasmata;o__Thermoplasmatales;f__GCA-001856825;g__GCA-001856825;s__
LMSG_G000007010.1	no	219_1	Pyrite-Copper	68.62	1.08	63.25	1	0	1	12	Medium quality	38569	866063	44.50	57	d__Archaea;p__Thermoplasmatota;c__Thermoplasmata;o__Thermoplasmatales;f__GCA-001856825;g__GCA-001856825;s__
LMSG_G000007011.1	no	219_1	Pyrite-Copper	62.50	0.00	62.50	1	2	1	11	Medium quality	55063	850291	44.50	37	d__Archaea;p__Thermoplasmatota;c__Thermoplasmata;o__Thermoplasmatales;f__GCA-001856825;g__GCA-001856825;s__
LMSG_G000007012.1	no	219_1	Lead-Zinc	85.08	1.61	77.02	1	0	1	19	Medium quality	46351	1752638	43.80	81	d__Archaea;p__Thermoplasmatota;c__Thermoplasmata;o__Thermoplasmatales;f__GCA-001856825;g__GCA-001856825;s__
LMSG_G000007013.1	no	219_1	Polymetallic	82.79	0.81	78.76	1	1	2	15	Medium quality	10790	1145202	44.80	127	d__Archaea;p__Thermoplasmatota;c__Thermoplasmata;o__Thermoplasmatales;f__GCA-001856825;g__GCA-001856825;s__
LMSG_G000007014.1	no	219_1	Polymetallic	76.20	0.81	72.17	1	1	0	14	Medium quality	13456	1059127	44.90	111	d__Archaea;p__Thermoplasmatota;c__Thermoplasmata;o__Thermoplasmatales;f__GCA-001856825;g__GCA-001856825;s__
LMSG_G000007015.1	no	219_1	Polymetallic	78.36	1.21	72.32	1	1	0	12	Medium quality	14014	1119291	44.90	107	d__Archaea;p__Thermoplasmatota;c__Thermoplasmata;o__Thermoplasmatales;f__GCA-001856825;g__GCA-001856825;s__
LMSG_G000007016.1	no	219_1	Polymetallic	65.32	0.00	65.32	1	2	0	9	Medium quality	13661	786589	45.50	83	d__Archaea;p__Thermoplasmatota;c__Thermoplasmata;o__Thermoplasmatales;f__GCA-001856825;g__GCA-001856825;s__
LMSG_G000007017.1	no	219_1	Polymetallic	94.35	0.81	90.32	1	6	2	19	High quality	45862	1378701	44.80	63	d__Archaea;p__Thermoplasmatota;c__Thermoplasmata;o__Thermoplasmatales;f__GCA-001856825;g__GCA-001856825;s__
LMSG_G000007018.1	no	219_1	Polymetallic	95.16	0.81	91.13	1	3	7	20	High quality	49096	1397357	44.90	75	d__Archaea;p__Thermoplasmatota;c__Thermoplasmata;o__Thermoplasmatales;f__GCA-001856825;g__GCA-001856825;s__
LMSG_G000007019.1	no	219_1	Polymetallic	91.12	0.81	87.09	1	2	7	17	Medium quality	35911	1355912	44.80	76	d__Archaea;p__Thermoplasmatota;c__Thermoplasmata;o__Thermoplasmatales;f__GCA-001856825;g__GCA-001856825;s__
LMSG_G000007020.1	no	219_1	Polymetallic	85.77	2.02	75.69	1	2	0	17	Medium quality	5263	1096828	44.90	302	d__Archaea;p__Thermoplasmatota;c__Thermoplasmata;o__Thermoplasmatales;f__GCA-001856825;g__GCA-001856825;s__
LMSG_G000007021.1	no	219_1	Polymetallic	79.47	0.81	75.44	1	0	1	16	Medium quality	17333	1244004	44.60	125	d__Archaea;p__Thermoplasmatota;c__Thermoplasmata;o__Thermoplasmatales;f__GCA-001856825;g__GCA-001856825;s__
LMSG_G000007022.1	no	219_1	Polymetallic	88.44	1.61	80.38	1	1	1	17	Medium quality	8248	1459322	44.50	215	d__Archaea;p__Thermoplasmatota;c__Thermoplasmata;o__Thermoplasmatales;f__GCA-001856825;g__GCA-001856825;s__

LMSG_G000007023.1	no	219_1	Polymetallic	56.93	0.27	55.59	1	0	1	10	Medium quality	8704	650726	45.20	96	d__Archaea;p__Thermoplasmatota;c__Thermoplasmata;o__Thermoplasmatales;f__GCA-001856825;g__GCA-001856825;s__
LMSG_G000007024.1	no	219_1	Tin-Zinc	94.35	0.27	93.01	1	1	1	19	High quality	24258	1397410	44.70	85	d__Archaea;p__Thermoplasmatota;c__Thermoplasmata;o__Thermoplasmatales;f__GCA-001856825;g__GCA-001856825;s__
LMSG_G000007025.1	no	219_1	Tin-Zinc	92.91	1.34	86.19	2	0	2	19	Medium quality	22955	1583592	44.50	168	d__Archaea;p__Thermoplasmatota;c__Thermoplasmata;o__Thermoplasmatales;f__GCA-001856825;g__GCA-001856825;s__
LMSG_G000007026.1	no	219_1	Lead-Zinc	75.13	1.61	67.07	1	1	0	14	Medium quality	9461	849661	45.70	127	d__Archaea;p__Thermoplasmatota;c__Thermoplasmata;o__Thermoplasmatales;f__GCA-001856825;g__GCA-001856825;s__
LMSG_G000007027.1	no	219_1	Lead-Zinc	89.92	2.28	78.50	1	2	2	19	Medium quality	12002	1416197	44.60	162	d__Archaea;p__Thermoplasmatota;c__Thermoplasmata;o__Thermoplasmatales;f__GCA-001856825;g__GCA-001856825;s__
LMSG_G000007028.1	no	219_1	Copper	62.50	2.08	52.11	1	0	2	14	Medium quality	8358	917161	44.60	129	d__Archaea;p__Thermoplasmatota;c__Thermoplasmata;o__Thermoplasmatales;f__GCA-001856825;g__GCA-001856825;s__
LMSG_G000007029.1	no	219_1	Lead-Zinc	87.90	0.00	87.90	1	1	1	18	Medium quality	26491	1299207	44.80	76	d__Archaea;p__Thermoplasmatota;c__Thermoplasmata;o__Thermoplasmatales;f__GCA-001856825;g__GCA-001856825;s__
LMSG_G000007030.1	no	219_1	Lead-Zinc	87.62	2.42	75.53	1	1	2	17	Medium quality	10658	1368219	44.50	160	d__Archaea;p__Thermoplasmatota;c__Thermoplasmata;o__Thermoplasmatales;f__GCA-001856825;g__GCA-001856825;s__
LMSG_G000007031.1	no	219_1	Arsenic	89.11	0.00	89.11	1	1	1	18	Medium quality	31877	1375022	44.60	105	d__Archaea;p__Thermoplasmatota;c__Thermoplasmata;o__Thermoplasmatales;f__GCA-001856825;g__GCA-001856825;s__
LMSG_G000007032.1	no	219_1	Pyrite-Copper	78.56	4.30	57.06	1	0	2	20	Medium quality	10234	1717523	44.60	343	d__Archaea;p__Thermoplasmatota;c__Thermoplasmata;o__Thermoplasmatales;f__GCA-001856825;g__GCA-001856825;s__
LMSG_G000007033.1	no	220_1	Lead-Zinc	84.81	1.61	76.75	1	0	0	15	Medium quality	57690	1248354	44.60	35	d__Archaea;p__Thermoplasmatota;c__Thermoplasmata;o__Thermoplasmatales;f__GCA-001856825;g__GCA-001856825;s__
LMSG_G000007034.1	no	220_1	Lead-Zinc	68.14	1.61	60.08	1	2	0	13	Medium quality	53361	966518	44.70	30	d__Archaea;p__Thermoplasmatota;c__Thermoplasmata;o__Thermoplasmatales;f__GCA-001856825;g__GCA-001856825;s__
LMSG_G000007035.1	no	221_1	Pyrite	60.62	0.40	58.61	1	1	1	10	Medium quality	10811	857724	45.20	104	d__Archaea;p__Thermoplasmatota;c__Thermoplasmata;o__Thermoplasmatales;f__GCA-001856825;g__GCA-001856825;s__GCA-001856825 sp001856825
LMSG_G000007036.1	no	221_1	Lead-Zinc	55.24	0.06	54.93	0	0	0	11	Medium quality	17648	728455	44.60	76	d__Archaea;p__Thermoplasmatota;c__Thermoplasmata;o__Thermoplasmatales;f__GCA-001856825;g__GCA-001856825;s__GCA-001856825 sp001856825
LMSG_G000007037.1	no	221_1	Polymetallic	89.65	3.36	72.85	1	2	1	15	Medium quality	15508	1257714	44.60	114	d__Archaea;p__Thermoplasmatota;c__Thermoplasmata;o__Thermoplasmatales;f__GCA-001856825;g__GCA-001856825;s__GCA-001856825 sp001856825
LMSG_G000007038.1	no	221_1	Polymetallic	86.69	2.69	73.25	1	2	1	17	Medium quality	20038	1250126	44.40	81	d__Archaea;p__Thermoplasmatota;c__Thermoplasmata;o__Thermoplasmatales;f__GCA-001856825;g__GCA-001856825;s__GCA-001856825 sp001856825
LMSG_G000007039.1	no	221_1	Polymetallic	83.06	2.15	72.31	1	1	1	19	Medium quality	32697	1441952	44.10	143	d__Archaea;p__Thermoplasmatota;c__Thermoplasmata;o__Thermoplasmatales;f__GCA-001856825;g__GCA-001856825;s__GCA-001856825 sp001856825
LMSG_G000007040.1	no	221_1	Polymetallic	85.88	1.61	77.82	0	2	1	17	Medium quality	22664	1471365	44.30	111	d__Archaea;p__Thermoplasmatota;c__Thermoplasmata;o__Thermoplasmatales;f__GCA-001856825;g__GCA-001856825;s__GCA-001856825 sp001856825
LMSG_G000007041.1	no	221_1	Polymetallic	85.48	1.88	76.08	1	2	1	17	Medium quality	14875	1536523	44.20	150	d__Archaea;p__Thermoplasmatota;c__Thermoplasmata;o__Thermoplasmatales;f__GCA-001856825;g__GCA-001856825;s__GCA-001856825 sp001856825
LMSG_G000007042.1	no	221_1	Polymetallic	58.46	0.00	58.46	1	3	6	14	Medium quality	33258	750146	45.00	49	d__Archaea;p__Thermoplasmatota;c__Thermoplasmata;o__Thermoplasmatales;f__GCA-001856825;g__GCA-001856825;s__GCA-001856825 sp001856825
LMSG_G000007043.1	no	221_1	Magnetite	80.24	2.42	68.15	1	2	1	15	Medium quality	18967	1161819	45.00	92	d__Archaea;p__Thermoplasmatota;c__Thermoplasmata;o__Thermoplasmatales;f__GCA-001856825;g__GCA-001856825;s__GCA-001856825 sp001856825
LMSG_G000007044.1	no	221_1	Copper	61.69	0.81	57.66	0	1	0	10	Medium quality	25523	898467	44.70	59	d__Archaea;p__Thermoplasmatota;c__Thermoplasmata;o__Thermoplasmatales;f__GCA-001856825;g__GCA-001856825;s__GCA-001856825 sp001856825
LMSG_G000007045.1	no	221_1	Copper	84.27	1.61	76.21	1	1	1	17	Medium quality	18234	1219979	44.40	92	d__Archaea;p__Thermoplasmatota;c__Thermoplasmata;o__Thermoplasmatales;f__GCA-001856825;g__GCA-001856825;s__GCA-001856825 sp001856825
LMSG_G000007046.1	no	221_1	Copper	62.86	0.81	58.83	1	1	0	11	Medium quality	25167	726270	44.70	47	d__Archaea;p__Thermoplasmatota;c__Thermoplasmata;o__Thermoplasmatales;f__GCA-001856825;g__GCA-001856825;s__GCA-001856825 sp001856825
LMSG_G000007047.1	no	221_1	Copper	81.04	0.81	77.01	1	1	1	18	Medium quality	20215	1217931	44.30	89	d__Archaea;p__Thermoplasmatota;c__Thermoplasmata;o__Thermoplasmatales;f__GCA-001856825;g__GCA-001856825;s__GCA-001856825 sp001856825
LMSG_G000007048.1	no	221_1	Lead-Zinc	88.18	2.10	77.68	2	1	10	20	Medium quality	12178	1417152	44.50	209	d__Archaea;p__Thermoplasmatota;c__Thermoplasmata;o__Thermoplasmatales;f__GCA-001856825;g__GCA-001856825;s__GCA-001856825 sp001856825
LMSG_G000007049.1	no	186_1	Lead-Zinc	95.92	1.61	87.86	1	2	1	19	High quality	13778	1983476	42.00	194	d__Archaea;p__Thermoplasmatota;c__Thermoplasmata;o__Thermoplasmatales;f__Thermoplasmataceae;g__;s__
LMSG_G000007050.1	no	186_1	Lead-Zinc	91.62	4.84	67.43	1	2	1	19	High quality	18142	1838934	42.30	139	d__Archaea;p__Thermoplasmatota;c__Thermoplasmata;o__Thermoplasmatales;f__Thermoplasmataceae;g__;s__
LMSG_G000007051.1	no	186_1	Pyrite	85.44	0.81	81.41	1	1	0	18	Medium quality	23067	1402325	42.40	79	d__Archaea;p__Thermoplasmatota;c__Thermoplasmata;o__Thermoplasmatales;f__Thermoplasmataceae;g__;s__
LMSG_G000007052.1	no	186_1	Pyrite	87.59	0.00	87.59	1	2	0	18	Medium quality	19116	1420902	42.30	104	d__Archaea;p__Thermoplasmatota;c__Thermoplasmata;o__Thermoplasmatales;f__Thermoplasmataceae;g__;s__
LMSG_G000007053.1	no	186_1	Copper	91.89	3.23	75.77	0	1	0	20	Medium quality	15540	2058005	42.30	204	d__Archaea;p__Thermoplasmatota;c__Thermoplasmata;o__Thermoplasmatales;f__Thermoplasmataceae;g__;s__
LMSG_G000007054.1	no	186_1	Lead-Zinc	64.11	1.80	55.12	0	0	0	11	Medium quality	18123	1097719	42.00	85	d__Archaea;p__Thermoplasmatota;c__Thermoplasmata;o__Thermoplasmatales;f__Thermoplasmataceae;g__;s__
LMSG_G000007055.1	no	186_1	Copper	76.57	1.61	68.51	1	1	0	12	Medium quality	14684	1210441	42.20	106	d__Archaea;p__Thermoplasmatota;c__Thermoplasmata;o__Thermoplasmatales;f__Thermoplasmataceae;g__;s__
LMSG_G000007056.1	no	186_1	Copper	79.79	0.00	79.79	1	0	0	13	Medium quality	15578	1244108	42.10	107	d__Archaea;p__Thermoplasmatota;c__Thermoplasmata;o__Thermoplasmatales;f__Thermoplasmataceae;g__;s__
LMSG_G000007057.1	no	186_1	Magnetite	83.11	4.03	62.95	1	0	0	15	Medium quality	15791	1445632	42.50	146	d__Archaea;p__Thermoplasmatota;c__Thermoplasmata;o__Thermoplasmatales;f__Thermoplasmataceae;g__;s__
LMSG_G000007058.1	no	186_1	Polymetallic	74.59	1.61	66.53	0	0	0	16	Medium quality	11895	1289662	41.90	131	d__Archaea;p__Thermoplasmatota;c__Thermoplasmata;o__Thermoplasmatales;f__Thermoplasmataceae;g__;s__
LMSG_G000007059.1	no	186_1	Copper	87.86	4.03	67.70	1	0	0	18	Medium quality	11955	1580922	42.20	159	d__Archaea;p__Thermoplasmatota;c__Thermoplasmata;o__Thermoplasmatales;f__Thermoplasmataceae;g__;s__
LMSG_G000007060.1	no	186_1	Copper	71.90	1.61	63.84	1	0	2	14	Medium quality	12498	1266276	42.10	134	d__Archaea;p__Thermoplasmatota;c__Thermoplasmata;o__Thermoplasmatales;f__Thermoplasmataceae;g__;s__
LMSG_G000007061.1	no	186_1	Copper	79.79	0.00	79.79	1	1	0	12	Medium quality	13084	1357655	42.30	125	d__Archaea;p__Thermoplasmatota;c__Thermoplasmata;o__Thermoplasmatales;f__Thermoplasmataceae;g__;s__
LMSG_G000007062.1	no	186_1	Arsenic	88.66	2.67	75.33	1	0	1	20	Medium quality	13661	1700508	42.10	165	d__Archaea;p__Thermoplasmatota;c__Thermoplasmata;o__Thermoplasmatales;f__Thermoplasmataceae;g__;s__
LMSG_G000007063.1	no	186_1	Pyrite	93.77	3.54	76.10	0	1	1	20	Medium quality	15680	1715167	42.30	148	d__Archaea;p__Thermoplasmatota;c__Thermoplasmata;o__Thermoplasmatales;f__Thermoplasmataceae;g__;s__
LMSG_G000007064.1	no	186_1	Lead-Zinc	94.44	0.00	94.44	1	1	0	20	Medium quality	24962	1799659	42.10	97	d__Archaea;p__Thermoplasmatota;c__Thermoplasmata;o__Thermoplasmatales;f__Thermoplasmataceae;g__;s__
LMSG_G000007065.1	no	186_1	Copper	93.23	2.36	81.45	1	2	0	18	Medium quality	16727	1881560	42.10	159	d__Archaea;p__Thermoplasmatota;c__Thermoplasmata;o__Thermoplasmatales;f__Thermoplasmataceae;g__;s__
LMSG_G000007066.1	no	186_1	Copper	93.23	0.00	93.23	1	2	0	17	Medium quality	15347	1735350	42.00	164	d__Archaea;p__Thermoplasmatota;c__Thermoplasmata;o__Thermoplasmatales;f__Thermoplasmataceae;g__;s__
LMSG_G000007067.1	no	186_1	Polymetallic	94.31	0.00	94.31	1	1	0	18	Medium quality	16437	1870513	42.10	175	d__Archaea;p__Thermoplasmatota;c__Thermoplasmata;o__Thermoplasmatales;f__Thermoplasmataceae;g__;s__
LMSG_G000007068.1	no	186_1	Polymetallic	95.11	2.05	84.88	1	1	0	17	Medium quality	12868	1906404	41.90	206	d__Archaea;p__Thermoplasmatota;c__Thermoplasmata;o__Thermoplasmatales;f__Thermoplasmataceae;g__;s__
LMSG_G000007069.1	no	186_1	Copper	95.11	2.42	83.02	1	1	0	19	Medium quality	12399	1870221	42.00	181	d__Archaea;p__Thermoplasmatota;c__Thermoplasmata;o__Thermoplasmatales;f__Thermoplasmataceae;g__;s__
LMSG_G000007070.1	no	186_1	Polymetallic	91.89	1.61	83.83	1	2	0	18	Medium quality	18252	1875431	42.10	174	d__Archaea;p__Thermoplasmatota;c__Thermoplasmata;o__Thermoplasmatales;f__Thermoplasmataceae;g__;s__
LMSG_G000007071.1	no	187_1	Pyrite	68.04	0.00	68.04	0	1	3	16	Medium quality	15240	1176057	44.00	111	d__Archaea;p__Thermoplasmatota;c__Thermoplasmata;o__Thermoplasmatales;f__Thermoplasmataceae;g__;s__
LMSG_G000007072.1	no	187_1	Lead-Zinc	77.37	1.21	71.33	0	0	1	11	Medium quality	13002	970795	44.00	91	d__Archaea;p__Thermoplasmatota;c__Thermoplasmata;o__Thermoplasmatales;f__Thermoplasmataceae;g__;s__
LMSG_G000007073.1	no	187_1	Lead-Zinc	87.59	1.13	81.95	0	1	1	14	Medium quality	8732	1268905	43.80	177	d__Archaea;p__Thermoplasmatota;c__Thermoplasmata;o__Thermoplasmatales;f__Thermoplasmataceae;g__;s__
LMSG_G000007074.1	no	212_1	Lead-Zinc	94.71	0.37	92.85	1	1	1	20	High quality	17843	1724593	40.90	151	d__Archaea;p__Thermoplasmatota;c__Thermoplasmata;o__Thermoplasmatales;f__Thermoplasmataceae;g__;s__
LMSG_G000007075.1	no	212_1	Copper	74.55	3.63	56.41	1	1	1	17	Medium quality	19344	1331973	40.90	110	d__Archaea;p__Thermoplasmatota;c__Thermoplasmata;o__Thermoplasmatales;f__Thermoplasmataceae;g__;s__
LMSG_G000007076.1	no	212_1	Lead-Zinc	96.32	0.81	92.29	2	0	4	20	Medium quality	28112	1836209	40.90	143	d__Archaea;p__Thermoplasmatota;c__Thermoplasmata;o__Thermoplasmatales;f__Thermoplasmataceae;g__;s__
LMSG_G000007077.1	no	212_1	Lead-Zinc	87.27	4.84	63.08	1	0	1	20	Medium quality	12029	1735484	40.60	202	d__Archaea;p__Thermoplasmatota;c__Thermoplasmata;o__Thermoplasmatales;f__Thermoplasmataceae;g__;s__
LMSG_G000007078.1	no	212_1	Copper	77.16	1.61	69.10	0	0	0	18	Medium quality	24505	1348076	40.90	86	d__Archaea;p__Thermoplasmatota;c__Thermoplasmata;o__Thermoplasmatales;f__Thermoplasmataceae;g__;s__
LMSG_G000007079.1	no	166_1	Copper	89.83	0.81	85.77	1	2	1	19	Medium quality	31398	1617804	32.90	66	d__Archaea;p__Thermoplasmatota;c__Thermoplasmata;o__Thermoplasmatales;f__Thermoplasmataceae;g__Acidiplasma;s__
LMSG_G000007080.1	no	166_1	Pyrite	85.77	1.36	79.00	1	0	1	18	Medium quality	9915	1565338	33.00	187	d__Archaea;p__Thermoplasmatota;c__Thermoplasmata;o__Thermoplasmatales;f__Thermoplasmataceae;g__Acidiplasma;s__
LMSG_G000007081.1	no	166_1	Pyrite	79.14	1.22	73.05	1	0	0	16	Medium quality	4241	1333536	33.20	370	d__Archaea;p__Thermoplasmatota;c__Thermoplasmata;o__Thermoplasmatales;f__Thermoplasmataceae;g__Acidiplasma;s__
LMSG_G000007082.1	no	166_1	Copper	91.46	1.63	83.33	1	2	0	16	Medium quality	29167	1700526	32.80	78	d__Archaea;p__Thermoplasmatota;c__Thermoplasmata;o__Thermoplasmatales;f__Thermoplasmataceae;g__Acidiplasma;s__
LMSG_G000007083.1	no	167_1	Lead-Zinc	97.15	4.07	76.83	1	2	2	19	High quality	73320	1764023	33.20	39	d__Archaea;p__Thermoplasmatota;c__Thermoplasmata;o__Thermoplasmatales;f__Thermoplasmataceae;g__Acidiplasma;s__

LMSG_G000007084.1	no	167_1	Copper	91.46	1.63	83.33	1	2	2	19	High quality	85753	1622175	33.40	42	d__Archaea;p__Thermoplasmatota;c__Thermoplasmata;o__Thermoplasmatales;f__Thermoplasmataceae;g__Acidiplasma;s__
LMSG_G000007085.1	no	167_1	Copper	91.86	0.81	87.80	3	1	2	18	High quality	77101	1441917	33.40	36	d__Archaea;p__Thermoplasmatota;c__Thermoplasmata;o__Thermoplasmatales;f__Thermoplasmataceae;g__Acidiplasma;s__
LMSG_G000007086.1	no	167_1	Copper	97.15	3.25	80.89	1	2	2	19	High quality	59018	1778574	33.50	60	d__Archaea;p__Thermoplasmatota;c__Thermoplasmata;o__Thermoplasmatales;f__Thermoplasmataceae;g__Acidiplasma;s__
LMSG_G000007087.1	no	167_1	Lead-Zinc	78.43	3.25	62.17	1	1	0	16	Medium quality	6748	1430147	33.60	248	d__Archaea;p__Thermoplasmatota;c__Thermoplasmata;o__Thermoplasmatales;f__Thermoplasmataceae;g__Acidiplasma;s__
LMSG_G000007088.1	no	167_1	Copper	90.52	3.29	74.05	0	2	0	19	Medium quality	6151	1714758	33.30	337	d__Archaea;p__Thermoplasmatota;c__Thermoplasmata;o__Thermoplasmatales;f__Thermoplasmataceae;g__Acidiplasma;s__
LMSG_G000007089.1	no	165_1	Copper	91.86	1.63	83.73	1	2	1	18	High quality	15632	1439796	34.30	121	d__Archaea;p__Thermoplasmatota;c__Thermoplasmata;o__Thermoplasmatales;f__Thermoplasmataceae;g__Acidiplasma;s__Acidiplasma aeolicum
LMSG_G000007090.1	no	165_1	Polymetallic	92.27	2.44	80.08	1	2	2	19	High quality	30476	1416784	34.20	74	d__Archaea;p__Thermoplasmatota;c__Thermoplasmata;o__Thermoplasmatales;f__Thermoplasmataceae;g__Acidiplasma;s__Acidiplasma aeolicum
LMSG_G000007091.1	no	165_1	Copper	95.52	2.44	83.33	1	2	2	18	High quality	24289	1469582	34.30	80	d__Archaea;p__Thermoplasmatota;c__Thermoplasmata;o__Thermoplasmatales;f__Thermoplasmataceae;g__Acidiplasma;s__Acidiplasma aeolicum
LMSG_G000007092.1	no	165_1	Copper	97.15	4.07	76.83	1	2	2	17	Medium quality	31444	1559814	34.10	83	d__Archaea;p__Thermoplasmatota;c__Thermoplasmata;o__Thermoplasmatales;f__Thermoplasmataceae;g__Acidiplasma;s__Acidiplasma aeolicum
LMSG_G000007093.1	no	165_1	Copper	94.71	3.25	78.45	1	1	1	17	Medium quality	26673	1601778	34.10	119	d__Archaea;p__Thermoplasmatota;c__Thermoplasmata;o__Thermoplasmatales;f__Thermoplasmataceae;g__Acidiplasma;s__Acidiplasma aeolicum
LMSG_G000007094.1	no	187_1	Lead-Zinc	72.25	0.81	68.22	0	0	2	13	Medium quality	9942	1107564	43.80	146	d__Archaea;p__Thermoplasmatota;c__Thermoplasmata;o__Thermoplasmatales;f__Thermoplasmataceae;g__B-DKE;s__
LMSG_G000007095.1	no	202_1	Lead-Zinc	61.24	1.30	54.73	1	2	2	16	Medium quality	39169	863363	47.80	34	d__Archaea;p__Thermoplasmatota;c__Thermoplasmata;o__Thermoplasmatales;f__Thermoplasmataceae;g__B-DKE;s__
LMSG_G000007096.1	no	207_1	Lead-Zinc	85.45	4.84	61.26	2	2	2	18	Medium quality	14801	1632199	45.90	153	d__Archaea;p__Thermoplasmatota;c__Thermoplasmata;o__Thermoplasmatales;f__Thermoplasmataceae;g__B-DKE;s__
LMSG_G000007097.1	no	207_1	Polymetallic	56.72	1.05	51.45	1	0	1	12	Medium quality	15262	1043732	45.90	146	d__Archaea;p__Thermoplasmatota;c__Thermoplasmata;o__Thermoplasmatales;f__Thermoplasmataceae;g__B-DKE;s__
LMSG_G000007098.1	no	207_1	Polymetallic	70.11	0.00	70.11	1	1	1	15	Medium quality	21411	1025235	46.30	81	d__Archaea;p__Thermoplasmatota;c__Thermoplasmata;o__Thermoplasmatales;f__Thermoplasmataceae;g__B-DKE;s__
LMSG_G000007099.1	no	207_1	Polymetallic	61.51	0.06	61.20	1	0	1	10	Medium quality	17149	958134	46.20	77	d__Archaea;p__Thermoplasmatota;c__Thermoplasmata;o__Thermoplasmatales;f__Thermoplasmataceae;g__B-DKE;s__
LMSG_G000007100.1	no	207_1	Copper	76.07	0.81	72.04	1	1	1	15	Medium quality	20967	1291577	46.30	91	d__Archaea;p__Thermoplasmatota;c__Thermoplasmata;o__Thermoplasmatales;f__Thermoplasmataceae;g__B-DKE;s__
LMSG_G000007101.1	no	207_1	Copper	73.97	3.23	57.85	2	0	1	14	Medium quality	13876	1065689	46.30	103	d__Archaea;p__Thermoplasmatota;c__Thermoplasmata;o__Thermoplasmatales;f__Thermoplasmataceae;g__B-DKE;s__
LMSG_G000007102.1	no	207_1	Copper	82.66	4.22	61.57	0	0	2	19	Medium quality	13037	1419818	46.00	146	d__Archaea;p__Thermoplasmatota;c__Thermoplasmata;o__Thermoplasmatales;f__Thermoplasmataceae;g__B-DKE;s__
LMSG_G000007103.1	no	207_1	Lead-Zinc	65.59	0.00	65.59	1	0	0	14	Medium quality	17887	938952	46.70	76	d__Archaea;p__Thermoplasmatota;c__Thermoplasmata;o__Thermoplasmatales;f__Thermoplasmataceae;g__B-DKE;s__
LMSG_G000007104.1	no	207_1	Pyrite-Copper	90.86	1.61	82.80	1	2	0	17	Medium quality	13674	1621960	45.90	177	d__Archaea;p__Thermoplasmatota;c__Thermoplasmata;o__Thermoplasmatales;f__Thermoplasmataceae;g__B-DKE;s__
LMSG_G000007105.1	no	207_1	Copper	84.55	0.00	84.55	1	0	1	18	Medium quality	42766	1300495	46.00	90	d__Archaea;p__Thermoplasmatota;c__Thermoplasmata;o__Thermoplasmatales;f__Thermoplasmataceae;g__B-DKE;s__
LMSG_G000007106.1	no	208_1	Copper	58.40	0.00	58.40	1	0	0	12	Medium quality	18338	1078511	46.30	94	d__Archaea;p__Thermoplasmatota;c__Thermoplasmata;o__Thermoplasmatales;f__Thermoplasmataceae;g__B-DKE;s__
LMSG_G000007107.1	no	208_1	Copper	65.81	0.00	65.81	0	0	0	16	Medium quality	12588	928394	46.30	90	d__Archaea;p__Thermoplasmatota;c__Thermoplasmata;o__Thermoplasmatales;f__Thermoplasmataceae;g__B-DKE;s__
LMSG_G000007108.1	no	208_1	Arsenic	92.20	1.05	86.93	1	1	0	19	Medium quality	19861	1397509	46.20	183	d__Archaea;p__Thermoplasmatota;c__Thermoplasmata;o__Thermoplasmatales;f__Thermoplasmataceae;g__B-DKE;s__
LMSG_G000007109.1	no	208_1	Lead-Zinc	68.88	2.82	54.77	1	0	0	13	Medium quality	9839	1372354	46.20	180	d__Archaea;p__Thermoplasmatota;c__Thermoplasmata;o__Thermoplasmatales;f__Thermoplasmataceae;g__B-DKE;s__
LMSG_G000007110.1	no	208_1	Copper	64.21	1.21	58.17	1	0	1	14	Medium quality	10019	1281826	45.80	308	d__Archaea;p__Thermoplasmatota;c__Thermoplasmata;o__Thermoplasmatales;f__Thermoplasmataceae;g__B-DKE;s__
LMSG_G000007111.1	no	208_1	Coal	96.50	0.40	94.49	1	1	1	18	High quality	17860	1352471	46.10	130	d__Archaea;p__Thermoplasmatota;c__Thermoplasmata;o__Thermoplasmatales;f__Thermoplasmataceae;g__B-DKE;s__
LMSG_G000007112.1	no	208_1	Coal	90.86	0.00	90.86	1	2	1	18	High quality	18492	1183718	46.20	102	d__Archaea;p__Thermoplasmatota;c__Thermoplasmata;o__Thermoplasmatales;f__Thermoplasmataceae;g__B-DKE;s__
LMSG_G000007113.1	no	208_1	Polymetallic	52.83	0.00	52.83	0	0	0	15	Medium quality	14469	885252	45.80	85	d__Archaea;p__Thermoplasmatota;c__Thermoplasmata;o__Thermoplasmatales;f__Thermoplasmataceae;g__B-DKE;s__
LMSG_G000007114.1	no	193_1	Pyrite	98.61	0.00	98.61	1	1	1	20	High quality	81433	1866750	44.40	47	d__Archaea;p__Thermoplasmatota;c__Thermoplasmata;o__Thermoplasmatales;f__Thermoplasmataceae;g__B-DKE;s__B-DKE sp002204705
LMSG_G000007115.1	no	169_1	Pyrite	96.32	0.00	96.32	1	2	2	20	High quality	1100711	1794012	38.00	10	d__Archaea;p__Thermoplasmatota;c__Thermoplasmata;o__Thermoplasmatales;f__Thermoplasmataceae;g__Cuniculiplasma;s__
LMSG_G000007116.1	no	179_1	Copper	73.18	1.61	65.12	1	2	2	15	Medium quality	127013	1381578	39.90	33	d__Archaea;p__Thermoplasmatota;c__Thermoplasmata;o__Thermoplasmatales;f__Thermoplasmataceae;g__Cuniculiplasma;s__
LMSG_G000007117.1	no	179_1	Copper	67.46	0.00	67.46	1	2	2	14	Medium quality	144672	1129226	40.10	17	d__Archaea;p__Thermoplasmatota;c__Thermoplasmata;o__Thermoplasmatales;f__Thermoplasmataceae;g__Cuniculiplasma;s__
LMSG_G000007118.1	no	182_1	Pyrite	90.68	0.27	89.34	1	1	1	18	High quality	25937	1274746	41.70	78	d__Archaea;p__Thermoplasmatota;c__Thermoplasmata;o__Thermoplasmatales;f__Thermoplasmataceae;g__Cuniculiplasma;s__
LMSG_G000007119.1	no	182_1	Pyrite	92.29	1.61	84.23	1	1	2	20	High quality	23873	1236640	41.60	81	d__Archaea;p__Thermoplasmatota;c__Thermoplasmata;o__Thermoplasmatales;f__Thermoplasmataceae;g__Cuniculiplasma;s__
LMSG_G000007120.1	no	182_1	Polymetallic	95.52	0.00	95.52	1	3	1	19	High quality	74881	1393215	41.30	48	d__Archaea;p__Thermoplasmatota;c__Thermoplasmata;o__Thermoplasmatales;f__Thermoplasmataceae;g__Cuniculiplasma;s__
LMSG_G000007121.1	no	182_1	Polymetallic	95.50	0.00	95.50	1	3	2	20	High quality	94111	1442325	41.40	58	d__Archaea;p__Thermoplasmatota;c__Thermoplasmata;o__Thermoplasmatales;f__Thermoplasmataceae;g__Cuniculiplasma;s__
LMSG_G000007122.1	no	182_1	Tin-Zinc	93.90	0.87	89.56	1	1	1	20	High quality	24050	1301654	41.50	98	d__Archaea;p__Thermoplasmatota;c__Thermoplasmata;o__Thermoplasmatales;f__Thermoplasmataceae;g__Cuniculiplasma;s__
LMSG_G000007123.1	no	182_1	Pyrite-Copper	94.71	0.81	90.68	1	2	1	19	High quality	68747	1388130	41.80	88	d__Archaea;p__Thermoplasmatota;c__Thermoplasmata;o__Thermoplasmatales;f__Thermoplasmataceae;g__Cuniculiplasma;s__
LMSG_G000007124.1	no	182_1	Pyrite-Copper	92.29	0.00	92.29	1	1	1	20	High quality	19517	1331366	41.70	95	d__Archaea;p__Thermoplasmatota;c__Thermoplasmata;o__Thermoplasmatales;f__Thermoplasmataceae;g__Cuniculiplasma;s__
LMSG_G000007125.1	no	182_1	Lead-Zinc	93.90	0.00	93.90	1	2	1	19	High quality	35273	1283355	41.70	64	d__Archaea;p__Thermoplasmatota;c__Thermoplasmata;o__Thermoplasmatales;f__Thermoplasmataceae;g__Cuniculiplasma;s__
LMSG_G000007126.1	no	182_1	Polymetallic	93.90	0.00	93.90	1	2	1	19	High quality	28847	1542474	41.00	90	d__Archaea;p__Thermoplasmatota;c__Thermoplasmata;o__Thermoplasmatales;f__Thermoplasmataceae;g__Cuniculiplasma;s__
LMSG_G000007127.1	no	182_1	Lead-Zinc	64.91	0.00	64.91	0	1	1	12	Medium quality	20141	806050	42.60	50	d__Archaea;p__Thermoplasmatota;c__Thermoplasmata;o__Thermoplasmatales;f__Thermoplasmataceae;g__Cuniculiplasma;s__
LMSG_G000007128.1	no	182_1	Copper	86.65	0.00	86.65	1	1	1	18	Medium quality	18550	1223404	41.70	104	d__Archaea;p__Thermoplasmatota;c__Thermoplasmata;o__Thermoplasmatales;f__Thermoplasmataceae;g__Cuniculiplasma;s__
LMSG_G000007129.1	no	182_1	Pyrite	60.03	0.81	56.00	1	2	1	11	Medium quality	27782	870262	42.60	58	d__Archaea;p__Thermoplasmatota;c__Thermoplasmata;o__Thermoplasmatales;f__Thermoplasmataceae;g__Cuniculiplasma;s__
LMSG_G000007130.1	no	182_1	Copper	76.16	0.17	75.32	1	1	1	17	Medium quality	30087	1215800	41.90	164	d__Archaea;p__Thermoplasmatota;c__Thermoplasmata;o__Thermoplasmatales;f__Thermoplasmataceae;g__Cuniculiplasma;s__
LMSG_G000007131.1	no	182_1	Copper	70.59	3.04	55.39	1	1	1	15	Medium quality	6678	991304	42.10	177	d__Archaea;p__Thermoplasmatota;c__Thermoplasmata;o__Thermoplasmatales;f__Thermoplasmataceae;g__Cuniculiplasma;s__
LMSG_G000007132.1	no	182_1	Copper	82.61	0.81	78.58	1	1	2	19	Medium quality	28434	1183303	41.60	84	d__Archaea;p__Thermoplasmatota;c__Thermoplasmata;o__Thermoplasmatales;f__Thermoplasmataceae;g__Cuniculiplasma;s__
LMSG_G000007133.1	no	182_1	Copper	79.30	0.00	79.30	1	1	0	12	Medium quality	4439	962711	42.30	285	d__Archaea;p__Thermoplasmatota;c__Thermoplasmata;o__Thermoplasmatales;f__Thermoplasmataceae;g__Cuniculiplasma;s__
LMSG_G000007134.1	no	182_1	Lead-Zinc	89.87	0.00	89.87	1	2	1	19	Medium quality	33724	1323408	41.60	64	d__Archaea;p__Thermoplasmatota;c__Thermoplasmata;o__Thermoplasmatales;f__Thermoplasmataceae;g__Cuniculiplasma;s__

LMSG_G000007135.1	no	182_1	Lead-Zinc	60.84	0.81	56.81	0	1	1	14	Medium quality	27571	761782	42.30	47	d__Archaea;p__Thermoplasmatota;c__Thermoplasmata;o__Thermoplasmatales;f__Thermoplasmataceae;g__Cuniculiplasma;s__
LMSG_G000007136.1	no	182_1	Lead-Zinc	73.74	3.23	57.62	0	2	1	14	Medium quality	15515	967414	42.40	91	d__Archaea;p__Thermoplasmatota;c__Thermoplasmata;o__Thermoplasmatales;f__Thermoplasmataceae;g__Cuniculiplasma;s__
LMSG_G000007137.1	no	182_1	Copper	73.74	0.00	73.74	1	2	1	16	Medium quality	33036	997962	42.30	45	d__Archaea;p__Thermoplasmatota;c__Thermoplasmata;o__Thermoplasmatales;f__Thermoplasmataceae;g__Cuniculiplasma;s__
LMSG_G000007138.1	no	182_1	Polymetallic	86.65	0.00	86.65	1	1	2	18	Medium quality	22129	1195198	41.60	81	d__Archaea;p__Thermoplasmatota;c__Thermoplasmata;o__Thermoplasmatales;f__Thermoplasmataceae;g__Cuniculiplasma;s__
LMSG_G000007139.1	no	182_1	Polymetallic	79.70	0.81	75.67	2	2	1	18	Medium quality	19821	1106267	41.60	76	d__Archaea;p__Thermoplasmatota;c__Thermoplasmata;o__Thermoplasmatales;f__Thermoplasmataceae;g__Cuniculiplasma;s__
LMSG_G000007140.1	no	182_1	Polymetallic	84.04	0.00	84.04	1	1	1	17	Medium quality	22578	1172183	41.70	82	d__Archaea;p__Thermoplasmatota;c__Thermoplasmata;o__Thermoplasmatales;f__Thermoplasmataceae;g__Cuniculiplasma;s__
LMSG_G000007141.1	no	182_1	Polymetallic	88.53	0.04	88.32	1	4	1	18	Medium quality	6642	1332976	41.40	282	d__Archaea;p__Thermoplasmatota;c__Thermoplasmata;o__Thermoplasmatales;f__Thermoplasmataceae;g__Cuniculiplasma;s__
LMSG_G000007142.1	no	182_1	Polymetallic	55.81	0.00	55.81	1	1	2	15	Medium quality	3970	821217	41.80	234	d__Archaea;p__Thermoplasmatota;c__Thermoplasmata;o__Thermoplasmatales;f__Thermoplasmataceae;g__Cuniculiplasma;s__
LMSG_G000007143.1	no	182_1	Polymetallic	68.65	0.85	64.41	0	2	1	11	Medium quality	4942	869300	42.00	211	d__Archaea;p__Thermoplasmatota;c__Thermoplasmata;o__Thermoplasmatales;f__Thermoplasmataceae;g__Cuniculiplasma;s__
LMSG_G000007144.1	no	182_1	Polymetallic	79.07	0.30	77.55	1	2	1	15	Medium quality	11756	1117619	41.70	143	d__Archaea;p__Thermoplasmatota;c__Thermoplasmata;o__Thermoplasmatales;f__Thermoplasmataceae;g__Cuniculiplasma;s__
LMSG_G000007145.1	no	182_1	Polymetallic	80.10	1.61	72.04	1	3	1	20	Medium quality	11233	1156028	41.60	141	d__Archaea;p__Thermoplasmatota;c__Thermoplasmata;o__Thermoplasmatales;f__Thermoplasmataceae;g__Cuniculiplasma;s__
LMSG_G000007146.1	no	182_1	Tin-Zinc	82.28	1.61	74.22	1	1	1	16	Medium quality	17528	1128394	42.00	117	d__Archaea;p__Thermoplasmatota;c__Thermoplasmata;o__Thermoplasmatales;f__Thermoplasmataceae;g__Cuniculiplasma;s__
LMSG_G000007147.1	no	182_1	Polymetallic	77.64	0.00	77.64	1	1	2	17	Medium quality	21692	992586	41.90	69	d__Archaea;p__Thermoplasmatota;c__Thermoplasmata;o__Thermoplasmatales;f__Thermoplasmataceae;g__Cuniculiplasma;s__
LMSG_G000007148.1	no	182_1	Copper	87.45	0.81	83.42	1	0	2	17	Medium quality	51205	1220066	41.90	49	d__Archaea;p__Thermoplasmatota;c__Thermoplasmata;o__Thermoplasmatales;f__Thermoplasmataceae;g__Cuniculiplasma;s__
LMSG_G000007149.1	no	182_1	Copper	66.12	0.00	66.12	1	1	1	15	Medium quality	39991	946138	41.50	40	d__Archaea;p__Thermoplasmatota;c__Thermoplasmata;o__Thermoplasmatales;f__Thermoplasmataceae;g__Cuniculiplasma;s__
LMSG_G000007150.1	no	182_1	Copper	67.29	1.61	59.23	0	0	0	15	Medium quality	46053	947768	41.20	39	d__Archaea;p__Thermoplasmatota;c__Thermoplasmata;o__Thermoplasmatales;f__Thermoplasmataceae;g__Cuniculiplasma;s__
LMSG_G000007151.1	no	182_1	Polymetallic	69.35	0.81	65.32	1	0	2	19	Medium quality	20505	935250	41.10	65	d__Archaea;p__Thermoplasmatota;c__Thermoplasmata;o__Thermoplasmatales;f__Thermoplasmataceae;g__Cuniculiplasma;s__
LMSG_G000007152.1	no	182_1	Magnetite	67.47	3.23	51.35	1	1	1	15	Medium quality	15096	1051128	41.20	84	d__Archaea;p__Thermoplasmatota;c__Thermoplasmata;o__Thermoplasmatales;f__Thermoplasmataceae;g__Cuniculiplasma;s__
LMSG_G000007153.1	no	182_1	Copper	86.65	0.00	86.65	1	1	2	17	Medium quality	33454	1269860	41.70	58	d__Archaea;p__Thermoplasmatota;c__Thermoplasmata;o__Thermoplasmatales;f__Thermoplasmataceae;g__Cuniculiplasma;s__
LMSG_G000007154.1	no	182_1	Lead-Zinc	82.61	0.81	78.58	1	2	1	18	Medium quality	17881	1163275	41.80	93	d__Archaea;p__Thermoplasmatota;c__Thermoplasmata;o__Thermoplasmatales;f__Thermoplasmataceae;g__Cuniculiplasma;s__
LMSG_G000007155.1	no	182_1	Lead-Zinc	89.87	0.00	89.87	1	1	1	19	Medium quality	20273	1216719	41.90	87	d__Archaea;p__Thermoplasmatota;c__Thermoplasmata;o__Thermoplasmatales;f__Thermoplasmataceae;g__Cuniculiplasma;s__
LMSG_G000007156.1	no	182_1	Pyrite-Copper	86.65	0.27	85.31	1	1	2	19	Medium quality	22532	1259915	41.40	81	d__Archaea;p__Thermoplasmatota;c__Thermoplasmata;o__Thermoplasmatales;f__Thermoplasmataceae;g__Cuniculiplasma;s__
LMSG_G000007157.1	no	182_1	Pyrite-Copper	88.66	2.42	76.57	1	2	2	20	Medium quality	17492	1459116	41.60	156	d__Archaea;p__Thermoplasmatota;c__Thermoplasmata;o__Thermoplasmatales;f__Thermoplasmataceae;g__Cuniculiplasma;s__
LMSG_G000007158.1	no	182_1	Pyrite-Copper	89.87	1.08	84.50	1	1	1	20	Medium quality	20370	1449813	41.30	137	d__Archaea;p__Thermoplasmatota;c__Thermoplasmata;o__Thermoplasmatales;f__Thermoplasmataceae;g__Cuniculiplasma;s__
LMSG_G000007159.1	no	182_1	Polymetallic	86.15	0.81	82.12	1	1	1	20	Medium quality	20609	1278284	41.10	89	d__Archaea;p__Thermoplasmatota;c__Thermoplasmata;o__Thermoplasmatales;f__Thermoplasmataceae;g__Cuniculiplasma;s__
LMSG_G000007160.1	no	182_1	Magnetite	80.19	4.84	56.00	1	2	1	17	Medium quality	13968	1221807	41.60	125	d__Archaea;p__Thermoplasmatota;c__Thermoplasmata;o__Thermoplasmatales;f__Thermoplasmataceae;g__Cuniculiplasma;s__
LMSG_G000007161.1	no	182_1	Magnetite	53.62	0.00	53.62	2	1	0	16	Medium quality	22476	878238	40.90	74	d__Archaea;p__Thermoplasmatota;c__Thermoplasmata;o__Thermoplasmatales;f__Thermoplasmataceae;g__Cuniculiplasma;s__
LMSG_G000007162.1	no	182_1	Pyrite-Copper	79.03	0.81	75.00	1	0	1	15	Medium quality	18289	1143576	41.60	102	d__Archaea;p__Thermoplasmatota;c__Thermoplasmata;o__Thermoplasmatales;f__Thermoplasmataceae;g__Cuniculiplasma;s__
LMSG_G000007163.1	no	182_1	Coal	83.68	0.00	83.68	1	1	0	18	Medium quality	3092	1201634	41.70	464	d__Archaea;p__Thermoplasmatota;c__Thermoplasmata;o__Thermoplasmatales;f__Thermoplasmataceae;g__Cuniculiplasma;s__
LMSG_G000007164.1	no	182_1	Coal	86.10	0.81	82.07	1	2	2	19	Medium quality	15082	1196280	41.50	114	d__Archaea;p__Thermoplasmatota;c__Thermoplasmata;o__Thermoplasmatales;f__Thermoplasmataceae;g__Cuniculiplasma;s__
LMSG_G000007165.1	no	183_1	Polymetallic	83.76	3.69	65.31	1	1	1	18	Medium quality	25147	1486480	40.30	114	d__Archaea;p__Thermoplasmatota;c__Thermoplasmata;o__Thermoplasmatales;f__Thermoplasmataceae;g__Cuniculiplasma;s__
LMSG_G000007166.1	no	183_1	Tin-Zinc	87.25	3.23	71.13	1	1	1	19	Medium quality	22910	1464061	40.40	128	d__Archaea;p__Thermoplasmatota;c__Thermoplasmata;o__Thermoplasmatales;f__Thermoplasmataceae;g__Cuniculiplasma;s__
LMSG_G000007167.1	no	184_1	Pyrite	55.16	0.27	53.82	2	1	1	7	Medium quality	5689	874567	43.70	178	d__Archaea;p__Thermoplasmatota;c__Thermoplasmata;o__Thermoplasmatales;f__Thermoplasmataceae;g__Cuniculiplasma;s__
LMSG_G000007168.1	no	184_1	Polymetallic	84.27	2.42	72.18	1	2	1	18	Medium quality	67244	1387107	42.60	31	d__Archaea;p__Thermoplasmatota;c__Thermoplasmata;o__Thermoplasmatales;f__Thermoplasmataceae;g__Cuniculiplasma;s__
LMSG_G000007169.1	no	184_1	Polymetallic	63.30	0.00	63.30	1	1	2	16	Medium quality	55231	892395	42.90	19	d__Archaea;p__Thermoplasmatota;c__Thermoplasmata;o__Thermoplasmatales;f__Thermoplasmataceae;g__Cuniculiplasma;s__
LMSG_G000007170.1	no	184_1	Polymetallic	64.11	0.00	64.11	0	0	1	17	Medium quality	67783	1029470	42.40	24	d__Archaea;p__Thermoplasmatota;c__Thermoplasmata;o__Thermoplasmatales;f__Thermoplasmataceae;g__Cuniculiplasma;s__
LMSG_G000007171.1	no	184_1	Polymetallic	86.69	0.81	82.66	1	2	2	19	Medium quality	86925	1437867	42.50	30	d__Archaea;p__Thermoplasmatota;c__Thermoplasmata;o__Thermoplasmatales;f__Thermoplasmataceae;g__Cuniculiplasma;s__
LMSG_G000007172.1	no	184_1	Copper	52.82	0.00	52.82	1	2	1	12	Medium quality	60750	836644	43.40	23	d__Archaea;p__Thermoplasmatota;c__Thermoplasmata;o__Thermoplasmatales;f__Thermoplasmataceae;g__Cuniculiplasma;s__
LMSG_G000007173.1	no	184_1	Copper	81.85	2.42	69.76	1	3	2	17	Medium quality	54342	1384198	42.60	50	d__Archaea;p__Thermoplasmatota;c__Thermoplasmata;o__Thermoplasmatales;f__Thermoplasmataceae;g__Cuniculiplasma;s__
LMSG_G000007174.1	no	188_1	Magnetite	90.62	4.03	70.46	1	2	1	19	High quality	12415	1546678	40.70	193	d__Archaea;p__Thermoplasmatota;c__Thermoplasmata;o__Thermoplasmatales;f__Thermoplasmataceae;g__Cuniculiplasma;s__
LMSG_G000007175.1	no	188_1	Lead-Zinc	75.00	1.61	66.94	1	0	0	14	Medium quality	6694	1411922	40.70	277	d__Archaea;p__Thermoplasmatota;c__Thermoplasmata;o__Thermoplasmatales;f__Thermoplasmataceae;g__Cuniculiplasma;s__
LMSG_G000007176.1	no	188_1	Lead-Zinc	78.66	2.42	66.57	1	1	1	16	Medium quality	32610	1138890	40.60	73	d__Archaea;p__Thermoplasmatota;c__Thermoplasmata;o__Thermoplasmatales;f__Thermoplasmataceae;g__Cuniculiplasma;s__
LMSG_G000007177.1	no	188_1	Copper	76.57	4.84	52.38	1	0	1	15	Medium quality	16160	1254409	40.40	126	d__Archaea;p__Thermoplasmatota;c__Thermoplasmata;o__Thermoplasmatales;f__Thermoplasmataceae;g__Cuniculiplasma;s__
LMSG_G000007178.1	no	188_1	Copper	76.97	0.81	72.94	1	0	2	16	Medium quality	27477	1178485	40.70	75	d__Archaea;p__Thermoplasmatota;c__Thermoplasmata;o__Thermoplasmatales;f__Thermoplasmataceae;g__Cuniculiplasma;s__
LMSG_G000007179.1	no	188_1	Polymetallic	75.18	0.81	71.15	1	1	1	16	Medium quality	8594	1155710	40.70	158	d__Archaea;p__Thermoplasmatota;c__Thermoplasmata;o__Thermoplasmatales;f__Thermoplasmataceae;g__Cuniculiplasma;s__
LMSG_G000007180.1	no	188_1	Magnetite	82.37	0.00	82.37	0	0	0	15	Medium quality	31078	1277058	40.60	81	d__Archaea;p__Thermoplasmatota;c__Thermoplasmata;o__Thermoplasmatales;f__Thermoplasmataceae;g__Cuniculiplasma;s__
LMSG_G000007181.1	no	206_1	Copper	72.17	1.61	64.11	1	1	1	15	Medium quality	46882	1391319	39.10	85	d__Archaea;p__Thermoplasmatota;c__Thermoplasmata;o__Thermoplasmatales;f__Thermoplasmataceae;g__Cuniculiplasma;s__
LMSG_G000007182.1	no	169_1	Iron	96.32	0.00	96.32	1	1	1	20	High quality	98413	2034572	37.10	121	d__Archaea;p__Thermoplasmatota;c__Thermoplasmata;o__Thermoplasmatales;f__Thermoplasmataceae;g__Cuniculiplasma;s__Cuniculiplasma divulgatum
LMSG_G000007183.1	no	169_1	Iron	96.32	0.00	96.32	1	1	1	20	High quality	106766	1727180	37.20	74	d__Archaea;p__Thermoplasmatota;c__Thermoplasmata;o__Thermoplasmatales;f__Thermoplasmataceae;g__Cuniculiplasma;s__Cuniculiplasma divulgatum
LMSG_G000007184.1	no	169_1	Iron	94.71	0.00	94.71	1	1	1	20	High quality	94414	1704827	37.20	61	d__Archaea;p__Thermoplasmatota;c__Thermoplasmata;o__Thermoplasmatales;f__Thermoplasmataceae;g__Cuniculiplasma;s__Cuniculiplasma divulgatum
LMSG_G000007185.1	no	169_1	Iron	96.32	0.00	96.32	1	1	2	19	High quality	54054	1790464	37.50	85	d__Archaea;p__Thermoplasmatota;c__Thermoplasmata;o__Thermoplasmatales;f__Thermoplasmataceae;g__Cuniculiplasma;s__Cuniculiplasma divulgatum

LMSG_G000007186.1	no	169_1	Iron	95.52	0.00	95.52	1	1	2	20	High quality	34577	1697159	37.20	80	d__Archaea;p__Thermoplasmatota;c__Thermoplasmata;o__Thermoplasmatales;f__Thermoplasmataceae;g__Cuniculiplasma;s__Cuniculiplasma divulgatum
LMSG_G000007187.1	no	169_1	Iron	97.13	1.88	87.73	1	1	1	19	High quality	92754	1791117	37.30	73	d__Archaea;p__Thermoplasmatota;c__Thermoplasmata;o__Thermoplasmatales;f__Thermoplasmataceae;g__Cuniculiplasma;s__Cuniculiplasma divulgatum
LMSG_G000007188.1	no	169_1	Iron	96.32	0.00	96.32	1	1	2	20	High quality	29389	1804572	37.10	69	d__Archaea;p__Thermoplasmatota;c__Thermoplasmata;o__Thermoplasmatales;f__Thermoplasmataceae;g__Cuniculiplasma;s__Cuniculiplasma divulgatum
LMSG_G000007189.1	no	169_1	Iron	96.32	0.00	96.32	1	1	2	18	High quality	89129	1668749	37.30	50	d__Archaea;p__Thermoplasmatota;c__Thermoplasmata;o__Thermoplasmatales;f__Thermoplasmataceae;g__Cuniculiplasma;s__Cuniculiplasma divulgatum
LMSG_G000007190.1	no	169_1	Iron	96.32	0.00	96.32	1	1	1	20	High quality	90149	1715447	37.30	40	d__Archaea;p__Thermoplasmatota;c__Thermoplasmata;o__Thermoplasmatales;f__Thermoplasmataceae;g__Cuniculiplasma;s__Cuniculiplasma divulgatum
LMSG_G000007191.1	no	169_1	Iron	96.32	0.00	96.32	1	1	2	19	High quality	113680	1795800	37.20	63	d__Archaea;p__Thermoplasmatota;c__Thermoplasmata;o__Thermoplasmatales;f__Thermoplasmataceae;g__Cuniculiplasma;s__Cuniculiplasma divulgatum
LMSG_G000007192.1	no	169_1	Iron	95.52	0.16	94.72	1	1	1	17	Medium quality	24819	1850779	37.60	128	d__Archaea;p__Thermoplasmatota;c__Thermoplasmata;o__Thermoplasmatales;f__Thermoplasmataceae;g__Cuniculiplasma;s__Cuniculiplasma divulgatum
LMSG_G000007193.1	no	169_1	Iron	75.95	1.23	69.80	1	0	2	16	Medium quality	4368	1459951	37.20	307	d__Archaea;p__Thermoplasmatota;c__Thermoplasmata;o__Thermoplasmatales;f__Thermoplasmataceae;g__Cuniculiplasma;s__Cuniculiplasma divulgatum
LMSG_G000007194.1	no	169_1	Iron	62.38	0.00	62.38	1	0	0	7	Medium quality	3397	1006582	36.90	277	d__Archaea;p__Thermoplasmatota;c__Thermoplasmata;o__Thermoplasmatales;f__Thermoplasmataceae;g__Cuniculiplasma;s__Cuniculiplasma divulgatum
LMSG_G000007195.1	no	169_1	Copper	96.32	0.00	96.32	1	1	2	20	High quality	492721	1916083	37.10	16	d__Archaea;p__Thermoplasmatota;c__Thermoplasmata;o__Thermoplasmatales;f__Thermoplasmataceae;g__Cuniculiplasma;s__Cuniculiplasma divulgatum
LMSG_G000007196.1	no	169_1	Copper	78.22	0.81	74.19	0	1	1	17	Medium quality	46908	1697097	37.10	83	d__Archaea;p__Thermoplasmatota;c__Thermoplasmata;o__Thermoplasmatales;f__Thermoplasmataceae;g__Cuniculiplasma;s__Cuniculiplasma divulgatum
LMSG_G000007197.1	no	169_1	Pyrite	93.90	0.81	89.87	1	2	2	19	High quality	42411	1726993	37.30	73	d__Archaea;p__Thermoplasmatota;c__Thermoplasmata;o__Thermoplasmatales;f__Thermoplasmataceae;g__Cuniculiplasma;s__Cuniculiplasma divulgatum
LMSG_G000007198.1	no	169_1	Pyrite	93.90	0.06	93.59	1	2	2	20	High quality	37795	1733055	37.20	102	d__Archaea;p__Thermoplasmatota;c__Thermoplasmata;o__Thermoplasmatales;f__Thermoplasmataceae;g__Cuniculiplasma;s__Cuniculiplasma divulgatum
LMSG_G000007199.1	no	169_1	Pyrite	72.67	1.51	65.11	1	1	1	18	Medium quality	5846	1479325	38.00	305	d__Archaea;p__Thermoplasmatota;c__Thermoplasmata;o__Thermoplasmatales;f__Thermoplasmataceae;g__Cuniculiplasma;s__Cuniculiplasma divulgatum
LMSG_G000007200.1	no	169_1	Lead-Zinc	93.90	1.13	88.26	1	1	1	15	Medium quality	39539	1787974	37.30	138	d__Archaea;p__Thermoplasmatota;c__Thermoplasmata;o__Thermoplasmatales;f__Thermoplasmataceae;g__Cuniculiplasma;s__Cuniculiplasma divulgatum
LMSG_G000007201.1	no	169_1	Lead-Zinc	93.90	4.84	69.71	2	0	2	19	Medium quality	15498	2007560	37.40	223	d__Archaea;p__Thermoplasmatota;c__Thermoplasmata;o__Thermoplasmatales;f__Thermoplasmataceae;g__Cuniculiplasma;s__Cuniculiplasma divulgatum
LMSG_G000007202.1	no	169_1	Lead-Zinc	85.03	4.03	64.87	1	0	0	17	Medium quality	16959	1369330	37.50	126	d__Archaea;p__Thermoplasmatota;c__Thermoplasmata;o__Thermoplasmatales;f__Thermoplasmataceae;g__Cuniculiplasma;s__Cuniculiplasma divulgatum
LMSG_G000007203.1	no	169_1	Pyrite	79.40	1.88	70.00	1	0	0	14	Medium quality	16719	1223657	37.60	115	d__Archaea;p__Thermoplasmatota;c__Thermoplasmata;o__Thermoplasmatales;f__Thermoplasmataceae;g__Cuniculiplasma;s__Cuniculiplasma divulgatum
LMSG_G000007204.1	no	169_1	Pyrite	84.09	1.61	76.03	1	1	1	15	Medium quality	13967	1313964	37.30	126	d__Archaea;p__Thermoplasmatota;c__Thermoplasmata;o__Thermoplasmatales;f__Thermoplasmataceae;g__Cuniculiplasma;s__Cuniculiplasma divulgatum
LMSG_G000007205.1	no	169_1	Lead-Zinc	92.02	0.00	92.02	1	0	2	19	Medium quality	41562	1901093	37.40	140	d__Archaea;p__Thermoplasmatota;c__Thermoplasmata;o__Thermoplasmatales;f__Thermoplasmataceae;g__Cuniculiplasma;s__Cuniculiplasma divulgatum
LMSG_G000007206.1	no	169_1	Arsenic	89.33	4.72	65.72	1	1	2	18	Medium quality	12798	2120947	37.40	273	d__Archaea;p__Thermoplasmatota;c__Thermoplasmata;o__Thermoplasmatales;f__Thermoplasmataceae;g__Cuniculiplasma;s__Cuniculiplasma divulgatum
LMSG_G000007207.1	no	169_1	Pyrite	80.07	0.97	75.24	1	0	2	16	Medium quality	14342	1398739	37.60	145	d__Archaea;p__Thermoplasmatota;c__Thermoplasmata;o__Thermoplasmatales;f__Thermoplasmataceae;g__Cuniculiplasma;s__Cuniculiplasma divulgatum
LMSG_G000007208.1	no	169_1	Polymetallic	81.81	3.40	64.81	1	0	2	14	Medium quality	14829	1356843	37.40	147	d__Archaea;p__Thermoplasmatota;c__Thermoplasmata;o__Thermoplasmatales;f__Thermoplasmataceae;g__Cuniculiplasma;s__Cuniculiplasma divulgatum
LMSG_G000007209.1	no	169_1	Copper	70.52	2.05	60.29	1	1	1	14	Medium quality	8962	1438023	37.60	213	d__Archaea;p__Thermoplasmatota;c__Thermoplasmata;o__Thermoplasmatales;f__Thermoplasmataceae;g__Cuniculiplasma;s__Cuniculiplasma divulgatum
LMSG_G000007210.1	no	169_1	Polymetallic	91.48	4.84	67.29	1	1	2	17	Medium quality	13055	2468840	37.60	309	d__Archaea;p__Thermoplasmatota;c__Thermoplasmata;o__Thermoplasmatales;f__Thermoplasmataceae;g__Cuniculiplasma;s__Cuniculiplasma divulgatum
LMSG_G000007211.1	no	169_1	Lead-Zinc	69.35	2.69	55.91	1	0	1	18	Medium quality	19319	1156247	37.40	88	d__Archaea;p__Thermoplasmatota;c__Thermoplasmata;o__Thermoplasmatales;f__Thermoplasmataceae;g__Cuniculiplasma;s__Cuniculiplasma divulgatum
LMSG_G000007212.1	no	169_1	Lead-Zinc	67.56	0.81	63.53	0	1	1	13	Medium quality	21592	958038	37.70	73	d__Archaea;p__Thermoplasmatota;c__Thermoplasmata;o__Thermoplasmatales;f__Thermoplasmataceae;g__Cuniculiplasma;s__Cuniculiplasma divulgatum
LMSG_G000007213.1	no	169_1	Copper	75.80	0.81	71.77	1	1	2	17	Medium quality	21543	1295768	37.30	91	d__Archaea;p__Thermoplasmatota;c__Thermoplasmata;o__Thermoplasmatales;f__Thermoplasmataceae;g__Cuniculiplasma;s__Cuniculiplasma divulgatum
LMSG_G000007214.1	no	169_1	Copper	85.10	2.42	73.01	1	1	2	18	Medium quality	20748	1396876	37.60	91	d__Archaea;p__Thermoplasmatota;c__Thermoplasmata;o__Thermoplasmatales;f__Thermoplasmataceae;g__Cuniculiplasma;s__Cuniculiplasma divulgatum
LMSG_G000007215.1	no	169_1	Polymetallic	91.48	0.81	87.45	1	0	1	17	Medium quality	29261	1653510	37.90	160	d__Archaea;p__Thermoplasmatota;c__Thermoplasmata;o__Thermoplasmatales;f__Thermoplasmataceae;g__Cuniculiplasma;s__Cuniculiplasma divulgatum
LMSG_G000007216.1	no	169_1	Copper	75.80	4.36	53.99	1	0	1	16	Medium quality	17317	1156868	37.70	92	d__Archaea;p__Thermoplasmatota;c__Thermoplasmata;o__Thermoplasmatales;f__Thermoplasmataceae;g__Cuniculiplasma;s__Cuniculiplasma divulgatum
LMSG_G000007217.1	no	169_1	Copper	88.66	0.00	88.66	1	0	1	17	Medium quality	26664	1474144	37.50	109	d__Archaea;p__Thermoplasmatota;c__Thermoplasmata;o__Thermoplasmatales;f__Thermoplasmataceae;g__Cuniculiplasma;s__Cuniculiplasma divulgatum
LMSG_G000007218.1	no	169_1	Copper	96.32	0.00	96.32	1	1	1	20	High quality	175521	1912806	37.10	36	d__Archaea;p__Thermoplasmatota;c__Thermoplasmata;o__Thermoplasmatales;f__Thermoplasmataceae;g__Cuniculiplasma;s__Cuniculiplasma divulgatum
LMSG_G000007219.1	no	158_1	Copper	96.29	3.25	80.03	4	1	1	20	High quality	30114	2145851	37.40	133	d__Archaea;p__Thermoplasmatota;c__Thermoplasmata;o__Thermoplasmatales;f__Thermoplasmataceae;g__Ferroplasma;s__
LMSG_G000007220.1	no	158_1	Polymetallic	95.21	2.44	83.02	1	1	2	16	Medium quality	41706	1395670	39.20	126	d__Archaea;p__Thermoplasmatota;c__Thermoplasmata;o__Thermoplasmatales;f__Thermoplasmataceae;g__Ferroplasma;s__
LMSG_G000007221.1	no	158_1	Lead-Zinc	81.72	1.67	73.38	1	1	1	14	Medium quality	4787	1302593	39.30	330	d__Archaea;p__Thermoplasmatota;c__Thermoplasmata;o__Thermoplasmatales;f__Thermoplasmataceae;g__Ferroplasma;s__
LMSG_G000007222.1	no	158_1	Pyrite	58.73	0.00	58.73	1	0	0	14	Medium quality	23500	606789	40.10	39	d__Archaea;p__Thermoplasmatota;c__Thermoplasmata;o__Thermoplasmatales;f__Thermoplasmataceae;g__Ferroplasma;s__
LMSG_G000007223.1	no	158_1	Copper	82.11	1.90	72.63	0	1	1	13	Medium quality	32789	1182381	39.00	66	d__Archaea;p__Thermoplasmatota;c__Thermoplasmata;o__Thermoplasmatales;f__Thermoplasmataceae;g__Ferroplasma;s__
LMSG_G000007224.1	no	158_1	Pyrite	81.01	1.63	72.88	0	1	0	15	Medium quality	6689	1280559	39.50	219	d__Archaea;p__Thermoplasmatota;c__Thermoplasmata;o__Thermoplasmatales;f__Thermoplasmataceae;g__Ferroplasma;s__
LMSG_G000007225.1	no	158_1	Pyrite	73.93	0.81	69.87	0	0	0	15	Medium quality	6617	1073836	40.20	206	d__Archaea;p__Thermoplasmatota;c__Thermoplasmata;o__Thermoplasmatales;f__Thermoplasmataceae;g__Ferroplasma;s__
LMSG_G000007226.1	no	159_1	Lead-Zinc	84.91	3.25	68.65	1	1	0	17	Medium quality	21940	1522367	38.40	135	d__Archaea;p__Thermoplasmatota;c__Thermoplasmata;o__Thermoplasmatales;f__Thermoplasmataceae;g__Ferroplasma;s__
LMSG_G000007227.1	no	159_1	Polymetallic	93.75	4.88	69.36	0	1	1	18	Medium quality	21770	1561153	38.40	121	d__Archaea;p__Thermoplasmatota;c__Thermoplasmata;o__Thermoplasmatales;f__Thermoplasmataceae;g__Ferroplasma;s__
LMSG_G000007228.1	no	159_1	Polymetallic	80.21	3.25	63.95	1	0	2	18	Medium quality	19708	1237483	38.30	93	d__Archaea;p__Thermoplasmatota;c__Thermoplasmata;o__Thermoplasmatales;f__Thermoplasmataceae;g__Ferroplasma;s__
LMSG_G000007229.1	no	159_1	Copper	74.07	2.44	61.88	1	1	2	13	Medium quality	15039	1465987	38.30	119	d__Archaea;p__Thermoplasmatota;c__Thermoplasmata;o__Thermoplasmatales;f__Thermoplasmataceae;g__Ferroplasma;s__
LMSG_G000007230.1	no	160_1	Pyrite	79.53	4.07	59.21	1	2	0	18	Medium quality	11392	1684376	37.10	241	d__Archaea;p__Thermoplasmatota;c__Thermoplasmata;o__Thermoplasmatales;f__Thermoplasmataceae;g__Ferroplasma;s__
LMSG_G000007231.1	no	160_1	Copper	59.95	0.00	59.95	0	0	1	10	Medium quality	19404	962524	36.20	80	d__Archaea;p__Thermoplasmatota;c__Thermoplasmata;o__Thermoplasmatales;f__Thermoplasmataceae;g__Ferroplasma;s__
LMSG_G000007232.1	no	163_1	Pyrite	98.73	0.81	94.67	1	2	2	20	High quality	42726	1848686	30.00	99	d__Archaea;p__Thermoplasmatota;c__Thermoplasmata;o__Thermoplasmatales;f__Thermoplasmataceae;g__Ferroplasma;s__
LMSG_G000007233.1	no	163_1	Pyrite	98.73	1.63	90.60	1	2	2	20	High quality	33230	2007660	30.00	117	d__Archaea;p__Thermoplasmatota;c__Thermoplasmata;o__Thermoplasmatales;f__Thermoplasmataceae;g__Ferroplasma;s__
LMSG_G000007234.1	no	163_1	Pyrite	98.73	3.25	82.47	1	2	1	20	High quality	27118	2015919	30.10	119	d__Archaea;p__Thermoplasmatota;c__Thermoplasmata;o__Thermoplasmatales;f__Thermoplasmataceae;g__Ferroplasma;s__
LMSG_G000007235.1	no	163_1	Pyrite	98.73	2.44	86.54	1	2	2	20	High quality	28520	1917270	30.10	114	d__Archaea;p__Thermoplasmatota;c__Thermoplasmata;o__Thermoplasmatales;f__Thermoplasmataceae;g__Ferroplasma;s__
LMSG_G000007236.1	no	163_1	Pyrite	85.39	2.44	73.20	1	0	1	16	Medium quality	7108	1601211	30.70	266	d__Archaea;p__Thermoplasmatota;c__Thermoplasmata;o__Thermoplasmatales;f__Thermoplasmataceae;g__Ferroplasma;s__

LMSG_G000007237.1	no	163_1	Polymetallic	96.29	0.41	94.26	1	1	1	19	High quality	14499	1927025	29.30	234	d__Archaea;p__Thermoplasmatota;c__Thermoplasmata;o__Thermoplasmatales;f__Thermoplasmataceae;g__Ferroplasma;s__
LMSG_G000007238.1	no	163_1	Polymetallic	97.92	2.03	87.76	1	2	2	19	High quality	41507	2137672	30.10	115	d__Archaea;p__Thermoplasmatota;c__Thermoplasmata;o__Thermoplasmatales;f__Thermoplasmataceae;g__Ferroplasma;s__
LMSG_G000007239.1	no	163_1	Polymetallic	56.46	0.00	56.46	1	0	1	18	Medium quality	73199	1451372	30.10	50	d__Archaea;p__Thermoplasmatota;c__Thermoplasmata;o__Thermoplasmatales;f__Thermoplasmataceae;g__Ferroplasma;s__
LMSG_G000007240.1	no	164_1	Copper	97.11	1.63	88.98	1	2	1	18	High quality	35994	2154019	32.60	106	d__Archaea;p__Thermoplasmatota;c__Thermoplasmata;o__Thermoplasmatales;f__Thermoplasmataceae;g__Ferroplasma;s__
LMSG_G000007241.1	no	164_1	Copper	87.82	0.81	83.76	1	1	1	19	Medium quality	39106	1756873	32.60	65	d__Archaea;p__Thermoplasmatota;c__Thermoplasmata;o__Thermoplasmatales;f__Thermoplasmataceae;g__Ferroplasma;s__
LMSG_G000007242.1	no	164_1	Copper	97.92	0.00	97.92	1	2	1	20	High quality	50544	2011108	32.60	109	d__Archaea;p__Thermoplasmatota;c__Thermoplasmata;o__Thermoplasmatales;f__Thermoplasmataceae;g__Ferroplasma;s__
LMSG_G000007243.1	no	164_1	Lead-Zinc	98.73	0.81	94.67	1	0	0	19	Medium quality	15581	2749404	32.80	272	d__Archaea;p__Thermoplasmatota;c__Thermoplasmata;o__Thermoplasmatales;f__Thermoplasmataceae;g__Ferroplasma;s__
LMSG_G000007244.1	no	164_1	Lead-Zinc	71.54	1.90	62.06	0	2	2	15	Medium quality	36956	1735146	32.10	83	d__Archaea;p__Thermoplasmatota;c__Thermoplasmata;o__Thermoplasmatales;f__Thermoplasmataceae;g__Ferroplasma;s__
LMSG_G000007245.1	no	164_1	Polymetallic	71.21	2.44	59.02	1	1	2	14	Medium quality	7722	1646053	32.70	236	d__Archaea;p__Thermoplasmatota;c__Thermoplasmata;o__Thermoplasmatales;f__Thermoplasmataceae;g__Ferroplasma;s__
LMSG_G000007246.1	no	164_1	Copper	77.43	1.63	69.30	1	0	0	16	Medium quality	22707	1766155	32.60	113	d__Archaea;p__Thermoplasmatota;c__Thermoplasmata;o__Thermoplasmatales;f__Thermoplasmataceae;g__Ferroplasma;s__
LMSG_G000007247.1	no	164_1	Copper	79.73	0.81	75.67	0	0	1	17	Medium quality	19772	2392826	32.60	164	d__Archaea;p__Thermoplasmatota;c__Thermoplasmata;o__Thermoplasmatales;f__Thermoplasmataceae;g__Ferroplasma;s__
LMSG_G000007248.1	no	161_1	Lead-Zinc	97.46	1.63	89.33	1	1	2	18	High quality	49419	1638859	36.90	96	d__Archaea;p__Thermoplasmatota;c__Thermoplasmata;o__Thermoplasmatales;f__Thermoplasmataceae;g__Ferroplasma;s__Ferroplasma acidiphilum
LMSG_G000007249.1	no	161_1	Lead-Zinc	96.02	1.36	89.25	2	3	2	18	High quality	30424	1452192	37.30	78	d__Archaea;p__Thermoplasmatota;c__Thermoplasmata;o__Thermoplasmatales;f__Thermoplasmataceae;g__Ferroplasma;s__Ferroplasma acidiphilum
LMSG_G000007250.1	no	161_1	Pyrite	94.79	0.81	90.73	1	1	1	20	High quality	19303	1565343	37.20	132	d__Archaea;p__Thermoplasmatota;c__Thermoplasmata;o__Thermoplasmatales;f__Thermoplasmataceae;g__Ferroplasma;s__Ferroplasma acidiphilum
LMSG_G000007251.1	no	161_1	Polymetallic	91.33	0.81	87.27	1	1	1	20	High quality	31192	1843944	36.30	245	d__Archaea;p__Thermoplasmatota;c__Thermoplasmata;o__Thermoplasmatales;f__Thermoplasmataceae;g__Ferroplasma;s__Ferroplasma acidiphilum
LMSG_G000007252.1	no	161_1	Polymetallic	90.74	3.25	74.48	1	1	2	15	Medium quality	23659	1272414	37.30	77	d__Archaea;p__Thermoplasmatota;c__Thermoplasmata;o__Thermoplasmatales;f__Thermoplasmataceae;g__Ferroplasma;s__Ferroplasma acidiphilum
LMSG_G000007253.1	no	161_1	Lead-Zinc	81.72	0.00	81.72	1	1	0	14	Medium quality	5191	1405225	37.50	334	d__Archaea;p__Thermoplasmatota;c__Thermoplasmata;o__Thermoplasmatales;f__Thermoplasmataceae;g__Ferroplasma;s__Ferroplasma acidiphilum
LMSG_G000007254.1	no	161_1	Lead-Zinc	88.52	0.00	88.52	1	1	1	13	Medium quality	25781	1186143	37.60	86	d__Archaea;p__Thermoplasmatota;c__Thermoplasmata;o__Thermoplasmatales;f__Thermoplasmataceae;g__Ferroplasma;s__Ferroplasma acidiphilum
LMSG_G000007255.1	no	161_1	Polymetallic	89.77	3.25	73.51	1	1	2	15	Medium quality	18451	1576104	37.20	137	d__Archaea;p__Thermoplasmatota;c__Thermoplasmata;o__Thermoplasmatales;f__Thermoplasmataceae;g__Ferroplasma;s__Ferroplasma acidiphilum
LMSG_G000007256.1	no	161_1	Polymetallic	87.53	1.63	79.40	1	0	3	18	Medium quality	27328	1449643	37.20	104	d__Archaea;p__Thermoplasmatota;c__Thermoplasmata;o__Thermoplasmatales;f__Thermoplasmataceae;g__Ferroplasma;s__Ferroplasma acidiphilum
LMSG_G000007257.1	no	161_1	Polymetallic	82.65	0.00	82.65	1	2	2	16	Medium quality	28865	1405319	36.60	82	d__Archaea;p__Thermoplasmatota;c__Thermoplasmata;o__Thermoplasmatales;f__Thermoplasmataceae;g__Ferroplasma;s__Ferroplasma acidiphilum
LMSG_G000007258.1	no	161_1	Copper	83.46	3.25	67.20	0	2	2	14	Medium quality	27901	1341123	37.30	80	d__Archaea;p__Thermoplasmatota;c__Thermoplasmata;o__Thermoplasmatales;f__Thermoplasmataceae;g__Ferroplasma;s__Ferroplasma acidiphilum
LMSG_G000007259.1	no	161_1	Copper	81.37	3.25	65.11	0	1	1	13	Medium quality	23405	1453459	37.10	100	d__Archaea;p__Thermoplasmatota;c__Thermoplasmata;o__Thermoplasmatales;f__Thermoplasmataceae;g__Ferroplasma;s__Ferroplasma acidiphilum
LMSG_G000007260.1	no	161_1	Polymetallic	86.00	0.00	86.00	1	1	2	16	Medium quality	56287	1157852	37.50	73	d__Archaea;p__Thermoplasmatota;c__Thermoplasmata;o__Thermoplasmatales;f__Thermoplasmataceae;g__Ferroplasma;s__Ferroplasma acidiphilum
LMSG_G000007261.1	no	161_1	Copper	85.09	0.81	81.03	0	1	2	17	Medium quality	24669	1199797	37.10	73	d__Archaea;p__Thermoplasmatota;c__Thermoplasmata;o__Thermoplasmatales;f__Thermoplasmataceae;g__Ferroplasma;s__Ferroplasma acidiphilum
LMSG_G000007262.1	no	161_1	Polymetallic	63.23	0.00	63.23	0	0	1	16	Medium quality	16667	1014923	37.20	89	d__Archaea;p__Thermoplasmatota;c__Thermoplasmata;o__Thermoplasmatales;f__Thermoplasmataceae;g__Ferroplasma;s__Ferroplasma acidiphilum
LMSG_G000007263.1	no	162_1	Iron	94.53	0.81	90.47	1	1	2	19	High quality	25753	2071597	36.90	171	d__Archaea;p__Thermoplasmatota;c__Thermoplasmata;o__Thermoplasmatales;f__Thermoplasmataceae;g__Ferroplasma;s__Ferroplasma sp002505185
LMSG_G000007264.1	no	162_1	Copper	88.97	1.63	80.84	1	1	2	15	Medium quality	8378	1404719	37.40	238	d__Archaea;p__Thermoplasmatota;c__Thermoplasmata;o__Thermoplasmatales;f__Thermoplasmataceae;g__Ferroplasma;s__Ferroplasma sp002505185
LMSG_G000007265.1	no	162_1	Polymetallic	67.45	2.57	54.62	1	3	6	13	Medium quality	3917	1054294	37.60	329	d__Archaea;p__Thermoplasmatota;c__Thermoplasmata;o__Thermoplasmatales;f__Thermoplasmataceae;g__Ferroplasma;s__Ferroplasma sp002505185
LMSG_G000007266.1	no	162_1	Lead-Zinc	74.93	4.88	50.54	0	1	2	13	Medium quality	6905	1277277	37.50	233	d__Archaea;p__Thermoplasmatota;c__Thermoplasmata;o__Thermoplasmatales;f__Thermoplasmataceae;g__Ferroplasma;s__Ferroplasma sp002505185
LMSG_G000007267.1	no	162_1	Polymetallic	67.23	0.81	63.17	1	1	1	12	Medium quality	9858	829827	37.70	108	d__Archaea;p__Thermoplasmatota;c__Thermoplasmata;o__Thermoplasmatales;f__Thermoplasmataceae;g__Ferroplasma;s__Ferroplasma sp002505185
LMSG_G000007268.1	no	162_1	Polymetallic	76.29	0.81	72.23	1	2	1	11	Medium quality	10391	1129506	37.20	133	d__Archaea;p__Thermoplasmatota;c__Thermoplasmata;o__Thermoplasmatales;f__Thermoplasmataceae;g__Ferroplasma;s__Ferroplasma sp002505185
LMSG_G000007269.1	no	162_1	Polymetallic	74.08	0.81	70.02	1	1	0	12	Medium quality	9462	1097679	37.10	142	d__Archaea;p__Thermoplasmatota;c__Thermoplasmata;o__Thermoplasmatales;f__Thermoplasmataceae;g__Ferroplasma;s__Ferroplasma sp002505185
LMSG_G000007270.1	no	162_1	Polymetallic	58.13	0.00	58.13	0	1	0	11	Medium quality	13069	681691	37.40	71	d__Archaea;p__Thermoplasmatota;c__Thermoplasmata;o__Thermoplasmatales;f__Thermoplasmataceae;g__Ferroplasma;s__Ferroplasma sp002505185
LMSG_G000007271.1	no	162_1	Polymetallic	86.14	0.81	82.08	1	1	1	13	Medium quality	10443	1274944	37.20	149	d__Archaea;p__Thermoplasmatota;c__Thermoplasmata;o__Thermoplasmatales;f__Thermoplasmataceae;g__Ferroplasma;s__Ferroplasma sp002505185
LMSG_G000007272.1	no	162_1	Polymetallic	69.87	3.66	51.58	1	0	3	9	Medium quality	3741	988955	37.30	325	d__Archaea;p__Thermoplasmatota;c__Thermoplasmata;o__Thermoplasmatales;f__Thermoplasmataceae;g__Ferroplasma;s__Ferroplasma sp002505185
LMSG_G000007273.1	no	162_1	Polymetallic	95.35	2.44	83.16	1	2	1	19	High quality	32084	1723340	37.10	120	d__Archaea;p__Thermoplasmatota;c__Thermoplasmata;o__Thermoplasmatales;f__Thermoplasmataceae;g__Ferroplasma;s__Ferroplasma sp002505185
LMSG_G000007274.1	no	162_1	Polymetallic	97.78	1.63	89.65	1	1	1	19	High quality	25053	1694028	37.10	153	d__Archaea;p__Thermoplasmatota;c__Thermoplasmata;o__Thermoplasmatales;f__Thermoplasmataceae;g__Ferroplasma;s__Ferroplasma sp002505185
LMSG_G000007275.1	no	162_1	Magnetite	91.28	2.44	79.09	1	1	1	18	High quality	14499	1544137	37.20	157	d__Archaea;p__Thermoplasmatota;c__Thermoplasmata;o__Thermoplasmatales;f__Thermoplasmataceae;g__Ferroplasma;s__Ferroplasma sp002505185
LMSG_G000007276.1	no	162_1	Lead-Zinc	91.28	2.03	81.12	1	2	1	17	Medium quality	14772	2289687	36.40	239	d__Archaea;p__Thermoplasmatota;c__Thermoplasmata;o__Thermoplasmatales;f__Thermoplasmataceae;g__Ferroplasma;s__Ferroplasma sp002505185
LMSG_G000007277.1	no	162_1	Pyrite	94.43	4.51	71.86	1	1	1	14	Medium quality	10527	1413469	37.30	178	d__Archaea;p__Thermoplasmatota;c__Thermoplasmata;o__Thermoplasmatales;f__Thermoplasmataceae;g__Ferroplasma;s__Ferroplasma sp002505185
LMSG_G000007278.1	no	162_1	Lead-Zinc	76.12	3.05	60.86	1	2	0	15	Medium quality	16182	1251007	37.50	143	d__Archaea;p__Thermoplasmatota;c__Thermoplasmata;o__Thermoplasmatales;f__Thermoplasmataceae;g__Ferroplasma;s__Ferroplasma sp002505185
LMSG_G000007279.1	no	162_1	Pyrite	89.47	1.63	81.34	0	2	1	16	Medium quality	18874	1584424	37.30	142	d__Archaea;p__Thermoplasmatota;c__Thermoplasmata;o__Thermoplasmatales;f__Thermoplasmataceae;g__Ferroplasma;s__Ferroplasma sp002505185
LMSG_G000007280.1	no	162_1	Pyrite	79.63	4.07	59.31	1	2	0	13	Medium quality	12842	1406267	37.30	142	d__Archaea;p__Thermoplasmatota;c__Thermoplasmata;o__Thermoplasmatales;f__Thermoplasmataceae;g__Ferroplasma;s__Ferroplasma sp002505185
LMSG_G000007281.1	no	162_1	Lead-Zinc	54.30	0.81	50.24	0	0	0	12	Medium quality	5655	729697	37.80	136	d__Archaea;p__Thermoplasmatota;c__Thermoplasmata;o__Thermoplasmatales;f__Thermoplasmataceae;g__Ferroplasma;s__Ferroplasma sp002505185
LMSG_G000007282.1	no	162_1	Polymetallic	75.51	1.79	66.57	1	2	0	16	Medium quality	2215	1188589	37.20	591	d__Archaea;p__Thermoplasmatota;c__Thermoplasmata;o__Thermoplasmatales;f__Thermoplasmataceae;g__Ferroplasma;s__Ferroplasma sp002505185
LMSG_G000007283.1	no	162_1	Copper	62.55	1.22	56.46	0	1	0	9	Medium quality	8292	900134	37.50	124	d__Archaea;p__Thermoplasmatota;c__Thermoplasmata;o__Thermoplasmatales;f__Thermoplasmataceae;g__Ferroplasma;s__Ferroplasma sp002505185
LMSG_G000007284.1	no	162_1	Polymetallic	80.97	4.07	60.65	1	2	1	13	Medium quality	7671	1377259	37.20	228	d__Archaea;p__Thermoplasmatota;c__Thermoplasmata;o__Thermoplasmatales;f__Thermoplasmataceae;g__Ferroplasma;s__Ferroplasma sp002505185
LMSG_G000007285.1	no	162_1	Magnetite	81.16	3.25	64.90	0	1	1	12	Medium quality	10711	1292067	37.20	150	d__Archaea;p__Thermoplasmatota;c__Thermoplasmata;o__Thermoplasmatales;f__Thermoplasmataceae;g__Ferroplasma;s__Ferroplasma sp002505185
LMSG_G000007286.1	no	162_1	Magnetite	85.08	4.07	64.76	1	0	0	16	Medium quality	8395	1519107	37.40	239	d__Archaea;p__Thermoplasmatota;c__Thermoplasmata;o__Thermoplasmatales;f__Thermoplasmataceae;g__Ferroplasma;s__Ferroplasma sp002505185
LMSG_G000007287.1	no	162_1	Magnetite	66.57	2.44	54.38	1	0	1	16	Medium quality	5601	1283689	37.40	272	d__Archaea;p__Thermoplasmatota;c__Thermoplasmata;o__Thermoplasmatales;f__Thermoplasmataceae;g__Ferroplasma;s__Ferroplasma sp002505185

LMSG_G000007288.1	no	162_1	Arsenic	82.72	4.07	62.40	1	1	1	14	Medium quality	9289	1329988	37.50	175	d__Archaea;p__Thermoplasmatota;c__Thermoplasmata;o__Thermoplasmatales;f__Thermoplasmataceae;g__Ferroplasma;s__Ferroplasma sp002505185
LMSG_G000007289.1	no	162_1	Lead-Zinc	92.50	3.25	76.24	1	2	0	17	Medium quality	22722	1504374	37.40	95	d__Archaea;p__Thermoplasmatota;c__Thermoplasmata;o__Thermoplasmatales;f__Thermoplasmataceae;g__Ferroplasma;s__Ferroplasma sp002505185
LMSG_G000007290.1	no	162_1	Polymetallic	97.51	2.44	85.32	1	1	0	19	Medium quality	15626	1672155	37.00	201	d__Archaea;p__Thermoplasmatota;c__Thermoplasmata;o__Thermoplasmatales;f__Thermoplasmataceae;g__Ferroplasma;s__Ferroplasma sp002505185
LMSG_G000007291.1	no	162_1	Pyrite	85.20	4.94	60.50	2	2	1	17	Medium quality	6490	1659085	37.70	311	d__Archaea;p__Thermoplasmatota;c__Thermoplasmata;o__Thermoplasmatales;f__Thermoplasmataceae;g__Ferroplasma;s__Ferroplasma sp002505185
LMSG_G000007292.1	no	162_1	Pyrite	88.95	4.42	66.85	1	1	1	17	Medium quality	7893	1344174	37.50	219	d__Archaea;p__Thermoplasmatota;c__Thermoplasmata;o__Thermoplasmatales;f__Thermoplasmataceae;g__Ferroplasma;s__Ferroplasma sp002505185
LMSG_G000007293.1	no	176_1	Copper	79.39	4.03	59.23	1	1	0	17	Medium quality	9769	1565048	41.10	214	d__Archaea;p__Thermoplasmatota;c__Thermoplasmata;o__Thermoplasmatales;f__Thermoplasmataceae;g__GCA-000496135;s__
LMSG_G000007294.1	no	177_1	Copper	72.36	0.81	68.33	1	0	2	17	Medium quality	28725	1190364	38.30	115	d__Archaea;p__Thermoplasmatota;c__Thermoplasmata;o__Thermoplasmatales;f__Thermoplasmataceae;g__GCA-000496135;s__
LMSG_G000007295.1	no	177_1	Polymetallic	53.75	0.00	53.75	0	0	0	11	Medium quality	15015	834242	38.70	78	d__Archaea;p__Thermoplasmatota;c__Thermoplasmata;o__Thermoplasmatales;f__Thermoplasmataceae;g__GCA-000496135;s__
LMSG_G000007296.1	no	178_1	Lead-Zinc	80.73	2.42	68.64	1	1	0	17	Medium quality	9363	1458162	38.80	208	d__Archaea;p__Thermoplasmatota;c__Thermoplasmata;o__Thermoplasmatales;f__Thermoplasmataceae;g__GCA-000496135;s__GCA-000496135 sp000496135
LMSG_G000007297.1	no	178_1	Lead-Zinc	69.50	1.77	60.63	1	1	0	14	Medium quality	12610	1116305	38.70	112	d__Archaea;p__Thermoplasmatota;c__Thermoplasmata;o__Thermoplasmatales;f__Thermoplasmataceae;g__GCA-000496135;s__GCA-000496135 sp000496135
LMSG_G000007298.1	no	178_1	Lead-Zinc	65.68	1.21	59.64	1	1	1	14	Medium quality	9893	1003267	38.50	128	d__Archaea;p__Thermoplasmatota;c__Thermoplasmata;o__Thermoplasmatales;f__Thermoplasmataceae;g__GCA-000496135;s__GCA-000496135 sp000496135
LMSG_G000007299.1	no	178_1	Pyrite-Copper	78.45	3.83	59.28	0	2	0	17	Medium quality	14370	1297445	38.60	113	d__Archaea;p__Thermoplasmatota;c__Thermoplasmata;o__Thermoplasmatales;f__Thermoplasmataceae;g__GCA-000496135;s__GCA-000496135 sp000496135
LMSG_G000007300.1	no	178_1	Pyrite-Copper	69.33	3.30	52.83	1	2	0	16	Medium quality	15688	1354405	37.80	124	d__Archaea;p__Thermoplasmatota;c__Thermoplasmata;o__Thermoplasmatales;f__Thermoplasmataceae;g__GCA-000496135;s__GCA-000496135 sp000496135
LMSG_G000007301.1	no	178_1	Tin-Zinc	79.06	2.42	66.97	1	1	0	17	Medium quality	12832	1336677	38.60	170	d__Archaea;p__Thermoplasmatota;c__Thermoplasmata;o__Thermoplasmatales;f__Thermoplasmataceae;g__GCA-000496135;s__GCA-000496135 sp000496135
LMSG_G000007302.1	no	178_1	Copper	67.30	2.42	55.21	1	0	0	16	Medium quality	9313	1170311	37.90	173	d__Archaea;p__Thermoplasmatota;c__Thermoplasmata;o__Thermoplasmatales;f__Thermoplasmataceae;g__GCA-000496135;s__GCA-000496135 sp000496135
LMSG_G000007303.1	no	178_1	Iron	78.22	0.81	74.19	0	0	0	16	Medium quality	4732	1565746	37.60	351	d__Archaea;p__Thermoplasmatota;c__Thermoplasmata;o__Thermoplasmatales;f__Thermoplasmataceae;g__GCA-000496135;s__GCA-000496135 sp000496135
LMSG_G000007304.1	no	178_1	Copper	53.98	0.00	53.98	1	1	0	14	Medium quality	35659	841602	38.20	27	d__Archaea;p__Thermoplasmatota;c__Thermoplasmata;o__Thermoplasmatales;f__Thermoplasmataceae;g__GCA-000496135;s__GCA-000496135 sp000496135
LMSG_G000007305.1	no	195_1	Lead-Zinc	89.24	1.61	81.18	1	2	1	14	Medium quality	32231	1198304	44.10	62	d__Archaea;p__Thermoplasmatota;c__Thermoplasmata;o__Thermoplasmatales;f__Thermoplasmataceae;g__UBA447;s__
LMSG_G000007306.1	no	196_1	Polymetallic	58.33	0.81	54.30	1	1	0	13	Medium quality	20973	715241	43.00	48	d__Archaea;p__Thermoplasmatota;c__Thermoplasmata;o__Thermoplasmatales;f__Thermoplasmataceae;g__UBA447;s__UBA447 sp002503205
LMSG_G000007307.1	no	196_1	Iron	77.56	0.00	77.56	0	0	0	15	Medium quality	5259	1435872	41.90	320	d__Archaea;p__Thermoplasmatota;c__Thermoplasmata;o__Thermoplasmatales;f__Thermoplasmataceae;g__UBA447;s__UBA447 sp002503205
LMSG_G000007308.1	no	196_1	Iron	79.75	2.42	67.66	1	2	1	14	Medium quality	9141	1472299	42.60	205	d__Archaea;p__Thermoplasmatota;c__Thermoplasmata;o__Thermoplasmatales;f__Thermoplasmataceae;g__UBA447;s__UBA447 sp002503205
LMSG_G000007309.1	no	196_1	Iron	88.19	0.00	88.19	1	1	1	16	Medium quality	8822	1829835	41.90	287	d__Archaea;p__Thermoplasmatota;c__Thermoplasmata;o__Thermoplasmatales;f__Thermoplasmataceae;g__UBA447;s__UBA447 sp002503205
LMSG_G000007310.1	no	196_1	Copper	53.87	0.00	53.87	1	0	1	10	Medium quality	20270	848764	43.00	68	d__Archaea;p__Thermoplasmatota;c__Thermoplasmata;o__Thermoplasmatales;f__Thermoplasmataceae;g__UBA447;s__UBA447 sp002503205
LMSG_G000007311.1	no	189_1	Copper	61.50	1.61	53.44	1	0	1	19	Medium quality	42385	868763	42.00	38	d__Archaea;p__Thermoplasmatota;c__Thermoplasmata;o__Thermoplasmatales;f__Thermoplasmataceae;g__UBA509;s__
LMSG_G000007312.1	no	189_1	Copper	57.35	0.00	57.35	1	0	0	14	Medium quality	46912	685928	42.00	20	d__Archaea;p__Thermoplasmatota;c__Thermoplasmata;o__Thermoplasmatales;f__Thermoplasmataceae;g__UBA509;s__
LMSG_G000007313.1	no	190_1	Copper	96.32	0.81	92.29	1	1	2	20	High quality	25299	1699199	45.20	96	d__Archaea;p__Thermoplasmatota;c__Thermoplasmata;o__Thermoplasmatales;f__Thermoplasmataceae;g__UBA509;s__
LMSG_G000007314.1	no	190_1	Lead-Zinc	99.55	3.23	83.43	1	2	2	20	High quality	66673	2098229	45.00	82	d__Archaea;p__Thermoplasmatota;c__Thermoplasmata;o__Thermoplasmatales;f__Thermoplasmataceae;g__UBA509;s__
LMSG_G000007315.1	no	190_1	Lead-Zinc	74.01	2.96	59.21	1	0	0	15	Medium quality	5029	1310970	45.40	280	d__Archaea;p__Thermoplasmatota;c__Thermoplasmata;o__Thermoplasmatales;f__Thermoplasmataceae;g__UBA509;s__
LMSG_G000007316.1	no	191_1	Lead-Zinc	80.19	2.23	69.05	1	2	0	16	Medium quality	18491	1140319	45.60	108	d__Archaea;p__Thermoplasmatota;c__Thermoplasmata;o__Thermoplasmatales;f__Thermoplasmataceae;g__UBA509;s__
LMSG_G000007317.1	no	191_1	Lead-Zinc	89.47	3.23	73.35	2	1	0	15	Medium quality	10129	1428578	45.50	192	d__Archaea;p__Thermoplasmatota;c__Thermoplasmata;o__Thermoplasmatales;f__Thermoplasmataceae;g__UBA509;s__
LMSG_G000007318.1	no	191_1	Polymetallic	85.84	0.81	81.81	1	1	0	18	Medium quality	14604	1400806	45.50	121	d__Archaea;p__Thermoplasmatota;c__Thermoplasmata;o__Thermoplasmatales;f__Thermoplasmataceae;g__UBA509;s__
LMSG_G000007319.1	no	191_1	Magnetite	66.12	0.00	66.12	1	1	0	11	Medium quality	20422	832072	45.60	61	d__Archaea;p__Thermoplasmatota;c__Thermoplasmata;o__Thermoplasmatales;f__Thermoplasmataceae;g__UBA509;s__
LMSG_G000007320.1	no	191_1	Lead-Zinc	76.78	2.87	62.46	1	2	0	16	Medium quality	6803	1360800	45.60	222	d__Archaea;p__Thermoplasmatota;c__Thermoplasmata;o__Thermoplasmatales;f__Thermoplasmataceae;g__UBA509;s__
LMSG_G000007321.1	no	191_1	Copper	59.23	1.37	52.38	1	1	0	13	Medium quality	11060	851642	45.20	98	d__Archaea;p__Thermoplasmatota;c__Thermoplasmata;o__Thermoplasmatales;f__Thermoplasmataceae;g__UBA509;s__
LMSG_G000007322.1	no	191_1	Copper	96.32	4.84	72.13	1	2	0	18	Medium quality	14924	1643155	45.50	154	d__Archaea;p__Thermoplasmatota;c__Thermoplasmata;o__Thermoplasmatales;f__Thermoplasmataceae;g__UBA509;s__
LMSG_G000007323.1	no	191_1	Lead-Zinc	96.32	0.81	92.29	1	1	0	17	Medium quality	26972	1448955	45.70	121	d__Archaea;p__Thermoplasmatota;c__Thermoplasmata;o__Thermoplasmatales;f__Thermoplasmataceae;g__UBA509;s__
LMSG_G000007324.1	no	191_1	Lead-Zinc	95.52	4.84	71.33	1	2	0	19	Medium quality	36677	1786522	45.10	93	d__Archaea;p__Thermoplasmatota;c__Thermoplasmata;o__Thermoplasmatales;f__Thermoplasmataceae;g__UBA509;s__
LMSG_G000007325.1	no	191_1	Magnetite	77.00	4.03	56.84	1	0	0	12	Medium quality	9586	1269599	45.40	172	d__Archaea;p__Thermoplasmatota;c__Thermoplasmata;o__Thermoplasmatales;f__Thermoplasmataceae;g__UBA509;s__
LMSG_G000007326.1	no	191_1	Pyrite-Copper	82.88	0.89	78.43	1	0	2	14	Medium quality	15870	1254810	45.50	148	d__Archaea;p__Thermoplasmatota;c__Thermoplasmata;o__Thermoplasmatales;f__Thermoplasmataceae;g__UBA509;s__
LMSG_G000007327.1	no	191_1	Pyrite-Copper	89.87	0.81	85.84	1	1	2	14	Medium quality	17642	1223867	45.60	131	d__Archaea;p__Thermoplasmatota;c__Thermoplasmata;o__Thermoplasmatales;f__Thermoplasmataceae;g__UBA509;s__
LMSG_G000007328.1	no	191_1	Copper	83.42	1.67	75.05	0	0	0	9	Medium quality	10239	1084876	45.60	121	d__Archaea;p__Thermoplasmatota;c__Thermoplasmata;o__Thermoplasmatales;f__Thermoplasmataceae;g__UBA509;s__
LMSG_G000007329.1	no	191_1	Polymetallic	93.15	4.90	68.65	0	0	1	18	Medium quality	10182	1595798	45.20	198	d__Archaea;p__Thermoplasmatota;c__Thermoplasmata;o__Thermoplasmatales;f__Thermoplasmataceae;g__UBA509;s__
LMSG_G000007330.1	no	191_1	Polymetallic	87.39	1.61	79.33	1	1	1	17	Medium quality	23474	1507402	45.30	99	d__Archaea;p__Thermoplasmatota;c__Thermoplasmata;o__Thermoplasmatales;f__Thermoplasmataceae;g__UBA509;s__
LMSG_G000007331.1	no	191_1	Copper	93.10	1.65	84.84	1	1	0	15	Medium quality	12636	1430789	45.40	145	d__Archaea;p__Thermoplasmatota;c__Thermoplasmata;o__Thermoplasmatales;f__Thermoplasmataceae;g__UBA509;s__
LMSG_G000007332.1	no	191_1	Copper	87.76	0.81	83.73	1	0	0	14	Medium quality	8423	1214456	45.50	158	d__Archaea;p__Thermoplasmatota;c__Thermoplasmata;o__Thermoplasmatales;f__Thermoplasmataceae;g__UBA509;s__
LMSG_G000007333.1	no	191_1	Copper	70.89	2.75	57.14	0	0	0	10	Medium quality	5464	1114639	45.60	217	d__Archaea;p__Thermoplasmatota;c__Thermoplasmata;o__Thermoplasmatales;f__Thermoplasmataceae;g__UBA509;s__
LMSG_G000007334.1	no	198_1	Pyrite	94.65	2.75	80.90	1	2	1	18	High quality	33534	1468321	47.20	119	d__Archaea;p__Thermoplasmatota;c__Thermoplasmata;o__Thermoplasmatales;f__Thermoplasmataceae;g__UBA509;s__
LMSG_G000007335.1	no	198_1	Pyrite	79.93	2.88	65.51	1	2	0	13	Medium quality	13228	1223296	47.50	161	d__Archaea;p__Thermoplasmatota;c__Thermoplasmata;o__Thermoplasmatales;f__Thermoplasmataceae;g__UBA509;s__
LMSG_G000007336.1	no	198_1	Pyrite	63.48	2.02	53.40	0	2	1	14	Medium quality	4923	1129879	47.30	253	d__Archaea;p__Thermoplasmatota;c__Thermoplasmata;o__Thermoplasmatales;f__Thermoplasmataceae;g__UBA509;s__
LMSG_G000007337.1	no	198_1	Copper	78.65	3.63	60.51	1	0	0	14	Medium quality	6315	1311948	47.50	247	d__Archaea;p__Thermoplasmatota;c__Thermoplasmata;o__Thermoplasmatales;f__Thermoplasmataceae;g__UBA509;s__
LMSG_G000007338.1	no	198_1	Copper	79.00	2.42	66.91	1	1	0	14	Medium quality	9525	1521057	47.20	211	d__Archaea;p__Thermoplasmatota;c__Thermoplasmata;o__Thermoplasmatales;f__Thermoplasmataceae;g__UBA509;s__

LMSG_G000007339.1	no	198_1	Copper	73.68	1.21	67.64	1	1	1	15	Medium quality	19056	1100874	47.40	87	d__Archaea;p__Thermoplasmatota;c__Thermoplasmata;o__Thermoplasmatales;f__Thermoplasmataceae;g__UBA509;s__
LMSG_G000007340.1	no	198_1	Lead-Zinc	68.82	2.84	54.61	1	1	0	14	Medium quality	13561	1345507	47.10	133	d__Archaea;p__Thermoplasmatota;c__Thermoplasmata;o__Thermoplasmatales;f__Thermoplasmataceae;g__UBA509;s__
LMSG_G000007341.1	no	198_1	Copper	68.95	0.06	68.64	1	1	1	10	Medium quality	25142	1224032	47.20	148	d__Archaea;p__Thermoplasmatota;c__Thermoplasmata;o__Thermoplasmatales;f__Thermoplasmataceae;g__UBA509;s__
LMSG_G000007342.1	no	212_1	Lead-Zinc	67.87	0.00	67.87	0	0	1	19	Medium quality	15320	1219734	40.70	114	d__Archaea;p__Thermoplasmatota;c__Thermoplasmata;o__Thermoplasmatales;f__Thermoplasmataceae;g__UBA509;s__
LMSG_G000007343.1	no	212_1	Copper	51.16	0.00	51.16	0	0	0	13	Medium quality	35602	773408	41.00	32	d__Archaea;p__Thermoplasmatota;c__Thermoplasmata;o__Thermoplasmatales;f__Thermoplasmataceae;g__UBA509;s__
LMSG_G000007344.1	no	198_2	Iron	93.14	0.00	93.14	1	1	2	19	High quality	63645	1647163	46.40	39	d__Archaea;p__Thermoplasmatota;c__Thermoplasmata;o__Thermoplasmatales;f__Thermoplasmataceae;g__UBA509;s__UBA509 sp002498845
LMSG_G000007345.1	no	198_2	Iron	95.56	0.00	95.56	1	2	1	20	High quality	54279	1723241	46.00	66	d__Archaea;p__Thermoplasmatota;c__Thermoplasmata;o__Thermoplasmatales;f__Thermoplasmataceae;g__UBA509;s__UBA509 sp002498845
LMSG_G000007346.1	no	198_2	Iron	96.37	0.00	96.37	1	1	2	20	High quality	19232	1781744	46.40	139	d__Archaea;p__Thermoplasmatota;c__Thermoplasmata;o__Thermoplasmatales;f__Thermoplasmataceae;g__UBA509;s__UBA509 sp002498845
LMSG_G000007347.1	no	198_2	Iron	98.79	0.00	98.79	1	1	1	20	High quality	22912	1872279	45.90	123	d__Archaea;p__Thermoplasmatota;c__Thermoplasmata;o__Thermoplasmatales;f__Thermoplasmataceae;g__UBA509;s__UBA509 sp002498845
LMSG_G000007348.1	no	198_2	Iron	89.91	0.00	89.91	1	1	2	20	Medium quality	97021	1719247	46.10	46	d__Archaea;p__Thermoplasmatota;c__Thermoplasmata;o__Thermoplasmatales;f__Thermoplasmataceae;g__UBA509;s__UBA509 sp002498845
LMSG_G000007349.1	no	198_2	Iron	89.65	0.00	89.65	1	1	2	19	Medium quality	8394	1563507	46.30	232	d__Archaea;p__Thermoplasmatota;c__Thermoplasmata;o__Thermoplasmatales;f__Thermoplasmataceae;g__UBA509;s__UBA509 sp002498845
LMSG_G000007350.1	no	198_2	Iron	80.34	0.85	76.10	1	0	3	15	Medium quality	3523	1440801	46.40	485	d__Archaea;p__Thermoplasmatota;c__Thermoplasmata;o__Thermoplasmatales;f__Thermoplasmataceae;g__UBA509;s__UBA509 sp002498845
LMSG_G000007351.1	no	198_2	Iron	83.74	0.25	82.50	0	0	2	17	Medium quality	9678	1532431	45.80	177	d__Archaea;p__Thermoplasmatota;c__Thermoplasmata;o__Thermoplasmatales;f__Thermoplasmataceae;g__UBA509;s__UBA509 sp002498845
LMSG_G000007352.1	no	198_2	Iron	85.80	0.00	85.80	1	1	2	19	Medium quality	8661	1609551	45.80	185	d__Archaea;p__Thermoplasmatota;c__Thermoplasmata;o__Thermoplasmatales;f__Thermoplasmataceae;g__UBA509;s__UBA509 sp002498845
LMSG_G000007353.1	no	198_2	Lead-Zinc	67.09	0.93	62.44	1	1	0	10	Medium quality	6525	1084071	47.50	188	d__Archaea;p__Thermoplasmatota;c__Thermoplasmata;o__Thermoplasmatales;f__Thermoplasmataceae;g__UBA509;s__UBA509 sp002498845
LMSG_G000007354.1	no	198_2	Copper	80.71	0.00	80.71	0	0	1	12	Medium quality	12939	1329105	46.70	130	d__Archaea;p__Thermoplasmatota;c__Thermoplasmata;o__Thermoplasmatales;f__Thermoplasmataceae;g__UBA509;s__UBA509 sp002498845
LMSG_G000007355.1	no	198_2	Polymetallic	56.39	0.81	52.36	1	0	0	10	Medium quality	4832	936245	47.50	241	d__Archaea;p__Thermoplasmatota;c__Thermoplasmata;o__Thermoplasmatales;f__Thermoplasmataceae;g__UBA509;s__UBA509 sp002498845
LMSG_G000007356.1	no	173_1	Polymetallic	94.17	0.00	94.17	1	3	2	18	High quality	19397	1455219	47.90	113	d__Archaea;p__Thermoplasmatota;c__Thermoplasmata;o__Thermoplasmatales;f__Thermoplasmataceae;g__UBA582;s__
LMSG_G000007357.1	no	173_1	Polymetallic	95.65	0.00	95.65	1	2	2	20	High quality	47910	1403565	48.00	63	d__Archaea;p__Thermoplasmatota;c__Thermoplasmata;o__Thermoplasmatales;f__Thermoplasmataceae;g__UBA582;s__
LMSG_G000007358.1	no	173_1	Polymetallic	72.67	0.00	72.67	1	2	1	19	Medium quality	103441	1006213	47.10	34	d__Archaea;p__Thermoplasmatota;c__Thermoplasmata;o__Thermoplasmatales;f__Thermoplasmataceae;g__UBA582;s__
LMSG_G000007359.1	no	173_1	Polymetallic	77.95	0.00	77.95	1	1	2	19	Medium quality	47758	1175670	48.40	42	d__Archaea;p__Thermoplasmatota;c__Thermoplasmata;o__Thermoplasmatales;f__Thermoplasmataceae;g__UBA582;s__
LMSG_G000007360.1	no	174_1	Copper	73.68	3.55	55.94	1	0	2	14	Medium quality	6474	1090463	48.70	178	d__Archaea;p__Thermoplasmatota;c__Thermoplasmata;o__Thermoplasmatales;f__Thermoplasmataceae;g__UBA582;s__
LMSG_G000007361.1	no	174_1	Magnetite	73.42	0.97	68.59	1	0	0	17	Medium quality	13138	1277512	48.60	145	d__Archaea;p__Thermoplasmatota;c__Thermoplasmata;o__Thermoplasmatales;f__Thermoplasmataceae;g__UBA582;s__
LMSG_G000007362.1	no	175_1	Copper	95.25	0.81	91.22	1	1	1	20	High quality	85010	1482881	48.70	41	d__Archaea;p__Thermoplasmatota;c__Thermoplasmata;o__Thermoplasmatales;f__Thermoplasmataceae;g__UBA582;s__
LMSG_G000007363.1	no	175_1	Copper	96.05	0.81	92.02	1	1	2	20	High quality	51137	1476837	48.60	40	d__Archaea;p__Thermoplasmatota;c__Thermoplasmata;o__Thermoplasmatales;f__Thermoplasmataceae;g__UBA582;s__
LMSG_G000007364.1	no	175_1	Polymetallic	96.05	2.42	83.96	1	1	3	20	High quality	54235	1543603	48.40	45	d__Archaea;p__Thermoplasmatota;c__Thermoplasmata;o__Thermoplasmatales;f__Thermoplasmataceae;g__UBA582;s__
LMSG_G000007365.1	no	175_1	Copper	92.02	0.00	92.02	1	1	1	16	Medium quality	57328	1362713	48.60	42	d__Archaea;p__Thermoplasmatota;c__Thermoplasmata;o__Thermoplasmatales;f__Thermoplasmataceae;g__UBA582;s__
LMSG_G000007366.1	no	175_1	Lead-Zinc	55.10	0.00	55.10	0	0	1	8	Medium quality	8110	714821	49.20	96	d__Archaea;p__Thermoplasmatota;c__Thermoplasmata;o__Thermoplasmatales;f__Thermoplasmataceae;g__UBA582;s__
LMSG_G000007367.1	no	175_1	Copper	87.99	0.00	87.99	1	1	2	20	Medium quality	33417	1394414	48.70	66	d__Archaea;p__Thermoplasmatota;c__Thermoplasmata;o__Thermoplasmatales;f__Thermoplasmataceae;g__UBA582;s__
LMSG_G000007368.1	no	175_1	Lead-Zinc	88.80	1.61	80.74	1	1	2	19	Medium quality	35522	1397103	48.70	63	d__Archaea;p__Thermoplasmatota;c__Thermoplasmata;o__Thermoplasmatales;f__Thermoplasmataceae;g__UBA582;s__
LMSG_G000007369.1	no	175_1	Polymetallic	73.93	2.42	61.84	0	1	2	16	Medium quality	7196	1206306	48.70	190	d__Archaea;p__Thermoplasmatota;c__Thermoplasmata;o__Thermoplasmatales;f__Thermoplasmataceae;g__UBA582;s__
LMSG_G000007370.1	no	175_1	Copper	89.62	4.03	69.46	1	1	1	17	Medium quality	12886	1322121	48.70	137	d__Archaea;p__Thermoplasmatota;c__Thermoplasmata;o__Thermoplasmatales;f__Thermoplasmataceae;g__UBA582;s__
LMSG_G000007371.1	no	175_1	Lead-Zinc	87.45	1.30	80.94	1	1	1	18	Medium quality	43073	1393294	48.70	50	d__Archaea;p__Thermoplasmatota;c__Thermoplasmata;o__Thermoplasmatales;f__Thermoplasmataceae;g__UBA582;s__
LMSG_G000007372.1	no	175_1	Lead-Zinc	79.21	0.81	75.18	1	1	1	19	Medium quality	18800	1153883	48.70	79	d__Archaea;p__Thermoplasmatota;c__Thermoplasmata;o__Thermoplasmatales;f__Thermoplasmataceae;g__UBA582;s__
LMSG_G000007373.1	no	175_1	Lead-Zinc	88.80	0.81	84.77	1	1	2	20	Medium quality	64042	1305370	48.70	43	d__Archaea;p__Thermoplasmatota;c__Thermoplasmata;o__Thermoplasmatales;f__Thermoplasmataceae;g__UBA582;s__
LMSG_G000007374.1	no	175_1	Pyrite-Copper	72.73	1.61	64.67	1	1	1	15	Medium quality	10120	1172156	49.00	145	d__Archaea;p__Thermoplasmatota;c__Thermoplasmata;o__Thermoplasmatales;f__Thermoplasmataceae;g__UBA582;s__
LMSG_G000007375.1	no	175_1	Pyrite-Copper	88.80	0.62	85.70	1	2	0	18	Medium quality	40387	1354235	48.80	58	d__Archaea;p__Thermoplasmatota;c__Thermoplasmata;o__Thermoplasmatales;f__Thermoplasmataceae;g__UBA582;s__
LMSG_G000007376.1	no	175_1	Lead-Zinc	87.99	3.66	69.69	1	1	2	18	Medium quality	13367	1405829	48.60	130	d__Archaea;p__Thermoplasmatota;c__Thermoplasmata;o__Thermoplasmatales;f__Thermoplasmataceae;g__UBA582;s__
LMSG_G000007377.1	no	175_1	Copper	93.91	0.81	89.88	1	0	2	18	Medium quality	49394	1495317	48.60	50	d__Archaea;p__Thermoplasmatota;c__Thermoplasmata;o__Thermoplasmatales;f__Thermoplasmataceae;g__UBA582;s__
LMSG_G000007378.1	no	175_1	Magnetite	81.54	4.29	60.11	1	1	0	14	Medium quality	22546	1102310	48.70	74	d__Archaea;p__Thermoplasmatota;c__Thermoplasmata;o__Thermoplasmatales;f__Thermoplasmataceae;g__UBA582;s__
LMSG_G000007379.1	no	181_1	Tin-Zinc	89.33	4.09	68.86	1	1	2	19	Medium quality	12423	1458098	48.00	182	d__Archaea;p__Thermoplasmatota;c__Thermoplasmata;o__Thermoplasmatales;f__Thermoplasmataceae;g__UBA582;s__
LMSG_G000007380.1	no	181_2	Copper	87.45	4.03	67.29	1	2	2	20	Medium quality	48017	1359763	48.40	72	d__Archaea;p__Thermoplasmatota;c__Thermoplasmata;o__Thermoplasmatales;f__Thermoplasmataceae;g__UBA582;s__
LMSG_G000007381.1	no	181_2	Copper	85.84	0.81	81.81	1	1	2	20	Medium quality	78417	1275765	48.30	46	d__Archaea;p__Thermoplasmatota;c__Thermoplasmata;o__Thermoplasmatales;f__Thermoplasmataceae;g__UBA582;s__
LMSG_G000007382.1	no	181_2	Copper	79.03	1.61	70.97	0	2	1	20	Medium quality	43421	1292400	48.40	55	d__Archaea;p__Thermoplasmatota;c__Thermoplasmata;o__Thermoplasmatales;f__Thermoplasmataceae;g__UBA582;s__
LMSG_G000007383.1	no	181_2	Lead-Zinc	87.94	2.67	74.61	0	1	2	20	Medium quality	31153	1349084	48.60	85	d__Archaea;p__Thermoplasmatota;c__Thermoplasmata;o__Thermoplasmatales;f__Thermoplasmataceae;g__UBA582;s__
LMSG_G000007384.1	no	185_1	Copper	62.09	1.61	54.03	1	1	0	12	Medium quality	13863	1113606	41.80	121	d__Archaea;p__Thermoplasmatota;c__Thermoplasmata;o__Thermoplasmatales;f__Thermoplasmataceae;g__UBA582;s__
LMSG_G000007385.1	no	185_1	Polymetallic	78.75	1.61	70.69	1	2	0	16	Medium quality	19328	1463653	41.80	109	d__Archaea;p__Thermoplasmatota;c__Thermoplasmata;o__Thermoplasmatales;f__Thermoplasmataceae;g__UBA582;s__
LMSG_G000007386.1	no	201_1	Pyrite	96.73	0.40	94.72	1	1	1	18	High quality	32198	1441816	49.50	75	d__Archaea;p__Thermoplasmatota;c__Thermoplasmata;o__Thermoplasmatales;f__Thermoplasmataceae;g__UBA582;s__
LMSG_G000007387.1	no	201_1	Lead-Zinc	90.28	0.00	90.28	1	2	1	19	High quality	43236	1380205	49.40	48	d__Archaea;p__Thermoplasmatota;c__Thermoplasmata;o__Thermoplasmatales;f__Thermoplasmataceae;g__UBA582;s__
LMSG_G000007388.1	no	201_1	Lead-Zinc	96.73	1.61	88.67	1	2	2	20	High quality	78120	1612067	49.20	28	d__Archaea;p__Thermoplasmatota;c__Thermoplasmata;o__Thermoplasmatales;f__Thermoplasmataceae;g__UBA582;s__
LMSG_G000007389.1	no	201_1	Lead-Zinc	93.50	0.56	90.71	1	2	1	19	High quality	28746	1407699	49.60	67	d__Archaea;p__Thermoplasmatota;c__Thermoplasmata;o__Thermoplasmatales;f__Thermoplasmataceae;g__UBA582;s__

LMSG_G000007390.1	no	201_1	Pyrite-Copper	94.31	0.81	90.28	1	2	2	20	High quality	50648	1596862	49.30	54	d__Archaea;p__Thermoplasmatota;c__Thermoplasmata;o__Thermoplasmatales;f__Thermoplasmataceae;g__UBA582;s__
LMSG_G000007391.1	no	201_1	Pyrite-Copper	97.53	2.82	83.42	1	2	2	20	High quality	51684	1587541	49.30	53	d__Archaea;p__Thermoplasmatota;c__Thermoplasmata;o__Thermoplasmatales;f__Thermoplasmataceae;g__UBA582;s__
LMSG_G000007392.1	no	201_1	Pyrite-Copper	95.11	0.00	95.11	1	2	2	19	High quality	47952	1509775	49.30	49	d__Archaea;p__Thermoplasmatota;c__Thermoplasmata;o__Thermoplasmatales;f__Thermoplasmataceae;g__UBA582;s__
LMSG_G000007393.1	no	201_1	Pyrite-Copper	98.34	4.33	76.68	1	5	4	20	High quality	92442	1580184	49.20	144	d__Archaea;p__Thermoplasmatota;c__Thermoplasmata;o__Thermoplasmatales;f__Thermoplasmataceae;g__UBA582;s__
LMSG_G000007394.1	no	201_1	Lead-Zinc	95.92	0.88	91.55	1	2	2	20	High quality	47113	1564677	49.40	56	d__Archaea;p__Thermoplasmatota;c__Thermoplasmata;o__Thermoplasmatales;f__Thermoplasmataceae;g__UBA582;s__
LMSG_G000007395.1	no	201_1	Pyrite-Copper	92.19	1.61	84.13	1	2	2	19	High quality	66995	1508828	49.00	49	d__Archaea;p__Thermoplasmatota;c__Thermoplasmata;o__Thermoplasmatales;f__Thermoplasmataceae;g__UBA582;s__
LMSG_G000007396.1	no	202_1	Copper	61.24	0.81	57.21	1	2	1	15	Medium quality	54668	1017331	48.70	35	d__Archaea;p__Thermoplasmatota;c__Thermoplasmata;o__Thermoplasmatales;f__Thermoplasmataceae;g__UBA582;s__
LMSG_G000007397.1	no	202_1	Copper	85.44	1.61	77.38	1	2	1	17	Medium quality	38992	1384851	48.70	59	d__Archaea;p__Thermoplasmatota;c__Thermoplasmata;o__Thermoplasmatales;f__Thermoplasmataceae;g__UBA582;s__
LMSG_G000007398.1	no	202_1	Copper	79.70	0.81	75.67	1	1	1	17	Medium quality	39886	1402878	48.00	55	d__Archaea;p__Thermoplasmatota;c__Thermoplasmata;o__Thermoplasmatales;f__Thermoplasmataceae;g__UBA582;s__
LMSG_G000007399.1	no	202_1	Lead-Zinc	76.57	0.81	72.54	1	2	2	16	Medium quality	40011	1103236	48.70	40	d__Archaea;p__Thermoplasmatota;c__Thermoplasmata;o__Thermoplasmatales;f__Thermoplasmataceae;g__UBA582;s__
LMSG_G000007400.1	no	202_1	Copper	65.28	0.81	61.25	1	2	1	15	Medium quality	54651	872323	48.20	21	d__Archaea;p__Thermoplasmatota;c__Thermoplasmata;o__Thermoplasmatales;f__Thermoplasmataceae;g__UBA582;s__
LMSG_G000007401.1	no	202_1	Lead-Zinc	74.55	0.00	74.55	1	0	0	17	Medium quality	46060	1133610	49.20	43	d__Archaea;p__Thermoplasmatota;c__Thermoplasmata;o__Thermoplasmatales;f__Thermoplasmataceae;g__UBA582;s__
LMSG_G000007402.1	no	203_1	Polymetallic	64.07	2.55	51.34	0	2	0	12	Medium quality	19587	785752	44.00	64	d__Archaea;p__Thermoplasmatota;c__Thermoplasmata;o__Thermoplasmatales;f__Thermoplasmataceae;g__UBA582;s__
LMSG_G000007403.1	no	203_1	Polymetallic	70.11	2.42	58.02	1	1	0	16	Medium quality	13771	1144018	44.70	137	d__Archaea;p__Thermoplasmatota;c__Thermoplasmata;o__Thermoplasmatales;f__Thermoplasmataceae;g__UBA582;s__
LMSG_G000007404.1	no	203_1	Polymetallic	78.26	3.76	59.45	1	0	1	19	Medium quality	9298	1369286	44.20	205	d__Archaea;p__Thermoplasmatota;c__Thermoplasmata;o__Thermoplasmatales;f__Thermoplasmataceae;g__UBA582;s__
LMSG_G000007405.1	no	203_1	Tin-Zinc	63.30	1.61	55.24	1	1	0	16	Medium quality	22844	911315	43.90	51	d__Archaea;p__Thermoplasmatota;c__Thermoplasmata;o__Thermoplasmatales;f__Thermoplasmataceae;g__UBA582;s__
LMSG_G000007406.1	no	203_1	Copper	77.14	2.42	65.05	1	1	2	13	Medium quality	17348	1175652	44.80	96	d__Archaea;p__Thermoplasmatota;c__Thermoplasmata;o__Thermoplasmatales;f__Thermoplasmataceae;g__UBA582;s__
LMSG_G000007407.1	no	203_1	Lead-Zinc	77.70	0.81	73.67	1	2	1	19	Medium quality	34072	1222239	44.60	64	d__Archaea;p__Thermoplasmatota;c__Thermoplasmata;o__Thermoplasmatales;f__Thermoplasmataceae;g__UBA582;s__
LMSG_G000007408.1	no	203_1	Pyrite-Copper	78.58	1.61	70.52	1	2	1	17	Medium quality	23113	1187771	44.40	77	d__Archaea;p__Thermoplasmatota;c__Thermoplasmata;o__Thermoplasmatales;f__Thermoplasmataceae;g__UBA582;s__
LMSG_G000007409.1	no	203_1	Pyrite-Copper	79.39	0.81	75.36	1	1	1	14	Medium quality	21059	892954	44.70	59	d__Archaea;p__Thermoplasmatota;c__Thermoplasmata;o__Thermoplasmatales;f__Thermoplasmataceae;g__UBA582;s__
LMSG_G000007410.1	no	203_1	Polymetallic	87.45	2.42	75.36	1	1	2	20	Medium quality	29339	1521135	44.30	79	d__Archaea;p__Thermoplasmatota;c__Thermoplasmata;o__Thermoplasmatales;f__Thermoplasmataceae;g__UBA582;s__
LMSG_G000007411.1	no	205_1	Copper	71.32	0.00	71.32	1	2	2	17	Medium quality	57146	1018312	44.70	24	d__Archaea;p__Thermoplasmatota;c__Thermoplasmata;o__Thermoplasmatales;f__Thermoplasmataceae;g__UBA582;s__
LMSG_G000007412.1	no	205_1	Copper	84.08	3.63	65.94	1	2	1	19	Medium quality	16093	1136703	44.70	113	d__Archaea;p__Thermoplasmatota;c__Thermoplasmata;o__Thermoplasmatales;f__Thermoplasmataceae;g__UBA582;s__
LMSG_G000007413.1	no	209_1	Copper	92.51	4.44	70.34	1	1	1	19	High quality	27456	1468374	44.80	89	d__Archaea;p__Thermoplasmatota;c__Thermoplasmata;o__Thermoplasmatales;f__Thermoplasmataceae;g__UBA582;s__
LMSG_G000007414.1	no	209_1	Lead-Zinc	72.15	0.00	72.15	0	0	1	14	Medium quality	16096	1278041	44.90	117	d__Archaea;p__Thermoplasmatota;c__Thermoplasmata;o__Thermoplasmatales;f__Thermoplasmataceae;g__UBA582;s__
LMSG_G000007415.1	no	209_1	Lead-Zinc	59.23	0.42	57.13	0	0	1	10	Medium quality	19430	840721	45.60	77	d__Archaea;p__Thermoplasmatota;c__Thermoplasmata;o__Thermoplasmatales;f__Thermoplasmataceae;g__UBA582;s__
LMSG_G000007416.1	no	209_1	Tin-Zinc	65.54	0.17	64.70	1	0	1	17	Medium quality	24412	1021577	45.00	71	d__Archaea;p__Thermoplasmatota;c__Thermoplasmata;o__Thermoplasmatales;f__Thermoplasmataceae;g__UBA582;s__
LMSG_G000007417.1	no	209_1	Copper	60.53	0.40	58.52	0	0	1	12	Medium quality	15123	866307	44.90	80	d__Archaea;p__Thermoplasmatota;c__Thermoplasmata;o__Thermoplasmatales;f__Thermoplasmataceae;g__UBA582;s__
LMSG_G000007418.1	no	209_1	Polymetallic	80.19	1.21	74.15	1	1	1	19	Medium quality	18982	1392554	44.50	100	d__Archaea;p__Thermoplasmatota;c__Thermoplasmata;o__Thermoplasmatales;f__Thermoplasmataceae;g__UBA582;s__
LMSG_G000007419.1	no	209_1	Magnetite	73.74	0.06	73.43	1	1	1	17	Medium quality	20524	1201067	44.70	81	d__Archaea;p__Thermoplasmatota;c__Thermoplasmata;o__Thermoplasmatales;f__Thermoplasmataceae;g__UBA582;s__
LMSG_G000007420.1	no	209_1	Copper	81.00	4.57	58.16	1	1	0	17	Medium quality	15379	1406340	44.80	141	d__Archaea;p__Thermoplasmatota;c__Thermoplasmata;o__Thermoplasmatales;f__Thermoplasmataceae;g__UBA582;s__
LMSG_G000007421.1	no	209_1	Coal	73.21	0.00	73.21	0	1	1	16	Medium quality	9497	1292485	44.90	183	d__Archaea;p__Thermoplasmatota;c__Thermoplasmata;o__Thermoplasmatales;f__Thermoplasmataceae;g__UBA582;s__
LMSG_G000007422.1	no	150_1	Pyrite-Copper	61.45	1.28	55.05	1	0	1	12	Medium quality	6402	1319613	52.90	262	d__Archaea;p__Thermoplasmatota;c__Thermoplasmata;o__UBA10834;f__;g__;s__
LMSG_G000007423.1	no	150_1	Polymetallic	56.43	0.80	52.43	1	0	0	11	Medium quality	7186	1114240	52.40	209	d__Archaea;p__Thermoplasmatota;c__Thermoplasmata;o__UBA10834;f__;g__;s__
LMSG_G000007424.1	no	152_1	Lead-Zinc	60.94	0.80	56.94	0	0	0	16	Medium quality	18363	1124399	48.90	104	d__Archaea;p__Thermoplasmatota;c__Thermoplasmata;o__UBA10834;f__;g__;s__
LMSG_G000007425.1	no	152_1	Copper	51.20	0.00	51.20	1	0	0	12	Medium quality	64387	1008137	49.40	40	d__Archaea;p__Thermoplasmatota;c__Thermoplasmata;o__UBA10834;f__;g__;s__
LMSG_G000007426.1	no	156_1	Magnetite	90.20	2.87	75.86	1	1	1	19	High quality	12712	1738386	52.00	183	d__Archaea;p__Thermoplasmatota;c__Thermoplasmata;o__UBA10834;f__;g__;s__
LMSG_G000007427.1	no	156_1	Magnetite	65.27	2.40	53.27	0	0	0	13	Medium quality	11166	1096757	52.50	132	d__Archaea;p__Thermoplasmatota;c__Thermoplasmata;o__UBA10834;f__;g__;s__
LMSG_G000007428.1	no	157_1	Polymetallic	90.20	0.16	89.40	2	1	1	18	High quality	17058	1343064	51.50	100	d__Archaea;p__Thermoplasmatota;c__Thermoplasmata;o__UBA10834;f__;g__;s__
LMSG_G000007429.1	no	157_1	Magnetite	88.60	1.60	80.60	1	2	2	15	Medium quality	17885	1598608	51.10	265	d__Archaea;p__Thermoplasmatota;c__Thermoplasmata;o__UBA10834;f__;g__;s__
LMSG_G000007430.1	no	157_1	Pyrite	64.89	1.60	56.89	0	0	1	13	Medium quality	4601	1099737	51.70	266	d__Archaea;p__Thermoplasmatota;c__Thermoplasmata;o__UBA10834;f__;g__;s__
LMSG_G000007431.1	no	157_1	Copper	93.32	1.76	84.52	1	1	0	17	Medium quality	7780	1672958	51.40	257	d__Archaea;p__Thermoplasmatota;c__Thermoplasmata;o__UBA10834;f__;g__;s__
LMSG_G000007432.1	no	168_1	Pyrite	96.26	0.00	96.26	1	1	1	19	High quality	25039	1621317	35.80	97	d__Archaea;p__Thermoplasmatota;c__Thermoplasmata;o__UBA184;f__;g__;s__
LMSG_G000007433.1	no	168_1	Pyrite	94.66	0.00	94.66	1	1	1	19	High quality	12912	1542472	35.80	186	d__Archaea;p__Thermoplasmatota;c__Thermoplasmata;o__UBA184;f__;g__;s__
LMSG_G000007434.1	no	168_1	Copper	93.06	0.00	93.06	1	1	1	19	High quality	11758	1530866	35.90	223	d__Archaea;p__Thermoplasmatota;c__Thermoplasmata;o__UBA184;f__;g__;s__
LMSG_G000007435.1	no	168_1	Lead-Zinc	94.66	0.00	94.66	1	1	1	19	High quality	24129	1487655	35.70	98	d__Archaea;p__Thermoplasmatota;c__Thermoplasmata;o__UBA184;f__;g__;s__
LMSG_G000007436.1	no	168_1	Pyrite	92.82	0.27	91.49	1	1	1	17	Medium quality	13452	1517413	35.70	162	d__Archaea;p__Thermoplasmatota;c__Thermoplasmata;o__UBA184;f__;g__;s__
LMSG_G000007437.1	no	168_1	Copper	88.71	0.00	88.71	1	1	1	18	Medium quality	5663	1612841	35.50	369	d__Archaea;p__Thermoplasmatota;c__Thermoplasmata;o__UBA184;f__;g__;s__
LMSG_G000007438.1	no	168_1	Polymetallic	79.64	0.80	75.64	1	1	1	17	Medium quality	5514	1286892	36.00	310	d__Archaea;p__Thermoplasmatota;c__Thermoplasmata;o__UBA184;f__;g__;s__
LMSG_G000007439.1	no	168_1	Polymetallic	79.93	0.00	79.93	1	0	1	13	Medium quality	4747	1258604	35.80	322	d__Archaea;p__Thermoplasmatota;c__Thermoplasmata;o__UBA184;f__;g__;s__
LMSG_G000007440.1	no	168_1	Copper	90.66	0.00	90.66	1	0	1	18	Medium quality	20440	1481694	35.50	98	d__Archaea;p__Thermoplasmatota;c__Thermoplasmata;o__UBA184;f__;g__;s__
LMSG_G000007441.1	no	168_1	Lead-Zinc	88.60	0.06	88.30	1	1	0	17	Medium quality	10412	1381699	35.60	198	d__Archaea;p__Thermoplasmatota;c__Thermoplasmata;o__UBA184;f__;g__;s__
LMSG_G000007442.1	no	168_1	Lead-Zinc	73.50	0.80	69.50	1	0	2	14	Medium quality	3332	1299481	35.00	437	d__Archaea;p__Thermoplasmatota;c__Thermoplasmata;o__UBA184;f__;g__;s__
LMSG_G000007443.1	no	168_1	Pyrite-Copper	55.30	0.00	55.30	0	0	2	11	Medium quality	2070	902891	36.90	451	d__Archaea;p__Thermoplasmatota;c__Thermoplasmata;o__UBA184;f__;g__;s__
LMSG_G000007444.1	no	168_1	Lead-Zinc	78.68	0.00	78.68	1	1	1	16	Medium quality	5473	1197528	35.50	249	d__Archaea;p__Thermoplasmatota;c__Thermoplasmata;o__UBA184;f__;g__;s__
LMSG_G000007445.1	no	168_1	Magnetite	63.73	0.00	63.73	1	0	1	14	Medium quality	10695	1141006	35.60	143	d__Archaea;p__Thermoplasmatota;c__Thermoplasmata;o__UBA184;f__;g__;s__
LMSG_G000007446.1	no	540_1	Pyrite-Copper	63.86	2.13	53.20	0	2	0	15	Medium quality	63477	1333677	69.70	124	d__Archaea;p__Thermoplasmatota;c__Thermoplasmata;o__UBA184;f__UBA184;g__;s__
LMSG_G000007447.1	no	540_1	Copper	69.87	0.80	65.87	1	1	2	15	Medium quality	27338	1265672	70.60	67	d__Archaea;p__Thermoplasmatota;c__Thermoplasmata;o__UBA184;f__UBA184;g__;s__
LMSG_G000007448.1	no	541_1	Copper	71.23	3.60	53.23	0	0	0	19	Medium quality	4788	1506814	69.30	427	d__Archaea;p__Thermoplasmatota;c__Thermoplasmata;o__UBA184;f__UBA184;g__;s__
LMSG_G000007449.1	no	542_1	Pyrite-Copper	96.66	2.00	86.66	1	1	2	19	High quality	17283	1906471	69.80	177	d__Archaea;p__Thermoplasmatota;c__Thermoplasmata;o__UBA184;f__UBA184;g__;s__
LMSG_G000007450.1	no	542_1	Pyrite-Copper	90.26	1.78	81.39	1	2	2	19	High quality	8403	1731791	70.00	284	d__Archaea;p__Thermoplasmatota;c__Thermoplasmata;o__UBA184;f__UBA184;g__;s__

LMSG_G000007451.1	no	542_1	Pyrite	94.02	2.40	82.02	2	0	1	18	Medium quality	33528	1907569	69.70	88	d__Archaea;p__Thermoplasmatota;c__Thermoplasmata;o__UBA184;f__UBA184;g__;s__
LMSG_G000007452.1	no	542_1	Pyrite	92.66	2.40	80.66	0	1	2	20	Medium quality	45640	1955092	69.60	145	d__Archaea;p__Thermoplasmatota;c__Thermoplasmata;o__UBA184;f__UBA184;g__;s__
LMSG_G000007453.1	no	542_1	Pyrite	76.85	3.20	60.85	1	1	0	16	Medium quality	7570	1587624	70.00	250	d__Archaea;p__Thermoplasmatota;c__Thermoplasmata;o__UBA184;f__UBA184;g__;s__
LMSG_G000007454.1	no	542_1	Pyrite	86.00	4.00	66.00	1	0	2	18	Medium quality	23137	1794756	69.80	134	d__Archaea;p__Thermoplasmatota;c__Thermoplasmata;o__UBA184;f__UBA184;g__;s__
LMSG_G000007455.1	no	542_1	Copper	90.62	1.60	82.62	1	0	2	17	Medium quality	14424	1749835	69.70	158	d__Archaea;p__Thermoplasmatota;c__Thermoplasmata;o__UBA184;f__UBA184;g__;s__
LMSG_G000007456.1	no	542_1	Copper	91.46	1.60	83.46	1	0	1	18	Medium quality	60301	1889940	69.60	43	d__Archaea;p__Thermoplasmatota;c__Thermoplasmata;o__UBA184;f__UBA184;g__;s__
LMSG_G000007457.1	no	542_1	Copper	71.86	3.51	54.33	1	2	2	15	Medium quality	29345	1328131	70.20	60	d__Archaea;p__Thermoplasmatota;c__Thermoplasmata;o__UBA184;f__UBA184;g__;s__
LMSG_G000007458.1	no	542_1	Pyrite-Copper	87.86	1.60	79.86	1	1	2	18	Medium quality	31237	1575856	70.30	111	d__Archaea;p__Thermoplasmatota;c__Thermoplasmata;o__UBA184;f__UBA184;g__;s__
LMSG_G000007459.1	no	542_1	Pyrite-Copper	89.30	2.82	75.19	2	1	1	19	Medium quality	67536	1853364	69.60	216	d__Archaea;p__Thermoplasmatota;c__Thermoplasmata;o__UBA184;f__UBA184;g__;s__
LMSG_G000007460.1	no	542_1	Lead-Zinc	85.06	0.80	81.06	1	1	1	19	Medium quality	59416	1751800	69.50	46	d__Archaea;p__Thermoplasmatota;c__Thermoplasmata;o__UBA184;f__UBA184;g__;s__
LMSG_G000007461.1	no	542_1	Lead-Zinc	66.51	3.20	50.51	1	1	1	18	Medium quality	2079	1276666	70.00	662	d__Archaea;p__Thermoplasmatota;c__Thermoplasmata;o__UBA184;f__UBA184;g__;s__
LMSG_G000007462.1	no	544_1	Pyrite	90.66	2.71	77.13	1	2	1	17	Medium quality	22737	1751715	68.60	119	d__Archaea;p__Thermoplasmatota;c__Thermoplasmata;o__UBA184;f__UBA184;g__;s__
LMSG_G000007463.1	no	544_1	Copper	91.06	0.98	86.14	1	1	1	15	Medium quality	35301	1641329	68.60	83	d__Archaea;p__Thermoplasmatota;c__Thermoplasmata;o__UBA184;f__UBA184;g__;s__
LMSG_G000007464.1	no	544_1	Copper	91.06	1.60	83.06	1	1	1	16	Medium quality	20804	1671145	68.70	129	d__Archaea;p__Thermoplasmatota;c__Thermoplasmata;o__UBA184;f__UBA184;g__;s__
LMSG_G000007465.1	no	544_1	Copper	94.26	1.73	85.63	1	2	1	15	Medium quality	42534	1782889	68.60	144	d__Archaea;p__Thermoplasmatota;c__Thermoplasmata;o__UBA184;f__UBA184;g__;s__
LMSG_G000007466.1	no	544_1	Polymetallic	91.86	3.38	74.94	1	1	1	15	Medium quality	38539	1769789	68.50	146	d__Archaea;p__Thermoplasmatota;c__Thermoplasmata;o__UBA184;f__UBA184;g__;s__
LMSG_G000007467.1	no	544_1	Copper	88.66	3.20	72.66	1	0	0	16	Medium quality	26097	1666028	68.80	114	d__Archaea;p__Thermoplasmatota;c__Thermoplasmata;o__UBA184;f__UBA184;g__;s__
LMSG_G000007468.1	no	544_1	Pyrite	86.26	1.66	77.96	1	0	0	16	Medium quality	19448	1636534	68.70	140	d__Archaea;p__Thermoplasmatota;c__Thermoplasmata;o__UBA184;f__UBA184;g__;s__
LMSG_G000007469.1	no	544_1	Copper	84.66	1.60	76.66	1	0	1	14	Medium quality	26154	1664517	68.70	112	d__Archaea;p__Thermoplasmatota;c__Thermoplasmata;o__UBA184;f__UBA184;g__;s__
LMSG_G000007470.1	no	544_1	Copper	87.42	1.60	79.42	1	1	1	16	Medium quality	7873	1652580	68.70	321	d__Archaea;p__Thermoplasmatota;c__Thermoplasmata;o__UBA184;f__UBA184;g__;s__
LMSG_G000007471.1	no	544_1	Magnetite	59.03	0.80	55.03	0	1	10	17	Medium quality	6078	1332088	67.70	349	d__Archaea;p__Thermoplasmatota;c__Thermoplasmata;o__UBA184;f__UBA184;g__;s__
LMSG_G000007472.1	no	544_1	Magnetite	83.04	1.60	75.04	1	0	1	13	Medium quality	22443	1659643	68.50	183	d__Archaea;p__Thermoplasmatota;c__Thermoplasmata;o__UBA184;f__UBA184;g__;s__
LMSG_G000007473.1	no	544_1	Magnetite	95.06	2.40	83.06	1	1	0	15	Medium quality	41347	1784463	68.50	135	d__Archaea;p__Thermoplasmatota;c__Thermoplasmata;o__UBA184;f__UBA184;g__;s__
LMSG_G000007474.1	no	544_1	Pyrite-Copper	54.60	0.86	50.30	1	0	1	14	Medium quality	5967	1180699	68.80	219	d__Archaea;p__Thermoplasmatota;c__Thermoplasmata;o__UBA184;f__UBA184;g__;s__
LMSG_G000007475.1	no	544_1	Pyrite-Copper	64.45	0.80	60.45	2	0	0	9	Medium quality	3585	1154870	68.60	348	d__Archaea;p__Thermoplasmatota;c__Thermoplasmata;o__UBA184;f__UBA184;g__;s__
LMSG_G000007476.1	no	546_1	Copper	96.26	2.40	84.26	1	2	2	20	High quality	90694	1610162	67.20	26	d__Archaea;p__Thermoplasmatota;c__Thermoplasmata;o__UBA184;f__UBA184;g__;s__
LMSG_G000007477.1	no	546_1	Copper	73.06	1.60	65.06	1	2	1	17	Medium quality	68182	1210946	67.60	24	d__Archaea;p__Thermoplasmatota;c__Thermoplasmata;o__UBA184;f__UBA184;g__;s__
LMSG_G000007478.1	no	546_1	Copper	87.86	2.40	75.86	0	1	2	19	Medium quality	43879	1452022	67.40	55	d__Archaea;p__Thermoplasmatota;c__Thermoplasmata;o__UBA184;f__UBA184;g__;s__
LMSG_G000007479.1	no	546_1	Copper	75.49	1.68	67.07	0	1	1	17	Medium quality	3211	1254245	67.70	441	d__Archaea;p__Thermoplasmatota;c__Thermoplasmata;o__UBA184;f__UBA184;g__;s__
LMSG_G000007480.1	no	546_1	Polymetallic	97.46	2.40	85.46	2	1	2	20	High quality	72268	1655028	67.10	80	d__Archaea;p__Thermoplasmatota;c__Thermoplasmata;o__UBA184;f__UBA184;g__;s__
LMSG_G000007481.1	no	546_1	Polymetallic	96.66	1.60	88.66	2	2	2	19	High quality	84426	1637719	67.20	80	d__Archaea;p__Thermoplasmatota;c__Thermoplasmata;o__UBA184;f__UBA184;g__;s__
LMSG_G000007482.1	no	546_1	Polymetallic	95.86	1.60	87.86	2	2	3	19	High quality	92546	1600283	67.30	94	d__Archaea;p__Thermoplasmatota;c__Thermoplasmata;o__UBA184;f__UBA184;g__;s__
LMSG_G000007483.1	no	546_1	Polymetallic	97.46	1.60	89.46	1	1	2	20	High quality	40117	1653874	67.30	142	d__Archaea;p__Thermoplasmatota;c__Thermoplasmata;o__UBA184;f__UBA184;g__;s__
LMSG_G000007484.1	no	546_1	Coal	95.06	2.40	83.06	1	1	2	20	High quality	81837	1586935	67.30	88	d__Archaea;p__Thermoplasmatota;c__Thermoplasmata;o__UBA184;f__UBA184;g__;s__
LMSG_G000007485.1	no	546_1	Coal	97.46	2.40	85.46	1	2	2	19	High quality	60432	1503334	67.50	83	d__Archaea;p__Thermoplasmatota;c__Thermoplasmata;o__UBA184;f__UBA184;g__;s__
LMSG_G000007486.1	no	546_1	Coal	84.54	0.00	84.54	1	1	2	18	Medium quality	38859	1310673	67.60	91	d__Archaea;p__Thermoplasmatota;c__Thermoplasmata;o__UBA184;f__UBA184;g__;s__
LMSG_G000007487.1	no	546_1	Coal	84.80	2.13	74.14	1	1	2	19	Medium quality	23113	1385681	67.40	85	d__Archaea;p__Thermoplasmatota;c__Thermoplasmata;o__UBA184;f__UBA184;g__;s__
LMSG_G000007488.1	no	546_1	Pyrite	73.64	1.85	64.41	1	2	1	19	Medium quality	99240	1130781	67.40	19	d__Archaea;p__Thermoplasmatota;c__Thermoplasmata;o__UBA184;f__UBA184;g__;s__
LMSG_G000007489.1	no	547_1	Lead-Zinc	97.06	2.00	87.06	1	1	3	19	High quality	32505	1486042	67.20	93	d__Archaea;p__Thermoplasmatota;c__Thermoplasmata;o__UBA184;f__UBA184;g__;s__
LMSG_G000007490.1	no	547_1	Pyrite	92.22	3.20	76.22	1	1	3	19	High quality	41064	1411034	67.40	92	d__Archaea;p__Thermoplasmatota;c__Thermoplasmata;o__UBA184;f__UBA184;g__;s__
LMSG_G000007491.1	no	547_1	Pyrite	96.57	2.40	84.57	1	1	1	20	High quality	38485	1466230	67.30	81	d__Archaea;p__Thermoplasmatota;c__Thermoplasmata;o__UBA184;f__UBA184;g__;s__
LMSG_G000007492.1	no	547_1	Pyrite	93.54	2.40	81.54	1	1	2	18	High quality	33724	1393656	67.30	99	d__Archaea;p__Thermoplasmatota;c__Thermoplasmata;o__UBA184;f__UBA184;g__;s__
LMSG_G000007493.1	no	547_1	Lead-Zinc	97.86	1.60	89.86	1	1	1	18	High quality	20794	1455965	67.40	115	d__Archaea;p__Thermoplasmatota;c__Thermoplasmata;o__UBA184;f__UBA184;g__;s__
LMSG_G000007494.1	no	547_1	Polymetallic	96.14	1.64	87.93	1	1	1	18	High quality	9633	1478063	67.20	265	d__Archaea;p__Thermoplasmatota;c__Thermoplasmata;o__UBA184;f__UBA184;g__;s__
LMSG_G000007495.1	no	547_1	Polymetallic	95.46	1.95	85.71	1	2	3	20	High quality	62014	1565621	67.10	79	d__Archaea;p__Thermoplasmatota;c__Thermoplasmata;o__UBA184;f__UBA184;g__;s__
LMSG_G000007496.1	no	547_1	Polymetallic	92.66	1.60	84.66	1	2	2	19	High quality	43289	1524617	67.20	93	d__Archaea;p__Thermoplasmatota;c__Thermoplasmata;o__UBA184;f__UBA184;g__;s__
LMSG_G000007497.1	no	547_1	Polymetallic	92.26	2.40	80.26	1	2	1	20	High quality	77712	1560225	67.10	88	d__Archaea;p__Thermoplasmatota;c__Thermoplasmata;o__UBA184;f__UBA184;g__;s__
LMSG_G000007498.1	no	547_1	Polymetallic	96.26	1.60	88.26	1	2	1	19	High quality	40382	1522869	67.20	75	d__Archaea;p__Thermoplasmatota;c__Thermoplasmata;o__UBA184;f__UBA184;g__;s__
LMSG_G000007499.1	no	547_1	Lead-Zinc	98.66	1.60	90.66	1	2	2	20	High quality	118534	1617501	67.00	43	d__Archaea;p__Thermoplasmatota;c__Thermoplasmata;o__UBA184;f__UBA184;g__;s__
LMSG_G000007500.1	no	547_1	Lead-Zinc	98.66	4.00	78.66	1	2	1	20	High quality	35793	1548746	67.20	70	d__Archaea;p__Thermoplasmatota;c__Thermoplasmata;o__UBA184;f__UBA184;g__;s__
LMSG_G000007501.1	no	547_1	Pyrite-Copper	98.66	1.60	90.66	1	2	2	20	High quality	71022	1453072	67.10	42	d__Archaea;p__Thermoplasmatota;c__Thermoplasmata;o__UBA184;f__UBA184;g__;s__
LMSG_G000007502.1	no	547_1	Lead-Zinc	97.06	1.60	89.06	1	2	2	19	High quality	91535	1485275	67.20	31	d__Archaea;p__Thermoplasmatota;c__Thermoplasmata;o__UBA184;f__UBA184;g__;s__
LMSG_G000007503.1	no	547_1	Pyrite	83.46	2.40	71.46	1	2	2	19	Medium quality	40280	1294977	67.30	75	d__Archaea;p__Thermoplasmatota;c__Thermoplasmata;o__UBA184;f__UBA184;g__;s__
LMSG_G000007504.1	no	547_1	Pyrite	79.97	2.40	67.97	1	0	1	16	Medium quality	10643	1207298	67.40	182	d__Archaea;p__Thermoplasmatota;c__Thermoplasmata;o__UBA184;f__UBA184;g__;s__
LMSG_G000007505.1	no	547_1	Copper	55.71	0.80	51.71	0	0	0	13	Medium quality	7427	891461	67.10	170	d__Archaea;p__Thermoplasmatota;c__Thermoplasmata;o__UBA184;f__UBA184;g__;s__
LMSG_G000007506.1	no	547_1	Copper	75.40	4.80	51.40	1	0	1	19	Medium quality	35696	1149393	67.20	87	d__Archaea;p__Thermoplasmatota;c__Thermoplasmata;o__UBA184;f__UBA184;g__;s__
LMSG_G000007507.1	no	547_1	Copper	76.40	2.05	66.14	1	0	1	13	Medium quality	22084	1075054	67.50	131	d__Archaea;p__Thermoplasmatota;c__Thermoplasmata;o__UBA184;f__UBA184;g__;s__
LMSG_G000007508.1	no	547_1	Copper	62.04	0.18	61.17	1	2	0	16	Medium quality	3107	816793	67.90	272	d__Archaea;p__Thermoplasmatota;c__Thermoplasmata;o__UBA184;f__UBA184;g__;s__
LMSG_G000007509.1	no	549_1	Polymetallic	94.66	3.20	78.66	1	2	2	20	High quality	50999	2111312	70.60	197	d__Archaea;p__Thermoplasmatota;c__Thermoplasmata;o__UBA184;f__UBA184;g__;s__
LMSG_G000007510.1	no	549_1	Polymetallic	91.46	1.60	83.46	1	1	1	15	Medium quality	19067	1597214	71.50	128	d__Archaea;p__Thermoplasmatota;c__Thermoplasmata;o__UBA184;f__UBA184;g__;s__
LMSG_G000007511.1	no	549_1	Polymetallic	63.42	0.93	58.76	1	0	1	14	Medium quality	2061	1295279	70.30	662	d__Archaea;p__Thermoplasmatota;c__Thermoplasmata;o__UBA184;f__UBA184;g__;s__
LMSG_G000007512.1	no	549_1	Coal	96.26	3.73	77.60	1	2	2	18	High quality	51271	1872420	71.10	93	d__Archaea;p__Thermoplasmatota;c__Thermoplasmata;o__UBA184;f__UBA184;g__;s__
LMSG_G000007513.1	no	549_1	Coal	96.00	1.60	88.00	1	2	2	19	High quality	50499	1899912	71.00	118	d__Archaea;p__Thermoplasmatota;c__Thermoplasmata;o__UBA184;f__UBA184;g__;s__
LMSG_G000007514.1	no	549_1	Coal	79.86	2.77	66.02	1	2	1	16	Medium quality	11977	1578740	71.10	161	d__Archaea;p__Thermoplasmatota;c__Thermoplasmata;o__UBA184;f__UBA184;g__;s__
LMSG_G000007515.1	no	549_1	Polymetallic	65.97	0.80	61.97	1	0	1	12	Medium quality	4158	1140105	71.30	293	d__Archaea;p__Thermoplasmatota;c__Thermoplasmata;o__UBA184;f__UBA184;g__;s__
LMSG_G000007516.1	no	549_1	Polymetallic	88.35	4.40	66.35	1	1	0	16	Medium quality	5551	1354644	71.80	293	d__Archaea;p__Thermoplasmatota;c__Thermoplasmata;o__UBA184;f__UBA184;g__;s__
LMSG_G000007517.1	no	550_1	Copper	50.77	0.00	50.77	1	0	1	11	Medium quality	1978	1164907	69.90	621	d__Archaea;p__Thermoplasmatota;c__Thermoplasmata;o__UBA184;f__UBA184;g__;s__
LMSG_G000007518.1	no	551_1	Copper	96.98	1.93	87.34	1	1	1	20	High quality	20312	2179960	68.30	154	d__Archaea;p__Thermoplasmatota;c__Thermoplasmata;o__UBA184;f__UBA184;g__;s__
LMSG_G000007519.1	no	551_1	Copper	97.46	1.87	88.13	1	1	1	20	High quality	23185	2151985	68.60	136	d__Archaea;p__Thermoplasmatota;c__Thermoplasmata;o__UBA184;f__UBA184;g__;s__
LMSG_G000007520.1	no	551_1	Copper	96.66	1.72	88.05	1	2	1	20	High quality	14080	2203876	68.20	214	d__Archaea;p__Thermoplasmatota;c__Thermoplasmata;o__UBA184;f__UBA184;g__;s__
LMSG_G000007521.1	no	551_1	Copper	97.86	1.64	89.65	1	1	0	20	Medium quality	25873	2037728	68.80	114	d__Archaea;p__Thermoplasmatota;c__Thermoplasmata;o__UBA184;f__UBA184;g__;s__
LMSG_G000007522.1	no	552_1	Pyrite	82.42	2.84	68.21	2	0	1	15	Medium quality	13356	1632395	69.70	202	d__Archaea;p__Thermoplasmatota;c__Thermoplasmata;o__UBA184;f__UBA184;g__;s__
LMSG_G000007523.1	no	552_1	Pyrite	80.29	1.60	72.29	0	1	1	15	Medium quality	26630	1280512	70.50	113	d__Archaea;p__Thermoplasmatota;c__Thermoplasmata;o__UBA184;f__UBA184;g__;s__
LMSG_G000007524.1	no	552_1	Pyrite-Copper	87.55	2.00	77.55	1	0	1	18	Medium quality	20976	1374423	70.40	119	d__Archaea;p__Thermoplasmatota;c__Thermoplasmata;o__UBA184;f__UBA184;g__;s__
LMSG_G000007525.1	no	552_1	Pyrite-Copper	89.03	3.52	71.43	1	1	2	18	Medium quality	34575	1346778	70.60	98	d__Archaea;p__Thermoplasmatota;c__Thermoplasmata;o__UBA184;f__UBA184;g__;s__
LMSG_G000007526.1	no	552_1	Pyrite	62.10	0.04	61.89	1	0	1	14	Medium quality	3290	1250877	69.80	427	d__Archaea;p__Thermoplasmatota;c__Thermoplasmata;o__UBA184;f__UBA184;g__;s__
LMSG_G000007527.1	no	552_1	Magnetite	96.66	1.60	88.66	1	1	2	20	High quality	51228	1750605	70.10	63	d__Archaea;p__Thermoplasmatota;c__Thermoplasmata;o__UBA184;f__UBA184;g__;s__

LMSG_G000007528.1	no	552_1	Copper	63.08	1.87	53.74	0	0	0	12	Medium quality	6371	855359	71.30	179	d__Archaea;p__Thermoplasmatota;c__Thermoplasmata;o__UBA184;f__UBA184;g__;s__
LMSG_G000007529.1	no	552_1	Polymetallic	97.46	3.67	79.11	1	2	0	20	Medium quality	46868	1793675	70.10	62	d__Archaea;p__Thermoplasmatota;c__Thermoplasmata;o__UBA184;f__UBA184;g__;s__
LMSG_G000007530.1	no	552_1	Copper	80.21	4.80	56.21	1	1	1	19	Medium quality	6327	1484278	70.20	267	d__Archaea;p__Thermoplasmatota;c__Thermoplasmata;o__UBA184;f__UBA184;g__;s__
LMSG_G000007531.1	no	552_1	Copper	87.06	1.60	79.06	1	2	1	18	Medium quality	50319	1535371	69.90	47	d__Archaea;p__Thermoplasmatota;c__Thermoplasmata;o__UBA184;f__UBA184;g__;s__
LMSG_G000007532.1	no	554_1	Copper	86.00	4.16	65.20	1	1	2	18	Medium quality	32581	2027674	69.70	100	d__Archaea;p__Thermoplasmatota;c__Thermoplasmata;o__UBA184;f__UBA184;g__;s__
LMSG_G000007533.1	no	555_1	Coal	91.46	2.40	79.46	1	1	1	20	High quality	89469	2096272	69.80	50	d__Archaea;p__Thermoplasmatota;c__Thermoplasmata;o__UBA184;f__UBA184;g__;s__
LMSG_G000007534.1	no	555_1	Pyrite-Copper	69.06	0.80	65.06	1	1	0	17	Medium quality	98086	1467720	70.00	28	d__Archaea;p__Thermoplasmatota;c__Thermoplasmata;o__UBA184;f__UBA184;g__;s__
LMSG_G000007535.1	no	555_1	Pyrite-Copper	64.86	1.60	56.86	1	1	1	15	Medium quality	9330	1349281	69.80	176	d__Archaea;p__Thermoplasmatota;c__Thermoplasmata;o__UBA184;f__UBA184;g__;s__
LMSG_G000007536.1	no	555_1	Coal	88.66	2.57	75.82	1	1	0	19	Medium quality	67988	1971063	69.90	39	d__Archaea;p__Thermoplasmatota;c__Thermoplasmata;o__UBA184;f__UBA184;g__;s__
LMSG_G000007537.1	no	556_1	Polymetallic	88.05	1.77	79.21	0	1	1	19	Medium quality	10576	1394318	71.10	189	d__Archaea;p__Thermoplasmatota;c__Thermoplasmata;o__UBA184;f__UBA184;g__;s__
LMSG_G000007538.1	no	558_1	Coal	95.42	3.20	79.42	1	1	2	19	High quality	18988	1770432	72.60	123	d__Archaea;p__Thermoplasmatota;c__Thermoplasmata;o__UBA184;f__UBA184;g__;s__
LMSG_G000007539.1	no	558_1	Polymetallic	85.30	1.60	77.30	1	1	1	14	Medium quality	14219	1492895	72.90	209	d__Archaea;p__Thermoplasmatota;c__Thermoplasmata;o__UBA184;f__UBA184;g__;s__
LMSG_G000007540.1	no	558_1	Coal	88.22	2.40	76.22	1	2	2	20	Medium quality	20868	1797728	72.10	162	d__Archaea;p__Thermoplasmatota;c__Thermoplasmata;o__UBA184;f__UBA184;g__;s__
LMSG_G000007541.1	no	558_1	Coal	90.66	2.40	78.66	1	2	0	18	Medium quality	24150	1673787	72.70	87	d__Archaea;p__Thermoplasmatota;c__Thermoplasmata;o__UBA184;f__UBA184;g__;s__
LMSG_G000007542.1	no	558_1	Copper	75.14	3.07	59.81	1	1	0	13	Medium quality	21409	1464147	72.50	109	d__Archaea;p__Thermoplasmatota;c__Thermoplasmata;o__UBA184;f__UBA184;g__;s__
LMSG_G000007543.1	no	558_1	Pyrite	80.74	2.40	68.74	1	1	1	16	Medium quality	17460	1637845	72.80	241	d__Archaea;p__Thermoplasmatota;c__Thermoplasmata;o__UBA184;f__UBA184;g__;s__
LMSG_G000007544.1	no	558_1	Copper	74.85	2.40	62.85	1	1	0	17	Medium quality	4908	1251544	73.30	374	d__Archaea;p__Thermoplasmatota;c__Thermoplasmata;o__UBA184;f__UBA184;g__;s__
LMSG_G000007545.1	no	559_1	Coal	61.06	1.60	53.06	1	1	0	12	Medium quality	7050	1040098	73.20	190	d__Archaea;p__Thermoplasmatota;c__Thermoplasmata;o__UBA184;f__UBA184;g__;s__
LMSG_G000007546.1	no	559_1	Coal	93.02	2.40	81.02	1	1	0	18	Medium quality	51155	1904546	72.20	57	d__Archaea;p__Thermoplasmatota;c__Thermoplasmata;o__UBA184;f__UBA184;g__;s__
LMSG_G000007547.1	no	562_1	Coal	80.75	4.00	60.75	1	2	0	17	Medium quality	57091	1810520	71.90	53	d__Archaea;p__Thermoplasmatota;c__Thermoplasmata;o__UBA184;f__UBA184;g__;s__
LMSG_G000007548.1	no	562_1	Coal	89.47	2.40	77.47	1	1	0	19	Medium quality	22331	1675956	72.40	105	d__Archaea;p__Thermoplasmatota;c__Thermoplasmata;o__UBA184;f__UBA184;g__;s__
LMSG_G000007549.1	no	563_1	Magnetite	94.66	2.40	82.66	1	1	2	19	High quality	40538	1689666	72.30	70	d__Archaea;p__Thermoplasmatota;c__Thermoplasmata;o__UBA184;f__UBA184;g__;s__
LMSG_G000007550.1	no	563_1	Lead-Zinc	97.06	4.00	77.06	1	2	1	18	High quality	34789	1810650	72.40	80	d__Archaea;p__Thermoplasmatota;c__Thermoplasmata;o__UBA184;f__UBA184;g__;s__
LMSG_G000007551.1	no	563_1	Coal	95.46	2.40	83.46	1	1	1	19	High quality	41159	1800684	72.10	59	d__Archaea;p__Thermoplasmatota;c__Thermoplasmata;o__UBA184;f__UBA184;g__;s__
LMSG_G000007552.1	no	563_1	Lead-Zinc	93.65	1.60	85.65	1	1	2	16	Medium quality	24632	1749449	72.70	98	d__Archaea;p__Thermoplasmatota;c__Thermoplasmata;o__UBA184;f__UBA184;g__;s__
LMSG_G000007553.1	no	563_1	Polymetallic	86.66	2.40	74.66	1	2	2	20	Medium quality	32857	1647266	72.50	81	d__Archaea;p__Thermoplasmatota;c__Thermoplasmata;o__UBA184;f__UBA184;g__;s__
LMSG_G000007554.1	no	563_1	Lead-Zinc	89.06	1.60	81.06	1	1	2	17	Medium quality	43174	1630536	72.80	60	d__Archaea;p__Thermoplasmatota;c__Thermoplasmata;o__UBA184;f__UBA184;g__;s__
LMSG_G000007555.1	no	563_1	Pyrite-Copper	87.00	2.40	75.00	1	0	1	17	Medium quality	6064	1652974	72.60	343	d__Archaea;p__Thermoplasmatota;c__Thermoplasmata;o__UBA184;f__UBA184;g__;s__
LMSG_G000007556.1	no	563_1	Coal	91.25	3.87	71.92	1	1	0	19	Medium quality	37685	1613811	72.40	57	d__Archaea;p__Thermoplasmatota;c__Thermoplasmata;o__UBA184;f__UBA184;g__;s__
LMSG_G000007557.1	no	564_1	Coal	95.42	4.80	71.42	1	1	2	19	High quality	29902	1832696	71.60	85	d__Archaea;p__Thermoplasmatota;c__Thermoplasmata;o__UBA184;f__UBA184;g__;s__
LMSG_G000007558.1	no	564_1	Polymetallic	84.85	1.60	76.85	1	0	0	19	Medium quality	5301	1288278	72.10	293	d__Archaea;p__Thermoplasmatota;c__Thermoplasmata;o__UBA184;f__UBA184;g__;s__
LMSG_G000007559.1	no	564_1	Coal	75.02	3.92	55.42	1	0	0	18	Medium quality	10715	1382927	71.30	146	d__Archaea;p__Thermoplasmatota;c__Thermoplasmata;o__UBA184;f__UBA184;g__;s__
LMSG_G000007560.1	no	564_1	Coal	82.62	0.80	78.62	1	0	1	19	Medium quality	36501	1434531	71.70	50	d__Archaea;p__Thermoplasmatota;c__Thermoplasmata;o__UBA184;f__UBA184;g__;s__
LMSG_G000007561.1	no	566_1	Copper	96.40	1.60	88.40	1	1	2	20	High quality	16697	1494474	70.20	157	d__Archaea;p__Thermoplasmatota;c__Thermoplasmata;o__UBA184;f__UBA184;g__;s__
LMSG_G000007562.1	no	566_1	Copper	93.20	2.67	79.87	1	2	1	20	High quality	29471	1477307	69.90	72	d__Archaea;p__Thermoplasmatota;c__Thermoplasmata;o__UBA184;f__UBA184;g__;s__
LMSG_G000007563.1	no	566_1	Copper	81.20	0.84	76.99	1	1	1	18	Medium quality	13952	1238035	70.60	117	d__Archaea;p__Thermoplasmatota;c__Thermoplasmata;o__UBA184;f__UBA184;g__;s__
LMSG_G000007564.1	no	566_1	Copper	87.60	0.80	83.60	1	1	1	18	Medium quality	23720	1255722	70.50	68	d__Archaea;p__Thermoplasmatota;c__Thermoplasmata;o__UBA184;f__UBA184;g__;s__
LMSG_G000007565.1	no	566_1	Pyrite	97.20	3.20	81.20	1	2	1	20	High quality	64280	1558083	69.80	70	d__Archaea;p__Thermoplasmatota;c__Thermoplasmata;o__UBA184;f__UBA184;g__;s__
LMSG_G000007566.1	no	566_1	Pyrite	98.00	1.91	88.46	2	2	2	20	High quality	57820	1552442	69.90	88	d__Archaea;p__Thermoplasmatota;c__Thermoplasmata;o__UBA184;f__UBA184;g__;s__
LMSG_G000007567.1	no	566_1	Pyrite	98.00	1.60	90.00	1	1	1	19	High quality	21341	1463054	70.30	119	d__Archaea;p__Thermoplasmatota;c__Thermoplasmata;o__UBA184;f__UBA184;g__;s__
LMSG_G000007568.1	no	566_1	Polymetallic	94.80	1.60	86.80	1	1	1	20	High quality	67091	1371629	70.20	70	d__Archaea;p__Thermoplasmatota;c__Thermoplasmata;o__UBA184;f__UBA184;g__;s__
LMSG_G000007569.1	no	566_1	Polymetallic	95.60	4.39	73.66	1	1	1	20	High quality	52119	1418384	70.10	97	d__Archaea;p__Thermoplasmatota;c__Thermoplasmata;o__UBA184;f__UBA184;g__;s__
LMSG_G000007570.1	no	566_1	Polymetallic	98.00	1.60	90.00	1	2	1	20	High quality	29369	1509559	70.00	81	d__Archaea;p__Thermoplasmatota;c__Thermoplasmata;o__UBA184;f__UBA184;g__;s__
LMSG_G000007571.1	no	566_1	Polymetallic	97.20	2.40	85.20	1	2	1	20	High quality	33116	1606601	69.90	85	d__Archaea;p__Thermoplasmatota;c__Thermoplasmata;o__UBA184;f__UBA184;g__;s__
LMSG_G000007572.1	no	566_1	Magnetite	96.40	1.60	88.40	1	2	3	20	High quality	56430	1536151	70.20	80	d__Archaea;p__Thermoplasmatota;c__Thermoplasmata;o__UBA184;f__UBA184;g__;s__
LMSG_G000007573.1	no	566_1	Lead-Zinc	94.00	2.40	82.00	1	2	1	19	High quality	47306	1524650	69.80	56	d__Archaea;p__Thermoplasmatota;c__Thermoplasmata;o__UBA184;f__UBA184;g__;s__
LMSG_G000007574.1	no	566_1	Pyrite-Copper	95.86	2.44	83.65	1	1	1	20	High quality	23123	1443035	70.20	102	d__Archaea;p__Thermoplasmatota;c__Thermoplasmata;o__UBA184;f__UBA184;g__;s__
LMSG_G000007575.1	no	566_1	Lead-Zinc	92.40	2.40	80.40	1	2	1	19	High quality	25656	1419423	70.10	75	d__Archaea;p__Thermoplasmatota;c__Thermoplasmata;o__UBA184;f__UBA184;g__;s__
LMSG_G000007576.1	no	566_1	Coal	94.80	1.60	86.80	1	1	1	20	High quality	35325	1494750	69.90	63	d__Archaea;p__Thermoplasmatota;c__Thermoplasmata;o__UBA184;f__UBA184;g__;s__
LMSG_G000007577.1	no	566_1	Coal	97.20	3.20	81.20	1	2	2	20	High quality	65327	1532544	70.00	50	d__Archaea;p__Thermoplasmatota;c__Thermoplasmata;o__UBA184;f__UBA184;g__;s__
LMSG_G000007578.1	no	566_1	Polymetallic	95.60	1.60	87.60	1	2	2	17	Medium quality	28041	1304769	70.30	61	d__Archaea;p__Thermoplasmatota;c__Thermoplasmata;o__UBA184;f__UBA184;g__;s__
LMSG_G000007579.1	no	566_1	Lead-Zinc	65.24	2.00	55.24	1	1	0	14	Medium quality	2454	1004082	70.40	458	d__Archaea;p__Thermoplasmatota;c__Thermoplasmata;o__UBA184;f__UBA184;g__;s__
LMSG_G000007580.1	no	566_1	Polymetallic	85.20	0.80	81.20	1	1	1	17	Medium quality	19660	1408082	70.40	199	d__Archaea;p__Thermoplasmatota;c__Thermoplasmata;o__UBA184;f__UBA184;g__;s__
LMSG_G000007581.1	no	566_1	Polymetallic	72.40	1.60	64.40	1	2	2	18	Medium quality	51339	1157502	70.70	63	d__Archaea;p__Thermoplasmatota;c__Thermoplasmata;o__UBA184;f__UBA184;g__;s__
LMSG_G000007582.1	no	566_1	Copper	89.20	1.68	80.78	1	2	0	18	Medium quality	30961	1409228	70.30	49	d__Archaea;p__Thermoplasmatota;c__Thermoplasmata;o__UBA184;f__UBA184;g__;s__
LMSG_G000007583.1	no	566_1	Lead-Zinc	71.47	1.87	62.13	1	1	1	13	Medium quality	3490	934052	70.50	294	d__Archaea;p__Thermoplasmatota;c__Thermoplasmata;o__UBA184;f__UBA184;g__;s__
LMSG_G000007584.1	no	566_1	Pyrite-Copper	89.20	2.44	76.99	1	2	1	19	Medium quality	23625	1377069	70.30	113	d__Archaea;p__Thermoplasmatota;c__Thermoplasmata;o__UBA184;f__UBA184;g__;s__
LMSG_G000007585.1	no	566_1	Pyrite-Copper	84.40	0.80	80.40	1	2	0	19	Medium quality	37656	1459269	69.90	60	d__Archaea;p__Thermoplasmatota;c__Thermoplasmata;o__UBA184;f__UBA184;g__;s__
LMSG_G000007586.1	no	566_1	Polymetallic	93.20	1.60	85.20	3	1	0	20	Medium quality	48354	1632185	69.60	118	d__Archaea;p__Thermoplasmatota;c__Thermoplasmata;o__UBA184;f__UBA184;g__;s__
LMSG_G000007587.1	no	566_1	Polymetallic	97.20	1.60	89.20	1	1	0	20	Medium quality	34630	1519008	70.00	66	d__Archaea;p__Thermoplasmatota;c__Thermoplasmata;o__UBA184;f__UBA184;g__;s__
LMSG_G000007588.1	no	566_1	Tin-Zinc	95.60	1.60	87.60	1	1	0	19	Medium quality	30129	1422021	70.30	83	d__Archaea;p__Thermoplasmatota;c__Thermoplasmata;o__UBA184;f__UBA184;g__;s__
LMSG_G000007589.1	no	566_1	Tin-Zinc	94.80	1.60	86.80	1	1	0	20	Medium quality	80895	1485379	70.00	116	d__Archaea;p__Thermoplasmatota;c__Thermoplasmata;o__UBA184;f__UBA184;g__;s__
LMSG_G000007590.1	no	566_1	Lead-Zinc	94.80	2.40	82.80	1	2	0	18	Medium quality	31602	1404535	70.10	66	d__Archaea;p__Thermoplasmatota;c__Thermoplasmata;o__UBA184;f__UBA184;g__;s__
LMSG_G000007591.1	no	566_1	Lead-Zinc	95.60	3.20	79.60	1	2	0	17	Medium quality	27169	1475247	69.90	72	d__Archaea;p__Thermoplasmatota;c__Thermoplasmata;o__UBA184;f__UBA184;g__;s__
LMSG_G000007592.1	no	566_1	Pyrite-Copper	94.80	1.60	86.80	1	2	0	20	Medium quality	26667	1421039	70.20	77	d__Archaea;p__Thermoplasmatota;c__Thermoplasmata;o__UBA184;f__UBA184;g__;s__
LMSG_G000007593.1	no	568_1	Coal	95.02	3.20	79.02	1	2	2	20	High quality	80628	2842390	68.80	114	d__Archaea;p__Thermoplasmatota;c__Thermoplasmata;o__UBA184;f__UBA184;g__;s__
LMSG_G000007594.1	no	569_1	Coal	92.26	1.60	84.26	1	1	2	18	High quality	30454	2611465	69.50	209	d__Archaea;p__Thermoplasmatota;c__Thermoplasmata;o__UBA184;f__UBA184;g__;s__
LMSG_G000007595.1	no	569_1	Coal	91.46	2.40	79.46	0	2	2	19	Medium quality	84435	2536526	69.50	96	d__Archaea;p__Thermoplasmatota;c__Thermoplasmata;o__UBA184;f__UBA184;g__;s__
LMSG_G000007596.1	no	569_1	Polymetallic	91.06	3.20	75.06	1	1	2	19	High quality	8153	2372564	69.30	390	d__Archaea;p__Thermoplasmatota;c__Thermoplasmata;o__UBA184;f__UBA184;g__;s__
LMSG_G000007597.1	no	569_1	Polymetallic	96.26	1.60	88.26	1	1	2	19	High quality	22642	2762740	69.10	227	d__Archaea;p__Thermoplasmatota;c__Thermoplasmata;o__UBA184;f__UBA184;g__;s__
LMSG_G000007598.1	no	569_1	Polymetallic	93.06	1.60	85.06	1	1	2	20	High quality	117385	2665464	69.10	59	d__Archaea;p__Thermoplasmatota;c__Thermoplasmata;o__UBA184;f__UBA184;g__;s__
LMSG_G000007599.1	no	569_1	Pyrite-Copper	92.26	1.60	84.26	1	1	2	19	High quality	37724	2550104	69.30	124	d__Archaea;p__Thermoplasmatota;c__Thermoplasmata;o__UBA184;f__UBA184;g__;s__
LMSG_G000007600.1	no	569_1	Pyrite-Copper	94.66	2.40	82.66	1	1	3	18	High quality	53523	2700301	69.10	93	d__Archaea;p__Thermoplasmatota;c__Thermoplasmata;o__UBA184;f__UBA184;g__;s__
LMSG_G000007601.1	no	569_1	Polymetallic	65.71	0.00	65.71	1	3	1	14	Medium quality	3786	1615471	69.40	484	d__Archaea;p__Thermoplasmatota;c__Thermoplasmata;o__UBA184;f__UBA184;g__;s__
LMSG_G000007602.1	no	569_1	Magnetite	95.46	1.60	87.46	0	1	2	19	Medium quality	90183	2611849	69.30	68	d__Archaea;p__Thermoplasmatota;c__Thermoplasmata;o__UBA184;f__UBA184;g__;s__
LMSG_G000007603.1	no	569_1	Magnetite	62.02	0.00	62.02	0	1	1	16	Medium quality	11681	1832589	68.50	202	d__Archaea;p__Thermoplasmatota;c__Thermoplasmata;o__UBA184;f__UBA184;g__;s__
LMSG_G000007604.1	no	569_1	Copper	94.66	2.67	81.33	1	2	1	20	High quality	40216	2785012	69.10	168	d__Archaea;p__Thermoplasmatota;c__Thermoplasmata;o__UBA184;f__UBA184;g__;s__

LMSG_G000007605.1	no	569_1	Copper	78.26	1.60	70.26	1	1	2	19	Medium quality	31063	2366729	69.20	142	d__Archaea;p__Thermoplasmatota;c__Thermoplasmata;o__UBA184;f__UBA184;g__;s__
LMSG_G000007606.1	no	569_1	Copper	71.46	3.68	53.04	0	0	1	16	Medium quality	3790	2306914	69.30	677	d__Archaea;p__Thermoplasmatota;c__Thermoplasmata;o__UBA184;f__UBA184;g__;s__
LMSG_G000007607.1	no	569_1	Copper	58.73	1.60	50.73	1	0	0	15	Medium quality	5091	1909092	68.90	469	d__Archaea;p__Thermoplasmatota;c__Thermoplasmata;o__UBA184;f__UBA184;g__;s__
LMSG_G000007608.1	no	576_1	Pyrite	96.80	3.60	78.80	1	1	3	20	High quality	105906	2129480	64.30	60	d__Archaea;p__Thermoplasmatota;c__Thermoplasmata;o__UBA184;f__UBA184;g__;s__
LMSG_G000007609.1	no	576_1	Pyrite	96.00	3.20	80.00	1	1	1	20	High quality	155987	2073990	64.40	34	d__Archaea;p__Thermoplasmatota;c__Thermoplasmata;o__UBA184;f__UBA184;g__;s__
LMSG_G000007610.1	no	576_1	Lead-Zinc	95.20	2.00	85.20	1	1	2	20	High quality	53896	2769077	62.90	149	d__Archaea;p__Thermoplasmatota;c__Thermoplasmata;o__UBA184;f__UBA184;g__;s__
LMSG_G000007611.1	no	576_1	Copper	97.60	3.20	81.60	1	1	3	20	High quality	108611	2213363	64.00	50	d__Archaea;p__Thermoplasmatota;c__Thermoplasmata;o__UBA184;f__UBA184;g__;s__
LMSG_G000007612.1	no	576_1	Lead-Zinc	97.60	3.20	81.60	1	1	1	20	High quality	90417	2183674	64.20	63	d__Archaea;p__Thermoplasmatota;c__Thermoplasmata;o__UBA184;f__UBA184;g__;s__
LMSG_G000007613.1	no	576_1	Copper	97.60	3.20	81.60	1	2	3	19	High quality	102836	2511269	63.50	96	d__Archaea;p__Thermoplasmatota;c__Thermoplasmata;o__UBA184;f__UBA184;g__;s__
LMSG_G000007614.1	no	576_1	Lead-Zinc	97.60	3.20	81.60	1	1	1	20	High quality	89366	2281019	64.00	57	d__Archaea;p__Thermoplasmatota;c__Thermoplasmata;o__UBA184;f__UBA184;g__;s__
LMSG_G000007615.1	no	576_1	Lead-Zinc	97.60	3.20	81.60	1	4	9	20	High quality	163373	2410676	63.70	77	d__Archaea;p__Thermoplasmatota;c__Thermoplasmata;o__UBA184;f__UBA184;g__;s__
LMSG_G000007616.1	no	576_1	Lead-Zinc	97.60	3.20	81.60	1	1	2	20	High quality	92733	2143984	64.20	47	d__Archaea;p__Thermoplasmatota;c__Thermoplasmata;o__UBA184;f__UBA184;g__;s__
LMSG_G000007617.1	no	576_1	Polymetallic	97.60	3.20	81.60	1	1	1	20	High quality	120940	2175511	64.10	47	d__Archaea;p__Thermoplasmatota;c__Thermoplasmata;o__UBA184;f__UBA184;g__;s__
LMSG_G000007618.1	no	576_1	Polymetallic	96.80	3.33	80.17	1	1	5	20	High quality	85233	2825099	64.00	180	d__Archaea;p__Thermoplasmatota;c__Thermoplasmata;o__UBA184;f__UBA184;g__;s__
LMSG_G000007619.1	no	576_1	Polymetallic	97.60	3.20	81.60	1	2	1	20	High quality	176324	2222369	64.00	35	d__Archaea;p__Thermoplasmatota;c__Thermoplasmata;o__UBA184;f__UBA184;g__;s__
LMSG_G000007620.1	no	576_1	Polymetallic	95.20	2.40	83.20	1	1	1	19	High quality	266711	2186724	64.00	51	d__Archaea;p__Thermoplasmatota;c__Thermoplasmata;o__UBA184;f__UBA184;g__;s__
LMSG_G000007621.1	no	576_1	Polymetallic	97.60	3.20	81.60	1	3	3	20	High quality	155524	2327044	63.70	53	d__Archaea;p__Thermoplasmatota;c__Thermoplasmata;o__UBA184;f__UBA184;g__;s__
LMSG_G000007622.1	no	576_1	Polymetallic	97.60	3.20	81.60	1	3	2	20	High quality	181747	2252137	63.90	43	d__Archaea;p__Thermoplasmatota;c__Thermoplasmata;o__UBA184;f__UBA184;g__;s__
LMSG_G000007623.1	no	576_1	Tin-Zinc	96.80	3.20	80.80	1	1	1	20	High quality	58034	2098312	64.30	69	d__Archaea;p__Thermoplasmatota;c__Thermoplasmata;o__UBA184;f__UBA184;g__;s__
LMSG_G000007624.1	no	576_1	Magnetite	96.80	3.20	80.80	1	1	2	20	High quality	77364	2139000	64.00	58	d__Archaea;p__Thermoplasmatota;c__Thermoplasmata;o__UBA184;f__UBA184;g__;s__
LMSG_G000007625.1	no	576_1	Lead-Zinc	96.80	4.80	72.80	1	2	1	20	High quality	56056	2528283	64.20	83	d__Archaea;p__Thermoplasmatota;c__Thermoplasmata;o__UBA184;f__UBA184;g__;s__
LMSG_G000007626.1	no	576_1	Pyrite-Copper	96.80	3.20	80.80	1	1	1	20	High quality	155999	2258138	63.90	65	d__Archaea;p__Thermoplasmatota;c__Thermoplasmata;o__UBA184;f__UBA184;g__;s__
LMSG_G000007627.1	no	576_1	Pyrite-Copper	97.60	2.40	85.60	1	2	2	20	High quality	60488	2179867	64.00	64	d__Archaea;p__Thermoplasmatota;c__Thermoplasmata;o__UBA184;f__UBA184;g__;s__
LMSG_G000007628.1	no	576_1	Magnetite	96.00	1.60	88.00	1	1	1	18	High quality	65119	2077836	64.10	46	d__Archaea;p__Thermoplasmatota;c__Thermoplasmata;o__UBA184;f__UBA184;g__;s__
LMSG_G000007629.1	no	576_1	Magnetite	97.60	4.00	77.60	1	2	1	20	High quality	70387	2168225	64.10	51	d__Archaea;p__Thermoplasmatota;c__Thermoplasmata;o__UBA184;f__UBA184;g__;s__
LMSG_G000007630.1	no	576_1	Magnetite	95.20	2.40	83.20	1	1	2	19	High quality	64786	2070883	64.10	49	d__Archaea;p__Thermoplasmatota;c__Thermoplasmata;o__UBA184;f__UBA184;g__;s__
LMSG_G000007631.1	no	576_1	Magnetite	93.60	1.66	85.30	1	2	1	18	High quality	79929	2247856	63.40	88	d__Archaea;p__Thermoplasmatota;c__Thermoplasmata;o__UBA184;f__UBA184;g__;s__
LMSG_G000007632.1	no	576_1	Pyrite-Copper	97.60	3.20	81.60	1	3	4	20	High quality	107222	2298698	63.90	110	d__Archaea;p__Thermoplasmatota;c__Thermoplasmata;o__UBA184;f__UBA184;g__;s__
LMSG_G000007633.1	no	576_1	Copper	75.20	2.40	63.20	1	1	1	20	Medium quality	77436	1632815	64.30	35	d__Archaea;p__Thermoplasmatota;c__Thermoplasmata;o__UBA184;f__UBA184;g__;s__
LMSG_G000007634.1	no	576_1	Lead-Zinc	67.14	1.60	59.14	0	1	0	15	Medium quality	2703	1440816	64.60	604	d__Archaea;p__Thermoplasmatota;c__Thermoplasmata;o__UBA184;f__UBA184;g__;s__
LMSG_G000007635.1	no	576_1	Pyrite	81.60	3.20	65.60	1	2	2	16	Medium quality	59977	1565532	63.80	45	d__Archaea;p__Thermoplasmatota;c__Thermoplasmata;o__UBA184;f__UBA184;g__;s__
LMSG_G000007636.1	no	576_1	Copper	72.80	0.80	68.80	1	0	0	20	Medium quality	121750	1482794	64.80	43	d__Archaea;p__Thermoplasmatota;c__Thermoplasmata;o__UBA184;f__UBA184;g__;s__
LMSG_G000007637.1	no	576_1	Copper	70.40	0.80	66.40	1	0	0	18	Medium quality	105983	1305546	64.90	29	d__Archaea;p__Thermoplasmatota;c__Thermoplasmata;o__UBA184;f__UBA184;g__;s__
LMSG_G000007638.1	no	576_1	Copper	76.80	2.40	64.80	0	2	1	19	Medium quality	72909	1596087	64.60	46	d__Archaea;p__Thermoplasmatota;c__Thermoplasmata;o__UBA184;f__UBA184;g__;s__
LMSG_G000007639.1	no	576_1	Copper	75.20	1.60	67.20	0	1	1	19	Medium quality	132495	1403099	64.50	53	d__Archaea;p__Thermoplasmatota;c__Thermoplasmata;o__UBA184;f__UBA184;g__;s__
LMSG_G000007640.1	no	576_1	Lead-Zinc	71.20	3.20	55.20	1	3	1	17	Medium quality	147236	1334524	63.60	28	d__Archaea;p__Thermoplasmatota;c__Thermoplasmata;o__UBA184;f__UBA184;g__;s__
LMSG_G000007641.1	no	576_1	Polymetallic	92.80	2.40	80.80	0	1	0	19	Medium quality	235366	2052910	64.20	49	d__Archaea;p__Thermoplasmatota;c__Thermoplasmata;o__UBA184;f__UBA184;g__;s__
LMSG_G000007642.1	no	576_1	Polymetallic	93.60	3.20	77.60	0	1	1	20	Medium quality	120834	1990092	64.30	37	d__Archaea;p__Thermoplasmatota;c__Thermoplasmata;o__UBA184;f__UBA184;g__;s__
LMSG_G000007643.1	no	576_1	Polymetallic	96.80	4.00	76.80	0	1	7	20	Medium quality	235180	2246568	63.90	94	d__Archaea;p__Thermoplasmatota;c__Thermoplasmata;o__UBA184;f__UBA184;g__;s__
LMSG_G000007644.1	no	576_1	Polymetallic	94.32	2.40	82.32	0	1	1	20	Medium quality	16009	1997124	64.60	235	d__Archaea;p__Thermoplasmatota;c__Thermoplasmata;o__UBA184;f__UBA184;g__;s__
LMSG_G000007645.1	no	576_1	Copper	78.40	1.60	70.40	1	1	1	20	Medium quality	55032	1980599	64.10	74	d__Archaea;p__Thermoplasmatota;c__Thermoplasmata;o__UBA184;f__UBA184;g__;s__
LMSG_G000007646.1	no	576_1	Polymetallic	72.00	1.60	64.00	0	1	0	16	Medium quality	86713	1740576	64.50	37	d__Archaea;p__Thermoplasmatota;c__Thermoplasmata;o__UBA184;f__UBA184;g__;s__
LMSG_G000007647.1	no	576_1	Lead-Zinc	85.60	0.80	81.60	0	1	1	19	Medium quality	76473	1818938	64.50	54	d__Archaea;p__Thermoplasmatota;c__Thermoplasmata;o__UBA184;f__UBA184;g__;s__
LMSG_G000007648.1	no	576_1	Pyrite-Copper	63.20	0.80	59.20	1	0	1	18	Medium quality	119455	1498750	64.30	64	d__Archaea;p__Thermoplasmatota;c__Thermoplasmata;o__UBA184;f__UBA184;g__;s__
LMSG_G000007649.1	no	576_1	Pyrite-Copper	81.20	3.20	65.20	1	1	1	19	Medium quality	94760	1793081	64.40	30	d__Archaea;p__Thermoplasmatota;c__Thermoplasmata;o__UBA184;f__UBA184;g__;s__
LMSG_G000007650.1	no	576_1	Lead-Zinc	77.60	4.80	53.60	0	1	0	20	Medium quality	77552	1724087	64.60	38	d__Archaea;p__Thermoplasmatota;c__Thermoplasmata;o__UBA184;f__UBA184;g__;s__
LMSG_G000007651.1	no	576_1	Polymetallic	97.60	3.20	81.60	1	1	0	20	Medium quality	108140	2138983	64.10	44	d__Archaea;p__Thermoplasmatota;c__Thermoplasmata;o__UBA184;f__UBA184;g__;s__
LMSG_G000007652.1	no	577_1	Pyrite-Copper	90.26	1.60	82.26	1	1	2	20	High quality	34812	2287810	61.60	120	d__Archaea;p__Thermoplasmatota;c__Thermoplasmata;o__UBA184;f__UBA184;g__;s__
LMSG_G000007653.1	no	577_1	Lead-Zinc	93.46	3.45	76.23	1	1	2	20	High quality	123914	2443853	61.90	156	d__Archaea;p__Thermoplasmatota;c__Thermoplasmata;o__UBA184;f__UBA184;g__;s__
LMSG_G000007654.1	no	577_1	Pyrite	88.40	1.60	80.40	0	1	2	20	Medium quality	20016	2700899	61.60	189	d__Archaea;p__Thermoplasmatota;c__Thermoplasmata;o__UBA184;f__UBA184;g__;s__
LMSG_G000007655.1	no	577_1	Lead-Zinc	80.26	1.20	74.26	1	1	2	18	Medium quality	56673	1939794	62.30	72	d__Archaea;p__Thermoplasmatota;c__Thermoplasmata;o__UBA184;f__UBA184;g__;s__
LMSG_G000007656.1	no	577_1	Lead-Zinc	91.06	2.27	79.73	0	3	3	18	Medium quality	114575	2897593	61.70	228	d__Archaea;p__Thermoplasmatota;c__Thermoplasmata;o__UBA184;f__UBA184;g__;s__
LMSG_G000007657.1	no	577_1	Copper	84.80	1.60	76.80	1	1	0	20	Medium quality	14896	2215596	62.70	258	d__Archaea;p__Thermoplasmatota;c__Thermoplasmata;o__UBA184;f__UBA184;g__;s__
LMSG_G000007658.1	no	577_1	Copper	72.96	2.40	60.96	0	2	0	20	Medium quality	6034	1865744	62.50	363	d__Archaea;p__Thermoplasmatota;c__Thermoplasmata;o__UBA184;f__UBA184;g__;s__
LMSG_G000007659.1	no	577_1	Copper	94.26	2.40	82.26	1	2	3	20	High quality	75332	2619537	61.60	78	d__Archaea;p__Thermoplasmatota;c__Thermoplasmata;o__UBA184;f__UBA184;g__;s__
LMSG_G000007660.1	no	238_1	Copper	72.73	0.00	72.73	1	0	0	14	Medium quality	2775	1329320	39.90	548	d__Archaea;p__Thermoproteota;c__Nitrososphaeria;o__Nitrososphaerales;f__Nitrosopumilaceae;g__Nitrosotalea;s__
LMSG_G000007661.1	no	238_1	Copper	70.71	1.29	64.24	1	2	0	16	Medium quality	3230	1344448	39.40	445	d__Archaea;p__Thermoproteota;c__Nitrososphaeria;o__Nitrososphaerales;f__Nitrosopumilaceae;g__Nitrosotalea;s__
LMSG_G000007662.1	no	239_1	Copper	56.50	0.00	56.50	0	2	0	12	Medium quality	1834	1221851	39.10	649	d__Archaea;p__Thermoproteota;c__Nitrososphaeria;o__Nitrososphaerales;f__Nitrosopumilaceae;g__Nitrosotalea;s__
LMSG_G000007663.1	no	213_1	Polymetallic	66.18	1.94	56.48	1	0	0	17	Medium quality	4143	863097	41.20	238	d__Archaea;p__Thermoproteota;c__Nitrososphaeria;o__Nitrososphaerales;f__Nitrosopumilaceae;g__Nitrosotenuis;s__
LMSG_G000007664.1	no	213_1	Polymetallic	58.22	0.00	58.22	0	1	1	16	Medium quality	3107	751441	41.00	257	d__Archaea;p__Thermoproteota;c__Nitrososphaeria;o__Nitrososphaerales;f__Nitrosopumilaceae;g__Nitrosotenuis;s__
LMSG_G000007665.1	no	514_1	Copper	56.60	0.00	56.60	1	0	7	15	Medium quality	5246	1910868	46.90	365	d__Archaea;p__Thermoproteota;c__Nitrososphaeria;o__Nitrososphaerales;f__UBA141;g__;s__
LMSG_G000007666.1	no	585_1	Copper	68.44	0.00	68.44	0	0	0	11	Medium quality	4029	1226596	65.60	342	d__Archaea;p__Thermoproteota;c__Nitrososphaeria;o__Nitrososphaerales;f__UBA183;g__BOG-1369;s__
LMSG_G000007667.1	no	585_1	Copper	85.69	0.97	80.84	1	1	0	15	Medium quality	10078	1818870	65.00	290	d__Archaea;p__Thermoproteota;c__Nitrososphaeria;o__Nitrososphaerales;f__UBA183;g__BOG-1369;s__
LMSG_G000007668.1	no	585_1	Magnetite	79.61	1.12	74.01	1	0	0	18	Medium quality	45831	1678331	65.00	60	d__Archaea;p__Thermoproteota;c__Nitrososphaeria;o__Nitrososphaerales;f__UBA183;g__BOG-1369;s__
LMSG_G000007669.1	no	585_1	Lead-Zinc	78.57	3.40	61.58	1	1	0	14	Medium quality	4461	1344325	65.60	340	d__Archaea;p__Thermoproteota;c__Nitrososphaeria;o__Nitrososphaerales;f__UBA183;g__BOG-1369;s__
LMSG_G000007670.1	no	585_1	Coal	83.00	2.02	72.92	1	1	1	19	Medium quality	31393	1730090	65.00	108	d__Archaea;p__Thermoproteota;c__Nitrososphaeria;o__Nitrososphaerales;f__UBA183;g__BOG-1369;s__
LMSG_G000007671.1	no	588_1	Polymetallic	78.31	2.91	63.75	0	0	2	17	Medium quality	46464	1517910	62.30	132	d__Archaea;p__Thermoproteota;c__Nitrososphaeria;o__Nitrososphaerales;f__UBA183;g__UBA183;s__
LMSG_G000007672.1	no	588_1	Magnetite	71.52	0.00	71.52	0	0	0	17	Medium quality	14865	1129240	62.20	127	d__Archaea;p__Thermoproteota;c__Nitrososphaeria;o__Nitrososphaerales;f__UBA183;g__UBA183;s__
LMSG_G000007673.1	no	588_1	Lead-Zinc	79.77	4.93	55.13	0	0	0	13	Medium quality	4662	1064538	62.10	258	d__Archaea;p__Thermoproteota;c__Nitrososphaeria;o__Nitrososphaerales;f__UBA183;g__UBA183;s__
LMSG_G000007674.1	no	588_1	Lead-Zinc	55.01	0.00	55.01	1	0	0	15	Medium quality	66772	586370	60.70	14	d__Archaea;p__Thermoproteota;c__Nitrososphaeria;o__Nitrososphaerales;f__UBA183;g__UBA183;s__
LMSG_G000007675.1	no	590_1	Lead-Zinc	54.85	0.00	54.85	1	0	0	16	Medium quality	386925	744511	58.90	36	d__Archaea;p__Thermoproteota;c__Nitrososphaeria;o__Nitrososphaerales;f__UBA183;g__UBA183;s__
LMSG_G000007676.1	no	590_1	Lead-Zinc	53.88	0.00	53.88	0	0	1	9	Medium quality	321196	541340	59.90	10	d__Archaea;p__Thermoproteota;c__Nitrososphaeria;o__Nitrososphaerales;f__UBA183;g__UBA183;s__
LMSG_G000007677.1	no	591_1	Copper	68.93	0.00	68.93	0	0	0	13	Medium quality	479770	888227	63.20	4	d__Archaea;p__Thermoproteota;c__Nitrososphaeria;o__Nitrososphaerales;f__UBA183;g__UBA183;s__
LMSG_G000007678.1	no	591_1	Copper	87.37	0.97	82.52	0	1	0	16	Medium quality	372304	1166093	62.60	9	d__Archaea;p__Thermoproteota;c__Nitrososphaeria;o__Nitrososphaerales;f__UBA183;g__UBA183;s__

LMSG_G000007679.1	no	591_1	Polymetallic	81.55	0.97	76.70	0	0	0	13	Medium quality	31888	1125175	64.20	65	d__Archaea;p__Thermoproteota;c__Nitrososphaeria;o__Nitrososphaerales;f__UBA183;g__UBA183;s__
LMSG_G000007680.1	no	591_1	Polymetallic	64.82	2.73	51.20	0	1	0	10	Medium quality	5120	784066	64.30	186	d__Archaea;p__Thermoproteota;c__Nitrososphaeria;o__Nitrososphaerales;f__UBA183;g__UBA183;s__
LMSG_G000007681.1	no	591_1	Polymetallic	70.87	0.00	70.87	0	0	0	13	Medium quality	21463	1073807	64.10	66	d__Archaea;p__Thermoproteota;c__Nitrososphaeria;o__Nitrososphaerales;f__UBA183;g__UBA183;s__
LMSG_G000007682.1	no	591_1	Polymetallic	74.75	0.97	69.90	0	0	0	11	Medium quality	34416	962433	64.70	47	d__Archaea;p__Thermoproteota;c__Nitrososphaeria;o__Nitrososphaerales;f__UBA183;g__UBA183;s__
LMSG_G000007683.1	no	591_1	Magnetite	89.80	0.97	84.95	1	0	0	20	Medium quality	82366	1847240	62.90	95	d__Archaea;p__Thermoproteota;c__Nitrososphaeria;o__Nitrososphaerales;f__UBA183;g__UBA183;s__
LMSG_G000007684.1	no	591_1	Pyrite-Copper	96.60	0.97	91.75	1	1	0	16	Medium quality	101695	1634493	63.00	52	d__Archaea;p__Thermoproteota;c__Nitrososphaeria;o__Nitrososphaerales;f__UBA183;g__UBA183;s__
LMSG_G000007685.1	no	591_1	Coal	70.47	1.46	63.19	0	0	0	10	Medium quality	5265	917869	64.00	217	d__Archaea;p__Thermoproteota;c__Nitrososphaeria;o__Nitrososphaerales;f__UBA183;g__UBA183;s__
LMSG_G000007686.1	no	591_1	Magnetite	75.24	1.94	65.54	1	1	0	15	Medium quality	39580	1362713	62.40	126	d__Archaea;p__Thermoproteota;c__Nitrososphaeria;o__Nitrososphaerales;f__UBA183;g__UBA183;s__
LMSG_G000007687.1	no	592_1	Pyrite	96.52	3.88	77.11	1	1	2	19	High quality	82338	1901119	61.60	104	d__Archaea;p__Thermoproteota;c__Nitrososphaeria;o__Nitrososphaerales;f__UBA183;g__UBA183;s__
LMSG_G000007688.1	no	592_1	Lead-Zinc	96.52	4.85	72.25	1	2	3	19	High quality	184215	1928403	61.40	68	d__Archaea;p__Thermoproteota;c__Nitrososphaeria;o__Nitrososphaerales;f__UBA183;g__UBA183;s__
LMSG_G000007689.1	no	592_1	Lead-Zinc	64.83	0.07	64.46	0	0	0	16	Medium quality	2210	1328951	61.40	649	d__Archaea;p__Thermoproteota;c__Nitrososphaeria;o__Nitrososphaerales;f__UBA183;g__UBA183;s__
LMSG_G000007690.1	no	592_1	Pyrite	65.94	1.94	56.24	0	0	0	12	Medium quality	57162	711410	61.80	17	d__Archaea;p__Thermoproteota;c__Nitrososphaeria;o__Nitrososphaerales;f__UBA183;g__UBA183;s__
LMSG_G000007691.1	no	592_1	Pyrite	81.47	0.97	76.62	0	0	0	15	Medium quality	222435	1382718	62.60	31	d__Archaea;p__Thermoproteota;c__Nitrososphaeria;o__Nitrososphaerales;f__UBA183;g__UBA183;s__
LMSG_G000007692.1	no	592_1	Pyrite	74.68	1.05	69.46	0	0	0	16	Medium quality	10604	1085604	62.60	156	d__Archaea;p__Thermoproteota;c__Nitrososphaeria;o__Nitrososphaerales;f__UBA183;g__UBA183;s__
LMSG_G000007693.1	no	592_1	Lead-Zinc	56.79	0.00	56.79	1	1	5	12	Medium quality	333237	611141	58.70	11	d__Archaea;p__Thermoproteota;c__Nitrososphaeria;o__Nitrososphaerales;f__UBA183;g__UBA183;s__
LMSG_G000007694.1	no	592_1	Copper	69.41	1.94	59.71	1	1	0	17	Medium quality	157016	1080632	59.70	59	d__Archaea;p__Thermoproteota;c__Nitrososphaeria;o__Nitrososphaerales;f__UBA183;g__UBA183;s__
LMSG_G000007695.1	no	592_1	Lead-Zinc	89.89	3.88	70.48	1	0	2	19	Medium quality	4975	1586106	61.60	416	d__Archaea;p__Thermoproteota;c__Nitrososphaeria;o__Nitrososphaerales;f__UBA183;g__UBA183;s__
LMSG_G000007696.1	no	592_1	Copper	62.13	0.00	62.13	0	0	0	9	Medium quality	64728	916530	60.90	69	d__Archaea;p__Thermoproteota;c__Nitrososphaeria;o__Nitrososphaerales;f__UBA183;g__UBA183;s__
LMSG_G000007697.1	no	592_1	Polymetallic	59.70	0.00	59.70	1	1	0	15	Medium quality	16800	923311	58.80	81	d__Archaea;p__Thermoproteota;c__Nitrososphaeria;o__Nitrososphaerales;f__UBA183;g__UBA183;s__
LMSG_G000007698.1	no	592_1	Copper	81.49	4.52	58.90	1	0	0	17	Medium quality	3038	1343627	62.00	515	d__Archaea;p__Thermoproteota;c__Nitrososphaeria;o__Nitrososphaerales;f__UBA183;g__UBA183;s__
LMSG_G000007699.1	no	592_1	Copper	59.12	0.00	59.12	0	1	0	12	Medium quality	1953	1059847	61.70	555	d__Archaea;p__Thermoproteota;c__Nitrososphaeria;o__Nitrososphaerales;f__UBA183;g__UBA183;s__
LMSG_G000007700.1	no	592_1	Polymetallic	77.10	1.94	67.40	2	1	0	19	Medium quality	145174	1286118	60.40	152	d__Archaea;p__Thermoproteota;c__Nitrososphaeria;o__Nitrososphaerales;f__UBA183;g__UBA183;s__
LMSG_G000007701.1	no	592_1	Magnetite	67.47	0.97	62.62	1	2	0	17	Medium quality	124426	882075	59.30	79	d__Archaea;p__Thermoproteota;c__Nitrososphaeria;o__Nitrososphaerales;f__UBA183;g__UBA183;s__
LMSG_G000007702.1	no	592_1	Pyrite-Copper	94.58	1.94	84.88	2	0	1	19	Medium quality	222164	1852074	61.70	97	d__Archaea;p__Thermoproteota;c__Nitrososphaeria;o__Nitrososphaerales;f__UBA183;g__UBA183;s__
LMSG_G000007703.1	no	592_1	Pyrite-Copper	87.78	1.94	78.08	1	1	2	18	Medium quality	260560	1257792	60.50	15	d__Archaea;p__Thermoproteota;c__Nitrososphaeria;o__Nitrososphaerales;f__UBA183;g__UBA183;s__
LMSG_G000007704.1	no	592_1	Pyrite-Copper	75.24	1.94	65.54	1	0	0	17	Medium quality	84525	1070817	59.40	70	d__Archaea;p__Thermoproteota;c__Nitrososphaeria;o__Nitrososphaerales;f__UBA183;g__UBA183;s__
LMSG_G000007705.1	no	592_1	Lead-Zinc	60.67	0.00	60.67	1	0	0	18	Medium quality	67112	732950	60.70	25	d__Archaea;p__Thermoproteota;c__Nitrososphaeria;o__Nitrososphaerales;f__UBA183;g__UBA183;s__
LMSG_G000007706.1	no	592_1	Magnetite	64.56	0.00	64.56	0	0	0	15	Medium quality	119791	839798	59.80	18	d__Archaea;p__Thermoproteota;c__Nitrososphaeria;o__Nitrososphaerales;f__UBA183;g__UBA183;s__
LMSG_G000007707.1	no	592_1	Magnetite	58.25	0.97	53.40	0	0	0	9	Medium quality	40551	520154	60.70	18	d__Archaea;p__Thermoproteota;c__Nitrososphaeria;o__Nitrososphaerales;f__UBA183;g__UBA183;s__
LMSG_G000007708.1	no	592_1	Pyrite-Copper	79.32	1.94	69.62	1	1	2	18	Medium quality	48022	1051345	59.50	45	d__Archaea;p__Thermoproteota;c__Nitrososphaeria;o__Nitrososphaerales;f__UBA183;g__UBA183;s__
LMSG_G000007709.1	no	592_1	Copper	96.52	3.88	77.11	1	1	0	19	Medium quality	153977	1875731	61.40	77	d__Archaea;p__Thermoproteota;c__Nitrososphaeria;o__Nitrososphaerales;f__UBA183;g__UBA183;s__
LMSG_G000007710.1	no	592_1	Magnetite	61.65	0.00	61.65	2	0	1	13	Medium quality	93647	640319	61.20	70	d__Archaea;p__Thermoproteota;c__Nitrososphaeria;o__Nitrososphaerales;f__UBA183;g__UBA183;s__
LMSG_G000007711.1	no	593_1	Lead-Zinc	56.31	0.97	51.46	0	0	0	10	Medium quality	57646	856931	61.50	82	d__Archaea;p__Thermoproteota;c__Nitrososphaeria;o__Nitrososphaerales;f__UBA183;g__UBA183;s__
LMSG_G000007712.1	no	593_1	Lead-Zinc	71.84	0.97	66.99	0	2	2	15	Medium quality	22835	1387905	60.10	82	d__Archaea;p__Thermoproteota;c__Nitrososphaeria;o__Nitrososphaerales;f__UBA183;g__UBA183;s__
LMSG_G000007713.1	no	593_1	Lead-Zinc	73.46	3.88	54.05	0	0	0	14	Medium quality	70427	1476227	61.30	192	d__Archaea;p__Thermoproteota;c__Nitrososphaeria;o__Nitrososphaerales;f__UBA183;g__UBA183;s__
LMSG_G000007714.1	no	593_1	Pyrite-Copper	82.03	1.94	72.33	0	1	0	13	Medium quality	51206	1124437	62.00	70	d__Archaea;p__Thermoproteota;c__Nitrososphaeria;o__Nitrososphaerales;f__UBA183;g__UBA183;s__
LMSG_G000007715.1	no	594_1	Copper	67.96	0.00	67.96	1	0	0	16	Medium quality	161294	990923	59.00	14	d__Archaea;p__Thermoproteota;c__Nitrososphaeria;o__Nitrososphaerales;f__UBA183;g__UBA183;s__
LMSG_G000007716.1	no	594_1	Copper	63.10	0.00	63.10	0	0	0	11	Medium quality	118781	797337	61.60	11	d__Archaea;p__Thermoproteota;c__Nitrososphaeria;o__Nitrososphaerales;f__UBA183;g__UBA183;s__
LMSG_G000007717.1	no	595_1	Copper	58.41	0.00	58.41	1	0	1	14	Medium quality	183288	882810	60.80	12	d__Archaea;p__Thermoproteota;c__Nitrososphaeria;o__Nitrososphaerales;f__UBA183;g__UBA183;s__
LMSG_G000007718.1	no	211_1	Lead-Zinc	90.77	0.97	85.92	1	3	2	20	High quality	11836	1403700	47.70	213	d__Archaea;p__Thermoproteota;c__Nitrososphaeria;o__UBA164;f__;g__;s__
LMSG_G000007719.1	no	211_1	Copper	93.20	1.19	87.23	1	1	2	20	High quality	109635	1657986	47.70	122	d__Archaea;p__Thermoproteota;c__Nitrososphaeria;o__UBA164;f__;g__;s__
LMSG_G000007720.1	no	211_2	Lead-Zinc	79.77	0.97	74.92	1	0	0	19	Medium quality	10941	1296720	47.10	197	d__Archaea;p__Thermoproteota;c__Nitrososphaeria;o__UBA164;f__;g__;s__
LMSG_G000007721.1	no	211_2	Lead-Zinc	61.32	0.00	61.32	1	3	7	18	Medium quality	16376	1029281	48.40	137	d__Archaea;p__Thermoproteota;c__Nitrososphaeria;o__UBA164;f__;g__;s__
LMSG_G000007722.1	no	211_2	Pyrite	70.38	0.00	70.38	1	0	0	18	Medium quality	52177	1111658	49.60	80	d__Archaea;p__Thermoproteota;c__Nitrososphaeria;o__UBA164;f__;g__;s__
LMSG_G000007723.1	no	582_1	Polymetallic	64.48	1.94	54.78	1	1	0	11	Medium quality	5862	1297815	64.80	318	d__Archaea;p__Thermoproteota;c__Nitrososphaeria;o__UBA164;f__;g__;s__
LMSG_G000007724.1	no	1677_1	Lead-Zinc	89.48	2.91	74.92	1	1	1	20	Medium quality	46379	1928418	45.10	147	d__Archaea;p__Thermoproteota;c__Nitrososphaeria;o__UBA164;f__UBA164;g__UBA160;s__
LMSG_G000007725.1	no	1677_1	Lead-Zinc	60.35	2.02	50.27	0	0	1	14	Medium quality	48856	1161834	46.10	57	d__Archaea;p__Thermoproteota;c__Nitrososphaeria;o__UBA164;f__UBA164;g__UBA160;s__
LMSG_G000007726.1	no	580_2	Lead-Zinc	86.35	0.93	81.68	1	7	5	19	Medium quality	4769	2221422	52.40	564	d__Archaea;p__Thermoproteota;c__Thermoproteia;o__Marsarchaeales;f__Marsarchaeaceae;g__;s__
LMSG_G000007727.1	no	580_1	Copper	90.42	2.80	76.41	1	4	10	20	High quality	62781	2017191	51.70	84	d__Archaea;p__Thermoproteota;c__Thermoproteia;o__Marsarchaeales;f__Marsarchaeaceae;g__ECH-B-1;s__
LMSG_G000007728.1	no	580_2	Pyrite-Copper	92.75	1.94	83.05	1	1	17	20	High quality	13691	2169660	51.50	309	d__Archaea;p__Thermoproteota;c__Thermoproteia;o__Marsarchaeales;f__Marsarchaeaceae;g__ECH-B-1;s__
LMSG_G000007729.1	no	580_2	Lead-Zinc	93.22	2.80	79.21	3	9	19	20	High quality	227279	2187559	51.60	81	d__Archaea;p__Thermoproteota;c__Thermoproteia;o__Marsarchaeales;f__Marsarchaeaceae;g__ECH-B-1;s__
LMSG_G000007730.1	no	580_2	Lead-Zinc	83.41	0.93	78.74	1	1	2	17	Medium quality	12839	1645017	51.20	155	d__Archaea;p__Thermoproteota;c__Thermoproteia;o__Marsarchaeales;f__Marsarchaeaceae;g__ECH-B-1;s__
LMSG_G000007731.1	no	1000_1	Antimony	97.41	2.59	84.48	1	3	2	20	High quality	150340	4032237	60.00	155	d__Bacteria;p__Acidobacteriota;c__Acidobacteriae;o__Acidobacteriales;f__Acidobacteriaceae;g__;s__
LMSG_G000007732.1	no	1000_1	Antimony	51.72	0.00	51.72	0	1	1	9	Medium quality	107860	1240982	61.20	21	d__Bacteria;p__Acidobacteriota;c__Acidobacteriae;o__Acidobacteriales;f__Acidobacteriaceae;g__;s__
LMSG_G000007733.1	no	1572_1	Copper	60.76	0.00	60.76	1	1	1	17	Medium quality	5023	2589375	53.40	603	d__Bacteria;p__Acidobacteriota;c__Acidobacteriae;o__Acidobacteriales;f__Acidobacteriaceae;g__;s__
LMSG_G000007734.1	no	1573_1	Antimony	73.10	2.16	62.33	0	0	0	13	Medium quality	5439	2264513	59.80	497	d__Bacteria;p__Acidobacteriota;c__Acidobacteriae;o__Acidobacteriales;f__Acidobacteriaceae;g__;s__
LMSG_G000007735.1	no	1573_1	Antimony	55.17	0.00	55.17	0	0	0	14	Medium quality	3528	1855650	59.80	573	d__Bacteria;p__Acidobacteriota;c__Acidobacteriae;o__Acidobacteriales;f__Acidobacteriaceae;g__;s__
LMSG_G000007736.1	no	884_1	Polymetallic	96.39	0.00	96.39	0	2	0	18	Medium quality	44282	2473422	57.40	88	d__Bacteria;p__Acidobacteriota;c__Acidobacteriae;o__Acidobacteriales;f__Acidobacteriaceae;g__;s__
LMSG_G000007737.1	no	884_1	Polymetallic	97.03	0.85	92.76	0	1	0	18	Medium quality	36889	2589431	57.30	105	d__Bacteria;p__Acidobacteriota;c__Acidobacteriae;o__Acidobacteriales;f__Acidobacteriaceae;g__;s__
LMSG_G000007738.1	no	884_1	Polymetallic	96.21	1.72	87.59	0	0	0	18	Medium quality	24138	2409464	57.40	140	d__Bacteria;p__Acidobacteriota;c__Acidobacteriae;o__Acidobacteriales;f__Acidobacteriaceae;g__;s__
LMSG_G000007739.1	no	884_1	Polymetallic	96.81	1.71	88.27	0	1	0	17	Medium quality	33676	2429305	57.40	108	d__Bacteria;p__Acidobacteriota;c__Acidobacteriae;o__Acidobacteriales;f__Acidobacteriaceae;g__;s__
LMSG_G000007740.1	no	884_1	Polymetallic	88.45	0.86	84.14	0	1	0	18	Medium quality	15105	2287191	57.50	210	d__Bacteria;p__Acidobacteriota;c__Acidobacteriae;o__Acidobacteriales;f__Acidobacteriaceae;g__;s__
LMSG_G000007741.1	no	884_1	Pyrite	91.06	0.43	88.93	0	0	0	18	Medium quality	8768	2217716	57.40	303	d__Bacteria;p__Acidobacteriota;c__Acidobacteriae;o__Acidobacteriales;f__Acidobacteriaceae;g__;s__
LMSG_G000007742.1	no	884_1	Pyrite	97.36	0.21	96.30	0	1	0	19	Medium quality	39918	2541840	57.20	152	d__Bacteria;p__Acidobacteriota;c__Acidobacteriae;o__Acidobacteriales;f__Acidobacteriaceae;g__;s__
LMSG_G000007743.1	no	884_1	Pyrite	91.54	0.85	87.27	0	0	0	16	Medium quality	13357	2536645	57.20	259	d__Bacteria;p__Acidobacteriota;c__Acidobacteriae;o__Acidobacteriales;f__Acidobacteriaceae;g__;s__
LMSG_G000007744.1	no	884_1	Pyrite	94.77	0.05	94.54	0	0	0	17	Medium quality	28075	2458753	57.40	213	d__Bacteria;p__Acidobacteriota;c__Acidobacteriae;o__Acidobacteriales;f__Acidobacteriaceae;g__;s__
LMSG_G000007745.1	no	885_1	Polymetallic	88.03	1.38	81.15	0	0	0	18	Medium quality	28182	2261390	59.00	218	d__Bacteria;p__Acidobacteriota;c__Acidobacteriae;o__Acidobacteriales;f__Acidobacteriaceae;g__;s__
LMSG_G000007746.1	no	886_1	Coal	66.28	0.43	64.13	0	0	0	15	Medium quality	9748	1792343	59.80	241	d__Bacteria;p__Acidobacteriota;c__Acidobacteriae;o__Acidobacteriales;f__Acidobacteriaceae;g__;s__
LMSG_G000007747.1	no	889_1	Copper	84.32	2.78	70.44	0	0	0	13	Medium quality	10735	3769623	57.40	469	d__Bacteria;p__Acidobacteriota;c__Acidobacteriae;o__Acidobacteriales;f__Acidobacteriaceae;g__;s__
LMSG_G000007748.1	no	889_1	Copper	66.37	0.85	62.10	0	0	0	9	Medium quality	4552	3295016	57.40	836	d__Bacteria;p__Acidobacteriota;c__Acidobacteriae;o__Acidobacteriales;f__Acidobacteriaceae;g__;s__
LMSG_G000007749.1	no	889_1	Copper	91.63	3.06	76.32	0	2	0	16	Medium quality	11086	4701078	57.00	555	d__Bacteria;p__Acidobacteriota;c__Acidobacteriae;o__Acidobacteriales;f__Acidobacteriaceae;g__;s__
LMSG_G000007750.1	no	889_1	Polymetallic	66.44	1.71	57.90	0	0	0	9	Medium quality	11108	2428679	57.20	283	d__Bacteria;p__Acidobacteriota;c__Acidobacteriae;o__Acidobacteriales;f__Acidobacteriaceae;g__;s__
LMSG_G000007751.1	no	891_1	Copper	85.64	0.00	85.64	0	0	0	16	Medium quality	20172	3241115	57.00	243	d__Bacteria;p__Acidobacteriota;c__Acidobacteriae;o__Acidobacteriales;f__Acidobacteriaceae;g__;s__
LMSG_G000007752.1	no	891_1	Copper	79.40	0.47	77.03	0	0	0	16	Medium quality	12082	4330134	56.70	609	d__Bacteria;p__Acidobacteriota;c__Acidobacteriae;o__Acidobacteriales;f__Acidobacteriaceae;g__;s__
LMSG_G000007753.1	no	891_1	Copper	62.01	1.46	54.70	0	1	0	15	Medium quality	10456	5359138	56.90	1238	d__Bacteria;p__Acidobacteriota;c__Acidobacteriae;o__Acidobacteriales;f__Acidobacteriaceae;g__;s__
LMSG_G000007754.1	no	891_1	Lead-Zinc	88.08	2.14	77.40	0	1	0	17	Medium quality	8143	4163404	56.70	636	d__Bacteria;p__Acidobacteriota;c__Acidobacteriae;o__Acidobacteriales;f__Acidobacteriaceae;g__;s__
LMSG_G000007755.1	no	891_1	Polymetallic	57.50	0.05	57.27	0	1	0	11	Medium quality	24772	1889427	57.60	147	d__Bacteria;p__Acidobacteriota;c__Acidobacteriae;o__Acidobacteriales;f__Acidobacteriaceae;g__;s__

LMSG_G000007756.1	no	891_1	Polymetallic	85.26	0.48	82.87	0	1	0	15	Medium quality	12543	3778929	56.60	367	d__Bacteria;p__Acidobacteriota;c__Acidobacteriae;o__Acidobacteriales;f__Acidobacteriaceae;g__;s__
LMSG_G000007757.1	no	891_1	Magnetite	55.17	0.00	55.17	0	0	0	15	Medium quality	22178	3400199	56.70	227	d__Bacteria;p__Acidobacteriota;c__Acidobacteriae;o__Acidobacteriales;f__Acidobacteriaceae;g__;s__
LMSG_G000007758.1	no	892_1	Lead-Zinc	87.43	1.62	79.32	0	2	0	14	Medium quality	11210	3869717	58.10	588	d__Bacteria;p__Acidobacteriota;c__Acidobacteriae;o__Acidobacteriales;f__Acidobacteriaceae;g__;s__
LMSG_G000007759.1	no	892_1	Lead-Zinc	76.72	1.28	70.31	0	0	0	17	Medium quality	6948	4157778	57.80	691	d__Bacteria;p__Acidobacteriota;c__Acidobacteriae;o__Acidobacteriales;f__Acidobacteriaceae;g__;s__
LMSG_G000007760.1	no	892_1	Pyrite-Copper	72.62	2.18	61.70	0	0	0	14	Medium quality	8391	2709420	58.50	447	d__Bacteria;p__Acidobacteriota;c__Acidobacteriae;o__Acidobacteriales;f__Acidobacteriaceae;g__;s__
LMSG_G000007761.1	no	892_2	Lead-Zinc	74.34	4.27	52.98	0	0	0	14	Medium quality	7662	3041905	58.30	484	d__Bacteria;p__Acidobacteriota;c__Acidobacteriae;o__Acidobacteriales;f__Acidobacteriaceae;g__;s__
LMSG_G000007762.1	no	894_1	Polymetallic	84.37	0.00	84.37	0	0	0	16	Medium quality	14614	3062620	56.10	293	d__Bacteria;p__Acidobacteriota;c__Acidobacteriae;o__Acidobacteriales;f__Acidobacteriaceae;g__;s__
LMSG_G000007763.1	no	894_1	Polymetallic	87.07	1.28	80.66	0	0	0	13	Medium quality	15510	2689223	56.30	243	d__Bacteria;p__Acidobacteriota;c__Acidobacteriae;o__Acidobacteriales;f__Acidobacteriaceae;g__;s__
LMSG_G000007764.1	no	894_1	Polymetallic	93.29	1.92	83.68	0	0	0	19	Medium quality	15440	3328880	56.20	318	d__Bacteria;p__Acidobacteriota;c__Acidobacteriae;o__Acidobacteriales;f__Acidobacteriaceae;g__;s__
LMSG_G000007765.1	no	894_1	Polymetallic	84.56	0.00	84.56	0	0	0	14	Medium quality	15506	2813865	56.20	259	d__Bacteria;p__Acidobacteriota;c__Acidobacteriae;o__Acidobacteriales;f__Acidobacteriaceae;g__;s__
LMSG_G000007766.1	no	894_1	Polymetallic	89.07	1.15	83.35	0	0	0	13	Medium quality	13929	3680952	56.10	381	d__Bacteria;p__Acidobacteriota;c__Acidobacteriae;o__Acidobacteriales;f__Acidobacteriaceae;g__;s__
LMSG_G000007767.1	no	894_1	Copper	91.71	0.00	91.71	0	0	0	16	Medium quality	27393	3001336	56.20	224	d__Bacteria;p__Acidobacteriota;c__Acidobacteriae;o__Acidobacteriales;f__Acidobacteriaceae;g__;s__
LMSG_G000007768.1	no	894_1	Polymetallic	77.57	2.61	64.52	0	0	0	16	Medium quality	3143	3116386	56.10	1162	d__Bacteria;p__Acidobacteriota;c__Acidobacteriae;o__Acidobacteriales;f__Acidobacteriaceae;g__;s__
LMSG_G000007769.1	no	894_1	Copper	86.01	0.00	86.01	1	1	1	18	Medium quality	29667	3474252	55.90	193	d__Bacteria;p__Acidobacteriota;c__Acidobacteriae;o__Acidobacteriales;f__Acidobacteriaceae;g__;s__
LMSG_G000007770.1	no	894_1	Copper	64.94	1.72	56.32	1	0	1	18	Medium quality	27922	3352933	55.90	183	d__Bacteria;p__Acidobacteriota;c__Acidobacteriae;o__Acidobacteriales;f__Acidobacteriaceae;g__;s__
LMSG_G000007771.1	no	894_1	Lead-Zinc	97.97	1.76	89.19	1	1	1	19	High quality	26694	3856286	56.00	227	d__Bacteria;p__Acidobacteriota;c__Acidobacteriae;o__Acidobacteriales;f__Acidobacteriaceae;g__;s__
LMSG_G000007772.1	no	894_1	Lead-Zinc	95.24	1.71	86.70	0	1	0	19	Medium quality	22328	3569227	56.00	246	d__Bacteria;p__Acidobacteriota;c__Acidobacteriae;o__Acidobacteriales;f__Acidobacteriaceae;g__;s__
LMSG_G000007773.1	no	894_1	Copper	59.01	0.31	57.45	0	0	0	13	Medium quality	12058	1660712	56.20	254	d__Bacteria;p__Acidobacteriota;c__Acidobacteriae;o__Acidobacteriales;f__Acidobacteriaceae;g__;s__
LMSG_G000007774.1	no	894_1	Copper	85.65	0.85	81.38	0	1	0	16	Medium quality	13796	3092593	56.20	364	d__Bacteria;p__Acidobacteriota;c__Acidobacteriae;o__Acidobacteriales;f__Acidobacteriaceae;g__;s__
LMSG_G000007775.1	no	894_1	Copper	85.67	0.00	85.67	0	0	0	17	Medium quality	19093	2882466	56.10	256	d__Bacteria;p__Acidobacteriota;c__Acidobacteriae;o__Acidobacteriales;f__Acidobacteriaceae;g__;s__
LMSG_G000007776.1	no	894_1	Copper	62.44	2.16	51.67	0	0	0	15	Medium quality	4995	2071039	56.60	470	d__Bacteria;p__Acidobacteriota;c__Acidobacteriae;o__Acidobacteriales;f__Acidobacteriaceae;g__;s__
LMSG_G000007777.1	no	916_1	Polymetallic	94.99	1.52	87.40	1	1	1	19	High quality	16793	3254908	61.20	277	d__Bacteria;p__Acidobacteriota;c__Acidobacteriae;o__Acidobacteriales;f__Acidobacteriaceae;g__;s__
LMSG_G000007778.1	no	916_1	Polymetallic	92.81	1.72	84.19	1	1	1	19	High quality	20338	3372384	61.20	242	d__Bacteria;p__Acidobacteriota;c__Acidobacteriae;o__Acidobacteriales;f__Acidobacteriaceae;g__;s__
LMSG_G000007779.1	no	916_1	Polymetallic	84.64	0.00	84.64	0	1	0	16	Medium quality	19688	2837822	61.40	197	d__Bacteria;p__Acidobacteriota;c__Acidobacteriae;o__Acidobacteriales;f__Acidobacteriaceae;g__;s__
LMSG_G000007780.1	no	916_1	Polymetallic	90.27	0.86	85.96	1	0	1	15	Medium quality	17404	3051256	61.20	245	d__Bacteria;p__Acidobacteriota;c__Acidobacteriae;o__Acidobacteriales;f__Acidobacteriaceae;g__;s__
LMSG_G000007781.1	no	916_1	Polymetallic	95.04	0.86	90.73	1	1	1	19	High quality	27128	3243223	61.00	195	d__Bacteria;p__Acidobacteriota;c__Acidobacteriae;o__Acidobacteriales;f__Acidobacteriaceae;g__;s__
LMSG_G000007782.1	no	916_1	Polymetallic	92.88	1.29	86.42	1	1	1	19	High quality	21939	3356738	61.00	230	d__Bacteria;p__Acidobacteriota;c__Acidobacteriae;o__Acidobacteriales;f__Acidobacteriaceae;g__;s__
LMSG_G000007783.1	no	916_1	Polymetallic	87.69	0.00	87.69	1	1	1	17	Medium quality	20459	3192794	61.00	239	d__Bacteria;p__Acidobacteriota;c__Acidobacteriae;o__Acidobacteriales;f__Acidobacteriaceae;g__;s__
LMSG_G000007784.1	no	916_1	Polymetallic	94.85	4.36	73.06	1	0	1	19	Medium quality	23006	3446496	61.00	234	d__Bacteria;p__Acidobacteriota;c__Acidobacteriae;o__Acidobacteriales;f__Acidobacteriaceae;g__;s__
LMSG_G000007785.1	no	916_1	Polymetallic	61.32	0.86	57.01	1	0	1	14	Medium quality	27391	2173129	61.40	113	d__Bacteria;p__Acidobacteriota;c__Acidobacteriae;o__Acidobacteriales;f__Acidobacteriaceae;g__;s__
LMSG_G000007786.1	no	916_1	Polymetallic	70.30	2.59	57.37	1	1	1	15	Medium quality	11544	2436438	61.50	284	d__Bacteria;p__Acidobacteriota;c__Acidobacteriae;o__Acidobacteriales;f__Acidobacteriaceae;g__;s__
LMSG_G000007787.1	no	917_1	Polymetallic	84.79	1.03	79.62	1	0	1	17	Medium quality	17875	2812555	62.70	218	d__Bacteria;p__Acidobacteriota;c__Acidobacteriae;o__Acidobacteriales;f__Acidobacteriaceae;g__;s__
LMSG_G000007788.1	no	917_1	Polymetallic	71.14	2.01	61.09	1	0	0	15	Medium quality	5803	2480866	62.50	536	d__Bacteria;p__Acidobacteriota;c__Acidobacteriae;o__Acidobacteriales;f__Acidobacteriaceae;g__;s__
LMSG_G000007789.1	no	917_1	Pyrite	88.79	3.50	71.28	1	1	1	15	Medium quality	109507	2866295	62.40	56	d__Bacteria;p__Acidobacteriota;c__Acidobacteriae;o__Acidobacteriales;f__Acidobacteriaceae;g__;s__
LMSG_G000007790.1	no	917_1	Antimony	80.58	3.07	65.26	0	1	0	16	Medium quality	6669	2598811	63.10	496	d__Bacteria;p__Acidobacteriota;c__Acidobacteriae;o__Acidobacteriales;f__Acidobacteriaceae;g__;s__
LMSG_G000007791.1	no	917_1	Copper	62.35	1.72	53.73	1	0	1	15	Medium quality	10671	2718688	63.20	466	d__Bacteria;p__Acidobacteriota;c__Acidobacteriae;o__Acidobacteriales;f__Acidobacteriaceae;g__;s__
LMSG_G000007792.1	no	917_1	Polymetallic	63.49	2.59	50.56	1	0	1	13	Medium quality	7847	2245577	62.60	340	d__Bacteria;p__Acidobacteriota;c__Acidobacteriae;o__Acidobacteriales;f__Acidobacteriaceae;g__;s__
LMSG_G000007793.1	no	919_1	Polymetallic	86.56	1.99	76.63	1	1	1	14	Medium quality	39577	2962067	59.80	316	d__Bacteria;p__Acidobacteriota;c__Acidobacteriae;o__Acidobacteriales;f__Acidobacteriaceae;g__;s__
LMSG_G000007794.1	no	919_1	Polymetallic	84.17	1.88	74.76	2	1	2	15	Medium quality	47388	2808474	59.90	255	d__Bacteria;p__Acidobacteriota;c__Acidobacteriae;o__Acidobacteriales;f__Acidobacteriaceae;g__;s__
LMSG_G000007795.1	no	919_1	Polymetallic	75.45	0.86	71.14	1	1	1	11	Medium quality	23264	2527410	60.10	162	d__Bacteria;p__Acidobacteriota;c__Acidobacteriae;o__Acidobacteriales;f__Acidobacteriaceae;g__;s__
LMSG_G000007796.1	no	919_1	Polymetallic	82.92	1.72	74.30	1	1	1	13	Medium quality	21990	2704690	60.00	193	d__Bacteria;p__Acidobacteriota;c__Acidobacteriae;o__Acidobacteriales;f__Acidobacteriaceae;g__;s__
LMSG_G000007797.1	no	919_1	Polymetallic	80.38	4.31	58.83	1	1	0	13	Medium quality	21349	2666876	60.20	197	d__Bacteria;p__Acidobacteriota;c__Acidobacteriae;o__Acidobacteriales;f__Acidobacteriaceae;g__;s__
LMSG_G000007798.1	no	919_1	Polymetallic	66.43	2.59	53.50	0	1	0	15	Medium quality	17703	2413350	60.00	191	d__Bacteria;p__Acidobacteriota;c__Acidobacteriae;o__Acidobacteriales;f__Acidobacteriaceae;g__;s__
LMSG_G000007799.1	no	919_1	Polymetallic	84.69	0.86	80.38	1	1	0	12	Medium quality	23157	2891528	60.10	177	d__Bacteria;p__Acidobacteriota;c__Acidobacteriae;o__Acidobacteriales;f__Acidobacteriaceae;g__;s__
LMSG_G000007800.1	no	919_1	Polymetallic	86.23	3.72	67.65	0	1	0	14	Medium quality	21662	2825036	60.10	196	d__Bacteria;p__Acidobacteriota;c__Acidobacteriae;o__Acidobacteriales;f__Acidobacteriaceae;g__;s__
LMSG_G000007801.1	no	919_1	Polymetallic	84.98	1.72	76.36	1	1	1	14	Medium quality	23098	2869132	60.00	176	d__Bacteria;p__Acidobacteriota;c__Acidobacteriae;o__Acidobacteriales;f__Acidobacteriaceae;g__;s__
LMSG_G000007802.1	no	919_1	Polymetallic	96.47	3.02	81.39	0	1	0	18	Medium quality	23502	3179849	60.20	222	d__Bacteria;p__Acidobacteriota;c__Acidobacteriae;o__Acidobacteriales;f__Acidobacteriaceae;g__;s__
LMSG_G000007803.1	no	920_1	Polymetallic	87.90	1.96	78.09	1	1	1	17	Medium quality	50175	2820520	59.90	186	d__Bacteria;p__Acidobacteriota;c__Acidobacteriae;o__Acidobacteriales;f__Acidobacteriaceae;g__;s__
LMSG_G000007804.1	no	920_1	Polymetallic	84.41	2.30	72.92	1	1	1	18	Medium quality	40227	2935745	59.60	156	d__Bacteria;p__Acidobacteriota;c__Acidobacteriae;o__Acidobacteriales;f__Acidobacteriaceae;g__;s__
LMSG_G000007805.1	no	922_1	Polymetallic	90.56	2.59	77.63	0	0	0	18	Medium quality	10671	3232623	62.80	560	d__Bacteria;p__Acidobacteriota;c__Acidobacteriae;o__Acidobacteriales;f__Acidobacteriaceae;g__;s__
LMSG_G000007806.1	no	923_1	Pyrite-Copper	90.54	2.16	79.77	1	1	1	16	Medium quality	76163	3379980	59.70	135	d__Bacteria;p__Acidobacteriota;c__Acidobacteriae;o__Acidobacteriales;f__Acidobacteriaceae;g__;s__
LMSG_G000007807.1	no	923_1	Lead-Zinc	91.16	2.16	80.39	1	0	1	19	Medium quality	68375	3514104	59.20	105	d__Bacteria;p__Acidobacteriota;c__Acidobacteriae;o__Acidobacteriales;f__Acidobacteriaceae;g__;s__
LMSG_G000007808.1	no	923_1	Pyrite	95.04	0.86	90.73	1	0	1	18	Medium quality	55388	4019807	59.40	168	d__Bacteria;p__Acidobacteriota;c__Acidobacteriae;o__Acidobacteriales;f__Acidobacteriaceae;g__;s__
LMSG_G000007809.1	no	923_1	Pyrite	95.04	1.72	86.42	1	0	1	18	Medium quality	33292	3284857	59.80	169	d__Bacteria;p__Acidobacteriota;c__Acidobacteriae;o__Acidobacteriales;f__Acidobacteriaceae;g__;s__
LMSG_G000007810.1	no	923_1	Pyrite	95.04	0.86	90.73	1	0	1	19	Medium quality	65612	3327303	59.60	82	d__Bacteria;p__Acidobacteriota;c__Acidobacteriae;o__Acidobacteriales;f__Acidobacteriaceae;g__;s__
LMSG_G000007811.1	no	923_1	Pyrite	95.23	0.86	90.92	1	0	1	19	Medium quality	60419	3897810	59.10	148	d__Bacteria;p__Acidobacteriota;c__Acidobacteriae;o__Acidobacteriales;f__Acidobacteriaceae;g__;s__
LMSG_G000007812.1	no	923_1	Pyrite	85.24	1.72	76.62	1	0	1	17	Medium quality	13286	3623655	59.50	461	d__Bacteria;p__Acidobacteriota;c__Acidobacteriae;o__Acidobacteriales;f__Acidobacteriaceae;g__;s__
LMSG_G000007813.1	no	923_1	Lead-Zinc	92.79	1.92	83.19	1	0	1	19	Medium quality	44372	3519122	59.40	212	d__Bacteria;p__Acidobacteriota;c__Acidobacteriae;o__Acidobacteriales;f__Acidobacteriaceae;g__;s__
LMSG_G000007814.1	no	923_1	Lead-Zinc	91.16	1.72	82.54	0	0	0	18	Medium quality	70110	3617785	59.10	110	d__Bacteria;p__Acidobacteriota;c__Acidobacteriae;o__Acidobacteriales;f__Acidobacteriaceae;g__;s__
LMSG_G000007815.1	no	923_1	Lead-Zinc	89.41	2.44	77.20	0	1	1	19	Medium quality	76131	3095893	59.80	79	d__Bacteria;p__Acidobacteriota;c__Acidobacteriae;o__Acidobacteriales;f__Acidobacteriaceae;g__;s__
LMSG_G000007816.1	no	923_1	Lead-Zinc	90.34	1.29	83.88	1	0	1	16	Medium quality	67480	3075248	59.70	72	d__Bacteria;p__Acidobacteriota;c__Acidobacteriae;o__Acidobacteriales;f__Acidobacteriaceae;g__;s__
LMSG_G000007817.1	no	923_1	Lead-Zinc	90.54	1.72	81.92	1	0	1	16	Medium quality	65392	3045525	59.80	78	d__Bacteria;p__Acidobacteriota;c__Acidobacteriae;o__Acidobacteriales;f__Acidobacteriaceae;g__;s__
LMSG_G000007818.1	no	923_1	Polymetallic	92.02	1.72	83.40	1	0	1	15	Medium quality	46730	3169890	59.70	128	d__Bacteria;p__Acidobacteriota;c__Acidobacteriae;o__Acidobacteriales;f__Acidobacteriaceae;g__;s__
LMSG_G000007819.1	no	923_1	Polymetallic	86.94	0.86	82.63	1	0	1	17	Medium quality	42295	3134437	59.60	119	d__Bacteria;p__Acidobacteriota;c__Acidobacteriae;o__Acidobacteriales;f__Acidobacteriaceae;g__;s__
LMSG_G000007820.1	no	923_1	Polymetallic	89.00	0.86	84.69	0	0	0	17	Medium quality	56873	3053219	59.70	98	d__Bacteria;p__Acidobacteriota;c__Acidobacteriae;o__Acidobacteriales;f__Acidobacteriaceae;g__;s__
LMSG_G000007821.1	no	923_1	Polymetallic	91.78	2.59	78.85	1	0	1	17	Medium quality	86157	3150562	59.80	74	d__Bacteria;p__Acidobacteriota;c__Acidobacteriae;o__Acidobacteriales;f__Acidobacteriaceae;g__;s__
LMSG_G000007822.1	no	923_1	Polymetallic	91.83	1.72	83.21	1	0	1	17	Medium quality	65002	3295355	59.60	99	d__Bacteria;p__Acidobacteriota;c__Acidobacteriae;o__Acidobacteriales;f__Acidobacteriaceae;g__;s__
LMSG_G000007823.1	no	923_1	Magnetite	91.40	1.72	82.78	1	0	1	16	Medium quality	74725	2946885	59.80	78	d__Bacteria;p__Acidobacteriota;c__Acidobacteriae;o__Acidobacteriales;f__Acidobacteriaceae;g__;s__
LMSG_G000007824.1	no	923_1	Pyrite-Copper	89.65	1.72	81.03	0	0	0	15	Medium quality	19051	2669552	60.20	220	d__Bacteria;p__Acidobacteriota;c__Acidobacteriae;o__Acidobacteriales;f__Acidobacteriaceae;g__;s__
LMSG_G000007825.1	no	923_1	Pyrite-Copper	88.35	1.03	83.18	1	0	1	16	Medium quality	82983	2886857	59.90	85	d__Bacteria;p__Acidobacteriota;c__Acidobacteriae;o__Acidobacteriales;f__Acidobacteriaceae;g__;s__
LMSG_G000007826.1	no	923_1	Pyrite-Copper	92.16	1.72	83.54	1	0	1	16	Medium quality	60827	3191796	59.50	80	d__Bacteria;p__Acidobacteriota;c__Acidobacteriae;o__Acidobacteriales;f__Acidobacteriaceae;g__;s__
LMSG_G000007827.1	no	923_1	Pyrite	89.24	0.86	84.93	1	0	1	18	Medium quality	65612	3251252	59.30	80	d__Bacteria;p__Acidobacteriota;c__Acidobacteriae;o__Acidobacteriales;f__Acidobacteriaceae;g__;s__
LMSG_G000007828.1	no	1028_1	Lead-Zinc	95.51	2.66	82.22	0	0	0	19	Medium quality	19312	4198911	59.80	366	d__Bacteria;p__Acidobacteriota;c__Acidobacteriae;o__Acidobacteriales;f__Acidobacteriaceae;g__Acidobacterium;s__
LMSG_G000007829.1	no	1028_1	Lead-Zinc	99.57	1.88	90.17	0	1	0	19	Medium quality	54446	3592706	60.30	104	d__Bacteria;p__Acidobacteriota;c__Acidobacteriae;o__Acidobacteriales;f__Acidobacteriaceae;g__Acidobacterium;s__
LMSG_G000007830.1	no	1028_1	Lead-Zinc	59.10	1.72	50.48	1	0	1	14	Medium quality	3452	3041912	60.10	940	d__Bacteria;p__Acidobacteriota;c__Acidobacteriae;o__Acidobacteriales;f__Acidobacteriaceae;g__Acidobacterium;s__

LMSG_G000007831.1	no	1028_1	Polymetallic	68.80	1.47	61.45	0	0	0	13	Medium quality	16554	2446632	60.90	225	d__Bacteria;p__Acidobacteriota;c__Acidobacteriae;o__Acidobacteriales;f__Acidobacteriaceae;g__Acidobacterium;s__
LMSG_G000007832.1	no	1028_1	Polymetallic	97.86	0.85	93.59	0	1	0	18	Medium quality	42328	3820418	59.80	150	d__Bacteria;p__Acidobacteriota;c__Acidobacteriae;o__Acidobacteriales;f__Acidobacteriaceae;g__Acidobacterium;s__
LMSG_G000007833.1	no	1028_1	Magnetite	93.87	1.71	85.33	0	1	0	19	Medium quality	38404	3616945	60.10	143	d__Bacteria;p__Acidobacteriota;c__Acidobacteriae;o__Acidobacteriales;f__Acidobacteriaceae;g__Acidobacterium;s__
LMSG_G000007834.1	no	1028_1	Lead-Zinc	94.44	1.28	88.03	0	0	0	19	Medium quality	39307	3866646	60.00	156	d__Bacteria;p__Acidobacteriota;c__Acidobacteriae;o__Acidobacteriales;f__Acidobacteriaceae;g__Acidobacterium;s__
LMSG_G000007835.1	no	900_1	Polymetallic	94.18	1.77	85.32	0	0	0	17	Medium quality	18184	3790474	61.90	283	d__Bacteria;p__Acidobacteriota;c__Acidobacteriae;o__Acidobacteriales;f__Acidobacteriaceae;g__Granulicella_A;s__
LMSG_G000007836.1	no	900_1	Polymetallic	83.81	1.72	75.19	0	0	0	18	Medium quality	10761	3533936	61.50	454	d__Bacteria;p__Acidobacteriota;c__Acidobacteriae;o__Acidobacteriales;f__Acidobacteriaceae;g__Granulicella_A;s__
LMSG_G000007837.1	no	900_1	Polymetallic	80.14	1.20	74.13	0	1	0	12	Medium quality	5143	3407924	61.90	776	d__Bacteria;p__Acidobacteriota;c__Acidobacteriae;o__Acidobacteriales;f__Acidobacteriaceae;g__Granulicella_A;s__
LMSG_G000007838.1	no	900_1	Polymetallic	95.04	0.14	94.33	0	1	0	18	Medium quality	46055	4019469	61.80	166	d__Bacteria;p__Acidobacteriota;c__Acidobacteriae;o__Acidobacteriales;f__Acidobacteriaceae;g__Granulicella_A;s__
LMSG_G000007839.1	no	900_1	Polymetallic	89.79	0.00	89.79	0	0	0	17	Medium quality	14481	3432940	61.90	328	d__Bacteria;p__Acidobacteriota;c__Acidobacteriae;o__Acidobacteriales;f__Acidobacteriaceae;g__Granulicella_A;s__
LMSG_G000007840.1	no	901_1	Lead-Zinc	98.92	2.21	87.88	1	1	1	18	High quality	44691	3703558	61.00	106	d__Bacteria;p__Acidobacteriota;c__Acidobacteriae;o__Acidobacteriales;f__Acidobacteriaceae;g__Granulicella_A;s__
LMSG_G000007841.1	no	901_1	Magnetite	80.24	0.00	80.24	0	1	0	19	Medium quality	36856	3319014	60.40	143	d__Bacteria;p__Acidobacteriota;c__Acidobacteriae;o__Acidobacteriales;f__Acidobacteriaceae;g__Granulicella_A;s__
LMSG_G000007842.1	no	901_1	Magnetite	94.61	1.72	85.99	1	1	0	19	Medium quality	55078	3725532	60.70	126	d__Bacteria;p__Acidobacteriota;c__Acidobacteriae;o__Acidobacteriales;f__Acidobacteriaceae;g__Granulicella_A;s__
LMSG_G000007843.1	no	901_1	Pyrite	66.91	1.72	58.29	1	0	0	13	Medium quality	2494	2167348	62.20	951	d__Bacteria;p__Acidobacteriota;c__Acidobacteriae;o__Acidobacteriales;f__Acidobacteriaceae;g__Granulicella_A;s__
LMSG_G000007844.1	no	901_1	Pyrite	51.39	0.00	51.39	0	0	0	10	Medium quality	4115	1608534	62.10	432	d__Bacteria;p__Acidobacteriota;c__Acidobacteriae;o__Acidobacteriales;f__Acidobacteriaceae;g__Granulicella_A;s__
LMSG_G000007845.1	no	901_1	Pyrite	83.99	0.31	82.44	1	1	0	19	Medium quality	9544	3342889	61.70	515	d__Bacteria;p__Acidobacteriota;c__Acidobacteriae;o__Acidobacteriales;f__Acidobacteriaceae;g__Granulicella_A;s__
LMSG_G000007846.1	no	901_1	Pyrite	88.36	2.49	75.91	1	1	1	15	Medium quality	12741	3520671	61.50	372	d__Bacteria;p__Acidobacteriota;c__Acidobacteriae;o__Acidobacteriales;f__Acidobacteriaceae;g__Granulicella_A;s__
LMSG_G000007847.1	no	901_1	Copper	61.48	0.00	61.48	0	0	0	16	Medium quality	4799	2144462	62.00	561	d__Bacteria;p__Acidobacteriota;c__Acidobacteriae;o__Acidobacteriales;f__Acidobacteriaceae;g__Granulicella_A;s__
LMSG_G000007848.1	no	901_1	Copper	70.83	1.72	62.21	1	0	0	15	Medium quality	4270	2596116	61.90	792	d__Bacteria;p__Acidobacteriota;c__Acidobacteriae;o__Acidobacteriales;f__Acidobacteriaceae;g__Granulicella_A;s__
LMSG_G000007849.1	no	902_1	Pyrite	90.15	0.57	87.28	1	1	2	17	Medium quality	25927	3919637	61.40	222	d__Bacteria;p__Acidobacteriota;c__Acidobacteriae;o__Acidobacteriales;f__Acidobacteriaceae;g__Granulicella_A;s__
LMSG_G000007850.1	no	902_1	Pyrite	93.10	0.86	88.79	0	0	0	19	Medium quality	29085	4005964	61.30	195	d__Bacteria;p__Acidobacteriota;c__Acidobacteriae;o__Acidobacteriales;f__Acidobacteriaceae;g__Granulicella_A;s__
LMSG_G000007851.1	no	902_1	Pyrite	93.63	3.97	73.79	1	0	2	17	Medium quality	24903	4150147	61.50	386	d__Bacteria;p__Acidobacteriota;c__Acidobacteriae;o__Acidobacteriales;f__Acidobacteriaceae;g__Granulicella_A;s__
LMSG_G000007852.1	no	902_1	Pyrite	93.60	3.45	76.36	0	1	0	18	Medium quality	27772	4370755	61.30	251	d__Bacteria;p__Acidobacteriota;c__Acidobacteriae;o__Acidobacteriales;f__Acidobacteriaceae;g__Granulicella_A;s__
LMSG_G000007853.1	no	902_1	Copper	95.85	0.43	93.70	0	0	0	18	Medium quality	15417	4127350	61.40	396	d__Bacteria;p__Acidobacteriota;c__Acidobacteriae;o__Acidobacteriales;f__Acidobacteriaceae;g__Granulicella_A;s__
LMSG_G000007854.1	no	926_1	Copper	67.95	0.22	66.88	1	0	1	9	Medium quality	2929	1993522	64.30	770	d__Bacteria;p__Acidobacteriota;c__Acidobacteriae;o__Acidobacteriales;f__Acidobacteriaceae;g__PALSA-350;s__
LMSG_G000007855.1	no	926_1	Pyrite-Copper	61.52	1.72	52.90	0	0	0	11	Medium quality	3309	2022747	64.60	668	d__Bacteria;p__Acidobacteriota;c__Acidobacteriae;o__Acidobacteriales;f__Acidobacteriaceae;g__PALSA-350;s__
LMSG_G000007856.1	no	926_1	Lead-Zinc	76.93	1.72	68.31	0	0	0	13	Medium quality	4789	3159845	63.70	789	d__Bacteria;p__Acidobacteriota;c__Acidobacteriae;o__Acidobacteriales;f__Acidobacteriaceae;g__PALSA-350;s__
LMSG_G000007857.1	no	1521_1	Polymetallic	94.82	2.21	83.78	1	1	1	18	High quality	76379	3700975	61.50	74	d__Bacteria;p__Acidobacteriota;c__Acidobacteriae;o__Acidobacteriales;f__Acidobacteriaceae;g__Terracidiphilus;s__
LMSG_G000007858.1	no	1521_1	Polymetallic	74.28	3.88	54.89	0	1	0	14	Medium quality	4390	2891581	62.20	837	d__Bacteria;p__Acidobacteriota;c__Acidobacteriae;o__Acidobacteriales;f__Acidobacteriaceae;g__Terracidiphilus;s__
LMSG_G000007859.1	no	1521_1	Magnetite	90.08	2.97	75.24	1	0	1	17	Medium quality	52854	3728193	61.40	141	d__Bacteria;p__Acidobacteriota;c__Acidobacteriae;o__Acidobacteriales;f__Acidobacteriaceae;g__Terracidiphilus;s__
LMSG_G000007860.1	no	909_1	Polymetallic	89.38	1.44	82.20	1	1	1	18	Medium quality	54778	3526011	60.10	292	d__Bacteria;p__Acidobacteriota;c__Acidobacteriae;o__Acidobacteriales;f__Acidobacteriaceae;g__Terracidiphilus;s__
LMSG_G000007861.1	no	909_1	Polymetallic	79.95	0.86	75.64	2	1	1	14	Medium quality	25424	2391478	60.90	144	d__Bacteria;p__Acidobacteriota;c__Acidobacteriae;o__Acidobacteriales;f__Acidobacteriaceae;g__Terracidiphilus;s__
LMSG_G000007862.1	no	910_1	Antimony	94.01	4.31	72.46	1	1	1	18	High quality	59996	3240579	61.70	95	d__Bacteria;p__Acidobacteriota;c__Acidobacteriae;o__Acidobacteriales;f__Acidobacteriaceae;g__Terracidiphilus;s__
LMSG_G000007863.1	no	910_1	Pyrite	85.20	2.43	73.06	1	0	1	15	Medium quality	66564	2919015	61.60	84	d__Bacteria;p__Acidobacteriota;c__Acidobacteriae;o__Acidobacteriales;f__Acidobacteriaceae;g__Terracidiphilus;s__
LMSG_G000007864.1	no	910_1	Lead-Zinc	55.24	0.00	55.24	1	0	1	12	Medium quality	3880	2351674	61.40	676	d__Bacteria;p__Acidobacteriota;c__Acidobacteriae;o__Acidobacteriales;f__Acidobacteriaceae;g__Terracidiphilus;s__
LMSG_G000007865.1	no	910_1	Antimony	89.65	3.50	72.14	1	1	0	19	Medium quality	56917	3246993	61.70	98	d__Bacteria;p__Acidobacteriota;c__Acidobacteriae;o__Acidobacteriales;f__Acidobacteriaceae;g__Terracidiphilus;s__
LMSG_G000007866.1	no	910_1	Antimony	93.48	1.77	84.62	1	1	0	19	Medium quality	51255	3695079	61.10	113	d__Bacteria;p__Acidobacteriota;c__Acidobacteriae;o__Acidobacteriales;f__Acidobacteriaceae;g__Terracidiphilus;s__
LMSG_G000007867.1	no	911_1	Polymetallic	63.71	2.59	50.78	1	0	1	9	Medium quality	22446	2178286	61.80	140	d__Bacteria;p__Acidobacteriota;c__Acidobacteriae;o__Acidobacteriales;f__Acidobacteriaceae;g__Terracidiphilus;s__
LMSG_G000007868.1	no	911_1	Polymetallic	60.95	1.72	52.33	0	0	0	11	Medium quality	9298	2026840	61.70	284	d__Bacteria;p__Acidobacteriota;c__Acidobacteriae;o__Acidobacteriales;f__Acidobacteriaceae;g__Terracidiphilus;s__
LMSG_G000007869.1	no	911_1	Polymetallic	72.87	4.31	51.32	1	1	1	12	Medium quality	22506	2851096	61.30	169	d__Bacteria;p__Acidobacteriota;c__Acidobacteriae;o__Acidobacteriales;f__Acidobacteriaceae;g__Terracidiphilus;s__
LMSG_G000007870.1	no	911_1	Coal	93.10	3.50	75.62	1	0	1	17	Medium quality	58413	4688855	60.50	293	d__Bacteria;p__Acidobacteriota;c__Acidobacteriae;o__Acidobacteriales;f__Acidobacteriaceae;g__Terracidiphilus;s__
LMSG_G000007871.1	no	911_1	Pyrite-Copper	94.82	2.59	81.89	0	1	0	19	Medium quality	60496	3936615	61.00	134	d__Bacteria;p__Acidobacteriota;c__Acidobacteriae;o__Acidobacteriales;f__Acidobacteriaceae;g__Terracidiphilus;s__
LMSG_G000007872.1	no	911_1	Pyrite-Copper	68.96	1.72	60.34	0	1	0	15	Medium quality	72275	2663328	61.30	83	d__Bacteria;p__Acidobacteriota;c__Acidobacteriae;o__Acidobacteriales;f__Acidobacteriaceae;g__Terracidiphilus;s__
LMSG_G000007873.1	no	911_1	Copper	81.70	4.74	58.00	1	0	1	13	Medium quality	35025	3656596	61.10	181	d__Bacteria;p__Acidobacteriota;c__Acidobacteriae;o__Acidobacteriales;f__Acidobacteriaceae;g__Terracidiphilus;s__
LMSG_G000007874.1	no	913_1	Polymetallic	54.88	0.00	54.88	0	0	0	14	Medium quality	38059	2533785	63.70	103	d__Bacteria;p__Acidobacteriota;c__Acidobacteriae;o__Acidobacteriales;f__Acidobacteriaceae;g__Terracidiphilus;s__
LMSG_G000007875.1	no	927_1	Lead-Zinc	95.68	2.59	82.75	1	1	2	20	High quality	38633	3279652	62.00	153	d__Bacteria;p__Acidobacteriota;c__Acidobacteriae;o__Acidobacteriales;f__Acidobacteriaceae;g__Terracidiphilus;s__
LMSG_G000007876.1	no	927_1	Pyrite-Copper	94.18	1.51	86.64	1	1	1	18	High quality	18219	3205555	62.00	227	d__Bacteria;p__Acidobacteriota;c__Acidobacteriae;o__Acidobacteriales;f__Acidobacteriaceae;g__Terracidiphilus;s__
LMSG_G000007877.1	no	927_1	Lead-Zinc	85.06	1.72	76.44	1	0	1	16	Medium quality	15994	2810346	62.50	252	d__Bacteria;p__Acidobacteriota;c__Acidobacteriae;o__Acidobacteriales;f__Acidobacteriaceae;g__Terracidiphilus;s__
LMSG_G000007878.1	no	927_1	Lead-Zinc	93.01	2.59	80.08	1	1	1	17	Medium quality	55066	3073246	62.30	155	d__Bacteria;p__Acidobacteriota;c__Acidobacteriae;o__Acidobacteriales;f__Acidobacteriaceae;g__Terracidiphilus;s__
LMSG_G000007879.1	no	1005_1	Magnetite	97.00	2.83	82.88	2	1	4	17	Medium quality	80240	2963199	62.10	157	d__Bacteria;p__Acidobacteriota;c__Acidobacteriae;o__Acidobacteriales;f__SCQP01;g__;s__
LMSG_G000007880.1	no	1005_1	Copper	79.81	1.85	70.56	1	0	1	15	Medium quality	3733	2312203	62.30	781	d__Bacteria;p__Acidobacteriota;c__Acidobacteriae;o__Acidobacteriales;f__SCQP01;g__;s__
LMSG_G000007881.1	no	1005_1	Copper	90.95	0.43	88.82	1	0	1	15	Medium quality	20415	2895908	62.00	219	d__Bacteria;p__Acidobacteriota;c__Acidobacteriae;o__Acidobacteriales;f__SCQP01;g__;s__
LMSG_G000007882.1	no	1005_1	Polymetallic	94.44	1.71	85.90	1	0	1	17	Medium quality	31434	2894384	62.10	153	d__Bacteria;p__Acidobacteriota;c__Acidobacteriae;o__Acidobacteriales;f__SCQP01;g__;s__
LMSG_G000007883.1	no	1005_1	Magnetite	65.51	2.14	54.83	1	1	1	14	Medium quality	10552	2835046	60.30	389	d__Bacteria;p__Acidobacteriota;c__Acidobacteriae;o__Acidobacteriales;f__SCQP01;g__;s__

LMSG_G000007884.1	no	1005_1	Magnetite	55.32	0.00	55.32	1	0	1	11	Medium quality	13136	1781873	62.10	205	d__Bacteria;p__Acidobacteriota;c__Acidobacteriae;o__Acidobacteriales;f__SCQP01;g__;s__
LMSG_G000007885.1	no	1005_1	Pyrite	87.41	0.85	83.14	1	0	1	17	Medium quality	5343	2674468	62.30	612	d__Bacteria;p__Acidobacteriota;c__Acidobacteriae;o__Acidobacteriales;f__SCQP01;g__;s__
LMSG_G000007886.1	no	1005_1	Copper	64.60	1.28	58.19	1	0	1	10	Medium quality	2862	1840396	62.90	827	d__Bacteria;p__Acidobacteriota;c__Acidobacteriae;o__Acidobacteriales;f__SCQP01;g__;s__
LMSG_G000007887.1	no	1005_1	Polymetallic	96.58	0.85	92.31	0	1	1	20	Medium quality	38202	2952613	62.00	137	d__Bacteria;p__Acidobacteriota;c__Acidobacteriae;o__Acidobacteriales;f__SCQP01;g__;s__
LMSG_G000007888.1	no	1005_1	Polymetallic	82.66	0.85	78.39	0	1	0	17	Medium quality	5630	2439896	62.40	519	d__Bacteria;p__Acidobacteriota;c__Acidobacteriae;o__Acidobacteriales;f__SCQP01;g__;s__
LMSG_G000007889.1	no	1005_1	Polymetallic	55.92	0.95	51.18	0	0	0	13	Medium quality	2657	1934237	62.40	796	d__Bacteria;p__Acidobacteriota;c__Acidobacteriae;o__Acidobacteriales;f__SCQP01;g__;s__
LMSG_G000007890.1	no	1005_1	Copper	62.75	0.85	58.48	0	0	0	18	Medium quality	10936	2041926	61.30	273	d__Bacteria;p__Acidobacteriota;c__Acidobacteriae;o__Acidobacteriales;f__SCQP01;g__;s__
LMSG_G000007891.1	no	1005_1	Coal	93.82	0.85	89.55	0	1	6	19	Medium quality	50564	2862437	62.10	135	d__Bacteria;p__Acidobacteriota;c__Acidobacteriae;o__Acidobacteriales;f__SCQP01;g__;s__
LMSG_G000007892.1	no	1005_1	Coal	93.16	2.99	78.21	0	0	0	16	Medium quality	35159	2800538	62.20	240	d__Bacteria;p__Acidobacteriota;c__Acidobacteriae;o__Acidobacteriales;f__SCQP01;g__;s__
LMSG_G000007893.1	no	1005_1	Antimony	76.01	1.72	67.39	0	1	0	17	Medium quality	6440	2390244	62.30	451	d__Bacteria;p__Acidobacteriota;c__Acidobacteriae;o__Acidobacteriales;f__SCQP01;g__;s__
LMSG_G000007894.1	no	1005_1	Copper	94.87	0.00	94.87	1	1	1	19	High quality	61711	3099621	61.60	110	d__Bacteria;p__Acidobacteriota;c__Acidobacteriae;o__Acidobacteriales;f__SCQP01;g__;s__
LMSG_G000007895.1	no	1033_1	Coal	90.88	1.07	85.52	1	1	1	20	High quality	87972	3801990	68.00	152	d__Bacteria;p__Acidobacteriota;c__Acidobacteriae;o__Acidobacteriales;f__SCQP01;g__;s__
LMSG_G000007896.1	no	1033_1	Coal	95.29	1.03	90.17	1	1	2	20	High quality	100676	3809711	68.00	75	d__Bacteria;p__Acidobacteriota;c__Acidobacteriae;o__Acidobacteriales;f__SCQP01;g__;s__
LMSG_G000007897.1	no	1033_1	Copper	81.31	0.85	77.04	0	0	0	16	Medium quality	11002	3059935	68.00	375	d__Bacteria;p__Acidobacteriota;c__Acidobacteriae;o__Acidobacteriales;f__SCQP01;g__;s__
LMSG_G000007898.1	no	1033_1	Polymetallic	92.59	2.74	78.92	1	0	1	15	Medium quality	10291	3350605	68.10	474	d__Bacteria;p__Acidobacteriota;c__Acidobacteriae;o__Acidobacteriales;f__SCQP01;g__;s__
LMSG_G000007899.1	no	1033_1	Magnetite	89.52	1.88	80.12	1	0	1	16	Medium quality	7324	3154990	68.20	586	d__Bacteria;p__Acidobacteriota;c__Acidobacteriae;o__Acidobacteriales;f__SCQP01;g__;s__
LMSG_G000007900.1	no	1033_1	Copper	91.45	4.27	70.09	1	0	1	17	Medium quality	17071	3756228	68.30	676	d__Bacteria;p__Acidobacteriota;c__Acidobacteriae;o__Acidobacteriales;f__SCQP01;g__;s__
LMSG_G000007901.1	no	1033_1	Copper	63.98	2.56	51.18	1	0	1	9	Medium quality	1989	2370590	67.70	1250	d__Bacteria;p__Acidobacteriota;c__Acidobacteriae;o__Acidobacteriales;f__SCQP01;g__;s__
LMSG_G000007902.1	no	1030_1	Coal	87.03	0.43	84.90	1	0	1	15	Medium quality	15764	2778739	69.80	258	d__Bacteria;p__Acidobacteriota;c__Acidobacteriae;o__Acidobacteriales;f__SCQP01;g__SCQP01;s__
LMSG_G000007903.1	no	1519_1	Polymetallic	95.65	1.79	86.69	1	1	1	19	High quality	25839	5308811	63.40	304	d__Bacteria;p__Acidobacteriota;c__Acidobacteriae;o__Bryobacterales;f__Bryobacteraceae;g__;s__
LMSG_G000007904.1	no	1519_1	Polymetallic	83.75	0.88	79.37	0	1	0	16	Medium quality	12365	4128167	63.30	410	d__Bacteria;p__Acidobacteriota;c__Acidobacteriae;o__Bryobacterales;f__Bryobacteraceae;g__;s__
LMSG_G000007905.1	no	1519_1	Polymetallic	58.98	0.92	54.37	0	1	1	14	Medium quality	4129	3662535	63.30	974	d__Bacteria;p__Acidobacteriota;c__Acidobacteriae;o__Bryobacterales;f__Bryobacteraceae;g__;s__
LMSG_G000007906.1	no	1520_1	Antimony	89.58	2.17	78.72	1	1	1	18	Medium quality	9340	4921277	63.50	617	d__Bacteria;p__Acidobacteriota;c__Acidobacteriae;o__Bryobacterales;f__Bryobacteraceae;g__;s__
LMSG_G000007907.1	no	1520_1	Antimony	80.58	1.79	71.65	1	0	1	18	Medium quality	7770	4741962	63.60	743	d__Bacteria;p__Acidobacteriota;c__Acidobacteriae;o__Bryobacterales;f__Bryobacteraceae;g__;s__
LMSG_G000007908.1	no	1400_1	Lead-Zinc	98.24	3.03	83.08	0	0	0	18	Medium quality	13435	6177066	66.80	686	d__Bacteria;p__Acidobacteriota;c__Acidobacteriae;o__Bryobacterales;f__Bryobacteraceae;g__UBA690;s__
LMSG_G000007909.1	no	997_1	Copper	92.09	2.56	79.27	0	0	1	20	Medium quality	59263	3070472	59.90	81	d__Bacteria;p__Acidobacteriota;c__Acidobacteriae;o__UBA7540;f__;g__;s__
LMSG_G000007910.1	no	995_1	Pyrite-Copper	84.44	3.42	67.35	1	1	2	17	Medium quality	8262	2764794	59.10	463	d__Bacteria;p__Acidobacteriota;c__Acidobacteriae;o__UBA7540;f__UBA7540;g__;s__
LMSG_G000007911.1	no	996_1	Pyrite-Copper	69.82	3.45	52.58	1	0	0	13	Medium quality	26797	2520708	61.30	211	d__Bacteria;p__Acidobacteriota;c__Acidobacteriae;o__UBA7540;f__UBA7540;g__;s__
LMSG_G000007912.1	no	998_1	Copper	94.60	1.71	86.06	1	2	1	20	High quality	119407	3347843	59.80	56	d__Bacteria;p__Acidobacteriota;c__Acidobacteriae;o__UBA7540;f__UBA7540;g__;s__
LMSG_G000007913.1	no	998_1	Copper	95.17	1.71	86.63	2	2	2	20	High quality	106745	3370394	59.90	62	d__Bacteria;p__Acidobacteriota;c__Acidobacteriae;o__UBA7540;f__UBA7540;g__;s__
LMSG_G000007914.1	no	998_1	Copper	95.45	1.71	86.91	2	1	1	20	High quality	111909	3333933	59.80	47	d__Bacteria;p__Acidobacteriota;c__Acidobacteriae;o__UBA7540;f__UBA7540;g__;s__
LMSG_G000007915.1	no	994_1	Copper	64.90	0.00	64.90	0	2	1	15	Medium quality	43315	2283103	58.60	92	d__Bacteria;p__Acidobacteriota;c__Acidobacteriae;o__UBA7540;f__UBA7540;g__RH2-MAG17b;s__
LMSG_G000007916.1	no	992_1	Pyrite-Copper	74.46	2.81	60.43	0	0	0	17	Medium quality	7052	2892498	57.70	515	d__Bacteria;p__Acidobacteriota;c__Acidobacteriae;o__UBA7540;f__UBA7540;g__SCRD01;s__
LMSG_G000007917.1	no	1207_1	Lead-Zinc	91.66	3.42	74.57	1	1	2	18	High quality	64182	3856476	62.90	96	d__Bacteria;p__Acidobacteriota;c__Acidobacteriae;o__UBA7541;f__;g__;s__
LMSG_G000007918.1	no	1207_1	Lead-Zinc	94.23	3.42	77.14	1	1	1	19	High quality	54273	3929394	63.00	102	d__Bacteria;p__Acidobacteriota;c__Acidobacteriae;o__UBA7541;f__;g__;s__
LMSG_G000007919.1	no	1207_1	Lead-Zinc	94.23	3.85	75.00	1	1	1	19	High quality	47547	3955565	63.00	112	d__Bacteria;p__Acidobacteriota;c__Acidobacteriae;o__UBA7541;f__;g__;s__
LMSG_G000007920.1	no	1207_1	Lead-Zinc	94.23	3.42	77.14	1	1	1	19	High quality	63341	4025201	63.00	94	d__Bacteria;p__Acidobacteriota;c__Acidobacteriae;o__UBA7541;f__;g__;s__
LMSG_G000007921.1	no	1207_1	Pyrite-Copper	94.23	3.42	77.14	1	1	1	19	High quality	64576	3949019	63.00	89	d__Bacteria;p__Acidobacteriota;c__Acidobacteriae;o__UBA7541;f__;g__;s__
LMSG_G000007922.1	no	293_1	Coal	80.50	0.57	77.66	0	1	1	19	Medium quality	8494	2841317	57.60	398	d__Bacteria;p__Acidobacteriota;c__Acidobacteriae;o__UBA7541;f__UBA7541;g__;s__
LMSG_G000007923.1	no	1348_1	Lead-Zinc	84.03	3.61	66.00	0	1	0	16	Medium quality	6667	2578473	62.20	489	d__Bacteria;p__Acidobacteriota;c__Holophagae;o__Holophagales;f__Holophagaceae;g__;s__
LMSG_G000007924.1	no	1348_1	Lead-Zinc	74.96	0.88	70.58	0	1	1	13	Medium quality	2785	2338018	62.00	944	d__Bacteria;p__Acidobacteriota;c__Holophagae;o__Holophagales;f__Holophagaceae;g__;s__
LMSG_G000007925.1	no	1348_1	Polymetallic	62.89	0.88	58.51	1	0	1	14	Medium quality	3587	1737449	62.20	601	d__Bacteria;p__Acidobacteriota;c__Holophagae;o__Holophagales;f__Holophagaceae;g__;s__
LMSG_G000007926.1	no	1348_1	Polymetallic	64.66	0.69	61.23	2	0	0	12	Medium quality	2796	2024008	62.20	809	d__Bacteria;p__Acidobacteriota;c__Holophagae;o__Holophagales;f__Holophagaceae;g__;s__
LMSG_G000007927.1	no	1349_1	Lead-Zinc	65.78	0.00	65.78	1	0	0	17	Medium quality	31899	2836431	70.00	311	d__Bacteria;p__Acidobacteriota;c__Holophagae;o__Holophagales;f__Holophagaceae;g__;s__
LMSG_G000007928.1	no	1350_1	Polymetallic	62.47	0.35	60.74	2	2	0	20	Medium quality	6645	2068738	67.90	378	d__Bacteria;p__Acidobacteriota;c__Holophagae;o__Holophagales;f__Holophagaceae;g__;s__
LMSG_G000007929.1	no	1164_1	Antimony	100.00	4.75	76.26	1	2	1	20	High quality	58765	4828491	64.50	120	d__Bacteria;p__Acidobacteriota;c__Thermoanaerobacula;o__Gp7-AA8;f__Gp7-AA8;g__;s__
LMSG_G000007930.1	no	1164_1	Antimony	83.97	2.99	69.02	1	1	2	19	Medium quality	7648	4253938	64.60	671	d__Bacteria;p__Acidobacteriota;c__Thermoanaerobacula;o__Gp7-AA8;f__Gp7-AA8;g__QHVT01;s__
LMSG_G000007931.1	no	1204_1	Polymetallic	73.27	3.45	56.03	0	0	0	14	Medium quality	6276	2699526	72.10	545	d__Bacteria;p__Acidobacteriota;c__Thermoanaerobacula;o__Thermoanaerobaculales;f__Thermoanaerobaculaceae;g__RBG-13-68-16;s__
LMSG_G000007932.1	no	1204_2	Antimony	76.49	3.85	57.26	0	1	0	11	Medium quality	6009	1840071	71.20	371	d__Bacteria;p__Acidobacteriota;c__Thermoanaerobacula;o__Thermoanaerobaculales;f__Thermoanaerobaculaceae;g__RBG-13-68-16;s__
LMSG_G000007933.1	no	1204_2	Antimony	82.26	4.62	59.15	1	0	0	15	Medium quality	5592	2360449	71.50	510	d__Bacteria;p__Acidobacteriota;c__Thermoanaerobacula;o__Thermoanaerobaculales;f__Thermoanaerobaculaceae;g__RBG-13-68-16;s__
LMSG_G000007934.1	no	1204_2	Antimony	50.99	0.00	50.99	0	0	0	3	Medium quality	5805	1070807	71.10	221	d__Bacteria;p__Acidobacteriota;c__Thermoanaerobacula;o__Thermoanaerobaculales;f__Thermoanaerobaculaceae;g__RBG-13-68-16;s__
LMSG_G000007935.1	no	1203_1	Nickel-Copper	80.76	3.99	60.82	2	1	2	19	Medium quality	10341	2989604	70.00	436	d__Bacteria;p__Acidobacteriota;c__Thermoanaerobacula;o__Thermoanaerobaculales;f__Thermoanaerobaculaceae;g__RBG-13-68-16;s__RBG-13-68-16 sp002279285
LMSG_G000007936.1	no	715_1	Coal	98.29	1.45	91.03	2	1	2	20	High quality	24519	4289760	70.00	272	d__Bacteria;p__Acidobacteriota;c__Thermoanaerobacula;o__UBA5066;f__Gp7-AA6;g__;s__
LMSG_G000007937.1	no	1394_1	Polymetallic	92.73	3.42	75.64	1	1	1	19	High quality	60937	3273083	65.50	258	d__Bacteria;p__Acidobacteriota;c__UBA4820;o__UBA4820;f__;g__;s__
LMSG_G000007938.1	no	1394_1	Lead-Zinc	88.46	2.56	75.64	0	0	0	19	Medium quality	43410	3278271	65.40	128	d__Bacteria;p__Acidobacteriota;c__UBA4820;o__UBA4820;f__;g__;s__
LMSG_G000007939.1	no	1394_1	Antimony	93.16	3.42	76.07	0	0	0	18	Medium quality	17969	3057580	65.50	235	d__Bacteria;p__Acidobacteriota;c__UBA4820;o__UBA4820;f__;g__;s__
LMSG_G000007940.1	no	1394_1	Antimony	82.78	1.99	72.81	0	0	0	15	Medium quality	4232	2710345	65.70	725	d__Bacteria;p__Acidobacteriota;c__UBA4820;o__UBA4820;f__;g__;s__
LMSG_G000007941.1	no	1395_1	Pyrite-Copper	77.77	0.00	77.77	1	1	1	18	Medium quality	35790	2596132	65.60	105	d__Bacteria;p__Acidobacteriota;c__UBA4820;o__UBA4820;f__;g__;s__
LMSG_G000007942.1	no	1395_2	Antimony	96.58	1.71	88.04	1	1	1	19	High quality	31286	3357361	67.20	190	d__Bacteria;p__Acidobacteriota;c__UBA4820;o__UBA4820;f__;g__;s__
LMSG_G000007943.1	no	1395_2	Antimony	94.87	3.42	77.78	1	1	1	18	High quality	26027	3464709	66.90	195	d__Bacteria;p__Acidobacteriota;c__UBA4820;o__UBA4820;f__;g__;s__
LMSG_G000007944.1	no	1395_2	Antimony	97.43	2.56	84.61	2	3	4	20	High quality	45761	3456631	67.10	295	d__Bacteria;p__Acidobacteriota;c__UBA4820;o__UBA4820;f__;g__;s__
LMSG_G000007945.1	no	1395_2	Antimony	97.43	2.56	84.61	0	1	1	19	Medium quality	35051	3455872	67.00	185	d__Bacteria;p__Acidobacteriota;c__UBA4820;o__UBA4820;f__;g__;s__
LMSG_G000007946.1	no	1395_2	Pyrite-Copper	50.86	0.00	50.86	1	0	0	14	Medium quality	9557	3113271	67.40	701	d__Bacteria;p__Acidobacteriota;c__UBA4820;o__UBA4820;f__;g__;s__
LMSG_G000007947.1	no	1395_2	Lead-Zinc	76.21	3.42	59.12	0	0	0	14	Medium quality	2826	2375997	67.00	971	d__Bacteria;p__Acidobacteriota;c__UBA4820;o__UBA4820;f__;g__;s__
LMSG_G000007948.1	no	1206_1	Antimony	76.49	1.03	71.37	0	0	0	14	Medium quality	11804	3259479	71.20	361	d__Bacteria;p__Acidobacteriota;c__Vicinamibacteria;o__Vicinamibacterales;f__Fen-181;g__FEN-299;s__
LMSG_G000007949.1	no	1206_1	Antimony	86.58	1.71	78.04	0	0	0	11	Medium quality	14794	3067012	71.40	264	d__Bacteria;p__Acidobacteriota;c__Vicinamibacteria;o__Vicinamibacterales;f__Fen-181;g__FEN-299;s__
LMSG_G000007950.1	no	1206_1	Antimony	93.92	4.44	71.70	0	2	1	20	Medium quality	11377	3906721	70.90	509	d__Bacteria;p__Acidobacteriota;c__Vicinamibacteria;o__Vicinamibacterales;f__Fen-181;g__FEN-299;s__
LMSG_G000007951.1	no	1_1	Tin-Zinc	97.43	1.28	91.02	1	1	1	20	High quality	27196	2099748	37.10	157	d__Bacteria;p__Actinobacteriota;c__Acidimicrobiia;o__Acidimicrobiales;f__;g__;s__
LMSG_G000007952.1	no	348_1	Lead-Zinc	100.00	2.14	89.32	1	1	1	20	High quality	81507	2580195	66.80	48	d__Bacteria;p__Actinobacteriota;c__Acidimicrobiia;o__Acidimicrobiales;f__;g__;s__
LMSG_G000007953.1	no	348_1	Lead-Zinc	86.34	2.99	71.39	0	0	0	16	Medium quality	4929	2164233	66.80	578	d__Bacteria;p__Actinobacteriota;c__Acidimicrobiia;o__Acidimicrobiales;f__;g__;s__
LMSG_G000007954.1	no	357_1	Copper	94.06	0.85	89.79	1	1	1	20	High quality	21914	2962298	64.80	192	d__Bacteria;p__Actinobacteriota;c__Acidimicrobiia;o__Acidimicrobiales;f__;g__;s__
LMSG_G000007955.1	no	357_1	Pyrite-Copper	95.72	2.99	80.77	2	1	1	19	High quality	26940	2756314	65.00	143	d__Bacteria;p__Actinobacteriota;c__Acidimicrobiia;o__Acidimicrobiales;f__;g__;s__
LMSG_G000007956.1	no	357_1	Pyrite-Copper	95.72	1.71	87.18	1	1	1	20	High quality	24669	2936208	64.80	169	d__Bacteria;p__Actinobacteriota;c__Acidimicrobiia;o__Acidimicrobiales;f__;g__;s__
LMSG_G000007957.1	no	357_1	Pyrite	74.58	1.80	65.56	1	1	1	14	Medium quality	3820	2272877	64.90	668	d__Bacteria;p__Actinobacteriota;c__Acidimicrobiia;o__Acidimicrobiales;f__;g__;s__

LMSG_G000007958.1	no	452_1	Lead-Zinc	94.87	2.14	84.19	1	1	7	20	High quality	73969	2634813	51.80	137	d__Bacteria;p__Actinobacteriota;c__Acidimicrobiia;o__Acidimicrobiales;f__;g__;s__
LMSG_G000007959.1	no	452_1	Lead-Zinc	94.87	1.28	88.46	1	1	1	20	High quality	59236	2641082	52.00	105	d__Bacteria;p__Actinobacteriota;c__Acidimicrobiia;o__Acidimicrobiales;f__;g__;s__
LMSG_G000007960.1	no	452_1	Lead-Zinc	92.30	1.38	85.42	2	1	2	19	High quality	47976	2340804	52.30	147	d__Bacteria;p__Actinobacteriota;c__Acidimicrobiia;o__Acidimicrobiales;f__;g__;s__
LMSG_G000007961.1	no	452_1	Lead-Zinc	90.59	1.28	84.18	1	1	1	19	High quality	84943	2363109	52.40	80	d__Bacteria;p__Actinobacteriota;c__Acidimicrobiia;o__Acidimicrobiales;f__;g__;s__
LMSG_G000007962.1	no	452_1	Pyrite-Copper	92.30	1.28	85.89	2	1	2	20	High quality	84599	2595422	52.10	124	d__Bacteria;p__Actinobacteriota;c__Acidimicrobiia;o__Acidimicrobiales;f__;g__;s__
LMSG_G000007963.1	no	452_1	Lead-Zinc	92.30	1.28	85.89	1	1	2	18	High quality	61737	2566049	51.90	110	d__Bacteria;p__Actinobacteriota;c__Acidimicrobiia;o__Acidimicrobiales;f__;g__;s__
LMSG_G000007964.1	no	452_1	Lead-Zinc	87.13	1.28	80.72	1	2	1	20	Medium quality	20723	2247227	52.10	221	d__Bacteria;p__Actinobacteriota;c__Acidimicrobiia;o__Acidimicrobiales;f__;g__;s__
LMSG_G000007965.1	no	452_1	Lead-Zinc	88.03	1.38	81.15	3	1	5	19	Medium quality	27129	2317453	52.20	188	d__Bacteria;p__Actinobacteriota;c__Acidimicrobiia;o__Acidimicrobiales;f__;g__;s__
LMSG_G000007966.1	no	452_1	Pyrite	84.84	2.50	72.36	0	0	0	15	Medium quality	5535	1941412	52.40	447	d__Bacteria;p__Actinobacteriota;c__Acidimicrobiia;o__Acidimicrobiales;f__;g__;s__
LMSG_G000007967.1	no	452_1	Pyrite	73.54	2.99	58.59	1	0	0	12	Medium quality	3468	1900646	52.50	639	d__Bacteria;p__Actinobacteriota;c__Acidimicrobiia;o__Acidimicrobiales;f__;g__;s__
LMSG_G000007968.1	no	452_1	Lead-Zinc	57.73	0.00	57.73	1	1	0	10	Medium quality	2550	1344266	52.50	600	d__Bacteria;p__Actinobacteriota;c__Acidimicrobiia;o__Acidimicrobiales;f__;g__;s__
LMSG_G000007969.1	no	453_1	Pyrite	88.03	2.14	77.35	0	1	0	18	Medium quality	48421	2270406	50.50	103	d__Bacteria;p__Actinobacteriota;c__Acidimicrobiia;o__Acidimicrobiales;f__;g__;s__
LMSG_G000007970.1	no	454_1	Lead-Zinc	65.42	2.56	52.60	0	0	0	16	Medium quality	15167	1396483	49.80	188	d__Bacteria;p__Actinobacteriota;c__Acidimicrobiia;o__Acidimicrobiales;f__;g__;s__
LMSG_G000007971.1	no	454_1	Lead-Zinc	82.90	2.12	72.33	0	0	0	19	Medium quality	17696	1832598	49.90	195	d__Bacteria;p__Actinobacteriota;c__Acidimicrobiia;o__Acidimicrobiales;f__;g__;s__
LMSG_G000007972.1	no	454_1	Tin-Zinc	89.74	1.28	83.33	0	0	0	19	Medium quality	10560	2132039	50.00	319	d__Bacteria;p__Actinobacteriota;c__Acidimicrobiia;o__Acidimicrobiales;f__;g__;s__
LMSG_G000007973.1	no	454_1	Tin-Zinc	88.17	1.28	81.76	1	6	8	18	Medium quality	15443	2021254	50.00	255	d__Bacteria;p__Actinobacteriota;c__Acidimicrobiia;o__Acidimicrobiales;f__;g__;s__
LMSG_G000007974.1	no	454_1	Lead-Zinc	86.32	1.28	79.91	0	0	0	15	Medium quality	25692	1739170	50.00	132	d__Bacteria;p__Actinobacteriota;c__Acidimicrobiia;o__Acidimicrobiales;f__;g__;s__
LMSG_G000007975.1	no	454_1	Polymetallic	83.76	1.28	77.35	0	2	0	12	Medium quality	20442	1719166	50.20	212	d__Bacteria;p__Actinobacteriota;c__Acidimicrobiia;o__Acidimicrobiales;f__;g__;s__
LMSG_G000007976.1	no	454_1	Polymetallic	88.03	1.28	81.62	1	0	1	16	Medium quality	24917	1883837	50.00	186	d__Bacteria;p__Actinobacteriota;c__Acidimicrobiia;o__Acidimicrobiales;f__;g__;s__
LMSG_G000007977.1	no	454_1	Polymetallic	82.47	0.85	78.20	0	1	0	13	Medium quality	12174	1479556	50.10	151	d__Bacteria;p__Actinobacteriota;c__Acidimicrobiia;o__Acidimicrobiales;f__;g__;s__
LMSG_G000007978.1	no	454_1	Polymetallic	88.31	1.28	81.90	0	0	0	16	Medium quality	31587	1873883	49.90	232	d__Bacteria;p__Actinobacteriota;c__Acidimicrobiia;o__Acidimicrobiales;f__;g__;s__
LMSG_G000007979.1	no	454_1	Polymetallic	92.30	1.28	85.89	1	0	1	17	Medium quality	27259	2075998	50.10	185	d__Bacteria;p__Actinobacteriota;c__Acidimicrobiia;o__Acidimicrobiales;f__;g__;s__
LMSG_G000007980.1	no	454_1	Copper	81.53	2.56	68.71	1	0	5	19	Medium quality	12505	2149570	49.90	268	d__Bacteria;p__Actinobacteriota;c__Acidimicrobiia;o__Acidimicrobiales;f__;g__;s__
LMSG_G000007981.1	no	454_1	Copper	86.32	1.28	79.91	1	0	1	19	Medium quality	27649	1965635	50.10	133	d__Bacteria;p__Actinobacteriota;c__Acidimicrobiia;o__Acidimicrobiales;f__;g__;s__
LMSG_G000007982.1	no	454_1	Copper	82.05	1.28	75.64	1	0	1	15	Medium quality	27139	1847511	50.10	135	d__Bacteria;p__Actinobacteriota;c__Acidimicrobiia;o__Acidimicrobiales;f__;g__;s__
LMSG_G000007983.1	no	454_1	Magnetite	94.01	2.14	83.33	1	1	1	20	High quality	37053	2407700	50.30	164	d__Bacteria;p__Actinobacteriota;c__Acidimicrobiia;o__Acidimicrobiales;f__;g__;s__
LMSG_G000007984.1	no	454_1	Polymetallic	87.17	1.28	80.76	1	0	1	14	Medium quality	23112	1837394	50.20	131	d__Bacteria;p__Actinobacteriota;c__Acidimicrobiia;o__Acidimicrobiales;f__;g__;s__
LMSG_G000007985.1	no	454_1	Magnetite	79.77	0.85	75.50	1	0	1	16	Medium quality	19016	1795229	50.30	233	d__Bacteria;p__Actinobacteriota;c__Acidimicrobiia;o__Acidimicrobiales;f__;g__;s__
LMSG_G000007986.1	no	454_1	Magnetite	94.01	1.28	87.60	0	0	1	19	Medium quality	22815	2348733	50.10	267	d__Bacteria;p__Actinobacteriota;c__Acidimicrobiia;o__Acidimicrobiales;f__;g__;s__
LMSG_G000007987.1	no	454_1	Magnetite	84.04	1.28	77.63	0	0	0	18	Medium quality	14989	1828184	50.20	182	d__Bacteria;p__Actinobacteriota;c__Acidimicrobiia;o__Acidimicrobiales;f__;g__;s__
LMSG_G000007988.1	no	454_1	Magnetite	81.70	1.36	74.91	1	0	1	15	Medium quality	8707	1709271	50.10	244	d__Bacteria;p__Actinobacteriota;c__Acidimicrobiia;o__Acidimicrobiales;f__;g__;s__
LMSG_G000007989.1	no	454_1	Copper	81.59	2.13	70.94	0	0	0	16	Medium quality	3772	2146193	50.30	696	d__Bacteria;p__Actinobacteriota;c__Acidimicrobiia;o__Acidimicrobiales;f__;g__;s__
LMSG_G000007990.1	no	454_1	Pyrite-Copper	62.46	1.49	55.03	0	0	0	17	Medium quality	6975	1972362	49.70	502	d__Bacteria;p__Actinobacteriota;c__Acidimicrobiia;o__Acidimicrobiales;f__;g__;s__
LMSG_G000007991.1	no	454_1	Copper	93.16	1.71	84.62	1	2	3	20	High quality	37391	2029040	50.20	133	d__Bacteria;p__Actinobacteriota;c__Acidimicrobiia;o__Acidimicrobiales;f__;g__;s__
LMSG_G000007992.1	no	454_1	Lead-Zinc	89.74	1.28	83.33	1	2	2	17	Medium quality	38351	2105292	50.30	110	d__Bacteria;p__Actinobacteriota;c__Acidimicrobiia;o__Acidimicrobiales;f__;g__;s__
LMSG_G000007993.1	no	454_1	Lead-Zinc	92.99	1.28	86.58	1	0	5	20	Medium quality	49456	2406160	50.20	160	d__Bacteria;p__Actinobacteriota;c__Acidimicrobiia;o__Acidimicrobiales;f__;g__;s__
LMSG_G000007994.1	no	454_1	Lead-Zinc	89.74	1.28	83.33	0	2	0	17	Medium quality	23238	1825512	50.00	122	d__Bacteria;p__Actinobacteriota;c__Acidimicrobiia;o__Acidimicrobiales;f__;g__;s__
LMSG_G000007995.1	no	454_1	Lead-Zinc	87.17	0.43	85.04	0	0	0	15	Medium quality	44291	1849421	50.00	104	d__Bacteria;p__Actinobacteriota;c__Acidimicrobiia;o__Acidimicrobiales;f__;g__;s__
LMSG_G000007996.1	no	454_1	Lead-Zinc	82.62	2.14	71.94	0	0	0	13	Medium quality	26061	1828758	50.10	154	d__Bacteria;p__Actinobacteriota;c__Acidimicrobiia;o__Acidimicrobiales;f__;g__;s__
LMSG_G000007997.1	no	454_1	Pyrite-Copper	88.03	0.85	83.76	0	1	0	16	Medium quality	30833	1901353	50.10	108	d__Bacteria;p__Actinobacteriota;c__Acidimicrobiia;o__Acidimicrobiales;f__;g__;s__
LMSG_G000007998.1	no	454_1	Lead-Zinc	91.88	1.28	85.47	1	0	1	18	Medium quality	26242	2343957	50.30	165	d__Bacteria;p__Actinobacteriota;c__Acidimicrobiia;o__Acidimicrobiales;f__;g__;s__
LMSG_G000007999.1	no	454_1	Lead-Zinc	90.59	1.28	84.18	1	1	4	18	High quality	32930	2088240	49.90	150	d__Bacteria;p__Actinobacteriota;c__Acidimicrobiia;o__Acidimicrobiales;f__;g__;s__
LMSG_G000008000.1	no	454_1	Copper	92.02	0.85	87.75	2	2	2	18	High quality	35423	2046785	50.30	146	d__Bacteria;p__Actinobacteriota;c__Acidimicrobiia;o__Acidimicrobiales;f__;g__;s__
LMSG_G000008001.1	no	454_1	Pyrite	83.76	2.99	68.81	0	0	0	11	Medium quality	27569	1676075	50.00	116	d__Bacteria;p__Actinobacteriota;c__Acidimicrobiia;o__Acidimicrobiales;f__;g__;s__
LMSG_G000008002.1	no	454_1	Pyrite	82.33	1.28	75.92	2	1	7	19	Medium quality	20262	1961368	50.20	154	d__Bacteria;p__Actinobacteriota;c__Acidimicrobiia;o__Acidimicrobiales;f__;g__;s__
LMSG_G000008003.1	no	454_1	Pyrite	83.76	1.28	77.35	1	0	1	15	Medium quality	16879	1791853	50.10	167	d__Bacteria;p__Actinobacteriota;c__Acidimicrobiia;o__Acidimicrobiales;f__;g__;s__
LMSG_G000008004.1	no	454_1	Pyrite	87.17	1.28	80.76	1	0	1	18	Medium quality	17953	1890556	50.20	162	d__Bacteria;p__Actinobacteriota;c__Acidimicrobiia;o__Acidimicrobiales;f__;g__;s__
LMSG_G000008005.1	no	454_1	Copper	90.59	1.71	82.05	2	0	2	17	Medium quality	21649	2309532	50.10	244	d__Bacteria;p__Actinobacteriota;c__Acidimicrobiia;o__Acidimicrobiales;f__;g__;s__
LMSG_G000008006.1	no	454_1	Copper	88.03	1.85	78.78	1	1	1	16	Medium quality	23231	2409465	50.10	220	d__Bacteria;p__Actinobacteriota;c__Acidimicrobiia;o__Acidimicrobiales;f__;g__;s__
LMSG_G000008007.1	no	454_1	Copper	81.19	1.28	74.78	1	2	2	14	Medium quality	22856	1650817	49.90	147	d__Bacteria;p__Actinobacteriota;c__Acidimicrobiia;o__Acidimicrobiales;f__;g__;s__
LMSG_G000008008.1	no	454_1	Copper	83.47	2.14	72.79	0	0	1	13	Medium quality	23340	1924732	49.70	180	d__Bacteria;p__Actinobacteriota;c__Acidimicrobiia;o__Acidimicrobiales;f__;g__;s__
LMSG_G000008009.1	no	454_1	Lead-Zinc	81.02	1.28	74.61	2	0	2	18	Medium quality	17433	1717153	50.00	196	d__Bacteria;p__Actinobacteriota;c__Acidimicrobiia;o__Acidimicrobiales;f__;g__;s__
LMSG_G000008010.1	no	456_1	Lead-Zinc	90.59	0.43	88.46	0	0	0	19	Medium quality	74215	2467872	56.30	128	d__Bacteria;p__Actinobacteriota;c__Acidimicrobiia;o__Acidimicrobiales;f__;g__;s__
LMSG_G000008011.1	no	456_1	Copper	91.45	0.64	88.25	0	1	0	20	Medium quality	39171	2867995	55.90	171	d__Bacteria;p__Actinobacteriota;c__Acidimicrobiia;o__Acidimicrobiales;f__;g__;s__
LMSG_G000008012.1	no	456_1	Lead-Zinc	88.03	1.28	81.62	0	0	0	19	Medium quality	67874	2351182	56.30	54	d__Bacteria;p__Actinobacteriota;c__Acidimicrobiia;o__Acidimicrobiales;f__;g__;s__
LMSG_G000008013.1	no	456_1	Polymetallic	88.88	0.52	86.27	0	0	0	19	Medium quality	102478	2374989	56.30	130	d__Bacteria;p__Actinobacteriota;c__Acidimicrobiia;o__Acidimicrobiales;f__;g__;s__
LMSG_G000008014.1	no	456_1	Polymetallic	90.59	0.43	88.46	0	1	0	20	Medium quality	79699	2441257	56.20	63	d__Bacteria;p__Actinobacteriota;c__Acidimicrobiia;o__Acidimicrobiales;f__;g__;s__
LMSG_G000008015.1	no	456_1	Polymetallic	88.03	0.43	85.90	0	0	0	19	Medium quality	79827	2308905	56.30	48	d__Bacteria;p__Actinobacteriota;c__Acidimicrobiia;o__Acidimicrobiales;f__;g__;s__
LMSG_G000008016.1	no	456_1	Polymetallic	88.88	2.14	78.20	0	0	0	19	Medium quality	80053	2382356	56.30	55	d__Bacteria;p__Actinobacteriota;c__Acidimicrobiia;o__Acidimicrobiales;f__;g__;s__
LMSG_G000008017.1	no	456_1	Copper	88.88	0.43	86.75	0	0	1	19	Medium quality	79169	2405253	56.30	101	d__Bacteria;p__Actinobacteriota;c__Acidimicrobiia;o__Acidimicrobiales;f__;g__;s__
LMSG_G000008018.1	no	456_1	Copper	88.03	0.43	85.90	0	0	0	19	Medium quality	101234	2465583	56.30	122	d__Bacteria;p__Actinobacteriota;c__Acidimicrobiia;o__Acidimicrobiales;f__;g__;s__
LMSG_G000008019.1	no	456_1	Copper	91.45	1.28	85.04	0	1	0	20	Medium quality	75888	2531164	56.20	60	d__Bacteria;p__Actinobacteriota;c__Acidimicrobiia;o__Acidimicrobiales;f__;g__;s__
LMSG_G000008020.1	no	456_1	Polymetallic	87.17	0.43	85.04	1	1	1	20	Medium quality	42583	2482321	56.20	126	d__Bacteria;p__Actinobacteriota;c__Acidimicrobiia;o__Acidimicrobiales;f__;g__;s__
LMSG_G000008021.1	no	456_1	Magnetite	85.53	0.43	83.40	1	2	0	17	Medium quality	15095	2369925	56.30	269	d__Bacteria;p__Actinobacteriota;c__Acidimicrobiia;o__Acidimicrobiales;f__;g__;s__
LMSG_G000008022.1	no	456_1	Copper	88.88	0.43	86.75	0	0	0	19	Medium quality	91230	2395739	56.30	86	d__Bacteria;p__Actinobacteriota;c__Acidimicrobiia;o__Acidimicrobiales;f__;g__;s__
LMSG_G000008023.1	no	456_1	Magnetite	69.08	1.45	61.82	1	0	1	19	Medium quality	12382	2157041	56.10	268	d__Bacteria;p__Actinobacteriota;c__Acidimicrobiia;o__Acidimicrobiales;f__;g__;s__
LMSG_G000008024.1	no	599_1	Copper	93.16	1.28	86.75	1	1	1	20	High quality	18571	2450993	47.20	197	d__Bacteria;p__Actinobacteriota;c__Acidimicrobiia;o__Acidimicrobiales;f__;g__;s__
LMSG_G000008025.1	no	599_1	Polymetallic	99.14	1.28	92.73	3	1	16	20	High quality	145969	2776696	47.30	124	d__Bacteria;p__Actinobacteriota;c__Acidimicrobiia;o__Acidimicrobiales;f__;g__;s__
LMSG_G000008026.1	no	599_1	Copper	96.58	1.85	87.33	0	1	0	19	Medium quality	13614	2773210	47.40	311	d__Bacteria;p__Actinobacteriota;c__Acidimicrobiia;o__Acidimicrobiales;f__;g__;s__
LMSG_G000008027.1	no	599_1	Polymetallic	94.01	2.14	83.33	1	0	1	20	Medium quality	115331	2304451	47.40	42	d__Bacteria;p__Actinobacteriota;c__Acidimicrobiia;o__Acidimicrobiales;f__;g__;s__
LMSG_G000008028.1	no	599_1	Polymetallic	99.14	2.14	88.46	0	1	0	20	Medium quality	93885	2730382	47.30	73	d__Bacteria;p__Actinobacteriota;c__Acidimicrobiia;o__Acidimicrobiales;f__;g__;s__
LMSG_G000008029.1	no	599_1	Copper	97.43	1.28	91.02	1	1	1	20	High quality	88764	2886584	47.40	72	d__Bacteria;p__Actinobacteriota;c__Acidimicrobiia;o__Acidimicrobiales;f__;g__;s__
LMSG_G000008030.1	no	599_1	Pyrite	77.92	0.43	75.79	0	0	0	15	Medium quality	6243	2155813	47.20	423	d__Bacteria;p__Actinobacteriota;c__Acidimicrobiia;o__Acidimicrobiales;f__;g__;s__
LMSG_G000008031.1	no	599_1	Lead-Zinc	69.84	2.28	58.45	0	0	0	13	Medium quality	2039	2077207	47.50	1082	d__Bacteria;p__Actinobacteriota;c__Acidimicrobiia;o__Acidimicrobiales;f__;g__;s__
LMSG_G000008032.1	no	599_1	Polymetallic	96.58	2.14	85.90	0	1	0	20	Medium quality	29946	2741517	47.30	146	d__Bacteria;p__Actinobacteriota;c__Acidimicrobiia;o__Acidimicrobiales;f__;g__;s__
LMSG_G000008033.1	no	599_1	Pyrite-Copper	93.50	1.28	87.09	0	1	0	19	Medium quality	27836	2359294	47.10	134	d__Bacteria;p__Actinobacteriota;c__Acidimicrobiia;o__Acidimicrobiales;f__;g__;s__
LMSG_G000008034.1	no	599_1	Pyrite-Copper	86.15	1.99	76.18	2	0	2	17	Medium quality	3763	2410339	47.20	733	d__Bacteria;p__Actinobacteriota;c__Acidimicrobiia;o__Acidimicrobiales;f__;g__;s__

LMSG_G000008035.1	no	599_1	Magnetite	96.58	1.57	88.75	0	0	0	20	Medium quality	39644	2748963	47.30	103	d__Bacteria;p__Actinobacteriota;c__Acidimicrobiia;o__Acidimicrobiales;f__;g__;s__
LMSG_G000008036.1	no	599_1	Magnetite	86.75	1.28	80.34	1	0	1	20	Medium quality	45141	2929764	47.40	184	d__Bacteria;p__Actinobacteriota;c__Acidimicrobiia;o__Acidimicrobiales;f__;g__;s__
LMSG_G000008037.1	no	599_1	Magnetite	97.77	2.14	87.09	2	0	3	20	Medium quality	103177	2906527	47.40	117	d__Bacteria;p__Actinobacteriota;c__Acidimicrobiia;o__Acidimicrobiales;f__;g__;s__
LMSG_G000008038.1	no	599_1	Magnetite	93.16	2.14	82.48	0	1	0	19	Medium quality	43159	2769432	47.30	121	d__Bacteria;p__Actinobacteriota;c__Acidimicrobiia;o__Acidimicrobiales;f__;g__;s__
LMSG_G000008039.1	no	599_1	Pyrite-Copper	93.58	2.14	82.90	2	0	2	19	Medium quality	19589	2821511	47.20	307	d__Bacteria;p__Actinobacteriota;c__Acidimicrobiia;o__Acidimicrobiales;f__;g__;s__
LMSG_G000008040.1	no	599_1	Pyrite-Copper	98.29	1.28	91.88	1	1	0	19	Medium quality	32473	2597012	47.40	165	d__Bacteria;p__Actinobacteriota;c__Acidimicrobiia;o__Acidimicrobiales;f__;g__;s__
LMSG_G000008041.1	no	683_1	Lead-Zinc	54.64	0.00	54.64	0	0	0	10	Medium quality	1886	1247041	41.80	684	d__Bacteria;p__Actinobacteriota;c__Acidimicrobiia;o__Acidimicrobiales;f__;g__;s__
LMSG_G000008042.1	no	684_1	Polymetallic	92.67	2.99	77.72	1	2	2	19	High quality	6047	2264431	37.80	474	d__Bacteria;p__Actinobacteriota;c__Acidimicrobiia;o__Acidimicrobiales;f__;g__;s__
LMSG_G000008043.1	no	684_1	Polymetallic	87.72	2.52	75.14	1	1	1	18	Medium quality	4229	2126370	37.80	598	d__Bacteria;p__Actinobacteriota;c__Acidimicrobiia;o__Acidimicrobiales;f__;g__;s__
LMSG_G000008044.1	no	684_1	Polymetallic	84.76	1.28	78.35	0	2	0	18	Medium quality	4101	2215171	37.80	664	d__Bacteria;p__Actinobacteriota;c__Acidimicrobiia;o__Acidimicrobiales;f__;g__;s__
LMSG_G000008045.1	no	684_1	Lead-Zinc	94.44	1.28	88.03	1	1	2	20	High quality	10142	2592762	37.90	375	d__Bacteria;p__Actinobacteriota;c__Acidimicrobiia;o__Acidimicrobiales;f__;g__;s__
LMSG_G000008046.1	no	684_1	Lead-Zinc	96.15	2.56	83.33	3	3	4	20	High quality	26495	2627129	37.70	161	d__Bacteria;p__Actinobacteriota;c__Acidimicrobiia;o__Acidimicrobiales;f__;g__;s__
LMSG_G000008047.1	no	684_1	Lead-Zinc	74.12	1.28	67.71	0	1	0	16	Medium quality	5698	2081872	37.70	430	d__Bacteria;p__Actinobacteriota;c__Acidimicrobiia;o__Acidimicrobiales;f__;g__;s__
LMSG_G000008048.1	no	685_1	Polymetallic	99.14	1.28	92.73	1	1	1	20	High quality	65317	2370928	40.60	76	d__Bacteria;p__Actinobacteriota;c__Acidimicrobiia;o__Acidimicrobiales;f__;g__;s__
LMSG_G000008049.1	no	685_1	Magnetite	98.29	1.28	91.88	1	4	7	20	High quality	62628	2394439	40.50	108	d__Bacteria;p__Actinobacteriota;c__Acidimicrobiia;o__Acidimicrobiales;f__;g__;s__
LMSG_G000008050.1	no	685_1	Magnetite	99.14	1.28	92.73	1	2	1	20	High quality	47575	2360959	40.50	69	d__Bacteria;p__Actinobacteriota;c__Acidimicrobiia;o__Acidimicrobiales;f__;g__;s__
LMSG_G000008051.1	no	685_1	Magnetite	98.29	1.28	91.88	1	0	1	20	Medium quality	46434	2319076	40.60	70	d__Bacteria;p__Actinobacteriota;c__Acidimicrobiia;o__Acidimicrobiales;f__;g__;s__
LMSG_G000008052.1	no	685_1	Magnetite	98.19	1.28	91.78	1	0	1	18	Medium quality	43548	2290572	40.50	76	d__Bacteria;p__Actinobacteriota;c__Acidimicrobiia;o__Acidimicrobiales;f__;g__;s__
LMSG_G000008053.1	no	686_1	Pyrite-Copper	97.43	1.28	91.02	1	2	1	20	High quality	76151	2736324	40.80	104	d__Bacteria;p__Actinobacteriota;c__Acidimicrobiia;o__Acidimicrobiales;f__;g__;s__
LMSG_G000008054.1	no	686_1	Copper	91.45	2.14	80.77	0	4	5	20	Medium quality	11831	2578785	40.80	289	d__Bacteria;p__Actinobacteriota;c__Acidimicrobiia;o__Acidimicrobiales;f__;g__;s__
LMSG_G000008055.1	no	686_1	Copper	98.14	1.38	91.26	0	1	0	20	Medium quality	10670	2484405	40.80	357	d__Bacteria;p__Actinobacteriota;c__Acidimicrobiia;o__Acidimicrobiales;f__;g__;s__
LMSG_G000008056.1	no	686_1	Pyrite-Copper	56.95	1.38	50.07	0	0	0	12	Medium quality	4073	1358108	40.80	374	d__Bacteria;p__Actinobacteriota;c__Acidimicrobiia;o__Acidimicrobiales;f__;g__;s__
LMSG_G000008057.1	no	708_1	Copper	65.38	1.28	58.97	0	0	0	11	Medium quality	13154	1809252	72.90	172	d__Bacteria;p__Actinobacteriota;c__Acidimicrobiia;o__Acidimicrobiales;f__;g__;s__
LMSG_G000008058.1	no	708_1	Pyrite	84.90	4.56	62.11	1	1	0	17	Medium quality	19851	2616142	72.60	188	d__Bacteria;p__Actinobacteriota;c__Acidimicrobiia;o__Acidimicrobiales;f__;g__;s__
LMSG_G000008059.1	no	708_1	Pyrite	77.44	1.88	68.04	0	0	0	15	Medium quality	9330	2091136	72.70	261	d__Bacteria;p__Actinobacteriota;c__Acidimicrobiia;o__Acidimicrobiales;f__;g__;s__
LMSG_G000008060.1	no	708_1	Lead-Zinc	82.33	1.71	73.79	1	0	1	18	Medium quality	17157	2706106	72.40	198	d__Bacteria;p__Actinobacteriota;c__Acidimicrobiia;o__Acidimicrobiales;f__;g__;s__
LMSG_G000008061.1	no	708_1	Lead-Zinc	83.76	2.14	73.08	1	0	1	20	Medium quality	30941	2846319	72.50	147	d__Bacteria;p__Actinobacteriota;c__Acidimicrobiia;o__Acidimicrobiales;f__;g__;s__
LMSG_G000008062.1	no	708_1	Lead-Zinc	83.11	2.14	72.43	1	1	1	18	Medium quality	31281	2812201	72.50	167	d__Bacteria;p__Actinobacteriota;c__Acidimicrobiia;o__Acidimicrobiales;f__;g__;s__
LMSG_G000008063.1	no	708_1	Lead-Zinc	87.93	4.70	64.42	1	1	1	19	Medium quality	33268	3114587	72.30	159	d__Bacteria;p__Actinobacteriota;c__Acidimicrobiia;o__Acidimicrobiales;f__;g__;s__
LMSG_G000008064.1	no	708_1	Lead-Zinc	92.30	3.09	76.87	0	0	0	20	Medium quality	33188	2885924	72.40	140	d__Bacteria;p__Actinobacteriota;c__Acidimicrobiia;o__Acidimicrobiales;f__;g__;s__
LMSG_G000008065.1	no	708_1	Copper	68.80	3.42	51.71	0	1	0	12	Medium quality	6373	2208023	73.00	465	d__Bacteria;p__Actinobacteriota;c__Acidimicrobiia;o__Acidimicrobiales;f__;g__;s__
LMSG_G000008066.1	no	708_1	Polymetallic	67.23	2.14	56.55	0	0	0	16	Medium quality	4510	2369841	72.60	612	d__Bacteria;p__Actinobacteriota;c__Acidimicrobiia;o__Acidimicrobiales;f__;g__;s__
LMSG_G000008067.1	no	719_1	Coal	72.83	2.99	57.88	0	0	0	16	Medium quality	4172	2565360	70.30	698	d__Bacteria;p__Actinobacteriota;c__Acidimicrobiia;o__Acidimicrobiales;f__;g__;s__
LMSG_G000008068.1	no	817_1	Copper	94.44	2.14	83.76	1	2	1	20	High quality	29709	3059169	60.80	275	d__Bacteria;p__Actinobacteriota;c__Acidimicrobiia;o__Acidimicrobiales;f__;g__;s__
LMSG_G000008069.1	no	817_1	Copper	69.84	2.14	59.16	0	0	0	15	Medium quality	4127	2211227	60.90	599	d__Bacteria;p__Actinobacteriota;c__Acidimicrobiia;o__Acidimicrobiales;f__;g__;s__
LMSG_G000008070.1	no	817_1	Copper	91.08	2.14	80.40	0	0	0	20	Medium quality	20027	2814362	60.70	198	d__Bacteria;p__Actinobacteriota;c__Acidimicrobiia;o__Acidimicrobiales;f__;g__;s__
LMSG_G000008071.1	no	817_1	Copper	88.11	2.14	77.43	1	0	1	20	Medium quality	22080	2903942	60.70	206	d__Bacteria;p__Actinobacteriota;c__Acidimicrobiia;o__Acidimicrobiales;f__;g__;s__
LMSG_G000008072.1	no	817_1	Polymetallic	88.46	4.37	66.62	1	0	0	20	Medium quality	9267	2656091	60.60	357	d__Bacteria;p__Actinobacteriota;c__Acidimicrobiia;o__Acidimicrobiales;f__;g__;s__
LMSG_G000008073.1	no	817_1	Polymetallic	89.74	2.14	79.06	0	0	0	20	Medium quality	12354	2668257	60.70	316	d__Bacteria;p__Actinobacteriota;c__Acidimicrobiia;o__Acidimicrobiales;f__;g__;s__
LMSG_G000008074.1	no	817_1	Polymetallic	79.62	1.57	71.79	0	0	0	16	Medium quality	7018	2395761	60.60	551	d__Bacteria;p__Actinobacteriota;c__Acidimicrobiia;o__Acidimicrobiales;f__;g__;s__
LMSG_G000008075.1	no	817_1	Polymetallic	79.44	3.16	63.63	0	0	0	19	Medium quality	4774	2828403	60.30	707	d__Bacteria;p__Actinobacteriota;c__Acidimicrobiia;o__Acidimicrobiales;f__;g__;s__
LMSG_G000008076.1	no	817_1	Polymetallic	88.88	2.14	78.20	1	1	1	20	Medium quality	22300	3171040	61.00	327	d__Bacteria;p__Actinobacteriota;c__Acidimicrobiia;o__Acidimicrobiales;f__;g__;s__
LMSG_G000008077.1	no	817_1	Tin-Zinc	92.22	2.14	81.54	0	1	0	20	Medium quality	14820	2959561	60.80	314	d__Bacteria;p__Actinobacteriota;c__Acidimicrobiia;o__Acidimicrobiales;f__;g__;s__
LMSG_G000008078.1	no	817_1	Tin-Zinc	91.49	2.14	80.81	0	0	0	20	Medium quality	25710	2814774	60.80	251	d__Bacteria;p__Actinobacteriota;c__Acidimicrobiia;o__Acidimicrobiales;f__;g__;s__
LMSG_G000008079.1	no	817_1	Polymetallic	74.70	1.99	64.73	0	0	0	15	Medium quality	5028	1883380	60.60	422	d__Bacteria;p__Actinobacteriota;c__Acidimicrobiia;o__Acidimicrobiales;f__;g__;s__
LMSG_G000008080.1	no	817_1	Copper	93.16	2.14	82.48	0	1	0	19	Medium quality	18593	3143404	60.90	259	d__Bacteria;p__Actinobacteriota;c__Acidimicrobiia;o__Acidimicrobiales;f__;g__;s__
LMSG_G000008081.1	no	818_1	Pyrite	88.03	1.71	79.49	0	1	0	19	Medium quality	10339	2688530	63.70	381	d__Bacteria;p__Actinobacteriota;c__Acidimicrobiia;o__Acidimicrobiales;f__;g__;s__
LMSG_G000008082.1	no	818_1	Pyrite	85.47	2.23	74.32	0	0	0	17	Medium quality	16021	3059748	63.90	405	d__Bacteria;p__Actinobacteriota;c__Acidimicrobiia;o__Acidimicrobiales;f__;g__;s__
LMSG_G000008083.1	no	818_1	Lead-Zinc	86.60	1.28	80.19	0	1	0	20	Medium quality	11950	2467495	63.80	256	d__Bacteria;p__Actinobacteriota;c__Acidimicrobiia;o__Acidimicrobiales;f__;g__;s__
LMSG_G000008084.1	no	818_1	Lead-Zinc	59.10	1.71	50.56	0	0	0	15	Medium quality	4443	1822142	63.10	459	d__Bacteria;p__Actinobacteriota;c__Acidimicrobiia;o__Acidimicrobiales;f__;g__;s__
LMSG_G000008085.1	no	819_1	Polymetallic	90.45	2.99	75.50	1	1	1	18	High quality	21796	2358342	69.20	140	d__Bacteria;p__Actinobacteriota;c__Acidimicrobiia;o__Acidimicrobiales;f__;g__;s__
LMSG_G000008086.1	no	819_1	Polymetallic	85.47	2.66	72.18	0	1	0	18	Medium quality	10916	2342375	69.20	256	d__Bacteria;p__Actinobacteriota;c__Acidimicrobiia;o__Acidimicrobiales;f__;g__;s__
LMSG_G000008087.1	no	819_1	Polymetallic	83.90	2.65	70.66	0	0	0	15	Medium quality	7290	2159001	69.20	388	d__Bacteria;p__Actinobacteriota;c__Acidimicrobiia;o__Acidimicrobiales;f__;g__;s__
LMSG_G000008088.1	no	819_1	Pyrite-Copper	96.58	2.99	81.63	1	1	1	19	High quality	50584	2422722	69.40	68	d__Bacteria;p__Actinobacteriota;c__Acidimicrobiia;o__Acidimicrobiales;f__;g__;s__
LMSG_G000008089.1	no	819_1	Copper	95.72	2.42	83.62	1	1	1	17	Medium quality	38451	2283647	69.40	232	d__Bacteria;p__Actinobacteriota;c__Acidimicrobiia;o__Acidimicrobiales;f__;g__;s__
LMSG_G000008090.1	no	819_1	Pyrite-Copper	96.58	4.61	73.56	0	1	0	20	Medium quality	49750	2443358	69.40	97	d__Bacteria;p__Actinobacteriota;c__Acidimicrobiia;o__Acidimicrobiales;f__;g__;s__
LMSG_G000008091.1	no	820_1	Copper	97.29	1.71	88.75	1	1	1	19	High quality	27222	3055249	68.00	206	d__Bacteria;p__Actinobacteriota;c__Acidimicrobiia;o__Acidimicrobiales;f__;g__;s__
LMSG_G000008092.1	no	820_1	Copper	94.44	4.70	70.94	1	2	1	19	High quality	24789	2995881	68.00	208	d__Bacteria;p__Actinobacteriota;c__Acidimicrobiia;o__Acidimicrobiales;f__;g__;s__
LMSG_G000008093.1	no	820_1	Copper	97.00	3.85	77.77	1	1	1	20	High quality	18214	4997212	67.80	502	d__Bacteria;p__Actinobacteriota;c__Acidimicrobiia;o__Acidimicrobiales;f__;g__;s__
LMSG_G000008094.1	no	820_1	Copper	95.63	2.14	84.95	1	1	1	18	High quality	23071	3293928	67.70	256	d__Bacteria;p__Actinobacteriota;c__Acidimicrobiia;o__Acidimicrobiales;f__;g__;s__
LMSG_G000008095.1	no	820_1	Copper	95.53	2.21	84.46	1	1	1	19	High quality	14080	3348987	67.90	343	d__Bacteria;p__Actinobacteriota;c__Acidimicrobiia;o__Acidimicrobiales;f__;g__;s__
LMSG_G000008096.1	no	820_1	Lead-Zinc	98.57	2.14	87.89	1	1	1	18	High quality	44159	3128444	68.00	119	d__Bacteria;p__Actinobacteriota;c__Acidimicrobiia;o__Acidimicrobiales;f__;g__;s__
LMSG_G000008097.1	no	820_1	Lead-Zinc	99.14	2.14	88.46	1	1	1	20	High quality	39358	3131356	67.90	114	d__Bacteria;p__Actinobacteriota;c__Acidimicrobiia;o__Acidimicrobiales;f__;g__;s__
LMSG_G000008098.1	no	820_1	Pyrite-Copper	97.43	4.08	77.04	1	1	2	20	High quality	24234	4647757	67.50	341	d__Bacteria;p__Actinobacteriota;c__Acidimicrobiia;o__Acidimicrobiales;f__;g__;s__
LMSG_G000008099.1	no	820_1	Copper	92.30	2.14	81.62	1	1	1	17	Medium quality	23454	2676811	68.10	150	d__Bacteria;p__Actinobacteriota;c__Acidimicrobiia;o__Acidimicrobiales;f__;g__;s__
LMSG_G000008100.1	no	820_1	Copper	81.62	1.88	72.22	0	0	0	13	Medium quality	9171	2267121	68.00	330	d__Bacteria;p__Actinobacteriota;c__Acidimicrobiia;o__Acidimicrobiales;f__;g__;s__
LMSG_G000008101.1	no	820_1	Lead-Zinc	85.37	1.80	76.35	1	1	0	18	Medium quality	9391	3014811	67.80	465	d__Bacteria;p__Actinobacteriota;c__Acidimicrobiia;o__Acidimicrobiales;f__;g__;s__
LMSG_G000008102.1	no	820_1	Lead-Zinc	94.01	4.70	70.51	1	0	1	18	Medium quality	29487	4323322	67.70	293	d__Bacteria;p__Actinobacteriota;c__Acidimicrobiia;o__Acidimicrobiales;f__;g__;s__
LMSG_G000008103.1	no	820_1	Polymetallic	52.54	0.00	52.54	1	0	1	12	Medium quality	4242	1869259	68.20	485	d__Bacteria;p__Actinobacteriota;c__Acidimicrobiia;o__Acidimicrobiales;f__;g__;s__
LMSG_G000008104.1	no	821_1	Polymetallic	94.44	1.79	85.51	1	1	1	18	High quality	9996	2773904	64.80	365	d__Bacteria;p__Actinobacteriota;c__Acidimicrobiia;o__Acidimicrobiales;f__;g__;s__
LMSG_G000008105.1	no	821_1	Polymetallic	79.74	3.42	62.65	0	0	0	13	Medium quality	8252	2221699	65.10	451	d__Bacteria;p__Actinobacteriota;c__Acidimicrobiia;o__Acidimicrobiales;f__;g__;s__
LMSG_G000008106.1	no	821_1	Polymetallic	73.59	1.71	65.05	1	0	1	16	Medium quality	6504	2323932	65.10	430	d__Bacteria;p__Actinobacteriota;c__Acidimicrobiia;o__Acidimicrobiales;f__;g__;s__
LMSG_G000008107.1	no	821_1	Polymetallic	58.99	1.28	52.58	0	0	0	13	Medium quality	3251	1899916	65.00	680	d__Bacteria;p__Actinobacteriota;c__Acidimicrobiia;o__Acidimicrobiales;f__;g__;s__
LMSG_G000008108.1	no	821_1	Copper	92.07	2.66	78.78	0	1	0	19	Medium quality	20720	3043855	65.10	415	d__Bacteria;p__Actinobacteriota;c__Acidimicrobiia;o__Acidimicrobiales;f__;g__;s__
LMSG_G000008109.1	no	823_1	Tin-Zinc	92.49	2.91	77.97	1	1	1	20	High quality	21667	2428060	68.00	144	d__Bacteria;p__Actinobacteriota;c__Acidimicrobiia;o__Acidimicrobiales;f__;g__;s__
LMSG_G000008110.1	no	824_1	Pyrite	65.21	2.14	54.53	0	0	0	13	Medium quality	2397	1556861	64.10	713	d__Bacteria;p__Actinobacteriota;c__Acidimicrobiia;o__Acidimicrobiales;f__;g__;s__
LMSG_G000008111.1	no	824_1	Pyrite	73.92	2.14	63.24	0	0	0	17	Medium quality	5568	1887585	64.00	421	d__Bacteria;p__Actinobacteriota;c__Acidimicrobiia;o__Acidimicrobiales;f__;g__;s__

LMSG_G000008112.1	no	876_1	Copper	78.30	0.43	76.17	1	1	1	18	Medium quality	8698	1824068	64.50	246	d__Bacteria;p__Actinobacteriota;c__Acidimicrobiia;o__Acidimicrobiales;f__;g__;s__
LMSG_G000008113.1	no	600_1	Pyrite-Copper	96.01	1.67	87.66	3	9	26	18	High quality	14884	3217862	53.60	443	d__Bacteria;p__Actinobacteriota;c__Acidimicrobiia;o__Acidimicrobiales;f__Acidimicrobiaceae;g__;s__
LMSG_G000008114.1	no	600_1	Pyrite	58.33	0.85	54.06	0	6	6	11	Medium quality	17560	1747338	52.70	202	d__Bacteria;p__Actinobacteriota;c__Acidimicrobiia;o__Acidimicrobiales;f__Acidimicrobiaceae;g__;s__
LMSG_G000008115.1	no	600_1	Pyrite	97.43	1.28	91.02	1	0	1	20	Medium quality	32892	2813683	53.30	208	d__Bacteria;p__Actinobacteriota;c__Acidimicrobiia;o__Acidimicrobiales;f__Acidimicrobiaceae;g__;s__
LMSG_G000008116.1	no	600_1	Pyrite-Copper	67.68	1.28	61.27	1	0	3	15	Medium quality	13778	2149675	53.20	268	d__Bacteria;p__Actinobacteriota;c__Acidimicrobiia;o__Acidimicrobiales;f__Acidimicrobiaceae;g__;s__
LMSG_G000008117.1	no	600_1	Pyrite-Copper	82.09	1.28	75.68	1	4	14	18	Medium quality	9113	2438467	53.90	456	d__Bacteria;p__Actinobacteriota;c__Acidimicrobiia;o__Acidimicrobiales;f__Acidimicrobiaceae;g__;s__
LMSG_G000008118.1	no	600_1	Lead-Zinc	93.73	1.28	87.32	1	1	1	18	High quality	12524	2448782	53.20	293	d__Bacteria;p__Actinobacteriota;c__Acidimicrobiia;o__Acidimicrobiales;f__Acidimicrobiaceae;g__;s__
LMSG_G000008119.1	no	600_1	Copper	96.29	1.28	89.88	2	1	1	19	High quality	33307	2640382	53.20	184	d__Bacteria;p__Actinobacteriota;c__Acidimicrobiia;o__Acidimicrobiales;f__Acidimicrobiaceae;g__;s__
LMSG_G000008120.1	no	600_1	Pyrite	93.82	2.85	79.58	2	2	2	18	High quality	22224	2539770	53.30	218	d__Bacteria;p__Actinobacteriota;c__Acidimicrobiia;o__Acidimicrobiales;f__Acidimicrobiaceae;g__;s__
LMSG_G000008121.1	no	600_1	Polymetallic	90.88	1.28	84.47	1	2	3	16	Medium quality	41788	2487351	53.30	153	d__Bacteria;p__Actinobacteriota;c__Acidimicrobiia;o__Acidimicrobiales;f__Acidimicrobiaceae;g__;s__
LMSG_G000008122.1	no	600_1	Pyrite	76.52	1.28	70.11	1	0	0	20	Medium quality	2776	2115943	53.40	853	d__Bacteria;p__Actinobacteriota;c__Acidimicrobiia;o__Acidimicrobiales;f__Acidimicrobiaceae;g__;s__
LMSG_G000008123.1	no	600_1	Lead-Zinc	85.75	1.28	79.34	1	0	1	15	Medium quality	11812	2210262	53.30	262	d__Bacteria;p__Actinobacteriota;c__Acidimicrobiia;o__Acidimicrobiales;f__Acidimicrobiaceae;g__;s__
LMSG_G000008124.1	no	600_1	Polymetallic	87.97	0.43	85.84	1	1	1	18	Medium quality	10212	2775235	53.20	377	d__Bacteria;p__Actinobacteriota;c__Acidimicrobiia;o__Acidimicrobiales;f__Acidimicrobiaceae;g__;s__
LMSG_G000008125.1	no	600_1	Polymetallic	68.18	0.00	68.18	0	0	0	12	Medium quality	4906	1840902	53.30	424	d__Bacteria;p__Actinobacteriota;c__Acidimicrobiia;o__Acidimicrobiales;f__Acidimicrobiaceae;g__;s__
LMSG_G000008126.1	no	600_1	Magnetite	79.05	0.43	76.92	0	3	0	18	Medium quality	44695	2372350	53.10	167	d__Bacteria;p__Actinobacteriota;c__Acidimicrobiia;o__Acidimicrobiales;f__Acidimicrobiaceae;g__;s__
LMSG_G000008127.1	no	600_1	Polymetallic	68.71	3.45	51.47	1	0	0	17	Medium quality	15897	2620201	53.00	258	d__Bacteria;p__Actinobacteriota;c__Acidimicrobiia;o__Acidimicrobiales;f__Acidimicrobiaceae;g__;s__
LMSG_G000008128.1	no	600_1	Polymetallic	70.00	0.00	70.00	1	1	1	16	Medium quality	34788	2278262	53.00	121	d__Bacteria;p__Actinobacteriota;c__Acidimicrobiia;o__Acidimicrobiales;f__Acidimicrobiaceae;g__;s__
LMSG_G000008129.1	no	600_1	Magnetite	70.94	0.00	70.94	3	2	3	17	Medium quality	26966	2151631	52.80	222	d__Bacteria;p__Actinobacteriota;c__Acidimicrobiia;o__Acidimicrobiales;f__Acidimicrobiaceae;g__;s__
LMSG_G000008130.1	no	600_1	Magnetite	86.32	2.14	75.64	1	2	1	19	Medium quality	33196	2635961	53.20	254	d__Bacteria;p__Actinobacteriota;c__Acidimicrobiia;o__Acidimicrobiales;f__Acidimicrobiaceae;g__;s__
LMSG_G000008131.1	no	600_1	Magnetite	77.92	0.43	75.79	0	1	0	14	Medium quality	27876	1638703	53.80	119	d__Bacteria;p__Actinobacteriota;c__Acidimicrobiia;o__Acidimicrobiales;f__Acidimicrobiaceae;g__;s__
LMSG_G000008132.1	no	600_1	Magnetite	59.48	0.00	59.48	1	1	2	16	Medium quality	27634	2024476	53.00	164	d__Bacteria;p__Actinobacteriota;c__Acidimicrobiia;o__Acidimicrobiales;f__Acidimicrobiaceae;g__;s__
LMSG_G000008133.1	no	600_1	Pyrite-Copper	74.87	1.28	68.46	1	1	2	17	Medium quality	23437	2306148	53.20	169	d__Bacteria;p__Actinobacteriota;c__Acidimicrobiia;o__Acidimicrobiales;f__Acidimicrobiaceae;g__;s__
LMSG_G000008134.1	no	600_1	Copper	98.29	2.14	87.61	2	3	0	20	Medium quality	39578	2870556	53.40	204	d__Bacteria;p__Actinobacteriota;c__Acidimicrobiia;o__Acidimicrobiales;f__Acidimicrobiaceae;g__;s__
LMSG_G000008135.1	no	600_1	Copper	58.75	0.85	54.48	0	0	0	15	Medium quality	6981	1372686	53.60	243	d__Bacteria;p__Actinobacteriota;c__Acidimicrobiia;o__Acidimicrobiales;f__Acidimicrobiaceae;g__;s__
LMSG_G000008136.1	no	600_1	Copper	68.31	0.52	65.70	0	0	0	18	Medium quality	6531	1671829	53.50	361	d__Bacteria;p__Actinobacteriota;c__Acidimicrobiia;o__Acidimicrobiales;f__Acidimicrobiaceae;g__;s__
LMSG_G000008137.1	no	601_1	Polymetallic	91.88	1.28	85.47	1	1	1	18	High quality	39957	2424678	52.10	162	d__Bacteria;p__Actinobacteriota;c__Acidimicrobiia;o__Acidimicrobiales;f__Acidimicrobiaceae;g__;s__
LMSG_G000008138.1	no	601_1	Polymetallic	96.15	1.71	87.61	0	2	0	19	Medium quality	17907	2435300	52.10	218	d__Bacteria;p__Actinobacteriota;c__Acidimicrobiia;o__Acidimicrobiales;f__Acidimicrobiaceae;g__;s__
LMSG_G000008139.1	no	601_1	Polymetallic	96.58	1.28	90.17	0	0	0	19	Medium quality	16759	2566682	52.20	235	d__Bacteria;p__Actinobacteriota;c__Acidimicrobiia;o__Acidimicrobiales;f__Acidimicrobiaceae;g__;s__
LMSG_G000008140.1	no	601_1	Polymetallic	84.18	0.00	84.18	1	1	1	20	Medium quality	34437	2378467	52.00	169	d__Bacteria;p__Actinobacteriota;c__Acidimicrobiia;o__Acidimicrobiales;f__Acidimicrobiaceae;g__;s__
LMSG_G000008141.1	no	601_1	Polymetallic	91.45	1.28	85.04	1	0	1	19	Medium quality	38907	2489985	52.20	164	d__Bacteria;p__Actinobacteriota;c__Acidimicrobiia;o__Acidimicrobiales;f__Acidimicrobiaceae;g__;s__
LMSG_G000008142.1	no	601_1	Polymetallic	96.15	1.28	89.74	0	1	0	20	Medium quality	35822	2572770	52.20	172	d__Bacteria;p__Actinobacteriota;c__Acidimicrobiia;o__Acidimicrobiales;f__Acidimicrobiaceae;g__;s__
LMSG_G000008143.1	no	601_1	Polymetallic	97.00	2.14	86.32	0	0	0	20	Medium quality	26096	2520885	52.20	189	d__Bacteria;p__Actinobacteriota;c__Acidimicrobiia;o__Acidimicrobiales;f__Acidimicrobiaceae;g__;s__
LMSG_G000008144.1	no	601_1	Polymetallic	96.15	1.28	89.74	1	0	1	19	Medium quality	15235	2535221	52.20	234	d__Bacteria;p__Actinobacteriota;c__Acidimicrobiia;o__Acidimicrobiales;f__Acidimicrobiaceae;g__;s__
LMSG_G000008145.1	no	601_1	Polymetallic	91.88	1.45	84.62	1	0	0	18	Medium quality	14038	2725737	52.20	512	d__Bacteria;p__Actinobacteriota;c__Acidimicrobiia;o__Acidimicrobiales;f__Acidimicrobiaceae;g__;s__
LMSG_G000008146.1	no	601_1	Polymetallic	65.94	2.23	54.79	3	13	26	10	Medium quality	2719	2474432	52.00	1086	d__Bacteria;p__Actinobacteriota;c__Acidimicrobiia;o__Acidimicrobiales;f__Acidimicrobiaceae;g__;s__
LMSG_G000008147.1	no	601_1	Copper	82.19	1.28	75.78	0	1	0	17	Medium quality	8035	2503375	52.10	419	d__Bacteria;p__Actinobacteriota;c__Acidimicrobiia;o__Acidimicrobiales;f__Acidimicrobiaceae;g__;s__
LMSG_G000008148.1	no	601_1	Pyrite-Copper	52.56	0.00	52.56	0	0	0	10	Medium quality	6670	1108732	52.00	203	d__Bacteria;p__Actinobacteriota;c__Acidimicrobiia;o__Acidimicrobiales;f__Acidimicrobiaceae;g__;s__
LMSG_G000008149.1	no	603_1	Polymetallic	95.72	2.99	80.77	1	1	1	18	High quality	64442	2231293	54.80	102	d__Bacteria;p__Actinobacteriota;c__Acidimicrobiia;o__Acidimicrobiales;f__Acidimicrobiaceae;g__;s__
LMSG_G000008150.1	no	603_1	Polymetallic	94.87	1.88	85.47	1	1	1	20	High quality	54483	2208273	54.90	108	d__Bacteria;p__Actinobacteriota;c__Acidimicrobiia;o__Acidimicrobiales;f__Acidimicrobiaceae;g__;s__
LMSG_G000008151.1	no	603_1	Polymetallic	92.30	1.28	85.89	1	1	1	16	Medium quality	21591	2421664	54.80	179	d__Bacteria;p__Actinobacteriota;c__Acidimicrobiia;o__Acidimicrobiales;f__Acidimicrobiaceae;g__;s__
LMSG_G000008152.1	no	603_1	Polymetallic	97.43	2.14	86.75	1	1	1	17	Medium quality	21986	2338440	54.80	154	d__Bacteria;p__Actinobacteriota;c__Acidimicrobiia;o__Acidimicrobiales;f__Acidimicrobiaceae;g__;s__
LMSG_G000008153.1	no	603_1	Polymetallic	93.16	1.45	85.90	0	2	0	20	Medium quality	32176	2349052	54.80	169	d__Bacteria;p__Actinobacteriota;c__Acidimicrobiia;o__Acidimicrobiales;f__Acidimicrobiaceae;g__;s__
LMSG_G000008154.1	no	603_1	Polymetallic	94.87	1.28	88.46	0	1	0	19	Medium quality	31406	2167235	54.90	135	d__Bacteria;p__Actinobacteriota;c__Acidimicrobiia;o__Acidimicrobiales;f__Acidimicrobiaceae;g__;s__
LMSG_G000008155.1	no	603_1	Copper	95.72	2.99	80.77	0	1	0	18	Medium quality	17613	2472011	54.90	248	d__Bacteria;p__Actinobacteriota;c__Acidimicrobiia;o__Acidimicrobiales;f__Acidimicrobiaceae;g__;s__
LMSG_G000008156.1	no	603_1	Polymetallic	96.58	1.28	90.17	1	1	1	20	High quality	34423	2722329	54.90	152	d__Bacteria;p__Actinobacteriota;c__Acidimicrobiia;o__Acidimicrobiales;f__Acidimicrobiaceae;g__;s__
LMSG_G000008157.1	no	603_1	Polymetallic	97.43	1.28	91.02	1	1	1	20	High quality	32544	2775500	55.00	166	d__Bacteria;p__Actinobacteriota;c__Acidimicrobiia;o__Acidimicrobiales;f__Acidimicrobiaceae;g__;s__
LMSG_G000008158.1	no	603_1	Polymetallic	97.43	1.28	91.02	1	1	1	18	High quality	45555	2496914	54.90	100	d__Bacteria;p__Actinobacteriota;c__Acidimicrobiia;o__Acidimicrobiales;f__Acidimicrobiaceae;g__;s__
LMSG_G000008159.1	no	603_1	Polymetallic	93.16	1.28	86.75	1	1	1	20	High quality	80362	2486496	55.00	101	d__Bacteria;p__Actinobacteriota;c__Acidimicrobiia;o__Acidimicrobiales;f__Acidimicrobiaceae;g__;s__
LMSG_G000008160.1	no	603_1	Polymetallic	98.29	1.71	89.75	1	2	1	20	High quality	82504	3187205	54.60	201	d__Bacteria;p__Actinobacteriota;c__Acidimicrobiia;o__Acidimicrobiales;f__Acidimicrobiaceae;g__;s__
LMSG_G000008161.1	no	603_1	Polymetallic	98.29	1.42	91.17	1	1	1	20	High quality	25936	2458259	54.90	137	d__Bacteria;p__Actinobacteriota;c__Acidimicrobiia;o__Acidimicrobiales;f__Acidimicrobiaceae;g__;s__
LMSG_G000008162.1	no	603_1	Polymetallic	97.43	2.14	86.75	1	1	1	20	High quality	33749	2646010	54.90	139	d__Bacteria;p__Actinobacteriota;c__Acidimicrobiia;o__Acidimicrobiales;f__Acidimicrobiaceae;g__;s__
LMSG_G000008163.1	no	603_1	Polymetallic	97.43	2.99	82.48	1	1	1	19	High quality	30566	2684574	54.90	150	d__Bacteria;p__Actinobacteriota;c__Acidimicrobiia;o__Acidimicrobiales;f__Acidimicrobiaceae;g__;s__
LMSG_G000008164.1	no	603_1	Polymetallic	70.68	0.00	70.68	1	1	1	16	Medium quality	40189	2584281	54.80	121	d__Bacteria;p__Actinobacteriota;c__Acidimicrobiia;o__Acidimicrobiales;f__Acidimicrobiaceae;g__;s__
LMSG_G000008165.1	no	603_1	Polymetallic	95.72	3.85	76.49	1	0	2	15	Medium quality	32785	2354583	54.80	115	d__Bacteria;p__Actinobacteriota;c__Acidimicrobiia;o__Acidimicrobiales;f__Acidimicrobiaceae;g__;s__
LMSG_G000008166.1	no	603_1	Pyrite-Copper	93.16	0.85	88.89	0	1	0	20	Medium quality	10587	2482819	54.90	304	d__Bacteria;p__Actinobacteriota;c__Acidimicrobiia;o__Acidimicrobiales;f__Acidimicrobiaceae;g__;s__
LMSG_G000008167.1	no	691_1	Tin-Zinc	86.15	1.28	79.74	0	0	0	17	Medium quality	26532	2331893	64.10	140	d__Bacteria;p__Actinobacteriota;c__Acidimicrobiia;o__Acidimicrobiales;f__Acidimicrobiaceae;g__Acidithrix;s__
LMSG_G000008168.1	no	691_1	Pyrite-Copper	74.35	2.14	63.67	1	1	0	13	Medium quality	8025	2063994	64.00	291	d__Bacteria;p__Actinobacteriota;c__Acidimicrobiia;o__Acidimicrobiales;f__Acidimicrobiaceae;g__Acidithrix;s__
LMSG_G000008169.1	no	691_1	Pyrite-Copper	81.19	1.38	74.31	0	0	0	13	Medium quality	7727	2031162	64.30	336	d__Bacteria;p__Actinobacteriota;c__Acidimicrobiia;o__Acidimicrobiales;f__Acidimicrobiaceae;g__Acidithrix;s__
LMSG_G000008170.1	no	691_1	Pyrite-Copper	79.48	4.11	58.95	0	0	0	12	Medium quality	14162	1901833	64.00	249	d__Bacteria;p__Actinobacteriota;c__Acidimicrobiia;o__Acidimicrobiales;f__Acidimicrobiaceae;g__Acidithrix;s__
LMSG_G000008171.1	no	691_1	Copper	67.34	0.43	65.21	0	0	0	10	Medium quality	6610	1659701	64.00	287	d__Bacteria;p__Actinobacteriota;c__Acidimicrobiia;o__Acidimicrobiales;f__Acidimicrobiaceae;g__Acidithrix;s__
LMSG_G000008172.1	no	692_1	Lead-Zinc	94.87	2.14	84.19	1	1	1	18	High quality	38769	3118646	64.20	121	d__Bacteria;p__Actinobacteriota;c__Acidimicrobiia;o__Acidimicrobiales;f__Acidimicrobiaceae;g__Acidithrix;s__
LMSG_G000008173.1	no	692_1	Lead-Zinc	100.00	2.99	85.05	0	1	0	19	Medium quality	33849	3114517	64.30	159	d__Bacteria;p__Actinobacteriota;c__Acidimicrobiia;o__Acidimicrobiales;f__Acidimicrobiaceae;g__Acidithrix;s__
LMSG_G000008174.1	no	692_1	Lead-Zinc	98.29	1.28	91.88	0	0	0	20	Medium quality	41563	2956089	64.20	130	d__Bacteria;p__Actinobacteriota;c__Acidimicrobiia;o__Acidimicrobiales;f__Acidimicrobiaceae;g__Acidithrix;s__
LMSG_G000008175.1	no	692_1	Lead-Zinc	60.47	1.72	51.85	0	0	0	15	Medium quality	3113	1916510	64.40	671	d__Bacteria;p__Actinobacteriota;c__Acidimicrobiia;o__Acidimicrobiales;f__Acidimicrobiaceae;g__Acidithrix;s__
LMSG_G000008176.1	no	692_1	Pyrite-Copper	90.59	3.85	71.36	0	0	0	17	Medium quality	32472	3035323	64.30	165	d__Bacteria;p__Actinobacteriota;c__Acidimicrobiia;o__Acidimicrobiales;f__Acidimicrobiaceae;g__Acidithrix;s__
LMSG_G000008177.1	no	692_1	Pyrite-Copper	96.58	1.28	90.17	0	0	0	19	Medium quality	43128	2707994	64.20	107	d__Bacteria;p__Actinobacteriota;c__Acidimicrobiia;o__Acidimicrobiales;f__Acidimicrobiaceae;g__Acidithrix;s__
LMSG_G000008178.1	no	692_1	Pyrite-Copper	98.29	4.70	74.79	1	0	1	18	Medium quality	28770	3852858	64.10	266	d__Bacteria;p__Actinobacteriota;c__Acidimicrobiia;o__Acidimicrobiales;f__Acidimicrobiaceae;g__Acidithrix;s__
LMSG_G000008179.1	no	692_2	Lead-Zinc	96.41	3.28	80.03	0	0	0	17	Medium quality	20015	3283089	64.20	398	d__Bacteria;p__Actinobacteriota;c__Acidimicrobiia;o__Acidimicrobiales;f__Acidimicrobiaceae;g__Acidithrix;s__
LMSG_G000008180.1	no	692_2	Lead-Zinc	92.20	3.28	75.82	0	0	0	17	Medium quality	7759	2875878	64.20	467	d__Bacteria;p__Actinobacteriota;c__Acidimicrobiia;o__Acidimicrobiales;f__Acidimicrobiaceae;g__Acidithrix;s__

LMSG_G000008181.1	no	692_2	Polymetallic	86.32	0.43	84.19	0	1	0	17	Medium quality	42933	2369402	64.00	84	d__Bacteria;p__Actinobacteriota;c__Acidimicrobiia;o__Acidimicrobiales;f__Acidimicrobiaceae;g__Acidithrix;s__
LMSG_G000008182.1	no	695_1	Polymetallic	94.01	3.85	74.78	2	1	1	20	High quality	215372	2730447	48.70	50	d__Bacteria;p__Actinobacteriota;c__Acidimicrobiia;o__Acidimicrobiales;f__Acidimicrobiaceae;g__Acidithrix;s__
LMSG_G000008183.1	no	695_1	Polymetallic	95.72	1.28	89.31	1	1	1	20	High quality	203810	2664416	48.80	77	d__Bacteria;p__Actinobacteriota;c__Acidimicrobiia;o__Acidimicrobiales;f__Acidimicrobiaceae;g__Acidithrix;s__
LMSG_G000008184.1	no	695_1	Copper	81.03	0.00	81.03	1	0	2	19	Medium quality	203682	2652929	48.70	97	d__Bacteria;p__Actinobacteriota;c__Acidimicrobiia;o__Acidimicrobiales;f__Acidimicrobiaceae;g__Acidithrix;s__
LMSG_G000008185.1	no	695_1	Lead-Zinc	98.29	1.28	91.88	1	0	0	20	Medium quality	214850	2772654	48.80	30	d__Bacteria;p__Actinobacteriota;c__Acidimicrobiia;o__Acidimicrobiales;f__Acidimicrobiaceae;g__Acidithrix;s__
LMSG_G000008186.1	no	695_1	Polymetallic	81.03	0.00	81.03	1	0	0	17	Medium quality	215117	2681107	48.80	73	d__Bacteria;p__Actinobacteriota;c__Acidimicrobiia;o__Acidimicrobiales;f__Acidimicrobiaceae;g__Acidithrix;s__
LMSG_G000008187.1	no	695_1	Lead-Zinc	97.43	1.28	91.02	1	1	0	20	Medium quality	117288	2828733	48.80	52	d__Bacteria;p__Actinobacteriota;c__Acidimicrobiia;o__Acidimicrobiales;f__Acidimicrobiaceae;g__Acidithrix;s__
LMSG_G000008188.1	no	696_1	Lead-Zinc	94.01	1.28	87.60	1	1	1	20	High quality	75558	2992943	49.60	140	d__Bacteria;p__Actinobacteriota;c__Acidimicrobiia;o__Acidimicrobiales;f__Acidimicrobiaceae;g__Acidithrix;s__
LMSG_G000008189.1	no	696_1	Lead-Zinc	95.72	1.28	89.31	2	0	7	20	Medium quality	26469	3061332	49.50	262	d__Bacteria;p__Actinobacteriota;c__Acidimicrobiia;o__Acidimicrobiales;f__Acidimicrobiaceae;g__Acidithrix;s__
LMSG_G000008190.1	no	696_1	Pyrite	83.86	0.71	80.30	0	0	1	16	Medium quality	3347	2183849	49.80	785	d__Bacteria;p__Actinobacteriota;c__Acidimicrobiia;o__Acidimicrobiales;f__Acidimicrobiaceae;g__Acidithrix;s__
LMSG_G000008191.1	no	696_1	Lead-Zinc	79.31	0.00	79.31	1	0	0	18	Medium quality	47700	2696508	49.60	124	d__Bacteria;p__Actinobacteriota;c__Acidimicrobiia;o__Acidimicrobiales;f__Acidimicrobiaceae;g__Acidithrix;s__
LMSG_G000008192.1	no	696_1	Polymetallic	95.72	1.28	89.31	1	0	0	20	Medium quality	108052	2703414	49.50	75	d__Bacteria;p__Actinobacteriota;c__Acidimicrobiia;o__Acidimicrobiales;f__Acidimicrobiaceae;g__Acidithrix;s__
LMSG_G000008193.1	no	696_1	Polymetallic	79.43	4.27	58.07	1	1	0	15	Medium quality	2925	2303220	49.50	899	d__Bacteria;p__Actinobacteriota;c__Acidimicrobiia;o__Acidimicrobiales;f__Acidimicrobiaceae;g__Acidithrix;s__
LMSG_G000008194.1	no	696_1	Polymetallic	93.16	4.70	69.66	1	0	0	20	Medium quality	83690	2754358	49.50	79	d__Bacteria;p__Actinobacteriota;c__Acidimicrobiia;o__Acidimicrobiales;f__Acidimicrobiaceae;g__Acidithrix;s__
LMSG_G000008195.1	no	696_1	Polymetallic	95.72	1.28	89.31	1	0	0	20	Medium quality	108672	2704928	49.60	63	d__Bacteria;p__Actinobacteriota;c__Acidimicrobiia;o__Acidimicrobiales;f__Acidimicrobiaceae;g__Acidithrix;s__
LMSG_G000008196.1	no	696_1	Polymetallic	95.72	2.14	85.04	1	0	0	20	Medium quality	107675	2719983	49.50	97	d__Bacteria;p__Actinobacteriota;c__Acidimicrobiia;o__Acidimicrobiales;f__Acidimicrobiaceae;g__Acidithrix;s__
LMSG_G000008197.1	no	696_1	Polymetallic	95.72	3.33	79.06	2	0	0	20	Medium quality	91159	2676371	49.50	77	d__Bacteria;p__Actinobacteriota;c__Acidimicrobiia;o__Acidimicrobiales;f__Acidimicrobiaceae;g__Acidithrix;s__
LMSG_G000008198.1	no	696_1	Polymetallic	95.72	1.38	88.84	1	0	4	20	Medium quality	18544	2765245	49.50	235	d__Bacteria;p__Actinobacteriota;c__Acidimicrobiia;o__Acidimicrobiales;f__Acidimicrobiaceae;g__Acidithrix;s__
LMSG_G000008199.1	no	696_1	Polymetallic	91.76	1.28	85.35	1	0	1	20	Medium quality	97633	2713640	49.50	84	d__Bacteria;p__Actinobacteriota;c__Acidimicrobiia;o__Acidimicrobiales;f__Acidimicrobiaceae;g__Acidithrix;s__
LMSG_G000008200.1	no	696_1	Polymetallic	78.57	2.99	63.62	2	14	11	19	Medium quality	10050	2717014	49.70	544	d__Bacteria;p__Actinobacteriota;c__Acidimicrobiia;o__Acidimicrobiales;f__Acidimicrobiaceae;g__Acidithrix;s__
LMSG_G000008201.1	no	696_1	Pyrite-Copper	79.59	0.00	79.59	0	1	0	18	Medium quality	33607	2543742	49.50	174	d__Bacteria;p__Actinobacteriota;c__Acidimicrobiia;o__Acidimicrobiales;f__Acidimicrobiaceae;g__Acidithrix;s__
LMSG_G000008202.1	no	697_1	Polymetallic	75.86	0.00	75.86	1	0	0	18	Medium quality	77689	2916897	49.50	120	d__Bacteria;p__Actinobacteriota;c__Acidimicrobiia;o__Acidimicrobiales;f__Acidimicrobiaceae;g__Acidithrix;s__
LMSG_G000008203.1	no	697_1	Polymetallic	89.40	2.14	78.72	1	0	0	17	Medium quality	64504	2883981	49.50	97	d__Bacteria;p__Actinobacteriota;c__Acidimicrobiia;o__Acidimicrobiales;f__Acidimicrobiaceae;g__Acidithrix;s__
LMSG_G000008204.1	no	697_1	Polymetallic	92.30	2.14	81.62	1	0	0	16	Medium quality	32108	2850109	49.50	152	d__Bacteria;p__Actinobacteriota;c__Acidimicrobiia;o__Acidimicrobiales;f__Acidimicrobiaceae;g__Acidithrix;s__
LMSG_G000008205.1	no	697_1	Polymetallic	86.32	0.43	84.19	1	0	0	16	Medium quality	35220	2771729	49.50	125	d__Bacteria;p__Actinobacteriota;c__Acidimicrobiia;o__Acidimicrobiales;f__Acidimicrobiaceae;g__Acidithrix;s__
LMSG_G000008206.1	no	697_1	Polymetallic	70.68	0.00	70.68	0	0	0	17	Medium quality	52024	2722730	49.60	103	d__Bacteria;p__Actinobacteriota;c__Acidimicrobiia;o__Acidimicrobiales;f__Acidimicrobiaceae;g__Acidithrix;s__
LMSG_G000008207.1	no	697_1	Polymetallic	93.16	1.28	86.75	2	0	0	18	Medium quality	38665	2897290	49.50	118	d__Bacteria;p__Actinobacteriota;c__Acidimicrobiia;o__Acidimicrobiales;f__Acidimicrobiaceae;g__Acidithrix;s__
LMSG_G000008208.1	no	697_1	Copper	73.44	1.72	64.82	1	1	2	15	Medium quality	61582	2702305	49.40	97	d__Bacteria;p__Actinobacteriota;c__Acidimicrobiia;o__Acidimicrobiales;f__Acidimicrobiaceae;g__Acidithrix;s__
LMSG_G000008209.1	no	697_1	Copper	94.01	1.28	87.60	1	0	1	20	Medium quality	82402	2953109	49.50	92	d__Bacteria;p__Actinobacteriota;c__Acidimicrobiia;o__Acidimicrobiales;f__Acidimicrobiaceae;g__Acidithrix;s__
LMSG_G000008210.1	no	697_1	Copper	93.16	1.28	86.75	1	0	1	20	Medium quality	65228	2940567	49.60	127	d__Bacteria;p__Actinobacteriota;c__Acidimicrobiia;o__Acidimicrobiales;f__Acidimicrobiaceae;g__Acidithrix;s__
LMSG_G000008211.1	no	697_1	Copper	78.66	1.04	73.44	0	0	0	15	Medium quality	3590	2191027	49.50	675	d__Bacteria;p__Actinobacteriota;c__Acidimicrobiia;o__Acidimicrobiales;f__Acidimicrobiaceae;g__Acidithrix;s__
LMSG_G000008212.1	no	697_1	Copper	95.29	1.36	88.50	0	0	0	17	Medium quality	6759	2694228	49.50	529	d__Bacteria;p__Actinobacteriota;c__Acidimicrobiia;o__Acidimicrobiales;f__Acidimicrobiaceae;g__Acidithrix;s__
LMSG_G000008213.1	no	604_1	Pyrite	95.72	4.70	72.22	0	1	0	19	Medium quality	36791	3162527	56.90	182	d__Bacteria;p__Actinobacteriota;c__Acidimicrobiia;o__Acidimicrobiales;f__Acidimicrobiaceae;g__Ferrimicrobium;s__
LMSG_G000008214.1	no	605_1	Pyrite	92.30	2.14	81.62	2	0	1	20	Medium quality	69292	2751886	57.30	81	d__Bacteria;p__Actinobacteriota;c__Acidimicrobiia;o__Acidimicrobiales;f__Acidimicrobiaceae;g__Ferrimicrobium;s__
LMSG_G000008215.1	no	605_1	Pyrite	92.30	2.99	77.35	2	0	1	20	Medium quality	69292	3211037	57.20	124	d__Bacteria;p__Actinobacteriota;c__Acidimicrobiia;o__Acidimicrobiales;f__Acidimicrobiaceae;g__Ferrimicrobium;s__
LMSG_G000008216.1	no	605_1	Pyrite	94.87	3.16	79.06	1	0	1	20	Medium quality	76273	3145428	57.20	136	d__Bacteria;p__Actinobacteriota;c__Acidimicrobiia;o__Acidimicrobiales;f__Acidimicrobiaceae;g__Ferrimicrobium;s__
LMSG_G000008217.1	no	606_1	Lead-Zinc	72.41	0.00	72.41	0	0	0	15	Medium quality	16416	2389599	58.60	199	d__Bacteria;p__Actinobacteriota;c__Acidimicrobiia;o__Acidimicrobiales;f__Acidimicrobiaceae;g__Ferrimicrobium;s__
LMSG_G000008218.1	no	606_1	Lead-Zinc	84.00	1.28	77.59	0	0	0	16	Medium quality	6624	2229832	59.00	395	d__Bacteria;p__Actinobacteriota;c__Acidimicrobiia;o__Acidimicrobiales;f__Acidimicrobiaceae;g__Ferrimicrobium;s__
LMSG_G000008219.1	no	606_1	Pyrite	97.43	2.14	86.75	0	0	1	18	Medium quality	26266	2770899	58.20	182	d__Bacteria;p__Actinobacteriota;c__Acidimicrobiia;o__Acidimicrobiales;f__Acidimicrobiaceae;g__Ferrimicrobium;s__
LMSG_G000008220.1	no	606_1	Pyrite	88.88	1.28	82.47	0	0	0	18	Medium quality	23200	2657283	58.20	179	d__Bacteria;p__Actinobacteriota;c__Acidimicrobiia;o__Acidimicrobiales;f__Acidimicrobiaceae;g__Ferrimicrobium;s__
LMSG_G000008221.1	no	606_1	Pyrite	95.72	1.28	89.31	0	0	0	18	Medium quality	27760	2403107	58.70	133	d__Bacteria;p__Actinobacteriota;c__Acidimicrobiia;o__Acidimicrobiales;f__Acidimicrobiaceae;g__Ferrimicrobium;s__
LMSG_G000008222.1	no	606_1	Lead-Zinc	94.87	2.14	84.19	0	0	0	19	Medium quality	35125	2400422	58.90	125	d__Bacteria;p__Actinobacteriota;c__Acidimicrobiia;o__Acidimicrobiales;f__Acidimicrobiaceae;g__Ferrimicrobium;s__
LMSG_G000008223.1	no	606_1	Lead-Zinc	90.59	0.85	86.32	0	1	0	18	Medium quality	21229	2313311	58.80	175	d__Bacteria;p__Actinobacteriota;c__Acidimicrobiia;o__Acidimicrobiales;f__Acidimicrobiaceae;g__Ferrimicrobium;s__
LMSG_G000008224.1	no	606_1	Polymetallic	96.58	1.28	90.17	0	0	0	18	Medium quality	24822	2668641	58.60	169	d__Bacteria;p__Actinobacteriota;c__Acidimicrobiia;o__Acidimicrobiales;f__Acidimicrobiaceae;g__Ferrimicrobium;s__
LMSG_G000008225.1	no	606_1	Polymetallic	94.87	1.28	88.46	0	0	0	19	Medium quality	25939	2582637	58.60	156	d__Bacteria;p__Actinobacteriota;c__Acidimicrobiia;o__Acidimicrobiales;f__Acidimicrobiaceae;g__Ferrimicrobium;s__
LMSG_G000008226.1	no	606_1	Polymetallic	96.58	1.28	90.17	0	1	0	17	Medium quality	43148	2639848	58.60	154	d__Bacteria;p__Actinobacteriota;c__Acidimicrobiia;o__Acidimicrobiales;f__Acidimicrobiaceae;g__Ferrimicrobium;s__
LMSG_G000008227.1	no	606_1	Polymetallic	96.58	1.28	90.17	0	0	0	18	Medium quality	37540	2727471	58.50	186	d__Bacteria;p__Actinobacteriota;c__Acidimicrobiia;o__Acidimicrobiales;f__Acidimicrobiaceae;g__Ferrimicrobium;s__
LMSG_G000008228.1	no	606_1	Tin-Zinc	96.58	2.14	85.90	0	1	0	19	Medium quality	15485	3009972	58.10	292	d__Bacteria;p__Actinobacteriota;c__Acidimicrobiia;o__Acidimicrobiales;f__Acidimicrobiaceae;g__Ferrimicrobium;s__
LMSG_G000008229.1	no	606_1	Polymetallic	97.43	1.28	91.02	0	0	0	19	Medium quality	28209	2836449	58.30	170	d__Bacteria;p__Actinobacteriota;c__Acidimicrobiia;o__Acidimicrobiales;f__Acidimicrobiaceae;g__Ferrimicrobium;s__
LMSG_G000008230.1	no	606_1	Copper	58.11	0.43	55.98	0	0	0	19	Medium quality	24948	1823899	58.10	105	d__Bacteria;p__Actinobacteriota;c__Acidimicrobiia;o__Acidimicrobiales;f__Acidimicrobiaceae;g__Ferrimicrobium;s__
LMSG_G000008231.1	no	606_1	Copper	97.43	1.28	91.02	0	0	0	20	Medium quality	29528	2770254	58.40	178	d__Bacteria;p__Actinobacteriota;c__Acidimicrobiia;o__Acidimicrobiales;f__Acidimicrobiaceae;g__Ferrimicrobium;s__

LMSG_G000008232.1	no	606_1	Copper	96.58	1.28	90.17	0	0	0	18	Medium quality	34557	2424263	58.90	139	d__Bacteria;p__Actinobacteriota;c__Acidimicrobiia;o__Acidimicrobiales;f__Acidimicrobiaceae;g__Ferrimicrobium;s__
LMSG_G000008233.1	no	606_1	Polymetallic	88.03	4.70	64.53	0	0	0	18	Medium quality	33763	2607270	58.60	127	d__Bacteria;p__Actinobacteriota;c__Acidimicrobiia;o__Acidimicrobiales;f__Acidimicrobiaceae;g__Ferrimicrobium;s__
LMSG_G000008234.1	no	606_1	Magnetite	88.88	4.27	67.52	0	0	0	19	Medium quality	11174	2251338	58.80	259	d__Bacteria;p__Actinobacteriota;c__Acidimicrobiia;o__Acidimicrobiales;f__Acidimicrobiaceae;g__Ferrimicrobium;s__
LMSG_G000008235.1	no	606_1	Copper	97.43	1.28	91.02	0	0	0	19	Medium quality	34296	2791209	58.60	203	d__Bacteria;p__Actinobacteriota;c__Acidimicrobiia;o__Acidimicrobiales;f__Acidimicrobiaceae;g__Ferrimicrobium;s__
LMSG_G000008236.1	no	606_1	Pyrite-Copper	94.01	1.71	85.47	0	0	0	18	Medium quality	9355	2730980	58.60	473	d__Bacteria;p__Actinobacteriota;c__Acidimicrobiia;o__Acidimicrobiales;f__Acidimicrobiaceae;g__Ferrimicrobium;s__
LMSG_G000008237.1	no	606_1	Pyrite-Copper	94.87	2.99	79.92	1	0	1	19	Medium quality	14132	2707666	58.60	278	d__Bacteria;p__Actinobacteriota;c__Acidimicrobiia;o__Acidimicrobiales;f__Acidimicrobiaceae;g__Ferrimicrobium;s__
LMSG_G000008238.1	no	606_1	Pyrite-Copper	97.43	1.28	91.02	0	0	0	19	Medium quality	27891	2565533	58.70	148	d__Bacteria;p__Actinobacteriota;c__Acidimicrobiia;o__Acidimicrobiales;f__Acidimicrobiaceae;g__Ferrimicrobium;s__
LMSG_G000008239.1	no	606_1	Copper	63.24	0.43	61.11	0	0	0	18	Medium quality	27737	1892927	58.30	104	d__Bacteria;p__Actinobacteriota;c__Acidimicrobiia;o__Acidimicrobiales;f__Acidimicrobiaceae;g__Ferrimicrobium;s__
LMSG_G000008240.1	no	606_1	Pyrite-Copper	75.63	1.28	69.22	1	0	1	16	Medium quality	10136	2909938	57.60	409	d__Bacteria;p__Actinobacteriota;c__Acidimicrobiia;o__Acidimicrobiales;f__Acidimicrobiaceae;g__Ferrimicrobium;s__
LMSG_G000008241.1	no	606_1	Pyrite	84.55	2.14	73.87	1	2	1	20	Medium quality	4755	2648883	58.00	649	d__Bacteria;p__Actinobacteriota;c__Acidimicrobiia;o__Acidimicrobiales;f__Acidimicrobiaceae;g__Ferrimicrobium;s__
LMSG_G000008242.1	no	606_1	Pyrite	97.43	3.85	78.20	0	0	0	20	Medium quality	25550	2999937	58.20	193	d__Bacteria;p__Actinobacteriota;c__Acidimicrobiia;o__Acidimicrobiales;f__Acidimicrobiaceae;g__Ferrimicrobium;s__
LMSG_G000008243.1	no	607_1	Magnetite	65.08	0.29	63.63	3	0	0	11	Medium quality	6891	3312805	55.50	528	d__Bacteria;p__Actinobacteriota;c__Acidimicrobiia;o__Acidimicrobiales;f__Acidimicrobiaceae;g__Ferrimicrobium;s__
LMSG_G000008244.1	no	781_1	Pyrite-Copper	86.24	2.85	72.00	1	0	0	11	Medium quality	9981	1657836	66.40	232	d__Bacteria;p__Actinobacteriota;c__Acidimicrobiia;o__Acidimicrobiales;f__Acidimicrobiaceae;g__Ferrimicrobium;s__
LMSG_G000008245.1	no	781_1	Pyrite-Copper	95.72	2.71	82.19	0	0	0	15	Medium quality	15787	1855349	66.30	144	d__Bacteria;p__Actinobacteriota;c__Acidimicrobiia;o__Acidimicrobiales;f__Acidimicrobiaceae;g__Ferrimicrobium;s__
LMSG_G000008246.1	no	781_1	Copper	72.71	0.69	69.25	0	0	0	10	Medium quality	2758	1406625	66.20	576	d__Bacteria;p__Actinobacteriota;c__Acidimicrobiia;o__Acidimicrobiales;f__Acidimicrobiaceae;g__Ferrimicrobium;s__
LMSG_G000008247.1	no	781_1	Copper	90.08	4.42	68.01	0	3	1	16	Medium quality	6662	1829066	66.50	521	d__Bacteria;p__Actinobacteriota;c__Acidimicrobiia;o__Acidimicrobiales;f__Acidimicrobiaceae;g__Ferrimicrobium;s__
LMSG_G000008248.1	no	782_1	Lead-Zinc	57.66	0.85	53.39	1	0	1	12	Medium quality	1979	1191572	61.40	629	d__Bacteria;p__Actinobacteriota;c__Acidimicrobiia;o__Acidimicrobiales;f__Acidimicrobiaceae;g__Ferrimicrobium;s__
LMSG_G000008249.1	no	782_1	Pyrite	94.87	0.85	90.60	2	0	2	20	Medium quality	61647	2051413	61.40	46	d__Bacteria;p__Actinobacteriota;c__Acidimicrobiia;o__Acidimicrobiales;f__Acidimicrobiaceae;g__Ferrimicrobium;s__
LMSG_G000008250.1	no	782_1	Pyrite	97.00	1.28	90.59	1	0	0	20	Medium quality	87225	2345960	61.50	95	d__Bacteria;p__Actinobacteriota;c__Acidimicrobiia;o__Acidimicrobiales;f__Acidimicrobiaceae;g__Ferrimicrobium;s__
LMSG_G000008251.1	no	782_1	Pyrite	97.00	0.85	92.73	4	0	2	20	Medium quality	63327	2399058	61.30	64	d__Bacteria;p__Actinobacteriota;c__Acidimicrobiia;o__Acidimicrobiales;f__Acidimicrobiaceae;g__Ferrimicrobium;s__
LMSG_G000008252.1	no	782_1	Pyrite-Copper	69.30	3.77	50.44	0	0	0	19	Medium quality	4778	1812402	61.60	482	d__Bacteria;p__Actinobacteriota;c__Acidimicrobiia;o__Acidimicrobiales;f__Acidimicrobiaceae;g__Ferrimicrobium;s__
LMSG_G000008253.1	no	782_1	Pyrite	97.00	0.85	92.73	2	0	0	20	Medium quality	60068	2243629	61.40	51	d__Bacteria;p__Actinobacteriota;c__Acidimicrobiia;o__Acidimicrobiales;f__Acidimicrobiaceae;g__Ferrimicrobium;s__
LMSG_G000008254.1	no	782_1	Pyrite	97.43	0.85	93.16	2	1	0	20	Medium quality	61524	2223577	61.40	50	d__Bacteria;p__Actinobacteriota;c__Acidimicrobiia;o__Acidimicrobiales;f__Acidimicrobiaceae;g__Ferrimicrobium;s__
LMSG_G000008255.1	no	607_1	Pyrite	92.30	1.28	85.89	2	2	2	19	High quality	32680	2601800	55.40	236	d__Bacteria;p__Actinobacteriota;c__Acidimicrobiia;o__Acidimicrobiales;f__Acidimicrobiaceae;g__Ferrimicrobium;s__Ferrimicrobium acidiphilum
LMSG_G000008256.1	no	607_1	Pyrite	91.45	1.28	85.04	1	6	8	20	High quality	33476	2407607	55.40	177	d__Bacteria;p__Actinobacteriota;c__Acidimicrobiia;o__Acidimicrobiales;f__Acidimicrobiaceae;g__Ferrimicrobium;s__Ferrimicrobium acidiphilum
LMSG_G000008257.1	no	607_1	Lead-Zinc	94.01	1.38	87.13	1	1	1	19	High quality	51195	2620603	55.50	141	d__Bacteria;p__Actinobacteriota;c__Acidimicrobiia;o__Acidimicrobiales;f__Acidimicrobiaceae;g__Ferrimicrobium;s__Ferrimicrobium acidiphilum
LMSG_G000008258.1	no	607_1	Copper	92.02	1.28	85.61	2	4	4	20	High quality	23066	3345667	55.20	344	d__Bacteria;p__Actinobacteriota;c__Acidimicrobiia;o__Acidimicrobiales;f__Acidimicrobiaceae;g__Ferrimicrobium;s__Ferrimicrobium acidiphilum
LMSG_G000008259.1	no	607_1	Lead-Zinc	86.32	3.85	67.09	1	6	2	20	Medium quality	61389	2812980	55.00	203	d__Bacteria;p__Actinobacteriota;c__Acidimicrobiia;o__Acidimicrobiales;f__Acidimicrobiaceae;g__Ferrimicrobium;s__Ferrimicrobium acidiphilum
LMSG_G000008260.1	no	607_1	Lead-Zinc	93.16	2.99	78.21	1	0	1	19	Medium quality	15328	2525724	55.40	235	d__Bacteria;p__Actinobacteriota;c__Acidimicrobiia;o__Acidimicrobiales;f__Acidimicrobiaceae;g__Ferrimicrobium;s__Ferrimicrobium acidiphilum
LMSG_G000008261.1	no	607_1	Pyrite	93.44	1.28	87.03	1	0	0	20	Medium quality	57510	2777654	55.40	157	d__Bacteria;p__Actinobacteriota;c__Acidimicrobiia;o__Acidimicrobiales;f__Acidimicrobiaceae;g__Ferrimicrobium;s__Ferrimicrobium acidiphilum
LMSG_G000008262.1	no	607_1	Pyrite	92.30	1.28	85.89	1	0	0	19	Medium quality	57644	2426807	55.40	138	d__Bacteria;p__Actinobacteriota;c__Acidimicrobiia;o__Acidimicrobiales;f__Acidimicrobiaceae;g__Ferrimicrobium;s__Ferrimicrobium acidiphilum
LMSG_G000008263.1	no	607_1	Lead-Zinc	88.60	1.28	82.19	2	0	1	20	Medium quality	24360	2772450	55.40	206	d__Bacteria;p__Actinobacteriota;c__Acidimicrobiia;o__Acidimicrobiales;f__Acidimicrobiaceae;g__Ferrimicrobium;s__Ferrimicrobium acidiphilum
LMSG_G000008264.1	no	607_1	Copper	92.30	1.28	85.89	1	0	1	20	Medium quality	40503	2634741	55.40	145	d__Bacteria;p__Actinobacteriota;c__Acidimicrobiia;o__Acidimicrobiales;f__Acidimicrobiaceae;g__Ferrimicrobium;s__Ferrimicrobium acidiphilum
LMSG_G000008265.1	no	607_1	Lead-Zinc	77.35	0.85	73.08	0	0	0	16	Medium quality	7265	2021482	55.50	340	d__Bacteria;p__Actinobacteriota;c__Acidimicrobiia;o__Acidimicrobiales;f__Acidimicrobiaceae;g__Ferrimicrobium;s__Ferrimicrobium acidiphilum
LMSG_G000008266.1	no	607_1	Lead-Zinc	91.45	2.14	80.77	2	0	0	20	Medium quality	24109	2572763	55.30	162	d__Bacteria;p__Actinobacteriota;c__Acidimicrobiia;o__Acidimicrobiales;f__Acidimicrobiaceae;g__Ferrimicrobium;s__Ferrimicrobium acidiphilum
LMSG_G000008267.1	no	607_1	Polymetallic	93.16	2.14	82.48	2	0	0	19	Medium quality	28647	2393840	55.40	140	d__Bacteria;p__Actinobacteriota;c__Acidimicrobiia;o__Acidimicrobiales;f__Acidimicrobiaceae;g__Ferrimicrobium;s__Ferrimicrobium acidiphilum
LMSG_G000008268.1	no	607_1	Polymetallic	89.74	1.28	83.33	2	0	0	20	Medium quality	29488	2591373	55.30	165	d__Bacteria;p__Actinobacteriota;c__Acidimicrobiia;o__Acidimicrobiales;f__Acidimicrobiaceae;g__Ferrimicrobium;s__Ferrimicrobium acidiphilum
LMSG_G000008269.1	no	607_1	Polymetallic	91.45	2.14	80.77	1	0	0	19	Medium quality	60728	2629971	55.50	187	d__Bacteria;p__Actinobacteriota;c__Acidimicrobiia;o__Acidimicrobiales;f__Acidimicrobiaceae;g__Ferrimicrobium;s__Ferrimicrobium acidiphilum
LMSG_G000008270.1	no	607_1	Polymetallic	93.16	1.28	86.75	1	0	0	18	Medium quality	62788	2367151	55.50	144	d__Bacteria;p__Actinobacteriota;c__Acidimicrobiia;o__Acidimicrobiales;f__Acidimicrobiaceae;g__Ferrimicrobium;s__Ferrimicrobium acidiphilum
LMSG_G000008271.1	no	607_1	Polymetallic	82.43	1.28	76.02	1	0	1	16	Medium quality	3943	2984951	55.20	969	d__Bacteria;p__Actinobacteriota;c__Acidimicrobiia;o__Acidimicrobiales;f__Acidimicrobiaceae;g__Ferrimicrobium;s__Ferrimicrobium acidiphilum
LMSG_G000008272.1	no	607_1	Polymetallic	63.19	0.85	58.92	1	2	2	14	Medium quality	2266	2104724	55.20	1013	d__Bacteria;p__Actinobacteriota;c__Acidimicrobiia;o__Acidimicrobiales;f__Acidimicrobiaceae;g__Ferrimicrobium;s__Ferrimicrobium acidiphilum
LMSG_G000008273.1	no	607_1	Tin-Zinc	92.30	1.28	85.89	2	0	0	19	Medium quality	33830	2449460	55.40	133	d__Bacteria;p__Actinobacteriota;c__Acidimicrobiia;o__Acidimicrobiales;f__Acidimicrobiaceae;g__Ferrimicrobium;s__Ferrimicrobium acidiphilum
LMSG_G000008274.1	no	607_1	Polymetallic	89.74	1.28	83.33	1	0	0	20	Medium quality	52189	2583688	55.50	192	d__Bacteria;p__Actinobacteriota;c__Acidimicrobiia;o__Acidimicrobiales;f__Acidimicrobiaceae;g__Ferrimicrobium;s__Ferrimicrobium acidiphilum
LMSG_G000008275.1	no	607_1	Copper	90.02	1.28	83.61	2	0	0	20	Medium quality	28865	2674048	55.20	156	d__Bacteria;p__Actinobacteriota;c__Acidimicrobiia;o__Acidimicrobiales;f__Acidimicrobiaceae;g__Ferrimicrobium;s__Ferrimicrobium acidiphilum
LMSG_G000008276.1	no	607_1	Polymetallic	87.17	0.43	85.04	2	0	0	20	Medium quality	21145	2571001	55.40	181	d__Bacteria;p__Actinobacteriota;c__Acidimicrobiia;o__Acidimicrobiales;f__Acidimicrobiaceae;g__Ferrimicrobium;s__Ferrimicrobium acidiphilum
LMSG_G000008277.1	no	607_1	Magnetite	93.73	1.28	87.32	2	0	0	19	Medium quality	26512	2725260	55.20	186	d__Bacteria;p__Actinobacteriota;c__Acidimicrobiia;o__Acidimicrobiales;f__Acidimicrobiaceae;g__Ferrimicrobium;s__Ferrimicrobium acidiphilum
LMSG_G000008278.1	no	607_1	Lead-Zinc	73.94	1.28	67.53	0	0	0	16	Medium quality	7299	1887731	55.50	316	d__Bacteria;p__Actinobacteriota;c__Acidimicrobiia;o__Acidimicrobiales;f__Acidimicrobiaceae;g__Ferrimicrobium;s__Ferrimicrobium acidiphilum
LMSG_G000008279.1	no	607_1	Polymetallic	92.59	2.14	81.91	1	0	0	19	Medium quality	32341	2791100	55.20	170	d__Bacteria;p__Actinobacteriota;c__Acidimicrobiia;o__Acidimicrobiales;f__Acidimicrobiaceae;g__Ferrimicrobium;s__Ferrimicrobium acidiphilum
LMSG_G000008280.1	no	607_1	Magnetite	91.45	2.14	80.77	1	0	0	20	Medium quality	31208	2683212	55.20	233	d__Bacteria;p__Actinobacteriota;c__Acidimicrobiia;o__Acidimicrobiales;f__Acidimicrobiaceae;g__Ferrimicrobium;s__Ferrimicrobium acidiphilum
LMSG_G000008281.1	no	607_1	Magnetite	78.67	2.56	65.85	1	0	0	18	Medium quality	12403	2491632	55.20	364	d__Bacteria;p__Actinobacteriota;c__Acidimicrobiia;o__Acidimicrobiales;f__Acidimicrobiaceae;g__Ferrimicrobium;s__Ferrimicrobium acidiphilum
LMSG_G000008282.1	no	607_1	Magnetite	63.29	1.57	55.46	1	0	1	18	Medium quality	7625	1876097	55.10	313	d__Bacteria;p__Actinobacteriota;c__Acidimicrobiia;o__Acidimicrobiales;f__Acidimicrobiaceae;g__Ferrimicrobium;s__Ferrimicrobium acidiphilum

LMSG_G000008283.1	no	607_1	Copper	82.05	1.28	75.64	2	0	0	20	Medium quality	29235	2367590	55.20	122	d__Bacteria;p__Actinobacteriota;c__Acidimicrobiia;o__Acidimicrobiales;f__Acidimicrobiaceae;g__Ferrimicrobium;s__Ferrimicrobium acidiphilum
LMSG_G000008284.1	no	607_1	Copper	91.45	2.14	80.77	1	0	1	19	Medium quality	26983	2562676	55.20	135	d__Bacteria;p__Actinobacteriota;c__Acidimicrobiia;o__Acidimicrobiales;f__Acidimicrobiaceae;g__Ferrimicrobium;s__Ferrimicrobium acidiphilum
LMSG_G000008285.1	no	607_1	Copper	85.47	2.99	70.52	1	0	0	18	Medium quality	27045	2371097	55.30	129	d__Bacteria;p__Actinobacteriota;c__Acidimicrobiia;o__Acidimicrobiales;f__Acidimicrobiaceae;g__Ferrimicrobium;s__Ferrimicrobium acidiphilum
LMSG_G000008286.1	no	607_1	Pyrite	87.17	1.28	80.76	1	0	0	17	Medium quality	60333	2493664	55.50	149	d__Bacteria;p__Actinobacteriota;c__Acidimicrobiia;o__Acidimicrobiales;f__Acidimicrobiaceae;g__Ferrimicrobium;s__Ferrimicrobium acidiphilum
LMSG_G000008287.1	no	607_1	Copper	91.45	0.43	89.32	2	1	0	20	Medium quality	34508	2563952	55.40	122	d__Bacteria;p__Actinobacteriota;c__Acidimicrobiia;o__Acidimicrobiales;f__Acidimicrobiaceae;g__Ferrimicrobium;s__Ferrimicrobium acidiphilum
LMSG_G000008288.1	no	607_1	Tin-Zinc	91.73	1.28	85.32	1	2	0	20	Medium quality	50285	2663317	55.40	157	d__Bacteria;p__Actinobacteriota;c__Acidimicrobiia;o__Acidimicrobiales;f__Acidimicrobiaceae;g__Ferrimicrobium;s__Ferrimicrobium acidiphilum
LMSG_G000008289.1	no	607_1	Copper	91.45	1.28	85.04	2	1	0	20	Medium quality	30500	2398419	55.30	116	d__Bacteria;p__Actinobacteriota;c__Acidimicrobiia;o__Acidimicrobiales;f__Acidimicrobiaceae;g__Ferrimicrobium;s__Ferrimicrobium acidiphilum
LMSG_G000008290.1	no	688_1	Lead-Zinc	88.46	1.28	82.05	0	0	0	15	Medium quality	8126	2114494	48.00	328	d__Bacteria;p__Actinobacteriota;c__Acidimicrobiia;o__Acidimicrobiales;f__Acidimicrobiaceae;g__Ferrithrix;s__
LMSG_G000008291.1	no	688_1	Tin-Zinc	97.00	1.28	90.59	0	0	0	19	Medium quality	19542	2578437	48.10	199	d__Bacteria;p__Actinobacteriota;c__Acidimicrobiia;o__Acidimicrobiales;f__Acidimicrobiaceae;g__Ferrithrix;s__
LMSG_G000008292.1	no	688_1	Tin-Zinc	94.01	2.99	79.06	0	1	0	18	Medium quality	12966	2261057	47.90	233	d__Bacteria;p__Actinobacteriota;c__Acidimicrobiia;o__Acidimicrobiales;f__Acidimicrobiaceae;g__Ferrithrix;s__
LMSG_G000008293.1	no	688_1	Pyrite-Copper	100.00	1.28	93.59	0	0	0	20	Medium quality	31338	2625000	48.10	137	d__Bacteria;p__Actinobacteriota;c__Acidimicrobiia;o__Acidimicrobiales;f__Acidimicrobiaceae;g__Ferrithrix;s__
LMSG_G000008294.1	no	689_1	Pyrite	98.29	3.85	79.06	1	1	1	19	High quality	22867	2562826	48.40	158	d__Bacteria;p__Actinobacteriota;c__Acidimicrobiia;o__Acidimicrobiales;f__Acidimicrobiaceae;g__Ferrithrix;s__
LMSG_G000008295.1	no	689_1	Pyrite	100.00	2.14	89.32	0	1	0	20	Medium quality	27745	2659889	48.50	166	d__Bacteria;p__Actinobacteriota;c__Acidimicrobiia;o__Acidimicrobiales;f__Acidimicrobiaceae;g__Ferrithrix;s__
LMSG_G000008296.1	no	689_1	Pyrite-Copper	89.74	1.28	83.33	0	0	0	15	Medium quality	50175	1972076	48.60	110	d__Bacteria;p__Actinobacteriota;c__Acidimicrobiia;o__Acidimicrobiales;f__Acidimicrobiaceae;g__Ferrithrix;s__
LMSG_G000008297.1	no	689_1	Pyrite-Copper	89.17	1.55	81.44	0	0	1	18	Medium quality	10927	2298186	48.50	290	d__Bacteria;p__Actinobacteriota;c__Acidimicrobiia;o__Acidimicrobiales;f__Acidimicrobiaceae;g__Ferrithrix;s__
LMSG_G000008298.1	no	689_1	Pyrite	98.29	1.28	91.88	0	1	0	20	Medium quality	36267	2673149	48.50	123	d__Bacteria;p__Actinobacteriota;c__Acidimicrobiia;o__Acidimicrobiales;f__Acidimicrobiaceae;g__Ferrithrix;s__
LMSG_G000008299.1	no	690_1	Pyrite	61.84	0.85	57.57	0	1	0	9	Medium quality	4504	1458631	48.30	355	d__Bacteria;p__Actinobacteriota;c__Acidimicrobiia;o__Acidimicrobiales;f__Acidimicrobiaceae;g__Ferrithrix;s__
LMSG_G000008300.1	no	690_1	Pyrite-Copper	97.77	3.85	78.54	0	0	0	18	Medium quality	20547	2853751	48.40	248	d__Bacteria;p__Actinobacteriota;c__Acidimicrobiia;o__Acidimicrobiales;f__Acidimicrobiaceae;g__Ferrithrix;s__
LMSG_G000008301.1	no	694_1	Lead-Zinc	92.02	2.14	81.34	1	0	0	19	Medium quality	10203	2380954	56.40	298	d__Bacteria;p__Actinobacteriota;c__Acidimicrobiia;o__Acidimicrobiales;f__Acidimicrobiaceae;g__Ferrithrix;s__
LMSG_G000008302.1	no	694_1	Lead-Zinc	99.14	2.21	88.07	1	0	1	20	Medium quality	27109	2571580	56.30	162	d__Bacteria;p__Actinobacteriota;c__Acidimicrobiia;o__Acidimicrobiales;f__Acidimicrobiaceae;g__Ferrithrix;s__
LMSG_G000008303.1	no	694_1	Polymetallic	97.01	1.28	90.60	2	1	0	19	Medium quality	45359	2629355	56.30	148	d__Bacteria;p__Actinobacteriota;c__Acidimicrobiia;o__Acidimicrobiales;f__Acidimicrobiaceae;g__Ferrithrix;s__
LMSG_G000008304.1	no	609_1	Lead-Zinc	96.58	1.38	89.70	1	1	1	20	High quality	16367	2741936	53.60	255	d__Bacteria;p__Actinobacteriota;c__Acidimicrobiia;o__Acidimicrobiales;f__Acidimicrobiaceae;g__SCQM01;s__
LMSG_G000008305.1	no	609_1	Lead-Zinc	99.14	1.38	92.26	1	1	1	19	High quality	27726	2949847	53.70	149	d__Bacteria;p__Actinobacteriota;c__Acidimicrobiia;o__Acidimicrobiales;f__Acidimicrobiaceae;g__SCQM01;s__
LMSG_G000008306.1	no	609_1	Lead-Zinc	56.75	1.28	50.34	1	0	1	13	Medium quality	45887	1301359	53.20	66	d__Bacteria;p__Actinobacteriota;c__Acidimicrobiia;o__Acidimicrobiales;f__Acidimicrobiaceae;g__SCQM01;s__
LMSG_G000008307.1	no	609_1	Lead-Zinc	72.35	1.03	67.23	0	1	0	16	Medium quality	5350	2041458	53.40	437	d__Bacteria;p__Actinobacteriota;c__Acidimicrobiia;o__Acidimicrobiales;f__Acidimicrobiaceae;g__SCQM01;s__
LMSG_G000008308.1	no	610_1	Tin-Zinc	60.65	1.71	52.11	0	0	0	14	Medium quality	5991	1430326	52.40	256	d__Bacteria;p__Actinobacteriota;c__Acidimicrobiia;o__Acidimicrobiales;f__Acidimicrobiaceae;g__SCQM01;s__
LMSG_G000008309.1	no	610_2	Arsenic	57.75	0.00	57.75	0	0	0	12	Medium quality	10135	1711622	52.40	265	d__Bacteria;p__Actinobacteriota;c__Acidimicrobiia;o__Acidimicrobiales;f__Acidimicrobiaceae;g__SCQM01;s__
LMSG_G000008310.1	no	610_2	Copper	73.06	2.36	61.24	1	0	0	16	Medium quality	9757	1634168	52.80	215	d__Bacteria;p__Actinobacteriota;c__Acidimicrobiia;o__Acidimicrobiales;f__Acidimicrobiaceae;g__SCQM01;s__
LMSG_G000008311.1	no	611_1	Polymetallic	94.27	1.28	87.86	1	1	1	18	High quality	17532	2814052	54.60	288	d__Bacteria;p__Actinobacteriota;c__Acidimicrobiia;o__Acidimicrobiales;f__Acidimicrobiaceae;g__SCQM01;s__
LMSG_G000008312.1	no	611_1	Polymetallic	94.48	1.28	88.07	1	1	1	19	High quality	22596	2980415	54.50	231	d__Bacteria;p__Actinobacteriota;c__Acidimicrobiia;o__Acidimicrobiales;f__Acidimicrobiaceae;g__SCQM01;s__
LMSG_G000008313.1	no	611_1	Polymetallic	94.87	2.14	84.19	0	0	0	19	Medium quality	44868	2785295	54.70	148	d__Bacteria;p__Actinobacteriota;c__Acidimicrobiia;o__Acidimicrobiales;f__Acidimicrobiaceae;g__SCQM01;s__
LMSG_G000008314.1	no	611_1	Polymetallic	95.01	2.28	83.62	1	1	0	20	Medium quality	22034	3130146	54.80	230	d__Bacteria;p__Actinobacteriota;c__Acidimicrobiia;o__Acidimicrobiales;f__Acidimicrobiaceae;g__SCQM01;s__
LMSG_G000008315.1	no	612_1	Lead-Zinc	69.84	1.28	63.43	0	0	0	11	Medium quality	4843	1445487	54.20	318	d__Bacteria;p__Actinobacteriota;c__Acidimicrobiia;o__Acidimicrobiales;f__Acidimicrobiaceae;g__SCQM01;s__
LMSG_G000008316.1	no	612_1	Magnetite	57.75	0.00	57.75	1	0	0	14	Medium quality	7025	1902848	54.00	342	d__Bacteria;p__Actinobacteriota;c__Acidimicrobiia;o__Acidimicrobiales;f__Acidimicrobiaceae;g__SCQM01;s__
LMSG_G000008317.1	no	613_1	Pyrite-Copper	99.14	1.71	90.60	1	0	0	18	Medium quality	42388	2390334	52.40	89	d__Bacteria;p__Actinobacteriota;c__Acidimicrobiia;o__Acidimicrobiales;f__Acidimicrobiaceae;g__SCQM01;s__
LMSG_G000008318.1	no	613_1	Pyrite-Copper	81.79	2.56	68.97	0	0	0	17	Medium quality	17351	2006149	52.30	195	d__Bacteria;p__Actinobacteriota;c__Acidimicrobiia;o__Acidimicrobiales;f__Acidimicrobiaceae;g__SCQM01;s__
LMSG_G000008319.1	no	616_1	Copper	97.48	1.28	91.07	1	4	5	19	High quality	42026	2786819	48.10	185	d__Bacteria;p__Actinobacteriota;c__Acidimicrobiia;o__Acidimicrobiales;f__Acidimicrobiaceae;g__SCQM01;s__
LMSG_G000008320.1	no	616_1	Polymetallic	100.00	1.28	93.59	1	0	0	19	Medium quality	50742	2685167	48.20	117	d__Bacteria;p__Actinobacteriota;c__Acidimicrobiia;o__Acidimicrobiales;f__Acidimicrobiaceae;g__SCQM01;s__
LMSG_G000008321.1	no	616_1	Polymetallic	100.00	1.28	93.59	1	0	0	19	Medium quality	45068	2719974	48.20	144	d__Bacteria;p__Actinobacteriota;c__Acidimicrobiia;o__Acidimicrobiales;f__Acidimicrobiaceae;g__SCQM01;s__
LMSG_G000008322.1	no	616_1	Polymetallic	100.00	1.28	93.59	1	0	0	20	Medium quality	50645	3027378	48.10	187	d__Bacteria;p__Actinobacteriota;c__Acidimicrobiia;o__Acidimicrobiales;f__Acidimicrobiaceae;g__SCQM01;s__
LMSG_G000008323.1	no	616_1	Polymetallic	100.00	1.28	93.59	1	0	0	20	Medium quality	44911	3215882	48.30	214	d__Bacteria;p__Actinobacteriota;c__Acidimicrobiia;o__Acidimicrobiales;f__Acidimicrobiaceae;g__SCQM01;s__
LMSG_G000008324.1	no	616_1	Polymetallic	99.14	1.28	92.73	3	0	3	20	Medium quality	55894	2826163	48.30	226	d__Bacteria;p__Actinobacteriota;c__Acidimicrobiia;o__Acidimicrobiales;f__Acidimicrobiaceae;g__SCQM01;s__
LMSG_G000008325.1	no	616_1	Polymetallic	99.14	1.28	92.73	0	0	0	20	Medium quality	33085	2648672	48.20	154	d__Bacteria;p__Actinobacteriota;c__Acidimicrobiia;o__Acidimicrobiales;f__Acidimicrobiaceae;g__SCQM01;s__
LMSG_G000008326.1	no	616_1	Magnetite	100.00	1.28	93.59	1	0	0	19	Medium quality	41304	2638949	48.10	105	d__Bacteria;p__Actinobacteriota;c__Acidimicrobiia;o__Acidimicrobiales;f__Acidimicrobiaceae;g__SCQM01;s__
LMSG_G000008327.1	no	616_1	Copper	66.95	0.00	66.95	1	4	2	20	Medium quality	36284	2884422	48.10	239	d__Bacteria;p__Actinobacteriota;c__Acidimicrobiia;o__Acidimicrobiales;f__Acidimicrobiaceae;g__SCQM01;s__
LMSG_G000008328.1	no	616_1	Copper	89.74	2.14	79.06	1	1	1	19	Medium quality	20170	3546240	48.10	260	d__Bacteria;p__Actinobacteriota;c__Acidimicrobiia;o__Acidimicrobiales;f__Acidimicrobiaceae;g__SCQM01;s__
LMSG_G000008329.1	no	616_1	Copper	68.26	0.00	68.26	1	1	0	19	Medium quality	18228	2647365	48.00	200	d__Bacteria;p__Actinobacteriota;c__Acidimicrobiia;o__Acidimicrobiales;f__Acidimicrobiaceae;g__SCQM01;s__
LMSG_G000008330.1	no	616_1	Magnetite	93.81	2.14	83.13	1	4	0	20	Medium quality	49888	3116524	48.20	266	d__Bacteria;p__Actinobacteriota;c__Acidimicrobiia;o__Acidimicrobiales;f__Acidimicrobiaceae;g__SCQM01;s__
LMSG_G000008331.1	no	506_1	Magnetite	96.58	1.00	91.60	0	0	0	20	Medium quality	11980	1783868	50.10	199	d__Bacteria;p__Actinobacteriota;c__Acidimicrobiia;o__Acidimicrobiales;f__Bog-793;g__;s__
LMSG_G000008332.1	no	506_1	Magnetite	59.08	0.00	59.08	0	0	0	13	Medium quality	48087	1093380	50.80	39	d__Bacteria;p__Actinobacteriota;c__Acidimicrobiia;o__Acidimicrobiales;f__Bog-793;g__;s__
LMSG_G000008333.1	no	506_1	Polymetallic	98.29	1.28	91.88	1	1	0	20	Medium quality	92072	1976609	50.20	69	d__Bacteria;p__Actinobacteriota;c__Acidimicrobiia;o__Acidimicrobiales;f__Bog-793;g__;s__
LMSG_G000008334.1	no	754_1	Polymetallic	94.87	1.28	88.46	1	1	1	19	High quality	65698	3098628	69.00	86	d__Bacteria;p__Actinobacteriota;c__Acidimicrobiia;o__Acidimicrobiales;f__Bog-793;g__;s__
LMSG_G000008335.1	no	754_1	Polymetallic	94.01	0.52	91.40	1	1	1	18	High quality	57229	2903125	69.10	87	d__Bacteria;p__Actinobacteriota;c__Acidimicrobiia;o__Acidimicrobiales;f__Bog-793;g__;s__

LMSG_G000008336.1	no	754_1	Polymetallic	97.43	2.56	84.61	1	1	1	20	High quality	44119	3210921	69.00	120	d__Bacteria;p__Actinobacteriota;c__Acidimicrobiia;o__Acidimicrobiales;f__Bog-793;g__;s__
LMSG_G000008337.1	no	754_1	Magnetite	95.72	0.43	93.59	1	1	1	19	High quality	18806	2659800	69.20	201	d__Bacteria;p__Actinobacteriota;c__Acidimicrobiia;o__Acidimicrobiales;f__Bog-793;g__;s__
LMSG_G000008338.1	no	754_1	Copper	90.78	1.71	82.24	1	1	1	16	Medium quality	6198	2548254	69.20	487	d__Bacteria;p__Actinobacteriota;c__Acidimicrobiia;o__Acidimicrobiales;f__Bog-793;g__;s__
LMSG_G000008339.1	no	754_1	Polymetallic	93.16	0.43	91.03	1	1	1	15	Medium quality	14585	2568412	69.10	224	d__Bacteria;p__Actinobacteriota;c__Acidimicrobiia;o__Acidimicrobiales;f__Bog-793;g__;s__
LMSG_G000008340.1	no	754_1	Polymetallic	87.17	1.28	80.76	1	1	1	11	Medium quality	55176	2690214	69.30	75	d__Bacteria;p__Actinobacteriota;c__Acidimicrobiia;o__Acidimicrobiales;f__Bog-793;g__;s__
LMSG_G000008341.1	no	754_1	Tin-Zinc	71.52	0.43	69.39	0	1	0	18	Medium quality	10348	2208915	69.10	245	d__Bacteria;p__Actinobacteriota;c__Acidimicrobiia;o__Acidimicrobiales;f__Bog-793;g__;s__
LMSG_G000008342.1	no	754_1	Tin-Zinc	74.44	0.43	72.31	1	0	1	14	Medium quality	10502	2221357	69.50	253	d__Bacteria;p__Actinobacteriota;c__Acidimicrobiia;o__Acidimicrobiales;f__Bog-793;g__;s__
LMSG_G000008343.1	no	754_1	Polymetallic	88.88	0.85	84.61	1	1	1	18	Medium quality	49490	2796448	69.20	100	d__Bacteria;p__Actinobacteriota;c__Acidimicrobiia;o__Acidimicrobiales;f__Bog-793;g__;s__
LMSG_G000008344.1	no	754_1	Copper	94.87	0.95	90.13	0	1	0	20	Medium quality	17920	3019226	69.10	228	d__Bacteria;p__Actinobacteriota;c__Acidimicrobiia;o__Acidimicrobiales;f__Bog-793;g__;s__
LMSG_G000008345.1	no	754_1	Copper	74.81	0.00	74.81	1	1	1	11	Medium quality	4803	2385631	69.40	566	d__Bacteria;p__Actinobacteriota;c__Acidimicrobiia;o__Acidimicrobiales;f__Bog-793;g__;s__
LMSG_G000008346.1	no	754_1	Copper	86.32	0.43	84.19	1	1	1	13	Medium quality	14704	2303244	69.30	198	d__Bacteria;p__Actinobacteriota;c__Acidimicrobiia;o__Acidimicrobiales;f__Bog-793;g__;s__
LMSG_G000008347.1	no	754_1	Lead-Zinc	64.03	1.28	57.62	0	0	0	16	Medium quality	6174	2136729	69.20	407	d__Bacteria;p__Actinobacteriota;c__Acidimicrobiia;o__Acidimicrobiales;f__Bog-793;g__;s__
LMSG_G000008348.1	no	754_1	Lead-Zinc	87.80	1.80	78.78	1	1	1	15	Medium quality	10018	2822043	69.30	405	d__Bacteria;p__Actinobacteriota;c__Acidimicrobiia;o__Acidimicrobiales;f__Bog-793;g__;s__
LMSG_G000008349.1	no	754_1	Copper	70.25	2.14	59.57	0	0	0	16	Medium quality	4304	2510814	68.80	629	d__Bacteria;p__Actinobacteriota;c__Acidimicrobiia;o__Acidimicrobiales;f__Bog-793;g__;s__
LMSG_G000008350.1	no	754_1	Copper	89.98	1.28	83.57	0	0	0	17	Medium quality	10792	2712408	69.40	324	d__Bacteria;p__Actinobacteriota;c__Acidimicrobiia;o__Acidimicrobiales;f__Bog-793;g__;s__
LMSG_G000008351.1	no	754_1	Copper	87.00	2.48	74.60	1	1	1	18	Medium quality	18263	3065220	69.20	414	d__Bacteria;p__Actinobacteriota;c__Acidimicrobiia;o__Acidimicrobiales;f__Bog-793;g__;s__
LMSG_G000008352.1	no	754_1	Lead-Zinc	84.18	2.87	69.81	2	1	2	15	Medium quality	5265	4180832	67.90	915	d__Bacteria;p__Actinobacteriota;c__Acidimicrobiia;o__Acidimicrobiales;f__Bog-793;g__;s__
LMSG_G000008353.1	no	754_1	Pyrite-Copper	65.02	0.00	65.02	1	0	1	13	Medium quality	5789	1442735	69.60	264	d__Bacteria;p__Actinobacteriota;c__Acidimicrobiia;o__Acidimicrobiales;f__Bog-793;g__;s__
LMSG_G000008354.1	no	767_1	Polymetallic	50.15	0.00	50.15	0	0	0	8	Medium quality	2618	1172373	73.70	447	d__Bacteria;p__Actinobacteriota;c__Acidimicrobiia;o__Acidimicrobiales;f__Bog-793;g__;s__
LMSG_G000008355.1	no	767_1	Polymetallic	68.30	3.00	53.30	0	0	0	8	Medium quality	2932	1768426	73.20	626	d__Bacteria;p__Actinobacteriota;c__Acidimicrobiia;o__Acidimicrobiales;f__Bog-793;g__;s__
LMSG_G000008356.1	no	768_1	Polymetallic	94.87	2.14	84.19	0	0	0	19	Medium quality	9300	3510458	74.00	522	d__Bacteria;p__Actinobacteriota;c__Acidimicrobiia;o__Acidimicrobiales;f__Bog-793;g__;s__
LMSG_G000008357.1	no	768_1	Polymetallic	96.58	3.21	80.56	0	1	0	19	Medium quality	16017	3225737	74.40	285	d__Bacteria;p__Actinobacteriota;c__Acidimicrobiia;o__Acidimicrobiales;f__Bog-793;g__;s__
LMSG_G000008358.1	no	768_1	Polymetallic	98.29	2.56	85.47	0	1	0	20	Medium quality	17663	3575727	74.20	298	d__Bacteria;p__Actinobacteriota;c__Acidimicrobiia;o__Acidimicrobiales;f__Bog-793;g__;s__
LMSG_G000008359.1	no	768_1	Polymetallic	87.74	1.28	81.33	1	1	1	19	Medium quality	18090	3170855	74.30	225	d__Bacteria;p__Actinobacteriota;c__Acidimicrobiia;o__Acidimicrobiales;f__Bog-793;g__;s__
LMSG_G000008360.1	no	768_1	Polymetallic	86.67	2.14	75.99	0	0	0	17	Medium quality	12803	2791102	74.50	342	d__Bacteria;p__Actinobacteriota;c__Acidimicrobiia;o__Acidimicrobiales;f__Bog-793;g__;s__
LMSG_G000008361.1	no	768_1	Polymetallic	55.75	0.00	55.75	0	0	0	11	Medium quality	2481	1681035	73.30	693	d__Bacteria;p__Actinobacteriota;c__Acidimicrobiia;o__Acidimicrobiales;f__Bog-793;g__;s__
LMSG_G000008362.1	no	770_1	Copper	96.58	3.28	80.20	1	1	1	19	High quality	36347	2881631	72.90	138	d__Bacteria;p__Actinobacteriota;c__Acidimicrobiia;o__Acidimicrobiales;f__Bog-793;g__;s__
LMSG_G000008363.1	no	770_1	Copper	96.58	2.14	85.90	1	1	1	17	Medium quality	31138	2773810	72.90	130	d__Bacteria;p__Actinobacteriota;c__Acidimicrobiia;o__Acidimicrobiales;f__Bog-793;g__;s__
LMSG_G000008364.1	no	770_1	Lead-Zinc	95.72	2.99	80.77	1	1	1	17	Medium quality	32225	2774717	72.90	132	d__Bacteria;p__Actinobacteriota;c__Acidimicrobiia;o__Acidimicrobiales;f__Bog-793;g__;s__
LMSG_G000008365.1	no	770_1	Lead-Zinc	90.59	3.85	71.36	1	1	1	16	Medium quality	33224	2439245	73.10	111	d__Bacteria;p__Actinobacteriota;c__Acidimicrobiia;o__Acidimicrobiales;f__Bog-793;g__;s__
LMSG_G000008366.1	no	770_1	Copper	92.22	2.56	79.40	0	0	0	19	Medium quality	15798	2846629	73.00	292	d__Bacteria;p__Actinobacteriota;c__Acidimicrobiia;o__Acidimicrobiales;f__Bog-793;g__;s__
LMSG_G000008367.1	no	770_1	Copper	76.53	1.71	67.99	0	0	0	10	Medium quality	9364	2166466	73.40	352	d__Bacteria;p__Actinobacteriota;c__Acidimicrobiia;o__Acidimicrobiales;f__Bog-793;g__;s__
LMSG_G000008368.1	no	770_1	Lead-Zinc	97.00	2.99	82.05	0	1	0	19	Medium quality	25942	3009795	72.80	186	d__Bacteria;p__Actinobacteriota;c__Acidimicrobiia;o__Acidimicrobiales;f__Bog-793;g__;s__
LMSG_G000008369.1	no	770_1	Lead-Zinc	89.36	1.71	80.82	0	1	0	15	Medium quality	34434	2294732	73.20	112	d__Bacteria;p__Actinobacteriota;c__Acidimicrobiia;o__Acidimicrobiales;f__Bog-793;g__;s__
LMSG_G000008370.1	no	770_1	Lead-Zinc	90.59	2.56	77.77	1	0	1	17	Medium quality	32617	2535942	73.00	104	d__Bacteria;p__Actinobacteriota;c__Acidimicrobiia;o__Acidimicrobiales;f__Bog-793;g__;s__
LMSG_G000008371.1	no	774_1	Lead-Zinc	66.04	1.88	56.64	0	0	0	9	Medium quality	4680	1414724	72.70	330	d__Bacteria;p__Actinobacteriota;c__Acidimicrobiia;o__Acidimicrobiales;f__Bog-793;g__;s__
LMSG_G000008372.1	no	775_1	Polymetallic	98.86	2.14	88.18	1	1	1	18	High quality	50890	2933499	70.60	81	d__Bacteria;p__Actinobacteriota;c__Acidimicrobiia;o__Acidimicrobiales;f__Bog-793;g__;s__
LMSG_G000008373.1	no	775_1	Coal	98.29	2.71	84.76	1	1	1	18	High quality	48212	2854223	70.80	107	d__Bacteria;p__Actinobacteriota;c__Acidimicrobiia;o__Acidimicrobiales;f__Bog-793;g__;s__
LMSG_G000008374.1	no	775_1	Coal	97.15	2.14	86.47	1	1	1	20	High quality	41690	3128206	70.40	170	d__Bacteria;p__Actinobacteriota;c__Acidimicrobiia;o__Acidimicrobiales;f__Bog-793;g__;s__
LMSG_G000008375.1	no	775_1	Coal	97.15	1.28	90.74	1	1	1	20	High quality	31765	2649821	70.80	125	d__Bacteria;p__Actinobacteriota;c__Acidimicrobiia;o__Acidimicrobiales;f__Bog-793;g__;s__
LMSG_G000008376.1	no	775_1	Polymetallic	98.86	2.14	88.18	1	1	1	17	Medium quality	88217	3160575	70.50	160	d__Bacteria;p__Actinobacteriota;c__Acidimicrobiia;o__Acidimicrobiales;f__Bog-793;g__;s__
LMSG_G000008377.1	no	775_1	Polymetallic	74.64	4.69	51.18	0	0	0	16	Medium quality	5045	2355407	70.40	515	d__Bacteria;p__Actinobacteriota;c__Acidimicrobiia;o__Acidimicrobiales;f__Bog-793;g__;s__
LMSG_G000008378.1	no	775_1	Polymetallic	84.39	3.42	67.30	0	0	0	17	Medium quality	7170	2342743	70.40	364	d__Bacteria;p__Actinobacteriota;c__Acidimicrobiia;o__Acidimicrobiales;f__Bog-793;g__;s__
LMSG_G000008379.1	no	776_1	Magnetite	69.15	0.00	69.15	0	1	0	11	Medium quality	10384	1784277	73.50	225	d__Bacteria;p__Actinobacteriota;c__Acidimicrobiia;o__Acidimicrobiales;f__Bog-793;g__;s__
LMSG_G000008380.1	no	778_1	Coal	75.40	4.06	55.11	0	0	0	15	Medium quality	2710	1561283	60.60	643	d__Bacteria;p__Actinobacteriota;c__Acidimicrobiia;o__Acidimicrobiales;f__Bog-793;g__;s__
LMSG_G000008381.1	no	815_1	Lead-Zinc	94.01	1.28	87.60	2	1	2	19	High quality	76995	1979388	45.80	63	d__Bacteria;p__Actinobacteriota;c__Acidimicrobiia;o__Acidimicrobiales;f__Bog-793;g__;s__
LMSG_G000008382.1	no	815_1	Polymetallic	98.29	1.45	91.03	1	1	1	20	High quality	73112	2211016	45.60	72	d__Bacteria;p__Actinobacteriota;c__Acidimicrobiia;o__Acidimicrobiales;f__Bog-793;g__;s__
LMSG_G000008383.1	no	815_1	Polymetallic	98.29	1.45	91.03	1	2	5	20	High quality	80021	2275061	46.00	83	d__Bacteria;p__Actinobacteriota;c__Acidimicrobiia;o__Acidimicrobiales;f__Bog-793;g__;s__
LMSG_G000008384.1	no	815_1	Polymetallic	97.43	1.28	91.02	1	1	2	20	High quality	55036	2108086	45.50	65	d__Bacteria;p__Actinobacteriota;c__Acidimicrobiia;o__Acidimicrobiales;f__Bog-793;g__;s__
LMSG_G000008385.1	no	815_1	Polymetallic	97.43	1.28	91.02	0	1	0	18	Medium quality	58515	1980275	45.60	48	d__Bacteria;p__Actinobacteriota;c__Acidimicrobiia;o__Acidimicrobiales;f__Bog-793;g__;s__
LMSG_G000008386.1	no	816_1	Lead-Zinc	92.30	1.47	84.95	2	0	2	20	Medium quality	9318	1956595	45.50	334	d__Bacteria;p__Actinobacteriota;c__Acidimicrobiia;o__Acidimicrobiales;f__Bog-793;g__;s__
LMSG_G000008387.1	no	816_1	Polymetallic	74.78	1.47	67.43	0	0	0	18	Medium quality	3403	1588481	45.90	567	d__Bacteria;p__Actinobacteriota;c__Acidimicrobiia;o__Acidimicrobiales;f__Bog-793;g__;s__
LMSG_G000008388.1	no	816_1	Magnetite	89.17	2.99	74.22	2	1	1	18	Medium quality	28780	1926706	45.40	118	d__Bacteria;p__Actinobacteriota;c__Acidimicrobiia;o__Acidimicrobiales;f__Bog-793;g__;s__
LMSG_G000008389.1	no	816_1	Pyrite-Copper	87.65	1.71	79.11	1	2	1	17	Medium quality	21960	1854769	45.50	145	d__Bacteria;p__Actinobacteriota;c__Acidimicrobiia;o__Acidimicrobiales;f__Bog-793;g__;s__
LMSG_G000008390.1	no	816_1	Polymetallic	75.78	3.85	56.55	1	0	1	18	Medium quality	21107	1954226	45.30	196	d__Bacteria;p__Actinobacteriota;c__Acidimicrobiia;o__Acidimicrobiales;f__Bog-793;g__;s__
LMSG_G000008391.1	no	816_1	Magnetite	83.23	2.14	72.55	0	0	0	16	Medium quality	15756	2078132	45.30	259	d__Bacteria;p__Actinobacteriota;c__Acidimicrobiia;o__Acidimicrobiales;f__Bog-793;g__;s__
LMSG_G000008392.1	no	816_1	Magnetite	58.62	0.00	58.62	0	0	0	18	Medium quality	36308	1281999	45.30	101	d__Bacteria;p__Actinobacteriota;c__Acidimicrobiia;o__Acidimicrobiales;f__Bog-793;g__;s__
LMSG_G000008393.1	no	816_1	Magnetite	89.89	2.14	79.21	0	0	0	20	Medium quality	32877	2064259	45.40	186	d__Bacteria;p__Actinobacteriota;c__Acidimicrobiia;o__Acidimicrobiales;f__Bog-793;g__;s__
LMSG_G000008394.1	no	816_1	Magnetite	69.84	0.00	69.84	0	0	0	16	Medium quality	29804	1523080	45.20	106	d__Bacteria;p__Actinobacteriota;c__Acidimicrobiia;o__Acidimicrobiales;f__Bog-793;g__;s__
LMSG_G000008395.1	no	717_1	Copper	94.01	3.42	76.92	1	1	1	20	High quality	15219	2858321	66.00	253	d__Bacteria;p__Actinobacteriota;c__Acidimicrobiia;o__Acidimicrobiales;f__Bog-793;g__Palsa-601;s__
LMSG_G000008396.1	no	717_1	Polymetallic	94.87	2.14	84.19	1	1	1	20	High quality	34162	4247805	66.20	238	d__Bacteria;p__Actinobacteriota;c__Acidimicrobiia;o__Acidimicrobiales;f__Bog-793;g__Palsa-601;s__
LMSG_G000008397.1	no	717_1	Polymetallic	91.54	4.27	70.18	1	1	1	18	High quality	33581	4065693	66.00	358	d__Bacteria;p__Actinobacteriota;c__Acidimicrobiia;o__Acidimicrobiales;f__Bog-793;g__Palsa-601;s__
LMSG_G000008398.1	no	717_1	Magnetite	97.43	2.56	84.61	1	1	1	20	High quality	49825	2552667	66.10	96	d__Bacteria;p__Actinobacteriota;c__Acidimicrobiia;o__Acidimicrobiales;f__Bog-793;g__Palsa-601;s__
LMSG_G000008399.1	no	717_1	Copper	69.36	1.71	60.82	1	1	1	16	Medium quality	4618	2431686	65.40	632	d__Bacteria;p__Actinobacteriota;c__Acidimicrobiia;o__Acidimicrobiales;f__Bog-793;g__Palsa-601;s__
LMSG_G000008400.1	no	717_1	Copper	89.74	1.71	81.20	1	1	1	20	Medium quality	64892	2414282	66.10	81	d__Bacteria;p__Actinobacteriota;c__Acidimicrobiia;o__Acidimicrobiales;f__Bog-793;g__Palsa-601;s__
LMSG_G000008401.1	no	717_1	Polymetallic	95.72	2.99	80.77	1	0	1	20	Medium quality	53567	3215300	65.90	149	d__Bacteria;p__Actinobacteriota;c__Acidimicrobiia;o__Acidimicrobiales;f__Bog-793;g__Palsa-601;s__
LMSG_G000008402.1	no	717_1	Copper	89.74	2.99	74.79	1	1	1	20	Medium quality	74201	3103713	66.00	87	d__Bacteria;p__Actinobacteriota;c__Acidimicrobiia;o__Acidimicrobiales;f__Bog-793;g__Palsa-601;s__
LMSG_G000008403.1	no	717_1	Pyrite-Copper	74.59	1.71	66.05	1	1	1	16	Medium quality	18391	1713501	65.80	128	d__Bacteria;p__Actinobacteriota;c__Acidimicrobiia;o__Acidimicrobiales;f__Bog-793;g__Palsa-601;s__
LMSG_G000008404.1	no	717_1	Pyrite-Copper	50.73	0.00	50.73	0	0	0	11	Medium quality	17724	1185735	65.70	93	d__Bacteria;p__Actinobacteriota;c__Acidimicrobiia;o__Acidimicrobiales;f__Bog-793;g__Palsa-601;s__
LMSG_G000008405.1	no	717_1	Copper	96.58	2.56	83.76	1	1	0	19	Medium quality	36471	2640599	66.10	119	d__Bacteria;p__Actinobacteriota;c__Acidimicrobiia;o__Acidimicrobiales;f__Bog-793;g__Palsa-601;s__
LMSG_G000008406.1	no	741_1	Copper	95.72	4.81	71.70	1	1	1	18	High quality	24704	2350346	69.10	130	d__Bacteria;p__Actinobacteriota;c__Acidimicrobiia;o__Acidimicrobiales;f__Bog-793;g__Palsa-601;s__
LMSG_G000008407.1	no	741_1	Copper	84.61	3.85	65.38	1	0	1	14	Medium quality	20799	2147098	69.20	146	d__Bacteria;p__Actinobacteriota;c__Acidimicrobiia;o__Acidimicrobiales;f__Bog-793;g__Palsa-601;s__
LMSG_G000008408.1	no	742_1	Copper	63.79	0.00	63.79	1	1	1	18	Medium quality	114877	2551610	69.30	54	d__Bacteria;p__Actinobacteriota;c__Acidimicrobiia;o__Acidimicrobiales;f__Bog-793;g__Palsa-601;s__
LMSG_G000008409.1	no	743_1	Pyrite	92.30	1.28	85.89	1	1	1	17	Medium quality	42654	3066827	71.80	108	d__Bacteria;p__Actinobacteriota;c__Acidimicrobiia;o__Acidimicrobiales;f__Bog-793;g__Palsa-601;s__
LMSG_G000008410.1	no	743_1	Copper	77.35	1.71	68.81	1	0	1	18	Medium quality	75292	2618616	71.90	54	d__Bacteria;p__Actinobacteriota;c__Acidimicrobiia;o__Acidimicrobiales;f__Bog-793;g__Palsa-601;s__
LMSG_G000008411.1	no	743_1	Pyrite-Copper	65.32	2.14	54.64	0	0	0	5	Medium quality	4196	1683990	72.80	451	d__Bacteria;p__Actinobacteriota;c__Acidimicrobiia;o__Acidimicrobiales;f__Bog-793;g__Palsa-601;s__
LMSG_G000008412.1	no	743_2	Magnetite	72.25	3.09	56.82	0	0	0	16	Medium quality	11748	2577965	72.70	270	d__Bacteria;p__Actinobacteriota;c__Acidimicrobiia;o__Acidimicrobiales;f__Bog-793;g__Palsa-601;s__

LMSG_G000008413.1	no	744_1	Tin-Zinc	95.72	3.42	78.63	1	1	1	20	High quality	28540	2646082	70.30	149	d__Bacteria;p__Actinobacteriota;c__Acidimicrobiia;o__Acidimicrobiales;f__Bog-793;g__Palsa-601;s__
LMSG_G000008414.1	no	744_1	Lead-Zinc	78.23	3.42	61.14	0	1	0	19	Medium quality	5394	2330540	70.40	525	d__Bacteria;p__Actinobacteriota;c__Acidimicrobiia;o__Acidimicrobiales;f__Bog-793;g__Palsa-601;s__
LMSG_G000008415.1	no	745_1	Polymetallic	94.77	2.21	83.70	1	1	1	20	High quality	15812	2596870	69.60	220	d__Bacteria;p__Actinobacteriota;c__Acidimicrobiia;o__Acidimicrobiales;f__Bog-793;g__Palsa-601;s__
LMSG_G000008416.1	no	745_1	Lead-Zinc	98.29	2.14	87.61	1	1	1	19	High quality	35750	2590311	69.60	110	d__Bacteria;p__Actinobacteriota;c__Acidimicrobiia;o__Acidimicrobiales;f__Bog-793;g__Palsa-601;s__
LMSG_G000008417.1	no	745_1	Lead-Zinc	93.16	2.56	80.34	1	1	1	19	High quality	16513	2498181	69.50	185	d__Bacteria;p__Actinobacteriota;c__Acidimicrobiia;o__Acidimicrobiales;f__Bog-793;g__Palsa-601;s__
LMSG_G000008418.1	no	745_1	Copper	85.47	4.00	65.47	1	1	1	17	Medium quality	32745	2530915	69.50	102	d__Bacteria;p__Actinobacteriota;c__Acidimicrobiia;o__Acidimicrobiales;f__Bog-793;g__Palsa-601;s__
LMSG_G000008419.1	no	745_1	Copper	87.42	1.36	80.63	0	1	0	7	Medium quality	3691	2007956	69.80	643	d__Bacteria;p__Actinobacteriota;c__Acidimicrobiia;o__Acidimicrobiales;f__Bog-793;g__Palsa-601;s__
LMSG_G000008420.1	no	745_1	Magnetite	85.80	1.36	79.01	1	1	1	17	Medium quality	15846	2308115	69.70	266	d__Bacteria;p__Actinobacteriota;c__Acidimicrobiia;o__Acidimicrobiales;f__Bog-793;g__Palsa-601;s__
LMSG_G000008421.1	no	745_1	Lead-Zinc	88.88	1.36	82.09	1	1	1	18	Medium quality	10429	2449503	69.70	325	d__Bacteria;p__Actinobacteriota;c__Acidimicrobiia;o__Acidimicrobiales;f__Bog-793;g__Palsa-601;s__
LMSG_G000008422.1	no	745_1	Pyrite-Copper	72.30	1.28	65.89	0	0	0	9	Medium quality	4093	1729931	69.90	476	d__Bacteria;p__Actinobacteriota;c__Acidimicrobiia;o__Acidimicrobiales;f__Bog-793;g__Palsa-601;s__
LMSG_G000008423.1	no	745_1	Lead-Zinc	91.85	2.78	77.97	1	0	1	17	Medium quality	10521	2473772	69.60	318	d__Bacteria;p__Actinobacteriota;c__Acidimicrobiia;o__Acidimicrobiales;f__Bog-793;g__Palsa-601;s__
LMSG_G000008424.1	no	698_1	Lead-Zinc	98.29	3.85	79.06	1	1	1	20	High quality	27892	4739466	66.30	294	d__Bacteria;p__Actinobacteriota;c__Acidimicrobiia;o__Acidimicrobiales;f__Palsa-688;g__;s__
LMSG_G000008425.1	no	698_1	Pyrite-Copper	94.01	4.70	70.51	1	1	1	20	High quality	40467	4273920	66.30	197	d__Bacteria;p__Actinobacteriota;c__Acidimicrobiia;o__Acidimicrobiales;f__Palsa-688;g__;s__
LMSG_G000008426.1	no	699_1	Lead-Zinc	90.59	3.85	71.36	1	2	1	19	High quality	43578	3507335	68.50	139	d__Bacteria;p__Actinobacteriota;c__Acidimicrobiia;o__Acidimicrobiales;f__Palsa-688;g__;s__
LMSG_G000008427.1	no	701_1	Polymetallic	98.29	2.14	87.61	1	1	1	20	High quality	71448	3790344	68.10	119	d__Bacteria;p__Actinobacteriota;c__Acidimicrobiia;o__Acidimicrobiales;f__Palsa-688;g__;s__
LMSG_G000008428.1	no	701_1	Polymetallic	98.29	1.57	90.46	1	1	1	19	High quality	85387	3596179	68.10	107	d__Bacteria;p__Actinobacteriota;c__Acidimicrobiia;o__Acidimicrobiales;f__Palsa-688;g__;s__
LMSG_G000008429.1	no	701_1	Polymetallic	97.60	2.14	86.92	1	1	1	17	Medium quality	53147	3728543	68.10	135	d__Bacteria;p__Actinobacteriota;c__Acidimicrobiia;o__Acidimicrobiales;f__Palsa-688;g__;s__
LMSG_G000008430.1	no	701_1	Copper	76.06	2.56	63.24	1	1	1	18	Medium quality	14553	3486944	67.80	329	d__Bacteria;p__Actinobacteriota;c__Acidimicrobiia;o__Acidimicrobiales;f__Palsa-688;g__;s__
LMSG_G000008431.1	no	702_1	Polymetallic	97.43	2.14	86.75	1	1	1	19	High quality	66652	3463383	70.40	97	d__Bacteria;p__Actinobacteriota;c__Acidimicrobiia;o__Acidimicrobiales;f__Palsa-688;g__;s__
LMSG_G000008432.1	no	702_1	Polymetallic	92.73	2.56	79.91	1	1	1	20	High quality	24260	3253611	70.40	199	d__Bacteria;p__Actinobacteriota;c__Acidimicrobiia;o__Acidimicrobiales;f__Palsa-688;g__;s__
LMSG_G000008433.1	no	702_1	Polymetallic	98.29	3.42	81.20	1	1	1	20	High quality	70978	3645591	70.30	98	d__Bacteria;p__Actinobacteriota;c__Acidimicrobiia;o__Acidimicrobiales;f__Palsa-688;g__;s__
LMSG_G000008434.1	no	702_1	Polymetallic	97.43	1.44	90.25	0	1	0	19	Medium quality	52304	3480649	70.40	103	d__Bacteria;p__Actinobacteriota;c__Acidimicrobiia;o__Acidimicrobiales;f__Palsa-688;g__;s__
LMSG_G000008435.1	no	703_1	Lead-Zinc	98.29	4.70	74.79	1	1	1	20	High quality	95228	3553955	71.20	66	d__Bacteria;p__Actinobacteriota;c__Acidimicrobiia;o__Acidimicrobiales;f__Palsa-688;g__;s__
LMSG_G000008436.1	no	703_1	Polymetallic	98.29	2.99	83.34	1	1	1	20	High quality	80221	3635938	71.10	86	d__Bacteria;p__Actinobacteriota;c__Acidimicrobiia;o__Acidimicrobiales;f__Palsa-688;g__;s__
LMSG_G000008437.1	no	703_1	Polymetallic	98.29	3.85	79.06	1	1	1	20	High quality	122659	3746231	71.00	72	d__Bacteria;p__Actinobacteriota;c__Acidimicrobiia;o__Acidimicrobiales;f__Palsa-688;g__;s__
LMSG_G000008438.1	no	703_1	Polymetallic	98.29	2.99	83.34	1	1	1	20	High quality	118415	3482947	71.20	51	d__Bacteria;p__Actinobacteriota;c__Acidimicrobiia;o__Acidimicrobiales;f__Palsa-688;g__;s__
LMSG_G000008439.1	no	703_1	Polymetallic	96.48	2.99	81.53	1	1	1	20	High quality	41500	3450984	71.20	149	d__Bacteria;p__Actinobacteriota;c__Acidimicrobiia;o__Acidimicrobiales;f__Palsa-688;g__;s__
LMSG_G000008440.1	no	703_1	Polymetallic	98.29	2.99	83.34	1	1	1	20	High quality	84520	3497899	71.20	90	d__Bacteria;p__Actinobacteriota;c__Acidimicrobiia;o__Acidimicrobiales;f__Palsa-688;g__;s__
LMSG_G000008441.1	no	703_1	Polymetallic	98.29	3.09	82.86	1	1	1	20	High quality	63967	3499968	71.20	99	d__Bacteria;p__Actinobacteriota;c__Acidimicrobiia;o__Acidimicrobiales;f__Palsa-688;g__;s__
LMSG_G000008442.1	no	703_1	Polymetallic	98.29	2.99	83.34	1	1	1	20	High quality	92819	3521122	71.20	59	d__Bacteria;p__Actinobacteriota;c__Acidimicrobiia;o__Acidimicrobiales;f__Palsa-688;g__;s__
LMSG_G000008443.1	no	703_1	Polymetallic	95.72	3.85	76.49	1	1	1	20	High quality	90925	3481040	71.20	62	d__Bacteria;p__Actinobacteriota;c__Acidimicrobiia;o__Acidimicrobiales;f__Palsa-688;g__;s__
LMSG_G000008444.1	no	703_1	Magnetite	98.29	3.15	82.56	1	1	1	20	High quality	91433	3544095	71.10	66	d__Bacteria;p__Actinobacteriota;c__Acidimicrobiia;o__Acidimicrobiales;f__Palsa-688;g__;s__
LMSG_G000008445.1	no	703_1	Copper	76.18	1.28	69.77	0	1	0	15	Medium quality	4249	2761218	70.90	734	d__Bacteria;p__Actinobacteriota;c__Acidimicrobiia;o__Acidimicrobiales;f__Palsa-688;g__;s__
LMSG_G000008446.1	no	703_1	Lead-Zinc	98.29	3.85	79.06	0	1	0	20	Medium quality	81453	3626293	71.10	84	d__Bacteria;p__Actinobacteriota;c__Acidimicrobiia;o__Acidimicrobiales;f__Palsa-688;g__;s__
LMSG_G000008447.1	no	703_1	Polymetallic	95.06	3.42	77.97	0	1	0	17	Medium quality	9731	3332832	71.20	494	d__Bacteria;p__Actinobacteriota;c__Acidimicrobiia;o__Acidimicrobiales;f__Palsa-688;g__;s__
LMSG_G000008448.1	no	703_1	Polymetallic	94.35	2.14	83.67	0	1	0	20	Medium quality	24906	3502000	71.10	230	d__Bacteria;p__Actinobacteriota;c__Acidimicrobiia;o__Acidimicrobiales;f__Palsa-688;g__;s__
LMSG_G000008449.1	no	703_1	Polymetallic	78.39	3.85	59.16	0	1	0	16	Medium quality	3890	3068136	71.00	904	d__Bacteria;p__Actinobacteriota;c__Acidimicrobiia;o__Acidimicrobiales;f__Palsa-688;g__;s__
LMSG_G000008450.1	no	703_1	Polymetallic	81.61	1.28	75.20	0	1	0	16	Medium quality	4555	2960349	71.20	809	d__Bacteria;p__Actinobacteriota;c__Acidimicrobiia;o__Acidimicrobiales;f__Palsa-688;g__;s__
LMSG_G000008451.1	no	703_1	Polymetallic	75.21	1.96	65.42	0	0	0	14	Medium quality	3912	2814916	71.30	808	d__Bacteria;p__Actinobacteriota;c__Acidimicrobiia;o__Acidimicrobiales;f__Palsa-688;g__;s__
LMSG_G000008452.1	no	703_1	Copper	80.28	0.00	80.28	0	0	0	16	Medium quality	6839	2980694	71.20	516	d__Bacteria;p__Actinobacteriota;c__Acidimicrobiia;o__Acidimicrobiales;f__Palsa-688;g__;s__
LMSG_G000008453.1	no	703_1	Pyrite-Copper	82.83	4.62	59.76	1	0	1	18	Medium quality	4092	2855336	71.30	811	d__Bacteria;p__Actinobacteriota;c__Acidimicrobiia;o__Acidimicrobiales;f__Palsa-688;g__;s__
LMSG_G000008454.1	no	721_1	Polymetallic	84.61	1.28	78.20	1	1	1	16	Medium quality	69089	2788515	70.00	62	d__Bacteria;p__Actinobacteriota;c__Acidimicrobiia;o__Acidimicrobiales;f__Palsa-688;g__;s__
LMSG_G000008455.1	no	721_1	Polymetallic	91.56	4.70	68.06	0	0	0	20	Medium quality	78997	4272136	69.60	94	d__Bacteria;p__Actinobacteriota;c__Acidimicrobiia;o__Acidimicrobiales;f__Palsa-688;g__;s__
LMSG_G000008456.1	no	721_1	Polymetallic	99.14	4.46	76.83	0	0	0	19	Medium quality	59874	4040261	69.80	109	d__Bacteria;p__Actinobacteriota;c__Acidimicrobiia;o__Acidimicrobiales;f__Palsa-688;g__;s__
LMSG_G000008457.1	no	721_1	Polymetallic	75.86	3.45	58.62	1	0	1	13	Medium quality	82602	2783869	70.20	53	d__Bacteria;p__Actinobacteriota;c__Acidimicrobiia;o__Acidimicrobiales;f__Palsa-688;g__;s__
LMSG_G000008458.1	no	722_1	Polymetallic	95.72	3.85	76.49	1	1	1	19	High quality	53402	4907565	69.70	138	d__Bacteria;p__Actinobacteriota;c__Acidimicrobiia;o__Acidimicrobiales;f__Palsa-688;g__;s__
LMSG_G000008459.1	no	722_1	Polymetallic	90.59	2.21	79.52	1	1	1	17	Medium quality	29198	3723968	70.20	182	d__Bacteria;p__Actinobacteriota;c__Acidimicrobiia;o__Acidimicrobiales;f__Palsa-688;g__;s__
LMSG_G000008460.1	no	722_1	Polymetallic	80.05	1.14	74.36	0	1	0	16	Medium quality	25731	2976816	70.60	146	d__Bacteria;p__Actinobacteriota;c__Acidimicrobiia;o__Acidimicrobiales;f__Palsa-688;g__;s__
LMSG_G000008461.1	no	722_1	Polymetallic	60.25	1.71	51.71	0	0	0	11	Medium quality	82645	2064169	70.70	62	d__Bacteria;p__Actinobacteriota;c__Acidimicrobiia;o__Acidimicrobiales;f__Palsa-688;g__;s__
LMSG_G000008462.1	no	723_1	Polymetallic	90.59	4.27	69.23	1	1	1	18	High quality	19834	4642561	64.10	416	d__Bacteria;p__Actinobacteriota;c__Acidimicrobiia;o__Acidimicrobiales;f__Palsa-688;g__;s__
LMSG_G000008463.1	no	723_1	Polymetallic	76.61	1.28	70.20	0	0	0	15	Medium quality	7134	2783779	64.10	435	d__Bacteria;p__Actinobacteriota;c__Acidimicrobiia;o__Acidimicrobiales;f__Palsa-688;g__;s__
LMSG_G000008464.1	no	723_1	Polymetallic	93.16	1.71	84.62	1	0	1	18	Medium quality	19254	3893223	64.30	300	d__Bacteria;p__Actinobacteriota;c__Acidimicrobiia;o__Acidimicrobiales;f__Palsa-688;g__;s__
LMSG_G000008465.1	no	724_1	Lead-Zinc	91.45	1.28	85.04	1	1	1	19	High quality	40412	2862466	64.40	150	d__Bacteria;p__Actinobacteriota;c__Acidimicrobiia;o__Acidimicrobiales;f__Palsa-688;g__;s__
LMSG_G000008466.1	no	724_1	Lead-Zinc	97.43	2.14	86.75	0	1	0	20	Medium quality	46105	3090011	64.50	150	d__Bacteria;p__Actinobacteriota;c__Acidimicrobiia;o__Acidimicrobiales;f__Palsa-688;g__;s__
LMSG_G000008467.1	no	724_1	Lead-Zinc	97.43	2.14	86.75	0	1	0	20	Medium quality	65701	3283139	64.50	124	d__Bacteria;p__Actinobacteriota;c__Acidimicrobiia;o__Acidimicrobiales;f__Palsa-688;g__;s__
LMSG_G000008468.1	no	725_1	Polymetallic	95.72	2.14	85.04	1	1	1	18	High quality	100906	3759510	64.20	142	d__Bacteria;p__Actinobacteriota;c__Acidimicrobiia;o__Acidimicrobiales;f__Palsa-688;g__;s__
LMSG_G000008469.1	no	725_1	Polymetallic	77.58	0.00	77.58	1	1	1	19	Medium quality	62441	3573377	64.00	136	d__Bacteria;p__Actinobacteriota;c__Acidimicrobiia;o__Acidimicrobiales;f__Palsa-688;g__;s__
LMSG_G000008470.1	no	725_1	Polymetallic	64.95	0.43	62.82	1	1	1	16	Medium quality	156075	2129893	63.80	38	d__Bacteria;p__Actinobacteriota;c__Acidimicrobiia;o__Acidimicrobiales;f__Palsa-688;g__;s__
LMSG_G000008471.1	no	749_1	Pyrite	83.90	4.70	60.40	1	0	1	19	Medium quality	10248	2234108	72.20	296	d__Bacteria;p__Actinobacteriota;c__Acidimicrobiia;o__Acidimicrobiales;f__Palsa-688;g__;s__
LMSG_G000008472.1	no	749_1	Pyrite	91.45	0.62	88.37	1	0	1	20	Medium quality	32284	2958579	71.90	130	d__Bacteria;p__Actinobacteriota;c__Acidimicrobiia;o__Acidimicrobiales;f__Palsa-688;g__;s__
LMSG_G000008473.1	no	749_2	Polymetallic	80.61	3.99	60.67	0	0	0	18	Medium quality	17804	2572587	71.90	165	d__Bacteria;p__Actinobacteriota;c__Acidimicrobiia;o__Acidimicrobiales;f__Palsa-688;g__;s__
LMSG_G000008474.1	no	749_2	Polymetallic	66.78	3.09	51.35	1	0	1	20	Medium quality	9599	2244824	71.80	242	d__Bacteria;p__Actinobacteriota;c__Acidimicrobiia;o__Acidimicrobiales;f__Palsa-688;g__;s__
LMSG_G000008475.1	no	750_1	Pyrite	53.76	0.00	53.76	0	0	0	17	Medium quality	4821	3175191	73.00	822	d__Bacteria;p__Actinobacteriota;c__Acidimicrobiia;o__Acidimicrobiales;f__Palsa-688;g__;s__
LMSG_G000008476.1	no	750_1	Pyrite	88.88	3.85	69.65	0	1	0	19	Medium quality	9516	2938132	72.30	406	d__Bacteria;p__Actinobacteriota;c__Acidimicrobiia;o__Acidimicrobiales;f__Palsa-688;g__;s__
LMSG_G000008477.1	no	750_1	Pyrite	56.06	0.85	51.79	0	0	0	15	Medium quality	9102	2142415	72.20	262	d__Bacteria;p__Actinobacteriota;c__Acidimicrobiia;o__Acidimicrobiales;f__Palsa-688;g__;s__
LMSG_G000008478.1	no	750_1	Lead-Zinc	62.24	0.86	57.93	0	1	0	13	Medium quality	3758	2035281	72.90	605	d__Bacteria;p__Actinobacteriota;c__Acidimicrobiia;o__Acidimicrobiales;f__Palsa-688;g__;s__
LMSG_G000008479.1	no	617_1	Tin-Zinc	77.12	3.28	60.74	2	4	8	17	Medium quality	2885	2358558	48.80	978	d__Bacteria;p__Actinobacteriota;c__Acidimicrobiia;o__Acidimicrobiales;f__RAAP-2;g__;s__
LMSG_G000008480.1	no	735_1	Polymetallic	97.15	2.23	86.00	1	1	1	15	Medium quality	12862	3108894	72.90	365	d__Bacteria;p__Actinobacteriota;c__Acidimicrobiia;o__Acidimicrobiales;f__RAAP-2;g__;s__
LMSG_G000008481.1	no	735_1	Lead-Zinc	53.44	0.00	53.44	0	1	0	13	Medium quality	79552	1955048	73.20	33	d__Bacteria;p__Actinobacteriota;c__Acidimicrobiia;o__Acidimicrobiales;f__RAAP-2;g__;s__
LMSG_G000008482.1	no	735_1	Copper	96.16	0.52	93.55	0	1	0	19	Medium quality	19086	2876440	73.20	204	d__Bacteria;p__Actinobacteriota;c__Acidimicrobiia;o__Acidimicrobiales;f__RAAP-2;g__;s__
LMSG_G000008483.1	no	735_1	Lead-Zinc	81.70	3.85	62.47	0	0	0	17	Medium quality	20987	2745803	73.30	331	d__Bacteria;p__Actinobacteriota;c__Acidimicrobiia;o__Acidimicrobiales;f__RAAP-2;g__;s__
LMSG_G000008484.1	no	735_1	Lead-Zinc	99.14	3.85	79.91	0	1	0	17	Medium quality	61179	3046230	73.20	89	d__Bacteria;p__Actinobacteriota;c__Acidimicrobiia;o__Acidimicrobiales;f__RAAP-2;g__;s__
LMSG_G000008485.1	no	735_1	Polymetallic	79.31	0.00	79.31	0	3	0	15	Medium quality	54780	2898597	73.10	415	d__Bacteria;p__Actinobacteriota;c__Acidimicrobiia;o__Acidimicrobiales;f__RAAP-2;g__;s__
LMSG_G000008486.1	no	735_1	Polymetallic	91.24	0.85	86.97	0	1	0	18	Medium quality	18253	2818811	73.30	244	d__Bacteria;p__Actinobacteriota;c__Acidimicrobiia;o__Acidimicrobiales;f__RAAP-2;g__;s__
LMSG_G000008487.1	no	735_1	Polymetallic	94.87	1.36	88.08	0	1	0	17	Medium quality	25192	2733254	73.30	187	d__Bacteria;p__Actinobacteriota;c__Acidimicrobiia;o__Acidimicrobiales;f__RAAP-2;g__;s__
LMSG_G000008488.1	no	735_1	Polymetallic	94.20	2.63	81.03	0	1	0	15	Medium quality	15444	2930519	73.10	289	d__Bacteria;p__Actinobacteriota;c__Acidimicrobiia;o__Acidimicrobiales;f__RAAP-2;g__;s__
LMSG_G000008489.1	no	735_1	Copper	85.85	1.28	79.44	0	1	0	15	Medium quality	13980	2613388	73.30	247	d__Bacteria;p__Actinobacteriota;c__Acidimicrobiia;o__Acidimicrobiales;f__RAAP-2;g__;s__

LMSG_G000008490.1	no	735_1	Copper	82.33	2.14	71.65	0	0	0	14	Medium quality	11991	2419812	73.60	309	d__Bacteria;p__Actinobacteriota;c__Acidimicrobiia; o__Acidimicrobiales;f__RAAP-2;g__;s__
LMSG_G000008491.1	no	735_1	Polymetallic	95.72	1.28	89.31	0	1	0	18	Medium quality	33645	2921945	73.20	124	d__Bacteria;p__Actinobacteriota;c__Acidimicrobiia; o__Acidimicrobiales;f__RAAP-2;g__;s__
LMSG_G000008492.1	no	735_1	Lead-Zinc	90.12	1.28	83.71	0	0	0	18	Medium quality	16032	2315011	73.40	168	d__Bacteria;p__Actinobacteriota;c__Acidimicrobiia; o__Acidimicrobiales;f__RAAP-2;g__;s__
LMSG_G000008493.1	no	735_1	Lead-Zinc	77.68	1.28	71.27	0	1	0	18	Medium quality	15697	2233777	73.40	169	d__Bacteria;p__Actinobacteriota;c__Acidimicrobiia; o__Acidimicrobiales;f__RAAP-2;g__;s__
LMSG_G000008494.1	no	736_1	Lead-Zinc	84.93	1.71	76.39	0	0	0	14	Medium quality	3884	2179840	71.10	670	d__Bacteria;p__Actinobacteriota;c__Acidimicrobiia; o__Acidimicrobiales;f__RAAP-2;g__;s__
LMSG_G000008495.1	no	736_1	Lead-Zinc	97.43	2.14	86.75	0	1	0	17	Medium quality	92307	2592758	71.00	43	d__Bacteria;p__Actinobacteriota;c__Acidimicrobiia; o__Acidimicrobiales;f__RAAP-2;g__;s__
LMSG_G000008496.1	no	736_1	Pyrite	97.43	3.42	80.34	1	1	2	19	High quality	32604	2832614	71.10	176	d__Bacteria;p__Actinobacteriota;c__Acidimicrobiia; o__Acidimicrobiales;f__RAAP-2;g__;s__
LMSG_G000008497.1	no	736_1	Pyrite	98.21	2.14	87.53	1	1	1	18	High quality	36199	2625201	71.20	104	d__Bacteria;p__Actinobacteriota;c__Acidimicrobiia; o__Acidimicrobiales;f__RAAP-2;g__;s__
LMSG_G000008498.1	no	736_1	Polymetallic	96.58	2.14	85.90	1	1	1	17	Medium quality	30958	2509815	71.20	122	d__Bacteria;p__Actinobacteriota;c__Acidimicrobiia; o__Acidimicrobiales;f__RAAP-2;g__;s__
LMSG_G000008499.1	no	736_1	Lead-Zinc	94.87	2.85	80.63	0	1	0	14	Medium quality	27892	2652163	71.10	174	d__Bacteria;p__Actinobacteriota;c__Acidimicrobiia; o__Acidimicrobiales;f__RAAP-2;g__;s__
LMSG_G000008500.1	no	736_1	Lead-Zinc	89.74	3.85	70.51	0	1	0	16	Medium quality	20077	2401619	71.30	156	d__Bacteria;p__Actinobacteriota;c__Acidimicrobiia; o__Acidimicrobiales;f__RAAP-2;g__;s__
LMSG_G000008501.1	no	736_1	Pyrite	97.43	2.99	82.48	0	1	0	18	Medium quality	31347	2712901	71.20	153	d__Bacteria;p__Actinobacteriota;c__Acidimicrobiia; o__Acidimicrobiales;f__RAAP-2;g__;s__
LMSG_G000008502.1	no	736_1	Pyrite	98.00	2.56	85.18	0	1	0	18	Medium quality	33058	2628803	71.20	118	d__Bacteria;p__Actinobacteriota;c__Acidimicrobiia; o__Acidimicrobiales;f__RAAP-2;g__;s__
LMSG_G000008503.1	no	736_1	Polymetallic	97.43	2.56	84.61	0	1	0	16	Medium quality	26517	2353586	71.20	117	d__Bacteria;p__Actinobacteriota;c__Acidimicrobiia; o__Acidimicrobiales;f__RAAP-2;g__;s__
LMSG_G000008504.1	no	736_1	Polymetallic	94.87	2.99	79.92	0	1	0	18	Medium quality	31354	2449375	71.10	116	d__Bacteria;p__Actinobacteriota;c__Acidimicrobiia; o__Acidimicrobiales;f__RAAP-2;g__;s__
LMSG_G000008505.1	no	736_1	Polymetallic	84.04	2.14	73.36	0	1	0	16	Medium quality	32736	2364998	71.10	108	d__Bacteria;p__Actinobacteriota;c__Acidimicrobiia; o__Acidimicrobiales;f__RAAP-2;g__;s__
LMSG_G000008506.1	no	736_1	Copper	65.51	0.00	65.51	0	1	1	12	Medium quality	19245	1803455	71.40	132	d__Bacteria;p__Actinobacteriota;c__Acidimicrobiia; o__Acidimicrobiales;f__RAAP-2;g__;s__
LMSG_G000008507.1	no	736_1	Magnetite	70.68	0.00	70.68	0	1	0	17	Medium quality	27003	2243722	71.20	122	d__Bacteria;p__Actinobacteriota;c__Acidimicrobiia; o__Acidimicrobiales;f__RAAP-2;g__;s__
LMSG_G000008508.1	no	736_1	Copper	94.87	2.14	84.19	0	1	0	18	Medium quality	23697	2401903	71.20	128	d__Bacteria;p__Actinobacteriota;c__Acidimicrobiia; o__Acidimicrobiales;f__RAAP-2;g__;s__
LMSG_G000008509.1	no	736_1	Lead-Zinc	97.43	2.14	86.75	0	1	0	16	Medium quality	27079	2317925	71.30	132	d__Bacteria;p__Actinobacteriota;c__Acidimicrobiia; o__Acidimicrobiales;f__RAAP-2;g__;s__
LMSG_G000008510.1	no	736_1	Lead-Zinc	96.58	1.95	86.85	1	1	0	18	Medium quality	25959	2543752	71.00	141	d__Bacteria;p__Actinobacteriota;c__Acidimicrobiia; o__Acidimicrobiales;f__RAAP-2;g__;s__
LMSG_G000008511.1	no	736_1	Copper	96.58	3.85	77.35	1	1	2	20	High quality	48435	2733714	70.90	103	d__Bacteria;p__Actinobacteriota;c__Acidimicrobiia; o__Acidimicrobiales;f__RAAP-2;g__;s__
LMSG_G000008512.1	no	737_1	Polymetallic	96.58	1.28	90.17	1	1	1	18	High quality	51108	2798040	71.00	88	d__Bacteria;p__Actinobacteriota;c__Acidimicrobiia; o__Acidimicrobiales;f__RAAP-2;g__;s__
LMSG_G000008513.1	no	737_1	Polymetallic	98.29	4.00	78.29	1	1	1	18	High quality	58449	2736646	71.00	73	d__Bacteria;p__Actinobacteriota;c__Acidimicrobiia; o__Acidimicrobiales;f__RAAP-2;g__;s__
LMSG_G000008514.1	no	737_1	Polymetallic	100.00	1.28	93.59	1	1	1	17	Medium quality	47209	3147906	70.70	140	d__Bacteria;p__Actinobacteriota;c__Acidimicrobiia; o__Acidimicrobiales;f__RAAP-2;g__;s__
LMSG_G000008515.1	no	737_1	Lead-Zinc	90.40	1.71	81.86	1	0	1	18	Medium quality	8715	2722798	70.70	537	d__Bacteria;p__Actinobacteriota;c__Acidimicrobiia; o__Acidimicrobiales;f__RAAP-2;g__;s__
LMSG_G000008516.1	no	737_1	Polymetallic	90.59	1.28	84.18	1	0	1	18	Medium quality	51788	2637110	71.00	77	d__Bacteria;p__Actinobacteriota;c__Acidimicrobiia; o__Acidimicrobiales;f__RAAP-2;g__;s__
LMSG_G000008517.1	no	737_1	Copper	97.24	1.28	90.83	0	1	0	15	Medium quality	57034	3029384	70.90	120	d__Bacteria;p__Actinobacteriota;c__Acidimicrobiia; o__Acidimicrobiales;f__RAAP-2;g__;s__
LMSG_G000008518.1	no	737_1	Copper	63.67	2.14	52.99	0	0	0	12	Medium quality	4850	1890596	71.20	429	d__Bacteria;p__Actinobacteriota;c__Acidimicrobiia; o__Acidimicrobiales;f__RAAP-2;g__;s__
LMSG_G000008519.1	no	737_1	Polymetallic	95.29	1.28	88.88	1	0	0	18	Medium quality	33664	2821304	71.10	166	d__Bacteria;p__Actinobacteriota;c__Acidimicrobiia; o__Acidimicrobiales;f__RAAP-2;g__;s__
LMSG_G000008520.1	no	737_1	Copper	85.28	2.42	73.18	0	0	0	19	Medium quality	4473	2856204	70.90	742	d__Bacteria;p__Actinobacteriota;c__Acidimicrobiia; o__Acidimicrobiales;f__RAAP-2;g__;s__
LMSG_G000008521.1	no	739_1	Pyrite-Copper	55.17	0.00	55.17	0	0	0	9	Medium quality	10809	2036566	73.50	253	d__Bacteria;p__Actinobacteriota;c__Acidimicrobiia; o__Acidimicrobiales;f__RAAP-2;g__;s__
LMSG_G000008522.1	no	740_1	Polymetallic	92.70	3.85	73.47	1	0	1	18	Medium quality	12020	3051886	72.10	328	d__Bacteria;p__Actinobacteriota;c__Acidimicrobiia; o__Acidimicrobiales;f__RAAP-2;g__;s__
LMSG_G000008523.1	no	740_1	Polymetallic	76.06	1.71	67.52	2	1	3	17	Medium quality	8344	2854843	71.60	536	d__Bacteria;p__Actinobacteriota;c__Acidimicrobiia; o__Acidimicrobiales;f__RAAP-2;g__;s__
LMSG_G000008524.1	no	740_1	Copper	75.48	3.42	58.39	0	0	0	14	Medium quality	7429	2766823	72.40	504	d__Bacteria;p__Actinobacteriota;c__Acidimicrobiia; o__Acidimicrobiales;f__RAAP-2;g__;s__
LMSG_G000008525.1	no	740_1	Polymetallic	73.50	0.43	71.37	0	0	0	13	Medium quality	11479	2356250	72.40	251	d__Bacteria;p__Actinobacteriota;c__Acidimicrobiia; o__Acidimicrobiales;f__RAAP-2;g__;s__
LMSG_G000008526.1	no	740_1	Copper	63.72	2.14	53.04	0	0	0	14	Medium quality	5305	2202383	71.90	467	d__Bacteria;p__Actinobacteriota;c__Acidimicrobiia; o__Acidimicrobiales;f__RAAP-2;g__;s__
LMSG_G000008527.1	no	746_1	Lead-Zinc	77.58	0.00	77.58	0	0	0	15	Medium quality	27674	2396226	71.90	286	d__Bacteria;p__Actinobacteriota;c__Acidimicrobiia; o__Acidimicrobiales;f__RAAP-2;g__;s__
LMSG_G000008528.1	no	746_1	Lead-Zinc	61.96	2.14	51.28	1	0	1	12	Medium quality	11435	1491726	71.60	174	d__Bacteria;p__Actinobacteriota;c__Acidimicrobiia; o__Acidimicrobiales;f__RAAP-2;g__;s__
LMSG_G000008529.1	no	756_1	Lead-Zinc	77.77	3.42	60.68	1	0	1	18	Medium quality	77131	2420403	69.20	70	d__Bacteria;p__Actinobacteriota;c__Acidimicrobiia; o__Acidimicrobiales;f__RAAP-2;g__;s__
LMSG_G000008530.1	no	758_1	Coal	89.12	3.99	69.18	1	1	1	17	Medium quality	10710	3748933	72.20	516	d__Bacteria;p__Actinobacteriota;c__Acidimicrobiia; o__Acidimicrobiales;f__RAAP-2;g__;s__
LMSG_G000008531.1	no	759_1	Copper	63.05	0.85	58.78	0	0	0	14	Medium quality	14838	1196287	70.50	97	d__Bacteria;p__Actinobacteriota;c__Acidimicrobiia; o__Acidimicrobiales;f__RAAP-2;g__;s__
LMSG_G000008532.1	no	759_1	Tin-Zinc	87.17	1.28	80.76	1	1	1	20	Medium quality	25244	2124889	70.50	110	d__Bacteria;p__Actinobacteriota;c__Acidimicrobiia; o__Acidimicrobiales;f__RAAP-2;g__;s__
LMSG_G000008533.1	no	759_1	Tin-Zinc	93.16	1.28	86.75	1	0	1	19	Medium quality	33675	2086612	70.80	93	d__Bacteria;p__Actinobacteriota;c__Acidimicrobiia; o__Acidimicrobiales;f__RAAP-2;g__;s__
LMSG_G000008534.1	no	760_1	Lead-Zinc	63.51	0.85	59.24	1	0	1	15	Medium quality	19262	1838515	71.00	179	d__Bacteria;p__Actinobacteriota;c__Acidimicrobiia; o__Acidimicrobiales;f__RAAP-2;g__;s__
LMSG_G000008535.1	no	762_1	Copper	95.64	4.19	74.70	1	1	1	20	High quality	18119	3373749	70.60	324	d__Bacteria;p__Actinobacteriota;c__Acidimicrobiia; o__Acidimicrobiales;f__RAAP-2;g__;s__
LMSG_G000008536.1	no	762_1	Copper	80.76	3.85	61.53	0	0	0	11	Medium quality	14470	2224748	71.10	195	d__Bacteria;p__Actinobacteriota;c__Acidimicrobiia; o__Acidimicrobiales;f__RAAP-2;g__;s__
LMSG_G000008537.1	no	762_1	Copper	69.87	1.85	60.62	4	0	2	19	Medium quality	13539	2133000	70.70	353	d__Bacteria;p__Actinobacteriota;c__Acidimicrobiia; o__Acidimicrobiales;f__RAAP-2;g__;s__
LMSG_G000008538.1	no	762_1	Magnetite	94.41	3.07	79.07	0	1	0	16	Medium quality	28788	3048086	70.30	197	d__Bacteria;p__Actinobacteriota;c__Acidimicrobiia; o__Acidimicrobiales;f__RAAP-2;g__;s__
LMSG_G000008539.1	no	763_1	Pyrite-Copper	66.91	1.88	57.51	0	0	0	10	Medium quality	8900	1471311	71.60	237	d__Bacteria;p__Actinobacteriota;c__Acidimicrobiia; o__Acidimicrobiales;f__RAAP-2;g__;s__
LMSG_G000008540.1	no	763_1	Lead-Zinc	85.32	4.27	63.96	1	1	0	20	Medium quality	7326	3241359	70.00	568	d__Bacteria;p__Actinobacteriota;c__Acidimicrobiia; o__Acidimicrobiales;f__RAAP-2;g__;s__
LMSG_G000008541.1	no	763_1	Pyrite	75.00	3.45	57.76	0	0	0	16	Medium quality	14927	2146395	71.20	182	d__Bacteria;p__Actinobacteriota;c__Acidimicrobiia; o__Acidimicrobiales;f__RAAP-2;g__;s__
LMSG_G000008542.1	no	809_1	Antimony	71.88	1.72	63.26	1	1	0	19	Medium quality	93822	1978553	71.00	40	d__Bacteria;p__Actinobacteriota;c__Acidimicrobiia; o__Acidimicrobiales;f__RAAP-2;g__;s__
LMSG_G000008543.1	no	809_1	Antimony	50.57	0.00	50.57	1	0	0	20	Medium quality	109513	979115	70.90	155	d__Bacteria;p__Actinobacteriota;c__Acidimicrobiia; o__Acidimicrobiales;f__RAAP-2;g__;s__
LMSG_G000008544.1	no	810_1	Pyrite	96.01	4.27	74.65	1	1	1	20	High quality	62764	1938329	70.90	55	d__Bacteria;p__Actinobacteriota;c__Acidimicrobiia; o__Acidimicrobiales;f__RAAP-2;g__;s__
LMSG_G000008545.1	no	810_1	Polymetallic	80.22	2.99	65.27	0	0	0	17	Medium quality	15995	1701123	71.30	167	d__Bacteria;p__Actinobacteriota;c__Acidimicrobiia; o__Acidimicrobiales;f__RAAP-2;g__;s__
LMSG_G000008546.1	no	810_1	Pyrite-Copper	83.07	3.51	65.51	1	1	0	18	Medium quality	25800	1694794	71.10	84	d__Bacteria;p__Actinobacteriota;c__Acidimicrobiia; o__Acidimicrobiales;f__RAAP-2;g__;s__
LMSG_G000008547.1	no	810_2	Pyrite-Copper	65.85	1.57	58.02	0	1	0	14	Medium quality	21732	1195303	71.70	97	d__Bacteria;p__Actinobacteriota;c__Acidimicrobiia; o__Acidimicrobiales;f__RAAP-2;g__;s__
LMSG_G000008548.1	no	810_2	Antimony	70.99	3.57	53.12	1	1	0	18	Medium quality	5986	1462278	71.40	508	d__Bacteria;p__Actinobacteriota;c__Acidimicrobiia; o__Acidimicrobiales;f__RAAP-2;g__;s__
LMSG_G000008549.1	no	810_2	Antimony	58.62	0.00	58.62	0	1	0	10	Medium quality	4230	1119630	72.20	295	d__Bacteria;p__Actinobacteriota;c__Acidimicrobiia; o__Acidimicrobiales;f__RAAP-2;g__;s__
LMSG_G000008550.1	no	813_1	Polymetallic	66.31	2.48	53.93	0	0	0	15	Medium quality	6498	1463642	64.30	278	d__Bacteria;p__Actinobacteriota;c__Acidimicrobiia; o__Acidimicrobiales;f__RAAP-2;g__;s__
LMSG_G000008551.1	no	814_1	Polymetallic	69.81	2.88	55.40	0	1	0	19	Medium quality	45876	1606487	65.00	108	d__Bacteria;p__Actinobacteriota;c__Acidimicrobiia; o__Acidimicrobiales;f__RAAP-2;g__;s__
LMSG_G000008552.1	no	814_1	Polymetallic	76.72	0.86	72.41	0	1	0	17	Medium quality	20579	1666409	64.80	135	d__Bacteria;p__Actinobacteriota;c__Acidimicrobiia; o__Acidimicrobiales;f__RAAP-2;g__;s__
LMSG_G000008553.1	no	825_1	Polymetallic	95.86	1.28	89.45	1	1	1	20	High quality	8907	3043321	60.70	451	d__Bacteria;p__Actinobacteriota;c__Acidimicrobiia; o__Acidimicrobiales;f__RAAP-2;g__;s__
LMSG_G000008554.1	no	825_1	Polymetallic	98.86	1.28	92.45	0	1	0	20	Medium quality	51895	2876341	60.60	186	d__Bacteria;p__Actinobacteriota;c__Acidimicrobiia; o__Acidimicrobiales;f__RAAP-2;g__;s__
LMSG_G000008555.1	no	825_1	Polymetallic	89.28	1.99	79.34	1	1	1	17	Medium quality	4500	2258178	60.80	704	d__Bacteria;p__Actinobacteriota;c__Acidimicrobiia; o__Acidimicrobiales;f__RAAP-2;g__;s__
LMSG_G000008556.1	no	825_1	Tin-Zinc	98.56	1.28	92.15	0	0	0	20	Medium quality	18367	2713948	60.60	205	d__Bacteria;p__Actinobacteriota;c__Acidimicrobiia; o__Acidimicrobiales;f__RAAP-2;g__;s__
LMSG_G000008557.1	no	825_1	Tin-Zinc	98.86	1.45	91.59	1	0	1	17	Medium quality	23607	2754770	60.60	183	d__Bacteria;p__Actinobacteriota;c__Acidimicrobiia; o__Acidimicrobiales;f__RAAP-2;g__;s__
LMSG_G000008558.1	no	825_1	Polymetallic	95.57	1.66	87.27	1	0	1	17	Medium quality	7782	2390375	60.70	419	d__Bacteria;p__Actinobacteriota;c__Acidimicrobiia; o__Acidimicrobiales;f__RAAP-2;g__;s__
LMSG_G000008559.1	no	825_1	Copper	97.15	2.14	86.47	1	1	1	20	High quality	37787	2958326	60.70	196	d__Bacteria;p__Actinobacteriota;c__Acidimicrobiia; o__Acidimicrobiales;f__RAAP-2;g__;s__
LMSG_G000008560.1	no	825_1	Copper	57.76	0.93	53.10	0	0	0	14	Medium quality	1919	1559588	60.70	845	d__Bacteria;p__Actinobacteriota;c__Acidimicrobiia; o__Acidimicrobiales;f__RAAP-2;g__;s__
LMSG_G000008561.1	no	733_1	Coal	90.97	3.85	71.74	1	0	1	15	Medium quality	46254	2525180	74.10	79	d__Bacteria;p__Actinobacteriota;c__Acidimicrobiia; o__Acidimicrobiales;f__RAAP-2;g__Bog-756;s__
LMSG_G000008562.1	no	731_1	Coal	98.29	3.42	81.20	1	1	1	19	High quality	74477	2641385	70.90	59	d__Bacteria;p__Actinobacteriota;c__Acidimicrobiia; o__Acidimicrobiales;f__RAAP-2;g__Palsa-461;s__
LMSG_G000008563.1	no	732_1	Coal	79.20	2.14	68.52	0	1	0	17	Medium quality	21471	1901346	69.80	125	d__Bacteria;p__Actinobacteriota;c__Acidimicrobiia; o__Acidimicrobiales;f__RAAP-2;g__Palsa-461;s__
LMSG_G000008564.1	no	753_1	Polymetallic	92.30	2.74	78.63	1	2	1	18	High quality	20312	1980062	64.00	218	d__Bacteria;p__Actinobacteriota;c__Acidimicrobiia; o__Acidimicrobiales;f__RAAP-2;g__RAAP-2;s__
LMSG_G000008565.1	no	753_1	Polymetallic	89.79	0.43	87.66	0	0	0	14	Medium quality	7411	1761395	64.30	289	d__Bacteria;p__Actinobacteriota;c__Acidimicrobiia; o__Acidimicrobiales;f__RAAP-2;g__RAAP-2;s__
LMSG_G000008566.1	no	753_1	Coal	67.95	2.66	54.66	0	0	0	11	Medium quality	2931	1584241	64.70	613	d__Bacteria;p__Actinobacteriota;c__Acidimicrobiia; o__Acidimicrobiales;f__RAAP-2;g__RAAP-2;s__

LMSG_G000008567.1	no	785_1	Antimony	81.09	1.28	74.68	1	0	1	15	Medium quality	6279	1556646	62.70	308	d__Bacteria;p__Actinobacteriota;c__Acidimicrobiia;o__Acidimicrobiales;f__RAAP-2;g__RAAP-2;s__
LMSG_G000008568.1	no	788_1	Copper	95.72	4.13	75.07	1	1	1	20	High quality	55133	2640129	61.10	295	d__Bacteria;p__Actinobacteriota;c__Acidimicrobiia;o__Acidimicrobiales;f__RAAP-2;g__RAAP-2;s__
LMSG_G000008569.1	no	788_1	Polymetallic	91.21	1.28	84.80	1	1	1	18	High quality	29308	2308048	61.10	129	d__Bacteria;p__Actinobacteriota;c__Acidimicrobiia;o__Acidimicrobiales;f__RAAP-2;g__RAAP-2;s__
LMSG_G000008570.1	no	788_1	Coal	96.15	4.70	72.65	1	1	1	20	High quality	15661	2204326	61.10	196	d__Bacteria;p__Actinobacteriota;c__Acidimicrobiia;o__Acidimicrobiales;f__RAAP-2;g__RAAP-2;s__
LMSG_G000008571.1	no	788_1	Polymetallic	88.88	0.43	86.75	1	1	1	17	Medium quality	56084	2042343	61.00	65	d__Bacteria;p__Actinobacteriota;c__Acidimicrobiia;o__Acidimicrobiales;f__RAAP-2;g__RAAP-2;s__
LMSG_G000008572.1	no	788_1	Polymetallic	81.81	1.72	73.19	1	1	1	15	Medium quality	37851	2141803	61.00	86	d__Bacteria;p__Actinobacteriota;c__Acidimicrobiia;o__Acidimicrobiales;f__RAAP-2;g__RAAP-2;s__
LMSG_G000008573.1	no	788_1	Polymetallic	77.01	0.86	72.70	0	0	0	17	Medium quality	12854	2154387	61.00	220	d__Bacteria;p__Actinobacteriota;c__Acidimicrobiia;o__Acidimicrobiales;f__RAAP-2;g__RAAP-2;s__
LMSG_G000008574.1	no	788_1	Polymetallic	72.44	2.30	60.96	1	1	1	17	Medium quality	15772	2181501	61.10	177	d__Bacteria;p__Actinobacteriota;c__Acidimicrobiia;o__Acidimicrobiales;f__RAAP-2;g__RAAP-2;s__
LMSG_G000008575.1	no	788_1	Antimony	89.36	1.42	82.24	0	1	0	19	Medium quality	8405	2335317	61.00	336	d__Bacteria;p__Actinobacteriota;c__Acidimicrobiia;o__Acidimicrobiales;f__RAAP-2;g__RAAP-2;s__
LMSG_G000008576.1	no	788_1	Antimony	99.14	2.23	87.99	1	0	1	20	Medium quality	49716	2586450	61.00	151	d__Bacteria;p__Actinobacteriota;c__Acidimicrobiia;o__Acidimicrobiales;f__RAAP-2;g__RAAP-2;s__
LMSG_G000008577.1	no	788_1	Antimony	86.67	2.93	72.02	0	0	0	17	Medium quality	67954	2455220	61.60	231	d__Bacteria;p__Actinobacteriota;c__Acidimicrobiia;o__Acidimicrobiales;f__RAAP-2;g__RAAP-2;s__
LMSG_G000008578.1	no	788_1	Antimony	93.64	2.14	82.96	0	0	0	16	Medium quality	50711	2267021	61.20	108	d__Bacteria;p__Actinobacteriota;c__Acidimicrobiia;o__Acidimicrobiales;f__RAAP-2;g__RAAP-2;s__
LMSG_G000008579.1	no	788_1	Polymetallic	89.74	3.85	70.51	1	2	1	20	Medium quality	37157	2400104	61.00	133	d__Bacteria;p__Actinobacteriota;c__Acidimicrobiia;o__Acidimicrobiales;f__RAAP-2;g__RAAP-2;s__
LMSG_G000008580.1	no	788_1	Polymetallic	92.15	1.28	85.74	1	1	0	20	Medium quality	56425	2142489	61.10	73	d__Bacteria;p__Actinobacteriota;c__Acidimicrobiia;o__Acidimicrobiales;f__RAAP-2;g__RAAP-2;s__
LMSG_G000008581.1	no	789_1	Antimony	62.69	0.00	62.69	1	0	1	17	Medium quality	5968	2010958	59.90	414	d__Bacteria;p__Actinobacteriota;c__Acidimicrobiia;o__Acidimicrobiales;f__RAAP-2;g__RAAP-2;s__
LMSG_G000008582.1	no	789_1	Antimony	81.03	1.72	72.41	1	0	0	19	Medium quality	29958	3425153	60.40	422	d__Bacteria;p__Actinobacteriota;c__Acidimicrobiia;o__Acidimicrobiales;f__RAAP-2;g__RAAP-2;s__
LMSG_G000008583.1	no	790_1	Lead-Zinc	97.43	4.70	73.93	1	1	1	20	High quality	275878	2001435	70.40	12	d__Bacteria;p__Actinobacteriota;c__Acidimicrobiia;o__Acidimicrobiales;f__RAAP-2;g__RAAP-2;s__
LMSG_G000008584.1	no	790_1	Lead-Zinc	82.90	4.96	58.12	1	1	1	19	Medium quality	137018	1852552	70.40	19	d__Bacteria;p__Actinobacteriota;c__Acidimicrobiia;o__Acidimicrobiales;f__RAAP-2;g__RAAP-2;s__
LMSG_G000008585.1	no	791_1	Polymetallic	62.06	0.00	62.06	1	0	1	13	Medium quality	36946	1796079	66.10	132	d__Bacteria;p__Actinobacteriota;c__Acidimicrobiia;o__Acidimicrobiales;f__RAAP-2;g__RAAP-2;s__
LMSG_G000008586.1	no	791_1	Pyrite	60.40	0.86	56.09	0	1	0	16	Medium quality	4968	1572407	65.00	353	d__Bacteria;p__Actinobacteriota;c__Acidimicrobiia;o__Acidimicrobiales;f__RAAP-2;g__RAAP-2;s__
LMSG_G000008587.1	no	792_1	Copper	53.44	0.00	53.44	0	0	0	2	Medium quality	46817	709856	65.30	25	d__Bacteria;p__Actinobacteriota;c__Acidimicrobiia;o__Acidimicrobiales;f__RAAP-2;g__RAAP-2;s__
LMSG_G000008588.1	no	792_1	Polymetallic	66.17	1.72	57.55	0	0	0	13	Medium quality	12268	1849041	66.50	182	d__Bacteria;p__Actinobacteriota;c__Acidimicrobiia;o__Acidimicrobiales;f__RAAP-2;g__RAAP-2;s__
LMSG_G000008589.1	no	792_1	Pyrite-Copper	51.72	0.00	51.72	0	0	0	12	Medium quality	9953	1490333	67.10	205	d__Bacteria;p__Actinobacteriota;c__Acidimicrobiia;o__Acidimicrobiales;f__RAAP-2;g__RAAP-2;s__
LMSG_G000008590.1	no	792_1	Pyrite-Copper	60.34	0.00	60.34	0	0	0	12	Medium quality	15030	1455041	66.10	119	d__Bacteria;p__Actinobacteriota;c__Acidimicrobiia;o__Acidimicrobiales;f__RAAP-2;g__RAAP-2;s__
LMSG_G000008591.1	no	792_1	Polymetallic	52.35	0.00	52.35	0	0	0	8	Medium quality	7707	1320290	67.70	237	d__Bacteria;p__Actinobacteriota;c__Acidimicrobiia;o__Acidimicrobiales;f__RAAP-2;g__RAAP-2;s__
LMSG_G000008592.1	no	792_1	Antimony	66.20	0.00	66.20	0	0	0	13	Medium quality	10733	1993970	66.80	479	d__Bacteria;p__Actinobacteriota;c__Acidimicrobiia;o__Acidimicrobiales;f__RAAP-2;g__RAAP-2;s__
LMSG_G000008593.1	no	793_1	Polymetallic	60.34	0.00	60.34	1	0	1	12	Medium quality	26106	1351894	68.10	122	d__Bacteria;p__Actinobacteriota;c__Acidimicrobiia;o__Acidimicrobiales;f__RAAP-2;g__RAAP-2;s__
LMSG_G000008594.1	no	793_1	Polymetallic	69.59	0.86	65.28	0	1	0	15	Medium quality	8506	1445921	66.80	195	d__Bacteria;p__Actinobacteriota;c__Acidimicrobiia;o__Acidimicrobiales;f__RAAP-2;g__RAAP-2;s__
LMSG_G000008595.1	no	793_1	Polymetallic	91.26	1.38	84.38	0	0	0	17	Medium quality	20494	2071973	67.20	188	d__Bacteria;p__Actinobacteriota;c__Acidimicrobiia;o__Acidimicrobiales;f__RAAP-2;g__RAAP-2;s__
LMSG_G000008596.1	no	793_1	Polymetallic	75.86	0.00	75.86	1	0	0	13	Medium quality	25938	1383107	66.50	70	d__Bacteria;p__Actinobacteriota;c__Acidimicrobiia;o__Acidimicrobiales;f__RAAP-2;g__RAAP-2;s__
LMSG_G000008597.1	no	793_1	Polymetallic	89.36	4.02	69.28	1	0	1	14	Medium quality	21144	1829062	67.30	155	d__Bacteria;p__Actinobacteriota;c__Acidimicrobiia;o__Acidimicrobiales;f__RAAP-2;g__RAAP-2;s__
LMSG_G000008598.1	no	793_1	Polymetallic	90.96	3.28	74.58	1	0	1	13	Medium quality	17053	1813094	67.40	162	d__Bacteria;p__Actinobacteriota;c__Acidimicrobiia;o__Acidimicrobiales;f__RAAP-2;g__RAAP-2;s__
LMSG_G000008599.1	no	793_1	Polymetallic	70.68	2.59	57.75	1	0	1	13	Medium quality	21828	1543656	67.00	110	d__Bacteria;p__Actinobacteriota;c__Acidimicrobiia;o__Acidimicrobiales;f__RAAP-2;g__RAAP-2;s__
LMSG_G000008600.1	no	793_1	Polymetallic	76.17	3.45	58.93	1	0	0	13	Medium quality	15747	1657981	67.50	165	d__Bacteria;p__Actinobacteriota;c__Acidimicrobiia;o__Acidimicrobiales;f__RAAP-2;g__RAAP-2;s__
LMSG_G000008601.1	no	793_1	Polymetallic	58.62	0.00	58.62	1	0	0	14	Medium quality	26097	1269407	66.70	179	d__Bacteria;p__Actinobacteriota;c__Acidimicrobiia;o__Acidimicrobiales;f__RAAP-2;g__RAAP-2;s__
LMSG_G000008602.1	no	794_1	Polymetallic	93.84	3.24	77.64	1	1	0	15	Medium quality	33620	1946545	68.40	111	d__Bacteria;p__Actinobacteriota;c__Acidimicrobiia;o__Acidimicrobiales;f__RAAP-2;g__RAAP-2;s__
LMSG_G000008603.1	no	794_1	Lead-Zinc	65.86	0.00	65.86	1	0	1	15	Medium quality	21853	1928876	68.80	177	d__Bacteria;p__Actinobacteriota;c__Acidimicrobiia;o__Acidimicrobiales;f__RAAP-2;g__RAAP-2;s__
LMSG_G000008604.1	no	794_1	Lead-Zinc	74.48	1.72	65.86	0	0	0	15	Medium quality	21280	2126909	68.30	139	d__Bacteria;p__Actinobacteriota;c__Acidimicrobiia;o__Acidimicrobiales;f__RAAP-2;g__RAAP-2;s__
LMSG_G000008605.1	no	794_1	Antimony	74.31	2.89	59.85	1	0	0	16	Medium quality	12127	1526876	68.50	186	d__Bacteria;p__Actinobacteriota;c__Acidimicrobiia;o__Acidimicrobiales;f__RAAP-2;g__RAAP-2;s__
LMSG_G000008606.1	no	794_1	Antimony	51.72	0.00	51.72	1	0	0	12	Medium quality	15244	1052581	68.50	79	d__Bacteria;p__Actinobacteriota;c__Acidimicrobiia;o__Acidimicrobiales;f__RAAP-2;g__RAAP-2;s__
LMSG_G000008607.1	no	796_1	Polymetallic	94.01	4.70	70.51	1	1	2	20	High quality	58201	2537820	66.00	119	d__Bacteria;p__Actinobacteriota;c__Acidimicrobiia;o__Acidimicrobiales;f__RAAP-2;g__RAAP-2;s__
LMSG_G000008608.1	no	796_1	Polymetallic	67.24	0.00	67.24	1	1	0	13	Medium quality	35713	1343954	65.00	93	d__Bacteria;p__Actinobacteriota;c__Acidimicrobiia;o__Acidimicrobiales;f__RAAP-2;g__RAAP-2;s__
LMSG_G000008609.1	no	796_1	Polymetallic	61.14	2.14	50.46	0	0	0	17	Medium quality	6094	1604309	66.60	307	d__Bacteria;p__Actinobacteriota;c__Acidimicrobiia;o__Acidimicrobiales;f__RAAP-2;g__RAAP-2;s__
LMSG_G000008610.1	no	796_1	Polymetallic	53.37	0.52	50.76	1	1	1	17	Medium quality	19537	1076407	65.20	111	d__Bacteria;p__Actinobacteriota;c__Acidimicrobiia;o__Acidimicrobiales;f__RAAP-2;g__RAAP-2;s__
LMSG_G000008611.1	no	796_1	Polymetallic	55.17	0.00	55.17	0	0	0	11	Medium quality	69189	928778	64.80	92	d__Bacteria;p__Actinobacteriota;c__Acidimicrobiia;o__Acidimicrobiales;f__RAAP-2;g__RAAP-2;s__
LMSG_G000008612.1	no	796_1	Polymetallic	74.95	1.57	67.12	0	0	0	13	Medium quality	16075	1554159	66.40	140	d__Bacteria;p__Actinobacteriota;c__Acidimicrobiia;o__Acidimicrobiales;f__RAAP-2;g__RAAP-2;s__
LMSG_G000008613.1	no	796_1	Polymetallic	60.34	1.72	51.72	1	0	1	14	Medium quality	34204	1177163	65.30	135	d__Bacteria;p__Actinobacteriota;c__Acidimicrobiia;o__Acidimicrobiales;f__RAAP-2;g__RAAP-2;s__
LMSG_G000008614.1	no	796_1	Polymetallic	88.08	3.56	70.28	0	0	0	16	Medium quality	21554	1956098	66.20	196	d__Bacteria;p__Actinobacteriota;c__Acidimicrobiia;o__Acidimicrobiales;f__RAAP-2;g__RAAP-2;s__
LMSG_G000008615.1	no	796_1	Polymetallic	62.06	1.72	53.44	1	0	1	13	Medium quality	27506	1248586	65.10	168	d__Bacteria;p__Actinobacteriota;c__Acidimicrobiia;o__Acidimicrobiales;f__RAAP-2;g__RAAP-2;s__
LMSG_G000008616.1	no	796_1	Polymetallic	80.79	1.72	72.17	0	0	0	17	Medium quality	4857	2109994	66.50	538	d__Bacteria;p__Actinobacteriota;c__Acidimicrobiia;o__Acidimicrobiales;f__RAAP-2;g__RAAP-2;s__
LMSG_G000008617.1	no	798_1	Polymetallic	54.13	0.00	54.13	0	0	0	12	Medium quality	11752	1216317	68.50	122	d__Bacteria;p__Actinobacteriota;c__Acidimicrobiia;o__Acidimicrobiales;f__RAAP-2;g__RAAP-2;s__
LMSG_G000008618.1	no	798_1	Polymetallic	73.19	2.42	61.09	0	1	0	17	Medium quality	22764	1705390	67.80	111	d__Bacteria;p__Actinobacteriota;c__Acidimicrobiia;o__Acidimicrobiales;f__RAAP-2;g__RAAP-2;s__
LMSG_G000008619.1	no	799_1	Polymetallic	73.59	2.99	58.64	1	0	1	15	Medium quality	39875	1786983	63.40	81	d__Bacteria;p__Actinobacteriota;c__Acidimicrobiia;o__Acidimicrobiales;f__RAAP-2;g__RAAP-2;s__
LMSG_G000008620.1	no	800_1	Polymetallic	81.81	2.14	71.13	1	1	0	17	Medium quality	16579	1837130	67.70	150	d__Bacteria;p__Actinobacteriota;c__Acidimicrobiia;o__Acidimicrobiales;f__RAAP-2;g__RAAP-2;s__
LMSG_G000008621.1	no	800_1	Polymetallic	85.70	4.84	61.49	1	1	1	18	Medium quality	17582	2049815	67.60	151	d__Bacteria;p__Actinobacteriota;c__Acidimicrobiia;o__Acidimicrobiales;f__RAAP-2;g__RAAP-2;s__
LMSG_G000008622.1	no	800_1	Polymetallic	82.64	0.85	78.37	1	0	1	17	Medium quality	15767	1803803	67.90	173	d__Bacteria;p__Actinobacteriota;c__Acidimicrobiia;o__Acidimicrobiales;f__RAAP-2;g__RAAP-2;s__
LMSG_G000008623.1	no	800_1	Copper	85.51	4.23	64.38	0	1	0	18	Medium quality	5586	2254760	67.60	497	d__Bacteria;p__Actinobacteriota;c__Acidimicrobiia;o__Acidimicrobiales;f__RAAP-2;g__RAAP-2;s__
LMSG_G000008624.1	no	801_1	Polymetallic	96.58	1.28	90.17	1	1	1	20	High quality	49006	2267286	69.90	127	d__Bacteria;p__Actinobacteriota;c__Acidimicrobiia;o__Acidimicrobiales;f__RAAP-2;g__RAAP-2;s__
LMSG_G000008625.1	no	801_1	Polymetallic	96.58	2.14	85.90	1	1	1	20	High quality	32840	2141217	69.80	110	d__Bacteria;p__Actinobacteriota;c__Acidimicrobiia;o__Acidimicrobiales;f__RAAP-2;g__RAAP-2;s__
LMSG_G000008626.1	no	801_1	Polymetallic	98.29	1.71	89.75	1	1	1	20	High quality	32462	2129276	70.00	119	d__Bacteria;p__Actinobacteriota;c__Acidimicrobiia;o__Acidimicrobiales;f__RAAP-2;g__RAAP-2;s__
LMSG_G000008627.1	no	801_1	Polymetallic	97.43	2.14	86.75	1	2	1	20	High quality	23765	2216303	69.60	152	d__Bacteria;p__Actinobacteriota;c__Acidimicrobiia;o__Acidimicrobiales;f__RAAP-2;g__RAAP-2;s__
LMSG_G000008628.1	no	801_1	Lead-Zinc	98.29	2.99	83.34	1	1	2	20	High quality	18956	2143323	70.00	178	d__Bacteria;p__Actinobacteriota;c__Acidimicrobiia;o__Acidimicrobiales;f__RAAP-2;g__RAAP-2;s__
LMSG_G000008629.1	no	801_1	Pyrite-Copper	96.48	3.70	77.97	1	1	1	20	High quality	42653	2059955	70.00	72	d__Bacteria;p__Actinobacteriota;c__Acidimicrobiia;o__Acidimicrobiales;f__RAAP-2;g__RAAP-2;s__
LMSG_G000008630.1	no	801_1	Lead-Zinc	97.43	2.74	83.76	1	1	1	17	Medium quality	25331	2080430	70.00	147	d__Bacteria;p__Actinobacteriota;c__Acidimicrobiia;o__Acidimicrobiales;f__RAAP-2;g__RAAP-2;s__
LMSG_G000008631.1	no	801_1	Copper	89.80	4.91	65.26	1	0	1	17	Medium quality	26573	1928224	69.80	113	d__Bacteria;p__Actinobacteriota;c__Acidimicrobiia;o__Acidimicrobiales;f__RAAP-2;g__RAAP-2;s__
LMSG_G000008632.1	no	801_1	Lead-Zinc	82.05	4.02	61.97	1	1	1	16	Medium quality	74951	2017944	69.70	60	d__Bacteria;p__Actinobacteriota;c__Acidimicrobiia;o__Acidimicrobiales;f__RAAP-2;g__RAAP-2;s__
LMSG_G000008633.1	no	801_1	Lead-Zinc	86.62	0.51	84.06	1	1	1	17	Medium quality	46291	1528676	70.00	53	d__Bacteria;p__Actinobacteriota;c__Acidimicrobiia;o__Acidimicrobiales;f__RAAP-2;g__RAAP-2;s__
LMSG_G000008634.1	no	801_1	Polymetallic	97.43	1.28	91.02	0	1	0	20	Medium quality	31478	2195782	69.70	130	d__Bacteria;p__Actinobacteriota;c__Acidimicrobiia;o__Acidimicrobiales;f__RAAP-2;g__RAAP-2;s__
LMSG_G000008635.1	no	801_1	Pyrite-Copper	64.20	1.71	55.66	0	0	0	10	Medium quality	29436	990533	70.20	60	d__Bacteria;p__Actinobacteriota;c__Acidimicrobiia;o__Acidimicrobiales;f__RAAP-2;g__RAAP-2;s__
LMSG_G000008636.1	no	801_1	Pyrite-Copper	66.14	2.99	51.19	1	0	1	12	Medium quality	27655	1160649	69.70	73	d__Bacteria;p__Actinobacteriota;c__Acidimicrobiia;o__Acidimicrobiales;f__RAAP-2;g__RAAP-2;s__
LMSG_G000008637.1	no	801_1	Pyrite	63.80	1.71	55.26	0	1	0	13	Medium quality	3035	1421370	70.20	495	d__Bacteria;p__Actinobacteriota;c__Acidimicrobiia;o__Acidimicrobiales;f__RAAP-2;g__RAAP-2;s__
LMSG_G000008638.1	no	802_1	Copper	57.60	0.51	55.08	0	0	0	15	Medium quality	2609	1273975	69.40	494	d__Bacteria;p__Actinobacteriota;c__Acidimicrobiia;o__Acidimicrobiales;f__RAAP-2;g__RAAP-2;s__
LMSG_G000008639.1	no	802_1	Pyrite-Copper	64.71	2.14	54.03	0	0	0	11	Medium quality	7198	1355021	69.80	218	d__Bacteria;p__Actinobacteriota;c__Acidimicrobiia;o__Acidimicrobiales;f__RAAP-2;g__RAAP-2;s__
LMSG_G000008640.1	no	802_1	Copper	59.78	1.71	51.24	1	0	0	16	Medium quality	30108	1130738	68.60	53	d__Bacteria;p__Actinobacteriota;c__Acidimicrobiia;o__Acidimicrobiales;f__RAAP-2;g__RAAP-2;s__
LMSG_G000008641.1	no	804_1	Antimony	56.89	0.00	56.89	0	1	0	14	Medium quality	11650	1444175	69.40	254	d__Bacteria;p__Actinobacteriota;c__Acidimicrobiia;o__Acidimicrobiales;f__RAAP-2;g__RAAP-2;s__
LMSG_G000008642.1	no	804_1	Antimony	68.28	1.71	59.74	0	0	0	14	Medium quality	5471	1450094	69.90	313	d__Bacteria;p__Actinobacteriota;c__Acidimicrobiia;o__Acidimicrobiales;f__RAAP-2;g__RAAP-2;s__
LMSG_G000008643.1	no	805_1	Polymetallic	76.03	1.72	67.41	0	0	0	18	Medium quality	16287	1809361	67.00	139	d__Bacteria;p__Actinobacteriota;c__Acidimicrobiia;o__Acidimicrobiales;f__RAAP-2;g__RAAP-2;s__

LMSG_G000008644.1	no	805_1	Antimony	84.70	3.42	67.61	0	1	0	19	Medium quality	27832	1664645	66.90	80	d__Bacteria;p__Actinobacteriota;c__Acidimicrobiia;o__Acidimicrobiales;f__RAAP-2;g__RAAP-2;s__
LMSG_G000008645.1	no	805_1	Antimony	65.67	0.00	65.67	1	1	0	15	Medium quality	36378	1416680	67.00	51	d__Bacteria;p__Actinobacteriota;c__Acidimicrobiia;o__Acidimicrobiales;f__RAAP-2;g__RAAP-2;s__
LMSG_G000008646.1	no	805_1	Antimony	79.45	2.56	66.63	0	1	0	18	Medium quality	48341	1664265	67.00	60	d__Bacteria;p__Actinobacteriota;c__Acidimicrobiia;o__Acidimicrobiales;f__RAAP-2;g__RAAP-2;s__
LMSG_G000008647.1	no	805_1	Antimony	86.15	3.85	66.92	0	1	0	18	Medium quality	39154	1822920	67.00	84	d__Bacteria;p__Actinobacteriota;c__Acidimicrobiia;o__Acidimicrobiales;f__RAAP-2;g__RAAP-2;s__
LMSG_G000008648.1	no	805_1	Antimony	78.67	3.85	59.44	1	1	1	16	Medium quality	42965	1609973	67.20	61	d__Bacteria;p__Actinobacteriota;c__Acidimicrobiia;o__Acidimicrobiales;f__RAAP-2;g__RAAP-2;s__
LMSG_G000008649.1	no	806_1	Antimony	58.71	0.86	54.40	1	0	1	16	Medium quality	22794	1596129	67.10	102	d__Bacteria;p__Actinobacteriota;c__Acidimicrobiia;o__Acidimicrobiales;f__RAAP-2;g__RAAP-2;s__
LMSG_G000008650.1	no	806_1	Antimony	64.60	1.72	55.98	1	0	1	16	Medium quality	20318	1612906	67.10	129	d__Bacteria;p__Actinobacteriota;c__Acidimicrobiia;o__Acidimicrobiales;f__RAAP-2;g__RAAP-2;s__
LMSG_G000008651.1	no	806_1	Pyrite-Copper	73.73	0.43	71.60	0	1	0	14	Medium quality	50365	1425284	67.30	50	d__Bacteria;p__Actinobacteriota;c__Acidimicrobiia;o__Acidimicrobiales;f__RAAP-2;g__RAAP-2;s__
LMSG_G000008652.1	no	807_1	Polymetallic	80.67	2.14	69.99	1	0	0	20	Medium quality	16559	1754249	72.50	166	d__Bacteria;p__Actinobacteriota;c__Acidimicrobiia;o__Acidimicrobiales;f__RAAP-2;g__RAAP-2;s__
LMSG_G000008653.1	no	807_2	Polymetallic	90.97	2.99	76.02	1	1	1	20	High quality	23739	1986307	72.00	135	d__Bacteria;p__Actinobacteriota;c__Acidimicrobiia;o__Acidimicrobiales;f__RAAP-2;g__RAAP-2;s__
LMSG_G000008654.1	no	808_1	Magnetite	89.36	3.93	69.71	1	0	1	19	Medium quality	11851	1817997	71.90	238	d__Bacteria;p__Actinobacteriota;c__Acidimicrobiia;o__Acidimicrobiales;f__RAAP-2;g__RAAP-2;s__
LMSG_G000008655.1	no	808_2	Polymetallic	56.88	0.43	54.75	1	0	1	16	Medium quality	83836	1187918	72.20	24	d__Bacteria;p__Actinobacteriota;c__Acidimicrobiia;o__Acidimicrobiales;f__RAAP-2;g__RAAP-2;s__
LMSG_G000008656.1	no	808_2	Polymetallic	76.92	2.62	63.82	1	1	1	20	Medium quality	23798	1911422	71.80	110	d__Bacteria;p__Actinobacteriota;c__Acidimicrobiia;o__Acidimicrobiales;f__RAAP-2;g__RAAP-2;s__
LMSG_G000008657.1	no	808_3	Antimony	83.56	2.56	70.74	0	1	0	19	Medium quality	24060	1807693	72.80	107	d__Bacteria;p__Actinobacteriota;c__Acidimicrobiia;o__Acidimicrobiales;f__RAAP-2;g__RAAP-2;s__
LMSG_G000008658.1	no	808_4	Polymetallic	67.98	1.99	58.01	0	1	0	17	Medium quality	4076	1347186	72.70	383	d__Bacteria;p__Actinobacteriota;c__Acidimicrobiia;o__Acidimicrobiales;f__RAAP-2;g__RAAP-2;s__
LMSG_G000008659.1	no	808_4	Polymetallic	81.11	2.78	67.23	0	1	0	16	Medium quality	10911	1597405	73.10	297	d__Bacteria;p__Actinobacteriota;c__Acidimicrobiia;o__Acidimicrobiales;f__RAAP-2;g__RAAP-2;s__
LMSG_G000008660.1	no	809_1	Antimony	84.06	2.99	69.11	1	1	0	19	Medium quality	75970	1727577	71.10	38	d__Bacteria;p__Actinobacteriota;c__Acidimicrobiia;o__Acidimicrobiales;f__RAAP-2;g__RAAP-2;s__
LMSG_G000008661.1	no	812_1	Copper	96.15	1.28	89.74	1	2	0	16	Medium quality	14060	1791766	63.90	194	d__Bacteria;p__Actinobacteriota;c__Acidimicrobiia;o__Acidimicrobiales;f__RAAP-2;g__RAAP-2;s__
LMSG_G000008662.1	no	813_1	Polymetallic	97.43	4.21	76.39	1	1	1	20	High quality	93336	1913033	63.80	151	d__Bacteria;p__Actinobacteriota;c__Acidimicrobiia;o__Acidimicrobiales;f__RAAP-2;g__RAAP-2;s__
LMSG_G000008663.1	no	813_1	Polymetallic	84.66	1.62	76.55	0	0	0	19	Medium quality	23575	1701974	64.10	111	d__Bacteria;p__Actinobacteriota;c__Acidimicrobiia;o__Acidimicrobiales;f__RAAP-2;g__RAAP-2;s__
LMSG_G000008664.1	no	813_1	Polymetallic	93.16	2.42	81.06	0	0	0	18	Medium quality	22395	1708237	63.90	117	d__Bacteria;p__Actinobacteriota;c__Acidimicrobiia;o__Acidimicrobiales;f__RAAP-2;g__RAAP-2;s__
LMSG_G000008665.1	no	813_1	Polymetallic	86.22	1.62	78.11	1	0	1	17	Medium quality	17973	1613759	63.90	114	d__Bacteria;p__Actinobacteriota;c__Acidimicrobiia;o__Acidimicrobiales;f__RAAP-2;g__RAAP-2;s__
LMSG_G000008666.1	no	814_1	Copper	92.30	2.14	81.62	0	1	0	19	Medium quality	23201	2037578	64.20	165	d__Bacteria;p__Actinobacteriota;c__Acidimicrobiia;o__Acidimicrobiales;f__RAAP-2;g__RAAP-2;s__
LMSG_G000008667.1	no	814_1	Antimony	91.45	1.38	84.57	0	0	0	19	Medium quality	8351	1910902	64.40	305	d__Bacteria;p__Actinobacteriota;c__Acidimicrobiia;o__Acidimicrobiales;f__RAAP-2;g__RAAP-2;s__
LMSG_G000008668.1	no	814_1	Antimony	68.25	2.14	57.57	1	0	1	13	Medium quality	2954	1457461	64.80	563	d__Bacteria;p__Actinobacteriota;c__Acidimicrobiia;o__Acidimicrobiales;f__RAAP-2;g__RAAP-2;s__
LMSG_G000008669.1	no	814_1	Polymetallic	93.58	1.97	83.73	1	7	5	20	High quality	99068	2417339	59.80	502	d__Bacteria;p__Actinobacteriota;c__Acidimicrobiia;o__Acidimicrobiales;f__RAAP-2;g__RAAP-2;s__
LMSG_G000008670.1	no	814_1	Polymetallic	78.20	3.13	62.54	0	1	0	17	Medium quality	40958	1786076	64.30	129	d__Bacteria;p__Actinobacteriota;c__Acidimicrobiia;o__Acidimicrobiales;f__RAAP-2;g__RAAP-2;s__
LMSG_G000008671.1	no	814_1	Polymetallic	81.62	2.23	70.47	0	1	0	18	Medium quality	36056	1596682	65.00	119	d__Bacteria;p__Actinobacteriota;c__Acidimicrobiia;o__Acidimicrobiales;f__RAAP-2;g__RAAP-2;s__
LMSG_G000008672.1	no	814_1	Polymetallic	89.74	3.70	71.23	1	1	0	18	Medium quality	21552	1981972	64.40	190	d__Bacteria;p__Actinobacteriota;c__Acidimicrobiia;o__Acidimicrobiales;f__RAAP-2;g__RAAP-2;s__
LMSG_G000008673.1	no	814_1	Polymetallic	73.12	1.28	66.71	0	0	0	16	Medium quality	36020	1394268	65.00	107	d__Bacteria;p__Actinobacteriota;c__Acidimicrobiia;o__Acidimicrobiales;f__RAAP-2;g__RAAP-2;s__
LMSG_G000008674.1	no	726_1	Polymetallic	93.58	3.07	78.24	0	1	0	19	Medium quality	30347	3662501	70.90	231	d__Bacteria;p__Actinobacteriota;c__Acidimicrobiia;o__Acidimicrobiales;f__UBA8190;g__;s__
LMSG_G000008675.1	no	726_1	Polymetallic	89.88	4.42	67.81	0	0	0	19	Medium quality	32617	3287903	71.20	206	d__Bacteria;p__Actinobacteriota;c__Acidimicrobiia;o__Acidimicrobiales;f__UBA8190;g__;s__
LMSG_G000008676.1	no	726_1	Polymetallic	91.59	2.14	80.91	0	0	0	18	Medium quality	31108	3165576	71.10	188	d__Bacteria;p__Actinobacteriota;c__Acidimicrobiia;o__Acidimicrobiales;f__UBA8190;g__;s__
LMSG_G000008677.1	no	726_1	Polymetallic	94.87	4.51	72.32	0	1	0	20	Medium quality	31334	3796907	70.90	237	d__Bacteria;p__Actinobacteriota;c__Acidimicrobiia;o__Acidimicrobiales;f__UBA8190;g__;s__
LMSG_G000008678.1	no	726_1	Polymetallic	91.45	2.14	80.77	0	1	0	17	Medium quality	27562	2787355	71.30	167	d__Bacteria;p__Actinobacteriota;c__Acidimicrobiia;o__Acidimicrobiales;f__UBA8190;g__;s__
LMSG_G000008679.1	no	726_1	Magnetite	89.97	4.27	68.61	0	1	0	19	Medium quality	23351	2977007	71.10	210	d__Bacteria;p__Actinobacteriota;c__Acidimicrobiia;o__Acidimicrobiales;f__UBA8190;g__;s__
LMSG_G000008680.1	no	728_1	Copper	83.76	1.28	77.35	0	0	0	17	Medium quality	24411	3070892	73.20	205	d__Bacteria;p__Actinobacteriota;c__Acidimicrobiia;o__Acidimicrobiales;f__UBA8190;g__;s__
LMSG_G000008681.1	no	728_1	Polymetallic	85.23	2.14	74.55	1	0	1	19	Medium quality	26556	3317327	73.10	172	d__Bacteria;p__Actinobacteriota;c__Acidimicrobiia;o__Acidimicrobiales;f__UBA8190;g__;s__
LMSG_G000008682.1	no	728_1	Magnetite	67.68	0.00	67.68	0	0	0	13	Medium quality	6526	2576797	73.80	495	d__Bacteria;p__Actinobacteriota;c__Acidimicrobiia;o__Acidimicrobiales;f__UBA8190;g__;s__
LMSG_G000008683.1	no	729_1	Pyrite	98.00	4.89	73.55	1	1	1	20	High quality	41663	2876739	70.50	94	d__Bacteria;p__Actinobacteriota;c__Acidimicrobiia;o__Acidimicrobiales;f__UBA8190;g__;s__
LMSG_G000008684.1	no	729_1	Polymetallic	94.58	4.70	71.08	1	1	1	19	High quality	39127	3001529	70.40	98	d__Bacteria;p__Actinobacteriota;c__Acidimicrobiia;o__Acidimicrobiales;f__UBA8190;g__;s__
LMSG_G000008685.1	no	729_1	Copper	96.86	4.70	73.36	1	1	1	19	High quality	35647	3137226	70.40	121	d__Bacteria;p__Actinobacteriota;c__Acidimicrobiia;o__Acidimicrobiales;f__UBA8190;g__;s__
LMSG_G000008686.1	no	729_1	Copper	90.31	4.65	67.05	1	1	1	19	High quality	28851	3742694	70.00	210	d__Bacteria;p__Actinobacteriota;c__Acidimicrobiia;o__Acidimicrobiales;f__UBA8190;g__;s__
LMSG_G000008687.1	no	729_1	Pyrite	92.21	4.02	72.13	1	1	1	17	Medium quality	43830	2833789	70.50	135	d__Bacteria;p__Actinobacteriota;c__Acidimicrobiia;o__Acidimicrobiales;f__UBA8190;g__;s__
LMSG_G000008688.1	no	729_1	Lead-Zinc	92.87	2.99	77.92	1	1	1	17	Medium quality	27128	2731253	70.40	140	d__Bacteria;p__Actinobacteriota;c__Acidimicrobiia;o__Acidimicrobiales;f__UBA8190;g__;s__
LMSG_G000008689.1	no	729_1	Polymetallic	81.98	4.56	59.19	0	0	0	18	Medium quality	7782	3252507	70.00	490	d__Bacteria;p__Actinobacteriota;c__Acidimicrobiia;o__Acidimicrobiales;f__UBA8190;g__;s__
LMSG_G000008690.1	no	729_1	Pyrite-Copper	76.44	3.42	59.35	1	0	1	15	Medium quality	7826	2093983	70.70	354	d__Bacteria;p__Actinobacteriota;c__Acidimicrobiia;o__Acidimicrobiales;f__UBA8190;g__;s__
LMSG_G000008691.1	no	877_1	Pyrite-Copper	84.62	1.89	75.17	0	1	0	15	Medium quality	12992	2095269	72.30	193	d__Bacteria;p__Actinobacteriota;c__Actinomycetia;o__Actinomycetales;f__;g__;s__
LMSG_G000008692.1	no	853_1	Antimony	88.01	2.24	76.79	0	0	0	19	Medium quality	26260	1628653	69.60	97	d__Bacteria;p__Actinobacteriota;c__Actinomycetia;o__Actinomycetales;f__Microbacteriaceae;g__Gryllotalpicola;s__
LMSG_G000008693.1	no	843_1	Lead-Zinc	94.87	1.35	88.12	1	1	1	20	High quality	82317	2074879	65.30	45	d__Bacteria;p__Actinobacteriota;c__Actinomycetia;o__Nanopelagicales;f__UBA5976;g__;s__
LMSG_G000008694.1	no	843_1	Polymetallic	93.25	0.81	89.20	1	1	1	20	High quality	122205	1909510	65.30	43	d__Bacteria;p__Actinobacteriota;c__Actinomycetia;o__Nanopelagicales;f__UBA5976;g__;s__
LMSG_G000008695.1	no	843_1	Lead-Zinc	94.33	1.88	84.92	0	1	0	20	Medium quality	133169	2121201	65.30	73	d__Bacteria;p__Actinobacteriota;c__Actinomycetia;o__Nanopelagicales;f__UBA5976;g__;s__
LMSG_G000008696.1	no	843_1	Polymetallic	93.79	0.54	91.09	0	0	0	20	Medium quality	70600	2027769	65.40	59	d__Bacteria;p__Actinobacteriota;c__Actinomycetia;o__Nanopelagicales;f__UBA5976;g__;s__
LMSG_G000008697.1	no	843_1	Polymetallic	92.57	2.54	79.87	1	0	0	19	Medium quality	15042	1938326	65.40	189	d__Bacteria;p__Actinobacteriota;c__Actinomycetia;o__Nanopelagicales;f__UBA5976;g__;s__
LMSG_G000008698.1	no	844_1	Polymetallic	91.72	4.32	70.10	1	1	1	19	High quality	23598	2058977	69.60	239	d__Bacteria;p__Actinobacteriota;c__Actinomycetia;o__Nanopelagicales;f__UBA5976;g__;s__
LMSG_G000008699.1	no	844_1	Polymetallic	86.25	3.14	70.55	1	0	0	19	Medium quality	13626	1954807	69.60	209	d__Bacteria;p__Actinobacteriota;c__Actinomycetia;o__Nanopelagicales;f__UBA5976;g__;s__
LMSG_G000008700.1	no	844_1	Polymetallic	81.69	4.56	58.89	0	1	0	18	Medium quality	6183	1725150	69.70	347	d__Bacteria;p__Actinobacteriota;c__Actinomycetia;o__Nanopelagicales;f__UBA5976;g__;s__
LMSG_G000008701.1	no	879_1	Tin-Zinc	52.02	0.00	52.02	0	0	0	13	Medium quality	6143	1839109	71.50	335	d__Bacteria;p__Actinobacteriota;c__Actinomycetia;o__Propionibacteriales;f__Propionibacteriaceae;g__;s__
LMSG_G000008702.1	no	880_1	Antimony	89.72	2.90	75.20	2	0	0	17	Medium quality	14475	3239558	69.80	278	d__Bacteria;p__Actinobacteriota;c__Actinomycetia;o__Propionibacteriales;f__Propionibacteriaceae;g__;s__
LMSG_G000008703.1	no	877_1	Lead-Zinc	66.99	1.96	57.19	0	1	0	11	Medium quality	3413	1965724	72.30	625	d__Bacteria;p__Actinobacteriota;c__Actinomycetia;o__RP-AC37;f__;g__;s__
LMSG_G000008704.1	no	869_1	Copper	80.74	2.64	67.55	0	1	0	16	Medium quality	7797	3964895	71.50	597	d__Bacteria;p__Actinobacteriota;c__Actinomycetia;o__Streptosporangiales;f__Streptosporangiaceae;g__;s__
LMSG_G000008705.1	no	869_1	Pyrite	72.05	3.07	56.68	0	1	0	14	Medium quality	2919	3441760	71.40	1221	d__Bacteria;p__Actinobacteriota;c__Actinomycetia;o__Streptosporangiales;f__Streptosporangiaceae;g__;s__
LMSG_G000008706.1	no	873_1	Polymetallic	61.48	1.53	53.82	0	0	0	14	Medium quality	2805	4164903	71.40	1532	d__Bacteria;p__Actinobacteriota;c__Actinomycetia;o__Streptosporangiales;f__Streptosporangiaceae;g__;s__
LMSG_G000008707.1	no	869_1	Copper	95.18	3.48	77.81	1	1	1	18	High quality	33357	4917561	71.30	307	d__Bacteria;p__Actinobacteriota;c__Actinomycetia;o__Streptosporangiales;f__Streptosporangiaceae;g__Palsa-506;s__
LMSG_G000008708.1	no	869_1	Lead-Zinc	94.26	3.86	74.94	1	0	1	19	Medium quality	8069	4648159	71.40	800	d__Bacteria;p__Actinobacteriota;c__Actinomycetia;o__Streptosporangiales;f__Streptosporangiaceae;g__Palsa-506;s__
LMSG_G000008709.1	no	869_1	Copper	89.70	3.65	71.43	0	0	0	18	Medium quality	26816	4675481	71.50	287	d__Bacteria;p__Actinobacteriota;c__Actinomycetia;o__Streptosporangiales;f__Streptosporangiaceae;g__Palsa-506;s__
LMSG_G000008710.1	no	869_1	Polymetallic	74.33	2.23	63.19	0	0	0	14	Medium quality	10362	3579416	71.50	410	d__Bacteria;p__Actinobacteriota;c__Actinomycetia;o__Streptosporangiales;f__Streptosporangiaceae;g__Palsa-506;s__
LMSG_G000008711.1	no	869_1	Magnetite	90.99	2.39	79.05	0	1	0	17	Medium quality	16939	4370660	71.40	374	d__Bacteria;p__Actinobacteriota;c__Actinomycetia;o__Streptosporangiales;f__Streptosporangiaceae;g__Palsa-506;s__
LMSG_G000008712.1	no	869_1	Pyrite-Copper	89.57	1.87	80.22	0	0	0	17	Medium quality	7012	4230503	71.60	784	d__Bacteria;p__Actinobacteriota;c__Actinomycetia;o__Streptosporangiales;f__Streptosporangiaceae;g__Palsa-506;s__
LMSG_G000008713.1	no	869_1	Pyrite-Copper	91.85	3.57	74.03	0	1	0	17	Medium quality	11633	4367453	71.30	522	d__Bacteria;p__Actinobacteriota;c__Actinomycetia;o__Streptosporangiales;f__Streptosporangiaceae;g__Palsa-506;s__

LMSG_G000008714.1	no	869_1	Pyrite-Copper	89.92	3.11	74.37	0	0	0	18	Medium quality	8750	4232207	71.30	588	d__Bacteria;p__Actinobacteriota;c__Actinomycetia;o__Streptosporangiales;f__Streptosporangiaceae;g__Palsa-506;s__
LMSG_G000008715.1	no	870_1	Coal	98.66	4.55	75.94	1	1	1	20	High quality	77629	5310122	67.00	117	d__Bacteria;p__Actinobacteriota;c__Actinomycetia;o__Streptosporangiales;f__Streptosporangiaceae;g__Palsa-506;s__
LMSG_G000008716.1	no	870_1	Coal	87.70	2.66	74.40	0	1	0	18	Medium quality	16864	4372692	67.10	383	d__Bacteria;p__Actinobacteriota;c__Actinomycetia;o__Streptosporangiales;f__Streptosporangiaceae;g__Palsa-506;s__
LMSG_G000008717.1	no	871_1	Lead-Zinc	97.86	3.27	81.50	1	2	1	20	High quality	52402	5069706	69.20	168	d__Bacteria;p__Actinobacteriota;c__Actinomycetia;o__Streptosporangiales;f__Streptosporangiaceae;g__Palsa-506;s__
LMSG_G000008718.1	no	871_1	Lead-Zinc	97.59	2.58	84.67	1	1	1	20	High quality	48502	5148039	69.10	193	d__Bacteria;p__Actinobacteriota;c__Actinomycetia;o__Streptosporangiales;f__Streptosporangiaceae;g__Palsa-506;s__
LMSG_G000008719.1	no	871_1	Pyrite-Copper	95.98	2.05	85.74	1	1	1	19	High quality	27062	5211748	69.10	388	d__Bacteria;p__Actinobacteriota;c__Actinomycetia;o__Streptosporangiales;f__Streptosporangiaceae;g__Palsa-506;s__
LMSG_G000008720.1	no	871_1	Lead-Zinc	97.45	3.79	78.52	0	1	1	20	Medium quality	19303	4930504	69.30	434	d__Bacteria;p__Actinobacteriota;c__Actinomycetia;o__Streptosporangiales;f__Streptosporangiaceae;g__Palsa-506;s__
LMSG_G000008721.1	no	871_1	Lead-Zinc	85.82	3.30	69.34	1	1	1	19	Medium quality	39060	4746097	69.20	238	d__Bacteria;p__Actinobacteriota;c__Actinomycetia;o__Streptosporangiales;f__Streptosporangiaceae;g__Palsa-506;s__
LMSG_G000008722.1	no	871_2	Coal	98.57	4.46	76.29	1	1	1	20	High quality	35326	5878913	69.30	299	d__Bacteria;p__Actinobacteriota;c__Actinomycetia;o__Streptosporangiales;f__Streptosporangiaceae;g__Palsa-506;s__
LMSG_G000008723.1	no	871_2	Coal	95.36	4.10	74.87	0	1	0	18	Medium quality	29648	5073113	69.50	300	d__Bacteria;p__Actinobacteriota;c__Actinomycetia;o__Streptosporangiales;f__Streptosporangiaceae;g__Palsa-506;s__
LMSG_G000008724.1	no	872_1	Coal	87.78	2.05	77.54	0	1	0	18	Medium quality	21976	3509153	70.30	254	d__Bacteria;p__Actinobacteriota;c__Actinomycetia;o__Streptosporangiales;f__Streptosporangiaceae;g__UBA9676;s__
LMSG_G000008725.1	no	1212_1	Lead-Zinc	93.96	1.72	85.34	0	2	3	20	Medium quality	155790	2100352	61.30	64	d__Bacteria;p__Actinobacteriota;c__Thermoleophilia;o__BMS3ABIN01;f__BMS3ABIN01;g__;s__
LMSG_G000008726.1	no	1212_1	Lead-Zinc	65.51	0.00	65.51	0	0	0	18	Medium quality	120610	1786671	61.40	24	d__Bacteria;p__Actinobacteriota;c__Thermoleophilia;o__BMS3ABIN01;f__BMS3ABIN01;g__;s__
LMSG_G000008727.1	no	1212_1	Lead-Zinc	82.32	2.16	71.55	0	1	1	19	Medium quality	50053	1893334	61.90	61	d__Bacteria;p__Actinobacteriota;c__Thermoleophilia;o__BMS3ABIN01;f__BMS3ABIN01;g__;s__
LMSG_G000008728.1	no	1212_1	Pyrite-Copper	84.34	2.06	74.03	0	0	0	16	Medium quality	32543	1697928	62.50	151	d__Bacteria;p__Actinobacteriota;c__Thermoleophilia;o__BMS3ABIN01;f__BMS3ABIN01;g__;s__
LMSG_G000008729.1	no	1212_1	Pyrite-Copper	89.22	2.16	78.45	0	1	1	16	Medium quality	40863	1742121	62.70	65	d__Bacteria;p__Actinobacteriota;c__Thermoleophilia;o__BMS3ABIN01;f__BMS3ABIN01;g__;s__
LMSG_G000008730.1	no	1212_1	Lead-Zinc	87.50	3.59	69.55	0	1	1	18	Medium quality	11615	1867321	62.40	211	d__Bacteria;p__Actinobacteriota;c__Thermoleophilia;o__BMS3ABIN01;f__BMS3ABIN01;g__;s__
LMSG_G000008731.1	no	1212_1	Pyrite-Copper	79.85	1.47	72.53	0	0	0	18	Medium quality	5505	1687072	63.00	378	d__Bacteria;p__Actinobacteriota;c__Thermoleophilia;o__BMS3ABIN01;f__BMS3ABIN01;g__;s__
LMSG_G000008732.1	no	1212_1	Pyrite-Copper	78.80	3.35	62.07	0	0	0	16	Medium quality	5846	1748262	62.40	373	d__Bacteria;p__Actinobacteriota;c__Thermoleophilia;o__BMS3ABIN01;f__BMS3ABIN01;g__;s__
LMSG_G000008733.1	no	828_1	Coal	69.31	2.56	56.49	0	1	0	16	Medium quality	4777	2421250	70.60	582	d__Bacteria;p__Actinobacteriota;c__Thermoleophilia;o__Solirubrobacterales;f__Solirubrobacteraceae;g__;s__
LMSG_G000008734.1	no	836_1	Pyrite	91.16	3.49	73.71	0	0	0	15	Medium quality	12877	2725420	69.40	309	d__Bacteria;p__Actinobacteriota;c__Thermoleophilia;o__Solirubrobacterales;f__Solirubrobacteraceae;g__Palsa-465;s__
LMSG_G000008735.1	no	836_1	Pyrite	71.08	0.28	69.66	0	1	0	11	Medium quality	14502	1673811	69.50	155	d__Bacteria;p__Actinobacteriota;c__Thermoleophilia;o__Solirubrobacterales;f__Solirubrobacteraceae;g__Palsa-465;s__
LMSG_G000008736.1	no	836_1	Pyrite-Copper	82.97	1.88	73.57	1	1	1	18	Medium quality	16824	2554897	68.80	199	d__Bacteria;p__Actinobacteriota;c__Thermoleophilia;o__Solirubrobacterales;f__Solirubrobacteraceae;g__Palsa-465;s__
LMSG_G000008737.1	no	836_1	Pyrite-Copper	66.06	2.03	55.93	0	0	0	17	Medium quality	5535	2021418	69.30	548	d__Bacteria;p__Actinobacteriota;c__Thermoleophilia;o__Solirubrobacterales;f__Solirubrobacteraceae;g__Palsa-465;s__
LMSG_G000008738.1	no	836_1	Pyrite	78.44	0.85	74.17	0	1	0	17	Medium quality	15583	2177765	69.50	322	d__Bacteria;p__Actinobacteriota;c__Thermoleophilia;o__Solirubrobacterales;f__Solirubrobacteraceae;g__Palsa-465;s__
LMSG_G000008739.1	no	837_1	Polymetallic	65.76	1.72	57.14	1	0	0	17	Medium quality	7682	2336727	68.40	417	d__Bacteria;p__Actinobacteriota;c__Thermoleophilia;o__Solirubrobacterales;f__Solirubrobacteraceae;g__Palsa-465;s__
LMSG_G000008740.1	no	837_2	Copper	84.61	1.42	77.49	2	1	7	20	Medium quality	46773	2887361	68.70	301	d__Bacteria;p__Actinobacteriota;c__Thermoleophilia;o__Solirubrobacterales;f__Solirubrobacteraceae;g__Palsa-465;s__
LMSG_G000008741.1	no	837_2	Magnetite	87.77	2.14	77.09	0	1	0	20	Medium quality	13390	2494650	68.70	368	d__Bacteria;p__Actinobacteriota;c__Thermoleophilia;o__Solirubrobacterales;f__Solirubrobacteraceae;g__Palsa-465;s__
LMSG_G000008742.1	no	837_2	Polymetallic	73.81	4.70	50.31	1	0	0	17	Medium quality	14118	2634150	69.00	284	d__Bacteria;p__Actinobacteriota;c__Thermoleophilia;o__Solirubrobacterales;f__Solirubrobacteraceae;g__Palsa-465;s__
LMSG_G000008743.1	no	837_2	Polymetallic	81.46	1.57	73.63	0	0	0	16	Medium quality	12203	2249918	68.50	268	d__Bacteria;p__Actinobacteriota;c__Thermoleophilia;o__Solirubrobacterales;f__Solirubrobacteraceae;g__Palsa-465;s__
LMSG_G000008744.1	no	837_2	Polymetallic	69.15	3.62	51.06	1	1	0	16	Medium quality	18873	2215018	68.40	174	d__Bacteria;p__Actinobacteriota;c__Thermoleophilia;o__Solirubrobacterales;f__Solirubrobacteraceae;g__Palsa-465;s__
LMSG_G000008745.1	no	783_1	Pyrite	56.89	0.00	56.89	1	0	0	15	Medium quality	4536	1617038	73.80	409	d__Bacteria;p__Actinobacteriota;c__UBA4738;o__UBA4738;f__;g__;s__
LMSG_G000008746.1	no	1368_1	Tin-Zinc	97.72	1.82	88.63	1	4	2	19	High quality	35157	2377112	46.20	128	d__Bacteria;p__Actinobacteriota;c__UBA9087;o__UBA10029;f__UBA10029;g__CG2-30-50-142;s__
LMSG_G000008747.1	no	1368_1	Lead-Zinc	59.97	0.33	58.31	0	0	1	11	Medium quality	1972	1242088	46.60	674	d__Bacteria;p__Actinobacteriota;c__UBA9087;o__UBA10029;f__UBA10029;g__CG2-30-50-142;s__
LMSG_G000008748.1	no	493_1	Antimony	84.38	0.51	81.84	0	0	0	16	Medium quality	6740	1293430	43.30	268	d__Bacteria;p__Aquificota;c__Aquificae;o__Aquificales;f__Aquificaceae;g__UBA11096;s__UBA11096 sp003534055
LMSG_G000008749.1	no	3_1	Pyrite-Copper	84.81	0.72	81.21	0	0	0	17	Medium quality	3217	3378604	50.70	1251	d__Bacteria;p__Armatimonadota;c__Chthonomonadetes;o__Chthonomonadales;f__;g__;s__
LMSG_G000008750.1	no	3_1	Antimony	56.07	0.93	51.45	0	0	0	9	Medium quality	1730	2087823	50.70	1239	d__Bacteria;p__Armatimonadota;c__Chthonomonadetes;o__Chthonomonadales;f__;g__;s__
LMSG_G000008751.1	no	3_1	Antimony	64.79	0.93	60.17	0	1	0	17	Medium quality	2621	3149819	50.70	1342	d__Bacteria;p__Armatimonadota;c__Chthonomonadetes;o__Chthonomonadales;f__;g__;s__
LMSG_G000008752.1	no	318_1	Polymetallic	94.44	0.37	92.59	1	3	1	20	High quality	23481	3524956	54.40	218	d__Bacteria;p__Armatimonadota;c__Chthonomonadetes;o__Chthonomonadales;f__;g__;s__
LMSG_G000008753.1	no	318_1	Polymetallic	94.44	0.19	93.52	1	1	1	18	High quality	14258	3488886	54.50	336	d__Bacteria;p__Armatimonadota;c__Chthonomonadetes;o__Chthonomonadales;f__;g__;s__
LMSG_G000008754.1	no	318_1	Polymetallic	93.51	0.19	92.59	1	2	1	20	High quality	34882	3555946	54.40	149	d__Bacteria;p__Armatimonadota;c__Chthonomonadetes;o__Chthonomonadales;f__;g__;s__
LMSG_G000008755.1	no	318_1	Pyrite-Copper	94.44	1.32	87.86	1	2	3	19	High quality	90281	3656370	54.50	127	d__Bacteria;p__Armatimonadota;c__Chthonomonadetes;o__Chthonomonadales;f__;g__;s__
LMSG_G000008756.1	no	318_1	Pyrite-Copper	91.29	0.27	89.95	1	1	2	18	High quality	31492	3516800	54.40	191	d__Bacteria;p__Armatimonadota;c__Chthonomonadetes;o__Chthonomonadales;f__;g__;s__
LMSG_G000008757.1	no	318_1	Polymetallic	81.38	0.44	79.19	0	0	0	19	Medium quality	4183	2913775	54.60	832	d__Bacteria;p__Armatimonadota;c__Chthonomonadetes;o__Chthonomonadales;f__;g__;s__
LMSG_G000008758.1	no	318_1	Polymetallic	63.65	1.03	58.51	0	1	1	15	Medium quality	2418	2062771	54.60	938	d__Bacteria;p__Armatimonadota;c__Chthonomonadetes;o__Chthonomonadales;f__;g__;s__
LMSG_G000008759.1	no	318_1	Pyrite-Copper	77.45	1.11	71.90	0	1	1	16	Medium quality	5220	2770366	54.90	637	d__Bacteria;p__Armatimonadota;c__Chthonomonadetes;o__Chthonomonadales;f__;g__;s__
LMSG_G000008760.1	no	309_1	Polymetallic	84.73	3.34	68.02	0	3	1	18	Medium quality	5197	3956219	45.00	972	d__Bacteria;p__Armatimonadota;c__Chthonomonadetes;o__Chthonomonadales;f__Chthonomonadaceae;g__;s__
LMSG_G000008761.1	no	309_1	Polymetallic	57.34	1.11	51.79	0	0	1	15	Medium quality	2338	2964967	44.90	1373	d__Bacteria;p__Armatimonadota;c__Chthonomonadetes;o__Chthonomonadales;f__Chthonomonadaceae;g__;s__
LMSG_G000008762.1	no	309_1	Polymetallic	83.12	0.93	78.50	0	2	0	17	Medium quality	4863	3945358	45.00	985	d__Bacteria;p__Armatimonadota;c__Chthonomonadetes;o__Chthonomonadales;f__Chthonomonadaceae;g__;s__
LMSG_G000008763.1	no	309_1	Polymetallic	55.30	0.93	50.68	0	0	0	11	Medium quality	1938	2639100	45.20	1422	d__Bacteria;p__Armatimonadota;c__Chthonomonadetes;o__Chthonomonadales;f__Chthonomonadaceae;g__;s__
LMSG_G000008764.1	no	310_1	Polymetallic	81.83	0.93	77.21	2	6	19	20	Medium quality	5145	3584752	52.10	857	d__Bacteria;p__Armatimonadota;c__Chthonomonadetes;o__Chthonomonadales;f__Chthonomonadaceae;g__;s__
LMSG_G000008765.1	no	310_1	Polymetallic	87.49	0.93	82.87	0	20	20	19	Medium quality	6578	3996392	52.00	775	d__Bacteria;p__Armatimonadota;c__Chthonomonadetes;o__Chthonomonadales;f__Chthonomonadaceae;g__;s__
LMSG_G000008766.1	no	310_1	Polymetallic	73.42	0.93	68.80	0	0	3	17	Medium quality	3857	3582460	52.10	1060	d__Bacteria;p__Armatimonadota;c__Chthonomonadetes;o__Chthonomonadales;f__Chthonomonadaceae;g__;s__
LMSG_G000008767.1	no	310_1	Polymetallic	74.73	1.08	69.33	0	0	0	18	Medium quality	4021	3675612	52.10	1063	d__Bacteria;p__Armatimonadota;c__Chthonomonadetes;o__Chthonomonadales;f__Chthonomonadaceae;g__;s__
LMSG_G000008768.1	no	311_1	Copper	90.53	0.93	85.91	1	2	1	19	High quality	49336	4259727	51.90	145	d__Bacteria;p__Armatimonadota;c__Chthonomonadetes;o__Chthonomonadales;f__Chthonomonadaceae;g__;s__
LMSG_G000008769.1	no	312_1	Antimony	78.60	2.78	64.72	0	1	1	17	Medium quality	6109	3294609	52.20	628	d__Bacteria;p__Armatimonadota;c__Chthonomonadetes;o__Chthonomonadales;f__Chthonomonadaceae;g__;s__
LMSG_G000008770.1	no	312_1	Antimony	94.44	1.85	85.19	0	3	1	19	Medium quality	23803	4208022	52.20	262	d__Bacteria;p__Armatimonadota;c__Chthonomonadetes;o__Chthonomonadales;f__Chthonomonadaceae;g__;s__
LMSG_G000008771.1	no	312_1	Antimony	56.47	1.23	50.30	0	2	1	15	Medium quality	2134	2437230	52.10	1210	d__Bacteria;p__Armatimonadota;c__Chthonomonadetes;o__Chthonomonadales;f__Chthonomonadaceae;g__;s__
LMSG_G000008772.1	no	312_1	Antimony	90.74	0.93	86.12	0	3	0	17	Medium quality	16049	4087300	52.20	349	d__Bacteria;p__Armatimonadota;c__Chthonomonadetes;o__Chthonomonadales;f__Chthonomonadaceae;g__;s__
LMSG_G000008773.1	no	312_1	Copper	94.90	2.31	83.33	0	2	10	20	Medium quality	18736	4031310	52.30	328	d__Bacteria;p__Armatimonadota;c__Chthonomonadetes;o__Chthonomonadales;f__Chthonomonadaceae;g__;s__
LMSG_G000008774.1	no	214_1	Copper	63.14	0.93	58.52	0	1	0	19	Medium quality	3637	1040497	38.80	336	d__Bacteria;p__Armatimonadota;c__Fimbriimonadia;o__Fimbriimonadales;f__Fimbriimonadaceae;g__;s__
LMSG_G000008775.1	no	402_1	Copper	96.09	1.48	88.69	1	1	1	20	High quality	66347	3344428	45.30	71	d__Bacteria;p__Bacteroidota;c__Bacteroidia;o__AKYH767;f__Palsa-948;g__;s__
LMSG_G000008776.1	no	1650_1	Polymetallic	68.66	2.06	58.35	0	1	0	16	Medium quality	4699	2691304	46.90	667	d__Bacteria;p__Bacteroidota;c__Bacteroidia;o__Bacteroidales;f__;g__;s__
LMSG_G000008777.1	no	535_1	Antimony	67.86	0.24	66.66	1	0	0	16	Medium quality	5587	1924327	36.10	398	d__Bacteria;p__Bacteroidota;c__Bacteroidia;o__Bacteroidales;f__Paludibacteraceae;g__Paludibacter;s__
LMSG_G000008778.1	no	535_1	Antimony	98.92	0.72	95.34	0	0	0	19	Medium quality	45463	3419430	36.60	108	d__Bacteria;p__Bacteroidota;c__Bacteroidia;o__Bacteroidales;f__Paludibacteraceae;g__Paludibacter;s__

LMSG_G000008779.1	no	535_1	Antimony	65.43	1.11	59.88	1	0	1	13	Medium quality	3332	2316710	36.50	786	d__Bacteria;p__Bacteroidota;c__Bacteroidia;o__Bacteroidales;f__Paludibacteraceae;g__Paludibacter;s__
LMSG_G000008780.1	no	350_1	Polymetallic	98.92	4.19	77.98	3	1	1	20	High quality	17218	2741326	39.80	247	d__Bacteria;p__Bacteroidota;c__Bacteroidia;o__Bacteroidales;f__Paludibacteraceae;g__Paludibacter_A;s__
LMSG_G000008781.1	no	536_1	Polymetallic	77.70	2.15	66.95	1	0	0	16	Medium quality	12904	2531510	43.60	325	d__Bacteria;p__Bacteroidota;c__Bacteroidia;o__Bacteroidales;f__Prolixibacteraceae;g__;s__
LMSG_G000008782.1	no	536_1	Polymetallic	77.34	2.15	66.59	0	0	0	18	Medium quality	9389	2892916	43.50	467	d__Bacteria;p__Bacteroidota;c__Bacteroidia;o__Bacteroidales;f__Prolixibacteraceae;g__;s__
LMSG_G000008783.1	no	536_1	Polymetallic	86.82	2.87	72.49	0	1	0	17	Medium quality	14100	3346071	43.40	367	d__Bacteria;p__Bacteroidota;c__Bacteroidia;o__Bacteroidales;f__Prolixibacteraceae;g__;s__
LMSG_G000008784.1	no	536_1	Polymetallic	79.03	1.48	71.62	1	0	0	18	Medium quality	7615	2867089	43.50	529	d__Bacteria;p__Bacteroidota;c__Bacteroidia;o__Bacteroidales;f__Prolixibacteraceae;g__;s__
LMSG_G000008785.1	no	536_1	Lead-Zinc	72.38	1.11	66.83	0	0	0	16	Medium quality	14604	2622905	43.70	333	d__Bacteria;p__Bacteroidota;c__Bacteroidia;o__Bacteroidales;f__Prolixibacteraceae;g__;s__
LMSG_G000008786.1	no	536_1	Lead-Zinc	80.55	0.72	76.97	0	0	0	17	Medium quality	14492	2727792	43.50	354	d__Bacteria;p__Bacteroidota;c__Bacteroidia;o__Bacteroidales;f__Prolixibacteraceae;g__;s__
LMSG_G000008787.1	no	536_1	Lead-Zinc	61.73	1.11	56.18	0	0	0	10	Medium quality	2366	2405233	43.50	1085	d__Bacteria;p__Bacteroidota;c__Bacteroidia;o__Bacteroidales;f__Prolixibacteraceae;g__;s__
LMSG_G000008788.1	no	536_1	Pyrite-Copper	86.02	1.34	79.30	1	1	1	16	Medium quality	6806	2993523	43.70	525	d__Bacteria;p__Bacteroidota;c__Bacteroidia;o__Bacteroidales;f__Prolixibacteraceae;g__;s__
LMSG_G000008789.1	no	536_1	Lead-Zinc	79.52	1.61	71.46	1	0	0	18	Medium quality	8248	2712011	43.90	425	d__Bacteria;p__Bacteroidota;c__Bacteroidia;o__Bacteroidales;f__Prolixibacteraceae;g__;s__
LMSG_G000008790.1	no	536_1	Pyrite-Copper	77.45	2.69	64.01	0	1	0	18	Medium quality	5834	3064450	43.40	618	d__Bacteria;p__Bacteroidota;c__Bacteroidia;o__Bacteroidales;f__Prolixibacteraceae;g__;s__
LMSG_G000008791.1	no	536_1	Lead-Zinc	63.28	1.92	53.70	1	0	0	15	Medium quality	2383	2845930	42.90	1286	d__Bacteria;p__Bacteroidota;c__Bacteroidia;o__Bacteroidales;f__Prolixibacteraceae;g__;s__
LMSG_G000008792.1	no	1650_1	Antimony	55.46	0.48	53.08	0	3	0	13	Medium quality	3759	1984226	46.70	560	d__Bacteria;p__Bacteroidota;c__Bacteroidia;o__Bacteroidales;f__UBA5072;g__;s__
LMSG_G000008793.1	no	460_1	Copper	62.76	1.46	55.49	0	0	0	5	Medium quality	2265	2800613	44.00	1262	d__Bacteria;p__Bacteroidota;c__Bacteroidia;o__Bacteroidales;f__VadinHA17;g__LD21;s__
LMSG_G000008794.1	no	460_1	Copper	55.37	0.00	55.37	1	0	1	6	Medium quality	2831	2412724	43.80	842	d__Bacteria;p__Bacteroidota;c__Bacteroidia;o__Bacteroidales;f__VadinHA17;g__LD21;s__
LMSG_G000008795.1	no	272_1	Polymetallic	96.32	1.25	90.09	1	1	1	19	High quality	20584	2700385	32.60	181	d__Bacteria;p__Bacteroidota;c__Bacteroidia;o__Chitinophagales;f__Chitinophagaceae;g__Hydrotalea;s__
LMSG_G000008796.1	no	272_1	Polymetallic	81.96	0.49	79.50	0	0	0	14	Medium quality	3669	2249931	32.40	705	d__Bacteria;p__Bacteroidota;c__Bacteroidia;o__Chitinophagales;f__Chitinophagaceae;g__Hydrotalea;s__
LMSG_G000008797.1	no	272_1	Polymetallic	91.10	0.99	86.18	1	0	1	17	Medium quality	7255	2409359	32.60	407	d__Bacteria;p__Bacteroidota;c__Bacteroidia;o__Chitinophagales;f__Chitinophagaceae;g__Hydrotalea;s__
LMSG_G000008798.1	no	272_1	Polymetallic	91.87	2.22	80.79	0	1	0	18	Medium quality	10394	2467253	32.50	295	d__Bacteria;p__Bacteroidota;c__Bacteroidia;o__Chitinophagales;f__Chitinophagaceae;g__Hydrotalea;s__
LMSG_G000008799.1	no	272_1	Polymetallic	57.90	1.48	50.52	1	0	1	11	Medium quality	3110	1690351	32.50	596	d__Bacteria;p__Bacteroidota;c__Bacteroidia;o__Chitinophagales;f__Chitinophagaceae;g__Hydrotalea;s__
LMSG_G000008800.1	no	272_1	Copper	99.23	2.22	88.15	1	1	1	19	High quality	14023	2809645	32.30	303	d__Bacteria;p__Bacteroidota;c__Bacteroidia;o__Chitinophagales;f__Chitinophagaceae;g__Hydrotalea;s__
LMSG_G000008801.1	no	275_1	Polymetallic	95.52	0.68	92.12	0	0	0	17	Medium quality	14892	2801888	37.30	278	d__Bacteria;p__Bacteroidota;c__Bacteroidia;o__Chitinophagales;f__Chitinophagaceae;g__Hydrotalea;s__
LMSG_G000008802.1	no	275_1	Polymetallic	99.75	0.43	97.62	0	1	0	18	Medium quality	17774	3086388	37.10	272	d__Bacteria;p__Bacteroidota;c__Bacteroidia;o__Chitinophagales;f__Chitinophagaceae;g__Hydrotalea;s__
LMSG_G000008803.1	no	276_1	Polymetallic	97.91	0.49	95.45	2	2	2	16	Medium quality	14271	3018707	37.20	350	d__Bacteria;p__Bacteroidota;c__Bacteroidia;o__Chitinophagales;f__Chitinophagaceae;g__Hydrotalea;s__Hydrotalea flava
LMSG_G000008804.1	no	276_1	Lead-Zinc	99.26	1.14	93.59	1	0	0	18	Medium quality	43281	3119040	37.10	159	d__Bacteria;p__Bacteroidota;c__Bacteroidia;o__Chitinophagales;f__Chitinophagaceae;g__Hydrotalea;s__Hydrotalea flava
LMSG_G000008805.1	no	276_1	Arsenic	73.36	1.48	65.98	0	0	0	12	Medium quality	10696	2490510	37.20	417	d__Bacteria;p__Bacteroidota;c__Bacteroidia;o__Chitinophagales;f__Chitinophagaceae;g__Hydrotalea;s__Hydrotalea flava
LMSG_G000008806.1	no	276_1	Copper	98.27	0.49	95.81	2	2	0	18	Medium quality	62628	3199365	37.00	85	d__Bacteria;p__Bacteroidota;c__Bacteroidia;o__Chitinophagales;f__Chitinophagaceae;g__Hydrotalea;s__Hydrotalea flava
LMSG_G000008807.1	no	282_1	Antimony	67.54	2.05	57.28	0	0	0	6	Medium quality	2652	2242143	48.30	936	d__Bacteria;p__Bacteroidota;c__Bacteroidia;o__Chitinophagales;f__Chitinophagaceae;g__Palsa-955;s__
LMSG_G000008808.1	no	274_1	Antimony	68.27	0.52	65.68	0	0	0	12	Medium quality	3886	1982838	45.00	562	d__Bacteria;p__Bacteroidota;c__Bacteroidia;o__Chitinophagales;f__Chitinophagaceae;g__Panacibacter;s__
LMSG_G000008809.1	no	283_1	Nickel-Copper	62.70	2.03	52.58	1	0	0	7	Medium quality	1850	1940097	39.80	1062	d__Bacteria;p__Bacteroidota;c__Bacteroidia;o__Chitinophagales;f__Chitinophagaceae;g__Sediminibacterium;s__Sediminibacterium sp002256765
LMSG_G000008810.1	no	284_1	Nickel-Copper	87.04	1.48	79.66	0	0	0	14	Medium quality	5403	2330728	36.50	536	d__Bacteria;p__Bacteroidota;c__Bacteroidia;o__Chitinophagales;f__Chitinophagaceae;g__Sediminibacterium;s__Sediminibacterium sp002281875
LMSG_G000008811.1	no	284_1	Nickel-Copper	87.83	1.38	80.92	0	0	0	12	Medium quality	3780	2439539	36.70	813	d__Bacteria;p__Bacteroidota;c__Bacteroidia;o__Chitinophagales;f__Chitinophagaceae;g__Sediminibacterium;s__Sediminibacterium sp002281875
LMSG_G000008812.1	no	32_1	Antimony	67.54	0.88	63.16	0	0	0	14	Medium quality	21823	2953501	33.50	196	d__Bacteria;p__Bacteroidota;c__Bacteroidia;o__Flavobacteriales;f__Flavobacteriaceae;g__Lutibacter;s__
LMSG_G000008813.1	no	534_1	Nickel-Copper	99.02	0.00	99.02	1	1	1	19	High quality	77703	4066944	39.40	116	d__Bacteria;p__Bacteroidota;c__Bacteroidia;o__Sphingobacteriales;f__Sphingobacteriaceae;g__Daejeonella;s__Daejeonella sp002257025
LMSG_G000008814.1	no	1662_1	Polymetallic	96.36	1.07	90.99	1	1	1	18	High quality	28816	3179040	37.50	176	d__Bacteria;p__Bacteroidota;c__Ignavibacteria;o__Ignavibacteriales;f__Ignavibacteriaceae;g__;s__
LMSG_G000008815.1	no	1662_1	Polymetallic	95.53	0.56	92.74	1	1	1	18	High quality	33411	3017125	37.50	143	d__Bacteria;p__Bacteroidota;c__Ignavibacteria;o__Ignavibacteriales;f__Ignavibacteriaceae;g__;s__
LMSG_G000008816.1	no	1662_1	Polymetallic	98.32	3.73	79.66	1	1	1	16	Medium quality	30848	3064799	37.50	138	d__Bacteria;p__Bacteroidota;c__Ignavibacteria;o__Ignavibacteriales;f__Ignavibacteriaceae;g__;s__
LMSG_G000008817.1	no	1662_1	Polymetallic	98.60	1.69	90.13	1	1	1	17	Medium quality	30468	3161968	37.40	173	d__Bacteria;p__Bacteroidota;c__Ignavibacteria;o__Ignavibacteriales;f__Ignavibacteriaceae;g__;s__
LMSG_G000008818.1	no	1660_1	Polymetallic	97.74	1.68	89.37	0	0	0	19	Medium quality	71718	4730670	40.10	102	d__Bacteria;p__Bacteroidota;c__Ignavibacteria;o__Ignavibacteriales;f__Ignavibacteriaceae;g__FEN-1297;s__
LMSG_G000008819.1	no	1660_1	Polymetallic	92.17	1.15	86.40	0	0	0	19	Medium quality	54918	4463145	40.30	119	d__Bacteria;p__Bacteroidota;c__Ignavibacteria;o__Ignavibacteriales;f__Ignavibacteriaceae;g__FEN-1297;s__
LMSG_G000008820.1	no	1660_1	Polymetallic	88.26	1.68	79.89	0	0	0	17	Medium quality	92945	4554506	40.30	254	d__Bacteria;p__Bacteroidota;c__Ignavibacteria;o__Ignavibacteriales;f__Ignavibacteriaceae;g__FEN-1297;s__
LMSG_G000008821.1	no	1660_1	Polymetallic	90.76	2.51	78.20	0	0	0	14	Medium quality	19157	4304836	40.60	355	d__Bacteria;p__Bacteroidota;c__Ignavibacteria;o__Ignavibacteriales;f__Ignavibacteriaceae;g__FEN-1297;s__
LMSG_G000008822.1	no	1661_1	Polymetallic	96.08	2.53	83.42	1	2	1	19	High quality	88958	4668801	36.70	192	d__Bacteria;p__Bacteroidota;c__Ignavibacteria;o__Ignavibacteriales;f__Ignavibacteriaceae;g__FEN-1297;s__
LMSG_G000008823.1	no	1663_1	Polymetallic	98.88	2.79	84.92	1	1	2	19	High quality	102880	4024720	32.20	63	d__Bacteria;p__Bacteroidota;c__Ignavibacteria;o__Ignavibacteriales;f__Ignavibacteriaceae;g__FEN-1297;s__
LMSG_G000008824.1	no	1663_1	Polymetallic	98.32	2.23	87.15	1	1	2	20	High quality	153479	3923778	32.20	71	d__Bacteria;p__Bacteroidota;c__Ignavibacteria;o__Ignavibacteriales;f__Ignavibacteriaceae;g__FEN-1297;s__
LMSG_G000008825.1	no	1663_1	Polymetallic	98.88	1.12	93.30	1	1	0	18	Medium quality	153296	3956163	32.10	62	d__Bacteria;p__Bacteroidota;c__Ignavibacteria;o__Ignavibacteriales;f__Ignavibacteriaceae;g__FEN-1297;s__
LMSG_G000008826.1	no	1665_1	Polymetallic	96.08	3.02	81.00	2	1	2	19	High quality	142252	4889024	36.10	66	d__Bacteria;p__Bacteroidota;c__Ignavibacteria;o__Ignavibacteriales;f__Ignavibacteriaceae;g__FEN-1297;s__
LMSG_G000008827.1	no	1665_1	Polymetallic	96.08	3.49	78.63	3	1	3	19	High quality	70237	5235487	36.20	174	d__Bacteria;p__Bacteroidota;c__Ignavibacteria;o__Ignavibacteriales;f__Ignavibacteriaceae;g__FEN-1297;s__
LMSG_G000008828.1	no	1665_1	Polymetallic	83.24	4.51	60.71	1	1	1	19	Medium quality	162487	4648881	36.20	55	d__Bacteria;p__Bacteroidota;c__Ignavibacteria;o__Ignavibacteriales;f__Ignavibacteriaceae;g__FEN-1297;s__
LMSG_G000008829.1	no	1666_1	Antimony	79.25	1.34	72.55	1	1	1	19	Medium quality	20978	2658070	32.80	235	d__Bacteria;p__Bacteroidota;c__Ignavibacteria;o__Ignavibacteriales;f__Ignavibacteriaceae;g__FEN-1297;s__
LMSG_G000008830.1	no	1666_1	Antimony	68.79	2.94	54.08	2	1	2	11	Medium quality	7314	2617389	32.90	494	d__Bacteria;p__Bacteroidota;c__Ignavibacteria;o__Ignavibacteriales;f__Ignavibacteriaceae;g__FEN-1297;s__
LMSG_G000008831.1	no	1668_1	Antimony	89.76	3.35	73.01	1	1	1	17	Medium quality	39112	3726882	34.70	214	d__Bacteria;p__Bacteroidota;c__Ignavibacteria;o__Ignavibacteriales;f__Ignavibacteriaceae;g__FEN-1297;s__
LMSG_G000008832.1	no	1668_1	Antimony	93.29	4.47	70.95	1	0	1	19	Medium quality	44219	3904126	34.90	166	d__Bacteria;p__Bacteroidota;c__Ignavibacteria;o__Ignavibacteriales;f__Ignavibacteriaceae;g__FEN-1297;s__
LMSG_G000008833.1	no	1669_1	Antimony	86.76	1.96	76.99	1	4	1	15	Medium quality	21357	2674515	35.40	306	d__Bacteria;p__Bacteroidota;c__Ignavibacteria;o__Ignavibacteriales;f__Ignavibacteriaceae;g__FEN-1297;s__
LMSG_G000008834.1	no	1669_1	Antimony	74.93	0.28	73.54	1	0	1	18	Medium quality	17007	2584629	35.50	220	d__Bacteria;p__Bacteroidota;c__Ignavibacteria;o__Ignavibacteriales;f__Ignavibacteriaceae;g__FEN-1297;s__
LMSG_G000008835.1	no	1669_1	Antimony	86.10	2.79	72.14	1	1	2	17	Medium quality	15486	2845427	35.50	263	d__Bacteria;p__Bacteroidota;c__Ignavibacteria;o__Ignavibacteriales;f__Ignavibacteriaceae;g__FEN-1297;s__
LMSG_G000008836.1	no	1669_1	Polymetallic	90.22	4.26	68.93	1	1	1	19	High quality	31729	3161850	35.20	190	d__Bacteria;p__Bacteroidota;c__Ignavibacteria;o__Ignavibacteriales;f__Ignavibacteriaceae;g__FEN-1297;s__
LMSG_G000008837.1	no	1669_1	Polymetallic	73.09	4.47	50.75	1	1	1	14	Medium quality	9967	4180418	35.20	493	d__Bacteria;p__Bacteroidota;c__Ignavibacteria;o__Ignavibacteriales;f__Ignavibacteriaceae;g__FEN-1297;s__
LMSG_G000008838.1	no	1669_1	Pyrite-Copper	81.28	0.56	78.49	0	1	1	19	Medium quality	67764	4041288	35.50	120	d__Bacteria;p__Bacteroidota;c__Ignavibacteria;o__Ignavibacteriales;f__Ignavibacteriaceae;g__FEN-1297;s__

LMSG_G000008839.1	no	1669_1	Pyrite-Copper	79.16	3.07	63.80	0	1	0	12	Medium quality	4376	1939442	35.20	531	d__Bacteria;p__Bacteroidota;c__Ignavibacteria;o__Ignavibacteriales;f__Ignavibacteriaceae;g__FEN-1297;s__
LMSG_G000008840.1	no	1669_1	Pyrite-Copper	67.09	1.71	58.53	1	2	1	16	Medium quality	2957	1771914	35.50	626	d__Bacteria;p__Bacteroidota;c__Ignavibacteria;o__Ignavibacteriales;f__Ignavibacteriaceae;g__FEN-1297;s__
LMSG_G000008841.1	no	1564_1	Polymetallic	88.32	2.01	78.29	0	0	0	16	Medium quality	7163	2950433	41.60	541	d__Bacteria;p__Bacteroidota;c__Ignavibacteria;o__Ignavibacteriales;f__Melioribacteraceae;g__;s__
LMSG_G000008842.1	no	1564_1	Polymetallic	94.69	2.74	81.01	1	1	1	17	Medium quality	14534	2933578	41.50	337	d__Bacteria;p__Bacteroidota;c__Ignavibacteria;o__Ignavibacteriales;f__Melioribacteraceae;g__;s__
LMSG_G000008843.1	no	1564_1	Polymetallic	76.31	2.29	64.89	0	0	0	14	Medium quality	2905	2569308	41.70	985	d__Bacteria;p__Bacteroidota;c__Ignavibacteria;o__Ignavibacteriales;f__Melioribacteraceae;g__;s__
LMSG_G000008844.1	no	1672_1	Lead-Zinc	70.42	0.56	67.63	0	0	0	12	Medium quality	4577	2000088	40.00	510	d__Bacteria;p__Bacteroidota;c__Ignavibacteria;o__Ignavibacteriales;f__Melioribacteraceae;g__;s__
LMSG_G000008845.1	no	1657_1	Polymetallic	92.54	3.91	72.99	1	1	1	15	Medium quality	14501	2803419	40.50	320	d__Bacteria;p__Bacteroidota;c__Ignavibacteria;o__Ignavibacteriales;f__Melioribacteraceae;g__Fen-1258;s__
LMSG_G000008846.1	no	1657_1	Polymetallic	90.65	3.17	74.83	0	0	0	17	Medium quality	13621	2871172	40.50	325	d__Bacteria;p__Bacteroidota;c__Ignavibacteria;o__Ignavibacteriales;f__Melioribacteraceae;g__Fen-1258;s__
LMSG_G000008847.1	no	1657_1	Polymetallic	85.84	2.23	74.67	0	0	0	14	Medium quality	14933	2784194	40.50	303	d__Bacteria;p__Bacteroidota;c__Ignavibacteria;o__Ignavibacteriales;f__Melioribacteraceae;g__Fen-1258;s__
LMSG_G000008848.1	no	1657_1	Polymetallic	87.53	1.06	82.23	0	0	0	15	Medium quality	17699	2837538	40.30	249	d__Bacteria;p__Bacteroidota;c__Ignavibacteria;o__Ignavibacteriales;f__Melioribacteraceae;g__Fen-1258;s__
LMSG_G000008849.1	no	1673_1	Pyrite-Copper	95.53	1.96	85.76	2	0	1	19	Medium quality	44165	3068491	36.50	140	d__Bacteria;p__Bacteroidota;c__Ignavibacteria;o__Ignavibacteriales;f__Melioribacteraceae;g__Fen-1258;s__
LMSG_G000008850.1	no	1674_1	Polymetallic	62.40	2.44	50.21	1	0	1	14	Medium quality	13419	1968369	36.90	349	d__Bacteria;p__Bacteroidota;c__Ignavibacteria;o__Ignavibacteriales;f__Melioribacteraceae;g__Fen-1258;s__
LMSG_G000008851.1	no	1674_1	Polymetallic	73.95	4.33	52.31	0	1	0	13	Medium quality	8673	2865524	36.10	434	d__Bacteria;p__Bacteroidota;c__Ignavibacteria;o__Ignavibacteriales;f__Melioribacteraceae;g__Fen-1258;s__
LMSG_G000008852.1	no	1658_1	Polymetallic	68.11	1.93	58.48	0	0	0	13	Medium quality	2263	1789712	29.20	836	d__Bacteria;p__Bacteroidota;c__Ignavibacteria;o__Ignavibacteriales;f__Melioribacteraceae;g__XYB12-FULL-38-5;s__
LMSG_G000008853.1	no	1369_1	Antimony	94.80	0.05	94.56	1	1	2	20	High quality	35173	2626781	30.10	120	d__Bacteria;p__Bacteroidota;c__Kapabacteria;o__Kapabacteriales;f__UBA2268;g__GWF2-33-9;s__
LMSG_G000008854.1	no	1369_1	Antimony	97.43	0.27	96.07	1	1	1	20	High quality	118798	2675472	30.30	42	d__Bacteria;p__Bacteroidota;c__Kapabacteria;o__Kapabacteriales;f__UBA2268;g__GWF2-33-9;s__
LMSG_G000008855.1	no	1369_1	Antimony	96.14	1.09	90.68	1	0	3	18	Medium quality	30238	2602011	30.20	171	d__Bacteria;p__Bacteroidota;c__Kapabacteria;o__Kapabacteriales;f__UBA2268;g__GWF2-33-9;s__
LMSG_G000008856.1	no	526_1	Polymetallic	75.31	1.73	66.66	0	0	1	16	Medium quality	3881	2852680	35.30	898	d__Bacteria;p__Bacteroidota;c__Kapabacteria;o__Kapabacteriales;f__XYC2-FULL-35-21;g__XYC2-FULL-35-21;s__
LMSG_G000008857.1	no	526_1	Polymetallic	65.94	1.82	56.86	0	0	0	12	Medium quality	2348	2358958	35.70	1118	d__Bacteria;p__Bacteroidota;c__Kapabacteria;o__Kapabacteriales;f__XYC2-FULL-35-21;g__XYC2-FULL-35-21;s__
LMSG_G000008858.1	no	526_1	Polymetallic	85.11	0.91	80.56	1	1	2	19	Medium quality	16248	3438313	35.30	322	d__Bacteria;p__Bacteroidota;c__Kapabacteria;o__Kapabacteriales;f__XYC2-FULL-35-21;g__XYC2-FULL-35-21;s__
LMSG_G000008859.1	no	1134_1	Polymetallic	78.51	0.82	74.42	0	3	0	18	Medium quality	6745	3330149	53.00	607	d__Bacteria;p__Bacteroidota;c__Kapabacteria;o__Palsa-1295;f__Palsa-1295;g__PALSA-1295;s__
LMSG_G000008860.1	no	1134_1	Polymetallic	81.00	1.37	74.17	0	3	0	18	Medium quality	5401	3452012	52.90	760	d__Bacteria;p__Bacteroidota;c__Kapabacteria;o__Palsa-1295;f__Palsa-1295;g__PALSA-1295;s__
LMSG_G000008861.1	no	1638_1	Polymetallic	98.88	4.37	77.03	1	1	1	19	High quality	109558	5101542	51.10	157	d__Bacteria;p__Bacteroidota;c__Kryptonia;o__;f__;g__;s__
LMSG_G000008862.1	no	1638_1	Polymetallic	95.06	3.83	75.94	1	1	1	19	High quality	125057	4279704	51.50	79	d__Bacteria;p__Bacteroidota;c__Kryptonia;o__;f__;g__;s__
LMSG_G000008863.1	no	1638_1	Lead-Zinc	98.88	3.28	82.49	1	1	1	18	High quality	71805	4318957	51.50	122	d__Bacteria;p__Bacteroidota;c__Kryptonia;o__;f__;g__;s__
LMSG_G000008864.1	no	1638_1	Lead-Zinc	76.70	2.02	66.60	1	3	1	16	Medium quality	9662	3068243	51.70	415	d__Bacteria;p__Bacteroidota;c__Kryptonia;o__;f__;g__;s__
LMSG_G000008865.1	no	1638_1	Polymetallic	84.13	3.28	67.74	1	0	1	19	Medium quality	122037	3752389	51.50	57	d__Bacteria;p__Bacteroidota;c__Kryptonia;o__;f__;g__;s__
LMSG_G000008866.1	no	1644_1	Copper	98.34	3.84	79.13	0	1	0	19	Medium quality	38462	4487661	51.10	191	d__Bacteria;p__Bacteroidota;c__Kryptonia;o__;f__;g__;s__
LMSG_G000008867.1	no	1639_1	Polymetallic	70.61	3.01	55.59	0	0	0	16	Medium quality	17134	1881242	47.00	199	d__Bacteria;p__Bacteroidota;c__Kryptonia;o__Kryptoniales;f__;g__;s__
LMSG_G000008868.1	no	1639_1	Polymetallic	85.36	3.28	68.97	0	0	0	11	Medium quality	23836	2190184	46.90	215	d__Bacteria;p__Bacteroidota;c__Kryptonia;o__Kryptoniales;f__;g__;s__
LMSG_G000008869.1	no	1639_1	Polymetallic	86.45	4.21	65.42	1	0	1	15	Medium quality	20050	2310096	46.90	267	d__Bacteria;p__Bacteroidota;c__Kryptonia;o__Kryptoniales;f__;g__;s__
LMSG_G000008870.1	no	1639_1	Polymetallic	86.73	3.01	71.71	1	0	0	16	Medium quality	10064	2340921	46.90	317	d__Bacteria;p__Bacteroidota;c__Kryptonia;o__Kryptoniales;f__;g__;s__
LMSG_G000008871.1	no	1639_1	Polymetallic	87.27	2.75	73.52	1	0	1	14	Medium quality	21562	2261345	46.90	237	d__Bacteria;p__Bacteroidota;c__Kryptonia;o__Kryptoniales;f__;g__;s__
LMSG_G000008872.1	no	1639_1	Lead-Zinc	96.29	4.10	75.80	1	1	1	20	High quality	33496	3501824	47.00	177	d__Bacteria;p__Bacteroidota;c__Kryptonia;o__Kryptoniales;f__;g__;s__
LMSG_G000008873.1	no	1639_1	Pyrite-Copper	71.13	1.82	62.03	0	0	0	12	Medium quality	4695	1583979	47.10	381	d__Bacteria;p__Bacteroidota;c__Kryptonia;o__Kryptoniales;f__;g__;s__
LMSG_G000008874.1	no	1639_1	Pyrite-Copper	64.94	2.73	51.28	0	0	0	12	Medium quality	4940	1760806	47.00	394	d__Bacteria;p__Bacteroidota;c__Kryptonia;o__Kryptoniales;f__;g__;s__
LMSG_G000008875.1	no	1639_1	Pyrite-Copper	83.43	3.57	65.61	1	0	1	12	Medium quality	4404	2040174	47.20	600	d__Bacteria;p__Bacteroidota;c__Kryptonia;o__Kryptoniales;f__;g__;s__
LMSG_G000008876.1	no	1640_1	Pyrite	73.70	4.10	53.21	0	0	0	14	Medium quality	7340	3361013	49.70	509	d__Bacteria;p__Bacteroidota;c__Kryptonia;o__Kryptoniales;f__;g__;s__
LMSG_G000008877.1	no	1640_1	Copper	72.51	2.42	60.43	0	0	2	13	Medium quality	2343	3282681	49.70	1500	d__Bacteria;p__Bacteroidota;c__Kryptonia;o__Kryptoniales;f__;g__;s__
LMSG_G000008878.1	no	1640_1	Copper	90.91	2.51	78.37	0	0	0	16	Medium quality	9418	4246926	49.50	566	d__Bacteria;p__Bacteroidota;c__Kryptonia;o__Kryptoniales;f__;g__;s__
LMSG_G000008879.1	no	1643_1	Copper	98.88	4.37	77.03	1	1	1	19	High quality	109548	4483613	51.50	96	d__Bacteria;p__Bacteroidota;c__Kryptonia;o__Kryptoniales;f__;g__;s__
LMSG_G000008880.1	no	1643_1	Polymetallic	98.88	3.53	81.25	1	6	4	18	High quality	82906	4307748	51.60	150	d__Bacteria;p__Bacteroidota;c__Kryptonia;o__Kryptoniales;f__;g__;s__
LMSG_G000008881.1	no	1643_1	Polymetallic	97.52	3.83	78.40	2	3	10	20	High quality	11696	4134765	51.60	521	d__Bacteria;p__Bacteroidota;c__Kryptonia;o__Kryptoniales;f__;g__;s__
LMSG_G000008882.1	no	1643_1	Tin-Zinc	98.88	3.55	81.13	1	1	1	19	High quality	52691	4472359	51.50	147	d__Bacteria;p__Bacteroidota;c__Kryptonia;o__Kryptoniales;f__;g__;s__
LMSG_G000008883.1	no	1643_1	Copper	98.34	2.57	85.50	0	2	3	19	Medium quality	21008	4280979	51.60	367	d__Bacteria;p__Bacteroidota;c__Kryptonia;o__Kryptoniales;f__;g__;s__
LMSG_G000008884.1	no	1643_1	Polymetallic	75.24	4.33	53.62	0	0	0	14	Medium quality	2617	3129349	51.70	1346	d__Bacteria;p__Bacteroidota;c__Kryptonia;o__Kryptoniales;f__;g__;s__
LMSG_G000008885.1	no	1643_1	Magnetite	98.34	4.37	76.49	1	0	1	18	Medium quality	89368	4218988	51.60	120	d__Bacteria;p__Bacteroidota;c__Kryptonia;o__Kryptoniales;f__;g__;s__
LMSG_G000008886.1	no	1643_1	Magnetite	64.13	1.72	55.51	1	0	1	16	Medium quality	17527	3804719	51.40	380	d__Bacteria;p__Bacteroidota;c__Kryptonia;o__Kryptoniales;f__;g__;s__
LMSG_G000008887.1	no	1643_1	Coal	84.76	3.01	69.74	0	1	0	16	Medium quality	5064	3538655	51.70	793	d__Bacteria;p__Bacteroidota;c__Kryptonia;o__Kryptoniales;f__;g__;s__
LMSG_G000008888.1	no	1643_1	Coal	86.72	4.37	64.87	1	2	7	17	Medium quality	4181	3524464	51.60	982	d__Bacteria;p__Bacteroidota;c__Kryptonia;o__Kryptoniales;f__;g__;s__
LMSG_G000008889.1	no	1644_1	Lead-Zinc	85.77	2.44	73.57	0	0	0	16	Medium quality	4684	3549524	51.40	972	d__Bacteria;p__Bacteroidota;c__Kryptonia;o__Kryptoniales;f__;g__;s__
LMSG_G000008890.1	no	1644_1	Magnetite	62.95	0.84	58.77	0	0	0	11	Medium quality	6060	2176016	51.30	400	d__Bacteria;p__Bacteroidota;c__Kryptonia;o__Kryptoniales;f__;g__;s__
LMSG_G000008891.1	no	1645_1	Pyrite-Copper	50.15	0.00	50.15	0	0	0	13	Medium quality	36923	2369948	52.20	84	d__Bacteria;p__Bacteroidota;c__Kryptonia;o__Kryptoniales;f__;g__;s__
LMSG_G000008892.1	no	1648_1	Copper	98.88	2.73	85.22	1	1	2	20	High quality	111702	3919351	52.80	74	d__Bacteria;p__Bacteroidota;c__Kryptonia;o__Kryptoniales;f__;g__;s__
LMSG_G000008893.1	no	1648_1	Polymetallic	98.88	2.73	85.22	1	1	1	19	High quality	131996	3695313	52.90	60	d__Bacteria;p__Bacteroidota;c__Kryptonia;o__Kryptoniales;f__;g__;s__
LMSG_G000008894.1	no	1648_1	Polymetallic	98.34	2.19	87.42	1	1	1	18	High quality	101429	3669913	53.00	65	d__Bacteria;p__Bacteroidota;c__Kryptonia;o__Kryptoniales;f__;g__;s__
LMSG_G000008895.1	no	1648_1	Polymetallic	98.34	1.64	90.15	1	1	1	19	High quality	215459	3603021	53.00	123	d__Bacteria;p__Bacteroidota;c__Kryptonia;o__Kryptoniales;f__;g__;s__
LMSG_G000008896.1	no	1648_1	Polymetallic	98.34	1.64	90.15	1	1	1	19	High quality	131987	3567199	53.10	54	d__Bacteria;p__Bacteroidota;c__Kryptonia;o__Kryptoniales;f__;g__;s__
LMSG_G000008897.1	no	1062_1	Polymetallic	97.81	3.19	81.88	1	2	1	19	High quality	23213	3205924	61.00	225	d__Bacteria;p__Bacteroidota;c__UBA10030;o__UBA10030;f__UBA10030;g__;s__
LMSG_G000008898.1	no	1062_1	Polymetallic	78.39	2.80	64.39	0	0	0	16	Medium quality	5409	2606475	61.30	604	d__Bacteria;p__Bacteroidota;c__UBA10030;o__UBA10030;f__UBA10030;g__;s__
LMSG_G000008899.1	no	1062_1	Polymetallic	96.61	3.02	81.50	0	1	0	18	Medium quality	40131	3125316	61.10	120	d__Bacteria;p__Bacteroidota;c__UBA10030;o__UBA10030;f__UBA10030;g__;s__
LMSG_G000008900.1	no	1062_1	Polymetallic	93.89	1.73	85.27	0	0	0	15	Medium quality	18780	3021702	61.10	217	d__Bacteria;p__Bacteroidota;c__UBA10030;o__UBA10030;f__UBA10030;g__;s__
LMSG_G000008901.1	no	298_1	Polymetallic	76.58	3.64	58.37	1	0	1	12	Medium quality	3063	2594923	47.30	1031	d__Bacteria;p__Bacteroidota;c__UBA10030;o__UBA10030;f__UBA8401;g__UBA8401;s__
LMSG_G000008902.1	no	1654_1	Polymetallic	67.12	2.73	53.45	0	0	0	12	Medium quality	2023	1689199	47.40	890	d__Bacteria;p__Bdellovibrionota;c__Bdellovibrionia;o__Bdellovibrionales;f__UBA1609;g__RBG-16-40-8;s__
LMSG_G000008903.1	no	1654_1	Polymetallic	84.80	4.20	63.82	0	0	0	17	Medium quality	5192	2348396	47.40	613	d__Bacteria;p__Bdellovibrionota;c__Bdellovibrionia;o__Bdellovibrionales;f__UBA1609;g__RBG-16-40-8;s__
LMSG_G000008904.1	no	1654_1	Polymetallic	79.78	0.30	78.30	0	0	0	17	Medium quality	4432	2189316	47.40	641	d__Bacteria;p__Bdellovibrionota;c__Bdellovibrionia;o__Bdellovibrionales;f__UBA1609;g__RBG-16-40-8;s__
LMSG_G000008905.1	no	1654_1	Polymetallic	84.69	1.05	79.46	1	1	1	16	Medium quality	3044	2298945	47.30	864	d__Bacteria;p__Bdellovibrionota;c__Bdellovibrionia;o__Bdellovibrionales;f__UBA1609;g__RBG-16-40-8;s__
LMSG_G000008906.1	no	1654_1	Pyrite	86.10	2.73	72.43	1	1	1	16	Medium quality	4406	2349279	47.40	684	d__Bacteria;p__Bdellovibrionota;c__Bdellovibrionia;o__Bdellovibrionales;f__UBA1609;g__RBG-16-40-8;s__

LMSG_G000008907.1	no	1654_1	Pyrite-Copper	67.31	0.00	67.31	0	1	0	13	Medium quality	4382	1555311	47.50	397	d__Bacteria;p__Bdellovibrionota;c__Bdellovibrionia;o__Bdellovibrionales;f__UBA1609;g__RBG-16-40-8;s__
LMSG_G000008908.1	no	1654_1	Pyrite-Copper	75.44	0.89	70.98	0	0	0	14	Medium quality	8484	2371420	47.40	430	d__Bacteria;p__Bdellovibrionota;c__Bdellovibrionia;o__Bdellovibrionales;f__UBA1609;g__RBG-16-40-8;s__
LMSG_G000008909.1	no	1027_1	Antimony	50.80	0.00	50.80	0	1	0	7	Medium quality	1994	1616446	53.70	850	d__Bacteria;p__Bdellovibrionota;c__Bdellovibrionia_A;o__UBA1018;f__UBA1018;g__;s__
LMSG_G000008910.1	no	269_1	Nickel-Copper	93.29	1.57	85.44	0	0	0	13	Medium quality	3926	2049140	40.30	644	d__Bacteria;p__Campylobacterota;c__Campylobacteria;o__Campylobacterales;f__Sulfurimonadaceae;g__Sulfuricurvum;s__Sulfuricurvum sp002282015
LMSG_G000008911.1	no	270_1	Antimony	83.59	0.82	79.51	0	0	0	16	Medium quality	17802	1665761	38.00	134	d__Bacteria;p__Campylobacterota;c__Campylobacteria;o__Campylobacterales;f__Sulfurimonadaceae;g__Sulfurimonas;s__
LMSG_G000008912.1	no	271_1	Antimony	71.53	0.82	67.45	3	0	1	17	Medium quality	19199	1423653	40.10	117	d__Bacteria;p__Campylobacterota;c__Campylobacteria;o__Campylobacterales;f__Sulfurimonadaceae;g__Sulfurimonas;s__
LMSG_G000008913.1	no	1731_1	Nickel-Copper	92.89	2.54	80.19	0	0	0	15	Medium quality	4618	1686434	42.60	473	d__Bacteria;p__Campylobacterota;c__Campylobacteria;o__Campylobacterales;f__Sulfurovaceae;g__Sulfurovum;s__Sulfurovum sp002281755
LMSG_G000008914.1	no	1731_1	Nickel-Copper	96.29	0.45	94.04	0	1	0	13	Medium quality	9571	1793723	42.50	248	d__Bacteria;p__Campylobacterota;c__Campylobacteria;o__Campylobacterales;f__Sulfurovaceae;g__Sulfurovum;s__Sulfurovum sp002281755
LMSG_G000008915.1	no	1731_1	Nickel-Copper	98.36	1.02	93.24	0	1	0	18	Medium quality	70282	2015658	41.60	56	d__Bacteria;p__Campylobacterota;c__Campylobacteria;o__Campylobacterales;f__Sulfurovaceae;g__Sulfurovum;s__Sulfurovum sp002281755
LMSG_G000008916.1	no	1731_1	Lead-Zinc	97.95	1.43	90.78	1	0	1	19	Medium quality	49936	1983034	41.60	74	d__Bacteria;p__Campylobacterota;c__Campylobacteria;o__Campylobacterales;f__Sulfurovaceae;g__Sulfurovum;s__Sulfurovum sp002281755
LMSG_G000008917.1	no	244_1	Lead-Zinc	85.45	4.55	62.73	1	1	1	15	Medium quality	27485	2369516	52.00	127	d__Bacteria;p__Chloroflexota;c__Anaerolineae;o__Anaerolineales;f__Anaerolineaceae;g__Bellilinea;s__
LMSG_G000008918.1	no	294_1	Antimony	57.94	1.09	52.49	0	0	0	11	Medium quality	11727	2133541	53.20	227	d__Bacteria;p__Chloroflexota;c__Anaerolineae;o__Anaerolineales;f__Anaerolineaceae;g__UBA6107;s__
LMSG_G000008919.1	no	232_1	Polymetallic	93.63	2.18	82.73	1	1	1	19	High quality	47222	3689942	51.80	134	d__Bacteria;p__Chloroflexota;c__Anaerolineae;o__Anaerolineales;f__EnvOPS12;g__Fen-1038;s__
LMSG_G000008920.1	no	232_1	Polymetallic	92.72	2.91	78.18	1	1	1	19	High quality	37279	3731506	51.80	152	d__Bacteria;p__Chloroflexota;c__Anaerolineae;o__Anaerolineales;f__EnvOPS12;g__Fen-1038;s__
LMSG_G000008921.1	no	232_1	Polymetallic	93.63	3.09	78.18	1	1	1	19	High quality	136640	3867032	51.80	194	d__Bacteria;p__Chloroflexota;c__Anaerolineae;o__Anaerolineales;f__EnvOPS12;g__Fen-1038;s__
LMSG_G000008922.1	no	1411_1	Polymetallic	81.67	1.09	76.22	0	0	0	17	Medium quality	4031	3350034	61.30	935	d__Bacteria;p__Chloroflexota;c__Anaerolineae;o__Thermoflexales;f__JO36;g__;s__
LMSG_G000008923.1	no	1411_1	Polymetallic	86.49	2.91	71.95	1	1	0	18	Medium quality	7699	3661862	61.30	609	d__Bacteria;p__Chloroflexota;c__Anaerolineae;o__Thermoflexales;f__JO36;g__;s__
LMSG_G000008924.1	no	1411_1	Polymetallic	85.18	3.18	69.28	1	0	0	14	Medium quality	12458	3608904	61.50	533	d__Bacteria;p__Chloroflexota;c__Anaerolineae;o__Thermoflexales;f__JO36;g__;s__
LMSG_G000008925.1	no	449_1	Copper	79.85	3.04	64.67	1	0	1	19	Medium quality	5154	4615355	58.30	925	d__Bacteria;p__Chloroflexota;c__Anaerolineae;o__UBA4142;f__UBA4142;g__UBA4142;s__
LMSG_G000008926.1	no	449_1	Copper	57.86	1.02	52.77	0	0	0	13	Medium quality	4257	2811763	60.20	710	d__Bacteria;p__Chloroflexota;c__Anaerolineae;o__UBA4142;f__UBA4142;g__UBA4142;s__
LMSG_G000008927.1	no	443_1	Lead-Zinc	89.76	3.42	72.66	2	1	4	17	Medium quality	7816	3627350	57.60	575	d__Bacteria;p__Chloroflexota;c__Dehalococcoidia;o__FW602-bin16;f__;g__;s__
LMSG_G000008928.1	no	443_1	Pyrite-Copper	96.69	2.27	85.35	1	0	3	19	Medium quality	27368	3841827	57.60	228	d__Bacteria;p__Chloroflexota;c__Dehalococcoidia;o__FW602-bin16;f__;g__;s__
LMSG_G000008929.1	no	667_1	Lead-Zinc	96.75	4.63	73.61	1	1	1	20	High quality	52505	2815619	74.60	85	d__Bacteria;p__Chloroflexota;c__Ellin6529;o__CSP1-4;f__CSP1-4;g__;s__
LMSG_G000008930.1	no	667_1	Polymetallic	94.90	1.85	85.65	1	1	1	19	High quality	22511	2671756	74.80	168	d__Bacteria;p__Chloroflexota;c__Ellin6529;o__CSP1-4;f__CSP1-4;g__;s__
LMSG_G000008931.1	no	667_1	Polymetallic	94.90	3.24	78.70	1	1	1	18	High quality	15527	2517206	74.80	255	d__Bacteria;p__Chloroflexota;c__Ellin6529;o__CSP1-4;f__CSP1-4;g__;s__
LMSG_G000008932.1	no	667_1	Lead-Zinc	93.98	1.85	84.73	1	1	1	18	High quality	7324	2728139	74.10	492	d__Bacteria;p__Chloroflexota;c__Ellin6529;o__CSP1-4;f__CSP1-4;g__;s__
LMSG_G000008933.1	no	667_1	Polymetallic	95.83	3.70	77.32	1	1	1	17	Medium quality	17705	2543716	74.90	223	d__Bacteria;p__Chloroflexota;c__Ellin6529;o__CSP1-4;f__CSP1-4;g__;s__
LMSG_G000008934.1	no	667_1	Lead-Zinc	77.80	3.06	62.51	0	1	0	14	Medium quality	5740	2149422	75.20	460	d__Bacteria;p__Chloroflexota;c__Ellin6529;o__CSP1-4;f__CSP1-4;g__;s__
LMSG_G000008935.1	no	667_1	Lead-Zinc	87.96	3.07	72.64	1	1	1	16	Medium quality	6057	2440181	74.80	500	d__Bacteria;p__Chloroflexota;c__Ellin6529;o__CSP1-4;f__CSP1-4;g__;s__
LMSG_G000008936.1	no	667_1	Polymetallic	86.11	3.76	67.34	0	1	0	19	Medium quality	6728	2469340	74.30	459	d__Bacteria;p__Chloroflexota;c__Ellin6529;o__CSP1-4;f__CSP1-4;g__;s__
LMSG_G000008937.1	no	667_1	Polymetallic	89.35	1.39	82.41	2	1	2	19	Medium quality	12931	2567060	74.70	278	d__Bacteria;p__Chloroflexota;c__Ellin6529;o__CSP1-4;f__CSP1-4;g__;s__
LMSG_G000008938.1	no	667_1	Pyrite-Copper	94.90	2.62	81.79	0	1	0	17	Medium quality	62570	2540968	74.80	56	d__Bacteria;p__Chloroflexota;c__Ellin6529;o__CSP1-4;f__CSP1-4;g__;s__
LMSG_G000008939.1	no	668_1	Polymetallic	73.08	3.67	54.74	0	0	0	15	Medium quality	7432	2200694	72.70	328	d__Bacteria;p__Chloroflexota;c__Ellin6529;o__CSP1-4;f__CSP1-4;g__;s__
LMSG_G000008940.1	no	668_1	Polymetallic	87.66	3.70	69.15	1	1	1	18	Medium quality	13710	2925549	72.50	316	d__Bacteria;p__Chloroflexota;c__Ellin6529;o__CSP1-4;f__CSP1-4;g__;s__
LMSG_G000008941.1	no	668_1	Polymetallic	87.30	3.67	68.96	1	1	1	17	Medium quality	41940	2777759	72.60	100	d__Bacteria;p__Chloroflexota;c__Ellin6529;o__CSP1-4;f__CSP1-4;g__;s__
LMSG_G000008942.1	no	668_1	Polymetallic	86.57	4.78	62.66	1	1	1	19	Medium quality	59401	3183992	72.50	78	d__Bacteria;p__Chloroflexota;c__Ellin6529;o__CSP1-4;f__CSP1-4;g__;s__
LMSG_G000008943.1	no	669_1	Lead-Zinc	90.63	2.14	79.93	1	1	1	18	High quality	40219	3050685	72.00	199	d__Bacteria;p__Chloroflexota;c__Ellin6529;o__CSP1-4;f__CSP1-4;g__;s__
LMSG_G000008944.1	no	669_1	Lead-Zinc	76.33	2.78	62.45	0	1	0	15	Medium quality	9532	2304741	72.50	359	d__Bacteria;p__Chloroflexota;c__Ellin6529;o__CSP1-4;f__CSP1-4;g__;s__
LMSG_G000008945.1	no	669_1	Polymetallic	79.35	2.29	67.89	0	0	0	14	Medium quality	14964	2312486	72.40	210	d__Bacteria;p__Chloroflexota;c__Ellin6529;o__CSP1-4;f__CSP1-4;g__;s__
LMSG_G000008946.1	no	669_1	Polymetallic	87.03	4.63	63.89	1	1	1	20	Medium quality	20522	2865828	72.00	189	d__Bacteria;p__Chloroflexota;c__Ellin6529;o__CSP1-4;f__CSP1-4;g__;s__
LMSG_G000008947.1	no	669_1	Polymetallic	88.88	1.85	79.63	0	0	0	20	Medium quality	30873	3283216	71.80	149	d__Bacteria;p__Chloroflexota;c__Ellin6529;o__CSP1-4;f__CSP1-4;g__;s__
LMSG_G000008948.1	no	669_1	Polymetallic	86.41	2.78	72.53	1	0	1	20	Medium quality	34819	2877676	72.00	115	d__Bacteria;p__Chloroflexota;c__Ellin6529;o__CSP1-4;f__CSP1-4;g__;s__
LMSG_G000008949.1	no	669_1	Pyrite-Copper	89.81	1.85	80.56	1	0	1	19	Medium quality	25867	2865376	72.10	194	d__Bacteria;p__Chloroflexota;c__Ellin6529;o__CSP1-4;f__CSP1-4;g__;s__
LMSG_G000008950.1	no	670_1	Pyrite-Copper	56.37	0.00	56.37	0	0	0	11	Medium quality	9832	1775677	72.80	236	d__Bacteria;p__Chloroflexota;c__Ellin6529;o__CSP1-4;f__CSP1-4;g__;s__
LMSG_G000008951.1	no	672_1	Polymetallic	92.75	2.78	78.87	0	0	0	17	Medium quality	12838	2639734	72.80	311	d__Bacteria;p__Chloroflexota;c__Ellin6529;o__CSP1-4;f__CSP1-4;g__;s__
LMSG_G000008952.1	no	672_1	Polymetallic	91.56	3.70	73.05	0	1	0	17	Medium quality	11916	2833063	72.60	337	d__Bacteria;p__Chloroflexota;c__Ellin6529;o__CSP1-4;f__CSP1-4;g__;s__
LMSG_G000008953.1	no	672_1	Polymetallic	89.96	2.78	76.08	0	0	0	18	Medium quality	16165	2647103	72.60	218	d__Bacteria;p__Chloroflexota;c__Ellin6529;o__CSP1-4;f__CSP1-4;g__;s__
LMSG_G000008954.1	no	673_1	Lead-Zinc	85.18	1.85	75.93	0	0	0	20	Medium quality	14007	2224494	71.50	193	d__Bacteria;p__Chloroflexota;c__Ellin6529;o__CSP1-4;f__CSP1-4;g__;s__
LMSG_G000008955.1	no	673_1	Pyrite-Copper	87.96	3.24	71.76	0	0	0	20	Medium quality	34731	2385262	71.50	95	d__Bacteria;p__Chloroflexota;c__Ellin6529;o__CSP1-4;f__CSP1-4;g__;s__
LMSG_G000008956.1	no	674_1	Copper	70.64	1.83	61.47	1	3	0	13	Medium quality	5432	2087866	72.70	475	d__Bacteria;p__Chloroflexota;c__Ellin6529;o__CSP1-4;f__CSP1-4;g__;s__
LMSG_G000008957.1	no	678_1	Antimony	76.43	3.70	57.92	0	0	0	17	Medium quality	10158	2130414	73.60	233	d__Bacteria;p__Chloroflexota;c__Ellin6529;o__CSP1-4;f__CSP1-4;g__;s__
LMSG_G000008958.1	no	680_1	Polymetallic	88.11	1.67	79.78	1	1	1	17	Medium quality	11317	2380856	72.00	296	d__Bacteria;p__Chloroflexota;c__Ellin6529;o__CSP1-4;f__CSP1-4;g__;s__
LMSG_G000008959.1	no	680_1	Polymetallic	90.74	1.39	83.80	1	0	1	17	Medium quality	20597	2406171	72.10	161	d__Bacteria;p__Chloroflexota;c__Ellin6529;o__CSP1-4;f__CSP1-4;g__;s__
LMSG_G000008960.1	no	680_1	Polymetallic	88.88	1.39	81.94	1	0	1	18	Medium quality	31136	2643887	71.90	132	d__Bacteria;p__Chloroflexota;c__Ellin6529;o__CSP1-4;f__CSP1-4;g__;s__
LMSG_G000008961.1	no	681_1	Pyrite-Copper	70.03	1.83	60.86	0	0	0	15	Medium quality	8513	1895209	71.80	303	d__Bacteria;p__Chloroflexota;c__Ellin6529;o__CSP1-4;f__CSP1-4;g__;s__
LMSG_G000008962.1	no	665_1	Copper	93.98	2.42	81.90	2	0	2	20	Medium quality	30793	3818980	68.20	194	d__Bacteria;p__Chloroflexota;c__Ellin6529;o__CSP1-4;f__CSP1-4;g__Fen-1039;s__
LMSG_G000008963.1	no	665_1	Copper	92.28	1.80	83.28	0	1	0	18	Medium quality	19277	3653220	68.30	291	d__Bacteria;p__Chloroflexota;c__Ellin6529;o__CSP1-4;f__CSP1-4;g__Fen-1039;s__
LMSG_G000008964.1	no	444_1	Polymetallic	53.67	0.38	51.77	1	0	1	11	Medium quality	2240	2254961	53.00	1069	d__Bacteria;p__Chloroflexota;c__FW602-bin22;o__FW602-bin22;f__;g__;s__
LMSG_G000008965.1	no	315_1	Pyrite	96.69	1.98	86.79	0	1	1	18	Medium quality	10251	3199965	46.40	446	d__Bacteria;p__Chloroflexota;c__Ktedonobacteria;o__Ktedonobacterales;f__;g__;s__
LMSG_G000008966.1	no	316_1	Antimony	85.72	2.18	74.83	0	0	0	17	Medium quality	3532	2853303	45.80	946	d__Bacteria;p__Chloroflexota;c__Ktedonobacteria;o__Ktedonobacterales;f__;g__;s__
LMSG_G000008967.1	no	316_1	Antimony	91.71	0.99	86.76	1	0	9	18	Medium quality	16431	3183661	45.70	335	d__Bacteria;p__Chloroflexota;c__Ktedonobacteria;o__Ktedonobacterales;f__;g__;s__
LMSG_G000008968.1	no	316_1	Antimony	77.86	0.99	72.91	1	0	7	12	Medium quality	5126	2333621	46.30	547	d__Bacteria;p__Chloroflexota;c__Ktedonobacteria;o__Ktedonobacterales;f__;g__;s__
LMSG_G000008969.1	no	316_1	Antimony	64.09	0.99	59.14	0	0	0	17	Medium quality	2301	2259275	46.10	1062	d__Bacteria;p__Chloroflexota;c__Ktedonobacteria;o__Ktedonobacterales;f__;g__;s__
LMSG_G000008970.1	no	520_1	Polymetallic	94.11	4.46	71.84	1	2	0	19	Medium quality	12492	3960644	65.50	456	d__Bacteria;p__Chloroflexota;c__Ktedonobacteria;o__Ktedonobacterales;f__;g__;s__
LMSG_G000008971.1	no	462_1	Lead-Zinc	63.15	1.75	54.38	1	0	1	14	Medium quality	13837	3298899	65.80	305	d__Bacteria;p__Chloroflexota;c__UBA6077;o__;f__;g__;s__
LMSG_G000008972.1	no	322_1	Pyrite	94.87	2.28	83.48	2	1	2	20	High quality	9274	3188307	37.30	544	d__Bacteria;p__Cyanobacteria;c__Vampirovibrionia;o__Obscuribacterales;f__;g__;s__
LMSG_G000008973.1	no	322_1	Pyrite	70.24	0.66	66.92	0	1	0	13	Medium quality	2140	2038965	38.00	1008	d__Bacteria;p__Cyanobacteria;c__Vampirovibrionia;o__Obscuribacterales;f__;g__;s__
LMSG_G000008974.1	no	322_1	Pyrite-Copper	87.17	1.33	80.53	0	3	1	19	Medium quality	5334	3056944	37.10	754	d__Bacteria;p__Cyanobacteria;c__Vampirovibrionia;o__Obscuribacterales;f__;g__;s__
LMSG_G000008975.1	no	322_1	Pyrite-Copper	77.78	1.45	70.52	0	0	0	15	Medium quality	3346	2139238	37.00	761	d__Bacteria;p__Cyanobacteria;c__Vampirovibrionia;o__Obscuribacterales;f__;g__;s__
LMSG_G000008976.1	no	322_1	Magnetite	66.96	1.04	61.74	0	0	0	15	Medium quality	3344	2169080	37.30	857	d__Bacteria;p__Cyanobacteria;c__Vampirovibrionia;o__Obscuribacterales;f__;g__;s__
LMSG_G000008977.1	no	324_1	Polymetallic	62.67	0.85	58.40	0	0	0	15	Medium quality	2533	1998179	33.00	902	d__Bacteria;p__Cyanobacteria;c__Vampirovibrionia;o__Obscuribacterales;f__;g__;s__
LMSG_G000008978.1	no	324_1	Polymetallic	79.99	1.14	74.30	1	0	1	17	Medium quality	9676	2669901	32.80	584	d__Bacteria;p__Cyanobacteria;c__Vampirovibrionia;o__Obscuribacterales;f__;g__;s__

LMSG_G000008979.1	no	324_2	Copper	95.72	0.57	92.88	1	1	2	20	High quality	56206	3366528	34.00	104	d__Bacteria;p__Cyanobacteria;c__Vampirovibrionia;o__Obscuribacterales;f__;g__;s__
LMSG_G000008980.1	no	324_2	Polymetallic	95.72	2.28	84.33	1	1	2	20	High quality	60773	3108139	33.70	130	d__Bacteria;p__Cyanobacteria;c__Vampirovibrionia;o__Obscuribacterales;f__;g__;s__
LMSG_G000008981.1	no	324_2	Polymetallic	74.02	1.03	68.89	0	0	1	17	Medium quality	2459	2289852	33.70	1045	d__Bacteria;p__Cyanobacteria;c__Vampirovibrionia;o__Obscuribacterales;f__;g__;s__
LMSG_G000008982.1	no	324_2	Polymetallic	85.28	1.04	80.06	1	2	2	18	Medium quality	5288	2940633	33.90	726	d__Bacteria;p__Cyanobacteria;c__Vampirovibrionia;o__Obscuribacterales;f__;g__;s__
LMSG_G000008983.1	no	324_2	Polymetallic	91.02	1.42	83.90	1	0	15	20	Medium quality	11051	2667955	33.20	372	d__Bacteria;p__Cyanobacteria;c__Vampirovibrionia;o__Obscuribacterales;f__;g__;s__
LMSG_G000008984.1	no	324_2	Polymetallic	89.23	0.81	85.20	0	0	1	19	Medium quality	4331	2926394	33.70	893	d__Bacteria;p__Cyanobacteria;c__Vampirovibrionia;o__Obscuribacterales;f__;g__;s__
LMSG_G000008985.1	no	324_2	Polymetallic	86.55	1.42	79.43	0	1	0	18	Medium quality	7540	2662950	33.20	455	d__Bacteria;p__Cyanobacteria;c__Vampirovibrionia;o__Obscuribacterales;f__;g__;s__
LMSG_G000008986.1	no	324_2	Polymetallic	93.39	0.85	89.12	1	2	0	19	Medium quality	14139	2826156	33.20	315	d__Bacteria;p__Cyanobacteria;c__Vampirovibrionia;o__Obscuribacterales;f__;g__;s__
LMSG_G000008987.1	no	326_1	Antimony	61.53	1.80	52.51	0	0	0	14	Medium quality	3626	1600935	39.60	486	d__Bacteria;p__Cyanobacteria;c__Vampirovibrionia;o__Obscuribacterales;f__;g__;s__
LMSG_G000008988.1	no	1016_1	Pyrite	93.43	0.64	90.26	1	1	1	16	Medium quality	8689	2166177	66.90	356	d__Bacteria;p__Deinococcota;c__Deinococci;o__Deinococcales;f__;g__;s__
LMSG_G000008989.1	no	1016_1	Pyrite	94.27	0.85	90.04	1	0	1	20	Medium quality	31816	2278584	66.80	121	d__Bacteria;p__Deinococcota;c__Deinococci;o__Deinococcales;f__;g__;s__
LMSG_G000008990.1	no	1016_1	Pyrite	94.43	0.00	94.43	0	1	0	20	Medium quality	40096	2246745	66.80	108	d__Bacteria;p__Deinococcota;c__Deinococci;o__Deinococcales;f__;g__;s__
LMSG_G000008991.1	no	1016_1	Pyrite	94.27	0.42	92.16	0	0	1	20	Medium quality	30966	2274928	66.90	119	d__Bacteria;p__Deinococcota;c__Deinococci;o__Deinococcales;f__;g__;s__
LMSG_G000008992.1	no	1016_1	Polymetallic	95.33	0.00	95.33	0	1	0	20	Medium quality	113455	2291067	66.80	36	d__Bacteria;p__Deinococcota;c__Deinococci;o__Deinococcales;f__;g__;s__
LMSG_G000008993.1	no	1016_1	Polymetallic	95.33	0.00	95.33	0	1	0	20	Medium quality	103454	2303354	66.80	37	d__Bacteria;p__Deinococcota;c__Deinococci;o__Deinococcales;f__;g__;s__
LMSG_G000008994.1	no	1016_1	Polymetallic	95.12	0.00	95.12	0	1	0	20	Medium quality	118255	2278489	66.80	31	d__Bacteria;p__Deinococcota;c__Deinococci;o__Deinococcales;f__;g__;s__
LMSG_G000008995.1	no	1016_1	Polymetallic	95.12	0.00	95.12	0	1	0	20	Medium quality	114486	2290420	66.80	35	d__Bacteria;p__Deinococcota;c__Deinococci;o__Deinococcales;f__;g__;s__
LMSG_G000008996.1	no	1016_1	Polymetallic	91.33	0.92	86.74	1	0	1	19	Medium quality	12176	2208254	66.90	259	d__Bacteria;p__Deinococcota;c__Deinococci;o__Deinococcales;f__;g__;s__
LMSG_G000008997.1	no	1016_1	Polymetallic	64.29	1.64	56.08	2	1	4	16	Medium quality	2234	1532269	67.10	750	d__Bacteria;p__Deinococcota;c__Deinococci;o__Deinococcales;f__;g__;s__
LMSG_G000008998.1	no	1016_1	Polymetallic	95.12	0.00	95.12	0	0	1	19	Medium quality	51587	2252480	66.90	61	d__Bacteria;p__Deinococcota;c__Deinococci;o__Deinococcales;f__;g__;s__
LMSG_G000008999.1	no	1016_1	Tin-Zinc	94.49	1.06	89.20	0	0	0	20	Medium quality	33569	2263331	66.90	107	d__Bacteria;p__Deinococcota;c__Deinococci;o__Deinococcales;f__;g__;s__
LMSG_G000009000.1	no	1016_1	Tin-Zinc	92.68	0.00	92.68	0	1	0	19	Medium quality	24397	2231190	66.80	139	d__Bacteria;p__Deinococcota;c__Deinococci;o__Deinococcales;f__;g__;s__
LMSG_G000009001.1	no	1016_1	Polymetallic	95.33	0.00	95.33	0	1	0	20	Medium quality	113964	2275484	66.80	31	d__Bacteria;p__Deinococcota;c__Deinococci;o__Deinococcales;f__;g__;s__
LMSG_G000009002.1	no	1016_1	Copper	95.12	0.00	95.12	0	1	0	20	Medium quality	117129	2269502	66.80	31	d__Bacteria;p__Deinococcota;c__Deinococci;o__Deinococcales;f__;g__;s__
LMSG_G000009003.1	no	1016_1	Copper	95.12	0.00	95.12	0	1	0	20	Medium quality	113455	2280369	66.80	32	d__Bacteria;p__Deinococcota;c__Deinococci;o__Deinococcales;f__;g__;s__
LMSG_G000009004.1	no	1016_1	Copper	95.12	0.42	93.01	0	1	0	20	Medium quality	96492	2318861	66.80	42	d__Bacteria;p__Deinococcota;c__Deinococci;o__Deinococcales;f__;g__;s__
LMSG_G000009005.1	no	1016_1	Polymetallic	86.44	0.00	86.44	0	1	0	20	Medium quality	104309	2163362	66.80	29	d__Bacteria;p__Deinococcota;c__Deinococci;o__Deinococcales;f__;g__;s__
LMSG_G000009006.1	no	1016_1	Magnetite	95.12	0.00	95.12	0	1	1	20	Medium quality	103454	2312778	66.80	41	d__Bacteria;p__Deinococcota;c__Deinococci;o__Deinococcales;f__;g__;s__
LMSG_G000009007.1	no	1016_1	Copper	95.12	0.00	95.12	0	0	0	20	Medium quality	108725	2292599	66.80	43	d__Bacteria;p__Deinococcota;c__Deinococci;o__Deinococcales;f__;g__;s__
LMSG_G000009008.1	no	1016_1	Lead-Zinc	94.49	0.00	94.49	0	0	0	20	Medium quality	68782	2252504	66.80	47	d__Bacteria;p__Deinococcota;c__Deinococci;o__Deinococcales;f__;g__;s__
LMSG_G000009009.1	no	1016_1	Lead-Zinc	95.12	0.00	95.12	0	1	0	20	Medium quality	113714	2273948	66.80	32	d__Bacteria;p__Deinococcota;c__Deinococci;o__Deinococcales;f__;g__;s__
LMSG_G000009010.1	no	1016_1	Lead-Zinc	93.00	0.21	91.95	1	0	1	19	Medium quality	16272	2230316	66.90	203	d__Bacteria;p__Deinococcota;c__Deinococci;o__Deinococcales;f__;g__;s__
LMSG_G000009011.1	no	1016_1	Pyrite-Copper	81.39	2.58	68.49	0	0	0	20	Medium quality	5625	2042179	66.90	488	d__Bacteria;p__Deinococcota;c__Deinococci;o__Deinococcales;f__;g__;s__
LMSG_G000009012.1	no	1016_1	Pyrite-Copper	94.27	0.00	94.27	0	0	0	20	Medium quality	23022	2245220	66.90	142	d__Bacteria;p__Deinococcota;c__Deinococci;o__Deinococcales;f__;g__;s__
LMSG_G000009013.1	no	1016_1	Lead-Zinc	94.27	0.00	94.27	0	0	0	20	Medium quality	66588	2288384	66.80	59	d__Bacteria;p__Deinococcota;c__Deinococci;o__Deinococcales;f__;g__;s__
LMSG_G000009014.1	no	1016_1	Polymetallic	61.52	1.27	55.17	1	0	1	14	Medium quality	7527	1636722	66.00	379	d__Bacteria;p__Deinococcota;c__Deinococci;o__Deinococcales;f__;g__;s__
LMSG_G000009015.1	no	1016_1	Magnetite	88.06	0.00	88.06	0	0	0	19	Medium quality	25152	2117435	66.50	203	d__Bacteria;p__Deinococcota;c__Deinococci;o__Deinococcales;f__;g__;s__
LMSG_G000009016.1	no	1016_1	Magnetite	66.44	0.99	61.50	1	0	1	17	Medium quality	7933	1639662	66.10	358	d__Bacteria;p__Deinococcota;c__Deinococci;o__Deinococcales;f__;g__;s__
LMSG_G000009017.1	no	408_1	Lead-Zinc	96.39	1.34	89.69	1	1	1	18	High quality	13392	2194678	59.10	237	d__Bacteria;p__Deinococcota;c__Deinococci;o__Deinococcales;f__Thermaceae;g__;s__
LMSG_G000009018.1	no	1730_1	Magnetite	60.57	0.00	60.57	1	1	1	14	Medium quality	4133	739471	39.30	222	d__Bacteria;p__Dependentiae;c__Babeliae;o__Babeliales;f__Babeliaceae;g__BOG-1370;s__
LMSG_G000009019.1	no	1631_1	Copper	59.14	1.63	51.01	2	5	20	20	Medium quality	52637	1301612	42.90	100	d__Bacteria;p__Dependentiae;c__Babeliae;o__Babeliales;f__Vermiphilaceae;g__;s__
LMSG_G000009020.1	no	1631_1	Pyrite-Copper	59.14	1.63	51.01	1	1	1	20	Medium quality	52757	1206326	42.70	45	d__Bacteria;p__Dependentiae;c__Babeliae;o__Babeliales;f__Vermiphilaceae;g__;s__
LMSG_G000009021.1	no	1728_1	Copper	56.91	0.81	52.85	2	2	5	20	Medium quality	34982	1279522	38.50	100	d__Bacteria;p__Dependentiae;c__Babeliae;o__Babeliales;f__Vermiphilaceae;g__;s__
LMSG_G000009022.1	no	1729_1	Antimony	58.13	1.02	53.05	3	5	7	20	Medium quality	111719	1269066	37.40	54	d__Bacteria;p__Dependentiae;c__Babeliae;o__Babeliales;f__Vermiphilaceae;g__;s__
LMSG_G000009023.1	no	1729_1	Antimony	58.13	1.42	51.02	3	0	1	19	Medium quality	85933	1266125	37.30	51	d__Bacteria;p__Dependentiae;c__Babeliae;o__Babeliales;f__Vermiphilaceae;g__;s__
LMSG_G000009024.1	no	1365_1	Polymetallic	83.89	3.62	65.79	0	1	0	16	Medium quality	5281	3573196	66.80	783	d__Bacteria;p__Desulfobacterota;c__Desulfarculia;o__Desulfarculales;f__Desulfarculaceae;g__;s__
LMSG_G000009025.1	no	1365_1	Polymetallic	71.84	2.28	60.44	0	1	0	8	Medium quality	2371	3093778	67.00	1446	d__Bacteria;p__Desulfobacterota;c__Desulfarculia;o__Desulfarculales;f__Desulfarculaceae;g__;s__
LMSG_G000009026.1	no	1365_1	Antimony	67.80	0.97	62.97	1	1	0	11	Medium quality	2963	2536436	67.10	895	d__Bacteria;p__Desulfobacterota;c__Desulfarculia;o__Desulfarculales;f__Desulfarculaceae;g__;s__
LMSG_G000009027.1	no	1363_1	Polymetallic	95.48	2.58	82.58	1	1	1	19	High quality	43717	4219172	58.20	149	d__Bacteria;p__Desulfobacterota;c__Desulfobaccia;o__Desulfobaccales;f__0-14-0-80-60-11;g__SURF-52;s__
LMSG_G000009028.1	no	1363_1	Polymetallic	88.44	1.78	79.56	0	0	0	20	Medium quality	15722	3927203	58.20	407	d__Bacteria;p__Desulfobacterota;c__Desulfobaccia;o__Desulfobaccales;f__0-14-0-80-60-11;g__SURF-52;s__
LMSG_G000009029.1	no	1363_1	Polymetallic	51.72	0.00	51.72	0	0	0	16	Medium quality	44130	2500528	57.00	77	d__Bacteria;p__Desulfobacterota;c__Desulfobaccia;o__Desulfobaccales;f__0-14-0-80-60-11;g__SURF-52;s__
LMSG_G000009030.1	no	1363_1	Polymetallic	92.41	3.32	75.83	1	0	1	18	Medium quality	23703	4048902	58.40	337	d__Bacteria;p__Desulfobacterota;c__Desulfobaccia;o__Desulfobaccales;f__0-14-0-80-60-11;g__SURF-52;s__
LMSG_G000009031.1	no	618_1	Antimony	62.28	0.88	57.90	0	0	0	12	Medium quality	6257	2187039	50.70	508	d__Bacteria;p__Desulfobacterota;c__Desulfobacteria;o__Desulfobacterales;f__Desulfatirhabdiaceae;g__RAAP-1;s__
LMSG_G000009032.1	no	1381_1	Polymetallic	92.73	1.29	86.28	0	1	1	20	Medium quality	18439	5869521	59.70	507	d__Bacteria;p__Desulfobacterota;c__Desulfobacteria;o__Desulfobacterales;f__Desulfosarcinaceae;g__Desulfatitalea;s__
LMSG_G000009033.1	no	1381_1	Polymetallic	90.94	1.29	84.49	0	0	0	18	Medium quality	18088	5392481	59.70	441	d__Bacteria;p__Desulfobacterota;c__Desulfobacteria;o__Desulfobacterales;f__Desulfosarcinaceae;g__Desulfatitalea;s__
LMSG_G000009034.1	no	1382_1	Polymetallic	94.84	0.00	94.84	1	1	1	18	High quality	14361	5798706	54.90	534	d__Bacteria;p__Desulfobacterota;c__Desulfobacteria;o__Desulfobacterales;f__Desulfosarcinaceae;g__Desulfatitalea;s__
LMSG_G000009035.1	no	1382_1	Polymetallic	94.60	1.61	86.54	1	0	1	19	Medium quality	22584	5926179	54.80	461	d__Bacteria;p__Desulfobacterota;c__Desulfobacteria;o__Desulfobacterales;f__Desulfosarcinaceae;g__Desulfatitalea;s__
LMSG_G000009036.1	no	1382_1	Polymetallic	75.26	0.20	74.28	0	0	0	18	Medium quality	5536	4526156	54.60	954	d__Bacteria;p__Desulfobacterota;c__Desulfobacteria;o__Desulfobacterales;f__Desulfosarcinaceae;g__Desulfatitalea;s__
LMSG_G000009037.1	no	1382_1	Polymetallic	91.61	0.00	91.61	0	1	1	20	Medium quality	148446	5836474	54.90	65	d__Bacteria;p__Desulfobacterota;c__Desulfobacteria;o__Desulfobacterales;f__Desulfosarcinaceae;g__Desulfatitalea;s__
LMSG_G000009038.1	no	1589_1	Antimony	63.35	2.10	52.87	0	0	0	13	Medium quality	3360	2154767	51.50	727	d__Bacteria;p__Desulfobacterota;c__Desulfobacteria;o__Desulfobacterales;f__SURF-3;g__SURF-3;s__
LMSG_G000009039.1	no	1383_1	Antimony	90.17	1.26	83.89	0	0	0	16	Medium quality	20826	2350471	63.40	166	d__Bacteria;p__Desulfobacterota;c__Desulfobulbia;o__Desulfobulbales;f__;g__;s__
LMSG_G000009040.1	no	1383_1	Antimony	93.78	3.11	78.24	0	1	0	19	Medium quality	9408	2347777	63.30	345	d__Bacteria;p__Desulfobacterota;c__Desulfobulbia;o__Desulfobulbales;f__;g__;s__
LMSG_G000009041.1	no	1354_1	Lead-Zinc	84.42	3.17	68.55	2	0	0	15	Medium quality	36508	2301828	62.50	100	d__Bacteria;p__Desulfobacterota;c__Desulfobulbia;o__Desulfobulbales;f__Desulfurivibrionaceae;g__;s__
LMSG_G000009042.1	no	1383_1	Antimony	85.72	4.30	64.23	1	0	1	14	Medium quality	6155	2187494	63.80	437	d__Bacteria;p__Desulfobacterota;c__Desulfobulbia;o__Desulfobulbales;f__Desulfurivibrionaceae;g__;s__
LMSG_G000009043.1	no	1383_2	Pyrite-Copper	75.62	2.41	63.55	1	0	1	18	Medium quality	6588	1973501	64.10	405	d__Bacteria;p__Desulfobacterota;c__Desulfobulbia;o__Desulfobulbales;f__Desulfurivibrionaceae;g__;s__
LMSG_G000009044.1	no	1383_2	Pyrite-Copper	79.82	3.51	62.28	0	0	0	14	Medium quality	5549	2015576	64.90	444	d__Bacteria;p__Desulfobacterota;c__Desulfobulbia;o__Desulfobulbales;f__Desulfurivibrionaceae;g__;s__
LMSG_G000009045.1	no	1383_2	Polymetallic	65.04	2.15	54.30	0	0	0	12	Medium quality	3280	1593810	64.60	508	d__Bacteria;p__Desulfobacterota;c__Desulfobulbia;o__Desulfobulbales;f__Desulfurivibrionaceae;g__;s__
LMSG_G000009046.1	no	1383_2	Pyrite-Copper	81.99	4.23	60.83	0	0	0	13	Medium quality	4331	2063198	64.70	543	d__Bacteria;p__Desulfobacterota;c__Desulfobulbia;o__Desulfobulbales;f__Desulfurivibrionaceae;g__;s__
LMSG_G000009047.1	no	1352_1	Pyrite-Copper	75.62	3.57	57.77	0	0	0	14	Medium quality	3100	2074219	58.40	762	d__Bacteria;p__Desulfobacterota;c__Desulfobulbia;o__Desulfobulbales;f__Desulfurivibrionaceae;g__UBA2262;s__
LMSG_G000009048.1	no	1636_1	Antimony	97.09	1.29	90.64	2	3	7	20	High quality	55456	3978233	46.20	192	d__Bacteria;p__Desulfobacterota;c__Desulfomonilia;o__Desulfomonilales;f__Desulfomonilaceae;g__;s__
LMSG_G000009049.1	no	1636_1	Antimony	97.09	0.00	97.09	1	5	1	19	High quality	66389	4006083	46.20	193	d__Bacteria;p__Desulfobacterota;c__Desulfomonilia;o__Desulfomonilales;f__Desulfomonilaceae;g__;s__

LMSG_G000009050.1	no	1636_1	Antimony	93.54	0.97	88.71	2	2	3	20	High quality	66275	4000207	46.40	212	d__Bacteria;p__Desulfobacterota;c__Desulfomonilia;o__Desulfomonilales;f__Desulfomonilaceae;g__;s__
LMSG_G000009051.1	no	1636_1	Antimony	90.00	0.65	86.78	2	2	5	19	High quality	25901	3475042	46.20	214	d__Bacteria;p__Desulfobacterota;c__Desulfomonilia;o__Desulfomonilales;f__Desulfomonilaceae;g__;s__
LMSG_G000009052.1	no	1636_1	Polymetallic	92.43	0.00	92.43	1	1	1	18	High quality	19700	3404768	46.10	259	d__Bacteria;p__Desulfobacterota;c__Desulfomonilia;o__Desulfomonilales;f__Desulfomonilaceae;g__;s__
LMSG_G000009053.1	no	1636_1	Polymetallic	97.09	0.00	97.09	4	2	1	19	High quality	58466	3771410	46.10	155	d__Bacteria;p__Desulfobacterota;c__Desulfomonilia;o__Desulfomonilales;f__Desulfomonilaceae;g__;s__
LMSG_G000009054.1	no	1636_1	Pyrite-Copper	96.45	0.00	96.45	1	1	2	19	High quality	40081	4125345	46.30	173	d__Bacteria;p__Desulfobacterota;c__Desulfomonilia;o__Desulfomonilales;f__Desulfomonilaceae;g__;s__
LMSG_G000009055.1	no	1636_1	Polymetallic	95.16	1.29	88.71	0	1	0	19	Medium quality	21713	3917405	46.10	357	d__Bacteria;p__Desulfobacterota;c__Desulfomonilia;o__Desulfomonilales;f__Desulfomonilaceae;g__;s__
LMSG_G000009056.1	no	1637_1	Pyrite-Copper	61.57	0.07	61.23	0	0	0	8	Medium quality	4565	2138321	45.30	482	d__Bacteria;p__Desulfobacterota;c__Desulfomonilia;o__Desulfomonilales;f__Desulfomonilaceae;g__;s__
LMSG_G000009057.1	no	1388_1	Antimony	93.54	1.29	87.09	1	0	1	17	Medium quality	71196	3481409	62.10	77	d__Bacteria;p__Desulfobacterota;c__Desulfuromonadia;o__Geobacterales;f__;g__;s__
LMSG_G000009058.1	no	1388_1	Antimony	95.42	1.94	85.75	1	0	1	19	Medium quality	71328	3489349	62.10	83	d__Bacteria;p__Desulfobacterota;c__Desulfuromonadia;o__Geobacterales;f__;g__;s__
LMSG_G000009059.1	no	1388_1	Antimony	75.78	1.75	67.01	1	0	1	14	Medium quality	25331	3035430	63.00	190	d__Bacteria;p__Desulfobacterota;c__Desulfuromonadia;o__Geobacterales;f__Geobacteraceae;g__;s__
LMSG_G000009060.1	no	1341_1	Antimony	52.63	0.00	52.63	0	0	0	10	Medium quality	120014	964862	51.80	10	d__Bacteria;p__Desulfobacterota;c__Desulfuromonadia;o__Geobacterales;f__Pseudopelobacteraceae;g__Pseudopelobacter;s__
LMSG_G000009061.1	no	1341_1	Antimony	77.41	0.65	74.19	1	1	0	16	Medium quality	344655	3000930	51.40	20	d__Bacteria;p__Desulfobacterota;c__Desulfuromonadia;o__Geobacterales;f__Pseudopelobacteraceae;g__Pseudopelobacter;s__
LMSG_G000009062.1	no	1341_1	Antimony	100.00	1.68	91.59	2	0	0	19	Medium quality	193130	3654244	51.20	40	d__Bacteria;p__Desulfobacterota;c__Desulfuromonadia;o__Geobacterales;f__Pseudopelobacteraceae;g__Pseudopelobacter;s__
LMSG_G000009063.1	no	1342_1	Lead-Zinc	91.87	1.29	85.42	2	1	1	17	Medium quality	22894	3296052	56.30	210	d__Bacteria;p__Desulfobacterota;c__Desulfuromonadia;o__Geobacterales;f__Pseudopelobacteraceae;g__Pseudopelobacter;s__
LMSG_G000009064.1	no	1343_1	Polymetallic	81.64	2.58	68.74	0	0	0	14	Medium quality	6066	2613392	60.70	498	d__Bacteria;p__Desulfobacterota;c__Desulfuromonadia;o__Geobacterales;f__Pseudopelobacteraceae;g__Pseudopelobacter;s__
LMSG_G000009065.1	no	1343_1	Polymetallic	73.68	1.75	64.91	0	1	0	17	Medium quality	7159	3118857	60.50	561	d__Bacteria;p__Desulfobacterota;c__Desulfuromonadia;o__Geobacterales;f__Pseudopelobacteraceae;g__Pseudopelobacter;s__
LMSG_G000009066.1	no	1343_1	Polymetallic	84.66	2.76	70.89	1	1	1	17	Medium quality	10075	3013853	60.70	541	d__Bacteria;p__Desulfobacterota;c__Desulfuromonadia;o__Geobacterales;f__Pseudopelobacteraceae;g__Pseudopelobacter;s__
LMSG_G000009067.1	no	1343_1	Polymetallic	70.71	0.32	69.10	0	0	0	17	Medium quality	6155	2286909	60.20	409	d__Bacteria;p__Desulfobacterota;c__Desulfuromonadia;o__Geobacterales;f__Pseudopelobacteraceae;g__Pseudopelobacter;s__
LMSG_G000009068.1	no	1343_1	Antimony	70.17	2.63	57.02	0	0	0	14	Medium quality	7399	2951573	60.50	716	d__Bacteria;p__Desulfobacterota;c__Desulfuromonadia;o__Geobacterales;f__Pseudopelobacteraceae;g__Pseudopelobacter;s__
LMSG_G000009069.1	no	1344_1	Antimony	90.69	4.19	69.73	1	1	0	17	Medium quality	22077	3335386	56.30	234	d__Bacteria;p__Desulfobacterota;c__Desulfuromonadia;o__Geobacterales;f__Pseudopelobacteraceae;g__Pseudopelobacter;s__
LMSG_G000009070.1	no	1345_1	Antimony	84.21	1.75	75.44	1	0	0	16	Medium quality	81666	4007064	55.00	341	d__Bacteria;p__Desulfobacterota;c__Desulfuromonadia;o__Geobacterales;f__Pseudopelobacteraceae;g__Pseudopelobacter;s__
LMSG_G000009071.1	no	1345_1	Antimony	69.12	0.00	69.12	2	0	0	15	Medium quality	83812	2605275	54.70	55	d__Bacteria;p__Desulfobacterota;c__Desulfuromonadia;o__Geobacterales;f__Pseudopelobacteraceae;g__Pseudopelobacter;s__
LMSG_G000009072.1	no	1385_1	Antimony	89.03	3.23	72.91	2	0	0	17	Medium quality	46609	2359060	60.90	81	d__Bacteria;p__Desulfobacterota;c__Desulfuromonadia;o__Geobacterales;f__Pseudopelobacteraceae;g__Trichlorobacter;s__
LMSG_G000009073.1	no	1070_1	Polymetallic	71.88	0.00	71.88	1	1	1	16	Medium quality	43110	2365619	62.90	117	d__Bacteria;p__Desulfobacterota;c__MBNT15;o__MBNT15;f__MBNT15;g__;s__
LMSG_G000009074.1	no	1070_1	Polymetallic	83.54	0.89	79.11	1	1	1	16	Medium quality	11490	2662609	63.40	291	d__Bacteria;p__Desulfobacterota;c__MBNT15;o__MBNT15;f__MBNT15;g__;s__
LMSG_G000009075.1	no	1071_1	Polymetallic	71.51	2.94	56.81	0	0	0	14	Medium quality	10594	1903508	65.30	205	d__Bacteria;p__Desulfobacterota;c__MBNT15;o__MBNT15;f__MBNT15;g__CG2-30-66-27;s__
LMSG_G000009076.1	no	1071_1	Polymetallic	66.45	1.05	61.20	0	1	0	15	Medium quality	7716	1503251	66.30	255	d__Bacteria;p__Desulfobacterota;c__MBNT15;o__MBNT15;f__MBNT15;g__CG2-30-66-27;s__
LMSG_G000009077.1	no	1071_1	Polymetallic	86.97	4.44	64.77	0	0	0	16	Medium quality	26072	2481183	65.80	292	d__Bacteria;p__Desulfobacterota;c__MBNT15;o__MBNT15;f__MBNT15;g__CG2-30-66-27;s__CG2-30-66-27 sp002279275
LMSG_G000009078.1	no	1071_1	Pyrite-Copper	88.23	3.78	69.33	4	2	3	16	Medium quality	33754	2585830	65.90	247	d__Bacteria;p__Desulfobacterota;c__MBNT15;o__MBNT15;f__MBNT15;g__CG2-30-66-27;s__CG2-30-66-27 sp002279275
LMSG_G000009079.1	no	1071_1	Nickel-Copper	60.82	1.14	55.11	0	1	0	7	Medium quality	1821	1109910	66.80	642	d__Bacteria;p__Desulfobacterota;c__MBNT15;o__MBNT15;f__MBNT15;g__CG2-30-66-27;s__CG2-30-66-27 sp002279275
LMSG_G000009080.1	no	1071_1	Lead-Zinc	73.40	2.52	60.80	0	0	0	14	Medium quality	3505	1796944	66.10	557	d__Bacteria;p__Desulfobacterota;c__MBNT15;o__MBNT15;f__MBNT15;g__CG2-30-66-27;s__CG2-30-66-27 sp002279275
LMSG_G000009081.1	no	463_1	Polymetallic	67.23	3.37	50.38	0	0	0	9	Medium quality	3815	2298982	57.70	676	d__Bacteria;p__Desulfobacterota;c__Syntrophia;o__Syntrophales;f__UBA5619;g__UBA5619;s__
LMSG_G000009082.1	no	502_1	Antimony	91.98	2.12	81.39	0	0	0	17	Medium quality	9563	3762092	55.90	484	d__Bacteria;p__Desulfobacterota;c__Syntrophobacteria;o__Syntrophobacterales;f__Syntrophobacteraceae;g__SbD1;s__
LMSG_G000009083.1	no	503_1	Copper	99.83	3.41	82.78	1	4	5	20	High quality	90195	5406443	54.20	163	d__Bacteria;p__Desulfobacterota;c__Syntrophobacteria;o__Syntrophobacterales;f__Syntrophobacteraceae;g__SbD1;s__
LMSG_G000009084.1	no	503_1	Lead-Zinc	96.26	2.58	83.36	1	1	1	18	High quality	13728	4777533	54.50	462	d__Bacteria;p__Desulfobacterota;c__Syntrophobacteria;o__Syntrophobacterales;f__Syntrophobacteraceae;g__SbD1;s__
LMSG_G000009085.1	no	503_1	Lead-Zinc	99.83	3.09	84.40	1	3	5	20	High quality	99512	5353137	54.20	169	d__Bacteria;p__Desulfobacterota;c__Syntrophobacteria;o__Syntrophobacterales;f__Syntrophobacteraceae;g__SbD1;s__
LMSG_G000009086.1	no	503_1	Pyrite-Copper	99.19	2.42	87.10	2	1	1	18	High quality	12230	4654516	54.50	532	d__Bacteria;p__Desulfobacterota;c__Syntrophobacteria;o__Syntrophobacterales;f__Syntrophobacteraceae;g__SbD1;s__
LMSG_G000009087.1	no	503_1	Magnetite	97.97	2.74	84.27	1	1	1	20	High quality	42504	5272910	54.20	219	d__Bacteria;p__Desulfobacterota;c__Syntrophobacteria;o__Syntrophobacterales;f__Syntrophobacteraceae;g__SbD1;s__
LMSG_G000009088.1	no	503_1	Copper	99.83	2.13	89.17	1	0	0	19	Medium quality	47133	5051197	54.40	193	d__Bacteria;p__Desulfobacterota;c__Syntrophobacteria;o__Syntrophobacterales;f__Syntrophobacteraceae;g__SbD1;s__
LMSG_G000009089.1	no	503_1	Polymetallic	71.95	2.97	57.08	1	0	0	14	Medium quality	2321	3470330	54.40	1622	d__Bacteria;p__Desulfobacterota;c__Syntrophobacteria;o__Syntrophobacterales;f__Syntrophobacteraceae;g__SbD1;s__
LMSG_G000009090.1	no	503_1	Polymetallic	99.83	2.10	89.35	1	0	0	19	Medium quality	57900	5282066	54.30	133	d__Bacteria;p__Desulfobacterota;c__Syntrophobacteria;o__Syntrophobacterales;f__Syntrophobacteraceae;g__SbD1;s__
LMSG_G000009091.1	no	503_1	Pyrite-Copper	80.59	3.89	61.12	0	0	0	13	Medium quality	5247	3397453	54.50	795	d__Bacteria;p__Desulfobacterota;c__Syntrophobacteria;o__Syntrophobacterales;f__Syntrophobacteraceae;g__SbD1;s__
LMSG_G000009092.1	no	503_1	Pyrite-Copper	76.58	3.39	59.65	0	0	0	17	Medium quality	5295	3541625	54.60	730	d__Bacteria;p__Desulfobacterota;c__Syntrophobacteria;o__Syntrophobacterales;f__Syntrophobacteraceae;g__SbD1;s__
LMSG_G000009093.1	no	503_1	Lead-Zinc	98.22	2.12	87.63	2	0	0	19	Medium quality	35627	4812226	54.40	223	d__Bacteria;p__Desulfobacterota;c__Syntrophobacteria;o__Syntrophobacterales;f__Syntrophobacteraceae;g__SbD1;s__
LMSG_G000009094.1	no	503_1	Magnetite	94.67	2.12	84.08	1	0	1	18	Medium quality	54892	5490555	54.10	281	d__Bacteria;p__Desulfobacterota;c__Syntrophobacteria;o__Syntrophobacterales;f__Syntrophobacteraceae;g__SbD1;s__
LMSG_G000009095.1	no	503_1	Magnetite	96.61	2.10	86.13	0	0	0	19	Medium quality	61876	5408701	54.20	251	d__Bacteria;p__Desulfobacterota;c__Syntrophobacteria;o__Syntrophobacterales;f__Syntrophobacteraceae;g__SbD1;s__
LMSG_G000009096.1	no	503_1	Magnetite	76.45	1.94	66.78	1	0	1	16	Medium quality	14243	4317901	54.10	497	d__Bacteria;p__Desulfobacterota;c__Syntrophobacteria;o__Syntrophobacterales;f__Syntrophobacteraceae;g__SbD1;s__
LMSG_G000009097.1	no	503_1	Pyrite	95.20	3.06	79.88	0	0	0	17	Medium quality	10530	4575761	54.40	556	d__Bacteria;p__Desulfobacterota;c__Syntrophobacteria;o__Syntrophobacterales;f__Syntrophobacteraceae;g__SbD1;s__
LMSG_G000009098.1	no	1552_1	Antimony	67.55	2.10	57.05	0	0	0	11	Medium quality	4865	2380926	63.30	583	d__Bacteria;p__Desulfobacterota_B;c__Binatia;o__Binatales;f__Binataceae;g__;s__
LMSG_G000009099.1	no	1553_1	Copper	87.78	4.78	63.89	0	0	0	18	Medium quality	5385	2489185	65.70	545	d__Bacteria;p__Desulfobacterota_B;c__Binatia;o__Binatales;f__Binataceae;g__;s__
LMSG_G000009100.1	no	1554_1	Antimony	60.28	0.00	60.28	0	0	0	16	Medium quality	39490	2292127	65.20	75	d__Bacteria;p__Desulfobacterota_B;c__Binatia;o__Binatales;f__Binataceae;g__;s__
LMSG_G000009101.1	no	1554_1	Antimony	91.59	2.61	78.52	0	1	0	18	Medium quality	35531	3575854	65.40	140	d__Bacteria;p__Desulfobacterota_B;c__Binatia;o__Binatales;f__Binataceae;g__;s__
LMSG_G000009102.1	no	1555_1	Lead-Zinc	88.46	0.89	84.00	1	1	1	18	Medium quality	11479	3416107	63.80	405	d__Bacteria;p__Desulfobacterota_B;c__Binatia;o__Binatales;f__Binataceae;g__;s__
LMSG_G000009103.1	no	1555_1	Lead-Zinc	89.91	0.00	89.91	1	0	1	17	Medium quality	89182	3350402	63.70	160	d__Bacteria;p__Desulfobacterota_B;c__Binatia;o__Binatales;f__Binataceae;g__;s__
LMSG_G000009104.1	no	1555_1	Copper	94.95	1.54	87.25	1	1	1	20	High quality	21791	3282971	63.80	230	d__Bacteria;p__Desulfobacterota_B;c__Binatia;o__Binatales;f__Binataceae;g__;s__
LMSG_G000009105.1	no	1558_1	Lead-Zinc	90.38	2.52	77.78	1	1	1	18	High quality	31200	3695733	61.50	242	d__Bacteria;p__Desulfobacterota_B;c__Binatia;o__Binatales;f__Binataceae;g__;s__
LMSG_G000009106.1	no	1558_1	Copper	80.81	3.36	64.01	1	1	1	17	Medium quality	16017	3520069	61.40	285	d__Bacteria;p__Desulfobacterota_B;c__Binatia;o__Binatales;f__Binataceae;g__;s__
LMSG_G000009107.1	no	1559_1	Copper	77.87	2.10	67.37	1	1	1	19	Medium quality	12467	3025982	65.40	291	d__Bacteria;p__Desulfobacterota_B;c__Binatia;o__Binatales;f__Binataceae;g__;s__
LMSG_G000009108.1	no	1560_1	Copper	86.55	1.79	77.63	0	1	0	15	Medium quality	22640	3441752	63.30	251	d__Bacteria;p__Desulfobacterota_B;c__Binatia;o__Binatales;f__Binataceae;g__;s__
LMSG_G000009109.1	no	1560_1	Lead-Zinc	96.63	4.20	75.63	0	1	0	19	Medium quality	35200	4293182	62.90	233	d__Bacteria;p__Desulfobacterota_B;c__Binatia;o__Binatales;f__Binataceae;g__;s__

LMSG_G000009110.1	no	1560_1	Lead-Zinc	96.21	3.41	79.17	0	1	0	19	Medium quality	66394	4666205	62.70	187	d__Bacteria;p__Desulfobacterota_B;c__Binatia;o__Binatales;f__Binataceae;g__;s__
LMSG_G000009111.1	no	1560_1	Lead-Zinc	90.75	4.20	69.75	0	1	0	16	Medium quality	28046	3917429	63.20	208	d__Bacteria;p__Desulfobacterota_B;c__Binatia;o__Binatales;f__Binataceae;g__;s__
LMSG_G000009112.1	no	1560_1	Pyrite-Copper	94.53	4.20	73.53	0	1	0	16	Medium quality	21449	3950213	63.20	288	d__Bacteria;p__Desulfobacterota_B;c__Binatia;o__Binatales;f__Binataceae;g__;s__
LMSG_G000009113.1	no	1560_1	Lead-Zinc	93.27	3.36	76.47	0	1	0	18	Medium quality	35172	3655933	63.30	151	d__Bacteria;p__Desulfobacterota_B;c__Binatia;o__Binatales;f__Binataceae;g__;s__
LMSG_G000009114.1	no	1566_1	Copper	93.90	3.53	76.26	1	1	1	20	High quality	78136	2969354	66.10	66	d__Bacteria;p__Desulfobacterota_B;c__Binatia;o__Binatales;f__Binataceae;g__;s__
LMSG_G000009115.1	no	1566_1	Copper	95.58	1.91	86.01	1	1	1	20	High quality	93467	3422610	66.10	62	d__Bacteria;p__Desulfobacterota_B;c__Binatia;o__Binatales;f__Binataceae;g__;s__
LMSG_G000009116.1	no	1566_1	Copper	95.58	0.84	91.38	1	1	1	19	High quality	79407	3337964	66.10	69	d__Bacteria;p__Desulfobacterota_B;c__Binatia;o__Binatales;f__Binataceae;g__;s__
LMSG_G000009117.1	no	1566_1	Copper	92.57	0.84	88.37	1	1	1	18	High quality	43358	3341339	66.10	145	d__Bacteria;p__Desulfobacterota_B;c__Binatia;o__Binatales;f__Binataceae;g__;s__
LMSG_G000009118.1	no	1566_1	Copper	95.79	1.75	87.04	1	1	1	19	High quality	65047	3163093	66.00	97	d__Bacteria;p__Desulfobacterota_B;c__Binatia;o__Binatales;f__Binataceae;g__;s__
LMSG_G000009119.1	no	1566_1	Copper	93.90	2.52	81.30	1	1	2	20	High quality	95805	3388636	66.00	102	d__Bacteria;p__Desulfobacterota_B;c__Binatia;o__Binatales;f__Binataceae;g__;s__
LMSG_G000009120.1	no	1566_1	Lead-Zinc	95.58	2.10	85.08	1	1	1	19	High quality	30030	2986013	66.30	151	d__Bacteria;p__Desulfobacterota_B;c__Binatia;o__Binatales;f__Binataceae;g__;s__
LMSG_G000009121.1	no	1566_1	Copper	95.58	0.84	91.38	1	1	1	17	Medium quality	32783	2916816	66.30	118	d__Bacteria;p__Desulfobacterota_B;c__Binatia;o__Binatales;f__Binataceae;g__;s__
LMSG_G000009122.1	no	1566_1	Pyrite	91.10	0.84	86.90	0	1	0	18	Medium quality	12533	2963649	66.30	322	d__Bacteria;p__Desulfobacterota_B;c__Binatia;o__Binatales;f__Binataceae;g__;s__
LMSG_G000009123.1	no	1566_1	Lead-Zinc	94.74	0.84	90.54	0	1	0	19	Medium quality	31437	2970302	66.30	137	d__Bacteria;p__Desulfobacterota_B;c__Binatia;o__Binatales;f__Binataceae;g__;s__
LMSG_G000009124.1	no	1566_1	Lead-Zinc	93.25	0.84	89.05	0	1	0	16	Medium quality	34557	2910560	66.30	119	d__Bacteria;p__Desulfobacterota_B;c__Binatia;o__Binatales;f__Binataceae;g__;s__
LMSG_G000009125.1	no	1566_1	Pyrite-Copper	79.48	0.84	75.28	0	1	0	16	Medium quality	5403	2383007	66.50	568	d__Bacteria;p__Desulfobacterota_B;c__Binatia;o__Binatales;f__Binataceae;g__;s__
LMSG_G000009126.1	no	1566_1	Lead-Zinc	93.90	0.84	89.70	0	1	1	20	Medium quality	35217	3065535	66.30	121	d__Bacteria;p__Desulfobacterota_B;c__Binatia;o__Binatales;f__Binataceae;g__;s__
LMSG_G000009127.1	no	1566_1	Lead-Zinc	95.58	0.84	91.38	1	1	0	19	Medium quality	31738	2962840	66.30	136	d__Bacteria;p__Desulfobacterota_B;c__Binatia;o__Binatales;f__Binataceae;g__;s__
LMSG_G000009128.1	no	1567_1	Lead-Zinc	93.85	1.68	85.45	1	1	1	19	High quality	138689	3787903	62.70	62	d__Bacteria;p__Desulfobacterota_B;c__Binatia;o__Binatales;f__Binataceae;g__;s__
LMSG_G000009129.1	no	1567_1	Lead-Zinc	93.85	1.68	85.45	1	1	1	19	High quality	119995	3914282	62.80	75	d__Bacteria;p__Desulfobacterota_B;c__Binatia;o__Binatales;f__Binataceae;g__;s__
LMSG_G000009130.1	no	1567_1	Pyrite-Copper	93.85	1.68	85.45	1	1	1	20	High quality	161506	3861997	62.70	52	d__Bacteria;p__Desulfobacterota_B;c__Binatia;o__Binatales;f__Binataceae;g__;s__
LMSG_G000009131.1	no	1567_1	Lead-Zinc	93.85	1.68	85.45	1	0	1	19	Medium quality	138689	3898690	62.70	63	d__Bacteria;p__Desulfobacterota_B;c__Binatia;o__Binatales;f__Binataceae;g__;s__
LMSG_G000009132.1	no	1567_1	Lead-Zinc	93.85	1.68	85.45	1	0	1	19	Medium quality	163884	3855704	62.70	52	d__Bacteria;p__Desulfobacterota_B;c__Binatia;o__Binatales;f__Binataceae;g__;s__
LMSG_G000009133.1	no	1568_1	Lead-Zinc	94.06	4.20	73.06	1	1	1	19	High quality	149385	3611467	60.80	58	d__Bacteria;p__Desulfobacterota_B;c__Binatia;o__Binatales;f__Binataceae;g__;s__
LMSG_G000009134.1	no	1568_1	Lead-Zinc	94.90	4.20	73.90	1	1	1	19	High quality	149359	3661414	60.80	59	d__Bacteria;p__Desulfobacterota_B;c__Binatia;o__Binatales;f__Binataceae;g__;s__
LMSG_G000009135.1	no	1568_1	Lead-Zinc	89.02	3.36	72.22	0	1	0	19	Medium quality	195792	3551292	60.70	94	d__Bacteria;p__Desulfobacterota_B;c__Binatia;o__Binatales;f__Binataceae;g__;s__
LMSG_G000009136.1	no	356_1	Copper	54.35	0.46	52.04	0	0	0	9	Medium quality	2173	1376385	63.10	669	d__Bacteria;p__Dormibacterota;c__Dormibacteria;o__Dormibacterales;f__;g__;s__
LMSG_G000009137.1	no	1680_1	Magnetite	82.87	1.08	77.47	0	1	0	19	Medium quality	15483	1914338	57.30	170	d__Bacteria;p__Dormibacterota;c__Dormibacteria;o__UBA8260;f__;g__;s__
LMSG_G000009138.1	no	620_1	Magnetite	61.18	0.00	61.18	0	0	0	12	Medium quality	2937	1492710	58.70	547	d__Bacteria;p__Dormibacterota;c__Dormibacteria;o__UBA8260;f__;g__;s__
LMSG_G000009139.1	no	620_1	Magnetite	71.34	0.00	71.34	0	0	0	15	Medium quality	7887	1700312	58.70	342	d__Bacteria;p__Dormibacterota;c__Dormibacteria;o__UBA8260;f__;g__;s__
LMSG_G000009140.1	no	620_1	Magnetite	85.03	0.31	83.49	1	9	6	20	Medium quality	23662	2054290	58.60	245	d__Bacteria;p__Dormibacterota;c__Dormibacteria;o__UBA8260;f__;g__;s__
LMSG_G000009141.1	no	621_1	Lead-Zinc	95.37	2.31	83.80	1	1	0	20	Medium quality	27687	2938696	66.80	181	d__Bacteria;p__Dormibacterota;c__Dormibacteria;o__UBA8260;f__Bog-877;g__;s__
LMSG_G000009142.1	no	623_1	Copper	91.20	2.88	76.80	1	1	1	19	High quality	23919	2584816	68.70	163	d__Bacteria;p__Dormibacterota;c__Dormibacteria;o__UBA8260;f__Bog-877;g__;s__
LMSG_G000009143.1	no	623_1	Copper	92.04	2.31	80.47	1	1	10	20	High quality	10642	2682105	68.80	422	d__Bacteria;p__Dormibacterota;c__Dormibacteria;o__UBA8260;f__Bog-877;g__;s__
LMSG_G000009144.1	no	623_1	Copper	89.81	2.62	76.70	0	0	0	18	Medium quality	29199	2644374	68.90	150	d__Bacteria;p__Dormibacterota;c__Dormibacteria;o__UBA8260;f__Bog-877;g__;s__
LMSG_G000009145.1	no	623_1	Copper	89.35	1.85	80.10	1	0	0	19	Medium quality	23420	2642442	68.90	187	d__Bacteria;p__Dormibacterota;c__Dormibacteria;o__UBA8260;f__Bog-877;g__;s__
LMSG_G000009146.1	no	623_1	Copper	59.72	0.83	55.56	0	1	0	14	Medium quality	14475	1268010	69.50	116	d__Bacteria;p__Dormibacterota;c__Dormibacteria;o__UBA8260;f__Bog-877;g__;s__
LMSG_G000009147.1	no	623_1	Lead-Zinc	82.87	4.63	59.73	1	2	1	19	Medium quality	20550	2812277	69.10	193	d__Bacteria;p__Dormibacterota;c__Dormibacteria;o__UBA8260;f__Bog-877;g__;s__
LMSG_G000009148.1	no	623_1	Lead-Zinc	72.68	0.93	68.06	1	1	1	14	Medium quality	16818	2065445	69.20	198	d__Bacteria;p__Dormibacterota;c__Dormibacteria;o__UBA8260;f__Bog-877;g__;s__
LMSG_G000009149.1	no	623_1	Lead-Zinc	65.27	0.93	60.65	1	0	1	13	Medium quality	8149	1588702	69.30	219	d__Bacteria;p__Dormibacterota;c__Dormibacteria;o__UBA8260;f__Bog-877;g__;s__
LMSG_G000009150.1	no	623_1	Pyrite	56.11	1.13	50.46	0	0	0	13	Medium quality	2343	1112206	69.10	468	d__Bacteria;p__Dormibacterota;c__Dormibacteria;o__UBA8260;f__Bog-877;g__;s__
LMSG_G000009151.1	no	624_1	Polymetallic	94.44	1.23	88.27	1	1	1	19	High quality	25037	2610729	67.20	164	d__Bacteria;p__Dormibacterota;c__Dormibacteria;o__UBA8260;f__Bog-877;g__;s__
LMSG_G000009152.1	no	624_1	Polymetallic	94.44	1.23	88.27	1	1	1	20	High quality	23664	2699349	67.20	178	d__Bacteria;p__Dormibacterota;c__Dormibacteria;o__UBA8260;f__Bog-877;g__;s__
LMSG_G000009153.1	no	624_1	Pyrite-Copper	91.82	2.16	81.02	1	1	1	17	Medium quality	21617	2728750	67.20	179	d__Bacteria;p__Dormibacterota;c__Dormibacteria;o__UBA8260;f__Bog-877;g__;s__
LMSG_G000009154.1	no	624_1	Copper	73.70	0.93	69.08	1	0	0	18	Medium quality	18270	2175302	66.70	198	d__Bacteria;p__Dormibacterota;c__Dormibacteria;o__UBA8260;f__Bog-877;g__;s__
LMSG_G000009155.1	no	624_1	Lead-Zinc	60.59	0.00	60.59	0	0	0	15	Medium quality	10735	1878295	67.20	225	d__Bacteria;p__Dormibacterota;c__Dormibacteria;o__UBA8260;f__Bog-877;g__;s__
LMSG_G000009156.1	no	624_1	Lead-Zinc	69.38	2.31	57.81	1	0	1	16	Medium quality	4396	1964173	67.10	568	d__Bacteria;p__Dormibacterota;c__Dormibacteria;o__UBA8260;f__Bog-877;g__;s__
LMSG_G000009157.1	no	624_1	Polymetallic	94.44	1.23	88.27	0	1	1	20	Medium quality	24024	2760081	67.10	169	d__Bacteria;p__Dormibacterota;c__Dormibacteria;o__UBA8260;f__Bog-877;g__;s__
LMSG_G000009158.1	no	624_1	Polymetallic	85.18	1.23	79.01	1	1	1	19	Medium quality	24046	2697140	67.00	171	d__Bacteria;p__Dormibacterota;c__Dormibacteria;o__UBA8260;f__Bog-877;g__;s__
LMSG_G000009159.1	no	624_1	Copper	80.08	3.70	61.57	1	0	0	15	Medium quality	7033	2170577	67.70	404	d__Bacteria;p__Dormibacterota;c__Dormibacteria;o__UBA8260;f__Bog-877;g__;s__
LMSG_G000009160.1	no	624_1	Copper	79.16	4.32	57.56	1	0	0	14	Medium quality	25907	1887428	67.80	105	d__Bacteria;p__Dormibacterota;c__Dormibacteria;o__UBA8260;f__Bog-877;g__;s__
LMSG_G000009161.1	no	624_1	Polymetallic	86.57	2.16	75.77	1	0	0	19	Medium quality	23817	2313571	67.30	168	d__Bacteria;p__Dormibacterota;c__Dormibacteria;o__UBA8260;f__Bog-877;g__;s__
LMSG_G000009162.1	no	624_1	Magnetite	87.80	2.16	77.00	1	0	1	17	Medium quality	21965	2087813	67.30	128	d__Bacteria;p__Dormibacterota;c__Dormibacteria;o__UBA8260;f__Bog-877;g__;s__
LMSG_G000009163.1	no	624_1	Pyrite-Copper	95.37	2.16	84.57	1	0	1	19	Medium quality	22088	2617128	67.20	188	d__Bacteria;p__Dormibacterota;c__Dormibacteria;o__UBA8260;f__Bog-877;g__;s__
LMSG_G000009164.1	no	624_1	Pyrite-Copper	93.98	3.09	78.55	1	0	1	19	Medium quality	21544	2596322	67.20	181	d__Bacteria;p__Dormibacterota;c__Dormibacteria;o__UBA8260;f__Bog-877;g__;s__
LMSG_G000009165.1	no	624_1	Magnetite	61.27	1.95	51.50	0	0	0	15	Medium quality	3742	1704711	66.80	517	d__Bacteria;p__Dormibacterota;c__Dormibacteria;o__UBA8260;f__Bog-877;g__;s__
LMSG_G000009166.1	no	627_1	Coal	94.44	0.00	94.44	1	1	1	19	High quality	25811	3392458	64.60	302	d__Bacteria;p__Dormibacterota;c__Dormibacteria;o__UBA8260;f__Bog-877;g__;s__
LMSG_G000009167.1	no	627_1	Coal	94.90	0.00	94.90	1	1	1	20	High quality	44857	3183131	64.60	277	d__Bacteria;p__Dormibacterota;c__Dormibacteria;o__UBA8260;f__Bog-877;g__;s__
LMSG_G000009168.1	no	627_1	Coal	89.94	0.08	89.52	1	0	1	19	Medium quality	10164	2642946	64.70	365	d__Bacteria;p__Dormibacterota;c__Dormibacteria;o__UBA8260;f__Bog-877;g__;s__
LMSG_G000009169.1	no	628_1	Copper	77.13	1.39	70.19	0	0	0	15	Medium quality	3937	1762844	68.40	541	d__Bacteria;p__Dormibacterota;c__Dormibacteria;o__UBA8260;f__Bog-877;g__;s__
LMSG_G000009170.1	no	628_1	Copper	83.57	2.59	70.64	1	0	1	19	Medium quality	6314	2365123	68.30	535	d__Bacteria;p__Dormibacterota;c__Dormibacteria;o__UBA8260;f__Bog-877;g__;s__
LMSG_G000009171.1	no	628_1	Polymetallic	68.96	1.72	60.34	1	1	1	15	Medium quality	52232	2377986	68.70	77	d__Bacteria;p__Dormibacterota;c__Dormibacteria;o__UBA8260;f__Bog-877;g__;s__
LMSG_G000009172.1	no	629_1	Coal	95.37	0.46	93.06	1	2	1	20	High quality	40881	2418981	71.00	187	d__Bacteria;p__Dormibacterota;c__Dormibacteria;o__UBA8260;f__Bog-877;g__;s__
LMSG_G000009173.1	no	629_1	Coal	97.68	3.96	77.88	1	0	1	19	Medium quality	23574	2308964	70.90	158	d__Bacteria;p__Dormibacterota;c__Dormibacteria;o__UBA8260;f__Bog-877;g__;s__
LMSG_G000009174.1	no	629_1	Coal	87.66	1.85	78.41	1	1	1	19	Medium quality	21611	2073982	71.10	127	d__Bacteria;p__Dormibacterota;c__Dormibacteria;o__UBA8260;f__Bog-877;g__;s__
LMSG_G000009175.1	no	630_1	Pyrite	82.87	1.39	75.93	1	1	1	14	Medium quality	5925	2065090	70.30	439	d__Bacteria;p__Dormibacterota;c__Dormibacteria;o__UBA8260;f__Bog-877;g__;s__
LMSG_G000009176.1	no	630_2	Lead-Zinc	64.81	0.00	64.81	0	0	0	15	Medium quality	33759	1251813	70.10	94	d__Bacteria;p__Dormibacterota;c__Dormibacteria;o__UBA8260;f__Bog-877;g__;s__
LMSG_G000009177.1	no	630_2	Lead-Zinc	78.05	1.85	68.80	0	0	0	14	Medium quality	23827	1778944	70.30	131	d__Bacteria;p__Dormibacterota;c__Dormibacteria;o__UBA8260;f__Bog-877;g__;s__
LMSG_G000009178.1	no	630_2	Copper	93.05	1.49	85.60	1	1	1	17	Medium quality	37485	2373117	69.90	126	d__Bacteria;p__Dormibacterota;c__Dormibacteria;o__UBA8260;f__Bog-877;g__;s__
LMSG_G000009179.1	no	631_1	Polymetallic	70.88	0.00	70.88	0	0	0	18	Medium quality	3313	1629835	70.70	531	d__Bacteria;p__Dormibacterota;c__Dormibacteria;o__UBA8260;f__Bog-877;g__;s__
LMSG_G000009180.1	no	631_2	Tin-Zinc	94.90	0.00	94.90	1	1	1	20	High quality	42376	2551099	70.50	101	d__Bacteria;p__Dormibacterota;c__Dormibacteria;o__UBA8260;f__Bog-877;g__;s__
LMSG_G000009181.1	no	631_2	Polymetallic	96.75	1.01	91.70	0	0	0	20	Medium quality	14736	2384732	70.80	247	d__Bacteria;p__Dormibacterota;c__Dormibacteria;o__UBA8260;f__Bog-877;g__;s__
LMSG_G000009182.1	no	631_2	Pyrite-Copper	91.39	1.85	82.14	1	0	0	18	Medium quality	54762	2428678	70.70	222	d__Bacteria;p__Dormibacterota;c__Dormibacteria;o__UBA8260;f__Bog-877;g__;s__
LMSG_G000009183.1	no	632_1	Lead-Zinc	93.98	4.17	73.15	2	1	2	18	High quality	29706	2123027	70.50	113	d__Bacteria;p__Dormibacterota;c__Dormibacteria;o__UBA8260;f__Bog-877;g__;s__
LMSG_G000009184.1	no	632_1	Magnetite	57.00	0.21	55.96	0	1	0	13	Medium quality	15750	1232617	70.50	187	d__Bacteria;p__Dormibacterota;c__Dormibacteria;o__UBA8260;f__Bog-877;g__;s__
LMSG_G000009185.1	no	632_1	Coal	58.22	0.00	58.22	1	0	0	17	Medium quality	9240	1216020	70.50	247	d__Bacteria;p__Dormibacterota;c__Dormibacteria;o__UBA8260;f__Bog-877;g__;s__
LMSG_G000009186.1	no	632_1	Polymetallic	53.90	0.00	53.90	1	0	0	13	Medium quality	11521	1803270	69.80	182	d__Bacteria;p__Dormibacterota;c__Dormibacteria;o__UBA8260;f__Bog-877;g__;s__

LMSG_G000009187.1	no	632_2	Magnetite	77.44	0.00	77.44	1	1	1	12	Medium quality	8614	1540936	71.00	233	d__Bacteria;p__Dormibacterota;c__Dormibacteria;o__UBA8260;f__Bog-877;g__;s__
LMSG_G000009188.1	no	632_2	Magnetite	72.80	0.46	70.49	0	1	0	16	Medium quality	7321	1233303	70.50	175	d__Bacteria;p__Dormibacterota;c__Dormibacteria;o__UBA8260;f__Bog-877;g__;s__
LMSG_G000009189.1	no	632_2	Copper	77.22	3.61	59.17	1	0	1	20	Medium quality	31200	2143982	70.60	311	d__Bacteria;p__Dormibacterota;c__Dormibacteria;o__UBA8260;f__Bog-877;g__;s__
LMSG_G000009190.1	no	632_2	Coal	79.78	2.78	65.90	1	1	1	16	Medium quality	8219	1807053	70.90	302	d__Bacteria;p__Dormibacterota;c__Dormibacteria;o__UBA8260;f__Bog-877;g__;s__
LMSG_G000009191.1	no	713_1	Coal	93.05	0.00	93.05	1	1	1	18	High quality	10012	2793226	73.80	509	d__Bacteria;p__Dormibacterota;c__Dormibacteria;o__UBA8260;f__Bog-877;g__;s__
LMSG_G000009192.1	no	713_1	Coal	89.62	1.03	84.48	0	1	0	19	Medium quality	15413	2522302	74.30	214	d__Bacteria;p__Dormibacterota;c__Dormibacteria;o__UBA8260;f__Bog-877;g__;s__
LMSG_G000009193.1	no	720_1	Copper	64.25	1.85	55.00	0	0	0	16	Medium quality	3521	2293156	73.20	709	d__Bacteria;p__Dormibacterota;c__Dormibacteria;o__UBA8260;f__Bog-877;g__;s__
LMSG_G000009194.1	no	720_1	Polymetallic	79.31	0.00	79.31	0	1	0	18	Medium quality	19866	3240527	73.60	302	d__Bacteria;p__Dormibacterota;c__Dormibacteria;o__UBA8260;f__Bog-877;g__;s__
LMSG_G000009195.1	no	636_1	Tin-Zinc	61.47	0.46	59.16	0	0	0	9	Medium quality	3537	1615244	73.00	501	d__Bacteria;p__Dormibacterota;c__Dormibacteria;o__UBA8260;f__UBA8260;g__;s__
LMSG_G000009196.1	no	636_1	Tin-Zinc	56.84	0.00	56.84	0	0	0	13	Medium quality	4438	1633232	73.40	440	d__Bacteria;p__Dormibacterota;c__Dormibacteria;o__UBA8260;f__UBA8260;g__;s__
LMSG_G000009197.1	no	636_1	Magnetite	93.82	0.93	89.20	1	0	1	19	Medium quality	21993	2454917	73.50	177	d__Bacteria;p__Dormibacterota;c__Dormibacteria;o__UBA8260;f__UBA8260;g__;s__
LMSG_G000009198.1	no	636_1	Lead-Zinc	80.23	0.15	79.46	0	0	0	17	Medium quality	7323	1955243	73.30	460	d__Bacteria;p__Dormibacterota;c__Dormibacteria;o__UBA8260;f__UBA8260;g__;s__
LMSG_G000009199.1	no	636_1	Copper	93.36	4.51	70.83	1	0	0	19	Medium quality	13653	2594622	73.30	293	d__Bacteria;p__Dormibacterota;c__Dormibacteria;o__UBA8260;f__UBA8260;g__;s__
LMSG_G000009200.1	no	636_1	Lead-Zinc	56.48	1.23	50.31	0	0	0	16	Medium quality	3356	1579895	73.30	518	d__Bacteria;p__Dormibacterota;c__Dormibacteria;o__UBA8260;f__UBA8260;g__;s__
LMSG_G000009201.1	no	636_1	Lead-Zinc	58.75	0.17	57.91	0	0	0	14	Medium quality	5176	1606576	73.00	422	d__Bacteria;p__Dormibacterota;c__Dormibacteria;o__UBA8260;f__UBA8260;g__;s__
LMSG_G000009202.1	no	636_1	Copper	51.37	0.15	50.60	0	0	0	16	Medium quality	5752	1310395	73.30	260	d__Bacteria;p__Dormibacterota;c__Dormibacteria;o__UBA8260;f__UBA8260;g__;s__
LMSG_G000009203.1	no	1178_1	Lead-Zinc	76.91	3.44	59.73	0	1	0	16	Medium quality	5722	3033716	59.40	721	d__Bacteria;p__Elusimicrobiota;c__Elusimicrobia;o__Elusimicrobiales;f__UBA9959;g__UBA9959;s__
LMSG_G000009204.1	no	1178_1	Lead-Zinc	61.68	1.12	56.07	0	1	0	11	Medium quality	5471	2526548	59.60	546	d__Bacteria;p__Elusimicrobiota;c__Elusimicrobia;o__Elusimicrobiales;f__UBA9959;g__UBA9959;s__
LMSG_G000009205.1	no	1180_1	Pyrite-Copper	92.49	3.37	75.64	1	1	3	19	High quality	132569	2523038	62.80	98	d__Bacteria;p__Elusimicrobiota;c__Elusimicrobia;o__Elusimicrobiales;f__UBA9959;g__UBA9959;s__
LMSG_G000009206.1	no	1183_1	Polymetallic	90.07	3.37	73.22	0	0	0	17	Medium quality	7945	2819045	63.70	480	d__Bacteria;p__Elusimicrobiota;c__Elusimicrobia;o__Elusimicrobiales;f__UBA9959;g__UBA9959;s__
LMSG_G000009207.1	no	1183_1	Polymetallic	84.01	3.44	66.83	0	0	0	16	Medium quality	22092	2482412	63.70	196	d__Bacteria;p__Elusimicrobiota;c__Elusimicrobia;o__Elusimicrobiales;f__UBA9959;g__UBA9959;s__
LMSG_G000009208.1	no	1175_1	Copper	87.57	0.10	87.06	0	0	0	18	Medium quality	8780	1805124	64.50	337	d__Bacteria;p__Elusimicrobiota;c__Elusimicrobia;o__UBA1565;f__;g__;s__
LMSG_G000009209.1	no	1175_1	Copper	92.06	1.53	84.40	0	0	0	16	Medium quality	10890	2002747	64.50	267	d__Bacteria;p__Elusimicrobiota;c__Elusimicrobia;o__UBA1565;f__;g__;s__
LMSG_G000009210.1	no	1175_1	Magnetite	74.65	0.00	74.65	1	1	1	19	Medium quality	5241	1904007	63.30	507	d__Bacteria;p__Elusimicrobiota;c__Elusimicrobia;o__UBA1565;f__;g__;s__
LMSG_G000009211.1	no	1176_1	Lead-Zinc	95.43	1.12	89.82	1	2	5	19	High quality	30730	2196115	63.50	147	d__Bacteria;p__Elusimicrobiota;c__Elusimicrobia;o__UBA1565;f__;g__;s__
LMSG_G000009212.1	no	1176_1	Lead-Zinc	94.31	0.00	94.31	1	1	1	20	High quality	51359	2306762	63.40	106	d__Bacteria;p__Elusimicrobiota;c__Elusimicrobia;o__UBA1565;f__;g__;s__
LMSG_G000009213.1	no	1176_1	Pyrite-Copper	50.55	0.00	50.55	0	0	0	12	Medium quality	2952	1110125	64.10	419	d__Bacteria;p__Elusimicrobiota;c__Elusimicrobia;o__UBA1565;f__;g__;s__
LMSG_G000009214.1	no	515_1	Pyrite	89.92	1.19	83.98	1	1	1	19	Medium quality	82582	1930448	49.50	104	d__Bacteria;p__Elusimicrobiota;c__Elusimicrobia;o__UBA1565;f__;g__;s__
LMSG_G000009215.1	no	516_1	Copper	95.43	0.00	95.43	1	3	3	19	High quality	102714	2048428	49.40	46	d__Bacteria;p__Elusimicrobiota;c__Elusimicrobia;o__UBA1565;f__;g__;s__
LMSG_G000009216.1	no	516_2	Polymetallic	92.06	0.00	92.06	1	2	5	20	High quality	25586	1945998	49.30	155	d__Bacteria;p__Elusimicrobiota;c__Elusimicrobia;o__UBA1565;f__;g__;s__
LMSG_G000009217.1	no	516_2	Polymetallic	91.50	2.25	80.27	1	2	8	20	High quality	42948	2070005	49.40	116	d__Bacteria;p__Elusimicrobiota;c__Elusimicrobia;o__UBA1565;f__;g__;s__
LMSG_G000009218.1	no	516_2	Polymetallic	93.19	1.12	87.58	1	1	4	20	High quality	28631	2076048	49.40	142	d__Bacteria;p__Elusimicrobiota;c__Elusimicrobia;o__UBA1565;f__;g__;s__
LMSG_G000009219.1	no	516_2	Pyrite	64.87	1.12	59.26	0	0	0	18	Medium quality	6320	1478340	49.30	258	d__Bacteria;p__Elusimicrobiota;c__Elusimicrobia;o__UBA1565;f__;g__;s__
LMSG_G000009220.1	no	517_1	Polymetallic	95.43	0.00	95.43	1	3	1	20	High quality	103135	2146215	50.50	75	d__Bacteria;p__Elusimicrobiota;c__Elusimicrobia;o__UBA1565;f__;g__;s__
LMSG_G000009221.1	no	517_1	Polymetallic	95.43	0.00	95.43	1	1	2	20	High quality	77171	2060330	50.50	63	d__Bacteria;p__Elusimicrobiota;c__Elusimicrobia;o__UBA1565;f__;g__;s__
LMSG_G000009222.1	no	517_1	Polymetallic	92.81	0.00	92.81	0	1	3	20	Medium quality	57007	2090961	50.40	121	d__Bacteria;p__Elusimicrobiota;c__Elusimicrobia;o__UBA1565;f__;g__;s__
LMSG_G000009223.1	no	518_1	Pyrite	96.56	2.25	85.33	0	0	1	18	Medium quality	22754	2163699	49.30	170	d__Bacteria;p__Elusimicrobiota;c__Elusimicrobia;o__UBA1565;f__;g__;s__
LMSG_G000009224.1	no	517_1	Polymetallic	69.30	2.78	55.43	0	0	0	15	Medium quality	4634	1416976	50.40	362	d__Bacteria;p__Elusimicrobiota;c__Elusimicrobia;o__UBA1565;f__UBA9628;g__;s__
LMSG_G000009225.1	no	1022_1	Polymetallic	89.88	3.75	71.16	1	2	1	17	Medium quality	10952	3175317	67.60	396	d__Bacteria;p__Elusimicrobiota;c__Elusimicrobia;o__UBA1565;f__UBA9628;g__GWA2-66-18;s__
LMSG_G000009226.1	no	1022_1	Antimony	87.82	2.62	74.72	0	1	0	18	Medium quality	21386	2997068	67.70	196	d__Bacteria;p__Elusimicrobiota;c__Elusimicrobia;o__UBA1565;f__UBA9628;g__GWA2-66-18;s__
LMSG_G000009227.1	no	1022_1	Antimony	92.32	1.50	84.83	0	1	0	18	Medium quality	12971	3072285	67.60	346	d__Bacteria;p__Elusimicrobiota;c__Elusimicrobia;o__UBA1565;f__UBA9628;g__GWA2-66-18;s__
LMSG_G000009228.1	no	1022_1	Antimony	93.63	1.50	86.14	0	1	1	20	Medium quality	10920	3087805	67.50	394	d__Bacteria;p__Elusimicrobiota;c__Elusimicrobia;o__UBA1565;f__UBA9628;g__GWA2-66-18;s__
LMSG_G000009229.1	no	1022_1	Antimony	95.31	2.62	82.21	0	1	1	19	Medium quality	16006	3199314	67.60	285	d__Bacteria;p__Elusimicrobiota;c__Elusimicrobia;o__UBA1565;f__UBA9628;g__GWA2-66-18;s__
LMSG_G000009230.1	no	1020_1	Antimony	64.35	0.00	64.35	0	0	0	14	Medium quality	4687	1596645	63.40	430	d__Bacteria;p__Elusimicrobiota;c__Elusimicrobia;o__UBA1565;f__UBA9628;g__PALSA-1176;s__
LMSG_G000009231.1	no	1125_1	Antimony	98.14	1.16	92.36	1	0	1	19	Medium quality	30848	2426706	64.50	127	d__Bacteria;p__Eremiobacterota;c__Eremiobacteria;o__UBP12;f__UBA5184;g__;s__
LMSG_G000009232.1	no	1125_1	Antimony	88.88	3.99	68.93	1	0	1	16	Medium quality	7841	2347137	64.70	385	d__Bacteria;p__Eremiobacterota;c__Eremiobacteria;o__UBP12;f__UBA5184;g__;s__
LMSG_G000009233.1	no	1125_1	Antimony	88.58	2.86	74.28	0	0	0	14	Medium quality	10286	2139887	64.80	261	d__Bacteria;p__Eremiobacterota;c__Eremiobacteria;o__UBP12;f__UBA5184;g__;s__
LMSG_G000009234.1	no	1125_1	Antimony	99.07	2.31	87.50	1	0	1	18	Medium quality	83492	2418768	64.80	165	d__Bacteria;p__Eremiobacterota;c__Eremiobacteria;o__UBP12;f__UBA5184;g__;s__
LMSG_G000009235.1	no	1125_2	Copper	99.07	0.93	94.45	1	2	1	19	High quality	81920	2639211	64.70	62	d__Bacteria;p__Eremiobacterota;c__Eremiobacteria;o__UBP12;f__UBA5184;g__;s__
LMSG_G000009236.1	no	1125_2	Lead-Zinc	97.22	0.93	92.60	1	1	1	19	High quality	67268	2723547	64.60	89	d__Bacteria;p__Eremiobacterota;c__Eremiobacteria;o__UBP12;f__UBA5184;g__;s__
LMSG_G000009237.1	no	1125_2	Tin-Zinc	98.14	1.01	93.09	1	3	7	18	High quality	59490	2720976	64.80	284	d__Bacteria;p__Eremiobacterota;c__Eremiobacteria;o__UBP12;f__UBA5184;g__;s__
LMSG_G000009238.1	no	1125_2	Lead-Zinc	99.07	1.85	89.82	1	1	1	19	High quality	130853	2747055	64.20	32	d__Bacteria;p__Eremiobacterota;c__Eremiobacteria;o__UBP12;f__UBA5184;g__;s__
LMSG_G000009239.1	no	1125_2	Pyrite-Copper	98.14	1.85	88.89	2	1	2	20	High quality	76614	2584238	64.60	66	d__Bacteria;p__Eremiobacterota;c__Eremiobacteria;o__UBP12;f__UBA5184;g__;s__
LMSG_G000009240.1	no	1125_2	Pyrite-Copper	98.14	2.21	87.09	1	1	3	19	High quality	113170	3035627	64.40	197	d__Bacteria;p__Eremiobacterota;c__Eremiobacteria;o__UBP12;f__UBA5184;g__;s__
LMSG_G000009241.1	no	1125_2	Copper	95.83	1.95	86.06	0	1	0	19	Medium quality	14361	2504653	64.90	276	d__Bacteria;p__Eremiobacterota;c__Eremiobacteria;o__UBP12;f__UBA5184;g__;s__
LMSG_G000009242.1	no	1125_2	Copper	92.59	0.00	92.59	0	0	0	18	Medium quality	11649	2191189	65.10	247	d__Bacteria;p__Eremiobacterota;c__Eremiobacteria;o__UBP12;f__UBA5184;g__;s__
LMSG_G000009243.1	no	1125_2	Tin-Zinc	97.58	2.40	85.59	1	0	1	19	Medium quality	30155	2586525	64.80	161	d__Bacteria;p__Eremiobacterota;c__Eremiobacteria;o__UBP12;f__UBA5184;g__;s__
LMSG_G000009244.1	no	1125_2	Pyrite-Copper	99.07	0.93	94.45	1	0	1	19	Medium quality	83974	2700878	64.50	54	d__Bacteria;p__Eremiobacterota;c__Eremiobacteria;o__UBP12;f__UBA5184;g__;s__
LMSG_G000009245.1	no	1125_2	Pyrite-Copper	97.22	3.70	78.71	2	0	2	17	Medium quality	63378	2555480	64.70	65	d__Bacteria;p__Eremiobacterota;c__Eremiobacteria;o__UBP12;f__UBA5184;g__;s__
LMSG_G000009246.1	no	1125_2	Polymetallic	64.70	2.34	52.99	0	1	0	11	Medium quality	4595	1641315	64.50	403	d__Bacteria;p__Eremiobacterota;c__Eremiobacteria;o__UBP12;f__UBA5184;g__;s__
LMSG_G000009247.1	no	1125_2	Polymetallic	65.73	0.28	64.31	0	0	0	10	Medium quality	2993	1770061	65.20	668	d__Bacteria;p__Eremiobacterota;c__Eremiobacteria;o__UBP12;f__UBA5184;g__;s__
LMSG_G000009248.1	no	1128_1	Pyrite-Copper	92.12	1.85	82.87	1	0	1	20	Medium quality	23851	2318724	63.30	153	d__Bacteria;p__Eremiobacterota;c__Eremiobacteria;o__UBP12;f__UBA5184;g__;s__
LMSG_G000009249.1	no	1128_2	Copper	61.65	2.01	51.62	1	0	0	14	Medium quality	11100	1656948	63.90	240	d__Bacteria;p__Eremiobacterota;c__Eremiobacteria;o__UBP12;f__UBA5184;g__;s__
LMSG_G000009250.1	no	1129_1	Antimony	85.54	2.50	73.04	1	1	1	16	Medium quality	13994	2068150	62.20	217	d__Bacteria;p__Eremiobacterota;c__Eremiobacteria;o__UBP12;f__UBA5184;g__;s__
LMSG_G000009251.1	no	1129_1	Antimony	89.36	1.39	82.42	0	0	0	20	Medium quality	58610	2135960	62.30	184	d__Bacteria;p__Eremiobacterota;c__Eremiobacteria;o__UBP12;f__UBA5184;g__;s__
LMSG_G000009252.1	no	1129_1	Antimony	89.58	2.41	77.55	1	0	1	19	Medium quality	36278	2269892	62.40	206	d__Bacteria;p__Eremiobacterota;c__Eremiobacteria;o__UBP12;f__UBA5184;g__;s__
LMSG_G000009253.1	no	1129_1	Pyrite-Copper	89.86	1.30	83.38	0	0	0	17	Medium quality	58125	2155182	62.20	134	d__Bacteria;p__Eremiobacterota;c__Eremiobacteria;o__UBP12;f__UBA5184;g__;s__
LMSG_G000009254.1	no	1130_1	Polymetallic	96.81	1.85	87.56	1	1	1	20	High quality	70975	2481584	61.80	64	d__Bacteria;p__Eremiobacterota;c__Eremiobacteria;o__UBP12;f__UBA5184;g__;s__
LMSG_G000009255.1	no	1130_1	Polymetallic	86.62	0.00	86.62	1	1	1	20	Medium quality	42758	2233212	61.60	83	d__Bacteria;p__Eremiobacterota;c__Eremiobacteria;o__UBP12;f__UBA5184;g__;s__
LMSG_G000009256.1	no	1130_2	Coal	85.59	2.35	73.87	0	0	0	17	Medium quality	4905	2004955	62.50	482	d__Bacteria;p__Eremiobacterota;c__Eremiobacteria;o__UBP12;f__UBA5184;g__;s__
LMSG_G000009257.1	no	1131_2	Copper	67.01	1.54	59.31	0	0	0	13	Medium quality	2173	1587506	62.80	785	d__Bacteria;p__Eremiobacterota;c__Eremiobacteria;o__UBP12;f__UBA5184;g__;s__
LMSG_G000009258.1	no	1132_1	Copper	98.14	0.00	98.14	1	1	1	20	High quality	131202	2193490	62.80	35	d__Bacteria;p__Eremiobacterota;c__Eremiobacteria;o__UBP12;f__UBA5184;g__;s__
LMSG_G000009259.1	no	1132_1	Copper	99.07	0.00	99.07	0	1	0	20	Medium quality	44110	2376372	62.80	89	d__Bacteria;p__Eremiobacterota;c__Eremiobacteria;o__UBP12;f__UBA5184;g__;s__
LMSG_G000009260.1	no	633_1	Coal	99.07	4.17	78.24	1	4	4	20	High quality	23324	2977619	65.90	232	d__Bacteria;p__Eremiobacterota;c__Eremiobacteria;o__UBP12;f__UBA5184;g__;s__
LMSG_G000009261.1	no	1131_1	Polymetallic	54.55	0.40	52.56	0	0	0	8	Medium quality	3001	1180870	63.50	457	d__Bacteria;p__Eremiobacterota;c__Eremiobacteria;o__UBP12;f__UBA5184;g__Bog-1527;s__
LMSG_G000009262.1	no	1128_2	Lead-Zinc	74.69	2.78	60.81	0	0	0	18	Medium quality	10081	2102903	64.00	260	d__Bacteria;p__Eremiobacterota;c__Eremiobacteria;o__UBP12;f__UBA5184;g__PALSA-1484;s__
LMSG_G000009263.1	no	1129_1	Polymetallic	59.75	1.36	52.94	0	0	0	9	Medium quality	5102	1806286	62.40	449	d__Bacteria;p__Eremiobacterota;c__Eremiobacteria;o__UBP12;f__UBA5184;g__PALSA-1484;s__

LMSG_G000009264.1	no	1129_2	Antimony	77.55	2.56	64.73	0	0	0	11	Medium quality	15408	1776968	62.20	226	d__Bacteria;p__Eremiobacterota;c__Eremiobacteria;o__UBP12;f__UBA5184;g__PALSA-1484;s__
LMSG_G000009265.1	no	1129_2	Pyrite-Copper	73.50	1.85	64.25	1	0	0	11	Medium quality	16516	1651849	62.10	168	d__Bacteria;p__Eremiobacterota;c__Eremiobacteria;o__UBP12;f__UBA5184;g__PALSA-1484;s__
LMSG_G000009266.1	no	1130_1	Polymetallic	95.88	1.39	88.94	2	1	2	20	High quality	133651	2672425	61.80	174	d__Bacteria;p__Eremiobacterota;c__Eremiobacteria;o__UBP12;f__UBA5184;g__PALSA-1484;s__
LMSG_G000009267.1	no	1130_1	Polymetallic	96.81	2.78	82.93	1	1	1	20	High quality	87756	2666320	61.70	52	d__Bacteria;p__Eremiobacterota;c__Eremiobacteria;o__UBP12;f__UBA5184;g__PALSA-1484;s__
LMSG_G000009268.1	no	1131_1	Polymetallic	52.03	0.00	52.03	0	0	0	5	Medium quality	2486	958119	63.40	386	d__Bacteria;p__Eremiobacterota;c__Eremiobacteria;o__UBP12;f__UBA5184;g__PALSA-1484;s__
LMSG_G000009269.1	no	1131_2	Copper	82.92	2.78	69.04	0	0	0	16	Medium quality	11734	2017360	62.80	287	d__Bacteria;p__Eremiobacterota;c__Eremiobacteria;o__UBP12;f__UBA5184;g__PALSA-1484;s__
LMSG_G000009270.1	no	299_1	Antimony	96.32	0.93	91.70	1	1	1	18	High quality	89762	2653569	56.60	83	d__Bacteria;p__Eremiobacterota;c__Eremiobacteria;o__UBP12;f__UBA5184;g__PALSA-1484;s__
LMSG_G000009271.1	no	299_1	Antimony	57.77	1.23	51.60	0	0	0	5	Medium quality	2162	1493629	56.90	739	d__Bacteria;p__Eremiobacterota;c__Eremiobacteria;o__UBP12;f__UBA5184;g__PALSA-1484;s__
LMSG_G000009272.1	no	299_1	Antimony	81.65	0.48	79.27	0	0	0	18	Medium quality	3869	2322515	56.90	779	d__Bacteria;p__Eremiobacterota;c__Eremiobacteria;o__UBP12;f__UBA5184;g__PALSA-1484;s__
LMSG_G000009273.1	no	637_1	Coal	96.29	3.50	78.79	2	2	1	20	High quality	49124	2779519	63.60	163	d__Bacteria;p__Eremiobacterota;c__Eremiobacteria;o__UBP12;f__UBA5184;g__PALSA-1484;s__
LMSG_G000009274.1	no	1123_1	Copper	95.60	1.85	86.35	0	1	1	20	Medium quality	22941	2220602	64.80	110	d__Bacteria;p__Eremiobacterota;c__Eremiobacteria;o__UBP12;f__UBA5184;g__PALSA-1504;s__
LMSG_G000009275.1	no	1123_1	Copper	96.36	1.85	87.11	0	0	0	20	Medium quality	21546	2105557	65.00	125	d__Bacteria;p__Eremiobacterota;c__Eremiobacteria;o__UBP12;f__UBA5184;g__PALSA-1504;s__
LMSG_G000009276.1	no	1155_1	Lead-Zinc	95.45	4.19	74.50	1	1	1	20	High quality	85415	2540739	40.80	84	d__Bacteria;p__Eremiobacterota;c__UBP9;o__;f__;g__;s__
LMSG_G000009277.1	no	1155_1	Pyrite	79.47	0.21	78.41	0	0	0	15	Medium quality	3827	1855379	40.80	523	d__Bacteria;p__Eremiobacterota;c__UBP9;o__;f__;g__;s__
LMSG_G000009278.1	no	1156_1	Copper	95.51	1.28	89.10	1	1	1	20	High quality	109572	2523610	40.80	81	d__Bacteria;p__Eremiobacterota;c__UBP9;o__;f__;g__;s__
LMSG_G000009279.1	no	1156_1	Copper	94.23	1.28	87.82	1	1	1	20	High quality	87181	2526739	41.00	75	d__Bacteria;p__Eremiobacterota;c__UBP9;o__;f__;g__;s__
LMSG_G000009280.1	no	1156_1	Copper	95.51	0.00	95.51	1	1	1	20	High quality	42894	2512289	40.90	105	d__Bacteria;p__Eremiobacterota;c__UBP9;o__;f__;g__;s__
LMSG_G000009281.1	no	1156_1	Copper	95.51	0.00	95.51	1	1	2	20	High quality	75759	2640574	40.90	100	d__Bacteria;p__Eremiobacterota;c__UBP9;o__;f__;g__;s__
LMSG_G000009282.1	no	1156_1	Pyrite-Copper	92.94	0.00	92.94	1	1	2	19	High quality	60004	2416143	40.80	90	d__Bacteria;p__Eremiobacterota;c__UBP9;o__;f__;g__;s__
LMSG_G000009283.1	no	1156_1	Magnetite	95.08	0.00	95.08	1	1	1	20	High quality	26141	2856252	40.90	268	d__Bacteria;p__Eremiobacterota;c__UBP9;o__;f__;g__;s__
LMSG_G000009284.1	no	1156_1	Magnetite	92.94	0.00	92.94	1	1	1	20	High quality	26673	2443057	40.80	140	d__Bacteria;p__Eremiobacterota;c__UBP9;o__;f__;g__;s__
LMSG_G000009285.1	no	1156_1	Magnetite	94.23	0.00	94.23	1	1	1	20	High quality	31998	2889721	40.80	254	d__Bacteria;p__Eremiobacterota;c__UBP9;o__;f__;g__;s__
LMSG_G000009286.1	no	1156_1	Copper	95.51	1.42	88.39	0	2	10	19	Medium quality	189400	2642927	41.00	71	d__Bacteria;p__Eremiobacterota;c__UBP9;o__;f__;g__;s__
LMSG_G000009287.1	no	1156_1	Pyrite	94.23	0.00	94.23	0	0	0	20	Medium quality	21500	2470287	40.70	161	d__Bacteria;p__Eremiobacterota;c__UBP9;o__;f__;g__;s__
LMSG_G000009288.1	no	1156_1	Copper	75.79	1.42	68.67	0	0	0	18	Medium quality	2729	1903511	40.70	807	d__Bacteria;p__Eremiobacterota;c__UBP9;o__;f__;g__;s__
LMSG_G000009289.1	no	1156_1	Polymetallic	95.51	0.00	95.51	1	0	0	20	Medium quality	32476	2408661	40.70	121	d__Bacteria;p__Eremiobacterota;c__UBP9;o__;f__;g__;s__
LMSG_G000009290.1	no	1156_1	Magnetite	94.23	0.50	91.74	1	0	0	20	Medium quality	54278	2524484	40.80	113	d__Bacteria;p__Eremiobacterota;c__UBP9;o__;f__;g__;s__
LMSG_G000009291.1	no	1156_1	Lead-Zinc	93.58	0.00	93.58	0	0	0	19	Medium quality	23542	2457991	40.70	162	d__Bacteria;p__Eremiobacterota;c__UBP9;o__;f__;g__;s__
LMSG_G000009292.1	no	1156_1	Lead-Zinc	94.87	0.00	94.87	0	1	0	18	Medium quality	25226	2395557	40.80	138	d__Bacteria;p__Eremiobacterota;c__UBP9;o__;f__;g__;s__
LMSG_G000009293.1	no	1156_1	Lead-Zinc	95.51	0.00	95.51	0	1	0	20	Medium quality	21698	2375106	40.70	187	d__Bacteria;p__Eremiobacterota;c__UBP9;o__;f__;g__;s__
LMSG_G000009294.1	no	1156_1	Lead-Zinc	95.51	0.64	92.31	0	1	0	20	Medium quality	24315	2404344	40.80	138	d__Bacteria;p__Eremiobacterota;c__UBP9;o__;f__;g__;s__
LMSG_G000009295.1	no	1156_1	Magnetite	94.23	0.00	94.23	0	1	0	20	Medium quality	20226	2352319	40.70	189	d__Bacteria;p__Eremiobacterota;c__UBP9;o__;f__;g__;s__
LMSG_G000009296.1	no	1156_1	Pyrite-Copper	96.29	1.44	89.09	1	0	1	20	Medium quality	67475	2602407	40.80	180	d__Bacteria;p__Eremiobacterota;c__UBP9;o__;f__;g__;s__
LMSG_G000009297.1	no	486_1	Polymetallic	93.84	1.62	85.77	3	1	1	20	High quality	51524	2676550	45.40	100	d__Bacteria;p__Firmicutes;c__Alicyclobacillia;o__Alicyclobacillales;f__;g__;s__
LMSG_G000009298.1	no	471_1	Copper	92.48	2.88	78.06	2	4	1	20	High quality	19421	3987337	56.80	410	d__Bacteria;p__Firmicutes;c__Alicyclobacillia;o__Alicyclobacillales;f__Acidibacillaceae;g__;s__
LMSG_G000009299.1	no	472_1	Polymetallic	71.02	1.92	61.41	2	0	1	11	Medium quality	3499	2503237	55.50	844	d__Bacteria;p__Firmicutes;c__Alicyclobacillia;o__Alicyclobacillales;f__Acidibacillaceae;g__;s__
LMSG_G000009300.1	no	474_1	Polymetallic	53.72	0.64	50.55	1	3	15	10	Medium quality	4300	1516276	44.50	428	d__Bacteria;p__Firmicutes;c__Alicyclobacillia;o__Alicyclobacillales;f__Acidibacillaceae;g__;s__
LMSG_G000009301.1	no	478_1	Polymetallic	97.30	1.28	90.89	4	4	1	20	High quality	181871	3331633	46.10	73	d__Bacteria;p__Firmicutes;c__Alicyclobacillia;o__Alicyclobacillales;f__Acidibacillaceae;g__;s__
LMSG_G000009302.1	no	478_1	Pyrite	89.67	1.33	83.01	3	1	1	20	Medium quality	26685	2911717	46.70	186	d__Bacteria;p__Firmicutes;c__Alicyclobacillia;o__Alicyclobacillales;f__Acidibacillaceae;g__;s__
LMSG_G000009303.1	no	478_1	Pyrite-Copper	54.97	0.00	54.97	1	1	0	12	Medium quality	2901	1727631	47.20	666	d__Bacteria;p__Firmicutes;c__Alicyclobacillia;o__Alicyclobacillales;f__Acidibacillaceae;g__;s__
LMSG_G000009304.1	no	478_2	Copper	57.09	0.54	54.40	2	0	0	6	Medium quality	5151	1653290	48.30	361	d__Bacteria;p__Firmicutes;c__Alicyclobacillia;o__Alicyclobacillales;f__Acidibacillaceae;g__;s__
LMSG_G000009305.1	no	486_1	Polymetallic	94.03	1.54	86.34	3	2	1	18	High quality	94519	2518966	45.40	63	d__Bacteria;p__Firmicutes;c__Alicyclobacillia;o__Alicyclobacillales;f__Acidibacillaceae;g__;s__
LMSG_G000009306.1	no	486_1	Polymetallic	94.10	1.18	88.21	2	1	1	19	High quality	35487	2701889	45.40	137	d__Bacteria;p__Firmicutes;c__Alicyclobacillia;o__Alicyclobacillales;f__Acidibacillaceae;g__;s__
LMSG_G000009307.1	no	486_1	Polymetallic	94.87	1.54	87.18	3	2	0	19	Medium quality	87705	2569327	45.30	64	d__Bacteria;p__Firmicutes;c__Alicyclobacillia;o__Alicyclobacillales;f__Acidibacillaceae;g__;s__
LMSG_G000009308.1	no	464_1	Tin-Zinc	86.35	2.76	72.58	2	1	0	17	Medium quality	16993	2272699	53.20	194	d__Bacteria;p__Firmicutes;c__Alicyclobacillia;o__Alicyclobacillales;f__Acidibacillaceae;g__Acidibacillus;s__Acidibacillus ferrooxidans
LMSG_G000009309.1	no	489_1	Lead-Zinc	95.43	2.36	83.62	1	1	0	19	Medium quality	48697	4263121	55.10	223	d__Bacteria;p__Firmicutes;c__Alicyclobacillia;o__Alicyclobacillales;f__Alicyclobacillaceae;g__;s__
LMSG_G000009310.1	no	489_1	Lead-Zinc	66.09	0.14	65.38	0	0	0	19	Medium quality	7554	2461221	55.50	420	d__Bacteria;p__Firmicutes;c__Alicyclobacillia;o__Alicyclobacillales;f__Alicyclobacillaceae;g__;s__
LMSG_G000009311.1	no	933_1	Lead-Zinc	94.63	1.99	84.68	1	2	2	12	Medium quality	7595	3498029	46.70	669	d__Bacteria;p__Firmicutes;c__Bacilli;o__Bacillales;f__Bacillaceae;g__Bacillus;s__Bacillus velezensis
LMSG_G000009312.1	no	1703_1	Copper	99.62	0.00	99.62	1	1	2	20	High quality	246261	3007764	37.10	37	d__Bacteria;p__Firmicutes;c__Bacilli;o__Lactobacillales;f__Enterococcaceae;g__Enterococcus;s__Enterococcus faecalis
LMSG_G000009313.1	no	1703_1	Lead-Zinc	97.37	2.06	87.08	0	0	0	20	Medium quality	119259	2889721	37.40	47	d__Bacteria;p__Firmicutes;c__Bacilli;o__Lactobacillales;f__Enterococcaceae;g__Enterococcus;s__Enterococcus faecalis
LMSG_G000009314.1	no	1703_1	Lead-Zinc	99.62	0.00	99.62	0	0	0	20	Medium quality	115245	2749366	37.50	33	d__Bacteria;p__Firmicutes;c__Bacilli;o__Lactobacillales;f__Enterococcaceae;g__Enterococcus;s__Enterococcus faecalis
LMSG_G000009315.1	no	1703_1	Polymetallic	77.69	2.45	65.46	1	1	1	15	Medium quality	3183	2115446	37.90	774	d__Bacteria;p__Firmicutes;c__Bacilli;o__Lactobacillales;f__Enterococcaceae;g__Enterococcus;s__Enterococcus faecalis
LMSG_G000009316.1	no	1703_1	Polymetallic	75.74	0.91	71.22	1	0	1	8	Medium quality	4967	2098613	37.50	510	d__Bacteria;p__Firmicutes;c__Bacilli;o__Lactobacillales;f__Enterococcaceae;g__Enterococcus;s__Enterococcus faecalis
LMSG_G000009317.1	no	1703_1	Polymetallic	92.91	1.92	83.31	1	0	1	7	Medium quality	16733	2476940	37.80	257	d__Bacteria;p__Firmicutes;c__Bacilli;o__Lactobacillales;f__Enterococcaceae;g__Enterococcus;s__Enterococcus faecalis
LMSG_G000009318.1	no	1703_1	Lead-Zinc	99.53	0.00	99.53	1	0	1	18	Medium quality	278486	2711409	37.50	47	d__Bacteria;p__Firmicutes;c__Bacilli;o__Lactobacillales;f__Enterococcaceae;g__Enterococcus;s__Enterococcus faecalis
LMSG_G000009319.1	no	229_1	Pyrite	94.61	0.00	94.61	1	1	3	20	High quality	23974	833784	29.50	65	d__Bacteria;p__Firmicutes;c__Bacilli;o__Mycoplasmatales;f__Metamycoplasmataceae;g__Mycoplasmopsis;s__Mycoplasmopsis bovis
LMSG_G000009320.1	no	229_1	Polymetallic	96.29	0.55	93.56	1	1	1	20	High quality	7793	1026000	29.90	185	d__Bacteria;p__Firmicutes;c__Bacilli;o__Mycoplasmatales;f__Metamycoplasmataceae;g__Mycoplasmopsis;s__Mycoplasmopsis bovis
LMSG_G000009321.1	no	229_1	Antimony	96.92	0.96	92.12	2	2	1	19	High quality	22549	858078	29.60	56	d__Bacteria;p__Firmicutes;c__Bacilli;o__Mycoplasmatales;f__Metamycoplasmataceae;g__Mycoplasmopsis;s__Mycoplasmopsis bovis
LMSG_G000009322.1	no	229_1	Polymetallic	96.92	0.00	96.92	2	1	1	20	High quality	22045	888238	29.20	63	d__Bacteria;p__Firmicutes;c__Bacilli;o__Mycoplasmatales;f__Metamycoplasmataceae;g__Mycoplasmopsis;s__Mycoplasmopsis bovis
LMSG_G000009323.1	no	229_1	Antimony	54.23	0.00	54.23	0	0	0	10	Medium quality	20047	515563	29.30	44	d__Bacteria;p__Firmicutes;c__Bacilli;o__Mycoplasmatales;f__Metamycoplasmataceae;g__Mycoplasmopsis;s__Mycoplasmopsis bovis
LMSG_G000009324.1	no	229_1	Antimony	84.61	0.00	84.61	0	2	4	20	Medium quality	20927	767199	29.40	64	d__Bacteria;p__Firmicutes;c__Bacilli;o__Mycoplasmatales;f__Metamycoplasmataceae;g__Mycoplasmopsis;s__Mycoplasmopsis bovis
LMSG_G000009325.1	no	230_1	Antimony	98.16	2.57	85.32	6	1	4	20	High quality	33747	3979971	29.70	200	d__Bacteria;p__Firmicutes_A;c__Clostridia;o__Clostridiales;f__Clostridiaceae;g__;s__
LMSG_G000009326.1	no	475_1	Antimony	97.95	2.66	84.67	2	13	2	18	High quality	77264	3306749	46.40	164	d__Bacteria;p__Firmicutes_B;c__Desulfitobacteriia;o__Desulfitobacteriales;f__;g__;s__
LMSG_G000009327.1	no	475_1	Antimony	81.74	1.79	72.77	1	0	0	16	Medium quality	8326	2233627	46.80	300	d__Bacteria;p__Firmicutes_B;c__Desulfitobacteriia;o__Desulfitobacteriales;f__;g__;s__
LMSG_G000009328.1	no	475_1	Antimony	61.75	1.72	53.15	0	0	0	11	Medium quality	4498	1634620	47.20	397	d__Bacteria;p__Firmicutes_B;c__Desulfitobacteriia;o__Desulfitobacteriales;f__;g__;s__
LMSG_G000009329.1	no	475_1	Antimony	77.05	4.63	53.88	1	0	0	16	Medium quality	6162	2242262	46.60	413	d__Bacteria;p__Firmicutes_B;c__Desulfitobacteriia;o__Desulfitobacteriales;f__;g__;s__
LMSG_G000009330.1	no	475_1	Antimony	98.46	2.89	84.01	5	0	0	17	Medium quality	132369	3096376	46.40	32	d__Bacteria;p__Firmicutes_B;c__Desulfitobacteriia;o__Desulfitobacteriales;f__;g__;s__
LMSG_G000009331.1	no	476_1	Lead-Zinc	93.18	2.76	79.37	0	2	1	17	Medium quality	39508	3161725	53.00	159	d__Bacteria;p__Firmicutes_B;c__Desulfitobacteriia;o__Desulfitobacteriales;f__;g__;s__
LMSG_G000009332.1	no	480_1	Pyrite-Copper	67.40	1.26	61.12	0	1	0	12	Medium quality	6519	1764657	51.50	303	d__Bacteria;p__Firmicutes_B;c__Desulfitobacteriia;o__Desulfitobacteriales;f__Desulfitobacteriaceae;g__;s__

LMSG_G000009333.1	no	468_1	Lead-Zinc	91.56	3.77	72.72	3	1	1	17	Medium quality	7668	4188604	60.30	697	d__Bacteria;p__Firmicutes_B;c__Desulfotomaculia;o__;f__;g__;s__
LMSG_G000009334.1	no	253_1	Polymetallic	86.72	2.77	72.86	0	0	0	19	Medium quality	7938	1816963	57.50	301	d__Bacteria;p__Firmicutes_B;c__Desulfotomaculia;o__Ammonifexales;f__Desulforudaceae;g__SURF-60;s__SURF-60 sp003604975
LMSG_G000009335.1	no	1099_1	Lead-Zinc	95.81	2.40	83.83	1	3	1	16	Medium quality	29185	3632553	49.40	257	d__Bacteria;p__Firmicutes_B;c__Thermincolia;o__Carboxydocellales;f__;g__;s__
LMSG_G000009336.1	no	1074_1	Lead-Zinc	86.44	2.40	74.44	4	3	11	17	Medium quality	28027	1962338	48.90	161	d__Bacteria;p__Firmicutes_D;c__Dethiobacteria;o__Dethiobacterales;f__Dethiobacteraceae;g__Dethiobacter;s__
LMSG_G000009337.1	no	1074_1	Lead-Zinc	64.30	2.40	52.30	0	0	0	14	Medium quality	4966	1222803	49.50	288	d__Bacteria;p__Firmicutes_D;c__Dethiobacteria;o__Dethiobacterales;f__Dethiobacteraceae;g__Dethiobacter;s__
LMSG_G000009338.1	no	1017_1	Polymetallic	93.06	0.99	88.11	1	2	1	19	High quality	19733	3036564	68.40	228	d__Bacteria;p__Firmicutes_E;c__DTU015;o__;f__;g__;s__
LMSG_G000009339.1	no	1017_1	Pyrite	54.52	0.50	52.05	0	0	0	12	Medium quality	2261	1928165	68.60	909	d__Bacteria;p__Firmicutes_E;c__DTU015;o__;f__;g__;s__
LMSG_G000009340.1	no	1017_1	Pyrite	94.55	0.99	89.60	0	2	0	19	Medium quality	41408	2983295	68.30	110	d__Bacteria;p__Firmicutes_E;c__DTU015;o__;f__;g__;s__
LMSG_G000009341.1	no	1017_1	Pyrite	82.66	0.45	80.41	0	0	0	15	Medium quality	4691	2336650	68.80	586	d__Bacteria;p__Firmicutes_E;c__DTU015;o__;f__;g__;s__
LMSG_G000009342.1	no	1017_1	Pyrite	84.27	0.86	80.00	0	0	0	11	Medium quality	3963	2718598	68.50	841	d__Bacteria;p__Firmicutes_E;c__DTU015;o__;f__;g__;s__
LMSG_G000009343.1	no	1017_1	Polymetallic	94.55	0.00	94.55	0	2	0	18	Medium quality	32325	3201449	68.10	165	d__Bacteria;p__Firmicutes_E;c__DTU015;o__;f__;g__;s__
LMSG_G000009344.1	no	1017_1	Polymetallic	94.55	0.00	94.55	0	2	0	19	Medium quality	33204	3362316	67.80	176	d__Bacteria;p__Firmicutes_E;c__DTU015;o__;f__;g__;s__
LMSG_G000009345.1	no	1017_1	Polymetallic	93.56	0.99	88.61	0	2	0	19	Medium quality	17476	3134130	68.10	269	d__Bacteria;p__Firmicutes_E;c__DTU015;o__;f__;g__;s__
LMSG_G000009346.1	no	1017_1	Polymetallic	94.22	0.00	94.22	0	2	0	19	Medium quality	31585	3344438	67.90	177	d__Bacteria;p__Firmicutes_E;c__DTU015;o__;f__;g__;s__
LMSG_G000009347.1	no	1017_1	Polymetallic	89.96	0.00	89.96	1	2	0	14	Medium quality	9320	2441700	69.20	355	d__Bacteria;p__Firmicutes_E;c__DTU015;o__;f__;g__;s__
LMSG_G000009348.1	no	482_1	Pyrite-Copper	92.81	2.45	80.56	2	0	2	19	Medium quality	164363	4119057	63.30	40	d__Bacteria;p__Firmicutes_E;c__DTU015;o__;f__;g__;s__
LMSG_G000009349.1	no	649_1	Lead-Zinc	60.49	0.50	58.02	0	0	0	9	Medium quality	5542	2530637	75.30	535	d__Bacteria;p__Firmicutes_E;c__Sulfobacillia;o__;f__;g__;s__
LMSG_G000009350.1	no	651_1	Polymetallic	85.14	0.36	83.32	0	1	0	13	Medium quality	8125	2336468	68.10	327	d__Bacteria;p__Firmicutes_E;c__Sulfobacillia;o__;f__;g__;s__
LMSG_G000009351.1	no	651_1	Pyrite-Copper	88.92	1.19	82.98	2	3	1	15	Medium quality	17118	2830436	67.70	236	d__Bacteria;p__Firmicutes_E;c__Sulfobacillia;o__;f__;g__;s__
LMSG_G000009352.1	no	651_1	Pyrite-Copper	88.11	1.37	81.27	0	1	0	12	Medium quality	19262	2606499	67.90	206	d__Bacteria;p__Firmicutes_E;c__Sulfobacillia;o__;f__;g__;s__
LMSG_G000009353.1	no	652_1	Lead-Zinc	71.55	4.23	50.40	0	1	0	17	Medium quality	28704	1981434	67.70	87	d__Bacteria;p__Firmicutes_E;c__Sulfobacillia;o__;f__;g__;s__
LMSG_G000009354.1	no	653_1	Polymetallic	72.62	0.00	72.62	1	0	0	14	Medium quality	11669	2408775	67.80	300	d__Bacteria;p__Firmicutes_E;c__Sulfobacillia;o__;f__;g__;s__
LMSG_G000009355.1	no	655_1	Copper	92.07	0.99	87.12	1	1	2	18	High quality	18892	3042050	68.70	236	d__Bacteria;p__Firmicutes_E;c__Sulfobacillia;o__;f__;g__;s__
LMSG_G000009356.1	no	659_1	Copper	91.74	2.97	76.89	1	2	2	20	High quality	195726	1837161	68.20	16	d__Bacteria;p__Firmicutes_E;c__Sulfobacillia;o__;f__;g__;s__
LMSG_G000009357.1	no	659_1	Copper	91.74	2.97	76.89	1	1	1	19	High quality	165802	1656196	68.80	22	d__Bacteria;p__Firmicutes_E;c__Sulfobacillia;o__;f__;g__;s__
LMSG_G000009358.1	no	659_1	Copper	90.09	1.98	80.19	2	1	1	17	Medium quality	52359	1468293	69.00	54	d__Bacteria;p__Firmicutes_E;c__Sulfobacillia;o__;f__;g__;s__
LMSG_G000009359.1	no	659_1	Copper	83.16	1.30	76.68	1	1	0	17	Medium quality	81826	1695137	68.10	32	d__Bacteria;p__Firmicutes_E;c__Sulfobacillia;o__;f__;g__;s__
LMSG_G000009360.1	no	659_1	Copper	90.75	3.00	75.73	0	1	0	17	Medium quality	104225	1617167	68.80	24	d__Bacteria;p__Firmicutes_E;c__Sulfobacillia;o__;f__;g__;s__
LMSG_G000009361.1	no	659_1	Copper	89.88	1.98	79.98	1	1	1	19	Medium quality	81290	1747986	68.30	41	d__Bacteria;p__Firmicutes_E;c__Sulfobacillia;o__;f__;g__;s__
LMSG_G000009362.1	no	659_1	Magnetite	90.75	2.97	75.90	1	1	0	14	Medium quality	65715	1652389	69.00	110	d__Bacteria;p__Firmicutes_E;c__Sulfobacillia;o__;f__;g__;s__
LMSG_G000009363.1	no	660_1	Magnetite	90.75	1.49	83.33	1	1	0	18	Medium quality	119139	1655451	64.20	62	d__Bacteria;p__Firmicutes_E;c__Sulfobacillia;o__;f__;g__;s__
LMSG_G000009364.1	no	661_1	Copper	90.75	2.64	77.55	1	1	1	15	Medium quality	113406	1636808	66.80	17	d__Bacteria;p__Firmicutes_E;c__Sulfobacillia;o__;f__;g__;s__
LMSG_G000009365.1	no	661_1	Copper	88.61	2.19	77.68	1	1	0	13	Medium quality	27963	1528397	66.90	135	d__Bacteria;p__Firmicutes_E;c__Sulfobacillia;o__;f__;g__;s__
LMSG_G000009366.1	no	661_1	Pyrite	74.75	0.03	74.58	0	0	0	12	Medium quality	5531	1204546	67.10	274	d__Bacteria;p__Firmicutes_E;c__Sulfobacillia;o__;f__;g__;s__
LMSG_G000009367.1	no	661_1	Copper	87.78	1.98	77.88	1	1	1	18	Medium quality	19879	1576608	66.80	140	d__Bacteria;p__Firmicutes_E;c__Sulfobacillia;o__;f__;g__;s__
LMSG_G000009368.1	no	661_1	Copper	57.24	0.50	54.77	0	0	0	7	Medium quality	2157	834556	67.20	430	d__Bacteria;p__Firmicutes_E;c__Sulfobacillia;o__;f__;g__;s__
LMSG_G000009369.1	no	661_1	Polymetallic	62.64	1.32	56.04	0	0	0	11	Medium quality	2797	1114267	66.90	467	d__Bacteria;p__Firmicutes_E;c__Sulfobacillia;o__;f__;g__;s__
LMSG_G000009370.1	no	661_1	Polymetallic	57.46	0.83	53.34	0	0	0	7	Medium quality	2803	878579	67.20	374	d__Bacteria;p__Firmicutes_E;c__Sulfobacillia;o__;f__;g__;s__
LMSG_G000009371.1	no	661_1	Tin-Zinc	69.73	0.78	65.85	0	0	0	12	Medium quality	3908	1186398	66.90	342	d__Bacteria;p__Firmicutes_E;c__Sulfobacillia;o__;f__;g__;s__
LMSG_G000009372.1	no	661_1	Tin-Zinc	73.81	0.99	68.86	1	0	0	12	Medium quality	3335	1045194	67.10	371	d__Bacteria;p__Firmicutes_E;c__Sulfobacillia;o__;f__;g__;s__
LMSG_G000009373.1	no	661_1	Pyrite-Copper	87.78	1.02	82.66	0	0	0	19	Medium quality	13503	1482350	66.80	172	d__Bacteria;p__Firmicutes_E;c__Sulfobacillia;o__;f__;g__;s__
LMSG_G000009374.1	no	661_1	Copper	90.75	0.99	85.80	1	1	0	15	Medium quality	75064	1626361	66.50	42	d__Bacteria;p__Firmicutes_E;c__Sulfobacillia;o__;f__;g__;s__
LMSG_G000009375.1	no	661_1	Magnetite	90.75	0.99	85.80	1	1	0	15	Medium quality	64113	1505650	66.90	37	d__Bacteria;p__Firmicutes_E;c__Sulfobacillia;o__;f__;g__;s__
LMSG_G000009376.1	no	1104_1	Copper	85.89	0.33	84.23	1	0	0	17	Medium quality	8632	2283857	56.60	293	d__Bacteria;p__Firmicutes_E;c__Sulfobacillia;o__Sulfobacillales;f__;g__;s__
LMSG_G000009377.1	no	1104_1	Copper	84.83	1.33	78.17	1	0	0	10	Medium quality	6503	2482469	56.70	427	d__Bacteria;p__Firmicutes_E;c__Sulfobacillia;o__Sulfobacillales;f__;g__;s__
LMSG_G000009378.1	no	1104_1	Copper	87.31	0.40	85.30	1	1	1	13	Medium quality	11765	2929699	56.70	434	d__Bacteria;p__Firmicutes_E;c__Sulfobacillia;o__Sulfobacillales;f__;g__;s__
LMSG_G000009379.1	no	1104_1	Copper	89.00	0.33	87.34	1	1	1	15	Medium quality	35158	3680768	56.30	284	d__Bacteria;p__Firmicutes_E;c__Sulfobacillia;o__Sulfobacillales;f__;g__;s__
LMSG_G000009380.1	no	1104_1	Copper	72.38	0.00	72.38	0	1	2	17	Medium quality	9485	2608694	56.40	432	d__Bacteria;p__Firmicutes_E;c__Sulfobacillia;o__Sulfobacillales;f__;g__;s__
LMSG_G000009381.1	no	1109_1	Polymetallic	93.23	2.64	80.03	4	1	1	16	Medium quality	14540	4765890	55.00	446	d__Bacteria;p__Firmicutes_E;c__Sulfobacillia;o__Sulfobacillales;f__;g__;s__
LMSG_G000009382.1	no	1109_1	Polymetallic	88.94	1.74	80.24	3	1	0	19	Medium quality	14943	5136456	55.00	501	d__Bacteria;p__Firmicutes_E;c__Sulfobacillia;o__Sulfobacillales;f__;g__;s__
LMSG_G000009383.1	no	1109_1	Polymetallic	76.49	2.91	61.94	1	1	2	14	Medium quality	6866	3763882	55.10	681	d__Bacteria;p__Firmicutes_E;c__Sulfobacillia;o__Sulfobacillales;f__;g__;s__
LMSG_G000009384.1	no	1109_1	Polymetallic	84.27	2.64	71.07	2	1	0	15	Medium quality	9268	4419710	55.00	585	d__Bacteria;p__Firmicutes_E;c__Sulfobacillia;o__Sulfobacillales;f__;g__;s__
LMSG_G000009385.1	no	1109_1	Polymetallic	88.94	1.65	80.69	1	1	1	17	Medium quality	13644	4793352	55.00	569	d__Bacteria;p__Firmicutes_E;c__Sulfobacillia;o__Sulfobacillales;f__;g__;s__
LMSG_G000009386.1	no	1109_1	Lead-Zinc	64.45	2.61	51.40	1	0	0	17	Medium quality	2706	3509542	55.30	1468	d__Bacteria;p__Firmicutes_E;c__Sulfobacillia;o__Sulfobacillales;f__;g__;s__
LMSG_G000009387.1	no	1147_1	Polymetallic	96.53	1.52	88.94	1	1	2	18	High quality	45726	3223548	65.40	145	d__Bacteria;p__Firmicutes_E;c__Sulfobacillia;o__Sulfobacillales;f__;g__;s__
LMSG_G000009388.1	no	1147_1	Magnetite	97.52	2.01	87.45	1	1	1	18	High quality	101146	3241786	65.40	122	d__Bacteria;p__Firmicutes_E;c__Sulfobacillia;o__Sulfobacillales;f__;g__;s__
LMSG_G000009389.1	no	1147_1	Copper	83.37	1.52	75.78	1	0	1	16	Medium quality	7672	2818064	65.70	433	d__Bacteria;p__Firmicutes_E;c__Sulfobacillia;o__Sulfobacillales;f__;g__;s__
LMSG_G000009390.1	no	1147_1	Polymetallic	75.84	3.04	60.65	0	0	0	12	Medium quality	6079	2770951	65.40	527	d__Bacteria;p__Firmicutes_E;c__Sulfobacillia;o__Sulfobacillales;f__;g__;s__
LMSG_G000009391.1	no	1148_1	Pyrite-Copper	97.52	1.98	87.62	1	3	3	18	High quality	47594	3079636	65.90	130	d__Bacteria;p__Firmicutes_E;c__Sulfobacillia;o__Sulfobacillales;f__;g__;s__
LMSG_G000009392.1	no	1148_1	Pyrite-Copper	97.52	0.99	92.57	1	3	3	18	High quality	46099	3777056	65.60	224	d__Bacteria;p__Firmicutes_E;c__Sulfobacillia;o__Sulfobacillales;f__;g__;s__
LMSG_G000009393.1	no	1148_1	Copper	97.52	0.99	92.57	1	1	3	17	Medium quality	73418	3025767	66.10	101	d__Bacteria;p__Firmicutes_E;c__Sulfobacillia;o__Sulfobacillales;f__;g__;s__
LMSG_G000009394.1	no	1148_1	Pyrite	97.52	0.99	92.57	1	3	3	17	Medium quality	61834	2992772	65.90	113	d__Bacteria;p__Firmicutes_E;c__Sulfobacillia;o__Sulfobacillales;f__;g__;s__
LMSG_G000009395.1	no	1148_1	Copper	97.52	1.52	89.93	1	2	2	17	Medium quality	39229	3083146	66.00	217	d__Bacteria;p__Firmicutes_E;c__Sulfobacillia;o__Sulfobacillales;f__;g__;s__
LMSG_G000009396.1	no	1148_1	Copper	97.52	0.99	92.57	1	1	3	16	Medium quality	62303	2988169	66.00	84	d__Bacteria;p__Firmicutes_E;c__Sulfobacillia;o__Sulfobacillales;f__;g__;s__
LMSG_G000009397.1	no	1148_1	Copper	97.52	0.99	92.57	1	3	3	16	Medium quality	49818	3020825	66.00	114	d__Bacteria;p__Firmicutes_E;c__Sulfobacillia;o__Sulfobacillales;f__;g__;s__
LMSG_G000009398.1	no	1148_1	Polymetallic	95.54	0.99	90.59	1	1	1	16	Medium quality	87660	2776412	66.20	111	d__Bacteria;p__Firmicutes_E;c__Sulfobacillia;o__Sulfobacillales;f__;g__;s__
LMSG_G000009399.1	no	1148_1	Magnetite	96.53	1.19	90.59	1	3	3	16	Medium quality	46968	2871594	66.00	119	d__Bacteria;p__Firmicutes_E;c__Sulfobacillia;o__Sulfobacillales;f__;g__;s__
LMSG_G000009400.1	no	1148_1	Pyrite-Copper	96.53	1.08	91.13	1	3	2	16	Medium quality	84249	2986525	66.10	124	d__Bacteria;p__Firmicutes_E;c__Sulfobacillia;o__Sulfobacillales;f__;g__;s__
LMSG_G000009401.1	no	1148_1	Pyrite	82.98	0.00	82.98	0	1	1	13	Medium quality	32399	2481154	65.90	122	d__Bacteria;p__Firmicutes_E;c__Sulfobacillia;o__Sulfobacillales;f__;g__;s__
LMSG_G000009402.1	no	1148_1	Pyrite	65.33	0.50	62.86	0	0	1	11	Medium quality	2895	2278757	66.20	888	d__Bacteria;p__Firmicutes_E;c__Sulfobacillia;o__Sulfobacillales;f__;g__;s__
LMSG_G000009403.1	no	1148_1	Copper	92.57	2.48	80.20	0	2	1	16	Medium quality	70335	2875014	66.10	153	d__Bacteria;p__Firmicutes_E;c__Sulfobacillia;o__Sulfobacillales;f__;g__;s__
LMSG_G000009404.1	no	1150_1	Copper	78.58	1.98	68.68	0	0	0	16	Medium quality	34644	2426470	68.90	102	d__Bacteria;p__Firmicutes_E;c__Sulfobacillia;o__Sulfobacillales;f__;g__;s__
LMSG_G000009405.1	no	1150_1	Polymetallic	79.03	0.33	77.38	0	0	1	17	Medium quality	6539	2324754	69.00	502	d__Bacteria;p__Firmicutes_E;c__Sulfobacillia;o__Sulfobacillales;f__;g__;s__
LMSG_G000009406.1	no	1151_1	Tin-Zinc	92.82	4.13	72.20	0	0	0	17	Medium quality	26345	3643108	68.50	225	d__Bacteria;p__Firmicutes_E;c__Sulfobacillia;o__Sulfobacillales;f__;g__;s__
LMSG_G000009407.1	no	1151_2	Pyrite	86.30	1.32	79.70	0	1	0	18	Medium quality	47948	3093274	67.40	229	d__Bacteria;p__Firmicutes_E;c__Sulfobacillia;o__Sulfobacillales;f__;g__;s__

LMSG_G000009408.1	no	1151_2	Copper	90.59	1.49	83.17	0	0	0	18	Medium quality	28061	3473237	67.50	182	d__Bacteria;p__Firmicutes_E;c__Sulfobacillia;o__Sulfobacillales;f__;g__;s__
LMSG_G000009409.1	no	1151_2	Copper	59.37	0.00	59.37	0	1	0	16	Medium quality	5050	2531732	67.80	578	d__Bacteria;p__Firmicutes_E;c__Sulfobacillia;o__Sulfobacillales;f__;g__;s__
LMSG_G000009410.1	no	1151_2	Copper	93.18	1.32	86.58	0	1	1	18	Medium quality	28884	3966605	67.30	379	d__Bacteria;p__Firmicutes_E;c__Sulfobacillia;o__Sulfobacillales;f__;g__;s__
LMSG_G000009411.1	no	1166_1	Copper	95.21	0.33	93.56	1	1	0	15	Medium quality	16888	3530542	59.50	432	d__Bacteria;p__Firmicutes_E;c__Sulfobacillia;o__Sulfobacillales;f__;g__;s__
LMSG_G000009412.1	no	1167_1	Copper	84.50	1.33	77.84	1	3	1	14	Medium quality	25729	3708025	59.90	211	d__Bacteria;p__Firmicutes_E;c__Sulfobacillia;o__Sulfobacillales;f__;g__;s__
LMSG_G000009413.1	no	1167_1	Copper	54.40	0.00	54.40	1	0	1	4	Medium quality	5484	2087523	61.00	537	d__Bacteria;p__Firmicutes_E;c__Sulfobacillia;o__Sulfobacillales;f__;g__;s__
LMSG_G000009414.1	no	1138_1	Tin-Zinc	93.00	1.00	88.00	1	3	2	18	High quality	35695	3820812	49.80	221	d__Bacteria;p__Firmicutes_E;c__Sulfobacillia;o__Sulfobacillales;f__Sulfobacillaceae;g__Sulfobacillus;s__
LMSG_G000009415.1	no	1138_1	Copper	88.83	0.00	88.83	0	2	0	18	Medium quality	10179	4600925	49.60	549	d__Bacteria;p__Firmicutes_E;c__Sulfobacillia;o__Sulfobacillales;f__Sulfobacillaceae;g__Sulfobacillus;s__
LMSG_G000009416.1	no	1138_1	Copper	64.89	0.00	64.89	0	1	1	10	Medium quality	7600	3398736	49.70	707	d__Bacteria;p__Firmicutes_E;c__Sulfobacillia;o__Sulfobacillales;f__Sulfobacillaceae;g__Sulfobacillus;s__
LMSG_G000009417.1	no	1139_1	Tin-Zinc	97.00	2.03	86.83	0	2	0	18	Medium quality	32320	4212802	49.60	208	d__Bacteria;p__Firmicutes_E;c__Sulfobacillia;o__Sulfobacillales;f__Sulfobacillaceae;g__Sulfobacillus;s__
LMSG_G000009418.1	no	1139_1	Copper	97.00	1.37	90.17	0	2	1	16	Medium quality	49557	3639734	49.70	177	d__Bacteria;p__Firmicutes_E;c__Sulfobacillia;o__Sulfobacillales;f__Sulfobacillaceae;g__Sulfobacillus;s__
LMSG_G000009419.1	no	1139_1	Copper	89.00	1.03	83.83	0	1	1	17	Medium quality	57741	3029478	49.60	180	d__Bacteria;p__Firmicutes_E;c__Sulfobacillia;o__Sulfobacillales;f__Sulfobacillaceae;g__Sulfobacillus;s__
LMSG_G000009420.1	no	1139_1	Copper	97.00	2.03	86.83	0	2	0	13	Medium quality	30387	3829379	49.70	189	d__Bacteria;p__Firmicutes_E;c__Sulfobacillia;o__Sulfobacillales;f__Sulfobacillaceae;g__Sulfobacillus;s__
LMSG_G000009421.1	no	1139_1	Copper	95.00	1.37	88.17	0	2	1	16	Medium quality	65285	3942809	49.80	218	d__Bacteria;p__Firmicutes_E;c__Sulfobacillia;o__Sulfobacillales;f__Sulfobacillaceae;g__Sulfobacillus;s__
LMSG_G000009422.1	no	1139_1	Pyrite	98.00	2.37	86.17	0	2	2	19	Medium quality	36814	4232282	49.80	313	d__Bacteria;p__Firmicutes_E;c__Sulfobacillia;o__Sulfobacillales;f__Sulfobacillaceae;g__Sulfobacillus;s__
LMSG_G000009423.1	no	1165_1	Polymetallic	83.49	4.00	63.49	0	2	0	16	Medium quality	8601	3044287	58.30	475	d__Bacteria;p__Firmicutes_E;c__Sulfobacillia;o__Sulfobacillales;f__Sulfobacillaceae;g__Sulfobacillus;s__
LMSG_G000009424.1	no	1165_1	Polymetallic	97.00	2.37	85.17	2	1	1	18	High quality	68047	4493897	57.40	172	d__Bacteria;p__Firmicutes_E;c__Sulfobacillia;o__Sulfobacillales;f__Sulfobacillaceae;g__Sulfobacillus;s__
LMSG_G000009425.1	no	1140_1	Lead-Zinc	98.50	0.00	98.50	1	1	15	20	High quality	87663	4147788	52.20	181	d__Bacteria;p__Firmicutes_E;c__Sulfobacillia;o__Sulfobacillales;f__Sulfobacillaceae;g__Sulfobacillus;s__Sulfobacillus benefaciens_A
LMSG_G000009426.1	no	1140_1	Lead-Zinc	71.00	0.03	70.83	0	1	0	15	Medium quality	5350	2971824	52.40	616	d__Bacteria;p__Firmicutes_E;c__Sulfobacillia;o__Sulfobacillales;f__Sulfobacillaceae;g__Sulfobacillus;s__Sulfobacillus benefaciens_A
LMSG_G000009427.1	no	1140_1	Pyrite	78.74	1.25	72.49	0	0	3	16	Medium quality	3328	3259002	52.30	1120	d__Bacteria;p__Firmicutes_E;c__Sulfobacillia;o__Sulfobacillales;f__Sulfobacillaceae;g__Sulfobacillus;s__Sulfobacillus benefaciens_A
LMSG_G000009428.1	no	1140_1	Pyrite	95.50	3.00	80.50	0	1	1	16	Medium quality	13101	3617301	52.20	396	d__Bacteria;p__Firmicutes_E;c__Sulfobacillia;o__Sulfobacillales;f__Sulfobacillaceae;g__Sulfobacillus;s__Sulfobacillus benefaciens_A
LMSG_G000009429.1	no	1140_1	Lead-Zinc	98.50	0.00	98.50	0	1	1	19	Medium quality	98988	4236620	52.10	131	d__Bacteria;p__Firmicutes_E;c__Sulfobacillia;o__Sulfobacillales;f__Sulfobacillaceae;g__Sulfobacillus;s__Sulfobacillus benefaciens_A
LMSG_G000009430.1	no	1140_1	Lead-Zinc	98.50	0.00	98.50	0	4	1	19	Medium quality	64886	4146137	52.20	117	d__Bacteria;p__Firmicutes_E;c__Sulfobacillia;o__Sulfobacillales;f__Sulfobacillaceae;g__Sulfobacillus;s__Sulfobacillus benefaciens_A
LMSG_G000009431.1	no	1140_1	Lead-Zinc	93.50	0.00	93.50	0	2	0	18	Medium quality	71383	4025962	52.20	118	d__Bacteria;p__Firmicutes_E;c__Sulfobacillia;o__Sulfobacillales;f__Sulfobacillaceae;g__Sulfobacillus;s__Sulfobacillus benefaciens_A
LMSG_G000009432.1	no	1140_1	Polymetallic	97.50	0.00	97.50	0	1	1	18	Medium quality	98651	4254355	52.20	142	d__Bacteria;p__Firmicutes_E;c__Sulfobacillia;o__Sulfobacillales;f__Sulfobacillaceae;g__Sulfobacillus;s__Sulfobacillus benefaciens_A
LMSG_G000009433.1	no	1140_1	Polymetallic	98.50	1.00	93.50	0	1	2	17	Medium quality	109040	4185648	52.20	140	d__Bacteria;p__Firmicutes_E;c__Sulfobacillia;o__Sulfobacillales;f__Sulfobacillaceae;g__Sulfobacillus;s__Sulfobacillus benefaciens_A
LMSG_G000009434.1	no	1140_1	Polymetallic	98.50	0.00	98.50	0	1	1	19	Medium quality	86736	4326994	52.20	174	d__Bacteria;p__Firmicutes_E;c__Sulfobacillia;o__Sulfobacillales;f__Sulfobacillaceae;g__Sulfobacillus;s__Sulfobacillus benefaciens_A
LMSG_G000009435.1	no	1140_1	Polymetallic	98.50	0.00	98.50	0	1	3	19	Medium quality	98783	4120997	52.20	144	d__Bacteria;p__Firmicutes_E;c__Sulfobacillia;o__Sulfobacillales;f__Sulfobacillaceae;g__Sulfobacillus;s__Sulfobacillus benefaciens_A
LMSG_G000009436.1	no	1140_1	Tin-Zinc	92.50	2.00	82.50	0	2	0	16	Medium quality	39841	4188815	52.00	169	d__Bacteria;p__Firmicutes_E;c__Sulfobacillia;o__Sulfobacillales;f__Sulfobacillaceae;g__Sulfobacillus;s__Sulfobacillus benefaciens_A
LMSG_G000009437.1	no	1140_1	Polymetallic	98.50	0.00	98.50	0	1	1	19	Medium quality	107698	4453406	52.30	159	d__Bacteria;p__Firmicutes_E;c__Sulfobacillia;o__Sulfobacillales;f__Sulfobacillaceae;g__Sulfobacillus;s__Sulfobacillus benefaciens_A
LMSG_G000009438.1	no	1140_1	Copper	94.00	0.00	94.00	0	1	3	17	Medium quality	98411	4067123	52.20	264	d__Bacteria;p__Firmicutes_E;c__Sulfobacillia;o__Sulfobacillales;f__Sulfobacillaceae;g__Sulfobacillus;s__Sulfobacillus benefaciens_A
LMSG_G000009439.1	no	1140_1	Copper	98.50	0.33	96.84	0	0	0	18	Medium quality	92542	4497316	52.40	195	d__Bacteria;p__Firmicutes_E;c__Sulfobacillia;o__Sulfobacillales;f__Sulfobacillaceae;g__Sulfobacillus;s__Sulfobacillus benefaciens_A
LMSG_G000009440.1	no	1140_1	Copper	97.50	3.50	80.00	0	2	1	17	Medium quality	54409	4089968	52.10	136	d__Bacteria;p__Firmicutes_E;c__Sulfobacillia;o__Sulfobacillales;f__Sulfobacillaceae;g__Sulfobacillus;s__Sulfobacillus benefaciens_A
LMSG_G000009441.1	no	1140_1	Copper	98.50	0.00	98.50	0	2	0	18	Medium quality	69494	4156860	52.10	124	d__Bacteria;p__Firmicutes_E;c__Sulfobacillia;o__Sulfobacillales;f__Sulfobacillaceae;g__Sulfobacillus;s__Sulfobacillus benefaciens_A
LMSG_G000009442.1	no	1140_1	Polymetallic	94.50	0.00	94.50	0	2	0	18	Medium quality	16337	3864484	52.20	316	d__Bacteria;p__Firmicutes_E;c__Sulfobacillia;o__Sulfobacillales;f__Sulfobacillaceae;g__Sulfobacillus;s__Sulfobacillus benefaciens_A
LMSG_G000009443.1	no	1140_1	Polymetallic	89.50	0.00	89.50	0	1	1	19	Medium quality	103236	4161187	52.20	177	d__Bacteria;p__Firmicutes_E;c__Sulfobacillia;o__Sulfobacillales;f__Sulfobacillaceae;g__Sulfobacillus;s__Sulfobacillus benefaciens_A
LMSG_G000009444.1	no	1140_1	Polymetallic	89.50	0.00	89.50	0	1	1	16	Medium quality	103325	3815040	52.20	160	d__Bacteria;p__Firmicutes_E;c__Sulfobacillia;o__Sulfobacillales;f__Sulfobacillaceae;g__Sulfobacillus;s__Sulfobacillus benefaciens_A
LMSG_G000009445.1	no	1140_1	Magnetite	80.68	0.33	79.02	0	1	1	15	Medium quality	6488	3516727	52.10	659	d__Bacteria;p__Firmicutes_E;c__Sulfobacillia;o__Sulfobacillales;f__Sulfobacillaceae;g__Sulfobacillus;s__Sulfobacillus benefaciens_A
LMSG_G000009446.1	no	1140_1	Copper	97.50	0.00	97.50	0	1	0	18	Medium quality	129011	3674593	52.20	180	d__Bacteria;p__Firmicutes_E;c__Sulfobacillia;o__Sulfobacillales;f__Sulfobacillaceae;g__Sulfobacillus;s__Sulfobacillus benefaciens_A
LMSG_G000009447.1	no	1140_1	Copper	98.50	1.00	93.50	0	2	0	17	Medium quality	53074	4106032	52.20	137	d__Bacteria;p__Firmicutes_E;c__Sulfobacillia;o__Sulfobacillales;f__Sulfobacillaceae;g__Sulfobacillus;s__Sulfobacillus benefaciens_A
LMSG_G000009448.1	no	1140_1	Polymetallic	98.50	0.50	96.00	0	1	1	20	Medium quality	97255	4342920	52.10	218	d__Bacteria;p__Firmicutes_E;c__Sulfobacillia;o__Sulfobacillales;f__Sulfobacillaceae;g__Sulfobacillus;s__Sulfobacillus benefaciens_A
LMSG_G000009449.1	no	1140_1	Polymetallic	98.00	0.00	98.00	0	1	4	20	Medium quality	114935	4171512	52.10	178	d__Bacteria;p__Firmicutes_E;c__Sulfobacillia;o__Sulfobacillales;f__Sulfobacillaceae;g__Sulfobacillus;s__Sulfobacillus benefaciens_A
LMSG_G000009450.1	no	1140_1	Copper	98.50	0.50	96.00	0	2	5	18	Medium quality	102349	4331741	52.10	175	d__Bacteria;p__Firmicutes_E;c__Sulfobacillia;o__Sulfobacillales;f__Sulfobacillaceae;g__Sulfobacillus;s__Sulfobacillus benefaciens_A
LMSG_G000009451.1	no	1140_1	Copper	92.50	0.00	92.50	0	1	1	19	Medium quality	76669	3531219	52.20	225	d__Bacteria;p__Firmicutes_E;c__Sulfobacillia;o__Sulfobacillales;f__Sulfobacillaceae;g__Sulfobacillus;s__Sulfobacillus benefaciens_A
LMSG_G000009452.1	no	1140_1	Copper	98.50	0.00	98.50	0	2	0	19	Medium quality	47512	4332887	52.00	142	d__Bacteria;p__Firmicutes_E;c__Sulfobacillia;o__Sulfobacillales;f__Sulfobacillaceae;g__Sulfobacillus;s__Sulfobacillus benefaciens_A
LMSG_G000009453.1	no	1105_1	Copper	97.00	3.87	77.67	0	2	0	18	Medium quality	17306	5468815	57.10	646	d__Bacteria;p__Firmicutes_E;c__Sulfobacillia;o__Sulfobacillales;f__Sulfobacillaceae;g__Sulfobacillus_B;s__
LMSG_G000009454.1	no	1105_1	Copper	78.99	1.78	70.08	0	0	0	13	Medium quality	5911	3400512	57.50	651	d__Bacteria;p__Firmicutes_E;c__Sulfobacillia;o__Sulfobacillales;f__Sulfobacillaceae;g__Sulfobacillus_B;s__
LMSG_G000009455.1	no	1105_1	Lead-Zinc	59.32	1.20	53.32	0	0	1	7	Medium quality	2643	2711600	58.80	1227	d__Bacteria;p__Firmicutes_E;c__Sulfobacillia;o__Sulfobacillales;f__Sulfobacillaceae;g__Sulfobacillus_B;s__
LMSG_G000009456.1	no	1105_1	Lead-Zinc	52.19	0.00	52.19	0	0	1	13	Medium quality	3785	2315446	57.40	656	d__Bacteria;p__Firmicutes_E;c__Sulfobacillia;o__Sulfobacillales;f__Sulfobacillaceae;g__Sulfobacillus_B;s__
LMSG_G000009457.1	no	1105_1	Copper	66.65	0.03	66.48	0	0	0	15	Medium quality	3669	2478010	57.70	737	d__Bacteria;p__Firmicutes_E;c__Sulfobacillia;o__Sulfobacillales;f__Sulfobacillaceae;g__Sulfobacillus_B;s__
LMSG_G000009458.1	no	1106_1	Lead-Zinc	81.87	3.13	66.25	0	1	1	15	Medium quality	8623	3156504	59.00	507	d__Bacteria;p__Firmicutes_E;c__Sulfobacillia;o__Sulfobacillales;f__Sulfobacillaceae;g__Sulfobacillus_B;s__
LMSG_G000009459.1	no	1106_1	Lead-Zinc	94.50	2.53	81.83	0	1	0	18	Medium quality	49903	3740291	59.10	219	d__Bacteria;p__Firmicutes_E;c__Sulfobacillia;o__Sulfobacillales;f__Sulfobacillaceae;g__Sulfobacillus_B;s__
LMSG_G000009460.1	no	1107_1	Lead-Zinc	76.51	1.00	71.51	0	1	0	15	Medium quality	6704	3584601	60.30	660	d__Bacteria;p__Firmicutes_E;c__Sulfobacillia;o__Sulfobacillales;f__Sulfobacillaceae;g__Sulfobacillus_B;s__

LMSG_G000009461.1	no	1107_1	Lead-Zinc	85.17	2.70	71.67	0	0	0	11	Medium quality	39997	3883710	60.20	245	d__Bacteria;p__Firmicutes_E;c__Sulfobacillia;o__Sulfobacillales;f__Sulfobacillaceae;g__Sulfobacillus_B;s__
LMSG_G000009462.1	no	1108_1	Polymetallic	72.60	1.37	65.77	0	2	1	16	Medium quality	7803	2682524	58.90	429	d__Bacteria;p__Firmicutes_E;c__Sulfobacillia;o__Sulfobacillales;f__Sulfobacillaceae;g__Sulfobacillus_B;s__
LMSG_G000009463.1	no	1108_1	Polymetallic	79.40	0.33	77.74	0	0	0	14	Medium quality	8355	2570071	58.80	393	d__Bacteria;p__Firmicutes_E;c__Sulfobacillia;o__Sulfobacillales;f__Sulfobacillaceae;g__Sulfobacillus_B;s__
LMSG_G000009464.1	no	1108_1	Polymetallic	92.01	4.19	71.06	0	1	0	16	Medium quality	8351	3346513	59.00	508	d__Bacteria;p__Firmicutes_E;c__Sulfobacillia;o__Sulfobacillales;f__Sulfobacillaceae;g__Sulfobacillus_B;s__
LMSG_G000009465.1	no	1108_1	Pyrite	68.52	0.60	65.51	0	1	0	11	Medium quality	4830	2674822	59.00	656	d__Bacteria;p__Firmicutes_E;c__Sulfobacillia;o__Sulfobacillales;f__Sulfobacillaceae;g__Sulfobacillus_B;s__
LMSG_G000009466.1	no	1108_1	Pyrite	89.86	0.90	85.35	0	1	1	17	Medium quality	6114	3238328	58.80	647	d__Bacteria;p__Firmicutes_E;c__Sulfobacillia;o__Sulfobacillales;f__Sulfobacillaceae;g__Sulfobacillus_B;s__
LMSG_G000009467.1	no	1108_1	Pyrite	55.36	0.09	54.91	0	0	2	16	Medium quality	4447	2055040	59.10	502	d__Bacteria;p__Firmicutes_E;c__Sulfobacillia;o__Sulfobacillales;f__Sulfobacillaceae;g__Sulfobacillus_B;s__
LMSG_G000009468.1	no	1143_1	Copper	90.59	2.00	80.59	0	1	2	16	Medium quality	9972	3523177	55.40	434	d__Bacteria;p__Firmicutes_E;c__Sulfobacillia;o__Sulfobacillales;f__Sulfobacillaceae;g__Sulfobacillus_B;s__
LMSG_G000009469.1	no	1143_1	Copper	91.50	0.33	89.84	0	3	1	15	Medium quality	21970	4273593	55.00	295	d__Bacteria;p__Firmicutes_E;c__Sulfobacillia;o__Sulfobacillales;f__Sulfobacillaceae;g__Sulfobacillus_B;s__
LMSG_G000009470.1	no	1141_1	Copper	91.00	0.33	89.34	0	1	1	16	Medium quality	24015	3730518	51.90	357	d__Bacteria;p__Firmicutes_E;c__Sulfobacillia;o__Sulfobacillales;f__Sulfobacillaceae;g__Sulfobacillus_C;s__
LMSG_G000009471.1	no	1141_1	Copper	90.50	0.33	88.84	0	3	1	19	Medium quality	30960	4455404	52.20	229	d__Bacteria;p__Firmicutes_E;c__Sulfobacillia;o__Sulfobacillales;f__Sulfobacillaceae;g__Sulfobacillus_C;s__
LMSG_G000009472.1	no	1145_1	Tin-Zinc	98.00	1.37	91.17	0	3	0	17	Medium quality	35652	3724207	53.60	166	d__Bacteria;p__Firmicutes_E;c__Sulfobacillia;o__Sulfobacillales;f__Sulfobacillaceae;g__Sulfobacillus_C;s__
LMSG_G000009473.1	no	1146_1	Pyrite-Copper	97.66	0.33	96.00	0	2	0	18	Medium quality	63278	3956322	52.50	123	d__Bacteria;p__Firmicutes_E;c__Sulfobacillia;o__Sulfobacillales;f__Sulfobacillaceae;g__Sulfobacillus_C;s__
LMSG_G000009474.1	no	1146_1	Pyrite-Copper	98.00	0.33	96.34	0	1	1	19	Medium quality	68921	3814005	52.50	114	d__Bacteria;p__Firmicutes_E;c__Sulfobacillia;o__Sulfobacillales;f__Sulfobacillaceae;g__Sulfobacillus_C;s__
LMSG_G000009475.1	no	1146_1	Polymetallic	93.90	0.92	89.28	0	3	1	16	Medium quality	28617	3544974	52.70	295	d__Bacteria;p__Firmicutes_E;c__Sulfobacillia;o__Sulfobacillales;f__Sulfobacillaceae;g__Sulfobacillus_C;s__
LMSG_G000009476.1	no	644_1	Copper	89.60	0.00	89.60	0	0	0	19	Medium quality	23676	3905296	70.20	300	d__Bacteria;p__Firmicutes_E;c__Thermaerobacteria;o__;f__;g__;s__
LMSG_G000009477.1	no	646_1	Pyrite	51.72	0.00	51.72	0	1	0	12	Medium quality	3125	2967576	69.70	997	d__Bacteria;p__Firmicutes_E;c__Thermaerobacteria;o__;f__;g__;s__
LMSG_G000009478.1	no	646_1	Pyrite	78.21	0.83	74.09	1	1	1	14	Medium quality	10204	3523769	69.30	480	d__Bacteria;p__Firmicutes_E;c__Thermaerobacteria;o__;f__;g__;s__
LMSG_G000009479.1	no	646_1	Pyrite	90.09	2.81	76.07	0	1	0	17	Medium quality	38196	3731561	69.00	204	d__Bacteria;p__Firmicutes_E;c__Thermaerobacteria;o__;f__;g__;s__
LMSG_G000009480.1	no	646_1	Copper	83.66	2.81	69.64	0	1	0	14	Medium quality	54214	3361052	69.00	153	d__Bacteria;p__Firmicutes_E;c__Thermaerobacteria;o__;f__;g__;s__
LMSG_G000009481.1	no	646_1	Polymetallic	81.68	3.80	62.71	0	1	0	13	Medium quality	57851	3720634	69.50	149	d__Bacteria;p__Firmicutes_E;c__Thermaerobacteria;o__;f__;g__;s__
LMSG_G000009482.1	no	711_1	Coal	61.75	0.00	61.75	0	1	0	15	Medium quality	5129	3207374	72.60	700	d__Bacteria;p__Firmicutes_E;c__Thermaerobacteria;o__;f__;g__;s__
LMSG_G000009483.1	no	711_1	Coal	80.99	1.08	75.59	0	1	0	18	Medium quality	8201	3919065	72.20	577	d__Bacteria;p__Firmicutes_E;c__Thermaerobacteria;o__;f__;g__;s__
LMSG_G000009484.1	no	461_1	Lead-Zinc	63.21	2.55	50.45	0	0	0	9	Medium quality	3290	2044277	62.20	733	d__Bacteria;p__Firmicutes_G;c__DTU065;o__;f__;g__;s__
LMSG_G000009485.1	no	1578_1	Polymetallic	93.40	4.40	71.43	0	1	0	16	Medium quality	37853	3208555	67.80	173	d__Bacteria;p__Gemmatimonadota;c__Gemmatimonadetes;o__Gemmatimonadales;f__Gemmatimonadaceae;g__Fen-1231;s__
LMSG_G000009486.1	no	1578_1	Polymetallic	87.91	4.40	65.94	0	0	0	19	Medium quality	41702	3205670	67.70	129	d__Bacteria;p__Gemmatimonadota;c__Gemmatimonadetes;o__Gemmatimonadales;f__Gemmatimonadaceae;g__Fen-1231;s__
LMSG_G000009487.1	no	1578_1	Polymetallic	90.65	4.95	65.93	0	1	0	16	Medium quality	55287	3262168	67.70	123	d__Bacteria;p__Gemmatimonadota;c__Gemmatimonadetes;o__Gemmatimonadales;f__Gemmatimonadaceae;g__Fen-1231;s__
LMSG_G000009488.1	no	1579_1	Polymetallic	58.02	0.86	53.71	1	1	2	15	Medium quality	3616	3384021	68.60	1012	d__Bacteria;p__Gemmatimonadota;c__Gemmatimonadetes;o__Gemmatimonadales;f__Gemmatimonadaceae;g__SCN-70-22;s__
LMSG_G000009489.1	no	1579_1	Polymetallic	61.37	1.72	52.75	1	4	1	19	Medium quality	5112	3368706	68.70	778	d__Bacteria;p__Gemmatimonadota;c__Gemmatimonadetes;o__Gemmatimonadales;f__Gemmatimonadaceae;g__SCN-70-22;s__
LMSG_G000009490.1	no	1378_1	Pyrite-Copper	90.32	1.29	83.87	1	1	2	19	High quality	27267	3566765	71.70	189	d__Bacteria;p__Myxococcota;c__Myxococcia;o__Myxococcales;f__;g__;s__
LMSG_G000009491.1	no	1378_1	Pyrite-Copper	80.43	0.00	80.43	1	0	1	17	Medium quality	17106	3140526	71.50	224	d__Bacteria;p__Myxococcota;c__Myxococcia;o__Myxococcales;f__;g__;s__
LMSG_G000009492.1	no	1379_1	Pyrite	93.22	0.65	90.00	1	1	1	18	High quality	37882	4368824	72.00	162	d__Bacteria;p__Myxococcota;c__Myxococcia;o__Myxococcales;f__;g__;s__
LMSG_G000009493.1	no	1379_1	Antimony	93.70	1.36	86.89	1	1	1	19	High quality	38727	4510795	71.90	202	d__Bacteria;p__Myxococcota;c__Myxococcia;o__Myxococcales;f__;g__;s__
LMSG_G000009494.1	no	1379_1	Antimony	91.29	1.42	84.20	1	1	2	20	High quality	36015	4345743	71.90	171	d__Bacteria;p__Myxococcota;c__Myxococcia;o__Myxococcales;f__;g__;s__
LMSG_G000009495.1	no	1379_1	Antimony	92.51	1.29	86.06	1	1	1	19	High quality	15529	4217381	71.80	422	d__Bacteria;p__Myxococcota;c__Myxococcia;o__Myxococcales;f__;g__;s__
LMSG_G000009496.1	no	1379_1	Antimony	91.77	1.94	82.10	1	1	1	19	High quality	34450	4375208	71.90	214	d__Bacteria;p__Myxococcota;c__Myxococcia;o__Myxococcales;f__;g__;s__
LMSG_G000009497.1	no	1379_1	Antimony	74.66	0.00	74.66	0	0	0	16	Medium quality	9438	3475850	71.80	442	d__Bacteria;p__Myxococcota;c__Myxococcia;o__Myxococcales;f__;g__;s__
LMSG_G000009498.1	no	1379_1	Antimony	83.40	0.00	83.40	1	0	1	19	Medium quality	12804	3965166	71.70	402	d__Bacteria;p__Myxococcota;c__Myxococcia;o__Myxococcales;f__;g__;s__
LMSG_G000009499.1	no	1379_1	Pyrite-Copper	87.90	0.00	87.90	1	1	1	19	Medium quality	48268	4066572	72.10	118	d__Bacteria;p__Myxococcota;c__Myxococcia;o__Myxococcales;f__;g__;s__
LMSG_G000009500.1	no	1379_1	Pyrite-Copper	71.04	2.90	56.53	0	0	0	14	Medium quality	4534	3104631	72.40	797	d__Bacteria;p__Myxococcota;c__Myxococcia;o__Myxococcales;f__;g__;s__
LMSG_G000009501.1	no	1373_1	Polymetallic	89.22	1.35	82.48	1	1	1	18	Medium quality	7691	3503104	74.40	608	d__Bacteria;p__Myxococcota;c__Myxococcia;o__Myxococcales;f__Anaeromyxobacteraceae;g__;s__
LMSG_G000009502.1	no	1372_1	Antimony	73.12	1.75	64.35	0	1	0	17	Medium quality	6823	2585988	73.20	479	d__Bacteria;p__Myxococcota;c__Myxococcia;o__Myxococcales;f__Anaeromyxobacteraceae;g__Anaeromyxobacter;s__
LMSG_G000009503.1	no	1373_1	Polymetallic	77.03	2.20	66.05	0	0	0	15	Medium quality	5000	3067875	73.90	726	d__Bacteria;p__Myxococcota;c__Myxococcia;o__Myxococcales;f__Anaeromyxobacteraceae;g__Anaeromyxobacter;s__
LMSG_G000009504.1	no	1373_1	Polymetallic	86.87	3.61	68.81	1	1	1	17	Medium quality	7813	3445685	74.40	593	d__Bacteria;p__Myxococcota;c__Myxococcia;o__Myxococcales;f__Anaeromyxobacteraceae;g__Anaeromyxobacter;s__
LMSG_G000009505.1	no	1373_1	Polymetallic	89.36	2.58	76.46	1	1	1	16	Medium quality	7482	3206549	74.70	562	d__Bacteria;p__Myxococcota;c__Myxococcia;o__Myxococcales;f__Anaeromyxobacteraceae;g__Anaeromyxobacter;s__
LMSG_G000009506.1	no	1374_1	Antimony	92.79	2.10	82.31	0	1	0	15	Medium quality	6639	2967260	75.50	583	d__Bacteria;p__Myxococcota;c__Myxococcia;o__Myxococcales;f__Anaeromyxobacteraceae;g__Anaeromyxobacter;s__
LMSG_G000009507.1	no	1374_1	Antimony	88.92	0.97	84.09	1	1	1	18	Medium quality	5072	2888814	75.00	677	d__Bacteria;p__Myxococcota;c__Myxococcia;o__Myxococcales;f__Anaeromyxobacteraceae;g__Anaeromyxobacter;s__
LMSG_G000009508.1	no	1374_1	Antimony	82.95	0.00	82.95	1	1	1	17	Medium quality	4924	2651774	75.00	650	d__Bacteria;p__Myxococcota;c__Myxococcia;o__Myxococcales;f__Anaeromyxobacteraceae;g__Anaeromyxobacter;s__
LMSG_G000009509.1	no	1374_1	Antimony	92.74	1.94	83.07	0	1	0	15	Medium quality	5726	2981262	75.30	642	d__Bacteria;p__Myxococcota;c__Myxococcia;o__Myxococcales;f__Anaeromyxobacteraceae;g__Anaeromyxobacter;s__
LMSG_G000009510.1	no	1374_1	Polymetallic	65.26	1.35	58.52	0	1	0	12	Medium quality	3546	2175523	75.00	662	d__Bacteria;p__Myxococcota;c__Myxococcia;o__Myxococcales;f__Anaeromyxobacteraceae;g__Anaeromyxobacter;s__
LMSG_G000009511.1	no	1374_1	Polymetallic	80.89	1.29	74.44	0	0	0	14	Medium quality	4340	2549635	75.10	683	d__Bacteria;p__Myxococcota;c__Myxococcia;o__Myxococcales;f__Anaeromyxobacteraceae;g__Anaeromyxobacter;s__
LMSG_G000009512.1	no	1374_1	Pyrite-Copper	88.07	2.47	75.71	0	0	0	14	Medium quality	4992	2618280	75.30	617	d__Bacteria;p__Myxococcota;c__Myxococcia;o__Myxococcales;f__Anaeromyxobacteraceae;g__Anaeromyxobacter;s__
LMSG_G000009513.1	no	1375_1	Antimony	86.18	1.65	77.94	1	1	1	18	Medium quality	5158	3566875	74.10	850	d__Bacteria;p__Myxococcota;c__Myxococcia;o__Myxococcales;f__Anaeromyxobacteraceae;g__Anaeromyxobacter;s__
LMSG_G000009514.1	no	1113_1	Polymetallic	75.63	1.76	66.82	0	7	13	19	Medium quality	5285	1968793	56.70	482	d__Bacteria;p__Nitrospinota_A;c__UBA7883;o__;f__;g__;s__
LMSG_G000009515.1	no	1113_1	Polymetallic	63.36	2.56	50.54	0	0	0	12	Medium quality	3745	1925264	56.00	625	d__Bacteria;p__Nitrospinota_A;c__UBA7883;o__;f__;g__;s__
LMSG_G000009516.1	no	1113_1	Polymetallic	66.60	1.98	56.72	0	0	0	10	Medium quality	2974	1738515	56.10	653	d__Bacteria;p__Nitrospinota_A;c__UBA7883;o__;f__;g__;s__
LMSG_G000009517.1	no	952_1	Lead-Zinc	71.05	1.75	62.28	0	0	0	15	Medium quality	32320	2141769	51.80	111	d__Bacteria;p__Nitrospirota;c__Leptospirillia;o__Leptospirillales;f__Leptospirillaceae;g__Leptospirillum;s__
LMSG_G000009518.1	no	952_1	Pyrite	89.96	1.82	80.87	0	0	0	17	Medium quality	28008	2420480	51.50	160	d__Bacteria;p__Nitrospirota;c__Leptospirillia;o__Leptospirillales;f__Leptospirillaceae;g__Leptospirillum;s__
LMSG_G000009519.1	no	952_1	Pyrite	89.03	2.05	78.81	0	1	0	17	Medium quality	38702	2477483	51.60	124	d__Bacteria;p__Nitrospirota;c__Leptospirillia;o__Leptospirillales;f__Leptospirillaceae;g__Leptospirillum;s__

LMSG_GO000009520.1	no	952_1	Pyrite	82.67	1.14	76.99	0	0	0	17	Medium quality	37117	2517313	51.20	136	d__Bacteria;p__Nitrospirota;c__Leptospirillia;o__Leptospirillales;f__Leptospirillaceae;g__Leptospirillum;s__
LMSG_GO000009521.1	no	952_1	Pyrite	61.40	1.75	52.63	1	0	1	9	Medium quality	7173	1722894	51.40	368	d__Bacteria;p__Nitrospirota;c__Leptospirillia;o__Leptospirillales;f__Leptospirillaceae;g__Leptospirillum;s__
LMSG_GO000009522.1	no	952_1	Lead-Zinc	94.48	2.73	80.85	1	0	1	19	Medium quality	35772	2315330	51.20	107	d__Bacteria;p__Nitrospirota;c__Leptospirillia;o__Leptospirillales;f__Leptospirillaceae;g__Leptospirillum;s__
LMSG_GO000009523.1	no	952_1	Tin-Zinc	87.21	1.82	78.12	0	0	0	18	Medium quality	33033	2006002	51.40	94	d__Bacteria;p__Nitrospirota;c__Leptospirillia;o__Leptospirillales;f__Leptospirillaceae;g__Leptospirillum;s__
LMSG_GO000009524.1	no	952_1	Polymetallic	81.76	1.82	72.67	0	0	0	16	Medium quality	42895	1928931	51.20	70	d__Bacteria;p__Nitrospirota;c__Leptospirillia;o__Leptospirillales;f__Leptospirillaceae;g__Leptospirillum;s__
LMSG_GO000009525.1	no	952_1	Pyrite-Copper	87.04	4.64	63.86	1	0	0	16	Medium quality	13659	2346416	51.50	233	d__Bacteria;p__Nitrospirota;c__Leptospirillia;o__Leptospirillales;f__Leptospirillaceae;g__Leptospirillum;s__
LMSG_GO000009526.1	no	952_1	Coal	85.45	1.82	76.36	1	1	1	17	Medium quality	37036	2158452	51.70	126	d__Bacteria;p__Nitrospirota;c__Leptospirillia;o__Leptospirillales;f__Leptospirillaceae;g__Leptospirillum;s__
LMSG_GO000009527.1	no	953_1	Lead-Zinc	89.03	1.82	79.94	1	1	0	17	Medium quality	57289	2034239	49.90	65	d__Bacteria;p__Nitrospirota;c__Leptospirillia;o__Leptospirillales;f__Leptospirillaceae;g__Leptospirillum;s__Leptospirillum ferrooxidans
LMSG_GO000009528.1	no	953_1	Copper	77.86	3.64	59.68	1	0	1	16	Medium quality	28822	1952981	50.30	159	d__Bacteria;p__Nitrospirota;c__Leptospirillia;o__Leptospirillales;f__Leptospirillaceae;g__Leptospirillum;s__Leptospirillum ferrooxidans
LMSG_GO000009529.1	no	953_1	Pyrite	85.00	1.82	75.91	1	0	0	16	Medium quality	53451	1990079	50.30	60	d__Bacteria;p__Nitrospirota;c__Leptospirillia;o__Leptospirillales;f__Leptospirillaceae;g__Leptospirillum;s__Leptospirillum ferrooxidans
LMSG_GO000009530.1	no	953_1	Pyrite	80.85	1.89	71.39	1	0	1	13	Medium quality	113461	2004772	50.20	78	d__Bacteria;p__Nitrospirota;c__Leptospirillia;o__Leptospirillales;f__Leptospirillaceae;g__Leptospirillum;s__Leptospirillum ferrooxidans
LMSG_GO000009531.1	no	953_1	Pyrite	80.85	0.91	76.31	1	0	0	13	Medium quality	65576	1876377	50.10	65	d__Bacteria;p__Nitrospirota;c__Leptospirillia;o__Leptospirillales;f__Leptospirillaceae;g__Leptospirillum;s__Leptospirillum ferrooxidans
LMSG_GO000009532.1	no	953_1	Lead-Zinc	80.85	1.82	71.76	1	2	1	15	Medium quality	106435	1850583	50.00	75	d__Bacteria;p__Nitrospirota;c__Leptospirillia;o__Leptospirillales;f__Leptospirillaceae;g__Leptospirillum;s__Leptospirillum ferrooxidans
LMSG_GO000009533.1	no	953_1	Lead-Zinc	60.52	0.00	60.52	1	1	1	16	Medium quality	70433	1381900	49.20	77	d__Bacteria;p__Nitrospirota;c__Leptospirillia;o__Leptospirillales;f__Leptospirillaceae;g__Leptospirillum;s__Leptospirillum ferrooxidans
LMSG_GO000009534.1	no	953_1	Polymetallic	51.75	0.00	51.75	1	0	0	16	Medium quality	49942	1468912	50.40	59	d__Bacteria;p__Nitrospirota;c__Leptospirillia;o__Leptospirillales;f__Leptospirillaceae;g__Leptospirillum;s__Leptospirillum ferrooxidans
LMSG_GO000009535.1	no	953_1	Polymetallic	86.76	1.82	77.67	1	1	0	18	Medium quality	78471	2034104	49.60	62	d__Bacteria;p__Nitrospirota;c__Leptospirillia;o__Leptospirillales;f__Leptospirillaceae;g__Leptospirillum;s__Leptospirillum ferrooxidans
LMSG_GO000009536.1	no	953_1	Pyrite-Copper	57.01	0.00	57.01	2	0	0	16	Medium quality	54569	1800345	50.80	72	d__Bacteria;p__Nitrospirota;c__Leptospirillia;o__Leptospirillales;f__Leptospirillaceae;g__Leptospirillum;s__Leptospirillum ferrooxidans
LMSG_GO000009537.1	no	953_1	Pyrite-Copper	86.36	0.91	81.82	1	0	0	18	Medium quality	94512	1794843	49.50	58	d__Bacteria;p__Nitrospirota;c__Leptospirillia;o__Leptospirillales;f__Leptospirillaceae;g__Leptospirillum;s__Leptospirillum ferrooxidans
LMSG_GO000009538.1	no	953_1	Pyrite-Copper	56.86	0.91	52.32	0	0	0	10	Medium quality	4655	1296812	50.70	319	d__Bacteria;p__Nitrospirota;c__Leptospirillia;o__Leptospirillales;f__Leptospirillaceae;g__Leptospirillum;s__Leptospirillum ferrooxidans
LMSG_GO000009539.1	no	953_1	Polymetallic	70.35	2.05	60.13	0	1	1	16	Medium quality	24110	1894769	50.60	123	d__Bacteria;p__Nitrospirota;c__Leptospirillia;o__Leptospirillales;f__Leptospirillaceae;g__Leptospirillum;s__Leptospirillum ferrooxidans
LMSG_GO000009540.1	no	953_1	Pyrite-Copper	69.03	1.82	59.94	1	0	1	14	Medium quality	72398	1257921	48.80	38	d__Bacteria;p__Nitrospirota;c__Leptospirillia;o__Leptospirillales;f__Leptospirillaceae;g__Leptospirillum;s__Leptospirillum ferrooxidans
LMSG_GO000009541.1	no	953_1	Pyrite	68.12	1.82	59.03	1	2	2	14	Medium quality	47888	1801895	49.90	79	d__Bacteria;p__Nitrospirota;c__Leptospirillia;o__Leptospirillales;f__Leptospirillaceae;g__Leptospirillum;s__Leptospirillum ferrooxidans
LMSG_GO000009542.1	no	1647_1	Coal	91.30	0.91	86.76	1	1	5	19	High quality	17901	2030706	56.90	225	d__Bacteria;p__Nitrospirota;c__Leptospirillia;o__Leptospirillales;f__Leptospirillaceae;g__Leptospirillum_A;s__
LMSG_GO000009543.1	no	1647_1	Coal	58.60	0.00	58.60	0	0	0	12	Medium quality	2026	1150961	57.20	633	d__Bacteria;p__Nitrospirota;c__Leptospirillia;o__Leptospirillales;f__Leptospirillaceae;g__Leptospirillum_A;s__
LMSG_GO000009544.1	no	1647_1	Coal	57.01	0.00	57.01	0	6	5	15	Medium quality	23203	1621642	57.50	108	d__Bacteria;p__Nitrospirota;c__Leptospirillia;o__Leptospirillales;f__Leptospirillaceae;g__Leptospirillum_A;s__
LMSG_GO000009545.1	no	967_1	Lead-Zinc	82.69	1.82	73.60	2	1	0	17	Medium quality	42874	2187971	56.90	103	d__Bacteria;p__Nitrospirota;c__Leptospirillia;o__Leptospirillales;f__Leptospirillaceae;g__Leptospirillum_A;s__
LMSG_GO000009546.1	no	967_1	Lead-Zinc	90.34	1.82	81.25	2	0	0	17	Medium quality	47909	2369711	56.40	123	d__Bacteria;p__Nitrospirota;c__Leptospirillia;o__Leptospirillales;f__Leptospirillaceae;g__Leptospirillum_A;s__
LMSG_GO000009547.1	no	967_1	Lead-Zinc	83.90	4.55	61.18	1	1	0	15	Medium quality	40234	2149272	56.70	87	d__Bacteria;p__Nitrospirota;c__Leptospirillia;o__Leptospirillales;f__Leptospirillaceae;g__Leptospirillum_A;s__
LMSG_GO000009548.1	no	967_1	Lead-Zinc	68.42	0.00	68.42	2	0	1	11	Medium quality	6135	1954694	56.40	450	d__Bacteria;p__Nitrospirota;c__Leptospirillia;o__Leptospirillales;f__Leptospirillaceae;g__Leptospirillum_A;s__
LMSG_GO000009549.1	no	967_1	Copper	78.45	1.82	69.36	2	1	0	15	Medium quality	48236	1953926	56.60	72	d__Bacteria;p__Nitrospirota;c__Leptospirillia;o__Leptospirillales;f__Leptospirillaceae;g__Leptospirillum_A;s__
LMSG_GO000009550.1	no	967_1	Copper	83.98	1.82	74.89	1	0	0	16	Medium quality	46094	2026146	55.90	80	d__Bacteria;p__Nitrospirota;c__Leptospirillia;o__Leptospirillales;f__Leptospirillaceae;g__Leptospirillum_A;s__
LMSG_GO000009551.1	no	967_1	Copper	80.37	1.82	71.28	2	0	0	15	Medium quality	61583	2118817	56.70	93	d__Bacteria;p__Nitrospirota;c__Leptospirillia;o__Leptospirillales;f__Leptospirillaceae;g__Leptospirillum_A;s__
LMSG_GO000009552.1	no	967_1	Copper	76.31	0.00	76.31	1	0	1	17	Medium quality	73626	2207751	56.80	313	d__Bacteria;p__Nitrospirota;c__Leptospirillia;o__Leptospirillales;f__Leptospirillaceae;g__Leptospirillum_A;s__
LMSG_GO000009553.1	no	967_1	Copper	64.91	1.75	56.14	1	0	1	16	Medium quality	14383	2258957	57.10	301	d__Bacteria;p__Nitrospirota;c__Leptospirillia;o__Leptospirillales;f__Leptospirillaceae;g__Leptospirillum_A;s__
LMSG_GO000009554.1	no	967_1	Pyrite	91.23	3.18	75.33	1	0	1	16	Medium quality	17490	2450585	56.20	196	d__Bacteria;p__Nitrospirota;c__Leptospirillia;o__Leptospirillales;f__Leptospirillaceae;g__Leptospirillum_A;s__
LMSG_GO000009555.1	no	967_1	Pyrite	93.08	1.82	83.99	2	0	0	17	Medium quality	47904	2734679	55.80	112	d__Bacteria;p__Nitrospirota;c__Leptospirillia;o__Leptospirillales;f__Leptospirillaceae;g__Leptospirillum_A;s__
LMSG_GO000009556.1	no	968_1	Copper	57.01	0.00	57.01	0	0	0	8	Medium quality	92414	924480	53.50	27	d__Bacteria;p__Nitrospirota;c__Leptospirillia;o__Leptospirillales;f__Leptospirillaceae;g__Leptospirillum_A;s__
LMSG_GO000009557.1	no	968_1	Copper	58.50	0.00	58.50	0	0	0	9	Medium quality	81385	970873	53.60	29	d__Bacteria;p__Nitrospirota;c__Leptospirillia;o__Leptospirillales;f__Leptospirillaceae;g__Leptospirillum_A;s__
LMSG_GO000009558.1	no	968_1	Copper	80.75	2.73	67.12	0	1	0	18	Medium quality	50035	2061504	55.70	80	d__Bacteria;p__Nitrospirota;c__Leptospirillia;o__Leptospirillales;f__Leptospirillaceae;g__Leptospirillum_A;s__
LMSG_GO000009559.1	no	968_1	Pyrite	88.35	3.18	72.45	0	0	0	18	Medium quality	16261	2080792	54.90	201	d__Bacteria;p__Nitrospirota;c__Leptospirillia;o__Leptospirillales;f__Leptospirillaceae;g__Leptospirillum_A;s__
LMSG_GO000009560.1	no	968_1	Polymetallic	53.50	0.00	53.50	0	0	0	14	Medium quality	7837	1126814	53.60	179	d__Bacteria;p__Nitrospirota;c__Leptospirillia;o__Leptospirillales;f__Leptospirillaceae;g__Leptospirillum_A;s__
LMSG_GO000009561.1	no	968_1	Pyrite-Copper	72.84	4.09	52.39	2	0	1	17	Medium quality	29188	2650371	55.70	327	d__Bacteria;p__Nitrospirota;c__Leptospirillia;o__Leptospirillales;f__Leptospirillaceae;g__Leptospirillum_A;s__
LMSG_GO000009562.1	no	968_1	Pyrite-Copper	82.82	2.27	71.46	1	0	0	18	Medium quality	15342	2352298	55.60	231	d__Bacteria;p__Nitrospirota;c__Leptospirillia;o__Leptospirillales;f__Leptospirillaceae;g__Leptospirillum_A;s__
LMSG_GO000009563.1	no	968_1	Lead-Zinc	64.03	1.75	55.26	1	0	1	16	Medium quality	10816	1651238	54.80	230	d__Bacteria;p__Nitrospirota;c__Leptospirillia;o__Leptospirillales;f__Leptospirillaceae;g__Leptospirillum_A;s__
LMSG_GO000009564.1	no	971_1	Pyrite-Copper	57.01	0.00	57.01	1	0	1	16	Medium quality	133755	1956211	54.50	73	d__Bacteria;p__Nitrospirota;c__Leptospirillia;o__Leptospirillales;f__Leptospirillaceae;g__Leptospirillum_A;s__
LMSG_GO000009565.1	no	972_1	Tin-Zinc	64.03	1.75	55.26	1	1	0	15	Medium quality	11803	2289527	56.50	290	d__Bacteria;p__Nitrospirota;c__Leptospirillia;o__Leptospirillales;f__Leptospirillaceae;g__Leptospirillum_A;s__
LMSG_GO000009566.1	no	972_1	Lead-Zinc	58.77	0.00	58.77	1	0	1	16	Medium quality	27098	2455853	56.40	158	d__Bacteria;p__Nitrospirota;c__Leptospirillia;o__Leptospirillales;f__Leptospirillaceae;g__Leptospirillum_A;s__
LMSG_GO000009567.1	no	966_1	Pyrite	50.17	0.00	50.17	1	0	0	15	Medium quality	75446	1623105	54.60	176	d__Bacteria;p__Nitrospirota;c__Leptospirillia;o__Leptospirillales;f__Leptospirillaceae;g__Leptospirillum_A;s__Leptospirillum_A ferriphilum
LMSG_GO000009568.1	no	966_1	Pyrite	67.19	0.00	67.19	2	0	2	15	Medium quality	51827	1260691	52.60	74	d__Bacteria;p__Nitrospirota;c__Leptospirillia;o__Leptospirillales;f__Leptospirillaceae;g__Leptospirillum_A;s__Leptospirillum_A ferriphilum
LMSG_GO000009569.1	no	966_2	Lead-Zinc	74.33	0.91	69.79	1	2	4	19	Medium quality	19540	1625663	52.60	231	d__Bacteria;p__Nitrospirota;c__Leptospirillia;o__Leptospirillales;f__Leptospirillaceae;g__Leptospirillum_A;s__Leptospirillum_A rubarum
LMSG_GO000009570.1	no	966_2	Copper	51.75	0.00	51.75	1	1	1	16	Medium quality	60055	1924182	56.00	188	d__Bacteria;p__Nitrospirota;c__Leptospirillia;o__Leptospirillales;f__Leptospirillaceae;g__Leptospirillum_A;s__Leptospirillum_A rubarum

LMSG_G000009571.1	no	966_2	Lead-Zinc	88.92	1.82	79.83	2	0	0	18	Medium quality	20674	2056411	54.90	150	d__Bacteria;p__Nitrospirota;c__Leptospirillia;o__Leptospirillales;f__Leptospirillaceae;g__Leptospirillum_A;s__Leptospirillum_A rubarum
LMSG_G000009572.1	no	966_2	Lead-Zinc	71.05	1.75	62.28	1	1	1	18	Medium quality	42019	2098426	55.30	236	d__Bacteria;p__Nitrospirota;c__Leptospirillia;o__Leptospirillales;f__Leptospirillaceae;g__Leptospirillum_A;s__Leptospirillum_A rubarum
LMSG_G000009573.1	no	966_2	Iron	93.22	1.82	84.13	1	1	1	19	High quality	42648	2579534	54.30	125	d__Bacteria;p__Nitrospirota;c__Leptospirillia;o__Leptospirillales;f__Leptospirillaceae;g__Leptospirillum_A;s__Leptospirillum_A rubarum
LMSG_G000009574.1	no	966_2	Iron	91.35	1.82	82.26	1	1	1	19	High quality	27657	2434433	54.10	148	d__Bacteria;p__Nitrospirota;c__Leptospirillia;o__Leptospirillales;f__Leptospirillaceae;g__Leptospirillum_A;s__Leptospirillum_A rubarum
LMSG_G000009575.1	no	966_2	Iron	93.22	1.82	84.13	1	1	1	20	High quality	30616	2568236	54.30	126	d__Bacteria;p__Nitrospirota;c__Leptospirillia;o__Leptospirillales;f__Leptospirillaceae;g__Leptospirillum_A;s__Leptospirillum_A rubarum
LMSG_G000009576.1	no	966_2	Iron	93.22	1.82	84.13	1	1	1	19	High quality	48442	2591667	54.30	104	d__Bacteria;p__Nitrospirota;c__Leptospirillia;o__Leptospirillales;f__Leptospirillaceae;g__Leptospirillum_A;s__Leptospirillum_A rubarum
LMSG_G000009577.1	no	966_2	Iron	93.22	1.82	84.13	1	1	1	19	High quality	44567	2575297	54.40	99	d__Bacteria;p__Nitrospirota;c__Leptospirillia;o__Leptospirillales;f__Leptospirillaceae;g__Leptospirillum_A;s__Leptospirillum_A rubarum
LMSG_G000009578.1	no	966_2	Iron	93.22	2.73	79.59	1	1	1	19	High quality	43772	2482989	54.40	134	d__Bacteria;p__Nitrospirota;c__Leptospirillia;o__Leptospirillales;f__Leptospirillaceae;g__Leptospirillum_A;s__Leptospirillum_A rubarum
LMSG_G000009579.1	no	966_2	Iron	93.22	2.73	79.59	1	1	1	20	High quality	35913	2405983	54.50	134	d__Bacteria;p__Nitrospirota;c__Leptospirillia;o__Leptospirillales;f__Leptospirillaceae;g__Leptospirillum_A;s__Leptospirillum_A rubarum
LMSG_G000009580.1	no	966_2	Iron	69.29	0.00	69.29	1	1	1	16	Medium quality	27322	2153214	55.10	139	d__Bacteria;p__Nitrospirota;c__Leptospirillia;o__Leptospirillales;f__Leptospirillaceae;g__Leptospirillum_A;s__Leptospirillum_A rubarum
LMSG_G000009581.1	no	966_2	Iron	93.22	1.82	84.13	0	0	0	19	Medium quality	50327	2587169	54.40	101	d__Bacteria;p__Nitrospirota;c__Leptospirillia;o__Leptospirillales;f__Leptospirillaceae;g__Leptospirillum_A;s__Leptospirillum_A rubarum
LMSG_G000009582.1	no	966_2	Iron	93.22	1.82	84.13	0	0	0	19	Medium quality	45786	2481529	54.60	91	d__Bacteria;p__Nitrospirota;c__Leptospirillia;o__Leptospirillales;f__Leptospirillaceae;g__Leptospirillum_A;s__Leptospirillum_A rubarum
LMSG_G000009583.1	no	966_2	Iron	93.22	1.82	84.13	0	0	0	19	Medium quality	49758	2601054	54.40	105	d__Bacteria;p__Nitrospirota;c__Leptospirillia;o__Leptospirillales;f__Leptospirillaceae;g__Leptospirillum_A;s__Leptospirillum_A rubarum
LMSG_G000009584.1	no	956_1	Copper	51.75	0.00	51.75	0	0	0	13	Medium quality	16303	1944262	60.60	151	d__Bacteria;p__Nitrospirota;c__Leptospirillia;o__Leptospirillales;f__Leptospirillaceae;g__UBA4572;s__
LMSG_G000009585.1	no	957_1	Lead-Zinc	54.38	0.00	54.38	0	0	0	11	Medium quality	40403	1025072	57.90	66	d__Bacteria;p__Nitrospirota;c__Leptospirillia;o__Leptospirillales;f__Leptospirillaceae;g__UBA4572;s__
LMSG_G000009586.1	no	957_1	Lead-Zinc	60.98	0.91	56.44	0	0	0	12	Medium quality	55807	1034581	57.00	31	d__Bacteria;p__Nitrospirota;c__Leptospirillia;o__Leptospirillales;f__Leptospirillaceae;g__UBA4572;s__
LMSG_G000009587.1	no	957_1	Pyrite	79.82	0.00	79.82	1	0	0	16	Medium quality	33089	2540231	60.20	323	d__Bacteria;p__Nitrospirota;c__Leptospirillia;o__Leptospirillales;f__Leptospirillaceae;g__UBA4572;s__
LMSG_G000009588.1	no	957_1	Pyrite	82.44	2.73	68.81	0	0	0	17	Medium quality	28736	2558446	60.10	191	d__Bacteria;p__Nitrospirota;c__Leptospirillia;o__Leptospirillales;f__Leptospirillaceae;g__UBA4572;s__
LMSG_G000009589.1	no	957_1	Pyrite	79.82	1.75	71.05	1	0	1	15	Medium quality	25739	2753811	60.20	401	d__Bacteria;p__Nitrospirota;c__Leptospirillia;o__Leptospirillales;f__Leptospirillaceae;g__UBA4572;s__
LMSG_G000009590.1	no	957_1	Lead-Zinc	72.80	3.51	55.26	1	1	0	15	Medium quality	47833	2552060	60.00	234	d__Bacteria;p__Nitrospirota;c__Leptospirillia;o__Leptospirillales;f__Leptospirillaceae;g__UBA4572;s__
LMSG_G000009591.1	no	957_1	Polymetallic	76.31	1.75	67.54	1	1	1	15	Medium quality	33031	2464853	60.00	178	d__Bacteria;p__Nitrospirota;c__Leptospirillia;o__Leptospirillales;f__Leptospirillaceae;g__UBA4572;s__
LMSG_G000009592.1	no	957_1	Copper	67.54	0.00	67.54	1	0	1	15	Medium quality	36833	1842345	60.40	77	d__Bacteria;p__Nitrospirota;c__Leptospirillia;o__Leptospirillales;f__Leptospirillaceae;g__UBA4572;s__
LMSG_G000009593.1	no	957_1	Copper	67.54	0.00	67.54	0	0	0	15	Medium quality	31322	1982640	60.20	96	d__Bacteria;p__Nitrospirota;c__Leptospirillia;o__Leptospirillales;f__Leptospirillaceae;g__UBA4572;s__
LMSG_G000009594.1	no	957_1	Copper	73.90	0.00	73.90	0	2	2	16	Medium quality	29500	2314933	59.70	198	d__Bacteria;p__Nitrospirota;c__Leptospirillia;o__Leptospirillales;f__Leptospirillaceae;g__UBA4572;s__
LMSG_G000009595.1	no	957_1	Polymetallic	53.50	0.00	53.50	0	1	0	10	Medium quality	31295	1896373	60.90	113	d__Bacteria;p__Nitrospirota;c__Leptospirillia;o__Leptospirillales;f__Leptospirillaceae;g__UBA4572;s__
LMSG_G000009596.1	no	957_1	Polymetallic	67.54	0.00	67.54	0	1	0	14	Medium quality	47926	2044545	60.30	104	d__Bacteria;p__Nitrospirota;c__Leptospirillia;o__Leptospirillales;f__Leptospirillaceae;g__UBA4572;s__
LMSG_G000009597.1	no	957_1	Magnetite	78.07	0.00	78.07	1	1	0	14	Medium quality	12930	2406232	60.10	283	d__Bacteria;p__Nitrospirota;c__Leptospirillia;o__Leptospirillales;f__Leptospirillaceae;g__UBA4572;s__
LMSG_G000009598.1	no	957_1	Copper	88.97	1.82	79.88	0	0	0	15	Medium quality	35479	2215147	59.80	92	d__Bacteria;p__Nitrospirota;c__Leptospirillia;o__Leptospirillales;f__Leptospirillaceae;g__UBA4572;s__
LMSG_G000009599.1	no	957_1	Copper	79.82	0.00	79.82	1	1	2	18	Medium quality	55615	2525577	59.80	212	d__Bacteria;p__Nitrospirota;c__Leptospirillia;o__Leptospirillales;f__Leptospirillaceae;g__UBA4572;s__
LMSG_G000009600.1	no	957_1	Copper	52.63	0.00	52.63	0	0	0	13	Medium quality	97550	963996	56.80	159	d__Bacteria;p__Nitrospirota;c__Leptospirillia;o__Leptospirillales;f__Leptospirillaceae;g__UBA4572;s__
LMSG_G000009601.1	no	957_1	Pyrite	78.07	1.75	69.30	0	2	0	18	Medium quality	40559	2403641	59.80	146	d__Bacteria;p__Nitrospirota;c__Leptospirillia;o__Leptospirillales;f__Leptospirillaceae;g__UBA4572;s__
LMSG_G000009602.1	no	957_1	Pyrite	89.05	2.73	75.42	0	0	0	18	Medium quality	23134	2914647	58.70	188	d__Bacteria;p__Nitrospirota;c__Leptospirillia;o__Leptospirillales;f__Leptospirillaceae;g__UBA4572;s__
LMSG_G000009603.1	no	957_1	Pyrite	84.43	4.55	61.71	0	0	0	17	Medium quality	35099	2356941	59.30	98	d__Bacteria;p__Nitrospirota;c__Leptospirillia;o__Leptospirillales;f__Leptospirillaceae;g__UBA4572;s__
LMSG_G000009604.1	no	958_1	Pyrite-Copper	90.00	1.82	80.91	1	1	1	17	Medium quality	64269	2285123	60.10	152	d__Bacteria;p__Nitrospirota;c__Leptospirillia;o__Leptospirillales;f__Leptospirillaceae;g__UBA4572;s__
LMSG_G000009605.1	no	958_1	Copper	84.54	1.82	75.45	1	1	1	14	Medium quality	67499	2115095	60.10	99	d__Bacteria;p__Nitrospirota;c__Leptospirillia;o__Leptospirillales;f__Leptospirillaceae;g__UBA4572;s__
LMSG_G000009606.1	no	958_1	Lead-Zinc	86.36	1.82	77.27	2	0	0	17	Medium quality	54484	2059104	59.30	83	d__Bacteria;p__Nitrospirota;c__Leptospirillia;o__Leptospirillales;f__Leptospirillaceae;g__UBA4572;s__
LMSG_G000009607.1	no	958_1	Pyrite	90.87	2.73	77.24	1	0	0	17	Medium quality	41219	2398753	59.80	125	d__Bacteria;p__Nitrospirota;c__Leptospirillia;o__Leptospirillales;f__Leptospirillaceae;g__UBA4572;s__
LMSG_G000009608.1	no	958_1	Pyrite	88.97	3.64	70.79	0	1	0	17	Medium quality	52434	2347873	60.00	119	d__Bacteria;p__Nitrospirota;c__Leptospirillia;o__Leptospirillales;f__Leptospirillaceae;g__UBA4572;s__
LMSG_G000009609.1	no	958_1	Pyrite	78.44	3.18	62.54	2	1	0	16	Medium quality	44470	2072104	60.20	107	d__Bacteria;p__Nitrospirota;c__Leptospirillia;o__Leptospirillales;f__Leptospirillaceae;g__UBA4572;s__
LMSG_G000009610.1	no	958_1	Pyrite	61.36	0.91	56.82	0	0	0	15	Medium quality	69409	1831339	60.70	265	d__Bacteria;p__Nitrospirota;c__Leptospirillia;o__Leptospirillales;f__Leptospirillaceae;g__UBA4572;s__
LMSG_G000009611.1	no	958_1	Copper	76.43	1.82	67.34	0	0	0	17	Medium quality	42182	1798727	59.40	87	d__Bacteria;p__Nitrospirota;c__Leptospirillia;o__Leptospirillales;f__Leptospirillaceae;g__UBA4572;s__
LMSG_G000009612.1	no	958_1	Copper	71.05	0.00	71.05	0	1	0	17	Medium quality	34861	2155929	60.00	120	d__Bacteria;p__Nitrospirota;c__Leptospirillia;o__Leptospirillales;f__Leptospirillaceae;g__UBA4572;s__
LMSG_G000009613.1	no	958_1	Polymetallic	59.54	0.00	59.54	0	0	0	12	Medium quality	37290	1666034	60.80	73	d__Bacteria;p__Nitrospirota;c__Leptospirillia;o__Leptospirillales;f__Leptospirillaceae;g__UBA4572;s__
LMSG_G000009614.1	no	958_1	Polymetallic	78.07	1.75	69.30	0	0	0	15	Medium quality	48573	2002745	59.00	91	d__Bacteria;p__Nitrospirota;c__Leptospirillia;o__Leptospirillales;f__Leptospirillaceae;g__UBA4572;s__
LMSG_G000009615.1	no	958_1	Magnetite	62.28	0.00	62.28	1	1	0	10	Medium quality	79326	934653	58.00	73	d__Bacteria;p__Nitrospirota;c__Leptospirillia;o__Leptospirillales;f__Leptospirillaceae;g__UBA4572;s__
LMSG_G000009616.1	no	958_1	Lead-Zinc	76.55	0.91	72.01	0	0	0	14	Medium quality	51320	1563279	58.70	61	d__Bacteria;p__Nitrospirota;c__Leptospirillia;o__Leptospirillales;f__Leptospirillaceae;g__UBA4572;s__
LMSG_G000009617.1	no	958_1	Pyrite-Copper	65.78	0.00	65.78	1	1	1	16	Medium quality	68140	2031582	60.70	125	d__Bacteria;p__Nitrospirota;c__Leptospirillia;o__Leptospirillales;f__Leptospirillaceae;g__UBA4572;s__
LMSG_G000009618.1	no	958_1	Pyrite-Copper	79.82	0.00	79.82	3	0	2	17	Medium quality	68085	2072003	59.20	135	d__Bacteria;p__Nitrospirota;c__Leptospirillia;o__Leptospirillales;f__Leptospirillaceae;g__UBA4572;s__
LMSG_G000009619.1	no	958_1	Pyrite-Copper	83.63	0.91	79.09	0	1	0	14	Medium quality	27248	1949813	59.90	104	d__Bacteria;p__Nitrospirota;c__Leptospirillia;o__Leptospirillales;f__Leptospirillaceae;g__UBA4572;s__
LMSG_G000009620.1	no	958_1	Pyrite-Copper	87.27	1.09	81.82	0	0	0	15	Medium quality	29275	1996944	60.10	112	d__Bacteria;p__Nitrospirota;c__Leptospirillia;o__Leptospirillales;f__Leptospirillaceae;g__UBA4572;s__
LMSG_G000009621.1	no	958_1	Magnetite	59.64	0.00	59.64	0	0	0	11	Medium quality	43815	1316059	58.70	54	d__Bacteria;p__Nitrospirota;c__Leptospirillia;o__Leptospirillales;f__Leptospirillaceae;g__UBA4572;s__

LMSG_G000009622.1	no	958_1	Pyrite	81.81	0.91	77.27	0	0	0	14	Medium quality	46378	1878270	60.10	71	d__Bacteria;p__Nitrospirota;c__Leptospirillia;o__Leptospirillales;f__Leptospirillaceae;g__UBA4572;s__
LMSG_G000009623.1	no	958_1	Copper	58.77	0.00	58.77	2	0	2	13	Medium quality	33799	904116	56.60	189	d__Bacteria;p__Nitrospirota;c__Leptospirillia;o__Leptospirillales;f__Leptospirillaceae;g__UBA4572;s__
LMSG_G000009624.1	no	958_1	Lead-Zinc	71.05	1.75	62.28	1	0	0	17	Medium quality	8877	2527630	59.40	625	d__Bacteria;p__Nitrospirota;c__Leptospirillia;o__Leptospirillales;f__Leptospirillaceae;g__UBA4572;s__
LMSG_G000009625.1	no	959_1	Copper	72.80	1.75	64.03	0	1	0	11	Medium quality	10983	1871046	60.20	197	d__Bacteria;p__Nitrospirota;c__Leptospirillia;o__Leptospirillales;f__Leptospirillaceae;g__UBA4572;s__
LMSG_G000009626.1	no	960_1	Polymetallic	79.82	3.51	62.28	1	1	1	17	Medium quality	66081	2170428	59.20	101	d__Bacteria;p__Nitrospirota;c__Leptospirillia;o__Leptospirillales;f__Leptospirillaceae;g__UBA4572;s__
LMSG_G000009627.1	no	960_1	Polymetallic	62.28	0.00	62.28	1	1	1	13	Medium quality	78699	1611977	59.90	123	d__Bacteria;p__Nitrospirota;c__Leptospirillia;o__Leptospirillales;f__Leptospirillaceae;g__UBA4572;s__
LMSG_G000009628.1	no	960_1	Polymetallic	78.07	1.75	69.30	1	1	1	16	Medium quality	40336	2119614	59.50	167	d__Bacteria;p__Nitrospirota;c__Leptospirillia;o__Leptospirillales;f__Leptospirillaceae;g__UBA4572;s__
LMSG_G000009629.1	no	960_1	Copper	56.14	0.00	56.14	0	0	0	14	Medium quality	35716	1040667	57.00	172	d__Bacteria;p__Nitrospirota;c__Leptospirillia;o__Leptospirillales;f__Leptospirillaceae;g__UBA4572;s__
LMSG_G000009630.1	no	960_1	Coal	72.80	0.00	72.80	2	1	0	16	Medium quality	4999	2133120	59.00	580	d__Bacteria;p__Nitrospirota;c__Leptospirillia;o__Leptospirillales;f__Leptospirillaceae;g__UBA4572;s__
LMSG_G000009631.1	no	960_1	Coal	81.57	0.00	81.57	1	1	1	17	Medium quality	32571	1995703	59.30	125	d__Bacteria;p__Nitrospirota;c__Leptospirillia;o__Leptospirillales;f__Leptospirillaceae;g__UBA4572;s__
LMSG_G000009632.1	no	960_1	Copper	64.03	0.00	64.03	2	0	0	17	Medium quality	14711	2009491	58.90	196	d__Bacteria;p__Nitrospirota;c__Leptospirillia;o__Leptospirillales;f__Leptospirillaceae;g__UBA4572;s__
LMSG_G000009633.1	no	960_2	Pyrite-Copper	70.17	0.00	70.17	2	0	2	15	Medium quality	13037	2009446	59.40	348	d__Bacteria;p__Nitrospirota;c__Leptospirillia;o__Leptospirillales;f__Leptospirillaceae;g__UBA4572;s__
LMSG_G000009634.1	no	960_2	Arsenic	50.87	0.00	50.87	0	0	0	13	Medium quality	11937	1218971	55.30	213	d__Bacteria;p__Nitrospirota;c__Leptospirillia;o__Leptospirillales;f__Leptospirillaceae;g__UBA4572;s__
LMSG_G000009635.1	no	960_2	Copper	60.52	0.00	60.52	1	0	1	15	Medium quality	12111	1648954	59.00	213	d__Bacteria;p__Nitrospirota;c__Leptospirillia;o__Leptospirillales;f__Leptospirillaceae;g__UBA4572;s__
LMSG_G000009636.1	no	963_1	Polymetallic	74.56	1.75	65.79	1	0	1	17	Medium quality	63261	1795454	57.90	92	d__Bacteria;p__Nitrospirota;c__Leptospirillia;o__Leptospirillales;f__Leptospirillaceae;g__UBA4572;s__
LMSG_G000009637.1	no	964_1	Copper	72.80	0.00	72.80	0	2	1	17	Medium quality	30178	2054708	59.70	123	d__Bacteria;p__Nitrospirota;c__Leptospirillia;o__Leptospirillales;f__Leptospirillaceae;g__UBA4572;s__
LMSG_G000009638.1	no	964_1	Lead-Zinc	72.80	2.63	59.65	0	1	0	13	Medium quality	9681	1899428	59.80	269	d__Bacteria;p__Nitrospirota;c__Leptospirillia;o__Leptospirillales;f__Leptospirillaceae;g__UBA4572;s__
LMSG_G000009639.1	no	964_1	Lead-Zinc	62.28	0.88	57.90	0	0	0	13	Medium quality	3879	1528185	60.40	435	d__Bacteria;p__Nitrospirota;c__Leptospirillia;o__Leptospirillales;f__Leptospirillaceae;g__UBA4572;s__
LMSG_G000009640.1	no	964_1	Polymetallic	67.54	0.00	67.54	0	0	0	16	Medium quality	79923	1340051	58.70	131	d__Bacteria;p__Nitrospirota;c__Leptospirillia;o__Leptospirillales;f__Leptospirillaceae;g__UBA4572;s__
LMSG_G000009641.1	no	964_1	Polymetallic	72.80	0.00	72.80	2	2	0	17	Medium quality	56627	2083086	60.10	114	d__Bacteria;p__Nitrospirota;c__Leptospirillia;o__Leptospirillales;f__Leptospirillaceae;g__UBA4572;s__
LMSG_G000009642.1	no	964_1	Polymetallic	78.23	1.36	71.42	1	1	0	15	Medium quality	68016	2003611	59.60	64	d__Bacteria;p__Nitrospirota;c__Leptospirillia;o__Leptospirillales;f__Leptospirillaceae;g__UBA4572;s__
LMSG_G000009643.1	no	964_1	Magnetite	75.98	2.73	62.35	0	0	0	16	Medium quality	27716	1531777	59.00	88	d__Bacteria;p__Nitrospirota;c__Leptospirillia;o__Leptospirillales;f__Leptospirillaceae;g__UBA4572;s__
LMSG_G000009644.1	no	964_1	Polymetallic	73.68	1.75	64.91	0	1	0	16	Medium quality	62139	1399270	58.50	116	d__Bacteria;p__Nitrospirota;c__Leptospirillia;o__Leptospirillales;f__Leptospirillaceae;g__UBA4572;s__
LMSG_G000009645.1	no	964_1	Magnetite	73.68	1.75	64.91	0	0	0	14	Medium quality	22721	1359844	58.30	95	d__Bacteria;p__Nitrospirota;c__Leptospirillia;o__Leptospirillales;f__Leptospirillaceae;g__UBA4572;s__
LMSG_G000009646.1	no	964_1	Pyrite-Copper	68.42	2.63	55.27	2	0	2	12	Medium quality	10344	1899309	60.30	365	d__Bacteria;p__Nitrospirota;c__Leptospirillia;o__Leptospirillales;f__Leptospirillaceae;g__UBA4572;s__
LMSG_G000009647.1	no	965_2	Pyrite	85.14	2.27	73.78	0	0	0	12	Medium quality	15815	1948983	60.40	191	d__Bacteria;p__Nitrospirota;c__Leptospirillia;o__Leptospirillales;f__Leptospirillaceae;g__UBA4572;s__
LMSG_G000009648.1	no	965_2	Pyrite	57.89	0.00	57.89	1	0	1	15	Medium quality	22664	1488571	59.80	157	d__Bacteria;p__Nitrospirota;c__Leptospirillia;o__Leptospirillales;f__Leptospirillaceae;g__UBA4572;s__
LMSG_G000009649.1	no	965_2	Pyrite	80.32	0.91	75.78	1	1	1	15	Medium quality	37135	1651197	58.70	122	d__Bacteria;p__Nitrospirota;c__Leptospirillia;o__Leptospirillales;f__Leptospirillaceae;g__UBA4572;s__
LMSG_G000009650.1	no	965_2	Pyrite	79.60	0.00	79.60	0	1	0	15	Medium quality	21781	1584689	58.70	98	d__Bacteria;p__Nitrospirota;c__Leptospirillia;o__Leptospirillales;f__Leptospirillaceae;g__UBA4572;s__
LMSG_G000009651.1	no	965_2	Lead-Zinc	67.54	1.75	58.77	0	0	0	13	Medium quality	5937	2289466	59.80	504	d__Bacteria;p__Nitrospirota;c__Leptospirillia;o__Leptospirillales;f__Leptospirillaceae;g__UBA4572;s__
LMSG_G000009652.1	no	970_1	Coal	88.63	1.82	79.54	1	0	1	16	Medium quality	57440	1847577	57.00	52	d__Bacteria;p__Nitrospirota;c__Leptospirillia;o__Leptospirillales;f__Leptospirillaceae;g__UBA4572;s__
LMSG_G000009653.1	no	965_1	Copper	83.33	1.82	74.24	1	1	2	18	Medium quality	58718	2387843	59.30	135	d__Bacteria;p__Nitrospirota;c__Leptospirillia;o__Leptospirillales;f__Leptospirillaceae;g__UBA4572;s__UBA4572 sp002387725
LMSG_G000009654.1	no	965_1	Iron	63.15	0.00	63.15	0	0	0	14	Medium quality	5870	1063648	57.20	200	d__Bacteria;p__Nitrospirota;c__Leptospirillia;o__Leptospirillales;f__Leptospirillaceae;g__UBA4572;s__UBA4572 sp002387725
LMSG_G000009655.1	no	965_1	Lead-Zinc	71.92	0.00	71.92	2	1	1	14	Medium quality	19317	1752832	58.40	327	d__Bacteria;p__Nitrospirota;c__Leptospirillia;o__Leptospirillales;f__Leptospirillaceae;g__UBA4572;s__UBA4572 sp002387725
LMSG_G000009656.1	no	513_1	Pyrite-Copper	61.02	1.82	51.93	0	0	0	11	Medium quality	3730	1197328	45.00	438	d__Bacteria;p__Nitrospirota;c__Thermodesulfovibrionia;o__;f__;g__;s__
LMSG_G000009657.1	no	945_1	Polymetallic	97.27	0.00	97.27	1	1	1	19	High quality	35078	2767891	53.20	110	d__Bacteria;p__Nitrospirota;c__Thermodesulfovibrionia;o__Thermodesulfovibrionales;f__JdFR-88;g__;s__
LMSG_G000009658.1	no	945_1	Polymetallic	98.18	0.00	98.18	1	2	1	19	High quality	73502	2694012	53.30	54	d__Bacteria;p__Nitrospirota;c__Thermodesulfovibrionia;o__Thermodesulfovibrionales;f__JdFR-88;g__;s__
LMSG_G000009659.1	no	945_1	Polymetallic	63.49	0.96	58.70	0	2	0	14	Medium quality	6130	1948410	53.90	462	d__Bacteria;p__Nitrospirota;c__Thermodesulfovibrionia;o__Thermodesulfovibrionales;f__JdFR-88;g__;s__
LMSG_G000009660.1	no	945_1	Polymetallic	77.61	1.36	70.80	0	0	0	14	Medium quality	11045	2191758	53.50	316	d__Bacteria;p__Nitrospirota;c__Thermodesulfovibrionia;o__Thermodesulfovibrionales;f__JdFR-88;g__;s__
LMSG_G000009661.1	no	946_1	Pyrite-Copper	88.18	1.82	79.09	0	2	1	17	Medium quality	59176	2446167	53.30	63	d__Bacteria;p__Nitrospirota;c__Thermodesulfovibrionia;o__Thermodesulfovibrionales;f__JdFR-88;g__;s__
LMSG_G000009662.1	no	947_1	Polymetallic	86.71	0.91	82.17	0	2	1	16	Medium quality	62524	2199959	55.40	76	d__Bacteria;p__Nitrospirota;c__Thermodesulfovibrionia;o__Thermodesulfovibrionales;f__JdFR-88;g__;s__
LMSG_G000009663.1	no	949_1	Polymetallic	94.88	2.39	82.95	1	2	1	18	High quality	58810	3015853	57.20	85	d__Bacteria;p__Nitrospirota;c__Thermodesulfovibrionia;o__Thermodesulfovibrionales;f__JdFR-88;g__;s__
LMSG_G000009664.1	no	949_1	Polymetallic	61.20	1.82	52.11	0	1	0	10	Medium quality	3782	1504858	58.50	443	d__Bacteria;p__Nitrospirota;c__Thermodesulfovibrionia;o__Thermodesulfovibrionales;f__JdFR-88;g__;s__
LMSG_G000009665.1	no	950_1	Lead-Zinc	94.69	1.82	85.60	1	1	1	18	High quality	44826	2611575	56.80	121	d__Bacteria;p__Nitrospirota;c__Thermodesulfovibrionia;o__Thermodesulfovibrionales;f__JdFR-88;g__;s__
LMSG_G000009666.1	no	950_1	Lead-Zinc	85.40	2.27	74.04	0	0	1	16	Medium quality	4508	2019091	57.70	548	d__Bacteria;p__Nitrospirota;c__Thermodesulfovibrionia;o__Thermodesulfovibrionales;f__JdFR-88;g__;s__
LMSG_G000009667.1	no	950_1	Lead-Zinc	79.87	0.64	76.69	0	0	0	17	Medium quality	6626	1952091	57.50	360	d__Bacteria;p__Nitrospirota;c__Thermodesulfovibrionia;o__Thermodesulfovibrionales;f__JdFR-88;g__;s__
LMSG_G000009668.1	no	950_1	Pyrite-Copper	99.09	2.84	84.89	0	1	1	19	Medium quality	25644	2691833	56.90	183	d__Bacteria;p__Nitrospirota;c__Thermodesulfovibrionia;o__Thermodesulfovibrionales;f__JdFR-88;g__;s__
LMSG_G000009669.1	no	950_1	Pyrite-Copper	57.96	0.45	55.69	0	0	0	13	Medium quality	4563	1314624	58.30	354	d__Bacteria;p__Nitrospirota;c__Thermodesulfovibrionia;o__Thermodesulfovibrionales;f__JdFR-88;g__;s__
LMSG_G000009670.1	no	934_1	Polymetallic	92.27	3.82	73.18	1	2	1	18	High quality	82406	3083186	55.60	96	d__Bacteria;p__Nitrospirota;c__Thermodesulfovibrionia;o__Thermodesulfovibrionales;f__UBA6898;g__PALSA-1316;s__
LMSG_G000009671.1	no	929_1	Polymetallic	83.51	0.91	78.97	0	2	0	17	Medium quality	96250	2881359	57.70	51	d__Bacteria;p__Nitrospirota;c__Thermodesulfovibrionia;o__Thermodesulfovibrionales;f__UBA6898;g__Sulfobium;s__
LMSG_G000009672.1	no	930_1	Polymetallic	95.89	3.64	77.71	0	1	1	17	Medium quality	41556	2813508	55.60	103	d__Bacteria;p__Nitrospirota;c__Thermodesulfovibrionia;o__Thermodesulfovibrionales;f__UBA6898;g__Sulfobium;s__
LMSG_G000009673.1	no	930_1	Polymetallic	70.05	0.00	70.05	0	1	0	10	Medium quality	61085	1624869	56.20	82	d__Bacteria;p__Nitrospirota;c__Thermodesulfovibrionia;o__Thermodesulfovibrionales;f__UBA6898;g__Sulfobium;s__
LMSG_G000009674.1	no	931_1	Antimony	68.54	3.21	52.48	0	1	0	17	Medium quality	81644	2664466	57.40	52	d__Bacteria;p__Nitrospirota;c__Thermodesulfovibrionia;o__Thermodesulfovibrionales;f__UBA6898;g__Sulfobium;s__
LMSG_G000009675.1	no	932_1	Antimony	74.59	2.95	59.82	0	2	1	11	Medium quality	7201	2135087	53.80	401	d__Bacteria;p__Nitrospirota;c__Thermodesulfovibrionia;o__Thermodesulfovibrionales;f__UBA6898;g__Sulfobium;s__
LMSG_G000009676.1	no	932_1	Antimony	78.43	4.73	54.80	0	3	1	11	Medium quality	26753	3078098	53.00	233	d__Bacteria;p__Nitrospirota;c__Thermodesulfovibrionia;o__Thermodesulfovibrionales;f__UBA6898;g__Sulfobium;s__
LMSG_G000009677.1	no	932_1	Antimony	73.71	1.53	66.09	0	1	1	12	Medium quality	18628	2621309	53.10	283	d__Bacteria;p__Nitrospirota;c__Thermodesulfovibrionia;o__Thermodesulfovibrionales;f__UBA6898;g__Sulfobium;s__

LMSG_G000009678.1	no	932_1	Polymetallic	77.74	2.51	65.22	0	0	0	9	Medium quality	8205	2011786	54.50	312	d__Bacteria;p__Nitrospirota;c__Thermodesulfovibrionia;o__Thermodesulfovibrionales;f__UBA6898;g__Sulfobium;s__
LMSG_G000009679.1	no	932_2	Pyrite-Copper	70.83	2.90	56.35	0	4	1	14	Medium quality	7775	2509834	53.50	411	d__Bacteria;p__Nitrospirota;c__Thermodesulfovibrionia;o__Thermodesulfovibrionales;f__UBA6898;g__Sulfobium;s__
LMSG_G000009680.1	no	1357_1	Pyrite-Copper	96.36	4.73	72.73	1	1	1	19	High quality	109425	3465872	51.70	143	d__Bacteria;p__Nitrospirota;c__Thermodesulfovibrionia;o__Thermodesulfovibrionales;f__UBA9159;g__;s__
LMSG_G000009681.1	no	936_1	Lead-Zinc	76.46	3.46	59.14	0	1	0	18	Medium quality	3148	2660446	49.40	946	d__Bacteria;p__Nitrospirota;c__Thermodesulfovibrionia;o__Thermodesulfovibrionales;f__UBA9935;g__;s__
LMSG_G000009682.1	no	936_1	Pyrite-Copper	58.52	0.23	57.36	1	0	0	10	Medium quality	2904	1782913	50.90	696	d__Bacteria;p__Nitrospirota;c__Thermodesulfovibrionia;o__Thermodesulfovibrionales;f__UBA9935;g__;s__
LMSG_G000009683.1	no	939_1	Pyrite-Copper	56.55	0.00	56.55	0	0	0	11	Medium quality	5153	1149232	46.30	276	d__Bacteria;p__Nitrospirota;c__Thermodesulfovibrionia;o__Thermodesulfovibrionales;f__UBA9935;g__GWB2-47-37;s__
LMSG_G000009684.1	no	940_1	Lead-Zinc	84.94	1.06	79.64	1	1	1	16	Medium quality	15497	1579023	43.60	177	d__Bacteria;p__Nitrospirota;c__Thermodesulfovibrionia;o__Thermodesulfovibrionales;f__UBA9935;g__GWB2-47-37;s__
LMSG_G000009685.1	no	940_1	Lead-Zinc	77.64	2.89	63.22	0	0	0	10	Medium quality	2871	1846537	43.60	724	d__Bacteria;p__Nitrospirota;c__Thermodesulfovibrionia;o__Thermodesulfovibrionales;f__UBA9935;g__GWB2-47-37;s__
LMSG_G000009686.1	no	940_1	Lead-Zinc	94.49	1.82	85.40	1	1	2	19	High quality	18930	2554676	43.10	189	d__Bacteria;p__Nitrospirota;c__Thermodesulfovibrionia;o__Thermodesulfovibrionales;f__UBA9935;g__GWB2-47-37;s__
LMSG_G000009687.1	no	940_1	Lead-Zinc	88.46	1.82	79.37	1	0	1	17	Medium quality	11810	2371793	43.40	326	d__Bacteria;p__Nitrospirota;c__Thermodesulfovibrionia;o__Thermodesulfovibrionales;f__UBA9935;g__GWB2-47-37;s__
LMSG_G000009688.1	no	940_1	Lead-Zinc	96.31	1.82	87.22	1	1	2	18	High quality	9926	2088860	43.50	271	d__Bacteria;p__Nitrospirota;c__Thermodesulfovibrionia;o__Thermodesulfovibrionales;f__UBA9935;g__GWB2-47-37;s__
LMSG_G000009689.1	no	940_1	Lead-Zinc	91.76	0.91	87.22	1	1	3	19	High quality	16487	2010559	43.50	214	d__Bacteria;p__Nitrospirota;c__Thermodesulfovibrionia;o__Thermodesulfovibrionales;f__UBA9935;g__GWB2-47-37;s__
LMSG_G000009690.1	no	940_1	Lead-Zinc	88.29	1.82	79.20	2	1	2	15	Medium quality	17408	1826430	43.60	196	d__Bacteria;p__Nitrospirota;c__Thermodesulfovibrionia;o__Thermodesulfovibrionales;f__UBA9935;g__GWB2-47-37;s__
LMSG_G000009691.1	no	940_1	Copper	89.64	1.92	80.05	1	2	2	18	Medium quality	7592	2257936	43.80	445	d__Bacteria;p__Nitrospirota;c__Thermodesulfovibrionia;o__Thermodesulfovibrionales;f__UBA9935;g__GWB2-47-37;s__
LMSG_G000009692.1	no	1358_1	Polymetallic	87.67	0.51	85.15	0	1	0	15	Medium quality	15094	3302198	59.70	295	d__Bacteria;p__Nitrospirota;c__UBA9217;o__UBA9217;f__;g__;s__
LMSG_G000009693.1	no	1358_1	Polymetallic	64.81	2.10	54.34	1	0	1	12	Medium quality	3825	2527340	60.20	742	d__Bacteria;p__Nitrospirota;c__UBA9217;o__UBA9217;f__;g__;s__
LMSG_G000009694.1	no	1358_1	Polymetallic	91.81	2.27	80.45	1	1	1	18	High quality	16504	3534600	59.40	285	d__Bacteria;p__Nitrospirota;c__UBA9217;o__UBA9217;f__UBA9217;g__;s__
LMSG_G000009695.1	no	1359_1	Copper	90.70	1.36	83.89	1	1	1	16	Medium quality	7963	2830258	55.20	462	d__Bacteria;p__Nitrospirota;c__UBA9217;o__UBA9217;f__UBA9217;g__;s__
LMSG_G000009696.1	no	1359_1	Copper	73.45	0.00	73.45	1	0	1	15	Medium quality	6069	2236046	55.20	411	d__Bacteria;p__Nitrospirota;c__UBA9217;o__UBA9217;f__UBA9217;g__;s__
LMSG_G000009697.1	no	1359_2	Pyrite-Copper	65.90	1.02	60.82	0	0	0	16	Medium quality	10827	2890272	54.50	497	d__Bacteria;p__Nitrospirota;c__UBA9217;o__UBA9217;f__UBA9217;g__;s__
LMSG_G000009698.1	no	1073_1	Polymetallic	83.17	3.23	67.05	0	0	0	16	Medium quality	10220	1230656	44.50	174	d__Bacteria;p__Omnitrophota;c__Koll11;o__GIF10;f__UBA6249;g__;s__
LMSG_G000009699.1	no	1073_1	Polymetallic	55.34	0.00	55.34	0	0	0	11	Medium quality	2892	771746	44.30	277	d__Bacteria;p__Omnitrophota;c__Koll11;o__GIF10;f__UBA6249;g__;s__
LMSG_G000009700.1	no	317_1	Antimony	55.83	1.08	50.46	0	0	0	14	Medium quality	3293	1288630	44.20	455	d__Bacteria;p__Omnitrophota;c__Koll11;o__UBA10015;f__GCA-002753745;g__;s__
LMSG_G000009701.1	no	1136_1	Lead-Zinc	93.48	0.00	93.48	1	1	2	19	High quality	92104	2099652	31.30	110	d__Bacteria;p__Omnitrophota;c__UBA8468;o__UBA8468;f__B48-G9;g__;s__
LMSG_G000009702.1	no	1136_1	Lead-Zinc	92.40	1.71	83.85	1	1	2	19	High quality	38453	2060195	31.30	121	d__Bacteria;p__Omnitrophota;c__UBA8468;o__UBA8468;f__B48-G9;g__;s__
LMSG_G000009703.1	no	1136_1	Lead-Zinc	93.54	0.00	93.54	1	1	1	19	High quality	33128	2025393	31.30	124	d__Bacteria;p__Omnitrophota;c__UBA8468;o__UBA8468;f__B48-G9;g__;s__
LMSG_G000009704.1	no	1136_1	Lead-Zinc	93.48	0.00	93.48	1	1	5	19	High quality	65108	2054214	31.20	130	d__Bacteria;p__Omnitrophota;c__UBA8468;o__UBA8468;f__B48-G9;g__;s__
LMSG_G000009705.1	no	1136_1	Pyrite-Copper	82.06	1.23	75.94	1	0	1	15	Medium quality	3642	1814432	31.70	617	d__Bacteria;p__Omnitrophota;c__UBA8468;o__UBA8468;f__B48-G9;g__;s__
LMSG_G000009706.1	no	398_1	Copper	63.63	0.16	62.85	1	0	1	17	Medium quality	4960	720920	29.80	178	d__Bacteria;p__Patescibacteria;c__;o__;f__;g__;s__
LMSG_G000009707.1	no	398_1	Copper	75.70	0.00	75.70	1	1	1	20	Medium quality	247953	768631	29.60	5	d__Bacteria;p__Patescibacteria;c__;o__;f__;g__;s__
LMSG_G000009708.1	no	404_1	Lead-Zinc	70.53	0.00	70.53	1	1	1	18	Medium quality	162353	560382	28.90	7	d__Bacteria;p__Patescibacteria;c__;o__;f__;g__;s__
LMSG_G000009709.1	no	404_1	Lead-Zinc	61.91	0.00	61.91	1	1	1	19	Medium quality	197564	578720	28.60	21	d__Bacteria;p__Patescibacteria;c__;o__;f__;g__;s__
LMSG_G000009710.1	no	404_1	Lead-Zinc	67.08	0.00	67.08	1	1	1	19	Medium quality	147822	554871	28.60	10	d__Bacteria;p__Patescibacteria;c__;o__;f__;g__;s__
LMSG_G000009711.1	no	405_1	Polymetallic	75.70	0.00	75.70	1	1	1	20	Medium quality	51276	733573	26.50	27	d__Bacteria;p__Patescibacteria;c__;o__;f__;g__;s__
LMSG_G000009712.1	no	405_1	Polymetallic	71.94	0.00	71.94	1	1	1	18	Medium quality	47171	705035	26.50	52	d__Bacteria;p__Patescibacteria;c__;o__;f__;g__;s__
LMSG_G000009713.1	no	407_1	Copper	68.65	3.45	51.41	1	1	1	15	Medium quality	38738	627412	27.00	29	d__Bacteria;p__Patescibacteria;c__;o__;f__;g__;s__
LMSG_G000009714.1	no	296_1	Polymetallic	62.37	1.10	56.87	1	1	1	18	Medium quality	13357	879929	46.20	109	d__Bacteria;p__Patescibacteria;c__ABY1;o__BM507;f__UBA12465;g__UBA12465;s__
LMSG_G000009715.1	no	296_1	Polymetallic	66.33	2.31	54.78	1	1	2	19	Medium quality	22279	896864	45.60	58	d__Bacteria;p__Patescibacteria;c__ABY1;o__BM507;f__UBA12465;g__UBA12465;s__
LMSG_G000009716.1	no	296_1	Polymetallic	69.30	2.97	54.45	1	1	2	18	Medium quality	24670	910197	46.00	68	d__Bacteria;p__Patescibacteria;c__ABY1;o__BM507;f__UBA12465;g__UBA12465;s__
LMSG_G000009717.1	no	1820_1	Polymetallic	55.94	0.50	53.47	1	1	5	15	Medium quality	22313	742284	45.60	68	d__Bacteria;p__Patescibacteria;c__ABY1;o__BM507;f__UBA917;g__;s__
LMSG_G000009718.1	no	1721_1	Polymetallic	55.63	0.38	53.74	0	0	0	17	Medium quality	31060	705854	45.30	64	d__Bacteria;p__Patescibacteria;c__ABY1;o__Magasanikbacterales;f__GWA2-37-8;g__2-02-FULL-45-10;s__
LMSG_G000006338.1	no	1818_1	Lead-Zinc	70.79	1.98	60.89	1	1	3	19	Medium quality	481287	1529883	53.90	20	d__Bacteria;p__Patescibacteria;c__ABY1;o__SG8-24;f__2-12-FULL-60-25;g__SZUA-46;s__
LMSG_G000006339.1	no	1818_1	Pyrite-Copper	70.79	1.98	60.89	1	1	1	19	Medium quality	480545	1526619	54.00	8	d__Bacteria;p__Patescibacteria;c__ABY1;o__SG8-24;f__2-12-FULL-60-25;g__SZUA-46;s__
LMSG_G000009719.1	no	409_1	Polymetallic	72.22	0.00	72.22	1	1	1	19	Medium quality	788857	1255068	50.00	4	d__Bacteria;p__Patescibacteria;c__ABY1;o__UBA10025;f__UBA10025;g__;s__
LMSG_G000009720.1	no	409_1	Polymetallic	71.29	0.00	71.29	1	1	1	19	Medium quality	877264	1238082	50.00	2	d__Bacteria;p__Patescibacteria;c__ABY1;o__UBA10025;f__UBA10025;g__;s__
LMSG_G000009721.1	no	1709_1	Pyrite-Copper	51.72	0.00	51.72	0	0	0	10	Medium quality	6924	539637	43.50	85	d__Bacteria;p__Patescibacteria;c__CPR2;o__UBA1875;f__;g__;s__
LMSG_G000009722.1	no	405_1	Lead-Zinc	73.98	0.00	73.98	1	1	4	20	Medium quality	15621	738091	26.80	82	d__Bacteria;p__Patescibacteria;c__Dojkabacteria;o__;f__;g__;s__
LMSG_G000009723.1	no	405_1	Polymetallic	73.11	0.00	73.11	0	0	0	18	Medium quality	12650	733359	26.40	100	d__Bacteria;p__Patescibacteria;c__Dojkabacteria;o__;f__;g__;s__
LMSG_G000009724.1	no	405_1	Polymetallic	72.25	0.16	71.47	1	1	1	19	Medium quality	22430	683371	26.40	41	d__Bacteria;p__Patescibacteria;c__Dojkabacteria;o__;f__;g__;s__
LMSG_G000009725.1	no	405_1	Magnetite	67.08	0.00	67.08	1	1	1	16	Medium quality	10026	736996	26.50	106	d__Bacteria;p__Patescibacteria;c__Dojkabacteria;o__;f__;g__;s__
LMSG_G000009726.1	no	406_1	Polymetallic	73.98	0.00	73.98	1	1	5	20	Medium quality	756931	882872	26.30	38	d__Bacteria;p__Patescibacteria;c__Dojkabacteria;o__;f__;g__;s__
LMSG_G000009727.1	no	406_1	Magnetite	72.25	0.00	72.25	1	1	1	20	Medium quality	166994	830520	26.20	7	d__Bacteria;p__Patescibacteria;c__Dojkabacteria;o__;f__;g__;s__
LMSG_G000009728.1	no	406_1	Magnetite	75.70	0.00	75.70	1	1	3	20	Medium quality	119181	920161	26.60	101	d__Bacteria;p__Patescibacteria;c__Dojkabacteria;o__;f__;g__;s__
LMSG_G000009729.1	no	406_1	Magnetite	72.25	0.00	72.25	1	1	1	20	Medium quality	196617	848135	26.30	10	d__Bacteria;p__Patescibacteria;c__Dojkabacteria;o__;f__;g__;s__
LMSG_G000009730.1	no	406_1	Magnetite	73.98	0.00	73.98	1	1	1	20	Medium quality	199165	850917	26.30	7	d__Bacteria;p__Patescibacteria;c__Dojkabacteria;o__;f__;g__;s__
LMSG_G000009731.1	no	407_1	Copper	73.98	0.57	71.11	2	3	13	20	Medium quality	15577	1003839	27.60	197	d__Bacteria;p__Patescibacteria;c__Dojkabacteria;o__;f__;g__;s__
LMSG_G000009732.1	no	1835_1	Polymetallic	63.36	1.98	53.46	0	1	0	16	Medium quality	47777	568663	39.00	30	d__Bacteria;p__Patescibacteria;c__Doudnabacteria;o__UBA920;f__;g__;s__
LMSG_G000009733.1	no	1724_1	Pyrite-Copper	56.00	0.00	56.00	1	1	2	19	Medium quality	16769	960279	43.30	75	d__Bacteria;p__Patescibacteria;c__Doudnabacteria;o__UBA920;f__UBA920;g__PALSA-1336;s__
LMSG_G000009734.1	no	422_1	Polymetallic	62.36	1.60	54.36	0	0	0	18	Medium quality	8205	701118	50.40	107	d__Bacteria;p__Patescibacteria;c__Doudnabacteria;o__UBA920;f__UBA920;g__PALSA-1336;s__
LMSG_G000009735.1	no	445_1	Antimony	72.99	1.35	66.23	0	1	0	18	Medium quality	5018	912980	49.10	232	d__Bacteria;p__Patescibacteria;c__Gracilibacteria;o__;f__;g__;s__
LMSG_G000009736.1	no	445_1	Antimony	72.24	1.19	66.30	0	1	0	17	Medium quality	4401	893014	49.10	265	d__Bacteria;p__Patescibacteria;c__Gracilibacteria;o__Peribacterales;f__;g__;s__
LMSG_G000009737.1	no	1838_1	Lead-Zinc	58.79	0.19	57.87	1	1	1	20	Medium quality	411291	747101	43.10	3	d__Bacteria;p__Patescibacteria;c__Microgenomatia;o__Daviesbacterales;f__UBA10151;g__GWC1-40-9;s__
LMSG_G000009738.1	no	1610_1	Pyrite-Copper	64.35	0.00	64.35	0	0	0	15	Medium quality	23162	899050	40.50	53	d__Bacteria;p__Patescibacteria;c__Microgenomatia;o__Daviesbacterales;f__UBA10151;g__UBA10151;s__
LMSG_G000009739.1	no	110_1	Polymetallic	62.59	0.50	60.12	1	10	0	17	Medium quality	7650	822403	47.30	173	d__Bacteria;p__Patescibacteria;c__Microgenomatia;o__Levybacterales;f__UBA12049;g__;s__
LMSG_G000009740.1	no	110_1	Polymetallic	57.68	0.99	52.73	1	0	1	13	Medium quality	7085	759085	47.30	135	d__Bacteria;p__Patescibacteria;c__Microgenomatia;o__Levybacterales;f__UBA12049;g__;s__
LMSG_G000009741.1	no	280_1	Polymetallic	59.18	1.08	53.78	1	1	1	19	Medium quality	21769	762729	37.90	60	d__Bacteria;p__Patescibacteria;c__Microgenomatia;o__Levybacterales;f__UBA12049;g__;s__
LMSG_G000009742.1	no	400_1	Pyrite	63.64	0.99	58.69	1	1	1	16	Medium quality	8833	1077770	39.10	164	d__Bacteria;p__Patescibacteria;c__Microgenomatia;o__Levybacterales;f__UBA12049;g__;s__
LMSG_G000009743.1	no	400_1	Magnetite	62.98	0.99	58.03	1	3	8	19	Medium quality	57546	1061409	39.30	67	d__Bacteria;p__Patescibacteria;c__Microgenomatia;o__Levybacterales;f__UBA12049;g__;s__
LMSG_G000009744.1	no	411_1	Pyrite-Copper	64.35	1.98	54.45	1	1	1	19	Medium quality	142788	1017628	40.60	31	d__Bacteria;p__Patescibacteria;c__Microgenomatia;o__Levybacterales;f__UBA12049;g__;s__
LMSG_G000009745.1	no	410_1	Antimony	59.50	0.99	54.55	0	3	0	17	Medium quality	26163	710970	42.70	55	d__Bacteria;p__Patescibacteria;c__Microgenomatia;o__Levybacterales;f__UBA12049;g__PJMG01;s__
LMSG_G000009746.1	no	328_1	Polymetallic	77.27	0.00	77.27	0	1	7	20	Medium quality	46830	881403	39.70	59	d__Bacteria;p__Patescibacteria;c__Microgenomatia;o__Shapirobacterales;f__UBA12405;g__;s__
LMSG_G000009747.1	no	328_1	Polymetallic	77.27	0.00	77.27	0	1	0	20	Medium quality	84114	922497	39.30	37	d__Bacteria;p__Patescibacteria;c__Microgenomatia;o__Shapirobacterales;f__UBA12405;g__;s__
LMSG_G000009748.1	no	328_1	Polymetallic	75.39	0.00	75.39	5	2	12	20	Medium quality	64245	961240	39.50	54	d__Bacteria;p__Patescibacteria;c__Microgenomatia;o__Shapirobacterales;f__UBA12405;g__;s__
LMSG_G000009749.1	no	328_1	Polymetallic	76.41	0.00	76.41	0	2	2	20	Medium quality	17148	972762	39.30	96	d__Bacteria;p__Patescibacteria;c__Microgenomatia;o__Shapirobacterales;f__UBA12405;g__;s__
LMSG_G000009750.1	no	1701_1	Polymetallic	57.59	0.00	57.59	1	1	2	17	Medium quality	18979	1159709	49.50	83	d__Bacteria;p__Patescibacteria;c__Microgenomatia;o__UBA10105;f__UBA927;g__UBA927;s__

LMSG_G000009751.1	no	1701_1	Polymetallic	63.53	2.18	52.64	1	2	1	19	Medium quality	62858	1139340	49.40	58	d__Bacteria;p__Patescibacteria;c__Microgenomatia;o__UBA10105;f__UBA927;g__UBA927;s__
LMSG_G000009752.1	no	1719_1	Pyrite-Copper	71.18	1.69	62.76	1	1	1	19	Medium quality	72331	978626	38.50	24	d__Bacteria;p__Patescibacteria;c__Microgenomatia;o__UBA1406;f__GWC2-37-13;g__;s__
LMSG_G000009753.1	no	301_1	Antimony	72.36	0.00	72.36	1	1	4	20	Medium quality	567797	1118329	41.50	7	d__Bacteria;p__Patescibacteria;c__Microgenomatia;o__UBA1406;f__GWC2-37-13;g__;s__
LMSG_G000009754.1	no	301_1	Antimony	72.36	0.00	72.36	1	2	7	20	Medium quality	114011	1067127	41.70	30	d__Bacteria;p__Patescibacteria;c__Microgenomatia;o__UBA1406;f__GWC2-37-13;g__;s__
LMSG_G000009755.1	no	301_1	Antimony	70.87	0.37	69.00	1	2	2	19	Medium quality	12729	921002	41.90	99	d__Bacteria;p__Patescibacteria;c__Microgenomatia;o__UBA1406;f__GWC2-37-13;g__;s__
LMSG_G000009756.1	no	1722_1	Polymetallic	61.22	0.86	56.91	1	1	3	19	Medium quality	82006	939556	33.40	62	d__Bacteria;p__Patescibacteria;c__Microgenomatia;o__UBA1406;f__GWC2-37-13;g__GWC2-37-13;s__
LMSG_G000009757.1	no	1679_1	Polymetallic	64.49	0.00	64.49	1	2	13	20	Medium quality	69274	1170862	46.90	184	d__Bacteria;p__Patescibacteria;c__Paceibacteria;o__;f__;g__;s__
LMSG_G000009758.1	no	1679_1	Magnetite	53.94	0.46	51.65	1	0	1	17	Medium quality	32162	581095	48.00	32	d__Bacteria;p__Patescibacteria;c__Paceibacteria;o__;f__;g__;s__
LMSG_G000009759.1	no	1679_1	Magnetite	57.43	0.00	57.43	0	0	0	17	Medium quality	11650	699517	47.70	128	d__Bacteria;p__Patescibacteria;c__Paceibacteria;o__;f__;g__;s__
LMSG_G000009760.1	no	1679_1	Magnetite	59.90	0.00	59.90	1	0	2	18	Medium quality	68480	692866	47.60	23	d__Bacteria;p__Patescibacteria;c__Paceibacteria;o__;f__;g__;s__
LMSG_G000009761.1	no	1679_1	Copper	64.49	0.00	64.49	1	1	1	20	Medium quality	46192	853189	47.00	58	d__Bacteria;p__Patescibacteria;c__Paceibacteria;o__;f__;g__;s__
LMSG_G000009762.1	no	1679_1	Copper	62.66	0.00	62.66	1	1	2	20	Medium quality	41864	698354	47.50	24	d__Bacteria;p__Patescibacteria;c__Paceibacteria;o__;f__;g__;s__
LMSG_G000009763.1	no	1679_1	Arsenic	61.43	0.20	60.42	1	0	2	17	Medium quality	22030	692968	47.30	77	d__Bacteria;p__Patescibacteria;c__Paceibacteria;o__;f__;g__;s__
LMSG_G000009764.1	no	1679_1	Copper	61.89	0.92	57.31	1	0	1	19	Medium quality	14481	709855	47.60	67	d__Bacteria;p__Patescibacteria;c__Paceibacteria;o__;f__;g__;s__
LMSG_G000009765.1	no	1679_1	Lead-Zinc	70.39	1.99	60.44	1	0	2	20	Medium quality	5718	692301	47.60	166	d__Bacteria;p__Patescibacteria;c__Paceibacteria;o__;f__;g__;s__
LMSG_G000006335.1	no	1786_1	Polymetallic	68.11	0.50	65.64	1	2	1	19	Medium quality	42414	678031	45.50	24	d__Bacteria;p__Patescibacteria;c__Paceibacteria;o__Portnoybacterales;f__;g__;s__
LMSG_G000006336.1	no	1786_1	Polymetallic	64.65	0.11	64.10	1	1	2	19	Medium quality	13208	658315	45.60	80	d__Bacteria;p__Patescibacteria;c__Paceibacteria;o__Portnoybacterales;f__;g__;s__
LMSG_G000009766.1	no	1786_1	Polymetallic	66.13	1.10	60.63	1	2	2	19	Medium quality	30610	647617	45.70	46	d__Bacteria;p__Patescibacteria;c__Paceibacteria;o__Portnoybacterales;f__;g__;s__
LMSG_G000009767.1	no	1714_1	Lead-Zinc	77.34	0.00	77.34	1	1	1	20	Medium quality	502876	634720	36.60	4	d__Bacteria;p__Patescibacteria;c__Paceibacteria;o__UBA6257;f__02-FULL-45-10c;g__;s__
LMSG_G000009768.1	no	1723_1	Polymetallic	68.23	3.44	51.05	1	0	3	18	Medium quality	7327	721294	42.60	122	d__Bacteria;p__Patescibacteria;c__Paceibacteria;o__UBA6257;f__2-01-FULL-56-20;g__;s__
LMSG_G000009769.1	no	1803_1	Lead-Zinc	60.79	0.11	60.24	1	0	2	18	Medium quality	9752	637021	56.60	105	d__Bacteria;p__Patescibacteria;c__Paceibacteria;o__UBA6257;f__2-01-FULL-56-20;g__;s__
LMSG_G000009770.1	no	1803_1	Lead-Zinc	55.74	0.09	55.29	0	0	1	18	Medium quality	9148	602684	56.90	108	d__Bacteria;p__Patescibacteria;c__Paceibacteria;o__UBA6257;f__2-01-FULL-56-20;g__;s__
LMSG_G000009771.1	no	1803_1	Lead-Zinc	64.65	0.16	63.87	2	1	5	15	Medium quality	11044	532786	56.90	83	d__Bacteria;p__Patescibacteria;c__Paceibacteria;o__UBA6257;f__2-01-FULL-56-20;g__;s__
LMSG_G000009772.1	no	1803_1	Lead-Zinc	70.97	2.35	59.22	0	1	0	14	Medium quality	3005	585663	57.20	228	d__Bacteria;p__Patescibacteria;c__Paceibacteria;o__UBA6257;f__2-01-FULL-56-20;g__;s__
LMSG_G000009773.1	no	1815_1	Copper	58.68	0.00	58.68	0	6	1	18	Medium quality	12126	662692	50.60	77	d__Bacteria;p__Patescibacteria;c__Paceibacteria;o__UBA6257;f__2-01-FULL-56-20;g__;s__
LMSG_G000009774.1	no	1815_1	Copper	57.99	0.00	57.99	0	1	0	20	Medium quality	8010	678944	50.60	97	d__Bacteria;p__Patescibacteria;c__Paceibacteria;o__UBA6257;f__2-01-FULL-56-20;g__;s__
LMSG_G000009775.1	no	1815_1	Copper	58.18	0.00	58.18	0	4	0	20	Medium quality	12536	700210	50.50	90	d__Bacteria;p__Patescibacteria;c__Paceibacteria;o__UBA6257;f__2-01-FULL-56-20;g__;s__
LMSG_G000009776.1	no	1815_1	Copper	58.95	0.00	58.95	2	7	13	19	Medium quality	21165	794064	50.50	92	d__Bacteria;p__Patescibacteria;c__Paceibacteria;o__UBA6257;f__2-01-FULL-56-20;g__;s__
LMSG_G000009777.1	no	1693_1	Pyrite-Copper	69.86	0.00	69.86	1	1	1	17	Medium quality	157597	611982	45.80	9	d__Bacteria;p__Patescibacteria;c__Paceibacteria;o__UBA6257;f__Colwellbacteraceae;g__;s__
LMSG_G000009778.1	no	1831_1	Magnetite	60.08	0.00	60.08	1	2	1	20	Medium quality	23880	592228	47.30	35	d__Bacteria;p__Patescibacteria;c__Paceibacteria;o__UBA6257;f__Colwellbacteraceae;g__;s__
LMSG_G000009779.1	no	1832_1	Polymetallic	53.70	0.00	53.70	1	0	0	20	Medium quality	6154	545149	51.00	117	d__Bacteria;p__Patescibacteria;c__Paceibacteria;o__UBA6257;f__Colwellbacteraceae;g__;s__
LMSG_G000009780.1	no	1832_2	Lead-Zinc	55.81	0.85	51.54	1	1	1	18	Medium quality	15019	538188	51.90	50	d__Bacteria;p__Patescibacteria;c__Paceibacteria;o__UBA6257;f__Colwellbacteraceae;g__;s__
LMSG_G000009781.1	no	1753_1	Polymetallic	78.85	2.81	64.81	2	2	2	20	Medium quality	599737	856109	49.10	24	d__Bacteria;p__Patescibacteria;c__Paceibacteria;o__UBA6257;f__GWB1-50-10;g__;s__
LMSG_G000009782.1	no	1753_1	Polymetallic	78.85	1.12	73.24	1	2	2	20	Medium quality	285560	824394	49.20	8	d__Bacteria;p__Patescibacteria;c__Paceibacteria;o__UBA6257;f__GWB1-50-10;g__;s__
LMSG_G000009783.1	no	1753_1	Polymetallic	59.75	1.12	54.14	1	2	1	17	Medium quality	212852	625063	48.90	6	d__Bacteria;p__Patescibacteria;c__Paceibacteria;o__UBA6257;f__GWB1-50-10;g__;s__
LMSG_G000009784.1	no	1753_1	Polymetallic	79.98	1.23	73.86	1	1	3	20	Medium quality	747876	835322	49.10	20	d__Bacteria;p__Patescibacteria;c__Paceibacteria;o__UBA6257;f__GWB1-50-10;g__;s__
LMSG_G000009785.1	no	1796_1	Polymetallic	73.24	1.69	64.82	1	2	3	19	Medium quality	345830	649157	43.00	32	d__Bacteria;p__Patescibacteria;c__Paceibacteria;o__UBA6257;f__GWB1-50-10;g__;s__
LMSG_G000009786.1	no	1796_1	Polymetallic	70.99	3.37	54.14	1	1	2	20	Medium quality	43163	632783	43.00	35	d__Bacteria;p__Patescibacteria;c__Paceibacteria;o__UBA6257;f__GWB1-50-10;g__;s__
LMSG_G000009787.1	no	1796_1	Polymetallic	73.24	2.25	62.01	2	1	2	19	Medium quality	157222	639316	42.60	49	d__Bacteria;p__Patescibacteria;c__Paceibacteria;o__UBA6257;f__GWB1-50-10;g__;s__
LMSG_G000009788.1	no	1796_1	Polymetallic	69.86	1.12	64.25	1	0	2	18	Medium quality	78496	570370	42.40	22	d__Bacteria;p__Patescibacteria;c__Paceibacteria;o__UBA6257;f__GWB1-50-10;g__;s__
LMSG_G000009789.1	no	1796_1	Lead-Zinc	67.14	1.69	58.72	1	2	2	20	Medium quality	12133	556629	42.50	57	d__Bacteria;p__Patescibacteria;c__Paceibacteria;o__UBA6257;f__GWB1-50-10;g__;s__
LMSG_G000009790.1	no	1797_1	Polymetallic	56.73	0.00	56.73	0	1	2	18	Medium quality	13084	617082	50.90	81	d__Bacteria;p__Patescibacteria;c__Paceibacteria;o__UBA6257;f__GWB1-50-10;g__;s__
LMSG_G000009791.1	no	1798_1	Polymetallic	65.54	1.12	59.93	1	0	2	18	Medium quality	36724	588606	51.70	39	d__Bacteria;p__Patescibacteria;c__Paceibacteria;o__UBA6257;f__GWB1-50-10;g__;s__
LMSG_G000009792.1	no	1798_1	Pyrite-Copper	72.21	1.12	66.60	0	4	0	19	Medium quality	50613	682890	51.50	48	d__Bacteria;p__Patescibacteria;c__Paceibacteria;o__UBA6257;f__GWB1-50-10;g__;s__
LMSG_G000009793.1	no	1798_1	Magnetite	53.86	0.20	52.84	0	1	1	13	Medium quality	2828	496100	51.70	202	d__Bacteria;p__Patescibacteria;c__Paceibacteria;o__UBA6257;f__GWB1-50-10;g__;s__
LMSG_G000009794.1	no	1798_1	Pyrite-Copper	67.01	1.72	58.39	1	0	0	15	Medium quality	7206	532051	51.40	105	d__Bacteria;p__Patescibacteria;c__Paceibacteria;o__UBA6257;f__GWB1-50-10;g__;s__
LMSG_G000009795.1	no	1798_1	Lead-Zinc	56.48	1.12	50.87	2	1	1	17	Medium quality	465406	602630	50.70	38	d__Bacteria;p__Patescibacteria;c__Paceibacteria;o__UBA6257;f__GWB1-50-10;g__;s__
LMSG_G000009796.1	no	1801_1	Polymetallic	79.48	0.56	76.68	1	1	1	20	Medium quality	31859	788050	42.50	62	d__Bacteria;p__Patescibacteria;c__Paceibacteria;o__UBA6257;f__GWB1-50-10;g__;s__
LMSG_G000009797.1	no	1802_1	Polymetallic	78.41	1.12	72.80	0	1	0	20	Medium quality	26056	777703	37.90	51	d__Bacteria;p__Patescibacteria;c__Paceibacteria;o__UBA6257;f__GWB1-50-10;g__;s__
LMSG_G000009798.1	no	1802_1	Lead-Zinc	80.14	3.37	63.29	0	1	0	19	Medium quality	78357	688747	38.00	20	d__Bacteria;p__Patescibacteria;c__Paceibacteria;o__UBA6257;f__GWB1-50-10;g__;s__
LMSG_G000009799.1	no	1802_1	Pyrite-Copper	79.02	0.00	79.02	1	1	1	19	Medium quality	17292	757077	37.80	65	d__Bacteria;p__Patescibacteria;c__Paceibacteria;o__UBA6257;f__GWB1-50-10;g__;s__
LMSG_G000009800.1	no	1805_1	Polymetallic	73.30	3.37	56.45	1	2	1	17	Medium quality	58140	727889	51.10	23	d__Bacteria;p__Patescibacteria;c__Paceibacteria;o__UBA6257;f__GWB1-50-10;g__;s__
LMSG_G000009801.1	no	1805_1	Polymetallic	74.99	1.12	69.38	1	1	2	18	Medium quality	100567	800859	50.80	65	d__Bacteria;p__Patescibacteria;c__Paceibacteria;o__UBA6257;f__GWB1-50-10;g__;s__
LMSG_G000009802.1	no	1805_1	Polymetallic	65.54	0.00	65.54	0	2	0	16	Medium quality	86054	682026	50.90	38	d__Bacteria;p__Patescibacteria;c__Paceibacteria;o__UBA6257;f__GWB1-50-10;g__;s__
LMSG_G000009803.1	no	1821_1	Polymetallic	65.16	1.12	59.55	1	0	1	20	Medium quality	32907	489921	54.90	23	d__Bacteria;p__Patescibacteria;c__Paceibacteria;o__UBA6257;f__GWB1-50-10;g__;s__
LMSG_G000009804.1	no	1821_1	Polymetallic	69.66	1.12	64.05	1	2	2	20	Medium quality	36712	561402	55.10	19	d__Bacteria;p__Patescibacteria;c__Paceibacteria;o__UBA6257;f__GWB1-50-10;g__;s__
LMSG_G000009805.1	no	1821_1	Polymetallic	62.92	1.12	57.31	1	1	1	20	Medium quality	44992	534467	55.00	18	d__Bacteria;p__Patescibacteria;c__Paceibacteria;o__UBA6257;f__GWB1-50-10;g__;s__
LMSG_G000009806.1	no	1822_1	Polymetallic	65.53	0.03	65.40	0	1	0	15	Medium quality	21243	564417	50.30	36	d__Bacteria;p__Patescibacteria;c__Paceibacteria;o__UBA6257;f__GWB1-50-10;g__;s__
LMSG_G000009807.1	no	1822_1	Polymetallic	59.55	0.00	59.55	1	1	1	16	Medium quality	29354	556684	50.40	57	d__Bacteria;p__Patescibacteria;c__Paceibacteria;o__UBA6257;f__GWB1-50-10;g__;s__
LMSG_G000009808.1	no	1828_1	Polymetallic	72.93	0.92	68.34	0	0	4	20	Medium quality	9573	670644	41.90	117	d__Bacteria;p__Patescibacteria;c__Paceibacteria;o__UBA6257;f__GWB1-50-10;g__;s__
LMSG_G000009809.1	no	1828_1	Polymetallic	66.50	0.31	64.94	0	3	0	19	Medium quality	10043	540281	42.30	89	d__Bacteria;p__Patescibacteria;c__Paceibacteria;o__UBA6257;f__GWB1-50-10;g__;s__
LMSG_G000009810.1	no	1828_1	Polymetallic	66.66	0.00	66.66	0	1	1	19	Medium quality	8060	511608	42.10	86	d__Bacteria;p__Patescibacteria;c__Paceibacteria;o__UBA6257;f__GWB1-50-10;g__;s__
LMSG_G000009811.1	no	1828_1	Polymetallic	73.64	1.88	64.24	1	1	5	20	Medium quality	13368	618519	42.40	75	d__Bacteria;p__Patescibacteria;c__Paceibacteria;o__UBA6257;f__GWB1-50-10;g__;s__
LMSG_G000009812.1	no	1800_1	Polymetallic	66.49	1.12	60.88	0	1	0	18	Medium quality	5727	566900	45.90	125	d__Bacteria;p__Patescibacteria;c__Paceibacteria;o__UBA6257;f__GWB1-50-10;g__GWA2-45-9;s__
LMSG_G000009813.1	no	1800_1	Polymetallic	67.73	0.66	64.42	1	1	1	16	Medium quality	5171	551663	46.10	127	d__Bacteria;p__Patescibacteria;c__Paceibacteria;o__UBA6257;f__GWB1-50-10;g__GWA2-45-9;s__
LMSG_G000009814.1	no	1779_1	Polymetallic	71.91	0.00	71.91	1	1	1	18	Medium quality	22455	758105	41.60	49	d__Bacteria;p__Patescibacteria;c__Paceibacteria;o__UBA6257;f__UBA9933;g__;s__
LMSG_G000009815.1	no	1779_1	Polymetallic	71.91	0.00	71.91	1	1	1	17	Medium quality	66073	717741	41.60	15	d__Bacteria;p__Patescibacteria;c__Paceibacteria;o__UBA6257;f__UBA9933;g__;s__
LMSG_G000009816.1	no	1779_1	Polymetallic	72.47	4.12	51.88	2	7	9	17	Medium quality	11676	954513	41.80	196	d__Bacteria;p__Patescibacteria;c__Paceibacteria;o__UBA6257;f__UBA9933;g__;s__
LMSG_G000009817.1	no	1779_1	Polymetallic	73.48	1.72	64.86	1	2	1	18	Medium quality	64577	752346	41.40	47	d__Bacteria;p__Patescibacteria;c__Paceibacteria;o__UBA6257;f__UBA9933;g__;s__
LMSG_G000009818.1	no	1780_1	Polymetallic	67.37	3.45	50.13	0	1	1	13	Medium quality	13666	545046	36.20	63	d__Bacteria;p__Patescibacteria;c__Paceibacteria;o__UBA6257;f__UBA9933;g__;s__
LMSG_G000009819.1	no	1780_1	Polymetallic	67.37	1.72	58.75	1	0	1	17	Medium quality	15282	570089	36.30	50	d__Bacteria;p__Patescibacteria;c__Paceibacteria;o__UBA6257;f__UBA9933;g__;s__
LMSG_G000009820.1	no	1781_1	Antimony	55.78	0.86	51.47	0	0	0	13	Medium quality	3393	383625	36.00	130	d__Bacteria;p__Patescibacteria;c__Paceibacteria;o__UBA6257;f__UBA9933;g__;s__
LMSG_G000009821.1	no	1781_1	Antimony	73.40	2.59	60.47	0	1	0	16	Medium quality	13423	651671	36.20	74	d__Bacteria;p__Patescibacteria;c__Paceibacteria;o__UBA6257;f__UBA9933;g__;s__
LMSG_G000009822.1	no	1784_1	Antimony	74.65	1.69	66.23	2	2	2	15	Medium quality	17063	712643	51.40	59	d__Bacteria;p__Patescibacteria;c__Paceibacteria;o__UBA6257;f__UBA9933;g__;s__
LMSG_G000009823.1	no	1787_1	Polymetallic	75.28	3.21	59.22	0	1	1	18	Medium quality	41800	673679	39.30	76	d__Bacteria;p__Patescibacteria;c__Paceibacteria;o__UBA6257;f__UBA9933;g__;s__
LMSG_G000009824.1	no	1787_1	Polymetallic	65.47	1.43	58.32	0	0	0	18	Medium quality	23467	510766	39.20	41	d__Bacteria;p__Patescibacteria;c__Paceibacteria;o__UBA6257;f__UBA9933;g__;s__
LMSG_G000009825.1	no	1787_1	Polymetallic	57.96	1.12	52.35	0	1	0	18	Medium quality	9287	525171	39.40	76	d__Bacteria;p__Patescibacteria;c__Paceibacteria;o__UBA6257;f__UBA9933;g__;s__

LMSG_G000009826.1	no	1787_1	Polymetallic	55.65	0.50	53.18	0	1	0	19	Medium quality	23272	514540	39.40	42	d__Bacteria;p__Patescibacteria;c__Paceibacteria;o__UBA6257;f__UBA9933;g__;s__
LMSG_G000009827.1	no	1787_1	Lead-Zinc	71.09	1.23	64.97	0	0	1	19	Medium quality	18653	556106	39.50	59	d__Bacteria;p__Patescibacteria;c__Paceibacteria;o__UBA6257;f__UBA9933;g__;s__
LMSG_G000009828.1	no	1787_1	Pyrite-Copper	66.60	0.00	66.60	1	1	1	19	Medium quality	19105	564770	39.40	44	d__Bacteria;p__Patescibacteria;c__Paceibacteria;o__UBA6257;f__UBA9933;g__;s__
LMSG_G000009829.1	no	1825_1	Polymetallic	66.18	1.12	60.57	0	1	0	15	Medium quality	24136	581433	39.60	55	d__Bacteria;p__Patescibacteria;c__Paceibacteria;o__UBA6257;f__UBA9933;g__;s__
LMSG_G000009830.1	no	1825_1	Polymetallic	71.80	0.00	71.80	1	3	3	19	Medium quality	27823	874313	39.80	112	d__Bacteria;p__Patescibacteria;c__Paceibacteria;o__UBA6257;f__UBA9933;g__;s__
LMSG_G000009831.1	no	1825_1	Polymetallic	68.24	1.12	62.63	0	0	0	16	Medium quality	30704	586922	39.70	58	d__Bacteria;p__Patescibacteria;c__Paceibacteria;o__UBA6257;f__UBA9933;g__;s__
LMSG_G000009832.1	no	1826_1	Lead-Zinc	69.62	0.00	69.62	1	0	1	17	Medium quality	34085	600419	38.90	28	d__Bacteria;p__Patescibacteria;c__Paceibacteria;o__UBA6257;f__UBA9933;g__;s__
LMSG_G000009833.1	no	1827_1	Polymetallic	74.53	2.58	61.62	4	2	1	19	Medium quality	24809	757714	38.90	77	d__Bacteria;p__Patescibacteria;c__Paceibacteria;o__UBA6257;f__UBA9933;g__;s__
LMSG_G000009834.1	no	1825_1	Polymetallic	70.97	0.00	70.97	0	0	3	19	Medium quality	34143	637224	39.50	63	d__Bacteria;p__Patescibacteria;c__Paceibacteria;o__UBA6257;f__UBA9933;g__W02-47-17b;s__
LMSG_G000009835.1	no	1825_1	Polymetallic	72.55	0.00	72.55	2	2	1	17	Medium quality	35931	639732	39.50	50	d__Bacteria;p__Patescibacteria;c__Paceibacteria;o__UBA6257;f__UBA9933;g__W02-47-17b;s__
LMSG_G000009836.1	no	1702_1	Antimony	60.22	0.00	60.22	1	0	6	18	Medium quality	16841	701485	50.00	70	d__Bacteria;p__Patescibacteria;c__Paceibacteria;o__UBA9983;f__UBA11359_A;g__;s__
LMSG_G000009837.1	no	1702_1	Polymetallic	61.93	0.00	61.93	1	1	1	20	Medium quality	12459	758817	49.90	76	d__Bacteria;p__Patescibacteria;c__Paceibacteria;o__UBA9983;f__UBA11359_A;g__;s__
LMSG_G000009838.1	no	1702_1	Polymetallic	60.65	0.00	60.65	1	1	1	20	Medium quality	64729	678044	50.10	17	d__Bacteria;p__Patescibacteria;c__Paceibacteria;o__UBA9983;f__UBA11359_A;g__;s__
LMSG_G000009839.1	no	1702_1	Polymetallic	62.79	0.00	62.79	1	1	1	20	Medium quality	43022	807601	49.80	29	d__Bacteria;p__Patescibacteria;c__Paceibacteria;o__UBA9983;f__UBA11359_A;g__;s__
LMSG_G000009840.1	no	1702_1	Polymetallic	63.64	0.00	63.64	1	1	2	20	Medium quality	42224	792139	50.00	24	d__Bacteria;p__Patescibacteria;c__Paceibacteria;o__UBA9983;f__UBA11359_A;g__;s__
LMSG_G000009841.1	no	1700_1	Polymetallic	60.08	0.85	55.81	1	2	2	20	Medium quality	99609	690323	45.50	9	d__Bacteria;p__Patescibacteria;c__Paceibacteria;o__UBA9983;f__UBA1539;g__UBA1550;s__
LMSG_G000009842.1	no	1700_1	Polymetallic	60.08	0.85	55.81	1	3	8	20	Medium quality	102966	699231	45.60	25	d__Bacteria;p__Patescibacteria;c__Paceibacteria;o__UBA9983;f__UBA1539;g__UBA1550;s__
LMSG_G000009843.1	no	1759_1	Copper	53.52	0.17	52.67	1	1	8	17	Medium quality	10633	617503	49.00	81	d__Bacteria;p__Patescibacteria;c__Paceibacteria;o__UBA9983;f__UBA2163;g__C7867-001;s__
LMSG_G000009844.1	no	1759_1	Copper	64.31	0.86	60.00	2	3	12	20	Medium quality	8149	802519	48.80	148	d__Bacteria;p__Patescibacteria;c__Paceibacteria;o__UBA9983;f__UBA2163;g__C7867-001;s__
LMSG_G000009845.1	no	1760_1	Antimony	56.38	0.00	56.38	1	2	1	19	Medium quality	56031	1012371	47.20	54	d__Bacteria;p__Patescibacteria;c__Paceibacteria;o__UBA9983;f__UBA2163;g__C7867-001;s__
LMSG_G000009846.1	no	1760_1	Antimony	77.01	1.72	68.39	2	4	2	17	Medium quality	19335	598834	47.40	36	d__Bacteria;p__Patescibacteria;c__Paceibacteria;o__UBA9983;f__UBA2163;g__C7867-001;s__
LMSG_G000009847.1	no	1760_1	Antimony	61.49	1.72	52.87	1	0	1	18	Medium quality	14653	487261	47.70	41	d__Bacteria;p__Patescibacteria;c__Paceibacteria;o__UBA9983;f__UBA2163;g__C7867-001;s__
LMSG_G000009848.1	no	1761_1	Polymetallic	66.92	1.72	58.30	1	1	1	18	Medium quality	12814	661106	61.00	78	d__Bacteria;p__Patescibacteria;c__Paceibacteria;o__UBA9983;f__UBA2163;g__C7867-001;s__
LMSG_G000009849.1	no	1762_1	Antimony	58.09	0.28	56.67	1	3	1	17	Medium quality	32275	564559	54.70	36	d__Bacteria;p__Patescibacteria;c__Paceibacteria;o__UBA9983;f__UBA2163;g__C7867-001;s__
LMSG_G000009850.1	no	1762_1	Antimony	54.50	0.85	50.23	1	1	1	16	Medium quality	14452	578801	54.70	46	d__Bacteria;p__Patescibacteria;c__Paceibacteria;o__UBA9983;f__UBA2163;g__C7867-001;s__
LMSG_G000009851.1	no	1765_1	Lead-Zinc	73.56	2.04	63.38	1	2	12	20	Medium quality	60922	637669	57.00	52	d__Bacteria;p__Patescibacteria;c__Paceibacteria;o__UBA9983;f__UBA2163;g__C7867-001;s__
LMSG_G000009852.1	no	1765_1	Polymetallic	65.11	0.00	65.11	0	1	1	19	Medium quality	14294	522504	57.10	54	d__Bacteria;p__Patescibacteria;c__Paceibacteria;o__UBA9983;f__UBA2163;g__C7867-001;s__
LMSG_G000009853.1	no	1766_1	Copper	51.44	0.00	51.44	0	0	0	7	Medium quality	1862	310655	56.20	172	d__Bacteria;p__Patescibacteria;c__Paceibacteria;o__UBA9983;f__UBA2163;g__C7867-001;s__
LMSG_G000009854.1	no	1766_1	Polymetallic	51.14	0.00	51.14	0	1	1	14	Medium quality	13966	380091	55.60	33	d__Bacteria;p__Patescibacteria;c__Paceibacteria;o__UBA9983;f__UBA2163;g__C7867-001;s__
LMSG_G000009855.1	no	1766_1	Polymetallic	52.10	0.00	52.10	1	2	2	19	Medium quality	10687	626694	55.70	97	d__Bacteria;p__Patescibacteria;c__Paceibacteria;o__UBA9983;f__UBA2163;g__C7867-001;s__
LMSG_G000009856.1	no	1770_1	Pyrite-Copper	57.18	0.00	57.18	1	1	3	20	Medium quality	25879	590959	58.90	54	d__Bacteria;p__Patescibacteria;c__Paceibacteria;o__UBA9983;f__UBA2163;g__C7867-001;s__
LMSG_G000009857.1	no	1008_1	Antimony	70.05	0.85	65.78	0	1	0	20	Medium quality	72722	790446	59.50	14	d__Bacteria;p__Patescibacteria;c__Paceibacteria;o__UBA9983;f__UBA2163;g__UBA10103;s__
LMSG_G000009858.1	no	1008_1	Antimony	70.05	0.85	65.78	1	1	1	20	Medium quality	76457	816387	59.40	16	d__Bacteria;p__Patescibacteria;c__Paceibacteria;o__UBA9983;f__UBA2163;g__UBA10103;s__
LMSG_G000009859.1	no	1008_1	Antimony	69.20	0.00	69.20	1	0	1	20	Medium quality	117948	775190	60.00	42	d__Bacteria;p__Patescibacteria;c__Paceibacteria;o__UBA9983;f__UBA2163;g__UBA10103;s__
LMSG_G000009860.1	no	1008_1	Antimony	62.36	0.00	62.36	0	1	0	15	Medium quality	45423	615074	60.40	24	d__Bacteria;p__Patescibacteria;c__Paceibacteria;o__UBA9983;f__UBA2163;g__UBA10103;s__
LMSG_G000009861.1	no	1008_1	Antimony	69.20	0.85	64.93	1	1	1	20	Medium quality	66919	791503	59.60	14	d__Bacteria;p__Patescibacteria;c__Paceibacteria;o__UBA9983;f__UBA2163;g__UBA10103;s__
LMSG_G000009862.1	no	1792_1	Polymetallic	74.29	0.00	74.29	1	0	3	19	Medium quality	26920	537299	46.80	53	d__Bacteria;p__Patescibacteria;c__Paceibacteria;o__UBA9983;f__UBA9973;g__;s__
LMSG_G000009863.1	no	1792_1	Polymetallic	74.29	0.00	74.29	1	0	3	20	Medium quality	18130	516812	46.80	37	d__Bacteria;p__Patescibacteria;c__Paceibacteria;o__UBA9983;f__UBA9973;g__;s__
LMSG_G000009864.1	no	1792_1	Polymetallic	75.42	0.61	72.36	2	0	5	18	Medium quality	13534	579883	46.90	81	d__Bacteria;p__Patescibacteria;c__Paceibacteria;o__UBA9983;f__UBA9973;g__;s__
LMSG_G000009865.1	no	1792_1	Polymetallic	76.54	0.39	74.59	1	2	12	18	Medium quality	59828	567713	46.90	59	d__Bacteria;p__Patescibacteria;c__Paceibacteria;o__UBA9983;f__UBA9973;g__;s__
LMSG_G000009866.1	no	1793_1	Polymetallic	81.16	0.00	81.16	1	2	2	18	Medium quality	125852	852662	46.60	12	d__Bacteria;p__Patescibacteria;c__Paceibacteria;o__UBA9983;f__UBA9973;g__;s__
LMSG_G000009867.1	no	1793_1	Polymetallic	81.16	0.00	81.16	1	3	2	19	Medium quality	219222	871593	46.70	47	d__Bacteria;p__Patescibacteria;c__Paceibacteria;o__UBA9983;f__UBA9973;g__;s__
LMSG_G000009868.1	no	1793_1	Polymetallic	57.54	0.00	57.54	0	1	1	16	Medium quality	125253	606198	45.80	9	d__Bacteria;p__Patescibacteria;c__Paceibacteria;o__UBA9983;f__UBA9973;g__;s__
LMSG_G000009869.1	no	1793_1	Polymetallic	81.16	0.00	81.16	0	2	1	18	Medium quality	68746	814302	47.00	24	d__Bacteria;p__Patescibacteria;c__Paceibacteria;o__UBA9983;f__UBA9973;g__;s__
LMSG_G000009870.1	no	1757_1	Pyrite-Copper	64.82	0.00	64.82	1	1	3	18	Medium quality	37779	912331	36.00	107	d__Bacteria;p__Patescibacteria;c__Paceibacteria;o__UBA9983;f__UBA9973;g__UBA8515;s__
LMSG_G000009871.1	no	1757_1	Lead-Zinc	70.58	1.12	64.97	1	1	1	17	Medium quality	38534	645213	35.90	46	d__Bacteria;p__Patescibacteria;c__Paceibacteria;o__UBA9983;f__UBA9973;g__UBA8515;s__
LMSG_G000009872.1	no	1782_1	Antimony	71.21	4.04	50.99	1	1	1	19	Medium quality	25682	1029139	37.10	70	d__Bacteria;p__Patescibacteria;c__Paceibacteria_A;o__;f__;g__;s__
LMSG_G000009873.1	no	1712_1	Pyrite-Copper	67.82	0.00	67.82	0	1	0	17	Medium quality	110579	850500	44.20	15	d__Bacteria;p__Patescibacteria;c__Paceibacteria_A;o__Moranbacterales;f__UBA2206;g__;s__
LMSG_G000009874.1	no	1712_1	Pyrite-Copper	65.34	0.00	65.34	1	0	1	17	Medium quality	20942	724697	44.50	84	d__Bacteria;p__Patescibacteria;c__Paceibacteria_A;o__Moranbacterales;f__UBA2206;g__;s__
LMSG_G000009875.1	no	1704_1	Antimony	68.70	0.07	68.37	0	0	3	16	Medium quality	4541	715821	35.70	193	d__Bacteria;p__Patescibacteria;c__Paceibacteria_A;o__Moranbacterales;f__UBA2206;g__UBA9630;s__
LMSG_G000009876.1	no	1704_1	Antimony	67.74	0.46	65.43	1	1	4	20	Medium quality	85394	939081	35.80	41	d__Bacteria;p__Patescibacteria;c__Paceibacteria_A;o__Moranbacterales;f__UBA2206;g__UBA9630;s__
LMSG_G000009877.1	no	1704_1	Antimony	67.74	0.00	67.74	1	1	4	20	Medium quality	136880	920981	35.70	28	d__Bacteria;p__Patescibacteria;c__Paceibacteria_A;o__Moranbacterales;f__UBA2206;g__UBA9630;s__
LMSG_G000009878.1	no	1704_1	Antimony	68.67	0.93	64.05	1	1	11	20	Medium quality	84022	955187	35.80	70	d__Bacteria;p__Patescibacteria;c__Paceibacteria_A;o__Moranbacterales;f__UBA2206;g__UBA9630;s__
LMSG_G000009879.1	no	1704_1	Antimony	66.73	2.57	53.87	2	2	3	20	Medium quality	33192	948763	35.70	57	d__Bacteria;p__Patescibacteria;c__Paceibacteria_A;o__Moranbacterales;f__UBA2206;g__UBA9630;s__
LMSG_G000009880.1	no	374_1	Magnetite	55.72	0.00	55.72	0	0	0	16	Medium quality	27459	522523	32.30	31	d__Bacteria;p__Patescibacteria;c__Saccharimonadia;o__Saccharimonadales;f__2-12-FULL-41-12;g__;s__
LMSG_G000009881.1	no	260_1	Antimony	56.89	0.00	56.89	1	3	1	15	Medium quality	10141	605670	51.00	76	d__Bacteria;p__Patescibacteria;c__Saccharimonadia;o__Saccharimonadales;f__Saccharimonadaceae;g__;s__
LMSG_G000009882.1	no	260_1	Antimony	61.68	0.85	57.41	1	2	4	19	Medium quality	58271	748335	50.80	36	d__Bacteria;p__Patescibacteria;c__Saccharimonadia;o__Saccharimonadales;f__Saccharimonadaceae;g__;s__
LMSG_G000009883.1	no	329_1	Antimony	60.89	1.85	51.64	1	1	0	19	Medium quality	77913	818031	46.40	20	d__Bacteria;p__Patescibacteria;c__Saccharimonadia;o__Saccharimonadales;f__Saccharimonadaceae;g__UBA1547;s__
LMSG_G000009884.1	no	330_1	Antimony	60.74	1.39	53.80	0	1	0	19	Medium quality	28012	756059	47.00	39	d__Bacteria;p__Patescibacteria;c__Saccharimonadia;o__Saccharimonadales;f__Saccharimonadaceae;g__UBA1547;s__
LMSG_G000009885.1	no	330_1	Antimony	52.88	0.00	52.88	0	1	0	16	Medium quality	37292	643754	47.00	38	d__Bacteria;p__Patescibacteria;c__Saccharimonadia;o__Saccharimonadales;f__Saccharimonadaceae;g__UBA1547;s__
LMSG_G000009886.1	no	330_1	Antimony	52.01	0.00	52.01	0	1	0	9	Medium quality	30606	438535	47.30	27	d__Bacteria;p__Patescibacteria;c__Saccharimonadia;o__Saccharimonadales;f__Saccharimonadaceae;g__UBA1547;s__
LMSG_G000009887.1	no	330_1	Antimony	64.96	2.55	52.23	1	2	1	19	Medium quality	23582	744769	46.80	61	d__Bacteria;p__Patescibacteria;c__Saccharimonadia;o__Saccharimonadales;f__Saccharimonadaceae;g__UBA1547;s__
LMSG_G000009888.1	no	331_1	Antimony	68.27	2.95	53.52	1	1	1	19	Medium quality	60843	768209	44.20	42	d__Bacteria;p__Patescibacteria;c__Saccharimonadia;o__Saccharimonadales;f__Saccharimonadaceae;g__UBA1547;s__
LMSG_G000009889.1	no	331_1	Antimony	52.83	0.00	52.83	1	1	1	18	Medium quality	29047	647560	43.90	35	d__Bacteria;p__Patescibacteria;c__Saccharimonadia;o__Saccharimonadales;f__Saccharimonadaceae;g__UBA1547;s__
LMSG_G000009890.1	no	353_1	Pyrite	58.20	0.10	57.69	0	0	0	10	Medium quality	3159	431644	30.00	151	d__Bacteria;p__Patescibacteria;c__Saccharimonadia;o__Saccharimonadales;f__SLHW01;g__;s__
LMSG_G000009891.1	no	353_1	Copper	58.38	1.12	52.77	0	0	0	17	Medium quality	6699	609017	29.50	124	d__Bacteria;p__Patescibacteria;c__Saccharimonadia;o__Saccharimonadales;f__SZUA-47;g__;s__
LMSG_G000009892.1	no	354_1	Pyrite	64.49	0.05	64.25	0	1	1	17	Medium quality	27587	742149	31.30	55	d__Bacteria;p__Patescibacteria;c__Saccharimonadia;o__Saccharimonadales;f__SZUA-47;g__;s__
LMSG_G000009893.1	no	354_1	Pyrite	68.66	1.58	60.78	0	1	2	16	Medium quality	11118	702229	31.20	86	d__Bacteria;p__Patescibacteria;c__Saccharimonadia;o__Saccharimonadales;f__SZUA-47;g__;s__
LMSG_G000009894.1	no	354_1	Lead-Zinc	69.36	0.00	69.36	0	0	0	18	Medium quality	120482	686819	31.50	15	d__Bacteria;p__Patescibacteria;c__Saccharimonadia;o__Saccharimonadales;f__SZUA-47;g__;s__
LMSG_G000009895.1	no	354_1	Polymetallic	73.71	0.00	73.71	0	0	0	18	Medium quality	12275	625931	31.10	92	d__Bacteria;p__Patescibacteria;c__Saccharimonadia;o__Saccharimonadales;f__SZUA-47;g__;s__
LMSG_G000009896.1	no	354_1	Polymetallic	55.51	0.89	51.05	1	0	1	13	Medium quality	3374	412670	30.70	131	d__Bacteria;p__Patescibacteria;c__Saccharimonadia;o__Saccharimonadales;f__SZUA-47;g__;s__
LMSG_G000009897.1	no	354_1	Copper	69.55	0.00	69.55	0	0	0	19	Medium quality	66790	633638	31.40	29	d__Bacteria;p__Patescibacteria;c__Saccharimonadia;o__Saccharimonadales;f__SZUA-47;g__;s__
LMSG_G000009898.1	no	354_1	Copper	70.11	0.00	70.11	0	0	0	17	Medium quality	69829	675694	31.40	23	d__Bacteria;p__Patescibacteria;c__Saccharimonadia;o__Saccharimonadales;f__SZUA-47;g__;s__

LMSG_G000009899.1	no	354_1	Copper	65.61	0.00	65.61	0	0	0	18	Medium quality	68056	615340	31.10	36	d__Bacteria;p__Patescibacteria;c__Saccharimonadia;o__Saccharimonadales;f__SZUA-47;g__;s__
LMSG_G000009900.1	no	354_1	Magnetite	68.13	0.90	63.64	0	0	0	18	Medium quality	8617	621142	31.10	156	d__Bacteria;p__Patescibacteria;c__Saccharimonadia;o__Saccharimonadales;f__SZUA-47;g__;s__
LMSG_G000009901.1	no	373_1	Pyrite	70.67	1.12	65.06	2	3	9	19	Medium quality	45520	763022	33.70	52	d__Bacteria;p__Patescibacteria;c__Saccharimonadia;o__Saccharimonadales;f__SZUA-47;g__;s__
LMSG_G000009902.1	no	373_1	Polymetallic	72.35	0.00	72.35	1	1	2	18	Medium quality	566997	770055	33.80	21	d__Bacteria;p__Patescibacteria;c__Saccharimonadia;o__Saccharimonadales;f__SZUA-47;g__;s__
LMSG_G000009903.1	no	373_1	Polymetallic	72.35	0.00	72.35	1	2	1	19	Medium quality	105801	715372	33.60	12	d__Bacteria;p__Patescibacteria;c__Saccharimonadia;o__Saccharimonadales;f__SZUA-47;g__;s__
LMSG_G000009904.1	no	373_1	Polymetallic	72.35	0.00	72.35	1	1	1	18	Medium quality	140022	693193	33.50	11	d__Bacteria;p__Patescibacteria;c__Saccharimonadia;o__Saccharimonadales;f__SZUA-47;g__;s__
LMSG_G000009905.1	no	373_1	Polymetallic	72.35	0.00	72.35	1	1	6	18	Medium quality	180003	751747	33.60	26	d__Bacteria;p__Patescibacteria;c__Saccharimonadia;o__Saccharimonadales;f__SZUA-47;g__;s__
LMSG_G000009906.1	no	373_1	Copper	72.35	0.00	72.35	1	1	7	19	Medium quality	110584	998698	33.20	81	d__Bacteria;p__Patescibacteria;c__Saccharimonadia;o__Saccharimonadales;f__SZUA-47;g__;s__
LMSG_G000009907.1	no	373_1	Copper	71.23	0.00	71.23	1	1	4	19	Medium quality	112631	686514	33.60	17	d__Bacteria;p__Patescibacteria;c__Saccharimonadia;o__Saccharimonadales;f__SZUA-47;g__;s__
LMSG_G000009908.1	no	373_1	Copper	72.35	0.00	72.35	1	1	1	18	Medium quality	125474	709158	33.40	9	d__Bacteria;p__Patescibacteria;c__Saccharimonadia;o__Saccharimonadales;f__SZUA-47;g__;s__
LMSG_G000009909.1	no	373_1	Polymetallic	72.35	0.00	72.35	1	2	1	19	Medium quality	112629	734709	33.70	28	d__Bacteria;p__Patescibacteria;c__Saccharimonadia;o__Saccharimonadales;f__SZUA-47;g__;s__
LMSG_G000009910.1	no	373_1	Magnetite	72.35	0.00	72.35	1	2	1	19	Medium quality	83585	740880	33.60	25	d__Bacteria;p__Patescibacteria;c__Saccharimonadia;o__Saccharimonadales;f__SZUA-47;g__;s__
LMSG_G000009911.1	no	373_1	Copper	72.35	0.00	72.35	2	3	16	18	Medium quality	138772	743105	33.90	35	d__Bacteria;p__Patescibacteria;c__Saccharimonadia;o__Saccharimonadales;f__SZUA-47;g__;s__
LMSG_G000009912.1	no	373_1	Lead-Zinc	70.11	1.12	64.50	1	1	1	18	Medium quality	45511	714058	33.60	28	d__Bacteria;p__Patescibacteria;c__Saccharimonadia;o__Saccharimonadales;f__SZUA-47;g__;s__
LMSG_G000009913.1	no	374_1	Polymetallic	72.24	3.09	56.80	2	3	2	20	Medium quality	35693	775138	32.00	43	d__Bacteria;p__Patescibacteria;c__Saccharimonadia;o__Saccharimonadales;f__SZUA-47;g__;s__
LMSG_G000009914.1	no	354_1	Pyrite	59.25	0.00	59.25	0	0	0	15	Medium quality	41626	558962	31.50	29	d__Bacteria;p__Patescibacteria;c__Saccharimonadia;o__Saccharimonadales;f__UBA4665;g__;s__
LMSG_G000009915.1	no	354_1	Polymetallic	60.56	0.05	60.32	0	0	0	18	Medium quality	6076	599438	31.10	120	d__Bacteria;p__Patescibacteria;c__Saccharimonadia;o__Saccharimonadales;f__UBA4665;g__;s__
LMSG_G000009916.1	no	359_1	Coal	59.59	0.99	54.64	0	0	0	17	Medium quality	8408	631628	40.30	86	d__Bacteria;p__Patescibacteria;c__Saccharimonadia;o__Saccharimonadales;f__UBA4665;g__;s__
LMSG_G000009917.1	no	359_1	Coal	66.54	1.66	58.25	0	1	0	16	Medium quality	10652	676896	40.50	103	d__Bacteria;p__Patescibacteria;c__Saccharimonadia;o__Saccharimonadales;f__UBA4665;g__;s__
LMSG_G000009918.1	no	362_1	Pyrite-Copper	71.79	0.00	71.79	1	0	1	18	Medium quality	118638	754855	37.50	20	d__Bacteria;p__Patescibacteria;c__Saccharimonadia;o__Saccharimonadales;f__UBA4665;g__;s__
LMSG_G000009919.1	no	362_1	Pyrite-Copper	63.93	0.00	63.93	1	0	1	17	Medium quality	59275	702470	38.00	27	d__Bacteria;p__Patescibacteria;c__Saccharimonadia;o__Saccharimonadales;f__UBA4665;g__;s__
LMSG_G000009920.1	no	363_1	Antimony	71.41	3.88	52.04	1	1	2	18	Medium quality	8106	671720	36.20	102	d__Bacteria;p__Patescibacteria;c__Saccharimonadia;o__Saccharimonadales;f__UBA4665;g__;s__
LMSG_G000009921.1	no	364_1	Polymetallic	62.62	1.12	57.01	0	0	0	18	Medium quality	27130	737105	33.00	46	d__Bacteria;p__Patescibacteria;c__Saccharimonadia;o__Saccharimonadales;f__UBA4665;g__;s__
LMSG_G000009922.1	no	365_1	Copper	50.78	0.00	50.78	0	0	0	11	Medium quality	3536	402289	30.60	127	d__Bacteria;p__Patescibacteria;c__Saccharimonadia;o__Saccharimonadales;f__UBA4665;g__;s__
LMSG_G000009923.1	no	365_1	Polymetallic	71.79	2.25	60.56	0	0	0	19	Medium quality	111995	851130	31.70	28	d__Bacteria;p__Patescibacteria;c__Saccharimonadia;o__Saccharimonadales;f__UBA4665;g__;s__
LMSG_G000009924.1	no	365_1	Magnetite	71.79	2.25	60.56	0	0	0	19	Medium quality	74447	767772	31.10	21	d__Bacteria;p__Patescibacteria;c__Saccharimonadia;o__Saccharimonadales;f__UBA4665;g__;s__
LMSG_G000009925.1	no	365_1	Magnetite	67.24	0.00	67.24	0	0	0	17	Medium quality	58439	851823	31.90	34	d__Bacteria;p__Patescibacteria;c__Saccharimonadia;o__Saccharimonadales;f__UBA4665;g__;s__
LMSG_G000009926.1	no	367_1	Polymetallic	74.04	1.12	68.43	1	1	1	19	Medium quality	115636	672586	35.90	22	d__Bacteria;p__Patescibacteria;c__Saccharimonadia;o__Saccharimonadales;f__UBA4665;g__;s__
LMSG_G000009927.1	no	367_1	Polymetallic	74.04	1.12	68.43	1	1	1	19	Medium quality	198064	690582	35.90	19	d__Bacteria;p__Patescibacteria;c__Saccharimonadia;o__Saccharimonadales;f__UBA4665;g__;s__
LMSG_G000009928.1	no	367_1	Polymetallic	53.99	0.17	53.14	0	0	0	16	Medium quality	10689	584592	35.60	82	d__Bacteria;p__Patescibacteria;c__Saccharimonadia;o__Saccharimonadales;f__UBA4665;g__;s__
LMSG_G000009929.1	no	367_1	Copper	74.04	1.12	68.43	2	1	6	20	Medium quality	114666	694432	36.00	20	d__Bacteria;p__Patescibacteria;c__Saccharimonadia;o__Saccharimonadales;f__UBA4665;g__;s__
LMSG_G000009930.1	no	368_1	Lead-Zinc	73.59	1.12	67.98	1	1	2	19	Medium quality	195144	744436	34.00	10	d__Bacteria;p__Patescibacteria;c__Saccharimonadia;o__Saccharimonadales;f__UBA4665;g__;s__
LMSG_G000009931.1	no	368_1	Pyrite	65.67	0.34	63.95	0	0	0	12	Medium quality	3933	552609	34.10	163	d__Bacteria;p__Patescibacteria;c__Saccharimonadia;o__Saccharimonadales;f__UBA4665;g__;s__
LMSG_G000009932.1	no	368_1	Copper	73.59	2.25	62.36	1	2	1	20	Medium quality	109529	784982	34.20	16	d__Bacteria;p__Patescibacteria;c__Saccharimonadia;o__Saccharimonadales;f__UBA4665;g__;s__
LMSG_G000009933.1	no	368_1	Polymetallic	72.47	1.12	66.86	1	3	1	18	Medium quality	142151	714287	34.50	15	d__Bacteria;p__Patescibacteria;c__Saccharimonadia;o__Saccharimonadales;f__UBA4665;g__;s__
LMSG_G000009934.1	no	370_1	Polymetallic	68.98	1.12	63.37	0	1	12	16	Medium quality	15616	955328	40.80	95	d__Bacteria;p__Patescibacteria;c__Saccharimonadia;o__Saccharimonadales;f__UBA4665;g__;s__
LMSG_G000009935.1	no	370_1	Magnetite	54.23	0.11	53.68	1	0	1	18	Medium quality	162811	693706	40.10	46	d__Bacteria;p__Patescibacteria;c__Saccharimonadia;o__Saccharimonadales;f__UBA4665;g__;s__
LMSG_G000009936.1	no	371_1	Lead-Zinc	66.81	0.99	61.86	0	1	0	16	Medium quality	21516	680309	44.30	46	d__Bacteria;p__Patescibacteria;c__Saccharimonadia;o__Saccharimonadales;f__UBA4665;g__;s__
LMSG_G000009937.1	no	371_1	Lead-Zinc	65.01	1.49	57.59	0	1	0	17	Medium quality	55065	659737	44.30	18	d__Bacteria;p__Patescibacteria;c__Saccharimonadia;o__Saccharimonadales;f__UBA4665;g__;s__
LMSG_G000009938.1	no	373_1	Copper	66.74	0.00	66.74	2	3	11	18	Medium quality	110392	663841	33.80	27	d__Bacteria;p__Patescibacteria;c__Saccharimonadia;o__Saccharimonadales;f__UBA4665;g__;s__
LMSG_G000009939.1	no	375_1	Polymetallic	74.60	2.25	63.37	1	2	1	19	Medium quality	45333	808758	35.30	41	d__Bacteria;p__Patescibacteria;c__Saccharimonadia;o__Saccharimonadales;f__UBA4665;g__;s__
LMSG_G000009940.1	no	375_1	Polymetallic	70.86	2.30	59.38	1	2	2	18	Medium quality	13700	773512	35.10	97	d__Bacteria;p__Patescibacteria;c__Saccharimonadia;o__Saccharimonadales;f__UBA4665;g__;s__
LMSG_G000009941.1	no	375_1	Polymetallic	73.48	1.12	67.87	1	0	1	19	Medium quality	53085	765829	35.30	36	d__Bacteria;p__Patescibacteria;c__Saccharimonadia;o__Saccharimonadales;f__UBA4665;g__;s__
LMSG_G000009942.1	no	375_1	Magnetite	59.74	1.12	54.13	1	1	1	17	Medium quality	29326	713064	35.50	71	d__Bacteria;p__Patescibacteria;c__Saccharimonadia;o__Saccharimonadales;f__UBA4665;g__;s__
LMSG_G000009943.1	no	376_1	Copper	59.72	0.46	57.41	2	1	2	19	Medium quality	96716	806236	36.40	36	d__Bacteria;p__Patescibacteria;c__Saccharimonadia;o__Saccharimonadales;f__UBA4665;g__;s__
LMSG_G000009944.1	no	380_1	Lead-Zinc	53.54	0.00	53.54	0	0	0	11	Medium quality	29775	545874	46.50	37	d__Bacteria;p__Patescibacteria;c__Saccharimonadia;o__Saccharimonadales;f__UBA4665;g__;s__
LMSG_G000009945.1	no	380_1	Lead-Zinc	65.74	0.93	61.12	1	1	1	16	Medium quality	49481	823237	45.50	31	d__Bacteria;p__Patescibacteria;c__Saccharimonadia;o__Saccharimonadales;f__UBA4665;g__;s__
LMSG_G000009946.1	no	380_1	Pyrite-Copper	51.71	0.14	51.00	0	0	0	14	Medium quality	15752	558935	46.40	52	d__Bacteria;p__Patescibacteria;c__Saccharimonadia;o__Saccharimonadales;f__UBA4665;g__;s__
LMSG_G000009947.1	no	382_1	Magnetite	64.81	1.80	55.81	1	0	1	18	Medium quality	45622	979043	45.00	27	d__Bacteria;p__Patescibacteria;c__Saccharimonadia;o__Saccharimonadales;f__UBA4665;g__;s__
LMSG_G000009948.1	no	384_1	Lead-Zinc	62.65	0.00	62.65	1	1	1	20	Medium quality	77561	832377	42.90	33	d__Bacteria;p__Patescibacteria;c__Saccharimonadia;o__Saccharimonadales;f__UBA4665;g__;s__
LMSG_G000009949.1	no	384_1	Lead-Zinc	62.93	0.00	62.93	1	0	1	15	Medium quality	65000	636496	43.60	16	d__Bacteria;p__Patescibacteria;c__Saccharimonadia;o__Saccharimonadales;f__UBA4665;g__;s__
LMSG_G000009950.1	no	384_1	Lead-Zinc	63.58	0.00	63.58	1	1	1	19	Medium quality	77146	845087	42.90	43	d__Bacteria;p__Patescibacteria;c__Saccharimonadia;o__Saccharimonadales;f__UBA4665;g__;s__
LMSG_G000009951.1	no	387_1	Pyrite-Copper	58.08	1.49	50.66	2	1	2	16	Medium quality	30651	617058	41.10	36	d__Bacteria;p__Patescibacteria;c__Saccharimonadia;o__Saccharimonadales;f__UBA4665;g__;s__
LMSG_G000009952.1	no	387_1	Pyrite-Copper	64.02	0.29	62.57	1	0	3	18	Medium quality	49146	677771	41.50	45	d__Bacteria;p__Patescibacteria;c__Saccharimonadia;o__Saccharimonadales;f__UBA4665;g__;s__
LMSG_G000009953.1	no	387_1	Pyrite-Copper	62.94	0.00	62.94	1	0	1	18	Medium quality	50374	646456	41.00	29	d__Bacteria;p__Patescibacteria;c__Saccharimonadia;o__Saccharimonadales;f__UBA4665;g__;s__
LMSG_G000009954.1	no	364_1	Polymetallic	68.98	1.12	63.37	0	0	0	18	Medium quality	25205	768694	33.10	49	d__Bacteria;p__Patescibacteria;c__Saccharimonadia;o__Saccharimonadales;f__UBA4665;g__UBA4729;s__
LMSG_G000009955.1	no	364_1	Polymetallic	69.55	1.24	63.38	0	0	1	19	Medium quality	17146	769039	33.20	92	d__Bacteria;p__Patescibacteria;c__Saccharimonadia;o__Saccharimonadales;f__UBA4665;g__UBA4729;s__
LMSG_G000009956.1	no	364_1	Polymetallic	65.05	1.12	59.44	0	0	0	18	Medium quality	35460	741623	33.10	38	d__Bacteria;p__Patescibacteria;c__Saccharimonadia;o__Saccharimonadales;f__UBA4665;g__UBA4729;s__
LMSG_G000009957.1	no	365_1	Magnetite	62.75	0.00	62.75	1	0	1	16	Medium quality	58439	709714	31.10	23	d__Bacteria;p__Patescibacteria;c__Saccharimonadia;o__Saccharimonadales;f__UBA4665;g__UBA4729;s__
LMSG_G000009958.1	no	376_1	Polymetallic	70.68	0.66	67.39	1	0	1	18	Medium quality	11228	739709	35.20	97	d__Bacteria;p__Patescibacteria;c__Saccharimonadia;o__Saccharimonadales;f__UBA4665;g__UBA4729;s__
LMSG_G000009959.1	no	234_1	Lead-Zinc	70.01	0.00	70.01	1	1	1	16	Medium quality	27341	843695	44.00	53	d__Bacteria;p__Patescibacteria;c__Saccharimonadia;o__Saccharimonadales;f__UBA4665;g__UBA5150;s__
LMSG_G000009960.1	no	360_1	Polymetallic	58.04	0.69	54.60	0	0	0	17	Medium quality	41479	622591	40.00	39	d__Bacteria;p__Patescibacteria;c__Saccharimonadia;o__Saccharimonadales;f__UBA4665;g__UBA5150;s__
LMSG_G000009961.1	no	360_1	Polymetallic	60.18	1.85	50.93	0	0	0	15	Medium quality	20213	570833	40.10	44	d__Bacteria;p__Patescibacteria;c__Saccharimonadia;o__Saccharimonadales;f__UBA4665;g__UBA5150;s__
LMSG_G000009962.1	no	344_1	Tin-Zinc	58.73	1.49	51.31	1	3	2	18	Medium quality	33561	723237	49.40	61	d__Bacteria;p__Patescibacteria;c__Saccharimonadia;o__Saccharimonadales;f__UBA4665;g__UBA6224;s__
LMSG_G000009963.1	no	1583_1	Polymetallic	62.54	0.00	62.54	1	0	2	18	Medium quality	12212	826152	45.80	91	d__Bacteria;p__Patescibacteria;c__UBA1384;o__;f__;g__;s__
LMSG_G000009964.1	no	1583_1	Polymetallic	69.96	1.49	62.54	1	1	1	18	Medium quality	20654	886038	46.10	60	d__Bacteria;p__Patescibacteria;c__UBA1384;o__;f__;g__;s__
LMSG_G000009965.1	no	428_2	Pyrite-Copper	81.53	1.29	75.08	0	1	1	17	Medium quality	19659	2222570	59.00	251	d__Bacteria;p__Planctomycetota;c__Phycisphaerae;o__;f__;g__;s__
LMSG_G000009966.1	no	429_1	Lead-Zinc	89.96	2.38	78.08	0	0	0	13	Medium quality	7021	2937119	52.20	497	d__Bacteria;p__Planctomycetota;c__Phycisphaerae;o__;f__;g__;s__
LMSG_G000009967.1	no	436_1	Polymetallic	71.90	0.07	71.57	1	0	1	20	Medium quality	15339	2621259	55.40	256	d__Bacteria;p__Planctomycetota;c__Phycisphaerae;o__;f__;g__;s__
LMSG_G000009968.1	no	436_1	Copper	82.56	3.98	62.68	0	0	0	18	Medium quality	30933	3174194	55.00	180	d__Bacteria;p__Planctomycetota;c__Phycisphaerae;o__;f__;g__;s__
LMSG_G000009969.1	no	437_1	Pyrite-Copper	80.18	1.24	73.99	0	1	0	15	Medium quality	11835	2492429	56.40	370	d__Bacteria;p__Planctomycetota;c__Phycisphaerae;o__;f__;g__;s__
LMSG_G000009970.1	no	437_2	Magnetite	70.04	2.46	57.76	0	0	0	15	Medium quality	9465	2669894	56.30	619	d__Bacteria;p__Planctomycetota;c__Phycisphaerae;o__;f__;g__;s__
LMSG_G000009971.1	no	438_1	Copper	57.03	0.59	54.11	0	0	0	11	Medium quality	2085	1758951	57.20	856	d__Bacteria;p__Planctomycetota;c__Phycisphaerae;o__;f__;g__;s__
LMSG_G000009972.1	no	446_1	Polymetallic	75.78	0.45	73.56	0	1	0	15	Medium quality	4418	2806527	57.30	786	d__Bacteria;p__Planctomycetota;c__Phycisphaerae;o__;f__;g__;s__
LMSG_G000009973.1	no	1065_1	Polymetallic	69.60	0.10	69.09	0	0	0	13	Medium quality	6968	3779110	59.60	746	d__Bacteria;p__Planctomycetota;c__Phycisphaerae;o__Phycisphaerales;f__;g__;s__
LMSG_G000009974.1	no	1569_1	Magnetite	73.46	1.68	65.08	1	1	2	18	Medium quality	27480	2881120	56.90	158	d__Bacteria;p__Planctomycetota;c__Phycisphaerae;o__Phycisphaerales;f__;g__;s__
LMSG_G000009975.1	no	1570_1	Magnetite	87.31	1.14	81.63	0	1	0	19	Medium quality	17122	3381290	55.20	330	d__Bacteria;p__Planctomycetota;c__Phycisphaerae;o__Phycisphaerales;f__;g__;s__

LMSG_G000009976.1	no	1571_1	Polymetallic	68.39	2.27	57.03	0	0	0	19	Medium quality	6536	2869685	55.80	470	d__Bacteria;p__Planctomycetota;c__Phycisphaerae;o__Phycisphaerales;f__;g__;s__
LMSG_G000009977.1	no	427_1	Copper	80.61	1.24	74.42	0	2	0	18	Medium quality	13944	3290593	61.50	460	d__Bacteria;p__Planctomycetota;c__Phycisphaerae;o__Phycisphaerales;f__;g__;s__
LMSG_G000009978.1	no	427_1	Lead-Zinc	61.95	0.07	61.62	0	0	0	18	Medium quality	3369	2220264	62.30	698	d__Bacteria;p__Planctomycetota;c__Phycisphaerae;o__Phycisphaerales;f__;g__;s__
LMSG_G000009979.1	no	428_1	Magnetite	74.02	0.33	72.35	0	1	1	16	Medium quality	37353	1958063	58.90	81	d__Bacteria;p__Planctomycetota;c__Phycisphaerae;o__Phycisphaerales;f__;g__;s__
LMSG_G000009980.1	no	429_1	Pyrite-Copper	73.45	0.00	73.45	0	1	1	19	Medium quality	12364	3092062	51.90	346	d__Bacteria;p__Planctomycetota;c__Phycisphaerae;o__Phycisphaerales;f__;g__;s__
LMSG_G000009981.1	no	429_1	Lead-Zinc	67.05	0.17	66.20	0	0	0	13	Medium quality	1949	2092385	53.00	1143	d__Bacteria;p__Planctomycetota;c__Phycisphaerae;o__Phycisphaerales;f__;g__;s__
LMSG_G000009982.1	no	429_1	Magnetite	70.07	1.14	64.39	0	0	0	14	Medium quality	9694	2947375	52.00	391	d__Bacteria;p__Planctomycetota;c__Phycisphaerae;o__Phycisphaerales;f__;g__;s__
LMSG_G000009983.1	no	434_1	Polymetallic	85.22	4.36	63.44	0	2	1	19	Medium quality	21579	3461475	54.80	222	d__Bacteria;p__Planctomycetota;c__Phycisphaerae;o__Phycisphaerales;f__;g__;s__
LMSG_G000009984.1	no	435_1	Lead-Zinc	70.90	0.00	70.90	0	0	0	17	Medium quality	3799	2502127	54.80	767	d__Bacteria;p__Planctomycetota;c__Phycisphaerae;o__Phycisphaerales;f__;g__;s__
LMSG_G000009985.1	no	435_1	Polymetallic	86.41	0.00	86.41	1	2	1	14	Medium quality	34663	3360549	54.50	219	d__Bacteria;p__Planctomycetota;c__Phycisphaerae;o__Phycisphaerales;f__;g__;s__
LMSG_G000009986.1	no	435_1	Polymetallic	92.11	2.27	80.75	1	0	2	20	Medium quality	22331	3627209	54.50	213	d__Bacteria;p__Planctomycetota;c__Phycisphaerae;o__Phycisphaerales;f__;g__;s__
LMSG_G000009987.1	no	437_1	Lead-Zinc	83.92	0.00	83.92	1	1	0	18	Medium quality	23847	2880292	56.30	240	d__Bacteria;p__Planctomycetota;c__Phycisphaerae;o__Phycisphaerales;f__;g__;s__
LMSG_G000009988.1	no	437_1	Lead-Zinc	88.06	0.76	84.28	1	1	4	17	Medium quality	28806	3117497	56.50	319	d__Bacteria;p__Planctomycetota;c__Phycisphaerae;o__Phycisphaerales;f__;g__;s__
LMSG_G000009989.1	no	437_1	Lead-Zinc	77.12	0.84	72.95	0	0	0	13	Medium quality	8935	2277920	56.30	321	d__Bacteria;p__Planctomycetota;c__Phycisphaerae;o__Phycisphaerales;f__;g__;s__
LMSG_G000009990.1	no	437_1	Lead-Zinc	78.83	3.48	61.46	0	0	1	17	Medium quality	7103	2528190	56.30	432	d__Bacteria;p__Planctomycetota;c__Phycisphaerae;o__Phycisphaerales;f__;g__;s__
LMSG_G000009991.1	no	1569_1	Polymetallic	96.21	1.82	87.12	1	2	2	20	High quality	54151	3850096	57.00	114	d__Bacteria;p__Planctomycetota;c__Phycisphaerae;o__UBA1161;f__;g__;s__
LMSG_G000009992.1	no	1569_1	Polymetallic	96.21	3.30	79.74	1	1	2	20	High quality	65139	3711948	57.00	103	d__Bacteria;p__Planctomycetota;c__Phycisphaerae;o__UBA1161;f__;g__;s__
LMSG_G000009993.1	no	1569_1	Polymetallic	95.64	0.64	92.47	1	2	2	20	High quality	41605	3629533	57.00	123	d__Bacteria;p__Planctomycetota;c__Phycisphaerae;o__UBA1161;f__;g__;s__
LMSG_G000009994.1	no	1569_1	Polymetallic	95.64	1.14	89.96	1	1	2	20	High quality	71791	3628590	57.00	88	d__Bacteria;p__Planctomycetota;c__Phycisphaerae;o__UBA1161;f__;g__;s__
LMSG_G000009995.1	no	1569_1	Polymetallic	68.65	2.69	55.21	0	0	0	15	Medium quality	6556	2226431	57.60	411	d__Bacteria;p__Planctomycetota;c__Phycisphaerae;o__UBA1161;f__;g__;s__
LMSG_G000009996.1	no	1569_1	Polymetallic	95.07	0.00	95.07	0	1	1	20	Medium quality	37587	3324182	57.00	137	d__Bacteria;p__Planctomycetota;c__Phycisphaerae;o__UBA1161;f__;g__;s__
LMSG_G000009997.1	no	1569_1	Polymetallic	87.14	0.00	87.14	0	1	0	19	Medium quality	28658	3047084	57.10	149	d__Bacteria;p__Planctomycetota;c__Phycisphaerae;o__UBA1161;f__;g__;s__
LMSG_G000009998.1	no	427_1	Pyrite-Copper	94.21	3.98	74.33	0	1	0	19	Medium quality	24965	4211699	61.50	236	d__Bacteria;p__Planctomycetota;c__Phycisphaerae;o__UBA1161;f__;g__;s__
LMSG_G000009999.1	no	427_1	Pyrite-Copper	68.11	0.00	68.11	0	0	0	16	Medium quality	8290	2810102	62.30	457	d__Bacteria;p__Planctomycetota;c__Phycisphaerae;o__UBA1161;f__;g__;s__
LMSG_G000010000.1	no	427_1	Copper	79.88	0.39	77.95	1	2	1	18	Medium quality	6028	3022551	61.60	595	d__Bacteria;p__Planctomycetota;c__Phycisphaerae;o__UBA1161;f__;g__;s__
LMSG_G000010001.1	no	427_1	Lead-Zinc	87.27	1.14	81.59	0	1	2	20	Medium quality	13639	3415633	61.60	428	d__Bacteria;p__Planctomycetota;c__Phycisphaerae;o__UBA1161;f__;g__;s__
LMSG_G000010002.1	no	427_1	Lead-Zinc	83.63	1.14	77.95	0	1	0	20	Medium quality	8388	3649492	61.60	556	d__Bacteria;p__Planctomycetota;c__Phycisphaerae;o__UBA1161;f__;g__;s__
LMSG_G000010003.1	no	427_1	Pyrite-Copper	83.29	0.77	79.42	0	0	0	17	Medium quality	8582	2934323	61.60	495	d__Bacteria;p__Planctomycetota;c__Phycisphaerae;o__UBA1161;f__;g__;s__
LMSG_G000010004.1	no	427_1	Lead-Zinc	88.63	2.27	77.27	0	3	0	20	Medium quality	21406	4224972	61.40	520	d__Bacteria;p__Planctomycetota;c__Phycisphaerae;o__UBA1161;f__;g__;s__
LMSG_G000010005.1	no	427_1	Magnetite	66.43	1.70	57.91	1	0	1	16	Medium quality	5662	2917437	61.60	641	d__Bacteria;p__Planctomycetota;c__Phycisphaerae;o__UBA1161;f__;g__;s__
LMSG_G000010006.1	no	428_1	Copper	88.97	1.24	82.78	0	2	1	19	Medium quality	17820	2604419	59.30	224	d__Bacteria;p__Planctomycetota;c__Phycisphaerae;o__UBA1161;f__;g__;s__
LMSG_G000010007.1	no	428_1	Copper	79.66	0.07	79.33	0	1	1	16	Medium quality	27764	2031903	59.00	121	d__Bacteria;p__Planctomycetota;c__Phycisphaerae;o__UBA1161;f__;g__;s__
LMSG_G000010008.1	no	428_1	Copper	56.89	0.00	56.89	0	0	0	10	Medium quality	2917	1438777	59.80	514	d__Bacteria;p__Planctomycetota;c__Phycisphaerae;o__UBA1161;f__;g__;s__
LMSG_G000010009.1	no	428_1	Copper	89.77	1.14	84.09	0	2	1	19	Medium quality	41293	2593623	58.90	111	d__Bacteria;p__Planctomycetota;c__Phycisphaerae;o__UBA1161;f__;g__;s__
LMSG_G000010010.1	no	428_1	Polymetallic	89.84	0.13	89.18	0	1	0	19	Medium quality	8773	2440221	59.40	338	d__Bacteria;p__Planctomycetota;c__Phycisphaerae;o__UBA1161;f__;g__;s__
LMSG_G000010011.1	no	428_1	Polymetallic	91.68	1.14	86.00	0	2	1	16	Medium quality	14343	2570313	59.20	289	d__Bacteria;p__Planctomycetota;c__Phycisphaerae;o__UBA1161;f__;g__;s__
LMSG_G000010012.1	no	428_1	Polymetallic	66.54	0.00	66.54	0	0	1	12	Medium quality	4435	1869642	59.40	480	d__Bacteria;p__Planctomycetota;c__Phycisphaerae;o__UBA1161;f__;g__;s__
LMSG_G000010013.1	no	428_1	Polymetallic	72.80	1.70	64.28	0	1	1	18	Medium quality	5472	1966166	59.50	462	d__Bacteria;p__Planctomycetota;c__Phycisphaerae;o__UBA1161;f__;g__;s__
LMSG_G000010014.1	no	428_1	Polymetallic	85.89	1.20	79.88	0	1	0	16	Medium quality	5141	2100672	59.50	480	d__Bacteria;p__Planctomycetota;c__Phycisphaerae;o__UBA1161;f__;g__;s__
LMSG_G000010015.1	no	428_1	Copper	67.36	0.00	67.36	0	2	0	16	Medium quality	7059	1799604	59.50	314	d__Bacteria;p__Planctomycetota;c__Phycisphaerae;o__UBA1161;f__;g__;s__
LMSG_G000010016.1	no	428_1	Polymetallic	95.97	0.00	95.97	0	2	1	20	Medium quality	30856	2553771	59.30	122	d__Bacteria;p__Planctomycetota;c__Phycisphaerae;o__UBA1161;f__;g__;s__
LMSG_G000010017.1	no	428_1	Magnetite	97.72	0.00	97.72	0	2	1	20	Medium quality	27750	2751508	59.10	146	d__Bacteria;p__Planctomycetota;c__Phycisphaerae;o__UBA1161;f__;g__;s__
LMSG_G000010018.1	no	428_1	Lead-Zinc	95.45	0.00	95.45	0	2	2	19	Medium quality	18516	2716852	59.10	212	d__Bacteria;p__Planctomycetota;c__Phycisphaerae;o__UBA1161;f__;g__;s__
LMSG_G000010019.1	no	428_1	Lead-Zinc	94.31	0.57	91.47	0	2	1	20	Medium quality	34172	2783290	59.10	167	d__Bacteria;p__Planctomycetota;c__Phycisphaerae;o__UBA1161;f__;g__;s__
LMSG_G000010020.1	no	428_1	Lead-Zinc	96.59	0.00	96.59	0	2	1	20	Medium quality	56412	2734087	59.10	107	d__Bacteria;p__Planctomycetota;c__Phycisphaerae;o__UBA1161;f__;g__;s__
LMSG_G000010021.1	no	428_1	Pyrite-Copper	96.59	1.36	89.78	0	2	2	20	Medium quality	37308	2728286	59.20	122	d__Bacteria;p__Planctomycetota;c__Phycisphaerae;o__UBA1161;f__;g__;s__
LMSG_G000010022.1	no	428_1	Lead-Zinc	96.59	0.00	96.59	0	2	1	20	Medium quality	37929	2693808	59.10	102	d__Bacteria;p__Planctomycetota;c__Phycisphaerae;o__UBA1161;f__;g__;s__
LMSG_G000010023.1	no	428_1	Magnetite	62.25	1.14	56.57	0	0	0	17	Medium quality	8980	1812879	59.00	272	d__Bacteria;p__Planctomycetota;c__Phycisphaerae;o__UBA1161;f__;g__;s__
LMSG_G000010024.1	no	428_1	Magnetite	65.00	0.33	63.33	0	0	0	16	Medium quality	13525	1754151	59.10	160	d__Bacteria;p__Planctomycetota;c__Phycisphaerae;o__UBA1161;f__;g__;s__
LMSG_G000010025.1	no	429_1	Pyrite	95.45	0.00	95.45	0	1	1	20	Medium quality	9692	4013676	52.00	697	d__Bacteria;p__Planctomycetota;c__Phycisphaerae;o__UBA1161;f__;g__;s__
LMSG_G000010026.1	no	429_1	Pyrite-Copper	96.59	0.00	96.59	0	1	0	20	Medium quality	16344	3717313	52.10	348	d__Bacteria;p__Planctomycetota;c__Phycisphaerae;o__UBA1161;f__;g__;s__
LMSG_G000010027.1	no	429_1	Lead-Zinc	73.20	0.00	73.20	0	0	0	10	Medium quality	5399	2416104	52.50	541	d__Bacteria;p__Planctomycetota;c__Phycisphaerae;o__UBA1161;f__;g__;s__
LMSG_G000010028.1	no	429_1	Copper	76.84	3.41	59.80	0	0	0	14	Medium quality	6022	2859955	52.40	596	d__Bacteria;p__Planctomycetota;c__Phycisphaerae;o__UBA1161;f__;g__;s__
LMSG_G000010029.1	no	429_1	Pyrite	96.59	0.00	96.59	0	1	0	20	Medium quality	13491	3816739	52.30	481	d__Bacteria;p__Planctomycetota;c__Phycisphaerae;o__UBA1161;f__;g__;s__
LMSG_G000010030.1	no	429_1	Pyrite	72.87	2.51	60.33	0	2	0	18	Medium quality	5866	2708223	52.30	583	d__Bacteria;p__Planctomycetota;c__Phycisphaerae;o__UBA1161;f__;g__;s__
LMSG_G000010031.1	no	429_1	Lead-Zinc	79.06	1.24	72.87	0	0	0	13	Medium quality	4229	3197227	52.30	861	d__Bacteria;p__Planctomycetota;c__Phycisphaerae;o__UBA1161;f__;g__;s__
LMSG_G000010032.1	no	429_1	Copper	97.15	0.00	97.15	0	2	0	18	Medium quality	14690	3752562	52.20	390	d__Bacteria;p__Planctomycetota;c__Phycisphaerae;o__UBA1161;f__;g__;s__
LMSG_G000010033.1	no	429_1	Lead-Zinc	83.92	0.07	83.59	0	1	0	14	Medium quality	10565	3006838	52.40	418	d__Bacteria;p__Planctomycetota;c__Phycisphaerae;o__UBA1161;f__;g__;s__
LMSG_G000010034.1	no	429_1	Lead-Zinc	92.89	0.00	92.89	0	1	0	17	Medium quality	9817	2920877	52.10	380	d__Bacteria;p__Planctomycetota;c__Phycisphaerae;o__UBA1161;f__;g__;s__
LMSG_G000010035.1	no	429_1	Magnetite	70.64	2.27	59.28	0	0	0	15	Medium quality	8570	2593136	52.00	387	d__Bacteria;p__Planctomycetota;c__Phycisphaerae;o__UBA1161;f__;g__;s__
LMSG_G000010036.1	no	430_1	Copper	87.44	2.61	74.41	0	0	1	17	Medium quality	11173	3493891	54.20	388	d__Bacteria;p__Planctomycetota;c__Phycisphaerae;o__UBA1161;f__;g__;s__
LMSG_G000010037.1	no	430_1	Copper	94.88	1.24	88.69	1	0	1	20	Medium quality	12840	4004880	54.10	412	d__Bacteria;p__Planctomycetota;c__Phycisphaerae;o__UBA1161;f__;g__;s__
LMSG_G000010038.1	no	430_1	Lead-Zinc	83.76	4.23	62.61	1	0	2	15	Medium quality	15711	3229791	54.00	471	d__Bacteria;p__Planctomycetota;c__Phycisphaerae;o__UBA1161;f__;g__;s__
LMSG_G000010039.1	no	430_1	Lead-Zinc	69.29	1.14	63.61	1	0	2	13	Medium quality	5820	2820337	54.00	528	d__Bacteria;p__Planctomycetota;c__Phycisphaerae;o__UBA1161;f__;g__;s__
LMSG_G000010040.1	no	430_1	Coal	95.45	2.27	84.09	1	1	1	19	High quality	19066	4432962	54.20	335	d__Bacteria;p__Planctomycetota;c__Phycisphaerae;o__UBA1161;f__;g__;s__
LMSG_G000010041.1	no	433_1	Copper	52.52	0.00	52.52	0	1	0	16	Medium quality	11419	1609983	52.70	226	d__Bacteria;p__Planctomycetota;c__Phycisphaerae;o__UBA1161;f__;g__;s__
LMSG_G000010042.1	no	434_1	Polymetallic	93.18	1.14	87.50	0	3	1	20	Medium quality	31333	3358007	54.70	219	d__Bacteria;p__Planctomycetota;c__Phycisphaerae;o__UBA1161;f__;g__;s__
LMSG_G000010043.1	no	435_1	Copper	95.38	0.52	92.80	1	1	2	19	High quality	25200	3888445	54.50	258	d__Bacteria;p__Planctomycetota;c__Phycisphaerae;o__UBA1161;f__;g__;s__
LMSG_G000010044.1	no	435_1	Polymetallic	97.15	0.00	97.15	1	2	2	20	High quality	23448	4347817	54.50	261	d__Bacteria;p__Planctomycetota;c__Phycisphaerae;o__UBA1161;f__;g__;s__
LMSG_G000010045.1	no	435_1	Polymetallic	97.72	1.70	89.20	1	1	2	19	High quality	39178	4117976	54.60	221	d__Bacteria;p__Planctomycetota;c__Phycisphaerae;o__UBA1161;f__;g__;s__
LMSG_G000010046.1	no	435_1	Polymetallic	96.02	2.27	84.66	1	1	2	20	High quality	26927	4194032	54.50	323	d__Bacteria;p__Planctomycetota;c__Phycisphaerae;o__UBA1161;f__;g__;s__
LMSG_G000010047.1	no	435_1	Polymetallic	97.72	1.14	92.04	1	1	2	20	High quality	53585	4500089	54.60	221	d__Bacteria;p__Planctomycetota;c__Phycisphaerae;o__UBA1161;f__;g__;s__
LMSG_G000010048.1	no	435_1	Polymetallic	96.21	0.00	96.21	1	1	2	20	High quality	17866	3798918	54.50	306	d__Bacteria;p__Planctomycetota;c__Phycisphaerae;o__UBA1161;f__;g__;s__
LMSG_G000010049.1	no	435_1	Polymetallic	93.93	1.70	85.41	1	2	2	19	High quality	25253	3534628	54.50	264	d__Bacteria;p__Planctomycetota;c__Phycisphaerae;o__UBA1161;f__;g__;s__
LMSG_G000010050.1	no	435_1	Polymetallic	95.07	0.57	92.23	1	2	2	17	Medium quality	21879	3776894	54.50	269	d__Bacteria;p__Planctomycetota;c__Phycisphaerae;o__UBA1161;f__;g__;s__
LMSG_G000010051.1	no	435_1	Polymetallic	90.90	4.99	65.97	1	2	2	17	Medium quality	19569	3929207	54.40	315	d__Bacteria;p__Planctomycetota;c__Phycisphaerae;o__UBA1161;f__;g__;s__
LMSG_G000010052.1	no	435_1	Pyrite	66.05	1.24	59.86	0	0	0	13	Medium quality	2484	2947917	54.70	1366	d__Bacteria;p__Planctomycetota;c__Phycisphaerae;o__UBA1161;f__;g__;s__

LMSG_G000010053.1	no	435_1	Pyrite	93.44	0.00	93.44	1	0	0	19	Medium quality	9596	3451816	54.60	507	d__Bacteria;p__Planctomycetota;c__Phycisphaerae;o__UBA1161;f__;g__;s__
LMSG_G000010054.1	no	435_1	Polymetallic	74.62	0.00	74.62	1	1	1	14	Medium quality	31529	2678368	54.40	238	d__Bacteria;p__Planctomycetota;c__Phycisphaerae;o__UBA1161;f__;g__;s__
LMSG_G000010055.1	no	435_1	Polymetallic	86.77	1.14	81.09	1	1	2	17	Medium quality	10165	3779617	54.50	575	d__Bacteria;p__Planctomycetota;c__Phycisphaerae;o__UBA1161;f__;g__;s__
LMSG_G000010056.1	no	435_1	Polymetallic	88.09	1.14	82.41	1	1	1	19	Medium quality	13193	3615856	54.50	408	d__Bacteria;p__Planctomycetota;c__Phycisphaerae;o__UBA1161;f__;g__;s__
LMSG_G000010057.1	no	435_1	Polymetallic	61.55	0.88	57.16	1	0	2	17	Medium quality	15456	3847601	54.10	421	d__Bacteria;p__Planctomycetota;c__Phycisphaerae;o__UBA1161;f__;g__;s__
LMSG_G000010058.1	no	435_1	Polymetallic	87.50	4.88	63.11	0	1	1	19	Medium quality	17998	3965616	54.40	334	d__Bacteria;p__Planctomycetota;c__Phycisphaerae;o__UBA1161;f__;g__;s__
LMSG_G000010059.1	no	436_1	Polymetallic	90.97	0.00	90.97	1	1	1	19	High quality	24980	3189053	55.20	220	d__Bacteria;p__Planctomycetota;c__Phycisphaerae;o__UBA1161;f__;g__;s__
LMSG_G000010060.1	no	436_1	Polymetallic	75.05	0.00	75.05	1	0	1	15	Medium quality	5546	2505206	55.20	512	d__Bacteria;p__Planctomycetota;c__Phycisphaerae;o__UBA1161;f__;g__;s__
LMSG_G000010061.1	no	437_1	Copper	73.72	2.38	61.84	1	1	2	14	Medium quality	5384	2357334	56.50	499	d__Bacteria;p__Planctomycetota;c__Phycisphaerae;o__UBA1161;f__;g__;s__
LMSG_G000010062.1	no	437_2	Magnetite	61.43	1.14	55.75	0	0	0	16	Medium quality	5372	2202402	56.40	598	d__Bacteria;p__Planctomycetota;c__Phycisphaerae;o__UBA1161;f__;g__;s__
LMSG_G000010063.1	no	438_1	Magnetite	69.03	2.27	57.67	0	1	0	13	Medium quality	6771	2345382	56.30	392	d__Bacteria;p__Planctomycetota;c__Phycisphaerae;o__UBA1161;f__;g__;s__
LMSG_G000010064.1	no	438_1	Magnetite	84.09	4.55	61.37	0	1	0	17	Medium quality	8074	3449161	56.40	577	d__Bacteria;p__Planctomycetota;c__Phycisphaerae;o__UBA1161;f__;g__;s__
LMSG_G000010065.1	no	438_1	Polymetallic	69.00	1.70	60.48	0	0	0	12	Medium quality	2527	2446473	56.80	1090	d__Bacteria;p__Planctomycetota;c__Phycisphaerae;o__UBA1161;f__;g__;s__
LMSG_G000010066.1	no	438_1	Polymetallic	71.49	0.31	69.95	0	1	0	16	Medium quality	4187	2493334	56.80	746	d__Bacteria;p__Planctomycetota;c__Phycisphaerae;o__UBA1161;f__;g__;s__
LMSG_G000010067.1	no	438_1	Antimony	62.89	1.24	56.70	0	0	0	6	Medium quality	2087	1577881	57.10	859	d__Bacteria;p__Planctomycetota;c__Phycisphaerae;o__UBA1161;f__;g__;s__
LMSG_G000010068.1	no	438_1	Copper	69.88	1.14	64.20	0	0	0	13	Medium quality	2529	2222096	56.90	1002	d__Bacteria;p__Planctomycetota;c__Phycisphaerae;o__UBA1161;f__;g__;s__
LMSG_G000010069.1	no	439_1	Lead-Zinc	92.20	1.14	86.52	1	1	1	20	High quality	13514	3638054	57.10	406	d__Bacteria;p__Planctomycetota;c__Phycisphaerae;o__UBA1161;f__;g__;s__
LMSG_G000010070.1	no	439_1	Lead-Zinc	95.45	0.00	95.45	1	1	2	20	High quality	54796	3841734	57.10	191	d__Bacteria;p__Planctomycetota;c__Phycisphaerae;o__UBA1161;f__;g__;s__
LMSG_G000010071.1	no	439_1	Lead-Zinc	87.50	1.14	81.82	1	1	2	18	Medium quality	13835	3717132	57.10	404	d__Bacteria;p__Planctomycetota;c__Phycisphaerae;o__UBA1161;f__;g__;s__
LMSG_G000010072.1	no	439_1	Copper	72.97	1.14	67.29	0	0	1	15	Medium quality	4699	2605642	57.30	621	d__Bacteria;p__Planctomycetota;c__Phycisphaerae;o__UBA1161;f__;g__;s__
LMSG_G000010073.1	no	440_1	Polymetallic	97.72	4.98	72.81	1	1	4	20	High quality	50261	4233084	56.80	327	d__Bacteria;p__Planctomycetota;c__Phycisphaerae;o__UBA1161;f__;g__;s__
LMSG_G000010074.1	no	441_1	Pyrite-Copper	97.72	0.00	97.72	1	2	2	20	High quality	45967	3741862	55.70	143	d__Bacteria;p__Planctomycetota;c__Phycisphaerae;o__UBA1161;f__;g__;s__
LMSG_G000010075.1	no	441_1	Pyrite-Copper	97.72	0.00	97.72	1	2	1	19	High quality	50034	3633259	55.70	125	d__Bacteria;p__Planctomycetota;c__Phycisphaerae;o__UBA1161;f__;g__;s__
LMSG_G000010076.1	no	441_1	Copper	71.55	3.74	52.84	0	1	0	12	Medium quality	3198	2517374	55.80	827	d__Bacteria;p__Planctomycetota;c__Phycisphaerae;o__UBA1161;f__;g__;s__
LMSG_G000010077.1	no	1837_1	Polymetallic	91.12	2.27	79.76	1	0	13	20	Medium quality	15390	5637188	41.50	585	d__Bacteria;p__Planctomycetota;c__Planctomycetes;o__Gemmatales;f__Gemmataceae;g__;s__
LMSG_G000010078.1	no	1837_1	Polymetallic	85.49	2.48	73.10	0	1	9	20	Medium quality	7502	4962927	41.30	838	d__Bacteria;p__Planctomycetota;c__Planctomycetes;o__Gemmatales;f__Gemmataceae;g__;s__
LMSG_G000010079.1	no	528_1	Polymetallic	96.02	3.41	78.98	1	2	2	20	High quality	39745	5867764	59.80	224	d__Bacteria;p__Planctomycetota;c__Planctomycetes;o__Gemmatales;f__Gemmataceae;g__;s__
LMSG_G000010080.1	no	528_1	Polymetallic	92.95	2.84	78.75	1	0	1	19	Medium quality	13998	5695111	59.90	545	d__Bacteria;p__Planctomycetota;c__Planctomycetes;o__Gemmatales;f__Gemmataceae;g__;s__
LMSG_G000010081.1	no	528_1	Copper	60.02	0.00	60.02	0	1	0	18	Medium quality	6684	4378622	59.60	904	d__Bacteria;p__Planctomycetota;c__Planctomycetes;o__Gemmatales;f__Gemmataceae;g__;s__
LMSG_G000010082.1	no	528_2	Antimony	94.31	2.58	81.40	1	0	1	20	Medium quality	35918	6444817	59.80	276	d__Bacteria;p__Planctomycetota;c__Planctomycetes;o__Gemmatales;f__Gemmataceae;g__;s__
LMSG_G000010083.1	no	528_2	Antimony	93.18	1.42	86.08	1	0	2	20	Medium quality	14135	6300536	59.80	661	d__Bacteria;p__Planctomycetota;c__Planctomycetes;o__Gemmatales;f__Gemmataceae;g__;s__
LMSG_G000010084.1	no	528_2	Antimony	93.75	2.27	82.39	1	0	1	20	Medium quality	17462	6481060	59.80	539	d__Bacteria;p__Planctomycetota;c__Planctomycetes;o__Gemmatales;f__Gemmataceae;g__;s__
LMSG_G000010085.1	no	528_2	Copper	95.45	2.27	84.09	1	0	1	20	Medium quality	94784	6710461	59.80	126	d__Bacteria;p__Planctomycetota;c__Planctomycetes;o__Gemmatales;f__Gemmataceae;g__;s__
LMSG_G000010086.1	no	1036_1	Lead-Zinc	90.09	2.33	78.47	1	0	1	18	Medium quality	5963	6055391	57.20	1355	d__Bacteria;p__Planctomycetota;c__Planctomycetes;o__Isosphaerales;f__Isosphaeraceae;g__;s__
LMSG_G000010087.1	no	1037_1	Polymetallic	51.17	0.11	50.65	1	0	1	8	Medium quality	1638	2719771	58.50	1671	d__Bacteria;p__Planctomycetota;c__Planctomycetes;o__Isosphaerales;f__Isosphaeraceae;g__;s__
LMSG_G000010088.1	no	1041_1	Antimony	83.19	1.48	75.80	2	0	0	19	Medium quality	8326	6029318	68.90	912	d__Bacteria;p__Planctomycetota;c__Planctomycetes;o__Isosphaerales;f__Isosphaeraceae;g__;s__
LMSG_G000010089.1	no	1042_1	Antimony	68.26	2.21	57.22	0	0	0	14	Medium quality	3646	4071890	62.60	1314	d__Bacteria;p__Planctomycetota;c__Planctomycetes;o__Isosphaerales;f__Isosphaeraceae;g__;s__
LMSG_G000010090.1	no	1043_1	Antimony	81.27	0.45	79.01	1	0	1	18	Medium quality	5304	4092694	65.00	921	d__Bacteria;p__Planctomycetota;c__Planctomycetes;o__Isosphaerales;f__Isosphaeraceae;g__;s__
LMSG_G000010091.1	no	1043_1	Antimony	93.79	1.32	87.19	2	0	0	20	Medium quality	9775	4838241	64.90	671	d__Bacteria;p__Planctomycetota;c__Planctomycetes;o__Isosphaerales;f__Isosphaeraceae;g__;s__
LMSG_G000010092.1	no	1043_1	Antimony	79.80	2.57	66.96	0	0	0	17	Medium quality	4119	3736137	65.00	1019	d__Bacteria;p__Planctomycetota;c__Planctomycetes;o__Isosphaerales;f__Isosphaeraceae;g__;s__
LMSG_G000010093.1	no	1043_1	Polymetallic	61.35	1.23	55.20	2	0	1	14	Medium quality	3195	3022455	65.20	1011	d__Bacteria;p__Planctomycetota;c__Planctomycetes;o__Isosphaerales;f__Isosphaeraceae;g__;s__
LMSG_G000010094.1	no	1043_1	Copper	99.61	1.16	93.80	2	0	0	20	Medium quality	79750	5250001	64.50	111	d__Bacteria;p__Planctomycetota;c__Planctomycetes;o__Isosphaerales;f__Isosphaeraceae;g__;s__
LMSG_G000010095.1	no	1044_1	Polymetallic	94.08	3.63	75.93	1	0	1	19	Medium quality	11964	5437933	64.10	624	d__Bacteria;p__Planctomycetota;c__Planctomycetes;o__Isosphaerales;f__Isosphaeraceae;g__;s__
LMSG_G000010096.1	no	1044_1	Polymetallic	91.98	1.27	85.64	0	0	0	20	Medium quality	51632	5409139	64.00	168	d__Bacteria;p__Planctomycetota;c__Planctomycetes;o__Isosphaerales;f__Isosphaeraceae;g__;s__
LMSG_G000010097.1	no	1045_1	Polymetallic	96.12	3.49	78.68	0	0	0	19	Medium quality	21450	6736843	65.50	415	d__Bacteria;p__Planctomycetota;c__Planctomycetes;o__Isosphaerales;f__Isosphaeraceae;g__;s__
LMSG_G000010098.1	no	1048_1	Polymetallic	80.84	2.39	68.88	1	0	0	17	Medium quality	3931	5722041	65.90	1648	d__Bacteria;p__Planctomycetota;c__Planctomycetes;o__Isosphaerales;f__Isosphaeraceae;g__;s__
LMSG_G000010099.1	no	1048_1	Polymetallic	91.72	2.33	80.10	1	0	1	19	Medium quality	7385	6117127	65.80	980	d__Bacteria;p__Planctomycetota;c__Planctomycetes;o__Isosphaerales;f__Isosphaeraceae;g__;s__
LMSG_G000010100.1	no	496_1	Pyrite-Copper	86.71	4.55	63.99	1	0	0	18	Medium quality	6871	5231855	63.30	1149	d__Bacteria;p__Planctomycetota;c__Planctomycetes;o__Pirellulales;f__UBA11386;g__;s__
LMSG_G000010101.1	no	498_1	Antimony	72.84	1.15	67.10	0	0	0	13	Medium quality	3121	4555381	62.40	1684	d__Bacteria;p__Planctomycetota;c__Planctomycetes;o__Planctomycetales;f__UBA10511;g__;s__
LMSG_G000010102.1	no	498_1	Antimony	94.25	1.25	87.99	0	1	0	19	Medium quality	12612	6026526	62.20	709	d__Bacteria;p__Planctomycetota;c__Planctomycetes;o__Planctomycetales;f__UBA10511;g__;s__
LMSG_G000010103.1	no	498_1	Antimony	91.95	0.00	91.95	0	0	1	20	Medium quality	23786	6023102	62.20	390	d__Bacteria;p__Planctomycetota;c__Planctomycetes;o__Planctomycetales;f__UBA10511;g__;s__
LMSG_G000010104.1	no	425_1	Nickel-Copper	96.77	0.00	96.77	1	1	2	19	High quality	15632	2019214	39.20	202	d__Bacteria;p__Proteobacteria;c__Alphaproteobacteria;o__16-39-46;f__16-39-46;g__16-39-46;s__16-39-46 sp002257235
LMSG_G000010105.1	no	1430_1	Polymetallic	97.51	1.71	88.98	0	0	0	19	Medium quality	17513	2945719	59.90	235	d__Bacteria;p__Proteobacteria;c__Alphaproteobacteria;o__Acetobacterales;f__Acetobacteraceae;g__;s__
LMSG_G000010106.1	no	1430_1	Polymetallic	95.60	0.25	94.36	0	0	0	18	Medium quality	24254	3156037	59.70	217	d__Bacteria;p__Proteobacteria;c__Alphaproteobacteria;o__Acetobacterales;f__Acetobacteraceae;g__;s__
LMSG_G000010107.1	no	1446_1	Copper	93.03	0.00	93.03	2	1	1	19	High quality	37391	4666988	66.80	253	d__Bacteria;p__Proteobacteria;c__Alphaproteobacteria;o__Acetobacterales;f__Acetobacteraceae;g__;s__
LMSG_G000010108.1	no	1446_1	Copper	86.34	0.25	85.10	0	1	0	17	Medium quality	13698	3632995	67.50	345	d__Bacteria;p__Proteobacteria;c__Alphaproteobacteria;o__Acetobacterales;f__Acetobacteraceae;g__;s__
LMSG_G000010109.1	no	1446_1	Copper	77.50	0.00	77.50	0	0	0	18	Medium quality	12332	3625415	67.30	417	d__Bacteria;p__Proteobacteria;c__Alphaproteobacteria;o__Acetobacterales;f__Acetobacteraceae;g__;s__
LMSG_G000010110.1	no	1446_1	Copper	71.37	0.28	69.98	0	0	0	15	Medium quality	6012	2950918	67.40	564	d__Bacteria;p__Proteobacteria;c__Alphaproteobacteria;o__Acetobacterales;f__Acetobacteraceae;g__;s__
LMSG_G000010111.1	no	1492_1	Polymetallic	90.89	1.62	82.81	0	0	0	19	Medium quality	64034	2515190	66.60	84	d__Bacteria;p__Proteobacteria;c__Alphaproteobacteria;o__Acetobacterales;f__Acetobacteraceae;g__;s__
LMSG_G000010112.1	no	1492_1	Polymetallic	97.82	3.23	81.66	0	0	0	19	Medium quality	54774	2661667	66.30	107	d__Bacteria;p__Proteobacteria;c__Alphaproteobacteria;o__Acetobacterales;f__Acetobacteraceae;g__;s__
LMSG_G000010113.1	no	1492_1	Polymetallic	62.06	0.00	62.06	0	0	0	13	Medium quality	47245	1834640	67.00	55	d__Bacteria;p__Proteobacteria;c__Alphaproteobacteria;o__Acetobacterales;f__Acetobacteraceae;g__;s__
LMSG_G000010114.1	no	1492_1	Polymetallic	79.31	0.00	79.31	0	0	0	18	Medium quality	46169	2326378	67.00	82	d__Bacteria;p__Proteobacteria;c__Alphaproteobacteria;o__Acetobacterales;f__Acetobacteraceae;g__;s__
LMSG_G000010115.1	no	1492_1	Polymetallic	87.02	0.92	82.42	0	0	0	19	Medium quality	47218	2352753	66.80	78	d__Bacteria;p__Proteobacteria;c__Alphaproteobacteria;o__Acetobacterales;f__Acetobacteraceae;g__;s__
LMSG_G000010116.1	no	1492_1	Antimony	79.31	1.72	70.69	0	0	0	18	Medium quality	65388	2467181	66.60	73	d__Bacteria;p__Proteobacteria;c__Alphaproteobacteria;o__Acetobacterales;f__Acetobacteraceae;g__;s__
LMSG_G000010117.1	no	1492_1	Antimony	79.31	1.72	70.69	1	0	1	18	Medium quality	34822	2398332	66.60	111	d__Bacteria;p__Proteobacteria;c__Alphaproteobacteria;o__Acetobacterales;f__Acetobacteraceae;g__;s__
LMSG_G000010118.1	no	1492_1	Antimony	81.03	1.72	72.41	0	0	0	19	Medium quality	54617	2490578	66.70	73	d__Bacteria;p__Proteobacteria;c__Alphaproteobacteria;o__Acetobacterales;f__Acetobacteraceae;g__;s__
LMSG_G000010119.1	no	1492_1	Antimony	96.01	2.58	83.11	0	0	0	19	Medium quality	49207	2721999	66.20	81	d__Bacteria;p__Proteobacteria;c__Alphaproteobacteria;o__Acetobacterales;f__Acetobacteraceae;g__;s__
LMSG_G000010120.1	no	1503_1	Antimony	89.53	2.36	77.72	1	0	1	17	Medium quality	23808	2801311	71.00	197	d__Bacteria;p__Proteobacteria;c__Alphaproteobacteria;o__Acetobacterales;f__Acetobacteraceae;g__;s__
LMSG_G000010121.1	no	1504_1	Lead-Zinc	93.53	1.49	86.07	0	1	1	19	Medium quality	56604	3290081	70.20	94	d__Bacteria;p__Proteobacteria;c__Alphaproteobacteria;o__Acetobacterales;f__Acetobacteraceae;g__;s__
LMSG_G000010122.1	no	1504_1	Lead-Zinc	84.87	1.00	79.90	1	1	1	17	Medium quality	29954	3127194	70.20	171	d__Bacteria;p__Proteobacteria;c__Alphaproteobacteria;o__Acetobacterales;f__Acetobacteraceae;g__;s__
LMSG_G000010123.1	no	1509_1	Polymetallic	67.43	0.00	67.43	0	0	0	14	Medium quality	9649	1864437	71.60	216	d__Bacteria;p__Proteobacteria;c__Alphaproteobacteria;o__Acetobacterales;f__Acetobacteraceae;g__;s__
LMSG_G000010124.1	no	1510_1	Polymetallic	73.89	1.22	67.78	0	0	0	15	Medium quality	6271	2949115	68.10	545	d__Bacteria;p__Proteobacteria;c__Alphaproteobacteria;o__Acetobacterales;f__Acetobacteraceae;g__;s__
LMSG_G000010125.1	no	1510_1	Polymetallic	53.08	0.50	50.60	0	0	0	10	Medium quality	4539	2174540	68.40	527	d__Bacteria;p__Proteobacteria;c__Alphaproteobacteria;o__Acetobacterales;f__Acetobacteraceae;g__;s__
LMSG_G000010126.1	no	1510_1	Polymetallic	53.44	0.00	53.44	0	1	0	13	Medium quality	5140	2835985	68.40	663	d__Bacteria;p__Proteobacteria;c__Alphaproteobacteria;o__Acetobacterales;f__Acetobacteraceae;g__;s__
LMSG_G000010127.1	no	1511_1	Polymetallic	74.45	1.72	65.83	0	0	0	17	Medium quality	14107	3036714	69.90	264	d__Bacteria;p__Proteobacteria;c__Alphaproteobacteria;o__Acetobacterales;f__Acetobacteraceae;g__;s__
LMSG_G000010128.1	no	1511_1	Polymetallic	56.03	0.00	56.03	0	0	0	12	Medium quality	24001	1976523	70.20	111	d__Bacteria;p__Proteobacteria;c__Alphaproteobacteria;o__Acetobacterales;f__Acetobacteraceae;g__;s__

LMSG_G000010129.1	no	1511_1	Polymetallic	89.55	3.65	71.31	0	1	0	17	Medium quality	17194	2984429	70.00	217	d__Bacteria;p__Proteobacteria;c__Alphaproteobacteria;o__Acetobacterales;f__Acetobacteraceae;g__;s__
LMSG_G000010130.1	no	1511_1	Polymetallic	69.52	2.49	57.09	0	0	0	16	Medium quality	11880	2829136	68.70	356	d__Bacteria;p__Proteobacteria;c__Alphaproteobacteria;o__Acetobacterales;f__Acetobacteraceae;g__;s__
LMSG_G000010131.1	no	1512_1	Polymetallic	96.26	4.60	73.26	0	0	0	19	Medium quality	45956	4343247	69.40	148	d__Bacteria;p__Proteobacteria;c__Alphaproteobacteria;o__Acetobacterales;f__Acetobacteraceae;g__;s__
LMSG_G000010132.1	no	1513_1	Polymetallic	98.50	1.83	89.33	0	0	0	19	Medium quality	57251	3966244	70.00	100	d__Bacteria;p__Proteobacteria;c__Alphaproteobacteria;o__Acetobacterales;f__Acetobacteraceae;g__;s__
LMSG_G000010133.1	no	1513_1	Polymetallic	96.96	4.23	75.82	1	0	1	18	Medium quality	61402	3793677	70.10	110	d__Bacteria;p__Proteobacteria;c__Alphaproteobacteria;o__Acetobacterales;f__Acetobacteraceae;g__;s__
LMSG_G000010134.1	no	1513_1	Polymetallic	94.52	3.57	76.70	0	0	0	19	Medium quality	44392	4765726	69.70	174	d__Bacteria;p__Proteobacteria;c__Alphaproteobacteria;o__Acetobacterales;f__Acetobacteraceae;g__;s__
LMSG_G000010135.1	no	1514_1	Polymetallic	63.04	2.49	50.61	0	0	0	15	Medium quality	11046	2855096	70.90	291	d__Bacteria;p__Proteobacteria;c__Alphaproteobacteria;o__Acetobacterales;f__Acetobacteraceae;g__;s__
LMSG_G000010136.1	no	1514_1	Polymetallic	52.93	0.00	52.93	0	1	0	14	Medium quality	13088	3332604	71.30	384	d__Bacteria;p__Proteobacteria;c__Alphaproteobacteria;o__Acetobacterales;f__Acetobacteraceae;g__;s__
LMSG_G000010137.1	no	1466_1	Lead-Zinc	55.32	0.00	55.32	0	0	0	16	Medium quality	5101	1809828	57.70	489	d__Bacteria;p__Proteobacteria;c__Alphaproteobacteria;o__Acetobacterales;f__Acetobacteraceae;g__Acidiphilium;s__
LMSG_G000010138.1	no	1466_1	Lead-Zinc	68.01	2.74	54.33	0	0	0	19	Medium quality	7885	3405929	57.60	616	d__Bacteria;p__Proteobacteria;c__Alphaproteobacteria;o__Acetobacterales;f__Acetobacteraceae;g__Acidiphilium;s__
LMSG_G000010139.1	no	1475_1	Polymetallic	68.41	0.25	67.17	0	0	0	17	Medium quality	19095	2164410	67.40	174	d__Bacteria;p__Proteobacteria;c__Alphaproteobacteria;o__Acetobacterales;f__Acetobacteraceae;g__Acidiphilium;s__
LMSG_G000010140.1	no	1477_1	Copper	74.61	0.26	73.29	0	1	0	14	Medium quality	12991	2162786	60.60	255	d__Bacteria;p__Proteobacteria;c__Alphaproteobacteria;o__Acetobacterales;f__Acetobacteraceae;g__Acidiphilium;s__
LMSG_G000010141.1	no	1477_1	Polymetallic	78.85	0.20	77.86	0	0	0	16	Medium quality	16199	2545572	60.70	248	d__Bacteria;p__Proteobacteria;c__Alphaproteobacteria;o__Acetobacterales;f__Acetobacteraceae;g__Acidiphilium;s__
LMSG_G000010142.1	no	1477_1	Polymetallic	86.77	1.16	80.97	0	0	0	17	Medium quality	14624	2621703	60.60	253	d__Bacteria;p__Proteobacteria;c__Alphaproteobacteria;o__Acetobacterales;f__Acetobacteraceae;g__Acidiphilium;s__
LMSG_G000010143.1	no	1477_1	Polymetallic	85.73	1.34	79.03	0	0	0	17	Medium quality	13795	2604222	60.80	245	d__Bacteria;p__Proteobacteria;c__Alphaproteobacteria;o__Acetobacterales;f__Acetobacteraceae;g__Acidiphilium;s__
LMSG_G000010144.1	no	1477_1	Polymetallic	86.61	1.89	77.16	0	0	0	18	Medium quality	13632	2555580	60.80	270	d__Bacteria;p__Proteobacteria;c__Alphaproteobacteria;o__Acetobacterales;f__Acetobacteraceae;g__Acidiphilium;s__
LMSG_G000010145.1	no	1477_1	Polymetallic	87.89	1.49	80.43	0	0	0	17	Medium quality	15779	2634694	60.70	270	d__Bacteria;p__Proteobacteria;c__Alphaproteobacteria;o__Acetobacterales;f__Acetobacteraceae;g__Acidiphilium;s__
LMSG_G000010146.1	no	1477_1	Copper	69.28	1.37	62.44	0	0	0	17	Medium quality	14523	2294108	60.20	239	d__Bacteria;p__Proteobacteria;c__Alphaproteobacteria;o__Acetobacterales;f__Acetobacteraceae;g__Acidiphilium;s__
LMSG_G000010147.1	no	1477_1	Pyrite	82.80	1.49	75.34	0	1	0	19	Medium quality	4383	2564371	61.10	808	d__Bacteria;p__Proteobacteria;c__Alphaproteobacteria;o__Acetobacterales;f__Acetobacteraceae;g__Acidiphilium;s__
LMSG_G000010148.1	no	1477_1	Pyrite	90.42	2.10	79.90	0	0	0	15	Medium quality	4165	2750390	60.90	846	d__Bacteria;p__Proteobacteria;c__Alphaproteobacteria;o__Acetobacterales;f__Acetobacteraceae;g__Acidiphilium;s__
LMSG_G000010149.1	no	1474_1	Pyrite	84.55	0.53	81.91	0	0	0	18	Medium quality	13171	2843569	68.60	287	d__Bacteria;p__Proteobacteria;c__Alphaproteobacteria;o__Acetobacterales;f__Acetobacteraceae;g__Acidiphilium;s__Acidiphilium multivorum
LMSG_G000010150.1	no	1474_1	Lead-Zinc	78.05	0.95	73.30	0	0	0	18	Medium quality	19729	2704184	68.80	250	d__Bacteria;p__Proteobacteria;c__Alphaproteobacteria;o__Acetobacterales;f__Acetobacteraceae;g__Acidiphilium;s__Acidiphilium multivorum
LMSG_G000010151.1	no	1474_1	Lead-Zinc	99.00	2.49	86.57	0	0	0	19	Medium quality	32685	3582259	68.10	164	d__Bacteria;p__Proteobacteria;c__Alphaproteobacteria;o__Acetobacterales;f__Acetobacteraceae;g__Acidiphilium;s__Acidiphilium multivorum
LMSG_G000010152.1	no	1474_1	Pyrite	59.46	0.78	55.58	0	0	0	12	Medium quality	3485	2094046	68.80	667	d__Bacteria;p__Proteobacteria;c__Alphaproteobacteria;o__Acetobacterales;f__Acetobacteraceae;g__Acidiphilium;s__Acidiphilium multivorum
LMSG_G000010153.1	no	1474_1	Pyrite	98.50	1.03	93.37	0	1	0	19	Medium quality	40018	3571051	68.20	140	d__Bacteria;p__Proteobacteria;c__Alphaproteobacteria;o__Acetobacterales;f__Acetobacteraceae;g__Acidiphilium;s__Acidiphilium multivorum
LMSG_G000010154.1	no	1474_1	Pyrite	63.86	1.16	58.08	0	0	0	11	Medium quality	3891	2279448	68.60	667	d__Bacteria;p__Proteobacteria;c__Alphaproteobacteria;o__Acetobacterales;f__Acetobacteraceae;g__Acidiphilium;s__Acidiphilium multivorum
LMSG_G000010155.1	no	1474_1	Lead-Zinc	94.52	0.10	94.03	0	0	0	19	Medium quality	39539	3495513	67.90	152	d__Bacteria;p__Proteobacteria;c__Alphaproteobacteria;o__Acetobacterales;f__Acetobacteraceae;g__Acidiphilium;s__Acidiphilium multivorum
LMSG_G000010156.1	no	1474_1	Lead-Zinc	97.26	0.87	92.91	0	1	0	19	Medium quality	37165	3353615	68.30	149	d__Bacteria;p__Proteobacteria;c__Alphaproteobacteria;o__Acetobacterales;f__Acetobacteraceae;g__Acidiphilium;s__Acidiphilium multivorum
LMSG_G000010157.1	no	1474_1	Lead-Zinc	96.76	1.08	91.35	0	0	0	18	Medium quality	33114	3651703	67.90	186	d__Bacteria;p__Proteobacteria;c__Alphaproteobacteria;o__Acetobacterales;f__Acetobacteraceae;g__Acidiphilium;s__Acidiphilium multivorum
LMSG_G000010158.1	no	1474_1	Lead-Zinc	99.41	1.00	94.44	0	0	0	19	Medium quality	42844	3535832	68.10	154	d__Bacteria;p__Proteobacteria;c__Alphaproteobacteria;o__Acetobacterales;f__Acetobacteraceae;g__Acidiphilium;s__Acidiphilium multivorum
LMSG_G000010159.1	no	1474_1	Lead-Zinc	79.20	0.00	79.20	0	0	0	15	Medium quality	17457	2589190	68.90	186	d__Bacteria;p__Proteobacteria;c__Alphaproteobacteria;o__Acetobacterales;f__Acetobacteraceae;g__Acidiphilium;s__Acidiphilium multivorum
LMSG_G000010160.1	no	1474_1	Lead-Zinc	88.59	0.35	86.86	0	0	0	16	Medium quality	12791	3310945	67.90	329	d__Bacteria;p__Proteobacteria;c__Alphaproteobacteria;o__Acetobacterales;f__Acetobacteraceae;g__Acidiphilium;s__Acidiphilium multivorum
LMSG_G000010161.1	no	1474_1	Polymetallic	87.56	2.90	73.05	0	0	0	16	Medium quality	5866	3248417	68.30	704	d__Bacteria;p__Proteobacteria;c__Alphaproteobacteria;o__Acetobacterales;f__Acetobacteraceae;g__Acidiphilium;s__Acidiphilium multivorum
LMSG_G000010162.1	no	1474_1	Polymetallic	55.49	1.03	50.36	0	0	0	11	Medium quality	6292	2078880	68.00	395	d__Bacteria;p__Proteobacteria;c__Alphaproteobacteria;o__Acetobacterales;f__Acetobacteraceae;g__Acidiphilium;s__Acidiphilium multivorum
LMSG_G000010163.1	no	1474_1	Polymetallic	99.37	0.75	95.64	0	0	0	19	Medium quality	40666	3178611	68.20	113	d__Bacteria;p__Proteobacteria;c__Alphaproteobacteria;o__Acetobacterales;f__Acetobacteraceae;g__Acidiphilium;s__Acidiphilium multivorum
LMSG_G000010164.1	no	1474_1	Antimony	64.01	2.16	53.24	0	0	0	16	Medium quality	20686	2276608	69.00	164	d__Bacteria;p__Proteobacteria;c__Alphaproteobacteria;o__Acetobacterales;f__Acetobacteraceae;g__Acidiphilium;s__Acidiphilium multivorum
LMSG_G000010165.1	no	1474_1	Polymetallic	97.38	0.50	94.90	0	0	0	19	Medium quality	36742	3207939	68.30	131	d__Bacteria;p__Proteobacteria;c__Alphaproteobacteria;o__Acetobacterales;f__Acetobacteraceae;g__Acidiphilium;s__Acidiphilium multivorum
LMSG_G000010166.1	no	1474_1	Copper	95.27	0.17	94.45	0	0	0	18	Medium quality	46099	3187369	68.20	114	d__Bacteria;p__Proteobacteria;c__Alphaproteobacteria;o__Acetobacterales;f__Acetobacteraceae;g__Acidiphilium;s__Acidiphilium multivorum
LMSG_G000010167.1	no	1474_1	Copper	85.82	0.50	83.34	0	0	0	18	Medium quality	42396	2925078	68.60	117	d__Bacteria;p__Proteobacteria;c__Alphaproteobacteria;o__Acetobacterales;f__Acetobacteraceae;g__Acidiphilium;s__Acidiphilium multivorum
LMSG_G000010168.1	no	1474_1	Polymetallic	97.23	0.50	94.75	0	0	0	19	Medium quality	28250	3328303	68.30	185	d__Bacteria;p__Proteobacteria;c__Alphaproteobacteria;o__Acetobacterales;f__Acetobacteraceae;g__Acidiphilium;s__Acidiphilium multivorum
LMSG_G000010169.1	no	1474_1	Polymetallic	98.38	0.50	95.90	0	1	0	19	Medium quality	30073	3261570	68.40	159	d__Bacteria;p__Proteobacteria;c__Alphaproteobacteria;o__Acetobacterales;f__Acetobacteraceae;g__Acidiphilium;s__Acidiphilium multivorum
LMSG_G000010170.1	no	1474_1	Magnetite	87.55	1.49	80.09	0	0	0	18	Medium quality	36978	2858897	69.00	125	d__Bacteria;p__Proteobacteria;c__Alphaproteobacteria;o__Acetobacterales;f__Acetobacteraceae;g__Acidiphilium;s__Acidiphilium multivorum
LMSG_G000010171.1	no	1474_1	Copper	93.03	0.25	91.79	0	0	0	19	Medium quality	52885	3423086	68.10	128	d__Bacteria;p__Proteobacteria;c__Alphaproteobacteria;o__Acetobacterales;f__Acetobacteraceae;g__Acidiphilium;s__Acidiphilium multivorum
LMSG_G000010172.1	no	1474_1	Copper	100.00	1.49	92.54	1	0	1	19	Medium quality	25645	6924430	64.80	601	d__Bacteria;p__Proteobacteria;c__Alphaproteobacteria;o__Acetobacterales;f__Acetobacteraceae;g__Acidiphilium;s__Acidiphilium multivorum
LMSG_G000010173.1	no	1474_1	Lead-Zinc	73.51	2.49	61.08	0	0	0	16	Medium quality	5567	3162904	66.90	665	d__Bacteria;p__Proteobacteria;c__Alphaproteobacteria;o__Acetobacterales;f__Acetobacteraceae;g__Acidiphilium;s__Acidiphilium multivorum
LMSG_G000010174.1	no	1474_1	Pyrite-Copper	74.50	0.17	73.68	0	0	0	18	Medium quality	15547	2639442	67.80	207	d__Bacteria;p__Proteobacteria;c__Alphaproteobacteria;o__Acetobacterales;f__Acetobacteraceae;g__Acidiphilium;s__Acidiphilium multivorum
LMSG_G000010175.1	no	1474_1	Pyrite-Copper	98.38	0.50	95.90	0	0	0	19	Medium quality	38878	3310257	68.40	132	d__Bacteria;p__Proteobacteria;c__Alphaproteobacteria;o__Acetobacterales;f__Acetobacteraceae;g__Acidiphilium;s__Acidiphilium multivorum
LMSG_G000010176.1	no	1474_1	Pyrite-Copper	73.88	0.41	71.81	0	0	0	14	Medium quality	4194	2423238	68.10	657	d__Bacteria;p__Proteobacteria;c__Alphaproteobacteria;o__Acetobacterales;f__Acetobacteraceae;g__Acidiphilium;s__Acidiphilium multivorum
LMSG_G000010177.1	no	1474_1	Pyrite	68.09	1.41	61.05	0	0	0	12	Medium quality	6977	2151060	69.70	396	d__Bacteria;p__Proteobacteria;c__Alphaproteobacteria;o__Acetobacterales;f__Acetobacteraceae;g__Acidiphilium;s__Acidiphilium multivorum
LMSG_G000010178.1	no	1474_1	Polymetallic	51.88	0.00	51.88	0	0	0	10	Medium quality	6536	1969589	69.50	377	d__Bacteria;p__Proteobacteria;c__Alphaproteobacteria;o__Acetobacterales;f__Acetobacteraceae;g__Acidiphilium;s__Acidiphilium multivorum
LMSG_G000010179.1	no	1473_1	Nickel-Copper	99.50	4.23	78.36	2	0	2	19	Medium quality	48223	4022156	63.80	246	d__Bacteria;p__Proteobacteria;c__Alphaproteobacteria;o__Acetobacterales;f__Acetobacteraceae;g__Acidiphilium;s__Acidiphilium rubrum
LMSG_G000010180.1	no	1475_2	Nickel-Copper	96.89	1.74	88.19	0	0	0	18	Medium quality	47478	3211479	67.10	219	d__Bacteria;p__Proteobacteria;c__Alphaproteobacteria;o__Acetobacterales;f__Acetobacteraceae;g__Acidiphilium;s__Acidiphilium sp002255515
LMSG_G000010181.1	no	1475_2	Nickel-Copper	94.65	0.68	91.26	0	2	0	19	Medium quality	37209	3236115	66.60	210	d__Bacteria;p__Proteobacteria;c__Alphaproteobacteria;o__Acetobacterales;f__Acetobacteraceae;g__Acidiphilium;s__Acidiphilium sp002255515
LMSG_G000010182.1	no	1476_1	Nickel-Copper	100.00	0.00	100.00	0	1	0	19	Medium quality	51219	3472944	59.80	163	d__Bacteria;p__Proteobacteria;c__Alphaproteobacteria;o__Acetobacterales;f__Acetobacteraceae;g__Acidiphilium;s__Acidiphilium sp002279355

LMSG_G000010183.1	no	1476_1	Nickel-Copper	99.00	0.17	98.18	0	0	0	19	Medium quality	57382	2770198	60.30	79	d__Bacteria;p__Proteobacteria;c__Alphaproteobacteria;o__Acetobacterales;f__Acetobacteraceae;g__Acidiphilium;s__Acidiphilium sp002279355
LMSG_G000010184.1	no	1469_1	Nickel-Copper	58.62	0.86	54.31	0	1	0	11	Medium quality	4014	1930593	58.60	552	d__Bacteria;p__Proteobacteria;c__Alphaproteobacteria;o__Acetobacterales;f__Acetobacteraceae;g__Acidocella;s__
LMSG_G000010185.1	no	1469_1	Copper	99.50	0.50	97.02	0	2	0	19	Medium quality	85475	2862762	57.20	73	d__Bacteria;p__Proteobacteria;c__Alphaproteobacteria;o__Acetobacterales;f__Acetobacteraceae;g__Acidocella;s__
LMSG_G000010186.1	no	1469_1	Copper	74.88	2.01	64.85	0	0	0	16	Medium quality	3724	2259861	57.50	551	d__Bacteria;p__Proteobacteria;c__Alphaproteobacteria;o__Acetobacterales;f__Acetobacteraceae;g__Acidocella;s__
LMSG_G000010187.1	no	1480_1	Polymetallic	86.35	0.50	83.87	0	0	0	17	Medium quality	20272	2227275	57.90	182	d__Bacteria;p__Proteobacteria;c__Alphaproteobacteria;o__Acetobacterales;f__Acetobacteraceae;g__Acidocella;s__
LMSG_G000010188.1	no	1481_1	Polymetallic	65.65	2.16	54.88	0	0	0	16	Medium quality	14283	1515104	60.40	125	d__Bacteria;p__Proteobacteria;c__Alphaproteobacteria;o__Acetobacterales;f__Acetobacteraceae;g__Acidocella;s__
LMSG_G000010189.1	no	1482_1	Polymetallic	89.11	1.00	84.14	0	0	0	18	Medium quality	30499	2342553	64.90	119	d__Bacteria;p__Proteobacteria;c__Alphaproteobacteria;o__Acetobacterales;f__Acetobacteraceae;g__Acidocella;s__
LMSG_G000010190.1	no	1482_1	Polymetallic	84.68	1.00	79.71	0	0	0	16	Medium quality	39419	2394928	64.90	93	d__Bacteria;p__Proteobacteria;c__Alphaproteobacteria;o__Acetobacterales;f__Acetobacteraceae;g__Acidocella;s__
LMSG_G000010191.1	no	1482_1	Polymetallic	91.90	1.49	84.44	0	0	0	17	Medium quality	55718	2399694	65.00	62	d__Bacteria;p__Proteobacteria;c__Alphaproteobacteria;o__Acetobacterales;f__Acetobacteraceae;g__Acidocella;s__
LMSG_G000010192.1	no	1482_1	Polymetallic	89.29	0.00	89.29	0	0	0	17	Medium quality	23025	2363679	64.90	142	d__Bacteria;p__Proteobacteria;c__Alphaproteobacteria;o__Acetobacterales;f__Acetobacteraceae;g__Acidocella;s__
LMSG_G000010193.1	no	1482_1	Polymetallic	90.90	0.29	89.44	0	0	0	17	Medium quality	17306	2370368	65.10	188	d__Bacteria;p__Proteobacteria;c__Alphaproteobacteria;o__Acetobacterales;f__Acetobacteraceae;g__Acidocella;s__
LMSG_G000010194.1	no	1482_2	Antimony	72.79	2.51	60.23	0	0	0	17	Medium quality	3392	1778173	65.90	569	d__Bacteria;p__Proteobacteria;c__Alphaproteobacteria;o__Acetobacterales;f__Acetobacteraceae;g__Acidocella;s__
LMSG_G000010195.1	no	1482_2	Antimony	58.62	0.00	58.62	0	0	0	18	Medium quality	7346	1925640	66.10	302	d__Bacteria;p__Proteobacteria;c__Alphaproteobacteria;o__Acetobacterales;f__Acetobacteraceae;g__Acidocella;s__
LMSG_G000010196.1	no	1482_2	Antimony	77.25	1.00	72.28	0	0	0	17	Medium quality	5802	2024166	66.00	393	d__Bacteria;p__Proteobacteria;c__Alphaproteobacteria;o__Acetobacterales;f__Acetobacteraceae;g__Acidocella;s__
LMSG_G000010197.1	no	1483_1	Polymetallic	94.69	1.00	89.72	0	2	0	17	Medium quality	29666	2258011	64.30	95	d__Bacteria;p__Proteobacteria;c__Alphaproteobacteria;o__Acetobacterales;f__Acetobacteraceae;g__Acidocella;s__
LMSG_G000010198.1	no	1484_1	Polymetallic	73.64	2.24	62.45	0	0	0	17	Medium quality	23920	2043879	65.30	116	d__Bacteria;p__Proteobacteria;c__Alphaproteobacteria;o__Acetobacterales;f__Acetobacteraceae;g__Acidocella;s__
LMSG_G000010199.1	no	1485_1	Polymetallic	95.46	3.86	76.19	0	0	0	18	Medium quality	22610	2509503	64.10	157	d__Bacteria;p__Proteobacteria;c__Alphaproteobacteria;o__Acetobacterales;f__Acetobacteraceae;g__Acidocella;s__
LMSG_G000010200.1	no	1485_1	Polymetallic	98.38	4.48	76.00	0	0	0	19	Medium quality	26068	2693757	63.70	168	d__Bacteria;p__Proteobacteria;c__Alphaproteobacteria;o__Acetobacterales;f__Acetobacteraceae;g__Acidocella;s__
LMSG_G000010201.1	no	1485_1	Polymetallic	97.85	0.50	95.37	0	0	0	19	Medium quality	70628	2665168	63.90	64	d__Bacteria;p__Proteobacteria;c__Alphaproteobacteria;o__Acetobacterales;f__Acetobacteraceae;g__Acidocella;s__
LMSG_G000010202.1	no	1485_1	Polymetallic	90.90	1.51	83.36	0	1	0	18	Medium quality	6508	2435550	64.10	457	d__Bacteria;p__Proteobacteria;c__Alphaproteobacteria;o__Acetobacterales;f__Acetobacteraceae;g__Acidocella;s__
LMSG_G000010203.1	no	1485_1	Polymetallic	98.50	0.50	96.02	1	0	1	19	Medium quality	93899	2807839	63.60	53	d__Bacteria;p__Proteobacteria;c__Alphaproteobacteria;o__Acetobacterales;f__Acetobacteraceae;g__Acidocella;s__
LMSG_G000010204.1	no	1485_1	Polymetallic	95.71	1.91	86.18	0	0	0	19	Medium quality	23644	2515018	64.00	165	d__Bacteria;p__Proteobacteria;c__Alphaproteobacteria;o__Acetobacterales;f__Acetobacteraceae;g__Acidocella;s__
LMSG_G000010205.1	no	1485_1	Polymetallic	98.00	2.34	86.31	0	0	0	19	Medium quality	24821	2576127	64.10	152	d__Bacteria;p__Proteobacteria;c__Alphaproteobacteria;o__Acetobacterales;f__Acetobacteraceae;g__Acidocella;s__
LMSG_G000010206.1	no	1485_1	Polymetallic	98.61	3.50	81.12	0	1	0	19	Medium quality	31057	2627442	63.90	155	d__Bacteria;p__Proteobacteria;c__Alphaproteobacteria;o__Acetobacterales;f__Acetobacteraceae;g__Acidocella;s__
LMSG_G000010207.1	no	1485_1	Polymetallic	75.84	2.74	62.16	0	0	0	15	Medium quality	21488	1908766	64.20	119	d__Bacteria;p__Proteobacteria;c__Alphaproteobacteria;o__Acetobacterales;f__Acetobacteraceae;g__Acidocella;s__
LMSG_G000010208.1	no	1487_1	Polymetallic	55.17	0.86	50.86	0	0	0	13	Medium quality	4700	1143883	67.50	262	d__Bacteria;p__Proteobacteria;c__Alphaproteobacteria;o__Acetobacterales;f__Acetobacteraceae;g__Acidocella;s__
LMSG_G000010209.1	no	1489_1	Polymetallic	75.18	2.74	61.50	1	0	1	15	Medium quality	7949	2349203	61.90	454	d__Bacteria;p__Proteobacteria;c__Alphaproteobacteria;o__Acetobacterales;f__Acetobacteraceae;g__Acidocella;s__
LMSG_G000010210.1	no	1489_1	Polymetallic	61.12	1.72	52.50	0	0	0	16	Medium quality	8454	2135423	62.20	326	d__Bacteria;p__Proteobacteria;c__Alphaproteobacteria;o__Acetobacterales;f__Acetobacteraceae;g__Acidocella;s__
LMSG_G000010211.1	no	1489_1	Polymetallic	51.72	0.00	51.72	0	1	0	12	Medium quality	7885	1864333	62.00	290	d__Bacteria;p__Proteobacteria;c__Alphaproteobacteria;o__Acetobacterales;f__Acetobacteraceae;g__Acidocella;s__
LMSG_G000010212.1	no	1490_1	Polymetallic	69.33	0.00	69.33	0	0	0	14	Medium quality	10913	2105094	64.10	224	d__Bacteria;p__Proteobacteria;c__Alphaproteobacteria;o__Acetobacterales;f__Acetobacteraceae;g__Acidocella;s__
LMSG_G000010213.1	no	1490_1	Polymetallic	74.13	0.00	74.13	0	0	0	18	Medium quality	37838	2592391	63.90	96	d__Bacteria;p__Proteobacteria;c__Alphaproteobacteria;o__Acetobacterales;f__Acetobacteraceae;g__Acidocella;s__
LMSG_G000010214.1	no	1490_1	Polymetallic	83.58	2.74	69.90	0	0	0	15	Medium quality	63321	2410364	63.90	64	d__Bacteria;p__Proteobacteria;c__Alphaproteobacteria;o__Acetobacterales;f__Acetobacteraceae;g__Acidocella;s__
LMSG_G000010215.1	no	1490_1	Polymetallic	98.88	1.99	88.93	0	0	0	19	Medium quality	48597	3079182	63.60	100	d__Bacteria;p__Proteobacteria;c__Alphaproteobacteria;o__Acetobacterales;f__Acetobacteraceae;g__Acidocella;s__
LMSG_G000010216.1	no	1490_1	Polymetallic	55.17	0.00	55.17	0	0	0	18	Medium quality	14153	2345057	63.70	267	d__Bacteria;p__Proteobacteria;c__Alphaproteobacteria;o__Acetobacterales;f__Acetobacteraceae;g__Acidocella;s__
LMSG_G000010217.1	no	1468_1	Nickel-Copper	72.13	0.50	69.65	1	1	1	17	Medium quality	160189	2165276	56.80	58	d__Bacteria;p__Proteobacteria;c__Alphaproteobacteria;o__Acetobacterales;f__Acetobacteraceae;g__Acidocella;s__Acidocella sp002255575
LMSG_G000010218.1	no	1510_1	Polymetallic	61.08	0.50	58.60	0	0	0	15	Medium quality	6316	2638259	67.50	484	d__Bacteria;p__Proteobacteria;c__Alphaproteobacteria;o__Acetobacterales;f__Acetobacteraceae;g__BOG-908;s__
LMSG_G000010219.1	no	1510_1	Polymetallic	71.54	2.24	60.35	0	0	0	15	Medium quality	5892	2994585	67.90	589	d__Bacteria;p__Proteobacteria;c__Alphaproteobacteria;o__Acetobacterales;f__Acetobacteraceae;g__BOG-908;s__
LMSG_G000010220.1	no	1510_1	Polymetallic	72.72	2.59	59.79	0	0	0	16	Medium quality	4334	2805246	68.90	751	d__Bacteria;p__Proteobacteria;c__Alphaproteobacteria;o__Acetobacterales;f__Acetobacteraceae;g__BOG-908;s__
LMSG_G000010221.1	no	1513_1	Polymetallic	95.77	2.65	82.51	0	0	0	18	Medium quality	64463	3588833	70.20	77	d__Bacteria;p__Proteobacteria;c__Alphaproteobacteria;o__Acetobacterales;f__Acetobacteraceae;g__BOG-930;s__
LMSG_G000010222.1	no	1513_1	Polymetallic	98.00	3.51	80.44	1	0	1	19	Medium quality	43373	3785808	70.10	148	d__Bacteria;p__Proteobacteria;c__Alphaproteobacteria;o__Acetobacterales;f__Acetobacteraceae;g__BOG-930;s__
LMSG_G000010223.1	no	1513_1	Polymetallic	97.46	2.24	86.27	0	0	0	19	Medium quality	45088	3777310	70.00	110	d__Bacteria;p__Proteobacteria;c__Alphaproteobacteria;o__Acetobacterales;f__Acetobacteraceae;g__BOG-930;s__
LMSG_G000010224.1	no	1513_1	Polymetallic	91.49	1.41	84.45	1	0	1	17	Medium quality	30755	3977821	70.10	190	d__Bacteria;p__Proteobacteria;c__Alphaproteobacteria;o__Acetobacterales;f__Acetobacteraceae;g__BOG-930;s__
LMSG_G000010225.1	no	1513_1	Polymetallic	89.82	3.35	73.08	0	0	0	17	Medium quality	15580	3679810	69.90	316	d__Bacteria;p__Proteobacteria;c__Alphaproteobacteria;o__Acetobacterales;f__Acetobacteraceae;g__BOG-930;s__
LMSG_G000010226.1	no	1513_1	Polymetallic	97.29	2.99	82.37	0	0	0	19	Medium quality	53149	3975309	70.10	115	d__Bacteria;p__Proteobacteria;c__Alphaproteobacteria;o__Acetobacterales;f__Acetobacteraceae;g__BOG-930;s__
LMSG_G000010227.1	no	1499_1	Lead-Zinc	92.41	0.25	91.17	0	0	0	19	Medium quality	43442	3012828	66.50	110	d__Bacteria;p__Proteobacteria;c__Alphaproteobacteria;o__Acetobacterales;f__Acetobacteraceae;g__G45-3;s__
LMSG_G000010228.1	no	1499_1	Pyrite	80.84	1.41	73.80	0	0	0	18	Medium quality	43701	2653427	66.50	90	d__Bacteria;p__Proteobacteria;c__Alphaproteobacteria;o__Acetobacterales;f__Acetobacteraceae;g__G45-3;s__
LMSG_G000010229.1	no	1499_1	Pyrite	66.12	1.74	57.42	0	0	0	16	Medium quality	19040	2495874	65.80	183	d__Bacteria;p__Proteobacteria;c__Alphaproteobacteria;o__Acetobacterales;f__Acetobacteraceae;g__G45-3;s__
LMSG_G000010230.1	no	1499_1	Copper	89.05	1.29	82.63	0	0	0	16	Medium quality	24851	2901714	66.50	165	d__Bacteria;p__Proteobacteria;c__Alphaproteobacteria;o__Acetobacterales;f__Acetobacteraceae;g__G45-3;s__
LMSG_G000010231.1	no	1499_1	Magnetite	94.15	0.75	90.42	0	0	0	19	Medium quality	49926	3055618	66.40	106	d__Bacteria;p__Proteobacteria;c__Alphaproteobacteria;o__Acetobacterales;f__Acetobacteraceae;g__G45-3;s__
LMSG_G000010232.1	no	1499_1	Lead-Zinc	98.17	0.50	95.69	0	0	0	19	Medium quality	48203	3106788	66.50	114	d__Bacteria;p__Proteobacteria;c__Alphaproteobacteria;o__Acetobacterales;f__Acetobacteraceae;g__G45-3;s__
LMSG_G000010233.1	no	1499_1	Lead-Zinc	99.00	1.74	90.30	0	0	0	20	Medium quality	46029	4300960	65.70	215	d__Bacteria;p__Proteobacteria;c__Alphaproteobacteria;o__Acetobacterales;f__Acetobacteraceae;g__G45-3;s__

LMSG_G000010234.1	no	1499_1	Lead-Zinc	94.02	1.74	85.32	0	0	0	19	Medium quality	54000	3026622	66.50	89	d__Bacteria;p__Proteobacteria;c__Alphaproteobacteria;o__Acetobacterales;f__Acetobacteraceae;g__G45-3;s__
LMSG_G000010235.1	no	1499_1	Lead-Zinc	97.01	2.36	85.20	0	0	0	19	Medium quality	49925	3197384	66.50	100	d__Bacteria;p__Proteobacteria;c__Alphaproteobacteria;o__Acetobacterales;f__Acetobacteraceae;g__G45-3;s__
LMSG_G000010236.1	no	1499_1	Pyrite-Copper	100.00	4.70	76.49	1	0	1	19	Medium quality	50321	3285602	66.50	104	d__Bacteria;p__Proteobacteria;c__Alphaproteobacteria;o__Acetobacterales;f__Acetobacteraceae;g__G45-3;s__
LMSG_G000010237.1	no	1499_1	Copper	96.26	2.74	82.58	0	0	0	17	Medium quality	46368	3191724	66.40	118	d__Bacteria;p__Proteobacteria;c__Alphaproteobacteria;o__Acetobacterales;f__Acetobacteraceae;g__G45-3;s__
LMSG_G000010238.1	no	1499_1	Copper	96.23	0.75	92.50	0	0	0	17	Medium quality	20851	3292098	66.30	246	d__Bacteria;p__Proteobacteria;c__Alphaproteobacteria;o__Acetobacterales;f__Acetobacteraceae;g__G45-3;s__
LMSG_G000010239.1	no	1500_1	Antimony	62.93	1.72	54.31	0	0	0	12	Medium quality	3495	1886286	67.20	621	d__Bacteria;p__Proteobacteria;c__Alphaproteobacteria;o__Acetobacterales;f__Acetobacteraceae;g__G45-3;s__
LMSG_G000010240.1	no	1500_1	Antimony	78.95	2.77	65.12	1	1	1	16	Medium quality	4324	2524775	66.20	705	d__Bacteria;p__Proteobacteria;c__Alphaproteobacteria;o__Acetobacterales;f__Acetobacteraceae;g__G45-3;s__
LMSG_G000010241.1	no	1500_1	Pyrite	55.17	0.00	55.17	0	0	0	16	Medium quality	27693	2018329	66.30	122	d__Bacteria;p__Proteobacteria;c__Alphaproteobacteria;o__Acetobacterales;f__Acetobacteraceae;g__G45-3;s__
LMSG_G000010242.1	no	1500_1	Pyrite	65.18	3.02	50.10	0	0	0	16	Medium quality	3307	2377032	66.10	762	d__Bacteria;p__Proteobacteria;c__Alphaproteobacteria;o__Acetobacterales;f__Acetobacteraceae;g__G45-3;s__
LMSG_G000010243.1	no	1500_1	Copper	86.46	2.86	72.16	0	1	0	19	Medium quality	10573	3160185	65.90	449	d__Bacteria;p__Proteobacteria;c__Alphaproteobacteria;o__Acetobacterales;f__Acetobacteraceae;g__G45-3;s__
LMSG_G000010244.1	no	1501_1	Lead-Zinc	87.65	1.74	78.95	0	0	0	16	Medium quality	17440	2894780	64.90	228	d__Bacteria;p__Proteobacteria;c__Alphaproteobacteria;o__Acetobacterales;f__Acetobacteraceae;g__G45-3;s__
LMSG_G000010245.1	no	1501_1	Pyrite	61.07	1.19	55.11	0	0	0	16	Medium quality	20022	2578677	64.70	481	d__Bacteria;p__Proteobacteria;c__Alphaproteobacteria;o__Acetobacterales;f__Acetobacteraceae;g__G45-3;s__
LMSG_G000010246.1	no	1501_1	Pyrite	70.09	1.57	62.24	0	1	0	15	Medium quality	26469	2707846	65.00	334	d__Bacteria;p__Proteobacteria;c__Alphaproteobacteria;o__Acetobacterales;f__Acetobacteraceae;g__G45-3;s__
LMSG_G000010247.1	no	1501_1	Lead-Zinc	80.26	1.11	74.72	0	2	0	17	Medium quality	14699	2900507	65.00	314	d__Bacteria;p__Proteobacteria;c__Alphaproteobacteria;o__Acetobacterales;f__Acetobacteraceae;g__G45-3;s__
LMSG_G000010248.1	no	1501_1	Lead-Zinc	83.29	0.58	80.39	0	2	0	16	Medium quality	15488	2900730	65.00	355	d__Bacteria;p__Proteobacteria;c__Alphaproteobacteria;o__Acetobacterales;f__Acetobacteraceae;g__G45-3;s__
LMSG_G000010249.1	no	1501_1	Pyrite-Copper	86.50	1.24	80.29	0	1	0	18	Medium quality	7825	3025539	65.10	505	d__Bacteria;p__Proteobacteria;c__Alphaproteobacteria;o__Acetobacterales;f__Acetobacteraceae;g__G45-3;s__
LMSG_G000010250.1	no	1502_1	Lead-Zinc	80.23	0.25	78.99	0	1	0	16	Medium quality	15757	2574155	67.60	206	d__Bacteria;p__Proteobacteria;c__Alphaproteobacteria;o__Acetobacterales;f__Acetobacteraceae;g__G45-3;s__
LMSG_G000010251.1	no	1502_1	Lead-Zinc	74.12	0.50	71.64	0	0	0	15	Medium quality	6778	2448204	68.00	466	d__Bacteria;p__Proteobacteria;c__Alphaproteobacteria;o__Acetobacterales;f__Acetobacteraceae;g__G45-3;s__
LMSG_G000010252.1	no	1502_1	Tin-Zinc	95.85	1.24	89.64	1	1	1	19	High quality	14163	3170973	67.50	343	d__Bacteria;p__Proteobacteria;c__Alphaproteobacteria;o__Acetobacterales;f__Acetobacteraceae;g__G45-3;s__
LMSG_G000010253.1	no	1502_1	Lead-Zinc	63.79	0.00	63.79	0	1	0	14	Medium quality	32551	2407989	67.90	109	d__Bacteria;p__Proteobacteria;c__Alphaproteobacteria;o__Acetobacterales;f__Acetobacteraceae;g__G45-3;s__
LMSG_G000010254.1	no	1502_1	Copper	95.52	1.24	89.31	0	1	0	19	Medium quality	50951	3194537	67.40	113	d__Bacteria;p__Proteobacteria;c__Alphaproteobacteria;o__Acetobacterales;f__Acetobacteraceae;g__G45-3;s__
LMSG_G000010255.1	no	1502_1	Lead-Zinc	81.31	2.49	68.88	0	1	0	14	Medium quality	29200	2613713	67.70	134	d__Bacteria;p__Proteobacteria;c__Alphaproteobacteria;o__Acetobacterales;f__Acetobacteraceae;g__G45-3;s__
LMSG_G000010256.1	no	1502_1	Pyrite	85.95	2.90	71.44	0	0	0	16	Medium quality	18701	2616785	68.00	208	d__Bacteria;p__Proteobacteria;c__Alphaproteobacteria;o__Acetobacterales;f__Acetobacteraceae;g__G45-3;s__
LMSG_G000010257.1	no	1502_1	Lead-Zinc	90.80	4.56	68.00	0	1	0	17	Medium quality	28877	2821767	67.70	135	d__Bacteria;p__Proteobacteria;c__Alphaproteobacteria;o__Acetobacterales;f__Acetobacteraceae;g__G45-3;s__
LMSG_G000010258.1	no	1502_1	Copper	99.00	1.24	92.79	0	1	1	20	Medium quality	36467	3203583	67.60	152	d__Bacteria;p__Proteobacteria;c__Alphaproteobacteria;o__Acetobacterales;f__Acetobacteraceae;g__G45-3;s__
LMSG_G000010259.1	no	1502_1	Copper	97.01	0.25	95.77	0	1	1	20	Medium quality	42369	2915183	67.80	115	d__Bacteria;p__Proteobacteria;c__Alphaproteobacteria;o__Acetobacterales;f__Acetobacteraceae;g__G45-3;s__
LMSG_G000010260.1	no	1502_1	Polymetallic	96.51	1.27	90.14	0	1	0	16	Medium quality	34707	2957457	67.60	139	d__Bacteria;p__Proteobacteria;c__Alphaproteobacteria;o__Acetobacterales;f__Acetobacteraceae;g__G45-3;s__
LMSG_G000010261.1	no	1502_1	Polymetallic	98.00	3.67	79.66	0	1	0	19	Medium quality	34835	3080825	67.40	154	d__Bacteria;p__Proteobacteria;c__Alphaproteobacteria;o__Acetobacterales;f__Acetobacteraceae;g__G45-3;s__
LMSG_G000010262.1	no	1502_1	Polymetallic	97.51	1.12	91.92	0	1	0	19	Medium quality	28814	3113064	67.60	161	d__Bacteria;p__Proteobacteria;c__Alphaproteobacteria;o__Acetobacterales;f__Acetobacteraceae;g__G45-3;s__
LMSG_G000010263.1	no	1502_1	Polymetallic	95.52	1.49	88.06	0	1	0	19	Medium quality	19755	2997654	67.60	228	d__Bacteria;p__Proteobacteria;c__Alphaproteobacteria;o__Acetobacterales;f__Acetobacteraceae;g__G45-3;s__
LMSG_G000010264.1	no	1502_1	Polymetallic	99.50	0.38	97.58	0	1	0	19	Medium quality	43350	3087284	67.40	130	d__Bacteria;p__Proteobacteria;c__Alphaproteobacteria;o__Acetobacterales;f__Acetobacteraceae;g__G45-3;s__
LMSG_G000010265.1	no	1502_1	Polymetallic	99.50	0.75	95.77	0	1	0	19	Medium quality	37717	3213373	67.30	142	d__Bacteria;p__Proteobacteria;c__Alphaproteobacteria;o__Acetobacterales;f__Acetobacteraceae;g__G45-3;s__
LMSG_G000010266.1	no	1502_1	Polymetallic	97.76	2.49	85.33	0	1	1	19	Medium quality	28226	3030715	67.40	177	d__Bacteria;p__Proteobacteria;c__Alphaproteobacteria;o__Acetobacterales;f__Acetobacteraceae;g__G45-3;s__
LMSG_G000010267.1	no	1502_1	Polymetallic	98.00	0.25	96.76	0	1	0	19	Medium quality	35701	3160446	67.30	180	d__Bacteria;p__Proteobacteria;c__Alphaproteobacteria;o__Acetobacterales;f__Acetobacteraceae;g__G45-3;s__
LMSG_G000010268.1	no	1502_1	Polymetallic	93.65	0.75	89.92	0	1	0	18	Medium quality	23074	2669326	67.90	165	d__Bacteria;p__Proteobacteria;c__Alphaproteobacteria;o__Acetobacterales;f__Acetobacteraceae;g__G45-3;s__
LMSG_G000010269.1	no	1502_1	Polymetallic	76.48	0.87	72.11	0	1	0	13	Medium quality	5225	2305615	68.00	499	d__Bacteria;p__Proteobacteria;c__Alphaproteobacteria;o__Acetobacterales;f__Acetobacteraceae;g__G45-3;s__
LMSG_G000010270.1	no	1502_1	Polymetallic	92.10	0.50	89.62	0	0	0	16	Medium quality	13125	2601404	68.00	270	d__Bacteria;p__Proteobacteria;c__Alphaproteobacteria;o__Acetobacterales;f__Acetobacteraceae;g__G45-3;s__
LMSG_G000010271.1	no	1502_1	Polymetallic	93.20	1.27	86.83	0	1	0	18	Medium quality	17334	2937412	67.70	236	d__Bacteria;p__Proteobacteria;c__Alphaproteobacteria;o__Acetobacterales;f__Acetobacteraceae;g__G45-3;s__
LMSG_G000010272.1	no	1502_1	Polymetallic	95.36	0.91	90.80	0	1	0	20	Medium quality	25303	2795804	67.80	164	d__Bacteria;p__Proteobacteria;c__Alphaproteobacteria;o__Acetobacterales;f__Acetobacteraceae;g__G45-3;s__
LMSG_G000010273.1	no	1502_1	Polymetallic	89.38	1.87	80.02	0	1	0	16	Medium quality	9572	2642183	67.90	346	d__Bacteria;p__Proteobacteria;c__Alphaproteobacteria;o__Acetobacterales;f__Acetobacteraceae;g__G45-3;s__
LMSG_G000010274.1	no	1502_1	Polymetallic	74.13	2.59	61.20	0	0	0	17	Medium quality	10669	2656395	67.70	331	d__Bacteria;p__Proteobacteria;c__Alphaproteobacteria;o__Acetobacterales;f__Acetobacteraceae;g__G45-3;s__
LMSG_G000010275.1	no	1502_1	Polymetallic	96.01	1.49	88.55	0	1	0	19	Medium quality	18849	2979834	67.50	227	d__Bacteria;p__Proteobacteria;c__Alphaproteobacteria;o__Acetobacterales;f__Acetobacteraceae;g__G45-3;s__
LMSG_G000010276.1	no	1502_1	Polymetallic	96.76	1.49	89.30	0	1	0	19	Medium quality	23020	2900216	67.80	181	d__Bacteria;p__Proteobacteria;c__Alphaproteobacteria;o__Acetobacterales;f__Acetobacteraceae;g__G45-3;s__
LMSG_G000010277.1	no	1502_1	Tin-Zinc	94.52	0.75	90.79	0	0	0	16	Medium quality	15958	2882063	67.70	264	d__Bacteria;p__Proteobacteria;c__Alphaproteobacteria;o__Acetobacterales;f__Acetobacteraceae;g__G45-3;s__
LMSG_G000010278.1	no	1502_1	Polymetallic	97.51	3.86	78.24	0	1	0	18	Medium quality	29418	3047212	67.70	161	d__Bacteria;p__Proteobacteria;c__Alphaproteobacteria;o__Acetobacterales;f__Acetobacteraceae;g__G45-3;s__
LMSG_G000010279.1	no	1502_1	Copper	93.24	2.49	80.81	0	1	0	19	Medium quality	7170	2820030	67.90	508	d__Bacteria;p__Proteobacteria;c__Alphaproteobacteria;o__Acetobacterales;f__Acetobacteraceae;g__G45-3;s__
LMSG_G000010280.1	no	1502_1	Copper	81.16	1.74	72.46	0	0	0	14	Medium quality	4424	2621943	67.90	682	d__Bacteria;p__Proteobacteria;c__Alphaproteobacteria;o__Acetobacterales;f__Acetobacteraceae;g__G45-3;s__
LMSG_G000010281.1	no	1502_1	Copper	84.82	1.99	74.87	0	1	0	18	Medium quality	41937	2624354	67.70	110	d__Bacteria;p__Proteobacteria;c__Alphaproteobacteria;o__Acetobacterales;f__Acetobacteraceae;g__G45-3;s__
LMSG_G000010282.1	no	1502_1	Polymetallic	54.79	0.26	53.47	0	0	0	10	Medium quality	2860	1847131	67.40	654	d__Bacteria;p__Proteobacteria;c__Alphaproteobacteria;o__Acetobacterales;f__Acetobacteraceae;g__G45-3;s__
LMSG_G000010283.1	no	1502_1	Copper	84.76	4.73	61.13	0	1	0	15	Medium quality	20594	2398174	68.20	167	d__Bacteria;p__Proteobacteria;c__Alphaproteobacteria;o__Acetobacterales;f__Acetobacteraceae;g__G45-3;s__
LMSG_G000010284.1	no	1502_1	Pyrite-Copper	99.25	4.00	79.25	0	1	0	18	Medium quality	28526	3253911	67.60	183	d__Bacteria;p__Proteobacteria;c__Alphaproteobacteria;o__Acetobacterales;f__Acetobacteraceae;g__G45-3;s__

LMSG_G000010285.1	no	1502_1	Pyrite-Copper	98.50	2.86	84.20	0	1	1	20	Medium quality	25905	3228196	67.50	174	d__Bacteria;p__Proteobacteria;c__Alphaproteobacteria;o__Acetobacterales;f__Acetobacteraceae;g__G45-3;s__
LMSG_G000010286.1	no	1502_1	Pyrite-Copper	98.26	2.66	84.98	0	1	0	18	Medium quality	25150	3053791	67.90	202	d__Bacteria;p__Proteobacteria;c__Alphaproteobacteria;o__Acetobacterales;f__Acetobacteraceae;g__G45-3;s__
LMSG_G000010287.1	no	1502_1	Pyrite-Copper	91.62	3.77	72.76	0	1	0	19	Medium quality	11687	2961070	68.00	567	d__Bacteria;p__Proteobacteria;c__Alphaproteobacteria;o__Acetobacterales;f__Acetobacteraceae;g__G45-3;s__
LMSG_G000010288.1	no	1502_1	Polymetallic	91.28	2.84	77.11	0	1	0	15	Medium quality	18535	2877008	67.30	249	d__Bacteria;p__Proteobacteria;c__Alphaproteobacteria;o__Acetobacterales;f__Acetobacteraceae;g__G45-3;s__
LMSG_G000010289.1	no	1502_1	Polymetallic	93.90	0.25	92.66	0	1	0	14	Medium quality	23182	3012822	67.30	265	d__Bacteria;p__Proteobacteria;c__Alphaproteobacteria;o__Acetobacterales;f__Acetobacteraceae;g__G45-3;s__
LMSG_G000010290.1	no	1501_2	Lead-Zinc	78.57	0.60	75.59	0	1	0	18	Medium quality	22965	2397975	66.20	145	d__Bacteria;p__Proteobacteria;c__Alphaproteobacteria;o__Acetobacterales;f__Acetobacteraceae;g__G45-3;s__G45-3 sp003352165
LMSG_G000010291.1	no	1501_2	Lead-Zinc	88.32	2.49	75.89	0	1	0	19	Medium quality	16455	2805379	66.30	249	d__Bacteria;p__Proteobacteria;c__Alphaproteobacteria;o__Acetobacterales;f__Acetobacteraceae;g__G45-3;s__G45-3 sp003352165
LMSG_G000010292.1	no	1501_2	Lead-Zinc	86.50	1.24	80.29	0	1	0	19	Medium quality	24018	2698195	66.20	162	d__Bacteria;p__Proteobacteria;c__Alphaproteobacteria;o__Acetobacterales;f__Acetobacteraceae;g__G45-3;s__G45-3 sp003352165
LMSG_G000010293.1	no	1501_2	Lead-Zinc	86.56	3.78	67.67	0	1	0	16	Medium quality	29203	2941756	66.30	133	d__Bacteria;p__Proteobacteria;c__Alphaproteobacteria;o__Acetobacterales;f__Acetobacteraceae;g__G45-3;s__G45-3 sp003352165
LMSG_G000010294.1	no	1501_2	Lead-Zinc	92.32	2.35	80.55	0	1	0	19	Medium quality	13387	2738659	66.30	241	d__Bacteria;p__Proteobacteria;c__Alphaproteobacteria;o__Acetobacterales;f__Acetobacteraceae;g__G45-3;s__G45-3 sp003352165
LMSG_G000010295.1	no	1501_2	Polymetallic	94.71	1.24	88.50	0	1	0	17	Medium quality	23107	2984414	66.20	180	d__Bacteria;p__Proteobacteria;c__Alphaproteobacteria;o__Acetobacterales;f__Acetobacteraceae;g__G45-3;s__G45-3 sp003352165
LMSG_G000010296.1	no	1501_2	Polymetallic	91.02	1.24	84.81	0	1	0	18	Medium quality	14967	2927579	66.10	278	d__Bacteria;p__Proteobacteria;c__Alphaproteobacteria;o__Acetobacterales;f__Acetobacteraceae;g__G45-3;s__G45-3 sp003352165
LMSG_G000010297.1	no	1501_2	Polymetallic	94.76	1.24	88.55	0	1	0	19	Medium quality	13410	3021969	66.10	339	d__Bacteria;p__Proteobacteria;c__Alphaproteobacteria;o__Acetobacterales;f__Acetobacteraceae;g__G45-3;s__G45-3 sp003352165
LMSG_G000010298.1	no	1501_2	Polymetallic	85.10	1.99	75.15	0	1	0	18	Medium quality	16418	2605597	66.30	250	d__Bacteria;p__Proteobacteria;c__Alphaproteobacteria;o__Acetobacterales;f__Acetobacteraceae;g__G45-3;s__G45-3 sp003352165
LMSG_G000010299.1	no	1501_2	Polymetallic	91.65	0.75	87.92	0	1	0	18	Medium quality	10406	2908214	66.10	399	d__Bacteria;p__Proteobacteria;c__Alphaproteobacteria;o__Acetobacterales;f__Acetobacteraceae;g__G45-3;s__G45-3 sp003352165
LMSG_G000010300.1	no	1501_2	Copper	94.27	1.52	86.66	0	1	1	20	Medium quality	24821	3144038	66.00	201	d__Bacteria;p__Proteobacteria;c__Alphaproteobacteria;o__Acetobacterales;f__Acetobacteraceae;g__G45-3;s__G45-3 sp003352165
LMSG_G000010301.1	no	1501_2	Copper	95.89	2.27	84.55	0	0	0	18	Medium quality	22291	3012467	66.20	190	d__Bacteria;p__Proteobacteria;c__Alphaproteobacteria;o__Acetobacterales;f__Acetobacteraceae;g__G45-3;s__G45-3 sp003352165
LMSG_G000010302.1	no	1501_2	Polymetallic	94.21	1.86	84.93	0	1	0	18	Medium quality	16286	2854232	66.20	248	d__Bacteria;p__Proteobacteria;c__Alphaproteobacteria;o__Acetobacterales;f__Acetobacteraceae;g__G45-3;s__G45-3 sp003352165
LMSG_G000010303.1	no	1501_2	Magnetite	52.60	0.00	52.60	0	1	0	11	Medium quality	24147	1548218	66.20	82	d__Bacteria;p__Proteobacteria;c__Alphaproteobacteria;o__Acetobacterales;f__Acetobacteraceae;g__G45-3;s__G45-3 sp003352165
LMSG_G000010304.1	no	1501_2	Copper	87.84	1.00	82.87	0	1	0	19	Medium quality	22893	2736840	66.20	173	d__Bacteria;p__Proteobacteria;c__Alphaproteobacteria;o__Acetobacterales;f__Acetobacteraceae;g__G45-3;s__G45-3 sp003352165
LMSG_G000010305.1	no	1501_2	Copper	89.92	4.98	65.05	0	1	0	18	Medium quality	26636	2784530	66.20	150	d__Bacteria;p__Proteobacteria;c__Alphaproteobacteria;o__Acetobacterales;f__Acetobacteraceae;g__G45-3;s__G45-3 sp003352165
LMSG_G000010306.1	no	1501_2	Lead-Zinc	83.17	2.24	71.98	0	1	0	16	Medium quality	5994	2642475	66.30	533	d__Bacteria;p__Proteobacteria;c__Alphaproteobacteria;o__Acetobacterales;f__Acetobacteraceae;g__G45-3;s__G45-3 sp003352165
LMSG_G000010307.1	no	1505_1	Lead-Zinc	72.13	0.75	68.40	0	0	0	16	Medium quality	14649	3158935	70.70	265	d__Bacteria;p__Proteobacteria;c__Alphaproteobacteria;o__Acetobacterales;f__Acetobacteraceae;g__Palsa-883;s__
LMSG_G000010308.1	no	1506_1	Coal	73.20	1.41	66.16	0	0	0	14	Medium quality	4726	2839599	70.70	677	d__Bacteria;p__Proteobacteria;c__Alphaproteobacteria;o__Acetobacterales;f__Acetobacteraceae;g__Palsa-883;s__
LMSG_G000010309.1	no	1507_1	Polymetallic	77.44	3.23	61.28	0	0	0	14	Medium quality	8858	2901181	69.40	400	d__Bacteria;p__Proteobacteria;c__Alphaproteobacteria;o__Acetobacterales;f__Acetobacteraceae;g__Palsa-883;s__
LMSG_G000010310.1	no	1507_1	Polymetallic	64.27	0.50	61.79	0	0	0	14	Medium quality	6800	2591778	69.70	451	d__Bacteria;p__Proteobacteria;c__Alphaproteobacteria;o__Acetobacterales;f__Acetobacteraceae;g__Palsa-883;s__
LMSG_G000010311.1	no	1526_1	Polymetallic	71.06	0.17	70.24	0	0	0	17	Medium quality	73254	3095932	63.50	65	d__Bacteria;p__Proteobacteria;c__Alphaproteobacteria;o__Acetobacterales;f__Acetobacteraceae;g__Rhodopila;s__
LMSG_G000010312.1	no	1527_1	Antimony	56.03	0.00	56.03	0	0	0	18	Medium quality	11130	4033440	63.10	512	d__Bacteria;p__Proteobacteria;c__Alphaproteobacteria;o__Acetobacterales;f__Acetobacteraceae;g__Rhodopila;s__
LMSG_G000010313.1	no	1528_1	Polymetallic	59.48	1.72	50.86	0	0	0	12	Medium quality	9826	3256818	64.80	725	d__Bacteria;p__Proteobacteria;c__Alphaproteobacteria;o__Acetobacterales;f__Acetobacteraceae;g__Rhodopila;s__
LMSG_G000010314.1	no	1495_1	Coal	68.91	1.00	63.93	0	0	0	13	Medium quality	4616	3270006	69.00	786	d__Bacteria;p__Proteobacteria;c__Alphaproteobacteria;o__ATCC43930;f__Stellaceae;g__;s__
LMSG_G000010315.1	no	1495_1	Coal	85.30	0.87	80.96	0	1	1	16	Medium quality	6456	3961186	68.90	721	d__Bacteria;p__Proteobacteria;c__Alphaproteobacteria;o__ATCC43930;f__Stellaceae;g__;s__
LMSG_G000010316.1	no	1495_1	Coal	65.23	0.92	60.62	0	0	0	14	Medium quality	4454	3453520	68.80	871	d__Bacteria;p__Proteobacteria;c__Alphaproteobacteria;o__ATCC43930;f__Stellaceae;g__;s__
LMSG_G000010317.1	no	1408_1	Copper	52.58	0.00	52.58	0	0	0	13	Medium quality	4611	2215612	69.70	516	d__Bacteria;p__Proteobacteria;c__Alphaproteobacteria;o__Caulobacterales;f__Caulobacteraceae;g__;s__
LMSG_G000010318.1	no	1205_1	Nickel-Copper	88.03	1.39	81.10	0	0	0	13	Medium quality	8057	2824678	68.70	435	d__Bacteria;p__Proteobacteria;c__Alphaproteobacteria;o__Caulobacterales;f__Caulobacteraceae;g__Brevundimonas;s__Brevundimonas subvibrioides_C
LMSG_G000010319.1	no	1409_1	Nickel-Copper	52.74	0.00	52.74	0	1	0	14	Medium quality	7372	3179816	67.10	445	d__Bacteria;p__Proteobacteria;c__Alphaproteobacteria;o__Caulobacterales;f__Caulobacteraceae;g__Caulobacter;s__Caulobacter sp002280875
LMSG_G000010320.1	no	1444_1	Polymetallic	97.06	2.37	85.24	1	1	1	19	High quality	26084	2977827	66.60	170	d__Bacteria;p__Proteobacteria;c__Alphaproteobacteria;o__Caulobacterales;f__Caulobacteraceae;g__Palsa-881;s__
LMSG_G000010321.1	no	1444_1	Polymetallic	97.45	1.49	89.99	1	1	1	19	High quality	108561	2995810	66.60	47	d__Bacteria;p__Proteobacteria;c__Alphaproteobacteria;o__Caulobacterales;f__Caulobacteraceae;g__Palsa-881;s__
LMSG_G000010322.1	no	1444_1	Copper	94.09	2.53	81.45	1	0	1	18	Medium quality	14134	3019148	66.50	334	d__Bacteria;p__Proteobacteria;c__Alphaproteobacteria;o__Caulobacterales;f__Caulobacteraceae;g__Palsa-881;s__
LMSG_G000010323.1	no	1444_1	Polymetallic	79.67	3.23	63.55	1	0	1	14	Medium quality	4484	2524183	66.70	683	d__Bacteria;p__Proteobacteria;c__Alphaproteobacteria;o__Caulobacterales;f__Caulobacteraceae;g__Palsa-881;s__
LMSG_G000010324.1	no	1444_1	Polymetallic	86.72	2.22	75.63	1	1	1	17	Medium quality	6654	2683956	66.60	497	d__Bacteria;p__Proteobacteria;c__Alphaproteobacteria;o__Caulobacterales;f__Caulobacteraceae;g__Palsa-881;s__
LMSG_G000010325.1	no	530_1	Polymetallic	90.81	2.61	77.77	1	2	1	20	High quality	58047	2143304	58.10	60	d__Bacteria;p__Proteobacteria;c__Alphaproteobacteria;o__Micavibrionales;f__UBA2020;g__PALSA-926;s__
LMSG_G000010326.1	no	530_1	Antimony	92.55	2.54	79.87	1	1	1	20	High quality	39971	2173160	58.20	80	d__Bacteria;p__Proteobacteria;c__Alphaproteobacteria;o__Micavibrionales;f__UBA2020;g__PALSA-926;s__
LMSG_G000010327.1	no	530_1	Polymetallic	92.99	4.57	70.17	1	1	1	19	High quality	73647	2353344	57.90	54	d__Bacteria;p__Proteobacteria;c__Alphaproteobacteria;o__Micavibrionales;f__UBA2020;g__PALSA-926;s__
LMSG_G000010328.1	no	530_1	Polymetallic	91.68	2.39	79.73	1	1	1	19	High quality	65154	2177379	58.00	56	d__Bacteria;p__Proteobacteria;c__Alphaproteobacteria;o__Micavibrionales;f__UBA2020;g__PALSA-926;s__
LMSG_G000010329.1	no	530_1	Lead-Zinc	86.46	1.96	76.68	1	1	0	17	Medium quality	76436	2149618	57.80	43	d__Bacteria;p__Proteobacteria;c__Alphaproteobacteria;o__Micavibrionales;f__UBA2020;g__PALSA-926;s__
LMSG_G000010330.1	no	530_1	Lead-Zinc	87.77	2.42	75.67	1	1	1	18	Medium quality	86228	2245816	58.00	41	d__Bacteria;p__Proteobacteria;c__Alphaproteobacteria;o__Micavibrionales;f__UBA2020;g__PALSA-926;s__
LMSG_G000010331.1	no	530_1	Lead-Zinc	76.97	3.32	60.36	0	1	0	13	Medium quality	4338	1947860	58.50	544	d__Bacteria;p__Proteobacteria;c__Alphaproteobacteria;o__Micavibrionales;f__UBA2020;g__PALSA-926;s__
LMSG_G000010332.1	no	530_1	Polymetallic	93.86	4.57	71.04	1	0	1	19	Medium quality	57157	2443625	57.90	96	d__Bacteria;p__Proteobacteria;c__Alphaproteobacteria;o__Micavibrionales;f__UBA2020;g__PALSA-926;s__
LMSG_G000010333.1	no	531_1	Polymetallic	74.11	3.57	56.29	1	0	1	16	Medium quality	4977	1969773	57.80	453	d__Bacteria;p__Proteobacteria;c__Alphaproteobacteria;o__Micavibrionales;f__UBA2020;g__PALSA-926;s__
LMSG_G000010334.1	no	1096_1	Polymetallic	83.19	0.00	83.19	1	1	4	19	Medium quality	8547	1173617	41.80	183	d__Bacteria;p__Proteobacteria;c__Alphaproteobacteria;o__Paracaedibacterales;f__;g__;s__
LMSG_G000010335.1	no	1096_1	Polymetallic	74.73	0.00	74.73	1	0	3	18	Medium quality	7568	1167648	42.00	206	d__Bacteria;p__Proteobacteria;c__Alphaproteobacteria;o__Paracaedibacterales;f__;g__;s__
LMSG_G000010336.1	no	1096_1	Polymetallic	73.50	0.10	73.02	0	0	0	17	Medium quality	6606	1074588	41.70	202	d__Bacteria;p__Proteobacteria;c__Alphaproteobacteria;o__Paracaedibacterales;f__;g__;s__
LMSG_G000010337.1	no	1096_1	Copper	55.09	0.00	55.09	1	0	1	12	Medium quality	7428	750348	41.90	134	d__Bacteria;p__Proteobacteria;c__Alphaproteobacteria;o__Paracaedibacterales;f__;g__;s__
LMSG_G000010338.1	no	1095_1	Lead-Zinc	81.85	1.20	75.85	2	0	1	16	Medium quality	5089	1813028	37.30	456	d__Bacteria;p__Proteobacteria;c__Alphaproteobacteria;o__Paracaedibacterales;f__UBA11393;g__;s__
LMSG_G000010339.1	no	1098_1	Polymetallic	83.61	1.71	75.08	0	0	0	15	Medium quality	5907	1202735	41.40	251	d__Bacteria;p__Proteobacteria;c__Alphaproteobacteria;o__Paracaedibacterales;f__UBA11393;g__Finniella;s__
LMSG_G000010340.1	no	1456_1	Nickel-Copper	98.39	1.52	90.79	0	1	0	19	Medium quality	114159	6393927	66.00	137	d__Bacteria;p__Proteobacteria;c__Alphaproteobacteria;o__Reyranellales;f__Reyranellaceae;g__Reyranella;s__Reyranella sp002281855
LMSG_G000010341.1	no	1456_1	Nickel-Copper	97.96	2.24	86.77	0	1	0	19	Medium quality	136258	6351986	66.00	130	d__Bacteria;p__Proteobacteria;c__Alphaproteobacteria;o__Reyranellales;f__Reyranellaceae;g__Reyranella;s__Reyranella sp002281855

LMSG_G000010342.1	no	1461_1	Antimony	91.37	0.94	86.67	0	1	0	18	Medium quality	125203	3810775	64.50	59	d__Bacteria;p__Proteobacteria;c__Alphaproteobacteria;o__Rhizobiales;f__Beijerinckiaceae;g__;s__
LMSG_G000010343.1	no	1461_1	Antimony	96.23	1.88	86.83	0	1	0	19	Medium quality	83386	3887409	64.50	70	d__Bacteria;p__Proteobacteria;c__Alphaproteobacteria;o__Rhizobiales;f__Beijerinckiaceae;g__;s__
LMSG_G000010344.1	no	1461_1	Antimony	89.70	3.29	73.25	0	1	0	19	Medium quality	75339	3827132	64.40	84	d__Bacteria;p__Proteobacteria;c__Alphaproteobacteria;o__Rhizobiales;f__Beijerinckiaceae;g__;s__
LMSG_G000010345.1	no	1461_1	Antimony	91.90	2.81	77.84	0	1	0	19	Medium quality	77183	3855483	64.60	99	d__Bacteria;p__Proteobacteria;c__Alphaproteobacteria;o__Rhizobiales;f__Beijerinckiaceae;g__;s__
LMSG_G000010346.1	no	1461_1	Antimony	94.51	1.90	84.99	0	0	0	19	Medium quality	83443	3813264	64.50	77	d__Bacteria;p__Proteobacteria;c__Alphaproteobacteria;o__Rhizobiales;f__Beijerinckiaceae;g__;s__
LMSG_G000010347.1	no	1461_1	Antimony	95.09	1.72	86.47	0	1	0	19	Medium quality	86486	3905545	64.40	82	d__Bacteria;p__Proteobacteria;c__Alphaproteobacteria;o__Rhizobiales;f__Beijerinckiaceae;g__;s__
LMSG_G000010348.1	no	1461_1	Polymetallic	64.56	0.78	60.65	0	0	0	16	Medium quality	6765	2441106	64.90	414	d__Bacteria;p__Proteobacteria;c__Alphaproteobacteria;o__Rhizobiales;f__Beijerinckiaceae;g__;s__
LMSG_G000010349.1	no	1066_1	Polymetallic	96.49	1.04	91.27	1	1	1	19	High quality	111923	2854422	60.90	57	d__Bacteria;p__Proteobacteria;c__Alphaproteobacteria;o__Rhizobiales;f__Beijerinckiaceae;g__Rhodoblastus;s__
LMSG_G000010350.1	no	1066_1	Polymetallic	98.22	0.94	93.52	0	0	0	19	Medium quality	71332	2977702	60.80	83	d__Bacteria;p__Proteobacteria;c__Alphaproteobacteria;o__Rhizobiales;f__Beijerinckiaceae;g__Rhodoblastus;s__
LMSG_G000010351.1	no	1066_1	Polymetallic	98.69	1.57	90.86	0	1	0	20	Medium quality	107195	2888486	60.90	52	d__Bacteria;p__Proteobacteria;c__Alphaproteobacteria;o__Rhizobiales;f__Beijerinckiaceae;g__Rhodoblastus;s__
LMSG_G000010352.1	no	1066_1	Polymetallic	99.00	1.57	91.17	0	1	0	19	Medium quality	58884	2866125	60.90	86	d__Bacteria;p__Proteobacteria;c__Alphaproteobacteria;o__Rhizobiales;f__Beijerinckiaceae;g__Rhodoblastus;s__
LMSG_G000010353.1	no	1461_1	Polymetallic	64.55	1.63	56.40	0	0	0	10	Medium quality	8453	2730168	64.80	397	d__Bacteria;p__Proteobacteria;c__Alphaproteobacteria;o__Rhizobiales;f__Beijerinckiaceae;g__Rhodoblastus;s__
LMSG_G000010354.1	no	1462_1	Pyrite	94.12	2.27	82.77	0	0	0	17	Medium quality	36140	3890483	64.00	182	d__Bacteria;p__Proteobacteria;c__Alphaproteobacteria;o__Rhizobiales;f__Beijerinckiaceae;g__Rhodoblastus;s__
LMSG_G000010355.1	no	1462_1	Antimony	74.73	1.32	68.15	0	0	0	13	Medium quality	33376	2537395	64.90	94	d__Bacteria;p__Proteobacteria;c__Alphaproteobacteria;o__Rhizobiales;f__Beijerinckiaceae;g__Rhodoblastus;s__
LMSG_G000010356.1	no	1462_1	Antimony	88.77	3.12	73.18	1	0	1	19	Medium quality	29507	3837212	64.10	190	d__Bacteria;p__Proteobacteria;c__Alphaproteobacteria;o__Rhizobiales;f__Beijerinckiaceae;g__Rhodoblastus;s__
LMSG_G000010357.1	no	1462_1	Antimony	87.72	3.59	69.77	0	0	0	17	Medium quality	34603	3102030	64.70	122	d__Bacteria;p__Proteobacteria;c__Alphaproteobacteria;o__Rhizobiales;f__Beijerinckiaceae;g__Rhodoblastus;s__
LMSG_G000010358.1	no	1462_1	Antimony	85.92	4.71	62.37	0	0	0	14	Medium quality	30825	3233377	64.50	145	d__Bacteria;p__Proteobacteria;c__Alphaproteobacteria;o__Rhizobiales;f__Beijerinckiaceae;g__Rhodoblastus;s__
LMSG_G000010359.1	no	1462_1	Antimony	90.83	2.73	77.20	0	0	0	17	Medium quality	35296	3041521	64.50	123	d__Bacteria;p__Proteobacteria;c__Alphaproteobacteria;o__Rhizobiales;f__Beijerinckiaceae;g__Rhodoblastus;s__
LMSG_G000010360.1	no	1462_1	Antimony	77.17	3.55	59.41	0	0	0	16	Medium quality	32740	2828593	64.90	119	d__Bacteria;p__Proteobacteria;c__Alphaproteobacteria;o__Rhizobiales;f__Beijerinckiaceae;g__Rhodoblastus;s__
LMSG_G000010361.1	no	1462_1	Polymetallic	61.21	0.94	56.51	0	0	0	14	Medium quality	8859	1885273	64.20	276	d__Bacteria;p__Proteobacteria;c__Alphaproteobacteria;o__Rhizobiales;f__Beijerinckiaceae;g__Rhodoblastus;s__
LMSG_G000010362.1	no	1462_1	Polymetallic	88.24	1.90	78.72	0	0	0	19	Medium quality	36297	3303279	64.50	129	d__Bacteria;p__Proteobacteria;c__Alphaproteobacteria;o__Rhizobiales;f__Beijerinckiaceae;g__Rhodoblastus;s__
LMSG_G000010363.1	no	1462_1	Pyrite-Copper	87.20	3.01	72.13	0	0	0	18	Medium quality	5789	3191034	64.30	703	d__Bacteria;p__Proteobacteria;c__Alphaproteobacteria;o__Rhizobiales;f__Beijerinckiaceae;g__Rhodoblastus;s__
LMSG_G000010364.1	no	1462_1	Pyrite-Copper	88.05	3.92	68.47	0	0	0	16	Medium quality	6847	3069440	64.50	639	d__Bacteria;p__Proteobacteria;c__Alphaproteobacteria;o__Rhizobiales;f__Beijerinckiaceae;g__Rhodoblastus;s__
LMSG_G000010365.1	no	1463_1	Lead-Zinc	75.78	4.66	52.48	1	0	0	18	Medium quality	4979	2921553	64.00	701	d__Bacteria;p__Proteobacteria;c__Alphaproteobacteria;o__Rhizobiales;f__Beijerinckiaceae;g__Rhodoblastus;s__
LMSG_G000010366.1	no	1463_1	Polymetallic	94.30	2.88	79.88	0	1	0	18	Medium quality	30642	3512956	64.30	181	d__Bacteria;p__Proteobacteria;c__Alphaproteobacteria;o__Rhizobiales;f__Beijerinckiaceae;g__Rhodoblastus;s__
LMSG_G000010367.1	no	1463_1	Polymetallic	92.08	2.59	79.11	0	0	0	19	Medium quality	26265	3515109	64.20	202	d__Bacteria;p__Proteobacteria;c__Alphaproteobacteria;o__Rhizobiales;f__Beijerinckiaceae;g__Rhodoblastus;s__
LMSG_G000010368.1	no	1463_2	Antimony	84.56	3.08	69.15	0	0	0	17	Medium quality	11352	3515720	64.00	364	d__Bacteria;p__Proteobacteria;c__Alphaproteobacteria;o__Rhizobiales;f__Beijerinckiaceae;g__Rhodoblastus;s__
LMSG_G000010369.1	no	1463_2	Antimony	84.35	3.24	68.16	0	0	0	16	Medium quality	13493	3077084	64.60	279	d__Bacteria;p__Proteobacteria;c__Alphaproteobacteria;o__Rhizobiales;f__Beijerinckiaceae;g__Rhodoblastus;s__
LMSG_G000010370.1	no	1463_2	Antimony	76.09	3.75	57.35	0	0	0	16	Medium quality	14552	2627814	65.10	226	d__Bacteria;p__Proteobacteria;c__Alphaproteobacteria;o__Rhizobiales;f__Beijerinckiaceae;g__Rhodoblastus;s__
LMSG_G000010371.1	no	1463_2	Antimony	75.77	4.15	55.01	0	0	0	16	Medium quality	14420	2548624	65.10	210	d__Bacteria;p__Proteobacteria;c__Alphaproteobacteria;o__Rhizobiales;f__Beijerinckiaceae;g__Rhodoblastus;s__
LMSG_G000010372.1	no	583_1	Nickel-Copper	92.33	1.08	86.93	2	1	2	18	High quality	153893	3742366	61.60	61	d__Bacteria;p__Proteobacteria;c__Alphaproteobacteria;o__Rhizobiales;f__Hyphomicrobiaceae;g__Hyphomicrobium;s__Hyphomicrobium sp002279935
LMSG_G000010373.1	no	1458_1	Nickel-Copper	77.89	2.11	67.35	1	1	0	15	Medium quality	8153	3704937	66.70	601	d__Bacteria;p__Proteobacteria;c__Alphaproteobacteria;o__Rhizobiales;f__Xanthobacteraceae;g__Aquabacter;s__
LMSG_G000010374.1	no	1457_1	Nickel-Copper	95.29	0.82	91.21	1	0	1	18	Medium quality	56548	4649427	67.50	216	d__Bacteria;p__Proteobacteria;c__Alphaproteobacteria;o__Rhizobiales;f__Xanthobacteraceae;g__Aquabacter;s__Aquabacter sp002279855
LMSG_G000010375.1	no	1535_1	Antimony	82.47	4.42	60.36	1	1	1	14	Medium quality	12870	5153928	62.00	586	d__Bacteria;p__Proteobacteria;c__Alphaproteobacteria;o__Rhizobiales;f__Xanthobacteraceae;g__Bradyrhizobium;s__
LMSG_G000010376.1	no	1535_1	Antimony	84.20	3.87	64.86	0	1	0	14	Medium quality	11050	5434197	61.90	671	d__Bacteria;p__Proteobacteria;c__Alphaproteobacteria;o__Rhizobiales;f__Xanthobacteraceae;g__Bradyrhizobium;s__
LMSG_G000010377.1	no	1535_1	Antimony	74.12	3.26	57.81	2	1	1	13	Medium quality	11549	4507475	61.80	480	d__Bacteria;p__Proteobacteria;c__Alphaproteobacteria;o__Rhizobiales;f__Xanthobacteraceae;g__Bradyrhizobium;s__
LMSG_G000010378.1	no	1535_1	Antimony	81.13	4.46	58.81	0	1	0	13	Medium quality	14038	4706542	61.80	475	d__Bacteria;p__Proteobacteria;c__Alphaproteobacteria;o__Rhizobiales;f__Xanthobacteraceae;g__Bradyrhizobium;s__
LMSG_G000010379.1	no	1535_1	Antimony	75.50	3.02	60.39	0	0	0	14	Medium quality	6831	4171388	61.90	682	d__Bacteria;p__Proteobacteria;c__Alphaproteobacteria;o__Rhizobiales;f__Xanthobacteraceae;g__Bradyrhizobium;s__
LMSG_G000010380.1	no	1433_1	Polymetallic	83.55	1.37	76.69	1	0	1	16	Medium quality	11640	2929420	66.60	301	d__Bacteria;p__Proteobacteria;c__Alphaproteobacteria;o__Rhodobacterales;f__Rhodobacteraceae;g__;s__
LMSG_G000010381.1	no	1433_1	Polymetallic	76.17	1.72	67.55	1	0	1	18	Medium quality	18701	3390316	66.60	283	d__Bacteria;p__Proteobacteria;c__Alphaproteobacteria;o__Rhodobacterales;f__Rhodobacteraceae;g__;s__
LMSG_G000010382.1	no	1434_1	Polymetallic	60.10	0.45	57.83	0	0	0	11	Medium quality	9168	1928370	68.40	266	d__Bacteria;p__Proteobacteria;c__Alphaproteobacteria;o__Rhodobacterales;f__Rhodobacteraceae;g__Allgaiera;s__
LMSG_G000010383.1	no	1435_1	Lead-Zinc	98.15	1.62	90.07	0	1	0	17	Medium quality	95081	3682016	69.10	97	d__Bacteria;p__Proteobacteria;c__Alphaproteobacteria;o__Rhodobacterales;f__Rhodobacteraceae;g__Allgaiera;s__
LMSG_G000010384.1	no	1435_2	Antimony	65.02	2.25	53.77	0	0	0	11	Medium quality	3072	2231589	68.60	798	d__Bacteria;p__Proteobacteria;c__Alphaproteobacteria;o__Rhodobacterales;f__Rhodobacteraceae;g__Allgaiera;s__
LMSG_G000010385.1	no	1437_1	Antimony	91.33	1.33	84.68	1	0	1	18	Medium quality	10656	3131132	63.00	387	d__Bacteria;p__Proteobacteria;c__Alphaproteobacteria;o__Rhodobacterales;f__Rhodobacteraceae;g__Allgaiera;s__
LMSG_G000010386.1	no	1437_1	Antimony	86.69	0.86	82.40	0	0	0	17	Medium quality	11864	2831248	63.20	306	d__Bacteria;p__Proteobacteria;c__Alphaproteobacteria;o__Rhodobacterales;f__Rhodobacteraceae;g__Allgaiera;s__
LMSG_G000010387.1	no	1396_1	Lead-Zinc	66.42	2.65	53.16	0	0	0	13	Medium quality	9127	3070223	68.70	393	d__Bacteria;p__Proteobacteria;c__Alphaproteobacteria;o__Rhodospirillales;f__Magnetospirillaceae;g__;s__
LMSG_G000010388.1	no	1396_1	Lead-Zinc	95.02	0.86	90.71	0	0	0	18	Medium quality	18201	4242339	68.60	389	d__Bacteria;p__Proteobacteria;c__Alphaproteobacteria;o__Rhodospirillales;f__Magnetospirillaceae;g__;s__
LMSG_G000010389.1	no	1396_1	Polymetallic	68.02	1.72	59.40	0	0	0	15	Medium quality	10683	3313693	68.80	362	d__Bacteria;p__Proteobacteria;c__Alphaproteobacteria;o__Rhodospirillales;f__Magnetospirillaceae;g__;s__
LMSG_G000010390.1	no	1397_1	Lead-Zinc	97.51	2.49	85.08	0	0	0	18	Medium quality	61549	3306319	65.30	96	d__Bacteria;p__Proteobacteria;c__Alphaproteobacteria;o__Rhodospirillales;f__Magnetospirillaceae;g__;s__
LMSG_G000010391.1	no	1397_1	Lead-Zinc	98.63	0.66	95.32	0	0	0	19	Medium quality	51456	3114909	65.40	109	d__Bacteria;p__Proteobacteria;c__Alphaproteobacteria;o__Rhodospirillales;f__Magnetospirillaceae;g__;s__
LMSG_G000010392.1	no	1397_1	Lead-Zinc	96.60	1.44	89.40	0	1	0	19	Medium quality	20155	3162460	65.50	247	d__Bacteria;p__Proteobacteria;c__Alphaproteobacteria;o__Rhodospirillales;f__Magnetospirillaceae;g__;s__
LMSG_G000010393.1	no	1397_1	Polymetallic	80.44	4.28	59.04	0	0	0	16	Medium quality	3533	2731052	66.00	894	d__Bacteria;p__Proteobacteria;c__Alphaproteobacteria;o__Rhodospirillales;f__Magnetospirillaceae;g__;s__
LMSG_G000010394.1	no	1398_1	Lead-Zinc	97.51	3.23	81.35	0	0	0	19	Medium quality	67704	3645237	67.00	125	d__Bacteria;p__Proteobacteria;c__Alphaproteobacteria;o__Rhodospirillales;f__Magnetospirillaceae;g__;s__
LMSG_G000010395.1	no	1159_1	Antimony	75.00	0.00	75.00	0	1	0	17	Medium quality	6786	3109816	66.80	587	d__Bacteria;p__Proteobacteria;c__Alphaproteobacteria;o__Rhodospirillales;f__Magnetospirillaceae;g__Telmatospirillum;s__

LMSG_G000010396.1	no	1159_2	Polymetallic	70.01	1.52	62.40	0	0	0	16	Medium quality	2947	2204442	66.20	780	d__Bacteria;p__Proteobacteria;c__Alphaproteobacteria;o__Rhodospirillales;f__Magnetospirillaceae;g__Telmatospirillum;s__
LMSG_G000010397.1	no	1159_2	Polymetallic	91.77	1.43	84.62	0	0	0	17	Medium quality	9139	3243993	66.50	453	d__Bacteria;p__Proteobacteria;c__Alphaproteobacteria;o__Rhodospirillales;f__Magnetospirillaceae;g__Telmatospirillum;s__
LMSG_G000010398.1	no	1159_2	Polymetallic	80.57	1.69	72.12	0	0	0	15	Medium quality	4720	2950738	66.70	734	d__Bacteria;p__Proteobacteria;c__Alphaproteobacteria;o__Rhodospirillales;f__Magnetospirillaceae;g__Telmatospirillum;s__
LMSG_G000010399.1	no	1160_1	Antimony	97.76	2.99	82.84	0	0	0	19	Medium quality	28200	4346678	64.30	235	d__Bacteria;p__Proteobacteria;c__Alphaproteobacteria;o__Rhodospirillales;f__Magnetospirillaceae;g__Telmatospirillum;s__
LMSG_G000010400.1	no	1428_1	Nickel-Copper	88.75	1.71	80.23	1	1	1	18	Medium quality	9694	2486407	68.00	305	d__Bacteria;p__Proteobacteria;c__Alphaproteobacteria;o__Sphingomonadales;f__Sphingomonadaceae;g__Croceibacterium;s__Croceibacterium sp002279825
LMSG_G000010401.1	no	1427_1	Tin-Zinc	96.93	4.00	76.95	1	1	1	19	High quality	41236	2458062	66.60	98	d__Bacteria;p__Proteobacteria;c__Alphaproteobacteria;o__Sphingomonadales;f__Sphingomonadaceae;g__Qipengyuania;s__
LMSG_G000010402.1	no	1422_1	Nickel-Copper	68.03	0.91	63.47	0	0	0	14	Medium quality	3356	2196523	65.20	636	d__Bacteria;p__Proteobacteria;c__Alphaproteobacteria;o__Sphingomonadales;f__Sphingomonadaceae;g__Sandarakinorhabdus;s__Sandarakinorhabdus sp002280855
LMSG_G000010403.1	no	1422_1	Nickel-Copper	96.07	3.58	78.18	1	1	1	19	High quality	215038	3412743	64.40	51	d__Bacteria;p__Proteobacteria;c__Alphaproteobacteria;o__Sphingomonadales;f__Sphingomonadaceae;g__Sandarakinorhabdus;s__Sandarakinorhabdus sp002280855
LMSG_G000010404.1	no	1417_1	Lead-Zinc	84.71	2.02	74.61	0	0	0	19	Medium quality	66059	4004271	62.00	97	d__Bacteria;p__Proteobacteria;c__Alphaproteobacteria;o__Sphingomonadales;f__Sphingomonadaceae;g__Sphingobium;s__
LMSG_G000010405.1	no	1418_1	Lead-Zinc	92.88	0.94	88.19	0	0	0	18	Medium quality	80420	3699925	63.50	84	d__Bacteria;p__Proteobacteria;c__Alphaproteobacteria;o__Sphingomonadales;f__Sphingomonadaceae;g__Sphingobium;s__
LMSG_G000010406.1	no	1415_1	Nickel-Copper	78.16	0.00	78.16	0	0	0	18	Medium quality	5508	2979780	62.90	708	d__Bacteria;p__Proteobacteria;c__Alphaproteobacteria;o__Sphingomonadales;f__Sphingomonadaceae;g__Sphingomonas;s__Sphingomonas sp002280555
LMSG_G000010407.1	no	1431_1	Tin-Zinc	50.28	0.00	50.28	0	0	0	13	Medium quality	2579	810953	66.70	323	d__Bacteria;p__Proteobacteria;c__Alphaproteobacteria;o__Sphingomonadales;f__Sphingomonadaceae;g__Sphingomonas_A;s__
LMSG_G000010408.1	no	1413_1	Nickel-Copper	95.85	1.42	88.74	0	0	0	19	Medium quality	26505	2248071	56.30	127	d__Bacteria;p__Proteobacteria;c__Alphaproteobacteria;o__Sphingomonadales;f__Sphingomonadaceae;g__Sphingorhabdus_B;s__Sphingorhabdus_B sp002282385
LMSG_G000010409.1	no	1413_1	Nickel-Copper	92.95	1.59	84.99	0	0	0	15	Medium quality	5882	2142803	56.50	486	d__Bacteria;p__Proteobacteria;c__Alphaproteobacteria;o__Sphingomonadales;f__Sphingomonadaceae;g__Sphingorhabdus_B;s__Sphingorhabdus_B sp002282385
LMSG_G000010410.1	no	1413_1	Nickel-Copper	90.70	1.79	81.75	0	0	0	19	Medium quality	87375	2172449	56.30	46	d__Bacteria;p__Proteobacteria;c__Alphaproteobacteria;o__Sphingomonadales;f__Sphingomonadaceae;g__Sphingorhabdus_B;s__Sphingorhabdus_B sp002282385
LMSG_G000010411.1	no	1055_1	Polymetallic	86.40	0.50	83.92	1	1	1	17	Medium quality	81877	3207055	60.50	96	d__Bacteria;p__Proteobacteria;c__Alphaproteobacteria;o__UBA1301;f__UBA1301;g__;s__
LMSG_G000010412.1	no	1054_1	Polymetallic	98.26	4.10	77.78	3	2	10	19	High quality	113389	4687197	58.80	451	d__Bacteria;p__Proteobacteria;c__Alphaproteobacteria;o__UBA1301;f__UBA1301;g__UBA6038;s__
LMSG_G000010413.1	no	1054_1	Polymetallic	98.91	2.28	87.50	1	1	2	20	High quality	94029	3860394	59.10	148	d__Bacteria;p__Proteobacteria;c__Alphaproteobacteria;o__UBA1301;f__UBA1301;g__UBA6038;s__
LMSG_G000010414.1	no	1054_1	Polymetallic	99.34	1.18	93.44	1	1	13	19	High quality	220012	3703909	59.30	222	d__Bacteria;p__Proteobacteria;c__Alphaproteobacteria;o__UBA1301;f__UBA1301;g__UBA6038;s__
LMSG_G000010415.1	no	1054_1	Polymetallic	70.68	0.00	70.68	2	0	2	13	Medium quality	148992	2970230	60.00	193	d__Bacteria;p__Proteobacteria;c__Alphaproteobacteria;o__UBA1301;f__UBA1301;g__UBA6038;s__
LMSG_G000010416.1	no	1055_1	Copper	98.69	1.74	90.02	1	2	1	19	High quality	214083	3761933	60.00	237	d__Bacteria;p__Proteobacteria;c__Alphaproteobacteria;o__UBA1301;f__UBA1301;g__UBA6038;s__
LMSG_G000010417.1	no	1055_1	Polymetallic	97.82	0.43	95.65	1	1	2	19	High quality	74228	3576381	59.80	102	d__Bacteria;p__Proteobacteria;c__Alphaproteobacteria;o__UBA1301;f__UBA1301;g__UBA6038;s__
LMSG_G000010418.1	no	1055_1	Pyrite-Copper	97.39	2.88	82.97	1	1	1	19	High quality	69023	3822058	59.80	226	d__Bacteria;p__Proteobacteria;c__Alphaproteobacteria;o__UBA1301;f__UBA1301;g__UBA6038;s__
LMSG_G000010419.1	no	1055_1	Lead-Zinc	96.73	0.87	92.39	1	2	1	17	Medium quality	43178	4034932	60.10	245	d__Bacteria;p__Proteobacteria;c__Alphaproteobacteria;o__UBA1301;f__UBA1301;g__UBA6038;s__
LMSG_G000010420.1	no	1055_1	Lead-Zinc	94.78	0.00	94.78	1	1	1	15	Medium quality	51826	3500158	60.20	168	d__Bacteria;p__Proteobacteria;c__Alphaproteobacteria;o__UBA1301;f__UBA1301;g__UBA6038;s__
LMSG_G000010421.1	no	1055_1	Polymetallic	95.65	1.30	89.13	1	1	2	17	Medium quality	28041	4173932	60.00	302	d__Bacteria;p__Proteobacteria;c__Alphaproteobacteria;o__UBA1301;f__UBA1301;g__UBA6038;s__
LMSG_G000010422.1	no	1055_1	Copper	74.74	1.30	68.22	1	0	2	13	Medium quality	4328	2631770	60.90	802	d__Bacteria;p__Proteobacteria;c__Alphaproteobacteria;o__UBA1301;f__UBA1301;g__UBA6038;s__
LMSG_G000010423.1	no	1055_1	Copper	71.55	0.86	67.24	0	0	0	6	Medium quality	4426	1606058	61.80	410	d__Bacteria;p__Proteobacteria;c__Alphaproteobacteria;o__UBA1301;f__UBA1301;g__UBA6038;s__
LMSG_G000010424.1	no	1055_1	Lead-Zinc	95.43	1.09	90.00	0	1	0	15	Medium quality	58256	3021971	60.20	94	d__Bacteria;p__Proteobacteria;c__Alphaproteobacteria;o__UBA1301;f__UBA1301;g__UBA6038;s__
LMSG_G000010425.1	no	1055_1	Lead-Zinc	50.86	0.00	50.86	0	0	0	4	Medium quality	4149	1395023	61.70	398	d__Bacteria;p__Proteobacteria;c__Alphaproteobacteria;o__UBA1301;f__UBA1301;g__UBA6038;s__
LMSG_G000010426.1	no	1055_1	Polymetallic	67.24	0.00	67.24	1	0	0	14	Medium quality	47803	2440661	60.60	109	d__Bacteria;p__Proteobacteria;c__Alphaproteobacteria;o__UBA1301;f__UBA1301;g__UBA6038;s__
LMSG_G000010427.1	no	1055_1	Antimony	70.68	0.00	70.68	0	0	0	13	Medium quality	31473	2490529	61.20	216	d__Bacteria;p__Proteobacteria;c__Alphaproteobacteria;o__UBA1301;f__UBA1301;g__UBA6038;s__
LMSG_G000010428.1	no	1055_1	Polymetallic	89.15	0.58	86.26	1	1	1	17	Medium quality	43065	3352780	60.20	210	d__Bacteria;p__Proteobacteria;c__Alphaproteobacteria;o__UBA1301;f__UBA1301;g__UBA6038;s__
LMSG_G000010429.1	no	1055_1	Pyrite-Copper	56.89	0.00	56.89	1	1	1	14	Medium quality	53073	2662159	60.70	120	d__Bacteria;p__Proteobacteria;c__Alphaproteobacteria;o__UBA1301;f__UBA1301;g__UBA6038;s__
LMSG_G000010430.1	no	1055_1	Polymetallic	93.49	2.16	82.68	1	1	0	15	Medium quality	41052	3223837	60.30	168	d__Bacteria;p__Proteobacteria;c__Alphaproteobacteria;o__UBA1301;f__UBA1301;g__UBA6038;s__
LMSG_G000010431.1	no	1523_1	Copper	66.76	0.14	66.04	1	1	1	17	Medium quality	15050	2355631	63.00	224	d__Bacteria;p__Proteobacteria;c__Alphaproteobacteria;o__UBA1301;f__UBA1301;g__UBA6038;s__
LMSG_G000010432.1	no	1189_2	Lead-Zinc	75.14	2.08	64.77	1	1	1	15	Medium quality	27655	1610740	66.30	166	d__Bacteria;p__Proteobacteria;c__Gammaproteobacteria;o__;f__;g__;s__
LMSG_G000010433.1	no	1189_2	Pyrite	62.02	1.15	56.28	0	0	0	14	Medium quality	28559	1224716	67.10	71	d__Bacteria;p__Proteobacteria;c__Gammaproteobacteria;o__;f__;g__;s__
LMSG_G000010434.1	no	1189_2	Pyrite	84.10	4.28	62.70	0	1	1	17	Medium quality	43748	1882932	66.00	121	d__Bacteria;p__Proteobacteria;c__Gammaproteobacteria;o__;f__;g__;s__
LMSG_G000010435.1	no	1189_2	Magnetite	72.70	2.28	61.29	1	1	1	14	Medium quality	23799	1432630	65.90	134	d__Bacteria;p__Proteobacteria;c__Gammaproteobacteria;o__;f__;g__;s__
LMSG_G000010436.1	no	1537_1	Pyrite-Copper	96.03	3.96	76.22	1	1	1	18	High quality	144526	2333136	63.90	62	d__Bacteria;p__Proteobacteria;c__Gammaproteobacteria;o__Acidiferrobacterales;f__Acidiferrobacteraceae;g__;s__
LMSG_G000010437.1	no	1537_1	Pyrite-Copper	90.24	1.22	84.15	1	0	1	15	Medium quality	21917	1988108	64.00	115	d__Bacteria;p__Proteobacteria;c__Gammaproteobacteria;o__Acidiferrobacterales;f__Acidiferrobacteraceae;g__;s__
LMSG_G000010438.1	no	1538_1	Tin-Zinc	92.68	1.22	86.59	0	1	0	16	Medium quality	41001	1943418	64.20	75	d__Bacteria;p__Proteobacteria;c__Gammaproteobacteria;o__Acidiferrobacterales;f__Acidiferrobacteraceae;g__;s__
LMSG_G000010439.1	no	1538_1	Tin-Zinc	92.68	1.05	87.43	0	1	0	16	Medium quality	25507	2039005	64.10	123	d__Bacteria;p__Proteobacteria;c__Gammaproteobacteria;o__Acidiferrobacterales;f__Acidiferrobacteraceae;g__;s__
LMSG_G000010440.1	no	1539_1	Pyrite-Copper	89.63	0.00	89.63	1	1	1	15	Medium quality	30532	1959395	63.40	97	d__Bacteria;p__Proteobacteria;c__Gammaproteobacteria;o__Acidiferrobacterales;f__Acidiferrobacteraceae;g__;s__
LMSG_G000010441.1	no	1539_1	Pyrite-Copper	80.89	1.22	74.80	1	1	1	16	Medium quality	38638	2130708	63.20	115	d__Bacteria;p__Proteobacteria;c__Gammaproteobacteria;o__Acidiferrobacterales;f__Acidiferrobacteraceae;g__;s__
LMSG_G000010442.1	no	602_1	Polymetallic	93.90	0.30	92.38	1	1	1	18	High quality	57126	2371504	60.50	96	d__Bacteria;p__Proteobacteria;c__Gammaproteobacteria;o__Acidiferrobacterales;f__Acidiferrobacteraceae;g__;s__
LMSG_G000010443.1	no	602_1	Pyrite-Copper	96.34	1.37	89.49	1	1	1	18	High quality	51143	2647069	60.30	146	d__Bacteria;p__Proteobacteria;c__Gammaproteobacteria;o__Acidiferrobacterales;f__Acidiferrobacteraceae;g__;s__
LMSG_G000010444.1	no	602_1	Pyrite-Copper	95.73	1.52	88.11	1	1	1	19	High quality	48282	2820969	60.40	167	d__Bacteria;p__Proteobacteria;c__Gammaproteobacteria;o__Acidiferrobacterales;f__Acidiferrobacteraceae;g__;s__
LMSG_G000010445.1	no	602_1	Polymetallic	86.19	0.95	81.45	0	0	0	14	Medium quality	9684	1721443	60.90	222	d__Bacteria;p__Proteobacteria;c__Gammaproteobacteria;o__Acidiferrobacterales;f__Acidiferrobacteraceae;g__;s__
LMSG_G000010446.1	no	602_1	Tin-Zinc	91.00	2.54	78.30	0	1	0	19	Medium quality	25785	1935680	60.80	116	d__Bacteria;p__Proteobacteria;c__Gammaproteobacteria;o__Acidiferrobacterales;f__Acidiferrobacteraceae;g__;s__
LMSG_G000010447.1	no	602_1	Tin-Zinc	93.66	2.13	82.99	0	1	0	17	Medium quality	20660	1912325	60.80	128	d__Bacteria;p__Proteobacteria;c__Gammaproteobacteria;o__Acidiferrobacterales;f__Acidiferrobacteraceae;g__;s__
LMSG_G000010448.1	no	602_1	Polymetallic	93.29	1.52	85.67	1	0	1	18	Medium quality	60053	2388035	61.00	96	d__Bacteria;p__Proteobacteria;c__Gammaproteobacteria;o__Acidiferrobacterales;f__Acidiferrobacteraceae;g__;s__
LMSG_G000010449.1	no	602_1	Pyrite-Copper	95.12	1.52	87.50	1	0	1	19	Medium quality	68026	2121590	60.70	72	d__Bacteria;p__Proteobacteria;c__Gammaproteobacteria;o__Acidiferrobacterales;f__Acidiferrobacteraceae;g__;s__
LMSG_G000010450.1	no	1540_1	Pyrite-Copper	83.53	0.61	80.49	1	1	1	18	Medium quality	40228	2346642	59.10	116	d__Bacteria;p__Proteobacteria;c__Gammaproteobacteria;o__Acidiferrobacterales;f__Acidiferrobacteraceae;g__Acidiferrobacter;s__
LMSG_G000010451.1	no	1540_1	Pyrite-Copper	92.68	3.39	75.71	1	1	0	16	Medium quality	32183	2239726	59.10	98	d__Bacteria;p__Proteobacteria;c__Gammaproteobacteria;o__Acidiferrobacterales;f__Acidiferrobacteraceae;g__Acidiferrobacter;s__
LMSG_G000010452.1	no	1544_1	Lead-Zinc	76.05	2.44	63.86	1	0	0	15	Medium quality	34382	1890291	66.50	89	d__Bacteria;p__Proteobacteria;c__Gammaproteobacteria;o__Acidiferrobacterales;f__Acidiferrobacteraceae;g__Acidiferrobacter;s__
LMSG_G000010453.1	no	1545_1	Lead-Zinc	62.93	1.72	54.31	1	0	0	15	Medium quality	32013	1957776	63.40	120	d__Bacteria;p__Proteobacteria;c__Gammaproteobacteria;o__Acidiferrobacterales;f__Acidiferrobacteraceae;g__Acidiferrobacter;s__
LMSG_G000010454.1	no	1546_1	Pyrite	78.65	1.22	72.56	0	0	0	12	Medium quality	6389	2013211	64.80	389	d__Bacteria;p__Proteobacteria;c__Gammaproteobacteria;o__Acidiferrobacterales;f__Acidiferrobacteraceae;g__Acidiferrobacter;s__

LMSG_G000010455.1	no	1546_1	Pyrite	81.70	0.61	78.66	1	0	1	16	Medium quality	66407	1968353	64.40	62	d__Bacteria;p__Proteobacteria;c__Gammaproteobacteria;o__Acidiferrobacterales;f__Acidiferrobacteraceae;g__Acidiferrobacter;s__
LMSG_G000010456.1	no	1546_1	Pyrite	84.14	1.58	76.27	1	0	1	19	Medium quality	57346	1989768	64.60	65	d__Bacteria;p__Proteobacteria;c__Gammaproteobacteria;o__Acidiferrobacterales;f__Acidiferrobacteraceae;g__Acidiferrobacter;s__
LMSG_G000010457.1	no	1546_1	Pyrite	85.36	1.22	79.27	1	1	1	18	Medium quality	62912	2653925	64.30	106	d__Bacteria;p__Proteobacteria;c__Gammaproteobacteria;o__Acidiferrobacterales;f__Acidiferrobacteraceae;g__Acidiferrobacter;s__
LMSG_G000010458.1	no	1547_1	Copper	96.90	2.17	86.06	1	1	1	18	High quality	20470	2449845	62.80	179	d__Bacteria;p__Proteobacteria;c__Gammaproteobacteria;o__Acidiferrobacterales;f__Acidiferrobacteraceae;g__Acidiferrobacter;s__
LMSG_G000010459.1	no	1547_1	Lead-Zinc	97.56	1.52	89.94	1	2	1	18	High quality	37372	2465770	62.70	122	d__Bacteria;p__Proteobacteria;c__Gammaproteobacteria;o__Acidiferrobacterales;f__Acidiferrobacteraceae;g__Acidiferrobacter;s__
LMSG_G000010460.1	no	1547_1	Pyrite-Copper	94.57	1.68	86.19	1	2	1	18	High quality	40234	2460440	62.70	107	d__Bacteria;p__Proteobacteria;c__Gammaproteobacteria;o__Acidiferrobacterales;f__Acidiferrobacteraceae;g__Acidiferrobacter;s__
LMSG_G000010461.1	no	1547_1	Copper	92.78	2.44	80.59	1	2	1	16	Medium quality	17309	2116442	63.10	176	d__Bacteria;p__Proteobacteria;c__Gammaproteobacteria;o__Acidiferrobacterales;f__Acidiferrobacteraceae;g__Acidiferrobacter;s__
LMSG_G000010462.1	no	1547_1	Copper	91.09	3.05	75.85	1	1	1	17	Medium quality	58041	2462315	62.90	180	d__Bacteria;p__Proteobacteria;c__Gammaproteobacteria;o__Acidiferrobacterales;f__Acidiferrobacteraceae;g__Acidiferrobacter;s__
LMSG_G000010463.1	no	1547_1	Lead-Zinc	75.20	0.91	70.63	0	0	0	12	Medium quality	25913	1704897	62.90	96	d__Bacteria;p__Proteobacteria;c__Gammaproteobacteria;o__Acidiferrobacterales;f__Acidiferrobacteraceae;g__Acidiferrobacter;s__
LMSG_G000010464.1	no	1547_1	Copper	89.53	0.95	84.79	0	1	0	14	Medium quality	36488	2062061	62.90	141	d__Bacteria;p__Proteobacteria;c__Gammaproteobacteria;o__Acidiferrobacterales;f__Acidiferrobacteraceae;g__Acidiferrobacter;s__
LMSG_G000010465.1	no	1547_1	Lead-Zinc	76.82	2.17	65.98	1	0	1	11	Medium quality	15008	1770200	63.30	174	d__Bacteria;p__Proteobacteria;c__Gammaproteobacteria;o__Acidiferrobacterales;f__Acidiferrobacteraceae;g__Acidiferrobacter;s__
LMSG_G000010466.1	no	1547_1	Polymetallic	65.86	0.00	65.86	1	0	1	12	Medium quality	18561	2034289	62.60	173	d__Bacteria;p__Proteobacteria;c__Gammaproteobacteria;o__Acidiferrobacterales;f__Acidiferrobacteraceae;g__Acidiferrobacter;s__
LMSG_G000010467.1	no	1547_1	Antimony	80.85	0.61	77.81	0	1	0	10	Medium quality	5058	1752551	63.30	398	d__Bacteria;p__Proteobacteria;c__Gammaproteobacteria;o__Acidiferrobacterales;f__Acidiferrobacteraceae;g__Acidiferrobacter;s__
LMSG_G000010468.1	no	1547_1	Antimony	83.45	3.66	65.16	1	1	1	15	Medium quality	4287	1995830	63.10	554	d__Bacteria;p__Proteobacteria;c__Gammaproteobacteria;o__Acidiferrobacterales;f__Acidiferrobacteraceae;g__Acidiferrobacter;s__
LMSG_G000010469.1	no	1547_1	Tin-Zinc	67.37	1.22	61.28	0	0	0	10	Medium quality	12652	1343282	64.00	173	d__Bacteria;p__Proteobacteria;c__Gammaproteobacteria;o__Acidiferrobacterales;f__Acidiferrobacteraceae;g__Acidiferrobacter;s__
LMSG_G000010470.1	no	1547_1	Tin-Zinc	56.89	0.00	56.89	1	0	0	9	Medium quality	10673	1764608	63.80	254	d__Bacteria;p__Proteobacteria;c__Gammaproteobacteria;o__Acidiferrobacterales;f__Acidiferrobacteraceae;g__Acidiferrobacter;s__
LMSG_G000010471.1	no	1547_1	Magnetite	95.73	4.57	72.87	0	0	0	17	Medium quality	29545	2318355	62.90	109	d__Bacteria;p__Proteobacteria;c__Gammaproteobacteria;o__Acidiferrobacterales;f__Acidiferrobacteraceae;g__Acidiferrobacter;s__
LMSG_G000010472.1	no	1547_1	Lead-Zinc	97.56	1.22	91.47	0	0	0	17	Medium quality	30906	2496410	62.70	157	d__Bacteria;p__Proteobacteria;c__Gammaproteobacteria;o__Acidiferrobacterales;f__Acidiferrobacteraceae;g__Acidiferrobacter;s__
LMSG_G000010473.1	no	1547_1	Pyrite-Copper	84.44	2.13	73.77	0	0	0	15	Medium quality	27458	2210979	62.90	120	d__Bacteria;p__Proteobacteria;c__Gammaproteobacteria;o__Acidiferrobacterales;f__Acidiferrobacteraceae;g__Acidiferrobacter;s__
LMSG_G000010474.1	no	1547_1	Pyrite-Copper	67.33	2.50	54.83	0	1	0	13	Medium quality	20539	1846924	62.90	158	d__Bacteria;p__Proteobacteria;c__Gammaproteobacteria;o__Acidiferrobacterales;f__Acidiferrobacteraceae;g__Acidiferrobacter;s__
LMSG_G000010475.1	no	1547_1	Pyrite-Copper	72.41	0.00	72.41	0	1	0	13	Medium quality	24498	1677800	63.00	102	d__Bacteria;p__Proteobacteria;c__Gammaproteobacteria;o__Acidiferrobacterales;f__Acidiferrobacteraceae;g__Acidiferrobacter;s__
LMSG_G000010476.1	no	1547_1	Lead-Zinc	75.20	4.57	52.34	1	1	1	17	Medium quality	34022	2233262	62.50	111	d__Bacteria;p__Proteobacteria;c__Gammaproteobacteria;o__Acidiferrobacterales;f__Acidiferrobacteraceae;g__Acidiferrobacter;s__
LMSG_G000010477.1	no	1547_1	Magnetite	67.61	2.03	57.45	1	0	3	17	Medium quality	14236	1818814	62.40	239	d__Bacteria;p__Proteobacteria;c__Gammaproteobacteria;o__Acidiferrobacterales;f__Acidiferrobacteraceae;g__Acidiferrobacter;s__
LMSG_G000010478.1	no	1547_1	Magnetite	77.58	0.00	77.58	0	1	0	14	Medium quality	31177	2025609	63.10	118	d__Bacteria;p__Proteobacteria;c__Gammaproteobacteria;o__Acidiferrobacterales;f__Acidiferrobacteraceae;g__Acidiferrobacter;s__
LMSG_G000010479.1	no	1547_1	Pyrite	63.10	0.91	58.53	1	0	0	7	Medium quality	19863	1382023	63.60	108	d__Bacteria;p__Proteobacteria;c__Gammaproteobacteria;o__Acidiferrobacterales;f__Acidiferrobacteraceae;g__Acidiferrobacter;s__
LMSG_G000010480.1	no	1547_1	Copper	90.83	2.95	76.10	0	0	0	14	Medium quality	20802	2195901	63.20	149	d__Bacteria;p__Proteobacteria;c__Gammaproteobacteria;o__Acidiferrobacterales;f__Acidiferrobacteraceae;g__Acidiferrobacter;s__
LMSG_G000010481.1	no	1547_1	Lead-Zinc	91.15	3.11	75.62	1	1	2	14	Medium quality	9749	2306541	62.70	379	d__Bacteria;p__Proteobacteria;c__Gammaproteobacteria;o__Acidiferrobacterales;f__Acidiferrobacteraceae;g__Acidiferrobacter;s__
LMSG_G000010482.1	no	1547_1	Copper	89.39	2.48	77.00	1	0	1	14	Medium quality	7564	2109318	62.80	362	d__Bacteria;p__Proteobacteria;c__Gammaproteobacteria;o__Acidiferrobacterales;f__Acidiferrobacteraceae;g__Acidiferrobacter;s__
LMSG_G000010483.1	no	1548_1	Lead-Zinc	62.22	0.00	62.22	1	1	0	15	Medium quality	15056	1285303	64.70	129	d__Bacteria;p__Proteobacteria;c__Gammaproteobacteria;o__Acidiferrobacterales;f__Acidiferrobacteraceae;g__Acidiferrobacter;s__
LMSG_G000010484.1	no	1548_1	Lead-Zinc	52.75	0.00	52.75	1	1	0	15	Medium quality	58136	1843599	64.20	214	d__Bacteria;p__Proteobacteria;c__Gammaproteobacteria;o__Acidiferrobacterales;f__Acidiferrobacteraceae;g__Acidiferrobacter;s__
LMSG_G000010485.1	no	1548_1	Lead-Zinc	53.44	0.00	53.44	0	1	0	14	Medium quality	49821	1916109	64.50	75	d__Bacteria;p__Proteobacteria;c__Gammaproteobacteria;o__Acidiferrobacterales;f__Acidiferrobacteraceae;g__Acidiferrobacter;s__
LMSG_G000010486.1	no	1548_1	Copper	62.26	0.51	59.72	1	1	0	15	Medium quality	9813	1775828	64.50	234	d__Bacteria;p__Proteobacteria;c__Gammaproteobacteria;o__Acidiferrobacterales;f__Acidiferrobacteraceae;g__Acidiferrobacter;s__
LMSG_G000010487.1	no	1548_1	Copper	91.95	3.20	75.95	1	1	0	17	Medium quality	39260	2666457	64.20	122	d__Bacteria;p__Proteobacteria;c__Gammaproteobacteria;o__Acidiferrobacterales;f__Acidiferrobacteraceae;g__Acidiferrobacter;s__
LMSG_G000010488.1	no	1548_1	Pyrite	76.74	4.27	55.40	0	2	1	16	Medium quality	21393	1826784	64.50	150	d__Bacteria;p__Proteobacteria;c__Gammaproteobacteria;o__Acidiferrobacterales;f__Acidiferrobacteraceae;g__Acidiferrobacter;s__
LMSG_G000010489.1	no	1549_1	Polymetallic	67.28	2.44	55.09	1	0	0	15	Medium quality	10544	2043574	64.80	256	d__Bacteria;p__Proteobacteria;c__Gammaproteobacteria;o__Acidiferrobacterales;f__Acidiferrobacteraceae;g__Acidiferrobacter;s__Acidiferrobacter sp003184265
LMSG_G000010490.1	no	1549_1	Copper	78.96	2.13	68.29	2	0	6	16	Medium quality	38241	2185566	64.30	239	d__Bacteria;p__Proteobacteria;c__Gammaproteobacteria;o__Acidiferrobacterales;f__Acidiferrobacteraceae;g__Acidiferrobacter;s__Acidiferrobacter sp003184265
LMSG_G000010491.1	no	1549_1	Polymetallic	61.28	1.22	55.19	0	0	0	13	Medium quality	23782	1637177	65.20	101	d__Bacteria;p__Proteobacteria;c__Gammaproteobacteria;o__Acidiferrobacterales;f__Acidiferrobacteraceae;g__Acidiferrobacter;s__Acidiferrobacter sp003184265
LMSG_G000010492.1	no	1549_1	Copper	58.93	0.00	58.93	1	0	0	14	Medium quality	9022	2298500	64.70	349	d__Bacteria;p__Proteobacteria;c__Gammaproteobacteria;o__Acidiferrobacterales;f__Acidiferrobacteraceae;g__Acidiferrobacter;s__Acidiferrobacter sp003184265
LMSG_G000010493.1	no	1549_1	Lead-Zinc	65.14	2.13	54.47	1	0	0	12	Medium quality	14860	1930181	64.40	165	d__Bacteria;p__Proteobacteria;c__Gammaproteobacteria;o__Acidiferrobacterales;f__Acidiferrobacteraceae;g__Acidiferrobacter;s__Acidiferrobacter sp003184265
LMSG_G000010494.1	no	1549_2	Copper	80.64	4.67	57.27	0	0	0	14	Medium quality	14091	2290092	64.40	206	d__Bacteria;p__Proteobacteria;c__Gammaproteobacteria;o__Acidiferrobacterales;f__Acidiferrobacteraceae;g__Acidiferrobacter;s__Acidiferrobacter thiooxydans
LMSG_G000010495.1	no	1549_2	Copper	66.07	2.13	55.40	0	0	0	14	Medium quality	11142	1905133	64.50	236	d__Bacteria;p__Proteobacteria;c__Gammaproteobacteria;o__Acidiferrobacterales;f__Acidiferrobacteraceae;g__Acidiferrobacter;s__Acidiferrobacter thiooxydans
LMSG_G000010496.1	no	1549_2	Lead-Zinc	89.39	3.60	71.41	1	1	1	15	Medium quality	17914	2367237	64.50	273	d__Bacteria;p__Proteobacteria;c__Gammaproteobacteria;o__Acidiferrobacterales;f__Acidiferrobacteraceae;g__Acidiferrobacter;s__Acidiferrobacter thiooxydans
LMSG_G000010497.1	no	1549_2	Lead-Zinc	64.22	1.83	55.08	0	0	0	14	Medium quality	14697	1841283	64.20	301	d__Bacteria;p__Proteobacteria;c__Gammaproteobacteria;o__Acidiferrobacterales;f__Acidiferrobacteraceae;g__Acidiferrobacter;s__Acidiferrobacter thiooxydans
LMSG_G000010498.1	no	1549_2	Lead-Zinc	54.64	0.61	51.60	1	0	0	12	Medium quality	17296	1476263	64.40	116	d__Bacteria;p__Proteobacteria;c__Gammaproteobacteria;o__Acidiferrobacterales;f__Acidiferrobacteraceae;g__Acidiferrobacter;s__Acidiferrobacter thiooxydans
LMSG_G000010499.1	no	1549_2	Polymetallic	55.40	0.00	55.40	1	0	0	15	Medium quality	6887	1997391	63.90	324	d__Bacteria;p__Proteobacteria;c__Gammaproteobacteria;o__Acidiferrobacterales;f__Acidiferrobacteraceae;g__Acidiferrobacter;s__Acidiferrobacter thiooxydans
LMSG_G000010500.1	no	1549_2	Polymetallic	75.47	2.44	63.28	0	1	0	16	Medium quality	8567	2290858	63.90	352	d__Bacteria;p__Proteobacteria;c__Gammaproteobacteria;o__Acidiferrobacterales;f__Acidiferrobacteraceae;g__Acidiferrobacter;s__Acidiferrobacter thiooxydans
LMSG_G000010501.1	no	1549_2	Magnetite	80.97	1.98	71.07	0	0	0	15	Medium quality	8621	2173769	64.30	296	d__Bacteria;p__Proteobacteria;c__Gammaproteobacteria;o__Acidiferrobacterales;f__Acidiferrobacteraceae;g__Acidiferrobacter;s__Acidiferrobacter thiooxydans

LMSG_G000010502.1	no	1549_2	Pyrite-Copper	54.82	0.86	50.51	0	0	0	16	Medium quality	32857	1650624	63.60	128	d__Bacteria;p__Proteobacteria;c__Gammaproteobacteria;o__Acidiferrobacterales;f__Acidiferrobacteraceae;g__Acidiferrobacter;s__Acidiferrobacter thiooxydans
LMSG_G000010503.1	no	1549_2	Polymetallic	62.43	0.61	59.39	0	1	0	18	Medium quality	15171	2425761	63.30	244	d__Bacteria;p__Proteobacteria;c__Gammaproteobacteria;o__Acidiferrobacterales;f__Acidiferrobacteraceae;g__Acidiferrobacter;s__Acidiferrobacter thiooxydans
LMSG_G000010504.1	no	1549_2	Pyrite	69.49	3.46	52.22	0	0	0	14	Medium quality	7773	1777606	64.40	256	d__Bacteria;p__Proteobacteria;c__Gammaproteobacteria;o__Acidiferrobacterales;f__Acidiferrobacteraceae;g__Acidiferrobacter;s__Acidiferrobacter thiooxydans
LMSG_G000010505.1	no	1549_2	Lead-Zinc	69.10	3.45	51.86	0	0	0	14	Medium quality	5451	2379054	64.60	529	d__Bacteria;p__Proteobacteria;c__Gammaproteobacteria;o__Acidiferrobacterales;f__Acidiferrobacteraceae;g__Acidiferrobacter;s__Acidiferrobacter thiooxydans
LMSG_G000010506.1	no	1549_2	Copper	67.21	0.00	67.21	0	1	0	12	Medium quality	7370	1714177	64.10	263	d__Bacteria;p__Proteobacteria;c__Gammaproteobacteria;o__Acidiferrobacterales;f__Acidiferrobacteraceae;g__Acidiferrobacter;s__Acidiferrobacter thiooxydans
LMSG_G000010507.1	no	1198_1	Copper	89.32	2.13	78.65	2	1	4	16	Medium quality	23979	2798632	59.50	230	d__Bacteria;p__Proteobacteria;c__Gammaproteobacteria;o__Acidiferrobacterales;f__Sulfurifustaceae;g__;s__
LMSG_G000010508.1	no	1199_1	Lead-Zinc	87.19	2.69	73.73	1	0	0	15	Medium quality	69813	2659341	61.70	173	d__Bacteria;p__Proteobacteria;c__Gammaproteobacteria;o__Acidiferrobacterales;f__Sulfurifustaceae;g__;s__
LMSG_G000010509.1	no	1199_1	Lead-Zinc	70.00	0.00	70.00	1	0	1	16	Medium quality	64262	2175751	62.40	220	d__Bacteria;p__Proteobacteria;c__Gammaproteobacteria;o__Acidiferrobacterales;f__Sulfurifustaceae;g__;s__
LMSG_G000010510.1	no	1199_2	Pyrite-Copper	52.75	0.00	52.75	0	0	0	15	Medium quality	20429	1526051	61.90	125	d__Bacteria;p__Proteobacteria;c__Gammaproteobacteria;o__Acidiferrobacterales;f__Sulfurifustaceae;g__;s__
LMSG_G000010511.1	no	1200_1	Pyrite-Copper	91.15	4.78	67.27	1	1	1	16	Medium quality	140470	2979941	61.10	92	d__Bacteria;p__Proteobacteria;c__Gammaproteobacteria;o__Acidiferrobacterales;f__Sulfurifustaceae;g__;s__
LMSG_G000010512.1	no	1201_1	Pyrite-Copper	94.82	3.82	75.70	1	0	1	14	Medium quality	10449	2196738	62.90	274	d__Bacteria;p__Proteobacteria;c__Gammaproteobacteria;o__Acidiferrobacterales;f__Sulfurifustaceae;g__;s__
LMSG_G000010513.1	no	1201_1	Pyrite-Copper	75.00	3.66	56.71	2	0	2	15	Medium quality	15471	1962279	63.00	178	d__Bacteria;p__Proteobacteria;c__Gammaproteobacteria;o__Acidiferrobacterales;f__Sulfurifustaceae;g__;s__
LMSG_G000010514.1	no	1201_1	Tin-Zinc	87.80	2.38	75.91	1	1	1	15	Medium quality	10455	2056212	63.00	265	d__Bacteria;p__Proteobacteria;c__Gammaproteobacteria;o__Acidiferrobacterales;f__Sulfurifustaceae;g__;s__
LMSG_G000010515.1	no	1201_1	Tin-Zinc	85.91	2.44	73.72	1	0	1	14	Medium quality	8946	1855816	63.00	248	d__Bacteria;p__Proteobacteria;c__Gammaproteobacteria;o__Acidiferrobacterales;f__Sulfurifustaceae;g__;s__
LMSG_G000010516.1	no	1201_1	Antimony	98.17	2.03	88.01	1	1	1	17	Medium quality	40581	2477424	62.70	111	d__Bacteria;p__Proteobacteria;c__Gammaproteobacteria;o__Acidiferrobacterales;f__Sulfurifustaceae;g__;s__
LMSG_G000010517.1	no	1201_1	Antimony	92.14	0.61	89.10	1	1	1	14	Medium quality	32552	2296210	62.90	141	d__Bacteria;p__Proteobacteria;c__Gammaproteobacteria;o__Acidiferrobacterales;f__Sulfurifustaceae;g__;s__
LMSG_G000010518.1	no	1201_1	Antimony	98.17	2.48	85.78	1	1	2	17	Medium quality	33645	2351363	62.90	111	d__Bacteria;p__Proteobacteria;c__Gammaproteobacteria;o__Acidiferrobacterales;f__Sulfurifustaceae;g__;s__
LMSG_G000010519.1	no	1201_1	Antimony	97.35	2.48	84.96	1	1	1	16	Medium quality	30136	2336967	62.80	117	d__Bacteria;p__Proteobacteria;c__Gammaproteobacteria;o__Acidiferrobacterales;f__Sulfurifustaceae;g__;s__
LMSG_G000010520.1	no	1201_1	Antimony	94.20	3.35	77.44	1	0	1	17	Medium quality	34726	2309310	62.90	113	d__Bacteria;p__Proteobacteria;c__Gammaproteobacteria;o__Acidiferrobacterales;f__Sulfurifustaceae;g__;s__
LMSG_G000010521.1	no	1201_1	Pyrite-Copper	95.96	1.83	86.82	0	1	0	15	Medium quality	20319	2144982	63.00	186	d__Bacteria;p__Proteobacteria;c__Gammaproteobacteria;o__Acidiferrobacterales;f__Sulfurifustaceae;g__;s__
LMSG_G000010522.1	no	978_1	Pyrite	86.41	3.11	70.89	0	1	0	13	Medium quality	7651	2269479	60.30	382	d__Bacteria;p__Proteobacteria;c__Gammaproteobacteria;o__Acidithiobacillales;f__Acidithiobacillaceae;g__Acidithiobacillus;s__
LMSG_G000010523.1	no	978_1	Pyrite-Copper	66.66	0.00	66.66	0	0	0	13	Medium quality	11754	1230322	60.60	113	d__Bacteria;p__Proteobacteria;c__Gammaproteobacteria;o__Acidithiobacillales;f__Acidithiobacillaceae;g__Acidithiobacillus;s__
LMSG_G000010524.1	no	978_1	Pyrite	76.50	2.21	65.46	2	0	0	19	Medium quality	6420	2712789	57.70	486	d__Bacteria;p__Proteobacteria;c__Gammaproteobacteria;o__Acidithiobacillales;f__Acidithiobacillaceae;g__Acidithiobacillus;s__
LMSG_G000010525.1	no	978_1	Lead-Zinc	62.45	0.00	62.45	1	0	1	12	Medium quality	5854	1388085	60.70	280	d__Bacteria;p__Proteobacteria;c__Gammaproteobacteria;o__Acidithiobacillales;f__Acidithiobacillaceae;g__Acidithiobacillus;s__
LMSG_G000010526.1	no	979_1	Antimony	98.66	3.52	81.07	1	1	1	18	High quality	39838	2038941	60.10	181	d__Bacteria;p__Proteobacteria;c__Gammaproteobacteria;o__Acidithiobacillales;f__Acidithiobacillaceae;g__Acidithiobacillus;s__
LMSG_G000010527.1	no	979_1	Antimony	78.96	4.35	57.23	0	0	0	18	Medium quality	13597	1603865	60.00	148	d__Bacteria;p__Proteobacteria;c__Gammaproteobacteria;o__Acidithiobacillales;f__Acidithiobacillaceae;g__Acidithiobacillus;s__
LMSG_G000010528.1	no	979_1	Antimony	98.94	4.87	74.62	2	1	0	16	Medium quality	16065	2086976	60.00	192	d__Bacteria;p__Proteobacteria;c__Gammaproteobacteria;o__Acidithiobacillales;f__Acidithiobacillaceae;g__Acidithiobacillus;s__
LMSG_G000010529.1	no	980_1	Copper	76.92	0.62	73.82	0	2	0	15	Medium quality	36998	2206488	57.70	117	d__Bacteria;p__Proteobacteria;c__Gammaproteobacteria;o__Acidithiobacillales;f__Acidithiobacillaceae;g__Acidithiobacillus;s__
LMSG_G000010530.1	no	981_1	Lead-Zinc	57.89	0.00	57.89	1	1	1	10	Medium quality	15506	1071405	58.80	105	d__Bacteria;p__Proteobacteria;c__Gammaproteobacteria;o__Acidithiobacillales;f__Acidithiobacillaceae;g__Acidithiobacillus;s__
LMSG_G000010531.1	no	981_1	Polymetallic	65.78	0.00	65.78	1	0	1	17	Medium quality	19569	1686624	58.10	164	d__Bacteria;p__Proteobacteria;c__Gammaproteobacteria;o__Acidithiobacillales;f__Acidithiobacillaceae;g__Acidithiobacillus;s__
LMSG_G000010532.1	no	981_1	Polymetallic	72.91	2.17	62.05	2	2	0	17	Medium quality	10190	1682607	57.90	183	d__Bacteria;p__Proteobacteria;c__Gammaproteobacteria;o__Acidithiobacillales;f__Acidithiobacillaceae;g__Acidithiobacillus;s__
LMSG_G000010533.1	no	981_1	Lead-Zinc	61.40	0.00	61.40	1	1	1	14	Medium quality	15917	1513328	58.40	207	d__Bacteria;p__Proteobacteria;c__Gammaproteobacteria;o__Acidithiobacillales;f__Acidithiobacillaceae;g__Acidithiobacillus;s__
LMSG_G000010534.1	no	981_1	Pyrite	54.38	0.00	54.38	0	0	0	16	Medium quality	11861	1344503	58.00	145	d__Bacteria;p__Proteobacteria;c__Gammaproteobacteria;o__Acidithiobacillales;f__Acidithiobacillaceae;g__Acidithiobacillus;s__
LMSG_G000010535.1	no	981_1	Pyrite	52.63	0.00	52.63	2	0	2	17	Medium quality	5820	2019495	57.80	538	d__Bacteria;p__Proteobacteria;c__Gammaproteobacteria;o__Acidithiobacillales;f__Acidithiobacillaceae;g__Acidithiobacillus;s__
LMSG_G000010536.1	no	981_1	Polymetallic	59.64	0.00	59.64	2	0	2	15	Medium quality	13978	1195649	59.50	181	d__Bacteria;p__Proteobacteria;c__Gammaproteobacteria;o__Acidithiobacillales;f__Acidithiobacillaceae;g__Acidithiobacillus;s__
LMSG_G000010537.1	no	982_1	Copper	68.42	3.51	50.88	0	0	0	14	Medium quality	6822	2116198	59.40	541	d__Bacteria;p__Proteobacteria;c__Gammaproteobacteria;o__Acidithiobacillales;f__Acidithiobacillaceae;g__Acidithiobacillus;s__
LMSG_G000010538.1	no	982_1	Polymetallic	73.68	0.00	73.68	0	1	0	14	Medium quality	18570	1637663	57.90	111	d__Bacteria;p__Proteobacteria;c__Gammaproteobacteria;o__Acidithiobacillales;f__Acidithiobacillaceae;g__Acidithiobacillus;s__
LMSG_G000010539.1	no	979_2	Lead-Zinc	82.80	0.35	81.05	1	3	2	15	Medium quality	17512	2227019	59.40	252	d__Bacteria;p__Proteobacteria;c__Gammaproteobacteria;o__Acidithiobacillales;f__Acidithiobacillaceae;g__Acidithiobacillus;s__Acidithiobacillus ferridurans
LMSG_G000010540.1	no	979_2	Copper	94.98	0.00	94.98	1	0	0	17	Medium quality	30380	2138155	59.50	97	d__Bacteria;p__Proteobacteria;c__Gammaproteobacteria;o__Acidithiobacillales;f__Acidithiobacillaceae;g__Acidithiobacillus;s__Acidithiobacillus ferrooxidans
LMSG_G000010541.1	no	979_2	Pyrite	98.09	1.24	91.88	0	0	0	18	Medium quality	73286	2596369	59.10	58	d__Bacteria;p__Proteobacteria;c__Gammaproteobacteria;o__Acidithiobacillales;f__Acidithiobacillaceae;g__Acidithiobacillus;s__Acidithiobacillus ferrooxidans
LMSG_G000010542.1	no	979_2	Pyrite	98.09	1.00	93.09	0	0	1	19	Medium quality	105745	3160419	57.30	427	d__Bacteria;p__Proteobacteria;c__Gammaproteobacteria;o__Acidithiobacillales;f__Acidithiobacillaceae;g__Acidithiobacillus;s__Acidithiobacillus ferrooxidans
LMSG_G000010543.1	no	979_2	Pyrite	73.87	4.04	53.69	0	0	0	18	Medium quality	49747	1615449	59.90	50	d__Bacteria;p__Proteobacteria;c__Gammaproteobacteria;o__Acidithiobacillales;f__Acidithiobacillaceae;g__Acidithiobacillus;s__Acidithiobacillus ferrooxidans
LMSG_G000010544.1	no	979_2	Lead-Zinc	75.97	2.69	62.52	2	1	0	14	Medium quality	22444	1930834	59.20	138	d__Bacteria;p__Proteobacteria;c__Gammaproteobacteria;o__Acidithiobacillales;f__Acidithiobacillaceae;g__Acidithiobacillus;s__Acidithiobacillus ferrooxidans
LMSG_G000010545.1	no	979_2	Copper	88.15	1.86	78.84	2	2	0	19	Medium quality	40877	2862240	58.70	128	d__Bacteria;p__Proteobacteria;c__Gammaproteobacteria;o__Acidithiobacillales;f__Acidithiobacillaceae;g__Acidithiobacillus;s__Acidithiobacillus ferrooxidans
LMSG_G000010546.1	no	979_2	Copper	81.70	0.62	78.60	1	1	0	17	Medium quality	41992	2522034	58.30	128	d__Bacteria;p__Proteobacteria;c__Gammaproteobacteria;o__Acidithiobacillales;f__Acidithiobacillaceae;g__Acidithiobacillus;s__Acidithiobacillus ferrooxidans
LMSG_G000010547.1	no	979_2	Copper	80.14	4.66	56.85	1	1	2	14	Medium quality	20736	1420241	60.90	145	d__Bacteria;p__Proteobacteria;c__Gammaproteobacteria;o__Acidithiobacillales;f__Acidithiobacillaceae;g__Acidithiobacillus;s__Acidithiobacillus ferrooxidans

LMSG_G000010548.1	no	979_2	Pyrite	64.91	1.75	56.14	1	1	0	13	Medium quality	13408	1342455	60.70	124	d__Bacteria;p__Proteobacteria;c__Gammaproteobacteria;o__Acidithiobacillales;f__Acidithiobacillaceae;g__Acidithiobacillus;s__Acidithiobacillus ferrooxidans
LMSG_G000010549.1	no	979_2	Lead-Zinc	60.61	1.09	55.18	0	0	0	14	Medium quality	7266	1378438	60.30	282	d__Bacteria;p__Proteobacteria;c__Gammaproteobacteria;o__Acidithiobacillales;f__Acidithiobacillaceae;g__Acidithiobacillus;s__Acidithiobacillus ferrooxidans
LMSG_G000010550.1	no	979_2	Lead-Zinc	98.61	0.62	95.51	0	0	0	20	Medium quality	44996	3762541	58.00	259	d__Bacteria;p__Proteobacteria;c__Gammaproteobacteria;o__Acidithiobacillales;f__Acidithiobacillaceae;g__Acidithiobacillus;s__Acidithiobacillus ferrooxidans
LMSG_G000010551.1	no	980_1	Polymetallic	97.97	1.24	91.76	2	1	2	19	High quality	49853	2924303	57.30	200	d__Bacteria;p__Proteobacteria;c__Gammaproteobacteria;o__Acidithiobacillales;f__Acidithiobacillaceae;g__Acidithiobacillus;s__Acidithiobacillus ferrooxidans_A
LMSG_G000010552.1	no	980_1	Polymetallic	79.38	1.24	73.17	1	1	1	16	Medium quality	40627	2086303	57.20	157	d__Bacteria;p__Proteobacteria;c__Gammaproteobacteria;o__Acidithiobacillales;f__Acidithiobacillaceae;g__Acidithiobacillus;s__Acidithiobacillus ferrooxidans_A
LMSG_G000010553.1	no	980_1	Polymetallic	65.26	1.75	56.49	1	1	1	13	Medium quality	44086	1812289	58.60	243	d__Bacteria;p__Proteobacteria;c__Gammaproteobacteria;o__Acidithiobacillales;f__Acidithiobacillaceae;g__Acidithiobacillus;s__Acidithiobacillus ferrooxidans_A
LMSG_G000010554.1	no	980_1	Polymetallic	89.64	1.55	81.88	1	5	1	18	Medium quality	69369	2715203	56.90	215	d__Bacteria;p__Proteobacteria;c__Gammaproteobacteria;o__Acidithiobacillales;f__Acidithiobacillaceae;g__Acidithiobacillus;s__Acidithiobacillus ferrooxidans_A
LMSG_G000010555.1	no	980_1	Pyrite-Copper	61.85	1.19	55.90	1	1	1	13	Medium quality	16600	1133912	57.20	129	d__Bacteria;p__Proteobacteria;c__Gammaproteobacteria;o__Acidithiobacillales;f__Acidithiobacillaceae;g__Acidithiobacillus;s__Acidithiobacillus ferrooxidans_A
LMSG_G000010556.1	no	980_1	Lead-Zinc	73.14	2.86	58.82	0	0	0	12	Medium quality	18639	1688889	57.60	131	d__Bacteria;p__Proteobacteria;c__Gammaproteobacteria;o__Acidithiobacillales;f__Acidithiobacillaceae;g__Acidithiobacillus;s__Acidithiobacillus ferrooxidans_A
LMSG_G000010557.1	no	980_1	Lead-Zinc	57.89	0.00	57.89	1	1	0	15	Medium quality	10892	1361282	58.10	350	d__Bacteria;p__Proteobacteria;c__Gammaproteobacteria;o__Acidithiobacillales;f__Acidithiobacillaceae;g__Acidithiobacillus;s__Acidithiobacillus ferrooxidans_A
LMSG_G000010558.1	no	980_1	Pyrite	73.97	2.90	59.48	1	1	0	14	Medium quality	23618	1548573	57.90	166	d__Bacteria;p__Proteobacteria;c__Gammaproteobacteria;o__Acidithiobacillales;f__Acidithiobacillaceae;g__Acidithiobacillus;s__Acidithiobacillus ferrooxidans_A
LMSG_G000010559.1	no	980_1	Copper	74.22	0.93	69.57	0	1	0	17	Medium quality	25274	1946530	57.30	117	d__Bacteria;p__Proteobacteria;c__Gammaproteobacteria;o__Acidithiobacillales;f__Acidithiobacillaceae;g__Acidithiobacillus;s__Acidithiobacillus ferrooxidans_A
LMSG_G000010560.1	no	980_1	Coal	99.33	1.86	90.02	3	4	4	20	High quality	76788	2928738	57.20	116	d__Bacteria;p__Proteobacteria;c__Gammaproteobacteria;o__Acidithiobacillales;f__Acidithiobacillaceae;g__Acidithiobacillus;s__Acidithiobacillus ferrooxidans_A
LMSG_G000010561.1	no	980_1	Copper	64.91	0.00	64.91	0	1	0	16	Medium quality	19769	1688087	57.50	110	d__Bacteria;p__Proteobacteria;c__Gammaproteobacteria;o__Acidithiobacillales;f__Acidithiobacillaceae;g__Acidithiobacillus;s__Acidithiobacillus ferrooxidans_A
LMSG_G000010562.1	no	980_1	Coal	81.15	0.62	78.05	1	1	1	15	Medium quality	11118	1693386	57.50	214	d__Bacteria;p__Proteobacteria;c__Gammaproteobacteria;o__Acidithiobacillales;f__Acidithiobacillaceae;g__Acidithiobacillus;s__Acidithiobacillus ferrooxidans_A
LMSG_G000010563.1	no	980_1	Copper	75.80	3.02	60.69	1	0	1	17	Medium quality	17403	1471715	57.80	110	d__Bacteria;p__Proteobacteria;c__Gammaproteobacteria;o__Acidithiobacillales;f__Acidithiobacillaceae;g__Acidithiobacillus;s__Acidithiobacillus ferrooxidans_A
LMSG_G000010564.1	no	980_1	Pyrite	66.87	0.21	65.84	0	0	0	18	Medium quality	13797	1219483	58.00	126	d__Bacteria;p__Proteobacteria;c__Gammaproteobacteria;o__Acidithiobacillales;f__Acidithiobacillaceae;g__Acidithiobacillus;s__Acidithiobacillus ferrooxidans_A
LMSG_G000010565.1	no	980_1	Pyrite	70.48	2.06	60.18	1	1	0	19	Medium quality	15502	1901708	57.20	334	d__Bacteria;p__Proteobacteria;c__Gammaproteobacteria;o__Acidithiobacillales;f__Acidithiobacillaceae;g__Acidithiobacillus;s__Acidithiobacillus ferrooxidans_A
LMSG_G000010566.1	no	980_1	Polymetallic	91.64	1.86	82.33	0	1	0	16	Medium quality	20343	1823435	57.70	129	d__Bacteria;p__Proteobacteria;c__Gammaproteobacteria;o__Acidithiobacillales;f__Acidithiobacillaceae;g__Acidithiobacillus;s__Acidithiobacillus ferrooxidans_A
LMSG_G000010567.1	no	980_1	Magnetite	83.78	0.00	83.78	0	0	0	16	Medium quality	50784	1801087	57.50	60	d__Bacteria;p__Proteobacteria;c__Gammaproteobacteria;o__Acidithiobacillales;f__Acidithiobacillaceae;g__Acidithiobacillus;s__Acidithiobacillus ferrooxidans_A
LMSG_G000010568.1	no	980_1	Magnetite	52.63	0.00	52.63	1	1	0	17	Medium quality	90064	1742200	56.60	48	d__Bacteria;p__Proteobacteria;c__Gammaproteobacteria;o__Acidithiobacillales;f__Acidithiobacillaceae;g__Acidithiobacillus;s__Acidithiobacillus ferrooxidans_A
LMSG_G000010569.1	no	980_1	Lead-Zinc	71.95	3.47	54.62	0	2	1	18	Medium quality	11835	1666880	58.10	248	d__Bacteria;p__Proteobacteria;c__Gammaproteobacteria;o__Acidithiobacillales;f__Acidithiobacillaceae;g__Acidithiobacillus;s__Acidithiobacillus ferrooxidans_A
LMSG_G000010570.1	no	980_1	Lead-Zinc	54.03	0.00	54.03	2	1	8	18	Medium quality	15475	1045212	57.30	230	d__Bacteria;p__Proteobacteria;c__Gammaproteobacteria;o__Acidithiobacillales;f__Acidithiobacillaceae;g__Acidithiobacillus;s__Acidithiobacillus ferrooxidans_A
LMSG_G000010571.1	no	980_1	Copper	71.38	0.00	71.38	1	1	1	13	Medium quality	28316	1291610	58.10	137	d__Bacteria;p__Proteobacteria;c__Gammaproteobacteria;o__Acidithiobacillales;f__Acidithiobacillaceae;g__Acidithiobacillus;s__Acidithiobacillus ferrooxidans_A
LMSG_G000010572.1	no	980_1	Copper	59.82	0.62	56.72	0	1	0	10	Medium quality	22070	1235382	57.50	79	d__Bacteria;p__Proteobacteria;c__Gammaproteobacteria;o__Acidithiobacillales;f__Acidithiobacillaceae;g__Acidithiobacillus;s__Acidithiobacillus ferrooxidans_A
LMSG_G000010573.1	no	980_1	Copper	57.72	0.00	57.72	1	1	1	15	Medium quality	22997	1035733	57.90	77	d__Bacteria;p__Proteobacteria;c__Gammaproteobacteria;o__Acidithiobacillales;f__Acidithiobacillaceae;g__Acidithiobacillus;s__Acidithiobacillus ferrooxidans_A
LMSG_G000010574.1	no	980_1	Pyrite	84.43	2.90	69.94	0	0	0	16	Medium quality	14207	1825816	57.60	226	d__Bacteria;p__Proteobacteria;c__Gammaproteobacteria;o__Acidithiobacillales;f__Acidithiobacillaceae;g__Acidithiobacillus;s__Acidithiobacillus ferrooxidans_A
LMSG_G000010575.1	no	976_1	Lead-Zinc	81.79	3.62	63.70	2	3	2	20	Medium quality	3897	3285022	52.90	1106	d__Bacteria;p__Proteobacteria;c__Gammaproteobacteria;o__Acidithiobacillales;f__Acidithiobacillaceae;g__Acidithiobacillus;s__Acidithiobacillus thiooxidans
LMSG_G000010576.1	no	976_1	Tin-Zinc	67.33	1.90	57.85	0	0	0	12	Medium quality	5654	1519211	53.90	312	d__Bacteria;p__Proteobacteria;c__Gammaproteobacteria;o__Acidithiobacillales;f__Acidithiobacillaceae;g__Acidithiobacillus;s__Acidithiobacillus thiooxidans
LMSG_G000010577.1	no	976_1	Polymetallic	78.84	1.24	72.63	2	1	0	15	Medium quality	35156	1833242	53.60	85	d__Bacteria;p__Proteobacteria;c__Gammaproteobacteria;o__Acidithiobacillales;f__Acidithiobacillaceae;g__Acidithiobacillus;s__Acidithiobacillus thiooxidans
LMSG_G000010578.1	no	976_1	Copper	90.02	0.62	86.92	2	0	0	17	Medium quality	50077	2221770	53.20	65	d__Bacteria;p__Proteobacteria;c__Gammaproteobacteria;o__Acidithiobacillales;f__Acidithiobacillaceae;g__Acidithiobacillus;s__Acidithiobacillus thiooxidans
LMSG_G000010579.1	no	976_1	Polymetallic	94.36	1.86	85.05	2	2	0	18	Medium quality	40710	2757671	53.10	140	d__Bacteria;p__Proteobacteria;c__Gammaproteobacteria;o__Acidithiobacillales;f__Acidithiobacillaceae;g__Acidithiobacillus;s__Acidithiobacillus thiooxidans
LMSG_G000010580.1	no	973_1	Lead-Zinc	54.38	0.00	54.38	0	1	0	15	Medium quality	5065	1560698	58.90	354	d__Bacteria;p__Proteobacteria;c__Gammaproteobacteria;o__Acidithiobacillales;f__Acidithiobacillaceae;g__Acidithiobacillus_A;s__
LMSG_G000010581.1	no	973_1	Polymetallic	79.81	0.00	79.81	1	2	0	17	Medium quality	42085	1922816	58.60	90	d__Bacteria;p__Proteobacteria;c__Gammaproteobacteria;o__Acidithiobacillales;f__Acidithiobacillaceae;g__Acidithiobacillus_A;s__
LMSG_G000010582.1	no	973_1	Pyrite-Copper	63.15	0.00	63.15	0	1	0	15	Medium quality	4841	1693878	58.70	406	d__Bacteria;p__Proteobacteria;c__Gammaproteobacteria;o__Acidithiobacillales;f__Acidithiobacillaceae;g__Acidithiobacillus_A;s__
LMSG_G000010583.1	no	973_1	Lead-Zinc	78.26	1.14	72.57	0	0	0	16	Medium quality	10382	1480855	58.90	202	d__Bacteria;p__Proteobacteria;c__Gammaproteobacteria;o__Acidithiobacillales;f__Acidithiobacillaceae;g__Acidithiobacillus_A;s__
LMSG_G000010584.1	no	973_1	Lead-Zinc	83.12	3.93	63.46	1	0	0	16	Medium quality	10349	1970465	58.50	465	d__Bacteria;p__Proteobacteria;c__Gammaproteobacteria;o__Acidithiobacillales;f__Acidithiobacillaceae;g__Acidithiobacillus_A;s__
LMSG_G000010585.1	no	973_1	Pyrite	70.17	1.75	61.40	1	1	0	14	Medium quality	9913	1451339	58.90	174	d__Bacteria;p__Proteobacteria;c__Gammaproteobacteria;o__Acidithiobacillales;f__Acidithiobacillaceae;g__Acidithiobacillus_A;s__
LMSG_G000010586.1	no	973_1	Pyrite	83.66	1.75	74.89	0	1	0	16	Medium quality	13356	2082640	58.90	297	d__Bacteria;p__Proteobacteria;c__Gammaproteobacteria;o__Acidithiobacillales;f__Acidithiobacillaceae;g__Acidithiobacillus_A;s__
LMSG_G000010587.1	no	973_1	Pyrite	81.60	4.09	61.16	2	1	0	15	Medium quality	6004	1751476	58.70	353	d__Bacteria;p__Proteobacteria;c__Gammaproteobacteria;o__Acidithiobacillales;f__Acidithiobacillaceae;g__Acidithiobacillus_A;s__

LMSG_G000010588.1	no	973_1	Pyrite	61.40	1.75	52.63	0	0	0	9	Medium quality	5651	990600	59.70	203	d__Bacteria;p__Proteobacteria;c__Gammaproteobacteria;o__Acidithiobacillales;f__Acidithiobacillaceae;g__Acidithiobacillus_A;s__
LMSG_G000010589.1	no	973_1	Polymetallic	86.11	2.54	73.43	1	0	0	16	Medium quality	20669	1700217	59.00	183	d__Bacteria;p__Proteobacteria;c__Gammaproteobacteria;o__Acidithiobacillales;f__Acidithiobacillaceae;g__Acidithiobacillus_A;s__
LMSG_G000010590.1	no	974_1	Pyrite	90.06	0.78	86.18	1	5	3	19	High quality	8564	1709647	60.10	322	d__Bacteria;p__Proteobacteria;c__Gammaproteobacteria;o__Acidithiobacillales;f__Acidithiobacillaceae;g__Acidithiobacillus_A;s__
LMSG_G000010591.1	no	974_1	Pyrite	94.36	0.00	94.36	2	0	0	17	Medium quality	59120	1865652	60.00	45	d__Bacteria;p__Proteobacteria;c__Gammaproteobacteria;o__Acidithiobacillales;f__Acidithiobacillaceae;g__Acidithiobacillus_A;s__
LMSG_G000010592.1	no	974_1	Pyrite	95.61	0.00	95.61	1	0	1	16	Medium quality	88938	1995488	59.90	111	d__Bacteria;p__Proteobacteria;c__Gammaproteobacteria;o__Acidithiobacillales;f__Acidithiobacillaceae;g__Acidithiobacillus_A;s__
LMSG_G000010593.1	no	974_1	Pyrite	95.61	0.00	95.61	2	0	0	17	Medium quality	70416	1923977	60.00	44	d__Bacteria;p__Proteobacteria;c__Gammaproteobacteria;o__Acidithiobacillales;f__Acidithiobacillaceae;g__Acidithiobacillus_A;s__
LMSG_G000010594.1	no	974_1	Pyrite-Copper	84.48	3.21	68.44	1	0	0	15	Medium quality	8611	1700360	60.20	304	d__Bacteria;p__Proteobacteria;c__Gammaproteobacteria;o__Acidithiobacillales;f__Acidithiobacillaceae;g__Acidithiobacillus_A;s__
LMSG_G000010595.1	no	974_1	Pyrite-Copper	76.97	0.62	73.87	1	1	0	14	Medium quality	62357	1507515	58.80	33	d__Bacteria;p__Proteobacteria;c__Gammaproteobacteria;o__Acidithiobacillales;f__Acidithiobacillaceae;g__Acidithiobacillus_A;s__
LMSG_G000010596.1	no	955_1	Pyrite	72.36	0.36	70.55	0	1	0	10	Medium quality	5317	1246408	63.60	282	d__Bacteria;p__Proteobacteria;c__Gammaproteobacteria;o__Acidithiobacillales;f__Acidithiobacillaceae;g__Acidithiobacillus_A;s__Acidithiobacillus_A caldus
LMSG_G000010597.1	no	955_1	Pyrite	93.63	4.08	73.24	1	0	1	14	Medium quality	5836	1941365	63.40	401	d__Bacteria;p__Proteobacteria;c__Gammaproteobacteria;o__Acidithiobacillales;f__Acidithiobacillaceae;g__Acidithiobacillus_A;s__Acidithiobacillus_A caldus
LMSG_G000010598.1	no	955_1	Lead-Zinc	56.14	0.00	56.14	0	1	0	7	Medium quality	6483	1109227	64.00	198	d__Bacteria;p__Proteobacteria;c__Gammaproteobacteria;o__Acidithiobacillales;f__Acidithiobacillaceae;g__Acidithiobacillus_A;s__Acidithiobacillus_A caldus
LMSG_G000010599.1	no	955_1	Polymetallic	58.77	0.88	54.39	1	2	0	13	Medium quality	6972	1481922	63.00	235	d__Bacteria;p__Proteobacteria;c__Gammaproteobacteria;o__Acidithiobacillales;f__Acidithiobacillaceae;g__Acidithiobacillus_A;s__Acidithiobacillus_A caldus
LMSG_G000010600.1	no	977_1	Lead-Zinc	64.03	0.00	64.03	1	0	1	11	Medium quality	4355	1624638	63.40	425	d__Bacteria;p__Proteobacteria;c__Gammaproteobacteria;o__Acidithiobacillales;f__Acidithiobacillaceae;g__RIZI01;s__RIZI01 sp003721225
LMSG_G000010601.1	no	977_1	Lead-Zinc	78.24	1.75	69.47	1	1	1	17	Medium quality	17892	2289846	62.20	180	d__Bacteria;p__Proteobacteria;c__Gammaproteobacteria;o__Acidithiobacillales;f__Acidithiobacillaceae;g__RIZI01;s__RIZI01 sp003721225
LMSG_G000010602.1	no	977_1	Lead-Zinc	83.14	2.06	72.86	1	0	0	14	Medium quality	5942	1842056	62.80	373	d__Bacteria;p__Proteobacteria;c__Gammaproteobacteria;o__Acidithiobacillales;f__Acidithiobacillaceae;g__RIZI01;s__RIZI01 sp003721225
LMSG_G000010603.1	no	977_1	Pyrite-Copper	95.06	3.40	78.09	2	1	0	15	Medium quality	25786	1997035	62.90	102	d__Bacteria;p__Proteobacteria;c__Gammaproteobacteria;o__Acidithiobacillales;f__Acidithiobacillaceae;g__RIZI01;s__RIZI01 sp003721225
LMSG_G000010604.1	no	977_1	Pyrite-Copper	74.03	1.75	65.26	1	0	1	13	Medium quality	4628	1590210	63.60	397	d__Bacteria;p__Proteobacteria;c__Gammaproteobacteria;o__Acidithiobacillales;f__Acidithiobacillaceae;g__RIZI01;s__RIZI01 sp003721225
LMSG_G000010605.1	no	1706_1	Lead-Zinc	81.63	2.33	70.01	0	0	0	15	Medium quality	14885	1398555	41.20	152	d__Bacteria;p__Proteobacteria;c__Gammaproteobacteria;o__Berkiellales;f__;g__;s__
LMSG_G000010606.1	no	1218_1	Lead-Zinc	64.65	0.00	64.65	0	3	0	11	Medium quality	3496	1707435	57.20	522	d__Bacteria;p__Proteobacteria;c__Gammaproteobacteria;o__Burkholderiales;f__;g__;s__
LMSG_G000010607.1	no	1222_1	Lead-Zinc	78.75	4.23	57.63	0	0	0	15	Medium quality	36440	2538647	57.50	101	d__Bacteria;p__Proteobacteria;c__Gammaproteobacteria;o__Burkholderiales;f__;g__;s__
LMSG_G000010608.1	no	1243_1	Lead-Zinc	67.58	0.00	67.58	1	1	1	16	Medium quality	31297	2132209	58.30	116	d__Bacteria;p__Proteobacteria;c__Gammaproteobacteria;o__Burkholderiales;f__;g__;s__
LMSG_G000010609.1	no	1243_1	Lead-Zinc	87.56	2.07	77.21	1	1	0	17	Medium quality	24706	2270604	58.30	135	d__Bacteria;p__Proteobacteria;c__Gammaproteobacteria;o__Burkholderiales;f__;g__;s__
LMSG_G000010610.1	no	1402_1	Polymetallic	84.68	0.46	82.37	2	0	0	19	Medium quality	9143	5274580	68.10	694	d__Bacteria;p__Proteobacteria;c__Gammaproteobacteria;o__Burkholderiales;f__Burkholderiaceae;g__Achromobacter;s__Achromobacter sp001299435
LMSG_G000010611.1	no	1402_1	Polymetallic	96.27	0.50	93.79	3	0	0	19	Medium quality	31417	6539770	68.30	302	d__Bacteria;p__Proteobacteria;c__Gammaproteobacteria;o__Burkholderiales;f__Burkholderiaceae;g__Achromobacter;s__Achromobacter sp001299435
LMSG_G000010612.1	no	1402_1	Polymetallic	71.75	1.29	65.32	0	0	0	14	Medium quality	2917	5039321	68.50	1927	d__Bacteria;p__Proteobacteria;c__Gammaproteobacteria;o__Burkholderiales;f__Burkholderiaceae;g__Achromobacter;s__Achromobacter sp001299435
LMSG_G000010613.1	no	1402_1	Polymetallic	93.76	0.93	89.09	3	0	0	17	Medium quality	34951	6487797	68.20	263	d__Bacteria;p__Proteobacteria;c__Gammaproteobacteria;o__Burkholderiales;f__Burkholderiaceae;g__Achromobacter;s__Achromobacter sp001299435
LMSG_G000010614.1	no	1402_1	Polymetallic	98.27	1.09	92.82	4	0	1	18	Medium quality	179885	6625482	68.20	75	d__Bacteria;p__Proteobacteria;c__Gammaproteobacteria;o__Burkholderiales;f__Burkholderiaceae;g__Achromobacter;s__Achromobacter sp001299435
LMSG_G000010615.1	no	1283_1	Nickel-Copper	72.12	1.73	63.47	0	0	0	11	Medium quality	5589	2981610	64.20	679	d__Bacteria;p__Proteobacteria;c__Gammaproteobacteria;o__Burkholderiales;f__Burkholderiaceae;g__Acidovorax_B;s__Acidovorax_B defluvii
LMSG_G000010616.1	no	1283_1	Nickel-Copper	90.80	4.98	65.90	1	1	0	18	Medium quality	41240	3605410	63.90	128	d__Bacteria;p__Proteobacteria;c__Gammaproteobacteria;o__Burkholderiales;f__Burkholderiaceae;g__Acidovorax_B;s__Acidovorax_B defluvii
LMSG_G000010617.1	no	1268_1	Polymetallic	66.94	2.20	55.94	0	0	0	17	Medium quality	48467	1673344	65.60	69	d__Bacteria;p__Proteobacteria;c__Gammaproteobacteria;o__Burkholderiales;f__Burkholderiaceae;g__GJ-E10;s__
LMSG_G000010618.1	no	1269_1	Polymetallic	63.63	0.12	63.02	1	0	0	17	Medium quality	10398	1624652	66.90	202	d__Bacteria;p__Proteobacteria;c__Gammaproteobacteria;o__Burkholderiales;f__Burkholderiaceae;g__GJ-E10;s__
LMSG_G000010619.1	no	1269_1	Polymetallic	79.73	2.88	65.33	1	1	0	17	Medium quality	34151	2318345	66.40	96	d__Bacteria;p__Proteobacteria;c__Gammaproteobacteria;o__Burkholderiales;f__Burkholderiaceae;g__GJ-E10;s__
LMSG_G000010620.1	no	1269_2	Lead-Zinc	97.22	1.28	90.85	1	1	1	19	High quality	24567	2606984	66.90	154	d__Bacteria;p__Proteobacteria;c__Gammaproteobacteria;o__Burkholderiales;f__Burkholderiaceae;g__GJ-E10;s__
LMSG_G000010621.1	no	1269_2	Lead-Zinc	95.67	1.85	86.42	1	1	1	16	Medium quality	41831	2644397	67.20	112	d__Bacteria;p__Proteobacteria;c__Gammaproteobacteria;o__Burkholderiales;f__Burkholderiaceae;g__GJ-E10;s__
LMSG_G000010622.1	no	1269_2	Lead-Zinc	93.41	1.23	87.24	1	1	1	17	Medium quality	63820	2552497	67.10	75	d__Bacteria;p__Proteobacteria;c__Gammaproteobacteria;o__Burkholderiales;f__Burkholderiaceae;g__GJ-E10;s__
LMSG_G000010623.1	no	1269_2	Lead-Zinc	91.47	1.95	81.70	1	1	1	16	Medium quality	7546	2417520	67.20	394	d__Bacteria;p__Proteobacteria;c__Gammaproteobacteria;o__Burkholderiales;f__Burkholderiaceae;g__GJ-E10;s__
LMSG_G000010624.1	no	1269_2	Copper	87.00	0.07	86.66	0	0	0	18	Medium quality	8454	2272016	67.10	340	d__Bacteria;p__Proteobacteria;c__Gammaproteobacteria;o__Burkholderiales;f__Burkholderiaceae;g__GJ-E10;s__
LMSG_G000010625.1	no	1269_2	Copper	72.71	2.48	60.32	1	0	1	12	Medium quality	4127	1963575	67.60	541	d__Bacteria;p__Proteobacteria;c__Gammaproteobacteria;o__Burkholderiales;f__Burkholderiaceae;g__GJ-E10;s__
LMSG_G000010626.1	no	1269_2	Copper	73.51	1.23	67.34	0	0	0	14	Medium quality	3502	1954574	67.60	613	d__Bacteria;p__Proteobacteria;c__Gammaproteobacteria;o__Burkholderiales;f__Burkholderiaceae;g__GJ-E10;s__
LMSG_G000010627.1	no	1269_2	Antimony	80.39	4.63	57.25	1	0	1	16	Medium quality	10008	1918575	67.70	237	d__Bacteria;p__Proteobacteria;c__Gammaproteobacteria;o__Burkholderiales;f__Burkholderiaceae;g__GJ-E10;s__
LMSG_G000010628.1	no	1269_2	Antimony	89.40	1.39	82.46	1	1	1	17	Medium quality	31316	2422592	66.90	112	d__Bacteria;p__Proteobacteria;c__Gammaproteobacteria;o__Burkholderiales;f__Burkholderiaceae;g__GJ-E10;s__
LMSG_G000010629.1	no	1269_2	Antimony	74.79	0.00	74.79	1	1	1	14	Medium quality	31341	1967511	66.80	101	d__Bacteria;p__Proteobacteria;c__Gammaproteobacteria;o__Burkholderiales;f__Burkholderiaceae;g__GJ-E10;s__
LMSG_G000010630.1	no	1269_2	Antimony	63.33	0.00	63.33	0	1	1	15	Medium quality	25501	1405967	67.60	76	d__Bacteria;p__Proteobacteria;c__Gammaproteobacteria;o__Burkholderiales;f__Burkholderiaceae;g__GJ-E10;s__
LMSG_G000010631.1	no	1269_2	Lead-Zinc	72.55	1.72	63.93	0	1	0	16	Medium quality	12983	1996412	67.60	362	d__Bacteria;p__Proteobacteria;c__Gammaproteobacteria;o__Burkholderiales;f__Burkholderiaceae;g__GJ-E10;s__
LMSG_G000010632.1	no	1269_2	Pyrite-Copper	66.27	1.63	58.15	0	0	0	14	Medium quality	5105	1500145	67.70	407	d__Bacteria;p__Proteobacteria;c__Gammaproteobacteria;o__Burkholderiales;f__Burkholderiaceae;g__GJ-E10;s__
LMSG_G000010633.1	no	1286_1	Lead-Zinc	78.73	0.55	75.98	1	0	0	19	Medium quality	23424	2766243	66.00	225	d__Bacteria;p__Proteobacteria;c__Gammaproteobacteria;o__Burkholderiales;f__Burkholderiaceae;g__Hylemonella;s__
LMSG_G000010634.1	no	306_1	Nickel-Copper	86.32	1.30	79.84	0	0	0	17	Medium quality	60823	2066384	55.20	50	d__Bacteria;p__Proteobacteria;c__Gammaproteobacteria;o__Burkholderiales;f__Burkholderiaceae;g__Limnohabitans;s__Limnohabitans sp002282445
LMSG_G000010635.1	no	306_1	Nickel-Copper	94.62	2.88	80.25	0	0	0	18	Medium quality	131004	2326812	55.00	40	d__Bacteria;p__Proteobacteria;c__Gammaproteobacteria;o__Burkholderiales;f__Burkholderiaceae;g__Limnohabitans;s__Limnohabitans sp002282445
LMSG_G000010636.1	no	1280_1	Nickel-Copper	52.68	0.47	50.35	0	0	0	11	Medium quality	27204	2279233	63.40	142	d__Bacteria;p__Proteobacteria;c__Gammaproteobacteria;o__Burkholderiales;f__Burkholderiaceae;g__Polaromonas;s__
LMSG_G000010637.1	no	1280_1	Nickel-Copper	92.51	4.30	71.02	0	1	0	18	Medium quality	19026	3796569	62.60	273	d__Bacteria;p__Proteobacteria;c__Gammaproteobacteria;o__Burkholderiales;f__Burkholderiaceae;g__Polaromonas;s__Polaromonas sp002256925
LMSG_G000010638.1	no	1279_1	Nickel-Copper	57.85	0.00	57.85	0	1	0	12	Medium quality	9006	3091839	64.00	448	d__Bacteria;p__Proteobacteria;c__Gammaproteobacteria;o__Burkholderiales;f__Burkholderiaceae;g__Polaromonas;s__Polaromonas sp002278925

LMSG_G000010639.1	no	308_1	Nickel-Copper	67.49	1.52	59.92	0	0	0	11	Medium quality	17978	1420944	45.40	141	d__Bacteria;p__Proteobacteria;c__Gammaproteobacteria;o__Burkholderiales;f__Burkholderiaceae;g__Polynucleobacter;s__
LMSG_G000010640.1	no	308_1	Nickel-Copper	63.10	0.00	63.10	0	1	0	16	Medium quality	26277	1728882	46.00	186	d__Bacteria;p__Proteobacteria;c__Gammaproteobacteria;o__Burkholderiales;f__Burkholderiaceae;g__Polynucleobacter;s__
LMSG_G000010641.1	no	308_1	Nickel-Copper	75.86	0.86	71.55	1	3	2	15	Medium quality	15430	1765859	45.70	271	d__Bacteria;p__Proteobacteria;c__Gammaproteobacteria;o__Burkholderiales;f__Burkholderiaceae;g__Polynucleobacter;s__
LMSG_G000010642.1	no	308_1	Nickel-Copper	76.46	0.10	75.95	0	1	0	16	Medium quality	45585	1516535	45.60	87	d__Bacteria;p__Proteobacteria;c__Gammaproteobacteria;o__Burkholderiales;f__Burkholderiaceae;g__Polynucleobacter;s__
LMSG_G000010643.1	no	308_1	Nickel-Copper	59.76	0.96	54.98	0	0	0	10	Medium quality	2317	1087780	46.20	498	d__Bacteria;p__Proteobacteria;c__Gammaproteobacteria;o__Burkholderiales;f__Burkholderiaceae;g__Polynucleobacter;s__
LMSG_G000010644.1	no	307_1	Antimony	93.54	0.81	89.52	0	0	0	17	Medium quality	48563	1901821	46.10	101	d__Bacteria;p__Proteobacteria;c__Gammaproteobacteria;o__Burkholderiales;f__Burkholderiaceae;g__Polynucleobacter;s__Polynucleobacter sp000342265
LMSG_G000010645.1	no	1287_1	Lead-Zinc	88.10	4.00	68.11	0	0	0	15	Medium quality	28160	2103437	66.80	231	d__Bacteria;p__Proteobacteria;c__Gammaproteobacteria;o__Burkholderiales;f__Burkholderiaceae;g__Serpentinomonas;s__Serpentinomonas mccroryi
LMSG_G000010646.1	no	1287_1	Pyrite-Copper	90.91	3.14	75.21	0	0	0	15	Medium quality	3768	2132234	66.60	703	d__Bacteria;p__Proteobacteria;c__Gammaproteobacteria;o__Burkholderiales;f__Burkholderiaceae;g__Serpentinomonas;s__Serpentinomonas mccroryi
LMSG_G000010647.1	no	1287_1	Nickel-Copper	89.74	1.20	83.76	2	0	2	17	Medium quality	18091	2022659	66.90	194	d__Bacteria;p__Proteobacteria;c__Gammaproteobacteria;o__Burkholderiales;f__Burkholderiaceae;g__Serpentinomonas;s__Serpentinomonas mccroryi
LMSG_G000010648.1	no	1293_1	Polymetallic	86.26	3.88	66.86	0	1	1	14	Medium quality	25529	2837468	65.60	163	d__Bacteria;p__Proteobacteria;c__Gammaproteobacteria;o__Burkholderiales;f__Burkholderiaceae;g__Thiomonas;s__
LMSG_G000010649.1	no	1293_1	Polymetallic	98.49	0.43	96.35	0	1	0	18	Medium quality	42640	3340728	65.30	126	d__Bacteria;p__Proteobacteria;c__Gammaproteobacteria;o__Burkholderiales;f__Burkholderiaceae;g__Thiomonas;s__
LMSG_G000010650.1	no	1293_1	Polymetallic	90.98	0.25	89.76	0	1	0	14	Medium quality	42925	2922828	65.80	99	d__Bacteria;p__Proteobacteria;c__Gammaproteobacteria;o__Burkholderiales;f__Burkholderiaceae;g__Thiomonas;s__
LMSG_G000010651.1	no	1293_1	Polymetallic	96.13	0.00	96.13	0	0	0	18	Medium quality	54477	3209098	65.50	102	d__Bacteria;p__Proteobacteria;c__Gammaproteobacteria;o__Burkholderiales;f__Burkholderiaceae;g__Thiomonas;s__
LMSG_G000010652.1	no	1293_1	Polymetallic	72.15	0.55	69.42	0	0	0	18	Medium quality	24841	2498635	65.60	178	d__Bacteria;p__Proteobacteria;c__Gammaproteobacteria;o__Burkholderiales;f__Burkholderiaceae;g__Thiomonas;s__
LMSG_G000010653.1	no	1293_1	Polymetallic	72.93	1.86	63.64	0	0	0	11	Medium quality	24612	2561114	65.90	155	d__Bacteria;p__Proteobacteria;c__Gammaproteobacteria;o__Burkholderiales;f__Burkholderiaceae;g__Thiomonas;s__
LMSG_G000010654.1	no	1293_1	Polymetallic	88.78	3.73	70.13	1	1	1	15	Medium quality	28200	2724103	65.70	152	d__Bacteria;p__Proteobacteria;c__Gammaproteobacteria;o__Burkholderiales;f__Burkholderiaceae;g__Thiomonas;s__
LMSG_G000010655.1	no	1293_1	Polymetallic	59.26	0.00	59.26	0	0	0	15	Medium quality	36111	3094908	66.40	816	d__Bacteria;p__Proteobacteria;c__Gammaproteobacteria;o__Burkholderiales;f__Burkholderiaceae;g__Thiomonas;s__
LMSG_G000010656.1	no	1293_1	Polymetallic	80.80	2.54	68.08	0	0	0	17	Medium quality	20432	2741088	65.90	184	d__Bacteria;p__Proteobacteria;c__Gammaproteobacteria;o__Burkholderiales;f__Burkholderiaceae;g__Thiomonas;s__
LMSG_G000010657.1	no	1293_1	Polymetallic	67.24	1.72	58.62	0	0	0	11	Medium quality	30016	2476529	66.10	126	d__Bacteria;p__Proteobacteria;c__Gammaproteobacteria;o__Burkholderiales;f__Burkholderiaceae;g__Thiomonas;s__
LMSG_G000010658.1	no	1293_1	Polymetallic	84.48	1.72	75.86	0	0	0	16	Medium quality	25499	3337594	65.60	210	d__Bacteria;p__Proteobacteria;c__Gammaproteobacteria;o__Burkholderiales;f__Burkholderiaceae;g__Thiomonas;s__
LMSG_G000010659.1	no	1293_1	Polymetallic	77.77	4.29	56.32	0	1	0	11	Medium quality	25230	2697211	65.70	158	d__Bacteria;p__Proteobacteria;c__Gammaproteobacteria;o__Burkholderiales;f__Burkholderiaceae;g__Thiomonas;s__
LMSG_G000010660.1	no	1293_1	Copper	55.09	0.43	52.95	0	0	0	12	Medium quality	38345	1218400	63.10	65	d__Bacteria;p__Proteobacteria;c__Gammaproteobacteria;o__Burkholderiales;f__Burkholderiaceae;g__Thiomonas;s__
LMSG_G000010661.1	no	1294_1	Polymetallic	75.05	4.08	54.67	0	1	0	14	Medium quality	6333	2535774	65.50	501	d__Bacteria;p__Proteobacteria;c__Gammaproteobacteria;o__Burkholderiales;f__Burkholderiaceae;g__Thiomonas;s__
LMSG_G000010662.1	no	1294_1	Polymetallic	74.03	3.14	58.35	0	0	0	13	Medium quality	5184	2339327	65.10	529	d__Bacteria;p__Proteobacteria;c__Gammaproteobacteria;o__Burkholderiales;f__Burkholderiaceae;g__Thiomonas;s__
LMSG_G000010663.1	no	1294_1	Polymetallic	79.64	2.28	68.25	0	1	0	16	Medium quality	10524	2334395	65.10	350	d__Bacteria;p__Proteobacteria;c__Gammaproteobacteria;o__Burkholderiales;f__Burkholderiaceae;g__Thiomonas;s__
LMSG_G000010664.1	no	1294_1	Polymetallic	63.89	0.60	60.91	0	1	0	13	Medium quality	6934	1744235	65.00	299	d__Bacteria;p__Proteobacteria;c__Gammaproteobacteria;o__Burkholderiales;f__Burkholderiaceae;g__Thiomonas;s__
LMSG_G000010665.1	no	1294_1	Polymetallic	81.91	4.82	57.81	0	1	0	16	Medium quality	5899	3724249	64.70	831	d__Bacteria;p__Proteobacteria;c__Gammaproteobacteria;o__Burkholderiales;f__Burkholderiaceae;g__Thiomonas;s__
LMSG_G000010666.1	no	1294_1	Polymetallic	58.80	1.72	50.18	0	0	0	16	Medium quality	8413	2833673	64.00	730	d__Bacteria;p__Proteobacteria;c__Gammaproteobacteria;o__Burkholderiales;f__Burkholderiaceae;g__Thiomonas;s__
LMSG_G000010667.1	no	1294_1	Polymetallic	69.86	2.43	57.70	0	0	0	18	Medium quality	5470	2080779	64.00	602	d__Bacteria;p__Proteobacteria;c__Gammaproteobacteria;o__Burkholderiales;f__Burkholderiaceae;g__Thiomonas;s__
LMSG_G000010668.1	no	1295_1	Polymetallic	94.23	1.76	85.41	1	2	1	18	High quality	48482	3070533	69.40	103	d__Bacteria;p__Proteobacteria;c__Gammaproteobacteria;o__Burkholderiales;f__Burkholderiaceae;g__Thiomonas;s__
LMSG_G000010669.1	no	1295_1	Polymetallic	92.50	4.14	71.81	1	0	0	17	Medium quality	32284	2951529	69.60	149	d__Bacteria;p__Proteobacteria;c__Gammaproteobacteria;o__Burkholderiales;f__Burkholderiaceae;g__Thiomonas;s__
LMSG_G000010670.1	no	1297_1	Polymetallic	92.43	4.85	68.17	1	0	0	16	Medium quality	22840	3849351	70.00	272	d__Bacteria;p__Proteobacteria;c__Gammaproteobacteria;o__Burkholderiales;f__Burkholderiaceae;g__Thiomonas;s__
LMSG_G000010671.1	no	1297_1	Polymetallic	76.83	3.45	59.59	0	0	0	16	Medium quality	22826	3274213	70.30	206	d__Bacteria;p__Proteobacteria;c__Gammaproteobacteria;o__Burkholderiales;f__Burkholderiaceae;g__Thiomonas;s__
LMSG_G000010672.1	no	1297_1	Antimony	68.44	1.72	59.82	1	0	0	12	Medium quality	10212	2879908	70.40	385	d__Bacteria;p__Proteobacteria;c__Gammaproteobacteria;o__Burkholderiales;f__Burkholderiaceae;g__Thiomonas;s__
LMSG_G000010673.1	no	1298_1	Polymetallic	70.00	3.45	52.76	1	0	0	17	Medium quality	11061	3187661	70.40	438	d__Bacteria;p__Proteobacteria;c__Gammaproteobacteria;o__Burkholderiales;f__Burkholderiaceae;g__Thiomonas;s__
LMSG_G000010674.1	no	1298_2	Lead-Zinc	71.62	2.02	61.54	0	0	0	16	Medium quality	46737	3370213	69.70	110	d__Bacteria;p__Proteobacteria;c__Gammaproteobacteria;o__Burkholderiales;f__Burkholderiaceae;g__Thiomonas;s__
LMSG_G000010675.1	no	1298_2	Copper	59.48	1.72	50.86	1	0	0	15	Medium quality	7293	2839826	70.30	519	d__Bacteria;p__Proteobacteria;c__Gammaproteobacteria;o__Burkholderiales;f__Burkholderiaceae;g__Thiomonas;s__
LMSG_G000010676.1	no	1299_1	Copper	82.45	1.43	75.30	0	0	0	15	Medium quality	8001	2880620	70.20	437	d__Bacteria;p__Proteobacteria;c__Gammaproteobacteria;o__Burkholderiales;f__Burkholderiaceae;g__Thiomonas;s__
LMSG_G000010677.1	no	1299_2	Lead-Zinc	85.69	1.58	77.81	1	2	0	13	Medium quality	31478	3164006	70.80	149	d__Bacteria;p__Proteobacteria;c__Gammaproteobacteria;o__Burkholderiales;f__Burkholderiaceae;g__Thiomonas;s__
LMSG_G000010678.1	no	1299_2	Lead-Zinc	89.37	1.06	84.09	1	2	1	15	Medium quality	30080	3248273	70.70	175	d__Bacteria;p__Proteobacteria;c__Gammaproteobacteria;o__Burkholderiales;f__Burkholderiaceae;g__Thiomonas;s__
LMSG_G000010679.1	no	1299_2	Pyrite	93.99	1.42	86.89	0	1	0	18	Medium quality	28028	3341085	70.50	160	d__Bacteria;p__Proteobacteria;c__Gammaproteobacteria;o__Burkholderiales;f__Burkholderiaceae;g__Thiomonas;s__
LMSG_G000010680.1	no	1299_2	Polymetallic	86.90	1.64	78.73	1	0	0	15	Medium quality	32398	2961622	70.90	139	d__Bacteria;p__Proteobacteria;c__Gammaproteobacteria;o__Burkholderiales;f__Burkholderiaceae;g__Thiomonas;s__
LMSG_G000010681.1	no	1299_2	Polymetallic	87.40	3.70	68.91	1	0	0	14	Medium quality	22009	3115536	70.80	212	d__Bacteria;p__Proteobacteria;c__Gammaproteobacteria;o__Burkholderiales;f__Burkholderiaceae;g__Thiomonas;s__
LMSG_G000010682.1	no	1299_2	Pyrite-Copper	93.56	2.71	80.02	1	0	0	16	Medium quality	50324	3359431	70.50	98	d__Bacteria;p__Proteobacteria;c__Gammaproteobacteria;o__Burkholderiales;f__Burkholderiaceae;g__Thiomonas;s__
LMSG_G000010683.1	no	1299_2	Pyrite	75.48	1.87	66.15	1	0	1	13	Medium quality	5406	2485059	70.80	531	d__Bacteria;p__Proteobacteria;c__Gammaproteobacteria;o__Burkholderiales;f__Burkholderiaceae;g__Thiomonas;s__
LMSG_G000010684.1	no	1299_2	Lead-Zinc	96.99	3.05	81.74	1	1	0	18	Medium quality	38797	3323462	70.70	126	d__Bacteria;p__Proteobacteria;c__Gammaproteobacteria;o__Burkholderiales;f__Burkholderiaceae;g__Thiomonas;s__
LMSG_G000010685.1	no	1301_1	Pyrite	92.81	4.62	69.70	0	0	0	20	Medium quality	29118	3280235	68.60	171	d__Bacteria;p__Proteobacteria;c__Gammaproteobacteria;o__Burkholderiales;f__Burkholderiaceae;g__Thiomonas;s__
LMSG_G000010686.1	no	1301_1	Antimony	89.03	1.18	83.13	0	1	0	17	Medium quality	40937	2728839	68.70	103	d__Bacteria;p__Proteobacteria;c__Gammaproteobacteria;o__Burkholderiales;f__Burkholderiaceae;g__Thiomonas;s__
LMSG_G000010687.1	no	1301_1	Antimony	86.00	0.28	84.63	0	1	0	16	Medium quality	26755	2464286	68.70	137	d__Bacteria;p__Proteobacteria;c__Gammaproteobacteria;o__Burkholderiales;f__Burkholderiaceae;g__Thiomonas;s__
LMSG_G000010688.1	no	1301_1	Antimony	94.84	3.09	79.39	0	0	0	18	Medium quality	33697	3099791	68.70	148	d__Bacteria;p__Proteobacteria;c__Gammaproteobacteria;o__Burkholderiales;f__Burkholderiaceae;g__Thiomonas;s__
LMSG_G000010689.1	no	1301_1	Antimony	82.69	0.14	81.98	0	1	0	15	Medium quality	23264	2164559	68.60	120	d__Bacteria;p__Proteobacteria;c__Gammaproteobacteria;o__Burkholderiales;f__Burkholderiaceae;g__Thiomonas;s__

LMSG_G000010690.1	no	1301_1	Polymetallic	87.67	2.29	76.23	0	0	0	19	Medium quality	19982	2952234	68.60	199	d__Bacteria;p__Proteobacteria;c__Gammaproteobacteria;o__Burkholderiales;f__Burkholderiaceae;g__Thiomonas;s__
LMSG_G000010691.1	no	1301_1	Polymetallic	91.78	1.44	84.56	0	1	0	19	Medium quality	22962	2990963	68.60	180	d__Bacteria;p__Proteobacteria;c__Gammaproteobacteria;o__Burkholderiales;f__Burkholderiaceae;g__Thiomonas;s__
LMSG_G000010692.1	no	1301_1	Polymetallic	94.23	3.56	76.44	0	0	0	17	Medium quality	26248	3140079	68.60	181	d__Bacteria;p__Proteobacteria;c__Gammaproteobacteria;o__Burkholderiales;f__Burkholderiaceae;g__Thiomonas;s__
LMSG_G000010693.1	no	1301_1	Polymetallic	93.57	3.46	76.28	0	1	0	19	Medium quality	18055	2809179	68.70	213	d__Bacteria;p__Proteobacteria;c__Gammaproteobacteria;o__Burkholderiales;f__Burkholderiaceae;g__Thiomonas;s__
LMSG_G000010694.1	no	1301_1	Polymetallic	92.39	2.07	82.02	0	0	0	19	Medium quality	17474	2861776	68.70	227	d__Bacteria;p__Proteobacteria;c__Gammaproteobacteria;o__Burkholderiales;f__Burkholderiaceae;g__Thiomonas;s__
LMSG_G000010695.1	no	1302_1	Polymetallic	83.02	4.48	60.63	0	0	0	13	Medium quality	13679	2680935	70.50	277	d__Bacteria;p__Proteobacteria;c__Gammaproteobacteria;o__Burkholderiales;f__Burkholderiaceae;g__Thiomonas;s__
LMSG_G000010696.1	no	1302_1	Polymetallic	62.16	1.72	53.54	0	1	0	13	Medium quality	6290	2600863	70.60	516	d__Bacteria;p__Proteobacteria;c__Gammaproteobacteria;o__Burkholderiales;f__Burkholderiaceae;g__Thiomonas;s__
LMSG_G000010697.1	no	1302_1	Polymetallic	64.26	0.00	64.26	0	0	0	13	Medium quality	11887	1925315	70.50	191	d__Bacteria;p__Proteobacteria;c__Gammaproteobacteria;o__Burkholderiales;f__Burkholderiaceae;g__Thiomonas;s__
LMSG_G000010698.1	no	1303_1	Copper	90.94	4.17	70.08	1	0	0	14	Medium quality	30500	2643918	69.20	113	d__Bacteria;p__Proteobacteria;c__Gammaproteobacteria;o__Burkholderiales;f__Burkholderiaceae;g__Thiomonas;s__
LMSG_G000010699.1	no	1303_1	Pyrite	98.43	1.84	89.23	2	0	2	19	Medium quality	27040	3077426	69.20	167	d__Bacteria;p__Proteobacteria;c__Gammaproteobacteria;o__Burkholderiales;f__Burkholderiaceae;g__Thiomonas;s__
LMSG_G000010700.1	no	1303_1	Copper	86.80	0.71	83.24	1	1	1	17	Medium quality	10651	2886718	69.30	358	d__Bacteria;p__Proteobacteria;c__Gammaproteobacteria;o__Burkholderiales;f__Burkholderiaceae;g__Thiomonas;s__
LMSG_G000010701.1	no	1303_1	Polymetallic	77.74	0.00	77.74	1	0	0	16	Medium quality	11759	2847160	69.50	337	d__Bacteria;p__Proteobacteria;c__Gammaproteobacteria;o__Burkholderiales;f__Burkholderiaceae;g__Thiomonas;s__
LMSG_G000010702.1	no	1303_1	Polymetallic	85.04	0.48	82.66	1	0	0	14	Medium quality	27378	2666516	69.40	133	d__Bacteria;p__Proteobacteria;c__Gammaproteobacteria;o__Burkholderiales;f__Burkholderiaceae;g__Thiomonas;s__
LMSG_G000010703.1	no	1303_1	Polymetallic	83.55	1.40	76.57	1	0	0	17	Medium quality	29786	2888785	69.30	172	d__Bacteria;p__Proteobacteria;c__Gammaproteobacteria;o__Burkholderiales;f__Burkholderiaceae;g__Thiomonas;s__
LMSG_G000010704.1	no	1303_1	Polymetallic	88.62	0.48	86.24	1	0	0	15	Medium quality	34153	2848141	69.20	141	d__Bacteria;p__Proteobacteria;c__Gammaproteobacteria;o__Burkholderiales;f__Burkholderiaceae;g__Thiomonas;s__
LMSG_G000010705.1	no	1303_1	Polymetallic	94.06	3.96	74.27	0	0	0	14	Medium quality	13423	2971250	69.20	335	d__Bacteria;p__Proteobacteria;c__Gammaproteobacteria;o__Burkholderiales;f__Burkholderiaceae;g__Thiomonas;s__
LMSG_G000010706.1	no	1303_1	Antimony	85.85	2.52	73.28	0	0	0	14	Medium quality	30876	2738474	69.30	127	d__Bacteria;p__Proteobacteria;c__Gammaproteobacteria;o__Burkholderiales;f__Burkholderiaceae;g__Thiomonas;s__
LMSG_G000010707.1	no	1303_1	Antimony	51.72	0.00	51.72	0	0	0	8	Medium quality	9620	1344731	69.10	149	d__Bacteria;p__Proteobacteria;c__Gammaproteobacteria;o__Burkholderiales;f__Burkholderiaceae;g__Thiomonas;s__
LMSG_G000010708.1	no	1303_1	Pyrite-Copper	96.90	0.05	96.67	0	0	0	18	Medium quality	33555	3040595	69.30	132	d__Bacteria;p__Proteobacteria;c__Gammaproteobacteria;o__Burkholderiales;f__Burkholderiaceae;g__Thiomonas;s__
LMSG_G000010709.1	no	1303_1	Polymetallic	90.89	0.12	90.28	1	1	0	18	Medium quality	11453	2884872	69.30	342	d__Bacteria;p__Proteobacteria;c__Gammaproteobacteria;o__Burkholderiales;f__Burkholderiaceae;g__Thiomonas;s__
LMSG_G000010710.1	no	1576_1	Copper	86.50	0.24	85.31	1	0	1	19	Medium quality	11181	2706270	64.20	272	d__Bacteria;p__Proteobacteria;c__Gammaproteobacteria;o__Burkholderiales;f__Burkholderiaceae;g__Thiomonas;s__
LMSG_G000010711.1	no	1306_1	Nickel-Copper	76.87	1.63	68.70	0	1	1	15	Medium quality	9311	2435999	64.20	409	d__Bacteria;p__Proteobacteria;c__Gammaproteobacteria;o__Burkholderiales;f__Burkholderiaceae;g__Thiomonas;s__Thiomonas arsenitoxydans
LMSG_G000010712.1	no	1306_1	Nickel-Copper	99.14	2.15	88.42	0	1	0	17	Medium quality	104455	3240393	63.90	68	d__Bacteria;p__Proteobacteria;c__Gammaproteobacteria;o__Burkholderiales;f__Burkholderiaceae;g__Thiomonas;s__Thiomonas arsenitoxydans
LMSG_G000010713.1	no	1306_1	Antimony	52.58	0.00	52.58	0	1	0	8	Medium quality	6320	1244196	63.70	246	d__Bacteria;p__Proteobacteria;c__Gammaproteobacteria;o__Burkholderiales;f__Burkholderiaceae;g__Thiomonas;s__Thiomonas arsenitoxydans
LMSG_G000010714.1	no	1305_1	Copper	61.59	0.21	60.52	0	1	0	13	Medium quality	21534	1423146	65.50	104	d__Bacteria;p__Proteobacteria;c__Gammaproteobacteria;o__Burkholderiales;f__Burkholderiaceae;g__Thiomonas;s__Thiomonas delicata
LMSG_G000010715.1	no	1305_1	Lead-Zinc	93.89	4.13	73.24	0	1	0	17	Medium quality	27346	2906256	66.30	157	d__Bacteria;p__Proteobacteria;c__Gammaproteobacteria;o__Burkholderiales;f__Burkholderiaceae;g__Thiomonas;s__Thiomonas delicata
LMSG_G000010716.1	no	1305_1	Pyrite	94.01	2.71	80.49	0	1	0	16	Medium quality	9944	2674450	66.60	355	d__Bacteria;p__Proteobacteria;c__Gammaproteobacteria;o__Burkholderiales;f__Burkholderiaceae;g__Thiomonas;s__Thiomonas delicata
LMSG_G000010717.1	no	1305_1	Lead-Zinc	64.95	0.00	64.95	1	0	0	12	Medium quality	4116	1645432	66.50	445	d__Bacteria;p__Proteobacteria;c__Gammaproteobacteria;o__Burkholderiales;f__Burkholderiaceae;g__Thiomonas;s__Thiomonas delicata
LMSG_G000010718.1	no	1305_1	Lead-Zinc	70.08	0.92	65.49	0	0	0	14	Medium quality	25700	2182322	66.70	133	d__Bacteria;p__Proteobacteria;c__Gammaproteobacteria;o__Burkholderiales;f__Burkholderiaceae;g__Thiomonas;s__Thiomonas delicata
LMSG_G000010719.1	no	1305_1	Lead-Zinc	91.20	0.12	90.59	0	1	0	16	Medium quality	25677	2467514	66.50	136	d__Bacteria;p__Proteobacteria;c__Gammaproteobacteria;o__Burkholderiales;f__Burkholderiaceae;g__Thiomonas;s__Thiomonas delicata
LMSG_G000010720.1	no	1305_1	Lead-Zinc	77.96	1.07	72.60	1	2	4	13	Medium quality	23220	2422378	66.20	189	d__Bacteria;p__Proteobacteria;c__Gammaproteobacteria;o__Burkholderiales;f__Burkholderiaceae;g__Thiomonas;s__Thiomonas delicata
LMSG_G000010721.1	no	1305_1	Polymetallic	89.24	4.71	65.67	0	1	0	16	Medium quality	7715	2889650	66.50	481	d__Bacteria;p__Proteobacteria;c__Gammaproteobacteria;o__Burkholderiales;f__Burkholderiaceae;g__Thiomonas;s__Thiomonas delicata
LMSG_G000010722.1	no	1305_1	Tin-Zinc	80.15	3.22	64.06	0	0	0	15	Medium quality	15939	2073438	66.20	165	d__Bacteria;p__Proteobacteria;c__Gammaproteobacteria;o__Burkholderiales;f__Burkholderiaceae;g__Thiomonas;s__Thiomonas delicata
LMSG_G000010723.1	no	1305_1	Polymetallic	87.55	1.77	78.72	0	1	0	16	Medium quality	43167	2653358	66.20	102	d__Bacteria;p__Proteobacteria;c__Gammaproteobacteria;o__Burkholderiales;f__Burkholderiaceae;g__Thiomonas;s__Thiomonas delicata
LMSG_G000010724.1	no	1305_1	Lead-Zinc	94.42	1.72	85.84	0	0	0	15	Medium quality	49941	2926971	66.40	120	d__Bacteria;p__Proteobacteria;c__Gammaproteobacteria;o__Burkholderiales;f__Burkholderiaceae;g__Thiomonas;s__Thiomonas delicata
LMSG_G000010725.1	no	1305_1	Lead-Zinc	97.17	0.86	92.88	0	1	1	18	Medium quality	67661	2955613	66.50	86	d__Bacteria;p__Proteobacteria;c__Gammaproteobacteria;o__Burkholderiales;f__Burkholderiaceae;g__Thiomonas;s__Thiomonas delicata
LMSG_G000010726.1	no	1305_1	Pyrite-Copper	90.55	1.29	84.10	0	1	1	16	Medium quality	31923	2946713	66.40	148	d__Bacteria;p__Proteobacteria;c__Gammaproteobacteria;o__Burkholderiales;f__Burkholderiaceae;g__Thiomonas;s__Thiomonas delicata
LMSG_G000010727.1	no	1305_1	Lead-Zinc	93.13	0.11	92.60	0	0	0	17	Medium quality	7030	2622484	66.50	464	d__Bacteria;p__Proteobacteria;c__Gammaproteobacteria;o__Burkholderiales;f__Burkholderiaceae;g__Thiomonas;s__Thiomonas delicata
LMSG_G000010728.1	no	1305_1	Nickel-Copper	96.59	0.86	92.30	0	1	0	19	Medium quality	40260	3321978	65.80	146	d__Bacteria;p__Proteobacteria;c__Gammaproteobacteria;o__Burkholderiales;f__Burkholderiaceae;g__Thiomonas;s__Thiomonas delicata
LMSG_G000010729.1	no	1305_1	Arsenic	66.05	1.85	56.82	0	0	0	14	Medium quality	5487	1914562	64.70	452	d__Bacteria;p__Proteobacteria;c__Gammaproteobacteria;o__Burkholderiales;f__Burkholderiaceae;g__Thiomonas;s__Thiomonas delicata
LMSG_G000010730.1	no	1327_1	Polymetallic	91.31	4.05	71.07	0	0	0	17	Medium quality	52363	2123954	59.30	75	d__Bacteria;p__Proteobacteria;c__Gammaproteobacteria;o__Burkholderiales;f__Ferrovaceae;g__;s__
LMSG_G000010731.1	no	1327_1	Polymetallic	94.46	4.52	71.88	1	0	1	18	Medium quality	25326	2251806	59.40	154	d__Bacteria;p__Proteobacteria;c__Gammaproteobacteria;o__Burkholderiales;f__Ferrovaceae;g__;s__
LMSG_G000010732.1	no	1328_1	Polymetallic	79.65	3.33	62.98	1	0	0	13	Medium quality	14998	1718441	59.20	235	d__Bacteria;p__Proteobacteria;c__Gammaproteobacteria;o__Burkholderiales;f__Ferrovaceae;g__;s__
LMSG_G000010733.1	no	1328_2	Copper	91.37	1.53	83.73	1	1	1	17	Medium quality	13062	1784470	60.10	195	d__Bacteria;p__Proteobacteria;c__Gammaproteobacteria;o__Burkholderiales;f__Ferrovaceae;g__;s__
LMSG_G000010734.1	no	1328_2	Copper	97.09	2.20	86.09	0	0	0	18	Medium quality	32721	2224066	59.40	122	d__Bacteria;p__Proteobacteria;c__Gammaproteobacteria;o__Burkholderiales;f__Ferrovaceae;g__;s__
LMSG_G000010735.1	no	1328_2	Copper	81.79	1.82	72.71	0	0	0	13	Medium quality	7892	1587004	59.80	255	d__Bacteria;p__Proteobacteria;c__Gammaproteobacteria;o__Burkholderiales;f__Ferrovaceae;g__;s__
LMSG_G000010736.1	no	1328_2	Copper	93.70	1.28	87.30	1	0	1	19	Medium quality	19442	1826300	60.00	149	d__Bacteria;p__Proteobacteria;c__Gammaproteobacteria;o__Burkholderiales;f__Ferrovaceae;g__;s__
LMSG_G000010737.1	no	1328_2	Pyrite	97.23	2.71	83.67	0	0	0	19	Medium quality	71721	2299243	59.20	67	d__Bacteria;p__Proteobacteria;c__Gammaproteobacteria;o__Burkholderiales;f__Ferrovaceae;g__;s__
LMSG_G000010738.1	no	1331_1	Polymetallic	96.05	1.45	88.81	2	0	0	17	Medium quality	94113	1969951	60.10	40	d__Bacteria;p__Proteobacteria;c__Gammaproteobacteria;o__Burkholderiales;f__Ferrovaceae;g__;s__
LMSG_G000010739.1	no	1331_1	Polymetallic	80.68	0.79	76.71	0	1	0	15	Medium quality	28561	1578358	60.10	101	d__Bacteria;p__Proteobacteria;c__Gammaproteobacteria;o__Burkholderiales;f__Ferrovaceae;g__;s__
LMSG_G000010740.1	no	1331_1	Antimony	92.70	2.41	80.66	0	2	0	16	Medium quality	12421	1964782	60.50	207	d__Bacteria;p__Proteobacteria;c__Gammaproteobacteria;o__Burkholderiales;f__Ferrovaceae;g__;s__
LMSG_G000010741.1	no	1331_1	Polymetallic	95.81	0.98	90.94	0	0	0	19	Medium quality	95494	1973399	60.60	36	d__Bacteria;p__Proteobacteria;c__Gammaproteobacteria;o__Burkholderiales;f__Ferrovaceae;g__;s__
LMSG_G000010742.1	no	1331_1	Polymetallic	96.31	0.47	93.95	2	0	0	18	Medium quality	94114	2035699	60.50	38	d__Bacteria;p__Proteobacteria;c__Gammaproteobacteria;o__Burkholderiales;f__Ferrovaceae;g__;s__
LMSG_G000010743.1	no	1331_1	Antimony	83.02	3.75	64.25	0	0	0	10	Medium quality	5324	1427624	60.60	307	d__Bacteria;p__Proteobacteria;c__Gammaproteobacteria;o__Burkholderiales;f__Ferrovaceae;g__;s__
LMSG_G000010744.1	no	1332_1	Lead-Zinc	97.56	1.80	88.58	0	0	0	17	Medium quality	29154	2215537	64.00	114	d__Bacteria;p__Proteobacteria;c__Gammaproteobacteria;o__Burkholderiales;f__Ferrovaceae;g__;s__
LMSG_G000010745.1	no	1332_1	Polymetallic	77.09	0.95	72.36	0	0	0	17	Medium quality	61661	1620613	63.80	45	d__Bacteria;p__Proteobacteria;c__Gammaproteobacteria;o__Burkholderiales;f__Ferrovaceae;g__;s__
LMSG_G000010746.1	no	1332_1	Polymetallic	67.08	0.24	65.90	1	0	1	14	Medium quality	82744	1317046	64.20	32	d__Bacteria;p__Proteobacteria;c__Gammaproteobacteria;o__Burkholderiales;f__Ferrovaceae;g__;s__

LMSG_G000010747.1	no	1332_1	Polymetallic	71.48	0.00	71.48	1	0	0	14	Medium quality	59341	1368392	63.70	31	d__Bacteria;p__Proteobacteria;c__Gammaproteobacteria;o__Burkholderiales;f__Ferrovaceae;g__;s__
LMSG_G000010748.1	no	1332_1	Polymetallic	74.48	0.47	72.12	0	0	0	14	Medium quality	57014	1540393	63.80	41	d__Bacteria;p__Proteobacteria;c__Gammaproteobacteria;o__Burkholderiales;f__Ferrovaceae;g__;s__
LMSG_G000010749.1	no	1332_1	Polymetallic	64.64	0.87	60.30	0	0	0	11	Medium quality	56364	1349982	64.50	35	d__Bacteria;p__Proteobacteria;c__Gammaproteobacteria;o__Burkholderiales;f__Ferrovaceae;g__;s__
LMSG_G000010750.1	no	1332_1	Polymetallic	83.14	1.42	76.04	0	0	0	15	Medium quality	58147	1666730	63.30	63	d__Bacteria;p__Proteobacteria;c__Gammaproteobacteria;o__Burkholderiales;f__Ferrovaceae;g__;s__
LMSG_G000010751.1	no	1335_1	Polymetallic	98.17	0.00	98.17	1	1	1	19	High quality	151190	1995102	53.00	63	d__Bacteria;p__Proteobacteria;c__Gammaproteobacteria;o__Burkholderiales;f__Ferrovaceae;g__Ferrovum;s__
LMSG_G000010752.1	no	1335_1	Polymetallic	99.39	0.00	99.39	1	3	1	20	High quality	120633	2401476	53.00	160	d__Bacteria;p__Proteobacteria;c__Gammaproteobacteria;o__Burkholderiales;f__Ferrovaceae;g__Ferrovum;s__
LMSG_G000010753.1	no	1335_1	Polymetallic	96.91	0.61	93.87	1	1	1	17	Medium quality	91916	1876984	53.10	65	d__Bacteria;p__Proteobacteria;c__Gammaproteobacteria;o__Burkholderiales;f__Ferrovaceae;g__Ferrovum;s__
LMSG_G000010754.1	no	1335_1	Polymetallic	98.10	0.20	97.09	1	0	0	17	Medium quality	34684	2031231	53.00	107	d__Bacteria;p__Proteobacteria;c__Gammaproteobacteria;o__Burkholderiales;f__Ferrovaceae;g__Ferrovum;s__
LMSG_G000010755.1	no	1335_1	Polymetallic	98.78	0.49	96.33	2	4	0	18	Medium quality	31083	2037534	53.00	195	d__Bacteria;p__Proteobacteria;c__Gammaproteobacteria;o__Burkholderiales;f__Ferrovaceae;g__Ferrovum;s__
LMSG_G000010756.1	no	1335_1	Pyrite	99.39	1.75	90.66	2	1	1	20	High quality	43798	3118172	53.00	225	d__Bacteria;p__Proteobacteria;c__Gammaproteobacteria;o__Burkholderiales;f__Ferrovaceae;g__Ferrovum;s__
LMSG_G000010757.1	no	1335_1	Pyrite	99.39	1.83	90.25	1	3	2	20	High quality	69345	3119492	53.40	308	d__Bacteria;p__Proteobacteria;c__Gammaproteobacteria;o__Burkholderiales;f__Ferrovaceae;g__Ferrovum;s__
LMSG_G000010758.1	no	1335_1	Polymetallic	99.39	4.88	75.00	1	2	1	20	High quality	153461	2408374	53.20	77	d__Bacteria;p__Proteobacteria;c__Gammaproteobacteria;o__Burkholderiales;f__Ferrovaceae;g__Ferrovum;s__
LMSG_G000010759.1	no	1335_1	Polymetallic	99.39	4.24	78.21	1	2	2	20	High quality	89671	2425480	53.10	72	d__Bacteria;p__Proteobacteria;c__Gammaproteobacteria;o__Burkholderiales;f__Ferrovaceae;g__Ferrovum;s__
LMSG_G000010760.1	no	1335_1	Polymetallic	99.39	3.05	84.15	1	1	1	19	High quality	108777	2314502	53.20	61	d__Bacteria;p__Proteobacteria;c__Gammaproteobacteria;o__Burkholderiales;f__Ferrovaceae;g__Ferrovum;s__
LMSG_G000010761.1	no	1335_1	Polymetallic	99.39	3.05	84.15	1	2	1	19	High quality	85201	1989720	53.20	50	d__Bacteria;p__Proteobacteria;c__Gammaproteobacteria;o__Burkholderiales;f__Ferrovaceae;g__Ferrovum;s__
LMSG_G000010762.1	no	1335_1	Antimony	92.68	0.00	92.68	1	2	1	18	High quality	79831	1746830	53.10	39	d__Bacteria;p__Proteobacteria;c__Gammaproteobacteria;o__Burkholderiales;f__Ferrovaceae;g__Ferrovum;s__
LMSG_G000010763.1	no	1335_1	Antimony	98.78	0.00	98.78	2	9	11	19	High quality	81403	2169508	53.10	129	d__Bacteria;p__Proteobacteria;c__Gammaproteobacteria;o__Burkholderiales;f__Ferrovaceae;g__Ferrovum;s__
LMSG_G000010764.1	no	1335_1	Polymetallic	96.95	4.27	75.61	1	3	2	20	High quality	76621	2365169	53.40	83	d__Bacteria;p__Proteobacteria;c__Gammaproteobacteria;o__Burkholderiales;f__Ferrovaceae;g__Ferrovum;s__
LMSG_G000010765.1	no	1335_1	Antimony	99.39	0.85	95.13	1	2	1	17	Medium quality	60500	1991270	53.20	73	d__Bacteria;p__Proteobacteria;c__Gammaproteobacteria;o__Burkholderiales;f__Ferrovaceae;g__Ferrovum;s__
LMSG_G000010766.1	no	1335_1	Pyrite	99.39	2.03	89.23	0	0	0	20	Medium quality	51424	3149635	53.40	204	d__Bacteria;p__Proteobacteria;c__Gammaproteobacteria;o__Burkholderiales;f__Ferrovaceae;g__Ferrovum;s__
LMSG_G000010767.1	no	1335_1	Pyrite	82.92	0.00	82.92	1	4	8	20	Medium quality	161948	1753481	53.10	105	d__Bacteria;p__Proteobacteria;c__Gammaproteobacteria;o__Burkholderiales;f__Ferrovaceae;g__Ferrovum;s__
LMSG_G000010768.1	no	1335_1	Polymetallic	98.17	0.00	98.17	0	1	0	19	Medium quality	155145	1891695	53.10	82	d__Bacteria;p__Proteobacteria;c__Gammaproteobacteria;o__Burkholderiales;f__Ferrovaceae;g__Ferrovum;s__
LMSG_G000010769.1	no	1335_1	Polymetallic	75.00	1.80	66.01	1	1	2	18	Medium quality	275967	1376295	52.60	90	d__Bacteria;p__Proteobacteria;c__Gammaproteobacteria;o__Burkholderiales;f__Ferrovaceae;g__Ferrovum;s__
LMSG_G000010770.1	no	1335_1	Polymetallic	76.21	1.83	67.07	0	1	1	16	Medium quality	116386	1375233	52.50	22	d__Bacteria;p__Proteobacteria;c__Gammaproteobacteria;o__Burkholderiales;f__Ferrovaceae;g__Ferrovum;s__
LMSG_G000010771.1	no	1335_1	Polymetallic	77.43	0.61	74.39	0	2	0	14	Medium quality	93427	1543370	53.10	35	d__Bacteria;p__Proteobacteria;c__Gammaproteobacteria;o__Burkholderiales;f__Ferrovaceae;g__Ferrovum;s__
LMSG_G000010772.1	no	1335_1	Polymetallic	85.97	3.66	67.68	0	1	1	19	Medium quality	93783	1751428	53.20	52	d__Bacteria;p__Proteobacteria;c__Gammaproteobacteria;o__Burkholderiales;f__Ferrovaceae;g__Ferrovum;s__
LMSG_G000010773.1	no	1335_1	Polymetallic	91.46	3.05	76.22	0	1	1	17	Medium quality	72646	1907453	53.10	63	d__Bacteria;p__Proteobacteria;c__Gammaproteobacteria;o__Burkholderiales;f__Ferrovaceae;g__Ferrovum;s__
LMSG_G000010774.1	no	1335_1	Polymetallic	58.53	1.22	52.44	0	1	1	14	Medium quality	129400	1131148	52.80	12	d__Bacteria;p__Proteobacteria;c__Gammaproteobacteria;o__Burkholderiales;f__Ferrovaceae;g__Ferrovum;s__
LMSG_G000010775.1	no	1335_1	Antimony	99.39	0.46	97.11	0	0	0	18	Medium quality	73658	2037311	53.00	80	d__Bacteria;p__Proteobacteria;c__Gammaproteobacteria;o__Burkholderiales;f__Ferrovaceae;g__Ferrovum;s__
LMSG_G000010776.1	no	1335_1	Antimony	98.78	0.17	97.94	0	0	0	19	Medium quality	78830	1960058	53.10	58	d__Bacteria;p__Proteobacteria;c__Gammaproteobacteria;o__Burkholderiales;f__Ferrovaceae;g__Ferrovum;s__
LMSG_G000010777.1	no	1335_1	Antimony	96.95	0.00	96.95	0	1	0	18	Medium quality	86447	1765359	52.90	40	d__Bacteria;p__Proteobacteria;c__Gammaproteobacteria;o__Burkholderiales;f__Ferrovaceae;g__Ferrovum;s__
LMSG_G000010778.1	no	1335_1	Polymetallic	99.39	1.22	93.30	0	0	0	17	Medium quality	92890	1958305	52.90	54	d__Bacteria;p__Proteobacteria;c__Gammaproteobacteria;o__Burkholderiales;f__Ferrovaceae;g__Ferrovum;s__
LMSG_G000010779.1	no	1335_1	Polymetallic	98.17	0.00	98.17	0	2	0	19	Medium quality	41836	2070855	53.20	96	d__Bacteria;p__Proteobacteria;c__Gammaproteobacteria;o__Burkholderiales;f__Ferrovaceae;g__Ferrovum;s__
LMSG_G000010780.1	no	1335_1	Polymetallic	96.34	0.00	96.34	0	2	0	16	Medium quality	54334	1795891	53.10	73	d__Bacteria;p__Proteobacteria;c__Gammaproteobacteria;o__Burkholderiales;f__Ferrovaceae;g__Ferrovum;s__
LMSG_G000010781.1	no	1335_1	Copper	83.88	1.37	77.03	1	0	1	11	Medium quality	5102	1621010	52.90	422	d__Bacteria;p__Proteobacteria;c__Gammaproteobacteria;o__Burkholderiales;f__Ferrovaceae;g__Ferrovum;s__
LMSG_G000010782.1	no	1335_1	Pyrite-Copper	99.39	0.00	99.39	2	0	0	18	Medium quality	89262	1935019	53.10	48	d__Bacteria;p__Proteobacteria;c__Gammaproteobacteria;o__Burkholderiales;f__Ferrovaceae;g__Ferrovum;s__
LMSG_G000010783.1	no	1335_1	Pyrite-Copper	92.68	3.66	74.39	0	2	0	19	Medium quality	59019	2403143	53.20	114	d__Bacteria;p__Proteobacteria;c__Gammaproteobacteria;o__Burkholderiales;f__Ferrovaceae;g__Ferrovum;s__
LMSG_G000010784.1	no	1335_1	Copper	89.63	0.61	86.59	0	0	0	17	Medium quality	78206	1800150	52.90	46	d__Bacteria;p__Proteobacteria;c__Gammaproteobacteria;o__Burkholderiales;f__Ferrovaceae;g__Ferrovum;s__
LMSG_G000010785.1	no	1335_1	Pyrite-Copper	84.14	0.00	84.14	1	2	1	17	Medium quality	75181	1647453	52.70	37	d__Bacteria;p__Proteobacteria;c__Gammaproteobacteria;o__Burkholderiales;f__Ferrovaceae;g__Ferrovum;s__
LMSG_G000010786.1	no	1335_1	Pyrite	99.39	1.83	90.25	1	1	0	20	Medium quality	74618	2626949	52.90	140	d__Bacteria;p__Proteobacteria;c__Gammaproteobacteria;o__Burkholderiales;f__Ferrovaceae;g__Ferrovum;s__
LMSG_G000010787.1	no	1335_1	Polymetallic	98.17	0.00	98.17	1	1	0	19	Medium quality	99810	1860963	53.20	96	d__Bacteria;p__Proteobacteria;c__Gammaproteobacteria;o__Burkholderiales;f__Ferrovaceae;g__Ferrovum;s__
LMSG_G000010788.1	no	1335_1	Pyrite-Copper	95.12	1.52	87.50	1	2	0	18	Medium quality	105505	1963982	53.20	57	d__Bacteria;p__Proteobacteria;c__Gammaproteobacteria;o__Burkholderiales;f__Ferrovaceae;g__Ferrovum;s__
LMSG_G000010789.1	no	1335_1	Pyrite-Copper	98.57	1.30	92.07	1	3	0	18	Medium quality	72103	2023086	53.10	125	d__Bacteria;p__Proteobacteria;c__Gammaproteobacteria;o__Burkholderiales;f__Ferrovaceae;g__Ferrovum;s__
LMSG_G000010790.1	no	1336_1	Copper	92.72	3.37	75.86	2	1	1	18	High quality	30228	2720608	56.70	221	d__Bacteria;p__Proteobacteria;c__Gammaproteobacteria;o__Burkholderiales;f__Ferrovaceae;g__Ferrovum;s__
LMSG_G000010791.1	no	1336_1	Copper	51.85	0.21	50.80	0	0	0	6	Medium quality	1756	913173	58.00	536	d__Bacteria;p__Proteobacteria;c__Gammaproteobacteria;o__Burkholderiales;f__Ferrovaceae;g__Ferrovum;s__
LMSG_G000010792.1	no	1336_1	Copper	94.39	1.45	87.15	0	0	0	19	Medium quality	59291	2329927	57.10	91	d__Bacteria;p__Proteobacteria;c__Gammaproteobacteria;o__Burkholderiales;f__Ferrovaceae;g__Ferrovum;s__
LMSG_G000010793.1	no	1336_1	Copper	90.35	1.58	82.44	0	1	0	18	Medium quality	34932	2131744	57.30	135	d__Bacteria;p__Proteobacteria;c__Gammaproteobacteria;o__Burkholderiales;f__Ferrovaceae;g__Ferrovum;s__
LMSG_G000010794.1	no	1336_1	Copper	71.12	0.68	67.70	1	0	0	14	Medium quality	63073	1341485	58.60	107	d__Bacteria;p__Proteobacteria;c__Gammaproteobacteria;o__Burkholderiales;f__Ferrovaceae;g__Ferrovum;s__
LMSG_G000010795.1	no	1336_1	Magnetite	90.44	0.00	90.44	0	0	0	19	Medium quality	36196	2151570	57.70	92	d__Bacteria;p__Proteobacteria;c__Gammaproteobacteria;o__Burkholderiales;f__Ferrovaceae;g__Ferrovum;s__
LMSG_G000010796.1	no	1336_1	Magnetite	86.69	1.30	80.17	1	1	1	20	Medium quality	14986	2129193	57.30	273	d__Bacteria;p__Proteobacteria;c__Gammaproteobacteria;o__Burkholderiales;f__Ferrovaceae;g__Ferrovum;s__
LMSG_G000010797.1	no	1336_1	Coal	93.04	2.24	81.83	1	1	0	17	Medium quality	35733	2393939	57.00	177	d__Bacteria;p__Proteobacteria;c__Gammaproteobacteria;o__Burkholderiales;f__Ferrovaceae;g__Ferrovum;s__
LMSG_G000010798.1	no	1336_1	Antimony	75.55	1.22	69.46	0	0	0	11	Medium quality	17293	1259356	59.10	151	d__Bacteria;p__Proteobacteria;c__Gammaproteobacteria;o__Burkholderiales;f__Ferrovaceae;g__Ferrovum;s__

LMSG_GO000010799.1	no	1336_1	Polymetallic	73.57	0.91	69.00	0	0	0	13	Medium quality	17958	1345689	59.00	183	d__Bacteria;p__Proteobacteria;c__Gammaproteobacteria;o__Burkholderiales;f__Ferrovaceae;g__Ferrovum;s__
LMSG_GO000010800.1	no	1336_1	Polymetallic	58.62	0.00	58.62	0	0	0	14	Medium quality	11208	1072635	58.40	127	d__Bacteria;p__Proteobacteria;c__Gammaproteobacteria;o__Burkholderiales;f__Ferrovaceae;g__Ferrovum;s__
LMSG_GO000010801.1	no	1336_1	Polymetallic	85.46	2.44	73.27	0	0	0	14	Medium quality	16996	1491090	58.40	156	d__Bacteria;p__Proteobacteria;c__Gammaproteobacteria;o__Burkholderiales;f__Ferrovaceae;g__Ferrovum;s__
LMSG_GO000010802.1	no	1336_1	Polymetallic	64.65	1.72	56.03	1	1	1	13	Medium quality	9173	1430291	58.80	187	d__Bacteria;p__Proteobacteria;c__Gammaproteobacteria;o__Burkholderiales;f__Ferrovaceae;g__Ferrovum;s__
LMSG_GO000010803.1	no	1336_1	Polymetallic	90.64	0.03	90.51	2	0	0	16	Medium quality	33884	1783904	58.20	93	d__Bacteria;p__Proteobacteria;c__Gammaproteobacteria;o__Burkholderiales;f__Ferrovaceae;g__Ferrovum;s__
LMSG_GO000010804.1	no	1336_1	Polymetallic	74.80	0.24	73.62	1	0	1	13	Medium quality	35488	1522305	58.20	85	d__Bacteria;p__Proteobacteria;c__Gammaproteobacteria;o__Burkholderiales;f__Ferrovaceae;g__Ferrovum;s__
LMSG_GO000010805.1	no	1336_1	Polymetallic	80.42	0.61	77.38	1	0	1	14	Medium quality	18933	1597236	58.40	124	d__Bacteria;p__Proteobacteria;c__Gammaproteobacteria;o__Burkholderiales;f__Ferrovaceae;g__Ferrovum;s__
LMSG_GO000010806.1	no	1336_1	Polymetallic	81.61	0.59	78.65	1	0	0	16	Medium quality	34751	1532477	58.80	103	d__Bacteria;p__Proteobacteria;c__Gammaproteobacteria;o__Burkholderiales;f__Ferrovaceae;g__Ferrovum;s__
LMSG_GO000010807.1	no	1336_1	Polymetallic	91.46	0.00	91.46	2	2	0	17	Medium quality	33788	1703406	58.10	74	d__Bacteria;p__Proteobacteria;c__Gammaproteobacteria;o__Burkholderiales;f__Ferrovaceae;g__Ferrovum;s__
LMSG_GO000010808.1	no	1336_1	Lead-Zinc	75.86	1.72	67.24	0	1	0	12	Medium quality	26703	1262331	59.00	101	d__Bacteria;p__Proteobacteria;c__Gammaproteobacteria;o__Burkholderiales;f__Ferrovaceae;g__Ferrovum;s__
LMSG_GO000010809.1	no	1336_1	Lead-Zinc	89.02	0.98	84.15	2	0	0	18	Medium quality	17384	1730637	58.40	139	d__Bacteria;p__Proteobacteria;c__Gammaproteobacteria;o__Burkholderiales;f__Ferrovaceae;g__Ferrovum;s__
LMSG_GO000010810.1	no	1336_1	Lead-Zinc	73.02	0.00	73.02	0	0	0	11	Medium quality	33235	1261579	58.60	75	d__Bacteria;p__Proteobacteria;c__Gammaproteobacteria;o__Burkholderiales;f__Ferrovaceae;g__Ferrovum;s__
LMSG_GO000010811.1	no	1336_1	Copper	85.66	1.83	76.52	2	1	0	9	Medium quality	9304	1487496	58.80	204	d__Bacteria;p__Proteobacteria;c__Gammaproteobacteria;o__Burkholderiales;f__Ferrovaceae;g__Ferrovum;s__
LMSG_GO000010812.1	no	1336_1	Copper	59.04	1.39	52.11	0	0	0	7	Medium quality	1907	1213241	58.50	688	d__Bacteria;p__Proteobacteria;c__Gammaproteobacteria;o__Burkholderiales;f__Ferrovaceae;g__Ferrovum;s__
LMSG_GO000010813.1	no	1337_1	Antimony	60.34	0.00	60.34	1	0	1	12	Medium quality	5491	1354793	57.40	340	d__Bacteria;p__Proteobacteria;c__Gammaproteobacteria;o__Burkholderiales;f__Ferrovaceae;g__Ferrovum;s__
LMSG_GO000010814.1	no	1337_1	Antimony	75.86	0.86	71.55	0	2	0	15	Medium quality	66308	1821297	56.80	51	d__Bacteria;p__Proteobacteria;c__Gammaproteobacteria;o__Burkholderiales;f__Ferrovaceae;g__Ferrovum;s__
LMSG_GO000010815.1	no	1337_1	Copper	86.59	2.36	74.80	0	0	0	16	Medium quality	10137	1917015	56.60	263	d__Bacteria;p__Proteobacteria;c__Gammaproteobacteria;o__Burkholderiales;f__Ferrovaceae;g__Ferrovum;s__
LMSG_GO000010816.1	no	1337_1	Lead-Zinc	84.01	0.82	79.93	2	0	1	17	Medium quality	62112	2214561	56.30	209	d__Bacteria;p__Proteobacteria;c__Gammaproteobacteria;o__Burkholderiales;f__Ferrovaceae;g__Ferrovum;s__
LMSG_GO000010817.1	no	1337_1	Pyrite	77.46	0.65	74.24	0	1	0	17	Medium quality	9077	1563947	56.90	244	d__Bacteria;p__Proteobacteria;c__Gammaproteobacteria;o__Burkholderiales;f__Ferrovaceae;g__Ferrovum;s__
LMSG_GO000010818.1	no	1337_1	Pyrite	92.33	1.86	83.05	1	0	1	17	Medium quality	56565	1876948	56.80	132	d__Bacteria;p__Proteobacteria;c__Gammaproteobacteria;o__Burkholderiales;f__Ferrovaceae;g__Ferrovum;s__
LMSG_GO000010819.1	no	1337_1	Pyrite	94.86	1.42	87.76	2	0	0	19	Medium quality	123231	2038961	56.60	50	d__Bacteria;p__Proteobacteria;c__Gammaproteobacteria;o__Burkholderiales;f__Ferrovaceae;g__Ferrovum;s__
LMSG_GO000010820.1	no	1337_1	Pyrite	80.50	1.30	74.02	1	0	1	12	Medium quality	4757	1672030	56.70	412	d__Bacteria;p__Proteobacteria;c__Gammaproteobacteria;o__Burkholderiales;f__Ferrovaceae;g__Ferrovum;s__
LMSG_GO000010821.1	no	1337_1	Lead-Zinc	77.81	0.00	77.81	0	0	0	14	Medium quality	32724	1581248	56.80	88	d__Bacteria;p__Proteobacteria;c__Gammaproteobacteria;o__Burkholderiales;f__Ferrovaceae;g__Ferrovum;s__
LMSG_GO000010822.1	no	1337_1	Copper	67.50	0.00	67.50	1	0	1	14	Medium quality	116397	1252399	56.60	57	d__Bacteria;p__Proteobacteria;c__Gammaproteobacteria;o__Burkholderiales;f__Ferrovaceae;g__Ferrovum;s__
LMSG_GO000010823.1	no	1337_1	Copper	84.79	0.00	84.79	2	1	0	17	Medium quality	61607	1799589	56.40	53	d__Bacteria;p__Proteobacteria;c__Gammaproteobacteria;o__Burkholderiales;f__Ferrovaceae;g__Ferrovum;s__
LMSG_GO000010824.1	no	1337_1	Pyrite-Copper	86.28	0.91	81.71	1	0	0	15	Medium quality	12524	1739704	56.70	176	d__Bacteria;p__Proteobacteria;c__Gammaproteobacteria;o__Burkholderiales;f__Ferrovaceae;g__Ferrovum;s__
LMSG_GO000010825.1	no	1337_1	Pyrite-Copper	92.49	0.00	92.49	2	0	0	19	Medium quality	121547	1989004	56.70	36	d__Bacteria;p__Proteobacteria;c__Gammaproteobacteria;o__Burkholderiales;f__Ferrovaceae;g__Ferrovum;s__
LMSG_GO000010826.1	no	1337_1	Pyrite	89.49	0.00	89.49	2	0	0	18	Medium quality	104636	1863538	56.60	48	d__Bacteria;p__Proteobacteria;c__Gammaproteobacteria;o__Burkholderiales;f__Ferrovaceae;g__Ferrovum;s__
LMSG_GO000010827.1	no	1337_2	Coal	63.90	1.66	55.61	1	2	1	16	Medium quality	105438	1681454	55.20	55	d__Bacteria;p__Proteobacteria;c__Gammaproteobacteria;o__Burkholderiales;f__Ferrovaceae;g__Ferrovum;s__
LMSG_GO000010828.1	no	1337_3	Polymetallic	94.62	2.95	79.86	3	1	14	18	High quality	84261	2125681	56.40	164	d__Bacteria;p__Proteobacteria;c__Gammaproteobacteria;o__Burkholderiales;f__Ferrovaceae;g__Ferrovum;s__
LMSG_GO000010829.1	no	1337_3	Polymetallic	92.49	1.50	84.99	0	1	0	18	Medium quality	37498	1925804	56.60	85	d__Bacteria;p__Proteobacteria;c__Gammaproteobacteria;o__Burkholderiales;f__Ferrovaceae;g__Ferrovum;s__
LMSG_GO000010830.1	no	1337_3	Polymetallic	59.43	0.03	59.27	0	0	0	12	Medium quality	18450	1044839	57.40	91	d__Bacteria;p__Proteobacteria;c__Gammaproteobacteria;o__Burkholderiales;f__Ferrovaceae;g__Ferrovum;s__
LMSG_GO000010831.1	no	1337_3	Polymetallic	71.07	1.83	61.93	1	1	0	17	Medium quality	18465	1376919	56.70	187	d__Bacteria;p__Proteobacteria;c__Gammaproteobacteria;o__Burkholderiales;f__Ferrovaceae;g__Ferrovum;s__
LMSG_GO000010832.1	no	1337_3	Polymetallic	73.59	0.00	73.59	2	0	0	16	Medium quality	18423	1359405	56.40	131	d__Bacteria;p__Proteobacteria;c__Gammaproteobacteria;o__Burkholderiales;f__Ferrovaceae;g__Ferrovum;s__
LMSG_GO000010833.1	no	1337_3	Polymetallic	92.32	1.23	86.19	0	1	0	17	Medium quality	45965	1891394	56.60	83	d__Bacteria;p__Proteobacteria;c__Gammaproteobacteria;o__Burkholderiales;f__Ferrovaceae;g__Ferrovum;s__
LMSG_GO000010834.1	no	1337_3	Polymetallic	86.33	1.08	80.92	2	1	0	16	Medium quality	39748	1794431	56.60	81	d__Bacteria;p__Proteobacteria;c__Gammaproteobacteria;o__Burkholderiales;f__Ferrovaceae;g__Ferrovum;s__
LMSG_GO000010835.1	no	1339_1	Pyrite	53.67	0.47	51.31	1	1	0	16	Medium quality	35509	1400316	56.10	76	d__Bacteria;p__Proteobacteria;c__Gammaproteobacteria;o__Burkholderiales;f__Ferrovaceae;g__Ferrovum;s__
LMSG_GO000010836.1	no	287_1	Lead-Zinc	97.70	1.45	90.46	1	3	5	19	High quality	125714	1968470	41.20	89	d__Bacteria;p__Proteobacteria;c__Gammaproteobacteria;o__Burkholderiales;f__Ferrovaceae;g__PN-J185;s__
LMSG_GO000010837.1	no	287_1	Polymetallic	92.28	2.78	78.36	1	5	5	15	Medium quality	10307	1868222	40.80	299	d__Bacteria;p__Proteobacteria;c__Gammaproteobacteria;o__Burkholderiales;f__Ferrovaceae;g__PN-J185;s__
LMSG_GO000010838.1	no	287_1	Polymetallic	92.04	0.00	92.04	2	1	1	15	Medium quality	91539	1603266	40.40	30	d__Bacteria;p__Proteobacteria;c__Gammaproteobacteria;o__Burkholderiales;f__Ferrovaceae;g__PN-J185;s__
LMSG_GO000010839.1	no	287_1	Polymetallic	96.87	0.98	92.00	1	2	3	17	Medium quality	48141	1937574	40.90	117	d__Bacteria;p__Proteobacteria;c__Gammaproteobacteria;o__Burkholderiales;f__Ferrovaceae;g__PN-J185;s__
LMSG_GO000010840.1	no	287_1	Pyrite	70.00	1.72	61.38	1	0	1	13	Medium quality	116775	1327374	41.30	28	d__Bacteria;p__Proteobacteria;c__Gammaproteobacteria;o__Burkholderiales;f__Ferrovaceae;g__PN-J185;s__
LMSG_GO000010841.1	no	287_1	Copper	87.55	1.42	80.45	1	3	0	14	Medium quality	120746	1603198	40.50	41	d__Bacteria;p__Proteobacteria;c__Gammaproteobacteria;o__Burkholderiales;f__Ferrovaceae;g__PN-J185;s__
LMSG_GO000010842.1	no	287_1	Lead-Zinc	90.08	1.45	82.84	0	1	0	15	Medium quality	105547	1602976	40.40	55	d__Bacteria;p__Proteobacteria;c__Gammaproteobacteria;o__Burkholderiales;f__Ferrovaceae;g__PN-J185;s__
LMSG_GO000010843.1	no	287_1	Copper	67.62	0.98	62.75	1	0	1	12	Medium quality	5660	1092791	40.90	227	d__Bacteria;p__Proteobacteria;c__Gammaproteobacteria;o__Burkholderiales;f__Ferrovaceae;g__PN-J185;s__
LMSG_GO000010844.1	no	287_1	Lead-Zinc	80.63	0.95	75.90	1	1	0	15	Medium quality	109260	1459151	40.80	40	d__Bacteria;p__Proteobacteria;c__Gammaproteobacteria;o__Burkholderiales;f__Ferrovaceae;g__PN-J185;s__
LMSG_GO000010845.1	no	287_1	Polymetallic	94.01	2.37	82.15	1	0	1	17	Medium quality	8910	1772334	41.00	293	d__Bacteria;p__Proteobacteria;c__Gammaproteobacteria;o__Burkholderiales;f__Ferrovaceae;g__PN-J185;s__
LMSG_GO000010846.1	no	287_1	Polymetallic	59.42	1.24	53.22	0	0	0	10	Medium quality	2097	1015319	41.20	520	d__Bacteria;p__Proteobacteria;c__Gammaproteobacteria;o__Burkholderiales;f__Ferrovaceae;g__PN-J185;s__
LMSG_GO000010847.1	no	287_1	Polymetallic	74.18	0.32	72.61	0	0	1	14	Medium quality	6017	1240660	40.60	252	d__Bacteria;p__Proteobacteria;c__Gammaproteobacteria;o__Burkholderiales;f__Ferrovaceae;g__PN-J185;s__
LMSG_GO000010848.1	no	287_1	Pyrite-Copper	55.55	0.71	52.00	0	1	0	5	Medium quality	1989	854726	41.30	448	d__Bacteria;p__Proteobacteria;c__Gammaproteobacteria;o__Burkholderiales;f__Ferrovaceae;g__PN-J185;s__
LMSG_GO000010849.1	no	287_1	Lead-Zinc	94.39	0.53	91.75	2	1	0	17	Medium quality	66054	1725244	41.20	97	d__Bacteria;p__Proteobacteria;c__Gammaproteobacteria;o__Burkholderiales;f__Ferrovaceae;g__PN-J185;s__

LMSG_G000010850.1	no	288_1	Nickel-Copper	97.23	0.98	92.36	1	1	2	18	High quality	110161	1919382	43.70	48	d__Bacteria;p__Proteobacteria;c__Gammaproteobacteria;o__Burkholderiales;f__Ferrovaceae;g__PN-J185;s__PN-J185 sp001431705
LMSG_G000010851.1	no	288_1	Nickel-Copper	80.38	0.50	77.88	0	1	0	14	Medium quality	108081	1399576	44.30	25	d__Bacteria;p__Proteobacteria;c__Gammaproteobacteria;o__Burkholderiales;f__Ferrovaceae;g__PN-J185;s__PN-J185 sp001431705
LMSG_G000010852.1	no	286_1	Lead-Zinc	96.98	2.63	83.86	3	2	2	18	High quality	45253	1922074	40.20	89	d__Bacteria;p__Proteobacteria;c__Gammaproteobacteria;o__Burkholderiales;f__Ferrovaceae;g__PN-J185;s__PN-J185 sp001581925
LMSG_G000010853.1	no	286_1	Pyrite	83.07	0.47	80.71	2	17	29	15	Medium quality	115198	1335749	41.30	92	d__Bacteria;p__Proteobacteria;c__Gammaproteobacteria;o__Burkholderiales;f__Ferrovaceae;g__PN-J185;s__PN-J185 sp001581925
LMSG_G000010854.1	no	286_1	Lead-Zinc	95.47	2.95	80.71	1	0	0	14	Medium quality	175656	1811668	39.60	63	d__Bacteria;p__Proteobacteria;c__Gammaproteobacteria;o__Burkholderiales;f__Ferrovaceae;g__PN-J185;s__PN-J185 sp001581925
LMSG_G000010855.1	no	1233_1	Nickel-Copper	99.52	1.46	92.24	1	0	1	18	Medium quality	230677	3273579	52.60	41	d__Bacteria;p__Proteobacteria;c__Gammaproteobacteria;o__Burkholderiales;f__Gallionellaceae;g__39-52-133;s__39-52-133 sp002278935
LMSG_G000010856.1	no	1233_1	Nickel-Copper	97.69	2.54	85.00	0	1	0	19	Medium quality	101700	3157518	52.80	44	d__Bacteria;p__Proteobacteria;c__Gammaproteobacteria;o__Burkholderiales;f__Gallionellaceae;g__39-52-133;s__39-52-133 sp002278935
LMSG_G000010857.1	no	1235_1	Lead-Zinc	98.33	2.22	87.22	0	3	0	17	Medium quality	45004	3230992	54.90	107	d__Bacteria;p__Proteobacteria;c__Gammaproteobacteria;o__Burkholderiales;f__Gallionellaceae;g__Gallionella;s__
LMSG_G000010858.1	no	1236_1	Antimony	79.72	1.79	70.79	0	1	0	13	Medium quality	43151	1997016	57.00	64	d__Bacteria;p__Proteobacteria;c__Gammaproteobacteria;o__Burkholderiales;f__Gallionellaceae;g__Gallionella;s__
LMSG_G000010859.1	no	1236_1	Antimony	67.61	1.94	57.92	0	1	0	13	Medium quality	6843	1472806	57.20	240	d__Bacteria;p__Proteobacteria;c__Gammaproteobacteria;o__Burkholderiales;f__Gallionellaceae;g__Gallionella;s__
LMSG_G000010860.1	no	1236_1	Antimony	67.06	2.36	55.27	0	0	0	12	Medium quality	6815	1438444	57.50	251	d__Bacteria;p__Proteobacteria;c__Gammaproteobacteria;o__Burkholderiales;f__Gallionellaceae;g__Gallionella;s__
LMSG_G000010861.1	no	1236_1	Coal	81.66	0.80	77.66	0	1	0	14	Medium quality	9174	1757846	57.50	256	d__Bacteria;p__Proteobacteria;c__Gammaproteobacteria;o__Burkholderiales;f__Gallionellaceae;g__Gallionella;s__
LMSG_G000010862.1	no	1532_1	Antimony	66.90	1.11	61.35	0	0	0	16	Medium quality	62340	1563927	57.00	37	d__Bacteria;p__Proteobacteria;c__Gammaproteobacteria;o__Burkholderiales;f__Gallionellaceae;g__Gallionella;s__
LMSG_G000010863.1	no	1533_1	Lead-Zinc	94.12	1.46	86.82	1	1	1	19	High quality	96778	2457907	58.40	34	d__Bacteria;p__Proteobacteria;c__Gammaproteobacteria;o__Burkholderiales;f__Gallionellaceae;g__Gallionella;s__
LMSG_G000010864.1	no	1533_1	Lead-Zinc	90.79	1.11	85.24	0	1	0	17	Medium quality	89481	2354219	58.30	48	d__Bacteria;p__Proteobacteria;c__Gammaproteobacteria;o__Burkholderiales;f__Gallionellaceae;g__Gallionella;s__
LMSG_G000010865.1	no	1533_1	Polymetallic	73.60	2.80	59.62	0	0	0	7	Medium quality	3572	1596230	59.00	523	d__Bacteria;p__Proteobacteria;c__Gammaproteobacteria;o__Burkholderiales;f__Gallionellaceae;g__Gallionella;s__
LMSG_G000010866.1	no	1533_1	Polymetallic	77.44	3.78	58.55	0	0	0	12	Medium quality	4410	1715349	59.00	449	d__Bacteria;p__Proteobacteria;c__Gammaproteobacteria;o__Burkholderiales;f__Gallionellaceae;g__Gallionella;s__
LMSG_G000010867.1	no	1533_1	Polymetallic	94.47	2.12	83.86	2	1	0	19	Medium quality	17497	2262602	58.50	174	d__Bacteria;p__Proteobacteria;c__Gammaproteobacteria;o__Burkholderiales;f__Gallionellaceae;g__Gallionella;s__
LMSG_G000010868.1	no	1221_1	Lead-Zinc	51.72	0.00	51.72	0	0	0	11	Medium quality	117839	2126157	58.10	25	d__Bacteria;p__Proteobacteria;c__Gammaproteobacteria;o__Burkholderiales;f__Gallionellaceae;g__PALSA-1006;s__
LMSG_G000010869.1	no	1221_1	Lead-Zinc	54.31	0.00	54.31	0	0	0	13	Medium quality	6459	2149164	58.70	406	d__Bacteria;p__Proteobacteria;c__Gammaproteobacteria;o__Burkholderiales;f__Gallionellaceae;g__PALSA-1006;s__
LMSG_G000010870.1	no	1229_1	Lead-Zinc	53.46	0.00	53.46	0	0	0	9	Medium quality	18543	1422348	60.70	99	d__Bacteria;p__Proteobacteria;c__Gammaproteobacteria;o__Burkholderiales;f__Gallionellaceae;g__Sideroxydans;s__
LMSG_G000010871.1	no	1230_1	Antimony	63.17	1.35	56.43	1	0	1	11	Medium quality	8903	1852616	59.60	242	d__Bacteria;p__Proteobacteria;c__Gammaproteobacteria;o__Burkholderiales;f__Gallionellaceae;g__Sideroxydans;s__
LMSG_G000010872.1	no	1216_1	Lead-Zinc	83.25	0.75	79.52	0	0	0	12	Medium quality	64057	2373282	56.20	57	d__Bacteria;p__Proteobacteria;c__Gammaproteobacteria;o__Burkholderiales;f__Nitrosomonadaceae;g__;s__
LMSG_G000010873.1	no	1216_1	Lead-Zinc	56.49	1.18	50.57	1	1	0	10	Medium quality	52156	1706683	56.30	44	d__Bacteria;p__Proteobacteria;c__Gammaproteobacteria;o__Burkholderiales;f__Nitrosomonadaceae;g__;s__
LMSG_G000010874.1	no	1218_1	Lead-Zinc	70.81	1.50	63.33	0	0	0	10	Medium quality	2516	1667542	57.00	722	d__Bacteria;p__Proteobacteria;c__Gammaproteobacteria;o__Burkholderiales;f__Nitrosomonadaceae;g__;s__
LMSG_G000010875.1	no	1218_2	Polymetallic	91.39	1.01	86.36	0	1	0	14	Medium quality	18716	2013229	58.20	157	d__Bacteria;p__Proteobacteria;c__Gammaproteobacteria;o__Burkholderiales;f__Nitrosomonadaceae;g__;s__
LMSG_G000010876.1	no	1218_2	Polymetallic	81.96	3.11	66.43	0	0	0	9	Medium quality	7543	1572765	58.50	301	d__Bacteria;p__Proteobacteria;c__Gammaproteobacteria;o__Burkholderiales;f__Nitrosomonadaceae;g__;s__
LMSG_G000010877.1	no	1218_2	Tin-Zinc	95.27	4.61	72.21	0	1	0	16	Medium quality	23444	2334438	57.70	147	d__Bacteria;p__Proteobacteria;c__Gammaproteobacteria;o__Burkholderiales;f__Nitrosomonadaceae;g__;s__
LMSG_G000010878.1	no	1309_1	Antimony	78.15	2.05	67.89	0	0	0	11	Medium quality	7007	2581730	68.70	420	d__Bacteria;p__Proteobacteria;c__Gammaproteobacteria;o__Burkholderiales;f__Palsa-1005;g__VBCG01;s__
LMSG_G000010879.1	no	1309_1	Antimony	83.26	1.28	76.89	0	0	0	14	Medium quality	10667	2606914	68.90	295	d__Bacteria;p__Proteobacteria;c__Gammaproteobacteria;o__Burkholderiales;f__Palsa-1005;g__VBCG01;s__
LMSG_G000010880.1	no	1322_1	Polymetallic	85.68	3.61	67.63	0	0	0	18	Medium quality	7775	2826292	56.90	460	d__Bacteria;p__Proteobacteria;c__Gammaproteobacteria;o__Burkholderiales;f__RBG-16-58-11;g__RBG-16-58-11;s__
LMSG_G000010881.1	no	1322_1	Polymetallic	94.46	2.34	82.78	1	0	1	18	Medium quality	28246	3267304	56.90	168	d__Bacteria;p__Proteobacteria;c__Gammaproteobacteria;o__Burkholderiales;f__RBG-16-58-11;g__RBG-16-58-11;s__
LMSG_G000010882.1	no	1323_1	Polymetallic	64.14	2.53	51.47	0	0	0	11	Medium quality	2595	1433766	58.30	617	d__Bacteria;p__Proteobacteria;c__Gammaproteobacteria;o__Burkholderiales;f__RBG-16-58-11;g__RBG-16-58-11;s__
LMSG_G000010883.1	no	1323_1	Polymetallic	68.04	1.38	61.13	1	0	0	14	Medium quality	3489	1560253	58.30	479	d__Bacteria;p__Proteobacteria;c__Gammaproteobacteria;o__Burkholderiales;f__RBG-16-58-11;g__RBG-16-58-11;s__
LMSG_G000010884.1	no	1323_1	Arsenic	74.01	1.83	64.85	0	0	1	12	Medium quality	5693	1748319	58.60	497	d__Bacteria;p__Proteobacteria;c__Gammaproteobacteria;o__Burkholderiales;f__RBG-16-58-11;g__RBG-16-58-11;s__
LMSG_G000010885.1	no	1323_1	Polymetallic	58.85	1.62	50.75	0	0	0	8	Medium quality	3026	1334136	58.60	463	d__Bacteria;p__Proteobacteria;c__Gammaproteobacteria;o__Burkholderiales;f__RBG-16-58-11;g__RBG-16-58-11;s__
LMSG_G000010886.1	no	1314_1	Antimony	68.96	1.45	61.72	0	0	0	13	Medium quality	4660	2367603	61.90	565	d__Bacteria;p__Proteobacteria;c__Gammaproteobacteria;o__Burkholderiales;f__Rhodocyclaceae;g__Azonexus;s__
LMSG_G000010887.1	no	1316_1	Polymetallic	71.02	2.51	58.50	1	0	0	15	Medium quality	11226	2371091	65.70	264	d__Bacteria;p__Proteobacteria;c__Gammaproteobacteria;o__Burkholderiales;f__Rhodocyclaceae;g__Sterolibacterium;s__
LMSG_G000010888.1	no	1316_1	Polymetallic	78.98	1.99	69.04	0	0	0	15	Medium quality	8749	2592130	65.90	353	d__Bacteria;p__Proteobacteria;c__Gammaproteobacteria;o__Burkholderiales;f__Rhodocyclaceae;g__Sterolibacterium;s__
LMSG_G000010889.1	no	1316_1	Lead-Zinc	67.24	0.00	67.24	1	0	0	19	Medium quality	17353	2580551	66.40	234	d__Bacteria;p__Proteobacteria;c__Gammaproteobacteria;o__Burkholderiales;f__Rhodocyclaceae;g__Sterolibacterium;s__
LMSG_G000010890.1	no	1316_1	Lead-Zinc	87.88	3.61	69.82	1	0	0	19	Medium quality	27765	3051938	65.70	167	d__Bacteria;p__Proteobacteria;c__Gammaproteobacteria;o__Burkholderiales;f__Rhodocyclaceae;g__Sterolibacterium;s__
LMSG_G000010891.1	no	1316_1	Lead-Zinc	65.51	0.00	65.51	0	0	0	15	Medium quality	26444	2656643	66.10	149	d__Bacteria;p__Proteobacteria;c__Gammaproteobacteria;o__Burkholderiales;f__Rhodocyclaceae;g__Sterolibacterium;s__
LMSG_G000010892.1	no	1316_1	Pyrite-Copper	94.80	4.66	71.50	2	0	0	18	Medium quality	37039	3620467	65.30	201	d__Bacteria;p__Proteobacteria;c__Gammaproteobacteria;o__Burkholderiales;f__Rhodocyclaceae;g__Sterolibacterium;s__
LMSG_G000010893.1	no	1311_1	Polymetallic	81.48	2.58	68.57	1	1	1	16	Medium quality	6730	3225807	69.90	620	d__Bacteria;p__Proteobacteria;c__Gammaproteobacteria;o__Burkholderiales;f__SG8-39;g__;s__
LMSG_G000010894.1	no	1311_1	Polymetallic	83.90	3.15	68.15	1	0	1	14	Medium quality	8243	3520709	69.80	536	d__Bacteria;p__Proteobacteria;c__Gammaproteobacteria;o__Burkholderiales;f__SG8-39;g__;s__
LMSG_G000010895.1	no	1251_1	Polymetallic	53.11	0.52	50.53	0	0	0	15	Medium quality	9575	2025775	64.10	226	d__Bacteria;p__Proteobacteria;c__Gammaproteobacteria;o__Burkholderiales;f__SG8-39;g__2-12-FULL-64-23;s__
LMSG_G000010896.1	no	1240_1	Copper	75.84	2.17	65.01	1	0	1	14	Medium quality	22503	2376765	64.70	148	d__Bacteria;p__Proteobacteria;c__Gammaproteobacteria;o__Burkholderiales;f__Sulfuricellaceae;g__;s__
LMSG_G000010897.1	no	1240_1	Polymetallic	90.04	2.92	75.43	3	0	0	18	Medium quality	104465	3367760	64.00	79	d__Bacteria;p__Proteobacteria;c__Gammaproteobacteria;o__Burkholderiales;f__Sulfuricellaceae;g__;s__
LMSG_G000010898.1	no	1240_1	Polymetallic	95.49	3.95	75.75	2	0	0	19	Medium quality	87771	3735796	63.70	74	d__Bacteria;p__Proteobacteria;c__Gammaproteobacteria;o__Burkholderiales;f__Sulfuricellaceae;g__;s__
LMSG_G000010899.1	no	1250_1	Lead-Zinc	80.77	2.33	69.11	1	0	1	17	Medium quality	20527	2480393	59.20	161	d__Bacteria;p__Proteobacteria;c__Gammaproteobacteria;o__Burkholderiales;f__Sulfuricellaceae;g__Sulfuricella;s__
LMSG_G000010900.1	no	1242_1	Lead-Zinc	91.01	3.59	73.05	0	0	0	16	Medium quality	21154	2759572	64.40	172	d__Bacteria;p__Proteobacteria;c__Gammaproteobacteria;o__Burkholderiales;f__Sulfuricellaceae;g__Sulfurirhabdus;s__
LMSG_G000010901.1	no	1242_1	Lead-Zinc	86.49	3.79	67.54	0	0	0	14	Medium quality	26396	2794096	64.30	151	d__Bacteria;p__Proteobacteria;c__Gammaproteobacteria;o__Burkholderiales;f__Sulfuricellaceae;g__Sulfurirhabdus;s__
LMSG_G000010902.1	no	1242_1	Lead-Zinc	87.51	4.34	65.79	0	0	0	16	Medium quality	18897	2890911	64.30	240	d__Bacteria;p__Proteobacteria;c__Gammaproteobacteria;o__Burkholderiales;f__Sulfuricellaceae;g__Sulfurirhabdus;s__
LMSG_G000010903.1	no	1242_1	Tin-Zinc	52.06	0.24	50.88	0	0	0	5	Medium quality	4366	1387141	65.10	350	d__Bacteria;p__Proteobacteria;c__Gammaproteobacteria;o__Burkholderiales;f__Sulfuricellaceae;g__Sulfurirhabdus;s__
LMSG_G000010904.1	no	1242_1	Lead-Zinc	81.46	3.24	65.27	0	0	0	12	Medium quality	21815	2582210	64.20	162	d__Bacteria;p__Proteobacteria;c__Gammaproteobacteria;o__Burkholderiales;f__Sulfuricellaceae;g__Sulfurirhabdus;s__

LMSG_G000010905.1	no	1242_1	Pyrite-Copper	86.75	2.65	73.52	0	0	0	13	Medium quality	21432	2663302	64.30	159	d__Bacteria;p__Proteobacteria;c__Gammaproteobacteria;o__Burkholderiales;f__Sulfuricellaceae;g__Sulfurirhabdus;s__
LMSG_G000010906.1	no	1241_1	Lead-Zinc	74.13	0.00	74.13	1	0	0	17	Medium quality	19980	2453045	63.90	172	d__Bacteria;p__Proteobacteria;c__Gammaproteobacteria;o__Burkholderiales;f__Sulfuricellaceae;g__UBA2239;s__
LMSG_G000010907.1	no	1241_1	Lead-Zinc	81.59	0.73	77.96	2	2	0	18	Medium quality	48194	2498665	64.00	91	d__Bacteria;p__Proteobacteria;c__Gammaproteobacteria;o__Burkholderiales;f__Sulfuricellaceae;g__UBA2239;s__
LMSG_G000010908.1	no	1241_1	Lead-Zinc	82.71	3.24	66.51	0	0	0	17	Medium quality	9834	2360140	64.30	333	d__Bacteria;p__Proteobacteria;c__Gammaproteobacteria;o__Burkholderiales;f__Sulfuricellaceae;g__UBA2239;s__
LMSG_G000010909.1	no	1223_1	Tin-Zinc	76.42	3.75	57.70	0	0	0	15	Medium quality	8079	1966260	53.40	281	d__Bacteria;p__Proteobacteria;c__Gammaproteobacteria;o__Burkholderiales;f__Sulfuriferulaceae;g__Sulfuriferula_A;s__
LMSG_G000010910.1	no	1226_1	Lead-Zinc	95.37	0.32	93.80	1	1	0	19	Medium quality	143591	2257495	57.70	20	d__Bacteria;p__Proteobacteria;c__Gammaproteobacteria;o__Burkholderiales;f__Sulfuriferulaceae;g__UBA2487;s__
LMSG_G000010911.1	no	1226_1	Lead-Zinc	96.32	1.42	89.22	1	1	0	19	Medium quality	150340	2272645	57.60	23	d__Bacteria;p__Proteobacteria;c__Gammaproteobacteria;o__Burkholderiales;f__Sulfuriferulaceae;g__UBA2487;s__
LMSG_G000010912.1	no	1226_1	Lead-Zinc	92.75	0.24	91.57	1	1	0	18	Medium quality	48531	2195728	57.80	82	d__Bacteria;p__Proteobacteria;c__Gammaproteobacteria;o__Burkholderiales;f__Sulfuriferulaceae;g__UBA2487;s__
LMSG_G000010913.1	no	1227_1	Pyrite-Copper	56.89	0.00	56.89	1	1	0	13	Medium quality	7095	1791126	53.20	338	d__Bacteria;p__Proteobacteria;c__Gammaproteobacteria;o__Burkholderiales;f__Sulfuriferulaceae;g__UBA2487;s__
LMSG_G000010914.1	no	1227_1	Arsenic	71.27	2.95	56.55	0	1	0	18	Medium quality	24875	1817774	52.90	104	d__Bacteria;p__Proteobacteria;c__Gammaproteobacteria;o__Burkholderiales;f__Sulfuriferulaceae;g__UBA2487;s__
LMSG_G000010915.1	no	1227_1	Copper	84.44	2.79	70.48	0	1	0	17	Medium quality	15131	2425968	53.00	305	d__Bacteria;p__Proteobacteria;c__Gammaproteobacteria;o__Burkholderiales;f__Sulfuriferulaceae;g__UBA2487;s__
LMSG_G000010916.1	no	1228_1	Antimony	91.39	2.19	80.44	0	1	0	19	Medium quality	19088	2359943	51.00	168	d__Bacteria;p__Proteobacteria;c__Gammaproteobacteria;o__Burkholderiales;f__Sulfuriferulaceae;g__UBA2487;s__
LMSG_G000010917.1	no	1228_1	Lead-Zinc	93.32	0.34	91.65	0	1	0	18	Medium quality	23081	2395984	50.90	164	d__Bacteria;p__Proteobacteria;c__Gammaproteobacteria;o__Burkholderiales;f__Sulfuriferulaceae;g__UBA2487;s__
LMSG_G000010918.1	no	1247_1	Tin-Zinc	95.37	0.47	93.01	1	1	1	18	High quality	45017	2218565	60.90	77	d__Bacteria;p__Proteobacteria;c__Gammaproteobacteria;o__Burkholderiales;f__Sulfuriferulaceae;g__UBA2487;s__
LMSG_G000010919.1	no	1247_1	Lead-Zinc	94.43	0.71	90.88	1	1	1	16	Medium quality	80334	2169209	60.90	51	d__Bacteria;p__Proteobacteria;c__Gammaproteobacteria;o__Burkholderiales;f__Sulfuriferulaceae;g__UBA2487;s__
LMSG_G000010920.1	no	1247_1	Copper	94.60	0.00	94.60	0	1	0	17	Medium quality	53532	2257000	60.90	62	d__Bacteria;p__Proteobacteria;c__Gammaproteobacteria;o__Burkholderiales;f__Sulfuriferulaceae;g__UBA2487;s__
LMSG_G000010921.1	no	1247_1	Lead-Zinc	94.90	0.09	94.43	0	2	0	17	Medium quality	54227	2147618	61.00	59	d__Bacteria;p__Proteobacteria;c__Gammaproteobacteria;o__Burkholderiales;f__Sulfuriferulaceae;g__UBA2487;s__
LMSG_G000010922.1	no	1247_1	Lead-Zinc	92.84	0.95	88.11	0	2	0	16	Medium quality	34041	2186357	60.90	90	d__Bacteria;p__Proteobacteria;c__Gammaproteobacteria;o__Burkholderiales;f__Sulfuriferulaceae;g__UBA2487;s__
LMSG_G000010923.1	no	1247_1	Lead-Zinc	90.22	1.52	82.65	0	0	0	15	Medium quality	12121	2160237	61.10	236	d__Bacteria;p__Proteobacteria;c__Gammaproteobacteria;o__Burkholderiales;f__Sulfuriferulaceae;g__UBA2487;s__
LMSG_G000010924.1	no	1247_1	Antimony	87.08	0.95	82.35	0	1	0	17	Medium quality	16341	2039770	60.90	164	d__Bacteria;p__Proteobacteria;c__Gammaproteobacteria;o__Burkholderiales;f__Sulfuriferulaceae;g__UBA2487;s__
LMSG_G000010925.1	no	1247_1	Polymetallic	56.89	0.00	56.89	1	0	1	10	Medium quality	3207	1374443	61.30	472	d__Bacteria;p__Proteobacteria;c__Gammaproteobacteria;o__Burkholderiales;f__Sulfuriferulaceae;g__UBA2487;s__
LMSG_G000010926.1	no	1247_1	Tin-Zinc	94.90	0.71	91.35	0	3	0	18	Medium quality	41876	2294029	60.80	91	d__Bacteria;p__Proteobacteria;c__Gammaproteobacteria;o__Burkholderiales;f__Sulfuriferulaceae;g__UBA2487;s__
LMSG_G000010927.1	no	1247_1	Pyrite-Copper	92.50	0.39	90.53	0	1	0	18	Medium quality	15341	2276405	60.80	212	d__Bacteria;p__Proteobacteria;c__Gammaproteobacteria;o__Burkholderiales;f__Sulfuriferulaceae;g__UBA2487;s__
LMSG_G000010928.1	no	1247_1	Pyrite-Copper	91.58	0.16	90.80	0	0	0	16	Medium quality	51108	2156464	61.00	79	d__Bacteria;p__Proteobacteria;c__Gammaproteobacteria;o__Burkholderiales;f__Sulfuriferulaceae;g__UBA2487;s__
LMSG_G000010929.1	no	1270_1	Lead-Zinc	89.68	2.90	75.20	0	1	0	14	Medium quality	52509	2466378	65.30	66	d__Bacteria;p__Proteobacteria;c__Gammaproteobacteria;o__Burkholderiales;f__Thiobacillaceae;g__;s__
LMSG_G000010930.1	no	1270_1	Lead-Zinc	86.07	2.05	75.81	0	0	0	16	Medium quality	30590	2573676	65.40	146	d__Bacteria;p__Proteobacteria;c__Gammaproteobacteria;o__Burkholderiales;f__Thiobacillaceae;g__;s__
LMSG_G000010931.1	no	1270_1	Polymetallic	55.17	0.00	55.17	0	0	0	9	Medium quality	27372	1179899	65.60	60	d__Bacteria;p__Proteobacteria;c__Gammaproteobacteria;o__Burkholderiales;f__Thiobacillaceae;g__;s__
LMSG_G000010932.1	no	1248_1	Lead-Zinc	62.51	2.13	51.84	0	0	0	8	Medium quality	6921	1368414	63.40	245	d__Bacteria;p__Proteobacteria;c__Gammaproteobacteria;o__Burkholderiales;f__Thiobacillaceae;g__Sulfuritortus;s__
LMSG_G000010933.1	no	1248_1	Lead-Zinc	64.93	1.65	56.70	2	0	2	18	Medium quality	9769	1807043	62.60	259	d__Bacteria;p__Proteobacteria;c__Gammaproteobacteria;o__Burkholderiales;f__Thiobacillaceae;g__Sulfuritortus;s__
LMSG_G000010934.1	no	1273_1	Lead-Zinc	70.39	1.52	62.82	0	0	0	15	Medium quality	17072	2084411	65.40	181	d__Bacteria;p__Proteobacteria;c__Gammaproteobacteria;o__Burkholderiales;f__Thiobacillaceae;g__Thiobacillus;s__
LMSG_G000010935.1	no	1277_1	Nickel-Copper	82.67	1.09	77.21	0	0	0	17	Medium quality	15160	2771800	61.90	247	d__Bacteria;p__Proteobacteria;c__Gammaproteobacteria;o__Burkholderiales;f__Thiobacillaceae;g__Thiobacillus;s__Thiobacillus sp002279795
LMSG_G000010936.1	no	1565_1	Polymetallic	92.02	2.17	81.18	1	2	0	19	Medium quality	48172	2482214	67.30	68	d__Bacteria;p__Proteobacteria;c__Gammaproteobacteria;o__Burkholderiales;f__Thiobacillaceae;g__UBA3361;s__
LMSG_G000010937.1	no	1565_1	Polymetallic	96.20	1.70	87.73	1	2	0	19	Medium quality	67594	2748344	67.20	71	d__Bacteria;p__Proteobacteria;c__Gammaproteobacteria;o__Burkholderiales;f__Thiobacillaceae;g__UBA3361;s__
LMSG_G000010938.1	no	1565_1	Polymetallic	98.34	3.56	80.55	1	2	0	17	Medium quality	54362	2753616	67.20	68	d__Bacteria;p__Proteobacteria;c__Gammaproteobacteria;o__Burkholderiales;f__Thiobacillaceae;g__UBA3361;s__
LMSG_G000010939.1	no	1565_1	Polymetallic	98.99	1.70	90.52	2	2	0	18	Medium quality	59179	2853747	67.20	78	d__Bacteria;p__Proteobacteria;c__Gammaproteobacteria;o__Burkholderiales;f__Thiobacillaceae;g__UBA3361;s__
LMSG_G000006326.1	no	1052_1	Polymetallic	84.20	0.00	84.20	1	0	0	18	Medium quality	24906	1698616	42.00	89	d__Bacteria;p__Proteobacteria;c__Gammaproteobacteria;o__Diplorickettsiales;f__Diplorickettsiaceae;g__;s__
LMSG_G000006327.1	no	1052_1	Polymetallic	89.82	0.50	87.31	2	4	6	20	Medium quality	22128	2506527	41.60	292	d__Bacteria;p__Proteobacteria;c__Gammaproteobacteria;o__Diplorickettsiales;f__Diplorickettsiaceae;g__;s__
LMSG_G000006328.1	no	1052_1	Polymetallic	94.57	0.00	94.57	1	0	0	18	Medium quality	25305	2117593	41.80	140	d__Bacteria;p__Proteobacteria;c__Gammaproteobacteria;o__Diplorickettsiales;f__Diplorickettsiaceae;g__;s__
LMSG_G000010940.1	no	1052_1	Copper	74.70	0.00	74.70	0	1	0	14	Medium quality	18966	1384975	42.40	106	d__Bacteria;p__Proteobacteria;c__Gammaproteobacteria;o__Diplorickettsiales;f__Diplorickettsiaceae;g__;s__
LMSG_G000010941.1	no	1588_1	Antimony	58.62	0.00	58.62	1	0	0	12	Medium quality	10693	3575050	33.90	445	d__Bacteria;p__Proteobacteria;c__Gammaproteobacteria;o__Diplorickettsiales;f__Diplorickettsiaceae;g__;s__
LMSG_G000006329.1	no	1588_1	Antimony	96.36	1.65	88.13	1	0	1	17	Medium quality	44323	3693116	34.00	168	d__Bacteria;p__Proteobacteria;c__Gammaproteobacteria;o__Diplorickettsiales;f__Diplorickettsiaceae;g__;s__
LMSG_G000006330.1	no	1588_1	Antimony	97.67	1.99	87.74	1	0	0	17	Medium quality	103046	3874368	33.90	101	d__Bacteria;p__Proteobacteria;c__Gammaproteobacteria;o__Diplorickettsiales;f__Diplorickettsiaceae;g__;s__
LMSG_G000006333.1	no	1588_1	Antimony	96.51	1.36	89.73	0	0	0	17	Medium quality	111353	3646305	34.10	135	d__Bacteria;p__Proteobacteria;c__Gammaproteobacteria;o__Diplorickettsiales;f__Diplorickettsiaceae;g__;s__
LMSG_G000010942.1	no	1052_1	Polymetallic	65.32	0.74	61.64	1	0	0	15	Medium quality	6548	1142956	42.60	219	d__Bacteria;p__Proteobacteria;c__Gammaproteobacteria;o__DSM-16500;f__DSM-16500;g__;s__
LMSG_G000010943.1	no	1587_1	Copper	83.95	3.10	68.45	0	0	0	11	Medium quality	28390	2264924	39.60	106	d__Bacteria;p__Proteobacteria;c__Gammaproteobacteria;o__DSM-16500;f__DSM-16500;g__SCGC-AG-212-F23;s__
LMSG_G000010944.1	no	1530_1	Polymetallic	97.91	0.04	97.73	8	2	1	18	High quality	323765	4921078	57.60	30	d__Bacteria;p__Proteobacteria;c__Gammaproteobacteria;o__Enterobacterales;f__Enterobacteriaceae;g__Klebsiella;s__Klebsiella pneumoniae
LMSG_G000010945.1	no	1530_1	Pyrite	99.30	0.21	98.25	7	0	0	18	Medium quality	247250	5213276	57.50	42	d__Bacteria;p__Proteobacteria;c__Gammaproteobacteria;o__Enterobacterales;f__Enterobacteriaceae;g__Klebsiella;s__Klebsiella pneumoniae
LMSG_G000010946.1	no	1530_1	Polymetallic	70.62	3.10	55.12	2	0	0	13	Medium quality	2316	3666442	58.20	1682	d__Bacteria;p__Proteobacteria;c__Gammaproteobacteria;o__Enterobacterales;f__Enterobacteriaceae;g__Klebsiella;s__Klebsiella pneumoniae
LMSG_G000010947.1	no	1530_1	Pyrite-Copper	98.76	0.76	94.98	3	0	1	18	Medium quality	359251	5161268	57.60	30	d__Bacteria;p__Proteobacteria;c__Gammaproteobacteria;o__Enterobacterales;f__Enterobacteriaceae;g__Klebsiella;s__Klebsiella pneumoniae
LMSG_G000010948.1	no	1530_1	Lead-Zinc	96.74	1.28	90.33	5	0	0	18	Medium quality	16044	4994622	57.90	442	d__Bacteria;p__Proteobacteria;c__Gammaproteobacteria;o__Enterobacterales;f__Enterobacteriaceae;g__Klebsiella;s__Klebsiella pneumoniae
LMSG_G000010949.1	no	1530_1	Lead-Zinc	99.55	0.12	98.96	7	1	0	18	Medium quality	148798	5424163	57.40	67	d__Bacteria;p__Proteobacteria;c__Gammaproteobacteria;o__Enterobacterales;f__Enterobacteriaceae;g__Klebsiella;s__Klebsiella pneumoniae
LMSG_G000010950.1	no	1530_1	Lead-Zinc	96.51	0.75	92.75	3	1	0	18	Medium quality	21407	4838100	58.10	304	d__Bacteria;p__Proteobacteria;c__Gammaproteobacteria;o__Enterobacterales;f__Enterobacteriaceae;g__Klebsiella;s__Klebsiella pneumoniae

LMSG_G000010951.1	no	262_1	Antimony	54.38	0.00	54.38	6	15	25	18	Medium quality	10443	3811958	47.00	726	d__Bacteria;p__Proteobacteria;c__Gammaproteobacteria;o__Enterobacterales;f__Shewanellaceae;g__Shewanella;s__
LMSG_G000010952.1	no	1115_1	Tin-Zinc	90.22	2.97	75.38	0	0	0	16	Medium quality	19391	2228064	55.50	152	d__Bacteria;p__Proteobacteria;c__Gammaproteobacteria;o__Halothiobacillales;f__Halothiobacillaceae;g__Halothiobacillus;s__
LMSG_G000010953.1	no	1171_1	Lead-Zinc	82.15	1.25	75.93	1	0	0	10	Medium quality	3746	1933433	53.40	639	d__Bacteria;p__Proteobacteria;c__Gammaproteobacteria;o__Halothiobacillales;f__Halothiobacillaceae;g__Halothiobacillus;s__
LMSG_G000010954.1	no	1171_1	Pyrite-Copper	98.85	0.77	95.02	2	0	0	18	Medium quality	376834	2597923	53.40	15	d__Bacteria;p__Proteobacteria;c__Gammaproteobacteria;o__Halothiobacillales;f__Halothiobacillaceae;g__Halothiobacillus;s__
LMSG_G000010955.1	no	1171_1	Nickel-Copper	74.95	3.75	56.22	1	0	1	8	Medium quality	2855	2125644	53.80	825	d__Bacteria;p__Proteobacteria;c__Gammaproteobacteria;o__Halothiobacillales;f__Halothiobacillaceae;g__Halothiobacillus;s__
LMSG_G000010956.1	no	1170_1	Nickel-Copper	98.85	1.92	89.28	1	1	1	19	High quality	42816	2531873	53.60	100	d__Bacteria;p__Proteobacteria;c__Gammaproteobacteria;o__Halothiobacillales;f__Halothiobacillaceae;g__Halothiobacillus;s__Halothiobacillus sp002281295
LMSG_G000010957.1	no	1170_1	Nickel-Copper	100.00	1.92	90.43	1	1	1	18	High quality	50024	2538161	53.50	107	d__Bacteria;p__Proteobacteria;c__Gammaproteobacteria;o__Halothiobacillales;f__Halothiobacillaceae;g__Halothiobacillus;s__Halothiobacillus sp002281295
LMSG_G000010958.1	no	1170_1	Nickel-Copper	100.00	1.92	90.43	1	0	0	17	Medium quality	41024	2527808	53.40	101	d__Bacteria;p__Proteobacteria;c__Gammaproteobacteria;o__Halothiobacillales;f__Halothiobacillaceae;g__Halothiobacillus;s__Halothiobacillus sp002281295
LMSG_G000010959.1	no	1115_2	Nickel-Copper	97.70	1.50	90.18	3	1	1	20	High quality	18543	2436009	55.60	209	d__Bacteria;p__Proteobacteria;c__Gammaproteobacteria;o__Halothiobacillales;f__Halothiobacillaceae;g__Halothiobacillus;s__Halothiobacillus sp002282715
LMSG_G000010960.1	no	1592_1	Lead-Zinc	77.58	0.00	77.58	0	0	0	15	Medium quality	14241	2206381	40.60	254	d__Bacteria;p__Proteobacteria;c__Gammaproteobacteria;o__Legionellales;f__Legionellaceae;g__DDPF01;s__
LMSG_G000010961.1	no	34_1	Lead-Zinc	74.13	3.45	56.89	1	1	0	17	Medium quality	24068	3509153	37.80	290	d__Bacteria;p__Proteobacteria;c__Gammaproteobacteria;o__Legionellales;f__Legionellaceae;g__Legionella;s__
LMSG_G000010962.1	no	1189_1	Lead-Zinc	70.68	0.00	70.68	1	0	1	13	Medium quality	38390	1407521	66.90	71	d__Bacteria;p__Proteobacteria;c__Gammaproteobacteria;o__Porisulfidales;f__Porisulfidaceae;g__;s__
LMSG_G000010963.1	no	1185_1	Polymetallic	91.98	0.93	87.36	2	1	1	16	Medium quality	31466	2470868	61.30	159	d__Bacteria;p__Proteobacteria;c__Gammaproteobacteria;o__Pseudomonadales;f__Moraxellaceae;g__;s__
LMSG_G000010964.1	no	1185_1	Antimony	89.75	0.56	86.98	0	0	0	17	Medium quality	21937	2344345	61.40	153	d__Bacteria;p__Proteobacteria;c__Gammaproteobacteria;o__Pseudomonadales;f__Moraxellaceae;g__;s__
LMSG_G000010965.1	no	1185_1	Polymetallic	57.72	0.00	57.72	1	0	0	14	Medium quality	3820	1795986	61.90	560	d__Bacteria;p__Proteobacteria;c__Gammaproteobacteria;o__Pseudomonadales;f__Moraxellaceae;g__;s__
LMSG_G000010966.1	no	1185_1	Polymetallic	81.27	2.19	70.32	0	0	0	16	Medium quality	4555	2044588	61.60	514	d__Bacteria;p__Proteobacteria;c__Gammaproteobacteria;o__Pseudomonadales;f__Moraxellaceae;g__;s__
LMSG_G000010967.1	no	1186_1	Polymetallic	91.47	1.30	84.99	0	0	0	14	Medium quality	20272	2359955	62.40	180	d__Bacteria;p__Proteobacteria;c__Gammaproteobacteria;o__Pseudomonadales;f__Moraxellaceae;g__;s__
LMSG_G000010968.1	no	1186_1	Polymetallic	94.81	3.66	76.53	1	0	0	15	Medium quality	18489	2410693	62.40	191	d__Bacteria;p__Proteobacteria;c__Gammaproteobacteria;o__Pseudomonadales;f__Moraxellaceae;g__;s__
LMSG_G000010969.1	no	1186_1	Polymetallic	86.54	2.17	75.70	0	0	0	11	Medium quality	19725	2238483	62.50	167	d__Bacteria;p__Proteobacteria;c__Gammaproteobacteria;o__Pseudomonadales;f__Moraxellaceae;g__;s__
LMSG_G000010970.1	no	1186_1	Polymetallic	89.07	0.93	84.45	0	2	0	13	Medium quality	20198	2168156	62.50	163	d__Bacteria;p__Proteobacteria;c__Gammaproteobacteria;o__Pseudomonadales;f__Moraxellaceae;g__;s__
LMSG_G000010971.1	no	1186_1	Polymetallic	91.97	0.74	88.27	2	0	0	17	Medium quality	19154	2414679	62.30	187	d__Bacteria;p__Proteobacteria;c__Gammaproteobacteria;o__Pseudomonadales;f__Moraxellaceae;g__;s__
LMSG_G000010972.1	no	1186_1	Antimony	69.82	0.00	69.82	1	1	0	13	Medium quality	4017	1764492	62.70	485	d__Bacteria;p__Proteobacteria;c__Gammaproteobacteria;o__Pseudomonadales;f__Moraxellaceae;g__;s__
LMSG_G000010973.1	no	1186_1	Antimony	94.32	1.15	88.55	0	1	0	19	Medium quality	41364	2471792	62.10	90	d__Bacteria;p__Proteobacteria;c__Gammaproteobacteria;o__Pseudomonadales;f__Moraxellaceae;g__;s__
LMSG_G000010974.1	no	1186_1	Antimony	95.37	0.80	91.36	0	1	0	18	Medium quality	23762	2576538	62.10	156	d__Bacteria;p__Proteobacteria;c__Gammaproteobacteria;o__Pseudomonadales;f__Moraxellaceae;g__;s__
LMSG_G000010975.1	no	1186_1	Antimony	93.66	0.90	89.19	0	1	0	18	Medium quality	23952	2513939	62.00	150	d__Bacteria;p__Proteobacteria;c__Gammaproteobacteria;o__Pseudomonadales;f__Moraxellaceae;g__;s__
LMSG_G000010976.1	no	1186_1	Antimony	94.38	1.05	89.14	1	0	0	17	Medium quality	15332	2414494	62.30	219	d__Bacteria;p__Proteobacteria;c__Gammaproteobacteria;o__Pseudomonadales;f__Moraxellaceae;g__;s__
LMSG_G000010977.1	no	1186_1	Polymetallic	90.00	2.73	76.35	1	1	0	17	Medium quality	20252	2291881	62.40	169	d__Bacteria;p__Proteobacteria;c__Gammaproteobacteria;o__Pseudomonadales;f__Moraxellaceae;g__;s__
LMSG_G000010978.1	no	1186_1	Pyrite	88.76	3.83	69.63	3	1	0	16	Medium quality	42946	2346119	62.50	99	d__Bacteria;p__Proteobacteria;c__Gammaproteobacteria;o__Pseudomonadales;f__Moraxellaceae;g__;s__
LMSG_G000010979.1	no	1187_1	Lead-Zinc	94.13	0.19	93.21	0	1	0	18	Medium quality	44626	2375705	61.80	77	d__Bacteria;p__Proteobacteria;c__Gammaproteobacteria;o__Pseudomonadales;f__Moraxellaceae;g__;s__
LMSG_G000010980.1	no	1187_1	Pyrite-Copper	64.13	2.59	51.20	0	0	0	13	Medium quality	16975	2016286	62.10	307	d__Bacteria;p__Proteobacteria;c__Gammaproteobacteria;o__Pseudomonadales;f__Moraxellaceae;g__;s__
LMSG_G000010981.1	no	1187_1	Pyrite-Copper	75.86	0.00	75.86	1	0	0	15	Medium quality	7742	1991489	62.30	322	d__Bacteria;p__Proteobacteria;c__Gammaproteobacteria;o__Pseudomonadales;f__Moraxellaceae;g__;s__
LMSG_G000010982.1	no	1187_1	Polymetallic	94.62	1.67	86.29	0	0	0	19	Medium quality	17269	2247844	62.10	174	d__Bacteria;p__Proteobacteria;c__Gammaproteobacteria;o__Pseudomonadales;f__Moraxellaceae;g__;s__
LMSG_G000010983.1	no	1187_1	Polymetallic	94.13	1.11	88.58	0	0	0	17	Medium quality	24720	2305666	62.00	124	d__Bacteria;p__Proteobacteria;c__Gammaproteobacteria;o__Pseudomonadales;f__Moraxellaceae;g__;s__
LMSG_G000010984.1	no	1187_1	Polymetallic	95.24	0.19	94.32	0	0	0	19	Medium quality	52986	2559704	61.60	86	d__Bacteria;p__Proteobacteria;c__Gammaproteobacteria;o__Pseudomonadales;f__Moraxellaceae;g__;s__
LMSG_G000010985.1	no	1187_1	Polymetallic	97.83	0.74	94.13	0	0	0	19	Medium quality	52633	2568126	61.50	72	d__Bacteria;p__Proteobacteria;c__Gammaproteobacteria;o__Pseudomonadales;f__Moraxellaceae;g__;s__
LMSG_G000010986.1	no	1187_1	Polymetallic	97.83	0.37	95.98	0	1	1	20	Medium quality	50834	2539042	61.70	84	d__Bacteria;p__Proteobacteria;c__Gammaproteobacteria;o__Pseudomonadales;f__Moraxellaceae;g__;s__
LMSG_G000010987.1	no	1187_1	Polymetallic	53.76	0.00	53.76	0	1	0	11	Medium quality	4976	1570382	62.50	369	d__Bacteria;p__Proteobacteria;c__Gammaproteobacteria;o__Pseudomonadales;f__Moraxellaceae;g__;s__
LMSG_G000010988.1	no	1187_1	Polymetallic	86.98	2.41	74.95	0	1	0	16	Medium quality	26805	2082266	62.20	141	d__Bacteria;p__Proteobacteria;c__Gammaproteobacteria;o__Pseudomonadales;f__Moraxellaceae;g__;s__
LMSG_G000010989.1	no	1187_1	Polymetallic	81.03	0.86	76.72	0	0	0	16	Medium quality	14875	1974957	62.10	199	d__Bacteria;p__Proteobacteria;c__Gammaproteobacteria;o__Pseudomonadales;f__Moraxellaceae;g__;s__
LMSG_G000010990.1	no	1187_1	Polymetallic	67.24	0.00	67.24	0	0	0	13	Medium quality	10081	1810480	62.30	235	d__Bacteria;p__Proteobacteria;c__Gammaproteobacteria;o__Pseudomonadales;f__Moraxellaceae;g__;s__
LMSG_G000010991.1	no	1187_1	Polymetallic	94.87	1.48	87.47	1	0	1	18	Medium quality	30111	2325253	61.90	115	d__Bacteria;p__Proteobacteria;c__Gammaproteobacteria;o__Pseudomonadales;f__Moraxellaceae;g__;s__
LMSG_G000010992.1	no	1187_1	Polymetallic	94.13	1.11	88.58	0	0	0	16	Medium quality	46736	2318637	61.90	72	d__Bacteria;p__Proteobacteria;c__Gammaproteobacteria;o__Pseudomonadales;f__Moraxellaceae;g__;s__
LMSG_G000010993.1	no	1187_1	Polymetallic	93.39	2.50	80.89	0	0	0	17	Medium quality	43222	2408934	61.70	90	d__Bacteria;p__Proteobacteria;c__Gammaproteobacteria;o__Pseudomonadales;f__Moraxellaceae;g__;s__
LMSG_G000010994.1	no	1187_1	Polymetallic	72.57	0.86	68.26	0	0	0	18	Medium quality	17101	2266482	61.50	235	d__Bacteria;p__Proteobacteria;c__Gammaproteobacteria;o__Pseudomonadales;f__Moraxellaceae;g__;s__
LMSG_G000010995.1	no	1187_1	Polymetallic	88.94	1.11	83.39	0	1	0	19	Medium quality	22136	2300411	61.60	179	d__Bacteria;p__Proteobacteria;c__Gammaproteobacteria;o__Pseudomonadales;f__Moraxellaceae;g__;s__
LMSG_G000010996.1	no	1187_1	Tin-Zinc	93.20	3.00	78.22	2	0	0	18	Medium quality	11766	2320906	62.10	283	d__Bacteria;p__Proteobacteria;c__Gammaproteobacteria;o__Pseudomonadales;f__Moraxellaceae;g__;s__
LMSG_G000010997.1	no	1187_1	Tin-Zinc	88.92	0.93	84.30	0	0	0	17	Medium quality	10354	2225644	62.10	312	d__Bacteria;p__Proteobacteria;c__Gammaproteobacteria;o__Pseudomonadales;f__Moraxellaceae;g__;s__
LMSG_G000010998.1	no	1192_1	Polymetallic	90.06	0.56	87.29	0	0	0	17	Medium quality	31512	2600991	52.10	112	d__Bacteria;p__Proteobacteria;c__Gammaproteobacteria;o__Pseudomonadales;f__Moraxellaceae;g__;s__
LMSG_G000010999.1	no	1193_1	Lead-Zinc	91.62	0.62	88.54	1	0	1	17	Medium quality	19089	2704083	50.40	200	d__Bacteria;p__Proteobacteria;c__Gammaproteobacteria;o__Pseudomonadales;f__Moraxellaceae;g__;s__
LMSG_G000011000.1	no	1193_1	Lead-Zinc	85.60	1.56	77.78	1	0	1	17	Medium quality	5438	2512403	50.50	535	d__Bacteria;p__Proteobacteria;c__Gammaproteobacteria;o__Pseudomonadales;f__Moraxellaceae;g__;s__
LMSG_G000011001.1	no	323_1	Polymetallic	83.49	3.20	67.50	0	0	0	19	Medium quality	4854	2407278	51.30	669	d__Bacteria;p__Proteobacteria;c__Gammaproteobacteria;o__Pseudomonadales;f__Moraxellaceae;g__;s__
LMSG_G000011002.1	no	323_1	Polymetallic	96.25	0.75	92.49	1	0	0	18	Medium quality	29475	2852155	51.00	173	d__Bacteria;p__Proteobacteria;c__Gammaproteobacteria;o__Pseudomonadales;f__Moraxellaceae;g__;s__
LMSG_G000011003.1	no	323_1	Polymetallic	95.92	0.56	93.15	0	0	0	18	Medium quality	30257	2743201	51.00	135	d__Bacteria;p__Proteobacteria;c__Gammaproteobacteria;o__Pseudomonadales;f__Moraxellaceae;g__;s__
LMSG_G000011004.1	no	323_1	Polymetallic	95.18	1.34	88.49	1	0	0	17	Medium quality	53016	2793198	51.20	126	d__Bacteria;p__Proteobacteria;c__Gammaproteobacteria;o__Pseudomonadales;f__Moraxellaceae;g__;s__
LMSG_G000011005.1	no	473_1	Polymetallic	71.19	0.65	67.95	0	0	0	14	Medium quality	5478	1906824	51.70	389	d__Bacteria;p__Proteobacteria;c__Gammaproteobacteria;o__Pseudomonadales;f__Moraxellaceae;g__;s__
LMSG_G000011006.1	no	473_1	Polymetallic	85.76	1.67	77.43	0	0	0	16	Medium quality	7920	2504617	51.60	453	d__Bacteria;p__Proteobacteria;c__Gammaproteobacteria;o__Pseudomonadales;f__Moraxellaceae;g__;s__
LMSG_G000011007.1	no	257_1	Polymetallic	83.92	1.14	78.22	1	5	5	18	Medium quality	9873	3368152	39.20	625	d__Bacteria;p__Proteobacteria;c__Gammaproteobacteria;o__Pseudomonadales;f__Moraxellaceae;g__Acinetobacter;s__Acinetobacter baumannii
LMSG_G000011008.1	no	1404_1	Copper	98.37	0.11	97.83	1	1	1	19	High quality	266032	6424814	66.20	49	d__Bacteria;p__Proteobacteria;c__Gammaproteobacteria;o__Pseudomonadales;f__Pseudomonadaceae;g__Pseudomonas;s__Pseudomonas aeruginosa
LMSG_G000011009.1	no	1404_1	Copper	94.80	0.11	94.26	1	1	1	18	High quality	209552	6320010	66.30	47	d__Bacteria;p__Proteobacteria;c__Gammaproteobacteria;o__Pseudomonadales;f__Pseudomonadaceae;g__Pseudomonas;s__Pseudomonas aeruginosa
LMSG_G000011010.1	no	1404_1	Polymetallic	99.02	0.95	94.27	2	1	1	19	High quality	216385	6982680	65.90	73	d__Bacteria;p__Proteobacteria;c__Gammaproteobacteria;o__Pseudomonadales;f__Pseudomonadaceae;g__Pseudomonas;s__Pseudomonas aeruginosa
LMSG_G000011011.1	no	1404_1	Polymetallic	98.05	0.69	94.61	2	1	1	19	High quality	212629	7217022	65.60	76	d__Bacteria;p__Proteobacteria;c__Gammaproteobacteria;o__Pseudomonadales;f__Pseudomonadaceae;g__Pseudomonas;s__Pseudomonas aeruginosa
LMSG_G000011012.1	no	1404_1	Polymetallic	99.02	0.64	95.82	2	1	1	19	High quality	216385	6973520	65.90	69	d__Bacteria;p__Proteobacteria;c__Gammaproteobacteria;o__Pseudomonadales;f__Pseudomonadaceae;g__Pseudomonas;s__Pseudomonas aeruginosa
LMSG_G000011013.1	no	1404_1	Polymetallic	99.02	1.00	94.01	2	1	1	19	High quality	201136	6938937	65.90	70	d__Bacteria;p__Proteobacteria;c__Gammaproteobacteria;o__Pseudomonadales;f__Pseudomonadaceae;g__Pseudomonas;s__Pseudomonas aeruginosa
LMSG_G000011014.1	no	1404_1	Copper	98.54	0.46	96.25	1	0	0	18	Medium quality	106262	6379627	66.30	108	d__Bacteria;p__Proteobacteria;c__Gammaproteobacteria;o__Pseudomonadales;f__Pseudomonadaceae;g__Pseudomonas;s__Pseudomonas aeruginosa
LMSG_G000011015.1	no	1404_1	Lead-Zinc	99.02	0.30	97.53	0	0	0	18	Medium quality	203398	6492537	66.20	56	d__Bacteria;p__Proteobacteria;c__Gammaproteobacteria;o__Pseudomonadales;f__Pseudomonadaceae;g__Pseudomonas;s__Pseudomonas aeruginosa
LMSG_G000011016.1	no	1404_1	Lead-Zinc	81.65	1.56	73.88	0	0	0	8	Medium quality	5664	5079390	67.00	1035	d__Bacteria;p__Proteobacteria;c__Gammaproteobacteria;o__Pseudomonadales;f__Pseudomonadaceae;g__Pseudomonas;s__Pseudomonas aeruginosa

LMSG_G000011017.1	no	1404_1	Copper	98.37	0.74	94.70	1	0	0	18	Medium quality	318171	6446544	66.30	49	d__Bacteria;p__Proteobacteria;c__Gammaproteobacteria;o__Pseudomonadales;f__Pseudomonadaceae;g__Pseudomonas;s__Pseudomonas aeruginosa
LMSG_G000011018.1	no	1404_1	Copper	94.78	1.77	85.92	0	1	0	16	Medium quality	117120	6176732	66.50	104	d__Bacteria;p__Proteobacteria;c__Gammaproteobacteria;o__Pseudomonadales;f__Pseudomonadaceae;g__Pseudomonas;s__Pseudomonas aeruginosa
LMSG_G000011019.1	no	1404_1	Lead-Zinc	85.53	1.47	78.20	1	0	0	14	Medium quality	8831	5462850	66.60	781	d__Bacteria;p__Proteobacteria;c__Gammaproteobacteria;o__Pseudomonadales;f__Pseudomonadaceae;g__Pseudomonas;s__Pseudomonas aeruginosa
LMSG_G000011020.1	no	1404_1	Polymetallic	96.68	0.91	92.12	2	0	0	17	Medium quality	217708	6803966	65.90	52	d__Bacteria;p__Proteobacteria;c__Gammaproteobacteria;o__Pseudomonadales;f__Pseudomonadaceae;g__Pseudomonas;s__Pseudomonas aeruginosa
LMSG_G000011021.1	no	1404_1	Polymetallic	63.15	0.00	63.15	2	1	0	14	Medium quality	121509	3927855	65.80	49	d__Bacteria;p__Proteobacteria;c__Gammaproteobacteria;o__Pseudomonadales;f__Pseudomonadaceae;g__Pseudomonas;s__Pseudomonas aeruginosa
LMSG_G000011022.1	no	1404_1	Polymetallic	96.99	0.95	92.26	2	1	0	16	Medium quality	180504	6707079	66.10	56	d__Bacteria;p__Proteobacteria;c__Gammaproteobacteria;o__Pseudomonadales;f__Pseudomonadaceae;g__Pseudomonas;s__Pseudomonas aeruginosa
LMSG_G000011023.1	no	1404_1	Polymetallic	93.12	0.76	89.35	2	1	0	19	Medium quality	26437	6108894	64.90	383	d__Bacteria;p__Proteobacteria;c__Gammaproteobacteria;o__Pseudomonadales;f__Pseudomonadaceae;g__Pseudomonas;s__Pseudomonas aeruginosa
LMSG_G000011024.1	no	1116_1	Polymetallic	66.51	2.93	51.87	2	1	2	14	Medium quality	13158	1637157	58.90	165	d__Bacteria;p__Proteobacteria;c__Gammaproteobacteria;o__SLND01;f__;g__;s__
LMSG_G000011025.1	no	1116_1	Coal	88.04	1.61	79.99	0	0	0	17	Medium quality	4879	2257128	58.40	560	d__Bacteria;p__Proteobacteria;c__Gammaproteobacteria;o__SLND01;f__;g__;s__
LMSG_G000011026.1	no	1117_1	Pyrite	95.85	2.36	84.03	1	2	1	18	High quality	29777	2484361	60.50	139	d__Bacteria;p__Proteobacteria;c__Gammaproteobacteria;o__SLND01;f__;g__;s__
LMSG_G000011027.1	no	1117_1	Copper	69.05	0.16	68.25	1	1	2	17	Medium quality	23241	1814731	60.60	122	d__Bacteria;p__Proteobacteria;c__Gammaproteobacteria;o__SLND01;f__;g__;s__
LMSG_G000011028.1	no	1117_1	Copper	60.14	0.45	57.88	1	1	2	12	Medium quality	45677	1618903	60.50	58	d__Bacteria;p__Proteobacteria;c__Gammaproteobacteria;o__SLND01;f__;g__;s__
LMSG_G000011029.1	no	1117_1	Copper	94.47	2.83	80.31	1	0	1	18	Medium quality	33554	2409885	60.20	128	d__Bacteria;p__Proteobacteria;c__Gammaproteobacteria;o__SLND01;f__;g__;s__
LMSG_G000011030.1	no	1117_1	Copper	83.15	2.02	73.07	1	0	1	17	Medium quality	28102	2083118	60.30	110	d__Bacteria;p__Proteobacteria;c__Gammaproteobacteria;o__SLND01;f__;g__;s__
LMSG_G000011031.1	no	1117_1	Copper	88.58	3.38	71.71	1	0	1	19	Medium quality	23382	2291705	60.60	126	d__Bacteria;p__Proteobacteria;c__Gammaproteobacteria;o__SLND01;f__;g__;s__
LMSG_G000011032.1	no	1117_1	Copper	87.71	1.18	81.83	1	1	1	18	Medium quality	23215	2310071	60.40	135	d__Bacteria;p__Proteobacteria;c__Gammaproteobacteria;o__SLND01;f__;g__;s__
LMSG_G000011033.1	no	1117_1	Copper	86.84	1.70	78.35	1	1	1	19	Medium quality	49066	2441192	60.10	105	d__Bacteria;p__Proteobacteria;c__Gammaproteobacteria;o__SLND01;f__;g__;s__
LMSG_G000011034.1	no	1117_1	Copper	71.31	2.54	58.63	0	0	0	13	Medium quality	67307	2122627	60.70	53	d__Bacteria;p__Proteobacteria;c__Gammaproteobacteria;o__SLND01;f__;g__;s__
LMSG_G000011035.1	no	1117_1	Pyrite-Copper	67.53	2.38	55.64	0	0	0	14	Medium quality	4453	1660710	61.20	419	d__Bacteria;p__Proteobacteria;c__Gammaproteobacteria;o__SLND01;f__;g__;s__
LMSG_G000011036.1	no	1117_1	Pyrite-Copper	57.21	1.26	50.89	0	0	0	14	Medium quality	3786	1401710	61.10	419	d__Bacteria;p__Proteobacteria;c__Gammaproteobacteria;o__SLND01;f__;g__;s__
LMSG_G000011037.1	no	1117_2	Copper	77.48	1.54	69.79	1	0	1	16	Medium quality	18989	1966748	60.70	157	d__Bacteria;p__Proteobacteria;c__Gammaproteobacteria;o__SLND01;f__;g__;s__
LMSG_G000011038.1	no	1117_2	Copper	83.89	1.20	77.89	1	0	1	16	Medium quality	20844	2152130	60.40	149	d__Bacteria;p__Proteobacteria;c__Gammaproteobacteria;o__SLND01;f__;g__;s__
LMSG_G000011039.1	no	1117_2	Copper	93.02	2.26	81.70	1	0	1	17	Medium quality	27383	2552128	60.50	144	d__Bacteria;p__Proteobacteria;c__Gammaproteobacteria;o__SLND01;f__;g__;s__
LMSG_G000011040.1	no	1117_2	Copper	90.50	3.01	75.44	1	0	1	17	Medium quality	23061	2500139	60.40	153	d__Bacteria;p__Proteobacteria;c__Gammaproteobacteria;o__SLND01;f__;g__;s__
LMSG_G000011041.1	no	1117_2	Copper	83.58	1.30	77.09	1	0	1	15	Medium quality	27426	2261195	60.40	119	d__Bacteria;p__Proteobacteria;c__Gammaproteobacteria;o__SLND01;f__;g__;s__
LMSG_G000011042.1	no	1117_2	Copper	66.84	0.00	66.84	1	0	0	15	Medium quality	11115	1960091	60.70	331	d__Bacteria;p__Proteobacteria;c__Gammaproteobacteria;o__SLND01;f__;g__;s__
LMSG_G000011043.1	no	1117_2	Copper	85.32	1.23	79.17	1	0	1	18	Medium quality	28581	2263145	60.40	125	d__Bacteria;p__Proteobacteria;c__Gammaproteobacteria;o__SLND01;f__;g__;s__
LMSG_G000011044.1	no	1117_2	Polymetallic	93.99	1.66	85.69	1	1	2	18	High quality	23794	2458967	60.60	169	d__Bacteria;p__Proteobacteria;c__Gammaproteobacteria;o__SLND01;f__;g__;s__
LMSG_G000011045.1	no	1117_2	Polymetallic	95.28	2.26	83.96	1	1	1	18	High quality	30042	2440877	60.70	137	d__Bacteria;p__Proteobacteria;c__Gammaproteobacteria;o__SLND01;f__;g__;s__
LMSG_G000011046.1	no	1117_2	Polymetallic	94.02	3.29	77.57	1	1	1	19	High quality	18829	2705604	60.40	223	d__Bacteria;p__Proteobacteria;c__Gammaproteobacteria;o__SLND01;f__;g__;s__
LMSG_G000011047.1	no	1117_2	Coal	95.19	2.63	82.06	1	1	1	20	High quality	23279	2611437	60.50	195	d__Bacteria;p__Proteobacteria;c__Gammaproteobacteria;o__SLND01;f__;g__;s__
LMSG_G000011048.1	no	1117_2	Coal	91.77	1.36	84.98	1	1	1	17	Medium quality	22065	2343540	60.70	158	d__Bacteria;p__Proteobacteria;c__Gammaproteobacteria;o__SLND01;f__;g__;s__
LMSG_G000011049.1	no	1117_2	Polymetallic	84.18	2.99	69.24	0	1	0	13	Medium quality	11332	1917545	60.80	206	d__Bacteria;p__Proteobacteria;c__Gammaproteobacteria;o__SLND01;f__;g__;s__
LMSG_G000011050.1	no	1117_2	Polymetallic	94.59	1.72	85.99	1	0	1	19	Medium quality	22635	2514907	60.70	177	d__Bacteria;p__Proteobacteria;c__Gammaproteobacteria;o__SLND01;f__;g__;s__
LMSG_G000011051.1	no	1117_2	Polymetallic	92.92	4.13	72.27	0	0	0	16	Medium quality	12243	2216746	61.10	261	d__Bacteria;p__Proteobacteria;c__Gammaproteobacteria;o__SLND01;f__;g__;s__
LMSG_G000011052.1	no	1117_2	Polymetallic	82.55	3.70	64.06	0	0	0	14	Medium quality	18569	2044116	60.80	172	d__Bacteria;p__Proteobacteria;c__Gammaproteobacteria;o__SLND01;f__;g__;s__
LMSG_G000011053.1	no	1117_2	Polymetallic	67.24	0.00	67.24	2	3	8	18	Medium quality	30578	1858768	60.30	275	d__Bacteria;p__Proteobacteria;c__Gammaproteobacteria;o__SLND01;f__;g__;s__
LMSG_G000011054.1	no	1117_2	Coal	93.65	2.08	83.24	0	0	0	15	Medium quality	24678	2177272	61.10	135	d__Bacteria;p__Proteobacteria;c__Gammaproteobacteria;o__SLND01;f__;g__;s__
LMSG_G000011055.1	no	1117_2	Tin-Zinc	62.24	0.00	62.24	0	0	0	13	Medium quality	5742	1650293	61.30	328	d__Bacteria;p__Proteobacteria;c__Gammaproteobacteria;o__SLND01;f__;g__;s__
LMSG_G000011056.1	no	1117_2	Tin-Zinc	67.16	2.10	56.67	0	0	0	13	Medium quality	5681	1527407	61.20	298	d__Bacteria;p__Proteobacteria;c__Gammaproteobacteria;o__SLND01;f__;g__;s__
LMSG_G000011057.1	no	575_1	Polymetallic	63.57	0.36	61.76	1	1	1	15	Medium quality	26300	1444188	60.90	78	d__Bacteria;p__Proteobacteria;c__Gammaproteobacteria;o__SLND01;f__;g__;s__
LMSG_G000011058.1	no	1076_1	Pyrite	68.42	1.54	60.73	0	1	0	13	Medium quality	90832	1707825	65.80	37	d__Bacteria;p__Proteobacteria;c__Gammaproteobacteria;o__Steroidobacterales;f__Steroidobacteraceae;g__;s__
LMSG_G000011059.1	no	1076_1	Lead-Zinc	85.14	3.15	69.39	1	0	1	17	Medium quality	47086	2718012	65.90	147	d__Bacteria;p__Proteobacteria;c__Gammaproteobacteria;o__Steroidobacterales;f__Steroidobacteraceae;g__;s__
LMSG_G000011060.1	no	1076_1	Copper	67.94	2.26	56.62	0	1	0	15	Medium quality	68508	2333458	65.00	65	d__Bacteria;p__Proteobacteria;c__Gammaproteobacteria;o__Steroidobacterales;f__Steroidobacteraceae;g__;s__
LMSG_G000011061.1	no	1077_1	Lead-Zinc	75.88	3.14	60.18	0	1	1	16	Medium quality	45498	2509300	67.00	80	d__Bacteria;p__Proteobacteria;c__Gammaproteobacteria;o__Steroidobacterales;f__Steroidobacteraceae;g__;s__
LMSG_G000011062.1	no	1077_1	Lead-Zinc	82.91	4.89	58.46	1	1	1	15	Medium quality	23996	2827686	67.00	159	d__Bacteria;p__Proteobacteria;c__Gammaproteobacteria;o__Steroidobacterales;f__Steroidobacteraceae;g__;s__
LMSG_G000011063.1	no	1077_1	Polymetallic	62.06	1.72	53.44	0	1	0	17	Medium quality	32646	2558241	66.80	113	d__Bacteria;p__Proteobacteria;c__Gammaproteobacteria;o__Steroidobacterales;f__Steroidobacteraceae;g__;s__
LMSG_G000011064.1	no	1077_1	Polymetallic	59.62	0.48	57.23	1	1	0	9	Medium quality	33040	1679549	67.60	84	d__Bacteria;p__Proteobacteria;c__Gammaproteobacteria;o__Steroidobacterales;f__Steroidobacteraceae;g__;s__
LMSG_G000011065.1	no	1077_1	Tin-Zinc	91.46	3.77	72.62	1	0	1	15	Medium quality	41890	3075383	67.10	127	d__Bacteria;p__Proteobacteria;c__Gammaproteobacteria;o__Steroidobacterales;f__Steroidobacteraceae;g__;s__
LMSG_G000011066.1	no	1077_1	Copper	55.67	0.80	51.69	0	0	0	16	Medium quality	32668	2113803	66.40	100	d__Bacteria;p__Proteobacteria;c__Gammaproteobacteria;o__Steroidobacterales;f__Steroidobacteraceae;g__;s__
LMSG_G000011067.1	no	1077_1	Copper	70.10	1.18	64.18	1	1	0	17	Medium quality	29398	2386672	66.70	129	d__Bacteria;p__Proteobacteria;c__Gammaproteobacteria;o__Steroidobacterales;f__Steroidobacteraceae;g__;s__
LMSG_G000011068.1	no	1077_1	Pyrite-Copper	74.73	2.82	60.65	1	0	0	13	Medium quality	26097	2124584	67.40	125	d__Bacteria;p__Proteobacteria;c__Gammaproteobacteria;o__Steroidobacterales;f__Steroidobacteraceae;g__;s__
LMSG_G000011069.1	no	1077_1	Pyrite	65.17	2.70	51.68	0	0	0	15	Medium quality	12856	3077385	66.40	284	d__Bacteria;p__Proteobacteria;c__Gammaproteobacteria;o__Steroidobacterales;f__Steroidobacteraceae;g__;s__
LMSG_G000011070.1	no	1077_1	Polymetallic	60.34	0.00	60.34	1	0	1	12	Medium quality	32177	2074298	67.40	119	d__Bacteria;p__Proteobacteria;c__Gammaproteobacteria;o__Steroidobacterales;f__Steroidobacteraceae;g__;s__
LMSG_G000011071.1	no	1077_1	Polymetallic	62.71	0.00	62.71	1	0	1	17	Medium quality	9881	3762395	65.90	467	d__Bacteria;p__Proteobacteria;c__Gammaproteobacteria;o__Steroidobacterales;f__Steroidobacteraceae;g__;s__
LMSG_G000011072.1	no	1078_1	Copper	51.72	0.00	51.72	0	1	0	17	Medium quality	38181	2458084	66.60	118	d__Bacteria;p__Proteobacteria;c__Gammaproteobacteria;o__Steroidobacterales;f__Steroidobacteraceae;g__;s__
LMSG_G000011073.1	no	1078_1	Polymetallic	74.26	2.88	59.89	0	0	0	17	Medium quality	14330	2733053	67.20	225	d__Bacteria;p__Proteobacteria;c__Gammaproteobacteria;o__Steroidobacterales;f__Steroidobacteraceae;g__;s__
LMSG_G000011074.1	no	1078_1	Copper	73.06	2.71	59.51	1	1	0	18	Medium quality	44046	2962144	66.40	132	d__Bacteria;p__Proteobacteria;c__Gammaproteobacteria;o__Steroidobacterales;f__Steroidobacteraceae;g__;s__
LMSG_G000011075.1	no	1078_1	Copper	67.54	3.20	51.55	1	1	1	14	Medium quality	20055	2631109	66.90	330	d__Bacteria;p__Proteobacteria;c__Gammaproteobacteria;o__Steroidobacterales;f__Steroidobacteraceae;g__;s__
LMSG_G000011076.1	no	1082_1	Lead-Zinc	88.58	4.45	66.33	1	1	1	17	Medium quality	91146	3213408	66.20	81	d__Bacteria;p__Proteobacteria;c__Gammaproteobacteria;o__Steroidobacterales;f__Steroidobacteraceae;g__;s__
LMSG_G000011077.1	no	1082_1	Pyrite	84.49	4.43	62.34	1	1	1	18	Medium quality	62606	3500923	66.30	105	d__Bacteria;p__Proteobacteria;c__Gammaproteobacteria;o__Steroidobacterales;f__Steroidobacteraceae;g__;s__
LMSG_G000011078.1	no	1082_1	Pyrite	60.34	0.00	60.34	0	0	0	11	Medium quality	61781	1448892	67.80	36	d__Bacteria;p__Proteobacteria;c__Gammaproteobacteria;o__Steroidobacterales;f__Steroidobacteraceae;g__;s__

LMSG_G000011079.1	no	1082_1	Polymetallic	79.08	3.45	61.81	1	1	1	16	Medium quality	41710	2726557	66.90	105	d__Bacteria;p__Proteobacteria;c__Gammaproteobacteria;o__Steroidobacterales;f__Steroidobacteraceae;g__;s__
LMSG_G000011080.1	no	1082_1	Polymetallic	54.34	0.73	50.68	1	1	1	12	Medium quality	22005	1806813	66.80	113	d__Bacteria;p__Proteobacteria;c__Gammaproteobacteria;o__Steroidobacterales;f__Steroidobacteraceae;g__;s__
LMSG_G000011081.1	no	1082_1	Polymetallic	68.16	0.37	66.30	0	1	0	17	Medium quality	34227	2444220	66.90	128	d__Bacteria;p__Proteobacteria;c__Gammaproteobacteria;o__Steroidobacterales;f__Steroidobacteraceae;g__;s__
LMSG_G000011082.1	no	1084_1	Copper	78.29	4.71	54.74	0	0	0	18	Medium quality	69135	3453876	65.60	120	d__Bacteria;p__Proteobacteria;c__Gammaproteobacteria;o__Steroidobacterales;f__Steroidobacteraceae;g__;s__
LMSG_G000011083.1	no	1088_1	Lead-Zinc	67.81	3.43	50.68	1	1	1	12	Medium quality	61108	2843882	67.10	74	d__Bacteria;p__Proteobacteria;c__Gammaproteobacteria;o__Steroidobacterales;f__Steroidobacteraceae;g__;s__
LMSG_G000011084.1	no	1088_1	Magnetite	58.96	1.72	50.34	1	0	1	16	Medium quality	38277	3103428	67.00	123	d__Bacteria;p__Proteobacteria;c__Gammaproteobacteria;o__Steroidobacterales;f__Steroidobacteraceae;g__;s__
LMSG_G000011085.1	no	1089_1	Antimony	72.41	3.45	55.17	1	1	1	14	Medium quality	85393	3076529	67.20	74	d__Bacteria;p__Proteobacteria;c__Gammaproteobacteria;o__Steroidobacterales;f__Steroidobacteraceae;g__;s__
LMSG_G000011086.1	no	1118_1	Lead-Zinc	68.48	3.64	50.31	0	1	0	15	Medium quality	4268	2325082	69.50	623	d__Bacteria;p__Proteobacteria;c__Gammaproteobacteria;o__Steroidobacterales;f__Steroidobacteraceae;g__;s__
LMSG_G000011087.1	no	1118_1	Pyrite-Copper	61.15	1.99	51.19	1	1	1	16	Medium quality	42288	2305837	68.60	111	d__Bacteria;p__Proteobacteria;c__Gammaproteobacteria;o__Steroidobacterales;f__Steroidobacteraceae;g__;s__
LMSG_G000011088.1	no	1119_1	Polymetallic	84.68	3.13	69.03	0	1	0	18	Medium quality	7040	2653557	69.30	481	d__Bacteria;p__Proteobacteria;c__Gammaproteobacteria;o__Steroidobacterales;f__Steroidobacteraceae;g__;s__
LMSG_G000011089.1	no	1119_1	Antimony	84.30	3.41	67.28	0	1	1	16	Medium quality	29458	2826428	69.30	153	d__Bacteria;p__Proteobacteria;c__Gammaproteobacteria;o__Steroidobacterales;f__Steroidobacteraceae;g__;s__
LMSG_G000011090.1	no	1119_1	Polymetallic	83.93	2.85	69.68	1	1	0	11	Medium quality	8285	2507055	69.80	434	d__Bacteria;p__Proteobacteria;c__Gammaproteobacteria;o__Steroidobacterales;f__Steroidobacteraceae;g__;s__
LMSG_G000011091.1	no	1119_1	Pyrite-Copper	78.62	2.29	67.15	0	1	0	12	Medium quality	17155	2385281	69.60	187	d__Bacteria;p__Proteobacteria;c__Gammaproteobacteria;o__Steroidobacterales;f__Steroidobacteraceae;g__;s__
LMSG_G000011092.1	no	1119_2	Polymetallic	76.17	4.08	55.79	1	1	0	14	Medium quality	15484	2726137	68.80	238	d__Bacteria;p__Proteobacteria;c__Gammaproteobacteria;o__Steroidobacterales;f__Steroidobacteraceae;g__;s__
LMSG_G000011093.1	no	1119_2	Pyrite-Copper	57.91	0.00	57.91	1	0	0	12	Medium quality	3332	1983883	69.40	639	d__Bacteria;p__Proteobacteria;c__Gammaproteobacteria;o__Steroidobacterales;f__Steroidobacteraceae;g__;s__
LMSG_G000011094.1	no	1120_1	Copper	77.59	2.84	63.40	0	0	0	17	Medium quality	14098	2464279	65.20	204	d__Bacteria;p__Proteobacteria;c__Gammaproteobacteria;o__Steroidobacterales;f__Steroidobacteraceae;g__;s__
LMSG_G000011095.1	no	1093_1	Coal	58.15	1.45	50.91	1	1	1	14	Medium quality	67285	1315781	69.30	26	d__Bacteria;p__Proteobacteria;c__Gammaproteobacteria;o__Steroidobacterales;f__Steroidobacteraceae;g__FEN-1191;s__
LMSG_G000011096.1	no	259_1	Nickel-Copper	93.90	0.00	93.90	0	0	0	17	Medium quality	41354	2020411	46.00	93	d__Bacteria;p__Proteobacteria;c__Gammaproteobacteria;o__Thiomicrospirales;f__Thiomicrospiraceae;g__GCA-002282575;s__GCA-002282575 sp002281095
LMSG_G000011097.1	no	259_1	Nickel-Copper	97.01	1.22	90.92	1	0	1	18	Medium quality	16067	1831110	46.30	152	d__Bacteria;p__Proteobacteria;c__Gammaproteobacteria;o__Thiomicrospirales;f__Thiomicrospiraceae;g__GCA-002282575;s__GCA-002282575 sp002281095
LMSG_G000011098.1	no	259_1	Nickel-Copper	74.82	0.00	74.82	1	0	1	11	Medium quality	4548	1170886	46.80	252	d__Bacteria;p__Proteobacteria;c__Gammaproteobacteria;o__Thiomicrospirales;f__Thiomicrospiraceae;g__GCA-002282575;s__GCA-002282575 sp002281095
LMSG_G000011099.1	no	259_1	Nickel-Copper	80.18	0.00	80.18	0	0	0	9	Medium quality	5878	1568834	46.40	347	d__Bacteria;p__Proteobacteria;c__Gammaproteobacteria;o__Thiomicrospirales;f__Thiomicrospiraceae;g__GCA-002282575;s__GCA-002282575 sp002281095
LMSG_G000011100.1	no	259_1	Lead-Zinc	75.86	2.51	63.33	2	0	0	16	Medium quality	10698	1575430	45.80	194	d__Bacteria;p__Proteobacteria;c__Gammaproteobacteria;o__Thiomicrospirales;f__Thiomicrospiraceae;g__GCA-002282575;s__GCA-002282575 sp002281095
LMSG_G000011101.1	no	259_1	Polymetallic	65.57	0.96	60.80	0	0	0	9	Medium quality	2007	1145959	46.20	624	d__Bacteria;p__Proteobacteria;c__Gammaproteobacteria;o__Thiomicrospirales;f__Thiomicrospiraceae;g__GCA-002282575;s__GCA-002282575 sp002281095
LMSG_G000011102.1	no	1584_1	Antimony	98.25	3.29	81.78	0	0	0	19	Medium quality	92625	1914212	41.80	45	d__Bacteria;p__Proteobacteria;c__Gammaproteobacteria;o__UBA1113;f__UBA1113;g__;s__
LMSG_G000006332.1	no	1585_1	Antimony	81.68	3.07	66.36	1	0	0	14	Medium quality	19505	1933382	41.30	193	d__Bacteria;p__Proteobacteria;c__Gammaproteobacteria;o__UBA1113;f__UBA1113;g__;s__
LMSG_G000011103.1	no	1586_1	Antimony	91.37	0.17	90.54	1	0	0	16	Medium quality	25372	2061621	41.20	154	d__Bacteria;p__Proteobacteria;c__Gammaproteobacteria;o__UBA1113;f__UBA1113;g__;s__
LMSG_G000006334.1	no	1586_1	Antimony	89.95	0.86	85.64	0	0	0	17	Medium quality	66600	1970068	41.10	71	d__Bacteria;p__Proteobacteria;c__Gammaproteobacteria;o__UBA1113;f__UBA1113;g__;s__
LMSG_G000011104.1	no	1586_1	Polymetallic	86.45	2.47	74.10	1	2	3	17	Medium quality	6073	1898914	41.70	426	d__Bacteria;p__Proteobacteria;c__Gammaproteobacteria;o__UBA1113;f__UBA1113;g__;s__
LMSG_G000011105.1	no	1586_1	Copper	88.66	2.66	75.34	0	0	0	17	Medium quality	18866	2127725	41.10	164	d__Bacteria;p__Proteobacteria;c__Gammaproteobacteria;o__UBA1113;f__UBA1113;g__;s__
LMSG_G000011106.1	no	1189_1	Lead-Zinc	81.60	1.72	72.98	1	1	2	17	Medium quality	54278	1722062	66.10	71	d__Bacteria;p__Proteobacteria;c__Gammaproteobacteria;o__UBA2770;f__UBA2770;g__;s__
LMSG_G000011107.1	no	1189_1	Pyrite-Copper	87.21	1.76	78.43	1	1	2	17	Medium quality	26224	1728921	66.70	94	d__Bacteria;p__Proteobacteria;c__Gammaproteobacteria;o__UBA2770;f__UBA2770;g__;s__
LMSG_G000011108.1	no	1189_1	Lead-Zinc	73.70	2.30	62.21	0	0	0	14	Medium quality	30132	1397642	67.50	70	d__Bacteria;p__Proteobacteria;c__Gammaproteobacteria;o__UBA2770;f__UBA2770;g__;s__
LMSG_G000011109.1	no	1189_2	Polymetallic	66.77	1.62	58.68	1	1	1	9	Medium quality	26390	1253200	66.70	63	d__Bacteria;p__Proteobacteria;c__Gammaproteobacteria;o__UBA2770;f__UBA2770;g__;s__
LMSG_G000011110.1	no	1189_2	Magnetite	64.84	1.71	56.30	0	1	2	17	Medium quality	5872	1627182	65.40	463	d__Bacteria;p__Proteobacteria;c__Gammaproteobacteria;o__UBA2770;f__UBA2770;g__;s__
LMSG_G000011111.1	no	1208_1	Lead-Zinc	62.49	1.57	54.64	1	1	1	10	Medium quality	3318	1357790	62.60	467	d__Bacteria;p__Proteobacteria;c__Gammaproteobacteria;o__UBA2770;f__UBA2770;g__;s__
LMSG_G000011112.1	no	571_1	Polymetallic	59.78	1.34	53.08	0	0	0	10	Medium quality	2502	1266482	61.20	555	d__Bacteria;p__Proteobacteria;c__Gammaproteobacteria;o__UBA2770;f__UBA2770;g__;s__
LMSG_G000011113.1	no	571_1	Polymetallic	86.89	1.54	79.20	1	0	1	17	Medium quality	19608	1960684	61.10	149	d__Bacteria;p__Proteobacteria;c__Gammaproteobacteria;o__UBA2770;f__UBA2770;g__;s__
LMSG_G000011114.1	no	571_1	Polymetallic	81.73	3.08	66.34	1	1	1	18	Medium quality	8992	1837238	61.30	284	d__Bacteria;p__Proteobacteria;c__Gammaproteobacteria;o__UBA2770;f__UBA2770;g__;s__
LMSG_G000011115.1	no	571_1	Tin-Zinc	72.83	1.34	66.14	0	1	0	14	Medium quality	5230	1624163	61.50	384	d__Bacteria;p__Proteobacteria;c__Gammaproteobacteria;o__UBA2770;f__UBA2770;g__;s__
LMSG_G000011116.1	no	571_1	Tin-Zinc	74.88	2.10	64.39	0	0	0	18	Medium quality	5581	1637444	61.30	355	d__Bacteria;p__Proteobacteria;c__Gammaproteobacteria;o__UBA2770;f__UBA2770;g__;s__
LMSG_G000011117.1	no	571_1	Polymetallic	67.64	2.29	56.17	1	0	0	14	Medium quality	3301	1487953	61.00	520	d__Bacteria;p__Proteobacteria;c__Gammaproteobacteria;o__UBA2770;f__UBA2770;g__;s__
LMSG_G000011118.1	no	571_1	Lead-Zinc	68.58	1.15	62.84	5	4	8	19	Medium quality	32791	2127927	62.10	221	d__Bacteria;p__Proteobacteria;c__Gammaproteobacteria;o__UBA2770;f__UBA2770;g__;s__
LMSG_G000011119.1	no	571_1	Lead-Zinc	77.58	0.00	77.58	0	1	0	17	Medium quality	52510	1677985	61.60	57	d__Bacteria;p__Proteobacteria;c__Gammaproteobacteria;o__UBA2770;f__UBA2770;g__;s__
LMSG_G000011120.1	no	571_1	Pyrite-Copper	87.04	1.43	79.90	1	1	1	19	Medium quality	39186	2000003	61.20	90	d__Bacteria;p__Proteobacteria;c__Gammaproteobacteria;o__UBA2770;f__UBA2770;g__;s__
LMSG_G000011121.1	no	571_1	Pyrite-Copper	87.06	0.14	86.35	1	1	1	15	Medium quality	17772	1925590	61.50	160	d__Bacteria;p__Proteobacteria;c__Gammaproteobacteria;o__UBA2770;f__UBA2770;g__;s__
LMSG_G000011122.1	no	572_1	Copper	80.31	0.57	77.44	1	1	0	19	Medium quality	106025	1794802	61.70	39	d__Bacteria;p__Proteobacteria;c__Gammaproteobacteria;o__UBA2770;f__UBA2770;g__;s__
LMSG_G000011123.1	no	572_1	Copper	87.35	1.15	81.61	1	1	1	18	Medium quality	71346	2031033	62.30	50	d__Bacteria;p__Proteobacteria;c__Gammaproteobacteria;o__UBA2770;f__UBA2770;g__;s__
LMSG_G000011124.1	no	572_1	Lead-Zinc	75.86	1.15	70.12	1	1	0	16	Medium quality	60636	1566111	63.00	45	d__Bacteria;p__Proteobacteria;c__Gammaproteobacteria;o__UBA2770;f__UBA2770;g__;s__
LMSG_G000011125.1	no	572_1	Lead-Zinc	77.01	0.57	74.14	1	2	0	17	Medium quality	57006	1607894	63.30	52	d__Bacteria;p__Proteobacteria;c__Gammaproteobacteria;o__UBA2770;f__UBA2770;g__;s__
LMSG_G000011126.1	no	572_1	Pyrite-Copper	64.49	0.72	60.87	1	1	0	16	Medium quality	56879	1415984	62.40	44	d__Bacteria;p__Proteobacteria;c__Gammaproteobacteria;o__UBA2770;f__UBA2770;g__;s__
LMSG_G000011127.1	no	574_1	Copper	95.35	0.57	92.48	1	1	1	20	High quality	57425	2111455	63.10	94	d__Bacteria;p__Proteobacteria;c__Gammaproteobacteria;o__UBA2770;f__UBA2770;g__;s__
LMSG_G000011128.1	no	574_1	Polymetallic	90.22	2.87	75.86	1	1	1	18	High quality	64158	1981902	63.30	60	d__Bacteria;p__Proteobacteria;c__Gammaproteobacteria;o__UBA2770;f__UBA2770;g__;s__
LMSG_G000011129.1	no	574_1	Copper	90.18	0.57	87.31	1	1	1	17	Medium quality	57551	1906988	63.10	55	d__Bacteria;p__Proteobacteria;c__Gammaproteobacteria;o__UBA2770;f__UBA2770;g__;s__
LMSG_G000011130.1	no	574_1	Copper	79.83	0.00	79.83	1	1	1	17	Medium quality	57551	1715147	63.60	46	d__Bacteria;p__Proteobacteria;c__Gammaproteobacteria;o__UBA2770;f__UBA2770;g__;s__
LMSG_G000011131.1	no	574_1	Polymetallic	89.65	1.44	82.47	1	2	2	18	Medium quality	64158	1896300	63.10	58	d__Bacteria;p__Proteobacteria;c__Gammaproteobacteria;o__UBA2770;f__UBA2770;g__;s__
LMSG_G000011132.1	no	574_1	Polymetallic	88.50	0.00	88.50	1	1	1	19	Medium quality	66231	1946793	63.20	54	d__Bacteria;p__Proteobacteria;c__Gammaproteobacteria;o__UBA2770;f__UBA2770;g__;s__
LMSG_G000011133.1	no	574_1	Polymetallic	86.78	0.00	86.78	2	4	7	17	Medium quality	61300	2135897	62.40	142	d__Bacteria;p__Proteobacteria;c__Gammaproteobacteria;o__UBA2770;f__UBA2770;g__;s__
LMSG_G000011134.1	no	575_1	Coal	80.79	0.95	76.06	2	3	3	18	Medium quality	307822	1917670	60.10	57	d__Bacteria;p__Proteobacteria;c__Gammaproteobacteria;o__UBA2770;f__UBA2770;g__;s__
LMSG_G000011135.1	no	575_1	Coal	89.08	4.89	64.66	1	1	1	19	Medium quality	29307	2432097	59.40	169	d__Bacteria;p__Proteobacteria;c__Gammaproteobacteria;o__UBA2770;f__UBA2770;g__;s__
LMSG_G000011136.1	no	575_1	Polymetallic	75.12	1.99	65.16	0	0	0	14	Medium quality	8397	1704787	60.00	267	d__Bacteria;p__Proteobacteria;c__Gammaproteobacteria;o__UBA2770;f__UBA2770;g__;s__
LMSG_G000011137.1	no	575_1	Polymetallic	71.43	0.36	69.62	1	1	2	16	Medium quality	35149	1697010	60.50	88	d__Bacteria;p__Proteobacteria;c__Gammaproteobacteria;o__UBA2770;f__UBA2770;g__;s__
LMSG_G000011138.1	no	575_1	Polymetallic	63.28	0.61	60.25	1	0	1	7	Medium quality	2641	1204019	60.60	505	d__Bacteria;p__Proteobacteria;c__Gammaproteobacteria;o__UBA2770;f__UBA2770;g__;s__
LMSG_G000011139.1	no	575_1	Polymetallic	67.11	1.04	61.94	0	1	0	15	Medium quality	26267	1585838	60.80	96	d__Bacteria;p__Proteobacteria;c__Gammaproteobacteria;o__UBA2770;f__UBA2770;g__;s__
LMSG_G000011140.1	no	575_1	Polymetallic	57.38	0.85	53.16	0	1	0	15	Medium quality	7366	1251704	60.40	217	d__Bacteria;p__Proteobacteria;c__Gammaproteobacteria;o__UBA2770;f__UBA2770;g__;s__
LMSG_G000011141.1	no	290_1	Lead-Zinc	58.65	1.49	51.23	2	0	1	14	Medium quality	3507	1485027	35.20	490	d__Bacteria;p__Proteobacteria;c__Gammaproteobacteria;o__UBA6186;f__UBA6186;g__;s__

LMSG_G000011142.1	no	1189_2	Lead-Zinc	69.87	1.07	64.51	0	0	0	11	Medium quality	2742	1210243	66.60	538	d__Bacteria;p__Proteobacteria;c__Gammaproteobacteria;o__UBA6729;f__UBA6729;g__;s__
LMSG_G000011143.1	no	292_1	Antimony	91.27	0.78	87.40	1	1	1	18	High quality	51757	1798465	38.30	60	d__Bacteria;p__Proteobacteria;c__Gammaproteobacteria;o__UBA9339;f__UBA9339;g__UBA9339;s__
LMSG_G000006331.1	no	292_1	Antimony	88.56	1.39	81.62	1	3	12	18	Medium quality	21264	1865162	38.30	169	d__Bacteria;p__Proteobacteria;c__Gammaproteobacteria;o__UBA9339;f__UBA9339;g__UBA9339;s__
LMSG_G000011144.1	no	292_1	Antimony	88.95	1.36	82.17	1	3	1	19	Medium quality	54774	1878347	38.20	92	d__Bacteria;p__Proteobacteria;c__Gammaproteobacteria;o__UBA9339;f__UBA9339;g__UBA9339;s__
LMSG_G000011145.1	no	1254_1	Polymetallic	73.97	2.73	60.35	0	1	0	14	Medium quality	17606	1797992	69.70	146	d__Bacteria;p__Proteobacteria;c__Gammaproteobacteria;o__Xanthomonadales;f__Rhodanobacteraceae;g__;s__
LMSG_G000011146.1	no	1254_1	Polymetallic	51.72	0.00	51.72	0	0	0	12	Medium quality	15676	1445331	69.50	121	d__Bacteria;p__Proteobacteria;c__Gammaproteobacteria;o__Xanthomonadales;f__Rhodanobacteraceae;g__;s__
LMSG_G000011147.1	no	1254_1	Polymetallic	69.19	2.93	54.53	0	0	0	13	Medium quality	13390	1621603	69.30	152	d__Bacteria;p__Proteobacteria;c__Gammaproteobacteria;o__Xanthomonadales;f__Rhodanobacteraceae;g__;s__
LMSG_G000011148.1	no	1254_1	Polymetallic	58.62	0.00	58.62	0	0	0	9	Medium quality	9594	1336310	69.80	180	d__Bacteria;p__Proteobacteria;c__Gammaproteobacteria;o__Xanthomonadales;f__Rhodanobacteraceae;g__;s__
LMSG_G000011149.1	no	1114_1	Antimony	79.17	2.08	68.79	0	1	0	15	Medium quality	25882	2177774	66.20	133	d__Bacteria;p__Proteobacteria;c__Gammaproteobacteria;o__Xanthomonadales;f__Rhodanobacteraceae;g__66-474;s__
LMSG_G000011150.1	no	1255_1	Lead-Zinc	92.65	1.62	84.54	2	0	1	18	Medium quality	21787	2798014	66.50	179	d__Bacteria;p__Proteobacteria;c__Gammaproteobacteria;o__Xanthomonadales;f__Rhodanobacteraceae;g__Metallibacterium;s__
LMSG_G000011151.1	no	1255_1	Copper	94.60	4.34	72.92	2	0	0	18	Medium quality	51093	3010659	66.50	87	d__Bacteria;p__Proteobacteria;c__Gammaproteobacteria;o__Xanthomonadales;f__Rhodanobacteraceae;g__Metallibacterium;s__
LMSG_G000011152.1	no	1255_1	Polymetallic	77.09	1.40	70.08	1	0	0	18	Medium quality	10298	2303243	66.40	290	d__Bacteria;p__Proteobacteria;c__Gammaproteobacteria;o__Xanthomonadales;f__Rhodanobacteraceae;g__Metallibacterium;s__
LMSG_G000011153.1	no	1255_1	Polymetallic	75.16	2.17	64.31	1	1	0	15	Medium quality	9583	2474461	66.10	326	d__Bacteria;p__Proteobacteria;c__Gammaproteobacteria;o__Xanthomonadales;f__Rhodanobacteraceae;g__Metallibacterium;s__
LMSG_G000011154.1	no	1255_1	Polymetallic	79.42	4.02	59.31	2	1	0	16	Medium quality	6395	2733134	66.60	580	d__Bacteria;p__Proteobacteria;c__Gammaproteobacteria;o__Xanthomonadales;f__Rhodanobacteraceae;g__Metallibacterium;s__
LMSG_G000011155.1	no	1255_1	Copper	61.55	1.72	52.93	0	0	0	16	Medium quality	15349	2460614	66.50	239	d__Bacteria;p__Proteobacteria;c__Gammaproteobacteria;o__Xanthomonadales;f__Rhodanobacteraceae;g__Metallibacterium;s__
LMSG_G000011156.1	no	1255_1	Copper	76.41	1.81	67.38	1	0	1	15	Medium quality	11846	2090446	66.80	204	d__Bacteria;p__Proteobacteria;c__Gammaproteobacteria;o__Xanthomonadales;f__Rhodanobacteraceae;g__Metallibacterium;s__
LMSG_G000011157.1	no	1255_1	Polymetallic	77.28	1.97	67.46	0	0	0	14	Medium quality	16144	2190744	67.10	255	d__Bacteria;p__Proteobacteria;c__Gammaproteobacteria;o__Xanthomonadales;f__Rhodanobacteraceae;g__Metallibacterium;s__
LMSG_G000011158.1	no	1255_1	Polymetallic	75.50	1.21	69.47	0	1	0	16	Medium quality	18239	1987484	66.90	146	d__Bacteria;p__Proteobacteria;c__Gammaproteobacteria;o__Xanthomonadales;f__Rhodanobacteraceae;g__Metallibacterium;s__
LMSG_G000011159.1	no	1255_1	Polymetallic	71.86	0.00	71.86	1	0	1	18	Medium quality	15986	2138492	66.90	178	d__Bacteria;p__Proteobacteria;c__Gammaproteobacteria;o__Xanthomonadales;f__Rhodanobacteraceae;g__Metallibacterium;s__
LMSG_G000011160.1	no	1255_1	Polymetallic	80.35	1.24	74.16	0	0	0	14	Medium quality	20908	2131941	67.10	151	d__Bacteria;p__Proteobacteria;c__Gammaproteobacteria;o__Xanthomonadales;f__Rhodanobacteraceae;g__Metallibacterium;s__
LMSG_G000011161.1	no	1255_1	Polymetallic	64.65	2.59	51.72	2	1	0	16	Medium quality	13376	1653947	67.10	145	d__Bacteria;p__Proteobacteria;c__Gammaproteobacteria;o__Xanthomonadales;f__Rhodanobacteraceae;g__Metallibacterium;s__
LMSG_G000011162.1	no	1255_1	Polymetallic	59.13	0.34	57.41	1	1	0	16	Medium quality	11653	1940399	66.90	214	d__Bacteria;p__Proteobacteria;c__Gammaproteobacteria;o__Xanthomonadales;f__Rhodanobacteraceae;g__Metallibacterium;s__
LMSG_G000011163.1	no	1255_1	Polymetallic	73.27	1.72	64.65	2	0	0	17	Medium quality	13932	2720189	66.90	329	d__Bacteria;p__Proteobacteria;c__Gammaproteobacteria;o__Xanthomonadales;f__Rhodanobacteraceae;g__Metallibacterium;s__
LMSG_G000011164.1	no	1255_1	Polymetallic	75.88	4.90	51.38	0	0	0	13	Medium quality	16521	1964576	67.10	151	d__Bacteria;p__Proteobacteria;c__Gammaproteobacteria;o__Xanthomonadales;f__Rhodanobacteraceae;g__Metallibacterium;s__
LMSG_G000011165.1	no	1255_1	Coal	95.03	4.29	73.58	2	0	0	17	Medium quality	16740	2674986	66.70	226	d__Bacteria;p__Proteobacteria;c__Gammaproteobacteria;o__Xanthomonadales;f__Rhodanobacteraceae;g__Metallibacterium;s__
LMSG_G000011166.1	no	1255_1	Coal	90.44	3.71	71.91	2	0	0	17	Medium quality	17115	2529886	66.80	202	d__Bacteria;p__Proteobacteria;c__Gammaproteobacteria;o__Xanthomonadales;f__Rhodanobacteraceae;g__Metallibacterium;s__
LMSG_G000011167.1	no	1255_1	Pyrite	96.76	3.39	79.83	0	0	0	17	Medium quality	38826	3134711	66.50	119	d__Bacteria;p__Proteobacteria;c__Gammaproteobacteria;o__Xanthomonadales;f__Rhodanobacteraceae;g__Metallibacterium;s__
LMSG_G000011168.1	no	1257_1	Lead-Zinc	62.75	1.72	54.13	0	0	0	15	Medium quality	3112	1897848	68.90	644	d__Bacteria;p__Proteobacteria;c__Gammaproteobacteria;o__Xanthomonadales;f__Rhodanobacteraceae;g__Metallibacterium;s__
LMSG_G000011169.1	no	1257_1	Copper	72.75	0.00	72.75	0	0	0	17	Medium quality	12221	2623686	68.30	299	d__Bacteria;p__Proteobacteria;c__Gammaproteobacteria;o__Xanthomonadales;f__Rhodanobacteraceae;g__Metallibacterium;s__
LMSG_G000011170.1	no	1257_1	Lead-Zinc	76.35	2.85	62.10	2	0	1	15	Medium quality	5485	1944389	68.60	549	d__Bacteria;p__Proteobacteria;c__Gammaproteobacteria;o__Xanthomonadales;f__Rhodanobacteraceae;g__Metallibacterium;s__
LMSG_G000011171.1	no	1257_1	Lead-Zinc	75.00	0.00	75.00	0	0	0	13	Medium quality	5319	2289916	68.90	516	d__Bacteria;p__Proteobacteria;c__Gammaproteobacteria;o__Xanthomonadales;f__Rhodanobacteraceae;g__Metallibacterium;s__
LMSG_G000011172.1	no	1257_1	Coal	56.89	0.00	56.89	0	0	0	10	Medium quality	5149	1719355	69.00	398	d__Bacteria;p__Proteobacteria;c__Gammaproteobacteria;o__Xanthomonadales;f__Rhodanobacteraceae;g__Metallibacterium;s__
LMSG_G000011173.1	no	1257_1	Coal	70.86	1.72	62.24	0	0	0	12	Medium quality	5203	2311316	68.90	553	d__Bacteria;p__Proteobacteria;c__Gammaproteobacteria;o__Xanthomonadales;f__Rhodanobacteraceae;g__Metallibacterium;s__
LMSG_G000011174.1	no	1257_1	Polymetallic	86.73	4.88	62.34	0	0	0	16	Medium quality	14407	2540661	68.20	224	d__Bacteria;p__Proteobacteria;c__Gammaproteobacteria;o__Xanthomonadales;f__Rhodanobacteraceae;g__Metallibacterium;s__
LMSG_G000011175.1	no	1257_1	Polymetallic	75.34	0.00	75.34	0	0	0	17	Medium quality	21365	2463991	68.40	209	d__Bacteria;p__Proteobacteria;c__Gammaproteobacteria;o__Xanthomonadales;f__Rhodanobacteraceae;g__Metallibacterium;s__
LMSG_G000011176.1	no	1257_1	Polymetallic	67.24	0.00	67.24	1	0	1	14	Medium quality	8861	1634769	68.90	250	d__Bacteria;p__Proteobacteria;c__Gammaproteobacteria;o__Xanthomonadales;f__Rhodanobacteraceae;g__Metallibacterium;s__
LMSG_G000011177.1	no	1257_1	Polymetallic	75.00	0.00	75.00	2	0	0	15	Medium quality	8129	2265611	68.50	365	d__Bacteria;p__Proteobacteria;c__Gammaproteobacteria;o__Xanthomonadales;f__Rhodanobacteraceae;g__Metallibacterium;s__
LMSG_G000011178.1	no	1257_1	Pyrite	69.86	3.86	50.58	1	0	1	14	Medium quality	3609	1845122	68.80	575	d__Bacteria;p__Proteobacteria;c__Gammaproteobacteria;o__Xanthomonadales;f__Rhodanobacteraceae;g__Metallibacterium;s__
LMSG_G000011179.1	no	1534_1	Tin-Zinc	73.07	0.69	69.63	0	0	0	20	Medium quality	55479	2044222	65.00	63	d__Bacteria;p__Proteobacteria;c__Gammaproteobacteria;o__Xanthomonadales;f__Rhodanobacteraceae;g__Metallibacterium;s__
LMSG_G000011180.1	no	1534_1	Pyrite-Copper	60.34	1.72	51.72	0	0	0	20	Medium quality	7238	3287918	63.70	555	d__Bacteria;p__Proteobacteria;c__Gammaproteobacteria;o__Xanthomonadales;f__Rhodanobacteraceae;g__Metallibacterium;s__
LMSG_G000011181.1	no	1534_1	Pyrite-Copper	77.58	0.86	73.27	2	0	1	15	Medium quality	21069	2440847	64.80	168	d__Bacteria;p__Proteobacteria;c__Gammaproteobacteria;o__Xanthomonadales;f__Rhodanobacteraceae;g__Metallibacterium;s__
LMSG_G000011182.1	no	1256_1	Copper	52.75	0.00	52.75	2	0	0	16	Medium quality	12118	1817813	66.90	194	d__Bacteria;p__Proteobacteria;c__Gammaproteobacteria;o__Xanthomonadales;f__Rhodanobacteraceae;g__Metallibacterium;s__Metallibacterium scheffleri
LMSG_G000011183.1	no	1256_1	Pyrite-Copper	71.55	0.86	67.24	0	0	0	18	Medium quality	22677	3155424	67.50	286	d__Bacteria;p__Proteobacteria;c__Gammaproteobacteria;o__Xanthomonadales;f__Rhodanobacteraceae;g__Metallibacterium;s__Metallibacterium scheffleri
LMSG_G000011184.1	no	1259_1	Coal	90.87	2.16	80.07	1	0	1	16	Medium quality	11932	3279600	64.50	370	d__Bacteria;p__Proteobacteria;c__Gammaproteobacteria;o__Xanthomonadales;f__Rhodanobacteraceae;g__Rhodanobacter;s__
LMSG_G000011185.1	no	1260_1	Antimony	60.34	1.72	51.72	0	0	0	14	Medium quality	8807	2206549	68.10	310	d__Bacteria;p__Proteobacteria;c__Gammaproteobacteria;o__Xanthomonadales;f__Rhodanobacteraceae;g__Rhodanobacter;s__
LMSG_G000011186.1	no	1260_1	Antimony	56.89	0.00	56.89	0	0	0	14	Medium quality	8867	2090220	68.00	255	d__Bacteria;p__Proteobacteria;c__Gammaproteobacteria;o__Xanthomonadales;f__Rhodanobacteraceae;g__Rhodanobacter;s__
LMSG_G000011187.1	no	1260_1	Antimony	85.49	4.81	61.44	1	0	0	13	Medium quality	15663	2802574	68.00	276	d__Bacteria;p__Proteobacteria;c__Gammaproteobacteria;o__Xanthomonadales;f__Rhodanobacteraceae;g__Rhodanobacter;s__
LMSG_G000011188.1	no	1260_1	Polymetallic	50.86	0.00	50.86	1	0	0	12	Medium quality	6067	1721779	66.20	377	d__Bacteria;p__Proteobacteria;c__Gammaproteobacteria;o__Xanthomonadales;f__Rhodanobacteraceae;g__Rhodanobacter;s__
LMSG_G000011189.1	no	1260_1	Polymetallic	59.48	1.72	50.86	1	0	0	17	Medium quality	3807	2928245	67.90	865	d__Bacteria;p__Proteobacteria;c__Gammaproteobacteria;o__Xanthomonadales;f__Rhodanobacteraceae;g__Rhodanobacter;s__
LMSG_G000011190.1	no	1063_1	Coal	76.47	0.21	75.43	1	0	0	18	Medium quality	2676	2976397	62.30	1270	d__Bacteria;p__Spirochaetota;c__Spirochaetia;o__Spirochaetales;f__;g__;s__
LMSG_G000011191.1	no	512_1	Antimony	88.30	2.51	75.77	1	0	0	18	Medium quality	13576	3792034	62.40	375	d__Bacteria;p__Spirochaetota;c__Spirochaetia;o__Treponematales;f__UBA8932;g__;s__
LMSG_G000011192.1	no	512_1	Antimony	74.65	0.57	71.78	1	1	0	15	Medium quality	7636	3509230	62.40	539	d__Bacteria;p__Spirochaetota;c__Spirochaetia;o__Treponematales;f__UBA8932;g__;s__
LMSG_G000011193.1	no	512_1	Antimony	68.58	1.15	62.84	1	0	0	13	Medium quality	9692	2812353	63.10	444	d__Bacteria;p__Spirochaetota;c__Spirochaetia;o__Treponematales;f__UBA8932;g__;s__
LMSG_G000011194.1	no	511_1	Antimony	56.82	0.00	56.82	1	0	0	13	Medium quality	5327	2317366	65.90	479	d__Bacteria;p__Spirochaetota;c__Spirochaetia;o__Treponematales;f__UBA8932;g__UBA1306;s__

LMSG_G000011195.1	no	1752_1	Lead-Zinc	97.70	1.15	91.96	2	1	2	18	High quality	71496	2591422	52.40	90	d__Bacteria;p__Spirochaetota;c__Spirochaetia;o__Treponematales;f__UBA8932;g__UBA8932;s__UBA8932 sp002427685
LMSG_G000011196.1	no	1752_1	Lead-Zinc	93.62	0.00	93.62	2	0	1	17	Medium quality	30989	2441607	52.40	135	d__Bacteria;p__Spirochaetota;c__Spirochaetia;o__Treponematales;f__UBA8932;g__UBA8932;s__UBA8932 sp002427685
LMSG_G000011197.1	no	1752_1	Polymetallic	55.88	0.00	55.88	0	1	0	12	Medium quality	2186	1408629	52.80	699	d__Bacteria;p__Spirochaetota;c__Spirochaetia;o__Treponematales;f__UBA8932;g__UBA8932;s__UBA8932 sp002427685
LMSG_G000011198.1	no	1752_1	Polymetallic	96.55	0.07	96.22	3	0	0	17	Medium quality	9141	2548349	52.10	383	d__Bacteria;p__Spirochaetota;c__Spirochaetia;o__Treponematales;f__UBA8932;g__UBA8932;s__UBA8932 sp002427685
LMSG_G000011199.1	no	983_1	Copper	78.08	2.68	64.67	0	1	0	10	Medium quality	5227	1902768	46.50	413	d__Bacteria;p__SZUA-79;c__;o__;f__;g__;s__
LMSG_G000011200.1	no	983_1	Copper	82.04	3.05	66.80	0	3	0	9	Medium quality	6290	2095953	46.40	386	d__Bacteria;p__SZUA-79;c__;o__;f__;g__;s__
LMSG_G000011201.1	no	984_1	Polymetallic	90.85	4.27	69.51	0	0	0	14	Medium quality	20701	2201478	47.10	191	d__Bacteria;p__SZUA-79;c__;o__;f__;g__;s__
LMSG_G000011202.1	no	984_1	Polymetallic	91.05	3.86	71.75	1	0	1	15	Medium quality	20092	2267640	47.10	205	d__Bacteria;p__SZUA-79;c__;o__;f__;g__;s__
LMSG_G000011203.1	no	984_1	Polymetallic	87.79	2.27	76.45	1	4	7	18	Medium quality	7686	2342575	47.20	367	d__Bacteria;p__SZUA-79;c__;o__;f__;g__;s__
LMSG_G000011204.1	no	984_1	Polymetallic	94.71	4.67	71.34	0	1	0	18	Medium quality	18985	2372499	47.30	224	d__Bacteria;p__SZUA-79;c__;o__;f__;g__;s__
LMSG_G000011205.1	no	984_1	Antimony	61.68	1.47	54.31	0	0	0	9	Medium quality	2227	1298580	47.70	630	d__Bacteria;p__SZUA-79;c__;o__;f__;g__;s__
LMSG_G000011206.1	no	984_1	Copper	95.12	3.66	76.83	1	5	7	18	High quality	87465	2584710	46.50	67	d__Bacteria;p__SZUA-79;c__;o__;f__;g__;s__
LMSG_G000011207.1	no	984_1	Copper	73.35	2.98	58.45	1	0	1	15	Medium quality	2726	1848846	47.20	770	d__Bacteria;p__SZUA-79;c__;o__;f__;g__;s__
LMSG_G000011208.1	no	984_1	Copper	61.59	1.10	56.08	0	0	0	12	Medium quality	2141	1582155	47.50	806	d__Bacteria;p__SZUA-79;c__;o__;f__;g__;s__
LMSG_G000011209.1	no	984_1	Pyrite-Copper	64.70	1.05	59.47	1	0	1	13	Medium quality	5984	1469247	47.10	303	d__Bacteria;p__SZUA-79;c__;o__;f__;g__;s__
LMSG_G000011210.1	no	984_1	Pyrite-Copper	63.86	1.73	55.20	1	0	1	12	Medium quality	13549	1906822	47.40	235	d__Bacteria;p__SZUA-79;c__;o__;f__;g__;s__
LMSG_G000011211.1	no	1746_1	Lead-Zinc	57.15	0.65	53.93	0	0	0	17	Medium quality	4262	1639505	30.20	437	d__Bacteria;p__SZUA-79;c__SZUA-79;o__Acidulodesulfobacterales;f__SZUA-79;g__Acididesulfobacter;s__
LMSG_G000011212.1	no	1746_1	Copper	75.99	4.37	54.12	0	0	0	16	Medium quality	3326	2634855	29.10	923	d__Bacteria;p__SZUA-79;c__SZUA-79;o__Acidulodesulfobacterales;f__SZUA-79;g__Acididesulfobacter;s__
LMSG_G000011213.1	no	1747_1	Polymetallic	80.96	3.30	64.48	1	0	0	17	Medium quality	44848	2482276	31.70	133	d__Bacteria;p__SZUA-79;c__SZUA-79;o__Acidulodesulfobacterales;f__SZUA-79;g__Acididesulfobacter;s__
LMSG_G000011214.1	no	1748_2	Polymetallic	70.93	1.28	64.52	0	1	0	17	Medium quality	12985	1689948	32.10	329	d__Bacteria;p__SZUA-79;c__SZUA-79;o__Acidulodesulfobacterales;f__SZUA-79;g__Acididesulfobacter;s__
LMSG_G000011215.1	no	1748_2	Tin-Zinc	87.64	4.63	64.51	0	0	0	17	Medium quality	8672	1893960	32.20	308	d__Bacteria;p__SZUA-79;c__SZUA-79;o__Acidulodesulfobacterales;f__SZUA-79;g__Acididesulfobacter;s__
LMSG_G000011216.1	no	1748_3	Lead-Zinc	80.39	2.76	66.59	2	0	1	16	Medium quality	12723	1789544	32.80	267	d__Bacteria;p__SZUA-79;c__SZUA-79;o__Acidulodesulfobacterales;f__SZUA-79;g__Acididesulfobacter;s__
LMSG_G000011217.1	no	1748_3	Lead-Zinc	72.02	2.74	58.35	0	0	0	17	Medium quality	6457	1709055	32.80	359	d__Bacteria;p__SZUA-79;c__SZUA-79;o__Acidulodesulfobacterales;f__SZUA-79;g__Acididesulfobacter;s__
LMSG_G000011218.1	no	1748_3	Polymetallic	77.10	1.71	68.54	0	0	0	17	Medium quality	10062	1612990	32.70	234	d__Bacteria;p__SZUA-79;c__SZUA-79;o__Acidulodesulfobacterales;f__SZUA-79;g__Acididesulfobacter;s__
LMSG_G000011219.1	no	1748_3	Polymetallic	80.23	3.93	60.57	0	1	0	16	Medium quality	12737	1837961	32.50	209	d__Bacteria;p__SZUA-79;c__SZUA-79;o__Acidulodesulfobacterales;f__SZUA-79;g__Acididesulfobacter;s__
LMSG_G000011220.1	no	1748_3	Polymetallic	81.41	2.99	66.45	0	0	0	17	Medium quality	11724	1916686	32.80	226	d__Bacteria;p__SZUA-79;c__SZUA-79;o__Acidulodesulfobacterales;f__SZUA-79;g__Acididesulfobacter;s__
LMSG_G000011221.1	no	1748_3	Polymetallic	83.23	2.20	72.25	0	0	0	18	Medium quality	18514	1641046	32.60	246	d__Bacteria;p__SZUA-79;c__SZUA-79;o__Acidulodesulfobacterales;f__SZUA-79;g__Acididesulfobacter;s__
LMSG_G000011222.1	no	1748_3	Polymetallic	91.09	3.02	76.01	0	1	0	19	Medium quality	34658	2262239	32.60	131	d__Bacteria;p__SZUA-79;c__SZUA-79;o__Acidulodesulfobacterales;f__SZUA-79;g__Acididesulfobacter;s__
LMSG_G000011223.1	no	1748_3	Polymetallic	89.32	1.12	83.71	0	0	0	18	Medium quality	27636	2469972	32.40	159	d__Bacteria;p__SZUA-79;c__SZUA-79;o__Acidulodesulfobacterales;f__SZUA-79;g__Acididesulfobacter;s__
LMSG_G000011224.1	no	1748_3	Polymetallic	89.32	1.12	83.71	0	1	0	18	Medium quality	42251	2040144	32.60	102	d__Bacteria;p__SZUA-79;c__SZUA-79;o__Acidulodesulfobacterales;f__SZUA-79;g__Acididesulfobacter;s__
LMSG_G000011225.1	no	1748_3	Polymetallic	91.75	1.20	85.76	0	1	0	17	Medium quality	58718	1895125	32.60	66	d__Bacteria;p__SZUA-79;c__SZUA-79;o__Acidulodesulfobacterales;f__SZUA-79;g__Acididesulfobacter;s__
LMSG_G000011226.1	no	1748_3	Tin-Zinc	66.44	1.12	60.83	0	0	0	16	Medium quality	7938	1498345	32.30	244	d__Bacteria;p__SZUA-79;c__SZUA-79;o__Acidulodesulfobacterales;f__SZUA-79;g__Acididesulfobacter;s__
LMSG_G000011227.1	no	1748_3	Polymetallic	77.04	1.87	67.68	0	0	0	16	Medium quality	21342	2026076	32.50	403	d__Bacteria;p__SZUA-79;c__SZUA-79;o__Acidulodesulfobacterales;f__SZUA-79;g__Acididesulfobacter;s__
LMSG_G000011228.1	no	1748_3	Copper	65.93	1.10	60.44	0	1	0	15	Medium quality	6556	1713609	31.70	460	d__Bacteria;p__SZUA-79;c__SZUA-79;o__Acidulodesulfobacterales;f__SZUA-79;g__Acididesulfobacter;s__
LMSG_G000011229.1	no	1746_2	Polymetallic	74.87	2.56	62.05	0	1	0	16	Medium quality	8617	2251295	29.60	309	d__Bacteria;p__SZUA-79;c__SZUA-79;o__Acidulodesulfobacterales;f__SZUA-79;g__Acididesulfobacter;s__Acididesulfobacter diazotrophicus
LMSG_G000011230.1	no	1746_2	Polymetallic	67.63	0.00	67.63	0	0	0	13	Medium quality	19407	2033424	28.70	476	d__Bacteria;p__SZUA-79;c__SZUA-79;o__Acidulodesulfobacterales;f__SZUA-79;g__Acididesulfobacter;s__Acididesulfobacter diazotrophicus
LMSG_G000011231.1	no	1746_2	Pyrite	56.16	0.00	56.16	0	0	0	8	Medium quality	5821	1400956	30.10	267	d__Bacteria;p__SZUA-79;c__SZUA-79;o__Acidulodesulfobacterales;f__SZUA-79;g__Acididesulfobacter;s__Acididesulfobacter diazotrophicus
LMSG_G000011232.1	no	1748_1	Pyrite	90.59	1.71	82.05	0	0	0	19	Medium quality	71798	2157346	32.50	52	d__Bacteria;p__SZUA-79;c__SZUA-79;o__Acidulodesulfobacterales;f__SZUA-79;g__Acididesulfobacter;s__Acididesulfobacter guangdongensis
LMSG_G000011233.1	no	1734_1	Pyrite	61.06	1.02	55.96	0	0	0	9	Medium quality	2375	913273	39.30	419	d__Bacteria;p__SZUA-79;c__SZUA-79;o__Acidulodesulfobacterales;f__SZUA-79;g__Acidulodesulfobacterium;s__
LMSG_G000011234.1	no	1735_1	Lead-Zinc	92.85	2.52	80.25	1	1	1	19	High quality	133262	1997570	37.30	21	d__Bacteria;p__SZUA-79;c__SZUA-79;o__Acidulodesulfobacterales;f__SZUA-79;g__Acidulodesulfobacterium;s__
LMSG_G000011235.1	no	1735_1	Lead-Zinc	88.65	1.68	80.25	1	1	1	18	Medium quality	62888	1953844	37.60	52	d__Bacteria;p__SZUA-79;c__SZUA-79;o__Acidulodesulfobacterales;f__SZUA-79;g__Acidulodesulfobacterium;s__
LMSG_G000011236.1	no	1735_1	Lead-Zinc	76.47	0.84	72.27	1	1	1	19	Medium quality	87306	1585204	37.50	53	d__Bacteria;p__SZUA-79;c__SZUA-79;o__Acidulodesulfobacterales;f__SZUA-79;g__Acidulodesulfobacterium;s__
LMSG_G000011237.1	no	1735_1	Pyrite-Copper	88.65	1.68	80.25	2	1	2	20	Medium quality	110889	1790643	37.60	28	d__Bacteria;p__SZUA-79;c__SZUA-79;o__Acidulodesulfobacterales;f__SZUA-79;g__Acidulodesulfobacterium;s__
LMSG_G000011238.1	no	1735_1	Lead-Zinc	84.45	2.52	71.85	1	1	1	18	Medium quality	94474	1791755	37.50	28	d__Bacteria;p__SZUA-79;c__SZUA-79;o__Acidulodesulfobacterales;f__SZUA-79;g__Acidulodesulfobacterium;s__
LMSG_G000011239.1	no	1735_2	Polymetallic	91.59	0.84	87.39	1	9	26	20	High quality	72955	1799066	37.40	156	d__Bacteria;p__SZUA-79;c__SZUA-79;o__Acidulodesulfobacterales;f__SZUA-79;g__Acidulodesulfobacterium;s__
LMSG_G000011240.1	no	1735_2	Polymetallic	91.17	0.84	86.97	1	11	23	18	High quality	62930	1760648	37.50	144	d__Bacteria;p__SZUA-79;c__SZUA-79;o__Acidulodesulfobacterales;f__SZUA-79;g__Acidulodesulfobacterium;s__
LMSG_G000011241.1	no	1735_2	Polymetallic	82.89	1.33	76.25	1	1	3	20	Medium quality	4897	1620246	36.70	509	d__Bacteria;p__SZUA-79;c__SZUA-79;o__Acidulodesulfobacterales;f__SZUA-79;g__Acidulodesulfobacterium;s__
LMSG_G000011242.1	no	1736_1	Lead-Zinc	70.83	1.72	62.21	1	1	0	18	Medium quality	34660	1610460	35.10	86	d__Bacteria;p__SZUA-79;c__SZUA-79;o__Acidulodesulfobacterales;f__SZUA-79;g__Acidulodesulfobacterium;s__
LMSG_G000011243.1	no	1737_1	Pyrite	78.60	3.78	59.69	1	1	0	17	Medium quality	9239	1616322	35.90	224	d__Bacteria;p__SZUA-79;c__SZUA-79;o__Acidulodesulfobacterales;f__SZUA-79;g__Acidulodesulfobacterium;s__
LMSG_G000011244.1	no	1737_1	Copper	58.13	0.43	56.00	1	1	0	12	Medium quality	4999	1097514	35.40	254	d__Bacteria;p__SZUA-79;c__SZUA-79;o__Acidulodesulfobacterales;f__SZUA-79;g__Acidulodesulfobacterium;s__
LMSG_G000011245.1	no	1737_2	Copper	90.51	3.66	72.20	1	1	1	19	High quality	18354	1650198	36.10	142	d__Bacteria;p__SZUA-79;c__SZUA-79;o__Acidulodesulfobacterales;f__SZUA-79;g__Acidulodesulfobacterium;s__
LMSG_G000011246.1	no	1737_2	Lead-Zinc	53.84	0.00	53.84	0	0	0	10	Medium quality	2089	1203043	37.10	638	d__Bacteria;p__SZUA-79;c__SZUA-79;o__Acidulodesulfobacterales;f__SZUA-79;g__Acidulodesulfobacterium;s__
LMSG_G000011247.1	no	1737_2	Lead-Zinc	81.46	0.00	81.46	1	0	0	18	Medium quality	22005	1561255	36.00	125	d__Bacteria;p__SZUA-79;c__SZUA-79;o__Acidulodesulfobacterales;f__SZUA-79;g__Acidulodesulfobacterium;s__
LMSG_G000011248.1	no	1737_2	Copper	84.48	0.00	84.48	0	1	0	14	Medium quality	19009	1401592	36.20	108	d__Bacteria;p__SZUA-79;c__SZUA-79;o__Acidulodesulfobacterales;f__SZUA-79;g__Acidulodesulfobacterium;s__
LMSG_G000011249.1	no	1737_2	Copper	75.43	0.00	75.43	0	1	0	13	Medium quality	29083	1322129	36.30	126	d__Bacteria;p__SZUA-79;c__SZUA-79;o__Acidulodesulfobacterales;f__SZUA-79;g__Acidulodesulfobacterium;s__
LMSG_G000011250.1	no	1737_2	Polymetallic	77.05	1.29	70.59	0	1	0	16	Medium quality	9295	1451351	35.90	288	d__Bacteria;p__SZUA-79;c__SZUA-79;o__Acidulodesulfobacterales;f__SZUA-79;g__Acidulodesulfobacterium;s__
LMSG_G000011251.1	no	1737_2	Copper	84.56	1.72	75.94	1	0	1	18	Medium quality	25320	1516397	35.80	194	d__Bacteria;p__SZUA-79;c__SZUA-79;o__Acidulodesulfobacterales;f__SZUA-79;g__Acidulodesulfobacterium;s__
LMSG_G000011252.1	no	1737_2	Lead-Zinc	88.79	0.86	84.48	1	1	1	17	Medium quality	35119	1880805	36.20	96	d__Bacteria;p__SZUA-79;c__SZUA-79;o__Acidulodesulfobacterales;f__SZUA-79;g__Acidulodesulfobacterium;s

LMSG_G000011253.1	no	1737_2	Lead-Zinc	86.96	3.33	70.30	1	1	1	16	Medium quality	18960	1712184	36.10	128	d__Bacteria;p__SZUA-79;c__SZUA-79;o__Acidulodesulfobacterales;f__SZUA-79;g__Acidulodesulfobacterium;s__
LMSG_G000011254.1	no	1737_2	Pyrite-Copper	65.94	1.08	60.56	1	1	1	16	Medium quality	12880	1184235	36.30	152	d__Bacteria;p__SZUA-79;c__SZUA-79;o__Acidulodesulfobacterales;f__SZUA-79;g__Acidulodesulfobacterium;s__
LMSG_G000011255.1	no	1737_2	Lead-Zinc	60.91	0.00	60.91	0	1	0	11	Medium quality	22824	915905	36.20	70	d__Bacteria;p__SZUA-79;c__SZUA-79;o__Acidulodesulfobacterales;f__SZUA-79;g__Acidulodesulfobacterium;s__
LMSG_G000011256.1	no	1737_2	Pyrite	84.05	0.86	79.74	1	1	2	19	Medium quality	10212	1707771	36.00	235	d__Bacteria;p__SZUA-79;c__SZUA-79;o__Acidulodesulfobacterales;f__SZUA-79;g__Acidulodesulfobacterium;s__
LMSG_G000011257.1	no	1737_2	Pyrite	80.67	1.14	74.96	1	1	1	19	Medium quality	12132	1457763	36.20	192	d__Bacteria;p__SZUA-79;c__SZUA-79;o__Acidulodesulfobacterales;f__SZUA-79;g__Acidulodesulfobacterium;s__
LMSG_G000011258.1	no	1737_2	Pyrite	60.27	0.97	55.43	0	1	2	13	Medium quality	13796	1123075	36.50	261	d__Bacteria;p__SZUA-79;c__SZUA-79;o__Acidulodesulfobacterales;f__SZUA-79;g__Acidulodesulfobacterium;s__
LMSG_G000011259.1	no	1737_2	Polymetallic	69.59	2.94	54.89	1	1	0	11	Medium quality	10041	1252507	36.00	284	d__Bacteria;p__SZUA-79;c__SZUA-79;o__Acidulodesulfobacterales;f__SZUA-79;g__Acidulodesulfobacterium;s__
LMSG_G000011260.1	no	1737_2	Pyrite	63.14	1.36	56.36	0	1	0	15	Medium quality	8468	1265074	35.80	248	d__Bacteria;p__SZUA-79;c__SZUA-79;o__Acidulodesulfobacterales;f__SZUA-79;g__Acidulodesulfobacterium;s__
LMSG_G000011261.1	no	1739_1	Pyrite-Copper	59.64	1.75	50.87	0	0	0	7	Medium quality	10127	1124429	40.70	153	d__Bacteria;p__SZUA-79;c__SZUA-79;o__Acidulodesulfobacterales;f__SZUA-79;g__Acidulodesulfobacterium;s__
LMSG_G000011262.1	no	1740_1	Pyrite-Copper	71.16	3.01	56.10	1	0	0	17	Medium quality	33600	1429986	35.10	221	d__Bacteria;p__SZUA-79;c__SZUA-79;o__Acidulodesulfobacterales;f__SZUA-79;g__Acidulodesulfobacterium;s__
LMSG_G000011263.1	no	1741_1	Lead-Zinc	84.61	3.30	68.13	1	1	1	17	Medium quality	163882	1530405	37.50	64	d__Bacteria;p__SZUA-79;c__SZUA-79;o__Acidulodesulfobacterales;f__SZUA-79;g__Acidulodesulfobacterium;s__
LMSG_G000011264.1	no	1741_1	Lead-Zinc	64.91	1.75	56.14	1	0	1	14	Medium quality	133984	1629684	36.90	41	d__Bacteria;p__SZUA-79;c__SZUA-79;o__Acidulodesulfobacterales;f__SZUA-79;g__Acidulodesulfobacterium;s__
LMSG_G000011265.1	no	1742_1	Pyrite-Copper	50.87	0.00	50.87	0	1	0	5	Medium quality	7969	797320	38.60	118	d__Bacteria;p__SZUA-79;c__SZUA-79;o__Acidulodesulfobacterales;f__SZUA-79;g__Acidulodesulfobacterium;s__
LMSG_G000011266.1	no	1743_1	Lead-Zinc	64.82	2.16	54.05	0	0	0	12	Medium quality	5434	939094	39.60	195	d__Bacteria;p__SZUA-79;c__SZUA-79;o__Acidulodesulfobacterales;f__SZUA-79;g__Acidulodesulfobacterium;s__
LMSG_G000011267.1	no	1743_1	Polymetallic	64.22	0.65	60.99	0	0	0	8	Medium quality	7013	877147	38.90	142	d__Bacteria;p__SZUA-79;c__SZUA-79;o__Acidulodesulfobacterales;f__SZUA-79;g__Acidulodesulfobacterium;s__
LMSG_G000011268.1	no	1743_1	Antimony	69.66	2.38	57.76	1	1	1	13	Medium quality	3999	1537150	38.30	478	d__Bacteria;p__SZUA-79;c__SZUA-79;o__Acidulodesulfobacterales;f__SZUA-79;g__Acidulodesulfobacterium;s__
LMSG_G000011269.1	no	1735_2	Lead-Zinc	76.64	2.61	63.57	0	0	0	14	Medium quality	3213	1370721	38.20	499	d__Bacteria;p__SZUA-79;c__SZUA-79;o__Acidulodesulfobacterales;f__SZUA-79;g__Acidulodesulfobacterium;s__Acidulodesulfobacterium ferriphilum
LMSG_G000011270.1	no	1735_2	Lead-Zinc	69.39	1.20	63.40	0	1	0	14	Medium quality	12623	1322560	37.90	141	d__Bacteria;p__SZUA-79;c__SZUA-79;o__Acidulodesulfobacterales;f__SZUA-79;g__Acidulodesulfobacterium;s__Acidulodesulfobacterium ferriphilum
LMSG_G000011271.1	no	1735_2	Tin-Zinc	69.88	0.21	68.83	0	0	0	10	Medium quality	6707	1072981	37.60	184	d__Bacteria;p__SZUA-79;c__SZUA-79;o__Acidulodesulfobacterales;f__SZUA-79;g__Acidulodesulfobacterium;s__Acidulodesulfobacterium ferriphilum
LMSG_G000011272.1	no	1735_2	Copper	88.23	0.84	84.03	0	0	0	19	Medium quality	43237	1635444	37.20	104	d__Bacteria;p__SZUA-79;c__SZUA-79;o__Acidulodesulfobacterales;f__SZUA-79;g__Acidulodesulfobacterium;s__Acidulodesulfobacterium ferriphilum
LMSG_G000011273.1	no	1735_2	Pyrite-Copper	66.38	2.28	55.01	1	0	1	16	Medium quality	8508	1400021	37.70	193	d__Bacteria;p__SZUA-79;c__SZUA-79;o__Acidulodesulfobacterales;f__SZUA-79;g__Acidulodesulfobacterium;s__Acidulodesulfobacterium ferriphilum
LMSG_G000011274.1	no	1735_2	Pyrite-Copper	88.21	0.91	83.67	2	1	2	18	Medium quality	16055	1749529	37.40	270	d__Bacteria;p__SZUA-79;c__SZUA-79;o__Acidulodesulfobacterales;f__SZUA-79;g__Acidulodesulfobacterium;s__Acidulodesulfobacterium ferriphilum
LMSG_G000011275.1	no	1735_2	Pyrite	80.25	3.57	62.40	0	0	0	17	Medium quality	11235	1694164	37.60	203	d__Bacteria;p__SZUA-79;c__SZUA-79;o__Acidulodesulfobacterales;f__SZUA-79;g__Acidulodesulfobacterium;s__Acidulodesulfobacterium ferriphilum
LMSG_G000011276.1	no	1735_2	Pyrite	91.81	1.08	86.40	1	1	0	19	Medium quality	24523	1789070	37.50	100	d__Bacteria;p__SZUA-79;c__SZUA-79;o__Acidulodesulfobacterales;f__SZUA-79;g__Acidulodesulfobacterium;s__Acidulodesulfobacterium ferriphilum
LMSG_G000011277.1	no	1735_2	Pyrite-Copper	72.45	1.72	63.83	0	0	0	9	Medium quality	6420	1253931	38.00	230	d__Bacteria;p__SZUA-79;c__SZUA-79;o__Acidulodesulfobacterales;f__SZUA-79;g__Acidulodesulfobacterium;s__Acidulodesulfobacterium ferriphilum
LMSG_G000011278.1	no	1735_2	Polymetallic	91.17	0.84	86.97	1	1	1	18	High quality	46052	1758502	37.50	85	d__Bacteria;p__SZUA-79;c__SZUA-79;o__Acidulodesulfobacterales;f__SZUA-79;g__Acidulodesulfobacterium;s__Acidulodesulfobacterium ferriphilum
LMSG_G000011279.1	no	1735_2	Polymetallic	86.76	0.84	82.56	0	0	0	13	Medium quality	25836	1413471	37.40	95	d__Bacteria;p__SZUA-79;c__SZUA-79;o__Acidulodesulfobacterales;f__SZUA-79;g__Acidulodesulfobacterium;s__Acidulodesulfobacterium ferriphilum
LMSG_G000011280.1	no	1735_2	Polymetallic	90.33	2.52	77.73	1	0	1	14	Medium quality	15763	1553242	37.40	152	d__Bacteria;p__SZUA-79;c__SZUA-79;o__Acidulodesulfobacterales;f__SZUA-79;g__Acidulodesulfobacterium;s__Acidulodesulfobacterium ferriphilum
LMSG_G000011281.1	no	1735_2	Polymetallic	75.49	1.68	67.09	0	0	0	14	Medium quality	8253	1179665	37.50	187	d__Bacteria;p__SZUA-79;c__SZUA-79;o__Acidulodesulfobacterales;f__SZUA-79;g__Acidulodesulfobacterium;s__Acidulodesulfobacterium ferriphilum
LMSG_G000011282.1	no	1735_2	Polymetallic	77.15	2.64	63.95	1	0	1	12	Medium quality	7620	1397083	37.30	244	d__Bacteria;p__SZUA-79;c__SZUA-79;o__Acidulodesulfobacterales;f__SZUA-79;g__Acidulodesulfobacterium;s__Acidulodesulfobacterium ferriphilum
LMSG_G000011283.1	no	1735_2	Lead-Zinc	90.33	0.84	86.13	1	1	1	17	Medium quality	21815	1648973	37.50	134	d__Bacteria;p__SZUA-79;c__SZUA-79;o__Acidulodesulfobacterales;f__SZUA-79;g__Acidulodesulfobacterium;s__Acidulodesulfobacterium ferriphilum
LMSG_G000011284.1	no	1735_2	Lead-Zinc	79.99	1.68	71.59	1	1	0	14	Medium quality	6934	1495424	37.60	290	d__Bacteria;p__SZUA-79;c__SZUA-79;o__Acidulodesulfobacterales;f__SZUA-79;g__Acidulodesulfobacterium;s__Acidulodesulfobacterium ferriphilum
LMSG_G000011285.1	no	1735_2	Lead-Zinc	88.79	1.94	79.10	1	1	1	16	Medium quality	17238	1404523	37.60	127	d__Bacteria;p__SZUA-79;c__SZUA-79;o__Acidulodesulfobacterales;f__SZUA-79;g__Acidulodesulfobacterium;s__Acidulodesulfobacterium ferriphilum
LMSG_G000011286.1	no	1735_2	Tin-Zinc	84.19	0.00	84.19	0	0	0	14	Medium quality	13208	1387462	37.70	150	d__Bacteria;p__SZUA-79;c__SZUA-79;o__Acidulodesulfobacterales;f__SZUA-79;g__Acidulodesulfobacterium;s__Acidulodesulfobacterium ferriphilum
LMSG_G000011287.1	no	1061_1	Antimony	72.69	2.59	59.76	0	0	0	16	Medium quality	4129	1561577	35.90	446	d__Bacteria;p__Thermodesulfobiota;c__Thermodesulfobiia;o__Thermodesulfobiales;f__Thermodesulfobiaceae;g__Thermodesulfobium;s__
LMSG_G000011288.1	no	1061_1	Antimony	66.06	1.72	57.44	0	0	0	16	Medium quality	5842	1244801	36.00	247	d__Bacteria;p__Thermodesulfobiota;c__Thermodesulfobiia;o__Thermodesulfobiales;f__Thermodesulfobiaceae;g__Thermodesulfobium;s__
LMSG_G000011289.1	no	1061_1	Antimony	59.34	0.00	59.34	0	0	0	12	Medium quality	4960	1222235	35.60	316	d__Bacteria;p__Thermodesulfobiota;c__Thermodesulfobiia;o__Thermodesulfobiales;f__Thermodesulfobiaceae;g__Thermodesulfobium;s__
LMSG_G000011290.1	no	1061_1	Antimony	64.94	0.00	64.94	0	0	0	12	Medium quality	5289	1156008	36.10	258	d__Bacteria;p__Thermodesulfobiota;c__Thermodesulfobiia;o__Thermodesulfobiales;f__Thermodesulfobiaceae;g__Thermodesulfobium;s__
LMSG_G000011291.1	no	1061_1	Antimony	74.21	4.14	53.53	0	0	0	13	Medium quality	3754	1508835	35.60	454	d__Bacteria;p__Thermodesulfobiota;c__Thermodesulfobiia;o__Thermodesulfobiales;f__Thermodesulfobiaceae;g__Thermodesulfobium;s__
LMSG_G000011292.1	no	426_1	Lead-Zinc	94.91	0.46	92.60	1	1	1	20	High quality	39935	2142445	39.10	109	d__Bacteria;p__Thermotogota;c__Thermotogae;o__Mesoaciditogales;f__Mesoaciditogaceae;g__ZAV-03;s__
LMSG_G000011293.1	no	426_1	Polymetallic	50.29	0.00	50.29	0	0	0	7	Medium quality	1730	930337	39.10	532	d__Bacteria;p__Thermotogota;c__Thermotogae;o__Mesoaciditogales;f__Mesoaciditogaceae;g__ZAV-03;s__
LMSG_G000011294.1	no	426_1	Polymetallic	61.87	0.15	61.10	0	1	0	13	Medium quality	2866	1139889	39.70	445	d__Bacteria;p__Thermotogota;c__Thermotogae;o__Mesoaciditogales;f__Mesoaciditogaceae;g__ZAV-03;s__
LMSG_G000011295.1	no	426_1	Polymetallic	62.63	1.00	57.63	0	0	0	10	Medium quality	2423	1271112	39.50	583	d__Bacteria;p__Thermotogota;c__Thermotogae;o__Mesoaciditogales;f__Mesoaciditogaceae;g__ZAV-03;s__
LMSG_G000011296.1	no	426_1	Lead-Zinc	80.89	0.62	77.81	0	0	0	16	Medium quality	3035	1480988	39.60	544	d__Bacteria;p__Thermotogota;c__Thermotogae;o__Mesoaciditogales;f__Mesoaciditogaceae;g__ZAV-03;s__
LMSG_G000011297.1	no	426_1	Pyrite-Copper	66.10	0.15	65.33	0	0	0	15	Medium quality	2718	1427248	40.00	589	d__Bacteria;p__Thermotogota;c__Thermotogae;o__Mesoaciditogales;f__Mesoaciditogaceae;g__ZAV-03;s__
LMSG_G000011298.1	no	426_1	Lead-Zinc	94.91	0.46	92.60	1	1	0	19	Medium quality	38069	2158946	39.10	101	d__Bacteria;p__Thermotogota;c__Thermotogae;o__Mesoaciditogales;f__Mesoaciditogaceae;g__ZAV-03;s__
LMSG_G000011299.1	no	938_1	Antimony	83.03	1.55	75.28	1	1	1	17	Medium quality	13214	2295075	38.10	243	d__Bacteria;p__Verrucomicrobiota;c__Lentisphaeria;o__Victivallales;f__GWF2-38-69;g__GWF2-38-69;s__
LMSG_G000011300.1	no	938_1	Antimony	88.81	3.38	71.92	0	0	0	19	Medium quality	24025	2437547	37.90	157	d__Bacteria;p__Verrucomicrobiota;c__Lentisphaeria;o__Victivallales;f__GWF2-38-69;g__GWF2-38-69;s__

LMSG_G000011301.1	no	1060_1	Lead-Zinc	98.64	0.93	93.99	2	1	1	20	High quality	81434	1701797	42.20	45	d__Bacteria;p__Verrucomicrobiota;c__Verrucomicrobiae;o__Chthoniobacterales;f__UBA6821;g__;s__
LMSG_G000011302.1	no	1060_1	Lead-Zinc	77.79	2.17	66.97	0	2	0	14	Medium quality	3018	1271345	42.60	493	d__Bacteria;p__Verrucomicrobiota;c__Verrucomicrobiae;o__Chthoniobacterales;f__UBA6821;g__;s__
LMSG_G000011303.1	no	1014_1	Antimony	74.71	1.72	66.09	0	0	0	15	Medium quality	11764	3326819	64.70	340	d__Bacteria;p__Verrucomicrobiota;c__Verrucomicrobiae;o__Pedosphaerales;f__;g__;s__
LMSG_G000011304.1	no	1018_1	Copper	57.13	0.72	53.56	0	0	0	15	Medium quality	3972	3658047	66.90	1087	d__Bacteria;p__Verrucomicrobiota;c__Verrucomicrobiae;o__Pedosphaerales;f__AV2;g__;s__
LMSG_G000011305.1	no	1015_1	Antimony	91.21	4.99	66.24	1	1	1	16	Medium quality	20332	5009082	65.30	323	d__Bacteria;p__Verrucomicrobiota;c__Verrucomicrobiae;o__Pedosphaerales;f__Pedosphaeraceae;g__;s__
LMSG_G000011306.1	no	1015_1	Antimony	81.52	3.82	62.44	1	0	1	16	Medium quality	4634	4460445	65.50	1115	d__Bacteria;p__Verrucomicrobiota;c__Verrucomicrobiae;o__Pedosphaerales;f__Pedosphaeraceae;g__;s__
LMSG_G000011307.1	no	1015_1	Antimony	80.41	3.88	61.01	0	0	0	15	Medium quality	7191	4282605	65.50	720	d__Bacteria;p__Verrucomicrobiota;c__Verrucomicrobiae;o__Pedosphaerales;f__Pedosphaeraceae;g__;s__
LMSG_G000011308.1	no	1015_1	Copper	94.25	4.43	72.10	0	1	0	17	Medium quality	22643	5312328	65.10	323	d__Bacteria;p__Verrucomicrobiota;c__Verrucomicrobiae;o__Pedosphaerales;f__Pedosphaeraceae;g__;s__
LMSG_G000011309.1	no	1149_1	Polymetallic	98.64	2.73	84.98	1	1	3	20	High quality	79653	3978469	54.80	221	d__Bacteria;p__Verrucomicrobiota;c__Verrucomicrobiae;o__Pedosphaerales;f__Pedosphaeraceae;g__UBA11358;s__
LMSG_G000011310.1	no	1149_1	Polymetallic	97.63	2.03	87.50	1	2	3	20	High quality	82672	3984583	54.80	155	d__Bacteria;p__Verrucomicrobiota;c__Verrucomicrobiae;o__Pedosphaerales;f__Pedosphaeraceae;g__UBA11358;s__
LMSG_G000011311.1	no	1149_1	Polymetallic	97.29	2.70	83.78	2	7	8	20	High quality	80882	4099198	54.80	188	d__Bacteria;p__Verrucomicrobiota;c__Verrucomicrobiae;o__Pedosphaerales;f__Pedosphaeraceae;g__UBA11358;s__
LMSG_G000011312.1	no	1149_1	Polymetallic	92.56	3.45	75.33	1	4	1	20	High quality	58195	3821552	54.80	247	d__Bacteria;p__Verrucomicrobiota;c__Verrucomicrobiae;o__Pedosphaerales;f__Pedosphaeraceae;g__UBA11358;s__
LMSG_G000011313.1	no	1149_1	Polymetallic	97.97	2.36	86.15	1	4	7	20	High quality	58169	3924861	54.90	207	d__Bacteria;p__Verrucomicrobiota;c__Verrucomicrobiae;o__Pedosphaerales;f__Pedosphaeraceae;g__UBA11358;s__
LMSG_G000011314.1	no	1149_1	Polymetallic	95.60	3.16	79.81	1	3	5	19	High quality	42259	3539576	55.10	199	d__Bacteria;p__Verrucomicrobiota;c__Verrucomicrobiae;o__Pedosphaerales;f__Pedosphaeraceae;g__UBA11358;s__
LMSG_G000011315.1	no	1149_1	Polymetallic	98.64	2.47	86.28	1	4	6	19	High quality	33639	3783710	55.00	299	d__Bacteria;p__Verrucomicrobiota;c__Verrucomicrobiae;o__Pedosphaerales;f__Pedosphaeraceae;g__UBA11358;s__
LMSG_G000011316.1	no	1149_1	Polymetallic	98.64	2.03	88.51	1	1	2	20	High quality	54711	3888516	54.90	108	d__Bacteria;p__Verrucomicrobiota;c__Verrucomicrobiae;o__Pedosphaerales;f__Pedosphaeraceae;g__UBA11358;s__
LMSG_G000011317.1	no	1149_1	Copper	93.60	3.38	76.71	1	1	1	19	High quality	20736	3436036	55.10	276	d__Bacteria;p__Verrucomicrobiota;c__Verrucomicrobiae;o__Pedosphaerales;f__Pedosphaeraceae;g__UBA11358;s__
LMSG_G000011318.1	no	1149_1	Polymetallic	93.24	2.03	83.11	1	1	1	17	Medium quality	23547	3449509	55.00	211	d__Bacteria;p__Verrucomicrobiota;c__Verrucomicrobiae;o__Pedosphaerales;f__Pedosphaeraceae;g__UBA11358;s__
LMSG_G000011319.1	no	1149_1	Polymetallic	94.25	4.09	73.79	1	0	1	18	Medium quality	20611	3551827	55.10	342	d__Bacteria;p__Verrucomicrobiota;c__Verrucomicrobiae;o__Pedosphaerales;f__Pedosphaeraceae;g__UBA11358;s__
LMSG_G000011320.1	no	1464_1	Polymetallic	62.07	0.34	60.39	0	0	0	9	Medium quality	2384	2444745	56.60	1149	d__Bacteria;p__Verrucomicrobiota;c__Verrucomicrobiae;o__Pedosphaerales;f__Pedosphaeraceae;g__UBA11358;s__
LMSG_G000011321.1	no	1464_1	Polymetallic	95.85	4.09	75.39	1	0	1	18	Medium quality	15472	4104923	56.40	360	d__Bacteria;p__Verrucomicrobiota;c__Verrucomicrobiae;o__Pedosphaerales;f__Pedosphaeraceae;g__UBA11358;s__
LMSG_G000011322.1	no	1464_1	Polymetallic	93.91	4.42	71.81	1	0	1	18	Medium quality	25212	4279114	56.40	241	d__Bacteria;p__Verrucomicrobiota;c__Verrucomicrobiae;o__Pedosphaerales;f__Pedosphaeraceae;g__UBA11358;s__
LMSG_G000011323.1	no	1464_1	Polymetallic	97.29	3.60	79.28	1	0	1	18	Medium quality	21245	4383270	56.20	304	d__Bacteria;p__Verrucomicrobiota;c__Verrucomicrobiae;o__Pedosphaerales;f__Pedosphaeraceae;g__UBA11358;s__
LMSG_G000011324.1	no	1602_1	Polymetallic	87.34	1.62	79.26	2	1	2	17	Medium quality	7750	1733231	43.00	286	d__Bacteria;p__Verrucomicrobiota_A;c__Chlamydiia;o__2-12-FULL-49-11;f__2-12-FULL-49-11;g__;s__
LMSG_G000011325.1	no	1607_1	Copper	67.17	0.68	63.80	0	1	1	15	Medium quality	6624	820283	44.50	213	d__Bacteria;p__Verrucomicrobiota_A;c__Chlamydiia;o__Parachlamydiales;f__Rhabdochlamydiaceae;g__;s__
LMSG_G000011326.1	no	1612_1	Lead-Zinc	88.85	0.68	85.48	3	1	1	20	Medium quality	44793	1463215	42.60	49	d__Bacteria;p__Verrucomicrobiota_A;c__Chlamydiia;o__Parachlamydiales;f__SM23-39;g__;s__
LMSG_G000011327.1	no	1617_1	Copper	91.72	3.27	75.40	1	1	2	20	High quality	21359	2238543	48.70	136	d__Bacteria;p__Verrucomicrobiota_A;c__Chlamydiia;o__Parachlamydiales;f__SM23-39;g__;s__
LMSG_G000011328.1	no	1617_1	Polymetallic	72.32	2.70	58.81	1	0	1	10	Medium quality	2711	1586438	49.40	650	d__Bacteria;p__Verrucomicrobiota_A;c__Chlamydiia;o__Parachlamydiales;f__SM23-39;g__;s__
LMSG_G000011329.1	no	1617_1	Polymetallic	79.11	4.13	58.45	0	0	0	17	Medium quality	3963	1714163	49.50	526	d__Bacteria;p__Verrucomicrobiota_A;c__Chlamydiia;o__Parachlamydiales;f__SM23-39;g__;s__
LMSG_G000011330.1	no	1617_1	Polymetallic	84.37	3.10	68.89	0	2	0	16	Medium quality	5718	1804673	49.40	411	d__Bacteria;p__Verrucomicrobiota_A;c__Chlamydiia;o__Parachlamydiales;f__SM23-39;g__;s__
LMSG_G000011331.1	no	1622_1	Copper	73.58	0.95	68.86	0	1	0	12	Medium quality	3863	1248702	40.30	361	d__Bacteria;p__Verrucomicrobiota_A;c__Chlamydiia;o__Parachlamydiales;f__SM23-39;g__;s__
LMSG_G000011332.1	no	1624_1	Polymetallic	62.06	0.00	62.06	0	0	0	14	Medium quality	5812	877229	43.70	159	d__Bacteria;p__Verrucomicrobiota_A;c__Chlamydiia;o__Parachlamydiales;f__SM23-39;g__PALSA-1448;s__
LMSG_G000011333.1	no	1624_1	Antimony	84.82	0.90	80.32	1	1	1	18	Medium quality	23679	1642660	43.70	119	d__Bacteria;p__Verrucomicrobiota_A;c__Chlamydiia;o__Parachlamydiales;f__SM23-39;g__PALSA-1448;s__
LMSG_G000011334.1	no	1624_1	Antimony	55.98	0.68	52.61	1	0	1	12	Medium quality	4096	900373	43.40	236	d__Bacteria;p__Verrucomicrobiota_A;c__Chlamydiia;o__Parachlamydiales;f__SM23-39;g__PALSA-1448;s__
LMSG_G000011335.1	no	1625_1	Antimony	89.86	0.81	85.81	1	1	0	18	Medium quality	14621	1779547	44.70	167	d__Bacteria;p__Verrucomicrobiota_A;c__Chlamydiia;o__Parachlamydiales;f__SM23-39;g__PALSA-1448;s__
LMSG_G000011336.1	no	1625_1	Antimony	93.24	0.68	89.87	1	2	0	19	Medium quality	24991	1959929	44.60	119	d__Bacteria;p__Verrucomicrobiota_A;c__Chlamydiia;o__Parachlamydiales;f__SM23-39;g__PALSA-1448;s__
LMSG_G000011337.1	no	1625_1	Antimony	93.24	0.68	89.87	2	2	0	19	Medium quality	37761	2002668	44.60	112	d__Bacteria;p__Verrucomicrobiota_A;c__Chlamydiia;o__Parachlamydiales;f__SM23-39;g__PALSA-1448;s__
LMSG_G000011338.1	no	1068_1	Polymetallic	98.83	0.37	97.00	1	1	1	20	High quality	48784	3863639	59.00	142	d__Bacteria;p__Zixibacteria;c__MSB-5A5;o__;f__;g__;s__
LMSG_G000011339.1	no	1068_1	Polymetallic	98.83	0.00	98.83	1	1	2	20	High quality	45185	3849117	58.90	146	d__Bacteria;p__Zixibacteria;c__MSB-5A5;o__;f__;g__;s__
LMSG_G000011340.1	no	1068_1	Polymetallic	96.08	0.00	96.08	1	1	2	20	High quality	30506	4117234	59.00	221	d__Bacteria;p__Zixibacteria;c__MSB-5A5;o__;f__;g__;s__
