# Supplementary material for: Mining of novel secondary metabolite biosynthetic gene clusters from acid mine drainage
Source: Sci Data. 2022 Dec 9;9:760. doi: 10.1038/s41597-022-01866-6 (PMC9734747; doi:10.1038/s41597-022-01866-6)
Supplement: Supplementary file 4 — Supplementary Table 3 [file 41597_2022_1866_MOESM4_ESM.pdf]

Supplementary Table 3. A total of 11,856 secondary metabolites biosynthetic gene clusters (smBGCs) in eight categories were obtained from 7,007 MAGs

smBGC file name	BGC_ID	Putative novel smBGCs	Genome ID	Sample ID	Product prediction	BiG-SCAPE class	Mineral type	smBGC length (kilo base pair)	Geographic location
CMS4_bin.10.orig.fa_k141_170603.region001.fasta	OEZ008529	yes	LMSG_G000004663.1	OES073660	bacteriocin	RiPPs	Copper	10.86	China: Jiangxi, ChengMenShan
CMS4_bin.10.orig.fa_k141_170603.region002.fasta	OEZ008529	yes	LMSG_G000004663.1	OES073660	terpene	Terpene	Copper	21.96	China: Jiangxi, ChengMenShan
CMS4_bin.100.permissive.fa_c00064_NODE_64...region001.fasta	OEZ008529	yes	LMSG_G000008773.1	OES073660	terpene	Terpene	Copper	18.66	China: Jiangxi, ChengMenShan
CMS4_bin.100.permissive.fa_c00076_NODE_76...region001.fasta	OEZ008529	yes	LMSG_G000008773.1	OES073660	bacteriocin	RiPPs	Copper	5.76	China: Jiangxi, ChengMenShan
CMS4_bin.100.permissive.fa_c00118_NODE_11...region001.fasta	OEZ008529	yes	LMSG_G000008773.1	OES073660	terpene	Terpene	Copper	11.21	China: Jiangxi, ChengMenShan
CMS4_bin.100.permissive.fa_c00128_NODE_12...region001.fasta	OEZ008529	yes	LMSG_G000008773.1	OES073660	NRPS-like	NRPS	Copper	9.93	China: Jiangxi, ChengMenShan
CMS4_bin.101.orig.fa_k141_1091949.region001.fasta	OEZ008529	yes	LMSG_G000004661.1	OES073660	terpene	Terpene	Copper	30.21	China: Jiangxi, ChengMenShan
CMS4_bin.101.orig.fa_k141_17055.region001.fasta	OEZ008529	yes	LMSG_G000004661.1	OES073660	bacteriocin	RiPPs	Copper	10.91	China: Jiangxi, ChengMenShan
CMS4_bin.101.orig.fa_k141_216209.region001.fasta	OEZ008529	yes	LMSG_G000004661.1	OES073660	terpene	Terpene	Copper	21.06	China: Jiangxi, ChengMenShan
CMS4_bin.102.orig.fa_k141_490233.region001.fasta	OEZ008529	yes	LMSG_G000004334.1	OES073660	terpene	Terpene	Copper	13.01	China: Jiangxi, ChengMenShan
CMS4_bin.102.orig.fa_k141_850999.region001.fasta	OEZ008529	yes	LMSG_G000004334.1	OES073660	terpene	Terpene	Copper	24.89	China: Jiangxi, ChengMenShan
CMS4_bin.102.orig.fa_k141_954451.region001.fasta	OEZ008529	yes	LMSG_G000004334.1	OES073660	T3PKS	PKSother	Copper	12.46	China: Jiangxi, ChengMenShan
CMS4_bin.104.orig.fa_k141_245105.region001.fasta	OEZ008529	yes	LMSG_G000004335.1	OES073660	NRPS-like	NRPS	Copper	27.57	China: Jiangxi, ChengMenShan
CMS4_bin.104.orig.fa_k141_427132.region001.fasta	OEZ008529	yes	LMSG_G000004335.1	OES073660	T3PKS	PKSother	Copper	41.14	China: Jiangxi, ChengMenShan
CMS4_bin.104.orig.fa_k141_427132.region002.fasta	OEZ008529	yes	LMSG_G000004335.1	OES073660	terpene	Terpene	Copper	19.41	China: Jiangxi, ChengMenShan
CMS4_bin.104.orig.fa_k141_804471.region001.fasta	OEZ008529	yes	LMSG_G000004335.1	OES073660	terpene	Terpene	Copper	18.38	China: Jiangxi, ChengMenShan
CMS4_bin.105.strict.fa_c00007_NODE_7_...region001.fasta	OEZ008529	yes	LMSG_G000011304.1	OES073660	NRPS	NRPS	Copper	11.09	China: Jiangxi, ChengMenShan
CMS4_bin.105.strict.fa_c00037_NODE_37...region001.fasta	OEZ008529	yes	LMSG_G000011304.1	OES073660	T1PKS	PKSI	Copper	8.35	China: Jiangxi, ChengMenShan
CMS4_bin.105.strict.fa_c00043_NODE_43...region001.fasta	OEZ008529	yes	LMSG_G000011304.1	OES073660	NRPS	NRPS	Copper	8.00	China: Jiangxi, ChengMenShan
CMS4_bin.105.strict.fa_c00069_NODE_69...region001.fasta	OEZ008529	yes	LMSG_G000011304.1	OES073660	transAT-PKS-like	PKSother	Copper	7.01	China: Jiangxi, ChengMenShan
CMS4_bin.105.strict.fa_c00114_NODE_11...region001.fasta	OEZ008529	yes	LMSG_G000011304.1	OES073660	transAT-PKS-like	PKSother	Copper	5.95	China: Jiangxi, ChengMenShan
CMS4_bin.105.strict.fa_c00182_NODE_18...region001.fasta	OEZ008529	yes	LMSG_G000011304.1	OES073660	terpene	Terpene	Copper	5.16	China: Jiangxi, ChengMenShan
CMS4_bin.106.orig.fa_k141_1517694.region001.fasta	OEZ008529	yes	LMSG_G000004336.1	OES073660	bacteriocin	RiPPs	Copper	8.48	China: Jiangxi, ChengMenShan
CMS4_bin.106.orig.fa_k141_771235.region001.fasta	OEZ008529	yes	LMSG_G000004336.1	OES073660	terpene	Terpene	Copper	21.72	China: Jiangxi, ChengMenShan
CMS4_bin.11.orig.fa_k141_1092915.region001.fasta	OEZ008529	yes	LMSG_G000004664.1	OES073660	NRPS-like	NRPS	Copper	17.90	China: Jiangxi, ChengMenShan
CMS4_bin.11.orig.fa_k141_114156.region001.fasta	OEZ008529	yes	LMSG_G000004664.1	OES073660	NRPS-like	NRPS	Copper	51.22	China: Jiangxi, ChengMenShan
CMS4_bin.11.orig.fa_k141_1207690.region001.fasta	OEZ008529	yes	LMSG_G000004664.1	OES073660	terpene	Terpene	Copper	29.74	China: Jiangxi, ChengMenShan
CMS4_bin.11.orig.fa_k141_319300.region001.fasta	OEZ008529	yes	LMSG_G000004664.1	OES073660	T3PKS	PKSother	Copper	41.05	China: Jiangxi, ChengMenShan
CMS4_bin.11.orig.fa_k141_809798.region001.fasta	OEZ008529	yes	LMSG_G000004664.1	OES073660	NRPS-like	NRPS	Copper	34.20	China: Jiangxi, ChengMenShan
CMS4_bin.12.orig.fa_k141_854505.region001.fasta	OEZ008529	yes	LMSG_G000004665.1	OES073660	TfuA-related	RiPPs	Copper	22.83	China: Jiangxi, ChengMenShan
CMS4_bin.13.orig.fa_k141_1394277.region001.fasta	OEZ008529	yes	LMSG_G000004666.1	OES073660	bacteriocin	RiPPs	Copper	10.88	China: Jiangxi, ChengMenShan
CMS4_bin.13.orig.fa_k141_1506728.region001.fasta	OEZ008529	yes	LMSG_G000004666.1	OES073660	NRPS-like	NRPS	Copper	33.40	China: Jiangxi, ChengMenShan
CMS4_bin.13.orig.fa_k141_20867.region001.fasta	OEZ008529	yes	LMSG_G000004666.1	OES073660	terpene	Terpene	Copper	29.20	China: Jiangxi, ChengMenShan
CMS4_bin.14.orig.fa_k141_1036481.region001.fasta	OEZ008529	yes	LMSG_G000004667.1	OES073660	terpene	Terpene	Copper	13.58	China: Jiangxi, ChengMenShan
CMS4_bin.14.orig.fa_k141_1281761.region001.fasta	OEZ008529	yes	LMSG_G000004667.1	OES073660	bacteriocin	RiPPs	Copper	10.65	China: Jiangxi, ChengMenShan
CMS4_bin.14.orig.fa_k141_1513986.region001.fasta	OEZ008529	yes	LMSG_G000004667.1	OES073660	NRPS-like	NRPS	Copper	14.45	China: Jiangxi, ChengMenShan
CMS4_bin.14.orig.fa_k141_701301.region001.fasta	OEZ008529	yes	LMSG_G000004667.1	OES073660	terpene	Terpene	Copper	19.64	China: Jiangxi, ChengMenShan
CMS4_bin.15.orig.fa_k141_219825.region001.fasta	OEZ008529	yes	LMSG_G000010094.1	OES073660	terpene	Terpene	Copper	22.15	China: Jiangxi, ChengMenShan
CMS4_bin.15.orig.fa_k141_515166.region001.fasta	OEZ008529	yes	LMSG_G000010094.1	OES073660	terpene	Terpene	Copper	21.65	China: Jiangxi, ChengMenShan
CMS4_bin.15.orig.fa_k141_603677.region001.fasta	OEZ008529	yes	LMSG_G000010094.1	OES073660	NRPS-like	NRPS	Copper	24.49	China: Jiangxi, ChengMenShan
CMS4_bin.17.orig.fa_k141_1062350.region001.fasta	OEZ008529	yes	LMSG_G000004669.1	OES073660	terpene	Terpene	Copper	22.04	China: Jiangxi, ChengMenShan
CMS4_bin.17.orig.fa_k141_1228477.region001.fasta	OEZ008529	yes	LMSG_G000004669.1	OES073660	bacteriocin	RiPPs	Copper	5.42	China: Jiangxi, ChengMenShan
CMS4_bin.17.orig.fa_k141_1392083.region001.fasta	OEZ008529	yes	LMSG_G000004669.1	OES073660	T1PKS.hglE-KS	PKSother	Copper	52.55	China: Jiangxi, ChengMenShan
CMS4_bin.17.orig.fa_k141_1424267.region001.fasta	OEZ008529	yes	LMSG_G000004669.1	OES073660	NRPS.T1PKS	PKS-NRP_Hybrids	Copper	43.23	China: Jiangxi, ChengMenShan
CMS4_bin.17.orig.fa_k141_245569.region001.fasta	OEZ008529	yes	LMSG_G000004669.1	OES073660	hglE-KS	PKSother	Copper	28.91	China: Jiangxi, ChengMenShan
CMS4_bin.17.orig.fa_k141_272210.region001.fasta	OEZ008529	yes	LMSG_G000004669.1	OES073660	NRPS-like	NRPS	Copper	22.84	China: Jiangxi, ChengMenShan
CMS4_bin.17.orig.fa_k141_375678.region001.fasta	OEZ008529	yes	LMSG_G000004669.1	OES073660	cyanobactin	RiPPs	Copper	9.71	China: Jiangxi, ChengMenShan
CMS4_bin.17.orig.fa_k141_71397.region001.fasta	OEZ008529	yes	LMSG_G000004669.1	OES073660	LAP	RiPPs	Copper	16.10	China: Jiangxi, ChengMenShan
CMS4_bin.17.orig.fa_k141_865535.region001.fasta	OEZ008529	yes	LMSG_G000004669.1	OES073660	terpene	Terpene	Copper	13.63	China: Jiangxi, ChengMenShan
CMS4_bin.17.orig.fa_k141_901264.region001.fasta	OEZ008529	yes	LMSG_G000004669.1	OES073660	T3PKS	PKSother	Copper	38.16	China: Jiangxi, ChengMenShan
CMS4_bin.17.orig.fa_k141_998831.region001.fasta	OEZ008529	yes	LMSG_G000004669.1	OES073660	NRPS-like	NRPS	Copper	28.45	China: Jiangxi, ChengMenShan
CMS4_bin.18.orig.fa_k141_1093620.region001.fasta	OEZ008529	yes	LMSG_G000004670.1	OES073660	NRPS.T1PKS	PKS-NRP_Hybrids	Copper	43.01	China: Jiangxi, ChengMenShan
CMS4_bin.18.orig.fa_k141_812655.region001.fasta	OEZ008529	yes	LMSG_G000004670.1	OES073660	terpene	Terpene	Copper	5.76	China: Jiangxi, ChengMenShan
CMS4_bin.18.orig.fa_k141_848870.region001.fasta	OEZ008529	yes	LMSG_G000004670.1	OES073660	NRPS	NRPS	Copper	9.58	China: Jiangxi, ChengMenShan
CMS4_bin.18.orig.fa_k141_897317.region001.fasta	OEZ008529	yes	LMSG_G000004670.1	OES073660	bacteriocin	RiPPs	Copper	6.05	China: Jiangxi, ChengMenShan
CMS4_bin.18.orig.fa_k141_909725.region001.fasta	OEZ008529	yes	LMSG_G000004670.1	OES073660	terpene	Terpene	Copper	22.39	China: Jiangxi, ChengMenShan
CMS4_bin.18.orig.fa_k141_914953.region001.fasta	OEZ008529	yes	LMSG_G000004670.1	OES073660	terpene	Terpene	Copper	12.42	China: Jiangxi, ChengMenShan
CMS4_bin.2.orig.fa_k141_1365552.region001.fasta	OEZ008529	yes	LMSG_G000004343.1	OES073660	bacteriocin	RiPPs	Copper	10.93	China: Jiangxi, ChengMenShan
CMS4_bin.2.orig.fa_k141_139517.region001.fasta	OEZ008529	yes	LMSG_G000004343.1	OES073660	NRPS-like	NRPS	Copper	25.82	China: Jiangxi, ChengMenShan
CMS4_bin.2.orig.fa_k141_314245.region001.fasta	OEZ008529	yes	LMSG_G000004343.1	OES073660	terpene	Terpene	Copper	20.96	China: Jiangxi, ChengMenShan
CMS4_bin.2.orig.fa_k141_833100.region001.fasta	OEZ008529	yes	LMSG_G000004343.1	OES073660	terpene	Terpene	Copper	7.74	China: Jiangxi, ChengMenShan
CMS4_bin.20.orig.fa_k141_1001312.region001.fasta	OEZ008529	yes	LMSG_G000004671.1	OES073660	terpene	Terpene	Copper	21.81	China: Jiangxi, ChengMenShan
CMS4_bin.20.orig.fa_k141_1102701.region001.fasta	OEZ008529	yes	LMSG_G000004671.1	OES073660	T3PKS	PKSother	Copper	36.74	China: Jiangxi, ChengMenShan
CMS4_bin.20.orig.fa_k141_3092.region001.fasta	OEZ008529	yes	LMSG_G000004671.1	OES073660	hglE-KS	PKSother	Copper	15.70	China: Jiangxi, ChengMenShan
CMS4_bin.20.orig.fa_k141_324201.region001.fasta	OEZ008529	yes	LMSG_G000004671.1	OES073660	cyanobactin	RiPPs	Copper	18.18	China: Jiangxi, ChengMenShan
CMS4_bin.20.orig.fa_k141_637635.region001.fasta	OEZ008529	yes	LMSG_G000004671.1	OES073660	T1PKS	PKSI	Copper	6.30	China: Jiangxi, ChengMenShan
CMS4_bin.20.orig.fa_k141_739185.region001.fasta	OEZ008529	yes	LMSG_G000004671.1	OES073660	terpene	Terpene	Copper	18.19	China: Jiangxi, ChengMenShan
CMS4_bin.20.orig.fa_k141_980494.region001.fasta	OEZ008529	yes	LMSG_G000004671.1	OES073660	hglE-KS	PKSother	Copper	13.34	China: Jiangxi, ChengMenShan
CMS4_bin.21.permissive.fa_c00022_NODE_22...region001.fasta	OEZ008529	yes	LMSG_G000004337.1	OES073660	lassopeptide	RiPPs	Copper	16.45	China: Jiangxi, ChengMenShan
CMS4_bin.21.permissive.fa_c00024_NODE_24...region001.fasta	OEZ008529	yes	LMSG_G000004337.1	OES073660	thiopeptide.LAP	RiPPs	Copper	26.57	China: Jiangxi, ChengMenShan
CMS4_bin.23.strict.fa_c00011_NODE_11...region001.fasta	OEZ008529	yes	LMSG_G000008568.1	OES073660	NRPS-like	NRPS	Copper	42.60	China: Jiangxi, ChengMenShan
CMS4_bin.23.strict.fa_c00013_NODE_13...region001.fasta	OEZ008529	yes	LMSG_G000008568.1	OES073660	other	Others	Copper	27.32	China: Jiangxi, ChengMenShan
CMS4_bin.24.orig.fa_k141_1047176.region001.fasta	OEZ008529	yes	LMSG_G000004673.1	OES073660	terpene	Terpene	Copper	23.10	China: Jiangxi, ChengMenShan
CMS4_bin.24.orig.fa_k141_1492108.region001.fasta	OEZ008529	yes	LMSG_G000004673.1	OES073660	lassopeptide	RiPPs	Copper	13.35	China: Jiangxi, ChengMenShan
CMS4_bin.24.orig.fa_k141_2461.region001.fasta	OEZ008529	yes	LMSG_G000004673.1	OES073660	bacteriocin	RiPPs	Copper	10.92	China: Jiangxi, ChengMenShan
CMS4_bin.24.orig.fa_k141_32287.region001.fasta	OEZ008529	yes	LMSG_G000004673.1	OES073660	NRPS-like	NRPS	Copper	23.19	China: Jiangxi, ChengMenShan
CMS4_bin.24.orig.fa_k141_393703.region001.fasta	OEZ008529	yes	LMSG_G000004673.1	OES073660	arylpolyene	Others	Copper	34.55	China: Jiangxi, ChengMenShan
CMS4_bin.25.orig.fa_k141_1202094.region001.fasta	OEZ008529	yes	LMSG_G000004674.1	OES073660	hglE-KS	PKSother	Copper	22.29	China: Jiangxi, ChengMenShan
CMS4_bin.25.orig.fa_k141_1285606.region001.fasta	OEZ008529	yes	LMSG_G000004674.1	OES073660	hglE-KS	PKSother	Copper	5.18	China: Jiangxi, ChengMenShan
CMS4_bin.25.orig.fa_k141_1402720.region001.fasta	OEZ008529	yes	LMSG_G000004674.1	OES073660	ladderane.bacteriocin	Others	Copper	41.20	China: Jiangxi, ChengMenShan
CMS4_bin.25.orig.fa_k141_1475394.region001.fasta	OEZ008529	yes	LMSG_G000004674.1	OES073660	bacteriocin	RiPPs	Copper	11.98	China: Jiangxi, ChengMenShan
CMS4_bin.25.orig.fa_k141_1550250.region001.fasta	OEZ008529	yes	LMSG_G000004674.1	OES073660	bacteriocin	RiPPs	Copper	11.98	China: Jiangxi, ChengMenShan
CMS4_bin.25.orig.fa_k141_17432.region001.fasta	OEZ008529	yes	LMSG_G000004674.1	OES073660	bacteriocin	RiPPs	Copper	11.86	China: Jiangxi, ChengMenShan
CMS4_bin.25.orig.fa_k141_192030.region001.fasta	OEZ008529	yes	LMSG_G000004674.1	OES073660	NRPS-like	NRPS	Copper	19.85	China: Jiangxi, ChengMenShan
CMS4_bin.25.orig.fa_k141_210459.region001.fasta	OEZ008529	yes	LMSG_G000004674.1	OES073660	T1PKS	PKSI	Copper	11.94	China: Jiangxi, ChengMenShan
CMS4_bin.25.orig.fa_k141_275961.region001.fasta	OEZ008529	yes	LMSG_G000004674.1	OES073660	terpene	Terpene	Copper	21.73	China: Jiangxi, ChengMenShan
CMS4_bin.25.orig.fa_k141_603904.region001.fasta	OEZ008529	yes	LMSG_G000004674.1	OES073660	ladderane	Others	Copper	24.14	China: Jiangxi, ChengMenShan
CMS4_bin.25.orig.fa_k141_901502.region001.fasta	OEZ008529	yes	LMSG_G000004674.1	OES073660	terpene	Terpene	Copper	22.04	China: Jiangxi, ChengMenShan
CMS4_bin.29.orig.fa_k141_1039324.region001.fasta	OEZ008529	yes	LMSG_G000004678.1	OES073660	T3PKS	PKSother	Copper	26.25	China: Jiangxi, ChengMenShan
CMS4_bin.29.orig.fa_k141_1101864.region001.fasta	OEZ008529	yes	LMSG_G000004678.1	OES073660	terpene	Terpene	Copper	14.40	China: Jiangxi, ChengMenShan
CMS4_bin.29.orig.fa_k141_1410860.region001.fasta	OEZ008529	yes	LMSG_G000004678.1	OES073660	bacteriocin	RiPPs	Copper	6.25	China: Jiangxi, ChengMenShan
CMS4_bin.29.orig.fa_k141_674312.region001.fasta	OEZ008529	yes	LMSG_G000004678.1	OES073660	terpene	Terpene	Copper	9.49	China: Jiangxi, ChengMenShan
CMS4_bin.3.strict.fa_c00006_NODE_6_...region001.fasta	OEZ008529	yes	LMSG_G000004345.1	OES073660	terpene	Terpene	Copper	21.81	China: Jiangxi, ChengMenShan
CMS4_bin.3.strict.fa_c00043_NODE_43...region001.fasta	OEZ008529	yes	LMSG_G000004345.1	OES073660	NRPS-like	NRPS	Copper	25.61	China: Jiangxi, ChengMenShan
CMS4_bin.3.strict.fa_c00046_NODE_46...region001.fasta	OEZ008529	yes	LMSG_G000004345.1	OES073660	terpene	Terpene	Copper	16.36	China: Jiangxi, ChengMenShan
CMS4_bin.33.strict.fa_c00104_NODE_10...region001.fasta	OEZ008529	yes	LMSG_G000008054.1	OES073660	T1PKS	PKSI	Copper	8.51	China: Jiangxi, ChengMenShan
CMS4_bin.34.strict.fa_c00001_NODE_1_...region001.fasta	OEZ008529	yes	LMSG_G000004680.1	OES073660	terpene	Terpene	Copper	21.79	China: Jiangxi, ChengMenShan
CMS4_bin.34.strict.fa_c00002_NODE_2_...region001.fasta	OEZ008529	yes	LMSG_G000004680.1	OES073660	terpene	Terpene	Copper	21.76	China: Jiangxi, ChengMenShan
CMS4_bin.34.strict.fa_c00048_NODE_48...region001.fasta	OEZ008529	yes	LMSG_G000004680.1	OES073660	terpene	Terpene	Copper	13.28	China: Jiangxi, ChengMenShan
CMS4_bin.35.orig.fa_k141_1222283.region001.fasta	OEZ008529	yes	LMSG_G000004681.1	OES073660	T3PKS	PKSother	Copper	41.05	China: Jiangxi, ChengMenShan
CMS4_bin.35.orig.fa_k141_1330015.region001.fasta	OEZ008529	yes	LMSG_G000004681.1	OES073660	TfuA-related	RiPPs	Copper	21.83	China: Jiangxi, ChengMenShan
CMS4_bin.36.strict.fa_c00010_NODE_10...region001.fasta	OEZ008529	yes	LMSG_G000004682.1	OES073660	bacteriocin	RiPPs	Copper	10.89	China: Jiangxi, ChengMenShan
CMS4_bin.36.strict.fa_c00018_NODE_18...region001.fasta	OEZ008529	yes	LMSG_G000004682.1	OES073660	bacteriocin	RiPPs	Copper	10.87	China: Jiangxi, ChengMenShan
CMS4_bin.37.strict.fa_c00012_NODE_12...region001.fasta	OEZ008529	yes	LMSG_G000004344.1	OES073660	terpene	Terpene	Copper	22.04	China: Jiangxi, ChengMenShan
CMS4_bin.37.strict.fa_c00014_NODE_14...region001.fasta	OEZ008529	yes	LMSG_G000004344.1	OES073660	lanthipeptide	RiPPs	Copper	23.14	China: Jiangxi, ChengMenShan
CMS4_bin.37.strict.fa_c00017_NODE_17...region001.fasta	OEZ008529	yes	LMSG_G000004344.1	OES073660	terpene	Terpene	Copper	13.65	China: Jiangxi, ChengMenShan
CMS4_bin.37.strict.fa_c00018_NODE_18...region001.fasta	OEZ008529	yes	LMSG_G000004344.1	OES073660	bacteriocin	RiPPs	Copper	12.63	China: Jiangxi, ChengMenShan
CMS4_bin.38.orig.fa_k141_1337036.region001.fasta	OEZ008529	yes	LMSG_G000006206.1	OES073660	terpene	Terpene	Copper	20.86	China: Jiangxi, ChengMenShan
CMS4_bin.38.orig.fa_k141_177980.region001.fasta	OEZ008529	yes	LMSG_G000006206.1	OES073660	betalactone	Others	Copper	30.62	China: Jiangxi, ChengMenShan
CMS4_bin.38.orig.fa_k141_177980.region002.fasta	OEZ008529	yes	LMSG_G000006206.1	OES073660	T3PKS	PKSother	Copper	41.13	China: Jiangxi, ChengMenShan
CMS4_bin.39.orig.fa_k141_1286878.region001.fasta	OEZ008529	yes	LMSG_G000004683.1	OES073660	terpene	Terpene	Copper	11.34	China: Jiangxi, ChengMenShan
CMS4_bin.39.orig.fa_k141_233533.region001.fasta	OEZ008529	yes	LMSG_G000004683.1	OES073660	T3PKS	PKSother	Copper	6.25	China: Jiangxi, ChengMenShan
CMS4_bin.39.orig.fa_k141_337614.region001.fasta	OEZ008529	yes	LMSG_G000004683.1	OES073660	arylpolyene	Others	Copper	10.98	China: Jiangxi, ChengMenShan
CMS4_bin.39.orig.fa_k141_719289.region001.fasta	OEZ008529	yes	LMSG_G000004683.1	OES073660	bacteriocin	RiPPs	Copper	6.59	China: Jiangxi, ChengMenShan
CMS4_bin.4.strict.fa_c00005_NODE_5_...region001.fasta	OEZ008529	yes	LMSG_G000004346.1	OES073660	terpene	Terpene	Copper	20.87	China: Jiangxi, ChengMenShan
CMS4_bin.40.strict.fa_c00001_NODE_1_...region001.fasta	OEZ008529	yes	LMSG_G000004684.1	OES073660	terpene	Terpene	Copper	19.83	China: Jiangxi, ChengMenShan
CMS4_bin.40.strict.fa_c00001_NODE_1_...region002.fasta	OEZ008529	yes	LMSG_G000004684.1	OES073660	arylpolyene	Others	Copper	41.08	China: Jiangxi, ChengMenShan
CMS4_bin.40.strict.fa_c00001_NODE_1_...region003.fasta	OEZ008529	yes	LMSG_G000004684.1	OES073660	terpene	Terpene	Copper	19.19	China: Jiangxi, ChengMenShan
CMS4_bin.40.strict.fa_c00001_NODE_1_...region004.fasta	OEZ008529	yes	LMSG_G000004684.1	OES073660	terpene	Terpene	Copper	21.91	China: Jiangxi, ChengMenShan
CMS4_bin.40.strict.fa_c00002_NODE_2_...region001.fasta	OEZ008529	yes	LMSG_G000004684.1	OES073660	hglE-KS	PKSother	Copper	52.18	China: Jiangxi, ChengMenShan
CMS4_bin.41.orig.fa_k141_110085.region001.fasta	OEZ008529	yes	LMSG_G000004685.1	OES073660	T1PKS	PKSI	Copper	40.98	China: Jiangxi, ChengMenShan
CMS4_bin.41.orig.fa_k141_1298918.region001.fasta	OEZ008529	yes	LMSG_G000004685.1	OES073660	terpene	Terpene	Copper	22.03	China: Jiangxi, ChengMenShan
CMS4_bin.41.orig.fa_k141_1409326.region001.fasta	OEZ008529	yes	LMSG_G000004685.1	OES073660	terpene	Terpene	Copper	18.65	China: Jiangxi, ChengMenShan
CMS4_bin.41.orig.fa_k141_1500598.region001.fasta	OEZ008529	yes	LMSG_G000004685.1	OES073660	hglE-KS	PKSother	Copper	27.98	China: Jiangxi, ChengMenShan
CMS4_bin.41.orig.fa_k141_204891.region001.fasta	OEZ008529	yes	LMSG_G000004685.1	OES073660	hglE-KS	PKSother	Copper	25.98	China: Jiangxi, ChengMenShan
CMS4_bin.41.orig.fa_k141_433139.region001.fasta	OEZ008529	yes	LMSG_G000004685.1	OES073660	cyanobactin	RiPPs	Copper	20.41	China: Jiangxi, ChengMenShan
CMS4_bin.41.orig.fa_k141_765529.region001.fasta	OEZ008529	yes	LMSG_G000004685.1	OES073660	T1PKS	PKSI	Copper	25.11	China: Jiangxi, ChengMenShan
CMS4_bin.42.orig.fa_k141_1141604.region001.fasta	OEZ008529	yes	LMSG_G000006323.1	OES073660	bacteriocin	RiPPs	Copper	10.24	China: Jiangxi, ChengMenShan
CMS4_bin.42.orig.fa_k141_1148487.region001.fasta	OEZ008529	yes	LMSG_G000006323.1	OES073660	NRPS-like	NRPS	Copper	42.82	China: Jiangxi, ChengMenShan
CMS4_bin.42.orig.fa_k141_1175746.region001.fasta	OEZ008529	yes	LMSG_G000006323.1	OES073660	CDPS	Others	Copper	20.71	China: Jiangxi, ChengMenShan
CMS4_bin.42.orig.fa_k141_1206102.region001.fasta	OEZ008529	yes	LMSG_G000006323.1	OES073660	CDPS	Others	Copper	9.09	China: Jiangxi, ChengMenShan
CMS4_bin.42.orig.fa_k141_1225833.region001.fasta	OEZ008529	yes	LMSG_G000006323.1	OES073660	transAT-PKS-like.NRPS-like	PKS-NRP_Hybrids	Copper	19.93	China: Jiangxi, ChengMenShan
CMS4_bin.42.orig.fa_k141_1239828.region001.fasta	OEZ008529	yes	LMSG_G000006323.1	OES073660	terpene	Terpene	Copper	21.00	China: Jiangxi, ChengMenShan
CMS4_bin.42.orig.fa_k141_1486270.region001.fasta	OEZ008529	yes	LMSG_G000006323.1	OES073660	TfuA-related	RiPPs	Copper	14.64	China: Jiangxi, ChengMenShan
CMS4_bin.42.orig.fa_k141_1512904.region001.fasta	OEZ008529	yes	LMSG_G000006323.1	OES073660	terpene	Terpene	Copper	20.86	China: Jiangxi, ChengMenShan
CMS4_bin.42.orig.fa_k141_1512904.region002.fasta	OEZ008529	yes	LMSG_G000006323.1	OES073660	T3PKS.bacteriocin	Others	Copper	36.27	China: Jiangxi, ChengMenShan
CMS4_bin.42.orig.fa_k141_213649.region001.fasta	OEZ008529	yes	LMSG_G000006323.1	OES073660	NRPS-like	NRPS	Copper	39.52	China: Jiangxi, ChengMenShan
CMS4_bin.42.orig.fa_k141_52971.region001.fasta	OEZ008529	yes	LMSG_G000006323.1	OES073660	terpene	Terpene	Copper	20.86	China: Jiangxi, ChengMenShan
CMS4_bin.42.orig.fa_k141_593061.region001.fasta	OEZ008529	yes	LMSG_G000006323.1	OES073660	transAT-PKS	PKSother	Copper	8.71	China: Jiangxi, ChengMenShan
CMS4_bin.42.orig.fa_k141_794522.region001.fasta	OEZ008529	yes	LMSG_G000006323.1	OES073660	bacteriocin	RiPPs	Copper	10.86	China: Jiangxi, ChengMenShan
CMS4_bin.43.orig.fa_k141_1009655.region001.fasta	OEZ008529	yes	LMSG_G000009104.1	OES073660	lassopeptide	RiPPs	Copper	14.07	China: Jiangxi, ChengMenShan
CMS4_bin.43.orig.fa_k141_1302522.region001.fasta	OEZ008529	yes	LMSG_G000009104.1	OES073660	terpene	Terpene	Copper	11.40	China: Jiangxi, ChengMenShan
CMS4_bin.43.orig.fa_k141_1492034.region001.fasta	OEZ008529	yes	LMSG_G000009104.1	OES073660	NRPS-like	NRPS	Copper	10.12	China: Jiangxi, ChengMenShan
CMS4_bin.43.orig.fa_k141_199913.region001.fasta	OEZ008529	yes	LMSG_G000009104.1	OES073660	NRPS-like	NRPS	Copper	13.37	China: Jiangxi, ChengMenShan
CMS4_bin.43.orig.fa_k141_912381.region001.fasta	OEZ008529	yes	LMSG_G000009104.1	OES073660	bacteriocin	RiPPs	Copper	5.39	China: Jiangxi, ChengMenShan
CMS4_bin.43.orig.fa_k141_942483.region001.fasta	OEZ008529	yes	LMSG_G000009104.1	OES073660	T3PKS	PKSother	Copper	40.69	China: Jiangxi, ChengMenShan
CMS4_bin.44.orig.fa_k141_185872.region001.fasta	OEZ008529	yes	LMSG_G000010140.1	OES073660	terpene	Terpene	Copper	20.13	China: Jiangxi, ChengMenShan
CMS4_bin.44.orig.fa_k141_224908.region001.fasta	OEZ008529	yes	LMSG_G000010140.1	OES073660	terpene	Terpene	Copper	6.73	China: Jiangxi, ChengMenShan
CMS4_bin.45.permissive.fa_c00001_NODE_1_...region001.fasta	OEZ008529	yes	LMSG_G000004686.1	OES073660	phosphonate	Others	Copper	26.72	China: Jiangxi, ChengMenShan
CMS4_bin.45.permissive.fa_c00007_NODE_7_...region001.fasta	OEZ008529	yes	LMSG_G000004686.1	OES073660	NRPS-like	NRPS	Copper	43.00	China: Jiangxi, ChengMenShan
CMS4_bin.45.permissive.fa_c00009_NODE_9_...region001.fasta	OEZ008529	yes	LMSG_G000004686.1	OES073660	LAP.bacteriocin	RiPPs	Copper	16.08	China: Jiangxi, ChengMenShan

CMS4_bin.45.permissive.fa_c00010_NODE_10...region001.fasta	OEZ008529	yes	LMSG_G000004686.1	OES073660	bacteriocin	RiPPs	Copper	10.28	China: Jiangxi, ChengMenShan
CMS4_bin.45.permissive.fa_c00023_NODE_23...region001.fasta	OEZ008529	yes	LMSG_G000004686.1	OES073660	NRPS-like	NRPS	Copper	25.90	China: Jiangxi, ChengMenShan
CMS4_bin.47.orig.fa_k141_472862.region001.fasta	OEZ008529	yes	LMSG_G000004687.1	OES073660	terpene	Terpene	Copper	10.46	China: Jiangxi, ChengMenShan
CMS4_bin.48.strict.fa_c00002_NODE_2_...region001.fasta	OEZ008529	yes	LMSG_G000004688.1	OES073660	NRPS-like	NRPS	Copper	42.83	China: Jiangxi, ChengMenShan
CMS4_bin.48.strict.fa_c00009_NODE_9_...region001.fasta	OEZ008529	yes	LMSG_G000004688.1	OES073660	bacteriocin	RiPPs	Copper	10.85	China: Jiangxi, ChengMenShan
CMS4_bin.48.strict.fa_c00033_NODE_33...region001.fasta	OEZ008529	yes	LMSG_G000004688.1	OES073660	bacteriocin	RiPPs	Copper	11.12	China: Jiangxi, ChengMenShan
CMS4_bin.48.strict.fa_c00110_NODE_11...region001.fasta	OEZ008529	yes	LMSG_G000004688.1	OES073660	T3PKS	PKSother	Copper	5.29	China: Jiangxi, ChengMenShan
CMS4_bin.49.orig.fa_k141_1297915.region001.fasta	OEZ008529	yes	LMSG_G000004338.1	OES073660	lanthipeptide	RiPPs	Copper	22.56	China: Jiangxi, ChengMenShan
CMS4_bin.49.orig.fa_k141_1458385.region001.fasta	OEZ008529	yes	LMSG_G000004338.1	OES073660	NRPS	NRPS	Copper	15.61	China: Jiangxi, ChengMenShan
CMS4_bin.49.orig.fa_k141_1515220.region001.fasta	OEZ008529	yes	LMSG_G000004338.1	OES073660	NRPS	NRPS	Copper	16.71	China: Jiangxi, ChengMenShan
CMS4_bin.49.orig.fa_k141_212425.region001.fasta	OEZ008529	yes	LMSG_G000004338.1	OES073660	terpene	Terpene	Copper	20.98	China: Jiangxi, ChengMenShan
CMS4_bin.49.orig.fa_k141_719208.region001.fasta	OEZ008529	yes	LMSG_G000004338.1	OES073660	NRPS	NRPS	Copper	13.70	China: Jiangxi, ChengMenShan
CMS4_bin.50.orig.fa_k141_1220875.region001.fasta	OEZ008529	yes	LMSG_G000010322.1	OES073660	terpene	Terpene	Copper	18.76	China: Jiangxi, ChengMenShan
CMS4_bin.50.orig.fa_k141_313813.region001.fasta	OEZ008529	yes	LMSG_G000010322.1	OES073660	terpene	Terpene	Copper	10.77	China: Jiangxi, ChengMenShan
CMS4_bin.50.orig.fa_k141_90653.region001.fasta	OEZ008529	yes	LMSG_G000010322.1	OES073660	bacteriocin	RiPPs	Copper	10.86	China: Jiangxi, ChengMenShan
CMS4_bin.51.orig.fa_k141_1288186.region001.fasta	OEZ008529	yes	LMSG_G000004689.1	OES073660	NRPS-like	NRPS	Copper	42.63	China: Jiangxi, ChengMenShan
CMS4_bin.52.orig.fa_k141_1429623.region001.fasta	OEZ008529	yes	LMSG_G000004347.1	OES073660	NRPS-like	NRPS	Copper	8.85	China: Jiangxi, ChengMenShan
CMS4_bin.54.orig.fa_k141_1148597.region001.fasta	OEZ008529	yes	LMSG_G000004690.1	OES073660	NRPS	NRPS	Copper	19.50	China: Jiangxi, ChengMenShan
CMS4_bin.54.orig.fa_k141_1216865.region001.fasta	OEZ008529	yes	LMSG_G000004690.1	OES073660	NRPS	NRPS	Copper	5.04	China: Jiangxi, ChengMenShan
CMS4_bin.54.orig.fa_k141_1480545.region001.fasta	OEZ008529	yes	LMSG_G000004690.1	OES073660	terpene	Terpene	Copper	24.84	China: Jiangxi, ChengMenShan
CMS4_bin.54.orig.fa_k141_1520249.region001.fasta	OEZ008529	yes	LMSG_G000004690.1	OES073660	terpene	Terpene	Copper	14.56	China: Jiangxi, ChengMenShan
CMS4_bin.54.orig.fa_k141_611829.region001.fasta	OEZ008529	yes	LMSG_G000004690.1	OES073660	NRPS-like	NRPS	Copper	9.25	China: Jiangxi, ChengMenShan
CMS4_bin.54.orig.fa_k141_629139.region001.fasta	OEZ008529	yes	LMSG_G000004690.1	OES073660	NRPS-like	NRPS	Copper	20.40	China: Jiangxi, ChengMenShan
CMS4_bin.54.orig.fa_k141_634341.region001.fasta	OEZ008529	yes	LMSG_G000004690.1	OES073660	bacteriocin	RiPPs	Copper	10.83	China: Jiangxi, ChengMenShan
CMS4_bin.54.orig.fa_k141_67096.region001.fasta	OEZ008529	yes	LMSG_G000004690.1	OES073660	T3PKS	PKSother	Copper	7.80	China: Jiangxi, ChengMenShan
CMS4_bin.57.orig.fa_k141_299628.region001.fasta	OEZ008529	yes	LMSG_G000009178.1	OES073660	terpene	Terpene	Copper	20.21	China: Jiangxi, ChengMenShan
CMS4_bin.57.orig.fa_k141_335939.region001.fasta	OEZ008529	yes	LMSG_G000009178.1	OES073660	terpene	Terpene	Copper	6.56	China: Jiangxi, ChengMenShan
CMS4_bin.6.strict.fa_c00001_NODE_1_...region001.fasta	OEZ008529	yes	LMSG_G000004700.1	OES073660	terpene	Terpene	Copper	22.14	China: Jiangxi, ChengMenShan
CMS4_bin.6.strict.fa_c00001_NODE_1_...region002.fasta	OEZ008529	yes	LMSG_G000004700.1	OES073660	T1PKS.hglE-KS	PKSother	Copper	54.96	China: Jiangxi, ChengMenShan
CMS4_bin.6.strict.fa_c00006_NODE_6_...region001.fasta	OEZ008529	yes	LMSG_G000004700.1	OES073660	NRPS	NRPS	Copper	44.41	China: Jiangxi, ChengMenShan
CMS4_bin.6.strict.fa_c00025_NODE_25...region001.fasta	OEZ008529	yes	LMSG_G000004700.1	OES073660	terpene	Terpene	Copper	21.98	China: Jiangxi, ChengMenShan
CMS4_bin.6.strict.fa_c00033_NODE_33...region001.fasta	OEZ008529	yes	LMSG_G000004700.1	OES073660	terpene	Terpene	Copper	19.83	China: Jiangxi, ChengMenShan
CMS4_bin.60.orig.fa_k141_1194365.region001.fasta	OEZ008529	yes	LMSG_G000010085.1	OES073660	NRPS-like	NRPS	Copper	30.42	China: Jiangxi, ChengMenShan
CMS4_bin.60.orig.fa_k141_199093.region001.fasta	OEZ008529	yes	LMSG_G000010085.1	OES073660	hglE-KS	PKSother	Copper	45.43	China: Jiangxi, ChengMenShan
CMS4_bin.60.orig.fa_k141_329817.region001.fasta	OEZ008529	yes	LMSG_G000010085.1	OES073660	terpene	Terpene	Copper	11.99	China: Jiangxi, ChengMenShan
CMS4_bin.60.orig.fa_k141_392470.region001.fasta	OEZ008529	yes	LMSG_G000010085.1	OES073660	acyl_amino_acids	Others	Copper	34.58	China: Jiangxi, ChengMenShan
CMS4_bin.60.orig.fa_k141_520409.region001.fasta	OEZ008529	yes	LMSG_G000010085.1	OES073660	T1PKS.hglE-KS	PKSother	Copper	47.32	China: Jiangxi, ChengMenShan
CMS4_bin.60.orig.fa_k141_596501.region001.fasta	OEZ008529	yes	LMSG_G000010085.1	OES073660	terpene	Terpene	Copper	14.92	China: Jiangxi, ChengMenShan
CMS4_bin.60.orig.fa_k141_712074.region001.fasta	OEZ008529	yes	LMSG_G000010085.1	OES073660	terpene	Terpene	Copper	14.91	China: Jiangxi, ChengMenShan
CMS4_bin.64.orig.fa_k141_1032731.region001.fasta	OEZ008529	yes	LMSG_G000004695.1	OES073660	NRPS-like	NRPS	Copper	11.22	China: Jiangxi, ChengMenShan
CMS4_bin.64.orig.fa_k141_1182972.region001.fasta	OEZ008529	yes	LMSG_G000004695.1	OES073660	bacteriocin	RiPPs	Copper	10.28	China: Jiangxi, ChengMenShan
CMS4_bin.64.orig.fa_k141_1508998.region001.fasta	OEZ008529	yes	LMSG_G000004695.1	OES073660	LAP.bacteriocin	RiPPs	Copper	11.57	China: Jiangxi, ChengMenShan
CMS4_bin.64.orig.fa_k141_732512.region001.fasta	OEZ008529	yes	LMSG_G000004695.1	OES073660	bacteriocin	RiPPs	Copper	6.45	China: Jiangxi, ChengMenShan
CMS4_bin.65.permissive.fa_c00066_NODE_66...region001.fasta	OEZ008529	yes	LMSG_G000004696.1	OES073660	NRPS	NRPS	Copper	5.45	China: Jiangxi, ChengMenShan
CMS4_bin.65.permissive.fa_c00086_NODE_86...region001.fasta	OEZ008529	yes	LMSG_G000004696.1	OES073660	terpene	Terpene	Copper	5.04	China: Jiangxi, ChengMenShan
CMS4_bin.67.strict.fa_c00002_NODE_2_...region001.fasta	OEZ008529	yes	LMSG_G000004349.1	OES073660	NRPS-like	NRPS	Copper	42.67	China: Jiangxi, ChengMenShan
CMS4_bin.67.strict.fa_c00003_NODE_3_...region001.fasta	OEZ008529	yes	LMSG_G000004349.1	OES073660	bacteriocin	RiPPs	Copper	10.86	China: Jiangxi, ChengMenShan
CMS4_bin.67.strict.fa_c00007_NODE_7_...region001.fasta	OEZ008529	yes	LMSG_G000004349.1	OES073660	hglE-KS	PKSother	Copper	48.86	China: Jiangxi, ChengMenShan
CMS4_bin.68.orig.fa_k141_1096533.region001.fasta	OEZ008529	yes	LMSG_G000004698.1	OES073660	terpene	Terpene	Copper	7.94	China: Jiangxi, ChengMenShan
CMS4_bin.68.orig.fa_k141_198508.region001.fasta	OEZ008529	yes	LMSG_G000004698.1	OES073660	T3PKS	PKSother	Copper	16.04	China: Jiangxi, ChengMenShan
CMS4_bin.68.orig.fa_k141_806463.region001.fasta	OEZ008529	yes	LMSG_G000004698.1	OES073660	terpene	Terpene	Copper	6.97	China: Jiangxi, ChengMenShan
CMS4_bin.68.orig.fa_k141_845999.region001.fasta	OEZ008529	yes	LMSG_G000004698.1	OES073660	terpene	Terpene	Copper	12.70	China: Jiangxi, ChengMenShan
CMS4_bin.7.orig.fa_k141_1237846.region001.fasta	OEZ008529	yes	LMSG_G000004707.1	OES073660	hglE-KS	PKSother	Copper	11.13	China: Jiangxi, ChengMenShan
CMS4_bin.7.orig.fa_k141_669309.region001.fasta	OEZ008529	yes	LMSG_G000004707.1	OES073660	T1PKS	PKSI	Copper	5.25	China: Jiangxi, ChengMenShan
CMS4_bin.71.orig.fa_k141_491565.region001.fasta	OEZ008529	yes	LMSG_G000004701.1	OES073660	arylpolyene	Others	Copper	18.54	China: Jiangxi, ChengMenShan
CMS4_bin.71.orig.fa_k141_709671.region001.fasta	OEZ008529	yes	LMSG_G000004701.1	OES073660	NRPS-like	NRPS	Copper	16.60	China: Jiangxi, ChengMenShan
CMS4_bin.71.orig.fa_k141_887920.region001.fasta	OEZ008529	yes	LMSG_G000004701.1	OES073660	terpene	Terpene	Copper	8.12	China: Jiangxi, ChengMenShan
CMS4_bin.72.strict.fa_c00013_NODE_13...region001.fasta	OEZ008529	yes	LMSG_G000004702.1	OES073660	NRPS-like	NRPS	Copper	9.09	China: Jiangxi, ChengMenShan
CMS4_bin.73.permissive.fa_c00043_NODE_43...region001.fasta	OEZ008529	yes	LMSG_G000008310.1	OES073660	terpene	Terpene	Copper	10.66	China: Jiangxi, ChengMenShan
CMS4_bin.74.orig.fa_k141_1280561.region001.fasta	OEZ008529	yes	LMSG_G000004703.1	OES073660	NRPS-like	NRPS	Copper	7.81	China: Jiangxi, ChengMenShan
CMS4_bin.74.orig.fa_k141_393432.region001.fasta	OEZ008529	yes	LMSG_G000004703.1	OES073660	terpene	Terpene	Copper	7.15	China: Jiangxi, ChengMenShan
CMS4_bin.74.orig.fa_k141_565650.region001.fasta	OEZ008529	yes	LMSG_G000004703.1	OES073660	terpene	Terpene	Copper	5.45	China: Jiangxi, ChengMenShan
CMS4_bin.78.orig.fa_k141_1101941.region001.fasta	OEZ008529	yes	LMSG_G000011308.1	OES073660	ladderane	Others	Copper	11.39	China: Jiangxi, ChengMenShan
CMS4_bin.78.orig.fa_k141_1197488.region001.fasta	OEZ008529	yes	LMSG_G000011308.1	OES073660	terpene	Terpene	Copper	20.87	China: Jiangxi, ChengMenShan
CMS4_bin.78.orig.fa_k141_1454245.region001.fasta	OEZ008529	yes	LMSG_G000011308.1	OES073660	terpene	Terpene	Copper	16.12	China: Jiangxi, ChengMenShan
CMS4_bin.78.orig.fa_k141_489677.region001.fasta	OEZ008529	yes	LMSG_G000011308.1	OES073660	terpene	Terpene	Copper	21.96	China: Jiangxi, ChengMenShan
CMS4_bin.78.orig.fa_k141_712999.region001.fasta	OEZ008529	yes	LMSG_G000011308.1	OES073660	terpene	Terpene	Copper	21.05	China: Jiangxi, ChengMenShan
CMS4_bin.79.orig.fa_k141_1183412.region001.fasta	OEZ008529	yes	LMSG_G000007873.1	OES073660	T3PKS	PKSother	Copper	16.38	China: Jiangxi, ChengMenShan
CMS4_bin.79.orig.fa_k141_1301763.region001.fasta	OEZ008529	yes	LMSG_G000007873.1	OES073660	NRPS-like	NRPS	Copper	23.02	China: Jiangxi, ChengMenShan
CMS4_bin.79.orig.fa_k141_1492007.region001.fasta	OEZ008529	yes	LMSG_G000007873.1	OES073660	terpene	Terpene	Copper	21.27	China: Jiangxi, ChengMenShan
CMS4_bin.80.orig.fa_k141_1023398.region001.fasta	OEZ008529	yes	LMSG_G000007751.1	OES073660	T1PKS	PKSI	Copper	9.64	China: Jiangxi, ChengMenShan
CMS4_bin.80.orig.fa_k141_1399197.region001.fasta	OEZ008529	yes	LMSG_G000007751.1	OES073660	terpene	Terpene	Copper	10.43	China: Jiangxi, ChengMenShan
CMS4_bin.80.orig.fa_k141_265758.region001.fasta	OEZ008529	yes	LMSG_G000007751.1	OES073660	T1PKS	PKSI	Copper	12.15	China: Jiangxi, ChengMenShan
CMS4_bin.80.orig.fa_k141_594547.region001.fasta	OEZ008529	yes	LMSG_G000007751.1	OES073660	T1PKS	PKSI	Copper	18.84	China: Jiangxi, ChengMenShan
CMS4_bin.80.orig.fa_k141_63424.region001.fasta	OEZ008529	yes	LMSG_G000007751.1	OES073660	terpene	Terpene	Copper	19.21	China: Jiangxi, ChengMenShan
CMS4_bin.80.orig.fa_k141_845038.region001.fasta	OEZ008529	yes	LMSG_G000007751.1	OES073660	betalactone.NRPS-like	Others	Copper	7.43	China: Jiangxi, ChengMenShan
CMS4_bin.80.orig.fa_k141_857148.region001.fasta	OEZ008529	yes	LMSG_G000007751.1	OES073660	NRPS.T1PKS	PKS-NRP_Hybrids	Copper	39.32	China: Jiangxi, ChengMenShan
CMS4_bin.81.orig.fa_k141_1539389.region001.fasta	OEZ008529	yes	LMSG_G000004708.1	OES073660	bacteriocin	RiPPs	Copper	10.89	China: Jiangxi, ChengMenShan
CMS4_bin.83.orig.fa_k141_1221128.region001.fasta	OEZ008529	yes	LMSG_G000004710.1	OES073660	bacteriocin	RiPPs	Copper	10.86	China: Jiangxi, ChengMenShan
CMS4_bin.83.orig.fa_k141_1530527.region001.fasta	OEZ008529	yes	LMSG_G000004710.1	OES073660	arylpolyene	Others	Copper	27.48	China: Jiangxi, ChengMenShan
CMS4_bin.83.orig.fa_k141_197745.region001.fasta	OEZ008529	yes	LMSG_G000004710.1	OES073660	NRPS-like	NRPS	Copper	39.10	China: Jiangxi, ChengMenShan
CMS4_bin.83.orig.fa_k141_77674.region001.fasta	OEZ008529	yes	LMSG_G000004710.1	OES073660	terpene	Terpene	Copper	23.08	China: Jiangxi, ChengMenShan
CMS4_bin.83.orig.fa_k141_936775.region001.fasta	OEZ008529	yes	LMSG_G000004710.1	OES073660	NRPS-like	NRPS	Copper	24.57	China: Jiangxi, ChengMenShan
CMS4_bin.85.strict.fa_c00010_NODE_10...region001.fasta	OEZ008529	yes	LMSG_G000006308.1	OES073660	terpene	Terpene	Copper	13.77	China: Jiangxi, ChengMenShan
CMS4_bin.85.strict.fa_c00069_NODE_69...region001.fasta	OEZ008529	yes	LMSG_G000006308.1	OES073660	NRPS	NRPS	Copper	12.45	China: Jiangxi, ChengMenShan
CMS4_bin.85.strict.fa_c00116_NODE_11...region001.fasta	OEZ008529	yes	LMSG_G000006308.1	OES073660	ladderane	Others	Copper	9.47	China: Jiangxi, ChengMenShan
CMS4_bin.85.strict.fa_c00153_NODE_15...region001.fasta	OEZ008529	yes	LMSG_G000006308.1	OES073660	terpene	Terpene	Copper	8.11	China: Jiangxi, ChengMenShan
CMS4_bin.86.orig.fa_k141_162875.region001.fasta	OEZ008529	yes	LMSG_G000004711.1	OES073660	arylpolyene	Others	Copper	13.83	China: Jiangxi, ChengMenShan
CMS4_bin.86.orig.fa_k141_216276.region001.fasta	OEZ008529	yes	LMSG_G000004711.1	OES073660	NRPS	NRPS	Copper	33.18	China: Jiangxi, ChengMenShan
CMS4_bin.86.orig.fa_k141_756595.region001.fasta	OEZ008529	yes	LMSG_G000004711.1	OES073660	terpene	Terpene	Copper	12.12	China: Jiangxi, ChengMenShan
CMS4_bin.86.orig.fa_k141_927074.region001.fasta	OEZ008529	yes	LMSG_G000004711.1	OES073660	terpene	Terpene	Copper	7.57	China: Jiangxi, ChengMenShan
CMS4_bin.87.orig.fa_k141_120596.region001.fasta	OEZ008529	yes	LMSG_G000004712.1	OES073660	T3PKS	PKSother	Copper	29.89	China: Jiangxi, ChengMenShan
CMS4_bin.87.orig.fa_k141_1384444.region001.fasta	OEZ008529	yes	LMSG_G000004712.1	OES073660	terpene	Terpene	Copper	25.15	China: Jiangxi, ChengMenShan
CMS4_bin.87.orig.fa_k141_238922.region001.fasta	OEZ008529	yes	LMSG_G000004712.1	OES073660	NRPS-like	NRPS	Copper	42.82	China: Jiangxi, ChengMenShan
CMS4_bin.87.orig.fa_k141_755174.region001.fasta	OEZ008529	yes	LMSG_G000004712.1	OES073660	LAP	RiPPs	Copper	23.76	China: Jiangxi, ChengMenShan
CMS4_bin.87.orig.fa_k141_787411.region001.fasta	OEZ008529	yes	LMSG_G000004712.1	OES073660	bacteriocin	RiPPs	Copper	10.92	China: Jiangxi, ChengMenShan
CMS4_bin.87.orig.fa_k141_896675.region001.fasta	OEZ008529	yes	LMSG_G000004712.1	OES073660	hserlactone	Others	Copper	20.66	China: Jiangxi, ChengMenShan
CMS4_bin.89.strict.fa_c00005_NODE_5_...region001.fasta	OEZ008529	yes	LMSG_G000004350.1	OES073660	NRPS-like	NRPS	Copper	41.47	China: Jiangxi, ChengMenShan
CMS4_bin.89.strict.fa_c00013_NODE_13...region001.fasta	OEZ008529	yes	LMSG_G000004350.1	OES073660	terpene	Terpene	Copper	20.92	China: Jiangxi, ChengMenShan
CMS4_bin.89.strict.fa_c00049_NODE_49...region001.fasta	OEZ008529	yes	LMSG_G000004350.1	OES073660	lanthipeptide	RiPPs	Copper	22.78	China: Jiangxi, ChengMenShan
CMS4_bin.89.strict.fa_c00055_NODE_55...region001.fasta	OEZ008529	yes	LMSG_G000004350.1	OES073660	T1PKS	PKSI	Copper	33.79	China: Jiangxi, ChengMenShan
CMS4_bin.89.strict.fa_c00061_NODE_61...region001.fasta	OEZ008529	yes	LMSG_G000004350.1	OES073660	betalactone	Others	Copper	27.80	China: Jiangxi, ChengMenShan
CMS4_bin.9.orig.fa_k141_1092237.region001.fasta	OEZ008529	yes	LMSG_G000004352.1	OES073660	NRPS	NRPS	Copper	51.33	China: Jiangxi, ChengMenShan
CMS4_bin.9.orig.fa_k141_1198945.region001.fasta	OEZ008529	yes	LMSG_G000004352.1	OES073660	NRPS	NRPS	Copper	13.99	China: Jiangxi, ChengMenShan
CMS4_bin.9.orig.fa_k141_1291929.region001.fasta	OEZ008529	yes	LMSG_G000004352.1	OES073660	lassopeptide	RiPPs	Copper	22.36	China: Jiangxi, ChengMenShan
CMS4_bin.9.orig.fa_k141_1312836.region001.fasta	OEZ008529	yes	LMSG_G000004352.1	OES073660	T1PKS.NRPS-like	PKS-NRP_Hybrids	Copper	48.43	China: Jiangxi, ChengMenShan
CMS4_bin.9.orig.fa_k141_1335756.region001.fasta	OEZ008529	yes	LMSG_G000004352.1	OES073660	LAP.bacteriocin	RiPPs	Copper	24.44	China: Jiangxi, ChengMenShan
CMS4_bin.9.orig.fa_k141_1376603.region001.fasta	OEZ008529	yes	LMSG_G000004352.1	OES073660	bacteriocin	RiPPs	Copper	10.47	China: Jiangxi, ChengMenShan
CMS4_bin.9.orig.fa_k141_1411601.region001.fasta	OEZ008529	yes	LMSG_G000004352.1	OES073660	T3PKS	PKSother	Copper	22.00	China: Jiangxi, ChengMenShan
CMS4_bin.9.orig.fa_k141_157401.region001.fasta	OEZ008529	yes	LMSG_G000004352.1	OES073660	ladderane	Others	Copper	33.30	China: Jiangxi, ChengMenShan
CMS4_bin.9.orig.fa_k141_31128.region001.fasta	OEZ008529	yes	LMSG_G000004352.1	OES073660	terpene	Terpene	Copper	21.96	China: Jiangxi, ChengMenShan
CMS4_bin.9.orig.fa_k141_491262.region001.fasta	OEZ008529	yes	LMSG_G000004352.1	OES073660	NRPS	NRPS	Copper	25.27	China: Jiangxi, ChengMenShan
CMS4_bin.9.orig.fa_k141_53988.region001.fasta	OEZ008529	yes	LMSG_G000004352.1	OES073660	NRPS	NRPS	Copper	10.54	China: Jiangxi, ChengMenShan
CMS4_bin.9.orig.fa_k141_5872.region001.fasta	OEZ008529	yes	LMSG_G000004352.1	OES073660	terpene	Terpene	Copper	22.03	China: Jiangxi, ChengMenShan
CMS4_bin.9.orig.fa_k141_762491.region001.fasta	OEZ008529	yes	LMSG_G000004352.1	OES073660	T1PKS	PKSI	Copper	6.42	China: Jiangxi, ChengMenShan
CMS4_bin.9.orig.fa_k141_939872.region001.fasta	OEZ008529	yes	LMSG_G000004352.1	OES073660	T1PKS	PKSI	Copper	8.50	China: Jiangxi, ChengMenShan
CMS4_bin.90.permissive.fa_c00096_NODE_96...region001.fasta	OEZ008529	yes	LMSG_G000004713.1	OES073660	bacteriocin	RiPPs	Copper	6.92	China: Jiangxi, ChengMenShan
CMS4_bin.91.permissive.fa_c00020_NODE_20...region001.fasta	OEZ008529	yes	LMSG_G000004714.1	OES073660	T1PKS.hglE-KS	PKSother	Copper	18.85	China: Jiangxi, ChengMenShan
CMS4_bin.91.permissive.fa_c00025_NODE_25...region001.fasta	OEZ008529	yes	LMSG_G000004714.1	OES073660	NRPS	NRPS	Copper	17.90	China: Jiangxi, ChengMenShan
CMS4_bin.91.permissive.fa_c00197_NODE_19...region001.fasta	OEZ008529	yes	LMSG_G000004714.1	OES073660	NRPS-like	NRPS	Copper	8.58	China: Jiangxi, ChengMenShan
CMS4_bin.91.permissive.fa_c00266_NODE_26...region001.fasta	OEZ008529	yes	LMSG_G000004714.1	OES073660	terpene	Terpene	Copper	7.41	China: Jiangxi, ChengMenShan
CMS4_bin.91.permissive.fa_c00270_NODE_27...region001.fasta	OEZ008529	yes	LMSG_G000004714.1	OES073660	T3PKS	PKSother	Copper	7.24	China: Jiangxi, ChengMenShan
CMS4_bin.91.permissive.fa_c00295_NODE_29...region001.fasta	OEZ008529	yes	LMSG_G000004714.1	OES073660	terpene	Terpene	Copper	6.86	China: Jiangxi, ChengMenShan
CMS4_bin.91.permissive.fa_c00374_NODE_37...region001.fasta	OEZ008529	yes	LMSG_G000004714.1	OES073660	indole	Others	Copper	5.84	China: Jiangxi, ChengMenShan
CMS4_bin.91.permissive.fa_c00420_NODE_42...region001.fasta	OEZ008529	yes	LMSG_G000004714.1	OES073660	NRPS	NRPS	Copper	5.39	China: Jiangxi, ChengMenShan
CMS4_bin.92.permissive.fa_c00019_NODE_19...region001.fasta	OEZ008529	yes	LMSG_G000004715.1	OES073660	terpene	Terpene	Copper	17.21	China: Jiangxi, ChengMenShan
CMS4_bin.92.permissive.fa_c00029_NODE_29...region001.fasta	OEZ008529	yes	LMSG_G000004715.1	OES073660	indole	Others	Copper	12.02	China: Jiangxi, ChengMenShan
CMS4_bin.92.permissive.fa_c00064_NODE_64...region001.fasta	OEZ008529	yes	LMSG_G000004715.1	OES073660	T3PKS	PKSother	Copper	14.04	China: Jiangxi, ChengMenShan
CMS4_bin.93.orig.fa_k141_114612.region001.fasta	OEZ008529	yes	LMSG_G000004716.1	OES073660	bacteriocin	RiPPs	Copper	9.11	China: Jiangxi, ChengMenShan
CMS4_bin.93.orig.fa_k141_1195766.region001.fasta	OEZ008529	yes	LMSG_G000004716.1	OES073660	NRPS-like	NRPS	Copper	6.47	China: Jiangxi, ChengMenShan
CMS4_bin.93.orig.fa_k141_1425943.region001.fasta	OEZ008529	yes	LMSG_G000004716.1	OES073660	proteusin	RiPPs	Copper	8.77	China: Jiangxi, ChengMenShan
CMS4_bin.93.orig.fa_k141_199749.region001.fasta	OEZ008529	yes	LMSG_G000004716.1	OES073660	T1PKS	PKSI	Copper	5.27	China: Jiangxi, ChengMenShan
CMS4_bin.93.orig.fa_k141_204195.region001.fasta	OEZ008529	yes	LMSG_G000004716.1	OES073660	terpene	Terpene	Copper	6.25	China: Jiangxi, ChengMenShan
CMS4_bin.93.orig.fa_k141_263637.region001.fasta	OEZ008529	yes	LMSG_G000004716.1	OES073660	bacteriocin	RiPPs	Copper	6.84	China: Jiangxi, ChengMenShan
CMS4_bin.93.orig.fa_k141_361204.region001.fasta	OEZ008529	yes	LMSG_G000004716.1	OES073660	NRPS	NRPS	Copper	21.46	China: Jiangxi, ChengMenShan
CMS4_bin.93.orig.fa_k141_670012.region001.fasta	OEZ008529	yes	LMSG_G000004716.1	OES073660	bacteriocin	RiPPs	Copper	5.33	China: Jiangxi, ChengMenShan
CMS4_bin.93.orig.fa_k141_712407.region001.fasta	OEZ008529	yes	LMSG_G000004716.1	OES073660	terpene	Terpene	Copper	20.83	China: Jiangxi, ChengMenShan
CMS4_bin.93.orig.fa_k141_909644.region001.fasta	OEZ008529	yes	LMSG_G000004716.1	OES073660	hglE-KS.NRPS-like	PKS-NRP_Hybrids	Copper	18.74	China: Jiangxi, ChengMenShan
CMS4_bin.95.orig.fa_k141_1082316.region001.fasta	OEZ008529	yes	LMSG_G000004718.1	OES073660	terpene	Terpene	Copper	17.30	China: Jiangxi, ChengMenShan
CMS4_bin.95.orig.fa_k141_1227405.region001.fasta	OEZ008529	yes	LMSG_G000004718.1	OES073660	terpene	Terpene	Copper	16.81	China: Jiangxi, ChengMenShan
CMS4_bin.95.orig.fa_k141_1385155.region001.fasta	OEZ008529	yes	LMSG_G000004718.1	OES073660	terpene	Terpene	Copper	18.47	China: Jiangxi, ChengMenShan
CMS4_bin.95.orig.fa_k141_617201.region001.fasta	OEZ008529	yes	LMSG_G000004718.1	OES073660	acyl_amino_acids	Others	Copper	12.53	China: Jiangxi, ChengMenShan
CMS4_bin.96.orig.fa_k141_1137780.region001.fasta	OEZ008529	yes	LMSG_G000004351.1	OES073660	terpene	Terpene	Copper	17.83	China: Jiangxi, ChengMenShan
CMS4_bin.96.orig.fa_k141_1525929.region001.fasta	OEZ008529	yes	LMSG_G000004351.1	OES073660	terpene	Terpene	Copper	15.00	China: Jiangxi, ChengMenShan
CMS4_bin.96.orig.fa_k141_199550.region001.fasta	OEZ008529	yes	LMSG_G000004351.1	OES073660	bacteriocin	RiPPs	Copper	5.85	China: Jiangxi, ChengMenShan
CMS4_bin.96.orig.fa_k141_317712.region001.fasta	OEZ008529	yes	LMSG_G000004351.1	OES073660	arylpolyene	Others	Copper	40.81	China: Jiangxi, ChengMenShan
CMS4_bin.96.orig.fa_k141_50594.region001.fasta	OEZ008529	yes	LMSG_G000004351.1	OES073660	NRPS-like	NRPS	Copper	29.69	China: Jiangxi, ChengMenShan
CMS4_bin.96.orig.fa_k141_532970.region001.fasta	OEZ008529	yes	LMSG_G000004351.1	OES073660	ladderane	Others	Copper	29.76	China: Jiangxi, ChengMenShan
CMS4_bin.96.orig.fa_k141_928496.region001.fasta	OEZ008529	yes	LMSG_G000004351.1	OES073660	arylpolyene	Others	Copper	27.91	China: Jiangxi, ChengMenShan
CMS4_bin.97.orig.fa_k141_1339926.region001.fasta	OEZ008529	yes	LMSG_G000004719.1	OES073660	NRPS-like	NRPS	Copper	18.22	China: Jiangxi, ChengMenShan
CMS4_bin.97.orig.fa_k141_351015.region001.fasta	OEZ008529	yes	LMSG_G000004719.1	OES073660	terpene	Terpene	Copper	14.89	China: Jiangxi, ChengMenShan
CMS4_bin.98.orig.fa_k141_1108164.region001.fasta	OEZ008529	yes	LMSG_G000004339.1	OES073660	other	Others	Copper	23.34	China: Jiangxi, ChengMenShan
CMS4_bin.98.orig.fa_k141_1214128.region001.fasta	OEZ008529	yes	LMSG_G000004339.1	OES073660	other	Others	Copper	35.70	China: Jiangxi, ChengMenShan
CMS4_bin.98.orig.fa_k141_1338162.region001.fasta	OEZ008529	yes	LMSG_G000004339.1	OES073660	betalactone	Others	Copper	17.08	China: Jiangxi, ChengMenShan
CMS4_bin.99.orig.fa_k141_1529038.region001.fasta	OEZ008529	yes	LMSG_G000008775.1	OES073660	lanthipeptide	RiPPs	Copper	17.85	China: Jiangxi, ChengMenShan
DBS5_bin.10.permissive.fa_c00074_NODE_74...region001.fasta	OEZ008529	yes	LMSG_G000004720.1	OES073661	terpene	Terpene	Pyrite	6.48	China: Guangdong, Daobaoshan
DBS5_bin.12.permissive.fa_c00010_NODE_10...region001.fasta	OEZ008529	yes	LMSG_G000004722.1	OES073661	betalactone	Others	Pyrite	22.54	China: Guangdong, Daobaoshan
DBS5_bin.12.permissive.fa_c00029_NODE_29...region001.fasta	OEZ008529	yes	LMSG_G000004722.1	OES073661	terpene	Terpene	Pyrite	14.70	China: Guangdong, Daobaoshan

DBS5_bin.12.permissive.fa_c00118_NODE_11...region001.fasta	OEZ008529	yes	LMSG_G000004722.1	OES073661	terpene	Terpene	Pyrite	7.99	China: Guangdong, Daobaoshan
DBS5_bin.13.orig.fa_k141_1271088.region001.fasta	OEZ008529	yes	LMSG_G000004723.1	OES073661	arylpolyene	Others	Pyrite	23.15	China: Guangdong, Daobaoshan
DBS5_bin.13.orig.fa_k141_1450140.region001.fasta	OEZ008529	yes	LMSG_G000004723.1	OES073661	terpene	Terpene	Pyrite	7.71	China: Guangdong, Daobaoshan
DBS5_bin.13.orig.fa_k141_574435.region001.fasta	OEZ008529	yes	LMSG_G000004723.1	OES073661	bacteriocin	RiPPs	Pyrite	10.85	China: Guangdong, Daobaoshan
DBS5_bin.14.orig.fa_k141_153616.region001.fasta	OEZ008529	yes	LMSG_G000004724.1	OES073661	T1PKS	PKSI	Pyrite	42.65	China: Guangdong, Daobaoshan
DBS5_bin.16.strict.fa_c00083_NODE_83...region001.fasta	OEZ008529	yes	LMSG_G000004726.1	OES073661	terpene	Terpene	Pyrite	11.73	China: Guangdong, Daobaoshan
DBS5_bin.16.strict.fa_c00095_NODE_95...region001.fasta	OEZ008529	yes	LMSG_G000004726.1	OES073661	terpene	Terpene	Pyrite	11.09	China: Guangdong, Daobaoshan
DBS5_bin.17.orig.fa_k141_1071077.region001.fasta	OEZ008529	yes	LMSG_G000010354.1	OES073661	NRPS-like	NRPS	Pyrite	12.82	China: Guangdong, Daobaoshan
DBS5_bin.17.orig.fa_k141_1360867.region001.fasta	OEZ008529	yes	LMSG_G000010354.1	OES073661	terpene	Terpene	Pyrite	20.85	China: Guangdong, Daobaoshan
DBS5_bin.17.orig.fa_k141_1495386.region001.fasta	OEZ008529	yes	LMSG_G000010354.1	OES073661	bacteriocin	RiPPs	Pyrite	10.86	China: Guangdong, Daobaoshan
DBS5_bin.17.orig.fa_k141_1495386.region002.fasta	OEZ008529	yes	LMSG_G000010354.1	OES073661	NRPS	NRPS	Pyrite	50.48	China: Guangdong, Daobaoshan
DBS5_bin.17.orig.fa_k141_448747.region001.fasta	OEZ008529	yes	LMSG_G000010354.1	OES073661	terpene	Terpene	Pyrite	22.06	China: Guangdong, Daobaoshan
DBS5_bin.17.orig.fa_k141_541274.region001.fasta	OEZ008529	yes	LMSG_G000010354.1	OES073661	NRPS-like	NRPS	Pyrite	44.07	China: Guangdong, Daobaoshan
DBS5_bin.17.orig.fa_k141_897871.region001.fasta	OEZ008529	yes	LMSG_G000010354.1	OES073661	terpene	Terpene	Pyrite	21.72	China: Guangdong, Daobaoshan
DBS5_bin.17.orig.fa_k141_957682.region001.fasta	OEZ008529	yes	LMSG_G000010354.1	OES073661	bacteriocin	RiPPs	Pyrite	11.72	China: Guangdong, Daobaoshan
DBS5_bin.2.orig.fa_k141_1001448.region001.fasta	OEZ008529	yes	LMSG_G000010737.1	OES073661	bacteriocin	RiPPs	Pyrite	10.84	China: Guangdong, Daobaoshan
DBS5_bin.20.orig.fa_k141_1198548.region001.fasta	OEZ008529	yes	LMSG_G000004728.1	OES073661	terpene	Terpene	Pyrite	21.00	China: Guangdong, Daobaoshan
DBS5_bin.20.orig.fa_k141_1394612.region001.fasta	OEZ008529	yes	LMSG_G000004728.1	OES073661	NRPS-like	NRPS	Pyrite	11.53	China: Guangdong, Daobaoshan
DBS5_bin.20.orig.fa_k141_876530.region001.fasta	OEZ008529	yes	LMSG_G000004728.1	OES073661	betalactone	Others	Pyrite	27.89	China: Guangdong, Daobaoshan
DBS5_bin.21.permissive.fa_c00019_NODE_19...region001.fasta	OEZ008529	yes	LMSG_G000008122.1	OES073661	terpene	Terpene	Pyrite	6.68	China: Guangdong, Daobaoshan
DBS5_bin.23.orig.fa_k141_1100793.region001.fasta	OEZ008529	yes	LMSG_G000010978.1	OES073661	T1PKS.hglE-KS	PKSother	Pyrite	39.13	China: Guangdong, Daobaoshan
DBS5_bin.24.orig.fa_k141_1114431.region001.fasta	OEZ008529	yes	LMSG_G000004730.1	OES073661	bacteriocin	RiPPs	Pyrite	5.43	China: Guangdong, Daobaoshan
DBS5_bin.24.orig.fa_k141_1625638.region001.fasta	OEZ008529	yes	LMSG_G000004730.1	OES073661	bacteriocin	RiPPs	Pyrite	10.05	China: Guangdong, Daobaoshan
DBS5_bin.24.orig.fa_k141_814545.region001.fasta	OEZ008529	yes	LMSG_G000004730.1	OES073661	bacteriocin	RiPPs	Pyrite	10.39	China: Guangdong, Daobaoshan
DBS5_bin.26.orig.fa_k141_1467848.region001.fasta	OEZ008529	yes	LMSG_G000006207.1	OES073661	bacteriocin	RiPPs	Pyrite	7.92	China: Guangdong, Daobaoshan
DBS5_bin.29.orig.fa_k141_1642425.region001.fasta	OEZ008529	yes	LMSG_G000007863.1	OES073661	T3PKS	PKSother	Pyrite	25.39	China: Guangdong, Daobaoshan
DBS5_bin.29.orig.fa_k141_476784.region001.fasta	OEZ008529	yes	LMSG_G000007863.1	OES073661	terpene	Terpene	Pyrite	30.07	China: Guangdong, Daobaoshan
DBS5_bin.29.orig.fa_k141_476784.region002.fasta	OEZ008529	yes	LMSG_G000007863.1	OES073661	bacteriocin	RiPPs	Pyrite	10.86	China: Guangdong, Daobaoshan
DBS5_bin.3.strict.fa_c00001_NODE_1_...region001.fasta	OEZ008529	yes	LMSG_G000004739.1	OES073661	NRPS-like.terpene	Others	Pyrite	48.38	China: Guangdong, Daobaoshan
DBS5_bin.3.strict.fa_c00020_NODE_20...region001.fasta	OEZ008529	yes	LMSG_G000004739.1	OES073661	T3PKS	PKSother	Pyrite	39.31	China: Guangdong, Daobaoshan
DBS5_bin.30.orig.fa_k141_1145116.region001.fasta	OEZ008529	yes	LMSG_G000004734.1	OES073661	ectoine	Others	Pyrite	6.67	China: Guangdong, Daobaoshan
DBS5_bin.30.orig.fa_k141_986568.region001.fasta	OEZ008529	no	LMSG_G000004734.1	OES073661	arylpolyene	Others	Pyrite	9.06	China: Guangdong, Daobaoshan
DBS5_bin.32.strict.fa_c00002_NODE_2_...region001.fasta	OEZ008529	yes	LMSG_G000009302.1	OES073661	betalactone	Others	Pyrite	28.23	China: Guangdong, Daobaoshan
DBS5_bin.32.strict.fa_c00044_NODE_44...region001.fasta	OEZ008529	yes	LMSG_G000009302.1	OES073661	betalactone	Others	Pyrite	15.44	China: Guangdong, Daobaoshan
DBS5_bin.33.strict.fa_c00001_NODE_1_...region001.fasta	OEZ008529	yes	LMSG_G000004735.1	OES073661	terpene	Terpene	Pyrite	20.29	China: Guangdong, Daobaoshan
DBS5_bin.33.strict.fa_c00001_NODE_1_...region002.fasta	OEZ008529	yes	LMSG_G000004735.1	OES073661	terpene	Terpene	Pyrite	12.57	China: Guangdong, Daobaoshan
DBS5_bin.33.strict.fa_c00020_NODE_20...region001.fasta	OEZ008529	yes	LMSG_G000004735.1	OES073661	NRPS-like	NRPS	Pyrite	23.72	China: Guangdong, Daobaoshan
DBS5_bin.33.strict.fa_c00096_NODE_96...region001.fasta	OEZ008529	yes	LMSG_G000004735.1	OES073661	bacteriocin	RiPPs	Pyrite	9.14	China: Guangdong, Daobaoshan
DBS5_bin.34.permissive.fa_c00014_NODE_14...region001.fasta	OEZ008529	no	LMSG_G000004736.1	OES073661	ectoine	Others	Pyrite	6.75	China: Guangdong, Daobaoshan
DBS5_bin.38.orig.fa_k141_1504539.region001.fasta	OEZ008529	yes	LMSG_G000010835.1	OES073661	bacteriocin	RiPPs	Pyrite	6.77	China: Guangdong, Daobaoshan
DBS5_bin.39.strict.fa_c00002_NODE_2_...region001.fasta	OEZ008529	yes	LMSG_G000004353.1	OES073661	acyl_amino_acids	Others	Pyrite	39.82	China: Guangdong, Daobaoshan
DBS5_bin.39.strict.fa_c00012_NODE_12...region001.fasta	OEZ008529	yes	LMSG_G000004353.1	OES073661	bacteriocin	RiPPs	Pyrite	10.14	China: Guangdong, Daobaoshan
DBS5_bin.39.strict.fa_c00016_NODE_16...region001.fasta	OEZ008529	yes	LMSG_G000004353.1	OES073661	arylpolyene	Others	Pyrite	33.34	China: Guangdong, Daobaoshan
DBS5_bin.4.orig.fa_k141_1506412.region001.fasta	OEZ008529	yes	LMSG_G000007789.1	OES073661	terpene	Terpene	Pyrite	19.68	China: Guangdong, Daobaoshan
DBS5_bin.4.orig.fa_k141_1542408.region001.fasta	OEZ008529	yes	LMSG_G000007789.1	OES073661	T3PKS	PKSother	Pyrite	31.76	China: Guangdong, Daobaoshan
DBS5_bin.4.orig.fa_k141_439323.region001.fasta	OEZ008529	yes	LMSG_G000007789.1	OES073661	bacteriocin	RiPPs	Pyrite	10.90	China: Guangdong, Daobaoshan
DBS5_bin.40.orig.fa_k141_1428282.region001.fasta	OEZ008529	yes	LMSG_G000010685.1	OES073661	bacteriocin	RiPPs	Pyrite	6.90	China: Guangdong, Daobaoshan
DBS5_bin.40.orig.fa_k141_1588447.region001.fasta	OEZ008529	yes	LMSG_G000010685.1	OES073661	terpene	Terpene	Pyrite	17.06	China: Guangdong, Daobaoshan
DBS5_bin.40.orig.fa_k141_18104.region001.fasta	OEZ008529	yes	LMSG_G000010685.1	OES073661	NRPS-like	NRPS	Pyrite	7.55	China: Guangdong, Daobaoshan
DBS5_bin.40.orig.fa_k141_989081.region001.fasta	OEZ008529	yes	LMSG_G000010685.1	OES073661	arylpolyene	Others	Pyrite	34.11	China: Guangdong, Daobaoshan
DBS5_bin.42.strict.fa_c00013_NODE_13...region001.fasta	OEZ008529	yes	LMSG_G000004740.1	OES073661	NRPS-like	NRPS	Pyrite	37.50	China: Guangdong, Daobaoshan
DBS5_bin.42.strict.fa_c00026_NODE_26...region001.fasta	OEZ008529	yes	LMSG_G000004740.1	OES073661	T3PKS	PKSother	Pyrite	22.44	China: Guangdong, Daobaoshan
DBS5_bin.5.orig.fa_k141_1017901.region001.fasta	OEZ008529	no	LMSG_G000010149.1	OES073661	ectoine	Others	Pyrite	10.40	China: Guangdong, Daobaoshan
DBS5_bin.5.orig.fa_k141_1022864.region001.fasta	OEZ008529	no	LMSG_G000010149.1	OES073661	terpene	Terpene	Pyrite	14.62	China: Guangdong, Daobaoshan
DBS5_bin.5.orig.fa_k141_1511964.region001.fasta	OEZ008529	no	LMSG_G000010149.1	OES073661	acyl_amino_acids	Others	Pyrite	15.27	China: Guangdong, Daobaoshan
DBS5_bin.6.orig.fa_k141_1288698.region001.fasta	OEZ008529	yes	LMSG_G000008586.1	OES073661	other	Others	Pyrite	7.14	China: Guangdong, Daobaoshan
DBS5_bin.7.orig.fa_k141_1150875.region001.fasta	OEZ008529	yes	LMSG_G000004741.1	OES073661	T1PKS	PKSI	Pyrite	47.19	China: Guangdong, Daobaoshan
DBS5_bin.7.orig.fa_k141_1324401.region001.fasta	OEZ008529	yes	LMSG_G000004741.1	OES073661	terpene	Terpene	Pyrite	12.09	China: Guangdong, Daobaoshan
DBS5_bin.7.orig.fa_k141_424289.region001.fasta	OEZ008529	yes	LMSG_G000004741.1	OES073661	betalactone	Others	Pyrite	26.70	China: Guangdong, Daobaoshan
DBS5_bin.9.orig.fa_k141_131463.region001.fasta	OEZ008529	yes	LMSG_G000004742.1	OES073661	terpene	Terpene	Pyrite	13.92	China: Guangdong, Daobaoshan
DBS5_bin.9.orig.fa_k141_1325657.region001.fasta	OEZ008529	yes	LMSG_G000004742.1	OES073661	arylpolyene	Others	Pyrite	14.00	China: Guangdong, Daobaoshan
DBS5_bin.9.orig.fa_k141_1586332.region001.fasta	OEZ008529	yes	LMSG_G000004742.1	OES073661	arylpolyene	Others	Pyrite	30.98	China: Guangdong, Daobaoshan
DBS5_bin.9.orig.fa_k141_366182.region001.fasta	OEZ008529	yes	LMSG_G000004742.1	OES073661	arylpolyene	Others	Pyrite	22.40	China: Guangdong, Daobaoshan
DBS5_bin.9.orig.fa_k141_651664.region001.fasta	OEZ008529	yes	LMSG_G000004742.1	OES073661	bacteriocin	RiPPs	Pyrite	10.86	China: Guangdong, Daobaoshan
DC1_bin.1.orig.fa_k141_100165.region001.fasta	OEZ008529	yes	LMSG_G000011182.1	OES073662	bacteriocin	RiPPs	Copper	5.26	China: Guangxi, Dachang
DC1_bin.1.orig.fa_k141_1349161.region001.fasta	OEZ008529	yes	LMSG_G000011182.1	OES073662	arylpolyene	Others	Copper	25.24	China: Guangxi, Dachang
DC1_bin.10.orig.fa_k141_1022647.region001.fasta	OEZ008529	yes	LMSG_G000010920.1	OES073662	thiopeptide	RiPPs	Copper	29.81	China: Guangxi, Dachang
DC1_bin.10.orig.fa_k141_1158177.region001.fasta	OEZ008529	yes	LMSG_G000010920.1	OES073662	arylpolyene	Others	Copper	41.19	China: Guangxi, Dachang
DC1_bin.10.orig.fa_k141_84108.region001.fasta	OEZ008529	yes	LMSG_G000010920.1	OES073662	terpene	Terpene	Copper	16.49	China: Guangxi, Dachang
DC1_bin.11.orig.fa_k141_1375197.region001.fasta	OEZ008529	yes	LMSG_G000006260.1	OES073662	T3PKS	PKSother	Copper	41.06	China: Guangxi, Dachang
DC1_bin.11.orig.fa_k141_872938.region001.fasta	OEZ008529	yes	LMSG_G000006260.1	OES073662	terpene	Terpene	Copper	24.11	China: Guangxi, Dachang
DC1_bin.11.orig.fa_k141_997177.region001.fasta	OEZ008529	yes	LMSG_G000006260.1	OES073662	bacteriocin	RiPPs	Copper	10.88	China: Guangxi, Dachang
DC1_bin.12.orig.fa_k141_392000.region001.fasta	OEZ008529	yes	LMSG_G000010896.1	OES073662	terpene	Terpene	Copper	12.99	China: Guangxi, Dachang
DC1_bin.12.orig.fa_k141_610266.region001.fasta	OEZ008529	yes	LMSG_G000010896.1	OES073662	acyl_amino_acids	Others	Copper	9.73	China: Guangxi, Dachang
DC1_bin.12.orig.fa_k141_745773.region001.fasta	OEZ008529	yes	LMSG_G000010896.1	OES073662	terpene	Terpene	Copper	11.75	China: Guangxi, Dachang
DC1_bin.13.strict.fa_c00045_NODE_45...region001.fasta	OEZ008529	yes	LMSG_G000010915.1	OES073662	T1PKS	PKSI	Copper	16.22	China: Guangxi, Dachang
DC1_bin.13.strict.fa_c00068_NODE_68...region001.fasta	OEZ008529	yes	LMSG_G000010915.1	OES073662	terpene	Terpene	Copper	11.85	China: Guangxi, Dachang
DC1_bin.13.strict.fa_c00071_NODE_71...region001.fasta	OEZ008529	yes	LMSG_G000010915.1	OES073662	bacteriocin	RiPPs	Copper	7.21	China: Guangxi, Dachang
DC1_bin.13.strict.fa_c00077_NODE_77...region001.fasta	OEZ008529	yes	LMSG_G000010915.1	OES073662	arylpolyene	Others	Copper	10.50	China: Guangxi, Dachang
DC1_bin.14.strict.fa_c00003_NODE_3_...region001.fasta	OEZ008529	yes	LMSG_G000008979.1	OES073662	bacteriocin	RiPPs	Copper	10.90	China: Guangxi, Dachang
DC1_bin.14.strict.fa_c00047_NODE_47...region001.fasta	OEZ008529	yes	LMSG_G000008979.1	OES073662	T1PKS.hglE-KS	PKSother	Copper	16.91	China: Guangxi, Dachang
DC1_bin.16.orig.fa_k141_901802.region001.fasta	OEZ008529	no	LMSG_G000010811.1	OES073662	acyl_amino_acids	Others	Copper	8.66	China: Guangxi, Dachang
DC1_bin.18.strict.fa_c00002_NODE_2_...region001.fasta	OEZ008529	yes	LMSG_G000010043.1	OES073662	NRPS-like.ectoine	Others	Copper	50.26	China: Guangxi, Dachang
DC1_bin.18.strict.fa_c00003_NODE_3_...region001.fasta	OEZ008529	yes	LMSG_G000010043.1	OES073662	ladderane	Others	Copper	41.26	China: Guangxi, Dachang
DC1_bin.18.strict.fa_c00003_NODE_3_...region002.fasta	OEZ008529	yes	LMSG_G000010043.1	OES073662	arylpolyene	Others	Copper	27.63	China: Guangxi, Dachang
DC1_bin.18.strict.fa_c00039_NODE_39...region001.fasta	OEZ008529	yes	LMSG_G000010043.1	OES073662	terpene	Terpene	Copper	14.10	China: Guangxi, Dachang
DC1_bin.18.strict.fa_c00091_NODE_91...region001.fasta	OEZ008529	yes	LMSG_G000010043.1	OES073662	bacteriocin	RiPPs	Copper	10.87	China: Guangxi, Dachang
DC1_bin.18.strict.fa_c00093_NODE_93...region001.fasta	OEZ008529	yes	LMSG_G000010043.1	OES073662	terpene	Terpene	Copper	13.48	China: Guangxi, Dachang
DC1_bin.18.strict.fa_c00108_NODE_10...region001.fasta	OEZ008529	yes	LMSG_G000010043.1	OES073662	arylpolyene	Others	Copper	12.92	China: Guangxi, Dachang
DC1_bin.18.strict.fa_c00134_NODE_13...region001.fasta	OEZ008529	yes	LMSG_G000010043.1	OES073662	T3PKS	PKSother	Copper	9.30	China: Guangxi, Dachang
DC1_bin.2.orig.fa_k141_348434.region001.fasta	OEZ008529	yes	LMSG_G000010317.1	OES073662	terpene	Terpene	Copper	5.18	China: Guangxi, Dachang
DC1_bin.2.orig.fa_k141_672917.region001.fasta	OEZ008529	yes	LMSG_G000010317.1	OES073662	ectoine	Others	Copper	6.60	China: Guangxi, Dachang
DC1_bin.21.orig.fa_k141_1038823.region001.fasta	OEZ008529	yes	LMSG_G000004743.1	OES073662	terpene	Terpene	Copper	18.55	China: Guangxi, Dachang
DC1_bin.21.orig.fa_k141_1522839.region001.fasta	OEZ008529	yes	LMSG_G000004743.1	OES073662	betalactone	Others	Copper	28.46	China: Guangxi, Dachang
DC1_bin.22.strict.fa_c00012_NODE_12...region001.fasta	OEZ008529	yes	LMSG_G000004354.1	OES073662	terpene	Terpene	Copper	20.36	China: Guangxi, Dachang
DC1_bin.23.orig.fa_k141_1146364.region001.fasta	OEZ008529	yes	LMSG_G000004340.1	OES073662	other	Others	Copper	40.44	China: Guangxi, Dachang
DC1_bin.23.orig.fa_k141_560508.region001.fasta	OEZ008529	yes	LMSG_G000004340.1	OES073662	other	Others	Copper	28.84	China: Guangxi, Dachang
DC1_bin.24.orig.fa_k141_1255480.region001.fasta	OEZ008529	yes	LMSG_G000004744.1	OES073662	NRPS	NRPS	Copper	13.39	China: Guangxi, Dachang
DC1_bin.24.orig.fa_k141_1360761.region001.fasta	OEZ008529	yes	LMSG_G000004744.1	OES073662	NRPS	NRPS	Copper	5.58	China: Guangxi, Dachang
DC1_bin.24.orig.fa_k141_141035.region001.fasta	OEZ008529	yes	LMSG_G000004744.1	OES073662	NRPS	NRPS	Copper	10.76	China: Guangxi, Dachang
DC1_bin.24.orig.fa_k141_3012.region001.fasta	OEZ008529	yes	LMSG_G000004744.1	OES073662	NRPS	NRPS	Copper	7.15	China: Guangxi, Dachang
DC1_bin.24.orig.fa_k141_578930.region001.fasta	OEZ008529	yes	LMSG_G000004744.1	OES073662	TfuA-related	RiPPs	Copper	26.18	China: Guangxi, Dachang
DC1_bin.24.orig.fa_k141_887631.region001.fasta	OEZ008529	yes	LMSG_G000004744.1	OES073662	NRPS-like	NRPS	Copper	14.75	China: Guangxi, Dachang
DC1_bin.26.orig.fa_k141_269467.region001.fasta	OEZ008529	yes	LMSG_G000004745.1	OES073662	NRPS.T1PKS	PKS-NRP_Hybrids	Copper	48.25	China: Guangxi, Dachang
DC1_bin.26.orig.fa_k141_323089.region001.fasta	OEZ008529	yes	LMSG_G000004745.1	OES073662	NRPS	NRPS	Copper	48.79	China: Guangxi, Dachang
DC1_bin.26.orig.fa_k141_50359.region001.fasta	OEZ008529	yes	LMSG_G000004745.1	OES073662	NRPS-like	NRPS	Copper	32.68	China: Guangxi, Dachang
DC1_bin.26.orig.fa_k141_545920.region001.fasta	OEZ008529	yes	LMSG_G000004745.1	OES073662	T1PKS.hglE-KS	PKSother	Copper	48.86	China: Guangxi, Dachang
DC1_bin.26.orig.fa_k141_555226.region001.fasta	OEZ008529	yes	LMSG_G000004745.1	OES073662	bacteriocin	RiPPs	Copper	7.88	China: Guangxi, Dachang
DC1_bin.26.orig.fa_k141_589389.region001.fasta	OEZ008529	yes	LMSG_G000004745.1	OES073662	NRPS	NRPS	Copper	47.07	China: Guangxi, Dachang
DC1_bin.26.orig.fa_k141_688104.region001.fasta	OEZ008529	yes	LMSG_G000004745.1	OES073662	T3PKS	PKSother	Copper	26.44	China: Guangxi, Dachang
DC1_bin.26.orig.fa_k141_783168.region001.fasta	OEZ008529	yes	LMSG_G000004745.1	OES073662	hglE-KS	PKSother	Copper	47.27	China: Guangxi, Dachang
DC1_bin.26.orig.fa_k141_840588.region001.fasta	OEZ008529	yes	LMSG_G000004745.1	OES073662	terpene	Terpene	Copper	21.87	China: Guangxi, Dachang
DC1_bin.26.orig.fa_k141_890808.region001.fasta	OEZ008529	yes	LMSG_G000004745.1	OES073662	linaridin	RiPPs	Copper	12.49	China: Guangxi, Dachang
DC1_bin.26.orig.fa_k141_981018.region001.fasta	OEZ008529	yes	LMSG_G000004745.1	OES073662	terpene	Terpene	Copper	22.03	China: Guangxi, Dachang
DC1_bin.27.permissive.fa_c00001_NODE_1_...region001.fasta	OEZ008529	yes	LMSG_G000011331.1	OES073662	NRPS	NRPS	Copper	23.94	China: Guangxi, Dachang
DC1_bin.28.strict.fa_c00005_NODE_5_...region001.fasta	OEZ008529	yes	LMSG_G000004746.1	OES073662	bacteriocin	RiPPs	Copper	8.06	China: Guangxi, Dachang
DC1_bin.28.strict.fa_c00008_NODE_8_...region001.fasta	OEZ008529	yes	LMSG_G000004746.1	OES073662	terpene	Terpene	Copper	17.67	China: Guangxi, Dachang
DC1_bin.28.strict.fa_c00016_NODE_16...region001.fasta	OEZ008529	yes	LMSG_G000004746.1	OES073662	T3PKS	PKSother	Copper	30.21	China: Guangxi, Dachang
DC1_bin.29.strict.fa_c00002_NODE_2_...region001.fasta	OEZ008529	yes	LMSG_G000009215.1	OES073662	bacteriocin	RiPPs	Copper	10.90	China: Guangxi, Dachang
DC1_bin.29.strict.fa_c00002_NODE_2_...region002.fasta	OEZ008529	yes	LMSG_G000009215.1	OES073662	T3PKS	PKSother	Copper	41.11	China: Guangxi, Dachang
DC1_bin.29.strict.fa_c00006_NODE_6_...region001.fasta	OEZ008529	yes	LMSG_G000009215.1	OES073662	terpene	Terpene	Copper	21.74	China: Guangxi, Dachang
DC1_bin.29.strict.fa_c00020_NODE_20...region001.fasta	OEZ008529	yes	LMSG_G000009215.1	OES073662	terpene	Terpene	Copper	21.42	China: Guangxi, Dachang
DC1_bin.3.orig.fa_k141_256523.region001.fasta	OEZ008529	yes	LMSG_G000004751.1	OES073662	NRPS-like	NRPS	Copper	42.66	China: Guangxi, Dachang
DC1_bin.3.orig.fa_k141_29480.region001.fasta	OEZ008529	yes	LMSG_G000004751.1	OES073662	terpene	Terpene	Copper	30.07	China: Guangxi, Dachang
DC1_bin.3.orig.fa_k141_29480.region002.fasta	OEZ008529	yes	LMSG_G000004751.1	OES073662	bacteriocin	RiPPs	Copper	10.86	China: Guangxi, Dachang
DC1_bin.3.orig.fa_k141_910894.region001.fasta	OEZ008529	yes	LMSG_G000004751.1	OES073662	T3PKS	PKSother	Copper	41.06	China: Guangxi, Dachang
DC1_bin.30.orig.fa_k141_783974.region001.fasta	OEZ008529	yes	LMSG_G000010698.1	OES073662	terpene	Terpene	Copper	12.59	China: Guangxi, Dachang
DC1_bin.30.orig.fa_k141_868484.region001.fasta	OEZ008529	yes	LMSG_G000010698.1	OES073662	bacteriocin	RiPPs	Copper	6.64	China: Guangxi, Dachang
DC1_bin.32.orig.fa_k141_1502952.region001.fasta	OEZ008529	yes	LMSG_G000010076.1	OES073662	terpene	Terpene	Copper	8.47	China: Guangxi, Dachang
DC1_bin.32.orig.fa_k141_857902.region001.fasta	OEZ008529	yes	LMSG_G000010076.1	OES073662	arylpolyene	Others	Copper	10.83	China: Guangxi, Dachang
DC1_bin.33.orig.fa_k141_1166272.region001.fasta	OEZ008529	yes	LMSG_G000004748.1	OES073662	arylpolyene	Others	Copper	6.66	China: Guangxi, Dachang
DC1_bin.33.orig.fa_k141_413225.region001.fasta	OEZ008529	yes	LMSG_G000004748.1	OES073662	terpene	Terpene	Copper	18.44	China: Guangxi, Dachang
DC1_bin.33.orig.fa_k141_491606.region001.fasta	OEZ008529	yes	LMSG_G000004748.1	OES073662	bacteriocin	RiPPs	Copper	6.45	China: Guangxi, Dachang
DC1_bin.35.permissive.fa_c00004_NODE_4_...region001.fasta	OEZ008529	yes	LMSG_G000004749.1	OES073662	NRPS-like	NRPS	Copper	42.67	China: Guangxi, Dachang
DC1_bin.35.permissive.fa_c00022_NODE_22...region001.fasta	OEZ008529	yes	LMSG_G000004749.1	OES073662	bacteriocin	RiPPs	Copper	5.90	China: Guangxi, Dachang
DC1_bin.36.orig.fa_k141_1248474.region001.fasta	OEZ008529	yes	LMSG_G000010714.1	OES073662	terpene	Terpene	Copper	11.43	China: Guangxi, Dachang
DC1_bin.36.orig.fa_k141_126781.region001.fasta	OEZ008529	yes	LMSG_G000010714.1	OES073662	bacteriocin	RiPPs	Copper	5.20	China: Guangxi, Dachang
DC1_bin.39.strict.fa_c00005_NODE_5_...region001.fasta	OEZ008529	yes	LMSG_G000008892.1	OES073662	terpene	Terpene	Copper	20.98	China: Guangxi, Dachang
DC1_bin.39.strict.fa_c00008_NODE_8_...region001.fasta	OEZ008529	yes	LMSG_G000008892.1	OES073662	terpene	Terpene	Copper	20.96	China: Guangxi, Dachang
DC1_bin.4.permissive.fa_c00005_NODE_5_...region001.fasta	OEZ008529	yes	LMSG_G000008768.1	OES073662	terpene	Terpene	Copper	22.07	China: Guangxi, Dachang
DC1_bin.4.permissive.fa_c00038_NODE_38...region001.fasta	OEZ008529	yes	LMSG_G000008768.1	OES073662	NRPS-like	NRPS	Copper	29.94	China: Guangxi, Dachang
DC1_bin.4.permissive.fa_c00048_NODE_48...region001.fasta	OEZ008529	yes	LMSG_G000008768.1	OES073662	bacteriocin	RiPPs	Copper	8.77	China: Guangxi, Dachang
DC1_bin.4.permissive.fa_c00051_NODE_51...region001.fasta	OEZ008529	yes	LMSG_G000008768.1	OES073662	terpene	Terpene	Copper	20.14	China: Guangxi, Dachang
DC1_bin.40.strict.fa_c00004_NODE_4_...region001.fasta	OEZ008529	yes	LMSG_G000004752.1	OES073662	bacteriocin	RiPPs	Copper	10.85	China: Guangxi, Dachang
DC1_bin.40.strict.fa_c00022_NODE_22...region001.fasta	OEZ008529	yes	LMSG_G000004752.1	OES073662	bacteriocin	RiPPs	Copper	9.15	China: Guangxi, Dachang
DC1_bin.41.strict.fa_c00002_NODE_2_...region001.fasta	OEZ008529	yes	LMSG_G000008184.1	OES073662	betalactone	Others	Copper	31.32	China: Guangxi, Dachang
DC1_bin.41.strict.fa_c00010_NODE_10...region001.fasta	OEZ008529	yes	LMSG_G000008184.1	OES073662	terpene	Terpene	Copper	20.86	China: Guangxi, Dachang
DC1_bin.42.orig.fa_k141_1492718.region001.fasta	OEZ008529	yes	LMSG_G000010675.1	OES073662	terpene	Terpene	Copper	7.15	China: Guangxi, Dachang
DC1_bin.42.orig.fa_k141_335401.region001.fasta	OEZ008529	yes	LMSG_G000010675.1	OES073662	arylpolyene	Others	Copper	7.90	China: Guangxi, Dachang
DC1_bin.42.orig.fa_k141_600416.region001.fasta	OEZ008529	yes	LMSG_G000010675.1	OES073662	NRPS-like	NRPS	Copper	6.89	China: Guangxi, Dachang
DC1_bin.45.orig.fa_k141_1253769.region001.fasta	OEZ008529	yes	LMSG_G000009637.1	OES073662	terpene	Terpene	Copper	18.21	China: Guangxi, Dachang
DC1_bin.45.orig.fa_k141_621258.region001.fasta	OEZ008529	yes	LMSG_G000009637.1	OES073662	NRPS-like	NRPS	Copper	16.80	China: Guangxi, Dachang
DC1_bin.45.orig.fa_k141_830017.region001.fasta	OEZ008529	yes	LMSG_G000009637.1	OES073662	terpene	Terpene	Copper	13.13	China: Guangxi, Dachang
DC1_bin.46.strict.fa_c00002_NODE_2_...region001.fasta	OEZ008529	yes	LMSG_G000008666.1	OES073662	NRPS-like	NRPS	Copper	35.05	China: Guangxi, Dachang
DC1_bin.47.strict.fa_c00004_NODE_4_...region001.fasta	OEZ008529	yes	LMSG_G000004754.1	OES073662	other	Others	Copper	67.80	China: Guangxi, Dachang
DC1_bin.5.orig.fa_k141_366720.region001.fasta	OEZ008529	yes	LMSG_G000004759.1	OES073662	hserlactone	Others	Copper	20.63	China: Guangxi, Dachang
DC1_bin.5.orig.fa_k141_911578.region001.fasta	OEZ008529	yes	LMSG_G000004759.1	OES073662	bacteriocin	RiPPs	Copper	10.87	China: Guangxi, Dachang
DC1_bin.51.orig.fa_k141_84582.region001.fasta	OEZ008529	yes	LMSG_G000008587.1	OES073662	other	Others	Copper	31.10	China: Guangxi, Dachang

DC1_bin.51.orig.fa_k141_889723.region001.fasta	OEZ008529	yes	LMSG_G000008587.1	OES073662	other	Others	Copper	11.45	China: Guangxi, Dachang
DC1_bin.53.strict.fa_c00002_NODE_2_...region001.fasta	OEZ008529	yes	LMSG_G000010416.1	OES073662	terpene	Terpene	Copper	25.02	China: Guangxi, Dachang
DC1_bin.53.strict.fa_c00007_NODE_7_...region001.fasta	OEZ008529	yes	LMSG_G000010416.1	OES073662	terpene	Terpene	Copper	20.84	China: Guangxi, Dachang
DC1_bin.53.strict.fa_c00026_NODE_26...region001.fasta	OEZ008529	yes	LMSG_G000010416.1	OES073662	bacteriocin	RiPPs	Copper	9.96	China: Guangxi, Dachang
DC1_bin.53.strict.fa_c00045_NODE_45...region001.fasta	OEZ008529	yes	LMSG_G000010416.1	OES073662	lanthipeptide	RiPPs	Copper	5.01	China: Guangxi, Dachang
DC1_bin.55.orig.fa_k141_1204375.region001.fasta	OEZ008529	yes	LMSG_G000004757.1	OES073662	T1PKS.NRPS-like	PKS-NRP_Hybrids	Copper	48.08	China: Guangxi, Dachang
DC1_bin.55.orig.fa_k141_292371.region001.fasta	OEZ008529	yes	LMSG_G000004757.1	OES073662	T3PKS	PKSother	Copper	25.07	China: Guangxi, Dachang
DC1_bin.55.orig.fa_k141_295464.region001.fasta	OEZ008529	yes	LMSG_G000004757.1	OES073662	terpene	Terpene	Copper	9.13	China: Guangxi, Dachang
DC1_bin.55.orig.fa_k141_659529.region001.fasta	OEZ008529	yes	LMSG_G000004757.1	OES073662	terpene	Terpene	Copper	15.54	China: Guangxi, Dachang
DC1_bin.55.orig.fa_k141_974092.region001.fasta	OEZ008529	yes	LMSG_G000004757.1	OES073662	NRPS-like	NRPS	Copper	25.67	China: Guangxi, Dachang
DC1_bin.58.orig.fa_k141_1361821.region001.fasta	OEZ008529	yes	LMSG_G000010815.1	OES073662	acyl_amino_acids	Others	Copper	13.38	China: Guangxi, Dachang
DC1_bin.58.orig.fa_k141_726663.region001.fasta	OEZ008529	yes	LMSG_G000010815.1	OES073662	NRPS.bacteriocin	Others	Copper	5.46	China: Guangxi, Dachang
DC1_bin.59.orig.fa_k141_693201.region001.fasta	OEZ008529	yes	LMSG_G000008623.1	OES073662	other	Others	Copper	5.50	China: Guangxi, Dachang
DC1_bin.6.strict.fa_c00002_NODE_2_...region001.fasta	OEZ008529	yes	LMSG_G000004760.1	OES073662	T3PKS	PKSother	Copper	30.19	China: Guangxi, Dachang
DC1_bin.62.strict.fa_c00046_NODE_46...region001.fasta	OEZ008529	yes	LMSG_G000009249.1	OES073662	TfuA-related	RiPPs	Copper	10.49	China: Guangxi, Dachang
DC1_bin.64.permissive.fa_c00009_NODE_9_...region001.fasta	OEZ008529	yes	LMSG_G000009971.1	OES073662	ladderane	Others	Copper	5.72	China: Guangxi, Dachang
DC1_bin.7.strict.fa_c00027_NODE_27...region001.fasta	OEZ008529	yes	LMSG_G000007791.1	OES073662	T3PKS	PKSother	Copper	21.01	China: Guangxi, Dachang
DC1_bin.7.strict.fa_c00120_NODE_12...region001.fasta	OEZ008529	yes	LMSG_G000007791.1	OES073662	bacteriocin	RiPPs	Copper	7.63	China: Guangxi, Dachang
DC1_bin.9.strict.fa_c00047_NODE_47...region001.fasta	OEZ008529	yes	LMSG_G000004761.1	OES073662	terpene	Terpene	Copper	6.97	China: Guangxi, Dachang
ERR225990_bin.1.strict.fa_c00001_NODE_1_...region001.fasta	OEZ008529	no	LMSG_G000004762.1	ERS211215	arylpolyene	Others	Lead-Zinc	41.18	China: Guangdong, Fankou
ERR225990_bin.1.strict.fa_c00004_NODE_4_...region001.fasta	OEZ008529	yes	LMSG_G000004762.1	ERS211215	terpene	Terpene	Lead-Zinc	22.97	China: Guangdong, Fankou
ERR225990_bin.1.strict.fa_c00008_NODE_8_...region001.fasta	OEZ008529	yes	LMSG_G000004762.1	ERS211215	bacteriocin	RiPPs	Lead-Zinc	8.61	China: Guangdong, Fankou
ERR225990_bin.1.strict.fa_c00074_NODE_74...region001.fasta	OEZ008529	yes	LMSG_G000004762.1	ERS211215	bacteriocin	RiPPs	Lead-Zinc	7.02	China: Guangdong, Fankou
ERR225990_bin.1.strict.fa_c00081_NODE_81...region001.fasta	OEZ008529	yes	LMSG_G000004762.1	ERS211215	bacteriocin	RiPPs	Lead-Zinc	7.45	China: Guangdong, Fankou
ERR225990_bin.11.strict.fa_c00003_NODE_3_...region001.fasta	OEZ008529	yes	LMSG_G000007064.1	ERS211215	bacteriocin	RiPPs	Lead-Zinc	11.01	China: Guangdong, Fankou
ERR225990_bin.5.strict.fa_c00060_NODE_60...region001.fasta	OEZ008529	no	LMSG_G000010575.1	ERS211215	bacteriocin	RiPPs	Lead-Zinc	7.98	China: Guangdong, Fankou
ERR225990_bin.9.strict.fa_c00077_NODE_77...region001.fasta	OEZ008529	yes	LMSG_G000007243.1	ERS211215	betalactone	Others	Lead-Zinc	10.32	China: Guangdong, Fankou
FK.Sedi.C1.2_bin.10.strict.fa_c00002_NODE_2_...region001.fas	OEZ008529	no	LMSG_G000009569.1	OES073649	NRPS-like	NRPS	Lead-Zinc	30.78	China: Guangdong, Fankou
FK.Sedi.C1.2_bin.10.strict.fa_c00028_NODE_28...region001.fas	OEZ008529	no	LMSG_G000009569.1	OES073649	terpene	Terpene	Lead-Zinc	11.83	China: Guangdong, Fankou
FK.Sedi.C1.2_bin.2.permissive.fa_c00001_NODE_1_...region001.	OEZ008529	yes	LMSG_G000007958.1	OES073649	betalactone	Others	Lead-Zinc	23.10	China: Guangdong, Fankou
FK.Sedi.C1.2_bin.2.permissive.fa_c00010_NODE_10...region001.	OEZ008529	yes	LMSG_G000007958.1	OES073649	terpene	Terpene	Lead-Zinc	26.80	China: Guangdong, Fankou
FK.Sedi.C1.3_bin.1.strict.fa_c00009_NODE_9_...region001.fast	OEZ008529	yes	LMSG_G000004780.1	OES073650	terpene	Terpene	Lead-Zinc	17.94	China: Guangdong, Fankou
FK.Sedi.C1.3_bin.1.strict.fa_c00010_NODE_10...region001.fast	OEZ008529	yes	LMSG_G000004780.1	OES073650	nucleoside	Others	Lead-Zinc	16.85	China: Guangdong, Fankou
FK.Sedi.C1.3_bin.1.strict.fa_c00016_NODE_16...region001.fast	OEZ008529	yes	LMSG_G000004780.1	OES073650	NRPS-like	NRPS	Lead-Zinc	36.32	China: Guangdong, Fankou
FK.Sedi.C1.3_bin.1.strict.fa_c00025_NODE_25...region001.fast	OEZ008529	yes	LMSG_G000004780.1	OES073650	terpene	Terpene	Lead-Zinc	22.14	China: Guangdong, Fankou
FK.Sedi.C1.3_bin.1.strict.fa_c00049_NODE_49...region001.fast	OEZ008529	yes	LMSG_G000004780.1	OES073650	acyl_amino_acids	Others	Lead-Zinc	14.58	China: Guangdong, Fankou
FK.Sedi.C1.3_bin.10.permissive.fa_c00007_NODE_7_...region001	OEZ008529	yes	LMSG_G000004779.1	OES073650	betalactone	Others	Lead-Zinc	23.53	China: Guangdong, Fankou
FK.Sedi.C1.3_bin.10.permissive.fa_c00023_NODE_23...region001	OEZ008529	yes	LMSG_G000004779.1	OES073650	betalactone	Others	Lead-Zinc	20.64	China: Guangdong, Fankou
FK.Sedi.C1.3_bin.2.orig.fa_k141_35420.region001.fasta	OEZ008529	yes	LMSG_G000007964.1	OES073650	terpene	Terpene	Lead-Zinc	26.23	China: Guangdong, Fankou
FK.Sedi.C1.3_bin.4.permissive.fa_c00002_NODE_2_...region001.	OEZ008529	yes	LMSG_G000007993.1	OES073650	TfuA-related.bacteriocin	RiPPs	Lead-Zinc	12.35	China: Guangdong, Fankou
FK.Sedi.C1.4_bin.1.strict.fa_c00002_NODE_2_...region001.fast	OEZ008529	yes	LMSG_G000010550.1	OES073651	bacteriocin.hserlactone	Others	Lead-Zinc	20.55	China: Guangdong, Fankou
FK.Sedi.C1.4_bin.1.strict.fa_c00004_NODE_4_...region001.fast	OEZ008529	no	LMSG_G000010550.1	OES073651	terpene	Terpene	Lead-Zinc	23.13	China: Guangdong, Fankou
FK.Sedi.C1.4_bin.1.strict.fa_c00005_NODE_5_...region001.fast	OEZ008529	yes	LMSG_G000010550.1	OES073651	hserlactone	Others	Lead-Zinc	20.55	China: Guangdong, Fankou
FK.Sedi.C1.4_bin.1.strict.fa_c00029_NODE_29...region001.fast	OEZ008529	yes	LMSG_G000010550.1	OES073651	arylpolyene	Others	Lead-Zinc	29.93	China: Guangdong, Fankou
FK.Sedi.C1.4_bin.1.strict.fa_c00036_NODE_36...region001.fast	OEZ008529	yes	LMSG_G000010550.1	OES073651	bacteriocin	RiPPs	Lead-Zinc	10.95	China: Guangdong, Fankou
FK.Sedi.C1.4_bin.6.orig.fa_k141_94326.region001.fasta	OEZ008529	yes	LMSG_G000009586.1	OES073651	terpene	Terpene	Lead-Zinc	22.15	China: Guangdong, Fankou
FK.Sedi.C1.4_bin.7.permissive.fa_c00010_NODE_10...region001.	OEZ008529	yes	LMSG_G000007965.1	OES073651	terpene	Terpene	Lead-Zinc	26.59	China: Guangdong, Fankou
FK.Sedi.C1.4_bin.7.permissive.fa_c00046_NODE_46...region001.	OEZ008529	yes	LMSG_G000007965.1	OES073651	betalactone	Others	Lead-Zinc	19.12	China: Guangdong, Fankou
FK.Sedi.C1.4_bin.9.strict.fa_c00006_NODE_6_...region001.fast	OEZ008529	yes	LMSG_G000009624.1	OES073651	acyl_amino_acids	Others	Lead-Zinc	30.64	China: Guangdong, Fankou
FK.Sedi.C1.4_bin.9.strict.fa_c00033_NODE_33...region001.fast	OEZ008529	yes	LMSG_G000009624.1	OES073651	NRPS-like	NRPS	Lead-Zinc	14.37	China: Guangdong, Fankou
FK.Sedi.C1.4_bin.9.strict.fa_c00039_NODE_39...region001.fast	OEZ008529	yes	LMSG_G000009624.1	OES073651	terpene	Terpene	Lead-Zinc	13.46	China: Guangdong, Fankou
FK.Sedi.C1.4_bin.9.strict.fa_c00105_NODE_10...region001.fast	OEZ008529	yes	LMSG_G000009624.1	OES073651	ectoine	Others	Lead-Zinc	6.15	China: Guangdong, Fankou
FK.Sedi.C1.4_bin.9.strict.fa_c00125_NODE_12...region001.fast	OEZ008529	yes	LMSG_G000009624.1	OES073651	acyl_amino_acids	Others	Lead-Zinc	5.44	China: Guangdong, Fankou
FK.Sedi.C1.5_bin.6.orig.fa_k141_133238.region001.fasta	OEZ008529	yes	LMSG_G000008872.1	OES073652	terpene	Terpene	Lead-Zinc	11.65	China: Guangdong, Fankou
FK.Sedi.C1.5_bin.6.orig.fa_k141_150790.region001.fasta	OEZ008529	yes	LMSG_G000008872.1	OES073652	terpene	Terpene	Lead-Zinc	12.83	China: Guangdong, Fankou
FK.Sedi.C1.5_bin.8.permissive.fa_c00020_NODE_20...region001.	OEZ008529	yes	LMSG_G000009687.1	OES073652	NRPS-like	NRPS	Lead-Zinc	22.56	China: Guangdong, Fankou
FK1_bin.1.strict.fa_c00006_NODE_6_...region001.fasta	OEZ008529	yes	LMSG_G000004355.1	OES073663	hserlactone	Others	Lead-Zinc	10.87	China: Guangdong, Fankou
FK1_bin.1.strict.fa_c00062_NODE_62...region001.fasta	OEZ008529	yes	LMSG_G000004355.1	OES073663	terpene	Terpene	Lead-Zinc	14.94	China: Guangdong, Fankou
FK1_bin.1.strict.fa_c00075_NODE_75...region001.fasta	OEZ008529	yes	LMSG_G000004355.1	OES073663	ladderane.bacteriocin	Others	Lead-Zinc	18.68	China: Guangdong, Fankou
FK1_bin.10.orig.fa_k141_511241.region001.fasta	OEZ008529	yes	LMSG_G000006261.1	OES073663	hserlactone	Others	Lead-Zinc	9.15	China: Guangdong, Fankou
FK1_bin.10.orig.fa_k141_526264.region001.fasta	OEZ008529	yes	LMSG_G000006261.1	OES073663	terpene	Terpene	Lead-Zinc	13.50	China: Guangdong, Fankou
FK1_bin.10.orig.fa_k141_753137.region001.fasta	OEZ008529	yes	LMSG_G000006261.1	OES073663	arylpolyene	Others	Lead-Zinc	38.22	China: Guangdong, Fankou
FK1_bin.10.orig.fa_k141_776011.region001.fasta	OEZ008529	yes	LMSG_G000006261.1	OES073663	bacteriocin	RiPPs	Lead-Zinc	10.77	China: Guangdong, Fankou
FK1_bin.12.orig.fa_k141_342658.region001.fasta	OEZ008529	yes	LMSG_G000008499.1	OES073663	lassopeptide	RiPPs	Lead-Zinc	23.45	China: Guangdong, Fankou
FK1_bin.12.orig.fa_k141_721514.region001.fasta	OEZ008529	yes	LMSG_G000008499.1	OES073663	terpene	Terpene	Lead-Zinc	17.37	China: Guangdong, Fankou
FK1_bin.13.orig.fa_k141_60946.region001.fasta	OEZ008529	no	LMSG_G000004763.1	OES073663	terpene	Terpene	Lead-Zinc	10.19	China: Guangdong, Fankou
FK1_bin.13.orig.fa_k141_635174.region001.fasta	OEZ008529	no	LMSG_G000004763.1	OES073663	ectoine	Others	Lead-Zinc	10.40	China: Guangdong, Fankou
FK1_bin.13.orig.fa_k141_706656.region001.fasta	OEZ008529	no	LMSG_G000004763.1	OES073663	terpene	Terpene	Lead-Zinc	6.64	China: Guangdong, Fankou
FK1_bin.13.orig.fa_k141_854224.region001.fasta	OEZ008529	no	LMSG_G000004763.1	OES073663	terpene	Terpene	Lead-Zinc	16.52	China: Guangdong, Fankou
FK1_bin.14.orig.fa_k141_689303.region001.fasta	OEZ008529	yes	LMSG_G000010227.1	OES073663	terpene	Terpene	Lead-Zinc	20.70	China: Guangdong, Fankou
FK1_bin.14.orig.fa_k141_820436.region001.fasta	OEZ008529	yes	LMSG_G000010227.1	OES073663	hserlactone	Others	Lead-Zinc	20.72	China: Guangdong, Fankou
FK1_bin.14.orig.fa_k141_878081.region001.fasta	OEZ008529	yes	LMSG_G000010227.1	OES073663	terpene.arylpolyene	Others	Lead-Zinc	42.19	China: Guangdong, Fankou
FK1_bin.16.strict.fa_c00022_NODE_22...region001.fasta	OEZ008529	yes	LMSG_G000004764.1	OES073663	bacteriocin	RiPPs	Lead-Zinc	10.84	China: Guangdong, Fankou
FK1_bin.17.strict.fa_c00007_NODE_7_...region001.fasta	OEZ008529	yes	LMSG_G000007994.1	OES073663	TfuA-related.bacteriocin	RiPPs	Lead-Zinc	15.73	China: Guangdong, Fankou
FK1_bin.19.strict.fa_c00001_NODE_1_...region001.fasta	OEZ008529	yes	LMSG_G000009894.1	OES073663	terpene	Terpene	Lead-Zinc	20.82	China: Guangdong, Fankou
FK1_bin.2.orig.fa_k141_171899.region001.fasta	OEZ008529	no	LMSG_G000009527.1	OES073663	terpene	Terpene	Lead-Zinc	23.29	China: Guangdong, Fankou
FK1_bin.2.orig.fa_k141_314648.region001.fasta	OEZ008529	no	LMSG_G000009527.1	OES073663	NRPS-like	NRPS	Lead-Zinc	39.09	China: Guangdong, Fankou
FK1_bin.2.orig.fa_k141_343734.region001.fasta	OEZ008529	no	LMSG_G000009527.1	OES073663	terpene	Terpene	Lead-Zinc	20.70	China: Guangdong, Fankou
FK1_bin.20.orig.fa_k141_415354.region001.fasta	OEZ008529	yes	LMSG_G000010244.1	OES073663	bacteriocin	RiPPs	Lead-Zinc	7.41	China: Guangdong, Fankou
FK1_bin.20.orig.fa_k141_452634.region001.fasta	OEZ008529	no	LMSG_G000010244.1	OES073663	terpene	Terpene	Lead-Zinc	7.13	China: Guangdong, Fankou
FK1_bin.20.orig.fa_k141_558100.region001.fasta	OEZ008529	yes	LMSG_G000010244.1	OES073663	T1PKS.hserlactone	Others	Lead-Zinc	50.13	China: Guangdong, Fankou
FK1_bin.20.orig.fa_k141_559338.region001.fasta	OEZ008529	yes	LMSG_G000010244.1	OES073663	terpene	Terpene	Lead-Zinc	21.11	China: Guangdong, Fankou
FK1_bin.20.orig.fa_k141_584946.region001.fasta	OEZ008529	yes	LMSG_G000010244.1	OES073663	arylpolyene	Others	Lead-Zinc	20.31	China: Guangdong, Fankou
FK1_bin.22.strict.fa_c00013_NODE_13...region001.fasta	OEZ008529	yes	LMSG_G000010432.1	OES073663	terpene	Terpene	Lead-Zinc	21.20	China: Guangdong, Fankou
FK1_bin.23.orig.fa_k141_618640.region001.fasta	OEZ008529	yes	LMSG_G000010253.1	OES073663	arylpolyene	Others	Lead-Zinc	41.19	China: Guangdong, Fankou
FK1_bin.24.orig.fa_k141_131695.region001.fasta	OEZ008529	yes	LMSG_G000004765.1	OES073663	bacteriocin	RiPPs	Lead-Zinc	6.12	China: Guangdong, Fankou
FK1_bin.24.orig.fa_k141_319760.region001.fasta	OEZ008529	no	LMSG_G000004765.1	OES073663	arylpolyene	Others	Lead-Zinc	8.18	China: Guangdong, Fankou
FK1_bin.25.strict.fa_c00022_NODE_22...region001.fasta	OEZ008529	yes	LMSG_G000004766.1	OES073663	bacteriocin	RiPPs	Lead-Zinc	8.68	China: Guangdong, Fankou
FK1_bin.25.strict.fa_c00138_NODE_13...region001.fasta	OEZ008529	yes	LMSG_G000004766.1	OES073663	NRPS	NRPS	Lead-Zinc	5.51	China: Guangdong, Fankou
FK1_bin.26.orig.fa_k141_2981.region001.fasta	OEZ008529	yes	LMSG_G000010463.1	OES073663	terpene	Terpene	Lead-Zinc	23.04	China: Guangdong, Fankou
FK1_bin.26.orig.fa_k141_450580.region001.fasta	OEZ008529	yes	LMSG_G000010463.1	OES073663	arylpolyene.hserlactone	Others	Lead-Zinc	37.69	China: Guangdong, Fankou
FK1_bin.27.orig.fa_k141_224897.region001.fasta	OEZ008529	yes	LMSG_G000011061.1	OES073663	terpene	Terpene	Lead-Zinc	15.08	China: Guangdong, Fankou
FK1_bin.27.orig.fa_k141_225121.region001.fasta	OEZ008529	yes	LMSG_G000011061.1	OES073663	NRPS-like	NRPS	Lead-Zinc	33.16	China: Guangdong, Fankou
FK1_bin.27.orig.fa_k141_313100.region001.fasta	OEZ008529	yes	LMSG_G000011061.1	OES073663	NRPS-like	NRPS	Lead-Zinc	26.94	China: Guangdong, Fankou
FK1_bin.27.orig.fa_k141_458294.region001.fasta	OEZ008529	yes	LMSG_G000011061.1	OES073663	NRPS-like	NRPS	Lead-Zinc	42.85	China: Guangdong, Fankou
FK1_bin.27.orig.fa_k141_704438.region001.fasta	OEZ008529	yes	LMSG_G000011061.1	OES073663	lassopeptide	RiPPs	Lead-Zinc	21.08	China: Guangdong, Fankou
FK1_bin.3.orig.fa_k141_442260.region001.fasta	OEZ008529	yes	LMSG_G000009517.1	OES073663	acyl_amino_acids	Others	Lead-Zinc	9.26	China: Guangdong, Fankou
FK1_bin.3.orig.fa_k141_699525.region001.fasta	OEZ008529	yes	LMSG_G000009517.1	OES073663	ectoine	Others	Lead-Zinc	10.40	China: Guangdong, Fankou
FK1_bin.3.orig.fa_k141_703932.region001.fasta	OEZ008529	yes	LMSG_G000009517.1	OES073663	NRPS-like	NRPS	Lead-Zinc	33.51	China: Guangdong, Fankou
FK1_bin.3.orig.fa_k141_730525.region001.fasta	OEZ008529	yes	LMSG_G000009517.1	OES073663	terpene	Terpene	Lead-Zinc	21.16	China: Guangdong, Fankou
FK1_bin.3.orig.fa_k141_761097.region001.fasta	OEZ008529	yes	LMSG_G000009517.1	OES073663	terpene	Terpene	Lead-Zinc	23.29	China: Guangdong, Fankou
FK1_bin.3.orig.fa_k141_783649.region001.fasta	OEZ008529	yes	LMSG_G000009517.1	OES073663	arylpolyene	Others	Lead-Zinc	29.03	China: Guangdong, Fankou
FK1_bin.30.strict.fa_c00011_NODE_11...region001.fasta	OEZ008529	yes	LMSG_G000004767.1	OES073663	terpene	Terpene	Lead-Zinc	23.45	China: Guangdong, Fankou
FK1_bin.32.strict.fa_c00026_NODE_26...region001.fasta	OEZ008529	yes	LMSG_G000004768.1	OES073663	bacteriocin	RiPPs	Lead-Zinc	10.84	China: Guangdong, Fankou
FK1_bin.33.strict.fa_c00019_NODE_19...region001.fasta	OEZ008529	no	LMSG_G000010533.1	OES073663	terpene	Terpene	Lead-Zinc	13.73	China: Guangdong, Fankou
FK1_bin.35.strict.fa_c00023_NODE_23...region001.fasta	OEZ008529	yes	LMSG_G000008259.1	OES073663	terpene	Terpene	Lead-Zinc	19.06	China: Guangdong, Fankou
FK1_bin.36.orig.fa_k141_282136.region001.fasta	OEZ008529	yes	LMSG_G000011076.1	OES073663	lassopeptide	RiPPs	Lead-Zinc	28.80	China: Guangdong, Fankou
FK1_bin.36.orig.fa_k141_282637.region001.fasta	OEZ008529	yes	LMSG_G000011076.1	OES073663	terpene	Terpene	Lead-Zinc	23.07	China: Guangdong, Fankou
FK1_bin.36.orig.fa_k141_361462.region001.fasta	OEZ008529	yes	LMSG_G000011076.1	OES073663	arylpolyene	Others	Lead-Zinc	41.20	China: Guangdong, Fankou
FK1_bin.36.orig.fa_k141_642886.region001.fasta	OEZ008529	yes	LMSG_G000011076.1	OES073663	NRPS-like	NRPS	Lead-Zinc	35.22	China: Guangdong, Fankou
FK1_bin.37.orig.fa_k141_115408.region001.fasta	OEZ008529	yes	LMSG_G000009585.1	OES073663	terpene	Terpene	Lead-Zinc	22.15	China: Guangdong, Fankou
FK1_bin.37.orig.fa_k141_170622.region001.fasta	OEZ008529	yes	LMSG_G000009585.1	OES073663	acyl_amino_acids	Others	Lead-Zinc	10.92	China: Guangdong, Fankou
FK1_bin.4.orig.fa_k141_122289.region001.fasta	OEZ008529	yes	LMSG_G000004771.1	OES073663	terpene	Terpene	Lead-Zinc	21.87	China: Guangdong, Fankou
FK1_bin.4.orig.fa_k141_143756.region001.fasta	OEZ008529	yes	LMSG_G000004771.1	OES073663	bacteriocin	RiPPs	Lead-Zinc	10.88	China: Guangdong, Fankou
FK1_bin.4.orig.fa_k141_24585.region001.fasta	OEZ008529	yes	LMSG_G000004771.1	OES073663	T3PKS.terpene	Others	Lead-Zinc	47.47	China: Guangdong, Fankou
FK1_bin.4.orig.fa_k141_836665.region001.fasta	OEZ008529	yes	LMSG_G000004771.1	OES073663	NRPS-like	NRPS	Lead-Zinc	33.09	China: Guangdong, Fankou
FK1_bin.40.orig.fa_k141_152112.region001.fasta	OEZ008529	yes	LMSG_G000011150.1	OES073663	bacteriocin	RiPPs	Lead-Zinc	10.98	China: Guangdong, Fankou
FK1_bin.40.orig.fa_k141_158572.region001.fasta	OEZ008529	yes	LMSG_G000011150.1	OES073663	terpene	Terpene	Lead-Zinc	13.62	China: Guangdong, Fankou
FK1_bin.40.orig.fa_k141_196256.region001.fasta	OEZ008529	yes	LMSG_G000011150.1	OES073663	arylpolyene	Others	Lead-Zinc	7.77	China: Guangdong, Fankou
FK1_bin.40.orig.fa_k141_405534.region001.fasta	OEZ008529	yes	LMSG_G000011150.1	OES073663	lanthipeptide	RiPPs	Lead-Zinc	13.43	China: Guangdong, Fankou
FK1_bin.41.orig.fa_k141_642879.region001.fasta	OEZ008529	yes	LMSG_G000008217.1	OES073663	terpene	Terpene	Lead-Zinc	16.59	China: Guangdong, Fankou
FK1_bin.43.strict.fa_c00008_NODE_8_...region001.fasta	OEZ008529	yes	LMSG_G000008515.1	OES073663	lassopeptide	RiPPs	Lead-Zinc	23.84	China: Guangdong, Fankou
FK1_bin.43.strict.fa_c00041_NODE_41...region001.fasta	OEZ008529	yes	LMSG_G000008515.1	OES073663	terpene	Terpene	Lead-Zinc	14.67	China: Guangdong, Fankou
FK1_bin.47.orig.fa_k141_726843.region001.fasta	OEZ008529	yes	LMSG_G000004769.1	OES073663	terpene	Terpene	Lead-Zinc	26.24	China: Guangdong, Fankou
FK1_bin.49.orig.fa_k141_182582.region001.fasta	OEZ008529	yes	LMSG_G000004770.1	OES073663	resorcinol	Others	Lead-Zinc	17.39	China: Guangdong, Fankou
FK1_bin.49.orig.fa_k141_230786.region001.fasta	OEZ008529	yes	LMSG_G000004770.1	OES073663	arylpolyene	Others	Lead-Zinc	7.45	China: Guangdong, Fankou
FK1_bin.49.orig.fa_k141_281328.region001.fasta	OEZ008529	yes	LMSG_G000004770.1	OES073663	arylpolyene.hserlactone	Others	Lead-Zinc	20.48	China: Guangdong, Fankou
FK1_bin.49.orig.fa_k141_344097.region001.fasta	OEZ008529	yes	LMSG_G000004770.1	OES073663	terpene	Terpene	Lead-Zinc	23.02	China: Guangdong, Fankou
FK1_bin.5.strict.fa_c00001_NODE_1_...region001.fasta	OEZ008529	yes	LMSG_G000008381.1	OES073663	T1PKS	PKSI	Lead-Zinc	43.02	China: Guangdong, Fankou
FK1_bin.5.strict.fa_c00004_NODE_4_...region001.fasta	OEZ008529	yes	LMSG_G000008381.1	OES073663	NRPS-like	NRPS	Lead-Zinc	44.28	China: Guangdong, Fankou
FK1_bin.5.strict.fa_c00031_NODE_31...region001.fasta	OEZ008529	yes	LMSG_G000008381.1	OES073663	terpene	Terpene	Lead-Zinc	14.29	China: Guangdong, Fankou
FK1_bin.50.orig.fa_k141_353879.region001.fasta	OEZ008529	yes	LMSG_G000008630.1	OES073663	other	Others	Lead-Zinc	40.72	China: Guangdong, Fankou
FK1_bin.50.orig.fa_k141_749560.region001.fasta	OEZ008529	yes	LMSG_G000008630.1	OES073663	NRPS-like	NRPS	Lead-Zinc	25.02	China: Guangdong, Fankou
FK1_bin.51.orig.fa_k141_149618.region001.fasta	OEZ008529	yes	LMSG_G000004772.1	OES073663	terpene	Terpene	Lead-Zinc	16.94	China: Guangdong, Fankou
FK1_bin.51.orig.fa_k141_589413.region001.fasta	OEZ008529	yes	LMSG_G000004772.1	OES073663	other	Others	Lead-Zinc	25.64	China: Guangdong, Fankou
FK1_bin.51.orig.fa_k141_615017.region001.fasta	OEZ008529	yes	LMSG_G000004772.1	OES073663	other	Others	Lead-Zinc	22.74	China: Guangdong, Fankou
FK1_bin.52.strict.fa_c00046_NODE_46...region001.fasta	OEZ008529	yes	LMSG_G000010027.1	OES073663	terpene	Terpene	Lead-Zinc	8.81	China: Guangdong, Fankou
FK1_bin.52.strict.fa_c00081_NODE_81...region001.fasta	OEZ008529	yes	LMSG_G000010027.1	OES073663	NRPS-like	NRPS	Lead-Zinc	7.29	China: Guangdong, Fankou
FK1_bin.52.strict.fa_c00117_NODE_11...region001.fasta	OEZ008529	yes	LMSG_G000010027.1	OES073663	terpene	Terpene	Lead-Zinc	5.96	China: Guangdong, Fankou
FK1_bin.54.orig.fa_k141_633392.region001.fasta	OEZ008529	yes	LMSG_G000006262.1	OES073663	bacteriocin	RiPPs	Lead-Zinc	10.88	China: Guangdong, Fankou
FK1_bin.54.orig.fa_k141_633392.region002.fasta	OEZ008529	yes	LMSG_G000006262.1	OES073663	terpene.arylpolyene	Others	Lead-Zinc	51.81	China: Guangdong, Fankou
FK1_bin.54.orig.fa_k141_688074.region001.fasta	OEZ008529	yes	LMSG_G000006262.1	OES073663	resorcinol.arylpolyene	Others	Lead-Zinc	30.34	China: Guangdong, Fankou
FK1_bin.55.permissive.fa_c00001_NODE_1_...region001.fasta	OEZ008529	yes	LMSG_G000008386.1	OES073663	NRPS-like	NRPS	Lead-Zinc	44.19	China: Guangdong, Fankou
FK1_bin.55.permissive.fa_c00129_NODE_12...region001.fasta	OEZ008529	yes	LMSG_G000008386.1	OES073663	terpene	Terpene	Lead-Zinc	5.20	China: Guangdong, Fankou
FK1_bin.56.permissive.fa_c00001_NODE_1_...region001.fasta	OEZ008529	yes	LMSG_G000004773.1	OES073663	NRPS-like	NRPS	Lead-Zinc	42.37	China: Guangdong, Fankou
FK1_bin.56.permissive.fa_c00005_NODE_5_...region001.fasta	OEZ008529	yes	LMSG_G000004773.1	OES073663	terpene	Terpene	Lead-Zinc	21.74	China: Guangdong, Fankou
FK1_bin.56.permissive.fa_c00060_NODE_60...region001.fasta	OEZ008529	yes	LMSG_G000004773.1	OES073663	ectoine	Others	Lead-Zinc	10.41	China: Guangdong, Fankou
FK1_bin.56.permissive.fa_c00158_NODE_15...region001.fasta	OEZ008529	yes	LMSG_G000004773.1	OES073663	terpene	Terpene	Lead-Zinc	5.23	China: Guangdong, Fankou
FK1_bin.6.orig.fa_k141_336601.region001.fasta	OEZ008529	yes	LMSG_G000004341.1	OES073663	NRPS-like	NRPS	Lead-Zinc	9.60	China: Guangdong, Fankou
FK1_bin.6.orig.fa_k141_348990.region001.fasta	OEZ008529	yes	LMSG_G000004341.1	OES073663	terpene	Terpene	Lead-Zinc	23.03	China: Guangdong, Fankou
FK1_bin.6.orig.fa_k141_451142.region001.fasta	OEZ008529	yes	LMSG_G000004341.1	OES073663	resorcinol.arylpolyene	Others	Lead-Zinc	29.97	China: Guangdong, Fankou
FK1_bin.6.orig.fa_k141_764834.region001.fasta	OEZ008529	yes	LMSG_G000004341.1	OES073663	arylpolyene	Others	Lead-Zinc	35.35	China: Guangdong, Fankou
FK1_bin.9.orig.fa_k141_283831.region001.fasta	OEZ008529	yes	LMSG_G000004775.1	OES073663	terpene	Terpene	Lead-Zinc	23.11	China: Guangdong, Fankou
FK1_bin.9.orig.fa_k141_573011.region001.fasta	OEZ008529	yes	LMSG_G000004775.1	OES073663	betalactone	Others	Lead-Zinc	21.42	China: Guangdong, Fankou
FK1_bin.9.orig.fa_k141_70125.region001.fasta	OEZ008529	yes	LMSG_G000004775.1	OES073663	NRPS-like	NRPS	Lead-Zinc	20.00	China: Guangdong, Fankou
FK1_bin.9.orig.fa_k141_803049.region001.fasta	OEZ008529	yes	LMSG_G000004775.1	OES073663	NRPS-like	NRPS	Lead-Zinc	40.62	China: Guangdong, Fankou
I1_bin.2.permissive.fa_c00002_NODE_2_...region001.fasta	OEZ008529	yes	LMSG_G000010951.1	OES073653	arylpolyene	Others	Antimony	21.28	China: Hunan
I1_bin.2.permissive.fa_c00029_NODE_29...region001.fasta	OEZ008529	no	LMSG_G000010951.1	OES073653	bacteriocin	RiPPs	Antimony	10.84	China: Hunan

I1_bin.2.permissive.fa_c00055_NODE_55...region001.fasta	OEZ008529	no	LMSG_G000010951.1	OES073653	arylpolyene	Others	Antimony	15.62	China: Hunan
I1_bin.2.permissive.fa_c00138_NODE_13...region001.fasta	OEZ008529	no	LMSG_G000010951.1	OES073653	hglE-KS.PUFA	Others	Antimony	8.48	China: Hunan
I1_bin.4.permissive.fa_c00017_NODE_17...region001.fasta	OEZ008529	yes	LMSG_G000010644.1	OES073653	NRPS.T1PKS	PKS-NRP_Hybrids	Antimony	45.29	China: Hunan
I1_bin.4.permissive.fa_c00042_NODE_42...region001.fasta	OEZ008529	no	LMSG_G000010644.1	OES073653	terpene	Terpene	Antimony	13.58	China: Hunan
I1_bin.5.orig.fa_k141_65179.region001.fasta	OEZ008529	yes	LMSG_G000008911.1	OES073653	hserlactone	Others	Antimony	15.29	China: Hunan
I1_bin.6.orig.fa_k141_122590.region001.fasta	OEZ008529	yes	LMSG_G000004783.1	OES073653	T3PKS	PKSother	Antimony	7.19	China: Hunan
I1_bin.7.permissive.fa_c00008_NODE_8_...region001.fasta	OEZ008529	yes	LMSG_G000004784.1	OES073653	NRPS-like	NRPS	Antimony	30.95	China: Hunan
I1_bin.7.permissive.fa_c00009_NODE_9_...region001.fasta	OEZ008529	yes	LMSG_G000004784.1	OES073653	bacteriocin	RiPPs	Antimony	10.85	China: Hunan
I1_bin.9.permissive.fa_c00012_NODE_12...region001.fasta	OEZ008529	yes	LMSG_G000004785.1	OES073653	betalactone.NRPS-like	Others	Antimony	25.25	China: Hunan
I2_bin.1.strict.fa_c00001_NODE_1_...region001.fasta	OEZ008529	yes	LMSG_G000004786.1	OES073654	siderophore	Others	Antimony	15.07	China: Hunan
I2_bin.1.strict.fa_c00013_NODE_13...region001.fasta	OEZ008529	yes	LMSG_G000004786.1	OES073654	arylpolyene	Others	Antimony	28.08	China: Hunan
I2_bin.1.strict.fa_c00021_NODE_21...region001.fasta	OEZ008529	yes	LMSG_G000004786.1	OES073654	thiopeptide	RiPPs	Antimony	24.98	China: Hunan
I2_bin.3.orig.fa_k141_30562.region001.fasta	OEZ008529	no	LMSG_G000004787.1	OES073654	hglE-KS	PKSother	Antimony	24.90	China: Hunan
I2_bin.3.orig.fa_k141_37929.region001.fasta	OEZ008529	no	LMSG_G000004787.1	OES073654	bacteriocin	RiPPs	Antimony	10.84	China: Hunan
I2_bin.3.orig.fa_k141_429489.region001.fasta	OEZ008529	yes	LMSG_G000004787.1	OES073654	arylpolyene	Others	Antimony	21.55	China: Hunan
I2_bin.4.orig.fa_k141_411067.region001.fasta	OEZ008529	yes	LMSG_G000004788.1	OES073654	terpene	Terpene	Antimony	21.61	China: Hunan
I2_bin.5.orig.fa_k141_434047.region001.fasta	OEZ008529	yes	LMSG_G000004789.1	OES073654	bacteriocin	RiPPs	Antimony	5.03	China: Hunan
I2_bin.6.strict.fa_c00006_NODE_6_...region001.fasta	OEZ008529	no	LMSG_G000004790.1	OES073654	terpene	Terpene	Antimony	20.98	China: Hunan
I2_bin.6.strict.fa_c00008_NODE_8_...region001.fasta	OEZ008529	yes	LMSG_G000004790.1	OES073654	NRPS.T1PKS	PKS-NRP_Hybrids	Antimony	79.01	China: Hunan
I2_bin.6.strict.fa_c00036_NODE_36...region001.fasta	OEZ008529	yes	LMSG_G000004790.1	OES073654	T1PKS	PKSI	Antimony	6.71	China: Hunan
I2_bin.7.strict.fa_c00042_NODE_42...region001.fasta	OEZ008529	yes	LMSG_G000008748.1	OES073654	bacteriocin	RiPPs	Antimony	6.39	China: Hunan
I2_bin.9.orig.fa_k141_269324.region001.fasta	OEZ008529	yes	LMSG_G000004792.1	OES073654	hserlactone	Others	Antimony	17.50	China: Hunan
L180206P-1-LD180201-0007_HK72WCCXY_L1_clean_bin.1.orig.fa_k1	OEZ008529	no	LMSG_G000010254.1	OES073742	terpene	Terpene	Copper	21.17	China: Jiangxi, Yongping
L180206P-1-LD180201-0007_HK72WCCXY_L1_clean_bin.1.orig.fa_k1	OEZ008529	yes	LMSG_G000010254.1	OES073742	hserlactone	Others	Copper	10.94	China: Jiangxi, Yongping
L180206P-1-LD180201-0007_HK72WCCXY_L1_clean_bin.1.orig.fa_k1	OEZ008529	yes	LMSG_G000010254.1	OES073742	arylpolyene	Others	Copper	21.39	China: Jiangxi, Yongping
L180206P-1-LD180201-0007_HK72WCCXY_L1_clean_bin.100.orig.fa_	OEZ008529	yes	LMSG_G000011027.1	OES073742	arylpolyene	Others	Copper	28.77	China: Jiangxi, Yongping
L180206P-1-LD180201-0007_HK72WCCXY_L1_clean_bin.100.orig.fa_	OEZ008529	yes	LMSG_G000011027.1	OES073742	resorcinol	Others	Copper	13.35	China: Jiangxi, Yongping
L180206P-1-LD180201-0007_HK72WCCXY_L1_clean_bin.100.orig.fa_	OEZ008529	yes	LMSG_G000011027.1	OES073742	bacteriocin	RiPPs	Copper	6.24	China: Jiangxi, Yongping
L180206P-1-LD180201-0007_HK72WCCXY_L1_clean_bin.104.orig.fa_	OEZ008529	yes	LMSG_G000010237.1	OES073742	terpene.arylpolyene	Others	Copper	24.79	China: Jiangxi, Yongping
L180206P-1-LD180201-0007_HK72WCCXY_L1_clean_bin.104.orig.fa_	OEZ008529	yes	LMSG_G000010237.1	OES073742	hserlactone	Others	Copper	11.29	China: Jiangxi, Yongping
L180206P-1-LD180201-0007_HK72WCCXY_L1_clean_bin.104.orig.fa_	OEZ008529	yes	LMSG_G000010237.1	OES073742	terpene	Terpene	Copper	17.54	China: Jiangxi, Yongping
L180206P-1-LD180201-0007_HK72WCCXY_L1_clean_bin.104.orig.fa_	OEZ008529	yes	LMSG_G000010237.1	OES073742	terpene	Terpene	Copper	15.68	China: Jiangxi, Yongping
L180206P-1-LD180201-0007_HK72WCCXY_L1_clean_bin.105.permissi	OEZ008529	yes	LMSG_G000009365.1	OES073742	betalactone	Others	Copper	32.65	China: Jiangxi, Yongping
L180206P-1-LD180201-0007_HK72WCCXY_L1_clean_bin.11.orig.fa_k	OEZ008529	yes	LMSG_G000004357.1	OES073742	terpene	Terpene	Copper	20.98	China: Jiangxi, Yongping
L180206P-1-LD180201-0007_HK72WCCXY_L1_clean_bin.11.orig.fa_k	OEZ008529	yes	LMSG_G000004357.1	OES073742	arylpolyene	Others	Copper	35.84	China: Jiangxi, Yongping
L180206P-1-LD180201-0007_HK72WCCXY_L1_clean_bin.11.orig.fa_k	OEZ008529	yes	LMSG_G000004357.1	OES073742	T3PKS	PKSother	Copper	41.07	China: Jiangxi, Yongping
L180206P-1-LD180201-0007_HK72WCCXY_L1_clean_bin.110.orig.fa_	OEZ008529	yes	LMSG_G000010537.1	OES073742	terpene	Terpene	Copper	12.06	China: Jiangxi, Yongping
L180206P-1-LD180201-0007_HK72WCCXY_L1_clean_bin.110.orig.fa_	OEZ008529	no	LMSG_G000010537.1	OES073742	bacteriocin	RiPPs	Copper	5.25	China: Jiangxi, Yongping
L180206P-1-LD180201-0007_HK72WCCXY_L1_clean_bin.110.orig.fa_	OEZ008529	no	LMSG_G000010537.1	OES073742	bacteriocin	RiPPs	Copper	10.86	China: Jiangxi, Yongping
L180206P-1-LD180201-0007_HK72WCCXY_L1_clean_bin.110.orig.fa_	OEZ008529	yes	LMSG_G000010537.1	OES073742	bacteriocin	RiPPs	Copper	6.47	China: Jiangxi, Yongping
L180206P-1-LD180201-0007_HK72WCCXY_L1_clean_bin.113.orig.fa_	OEZ008529	yes	LMSG_G000009114.1	OES073742	NRPS-like	NRPS	Copper	26.72	China: Jiangxi, Yongping
L180206P-1-LD180201-0007_HK72WCCXY_L1_clean_bin.113.orig.fa_	OEZ008529	yes	LMSG_G000009114.1	OES073742	terpene	Terpene	Copper	25.07	China: Jiangxi, Yongping
L180206P-1-LD180201-0007_HK72WCCXY_L1_clean_bin.113.orig.fa_	OEZ008529	yes	LMSG_G000009114.1	OES073742	bacteriocin	RiPPs	Copper	10.87	China: Jiangxi, Yongping
L180206P-1-LD180201-0007_HK72WCCXY_L1_clean_bin.114.orig.fa_	OEZ008529	yes	LMSG_G000011037.1	OES073742	arylpolyene	Others	Copper	7.89	China: Jiangxi, Yongping
L180206P-1-LD180201-0007_HK72WCCXY_L1_clean_bin.114.orig.fa_	OEZ008529	yes	LMSG_G000011037.1	OES073742	arylpolyene	Others	Copper	14.91	China: Jiangxi, Yongping
L180206P-1-LD180201-0007_HK72WCCXY_L1_clean_bin.114.orig.fa_	OEZ008529	yes	LMSG_G000011037.1	OES073742	terpene	Terpene	Copper	11.85	China: Jiangxi, Yongping
L180206P-1-LD180201-0007_HK72WCCXY_L1_clean_bin.114.orig.fa_	OEZ008529	yes	LMSG_G000011037.1	OES073742	bacteriocin	RiPPs	Copper	5.95	China: Jiangxi, Yongping
L180206P-1-LD180201-0007_HK72WCCXY_L1_clean_bin.114.orig.fa_	OEZ008529	yes	LMSG_G000011037.1	OES073742	terpene	Terpene	Copper	23.08	China: Jiangxi, Yongping
L180206P-1-LD180201-0007_HK72WCCXY_L1_clean_bin.114.orig.fa_	OEZ008529	yes	LMSG_G000011037.1	OES073742	NRPS-like	NRPS	Copper	15.67	China: Jiangxi, Yongping
L180206P-1-LD180201-0007_HK72WCCXY_L1_clean_bin.114.orig.fa_	OEZ008529	yes	LMSG_G000011037.1	OES073742	arylpolyene	Others	Copper	20.47	China: Jiangxi, Yongping
L180206P-1-LD180201-0007_HK72WCCXY_L1_clean_bin.115.strict.f	OEZ008529	yes	LMSG_G000008135.1	OES073742	terpene	Terpene	Copper	13.00	China: Jiangxi, Yongping
L180206P-1-LD180201-0007_HK72WCCXY_L1_clean_bin.117.orig.fa_	OEZ008529	yes	LMSG_G000004794.1	OES073742	NRPS-like	NRPS	Copper	42.62	China: Jiangxi, Yongping
L180206P-1-LD180201-0007_HK72WCCXY_L1_clean_bin.117.orig.fa_	OEZ008529	yes	LMSG_G000004794.1	OES073742	terpene	Terpene	Copper	21.76	China: Jiangxi, Yongping
L180206P-1-LD180201-0007_HK72WCCXY_L1_clean_bin.117.orig.fa_	OEZ008529	yes	LMSG_G000004794.1	OES073742	NRPS-like	NRPS	Copper	42.78	China: Jiangxi, Yongping
L180206P-1-LD180201-0007_HK72WCCXY_L1_clean_bin.118.orig.fa_	OEZ008529	yes	LMSG_G000008057.1	OES073742	terpene	Terpene	Copper	18.45	China: Jiangxi, Yongping
L180206P-1-LD180201-0007_HK72WCCXY_L1_clean_bin.118.orig.fa_	OEZ008529	yes	LMSG_G000008057.1	OES073742	lassopeptide	RiPPs	Copper	6.30	China: Jiangxi, Yongping
L180206P-1-LD180201-0007_HK72WCCXY_L1_clean_bin.119.permissi	OEZ008529	yes	LMSG_G000009286.1	OES073742	terpene	Terpene	Copper	22.06	China: Jiangxi, Yongping
L180206P-1-LD180201-0007_HK72WCCXY_L1_clean_bin.119.permissi	OEZ008529	yes	LMSG_G000009286.1	OES073742	terpene	Terpene	Copper	21.73	China: Jiangxi, Yongping
L180206P-1-LD180201-0007_HK72WCCXY_L1_clean_bin.14.orig.fa_k	OEZ008529	yes	LMSG_G000004358.1	OES073742	lanthipeptide	RiPPs	Copper	21.38	China: Jiangxi, Yongping
L180206P-1-LD180201-0007_HK72WCCXY_L1_clean_bin.2.orig.fa_k1	OEZ008529	yes	LMSG_G000008405.1	OES073742	terpene	Terpene	Copper	20.77	China: Jiangxi, Yongping
L180206P-1-LD180201-0007_HK72WCCXY_L1_clean_bin.20.orig.fa_k	OEZ008529	yes	LMSG_G000004797.1	OES073742	bacteriocin	RiPPs	Copper	9.71	China: Jiangxi, Yongping
L180206P-1-LD180201-0007_HK72WCCXY_L1_clean_bin.20.orig.fa_k	OEZ008529	yes	LMSG_G000004797.1	OES073742	terpene	Terpene	Copper	6.47	China: Jiangxi, Yongping
L180206P-1-LD180201-0007_HK72WCCXY_L1_clean_bin.20.orig.fa_k	OEZ008529	yes	LMSG_G000004797.1	OES073742	NRPS-like	NRPS	Copper	5.57	China: Jiangxi, Yongping
L180206P-1-LD180201-0007_HK72WCCXY_L1_clean_bin.22.strict.fa	OEZ008529	yes	LMSG_G000006209.1	OES073742	terpene	Terpene	Copper	20.83	China: Jiangxi, Yongping
L180206P-1-LD180201-0007_HK72WCCXY_L1_clean_bin.22.strict.fa	OEZ008529	yes	LMSG_G000006209.1	OES073742	T3PKS	PKSother	Copper	41.08	China: Jiangxi, Yongping
L180206P-1-LD180201-0007_HK72WCCXY_L1_clean_bin.23.permissiv	OEZ008529	yes	LMSG_G000009208.1	OES073742	terpene	Terpene	Copper	11.05	China: Jiangxi, Yongping
L180206P-1-LD180201-0007_HK72WCCXY_L1_clean_bin.23.permissiv	OEZ008529	yes	LMSG_G000009208.1	OES073742	terpene	Terpene	Copper	10.62	China: Jiangxi, Yongping
L180206P-1-LD180201-0007_HK72WCCXY_L1_clean_bin.23.permissiv	OEZ008529	yes	LMSG_G000009208.1	OES073742	T3PKS	PKSother	Copper	17.25	China: Jiangxi, Yongping
L180206P-1-LD180201-0007_HK72WCCXY_L1_clean_bin.23.permissiv	OEZ008529	yes	LMSG_G000009208.1	OES073742	terpene	Terpene	Copper	9.75	China: Jiangxi, Yongping
L180206P-1-LD180201-0007_HK72WCCXY_L1_clean_bin.28.orig.fa_k	OEZ008529	yes	LMSG_G000010107.1	OES073742	betalactone	Others	Copper	22.63	China: Jiangxi, Yongping
L180206P-1-LD180201-0007_HK72WCCXY_L1_clean_bin.28.orig.fa_k	OEZ008529	yes	LMSG_G000010107.1	OES073742	hserlactone	Others	Copper	11.44	China: Jiangxi, Yongping
L180206P-1-LD180201-0007_HK72WCCXY_L1_clean_bin.28.orig.fa_k	OEZ008529	yes	LMSG_G000010107.1	OES073742	terpene	Terpene	Copper	20.79	China: Jiangxi, Yongping
L180206P-1-LD180201-0007_HK72WCCXY_L1_clean_bin.28.orig.fa_k	OEZ008529	yes	LMSG_G000010107.1	OES073742	bacteriocin	RiPPs	Copper	10.89	China: Jiangxi, Yongping
L180206P-1-LD180201-0007_HK72WCCXY_L1_clean_bin.28.orig.fa_k	OEZ008529	yes	LMSG_G000010107.1	OES073742	phosphonate	Others	Copper	40.87	China: Jiangxi, Yongping
L180206P-1-LD180201-0007_HK72WCCXY_L1_clean_bin.28.orig.fa_k	OEZ008529	yes	LMSG_G000010107.1	OES073742	terpene	Terpene	Copper	18.71	China: Jiangxi, Yongping
L180206P-1-LD180201-0007_HK72WCCXY_L1_clean_bin.28.orig.fa_k	OEZ008529	yes	LMSG_G000010107.1	OES073742	NRPS-like	NRPS	Copper	28.55	China: Jiangxi, Yongping
L180206P-1-LD180201-0007_HK72WCCXY_L1_clean_bin.30.orig.fa_k	OEZ008529	yes	LMSG_G000008407.1	OES073742	NRPS-like	NRPS	Copper	42.96	China: Jiangxi, Yongping
L180206P-1-LD180201-0007_HK72WCCXY_L1_clean_bin.38.orig.fa_k	OEZ008529	yes	LMSG_G000008631.1	OES073742	other	Others	Copper	40.67	China: Jiangxi, Yongping
L180206P-1-LD180201-0007_HK72WCCXY_L1_clean_bin.38.orig.fa_k	OEZ008529	yes	LMSG_G000008631.1	OES073742	NRPS-like	NRPS	Copper	22.80	China: Jiangxi, Yongping
L180206P-1-LD180201-0007_HK72WCCXY_L1_clean_bin.41.permissiv	OEZ008529	yes	LMSG_G000009528.1	OES073742	acyl_amino_acids	Others	Copper	38.52	China: Jiangxi, Yongping
L180206P-1-LD180201-0007_HK72WCCXY_L1_clean_bin.41.permissiv	OEZ008529	no	LMSG_G000009528.1	OES073742	terpene	Terpene	Copper	20.42	China: Jiangxi, Yongping
L180206P-1-LD180201-0007_HK72WCCXY_L1_clean_bin.41.permissiv	OEZ008529	no	LMSG_G000009528.1	OES073742	NRPS-like	NRPS	Copper	35.43	China: Jiangxi, Yongping
L180206P-1-LD180201-0007_HK72WCCXY_L1_clean_bin.41.permissiv	OEZ008529	no	LMSG_G000009528.1	OES073742	terpene	Terpene	Copper	13.51	China: Jiangxi, Yongping
L180206P-1-LD180201-0007_HK72WCCXY_L1_clean_bin.42.orig.fa_k	OEZ008529	yes	LMSG_G000006502.1	OES073742	thiopeptide.LAP	RiPPs	Copper	33.96	China: Jiangxi, Yongping
L180206P-1-LD180201-0007_HK72WCCXY_L1_clean_bin.43.orig.fa_k	OEZ008529	yes	LMSG_G000011127.1	OES073742	terpene	Terpene	Copper	22.80	China: Jiangxi, Yongping
L180206P-1-LD180201-0007_HK72WCCXY_L1_clean_bin.43.orig.fa_k	OEZ008529	yes	LMSG_G000011127.1	OES073742	arylpolyene	Others	Copper	41.16	China: Jiangxi, Yongping
L180206P-1-LD180201-0007_HK72WCCXY_L1_clean_bin.44.strict.fa	OEZ008529	yes	LMSG_G000004801.1	OES073742	terpene	Terpene	Copper	23.04	China: Jiangxi, Yongping
L180206P-1-LD180201-0007_HK72WCCXY_L1_clean_bin.44.strict.fa	OEZ008529	yes	LMSG_G000004801.1	OES073742	arylpolyene.hserlactone	Others	Copper	41.20	China: Jiangxi, Yongping
L180206P-1-LD180201-0007_HK72WCCXY_L1_clean_bin.45.orig.fa_k	OEZ008529	yes	LMSG_G000009632.1	OES073742	terpene	Terpene	Copper	20.94	China: Jiangxi, Yongping
L180206P-1-LD180201-0007_HK72WCCXY_L1_clean_bin.45.orig.fa_k	OEZ008529	yes	LMSG_G000009632.1	OES073742	acyl_amino_acids	Others	Copper	5.92	China: Jiangxi, Yongping
L180206P-1-LD180201-0007_HK72WCCXY_L1_clean_bin.45.orig.fa_k	OEZ008529	yes	LMSG_G000009632.1	OES073742	terpene	Terpene	Copper	13.01	China: Jiangxi, Yongping
L180206P-1-LD180201-0007_HK72WCCXY_L1_clean_bin.45.orig.fa_k	OEZ008529	yes	LMSG_G000009632.1	OES073742	NRPS-like	NRPS	Copper	10.31	China: Jiangxi, Yongping
L180206P-1-LD180201-0007_HK72WCCXY_L1_clean_bin.46.strict.fa	OEZ008529	yes	LMSG_G000010028.1	OES073742	T3PKS	PKSother	Copper	9.51	China: Jiangxi, Yongping
L180206P-1-LD180201-0007_HK72WCCXY_L1_clean_bin.46.strict.fa	OEZ008529	yes	LMSG_G000010028.1	OES073742	NRPS-like	NRPS	Copper	8.60	China: Jiangxi, Yongping
L180206P-1-LD180201-0007_HK72WCCXY_L1_clean_bin.46.strict.fa	OEZ008529	yes	LMSG_G000010028.1	OES073742	terpene	Terpene	Copper	5.03	China: Jiangxi, Yongping
L180206P-1-LD180201-0007_HK72WCCXY_L1_clean_bin.47.strict.fa	OEZ008529	yes	LMSG_G000010464.1	OES073742	arylpolyene	Others	Copper	25.52	China: Jiangxi, Yongping
L180206P-1-LD180201-0007_HK72WCCXY_L1_clean_bin.47.strict.fa	OEZ008529	yes	LMSG_G000010464.1	OES073742	terpene	Terpene	Copper	16.09	China: Jiangxi, Yongping
L180206P-1-LD180201-0007_HK72WCCXY_L1_clean_bin.47.strict.fa	OEZ008529	yes	LMSG_G000010464.1	OES073742	NRPS-like	NRPS	Copper	17.03	China: Jiangxi, Yongping
L180206P-1-LD180201-0007_HK72WCCXY_L1_clean_bin.48.orig.fa_k	OEZ008529	yes	LMSG_G000009154.1	OES073742	T3PKS	PKSother	Copper	16.88	China: Jiangxi, Yongping
L180206P-1-LD180201-0007_HK72WCCXY_L1_clean_bin.49.orig.fa_k	OEZ008529	yes	LMSG_G000007747.1	OES073742	NRPS-like	NRPS	Copper	24.68	China: Jiangxi, Yongping
L180206P-1-LD180201-0007_HK72WCCXY_L1_clean_bin.49.orig.fa_k	OEZ008529	yes	LMSG_G000007747.1	OES073742	terpene	Terpene	Copper	13.90	China: Jiangxi, Yongping
L180206P-1-LD180201-0007_HK72WCCXY_L1_clean_bin.5.orig.fa_k1	OEZ008529	yes	LMSG_G000008362.1	OES073742	terpene	Terpene	Copper	18.13	China: Jiangxi, Yongping
L180206P-1-LD180201-0007_HK72WCCXY_L1_clean_bin.51.orig.fa_k	OEZ008529	yes	LMSG_G000004802.1	OES073742	terpene	Terpene	Copper	17.52	China: Jiangxi, Yongping
L180206P-1-LD180201-0007_HK72WCCXY_L1_clean_bin.53.orig.fa_k	OEZ008529	yes	LMSG_G000004803.1	OES073742	arylpolyene	Others	Copper	21.29	China: Jiangxi, Yongping
L180206P-1-LD180201-0007_HK72WCCXY_L1_clean_bin.53.orig.fa_k	OEZ008529	yes	LMSG_G000004803.1	OES073742	terpene	Terpene	Copper	7.56	China: Jiangxi, Yongping
L180206P-1-LD180201-0007_HK72WCCXY_L1_clean_bin.53.orig.fa_k	OEZ008529	yes	LMSG_G000004803.1	OES073742	bacteriocin	RiPPs	Copper	10.86	China: Jiangxi, Yongping
L180206P-1-LD180201-0007_HK72WCCXY_L1_clean_bin.53.orig.fa_k	OEZ008529	yes	LMSG_G000004803.1	OES073742	lassopeptide	RiPPs	Copper	18.97	China: Jiangxi, Yongping
L180206P-1-LD180201-0007_HK72WCCXY_L1_clean_bin.55.orig.fa_k	OEZ008529	yes	LMSG_G000009356.1	OES073742	NRPS	NRPS	Copper	49.31	China: Jiangxi, Yongping
L180206P-1-LD180201-0007_HK72WCCXY_L1_clean_bin.55.orig.fa_k	OEZ008529	yes	LMSG_G000009356.1	OES073742	betalactone	Others	Copper	31.59	China: Jiangxi, Yongping
L180206P-1-LD180201-0007_HK72WCCXY_L1_clean_bin.56.strict.fa	OEZ008529	yes	LMSG_G000010041.1	OES073742	ladderane	Others	Copper	27.80	China: Jiangxi, Yongping
L180206P-1-LD180201-0007_HK72WCCXY_L1_clean_bin.56.strict.fa	OEZ008529	yes	LMSG_G000010041.1	OES073742	terpene	Terpene	Copper	14.26	China: Jiangxi, Yongping
L180206P-1-LD180201-0007_HK72WCCXY_L1_clean_bin.56.strict.fa	OEZ008529	yes	LMSG_G000010041.1	OES073742	ectoine	Others	Copper	9.35	China: Jiangxi, Yongping
L180206P-1-LD180201-0007_HK72WCCXY_L1_clean_bin.59.orig.fa_k	OEZ008529	yes	LMSG_G000004804.1	OES073742	bacteriocin	RiPPs	Copper	10.88	China: Jiangxi, Yongping
L180206P-1-LD180201-0007_HK72WCCXY_L1_clean_bin.59.orig.fa_k	OEZ008529	yes	LMSG_G000004804.1	OES073742	NRPS-like	NRPS	Copper	10.07	China: Jiangxi, Yongping
L180206P-1-LD180201-0007_HK72WCCXY_L1_clean_bin.59.orig.fa_k	OEZ008529	yes	LMSG_G000004804.1	OES073742	terpene	Terpene	Copper	17.19	China: Jiangxi, Yongping
L180206P-1-LD180201-0007_HK72WCCXY_L1_clean_bin.62.permissiv	OEZ008529	yes	LMSG_G000007847.1	OES073742	NRPS-like	NRPS	Copper	5.13	China: Jiangxi, Yongping
L180206P-1-LD180201-0007_HK72WCCXY_L1_clean_bin.65.strict.fa	OEZ008529	yes	LMSG_G000007767.1	OES073742	terpene	Terpene	Copper	29.77	China: Jiangxi, Yongping
L180206P-1-LD180201-0007_HK72WCCXY_L1_clean_bin.65.strict.fa	OEZ008529	yes	LMSG_G000007767.1	OES073742	T3PKS	PKSother	Copper	30.28	China: Jiangxi, Yongping
L180206P-1-LD180201-0007_HK72WCCXY_L1_clean_bin.65.strict.fa	OEZ008529	yes	LMSG_G000007767.1	OES073742	NRPS-like	NRPS	Copper	9.73	China: Jiangxi, Yongping
L180206P-1-LD180201-0007_HK72WCCXY_L1_clean_bin.67.orig.fa_k	OEZ008529	yes	LMSG_G000011151.1	OES073742	terpene	Terpene	Copper	23.13	China: Jiangxi, Yongping
L180206P-1-LD180201-0007_HK72WCCXY_L1_clean_bin.67.orig.fa_k	OEZ008529	yes	LMSG_G000011151.1	OES073742	arylpolyene	Others	Copper	38.98	China: Jiangxi, Yongping
L180206P-1-LD180201-0007_HK72WCCXY_L1_clean_bin.67.orig.fa_k	OEZ008529	yes	LMSG_G000011151.1	OES073742	arylpolyene	Others	Copper	25.49	China: Jiangxi, Yongping
L180206P-1-LD180201-0007_HK72WCCXY_L1_clean_bin.67.orig.fa_k	OEZ008529	yes	LMSG_G000011151.1	OES073742	bacteriocin	RiPPs	Copper	10.92	China: Jiangxi, Yongping
L180206P-1-LD180201-0007_HK72WCCXY_L1_clean_bin.69.orig.fa_k	OEZ008529	yes	LMSG_G000011122.1	OES073742	terpene	Terpene	Copper	16.14	China: Jiangxi, Yongping
L180206P-1-LD180201-0007_HK72WCCXY_L1_clean_bin.69.orig.fa_k	OEZ008529	yes	LMSG_G000011122.1	OES073742	arylpolyene	Others	Copper	41.16	China: Jiangxi, Yongping
L180206P-1-LD180201-0007_HK72WCCXY_L1_clean_bin.7.strict.fa_	OEZ008529	yes	LMSG_G000007752.1	OES073742	NRPS.T1PKS	PKS-NRP_Hybrids	Copper	41.92	China: Jiangxi, Yongping
L180206P-1-LD180201-0007_HK72WCCXY_L1_clean_bin.7.strict.fa_	OEZ008529	yes	LMSG_G000007752.1	OES073742	terpene	Terpene	Copper	22.04	China: Jiangxi, Yongping
L180206P-1-LD180201-0007_HK72WCCXY_L1_clean_bin.7.strict.fa_	OEZ008529	yes	LMSG_G000007752.1	OES073742	T1PKS	PKSI	Copper	15.31	China: Jiangxi, Yongping
L180206P-1-LD180201-0007_HK72WCCXY_L1_clean_bin.7.strict.fa_	OEZ008529	yes	LMSG_G000007752.1	OES073742	NRPS	NRPS	Copper	9.64	China: Jiangxi, Yongping
L180206P-1-LD180201-0007_HK72WCCXY_L1_clean_bin.7.strict.fa_	OEZ008529	yes	LMSG_G000007752.1	OES073742	betalactone.NRPS-like	Others	Copper	6.49	China: Jiangxi, Yongping
L180206P-1-LD180201-0007_HK72WCCXY_L1_clean_bin.7.strict.fa_	OEZ008529	yes	LMSG_G000007752.1	OES073742	T1PKS	PKSI	Copper	5.86	China: Jiangxi, Yongping
L180206P-1-LD180201-0007_HK72WCCXY_L1_clean_bin.72.orig.fa_k	OEZ008529	yes	LMSG_G000004806.1	OES073742	linaridin	RiPPs	Copper	17.18	China: Jiangxi, Yongping
L180206P-1-LD180201-0007_HK72WCCXY_L1_clean_bin.72.orig.fa_k	OEZ008529	yes	LMSG_G000004806.1	OES073742	terpene	Terpene	Copper	14.07	China: Jiangxi, Yongping
L180206P-1-LD180201-0007_HK72WCCXY_L1_clean_bin.72.orig.fa_k	OEZ008529	yes	LMSG_G000004806.1	OES073742	terpene	Terpene	Copper	21.88	China: Jiangxi, Yongping
L180206P-1-LD180201-0007_HK72WCCXY_L1_clean_bin.72.orig.fa_k	OEZ008529	yes	LMSG_G000004806.1	OES073742	NRPS-like	NRPS	Copper	42.85	China: Jiangxi, Yongping
L180206P-1-LD180201-0007_HK72WCCXY_L1_clean_bin.72.orig.fa_k	OEZ008529	yes	LMSG_G000004806.1	OES073742	T3PKS	PKSother	Copper	31.06	China: Jiangxi, Yongping
L180206P-1-LD180201-0007_HK72WCCXY_L1_clean_bin.73.orig.fa_k	OEZ008529	yes	LMSG_G000007954.1	OES073742	lassopeptide	RiPPs	Copper	10.31	China: Jiangxi, Yongping
L180206P-1-LD180201-0007_HK72WCCXY_L1_clean_bin.73.orig.fa_k	OEZ008529	yes	LMSG_G000007954.1	OES073742	NRPS	NRPS	Copper	14.89	China: Jiangxi, Yongping
L180206P-1-LD180201-0007_HK72WCCXY_L1_clean_bin.76.orig.fa_k	OEZ008529	no	LMSG_G000011014.1	OES073742	NRPS	NRPS	Copper	62.35	China: Jiangxi, Yongping
L180206P-1-LD180201-0007_HK72WCCXY_L1_clean_bin.76.orig.fa_k	OEZ008529	no	LMSG_G000011014.1	OES073742	NRPS	NRPS	Copper	27.10	China: Jiangxi, Yongping
L180206P-1-LD180201-0007_HK72WCCXY_L1_clean_bin.76.orig.fa_k	OEZ008529	no	LMSG_G000011014.1	OES073742	NAGGN	Others	Copper	14.76	China: Jiangxi, Yongping
L180206P-1-LD180201-0007_HK72WCCXY_L1_clean_bin.76.orig.fa_k	OEZ008529	no	LMSG_G000011014.1	OES073742	hserlactone	Others	Copper	20.61	China: Jiangxi, Yongping
L180206P-1-LD180201-0007_HK72WCCXY_L1_clean_bin.76.orig.fa_k	OEZ008529	no	LMSG_G000011014.1	OES073742	NRPS	NRPS	Copper	42.73	China: Jiangxi, Yongping
L180206P-1-LD180201-0007_HK72WCCXY_L1_clean_bin.76.orig.fa_k	OEZ008529	no	LMSG_G000011014.1	OES073742	phenazine	Others	Copper	10.23	China: Jiangxi, Yongping
L180206P-1-LD180201-0007_HK72WCCXY_L1_clean_bin.76.orig.fa_k	OEZ008529	no	LMSG_G000011014.1	OES073742	NRPS	NRPS	Copper	34.30	China: Jiangxi, Yongping
L180206P-1-LD180201-0007_HK72WCCXY_L1_clean_bin.76.orig.fa_k	OEZ008529	no	LMSG_G000011014.1	OES073742	bacteriocin	RiPPs	Copper	10.86	China: Jiangxi, Yongping
L180206P-1-LD180201-0007_HK72WCCXY_L1_clean_bin.76.orig.fa_k	OEZ008529	no	LMSG_G000011014.1	OES073742	thiopeptide	RiPPs	Copper	33.00	China: Jiangxi, Yongping
L180206P-1-LD180201-0007_HK72WCCXY_L1_clean_bin.76.orig.fa_k	OEZ008529	no	LMSG_G000011014.1	OES073742	bacteriocin	RiPPs	Copper	10.83	China: Jiangxi, Yongping
L180206P-1-LD180201-0007_HK72WCCXY_L1_clean_bin.76.orig.fa_k	OEZ008529	no	LMSG_G000011014.1	OES073742	NRPS-like	NRPS	Copper	42.98	China: Jiangxi, Yongping
L180206P-1-LD180201-0007_HK72WCCXY_L1_clean_bin.76.orig.fa_k	OEZ008529	no	LMSG_G000011014.1	OES073742	betalactone.NRPS-like	Others	Copper	24.24	China: Jiangxi, Yongping
L180206P-1-LD180201-0007_HK72WCCXY_L1_clean_bin.76.orig.fa_k	OEZ008529	no	LMSG_G000011014.1	OES073742	hserlactone	Others	Copper	20.61	China: Jiangxi, Yongping
L180206P-1-LD180201-0007_HK72WCCXY_L1_clean_bin.76.orig.fa_k	OEZ008529	no	LMSG_G000011014.1	OES073742	phenazine	Others	Copper	10.23	China: Jiangxi, Yongping
L180206P-1-LD180201-0007_HK72WCCXY_L1_clean_bin.78.orig.fa_k	OEZ008529	yes	LMSG_G000007912.1	OES073742	NRPS-like	NRPS	Copper	42.82	China: Jiangxi, Yongping
L180206P-1-LD180201-0007_HK72WCCXY_L1_clean_bin.78.orig.fa_k	OEZ008529	yes	LMSG_G000007912.1	OES073742	bacteriocin	RiPPs	Copper	10.81	China: Jiangxi, Yongping
L180206P-1-LD180201-0007_HK72WCCXY_L1_clean_bin.78.orig.fa_k	OEZ008529	yes	LMSG_G000007912.1	OES073742	terpene	Terpene	Copper	20.88	China: Jiangxi, Yongping
L180206P-1-LD180201-0007_HK72WCCXY_L1_clean_bin.80.orig.fa_k	OEZ008529	yes	LMSG_G000009393.1	OES073742	NRPS	NRPS	Copper	21.96	China: Jiangxi, Yongping
L180206P-1-LD180201-0007_HK72WCCXY_L1_clean_bin.80.orig.fa_k	OEZ008529	yes	LMSG_G000009393.1	OES073742	terpene	Terpene	Copper	21.68	China: Jiangxi, Yongping
L180206P-1-LD180201-0007_HK72WCCXY_L1_clean_bin.80.orig.fa_k	OEZ008529	yes	LMSG_G000009393.1	OES073742	bacteriocin	RiPPs	Copper	10.81	China: Jiangxi, Yongping

L180206P-1-LD180201-0007_HK72WCCXY_L1_clean_bin.80.orig.fa_k	OEZ008529	yes	LMSG_G000009393.1	OES073742	NRPS-like	NRPS	Copper	22.21	China: Jiangxi, Yongping
L180206P-1-LD180201-0007_HK72WCCXY_L1_clean_bin.81.orig.fa_k	OEZ008529	yes	LMSG_G000010006.1	OES073742	terpene	Terpene	Copper	21.87	China: Jiangxi, Yongping
L180206P-1-LD180201-0007_HK72WCCXY_L1_clean_bin.81.orig.fa_k	OEZ008529	yes	LMSG_G000010006.1	OES073742	arylpolyene	Others	Copper	22.60	China: Jiangxi, Yongping
L180206P-1-LD180201-0007_HK72WCCXY_L1_clean_bin.81.orig.fa_k	OEZ008529	yes	LMSG_G000010006.1	OES073742	arylpolyene	Others	Copper	8.70	China: Jiangxi, Yongping
L180206P-1-LD180201-0007_HK72WCCXY_L1_clean_bin.83.orig.fa_k	OEZ008529	yes	LMSG_G000004362.1	OES073742	T3PKS	PKSother	Copper	41.07	China: Jiangxi, Yongping
L180206P-1-LD180201-0007_HK72WCCXY_L1_clean_bin.83.orig.fa_k	OEZ008529	yes	LMSG_G000004362.1	OES073742	terpene	Terpene	Copper	21.07	China: Jiangxi, Yongping
L180206P-1-LD180201-0007_HK72WCCXY_L1_clean_bin.86.strict.fa	OEZ008529	yes	LMSG_G000009605.1	OES073742	terpene	Terpene	Copper	21.99	China: Jiangxi, Yongping
L180206P-1-LD180201-0007_HK72WCCXY_L1_clean_bin.86.strict.fa	OEZ008529	yes	LMSG_G000009605.1	OES073742	NRPS-like	NRPS	Copper	25.63	China: Jiangxi, Yongping
L180206P-1-LD180201-0007_HK72WCCXY_L1_clean_bin.86.strict.fa	OEZ008529	yes	LMSG_G000009605.1	OES073742	terpene	Terpene	Copper	19.54	China: Jiangxi, Yongping
L180206P-1-LD180201-0007_HK72WCCXY_L1_clean_bin.87.orig.fa_k	OEZ008529	yes	LMSG_G000008171.1	OES073742	terpene	Terpene	Copper	8.18	China: Jiangxi, Yongping
L180206P-1-LD180201-0007_HK72WCCXY_L1_clean_bin.9.orig.fa_kl	OEZ008529	yes	LMSG_G000004810.1	OES073742	T3PKS.terpene	Others	Copper	35.83	China: Jiangxi, Yongping
L180206P-1-LD180201-0007_HK72WCCXY_L1_clean_bin.94.permissiv	OEZ008529	yes	LMSG_G000009635.1	OES073742	terpene	Terpene	Copper	20.86	China: Jiangxi, Yongping
L180206P-1-LD180201-0007_HK72WCCXY_L1_clean_bin.94.permissiv	OEZ008529	yes	LMSG_G000009635.1	OES073742	NRPS-like	NRPS	Copper	20.93	China: Jiangxi, Yongping
L180206P-1-LD180201-0007_HK72WCCXY_L1_clean_bin.94.permissiv	OEZ008529	yes	LMSG_G000009635.1	OES073742	terpene	Terpene	Copper	12.47	China: Jiangxi, Yongping
L180206P-1-LD180201-0007_HK72WCCXY_L1_clean_bin.94.permissiv	OEZ008529	yes	LMSG_G000009635.1	OES073742	acyl_amino_acids	Others	Copper	16.74	China: Jiangxi, Yongping
L180206P-1-LD180201-0007_HK72WCCXY_L1_clean_bin.94.permissiv	OEZ008529	yes	LMSG_G000009635.1	OES073742	nucleoside	Others	Copper	13.18	China: Jiangxi, Yongping
L180206P-1-LD180201-0007_HK72WCCXY_L1_clean_bin.94.permissiv	OEZ008529	yes	LMSG_G000009635.1	OES073742	acyl_amino_acids	Others	Copper	14.23	China: Jiangxi, Yongping
L180206P-1-LD180201-0007_HK72WCCXY_L1_clean_bin.98.orig.fa_k	OEZ008529	yes	LMSG_G000004809.1	OES073742	bacteriocin	RiPPs	Copper	10.81	China: Jiangxi, Yongping
L180206P-1-LD180201-0007_HK72WCCXY_L1_clean_bin.98.orig.fa_k	OEZ008529	yes	LMSG_G000004809.1	OES073742	terpene	Terpene	Copper	11.73	China: Jiangxi, Yongping
L180206P-1-LD180201-0016_HK72WCCXY_L1_clean_bin.1.orig.fa_kl	OEZ008529	yes	LMSG_G000010900.1	OES073711	NRPS-like	NRPS	Lead-Zinc	26.21	China: Hunan, Shuikoushan
L180206P-1-LD180201-0016_HK72WCCXY_L1_clean_bin.1.orig.fa_kl	OEZ008529	yes	LMSG_G000010900.1	OES073711	acyl_amino_acids	Others	Lead-Zinc	5.33	China: Hunan, Shuikoushan
L180206P-1-LD180201-0016_HK72WCCXY_L1_clean_bin.1.orig.fa_kl	OEZ008529	yes	LMSG_G000010900.1	OES073711	arylpolyene	Others	Lead-Zinc	27.84	China: Hunan, Shuikoushan
L180206P-1-LD180201-0016_HK72WCCXY_L1_clean_bin.1.orig.fa_kl	OEZ008529	yes	LMSG_G000010900.1	OES073711	terpene	Terpene	Lead-Zinc	21.68	China: Hunan, Shuikoushan
L180206P-1-LD180201-0016_HK72WCCXY_L1_clean_bin.10.strict.fa	OEZ008529	yes	LMSG_G000008185.1	OES073711	betalactone	Others	Lead-Zinc	31.32	China: Hunan, Shuikoushan
L180206P-1-LD180201-0016_HK72WCCXY_L1_clean_bin.10.strict.fa	OEZ008529	yes	LMSG_G000008185.1	OES073711	terpene	Terpene	Lead-Zinc	20.86	China: Hunan, Shuikoushan
L180206P-1-LD180201-0016_HK72WCCXY_L1_clean_bin.100.orig.fa_	OEZ008529	yes	LMSG_G000010677.1	OES073711	betalactone	Others	Lead-Zinc	28.50	China: Hunan, Shuikoushan
L180206P-1-LD180201-0016_HK72WCCXY_L1_clean_bin.100.orig.fa_	OEZ008529	yes	LMSG_G000010677.1	OES073711	arylpolyene	Others	Lead-Zinc	35.66	China: Hunan, Shuikoushan
L180206P-1-LD180201-0016_HK72WCCXY_L1_clean_bin.100.orig.fa_	OEZ008529	yes	LMSG_G000010677.1	OES073711	NRPS-like	NRPS	Lead-Zinc	12.55	China: Hunan, Shuikoushan
L180206P-1-LD180201-0016_HK72WCCXY_L1_clean_bin.11.orig.fa_k	OEZ008529	yes	LMSG_G000004811.1	OES073711	terpene	Terpene	Lead-Zinc	19.70	China: Hunan, Shuikoushan
L180206P-1-LD180201-0016_HK72WCCXY_L1_clean_bin.11.orig.fa_k	OEZ008529	yes	LMSG_G000004811.1	OES073711	terpene	Terpene	Lead-Zinc	6.81	China: Hunan, Shuikoushan
L180206P-1-LD180201-0016_HK72WCCXY_L1_clean_bin.11.orig.fa_k	OEZ008529	yes	LMSG_G000004811.1	OES073711	ectoine	Others	Lead-Zinc	10.40	China: Hunan, Shuikoushan
L180206P-1-LD180201-0016_HK72WCCXY_L1_clean_bin.12.orig.fa_k	OEZ008529	yes	LMSG_G000004812.1	OES073711	hglE-KS	PKSother	Lead-Zinc	30.14	China: Hunan, Shuikoushan
L180206P-1-LD180201-0016_HK72WCCXY_L1_clean_bin.14.orig.fa_k	OEZ008529	yes	LMSG_G000010715.1	OES073711	NRPS	NRPS	Lead-Zinc	23.63	China: Hunan, Shuikoushan
L180206P-1-LD180201-0016_HK72WCCXY_L1_clean_bin.14.orig.fa_k	OEZ008529	yes	LMSG_G000010715.1	OES073711	NRPS.T1PKS	PKS-NRP_Hybrids	Lead-Zinc	18.06	China: Hunan, Shuikoushan
L180206P-1-LD180201-0016_HK72WCCXY_L1_clean_bin.14.orig.fa_k	OEZ008529	yes	LMSG_G000010715.1	OES073711	arylpolyene	Others	Lead-Zinc	32.58	China: Hunan, Shuikoushan
L180206P-1-LD180201-0016_HK72WCCXY_L1_clean_bin.14.orig.fa_k	OEZ008529	yes	LMSG_G000010715.1	OES073711	terpene	Terpene	Lead-Zinc	16.14	China: Hunan, Shuikoushan
L180206P-1-LD180201-0016_HK72WCCXY_L1_clean_bin.15.orig.fa_k	OEZ008529	yes	LMSG_G000006263.1	OES073711	betalactone	Others	Lead-Zinc	23.34	China: Hunan, Shuikoushan
L180206P-1-LD180201-0016_HK72WCCXY_L1_clean_bin.15.orig.fa_k	OEZ008529	yes	LMSG_G000006263.1	OES073711	terpene	Terpene	Lead-Zinc	24.42	China: Hunan, Shuikoushan
L180206P-1-LD180201-0016_HK72WCCXY_L1_clean_bin.16.strict.fa	OEZ008529	yes	LMSG_G000010496.1	OES073711	arylpolyene	Others	Lead-Zinc	21.63	China: Hunan, Shuikoushan
L180206P-1-LD180201-0016_HK72WCCXY_L1_clean_bin.16.strict.fa	OEZ008529	yes	LMSG_G000010496.1	OES073711	terpene	Terpene	Lead-Zinc	12.34	China: Hunan, Shuikoushan
L180206P-1-LD180201-0016_HK72WCCXY_L1_clean_bin.16.strict.fa	OEZ008529	yes	LMSG_G000010496.1	OES073711	hserlactone	Others	Lead-Zinc	9.07	China: Hunan, Shuikoushan
L180206P-1-LD180201-0016_HK72WCCXY_L1_clean_bin.17.permissiv	OEZ008529	yes	LMSG_G000008118.1	OES073711	terpene	Terpene	Lead-Zinc	20.40	China: Hunan, Shuikoushan
L180206P-1-LD180201-0016_HK72WCCXY_L1_clean_bin.18.orig.fa_k	OEZ008529	yes	LMSG_G000004813.1	OES073711	terpene	Terpene	Lead-Zinc	21.89	China: Hunan, Shuikoushan
L180206P-1-LD180201-0016_HK72WCCXY_L1_clean_bin.18.orig.fa_k	OEZ008529	yes	LMSG_G000004813.1	OES073711	arylpolyene	Others	Lead-Zinc	15.08	China: Hunan, Shuikoushan
L180206P-1-LD180201-0016_HK72WCCXY_L1_clean_bin.18.orig.fa_k	OEZ008529	yes	LMSG_G000004813.1	OES073711	bacteriocin	RiPPs	Lead-Zinc	9.46	China: Hunan, Shuikoushan
L180206P-1-LD180201-0016_HK72WCCXY_L1_clean_bin.2.orig.fa_kl	OEZ008529	yes	LMSG_G000004816.1	OES073711	bacteriocin	RiPPs	Lead-Zinc	7.51	China: Hunan, Shuikoushan
L180206P-1-LD180201-0016_HK72WCCXY_L1_clean_bin.2.orig.fa_kl	OEZ008529	yes	LMSG_G000004816.1	OES073711	NRPS-like	NRPS	Lead-Zinc	28.23	China: Hunan, Shuikoushan
L180206P-1-LD180201-0016_HK72WCCXY_L1_clean_bin.2.orig.fa_kl	OEZ008529	yes	LMSG_G000004816.1	OES073711	terpene	Terpene	Lead-Zinc	10.42	China: Hunan, Shuikoushan
L180206P-1-LD180201-0016_HK72WCCXY_L1_clean_bin.2.orig.fa_kl	OEZ008529	yes	LMSG_G000004816.1	OES073711	hserlactone	Others	Lead-Zinc	10.97	China: Hunan, Shuikoushan
L180206P-1-LD180201-0016_HK72WCCXY_L1_clean_bin.20.orig.fa_k	OEZ008529	yes	LMSG_G000010872.1	OES073711	terpene	Terpene	Lead-Zinc	20.80	China: Hunan, Shuikoushan
L180206P-1-LD180201-0016_HK72WCCXY_L1_clean_bin.20.orig.fa_k	OEZ008529	yes	LMSG_G000010872.1	OES073711	terpene	Terpene	Lead-Zinc	15.34	China: Hunan, Shuikoushan
L180206P-1-LD180201-0016_HK72WCCXY_L1_clean_bin.20.orig.fa_k	OEZ008529	yes	LMSG_G000010872.1	OES073711	bacteriocin	RiPPs	Lead-Zinc	11.37	China: Hunan, Shuikoushan
L180206P-1-LD180201-0016_HK72WCCXY_L1_clean_bin.20.orig.fa_k	OEZ008529	yes	LMSG_G000010872.1	OES073711	acyl_amino_acids	Others	Lead-Zinc	34.17	China: Hunan, Shuikoushan
L180206P-1-LD180201-0016_HK72WCCXY_L1_clean_bin.22.orig.fa_k	OEZ008529	yes	LMSG_G000004363.1	OES073711	other	Others	Lead-Zinc	41.18	China: Hunan, Shuikoushan
L180206P-1-LD180201-0016_HK72WCCXY_L1_clean_bin.22.orig.fa_k	OEZ008529	yes	LMSG_G000004363.1	OES073711	other	Others	Lead-Zinc	40.41	China: Hunan, Shuikoushan
L180206P-1-LD180201-0016_HK72WCCXY_L1_clean_bin.25.orig.fa_k	OEZ008529	yes	LMSG_G000010910.1	OES073711	hserlactone	Others	Lead-Zinc	20.66	China: Hunan, Shuikoushan
L180206P-1-LD180201-0016_HK72WCCXY_L1_clean_bin.25.orig.fa_k	OEZ008529	yes	LMSG_G000010910.1	OES073711	thiopeptide	RiPPs	Lead-Zinc	23.84	China: Hunan, Shuikoushan
L180206P-1-LD180201-0016_HK72WCCXY_L1_clean_bin.25.orig.fa_k	OEZ008529	yes	LMSG_G000010910.1	OES073711	T1PKS	PKSI	Lead-Zinc	43.55	China: Hunan, Shuikoushan
L180206P-1-LD180201-0016_HK72WCCXY_L1_clean_bin.25.orig.fa_k	OEZ008529	yes	LMSG_G000010910.1	OES073711	terpene	Terpene	Lead-Zinc	18.35	China: Hunan, Shuikoushan
L180206P-1-LD180201-0016_HK72WCCXY_L1_clean_bin.28.orig.fa_k	OEZ008529	yes	LMSG_G000010405.1	OES073711	terpene	Terpene	Lead-Zinc	8.56	China: Hunan, Shuikoushan
L180206P-1-LD180201-0016_HK72WCCXY_L1_clean_bin.3.orig.fa_kl	OEZ008529	yes	LMSG_G000010365.1	OES073711	bacteriocin	RiPPs	Lead-Zinc	5.12	China: Hunan, Shuikoushan
L180206P-1-LD180201-0016_HK72WCCXY_L1_clean_bin.3.orig.fa_kl	OEZ008529	yes	LMSG_G000010365.1	OES073711	NRPS-like	NRPS	Lead-Zinc	6.17	China: Hunan, Shuikoushan
L180206P-1-LD180201-0016_HK72WCCXY_L1_clean_bin.30.strict.fa	OEZ008529	yes	LMSG_G000004817.1	OES073711	terpene	Terpene	Lead-Zinc	20.82	China: Hunan, Shuikoushan
L180206P-1-LD180201-0016_HK72WCCXY_L1_clean_bin.30.strict.fa	OEZ008529	yes	LMSG_G000004817.1	OES073711	terpene	Terpene	Lead-Zinc	20.83	China: Hunan, Shuikoushan
L180206P-1-LD180201-0016_HK72WCCXY_L1_clean_bin.30.strict.fa	OEZ008529	yes	LMSG_G000004817.1	OES073711	bacteriocin	RiPPs	Lead-Zinc	9.02	China: Hunan, Shuikoushan
L180206P-1-LD180201-0016_HK72WCCXY_L1_clean_bin.30.strict.fa	OEZ008529	yes	LMSG_G000004817.1	OES073711	lassopeptide.bacteriocin	RiPPs	Lead-Zinc	29.38	China: Hunan, Shuikoushan
L180206P-1-LD180201-0016_HK72WCCXY_L1_clean_bin.30.strict.fa	OEZ008529	yes	LMSG_G000004817.1	OES073711	hserlactone	Others	Lead-Zinc	11.66	China: Hunan, Shuikoushan
L180206P-1-LD180201-0016_HK72WCCXY_L1_clean_bin.31.strict.fa	OEZ008529	yes	LMSG_G000010854.1	OES073711	bacteriocin	RiPPs	Lead-Zinc	6.70	China: Hunan, Shuikoushan
L180206P-1-LD180201-0016_HK72WCCXY_L1_clean_bin.32.orig.fa_k	OEZ008529	yes	LMSG_G000010889.1	OES073711	acyl_amino_acids	Others	Lead-Zinc	13.84	China: Hunan, Shuikoushan
L180206P-1-LD180201-0016_HK72WCCXY_L1_clean_bin.32.orig.fa_k	OEZ008529	yes	LMSG_G000010889.1	OES073711	terpene	Terpene	Lead-Zinc	6.38	China: Hunan, Shuikoushan
L180206P-1-LD180201-0016_HK72WCCXY_L1_clean_bin.32.orig.fa_k	OEZ008529	yes	LMSG_G000010889.1	OES073711	betalactone	Others	Lead-Zinc	14.50	China: Hunan, Shuikoushan
L180206P-1-LD180201-0016_HK72WCCXY_L1_clean_bin.33.orig.fa_k	OEZ008529	yes	LMSG_G000010906.1	OES073711	terpene	Terpene	Lead-Zinc	6.46	China: Hunan, Shuikoushan
L180206P-1-LD180201-0016_HK72WCCXY_L1_clean_bin.34.permissiv	OEZ008529	yes	LMSG_G000009690.1	OES073711	NRPS-like	NRPS	Lead-Zinc	23.25	China: Hunan, Shuikoushan
L180206P-1-LD180201-0016_HK72WCCXY_L1_clean_bin.35.orig.fa_k	OEZ008529	yes	LMSG_G000004818.1	OES073711	bacteriocin	RiPPs	Lead-Zinc	10.80	China: Hunan, Shuikoushan
L180206P-1-LD180201-0016_HK72WCCXY_L1_clean_bin.35.orig.fa_k	OEZ008529	yes	LMSG_G000004818.1	OES073711	betalactone	Others	Lead-Zinc	17.96	China: Hunan, Shuikoushan
L180206P-1-LD180201-0016_HK72WCCXY_L1_clean_bin.36.orig.fa_k	OEZ008529	yes	LMSG_G000004819.1	OES073711	arylpolyene	Others	Lead-Zinc	29.07	China: Hunan, Shuikoushan
L180206P-1-LD180201-0016_HK72WCCXY_L1_clean_bin.36.orig.fa_k	OEZ008529	yes	LMSG_G000004819.1	OES073711	acyl_amino_acids	Others	Lead-Zinc	56.98	China: Hunan, Shuikoushan
L180206P-1-LD180201-0016_HK72WCCXY_L1_clean_bin.36.orig.fa_k	OEZ008529	yes	LMSG_G000004819.1	OES073711	acyl_amino_acids	Others	Lead-Zinc	47.62	China: Hunan, Shuikoushan
L180206P-1-LD180201-0016_HK72WCCXY_L1_clean_bin.36.orig.fa_k	OEZ008529	yes	LMSG_G000004819.1	OES073711	resorcinol.arylpolyene	Others	Lead-Zinc	26.16	China: Hunan, Shuikoushan
L180206P-1-LD180201-0016_HK72WCCXY_L1_clean_bin.37.orig.fa_k	OEZ008529	yes	LMSG_G000010933.1	OES073711	bacteriocin	RiPPs	Lead-Zinc	8.75	China: Hunan, Shuikoushan
L180206P-1-LD180201-0016_HK72WCCXY_L1_clean_bin.4.orig.fa_kl	OEZ008529	yes	LMSG_G000010633.1	OES073711	arylpolyene	Others	Lead-Zinc	11.94	China: Hunan, Shuikoushan
L180206P-1-LD180201-0016_HK72WCCXY_L1_clean_bin.4.orig.fa_kl	OEZ008529	yes	LMSG_G000010633.1	OES073711	terpene	Terpene	Lead-Zinc	16.80	China: Hunan, Shuikoushan
L180206P-1-LD180201-0016_HK72WCCXY_L1_clean_bin.40.orig.fa_k	OEZ008529	yes	LMSG_G000004821.1	OES073711	bacteriocin	RiPPs	Lead-Zinc	10.89	China: Hunan, Shuikoushan
L180206P-1-LD180201-0016_HK72WCCXY_L1_clean_bin.40.orig.fa_k	OEZ008529	yes	LMSG_G000004821.1	OES073711	betalactone	Others	Lead-Zinc	16.93	China: Hunan, Shuikoushan
L180206P-1-LD180201-0016_HK72WCCXY_L1_clean_bin.40.orig.fa_k	OEZ008529	yes	LMSG_G000004821.1	OES073711	NRPS.T1PKS	PKS-NRP_Hybrids	Lead-Zinc	52.28	China: Hunan, Shuikoushan
L180206P-1-LD180201-0016_HK72WCCXY_L1_clean_bin.40.orig.fa_k	OEZ008529	yes	LMSG_G000004821.1	OES073711	terpene	Terpene	Lead-Zinc	15.57	China: Hunan, Shuikoushan
L180206P-1-LD180201-0016_HK72WCCXY_L1_clean_bin.40.orig.fa_k	OEZ008529	yes	LMSG_G000004821.1	OES073711	arylpolyene	Others	Lead-Zinc	41.28	China: Hunan, Shuikoushan
L180206P-1-LD180201-0016_HK72WCCXY_L1_clean_bin.41.orig.fa_k	OEZ008529	yes	LMSG_G000004366.1	OES073711	terpene	Terpene	Lead-Zinc	18.34	China: Hunan, Shuikoushan
L180206P-1-LD180201-0016_HK72WCCXY_L1_clean_bin.41.orig.fa_k	OEZ008529	yes	LMSG_G000004366.1	OES073711	bacteriocin	RiPPs	Lead-Zinc	10.84	China: Hunan, Shuikoushan
L180206P-1-LD180201-0016_HK72WCCXY_L1_clean_bin.42.orig.fa_k	OEZ008529	yes	LMSG_G000010329.1	OES073711	NRPS-like	NRPS	Lead-Zinc	30.11	China: Hunan, Shuikoushan
L180206P-1-LD180201-0016_HK72WCCXY_L1_clean_bin.43.strict.fa	OEZ008529	yes	LMSG_G000009336.1	OES073711	betalactone	Others	Lead-Zinc	24.46	China: Hunan, Shuikoushan
L180206P-1-LD180201-0016_HK72WCCXY_L1_clean_bin.43.strict.fa	OEZ008529	yes	LMSG_G000009336.1	OES073711	betalactone	Others	Lead-Zinc	24.70	China: Hunan, Shuikoushan
L180206P-1-LD180201-0016_HK72WCCXY_L1_clean_bin.44.orig.fa_k	OEZ008529	yes	LMSG_G000006210.1	OES073711	NRPS-like	NRPS	Lead-Zinc	37.21	China: Hunan, Shuikoushan
L180206P-1-LD180201-0016_HK72WCCXY_L1_clean_bin.44.orig.fa_k	OEZ008529	yes	LMSG_G000006210.1	OES073711	terpene	Terpene	Lead-Zinc	18.63	China: Hunan, Shuikoushan
L180206P-1-LD180201-0016_HK72WCCXY_L1_clean_bin.44.orig.fa_k	OEZ008529	yes	LMSG_G000006210.1	OES073711	acyl_amino_acids	Others	Lead-Zinc	59.44	China: Hunan, Shuikoushan
L180206P-1-LD180201-0016_HK72WCCXY_L1_clean_bin.44.orig.fa_k	OEZ008529	yes	LMSG_G000006210.1	OES073711	betalactone.arylpolyene	Others	Lead-Zinc	47.16	China: Hunan, Shuikoushan
L180206P-1-LD180201-0016_HK72WCCXY_L1_clean_bin.45.orig.fa_k	OEZ008529	yes	LMSG_G000004822.1	OES073711	betalactone	Others	Lead-Zinc	7.04	China: Hunan, Shuikoushan
L180206P-1-LD180201-0016_HK72WCCXY_L1_clean_bin.45.orig.fa_k	OEZ008529	yes	LMSG_G000004822.1	OES073711	T1PKS.hglE-KS	PKSother	Lead-Zinc	33.25	China: Hunan, Shuikoushan
L180206P-1-LD180201-0016_HK72WCCXY_L1_clean_bin.45.orig.fa_k	OEZ008529	yes	LMSG_G000004822.1	OES073711	hserlactone	Others	Lead-Zinc	20.58	China: Hunan, Shuikoushan
L180206P-1-LD180201-0016_HK72WCCXY_L1_clean_bin.47.orig.fa_k	OEZ008529	yes	LMSG_G000007908.1	OES073711	terpene	Terpene	Lead-Zinc	16.76	China: Hunan, Shuikoushan
L180206P-1-LD180201-0016_HK72WCCXY_L1_clean_bin.47.orig.fa_k	OEZ008529	yes	LMSG_G000007908.1	OES073711	NRPS	NRPS	Lead-Zinc	23.25	China: Hunan, Shuikoushan
L180206P-1-LD180201-0016_HK72WCCXY_L1_clean_bin.47.orig.fa_k	OEZ008529	yes	LMSG_G000007908.1	OES073711	NRPS-like	NRPS	Lead-Zinc	10.49	China: Hunan, Shuikoushan
L180206P-1-LD180201-0016_HK72WCCXY_L1_clean_bin.47.orig.fa_k	OEZ008529	yes	LMSG_G000007908.1	OES073711	terpene	Terpene	Lead-Zinc	22.00	China: Hunan, Shuikoushan
L180206P-1-LD180201-0016_HK72WCCXY_L1_clean_bin.47.orig.fa_k	OEZ008529	yes	LMSG_G000007908.1	OES073711	NRPS-like	NRPS	Lead-Zinc	27.75	China: Hunan, Shuikoushan
L180206P-1-LD180201-0016_HK72WCCXY_L1_clean_bin.48.orig.fa_k	OEZ008529	yes	LMSG_G000010921.1	OES073711	arylpolyene	Others	Lead-Zinc	41.19	China: Hunan, Shuikoushan
L180206P-1-LD180201-0016_HK72WCCXY_L1_clean_bin.48.orig.fa_k	OEZ008529	yes	LMSG_G000010921.1	OES073711	terpene	Terpene	Lead-Zinc	21.66	China: Hunan, Shuikoushan
L180206P-1-LD180201-0016_HK72WCCXY_L1_clean_bin.48.orig.fa_k	OEZ008529	yes	LMSG_G000010921.1	OES073711	thiopeptide	RiPPs	Lead-Zinc	34.34	China: Hunan, Shuikoushan
L180206P-1-LD180201-0016_HK72WCCXY_L1_clean_bin.49.orig.fa_k	OEZ008529	no	LMSG_G000004824.1	OES073711	terpene	Terpene	Lead-Zinc	12.43	China: Hunan, Shuikoushan
L180206P-1-LD180201-0016_HK72WCCXY_L1_clean_bin.49.orig.fa_k	OEZ008529	yes	LMSG_G000004824.1	OES073711	acyl_amino_acids	Others	Lead-Zinc	30.70	China: Hunan, Shuikoushan
L180206P-1-LD180201-0016_HK72WCCXY_L1_clean_bin.49.orig.fa_k	OEZ008529	yes	LMSG_G000004824.1	OES073711	terpene	Terpene	Lead-Zinc	20.85	China: Hunan, Shuikoushan
L180206P-1-LD180201-0016_HK72WCCXY_L1_clean_bin.49.orig.fa_k	OEZ008529	yes	LMSG_G000004824.1	OES073711	bacteriocin	RiPPs	Lead-Zinc	10.74	China: Hunan, Shuikoushan
L180206P-1-LD180201-0016_HK72WCCXY_L1_clean_bin.49.orig.fa_k	OEZ008529	yes	LMSG_G000004824.1	OES073711	bacteriocin	RiPPs	Lead-Zinc	8.33	China: Hunan, Shuikoushan
L180206P-1-LD180201-0016_HK72WCCXY_L1_clean_bin.49.orig.fa_k	OEZ008529	yes	LMSG_G000004824.1	OES073711	arylpolyene	Others	Lead-Zinc	7.37	China: Hunan, Shuikoushan
L180206P-1-LD180201-0016_HK72WCCXY_L1_clean_bin.5.strict.fa_	OEZ008529	no	LMSG_G000010645.1	OES073711	terpene	Terpene	Lead-Zinc	25.95	China: Hunan, Shuikoushan
L180206P-1-LD180201-0016_HK72WCCXY_L1_clean_bin.5.strict.fa_	OEZ008529	no	LMSG_G000010645.1	OES073711	bacteriocin	RiPPs	Lead-Zinc	11.01	China: Hunan, Shuikoushan
L180206P-1-LD180201-0016_HK72WCCXY_L1_clean_bin.53.orig.fa_k	OEZ008529	no	LMSG_G000004825.1	OES073711	arylpolyene	Others	Lead-Zinc	26.61	China: Hunan, Shuikoushan
L180206P-1-LD180201-0016_HK72WCCXY_L1_clean_bin.53.orig.fa_k	OEZ008529	no	LMSG_G000004825.1	OES073711	siderophore	Others	Lead-Zinc	8.65	China: Hunan, Shuikoushan
L180206P-1-LD180201-0016_HK72WCCXY_L1_clean_bin.53.orig.fa_k	OEZ008529	no	LMSG_G000004825.1	OES073711	bacteriocin	RiPPs	Lead-Zinc	10.87	China: Hunan, Shuikoushan
L180206P-1-LD180201-0016_HK72WCCXY_L1_clean_bin.54.orig.fa_k	OEZ008529	yes	LMSG_G000004826.1	OES073711	lassopeptide	RiPPs	Lead-Zinc	15.22	China: Hunan, Shuikoushan
L180206P-1-LD180201-0016_HK72WCCXY_L1_clean_bin.54.orig.fa_k	OEZ008529	yes	LMSG_G000004826.1	OES073711	bacteriocin	RiPPs	Lead-Zinc	10.90	China: Hunan, Shuikoushan
L180206P-1-LD180201-0016_HK72WCCXY_L1_clean_bin.54.orig.fa_k	OEZ008529	yes	LMSG_G000004826.1	OES073711	arylpolyene	Others	Lead-Zinc	37.55	China: Hunan, Shuikoushan
L180206P-1-LD180201-0016_HK72WCCXY_L1_clean_bin.54.orig.fa_k	OEZ008529	yes	LMSG_G000004826.1	OES073711	NRPS-like	NRPS	Lead-Zinc	27.05	China: Hunan, Shuikoushan
L180206P-1-LD180201-0016_HK72WCCXY_L1_clean_bin.54.orig.fa_k	OEZ008529	yes	LMSG_G000004826.1	OES073711	terpene	Terpene	Lead-Zinc	22.64	China: Hunan, Shuikoushan
L180206P-1-LD180201-0016_HK72WCCXY_L1_clean_bin.55.orig.fa_k	OEZ008529	yes	LMSG_G000004827.1	OES073711	terpene	Terpene	Lead-Zinc	15.54	China: Hunan, Shuikoushan
L180206P-1-LD180201-0016_HK72WCCXY_L1_clean_bin.55.orig.fa_k	OEZ008529	yes	LMSG_G000004827.1	OES073711	NRPS-like	NRPS	Lead-Zinc	25.48	China: Hunan, Shuikoushan
L180206P-1-LD180201-0016_HK72WCCXY_L1_clean_bin.55.orig.fa_k	OEZ008529	yes	LMSG_G000004827.1	OES073711	bacteriocin	RiPPs	Lead-Zinc	10.13	China: Hunan, Shuikoushan
L180206P-1-LD180201-0016_HK72WCCXY_L1_clean_bin.55.orig.fa_k	OEZ008529	yes	LMSG_G000004827.1	OES073711	arylpolyene	Others	Lead-Zinc	41.24	China: Hunan, Shuikoushan
L180206P-1-LD180201-0016_HK72WCCXY_L1_clean_bin.55.orig.fa_k	OEZ008529	yes	LMSG_G000004827.1	OES073711	betalactone	Others	Lead-Zinc	22.37	China: Hunan, Shuikoushan
L180206P-1-LD180201-0016_HK72WCCXY_L1_clean_bin.56.strict.fa	OEZ008529	yes	LMSG_G000010961.1	OES073711	NRPS-like	NRPS	Lead-Zinc	32.62	China: Hunan, Shuikoushan
L180206P-1-LD180201-0016_HK72WCCXY_L1_clean_bin.56.strict.fa	OEZ008529	yes	LMSG_G000010961.1	OES073711	betalactone	Others	Lead-Zinc	23.03	China: Hunan, Shuikoushan
L180206P-1-LD180201-0016_HK72WCCXY_L1_clean_bin.56.strict.fa	OEZ008529	yes	LMSG_G000010961.1	OES073711	siderophore	Others	Lead-Zinc	11.74	China: Hunan, Shuikoushan
L180206P-1-LD180201-0016_HK72WCCXY_L1_clean_bin.56.strict.fa	OEZ008529	yes	LMSG_G000010961.1	OES073711	NRPS-like	NRPS	Lead-Zinc	35.62	China: Hunan, Shuikoushan
L180206P-1-LD180201-0016_HK72WCCXY_L1_clean_bin.56.strict.fa	OEZ008529	yes	LMSG_G000010961.1	OES073711	terpene	Terpene	Lead-Zinc	23.97	China: Hunan, Shuikoushan
L180206P-1-LD180201-0016_HK72WCCXY_L1_clean_bin.56.strict.fa	OEZ008529	yes	LMSG_G000010961.1	OES073711	bacteriocin	RiPPs	Lead-Zinc	5.64	China: Hunan, Shuikoushan
L180206P-1-LD180201-0016_HK72WCCXY_L1_clean_bin.56.strict.fa	OEZ008529	yes	LMSG_G000010961.1	OES073711	NRPS-like	NRPS	Lead-Zinc	26.25	China: Hunan, Shuikoushan
L180206P-1-LD180201-0016_HK72WCCXY_L1_clean_bin.56.strict.fa	OEZ008529	yes	LMSG_G000010961.1	OES073711	NRPS.hglE-KS	PKS-NRP_Hybrids	Lead-Zinc	25.85	China: Hunan, Shuikoushan
L180206P-1-LD180201-0016_HK72WCCXY_L1_clean_bin.56.strict.fa	OEZ008529	yes	LMSG_G000010961.1	OES073711	NRPS	NRPS	Lead-Zinc	24.03	China: Hunan, Shuikoushan
L180206P-1-LD180201-0016_HK72WCCXY_L1_clean_bin.56.strict.fa	OEZ008529	yes	LMSG_G000010961.1	OES073711	NRPS-like	NRPS	Lead-Zinc	21.09	China: Hunan, Shuikoushan
L180206P-1-LD180201-0016_HK72WCCXY_L1_clean_bin.56.strict.fa	OEZ008529	yes	LMSG_G000010961.1	OES073711	NRPS-like	NRPS	Lead-Zinc	18.83	China: Hunan, Shuikoushan
L180206P-1-LD180201-0016_HK72WCCXY_L1_clean_bin.56.strict.fa	OEZ008529	yes	LMSG_G000010961.1	OES073711	T3PKS	PKSother	Lead-Zinc	18.68	China: Hunan, Shuikoushan
L180206P-1-LD180201-0016_HK72WCCXY_L1_clean_bin.56.strict.fa	OEZ008529	yes	LMSG_G000010961.1	OES073711	NRPS-like	NRPS	Lead-Zinc	17.94	China: Hunan, Shuikoushan
L180206P-1-LD180201-0016_HK72WCCXY_L1_clean_bin.56.strict.fa	OEZ008529	yes	LMSG_G000010961.1	OES073711	NRPS	NRPS	Lead-Zinc	11.96	China: Hunan, Shuikoushan
L180206P-1-LD180201-0016_HK72WCCXY_L1_clean_bin.56.strict.fa	OEZ008529	yes	LMSG_G000010961.1	OES073711	bacteriocin	RiPPs	Lead-Zinc	5.19	China: Hunan, Shuikoushan
L180206P-1-LD180201-0016_HK72WCCXY_L1_clean_bin.57.strict.fa	OEZ008529	yes	LMSG_G000004828.1	OES073711	NRPS-like	NRPS	Lead-Zinc	7.81	China: Hunan, Shuikoushan
L180206P-1-LD180201-0016_HK72WCCXY_L1_clean_bin.59.strict.fa	OEZ008529	yes	LMSG_G000008785.1	OES073711	bacteriocin	RiPPs	Lead-Zinc	12.21	China: Hunan, Shuikoushan
L180206P-1-LD180201-0016_HK72WCCXY_L1_clean_bin.59.strict.fa	OEZ008529	yes	LMSG_G000008785.1	OES073711	resorcinol	Others	Lead-Zinc	29.05	China: Hunan, Shuikoushan
L180206P-1-LD180201-0016_HK72WCCXY_L1_clean_bin.59.strict.fa	OEZ008529	yes	LMSG_G000008785.1	OES073711	NRPS-like	NRPS	Lead-Zinc	15.58	China: Hunan, Shuikoushan
L180206P-1-LD180201-0016_HK72WCCXY_L1_clean_bin.59.strict.fa	OEZ008529	yes	LMSG_G000008785.1	OES073711	resorcinol	Others	Lead-Zinc	13.06	China: Hunan, Shuikoushan
L180206P-1-LD180201-0016_HK72WCCXY_L1_clean_bin.59.strict.fa	OEZ008529	yes	LMSG_G000008785.1	OES073711	NRPS-like	NRPS	Lead-Zinc	7.60	China: Hunan, Shuikoushan
L180206P-1-LD180201-0016_HK72WCCXY_L1_clean_bin.59.strict.fa	OEZ008529	yes	LMSG_G000008785.1	OES073711	resorcinol	Others	Lead-Zinc	6.89	China: Hunan, Shuikoushan
L180206P-1-LD180201-0016_HK72WCCXY_L1_clean_bin.6.orig.fa_kl	OEZ008529	yes	LMSG_G000004833.1	OES073711	bacteriocin	RiPPs	Lead-Zinc	10.81	China: Hunan, Shuikoushan
L180206P-1-LD180201-0016_HK72WCCXY_L1_clean_bin.60.strict.fa	OEZ008529	yes	LMSG_G000008189.1	OES073711	terpene	Terpene	Lead-Zinc	20.86	China: Hunan, Shuikoushan
L180206P-1-LD180201-0016_HK72WCCXY_L1_clean_bin.61.permissiv	OEZ008529	yes	LMSG_G000009771.1	OES073711	LAP	RiPPs	Lead-Zinc	11.48	China: Hunan, Shuikoushan
L180206P-1-LD180201-0016_HK72WCCXY_L1_clean_bin.62.orig.fa_k	OEZ008529	yes	LMSG_G000004829.1	OES073711	NRPS-like	NRPS	Lead-Zinc	42.61	China: Hunan, Shuikoushan
L180206P-1-LD180201-0016_HK72WCCXY_L1_clean_bin.63.strict.fa	OEZ008529	yes	LMSG_G000010960.1	OES073711	T3PKS	PKSother	Lead-Zinc	15.92	China: Hunan, Shuikoushan
L180206P-1-LD180201-0016_HK72WCCXY_L1_clean_bin.63.strict.fa	OEZ008529	yes	LMSG_G000010960.1	OES073711	NRPS-like	NRPS	Lead-Zinc	12.58	China: Hunan, Shuikoushan
L180206P-1-LD180201-0016_HK72WCCXY_L1_clean_bin.64.orig.fa_k	OEZ008529	no	LMSG_G000010150.1	OES073711	terpene	Terpene	Lead-Zinc	10.70	China: Hunan, Shuikoushan
L180206P-1-LD180201-0016_HK72WCCXY_L1_clean_bin.64.orig.fa_k	OEZ008529	no	LMSG_G000010150.1	OES073711	ectoine	Others	Lead-Zinc	10.40	China: Hunan, Shuikoushan

L180206P-1-LD180201-0016_HK72WCCXY_L1_clean_bin.64.orig.fa_k	OEZ008529	no	LMSG_G000010150.1	OES073711	terpene	Terpene	Lead-Zinc	21.71	China: Hunan, Shuikoushan
L180206P-1-LD180201-0016_HK72WCCXY_L1_clean_bin.65.orig.fa_k	OEZ008529	yes	LMSG_G000010608.1	OES073711	arylpolyene	Others	Lead-Zinc	27.22	China: Hunan, Shuikoushan
L180206P-1-LD180201-0016_HK72WCCXY_L1_clean_bin.65.orig.fa_k	OEZ008529	yes	LMSG_G000010608.1	OES073711	terpene	Terpene	Lead-Zinc	15.45	China: Hunan, Shuikoushan
L180206P-1-LD180201-0016_HK72WCCXY_L1_clean_bin.65.orig.fa_k	OEZ008529	yes	LMSG_G000010608.1	OES073711	hserlactone	Others	Lead-Zinc	17.10	China: Hunan, Shuikoushan
L180206P-1-LD180201-0016_HK72WCCXY_L1_clean_bin.66.orig.fa_k	OEZ008529	yes	LMSG_G000004830.1	OES073711	butyrolactone	Others	Lead-Zinc	9.91	China: Hunan, Shuikoushan
L180206P-1-LD180201-0016_HK72WCCXY_L1_clean_bin.66.orig.fa_k	OEZ008529	yes	LMSG_G000004830.1	OES073711	hserlactone	Others	Lead-Zinc	10.79	China: Hunan, Shuikoushan
L180206P-1-LD180201-0016_HK72WCCXY_L1_clean_bin.66.orig.fa_k	OEZ008529	yes	LMSG_G000004830.1	OES073711	bacteriocin	RiPPs	Lead-Zinc	6.67	China: Hunan, Shuikoushan
L180206P-1-LD180201-0016_HK72WCCXY_L1_clean_bin.67.strict.fa	OEZ008529	yes	LMSG_G000004831.1	OES073711	arylpolyene	Others	Lead-Zinc	22.68	China: Hunan, Shuikoushan
L180206P-1-LD180201-0016_HK72WCCXY_L1_clean_bin.67.strict.fa	OEZ008529	yes	LMSG_G000004831.1	OES073711	bacteriocin	RiPPs	Lead-Zinc	11.84	China: Hunan, Shuikoushan
L180206P-1-LD180201-0016_HK72WCCXY_L1_clean_bin.67.strict.fa	OEZ008529	yes	LMSG_G000004831.1	OES073711	resorcinol.arylpolyene	Others	Lead-Zinc	23.15	China: Hunan, Shuikoushan
L180206P-1-LD180201-0016_HK72WCCXY_L1_clean_bin.67.strict.fa	OEZ008529	yes	LMSG_G000004831.1	OES073711	arylpolyene	Others	Lead-Zinc	5.69	China: Hunan, Shuikoushan
L180206P-1-LD180201-0016_HK72WCCXY_L1_clean_bin.67.strict.fa	OEZ008529	yes	LMSG_G000004831.1	OES073711	resorcinol	Others	Lead-Zinc	5.51	China: Hunan, Shuikoushan
L180206P-1-LD180201-0016_HK72WCCXY_L1_clean_bin.68.orig.fa_k	OEZ008529	yes	LMSG_G000011100.1	OES073711	T3PKS	PKSother	Lead-Zinc	6.41	China: Hunan, Shuikoushan
L180206P-1-LD180201-0016_HK72WCCXY_L1_clean_bin.69.strict.fa	OEZ008529	yes	LMSG_G000004832.1	OES073711	NRPS-like	NRPS	Lead-Zinc	12.05	China: Hunan, Shuikoushan
L180206P-1-LD180201-0016_HK72WCCXY_L1_clean_bin.69.strict.fa	OEZ008529	yes	LMSG_G000004832.1	OES073711	terpene	Terpene	Lead-Zinc	10.40	China: Hunan, Shuikoushan
L180206P-1-LD180201-0016_HK72WCCXY_L1_clean_bin.70.strict.fa	OEZ008529	yes	LMSG_G000006264.1	OES073711	T1PKS.hglE-KS	PKSother	Lead-Zinc	32.71	China: Hunan, Shuikoushan
L180206P-1-LD180201-0016_HK72WCCXY_L1_clean_bin.70.strict.fa	OEZ008529	no	LMSG_G000006264.1	OES073711	terpene	Terpene	Lead-Zinc	19.63	China: Hunan, Shuikoushan
L180206P-1-LD180201-0016_HK72WCCXY_L1_clean_bin.71.orig.fa_k	OEZ008529	yes	LMSG_G000004834.1	OES073711	terpene	Terpene	Lead-Zinc	12.40	China: Hunan, Shuikoushan
L180206P-1-LD180201-0016_HK72WCCXY_L1_clean_bin.71.orig.fa_k	OEZ008529	yes	LMSG_G000004834.1	OES073711	hserlactone	Others	Lead-Zinc	20.63	China: Hunan, Shuikoushan
L180206P-1-LD180201-0016_HK72WCCXY_L1_clean_bin.71.orig.fa_k	OEZ008529	yes	LMSG_G000004834.1	OES073711	terpene	Terpene	Lead-Zinc	19.82	China: Hunan, Shuikoushan
L180206P-1-LD180201-0016_HK72WCCXY_L1_clean_bin.72.orig.fa_k	OEZ008529	yes	LMSG_G000004367.1	OES073711	ladderane	Others	Lead-Zinc	34.06	China: Hunan, Shuikoushan
L180206P-1-LD180201-0016_HK72WCCXY_L1_clean_bin.72.orig.fa_k	OEZ008529	yes	LMSG_G000004367.1	OES073711	terpene	Terpene	Lead-Zinc	20.79	China: Hunan, Shuikoushan
L180206P-1-LD180201-0016_HK72WCCXY_L1_clean_bin.72.orig.fa_k	OEZ008529	yes	LMSG_G000004367.1	OES073711	terpene	Terpene	Lead-Zinc	14.66	China: Hunan, Shuikoushan
L180206P-1-LD180201-0016_HK72WCCXY_L1_clean_bin.74.orig.fa_k	OEZ008529	yes	LMSG_G000010870.1	OES073711	terpene	Terpene	Lead-Zinc	21.65	China: Hunan, Shuikoushan
L180206P-1-LD180201-0016_HK72WCCXY_L1_clean_bin.74.orig.fa_k	OEZ008529	yes	LMSG_G000010870.1	OES073711	bacteriocin	RiPPs	Lead-Zinc	10.22	China: Hunan, Shuikoushan
L180206P-1-LD180201-0016_HK72WCCXY_L1_clean_bin.75.orig.fa_k	OEZ008529	yes	LMSG_G000010390.1	OES073711	terpene	Terpene	Lead-Zinc	21.77	China: Hunan, Shuikoushan
L180206P-1-LD180201-0016_HK72WCCXY_L1_clean_bin.75.orig.fa_k	OEZ008529	yes	LMSG_G000010390.1	OES073711	NRPS.T1PKS	PKS-NRP_Hybrids	Lead-Zinc	96.78	China: Hunan, Shuikoushan
L180206P-1-LD180201-0016_HK72WCCXY_L1_clean_bin.75.orig.fa_k	OEZ008529	yes	LMSG_G000010390.1	OES073711	NRPS-like	NRPS	Lead-Zinc	25.78	China: Hunan, Shuikoushan
L180206P-1-LD180201-0016_HK72WCCXY_L1_clean_bin.75.orig.fa_k	OEZ008529	yes	LMSG_G000010390.1	OES073711	cyanobactin	RiPPs	Lead-Zinc	12.50	China: Hunan, Shuikoushan
L180206P-1-LD180201-0016_HK72WCCXY_L1_clean_bin.75.orig.fa_k	OEZ008529	yes	LMSG_G000010390.1	OES073711	bacteriocin	RiPPs	Lead-Zinc	10.89	China: Hunan, Shuikoushan
L180206P-1-LD180201-0016_HK72WCCXY_L1_clean_bin.75.orig.fa_k	OEZ008529	yes	LMSG_G000010390.1	OES073711	terpene	Terpene	Lead-Zinc	16.17	China: Hunan, Shuikoushan
L180206P-1-LD180201-0016_HK72WCCXY_L1_clean_bin.75.orig.fa_k	OEZ008529	yes	LMSG_G000010390.1	OES073711	ectoine	Others	Lead-Zinc	10.39	China: Hunan, Shuikoushan
L180206P-1-LD180201-0016_HK72WCCXY_L1_clean_bin.78.orig.fa_k	OEZ008529	yes	LMSG_G000004368.1	OES073711	T3PKS	PKSother	Lead-Zinc	25.30	China: Hunan, Shuikoushan
L180206P-1-LD180201-0016_HK72WCCXY_L1_clean_bin.78.orig.fa_k	OEZ008529	yes	LMSG_G000004368.1	OES073711	bacteriocin	RiPPs	Lead-Zinc	9.29	China: Hunan, Shuikoushan
L180206P-1-LD180201-0016_HK72WCCXY_L1_clean_bin.78.orig.fa_k	OEZ008529	yes	LMSG_G000004368.1	OES073711	terpene	Terpene	Lead-Zinc	29.81	China: Hunan, Shuikoushan
L180206P-1-LD180201-0016_HK72WCCXY_L1_clean_bin.79.strict.fa	OEZ008529	yes	LMSG_G000008916.1	OES073711	lassopeptide	RiPPs	Lead-Zinc	23.26	China: Hunan, Shuikoushan
L180206P-1-LD180201-0016_HK72WCCXY_L1_clean_bin.81.orig.fa_k	OEZ008529	yes	LMSG_G000004837.1	OES073711	NRPS	NRPS	Lead-Zinc	37.67	China: Hunan, Shuikoushan
L180206P-1-LD180201-0016_HK72WCCXY_L1_clean_bin.81.orig.fa_k	OEZ008529	yes	LMSG_G000004837.1	OES073711	butyrolactone	Others	Lead-Zinc	10.73	China: Hunan, Shuikoushan
L180206P-1-LD180201-0016_HK72WCCXY_L1_clean_bin.81.orig.fa_k	OEZ008529	yes	LMSG_G000004837.1	OES073711	bacteriocin	RiPPs	Lead-Zinc	6.11	China: Hunan, Shuikoushan
L180206P-1-LD180201-0016_HK72WCCXY_L1_clean_bin.81.orig.fa_k	OEZ008529	yes	LMSG_G000004837.1	OES073711	NRPS	NRPS	Lead-Zinc	12.48	China: Hunan, Shuikoushan
L180206P-1-LD180201-0016_HK72WCCXY_L1_clean_bin.81.orig.fa_k	OEZ008529	yes	LMSG_G000004837.1	OES073711	NRPS	NRPS	Lead-Zinc	34.84	China: Hunan, Shuikoushan
L180206P-1-LD180201-0016_HK72WCCXY_L1_clean_bin.81.orig.fa_k	OEZ008529	yes	LMSG_G000004837.1	OES073711	NRPS	NRPS	Lead-Zinc	14.23	China: Hunan, Shuikoushan
L180206P-1-LD180201-0016_HK72WCCXY_L1_clean_bin.81.orig.fa_k	OEZ008529	yes	LMSG_G000004837.1	OES073711	NRPS	NRPS	Lead-Zinc	44.29	China: Hunan, Shuikoushan
L180206P-1-LD180201-0016_HK72WCCXY_L1_clean_bin.81.orig.fa_k	OEZ008529	yes	LMSG_G000004837.1	OES073711	NRPS-like	NRPS	Lead-Zinc	21.21	China: Hunan, Shuikoushan
L180206P-1-LD180201-0016_HK72WCCXY_L1_clean_bin.81.orig.fa_k	OEZ008529	yes	LMSG_G000004837.1	OES073711	NRPS	NRPS	Lead-Zinc	21.27	China: Hunan, Shuikoushan
L180206P-1-LD180201-0016_HK72WCCXY_L1_clean_bin.81.orig.fa_k	OEZ008529	yes	LMSG_G000004837.1	OES073711	NRPS.butyrolactone	Others	Lead-Zinc	43.13	China: Hunan, Shuikoushan
L180206P-1-LD180201-0016_HK72WCCXY_L1_clean_bin.81.orig.fa_k	OEZ008529	yes	LMSG_G000004837.1	OES073711	NRPS	NRPS	Lead-Zinc	22.83	China: Hunan, Shuikoushan
L180206P-1-LD180201-0016_HK72WCCXY_L1_clean_bin.81.orig.fa_k	OEZ008529	yes	LMSG_G000004837.1	OES073711	phosphonate	Others	Lead-Zinc	11.72	China: Hunan, Shuikoushan
L180206P-1-LD180201-0016_HK72WCCXY_L1_clean_bin.82.strict.fa	OEZ008529	yes	LMSG_G000009335.1	OES073711	betalactone	Others	Lead-Zinc	26.16	China: Hunan, Shuikoushan
L180206P-1-LD180201-0016_HK72WCCXY_L1_clean_bin.82.strict.fa	OEZ008529	yes	LMSG_G000009335.1	OES073711	betalactone	Others	Lead-Zinc	15.07	China: Hunan, Shuikoushan
L180206P-1-LD180201-0016_HK72WCCXY_L1_clean_bin.82.strict.fa	OEZ008529	yes	LMSG_G000009335.1	OES073711	betalactone	Others	Lead-Zinc	19.59	China: Hunan, Shuikoushan
L180206P-1-LD180201-0016_HK72WCCXY_L1_clean_bin.84.orig.fa_k	OEZ008529	yes	LMSG_G000010419.1	OES073711	terpene	Terpene	Lead-Zinc	20.62	China: Hunan, Shuikoushan
L180206P-1-LD180201-0016_HK72WCCXY_L1_clean_bin.84.orig.fa_k	OEZ008529	yes	LMSG_G000010419.1	OES073711	terpene	Terpene	Lead-Zinc	25.02	China: Hunan, Shuikoushan
L180206P-1-LD180201-0016_HK72WCCXY_L1_clean_bin.85.orig.fa_k	OEZ008529	yes	LMSG_G000010404.1	OES073711	hserlactone	Others	Lead-Zinc	15.62	China: Hunan, Shuikoushan
L180206P-1-LD180201-0016_HK72WCCXY_L1_clean_bin.85.orig.fa_k	OEZ008529	no	LMSG_G000010404.1	OES073711	lassopeptide	RiPPs	Lead-Zinc	12.02	China: Hunan, Shuikoushan
L180206P-1-LD180201-0016_HK72WCCXY_L1_clean_bin.85.orig.fa_k	OEZ008529	yes	LMSG_G000010404.1	OES073711	terpene	Terpene	Lead-Zinc	24.24	China: Hunan, Shuikoushan
L180206P-1-LD180201-0016_HK72WCCXY_L1_clean_bin.87.orig.fa_k	OEZ008529	yes	LMSG_G000004839.1	OES073711	bacteriocin	RiPPs	Lead-Zinc	10.22	China: Hunan, Shuikoushan
L180206P-1-LD180201-0016_HK72WCCXY_L1_clean_bin.87.orig.fa_k	OEZ008529	no	LMSG_G000004839.1	OES073711	terpene	Terpene	Lead-Zinc	9.67	China: Hunan, Shuikoushan
L180206P-1-LD180201-0016_HK72WCCXY_L1_clean_bin.87.orig.fa_k	OEZ008529	yes	LMSG_G000004839.1	OES073711	bacteriocin	RiPPs	Lead-Zinc	8.80	China: Hunan, Shuikoushan
L180206P-1-LD180201-0016_HK72WCCXY_L1_clean_bin.88.orig.fa_k	OEZ008529	yes	LMSG_G000004840.1	OES073711	NRPS	NRPS	Lead-Zinc	20.43	China: Hunan, Shuikoushan
L180206P-1-LD180201-0016_HK72WCCXY_L1_clean_bin.88.orig.fa_k	OEZ008529	yes	LMSG_G000004840.1	OES073711	NRPS	NRPS	Lead-Zinc	5.79	China: Hunan, Shuikoushan
L180206P-1-LD180201-0016_HK72WCCXY_L1_clean_bin.88.orig.fa_k	OEZ008529	yes	LMSG_G000004840.1	OES073711	terpene	Terpene	Lead-Zinc	6.06	China: Hunan, Shuikoushan
L180206P-1-LD180201-0016_HK72WCCXY_L1_clean_bin.88.orig.fa_k	OEZ008529	yes	LMSG_G000004840.1	OES073711	hserlactone	Others	Lead-Zinc	19.77	China: Hunan, Shuikoushan
L180206P-1-LD180201-0016_HK72WCCXY_L1_clean_bin.88.orig.fa_k	OEZ008529	yes	LMSG_G000004840.1	OES073711	bacteriocin	RiPPs	Lead-Zinc	8.36	China: Hunan, Shuikoushan
L180206P-1-LD180201-0016_HK72WCCXY_L1_clean_bin.88.orig.fa_k	OEZ008529	yes	LMSG_G000004840.1	OES073711	NRPS-like	NRPS	Lead-Zinc	22.67	China: Hunan, Shuikoushan
L180206P-1-LD180201-0016_HK72WCCXY_L1_clean_bin.91.orig.fa_k	OEZ008529	yes	LMSG_G000004370.1	OES073711	hserlactone	Others	Lead-Zinc	12.43	China: Hunan, Shuikoushan
L180206P-1-LD180201-0016_HK72WCCXY_L1_clean_bin.91.orig.fa_k	OEZ008529	yes	LMSG_G000004370.1	OES073711	terpene	Terpene	Lead-Zinc	12.19	China: Hunan, Shuikoushan
L180206P-1-LD180201-0016_HK72WCCXY_L1_clean_bin.91.orig.fa_k	OEZ008529	yes	LMSG_G000004370.1	OES073711	T1PKS.hglE-KS	PKSother	Lead-Zinc	25.32	China: Hunan, Shuikoushan
L180206P-1-LD180201-0016_HK72WCCXY_L1_clean_bin.91.orig.fa_k	OEZ008529	yes	LMSG_G000004370.1	OES073711	NRPS-like	NRPS	Lead-Zinc	25.80	China: Hunan, Shuikoushan
L180206P-1-LD180201-0016_HK72WCCXY_L1_clean_bin.94.orig.fa_k	OEZ008529	yes	LMSG_G000006211.1	OES073711	T3PKS	PKSother	Lead-Zinc	33.11	China: Hunan, Shuikoushan
L180206P-1-LD180201-0016_HK72WCCXY_L1_clean_bin.94.orig.fa_k	OEZ008529	yes	LMSG_G000006211.1	OES073711	bacteriocin	RiPPs	Lead-Zinc	10.83	China: Hunan, Shuikoushan
L180206P-1-LD180201-0016_HK72WCCXY_L1_clean_bin.94.orig.fa_k	OEZ008529	yes	LMSG_G000006211.1	OES073711	terpene	Terpene	Lead-Zinc	23.17	China: Hunan, Shuikoushan
L180206P-1-LD180201-0016_HK72WCCXY_L1_clean_bin.95.strict.fa	OEZ008529	yes	LMSG_G000004841.1	OES073711	sactipeptide	RiPPs	Lead-Zinc	8.31	China: Hunan, Shuikoushan
L180206P-1-LD180201-0016_HK72WCCXY_L1_clean_bin.96.orig.fa_k	OEZ008529	no	LMSG_G000006265.1	OES073711	NRPS	NRPS	Lead-Zinc	6.55	China: Hunan, Shuikoushan
L180206P-1-LD180201-0016_HK72WCCXY_L1_clean_bin.96.orig.fa_k	OEZ008529	yes	LMSG_G000006265.1	OES073711	NRPS-like	NRPS	Lead-Zinc	21.19	China: Hunan, Shuikoushan
L180206P-1-LD180201-0016_HK72WCCXY_L1_clean_bin.98.strict.fa	OEZ008529	yes	LMSG_G000010508.1	OES073711	NRPS-like	NRPS	Lead-Zinc	42.86	China: Hunan, Shuikoushan
L180206P-1-LD180201-0016_HK72WCCXY_L1_clean_bin.98.strict.fa	OEZ008529	yes	LMSG_G000010508.1	OES073711	arylpolyene	Others	Lead-Zinc	21.85	China: Hunan, Shuikoushan
L180206P-1-LD180201-0016_HK72WCCXY_L1_clean_bin.98.strict.fa	OEZ008529	yes	LMSG_G000010508.1	OES073711	acyl_amino_acids	Others	Lead-Zinc	24.10	China: Hunan, Shuikoushan
L180206P-2-LD180205-0002_HK72WCCXY_L2_clean_bin.1.strict.fa_	OEZ008529	yes	LMSG_G000010816.1	OES073745	acyl_amino_acids	Others	Lead-Zinc	28.72	China: Jiangxi, Yinshan
L180206P-2-LD180205-0002_HK72WCCXY_L2_clean_bin.1.strict.fa_	OEZ008529	yes	LMSG_G000010816.1	OES073745	acyl_amino_acids	Others	Lead-Zinc	14.50	China: Jiangxi, Yinshan
L180206P-2-LD180205-0002_HK72WCCXY_L2_clean_bin.10.orig.fa_k	OEZ008529	no	LMSG_G000010255.1	OES073745	terpene	Terpene	Lead-Zinc	21.53	China: Jiangxi, Yinshan
L180206P-2-LD180205-0002_HK72WCCXY_L2_clean_bin.10.orig.fa_k	OEZ008529	yes	LMSG_G000010255.1	OES073745	arylpolyene	Others	Lead-Zinc	35.73	China: Jiangxi, Yinshan
L180206P-2-LD180205-0002_HK72WCCXY_L2_clean_bin.12.orig.fa_k	OEZ008529	no	LMSG_G000010151.1	OES073745	terpene	Terpene	Lead-Zinc	16.50	China: Jiangxi, Yinshan
L180206P-2-LD180205-0002_HK72WCCXY_L2_clean_bin.12.orig.fa_k	OEZ008529	no	LMSG_G000010151.1	OES073745	ectoine	Others	Lead-Zinc	10.40	China: Jiangxi, Yinshan
L180206P-2-LD180205-0002_HK72WCCXY_L2_clean_bin.12.orig.fa_k	OEZ008529	no	LMSG_G000010151.1	OES073745	acyl_amino_acids	Others	Lead-Zinc	27.04	China: Jiangxi, Yinshan
L180206P-2-LD180205-0002_HK72WCCXY_L2_clean_bin.12.orig.fa_k	OEZ008529	no	LMSG_G000010151.1	OES073745	terpene	Terpene	Lead-Zinc	15.94	China: Jiangxi, Yinshan
L180206P-2-LD180205-0002_HK72WCCXY_L2_clean_bin.12.orig.fa_k	OEZ008529	no	LMSG_G000010151.1	OES073745	terpene	Terpene	Lead-Zinc	21.06	China: Jiangxi, Yinshan
L180206P-2-LD180205-0002_HK72WCCXY_L2_clean_bin.14.orig.fa_k	OEZ008529	yes	LMSG_G000008500.1	OES073745	terpene	Terpene	Lead-Zinc	24.03	China: Jiangxi, Yinshan
L180206P-2-LD180205-0002_HK72WCCXY_L2_clean_bin.14.orig.fa_k	OEZ008529	yes	LMSG_G000008500.1	OES073745	lassopeptide	RiPPs	Lead-Zinc	23.45	China: Jiangxi, Yinshan
L180206P-2-LD180205-0002_HK72WCCXY_L2_clean_bin.16.orig.fa_k	OEZ008529	yes	LMSG_G000010453.1	OES073745	terpene	Terpene	Lead-Zinc	23.04	China: Jiangxi, Yinshan
L180206P-2-LD180205-0002_HK72WCCXY_L2_clean_bin.16.orig.fa_k	OEZ008529	yes	LMSG_G000010453.1	OES073745	resorcinol.arylpolyene	Others	Lead-Zinc	38.50	China: Jiangxi, Yinshan
L180206P-2-LD180205-0002_HK72WCCXY_L2_clean_bin.16.orig.fa_k	OEZ008529	yes	LMSG_G000010453.1	OES073745	arylpolyene.hserlactone	Others	Lead-Zinc	30.27	China: Jiangxi, Yinshan
L180206P-2-LD180205-0002_HK72WCCXY_L2_clean_bin.18.orig.fa_k	OEZ008529	yes	LMSG_G000007807.1	OES073745	T3PKS	PKSother	Lead-Zinc	41.09	China: Jiangxi, Yinshan
L180206P-2-LD180205-0002_HK72WCCXY_L2_clean_bin.19.orig.fa_k	OEZ008529	yes	LMSG_G000009606.1	OES073745	terpene	Terpene	Lead-Zinc	12.93	China: Jiangxi, Yinshan
L180206P-2-LD180205-0002_HK72WCCXY_L2_clean_bin.19.orig.fa_k	OEZ008529	yes	LMSG_G000009606.1	OES073745	terpene	Terpene	Lead-Zinc	22.09	China: Jiangxi, Yinshan
L180206P-2-LD180205-0002_HK72WCCXY_L2_clean_bin.20.orig.fa_k	OEZ008529	yes	LMSG_G000010621.1	OES073745	terpene	Terpene	Lead-Zinc	20.85	China: Jiangxi, Yinshan
L180206P-2-LD180205-0002_HK72WCCXY_L2_clean_bin.20.orig.fa_k	OEZ008529	yes	LMSG_G000010621.1	OES073745	arylpolyene	Others	Lead-Zinc	32.16	China: Jiangxi, Yinshan
L180206P-2-LD180205-0002_HK72WCCXY_L2_clean_bin.20.orig.fa_k	OEZ008529	no	LMSG_G000010621.1	OES073745	terpene	Terpene	Lead-Zinc	18.33	China: Jiangxi, Yinshan
L180206P-2-LD180205-0002_HK72WCCXY_L2_clean_bin.20.orig.fa_k	OEZ008529	yes	LMSG_G000010621.1	OES073745	bacteriocin	RiPPs	Lead-Zinc	10.82	China: Jiangxi, Yinshan
L180206P-2-LD180205-0002_HK72WCCXY_L2_clean_bin.21.orig.fa_k	OEZ008529	no	LMSG_G000010290.1	OES073745	arylpolyene	Others	Lead-Zinc	32.25	China: Jiangxi, Yinshan
L180206P-2-LD180205-0002_HK72WCCXY_L2_clean_bin.21.orig.fa_k	OEZ008529	no	LMSG_G000010290.1	OES073745	terpene	Terpene	Lead-Zinc	18.49	China: Jiangxi, Yinshan
L180206P-2-LD180205-0002_HK72WCCXY_L2_clean_bin.24.orig.fa_k	OEZ008529	yes	LMSG_G000007828.1	OES073745	bacteriocin	RiPPs	Lead-Zinc	7.61	China: Jiangxi, Yinshan
L180206P-2-LD180205-0002_HK72WCCXY_L2_clean_bin.24.orig.fa_k	OEZ008529	yes	LMSG_G000007828.1	OES073745	NRPS-like	NRPS	Lead-Zinc	11.85	China: Jiangxi, Yinshan
L180206P-2-LD180205-0002_HK72WCCXY_L2_clean_bin.24.orig.fa_k	OEZ008529	yes	LMSG_G000007828.1	OES073745	T3PKS	PKSother	Lead-Zinc	22.10	China: Jiangxi, Yinshan
L180206P-2-LD180205-0002_HK72WCCXY_L2_clean_bin.24.orig.fa_k	OEZ008529	yes	LMSG_G000007828.1	OES073745	terpene	Terpene	Lead-Zinc	21.87	China: Jiangxi, Yinshan
L180206P-2-LD180205-0002_HK72WCCXY_L2_clean_bin.24.orig.fa_k	OEZ008529	yes	LMSG_G000007828.1	OES073745	terpene	Terpene	Lead-Zinc	19.25	China: Jiangxi, Yinshan
L180206P-2-LD180205-0002_HK72WCCXY_L2_clean_bin.25.orig.fa_k	OEZ008529	yes	LMSG_G000008218.1	OES073745	terpene	Terpene	Lead-Zinc	9.27	China: Jiangxi, Yinshan
L180206P-2-LD180205-0002_HK72WCCXY_L2_clean_bin.26.orig.fa_k	OEZ008529	yes	LMSG_G000010556.1	OES073745	arylpolyene	Others	Lead-Zinc	25.30	China: Jiangxi, Yinshan
L180206P-2-LD180205-0002_HK72WCCXY_L2_clean_bin.26.orig.fa_k	OEZ008529	yes	LMSG_G000010556.1	OES073745	bacteriocin	RiPPs	Lead-Zinc	6.80	China: Jiangxi, Yinshan
L180206P-2-LD180205-0002_HK72WCCXY_L2_clean_bin.26.orig.fa_k	OEZ008529	yes	LMSG_G000010556.1	OES073745	terpene	Terpene	Lead-Zinc	7.59	China: Jiangxi, Yinshan
L180206P-2-LD180205-0002_HK72WCCXY_L2_clean_bin.27.orig.fa_k	OEZ008529	yes	LMSG_G000008260.1	OES073745	terpene	Terpene	Lead-Zinc	19.12	China: Jiangxi, Yinshan
L180206P-2-LD180205-0002_HK72WCCXY_L2_clean_bin.29.orig.fa_k	OEZ008529	yes	LMSG_G000009638.1	OES073745	terpene	Terpene	Lead-Zinc	12.17	China: Jiangxi, Yinshan
L180206P-2-LD180205-0002_HK72WCCXY_L2_clean_bin.29.orig.fa_k	OEZ008529	yes	LMSG_G000009638.1	OES073745	terpene	Terpene	Lead-Zinc	8.16	China: Jiangxi, Yinshan
L180206P-2-LD180205-0002_HK72WCCXY_L2_clean_bin.29.orig.fa_k	OEZ008529	yes	LMSG_G000009638.1	OES073745	NRPS-like	NRPS	Lead-Zinc	5.59	China: Jiangxi, Yinshan
L180206P-2-LD180205-0002_HK72WCCXY_L2_clean_bin.3.orig.fa_k1	OEZ008529	yes	LMSG_G000008708.1	OES073745	terpene	Terpene	Lead-Zinc	10.93	China: Jiangxi, Yinshan
L180206P-2-LD180205-0002_HK72WCCXY_L2_clean_bin.3.orig.fa_k1	OEZ008529	yes	LMSG_G000008708.1	OES073745	betalactone	Others	Lead-Zinc	16.47	China: Jiangxi, Yinshan
L180206P-2-LD180205-0002_HK72WCCXY_L2_clean_bin.3.orig.fa_k1	OEZ008529	yes	LMSG_G000008708.1	OES073745	bacteriocin	RiPPs	Lead-Zinc	6.10	China: Jiangxi, Yinshan
L180206P-2-LD180205-0002_HK72WCCXY_L2_clean_bin.3.orig.fa_k1	OEZ008529	yes	LMSG_G000008708.1	OES073745	terpene	Terpene	Lead-Zinc	10.44	China: Jiangxi, Yinshan
L180206P-2-LD180205-0002_HK72WCCXY_L2_clean_bin.30.orig.fa_k	OEZ008529	no	LMSG_G000011015.1	OES073745	NRPS	NRPS	Lead-Zinc	25.01	China: Jiangxi, Yinshan
L180206P-2-LD180205-0002_HK72WCCXY_L2_clean_bin.30.orig.fa_k	OEZ008529	no	LMSG_G000011015.1	OES073745	NRPS	NRPS	Lead-Zinc	42.73	China: Jiangxi, Yinshan
L180206P-2-LD180205-0002_HK72WCCXY_L2_clean_bin.30.orig.fa_k	OEZ008529	no	LMSG_G000011015.1	OES073745	NRPS	NRPS	Lead-Zinc	62.35	China: Jiangxi, Yinshan
L180206P-2-LD180205-0002_HK72WCCXY_L2_clean_bin.30.orig.fa_k	OEZ008529	no	LMSG_G000011015.1	OES073745	hserlactone	Others	Lead-Zinc	20.61	China: Jiangxi, Yinshan
L180206P-2-LD180205-0002_HK72WCCXY_L2_clean_bin.30.orig.fa_k	OEZ008529	no	LMSG_G000011015.1	OES073745	thiopeptide	RiPPs	Lead-Zinc	33.00	China: Jiangxi, Yinshan
L180206P-2-LD180205-0002_HK72WCCXY_L2_clean_bin.30.orig.fa_k	OEZ008529	no	LMSG_G000011015.1	OES073745	phenazine	Others	Lead-Zinc	10.23	China: Jiangxi, Yinshan
L180206P-2-LD180205-0002_HK72WCCXY_L2_clean_bin.30.orig.fa_k	OEZ008529	no	LMSG_G000011015.1	OES073745	betalactone.NRPS-like	Others	Lead-Zinc	41.82	China: Jiangxi, Yinshan
L180206P-2-LD180205-0002_HK72WCCXY_L2_clean_bin.30.orig.fa_k	OEZ008529	no	LMSG_G000011015.1	OES073745	NRPS	NRPS	Lead-Zinc	33.14	China: Jiangxi, Yinshan
L180206P-2-LD180205-0002_HK72WCCXY_L2_clean_bin.30.orig.fa_k	OEZ008529	no	LMSG_G000011015.1	OES073745	hserlactone	Others	Lead-Zinc	20.61	China: Jiangxi, Yinshan
L180206P-2-LD180205-0002_HK72WCCXY_L2_clean_bin.30.orig.fa_k	OEZ008529	no	LMSG_G000011015.1	OES073745	NAGGN	Others	Lead-Zinc	14.76	China: Jiangxi, Yinshan
L180206P-2-LD180205-0002_HK72WCCXY_L2_clean_bin.30.orig.fa_k	OEZ008529	no	LMSG_G000011015.1	OES073745	bacteriocin	RiPPs	Lead-Zinc	10.86	China: Jiangxi, Yinshan
L180206P-2-LD180205-0002_HK72WCCXY_L2_clean_bin.30.orig.fa_k	OEZ008529	no	LMSG_G000011015.1	OES073745	NRPS	NRPS	Lead-Zinc	49.55	China: Jiangxi, Yinshan
L180206P-2-LD180205-0002_HK72WCCXY_L2_clean_bin.30.orig.fa_k	OEZ008529	no	LMSG_G000011015.1	OES073745	phenazine	Others	Lead-Zinc	10.23	China: Jiangxi, Yinshan
L180206P-2-LD180205-0002_HK72WCCXY_L2_clean_bin.30.orig.fa_k	OEZ008529	no	LMSG_G000011015.1	OES073745	bacteriocin	RiPPs	Lead-Zinc	10.83	China: Jiangxi, Yinshan
L180206P-2-LD180205-0002_HK72WCCXY_L2_clean_bin.30.orig.fa_k	OEZ008529	no	LMSG_G000011015.1	OES073745	NRPS-like	NRPS	Lead-Zinc	42.98	China: Jiangxi, Yinshan
L180206P-2-LD180205-0002_HK72WCCXY_L2_clean_bin.32.orig.fa_k	OEZ008529	yes	LMSG_G000004845.1	OES073745	NRPS	NRPS	Lead-Zinc	7.23	China: Jiangxi, Yinshan
L180206P-2-LD180205-0002_HK72WCCXY_L2_clean_bin.34.strict.fa	OEZ008529	yes	LMSG_G000008306.1	OES073745	NRPS-like	NRPS	Lead-Zinc	37.99	China: Jiangxi, Yinshan
L180206P-2-LD180205-0002_HK72WCCXY_L2_clean_bin.34.strict.fa	OEZ008529	yes	LMSG_G000008306.1	OES073745	terpene	Terpene	Lead-Zinc	15.96	China: Jiangxi, Yinshan
L180206P-2-LD180205-0002_HK72WCCXY_L2_clean_bin.35.orig.fa_k	OEZ008529	yes	LMSG_G000008347.1	OES073745	bacteriocin	RiPPs	Lead-Zinc	6.58	China: Jiangxi, Yinshan
L180206P-2-LD180205-0002_HK72WCCXY_L2_clean_bin.35.orig.fa_k	OEZ008529	yes	LMSG_G000008347.1	OES073745	terpene	Terpene	Lead-Zinc	5.80	China: Jiangxi, Yinshan
L180206P-2-LD180205-0002_HK72WCCXY_L2_clean_bin.35.orig.fa_k	OEZ008529	yes	LMSG_G000008347.1	OES073745	terpene	Terpene	Lead-Zinc	12.17	China: Jiangxi, Yinshan
L180206P-2-LD180205-0002_HK72WCCXY_L2_clean_bin.37.orig.fa_k	OEZ008529	yes	LMSG_G000004847.1	OES073745	T1PKS	PKSI	Lead-Zinc	12.37	China: Jiangxi, Yinshan
L180206P-2-LD180205-0002_HK72WCCXY_L2_clean_bin.37.orig.fa_k	OEZ008529	yes	LMSG_G000004847.1	OES073745	NRPS-like	NRPS	Lead-Zinc	9.60	China: Jiangxi, Yinshan
L180206P-2-LD180205-0002_HK72WCCXY_L2_clean_bin.38.strict.fa	OEZ008529	yes	LMSG_G000010808.1	OES073745	acyl_amino_acids	Others	Lead-Zinc	30.86	China: Jiangxi, Yinshan
L180206P-2-LD180205-0002_HK72WCCXY_L2_clean_bin.39.orig.fa_k	OEZ008529	yes	LMSG_G000004848.1	OES073745	NRPS-like	NRPS	Lead-Zinc	25.91	China: Jiangxi, Yinshan
L180206P-2-LD180205-0002_HK72WCCXY_L2_clean_bin.39.orig.fa_k	OEZ008529	yes	LMSG_G000004848.1	OES073745	betalactone	Others	Lead-Zinc	18.75	China: Jiangxi, Yinshan
L180206P-2-LD180205-0002_HK72WCCXY_L2_clean_bin.39.orig.fa_k	OEZ008529	yes	LMSG_G000004848.1	OES073745	NRPS-like	NRPS	Lead-Zinc	26.11	China: Jiangxi, Yinshan
L180206P-2-LD180205-0002_HK72WCCXY_L2_clean_bin.4.permissive	OEZ008529	yes	LMSG_G000009984.1	OES073745	NRPS-like	NRPS	Lead-Zinc	12.16	China: Jiangxi, Yinshan
L180206P-2-LD180205-0002_HK72WCCXY_L2_clean_bin.4.permissive	OEZ008529	yes	LMSG_G000009984.1	OES073745	ectoine	Others	Lead-Zinc	6.49	China: Jiangxi, Yinshan
L180206P-2-LD180205-0002_HK72WCCXY_L2_clean_bin.5.orig.fa_k1	OEZ008529	yes	LMSG_G000010452.1	OES073745	arylpolyene.hserlactone	Others	Lead-Zinc	34.62	China: Jiangxi, Yinshan
L180206P-2-LD180205-0002_HK72WCCXY_L2_clean_bin.5.orig.fa_k1	OEZ008529	yes	LMSG_G000010452.1	OES073745	resorcinol	Others	Lead-Zinc	20.43	China: Jiangxi, Yinshan
L180206P-2-LD180205-0002_HK72WCCXY_L2_clean_bin.8.strict.fa_	OEZ008529	yes	LMSG_G000008179.1	OES073745	terpene	Terpene	Lead-Zinc	20.94	China: Jiangxi, Yinshan
L180206P-2-LD180205-0002_HK72WCCXY_L2_clean_bin.8.strict.fa_	OEZ008529	yes	LMSG_G000008179.1	OES073745	betalactone	Others	Lead-Zinc	10.34	China: Jiangxi, Yinshan
L180206P-2-LD180205-0002_HK72WCCXY_L2_clean_bin.9.orig.fa_k1	OEZ008529	yes	LMSG_G000010678.1	OES073745	NRPS-like	NRPS	Lead-Zinc	9.54	China: Jiangxi, Yinshan
L180206P-2-LD180205-0002_HK72WCCXY_L2_clean_bin.9.orig.fa_k1	OEZ008529	yes	LMSG_G000010678.1	OES073745	betalactone	Others	Lead-Zinc	17.73	China: Jiangxi, Yinshan
L180206P-2-LD180205-0002_HK72WCCXY_L2_clean_bin.9.orig.fa_k1	OEZ008529	yes	LMSG_G000010678.1	OES073745	betalactone	Others	Lead-Zinc	24.36	China: Jiangxi, Yinshan
L180206P-2-LD180205-0002_HK72WCCXY_L2_clean_bin.9.orig.fa_k1	OEZ008529	yes	LMSG_G000010678.1	OES073745	arylpolyene	Others	Lead-Zinc	13.28	China: Jiangxi, Yinshan
L180206P-2-LD180205-0003_HK72WCCXY_L2_clean_bin.100.orig.fa_	OEZ008529	yes	LMSG_G000004849.1	OES073734	PKS-like	PKSother	Pyrite	7.43	China: Guangdong, Yunfu
L180206P-2-LD180205-0003_HK72WCCXY_L2_clean_bin.101.orig.fa_	OEZ008529	yes	LMSG_G000007957.1	OES073734	NRPS	NRPS	Pyrite	8.72	China: Guangdong, Yunfu
L180206P-2-LD180205-0003_HK72WCCXY_L2_clean_bin.102.orig.fa_	OEZ008529	yes	LMSG_G000010587.1	OES073734	terpene	Terpene	Pyrite	5.95	China: Guangdong, Yunfu

L180206P-2-LD180205-0003_HK72WCCXY_L2_clean_bin.102.orig.fa_	OEZ008529	yes	LMSG_G000010587.1	OES073734	bacteriocin	RiPPs	Pyrite	5.70	China: Guangdong, Yunfu
L180206P-2-LD180205-0003_HK72WCCXY_L2_clean_bin.102.orig.fa_	OEZ008529	yes	LMSG_G000010587.1	OES073734	bacteriocin	RiPPs	Pyrite	5.30	China: Guangdong, Yunfu
L180206P-2-LD180205-0003_HK72WCCXY_L2_clean_bin.104.orig.fa_	OEZ008529	yes	LMSG_G000010504.1	OES073734	arylpolyene	Others	Pyrite	23.17	China: Guangdong, Yunfu
L180206P-2-LD180205-0003_HK72WCCXY_L2_clean_bin.105.orig.fa_	OEZ008529	yes	LMSG_G000004850.1	OES073734	NRPS-like	NRPS	Pyrite	44.50	China: Guangdong, Yunfu
L180206P-2-LD180205-0003_HK72WCCXY_L2_clean_bin.105.orig.fa_	OEZ008529	yes	LMSG_G000004850.1	OES073734	T1PKS.hglE-KS	PKSother	Pyrite	27.82	China: Guangdong, Yunfu
L180206P-2-LD180205-0003_HK72WCCXY_L2_clean_bin.107.orig.fa_	OEZ008529	yes	LMSG_G000004851.1	OES073734	terpene	Terpene	Pyrite	22.87	China: Guangdong, Yunfu
L180206P-2-LD180205-0003_HK72WCCXY_L2_clean_bin.109.orig.fa_	OEZ008529	yes	LMSG_G000008409.1	OES073734	terpene	Terpene	Pyrite	11.20	China: Guangdong, Yunfu
L180206P-2-LD180205-0003_HK72WCCXY_L2_clean_bin.109.orig.fa_	OEZ008529	yes	LMSG_G000008409.1	OES073734	betalactone	Others	Pyrite	33.89	China: Guangdong, Yunfu
L180206P-2-LD180205-0003_HK72WCCXY_L2_clean_bin.114.orig.fa_	OEZ008529	yes	LMSG_G000010756.1	OES073734	acyl_amino_acids	Others	Pyrite	33.28	China: Guangdong, Yunfu
L180206P-2-LD180205-0003_HK72WCCXY_L2_clean_bin.114.orig.fa_	OEZ008529	yes	LMSG_G000010756.1	OES073734	bacteriocin	RiPPs	Pyrite	9.36	China: Guangdong, Yunfu
L180206P-2-LD180205-0003_HK72WCCXY_L2_clean_bin.13.permissiv	OEZ008529	yes	LMSG_G000006317.1	OES073734	CDPS	Others	Pyrite	11.99	China: Guangdong, Yunfu
L180206P-2-LD180205-0003_HK72WCCXY_L2_clean_bin.13.permissiv	OEZ008529	no	LMSG_G000006317.1	OES073734	NRPS.T1PKS	PKS-NRP_Hybrids	Pyrite	9.97	China: Guangdong, Yunfu
L180206P-2-LD180205-0003_HK72WCCXY_L2_clean_bin.13.permissiv	OEZ008529	yes	LMSG_G000006317.1	OES073734	hglE-KS	PKSother	Pyrite	8.22	China: Guangdong, Yunfu
L180206P-2-LD180205-0003_HK72WCCXY_L2_clean_bin.13.permissiv	OEZ008529	yes	LMSG_G000006317.1	OES073734	NRPS-like	NRPS	Pyrite	7.93	China: Guangdong, Yunfu
L180206P-2-LD180205-0003_HK72WCCXY_L2_clean_bin.13.permissiv	OEZ008529	yes	LMSG_G000006317.1	OES073734	NRPS	NRPS	Pyrite	7.18	China: Guangdong, Yunfu
L180206P-2-LD180205-0003_HK72WCCXY_L2_clean_bin.17.orig.fa_k	OEZ008529	yes	LMSG_G000008965.1	OES073734	lanthipeptide	RiPPs	Pyrite	19.16	China: Guangdong, Yunfu
L180206P-2-LD180205-0003_HK72WCCXY_L2_clean_bin.17.orig.fa_k	OEZ008529	yes	LMSG_G000008965.1	OES073734	terpene	Terpene	Pyrite	7.32	China: Guangdong, Yunfu
L180206P-2-LD180205-0003_HK72WCCXY_L2_clean_bin.17.orig.fa_k	OEZ008529	yes	LMSG_G000008965.1	OES073734	NRPS	NRPS	Pyrite	31.31	China: Guangdong, Yunfu
L180206P-2-LD180205-0003_HK72WCCXY_L2_clean_bin.17.orig.fa_k	OEZ008529	yes	LMSG_G000008965.1	OES073734	NRPS	NRPS	Pyrite	24.63	China: Guangdong, Yunfu
L180206P-2-LD180205-0003_HK72WCCXY_L2_clean_bin.17.orig.fa_k	OEZ008529	yes	LMSG_G000008965.1	OES073734	NRPS	NRPS	Pyrite	12.40	China: Guangdong, Yunfu
L180206P-2-LD180205-0003_HK72WCCXY_L2_clean_bin.20.orig.fa_k	OEZ008529	yes	LMSG_G000007808.1	OES073734	T1PKS	PKSI	Pyrite	19.06	China: Guangdong, Yunfu
L180206P-2-LD180205-0003_HK72WCCXY_L2_clean_bin.20.orig.fa_k	OEZ008529	yes	LMSG_G000007808.1	OES073734	terpene	Terpene	Pyrite	29.74	China: Guangdong, Yunfu
L180206P-2-LD180205-0003_HK72WCCXY_L2_clean_bin.20.orig.fa_k	OEZ008529	yes	LMSG_G000007808.1	OES073734	T3PKS	PKSother	Pyrite	12.57	China: Guangdong, Yunfu
L180206P-2-LD180205-0003_HK72WCCXY_L2_clean_bin.22.orig.fa_k	OEZ008529	yes	LMSG_G000004860.1	OES073734	other	Others	Pyrite	40.15	China: Guangdong, Yunfu
L180206P-2-LD180205-0003_HK72WCCXY_L2_clean_bin.23.permissiv	OEZ008529	yes	LMSG_G000009427.1	OES073734	bacteriocin	RiPPs	Pyrite	6.24	China: Guangdong, Yunfu
L180206P-2-LD180205-0003_HK72WCCXY_L2_clean_bin.23.permissiv	OEZ008529	yes	LMSG_G000009427.1	OES073734	betalactone	Others	Pyrite	5.98	China: Guangdong, Yunfu
L180206P-2-LD180205-0003_HK72WCCXY_L2_clean_bin.29.strict.fa	OEZ008529	yes	LMSG_G000009277.1	OES073734	terpene	Terpene	Pyrite	6.25	China: Guangdong, Yunfu
L180206P-2-LD180205-0003_HK72WCCXY_L2_clean_bin.30.orig.fa_k	OEZ008529	yes	LMSG_G000004372.1	OES073734	other	Others	Pyrite	31.22	China: Guangdong, Yunfu
L180206P-2-LD180205-0003_HK72WCCXY_L2_clean_bin.30.orig.fa_k	OEZ008529	yes	LMSG_G000004372.1	OES073734	betalactone	Others	Pyrite	20.90	China: Guangdong, Yunfu
L180206P-2-LD180205-0003_HK72WCCXY_L2_clean_bin.31.orig.fa_k	OEZ008529	yes	LMSG_G000010025.1	OES073734	terpene	Terpene	Pyrite	16.70	China: Guangdong, Yunfu
L180206P-2-LD180205-0003_HK72WCCXY_L2_clean_bin.31.orig.fa_k	OEZ008529	yes	LMSG_G000010025.1	OES073734	terpene	Terpene	Pyrite	16.02	China: Guangdong, Yunfu
L180206P-2-LD180205-0003_HK72WCCXY_L2_clean_bin.31.orig.fa_k	OEZ008529	yes	LMSG_G000010025.1	OES073734	NRPS-like	NRPS	Pyrite	10.82	China: Guangdong, Yunfu
L180206P-2-LD180205-0003_HK72WCCXY_L2_clean_bin.31.orig.fa_k	OEZ008529	yes	LMSG_G000010025.1	OES073734	T3PKS	PKSother	Pyrite	16.58	China: Guangdong, Yunfu
L180206P-2-LD180205-0003_HK72WCCXY_L2_clean_bin.34.strict.fa	OEZ008529	yes	LMSG_G000004865.1	OES073734	T3PKS	PKSother	Pyrite	41.07	China: Guangdong, Yunfu
L180206P-2-LD180205-0003_HK72WCCXY_L2_clean_bin.34.strict.fa	OEZ008529	yes	LMSG_G000004865.1	OES073734	terpene	Terpene	Pyrite	20.98	China: Guangdong, Yunfu
L180206P-2-LD180205-0003_HK72WCCXY_L2_clean_bin.34.strict.fa	OEZ008529	yes	LMSG_G000004865.1	OES073734	terpene	Terpene	Pyrite	20.96	China: Guangdong, Yunfu
L180206P-2-LD180205-0003_HK72WCCXY_L2_clean_bin.35.orig.fa_k	OEZ008529	yes	LMSG_G000010256.1	OES073734	arylpolyene	Others	Pyrite	34.23	China: Guangdong, Yunfu
L180206P-2-LD180205-0003_HK72WCCXY_L2_clean_bin.35.orig.fa_k	OEZ008529	no	LMSG_G000010256.1	OES073734	terpene	Terpene	Pyrite	21.20	China: Guangdong, Yunfu
L180206P-2-LD180205-0003_HK72WCCXY_L2_clean_bin.36.strict.fa	OEZ008529	yes	LMSG_G000008876.1	OES073734	terpene	Terpene	Pyrite	7.91	China: Guangdong, Yunfu
L180206P-2-LD180205-0003_HK72WCCXY_L2_clean_bin.41.orig.fa_k	OEZ008529	yes	LMSG_G000009122.1	OES073734	bacteriocin	RiPPs	Pyrite	10.87	China: Guangdong, Yunfu
L180206P-2-LD180205-0003_HK72WCCXY_L2_clean_bin.41.orig.fa_k	OEZ008529	yes	LMSG_G000009122.1	OES073734	terpene	Terpene	Pyrite	25.09	China: Guangdong, Yunfu
L180206P-2-LD180205-0003_HK72WCCXY_L2_clean_bin.46.orig.fa_k	OEZ008529	yes	LMSG_G000004866.1	OES073734	T3PKS	PKSother	Pyrite	14.10	China: Guangdong, Yunfu
L180206P-2-LD180205-0003_HK72WCCXY_L2_clean_bin.47.orig.fa_k	OEZ008529	yes	LMSG_G000009492.1	OES073734	T1PKS.hglE-KS	PKSother	Pyrite	32.15	China: Guangdong, Yunfu
L180206P-2-LD180205-0003_HK72WCCXY_L2_clean_bin.47.orig.fa_k	OEZ008529	yes	LMSG_G000009492.1	OES073734	bacteriocin	RiPPs	Pyrite	8.58	China: Guangdong, Yunfu
L180206P-2-LD180205-0003_HK72WCCXY_L2_clean_bin.47.orig.fa_k	OEZ008529	yes	LMSG_G000009492.1	OES073734	bacteriocin	RiPPs	Pyrite	11.17	China: Guangdong, Yunfu
L180206P-2-LD180205-0003_HK72WCCXY_L2_clean_bin.47.orig.fa_k	OEZ008529	yes	LMSG_G000009492.1	OES073734	T3PKS	PKSother	Pyrite	41.05	China: Guangdong, Yunfu
L180206P-2-LD180205-0003_HK72WCCXY_L2_clean_bin.47.orig.fa_k	OEZ008529	yes	LMSG_G000009492.1	OES073734	NRPS-like	NRPS	Pyrite	28.81	China: Guangdong, Yunfu
L180206P-2-LD180205-0003_HK72WCCXY_L2_clean_bin.47.orig.fa_k	OEZ008529	yes	LMSG_G000009492.1	OES073734	TfuA-related	RiPPs	Pyrite	12.52	China: Guangdong, Yunfu
L180206P-2-LD180205-0003_HK72WCCXY_L2_clean_bin.48.orig.fa_k	OEZ008529	yes	LMSG_G000010454.1	OES073734	arylpolyene.hserlactone	Others	Pyrite	10.14	China: Guangdong, Yunfu
L180206P-2-LD180205-0003_HK72WCCXY_L2_clean_bin.48.orig.fa_k	OEZ008529	yes	LMSG_G000010454.1	OES073734	terpene	Terpene	Pyrite	15.42	China: Guangdong, Yunfu
L180206P-2-LD180205-0003_HK72WCCXY_L2_clean_bin.48.orig.fa_k	OEZ008529	yes	LMSG_G000010454.1	OES073734	NRPS-like	NRPS	Pyrite	14.76	China: Guangdong, Yunfu
L180206P-2-LD180205-0003_HK72WCCXY_L2_clean_bin.56.orig.fa_k	OEZ008529	yes	LMSG_G000009287.1	OES073734	terpene	Terpene	Pyrite	12.70	China: Guangdong, Yunfu
L180206P-2-LD180205-0003_HK72WCCXY_L2_clean_bin.56.orig.fa_k	OEZ008529	yes	LMSG_G000009287.1	OES073734	terpene	Terpene	Pyrite	22.06	China: Guangdong, Yunfu
L180206P-2-LD180205-0003_HK72WCCXY_L2_clean_bin.57.orig.fa_k	OEZ008529	yes	LMSG_G000004869.1	OES073734	terpene	Terpene	Pyrite	8.48	China: Guangdong, Yunfu
L180206P-2-LD180205-0003_HK72WCCXY_L2_clean_bin.63.orig.fa_k	OEZ008529	yes	LMSG_G000004374.1	OES073734	terpene	Terpene	Pyrite	23.11	China: Guangdong, Yunfu
L180206P-2-LD180205-0003_HK72WCCXY_L2_clean_bin.63.orig.fa_k	OEZ008529	yes	LMSG_G000004374.1	OES073734	arylpolyene	Others	Pyrite	38.38	China: Guangdong, Yunfu
L180206P-2-LD180205-0003_HK72WCCXY_L2_clean_bin.64.orig.fa_k	OEZ008529	yes	LMSG_G000004871.1	OES073734	terpene	Terpene	Pyrite	26.28	China: Guangdong, Yunfu
L180206P-2-LD180205-0003_HK72WCCXY_L2_clean_bin.64.orig.fa_k	OEZ008529	yes	LMSG_G000004871.1	OES073734	betalactone	Others	Pyrite	26.35	China: Guangdong, Yunfu
L180206P-2-LD180205-0003_HK72WCCXY_L2_clean_bin.67.orig.fa_k	OEZ008529	yes	LMSG_G000011026.1	OES073734	resorcinol.arylpolyene	Others	Pyrite	16.93	China: Guangdong, Yunfu
L180206P-2-LD180205-0003_HK72WCCXY_L2_clean_bin.67.orig.fa_k	OEZ008529	yes	LMSG_G000011026.1	OES073734	terpene	Terpene	Pyrite	20.54	China: Guangdong, Yunfu
L180206P-2-LD180205-0003_HK72WCCXY_L2_clean_bin.67.orig.fa_k	OEZ008529	yes	LMSG_G000011026.1	OES073734	bacteriocin	RiPPs	Pyrite	6.99	China: Guangdong, Yunfu
L180206P-2-LD180205-0003_HK72WCCXY_L2_clean_bin.67.orig.fa_k	OEZ008529	yes	LMSG_G000011026.1	OES073734	arylpolyene	Others	Pyrite	42.42	China: Guangdong, Yunfu
L180206P-2-LD180205-0003_HK72WCCXY_L2_clean_bin.68.orig.fa_k	OEZ008529	yes	LMSG_G000008734.1	OES073734	terpene	Terpene	Pyrite	21.24	China: Guangdong, Yunfu
L180206P-2-LD180205-0003_HK72WCCXY_L2_clean_bin.7.orig.fa_kl	OEZ008529	yes	LMSG_G000008544.1	OES073734	betalactone	Others	Pyrite	26.68	China: Guangdong, Yunfu
L180206P-2-LD180205-0003_HK72WCCXY_L2_clean_bin.7.orig.fa_kl	OEZ008529	yes	LMSG_G000008544.1	OES073734	other	Others	Pyrite	7.60	China: Guangdong, Yunfu
L180206P-2-LD180205-0003_HK72WCCXY_L2_clean_bin.74.orig.fa_k	OEZ008529	yes	LMSG_G000007051.1	OES073734	bacteriocin	RiPPs	Pyrite	6.60	China: Guangdong, Yunfu
L180206P-2-LD180205-0003_HK72WCCXY_L2_clean_bin.75.orig.fa_k	OEZ008529	yes	LMSG_G000010716.1	OES073734	arylpolyene	Others	Pyrite	10.97	China: Guangdong, Yunfu
L180206P-2-LD180205-0003_HK72WCCXY_L2_clean_bin.75.orig.fa_k	OEZ008529	yes	LMSG_G000010716.1	OES073734	NRPS	NRPS	Pyrite	14.48	China: Guangdong, Yunfu
L180206P-2-LD180205-0003_HK72WCCXY_L2_clean_bin.75.orig.fa_k	OEZ008529	yes	LMSG_G000010716.1	OES073734	bacteriocin	RiPPs	Pyrite	10.79	China: Guangdong, Yunfu
L180206P-2-LD180205-0003_HK72WCCXY_L2_clean_bin.75.orig.fa_k	OEZ008529	yes	LMSG_G000010716.1	OES073734	terpene	Terpene	Pyrite	17.53	China: Guangdong, Yunfu
L180206P-2-LD180205-0003_HK72WCCXY_L2_clean_bin.75.orig.fa_k	OEZ008529	yes	LMSG_G000010716.1	OES073734	NRPS	NRPS	Pyrite	9.64	China: Guangdong, Yunfu
L180206P-2-LD180205-0003_HK72WCCXY_L2_clean_bin.76.orig.fa_k	OEZ008529	yes	LMSG_G000004874.1	OES073734	hserlactone	Others	Pyrite	8.81	China: Guangdong, Yunfu
L180206P-2-LD180205-0003_HK72WCCXY_L2_clean_bin.76.orig.fa_k	OEZ008529	yes	LMSG_G000004874.1	OES073734	LAP	RiPPs	Pyrite	7.04	China: Guangdong, Yunfu
L180206P-2-LD180205-0003_HK72WCCXY_L2_clean_bin.76.orig.fa_k	OEZ008529	yes	LMSG_G000004874.1	OES073734	terpene	Terpene	Pyrite	5.83	China: Guangdong, Yunfu
L180206P-2-LD180205-0003_HK72WCCXY_L2_clean_bin.76.orig.fa_k	OEZ008529	yes	LMSG_G000004874.1	OES073734	betalactone	Others	Pyrite	26.24	China: Guangdong, Yunfu
L180206P-2-LD180205-0003_HK72WCCXY_L2_clean_bin.76.orig.fa_k	OEZ008529	yes	LMSG_G000004874.1	OES073734	terpene	Terpene	Pyrite	13.32	China: Guangdong, Yunfu
L180206P-2-LD180205-0003_HK72WCCXY_L2_clean_bin.77.orig.fa_k	OEZ008529	yes	LMSG_G000004375.1	OES073734	betalactone	Others	Pyrite	10.18	China: Guangdong, Yunfu
L180206P-2-LD180205-0003_HK72WCCXY_L2_clean_bin.77.orig.fa_k	OEZ008529	yes	LMSG_G000004375.1	OES073734	terpene	Terpene	Pyrite	6.74	China: Guangdong, Yunfu
L180206P-2-LD180205-0003_HK72WCCXY_L2_clean_bin.79.orig.fa_k	OEZ008529	yes	LMSG_G000007230.1	OES073734	ectoine	Others	Pyrite	8.75	China: Guangdong, Yunfu
L180206P-2-LD180205-0003_HK72WCCXY_L2_clean_bin.79.orig.fa_k	OEZ008529	yes	LMSG_G000007230.1	OES073734	ectoine	Others	Pyrite	7.10	China: Guangdong, Yunfu
L180206P-2-LD180205-0003_HK72WCCXY_L2_clean_bin.80.strict.fa	OEZ008529	yes	LMSG_G000004875.1	OES073734	arylpolyene	Others	Pyrite	49.94	China: Guangdong, Yunfu
L180206P-2-LD180205-0003_HK72WCCXY_L2_clean_bin.82.orig.fa_k	OEZ008529	yes	LMSG_G000004376.1	OES073734	terpene	Terpene	Pyrite	22.00	China: Guangdong, Yunfu
L180206P-2-LD180205-0003_HK72WCCXY_L2_clean_bin.82.orig.fa_k	OEZ008529	yes	LMSG_G000004376.1	OES073734	terpene	Terpene	Pyrite	17.67	China: Guangdong, Yunfu
L180206P-2-LD180205-0003_HK72WCCXY_L2_clean_bin.83.orig.fa_k	OEZ008529	yes	LMSG_G000010699.1	OES073734	bacteriocin	RiPPs	Pyrite	5.48	China: Guangdong, Yunfu
L180206P-2-LD180205-0003_HK72WCCXY_L2_clean_bin.83.orig.fa_k	OEZ008529	yes	LMSG_G000010699.1	OES073734	terpene	Terpene	Pyrite	18.18	China: Guangdong, Yunfu
L180206P-2-LD180205-0003_HK72WCCXY_L2_clean_bin.83.orig.fa_k	OEZ008529	yes	LMSG_G000010699.1	OES073734	betalactone	Others	Pyrite	20.49	China: Guangdong, Yunfu
L180206P-2-LD180205-0003_HK72WCCXY_L2_clean_bin.83.orig.fa_k	OEZ008529	yes	LMSG_G000010699.1	OES073734	arylpolyene	Others	Pyrite	11.76	China: Guangdong, Yunfu
L180206P-2-LD180205-0003_HK72WCCXY_L2_clean_bin.83.orig.fa_k	OEZ008529	yes	LMSG_G000010699.1	OES073734	NRPS.T1PKS	PKS-NRP_Hybrids	Pyrite	35.27	China: Guangdong, Yunfu
L180206P-2-LD180205-0003_HK72WCCXY_L2_clean_bin.83.orig.fa_k	OEZ008529	yes	LMSG_G000010699.1	OES073734	arylpolyene	Others	Pyrite	20.46	China: Guangdong, Yunfu
L180206P-2-LD180205-0003_HK72WCCXY_L2_clean_bin.85.strict.fa	OEZ008529	yes	LMSG_G000010817.1	OES073734	acyl_amino_acids	Others	Pyrite	8.77	China: Guangdong, Yunfu
L180206P-2-LD180205-0003_HK72WCCXY_L2_clean_bin.95.orig.fa_k	OEZ008529	yes	LMSG_G000004881.1	OES073734	NRPS-like	NRPS	Pyrite	7.66	China: Guangdong, Yunfu
L180206P-2-LD180205-0003_HK72WCCXY_L2_clean_bin.96.orig.fa_k	OEZ008529	yes	LMSG_G000009394.1	OES073734	NRPS	NRPS	Pyrite	23.91	China: Guangdong, Yunfu
L180206P-2-LD180205-0003_HK72WCCXY_L2_clean_bin.96.orig.fa_k	OEZ008529	yes	LMSG_G000009394.1	OES073734	terpene	Terpene	Pyrite	21.68	China: Guangdong, Yunfu
L180206P-2-LD180205-0003_HK72WCCXY_L2_clean_bin.96.orig.fa_k	OEZ008529	yes	LMSG_G000009394.1	OES073734	NRPS-like	NRPS	Pyrite	22.21	China: Guangdong, Yunfu
L180206P-2-LD180205-0003_HK72WCCXY_L2_clean_bin.97.permissiv	OEZ008529	yes	LMSG_G000009097.1	OES073734	betalactone.bacteriocin	Others	Pyrite	29.69	China: Guangdong, Yunfu
L180206P-2-LD180205-0003_HK72WCCXY_L2_clean_bin.97.permissiv	OEZ008529	yes	LMSG_G000009097.1	OES073734	acyl_amino_acids	Others	Pyrite	23.99	China: Guangdong, Yunfu
L180206P-2-LD180205-0003_HK72WCCXY_L2_clean_bin.97.permissiv	OEZ008529	yes	LMSG_G000009097.1	OES073734	betalactone	Others	Pyrite	21.84	China: Guangdong, Yunfu
L180206P-2-LD180205-0003_HK72WCCXY_L2_clean_bin.97.permissiv	OEZ008529	yes	LMSG_G000009097.1	OES073734	betalactone	Others	Pyrite	14.04	China: Guangdong, Yunfu
L180206P-2-LD180205-0003_HK72WCCXY_L2_clean_bin.98.orig.fa_k	OEZ008529	yes	LMSG_G000008249.1	OES073734	other	Others	Pyrite	21.43	China: Guangdong, Yunfu
L180206P-2-LD180205-0004_HK72WCCXY_L2_clean_bin.1.orig.fa_kl	OEZ008529	yes	LMSG_G000009175.1	OES073735	terpene	Terpene	Pyrite	5.25	China: Guangdong, Yunfu
L180206P-2-LD180205-0004_HK72WCCXY_L2_clean_bin.10.orig.fa_k	OEZ008529	yes	LMSG_G000007809.1	OES073735	terpene	Terpene	Pyrite	29.86	China: Guangdong, Yunfu
L180206P-2-LD180205-0004_HK72WCCXY_L2_clean_bin.10.orig.fa_k	OEZ008529	yes	LMSG_G000007809.1	OES073735	T3PKS	PKSother	Pyrite	6.82	China: Guangdong, Yunfu
L180206P-2-LD180205-0004_HK72WCCXY_L2_clean_bin.13.orig.fa_k	OEZ008529	yes	LMSG_G000009478.1	OES073735	betalactone	Others	Pyrite	12.15	China: Guangdong, Yunfu
L180206P-2-LD180205-0004_HK72WCCXY_L2_clean_bin.18.orig.fa_k	OEZ008529	yes	LMSG_G000004883.1	OES073735	bacteriocin	RiPPs	Pyrite	11.01	China: Guangdong, Yunfu
L180206P-2-LD180205-0004_HK72WCCXY_L2_clean_bin.2.orig.fa_kl	OEZ008529	no	LMSG_G000004888.1	OES073735	bacteriocin	RiPPs	Pyrite	9.32	China: Guangdong, Yunfu
L180206P-2-LD180205-0004_HK72WCCXY_L2_clean_bin.2.orig.fa_kl	OEZ008529	no	LMSG_G000004888.1	OES073735	bacteriocin	RiPPs	Pyrite	8.45	China: Guangdong, Yunfu
L180206P-2-LD180205-0004_HK72WCCXY_L2_clean_bin.2.orig.fa_kl	OEZ008529	no	LMSG_G000004888.1	OES073735	NRPS.T1PKS	PKS-NRP_Hybrids	Pyrite	59.17	China: Guangdong, Yunfu
L180206P-2-LD180205-0004_HK72WCCXY_L2_clean_bin.2.orig.fa_kl	OEZ008529	no	LMSG_G000004888.1	OES073735	thiopeptide	RiPPs	Pyrite	18.83	China: Guangdong, Yunfu
L180206P-2-LD180205-0004_HK72WCCXY_L2_clean_bin.2.orig.fa_kl	OEZ008529	no	LMSG_G000004888.1	OES073735	NRPS	NRPS	Pyrite	52.29	China: Guangdong, Yunfu
L180206P-2-LD180205-0004_HK72WCCXY_L2_clean_bin.23.orig.fa_k	OEZ008529	yes	LMSG_G000004886.1	OES073735	terpene	Terpene	Pyrite	14.47	China: Guangdong, Yunfu
L180206P-2-LD180205-0004_HK72WCCXY_L2_clean_bin.23.orig.fa_k	OEZ008529	yes	LMSG_G000004886.1	OES073735	bacteriocin	RiPPs	Pyrite	10.80	China: Guangdong, Yunfu
L180206P-2-LD180205-0004_HK72WCCXY_L2_clean_bin.23.orig.fa_k	OEZ008529	yes	LMSG_G000004886.1	OES073735	terpene	Terpene	Pyrite	5.16	China: Guangdong, Yunfu
L180206P-2-LD180205-0004_HK72WCCXY_L2_clean_bin.23.orig.fa_k	OEZ008529	yes	LMSG_G000004886.1	OES073735	NRPS-like	NRPS	Pyrite	19.30	China: Guangdong, Yunfu
L180206P-2-LD180205-0004_HK72WCCXY_L2_clean_bin.26.permissiv	OEZ008529	yes	LMSG_G000009901.1	OES073735	hserlactone	Others	Pyrite	5.58	China: Guangdong, Yunfu
L180206P-2-LD180205-0004_HK72WCCXY_L2_clean_bin.28.orig.fa_k	OEZ008529	yes	LMSG_G000004887.1	OES073735	terpene	Terpene	Pyrite	22.05	China: Guangdong, Yunfu
L180206P-2-LD180205-0004_HK72WCCXY_L2_clean_bin.28.orig.fa_k	OEZ008529	yes	LMSG_G000004887.1	OES073735	terpene	Terpene	Pyrite	21.74	China: Guangdong, Yunfu
L180206P-2-LD180205-0004_HK72WCCXY_L2_clean_bin.28.orig.fa_k	OEZ008529	yes	LMSG_G000004887.1	OES073735	acyl_amino_acids.hserlactone	Others	Pyrite	40.30	China: Guangdong, Yunfu
L180206P-2-LD180205-0004_HK72WCCXY_L2_clean_bin.29.orig.fa_k	OEZ008529	yes	LMSG_G000009647.1	OES073735	terpene	Terpene	Pyrite	21.10	China: Guangdong, Yunfu
L180206P-2-LD180205-0004_HK72WCCXY_L2_clean_bin.29.orig.fa_k	OEZ008529	yes	LMSG_G000009647.1	OES073735	NRPS-like	NRPS	Pyrite	8.90	China: Guangdong, Yunfu
L180206P-2-LD180205-0004_HK72WCCXY_L2_clean_bin.29.orig.fa_k	OEZ008529	yes	LMSG_G000009647.1	OES073735	terpene	Terpene	Pyrite	6.36	China: Guangdong, Yunfu
L180206P-2-LD180205-0004_HK72WCCXY_L2_clean_bin.29.orig.fa_k	OEZ008529	yes	LMSG_G000009647.1	OES073735	nucleoside	Others	Pyrite	7.51	China: Guangdong, Yunfu
L180206P-2-LD180205-0004_HK72WCCXY_L2_clean_bin.31.orig.fa_k	OEZ008529	yes	LMSG_G000004889.1	OES073735	terpene	Terpene	Pyrite	6.71	China: Guangdong, Yunfu
L180206P-2-LD180205-0004_HK72WCCXY_L2_clean_bin.34.orig.fa_k	OEZ008529	yes	LMSG_G000009529.1	OES073735	acyl_amino_acids	Others	Pyrite	7.54	China: Guangdong, Yunfu
L180206P-2-LD180205-0004_HK72WCCXY_L2_clean_bin.34.orig.fa_k	OEZ008529	no	LMSG_G000009529.1	OES073735	terpene	Terpene	Pyrite	23.29	China: Guangdong, Yunfu
L180206P-2-LD180205-0004_HK72WCCXY_L2_clean_bin.34.orig.fa_k	OEZ008529	no	LMSG_G000009529.1	OES073735	NRPS-like	NRPS	Pyrite	39.09	China: Guangdong, Yunfu
L180206P-2-LD180205-0004_HK72WCCXY_L2_clean_bin.34.orig.fa_k	OEZ008529	no	LMSG_G000009529.1	OES073735	terpene	Terpene	Pyrite	13.92	China: Guangdong, Yunfu
L180206P-2-LD180205-0004_HK72WCCXY_L2_clean_bin.38.orig.fa_k	OEZ008529	yes	LMSG_G000010585.1	OES073735	arylpolyene	Others	Pyrite	31.19	China: Guangdong, Yunfu
L180206P-2-LD180205-0004_HK72WCCXY_L2_clean_bin.38.orig.fa_k	OEZ008529	yes	LMSG_G000010585.1	OES073735	terpene	Terpene	Pyrite	11.53	China: Guangdong, Yunfu
L180206P-2-LD180205-0004_HK72WCCXY_L2_clean_bin.38.orig.fa_k	OEZ008529	yes	LMSG_G000010585.1	OES073735	terpene	Terpene	Pyrite	5.98	China: Guangdong, Yunfu
L180206P-2-LD180205-0004_HK72WCCXY_L2_clean_bin.39.orig.fa_k	OEZ008529	yes	LMSG_G000010591.1	OES073735	arylpolyene	Others	Pyrite	26.18	China: Guangdong, Yunfu
L180206P-2-LD180205-0004_HK72WCCXY_L2_clean_bin.39.orig.fa_k	OEZ008529	yes	LMSG_G000010591.1	OES073735	terpene	Terpene	Pyrite	23.02	China: Guangdong, Yunfu
L180206P-2-LD180205-0004_HK72WCCXY_L2_clean_bin.40.strict.fa	OEZ008529	yes	LMSG_G000008250.1	OES073735	other	Others	Pyrite	21.37	China: Guangdong, Yunfu
L180206P-2-LD180205-0004_HK72WCCXY_L2_clean_bin.41.strict.fa	OEZ008529	yes	LMSG_G000010053.1	OES073735	ladderane	Others	Pyrite	37.54	China: Guangdong, Yunfu
L180206P-2-LD180205-0004_HK72WCCXY_L2_clean_bin.41.strict.fa	OEZ008529	yes	LMSG_G000010053.1	OES073735	ectoine	Others	Pyrite	10.40	China: Guangdong, Yunfu
L180206P-2-LD180205-0004_HK72WCCXY_L2_clean_bin.41.strict.fa	OEZ008529	yes	LMSG_G000010053.1	OES073735	NRPS-like	NRPS	Pyrite	21.69	China: Guangdong, Yunfu
L180206P-2-LD180205-0004_HK72WCCXY_L2_clean_bin.41.strict.fa	OEZ008529	yes	LMSG_G000010053.1	OES073735	terpene	Terpene	Pyrite	15.07	China: Guangdong, Yunfu
L180206P-2-LD180205-0004_HK72WCCXY_L2_clean_bin.41.strict.fa	OEZ008529	yes	LMSG_G000010053.1	OES073735	bacteriocin	RiPPs	Pyrite	7.91	China: Guangdong, Yunfu
L180206P-2-LD180205-0004_HK72WCCXY_L2_clean_bin.41.strict.fa	OEZ008529	yes	LMSG_G000010053.1	OES073735	T3PKS	PKSother	Pyrite	6.47	China: Guangdong, Yunfu
L180206P-2-LD180205-0004_HK72WCCXY_L2_clean_bin.44.orig.fa_k	OEZ008529	yes	LMSG_G000009340.1	OES073735	bacteriocin	RiPPs	Pyrite	10.81	China: Guangdong, Yunfu
L180206P-2-LD180205-0004_HK72WCCXY_L2_clean_bin.45.orig.fa_k	OEZ008529	yes	LMSG_G000004891.1	OES073735	arylpolyene.hserlactone	Others	Pyrite	27.35	China: Guangdong, Yunfu
L180206P-2-LD180205-0004_HK72WCCXY_L2_clean_bin.45.orig.fa_k	OEZ008529	yes	LMSG_G000004891.1	OES073735	terpene	Terpene	Pyrite	23.02	China: Guangdong, Yunfu
L180206P-2-LD180205-0004_HK72WCCXY_L2_clean_bin.5.strict.fa_	OEZ008529	yes	LMSG_G000008001.1	OES073735	TfuA-related.bacteriocin	RiPPs	Pyrite	7.61	China: Guangdong, Yunfu
L180206P-2-LD180205-0004_HK72WCCXY_L2_clean_bin.52.orig.fa_k	OEZ008529	yes	LMSG_G000009607.1	OES073735	terpene	Terpene	Pyrite	21.99	China: Guangdong, Yunfu
L180206P-2-LD180205-0004_HK72WCCXY_L2_clean_bin.52.orig.fa_k	OEZ008529	yes	LMSG_G000009607.1	OES073735	terpene	Terpene	Pyrite	10.21	China: Guangdong, Yunfu
L180206P-2-LD180205-0004_HK72WCCXY_L2_clean_bin.52.orig.fa_k	OEZ008529	yes	LMSG_G000009607.1	OES073735	NRPS-like	NRPS	Pyrite	29.23	China: Guangdong, Yunfu
L180206P-2-LD180205-0004_HK72WCCXY_L2_clean_bin.54.orig.fa_k	OEZ008529	yes	LMSG_G000004892.1	OES073735	resorcinol.arylpolyene	Others	Pyrite	41.44	China: Guangdong, Yunfu
L180206P-2-LD180205-0004_HK72WCCXY_L2_clean_bin.54.orig.fa_k	OEZ008529	yes	LMSG_G000004892.1	OES073735	bacteriocin	RiPPs	Pyrite	10.79	China: Guangdong, Yunfu
L180206P-2-LD180205-0004_HK72WCCXY_L2_clean_bin.54.orig.fa_k	OEZ008529	yes	LMSG_G000004892.1	OES073735	terpene	Terpene	Pyrite	20.85	China: Guangdong, Yunfu
L180206P-2-LD180205-0004_HK72WCCXY_L2_clean_bin.54.orig.fa_k	OEZ008529	yes	LMSG_G000004892.1	OES073735	arylpolyene	Others	Pyrite	43.59	China: Guangdong, Yunfu
L180206P-2-LD180205-0004_HK72WCCXY_L2_clean_bin.55.orig.fa_k	OEZ008529	yes	LMSG_G000008735.1	OES073735	terpene	Terpene	Pyrite	17.11	China: Guangdong, Yunfu
L180206P-2-LD180205-0004_HK72WCCXY_L2_clean_bin.56.strict.fa	OEZ008529	yes	LMSG_G000009587.1	OES073735	terpene	Terpene	Pyrite	22.15	China: Guangdong, Yunfu
L180206P-2-LD180205-0004_HK72WCCXY_L2_clean_bin.56.strict.fa	OEZ008529	yes	LMSG_G000009587.1	OES073735	NRPS-like	NRPS	Pyrite	17.34	China: Guangdong, Yunfu
L180206P-2-LD180205-0004_HK72WCCXY_L2_clean_bin.56.strict.fa	OEZ008529	yes	LMSG_G000009587.1	OES073735	terpene	Terpene	Pyrite	13.57	China: Guangdong, Yunfu
L180206P-2-LD180205-0004_HK72WCCXY_L2_clean_bin.57.strict.fa	OEZ008529	yes	LMSG_G000004377.1	OES073735	terpene	Terpene	Pyrite	20.83	China: Guangdong, Yunfu
L180206P-2-LD180205-0004_HK72WCCXY_L2_clean_bin.57.strict.fa	OEZ008529	yes	LMSG_G000004377.1	OES073735	terpene	Terpene	Pyrite	19.82	China: Guangdong, Yunfu
L180206P-2-LD180205-0004_HK72WCCXY_L2_clean_bin.57.strict.fa	OEZ008529	yes	LMSG_G000004377.1	OES073735	T3PKS	PKSother	Pyrite	21.84	China: Guangdong, Yunfu
L180206P-2-LD180205-0004_HK72WCCXY_L2_clean_bin.61.orig.fa_k	OEZ008529	yes	LMSG_G000007741.1	OES073735	T3PKS	PKSother	Pyrite	6.77	China: Guangdong, Yunfu
L180206P-2-LD180205-0004_HK72WCCXY_L2_clean_bin.61.orig.fa_k	OEZ008529	yes	LMSG_G000007741.1	OES073735	terpene	Terpene	Pyrite	12.60	China: Guangdong, Yunfu

L180206P-2-LD180205-0004_HK72WCCXY_L2_clean_bin.62.orig.fa_k	OEZ008529	yes	LMSG_G000007850.1	OES073735	T3PKS	PKSother	Pyrite	14.13	China: Guangdong, Yunfu
L180206P-2-LD180205-0004_HK72WCCXY_L2_clean_bin.62.orig.fa_k	OEZ008529	yes	LMSG_G000007850.1	OES073735	NRPS-like	NRPS	Pyrite	40.23	China: Guangdong, Yunfu
L180206P-2-LD180205-0004_HK72WCCXY_L2_clean_bin.62.orig.fa_k	OEZ008529	yes	LMSG_G000007850.1	OES073735	NRPS-like.terpene	Others	Pyrite	47.83	China: Guangdong, Yunfu
L180206P-2-LD180205-0004_HK72WCCXY_L2_clean_bin.62.orig.fa_k	OEZ008529	yes	LMSG_G000007850.1	OES073735	terpene	Terpene	Pyrite	12.00	China: Guangdong, Yunfu
L180206P-2-LD180205-0004_HK72WCCXY_L2_clean_bin.63.strict.fa	OEZ008529	yes	LMSG_G000007966.1	OES073735	terpene	Terpene	Pyrite	16.69	China: Guangdong, Yunfu
L180206P-2-LD180205-0004_HK72WCCXY_L2_clean_bin.64.orig.fa_k	OEZ008529	yes	LMSG_G000010153.1	OES073735	betalactone	Others	Pyrite	21.46	China: Guangdong, Yunfu
L180206P-2-LD180205-0004_HK72WCCXY_L2_clean_bin.64.orig.fa_k	OEZ008529	no	LMSG_G000010153.1	OES073735	terpene	Terpene	Pyrite	21.71	China: Guangdong, Yunfu
L180206P-2-LD180205-0004_HK72WCCXY_L2_clean_bin.64.orig.fa_k	OEZ008529	no	LMSG_G000010153.1	OES073735	acyl_amino_acids	Others	Pyrite	23.75	China: Guangdong, Yunfu
L180206P-2-LD180205-0004_HK72WCCXY_L2_clean_bin.64.orig.fa_k	OEZ008529	no	LMSG_G000010153.1	OES073735	ectoine	Others	Pyrite	10.40	China: Guangdong, Yunfu
L180206P-2-LD180205-0004_HK72WCCXY_L2_clean_bin.64.orig.fa_k	OEZ008529	no	LMSG_G000010153.1	OES073735	terpene	Terpene	Pyrite	21.95	China: Guangdong, Yunfu
L180206P-2-LD180205-0004_HK72WCCXY_L2_clean_bin.64.orig.fa_k	OEZ008529	no	LMSG_G000010153.1	OES073735	terpene	Terpene	Pyrite	6.66	China: Guangdong, Yunfu
L180206P-2-LD180205-0004_HK72WCCXY_L2_clean_bin.65.orig.fa_k	OEZ008529	yes	LMSG_G000004893.1	OES073735	terpene	Terpene	Pyrite	17.54	China: Guangdong, Yunfu
L180206P-2-LD180205-0004_HK72WCCXY_L2_clean_bin.67.orig.fa_k	OEZ008529	yes	LMSG_G000008058.1	OES073735	terpene	Terpene	Pyrite	22.29	China: Guangdong, Yunfu
L180206P-2-LD180205-0004_HK72WCCXY_L2_clean_bin.67.orig.fa_k	OEZ008529	yes	LMSG_G000008058.1	OES073735	terpene	Terpene	Pyrite	13.21	China: Guangdong, Yunfu
L180206P-2-LD180205-0004_HK72WCCXY_L2_clean_bin.68.orig.fa_k	OEZ008529	yes	LMSG_G000009401.1	OES073735	NRPS-like	NRPS	Pyrite	22.18	China: Guangdong, Yunfu
L180206P-2-LD180205-0004_HK72WCCXY_L2_clean_bin.68.orig.fa_k	OEZ008529	yes	LMSG_G000009401.1	OES073735	terpene	Terpene	Pyrite	20.22	China: Guangdong, Yunfu
L180206P-2-LD180205-0004_HK72WCCXY_L2_clean_bin.69.strict.fa	OEZ008529	yes	LMSG_G000007334.1	OES073735	bacteriocin	RiPPs	Pyrite	5.97	China: Guangdong, Yunfu
L180206P-2-LD180205-0004_HK72WCCXY_L2_clean_bin.7.orig.fa_kl	OEZ008529	yes	LMSG_G000008081.1	OES073735	T1PKS.hglE-KS	PKSother	Pyrite	23.48	China: Guangdong, Yunfu
L180206P-2-LD180205-0004_HK72WCCXY_L2_clean_bin.7.orig.fa_kl	OEZ008529	yes	LMSG_G000008081.1	OES073735	arylpolyene	Others	Pyrite	21.41	China: Guangdong, Yunfu
L180206P-2-LD180205-0004_HK72WCCXY_L2_clean_bin.70.orig.fa_k	OEZ008529	yes	LMSG_G000008475.1	OES073735	terpene	Terpene	Pyrite	6.01	China: Guangdong, Yunfu
L180206P-2-LD180205-0004_HK72WCCXY_L2_clean_bin.70.orig.fa_k	OEZ008529	yes	LMSG_G000008475.1	OES073735	betalactone	Others	Pyrite	6.47	China: Guangdong, Yunfu
L180206P-2-LD180205-0004_HK72WCCXY_L2_clean_bin.71.orig.fa_k	OEZ008529	yes	LMSG_G000008255.1	OES073735	terpene	Terpene	Pyrite	19.11	China: Guangdong, Yunfu
L180206P-2-LD180205-0004_HK72WCCXY_L2_clean_bin.72.orig.fa_k	OEZ008529	yes	LMSG_G000007052.1	OES073735	bacteriocin	RiPPs	Pyrite	11.01	China: Guangdong, Yunfu
L180206P-2-LD180205-0004_HK72WCCXY_L2_clean_bin.73.orig.fa_k	OEZ008529	yes	LMSG_G000008989.1	OES073735	terpene	Terpene	Pyrite	15.83	China: Guangdong, Yunfu
L180206P-2-LD180205-0004_HK72WCCXY_L2_clean_bin.73.orig.fa_k	OEZ008529	yes	LMSG_G000008989.1	OES073735	bacteriocin	RiPPs	Pyrite	9.77	China: Guangdong, Yunfu
L180206P-2-LD180205-0004_HK72WCCXY_L2_clean_bin.74.permissiv	OEZ008529	yes	LMSG_G000004894.1	OES073735	terpene	Terpene	Pyrite	22.05	China: Guangdong, Yunfu
L180206P-2-LD180205-0004_HK72WCCXY_L2_clean_bin.74.permissiv	OEZ008529	yes	LMSG_G000004894.1	OES073735	terpene	Terpene	Pyrite	21.74	China: Guangdong, Yunfu
L180206P-2-LD180205-0004_HK72WCCXY_L2_clean_bin.74.permissiv	OEZ008529	yes	LMSG_G000004894.1	OES073735	bacteriocin	RiPPs	Pyrite	10.90	China: Guangdong, Yunfu
L180206P-2-LD180205-0004_HK72WCCXY_L2_clean_bin.74.permissiv	OEZ008529	yes	LMSG_G000004894.1	OES073735	hserlactone	Others	Pyrite	14.57	China: Guangdong, Yunfu
L180206P-2-LD180205-0004_HK72WCCXY_L2_clean_bin.75.strict.fa	OEZ008529	yes	LMSG_G000008972.1	OES073735	T1PKS	PKSI	Pyrite	7.14	China: Guangdong, Yunfu
L180206P-2-LD180205-0004_HK72WCCXY_L2_clean_bin.76.orig.fa_k	OEZ008529	yes	LMSG_G000008683.1	OES073735	terpene	Terpene	Pyrite	17.86	China: Guangdong, Yunfu
L180206P-2-LD180205-0004_HK72WCCXY_L2_clean_bin.76.orig.fa_k	OEZ008529	yes	LMSG_G000008683.1	OES073735	NRPS-like	NRPS	Pyrite	34.67	China: Guangdong, Yunfu
L180206P-2-LD180205-0004_HK72WCCXY_L2_clean_bin.76.orig.fa_k	OEZ008529	yes	LMSG_G000008683.1	OES073735	betalactone	Others	Pyrite	19.24	China: Guangdong, Yunfu
L180206P-2-LD180205-0004_HK72WCCXY_L2_clean_bin.76.orig.fa_k	OEZ008529	yes	LMSG_G000008683.1	OES073735	bacteriocin	RiPPs	Pyrite	10.16	China: Guangdong, Yunfu
L180206P-2-LD180205-0004_HK72WCCXY_L2_clean_bin.76.orig.fa_k	OEZ008529	yes	LMSG_G000008683.1	OES073735	ectoine	Others	Pyrite	10.42	China: Guangdong, Yunfu
L180206P-2-LD180205-0004_HK72WCCXY_L2_clean_bin.8.orig.fa_kl	OEZ008529	yes	LMSG_G000006214.1	OES073735	terpene	Terpene	Pyrite	15.40	China: Guangdong, Yunfu
L180206P-2-LD180205-0004_HK72WCCXY_L2_clean_bin.84.strict.fa	OEZ008529	yes	LMSG_G000010818.1	OES073735	acyl_amino_acids	Others	Pyrite	42.36	China: Guangdong, Yunfu
L180206P-2-LD180205-0004_HK72WCCXY_L2_clean_bin.85.orig.fa_k	OEZ008529	yes	LMSG_G000011077.1	OES073735	terpene	Terpene	Pyrite	23.07	China: Guangdong, Yunfu
L180206P-2-LD180205-0004_HK72WCCXY_L2_clean_bin.85.orig.fa_k	OEZ008529	yes	LMSG_G000011077.1	OES073735	lassopeptide	RiPPs	Pyrite	28.80	China: Guangdong, Yunfu
L180206P-2-LD180205-0004_HK72WCCXY_L2_clean_bin.85.orig.fa_k	OEZ008529	yes	LMSG_G000011077.1	OES073735	bacteriocin	RiPPs	Pyrite	10.90	China: Guangdong, Yunfu
L180206P-2-LD180205-0004_HK72WCCXY_L2_clean_bin.85.orig.fa_k	OEZ008529	yes	LMSG_G000011077.1	OES073735	NRPS-like	NRPS	Pyrite	38.63	China: Guangdong, Yunfu
L180206P-2-LD180205-0004_HK72WCCXY_L2_clean_bin.85.orig.fa_k	OEZ008529	yes	LMSG_G000011077.1	OES073735	NRPS-like	NRPS	Pyrite	29.01	China: Guangdong, Yunfu
L180206P-2-LD180205-0004_HK72WCCXY_L2_clean_bin.85.orig.fa_k	OEZ008529	yes	LMSG_G000011077.1	OES073735	arylpolyene	Others	Pyrite	35.21	China: Guangdong, Yunfu
L180206P-2-LD180205-0004_HK72WCCXY_L2_clean_bin.87.orig.fa_k	OEZ008529	yes	LMSG_G000006446.1	OES073735	nucleoside	Others	Pyrite	21.35	China: Guangdong, Yunfu
L180206P-2-LD180205-0004_HK72WCCXY_L2_clean_bin.89.orig.fa_k	OEZ008529	yes	LMSG_G000009518.1	OES073735	arylpolyene	Others	Pyrite	34.09	China: Guangdong, Yunfu
L180206P-2-LD180205-0004_HK72WCCXY_L2_clean_bin.89.orig.fa_k	OEZ008529	yes	LMSG_G000009518.1	OES073735	NRPS-like	NRPS	Pyrite	24.96	China: Guangdong, Yunfu
L180206P-2-LD180205-0004_HK72WCCXY_L2_clean_bin.89.orig.fa_k	OEZ008529	yes	LMSG_G000009518.1	OES073735	ectoine	Others	Pyrite	7.62	China: Guangdong, Yunfu
L180206P-2-LD180205-0004_HK72WCCXY_L2_clean_bin.89.orig.fa_k	OEZ008529	yes	LMSG_G000009518.1	OES073735	acyl_amino_acids	Others	Pyrite	10.23	China: Guangdong, Yunfu
L180206P-2-LD180205-0004_HK72WCCXY_L2_clean_bin.89.orig.fa_k	OEZ008529	yes	LMSG_G000009518.1	OES073735	terpene	Terpene	Pyrite	23.29	China: Guangdong, Yunfu
L180206P-2-LD180205-0004_HK72WCCXY_L2_clean_bin.89.orig.fa_k	OEZ008529	yes	LMSG_G000009518.1	OES073735	terpene	Terpene	Pyrite	15.37	China: Guangdong, Yunfu
L180206P-2-LD180205-0004_HK72WCCXY_L2_clean_bin.9.orig.fa_kl	OEZ008529	yes	LMSG_G000010228.1	OES073735	terpene.arylpolyene	Others	Pyrite	42.19	China: Guangdong, Yunfu
L180206P-2-LD180205-0004_HK72WCCXY_L2_clean_bin.9.orig.fa_kl	OEZ008529	yes	LMSG_G000010228.1	OES073735	terpene	Terpene	Pyrite	20.77	China: Guangdong, Yunfu
L180206P-2-LD180205-0004_HK72WCCXY_L2_clean_bin.9.orig.fa_kl	OEZ008529	yes	LMSG_G000010228.1	OES073735	hserlactone	Others	Pyrite	13.01	China: Guangdong, Yunfu
L180206P-2-LD180205-0004_HK72WCCXY_L2_clean_bin.90.strict.fa	OEZ008529	yes	LMSG_G000009428.1	OES073735	ectoine	Others	Pyrite	7.88	China: Guangdong, Yunfu
L180206P-2-LD180205-0004_HK72WCCXY_L2_clean_bin.90.strict.fa	OEZ008529	yes	LMSG_G000009428.1	OES073735	bacteriocin	RiPPs	Pyrite	5.53	China: Guangdong, Yunfu
L180206P-2-LD180205-0004_HK72WCCXY_L2_clean_bin.93.strict.fa	OEZ008529	yes	LMSG_G000010029.1	OES073735	NRPS-like	NRPS	Pyrite	30.09	China: Guangdong, Yunfu
L180206P-2-LD180205-0004_HK72WCCXY_L2_clean_bin.93.strict.fa	OEZ008529	yes	LMSG_G000010029.1	OES073735	T3PKS	PKSother	Pyrite	27.81	China: Guangdong, Yunfu
L180206P-2-LD180205-0004_HK72WCCXY_L2_clean_bin.93.strict.fa	OEZ008529	yes	LMSG_G000010029.1	OES073735	terpene	Terpene	Pyrite	12.33	China: Guangdong, Yunfu
L180206P-2-LD180205-0004_HK72WCCXY_L2_clean_bin.93.strict.fa	OEZ008529	yes	LMSG_G000010029.1	OES073735	terpene	Terpene	Pyrite	11.25	China: Guangdong, Yunfu
L180206P-2-LD180205-0004_HK72WCCXY_L2_clean_bin.96.orig.fa_k	OEZ008529	yes	LMSG_G000010786.1	OES073735	arylpolyene	Others	Pyrite	10.39	China: Guangdong, Yunfu
L180206P-2-LD180205-0004_HK72WCCXY_L2_clean_bin.96.orig.fa_k	OEZ008529	yes	LMSG_G000010786.1	OES073735	acyl_amino_acids	Others	Pyrite	43.28	China: Guangdong, Yunfu
L180206P-2-LD180205-0004_HK72WCCXY_L2_clean_bin.97.orig.fa_k	OEZ008529	yes	LMSG_G000008501.1	OES073735	LAP	RiPPs	Pyrite	9.88	China: Guangdong, Yunfu
L180206P-2-LD180205-0004_HK72WCCXY_L2_clean_bin.97.orig.fa_k	OEZ008529	yes	LMSG_G000008501.1	OES073735	lassopeptide	RiPPs	Pyrite	23.45	China: Guangdong, Yunfu
L180206P-2-LD180205-0004_HK72WCCXY_L2_clean_bin.97.orig.fa_k	OEZ008529	yes	LMSG_G000008501.1	OES073735	terpene	Terpene	Pyrite	17.08	China: Guangdong, Yunfu
L180206P-3-LD180205-0005_HK72WCCXY_L3_clean_bin.1.orig.fa_kl	OEZ008529	yes	LMSG_G000007844.1	OES073736	T3PKS	PKSother	Pyrite	5.01	China: Guangdong, Yunfu
L180206P-3-LD180205-0005_HK72WCCXY_L3_clean_bin.10.orig.fa_k	OEZ008529	yes	LMSG_G000004896.1	OES073736	other	Others	Pyrite	21.43	China: Guangdong, Yunfu
L180206P-3-LD180205-0005_HK72WCCXY_L3_clean_bin.12.strict.fa	OEZ008529	yes	LMSG_G000008002.1	OES073736	TfuA-related.bacteriocin	RiPPs	Pyrite	9.26	China: Guangdong, Yunfu
L180206P-3-LD180205-0005_HK72WCCXY_L3_clean_bin.13.orig.fa_k	OEZ008529	yes	LMSG_G000008476.1	OES073736	betalactone	Others	Pyrite	12.16	China: Guangdong, Yunfu
L180206P-3-LD180205-0005_HK72WCCXY_L3_clean_bin.13.orig.fa_k	OEZ008529	yes	LMSG_G000008476.1	OES073736	terpene	Terpene	Pyrite	16.69	China: Guangdong, Yunfu
L180206P-3-LD180205-0005_HK72WCCXY_L3_clean_bin.13.orig.fa_k	OEZ008529	yes	LMSG_G000008476.1	OES073736	terpene	Terpene	Pyrite	5.29	China: Guangdong, Yunfu
L180206P-3-LD180205-0005_HK72WCCXY_L3_clean_bin.13.orig.fa_k	OEZ008529	yes	LMSG_G000008476.1	OES073736	terpene	Terpene	Pyrite	5.32	China: Guangdong, Yunfu
L180206P-3-LD180205-0005_HK72WCCXY_L3_clean_bin.16.orig.fa_k	OEZ008529	yes	LMSG_G000010522.1	OES073736	arylpolyene	Others	Pyrite	27.61	China: Guangdong, Yunfu
L180206P-3-LD180205-0005_HK72WCCXY_L3_clean_bin.16.orig.fa_k	OEZ008529	no	LMSG_G000010522.1	OES073736	arylpolyene	Others	Pyrite	8.73	China: Guangdong, Yunfu
L180206P-3-LD180205-0005_HK72WCCXY_L3_clean_bin.16.orig.fa_k	OEZ008529	no	LMSG_G000010522.1	OES073736	terpene	Terpene	Pyrite	13.29	China: Guangdong, Yunfu
L180206P-3-LD180205-0005_HK72WCCXY_L3_clean_bin.16.orig.fa_k	OEZ008529	yes	LMSG_G000010522.1	OES073736	bacteriocin	RiPPs	Pyrite	7.29	China: Guangdong, Yunfu
L180206P-3-LD180205-0005_HK72WCCXY_L3_clean_bin.18.orig.fa_k	OEZ008529	yes	LMSG_G000010564.1	OES073736	T1PKS	PKSI	Pyrite	29.91	China: Guangdong, Yunfu
L180206P-3-LD180205-0005_HK72WCCXY_L3_clean_bin.18.orig.fa_k	OEZ008529	no	LMSG_G000010564.1	OES073736	terpene	Terpene	Pyrite	14.83	China: Guangdong, Yunfu
L180206P-3-LD180205-0005_HK72WCCXY_L3_clean_bin.18.orig.fa_k	OEZ008529	yes	LMSG_G000010564.1	OES073736	arylpolyene	Others	Pyrite	40.67	China: Guangdong, Yunfu
L180206P-3-LD180205-0005_HK72WCCXY_L3_clean_bin.19.orig.fa_k	OEZ008529	yes	LMSG_G000009588.1	OES073736	acyl_amino_acids	Others	Pyrite	14.99	China: Guangdong, Yunfu
L180206P-3-LD180205-0005_HK72WCCXY_L3_clean_bin.19.orig.fa_k	OEZ008529	yes	LMSG_G000009588.1	OES073736	terpene	Terpene	Pyrite	14.40	China: Guangdong, Yunfu
L180206P-3-LD180205-0005_HK72WCCXY_L3_clean_bin.19.orig.fa_k	OEZ008529	yes	LMSG_G000009588.1	OES073736	terpene	Terpene	Pyrite	22.15	China: Guangdong, Yunfu
L180206P-3-LD180205-0005_HK72WCCXY_L3_clean_bin.19.orig.fa_k	OEZ008529	yes	LMSG_G000009588.1	OES073736	NRPS-like	NRPS	Pyrite	12.82	China: Guangdong, Yunfu
L180206P-3-LD180205-0005_HK72WCCXY_L3_clean_bin.2.orig.fa_kl	OEZ008529	yes	LMSG_G000008502.1	OES073736	terpene	Terpene	Pyrite	26.16	China: Guangdong, Yunfu
L180206P-3-LD180205-0005_HK72WCCXY_L3_clean_bin.2.orig.fa_kl	OEZ008529	yes	LMSG_G000008502.1	OES073736	arylpolyene	Others	Pyrite	15.74	China: Guangdong, Yunfu
L180206P-3-LD180205-0005_HK72WCCXY_L3_clean_bin.2.orig.fa_kl	OEZ008529	yes	LMSG_G000008502.1	OES073736	lassopeptide	RiPPs	Pyrite	23.45	China: Guangdong, Yunfu
L180206P-3-LD180205-0005_HK72WCCXY_L3_clean_bin.20.orig.fa_k	OEZ008529	yes	LMSG_G000008294.1	OES073736	terpene	Terpene	Pyrite	23.48	China: Guangdong, Yunfu
L180206P-3-LD180205-0005_HK72WCCXY_L3_clean_bin.22.orig.fa_k	OEZ008529	no	LMSG_G000010154.1	OES073736	ectoine	Others	Pyrite	7.59	China: Guangdong, Yunfu
L180206P-3-LD180205-0005_HK72WCCXY_L3_clean_bin.23.orig.fa_k	OEZ008529	yes	LMSG_G000007810.1	OES073736	terpene	Terpene	Pyrite	29.81	China: Guangdong, Yunfu
L180206P-3-LD180205-0005_HK72WCCXY_L3_clean_bin.23.orig.fa_k	OEZ008529	yes	LMSG_G000007810.1	OES073736	T3PKS	PKSother	Pyrite	12.57	China: Guangdong, Yunfu
L180206P-3-LD180205-0005_HK72WCCXY_L3_clean_bin.25.orig.fa_k	OEZ008529	no	LMSG_G000010596.1	OES073736	bacteriocin	RiPPs	Pyrite	5.45	China: Guangdong, Yunfu
L180206P-3-LD180205-0005_HK72WCCXY_L3_clean_bin.27.orig.fa_k	OEZ008529	yes	LMSG_G000004899.1	OES073736	butyrolactone	Others	Pyrite	13.50	China: Guangdong, Yunfu
L180206P-3-LD180205-0005_HK72WCCXY_L3_clean_bin.27.orig.fa_k	OEZ008529	yes	LMSG_G000004899.1	OES073736	NRPS.T1PKS	PKS-NRP_Hybrids	Pyrite	53.03	China: Guangdong, Yunfu
L180206P-3-LD180205-0005_HK72WCCXY_L3_clean_bin.27.orig.fa_k	OEZ008529	yes	LMSG_G000004899.1	OES073736	NRPS	NRPS	Pyrite	74.78	China: Guangdong, Yunfu
L180206P-3-LD180205-0005_HK72WCCXY_L3_clean_bin.27.orig.fa_k	OEZ008529	yes	LMSG_G000004899.1	OES073736	arylpolyene.hserlactone	Others	Pyrite	25.50	China: Guangdong, Yunfu
L180206P-3-LD180205-0005_HK72WCCXY_L3_clean_bin.27.orig.fa_k	OEZ008529	yes	LMSG_G000004899.1	OES073736	arylpolyene	Others	Pyrite	41.22	China: Guangdong, Yunfu
L180206P-3-LD180205-0005_HK72WCCXY_L3_clean_bin.27.orig.fa_k	OEZ008529	yes	LMSG_G000004899.1	OES073736	bacteriocin	RiPPs	Pyrite	10.79	China: Guangdong, Yunfu
L180206P-3-LD180205-0005_HK72WCCXY_L3_clean_bin.27.orig.fa_k	OEZ008529	yes	LMSG_G000004899.1	OES073736	NRPS	NRPS	Pyrite	38.42	China: Guangdong, Yunfu
L180206P-3-LD180205-0005_HK72WCCXY_L3_clean_bin.27.orig.fa_k	OEZ008529	yes	LMSG_G000004899.1	OES073736	NRPS	NRPS	Pyrite	9.53	China: Guangdong, Yunfu
L180206P-3-LD180205-0005_HK72WCCXY_L3_clean_bin.28.strict.fa	OEZ008529	yes	LMSG_G000007851.1	OES073736	NRPS-like	NRPS	Pyrite	33.16	China: Guangdong, Yunfu
L180206P-3-LD180205-0005_HK72WCCXY_L3_clean_bin.28.strict.fa	OEZ008529	yes	LMSG_G000007851.1	OES073736	terpene	Terpene	Pyrite	22.05	China: Guangdong, Yunfu
L180206P-3-LD180205-0005_HK72WCCXY_L3_clean_bin.28.strict.fa	OEZ008529	yes	LMSG_G000007851.1	OES073736	terpene	Terpene	Pyrite	14.01	China: Guangdong, Yunfu
L180206P-3-LD180205-0005_HK72WCCXY_L3_clean_bin.28.strict.fa	OEZ008529	yes	LMSG_G000007851.1	OES073736	T3PKS	PKSother	Pyrite	6.83	China: Guangdong, Yunfu
L180206P-3-LD180205-0005_HK72WCCXY_L3_clean_bin.29.strict.fa	OEZ008529	no	LMSG_G000009530.1	OES073736	terpene	Terpene	Pyrite	23.29	China: Guangdong, Yunfu
L180206P-3-LD180205-0005_HK72WCCXY_L3_clean_bin.29.strict.fa	OEZ008529	no	LMSG_G000009530.1	OES073736	terpene	Terpene	Pyrite	22.05	China: Guangdong, Yunfu
L180206P-3-LD180205-0005_HK72WCCXY_L3_clean_bin.31.permissiv	OEZ008529	yes	LMSG_G000009567.1	OES073736	terpene	Terpene	Pyrite	22.05	China: Guangdong, Yunfu
L180206P-3-LD180205-0005_HK72WCCXY_L3_clean_bin.31.permissiv	OEZ008529	no	LMSG_G000009567.1	OES073736	NRPS-like	NRPS	Pyrite	42.70	China: Guangdong, Yunfu
L180206P-3-LD180205-0005_HK72WCCXY_L3_clean_bin.31.permissiv	OEZ008529	no	LMSG_G000009567.1	OES073736	terpene	Terpene	Pyrite	23.38	China: Guangdong, Yunfu
L180206P-3-LD180205-0005_HK72WCCXY_L3_clean_bin.31.permissiv	OEZ008529	no	LMSG_G000009567.1	OES073736	ectoine	Others	Pyrite	10.40	China: Guangdong, Yunfu
L180206P-3-LD180205-0005_HK72WCCXY_L3_clean_bin.32.orig.fa_k	OEZ008529	yes	LMSG_G000009466.1	OES073736	bacteriocin	RiPPs	Pyrite	5.63	China: Guangdong, Yunfu
L180206P-3-LD180205-0005_HK72WCCXY_L3_clean_bin.33.orig.fa_k	OEZ008529	yes	LMSG_G000010679.1	OES073736	terpene	Terpene	Pyrite	20.84	China: Guangdong, Yunfu
L180206P-3-LD180205-0005_HK72WCCXY_L3_clean_bin.33.orig.fa_k	OEZ008529	yes	LMSG_G000010679.1	OES073736	NRPS-like	NRPS	Pyrite	26.72	China: Guangdong, Yunfu
L180206P-3-LD180205-0005_HK72WCCXY_L3_clean_bin.33.orig.fa_k	OEZ008529	yes	LMSG_G000010679.1	OES073736	betalactone	Others	Pyrite	24.16	China: Guangdong, Yunfu
L180206P-3-LD180205-0005_HK72WCCXY_L3_clean_bin.33.orig.fa_k	OEZ008529	yes	LMSG_G000010679.1	OES073736	arylpolyene	Others	Pyrite	21.38	China: Guangdong, Yunfu
L180206P-3-LD180205-0005_HK72WCCXY_L3_clean_bin.33.orig.fa_k	OEZ008529	yes	LMSG_G000010679.1	OES073736	NRPS-like	NRPS	Pyrite	6.03	China: Guangdong, Yunfu
L180206P-3-LD180205-0005_HK72WCCXY_L3_clean_bin.34.orig.fa_k	OEZ008529	yes	LMSG_G000004378.1	OES073736	terpene	Terpene	Pyrite	20.86	China: Guangdong, Yunfu
L180206P-3-LD180205-0005_HK72WCCXY_L3_clean_bin.35.orig.fa_k	OEZ008529	yes	LMSG_G000004379.1	OES073736	terpene	Terpene	Pyrite	23.02	China: Guangdong, Yunfu
L180206P-3-LD180205-0005_HK72WCCXY_L3_clean_bin.35.orig.fa_k	OEZ008529	yes	LMSG_G000004379.1	OES073736	T1PKS	PKSI	Pyrite	17.84	China: Guangdong, Yunfu
L180206P-3-LD180205-0005_HK72WCCXY_L3_clean_bin.35.orig.fa_k	OEZ008529	yes	LMSG_G000004379.1	OES073736	arylpolyene	Others	Pyrite	27.18	China: Guangdong, Yunfu
L180206P-3-LD180205-0005_HK72WCCXY_L3_clean_bin.4.strict.fa_	OEZ008529	yes	LMSG_G000010147.1	OES073736	terpene	Terpene	Pyrite	19.03	China: Guangdong, Yunfu
L180206P-3-LD180205-0005_HK72WCCXY_L3_clean_bin.4.strict.fa_	OEZ008529	yes	LMSG_G000010147.1	OES073736	T3PKS	PKSother	Pyrite	15.57	China: Guangdong, Yunfu
L180206P-3-LD180205-0005_HK72WCCXY_L3_clean_bin.40.strict.fa	OEZ008529	yes	LMSG_G000009892.1	OES073736	terpene	Terpene	Pyrite	16.21	China: Guangdong, Yunfu
L180206P-3-LD180205-0005_HK72WCCXY_L3_clean_bin.41.strict.fa	OEZ008529	yes	LMSG_G000010245.1	OES073736	T1PKS.terpene.hserlactone	Others	Pyrite	50.49	China: Guangdong, Yunfu
L180206P-3-LD180205-0005_HK72WCCXY_L3_clean_bin.41.strict.fa	OEZ008529	yes	LMSG_G000010245.1	OES073736	terpene	Terpene	Pyrite	15.22	China: Guangdong, Yunfu
L180206P-3-LD180205-0005_HK72WCCXY_L3_clean_bin.41.strict.fa	OEZ008529	yes	LMSG_G000010245.1	OES073736	bacteriocin	RiPPs	Pyrite	7.83	China: Guangdong, Yunfu
L180206P-3-LD180205-0005_HK72WCCXY_L3_clean_bin.41.strict.fa	OEZ008529	yes	LMSG_G000010245.1	OES073736	arylpolyene	Others	Pyrite	10.91	China: Guangdong, Yunfu
L180206P-3-LD180205-0005_HK72WCCXY_L3_clean_bin.42.orig.fa_k	OEZ008529	yes	LMSG_G000004900.1	OES073736	terpene	Terpene	Pyrite	16.53	China: Guangdong, Yunfu
L180206P-3-LD180205-0005_HK72WCCXY_L3_clean_bin.42.orig.fa_k	OEZ008529	yes	LMSG_G000004900.1	OES073736	bacteriocin	RiPPs	Pyrite	10.83	China: Guangdong, Yunfu
L180206P-3-LD180205-0005_HK72WCCXY_L3_clean_bin.42.orig.fa_k	OEZ008529	yes	LMSG_G000004900.1	OES073736	NRPS-like	NRPS	Pyrite	29.52	China: Guangdong, Yunfu
L180206P-3-LD180205-0005_HK72WCCXY_L3_clean_bin.42.orig.fa_k	OEZ008529	yes	LMSG_G000004900.1	OES073736	lassopeptide.hserlactone	Others	Pyrite	29.96	China: Guangdong, Yunfu
L180206P-3-LD180205-0005_HK72WCCXY_L3_clean_bin.42.orig.fa_k	OEZ008529	yes	LMSG_G000004900.1	OES073736	arylpolyene	Others	Pyrite	23.26	China: Guangdong, Yunfu
L180206P-3-LD180205-0005_HK72WCCXY_L3_clean_bin.43.orig.fa_k	OEZ008529	no	LMSG_G000004901.1	OES073736	bacteriocin	RiPPs	Pyrite	10.87	China: Guangdong, Yunfu
L180206P-3-LD180205-0005_HK72WCCXY_L3_clean_bin.43.orig.fa_k	OEZ008529	no	LMSG_G000004901.1	OES073736	arylpolyene	Others	Pyrite	41.20	China: Guangdong, Yunfu
L180206P-3-LD180205-0005_HK72WCCXY_L3_clean_bin.43.orig.fa_k	OEZ008529	no	LMSG_G000004901.1	OES073736	arylpolyene	Others	Pyrite	44.87	China: Guangdong, Yunfu
L180206P-3-LD180205-0005_HK72WCCXY_L3_clean_bin.43.orig.fa_k	OEZ008529	no	LMSG_G000004901.1	OES073736	siderophore	Others	Pyrite	11.88	China: Guangdong, Yunfu
L180206P-3-LD180205-0005_HK72WCCXY_L3_clean_bin.43.orig.fa_k	OEZ008529	no	LMSG_G000004901.1	OES073736	terpene	Terpene	Pyrite	20.87	China: Guangdong, Yunfu
L180206P-3-LD180205-0005_HK72WCCXY_L3_clean_bin.46.orig.fa_k	OEZ008529	yes	LMSG_G000009608.1	OES073736	NRPS-like	NRPS	Pyrite	34.82	China: Guangdong, Yunfu
L180206P-3-LD180205-0005_HK72WCCXY_L3_clean_bin.46.orig.fa_k	OEZ008529	yes	LMSG_G000009608.1	OES073736	terpene	Terpene	Pyrite	22.09	China: Guangdong, Yunfu
L180206P-3-LD180205-0005_HK72WCCXY_L3_clean_bin.47.orig.fa_k	OEZ008529	yes	LMSG_G000010819.1	OES073736	acyl_amino_acids	Others	Pyrite	31.99	China: Guangdong, Yunfu
L180206P-3-LD180205-0005_HK72WCCXY_L3_clean_bin.48.orig.fa_k	OEZ008529	yes	LMSG_G000004902.1	OES073736	arylpolyene	Others	Pyrite	6.15	China: Guangdong, Yunfu
L180206P-3-LD180205-0005_HK72WCCXY_L3_clean_bin.49.orig.fa_k	OEZ008529	yes	LMSG_G000008990.1	OES073736	bacteriocin	RiPPs	Pyrite	9.79	China: Guangdong, Yunfu
L180206P-3-LD180205-0005_HK72WCCXY_L3_clean_bin.49.orig.fa_k	OEZ008529	yes	LMSG_G000008990.1	OES073736	terpene	Terpene	Pyrite	15.83	China: Guangdong, Yunfu
L180206P-3-LD180205-0005_HK72WCCXY_L3_clean_bin.5.orig.fa_kl	OEZ008529	yes	LMSG_G000008219.1	OES073736	terpene	Terpene	Pyrite	14.38	China: Guangdong, Yunfu
L180206P-3-LD180205-0005_HK72WCCXY_L3_clean_bin.51.orig.fa_k	OEZ008529	yes	LMSG_G000011078.1	OES073736	arylpolyene	Others	Pyrite	31.20	China: Guangdong, Yunfu
L180206P-3-LD180205-0005_HK72WCCXY_L3_clean_bin.51.orig.fa_k	OEZ008529	yes	LMSG_G000011078.1	OES073736	NRPS-like	NRPS	Pyrite	31.28	China: Guangdong, Yunfu
L180206P-3-LD180205-0005_HK72WCCXY_L3_clean_bin.52.orig.fa_k	OEZ008529	yes	LMSG_G000010455.1	OES073736	terpene	Terpene	Pyrite	23.02	China: Guangdong, Yunfu
L180206P-3-LD180205-0005_HK72WCCXY_L3_clean_bin.52.orig.fa_k	OEZ008529	yes	LMSG_G000010455.1	OES073736	arylpolyene.hserlactone	Others	Pyrite	27.35	China: Guangdong, Yunfu
L180206P-3-LD180205-0005_HK72WCCXY_L3_clean_bin.55.orig.fa_k	OEZ008529	yes	LMSG_G000009519.1	OES073736	terpene	Terpene	Pyrite	21.16	China: Guangdong, Yunfu
L180206P-3-LD180205-0005_HK72WCCXY_L3_clean_bin.55.orig.fa_k	OEZ008529	yes	LMSG_G000009519.1	OES073736	arylpolyene	Others	Pyrite	30.85	China: Guangdong, Yunfu
L180206P-3-LD180205-0005_HK72WCCXY_L3_clean_bin.55.orig.fa_k	OEZ008529	yes	LMSG_G000009519.1	OES073736	ectoine	Others	Pyrite	10.40	China: Guangdong, Yunfu
L180206P-3-LD180205-0005_HK72WCCXY_L3_clean_bin.55.orig.fa_k	OEZ008529	yes	LMSG_G000009519.1	OES073736	NRPS-like	NRPS	Pyrite	33.51	China: Guangdong, Yunfu
L180206P-3-LD180205-0005_HK72WCCXY_L3_clean_bin.55.orig.fa_k	OEZ008529	yes	LMSG_G000009519.1	OES073736	acyl_amino_acids	Others	Pyrite	7.41	China: Guangdong, Yunfu
L180206P-3-LD180205-0005_HK72WCCXY_L3_clean_bin.55.orig.fa_k	OEZ008529	yes	LMSG_G000009519.1	OES073736	terpene	Terpene	Pyrite	23.29	China: Guangdong, Yunfu
L180206P-3-LD180205-0005_HK72WCCXY_L3_clean_bin.56.strict.fa	OEZ008529	yes	LMSG_G000008261.1	OES073736	terpene	Terpene	Pyrite	20.86	China: Guangdong, Yunfu
L180206P-3-LD180205-0005_HK72WCCXY_L3_clean_bin.58.orig.fa_k	OEZ008529	yes	LMSG_G000004903.1	OES073736	terpene	Terpene	Pyrite	15.24	China: Guangdong, Yunfu
L180206P-3-LD180205-0005_HK72WCCXY_L3_clean_bin.58.orig.fa_k	OEZ008529	yes	LMSG_G000004903.1	OES073736	bacteriocin	RiPPs	Pyrite	9.46	China: Guangdong, Yunfu
L180206P-3-LD180205-0005_HK72WCCXY_L3_clean_bin.59.orig.fa_k	OEZ008529	no	LMSG_G000010945.1	OES073736	NRPS	NRPS	Pyrite	52.29	China: Guangdong, Yunfu
L180206P-3-LD180205-0005_HK72WCCXY_L3_clean_bin.59.orig.fa_k	OEZ008529	no	LMSG_G000010945.1	OES073736	bacteriocin	RiPPs	Pyrite	8.45	China: Guangdong, Yunfu
L180206P-3-LD180205-0005_HK72WCCXY_L3_clean_bin.59.orig.fa_k	OEZ008529	no	LMSG_G000010945.1	OES073736	NRPS.T1PKS	PKS-NRP_Hybrids	Pyrite	59.17	China: Guangdong, Yunfu

L180206P-3-LD180205-0005_HK72WCCXY_L3_clean_bin.59.orig.fa_k	OEZ008529	no	LMSG_G000010945.1	OES073736	thiopeptide	RiPPs	Pyrite	18.83	China: Guangdong, Yunfu
L180206P-3-LD180205-0005_HK72WCCXY_L3_clean_bin.61.strict.fa	OEZ008529	yes	LMSG_G000010757.1	OES073736	bacteriocin	RiPPs	Pyrite	11.75	China: Guangdong, Yunfu
L180206P-3-LD180205-0005_HK72WCCXY_L3_clean_bin.61.strict.fa	OEZ008529	yes	LMSG_G000010757.1	OES073736	acyl_amino_acids	Others	Pyrite	39.24	China: Guangdong, Yunfu
L180206P-3-LD180205-0005_HK72WCCXY_L3_clean_bin.7.strict.fa_	OEZ008529	yes	LMSG_G000007742.1	OES073736	terpene	Terpene	Pyrite	24.91	China: Guangdong, Yunfu
L180206P-3-LD180205-0005_HK72WCCXY_L3_clean_bin.7.strict.fa_	OEZ008529	yes	LMSG_G000007742.1	OES073736	T3PKS	PKSother	Pyrite	17.11	China: Guangdong, Yunfu
L180206P-3-LD180205-0005_HK72WCCXY_L3_clean_bin.8.orig.fa_kl	OEZ008529	yes	LMSG_G000004381.1	OES073736	terpene	Terpene	Pyrite	20.86	China: Guangdong, Yunfu
L180206P-3-LD180205-0005_HK72WCCXY_L3_clean_bin.9.strict.fa_	OEZ008529	yes	LMSG_G000007335.1	OES073736	bacteriocin	RiPPs	Pyrite	5.54	China: Guangdong, Yunfu
L180206P-3-LD180205-0006_HK72WCCXY_L3_clean_bin.1.orig.fa_kl	OEZ008529	yes	LMSG_G000008220.1	OES073737	terpene	Terpene	Pyrite	14.38	China: Guangdong, Yunfu
L180206P-3-LD180205-0006_HK72WCCXY_L3_clean_bin.10.orig.fa_k	OEZ008529	yes	LMSG_G000008254.1	OES073737	other	Others	Pyrite	21.43	China: Guangdong, Yunfu
L180206P-3-LD180205-0006_HK72WCCXY_L3_clean_bin.12.orig.fa_k	OEZ008529	yes	LMSG_G000011058.1	OES073737	bacteriocin	RiPPs	Pyrite	5.42	China: Guangdong, Yunfu
L180206P-3-LD180205-0006_HK72WCCXY_L3_clean_bin.12.orig.fa_k	OEZ008529	yes	LMSG_G000011058.1	OES073737	NRPS-like	NRPS	Pyrite	24.18	China: Guangdong, Yunfu
L180206P-3-LD180205-0006_HK72WCCXY_L3_clean_bin.13.orig.fa_k	OEZ008529	yes	LMSG_G000010488.1	OES073737	terpene	Terpene	Pyrite	7.32	China: Guangdong, Yunfu
L180206P-3-LD180205-0006_HK72WCCXY_L3_clean_bin.13.orig.fa_k	OEZ008529	yes	LMSG_G000010488.1	OES073737	arylpolyene	Others	Pyrite	17.19	China: Guangdong, Yunfu
L180206P-3-LD180205-0006_HK72WCCXY_L3_clean_bin.13.orig.fa_k	OEZ008529	yes	LMSG_G000010488.1	OES073737	hserlactone	Others	Pyrite	8.82	China: Guangdong, Yunfu
L180206P-3-LD180205-0006_HK72WCCXY_L3_clean_bin.17.orig.fa_k	OEZ008529	yes	LMSG_G000007811.1	OES073737	bacteriocin	RiPPs	Pyrite	10.96	China: Guangdong, Yunfu
L180206P-3-LD180205-0006_HK72WCCXY_L3_clean_bin.17.orig.fa_k	OEZ008529	yes	LMSG_G000007811.1	OES073737	terpene	Terpene	Pyrite	29.81	China: Guangdong, Yunfu
L180206P-3-LD180205-0006_HK72WCCXY_L3_clean_bin.17.orig.fa_k	OEZ008529	yes	LMSG_G000007811.1	OES073737	T3PKS	PKSother	Pyrite	12.57	China: Guangdong, Yunfu
L180206P-3-LD180205-0006_HK72WCCXY_L3_clean_bin.22.orig.fa_k	OEZ008529	yes	LMSG_G000004905.1	OES073737	terpene	Terpene	Pyrite	22.04	China: Guangdong, Yunfu
L180206P-3-LD180205-0006_HK72WCCXY_L3_clean_bin.23.orig.fa_k	OEZ008529	yes	LMSG_G000008687.1	OES073737	terpene	Terpene	Pyrite	19.67	China: Guangdong, Yunfu
L180206P-3-LD180205-0006_HK72WCCXY_L3_clean_bin.23.orig.fa_k	OEZ008529	yes	LMSG_G000008687.1	OES073737	betalactone	Others	Pyrite	19.45	China: Guangdong, Yunfu
L180206P-3-LD180205-0006_HK72WCCXY_L3_clean_bin.23.orig.fa_k	OEZ008529	yes	LMSG_G000008687.1	OES073737	NRPS-like	NRPS	Pyrite	26.89	China: Guangdong, Yunfu
L180206P-3-LD180205-0006_HK72WCCXY_L3_clean_bin.23.orig.fa_k	OEZ008529	yes	LMSG_G000008687.1	OES073737	bacteriocin	RiPPs	Pyrite	10.50	China: Guangdong, Yunfu
L180206P-3-LD180205-0006_HK72WCCXY_L3_clean_bin.23.orig.fa_k	OEZ008529	yes	LMSG_G000008687.1	OES073737	ectoine	Others	Pyrite	10.42	China: Guangdong, Yunfu
L180206P-3-LD180205-0006_HK72WCCXY_L3_clean_bin.24.orig.fa_k	OEZ008529	yes	LMSG_G000007336.1	OES073737	bacteriocin	RiPPs	Pyrite	5.02	China: Guangdong, Yunfu
L180206P-3-LD180205-0006_HK72WCCXY_L3_clean_bin.25.orig.fa_k	OEZ008529	yes	LMSG_G000008496.1	OES073737	lassopeptide	RiPPs	Pyrite	23.45	China: Guangdong, Yunfu
L180206P-3-LD180205-0006_HK72WCCXY_L3_clean_bin.25.orig.fa_k	OEZ008529	yes	LMSG_G000008496.1	OES073737	terpene	Terpene	Pyrite	26.16	China: Guangdong, Yunfu
L180206P-3-LD180205-0006_HK72WCCXY_L3_clean_bin.25.orig.fa_k	OEZ008529	yes	LMSG_G000008496.1	OES073737	LAP	RiPPs	Pyrite	9.35	China: Guangdong, Yunfu
L180206P-3-LD180205-0006_HK72WCCXY_L3_clean_bin.28.orig.fa_k	OEZ008529	yes	LMSG_G000007849.1	OES073737	T3PKS	PKSother	Pyrite	12.12	China: Guangdong, Yunfu
L180206P-3-LD180205-0006_HK72WCCXY_L3_clean_bin.28.orig.fa_k	OEZ008529	yes	LMSG_G000007849.1	OES073737	terpene	Terpene	Pyrite	17.88	China: Guangdong, Yunfu
L180206P-3-LD180205-0006_HK72WCCXY_L3_clean_bin.28.orig.fa_k	OEZ008529	yes	LMSG_G000007849.1	OES073737	NRPS-like.terpene	Others	Pyrite	47.42	China: Guangdong, Yunfu
L180206P-3-LD180205-0006_HK72WCCXY_L3_clean_bin.29.orig.fa_k	OEZ008529	yes	LMSG_G000007743.1	OES073737	bacteriocin	RiPPs	Pyrite	10.88	China: Guangdong, Yunfu
L180206P-3-LD180205-0006_HK72WCCXY_L3_clean_bin.29.orig.fa_k	OEZ008529	yes	LMSG_G000007743.1	OES073737	terpene	Terpene	Pyrite	7.06	China: Guangdong, Yunfu
L180206P-3-LD180205-0006_HK72WCCXY_L3_clean_bin.29.orig.fa_k	OEZ008529	yes	LMSG_G000007743.1	OES073737	terpene	Terpene	Pyrite	14.15	China: Guangdong, Yunfu
L180206P-3-LD180205-0006_HK72WCCXY_L3_clean_bin.29.orig.fa_k	OEZ008529	yes	LMSG_G000007743.1	OES073737	T3PKS	PKSother	Pyrite	16.19	China: Guangdong, Yunfu
L180206P-3-LD180205-0006_HK72WCCXY_L3_clean_bin.3.permissive	OEZ008529	yes	LMSG_G000008299.1	OES073737	terpene	Terpene	Pyrite	10.28	China: Guangdong, Yunfu
L180206P-3-LD180205-0006_HK72WCCXY_L3_clean_bin.32.permissiv	OEZ008529	yes	LMSG_G000008114.1	OES073737	betalactone	Others	Pyrite	28.89	China: Guangdong, Yunfu
L180206P-3-LD180205-0006_HK72WCCXY_L3_clean_bin.32.permissiv	OEZ008529	yes	LMSG_G000008114.1	OES073737	terpene	Terpene	Pyrite	13.47	China: Guangdong, Yunfu
L180206P-3-LD180205-0006_HK72WCCXY_L3_clean_bin.33.orig.fa_k	OEZ008529	yes	LMSG_G000004906.1	OES073737	betalactone	Others	Pyrite	20.93	China: Guangdong, Yunfu
L180206P-3-LD180205-0006_HK72WCCXY_L3_clean_bin.33.orig.fa_k	OEZ008529	yes	LMSG_G000004906.1	OES073737	terpene	Terpene	Pyrite	20.84	China: Guangdong, Yunfu
L180206P-3-LD180205-0006_HK72WCCXY_L3_clean_bin.33.orig.fa_k	OEZ008529	yes	LMSG_G000004906.1	OES073737	betalactone	Others	Pyrite	25.59	China: Guangdong, Yunfu
L180206P-3-LD180205-0006_HK72WCCXY_L3_clean_bin.33.orig.fa_k	OEZ008529	yes	LMSG_G000004906.1	OES073737	arylpolyene	Others	Pyrite	28.81	China: Guangdong, Yunfu
L180206P-3-LD180205-0006_HK72WCCXY_L3_clean_bin.33.orig.fa_k	OEZ008529	yes	LMSG_G000004906.1	OES073737	NRPS-like	NRPS	Pyrite	40.38	China: Guangdong, Yunfu
L180206P-3-LD180205-0006_HK72WCCXY_L3_clean_bin.33.orig.fa_k	OEZ008529	yes	LMSG_G000004906.1	OES073737	NRPS-like	NRPS	Pyrite	41.26	China: Guangdong, Yunfu
L180206P-3-LD180205-0006_HK72WCCXY_L3_clean_bin.34.strict.fa	OEZ008529	yes	LMSG_G000009648.1	OES073737	terpene	Terpene	Pyrite	21.70	China: Guangdong, Yunfu
L180206P-3-LD180205-0006_HK72WCCXY_L3_clean_bin.34.strict.fa	OEZ008529	yes	LMSG_G000009648.1	OES073737	nucleoside	Others	Pyrite	14.03	China: Guangdong, Yunfu
L180206P-3-LD180205-0006_HK72WCCXY_L3_clean_bin.34.strict.fa	OEZ008529	yes	LMSG_G000009648.1	OES073737	terpene	Terpene	Pyrite	5.72	China: Guangdong, Yunfu
L180206P-3-LD180205-0006_HK72WCCXY_L3_clean_bin.36.orig.fa_k	OEZ008529	yes	LMSG_G000008991.1	OES073737	terpene	Terpene	Pyrite	12.24	China: Guangdong, Yunfu
L180206P-3-LD180205-0006_HK72WCCXY_L3_clean_bin.36.orig.fa_k	OEZ008529	yes	LMSG_G000008991.1	OES073737	bacteriocin	RiPPs	Pyrite	10.80	China: Guangdong, Yunfu
L180206P-3-LD180205-0006_HK72WCCXY_L3_clean_bin.37.orig.fa_k	OEZ008529	yes	LMSG_G000010148.1	OES073737	T3PKS	PKSother	Pyrite	6.65	China: Guangdong, Yunfu
L180206P-3-LD180205-0006_HK72WCCXY_L3_clean_bin.37.orig.fa_k	OEZ008529	yes	LMSG_G000010148.1	OES073737	terpene	Terpene	Pyrite	6.70	China: Guangdong, Yunfu
L180206P-3-LD180205-0006_HK72WCCXY_L3_clean_bin.38.strict.fa	OEZ008529	yes	LMSG_G000010030.1	OES073737	NRPS-like	NRPS	Pyrite	11.27	China: Guangdong, Yunfu
L180206P-3-LD180205-0006_HK72WCCXY_L3_clean_bin.38.strict.fa	OEZ008529	yes	LMSG_G000010030.1	OES073737	terpene	Terpene	Pyrite	6.70	China: Guangdong, Yunfu
L180206P-3-LD180205-0006_HK72WCCXY_L3_clean_bin.38.strict.fa	OEZ008529	yes	LMSG_G000010030.1	OES073737	T3PKS	PKSother	Pyrite	5.43	China: Guangdong, Yunfu
L180206P-3-LD180205-0006_HK72WCCXY_L3_clean_bin.38.strict.fa	OEZ008529	yes	LMSG_G000010030.1	OES073737	terpene	Terpene	Pyrite	5.29	China: Guangdong, Yunfu
L180206P-3-LD180205-0006_HK72WCCXY_L3_clean_bin.39.orig.fa_k	OEZ008529	no	LMSG_G000010548.1	OES073737	T1PKS	PKSI	Pyrite	10.00	China: Guangdong, Yunfu
L180206P-3-LD180205-0006_HK72WCCXY_L3_clean_bin.39.orig.fa_k	OEZ008529	no	LMSG_G000010548.1	OES073737	terpene	Terpene	Pyrite	17.01	China: Guangdong, Yunfu
L180206P-3-LD180205-0006_HK72WCCXY_L3_clean_bin.39.orig.fa_k	OEZ008529	no	LMSG_G000010548.1	OES073737	arylpolyene	Others	Pyrite	30.80	China: Guangdong, Yunfu
L180206P-3-LD180205-0006_HK72WCCXY_L3_clean_bin.40.strict.fa	OEZ008529	yes	LMSG_G000009589.1	OES073737	terpene	Terpene	Pyrite	22.15	China: Guangdong, Yunfu
L180206P-3-LD180205-0006_HK72WCCXY_L3_clean_bin.40.strict.fa	OEZ008529	yes	LMSG_G000009589.1	OES073737	acyl_amino_acids	Others	Pyrite	28.14	China: Guangdong, Yunfu
L180206P-3-LD180205-0006_HK72WCCXY_L3_clean_bin.40.strict.fa	OEZ008529	yes	LMSG_G000009589.1	OES073737	terpene	Terpene	Pyrite	18.32	China: Guangdong, Yunfu
L180206P-3-LD180205-0006_HK72WCCXY_L3_clean_bin.40.strict.fa	OEZ008529	yes	LMSG_G000009589.1	OES073737	acyl_amino_acids	Others	Pyrite	15.71	China: Guangdong, Yunfu
L180206P-3-LD180205-0006_HK72WCCXY_L3_clean_bin.43.orig.fa_k	OEZ008529	yes	LMSG_G000010766.1	OES073737	bacteriocin	RiPPs	Pyrite	11.75	China: Guangdong, Yunfu
L180206P-3-LD180205-0006_HK72WCCXY_L3_clean_bin.43.orig.fa_k	OEZ008529	yes	LMSG_G000010766.1	OES073737	acyl_amino_acids	Others	Pyrite	39.22	China: Guangdong, Yunfu
L180206P-3-LD180205-0006_HK72WCCXY_L3_clean_bin.45.orig.fa_k	OEZ008529	yes	LMSG_G000010534.1	OES073737	terpene	Terpene	Pyrite	22.73	China: Guangdong, Yunfu
L180206P-3-LD180205-0006_HK72WCCXY_L3_clean_bin.49.orig.fa_k	OEZ008529	yes	LMSG_G000008256.1	OES073737	terpene	Terpene	Pyrite	19.12	China: Guangdong, Yunfu
L180206P-3-LD180205-0006_HK72WCCXY_L3_clean_bin.5.permissive	OEZ008529	yes	LMSG_G000008003.1	OES073737	TfuA-related.bacteriocin	RiPPs	Pyrite	23.52	China: Guangdong, Yunfu
L180206P-3-LD180205-0006_HK72WCCXY_L3_clean_bin.50.strict.fa	OEZ008529	yes	LMSG_G000010586.1	OES073737	terpene	Terpene	Pyrite	14.31	China: Guangdong, Yunfu
L180206P-3-LD180205-0006_HK72WCCXY_L3_clean_bin.52.strict.fa	OEZ008529	yes	LMSG_G000010558.1	OES073737	arylpolyene	Others	Pyrite	40.67	China: Guangdong, Yunfu
L180206P-3-LD180205-0006_HK72WCCXY_L3_clean_bin.52.strict.fa	OEZ008529	yes	LMSG_G000010558.1	OES073737	terpene	Terpene	Pyrite	19.67	China: Guangdong, Yunfu
L180206P-3-LD180205-0006_HK72WCCXY_L3_clean_bin.55.strict.fa	OEZ008529	no	LMSG_G000004382.1	OES073737	thiopeptide	RiPPs	Pyrite	40.35	China: Guangdong, Yunfu
L180206P-3-LD180205-0006_HK72WCCXY_L3_clean_bin.55.strict.fa	OEZ008529	no	LMSG_G000004382.1	OES073737	arylpolyene	Others	Pyrite	26.66	China: Guangdong, Yunfu
L180206P-3-LD180205-0006_HK72WCCXY_L3_clean_bin.55.strict.fa	OEZ008529	yes	LMSG_G000004382.1	OES073737	betalactone	Others	Pyrite	23.22	China: Guangdong, Yunfu
L180206P-3-LD180205-0006_HK72WCCXY_L3_clean_bin.57.orig.fa_k	OEZ008529	yes	LMSG_G000004907.1	OES073737	acyl_amino_acids	Others	Pyrite	33.15	China: Guangdong, Yunfu
L180206P-3-LD180205-0006_HK72WCCXY_L3_clean_bin.58.orig.fa_k	OEZ008529	yes	LMSG_G000009219.1	OES073737	T3PKS	PKSother	Pyrite	5.09	China: Guangdong, Yunfu
L180206P-3-LD180205-0006_HK72WCCXY_L3_clean_bin.59.orig.fa_k	OEZ008529	yes	LMSG_G000008214.1	OES073737	terpene	Terpene	Pyrite	20.86	China: Guangdong, Yunfu
L180206P-3-LD180205-0006_HK72WCCXY_L3_clean_bin.6.orig.fa_kl	OEZ008529	yes	LMSG_G000008541.1	OES073737	terpene	Terpene	Pyrite	23.58	China: Guangdong, Yunfu
L180206P-3-LD180205-0006_HK72WCCXY_L3_clean_bin.6.orig.fa_kl	OEZ008529	yes	LMSG_G000008541.1	OES073737	bacteriocin	RiPPs	Pyrite	10.80	China: Guangdong, Yunfu
L180206P-3-LD180205-0006_HK72WCCXY_L3_clean_bin.60.orig.fa_k	OEZ008529	no	LMSG_G000009531.1	OES073737	terpene	Terpene	Pyrite	22.05	China: Guangdong, Yunfu
L180206P-3-LD180205-0006_HK72WCCXY_L3_clean_bin.60.orig.fa_k	OEZ008529	no	LMSG_G000009531.1	OES073737	terpene	Terpene	Pyrite	23.29	China: Guangdong, Yunfu
L180206P-3-LD180205-0006_HK72WCCXY_L3_clean_bin.61.orig.fa_k	OEZ008529	yes	LMSG_G000009520.1	OES073737	arylpolyene	Others	Pyrite	41.19	China: Guangdong, Yunfu
L180206P-3-LD180205-0006_HK72WCCXY_L3_clean_bin.61.orig.fa_k	OEZ008529	yes	LMSG_G000009520.1	OES073737	terpene	Terpene	Pyrite	23.29	China: Guangdong, Yunfu
L180206P-3-LD180205-0006_HK72WCCXY_L3_clean_bin.61.orig.fa_k	OEZ008529	yes	LMSG_G000009520.1	OES073737	ectoine	Others	Pyrite	7.05	China: Guangdong, Yunfu
L180206P-3-LD180205-0006_HK72WCCXY_L3_clean_bin.61.orig.fa_k	OEZ008529	yes	LMSG_G000009520.1	OES073737	terpene	Terpene	Pyrite	21.16	China: Guangdong, Yunfu
L180206P-3-LD180205-0006_HK72WCCXY_L3_clean_bin.61.orig.fa_k	OEZ008529	yes	LMSG_G000009520.1	OES073737	acyl_amino_acids	Others	Pyrite	9.12	China: Guangdong, Yunfu
L180206P-3-LD180205-0006_HK72WCCXY_L3_clean_bin.61.orig.fa_k	OEZ008529	yes	LMSG_G000009520.1	OES073737	NRPS-like	NRPS	Pyrite	33.51	China: Guangdong, Yunfu
L180206P-3-LD180205-0006_HK72WCCXY_L3_clean_bin.63.orig.fa_k	OEZ008529	yes	LMSG_G000010241.1	OES073737	arylpolyene	Others	Pyrite	40.68	China: Guangdong, Yunfu
L180206P-3-LD180205-0006_HK72WCCXY_L3_clean_bin.63.orig.fa_k	OEZ008529	yes	LMSG_G000010241.1	OES073737	terpene	Terpene	Pyrite	12.34	China: Guangdong, Yunfu
L180206P-3-LD180205-0006_HK72WCCXY_L3_clean_bin.63.orig.fa_k	OEZ008529	yes	LMSG_G000010241.1	OES073737	terpene	Terpene	Pyrite	12.66	China: Guangdong, Yunfu
L180206P-3-LD180205-0006_HK72WCCXY_L3_clean_bin.64.orig.fa_k	OEZ008529	no	LMSG_G000010597.1	OES073737	bacteriocin	RiPPs	Pyrite	7.22	China: Guangdong, Yunfu
L180206P-3-LD180205-0006_HK72WCCXY_L3_clean_bin.66.strict.fa	OEZ008529	yes	LMSG_G000009893.1	OES073737	terpene	Terpene	Pyrite	20.71	China: Guangdong, Yunfu
L180206P-3-LD180205-0006_HK72WCCXY_L3_clean_bin.67.orig.fa_k	OEZ008529	yes	LMSG_G000010456.1	OES073737	arylpolyene.hserlactone	Others	Pyrite	27.35	China: Guangdong, Yunfu
L180206P-3-LD180205-0006_HK72WCCXY_L3_clean_bin.68.strict.fa	OEZ008529	yes	LMSG_G000010592.1	OES073737	terpene	Terpene	Pyrite	23.02	China: Guangdong, Yunfu
L180206P-3-LD180205-0006_HK72WCCXY_L3_clean_bin.68.strict.fa	OEZ008529	yes	LMSG_G000010592.1	OES073737	arylpolyene	Others	Pyrite	26.57	China: Guangdong, Yunfu
L180206P-3-LD180205-0006_HK72WCCXY_L3_clean_bin.69.orig.fa_k	OEZ008529	yes	LMSG_G000009609.1	OES073737	terpene	Terpene	Pyrite	19.78	China: Guangdong, Yunfu
L180206P-3-LD180205-0006_HK72WCCXY_L3_clean_bin.69.orig.fa_k	OEZ008529	yes	LMSG_G000009609.1	OES073737	NRPS-like	NRPS	Pyrite	31.04	China: Guangdong, Yunfu
L180206P-3-LD180205-0006_HK72WCCXY_L3_clean_bin.69.orig.fa_k	OEZ008529	yes	LMSG_G000009609.1	OES073737	terpene	Terpene	Pyrite	21.99	China: Guangdong, Yunfu
L180206P-3-LD180205-0006_HK72WCCXY_L3_clean_bin.9.orig.fa_kl	OEZ008529	yes	LMSG_G000008477.1	OES073737	betalactone	Others	Pyrite	24.25	China: Guangdong, Yunfu
L180206P-3-LD180205-0006_HK72WCCXY_L3_clean_bin.9.orig.fa_kl	OEZ008529	yes	LMSG_G000008477.1	OES073737	terpene	Terpene	Pyrite	13.05	China: Guangdong, Yunfu
L180206P-3-LD180205-0007_HK72WCCXY_L3_clean_bin.1.orig.fa_kl	OEZ008529	yes	LMSG_G000008988.1	OES073738	bacteriocin	RiPPs	Pyrite	8.05	China: Guangdong, Yunfu
L180206P-3-LD180205-0007_HK72WCCXY_L3_clean_bin.1.orig.fa_kl	OEZ008529	yes	LMSG_G000008988.1	OES073738	terpene	Terpene	Pyrite	12.17	China: Guangdong, Yunfu
L180206P-3-LD180205-0007_HK72WCCXY_L3_clean_bin.10.permissiv	OEZ008529	yes	LMSG_G000009610.1	OES073738	terpene	Terpene	Pyrite	23.28	China: Guangdong, Yunfu
L180206P-3-LD180205-0007_HK72WCCXY_L3_clean_bin.10.permissiv	OEZ008529	yes	LMSG_G000009610.1	OES073738	terpene	Terpene	Pyrite	21.99	China: Guangdong, Yunfu
L180206P-3-LD180205-0007_HK72WCCXY_L3_clean_bin.10.permissiv	OEZ008529	yes	LMSG_G000009610.1	OES073738	NRPS-like	NRPS	Pyrite	28.41	China: Guangdong, Yunfu
L180206P-3-LD180205-0007_HK72WCCXY_L3_clean_bin.12.permissiv	OEZ008529	yes	LMSG_G000010767.1	OES073738	acyl_amino_acids	Others	Pyrite	43.86	China: Guangdong, Yunfu
L180206P-3-LD180205-0007_HK72WCCXY_L3_clean_bin.17.orig.fa_k	OEZ008529	yes	LMSG_G000008472.1	OES073738	terpene	Terpene	Pyrite	12.02	China: Guangdong, Yunfu
L180206P-3-LD180205-0007_HK72WCCXY_L3_clean_bin.18.orig.fa_k	OEZ008529	yes	LMSG_G000010574.1	OES073738	bacteriocin	RiPPs	Pyrite	10.85	China: Guangdong, Yunfu
L180206P-3-LD180205-0007_HK72WCCXY_L3_clean_bin.18.orig.fa_k	OEZ008529	yes	LMSG_G000010574.1	OES073738	terpene	Terpene	Pyrite	8.81	China: Guangdong, Yunfu
L180206P-3-LD180205-0007_HK72WCCXY_L3_clean_bin.18.orig.fa_k	OEZ008529	no	LMSG_G000010574.1	OES073738	terpene	Terpene	Pyrite	11.50	China: Guangdong, Yunfu
L180206P-3-LD180205-0007_HK72WCCXY_L3_clean_bin.2.orig.fa_kl	OEZ008529	yes	LMSG_G000008215.1	OES073738	terpene	Terpene	Pyrite	20.86	China: Guangdong, Yunfu
L180206P-3-LD180205-0007_HK72WCCXY_L3_clean_bin.22.orig.fa_k	OEZ008529	yes	LMSG_G000010588.1	OES073738	terpene	Terpene	Pyrite	14.72	China: Guangdong, Yunfu
L180206P-3-LD180205-0007_HK72WCCXY_L3_clean_bin.23.orig.fa_k	OEZ008529	yes	LMSG_G000010434.1	OES073738	terpene	Terpene	Pyrite	23.10	China: Guangdong, Yunfu
L180206P-3-LD180205-0007_HK72WCCXY_L3_clean_bin.24.orig.fa_k	OEZ008529	yes	LMSG_G000008251.1	OES073738	other	Others	Pyrite	21.43	China: Guangdong, Yunfu
L180206P-3-LD180205-0007_HK72WCCXY_L3_clean_bin.25.orig.fa_k	OEZ008529	yes	LMSG_G000010820.1	OES073738	acyl_amino_acids	Others	Pyrite	14.60	China: Guangdong, Yunfu
L180206P-3-LD180205-0007_HK72WCCXY_L3_clean_bin.26.orig.fa_k	OEZ008529	yes	LMSG_G000004383.1	OES073738	bacteriocin	RiPPs	Pyrite	10.46	China: Guangdong, Yunfu
L180206P-3-LD180205-0007_HK72WCCXY_L3_clean_bin.26.orig.fa_k	OEZ008529	yes	LMSG_G000004383.1	OES073738	terpene	Terpene	Pyrite	17.86	China: Guangdong, Yunfu
L180206P-3-LD180205-0007_HK72WCCXY_L3_clean_bin.26.orig.fa_k	OEZ008529	yes	LMSG_G000004383.1	OES073738	betalactone	Others	Pyrite	27.15	China: Guangdong, Yunfu
L180206P-3-LD180205-0007_HK72WCCXY_L3_clean_bin.26.orig.fa_k	OEZ008529	yes	LMSG_G000004383.1	OES073738	ectoine	Others	Pyrite	6.12	China: Guangdong, Yunfu
L180206P-3-LD180205-0007_HK72WCCXY_L3_clean_bin.26.orig.fa_k	OEZ008529	yes	LMSG_G000004383.1	OES073738	NRPS-like	NRPS	Pyrite	39.30	China: Guangdong, Yunfu
L180206P-3-LD180205-0007_HK72WCCXY_L3_clean_bin.28.orig.fa_k	OEZ008529	yes	LMSG_G000010242.1	OES073738	arylpolyene	Others	Pyrite	5.43	China: Guangdong, Yunfu
L180206P-3-LD180205-0007_HK72WCCXY_L3_clean_bin.28.orig.fa_k	OEZ008529	yes	LMSG_G000010242.1	OES073738	bacteriocin	RiPPs	Pyrite	8.27	China: Guangdong, Yunfu
L180206P-3-LD180205-0007_HK72WCCXY_L3_clean_bin.30.strict.fa	OEZ008529	yes	LMSG_G000007812.1	OES073738	terpene	Terpene	Pyrite	15.06	China: Guangdong, Yunfu
L180206P-3-LD180205-0007_HK72WCCXY_L3_clean_bin.30.strict.fa	OEZ008529	yes	LMSG_G000007812.1	OES073738	terpene	Terpene	Pyrite	13.44	China: Guangdong, Yunfu
L180206P-3-LD180205-0007_HK72WCCXY_L3_clean_bin.30.strict.fa	OEZ008529	yes	LMSG_G000007812.1	OES073738	T3PKS	PKSother	Pyrite	7.90	China: Guangdong, Yunfu
L180206P-3-LD180205-0007_HK72WCCXY_L3_clean_bin.32.strict.fa	OEZ008529	yes	LMSG_G000008295.1	OES073738	terpene	Terpene	Pyrite	23.48	China: Guangdong, Yunfu
L180206P-3-LD180205-0007_HK72WCCXY_L3_clean_bin.33.strict.fa	OEZ008529	yes	LMSG_G000008738.1	OES073738	terpene	Terpene	Pyrite	6.73	China: Guangdong, Yunfu
L180206P-3-LD180205-0007_HK72WCCXY_L3_clean_bin.34.strict.fa	OEZ008529	yes	LMSG_G000009649.1	OES073738	terpene	Terpene	Pyrite	21.89	China: Guangdong, Yunfu
L180206P-3-LD180205-0007_HK72WCCXY_L3_clean_bin.34.strict.fa	OEZ008529	yes	LMSG_G000009649.1	OES073738	terpene	Terpene	Pyrite	12.28	China: Guangdong, Yunfu
L180206P-3-LD180205-0007_HK72WCCXY_L3_clean_bin.36.strict.fa	OEZ008529	yes	LMSG_G000010246.1	OES073738	terpene	Terpene	Pyrite	21.11	China: Guangdong, Yunfu
L180206P-3-LD180205-0007_HK72WCCXY_L3_clean_bin.36.strict.fa	OEZ008529	yes	LMSG_G000010246.1	OES073738	T1PKS.hserlactone	Others	Pyrite	57.71	China: Guangdong, Yunfu
L180206P-3-LD180205-0007_HK72WCCXY_L3_clean_bin.36.strict.fa	OEZ008529	yes	LMSG_G000010246.1	OES073738	terpene	Terpene	Pyrite	24.88	China: Guangdong, Yunfu
L180206P-3-LD180205-0007_HK72WCCXY_L3_clean_bin.36.strict.fa	OEZ008529	yes	LMSG_G000010246.1	OES073738	betalactone	Others	Pyrite	26.48	China: Guangdong, Yunfu
L180206P-3-LD180205-0007_HK72WCCXY_L3_clean_bin.36.strict.fa	OEZ008529	yes	LMSG_G000010246.1	OES073738	bacteriocin	RiPPs	Pyrite	10.89	China: Guangdong, Yunfu
L180206P-3-LD180205-0007_HK72WCCXY_L3_clean_bin.39.permissiv	OEZ008529	yes	LMSG_G000004912.1	OES073738	terpene	Terpene	Pyrite	17.83	China: Guangdong, Yunfu
L180206P-3-LD180205-0007_HK72WCCXY_L3_clean_bin.39.permissiv	OEZ008529	yes	LMSG_G000004912.1	OES073738	NRPS-like	NRPS	Pyrite	11.98	China: Guangdong, Yunfu
L180206P-3-LD180205-0007_HK72WCCXY_L3_clean_bin.39.permissiv	OEZ008529	yes	LMSG_G000004912.1	OES073738	terpene	Terpene	Pyrite	10.91	China: Guangdong, Yunfu
L180206P-3-LD180205-0007_HK72WCCXY_L3_clean_bin.39.permissiv	OEZ008529	yes	LMSG_G000004912.1	OES073738	T3PKS	PKSother	Pyrite	5.48	China: Guangdong, Yunfu
L180206P-3-LD180205-0007_HK72WCCXY_L3_clean_bin.40.orig.fa_k	OEZ008529	yes	LMSG_G000010479.1	OES073738	terpene	Terpene	Pyrite	20.51	China: Guangdong, Yunfu
L180206P-3-LD180205-0007_HK72WCCXY_L3_clean_bin.42.orig.fa_k	OEZ008529	yes	LMSG_G000008637.1	OES073738	other	Others	Pyrite	7.88	China: Guangdong, Yunfu
L180206P-3-LD180205-0007_HK72WCCXY_L3_clean_bin.43.strict.fa	OEZ008529	yes	LMSG_G000008004.1	OES073738	TfuA-related.bacteriocin	RiPPs	Pyrite	13.33	China: Guangdong, Yunfu
L180206P-3-LD180205-0007_HK72WCCXY_L3_clean_bin.44.orig.fa_k	OEZ008529	yes	LMSG_G000008059.1	OES073738	terpene	Terpene	Pyrite	9.55	China: Guangdong, Yunfu
L180206P-3-LD180205-0007_HK72WCCXY_L3_clean_bin.44.orig.fa_k	OEZ008529	yes	LMSG_G000008059.1	OES073738	terpene	Terpene	Pyrite	8.11	China: Guangdong, Yunfu
L180206P-3-LD180205-0007_HK72WCCXY_L3_clean_bin.46.orig.fa_k	OEZ008529	yes	LMSG_G000006315.1	OES073738	T3PKS	PKSother	Pyrite	9.30	China: Guangdong, Yunfu
L180206P-3-LD180205-0007_HK72WCCXY_L3_clean_bin.46.orig.fa_k	OEZ008529	yes	LMSG_G000006315.1	OES073738	betalactone	Others	Pyrite	5.63	China: Guangdong, Yunfu
L180206P-3-LD180205-0007_HK72WCCXY_L3_clean_bin.46.orig.fa_k	OEZ008529	yes	LMSG_G000006315.1	OES073738	terpene	Terpene	Pyrite	26.51	China: Guangdong, Yunfu
L180206P-3-LD180205-0007_HK72WCCXY_L3_clean_bin.46.orig.fa_k	OEZ008529	yes	LMSG_G000006315.1	OES073738	terpene	Terpene	Pyrite	14.34	China: Guangdong, Yunfu
L180206P-3-LD180205-0007_HK72WCCXY_L3_clean_bin.47.strict.fa	OEZ008529	yes	LMSG_G000006215.1	OES073738	bacteriocin	RiPPs	Pyrite	11.01	China: Guangdong, Yunfu
L180206P-3-LD180205-0007_HK72WCCXY_L3_clean_bin.5.strict.fa_	OEZ008529	yes	LMSG_G000008082.1	OES073738	T1PKS.hglE-KS	PKSother	Pyrite	17.14	China: Guangdong, Yunfu
L180206P-3-LD180205-0007_HK72WCCXY_L3_clean_bin.5.strict.fa_	OEZ008529	yes	LMSG_G000008082.1	OES073738	arylpolyene	Others	Pyrite	16.52	China: Guangdong, Yunfu
L180206P-3-LD180205-0007_HK72WCCXY_L3_clean_bin.51.permissiv	OEZ008529	yes	LMSG_G000009521.1	OES073738	terpene	Terpene	Pyrite	8.97	China: Guangdong, Yunfu
L180206P-3-LD180205-0007_HK72WCCXY_L3_clean_bin.51.permissiv	OEZ008529	yes	LMSG_G000009521.1	OES073738	arylpolyene	Others	Pyrite	8.07	China: Guangdong, Yunfu
L180206P-3-LD180205-0007_HK72WCCXY_L3_clean_bin.53.orig.fa_k	OEZ008529	yes	LMSG_G000004913.1	OES073738	siderophore	Others	Pyrite	11.80	China: Guangdong, Yunfu
L180206P-3-LD180205-0007_HK72WCCXY_L3_clean_bin.53.orig.fa_k	OEZ008529	yes	LMSG_G000004913.1	OES073738	bacteriocin	RiPPs	Pyrite	10.31	China: Guangdong, Yunfu
L180206P-3-LD180205-0007_HK72WCCXY_L3_clean_bin.53.orig.fa_k	OEZ008529	yes	LMSG_G000004913.1	OES073738	NRPS-like	NRPS	Pyrite	42.53	China: Guangdong, Yunfu
L180206P-3-LD180205-0007_HK72WCCXY_L3_clean_bin.54.orig.fa_k	OEZ008529	yes	LMSG_G000010229.1	OES073738	hserlactone	Others	Pyrite	14.09	China: Guangdong, Yunfu
L180206P-3-LD180205-0007_HK72WCCXY_L3_clean_bin.54.orig.fa_k	OEZ008529	yes	LMSG_G000010229.1	OES073738	terpene	Terpene	Pyrite	11.65	China: Guangdong, Yunfu
L180206P-3-LD180205-0007_HK72WCCXY_L3_clean_bin.54.orig.fa_k	OEZ008529	yes	LMSG_G000010229.1	OES073738	terpene.arylpolyene	Others	Pyrite	41.94	China: Guangdong, Yunfu
L180206P-3-LD180205-0007_HK72WCCXY_L3_clean_bin.57.orig.fa_k	OEZ008529	yes	LMSG_G000004914.1	OES073738	bacteriocin	RiPPs	Pyrite	10.81	China: Guangdong, Yunfu
L180206P-3-LD180205-0007_HK72WCCXY_L3_clean_bin.59.orig.fa_k	OEZ008529	yes	LMSG_G000004915.1	OES073738	betalactone	Others	Pyrite	22.42	China: Guangdong, Yunfu

L180206P-3-LD180205-0007_HK72WCCXY_L3_clean_bin.6.orig.fa_kl	OEZ008529	yes	LMSG_G000008221.1	OES073738	terpene	Terpene	Pyrite	14.38	China: Guangdong, Yunfu
L180206P-3-LD180205-0007_HK72WCCXY_L3_clean_bin.61.strict.fa	OEZ008529	yes	LMSG_G000004916.1	OES073738	bacteriocin	RiPPs	Pyrite	8.58	China: Guangdong, Yunfu
L180206P-3-LD180205-0007_HK72WCCXY_L3_clean_bin.63.orig.fa_k	OEZ008529	yes	LMSG_G000008705.1	OES073738	bacteriocin	RiPPs	Pyrite	6.32	China: Guangdong, Yunfu
L180206P-3-LD180205-0007_HK72WCCXY_L3_clean_bin.63.orig.fa_k	OEZ008529	no	LMSG_G000008705.1	OES073738	betalactone	Others	Pyrite	6.93	China: Guangdong, Yunfu
L180206P-3-LD180205-0007_HK72WCCXY_L3_clean_bin.63.orig.fa_k	OEZ008529	yes	LMSG_G000008705.1	OES073738	bacteriocin	RiPPs	Pyrite	7.26	China: Guangdong, Yunfu
L180206P-3-LD180205-0007_HK72WCCXY_L3_clean_bin.66.strict.fa	OEZ008529	yes	LMSG_G000007845.1	OES073738	T3PKS	PKSother	Pyrite	15.17	China: Guangdong, Yunfu
L180206P-3-LD180205-0007_HK72WCCXY_L3_clean_bin.66.strict.fa	OEZ008529	yes	LMSG_G000007845.1	OES073738	terpene	Terpene	Pyrite	11.55	China: Guangdong, Yunfu
L180206P-3-LD180205-0007_HK72WCCXY_L3_clean_bin.66.strict.fa	OEZ008529	yes	LMSG_G000007845.1	OES073738	NRPS-like	NRPS	Pyrite	9.88	China: Guangdong, Yunfu
L180206P-3-LD180205-0007_HK72WCCXY_L3_clean_bin.66.strict.fa	OEZ008529	yes	LMSG_G000007845.1	OES073738	terpene	Terpene	Pyrite	9.82	China: Guangdong, Yunfu
L180206P-3-LD180205-0007_HK72WCCXY_L3_clean_bin.67.strict.fa	OEZ008529	yes	LMSG_G000004384.1	OES073738	terpene	Terpene	Pyrite	21.68	China: Guangdong, Yunfu
L180206P-3-LD180205-0007_HK72WCCXY_L3_clean_bin.68.orig.fa_k	OEZ008529	no	LMSG_G000006216.1	OES073738	NRPS-like	NRPS	Pyrite	23.77	China: Guangdong, Yunfu
L180206P-3-LD180205-0007_HK72WCCXY_L3_clean_bin.68.orig.fa_k	OEZ008529	no	LMSG_G000006216.1	OES073738	terpene	Terpene	Pyrite	13.30	China: Guangdong, Yunfu
L180206P-3-LD180205-0007_HK72WCCXY_L3_clean_bin.68.orig.fa_k	OEZ008529	no	LMSG_G000006216.1	OES073738	terpene	Terpene	Pyrite	23.29	China: Guangdong, Yunfu
L180206P-3-LD180205-0007_HK72WCCXY_L3_clean_bin.69.permissiv	OEZ008529	yes	LMSG_G000008262.1	OES073738	terpene	Terpene	Pyrite	20.86	China: Guangdong, Yunfu
L180206P-3-LD180205-0007_HK72WCCXY_L3_clean_bin.7.strict.fa_	OEZ008529	yes	LMSG_G000007967.1	OES073738	terpene	Terpene	Pyrite	5.08	China: Guangdong, Yunfu
L180206P-3-LD180205-0007_HK72WCCXY_L3_clean_bin.71.orig.fa_k	OEZ008529	yes	LMSG_G000009479.1	OES073738	betalactone	Others	Pyrite	19.49	China: Guangdong, Yunfu
L180206P-3-LD180205-0007_HK72WCCXY_L3_clean_bin.73.strict.fa	OEZ008529	yes	LMSG_G000009214.1	OES073738	terpene	Terpene	Pyrite	14.12	China: Guangdong, Yunfu
L180206P-3-LD180205-0007_HK72WCCXY_L3_clean_bin.73.strict.fa	OEZ008529	yes	LMSG_G000009214.1	OES073738	hserlactone	Others	Pyrite	19.23	China: Guangdong, Yunfu
L180206P-3-LD180205-0007_HK72WCCXY_L3_clean_bin.73.strict.fa	OEZ008529	yes	LMSG_G000009214.1	OES073738	bacteriocin	RiPPs	Pyrite	10.90	China: Guangdong, Yunfu
L180206P-3-LD180205-0007_HK72WCCXY_L3_clean_bin.73.strict.fa	OEZ008529	yes	LMSG_G000009214.1	OES073738	terpene	Terpene	Pyrite	22.05	China: Guangdong, Yunfu
L180206P-3-LD180205-0007_HK72WCCXY_L3_clean_bin.74.orig.fa_k	OEZ008529	yes	LMSG_G000007852.1	OES073738	NRPS-like	NRPS	Pyrite	11.35	China: Guangdong, Yunfu
L180206P-3-LD180205-0007_HK72WCCXY_L3_clean_bin.74.orig.fa_k	OEZ008529	yes	LMSG_G000007852.1	OES073738	terpene	Terpene	Pyrite	22.04	China: Guangdong, Yunfu
L180206P-3-LD180205-0007_HK72WCCXY_L3_clean_bin.74.orig.fa_k	OEZ008529	yes	LMSG_G000007852.1	OES073738	terpene	Terpene	Pyrite	14.20	China: Guangdong, Yunfu
L180206P-3-LD180205-0007_HK72WCCXY_L3_clean_bin.74.orig.fa_k	OEZ008529	yes	LMSG_G000007852.1	OES073738	NRPS-like	NRPS	Pyrite	43.27	China: Guangdong, Yunfu
L180206P-3-LD180205-0007_HK72WCCXY_L3_clean_bin.74.orig.fa_k	OEZ008529	yes	LMSG_G000007852.1	OES073738	T3PKS	PKSother	Pyrite	14.39	China: Guangdong, Yunfu
L180206P-3-LD180205-0007_HK72WCCXY_L3_clean_bin.76.permissiv	OEZ008529	yes	LMSG_G000007744.1	OES073738	bacteriocin	RiPPs	Pyrite	10.88	China: Guangdong, Yunfu
L180206P-3-LD180205-0007_HK72WCCXY_L3_clean_bin.76.permissiv	OEZ008529	yes	LMSG_G000007744.1	OES073738	terpene	Terpene	Pyrite	17.33	China: Guangdong, Yunfu
L180206P-3-LD180205-0007_HK72WCCXY_L3_clean_bin.76.permissiv	OEZ008529	yes	LMSG_G000007744.1	OES073738	T3PKS	PKSother	Pyrite	16.32	China: Guangdong, Yunfu
L180206P-3-LD180205-0007_HK72WCCXY_L3_clean_bin.76.permissiv	OEZ008529	yes	LMSG_G000007744.1	OES073738	terpene	Terpene	Pyrite	6.76	China: Guangdong, Yunfu
L180206P-3-LD180205-0007_HK72WCCXY_L3_clean_bin.77.orig.fa_k	OEZ008529	yes	LMSG_G000010593.1	OES073738	terpene	Terpene	Pyrite	23.02	China: Guangdong, Yunfu
L180206P-3-LD180205-0007_HK72WCCXY_L3_clean_bin.77.orig.fa_k	OEZ008529	yes	LMSG_G000010593.1	OES073738	arylpolyene	Others	Pyrite	26.02	China: Guangdong, Yunfu
L180206P-3-LD180205-0007_HK72WCCXY_L3_clean_bin.79.orig.fa_k	OEZ008529	yes	LMSG_G000008497.1	OES073738	LAP	RiPPs	Pyrite	9.64	China: Guangdong, Yunfu
L180206P-3-LD180205-0007_HK72WCCXY_L3_clean_bin.79.orig.fa_k	OEZ008529	yes	LMSG_G000008497.1	OES073738	terpene	Terpene	Pyrite	26.16	China: Guangdong, Yunfu
L180206P-3-LD180205-0007_HK72WCCXY_L3_clean_bin.80.permissiv	OEZ008529	yes	LMSG_G000010535.1	OES073738	T1PKS	PKSI	Pyrite	16.89	China: Guangdong, Yunfu
L180206P-3-LD180205-0007_HK72WCCXY_L3_clean_bin.80.permissiv	OEZ008529	no	LMSG_G000010535.1	OES073738	terpene	Terpene	Pyrite	7.70	China: Guangdong, Yunfu
L180206P-3-LD180205-0007_HK72WCCXY_L3_clean_bin.81.strict.fa	OEZ008529	yes	LMSG_G000008213.1	OES073738	terpene	Terpene	Pyrite	13.96	China: Guangdong, Yunfu
L180206P-3-LD180205-0007_HK72WCCXY_L3_clean_bin.82.permissiv	OEZ008529	yes	LMSG_G000009568.1	OES073738	terpene	Terpene	Pyrite	22.05	China: Guangdong, Yunfu
L180206P-3-LD180205-0007_HK72WCCXY_L3_clean_bin.82.permissiv	OEZ008529	no	LMSG_G000009568.1	OES073738	NRPS-like	NRPS	Pyrite	33.69	China: Guangdong, Yunfu
L180206P-3-LD180205-0007_HK72WCCXY_L3_clean_bin.82.permissiv	OEZ008529	yes	LMSG_G000009568.1	OES073738	acyl_amino_acids	Others	Pyrite	40.16	China: Guangdong, Yunfu
L180206P-3-LD180205-0007_HK72WCCXY_L3_clean_bin.82.permissiv	OEZ008529	yes	LMSG_G000009568.1	OES073738	acyl_amino_acids	Others	Pyrite	32.81	China: Guangdong, Yunfu
L180206P-3-LD180205-0007_HK72WCCXY_L3_clean_bin.82.permissiv	OEZ008529	yes	LMSG_G000009568.1	OES073738	acyl_amino_acids	Others	Pyrite	9.38	China: Guangdong, Yunfu
L180206P-3-LD180205-0007_HK72WCCXY_L3_clean_bin.9.strict.fa_	OEZ008529	yes	LMSG_G000008115.1	OES073738	terpene	Terpene	Pyrite	10.86	China: Guangdong, Yunfu
L180206P-4-LD180201-0001_HK72WCCXY_L4_clean_bin.13.orig.fa_k	OEZ008529	yes	LMSG_G000004918.1	OES073748	LAP	RiPPs	Lead-Zinc	8.55	China: Jiangxi, Yinshan
L180206P-4-LD180201-0001_HK72WCCXY_L4_clean_bin.13.orig.fa_k	OEZ008529	yes	LMSG_G000004918.1	OES073748	terpene	Terpene	Lead-Zinc	24.01	China: Jiangxi, Yinshan
L180206P-4-LD180201-0001_HK72WCCXY_L4_clean_bin.13.orig.fa_k	OEZ008529	yes	LMSG_G000004918.1	OES073748	arylpolyene	Others	Lead-Zinc	10.03	China: Jiangxi, Yinshan
L180206P-4-LD180201-0001_HK72WCCXY_L4_clean_bin.13.orig.fa_k	OEZ008529	yes	LMSG_G000004918.1	OES073748	T1PKS	PKSI	Lead-Zinc	10.22	China: Jiangxi, Yinshan
L180206P-4-LD180201-0001_HK72WCCXY_L4_clean_bin.13.orig.fa_k	OEZ008529	yes	LMSG_G000004918.1	OES073748	T1PKS	PKSI	Lead-Zinc	9.79	China: Jiangxi, Yinshan
L180206P-4-LD180201-0001_HK72WCCXY_L4_clean_bin.14.orig.fa_k	OEZ008529	no	LMSG_G000010155.1	OES073748	acyl_amino_acids	Others	Lead-Zinc	53.19	China: Jiangxi, Yinshan
L180206P-4-LD180201-0001_HK72WCCXY_L4_clean_bin.14.orig.fa_k	OEZ008529	no	LMSG_G000010155.1	OES073748	terpene	Terpene	Lead-Zinc	16.16	China: Jiangxi, Yinshan
L180206P-4-LD180201-0001_HK72WCCXY_L4_clean_bin.14.orig.fa_k	OEZ008529	no	LMSG_G000010155.1	OES073748	ectoine	Others	Lead-Zinc	10.40	China: Jiangxi, Yinshan
L180206P-4-LD180201-0001_HK72WCCXY_L4_clean_bin.14.orig.fa_k	OEZ008529	no	LMSG_G000010155.1	OES073748	terpene	Terpene	Lead-Zinc	21.71	China: Jiangxi, Yinshan
L180206P-4-LD180201-0001_HK72WCCXY_L4_clean_bin.14.orig.fa_k	OEZ008529	no	LMSG_G000010155.1	OES073748	terpene	Terpene	Lead-Zinc	21.07	China: Jiangxi, Yinshan
L180206P-4-LD180201-0001_HK72WCCXY_L4_clean_bin.15.orig.fa_k	OEZ008529	yes	LMSG_G000010809.1	OES073748	acyl_amino_acids	Others	Lead-Zinc	23.79	China: Jiangxi, Yinshan
L180206P-4-LD180201-0001_HK72WCCXY_L4_clean_bin.15.orig.fa_k	OEZ008529	yes	LMSG_G000010809.1	OES073748	NRPS-like	NRPS	Lead-Zinc	16.96	China: Jiangxi, Yinshan
L180206P-4-LD180201-0001_HK72WCCXY_L4_clean_bin.16.orig.fa_k	OEZ008529	yes	LMSG_G000008481.1	OES073748	terpene	Terpene	Lead-Zinc	26.09	China: Jiangxi, Yinshan
L180206P-4-LD180201-0001_HK72WCCXY_L4_clean_bin.16.orig.fa_k	OEZ008529	yes	LMSG_G000008481.1	OES073748	lassopeptide	RiPPs	Lead-Zinc	7.15	China: Jiangxi, Yinshan
L180206P-4-LD180201-0001_HK72WCCXY_L4_clean_bin.2.orig.fa_kl	OEZ008529	yes	LMSG_G000010569.1	OES073748	bacteriocin	RiPPs	Lead-Zinc	10.84	China: Jiangxi, Yinshan
L180206P-4-LD180201-0001_HK72WCCXY_L4_clean_bin.2.orig.fa_kl	OEZ008529	no	LMSG_G000010569.1	OES073748	arylpolyene	Others	Lead-Zinc	16.24	China: Jiangxi, Yinshan
L180206P-4-LD180201-0001_HK72WCCXY_L4_clean_bin.2.orig.fa_kl	OEZ008529	no	LMSG_G000010569.1	OES073748	terpene	Terpene	Lead-Zinc	14.73	China: Jiangxi, Yinshan
L180206P-4-LD180201-0001_HK72WCCXY_L4_clean_bin.21.orig.fa_k	OEZ008529	yes	LMSG_G000004919.1	OES073748	arylpolyene	Others	Lead-Zinc	20.52	China: Jiangxi, Yinshan
L180206P-4-LD180201-0001_HK72WCCXY_L4_clean_bin.21.orig.fa_k	OEZ008529	yes	LMSG_G000004919.1	OES073748	NRPS	NRPS	Lead-Zinc	34.61	China: Jiangxi, Yinshan
L180206P-4-LD180201-0001_HK72WCCXY_L4_clean_bin.21.orig.fa_k	OEZ008529	yes	LMSG_G000004919.1	OES073748	betalactone	Others	Lead-Zinc	32.33	China: Jiangxi, Yinshan
L180206P-4-LD180201-0001_HK72WCCXY_L4_clean_bin.21.orig.fa_k	OEZ008529	yes	LMSG_G000004919.1	OES073748	bacteriocin	RiPPs	Lead-Zinc	10.54	China: Jiangxi, Yinshan
L180206P-4-LD180201-0001_HK72WCCXY_L4_clean_bin.21.orig.fa_k	OEZ008529	yes	LMSG_G000004919.1	OES073748	NRPS.T1PKS	PKS-NRP_Hybrids	Lead-Zinc	25.24	China: Jiangxi, Yinshan
L180206P-4-LD180201-0001_HK72WCCXY_L4_clean_bin.21.orig.fa_k	OEZ008529	yes	LMSG_G000004919.1	OES073748	terpene	Terpene	Lead-Zinc	16.35	China: Jiangxi, Yinshan
L180206P-4-LD180201-0001_HK72WCCXY_L4_clean_bin.21.orig.fa_k	OEZ008529	yes	LMSG_G000004919.1	OES073748	NRPS-like	NRPS	Lead-Zinc	38.97	China: Jiangxi, Yinshan
L180206P-4-LD180201-0001_HK72WCCXY_L4_clean_bin.21.orig.fa_k	OEZ008529	yes	LMSG_G000004919.1	OES073748	arylpolyene	Others	Lead-Zinc	15.76	China: Jiangxi, Yinshan
L180206P-4-LD180201-0001_HK72WCCXY_L4_clean_bin.23.orig.fa_k	OEZ008529	yes	LMSG_G000009545.1	OES073748	ectoine	Others	Lead-Zinc	6.77	China: Jiangxi, Yinshan
L180206P-4-LD180201-0001_HK72WCCXY_L4_clean_bin.23.orig.fa_k	OEZ008529	no	LMSG_G000009545.1	OES073748	terpene	Terpene	Lead-Zinc	23.38	China: Jiangxi, Yinshan
L180206P-4-LD180201-0001_HK72WCCXY_L4_clean_bin.23.orig.fa_k	OEZ008529	yes	LMSG_G000009545.1	OES073748	terpene	Terpene	Lead-Zinc	22.06	China: Jiangxi, Yinshan
L180206P-4-LD180201-0001_HK72WCCXY_L4_clean_bin.23.orig.fa_k	OEZ008529	yes	LMSG_G000009545.1	OES073748	NRPS-like	NRPS	Lead-Zinc	40.72	China: Jiangxi, Yinshan
L180206P-4-LD180201-0001_HK72WCCXY_L4_clean_bin.24.orig.fa_k	OEZ008529	yes	LMSG_G000008263.1	OES073748	terpene	Terpene	Lead-Zinc	19.17	China: Jiangxi, Yinshan
L180206P-4-LD180201-0001_HK72WCCXY_L4_clean_bin.25.orig.fa_k	OEZ008529	no	LMSG_G000011016.1	OES073748	NRPS	NRPS	Lead-Zinc	7.27	China: Jiangxi, Yinshan
L180206P-4-LD180201-0001_HK72WCCXY_L4_clean_bin.25.orig.fa_k	OEZ008529	no	LMSG_G000011016.1	OES073748	bacteriocin	RiPPs	Lead-Zinc	5.77	China: Jiangxi, Yinshan
L180206P-4-LD180201-0001_HK72WCCXY_L4_clean_bin.25.orig.fa_k	OEZ008529	no	LMSG_G000011016.1	OES073748	phenazine	Others	Lead-Zinc	17.24	China: Jiangxi, Yinshan
L180206P-4-LD180201-0001_HK72WCCXY_L4_clean_bin.25.orig.fa_k	OEZ008529	no	LMSG_G000011016.1	OES073748	hserlactone	Others	Lead-Zinc	10.08	China: Jiangxi, Yinshan
L180206P-4-LD180201-0001_HK72WCCXY_L4_clean_bin.25.orig.fa_k	OEZ008529	no	LMSG_G000011016.1	OES073748	hserlactone	Others	Lead-Zinc	11.08	China: Jiangxi, Yinshan
L180206P-4-LD180201-0001_HK72WCCXY_L4_clean_bin.25.orig.fa_k	OEZ008529	no	LMSG_G000011016.1	OES073748	NRPS-like	NRPS	Lead-Zinc	8.27	China: Jiangxi, Yinshan
L180206P-4-LD180201-0001_HK72WCCXY_L4_clean_bin.25.orig.fa_k	OEZ008529	no	LMSG_G000011016.1	OES073748	NRPS-like	NRPS	Lead-Zinc	5.97	China: Jiangxi, Yinshan
L180206P-4-LD180201-0001_HK72WCCXY_L4_clean_bin.25.orig.fa_k	OEZ008529	no	LMSG_G000011016.1	OES073748	NRPS	NRPS	Lead-Zinc	12.65	China: Jiangxi, Yinshan
L180206P-4-LD180201-0001_HK72WCCXY_L4_clean_bin.25.orig.fa_k	OEZ008529	no	LMSG_G000011016.1	OES073748	NRPS	NRPS	Lead-Zinc	9.25	China: Jiangxi, Yinshan
L180206P-4-LD180201-0001_HK72WCCXY_L4_clean_bin.25.orig.fa_k	OEZ008529	no	LMSG_G000011016.1	OES073748	NRPS	NRPS	Lead-Zinc	5.02	China: Jiangxi, Yinshan
L180206P-4-LD180201-0001_HK72WCCXY_L4_clean_bin.25.orig.fa_k	OEZ008529	no	LMSG_G000011016.1	OES073748	NRPS	NRPS	Lead-Zinc	7.16	China: Jiangxi, Yinshan
L180206P-4-LD180201-0001_HK72WCCXY_L4_clean_bin.27.strict.fa	OEZ008529	yes	LMSG_G000008222.1	OES073748	terpene	Terpene	Lead-Zinc	18.54	China: Jiangxi, Yinshan
L180206P-4-LD180201-0001_HK72WCCXY_L4_clean_bin.3.orig.fa_kl	OEZ008529	yes	LMSG_G000008348.1	OES073748	terpene	Terpene	Lead-Zinc	7.16	China: Jiangxi, Yinshan
L180206P-4-LD180201-0001_HK72WCCXY_L4_clean_bin.3.orig.fa_kl	OEZ008529	yes	LMSG_G000008348.1	OES073748	butyrolactone	Others	Lead-Zinc	7.72	China: Jiangxi, Yinshan
L180206P-4-LD180201-0001_HK72WCCXY_L4_clean_bin.3.orig.fa_kl	OEZ008529	yes	LMSG_G000008348.1	OES073748	terpene	Terpene	Lead-Zinc	5.61	China: Jiangxi, Yinshan
L180206P-4-LD180201-0001_HK72WCCXY_L4_clean_bin.3.orig.fa_kl	OEZ008529	yes	LMSG_G000008348.1	OES073748	T3PKS	PKSother	Lead-Zinc	8.00	China: Jiangxi, Yinshan
L180206P-4-LD180201-0001_HK72WCCXY_L4_clean_bin.3.orig.fa_kl	OEZ008529	yes	LMSG_G000008348.1	OES073748	bacteriocin	RiPPs	Lead-Zinc	6.04	China: Jiangxi, Yinshan
L180206P-4-LD180201-0001_HK72WCCXY_L4_clean_bin.30.orig.fa_k	OEZ008529	yes	LMSG_G000008688.1	OES073748	bacteriocin	RiPPs	Lead-Zinc	10.77	China: Jiangxi, Yinshan
L180206P-4-LD180201-0001_HK72WCCXY_L4_clean_bin.30.orig.fa_k	OEZ008529	yes	LMSG_G000008688.1	OES073748	betalactone	Others	Lead-Zinc	9.22	China: Jiangxi, Yinshan
L180206P-4-LD180201-0001_HK72WCCXY_L4_clean_bin.30.orig.fa_k	OEZ008529	yes	LMSG_G000008688.1	OES073748	NRPS-like	NRPS	Lead-Zinc	23.60	China: Jiangxi, Yinshan
L180206P-4-LD180201-0001_HK72WCCXY_L4_clean_bin.30.orig.fa_k	OEZ008529	yes	LMSG_G000008688.1	OES073748	terpene	Terpene	Lead-Zinc	19.44	China: Jiangxi, Yinshan
L180206P-4-LD180201-0001_HK72WCCXY_L4_clean_bin.34.orig.fa_k	OEZ008529	no	LMSG_G000010622.1	OES073748	arylpolyene	Others	Lead-Zinc	7.32	China: Jiangxi, Yinshan
L180206P-4-LD180201-0001_HK72WCCXY_L4_clean_bin.34.orig.fa_k	OEZ008529	yes	LMSG_G000010622.1	OES073748	bacteriocin	RiPPs	Lead-Zinc	10.99	China: Jiangxi, Yinshan
L180206P-4-LD180201-0001_HK72WCCXY_L4_clean_bin.34.orig.fa_k	OEZ008529	yes	LMSG_G000010622.1	OES073748	terpene	Terpene	Lead-Zinc	23.00	China: Jiangxi, Yinshan
L180206P-4-LD180201-0001_HK72WCCXY_L4_clean_bin.34.orig.fa_k	OEZ008529	yes	LMSG_G000010622.1	OES073748	arylpolyene	Others	Lead-Zinc	41.19	China: Jiangxi, Yinshan
L180206P-4-LD180201-0001_HK72WCCXY_L4_clean_bin.35.orig.fa_k	OEZ008529	yes	LMSG_G000009566.1	OES073748	NRPS-like	NRPS	Lead-Zinc	20.02	China: Jiangxi, Yinshan
L180206P-4-LD180201-0001_HK72WCCXY_L4_clean_bin.35.orig.fa_k	OEZ008529	yes	LMSG_G000009566.1	OES073748	terpene	Terpene	Lead-Zinc	23.40	China: Jiangxi, Yinshan
L180206P-4-LD180201-0001_HK72WCCXY_L4_clean_bin.35.orig.fa_k	OEZ008529	yes	LMSG_G000009566.1	OES073748	terpene	Terpene	Lead-Zinc	11.49	China: Jiangxi, Yinshan
L180206P-4-LD180201-0001_HK72WCCXY_L4_clean_bin.37.orig.fa_k	OEZ008529	no	LMSG_G000010483.1	OES073748	arylpolyene	Others	Lead-Zinc	5.50	China: Jiangxi, Yinshan
L180206P-4-LD180201-0001_HK72WCCXY_L4_clean_bin.37.orig.fa_k	OEZ008529	yes	LMSG_G000010483.1	OES073748	terpene	Terpene	Lead-Zinc	15.69	China: Jiangxi, Yinshan
L180206P-4-LD180201-0001_HK72WCCXY_L4_clean_bin.38.orig.fa_k	OEZ008529	yes	LMSG_G000009155.1	OES073748	terpene	Terpene	Lead-Zinc	13.89	China: Jiangxi, Yinshan
L180206P-4-LD180201-0001_HK72WCCXY_L4_clean_bin.38.orig.fa_k	OEZ008529	yes	LMSG_G000009155.1	OES073748	T3PKS	PKSother	Lead-Zinc	12.08	China: Jiangxi, Yinshan
L180206P-4-LD180201-0001_HK72WCCXY_L4_clean_bin.4.strict.fa_	OEZ008529	yes	LMSG_G000007813.1	OES073748	T3PKS	PKSother	Lead-Zinc	32.17	China: Jiangxi, Yinshan
L180206P-4-LD180201-0001_HK72WCCXY_L4_clean_bin.40.strict.fa	OEZ008529	yes	LMSG_G000009429.1	OES073748	betalactone	Others	Lead-Zinc	30.86	China: Jiangxi, Yinshan
L180206P-4-LD180201-0001_HK72WCCXY_L4_clean_bin.40.strict.fa	OEZ008529	yes	LMSG_G000009429.1	OES073748	bacteriocin	RiPPs	Lead-Zinc	10.85	China: Jiangxi, Yinshan
L180206P-4-LD180201-0001_HK72WCCXY_L4_clean_bin.40.strict.fa	OEZ008529	yes	LMSG_G000009429.1	OES073748	betalactone	Others	Lead-Zinc	22.80	China: Jiangxi, Yinshan
L180206P-4-LD180201-0001_HK72WCCXY_L4_clean_bin.40.strict.fa	OEZ008529	yes	LMSG_G000009429.1	OES073748	ectoine	Others	Lead-Zinc	10.40	China: Jiangxi, Yinshan
L180206P-4-LD180201-0001_HK72WCCXY_L4_clean_bin.41.permissiv	OEZ008529	yes	LMSG_G000008009.1	OES073748	bacteriocin	RiPPs	Lead-Zinc	10.32	China: Jiangxi, Yinshan
L180206P-4-LD180201-0001_HK72WCCXY_L4_clean_bin.41.permissiv	OEZ008529	yes	LMSG_G000008009.1	OES073748	TfuA-related.bacteriocin	RiPPs	Lead-Zinc	13.14	China: Jiangxi, Yinshan
L180206P-4-LD180201-0001_HK72WCCXY_L4_clean_bin.42.orig.fa_k	OEZ008529	no	LMSG_G000010291.1	OES073748	terpene	Terpene	Lead-Zinc	21.15	China: Jiangxi, Yinshan
L180206P-4-LD180201-0001_HK72WCCXY_L4_clean_bin.42.orig.fa_k	OEZ008529	no	LMSG_G000010291.1	OES073748	arylpolyene	Others	Lead-Zinc	35.38	China: Jiangxi, Yinshan
L180206P-4-LD180201-0001_HK72WCCXY_L4_clean_bin.44.orig.fa_k	OEZ008529	yes	LMSG_G000004920.1	OES073748	NRPS-like	NRPS	Lead-Zinc	19.19	China: Jiangxi, Yinshan
L180206P-4-LD180201-0001_HK72WCCXY_L4_clean_bin.44.orig.fa_k	OEZ008529	yes	LMSG_G000004920.1	OES073748	terpene	Terpene	Lead-Zinc	9.97	China: Jiangxi, Yinshan
L180206P-4-LD180201-0001_HK72WCCXY_L4_clean_bin.44.orig.fa_k	OEZ008529	yes	LMSG_G000004920.1	OES073748	resorcinol.arylpolyene	Others	Lead-Zinc	14.40	China: Jiangxi, Yinshan
L180206P-4-LD180201-0001_HK72WCCXY_L4_clean_bin.45.permissiv	OEZ008529	yes	LMSG_G000008010.1	OES073748	betalactone	Others	Lead-Zinc	23.53	China: Jiangxi, Yinshan
L180206P-4-LD180201-0001_HK72WCCXY_L4_clean_bin.45.permissiv	OEZ008529	yes	LMSG_G000008010.1	OES073748	betalactone	Others	Lead-Zinc	23.00	China: Jiangxi, Yinshan
L180206P-4-LD180201-0001_HK72WCCXY_L4_clean_bin.45.permissiv	OEZ008529	yes	LMSG_G000008010.1	OES073748	TfuA-related.bacteriocin	RiPPs	Lead-Zinc	25.71	China: Jiangxi, Yinshan
L180206P-4-LD180201-0001_HK72WCCXY_L4_clean_bin.9.orig.fa_kl	OEZ008529	yes	LMSG_G000004921.1	OES073748	terpene	Terpene	Lead-Zinc	29.77	China: Jiangxi, Yinshan
L180206P-4-LD180201-0001_HK72WCCXY_L4_clean_bin.9.orig.fa_kl	OEZ008529	yes	LMSG_G000004921.1	OES073748	T3PKS	PKSother	Lead-Zinc	24.18	China: Jiangxi, Yinshan
L180206P-4-LD180201-0001_HK72WCCXY_L4_clean_bin.9.orig.fa_kl	OEZ008529	yes	LMSG_G000004921.1	OES073748	NRPS-like	NRPS	Lead-Zinc	9.73	China: Jiangxi, Yinshan
L180206P-4-LD180201-0001_HK72WCCXY_L4_clean_bin.9.orig.fa_kl	OEZ008529	yes	LMSG_G000004921.1	OES073748	NRPS	NRPS	Lead-Zinc	16.75	China: Jiangxi, Yinshan
L180206P-4-LD180201-0005_HK72WCCXY_L4_clean_bin.1.orig.fa_kl	OEZ008529	yes	LMSG_G000004386.1	OES073744	terpene	Terpene	Lead-Zinc	23.07	China: Jiangxi, Yinshan
L180206P-4-LD180201-0005_HK72WCCXY_L4_clean_bin.1.orig.fa_kl	OEZ008529	yes	LMSG_G000004386.1	OES073744	NRPS-like	NRPS	Lead-Zinc	29.27	China: Jiangxi, Yinshan
L180206P-4-LD180201-0005_HK72WCCXY_L4_clean_bin.1.orig.fa_kl	OEZ008529	yes	LMSG_G000004386.1	OES073744	lassopeptide	RiPPs	Lead-Zinc	28.80	China: Jiangxi, Yinshan
L180206P-4-LD180201-0005_HK72WCCXY_L4_clean_bin.1.orig.fa_kl	OEZ008529	yes	LMSG_G000004386.1	OES073744	arylpolyene	Others	Lead-Zinc	31.34	China: Jiangxi, Yinshan
L180206P-4-LD180201-0005_HK72WCCXY_L4_clean_bin.1.orig.fa_kl	OEZ008529	yes	LMSG_G000004386.1	OES073744	bacteriocin	RiPPs	Lead-Zinc	10.51	China: Jiangxi, Yinshan
L180206P-4-LD180201-0005_HK72WCCXY_L4_clean_bin.10.orig.fa_k	OEZ008529	no	LMSG_G000010156.1	OES073744	terpene	Terpene	Lead-Zinc	18.72	China: Jiangxi, Yinshan
L180206P-4-LD180201-0005_HK72WCCXY_L4_clean_bin.10.orig.fa_k	OEZ008529	no	LMSG_G000010156.1	OES073744	terpene	Terpene	Lead-Zinc	21.06	China: Jiangxi, Yinshan
L180206P-4-LD180201-0005_HK72WCCXY_L4_clean_bin.10.orig.fa_k	OEZ008529	no	LMSG_G000010156.1	OES073744	terpene	Terpene	Lead-Zinc	21.95	China: Jiangxi, Yinshan
L180206P-4-LD180201-0005_HK72WCCXY_L4_clean_bin.10.orig.fa_k	OEZ008529	no	LMSG_G000010156.1	OES073744	ectoine	Others	Lead-Zinc	10.40	China: Jiangxi, Yinshan
L180206P-4-LD180201-0005_HK72WCCXY_L4_clean_bin.13.strict.fa	OEZ008529	no	LMSG_G000009532.1	OES073744	terpene	Terpene	Lead-Zinc	21.49	China: Jiangxi, Yinshan
L180206P-4-LD180201-0005_HK72WCCXY_L4_clean_bin.14.strict.fa	OEZ008529	no	LMSG_G000010570.1	OES073744	arylpolyene	Others	Lead-Zinc	29.83	China: Jiangxi, Yinshan
L180206P-4-LD180201-0005_HK72WCCXY_L4_clean_bin.14.strict.fa	OEZ008529	no	LMSG_G000010570.1	OES073744	bacteriocin	RiPPs	Lead-Zinc	6.88	China: Jiangxi, Yinshan
L180206P-4-LD180201-0005_HK72WCCXY_L4_clean_bin.15.orig.fa_k	OEZ008529	yes	LMSG_G000006266.1	OES073744	lassopeptide	RiPPs	Lead-Zinc	22.48	China: Jiangxi, Yinshan
L180206P-4-LD180201-0005_HK72WCCXY_L4_clean_bin.15.orig.fa_k	OEZ008529	yes	LMSG_G000006266.1	OES073744	NRPS-like	NRPS	Lead-Zinc	39.39	China: Jiangxi, Yinshan
L180206P-4-LD180201-0005_HK72WCCXY_L4_clean_bin.15.orig.fa_k	OEZ008529	yes	LMSG_G000006266.1	OES073744	NRPS-like	NRPS	Lead-Zinc	40.82	China: Jiangxi, Yinshan
L180206P-4-LD180201-0005_HK72WCCXY_L4_clean_bin.15.orig.fa_k	OEZ008529	yes	LMSG_G000006266.1	OES073744	NRPS-like	NRPS	Lead-Zinc	42.85	China: Jiangxi, Yinshan
L180206P-4-LD180201-0005_HK72WCCXY_L4_clean_bin.15.orig.fa_k	OEZ008529	yes	LMSG_G000006266.1	OES073744	terpene	Terpene	Lead-Zinc	21.21	China: Jiangxi, Yinshan
L180206P-4-LD180201-0005_HK72WCCXY_L4_clean_bin.15.orig.fa_k	OEZ008529	yes	LMSG_G000006266.1	OES073744	arylpolyene	Others	Lead-Zinc	23.01	China: Jiangxi, Yinshan
L180206P-4-LD180201-0005_HK72WCCXY_L4_clean_bin.15.orig.fa_k	OEZ008529	yes	LMSG_G000006266.1	OES073744	bacteriocin	RiPPs	Lead-Zinc	10.20	China: Jiangxi, Yinshan
L180206P-4-LD180201-0005_HK72WCCXY_L4_clean_bin.15.orig.fa_k	OEZ008529	yes	LMSG_G000006266.1	OES073744	hserlactone	Others	Lead-Zinc	18.30	China: Jiangxi, Yinshan
L180206P-4-LD180201-0005_HK72WCCXY_L4_clean_bin.16.orig.fa_k	OEZ008529	no	LMSG_G000004923.1	OES073744	betalactone.NRPS-like	Others	Lead-Zinc	41.82	China: Jiangxi, Yinshan
L180206P-4-LD180201-0005_HK72WCCXY_L4_clean_bin.16.orig.fa_k	OEZ008529	no	LMSG_G000004923.1	OES073744	NRPS	NRPS	Lead-Zinc	44.23	China: Jiangxi, Yinshan
L180206P-4-LD180201-0005_HK72WCCXY_L4_clean_bin.16.orig.fa_k	OEZ008529	no	LMSG_G000004923.1	OES073744	NAGGN	Others	Lead-Zinc	14.76	China: Jiangxi, Yinshan
L180206P-4-LD180201-0005_HK72WCCXY_L4_clean_bin.16.orig.fa_k	OEZ008529	no	LMSG_G000004923.1	OES073744	hserlactone	Others	Lead-Zinc	20.61	China: Jiangxi, Yinshan
L180206P-4-LD180201-0005_HK72WCCXY_L4_clean_bin.16.orig.fa_k	OEZ008529	no	LMSG_G000004923.1	OES073744	bacteriocin	RiPPs	Lead-Zinc	10.86	China: Jiangxi, Yinshan
L180206P-4-LD180201-0005_HK72WCCXY_L4_clean_bin.16.orig.fa_k	OEZ008529	no	LMSG_G000004923.1	OES073744	NRPS	NRPS	Lead-Zinc	49.55	China: Jiangxi, Yinshan
L180206P-4-LD180201-0005_HK72WCCXY_L4_clean_bin.16.orig.fa_k	OEZ008529	no	LMSG_G000004923.1	OES073744	hserlactone	Others	Lead-Zinc	20.61	China: Jiangxi, Yinshan
L180206P-4-LD180201-0005_HK72WCCXY_L4_clean_bin.16.orig.fa_k	OEZ008529	no	LMSG_G000004923.1	OES073744	thiopeptide	RiPPs	Lead-Zinc	33.00	China: Jiangxi, Yinshan
L180206P-4-LD180201-0005_HK72WCCXY_L4_clean_bin.16.orig.fa_k	OEZ008529	no	LMSG_G000004923.1	OES073744	NRPS	NRPS	Lead-Zinc	25.01	China: Jiangxi, Yinshan
L180206P-4-LD180201-0005_HK72WCCXY_L4_clean_bin.16.orig.fa_k	OEZ008529	no	LMSG_G000004923.1	OES073744	NRPS	NRPS	Lead-Zinc	42.73	China: Jiangxi, Yinshan
L180206P-4-LD180201-0005_HK72WCCXY_L4_clean_bin.16.orig.fa_k	OEZ008529	no	LMSG_G000004923.1	OES073744	phenazine	Others	Lead-Zinc	10.23	China: Jiangxi, Yinshan
L180206P-4-LD180201-0005_HK72WCCXY_L4_clean_bin.16.orig.fa_k	OEZ008529	no	LMSG_G000004923.1	OES073744	bacteriocin	RiPPs	Lead-Zinc	10.83	China: Jiangxi, Yinshan
L180206P-4-LD180201-0005_HK72WCCXY_L4_clean_bin.16.orig.fa_k	OEZ008529	no	LMSG_G000004923.1	OES073744	NRPS-like	NRPS	Lead-Zinc	42.98	China: Jiangxi, Yinshan
L180206P-4-LD180201-0005_HK72WCCXY_L4_clean_bin.16.orig.fa_k	OEZ008529	no	LMSG_G000004923.1	OES073744	phenazine	Others	Lead-Zinc	10.23	China: Jiangxi, Yinshan
L180206P-4-LD180201-0005_HK72WCCXY_L4_clean_bin.16.orig.fa_k	OEZ008529	no	LMSG_G000004923.1	OES073744	NRPS	NRPS	Lead-Zinc	62.35	China: Jiangxi, Yinshan

L180206P-4-LD180201-0005_HK72WCCXY_L4_clean_bin.17.orig.fa_k	OEZ008529	no	LMSG_G000010544.1	OES073744	hserlactone	Others	Lead-Zinc	11.46	China: Jiangxi, Yinshan
L180206P-4-LD180201-0005_HK72WCCXY_L4_clean_bin.17.orig.fa_k	OEZ008529	no	LMSG_G000010544.1	OES073744	arylpolyene	Others	Lead-Zinc	35.11	China: Jiangxi, Yinshan
L180206P-4-LD180201-0005_HK72WCCXY_L4_clean_bin.17.orig.fa_k	OEZ008529	no	LMSG_G000010544.1	OES073744	terpene	Terpene	Lead-Zinc	16.24	China: Jiangxi, Yinshan
L180206P-4-LD180201-0005_HK72WCCXY_L4_clean_bin.20.orig.fa_k	OEZ008529	yes	LMSG_G000010684.1	OES073744	NRPS-like	NRPS	Lead-Zinc	31.89	China: Jiangxi, Yinshan
L180206P-4-LD180201-0005_HK72WCCXY_L4_clean_bin.20.orig.fa_k	OEZ008529	yes	LMSG_G000010684.1	OES073744	arylpolyene	Others	Lead-Zinc	9.78	China: Jiangxi, Yinshan
L180206P-4-LD180201-0005_HK72WCCXY_L4_clean_bin.20.orig.fa_k	OEZ008529	yes	LMSG_G000010684.1	OES073744	terpene	Terpene	Lead-Zinc	20.84	China: Jiangxi, Yinshan
L180206P-4-LD180201-0005_HK72WCCXY_L4_clean_bin.20.orig.fa_k	OEZ008529	yes	LMSG_G000010684.1	OES073744	betalactone	Others	Lead-Zinc	28.50	China: Jiangxi, Yinshan
L180206P-4-LD180201-0005_HK72WCCXY_L4_clean_bin.20.orig.fa_k	OEZ008529	yes	LMSG_G000010684.1	OES073744	betalactone	Others	Lead-Zinc	24.97	China: Jiangxi, Yinshan
L180206P-4-LD180201-0005_HK72WCCXY_L4_clean_bin.21.orig.fa_k	OEZ008529	yes	LMSG_G000008414.1	OES073744	TfuA-related	RiPPs	Lead-Zinc	8.63	China: Jiangxi, Yinshan
L180206P-4-LD180201-0005_HK72WCCXY_L4_clean_bin.21.orig.fa_k	OEZ008529	yes	LMSG_G000008414.1	OES073744	TfuA-related.bacteriocin	RiPPs	Lead-Zinc	5.93	China: Jiangxi, Yinshan
L180206P-4-LD180201-0005_HK72WCCXY_L4_clean_bin.21.orig.fa_k	OEZ008529	yes	LMSG_G000008414.1	OES073744	terpene	Terpene	Lead-Zinc	8.91	China: Jiangxi, Yinshan
L180206P-4-LD180201-0005_HK72WCCXY_L4_clean_bin.24.orig.fa_k	OEZ008529	yes	LMSG_G000010257.1	OES073744	arylpolyene	Others	Lead-Zinc	41.19	China: Jiangxi, Yinshan
L180206P-4-LD180201-0005_HK72WCCXY_L4_clean_bin.24.orig.fa_k	OEZ008529	yes	LMSG_G000010257.1	OES073744	terpene	Terpene	Lead-Zinc	24.87	China: Jiangxi, Yinshan
L180206P-4-LD180201-0005_HK72WCCXY_L4_clean_bin.25.orig.fa_k	OEZ008529	yes	LMSG_G000011171.1	OES073744	arylpolyene	Others	Lead-Zinc	6.97	China: Jiangxi, Yinshan
L180206P-4-LD180201-0005_HK72WCCXY_L4_clean_bin.29.strict.fa	OEZ008529	yes	LMSG_G000004387.1	OES073744	NRPS-like	NRPS	Lead-Zinc	42.77	China: Jiangxi, Yinshan
L180206P-4-LD180201-0005_HK72WCCXY_L4_clean_bin.29.strict.fa	OEZ008529	yes	LMSG_G000004387.1	OES073744	terpene	Terpene	Lead-Zinc	17.01	China: Jiangxi, Yinshan
L180206P-4-LD180201-0005_HK72WCCXY_L4_clean_bin.30.strict.fa	OEZ008529	yes	LMSG_G000010138.1	OES073744	NRPS-like	NRPS	Lead-Zinc	14.51	China: Jiangxi, Yinshan
L180206P-4-LD180201-0005_HK72WCCXY_L4_clean_bin.30.strict.fa	OEZ008529	yes	LMSG_G000010138.1	OES073744	terpene	Terpene	Lead-Zinc	9.50	China: Jiangxi, Yinshan
L180206P-4-LD180201-0005_HK72WCCXY_L4_clean_bin.30.strict.fa	OEZ008529	yes	LMSG_G000010138.1	OES073744	terpene	Terpene	Lead-Zinc	9.16	China: Jiangxi, Yinshan
L180206P-4-LD180201-0005_HK72WCCXY_L4_clean_bin.31.orig.fa_k	OEZ008529	yes	LMSG_G000006267.1	OES073744	terpene	Terpene	Lead-Zinc	13.65	China: Jiangxi, Yinshan
L180206P-4-LD180201-0005_HK72WCCXY_L4_clean_bin.31.orig.fa_k	OEZ008529	yes	LMSG_G000006267.1	OES073744	arylpolyene	Others	Lead-Zinc	22.00	China: Jiangxi, Yinshan
L180206P-4-LD180201-0005_HK72WCCXY_L4_clean_bin.31.orig.fa_k	OEZ008529	yes	LMSG_G000006267.1	OES073744	arylpolyene	Others	Lead-Zinc	32.56	China: Jiangxi, Yinshan
L180206P-4-LD180201-0005_HK72WCCXY_L4_clean_bin.31.orig.fa_k	OEZ008529	yes	LMSG_G000006267.1	OES073744	bacteriocin	RiPPs	Lead-Zinc	10.99	China: Jiangxi, Yinshan
L180206P-4-LD180201-0005_HK72WCCXY_L4_clean_bin.31.orig.fa_k	OEZ008529	no	LMSG_G000006267.1	OES073744	terpene	Terpene	Lead-Zinc	15.79	China: Jiangxi, Yinshan
L180206P-4-LD180201-0005_HK72WCCXY_L4_clean_bin.32.orig.fa_k	OEZ008529	yes	LMSG_G000007771.1	OES073744	T3PKS	PKSother	Lead-Zinc	36.92	China: Jiangxi, Yinshan
L180206P-4-LD180201-0005_HK72WCCXY_L4_clean_bin.32.orig.fa_k	OEZ008529	yes	LMSG_G000007771.1	OES073744	NRPS-like	NRPS	Lead-Zinc	23.90	China: Jiangxi, Yinshan
L180206P-4-LD180201-0005_HK72WCCXY_L4_clean_bin.32.orig.fa_k	OEZ008529	yes	LMSG_G000007771.1	OES073744	terpene	Terpene	Lead-Zinc	29.77	China: Jiangxi, Yinshan
L180206P-4-LD180201-0005_HK72WCCXY_L4_clean_bin.36.orig.fa_k	OEZ008529	no	LMSG_G000010821.1	OES073744	acyl_amino_acids	Others	Lead-Zinc	5.24	China: Jiangxi, Yinshan
L180206P-4-LD180201-0005_HK72WCCXY_L4_clean_bin.36.orig.fa_k	OEZ008529	yes	LMSG_G000010821.1	OES073744	acyl_amino_acids	Others	Lead-Zinc	24.66	China: Jiangxi, Yinshan
L180206P-4-LD180201-0005_HK72WCCXY_L4_clean_bin.37.orig.fa_k	OEZ008529	yes	LMSG_G000004928.1	OES073744	terpene	Terpene	Lead-Zinc	22.91	China: Jiangxi, Yinshan
L180206P-4-LD180201-0005_HK72WCCXY_L4_clean_bin.38.orig.fa_k	OEZ008529	yes	LMSG_G000008304.1	OES073744	NRPS-like	NRPS	Lead-Zinc	17.70	China: Jiangxi, Yinshan
L180206P-4-LD180201-0005_HK72WCCXY_L4_clean_bin.38.orig.fa_k	OEZ008529	yes	LMSG_G000008304.1	OES073744	terpene	Terpene	Lead-Zinc	15.29	China: Jiangxi, Yinshan
L180206P-4-LD180201-0005_HK72WCCXY_L4_clean_bin.6.orig.fa_kl	OEZ008529	yes	LMSG_G000007814.1	OES073744	T3PKS	PKSother	Lead-Zinc	41.09	China: Jiangxi, Yinshan
L180206P-4-LD180201-0005_HK72WCCXY_L4_clean_bin.7.strict.fa_	OEZ008529	yes	LMSG_G000010810.1	OES073744	acyl_amino_acids	Others	Lead-Zinc	47.26	China: Jiangxi, Yinshan
L180206P-4-LD180201-0005_HK72WCCXY_L4_clean_bin.8.orig.fa_kl	OEZ008529	yes	LMSG_G000004931.1	OES073744	terpene	Terpene	Lead-Zinc	23.20	China: Jiangxi, Yinshan
L180206P-4-LD180201-0005_HK72WCCXY_L4_clean_bin.8.orig.fa_kl	OEZ008529	yes	LMSG_G000004931.1	OES073744	bacteriocin	RiPPs	Lead-Zinc	10.87	China: Jiangxi, Yinshan
L180206P-4-LD180201-0005_HK72WCCXY_L4_clean_bin.8.orig.fa_kl	OEZ008529	yes	LMSG_G000004931.1	OES073744	arylpolyene	Others	Lead-Zinc	9.90	China: Jiangxi, Yinshan
L180206P-4-LD180201-0005_HK72WCCXY_L4_clean_bin.8.orig.fa_kl	OEZ008529	yes	LMSG_G000004931.1	OES073744	hserlactone	Others	Lead-Zinc	20.68	China: Jiangxi, Yinshan
L180206P-4-LD180201-0008_HK72WCCXY_L4_clean_bin.103.orig.fa_	OEZ008529	yes	LMSG_G000004932.1	OES073741	NRPS-like	NRPS	Copper	34.29	China: Jiangxi, Yongping
L180206P-4-LD180201-0008_HK72WCCXY_L4_clean_bin.103.orig.fa_	OEZ008529	yes	LMSG_G000004932.1	OES073741	terpene	Terpene	Copper	42.65	China: Jiangxi, Yongping
L180206P-4-LD180201-0008_HK72WCCXY_L4_clean_bin.105.orig.fa_	OEZ008529	yes	LMSG_G000009115.1	OES073741	terpene	Terpene	Copper	25.09	China: Jiangxi, Yongping
L180206P-4-LD180201-0008_HK72WCCXY_L4_clean_bin.105.orig.fa_	OEZ008529	yes	LMSG_G000009115.1	OES073741	NRPS-like	NRPS	Copper	27.74	China: Jiangxi, Yongping
L180206P-4-LD180201-0008_HK72WCCXY_L4_clean_bin.105.orig.fa_	OEZ008529	yes	LMSG_G000009115.1	OES073741	bacteriocin	RiPPs	Copper	10.87	China: Jiangxi, Yongping
L180206P-4-LD180201-0008_HK72WCCXY_L4_clean_bin.106.orig.fa_	OEZ008529	yes	LMSG_G000009146.1	OES073741	terpene	Terpene	Copper	11.60	China: Jiangxi, Yongping
L180206P-4-LD180201-0008_HK72WCCXY_L4_clean_bin.107.strict.f	OEZ008529	yes	LMSG_G000004934.1	OES073741	NRPS.T1PKS	PKS-NRP_Hybrids	Copper	34.53	China: Jiangxi, Yongping
L180206P-4-LD180201-0008_HK72WCCXY_L4_clean_bin.107.strict.f	OEZ008529	yes	LMSG_G000004934.1	OES073741	T3PKS	PKSother	Copper	21.07	China: Jiangxi, Yongping
L180206P-4-LD180201-0008_HK72WCCXY_L4_clean_bin.107.strict.f	OEZ008529	yes	LMSG_G000004934.1	OES073741	NRPS	NRPS	Copper	17.81	China: Jiangxi, Yongping
L180206P-4-LD180201-0008_HK72WCCXY_L4_clean_bin.107.strict.f	OEZ008529	yes	LMSG_G000004934.1	OES073741	NRPS-like	NRPS	Copper	12.10	China: Jiangxi, Yongping
L180206P-4-LD180201-0008_HK72WCCXY_L4_clean_bin.107.strict.f	OEZ008529	yes	LMSG_G000004934.1	OES073741	terpene	Terpene	Copper	11.24	China: Jiangxi, Yongping
L180206P-4-LD180201-0008_HK72WCCXY_L4_clean_bin.107.strict.f	OEZ008529	yes	LMSG_G000004934.1	OES073741	T1PKS	PKSI	Copper	9.43	China: Jiangxi, Yongping
L180206P-4-LD180201-0008_HK72WCCXY_L4_clean_bin.11.orig.fa_k	OEZ008529	no	LMSG_G000011017.1	OES073741	NRPS	NRPS	Copper	62.35	China: Jiangxi, Yongping
L180206P-4-LD180201-0008_HK72WCCXY_L4_clean_bin.11.orig.fa_k	OEZ008529	no	LMSG_G000011017.1	OES073741	NRPS	NRPS	Copper	44.23	China: Jiangxi, Yongping
L180206P-4-LD180201-0008_HK72WCCXY_L4_clean_bin.11.orig.fa_k	OEZ008529	no	LMSG_G000011017.1	OES073741	hserlactone	Others	Copper	20.61	China: Jiangxi, Yongping
L180206P-4-LD180201-0008_HK72WCCXY_L4_clean_bin.11.orig.fa_k	OEZ008529	no	LMSG_G000011017.1	OES073741	NRPS	NRPS	Copper	25.01	China: Jiangxi, Yongping
L180206P-4-LD180201-0008_HK72WCCXY_L4_clean_bin.11.orig.fa_k	OEZ008529	no	LMSG_G000011017.1	OES073741	NRPS	NRPS	Copper	43.60	China: Jiangxi, Yongping
L180206P-4-LD180201-0008_HK72WCCXY_L4_clean_bin.11.orig.fa_k	OEZ008529	no	LMSG_G000011017.1	OES073741	NAGGN	Others	Copper	14.76	China: Jiangxi, Yongping
L180206P-4-LD180201-0008_HK72WCCXY_L4_clean_bin.11.orig.fa_k	OEZ008529	no	LMSG_G000011017.1	OES073741	hserlactone	Others	Copper	20.61	China: Jiangxi, Yongping
L180206P-4-LD180201-0008_HK72WCCXY_L4_clean_bin.11.orig.fa_k	OEZ008529	no	LMSG_G000011017.1	OES073741	betalactone.NRPS-like	Others	Copper	41.82	China: Jiangxi, Yongping
L180206P-4-LD180201-0008_HK72WCCXY_L4_clean_bin.11.orig.fa_k	OEZ008529	no	LMSG_G000011017.1	OES073741	phenazine	Others	Copper	10.23	China: Jiangxi, Yongping
L180206P-4-LD180201-0008_HK72WCCXY_L4_clean_bin.11.orig.fa_k	OEZ008529	no	LMSG_G000011017.1	OES073741	thiopeptide	RiPPs	Copper	33.00	China: Jiangxi, Yongping
L180206P-4-LD180201-0008_HK72WCCXY_L4_clean_bin.11.orig.fa_k	OEZ008529	no	LMSG_G000011017.1	OES073741	phenazine	Others	Copper	10.23	China: Jiangxi, Yongping
L180206P-4-LD180201-0008_HK72WCCXY_L4_clean_bin.11.orig.fa_k	OEZ008529	no	LMSG_G000011017.1	OES073741	bacteriocin	RiPPs	Copper	10.83	China: Jiangxi, Yongping
L180206P-4-LD180201-0008_HK72WCCXY_L4_clean_bin.11.orig.fa_k	OEZ008529	no	LMSG_G000011017.1	OES073741	NRPS-like	NRPS	Copper	42.98	China: Jiangxi, Yongping
L180206P-4-LD180201-0008_HK72WCCXY_L4_clean_bin.11.orig.fa_k	OEZ008529	no	LMSG_G000011017.1	OES073741	bacteriocin	RiPPs	Copper	10.86	China: Jiangxi, Yongping
L180206P-4-LD180201-0008_HK72WCCXY_L4_clean_bin.11.orig.fa_k	OEZ008529	no	LMSG_G000011017.1	OES073741	NRPS	NRPS	Copper	49.55	China: Jiangxi, Yongping
L180206P-4-LD180201-0008_HK72WCCXY_L4_clean_bin.110.orig.fa_	OEZ008529	yes	LMSG_G000004936.1	OES073741	T1PKS	PKSI	Copper	5.93	China: Jiangxi, Yongping
L180206P-4-LD180201-0008_HK72WCCXY_L4_clean_bin.110.orig.fa_	OEZ008529	yes	LMSG_G000004936.1	OES073741	LAP	RiPPs	Copper	6.49	China: Jiangxi, Yongping
L180206P-4-LD180201-0008_HK72WCCXY_L4_clean_bin.110.orig.fa_	OEZ008529	yes	LMSG_G000004936.1	OES073741	cyanobactin	RiPPs	Copper	6.45	China: Jiangxi, Yongping
L180206P-4-LD180201-0008_HK72WCCXY_L4_clean_bin.110.orig.fa_	OEZ008529	yes	LMSG_G000004936.1	OES073741	bacteriocin	RiPPs	Copper	5.77	China: Jiangxi, Yongping
L180206P-4-LD180201-0008_HK72WCCXY_L4_clean_bin.110.orig.fa_	OEZ008529	yes	LMSG_G000004936.1	OES073741	T3PKS	PKSother	Copper	5.75	China: Jiangxi, Yongping
L180206P-4-LD180201-0008_HK72WCCXY_L4_clean_bin.110.orig.fa_	OEZ008529	yes	LMSG_G000004936.1	OES073741	T1PKS.hglE-KS	PKSother	Copper	19.99	China: Jiangxi, Yongping
L180206P-4-LD180201-0008_HK72WCCXY_L4_clean_bin.111.orig.fa_	OEZ008529	yes	LMSG_G000009374.1	OES073741	betalactone	Others	Copper	32.65	China: Jiangxi, Yongping
L180206P-4-LD180201-0008_HK72WCCXY_L4_clean_bin.112.strict.f	OEZ008529	yes	LMSG_G000008879.1	OES073741	arylpolyene	Others	Copper	41.11	China: Jiangxi, Yongping
L180206P-4-LD180201-0008_HK72WCCXY_L4_clean_bin.112.strict.f	OEZ008529	yes	LMSG_G000008879.1	OES073741	terpene	Terpene	Copper	20.98	China: Jiangxi, Yongping
L180206P-4-LD180201-0008_HK72WCCXY_L4_clean_bin.112.strict.f	OEZ008529	yes	LMSG_G000008879.1	OES073741	T3PKS	PKSother	Copper	41.07	China: Jiangxi, Yongping
L180206P-4-LD180201-0008_HK72WCCXY_L4_clean_bin.114.orig.fa_	OEZ008529	yes	LMSG_G000011038.1	OES073741	arylpolyene	Others	Copper	20.43	China: Jiangxi, Yongping
L180206P-4-LD180201-0008_HK72WCCXY_L4_clean_bin.114.orig.fa_	OEZ008529	yes	LMSG_G000011038.1	OES073741	bacteriocin	RiPPs	Copper	7.83	China: Jiangxi, Yongping
L180206P-4-LD180201-0008_HK72WCCXY_L4_clean_bin.114.orig.fa_	OEZ008529	yes	LMSG_G000011038.1	OES073741	NRPS-like	NRPS	Copper	17.44	China: Jiangxi, Yongping
L180206P-4-LD180201-0008_HK72WCCXY_L4_clean_bin.114.orig.fa_	OEZ008529	yes	LMSG_G000011038.1	OES073741	arylpolyene	Others	Copper	17.61	China: Jiangxi, Yongping
L180206P-4-LD180201-0008_HK72WCCXY_L4_clean_bin.114.orig.fa_	OEZ008529	yes	LMSG_G000011038.1	OES073741	terpene	Terpene	Copper	23.08	China: Jiangxi, Yongping
L180206P-4-LD180201-0008_HK72WCCXY_L4_clean_bin.118.strict.f	OEZ008529	yes	LMSG_G000010068.1	OES073741	terpene	Terpene	Copper	7.61	China: Jiangxi, Yongping
L180206P-4-LD180201-0008_HK72WCCXY_L4_clean_bin.119.orig.fa_	OEZ008529	yes	LMSG_G000008406.1	OES073741	NRPS-like	NRPS	Copper	30.35	China: Jiangxi, Yongping
L180206P-4-LD180201-0008_HK72WCCXY_L4_clean_bin.121.orig.fa_	OEZ008529	yes	LMSG_G000004388.1	OES073741	betalactone	Others	Copper	15.49	China: Jiangxi, Yongping
L180206P-4-LD180201-0008_HK72WCCXY_L4_clean_bin.121.orig.fa_	OEZ008529	yes	LMSG_G000004388.1	OES073741	other	Others	Copper	23.71	China: Jiangxi, Yongping
L180206P-4-LD180201-0008_HK72WCCXY_L4_clean_bin.123.orig.fa_	OEZ008529	no	LMSG_G000004939.1	OES073741	thiopeptide	RiPPs	Copper	26.29	China: Jiangxi, Yongping
L180206P-4-LD180201-0008_HK72WCCXY_L4_clean_bin.123.orig.fa_	OEZ008529	no	LMSG_G000004939.1	OES073741	NRPS	NRPS	Copper	53.46	China: Jiangxi, Yongping
L180206P-4-LD180201-0008_HK72WCCXY_L4_clean_bin.14.permissiv	OEZ008529	yes	LMSG_G000008055.1	OES073741	T1PKS	PKSI	Copper	18.16	China: Jiangxi, Yongping
L180206P-4-LD180201-0008_HK72WCCXY_L4_clean_bin.16.orig.fa_k	OEZ008529	yes	LMSG_G000010007.1	OES073741	arylpolyene	Others	Copper	14.30	China: Jiangxi, Yongping
L180206P-4-LD180201-0008_HK72WCCXY_L4_clean_bin.16.orig.fa_k	OEZ008529	yes	LMSG_G000010007.1	OES073741	terpene	Terpene	Copper	21.87	China: Jiangxi, Yongping
L180206P-4-LD180201-0008_HK72WCCXY_L4_clean_bin.18.strict.fa	OEZ008529	yes	LMSG_G000004389.1	OES073741	lassopeptide	RiPPs	Copper	22.75	China: Jiangxi, Yongping
L180206P-4-LD180201-0008_HK72WCCXY_L4_clean_bin.18.strict.fa	OEZ008529	yes	LMSG_G000004389.1	OES073741	NRPS	NRPS	Copper	44.20	China: Jiangxi, Yongping
L180206P-4-LD180201-0008_HK72WCCXY_L4_clean_bin.19.strict.fa	OEZ008529	yes	LMSG_G000008029.1	OES073741	LAP.thiopeptide	RiPPs	Copper	19.56	China: Jiangxi, Yongping
L180206P-4-LD180201-0008_HK72WCCXY_L4_clean_bin.19.strict.fa	OEZ008529	yes	LMSG_G000008029.1	OES073741	lassopeptide	RiPPs	Copper	12.41	China: Jiangxi, Yongping
L180206P-4-LD180201-0008_HK72WCCXY_L4_clean_bin.2.orig.fa_kl	OEZ008529	yes	LMSG_G000010108.1	OES073741	bacteriocin	RiPPs	Copper	7.70	China: Jiangxi, Yongping
L180206P-4-LD180201-0008_HK72WCCXY_L4_clean_bin.2.orig.fa_kl	OEZ008529	yes	LMSG_G000010108.1	OES073741	TfuA-related	RiPPs	Copper	6.85	China: Jiangxi, Yongping
L180206P-4-LD180201-0008_HK72WCCXY_L4_clean_bin.2.orig.fa_kl	OEZ008529	yes	LMSG_G000010108.1	OES073741	terpene	Terpene	Copper	9.00	China: Jiangxi, Yongping
L180206P-4-LD180201-0008_HK72WCCXY_L4_clean_bin.2.orig.fa_kl	OEZ008529	yes	LMSG_G000010108.1	OES073741	terpene	Terpene	Copper	6.05	China: Jiangxi, Yongping
L180206P-4-LD180201-0008_HK72WCCXY_L4_clean_bin.2.orig.fa_kl	OEZ008529	yes	LMSG_G000010108.1	OES073741	phosphonate	Others	Copper	15.82	China: Jiangxi, Yongping
L180206P-4-LD180201-0008_HK72WCCXY_L4_clean_bin.2.orig.fa_kl	OEZ008529	yes	LMSG_G000010108.1	OES073741	terpene	Terpene	Copper	20.79	China: Jiangxi, Yongping
L180206P-4-LD180201-0008_HK72WCCXY_L4_clean_bin.22.permissiv	OEZ008529	yes	LMSG_G000007991.1	OES073741	bacteriocin	RiPPs	Copper	7.42	China: Jiangxi, Yongping
L180206P-4-LD180201-0008_HK72WCCXY_L4_clean_bin.22.permissiv	OEZ008529	yes	LMSG_G000007991.1	OES073741	TfuA-related.bacteriocin	RiPPs	Copper	13.45	China: Jiangxi, Yongping
L180206P-4-LD180201-0008_HK72WCCXY_L4_clean_bin.24.permissiv	OEZ008529	yes	LMSG_G000007753.1	OES073741	NRPS.T1PKS	PKS-NRP_Hybrids	Copper	32.25	China: Jiangxi, Yongping
L180206P-4-LD180201-0008_HK72WCCXY_L4_clean_bin.24.permissiv	OEZ008529	yes	LMSG_G000007753.1	OES073741	terpene	Terpene	Copper	23.30	China: Jiangxi, Yongping
L180206P-4-LD180201-0008_HK72WCCXY_L4_clean_bin.24.permissiv	OEZ008529	yes	LMSG_G000007753.1	OES073741	NRPS	NRPS	Copper	20.05	China: Jiangxi, Yongping
L180206P-4-LD180201-0008_HK72WCCXY_L4_clean_bin.24.permissiv	OEZ008529	yes	LMSG_G000007753.1	OES073741	phosphonate	Others	Copper	15.26	China: Jiangxi, Yongping
L180206P-4-LD180201-0008_HK72WCCXY_L4_clean_bin.24.permissiv	OEZ008529	yes	LMSG_G000007753.1	OES073741	betalactone.NRPS-like	Others	Copper	14.14	China: Jiangxi, Yongping
L180206P-4-LD180201-0008_HK72WCCXY_L4_clean_bin.24.permissiv	OEZ008529	yes	LMSG_G000007753.1	OES073741	lassopeptide	RiPPs	Copper	12.84	China: Jiangxi, Yongping
L180206P-4-LD180201-0008_HK72WCCXY_L4_clean_bin.24.permissiv	OEZ008529	yes	LMSG_G000007753.1	OES073741	NRPS	NRPS	Copper	10.89	China: Jiangxi, Yongping
L180206P-4-LD180201-0008_HK72WCCXY_L4_clean_bin.25.orig.fa_k	OEZ008529	yes	LMSG_G000011129.1	OES073741	terpene	Terpene	Copper	22.80	China: Jiangxi, Yongping
L180206P-4-LD180201-0008_HK72WCCXY_L4_clean_bin.25.orig.fa_k	OEZ008529	yes	LMSG_G000011129.1	OES073741	arylpolyene	Others	Copper	41.16	China: Jiangxi, Yongping
L180206P-4-LD180201-0008_HK72WCCXY_L4_clean_bin.3.strict.fa_	OEZ008529	yes	LMSG_G000004390.1	OES073741	terpene	Terpene	Copper	21.15	China: Jiangxi, Yongping
L180206P-4-LD180201-0008_HK72WCCXY_L4_clean_bin.3.strict.fa_	OEZ008529	yes	LMSG_G000004390.1	OES073741	terpene	Terpene	Copper	21.10	China: Jiangxi, Yongping
L180206P-4-LD180201-0008_HK72WCCXY_L4_clean_bin.3.strict.fa_	OEZ008529	yes	LMSG_G000004390.1	OES073741	arylpolyene	Others	Copper	34.53	China: Jiangxi, Yongping
L180206P-4-LD180201-0008_HK72WCCXY_L4_clean_bin.3.strict.fa_	OEZ008529	yes	LMSG_G000004390.1	OES073741	ectoine	Others	Copper	10.40	China: Jiangxi, Yongping
L180206P-4-LD180201-0008_HK72WCCXY_L4_clean_bin.3.strict.fa_	OEZ008529	yes	LMSG_G000004390.1	OES073741	arylpolyene	Others	Copper	12.29	China: Jiangxi, Yongping
L180206P-4-LD180201-0008_HK72WCCXY_L4_clean_bin.33.strict.fa	OEZ008529	yes	LMSG_G000007848.1	OES073741	NRPS-like	NRPS	Copper	9.23	China: Jiangxi, Yongping
L180206P-4-LD180201-0008_HK72WCCXY_L4_clean_bin.33.strict.fa	OEZ008529	yes	LMSG_G000007848.1	OES073741	terpene	Terpene	Copper	5.38	China: Jiangxi, Yongping
L180206P-4-LD180201-0008_HK72WCCXY_L4_clean_bin.35.orig.fa_k	OEZ008529	yes	LMSG_G000009241.1	OES073741	T3PKS	PKSother	Copper	21.06	China: Jiangxi, Yongping
L180206P-4-LD180201-0008_HK72WCCXY_L4_clean_bin.35.orig.fa_k	OEZ008529	yes	LMSG_G000009241.1	OES073741	terpene	Terpene	Copper	14.63	China: Jiangxi, Yongping
L180206P-4-LD180201-0008_HK72WCCXY_L4_clean_bin.35.orig.fa_k	OEZ008529	yes	LMSG_G000009241.1	OES073741	terpene	Terpene	Copper	10.19	China: Jiangxi, Yongping
L180206P-4-LD180201-0008_HK72WCCXY_L4_clean_bin.44.orig.fa_k	OEZ008529	yes	LMSG_G000007897.1	OES073741	NRPS-like	NRPS	Copper	19.42	China: Jiangxi, Yongping
L180206P-4-LD180201-0008_HK72WCCXY_L4_clean_bin.44.orig.fa_k	OEZ008529	yes	LMSG_G000007897.1	OES073741	NRPS-like	NRPS	Copper	6.48	China: Jiangxi, Yongping
L180206P-4-LD180201-0008_HK72WCCXY_L4_clean_bin.44.orig.fa_k	OEZ008529	yes	LMSG_G000007897.1	OES073741	NRPS	NRPS	Copper	10.10	China: Jiangxi, Yongping
L180206P-4-LD180201-0008_HK72WCCXY_L4_clean_bin.46.orig.fa_k	OEZ008529	yes	LMSG_G000009209.1	OES073741	T3PKS	PKSother	Copper	25.25	China: Jiangxi, Yongping
L180206P-4-LD180201-0008_HK72WCCXY_L4_clean_bin.46.orig.fa_k	OEZ008529	yes	LMSG_G000009209.1	OES073741	terpene	Terpene	Copper	21.73	China: Jiangxi, Yongping
L180206P-4-LD180201-0008_HK72WCCXY_L4_clean_bin.46.orig.fa_k	OEZ008529	yes	LMSG_G000009209.1	OES073741	terpene	Terpene	Copper	15.22	China: Jiangxi, Yongping
L180206P-4-LD180201-0008_HK72WCCXY_L4_clean_bin.47.orig.fa_k	OEZ008529	yes	LMSG_G000004944.1	OES073741	other	Others	Copper	8.35	China: Jiangxi, Yongping
L180206P-4-LD180201-0008_HK72WCCXY_L4_clean_bin.47.orig.fa_k	OEZ008529	yes	LMSG_G000004944.1	OES073741	other	Others	Copper	14.45	China: Jiangxi, Yongping
L180206P-4-LD180201-0008_HK72WCCXY_L4_clean_bin.48.strict.fa	OEZ008529	yes	LMSG_G000007773.1	OES073741	terpene	Terpene	Copper	20.59	China: Jiangxi, Yongping
L180206P-4-LD180201-0008_HK72WCCXY_L4_clean_bin.48.strict.fa	OEZ008529	yes	LMSG_G000007773.1	OES073741	T3PKS	PKSother	Copper	18.49	China: Jiangxi, Yongping
L180206P-4-LD180201-0008_HK72WCCXY_L4_clean_bin.5.permissive	OEZ008529	yes	LMSG_G000008136.1	OES073741	terpene	Terpene	Copper	9.11	China: Jiangxi, Yongping
L180206P-4-LD180201-0008_HK72WCCXY_L4_clean_bin.51.orig.fa_k	OEZ008529	yes	LMSG_G000011072.1	OES073741	lassopeptide	RiPPs	Copper	12.41	China: Jiangxi, Yongping
L180206P-4-LD180201-0008_HK72WCCXY_L4_clean_bin.51.orig.fa_k	OEZ008529	yes	LMSG_G000011072.1	OES073741	NRPS-like	NRPS	Copper	42.82	China: Jiangxi, Yongping
L180206P-4-LD180201-0008_HK72WCCXY_L4_clean_bin.51.orig.fa_k	OEZ008529	yes	LMSG_G000011072.1	OES073741	terpene	Terpene	Copper	23.25	China: Jiangxi, Yongping
L180206P-4-LD180201-0008_HK72WCCXY_L4_clean_bin.51.orig.fa_k	OEZ008529	yes	LMSG_G000011072.1	OES073741	hserlactone	Others	Copper	17.21	China: Jiangxi, Yongping
L180206P-4-LD180201-0008_HK72WCCXY_L4_clean_bin.51.orig.fa_k	OEZ008529	yes	LMSG_G000011072.1	OES073741	bacteriocin	RiPPs	Copper	10.33	China: Jiangxi, Yongping
L180206P-4-LD180201-0008_HK72WCCXY_L4_clean_bin.56.orig.fa_k	OEZ008529	yes	LMSG_G000004391.1	OES073741	terpene	Terpene	Copper	18.11	China: Jiangxi, Yongping
L180206P-4-LD180201-0008_HK72WCCXY_L4_clean_bin.57.orig.fa_k	OEZ008529	yes	LMSG_G000008395.1	OES073741	terpene	Terpene	Copper	12.18	China: Jiangxi, Yongping
L180206P-4-LD180201-0008_HK72WCCXY_L4_clean_bin.58.orig.fa_k	OEZ008529	yes	LMSG_G000009099.1	OES073741	terpene	Terpene	Copper	19.96	China: Jiangxi, Yongping
L180206P-4-LD180201-0008_HK72WCCXY_L4_clean_bin.58.orig.fa_k	OEZ008529	yes	LMSG_G000009099.1	OES073741	lassopeptide	RiPPs	Copper	8.60	China: Jiangxi, Yongping
L180206P-4-LD180201-0008_HK72WCCXY_L4_clean_bin.6.orig.fa_kl	OEZ008529	yes	LMSG_G000004949.1	OES073741	bacteriocin	RiPPs	Copper	10.91	China: Jiangxi, Yongping
L180206P-4-LD180201-0008_HK72WCCXY_L4_clean_bin.6.orig.fa_kl	OEZ008529	yes	LMSG_G000004949.1	OES073741	terpene	Terpene	Copper	21.23	China: Jiangxi, Yongping
L180206P-4-LD180201-0008_HK72WCCXY_L4_clean_bin.6.orig.fa_kl	OEZ008529	yes	LMSG_G000004949.1	OES073741	terpene	Terpene	Copper	24.97	China: Jiangxi, Yongping
L180206P-4-LD180201-0008_HK72WCCXY_L4_clean_bin.6.orig.fa_kl	OEZ008529	yes	LMSG_G000004949.1	OES073741	T3PKS	PKSother	Copper	9.65	China: Jiangxi, Yongping
L180206P-4-LD180201-0008_HK72WCCXY_L4_clean_bin.6.orig.fa_kl	OEZ008529	yes	LMSG_G000004949.1	OES073741	NRPS-like	NRPS	Copper	30.37	China: Jiangxi, Yongping
L180206P-4-LD180201-0008_HK72WCCXY_L4_clean_bin.60.orig.fa_k	OEZ008529	yes	LMSG_G000009359.1	OES073741	betalactone	Others	Copper	31.59	China: Jiangxi, Yongping
L180206P-4-LD180201-0008_HK72WCCXY_L4_clean_bin.62.orig.fa_k	OEZ008529	yes	LMSG_G000004392.1	OES073741	terpene	Terpene	Copper	20.88	China: Jiangxi, Yongping
L180206P-4-LD180201-0008_HK72WCCXY_L4_clean_bin.62.orig.fa_k	OEZ008529	yes	LMSG_G000004392.1	OES073741	bacteriocin	RiPPs	Copper	10.81	China: Jiangxi, Yongping
L180206P-4-LD180201-0008_HK72WCCXY_L4_clean_bin.62.orig.fa_k	OEZ008529	yes	LMSG_G000004392.1	OES073741	NRPS-like	NRPS	Copper	42.82	China: Jiangxi, Yongping
L180206P-4-LD180201-0008_HK72WCCXY_L4_clean_bin.66.orig.fa_k	OEZ008529	yes	LMSG_G000010792.1	OES073741	NRPS-like	NRPS	Copper	30.79	China: Jiangxi, Yongping
L180206P-4-LD180201-0008_HK72WCCXY_L4_clean_bin.66.orig.fa_k	OEZ008529	yes	LMSG_G000010792.1	OES073741	bacteriocin	RiPPs	Copper	5.52	China: Jiangxi, Yongping
L180206P-4-LD180201-0008_HK72WCCXY_L4_clean_bin.66.orig.fa_k	OEZ008529	yes	LMSG_G000010792.1	OES073741	acyl_amino_acids	Others	Copper	16.40	China: Jiangxi, Yongping
L180206P-4-LD180201-0008_HK72WCCXY_L4_clean_bin.67.orig.fa_k	OEZ008529	yes	LMSG_G000008640.1	OES073741	other	Others	Copper	6.13	China: Jiangxi, Yongping
L180206P-4-LD180201-0008_HK72WCCXY_L4_clean_bin.69.orig.fa_k	OEZ008529	yes	LMSG_G000011028.1	OES073741	resorcinol.arylpolyene	Others	Copper	41.52	China: Jiangxi, Yongping
L180206P-4-LD180201-0008_HK72WCCXY_L4_clean_bin.69.orig.fa_k	OEZ008529	yes	LMSG_G000011028.1	OES073741	bacteriocin	RiPPs	Copper	7.79	China: Jiangxi, Yongping
L180206P-4-LD180201-0008_HK72WCCXY_L4_clean_bin.73.strict.fa	OEZ008529	yes	LMSG_G000009395.1	OES073741	terpene	Terpene	Copper	21.75	China: Jiangxi, Yongping
L180206P-4-LD180201-0008_HK72WCCXY_L4_clean_bin.73.strict.fa	OEZ008529	yes	LMSG_G000009395.1	OES073741	NRPS	NRPS	Copper	26.89	China: Jiangxi, Yongping
L180206P-4-LD180201-0008_HK72WCCXY_L4_clean_bin.73.strict.fa	OEZ008529	yes	LMSG_G000009395.1	OES073741	bacteriocin	RiPPs	Copper	8.66	China: Jiangxi, Yongping

L180206P-4-LD180201-0008_HK72WCCXY_L4_clean_bin.77.orig.fa_k	OEZ008529	yes	LMSG_G000007362.1	OES073741	bacteriocin	RiPPs	Copper	11.97	China: Jiangxi, Yongping
L180206P-4-LD180201-0008_HK72WCCXY_L4_clean_bin.82.orig.fa_k	OEZ008529	yes	LMSG_G000009108.1	OES073741	NRPS-like	NRPS	Copper	27.23	China: Jiangxi, Yongping
L180206P-4-LD180201-0008_HK72WCCXY_L4_clean_bin.82.orig.fa_k	OEZ008529	yes	LMSG_G000009108.1	OES073741	T3PKS	PKSother	Copper	41.01	China: Jiangxi, Yongping
L180206P-4-LD180201-0008_HK72WCCXY_L4_clean_bin.82.orig.fa_k	OEZ008529	yes	LMSG_G000009108.1	OES073741	terpene	Terpene	Copper	17.65	China: Jiangxi, Yongping
L180206P-4-LD180201-0008_HK72WCCXY_L4_clean_bin.82.orig.fa_k	OEZ008529	yes	LMSG_G000009108.1	OES073741	terpene	Terpene	Copper	21.97	China: Jiangxi, Yongping
L180206P-4-LD180201-0008_HK72WCCXY_L4_clean_bin.84.orig.fa_k	OEZ008529	yes	LMSG_G000010461.1	OES073741	terpene	Terpene	Copper	10.83	China: Jiangxi, Yongping
L180206P-4-LD180201-0008_HK72WCCXY_L4_clean_bin.84.orig.fa_k	OEZ008529	yes	LMSG_G000010461.1	OES073741	arylpolyene.hserlactone	Others	Copper	34.39	China: Jiangxi, Yongping
L180206P-4-LD180201-0008_HK72WCCXY_L4_clean_bin.85.orig.fa_k	OEZ008529	yes	LMSG_G000004950.1	OES073741	T3PKS.terpene	Others	Copper	48.14	China: Jiangxi, Yongping
L180206P-4-LD180201-0008_HK72WCCXY_L4_clean_bin.85.orig.fa_k	OEZ008529	yes	LMSG_G000004950.1	OES073741	NRPS-like.bacteriocin	Others	Copper	37.56	China: Jiangxi, Yongping
L180206P-4-LD180201-0008_HK72WCCXY_L4_clean_bin.88.strict.fa	OEZ008529	yes	LMSG_G000009278.1	OES073741	terpene	Terpene	Copper	21.73	China: Jiangxi, Yongping
L180206P-4-LD180201-0008_HK72WCCXY_L4_clean_bin.88.strict.fa	OEZ008529	yes	LMSG_G000009278.1	OES073741	terpene	Terpene	Copper	22.06	China: Jiangxi, Yongping
L180206P-4-LD180201-0008_HK72WCCXY_L4_clean_bin.9.orig.fa_kl	OEZ008529	yes	LMSG_G000004953.1	OES073741	T3PKS	PKSother	Copper	13.57	China: Jiangxi, Yongping
L180206P-4-LD180201-0008_HK72WCCXY_L4_clean_bin.94.orig.fa_k	OEZ008529	yes	LMSG_G000010036.1	OES073741	arylpolyene	Others	Copper	38.43	China: Jiangxi, Yongping
L180206P-4-LD180201-0008_HK72WCCXY_L4_clean_bin.94.orig.fa_k	OEZ008529	yes	LMSG_G000010036.1	OES073741	T3PKS	PKSother	Copper	7.90	China: Jiangxi, Yongping
L180206P-4-LD180201-0008_HK72WCCXY_L4_clean_bin.94.orig.fa_k	OEZ008529	yes	LMSG_G000010036.1	OES073741	NRPS-like	NRPS	Copper	6.03	China: Jiangxi, Yongping
L180206P-4-LD180201-0008_HK72WCCXY_L4_clean_bin.94.orig.fa_k	OEZ008529	yes	LMSG_G000010036.1	OES073741	T3PKS	PKSother	Copper	14.40	China: Jiangxi, Yongping
L180206P-4-LD180201-0008_HK72WCCXY_L4_clean_bin.94.orig.fa_k	OEZ008529	yes	LMSG_G000010036.1	OES073741	ectoine	Others	Copper	10.04	China: Jiangxi, Yongping
L180206P-4-LD180201-0008_HK72WCCXY_L4_clean_bin.94.orig.fa_k	OEZ008529	yes	LMSG_G000010036.1	OES073741	terpene	Terpene	Copper	21.34	China: Jiangxi, Yongping
L180206P-4-LD180201-0008_HK72WCCXY_L4_clean_bin.95.orig.fa_k	OEZ008529	yes	LMSG_G000008704.1	OES073741	terpene	Terpene	Copper	11.26	China: Jiangxi, Yongping
L180206P-4-LD180201-0008_HK72WCCXY_L4_clean_bin.95.orig.fa_k	OEZ008529	yes	LMSG_G000008704.1	OES073741	terpene	Terpene	Copper	13.37	China: Jiangxi, Yongping
L180206P-4-LD180201-0008_HK72WCCXY_L4_clean_bin.95.orig.fa_k	OEZ008529	yes	LMSG_G000008704.1	OES073741	LAP	RiPPs	Copper	6.77	China: Jiangxi, Yongping
L180206P-4-LD180201-0008_HK72WCCXY_L4_clean_bin.95.orig.fa_k	OEZ008529	yes	LMSG_G000008704.1	OES073741	bacteriocin	RiPPs	Copper	9.85	China: Jiangxi, Yongping
L180206P-4-LD180201-0008_HK72WCCXY_L4_clean_bin.96.orig.fa_k	OEZ008529	yes	LMSG_G000008531.1	OES073741	terpene	Terpene	Copper	11.19	China: Jiangxi, Yongping
L180206P-4-LD180201-0008_HK72WCCXY_L4_clean_bin.96.orig.fa_k	OEZ008529	yes	LMSG_G000008531.1	OES073741	terpene	Terpene	Copper	12.78	China: Jiangxi, Yongping
L180206P-5-LD180201-0011_HK72WCCXY_L5_clean_bin.10.orig.fa_k	OEZ008529	yes	LMSG_G000010676.1	OES073681	terpene	Terpene	Copper	11.15	China: Jiangxi, Dexing
L180206P-5-LD180201-0011_HK72WCCXY_L5_clean_bin.10.orig.fa_k	OEZ008529	yes	LMSG_G000010676.1	OES073681	arylpolyene	Others	Copper	15.73	China: Jiangxi, Dexing
L180206P-5-LD180201-0011_HK72WCCXY_L5_clean_bin.10.orig.fa_k	OEZ008529	yes	LMSG_G000010676.1	OES073681	bacteriocin	RiPPs	Copper	8.72	China: Jiangxi, Dexing
L180206P-5-LD180201-0011_HK72WCCXY_L5_clean_bin.10.orig.fa_k	OEZ008529	yes	LMSG_G000010676.1	OES073681	NRPS	NRPS	Copper	29.26	China: Jiangxi, Dexing
L180206P-5-LD180201-0011_HK72WCCXY_L5_clean_bin.10.orig.fa_k	OEZ008529	yes	LMSG_G000010676.1	OES073681	NRPS	NRPS	Copper	13.90	China: Jiangxi, Dexing
L180206P-5-LD180201-0011_HK72WCCXY_L5_clean_bin.11.orig.fa_k	OEZ008529	yes	LMSG_G000008069.1	OES073681	other	Others	Copper	6.17	China: Jiangxi, Dexing
L180206P-5-LD180201-0011_HK72WCCXY_L5_clean_bin.11.orig.fa_k	OEZ008529	yes	LMSG_G000008069.1	OES073681	hglE-KS	PKSother	Copper	5.43	China: Jiangxi, Dexing
L180206P-5-LD180201-0011_HK72WCCXY_L5_clean_bin.12.orig.fa_k	OEZ008529	yes	LMSG_G000010624.1	OES073681	bacteriocin	RiPPs	Copper	6.58	China: Jiangxi, Dexing
L180206P-5-LD180201-0011_HK72WCCXY_L5_clean_bin.12.orig.fa_k	OEZ008529	no	LMSG_G000010624.1	OES073681	arylpolyene	Others	Copper	7.30	China: Jiangxi, Dexing
L180206P-5-LD180201-0011_HK72WCCXY_L5_clean_bin.12.orig.fa_k	OEZ008529	no	LMSG_G000010624.1	OES073681	terpene	Terpene	Copper	12.24	China: Jiangxi, Dexing
L180206P-5-LD180201-0011_HK72WCCXY_L5_clean_bin.12.orig.fa_k	OEZ008529	no	LMSG_G000010624.1	OES073681	terpene	Terpene	Copper	9.14	China: Jiangxi, Dexing
L180206P-5-LD180201-0011_HK72WCCXY_L5_clean_bin.12.orig.fa_k	OEZ008529	no	LMSG_G000010624.1	OES073681	arylpolyene	Others	Copper	6.99	China: Jiangxi, Dexing
L180206P-5-LD180201-0011_HK72WCCXY_L5_clean_bin.13.orig.fa_k	OEZ008529	yes	LMSG_G000011039.1	OES073681	terpene	Terpene	Copper	11.85	China: Jiangxi, Dexing
L180206P-5-LD180201-0011_HK72WCCXY_L5_clean_bin.13.orig.fa_k	OEZ008529	yes	LMSG_G000011039.1	OES073681	bacteriocin	RiPPs	Copper	10.71	China: Jiangxi, Dexing
L180206P-5-LD180201-0011_HK72WCCXY_L5_clean_bin.13.orig.fa_k	OEZ008529	yes	LMSG_G000011039.1	OES073681	arylpolyene	Others	Copper	20.45	China: Jiangxi, Dexing
L180206P-5-LD180201-0011_HK72WCCXY_L5_clean_bin.13.orig.fa_k	OEZ008529	yes	LMSG_G000011039.1	OES073681	terpene	Terpene	Copper	23.08	China: Jiangxi, Dexing
L180206P-5-LD180201-0011_HK72WCCXY_L5_clean_bin.13.orig.fa_k	OEZ008529	yes	LMSG_G000011039.1	OES073681	NRPS-like	NRPS	Copper	23.73	China: Jiangxi, Dexing
L180206P-5-LD180201-0011_HK72WCCXY_L5_clean_bin.13.orig.fa_k	OEZ008529	yes	LMSG_G000011039.1	OES073681	arylpolyene	Others	Copper	28.44	China: Jiangxi, Dexing
L180206P-5-LD180201-0011_HK72WCCXY_L5_clean_bin.15.orig.fa_k	OEZ008529	yes	LMSG_G000008349.1	OES073681	bacteriocin	RiPPs	Copper	10.60	China: Jiangxi, Dexing
L180206P-5-LD180201-0011_HK72WCCXY_L5_clean_bin.15.orig.fa_k	OEZ008529	yes	LMSG_G000008349.1	OES073681	other	Others	Copper	12.33	China: Jiangxi, Dexing
L180206P-5-LD180201-0011_HK72WCCXY_L5_clean_bin.15.orig.fa_k	OEZ008529	yes	LMSG_G000008349.1	OES073681	butyrolactone	Others	Copper	6.18	China: Jiangxi, Dexing
L180206P-5-LD180201-0011_HK72WCCXY_L5_clean_bin.15.orig.fa_k	OEZ008529	yes	LMSG_G000008349.1	OES073681	terpene	Terpene	Copper	8.99	China: Jiangxi, Dexing
L180206P-5-LD180201-0011_HK72WCCXY_L5_clean_bin.17.orig.fa_k	OEZ008529	no	LMSG_G000004954.1	OES073681	terpene	Terpene	Copper	21.22	China: Jiangxi, Dexing
L180206P-5-LD180201-0011_HK72WCCXY_L5_clean_bin.17.orig.fa_k	OEZ008529	yes	LMSG_G000004954.1	OES073681	arylpolyene	Others	Copper	23.27	China: Jiangxi, Dexing
L180206P-5-LD180201-0011_HK72WCCXY_L5_clean_bin.18.strict.fa	OEZ008529	yes	LMSG_G000011206.1	OES073681	arylpolyene	Others	Copper	47.93	China: Jiangxi, Dexing
L180206P-5-LD180201-0011_HK72WCCXY_L5_clean_bin.19.orig.fa_k	OEZ008529	yes	LMSG_G000011155.1	OES073681	arylpolyene	Others	Copper	22.32	China: Jiangxi, Dexing
L180206P-5-LD180201-0011_HK72WCCXY_L5_clean_bin.19.orig.fa_k	OEZ008529	yes	LMSG_G000011155.1	OES073681	bacteriocin	RiPPs	Copper	7.88	China: Jiangxi, Dexing
L180206P-5-LD180201-0011_HK72WCCXY_L5_clean_bin.19.orig.fa_k	OEZ008529	yes	LMSG_G000011155.1	OES073681	terpene	Terpene	Copper	13.52	China: Jiangxi, Dexing
L180206P-5-LD180201-0011_HK72WCCXY_L5_clean_bin.19.orig.fa_k	OEZ008529	yes	LMSG_G000011155.1	OES073681	NRPS.T1PKS	PKS-NRP_Hybrids	Copper	44.55	China: Jiangxi, Dexing
L180206P-5-LD180201-0011_HK72WCCXY_L5_clean_bin.19.orig.fa_k	OEZ008529	yes	LMSG_G000011155.1	OES073681	arylpolyene	Others	Copper	21.27	China: Jiangxi, Dexing
L180206P-5-LD180201-0011_HK72WCCXY_L5_clean_bin.21.orig.fa_k	OEZ008529	yes	LMSG_G000011029.1	OES073681	arylpolyene	Others	Copper	28.65	China: Jiangxi, Dexing
L180206P-5-LD180201-0011_HK72WCCXY_L5_clean_bin.21.orig.fa_k	OEZ008529	yes	LMSG_G000011029.1	OES073681	bacteriocin	RiPPs	Copper	8.56	China: Jiangxi, Dexing
L180206P-5-LD180201-0011_HK72WCCXY_L5_clean_bin.21.orig.fa_k	OEZ008529	yes	LMSG_G000011029.1	OES073681	arylpolyene	Others	Copper	23.21	China: Jiangxi, Dexing
L180206P-5-LD180201-0011_HK72WCCXY_L5_clean_bin.21.orig.fa_k	OEZ008529	yes	LMSG_G000011029.1	OES073681	terpene	Terpene	Copper	20.85	China: Jiangxi, Dexing
L180206P-5-LD180201-0011_HK72WCCXY_L5_clean_bin.21.orig.fa_k	OEZ008529	yes	LMSG_G000011029.1	OES073681	terpene	Terpene	Copper	17.69	China: Jiangxi, Dexing
L180206P-5-LD180201-0011_HK72WCCXY_L5_clean_bin.22.strict.fa	OEZ008529	no	LMSG_G000009312.1	OES073681	lassopeptide	RiPPs	Copper	24.33	China: Jiangxi, Dexing
L180206P-5-LD180201-0011_HK72WCCXY_L5_clean_bin.22.strict.fa	OEZ008529	no	LMSG_G000009312.1	OES073681	lanthipeptide	RiPPs	Copper	8.34	China: Jiangxi, Dexing
L180206P-5-LD180201-0011_HK72WCCXY_L5_clean_bin.23.orig.fa_k	OEZ008529	yes	LMSG_G000009259.1	OES073681	TfuA-related	RiPPs	Copper	21.93	China: Jiangxi, Dexing
L180206P-5-LD180201-0011_HK72WCCXY_L5_clean_bin.23.orig.fa_k	OEZ008529	yes	LMSG_G000009259.1	OES073681	T3PKS	PKSother	Copper	5.17	China: Jiangxi, Dexing
L180206P-5-LD180201-0011_HK72WCCXY_L5_clean_bin.24.orig.fa_k	OEZ008529	yes	LMSG_G000007989.1	OES073681	bacteriocin	RiPPs	Copper	10.32	China: Jiangxi, Dexing
L180206P-5-LD180201-0011_HK72WCCXY_L5_clean_bin.24.orig.fa_k	OEZ008529	yes	LMSG_G000007989.1	OES073681	TfuA-related.bacteriocin	RiPPs	Copper	8.21	China: Jiangxi, Dexing
L180206P-5-LD180201-0011_HK72WCCXY_L5_clean_bin.25.strict.fa	OEZ008529	yes	LMSG_G000009269.1	OES073681	terpene	Terpene	Copper	16.39	China: Jiangxi, Dexing
L180206P-5-LD180201-0011_HK72WCCXY_L5_clean_bin.25.strict.fa	OEZ008529	yes	LMSG_G000009269.1	OES073681	TfuA-related	RiPPs	Copper	5.69	China: Jiangxi, Dexing
L180206P-5-LD180201-0011_HK72WCCXY_L5_clean_bin.27.orig.fa_k	OEZ008529	yes	LMSG_G000004955.1	OES073681	bacteriocin	RiPPs	Copper	8.36	China: Jiangxi, Dexing
L180206P-5-LD180201-0011_HK72WCCXY_L5_clean_bin.27.orig.fa_k	OEZ008529	yes	LMSG_G000004955.1	OES073681	betalactone	Others	Copper	18.18	China: Jiangxi, Dexing
L180206P-5-LD180201-0011_HK72WCCXY_L5_clean_bin.27.orig.fa_k	OEZ008529	yes	LMSG_G000004955.1	OES073681	terpene	Terpene	Copper	26.52	China: Jiangxi, Dexing
L180206P-5-LD180201-0011_HK72WCCXY_L5_clean_bin.28.orig.fa_k	OEZ008529	yes	LMSG_G000008482.1	OES073681	arylpolyene	Others	Copper	8.49	China: Jiangxi, Dexing
L180206P-5-LD180201-0011_HK72WCCXY_L5_clean_bin.28.orig.fa_k	OEZ008529	yes	LMSG_G000008482.1	OES073681	terpene	Terpene	Copper	17.00	China: Jiangxi, Dexing
L180206P-5-LD180201-0011_HK72WCCXY_L5_clean_bin.3.orig.fa_kl	OEZ008529	yes	LMSG_G000004957.1	OES073681	NRPS-like	NRPS	Copper	17.37	China: Jiangxi, Dexing
L180206P-5-LD180201-0011_HK72WCCXY_L5_clean_bin.3.orig.fa_kl	OEZ008529	yes	LMSG_G000004957.1	OES073681	terpene	Terpene	Copper	20.31	China: Jiangxi, Dexing
L180206P-5-LD180201-0011_HK72WCCXY_L5_clean_bin.3.orig.fa_kl	OEZ008529	yes	LMSG_G000004957.1	OES073681	lassopeptide	RiPPs	Copper	14.91	China: Jiangxi, Dexing
L180206P-5-LD180201-0011_HK72WCCXY_L5_clean_bin.3.orig.fa_kl	OEZ008529	yes	LMSG_G000004957.1	OES073681	arylpolyene	Others	Copper	10.94	China: Jiangxi, Dexing
L180206P-5-LD180201-0011_HK72WCCXY_L5_clean_bin.30.strict.fa	OEZ008529	yes	LMSG_G000010571.1	OES073681	arylpolyene	Others	Copper	41.19	China: Jiangxi, Dexing
L180206P-5-LD180201-0011_HK72WCCXY_L5_clean_bin.30.strict.fa	OEZ008529	yes	LMSG_G000010571.1	OES073681	bacteriocin	RiPPs	Copper	10.84	China: Jiangxi, Dexing
L180206P-5-LD180201-0011_HK72WCCXY_L5_clean_bin.31.permissiv	OEZ008529	yes	LMSG_G000009298.1	OES073681	betalactone	Others	Copper	25.70	China: Jiangxi, Dexing
L180206P-5-LD180201-0011_HK72WCCXY_L5_clean_bin.31.permissiv	OEZ008529	yes	LMSG_G000009298.1	OES073681	betalactone	Others	Copper	23.83	China: Jiangxi, Dexing
L180206P-5-LD180201-0011_HK72WCCXY_L5_clean_bin.32.strict.fa	OEZ008529	yes	LMSG_G000010822.1	OES073681	acyl_amino_acids	Others	Copper	60.85	China: Jiangxi, Dexing
L180206P-5-LD180201-0011_HK72WCCXY_L5_clean_bin.34.strict.fa	OEZ008529	yes	LMSG_G000004956.1	OES073681	terpene	Terpene	Copper	22.05	China: Jiangxi, Dexing
L180206P-5-LD180201-0011_HK72WCCXY_L5_clean_bin.34.strict.fa	OEZ008529	yes	LMSG_G000004956.1	OES073681	acyl_amino_acids	Others	Copper	67.88	China: Jiangxi, Dexing
L180206P-5-LD180201-0011_HK72WCCXY_L5_clean_bin.34.strict.fa	OEZ008529	yes	LMSG_G000004956.1	OES073681	nucleoside	Others	Copper	18.95	China: Jiangxi, Dexing
L180206P-5-LD180201-0011_HK72WCCXY_L5_clean_bin.34.strict.fa	OEZ008529	yes	LMSG_G000004956.1	OES073681	NRPS-like	NRPS	Copper	31.13	China: Jiangxi, Dexing
L180206P-5-LD180201-0011_HK72WCCXY_L5_clean_bin.34.strict.fa	OEZ008529	yes	LMSG_G000004956.1	OES073681	terpene	Terpene	Copper	11.04	China: Jiangxi, Dexing
L180206P-5-LD180201-0011_HK72WCCXY_L5_clean_bin.35.orig.fa_k	OEZ008529	yes	LMSG_G000010793.1	OES073681	acyl_amino_acids	Others	Copper	23.61	China: Jiangxi, Dexing
L180206P-5-LD180201-0011_HK72WCCXY_L5_clean_bin.35.orig.fa_k	OEZ008529	yes	LMSG_G000010793.1	OES073681	NRPS-like	NRPS	Copper	25.13	China: Jiangxi, Dexing
L180206P-5-LD180201-0011_HK72WCCXY_L5_clean_bin.4.orig.fa_kl	OEZ008529	yes	LMSG_G000009355.1	OES073681	betalactone	Others	Copper	22.55	China: Jiangxi, Dexing
L180206P-5-LD180201-0011_HK72WCCXY_L5_clean_bin.4.orig.fa_kl	OEZ008529	yes	LMSG_G000009355.1	OES073681	betalactone	Others	Copper	25.88	China: Jiangxi, Dexing
L180206P-5-LD180201-0011_HK72WCCXY_L5_clean_bin.5.strict.fa_	OEZ008529	yes	LMSG_G000008264.1	OES073681	terpene	Terpene	Copper	19.06	China: Jiangxi, Dexing
L180206P-5-LD180201-0011_HK72WCCXY_L5_clean_bin.7.orig.fa_kl	OEZ008529	yes	LMSG_G000004958.1	OES073681	NRPS-like	NRPS	Copper	31.95	China: Jiangxi, Dexing
L180206P-5-LD180201-0011_HK72WCCXY_L5_clean_bin.7.orig.fa_kl	OEZ008529	yes	LMSG_G000004958.1	OES073681	arylpolyene	Others	Copper	12.26	China: Jiangxi, Dexing
L180206P-5-LD180201-0011_HK72WCCXY_L5_clean_bin.7.orig.fa_kl	OEZ008529	yes	LMSG_G000004958.1	OES073681	betalactone	Others	Copper	22.94	China: Jiangxi, Dexing
L180206P-5-LD180201-0011_HK72WCCXY_L5_clean_bin.7.orig.fa_kl	OEZ008529	yes	LMSG_G000004958.1	OES073681	terpene	Terpene	Copper	23.09	China: Jiangxi, Dexing
L180206P-5-LD180201-0011_HK72WCCXY_L5_clean_bin.8.permissive	OEZ008529	yes	LMSG_G000010422.1	OES073681	terpene	Terpene	Copper	7.18	China: Jiangxi, Dexing
L180206P-5-LD180201-0011_HK72WCCXY_L5_clean_bin.8.permissive	OEZ008529	yes	LMSG_G000010422.1	OES073681	hserlactone	Others	Copper	5.96	China: Jiangxi, Dexing
L180206P-5-LD180201-0011_HK72WCCXY_L5_clean_bin.8.permissive	OEZ008529	yes	LMSG_G000010422.1	OES073681	terpene	Terpene	Copper	5.39	China: Jiangxi, Dexing
L180206P-5-LD180201-0012_HK72WCCXY_L5_clean_bin.11.orig.fa_k	OEZ008529	yes	LMSG_G000008070.1	OES073679	T1PKS.hglE-KS	PKSother	Copper	26.24	China: Jiangxi, Dexing
L180206P-5-LD180201-0012_HK72WCCXY_L5_clean_bin.11.orig.fa_k	OEZ008529	yes	LMSG_G000008070.1	OES073679	other	Others	Copper	7.52	China: Jiangxi, Dexing
L180206P-5-LD180201-0012_HK72WCCXY_L5_clean_bin.12.orig.fa_k	OEZ008529	yes	LMSG_G000004959.1	OES073679	T1PKS	PKSI	Copper	32.47	China: Jiangxi, Dexing
L180206P-5-LD180201-0012_HK72WCCXY_L5_clean_bin.12.orig.fa_k	OEZ008529	yes	LMSG_G000004959.1	OES073679	terpene	Terpene	Copper	11.40	China: Jiangxi, Dexing
L180206P-5-LD180201-0012_HK72WCCXY_L5_clean_bin.12.orig.fa_k	OEZ008529	yes	LMSG_G000004959.1	OES073679	bacteriocin	RiPPs	Copper	10.80	China: Jiangxi, Dexing
L180206P-5-LD180201-0012_HK72WCCXY_L5_clean_bin.12.orig.fa_k	OEZ008529	yes	LMSG_G000004959.1	OES073679	terpene	Terpene	Copper	6.29	China: Jiangxi, Dexing
L180206P-5-LD180201-0012_HK72WCCXY_L5_clean_bin.13.strict.fa	OEZ008529	yes	LMSG_G000007981.1	OES073679	bacteriocin	RiPPs	Copper	10.32	China: Jiangxi, Dexing
L180206P-5-LD180201-0012_HK72WCCXY_L5_clean_bin.13.strict.fa	OEZ008529	yes	LMSG_G000007981.1	OES073679	TfuA-related.bacteriocin	RiPPs	Copper	12.36	China: Jiangxi, Dexing
L180206P-5-LD180201-0012_HK72WCCXY_L5_clean_bin.14.orig.fa_k	OEZ008529	no	LMSG_G000010625.1	OES073679	arylpolyene	Others	Copper	8.80	China: Jiangxi, Dexing
L180206P-5-LD180201-0012_HK72WCCXY_L5_clean_bin.14.orig.fa_k	OEZ008529	yes	LMSG_G000010625.1	OES073679	bacteriocin	RiPPs	Copper	5.60	China: Jiangxi, Dexing
L180206P-5-LD180201-0012_HK72WCCXY_L5_clean_bin.14.orig.fa_k	OEZ008529	no	LMSG_G000010625.1	OES073679	terpene	Terpene	Copper	5.43	China: Jiangxi, Dexing
L180206P-5-LD180201-0012_HK72WCCXY_L5_clean_bin.17.orig.fa_k	OEZ008529	yes	LMSG_G000004961.1	OES073679	terpene	Terpene	Copper	26.04	China: Jiangxi, Dexing
L180206P-5-LD180201-0012_HK72WCCXY_L5_clean_bin.17.orig.fa_k	OEZ008529	yes	LMSG_G000004961.1	OES073679	T2PKS	PKSother	Copper	68.73	China: Jiangxi, Dexing
L180206P-5-LD180201-0012_HK72WCCXY_L5_clean_bin.17.orig.fa_k	OEZ008529	yes	LMSG_G000004961.1	OES073679	T3PKS	PKSother	Copper	13.37	China: Jiangxi, Dexing
L180206P-5-LD180201-0012_HK72WCCXY_L5_clean_bin.17.orig.fa_k	OEZ008529	yes	LMSG_G000004961.1	OES073679	arylpolyene	Others	Copper	28.68	China: Jiangxi, Dexing
L180206P-5-LD180201-0012_HK72WCCXY_L5_clean_bin.17.orig.fa_k	OEZ008529	yes	LMSG_G000004961.1	OES073679	ectoine	Others	Copper	8.14	China: Jiangxi, Dexing
L180206P-5-LD180201-0012_HK72WCCXY_L5_clean_bin.17.orig.fa_k	OEZ008529	yes	LMSG_G000004961.1	OES073679	indole	Others	Copper	21.11	China: Jiangxi, Dexing
L180206P-5-LD180201-0012_HK72WCCXY_L5_clean_bin.17.orig.fa_k	OEZ008529	yes	LMSG_G000004961.1	OES073679	NRPS	NRPS	Copper	31.21	China: Jiangxi, Dexing
L180206P-5-LD180201-0012_HK72WCCXY_L5_clean_bin.17.orig.fa_k	OEZ008529	yes	LMSG_G000004961.1	OES073679	linaridin	RiPPs	Copper	26.46	China: Jiangxi, Dexing
L180206P-5-LD180201-0012_HK72WCCXY_L5_clean_bin.17.orig.fa_k	OEZ008529	yes	LMSG_G000004961.1	OES073679	ladderane	Others	Copper	7.30	China: Jiangxi, Dexing
L180206P-5-LD180201-0012_HK72WCCXY_L5_clean_bin.17.orig.fa_k	OEZ008529	yes	LMSG_G000004961.1	OES073679	linaridin	RiPPs	Copper	14.22	China: Jiangxi, Dexing
L180206P-5-LD180201-0012_HK72WCCXY_L5_clean_bin.17.orig.fa_k	OEZ008529	yes	LMSG_G000004961.1	OES073679	T1PKS.lanthipeptide	Others	Copper	45.50	China: Jiangxi, Dexing
L180206P-5-LD180201-0012_HK72WCCXY_L5_clean_bin.17.orig.fa_k	OEZ008529	yes	LMSG_G000004961.1	OES073679	terpene	Terpene	Copper	20.97	China: Jiangxi, Dexing
L180206P-5-LD180201-0012_HK72WCCXY_L5_clean_bin.18.orig.fa_k	OEZ008529	yes	LMSG_G000009116.1	OES073679	terpene	Terpene	Copper	25.09	China: Jiangxi, Dexing
L180206P-5-LD180201-0012_HK72WCCXY_L5_clean_bin.18.orig.fa_k	OEZ008529	yes	LMSG_G000009116.1	OES073679	bacteriocin	RiPPs	Copper	10.87	China: Jiangxi, Dexing
L180206P-5-LD180201-0012_HK72WCCXY_L5_clean_bin.18.orig.fa_k	OEZ008529	yes	LMSG_G000009116.1	OES073679	NRPS-like	NRPS	Copper	25.94	China: Jiangxi, Dexing
L180206P-5-LD180201-0012_HK72WCCXY_L5_clean_bin.19.orig.fa_k	OEZ008529	yes	LMSG_G000004962.1	OES073679	terpene	Terpene	Copper	7.91	China: Jiangxi, Dexing
L180206P-5-LD180201-0012_HK72WCCXY_L5_clean_bin.2.orig.fa_kl	OEZ008529	yes	LMSG_G000004965.1	OES073679	resorcinol.arylpolyene	Others	Copper	69.35	China: Jiangxi, Dexing
L180206P-5-LD180201-0012_HK72WCCXY_L5_clean_bin.20.strict.fa	OEZ008529	yes	LMSG_G000006218.1	OES073679	ectoine	Others	Copper	9.69	China: Jiangxi, Dexing
L180206P-5-LD180201-0012_HK72WCCXY_L5_clean_bin.20.strict.fa	OEZ008529	yes	LMSG_G000006218.1	OES073679	terpene	Terpene	Copper	21.79	China: Jiangxi, Dexing
L180206P-5-LD180201-0012_HK72WCCXY_L5_clean_bin.22.strict.fa	OEZ008529	yes	LMSG_G000004963.1	OES073679	bacteriocin	RiPPs	Copper	10.59	China: Jiangxi, Dexing
L180206P-5-LD180201-0012_HK72WCCXY_L5_clean_bin.24.orig.fa_k	OEZ008529	yes	LMSG_G000004394.1	OES073679	ectoine	Others	Copper	10.13	China: Jiangxi, Dexing
L180206P-5-LD180201-0012_HK72WCCXY_L5_clean_bin.24.orig.fa_k	OEZ008529	no	LMSG_G000004394.1	OES073679	NRPS	NRPS	Copper	7.14	China: Jiangxi, Dexing
L180206P-5-LD180201-0012_HK72WCCXY_L5_clean_bin.24.orig.fa_k	OEZ008529	yes	LMSG_G000004394.1	OES073679	terpene	Terpene	Copper	15.66	China: Jiangxi, Dexing
L180206P-5-LD180201-0012_HK72WCCXY_L5_clean_bin.24.orig.fa_k	OEZ008529	yes	LMSG_G000004394.1	OES073679	terpene	Terpene	Copper	12.88	China: Jiangxi, Dexing
L180206P-5-LD180201-0012_HK72WCCXY_L5_clean_bin.24.orig.fa_k	OEZ008529	yes	LMSG_G000004394.1	OES073679	terpene	Terpene	Copper	26.11	China: Jiangxi, Dexing
L180206P-5-LD180201-0012_HK72WCCXY_L5_clean_bin.24.orig.fa_k	OEZ008529	yes	LMSG_G000004394.1	OES073679	T1PKS	PKSI	Copper	51.38	China: Jiangxi, Dexing
L180206P-5-LD180201-0012_HK72WCCXY_L5_clean_bin.24.orig.fa_k	OEZ008529	yes	LMSG_G000004394.1	OES073679	betalactone	Others	Copper	37.89	China: Jiangxi, Dexing
L180206P-5-LD180201-0012_HK72WCCXY_L5_clean_bin.24.orig.fa_k	OEZ008529	yes	LMSG_G000004394.1	OES073679	terpene	Terpene	Copper	22.53	China: Jiangxi, Dexing
L180206P-5-LD180201-0012_HK72WCCXY_L5_clean_bin.24.orig.fa_k	OEZ008529	yes	LMSG_G000004394.1	OES073679	NRPS	NRPS	Copper	29.36	China: Jiangxi, Dexing
L180206P-5-LD180201-0012_HK72WCCXY_L5_clean_bin.24.orig.fa_k	OEZ008529	yes	LMSG_G000004394.1	OES073679	T1PKS	PKSI	Copper	25.64	China: Jiangxi, Dexing
L180206P-5-LD180201-0012_HK72WCCXY_L5_clean_bin.24.orig.fa_k	OEZ008529	no	LMSG_G000004394.1	OES073679	NRPS	NRPS	Copper	8.77	China: Jiangxi, Dexing
L180206P-5-LD180201-0012_HK72WCCXY_L5_clean_bin.24.orig.fa_k	OEZ008529	yes	LMSG_G000004394.1	OES073679	siderophore	Others	Copper	13.43	China: Jiangxi, Dexing
L180206P-5-LD180201-0012_HK72WCCXY_L5_clean_bin.24.orig.fa_k	OEZ008529	yes	LMSG_G000004394.1	OES073679	terpene	Terpene	Copper	19.45	China: Jiangxi, Dexing
L180206P-5-LD180201-0012_HK72WCCXY_L5_clean_bin.24.orig.fa_k	OEZ008529	yes	LMSG_G000004394.1	OES073679	T3PKS	PKSother	Copper	21.88	China: Jiangxi, Dexing
L180206P-5-LD180201-0012_HK72WCCXY_L5_clean_bin.24.orig.fa_k	OEZ008529	yes	LMSG_G000004394.1	OES073679	terpene	Terpene	Copper	17.45	China: Jiangxi, Dexing
L180206P-5-LD180201-0012_HK72WCCXY_L5_clean_bin.24.orig.fa_k	OEZ008529	yes	LMSG_G000004394.1	OES073679	NRPS	NRPS	Copper	45.46	China: Jiangxi, Dexing
L180206P-5-LD180201-0012_HK72WCCXY_L5_clean_bin.24.orig.fa_k	OEZ008529	yes	LMSG_G000004394.1	OES073679	butyrolactone	Others	Copper	10.82	China: Jiangxi, Dexing
L180206P-5-LD180201-0012_HK72WCCXY_L5_clean_bin.25.orig.fa_k	OEZ008529	yes	LMSG_G000008350.1	OES073679	bacteriocin	RiPPs	Copper	7.47	China: Jiangxi, Dexing
L180206P-5-LD180201-0012_HK72WCCXY_L5_clean_bin.25.orig.fa_k	OEZ008529	yes	LMSG_G000008350.1	OES073679	terpene	Terpene	Copper	9.91	China: Jiangxi, Dexing
L180206P-5-LD180201-0012_HK72WCCXY_L5_clean_bin.25.orig.fa_k	OEZ008529	yes	LMSG_G000008350.1	OES073679	butyrolactone	Others	Copper	11.01	China: Jiangxi, Dexing
L180206P-5-LD180201-0012_HK72WCCXY_L5_clean_bin.25.orig.fa_k	OEZ008529	yes	LMSG_G000008350.1	OES073679	T3PKS	PKSother	Copper	12.90	China: Jiangxi, Dexing
L180206P-5-LD180201-0012_HK72WCCXY_L5_clean_bin.26.orig.fa_k	OEZ008529	yes	LMSG_G000010258.1	OES073679	arylpolyene	Others	Copper	23.40	China: Jiangxi, Dexing
L180206P-5-LD180201-0012_HK72WCCXY_L5_clean_bin.26.orig.fa_k	OEZ008529	no	LMSG_G000010258.1	OES073679	terpene	Terpene	Copper	21.20	China: Jiangxi, Dexing
L180206P-5-LD180201-0012_HK72WCCXY_L5_clean_bin.27.orig.fa_k	OEZ008529	yes	LMSG_G000008524.1	OES073679	hglE-KS	PKSother	Copper	5.67	China: Jiangxi, Dexing
L180206P-5-LD180201-0012_HK72WCCXY_L5_clean_bin.27.orig.fa_k	OEZ008529	yes	LMSG_G000008524.1	OES073679	LAP	RiPPs	Copper	8.66	China: Jiangxi, Dexing
L180206P-5-LD180201-0012_HK72WCCXY_L5_clean_bin.28.strict.fa	OEZ008529	yes	LMSG_G000004964.1	OES073679	ectoine	Others	Copper	5.33	China: Jiangxi, Dexing
L180206P-5-LD180201-0012_HK72WCCXY_L5_clean_bin.29.orig.fa_k	OEZ008529	yes	LMSG_G000009242.1	OES073679	T3PKS	PKSother	Copper	11.17	China: Jiangxi, Dexing
L180206P-5-LD180201-0012_HK72WCCXY_L5_clean_bin.29.orig.fa_k	OEZ008529	yes	LMSG_G000009242.1	OES073679	terpene	Terpene	Copper	10.67	China: Jiangxi, Dexing
L180206P-5-LD180201-0012_HK72WCCXY_L5_clean_bin.3.orig.fa_kl	OEZ008529	no	LMSG_G000004973.1	OES073679	terpene	Terpene	Copper	20.97	China: Jiangxi, Dexing

L180206P-5-LD180201-0012_HK72WCCXY_L5_clean_bin.3.orig.fa_kl	OEZ008529	yes	LMSG_G000004973.1	OES073679	ectoine	Others	Copper	8.11	China: Jiangxi, Dexing
L180206P-5-LD180201-0012_HK72WCCXY_L5_clean_bin.3.orig.fa_kl	OEZ008529	no	LMSG_G000004973.1	OES073679	T2PKS.blactam	Others	Copper	81.23	China: Jiangxi, Dexing
L180206P-5-LD180201-0012_HK72WCCXY_L5_clean_bin.3.orig.fa_kl	OEZ008529	no	LMSG_G000004973.1	OES073679	arylpolyene	Others	Copper	28.88	China: Jiangxi, Dexing
L180206P-5-LD180201-0012_HK72WCCXY_L5_clean_bin.3.orig.fa_kl	OEZ008529	no	LMSG_G000004973.1	OES073679	NRPS	NRPS	Copper	31.37	China: Jiangxi, Dexing
L180206P-5-LD180201-0012_HK72WCCXY_L5_clean_bin.3.orig.fa_kl	OEZ008529	no	LMSG_G000004973.1	OES073679	ladderane	Others	Copper	38.26	China: Jiangxi, Dexing
L180206P-5-LD180201-0012_HK72WCCXY_L5_clean_bin.3.orig.fa_kl	OEZ008529	no	LMSG_G000004973.1	OES073679	terpene	Terpene	Copper	26.07	China: Jiangxi, Dexing
L180206P-5-LD180201-0012_HK72WCCXY_L5_clean_bin.3.orig.fa_kl	OEZ008529	no	LMSG_G000004973.1	OES073679	T3PKS	PKSother	Copper	13.28	China: Jiangxi, Dexing
L180206P-5-LD180201-0012_HK72WCCXY_L5_clean_bin.3.orig.fa_kl	OEZ008529	no	LMSG_G000004973.1	OES073679	indole	Others	Copper	21.09	China: Jiangxi, Dexing
L180206P-5-LD180201-0012_HK72WCCXY_L5_clean_bin.30.orig.fa_k	OEZ008529	yes	LMSG_G000010000.1	OES073679	terpene	Terpene	Copper	5.96	China: Jiangxi, Dexing
L180206P-5-LD180201-0012_HK72WCCXY_L5_clean_bin.30.orig.fa_k	OEZ008529	yes	LMSG_G000010000.1	OES073679	ectoine	Others	Copper	8.46	China: Jiangxi, Dexing
L180206P-5-LD180201-0012_HK72WCCXY_L5_clean_bin.30.orig.fa_k	OEZ008529	yes	LMSG_G000010000.1	OES073679	terpene	Terpene	Copper	13.13	China: Jiangxi, Dexing
L180206P-5-LD180201-0012_HK72WCCXY_L5_clean_bin.30.orig.fa_k	OEZ008529	yes	LMSG_G000010000.1	OES073679	arylpolyene	Others	Copper	5.32	China: Jiangxi, Dexing
L180206P-5-LD180201-0012_HK72WCCXY_L5_clean_bin.32.orig.fa_k	OEZ008529	yes	LMSG_G000004967.1	OES073679	betalactone	Others	Copper	29.61	China: Jiangxi, Dexing
L180206P-5-LD180201-0012_HK72WCCXY_L5_clean_bin.33.strict.fa	OEZ008529	yes	LMSG_G000004395.1	OES073679	betalactone	Others	Copper	29.11	China: Jiangxi, Dexing
L180206P-5-LD180201-0012_HK72WCCXY_L5_clean_bin.34.strict.fa	OEZ008529	yes	LMSG_G000004968.1	OES073679	other	Others	Copper	31.70	China: Jiangxi, Dexing
L180206P-5-LD180201-0012_HK72WCCXY_L5_clean_bin.34.strict.fa	OEZ008529	yes	LMSG_G000004968.1	OES073679	NRPS-like	NRPS	Copper	21.19	China: Jiangxi, Dexing
L180206P-5-LD180201-0012_HK72WCCXY_L5_clean_bin.36.orig.fa_k	OEZ008529	yes	LMSG_G000004969.1	OES073679	NRPS.T1PKS	PKS-NRP_Hybrids	Copper	31.55	China: Jiangxi, Dexing
L180206P-5-LD180201-0012_HK72WCCXY_L5_clean_bin.36.orig.fa_k	OEZ008529	yes	LMSG_G000004969.1	OES073679	terpene	Terpene	Copper	29.71	China: Jiangxi, Dexing
L180206P-5-LD180201-0012_HK72WCCXY_L5_clean_bin.36.orig.fa_k	OEZ008529	yes	LMSG_G000004969.1	OES073679	NRPS-like	NRPS	Copper	22.94	China: Jiangxi, Dexing
L180206P-5-LD180201-0012_HK72WCCXY_L5_clean_bin.36.orig.fa_k	OEZ008529	yes	LMSG_G000004969.1	OES073679	T3PKS	PKSother	Copper	15.76	China: Jiangxi, Dexing
L180206P-5-LD180201-0012_HK72WCCXY_L5_clean_bin.36.orig.fa_k	OEZ008529	yes	LMSG_G000004969.1	OES073679	NRPS	NRPS	Copper	16.36	China: Jiangxi, Dexing
L180206P-5-LD180201-0012_HK72WCCXY_L5_clean_bin.36.orig.fa_k	OEZ008529	yes	LMSG_G000004969.1	OES073679	NRPS	NRPS	Copper	38.22	China: Jiangxi, Dexing
L180206P-5-LD180201-0012_HK72WCCXY_L5_clean_bin.37.orig.fa_k	OEZ008529	yes	LMSG_G000004970.1	OES073679	ectoine	Others	Copper	6.01	China: Jiangxi, Dexing
L180206P-5-LD180201-0012_HK72WCCXY_L5_clean_bin.38.strict.fa	OEZ008529	yes	LMSG_G000004971.1	OES073679	ectoine	Others	Copper	6.96	China: Jiangxi, Dexing
L180206P-5-LD180201-0012_HK72WCCXY_L5_clean_bin.39.orig.fa_k	OEZ008529	yes	LMSG_G000004972.1	OES073679	NRPS	NRPS	Copper	26.19	China: Jiangxi, Dexing
L180206P-5-LD180201-0012_HK72WCCXY_L5_clean_bin.39.orig.fa_k	OEZ008529	yes	LMSG_G000004972.1	OES073679	T3PKS	PKSother	Copper	22.84	China: Jiangxi, Dexing
L180206P-5-LD180201-0012_HK72WCCXY_L5_clean_bin.39.orig.fa_k	OEZ008529	yes	LMSG_G000004972.1	OES073679	ectoine	Others	Copper	10.40	China: Jiangxi, Dexing
L180206P-5-LD180201-0012_HK72WCCXY_L5_clean_bin.39.orig.fa_k	OEZ008529	yes	LMSG_G000004972.1	OES073679	terpene	Terpene	Copper	22.23	China: Jiangxi, Dexing
L180206P-5-LD180201-0012_HK72WCCXY_L5_clean_bin.39.orig.fa_k	OEZ008529	yes	LMSG_G000004972.1	OES073679	T1PKS	PKSI	Copper	39.01	China: Jiangxi, Dexing
L180206P-5-LD180201-0012_HK72WCCXY_L5_clean_bin.39.orig.fa_k	OEZ008529	yes	LMSG_G000004972.1	OES073679	NRPS	NRPS	Copper	29.45	China: Jiangxi, Dexing
L180206P-5-LD180201-0012_HK72WCCXY_L5_clean_bin.40.orig.fa_k	OEZ008529	yes	LMSG_G000009258.1	OES073679	TfuA-related	RiPPs	Copper	21.93	China: Jiangxi, Dexing
L180206P-5-LD180201-0012_HK72WCCXY_L5_clean_bin.41.orig.fa_k	OEZ008529	yes	LMSG_G000004974.1	OES073679	T3PKS	PKSother	Copper	8.80	China: Jiangxi, Dexing
L180206P-5-LD180201-0012_HK72WCCXY_L5_clean_bin.41.orig.fa_k	OEZ008529	no	LMSG_G000004974.1	OES073679	terpene	Terpene	Copper	6.25	China: Jiangxi, Dexing
L180206P-5-LD180201-0012_HK72WCCXY_L5_clean_bin.41.orig.fa_k	OEZ008529	yes	LMSG_G000004974.1	OES073679	NRPS-like	NRPS	Copper	10.37	China: Jiangxi, Dexing
L180206P-5-LD180201-0012_HK72WCCXY_L5_clean_bin.42.orig.fa_k	OEZ008529	yes	LMSG_G000004975.1	OES073679	bacteriocin	RiPPs	Copper	10.84	China: Jiangxi, Dexing
L180206P-5-LD180201-0012_HK72WCCXY_L5_clean_bin.43.orig.fa_k	OEZ008529	yes	LMSG_G000011030.1	OES073679	arylpolyene	Others	Copper	23.21	China: Jiangxi, Dexing
L180206P-5-LD180201-0012_HK72WCCXY_L5_clean_bin.43.orig.fa_k	OEZ008529	yes	LMSG_G000011030.1	OES073679	terpene	Terpene	Copper	20.85	China: Jiangxi, Dexing
L180206P-5-LD180201-0012_HK72WCCXY_L5_clean_bin.46.strict.fa	OEZ008529	yes	LMSG_G000004976.1	OES073679	T3PKS	PKSother	Copper	9.47	China: Jiangxi, Dexing
L180206P-5-LD180201-0012_HK72WCCXY_L5_clean_bin.47.strict.fa	OEZ008529	yes	LMSG_G000008024.1	OES073679	thiopeptide.LAP	RiPPs	Copper	17.71	China: Jiangxi, Dexing
L180206P-5-LD180201-0012_HK72WCCXY_L5_clean_bin.5.strict.fa_	OEZ008529	yes	LMSG_G000008089.1	OES073679	T1PKS.hglE-KS	PKSother	Copper	31.67	China: Jiangxi, Dexing
L180206P-5-LD180201-0012_HK72WCCXY_L5_clean_bin.52.orig.fa_k	OEZ008529	yes	LMSG_G000004979.1	OES073679	NRPS.T1PKS	PKS-NRP_Hybrids	Copper	16.33	China: Jiangxi, Dexing
L180206P-5-LD180201-0012_HK72WCCXY_L5_clean_bin.52.orig.fa_k	OEZ008529	yes	LMSG_G000004979.1	OES073679	terpene	Terpene	Copper	17.18	China: Jiangxi, Dexing
L180206P-5-LD180201-0012_HK72WCCXY_L5_clean_bin.52.orig.fa_k	OEZ008529	yes	LMSG_G000004979.1	OES073679	siderophore	Others	Copper	12.95	China: Jiangxi, Dexing
L180206P-5-LD180201-0012_HK72WCCXY_L5_clean_bin.52.orig.fa_k	OEZ008529	yes	LMSG_G000004979.1	OES073679	T1PKS.hglE-KS.PUFA	Others	Copper	47.38	China: Jiangxi, Dexing
L180206P-5-LD180201-0012_HK72WCCXY_L5_clean_bin.52.orig.fa_k	OEZ008529	yes	LMSG_G000004979.1	OES073679	T3PKS	PKSother	Copper	41.04	China: Jiangxi, Dexing
L180206P-5-LD180201-0012_HK72WCCXY_L5_clean_bin.52.orig.fa_k	OEZ008529	yes	LMSG_G000004979.1	OES073679	siderophore	Others	Copper	11.76	China: Jiangxi, Dexing
L180206P-5-LD180201-0012_HK72WCCXY_L5_clean_bin.52.orig.fa_k	OEZ008529	yes	LMSG_G000004979.1	OES073679	NRPS	NRPS	Copper	32.75	China: Jiangxi, Dexing
L180206P-5-LD180201-0012_HK72WCCXY_L5_clean_bin.54.orig.fa_k	OEZ008529	yes	LMSG_G000010423.1	OES073679	terpene	Terpene	Copper	6.67	China: Jiangxi, Dexing
L180206P-5-LD180201-0012_HK72WCCXY_L5_clean_bin.55.orig.fa_k	OEZ008529	yes	LMSG_G000004396.1	OES073679	bacteriocin	RiPPs	Copper	11.97	China: Jiangxi, Dexing
L180206P-5-LD180201-0012_HK72WCCXY_L5_clean_bin.57.orig.fa_k	OEZ008529	yes	LMSG_G000004981.1	OES073679	ectoine	Others	Copper	6.22	China: Jiangxi, Dexing
L180206P-5-LD180201-0012_HK72WCCXY_L5_clean_bin.58.strict.fa	OEZ008529	yes	LMSG_G000004982.1	OES073679	TfuA-related	RiPPs	Copper	21.95	China: Jiangxi, Dexing
L180206P-5-LD180201-0012_HK72WCCXY_L5_clean_bin.58.strict.fa	OEZ008529	yes	LMSG_G000004982.1	OES073679	terpene	Terpene	Copper	20.85	China: Jiangxi, Dexing
L180206P-5-LD180201-0012_HK72WCCXY_L5_clean_bin.60.orig.fa_k	OEZ008529	yes	LMSG_G000004983.1	OES073679	ectoine	Others	Copper	7.13	China: Jiangxi, Dexing
L180206P-5-LD180201-0012_HK72WCCXY_L5_clean_bin.60.orig.fa_k	OEZ008529	yes	LMSG_G000004983.1	OES073679	terpene	Terpene	Copper	8.84	China: Jiangxi, Dexing
L180206P-5-LD180201-0012_HK72WCCXY_L5_clean_bin.61.orig.fa_k	OEZ008529	yes	LMSG_G000008866.1	OES073679	T3PKS	PKSother	Copper	24.78	China: Jiangxi, Dexing
L180206P-5-LD180201-0012_HK72WCCXY_L5_clean_bin.61.orig.fa_k	OEZ008529	yes	LMSG_G000008866.1	OES073679	terpene	Terpene	Copper	20.96	China: Jiangxi, Dexing
L180206P-5-LD180201-0012_HK72WCCXY_L5_clean_bin.61.orig.fa_k	OEZ008529	yes	LMSG_G000008866.1	OES073679	terpene	Terpene	Copper	11.72	China: Jiangxi, Dexing
L180206P-5-LD180201-0012_HK72WCCXY_L5_clean_bin.62.orig.fa_k	OEZ008529	yes	LMSG_G000011040.1	OES073679	arylpolyene	Others	Copper	28.30	China: Jiangxi, Dexing
L180206P-5-LD180201-0012_HK72WCCXY_L5_clean_bin.62.orig.fa_k	OEZ008529	yes	LMSG_G000011040.1	OES073679	bacteriocin	RiPPs	Copper	9.82	China: Jiangxi, Dexing
L180206P-5-LD180201-0012_HK72WCCXY_L5_clean_bin.62.orig.fa_k	OEZ008529	yes	LMSG_G000011040.1	OES073679	NRPS-like	NRPS	Copper	11.46	China: Jiangxi, Dexing
L180206P-5-LD180201-0012_HK72WCCXY_L5_clean_bin.62.orig.fa_k	OEZ008529	yes	LMSG_G000011040.1	OES073679	resorcinol	Others	Copper	7.08	China: Jiangxi, Dexing
L180206P-5-LD180201-0012_HK72WCCXY_L5_clean_bin.62.orig.fa_k	OEZ008529	yes	LMSG_G000011040.1	OES073679	terpene	Terpene	Copper	23.08	China: Jiangxi, Dexing
L180206P-5-LD180201-0012_HK72WCCXY_L5_clean_bin.62.orig.fa_k	OEZ008529	yes	LMSG_G000011040.1	OES073679	arylpolyene	Others	Copper	20.45	China: Jiangxi, Dexing
L180206P-5-LD180201-0012_HK72WCCXY_L5_clean_bin.62.orig.fa_k	OEZ008529	yes	LMSG_G000011040.1	OES073679	terpene	Terpene	Copper	11.85	China: Jiangxi, Dexing
L180206P-5-LD180201-0012_HK72WCCXY_L5_clean_bin.63.orig.fa_k	OEZ008529	yes	LMSG_G000004397.1	OES073679	T3PKS	PKSother	Copper	20.79	China: Jiangxi, Dexing
L180206P-5-LD180201-0012_HK72WCCXY_L5_clean_bin.63.orig.fa_k	OEZ008529	yes	LMSG_G000004397.1	OES073679	betalactone	Others	Copper	25.47	China: Jiangxi, Dexing
L180206P-5-LD180201-0012_HK72WCCXY_L5_clean_bin.63.orig.fa_k	OEZ008529	yes	LMSG_G000004397.1	OES073679	terpene	Terpene	Copper	22.22	China: Jiangxi, Dexing
L180206P-5-LD180201-0012_HK72WCCXY_L5_clean_bin.63.orig.fa_k	OEZ008529	yes	LMSG_G000004397.1	OES073679	siderophore	Others	Copper	13.60	China: Jiangxi, Dexing
L180206P-5-LD180201-0012_HK72WCCXY_L5_clean_bin.63.orig.fa_k	OEZ008529	yes	LMSG_G000004397.1	OES073679	ectoine	Others	Copper	10.38	China: Jiangxi, Dexing
L180206P-5-LD180201-0012_HK72WCCXY_L5_clean_bin.65.orig.fa_k	OEZ008529	no	LMSG_G000004984.1	OES073679	terpene	Terpene	Copper	8.17	China: Jiangxi, Dexing
L180206P-5-LD180201-0012_HK72WCCXY_L5_clean_bin.68.permissiv	OEZ008529	yes	LMSG_G000004398.1	OES073679	terpene	Terpene	Copper	14.47	China: Jiangxi, Dexing
L180206P-5-LD180201-0012_HK72WCCXY_L5_clean_bin.68.permissiv	OEZ008529	yes	LMSG_G000004398.1	OES073679	ectoine	Others	Copper	10.40	China: Jiangxi, Dexing
L180206P-5-LD180201-0012_HK72WCCXY_L5_clean_bin.68.permissiv	OEZ008529	yes	LMSG_G000004398.1	OES073679	betalactone	Others	Copper	16.42	China: Jiangxi, Dexing
L180206P-5-LD180201-0012_HK72WCCXY_L5_clean_bin.69.orig.fa_k	OEZ008529	yes	LMSG_G000004985.1	OES073679	ectoine	Others	Copper	10.39	China: Jiangxi, Dexing
L180206P-5-LD180201-0012_HK72WCCXY_L5_clean_bin.7.orig.fa_kl	OEZ008529	yes	LMSG_G000009088.1	OES073679	betalactone	Others	Copper	31.07	China: Jiangxi, Dexing
L180206P-5-LD180201-0012_HK72WCCXY_L5_clean_bin.7.orig.fa_kl	OEZ008529	yes	LMSG_G000009088.1	OES073679	thiopeptide	RiPPs	Copper	31.20	China: Jiangxi, Dexing
L180206P-5-LD180201-0012_HK72WCCXY_L5_clean_bin.7.orig.fa_kl	OEZ008529	yes	LMSG_G000009088.1	OES073679	betalactone	Others	Copper	24.61	China: Jiangxi, Dexing
L180206P-5-LD180201-0012_HK72WCCXY_L5_clean_bin.7.orig.fa_kl	OEZ008529	yes	LMSG_G000009088.1	OES073679	terpene	Terpene	Copper	22.04	China: Jiangxi, Dexing
L180206P-5-LD180201-0012_HK72WCCXY_L5_clean_bin.7.orig.fa_kl	OEZ008529	yes	LMSG_G000009088.1	OES073679	betalactone	Others	Copper	27.73	China: Jiangxi, Dexing
L180206P-5-LD180201-0012_HK72WCCXY_L5_clean_bin.70.strict.fa	OEZ008529	yes	LMSG_G000004987.1	OES073679	terpene	Terpene	Copper	21.09	China: Jiangxi, Dexing
L180206P-5-LD180201-0012_HK72WCCXY_L5_clean_bin.70.strict.fa	OEZ008529	yes	LMSG_G000004987.1	OES073679	other	Others	Copper	24.69	China: Jiangxi, Dexing
L180206P-5-LD180201-0012_HK72WCCXY_L5_clean_bin.71.orig.fa_k	OEZ008529	no	LMSG_G000010545.1	OES073679	hserlactone	Others	Copper	15.13	China: Jiangxi, Dexing
L180206P-5-LD180201-0012_HK72WCCXY_L5_clean_bin.71.orig.fa_k	OEZ008529	no	LMSG_G000010545.1	OES073679	T1PKS	PKSI	Copper	45.12	China: Jiangxi, Dexing
L180206P-5-LD180201-0012_HK72WCCXY_L5_clean_bin.71.orig.fa_k	OEZ008529	no	LMSG_G000010545.1	OES073679	terpene.thiopeptide.LAP	Others	Copper	39.52	China: Jiangxi, Dexing
L180206P-5-LD180201-0012_HK72WCCXY_L5_clean_bin.71.orig.fa_k	OEZ008529	no	LMSG_G000010545.1	OES073679	arylpolyene	Others	Copper	41.19	China: Jiangxi, Dexing
L180206P-5-LD180201-0012_HK72WCCXY_L5_clean_bin.72.strict.fa	OEZ008529	yes	LMSG_G000004988.1	OES073679	resorcinol.arylpolyene	Others	Copper	12.30	China: Jiangxi, Dexing
L180206P-5-LD180201-0012_HK72WCCXY_L5_clean_bin.72.strict.fa	OEZ008529	yes	LMSG_G000004988.1	OES073679	arylpolyene	Others	Copper	12.04	China: Jiangxi, Dexing
L180206P-5-LD180201-0012_HK72WCCXY_L5_clean_bin.72.strict.fa	OEZ008529	yes	LMSG_G000004988.1	OES073679	butyrolactone	Others	Copper	8.20	China: Jiangxi, Dexing
L180206P-5-LD180201-0012_HK72WCCXY_L5_clean_bin.72.strict.fa	OEZ008529	yes	LMSG_G000004988.1	OES073679	ectoine	Others	Copper	5.40	China: Jiangxi, Dexing
L180206P-5-LD180201-0012_HK72WCCXY_L5_clean_bin.73.strict.fa	OEZ008529	yes	LMSG_G000010794.1	OES073679	acyl_amino_acids	Others	Copper	43.61	China: Jiangxi, Dexing
L180206P-5-LD180201-0012_HK72WCCXY_L5_clean_bin.77.orig.fa_k	OEZ008529	yes	LMSG_G000004399.1	OES073679	NRPS-like	NRPS	Copper	32.18	China: Jiangxi, Dexing
L180206P-5-LD180201-0012_HK72WCCXY_L5_clean_bin.77.orig.fa_k	OEZ008529	yes	LMSG_G000004399.1	OES073679	bacteriocin	RiPPs	Copper	10.86	China: Jiangxi, Dexing
L180206P-5-LD180201-0012_HK72WCCXY_L5_clean_bin.77.orig.fa_k	OEZ008529	yes	LMSG_G000004399.1	OES073679	betalactone	Others	Copper	27.11	China: Jiangxi, Dexing
L180206P-5-LD180201-0012_HK72WCCXY_L5_clean_bin.77.orig.fa_k	OEZ008529	yes	LMSG_G000004399.1	OES073679	T3PKS	PKSother	Copper	41.04	China: Jiangxi, Dexing
L180206P-5-LD180201-0012_HK72WCCXY_L5_clean_bin.77.orig.fa_k	OEZ008529	yes	LMSG_G000004399.1	OES073679	terpene	Terpene	Copper	16.38	China: Jiangxi, Dexing
L180206P-5-LD180201-0012_HK72WCCXY_L5_clean_bin.77.orig.fa_k	OEZ008529	yes	LMSG_G000004399.1	OES073679	hserlactone	Others	Copper	12.08	China: Jiangxi, Dexing
L180206P-5-LD180201-0012_HK72WCCXY_L5_clean_bin.77.orig.fa_k	OEZ008529	yes	LMSG_G000004399.1	OES073679	betalactone	Others	Copper	30.07	China: Jiangxi, Dexing
L180206P-5-LD180201-0012_HK72WCCXY_L5_clean_bin.77.orig.fa_k	OEZ008529	yes	LMSG_G000004399.1	OES073679	terpene	Terpene	Copper	14.34	China: Jiangxi, Dexing
L180206P-5-LD180201-0012_HK72WCCXY_L5_clean_bin.77.orig.fa_k	OEZ008529	yes	LMSG_G000004399.1	OES073679	arylpolyene	Others	Copper	41.55	China: Jiangxi, Dexing
L180206P-5-LD180201-0012_HK72WCCXY_L5_clean_bin.78.orig.fa_k	OEZ008529	yes	LMSG_G000004400.1	OES073679	ectoine	Others	Copper	8.40	China: Jiangxi, Dexing
L180206P-5-LD180201-0012_HK72WCCXY_L5_clean_bin.78.orig.fa_k	OEZ008529	yes	LMSG_G000004400.1	OES073679	siderophore	Others	Copper	6.51	China: Jiangxi, Dexing
L180206P-5-LD180201-0012_HK72WCCXY_L5_clean_bin.78.orig.fa_k	OEZ008529	yes	LMSG_G000004400.1	OES073679	T3PKS	PKSother	Copper	41.26	China: Jiangxi, Dexing
L180206P-5-LD180201-0012_HK72WCCXY_L5_clean_bin.81.orig.fa_k	OEZ008529	yes	LMSG_G000010572.1	OES073679	terpene	Terpene	Copper	19.52	China: Jiangxi, Dexing
L180206P-5-LD180201-0012_HK72WCCXY_L5_clean_bin.81.orig.fa_k	OEZ008529	yes	LMSG_G000010572.1	OES073679	bacteriocin	RiPPs	Copper	7.73	China: Jiangxi, Dexing
L180206P-5-LD180201-0012_HK72WCCXY_L5_clean_bin.81.orig.fa_k	OEZ008529	yes	LMSG_G000010572.1	OES073679	bacteriocin	RiPPs	Copper	10.84	China: Jiangxi, Dexing
L180206P-5-LD180201-0012_HK72WCCXY_L5_clean_bin.82.orig.fa_k	OEZ008529	yes	LMSG_G000010823.1	OES073679	acyl_amino_acids	Others	Copper	37.54	China: Jiangxi, Dexing
L180206P-5-LD180201-0012_HK72WCCXY_L5_clean_bin.83.strict.fa	OEZ008529	yes	LMSG_G000006269.1	OES073679	ectoine	Others	Copper	5.51	China: Jiangxi, Dexing
L180206P-5-LD180201-0012_HK72WCCXY_L5_clean_bin.84.orig.fa_k	OEZ008529	yes	LMSG_G000006324.1	OES073679	CDPS	Others	Copper	9.47	China: Jiangxi, Dexing
L180206P-5-LD180201-0012_HK72WCCXY_L5_clean_bin.85.orig.fa_k	OEZ008529	yes	LMSG_G000008709.1	OES073679	bacteriocin	RiPPs	Copper	8.18	China: Jiangxi, Dexing
L180206P-5-LD180201-0012_HK72WCCXY_L5_clean_bin.85.orig.fa_k	OEZ008529	yes	LMSG_G000008709.1	OES073679	terpene	Terpene	Copper	26.50	China: Jiangxi, Dexing
L180206P-5-LD180201-0012_HK72WCCXY_L5_clean_bin.85.orig.fa_k	OEZ008529	yes	LMSG_G000008709.1	OES073679	LAP	RiPPs	Copper	15.16	China: Jiangxi, Dexing
L180206P-5-LD180201-0012_HK72WCCXY_L5_clean_bin.85.orig.fa_k	OEZ008529	yes	LMSG_G000008709.1	OES073679	betalactone	Others	Copper	25.38	China: Jiangxi, Dexing
L180206P-5-LD180201-0012_HK72WCCXY_L5_clean_bin.86.strict.fa	OEZ008529	yes	LMSG_G000004401.1	OES073679	LAP.thiopeptide	RiPPs	Copper	27.79	China: Jiangxi, Dexing
L180206P-5-LD180201-0012_HK72WCCXY_L5_clean_bin.86.strict.fa	OEZ008529	no	LMSG_G000004401.1	OES073679	lanthipeptide	RiPPs	Copper	13.26	China: Jiangxi, Dexing
L180206P-5-LD180201-0012_HK72WCCXY_L5_clean_bin.86.strict.fa	OEZ008529	no	LMSG_G000004401.1	OES073679	ectoine	Others	Copper	10.39	China: Jiangxi, Dexing
L180206P-5-LD180201-0012_HK72WCCXY_L5_clean_bin.86.strict.fa	OEZ008529	yes	LMSG_G000004401.1	OES073679	betalactone	Others	Copper	26.62	China: Jiangxi, Dexing
L180206P-5-LD180201-0012_HK72WCCXY_L5_clean_bin.86.strict.fa	OEZ008529	no	LMSG_G000004401.1	OES073679	T3PKS	PKSother	Copper	26.64	China: Jiangxi, Dexing
L180206P-5-LD180201-0012_HK72WCCXY_L5_clean_bin.86.strict.fa	OEZ008529	no	LMSG_G000004401.1	OES073679	lanthipeptide	RiPPs	Copper	8.61	China: Jiangxi, Dexing
L180206P-5-LD180201-0012_HK72WCCXY_L5_clean_bin.87.orig.fa_k	OEZ008529	yes	LMSG_G000004991.1	OES073679	ectoine	Others	Copper	7.61	China: Jiangxi, Dexing
L180206P-5-LD180201-0012_HK72WCCXY_L5_clean_bin.87.orig.fa_k	OEZ008529	yes	LMSG_G000004991.1	OES073679	T1PKS	PKSI	Copper	15.65	China: Jiangxi, Dexing
L180206P-5-LD180201-0012_HK72WCCXY_L5_clean_bin.87.orig.fa_k	OEZ008529	yes	LMSG_G000004991.1	OES073679	NRPS	NRPS	Copper	8.49	China: Jiangxi, Dexing
L180206P-5-LD180201-0012_HK72WCCXY_L5_clean_bin.87.orig.fa_k	OEZ008529	yes	LMSG_G000004991.1	OES073679	terpene	Terpene	Copper	11.91	China: Jiangxi, Dexing
L180206P-5-LD180201-0012_HK72WCCXY_L5_clean_bin.87.orig.fa_k	OEZ008529	yes	LMSG_G000004991.1	OES073679	terpene	Terpene	Copper	13.83	China: Jiangxi, Dexing
L180206P-5-LD180201-0012_HK72WCCXY_L5_clean_bin.87.orig.fa_k	OEZ008529	yes	LMSG_G000004991.1	OES073679	T3PKS	PKSother	Copper	33.10	China: Jiangxi, Dexing
L180206P-5-LD180201-0012_HK72WCCXY_L5_clean_bin.87.orig.fa_k	OEZ008529	yes	LMSG_G000004991.1	OES073679	NRPS	NRPS	Copper	28.33	China: Jiangxi, Dexing
L180206P-5-LD180201-0012_HK72WCCXY_L5_clean_bin.87.orig.fa_k	OEZ008529	yes	LMSG_G000004991.1	OES073679	betalactone	Others	Copper	26.01	China: Jiangxi, Dexing
L180206P-5-LD180201-0012_HK72WCCXY_L5_clean_bin.87.orig.fa_k	OEZ008529	yes	LMSG_G000004991.1	OES073679	bacteriocin	RiPPs	Copper	8.32	China: Jiangxi, Dexing
L180206P-5-LD180201-0012_HK72WCCXY_L5_clean_bin.87.orig.fa_k	OEZ008529	yes	LMSG_G000004991.1	OES073679	betalactone	Others	Copper	28.47	China: Jiangxi, Dexing
L180206P-5-LD180201-0012_HK72WCCXY_L5_clean_bin.87.orig.fa_k	OEZ008529	yes	LMSG_G000004991.1	OES073679	butyrolactone	Others	Copper	10.80	China: Jiangxi, Dexing
L180206P-5-LD180201-0012_HK72WCCXY_L5_clean_bin.87.orig.fa_k	OEZ008529	yes	LMSG_G000004991.1	OES073679	LAP.thiopeptide	RiPPs	Copper	27.99	China: Jiangxi, Dexing
L180206P-5-LD180201-0012_HK72WCCXY_L5_clean_bin.87.orig.fa_k	OEZ008529	yes	LMSG_G000004991.1	OES073679	lassopeptide	RiPPs	Copper	7.57	China: Jiangxi, Dexing
L180206P-5-LD180201-0012_HK72WCCXY_L5_clean_bin.87.orig.fa_k	OEZ008529	yes	LMSG_G000004991.1	OES073679	T1PKS	PKSI	Copper	41.25	China: Jiangxi, Dexing
L180206P-5-LD180201-0012_HK72WCCXY_L5_clean_bin.87.orig.fa_k	OEZ008529	yes	LMSG_G000004991.1	OES073679	NRPS	NRPS	Copper	59.24	China: Jiangxi, Dexing
L180206P-5-LD180201-0012_HK72WCCXY_L5_clean_bin.9.orig.fa_kl	OEZ008529	yes	LMSG_G000006219.1	OES073679	PKS-like	PKSother	Copper	41.00	China: Jiangxi, Dexing
L180206P-5-LD180201-0012_HK72WCCXY_L5_clean_bin.9.orig.fa_kl	OEZ008529	yes	LMSG_G000006219.1	OES073679	betalactone	Others	Copper	25.70	China: Jiangxi, Dexing
L180206P-5-LD180201-0012_HK72WCCXY_L5_clean_bin.91.orig.fa_k	OEZ008529	yes	LMSG_G000004993.1	OES073679	T2PKS	PKSother	Copper	38.08	China: Jiangxi, Dexing
L180206P-5-LD180201-0012_HK72WCCXY_L5_clean_bin.91.orig.fa_k	OEZ008529	yes	LMSG_G000004993.1	OES073679	NAGGN	Others	Copper	13.72	China: Jiangxi, Dexing
L180206P-5-LD180201-0012_HK72WCCXY_L5_clean_bin.91.orig.fa_k	OEZ008529	yes	LMSG_G000004993.1	OES073679	siderophore	Others	Copper	15.53	China: Jiangxi, Dexing
L180206P-5-LD180201-0012_HK72WCCXY_L5_clean_bin.91.orig.fa_k	OEZ008529	yes	LMSG_G000004993.1	OES073679	T3PKS	PKSother	Copper	37.25	China: Jiangxi, Dexing
L180206P-5-LD180201-0012_HK72WCCXY_L5_clean_bin.91.orig.fa_k	OEZ008529	yes	LMSG_G000004993.1	OES073679	lassopeptide	RiPPs	Copper	9.09	China: Jiangxi, Dexing
L180206P-5-LD180201-0012_HK72WCCXY_L5_clean_bin.92.orig.fa_k	OEZ008529	yes	LMSG_G000010700.1	OES073679	NRPS	NRPS	Copper	25.85	China: Jiangxi, Dexing
L180206P-5-LD180201-0012_HK72WCCXY_L5_clean_bin.92.orig.fa_k	OEZ008529	yes	LMSG_G000010700.1	OES073679	T1PKS.NRPS-like	PKS-NRP_Hybrids	Copper	16.63	China: Jiangxi, Dexing
L180206P-5-LD180201-0012_HK72WCCXY_L5_clean_bin.92.orig.fa_k	OEZ008529	yes	LMSG_G000010700.1	OES073679	terpene	Terpene	Copper	12.59	China: Jiangxi, Dexing
L180206P-5-LD180201-0012_HK72WCCXY_L5_clean_bin.93.orig.fa_k	OEZ008529	yes	LMSG_G000004994.1	OES073679	betalactone	Others	Copper	5.05	China: Jiangxi, Dexing
L180206P-5-LD180201-0012_HK72WCCXY_L5_clean_bin.93.orig.fa_k	OEZ008529	yes	LMSG_G000004994.1	OES073679	terpene	Terpene	Copper	15.65	China: Jiangxi, Dexing
L180206P-5-LD180201-0013_HK72WCCXY_L5_clean_bin.10.orig.fa_k	OEZ008529	yes	LMSG_G000008026.1	OES073680	lassopeptide	RiPPs	Copper	15.62	China: Jiangxi, Dexing
L180206P-5-LD180201-0013_HK72WCCXY_L5_clean_bin.10.orig.fa_k	OEZ008529	yes	LMSG_G000008026.1	OES073680	thiopeptide	RiPPs	Copper	7.55	China: Jiangxi, Dexing
L180206P-5-LD180201-0013_HK72WCCXY_L5_clean_bin.10.orig.fa_k	OEZ008529	yes	LMSG_G000008026.1	OES073680	LAP	RiPPs	Copper	11.96	China: Jiangxi, Dexing
L180206P-5-LD180201-0013_HK72WCCXY_L5_clean_bin.11.orig.fa_k	OEZ008529	no	LMSG_G000004995.1	OES073680	bacteriocin	RiPPs	Copper	10.88	China: Jiangxi, Dexing
L180206P-5-LD180201-0013_HK72WCCXY_L5_clean_bin.11.orig.fa_k	OEZ008529	yes	LMSG_G000004995.1	OES073680	NRPS	NRPS	Copper	45.11	China: Jiangxi, Dexing
L180206P-5-LD180201-0013_HK72WCCXY_L5_clean_bin.11.orig.fa_k	OEZ008529	no	LMSG_G000004995.1	OES073680	bacteriocin	RiPPs	Copper	10.85	China: Jiangxi, Dexing
L180206P-5-LD180201-0013_HK72WCCXY_L5_clean_bin.15.strict.fa	OEZ008529	yes	LMSG_G000008011.1	OES073680	betalactone	Others	Copper	23.53	China: Jiangxi, Dexing
L180206P-5-LD180201-0013_HK72WCCXY_L5_clean_bin.15.strict.fa	OEZ008529	yes	LMSG_G000008011.1	OES073680	betalactone	Others	Copper	24.98	China: Jiangxi, Dexing
L180206P-5-LD180201-0013_HK72WCCXY_L5_clean_bin.15.strict.fa	OEZ008529	yes	LMSG_G000008011.1	OES073680	TfuA-related	RiPPs	Copper	22.83	China: Jiangxi, Dexing
L180206P-5-LD180201-0013_HK72WCCXY_L5_clean_bin.15.strict.fa	OEZ008529	yes	LMSG_G000008011.1	OES073680	bacteriocin	RiPPs	Copper	10.30	China: Jiangxi, Dexing
L180206P-5-LD180201-0013_HK72WCCXY_L5_clean_bin.15.strict.fa	OEZ008529	yes	LMSG_G000008011.1	OES073680	bacteriocin	RiPPs	Copper	8.59	China: Jiangxi, Dexing
L180206P-5-LD180201-0013_HK72WCCXY_L5_clean_bin.16.orig.fa_k	OEZ008529	yes	LMSG_G000011031.1	OES073680	arylpolyene	Others	Copper	28.65	China: Jiangxi, Dexing
L180206P-5-LD180201-0013_HK72WCCXY_L5_clean_bin.16.orig.fa_k	OEZ008529	yes	LMSG_G000011031.1	OES073680	bacteriocin	RiPPs	Copper	6.73	China: Jiangxi, Dexing

L180206P-5-LD180201-0013_HK72WCCXY_L5_clean_bin.16.orig.fa_k	OEZ008529	yes	LMSG_G000011031.1	OES073680	arylpolyene	Others	Copper	23.21	China: Jiangxi, Dexing
L180206P-5-LD180201-0013_HK72WCCXY_L5_clean_bin.17.orig.fa_k	OEZ008529	yes	LMSG_G000010790.1	OES073680	NRPS-like	NRPS	Copper	22.78	China: Jiangxi, Dexing
L180206P-5-LD180201-0013_HK72WCCXY_L5_clean_bin.17.orig.fa_k	OEZ008529	yes	LMSG_G000010790.1	OES073680	bacteriocin	RiPPs	Copper	10.80	China: Jiangxi, Dexing
L180206P-5-LD180201-0013_HK72WCCXY_L5_clean_bin.19.strict.fa	OEZ008529	yes	LMSG_G000004402.1	OES073680	TfuA-related	RiPPs	Copper	21.93	China: Jiangxi, Dexing
L180206P-5-LD180201-0013_HK72WCCXY_L5_clean_bin.2.orig.fa_kl	OEZ008529	yes	LMSG_G000004998.1	OES073680	arylpolyene	Others	Copper	28.92	China: Jiangxi, Dexing
L180206P-5-LD180201-0013_HK72WCCXY_L5_clean_bin.2.orig.fa_kl	OEZ008529	yes	LMSG_G000004998.1	OES073680	bacteriocin	RiPPs	Copper	10.89	China: Jiangxi, Dexing
L180206P-5-LD180201-0013_HK72WCCXY_L5_clean_bin.20.orig.fa_k	OEZ008529	yes	LMSG_G000008526.1	OES073680	LAP	RiPPs	Copper	8.56	China: Jiangxi, Dexing
L180206P-5-LD180201-0013_HK72WCCXY_L5_clean_bin.20.orig.fa_k	OEZ008529	yes	LMSG_G000008526.1	OES073680	ectoine	Others	Copper	5.11	China: Jiangxi, Dexing
L180206P-5-LD180201-0013_HK72WCCXY_L5_clean_bin.22.orig.fa_k	OEZ008529	yes	LMSG_G000011041.1	OES073680	NRPS-like	NRPS	Copper	14.91	China: Jiangxi, Dexing
L180206P-5-LD180201-0013_HK72WCCXY_L5_clean_bin.22.orig.fa_k	OEZ008529	yes	LMSG_G000011041.1	OES073680	terpene	Terpene	Copper	11.85	China: Jiangxi, Dexing
L180206P-5-LD180201-0013_HK72WCCXY_L5_clean_bin.22.orig.fa_k	OEZ008529	yes	LMSG_G000011041.1	OES073680	terpene	Terpene	Copper	23.08	China: Jiangxi, Dexing
L180206P-5-LD180201-0013_HK72WCCXY_L5_clean_bin.22.orig.fa_k	OEZ008529	yes	LMSG_G000011041.1	OES073680	arylpolyene	Others	Copper	20.45	China: Jiangxi, Dexing
L180206P-5-LD180201-0013_HK72WCCXY_L5_clean_bin.22.orig.fa_k	OEZ008529	yes	LMSG_G000011041.1	OES073680	bacteriocin	RiPPs	Copper	10.71	China: Jiangxi, Dexing
L180206P-5-LD180201-0013_HK72WCCXY_L5_clean_bin.23.permissiv	OEZ008529	yes	LMSG_G000007982.1	OES073680	TfuA-related.bacteriocin	RiPPs	Copper	8.34	China: Jiangxi, Dexing
L180206P-5-LD180201-0013_HK72WCCXY_L5_clean_bin.24.orig.fa_k	OEZ008529	yes	LMSG_G000009117.1	OES073680	NRPS-like	NRPS	Copper	25.88	China: Jiangxi, Dexing
L180206P-5-LD180201-0013_HK72WCCXY_L5_clean_bin.24.orig.fa_k	OEZ008529	yes	LMSG_G000009117.1	OES073680	bacteriocin	RiPPs	Copper	8.28	China: Jiangxi, Dexing
L180206P-5-LD180201-0013_HK72WCCXY_L5_clean_bin.24.orig.fa_k	OEZ008529	yes	LMSG_G000009117.1	OES073680	terpene	Terpene	Copper	25.09	China: Jiangxi, Dexing
L180206P-5-LD180201-0013_HK72WCCXY_L5_clean_bin.25.strict.fa	OEZ008529	yes	LMSG_G000009653.1	OES073680	acyl_amino_acids	Others	Copper	67.94	China: Jiangxi, Dexing
L180206P-5-LD180201-0013_HK72WCCXY_L5_clean_bin.25.strict.fa	OEZ008529	yes	LMSG_G000009653.1	OES073680	NRPS-like	NRPS	Copper	30.60	China: Jiangxi, Dexing
L180206P-5-LD180201-0013_HK72WCCXY_L5_clean_bin.25.strict.fa	OEZ008529	yes	LMSG_G000009653.1	OES073680	terpene	Terpene	Copper	21.89	China: Jiangxi, Dexing
L180206P-5-LD180201-0013_HK72WCCXY_L5_clean_bin.25.strict.fa	OEZ008529	yes	LMSG_G000009653.1	OES073680	terpene	Terpene	Copper	12.60	China: Jiangxi, Dexing
L180206P-5-LD180201-0013_HK72WCCXY_L5_clean_bin.25.strict.fa	OEZ008529	yes	LMSG_G000009653.1	OES073680	nucleoside	Others	Copper	8.77	China: Jiangxi, Dexing
L180206P-5-LD180201-0013_HK72WCCXY_L5_clean_bin.26.strict.fa	OEZ008529	yes	LMSG_G000004403.1	OES073680	betalactone	Others	Copper	15.94	China: Jiangxi, Dexing
L180206P-5-LD180201-0013_HK72WCCXY_L5_clean_bin.26.strict.fa	OEZ008529	yes	LMSG_G000004403.1	OES073680	betalactone	Others	Copper	17.58	China: Jiangxi, Dexing
L180206P-5-LD180201-0013_HK72WCCXY_L5_clean_bin.28.orig.fa_k	OEZ008529	yes	LMSG_G000008287.1	OES073680	terpene	Terpene	Copper	19.09	China: Jiangxi, Dexing
L180206P-5-LD180201-0013_HK72WCCXY_L5_clean_bin.29.orig.fa_k	OEZ008529	yes	LMSG_G000008071.1	OES073680	T1PKS.hglE-KS	PKSother	Copper	26.40	China: Jiangxi, Dexing
L180206P-5-LD180201-0013_HK72WCCXY_L5_clean_bin.29.orig.fa_k	OEZ008529	yes	LMSG_G000008071.1	OES073680	arylpolyene	Others	Copper	5.91	China: Jiangxi, Dexing
L180206P-5-LD180201-0013_HK72WCCXY_L5_clean_bin.29.orig.fa_k	OEZ008529	yes	LMSG_G000008071.1	OES073680	other	Others	Copper	7.52	China: Jiangxi, Dexing
L180206P-5-LD180201-0013_HK72WCCXY_L5_clean_bin.31.orig.fa_k	OEZ008529	yes	LMSG_G000004999.1	OES073680	arylpolyene	Others	Copper	15.17	China: Jiangxi, Dexing
L180206P-5-LD180201-0013_HK72WCCXY_L5_clean_bin.31.orig.fa_k	OEZ008529	yes	LMSG_G000004999.1	OES073680	lassopeptide	RiPPs	Copper	7.00	China: Jiangxi, Dexing
L180206P-5-LD180201-0013_HK72WCCXY_L5_clean_bin.31.orig.fa_k	OEZ008529	yes	LMSG_G000004999.1	OES073680	terpene	Terpene	Copper	26.09	China: Jiangxi, Dexing
L180206P-5-LD180201-0013_HK72WCCXY_L5_clean_bin.32.orig.fa_k	OEZ008529	yes	LMSG_G000005000.1	OES073680	NRPS-like	NRPS	Copper	6.12	China: Jiangxi, Dexing
L180206P-5-LD180201-0013_HK72WCCXY_L5_clean_bin.32.orig.fa_k	OEZ008529	yes	LMSG_G000005000.1	OES073680	terpene	Terpene	Copper	6.31	China: Jiangxi, Dexing
L180206P-5-LD180201-0013_HK72WCCXY_L5_clean_bin.32.orig.fa_k	OEZ008529	yes	LMSG_G000005000.1	OES073680	terpene	Terpene	Copper	6.01	China: Jiangxi, Dexing
L180206P-5-LD180201-0013_HK72WCCXY_L5_clean_bin.32.orig.fa_k	OEZ008529	yes	LMSG_G000005000.1	OES073680	bacteriocin	RiPPs	Copper	6.27	China: Jiangxi, Dexing
L180206P-5-LD180201-0013_HK72WCCXY_L5_clean_bin.33.strict.fa	OEZ008529	yes	LMSG_G000008351.1	OES073680	bacteriocin	RiPPs	Copper	10.80	China: Jiangxi, Dexing
L180206P-5-LD180201-0013_HK72WCCXY_L5_clean_bin.33.strict.fa	OEZ008529	yes	LMSG_G000008351.1	OES073680	terpene	Terpene	Copper	18.60	China: Jiangxi, Dexing
L180206P-5-LD180201-0013_HK72WCCXY_L5_clean_bin.33.strict.fa	OEZ008529	yes	LMSG_G000008351.1	OES073680	butyrolactone	Others	Copper	10.39	China: Jiangxi, Dexing
L180206P-5-LD180201-0013_HK72WCCXY_L5_clean_bin.33.strict.fa	OEZ008529	yes	LMSG_G000008351.1	OES073680	T3PKS	PKSother	Copper	13.07	China: Jiangxi, Dexing
L180206P-5-LD180201-0013_HK72WCCXY_L5_clean_bin.34.orig.fa_k	OEZ008529	yes	LMSG_G000005001.1	OES073680	betalactone	Others	Copper	18.37	China: Jiangxi, Dexing
L180206P-5-LD180201-0013_HK72WCCXY_L5_clean_bin.34.orig.fa_k	OEZ008529	yes	LMSG_G000005001.1	OES073680	betalactone	Others	Copper	22.44	China: Jiangxi, Dexing
L180206P-5-LD180201-0013_HK72WCCXY_L5_clean_bin.35.orig.fa_k	OEZ008529	yes	LMSG_G000011082.1	OES073680	NRPS-like	NRPS	Copper	20.84	China: Jiangxi, Dexing
L180206P-5-LD180201-0013_HK72WCCXY_L5_clean_bin.35.orig.fa_k	OEZ008529	yes	LMSG_G000011082.1	OES073680	NRPS-like	NRPS	Copper	21.56	China: Jiangxi, Dexing
L180206P-5-LD180201-0013_HK72WCCXY_L5_clean_bin.35.orig.fa_k	OEZ008529	yes	LMSG_G000011082.1	OES073680	terpene	Terpene	Copper	23.10	China: Jiangxi, Dexing
L180206P-5-LD180201-0013_HK72WCCXY_L5_clean_bin.35.orig.fa_k	OEZ008529	yes	LMSG_G000011082.1	OES073680	betalactone	Others	Copper	23.04	China: Jiangxi, Dexing
L180206P-5-LD180201-0013_HK72WCCXY_L5_clean_bin.35.orig.fa_k	OEZ008529	yes	LMSG_G000011082.1	OES073680	lassopeptide	RiPPs	Copper	22.55	China: Jiangxi, Dexing
L180206P-5-LD180201-0013_HK72WCCXY_L5_clean_bin.35.orig.fa_k	OEZ008529	yes	LMSG_G000011082.1	OES073680	NRPS-like.terpene	Others	Copper	22.16	China: Jiangxi, Dexing
L180206P-5-LD180201-0013_HK72WCCXY_L5_clean_bin.38.permissiv	OEZ008529	yes	LMSG_G000009977.1	OES073680	terpene	Terpene	Copper	19.95	China: Jiangxi, Dexing
L180206P-5-LD180201-0013_HK72WCCXY_L5_clean_bin.38.permissiv	OEZ008529	yes	LMSG_G000009977.1	OES073680	arylpolyene	Others	Copper	15.09	China: Jiangxi, Dexing
L180206P-5-LD180201-0013_HK72WCCXY_L5_clean_bin.38.permissiv	OEZ008529	yes	LMSG_G000009977.1	OES073680	terpene	Terpene	Copper	5.25	China: Jiangxi, Dexing
L180206P-5-LD180201-0013_HK72WCCXY_L5_clean_bin.39.orig.fa_k	OEZ008529	yes	LMSG_G000011156.1	OES073680	bacteriocin	RiPPs	Copper	7.87	China: Jiangxi, Dexing
L180206P-5-LD180201-0013_HK72WCCXY_L5_clean_bin.39.orig.fa_k	OEZ008529	yes	LMSG_G000011156.1	OES073680	arylpolyene	Others	Copper	9.81	China: Jiangxi, Dexing
L180206P-5-LD180201-0013_HK72WCCXY_L5_clean_bin.39.orig.fa_k	OEZ008529	yes	LMSG_G000011156.1	OES073680	arylpolyene	Others	Copper	20.95	China: Jiangxi, Dexing
L180206P-5-LD180201-0013_HK72WCCXY_L5_clean_bin.40.orig.fa_k	OEZ008529	yes	LMSG_G000008707.1	OES073680	bacteriocin	RiPPs	Copper	5.54	China: Jiangxi, Dexing
L180206P-5-LD180201-0013_HK72WCCXY_L5_clean_bin.40.orig.fa_k	OEZ008529	yes	LMSG_G000008707.1	OES073680	betalactone	Others	Copper	24.82	China: Jiangxi, Dexing
L180206P-5-LD180201-0013_HK72WCCXY_L5_clean_bin.40.orig.fa_k	OEZ008529	yes	LMSG_G000008707.1	OES073680	terpene	Terpene	Copper	26.30	China: Jiangxi, Dexing
L180206P-5-LD180201-0013_HK72WCCXY_L5_clean_bin.40.orig.fa_k	OEZ008529	yes	LMSG_G000008707.1	OES073680	bacteriocin	RiPPs	Copper	6.19	China: Jiangxi, Dexing
L180206P-5-LD180201-0013_HK72WCCXY_L5_clean_bin.40.orig.fa_k	OEZ008529	yes	LMSG_G000008707.1	OES073680	LAP	RiPPs	Copper	10.55	China: Jiangxi, Dexing
L180206P-5-LD180201-0013_HK72WCCXY_L5_clean_bin.42.orig.fa_k	OEZ008529	no	LMSG_G000010259.1	OES073680	terpene	Terpene	Copper	21.20	China: Jiangxi, Dexing
L180206P-5-LD180201-0013_HK72WCCXY_L5_clean_bin.42.orig.fa_k	OEZ008529	yes	LMSG_G000010259.1	OES073680	arylpolyene	Others	Copper	40.92	China: Jiangxi, Dexing
L180206P-5-LD180201-0013_HK72WCCXY_L5_clean_bin.45.permissiv	OEZ008529	yes	LMSG_G000008559.1	OES073680	hglE-KS	PKSother	Copper	22.65	China: Jiangxi, Dexing
L180206P-5-LD180201-0013_HK72WCCXY_L5_clean_bin.47.orig.fa_k	OEZ008529	yes	LMSG_G000007367.1	OES073680	bacteriocin	RiPPs	Copper	11.18	China: Jiangxi, Dexing
L180206P-5-LD180201-0013_HK72WCCXY_L5_clean_bin.48.strict.fa	OEZ008529	yes	LMSG_G000009170.1	OES073680	terpene	Terpene	Copper	7.18	China: Jiangxi, Dexing
L180206P-5-LD180201-0013_HK72WCCXY_L5_clean_bin.49.permissiv	OEZ008529	yes	LMSG_G000008119.1	OES073680	terpene	Terpene	Copper	20.90	China: Jiangxi, Dexing
L180206P-5-LD180201-0013_HK72WCCXY_L5_clean_bin.5.orig.fa_kl	OEZ008529	yes	LMSG_G000007749.1	OES073680	NRPS.butyrolactone	Others	Copper	15.16	China: Jiangxi, Dexing
L180206P-5-LD180201-0013_HK72WCCXY_L5_clean_bin.5.orig.fa_kl	OEZ008529	yes	LMSG_G000007749.1	OES073680	NRPS	NRPS	Copper	5.44	China: Jiangxi, Dexing
L180206P-5-LD180201-0013_HK72WCCXY_L5_clean_bin.5.orig.fa_kl	OEZ008529	yes	LMSG_G000007749.1	OES073680	terpene	Terpene	Copper	12.45	China: Jiangxi, Dexing
L180206P-5-LD180201-0013_HK72WCCXY_L5_clean_bin.5.orig.fa_kl	OEZ008529	yes	LMSG_G000007749.1	OES073680	terpene	Terpene	Copper	11.22	China: Jiangxi, Dexing
L180206P-5-LD180201-0013_HK72WCCXY_L5_clean_bin.5.orig.fa_kl	OEZ008529	yes	LMSG_G000007749.1	OES073680	terpene	Terpene	Copper	7.49	China: Jiangxi, Dexing
L180206P-5-LD180201-0013_HK72WCCXY_L5_clean_bin.5.orig.fa_kl	OEZ008529	yes	LMSG_G000007749.1	OES073680	T3PKS	PKSother	Copper	8.09	China: Jiangxi, Dexing
L180206P-5-LD180201-0013_HK72WCCXY_L5_clean_bin.50.orig.fa_k	OEZ008529	no	LMSG_G000010546.1	OES073680	terpene.thiopeptide.LAP	Others	Copper	46.41	China: Jiangxi, Dexing
L180206P-5-LD180201-0013_HK72WCCXY_L5_clean_bin.50.orig.fa_k	OEZ008529	no	LMSG_G000010546.1	OES073680	arylpolyene	Others	Copper	41.19	China: Jiangxi, Dexing
L180206P-5-LD180201-0013_HK72WCCXY_L5_clean_bin.50.orig.fa_k	OEZ008529	no	LMSG_G000010546.1	OES073680	T1PKS	PKSI	Copper	45.12	China: Jiangxi, Dexing
L180206P-5-LD180201-0013_HK72WCCXY_L5_clean_bin.52.strict.fa	OEZ008529	yes	LMSG_G000009083.1	OES073680	betalactone	Others	Copper	31.07	China: Jiangxi, Dexing
L180206P-5-LD180201-0013_HK72WCCXY_L5_clean_bin.52.strict.fa	OEZ008529	yes	LMSG_G000009083.1	OES073680	betalactone	Others	Copper	27.62	China: Jiangxi, Dexing
L180206P-5-LD180201-0013_HK72WCCXY_L5_clean_bin.52.strict.fa	OEZ008529	yes	LMSG_G000009083.1	OES073680	thiopeptide	RiPPs	Copper	31.20	China: Jiangxi, Dexing
L180206P-5-LD180201-0013_HK72WCCXY_L5_clean_bin.52.strict.fa	OEZ008529	yes	LMSG_G000009083.1	OES073680	acyl_amino_acids.betalactone	Others	Copper	60.19	China: Jiangxi, Dexing
L180206P-5-LD180201-0013_HK72WCCXY_L5_clean_bin.52.strict.fa	OEZ008529	yes	LMSG_G000009083.1	OES073680	betalactone.bacteriocin	Others	Copper	29.69	China: Jiangxi, Dexing
L180206P-5-LD180201-0013_HK72WCCXY_L5_clean_bin.52.strict.fa	OEZ008529	yes	LMSG_G000009083.1	OES073680	ladderane	Others	Copper	41.18	China: Jiangxi, Dexing
L180206P-5-LD180201-0013_HK72WCCXY_L5_clean_bin.52.strict.fa	OEZ008529	yes	LMSG_G000009083.1	OES073680	terpene	Terpene	Copper	22.04	China: Jiangxi, Dexing
L180206P-5-LD180201-0013_HK72WCCXY_L5_clean_bin.55.orig.fa_k	OEZ008529	yes	LMSG_G000011169.1	OES073680	arylpolyene	Others	Copper	10.21	China: Jiangxi, Dexing
L180206P-5-LD180201-0013_HK72WCCXY_L5_clean_bin.7.orig.fa_kl	OEZ008529	yes	LMSG_G000005003.1	OES073680	arylpolyene	Others	Copper	30.83	China: Jiangxi, Dexing
L180206P-5-LD180201-0013_HK72WCCXY_L5_clean_bin.7.orig.fa_kl	OEZ008529	yes	LMSG_G000005003.1	OES073680	NRPS	NRPS	Copper	16.27	China: Jiangxi, Dexing
L180206P-5-LD180201-0013_HK72WCCXY_L5_clean_bin.7.orig.fa_kl	OEZ008529	yes	LMSG_G000005003.1	OES073680	NRPS	NRPS	Copper	20.07	China: Jiangxi, Dexing
L180206P-5-LD180201-0013_HK72WCCXY_L5_clean_bin.8.orig.fa_kl	OEZ008529	yes	LMSG_G000010573.1	OES073680	bacteriocin	RiPPs	Copper	10.84	China: Jiangxi, Dexing
L180206P-5-LD180201-0013_HK72WCCXY_L5_clean_bin.8.orig.fa_kl	OEZ008529	yes	LMSG_G000010573.1	OES073680	arylpolyene	Others	Copper	41.19	China: Jiangxi, Dexing
L180206P-6-LD180201-0014_HK72WCCXY_L6_clean_bin.1.orig.fa_kl	OEZ008529	yes	LMSG_G000009522.1	OES073687	arylpolyene	Others	Lead-Zinc	32.10	China: Hunan, Huangshaping
L180206P-6-LD180201-0014_HK72WCCXY_L6_clean_bin.1.orig.fa_kl	OEZ008529	yes	LMSG_G000009522.1	OES073687	terpene	Terpene	Lead-Zinc	23.29	China: Hunan, Huangshaping
L180206P-6-LD180201-0014_HK72WCCXY_L6_clean_bin.1.orig.fa_kl	OEZ008529	yes	LMSG_G000009522.1	OES073687	NRPS-like	NRPS	Lead-Zinc	33.51	China: Hunan, Huangshaping
L180206P-6-LD180201-0014_HK72WCCXY_L6_clean_bin.1.orig.fa_kl	OEZ008529	yes	LMSG_G000009522.1	OES073687	terpene	Terpene	Lead-Zinc	21.16	China: Hunan, Huangshaping
L180206P-6-LD180201-0014_HK72WCCXY_L6_clean_bin.1.orig.fa_kl	OEZ008529	yes	LMSG_G000009522.1	OES073687	ectoine	Others	Lead-Zinc	10.40	China: Hunan, Huangshaping
L180206P-6-LD180201-0014_HK72WCCXY_L6_clean_bin.12.orig.fa_k	OEZ008529	yes	LMSG_G000008305.1	OES073687	NRPS-like	NRPS	Lead-Zinc	29.12	China: Hunan, Huangshaping
L180206P-6-LD180201-0014_HK72WCCXY_L6_clean_bin.12.orig.fa_k	OEZ008529	yes	LMSG_G000008305.1	OES073687	betalactone	Others	Lead-Zinc	20.28	China: Hunan, Huangshaping
L180206P-6-LD180201-0014_HK72WCCXY_L6_clean_bin.12.orig.fa_k	OEZ008529	yes	LMSG_G000008305.1	OES073687	terpene	Terpene	Lead-Zinc	16.10	China: Hunan, Huangshaping
L180206P-6-LD180201-0014_HK72WCCXY_L6_clean_bin.13.orig.fa_k	OEZ008529	yes	LMSG_G000007049.1	OES073687	bacteriocin	RiPPs	Lead-Zinc	11.01	China: Hunan, Huangshaping
L180206P-6-LD180201-0014_HK72WCCXY_L6_clean_bin.14.orig.fa_k	OEZ008529	no	LMSG_G000010293.1	OES073687	arylpolyene	Others	Lead-Zinc	40.79	China: Hunan, Huangshaping
L180206P-6-LD180201-0014_HK72WCCXY_L6_clean_bin.14.orig.fa_k	OEZ008529	yes	LMSG_G000010293.1	OES073687	terpene	Terpene	Lead-Zinc	24.86	China: Hunan, Huangshaping
L180206P-6-LD180201-0014_HK72WCCXY_L6_clean_bin.14.orig.fa_k	OEZ008529	no	LMSG_G000010293.1	OES073687	terpene	Terpene	Lead-Zinc	21.34	China: Hunan, Huangshaping
L180206P-6-LD180201-0014_HK72WCCXY_L6_clean_bin.17.orig.fa_k	OEZ008529	yes	LMSG_G000010498.1	OES073687	arylpolyene	Others	Lead-Zinc	16.96	China: Hunan, Huangshaping
L180206P-6-LD180201-0014_HK72WCCXY_L6_clean_bin.2.permissive	OEZ008529	yes	LMSG_G000009425.1	OES073687	betalactone	Others	Lead-Zinc	22.78	China: Hunan, Huangshaping
L180206P-6-LD180201-0014_HK72WCCXY_L6_clean_bin.2.permissive	OEZ008529	yes	LMSG_G000009425.1	OES073687	bacteriocin	RiPPs	Lead-Zinc	10.85	China: Hunan, Huangshaping
L180206P-6-LD180201-0014_HK72WCCXY_L6_clean_bin.2.permissive	OEZ008529	yes	LMSG_G000009425.1	OES073687	betalactone	Others	Lead-Zinc	30.86	China: Hunan, Huangshaping
L180206P-6-LD180201-0014_HK72WCCXY_L6_clean_bin.2.permissive	OEZ008529	yes	LMSG_G000009425.1	OES073687	ectoine	Others	Lead-Zinc	9.42	China: Hunan, Huangshaping
L180206P-6-LD180201-0014_HK72WCCXY_L6_clean_bin.20.orig.fa_k	OEZ008529	yes	LMSG_G000010493.1	OES073687	terpene	Terpene	Lead-Zinc	7.89	China: Hunan, Huangshaping
L180206P-6-LD180201-0014_HK72WCCXY_L6_clean_bin.23.orig.fa_k	OEZ008529	yes	LMSG_G000005005.1	OES073687	betalactone	Others	Lead-Zinc	19.30	China: Hunan, Huangshaping
L180206P-6-LD180201-0014_HK72WCCXY_L6_clean_bin.23.orig.fa_k	OEZ008529	yes	LMSG_G000005005.1	OES073687	betalactone	Others	Lead-Zinc	11.44	China: Hunan, Huangshaping
L180206P-6-LD180201-0014_HK72WCCXY_L6_clean_bin.24.orig.fa_k	OEZ008529	yes	LMSG_G000010583.1	OES073687	terpene	Terpene	Lead-Zinc	15.69	China: Hunan, Huangshaping
L180206P-6-LD180201-0014_HK72WCCXY_L6_clean_bin.25.orig.fa_k	OEZ008529	yes	LMSG_G000006220.1	OES073687	betalactone	Others	Lead-Zinc	28.49	China: Hunan, Huangshaping
L180206P-6-LD180201-0014_HK72WCCXY_L6_clean_bin.26.permissiv	OEZ008529	yes	LMSG_G000010137.1	OES073687	arylpolyene	Others	Lead-Zinc	11.62	China: Hunan, Huangshaping
L180206P-6-LD180201-0014_HK72WCCXY_L6_clean_bin.26.permissiv	OEZ008529	yes	LMSG_G000010137.1	OES073687	acyl_amino_acids	Others	Lead-Zinc	8.68	China: Hunan, Huangshaping
L180206P-6-LD180201-0014_HK72WCCXY_L6_clean_bin.27.strict.fa	OEZ008529	yes	LMSG_G000009590.1	OES073687	acyl_amino_acids	Others	Lead-Zinc	46.80	China: Hunan, Huangshaping
L180206P-6-LD180201-0014_HK72WCCXY_L6_clean_bin.27.strict.fa	OEZ008529	yes	LMSG_G000009590.1	OES073687	terpene	Terpene	Lead-Zinc	22.15	China: Hunan, Huangshaping
L180206P-6-LD180201-0014_HK72WCCXY_L6_clean_bin.27.strict.fa	OEZ008529	yes	LMSG_G000009590.1	OES073687	NRPS-like	NRPS	Lead-Zinc	42.62	China: Hunan, Huangshaping
L180206P-6-LD180201-0014_HK72WCCXY_L6_clean_bin.27.strict.fa	OEZ008529	yes	LMSG_G000009590.1	OES073687	terpene	Terpene	Lead-Zinc	22.86	China: Hunan, Huangshaping
L180206P-6-LD180201-0014_HK72WCCXY_L6_clean_bin.28.orig.fa_k	OEZ008529	yes	LMSG_G000005006.1	OES073687	betalactone	Others	Lead-Zinc	23.24	China: Hunan, Huangshaping
L180206P-6-LD180201-0014_HK72WCCXY_L6_clean_bin.28.orig.fa_k	OEZ008529	yes	LMSG_G000005006.1	OES073687	T1PKS	PKSI	Lead-Zinc	28.58	China: Hunan, Huangshaping
L180206P-6-LD180201-0014_HK72WCCXY_L6_clean_bin.28.orig.fa_k	OEZ008529	yes	LMSG_G000005006.1	OES073687	NRPS.T1PKS	PKS-NRP_Hybrids	Lead-Zinc	52.27	China: Hunan, Huangshaping
L180206P-6-LD180201-0014_HK72WCCXY_L6_clean_bin.28.orig.fa_k	OEZ008529	yes	LMSG_G000005006.1	OES073687	NRPS	NRPS	Lead-Zinc	46.34	China: Hunan, Huangshaping
L180206P-6-LD180201-0014_HK72WCCXY_L6_clean_bin.29.permissiv	OEZ008529	yes	LMSG_G000005007.1	OES073687	transAT-PKS-like.NRPS-like	PKS-NRP_Hybrids	Lead-Zinc	22.68	China: Hunan, Huangshaping
L180206P-6-LD180201-0014_HK72WCCXY_L6_clean_bin.29.permissiv	OEZ008529	yes	LMSG_G000005007.1	OES073687	phosphonate	Others	Lead-Zinc	17.57	China: Hunan, Huangshaping
L180206P-6-LD180201-0014_HK72WCCXY_L6_clean_bin.29.permissiv	OEZ008529	yes	LMSG_G000005007.1	OES073687	hserlactone	Others	Lead-Zinc	9.56	China: Hunan, Huangshaping
L180206P-6-LD180201-0014_HK72WCCXY_L6_clean_bin.31.orig.fa_k	OEZ008529	yes	LMSG_G000005009.1	OES073687	arylpolyene	Others	Lead-Zinc	41.18	China: Hunan, Huangshaping
L180206P-6-LD180201-0014_HK72WCCXY_L6_clean_bin.34.permissiv	OEZ008529	yes	LMSG_G000008265.1	OES073687	terpene	Terpene	Lead-Zinc	11.54	China: Hunan, Huangshaping
L180206P-6-LD180201-0014_HK72WCCXY_L6_clean_bin.37.strict.fa	OEZ008529	no	LMSG_G000006270.1	OES073687	NRPS.bacteriocin	Others	Lead-Zinc	51.79	China: Hunan, Huangshaping
L180206P-6-LD180201-0014_HK72WCCXY_L6_clean_bin.37.strict.fa	OEZ008529	no	LMSG_G000006270.1	OES073687	NRPS	NRPS	Lead-Zinc	65.41	China: Hunan, Huangshaping
L180206P-6-LD180201-0014_HK72WCCXY_L6_clean_bin.37.strict.fa	OEZ008529	no	LMSG_G000006270.1	OES073687	transAT-PKS-like	PKSother	Lead-Zinc	33.84	China: Hunan, Huangshaping
L180206P-6-LD180201-0014_HK72WCCXY_L6_clean_bin.37.strict.fa	OEZ008529	yes	LMSG_G000006270.1	OES073687	NRPS.T3PKS.transAT-PKS	PKS-NRP_Hybrids	Lead-Zinc	49.07	China: Hunan, Huangshaping
L180206P-6-LD180201-0014_HK72WCCXY_L6_clean_bin.37.strict.fa	OEZ008529	no	LMSG_G000006270.1	OES073687	PKS-like	PKSother	Lead-Zinc	21.66	China: Hunan, Huangshaping
L180206P-6-LD180201-0014_HK72WCCXY_L6_clean_bin.37.strict.fa	OEZ008529	no	LMSG_G000006270.1	OES073687	terpene	Terpene	Lead-Zinc	20.74	China: Hunan, Huangshaping
L180206P-6-LD180201-0014_HK72WCCXY_L6_clean_bin.37.strict.fa	OEZ008529	no	LMSG_G000006270.1	OES073687	other	Others	Lead-Zinc	29.31	China: Hunan, Huangshaping
L180206P-6-LD180201-0014_HK72WCCXY_L6_clean_bin.37.strict.fa	OEZ008529	no	LMSG_G000006270.1	OES073687	NRPS	NRPS	Lead-Zinc	22.16	China: Hunan, Huangshaping
L180206P-6-LD180201-0014_HK72WCCXY_L6_clean_bin.37.strict.fa	OEZ008529	no	LMSG_G000006270.1	OES073687	terpene	Terpene	Lead-Zinc	12.57	China: Hunan, Huangshaping
L180206P-6-LD180201-0014_HK72WCCXY_L6_clean_bin.37.strict.fa	OEZ008529	no	LMSG_G000006270.1	OES073687	NRPS	NRPS	Lead-Zinc	28.95	China: Hunan, Huangshaping
L180206P-6-LD180201-0014_HK72WCCXY_L6_clean_bin.37.strict.fa	OEZ008529	yes	LMSG_G000006270.1	OES073687	transAT-PKS-like	PKSother	Lead-Zinc	24.64	China: Hunan, Huangshaping
L180206P-6-LD180201-0014_HK72WCCXY_L6_clean_bin.37.strict.fa	OEZ008529	no	LMSG_G000006270.1	OES073687	transAT-PKS-like	PKSother	Lead-Zinc	20.03	China: Hunan, Huangshaping
L180206P-6-LD180201-0014_HK72WCCXY_L6_clean_bin.37.strict.fa	OEZ008529	no	LMSG_G000006270.1	OES073687	NRPS	NRPS	Lead-Zinc	16.60	China: Hunan, Huangshaping
L180206P-6-LD180201-0014_HK72WCCXY_L6_clean_bin.37.strict.fa	OEZ008529	no	LMSG_G000006270.1	OES073687	betalactone	Others	Lead-Zinc	14.81	China: Hunan, Huangshaping
L180206P-6-LD180201-0014_HK72WCCXY_L6_clean_bin.37.strict.fa	OEZ008529	no	LMSG_G000006270.1	OES073687	transAT-PKS-like	PKSother	Lead-Zinc	13.10	China: Hunan, Huangshaping
L180206P-6-LD180201-0014_HK72WCCXY_L6_clean_bin.37.strict.fa	OEZ008529	no	LMSG_G000006270.1	OES073687	lanthipeptide	RiPPs	Lead-Zinc	12.48	China: Hunan, Huangshaping
L180206P-6-LD180201-0014_HK72WCCXY_L6_clean_bin.37.strict.fa	OEZ008529	no	LMSG_G000006270.1	OES073687	NRPS	NRPS	Lead-Zinc	10.63	China: Hunan, Huangshaping
L180206P-6-LD180201-0014_HK72WCCXY_L6_clean_bin.37.strict.fa	OEZ008529	no	LMSG_G000006270.1	OES073687	transAT-PKS-like	PKSother	Lead-Zinc	9.20	China: Hunan, Huangshaping
L180206P-6-LD180201-0014_HK72WCCXY_L6_clean_bin.37.strict.fa	OEZ008529	no	LMSG_G000006270.1	OES073687	lanthipeptide	RiPPs	Lead-Zinc	8.76	China: Hunan, Huangshaping
L180206P-6-LD180201-0014_HK72WCCXY_L6_clean_bin.37.strict.fa	OEZ008529	no	LMSG_G000006270.1	OES073687	transAT-PKS-like	PKSother	Lead-Zinc	8.14	China: Hunan, Huangshaping
L180206P-6-LD180201-0014_HK72WCCXY_L6_clean_bin.37.strict.fa	OEZ008529	no	LMSG_G000006270.1	OES073687	NRPS-like	NRPS	Lead-Zinc	6.06	China: Hunan, Huangshaping
L180206P-6-LD180201-0014_HK72WCCXY_L6_clean_bin.37.strict.fa	OEZ008529	no	LMSG_G000006270.1	OES073687	NRPS	NRPS	Lead-Zinc	5.99	China: Hunan, Huangshaping
L180206P-6-LD180201-0014_HK72WCCXY_L6_clean_bin.37.strict.fa	OEZ008529	no	LMSG_G000006270.1	OES073687	transAT-PKS-like	PKSother	Lead-Zinc	5.10	China: Hunan, Huangshaping
L180206P-6-LD180201-0014_HK72WCCXY_L6_clean_bin.38.orig.fa_k	OEZ008529	no	LMSG_G000010250.1	OES073687	terpene	Terpene	Lead-Zinc	21.09	China: Hunan, Huangshaping
L180206P-6-LD180201-0014_HK72WCCXY_L6_clean_bin.38.orig.fa_k	OEZ008529	yes	LMSG_G000010250.1	OES073687	arylpolyene	Others	Lead-Zinc	18.92	China: Hunan, Huangshaping
L180206P-6-LD180201-0014_HK72WCCXY_L6_clean_bin.42.orig.fa_k	OEZ008529	yes	LMSG_G000009459.1	OES073687	lanthipeptide	RiPPs	Lead-Zinc	11.07	China: Hunan, Huangshaping
L180206P-6-LD180201-0014_HK72WCCXY_L6_clean_bin.42.orig.fa_k	OEZ008529	yes	LMSG_G000009459.1	OES073687	bacteriocin	RiPPs	Lead-Zinc	10.84	China: Hunan, Huangshaping
L180206P-6-LD180201-0014_HK72WCCXY_L6_clean_bin.44.orig.fa_k	OEZ008529	yes	LMSG_G000009460.1	OES073687	bacteriocin	RiPPs	Lead-Zinc	8.55	China: Hunan, Huangshaping
L180206P-6-LD180201-0014_HK72WCCXY_L6_clean_bin.6.orig.fa_kl	OEZ008529	no	LMSG_G000010157.1	OES073687	ectoine	Others	Lead-Zinc	9.55	China: Hunan, Huangshaping
L180206P-6-LD180201-0014_HK72WCCXY_L6_clean_bin.6.orig.fa_kl	OEZ008529	no	LMSG_G000010157.1	OES073687	terpene	Terpene	Lead-Zinc	21.71	China: Hunan, Huangshaping
L180206P-6-LD180201-0014_HK72WCCXY_L6_clean_bin.6.orig.fa_kl	OEZ008529	no	LMSG_G000010157.1	OES073687	terpene	Terpene	Lead-Zinc	21.95	China: Hunan, Huangshaping
L180206P-6-LD180201-0014_HK72WCCXY_L6_clean_bin.6.orig.fa_kl	OEZ008529	no	LMSG_G000010157.1	OES073687	terpene	Terpene	Lead-Zinc	16.15	China: Hunan, Huangshaping
L180206P-6-LD180201-0014_HK72WCCXY_L6_clean_bin.8.orig.fa_kl	OEZ008529	yes	LMSG_G000010717.1	OES073687	T1PKS.NRPS-like	PKS-NRP_Hybrids	Lead-Zinc	8.96	China: Hunan, Huangshaping
L180206P-6-LD180201-0018_HK72WCCXY_L6_clean_bin.10.orig.fa_k	OEZ008529	yes	LMSG_G000005012.1	OES073712	terpene	Terpene	Lead-Zinc	21.65	China: Hunan, Shuikoushan
L180206P-6-LD180201-0018_HK72WCCXY_L6_clean_bin.10.orig.fa_k	OEZ008529	no	LMSG_G000005012.1	OES073712	resorcinol.arylpolyene	Others	Lead-Zinc	8.86	China: Hunan, Shuikoushan

L180206P-6-LD180201-0018_HK72WCCXY_L6_clean_bin.10.orig.fa_k	OEZ008529	yes	LMSG_G000005012.1	OES073712	bacteriocin	RiPPs	Lead-Zinc	10.31	China: Hunan, Shuikoushan
L180206P-6-LD180201-0018_HK72WCCXY_L6_clean_bin.12.strict.fa	OEZ008529	yes	LMSG_G000005013.1	OES073712	betalactone	Others	Lead-Zinc	8.45	China: Hunan, Shuikoushan
L180206P-6-LD180201-0018_HK72WCCXY_L6_clean_bin.12.strict.fa	OEZ008529	yes	LMSG_G000005013.1	OES073712	NRPS-like	NRPS	Lead-Zinc	7.58	China: Hunan, Shuikoushan
L180206P-6-LD180201-0018_HK72WCCXY_L6_clean_bin.12.strict.fa	OEZ008529	yes	LMSG_G000005013.1	OES073712	bacteriocin	RiPPs	Lead-Zinc	5.65	China: Hunan, Shuikoushan
L180206P-6-LD180201-0018_HK72WCCXY_L6_clean_bin.12.strict.fa	OEZ008529	yes	LMSG_G000005013.1	OES073712	terpene	Terpene	Lead-Zinc	5.82	China: Hunan, Shuikoushan
L180206P-6-LD180201-0018_HK72WCCXY_L6_clean_bin.12.strict.fa	OEZ008529	yes	LMSG_G000005013.1	OES073712	NRPS-like	NRPS	Lead-Zinc	5.51	China: Hunan, Shuikoushan
L180206P-6-LD180201-0018_HK72WCCXY_L6_clean_bin.12.strict.fa	OEZ008529	yes	LMSG_G000005013.1	OES073712	T3PKS	PKSother	Lead-Zinc	5.36	China: Hunan, Shuikoushan
L180206P-6-LD180201-0018_HK72WCCXY_L6_clean_bin.13.orig.fa_k	OEZ008529	yes	LMSG_G000005014.1	OES073712	bacteriocin	RiPPs	Lead-Zinc	10.89	China: Hunan, Shuikoushan
L180206P-6-LD180201-0018_HK72WCCXY_L6_clean_bin.13.orig.fa_k	OEZ008529	yes	LMSG_G000005014.1	OES073712	terpene	Terpene	Lead-Zinc	21.77	China: Hunan, Shuikoushan
L180206P-6-LD180201-0018_HK72WCCXY_L6_clean_bin.13.orig.fa_k	OEZ008529	yes	LMSG_G000005014.1	OES073712	ectoine	Others	Lead-Zinc	5.50	China: Hunan, Shuikoushan
L180206P-6-LD180201-0018_HK72WCCXY_L6_clean_bin.13.orig.fa_k	OEZ008529	yes	LMSG_G000005014.1	OES073712	NRPS.T1PKS	PKS-NRP_Hybrids	Lead-Zinc	38.50	China: Hunan, Shuikoushan
L180206P-6-LD180201-0018_HK72WCCXY_L6_clean_bin.13.orig.fa_k	OEZ008529	yes	LMSG_G000005014.1	OES073712	terpene	Terpene	Lead-Zinc	23.70	China: Hunan, Shuikoushan
L180206P-6-LD180201-0018_HK72WCCXY_L6_clean_bin.13.orig.fa_k	OEZ008529	yes	LMSG_G000005014.1	OES073712	cyanobactin	RiPPs	Lead-Zinc	21.89	China: Hunan, Shuikoushan
L180206P-6-LD180201-0018_HK72WCCXY_L6_clean_bin.13.orig.fa_k	OEZ008529	yes	LMSG_G000005014.1	OES073712	NRPS-like	NRPS	Lead-Zinc	22.69	China: Hunan, Shuikoushan
L180206P-6-LD180201-0018_HK72WCCXY_L6_clean_bin.14.orig.fa_k	OEZ008529	yes	LMSG_G000010868.1	OES073712	terpene	Terpene	Lead-Zinc	21.65	China: Hunan, Shuikoushan
L180206P-6-LD180201-0018_HK72WCCXY_L6_clean_bin.14.orig.fa_k	OEZ008529	yes	LMSG_G000010868.1	OES073712	hserlactone	Others	Lead-Zinc	16.29	China: Hunan, Shuikoushan
L180206P-6-LD180201-0018_HK72WCCXY_L6_clean_bin.15.strict.fa	OEZ008529	yes	LMSG_G000008804.1	OES073712	terpene	Terpene	Lead-Zinc	23.46	China: Hunan, Shuikoushan
L180206P-6-LD180201-0018_HK72WCCXY_L6_clean_bin.15.strict.fa	OEZ008529	yes	LMSG_G000008804.1	OES073712	T3PKS	PKSother	Lead-Zinc	24.26	China: Hunan, Shuikoushan
L180206P-6-LD180201-0018_HK72WCCXY_L6_clean_bin.15.strict.fa	OEZ008529	yes	LMSG_G000008804.1	OES073712	bacteriocin	RiPPs	Lead-Zinc	6.97	China: Hunan, Shuikoushan
L180206P-6-LD180201-0018_HK72WCCXY_L6_clean_bin.17.orig.fa_k	OEZ008529	yes	LMSG_G000005015.1	OES073712	NRPS-like	NRPS	Lead-Zinc	6.03	China: Hunan, Shuikoushan
L180206P-6-LD180201-0018_HK72WCCXY_L6_clean_bin.17.orig.fa_k	OEZ008529	yes	LMSG_G000005015.1	OES073712	terpene	Terpene	Lead-Zinc	11.36	China: Hunan, Shuikoushan
L180206P-6-LD180201-0018_HK72WCCXY_L6_clean_bin.17.orig.fa_k	OEZ008529	yes	LMSG_G000005015.1	OES073712	NRPS-like	NRPS	Lead-Zinc	37.35	China: Hunan, Shuikoushan
L180206P-6-LD180201-0018_HK72WCCXY_L6_clean_bin.17.orig.fa_k	OEZ008529	yes	LMSG_G000005015.1	OES073712	NRPS	NRPS	Lead-Zinc	14.06	China: Hunan, Shuikoushan
L180206P-6-LD180201-0018_HK72WCCXY_L6_clean_bin.17.orig.fa_k	OEZ008529	yes	LMSG_G000005015.1	OES073712	T1PKS	PKSI	Lead-Zinc	21.10	China: Hunan, Shuikoushan
L180206P-6-LD180201-0018_HK72WCCXY_L6_clean_bin.17.orig.fa_k	OEZ008529	yes	LMSG_G000005015.1	OES073712	terpene	Terpene	Lead-Zinc	16.58	China: Hunan, Shuikoushan
L180206P-6-LD180201-0018_HK72WCCXY_L6_clean_bin.17.orig.fa_k	OEZ008529	yes	LMSG_G000005015.1	OES073712	NRPS-like	NRPS	Lead-Zinc	13.05	China: Hunan, Shuikoushan
L180206P-6-LD180201-0018_HK72WCCXY_L6_clean_bin.18.orig.fa_k	OEZ008529	yes	LMSG_G000005016.1	OES073712	NRPS-like	NRPS	Lead-Zinc	42.43	China: Hunan, Shuikoushan
L180206P-6-LD180201-0018_HK72WCCXY_L6_clean_bin.18.orig.fa_k	OEZ008529	yes	LMSG_G000005016.1	OES073712	terpene.hserlactone	Others	Lead-Zinc	14.17	China: Hunan, Shuikoushan
L180206P-6-LD180201-0018_HK72WCCXY_L6_clean_bin.18.orig.fa_k	OEZ008529	yes	LMSG_G000005016.1	OES073712	bacteriocin	RiPPs	Lead-Zinc	11.18	China: Hunan, Shuikoushan
L180206P-6-LD180201-0018_HK72WCCXY_L6_clean_bin.18.orig.fa_k	OEZ008529	yes	LMSG_G000005016.1	OES073712	terpene	Terpene	Lead-Zinc	18.98	China: Hunan, Shuikoushan
L180206P-6-LD180201-0018_HK72WCCXY_L6_clean_bin.19.orig.fa_k	OEZ008529	yes	LMSG_G000005017.1	OES073712	siderophore	Others	Lead-Zinc	13.51	China: Hunan, Shuikoushan
L180206P-6-LD180201-0018_HK72WCCXY_L6_clean_bin.19.orig.fa_k	OEZ008529	yes	LMSG_G000005017.1	OES073712	terpene	Terpene	Lead-Zinc	20.85	China: Hunan, Shuikoushan
L180206P-6-LD180201-0018_HK72WCCXY_L6_clean_bin.19.orig.fa_k	OEZ008529	yes	LMSG_G000005017.1	OES073712	terpene	Terpene	Lead-Zinc	24.97	China: Hunan, Shuikoushan
L180206P-6-LD180201-0018_HK72WCCXY_L6_clean_bin.19.orig.fa_k	OEZ008529	yes	LMSG_G000005017.1	OES073712	NRPS-like	NRPS	Lead-Zinc	42.50	China: Hunan, Shuikoushan
L180206P-6-LD180201-0018_HK72WCCXY_L6_clean_bin.2.orig.fa_kl	OEZ008529	yes	LMSG_G000005023.1	OES073712	terpene	Terpene	Lead-Zinc	22.03	China: Hunan, Shuikoushan
L180206P-6-LD180201-0018_HK72WCCXY_L6_clean_bin.2.orig.fa_kl	OEZ008529	yes	LMSG_G000005023.1	OES073712	NRPS-like	NRPS	Lead-Zinc	23.49	China: Hunan, Shuikoushan
L180206P-6-LD180201-0018_HK72WCCXY_L6_clean_bin.21.orig.fa_k	OEZ008529	yes	LMSG_G000005018.1	OES073712	arylpolyene	Others	Lead-Zinc	27.81	China: Hunan, Shuikoushan
L180206P-6-LD180201-0018_HK72WCCXY_L6_clean_bin.21.orig.fa_k	OEZ008529	yes	LMSG_G000005018.1	OES073712	NRPS-like	NRPS	Lead-Zinc	20.88	China: Hunan, Shuikoushan
L180206P-6-LD180201-0018_HK72WCCXY_L6_clean_bin.21.orig.fa_k	OEZ008529	yes	LMSG_G000005018.1	OES073712	terpene	Terpene	Lead-Zinc	23.10	China: Hunan, Shuikoushan
L180206P-6-LD180201-0018_HK72WCCXY_L6_clean_bin.21.orig.fa_k	OEZ008529	yes	LMSG_G000005018.1	OES073712	betalactone	Others	Lead-Zinc	32.30	China: Hunan, Shuikoushan
L180206P-6-LD180201-0018_HK72WCCXY_L6_clean_bin.21.orig.fa_k	OEZ008529	yes	LMSG_G000005018.1	OES073712	bacteriocin	RiPPs	Lead-Zinc	10.90	China: Hunan, Shuikoushan
L180206P-6-LD180201-0018_HK72WCCXY_L6_clean_bin.21.orig.fa_k	OEZ008529	yes	LMSG_G000005018.1	OES073712	NRPS-like	NRPS	Lead-Zinc	5.79	China: Hunan, Shuikoushan
L180206P-6-LD180201-0018_HK72WCCXY_L6_clean_bin.22.orig.fa_k	OEZ008529	yes	LMSG_G000005019.1	OES073712	terpene	Terpene	Lead-Zinc	14.62	China: Hunan, Shuikoushan
L180206P-6-LD180201-0018_HK72WCCXY_L6_clean_bin.22.orig.fa_k	OEZ008529	yes	LMSG_G000005019.1	OES073712	butyrolactone	Others	Lead-Zinc	6.57	China: Hunan, Shuikoushan
L180206P-6-LD180201-0018_HK72WCCXY_L6_clean_bin.22.orig.fa_k	OEZ008529	yes	LMSG_G000005019.1	OES073712	hserlactone	Others	Lead-Zinc	11.31	China: Hunan, Shuikoushan
L180206P-6-LD180201-0018_HK72WCCXY_L6_clean_bin.22.orig.fa_k	OEZ008529	yes	LMSG_G000005019.1	OES073712	T3PKS	PKSother	Lead-Zinc	41.04	China: Hunan, Shuikoushan
L180206P-6-LD180201-0018_HK72WCCXY_L6_clean_bin.22.orig.fa_k	OEZ008529	yes	LMSG_G000005019.1	OES073712	arylpolyene	Others	Lead-Zinc	29.45	China: Hunan, Shuikoushan
L180206P-6-LD180201-0018_HK72WCCXY_L6_clean_bin.22.orig.fa_k	OEZ008529	yes	LMSG_G000005019.1	OES073712	terpene	Terpene	Lead-Zinc	20.83	China: Hunan, Shuikoushan
L180206P-6-LD180201-0018_HK72WCCXY_L6_clean_bin.22.orig.fa_k	OEZ008529	yes	LMSG_G000005019.1	OES073712	NRPS-like	NRPS	Lead-Zinc	44.05	China: Hunan, Shuikoushan
L180206P-6-LD180201-0018_HK72WCCXY_L6_clean_bin.22.orig.fa_k	OEZ008529	yes	LMSG_G000005019.1	OES073712	butyrolactone	Others	Lead-Zinc	10.71	China: Hunan, Shuikoushan
L180206P-6-LD180201-0018_HK72WCCXY_L6_clean_bin.22.orig.fa_k	OEZ008529	yes	LMSG_G000005019.1	OES073712	bacteriocin	RiPPs	Lead-Zinc	10.81	China: Hunan, Shuikoushan
L180206P-6-LD180201-0018_HK72WCCXY_L6_clean_bin.23.orig.fa_k	OEZ008529	yes	LMSG_G000005020.1	OES073712	bacteriocin	RiPPs	Lead-Zinc	10.90	China: Hunan, Shuikoushan
L180206P-6-LD180201-0018_HK72WCCXY_L6_clean_bin.23.orig.fa_k	OEZ008529	yes	LMSG_G000005020.1	OES073712	betalactone	Others	Lead-Zinc	32.27	China: Hunan, Shuikoushan
L180206P-6-LD180201-0018_HK72WCCXY_L6_clean_bin.23.orig.fa_k	OEZ008529	yes	LMSG_G000005020.1	OES073712	NRPS-like.bacteriocin	Others	Lead-Zinc	41.97	China: Hunan, Shuikoushan
L180206P-6-LD180201-0018_HK72WCCXY_L6_clean_bin.23.orig.fa_k	OEZ008529	yes	LMSG_G000005020.1	OES073712	NRPS-like	NRPS	Lead-Zinc	16.11	China: Hunan, Shuikoushan
L180206P-6-LD180201-0018_HK72WCCXY_L6_clean_bin.23.orig.fa_k	OEZ008529	yes	LMSG_G000005020.1	OES073712	lassopeptide	RiPPs	Lead-Zinc	22.64	China: Hunan, Shuikoushan
L180206P-6-LD180201-0018_HK72WCCXY_L6_clean_bin.23.orig.fa_k	OEZ008529	yes	LMSG_G000005020.1	OES073712	arylpolyene	Others	Lead-Zinc	41.21	China: Hunan, Shuikoushan
L180206P-6-LD180201-0018_HK72WCCXY_L6_clean_bin.23.orig.fa_k	OEZ008529	yes	LMSG_G000005020.1	OES073712	terpene	Terpene	Lead-Zinc	23.09	China: Hunan, Shuikoushan
L180206P-6-LD180201-0018_HK72WCCXY_L6_clean_bin.25.orig.fa_k	OEZ008529	yes	LMSG_G000005021.1	OES073712	terpene	Terpene	Lead-Zinc	16.80	China: Hunan, Shuikoushan
L180206P-6-LD180201-0018_HK72WCCXY_L6_clean_bin.25.orig.fa_k	OEZ008529	yes	LMSG_G000005021.1	OES073712	bacteriocin	RiPPs	Lead-Zinc	6.59	China: Hunan, Shuikoushan
L180206P-6-LD180201-0018_HK72WCCXY_L6_clean_bin.26.orig.fa_k	OEZ008529	yes	LMSG_G000005022.1	OES073712	terpene	Terpene	Lead-Zinc	21.69	China: Hunan, Shuikoushan
L180206P-6-LD180201-0018_HK72WCCXY_L6_clean_bin.26.orig.fa_k	OEZ008529	yes	LMSG_G000005022.1	OES073712	bacteriocin	RiPPs	Lead-Zinc	9.82	China: Hunan, Shuikoushan
L180206P-6-LD180201-0018_HK72WCCXY_L6_clean_bin.27.strict.fa	OEZ008529	yes	LMSG_G000008789.1	OES073712	NRPS-like	NRPS	Lead-Zinc	20.03	China: Hunan, Shuikoushan
L180206P-6-LD180201-0018_HK72WCCXY_L6_clean_bin.27.strict.fa	OEZ008529	yes	LMSG_G000008789.1	OES073712	bacteriocin	RiPPs	Lead-Zinc	5.42	China: Hunan, Shuikoushan
L180206P-6-LD180201-0018_HK72WCCXY_L6_clean_bin.29.orig.fa_k	OEZ008529	yes	LMSG_G000010863.1	OES073712	betalactone.arylpolyene	Others	Lead-Zinc	35.53	China: Hunan, Shuikoushan
L180206P-6-LD180201-0018_HK72WCCXY_L6_clean_bin.29.orig.fa_k	OEZ008529	yes	LMSG_G000010863.1	OES073712	terpene	Terpene	Lead-Zinc	18.63	China: Hunan, Shuikoushan
L180206P-6-LD180201-0018_HK72WCCXY_L6_clean_bin.29.orig.fa_k	OEZ008529	yes	LMSG_G000010863.1	OES073712	acyl_amino_acids	Others	Lead-Zinc	61.81	China: Hunan, Shuikoushan
L180206P-6-LD180201-0018_HK72WCCXY_L6_clean_bin.29.orig.fa_k	OEZ008529	yes	LMSG_G000010863.1	OES073712	NRPS-like	NRPS	Lead-Zinc	37.98	China: Hunan, Shuikoushan
L180206P-6-LD180201-0018_HK72WCCXY_L6_clean_bin.3.orig.fa_kl	OEZ008529	yes	LMSG_G000005024.1	OES073712	terpene	Terpene	Lead-Zinc	11.45	China: Hunan, Shuikoushan
L180206P-6-LD180201-0018_HK72WCCXY_L6_clean_bin.3.orig.fa_kl	OEZ008529	yes	LMSG_G000005024.1	OES073712	NRPS-like	NRPS	Lead-Zinc	19.55	China: Hunan, Shuikoushan
L180206P-6-LD180201-0018_HK72WCCXY_L6_clean_bin.3.orig.fa_kl	OEZ008529	yes	LMSG_G000005024.1	OES073712	ladderane	Others	Lead-Zinc	31.12	China: Hunan, Shuikoushan
L180206P-6-LD180201-0018_HK72WCCXY_L6_clean_bin.30.orig.fa_k	OEZ008529	yes	LMSG_G000010890.1	OES073712	betalactone	Others	Lead-Zinc	25.35	China: Hunan, Shuikoushan
L180206P-6-LD180201-0018_HK72WCCXY_L6_clean_bin.30.orig.fa_k	OEZ008529	yes	LMSG_G000010890.1	OES073712	terpene	Terpene	Lead-Zinc	14.62	China: Hunan, Shuikoushan
L180206P-6-LD180201-0018_HK72WCCXY_L6_clean_bin.30.orig.fa_k	OEZ008529	yes	LMSG_G000010890.1	OES073712	acyl_amino_acids	Others	Lead-Zinc	13.44	China: Hunan, Shuikoushan
L180206P-6-LD180201-0018_HK72WCCXY_L6_clean_bin.31.orig.fa_k	OEZ008529	yes	LMSG_G000010383.1	OES073712	NRPS-like	NRPS	Lead-Zinc	13.61	China: Hunan, Shuikoushan
L180206P-6-LD180201-0018_HK72WCCXY_L6_clean_bin.31.orig.fa_k	OEZ008529	yes	LMSG_G000010383.1	OES073712	terpene	Terpene	Lead-Zinc	21.01	China: Hunan, Shuikoushan
L180206P-6-LD180201-0018_HK72WCCXY_L6_clean_bin.31.orig.fa_k	OEZ008529	yes	LMSG_G000010383.1	OES073712	hserlactone	Others	Lead-Zinc	20.59	China: Hunan, Shuikoushan
L180206P-6-LD180201-0018_HK72WCCXY_L6_clean_bin.31.orig.fa_k	OEZ008529	yes	LMSG_G000010383.1	OES073712	NRPS.T1PKS	PKS-NRP_Hybrids	Lead-Zinc	52.41	China: Hunan, Shuikoushan
L180206P-6-LD180201-0018_HK72WCCXY_L6_clean_bin.31.orig.fa_k	OEZ008529	yes	LMSG_G000010383.1	OES073712	bacteriocin	RiPPs	Lead-Zinc	10.89	China: Hunan, Shuikoushan
L180206P-6-LD180201-0018_HK72WCCXY_L6_clean_bin.31.orig.fa_k	OEZ008529	yes	LMSG_G000010383.1	OES073712	hserlactone	Others	Lead-Zinc	19.45	China: Hunan, Shuikoushan
L180206P-6-LD180201-0018_HK72WCCXY_L6_clean_bin.32.orig.fa_k	OEZ008529	yes	LMSG_G000010999.1	OES073712	bacteriocin	RiPPs	Lead-Zinc	8.05	China: Hunan, Shuikoushan
L180206P-6-LD180201-0018_HK72WCCXY_L6_clean_bin.37.orig.fa_k	OEZ008529	yes	LMSG_G000007815.1	OES073712	terpene	Terpene	Lead-Zinc	29.81	China: Hunan, Shuikoushan
L180206P-6-LD180201-0018_HK72WCCXY_L6_clean_bin.37.orig.fa_k	OEZ008529	yes	LMSG_G000007815.1	OES073712	T3PKS	PKSother	Lead-Zinc	32.06	China: Hunan, Shuikoushan
L180206P-6-LD180201-0018_HK72WCCXY_L6_clean_bin.38.orig.fa_k	OEZ008529	yes	LMSG_G000010607.1	OES073712	terpene	Terpene	Lead-Zinc	20.83	China: Hunan, Shuikoushan
L180206P-6-LD180201-0018_HK72WCCXY_L6_clean_bin.38.orig.fa_k	OEZ008529	yes	LMSG_G000010607.1	OES073712	bacteriocin	RiPPs	Lead-Zinc	8.65	China: Hunan, Shuikoushan
L180206P-6-LD180201-0018_HK72WCCXY_L6_clean_bin.38.orig.fa_k	OEZ008529	yes	LMSG_G000010607.1	OES073712	terpene	Terpene	Lead-Zinc	19.48	China: Hunan, Shuikoushan
L180206P-6-LD180201-0018_HK72WCCXY_L6_clean_bin.38.orig.fa_k	OEZ008529	yes	LMSG_G000010607.1	OES073712	arylpolyene	Others	Lead-Zinc	12.35	China: Hunan, Shuikoushan
L180206P-6-LD180201-0018_HK72WCCXY_L6_clean_bin.39.permissiv	OEZ008529	yes	LMSG_G000008188.1	OES073712	terpene	Terpene	Lead-Zinc	20.86	China: Hunan, Shuikoushan
L180206P-6-LD180201-0018_HK72WCCXY_L6_clean_bin.39.permissiv	OEZ008529	yes	LMSG_G000008188.1	OES073712	betalactone	Others	Lead-Zinc	21.04	China: Hunan, Shuikoushan
L180206P-6-LD180201-0018_HK72WCCXY_L6_clean_bin.4.orig.fa_kl	OEZ008529	yes	LMSG_G000005027.1	OES073712	terpene	Terpene	Lead-Zinc	10.45	China: Hunan, Shuikoushan
L180206P-6-LD180201-0018_HK72WCCXY_L6_clean_bin.4.orig.fa_kl	OEZ008529	yes	LMSG_G000005027.1	OES073712	resorcinol	Others	Lead-Zinc	5.47	China: Hunan, Shuikoushan
L180206P-6-LD180201-0018_HK72WCCXY_L6_clean_bin.46.orig.fa_k	OEZ008529	yes	LMSG_G000010420.1	OES073712	terpene	Terpene	Lead-Zinc	15.47	China: Hunan, Shuikoushan
L180206P-6-LD180201-0018_HK72WCCXY_L6_clean_bin.46.orig.fa_k	OEZ008529	yes	LMSG_G000010420.1	OES073712	terpene	Terpene	Lead-Zinc	15.57	China: Hunan, Shuikoushan
L180206P-6-LD180201-0018_HK72WCCXY_L6_clean_bin.47.strict.fa	OEZ008529	yes	LMSG_G000005026.1	OES073712	bacteriocin	RiPPs	Lead-Zinc	10.80	China: Hunan, Shuikoushan
L180206P-6-LD180201-0018_HK72WCCXY_L6_clean_bin.47.strict.fa	OEZ008529	yes	LMSG_G000005026.1	OES073712	NRPS-like.ladderane	Others	Lead-Zinc	17.31	China: Hunan, Shuikoushan
L180206P-6-LD180201-0018_HK72WCCXY_L6_clean_bin.47.strict.fa	OEZ008529	yes	LMSG_G000005026.1	OES073712	NRPS	NRPS	Lead-Zinc	11.52	China: Hunan, Shuikoushan
L180206P-6-LD180201-0018_HK72WCCXY_L6_clean_bin.53.orig.fa_k	OEZ008529	yes	LMSG_G000011086.1	OES073712	arylpolyene	Others	Lead-Zinc	7.41	China: Hunan, Shuikoushan
L180206P-6-LD180201-0018_HK72WCCXY_L6_clean_bin.53.orig.fa_k	OEZ008529	yes	LMSG_G000011086.1	OES073712	terpene	Terpene	Lead-Zinc	5.51	China: Hunan, Shuikoushan
L180206P-6-LD180201-0018_HK72WCCXY_L6_clean_bin.54.permissiv	OEZ008529	yes	LMSG_G000008889.1	OES073712	T3PKS	PKSother	Lead-Zinc	6.20	China: Hunan, Shuikoushan
L180206P-6-LD180201-0018_HK72WCCXY_L6_clean_bin.55.orig.fa_k	OEZ008529	yes	LMSG_G000009688.1	OES073712	NRPS-like	NRPS	Lead-Zinc	15.82	China: Hunan, Shuikoushan
L180206P-6-LD180201-0018_HK72WCCXY_L6_clean_bin.56.orig.fa_k	OEZ008529	yes	LMSG_G000010907.1	OES073712	terpene	Terpene	Lead-Zinc	9.51	China: Hunan, Shuikoushan
L180206P-6-LD180201-0018_HK72WCCXY_L6_clean_bin.57.orig.fa_k	OEZ008529	yes	LMSG_G000010911.1	OES073712	terpene	Terpene	Lead-Zinc	18.35	China: Hunan, Shuikoushan
L180206P-6-LD180201-0018_HK72WCCXY_L6_clean_bin.57.orig.fa_k	OEZ008529	yes	LMSG_G000010911.1	OES073712	T1PKS	PKSI	Lead-Zinc	43.37	China: Hunan, Shuikoushan
L180206P-6-LD180201-0018_HK72WCCXY_L6_clean_bin.57.orig.fa_k	OEZ008529	yes	LMSG_G000010911.1	OES073712	hserlactone	Others	Lead-Zinc	20.66	China: Hunan, Shuikoushan
L180206P-6-LD180201-0018_HK72WCCXY_L6_clean_bin.57.orig.fa_k	OEZ008529	yes	LMSG_G000010911.1	OES073712	thiopeptide	RiPPs	Lead-Zinc	23.84	China: Hunan, Shuikoushan
L180206P-6-LD180201-0018_HK72WCCXY_L6_clean_bin.58.orig.fa_k	OEZ008529	yes	LMSG_G000008632.1	OES073712	NRPS-like	NRPS	Lead-Zinc	23.51	China: Hunan, Shuikoushan
L180206P-6-LD180201-0018_HK72WCCXY_L6_clean_bin.58.orig.fa_k	OEZ008529	yes	LMSG_G000008632.1	OES073712	other	Others	Lead-Zinc	40.72	China: Hunan, Shuikoushan
L180206P-6-LD180201-0018_HK72WCCXY_L6_clean_bin.59.orig.fa_k	OEZ008529	yes	LMSG_G000010901.1	OES073712	NRPS-like	NRPS	Lead-Zinc	23.84	China: Hunan, Shuikoushan
L180206P-6-LD180201-0018_HK72WCCXY_L6_clean_bin.59.orig.fa_k	OEZ008529	yes	LMSG_G000010901.1	OES073712	hserlactone	Others	Lead-Zinc	12.91	China: Hunan, Shuikoushan
L180206P-6-LD180201-0018_HK72WCCXY_L6_clean_bin.59.orig.fa_k	OEZ008529	yes	LMSG_G000010901.1	OES073712	acyl_amino_acids	Others	Lead-Zinc	7.19	China: Hunan, Shuikoushan
L180206P-6-LD180201-0018_HK72WCCXY_L6_clean_bin.59.orig.fa_k	OEZ008529	yes	LMSG_G000010901.1	OES073712	terpene	Terpene	Lead-Zinc	21.68	China: Hunan, Shuikoushan
L180206P-6-LD180201-0018_HK72WCCXY_L6_clean_bin.60.orig.fa_k	OEZ008529	yes	LMSG_G000010330.1	OES073712	NRPS-like	NRPS	Lead-Zinc	30.11	China: Hunan, Shuikoushan
L180206P-6-LD180201-0018_HK72WCCXY_L6_clean_bin.61.orig.fa_k	OEZ008529	yes	LMSG_G000008446.1	OES073712	terpene	Terpene	Lead-Zinc	26.50	China: Hunan, Shuikoushan
L180206P-6-LD180201-0018_HK72WCCXY_L6_clean_bin.63.strict.fa	OEZ008529	yes	LMSG_G000010509.1	OES073712	arylpolyene	Others	Lead-Zinc	21.61	China: Hunan, Shuikoushan
L180206P-6-LD180201-0018_HK72WCCXY_L6_clean_bin.63.strict.fa	OEZ008529	yes	LMSG_G000010509.1	OES073712	NRPS-like	NRPS	Lead-Zinc	23.05	China: Hunan, Shuikoushan
L180206P-6-LD180201-0018_HK72WCCXY_L6_clean_bin.64.orig.fa_k	OEZ008529	yes	LMSG_G000010387.1	OES073712	T3PKS	PKSother	Lead-Zinc	6.53	China: Hunan, Shuikoushan
L180206P-6-LD180201-0018_HK72WCCXY_L6_clean_bin.64.orig.fa_k	OEZ008529	yes	LMSG_G000010387.1	OES073712	NRPS-like.bacteriocin	Others	Lead-Zinc	10.61	China: Hunan, Shuikoushan
L180206P-6-LD180201-0018_HK72WCCXY_L6_clean_bin.64.orig.fa_k	OEZ008529	yes	LMSG_G000010387.1	OES073712	terpene	Terpene	Lead-Zinc	5.91	China: Hunan, Shuikoushan
L180206P-6-LD180201-0018_HK72WCCXY_L6_clean_bin.64.orig.fa_k	OEZ008529	yes	LMSG_G000010387.1	OES073712	terpene	Terpene	Lead-Zinc	6.96	China: Hunan, Shuikoushan
L180206P-6-LD180201-0018_HK72WCCXY_L6_clean_bin.65.orig.fa_k	OEZ008529	yes	LMSG_G000010718.1	OES073712	terpene	Terpene	Lead-Zinc	7.11	China: Hunan, Shuikoushan
L180206P-6-LD180201-0018_HK72WCCXY_L6_clean_bin.65.orig.fa_k	OEZ008529	yes	LMSG_G000010718.1	OES073712	arylpolyene	Others	Lead-Zinc	31.03	China: Hunan, Shuikoushan
L180206P-6-LD180201-0018_HK72WCCXY_L6_clean_bin.65.orig.fa_k	OEZ008529	yes	LMSG_G000010718.1	OES073712	NRPS	NRPS	Lead-Zinc	21.45	China: Hunan, Shuikoushan
L180206P-6-LD180201-0018_HK72WCCXY_L6_clean_bin.65.orig.fa_k	OEZ008529	yes	LMSG_G000010718.1	OES073712	NRPS.T1PKS	PKS-NRP_Hybrids	Lead-Zinc	9.38	China: Hunan, Shuikoushan
L180206P-6-LD180201-0018_HK72WCCXY_L6_clean_bin.66.orig.fa_k	OEZ008529	yes	LMSG_G000005029.1	OES073712	T1PKS	PKSI	Lead-Zinc	9.45	China: Hunan, Shuikoushan
L180206P-6-LD180201-0018_HK72WCCXY_L6_clean_bin.66.orig.fa_k	OEZ008529	yes	LMSG_G000005029.1	OES073712	betalactone.arylpolyene	Others	Lead-Zinc	47.01	China: Hunan, Shuikoushan
L180206P-6-LD180201-0018_HK72WCCXY_L6_clean_bin.66.orig.fa_k	OEZ008529	yes	LMSG_G000005029.1	OES073712	NRPS-like	NRPS	Lead-Zinc	42.84	China: Hunan, Shuikoushan
L180206P-6-LD180201-0018_HK72WCCXY_L6_clean_bin.66.orig.fa_k	OEZ008529	yes	LMSG_G000005029.1	OES073712	terpene	Terpene	Lead-Zinc	20.86	China: Hunan, Shuikoushan
L180206P-6-LD180201-0018_HK72WCCXY_L6_clean_bin.66.orig.fa_k	OEZ008529	yes	LMSG_G000005029.1	OES073712	bacteriocin	RiPPs	Lead-Zinc	10.90	China: Hunan, Shuikoushan
L180206P-6-LD180201-0018_HK72WCCXY_L6_clean_bin.67.orig.fa_k	OEZ008529	yes	LMSG_G000010852.1	OES073712	bacteriocin	RiPPs	Lead-Zinc	6.77	China: Hunan, Shuikoushan
L180206P-6-LD180201-0018_HK72WCCXY_L6_clean_bin.68.strict.fa	OEZ008529	yes	LMSG_G000010605.1	OES073712	terpene	Terpene	Lead-Zinc	19.47	China: Hunan, Shuikoushan
L180206P-6-LD180201-0018_HK72WCCXY_L6_clean_bin.68.strict.fa	OEZ008529	yes	LMSG_G000010605.1	OES073712	NRPS-like	NRPS	Lead-Zinc	16.25	China: Hunan, Shuikoushan
L180206P-6-LD180201-0018_HK72WCCXY_L6_clean_bin.69.strict.fa	OEZ008529	yes	LMSG_G000006311.1	OES073712	NRPS-like	NRPS	Lead-Zinc	24.66	China: Hunan, Shuikoushan
L180206P-6-LD180201-0018_HK72WCCXY_L6_clean_bin.69.strict.fa	OEZ008529	yes	LMSG_G000006311.1	OES073712	TfuA-related	RiPPs	Lead-Zinc	12.12	China: Hunan, Shuikoushan
L180206P-6-LD180201-0018_HK72WCCXY_L6_clean_bin.69.strict.fa	OEZ008529	yes	LMSG_G000006311.1	OES073712	T3PKS	PKSother	Lead-Zinc	15.69	China: Hunan, Shuikoushan
L180206P-6-LD180201-0018_HK72WCCXY_L6_clean_bin.71.permissiv	OEZ008529	yes	LMSG_G000005031.1	OES073712	terpene	Terpene	Lead-Zinc	11.60	China: Hunan, Shuikoushan
L180206P-6-LD180201-0018_HK72WCCXY_L6_clean_bin.72.orig.fa_k	OEZ008529	yes	LMSG_G000005032.1	OES073712	arylpolyene	Others	Lead-Zinc	12.25	China: Hunan, Shuikoushan
L180206P-6-LD180201-0018_HK72WCCXY_L6_clean_bin.72.orig.fa_k	OEZ008529	yes	LMSG_G000005032.1	OES073712	acyl_amino_acids	Others	Lead-Zinc	10.89	China: Hunan, Shuikoushan
L180206P-6-LD180201-0018_HK72WCCXY_L6_clean_bin.72.orig.fa_k	OEZ008529	no	LMSG_G000005032.1	OES073712	terpene	Terpene	Lead-Zinc	11.82	China: Hunan, Shuikoushan
L180206P-6-LD180201-0018_HK72WCCXY_L6_clean_bin.72.orig.fa_k	OEZ008529	yes	LMSG_G000005032.1	OES073712	bacteriocin	RiPPs	Lead-Zinc	10.89	China: Hunan, Shuikoushan
L180206P-6-LD180201-0018_HK72WCCXY_L6_clean_bin.73.orig.fa_k	OEZ008529	no	LMSG_G000010294.1	OES073712	arylpolyene	Others	Lead-Zinc	18.63	China: Hunan, Shuikoushan
L180206P-6-LD180201-0018_HK72WCCXY_L6_clean_bin.73.orig.fa_k	OEZ008529	no	LMSG_G000010294.1	OES073712	terpene	Terpene	Lead-Zinc	9.40	China: Hunan, Shuikoushan
L180206P-6-LD180201-0018_HK72WCCXY_L6_clean_bin.73.orig.fa_k	OEZ008529	no	LMSG_G000010294.1	OES073712	terpene	Terpene	Lead-Zinc	8.28	China: Hunan, Shuikoushan
L180206P-6-LD180201-0018_HK72WCCXY_L6_clean_bin.74.orig.fa_k	OEZ008529	yes	LMSG_G000011301.1	OES073712	terpene	Terpene	Lead-Zinc	20.79	China: Hunan, Shuikoushan
L180206P-6-LD180201-0018_HK72WCCXY_L6_clean_bin.74.orig.fa_k	OEZ008529	yes	LMSG_G000011301.1	OES073712	ladderane	Others	Lead-Zinc	34.14	China: Hunan, Shuikoushan
L180206P-6-LD180201-0018_HK72WCCXY_L6_clean_bin.74.orig.fa_k	OEZ008529	yes	LMSG_G000011301.1	OES073712	terpene	Terpene	Lead-Zinc	20.78	China: Hunan, Shuikoushan
L180206P-6-LD180201-0018_HK72WCCXY_L6_clean_bin.75.orig.fa_k	OEZ008529	yes	LMSG_G000008187.1	OES073712	betalactone	Others	Lead-Zinc	31.32	China: Hunan, Shuikoushan
L180206P-6-LD180201-0018_HK72WCCXY_L6_clean_bin.75.orig.fa_k	OEZ008529	yes	LMSG_G000008187.1	OES073712	terpene	Terpene	Lead-Zinc	20.86	China: Hunan, Shuikoushan
L180206P-6-LD180201-0018_HK72WCCXY_L6_clean_bin.76.orig.fa_k	OEZ008529	yes	LMSG_G000010873.1	OES073712	bacteriocin	RiPPs	Lead-Zinc	11.37	China: Hunan, Shuikoushan
L180206P-6-LD180201-0018_HK72WCCXY_L6_clean_bin.76.orig.fa_k	OEZ008529	yes	LMSG_G000010873.1	OES073712	terpene	Terpene	Lead-Zinc	20.80	China: Hunan, Shuikoushan
L180206P-6-LD180201-0018_HK72WCCXY_L6_clean_bin.76.orig.fa_k	OEZ008529	yes	LMSG_G000010873.1	OES073712	terpene	Terpene	Lead-Zinc	15.34	China: Hunan, Shuikoushan
L180206P-6-LD180201-0018_HK72WCCXY_L6_clean_bin.76.orig.fa_k	OEZ008529	yes	LMSG_G000010873.1	OES073712	NRPS-like	NRPS	Lead-Zinc	33.02	China: Hunan, Shuikoushan
L180206P-6-LD180201-0018_HK72WCCXY_L6_clean_bin.76.orig.fa_k	OEZ008529	yes	LMSG_G000010873.1	OES073712	acyl_amino_acids	Others	Lead-Zinc	34.17	China: Hunan, Shuikoushan
L180206P-6-LD180201-0018_HK72WCCXY_L6_clean_bin.76.orig.fa_k	OEZ008529	yes	LMSG_G000010873.1	OES073712	hserlactone	Others	Lead-Zinc	19.82	China: Hunan, Shuikoushan
L180206P-6-LD180201-0018_HK72WCCXY_L6_clean_bin.77.strict.fa	OEZ008529	yes	LMSG_G000011216.1	OES073712	hserlactone	Others	Lead-Zinc	6.84	China: Hunan, Shuikoushan
L180206P-6-LD180201-0018_HK72WCCXY_L6_clean_bin.78.strict.fa	OEZ008529	no	LMSG_G000010549.1	OES073712	arylpolyene	Others	Lead-Zinc	11.16	China: Hunan, Shuikoushan
L180206P-6-LD180201-0018_HK72WCCXY_L6_clean_bin.79.orig.fa_k	OEZ008529	yes	LMSG_G000010922.1	OES073712	terpene	Terpene	Lead-Zinc	19.63	China: Hunan, Shuikoushan
L180206P-6-LD180201-0018_HK72WCCXY_L6_clean_bin.79.orig.fa_k	OEZ008529	yes	LMSG_G000010922.1	OES073712	arylpolyene	Others	Lead-Zinc	41.19	China: Hunan, Shuikoushan
L180206P-6-LD180201-0018_HK72WCCXY_L6_clean_bin.79.orig.fa_k	OEZ008529	yes	LMSG_G000010922.1	OES073712	thiopeptide	RiPPs	Lead-Zinc	27.40	China: Hunan, Shuikoushan

L180206P-6-LD180201-0018_HK72WCCXY_L6_clean_bin.8.orig.fa_k1	OEZ008529	yes	LMSG_G000010929.1	OES073712	arylpolyene	Others	Lead-Zinc	41.22	China: Hunan, Shuikoushan
L180206P-6-LD180201-0018_HK72WCCXY_L6_clean_bin.8.orig.fa_k1	OEZ008529	yes	LMSG_G000010929.1	OES073712	bacteriocin	RiPPs	Lead-Zinc	10.88	China: Hunan, Shuikoushan
L180206P-6-LD180201-0018_HK72WCCXY_L6_clean_bin.8.orig.fa_k1	OEZ008529	yes	LMSG_G000010929.1	OES073712	terpene	Terpene	Lead-Zinc	23.01	China: Hunan, Shuikoushan
L180206P-6-LD180201-0018_HK72WCCXY_L6_clean_bin.8.orig.fa_k1	OEZ008529	yes	LMSG_G000010929.1	OES073712	acyl_amino_acids	Others	Lead-Zinc	56.89	China: Hunan, Shuikoushan
L180206P-6-LD180201-0018_HK72WCCXY_L6_clean_bin.8.orig.fa_k1	OEZ008529	yes	LMSG_G000010929.1	OES073712	terpene	Terpene	Lead-Zinc	15.52	China: Hunan, Shuikoushan
L180206P-6-LD180201-0018_HK72WCCXY_L6_clean_bin.8.orig.fa_k1	OEZ008529	yes	LMSG_G000010929.1	OES073712	bacteriocin	RiPPs	Lead-Zinc	10.17	China: Hunan, Shuikoushan
L180206P-6-LD180201-0018_HK72WCCXY_L6_clean_bin.80.strict.fa	OEZ008529	yes	LMSG_G000004405.1	OES073712	betalactone	Others	Lead-Zinc	26.16	China: Hunan, Shuikoushan
L180206P-6-LD180201-0018_HK72WCCXY_L6_clean_bin.80.strict.fa	OEZ008529	yes	LMSG_G000004405.1	OES073712	betalactone	Others	Lead-Zinc	16.56	China: Hunan, Shuikoushan
L180206P-6-LD180201-0018_HK72WCCXY_L6_clean_bin.80.strict.fa	OEZ008529	yes	LMSG_G000004405.1	OES073712	betalactone	Others	Lead-Zinc	26.22	China: Hunan, Shuikoushan
L180206P-6-LD180201-0018_HK72WCCXY_L6_clean_bin.81.orig.fa_k	OEZ008529	yes	LMSG_G000010609.1	OES073712	arylpolyene	Others	Lead-Zinc	20.92	China: Hunan, Shuikoushan
L180206P-6-LD180201-0018_HK72WCCXY_L6_clean_bin.81.orig.fa_k	OEZ008529	yes	LMSG_G000010609.1	OES073712	terpene	Terpene	Lead-Zinc	20.46	China: Hunan, Shuikoushan
L180206P-6-LD180201-0018_HK72WCCXY_L6_clean_bin.81.orig.fa_k	OEZ008529	yes	LMSG_G000010609.1	OES073712	hserlactone	Others	Lead-Zinc	17.99	China: Hunan, Shuikoushan
L180206P-6-LD180201-0019_HK72WCCXY_L6_clean_bin.1.orig.fa_k1	OEZ008529	yes	LMSG_G000009430.1	OES073688	bacteriocin	RiPPs	Lead-Zinc	10.85	China: Hunan, Huangshaping
L180206P-6-LD180201-0019_HK72WCCXY_L6_clean_bin.1.orig.fa_k1	OEZ008529	yes	LMSG_G000009430.1	OES073688	ectoine	Others	Lead-Zinc	10.40	China: Hunan, Huangshaping
L180206P-6-LD180201-0019_HK72WCCXY_L6_clean_bin.1.orig.fa_k1	OEZ008529	yes	LMSG_G000009430.1	OES073688	betalactone	Others	Lead-Zinc	28.37	China: Hunan, Huangshaping
L180206P-6-LD180201-0019_HK72WCCXY_L6_clean_bin.1.orig.fa_k1	OEZ008529	yes	LMSG_G000009430.1	OES073688	betalactone	Others	Lead-Zinc	22.80	China: Hunan, Huangshaping
L180206P-6-LD180201-0019_HK72WCCXY_L6_clean_bin.11.orig.fa_k	OEZ008529	no	LMSG_G000010598.1	OES073688	terpene	Terpene	Lead-Zinc	5.68	China: Hunan, Huangshaping
L180206P-6-LD180201-0019_HK72WCCXY_L6_clean_bin.12.orig.fa_k	OEZ008529	yes	LMSG_G000011168.1	OES073688	arylpolyene	Others	Lead-Zinc	10.55	China: Hunan, Huangshaping
L180206P-6-LD180201-0019_HK72WCCXY_L6_clean_bin.13.orig.fa_k	OEZ008529	no	LMSG_G000010525.1	OES073688	terpene	Terpene	Lead-Zinc	11.36	China: Hunan, Huangshaping
L180206P-6-LD180201-0019_HK72WCCXY_L6_clean_bin.15.orig.fa_k	OEZ008529	yes	LMSG_G000005033.1	OES073688	bacteriocin	RiPPs	Lead-Zinc	11.03	China: Hunan, Huangshaping
L180206P-6-LD180201-0019_HK72WCCXY_L6_clean_bin.15.orig.fa_k	OEZ008529	yes	LMSG_G000005033.1	OES073688	thiopeptide.LAP	RiPPs	Lead-Zinc	38.53	China: Hunan, Huangshaping
L180206P-6-LD180201-0019_HK72WCCXY_L6_clean_bin.20.strict.fa	OEZ008529	yes	LMSG_G000010584.1	OES073688	bacteriocin	RiPPs	Lead-Zinc	5.44	China: Hunan, Huangshaping
L180206P-6-LD180201-0019_HK72WCCXY_L6_clean_bin.20.strict.fa	OEZ008529	yes	LMSG_G000010584.1	OES073688	terpene	Terpene	Lead-Zinc	5.05	China: Hunan, Huangshaping
L180206P-6-LD180201-0019_HK72WCCXY_L6_clean_bin.23.strict.fa	OEZ008529	yes	LMSG_G000010557.1	OES073688	terpene	Terpene	Lead-Zinc	23.03	China: Hunan, Huangshaping
L180206P-6-LD180201-0019_HK72WCCXY_L6_clean_bin.23.strict.fa	OEZ008529	yes	LMSG_G000010557.1	OES073688	arylpolyene	Others	Lead-Zinc	25.35	China: Hunan, Huangshaping
L180206P-6-LD180201-0019_HK72WCCXY_L6_clean_bin.31.orig.fa_k	OEZ008529	yes	LMSG_G000010251.1	OES073688	betalactone	Others	Lead-Zinc	22.98	China: Hunan, Huangshaping
L180206P-6-LD180201-0019_HK72WCCXY_L6_clean_bin.31.orig.fa_k	OEZ008529	no	LMSG_G000010251.1	OES073688	terpene	Terpene	Lead-Zinc	9.65	China: Hunan, Huangshaping
L180206P-6-LD180201-0019_HK72WCCXY_L6_clean_bin.31.orig.fa_k	OEZ008529	yes	LMSG_G000010251.1	OES073688	arylpolyene	Others	Lead-Zinc	13.22	China: Hunan, Huangshaping
L180206P-6-LD180201-0019_HK72WCCXY_L6_clean_bin.32.orig.fa_k	OEZ008529	yes	LMSG_G000009461.1	OES073688	bacteriocin	RiPPs	Lead-Zinc	8.55	China: Hunan, Huangshaping
L180206P-6-LD180201-0019_HK72WCCXY_L6_clean_bin.32.orig.fa_k	OEZ008529	yes	LMSG_G000009461.1	OES073688	lanthipeptide	RiPPs	Lead-Zinc	11.98	China: Hunan, Huangshaping
L180206P-6-LD180201-0019_HK72WCCXY_L6_clean_bin.33.orig.fa_k	OEZ008529	yes	LMSG_G000007829.1	OES073688	NRPS-like	NRPS	Lead-Zinc	15.02	China: Hunan, Huangshaping
L180206P-6-LD180201-0019_HK72WCCXY_L6_clean_bin.33.orig.fa_k	OEZ008529	yes	LMSG_G000007829.1	OES073688	bacteriocin	RiPPs	Lead-Zinc	10.88	China: Hunan, Huangshaping
L180206P-6-LD180201-0019_HK72WCCXY_L6_clean_bin.33.orig.fa_k	OEZ008529	yes	LMSG_G000007829.1	OES073688	terpene	Terpene	Lead-Zinc	21.87	China: Hunan, Huangshaping
L180206P-6-LD180201-0019_HK72WCCXY_L6_clean_bin.33.orig.fa_k	OEZ008529	yes	LMSG_G000007829.1	OES073688	T3PKS	PKSother	Lead-Zinc	16.01	China: Hunan, Huangshaping
L180206P-6-LD180201-0019_HK72WCCXY_L6_clean_bin.33.orig.fa_k	OEZ008529	yes	LMSG_G000007829.1	OES073688	bacteriocin	RiPPs	Lead-Zinc	10.95	China: Hunan, Huangshaping
L180206P-6-LD180201-0019_HK72WCCXY_L6_clean_bin.33.orig.fa_k	OEZ008529	yes	LMSG_G000007829.1	OES073688	terpene	Terpene	Lead-Zinc	16.18	China: Hunan, Huangshaping
L180206P-6-LD180201-0019_HK72WCCXY_L6_clean_bin.34.strict.fa	OEZ008529	yes	LMSG_G000009310.1	OES073688	terpene	Terpene	Lead-Zinc	8.15	China: Hunan, Huangshaping
L180206P-6-LD180201-0019_HK72WCCXY_L6_clean_bin.35.orig.fa_k	OEZ008529	yes	LMSG_G000004408.1	OES073688	terpene	Terpene	Lead-Zinc	21.27	China: Hunan, Huangshaping
L180206P-6-LD180201-0019_HK72WCCXY_L6_clean_bin.35.orig.fa_k	OEZ008529	yes	LMSG_G000004408.1	OES073688	T3PKS	PKSother	Lead-Zinc	27.91	China: Hunan, Huangshaping
L180206P-6-LD180201-0019_HK72WCCXY_L6_clean_bin.35.orig.fa_k	OEZ008529	yes	LMSG_G000004408.1	OES073688	other	Others	Lead-Zinc	36.37	China: Hunan, Huangshaping
L180206P-6-LD180201-0019_HK72WCCXY_L6_clean_bin.35.orig.fa_k	OEZ008529	yes	LMSG_G000004408.1	OES073688	TfuA-related	RiPPs	Lead-Zinc	23.73	China: Hunan, Huangshaping
L180206P-6-LD180201-0019_HK72WCCXY_L6_clean_bin.35.orig.fa_k	OEZ008529	yes	LMSG_G000004408.1	OES073688	butyrolactone	Others	Lead-Zinc	6.16	China: Hunan, Huangshaping
L180206P-6-LD180201-0019_HK72WCCXY_L6_clean_bin.39.strict.fa	OEZ008529	yes	LMSG_G000010484.1	OES073688	terpene	Terpene	Lead-Zinc	23.04	China: Hunan, Huangshaping
L180206P-6-LD180201-0019_HK72WCCXY_L6_clean_bin.39.strict.fa	OEZ008529	yes	LMSG_G000010484.1	OES073688	arylpolyene	Others	Lead-Zinc	22.24	China: Hunan, Huangshaping
L180206P-6-LD180201-0019_HK72WCCXY_L6_clean_bin.39.strict.fa	OEZ008529	yes	LMSG_G000010484.1	OES073688	hserlactone	Others	Lead-Zinc	12.58	China: Hunan, Huangshaping
L180206P-6-LD180201-0019_HK72WCCXY_L6_clean_bin.4.permissive	OEZ008529	yes	LMSG_G000008257.1	OES073688	terpene	Terpene	Lead-Zinc	19.06	China: Hunan, Huangshaping
L180206P-6-LD180201-0019_HK72WCCXY_L6_clean_bin.40.orig.fa_k	OEZ008529	yes	LMSG_G000005034.1	OES073688	ectoine	Others	Lead-Zinc	6.48	China: Hunan, Huangshaping
L180206P-6-LD180201-0019_HK72WCCXY_L6_clean_bin.40.orig.fa_k	OEZ008529	yes	LMSG_G000005034.1	OES073688	acyl_amino_acids	Others	Lead-Zinc	19.43	China: Hunan, Huangshaping
L180206P-6-LD180201-0019_HK72WCCXY_L6_clean_bin.40.orig.fa_k	OEZ008529	yes	LMSG_G000005034.1	OES073688	terpene	Terpene	Lead-Zinc	21.16	China: Hunan, Huangshaping
L180206P-6-LD180201-0019_HK72WCCXY_L6_clean_bin.40.orig.fa_k	OEZ008529	yes	LMSG_G000005034.1	OES073688	terpene	Terpene	Lead-Zinc	23.29	China: Hunan, Huangshaping
L180206P-6-LD180201-0019_HK72WCCXY_L6_clean_bin.40.orig.fa_k	OEZ008529	yes	LMSG_G000005034.1	OES073688	arylpolyene	Others	Lead-Zinc	10.50	China: Hunan, Huangshaping
L180206P-6-LD180201-0019_HK72WCCXY_L6_clean_bin.40.orig.fa_k	OEZ008529	yes	LMSG_G000005034.1	OES073688	NRPS-like	NRPS	Lead-Zinc	25.48	China: Hunan, Huangshaping
L180206P-6-LD180201-0019_HK72WCCXY_L6_clean_bin.41.orig.fa_k	OEZ008529	yes	LMSG_G000005035.1	OES073688	NRPS-like	NRPS	Lead-Zinc	38.12	China: Hunan, Huangshaping
L180206P-6-LD180201-0019_HK72WCCXY_L6_clean_bin.41.orig.fa_k	OEZ008529	yes	LMSG_G000005035.1	OES073688	betalactone	Others	Lead-Zinc	21.30	China: Hunan, Huangshaping
L180206P-6-LD180201-0019_HK72WCCXY_L6_clean_bin.41.orig.fa_k	OEZ008529	yes	LMSG_G000005035.1	OES073688	terpene	Terpene	Lead-Zinc	16.93	China: Hunan, Huangshaping
L180206P-6-LD180201-0019_HK72WCCXY_L6_clean_bin.43.orig.fa_k	OEZ008529	no	LMSG_G000010158.1	OES073688	terpene	Terpene	Lead-Zinc	11.40	China: Hunan, Huangshaping
L180206P-6-LD180201-0019_HK72WCCXY_L6_clean_bin.43.orig.fa_k	OEZ008529	no	LMSG_G000010158.1	OES073688	terpene	Terpene	Lead-Zinc	21.07	China: Hunan, Huangshaping
L180206P-6-LD180201-0019_HK72WCCXY_L6_clean_bin.43.orig.fa_k	OEZ008529	no	LMSG_G000010158.1	OES073688	ectoine	Others	Lead-Zinc	10.40	China: Hunan, Huangshaping
L180206P-6-LD180201-0019_HK72WCCXY_L6_clean_bin.43.orig.fa_k	OEZ008529	no	LMSG_G000010158.1	OES073688	terpene	Terpene	Lead-Zinc	21.95	China: Hunan, Huangshaping
L180206P-6-LD180201-0019_HK72WCCXY_L6_clean_bin.5.orig.fa_k1	OEZ008529	yes	LMSG_G000008495.1	OES073688	lassopeptide	RiPPs	Lead-Zinc	23.32	China: Hunan, Huangshaping
L180206P-6-LD180201-0019_HK72WCCXY_L6_clean_bin.5.orig.fa_k1	OEZ008529	yes	LMSG_G000008495.1	OES073688	arylpolyene	Others	Lead-Zinc	35.77	China: Hunan, Huangshaping
L180206P-6-LD180201-0019_HK72WCCXY_L6_clean_bin.5.orig.fa_k1	OEZ008529	yes	LMSG_G000008495.1	OES073688	terpene	Terpene	Lead-Zinc	26.09	China: Hunan, Huangshaping
L180206P-6-LD180201-0019_HK72WCCXY_L6_clean_bin.8.strict.fa_	OEZ008529	yes	LMSG_G000005037.1	OES073688	terpene	Terpene	Lead-Zinc	21.74	China: Hunan, Huangshaping
L180206P-6-LD180201-0019_HK72WCCXY_L6_clean_bin.8.strict.fa_	OEZ008529	yes	LMSG_G000005037.1	OES073688	NRPS-like	NRPS	Lead-Zinc	41.06	China: Hunan, Huangshaping
L180206P-6-LD180201-0019_HK72WCCXY_L6_clean_bin.8.strict.fa_	OEZ008529	yes	LMSG_G000005037.1	OES073688	terpene	Terpene	Lead-Zinc	13.68	China: Hunan, Huangshaping
L180206P-6-LD180201-0019_HK72WCCXY_L6_clean_bin.8.strict.fa_	OEZ008529	yes	LMSG_G000005037.1	OES073688	acyl_amino_acids	Others	Lead-Zinc	9.66	China: Hunan, Huangshaping
L180206P-6-LD180201-0019_HK72WCCXY_L6_clean_bin.8.strict.fa_	OEZ008529	yes	LMSG_G000005037.1	OES073688	ectoine	Others	Lead-Zinc	7.25	China: Hunan, Huangshaping
L180206P-6-LD180201-0019_HK72WCCXY_L6_clean_bin.9.strict.fa_	OEZ008529	no	LMSG_G000009311.1	OES073688	NRPS	NRPS	Lead-Zinc	34.44	China: Hunan, Huangshaping
L180206P-6-LD180201-0019_HK72WCCXY_L6_clean_bin.9.strict.fa_	OEZ008529	no	LMSG_G000009311.1	OES073688	transAT-PKS	PKSother	Lead-Zinc	11.51	China: Hunan, Huangshaping
L180206P-6-LD180201-0019_HK72WCCXY_L6_clean_bin.9.strict.fa_	OEZ008529	no	LMSG_G000009311.1	OES073688	NRPS	NRPS	Lead-Zinc	10.94	China: Hunan, Huangshaping
L180206P-6-LD180201-0019_HK72WCCXY_L6_clean_bin.9.strict.fa_	OEZ008529	no	LMSG_G000009311.1	OES073688	transAT-PKS-like	PKSother	Lead-Zinc	9.89	China: Hunan, Huangshaping
L180206P-6-LD180201-0019_HK72WCCXY_L6_clean_bin.9.strict.fa_	OEZ008529	no	LMSG_G000009311.1	OES073688	NRPS	NRPS	Lead-Zinc	9.13	China: Hunan, Huangshaping
L180206P-6-LD180201-0019_HK72WCCXY_L6_clean_bin.9.strict.fa_	OEZ008529	no	LMSG_G000009311.1	OES073688	PKS-like	PKSother	Lead-Zinc	8.44	China: Hunan, Huangshaping
L180206P-6-LD180201-0019_HK72WCCXY_L6_clean_bin.9.strict.fa_	OEZ008529	no	LMSG_G000009311.1	OES073688	betalactone	Others	Lead-Zinc	8.21	China: Hunan, Huangshaping
L180206P-6-LD180201-0019_HK72WCCXY_L6_clean_bin.9.strict.fa_	OEZ008529	no	LMSG_G000009311.1	OES073688	NRPS	NRPS	Lead-Zinc	8.07	China: Hunan, Huangshaping
L180206P-6-LD180201-0019_HK72WCCXY_L6_clean_bin.9.strict.fa_	OEZ008529	no	LMSG_G000009311.1	OES073688	transAT-PKS-like	PKSother	Lead-Zinc	7.74	China: Hunan, Huangshaping
L180206P-6-LD180201-0019_HK72WCCXY_L6_clean_bin.9.strict.fa_	OEZ008529	no	LMSG_G000009311.1	OES073688	bacteriocin	RiPPs	Lead-Zinc	6.46	China: Hunan, Huangshaping
L180206P-6-LD180201-0019_HK72WCCXY_L6_clean_bin.9.strict.fa_	OEZ008529	no	LMSG_G000009311.1	OES073688	terpene	Terpene	Lead-Zinc	7.58	China: Hunan, Huangshaping
L180206P-6-LD180201-0019_HK72WCCXY_L6_clean_bin.9.strict.fa_	OEZ008529	no	LMSG_G000009311.1	OES073688	transAT-PKS-like	PKSother	Lead-Zinc	7.40	China: Hunan, Huangshaping
L180206P-6-LD180201-0019_HK72WCCXY_L6_clean_bin.9.strict.fa_	OEZ008529	no	LMSG_G000009311.1	OES073688	transAT-PKS-like	PKSother	Lead-Zinc	7.08	China: Hunan, Huangshaping
L180206P-6-LD180201-0019_HK72WCCXY_L6_clean_bin.9.strict.fa_	OEZ008529	no	LMSG_G000009311.1	OES073688	transAT-PKS-like	PKSother	Lead-Zinc	6.11	China: Hunan, Huangshaping
L180206P-6-LD180201-0019_HK72WCCXY_L6_clean_bin.9.strict.fa_	OEZ008529	no	LMSG_G000009311.1	OES073688	transAT-PKS-like	PKSother	Lead-Zinc	5.95	China: Hunan, Huangshaping
L180206P-6-LD180201-0019_HK72WCCXY_L6_clean_bin.9.strict.fa_	OEZ008529	no	LMSG_G000009311.1	OES073688	T3PKS	PKSother	Lead-Zinc	5.39	China: Hunan, Huangshaping
L180206P-6-LD180201-0019_HK72WCCXY_L6_clean_bin.9.strict.fa_	OEZ008529	yes	LMSG_G000009311.1	OES073688	NRPS.transAT-PKS-like	PKS-NRP_Hybrids	Lead-Zinc	5.10	China: Hunan, Huangshaping
L180206P-7-LD180201-0009_HK72WCCXY_L7_clean_bin.10.orig.fa_k	OEZ008529	yes	LMSG_G000005038.1	OES073739	acyl_amino_acids	Others	Copper	28.77	China: Jiangxi, Yongping
L180206P-7-LD180201-0009_HK72WCCXY_L7_clean_bin.10.orig.fa_k	OEZ008529	yes	LMSG_G000005038.1	OES073739	arylpolyene	Others	Copper	21.42	China: Jiangxi, Yongping
L180206P-7-LD180201-0009_HK72WCCXY_L7_clean_bin.12.strict.fa	OEZ008529	yes	LMSG_G000006857.1	OES073739	LAP	RiPPs	Copper	5.82	China: Jiangxi, Yongping
L180206P-7-LD180201-0009_HK72WCCXY_L7_clean_bin.14.strict.fa	OEZ008529	yes	LMSG_G000005039.1	OES073739	terpene	Terpene	Copper	11.57	China: Jiangxi, Yongping
L180206P-7-LD180201-0009_HK72WCCXY_L7_clean_bin.20.orig.fa_k	OEZ008529	yes	LMSG_G000011032.1	OES073739	arylpolyene	Others	Copper	40.80	China: Jiangxi, Yongping
L180206P-7-LD180201-0009_HK72WCCXY_L7_clean_bin.20.orig.fa_k	OEZ008529	yes	LMSG_G000011032.1	OES073739	terpene	Terpene	Copper	20.85	China: Jiangxi, Yongping
L180206P-7-LD180201-0009_HK72WCCXY_L7_clean_bin.20.orig.fa_k	OEZ008529	yes	LMSG_G000011032.1	OES073739	bacteriocin	RiPPs	Copper	7.65	China: Jiangxi, Yongping
L180206P-7-LD180201-0009_HK72WCCXY_L7_clean_bin.20.orig.fa_k	OEZ008529	yes	LMSG_G000011032.1	OES073739	resorcinol.arylpolyene	Others	Copper	44.56	China: Jiangxi, Yongping
L180206P-7-LD180201-0009_HK72WCCXY_L7_clean_bin.20.orig.fa_k	OEZ008529	yes	LMSG_G000011032.1	OES073739	terpene	Terpene	Copper	12.01	China: Jiangxi, Yongping
L180206P-7-LD180201-0009_HK72WCCXY_L7_clean_bin.21.orig.fa_k	OEZ008529	yes	LMSG_G000004410.1	OES073739	NRPS-like	NRPS	Copper	38.18	China: Jiangxi, Yongping
L180206P-7-LD180201-0009_HK72WCCXY_L7_clean_bin.23.orig.fa_k	OEZ008529	yes	LMSG_G000008366.1	OES073739	terpene	Terpene	Copper	9.16	China: Jiangxi, Yongping
L180206P-7-LD180201-0009_HK72WCCXY_L7_clean_bin.25.strict.fa	OEZ008529	yes	LMSG_G000009367.1	OES073739	betalactone	Others	Copper	32.65	China: Jiangxi, Yongping
L180206P-7-LD180201-0009_HK72WCCXY_L7_clean_bin.26.permissiv	OEZ008529	yes	LMSG_G000008878.1	OES073739	T3PKS	PKSother	Copper	21.18	China: Jiangxi, Yongping
L180206P-7-LD180201-0009_HK72WCCXY_L7_clean_bin.26.permissiv	OEZ008529	yes	LMSG_G000008878.1	OES073739	terpene	Terpene	Copper	6.74	China: Jiangxi, Yongping
L180206P-7-LD180201-0009_HK72WCCXY_L7_clean_bin.28.orig.fa_k	OEZ008529	yes	LMSG_G000009360.1	OES073739	betalactone	Others	Copper	31.59	China: Jiangxi, Yongping
L180206P-7-LD180201-0009_HK72WCCXY_L7_clean_bin.29.orig.fa_k	OEZ008529	yes	LMSG_G000007909.1	OES073739	NRPS-like	NRPS	Copper	42.62	China: Jiangxi, Yongping
L180206P-7-LD180201-0009_HK72WCCXY_L7_clean_bin.29.orig.fa_k	OEZ008529	yes	LMSG_G000007909.1	OES073739	terpene	Terpene	Copper	21.80	China: Jiangxi, Yongping
L180206P-7-LD180201-0009_HK72WCCXY_L7_clean_bin.29.orig.fa_k	OEZ008529	yes	LMSG_G000007909.1	OES073739	NRPS-like	NRPS	Copper	38.59	China: Jiangxi, Yongping
L180206P-7-LD180201-0009_HK72WCCXY_L7_clean_bin.31.strict.fa	OEZ008529	yes	LMSG_G000005041.1	OES073739	terpene.arylpolyene	Others	Copper	29.42	China: Jiangxi, Yongping
L180206P-7-LD180201-0009_HK72WCCXY_L7_clean_bin.31.strict.fa	OEZ008529	yes	LMSG_G000005041.1	OES073739	terpene	Terpene	Copper	19.32	China: Jiangxi, Yongping
L180206P-7-LD180201-0009_HK72WCCXY_L7_clean_bin.31.strict.fa	OEZ008529	yes	LMSG_G000005041.1	OES073739	NRPS-like	NRPS	Copper	12.28	China: Jiangxi, Yongping
L180206P-7-LD180201-0009_HK72WCCXY_L7_clean_bin.31.strict.fa	OEZ008529	yes	LMSG_G000005041.1	OES073739	ladderane	Others	Copper	11.04	China: Jiangxi, Yongping
L180206P-7-LD180201-0009_HK72WCCXY_L7_clean_bin.32.orig.fa_k	OEZ008529	yes	LMSG_G000007053.1	OES073739	bacteriocin	RiPPs	Copper	8.23	China: Jiangxi, Yongping
L180206P-7-LD180201-0009_HK72WCCXY_L7_clean_bin.35.strict.fa	OEZ008529	yes	LMSG_G000007775.1	OES073739	T3PKS	PKSother	Copper	16.80	China: Jiangxi, Yongping
L180206P-7-LD180201-0009_HK72WCCXY_L7_clean_bin.35.strict.fa	OEZ008529	yes	LMSG_G000007775.1	OES073739	terpene	Terpene	Copper	11.18	China: Jiangxi, Yongping
L180206P-7-LD180201-0009_HK72WCCXY_L7_clean_bin.36.orig.fa_k	OEZ008529	yes	LMSG_G000007915.1	OES073739	T3PKS	PKSother	Copper	31.06	China: Jiangxi, Yongping
L180206P-7-LD180201-0009_HK72WCCXY_L7_clean_bin.36.orig.fa_k	OEZ008529	yes	LMSG_G000007915.1	OES073739	terpene	Terpene	Copper	12.59	China: Jiangxi, Yongping
L180206P-7-LD180201-0009_HK72WCCXY_L7_clean_bin.39.orig.fa_k	OEZ008529	yes	LMSG_G000006271.1	OES073739	NRPS-like	NRPS	Copper	21.99	China: Jiangxi, Yongping
L180206P-7-LD180201-0009_HK72WCCXY_L7_clean_bin.39.orig.fa_k	OEZ008529	yes	LMSG_G000006271.1	OES073739	terpene	Terpene	Copper	8.14	China: Jiangxi, Yongping
L180206P-7-LD180201-0009_HK72WCCXY_L7_clean_bin.41.orig.fa_k	OEZ008529	yes	LMSG_G000010008.1	OES073739	ladderane	Others	Copper	6.25	China: Jiangxi, Yongping
L180206P-7-LD180201-0009_HK72WCCXY_L7_clean_bin.44.strict.fa	OEZ008529	yes	LMSG_G000011042.1	OES073739	arylpolyene	Others	Copper	14.33	China: Jiangxi, Yongping
L180206P-7-LD180201-0009_HK72WCCXY_L7_clean_bin.44.strict.fa	OEZ008529	yes	LMSG_G000011042.1	OES073739	terpene	Terpene	Copper	11.47	China: Jiangxi, Yongping
L180206P-7-LD180201-0009_HK72WCCXY_L7_clean_bin.44.strict.fa	OEZ008529	yes	LMSG_G000011042.1	OES073739	bacteriocin	RiPPs	Copper	8.09	China: Jiangxi, Yongping
L180206P-7-LD180201-0009_HK72WCCXY_L7_clean_bin.44.strict.fa	OEZ008529	yes	LMSG_G000011042.1	OES073739	NRPS-like	NRPS	Copper	6.08	China: Jiangxi, Yongping
L180206P-7-LD180201-0009_HK72WCCXY_L7_clean_bin.45.orig.fa_k	OEZ008529	no	LMSG_G000011008.1	OES073739	NRPS	NRPS	Copper	44.23	China: Jiangxi, Yongping
L180206P-7-LD180201-0009_HK72WCCXY_L7_clean_bin.45.orig.fa_k	OEZ008529	no	LMSG_G000011008.1	OES073739	phenazine	Others	Copper	10.23	China: Jiangxi, Yongping
L180206P-7-LD180201-0009_HK72WCCXY_L7_clean_bin.45.orig.fa_k	OEZ008529	no	LMSG_G000011008.1	OES073739	NRPS	NRPS	Copper	25.01	China: Jiangxi, Yongping
L180206P-7-LD180201-0009_HK72WCCXY_L7_clean_bin.45.orig.fa_k	OEZ008529	no	LMSG_G000011008.1	OES073739	NRPS	NRPS	Copper	43.60	China: Jiangxi, Yongping
L180206P-7-LD180201-0009_HK72WCCXY_L7_clean_bin.45.orig.fa_k	OEZ008529	no	LMSG_G000011008.1	OES073739	betalactone.NRPS-like	Others	Copper	41.82	China: Jiangxi, Yongping
L180206P-7-LD180201-0009_HK72WCCXY_L7_clean_bin.45.orig.fa_k	OEZ008529	no	LMSG_G000011008.1	OES073739	phenazine	Others	Copper	10.23	China: Jiangxi, Yongping
L180206P-7-LD180201-0009_HK72WCCXY_L7_clean_bin.45.orig.fa_k	OEZ008529	no	LMSG_G000011008.1	OES073739	bacteriocin	RiPPs	Copper	10.83	China: Jiangxi, Yongping
L180206P-7-LD180201-0009_HK72WCCXY_L7_clean_bin.45.orig.fa_k	OEZ008529	no	LMSG_G000011008.1	OES073739	NRPS-like	NRPS	Copper	42.98	China: Jiangxi, Yongping
L180206P-7-LD180201-0009_HK72WCCXY_L7_clean_bin.45.orig.fa_k	OEZ008529	no	LMSG_G000011008.1	OES073739	thiopeptide	RiPPs	Copper	33.00	China: Jiangxi, Yongping
L180206P-7-LD180201-0009_HK72WCCXY_L7_clean_bin.45.orig.fa_k	OEZ008529	no	LMSG_G000011008.1	OES073739	hserlactone	Others	Copper	20.61	China: Jiangxi, Yongping
L180206P-7-LD180201-0009_HK72WCCXY_L7_clean_bin.45.orig.fa_k	OEZ008529	no	LMSG_G000011008.1	OES073739	hserlactone	Others	Copper	20.61	China: Jiangxi, Yongping
L180206P-7-LD180201-0009_HK72WCCXY_L7_clean_bin.45.orig.fa_k	OEZ008529	no	LMSG_G000011008.1	OES073739	NAGGN	Others	Copper	14.76	China: Jiangxi, Yongping
L180206P-7-LD180201-0009_HK72WCCXY_L7_clean_bin.45.orig.fa_k	OEZ008529	no	LMSG_G000011008.1	OES073739	bacteriocin	RiPPs	Copper	10.86	China: Jiangxi, Yongping
L180206P-7-LD180201-0009_HK72WCCXY_L7_clean_bin.45.orig.fa_k	OEZ008529	no	LMSG_G000011008.1	OES073739	NRPS	NRPS	Copper	49.55	China: Jiangxi, Yongping
L180206P-7-LD180201-0009_HK72WCCXY_L7_clean_bin.45.orig.fa_k	OEZ008529	no	LMSG_G000011008.1	OES073739	NRPS	NRPS	Copper	62.35	China: Jiangxi, Yongping
L180206P-7-LD180201-0009_HK72WCCXY_L7_clean_bin.50.orig.fa_k	OEZ008529	yes	LMSG_G000005047.1	OES073739	terpene	Terpene	Copper	20.79	China: Jiangxi, Yongping
L180206P-7-LD180201-0009_HK72WCCXY_L7_clean_bin.50.orig.fa_k	OEZ008529	yes	LMSG_G000005047.1	OES073739	phosphonate	Others	Copper	21.35	China: Jiangxi, Yongping
L180206P-7-LD180201-0009_HK72WCCXY_L7_clean_bin.50.orig.fa_k	OEZ008529	yes	LMSG_G000005047.1	OES073739	bacteriocin	RiPPs	Copper	10.94	China: Jiangxi, Yongping
L180206P-7-LD180201-0009_HK72WCCXY_L7_clean_bin.50.orig.fa_k	OEZ008529	yes	LMSG_G000005047.1	OES073739	NRPS-like	NRPS	Copper	35.86	China: Jiangxi, Yongping
L180206P-7-LD180201-0009_HK72WCCXY_L7_clean_bin.50.orig.fa_k	OEZ008529	yes	LMSG_G000005047.1	OES073739	betalactone	Others	Copper	13.14	China: Jiangxi, Yongping
L180206P-7-LD180201-0009_HK72WCCXY_L7_clean_bin.50.orig.fa_k	OEZ008529	yes	LMSG_G000005047.1	OES073739	terpene	Terpene	Copper	18.63	China: Jiangxi, Yongping
L180206P-7-LD180201-0009_HK72WCCXY_L7_clean_bin.53.orig.fa_k	OEZ008529	yes	LMSG_G000009107.1	OES073739	lassopeptide	RiPPs	Copper	7.18	China: Jiangxi, Yongping
L180206P-7-LD180201-0009_HK72WCCXY_L7_clean_bin.53.orig.fa_k	OEZ008529	yes	LMSG_G000009107.1	OES073739	NRPS-like	NRPS	Copper	35.64	China: Jiangxi, Yongping
L180206P-7-LD180201-0009_HK72WCCXY_L7_clean_bin.6.orig.fa_k1	OEZ008529	yes	LMSG_G000005055.1	OES073739	terpene	Terpene	Copper	23.68	China: Jiangxi, Yongping
L180206P-7-LD180201-0009_HK72WCCXY_L7_clean_bin.60.orig.fa_k	OEZ008529	yes	LMSG_G000009235.1	OES073739	T3PKS.terpene	Others	Copper	46.02	China: Jiangxi, Yongping
L180206P-7-LD180201-0009_HK72WCCXY_L7_clean_bin.61.strict.fa	OEZ008529	yes	LMSG_G000005051.1	OES073739	NRPS-like	NRPS	Copper	25.92	China: Jiangxi, Yongping
L180206P-7-LD180201-0009_HK72WCCXY_L7_clean_bin.61.strict.fa	OEZ008529	yes	LMSG_G000005051.1	OES073739	terpene	Terpene	Copper	25.07	China: Jiangxi, Yongping
L180206P-7-LD180201-0009_HK72WCCXY_L7_clean_bin.61.strict.fa	OEZ008529	yes	LMSG_G000005051.1	OES073739	bacteriocin	RiPPs	Copper	10.87	China: Jiangxi, Yongping
L180206P-7-LD180201-0009_HK72WCCXY_L7_clean_bin.65.orig.fa_k	OEZ008529	yes	LMSG_G000005053.1	OES073739	hserlactone	Others	Copper	7.33	China: Jiangxi, Yongping
L180206P-7-LD180201-0009_HK72WCCXY_L7_clean_bin.66.strict.fa	OEZ008529	no	LMSG_G000005054.1	OES073739	lassopeptide	RiPPs	Copper	24.33	China: Jiangxi, Yongping
L180206P-7-LD180201-0009_HK72WCCXY_L7_clean_bin.71.permissiv	OEZ008529	yes	LMSG_G000004411.1	OES073739	betalactone	Others	Copper	19.18	China: Jiangxi, Yongping
L180206P-7-LD180201-0009_HK72WCCXY_L7_clean_bin.71.permissiv	OEZ008529	yes	LMSG_G000004411.1	OES073739	terpene	Terpene	Copper	12.69	China: Jiangxi, Yongping
L180206P-7-LD180201-0009_HK72WCCXY_L7_clean_bin.71.permissiv	OEZ008529	yes	LMSG_G000004411.1	OES073739	NRPS-like	NRPS	Copper	10.65	China: Jiangxi, Yongping
L180206P-7-LD180201-0009_HK72WCCXY_L7_clean_bin.71.permissiv	OEZ008529	yes	LMSG_G000004411.1	OES073739	betalactone	Others	Copper	7.97	China: Jiangxi, Yongping
L180206P-7-LD180201-0009_HK72WCCXY_L7_clean_bin.74.orig.fa_k	OEZ008529	yes	LMSG_G000010487.1	OES073739	arylpolyene	Others	Copper	21.91	China: Jiangxi, Yongping
L180206P-7-LD180201-0009_HK72WCCXY_L7_clean_bin.74.orig.fa_k	OEZ008529	yes	LMSG_G000010487.1	OES073739	hserlactone	Others	Copper	13.45	China: Jiangxi, Yongping
L180206P-7-LD180201-0009_HK72WCCXY_L7_clean_bin.75.strict.fa	OEZ008529	yes	LMSG_G000010462.1	OES073739	NRPS-like.terpene	Others	Copper	56.01	China: Jiangxi, Yongping
L180206P-7-LD180201-0009_HK72WCCXY_L7_clean_bin.75.strict.fa	OEZ008529	yes	LMSG_G000010462.1	OES073739	arylpolyene	Others	Copper	41.23	China: Jiangxi, Yongping
L180206P-7-LD180201-0009_HK72WCCXY_L7_clean_bin.76.orig.fa_k	OEZ008529	yes	LMSG_G000008399.1	OES073739	terpene	Terpene	Copper	11.15	China: Jiangxi, Yongping
L180206P-7-LD180201-0009_HK72WCCXY_L7_clean_bin.82.strict.fa	OEZ008529	yes	LMSG_G000009279.1	OES073739	terpene	Terpene	Copper	21.73	China: Jiangxi, Yongping

L180206P-7-LD180201-0009_HK72WCCXY_L7_clean_bin.82.strict.fa	OEZ008529	yes	LMSG_G000009279.1	OES073739	terpene	Terpene	Copper	22.06	China: Jiangxi, Yongping
L180206P-7-LD180201-0009_HK72WCCXY_L7_clean_bin.82.strict.fa	OEZ008529	yes	LMSG_G000009279.1	OES073739	thiopeptide	RiPPs	Copper	38.03	China: Jiangxi, Yongping
L180206P-7-LD180201-0010_HK72WCCXY_L7_L8_clean_R_bin.15.orig	OEZ008529	yes	LMSG_G000011033.1	OES073743	resorcinol.arylpolyene	Others	Copper	44.56	China: Jiangxi, Yongping
L180206P-7-LD180201-0010_HK72WCCXY_L7_L8_clean_R_bin.15.orig	OEZ008529	yes	LMSG_G000011033.1	OES073743	bacteriocin	RiPPs	Copper	7.67	China: Jiangxi, Yongping
L180206P-7-LD180201-0010_HK72WCCXY_L7_L8_clean_R_bin.15.orig	OEZ008529	yes	LMSG_G000011033.1	OES073743	arylpolyene	Others	Copper	36.71	China: Jiangxi, Yongping
L180206P-7-LD180201-0010_HK72WCCXY_L7_L8_clean_R_bin.15.orig	OEZ008529	yes	LMSG_G000011033.1	OES073743	terpene	Terpene	Copper	23.08	China: Jiangxi, Yongping
L180206P-7-LD180201-0010_HK72WCCXY_L7_L8_clean_R_bin.16.stri	OEZ008529	yes	LMSG_G000010109.1	OES073743	TfuA-related	RiPPs	Copper	21.98	China: Jiangxi, Yongping
L180206P-7-LD180201-0010_HK72WCCXY_L7_L8_clean_R_bin.16.stri	OEZ008529	yes	LMSG_G000010109.1	OES073743	betalactone	Others	Copper	13.28	China: Jiangxi, Yongping
L180206P-7-LD180201-0010_HK72WCCXY_L7_L8_clean_R_bin.16.stri	OEZ008529	yes	LMSG_G000010109.1	OES073743	bacteriocin	RiPPs	Copper	9.27	China: Jiangxi, Yongping
L180206P-7-LD180201-0010_HK72WCCXY_L7_L8_clean_R_bin.16.stri	OEZ008529	yes	LMSG_G000010109.1	OES073743	NRPS-like	NRPS	Copper	14.86	China: Jiangxi, Yongping
L180206P-7-LD180201-0010_HK72WCCXY_L7_L8_clean_R_bin.16.stri	OEZ008529	yes	LMSG_G000010109.1	OES073743	terpene	Terpene	Copper	10.86	China: Jiangxi, Yongping
L180206P-7-LD180201-0010_HK72WCCXY_L7_L8_clean_R_bin.16.stri	OEZ008529	yes	LMSG_G000010109.1	OES073743	phosphonate	Others	Copper	9.78	China: Jiangxi, Yongping
L180206P-7-LD180201-0010_HK72WCCXY_L7_L8_clean_R_bin.19.stri	OEZ008529	yes	LMSG_G000010482.1	OES073743	hserlactone	Others	Copper	10.61	China: Jiangxi, Yongping
L180206P-7-LD180201-0010_HK72WCCXY_L7_L8_clean_R_bin.19.stri	OEZ008529	yes	LMSG_G000010482.1	OES073743	terpene	Terpene	Copper	11.20	China: Jiangxi, Yongping
L180206P-7-LD180201-0010_HK72WCCXY_L7_L8_clean_R_bin.19.stri	OEZ008529	yes	LMSG_G000010482.1	OES073743	arylpolyene	Others	Copper	10.37	China: Jiangxi, Yongping
L180206P-7-LD180201-0010_HK72WCCXY_L7_L8_clean_R_bin.19.stri	OEZ008529	yes	LMSG_G000010482.1	OES073743	bacteriocin	RiPPs	Copper	7.18	China: Jiangxi, Yongping
L180206P-7-LD180201-0010_HK72WCCXY_L7_L8_clean_R_bin.20.stri	OEZ008529	yes	LMSG_G000009118.1	OES073743	NRPS-like	NRPS	Copper	25.81	China: Jiangxi, Yongping
L180206P-7-LD180201-0010_HK72WCCXY_L7_L8_clean_R_bin.20.stri	OEZ008529	yes	LMSG_G000009118.1	OES073743	bacteriocin	RiPPs	Copper	10.87	China: Jiangxi, Yongping
L180206P-7-LD180201-0010_HK72WCCXY_L7_L8_clean_R_bin.20.stri	OEZ008529	yes	LMSG_G000009118.1	OES073743	terpene	Terpene	Copper	25.09	China: Jiangxi, Yongping
L180206P-7-LD180201-0010_HK72WCCXY_L7_L8_clean_R_bin.21.stri	OEZ008529	yes	LMSG_G000007913.1	OES073743	bacteriocin	RiPPs	Copper	10.81	China: Jiangxi, Yongping
L180206P-7-LD180201-0010_HK72WCCXY_L7_L8_clean_R_bin.21.stri	OEZ008529	yes	LMSG_G000007913.1	OES073743	NRPS-like	NRPS	Copper	42.82	China: Jiangxi, Yongping
L180206P-7-LD180201-0010_HK72WCCXY_L7_L8_clean_R_bin.21.stri	OEZ008529	yes	LMSG_G000007913.1	OES073743	terpene	Terpene	Copper	20.55	China: Jiangxi, Yongping
L180206P-7-LD180201-0010_HK72WCCXY_L7_L8_clean_R_bin.22.perm	OEZ008529	yes	LMSG_G000008367.1	OES073743	terpene	Terpene	Copper	8.91	China: Jiangxi, Yongping
L180206P-7-LD180201-0010_HK72WCCXY_L7_L8_clean_R_bin.5.stric	OEZ008529	yes	LMSG_G000009280.1	OES073743	terpene	Terpene	Copper	22.06	China: Jiangxi, Yongping
L180206P-7-LD180201-0010_HK72WCCXY_L7_L8_clean_R_bin.5.stric	OEZ008529	yes	LMSG_G000009280.1	OES073743	terpene	Terpene	Copper	15.04	China: Jiangxi, Yongping
L180206P-7-LD180201-0010_HK72WCCXY_L7_L8_clean_R_bin.6.stric	OEZ008529	no	LMSG_G000011018.1	OES073743	betalactone.NRPS-like	Others	Copper	41.86	China: Jiangxi, Yongping
L180206P-7-LD180201-0010_HK72WCCXY_L7_L8_clean_R_bin.6.stric	OEZ008529	no	LMSG_G000011018.1	OES073743	bacteriocin	RiPPs	Copper	10.83	China: Jiangxi, Yongping
L180206P-7-LD180201-0010_HK72WCCXY_L7_L8_clean_R_bin.6.stric	OEZ008529	no	LMSG_G000011018.1	OES073743	NRPS-like	NRPS	Copper	42.98	China: Jiangxi, Yongping
L180206P-7-LD180201-0010_HK72WCCXY_L7_L8_clean_R_bin.6.stric	OEZ008529	no	LMSG_G000011018.1	OES073743	NAGGN	Others	Copper	14.76	China: Jiangxi, Yongping
L180206P-7-LD180201-0010_HK72WCCXY_L7_L8_clean_R_bin.6.stric	OEZ008529	no	LMSG_G000011018.1	OES073743	hserlactone	Others	Copper	20.61	China: Jiangxi, Yongping
L180206P-7-LD180201-0010_HK72WCCXY_L7_L8_clean_R_bin.6.stric	OEZ008529	no	LMSG_G000011018.1	OES073743	hserlactone	Others	Copper	20.61	China: Jiangxi, Yongping
L180206P-7-LD180201-0010_HK72WCCXY_L7_L8_clean_R_bin.6.stric	OEZ008529	no	LMSG_G000011018.1	OES073743	NRPS	NRPS	Copper	24.83	China: Jiangxi, Yongping
L180206P-7-LD180201-0010_HK72WCCXY_L7_L8_clean_R_bin.6.stric	OEZ008529	no	LMSG_G000011018.1	OES073743	NRPS	NRPS	Copper	42.51	China: Jiangxi, Yongping
L180206P-7-LD180201-0010_HK72WCCXY_L7_L8_clean_R_bin.6.stric	OEZ008529	no	LMSG_G000011018.1	OES073743	NRPS	NRPS	Copper	49.55	China: Jiangxi, Yongping
L180206P-7-LD180201-0010_HK72WCCXY_L7_L8_clean_R_bin.6.stric	OEZ008529	no	LMSG_G000011018.1	OES073743	bacteriocin	RiPPs	Copper	10.86	China: Jiangxi, Yongping
L180206P-7-LD180201-0010_HK72WCCXY_L7_L8_clean_R_bin.6.stric	OEZ008529	no	LMSG_G000011018.1	OES073743	thiopeptide	RiPPs	Copper	33.00	China: Jiangxi, Yongping
L180206P-7-LD180201-0010_HK72WCCXY_L7_L8_clean_R_bin.6.stric	OEZ008529	no	LMSG_G000011018.1	OES073743	NRPS.phenazine	Others	Copper	60.22	China: Jiangxi, Yongping
L180206P-7-LD180201-0010_HK72WCCXY_L7_L8_clean_R_bin.6.stric	OEZ008529	no	LMSG_G000011018.1	OES073743	NRPS	NRPS	Copper	61.47	China: Jiangxi, Yongping
L180206P-7-LD180201-0010_HK72WCCXY_L7_L8_clean_R_bin.6.stric	OEZ008529	no	LMSG_G000011018.1	OES073743	phenazine	Others	Copper	10.16	China: Jiangxi, Yongping
L180206P-7-LD180201-0015_HK72WCCXY_L7_clean_bin.11.permissiv	OEZ008529	yes	LMSG_G000008191.1	OES073713	terpene	Terpene	Lead-Zinc	20.86	China: Hunan, Shuikoushan
L180206P-7-LD180201-0015_HK72WCCXY_L7_clean_bin.12.orig.fa_k	OEZ008529	yes	LMSG_G000009203.1	OES073713	hglE-KS	PKSother	Lead-Zinc	9.37	China: Hunan, Shuikoushan
L180206P-7-LD180201-0015_HK72WCCXY_L7_clean_bin.12.orig.fa_k	OEZ008529	yes	LMSG_G000009203.1	OES073713	lanthipeptide	RiPPs	Lead-Zinc	9.51	China: Hunan, Shuikoushan
L180206P-7-LD180201-0015_HK72WCCXY_L7_clean_bin.14.orig.fa_k	OEZ008529	yes	LMSG_G000010424.1	OES073713	NRPS-like	NRPS	Lead-Zinc	25.78	China: Hunan, Shuikoushan
L180206P-7-LD180201-0015_HK72WCCXY_L7_clean_bin.14.orig.fa_k	OEZ008529	yes	LMSG_G000010424.1	OES073713	terpene	Terpene	Lead-Zinc	15.42	China: Hunan, Shuikoushan
L180206P-7-LD180201-0015_HK72WCCXY_L7_clean_bin.14.orig.fa_k	OEZ008529	yes	LMSG_G000010424.1	OES073713	terpene	Terpene	Lead-Zinc	21.95	China: Hunan, Shuikoushan
L180206P-7-LD180201-0015_HK72WCCXY_L7_clean_bin.14.orig.fa_k	OEZ008529	yes	LMSG_G000010424.1	OES073713	hserlactone	Others	Lead-Zinc	7.82	China: Hunan, Shuikoushan
L180206P-7-LD180201-0015_HK72WCCXY_L7_clean_bin.16.orig.fa_k	OEZ008529	yes	LMSG_G000005062.1	OES073713	hserlactone	Others	Lead-Zinc	13.36	China: Hunan, Shuikoushan
L180206P-7-LD180201-0015_HK72WCCXY_L7_clean_bin.16.orig.fa_k	OEZ008529	yes	LMSG_G000005062.1	OES073713	hserlactone	Others	Lead-Zinc	20.69	China: Hunan, Shuikoushan
L180206P-7-LD180201-0015_HK72WCCXY_L7_clean_bin.16.orig.fa_k	OEZ008529	yes	LMSG_G000005062.1	OES073713	betalactone	Others	Lead-Zinc	32.92	China: Hunan, Shuikoushan
L180206P-7-LD180201-0015_HK72WCCXY_L7_clean_bin.16.orig.fa_k	OEZ008529	yes	LMSG_G000005062.1	OES073713	bacteriocin	RiPPs	Lead-Zinc	10.88	China: Hunan, Shuikoushan
L180206P-7-LD180201-0015_HK72WCCXY_L7_clean_bin.16.orig.fa_k	OEZ008529	yes	LMSG_G000005062.1	OES073713	terpene	Terpene	Lead-Zinc	16.09	China: Hunan, Shuikoushan
L180206P-7-LD180201-0015_HK72WCCXY_L7_clean_bin.16.orig.fa_k	OEZ008529	yes	LMSG_G000005062.1	OES073713	terpene	Terpene	Lead-Zinc	20.81	China: Hunan, Shuikoushan
L180206P-7-LD180201-0015_HK72WCCXY_L7_clean_bin.17.strict.fa	OEZ008529	yes	LMSG_G000006222.1	OES073713	betalactone	Others	Lead-Zinc	31.32	China: Hunan, Shuikoushan
L180206P-7-LD180201-0015_HK72WCCXY_L7_clean_bin.17.strict.fa	OEZ008529	yes	LMSG_G000006222.1	OES073713	terpene	Terpene	Lead-Zinc	20.86	China: Hunan, Shuikoushan
L180206P-7-LD180201-0015_HK72WCCXY_L7_clean_bin.19.orig.fa_k	OEZ008529	yes	LMSG_G000007754.1	OES073713	NRPS.T1PKS	PKS-NRP_Hybrids	Lead-Zinc	10.53	China: Hunan, Shuikoushan
L180206P-7-LD180201-0015_HK72WCCXY_L7_clean_bin.19.orig.fa_k	OEZ008529	yes	LMSG_G000007754.1	OES073713	terpene	Terpene	Lead-Zinc	14.97	China: Hunan, Shuikoushan
L180206P-7-LD180201-0015_HK72WCCXY_L7_clean_bin.19.orig.fa_k	OEZ008529	yes	LMSG_G000007754.1	OES073713	NRPS	NRPS	Lead-Zinc	5.34	China: Hunan, Shuikoushan
L180206P-7-LD180201-0015_HK72WCCXY_L7_clean_bin.19.orig.fa_k	OEZ008529	yes	LMSG_G000007754.1	OES073713	betalactone.NRPS-like	Others	Lead-Zinc	18.15	China: Hunan, Shuikoushan
L180206P-7-LD180201-0015_HK72WCCXY_L7_clean_bin.19.orig.fa_k	OEZ008529	yes	LMSG_G000007754.1	OES073713	NRPS-like	NRPS	Lead-Zinc	8.15	China: Hunan, Shuikoushan
L180206P-7-LD180201-0015_HK72WCCXY_L7_clean_bin.2.orig.fa_k1	OEZ008529	yes	LMSG_G000010674.1	OES073713	arylpolyene	Others	Lead-Zinc	41.28	China: Hunan, Shuikoushan
L180206P-7-LD180201-0015_HK72WCCXY_L7_clean_bin.2.orig.fa_k1	OEZ008529	yes	LMSG_G000010674.1	OES073713	betalactone	Others	Lead-Zinc	24.71	China: Hunan, Shuikoushan
L180206P-7-LD180201-0015_HK72WCCXY_L7_clean_bin.2.orig.fa_k1	OEZ008529	yes	LMSG_G000010674.1	OES073713	terpene	Terpene	Lead-Zinc	13.56	China: Hunan, Shuikoushan
L180206P-7-LD180201-0015_HK72WCCXY_L7_clean_bin.2.orig.fa_k1	OEZ008529	yes	LMSG_G000010674.1	OES073713	bacteriocin	RiPPs	Lead-Zinc	10.89	China: Hunan, Shuikoushan
L180206P-7-LD180201-0015_HK72WCCXY_L7_clean_bin.2.orig.fa_k1	OEZ008529	yes	LMSG_G000010674.1	OES073713	NRPS.T1PKS	PKS-NRP_Hybrids	Lead-Zinc	52.10	China: Hunan, Shuikoushan
L180206P-7-LD180201-0015_HK72WCCXY_L7_clean_bin.21.orig.fa_k	OEZ008529	yes	LMSG_G000005063.1	OES073713	acyl_amino_acids	Others	Lead-Zinc	12.90	China: Hunan, Shuikoushan
L180206P-7-LD180201-0015_HK72WCCXY_L7_clean_bin.21.orig.fa_k	OEZ008529	yes	LMSG_G000005063.1	OES073713	terpene	Terpene	Lead-Zinc	21.68	China: Hunan, Shuikoushan
L180206P-7-LD180201-0015_HK72WCCXY_L7_clean_bin.21.orig.fa_k	OEZ008529	yes	LMSG_G000005063.1	OES073713	hserlactone	Others	Lead-Zinc	12.75	China: Hunan, Shuikoushan
L180206P-7-LD180201-0015_HK72WCCXY_L7_clean_bin.21.orig.fa_k	OEZ008529	yes	LMSG_G000005063.1	OES073713	NRPS-like	NRPS	Lead-Zinc	15.09	China: Hunan, Shuikoushan
L180206P-7-LD180201-0015_HK72WCCXY_L7_clean_bin.22.orig.fa_k	OEZ008529	yes	LMSG_G000009084.1	OES073713	betalactone	Others	Lead-Zinc	12.84	China: Hunan, Shuikoushan
L180206P-7-LD180201-0015_HK72WCCXY_L7_clean_bin.22.orig.fa_k	OEZ008529	yes	LMSG_G000009084.1	OES073713	terpene	Terpene	Lead-Zinc	11.87	China: Hunan, Shuikoushan
L180206P-7-LD180201-0015_HK72WCCXY_L7_clean_bin.22.orig.fa_k	OEZ008529	yes	LMSG_G000009084.1	OES073713	betalactone	Others	Lead-Zinc	7.03	China: Hunan, Shuikoushan
L180206P-7-LD180201-0015_HK72WCCXY_L7_clean_bin.23.orig.fa_k	OEZ008529	no	LMSG_G000010505.1	OES073713	arylpolyene	Others	Lead-Zinc	6.80	China: Hunan, Shuikoushan
L180206P-7-LD180201-0015_HK72WCCXY_L7_clean_bin.23.orig.fa_k	OEZ008529	yes	LMSG_G000010505.1	OES073713	terpene	Terpene	Lead-Zinc	6.08	China: Hunan, Shuikoushan
L180206P-7-LD180201-0015_HK72WCCXY_L7_clean_bin.24.orig.fa_k	OEZ008529	yes	LMSG_G000005064.1	OES073713	terpene	Terpene	Lead-Zinc	23.42	China: Hunan, Shuikoushan
L180206P-7-LD180201-0015_HK72WCCXY_L7_clean_bin.24.orig.fa_k	OEZ008529	yes	LMSG_G000005064.1	OES073713	NRPS-like	NRPS	Lead-Zinc	43.86	China: Hunan, Shuikoushan
L180206P-7-LD180201-0015_HK72WCCXY_L7_clean_bin.24.orig.fa_k	OEZ008529	yes	LMSG_G000005064.1	OES073713	betalactone	Others	Lead-Zinc	28.19	China: Hunan, Shuikoushan
L180206P-7-LD180201-0015_HK72WCCXY_L7_clean_bin.25.orig.fa_k	OEZ008529	yes	LMSG_G000005065.1	OES073713	NRPS-like	NRPS	Lead-Zinc	10.67	China: Hunan, Shuikoushan
L180206P-7-LD180201-0015_HK72WCCXY_L7_clean_bin.25.orig.fa_k	OEZ008529	yes	LMSG_G000005065.1	OES073713	betalactone	Others	Lead-Zinc	24.27	China: Hunan, Shuikoushan
L180206P-7-LD180201-0015_HK72WCCXY_L7_clean_bin.26.orig.fa_k	OEZ008529	no	LMSG_G000010719.1	OES073713	T1PKS	PKSI	Lead-Zinc	5.26	China: Hunan, Shuikoushan
L180206P-7-LD180201-0015_HK72WCCXY_L7_clean_bin.26.orig.fa_k	OEZ008529	yes	LMSG_G000010719.1	OES073713	bacteriocin	RiPPs	Lead-Zinc	10.93	China: Hunan, Shuikoushan
L180206P-7-LD180201-0015_HK72WCCXY_L7_clean_bin.26.orig.fa_k	OEZ008529	yes	LMSG_G000010719.1	OES073713	arylpolyene	Others	Lead-Zinc	14.68	China: Hunan, Shuikoushan
L180206P-7-LD180201-0015_HK72WCCXY_L7_clean_bin.26.orig.fa_k	OEZ008529	yes	LMSG_G000010719.1	OES073713	NRPS-like	NRPS	Lead-Zinc	19.53	China: Hunan, Shuikoushan
L180206P-7-LD180201-0015_HK72WCCXY_L7_clean_bin.26.orig.fa_k	OEZ008529	yes	LMSG_G000010719.1	OES073713	NRPS	NRPS	Lead-Zinc	5.02	China: Hunan, Shuikoushan
L180206P-7-LD180201-0015_HK72WCCXY_L7_clean_bin.26.orig.fa_k	OEZ008529	yes	LMSG_G000010719.1	OES073713	NRPS	NRPS	Lead-Zinc	17.34	China: Hunan, Shuikoushan
L180206P-7-LD180201-0015_HK72WCCXY_L7_clean_bin.26.orig.fa_k	OEZ008529	yes	LMSG_G000010719.1	OES073713	terpene	Terpene	Lead-Zinc	10.93	China: Hunan, Shuikoushan
L180206P-7-LD180201-0015_HK72WCCXY_L7_clean_bin.28.orig.fa_k	OEZ008529	yes	LMSG_G000005066.1	OES073713	terpene	Terpene	Lead-Zinc	18.78	China: Hunan, Shuikoushan
L180206P-7-LD180201-0015_HK72WCCXY_L7_clean_bin.28.orig.fa_k	OEZ008529	yes	LMSG_G000005066.1	OES073713	T1PKS	PKSI	Lead-Zinc	8.23	China: Hunan, Shuikoushan
L180206P-7-LD180201-0015_HK72WCCXY_L7_clean_bin.28.orig.fa_k	OEZ008529	yes	LMSG_G000005066.1	OES073713	terpene	Terpene	Lead-Zinc	19.50	China: Hunan, Shuikoushan
L180206P-7-LD180201-0015_HK72WCCXY_L7_clean_bin.29.orig.fa_k	OEZ008529	yes	LMSG_G000010394.1	OES073713	hserlactone	Others	Lead-Zinc	13.36	China: Hunan, Shuikoushan
L180206P-7-LD180201-0015_HK72WCCXY_L7_clean_bin.29.orig.fa_k	OEZ008529	yes	LMSG_G000010394.1	OES073713	bacteriocin	RiPPs	Lead-Zinc	10.81	China: Hunan, Shuikoushan
L180206P-7-LD180201-0015_HK72WCCXY_L7_clean_bin.29.orig.fa_k	OEZ008529	yes	LMSG_G000010394.1	OES073713	terpene	Terpene	Lead-Zinc	14.62	China: Hunan, Shuikoushan
L180206P-7-LD180201-0015_HK72WCCXY_L7_clean_bin.29.orig.fa_k	OEZ008529	yes	LMSG_G000010394.1	OES073713	T3PKS	PKSother	Lead-Zinc	32.00	China: Hunan, Shuikoushan
L180206P-7-LD180201-0015_HK72WCCXY_L7_clean_bin.29.orig.fa_k	OEZ008529	yes	LMSG_G000010394.1	OES073713	butyrolactone	Others	Lead-Zinc	6.57	China: Hunan, Shuikoushan
L180206P-7-LD180201-0015_HK72WCCXY_L7_clean_bin.29.orig.fa_k	OEZ008529	yes	LMSG_G000010394.1	OES073713	hserlactone	Others	Lead-Zinc	11.31	China: Hunan, Shuikoushan
L180206P-7-LD180201-0015_HK72WCCXY_L7_clean_bin.29.orig.fa_k	OEZ008529	yes	LMSG_G000010394.1	OES073713	arylpolyene	Others	Lead-Zinc	29.45	China: Hunan, Shuikoushan
L180206P-7-LD180201-0015_HK72WCCXY_L7_clean_bin.29.orig.fa_k	OEZ008529	yes	LMSG_G000010394.1	OES073713	NRPS-like	NRPS	Lead-Zinc	44.05	China: Hunan, Shuikoushan
L180206P-7-LD180201-0015_HK72WCCXY_L7_clean_bin.29.orig.fa_k	OEZ008529	yes	LMSG_G000010394.1	OES073713	butyrolactone	Others	Lead-Zinc	10.71	China: Hunan, Shuikoushan
L180206P-7-LD180201-0015_HK72WCCXY_L7_clean_bin.29.orig.fa_k	OEZ008529	yes	LMSG_G000010394.1	OES073713	terpene	Terpene	Lead-Zinc	14.55	China: Hunan, Shuikoushan
L180206P-7-LD180201-0015_HK72WCCXY_L7_clean_bin.3.strict.fa_	OEZ008529	yes	LMSG_G000007974.1	OES073713	bacteriocin	RiPPs	Lead-Zinc	5.50	China: Hunan, Shuikoushan
L180206P-7-LD180201-0015_HK72WCCXY_L7_clean_bin.31.orig.fa_k	OEZ008529	yes	LMSG_G000007816.1	OES073713	T3PKS	PKSother	Lead-Zinc	32.06	China: Hunan, Shuikoushan
L180206P-7-LD180201-0015_HK72WCCXY_L7_clean_bin.31.orig.fa_k	OEZ008529	yes	LMSG_G000007816.1	OES073713	terpene	Terpene	Lead-Zinc	28.83	China: Hunan, Shuikoushan
L180206P-7-LD180201-0015_HK72WCCXY_L7_clean_bin.33.orig.fa_k	OEZ008529	yes	LMSG_G000010869.1	OES073713	terpene	Terpene	Lead-Zinc	7.42	China: Hunan, Shuikoushan
L180206P-7-LD180201-0015_HK72WCCXY_L7_clean_bin.33.orig.fa_k	OEZ008529	yes	LMSG_G000010869.1	OES073713	bacteriocin	RiPPs	Lead-Zinc	7.34	China: Hunan, Shuikoushan
L180206P-7-LD180201-0015_HK72WCCXY_L7_clean_bin.34.orig.fa_k	OEZ008529	yes	LMSG_G000008173.1	OES073713	terpene	Terpene	Lead-Zinc	21.08	China: Hunan, Shuikoushan
L180206P-7-LD180201-0015_HK72WCCXY_L7_clean_bin.35.orig.fa_k	OEZ008529	yes	LMSG_G000010744.1	OES073713	bacteriocin	RiPPs	Lead-Zinc	10.84	China: Hunan, Shuikoushan
L180206P-7-LD180201-0015_HK72WCCXY_L7_clean_bin.37.orig.fa_k	OEZ008529	yes	LMSG_G000007050.1	OES073713	bacteriocin	RiPPs	Lead-Zinc	9.93	China: Hunan, Shuikoushan
L180206P-7-LD180201-0015_HK72WCCXY_L7_clean_bin.38.strict.fa	OEZ008529	yes	LMSG_G000009331.1	OES073713	sactipeptide	RiPPs	Lead-Zinc	20.14	China: Hunan, Shuikoushan
L180206P-7-LD180201-0015_HK72WCCXY_L7_clean_bin.38.strict.fa	OEZ008529	yes	LMSG_G000009331.1	OES073713	betalactone	Others	Lead-Zinc	24.45	China: Hunan, Shuikoushan
L180206P-7-LD180201-0015_HK72WCCXY_L7_clean_bin.38.strict.fa	OEZ008529	yes	LMSG_G000009331.1	OES073713	betalactone	Others	Lead-Zinc	20.71	China: Hunan, Shuikoushan
L180206P-7-LD180201-0015_HK72WCCXY_L7_clean_bin.4.orig.fa_k1	OEZ008529	yes	LMSG_G000006313.1	OES073713	T1PKS	PKSI	Lead-Zinc	43.37	China: Hunan, Shuikoushan
L180206P-7-LD180201-0015_HK72WCCXY_L7_clean_bin.4.orig.fa_k1	OEZ008529	yes	LMSG_G000006313.1	OES073713	hserlactone	Others	Lead-Zinc	20.66	China: Hunan, Shuikoushan
L180206P-7-LD180201-0015_HK72WCCXY_L7_clean_bin.4.orig.fa_k1	OEZ008529	yes	LMSG_G000006313.1	OES073713	terpene	Terpene	Lead-Zinc	18.35	China: Hunan, Shuikoushan
L180206P-7-LD180201-0015_HK72WCCXY_L7_clean_bin.4.orig.fa_k1	OEZ008529	yes	LMSG_G000006313.1	OES073713	thiopeptide	RiPPs	Lead-Zinc	23.84	China: Hunan, Shuikoushan
L180206P-7-LD180201-0015_HK72WCCXY_L7_clean_bin.41.permissiv	OEZ008529	yes	LMSG_G000009655.1	OES073713	terpene	Terpene	Lead-Zinc	17.11	China: Hunan, Shuikoushan
L180206P-7-LD180201-0015_HK72WCCXY_L7_clean_bin.42.orig.fa_k	OEZ008529	yes	LMSG_G000010979.1	OES073713	T1PKS	PKSI	Lead-Zinc	23.46	China: Hunan, Shuikoushan
L180206P-7-LD180201-0015_HK72WCCXY_L7_clean_bin.43.strict.fa	OEZ008529	yes	LMSG_G000005067.1	OES073713	T1PKS	PKSI	Lead-Zinc	25.60	China: Hunan, Shuikoushan
L180206P-7-LD180201-0015_HK72WCCXY_L7_clean_bin.43.strict.fa	OEZ008529	yes	LMSG_G000005067.1	OES073713	bacteriocin	RiPPs	Lead-Zinc	10.74	China: Hunan, Shuikoushan
L180206P-7-LD180201-0015_HK72WCCXY_L7_clean_bin.43.strict.fa	OEZ008529	yes	LMSG_G000005067.1	OES073713	arylpolyene	Others	Lead-Zinc	21.01	China: Hunan, Shuikoushan
L180206P-7-LD180201-0015_HK72WCCXY_L7_clean_bin.43.strict.fa	OEZ008529	yes	LMSG_G000005067.1	OES073713	terpene	Terpene	Lead-Zinc	9.32	China: Hunan, Shuikoushan
L180206P-7-LD180201-0015_HK72WCCXY_L7_clean_bin.46.orig.fa_k	OEZ008529	yes	LMSG_G000008603.1	OES073713	other	Others	Lead-Zinc	22.63	China: Hunan, Shuikoushan
L180206P-7-LD180201-0015_HK72WCCXY_L7_clean_bin.46.orig.fa_k	OEZ008529	yes	LMSG_G000008603.1	OES073713	other	Others	Lead-Zinc	23.10	China: Hunan, Shuikoushan
L180206P-7-LD180201-0015_HK72WCCXY_L7_clean_bin.47.orig.fa_k	OEZ008529	yes	LMSG_G000005068.1	OES073713	NRPS-like	NRPS	Lead-Zinc	18.75	China: Hunan, Shuikoushan
L180206P-7-LD180201-0015_HK72WCCXY_L7_clean_bin.52.strict.fa	OEZ008529	yes	LMSG_G000005069.1	OES073713	terpene	Terpene	Lead-Zinc	11.42	China: Hunan, Shuikoushan
L180206P-7-LD180201-0015_HK72WCCXY_L7_clean_bin.52.strict.fa	OEZ008529	yes	LMSG_G000005069.1	OES073713	ectoine	Others	Lead-Zinc	10.40	China: Hunan, Shuikoushan
L180206P-7-LD180201-0015_HK72WCCXY_L7_clean_bin.52.strict.fa	OEZ008529	yes	LMSG_G000005069.1	OES073713	T3PKS	PKSother	Lead-Zinc	23.96	China: Hunan, Shuikoushan
L180206P-7-LD180201-0015_HK72WCCXY_L7_clean_bin.52.strict.fa	OEZ008529	yes	LMSG_G000005069.1	OES073713	ladderane	Others	Lead-Zinc	26.66	China: Hunan, Shuikoushan
L180206P-7-LD180201-0015_HK72WCCXY_L7_clean_bin.52.strict.fa	OEZ008529	yes	LMSG_G000005069.1	OES073713	terpene	Terpene	Lead-Zinc	12.58	China: Hunan, Shuikoushan
L180206P-7-LD180201-0015_HK72WCCXY_L7_clean_bin.52.strict.fa	OEZ008529	yes	LMSG_G000005069.1	OES073713	arylpolyene	Others	Lead-Zinc	23.31	China: Hunan, Shuikoushan
L180206P-7-LD180201-0015_HK72WCCXY_L7_clean_bin.52.strict.fa	OEZ008529	yes	LMSG_G000005069.1	OES073713	terpene	Terpene	Lead-Zinc	13.49	China: Hunan, Shuikoushan
L180206P-7-LD180201-0015_HK72WCCXY_L7_clean_bin.53.orig.fa_k	OEZ008529	yes	LMSG_G000004412.1	OES073713	T3PKS	PKSother	Lead-Zinc	10.68	China: Hunan, Shuikoushan
L180206P-7-LD180201-0015_HK72WCCXY_L7_clean_bin.53.orig.fa_k	OEZ008529	yes	LMSG_G000004412.1	OES073713	butyrolactone	Others	Lead-Zinc	10.88	China: Hunan, Shuikoushan
L180206P-7-LD180201-0015_HK72WCCXY_L7_clean_bin.53.orig.fa_k	OEZ008529	yes	LMSG_G000004412.1	OES073713	bacteriocin	RiPPs	Lead-Zinc	7.70	China: Hunan, Shuikoushan
L180206P-7-LD180201-0015_HK72WCCXY_L7_clean_bin.55.orig.fa_k	OEZ008529	yes	LMSG_G000008435.1	OES073713	terpene	Terpene	Lead-Zinc	26.50	China: Hunan, Shuikoushan
L180206P-7-LD180201-0015_HK72WCCXY_L7_clean_bin.57.orig.fa_k	OEZ008529	yes	LMSG_G000010388.1	OES073713	betalactone	Others	Lead-Zinc	10.67	China: Hunan, Shuikoushan
L180206P-7-LD180201-0015_HK72WCCXY_L7_clean_bin.57.orig.fa_k	OEZ008529	yes	LMSG_G000010388.1	OES073713	T3PKS	PKSother	Lead-Zinc	13.28	China: Hunan, Shuikoushan
L180206P-7-LD180201-0015_HK72WCCXY_L7_clean_bin.57.orig.fa_k	OEZ008529	yes	LMSG_G000010388.1	OES073713	terpene	Terpene	Lead-Zinc	8.74	China: Hunan, Shuikoushan
L180206P-7-LD180201-0015_HK72WCCXY_L7_clean_bin.57.orig.fa_k	OEZ008529	yes	LMSG_G000010388.1	OES073713	terpene	Terpene	Lead-Zinc	14.79	China: Hunan, Shuikoushan
L180206P-7-LD180201-0015_HK72WCCXY_L7_clean_bin.57.orig.fa_k	OEZ008529	yes	LMSG_G000010388.1	OES073713	NRPS-like.bacteriocin	Others	Lead-Zinc	12.16	China: Hunan, Shuikoushan
L180206P-7-LD180201-0015_HK72WCCXY_L7_clean_bin.6.permissive	OEZ008529	yes	LMSG_G000008123.1	OES073713	terpene	Terpene	Lead-Zinc	11.42	China: Hunan, Shuikoushan
L180206P-7-LD180201-0015_HK72WCCXY_L7_clean_bin.6.permissive	OEZ008529	yes	LMSG_G000008123.1	OES073713	terpene	Terpene	Lead-Zinc	6.58	China: Hunan, Shuikoushan
L180206P-7-LD180201-0015_HK72WCCXY_L7_clean_bin.60.orig.fa_k	OEZ008529	yes	LMSG_G000010121.1	OES073713	terpene	Terpene	Lead-Zinc	18.34	China: Hunan, Shuikoushan
L180206P-7-LD180201-0015_HK72WCCXY_L7_clean_bin.60.orig.fa_k	OEZ008529	yes	LMSG_G000010121.1	OES073713	bacteriocin	RiPPs	Lead-Zinc	10.84	China: Hunan, Shuikoushan
L180206P-7-LD180201-0015_HK72WCCXY_L7_clean_bin.60.orig.fa_k	OEZ008529	yes	LMSG_G000010121.1	OES073713	arylpolyene	Others	Lead-Zinc	29.34	China: Hunan, Shuikoushan
L180206P-7-LD180201-0015_HK72WCCXY_L7_clean_bin.62.orig.fa_k	OEZ008529	yes	LMSG_G000010908.1	OES073713	terpene	Terpene	Lead-Zinc	5.77	China: Hunan, Shuikoushan
L180206P-7-LD180201-0015_HK72WCCXY_L7_clean_bin.63.orig.fa_k	OEZ008529	no	LMSG_G000010949.1	OES073713	bacteriocin	RiPPs	Lead-Zinc	7.07	China: Hunan, Shuikoushan
L180206P-7-LD180201-0015_HK72WCCXY_L7_clean_bin.63.orig.fa_k	OEZ008529	no	LMSG_G000010949.1	OES073713	bacteriocin	RiPPs	Lead-Zinc	8.45	China: Hunan, Shuikoushan
L180206P-7-LD180201-0015_HK72WCCXY_L7_clean_bin.63.orig.fa_k	OEZ008529	no	LMSG_G000010949.1	OES073713	NRPS	NRPS	Lead-Zinc	52.29	China: Hunan, Shuikoushan
L180206P-7-LD180201-0015_HK72WCCXY_L7_clean_bin.63.orig.fa_k	OEZ008529	no	LMSG_G000010949.1	OES073713	NRPS.T1PKS	PKS-NRP_Hybrids	Lead-Zinc	59.17	China: Hunan, Shuikoushan
L180206P-7-LD180201-0015_HK72WCCXY_L7_clean_bin.63.orig.fa_k	OEZ008529	no	LMSG_G000010949.1	OES073713	thiopeptide	RiPPs	Lead-Zinc	18.83	China: Hunan, Shuikoushan
L180206P-7-LD180201-0015_HK72WCCXY_L7_clean_bin.64.orig.fa_k	OEZ008529	yes	LMSG_G000010923.1	OES073713	terpene	Terpene	Lead-Zinc	18.81	China: Hunan, Shuikoushan
L180206P-7-LD180201-0015_HK72WCCXY_L7_clean_bin.64.orig.fa_k	OEZ008529	yes	LMSG_G000010923.1	OES073713	arylpolyene	Others	Lead-Zinc	29.67	China: Hunan, Shuikoushan
L180206P-7-LD180201-0015_HK72WCCXY_L7_clean_bin.65.orig.fa_k	OEZ008529	yes	LMSG_G000010391.1	OES073713	terpene	Terpene	Lead-Zinc	21.77	China: Hunan, Shuikoushan
L180206P-7-LD180201-0015_HK72WCCXY_L7_clean_bin.65.orig.fa_k	OEZ008529	yes	LMSG_G000010391.1	OES073713	terpene	Terpene	Lead-Zinc	23.70	China: Hunan, Shuikoushan
L180206P-7-LD180201-0015_HK72WCCXY_L7_clean_bin.65.orig.fa_k	OEZ008529	yes	LMSG_G000010391.1	OES073713	ectoine	Others	Lead-Zinc	10.39	China: Hunan, Shuikoushan
L180206P-7-LD180201-0015_HK72WCCXY_L7_clean_bin.65.orig.fa_k	OEZ008529	yes	LMSG_G000010391.1	OES073713	cyanobactin	RiPPs	Lead-Zinc	21.89	China: Hunan, Shuikoushan
L180206P-7-LD180201-0015_HK72WCCXY_L7_clean_bin.65.orig.fa_k	OEZ008529	yes	LMSG_G000010391.1	OES073713	bacteriocin	RiPPs	Lead-Zinc	10.89	China: Hunan, Shuikoushan
L180206P-7-LD180201-0015_HK72WCCXY_L7_clean_bin.65.orig.fa_k	OEZ008529	yes	LMSG_G000010391.1	OES073713	NRPS.T1PKS	PKS-NRP_Hybrids	Lead-Zinc	92.33	China: Hunan, Shuikoushan
L180206P-7-LD180201-0015_HK72WCCXY_L7_clean_bin.67.orig.fa_k	OEZ008529	yes	LMSG_G000005071.1	OES073713	terpene	Terpene	Lead-Zinc	22.01	China: Hunan, Shuikoushan

L180206P-7-LD180201-0015_HK72WCCXY_L7_clean_bin.67.orig.fa_k	OEZ008529	yes	LMSG_G000005071.1	OES073713	terpene	Terpene	Lead-Zinc	16.42	China: Hunan, Shuikoushan
L180206P-7-LD180201-0015_HK72WCCXY_L7_clean_bin.67.orig.fa_k	OEZ008529	yes	LMSG_G000005071.1	OES073713	NRPS-like	NRPS	Lead-Zinc	42.63	China: Hunan, Shuikoushan
L180206P-7-LD180201-0015_HK72WCCXY_L7_clean_bin.68.orig.fa_k	OEZ008529	yes	LMSG_G000009262.1	OES073713	terpene	Terpene	Lead-Zinc	11.78	China: Hunan, Shuikoushan
L180206P-7-LD180201-0015_HK72WCCXY_L7_clean_bin.68.orig.fa_k	OEZ008529	yes	LMSG_G000009262.1	OES073713	TfuA-related	RiPPs	Lead-Zinc	12.36	China: Hunan, Shuikoushan
L180206P-7-LD180201-0015_HK72WCCXY_L7_clean_bin.71.orig.fa_k	OEZ008529	yes	LMSG_G000005072.1	OES073713	terpene	Terpene	Lead-Zinc	17.98	China: Hunan, Shuikoushan
L180206P-7-LD180201-0015_HK72WCCXY_L7_clean_bin.71.orig.fa_k	OEZ008529	yes	LMSG_G000005072.1	OES073713	arylpolyene	Others	Lead-Zinc	16.86	China: Hunan, Shuikoushan
L180206P-7-LD180201-0015_HK72WCCXY_L7_clean_bin.71.orig.fa_k	OEZ008529	yes	LMSG_G000005072.1	OES073713	NRPS.T1PKS	PKS-NRP_Hybrids	Lead-Zinc	34.45	China: Hunan, Shuikoushan
L180206P-7-LD180201-0015_HK72WCCXY_L7_clean_bin.71.orig.fa_k	OEZ008529	yes	LMSG_G000005072.1	OES073713	bacteriocin	RiPPs	Lead-Zinc	8.06	China: Hunan, Shuikoushan
L180206P-7-LD180201-0015_HK72WCCXY_L7_clean_bin.71.orig.fa_k	OEZ008529	yes	LMSG_G000005072.1	OES073713	terpene	Terpene	Lead-Zinc	10.65	China: Hunan, Shuikoushan
L180206P-7-LD180201-0015_HK72WCCXY_L7_clean_bin.71.orig.fa_k	OEZ008529	yes	LMSG_G000005072.1	OES073713	NRPS	NRPS	Lead-Zinc	7.50	China: Hunan, Shuikoushan
L180206P-7-LD180201-0015_HK72WCCXY_L7_clean_bin.71.orig.fa_k	OEZ008529	yes	LMSG_G000005072.1	OES073713	terpene	Terpene	Lead-Zinc	13.87	China: Hunan, Shuikoushan
L180206P-7-LD180201-0015_HK72WCCXY_L7_clean_bin.72.orig.fa_k	OEZ008529	yes	LMSG_G000008584.1	OES073713	other	Others	Lead-Zinc	40.41	China: Hunan, Shuikoushan
L180206P-7-LD180201-0015_HK72WCCXY_L7_clean_bin.72.orig.fa_k	OEZ008529	yes	LMSG_G000008584.1	OES073713	other	Others	Lead-Zinc	29.65	China: Hunan, Shuikoushan
L180206P-7-LD180201-0015_HK72WCCXY_L7_clean_bin.73.orig.fa_k	OEZ008529	yes	LMSG_G000008633.1	OES073713	NRPS-like	NRPS	Lead-Zinc	23.36	China: Hunan, Shuikoushan
L180206P-7-LD180201-0015_HK72WCCXY_L7_clean_bin.73.orig.fa_k	OEZ008529	yes	LMSG_G000008633.1	OES073713	other	Others	Lead-Zinc	22.85	China: Hunan, Shuikoushan
L180206P-7-LD180201-0015_HK72WCCXY_L7_clean_bin.76.orig.fa_k	OEZ008529	yes	LMSG_G000005074.1	OES073713	terpene	Terpene	Lead-Zinc	23.27	China: Hunan, Shuikoushan
L180206P-7-LD180201-0015_HK72WCCXY_L7_clean_bin.76.orig.fa_k	OEZ008529	yes	LMSG_G000005074.1	OES073713	NRPS-like	NRPS	Lead-Zinc	5.10	China: Hunan, Shuikoushan
L180206P-7-LD180201-0015_HK72WCCXY_L7_clean_bin.76.orig.fa_k	OEZ008529	yes	LMSG_G000005074.1	OES073713	terpene	Terpene	Lead-Zinc	9.87	China: Hunan, Shuikoushan
L180206P-7-LD180201-0015_HK72WCCXY_L7_clean_bin.76.orig.fa_k	OEZ008529	yes	LMSG_G000005074.1	OES073713	NRPS-like	NRPS	Lead-Zinc	37.36	China: Hunan, Shuikoushan
L180206P-7-LD180201-0015_HK72WCCXY_L7_clean_bin.76.orig.fa_k	OEZ008529	yes	LMSG_G000005074.1	OES073713	T3PKS	PKSother	Lead-Zinc	21.64	China: Hunan, Shuikoushan
L180206P-7-LD180201-0015_HK72WCCXY_L7_clean_bin.77.orig.fa_k	OEZ008529	yes	LMSG_G000005075.1	OES073713	terpene	Terpene	Lead-Zinc	11.13	China: Hunan, Shuikoushan
L180206P-7-LD180201-0015_HK72WCCXY_L7_clean_bin.77.orig.fa_k	OEZ008529	yes	LMSG_G000005075.1	OES073713	NRPS	NRPS	Lead-Zinc	19.64	China: Hunan, Shuikoushan
L180206P-7-LD180201-0015_HK72WCCXY_L7_clean_bin.77.orig.fa_k	OEZ008529	yes	LMSG_G000005075.1	OES073713	bacteriocin	RiPPs	Lead-Zinc	6.50	China: Hunan, Shuikoushan
L180206P-7-LD180201-0015_HK72WCCXY_L7_clean_bin.77.orig.fa_k	OEZ008529	yes	LMSG_G000005075.1	OES073713	arylpolyene	Others	Lead-Zinc	41.27	China: Hunan, Shuikoushan
L180206P-7-LD180201-0015_HK72WCCXY_L7_clean_bin.78.orig.fa_k	OEZ008529	yes	LMSG_G000010600.1	OES073713	terpene	Terpene	Lead-Zinc	5.08	China: Hunan, Shuikoushan
L180206P-7-LD180201-0015_HK72WCCXY_L7_clean_bin.80.orig.fa_k	OEZ008529	yes	LMSG_G000005077.1	OES073713	acyl_amino_acids	Others	Lead-Zinc	28.21	China: Hunan, Shuikoushan
L180206P-7-LD180201-0015_HK72WCCXY_L7_clean_bin.80.orig.fa_k	OEZ008529	yes	LMSG_G000005077.1	OES073713	bacteriocin	RiPPs	Lead-Zinc	11.37	China: Hunan, Shuikoushan
L180206P-7-LD180201-0015_HK72WCCXY_L7_clean_bin.80.orig.fa_k	OEZ008529	yes	LMSG_G000005077.1	OES073713	acyl_amino_acids	Others	Lead-Zinc	40.34	China: Hunan, Shuikoushan
L180206P-7-LD180201-0015_HK72WCCXY_L7_clean_bin.80.orig.fa_k	OEZ008529	yes	LMSG_G000005077.1	OES073713	NRPS-like.hserlactone	Others	Lead-Zinc	45.08	China: Hunan, Shuikoushan
L180206P-7-LD180201-0015_HK72WCCXY_L7_clean_bin.80.orig.fa_k	OEZ008529	yes	LMSG_G000005077.1	OES073713	bacteriocin	RiPPs	Lead-Zinc	10.27	China: Hunan, Shuikoushan
L180206P-7-LD180201-0015_HK72WCCXY_L7_clean_bin.80.orig.fa_k	OEZ008529	yes	LMSG_G000005077.1	OES073713	terpene	Terpene	Lead-Zinc	15.34	China: Hunan, Shuikoushan
L180206P-7-LD180201-0015_HK72WCCXY_L7_clean_bin.80.orig.fa_k	OEZ008529	yes	LMSG_G000005077.1	OES073713	terpene	Terpene	Lead-Zinc	20.80	China: Hunan, Shuikoushan
L180206P-7-LD180201-0015_HK72WCCXY_L7_clean_bin.81.strict.fa	OEZ008529	yes	LMSG_G000009770.1	OES073713	LAP	RiPPs	Lead-Zinc	6.88	China: Hunan, Shuikoushan
L180206P-7-LD180201-0015_HK72WCCXY_L7_clean_bin.9.orig.fa_k1	OEZ008529	yes	LMSG_G000010891.1	OES073713	arylpolyene	Others	Lead-Zinc	5.78	China: Hunan, Shuikoushan
L180206P-7-LD180201-0015_HK72WCCXY_L7_clean_bin.9.orig.fa_k1	OEZ008529	yes	LMSG_G000010891.1	OES073713	terpene	Terpene	Lead-Zinc	13.40	China: Hunan, Shuikoushan
L180206P-7-LD180201-0015_HK72WCCXY_L7_clean_bin.9.orig.fa_k1	OEZ008529	yes	LMSG_G000010891.1	OES073713	arylpolyene	Others	Lead-Zinc	17.77	China: Hunan, Shuikoushan
L180206P-7-LD180205-0001_HK72WCCXY_L7_clean_bin.1.orig.fa_k1	OEZ008529	no	LMSG_G000010623.1	OES073746	terpene	Terpene	Lead-Zinc	6.43	China: Jiangxi, Yinshan
L180206P-7-LD180205-0001_HK72WCCXY_L7_clean_bin.1.orig.fa_k1	OEZ008529	yes	LMSG_G000010623.1	OES073746	arylpolyene	Others	Lead-Zinc	9.46	China: Jiangxi, Yinshan
L180206P-7-LD180205-0001_HK72WCCXY_L7_clean_bin.1.orig.fa_k1	OEZ008529	no	LMSG_G000010623.1	OES073746	terpene	Terpene	Lead-Zinc	6.54	China: Jiangxi, Yinshan
L180206P-7-LD180205-0001_HK72WCCXY_L7_clean_bin.1.orig.fa_k1	OEZ008529	yes	LMSG_G000010623.1	OES073746	bacteriocin	RiPPs	Lead-Zinc	10.99	China: Jiangxi, Yinshan
L180206P-7-LD180205-0001_HK72WCCXY_L7_clean_bin.10.strict.fa	OEZ008529	no	LMSG_G000009563.1	OES073746	terpene	Terpene	Lead-Zinc	20.61	China: Jiangxi, Yinshan
L180206P-7-LD180205-0001_HK72WCCXY_L7_clean_bin.12.orig.fa_k	OEZ008529	yes	LMSG_G000010465.1	OES073746	arylpolyene	Others	Lead-Zinc	19.45	China: Jiangxi, Yinshan
L180206P-7-LD180205-0001_HK72WCCXY_L7_clean_bin.12.orig.fa_k	OEZ008529	yes	LMSG_G000010465.1	OES073746	terpene	Terpene	Lead-Zinc	18.90	China: Jiangxi, Yinshan
L180206P-7-LD180205-0001_HK72WCCXY_L7_clean_bin.15.strict.fa	OEZ008529	yes	LMSG_G000008483.1	OES073746	terpene	Terpene	Lead-Zinc	26.09	China: Jiangxi, Yinshan
L180206P-7-LD180205-0001_HK72WCCXY_L7_clean_bin.15.strict.fa	OEZ008529	yes	LMSG_G000008483.1	OES073746	lassopeptide	RiPPs	Lead-Zinc	13.40	China: Jiangxi, Yinshan
L180206P-7-LD180205-0001_HK72WCCXY_L7_clean_bin.16.orig.fa_k	OEZ008529	yes	LMSG_G000009651.1	OES073746	terpene	Terpene	Lead-Zinc	8.90	China: Jiangxi, Yinshan
L180206P-7-LD180205-0001_HK72WCCXY_L7_clean_bin.16.orig.fa_k	OEZ008529	yes	LMSG_G000009651.1	OES073746	NRPS-like	NRPS	Lead-Zinc	6.74	China: Jiangxi, Yinshan
L180206P-7-LD180205-0001_HK72WCCXY_L7_clean_bin.16.orig.fa_k	OEZ008529	yes	LMSG_G000009651.1	OES073746	terpene	Terpene	Lead-Zinc	10.73	China: Jiangxi, Yinshan
L180206P-7-LD180205-0001_HK72WCCXY_L7_clean_bin.16.orig.fa_k	OEZ008529	yes	LMSG_G000009651.1	OES073746	terpene	Terpene	Lead-Zinc	8.71	China: Jiangxi, Yinshan
L180206P-7-LD180205-0001_HK72WCCXY_L7_clean_bin.19.orig.fa_k	OEZ008529	yes	LMSG_G000004414.1	OES073746	terpene	Terpene	Lead-Zinc	20.99	China: Jiangxi, Yinshan
L180206P-7-LD180205-0001_HK72WCCXY_L7_clean_bin.19.orig.fa_k	OEZ008529	no	LMSG_G000004414.1	OES073746	T3PKS	PKSother	Lead-Zinc	22.75	China: Jiangxi, Yinshan
L180206P-7-LD180205-0001_HK72WCCXY_L7_clean_bin.19.orig.fa_k	OEZ008529	no	LMSG_G000004414.1	OES073746	terpene	Terpene	Lead-Zinc	21.18	China: Jiangxi, Yinshan
L180206P-7-LD180205-0001_HK72WCCXY_L7_clean_bin.19.orig.fa_k	OEZ008529	no	LMSG_G000004414.1	OES073746	bacteriocin	RiPPs	Lead-Zinc	10.88	China: Jiangxi, Yinshan
L180206P-7-LD180205-0001_HK72WCCXY_L7_clean_bin.2.orig.fa_k1	OEZ008529	yes	LMSG_G000009546.1	OES073746	ectoine	Others	Lead-Zinc	7.28	China: Jiangxi, Yinshan
L180206P-7-LD180205-0001_HK72WCCXY_L7_clean_bin.2.orig.fa_k1	OEZ008529	no	LMSG_G000009546.1	OES073746	terpene	Terpene	Lead-Zinc	20.96	China: Jiangxi, Yinshan
L180206P-7-LD180205-0001_HK72WCCXY_L7_clean_bin.2.orig.fa_k1	OEZ008529	yes	LMSG_G000009546.1	OES073746	NRPS-like	NRPS	Lead-Zinc	42.73	China: Jiangxi, Yinshan
L180206P-7-LD180205-0001_HK72WCCXY_L7_clean_bin.2.orig.fa_k1	OEZ008529	yes	LMSG_G000009546.1	OES073746	ectoine	Others	Lead-Zinc	6.73	China: Jiangxi, Yinshan
L180206P-7-LD180205-0001_HK72WCCXY_L7_clean_bin.2.orig.fa_k1	OEZ008529	yes	LMSG_G000009546.1	OES073746	terpene	Terpene	Lead-Zinc	22.06	China: Jiangxi, Yinshan
L180206P-7-LD180205-0001_HK72WCCXY_L7_clean_bin.22.strict.fa	OEZ008529	yes	LMSG_G000011170.1	OES073746	arylpolyene	Others	Lead-Zinc	14.51	China: Jiangxi, Yinshan
L180206P-7-LD180205-0001_HK72WCCXY_L7_clean_bin.27.orig.fa_k	OEZ008529	no	LMSG_G000010159.1	OES073746	ectoine	Others	Lead-Zinc	7.54	China: Jiangxi, Yinshan
L180206P-7-LD180205-0001_HK72WCCXY_L7_clean_bin.27.orig.fa_k	OEZ008529	no	LMSG_G000010159.1	OES073746	terpene	Terpene	Lead-Zinc	11.44	China: Jiangxi, Yinshan
L180206P-7-LD180205-0001_HK72WCCXY_L7_clean_bin.27.orig.fa_k	OEZ008529	no	LMSG_G000010159.1	OES073746	acyl_amino_acids	Others	Lead-Zinc	15.42	China: Jiangxi, Yinshan
L180206P-7-LD180205-0001_HK72WCCXY_L7_clean_bin.3.strict.fa_	OEZ008529	no	LMSG_G000009533.1	OES073746	terpene	Terpene	Lead-Zinc	22.05	China: Jiangxi, Yinshan
L180206P-7-LD180205-0001_HK72WCCXY_L7_clean_bin.4.orig.fa_k1	OEZ008529	yes	LMSG_G000007830.1	OES073746	T3PKS	PKSother	Lead-Zinc	6.23	China: Jiangxi, Yinshan
L180206P-7-LD180205-0001_HK72WCCXY_L7_clean_bin.4.orig.fa_k1	OEZ008529	yes	LMSG_G000007830.1	OES073746	terpene	Terpene	Lead-Zinc	7.62	China: Jiangxi, Yinshan
L180206P-8-LD180201-0003_HK72WCCXY_L8_clean_bin.1.orig.fa_k1	OEZ008529	yes	LMSG_G000007368.1	OES073747	bacteriocin	RiPPs	Lead-Zinc	11.97	China: Jiangxi, Yinshan
L180206P-8-LD180201-0003_HK72WCCXY_L8_clean_bin.10.orig.fa_k	OEZ008529	yes	LMSG_G000008012.1	OES073747	betalactone	Others	Lead-Zinc	22.75	China: Jiangxi, Yinshan
L180206P-8-LD180201-0003_HK72WCCXY_L8_clean_bin.10.orig.fa_k	OEZ008529	yes	LMSG_G000008012.1	OES073747	bacteriocin	RiPPs	Lead-Zinc	5.13	China: Jiangxi, Yinshan
L180206P-8-LD180201-0003_HK72WCCXY_L8_clean_bin.10.orig.fa_k	OEZ008529	yes	LMSG_G000008012.1	OES073747	bacteriocin	RiPPs	Lead-Zinc	10.30	China: Jiangxi, Yinshan
L180206P-8-LD180201-0003_HK72WCCXY_L8_clean_bin.14.orig.fa_k	OEZ008529	yes	LMSG_G000010031.1	OES073747	terpene	Terpene	Lead-Zinc	5.14	China: Jiangxi, Yinshan
L180206P-8-LD180201-0003_HK72WCCXY_L8_clean_bin.14.orig.fa_k	OEZ008529	yes	LMSG_G000010031.1	OES073747	terpene	Terpene	Lead-Zinc	5.06	China: Jiangxi, Yinshan
L180206P-8-LD180201-0003_HK72WCCXY_L8_clean_bin.15.orig.fa_k	OEZ008529	yes	LMSG_G000008352.1	OES073747	butyrolactone	Others	Lead-Zinc	9.93	China: Jiangxi, Yinshan
L180206P-8-LD180201-0003_HK72WCCXY_L8_clean_bin.15.orig.fa_k	OEZ008529	yes	LMSG_G000008352.1	OES073747	terpene	Terpene	Lead-Zinc	5.87	China: Jiangxi, Yinshan
L180206P-8-LD180201-0003_HK72WCCXY_L8_clean_bin.15.orig.fa_k	OEZ008529	yes	LMSG_G000008352.1	OES073747	bacteriocin	RiPPs	Lead-Zinc	8.92	China: Jiangxi, Yinshan
L180206P-8-LD180201-0003_HK72WCCXY_L8_clean_bin.15.orig.fa_k	OEZ008529	yes	LMSG_G000008352.1	OES073747	terpene	Terpene	Lead-Zinc	7.33	China: Jiangxi, Yinshan
L180206P-8-LD180201-0003_HK72WCCXY_L8_clean_bin.15.orig.fa_k	OEZ008529	yes	LMSG_G000008352.1	OES073747	T3PKS	PKSother	Lead-Zinc	6.02	China: Jiangxi, Yinshan
L180206P-8-LD180201-0003_HK72WCCXY_L8_clean_bin.16.orig.fa_k	OEZ008529	yes	LMSG_G000008484.1	OES073747	terpene	Terpene	Lead-Zinc	26.09	China: Jiangxi, Yinshan
L180206P-8-LD180201-0003_HK72WCCXY_L8_clean_bin.16.orig.fa_k	OEZ008529	yes	LMSG_G000008484.1	OES073747	lassopeptide	RiPPs	Lead-Zinc	7.09	China: Jiangxi, Yinshan
L180206P-8-LD180201-0003_HK72WCCXY_L8_clean_bin.17.orig.fa_k	OEZ008529	yes	LMSG_G000007054.1	OES073747	bacteriocin	RiPPs	Lead-Zinc	10.87	China: Jiangxi, Yinshan
L180206P-8-LD180201-0003_HK72WCCXY_L8_clean_bin.18.orig.fa_k	OEZ008529	yes	LMSG_G000008510.1	OES073747	terpene	Terpene	Lead-Zinc	17.07	China: Jiangxi, Yinshan
L180206P-8-LD180201-0003_HK72WCCXY_L8_clean_bin.18.orig.fa_k	OEZ008529	yes	LMSG_G000008510.1	OES073747	lassopeptide	RiPPs	Lead-Zinc	16.82	China: Jiangxi, Yinshan
L180206P-8-LD180201-0003_HK72WCCXY_L8_clean_bin.2.strict.fa_	OEZ008529	yes	LMSG_G000006787.1	OES073747	hserlactone	Others	Lead-Zinc	7.27	China: Jiangxi, Yinshan
L180206P-8-LD180201-0003_HK72WCCXY_L8_clean_bin.22.orig.fa_k	OEZ008529	yes	LMSG_G000007244.1	OES073747	betalactone	Others	Lead-Zinc	11.32	China: Jiangxi, Yinshan
L180206P-8-LD180201-0003_HK72WCCXY_L8_clean_bin.28.orig.fa_k	OEZ008529	no	LMSG_G000004416.1	OES073747	arylpolyene	Others	Lead-Zinc	26.60	China: Jiangxi, Yinshan
L180206P-8-LD180201-0003_HK72WCCXY_L8_clean_bin.28.orig.fa_k	OEZ008529	no	LMSG_G000004416.1	OES073747	siderophore	Others	Lead-Zinc	18.44	China: Jiangxi, Yinshan
L180206P-8-LD180201-0003_HK72WCCXY_L8_clean_bin.28.orig.fa_k	OEZ008529	no	LMSG_G000004416.1	OES073747	betalactone	Others	Lead-Zinc	29.08	China: Jiangxi, Yinshan
L180206P-8-LD180201-0003_HK72WCCXY_L8_clean_bin.28.orig.fa_k	OEZ008529	no	LMSG_G000004416.1	OES073747	NRPS.arylpolyene.hserlactone	Others	Lead-Zinc	83.19	China: Jiangxi, Yinshan
L180206P-8-LD180201-0003_HK72WCCXY_L8_clean_bin.28.orig.fa_k	OEZ008529	no	LMSG_G000004416.1	OES073747	NRPS	NRPS	Lead-Zinc	56.75	China: Jiangxi, Yinshan
L180206P-8-LD180201-0003_HK72WCCXY_L8_clean_bin.28.orig.fa_k	OEZ008529	no	LMSG_G000004416.1	OES073747	NRPS-like	NRPS	Lead-Zinc	44.00	China: Jiangxi, Yinshan
L180206P-8-LD180201-0003_HK72WCCXY_L8_clean_bin.3.strict.fa_	OEZ008529	yes	LMSG_G000009156.1	OES073747	T3PKS	PKSother	Lead-Zinc	13.77	China: Jiangxi, Yinshan
L180206P-8-LD180201-0003_HK72WCCXY_L8_clean_bin.3.strict.fa_	OEZ008529	yes	LMSG_G000009156.1	OES073747	terpene	Terpene	Lead-Zinc	6.19	China: Jiangxi, Yinshan
L180206P-8-LD180201-0003_HK72WCCXY_L8_clean_bin.31.orig.fa_k	OEZ008529	yes	LMSG_G000008307.1	OES073747	NRPS-like	NRPS	Lead-Zinc	11.26	China: Jiangxi, Yinshan
L180206P-8-LD180201-0003_HK72WCCXY_L8_clean_bin.34.orig.fa_k	OEZ008529	yes	LMSG_G000011062.1	OES073747	NRPS-like.hserlactone	Others	Lead-Zinc	17.26	China: Jiangxi, Yinshan
L180206P-8-LD180201-0003_HK72WCCXY_L8_clean_bin.34.orig.fa_k	OEZ008529	yes	LMSG_G000011062.1	OES073747	lassopeptide	RiPPs	Lead-Zinc	19.14	China: Jiangxi, Yinshan
L180206P-8-LD180201-0003_HK72WCCXY_L8_clean_bin.34.orig.fa_k	OEZ008529	yes	LMSG_G000011062.1	OES073747	NRPS-like	NRPS	Lead-Zinc	8.43	China: Jiangxi, Yinshan
L180206P-8-LD180201-0003_HK72WCCXY_L8_clean_bin.34.orig.fa_k	OEZ008529	yes	LMSG_G000011062.1	OES073747	NRPS-like	NRPS	Lead-Zinc	22.46	China: Jiangxi, Yinshan
L180206P-8-LD180201-0003_HK72WCCXY_L8_clean_bin.34.orig.fa_k	OEZ008529	yes	LMSG_G000011062.1	OES073747	NRPS-like	NRPS	Lead-Zinc	31.17	China: Jiangxi, Yinshan
L180206P-8-LD180201-0003_HK72WCCXY_L8_clean_bin.34.orig.fa_k	OEZ008529	yes	LMSG_G000011062.1	OES073747	NRPS-like	NRPS	Lead-Zinc	31.11	China: Jiangxi, Yinshan
L180206P-8-LD180201-0003_HK72WCCXY_L8_clean_bin.34.orig.fa_k	OEZ008529	yes	LMSG_G000011062.1	OES073747	arylpolyene	Others	Lead-Zinc	23.32	China: Jiangxi, Yinshan
L180206P-8-LD180201-0003_HK72WCCXY_L8_clean_bin.34.orig.fa_k	OEZ008529	yes	LMSG_G000011062.1	OES073747	terpene	Terpene	Lead-Zinc	5.25	China: Jiangxi, Yinshan
L180206P-8-LD180201-0003_HK72WCCXY_L8_clean_bin.34.orig.fa_k	OEZ008529	yes	LMSG_G000011062.1	OES073747	NRPS-like	NRPS	Lead-Zinc	22.20	China: Jiangxi, Yinshan
L180206P-8-LD180201-0003_HK72WCCXY_L8_clean_bin.34.orig.fa_k	OEZ008529	yes	LMSG_G000011062.1	OES073747	bacteriocin	RiPPs	Lead-Zinc	8.13	China: Jiangxi, Yinshan
L180206P-8-LD180201-0003_HK72WCCXY_L8_clean_bin.38.orig.fa_k	OEZ008529	yes	LMSG_G000009431.1	OES073747	betalactone	Others	Lead-Zinc	22.78	China: Jiangxi, Yinshan
L180206P-8-LD180201-0003_HK72WCCXY_L8_clean_bin.38.orig.fa_k	OEZ008529	yes	LMSG_G000009431.1	OES073747	bacteriocin	RiPPs	Lead-Zinc	10.85	China: Jiangxi, Yinshan
L180206P-8-LD180201-0003_HK72WCCXY_L8_clean_bin.38.orig.fa_k	OEZ008529	yes	LMSG_G000009431.1	OES073747	betalactone	Others	Lead-Zinc	30.86	China: Jiangxi, Yinshan
L180206P-8-LD180201-0003_HK72WCCXY_L8_clean_bin.38.orig.fa_k	OEZ008529	yes	LMSG_G000009431.1	OES073747	ectoine	Others	Lead-Zinc	10.32	China: Jiangxi, Yinshan
L180206P-8-LD180201-0003_HK72WCCXY_L8_clean_bin.39.orig.fa_k	OEZ008529	yes	LMSG_G000009547.1	OES073747	NRPS-like	NRPS	Lead-Zinc	42.73	China: Jiangxi, Yinshan
L180206P-8-LD180201-0003_HK72WCCXY_L8_clean_bin.39.orig.fa_k	OEZ008529	yes	LMSG_G000009547.1	OES073747	terpene	Terpene	Lead-Zinc	22.06	China: Jiangxi, Yinshan
L180206P-8-LD180201-0003_HK72WCCXY_L8_clean_bin.39.orig.fa_k	OEZ008529	yes	LMSG_G000009547.1	OES073747	ectoine	Others	Lead-Zinc	7.57	China: Jiangxi, Yinshan
L180206P-8-LD180201-0003_HK72WCCXY_L8_clean_bin.39.orig.fa_k	OEZ008529	no	LMSG_G000009547.1	OES073747	terpene	Terpene	Lead-Zinc	23.38	China: Jiangxi, Yinshan
L180206P-8-LD180201-0003_HK72WCCXY_L8_clean_bin.39.orig.fa_k	OEZ008529	yes	LMSG_G000009547.1	OES073747	ectoine	Others	Lead-Zinc	7.21	China: Jiangxi, Yinshan
L180206P-8-LD180201-0003_HK72WCCXY_L8_clean_bin.42.strict.fa	OEZ008529	yes	LMSG_G000008223.1	OES073747	terpene	Terpene	Lead-Zinc	10.43	China: Jiangxi, Yinshan
L180206P-8-LD180201-0003_HK72WCCXY_L8_clean_bin.42.strict.fa	OEZ008529	yes	LMSG_G000008223.1	OES073747	terpene	Terpene	Lead-Zinc	7.38	China: Jiangxi, Yinshan
L180206P-8-LD180201-0003_HK72WCCXY_L8_clean_bin.44.orig.fa_k	OEZ008529	yes	LMSG_G000007772.1	OES073747	terpene	Terpene	Lead-Zinc	29.77	China: Jiangxi, Yinshan
L180206P-8-LD180201-0003_HK72WCCXY_L8_clean_bin.44.orig.fa_k	OEZ008529	yes	LMSG_G000007772.1	OES073747	T3PKS	PKSother	Lead-Zinc	24.42	China: Jiangxi, Yinshan
L180206P-8-LD180201-0003_HK72WCCXY_L8_clean_bin.44.orig.fa_k	OEZ008529	yes	LMSG_G000007772.1	OES073747	NRPS-like	NRPS	Lead-Zinc	10.01	China: Jiangxi, Yinshan
L180206P-8-LD180201-0003_HK72WCCXY_L8_clean_bin.52.orig.fa_k	OEZ008529	no	LMSG_G000010160.1	OES073747	ectoine	Others	Lead-Zinc	9.50	China: Jiangxi, Yinshan
L180206P-8-LD180201-0003_HK72WCCXY_L8_clean_bin.52.orig.fa_k	OEZ008529	no	LMSG_G000010160.1	OES073747	terpene	Terpene	Lead-Zinc	11.69	China: Jiangxi, Yinshan
L180206P-8-LD180201-0003_HK72WCCXY_L8_clean_bin.52.orig.fa_k	OEZ008529	no	LMSG_G000010160.1	OES073747	acyl_amino_acids	Others	Lead-Zinc	8.42	China: Jiangxi, Yinshan
L180206P-8-LD180201-0003_HK72WCCXY_L8_clean_bin.52.orig.fa_k	OEZ008529	no	LMSG_G000010160.1	OES073747	terpene	Terpene	Lead-Zinc	20.60	China: Jiangxi, Yinshan
L180206P-8-LD180201-0003_HK72WCCXY_L8_clean_bin.54.orig.fa_k	OEZ008529	no	LMSG_G000010481.1	OES073747	arylpolyene	Others	Lead-Zinc	17.04	China: Jiangxi, Yinshan
L180206P-8-LD180201-0003_HK72WCCXY_L8_clean_bin.54.orig.fa_k	OEZ008529	yes	LMSG_G000010481.1	OES073747	hserlactone	Others	Lead-Zinc	11.18	China: Jiangxi, Yinshan
L180206P-8-LD180201-0003_HK72WCCXY_L8_clean_bin.55.orig.fa_k	OEZ008529	yes	LMSG_G000010620.1	OES073747	terpene	Terpene	Lead-Zinc	23.00	China: Jiangxi, Yinshan
L180206P-8-LD180201-0003_HK72WCCXY_L8_clean_bin.55.orig.fa_k	OEZ008529	yes	LMSG_G000010620.1	OES073747	bacteriocin	RiPPs	Lead-Zinc	10.99	China: Jiangxi, Yinshan
L180206P-8-LD180201-0003_HK72WCCXY_L8_clean_bin.55.orig.fa_k	OEZ008529	no	LMSG_G000010620.1	OES073747	arylpolyene	Others	Lead-Zinc	5.52	China: Jiangxi, Yinshan
L180206P-8-LD180201-0003_HK72WCCXY_L8_clean_bin.55.orig.fa_k	OEZ008529	yes	LMSG_G000010620.1	OES073747	arylpolyene	Others	Lead-Zinc	25.53	China: Jiangxi, Yinshan
L180206P-8-LD180201-0003_HK72WCCXY_L8_clean_bin.7.orig.fa_k1	OEZ008529	no	LMSG_G000011019.1	OES073747	NRPS	NRPS	Lead-Zinc	10.77	China: Jiangxi, Yinshan
L180206P-8-LD180201-0003_HK72WCCXY_L8_clean_bin.7.orig.fa_k1	OEZ008529	no	LMSG_G000011019.1	OES073747	NRPS-like	NRPS	Lead-Zinc	12.75	China: Jiangxi, Yinshan
L180206P-8-LD180201-0003_HK72WCCXY_L8_clean_bin.7.orig.fa_k1	OEZ008529	no	LMSG_G000011019.1	OES073747	betalactone.NRPS-like	Others	Lead-Zinc	17.85	China: Jiangxi, Yinshan
L180206P-8-LD180201-0003_HK72WCCXY_L8_clean_bin.7.orig.fa_k1	OEZ008529	no	LMSG_G000011019.1	OES073747	thiopeptide	RiPPs	Lead-Zinc	19.87	China: Jiangxi, Yinshan
L180206P-8-LD180201-0003_HK72WCCXY_L8_clean_bin.7.orig.fa_k1	OEZ008529	no	LMSG_G000011019.1	OES073747	NRPS	NRPS	Lead-Zinc	7.20	China: Jiangxi, Yinshan
L180206P-8-LD180201-0003_HK72WCCXY_L8_clean_bin.7.orig.fa_k1	OEZ008529	no	LMSG_G000011019.1	OES073747	bacteriocin	RiPPs	Lead-Zinc	8.61	China: Jiangxi, Yinshan
L180206P-8-LD180201-0003_HK72WCCXY_L8_clean_bin.7.orig.fa_k1	OEZ008529	no	LMSG_G000011019.1	OES073747	NRPS	NRPS	Lead-Zinc	23.54	China: Jiangxi, Yinshan
L180206P-8-LD180201-0003_HK72WCCXY_L8_clean_bin.7.orig.fa_k1	OEZ008529	no	LMSG_G000011019.1	OES073747	NRPS	NRPS	Lead-Zinc	8.61	China: Jiangxi, Yinshan
L180206P-8-LD180201-0003_HK72WCCXY_L8_clean_bin.7.orig.fa_k1	OEZ008529	no	LMSG_G000011019.1	OES073747	NRPS	NRPS	Lead-Zinc	11.91	China: Jiangxi, Yinshan
L180206P-8-LD180201-0003_HK72WCCXY_L8_clean_bin.7.orig.fa_k1	OEZ008529	no	LMSG_G000011019.1	OES073747	NRPS	NRPS	Lead-Zinc	20.29	China: Jiangxi, Yinshan
L180206P-8-LD180201-0003_HK72WCCXY_L8_clean_bin.7.orig.fa_k1	OEZ008529	no	LMSG_G000011019.1	OES073747	phenazine	Others	Lead-Zinc	10.23	China: Jiangxi, Yinshan
L180206P-8-LD180201-0003_HK72WCCXY_L8_clean_bin.7.orig.fa_k1	OEZ008529	no	LMSG_G000011019.1	OES073747	NRPS	NRPS	Lead-Zinc	10.58	China: Jiangxi, Yinshan
L180206P-8-LD180201-0003_HK72WCCXY_L8_clean_bin.7.orig.fa_k1	OEZ008529	no	LMSG_G000011019.1	OES073747	NRPS-like	NRPS	Lead-Zinc	6.64	China: Jiangxi, Yinshan
L180206P-8-LD180201-0003_HK72WCCXY_L8_clean_bin.7.orig.fa_k1	OEZ008529	no	LMSG_G000011019.1	OES073747	bacteriocin	RiPPs	Lead-Zinc	6.20	China: Jiangxi, Yinshan
L180206P-8-LD180201-0003_HK72WCCXY_L8_clean_bin.7.orig.fa_k1	OEZ008529	no	LMSG_G000011019.1	OES073747	NRPS	NRPS	Lead-Zinc	6.79	China: Jiangxi, Yinshan
L180206P-8-LD180201-0003_HK72WCCXY_L8_clean_bin.7.orig.fa_k1	OEZ008529	no	LMSG_G000011019.1	OES073747	NRPS	NRPS	Lead-Zinc	8.46	China: Jiangxi, Yinshan
L180206P-8-LD180201-0003_HK72WCCXY_L8_clean_bin.7.orig.fa_k1	OEZ008529	no	LMSG_G000011019.1	OES073747	hserlactone	Others	Lead-Zinc	6.10	China: Jiangxi, Yinshan
L180206P-8-LD180201-0003_HK72WCCXY_L8_clean_bin.8.orig.fa_k1	OEZ008529	yes	LMSG_G000008266.1	OES073747	terpene	Terpene	Lead-Zinc	19.09	China: Jiangxi, Yinshan
L180206P-8-LD180201-0006_HK72WCCXY_L8_clean_bin.13.orig.fa_k	OEZ008529	yes	LMSG_G000008638.1	OES073740	other	Others	Copper	5.67	China: Jiangxi, Yongping
L180206P-8-LD180201-0006_HK72WCCXY_L8_clean_bin.19.orig.fa_k	OEZ008529	yes	LMSG_G000011130.1	OES073740	terpene	Terpene	Copper	22.80	China: Jiangxi, Yongping
L180206P-8-LD180201-0006_HK72WCCXY_L8_clean_bin.19.orig.fa_k	OEZ008529	yes	LMSG_G000011130.1	OES073740	arylpolyene	Others	Copper	41.16	China: Jiangxi, Yongping
L180206P-8-LD180201-0006_HK72WCCXY_L8_clean_bin.2.orig.fa_k1	OEZ008529	yes	LMSG_G000008418.1	OES073740	terpene	Terpene	Copper	8.67	China: Jiangxi, Yongping
L180206P-8-LD180201-0006_HK72WCCXY_L8_clean_bin.2.orig.fa_k1	OEZ008529	yes	LMSG_G000008418.1	OES073740	TfuA-related	RiPPs	Copper	18.46	China: Jiangxi, Yongping
L180206P-8-LD180201-0006_HK72WCCXY_L8_clean_bin.2.orig.fa_k1	OEZ008529	yes	LMSG_G000008418.1	OES073740	betalactone	Others	Copper	33.95	China: Jiangxi, Yongping
L180206P-8-LD180201-0006_HK72WCCXY_L8_clean_bin.20.strict.fa	OEZ008529	yes	LMSG_G000005082.1	OES073740	betalactone	Others	Copper	10.54	China: Jiangxi, Yongping
L180206P-8-LD180201-0006_HK72WCCXY_L8_clean_bin.20.strict.fa	OEZ008529	yes	LMSG_G000005082.1	OES073740	terpene	Terpene	Copper	9.02	China: Jiangxi, Yongping
L180206P-8-LD180201-0006_HK72WCCXY_L8_clean_bin.20.strict.fa	OEZ008529	yes	LMSG_G000005082.1	OES073740	bacteriocin	RiPPs	Copper	5.71	China: Jiangxi, Yongping
L180206P-8-LD180201-0006_HK72WCCXY_L8_clean_bin.21.strict.fa	OEZ008529	yes	LMSG_G000007900.1	OES073740	NRPS	NRPS	Copper	34.28	China: Jiangxi, Yongping
L180206P-8-LD180201-0006_HK72WCCXY_L8_clean_bin.21.strict.fa	OEZ008529	yes	LMSG_G000007900.1	OES073740	NRPS-like	NRPS	Copper	24.83	China: Jiangxi, Yongping
L180206P-8-LD180201-0006_HK72WCCXY_L8_clean_bin.21.strict.fa	OEZ008529	yes	LMSG_G000007900.1	OES073740	NRPS-like	NRPS	Copper	19.54	China: Jiangxi, Yongping
L180206P-8-LD180201-0006_HK72WCCXY_L8_clean_bin.22.orig.fa_k	OEZ008529	yes	LMSG_G000005083.1	OES073740	NRPS-like	NRPS	Copper	5.66	China: Jiangxi, Yongping
L180206P-8-LD180201-0006_HK72WCCXY_L8_clean_bin.22.orig.fa_k	OEZ008529	yes	LMSG_G000005083.1	OES073740	terpene	Terpene	Copper	5.35	China: Jiangxi, Yongping

L180206P-8-LD180201-0006_HK72WCCXY_L8_clean_bin.23.orig.fa_k	OEZ008529	yes	LMSG_G000009357.1	OES073740	betalactone	Others	Copper	31.59	China: Jiangxi, Yongping
L180206P-8-LD180201-0006_HK72WCCXY_L8_clean_bin.29.orig.fa_k	OEZ008529	yes	LMSG_G000011123.1	OES073740	arylpolyene	Others	Copper	41.16	China: Jiangxi, Yongping
L180206P-8-LD180201-0006_HK72WCCXY_L8_clean_bin.29.orig.fa_k	OEZ008529	yes	LMSG_G000011123.1	OES073740	terpene	Terpene	Copper	23.11	China: Jiangxi, Yongping
L180206P-8-LD180201-0006_HK72WCCXY_L8_clean_bin.31.strict.fa	OEZ008529	yes	LMSG_G000005085.1	OES073740	terpene	Terpene	Copper	21.73	China: Jiangxi, Yongping
L180206P-8-LD180201-0006_HK72WCCXY_L8_clean_bin.31.strict.fa	OEZ008529	yes	LMSG_G000005085.1	OES073740	T3PKS	PKSother	Copper	25.24	China: Jiangxi, Yongping
L180206P-8-LD180201-0006_HK72WCCXY_L8_clean_bin.31.strict.fa	OEZ008529	yes	LMSG_G000005085.1	OES073740	terpene	Terpene	Copper	15.16	China: Jiangxi, Yongping
L180206P-8-LD180201-0006_HK72WCCXY_L8_clean_bin.35.permissiv	OEZ008529	yes	LMSG_G000008883.1	OES073740	arylpolyene	Others	Copper	21.81	China: Jiangxi, Yongping
L180206P-8-LD180201-0006_HK72WCCXY_L8_clean_bin.35.permissiv	OEZ008529	yes	LMSG_G000008883.1	OES073740	T3PKS	PKSother	Copper	21.20	China: Jiangxi, Yongping
L180206P-8-LD180201-0006_HK72WCCXY_L8_clean_bin.35.permissiv	OEZ008529	yes	LMSG_G000008883.1	OES073740	terpene	Terpene	Copper	20.72	China: Jiangxi, Yongping
L180206P-8-LD180201-0006_HK72WCCXY_L8_clean_bin.36.orig.fa_k	OEZ008529	yes	LMSG_G000010072.1	OES073740	terpene	Terpene	Copper	13.62	China: Jiangxi, Yongping
L180206P-8-LD180201-0006_HK72WCCXY_L8_clean_bin.36.orig.fa_k	OEZ008529	yes	LMSG_G000010072.1	OES073740	arylpolyene	Others	Copper	8.10	China: Jiangxi, Yongping
L180206P-8-LD180201-0006_HK72WCCXY_L8_clean_bin.36.orig.fa_k	OEZ008529	yes	LMSG_G000010072.1	OES073740	terpene	Terpene	Copper	5.19	China: Jiangxi, Yongping
L180206P-8-LD180201-0006_HK72WCCXY_L8_clean_bin.36.orig.fa_k	OEZ008529	yes	LMSG_G000010072.1	OES073740	arylpolyene	Others	Copper	6.38	China: Jiangxi, Yongping
L180206P-8-LD180201-0006_HK72WCCXY_L8_clean_bin.37.orig.fa_k	OEZ008529	yes	LMSG_G000004418.1	OES073740	terpene	Terpene	Copper	12.25	China: Jiangxi, Yongping
L180206P-8-LD180201-0006_HK72WCCXY_L8_clean_bin.37.orig.fa_k	OEZ008529	yes	LMSG_G000004418.1	OES073740	bacteriocin	RiPPs	Copper	10.51	China: Jiangxi, Yongping
L180206P-8-LD180201-0006_HK72WCCXY_L8_clean_bin.38.orig.fa_k	OEZ008529	yes	LMSG_G000005087.1	OES073740	arylpolyene	Others	Copper	24.96	China: Jiangxi, Yongping
L180206P-8-LD180201-0006_HK72WCCXY_L8_clean_bin.38.orig.fa_k	OEZ008529	yes	LMSG_G000005087.1	OES073740	bacteriocin	RiPPs	Copper	10.92	China: Jiangxi, Yongping
L180206P-8-LD180201-0006_HK72WCCXY_L8_clean_bin.38.orig.fa_k	OEZ008529	yes	LMSG_G000005087.1	OES073740	terpene	Terpene	Copper	14.64	China: Jiangxi, Yongping
L180206P-8-LD180201-0006_HK72WCCXY_L8_clean_bin.38.orig.fa_k	OEZ008529	yes	LMSG_G000005087.1	OES073740	arylpolyene	Others	Copper	41.15	China: Jiangxi, Yongping
L180206P-8-LD180201-0006_HK72WCCXY_L8_clean_bin.39.orig.fa_k	OEZ008529	yes	LMSG_G000010110.1	OES073740	NRPS-like	NRPS	Copper	7.74	China: Jiangxi, Yongping
L180206P-8-LD180201-0006_HK72WCCXY_L8_clean_bin.39.orig.fa_k	OEZ008529	yes	LMSG_G000010110.1	OES073740	terpene	Terpene	Copper	6.29	China: Jiangxi, Yongping
L180206P-8-LD180201-0006_HK72WCCXY_L8_clean_bin.39.orig.fa_k	OEZ008529	yes	LMSG_G000010110.1	OES073740	bacteriocin	RiPPs	Copper	10.89	China: Jiangxi, Yongping
L180206P-8-LD180201-0006_HK72WCCXY_L8_clean_bin.39.orig.fa_k	OEZ008529	yes	LMSG_G000010110.1	OES073740	TfuA-related	RiPPs	Copper	11.78	China: Jiangxi, Yongping
L180206P-8-LD180201-0006_HK72WCCXY_L8_clean_bin.40.orig.fa_k	OEZ008529	yes	LMSG_G000011043.1	OES073740	NRPS-like	NRPS	Copper	9.65	China: Jiangxi, Yongping
L180206P-8-LD180201-0006_HK72WCCXY_L8_clean_bin.40.orig.fa_k	OEZ008529	yes	LMSG_G000011043.1	OES073740	terpene	Terpene	Copper	23.08	China: Jiangxi, Yongping
L180206P-8-LD180201-0006_HK72WCCXY_L8_clean_bin.40.orig.fa_k	OEZ008529	yes	LMSG_G000011043.1	OES073740	arylpolyene	Others	Copper	19.75	China: Jiangxi, Yongping
L180206P-8-LD180201-0006_HK72WCCXY_L8_clean_bin.40.orig.fa_k	OEZ008529	yes	LMSG_G000011043.1	OES073740	arylpolyene	Others	Copper	28.11	China: Jiangxi, Yongping
L180206P-8-LD180201-0006_HK72WCCXY_L8_clean_bin.40.orig.fa_k	OEZ008529	yes	LMSG_G000011043.1	OES073740	bacteriocin	RiPPs	Copper	10.71	China: Jiangxi, Yongping
L180206P-8-LD180201-0006_HK72WCCXY_L8_clean_bin.43.orig.fa_k	OEZ008529	yes	LMSG_G000005089.1	OES073740	NRPS-like	NRPS	Copper	12.12	China: Jiangxi, Yongping
L180206P-8-LD180201-0006_HK72WCCXY_L8_clean_bin.47.orig.fa_k	OEZ008529	yes	LMSG_G000007748.1	OES073740	NRPS-like	NRPS	Copper	6.08	China: Jiangxi, Yongping
L180206P-8-LD180201-0006_HK72WCCXY_L8_clean_bin.47.orig.fa_k	OEZ008529	yes	LMSG_G000007748.1	OES073740	terpene	Terpene	Copper	7.00	China: Jiangxi, Yongping
L180206P-8-LD180201-0006_HK72WCCXY_L8_clean_bin.5.orig.fa_kl	OEZ008529	yes	LMSG_G000009106.1	OES073740	lassopeptide	RiPPs	Copper	13.72	China: Jiangxi, Yongping
L180206P-8-LD180201-0006_HK72WCCXY_L8_clean_bin.5.orig.fa_kl	OEZ008529	yes	LMSG_G000009106.1	OES073740	NRPS-like	NRPS	Copper	8.53	China: Jiangxi, Yongping
L180206P-8-LD180201-0006_HK72WCCXY_L8_clean_bin.5.orig.fa_kl	OEZ008529	yes	LMSG_G000009106.1	OES073740	T3PKS	PKSother	Copper	35.37	China: Jiangxi, Yongping
L180206P-8-LD180201-0006_HK72WCCXY_L8_clean_bin.5.orig.fa_kl	OEZ008529	yes	LMSG_G000009106.1	OES073740	terpene	Terpene	Copper	22.17	China: Jiangxi, Yongping
L180206P-8-LD180201-0006_HK72WCCXY_L8_clean_bin.5.orig.fa_kl	OEZ008529	yes	LMSG_G000009106.1	OES073740	bacteriocin	RiPPs	Copper	6.29	China: Jiangxi, Yongping
L180206P-8-LD180201-0006_HK72WCCXY_L8_clean_bin.51.strict.fa	OEZ008529	yes	LMSG_G000009189.1	OES073740	terpene	Terpene	Copper	16.52	China: Jiangxi, Yongping
L180206P-8-LD180201-0006_HK72WCCXY_L8_clean_bin.52.orig.fa_k	OEZ008529	yes	LMSG_G000007914.1	OES073740	NRPS-like	NRPS	Copper	42.82	China: Jiangxi, Yongping
L180206P-8-LD180201-0006_HK72WCCXY_L8_clean_bin.52.orig.fa_k	OEZ008529	yes	LMSG_G000007914.1	OES073740	terpene	Terpene	Copper	20.88	China: Jiangxi, Yongping
L180206P-8-LD180201-0006_HK72WCCXY_L8_clean_bin.52.orig.fa_k	OEZ008529	yes	LMSG_G000007914.1	OES073740	bacteriocin	RiPPs	Copper	10.81	China: Jiangxi, Yongping
L180206P-8-LD180201-0006_HK72WCCXY_L8_clean_bin.53.orig.fa_k	OEZ008529	yes	LMSG_G000010009.1	OES073740	terpene	Terpene	Copper	21.87	China: Jiangxi, Yongping
L180206P-8-LD180201-0006_HK72WCCXY_L8_clean_bin.54.strict.fa	OEZ008529	yes	LMSG_G000006859.1	OES073740	LAP	RiPPs	Copper	6.65	China: Jiangxi, Yongping
L180206P-8-LD180201-0006_HK72WCCXY_L8_clean_bin.59.orig.fa_k	OEZ008529	yes	LMSG_G000008410.1	OES073740	terpene	Terpene	Copper	9.90	China: Jiangxi, Yongping
L180206P-8-LD180201-0006_HK72WCCXY_L8_clean_bin.59.orig.fa_k	OEZ008529	yes	LMSG_G000008410.1	OES073740	betalactone	Others	Copper	18.89	China: Jiangxi, Yongping
L180206P-8-LD180201-0006_HK72WCCXY_L8_clean_bin.61.orig.fa_k	OEZ008529	yes	LMSG_G000009119.1	OES073740	NRPS-like	NRPS	Copper	25.93	China: Jiangxi, Yongping
L180206P-8-LD180201-0006_HK72WCCXY_L8_clean_bin.61.orig.fa_k	OEZ008529	yes	LMSG_G000009119.1	OES073740	bacteriocin	RiPPs	Copper	10.87	China: Jiangxi, Yongping
L180206P-8-LD180201-0006_HK72WCCXY_L8_clean_bin.61.orig.fa_k	OEZ008529	yes	LMSG_G000009119.1	OES073740	terpene	Terpene	Copper	25.09	China: Jiangxi, Yongping
L180206P-8-LD180201-0006_HK72WCCXY_L8_clean_bin.64.permissiv	OEZ008529	yes	LMSG_G000009281.1	OES073740	terpene	Terpene	Copper	21.73	China: Jiangxi, Yongping
L180206P-8-LD180201-0006_HK72WCCXY_L8_clean_bin.64.permissiv	OEZ008529	yes	LMSG_G000009281.1	OES073740	terpene	Terpene	Copper	22.06	China: Jiangxi, Yongping
L180206P-8-LD180201-0006_HK72WCCXY_L8_clean_bin.65.orig.fa_k	OEZ008529	yes	LMSG_G000010037.1	OES073740	ladderane	Others	Copper	19.33	China: Jiangxi, Yongping
L180206P-8-LD180201-0006_HK72WCCXY_L8_clean_bin.65.orig.fa_k	OEZ008529	yes	LMSG_G000010037.1	OES073740	terpene	Terpene	Copper	12.72	China: Jiangxi, Yongping
L180206P-8-LD180201-0006_HK72WCCXY_L8_clean_bin.65.orig.fa_k	OEZ008529	yes	LMSG_G000010037.1	OES073740	ectoine	Others	Copper	6.70	China: Jiangxi, Yongping
L180206P-8-LD180201-0006_HK72WCCXY_L8_clean_bin.65.orig.fa_k	OEZ008529	yes	LMSG_G000010037.1	OES073740	terpene	Terpene	Copper	20.80	China: Jiangxi, Yongping
L180206P-8-LD180201-0006_HK72WCCXY_L8_clean_bin.65.orig.fa_k	OEZ008529	yes	LMSG_G000010037.1	OES073740	T3PKS	PKSother	Copper	14.42	China: Jiangxi, Yongping
L180206P-8-LD180201-0006_HK72WCCXY_L8_clean_bin.67.orig.fa_k	OEZ008529	yes	LMSG_G000010238.1	OES073740	hserlactone	Others	Copper	17.16	China: Jiangxi, Yongping
L180206P-8-LD180201-0006_HK72WCCXY_L8_clean_bin.67.orig.fa_k	OEZ008529	yes	LMSG_G000010238.1	OES073740	terpene	Terpene	Copper	18.45	China: Jiangxi, Yongping
L180206P-8-LD180201-0006_HK72WCCXY_L8_clean_bin.67.orig.fa_k	OEZ008529	yes	LMSG_G000010238.1	OES073740	terpene	Terpene	Copper	18.81	China: Jiangxi, Yongping
L180206P-8-LD180201-0006_HK72WCCXY_L8_clean_bin.67.orig.fa_k	OEZ008529	yes	LMSG_G000010238.1	OES073740	arylpolyene	Others	Copper	10.57	China: Jiangxi, Yongping
L180206P-8-LD180201-0006_HK72WCCXY_L8_clean_bin.67.orig.fa_k	OEZ008529	yes	LMSG_G000010238.1	OES073740	terpene	Terpene	Copper	12.04	China: Jiangxi, Yongping
L180206P-8-LD180201-0006_HK72WCCXY_L8_clean_bin.68.strict.fa	OEZ008529	yes	LMSG_G000007774.1	OES073740	T3PKS	PKSother	Copper	19.44	China: Jiangxi, Yongping
L180206P-8-LD180201-0006_HK72WCCXY_L8_clean_bin.68.strict.fa	OEZ008529	yes	LMSG_G000007774.1	OES073740	terpene	Terpene	Copper	18.31	China: Jiangxi, Yongping
L180206P-8-LD180201-0006_HK72WCCXY_L8_clean_bin.68.strict.fa	OEZ008529	yes	LMSG_G000007774.1	OES073740	NRPS-like	NRPS	Copper	16.78	China: Jiangxi, Yongping
L180206P-8-LD180201-0006_HK72WCCXY_L8_clean_bin.70.orig.fa_k	OEZ008529	yes	LMSG_G000008400.1	OES073740	terpene	Terpene	Copper	20.99	China: Jiangxi, Yongping
L180206P-8-LD180201-0006_HK72WCCXY_L8_clean_bin.71.orig.fa_k	OEZ008529	yes	LMSG_G000004420.1	OES073740	TfuA-related	RiPPs	Copper	21.88	China: Jiangxi, Yongping
L180206P-8-LD180201-0006_HK72WCCXY_L8_clean_bin.71.orig.fa_k	OEZ008529	yes	LMSG_G000004420.1	OES073740	ectoine	Others	Copper	10.38	China: Jiangxi, Yongping
L180206P-8-LD180201-0006_HK72WCCXY_L8_clean_bin.71.orig.fa_k	OEZ008529	no	LMSG_G000004420.1	OES073740	bacteriocin	RiPPs	Copper	10.95	China: Jiangxi, Yongping
L180206P-8-LD180201-0006_HK72WCCXY_L8_clean_bin.71.orig.fa_k	OEZ008529	yes	LMSG_G000004420.1	OES073740	bacteriocin	RiPPs	Copper	10.81	China: Jiangxi, Yongping
L180206P-8-LD180201-0006_HK72WCCXY_L8_clean_bin.71.orig.fa_k	OEZ008529	yes	LMSG_G000004420.1	OES073740	lanthipeptide	RiPPs	Copper	24.33	China: Jiangxi, Yongping
L180206P-8-LD180201-0006_HK72WCCXY_L8_clean_bin.71.orig.fa_k	OEZ008529	yes	LMSG_G000004420.1	OES073740	terpene	Terpene	Copper	20.83	China: Jiangxi, Yongping
L180206P-8-LD180201-0006_HK72WCCXY_L8_clean_bin.71.orig.fa_k	OEZ008529	yes	LMSG_G000004420.1	OES073740	T3PKS	PKSother	Copper	29.46	China: Jiangxi, Yongping
L180206P-8-LD180201-0006_HK72WCCXY_L8_clean_bin.71.orig.fa_k	OEZ008529	yes	LMSG_G000004420.1	OES073740	bacteriocin	RiPPs	Copper	10.86	China: Jiangxi, Yongping
L180206P-8-LD180201-0006_HK72WCCXY_L8_clean_bin.73.orig.fa_k	OEZ008529	yes	LMSG_G000008408.1	OES073740	terpene	Terpene	Copper	16.38	China: Jiangxi, Yongping
L180206P-8-LD180201-0006_HK72WCCXY_L8_clean_bin.74.orig.fa_k	OEZ008529	yes	LMSG_G000010458.1	OES073740	arylpolyene	Others	Copper	30.81	China: Jiangxi, Yongping
L180206P-8-LD180201-0006_HK72WCCXY_L8_clean_bin.74.orig.fa_k	OEZ008529	yes	LMSG_G000010458.1	OES073740	terpene	Terpene	Copper	18.79	China: Jiangxi, Yongping
L180206P-8-LD180201-0006_HK72WCCXY_L8_clean_bin.75.orig.fa_k	OEZ008529	yes	LMSG_G000008065.1	OES073740	terpene	Terpene	Copper	11.30	China: Jiangxi, Yongping
L180206P-8-LD180201-0006_HK72WCCXY_L8_clean_bin.76.orig.fa_k	OEZ008529	yes	LMSG_G000007365.1	OES073740	bacteriocin	RiPPs	Copper	11.97	China: Jiangxi, Yongping
L180206P-8-LD180201-0006_HK72WCCXY_L8_clean_bin.82.orig.fa_k	OEZ008529	yes	LMSG_G000011034.1	OES073740	terpene	Terpene	Copper	23.08	China: Jiangxi, Yongping
L180206P-8-LD180201-0006_HK72WCCXY_L8_clean_bin.82.orig.fa_k	OEZ008529	yes	LMSG_G000011034.1	OES073740	NRPS-like	NRPS	Copper	20.97	China: Jiangxi, Yongping
L180206P-8-LD180201-0006_HK72WCCXY_L8_clean_bin.82.orig.fa_k	OEZ008529	yes	LMSG_G000011034.1	OES073740	resorcinol.arylpolyene	Others	Copper	41.96	China: Jiangxi, Yongping
L180206P-8-LD180201-0006_HK72WCCXY_L8_clean_bin.82.orig.fa_k	OEZ008529	yes	LMSG_G000011034.1	OES073740	terpene	Terpene	Copper	20.85	China: Jiangxi, Yongping
L180206P-8-LD180201-0006_HK72WCCXY_L8_clean_bin.82.orig.fa_k	OEZ008529	yes	LMSG_G000011034.1	OES073740	bacteriocin	RiPPs	Copper	7.67	China: Jiangxi, Yongping
L180206P-8-LD180201-0006_HK72WCCXY_L8_clean_bin.82.orig.fa_k	OEZ008529	yes	LMSG_G000011034.1	OES073740	arylpolyene	Others	Copper	43.59	China: Jiangxi, Yongping
L180206P-8-LD180201-0006_HK72WCCXY_L8_clean_bin.83.orig.fa_k	OEZ008529	no	LMSG_G000011009.1	OES073740	betalactone.NRPS-like	Others	Copper	41.82	China: Jiangxi, Yongping
L180206P-8-LD180201-0006_HK72WCCXY_L8_clean_bin.83.orig.fa_k	OEZ008529	no	LMSG_G000011009.1	OES073740	NRPS	NRPS	Copper	44.23	China: Jiangxi, Yongping
L180206P-8-LD180201-0006_HK72WCCXY_L8_clean_bin.83.orig.fa_k	OEZ008529	no	LMSG_G000011009.1	OES073740	NRPS	NRPS	Copper	62.35	China: Jiangxi, Yongping
L180206P-8-LD180201-0006_HK72WCCXY_L8_clean_bin.83.orig.fa_k	OEZ008529	no	LMSG_G000011009.1	OES073740	NRPS	NRPS	Copper	25.01	China: Jiangxi, Yongping
L180206P-8-LD180201-0006_HK72WCCXY_L8_clean_bin.83.orig.fa_k	OEZ008529	no	LMSG_G000011009.1	OES073740	NRPS	NRPS	Copper	42.73	China: Jiangxi, Yongping
L180206P-8-LD180201-0006_HK72WCCXY_L8_clean_bin.83.orig.fa_k	OEZ008529	no	LMSG_G000011009.1	OES073740	phenazine	Others	Copper	10.23	China: Jiangxi, Yongping
L180206P-8-LD180201-0006_HK72WCCXY_L8_clean_bin.83.orig.fa_k	OEZ008529	no	LMSG_G000011009.1	OES073740	hserlactone	Others	Copper	20.61	China: Jiangxi, Yongping
L180206P-8-LD180201-0006_HK72WCCXY_L8_clean_bin.83.orig.fa_k	OEZ008529	no	LMSG_G000011009.1	OES073740	thiopeptide	RiPPs	Copper	33.00	China: Jiangxi, Yongping
L180206P-8-LD180201-0006_HK72WCCXY_L8_clean_bin.83.orig.fa_k	OEZ008529	no	LMSG_G000011009.1	OES073740	hserlactone	Others	Copper	20.61	China: Jiangxi, Yongping
L180206P-8-LD180201-0006_HK72WCCXY_L8_clean_bin.83.orig.fa_k	OEZ008529	no	LMSG_G000011009.1	OES073740	NAGGN	Others	Copper	14.76	China: Jiangxi, Yongping
L180206P-8-LD180201-0006_HK72WCCXY_L8_clean_bin.83.orig.fa_k	OEZ008529	no	LMSG_G000011009.1	OES073740	phenazine	Others	Copper	10.23	China: Jiangxi, Yongping
L180206P-8-LD180201-0006_HK72WCCXY_L8_clean_bin.83.orig.fa_k	OEZ008529	no	LMSG_G000011009.1	OES073740	bacteriocin	RiPPs	Copper	10.83	China: Jiangxi, Yongping
L180206P-8-LD180201-0006_HK72WCCXY_L8_clean_bin.83.orig.fa_k	OEZ008529	no	LMSG_G000011009.1	OES073740	NRPS-like	NRPS	Copper	42.98	China: Jiangxi, Yongping
L180206P-8-LD180201-0006_HK72WCCXY_L8_clean_bin.83.orig.fa_k	OEZ008529	no	LMSG_G000011009.1	OES073740	bacteriocin	RiPPs	Copper	10.86	China: Jiangxi, Yongping
L180206P-8-LD180201-0006_HK72WCCXY_L8_clean_bin.83.orig.fa_k	OEZ008529	no	LMSG_G000011009.1	OES073740	NRPS	NRPS	Copper	49.55	China: Jiangxi, Yongping
L180206P-8-LD180201-0006_HK72WCCXY_L8_clean_bin.9.orig.fa_kl	OEZ008529	yes	LMSG_G000010061.1	OES073740	arylpolyene	Others	Copper	7.87	China: Jiangxi, Yongping
L180206P-8-LD180201-0006_HK72WCCXY_L8_clean_bin.9.orig.fa_kl	OEZ008529	yes	LMSG_G000010061.1	OES073740	NRPS-like	NRPS	Copper	13.78	China: Jiangxi, Yongping
L180206P-8-LD180201-0006_HK72WCCXY_L8_clean_bin.9.orig.fa_kl	OEZ008529	yes	LMSG_G000010061.1	OES073740	ectoine	Others	Copper	8.49	China: Jiangxi, Yongping
L180206P-8-LD180201-0006_HK72WCCXY_L8_clean_bin.9.orig.fa_kl	OEZ008529	yes	LMSG_G000010061.1	OES073740	terpene	Terpene	Copper	5.27	China: Jiangxi, Yongping
L180206P-8-LD180201-0017_HK72WCCXY_L8_clean_bin.1.strict.fa_	OEZ008529	yes	LMSG_G000005107.1	OES073710	bacteriocin	RiPPs	Lead-Zinc	10.87	China: Hunan, Shuikoushan
L180206P-8-LD180201-0017_HK72WCCXY_L8_clean_bin.1.strict.fa_	OEZ008529	yes	LMSG_G000005107.1	OES073710	betalactone	Others	Lead-Zinc	30.73	China: Hunan, Shuikoushan
L180206P-8-LD180201-0017_HK72WCCXY_L8_clean_bin.10.orig.fa_k	OEZ008529	yes	LMSG_G000005095.1	OES073710	NRPS.T1PKS	PKS-NRP_Hybrids	Lead-Zinc	13.87	China: Hunan, Shuikoushan
L180206P-8-LD180201-0017_HK72WCCXY_L8_clean_bin.103.orig.fa_	OEZ008529	yes	LMSG_G000010912.1	OES073710	thiopeptide	RiPPs	Lead-Zinc	18.29	China: Hunan, Shuikoushan
L180206P-8-LD180201-0017_HK72WCCXY_L8_clean_bin.103.orig.fa_	OEZ008529	yes	LMSG_G000010912.1	OES073710	T1PKS	PKSI	Lead-Zinc	14.26	China: Hunan, Shuikoushan
L180206P-8-LD180201-0017_HK72WCCXY_L8_clean_bin.103.orig.fa_	OEZ008529	yes	LMSG_G000010912.1	OES073710	terpene	Terpene	Lead-Zinc	9.51	China: Hunan, Shuikoushan
L180206P-8-LD180201-0017_HK72WCCXY_L8_clean_bin.103.orig.fa_	OEZ008529	yes	LMSG_G000010912.1	OES073710	hserlactone	Others	Lead-Zinc	13.44	China: Hunan, Shuikoushan
L180206P-8-LD180201-0017_HK72WCCXY_L8_clean_bin.104.orig.fa_	OEZ008529	yes	LMSG_G000011326.1	OES073710	NRPS-like	NRPS	Lead-Zinc	33.06	China: Hunan, Shuikoushan
L180206P-8-LD180201-0017_HK72WCCXY_L8_clean_bin.107.orig.fa_	OEZ008529	yes	LMSG_G000005092.1	OES073710	terpene	Terpene	Lead-Zinc	13.76	China: Hunan, Shuikoushan
L180206P-8-LD180201-0017_HK72WCCXY_L8_clean_bin.107.orig.fa_	OEZ008529	yes	LMSG_G000005092.1	OES073710	NRPS-like	NRPS	Lead-Zinc	28.66	China: Hunan, Shuikoushan
L180206P-8-LD180201-0017_HK72WCCXY_L8_clean_bin.108.orig.fa_	OEZ008529	yes	LMSG_G000005093.1	OES073710	hglE-KS	PKSother	Lead-Zinc	28.45	China: Hunan, Shuikoushan
L180206P-8-LD180201-0017_HK72WCCXY_L8_clean_bin.108.orig.fa_	OEZ008529	yes	LMSG_G000005093.1	OES073710	arylpolyene	Others	Lead-Zinc	13.67	China: Hunan, Shuikoushan
L180206P-8-LD180201-0017_HK72WCCXY_L8_clean_bin.108.orig.fa_	OEZ008529	yes	LMSG_G000005093.1	OES073710	lassopeptide	RiPPs	Lead-Zinc	13.24	China: Hunan, Shuikoushan
L180206P-8-LD180201-0017_HK72WCCXY_L8_clean_bin.108.orig.fa_	OEZ008529	yes	LMSG_G000005093.1	OES073710	arylpolyene	Others	Lead-Zinc	42.19	China: Hunan, Shuikoushan
L180206P-8-LD180201-0017_HK72WCCXY_L8_clean_bin.109.orig.fa_	OEZ008529	no	LMSG_G000005094.1	OES073710	terpene	Terpene	Lead-Zinc	21.05	China: Hunan, Shuikoushan
L180206P-8-LD180201-0017_HK72WCCXY_L8_clean_bin.109.orig.fa_	OEZ008529	no	LMSG_G000005094.1	OES073710	bacteriocin	RiPPs	Lead-Zinc	11.01	China: Hunan, Shuikoushan
L180206P-8-LD180201-0017_HK72WCCXY_L8_clean_bin.11.orig.fa_k	OEZ008529	yes	LMSG_G000007817.1	OES073710	terpene	Terpene	Lead-Zinc	29.46	China: Hunan, Shuikoushan
L180206P-8-LD180201-0017_HK72WCCXY_L8_clean_bin.11.orig.fa_k	OEZ008529	yes	LMSG_G000007817.1	OES073710	T3PKS	PKSother	Lead-Zinc	24.39	China: Hunan, Shuikoushan
L180206P-8-LD180201-0017_HK72WCCXY_L8_clean_bin.111.strict.f	OEZ008529	yes	LMSG_G000007927.1	OES073710	bacteriocin	RiPPs	Lead-Zinc	10.56	China: Hunan, Shuikoushan
L180206P-8-LD180201-0017_HK72WCCXY_L8_clean_bin.112.orig.fa_	OEZ008529	yes	LMSG_G000009041.1	OES073710	NRPS-like	NRPS	Lead-Zinc	27.60	China: Hunan, Shuikoushan
L180206P-8-LD180201-0017_HK72WCCXY_L8_clean_bin.112.orig.fa_	OEZ008529	yes	LMSG_G000009041.1	OES073710	bacteriocin	RiPPs	Lead-Zinc	6.22	China: Hunan, Shuikoushan
L180206P-8-LD180201-0017_HK72WCCXY_L8_clean_bin.112.orig.fa_	OEZ008529	yes	LMSG_G000009041.1	OES073710	thiopeptide	RiPPs	Lead-Zinc	20.69	China: Hunan, Shuikoushan
L180206P-8-LD180201-0017_HK72WCCXY_L8_clean_bin.112.orig.fa_	OEZ008529	yes	LMSG_G000009041.1	OES073710	arylpolyene	Others	Lead-Zinc	18.34	China: Hunan, Shuikoushan
L180206P-8-LD180201-0017_HK72WCCXY_L8_clean_bin.113.orig.fa_	OEZ008529	yes	LMSG_G000005096.1	OES073710	ectoine	Others	Lead-Zinc	10.41	China: Hunan, Shuikoushan
L180206P-8-LD180201-0017_HK72WCCXY_L8_clean_bin.113.orig.fa_	OEZ008529	yes	LMSG_G000005096.1	OES073710	terpene	Terpene	Lead-Zinc	14.41	China: Hunan, Shuikoushan
L180206P-8-LD180201-0017_HK72WCCXY_L8_clean_bin.117.orig.fa_	OEZ008529	yes	LMSG_G000005100.1	OES073710	arylpolyene	Others	Lead-Zinc	26.57	China: Hunan, Shuikoushan
L180206P-8-LD180201-0017_HK72WCCXY_L8_clean_bin.117.orig.fa_	OEZ008529	yes	LMSG_G000005100.1	OES073710	terpene	Terpene	Lead-Zinc	13.96	China: Hunan, Shuikoushan
L180206P-8-LD180201-0017_HK72WCCXY_L8_clean_bin.117.orig.fa_	OEZ008529	yes	LMSG_G000005100.1	OES073710	hserlactone	Others	Lead-Zinc	17.10	China: Hunan, Shuikoushan
L180206P-8-LD180201-0017_HK72WCCXY_L8_clean_bin.117.orig.fa_	OEZ008529	yes	LMSG_G000005100.1	OES073710	hserlactone	Others	Lead-Zinc	12.31	China: Hunan, Shuikoushan
L180206P-8-LD180201-0017_HK72WCCXY_L8_clean_bin.118.orig.fa_	OEZ008529	no	LMSG_G000010306.1	OES073710	arylpolyene	Others	Lead-Zinc	8.39	China: Hunan, Shuikoushan
L180206P-8-LD180201-0017_HK72WCCXY_L8_clean_bin.118.orig.fa_	OEZ008529	no	LMSG_G000010306.1	OES073710	terpene	Terpene	Lead-Zinc	7.54	China: Hunan, Shuikoushan
L180206P-8-LD180201-0017_HK72WCCXY_L8_clean_bin.120.orig.fa_	OEZ008529	yes	LMSG_G000009063.1	OES073710	hserlactone	Others	Lead-Zinc	12.91	China: Hunan, Shuikoushan
L180206P-8-LD180201-0017_HK72WCCXY_L8_clean_bin.120.orig.fa_	OEZ008529	yes	LMSG_G000009063.1	OES073710	hglE-KS	PKSother	Lead-Zinc	8.57	China: Hunan, Shuikoushan
L180206P-8-LD180201-0017_HK72WCCXY_L8_clean_bin.120.orig.fa_	OEZ008529	yes	LMSG_G000009063.1	OES073710	NRPS-like	NRPS	Lead-Zinc	10.53	China: Hunan, Shuikoushan
L180206P-8-LD180201-0017_HK72WCCXY_L8_clean_bin.120.orig.fa_	OEZ008529	yes	LMSG_G000009063.1	OES073710	T1PKS.hglE-KS	PKSother	Lead-Zinc	16.59	China: Hunan, Shuikoushan
L180206P-8-LD180201-0017_HK72WCCXY_L8_clean_bin.122.orig.fa_	OEZ008529	yes	LMSG_G000006224.1	OES073710	betalactone	Others	Lead-Zinc	26.19	China: Hunan, Shuikoushan
L180206P-8-LD180201-0017_HK72WCCXY_L8_clean_bin.122.orig.fa_	OEZ008529	yes	LMSG_G000006224.1	OES073710	betalactone	Others	Lead-Zinc	20.21	China: Hunan, Shuikoushan
L180206P-8-LD180201-0017_HK72WCCXY_L8_clean_bin.123.strict.f	OEZ008529	yes	LMSG_G000005102.1	OES073710	resorcinol.arylpolyene	Others	Lead-Zinc	20.53	China: Hunan, Shuikoushan
L180206P-8-LD180201-0017_HK72WCCXY_L8_clean_bin.123.strict.f	OEZ008529	yes	LMSG_G000005102.1	OES073710	T3PKS	PKSother	Lead-Zinc	10.43	China: Hunan, Shuikoushan
L180206P-8-LD180201-0017_HK72WCCXY_L8_clean_bin.123.strict.f	OEZ008529	yes	LMSG_G000005102.1	OES073710	arylpolyene	Others	Lead-Zinc	7.77	China: Hunan, Shuikoushan
L180206P-8-LD180201-0017_HK72WCCXY_L8_clean_bin.125.orig.fa_	OEZ008529	yes	LMSG_G000010392.1	OES073710	bacteriocin	RiPPs	Lead-Zinc	6.58	China: Hunan, Shuikoushan
L180206P-8-LD180201-0017_HK72WCCXY_L8_clean_bin.125.orig.fa_	OEZ008529	yes	LMSG_G000010392.1	OES073710	ectoine	Others	Lead-Zinc	10.39	China: Hunan, Shuikoushan
L180206P-8-LD180201-0017_HK72WCCXY_L8_clean_bin.125.orig.fa_	OEZ008529	yes	LMSG_G000010392.1	OES073710	NRPS.T1PKS	PKS-NRP_Hybrids	Lead-Zinc	38.71	China: Hunan, Shuikoushan
L180206P-8-LD180201-0017_HK72WCCXY_L8_clean_bin.125.orig.fa_	OEZ008529	yes	LMSG_G000010392.1	OES073710	NRPS.T1PKS	PKS-NRP_Hybrids	Lead-Zinc	20.30	China: Hunan, Shuikoushan
L180206P-8-LD180201-0017_HK72WCCXY_L8_clean_bin.125.orig.fa_	OEZ008529	yes	LMSG_G000010392.1	OES073710	terpene	Terpene	Lead-Zinc	12.26	China: Hunan, Shuikoushan
L180206P-8-LD180201-0017_HK72WCCXY_L8_clean_bin.125.orig.fa_	OEZ008529	yes	LMSG_G000010392.1	OES073710	cyanobactin	RiPPs	Lead-Zinc	21.75	China: Hunan, Shuikoushan
L180206P-8-LD180201-0017_HK72WCCXY_L8_clean_bin.125.orig.fa_	OEZ008529	yes	LMSG_G000010392.1	OES073710	NRPS-like	NRPS	Lead-Zinc	13.14	China: Hunan, Shuikoushan
L180206P-8-LD180201-0017_HK72WCCXY_L8_clean_bin.125.orig.fa_	OEZ008529	yes	LMSG_G000010392.1	OES073710	terpene	Terpene	Lead-Zinc	13.12	China: Hunan, Shuikoushan
L180206P-8-LD180201-0017_HK72WCCXY_L8_clean_bin.126.orig.fa_	OEZ008529	yes	LMSG_G000008583.1	OES073710	other	Others	Lead-Zinc	40.41	China: Hunan, Shuikoushan
L180206P-8-LD180201-0017_HK72WCCXY_L8_clean_bin.126.orig.fa_	OEZ008529	yes	LMSG_G000008583.1	OES073710	other	Others	Lead-Zinc	41.18	China: Hunan, Shuikoushan
L180206P-8-LD180201-0017_HK72WCCXY_L8_clean_bin.127.orig.fa_	OEZ008529	yes	LMSG_G000005103.1	OES073710	terpene	Terpene	Lead-Zinc	15.47	China: Hunan, Shuikoushan
L180206P-8-LD180201-0017_HK72WCCXY_L8_clean_bin.127.orig.fa_	OEZ008529	yes	LMSG_G000005103.1	OES073710	hserlactone	Others	Lead-Zinc	20.45	China: Hunan, Shuikoushan
L180206P-8-LD180201-0017_HK72WCCXY_L8_clean_bin.13.orig.fa_k	OEZ008529	yes	LMSG_G000010122.1	OES073710	bacteriocin	RiPPs	Lead-Zinc	10.84	China: Hunan, Shuikoushan
L180206P-8-LD180201-0017_HK72WCCXY_L8_clean_bin.13.orig.fa_k	OEZ008529	yes	LMSG_G000010122.1	OES073710	terpene	Terpene	Lead-Zinc	18.34	China: Hunan, Shuikoushan
L180206P-8-LD180201-0017_HK72WCCXY_L8_clean_bin.131.orig.fa_	OEZ008529	yes	LMSG_G000007938.1	OES073710	bacteriocin	RiPPs	Lead-Zinc	10.85	China: Hunan, Shuikoushan
L180206P-8-LD180201-0017_HK72WCCXY_L8_clean_bin.131.orig.fa_	OEZ008529	yes	LMSG_G000007938.1	OES073710	bacteriocin	RiPPs	Lead-Zinc	10.85	China: Hunan, Shuikoushan
L180206P-8-LD180201-0017_HK72WCCXY_L8_clean_bin.131.orig.fa_	OEZ008529	yes	LMSG_G000007938.1	OES073710	T3PKS	PKSother	Lead-Zinc	25.21	China: Hunan, Shuikoushan

L180206P-8-LD180201-0017_HK72WCCXY_L8_clean_bin.131.orig.fa_	OEZ008529	yes	LMSG_G000007938.1	OES073710	terpene	Terpene	Lead-Zinc	15.34	China: Hunan, Shuikoushan
L180206P-8-LD180201-0017_HK72WCCXY_L8_clean_bin.132.orig.fa_	OEZ008529	yes	LMSG_G000005104.1	OES073710	bacteriocin	RiPPs	Lead-Zinc	7.66	China: Hunan, Shuikoushan
L180206P-8-LD180201-0017_HK72WCCXY_L8_clean_bin.133.orig.fa_	OEZ008529	yes	LMSG_G000004423.1	OES073710	terpene	Terpene	Lead-Zinc	19.23	China: Hunan, Shuikoushan
L180206P-8-LD180201-0017_HK72WCCXY_L8_clean_bin.133.orig.fa_	OEZ008529	yes	LMSG_G000004423.1	OES073710	NRPS-like	NRPS	Lead-Zinc	34.69	China: Hunan, Shuikoushan
L180206P-8-LD180201-0017_HK72WCCXY_L8_clean_bin.133.orig.fa_	OEZ008529	yes	LMSG_G000004423.1	OES073710	T1PKS	PKSI	Lead-Zinc	30.65	China: Hunan, Shuikoushan
L180206P-8-LD180201-0017_HK72WCCXY_L8_clean_bin.17.orig.fa_k	OEZ008529	yes	LMSG_G000004424.1	OES073710	proteusin.bacteriocin	RiPPs	Lead-Zinc	13.03	China: Hunan, Shuikoushan
L180206P-8-LD180201-0017_HK72WCCXY_L8_clean_bin.17.orig.fa_k	OEZ008529	yes	LMSG_G000004424.1	OES073710	betalactone	Others	Lead-Zinc	18.51	China: Hunan, Shuikoushan
L180206P-8-LD180201-0017_HK72WCCXY_L8_clean_bin.17.orig.fa_k	OEZ008529	yes	LMSG_G000004424.1	OES073710	thiopeptide.LAP.bacteriocin	RiPPs	Lead-Zinc	16.87	China: Hunan, Shuikoushan
L180206P-8-LD180201-0017_HK72WCCXY_L8_clean_bin.18.permissiv	OEZ008529	yes	LMSG_G000005106.1	OES073710	betalactone	Others	Lead-Zinc	25.61	China: Hunan, Shuikoushan
L180206P-8-LD180201-0017_HK72WCCXY_L8_clean_bin.18.permissiv	OEZ008529	yes	LMSG_G000005106.1	OES073710	betalactone	Others	Lead-Zinc	18.31	China: Hunan, Shuikoushan
L180206P-8-LD180201-0017_HK72WCCXY_L8_clean_bin.19.orig.fa_k	OEZ008529	yes	LMSG_G000009236.1	OES073710	T3PKS.terpene	Others	Lead-Zinc	46.02	China: Hunan, Shuikoushan
L180206P-8-LD180201-0017_HK72WCCXY_L8_clean_bin.20.orig.fa_k	OEZ008529	yes	LMSG_G000010307.1	OES073710	terpene	Terpene	Lead-Zinc	13.69	China: Hunan, Shuikoushan
L180206P-8-LD180201-0017_HK72WCCXY_L8_clean_bin.20.orig.fa_k	OEZ008529	yes	LMSG_G000010307.1	OES073710	bacteriocin	RiPPs	Lead-Zinc	7.99	China: Hunan, Shuikoushan
L180206P-8-LD180201-0017_HK72WCCXY_L8_clean_bin.21.orig.fa_k	OEZ008529	yes	LMSG_G000005108.1	OES073710	terpene	Terpene	Lead-Zinc	9.39	China: Hunan, Shuikoushan
L180206P-8-LD180201-0017_HK72WCCXY_L8_clean_bin.21.orig.fa_k	OEZ008529	yes	LMSG_G000005108.1	OES073710	bacteriocin	RiPPs	Lead-Zinc	9.42	China: Hunan, Shuikoushan
L180206P-8-LD180201-0017_HK72WCCXY_L8_clean_bin.27.orig.fa_k	OEZ008529	yes	LMSG_G000004425.1	OES073710	bacteriocin	RiPPs	Lead-Zinc	10.29	China: Hunan, Shuikoushan
L180206P-8-LD180201-0017_HK72WCCXY_L8_clean_bin.28.orig.fa_k	OEZ008529	yes	LMSG_G000005111.1	OES073710	terpene	Terpene	Lead-Zinc	7.35	China: Hunan, Shuikoushan
L180206P-8-LD180201-0017_HK72WCCXY_L8_clean_bin.28.orig.fa_k	OEZ008529	yes	LMSG_G000005111.1	OES073710	hserlactone	Others	Lead-Zinc	10.60	China: Hunan, Shuikoushan
L180206P-8-LD180201-0017_HK72WCCXY_L8_clean_bin.28.orig.fa_k	OEZ008529	yes	LMSG_G000005111.1	OES073710	NRPS-like	NRPS	Lead-Zinc	39.48	China: Hunan, Shuikoushan
L180206P-8-LD180201-0017_HK72WCCXY_L8_clean_bin.28.orig.fa_k	OEZ008529	yes	LMSG_G000005111.1	OES073710	terpene	Terpene	Lead-Zinc	12.36	China: Hunan, Shuikoushan
L180206P-8-LD180201-0017_HK72WCCXY_L8_clean_bin.28.orig.fa_k	OEZ008529	yes	LMSG_G000005111.1	OES073710	terpene	Terpene	Lead-Zinc	13.72	China: Hunan, Shuikoushan
L180206P-8-LD180201-0017_HK72WCCXY_L8_clean_bin.29.orig.fa_k	OEZ008529	yes	LMSG_G000005112.1	OES073710	bacteriocin	RiPPs	Lead-Zinc	10.90	China: Hunan, Shuikoushan
L180206P-8-LD180201-0017_HK72WCCXY_L8_clean_bin.29.orig.fa_k	OEZ008529	yes	LMSG_G000005112.1	OES073710	sactipeptide	RiPPs	Lead-Zinc	20.16	China: Hunan, Shuikoushan
L180206P-8-LD180201-0017_HK72WCCXY_L8_clean_bin.3.orig.fa_k1	OEZ008529	yes	LMSG_G000005118.1	OES073710	terpene	Terpene	Lead-Zinc	14.94	China: Hunan, Shuikoushan
L180206P-8-LD180201-0017_HK72WCCXY_L8_clean_bin.3.orig.fa_k1	OEZ008529	yes	LMSG_G000005118.1	OES073710	NRPS-like	NRPS	Lead-Zinc	22.22	China: Hunan, Shuikoushan
L180206P-8-LD180201-0017_HK72WCCXY_L8_clean_bin.3.orig.fa_k1	OEZ008529	no	LMSG_G000005118.1	OES073710	ladderane	Others	Lead-Zinc	22.00	China: Hunan, Shuikoushan
L180206P-8-LD180201-0017_HK72WCCXY_L8_clean_bin.30.orig.fa_k	OEZ008529	yes	LMSG_G000004426.1	OES073710	T3PKS	PKSother	Lead-Zinc	40.25	China: Hunan, Shuikoushan
L180206P-8-LD180201-0017_HK72WCCXY_L8_clean_bin.30.orig.fa_k	OEZ008529	yes	LMSG_G000004426.1	OES073710	bacteriocin	RiPPs	Lead-Zinc	11.88	China: Hunan, Shuikoushan
L180206P-8-LD180201-0017_HK72WCCXY_L8_clean_bin.30.orig.fa_k	OEZ008529	yes	LMSG_G000004426.1	OES073710	bacteriocin	RiPPs	Lead-Zinc	10.81	China: Hunan, Shuikoushan
L180206P-8-LD180201-0017_HK72WCCXY_L8_clean_bin.30.orig.fa_k	OEZ008529	yes	LMSG_G000004426.1	OES073710	betalactone	Others	Lead-Zinc	17.91	China: Hunan, Shuikoushan
L180206P-8-LD180201-0017_HK72WCCXY_L8_clean_bin.30.orig.fa_k	OEZ008529	yes	LMSG_G000004426.1	OES073710	T3PKS	PKSother	Lead-Zinc	25.23	China: Hunan, Shuikoushan
L180206P-8-LD180201-0017_HK72WCCXY_L8_clean_bin.30.orig.fa_k	OEZ008529	yes	LMSG_G000004426.1	OES073710	TfuA-related	RiPPs	Lead-Zinc	22.34	China: Hunan, Shuikoushan
L180206P-8-LD180201-0017_HK72WCCXY_L8_clean_bin.31.orig.fa_k	OEZ008529	yes	LMSG_G000005114.1	OES073710	bacteriocin	RiPPs	Lead-Zinc	11.79	China: Hunan, Shuikoushan
L180206P-8-LD180201-0017_HK72WCCXY_L8_clean_bin.37.orig.fa_k	OEZ008529	yes	LMSG_G000004428.1	OES073710	terpene	Terpene	Lead-Zinc	22.23	China: Hunan, Shuikoushan
L180206P-8-LD180201-0017_HK72WCCXY_L8_clean_bin.37.orig.fa_k	OEZ008529	yes	LMSG_G000004428.1	OES073710	transAT-PKS-like	PKSother	Lead-Zinc	38.22	China: Hunan, Shuikoushan
L180206P-8-LD180201-0017_HK72WCCXY_L8_clean_bin.37.orig.fa_k	OEZ008529	yes	LMSG_G000004428.1	OES073710	NRPS	NRPS	Lead-Zinc	26.40	China: Hunan, Shuikoushan
L180206P-8-LD180201-0017_HK72WCCXY_L8_clean_bin.38.orig.fa_k	OEZ008529	yes	LMSG_G000010485.1	OES073710	hserlactone	Others	Lead-Zinc	13.75	China: Hunan, Shuikoushan
L180206P-8-LD180201-0017_HK72WCCXY_L8_clean_bin.38.orig.fa_k	OEZ008529	yes	LMSG_G000010485.1	OES073710	terpene	Terpene	Lead-Zinc	23.04	China: Hunan, Shuikoushan
L180206P-8-LD180201-0017_HK72WCCXY_L8_clean_bin.38.orig.fa_k	OEZ008529	yes	LMSG_G000010485.1	OES073710	arylpolyene	Others	Lead-Zinc	21.79	China: Hunan, Shuikoushan
L180206P-8-LD180201-0017_HK72WCCXY_L8_clean_bin.4.orig.fa_k1	OEZ008529	yes	LMSG_G000010919.1	OES073710	arylpolyene	Others	Lead-Zinc	34.86	China: Hunan, Shuikoushan
L180206P-8-LD180201-0017_HK72WCCXY_L8_clean_bin.4.orig.fa_k1	OEZ008529	yes	LMSG_G000010919.1	OES073710	terpene	Terpene	Lead-Zinc	21.66	China: Hunan, Shuikoushan
L180206P-8-LD180201-0017_HK72WCCXY_L8_clean_bin.4.orig.fa_k1	OEZ008529	yes	LMSG_G000010919.1	OES073710	thiopeptide	RiPPs	Lead-Zinc	27.43	China: Hunan, Shuikoushan
L180206P-8-LD180201-0017_HK72WCCXY_L8_clean_bin.40.orig.fa_k	OEZ008529	yes	LMSG_G000008604.1	OES073710	other	Others	Lead-Zinc	13.15	China: Hunan, Shuikoushan
L180206P-8-LD180201-0017_HK72WCCXY_L8_clean_bin.40.orig.fa_k	OEZ008529	yes	LMSG_G000008604.1	OES073710	other	Others	Lead-Zinc	17.79	China: Hunan, Shuikoushan
L180206P-8-LD180201-0017_HK72WCCXY_L8_clean_bin.41.orig.fa_k	OEZ008529	yes	LMSG_G000004429.1	OES073710	terpene	Terpene	Lead-Zinc	20.91	China: Hunan, Shuikoushan
L180206P-8-LD180201-0017_HK72WCCXY_L8_clean_bin.43.orig.fa_k	OEZ008529	yes	LMSG_G000005119.1	OES073710	terpene	Terpene	Lead-Zinc	11.38	China: Hunan, Shuikoushan
L180206P-8-LD180201-0017_HK72WCCXY_L8_clean_bin.43.orig.fa_k	OEZ008529	yes	LMSG_G000005119.1	OES073710	lanthipeptide	RiPPs	Lead-Zinc	16.81	China: Hunan, Shuikoushan
L180206P-8-LD180201-0017_HK72WCCXY_L8_clean_bin.43.orig.fa_k	OEZ008529	yes	LMSG_G000005119.1	OES073710	T1PKS	PKSI	Lead-Zinc	45.00	China: Hunan, Shuikoushan
L180206P-8-LD180201-0017_HK72WCCXY_L8_clean_bin.43.orig.fa_k	OEZ008529	yes	LMSG_G000005119.1	OES073710	terpene	Terpene	Lead-Zinc	17.08	China: Hunan, Shuikoushan
L180206P-8-LD180201-0017_HK72WCCXY_L8_clean_bin.43.orig.fa_k	OEZ008529	yes	LMSG_G000005119.1	OES073710	T1PKS.hglE-KS	PKSother	Lead-Zinc	32.52	China: Hunan, Shuikoushan
L180206P-8-LD180201-0017_HK72WCCXY_L8_clean_bin.44.strict.fa	OEZ008529	yes	LMSG_G000005120.1	OES073710	arylpolyene	Others	Lead-Zinc	47.18	China: Hunan, Shuikoushan
L180206P-8-LD180201-0017_HK72WCCXY_L8_clean_bin.46.orig.fa_k	OEZ008529	yes	LMSG_G000010864.1	OES073710	betalactone.arylpolyene	Others	Lead-Zinc	47.16	China: Hunan, Shuikoushan
L180206P-8-LD180201-0017_HK72WCCXY_L8_clean_bin.46.orig.fa_k	OEZ008529	yes	LMSG_G000010864.1	OES073710	acyl_amino_acids	Others	Lead-Zinc	30.67	China: Hunan, Shuikoushan
L180206P-8-LD180201-0017_HK72WCCXY_L8_clean_bin.46.orig.fa_k	OEZ008529	yes	LMSG_G000010864.1	OES073710	acyl_amino_acids	Others	Lead-Zinc	23.92	China: Hunan, Shuikoushan
L180206P-8-LD180201-0017_HK72WCCXY_L8_clean_bin.46.orig.fa_k	OEZ008529	yes	LMSG_G000010864.1	OES073710	terpene	Terpene	Lead-Zinc	18.63	China: Hunan, Shuikoushan
L180206P-8-LD180201-0017_HK72WCCXY_L8_clean_bin.48.orig.fa_k	OEZ008529	yes	LMSG_G000010720.1	OES073710	arylpolyene	Others	Lead-Zinc	10.65	China: Hunan, Shuikoushan
L180206P-8-LD180201-0017_HK72WCCXY_L8_clean_bin.48.orig.fa_k	OEZ008529	yes	LMSG_G000010720.1	OES073710	bacteriocin	RiPPs	Lead-Zinc	10.10	China: Hunan, Shuikoushan
L180206P-8-LD180201-0017_HK72WCCXY_L8_clean_bin.48.orig.fa_k	OEZ008529	yes	LMSG_G000010720.1	OES073710	NRPS.T1PKS	PKS-NRP_Hybrids	Lead-Zinc	7.03	China: Hunan, Shuikoushan
L180206P-8-LD180201-0017_HK72WCCXY_L8_clean_bin.48.orig.fa_k	OEZ008529	yes	LMSG_G000010720.1	OES073710	NRPS	NRPS	Lead-Zinc	28.24	China: Hunan, Shuikoushan
L180206P-8-LD180201-0017_HK72WCCXY_L8_clean_bin.48.orig.fa_k	OEZ008529	yes	LMSG_G000010720.1	OES073710	terpene	Terpene	Lead-Zinc	12.52	China: Hunan, Shuikoushan
L180206P-8-LD180201-0017_HK72WCCXY_L8_clean_bin.48.orig.fa_k	OEZ008529	yes	LMSG_G000010720.1	OES073710	arylpolyene	Others	Lead-Zinc	20.34	China: Hunan, Shuikoushan
L180206P-8-LD180201-0017_HK72WCCXY_L8_clean_bin.48.orig.fa_k	OEZ008529	yes	LMSG_G000010720.1	OES073710	NRPS	NRPS	Lead-Zinc	5.11	China: Hunan, Shuikoushan
L180206P-8-LD180201-0017_HK72WCCXY_L8_clean_bin.48.orig.fa_k	OEZ008529	yes	LMSG_G000010720.1	OES073710	NRPS-like	NRPS	Lead-Zinc	42.86	China: Hunan, Shuikoushan
L180206P-8-LD180201-0017_HK72WCCXY_L8_clean_bin.5.strict.fa_	OEZ008529	yes	LMSG_G000005125.1	OES073710	hglE-KS	PKSother	Lead-Zinc	21.21	China: Hunan, Shuikoushan
L180206P-8-LD180201-0017_HK72WCCXY_L8_clean_bin.5.strict.fa_	OEZ008529	yes	LMSG_G000005125.1	OES073710	terpene	Terpene	Lead-Zinc	19.28	China: Hunan, Shuikoushan
L180206P-8-LD180201-0017_HK72WCCXY_L8_clean_bin.50.strict.fa	OEZ008529	no	LMSG_G000010530.1	OES073710	terpene	Terpene	Lead-Zinc	9.66	China: Hunan, Shuikoushan
L180206P-8-LD180201-0017_HK72WCCXY_L8_clean_bin.51.orig.fa_k	OEZ008529	yes	LMSG_G000005121.1	OES073710	T1PKS.hglE-KS	PKSother	Lead-Zinc	31.58	China: Hunan, Shuikoushan
L180206P-8-LD180201-0017_HK72WCCXY_L8_clean_bin.51.orig.fa_k	OEZ008529	yes	LMSG_G000005121.1	OES073710	NRPS	NRPS	Lead-Zinc	42.59	China: Hunan, Shuikoushan
L180206P-8-LD180201-0017_HK72WCCXY_L8_clean_bin.51.orig.fa_k	OEZ008529	yes	LMSG_G000005121.1	OES073710	lanthipeptide	RiPPs	Lead-Zinc	22.66	China: Hunan, Shuikoushan
L180206P-8-LD180201-0017_HK72WCCXY_L8_clean_bin.51.orig.fa_k	OEZ008529	yes	LMSG_G000005121.1	OES073710	T1PKS	PKSI	Lead-Zinc	30.07	China: Hunan, Shuikoushan
L180206P-8-LD180201-0017_HK72WCCXY_L8_clean_bin.51.orig.fa_k	OEZ008529	yes	LMSG_G000005121.1	OES073710	terpene	Terpene	Lead-Zinc	17.38	China: Hunan, Shuikoushan
L180206P-8-LD180201-0017_HK72WCCXY_L8_clean_bin.52.orig.fa_k	OEZ008529	yes	LMSG_G000006225.1	OES073710	T3PKS	PKSother	Lead-Zinc	21.00	China: Hunan, Shuikoushan
L180206P-8-LD180201-0017_HK72WCCXY_L8_clean_bin.52.orig.fa_k	OEZ008529	yes	LMSG_G000006225.1	OES073710	terpene	Terpene	Lead-Zinc	22.01	China: Hunan, Shuikoushan
L180206P-8-LD180201-0017_HK72WCCXY_L8_clean_bin.52.orig.fa_k	OEZ008529	yes	LMSG_G000006225.1	OES073710	NRPS-like	NRPS	Lead-Zinc	14.64	China: Hunan, Shuikoushan
L180206P-8-LD180201-0017_HK72WCCXY_L8_clean_bin.53.orig.fa_k	OEZ008529	yes	LMSG_G000009204.1	OES073710	terpene	Terpene	Lead-Zinc	5.83	China: Hunan, Shuikoushan
L180206P-8-LD180201-0017_HK72WCCXY_L8_clean_bin.55.orig.fa_k	OEZ008529	yes	LMSG_G000010932.1	OES073710	terpene	Terpene	Lead-Zinc	9.26	China: Hunan, Shuikoushan
L180206P-8-LD180201-0017_HK72WCCXY_L8_clean_bin.56.strict.fa	OEZ008529	yes	LMSG_G000005123.1	OES073710	T3PKS	PKSother	Lead-Zinc	23.12	China: Hunan, Shuikoushan
L180206P-8-LD180201-0017_HK72WCCXY_L8_clean_bin.6.permissive	OEZ008529	yes	LMSG_G000008864.1	OES073710	terpene	Terpene	Lead-Zinc	18.23	China: Hunan, Shuikoushan
L180206P-8-LD180201-0017_HK72WCCXY_L8_clean_bin.6.permissive	OEZ008529	yes	LMSG_G000008864.1	OES073710	T3PKS	PKSother	Lead-Zinc	13.61	China: Hunan, Shuikoushan
L180206P-8-LD180201-0017_HK72WCCXY_L8_clean_bin.61.orig.fa_k	OEZ008529	yes	LMSG_G000005127.1	OES073710	bacteriocin.arylpolyene	Others	Lead-Zinc	41.19	China: Hunan, Shuikoushan
L180206P-8-LD180201-0017_HK72WCCXY_L8_clean_bin.61.orig.fa_k	OEZ008529	yes	LMSG_G000005127.1	OES073710	acyl_amino_acids	Others	Lead-Zinc	28.21	China: Hunan, Shuikoushan
L180206P-8-LD180201-0017_HK72WCCXY_L8_clean_bin.62.orig.fa_k	OEZ008529	yes	LMSG_G000005128.1	OES073710	betalactone	Others	Lead-Zinc	22.58	China: Hunan, Shuikoushan
L180206P-8-LD180201-0017_HK72WCCXY_L8_clean_bin.63.orig.fa_k	OEZ008529	yes	LMSG_G000007923.1	OES073710	bacteriocin	RiPPs	Lead-Zinc	10.80	China: Hunan, Shuikoushan
L180206P-8-LD180201-0017_HK72WCCXY_L8_clean_bin.64.strict.fa	OEZ008529	yes	LMSG_G000007992.1	OES073710	TfuA-related.bacteriocin	RiPPs	Lead-Zinc	15.15	China: Hunan, Shuikoushan
L180206P-8-LD180201-0017_HK72WCCXY_L8_clean_bin.64.strict.fa	OEZ008529	yes	LMSG_G000007992.1	OES073710	bacteriocin	RiPPs	Lead-Zinc	5.26	China: Hunan, Shuikoushan
L180206P-8-LD180201-0017_HK72WCCXY_L8_clean_bin.67.orig.fa_k	OEZ008529	yes	LMSG_G000005129.1	OES073710	arylpolyene	Others	Lead-Zinc	7.02	China: Hunan, Shuikoushan
L180206P-8-LD180201-0017_HK72WCCXY_L8_clean_bin.67.orig.fa_k	OEZ008529	yes	LMSG_G000005129.1	OES073710	arylpolyene.bacteriocin	Others	Lead-Zinc	6.83	China: Hunan, Shuikoushan
L180206P-8-LD180201-0017_HK72WCCXY_L8_clean_bin.69.orig.fa_k	OEZ008529	yes	LMSG_G000005130.1	OES073710	terpene	Terpene	Lead-Zinc	24.24	China: Hunan, Shuikoushan
L180206P-8-LD180201-0017_HK72WCCXY_L8_clean_bin.7.orig.fa_k1	OEZ008529	yes	LMSG_G000006226.1	OES073710	terpene	Terpene	Lead-Zinc	17.11	China: Hunan, Shuikoushan
L180206P-8-LD180201-0017_HK72WCCXY_L8_clean_bin.70.permissiv	OEZ008529	yes	LMSG_G000005131.1	OES073710	LAP	RiPPs	Lead-Zinc	22.15	China: Hunan, Shuikoushan
L180206P-8-LD180201-0017_HK72WCCXY_L8_clean_bin.70.permissiv	OEZ008529	yes	LMSG_G000005131.1	OES073710	acyl_amino_acids	Others	Lead-Zinc	60.72	China: Hunan, Shuikoushan
L180206P-8-LD180201-0017_HK72WCCXY_L8_clean_bin.71.orig.fa_k	OEZ008529	yes	LMSG_G000010331.1	OES073710	T1PKS	PKSI	Lead-Zinc	27.46	China: Hunan, Shuikoushan
L180206P-8-LD180201-0017_HK72WCCXY_L8_clean_bin.72.orig.fa_k	OEZ008529	yes	LMSG_G000008917.1	OES073710	hglE-KS	PKSother	Lead-Zinc	20.41	China: Hunan, Shuikoushan
L180206P-8-LD180201-0017_HK72WCCXY_L8_clean_bin.73.orig.fa_k	OEZ008529	yes	LMSG_G000008971.1	OES073710	NRPS-like	NRPS	Lead-Zinc	13.68	China: Hunan, Shuikoushan
L180206P-8-LD180201-0017_HK72WCCXY_L8_clean_bin.75.orig.fa_k	OEZ008529	yes	LMSG_G000005132.1	OES073710	TfuA-related	RiPPs	Lead-Zinc	7.95	China: Hunan, Shuikoushan
L180206P-8-LD180201-0017_HK72WCCXY_L8_clean_bin.78.permissiv	OEZ008529	yes	LMSG_G000005134.1	OES073710	T3PKS	PKSother	Lead-Zinc	5.49	China: Hunan, Shuikoushan
L180206P-8-LD180201-0017_HK72WCCXY_L8_clean_bin.80.orig.fa_k	OEZ008529	yes	LMSG_G000010930.1	OES073710	arylpolyene	Others	Lead-Zinc	38.88	China: Hunan, Shuikoushan
L180206P-8-LD180201-0017_HK72WCCXY_L8_clean_bin.80.orig.fa_k	OEZ008529	yes	LMSG_G000010930.1	OES073710	acyl_amino_acids	Others	Lead-Zinc	15.79	China: Hunan, Shuikoushan
L180206P-8-LD180201-0017_HK72WCCXY_L8_clean_bin.80.orig.fa_k	OEZ008529	yes	LMSG_G000010930.1	OES073710	bacteriocin	RiPPs	Lead-Zinc	9.96	China: Hunan, Shuikoushan
L180206P-8-LD180201-0017_HK72WCCXY_L8_clean_bin.80.orig.fa_k	OEZ008529	no	LMSG_G000010930.1	OES073710	terpene	Terpene	Lead-Zinc	11.96	China: Hunan, Shuikoushan
L180206P-8-LD180201-0017_HK72WCCXY_L8_clean_bin.80.orig.fa_k	OEZ008529	yes	LMSG_G000010930.1	OES073710	bacteriocin	RiPPs	Lead-Zinc	10.74	China: Hunan, Shuikoushan
L180206P-8-LD180201-0017_HK72WCCXY_L8_clean_bin.80.orig.fa_k	OEZ008529	yes	LMSG_G000010930.1	OES073710	terpene	Terpene	Lead-Zinc	13.32	China: Hunan, Shuikoushan
L180206P-8-LD180201-0017_HK72WCCXY_L8_clean_bin.81.orig.fa_k	OEZ008529	yes	LMSG_G000008172.1	OES073710	terpene	Terpene	Lead-Zinc	21.08	China: Hunan, Shuikoushan
L180206P-8-LD180201-0017_HK72WCCXY_L8_clean_bin.84.permissiv	OEZ008529	yes	LMSG_G000005135.1	OES073710	hserlactone	Others	Lead-Zinc	20.50	China: Hunan, Shuikoushan
L180206P-8-LD180201-0017_HK72WCCXY_L8_clean_bin.86.permissiv	OEZ008529	yes	LMSG_G000010425.1	OES073710	hserlactone	Others	Lead-Zinc	5.54	China: Hunan, Shuikoushan
L180206P-8-LD180201-0017_HK72WCCXY_L8_clean_bin.87.orig.fa_k	OEZ008529	yes	LMSG_G000005137.1	OES073710	bacteriocin	RiPPs	Lead-Zinc	6.36	China: Hunan, Shuikoushan
L180206P-8-LD180201-0017_HK72WCCXY_L8_clean_bin.87.orig.fa_k	OEZ008529	yes	LMSG_G000005137.1	OES073710	terpene	Terpene	Lead-Zinc	12.75	China: Hunan, Shuikoushan
L180206P-8-LD180201-0017_HK72WCCXY_L8_clean_bin.87.orig.fa_k	OEZ008529	yes	LMSG_G000005137.1	OES073710	T3PKS	PKSother	Lead-Zinc	32.95	China: Hunan, Shuikoushan
L180206P-8-LD180201-0017_HK72WCCXY_L8_clean_bin.87.orig.fa_k	OEZ008529	yes	LMSG_G000005137.1	OES073710	T1PKS.hglE-KS	PKSother	Lead-Zinc	34.36	China: Hunan, Shuikoushan
L180206P-8-LD180201-0017_HK72WCCXY_L8_clean_bin.87.orig.fa_k	OEZ008529	yes	LMSG_G000005137.1	OES073710	terpene	Terpene	Lead-Zinc	22.03	China: Hunan, Shuikoushan
L180206P-8-LD180201-0017_HK72WCCXY_L8_clean_bin.88.strict.fa	OEZ008529	yes	LMSG_G000010631.1	OES073710	bacteriocin	RiPPs	Lead-Zinc	10.99	China: Hunan, Shuikoushan
L180206P-8-LD180201-0017_HK72WCCXY_L8_clean_bin.88.strict.fa	OEZ008529	yes	LMSG_G000010631.1	OES073710	terpene	Terpene	Lead-Zinc	14.29	China: Hunan, Shuikoushan
L180206P-8-LD180201-0017_HK72WCCXY_L8_clean_bin.88.strict.fa	OEZ008529	yes	LMSG_G000010631.1	OES073710	arylpolyene	Others	Lead-Zinc	17.58	China: Hunan, Shuikoushan
L180206P-8-LD180201-0017_HK72WCCXY_L8_clean_bin.88.strict.fa	OEZ008529	no	LMSG_G000010631.1	OES073710	terpene	Terpene	Lead-Zinc	6.16	China: Hunan, Shuikoushan
L180206P-8-LD180201-0017_HK72WCCXY_L8_clean_bin.89.orig.fa_k	OEZ008529	yes	LMSG_G000005138.1	OES073710	terpene	Terpene	Lead-Zinc	8.54	China: Hunan, Shuikoushan
L180206P-8-LD180201-0017_HK72WCCXY_L8_clean_bin.89.orig.fa_k	OEZ008529	yes	LMSG_G000005138.1	OES073710	terpene	Terpene	Lead-Zinc	15.22	China: Hunan, Shuikoushan
L180206P-8-LD180201-0017_HK72WCCXY_L8_clean_bin.89.orig.fa_k	OEZ008529	yes	LMSG_G000005138.1	OES073710	NRPS.T1PKS	PKS-NRP_Hybrids	Lead-Zinc	27.82	China: Hunan, Shuikoushan
L180206P-8-LD180201-0017_HK72WCCXY_L8_clean_bin.90.orig.fa_k	OEZ008529	yes	LMSG_G000010902.1	OES073710	NRPS-like	NRPS	Lead-Zinc	16.62	China: Hunan, Shuikoushan
L180206P-8-LD180201-0017_HK72WCCXY_L8_clean_bin.90.orig.fa_k	OEZ008529	yes	LMSG_G000010902.1	OES073710	arylpolyene	Others	Lead-Zinc	19.00	China: Hunan, Shuikoushan
L180206P-8-LD180201-0017_HK72WCCXY_L8_clean_bin.90.orig.fa_k	OEZ008529	yes	LMSG_G000010902.1	OES073710	hserlactone	Others	Lead-Zinc	12.75	China: Hunan, Shuikoushan
L180206P-8-LD180201-0017_HK72WCCXY_L8_clean_bin.90.orig.fa_k	OEZ008529	yes	LMSG_G000010902.1	OES073710	terpene	Terpene	Lead-Zinc	21.68	China: Hunan, Shuikoushan
L180206P-8-LD180201-0017_HK72WCCXY_L8_clean_bin.93.strict.fa	OEZ008529	yes	LMSG_G000008786.1	OES073710	bacteriocin	RiPPs	Lead-Zinc	12.21	China: Hunan, Shuikoushan
L180206P-8-LD180201-0017_HK72WCCXY_L8_clean_bin.93.strict.fa	OEZ008529	yes	LMSG_G000008786.1	OES073710	NRPS-like	NRPS	Lead-Zinc	22.59	China: Hunan, Shuikoushan
L180206P-8-LD180201-0017_HK72WCCXY_L8_clean_bin.93.strict.fa	OEZ008529	yes	LMSG_G000008786.1	OES073710	NRPS-like	NRPS	Lead-Zinc	18.60	China: Hunan, Shuikoushan
L180206P-8-LD180201-0017_HK72WCCXY_L8_clean_bin.94.orig.fa_k	OEZ008529	yes	LMSG_G000005140.1	OES073710	betalactone	Others	Lead-Zinc	13.58	China: Hunan, Shuikoushan
L180206P-8-LD180201-0017_HK72WCCXY_L8_clean_bin.94.orig.fa_k	OEZ008529	yes	LMSG_G000005140.1	OES073710	terpene	Terpene	Lead-Zinc	18.84	China: Hunan, Shuikoushan
L180206P-8-LD180201-0017_HK72WCCXY_L8_clean_bin.94.orig.fa_k	OEZ008529	yes	LMSG_G000005140.1	OES073710	betalactone	Others	Lead-Zinc	8.89	China: Hunan, Shuikoushan
L180206P-8-LD180201-0017_HK72WCCXY_L8_clean_bin.97.strict.fa	OEZ008529	yes	LMSG_G000009689.1	OES073710	NRPS-like	NRPS	Lead-Zinc	21.56	China: Hunan, Shuikoushan
L180206P-8-LD180201-0017_HK72WCCXY_L8_clean_bin.98.permissiv	OEZ008529	no	LMSG_G000009548.1	OES073710	terpene	Terpene	Lead-Zinc	14.86	China: Hunan, Shuikoushan
L180206P-8-LD180201-0017_HK72WCCXY_L8_clean_bin.98.permissiv	OEZ008529	no	LMSG_G000009548.1	OES073710	terpene	Terpene	Lead-Zinc	13.19	China: Hunan, Shuikoushan
L180206P-8-LD180201-0017_HK72WCCXY_L8_clean_bin.98.permissiv	OEZ008529	no	LMSG_G000009548.1	OES073710	NRPS-like	NRPS	Lead-Zinc	12.07	China: Hunan, Shuikoushan
L180206P-8-LD180201-0017_HK72WCCXY_L8_clean_bin.98.permissiv	OEZ008529	yes	LMSG_G000009548.1	OES073710	ectoine	Others	Lead-Zinc	10.40	China: Hunan, Shuikoushan
L180322-P-10-LD180320-0031_HL3MGCCXY_L6_clean_bin.13.orig.fa	OEZ008529	yes	LMSG_G000007762.1	OES073701	terpene	Terpene	Polymetallic	29.77	China: Guangxi, Longshenao
L180322-P-10-LD180320-0031_HL3MGCCXY_L6_clean_bin.13.orig.fa	OEZ008529	yes	LMSG_G000007762.1	OES073701	T3PKS	PKSother	Polymetallic	11.81	China: Guangxi, Longshenao
L180322-P-10-LD180320-0031_HL3MGCCXY_L6_clean_bin.13.orig.fa	OEZ008529	yes	LMSG_G000007762.1	OES073701	NRPS-like	NRPS	Polymetallic	10.20	China: Guangxi, Longshenao
L180322-P-10-LD180320-0031_HL3MGCCXY_L6_clean_bin.15.orig.fa	OEZ008529	yes	LMSG_G000010010.1	OES073701	arylpolyene	Others	Polymetallic	6.37	China: Guangxi, Longshenao
L180322-P-10-LD180320-0031_HL3MGCCXY_L6_clean_bin.15.orig.fa	OEZ008529	yes	LMSG_G000010010.1	OES073701	terpene	Terpene	Polymetallic	7.14	China: Guangxi, Longshenao
L180322-P-10-LD180320-0031_HL3MGCCXY_L6_clean_bin.15.orig.fa	OEZ008529	yes	LMSG_G000010010.1	OES073701	terpene	Terpene	Polymetallic	15.45	China: Guangxi, Longshenao
L180322-P-10-LD180320-0031_HL3MGCCXY_L6_clean_bin.15.orig.fa	OEZ008529	yes	LMSG_G000010010.1	OES073701	ladderane	Others	Polymetallic	8.77	China: Guangxi, Longshenao
L180322-P-10-LD180320-0031_HL3MGCCXY_L6_clean_bin.16.orig.fa	OEZ008529	yes	LMSG_G000008267.1	OES073701	terpene	Terpene	Polymetallic	19.12	China: Guangxi, Longshenao
L180322-P-10-LD180320-0031_HL3MGCCXY_L6_clean_bin.18.orig.fa	OEZ008529	yes	LMSG_G000005147.1	OES073701	T3PKS	PKSother	Polymetallic	24.23	China: Guangxi, Longshenao
L180322-P-10-LD180320-0031_HL3MGCCXY_L6_clean_bin.2.orig.fa_	OEZ008529	yes	LMSG_G000008224.1	OES073701	terpene	Terpene	Polymetallic	14.59	China: Guangxi, Longshenao
L180322-P-10-LD180320-0031_HL3MGCCXY_L6_clean_bin.20.orig.fa	OEZ008529	no	LMSG_G000010295.1	OES073701	terpene	Terpene	Polymetallic	15.39	China: Guangxi, Longshenao
L180322-P-10-LD180320-0031_HL3MGCCXY_L6_clean_bin.20.orig.fa	OEZ008529	no	LMSG_G000010295.1	OES073701	arylpolyene	Others	Polymetallic	16.85	China: Guangxi, Longshenao
L180322-P-10-LD180320-0031_HL3MGCCXY_L6_clean_bin.21.orig.fa	OEZ008529	yes	LMSG_G000008742.1	OES073701	terpene	Terpene	Polymetallic	19.24	China: Guangxi, Longshenao
L180322-P-10-LD180320-0031_HL3MGCCXY_L6_clean_bin.21.orig.fa	OEZ008529	yes	LMSG_G000008742.1	OES073701	NRPS-like	NRPS	Polymetallic	7.14	China: Guangxi, Longshenao
L180322-P-10-LD180320-0031_HL3MGCCXY_L6_clean_bin.23.orig.fa	OEZ008529	yes	LMSG_G000008553.1	OES073701	hglE-KS	PKSother	Polymetallic	17.52	China: Guangxi, Longshenao
L180322-P-10-LD180320-0031_HL3MGCCXY_L6_clean_bin.24.orig.fa	OEZ008529	yes	LMSG_G000004430.1	OES073701	T1PKS	PKSI	Polymetallic	22.44	China: Guangxi, Longshenao
L180322-P-10-LD180320-0031_HL3MGCCXY_L6_clean_bin.24.orig.fa	OEZ008529	yes	LMSG_G000004430.1	OES073701	terpene	Terpene	Polymetallic	15.12	China: Guangxi, Longshenao
L180322-P-10-LD180320-0031_HL3MGCCXY_L6_clean_bin.24.orig.fa	OEZ008529	yes	LMSG_G000004430.1	OES073701	NRPS-like	NRPS	Polymetallic	31.31	China: Guangxi, Longshenao
L180322-P-10-LD180320-0031_HL3MGCCXY_L6_clean_bin.25.orig.fa	OEZ008529	yes	LMSG_G000005148.1	OES073701	T1PKS	PKSI	Polymetallic	15.77	China: Guangxi, Longshenao
L180322-P-10-LD180320-0031_HL3MGCCXY_L6_clean_bin.25.orig.fa	OEZ008529	yes	LMSG_G000005148.1	OES073701	T1PKS	PKSI	Polymetallic	10.21	China: Guangxi, Longshenao
L180322-P-10-LD180320-0031_HL3MGCCXY_L6_clean_bin.25.orig.fa	OEZ008529	yes	LMSG_G000005148.1	OES073701	terpene	Terpene	Polymetallic	16.88	China: Guangxi, Longshenao
L180322-P-10-LD180320-0031_HL3MGCCXY_L6_clean_bin.25.orig.fa	OEZ008529	yes	LMSG_G000005148.1	OES073701	arylpolyene	Others	Polymetallic	24.65	China: Guangxi, Longshenao
L180322-P-10-LD180320-0031_HL3MGCCXY_L6_clean_bin.26.orig.fa	OEZ008529	yes	LMSG_G000005149.1	OES073701	terpene	Terpene	Polymetallic	19.62	China: Guangxi, Longshenao
L180322-P-10-LD180320-0031_HL3MGCCXY_L6_clean_bin.3.strict.f	OEZ008529	yes	LMSG_G000007858.1	OES073701	T3PKS	PKSother	Polymetallic	9.28	China: Guangxi, Longshenao
L180322-P-10-LD180320-0031_HL3MGCCXY_L6_clean_bin.3.strict.f	OEZ008529	yes	LMSG_G000007858.1	OES073701	terpene	Terpene	Polymetallic	7.89	China: Guangxi, Longshenao
L180322-P-10-LD180320-0031_HL3MGCCXY_L6_clean_bin.33.orig.fa	OEZ008529	yes	LMSG_G000004431.1	OES073701	terpene	Terpene	Polymetallic	21.92	China: Guangxi, Longshenao

L180322-P-10-LD180320-0031_HL3MGCCXY_L6_clean_bin.33.orig.fa	OEZ008529	yes	LMSG_G000004431.1	OES073701	T3PKS	PKSother	Polymetallic	16.88	China: Guangxi, Longshenao
L180322-P-10-LD180320-0031_HL3MGCCXY_L6_clean_bin.34.orig.fa	OEZ008529	yes	LMSG_G000008072.1	OES073701	T1PKS.hglE-KS	PKSother	Polymetallic	15.87	China: Guangxi, Longshenao
L180322-P-10-LD180320-0031_HL3MGCCXY_L6_clean_bin.34.orig.fa	OEZ008529	yes	LMSG_G000008072.1	OES073701	other	Others	Polymetallic	9.06	China: Guangxi, Longshenao
L180322-P-10-LD180320-0031_HL3MGCCXY_L6_clean_bin.34.orig.fa	OEZ008529	yes	LMSG_G000008072.1	OES073701	arylpolyene	Others	Polymetallic	7.22	China: Guangxi, Longshenao
L180322-P-10-LD180320-0031_HL3MGCCXY_L6_clean_bin.39.permiss	OEZ008529	yes	LMSG_G000007975.1	OES073701	TfuA-related.bacteriocin	RiPPs	Polymetallic	13.29	China: Guangxi, Longshenao
L180322-P-10-LD180320-0031_HL3MGCCXY_L6_clean_bin.39.permiss	OEZ008529	yes	LMSG_G000007975.1	OES073701	bacteriocin	RiPPs	Polymetallic	10.22	China: Guangxi, Longshenao
L180322-P-10-LD180320-0031_HL3MGCCXY_L6_clean_bin.40.orig.fa	OEZ008529	yes	LMSG_G000010044.1	OES073701	ladderane	Others	Polymetallic	14.47	China: Guangxi, Longshenao
L180322-P-10-LD180320-0031_HL3MGCCXY_L6_clean_bin.40.orig.fa	OEZ008529	yes	LMSG_G000010044.1	OES073701	T3PKS	PKSother	Polymetallic	13.54	China: Guangxi, Longshenao
L180322-P-10-LD180320-0031_HL3MGCCXY_L6_clean_bin.40.orig.fa	OEZ008529	yes	LMSG_G000010044.1	OES073701	arylpolyene	Others	Polymetallic	26.93	China: Guangxi, Longshenao
L180322-P-10-LD180320-0031_HL3MGCCXY_L6_clean_bin.40.orig.fa	OEZ008529	yes	LMSG_G000010044.1	OES073701	NRPS-like.ectoine	Others	Polymetallic	49.56	China: Guangxi, Longshenao
L180322-P-10-LD180320-0031_HL3MGCCXY_L6_clean_bin.40.orig.fa	OEZ008529	yes	LMSG_G000010044.1	OES073701	arylpolyene	Others	Polymetallic	17.51	China: Guangxi, Longshenao
L180322-P-10-LD180320-0031_HL3MGCCXY_L6_clean_bin.40.orig.fa	OEZ008529	yes	LMSG_G000010044.1	OES073701	terpene	Terpene	Polymetallic	9.10	China: Guangxi, Longshenao
L180322-P-10-LD180320-0031_HL3MGCCXY_L6_clean_bin.40.orig.fa	OEZ008529	yes	LMSG_G000010044.1	OES073701	bacteriocin	RiPPs	Polymetallic	10.87	China: Guangxi, Longshenao
L180322-P-10-LD180320-0031_HL3MGCCXY_L6_clean_bin.40.orig.fa	OEZ008529	yes	LMSG_G000010044.1	OES073701	terpene	Terpene	Polymetallic	19.46	China: Guangxi, Longshenao
L180322-P-10-LD180320-0031_HL3MGCCXY_L6_clean_bin.41.orig.fa	OEZ008529	yes	LMSG_G000008431.1	OES073701	terpene	Terpene	Polymetallic	26.48	China: Guangxi, Longshenao
L180322-P-10-LD180320-0031_HL3MGCCXY_L6_clean_bin.42.orig.fa	OEZ008529	yes	LMSG_G000008447.1	OES073701	terpene	Terpene	Polymetallic	8.19	China: Guangxi, Longshenao
L180322-P-10-LD180320-0031_HL3MGCCXY_L6_clean_bin.44.orig.fa	OEZ008529	yes	LMSG_G000011063.1	OES073701	bacteriocin	RiPPs	Polymetallic	6.91	China: Guangxi, Longshenao
L180322-P-10-LD180320-0031_HL3MGCCXY_L6_clean_bin.44.orig.fa	OEZ008529	yes	LMSG_G000011063.1	OES073701	NRPS-like	NRPS	Polymetallic	28.06	China: Guangxi, Longshenao
L180322-P-10-LD180320-0031_HL3MGCCXY_L6_clean_bin.44.orig.fa	OEZ008529	yes	LMSG_G000011063.1	OES073701	NRPS-like	NRPS	Polymetallic	27.17	China: Guangxi, Longshenao
L180322-P-10-LD180320-0031_HL3MGCCXY_L6_clean_bin.44.orig.fa	OEZ008529	yes	LMSG_G000011063.1	OES073701	lassopeptide.hserlactone	Others	Polymetallic	20.35	China: Guangxi, Longshenao
L180322-P-10-LD180320-0031_HL3MGCCXY_L6_clean_bin.44.orig.fa	OEZ008529	yes	LMSG_G000011063.1	OES073701	terpene	Terpene	Polymetallic	23.19	China: Guangxi, Longshenao
L180322-P-10-LD180320-0031_HL3MGCCXY_L6_clean_bin.48.orig.fa	OEZ008529	yes	LMSG_G000011131.1	OES073701	terpene	Terpene	Polymetallic	23.11	China: Guangxi, Longshenao
L180322-P-10-LD180320-0031_HL3MGCCXY_L6_clean_bin.48.orig.fa	OEZ008529	yes	LMSG_G000011131.1	OES073701	arylpolyene	Others	Polymetallic	41.16	China: Guangxi, Longshenao
L180322-P-10-LD180320-0031_HL3MGCCXY_L6_clean_bin.49.orig.fa	OEZ008529	no	LMSG_G000010260.1	OES073701	terpene	Terpene	Polymetallic	21.16	China: Guangxi, Longshenao
L180322-P-10-LD180320-0031_HL3MGCCXY_L6_clean_bin.49.orig.fa	OEZ008529	yes	LMSG_G000010260.1	OES073701	arylpolyene	Others	Polymetallic	41.19	China: Guangxi, Longshenao
L180322-P-10-LD180320-0031_HL3MGCCXY_L6_clean_bin.51.permiss	OEZ008529	yes	LMSG_G000008105.1	OES073701	betalactone	Others	Polymetallic	16.59	China: Guangxi, Longshenao
L180322-P-10-LD180320-0031_HL3MGCCXY_L6_clean_bin.55.orig.fa	OEZ008529	yes	LMSG_G000006497.1	OES073701	terpene	Terpene	Polymetallic	6.53	China: Guangxi, Longshenao
L180322-P-10-LD180320-0031_HL3MGCCXY_L6_clean_bin.57.orig.fa	OEZ008529	yes	LMSG_G000005151.1	OES073701	T3PKS	PKSother	Polymetallic	14.47	China: Guangxi, Longshenao
L180322-P-10-LD180320-0031_HL3MGCCXY_L6_clean_bin.57.orig.fa	OEZ008529	yes	LMSG_G000005151.1	OES073701	NRPS-like	NRPS	Polymetallic	22.20	China: Guangxi, Longshenao
L180322-P-10-LD180320-0031_HL3MGCCXY_L6_clean_bin.58.strict.	OEZ008529	yes	LMSG_G000009723.1	OES073701	LAP	RiPPs	Polymetallic	12.65	China: Guangxi, Longshenao
L180322-P-10-LD180320-0031_HL3MGCCXY_L6_clean_bin.59.orig.fa	OEZ008529	yes	LMSG_G000005152.1	OES073701	terpene	Terpene	Polymetallic	23.16	China: Guangxi, Longshenao
L180322-P-10-LD180320-0031_HL3MGCCXY_L6_clean_bin.59.orig.fa	OEZ008529	yes	LMSG_G000005152.1	OES073701	bacteriocin	RiPPs	Polymetallic	8.80	China: Guangxi, Longshenao
L180322-P-10-LD180320-0031_HL3MGCCXY_L6_clean_bin.59.orig.fa	OEZ008529	yes	LMSG_G000005152.1	OES073701	NRPS-like	NRPS	Polymetallic	38.68	China: Guangxi, Longshenao
L180322-P-10-LD180320-0031_HL3MGCCXY_L6_clean_bin.60.orig.fa	OEZ008529	yes	LMSG_G000008334.1	OES073701	bacteriocin	RiPPs	Polymetallic	10.80	China: Guangxi, Longshenao
L180322-P-10-LD180320-0031_HL3MGCCXY_L6_clean_bin.60.orig.fa	OEZ008529	yes	LMSG_G000008334.1	OES073701	butyrolactone	Others	Polymetallic	11.01	China: Guangxi, Longshenao
L180322-P-10-LD180320-0031_HL3MGCCXY_L6_clean_bin.60.orig.fa	OEZ008529	yes	LMSG_G000008334.1	OES073701	T3PKS	PKSother	Polymetallic	41.06	China: Guangxi, Longshenao
L180322-P-10-LD180320-0031_HL3MGCCXY_L6_clean_bin.60.orig.fa	OEZ008529	yes	LMSG_G000008334.1	OES073701	terpene	Terpene	Polymetallic	18.34	China: Guangxi, Longshenao
L180322-P-10-LD180320-0031_HL3MGCCXY_L6_clean_bin.62.orig.fa	OEZ008529	yes	LMSG_G000008498.1	OES073701	lassopeptide	RiPPs	Polymetallic	23.45	China: Guangxi, Longshenao
L180322-P-10-LD180320-0031_HL3MGCCXY_L6_clean_bin.62.orig.fa	OEZ008529	yes	LMSG_G000008498.1	OES073701	terpene	Terpene	Polymetallic	17.08	China: Guangxi, Longshenao
L180322-P-10-LD180320-0031_HL3MGCCXY_L6_clean_bin.63.orig.fa	OEZ008529	yes	LMSG_G000006432.1	OES073701	terpene	Terpene	Polymetallic	6.93	China: Guangxi, Longshenao
L180322-P-10-LD180320-0031_HL3MGCCXY_L6_clean_bin.65.orig.fa	OEZ008529	no	LMSG_G000010161.1	OES073701	acyl_amino_acids	Others	Polymetallic	6.60	China: Guangxi, Longshenao
L180322-P-10-LD180320-0031_HL3MGCCXY_L6_clean_bin.65.orig.fa	OEZ008529	no	LMSG_G000010161.1	OES073701	ectoine	Others	Polymetallic	5.74	China: Guangxi, Longshenao
L180322-P-10-LD180320-0031_HL3MGCCXY_L6_clean_bin.65.orig.fa	OEZ008529	no	LMSG_G000010161.1	OES073701	terpene	Terpene	Polymetallic	14.07	China: Guangxi, Longshenao
L180322-P-10-LD180320-0031_HL3MGCCXY_L6_clean_bin.65.orig.fa	OEZ008529	no	LMSG_G000010161.1	OES073701	terpene	Terpene	Polymetallic	5.44	China: Guangxi, Longshenao
L180322-P-10-LD180320-0031_HL3MGCCXY_L6_clean_bin.69.strict.	OEZ008529	yes	LMSG_G000008485.1	OES073701	terpene	Terpene	Polymetallic	26.09	China: Guangxi, Longshenao
L180322-P-10-LD180320-0031_HL3MGCCXY_L6_clean_bin.69.strict.	OEZ008529	yes	LMSG_G000008485.1	OES073701	lassopeptide	RiPPs	Polymetallic	7.64	China: Guangxi, Longshenao
L180322-P-10-LD180320-0031_HL3MGCCXY_L6_clean_bin.7.orig.fa_	OEZ008529	yes	LMSG_G000005156.1	OES073701	NRPS-like	NRPS	Polymetallic	17.99	China: Guangxi, Longshenao
L180322-P-10-LD180320-0031_HL3MGCCXY_L6_clean_bin.7.orig.fa_	OEZ008529	yes	LMSG_G000005156.1	OES073701	terpene	Terpene	Polymetallic	23.78	China: Guangxi, Longshenao
L180322-P-10-LD180320-0031_HL3MGCCXY_L6_clean_bin.7.orig.fa_	OEZ008529	yes	LMSG_G000005156.1	OES073701	betalactone	Others	Polymetallic	22.33	China: Guangxi, Longshenao
L180322-P-10-LD180320-0031_HL3MGCCXY_L6_clean_bin.7.orig.fa_	OEZ008529	yes	LMSG_G000005156.1	OES073701	other	Others	Polymetallic	17.45	China: Guangxi, Longshenao
L180322-P-10-LD180320-0031_HL3MGCCXY_L6_clean_bin.7.orig.fa_	OEZ008529	yes	LMSG_G000005156.1	OES073701	terpene	Terpene	Polymetallic	14.99	China: Guangxi, Longshenao
L180322-P-10-LD180320-0031_HL3MGCCXY_L6_clean_bin.71.orig.fa	OEZ008529	yes	LMSG_G000008739.1	OES073701	NRPS-like	NRPS	Polymetallic	14.40	China: Guangxi, Longshenao
L180322-P-10-LD180320-0031_HL3MGCCXY_L6_clean_bin.71.orig.fa	OEZ008529	yes	LMSG_G000008739.1	OES073701	terpene	Terpene	Polymetallic	19.72	China: Guangxi, Longshenao
L180322-P-10-LD180320-0031_HL3MGCCXY_L6_clean_bin.72.orig.fa	OEZ008529	yes	LMSG_G000008992.1	OES073701	terpene	Terpene	Polymetallic	20.91	China: Guangxi, Longshenao
L180322-P-10-LD180320-0031_HL3MGCCXY_L6_clean_bin.72.orig.fa	OEZ008529	yes	LMSG_G000008992.1	OES073701	bacteriocin	RiPPs	Polymetallic	10.80	China: Guangxi, Longshenao
L180322-P-10-LD180320-0031_HL3MGCCXY_L6_clean_bin.74.strict.	OEZ008529	yes	LMSG_G000009895.1	OES073701	terpene	Terpene	Polymetallic	12.94	China: Guangxi, Longshenao
L180322-P-10-LD180320-0031_HL3MGCCXY_L6_clean_bin.78.strict.	OEZ008529	yes	LMSG_G000008320.1	OES073701	other	Others	Polymetallic	30.19	China: Guangxi, Longshenao
L180322-P-10-LD180320-0031_HL3MGCCXY_L6_clean_bin.78.strict.	OEZ008529	yes	LMSG_G000008320.1	OES073701	terpene	Terpene	Polymetallic	21.09	China: Guangxi, Longshenao
L180322-P-10-LD180320-0031_HL3MGCCXY_L6_clean_bin.79.strict.	OEZ008529	yes	LMSG_G000008013.1	OES073701	betalactone	Others	Polymetallic	23.60	China: Guangxi, Longshenao
L180322-P-10-LD180320-0031_HL3MGCCXY_L6_clean_bin.79.strict.	OEZ008529	yes	LMSG_G000008013.1	OES073701	betalactone	Others	Polymetallic	30.02	China: Guangxi, Longshenao
L180322-P-10-LD180320-0031_HL3MGCCXY_L6_clean_bin.79.strict.	OEZ008529	yes	LMSG_G000008013.1	OES073701	bacteriocin	RiPPs	Polymetallic	10.30	China: Guangxi, Longshenao
L180322-P-10-LD180320-0031_HL3MGCCXY_L6_clean_bin.80.orig.fa	OEZ008529	yes	LMSG_G000007736.1	OES073701	terpene	Terpene	Polymetallic	29.47	China: Guangxi, Longshenao
L180322-P-10-LD180320-0031_HL3MGCCXY_L6_clean_bin.82.orig.fa	OEZ008529	yes	LMSG_G000008473.1	OES073701	bacteriocin	RiPPs	Polymetallic	10.47	China: Guangxi, Longshenao
L180322-P-10-LD180320-0031_HL3MGCCXY_L6_clean_bin.82.orig.fa	OEZ008529	yes	LMSG_G000008473.1	OES073701	terpene	Terpene	Polymetallic	11.72	China: Guangxi, Longshenao
L180322-P-10-LD180320-0031_HL3MGCCXY_L6_clean_bin.84.orig.fa	OEZ008529	yes	LMSG_G000006828.1	OES073701	lanthipeptide	RiPPs	Polymetallic	6.89	China: Guangxi, Longshenao
L180322-P-10-LD180320-0031_HL3MGCCXY_L6_clean_bin.85.orig.fa	OEZ008529	yes	LMSG_G000008624.1	OES073701	other	Others	Polymetallic	40.72	China: Guangxi, Longshenao
L180322-P-10-LD180320-0031_HL3MGCCXY_L6_clean_bin.86.orig.fa	OEZ008529	yes	LMSG_G000009382.1	OES073701	ladderane	Others	Polymetallic	5.99	China: Guangxi, Longshenao
L180322-P-10-LD180320-0031_HL3MGCCXY_L6_clean_bin.86.orig.fa	OEZ008529	yes	LMSG_G000009382.1	OES073701	terpene	Terpene	Polymetallic	21.41	China: Guangxi, Longshenao
L180322-P-10-LD180320-0031_HL3MGCCXY_L6_clean_bin.86.orig.fa	OEZ008529	yes	LMSG_G000009382.1	OES073701	bacteriocin	RiPPs	Polymetallic	5.45	China: Guangxi, Longshenao
L180322-P-10-LD180320-0031_HL3MGCCXY_L6_clean_bin.88.orig.fa	OEZ008529	yes	LMSG_G000011152.1	OES073701	terpene	Terpene	Polymetallic	7.96	China: Guangxi, Longshenao
L180322-P-10-LD180320-0031_HL3MGCCXY_L6_clean_bin.88.orig.fa	OEZ008529	yes	LMSG_G000011152.1	OES073701	arylpolyene	Others	Polymetallic	12.52	China: Guangxi, Longshenao
L180322-P-10-LD180320-0031_HL3MGCCXY_L6_clean_bin.92.strict.	OEZ008529	yes	LMSG_G000009432.1	OES073701	betalactone	Others	Polymetallic	30.86	China: Guangxi, Longshenao
L180322-P-10-LD180320-0031_HL3MGCCXY_L6_clean_bin.92.strict.	OEZ008529	yes	LMSG_G000009432.1	OES073701	bacteriocin	RiPPs	Polymetallic	10.85	China: Guangxi, Longshenao
L180322-P-10-LD180320-0031_HL3MGCCXY_L6_clean_bin.92.strict.	OEZ008529	yes	LMSG_G000009432.1	OES073701	ectoine	Others	Polymetallic	10.40	China: Guangxi, Longshenao
L180322-P-10-LD180320-0031_HL3MGCCXY_L6_clean_bin.92.strict.	OEZ008529	yes	LMSG_G000009432.1	OES073701	betalactone	Others	Polymetallic	22.78	China: Guangxi, Longshenao
L180322-P-10-LD180320-0031_HL3MGCCXY_L6_clean_bin.92.strict.	OEZ008529	yes	LMSG_G000009432.1	OES073701	LAP	RiPPs	Polymetallic	18.55	China: Guangxi, Longshenao
L180322-P-10-LD180320-0031_HL3MGCCXY_L6_clean_bin.95.orig.fa	OEZ008529	yes	LMSG_G000009343.1	OES073701	bacteriocin	RiPPs	Polymetallic	10.81	China: Guangxi, Longshenao
L180322-P-10-LD180320-0031_HL3MGCCXY_L6_clean_bin.96.orig.fa	OEZ008529	yes	LMSG_G000009534.1	OES073701	acyl_amino_acids	Others	Polymetallic	7.40	China: Guangxi, Longshenao
L180322-P-10-LD180320-0031_HL3MGCCXY_L6_clean_bin.96.orig.fa	OEZ008529	no	LMSG_G000009534.1	OES073701	terpene	Terpene	Polymetallic	23.29	China: Guangxi, Longshenao
L180322-P-10-LD180320-0031_HL3MGCCXY_L6_clean_bin.96.orig.fa	OEZ008529	no	LMSG_G000009534.1	OES073701	terpene	Terpene	Polymetallic	21.75	China: Guangxi, Longshenao
L180322-P-10-LD180320-0031_HL3MGCCXY_L6_clean_bin.97.strict.	OEZ008529	yes	LMSG_G000009462.1	OES073701	lanthipeptide	RiPPs	Polymetallic	14.17	China: Guangxi, Longshenao
L180322-P-10-LD180320-0031_HL3MGCCXY_L6_clean_bin.97.strict.	OEZ008529	yes	LMSG_G000009462.1	OES073701	bacteriocin	RiPPs	Polymetallic	6.35	China: Guangxi, Longshenao
L180322-P-10-LD180320-0032_HL3MGCCXY_L6_clean_bin.1.strict.f	OEZ008529	yes	LMSG_G000010011.1	OES073702	terpene	Terpene	Polymetallic	21.87	China: Guangxi, Longshenao
L180322-P-10-LD180320-0032_HL3MGCCXY_L6_clean_bin.1.strict.f	OEZ008529	yes	LMSG_G000010011.1	OES073702	arylpolyene	Others	Polymetallic	20.08	China: Guangxi, Longshenao
L180322-P-10-LD180320-0032_HL3MGCCXY_L6_clean_bin.1.strict.f	OEZ008529	yes	LMSG_G000010011.1	OES073702	terpene	Terpene	Polymetallic	14.24	China: Guangxi, Longshenao
L180322-P-10-LD180320-0032_HL3MGCCXY_L6_clean_bin.1.strict.f	OEZ008529	yes	LMSG_G000010011.1	OES073702	ladderane	Others	Polymetallic	7.93	China: Guangxi, Longshenao
L180322-P-10-LD180320-0032_HL3MGCCXY_L6_clean_bin.10.orig.fa	OEZ008529	yes	LMSG_G000009344.1	OES073702	bacteriocin	RiPPs	Polymetallic	10.81	China: Guangxi, Longshenao
L180322-P-10-LD180320-0032_HL3MGCCXY_L6_clean_bin.13.orig.fa	OEZ008529	yes	LMSG_G000011153.1	OES073702	arylpolyene	Others	Polymetallic	10.65	China: Guangxi, Longshenao
L180322-P-10-LD180320-0032_HL3MGCCXY_L6_clean_bin.13.orig.fa	OEZ008529	yes	LMSG_G000011153.1	OES073702	terpene	Terpene	Polymetallic	13.95	China: Guangxi, Longshenao
L180322-P-10-LD180320-0032_HL3MGCCXY_L6_clean_bin.13.orig.fa	OEZ008529	yes	LMSG_G000011153.1	OES073702	arylpolyene	Others	Polymetallic	7.16	China: Guangxi, Longshenao
L180322-P-10-LD180320-0032_HL3MGCCXY_L6_clean_bin.15.orig.fa	OEZ008529	yes	LMSG_G000008434.1	OES073702	terpene	Terpene	Polymetallic	25.04	China: Guangxi, Longshenao
L180322-P-10-LD180320-0032_HL3MGCCXY_L6_clean_bin.17.orig.fa	OEZ008529	yes	LMSG_G000006433.1	OES073702	terpene	Terpene	Polymetallic	12.77	China: Guangxi, Longshenao
L180322-P-10-LD180320-0032_HL3MGCCXY_L6_clean_bin.18.orig.fa	OEZ008529	yes	LMSG_G000009381.1	OES073702	terpene	Terpene	Polymetallic	21.71	China: Guangxi, Longshenao
L180322-P-10-LD180320-0032_HL3MGCCXY_L6_clean_bin.18.orig.fa	OEZ008529	yes	LMSG_G000009381.1	OES073702	bacteriocin	RiPPs	Polymetallic	6.41	China: Guangxi, Longshenao
L180322-P-10-LD180320-0032_HL3MGCCXY_L6_clean_bin.19.strict.	OEZ008529	yes	LMSG_G000008313.1	OES073702	terpene	Terpene	Polymetallic	21.99	China: Guangxi, Longshenao
L180322-P-10-LD180320-0032_HL3MGCCXY_L6_clean_bin.19.strict.	OEZ008529	yes	LMSG_G000008313.1	OES073702	terpene	Terpene	Polymetallic	21.10	China: Guangxi, Longshenao
L180322-P-10-LD180320-0032_HL3MGCCXY_L6_clean_bin.19.strict.	OEZ008529	yes	LMSG_G000008313.1	OES073702	other	Others	Polymetallic	22.43	China: Guangxi, Longshenao
L180322-P-10-LD180320-0032_HL3MGCCXY_L6_clean_bin.19.strict.	OEZ008529	yes	LMSG_G000008313.1	OES073702	betalactone	Others	Polymetallic	19.97	China: Guangxi, Longshenao
L180322-P-10-LD180320-0032_HL3MGCCXY_L6_clean_bin.19.strict.	OEZ008529	yes	LMSG_G000008313.1	OES073702	NRPS-like	NRPS	Polymetallic	30.66	China: Guangxi, Longshenao
L180322-P-10-LD180320-0032_HL3MGCCXY_L6_clean_bin.20.orig.fa	OEZ008529	yes	LMSG_G000009151.1	OES073702	T3PKS	PKSother	Polymetallic	17.01	China: Guangxi, Longshenao
L180322-P-10-LD180320-0032_HL3MGCCXY_L6_clean_bin.20.orig.fa	OEZ008529	yes	LMSG_G000009151.1	OES073702	terpene	Terpene	Polymetallic	20.58	China: Guangxi, Longshenao
L180322-P-10-LD180320-0032_HL3MGCCXY_L6_clean_bin.28.orig.fa	OEZ008529	yes	LMSG_G000011064.1	OES073702	terpene	Terpene	Polymetallic	21.21	China: Guangxi, Longshenao
L180322-P-10-LD180320-0032_HL3MGCCXY_L6_clean_bin.28.orig.fa	OEZ008529	yes	LMSG_G000011064.1	OES073702	hserlactone	Others	Polymetallic	17.73	China: Guangxi, Longshenao
L180322-P-10-LD180320-0032_HL3MGCCXY_L6_clean_bin.28.orig.fa	OEZ008529	yes	LMSG_G000011064.1	OES073702	NRPS-like	NRPS	Polymetallic	31.10	China: Guangxi, Longshenao
L180322-P-10-LD180320-0032_HL3MGCCXY_L6_clean_bin.28.orig.fa	OEZ008529	yes	LMSG_G000011064.1	OES073702	NRPS-like	NRPS	Polymetallic	41.97	China: Guangxi, Longshenao
L180322-P-10-LD180320-0032_HL3MGCCXY_L6_clean_bin.28.orig.fa	OEZ008529	yes	LMSG_G000011064.1	OES073702	NRPS-like	NRPS	Polymetallic	25.05	China: Guangxi, Longshenao
L180322-P-10-LD180320-0032_HL3MGCCXY_L6_clean_bin.3.strict.f	OEZ008529	yes	LMSG_G000008554.1	OES073702	hglE-KS	PKSother	Polymetallic	36.83	China: Guangxi, Longshenao
L180322-P-10-LD180320-0032_HL3MGCCXY_L6_clean_bin.30.orig.fa	OEZ008529	yes	LMSG_G000005159.1	OES073702	terpene	Terpene	Polymetallic	23.49	China: Guangxi, Longshenao
L180322-P-10-LD180320-0032_HL3MGCCXY_L6_clean_bin.30.orig.fa	OEZ008529	yes	LMSG_G000005159.1	OES073702	terpene	Terpene	Polymetallic	22.03	China: Guangxi, Longshenao
L180322-P-10-LD180320-0032_HL3MGCCXY_L6_clean_bin.31.orig.fa	OEZ008529	yes	LMSG_G000009915.1	OES073702	terpene	Terpene	Polymetallic	10.92	China: Guangxi, Longshenao
L180322-P-10-LD180320-0032_HL3MGCCXY_L6_clean_bin.32.orig.fa	OEZ008529	yes	LMSG_G000008335.1	OES073702	bacteriocin	RiPPs	Polymetallic	10.80	China: Guangxi, Longshenao
L180322-P-10-LD180320-0032_HL3MGCCXY_L6_clean_bin.32.orig.fa	OEZ008529	yes	LMSG_G000008335.1	OES073702	T3PKS	PKSother	Polymetallic	41.06	China: Guangxi, Longshenao
L180322-P-10-LD180320-0032_HL3MGCCXY_L6_clean_bin.35.orig.fa	OEZ008529	yes	LMSG_G000008073.1	OES073702	T1PKS.hglE-KS	PKSother	Polymetallic	16.87	China: Guangxi, Longshenao
L180322-P-10-LD180320-0032_HL3MGCCXY_L6_clean_bin.35.orig.fa	OEZ008529	yes	LMSG_G000008073.1	OES073702	arylpolyene	Others	Polymetallic	22.81	China: Guangxi, Longshenao
L180322-P-10-LD180320-0032_HL3MGCCXY_L6_clean_bin.38.orig.fa	OEZ008529	yes	LMSG_G000009711.1	OES073702	LAP	RiPPs	Polymetallic	13.87	China: Guangxi, Longshenao
L180322-P-10-LD180320-0032_HL3MGCCXY_L6_clean_bin.39.orig.fa	OEZ008529	yes	LMSG_G000011132.1	OES073702	terpene	Terpene	Polymetallic	23.01	China: Guangxi, Longshenao
L180322-P-10-LD180320-0032_HL3MGCCXY_L6_clean_bin.40.orig.fa	OEZ008529	yes	LMSG_G000006498.1	OES073702	terpene	Terpene	Polymetallic	15.18	China: Guangxi, Longshenao
L180322-P-10-LD180320-0032_HL3MGCCXY_L6_clean_bin.41.strict.	OEZ008529	yes	LMSG_G000008321.1	OES073702	terpene	Terpene	Polymetallic	21.09	China: Guangxi, Longshenao
L180322-P-10-LD180320-0032_HL3MGCCXY_L6_clean_bin.45.orig.fa	OEZ008529	yes	LMSG_G000010261.1	OES073702	arylpolyene	Others	Polymetallic	41.19	China: Guangxi, Longshenao
L180322-P-10-LD180320-0032_HL3MGCCXY_L6_clean_bin.45.orig.fa	OEZ008529	no	LMSG_G000010261.1	OES073702	terpene	Terpene	Polymetallic	21.16	China: Guangxi, Longshenao
L180322-P-10-LD180320-0032_HL3MGCCXY_L6_clean_bin.47.orig.fa	OEZ008529	yes	LMSG_G000008503.1	OES073702	lassopeptide	RiPPs	Polymetallic	23.45	China: Guangxi, Longshenao
L180322-P-10-LD180320-0032_HL3MGCCXY_L6_clean_bin.47.orig.fa	OEZ008529	yes	LMSG_G000008503.1	OES073702	terpene	Terpene	Polymetallic	17.08	China: Guangxi, Longshenao
L180322-P-10-LD180320-0032_HL3MGCCXY_L6_clean_bin.5.orig.fa_	OEZ008529	yes	LMSG_G000010442.1	OES073702	NRPS-like	NRPS	Polymetallic	42.80	China: Guangxi, Longshenao
L180322-P-10-LD180320-0032_HL3MGCCXY_L6_clean_bin.5.orig.fa_	OEZ008529	yes	LMSG_G000010442.1	OES073702	bacteriocin	RiPPs	Polymetallic	8.80	China: Guangxi, Longshenao
L180322-P-10-LD180320-0032_HL3MGCCXY_L6_clean_bin.5.orig.fa_	OEZ008529	yes	LMSG_G000010442.1	OES073702	terpene	Terpene	Polymetallic	23.16	China: Guangxi, Longshenao
L180322-P-10-LD180320-0032_HL3MGCCXY_L6_clean_bin.50.strict.	OEZ008529	yes	LMSG_G000010045.1	OES073702	ectoine	Others	Polymetallic	10.40	China: Guangxi, Longshenao
L180322-P-10-LD180320-0032_HL3MGCCXY_L6_clean_bin.50.strict.	OEZ008529	yes	LMSG_G000010045.1	OES073702	terpene	Terpene	Polymetallic	20.43	China: Guangxi, Longshenao
L180322-P-10-LD180320-0032_HL3MGCCXY_L6_clean_bin.50.strict.	OEZ008529	yes	LMSG_G000010045.1	OES073702	ladderane	Others	Polymetallic	41.26	China: Guangxi, Longshenao
L180322-P-10-LD180320-0032_HL3MGCCXY_L6_clean_bin.50.strict.	OEZ008529	yes	LMSG_G000010045.1	OES073702	bacteriocin	RiPPs	Polymetallic	10.66	China: Guangxi, Longshenao
L180322-P-10-LD180320-0032_HL3MGCCXY_L6_clean_bin.50.strict.	OEZ008529	yes	LMSG_G000010045.1	OES073702	arylpolyene	Others	Polymetallic	26.82	China: Guangxi, Longshenao
L180322-P-10-LD180320-0032_HL3MGCCXY_L6_clean_bin.50.strict.	OEZ008529	yes	LMSG_G000010045.1	OES073702	T3PKS	PKSother	Polymetallic	18.82	China: Guangxi, Longshenao
L180322-P-10-LD180320-0032_HL3MGCCXY_L6_clean_bin.50.strict.	OEZ008529	yes	LMSG_G000010045.1	OES073702	terpene	Terpene	Polymetallic	15.92	China: Guangxi, Longshenao
L180322-P-10-LD180320-0032_HL3MGCCXY_L6_clean_bin.50.strict.	OEZ008529	yes	LMSG_G000010045.1	OES073702	NRPS-like	NRPS	Polymetallic	14.15	China: Guangxi, Longshenao
L180322-P-10-LD180320-0032_HL3MGCCXY_L6_clean_bin.50.strict.	OEZ008529	yes	LMSG_G000010045.1	OES073702	arylpolyene	Others	Polymetallic	13.38	China: Guangxi, Longshenao
L180322-P-10-LD180320-0032_HL3MGCCXY_L6_clean_bin.51.orig.fa	OEZ008529	yes	LMSG_G000011113.1	OES073702	arylpolyene	Others	Polymetallic	37.62	China: Guangxi, Longshenao
L180322-P-10-LD180320-0032_HL3MGCCXY_L6_clean_bin.51.orig.fa	OEZ008529	yes	LMSG_G000011113.1	OES073702	terpene	Terpene	Polymetallic	19.63	China: Guangxi, Longshenao
L180322-P-10-LD180320-0032_HL3MGCCXY_L6_clean_bin.52.orig.fa	OEZ008529	yes	LMSG_G000008225.1	OES073702	terpene	Terpene	Polymetallic	20.86	China: Guangxi, Longshenao
L180322-P-10-LD180320-0032_HL3MGCCXY_L6_clean_bin.56.orig.fa	OEZ008529	yes	LMSG_G000011073.1	OES073702	bacteriocin	RiPPs	Polymetallic	9.11	China: Guangxi, Longshenao
L180322-P-10-LD180320-0032_HL3MGCCXY_L6_clean_bin.56.orig.fa	OEZ008529	yes	LMSG_G000011073.1	OES073702	terpene	Terpene	Polymetallic	23.25	China: Guangxi, Longshenao
L180322-P-10-LD180320-0032_HL3MGCCXY_L6_clean_bin.56.orig.fa	OEZ008529	yes	LMSG_G000011073.1	OES073702	NRPS-like	NRPS	Polymetallic	8.69	China: Guangxi, Longshenao
L180322-P-10-LD180320-0032_HL3MGCCXY_L6_clean_bin.56.orig.fa	OEZ008529	yes	LMSG_G000011073.1	OES073702	NRPS-like	NRPS	Polymetallic	27.08	China: Guangxi, Longshenao
L180322-P-10-LD180320-0032_HL3MGCCXY_L6_clean_bin.56.orig.fa	OEZ008529	yes	LMSG_G000011073.1	OES073702	lassopeptide.hserlactone	Others	Polymetallic	23.62	China: Guangxi, Longshenao
L180322-P-10-LD180320-0032_HL3MGCCXY_L6_clean_bin.57.strict.	OEZ008529	yes	LMSG_G000007976.1	OES073702	bacteriocin	RiPPs	Polymetallic	5.68	China: Guangxi, Longshenao
L180322-P-10-LD180320-0032_HL3MGCCXY_L6_clean_bin.58.orig.fa	OEZ008529	yes	LMSG_G000008993.1	OES073702	bacteriocin	RiPPs	Polymetallic	10.80	China: Guangxi, Longshenao
L180322-P-10-LD180320-0032_HL3MGCCXY_L6_clean_bin.58.orig.fa	OEZ008529	yes	LMSG_G000008993.1	OES073702	terpene	Terpene	Polymetallic	20.91	China: Guangxi, Longshenao
L180322-P-10-LD180320-0032_HL3MGCCXY_L6_clean_bin.59.strict.	OEZ008529	yes	LMSG_G000009433.1	OES073702	bacteriocin	RiPPs	Polymetallic	10.85	China: Guangxi, Longshenao
L180322-P-10-LD180320-0032_HL3MGCCXY_L6_clean_bin.59.strict.	OEZ008529	yes	LMSG_G000009433.1	OES073702	betalactone	Others	Polymetallic	30.86	China: Guangxi, Longshenao
L180322-P-10-LD180320-0032_HL3MGCCXY_L6_clean_bin.59.strict.	OEZ008529	yes	LMSG_G000009433.1	OES073702	betalactone	Others	Polymetallic	22.78	China: Guangxi, Longshenao
L180322-P-10-LD180320-0032_HL3MGCCXY_L6_clean_bin.59.strict.	OEZ008529	yes	LMSG_G000009433.1	OES073702	ectoine	Others	Polymetallic	9.58	China: Guangxi, Longshenao
L180322-P-10-LD180320-0032_HL3MGCCXY_L6_clean_bin.60.orig.fa	OEZ008529	yes	LMSG_G000008014.1	OES073702	betalactone	Others	Polymetallic	23.60	China: Guangxi, Longshenao
L180322-P-10-LD180320-0032_HL3MGCCXY_L6_clean_bin.60.orig.fa	OEZ008529	yes	LMSG_G000008014.1	OES073702	betalactone	Others	Polymetallic	30.02	China: Guangxi, Longshenao
L180322-P-10-LD180320-0032_HL3MGCCXY_L6_clean_bin.60.orig.fa	OEZ008529	yes	LMSG_G000008014.1	OES073702	bacteriocin	RiPPs	Polymetallic	10.30	China: Guangxi, Longshenao
L180322-P-10-LD180320-0032_HL3MGCCXY_L6_clean_bin.60.orig.fa	OEZ008529	yes	LMSG_G000008014.1	OES073702	bacteriocin	RiPPs	Polymetallic	5.13	China: Guangxi, Longshenao
L180322-P-10-LD180320-0032_HL3MGCCXY_L6_clean_bin.61.orig.fa	OEZ008529	yes	LMSG_G000008625.1	OES073702	other	Others	Polymetallic	35.86	China: Guangxi, Longshenao
L180322-P-10-LD180320-0032_HL3MGCCXY_L6_clean_bin.61.orig.fa	OEZ008529	yes	LMSG_G000008625.1	OES073702	NRPS-like	NRPS	Polymetallic	34.62	China: Guangxi, Longshenao
L180322-P-10-LD180320-0032_HL3MGCCXY_L6_clean_bin.67.orig.fa	OEZ008529	yes	LMSG_G000008674.1	OES073702	arylpolyene	Others	Polymetallic	41.21	China: Guangxi, Longshenao
L180322-P-10-LD180320-0032_HL3MGCCXY_L6_clean_bin.67.orig.fa	OEZ008529	yes	LMSG_G000008674.1	OES073702	terpene	Terpene	Polymetallic	26.42	China: Guangxi, Longshenao
L180322-P-10-LD180320-0032_HL3MGCCXY_L6_clean_bin.67.orig.fa	OEZ008529	yes	LMSG_G000008674.1	OES073702	LAP	RiPPs	Polymetallic	11.24	China: Guangxi, Longshenao

L180322-P-10-LD180320-0032_HL3MGCCXY_L6_clean_bin.67.orig.fa	OEZ008529	yes	LMSG_G000008674.1	OES073702	T1PKS	PKSI	Polymetallic	9.61	China: Guangxi, Longshenao
L180322-P-10-LD180320-0032_HL3MGCCXY_L6_clean_bin.67.orig.fa	OEZ008529	yes	LMSG_G000008674.1	OES073702	betalactone	Others	Polymetallic	36.22	China: Guangxi, Longshenao
L180322-P-10-LD180320-0032_HL3MGCCXY_L6_clean_bin.67.orig.fa	OEZ008529	yes	LMSG_G000008674.1	OES073702	T1PKS	PKSI	Polymetallic	16.02	China: Guangxi, Longshenao
L180322-P-10-LD180320-0032_HL3MGCCXY_L6_clean_bin.68.orig.fa	OEZ008529	yes	LMSG_G000010466.1	OES073702	arylpolyene	Others	Polymetallic	29.74	China: Guangxi, Longshenao
L180322-P-10-LD180320-0032_HL3MGCCXY_L6_clean_bin.68.orig.fa	OEZ008529	yes	LMSG_G000010466.1	OES073702	terpene	Terpene	Polymetallic	23.04	China: Guangxi, Longshenao
L180322-P-10-LD180320-0032_HL3MGCCXY_L6_clean_bin.7.strict.f	OEZ008529	yes	LMSG_G000009463.1	OES073702	bacteriocin	RiPPs	Polymetallic	9.95	China: Guangxi, Longshenao
L180322-P-10-LD180320-0032_HL3MGCCXY_L6_clean_bin.7.strict.f	OEZ008529	yes	LMSG_G000009463.1	OES073702	NRPS	NRPS	Polymetallic	5.57	China: Guangxi, Longshenao
L180322-P-10-LD180320-0032_HL3MGCCXY_L6_clean_bin.70.orig.fa	OEZ008529	yes	LMSG_G000007831.1	OES073702	T3PKS	PKSother	Polymetallic	23.29	China: Guangxi, Longshenao
L180322-P-10-LD180320-0032_HL3MGCCXY_L6_clean_bin.70.orig.fa	OEZ008529	yes	LMSG_G000007831.1	OES073702	bacteriocin	RiPPs	Polymetallic	5.46	China: Guangxi, Longshenao
L180322-P-10-LD180320-0032_HL3MGCCXY_L6_clean_bin.70.orig.fa	OEZ008529	yes	LMSG_G000007831.1	OES073702	terpene	Terpene	Polymetallic	8.78	China: Guangxi, Longshenao
L180322-P-10-LD180320-0032_HL3MGCCXY_L6_clean_bin.70.orig.fa	OEZ008529	yes	LMSG_G000007831.1	OES073702	bacteriocin	RiPPs	Polymetallic	5.57	China: Guangxi, Longshenao
L180322-P-10-LD180320-0032_HL3MGCCXY_L6_clean_bin.70.orig.fa	OEZ008529	yes	LMSG_G000007831.1	OES073702	terpene	Terpene	Polymetallic	17.29	China: Guangxi, Longshenao
L180322-P-10-LD180320-0032_HL3MGCCXY_L6_clean_bin.71.strict.	OEZ008529	yes	LMSG_G000009216.1	OES073702	terpene	Terpene	Polymetallic	21.74	China: Guangxi, Longshenao
L180322-P-10-LD180320-0032_HL3MGCCXY_L6_clean_bin.71.strict.	OEZ008529	yes	LMSG_G000009216.1	OES073702	hserlactone	Others	Polymetallic	21.31	China: Guangxi, Longshenao
L180322-P-10-LD180320-0032_HL3MGCCXY_L6_clean_bin.71.strict.	OEZ008529	yes	LMSG_G000009216.1	OES073702	terpene	Terpene	Polymetallic	21.66	China: Guangxi, Longshenao
L180322-P-10-LD180320-0032_HL3MGCCXY_L6_clean_bin.73.orig.fa	OEZ008529	yes	LMSG_G000008106.1	OES073702	betalactone	Others	Polymetallic	6.70	China: Guangxi, Longshenao
L180322-P-10-LD180320-0032_HL3MGCCXY_L6_clean_bin.73.orig.fa	OEZ008529	yes	LMSG_G000008106.1	OES073702	NRPS-like	NRPS	Polymetallic	10.57	China: Guangxi, Longshenao
L180322-P-10-LD180320-0032_HL3MGCCXY_L6_clean_bin.77.orig.fa	OEZ008529	yes	LMSG_G000008448.1	OES073702	terpene	Terpene	Polymetallic	26.50	China: Guangxi, Longshenao
L180322-P-10-LD180320-0032_HL3MGCCXY_L6_clean_bin.77.orig.fa	OEZ008529	yes	LMSG_G000008448.1	OES073702	lassopeptide	RiPPs	Polymetallic	6.57	China: Guangxi, Longshenao
L180322-P-10-LD180320-0032_HL3MGCCXY_L6_clean_bin.8.orig.fa_	OEZ008529	yes	LMSG_G000008268.1	OES073702	terpene	Terpene	Polymetallic	19.12	China: Guangxi, Longshenao
L180322-P-10-LD180320-0032_HL3MGCCXY_L6_clean_bin.80.orig.fa	OEZ008529	yes	LMSG_G000005164.1	OES073702	terpene	Terpene	Polymetallic	19.51	China: Guangxi, Longshenao
L180322-P-10-LD180320-0032_HL3MGCCXY_L6_clean_bin.80.orig.fa	OEZ008529	yes	LMSG_G000005164.1	OES073702	NRPS-like	NRPS	Polymetallic	14.15	China: Guangxi, Longshenao
L180322-P-10-LD180320-0032_HL3MGCCXY_L6_clean_bin.83.strict.	OEZ008529	yes	LMSG_G000008382.1	OES073702	T1PKS	PKSI	Polymetallic	43.02	China: Guangxi, Longshenao
L180322-P-10-LD180320-0032_HL3MGCCXY_L6_clean_bin.83.strict.	OEZ008529	yes	LMSG_G000008382.1	OES073702	NRPS-like	NRPS	Polymetallic	44.28	China: Guangxi, Longshenao
L180322-P-10-LD180320-0032_HL3MGCCXY_L6_clean_bin.83.strict.	OEZ008529	yes	LMSG_G000008382.1	OES073702	terpene	Terpene	Polymetallic	16.86	China: Guangxi, Longshenao
L180322-P-10-LD180320-0032_HL3MGCCXY_L6_clean_bin.85.orig.fa	OEZ008529	yes	LMSG_G000007763.1	OES073702	terpene	Terpene	Polymetallic	29.77	China: Guangxi, Longshenao
L180322-P-10-LD180320-0032_HL3MGCCXY_L6_clean_bin.85.orig.fa	OEZ008529	yes	LMSG_G000007763.1	OES073702	T3PKS	PKSother	Polymetallic	27.27	China: Guangxi, Longshenao
L180322-P-10-LD180320-0032_HL3MGCCXY_L6_clean_bin.86.orig.fa	OEZ008529	yes	LMSG_G000008684.1	OES073702	bacteriocin	RiPPs	Polymetallic	10.77	China: Guangxi, Longshenao
L180322-P-10-LD180320-0032_HL3MGCCXY_L6_clean_bin.86.orig.fa	OEZ008529	yes	LMSG_G000008684.1	OES073702	betalactone	Others	Polymetallic	21.54	China: Guangxi, Longshenao
L180322-P-10-LD180320-0032_HL3MGCCXY_L6_clean_bin.86.orig.fa	OEZ008529	yes	LMSG_G000008684.1	OES073702	NRPS-like	NRPS	Polymetallic	39.32	China: Guangxi, Longshenao
L180322-P-10-LD180320-0032_HL3MGCCXY_L6_clean_bin.86.orig.fa	OEZ008529	yes	LMSG_G000008684.1	OES073702	ectoine	Others	Polymetallic	10.23	China: Guangxi, Longshenao
L180322-P-10-LD180320-0032_HL3MGCCXY_L6_clean_bin.86.orig.fa	OEZ008529	yes	LMSG_G000008684.1	OES073702	terpene	Terpene	Polymetallic	26.85	China: Guangxi, Longshenao
L180322-P-10-LD180320-0032_HL3MGCCXY_L6_clean_bin.87.orig.fa	OEZ008529	yes	LMSG_G000007737.1	OES073702	terpene	Terpene	Polymetallic	25.65	China: Guangxi, Longshenao
L180322-P-10-LD180320-0032_HL3MGCCXY_L6_clean_bin.89.orig.fa	OEZ008529	yes	LMSG_G000008486.1	OES073702	NRPS-like	NRPS	Polymetallic	7.01	China: Guangxi, Longshenao
L180322-P-10-LD180320-0032_HL3MGCCXY_L6_clean_bin.89.orig.fa	OEZ008529	yes	LMSG_G000008486.1	OES073702	terpene	Terpene	Polymetallic	13.02	China: Guangxi, Longshenao
L180322-P-10-LD180320-0032_HL3MGCCXY_L6_clean_bin.89.orig.fa	OEZ008529	yes	LMSG_G000008486.1	OES073702	lassopeptide	RiPPs	Polymetallic	7.10	China: Guangxi, Longshenao
L180322-P-10-LD180320-0032_HL3MGCCXY_L6_clean_bin.91.orig.fa	OEZ008529	no	LMSG_G000010296.1	OES073702	arylpolyene	Others	Polymetallic	14.71	China: Guangxi, Longshenao
L180322-P-10-LD180320-0032_HL3MGCCXY_L6_clean_bin.91.orig.fa	OEZ008529	no	LMSG_G000010296.1	OES073702	terpene	Terpene	Polymetallic	21.75	China: Guangxi, Longshenao
L180322-P-10-LD180320-0032_HL3MGCCXY_L6_clean_bin.92.orig.fa	OEZ008529	yes	LMSG_G000008512.1	OES073702	terpene	Terpene	Polymetallic	21.37	China: Guangxi, Longshenao
L180322-P-10-LD180320-0032_HL3MGCCXY_L6_clean_bin.97.permiss	OEZ008529	yes	LMSG_G000008884.1	OES073702	T3PKS	PKSother	Polymetallic	5.78	China: Guangxi, Longshenao
L180322-P-10-LD180320-0033_HL3MGCCXY_L6_clean_bin.10.orig.fa	OEZ008529	yes	LMSG_G000008432.1	OES073703	terpene	Terpene	Polymetallic	26.56	China: Guangxi, Longshenao
L180322-P-10-LD180320-0033_HL3MGCCXY_L6_clean_bin.15.orig.fa	OEZ008529	yes	LMSG_G000009152.1	OES073703	terpene	Terpene	Polymetallic	19.37	China: Guangxi, Longshenao
L180322-P-10-LD180320-0033_HL3MGCCXY_L6_clean_bin.15.orig.fa	OEZ008529	yes	LMSG_G000009152.1	OES073703	T3PKS	PKSother	Polymetallic	16.88	China: Guangxi, Longshenao
L180322-P-10-LD180320-0033_HL3MGCCXY_L6_clean_bin.18.permiss	OEZ008529	yes	LMSG_G000008074.1	OES073703	T1PKS.hglE-KS	PKSother	Polymetallic	13.50	China: Guangxi, Longshenao
L180322-P-10-LD180320-0033_HL3MGCCXY_L6_clean_bin.18.permiss	OEZ008529	yes	LMSG_G000008074.1	OES073703	other	Others	Polymetallic	7.75	China: Guangxi, Longshenao
L180322-P-10-LD180320-0033_HL3MGCCXY_L6_clean_bin.18.permiss	OEZ008529	yes	LMSG_G000008074.1	OES073703	arylpolyene	Others	Polymetallic	5.63	China: Guangxi, Longshenao
L180322-P-10-LD180320-0033_HL3MGCCXY_L6_clean_bin.2.orig.fa_	OEZ008529	no	LMSG_G000010297.1	OES073703	arylpolyene	Others	Polymetallic	31.97	China: Guangxi, Longshenao
L180322-P-10-LD180320-0033_HL3MGCCXY_L6_clean_bin.2.orig.fa_	OEZ008529	no	LMSG_G000010297.1	OES073703	terpene	Terpene	Polymetallic	7.41	China: Guangxi, Longshenao
L180322-P-10-LD180320-0033_HL3MGCCXY_L6_clean_bin.2.orig.fa_	OEZ008529	no	LMSG_G000010297.1	OES073703	terpene	Terpene	Polymetallic	11.84	China: Guangxi, Longshenao
L180322-P-10-LD180320-0033_HL3MGCCXY_L6_clean_bin.21.strict.	OEZ008529	yes	LMSG_G000008226.1	OES073703	terpene	Terpene	Polymetallic	14.38	China: Guangxi, Longshenao
L180322-P-10-LD180320-0033_HL3MGCCXY_L6_clean_bin.29.orig.fa	OEZ008529	yes	LMSG_G000008743.1	OES073703	NRPS-like	NRPS	Polymetallic	5.78	China: Guangxi, Longshenao
L180322-P-10-LD180320-0033_HL3MGCCXY_L6_clean_bin.29.orig.fa	OEZ008529	yes	LMSG_G000008743.1	OES073703	terpene	Terpene	Polymetallic	13.13	China: Guangxi, Longshenao
L180322-P-10-LD180320-0033_HL3MGCCXY_L6_clean_bin.3.orig.fa_	OEZ008529	yes	LMSG_G000007977.1	OES073703	TfuA-related.bacteriocin	RiPPs	Polymetallic	5.07	China: Guangxi, Longshenao
L180322-P-10-LD180320-0033_HL3MGCCXY_L6_clean_bin.30.orig.fa	OEZ008529	yes	LMSG_G000005166.1	OES073703	hglE-KS	PKSother	Polymetallic	21.16	China: Guangxi, Longshenao
L180322-P-10-LD180320-0033_HL3MGCCXY_L6_clean_bin.31.orig.fa	OEZ008529	yes	LMSG_G000008340.1	OES073703	T3PKS	PKSother	Polymetallic	41.06	China: Guangxi, Longshenao
L180322-P-10-LD180320-0033_HL3MGCCXY_L6_clean_bin.31.orig.fa	OEZ008529	yes	LMSG_G000008340.1	OES073703	bacteriocin	RiPPs	Polymetallic	10.80	China: Guangxi, Longshenao
L180322-P-10-LD180320-0033_HL3MGCCXY_L6_clean_bin.32.orig.fa	OEZ008529	yes	LMSG_G000008626.1	OES073703	NRPS-like	NRPS	Polymetallic	18.39	China: Guangxi, Longshenao
L180322-P-10-LD180320-0033_HL3MGCCXY_L6_clean_bin.32.orig.fa	OEZ008529	yes	LMSG_G000008626.1	OES073703	other	Others	Polymetallic	40.72	China: Guangxi, Longshenao
L180322-P-10-LD180320-0033_HL3MGCCXY_L6_clean_bin.34.strict.	OEZ008529	yes	LMSG_G000005168.1	OES073703	terpene	Terpene	Polymetallic	21.09	China: Guangxi, Longshenao
L180322-P-10-LD180320-0033_HL3MGCCXY_L6_clean_bin.34.strict.	OEZ008529	yes	LMSG_G000005168.1	OES073703	other	Others	Polymetallic	9.49	China: Guangxi, Longshenao
L180322-P-10-LD180320-0033_HL3MGCCXY_L6_clean_bin.4.strict.f	OEZ008529	yes	LMSG_G000008383.1	OES073703	T1PKS	PKSI	Polymetallic	43.02	China: Guangxi, Longshenao
L180322-P-10-LD180320-0033_HL3MGCCXY_L6_clean_bin.4.strict.f	OEZ008529	yes	LMSG_G000008383.1	OES073703	NRPS-like	NRPS	Polymetallic	44.28	China: Guangxi, Longshenao
L180322-P-10-LD180320-0033_HL3MGCCXY_L6_clean_bin.4.strict.f	OEZ008529	yes	LMSG_G000008383.1	OES073703	terpene	Terpene	Polymetallic	15.07	China: Guangxi, Longshenao
L180322-P-10-LD180320-0033_HL3MGCCXY_L6_clean_bin.40.orig.fa	OEZ008529	yes	LMSG_G000008994.1	OES073703	terpene	Terpene	Polymetallic	20.91	China: Guangxi, Longshenao
L180322-P-10-LD180320-0033_HL3MGCCXY_L6_clean_bin.40.orig.fa	OEZ008529	yes	LMSG_G000008994.1	OES073703	bacteriocin	RiPPs	Polymetallic	10.80	China: Guangxi, Longshenao
L180322-P-10-LD180320-0033_HL3MGCCXY_L6_clean_bin.43.orig.fa	OEZ008529	yes	LMSG_G000007738.1	OES073703	terpene	Terpene	Polymetallic	22.81	China: Guangxi, Longshenao
L180322-P-10-LD180320-0033_HL3MGCCXY_L6_clean_bin.5.orig.fa_	OEZ008529	no	LMSG_G000008474.1	OES073703	terpene	Terpene	Polymetallic	6.46	China: Guangxi, Longshenao
L180322-P-10-LD180320-0033_HL3MGCCXY_L6_clean_bin.5.orig.fa_	OEZ008529	yes	LMSG_G000008474.1	OES073703	bacteriocin	RiPPs	Polymetallic	5.27	China: Guangxi, Longshenao
L180322-P-10-LD180320-0033_HL3MGCCXY_L6_clean_bin.5.orig.fa_	OEZ008529	yes	LMSG_G000008474.1	OES073703	T3PKS	PKSother	Polymetallic	17.15	China: Guangxi, Longshenao
L180322-P-10-LD180320-0033_HL3MGCCXY_L6_clean_bin.5.orig.fa_	OEZ008529	yes	LMSG_G000008474.1	OES073703	LAP	RiPPs	Polymetallic	8.54	China: Guangxi, Longshenao
L180322-P-10-LD180320-0033_HL3MGCCXY_L6_clean_bin.50.orig.fa	OEZ008529	yes	LMSG_G000006435.1	OES073703	nucleoside	Others	Polymetallic	15.37	China: Guangxi, Longshenao
L180322-P-10-LD180320-0033_HL3MGCCXY_L6_clean_bin.52.orig.fa	OEZ008529	yes	LMSG_G000007764.1	OES073703	terpene	Terpene	Polymetallic	27.19	China: Guangxi, Longshenao
L180322-P-10-LD180320-0033_HL3MGCCXY_L6_clean_bin.52.orig.fa	OEZ008529	yes	LMSG_G000007764.1	OES073703	T3PKS	PKSother	Polymetallic	26.87	China: Guangxi, Longshenao
L180322-P-10-LD180320-0033_HL3MGCCXY_L6_clean_bin.52.orig.fa	OEZ008529	yes	LMSG_G000007764.1	OES073703	NRPS-like	NRPS	Polymetallic	12.34	China: Guangxi, Longshenao
L180322-P-10-LD180320-0033_HL3MGCCXY_L6_clean_bin.56.orig.fa	OEZ008529	yes	LMSG_G000008504.1	OES073703	terpene	Terpene	Polymetallic	17.08	China: Guangxi, Longshenao
L180322-P-10-LD180320-0033_HL3MGCCXY_L6_clean_bin.56.orig.fa	OEZ008529	yes	LMSG_G000008504.1	OES073703	lassopeptide	RiPPs	Polymetallic	23.45	China: Guangxi, Longshenao
L180322-P-10-LD180320-0033_HL3MGCCXY_L6_clean_bin.58.orig.fa	OEZ008529	yes	LMSG_G000009903.1	OES073703	hserlactone	Others	Polymetallic	5.55	China: Guangxi, Longshenao
L180322-P-10-LD180320-0033_HL3MGCCXY_L6_clean_bin.59.orig.fa	OEZ008529	yes	LMSG_G000008675.1	OES073703	T1PKS	PKSI	Polymetallic	25.53	China: Guangxi, Longshenao
L180322-P-10-LD180320-0033_HL3MGCCXY_L6_clean_bin.59.orig.fa	OEZ008529	yes	LMSG_G000008675.1	OES073703	arylpolyene	Others	Polymetallic	34.24	China: Guangxi, Longshenao
L180322-P-10-LD180320-0033_HL3MGCCXY_L6_clean_bin.59.orig.fa	OEZ008529	yes	LMSG_G000008675.1	OES073703	terpene	Terpene	Polymetallic	26.44	China: Guangxi, Longshenao
L180322-P-10-LD180320-0033_HL3MGCCXY_L6_clean_bin.6.orig.fa_	OEZ008529	yes	LMSG_G000009338.1	OES073703	bacteriocin	RiPPs	Polymetallic	10.81	China: Guangxi, Longshenao
L180322-P-10-LD180320-0033_HL3MGCCXY_L6_clean_bin.60.strict.	OEZ008529	yes	LMSG_G000009217.1	OES073703	terpene	Terpene	Polymetallic	21.74	China: Guangxi, Longshenao
L180322-P-10-LD180320-0033_HL3MGCCXY_L6_clean_bin.60.strict.	OEZ008529	yes	LMSG_G000009217.1	OES073703	hserlactone	Others	Polymetallic	21.31	China: Guangxi, Longshenao
L180322-P-10-LD180320-0033_HL3MGCCXY_L6_clean_bin.60.strict.	OEZ008529	yes	LMSG_G000009217.1	OES073703	terpene	Terpene	Polymetallic	22.05	China: Guangxi, Longshenao
L180322-P-10-LD180320-0033_HL3MGCCXY_L6_clean_bin.61.permiss	OEZ008529	yes	LMSG_G000008269.1	OES073703	terpene	Terpene	Polymetallic	20.86	China: Guangxi, Longshenao
L180322-P-10-LD180320-0033_HL3MGCCXY_L6_clean_bin.64.orig.fa	OEZ008529	yes	LMSG_G000011079.1	OES073703	terpene	Terpene	Polymetallic	10.39	China: Guangxi, Longshenao
L180322-P-10-LD180320-0033_HL3MGCCXY_L6_clean_bin.64.orig.fa	OEZ008529	yes	LMSG_G000011079.1	OES073703	terpene	Terpene	Polymetallic	11.33	China: Guangxi, Longshenao
L180322-P-10-LD180320-0033_HL3MGCCXY_L6_clean_bin.64.orig.fa	OEZ008529	yes	LMSG_G000011079.1	OES073703	NRPS-like	NRPS	Polymetallic	41.96	China: Guangxi, Longshenao
L180322-P-10-LD180320-0033_HL3MGCCXY_L6_clean_bin.64.orig.fa	OEZ008529	yes	LMSG_G000011079.1	OES073703	bacteriocin	RiPPs	Polymetallic	9.79	China: Guangxi, Longshenao
L180322-P-10-LD180320-0033_HL3MGCCXY_L6_clean_bin.67.orig.fa	OEZ008529	yes	LMSG_G000010262.1	OES073703	arylpolyene	Others	Polymetallic	35.55	China: Guangxi, Longshenao
L180322-P-10-LD180320-0033_HL3MGCCXY_L6_clean_bin.67.orig.fa	OEZ008529	no	LMSG_G000010262.1	OES073703	terpene	Terpene	Polymetallic	21.20	China: Guangxi, Longshenao
L180322-P-10-LD180320-0033_HL3MGCCXY_L6_clean_bin.69.orig.fa	OEZ008529	yes	LMSG_G000008487.1	OES073703	arylpolyene	Others	Polymetallic	10.96	China: Guangxi, Longshenao
L180322-P-10-LD180320-0033_HL3MGCCXY_L6_clean_bin.69.orig.fa	OEZ008529	yes	LMSG_G000008487.1	OES073703	lassopeptide	RiPPs	Polymetallic	7.07	China: Guangxi, Longshenao
L180322-P-10-LD180320-0033_HL3MGCCXY_L6_clean_bin.70.orig.fa	OEZ008529	yes	LMSG_G000011128.1	OES073703	terpene	Terpene	Polymetallic	23.01	China: Guangxi, Longshenao
L180322-P-10-LD180320-0033_HL3MGCCXY_L6_clean_bin.71.strict.	OEZ008529	yes	LMSG_G000009383.1	OES073703	terpene	Terpene	Polymetallic	9.42	China: Guangxi, Longshenao
L180322-P-10-LD180320-0033_HL3MGCCXY_L6_clean_bin.74.strict.	OEZ008529	yes	LMSG_G000009640.1	OES073703	terpene	Terpene	Polymetallic	22.03	China: Guangxi, Longshenao
L180322-P-10-LD180320-0033_HL3MGCCXY_L6_clean_bin.74.strict.	OEZ008529	yes	LMSG_G000009640.1	OES073703	terpene	Terpene	Polymetallic	23.48	China: Guangxi, Longshenao
L180322-P-10-LD180320-0033_HL3MGCCXY_L6_clean_bin.75.orig.fa	OEZ008529	yes	LMSG_G000010012.1	OES073703	ladderane	Others	Polymetallic	8.49	China: Guangxi, Longshenao
L180322-P-10-LD180320-0033_HL3MGCCXY_L6_clean_bin.75.orig.fa	OEZ008529	yes	LMSG_G000010012.1	OES073703	terpene	Terpene	Polymetallic	5.13	China: Guangxi, Longshenao
L180322-P-10-LD180320-0033_HL3MGCCXY_L6_clean_bin.77.orig.fa	OEZ008529	yes	LMSG_G000008401.1	OES073703	terpene	Terpene	Polymetallic	26.09	China: Guangxi, Longshenao
L180322-P-10-LD180320-0033_HL3MGCCXY_L6_clean_bin.79.orig.fa	OEZ008529	yes	LMSG_G000007818.1	OES073703	T3PKS	PKSother	Polymetallic	33.06	China: Guangxi, Longshenao
L180322-P-10-LD180320-0033_HL3MGCCXY_L6_clean_bin.79.orig.fa	OEZ008529	yes	LMSG_G000007818.1	OES073703	bacteriocin	RiPPs	Polymetallic	10.88	China: Guangxi, Longshenao
L180322-P-10-LD180320-0033_HL3MGCCXY_L6_clean_bin.79.orig.fa	OEZ008529	yes	LMSG_G000007818.1	OES073703	terpene	Terpene	Polymetallic	29.81	China: Guangxi, Longshenao
L180322-P-10-LD180320-0033_HL3MGCCXY_L6_clean_bin.8.orig.fa_	OEZ008529	yes	LMSG_G000008015.1	OES073703	bacteriocin	RiPPs	Polymetallic	10.30	China: Guangxi, Longshenao
L180322-P-10-LD180320-0033_HL3MGCCXY_L6_clean_bin.8.orig.fa_	OEZ008529	yes	LMSG_G000008015.1	OES073703	betalactone	Others	Polymetallic	30.02	China: Guangxi, Longshenao
L180322-P-10-LD180320-0033_HL3MGCCXY_L6_clean_bin.8.orig.fa_	OEZ008529	yes	LMSG_G000008015.1	OES073703	betalactone	Others	Polymetallic	23.60	China: Guangxi, Longshenao
L180322-P-10-LD180320-0033_HL3MGCCXY_L6_clean_bin.81.orig.fa	OEZ008529	yes	LMSG_G000006830.1	OES073703	lanthipeptide	RiPPs	Polymetallic	14.65	China: Guangxi, Longshenao
L180322-P-10-LD180320-0033_HL3MGCCXY_L6_clean_bin.84.strict.	OEZ008529	yes	LMSG_G000010046.1	OES073703	NRPS-like.ectoine	Others	Polymetallic	50.26	China: Guangxi, Longshenao
L180322-P-10-LD180320-0033_HL3MGCCXY_L6_clean_bin.84.strict.	OEZ008529	yes	LMSG_G000010046.1	OES073703	terpene	Terpene	Polymetallic	20.42	China: Guangxi, Longshenao
L180322-P-10-LD180320-0033_HL3MGCCXY_L6_clean_bin.84.strict.	OEZ008529	yes	LMSG_G000010046.1	OES073703	ladderane	Others	Polymetallic	41.26	China: Guangxi, Longshenao
L180322-P-10-LD180320-0033_HL3MGCCXY_L6_clean_bin.84.strict.	OEZ008529	yes	LMSG_G000010046.1	OES073703	bacteriocin	RiPPs	Polymetallic	6.64	China: Guangxi, Longshenao
L180322-P-10-LD180320-0033_HL3MGCCXY_L6_clean_bin.84.strict.	OEZ008529	yes	LMSG_G000010046.1	OES073703	terpene	Terpene	Polymetallic	13.04	China: Guangxi, Longshenao
L180322-P-10-LD180320-0033_HL3MGCCXY_L6_clean_bin.84.strict.	OEZ008529	yes	LMSG_G000010046.1	OES073703	T3PKS	PKSother	Polymetallic	16.63	China: Guangxi, Longshenao
L180322-P-10-LD180320-0033_HL3MGCCXY_L6_clean_bin.84.strict.	OEZ008529	yes	LMSG_G000010046.1	OES073703	arylpolyene	Others	Polymetallic	16.10	China: Guangxi, Longshenao
L180322-P-10-LD180320-0033_HL3MGCCXY_L6_clean_bin.85.permiss	OEZ008529	yes	LMSG_G000008311.1	OES073703	other	Others	Polymetallic	22.17	China: Guangxi, Longshenao
L180322-P-10-LD180320-0033_HL3MGCCXY_L6_clean_bin.85.permiss	OEZ008529	yes	LMSG_G000008311.1	OES073703	terpene	Terpene	Polymetallic	14.18	China: Guangxi, Longshenao
L180322-P-10-LD180320-0033_HL3MGCCXY_L6_clean_bin.85.permiss	OEZ008529	yes	LMSG_G000008311.1	OES073703	NRPS-like	NRPS	Polymetallic	27.71	China: Guangxi, Longshenao
L180322-P-10-LD180320-0033_HL3MGCCXY_L6_clean_bin.85.permiss	OEZ008529	yes	LMSG_G000008311.1	OES073703	terpene	Terpene	Polymetallic	12.43	China: Guangxi, Longshenao
L180322-P-10-LD180320-0033_HL3MGCCXY_L6_clean_bin.86.strict.	OEZ008529	yes	LMSG_G000009434.1	OES073703	bacteriocin	RiPPs	Polymetallic	10.85	China: Guangxi, Longshenao
L180322-P-10-LD180320-0033_HL3MGCCXY_L6_clean_bin.86.strict.	OEZ008529	yes	LMSG_G000009434.1	OES073703	betalactone	Others	Polymetallic	30.86	China: Guangxi, Longshenao
L180322-P-10-LD180320-0033_HL3MGCCXY_L6_clean_bin.86.strict.	OEZ008529	yes	LMSG_G000009434.1	OES073703	ectoine	Others	Polymetallic	10.06	China: Guangxi, Longshenao
L180322-P-10-LD180320-0033_HL3MGCCXY_L6_clean_bin.86.strict.	OEZ008529	yes	LMSG_G000009434.1	OES073703	betalactone	Others	Polymetallic	22.78	China: Guangxi, Longshenao
L180322-P-10-LD180320-0033_HL3MGCCXY_L6_clean_bin.9.orig.fa_	OEZ008529	yes	LMSG_G000004432.1	OES073703	lassopeptide	RiPPs	Polymetallic	15.57	China: Guangxi, Longshenao
L180322-P-10-LD180320-0033_HL3MGCCXY_L6_clean_bin.9.orig.fa_	OEZ008529	yes	LMSG_G000004432.1	OES073703	terpene	Terpene	Polymetallic	26.33	China: Guangxi, Longshenao
L180322-P-11-LD180320-0034_HL3MGCCXY_L7_clean_bin.1.orig.fa_	OEZ008529	yes	LMSG_G000006504.1	OES073704	thiopeptide	RiPPs	Polymetallic	25.09	China: Guangxi, Longshenao
L180322-P-11-LD180320-0034_HL3MGCCXY_L7_clean_bin.11.strict.	OEZ008529	yes	LMSG_G000008227.1	OES073704	terpene	Terpene	Polymetallic	15.17	China: Guangxi, Longshenao
L180322-P-11-LD180320-0034_HL3MGCCXY_L7_clean_bin.12.orig.fa	OEZ008529	yes	LMSG_G000007765.1	OES073704	NRPS-like	NRPS	Polymetallic	6.28	China: Guangxi, Longshenao
L180322-P-11-LD180320-0034_HL3MGCCXY_L7_clean_bin.12.orig.fa	OEZ008529	yes	LMSG_G000007765.1	OES073704	terpene	Terpene	Polymetallic	17.91	China: Guangxi, Longshenao
L180322-P-11-LD180320-0034_HL3MGCCXY_L7_clean_bin.12.orig.fa	OEZ008529	yes	LMSG_G000007765.1	OES073704	T3PKS	PKSother	Polymetallic	21.16	China: Guangxi, Longshenao
L180322-P-11-LD180320-0034_HL3MGCCXY_L7_clean_bin.12.orig.fa	OEZ008529	yes	LMSG_G000007765.1	OES073704	terpene	Terpene	Polymetallic	11.57	China: Guangxi, Longshenao
L180322-P-11-LD180320-0034_HL3MGCCXY_L7_clean_bin.17.orig.fa	OEZ008529	yes	LMSG_G000010445.1	OES073704	NRPS-like	NRPS	Polymetallic	8.86	China: Guangxi, Longshenao
L180322-P-11-LD180320-0034_HL3MGCCXY_L7_clean_bin.17.orig.fa	OEZ008529	yes	LMSG_G000010445.1	OES073704	terpene	Terpene	Polymetallic	10.34	China: Guangxi, Longshenao
L180322-P-11-LD180320-0034_HL3MGCCXY_L7_clean_bin.17.orig.fa	OEZ008529	yes	LMSG_G000010445.1	OES073704	bacteriocin	RiPPs	Polymetallic	8.80	China: Guangxi, Longshenao
L180322-P-11-LD180320-0034_HL3MGCCXY_L7_clean_bin.18.orig.fa	OEZ008529	yes	LMSG_G000008480.1	OES073704	terpene	Terpene	Polymetallic	10.86	China: Guangxi, Longshenao
L180322-P-11-LD180320-0034_HL3MGCCXY_L7_clean_bin.2.strict.f	OEZ008529	yes	LMSG_G000010013.1	OES073704	terpene	Terpene	Polymetallic	12.58	China: Guangxi, Longshenao
L180322-P-11-LD180320-0034_HL3MGCCXY_L7_clean_bin.2.strict.f	OEZ008529	yes	LMSG_G000010013.1	OES073704	arylpolyene	Others	Polymetallic	5.07	China: Guangxi, Longshenao
L180322-P-11-LD180320-0034_HL3MGCCXY_L7_clean_bin.20.orig.fa	OEZ008529	yes	LMSG_G000008385.1	OES073704	terpene	Terpene	Polymetallic	14.38	China: Guangxi, Longshenao
L180322-P-11-LD180320-0034_HL3MGCCXY_L7_clean_bin.20.orig.fa	OEZ008529	yes	LMSG_G000008385.1	OES073704	T1PKS	PKSI	Polymetallic	22.44	China: Guangxi, Longshenao
L180322-P-11-LD180320-0034_HL3MGCCXY_L7_clean_bin.22.orig.fa	OEZ008529	yes	LMSG_G000008396.1	OES073704	terpene	Terpene	Polymetallic	26.09	China: Guangxi, Longshenao
L180322-P-11-LD180320-0034_HL3MGCCXY_L7_clean_bin.26.permiss	OEZ008529	yes	LMSG_G000007978.1	OES073704	TfuA-related.bacteriocin	RiPPs	Polymetallic	19.26	China: Guangxi, Longshenao
L180322-P-11-LD180320-0034_HL3MGCCXY_L7_clean_bin.27.orig.fa	OEZ008529	yes	LMSG_G000004433.1	OES073704	terpene	Terpene	Polymetallic	26.48	China: Guangxi, Longshenao
L180322-P-11-LD180320-0034_HL3MGCCXY_L7_clean_bin.3.strict.f	OEZ008529	yes	LMSG_G000008880.1	OES073704	terpene	Terpene	Polymetallic	20.98	China: Guangxi, Longshenao
L180322-P-11-LD180320-0034_HL3MGCCXY_L7_clean_bin.3.strict.f	OEZ008529	yes	LMSG_G000008880.1	OES073704	T3PKS	PKSother	Polymetallic	41.07	China: Guangxi, Longshenao
L180322-P-11-LD180320-0034_HL3MGCCXY_L7_clean_bin.3.strict.f	OEZ008529	yes	LMSG_G000008880.1	OES073704	arylpolyene	Others	Polymetallic	41.22	China: Guangxi, Longshenao
L180322-P-11-LD180320-0034_HL3MGCCXY_L7_clean_bin.30.orig.fa	OEZ008529	yes	LMSG_G000009345.1	OES073704	bacteriocin	RiPPs	Polymetallic	10.81	China: Guangxi, Longshenao
L180322-P-11-LD180320-0034_HL3MGCCXY_L7_clean_bin.33.orig.fa	OEZ008529	yes	LMSG_G000008075.1	OES073704	T1PKS.hglE-KS	PKSother	Polymetallic	13.57	China: Guangxi, Longshenao
L180322-P-11-LD180320-0034_HL3MGCCXY_L7_clean_bin.34.orig.fa	OEZ008529	yes	LMSG_G000008449.1	OES073704	terpene	Terpene	Polymetallic	11.51	China: Guangxi, Longshenao
L180322-P-11-LD180320-0034_HL3MGCCXY_L7_clean_bin.35.orig.fa	OEZ008529	yes	LMSG_G000008336.1	OES073704	terpene	Terpene	Polymetallic	26.24	China: Guangxi, Longshenao
L180322-P-11-LD180320-0034_HL3MGCCXY_L7_clean_bin.35.orig.fa	OEZ008529	yes	LMSG_G000008336.1	OES073704	T3PKS	PKSother	Polymetallic	40.71	China: Guangxi, Longshenao
L180322-P-11-LD180320-0034_HL3MGCCXY_L7_clean_bin.35.orig.fa	OEZ008529	yes	LMSG_G000008336.1	OES073704	butyrolactone	Others	Polymetallic	11.01	China: Guangxi, Longshenao
L180322-P-11-LD180320-0034_HL3MGCCXY_L7_clean_bin.35.orig.fa	OEZ008529	yes	LMSG_G000008336.1	OES073704	bacteriocin	RiPPs	Polymetallic	10.80	China: Guangxi, Longshenao
L180322-P-11-LD180320-0034_HL3MGCCXY_L7_clean_bin.37.strict.	OEZ008529	yes	LMSG_G000010047.1	OES073704	T3PKS	PKSother	Polymetallic	30.96	China: Guangxi, Longshenao
L180322-P-11-LD180320-0034_HL3MGCCXY_L7_clean_bin.37.strict.	OEZ008529	yes	LMSG_G000010047.1	OES073704	ladderane	Others	Polymetallic	41.26	China: Guangxi, Longshenao
L180322-P-11-LD180320-0034_HL3MGCCXY_L7_clean_bin.37.strict.	OEZ008529	yes	LMSG_G000010047.1	OES073704	NRPS-like.ectoine	Others	Polymetallic	49.33	China: Guangxi, Longshenao
L180322-P-11-LD180320-0034_HL3MGCCXY_L7_clean_bin.37.strict.	OEZ008529	yes	LMSG_G000010047.1	OES073704	terpene	Terpene	Polymetallic	21.90	China: Guangxi, Longshenao
L180322-P-11-LD180320-0034_HL3MGCCXY_L7_clean_bin.37.strict.	OEZ008529	yes	LMSG_G000010047.1	OES073704	terpene	Terpene	Polymetallic	22.00	China: Guangxi, Longshenao
L180322-P-11-LD180320-0034_HL3MGCCXY_L7_clean_bin.37.strict.	OEZ008529	yes	LMSG_G000010047.1	OES073704	arylpolyene	Others	Polymetallic	24.09	China: Guangxi, Longshenao
L180322-P-11-LD180320-0034_HL3MGCCXY_L7_clean_bin.37.strict.	OEZ008529	yes	LMSG_G000010047.1	OES073704	arylpolyene	Others	Polymetallic	16.03	China: Guangxi, Longshenao

L180322-P-11-LD180320-0034_HL3MGCCXY_L7_clean_bin.37.strict. OEZ008529	yes	LMSG_G000010047.1	OES073704	bacteriocin	RiPPs	Polymetallic	6.92	China: Guangxi, Longshenao
L180322-P-11-LD180320-0034_HL3MGCCXY_L7_clean_bin.39.orig.fa OEZ008529	yes	LMSG_G000009157.1	OES073704	T3PKS	PKSother	Polymetallic	16.88	China: Guangxi, Longshenao
L180322-P-11-LD180320-0034_HL3MGCCXY_L7_clean_bin.39.orig.fa OEZ008529	yes	LMSG_G000009157.1	OES073704	terpene	Terpene	Polymetallic	17.99	China: Guangxi, Longshenao
L180322-P-11-LD180320-0034_HL3MGCCXY_L7_clean_bin.4.orig.fa_ OEZ008529	yes	LMSG_G000011114.1	OES073704	arylpolyene	Others	Polymetallic	8.11	China: Guangxi, Longshenao
L180322-P-11-LD180320-0034_HL3MGCCXY_L7_clean_bin.40.orig.fa OEZ008529	yes	LMSG_G000005173.1	OES073704	bacteriocin	RiPPs	Polymetallic	5.21	China: Guangxi, Longshenao
L180322-P-11-LD180320-0034_HL3MGCCXY_L7_clean_bin.40.orig.fa OEZ008529	yes	LMSG_G000005173.1	OES073704	bacteriocin	RiPPs	Polymetallic	10.30	China: Guangxi, Longshenao
L180322-P-11-LD180320-0034_HL3MGCCXY_L7_clean_bin.40.orig.fa OEZ008529	yes	LMSG_G000005173.1	OES073704	betalactone	Others	Polymetallic	30.02	China: Guangxi, Longshenao
L180322-P-11-LD180320-0034_HL3MGCCXY_L7_clean_bin.40.orig.fa OEZ008529	yes	LMSG_G000005173.1	OES073704	betalactone	Others	Polymetallic	23.60	China: Guangxi, Longshenao
L180322-P-11-LD180320-0034_HL3MGCCXY_L7_clean_bin.41.strict. OEZ008529	yes	LMSG_G000008322.1	OES073704	terpene	Terpene	Polymetallic	21.09	China: Guangxi, Longshenao
L180322-P-11-LD180320-0034_HL3MGCCXY_L7_clean_bin.41.strict. OEZ008529	yes	LMSG_G000008322.1	OES073704	other	Others	Polymetallic	11.04	China: Guangxi, Longshenao
L180322-P-11-LD180320-0034_HL3MGCCXY_L7_clean_bin.43.orig.fa OEZ008529	yes	LMSG_G000008634.1	OES073704	NRPS-like	NRPS	Polymetallic	42.47	China: Guangxi, Longshenao
L180322-P-11-LD180320-0034_HL3MGCCXY_L7_clean_bin.43.orig.fa OEZ008529	yes	LMSG_G000008634.1	OES073704	other	Others	Polymetallic	40.72	China: Guangxi, Longshenao
L180322-P-11-LD180320-0034_HL3MGCCXY_L7_clean_bin.45.orig.fa OEZ008529	no	LMSG_G000010298.1	OES073704	terpene	Terpene	Polymetallic	21.25	China: Guangxi, Longshenao
L180322-P-11-LD180320-0034_HL3MGCCXY_L7_clean_bin.47.strict. OEZ008529	yes	LMSG_G000005174.1	OES073704	terpene	Terpene	Polymetallic	20.82	China: Guangxi, Longshenao
L180322-P-11-LD180320-0034_HL3MGCCXY_L7_clean_bin.49.orig.fa OEZ008529	yes	LMSG_G000004434.1	OES073704	arylpolyene	Others	Polymetallic	41.16	China: Guangxi, Longshenao
L180322-P-11-LD180320-0034_HL3MGCCXY_L7_clean_bin.49.orig.fa OEZ008529	yes	LMSG_G000004434.1	OES073704	terpene	Terpene	Polymetallic	22.73	China: Guangxi, Longshenao
L180322-P-11-LD180320-0034_HL3MGCCXY_L7_clean_bin.5.orig.fa_ OEZ008529	yes	LMSG_G000009384.1	OES073704	bacteriocin	RiPPs	Polymetallic	5.27	China: Guangxi, Longshenao
L180322-P-11-LD180320-0034_HL3MGCCXY_L7_clean_bin.5.orig.fa_ OEZ008529	yes	LMSG_G000009384.1	OES073704	terpene	Terpene	Polymetallic	11.49	China: Guangxi, Longshenao
L180322-P-11-LD180320-0034_HL3MGCCXY_L7_clean_bin.50.orig.fa OEZ008529	yes	LMSG_G000009464.1	OES073704	bacteriocin	RiPPs	Polymetallic	9.85	China: Guangxi, Longshenao
L180322-P-11-LD180320-0034_HL3MGCCXY_L7_clean_bin.50.orig.fa OEZ008529	yes	LMSG_G000009464.1	OES073704	NRPS	NRPS	Polymetallic	11.00	China: Guangxi, Longshenao
L180322-P-11-LD180320-0034_HL3MGCCXY_L7_clean_bin.51.orig.fa OEZ008529	yes	LMSG_G000008995.1	OES073704	bacteriocin	RiPPs	Polymetallic	10.80	China: Guangxi, Longshenao
L180322-P-11-LD180320-0034_HL3MGCCXY_L7_clean_bin.51.orig.fa OEZ008529	yes	LMSG_G000008995.1	OES073704	terpene	Terpene	Polymetallic	20.91	China: Guangxi, Longshenao
L180322-P-11-LD180320-0034_HL3MGCCXY_L7_clean_bin.58.strict. OEZ008529	yes	LMSG_G000009435.1	OES073704	bacteriocin	RiPPs	Polymetallic	10.85	China: Guangxi, Longshenao
L180322-P-11-LD180320-0034_HL3MGCCXY_L7_clean_bin.58.strict. OEZ008529	yes	LMSG_G000009435.1	OES073704	betalactone	Others	Polymetallic	30.86	China: Guangxi, Longshenao
L180322-P-11-LD180320-0034_HL3MGCCXY_L7_clean_bin.58.strict. OEZ008529	yes	LMSG_G000009435.1	OES073704	ectoine	Others	Polymetallic	10.40	China: Guangxi, Longshenao
L180322-P-11-LD180320-0034_HL3MGCCXY_L7_clean_bin.58.strict. OEZ008529	yes	LMSG_G000009435.1	OES073704	betalactone	Others	Polymetallic	22.78	China: Guangxi, Longshenao
L180322-P-11-LD180320-0034_HL3MGCCXY_L7_clean_bin.59.orig.fa OEZ008529	yes	LMSG_G000008104.1	OES073704	NRPS-like	NRPS	Polymetallic	6.33	China: Guangxi, Longshenao
L180322-P-11-LD180320-0034_HL3MGCCXY_L7_clean_bin.59.orig.fa OEZ008529	yes	LMSG_G000008104.1	OES073704	betalactone	Others	Polymetallic	21.16	China: Guangxi, Longshenao
L180322-P-11-LD180320-0034_HL3MGCCXY_L7_clean_bin.59.orig.fa OEZ008529	yes	LMSG_G000008104.1	OES073704	betalactone	Others	Polymetallic	20.51	China: Guangxi, Longshenao
L180322-P-11-LD180320-0034_HL3MGCCXY_L7_clean_bin.61.orig.fa OEZ008529	yes	LMSG_G000008676.1	OES073704	arylpolyene	Others	Polymetallic	11.63	China: Guangxi, Longshenao
L180322-P-11-LD180320-0034_HL3MGCCXY_L7_clean_bin.61.orig.fa OEZ008529	yes	LMSG_G000008676.1	OES073704	T1PKS	PKSI	Polymetallic	10.22	China: Guangxi, Longshenao
L180322-P-11-LD180320-0034_HL3MGCCXY_L7_clean_bin.61.orig.fa OEZ008529	yes	LMSG_G000008676.1	OES073704	terpene	Terpene	Polymetallic	26.43	China: Guangxi, Longshenao
L180322-P-11-LD180320-0034_HL3MGCCXY_L7_clean_bin.61.orig.fa OEZ008529	yes	LMSG_G000008676.1	OES073704	T1PKS	PKSI	Polymetallic	7.86	China: Guangxi, Longshenao
L180322-P-11-LD180320-0034_HL3MGCCXY_L7_clean_bin.64.permiss OEZ008529	yes	LMSG_G000004435.1	OES073704	terpene	Terpene	Polymetallic	22.05	China: Guangxi, Longshenao
L180322-P-11-LD180320-0034_HL3MGCCXY_L7_clean_bin.64.permiss OEZ008529	yes	LMSG_G000004435.1	OES073704	terpene	Terpene	Polymetallic	21.74	China: Guangxi, Longshenao
L180322-P-11-LD180320-0034_HL3MGCCXY_L7_clean_bin.64.permiss OEZ008529	yes	LMSG_G000004435.1	OES073704	hserlactone	Others	Polymetallic	21.31	China: Guangxi, Longshenao
L180322-P-11-LD180320-0034_HL3MGCCXY_L7_clean_bin.67.strict. OEZ008529	yes	LMSG_G000008312.1	OES073704	terpene	Terpene	Polymetallic	23.75	China: Guangxi, Longshenao
L180322-P-11-LD180320-0034_HL3MGCCXY_L7_clean_bin.67.strict. OEZ008529	yes	LMSG_G000008312.1	OES073704	other	Others	Polymetallic	40.37	China: Guangxi, Longshenao
L180322-P-11-LD180320-0034_HL3MGCCXY_L7_clean_bin.67.strict. OEZ008529	yes	LMSG_G000008312.1	OES073704	terpene	Terpene	Polymetallic	17.29	China: Guangxi, Longshenao
L180322-P-11-LD180320-0034_HL3MGCCXY_L7_clean_bin.67.strict. OEZ008529	yes	LMSG_G000008312.1	OES073704	NRPS-like	NRPS	Polymetallic	27.58	China: Guangxi, Longshenao
L180322-P-11-LD180320-0034_HL3MGCCXY_L7_clean_bin.69.orig.fa OEZ008529	yes	LMSG_G000007739.1	OES073704	terpene	Terpene	Polymetallic	29.47	China: Guangxi, Longshenao
L180322-P-11-LD180320-0034_HL3MGCCXY_L7_clean_bin.7.orig.fa_ OEZ008529	yes	LMSG_G000006499.1	OES073704	terpene	Terpene	Polymetallic	15.87	China: Guangxi, Longshenao
L180322-P-11-LD180320-0034_HL3MGCCXY_L7_clean_bin.71.orig.fa OEZ008529	yes	LMSG_G000008516.1	OES073704	terpene	Terpene	Polymetallic	26.31	China: Guangxi, Longshenao
L180322-P-11-LD180320-0034_HL3MGCCXY_L7_clean_bin.72.permiss OEZ008529	yes	LMSG_G000008270.1	OES073704	terpene	Terpene	Polymetallic	19.06	China: Guangxi, Longshenao
L180322-P-11-LD180320-0034_HL3MGCCXY_L7_clean_bin.73.orig.fa OEZ008529	yes	LMSG_G000011154.1	OES073704	terpene	Terpene	Polymetallic	14.22	China: Guangxi, Longshenao
L180322-P-11-LD180320-0034_HL3MGCCXY_L7_clean_bin.73.orig.fa OEZ008529	yes	LMSG_G000011154.1	OES073704	bacteriocin	RiPPs	Polymetallic	5.89	China: Guangxi, Longshenao
L180322-P-11-LD180320-0034_HL3MGCCXY_L7_clean_bin.73.orig.fa OEZ008529	yes	LMSG_G000011154.1	OES073704	arylpolyene	Others	Polymetallic	12.26	China: Guangxi, Longshenao
L180322-P-11-LD180320-0034_HL3MGCCXY_L7_clean_bin.76.permiss OEZ008529	yes	LMSG_G000008555.1	OES073704	hglE-KS	PKSother	Polymetallic	5.92	China: Guangxi, Longshenao
L180322-P-11-LD180320-0034_HL3MGCCXY_L7_clean_bin.80.orig.fa OEZ008529	yes	LMSG_G000009641.1	OES073704	terpene	Terpene	Polymetallic	22.03	China: Guangxi, Longshenao
L180322-P-11-LD180320-0034_HL3MGCCXY_L7_clean_bin.80.orig.fa OEZ008529	yes	LMSG_G000009641.1	OES073704	NRPS-like	NRPS	Polymetallic	42.65	China: Guangxi, Longshenao
L180322-P-11-LD180320-0034_HL3MGCCXY_L7_clean_bin.80.orig.fa OEZ008529	yes	LMSG_G000009641.1	OES073704	terpene	Terpene	Polymetallic	23.49	China: Guangxi, Longshenao
L180322-P-11-LD180320-0034_HL3MGCCXY_L7_clean_bin.88.orig.fa OEZ008529	yes	LMSG_G000007819.1	OES073704	terpene	Terpene	Polymetallic	29.81	China: Guangxi, Longshenao
L180322-P-11-LD180320-0034_HL3MGCCXY_L7_clean_bin.88.orig.fa OEZ008529	yes	LMSG_G000007819.1	OES073704	T3PKS	PKSother	Polymetallic	41.09	China: Guangxi, Longshenao
L180322-P-11-LD180320-0034_HL3MGCCXY_L7_clean_bin.9.orig.fa_ OEZ008529	yes	LMSG_G000005177.1	OES073704	betalactone	Others	Polymetallic	16.99	China: Guangxi, Longshenao
L180322-P-11-LD180320-0034_HL3MGCCXY_L7_clean_bin.9.orig.fa_ OEZ008529	yes	LMSG_G000005177.1	OES073704	other	Others	Polymetallic	20.98	China: Guangxi, Longshenao
L180322-P-11-LD180320-0034_HL3MGCCXY_L7_clean_bin.9.orig.fa_ OEZ008529	yes	LMSG_G000005177.1	OES073704	betalactone	Others	Polymetallic	33.33	China: Guangxi, Longshenao
L180322-P-11-LD180320-0034_HL3MGCCXY_L7_clean_bin.9.orig.fa_ OEZ008529	yes	LMSG_G000005177.1	OES073704	terpene	Terpene	Polymetallic	26.16	China: Guangxi, Longshenao
L180322-P-11-LD180320-0034_HL3MGCCXY_L7_clean_bin.9.orig.fa_ OEZ008529	yes	LMSG_G000005177.1	OES073704	NRPS	NRPS	Polymetallic	34.39	China: Guangxi, Longshenao
L180322-P-11-LD180320-0034_HL3MGCCXY_L7_clean_bin.9.orig.fa_ OEZ008529	yes	LMSG_G000005177.1	OES073704	bacteriocin	RiPPs	Polymetallic	10.80	China: Guangxi, Longshenao
L180322-P-11-LD180321-0001_HL3MGCCXY_L7_clean_bin.1.orig.fa_ OEZ008529	yes	LMSG_G000007835.1	OES073695	terpene	Terpene	Polymetallic	10.79	China: Guizhou, Longli
L180322-P-11-LD180321-0001_HL3MGCCXY_L7_clean_bin.1.orig.fa_ OEZ008529	yes	LMSG_G000007835.1	OES073695	terpene	Terpene	Polymetallic	22.04	China: Guizhou, Longli
L180322-P-11-LD180321-0001_HL3MGCCXY_L7_clean_bin.1.orig.fa_ OEZ008529	yes	LMSG_G000007835.1	OES073695	terpene	Terpene	Polymetallic	10.85	China: Guizhou, Longli
L180322-P-11-LD180321-0001_HL3MGCCXY_L7_clean_bin.1.orig.fa_ OEZ008529	yes	LMSG_G000007835.1	OES073695	NRPS-like	NRPS	Polymetallic	38.29	China: Guizhou, Longli
L180322-P-11-LD180321-0001_HL3MGCCXY_L7_clean_bin.1.orig.fa_ OEZ008529	yes	LMSG_G000007835.1	OES073695	T3PKS	PKSother	Polymetallic	33.98	China: Guizhou, Longli
L180322-P-11-LD180321-0001_HL3MGCCXY_L7_clean_bin.1.orig.fa_ OEZ008529	yes	LMSG_G000007835.1	OES073695	NRPS-like	NRPS	Polymetallic	21.59	China: Guizhou, Longli
L180322-P-11-LD180321-0001_HL3MGCCXY_L7_clean_bin.10.orig.fa OEZ008529	yes	LMSG_G000010648.1	OES073695	NRPS	NRPS	Polymetallic	25.50	China: Guizhou, Longli
L180322-P-11-LD180321-0001_HL3MGCCXY_L7_clean_bin.10.orig.fa OEZ008529	yes	LMSG_G000010648.1	OES073695	NRPS-like	NRPS	Polymetallic	24.83	China: Guizhou, Longli
L180322-P-11-LD180321-0001_HL3MGCCXY_L7_clean_bin.10.orig.fa OEZ008529	yes	LMSG_G000010648.1	OES073695	T1PKS	PKSI	Polymetallic	5.71	China: Guizhou, Longli
L180322-P-11-LD180321-0001_HL3MGCCXY_L7_clean_bin.10.orig.fa OEZ008529	yes	LMSG_G000010648.1	OES073695	NRPS	NRPS	Polymetallic	6.68	China: Guizhou, Longli
L180322-P-11-LD180321-0001_HL3MGCCXY_L7_clean_bin.10.orig.fa OEZ008529	yes	LMSG_G000010648.1	OES073695	arylpolyene	Others	Polymetallic	41.29	China: Guizhou, Longli
L180322-P-11-LD180321-0001_HL3MGCCXY_L7_clean_bin.10.orig.fa OEZ008529	yes	LMSG_G000010648.1	OES073695	terpene	Terpene	Polymetallic	13.11	China: Guizhou, Longli
L180322-P-11-LD180321-0001_HL3MGCCXY_L7_clean_bin.10.orig.fa OEZ008529	yes	LMSG_G000010648.1	OES073695	acyl_amino_acids	Others	Polymetallic	26.90	China: Guizhou, Longli
L180322-P-11-LD180321-0001_HL3MGCCXY_L7_clean_bin.11.orig.fa OEZ008529	yes	LMSG_G000005178.1	OES073695	terpene	Terpene	Polymetallic	11.33	China: Guizhou, Longli
L180322-P-11-LD180321-0001_HL3MGCCXY_L7_clean_bin.11.orig.fa OEZ008529	yes	LMSG_G000005178.1	OES073695	arylpolyene	Others	Polymetallic	33.35	China: Guizhou, Longli
L180322-P-11-LD180321-0001_HL3MGCCXY_L7_clean_bin.11.orig.fa OEZ008529	yes	LMSG_G000005178.1	OES073695	NRPS	NRPS	Polymetallic	16.12	China: Guizhou, Longli
L180322-P-11-LD180321-0001_HL3MGCCXY_L7_clean_bin.12.orig.fa OEZ008529	yes	LMSG_G000010882.1	OES073695	terpene	Terpene	Polymetallic	6.09	China: Guizhou, Longli
L180322-P-11-LD180321-0001_HL3MGCCXY_L7_clean_bin.14.strict. OEZ008529	yes	LMSG_G000007793.1	OES073695	terpene	Terpene	Polymetallic	13.67	China: Guizhou, Longli
L180322-P-11-LD180321-0001_HL3MGCCXY_L7_clean_bin.15.orig.fa OEZ008529	yes	LMSG_G000005179.1	OES073695	NRPS.T1PKS	PKS-NRP_Hybrids	Polymetallic	17.30	China: Guizhou, Longli
L180322-P-11-LD180321-0001_HL3MGCCXY_L7_clean_bin.16.permiss OEZ008529	yes	LMSG_G000005180.1	OES073695	T2PKS	PKSother	Polymetallic	23.81	China: Guizhou, Longli
L180322-P-11-LD180321-0001_HL3MGCCXY_L7_clean_bin.16.permiss OEZ008529	yes	LMSG_G000005180.1	OES073695	terpene	Terpene	Polymetallic	9.34	China: Guizhou, Longli
L180322-P-11-LD180321-0001_HL3MGCCXY_L7_clean_bin.16.permiss OEZ008529	yes	LMSG_G000005180.1	OES073695	T2PKS	PKSother	Polymetallic	7.88	China: Guizhou, Longli
L180322-P-11-LD180321-0001_HL3MGCCXY_L7_clean_bin.16.permiss OEZ008529	yes	LMSG_G000005180.1	OES073695	T1PKS	PKSI	Polymetallic	7.32	China: Guizhou, Longli
L180322-P-11-LD180321-0001_HL3MGCCXY_L7_clean_bin.16.permiss OEZ008529	yes	LMSG_G000005180.1	OES073695	NRPS-like	NRPS	Polymetallic	6.31	China: Guizhou, Longli
L180322-P-11-LD180321-0001_HL3MGCCXY_L7_clean_bin.16.permiss OEZ008529	yes	LMSG_G000005180.1	OES073695	NRPS	NRPS	Polymetallic	5.51	China: Guizhou, Longli
L180322-P-11-LD180321-0001_HL3MGCCXY_L7_clean_bin.17.orig.fa OEZ008529	yes	LMSG_G000008138.1	OES073695	terpene	Terpene	Polymetallic	11.89	China: Guizhou, Longli
L180322-P-11-LD180321-0001_HL3MGCCXY_L7_clean_bin.18.orig.fa OEZ008529	yes	LMSG_G000010349.1	OES073695	hserlactone	Others	Polymetallic	20.64	China: Guizhou, Longli
L180322-P-11-LD180321-0001_HL3MGCCXY_L7_clean_bin.18.orig.fa OEZ008529	yes	LMSG_G000010349.1	OES073695	bacteriocin	RiPPs	Polymetallic	10.91	China: Guizhou, Longli
L180322-P-11-LD180321-0001_HL3MGCCXY_L7_clean_bin.18.orig.fa OEZ008529	yes	LMSG_G000010349.1	OES073695	NRPS-like	NRPS	Polymetallic	9.63	China: Guizhou, Longli
L180322-P-11-LD180321-0001_HL3MGCCXY_L7_clean_bin.18.orig.fa OEZ008529	yes	LMSG_G000010349.1	OES073695	betalactone	Others	Polymetallic	22.21	China: Guizhou, Longli
L180322-P-11-LD180321-0001_HL3MGCCXY_L7_clean_bin.18.orig.fa OEZ008529	yes	LMSG_G000010349.1	OES073695	terpene	Terpene	Polymetallic	20.73	China: Guizhou, Longli
L180322-P-11-LD180321-0001_HL3MGCCXY_L7_clean_bin.22.permiss OEZ008529	yes	LMSG_G000008764.1	OES073695	terpene	Terpene	Polymetallic	18.50	China: Guizhou, Longli
L180322-P-11-LD180321-0001_HL3MGCCXY_L7_clean_bin.22.permiss OEZ008529	yes	LMSG_G000008764.1	OES073695	terpene	Terpene	Polymetallic	12.01	China: Guizhou, Longli
L180322-P-11-LD180321-0001_HL3MGCCXY_L7_clean_bin.22.permiss OEZ008529	yes	LMSG_G000008764.1	OES073695	bacteriocin	RiPPs	Polymetallic	8.20	China: Guizhou, Longli
L180322-P-11-LD180321-0001_HL3MGCCXY_L7_clean_bin.24.orig.fa OEZ008529	yes	LMSG_G000009991.1	OES073695	NRPS-like	NRPS	Polymetallic	44.04	China: Guizhou, Longli
L180322-P-11-LD180321-0001_HL3MGCCXY_L7_clean_bin.24.orig.fa OEZ008529	yes	LMSG_G000009991.1	OES073695	arylpolyene	Others	Polymetallic	42.25	China: Guizhou, Longli
L180322-P-11-LD180321-0001_HL3MGCCXY_L7_clean_bin.24.orig.fa OEZ008529	yes	LMSG_G000009991.1	OES073695	terpene	Terpene	Polymetallic	18.45	China: Guizhou, Longli
L180322-P-11-LD180321-0001_HL3MGCCXY_L7_clean_bin.24.orig.fa OEZ008529	yes	LMSG_G000009991.1	OES073695	arylpolyene	Others	Polymetallic	21.37	China: Guizhou, Longli
L180322-P-11-LD180321-0001_HL3MGCCXY_L7_clean_bin.24.orig.fa OEZ008529	yes	LMSG_G000009991.1	OES073695	ectoine	Others	Polymetallic	10.40	China: Guizhou, Longli
L180322-P-11-LD180321-0001_HL3MGCCXY_L7_clean_bin.24.orig.fa OEZ008529	yes	LMSG_G000009991.1	OES073695	arylpolyene	Others	Polymetallic	33.78	China: Guizhou, Longli
L180322-P-11-LD180321-0001_HL3MGCCXY_L7_clean_bin.24.orig.fa OEZ008529	yes	LMSG_G000009991.1	OES073695	terpene	Terpene	Polymetallic	21.84	China: Guizhou, Longli
L180322-P-11-LD180321-0001_HL3MGCCXY_L7_clean_bin.25.orig.fa OEZ008529	yes	LMSG_G000005181.1	OES073695	terpene	Terpene	Polymetallic	21.94	China: Guizhou, Longli
L180322-P-11-LD180321-0001_HL3MGCCXY_L7_clean_bin.26.orig.fa OEZ008529	yes	LMSG_G000005182.1	OES073695	acyl_amino_acids	Others	Polymetallic	5.58	China: Guizhou, Longli
L180322-P-11-LD180321-0001_HL3MGCCXY_L7_clean_bin.27.orig.fa OEZ008529	yes	LMSG_G000009179.1	OES073695	terpene	Terpene	Polymetallic	5.85	China: Guizhou, Longli
L180322-P-11-LD180321-0001_HL3MGCCXY_L7_clean_bin.29.orig.fa OEZ008529	yes	LMSG_G000008571.1	OES073695	NRPS-like	NRPS	Polymetallic	25.88	China: Guizhou, Longli
L180322-P-11-LD180321-0001_HL3MGCCXY_L7_clean_bin.3.orig.fa_ OEZ008529	yes	LMSG_G000010199.1	OES073695	terpene	Terpene	Polymetallic	21.91	China: Guizhou, Longli
L180322-P-11-LD180321-0001_HL3MGCCXY_L7_clean_bin.3.orig.fa_ OEZ008529	yes	LMSG_G000010199.1	OES073695	terpene	Terpene	Polymetallic	21.75	China: Guizhou, Longli
L180322-P-11-LD180321-0001_HL3MGCCXY_L7_clean_bin.3.orig.fa_ OEZ008529	yes	LMSG_G000010199.1	OES073695	arylpolyene	Others	Polymetallic	15.50	China: Guizhou, Longli
L180322-P-11-LD180321-0001_HL3MGCCXY_L7_clean_bin.31.strict. OEZ008529	yes	LMSG_G000011309.1	OES073695	terpene	Terpene	Polymetallic	20.88	China: Guizhou, Longli
L180322-P-11-LD180321-0001_HL3MGCCXY_L7_clean_bin.31.strict. OEZ008529	yes	LMSG_G000011309.1	OES073695	terpene	Terpene	Polymetallic	21.06	China: Guizhou, Longli
L180322-P-11-LD180321-0001_HL3MGCCXY_L7_clean_bin.31.strict. OEZ008529	yes	LMSG_G000011309.1	OES073695	ladderane	Others	Polymetallic	31.47	China: Guizhou, Longli
L180322-P-11-LD180321-0001_HL3MGCCXY_L7_clean_bin.31.strict. OEZ008529	yes	LMSG_G000011309.1	OES073695	NRPS	NRPS	Polymetallic	25.75	China: Guizhou, Longli
L180322-P-11-LD180321-0001_HL3MGCCXY_L7_clean_bin.31.strict. OEZ008529	yes	LMSG_G000011309.1	OES073695	terpene	Terpene	Polymetallic	9.49	China: Guizhou, Longli
L180322-P-11-LD180321-0001_HL3MGCCXY_L7_clean_bin.32.orig.fa OEZ008529	yes	LMSG_G000009976.1	OES073695	arylpolyene	Others	Polymetallic	6.41	China: Guizhou, Longli
L180322-P-11-LD180321-0001_HL3MGCCXY_L7_clean_bin.32.orig.fa OEZ008529	yes	LMSG_G000009976.1	OES073695	ladderane	Others	Polymetallic	6.14	China: Guizhou, Longli
L180322-P-11-LD180321-0001_HL3MGCCXY_L7_clean_bin.32.orig.fa OEZ008529	yes	LMSG_G000009976.1	OES073695	NRPS-like	NRPS	Polymetallic	16.38	China: Guizhou, Longli
L180322-P-11-LD180321-0001_HL3MGCCXY_L7_clean_bin.32.orig.fa OEZ008529	yes	LMSG_G000009976.1	OES073695	bacteriocin	RiPPs	Polymetallic	7.13	China: Guizhou, Longli
L180322-P-11-LD180321-0001_HL3MGCCXY_L7_clean_bin.32.orig.fa OEZ008529	yes	LMSG_G000009976.1	OES073695	terpene	Terpene	Polymetallic	14.41	China: Guizhou, Longli
L180322-P-11-LD180321-0001_HL3MGCCXY_L7_clean_bin.33.orig.fa OEZ008529	yes	LMSG_G000010665.1	OES073695	hserlactone	Others	Polymetallic	20.58	China: Guizhou, Longli
L180322-P-11-LD180321-0001_HL3MGCCXY_L7_clean_bin.35.strict. OEZ008529	yes	LMSG_G000009972.1	OES073695	arylpolyene	Others	Polymetallic	9.66	China: Guizhou, Longli
L180322-P-11-LD180321-0001_HL3MGCCXY_L7_clean_bin.36.strict. OEZ008529	yes	LMSG_G000009305.1	OES073695	terpene	Terpene	Polymetallic	20.89	China: Guizhou, Longli
L180322-P-11-LD180321-0001_HL3MGCCXY_L7_clean_bin.39.orig.fa OEZ008529	yes	LMSG_G000010197.1	OES073695	T3PKS	PKSother	Polymetallic	31.60	China: Guizhou, Longli
L180322-P-11-LD180321-0001_HL3MGCCXY_L7_clean_bin.39.orig.fa OEZ008529	yes	LMSG_G000010197.1	OES073695	terpene	Terpene	Polymetallic	24.81	China: Guizhou, Longli
L180322-P-11-LD180321-0001_HL3MGCCXY_L7_clean_bin.39.orig.fa OEZ008529	yes	LMSG_G000010197.1	OES073695	arylpolyene	Others	Polymetallic	23.57	China: Guizhou, Longli
L180322-P-11-LD180321-0001_HL3MGCCXY_L7_clean_bin.39.orig.fa OEZ008529	yes	LMSG_G000010197.1	OES073695	terpene	Terpene	Polymetallic	11.64	China: Guizhou, Longli
L180322-P-11-LD180321-0001_HL3MGCCXY_L7_clean_bin.40.orig.fa OEZ008529	yes	LMSG_G000007867.1	OES073695	terpene	Terpene	Polymetallic	11.43	China: Guizhou, Longli
L180322-P-11-LD180321-0001_HL3MGCCXY_L7_clean_bin.40.orig.fa OEZ008529	yes	LMSG_G000007867.1	OES073695	NRPS-like	NRPS	Polymetallic	14.32	China: Guizhou, Longli
L180322-P-11-LD180321-0001_HL3MGCCXY_L7_clean_bin.40.orig.fa OEZ008529	yes	LMSG_G000007867.1	OES073695	T3PKS	PKSother	Polymetallic	8.22	China: Guizhou, Longli
L180322-P-11-LD180321-0001_HL3MGCCXY_L7_clean_bin.41.strict. OEZ008529	yes	LMSG_G000010787.1	OES073695	bacteriocin	RiPPs	Polymetallic	9.36	China: Guizhou, Longli
L180322-P-11-LD180321-0001_HL3MGCCXY_L7_clean_bin.42.orig.fa OEZ008529	yes	LMSG_G000010325.1	OES073695	NRPS-like	NRPS	Polymetallic	30.04	China: Guizhou, Longli
L180322-P-11-LD180321-0001_HL3MGCCXY_L7_clean_bin.42.orig.fa OEZ008529	yes	LMSG_G000010325.1	OES073695	T1PKS	PKSI	Polymetallic	33.85	China: Guizhou, Longli
L180322-P-11-LD180321-0001_HL3MGCCXY_L7_clean_bin.43.strict. OEZ008529	yes	LMSG_G000005183.1	OES073695	terpene	Terpene	Polymetallic	20.86	China: Guizhou, Longli
L180322-P-11-LD180321-0001_HL3MGCCXY_L7_clean_bin.44.orig.fa OEZ008529	yes	LMSG_G000011176.1	OES073695	arylpolyene	Others	Polymetallic	7.90	China: Guizhou, Longli
L180322-P-11-LD180321-0001_HL3MGCCXY_L7_clean_bin.45.orig.fa OEZ008529	yes	LMSG_G000007777.1	OES073695	hglE-KS	PKSother	Polymetallic	41.46	China: Guizhou, Longli
L180322-P-11-LD180321-0001_HL3MGCCXY_L7_clean_bin.45.orig.fa OEZ008529	yes	LMSG_G000007777.1	OES073695	T3PKS	PKSother	Polymetallic	25.80	China: Guizhou, Longli
L180322-P-11-LD180321-0001_HL3MGCCXY_L7_clean_bin.45.orig.fa OEZ008529	yes	LMSG_G000007777.1	OES073695	bacteriocin	RiPPs	Polymetallic	7.87	China: Guizhou, Longli
L180322-P-11-LD180321-0001_HL3MGCCXY_L7_clean_bin.45.orig.fa OEZ008529	yes	LMSG_G000007777.1	OES073695	terpene	Terpene	Polymetallic	20.96	China: Guizhou, Longli
L180322-P-11-LD180321-0001_HL3MGCCXY_L7_clean_bin.46.orig.fa OEZ008529	yes	LMSG_G000010132.1	OES073695	terpene	Terpene	Polymetallic	14.13	China: Guizhou, Longli
L180322-P-11-LD180321-0001_HL3MGCCXY_L7_clean_bin.46.orig.fa OEZ008529	yes	LMSG_G000010132.1	OES073695	terpene	Terpene	Polymetallic	23.31	China: Guizhou, Longli
L180322-P-11-LD180321-0001_HL3MGCCXY_L7_clean_bin.46.orig.fa OEZ008529	yes	LMSG_G000010132.1	OES073695	betalactone	Others	Polymetallic	30.93	China: Guizhou, Longli
L180322-P-11-LD180321-0001_HL3MGCCXY_L7_clean_bin.47.orig.fa OEZ008529	yes	LMSG_G000011145.1	OES073695	arylpolyene	Others	Polymetallic	23.54	China: Guizhou, Longli
L180322-P-11-LD180321-0001_HL3MGCCXY_L7_clean_bin.47.orig.fa OEZ008529	yes	LMSG_G000011145.1	OES073695	bacteriocin	RiPPs	Polymetallic	9.54	China: Guizhou, Longli
L180322-P-11-LD180321-0001_HL3MGCCXY_L7_clean_bin.48.orig.fa OEZ008529	yes	LMSG_G000008616.1	OES073695	other	Others	Polymetallic	5.34	China: Guizhou, Longli
L180322-P-11-LD180321-0001_HL3MGCCXY_L7_clean_bin.49.orig.fa OEZ008529	yes	LMSG_G000005184.1	OES073695	terpene	Terpene	Polymetallic	20.79	China: Guizhou, Longli
L180322-P-11-LD180321-0001_HL3MGCCXY_L7_clean_bin.49.orig.fa OEZ008529	yes	LMSG_G000005184.1	OES073695	T3PKS	PKSother	Polymetallic	41.06	China: Guizhou, Longli
L180322-P-11-LD180321-0001_HL3MGCCXY_L7_clean_bin.49.orig.fa OEZ008529	yes	LMSG_G000005184.1	OES073695	terpene	Terpene	Polymetallic	16.96	China: Guizhou, Longli
L180322-P-11-LD180321-0001_HL3MGCCXY_L7_clean_bin.49.orig.fa OEZ008529	yes	LMSG_G000005184.1	OES073695	terpene	Terpene	Polymetallic	21.93	China: Guizhou, Longli
L180322-P-11-LD180321-0001_HL3MGCCXY_L7_clean_bin.49.orig.fa OEZ008529	yes	LMSG_G000005184.1	OES073695	hserlactone	Others	Polymetallic	17.65	China: Guizhou, Longli
L180322-P-11-LD180321-0001_HL3MGCCXY_L7_clean_bin.49.orig.fa OEZ008529	yes	LMSG_G000005184.1	OES073695	terpene	Terpene	Polymetallic	20.92	China: Guizhou, Longli
L180322-P-11-LD180321-0001_HL3MGCCXY_L7_clean_bin.50.orig.fa OEZ008529	yes	LMSG_G000005185.1	OES073695	terpene	Terpene	Polymetallic	14.76	China: Guizhou, Longli
L180322-P-11-LD180321-0001_HL3MGCCXY_L7_clean_bin.50.orig.fa OEZ008529	yes	LMSG_G000005185.1	OES073695	arylpolyene	Others	Polymetallic	5.65	China: Guizhou, Longli
L180322-P-11-LD180321-0001_HL3MGCCXY_L7_clean_bin.50.orig.fa OEZ008529	yes	LMSG_G000005185.1	OES073695	terpene	Terpene	Polymetallic	6.44	China: Guizhou, Longli
L180322-P-11-LD180321-0001_HL3MGCCXY_L7_clean_bin.51.orig.fa OEZ008529	yes	LMSG_G000011049.1	OES073695	NRPS-like	NRPS	Polymetallic	9.08	China: Guizhou, Longli
L180322-P-11-LD180321-0001_HL3MGCCXY_L7_clean_bin.51.orig.fa OEZ008529	yes	LMSG_G000011049.1	OES073695	bacteriocin	RiPPs	Polymetallic	7.51	China: Guizhou, Longli
L180322-P-11-LD180321-0001_HL3MGCCXY_L7_clean_bin.51.orig.fa OEZ008529	yes	LMSG_G000011049.1	OES073695	resorcinol.arylpolyene	Others	Polymetallic	16.88	China: Guizhou, Longli
L180322-P-11-LD180321-0001_HL3MGCCXY_L7_clean_bin.51.orig.fa OEZ008529	yes	LMSG_G000011049.1	OES073695	arylpolyene	Others	Polymetallic	26.78	China: Guizhou, Longli
L180322-P-11-LD180321-0001_HL3MGCCXY_L7_clean_bin.51.orig.fa OEZ008529	yes	LMSG_G000011049.1	OES073695	terpene	Terpene	Polymetallic	11.75	China: Guizhou, Longli
L180322-P-11-LD180321-0001_HL3MGCCXY_L7_clean_bin.53.permiss OEZ008529	yes	LMSG_G000008760.1	OES073695	terpene	Terpene	Polymetallic	9.96	China: Guizhou, Longli
L180322-P-11-LD180321-0001_HL3MGCCXY_L7_clean_bin.53.permiss OEZ008529	yes	LMSG_G000008760.1	OES073695	terpene	Terpene	Polymetallic	5.71	China: Guizhou, Longli

L180322-P-11-LD180321-0001_HL3MGCCXY_L7_clean_bin.55.strict.	OEZ008529	yes	LMSG_G000010552.1	OES073695	arylpolyene	Others	Polymetallic	41.19	China: Guizhou, Longli
L180322-P-11-LD180321-0001_HL3MGCCXY_L7_clean_bin.55.strict.	OEZ008529	yes	LMSG_G000010552.1	OES073695	bacteriocin	RiPPs	Polymetallic	8.84	China: Guizhou, Longli
L180322-P-11-LD180321-0001_HL3MGCCXY_L7_clean_bin.56.strict.	OEZ008529	yes	LMSG_G000010412.1	OES073695	terpene	Terpene	Polymetallic	25.01	China: Guizhou, Longli
L180322-P-11-LD180321-0001_HL3MGCCXY_L7_clean_bin.56.strict.	OEZ008529	yes	LMSG_G000010412.1	OES073695	terpene	Terpene	Polymetallic	20.85	China: Guizhou, Longli
L180322-P-11-LD180321-0001_HL3MGCCXY_L7_clean_bin.56.strict.	OEZ008529	yes	LMSG_G000010412.1	OES073695	NRPS-like	NRPS	Polymetallic	26.06	China: Guizhou, Longli
L180322-P-11-LD180321-0001_HL3MGCCXY_L7_clean_bin.56.strict.	OEZ008529	yes	LMSG_G000010412.1	OES073695	hserlactone	Others	Polymetallic	13.57	China: Guizhou, Longli
L180322-P-11-LD180321-0001_HL3MGCCXY_L7_clean_bin.57.orig.fa	OEZ008529	no	LMSG_G000010829.1	OES073695	acyl_amino_acids	Others	Polymetallic	12.75	China: Guizhou, Longli
L180322-P-11-LD180321-0001_HL3MGCCXY_L7_clean_bin.57.orig.fa	OEZ008529	yes	LMSG_G000010829.1	OES073695	T1PKS.hserlactone	Others	Polymetallic	15.08	China: Guizhou, Longli
L180322-P-11-LD180321-0001_HL3MGCCXY_L7_clean_bin.58.orig.fa	OEZ008529	yes	LMSG_G000010670.1	OES073695	bacteriocin	RiPPs	Polymetallic	10.92	China: Guizhou, Longli
L180322-P-11-LD180321-0001_HL3MGCCXY_L7_clean_bin.58.orig.fa	OEZ008529	yes	LMSG_G000010670.1	OES073695	betalactone	Others	Polymetallic	23.96	China: Guizhou, Longli
L180322-P-11-LD180321-0001_HL3MGCCXY_L7_clean_bin.58.orig.fa	OEZ008529	yes	LMSG_G000010670.1	OES073695	terpene	Terpene	Polymetallic	16.49	China: Guizhou, Longli
L180322-P-11-LD180321-0001_HL3MGCCXY_L7_clean_bin.58.orig.fa	OEZ008529	yes	LMSG_G000010670.1	OES073695	arylpolyene	Others	Polymetallic	26.77	China: Guizhou, Longli
L180322-P-11-LD180321-0001_HL3MGCCXY_L7_clean_bin.58.orig.fa	OEZ008529	yes	LMSG_G000010670.1	OES073695	NRPS	NRPS	Polymetallic	46.40	China: Guizhou, Longli
L180322-P-11-LD180321-0001_HL3MGCCXY_L7_clean_bin.59.strict.	OEZ008529	yes	LMSG_G000009626.1	OES073695	terpene	Terpene	Polymetallic	22.09	China: Guizhou, Longli
L180322-P-11-LD180321-0001_HL3MGCCXY_L7_clean_bin.59.strict.	OEZ008529	yes	LMSG_G000009626.1	OES073695	terpene	Terpene	Polymetallic	23.33	China: Guizhou, Longli
L180322-P-11-LD180321-0001_HL3MGCCXY_L7_clean_bin.59.strict.	OEZ008529	yes	LMSG_G000009626.1	OES073695	nucleoside	Others	Polymetallic	12.87	China: Guizhou, Longli
L180322-P-11-LD180321-0001_HL3MGCCXY_L7_clean_bin.59.strict.	OEZ008529	yes	LMSG_G000009626.1	OES073695	terpene	Terpene	Polymetallic	11.99	China: Guizhou, Longli
L180322-P-11-LD180321-0001_HL3MGCCXY_L7_clean_bin.59.strict.	OEZ008529	yes	LMSG_G000009626.1	OES073695	NRPS-like	NRPS	Polymetallic	9.60	China: Guizhou, Longli
L180322-P-11-LD180321-0001_HL3MGCCXY_L7_clean_bin.60.orig.fa	OEZ008529	yes	LMSG_G000005186.1	OES073695	betalactone	Others	Polymetallic	14.37	China: Guizhou, Longli
L180322-P-11-LD180321-0001_HL3MGCCXY_L7_clean_bin.61.orig.fa	OEZ008529	yes	LMSG_G000008156.1	OES073695	terpene	Terpene	Polymetallic	19.29	China: Guizhou, Longli
L180322-P-11-LD180321-0001_HL3MGCCXY_L7_clean_bin.63.strict.	OEZ008529	no	LMSG_G000005187.1	OES073695	arylpolyene	Others	Polymetallic	27.72	China: Guizhou, Longli
L180322-P-11-LD180321-0001_HL3MGCCXY_L7_clean_bin.63.strict.	OEZ008529	no	LMSG_G000005187.1	OES073695	bacteriocin	RiPPs	Polymetallic	10.36	China: Guizhou, Longli
L180322-P-11-LD180321-0001_HL3MGCCXY_L7_clean_bin.63.strict.	OEZ008529	yes	LMSG_G000005187.1	OES073695	bacteriocin	RiPPs	Polymetallic	10.30	China: Guizhou, Longli
L180322-P-11-LD180321-0001_HL3MGCCXY_L7_clean_bin.63.strict.	OEZ008529	no	LMSG_G000005187.1	OES073695	terpene	Terpene	Polymetallic	11.92	China: Guizhou, Longli
L180322-P-11-LD180321-0001_HL3MGCCXY_L7_clean_bin.63.strict.	OEZ008529	yes	LMSG_G000005187.1	OES073695	bacteriocin	RiPPs	Polymetallic	7.42	China: Guizhou, Longli
L180322-P-11-LD180321-0001_HL3MGCCXY_L7_clean_bin.8.strict.f	OEZ008529	yes	LMSG_G000008669.1	OES073695	NRPS-like	NRPS	Polymetallic	43.81	China: Guizhou, Longli
L180322-P-11-LD180321-0002_HL3MGCCXY_L7_clean_bin.10.orig.fa	OEZ008529	yes	LMSG_G000010883.1	OES073696	bacteriocin	RiPPs	Polymetallic	5.34	China: Guizhou, Longli
L180322-P-11-LD180321-0002_HL3MGCCXY_L7_clean_bin.11.orig.fa	OEZ008529	yes	LMSG_G000010396.1	OES073696	terpene	Terpene	Polymetallic	6.21	China: Guizhou, Longli
L180322-P-11-LD180321-0002_HL3MGCCXY_L7_clean_bin.11.orig.fa	OEZ008529	yes	LMSG_G000010396.1	OES073696	hserlactone	Others	Polymetallic	11.34	China: Guizhou, Longli
L180322-P-11-LD180321-0002_HL3MGCCXY_L7_clean_bin.17.orig.fa	OEZ008529	yes	LMSG_G000010310.1	OES073696	arylpolyene	Others	Polymetallic	6.17	China: Guizhou, Longli
L180322-P-11-LD180321-0002_HL3MGCCXY_L7_clean_bin.17.orig.fa	OEZ008529	yes	LMSG_G000010310.1	OES073696	T3PKS	PKSother	Polymetallic	16.07	China: Guizhou, Longli
L180322-P-11-LD180321-0002_HL3MGCCXY_L7_clean_bin.17.orig.fa	OEZ008529	yes	LMSG_G000010310.1	OES073696	terpene	Terpene	Polymetallic	11.56	China: Guizhou, Longli
L180322-P-11-LD180321-0002_HL3MGCCXY_L7_clean_bin.17.orig.fa	OEZ008529	yes	LMSG_G000010310.1	OES073696	terpene	Terpene	Polymetallic	14.42	China: Guizhou, Longli
L180322-P-11-LD180321-0002_HL3MGCCXY_L7_clean_bin.17.orig.fa	OEZ008529	yes	LMSG_G000010310.1	OES073696	terpene	Terpene	Polymetallic	9.33	China: Guizhou, Longli
L180322-P-11-LD180321-0002_HL3MGCCXY_L7_clean_bin.18.orig.fa	OEZ008529	yes	LMSG_G000008157.1	OES073696	terpene	Terpene	Polymetallic	7.45	China: Guizhou, Longli
L180322-P-11-LD180321-0002_HL3MGCCXY_L7_clean_bin.2.orig.fa_	OEZ008529	yes	LMSG_G000008619.1	OES073696	other	Others	Polymetallic	11.08	China: Guizhou, Longli
L180322-P-11-LD180321-0002_HL3MGCCXY_L7_clean_bin.2.orig.fa_	OEZ008529	yes	LMSG_G000008619.1	OES073696	NRPS-like	NRPS	Polymetallic	18.13	China: Guizhou, Longli
L180322-P-11-LD180321-0002_HL3MGCCXY_L7_clean_bin.20.orig.fa	OEZ008529	yes	LMSG_G000005189.1	OES073696	terpene	Terpene	Polymetallic	20.92	China: Guizhou, Longli
L180322-P-11-LD180321-0002_HL3MGCCXY_L7_clean_bin.20.orig.fa	OEZ008529	yes	LMSG_G000005189.1	OES073696	phosphonate	Others	Polymetallic	36.44	China: Guizhou, Longli
L180322-P-11-LD180321-0002_HL3MGCCXY_L7_clean_bin.20.orig.fa	OEZ008529	yes	LMSG_G000005189.1	OES073696	betalactone	Others	Polymetallic	29.74	China: Guizhou, Longli
L180322-P-11-LD180321-0002_HL3MGCCXY_L7_clean_bin.20.orig.fa	OEZ008529	yes	LMSG_G000005189.1	OES073696	T1PKS	PKSI	Polymetallic	37.29	China: Guizhou, Longli
L180322-P-11-LD180321-0002_HL3MGCCXY_L7_clean_bin.20.orig.fa	OEZ008529	yes	LMSG_G000005189.1	OES073696	T3PKS	PKSother	Polymetallic	41.06	China: Guizhou, Longli
L180322-P-11-LD180321-0002_HL3MGCCXY_L7_clean_bin.20.orig.fa	OEZ008529	yes	LMSG_G000005189.1	OES073696	bacteriocin	RiPPs	Polymetallic	10.82	China: Guizhou, Longli
L180322-P-11-LD180321-0002_HL3MGCCXY_L7_clean_bin.20.orig.fa	OEZ008529	yes	LMSG_G000005189.1	OES073696	bacteriocin	RiPPs	Polymetallic	10.90	China: Guizhou, Longli
L180322-P-11-LD180321-0002_HL3MGCCXY_L7_clean_bin.20.orig.fa	OEZ008529	yes	LMSG_G000005189.1	OES073696	NRPS-like	NRPS	Polymetallic	24.32	China: Guizhou, Longli
L180322-P-11-LD180321-0002_HL3MGCCXY_L7_clean_bin.20.orig.fa	OEZ008529	yes	LMSG_G000005189.1	OES073696	bacteriocin	RiPPs	Polymetallic	10.83	China: Guizhou, Longli
L180322-P-11-LD180321-0002_HL3MGCCXY_L7_clean_bin.21.strict.	OEZ008529	yes	LMSG_G000009307.1	OES073696	terpene	Terpene	Polymetallic	20.89	China: Guizhou, Longli
L180322-P-11-LD180321-0002_HL3MGCCXY_L7_clean_bin.22.strict.	OEZ008529	yes	LMSG_G000010804.1	OES073696	acyl_amino_acids	Others	Polymetallic	35.49	China: Guizhou, Longli
L180322-P-11-LD180321-0002_HL3MGCCXY_L7_clean_bin.22.strict.	OEZ008529	yes	LMSG_G000010804.1	OES073696	acyl_amino_acids	Others	Polymetallic	11.68	China: Guizhou, Longli
L180322-P-11-LD180321-0002_HL3MGCCXY_L7_clean_bin.26.orig.fa	OEZ008529	yes	LMSG_G000010350.1	OES073696	hserlactone	Others	Polymetallic	12.16	China: Guizhou, Longli
L180322-P-11-LD180321-0002_HL3MGCCXY_L7_clean_bin.26.orig.fa	OEZ008529	yes	LMSG_G000010350.1	OES073696	bacteriocin	RiPPs	Polymetallic	10.91	China: Guizhou, Longli
L180322-P-11-LD180321-0002_HL3MGCCXY_L7_clean_bin.26.orig.fa	OEZ008529	yes	LMSG_G000010350.1	OES073696	betalactone	Others	Polymetallic	24.97	China: Guizhou, Longli
L180322-P-11-LD180321-0002_HL3MGCCXY_L7_clean_bin.26.orig.fa	OEZ008529	yes	LMSG_G000010350.1	OES073696	terpene	Terpene	Polymetallic	20.66	China: Guizhou, Longli
L180322-P-11-LD180321-0002_HL3MGCCXY_L7_clean_bin.26.orig.fa	OEZ008529	yes	LMSG_G000010350.1	OES073696	NRPS-like	NRPS	Polymetallic	37.93	China: Guizhou, Longli
L180322-P-11-LD180321-0002_HL3MGCCXY_L7_clean_bin.27.orig.fa	OEZ008529	yes	LMSG_G000011146.1	OES073696	bacteriocin	RiPPs	Polymetallic	10.87	China: Guizhou, Longli
L180322-P-11-LD180321-0002_HL3MGCCXY_L7_clean_bin.28.strict.	OEZ008529	yes	LMSG_G000010768.1	OES073696	acyl_amino_acids	Others	Polymetallic	34.69	China: Guizhou, Longli
L180322-P-11-LD180321-0002_HL3MGCCXY_L7_clean_bin.28.strict.	OEZ008529	yes	LMSG_G000010768.1	OES073696	bacteriocin	RiPPs	Polymetallic	11.75	China: Guizhou, Longli
L180322-P-11-LD180321-0002_HL3MGCCXY_L7_clean_bin.29.permiss	OEZ008529	yes	LMSG_G000005190.1	OES073696	terpene	Terpene	Polymetallic	11.80	China: Guizhou, Longli
L180322-P-11-LD180321-0002_HL3MGCCXY_L7_clean_bin.29.permiss	OEZ008529	yes	LMSG_G000005190.1	OES073696	terpene	Terpene	Polymetallic	8.78	China: Guizhou, Longli
L180322-P-11-LD180321-0002_HL3MGCCXY_L7_clean_bin.3.orig.fa_	OEZ008529	yes	LMSG_G000005192.1	OES073696	bacteriocin	RiPPs	Polymetallic	5.61	China: Guizhou, Longli
L180322-P-11-LD180321-0002_HL3MGCCXY_L7_clean_bin.3.orig.fa_	OEZ008529	yes	LMSG_G000005192.1	OES073696	bacteriocin	RiPPs	Polymetallic	8.92	China: Guizhou, Longli
L180322-P-11-LD180321-0002_HL3MGCCXY_L7_clean_bin.3.orig.fa_	OEZ008529	yes	LMSG_G000005192.1	OES073696	betalactone	Others	Polymetallic	31.00	China: Guizhou, Longli
L180322-P-11-LD180321-0002_HL3MGCCXY_L7_clean_bin.3.orig.fa_	OEZ008529	yes	LMSG_G000005192.1	OES073696	terpene	Terpene	Polymetallic	23.10	China: Guizhou, Longli
L180322-P-11-LD180321-0002_HL3MGCCXY_L7_clean_bin.3.orig.fa_	OEZ008529	yes	LMSG_G000005192.1	OES073696	arylpolyene	Others	Polymetallic	22.65	China: Guizhou, Longli
L180322-P-11-LD180321-0002_HL3MGCCXY_L7_clean_bin.3.orig.fa_	OEZ008529	yes	LMSG_G000005192.1	OES073696	lassopeptide	RiPPs	Polymetallic	11.10	China: Guizhou, Longli
L180322-P-11-LD180321-0002_HL3MGCCXY_L7_clean_bin.30.orig.fa	OEZ008529	yes	LMSG_G000007779.1	OES073696	terpene	Terpene	Polymetallic	6.11	China: Guizhou, Longli
L180322-P-11-LD180321-0002_HL3MGCCXY_L7_clean_bin.30.orig.fa	OEZ008529	yes	LMSG_G000007779.1	OES073696	bacteriocin	RiPPs	Polymetallic	10.88	China: Guizhou, Longli
L180322-P-11-LD180321-0002_HL3MGCCXY_L7_clean_bin.30.orig.fa	OEZ008529	yes	LMSG_G000007779.1	OES073696	terpene	Terpene	Polymetallic	17.29	China: Guizhou, Longli
L180322-P-11-LD180321-0002_HL3MGCCXY_L7_clean_bin.30.orig.fa	OEZ008529	yes	LMSG_G000007779.1	OES073696	T3PKS	PKSother	Polymetallic	25.80	China: Guizhou, Longli
L180322-P-11-LD180321-0002_HL3MGCCXY_L7_clean_bin.31.orig.fa	OEZ008529	yes	LMSG_G000010671.1	OES073696	bacteriocin	RiPPs	Polymetallic	10.92	China: Guizhou, Longli
L180322-P-11-LD180321-0002_HL3MGCCXY_L7_clean_bin.31.orig.fa	OEZ008529	yes	LMSG_G000010671.1	OES073696	NRPS	NRPS	Polymetallic	20.15	China: Guizhou, Longli
L180322-P-11-LD180321-0002_HL3MGCCXY_L7_clean_bin.31.orig.fa	OEZ008529	yes	LMSG_G000010671.1	OES073696	betalactone	Others	Polymetallic	23.91	China: Guizhou, Longli
L180322-P-11-LD180321-0002_HL3MGCCXY_L7_clean_bin.31.orig.fa	OEZ008529	yes	LMSG_G000010671.1	OES073696	arylpolyene	Others	Polymetallic	41.28	China: Guizhou, Longli
L180322-P-11-LD180321-0002_HL3MGCCXY_L7_clean_bin.31.orig.fa	OEZ008529	yes	LMSG_G000010671.1	OES073696	terpene	Terpene	Polymetallic	11.36	China: Guizhou, Longli
L180322-P-11-LD180321-0002_HL3MGCCXY_L7_clean_bin.32.strict.	OEZ008529	yes	LMSG_G000010551.1	OES073696	arylpolyene	Others	Polymetallic	41.19	China: Guizhou, Longli
L180322-P-11-LD180321-0002_HL3MGCCXY_L7_clean_bin.32.strict.	OEZ008529	yes	LMSG_G000010551.1	OES073696	terpene	Terpene	Polymetallic	23.03	China: Guizhou, Longli
L180322-P-11-LD180321-0002_HL3MGCCXY_L7_clean_bin.32.strict.	OEZ008529	yes	LMSG_G000010551.1	OES073696	bacteriocin	RiPPs	Polymetallic	10.84	China: Guizhou, Longli
L180322-P-11-LD180321-0002_HL3MGCCXY_L7_clean_bin.32.strict.	OEZ008529	yes	LMSG_G000010551.1	OES073696	hserlactone	Others	Polymetallic	11.56	China: Guizhou, Longli
L180322-P-11-LD180321-0002_HL3MGCCXY_L7_clean_bin.32.strict.	OEZ008529	yes	LMSG_G000010551.1	OES073696	hserlactone	Others	Polymetallic	5.14	China: Guizhou, Longli
L180322-P-11-LD180321-0002_HL3MGCCXY_L7_clean_bin.33.strict.	OEZ008529	yes	LMSG_G000007794.1	OES073696	terpene	Terpene	Polymetallic	16.55	China: Guizhou, Longli
L180322-P-11-LD180321-0002_HL3MGCCXY_L7_clean_bin.33.strict.	OEZ008529	yes	LMSG_G000007794.1	OES073696	T3PKS	PKSother	Polymetallic	9.24	China: Guizhou, Longli
L180322-P-11-LD180321-0002_HL3MGCCXY_L7_clean_bin.33.strict.	OEZ008529	yes	LMSG_G000007794.1	OES073696	terpene	Terpene	Polymetallic	8.20	China: Guizhou, Longli
L180322-P-11-LD180321-0002_HL3MGCCXY_L7_clean_bin.34.strict.	OEZ008529	no	LMSG_G000005191.1	OES073696	terpene	Terpene	Polymetallic	14.58	China: Guizhou, Longli
L180322-P-11-LD180321-0002_HL3MGCCXY_L7_clean_bin.34.strict.	OEZ008529	yes	LMSG_G000005191.1	OES073696	NRPS-like	NRPS	Polymetallic	17.25	China: Guizhou, Longli
L180322-P-11-LD180321-0002_HL3MGCCXY_L7_clean_bin.34.strict.	OEZ008529	no	LMSG_G000005191.1	OES073696	terpene	Terpene	Polymetallic	13.46	China: Guizhou, Longli
L180322-P-11-LD180321-0002_HL3MGCCXY_L7_clean_bin.39.orig.fa	OEZ008529	yes	LMSG_G000008580.1	OES073696	other	Others	Polymetallic	26.10	China: Guizhou, Longli
L180322-P-11-LD180321-0002_HL3MGCCXY_L7_clean_bin.39.orig.fa	OEZ008529	yes	LMSG_G000008580.1	OES073696	NRPS-like	NRPS	Polymetallic	42.81	China: Guizhou, Longli
L180322-P-11-LD180321-0002_HL3MGCCXY_L7_clean_bin.4.orig.fa_	OEZ008529	yes	LMSG_G000007836.1	OES073696	T3PKS	PKSother	Polymetallic	23.75	China: Guizhou, Longli
L180322-P-11-LD180321-0002_HL3MGCCXY_L7_clean_bin.4.orig.fa_	OEZ008529	yes	LMSG_G000007836.1	OES073696	betalactone	Others	Polymetallic	10.72	China: Guizhou, Longli
L180322-P-11-LD180321-0002_HL3MGCCXY_L7_clean_bin.4.orig.fa_	OEZ008529	yes	LMSG_G000007836.1	OES073696	terpene	Terpene	Polymetallic	16.15	China: Guizhou, Longli
L180322-P-11-LD180321-0002_HL3MGCCXY_L7_clean_bin.4.orig.fa_	OEZ008529	yes	LMSG_G000007836.1	OES073696	terpene	Terpene	Polymetallic	11.10	China: Guizhou, Longli
L180322-P-11-LD180321-0002_HL3MGCCXY_L7_clean_bin.40.orig.fa	OEZ008529	yes	LMSG_G000010200.1	OES073696	terpene	Terpene	Polymetallic	13.20	China: Guizhou, Longli
L180322-P-11-LD180321-0002_HL3MGCCXY_L7_clean_bin.40.orig.fa	OEZ008529	yes	LMSG_G000010200.1	OES073696	terpene	Terpene	Polymetallic	20.51	China: Guizhou, Longli
L180322-P-11-LD180321-0002_HL3MGCCXY_L7_clean_bin.40.orig.fa	OEZ008529	yes	LMSG_G000010200.1	OES073696	arylpolyene	Others	Polymetallic	29.84	China: Guizhou, Longli
L180322-P-11-LD180321-0002_HL3MGCCXY_L7_clean_bin.41.strict.	OEZ008529	yes	LMSG_G000011310.1	OES073696	terpene	Terpene	Polymetallic	21.06	China: Guizhou, Longli
L180322-P-11-LD180321-0002_HL3MGCCXY_L7_clean_bin.41.strict.	OEZ008529	yes	LMSG_G000011310.1	OES073696	NRPS	NRPS	Polymetallic	42.87	China: Guizhou, Longli
L180322-P-11-LD180321-0002_HL3MGCCXY_L7_clean_bin.41.strict.	OEZ008529	yes	LMSG_G000011310.1	OES073696	ladderane	Others	Polymetallic	37.36	China: Guizhou, Longli
L180322-P-11-LD180321-0002_HL3MGCCXY_L7_clean_bin.41.strict.	OEZ008529	yes	LMSG_G000011310.1	OES073696	terpene	Terpene	Polymetallic	20.53	China: Guizhou, Longli
L180322-P-11-LD180321-0002_HL3MGCCXY_L7_clean_bin.41.strict.	OEZ008529	yes	LMSG_G000011310.1	OES073696	terpene	Terpene	Polymetallic	9.45	China: Guizhou, Longli
L180322-P-11-LD180321-0002_HL3MGCCXY_L7_clean_bin.44.permiss	OEZ008529	yes	LMSG_G000008765.1	OES073696	terpene	Terpene	Polymetallic	8.99	China: Guizhou, Longli
L180322-P-11-LD180321-0002_HL3MGCCXY_L7_clean_bin.44.permiss	OEZ008529	yes	LMSG_G000008765.1	OES073696	terpene	Terpene	Polymetallic	6.43	China: Guizhou, Longli
L180322-P-11-LD180321-0002_HL3MGCCXY_L7_clean_bin.44.permiss	OEZ008529	yes	LMSG_G000008765.1	OES073696	NRPS-like	NRPS	Polymetallic	5.62	China: Guizhou, Longli
L180322-P-11-LD180321-0002_HL3MGCCXY_L7_clean_bin.45.strict.	OEZ008529	yes	LMSG_G000005193.1	OES073696	bacteriocin	RiPPs	Polymetallic	7.53	China: Guizhou, Longli
L180322-P-11-LD180321-0002_HL3MGCCXY_L7_clean_bin.45.strict.	OEZ008529	yes	LMSG_G000005193.1	OES073696	T1PKS	PKSI	Polymetallic	6.44	China: Guizhou, Longli
L180322-P-11-LD180321-0002_HL3MGCCXY_L7_clean_bin.47.permiss	OEZ008529	yes	LMSG_G000009300.1	OES073696	betalactone	Others	Polymetallic	8.22	China: Guizhou, Longli
L180322-P-11-LD180321-0002_HL3MGCCXY_L7_clean_bin.48.orig.fa	OEZ008529	yes	LMSG_G000010263.1	OES073696	arylpolyene	Others	Polymetallic	21.02	China: Guizhou, Longli
L180322-P-11-LD180321-0002_HL3MGCCXY_L7_clean_bin.48.orig.fa	OEZ008529	no	LMSG_G000010263.1	OES073696	terpene	Terpene	Polymetallic	14.26	China: Guizhou, Longli
L180322-P-11-LD180321-0002_HL3MGCCXY_L7_clean_bin.49.orig.fa	OEZ008529	yes	LMSG_G000008139.1	OES073696	terpene	Terpene	Polymetallic	11.56	China: Guizhou, Longli
L180322-P-11-LD180321-0002_HL3MGCCXY_L7_clean_bin.50.orig.fa	OEZ008529	yes	LMSG_G000004436.1	OES073696	terpene	Terpene	Polymetallic	22.07	China: Guizhou, Longli
L180322-P-11-LD180321-0002_HL3MGCCXY_L7_clean_bin.50.orig.fa	OEZ008529	yes	LMSG_G000004436.1	OES073696	arylpolyene	Others	Polymetallic	42.25	China: Guizhou, Longli
L180322-P-11-LD180321-0002_HL3MGCCXY_L7_clean_bin.50.orig.fa	OEZ008529	yes	LMSG_G000004436.1	OES073696	terpene	Terpene	Polymetallic	21.84	China: Guizhou, Longli
L180322-P-11-LD180321-0002_HL3MGCCXY_L7_clean_bin.50.orig.fa	OEZ008529	yes	LMSG_G000004436.1	OES073696	NRPS-like	NRPS	Polymetallic	33.30	China: Guizhou, Longli
L180322-P-11-LD180321-0002_HL3MGCCXY_L7_clean_bin.50.orig.fa	OEZ008529	yes	LMSG_G000004436.1	OES073696	arylpolyene	Others	Polymetallic	21.30	China: Guizhou, Longli
L180322-P-11-LD180321-0002_HL3MGCCXY_L7_clean_bin.50.orig.fa	OEZ008529	yes	LMSG_G000004436.1	OES073696	ectoine	Others	Polymetallic	10.40	China: Guizhou, Longli
L180322-P-11-LD180321-0002_HL3MGCCXY_L7_clean_bin.50.orig.fa	OEZ008529	yes	LMSG_G000004436.1	OES073696	arylpolyene	Others	Polymetallic	33.78	China: Guizhou, Longli
L180322-P-11-LD180321-0002_HL3MGCCXY_L7_clean_bin.51.strict.	OEZ008529	yes	LMSG_G000004437.1	OES073696	terpene	Terpene	Polymetallic	20.85	China: Guizhou, Longli
L180322-P-11-LD180321-0002_HL3MGCCXY_L7_clean_bin.51.strict.	OEZ008529	yes	LMSG_G000004437.1	OES073696	terpene	Terpene	Polymetallic	25.01	China: Guizhou, Longli
L180322-P-11-LD180321-0002_HL3MGCCXY_L7_clean_bin.51.strict.	OEZ008529	yes	LMSG_G000004437.1	OES073696	hserlactone	Others	Polymetallic	20.63	China: Guizhou, Longli
L180322-P-11-LD180321-0002_HL3MGCCXY_L7_clean_bin.51.strict.	OEZ008529	yes	LMSG_G000004437.1	OES073696	betalactone	Others	Polymetallic	18.84	China: Guizhou, Longli
L180322-P-11-LD180321-0002_HL3MGCCXY_L7_clean_bin.55.orig.fa	OEZ008529	yes	LMSG_G000008593.1	OES073696	betalactone	Others	Polymetallic	5.78	China: Guizhou, Longli
L180322-P-11-LD180321-0002_HL3MGCCXY_L7_clean_bin.56.strict.	OEZ008529	yes	LMSG_G000008192.1	OES073696	terpene	Terpene	Polymetallic	20.86	China: Guizhou, Longli
L180322-P-11-LD180321-0002_HL3MGCCXY_L7_clean_bin.57.orig.fa	OEZ008529	yes	LMSG_G000010695.1	OES073696	bacteriocin	RiPPs	Polymetallic	6.69	China: Guizhou, Longli
L180322-P-11-LD180321-0002_HL3MGCCXY_L7_clean_bin.57.orig.fa	OEZ008529	yes	LMSG_G000010695.1	OES073696	NRPS	NRPS	Polymetallic	5.43	China: Guizhou, Longli
L180322-P-11-LD180321-0002_HL3MGCCXY_L7_clean_bin.57.orig.fa	OEZ008529	yes	LMSG_G000010695.1	OES073696	arylpolyene	Others	Polymetallic	23.47	China: Guizhou, Longli
L180322-P-11-LD180321-0002_HL3MGCCXY_L7_clean_bin.57.orig.fa	OEZ008529	yes	LMSG_G000010695.1	OES073696	betalactone	Others	Polymetallic	14.39	China: Guizhou, Longli
L180322-P-11-LD180321-0002_HL3MGCCXY_L7_clean_bin.7.strict.f	OEZ008529	yes	LMSG_G000010828.1	OES073696	acyl_amino_acids	Others	Polymetallic	41.12	China: Guizhou, Longli
L180322-P-11-LD180321-0002_HL3MGCCXY_L7_clean_bin.7.strict.f	OEZ008529	yes	LMSG_G000010828.1	OES073696	T1PKS.hserlactone	Others	Polymetallic	23.43	China: Guizhou, Longli
L180322-P-11-LD180321-0002_HL3MGCCXY_L7_clean_bin.9.orig.fa_	OEZ008529	yes	LMSG_G000005196.1	OES073696	terpene	Terpene	Polymetallic	13.99	China: Guizhou, Longli
L180322-P-11-LD180321-0002_HL3MGCCXY_L7_clean_bin.9.orig.fa_	OEZ008529	yes	LMSG_G000005196.1	OES073696	terpene	Terpene	Polymetallic	8.45	China: Guizhou, Longli
L180322-P-11-LD180321-0002_HL3MGCCXY_L7_clean_bin.9.orig.fa_	OEZ008529	yes	LMSG_G000005196.1	OES073696	T3PKS	PKSother	Polymetallic	27.94	China: Guizhou, Longli
L180322-P-11-LD180321-0002_HL3MGCCXY_L7_clean_bin.9.orig.fa_	OEZ008529	yes	LMSG_G000005196.1	OES073696	terpene	Terpene	Polymetallic	7.89	China: Guizhou, Longli
L180322-P-1-LD180319-0001_HL3GTCCXY_L1_clean_bin.1.strict.fa	OEZ008529	yes	LMSG_G000007803.1	OES073689	hglE-KS	PKSother	Polymetallic	43.68	China: Guizhou, Jiuan
L180322-P-1-LD180319-0001_HL3GTCCXY_L1_clean_bin.1.strict.fa	OEZ008529	yes	LMSG_G000007803.1	OES073689	T3PKS	PKSother	Polymetallic	41.10	China: Guizhou, Jiuan
L180322-P-1-LD180319-0001_HL3GTCCXY_L1_clean_bin.1.strict.fa	OEZ008529	yes	LMSG_G000007803.1	OES073689	terpene	Terpene	Polymetallic	10.89	China: Guizhou, Jiuan
L180322-P-1-LD180319-0001_HL3GTCCXY_L1_clean_bin.13.strict.f	OEZ008529	yes	LMSG_G000008076.1	OES073689	T1PKS.hglE-KS	PKSother	Polymetallic	26.79	China: Guizhou, Jiuan
L180322-P-1-LD180319-0001_HL3GTCCXY_L1_clean_bin.14.strict.f	OEZ008529	yes	LMSG_G000005197.1	OES073689	sactipeptide	RiPPs	Polymetallic	9.32	China: Guizhou, Jiuan
L180322-P-1-LD180319-0001_HL3GTCCXY_L1_clean_bin.17.permissi	OEZ008529	yes	LMSG_G000008271.1	OES073689	terpene	Terpene	Polymetallic	6.49	China: Guizhou, Jiuan
L180322-P-1-LD180319-0001_HL3GTCCXY_L1_clean_bin.19.orig.fa_	OEZ008529	no	LMSG_G000011010.1	OES073689	thiopeptide	RiPPs	Polymetallic	33.00	China: Guizhou, Jiuan
L180322-P-1-LD180319-0001_HL3GTCCXY_L1_clean_bin.19.orig.fa_	OEZ008529	no	LMSG_G000011010.1	OES073689	NRPS	NRPS	Polymetallic	53.04	China: Guizhou, Jiuan
L180322-P-1-LD180319-0001_HL3GTCCXY_L1_clean_bin.19.orig.fa_	OEZ008529	no	LMSG_G000011010.1	OES073689	hserlactone	Others	Polymetallic	20.61	China: Guizhou, Jiuan
L180322-P-1-LD180319-0001_HL3GTCCXY_L1_clean_bin.19.orig.fa_	OEZ008529	no	LMSG_G000011010.1	OES073689	NAGGN	Others	Polymetallic	14.76	China: Guizhou, Jiuan
L180322-P-1-LD180319-0001_HL3GTCCXY_L1_clean_bin.19.orig.fa_	OEZ008529	no	LMSG_G000011010.1	OES073689	NRPS	NRPS	Polymetallic	5.44	China: Guizhou, Jiuan
L180322-P-1-LD180319-0001_HL3GTCCXY_L1_clean_bin.19.orig.fa_	OEZ008529	no	LMSG_G000011010.1	OES073689	NRPS	NRPS	Polymetallic	6.21	China: Guizhou, Jiuan
L180322-P-1-LD180319-0001_HL3GTCCXY_L1_clean_bin.19.orig.fa_	OEZ008529	no	LMSG_G000011010.1	OES073689	NRPS	NRPS	Polymetallic	31.78	China: Guizhou, Jiuan
L180322-P-1-LD180319-0001_HL3GTCCXY_L1_clean_bin.19.orig.fa_	OEZ008529	no	LMSG_G000011010.1	OES073689	betalactone.NRPS-like	Others	Polymetallic	37.35	China: Guizhou, Jiuan
L180322-P-1-LD180319-0001_HL3GTCCXY_L1_clean_bin.19.orig.fa_	OEZ008529	no	LMSG_G000011010.1	OES073689	NRPS-like	NRPS	Polymetallic	42.98	China: Guizhou, Jiuan
L180322-P-1-LD180319-0001_HL3GTCCXY_L1_clean_bin.19.orig.fa_	OEZ008529	no	LMSG_G000011010.1	OES073689	bacteriocin	RiPPs	Polymetallic	10.83	China: Guizhou, Jiuan
L180322-P-1-LD180319-0001_HL3GTCCXY_L1_clean_bin.19.orig.fa_	OEZ008529	no	LMSG_G000011010.1	OES073689	phenazine	Others	Polymetallic	10.23	China: Guizhou, Jiuan
L180322-P-1-LD180319-0001_HL3GTCCXY_L1_clean_bin.19.orig.fa_	OEZ008529	no	LMSG_G000011010.1	OES073689	phenazine	Others	Polymetallic	10.23	China: Guizhou, Jiuan
L180322-P-1-LD180319-0001_HL3GTCCXY_L1_clean_bin.19.orig.fa_	OEZ008529	no	LMSG_G000011010.1	OES073689	hserlactone	Others	Polymetallic	20.61	China: Guizhou, Jiuan
L180322-P-1-LD180319-0001_HL3GTCCXY_L1_clean_bin.19.orig.fa_	OEZ008529	no	LMSG_G000011010.1	OES073689	NRPS	NRPS	Polymetallic	49.55	China: Guizhou, Jiuan
L180322-P-1-LD180319-0001_HL3GTCCXY_L1_clean_bin.19.orig.fa_	OEZ008529	no	LMSG_G000011010.1	OES073689	bacteriocin	RiPPs	Polymetallic	10.86	China: Guizhou, Jiuan
L180322-P-1-LD180319-0001_HL3GTCCXY_L1_clean_bin.19.orig.fa_	OEZ008529	no	LMSG_G000011010.1	OES073689	NRPS	NRPS	Polymetallic	42.58	China: Guizhou, Jiuan
L180322-P-1-LD180319-0001_HL3GTCCXY_L1_clean_bin.20.orig.fa_	OEZ008529	yes	LMSG_G000008607.1	OES073689	other	Others	Polymetallic	28.51	China: Guizhou, Jiuan
L180322-P-1-LD180319-0001_HL3GTCCXY_L1_clean_bin.21.orig.fa_	OEZ008529	yes	LMSG_G000005199.1	OES073689	terpene	Terpene	Polymetallic	21.73	China: Guizhou, Jiuan
L180322-P-1-LD180319-0001_HL3GTCCXY_L1_clean_bin.21.orig.fa_	OEZ008529	yes	LMSG_G000005199.1	OES073689	arylpolyene	Others	Polymetallic	25.24	China: Guizhou, Jiuan
L180322-P-1-LD180319-0001_HL3GTCCXY_L1_clean_bin.21.orig.fa_	OEZ008529	yes	LMSG_G000005199.1	OES073689	terpene	Terpene	Polymetallic	21.91	China: Guizhou, Jiuan
L180322-P-1-LD180319-0001_HL3GTCCXY_L1_clean_bin.22.orig.fa_	OEZ008529	yes	LMSG_G000010758.1	OES073689	bacteriocin	RiPPs	Polymetallic	11.75	China: Guizhou, Jiuan

L180322-P-1-LD180319-0001_HL3GTCCXY_L1_clean_bin.22.orig.fa_	OEZ008529	yes	LMSG_G000010758.1	OES073689	acyl_amino_acids	Others	Polymetallic	16.62	China: Guizhou, Jiuan
L180322-P-1-LD180319-0001_HL3GTCCXY_L1_clean_bin.23.orig.fa_	OEZ008529	yes	LMSG_G000010421.1	OES073689	terpene	Terpene	Polymetallic	19.30	China: Guizhou, Jiuan
L180322-P-1-LD180319-0001_HL3GTCCXY_L1_clean_bin.23.orig.fa_	OEZ008529	yes	LMSG_G000010421.1	OES073689	terpene	Terpene	Polymetallic	25.02	China: Guizhou, Jiuan
L180322-P-1-LD180319-0001_HL3GTCCXY_L1_clean_bin.23.orig.fa_	OEZ008529	yes	LMSG_G000010421.1	OES073689	hserlactone	Others	Polymetallic	14.81	China: Guizhou, Jiuan
L180322-P-1-LD180319-0001_HL3GTCCXY_L1_clean_bin.23.orig.fa_	OEZ008529	yes	LMSG_G000010421.1	OES073689	hserlactone	Others	Polymetallic	5.94	China: Guizhou, Jiuan
L180322-P-1-LD180319-0001_HL3GTCCXY_L1_clean_bin.23.orig.fa_	OEZ008529	yes	LMSG_G000010421.1	OES073689	bacteriocin	RiPPs	Polymetallic	10.88	China: Guizhou, Jiuan
L180322-P-1-LD180319-0001_HL3GTCCXY_L1_clean_bin.24.strict.f	OEZ008529	yes	LMSG_G000009220.1	OES073689	terpene	Terpene	Polymetallic	21.74	China: Guizhou, Jiuan
L180322-P-1-LD180319-0001_HL3GTCCXY_L1_clean_bin.24.strict.f	OEZ008529	yes	LMSG_G000009220.1	OES073689	hserlactone	Others	Polymetallic	19.74	China: Guizhou, Jiuan
L180322-P-1-LD180319-0001_HL3GTCCXY_L1_clean_bin.24.strict.f	OEZ008529	yes	LMSG_G000009220.1	OES073689	terpene	Terpene	Polymetallic	19.59	China: Guizhou, Jiuan
L180322-P-1-LD180319-0001_HL3GTCCXY_L1_clean_bin.25.orig.fa_	OEZ008529	yes	LMSG_G000008522.1	OES073689	T1PKS.hglE-KS	PKSother	Polymetallic	12.93	China: Guizhou, Jiuan
L180322-P-1-LD180319-0001_HL3GTCCXY_L1_clean_bin.25.orig.fa_	OEZ008529	yes	LMSG_G000008522.1	OES073689	lassopeptide	RiPPs	Polymetallic	7.64	China: Guizhou, Jiuan
L180322-P-1-LD180319-0001_HL3GTCCXY_L1_clean_bin.26.permissi	OEZ008529	yes	LMSG_G000008881.1	OES073689	T3PKS	PKSother	Polymetallic	27.16	China: Guizhou, Jiuan
L180322-P-1-LD180319-0001_HL3GTCCXY_L1_clean_bin.26.permissi	OEZ008529	yes	LMSG_G000008881.1	OES073689	arylpolyene	Others	Polymetallic	15.28	China: Guizhou, Jiuan
L180322-P-1-LD180319-0001_HL3GTCCXY_L1_clean_bin.26.permissi	OEZ008529	yes	LMSG_G000008881.1	OES073689	terpene	Terpene	Polymetallic	8.90	China: Guizhou, Jiuan
L180322-P-1-LD180319-0001_HL3GTCCXY_L1_clean_bin.26.permissi	OEZ008529	yes	LMSG_G000008881.1	OES073689	NRPS	NRPS	Polymetallic	7.46	China: Guizhou, Jiuan
L180322-P-1-LD180319-0001_HL3GTCCXY_L1_clean_bin.27.orig.fa_	OEZ008529	yes	LMSG_G000009089.1	OES073689	betalactone	Others	Polymetallic	22.77	China: Guizhou, Jiuan
L180322-P-1-LD180319-0001_HL3GTCCXY_L1_clean_bin.27.orig.fa_	OEZ008529	yes	LMSG_G000009089.1	OES073689	bacteriocin	RiPPs	Polymetallic	6.58	China: Guizhou, Jiuan
L180322-P-1-LD180319-0001_HL3GTCCXY_L1_clean_bin.27.orig.fa_	OEZ008529	yes	LMSG_G000009089.1	OES073689	betalactone	Others	Polymetallic	5.69	China: Guizhou, Jiuan
L180322-P-1-LD180319-0001_HL3GTCCXY_L1_clean_bin.28.orig.fa_	OEZ008529	yes	LMSG_G000010264.1	OES073689	arylpolyene	Others	Polymetallic	41.19	China: Guizhou, Jiuan
L180322-P-1-LD180319-0001_HL3GTCCXY_L1_clean_bin.28.orig.fa_	OEZ008529	no	LMSG_G000010264.1	OES073689	terpene	Terpene	Polymetallic	21.52	China: Guizhou, Jiuan
L180322-P-1-LD180319-0001_HL3GTCCXY_L1_clean_bin.3.orig.fa_k	OEZ008529	yes	LMSG_G000008996.1	OES073689	bacteriocin	RiPPs	Polymetallic	10.32	China: Guizhou, Jiuan
L180322-P-1-LD180319-0001_HL3GTCCXY_L1_clean_bin.30.strict.f	OEZ008529	yes	LMSG_G000010054.1	OES073689	terpene	Terpene	Polymetallic	22.10	China: Guizhou, Jiuan
L180322-P-1-LD180319-0001_HL3GTCCXY_L1_clean_bin.30.strict.f	OEZ008529	yes	LMSG_G000010054.1	OES073689	ladderane	Others	Polymetallic	41.26	China: Guizhou, Jiuan
L180322-P-1-LD180319-0001_HL3GTCCXY_L1_clean_bin.30.strict.f	OEZ008529	yes	LMSG_G000010054.1	OES073689	NRPS-like.ectoine	Others	Polymetallic	31.11	China: Guizhou, Jiuan
L180322-P-1-LD180319-0001_HL3GTCCXY_L1_clean_bin.30.strict.f	OEZ008529	yes	LMSG_G000010054.1	OES073689	bacteriocin	RiPPs	Polymetallic	10.68	China: Guizhou, Jiuan
L180322-P-1-LD180319-0001_HL3GTCCXY_L1_clean_bin.30.strict.f	OEZ008529	yes	LMSG_G000010054.1	OES073689	arylpolyene	Others	Polymetallic	25.55	China: Guizhou, Jiuan
L180322-P-1-LD180319-0001_HL3GTCCXY_L1_clean_bin.30.strict.f	OEZ008529	yes	LMSG_G000010054.1	OES073689	arylpolyene	Others	Polymetallic	15.14	China: Guizhou, Jiuan
L180322-P-1-LD180319-0001_HL3GTCCXY_L1_clean_bin.30.strict.f	OEZ008529	yes	LMSG_G000010054.1	OES073689	terpene	Terpene	Polymetallic	5.62	China: Guizhou, Jiuan
L180322-P-1-LD180319-0001_HL3GTCCXY_L1_clean_bin.31.strict.f	OEZ008529	yes	LMSG_G000007755.1	OES073689	betalactone.NRPS-like	Others	Polymetallic	25.47	China: Guizhou, Jiuan
L180322-P-1-LD180319-0001_HL3GTCCXY_L1_clean_bin.31.strict.f	OEZ008529	yes	LMSG_G000007755.1	OES073689	T1PKS	PKSI	Polymetallic	17.94	China: Guizhou, Jiuan
L180322-P-1-LD180319-0001_HL3GTCCXY_L1_clean_bin.31.strict.f	OEZ008529	yes	LMSG_G000007755.1	OES073689	T3PKS	PKSother	Polymetallic	10.95	China: Guizhou, Jiuan
L180322-P-1-LD180319-0001_HL3GTCCXY_L1_clean_bin.32.orig.fa_	OEZ008529	yes	LMSG_G000008158.1	OES073689	terpene	Terpene	Polymetallic	23.44	China: Guizhou, Jiuan
L180322-P-1-LD180319-0001_HL3GTCCXY_L1_clean_bin.34.orig.fa_	OEZ008529	yes	LMSG_G000007795.1	OES073689	T3PKS	PKSother	Polymetallic	26.43	China: Guizhou, Jiuan
L180322-P-1-LD180319-0001_HL3GTCCXY_L1_clean_bin.34.orig.fa_	OEZ008529	yes	LMSG_G000007795.1	OES073689	terpene	Terpene	Polymetallic	10.59	China: Guizhou, Jiuan
L180322-P-1-LD180319-0001_HL3GTCCXY_L1_clean_bin.35.orig.fa_	OEZ008529	yes	LMSG_G000010649.1	OES073689	NRPS.T1PKS	PKS-NRP_Hybrids	Polymetallic	43.86	China: Guizhou, Jiuan
L180322-P-1-LD180319-0001_HL3GTCCXY_L1_clean_bin.35.orig.fa_	OEZ008529	yes	LMSG_G000010649.1	OES073689	NRPS.T1PKS	PKS-NRP_Hybrids	Polymetallic	42.64	China: Guizhou, Jiuan
L180322-P-1-LD180319-0001_HL3GTCCXY_L1_clean_bin.35.orig.fa_	OEZ008529	yes	LMSG_G000010649.1	OES073689	NRPS-like	NRPS	Polymetallic	21.27	China: Guizhou, Jiuan
L180322-P-1-LD180319-0001_HL3GTCCXY_L1_clean_bin.35.orig.fa_	OEZ008529	yes	LMSG_G000010649.1	OES073689	NRPS	NRPS	Polymetallic	24.74	China: Guizhou, Jiuan
L180322-P-1-LD180319-0001_HL3GTCCXY_L1_clean_bin.35.orig.fa_	OEZ008529	yes	LMSG_G000010649.1	OES073689	NRPS-like	NRPS	Polymetallic	22.95	China: Guizhou, Jiuan
L180322-P-1-LD180319-0001_HL3GTCCXY_L1_clean_bin.35.orig.fa_	OEZ008529	yes	LMSG_G000010649.1	OES073689	arylpolyene	Others	Polymetallic	41.29	China: Guizhou, Jiuan
L180322-P-1-LD180319-0001_HL3GTCCXY_L1_clean_bin.37.orig.fa_	OEZ008529	yes	LMSG_G000011157.1	OES073689	arylpolyene	Others	Polymetallic	22.10	China: Guizhou, Jiuan
L180322-P-1-LD180319-0001_HL3GTCCXY_L1_clean_bin.38.orig.fa_	OEZ008529	yes	LMSG_G000006272.1	OES073689	terpene	Terpene	Polymetallic	29.66	China: Guizhou, Jiuan
L180322-P-1-LD180319-0001_HL3GTCCXY_L1_clean_bin.38.orig.fa_	OEZ008529	yes	LMSG_G000006272.1	OES073689	T3PKS	PKSother	Polymetallic	34.11	China: Guizhou, Jiuan
L180322-P-1-LD180319-0001_HL3GTCCXY_L1_clean_bin.40.orig.fa_	OEZ008529	yes	LMSG_G000011044.1	OES073689	NRPS-like	NRPS	Polymetallic	14.03	China: Guizhou, Jiuan
L180322-P-1-LD180319-0001_HL3GTCCXY_L1_clean_bin.40.orig.fa_	OEZ008529	yes	LMSG_G000011044.1	OES073689	terpene	Terpene	Polymetallic	11.59	China: Guizhou, Jiuan
L180322-P-1-LD180319-0001_HL3GTCCXY_L1_clean_bin.40.orig.fa_	OEZ008529	yes	LMSG_G000011044.1	OES073689	bacteriocin	RiPPs	Polymetallic	6.74	China: Guizhou, Jiuan
L180322-P-1-LD180319-0001_HL3GTCCXY_L1_clean_bin.40.orig.fa_	OEZ008529	yes	LMSG_G000011044.1	OES073689	resorcinol.arylpolyene	Others	Polymetallic	20.99	China: Guizhou, Jiuan
L180322-P-1-LD180319-0001_HL3GTCCXY_L1_clean_bin.40.orig.fa_	OEZ008529	yes	LMSG_G000011044.1	OES073689	terpene	Terpene	Polymetallic	20.85	China: Guizhou, Jiuan
L180322-P-1-LD180319-0001_HL3GTCCXY_L1_clean_bin.40.orig.fa_	OEZ008529	yes	LMSG_G000011044.1	OES073689	arylpolyene	Others	Polymetallic	38.54	China: Guizhou, Jiuan
L180322-P-1-LD180319-0001_HL3GTCCXY_L1_clean_bin.42.orig.fa_	OEZ008529	yes	LMSG_G000005201.1	OES073689	T1PKS.hglE-KS	PKSother	Polymetallic	19.21	China: Guizhou, Jiuan
L180322-P-1-LD180319-0001_HL3GTCCXY_L1_clean_bin.43.strict.f	OEZ008529	yes	LMSG_G000009266.1	OES073689	terpene	Terpene	Polymetallic	20.85	China: Guizhou, Jiuan
L180322-P-1-LD180319-0001_HL3GTCCXY_L1_clean_bin.43.strict.f	OEZ008529	yes	LMSG_G000009266.1	OES073689	TfuA-related	RiPPs	Polymetallic	19.41	China: Guizhou, Jiuan
L180322-P-1-LD180319-0001_HL3GTCCXY_L1_clean_bin.43.strict.f	OEZ008529	yes	LMSG_G000009266.1	OES073689	LAP	RiPPs	Polymetallic	14.58	China: Guizhou, Jiuan
L180322-P-1-LD180319-0001_HL3GTCCXY_L1_clean_bin.47.strict.f	OEZ008529	yes	LMSG_G000005203.1	OES073689	NRPS-like.terpene	Others	Polymetallic	22.99	China: Guizhou, Jiuan
L180322-P-1-LD180319-0001_HL3GTCCXY_L1_clean_bin.48.strict.f	OEZ008529	yes	LMSG_G000008140.1	OES073689	terpene	Terpene	Polymetallic	11.31	China: Guizhou, Jiuan
L180322-P-1-LD180319-0001_HL3GTCCXY_L1_clean_bin.48.strict.f	OEZ008529	yes	LMSG_G000008140.1	OES073689	terpene	Terpene	Polymetallic	11.44	China: Guizhou, Jiuan
L180322-P-1-LD180319-0001_HL3GTCCXY_L1_clean_bin.49.orig.fa_	OEZ008529	yes	LMSG_G000011070.1	OES073689	NRPS-like	NRPS	Polymetallic	41.97	China: Guizhou, Jiuan
L180322-P-1-LD180319-0001_HL3GTCCXY_L1_clean_bin.49.orig.fa_	OEZ008529	yes	LMSG_G000011070.1	OES073689	siderophore.hserlactone	Others	Polymetallic	10.17	China: Guizhou, Jiuan
L180322-P-1-LD180319-0001_HL3GTCCXY_L1_clean_bin.49.orig.fa_	OEZ008529	yes	LMSG_G000011070.1	OES073689	lassopeptide	RiPPs	Polymetallic	15.94	China: Guizhou, Jiuan
L180322-P-1-LD180319-0001_HL3GTCCXY_L1_clean_bin.49.orig.fa_	OEZ008529	yes	LMSG_G000011070.1	OES073689	NRPS-like	NRPS	Polymetallic	21.00	China: Guizhou, Jiuan
L180322-P-1-LD180319-0001_HL3GTCCXY_L1_clean_bin.50.orig.fa_	OEZ008529	yes	LMSG_G000007820.1	OES073689	T3PKS	PKSother	Polymetallic	22.50	China: Guizhou, Jiuan
L180322-P-1-LD180319-0001_HL3GTCCXY_L1_clean_bin.50.orig.fa_	OEZ008529	yes	LMSG_G000007820.1	OES073689	terpene	Terpene	Polymetallic	29.81	China: Guizhou, Jiuan
L180322-P-1-LD180319-0001_HL3GTCCXY_L1_clean_bin.51.orig.fa_	OEZ008529	no	LMSG_G000010610.1	OES073689	ectoine	Others	Polymetallic	8.32	China: Guizhou, Jiuan
L180322-P-1-LD180319-0001_HL3GTCCXY_L1_clean_bin.51.orig.fa_	OEZ008529	yes	LMSG_G000010610.1	OES073689	betalactone	Others	Polymetallic	19.10	China: Guizhou, Jiuan
L180322-P-1-LD180319-0001_HL3GTCCXY_L1_clean_bin.51.orig.fa_	OEZ008529	no	LMSG_G000010610.1	OES073689	phosphonate	Others	Polymetallic	13.93	China: Guizhou, Jiuan
L180322-P-1-LD180319-0001_HL3GTCCXY_L1_clean_bin.51.orig.fa_	OEZ008529	yes	LMSG_G000010610.1	OES073689	resorcinol	Others	Polymetallic	24.71	China: Guizhou, Jiuan
L180322-P-1-LD180319-0001_HL3GTCCXY_L1_clean_bin.51.orig.fa_	OEZ008529	yes	LMSG_G000010610.1	OES073689	terpene	Terpene	Polymetallic	12.47	China: Guizhou, Jiuan
L180322-P-1-LD180319-0001_HL3GTCCXY_L1_clean_bin.51.orig.fa_	OEZ008529	no	LMSG_G000010610.1	OES073689	NRPS-like	NRPS	Polymetallic	6.66	China: Guizhou, Jiuan
L180322-P-1-LD180319-0001_HL3GTCCXY_L1_clean_bin.52.orig.fa_	OEZ008529	yes	LMSG_G000008372.1	OES073689	terpene	Terpene	Polymetallic	9.02	China: Guizhou, Jiuan
L180322-P-1-LD180319-0001_HL3GTCCXY_L1_clean_bin.53.orig.fa_	OEZ008529	yes	LMSG_G000008463.1	OES073689	terpene	Terpene	Polymetallic	8.96	China: Guizhou, Jiuan
L180322-P-1-LD180319-0001_HL3GTCCXY_L1_clean_bin.53.orig.fa_	OEZ008529	yes	LMSG_G000008463.1	OES073689	betalactone	Others	Polymetallic	9.94	China: Guizhou, Jiuan
L180322-P-1-LD180319-0001_HL3GTCCXY_L1_clean_bin.54.orig.fa_	OEZ008529	yes	LMSG_G000008436.1	OES073689	terpene	Terpene	Polymetallic	26.50	China: Guizhou, Jiuan
L180322-P-1-LD180319-0001_HL3GTCCXY_L1_clean_bin.55.strict.f	OEZ008529	yes	LMSG_G000009263.1	OES073689	TfuA-related	RiPPs	Polymetallic	12.62	China: Guizhou, Jiuan
L180322-P-1-LD180319-0001_HL3GTCCXY_L1_clean_bin.57.orig.fa_	OEZ008529	yes	LMSG_G000007837.1	OES073689	terpene	Terpene	Polymetallic	6.48	China: Guizhou, Jiuan
L180322-P-1-LD180319-0001_HL3GTCCXY_L1_clean_bin.57.orig.fa_	OEZ008529	yes	LMSG_G000007837.1	OES073689	terpene	Terpene	Polymetallic	10.98	China: Guizhou, Jiuan
L180322-P-1-LD180319-0001_HL3GTCCXY_L1_clean_bin.57.orig.fa_	OEZ008529	yes	LMSG_G000007837.1	OES073689	NRPS-like	NRPS	Polymetallic	6.54	China: Guizhou, Jiuan
L180322-P-1-LD180319-0001_HL3GTCCXY_L1_clean_bin.6.orig.fa_k	OEZ008529	yes	LMSG_G000006227.1	OES073689	betalactone	Others	Polymetallic	16.65	China: Guizhou, Jiuan
L180322-P-1-LD180319-0001_HL3GTCCXY_L1_clean_bin.6.orig.fa_k	OEZ008529	yes	LMSG_G000006227.1	OES073689	ladderane	Others	Polymetallic	36.75	China: Guizhou, Jiuan
L180322-P-1-LD180319-0001_HL3GTCCXY_L1_clean_bin.8.orig.fa_k	OEZ008529	yes	LMSG_G000010984.1	OES073689	T1PKS	PKSI	Polymetallic	26.59	China: Guizhou, Jiuan
L180322-P-1-LD180319-0002_HL3GTCCXY_L1_clean_bin.1.strict.fa	OEZ008529	yes	LMSG_G000009221.1	OES073690	terpene	Terpene	Polymetallic	21.74	China: Guizhou, Jiuan
L180322-P-1-LD180319-0002_HL3GTCCXY_L1_clean_bin.1.strict.fa	OEZ008529	yes	LMSG_G000009221.1	OES073690	hserlactone	Others	Polymetallic	14.43	China: Guizhou, Jiuan
L180322-P-1-LD180319-0002_HL3GTCCXY_L1_clean_bin.1.strict.fa	OEZ008529	yes	LMSG_G000009221.1	OES073690	terpene	Terpene	Polymetallic	12.90	China: Guizhou, Jiuan
L180322-P-1-LD180319-0002_HL3GTCCXY_L1_clean_bin.11.orig.fa_	OEZ008529	yes	LMSG_G000011071.1	OES073690	bacteriocin	RiPPs	Polymetallic	5.55	China: Guizhou, Jiuan
L180322-P-1-LD180319-0002_HL3GTCCXY_L1_clean_bin.11.orig.fa_	OEZ008529	yes	LMSG_G000011071.1	OES073690	NRPS-like	NRPS	Polymetallic	6.94	China: Guizhou, Jiuan
L180322-P-1-LD180319-0002_HL3GTCCXY_L1_clean_bin.11.orig.fa_	OEZ008529	yes	LMSG_G000011071.1	OES073690	arylpolyene	Others	Polymetallic	12.95	China: Guizhou, Jiuan
L180322-P-1-LD180319-0002_HL3GTCCXY_L1_clean_bin.11.orig.fa_	OEZ008529	yes	LMSG_G000011071.1	OES073690	hserlactone	Others	Polymetallic	6.22	China: Guizhou, Jiuan
L180322-P-1-LD180319-0002_HL3GTCCXY_L1_clean_bin.11.orig.fa_	OEZ008529	yes	LMSG_G000011071.1	OES073690	bacteriocin	RiPPs	Polymetallic	10.90	China: Guizhou, Jiuan
L180322-P-1-LD180319-0002_HL3GTCCXY_L1_clean_bin.11.orig.fa_	OEZ008529	yes	LMSG_G000011071.1	OES073690	terpene	Terpene	Polymetallic	11.69	China: Guizhou, Jiuan
L180322-P-1-LD180319-0002_HL3GTCCXY_L1_clean_bin.11.orig.fa_	OEZ008529	yes	LMSG_G000011071.1	OES073690	lassopeptide.siderophore	Others	Polymetallic	5.98	China: Guizhou, Jiuan
L180322-P-1-LD180319-0002_HL3GTCCXY_L1_clean_bin.11.orig.fa_	OEZ008529	yes	LMSG_G000011071.1	OES073690	NRPS-like	NRPS	Polymetallic	5.67	China: Guizhou, Jiuan
L180322-P-1-LD180319-0002_HL3GTCCXY_L1_clean_bin.12.orig.fa_	OEZ008529	yes	LMSG_G000008437.1	OES073690	terpene	Terpene	Polymetallic	26.50	China: Guizhou, Jiuan
L180322-P-1-LD180319-0002_HL3GTCCXY_L1_clean_bin.13.orig.fa_	OEZ008529	yes	LMSG_G000007804.1	OES073690	hglE-KS	PKSother	Polymetallic	43.68	China: Guizhou, Jiuan
L180322-P-1-LD180319-0002_HL3GTCCXY_L1_clean_bin.13.orig.fa_	OEZ008529	yes	LMSG_G000007804.1	OES073690	T3PKS	PKSother	Polymetallic	41.10	China: Guizhou, Jiuan
L180322-P-1-LD180319-0002_HL3GTCCXY_L1_clean_bin.13.orig.fa_	OEZ008529	yes	LMSG_G000007804.1	OES073690	terpene	Terpene	Polymetallic	10.57	China: Guizhou, Jiuan
L180322-P-1-LD180319-0002_HL3GTCCXY_L1_clean_bin.15.orig.fa_	OEZ008529	yes	LMSG_G000010201.1	OES073690	terpene	Terpene	Polymetallic	21.91	China: Guizhou, Jiuan
L180322-P-1-LD180319-0002_HL3GTCCXY_L1_clean_bin.15.orig.fa_	OEZ008529	yes	LMSG_G000010201.1	OES073690	terpene	Terpene	Polymetallic	12.89	China: Guizhou, Jiuan
L180322-P-1-LD180319-0002_HL3GTCCXY_L1_clean_bin.15.orig.fa_	OEZ008529	yes	LMSG_G000010201.1	OES073690	arylpolyene	Others	Polymetallic	25.52	China: Guizhou, Jiuan
L180322-P-1-LD180319-0002_HL3GTCCXY_L1_clean_bin.16.orig.fa_	OEZ008529	yes	LMSG_G000010709.1	OES073690	arylpolyene	Others	Polymetallic	22.76	China: Guizhou, Jiuan
L180322-P-1-LD180319-0002_HL3GTCCXY_L1_clean_bin.16.orig.fa_	OEZ008529	yes	LMSG_G000010709.1	OES073690	bacteriocin	RiPPs	Polymetallic	9.18	China: Guizhou, Jiuan
L180322-P-1-LD180319-0002_HL3GTCCXY_L1_clean_bin.16.orig.fa_	OEZ008529	yes	LMSG_G000010709.1	OES073690	T1PKS.NRPS-like	PKS-NRP_Hybrids	Polymetallic	8.35	China: Guizhou, Jiuan
L180322-P-1-LD180319-0002_HL3GTCCXY_L1_clean_bin.16.orig.fa_	OEZ008529	yes	LMSG_G000010709.1	OES073690	terpene	Terpene	Polymetallic	14.31	China: Guizhou, Jiuan
L180322-P-1-LD180319-0002_HL3GTCCXY_L1_clean_bin.16.orig.fa_	OEZ008529	yes	LMSG_G000010709.1	OES073690	NRPS	NRPS	Polymetallic	19.48	China: Guizhou, Jiuan
L180322-P-1-LD180319-0002_HL3GTCCXY_L1_clean_bin.18.strict.f	OEZ008529	no	LMSG_G000010830.1	OES073690	acyl_amino_acids	Others	Polymetallic	5.08	China: Guizhou, Jiuan
L180322-P-1-LD180319-0002_HL3GTCCXY_L1_clean_bin.2.orig.fa_k	OEZ008529	yes	LMSG_G000011045.1	OES073690	resorcinol.arylpolyene	Others	Polymetallic	22.20	China: Guizhou, Jiuan
L180322-P-1-LD180319-0002_HL3GTCCXY_L1_clean_bin.2.orig.fa_k	OEZ008529	yes	LMSG_G000011045.1	OES073690	bacteriocin	RiPPs	Polymetallic	9.45	China: Guizhou, Jiuan
L180322-P-1-LD180319-0002_HL3GTCCXY_L1_clean_bin.2.orig.fa_k	OEZ008529	yes	LMSG_G000011045.1	OES073690	arylpolyene	Others	Polymetallic	43.14	China: Guizhou, Jiuan
L180322-P-1-LD180319-0002_HL3GTCCXY_L1_clean_bin.2.orig.fa_k	OEZ008529	yes	LMSG_G000011045.1	OES073690	terpene	Terpene	Polymetallic	23.08	China: Guizhou, Jiuan
L180322-P-1-LD180319-0002_HL3GTCCXY_L1_clean_bin.2.orig.fa_k	OEZ008529	yes	LMSG_G000011045.1	OES073690	terpene	Terpene	Polymetallic	18.03	China: Guizhou, Jiuan
L180322-P-1-LD180319-0002_HL3GTCCXY_L1_clean_bin.2.orig.fa_k	OEZ008529	yes	LMSG_G000011045.1	OES073690	NRPS-like	NRPS	Polymetallic	15.11	China: Guizhou, Jiuan
L180322-P-1-LD180319-0002_HL3GTCCXY_L1_clean_bin.20.orig.fa_	OEZ008529	yes	LMSG_G000010411.1	OES073690	bacteriocin	RiPPs	Polymetallic	10.62	China: Guizhou, Jiuan
L180322-P-1-LD180319-0002_HL3GTCCXY_L1_clean_bin.20.orig.fa_	OEZ008529	yes	LMSG_G000010411.1	OES073690	terpene	Terpene	Polymetallic	18.90	China: Guizhou, Jiuan
L180322-P-1-LD180319-0002_HL3GTCCXY_L1_clean_bin.20.orig.fa_	OEZ008529	yes	LMSG_G000010411.1	OES073690	terpene	Terpene	Polymetallic	25.02	China: Guizhou, Jiuan
L180322-P-1-LD180319-0002_HL3GTCCXY_L1_clean_bin.20.orig.fa_	OEZ008529	yes	LMSG_G000010411.1	OES073690	hserlactone	Others	Polymetallic	14.34	China: Guizhou, Jiuan
L180322-P-1-LD180319-0002_HL3GTCCXY_L1_clean_bin.21.orig.fa_	OEZ008529	yes	LMSG_G000008464.1	OES073690	bacteriocin	RiPPs	Polymetallic	10.80	China: Guizhou, Jiuan
L180322-P-1-LD180319-0002_HL3GTCCXY_L1_clean_bin.21.orig.fa_	OEZ008529	yes	LMSG_G000008464.1	OES073690	NRPS-like	NRPS	Polymetallic	5.39	China: Guizhou, Jiuan
L180322-P-1-LD180319-0002_HL3GTCCXY_L1_clean_bin.21.orig.fa_	OEZ008529	yes	LMSG_G000008464.1	OES073690	terpene	Terpene	Polymetallic	26.38	China: Guizhou, Jiuan
L180322-P-1-LD180319-0002_HL3GTCCXY_L1_clean_bin.21.orig.fa_	OEZ008529	yes	LMSG_G000008464.1	OES073690	betalactone	Others	Polymetallic	14.24	China: Guizhou, Jiuan
L180322-P-1-LD180319-0002_HL3GTCCXY_L1_clean_bin.22.strict.f	OEZ008529	yes	LMSG_G000008141.1	OES073690	terpene	Terpene	Polymetallic	20.85	China: Guizhou, Jiuan
L180322-P-1-LD180319-0002_HL3GTCCXY_L1_clean_bin.24.orig.fa_	OEZ008529	yes	LMSG_G000007821.1	OES073690	T3PKS	PKSother	Polymetallic	30.49	China: Guizhou, Jiuan
L180322-P-1-LD180319-0002_HL3GTCCXY_L1_clean_bin.24.orig.fa_	OEZ008529	yes	LMSG_G000007821.1	OES073690	terpene	Terpene	Polymetallic	29.77	China: Guizhou, Jiuan
L180322-P-1-LD180319-0002_HL3GTCCXY_L1_clean_bin.28.orig.fa_	OEZ008529	yes	LMSG_G000010669.1	OES073690	T1PKS	PKSI	Polymetallic	34.07	China: Guizhou, Jiuan
L180322-P-1-LD180319-0002_HL3GTCCXY_L1_clean_bin.28.orig.fa_	OEZ008529	yes	LMSG_G000010669.1	OES073690	bacteriocin	RiPPs	Polymetallic	10.90	China: Guizhou, Jiuan
L180322-P-1-LD180319-0002_HL3GTCCXY_L1_clean_bin.28.orig.fa_	OEZ008529	yes	LMSG_G000010669.1	OES073690	NRPS.T1PKS	PKS-NRP_Hybrids	Polymetallic	53.43	China: Guizhou, Jiuan
L180322-P-1-LD180319-0002_HL3GTCCXY_L1_clean_bin.28.orig.fa_	OEZ008529	yes	LMSG_G000010669.1	OES073690	betalactone	Others	Polymetallic	23.37	China: Guizhou, Jiuan
L180322-P-1-LD180319-0002_HL3GTCCXY_L1_clean_bin.28.orig.fa_	OEZ008529	yes	LMSG_G000010669.1	OES073690	betalactone	Others	Polymetallic	18.19	China: Guizhou, Jiuan
L180322-P-1-LD180319-0002_HL3GTCCXY_L1_clean_bin.29.orig.fa_	OEZ008529	yes	LMSG_G000007796.1	OES073690	T3PKS	PKSother	Polymetallic	14.37	China: Guizhou, Jiuan
L180322-P-1-LD180319-0002_HL3GTCCXY_L1_clean_bin.29.orig.fa_	OEZ008529	yes	LMSG_G000007796.1	OES073690	terpene	Terpene	Polymetallic	10.59	China: Guizhou, Jiuan
L180322-P-1-LD180319-0002_HL3GTCCXY_L1_clean_bin.3.orig.fa_k	OEZ008529	no	LMSG_G000011020.1	OES073690	NRPS	NRPS	Polymetallic	49.55	China: Guizhou, Jiuan
L180322-P-1-LD180319-0002_HL3GTCCXY_L1_clean_bin.3.orig.fa_k	OEZ008529	no	LMSG_G000011020.1	OES073690	bacteriocin	RiPPs	Polymetallic	10.86	China: Guizhou, Jiuan
L180322-P-1-LD180319-0002_HL3GTCCXY_L1_clean_bin.3.orig.fa_k	OEZ008529	no	LMSG_G000011020.1	OES073690	NRPS	NRPS	Polymetallic	44.23	China: Guizhou, Jiuan
L180322-P-1-LD180319-0002_HL3GTCCXY_L1_clean_bin.3.orig.fa_k	OEZ008529	no	LMSG_G000011020.1	OES073690	NRPS	NRPS	Polymetallic	6.21	China: Guizhou, Jiuan
L180322-P-1-LD180319-0002_HL3GTCCXY_L1_clean_bin.3.orig.fa_k	OEZ008529	no	LMSG_G000011020.1	OES073690	betalactone.NRPS-like	Others	Polymetallic	41.86	China: Guizhou, Jiuan
L180322-P-1-LD180319-0002_HL3GTCCXY_L1_clean_bin.3.orig.fa_k	OEZ008529	no	LMSG_G000011020.1	OES073690	hserlactone	Others	Polymetallic	20.61	China: Guizhou, Jiuan
L180322-P-1-LD180319-0002_HL3GTCCXY_L1_clean_bin.3.orig.fa_k	OEZ008529	no	LMSG_G000011020.1	OES073690	NRPS-like	NRPS	Polymetallic	42.98	China: Guizhou, Jiuan
L180322-P-1-LD180319-0002_HL3GTCCXY_L1_clean_bin.3.orig.fa_k	OEZ008529	no	LMSG_G000011020.1	OES073690	bacteriocin	RiPPs	Polymetallic	10.83	China: Guizhou, Jiuan
L180322-P-1-LD180319-0002_HL3GTCCXY_L1_clean_bin.3.orig.fa_k	OEZ008529	no	LMSG_G000011020.1	OES073690	phenazine	Others	Polymetallic	10.23	China: Guizhou, Jiuan
L180322-P-1-LD180319-0002_HL3GTCCXY_L1_clean_bin.3.orig.fa_k	OEZ008529	no	LMSG_G000011020.1	OES073690	NRPS	NRPS	Polymetallic	5.44	China: Guizhou, Jiuan
L180322-P-1-LD180319-0002_HL3GTCCXY_L1_clean_bin.3.orig.fa_k	OEZ008529	no	LMSG_G000011020.1	OES073690	thiopeptide	RiPPs	Polymetallic	33.00	China: Guizhou, Jiuan
L180322-P-1-LD180319-0002_HL3GTCCXY_L1_clean_bin.3.orig.fa_k	OEZ008529	no	LMSG_G000011020.1	OES073690	NRPS	NRPS	Polymetallic	53.04	China: Guizhou, Jiuan
L180322-P-1-LD180319-0002_HL3GTCCXY_L1_clean_bin.3.orig.fa_k	OEZ008529	no	LMSG_G000011020.1	OES073690	NRPS	NRPS	Polymetallic	42.58	China: Guizhou, Jiuan
L180322-P-1-LD180319-0002_HL3GTCCXY_L1_clean_bin.3.orig.fa_k	OEZ008529	no	LMSG_G000011020.1	OES073690	phenazine	Others	Polymetallic	10.23	China: Guizhou, Jiuan
L180322-P-1-LD180319-0002_HL3GTCCXY_L1_clean_bin.3.orig.fa_k	OEZ008529	no	LMSG_G000011020.1	OES073690	hserlactone	Others	Polymetallic	20.61	China: Guizhou, Jiuan
L180322-P-1-LD180319-0002_HL3GTCCXY_L1_clean_bin.3.orig.fa_k	OEZ008529	no	LMSG_G000011020.1	OES073690	NAGGN	Others	Polymetallic	14.76	China: Guizhou, Jiuan
L180322-P-1-LD180319-0002_HL3GTCCXY_L1_clean_bin.31.orig.fa_	OEZ008529	yes	LMSG_G000010985.1	OES073690	T1PKS.hglE-KS	PKSother	Polymetallic	32.39	China: Guizhou, Jiuan
L180322-P-1-LD180319-0002_HL3GTCCXY_L1_clean_bin.32.orig.fa_	OEZ008529	yes	LMSG_G000005204.1	OES073690	ladderane	Others	Polymetallic	5.45	China: Guizhou, Jiuan
L180322-P-1-LD180319-0002_HL3GTCCXY_L1_clean_bin.33.orig.fa_	OEZ008529	no	LMSG_G000010162.1	OES073690	acyl_amino_acids	Others	Polymetallic	5.50	China: Guizhou, Jiuan
L180322-P-1-LD180319-0002_HL3GTCCXY_L1_clean_bin.33.orig.fa_	OEZ008529	no	LMSG_G000010162.1	OES073690	ectoine	Others	Polymetallic	7.58	China: Guizhou, Jiuan
L180322-P-1-LD180319-0002_HL3GTCCXY_L1_clean_bin.34.orig.fa_	OEZ008529	yes	LMSG_G000009254.1	OES073690	terpene	Terpene	Polymetallic	20.85	China: Guizhou, Jiuan
L180322-P-1-LD180319-0002_HL3GTCCXY_L1_clean_bin.34.orig.fa_	OEZ008529	yes	LMSG_G000009254.1	OES073690	TfuA-related	RiPPs	Polymetallic	21.72	China: Guizhou, Jiuan
L180322-P-1-LD180319-0002_HL3GTCCXY_L1_clean_bin.36.orig.fa_	OEZ008529	yes	LMSG_G000008086.1	OES073690	hglE-KS	PKSother	Polymetallic	11.15	China: Guizhou, Jiuan
L180322-P-1-LD180319-0002_HL3GTCCXY_L1_clean_bin.37.orig.fa_	OEZ008529	yes	LMSG_G000010650.1	OES073690	arylpolyene	Others	Polymetallic	37.51	China: Guizhou, Jiuan
L180322-P-1-LD180319-0002_HL3GTCCXY_L1_clean_bin.37.orig.fa_	OEZ008529	yes	LMSG_G000010650.1	OES073690	NRPS-like	NRPS	Polymetallic	24.42	China: Guizhou, Jiuan
L180322-P-1-LD180319-0002_HL3GTCCXY_L1_clean_bin.37.orig.fa_	OEZ008529	yes	LMSG_G000010650.1	OES073690	NRPS	NRPS	Polymetallic	24.83	China: Guizhou, Jiuan
L180322-P-1-LD180319-0002_HL3GTCCXY_L1_clean_bin.37.orig.fa_	OEZ008529	yes	LMSG_G000010650.1	OES073690	NRPS.T1PKS	PKS-NRP_Hybrids	Polymetallic	44.07	China: Guizhou, Jiuan
L180322-P-1-LD180319-0002_HL3GTCCXY_L1_clean_bin.37.orig.fa_	OEZ008529	yes	LMSG_G000010650.1	OES073690	terpene	Terpene	Polymetallic	11.15	China: Guizhou, Jiuan
L180322-P-1-LD180319-0002_HL3GTCCXY_L1_clean_bin.38.orig.fa_	OEZ008529	yes	LMSG_G000008164.1	OES073690	terpene	Terpene	Polymetallic	23.44	China: Guizhou, Jiuan
L180322-P-1-LD180319-0002_HL3GTCCXY_L1_clean_bin.4.orig.fa_k	OEZ008529	yes	LMSG_G000010611.1	OES073690	terpene	Terpene	Polymetallic	21.71	China: Guizhou, Jiuan
L180322-P-1-LD180319-0002_HL3GTCCXY_L1_clean_bin.4.orig.fa_k	OEZ008529	no	LMSG_G000010611.1	OES073690	phosphonate	Others	Polymetallic	6.51	China: Guizhou, Jiuan

L180322-P-1-LD180319-0002_HL3GTCCXY_L1_clean_bin.4.orig.fa_k	OEZ008529	no	LMSG_G000010611.1	OES073690	siderophore	Others	Polymetallic	11.85	China: Guizhou, Jiuan
L180322-P-1-LD180319-0002_HL3GTCCXY_L1_clean_bin.4.orig.fa_k	OEZ008529	no	LMSG_G000010611.1	OES073690	ectoine	Others	Polymetallic	10.41	China: Guizhou, Jiuan
L180322-P-1-LD180319-0002_HL3GTCCXY_L1_clean_bin.4.orig.fa_k	OEZ008529	no	LMSG_G000010611.1	OES073690	betalactone	Others	Polymetallic	12.31	China: Guizhou, Jiuan
L180322-P-1-LD180319-0002_HL3GTCCXY_L1_clean_bin.4.orig.fa_k	OEZ008529	yes	LMSG_G000010611.1	OES073690	resorcinol	Others	Polymetallic	41.93	China: Guizhou, Jiuan
L180322-P-1-LD180319-0002_HL3GTCCXY_L1_clean_bin.4.orig.fa_k	OEZ008529	no	LMSG_G000010611.1	OES073690	NRPS-like	NRPS	Polymetallic	43.97	China: Guizhou, Jiuan
L180322-P-1-LD180319-0002_HL3GTCCXY_L1_clean_bin.40.orig.fa_	OEZ008529	yes	LMSG_G000010265.1	OES073690	arylpolyene	Others	Polymetallic	35.55	China: Guizhou, Jiuan
L180322-P-1-LD180319-0002_HL3GTCCXY_L1_clean_bin.40.orig.fa_	OEZ008529	no	LMSG_G000010265.1	OES073690	terpene	Terpene	Polymetallic	21.16	China: Guizhou, Jiuan
L180322-P-1-LD180319-0002_HL3GTCCXY_L1_clean_bin.41.orig.fa_	OEZ008529	yes	LMSG_G000011158.1	OES073690	bacteriocin	RiPPs	Polymetallic	7.89	China: Guizhou, Jiuan
L180322-P-1-LD180319-0002_HL3GTCCXY_L1_clean_bin.41.orig.fa_	OEZ008529	yes	LMSG_G000011158.1	OES073690	terpene	Terpene	Polymetallic	13.22	China: Guizhou, Jiuan
L180322-P-1-LD180319-0002_HL3GTCCXY_L1_clean_bin.41.orig.fa_	OEZ008529	yes	LMSG_G000011158.1	OES073690	arylpolyene	Others	Polymetallic	23.50	China: Guizhou, Jiuan
L180322-P-1-LD180319-0002_HL3GTCCXY_L1_clean_bin.42.orig.fa_	OEZ008529	yes	LMSG_G000010759.1	OES073690	terpene	Terpene	Polymetallic	14.72	China: Guizhou, Jiuan
L180322-P-1-LD180319-0002_HL3GTCCXY_L1_clean_bin.42.orig.fa_	OEZ008529	yes	LMSG_G000010759.1	OES073690	bacteriocin	RiPPs	Polymetallic	11.75	China: Guizhou, Jiuan
L180322-P-1-LD180319-0002_HL3GTCCXY_L1_clean_bin.42.orig.fa_	OEZ008529	yes	LMSG_G000010759.1	OES073690	acyl_amino_acids	Others	Polymetallic	42.27	China: Guizhou, Jiuan
L180322-P-1-LD180319-0002_HL3GTCCXY_L1_clean_bin.5.orig.fa_k	OEZ008529	yes	LMSG_G000010048.1	OES073690	T3PKS	PKSother	Polymetallic	6.96	China: Guizhou, Jiuan
L180322-P-1-LD180319-0002_HL3GTCCXY_L1_clean_bin.5.orig.fa_k	OEZ008529	yes	LMSG_G000010048.1	OES073690	terpene	Terpene	Polymetallic	10.04	China: Guizhou, Jiuan
L180322-P-1-LD180319-0002_HL3GTCCXY_L1_clean_bin.5.orig.fa_k	OEZ008529	yes	LMSG_G000010048.1	OES073690	ladderane	Others	Polymetallic	28.76	China: Guizhou, Jiuan
L180322-P-1-LD180319-0002_HL3GTCCXY_L1_clean_bin.5.orig.fa_k	OEZ008529	yes	LMSG_G000010048.1	OES073690	NRPS-like.ectoine	Others	Polymetallic	49.56	China: Guizhou, Jiuan
L180322-P-1-LD180319-0002_HL3GTCCXY_L1_clean_bin.5.orig.fa_k	OEZ008529	yes	LMSG_G000010048.1	OES073690	arylpolyene	Others	Polymetallic	8.03	China: Guizhou, Jiuan
L180322-P-1-LD180319-0002_HL3GTCCXY_L1_clean_bin.5.orig.fa_k	OEZ008529	yes	LMSG_G000010048.1	OES073690	bacteriocin	RiPPs	Polymetallic	10.68	China: Guizhou, Jiuan
L180322-P-1-LD180319-0002_HL3GTCCXY_L1_clean_bin.7.orig.fa_k	OEZ008529	yes	LMSG_G000007756.1	OES073690	terpene	Terpene	Polymetallic	20.21	China: Guizhou, Jiuan
L180322-P-1-LD180319-0002_HL3GTCCXY_L1_clean_bin.7.orig.fa_k	OEZ008529	yes	LMSG_G000007756.1	OES073690	NRPS.T1PKS	PKS-NRP_Hybrids	Polymetallic	20.89	China: Guizhou, Jiuan
L180322-P-1-LD180319-0002_HL3GTCCXY_L1_clean_bin.7.orig.fa_k	OEZ008529	yes	LMSG_G000007756.1	OES073690	NRPS.T1PKS	PKS-NRP_Hybrids	Polymetallic	18.23	China: Guizhou, Jiuan
L180322-P-1-LD180319-0002_HL3GTCCXY_L1_clean_bin.9.orig.fa_k	OEZ008529	yes	LMSG_G000008608.1	OES073690	other	Others	Polymetallic	27.37	China: Guizhou, Jiuan
L180322-P-1-LD180319-0002_HL3GTCCXY_L1_clean_bin.9.orig.fa_k	OEZ008529	yes	LMSG_G000008608.1	OES073690	other	Others	Polymetallic	5.03	China: Guizhou, Jiuan
L180322-P-1-LD180319-0002_HL3GTCCXY_L1_clean_bin.9.orig.fa_k	OEZ008529	yes	LMSG_G000008608.1	OES073690	other	Others	Polymetallic	28.51	China: Guizhou, Jiuan
L180322-P-1-LD180319-0003_HL3GTCCXY_L1_clean_bin.10.orig.fa_	OEZ008529	yes	LMSG_G000005205.1	OES073692	other	Others	Polymetallic	19.91	China: Guizhou, Jiuan
L180322-P-1-LD180319-0003_HL3GTCCXY_L1_clean_bin.10.orig.fa_	OEZ008529	yes	LMSG_G000005205.1	OES073692	other	Others	Polymetallic	6.86	China: Guizhou, Jiuan
L180322-P-1-LD180319-0003_HL3GTCCXY_L1_clean_bin.11.orig.fa_	OEZ008529	yes	LMSG_G000005206.1	OES073692	betalactone	Others	Polymetallic	30.48	China: Guizhou, Jiuan
L180322-P-1-LD180319-0003_HL3GTCCXY_L1_clean_bin.11.orig.fa_	OEZ008529	yes	LMSG_G000005206.1	OES073692	terpene	Terpene	Polymetallic	22.05	China: Guizhou, Jiuan
L180322-P-1-LD180319-0003_HL3GTCCXY_L1_clean_bin.11.orig.fa_	OEZ008529	yes	LMSG_G000005206.1	OES073692	NRPS-like	NRPS	Polymetallic	42.62	China: Guizhou, Jiuan
L180322-P-1-LD180319-0003_HL3GTCCXY_L1_clean_bin.11.orig.fa_	OEZ008529	yes	LMSG_G000005206.1	OES073692	T3PKS	PKSother	Polymetallic	41.06	China: Guizhou, Jiuan
L180322-P-1-LD180319-0003_HL3GTCCXY_L1_clean_bin.11.orig.fa_	OEZ008529	yes	LMSG_G000005206.1	OES073692	terpene	Terpene	Polymetallic	13.85	China: Guizhou, Jiuan
L180322-P-1-LD180319-0003_HL3GTCCXY_L1_clean_bin.11.orig.fa_	OEZ008529	yes	LMSG_G000005206.1	OES073692	NRPS-like	NRPS	Polymetallic	43.38	China: Guizhou, Jiuan
L180322-P-1-LD180319-0003_HL3GTCCXY_L1_clean_bin.12.permissi	OEZ008529	yes	LMSG_G000009301.1	OES073692	betalactone	Others	Polymetallic	18.61	China: Guizhou, Jiuan
L180322-P-1-LD180319-0003_HL3GTCCXY_L1_clean_bin.12.permissi	OEZ008529	yes	LMSG_G000009301.1	OES073692	betalactone	Others	Polymetallic	17.23	China: Guizhou, Jiuan
L180322-P-1-LD180319-0003_HL3GTCCXY_L1_clean_bin.14.orig.fa_	OEZ008529	no	LMSG_G000010266.1	OES073692	terpene	Terpene	Polymetallic	21.52	China: Guizhou, Jiuan
L180322-P-1-LD180319-0003_HL3GTCCXY_L1_clean_bin.14.orig.fa_	OEZ008529	yes	LMSG_G000010266.1	OES073692	arylpolyene	Others	Polymetallic	35.55	China: Guizhou, Jiuan
L180322-P-1-LD180319-0003_HL3GTCCXY_L1_clean_bin.16.orig.fa_	OEZ008529	yes	LMSG_G000009181.1	OES073692	T3PKS	PKSother	Polymetallic	21.53	China: Guizhou, Jiuan
L180322-P-1-LD180319-0003_HL3GTCCXY_L1_clean_bin.16.orig.fa_	OEZ008529	yes	LMSG_G000009181.1	OES073692	terpene	Terpene	Polymetallic	15.14	China: Guizhou, Jiuan
L180322-P-1-LD180319-0003_HL3GTCCXY_L1_clean_bin.18.orig.fa_	OEZ008529	yes	LMSG_G000004439.1	OES073692	betalactone	Others	Polymetallic	14.24	China: Guizhou, Jiuan
L180322-P-1-LD180319-0003_HL3GTCCXY_L1_clean_bin.18.orig.fa_	OEZ008529	yes	LMSG_G000004439.1	OES073692	bacteriocin	RiPPs	Polymetallic	10.19	China: Guizhou, Jiuan
L180322-P-1-LD180319-0003_HL3GTCCXY_L1_clean_bin.18.orig.fa_	OEZ008529	yes	LMSG_G000004439.1	OES073692	terpene	Terpene	Polymetallic	25.32	China: Guizhou, Jiuan
L180322-P-1-LD180319-0003_HL3GTCCXY_L1_clean_bin.19.orig.fa_	OEZ008529	yes	LMSG_G000008124.1	OES073692	terpene	Terpene	Polymetallic	6.54	China: Guizhou, Jiuan
L180322-P-1-LD180319-0003_HL3GTCCXY_L1_clean_bin.21.orig.fa_	OEZ008529	yes	LMSG_G000004440.1	OES073692	betalactone	Others	Polymetallic	18.43	China: Guizhou, Jiuan
L180322-P-1-LD180319-0003_HL3GTCCXY_L1_clean_bin.21.orig.fa_	OEZ008529	yes	LMSG_G000004440.1	OES073692	NRPS-like	NRPS	Polymetallic	5.89	China: Guizhou, Jiuan
L180322-P-1-LD180319-0003_HL3GTCCXY_L1_clean_bin.22.strict.f	OEZ008529	yes	LMSG_G000006438.1	OES073692	nucleoside	Others	Polymetallic	13.06	China: Guizhou, Jiuan
L180322-P-1-LD180319-0003_HL3GTCCXY_L1_clean_bin.22.strict.f	OEZ008529	yes	LMSG_G000006438.1	OES073692	terpene	Terpene	Polymetallic	6.81	China: Guizhou, Jiuan
L180322-P-1-LD180319-0003_HL3GTCCXY_L1_clean_bin.24.strict.f	OEZ008529	yes	LMSG_G000005208.1	OES073692	T3PKS	PKSother	Polymetallic	40.70	China: Guizhou, Jiuan
L180322-P-1-LD180319-0003_HL3GTCCXY_L1_clean_bin.24.strict.f	OEZ008529	yes	LMSG_G000005208.1	OES073692	arylpolyene	Others	Polymetallic	27.04	China: Guizhou, Jiuan
L180322-P-1-LD180319-0003_HL3GTCCXY_L1_clean_bin.24.strict.f	OEZ008529	yes	LMSG_G000005208.1	OES073692	terpene	Terpene	Polymetallic	21.91	China: Guizhou, Jiuan
L180322-P-1-LD180319-0003_HL3GTCCXY_L1_clean_bin.24.strict.f	OEZ008529	yes	LMSG_G000005208.1	OES073692	terpene	Terpene	Polymetallic	14.20	China: Guizhou, Jiuan
L180322-P-1-LD180319-0003_HL3GTCCXY_L1_clean_bin.24.strict.f	OEZ008529	yes	LMSG_G000005208.1	OES073692	NRPS-like	NRPS	Polymetallic	30.46	China: Guizhou, Jiuan
L180322-P-1-LD180319-0003_HL3GTCCXY_L1_clean_bin.25.orig.fa_	OEZ008529	yes	LMSG_G000008355.1	OES073692	NRPS-like	NRPS	Polymetallic	5.10	China: Guizhou, Jiuan
L180322-P-1-LD180319-0003_HL3GTCCXY_L1_clean_bin.27.orig.fa_	OEZ008529	yes	LMSG_G000004441.1	OES073692	TfuA-related	RiPPs	Polymetallic	19.77	China: Guizhou, Jiuan
L180322-P-1-LD180319-0003_HL3GTCCXY_L1_clean_bin.27.orig.fa_	OEZ008529	yes	LMSG_G000004441.1	OES073692	terpene	Terpene	Polymetallic	20.85	China: Guizhou, Jiuan
L180322-P-1-LD180319-0003_HL3GTCCXY_L1_clean_bin.28.permissi	OEZ008529	yes	LMSG_G000009224.1	OES073692	terpene	Terpene	Polymetallic	5.42	China: Guizhou, Jiuan
L180322-P-1-LD180319-0003_HL3GTCCXY_L1_clean_bin.29.orig.fa_	OEZ008529	yes	LMSG_G000004442.1	OES073692	terpene	Terpene	Polymetallic	8.87	China: Guizhou, Jiuan
L180322-P-1-LD180319-0003_HL3GTCCXY_L1_clean_bin.3.orig.fa_k	OEZ008529	no	LMSG_G000010163.1	OES073692	terpene	Terpene	Polymetallic	21.85	China: Guizhou, Jiuan
L180322-P-1-LD180319-0003_HL3GTCCXY_L1_clean_bin.3.orig.fa_k	OEZ008529	no	LMSG_G000010163.1	OES073692	acyl_amino_acids	Others	Polymetallic	35.17	China: Guizhou, Jiuan
L180322-P-1-LD180319-0003_HL3GTCCXY_L1_clean_bin.3.orig.fa_k	OEZ008529	no	LMSG_G000010163.1	OES073692	terpene	Terpene	Polymetallic	21.71	China: Guizhou, Jiuan
L180322-P-1-LD180319-0003_HL3GTCCXY_L1_clean_bin.3.orig.fa_k	OEZ008529	no	LMSG_G000010163.1	OES073692	ectoine	Others	Polymetallic	10.40	China: Guizhou, Jiuan
L180322-P-1-LD180319-0003_HL3GTCCXY_L1_clean_bin.30.strict.f	OEZ008529	yes	LMSG_G000008159.1	OES073692	terpene	Terpene	Polymetallic	23.28	China: Guizhou, Jiuan
L180322-P-1-LD180319-0003_HL3GTCCXY_L1_clean_bin.31.orig.fa_	OEZ008529	yes	LMSG_G000005209.1	OES073692	T3PKS	PKSother	Polymetallic	22.86	China: Guizhou, Jiuan
L180322-P-1-LD180319-0003_HL3GTCCXY_L1_clean_bin.31.orig.fa_	OEZ008529	yes	LMSG_G000005209.1	OES073692	NRPS-like	NRPS	Polymetallic	5.43	China: Guizhou, Jiuan
L180322-P-1-LD180319-0003_HL3GTCCXY_L1_clean_bin.31.orig.fa_	OEZ008529	yes	LMSG_G000005209.1	OES073692	lassopeptide	RiPPs	Polymetallic	5.84	China: Guizhou, Jiuan
L180322-P-1-LD180319-0003_HL3GTCCXY_L1_clean_bin.31.orig.fa_	OEZ008529	yes	LMSG_G000005209.1	OES073692	terpene	Terpene	Polymetallic	13.84	China: Guizhou, Jiuan
L180322-P-1-LD180319-0003_HL3GTCCXY_L1_clean_bin.31.orig.fa_	OEZ008529	yes	LMSG_G000005209.1	OES073692	terpene	Terpene	Polymetallic	15.51	China: Guizhou, Jiuan
L180322-P-1-LD180319-0003_HL3GTCCXY_L1_clean_bin.35.orig.fa_	OEZ008529	yes	LMSG_G000010986.1	OES073692	T1PKS.hglE-KS	PKSother	Polymetallic	32.93	China: Guizhou, Jiuan
L180322-P-1-LD180319-0003_HL3GTCCXY_L1_clean_bin.36.strict.f	OEZ008529	yes	LMSG_G000010769.1	OES073692	bacteriocin	RiPPs	Polymetallic	11.75	China: Guizhou, Jiuan
L180322-P-1-LD180319-0003_HL3GTCCXY_L1_clean_bin.37.orig.fa_	OEZ008529	yes	LMSG_G000011050.1	OES073692	NRPS-like	NRPS	Polymetallic	5.43	China: Guizhou, Jiuan
L180322-P-1-LD180319-0003_HL3GTCCXY_L1_clean_bin.37.orig.fa_	OEZ008529	yes	LMSG_G000011050.1	OES073692	resorcinol.arylpolyene	Others	Polymetallic	22.32	China: Guizhou, Jiuan
L180322-P-1-LD180319-0003_HL3GTCCXY_L1_clean_bin.37.orig.fa_	OEZ008529	yes	LMSG_G000011050.1	OES073692	terpene	Terpene	Polymetallic	20.85	China: Guizhou, Jiuan
L180322-P-1-LD180319-0003_HL3GTCCXY_L1_clean_bin.37.orig.fa_	OEZ008529	yes	LMSG_G000011050.1	OES073692	bacteriocin	RiPPs	Polymetallic	9.28	China: Guizhou, Jiuan
L180322-P-1-LD180319-0003_HL3GTCCXY_L1_clean_bin.37.orig.fa_	OEZ008529	yes	LMSG_G000011050.1	OES073692	arylpolyene	Others	Polymetallic	42.98	China: Guizhou, Jiuan
L180322-P-1-LD180319-0003_HL3GTCCXY_L1_clean_bin.37.orig.fa_	OEZ008529	yes	LMSG_G000011050.1	OES073692	terpene	Terpene	Polymetallic	23.09	China: Guizhou, Jiuan
L180322-P-1-LD180319-0003_HL3GTCCXY_L1_clean_bin.39.orig.fa_	OEZ008529	yes	LMSG_G000005210.1	OES073692	betalactone.NRPS-like	Others	Polymetallic	27.00	China: Guizhou, Jiuan
L180322-P-1-LD180319-0003_HL3GTCCXY_L1_clean_bin.39.orig.fa_	OEZ008529	yes	LMSG_G000005210.1	OES073692	terpene	Terpene	Polymetallic	29.64	China: Guizhou, Jiuan
L180322-P-1-LD180319-0003_HL3GTCCXY_L1_clean_bin.39.orig.fa_	OEZ008529	yes	LMSG_G000005210.1	OES073692	NRPS.T1PKS	PKS-NRP_Hybrids	Polymetallic	52.71	China: Guizhou, Jiuan
L180322-P-1-LD180319-0003_HL3GTCCXY_L1_clean_bin.39.orig.fa_	OEZ008529	yes	LMSG_G000005210.1	OES073692	NRPS.T1PKS	PKS-NRP_Hybrids	Polymetallic	24.89	China: Guizhou, Jiuan
L180322-P-1-LD180319-0003_HL3GTCCXY_L1_clean_bin.39.orig.fa_	OEZ008529	yes	LMSG_G000005210.1	OES073692	T1PKS	PKSI	Polymetallic	19.86	China: Guizhou, Jiuan
L180322-P-1-LD180319-0003_HL3GTCCXY_L1_clean_bin.4.orig.fa_k	OEZ008529	yes	LMSG_G000005214.1	OES073692	T3PKS	PKSother	Polymetallic	12.02	China: Guizhou, Jiuan
L180322-P-1-LD180319-0003_HL3GTCCXY_L1_clean_bin.4.orig.fa_k	OEZ008529	yes	LMSG_G000005214.1	OES073692	terpene	Terpene	Polymetallic	8.78	China: Guizhou, Jiuan
L180322-P-1-LD180319-0003_HL3GTCCXY_L1_clean_bin.4.orig.fa_k	OEZ008529	yes	LMSG_G000005214.1	OES073692	bacteriocin	RiPPs	Polymetallic	7.11	China: Guizhou, Jiuan
L180322-P-1-LD180319-0003_HL3GTCCXY_L1_clean_bin.40.orig.fa_	OEZ008529	no	LMSG_G000011011.1	OES073692	NRPS	NRPS	Polymetallic	49.55	China: Guizhou, Jiuan
L180322-P-1-LD180319-0003_HL3GTCCXY_L1_clean_bin.40.orig.fa_	OEZ008529	no	LMSG_G000011011.1	OES073692	bacteriocin	RiPPs	Polymetallic	10.86	China: Guizhou, Jiuan
L180322-P-1-LD180319-0003_HL3GTCCXY_L1_clean_bin.40.orig.fa_	OEZ008529	no	LMSG_G000011011.1	OES073692	NRPS	NRPS	Polymetallic	6.21	China: Guizhou, Jiuan
L180322-P-1-LD180319-0003_HL3GTCCXY_L1_clean_bin.40.orig.fa_	OEZ008529	no	LMSG_G000011011.1	OES073692	hserlactone	Others	Polymetallic	20.61	China: Guizhou, Jiuan
L180322-P-1-LD180319-0003_HL3GTCCXY_L1_clean_bin.40.orig.fa_	OEZ008529	no	LMSG_G000011011.1	OES073692	betalactone.NRPS-like	Others	Polymetallic	41.86	China: Guizhou, Jiuan
L180322-P-1-LD180319-0003_HL3GTCCXY_L1_clean_bin.40.orig.fa_	OEZ008529	no	LMSG_G000011011.1	OES073692	NRPS-like	NRPS	Polymetallic	42.98	China: Guizhou, Jiuan
L180322-P-1-LD180319-0003_HL3GTCCXY_L1_clean_bin.40.orig.fa_	OEZ008529	no	LMSG_G000011011.1	OES073692	bacteriocin	RiPPs	Polymetallic	10.83	China: Guizhou, Jiuan
L180322-P-1-LD180319-0003_HL3GTCCXY_L1_clean_bin.40.orig.fa_	OEZ008529	no	LMSG_G000011011.1	OES073692	phenazine	Others	Polymetallic	10.23	China: Guizhou, Jiuan
L180322-P-1-LD180319-0003_HL3GTCCXY_L1_clean_bin.40.orig.fa_	OEZ008529	no	LMSG_G000011011.1	OES073692	NRPS	NRPS	Polymetallic	5.44	China: Guizhou, Jiuan
L180322-P-1-LD180319-0003_HL3GTCCXY_L1_clean_bin.40.orig.fa_	OEZ008529	no	LMSG_G000011011.1	OES073692	thiopeptide	RiPPs	Polymetallic	33.00	China: Guizhou, Jiuan
L180322-P-1-LD180319-0003_HL3GTCCXY_L1_clean_bin.40.orig.fa_	OEZ008529	no	LMSG_G000011011.1	OES073692	NRPS	NRPS	Polymetallic	53.04	China: Guizhou, Jiuan
L180322-P-1-LD180319-0003_HL3GTCCXY_L1_clean_bin.40.orig.fa_	OEZ008529	no	LMSG_G000011011.1	OES073692	NRPS	NRPS	Polymetallic	44.29	China: Guizhou, Jiuan
L180322-P-1-LD180319-0003_HL3GTCCXY_L1_clean_bin.40.orig.fa_	OEZ008529	no	LMSG_G000011011.1	OES073692	NRPS	NRPS	Polymetallic	42.58	China: Guizhou, Jiuan
L180322-P-1-LD180319-0003_HL3GTCCXY_L1_clean_bin.40.orig.fa_	OEZ008529	no	LMSG_G000011011.1	OES073692	phenazine	Others	Polymetallic	10.23	China: Guizhou, Jiuan
L180322-P-1-LD180319-0003_HL3GTCCXY_L1_clean_bin.40.orig.fa_	OEZ008529	no	LMSG_G000011011.1	OES073692	hserlactone	Others	Polymetallic	20.61	China: Guizhou, Jiuan
L180322-P-1-LD180319-0003_HL3GTCCXY_L1_clean_bin.40.orig.fa_	OEZ008529	no	LMSG_G000011011.1	OES073692	NAGGN	Others	Polymetallic	14.76	China: Guizhou, Jiuan
L180322-P-1-LD180319-0003_HL3GTCCXY_L1_clean_bin.41.orig.fa_	OEZ008529	yes	LMSG_G000010202.1	OES073692	arylpolyene	Others	Polymetallic	10.89	China: Guizhou, Jiuan
L180322-P-1-LD180319-0003_HL3GTCCXY_L1_clean_bin.41.orig.fa_	OEZ008529	yes	LMSG_G000010202.1	OES073692	terpene	Terpene	Polymetallic	6.29	China: Guizhou, Jiuan
L180322-P-1-LD180319-0003_HL3GTCCXY_L1_clean_bin.41.orig.fa_	OEZ008529	yes	LMSG_G000010202.1	OES073692	terpene	Terpene	Polymetallic	13.86	China: Guizhou, Jiuan
L180322-P-1-LD180319-0003_HL3GTCCXY_L1_clean_bin.42.orig.fa_	OEZ008529	no	LMSG_G000005211.1	OES073692	NRPS-like	NRPS	Polymetallic	15.49	China: Guizhou, Jiuan
L180322-P-1-LD180319-0003_HL3GTCCXY_L1_clean_bin.42.orig.fa_	OEZ008529	yes	LMSG_G000005211.1	OES073692	terpene	Terpene	Polymetallic	21.71	China: Guizhou, Jiuan
L180322-P-1-LD180319-0003_HL3GTCCXY_L1_clean_bin.42.orig.fa_	OEZ008529	yes	LMSG_G000005211.1	OES073692	betalactone	Others	Polymetallic	22.57	China: Guizhou, Jiuan
L180322-P-1-LD180319-0003_HL3GTCCXY_L1_clean_bin.42.orig.fa_	OEZ008529	yes	LMSG_G000005211.1	OES073692	resorcinol	Others	Polymetallic	38.01	China: Guizhou, Jiuan
L180322-P-1-LD180319-0003_HL3GTCCXY_L1_clean_bin.42.orig.fa_	OEZ008529	no	LMSG_G000005211.1	OES073692	siderophore	Others	Polymetallic	11.85	China: Guizhou, Jiuan
L180322-P-1-LD180319-0003_HL3GTCCXY_L1_clean_bin.42.orig.fa_	OEZ008529	no	LMSG_G000005211.1	OES073692	phosphonate	Others	Polymetallic	40.87	China: Guizhou, Jiuan
L180322-P-1-LD180319-0003_HL3GTCCXY_L1_clean_bin.42.orig.fa_	OEZ008529	no	LMSG_G000005211.1	OES073692	ectoine	Others	Polymetallic	10.41	China: Guizhou, Jiuan
L180322-P-1-LD180319-0003_HL3GTCCXY_L1_clean_bin.43.orig.fa_	OEZ008529	yes	LMSG_G000005212.1	OES073692	terpene	Terpene	Polymetallic	11.41	China: Guizhou, Jiuan
L180322-P-1-LD180319-0003_HL3GTCCXY_L1_clean_bin.43.orig.fa_	OEZ008529	yes	LMSG_G000005212.1	OES073692	NRPS-like	NRPS	Polymetallic	14.06	China: Guizhou, Jiuan
L180322-P-1-LD180319-0003_HL3GTCCXY_L1_clean_bin.43.orig.fa_	OEZ008529	yes	LMSG_G000005212.1	OES073692	T1PKS.NRPS-like	PKS-NRP_Hybrids	Polymetallic	31.35	China: Guizhou, Jiuan
L180322-P-1-LD180319-0003_HL3GTCCXY_L1_clean_bin.43.orig.fa_	OEZ008529	yes	LMSG_G000005212.1	OES073692	terpene	Terpene	Polymetallic	5.02	China: Guizhou, Jiuan
L180322-P-1-LD180319-0003_HL3GTCCXY_L1_clean_bin.45.orig.fa_	OEZ008529	yes	LMSG_G000008998.1	OES073692	terpene	Terpene	Polymetallic	20.91	China: Guizhou, Jiuan
L180322-P-1-LD180319-0003_HL3GTCCXY_L1_clean_bin.45.orig.fa_	OEZ008529	yes	LMSG_G000008998.1	OES073692	bacteriocin	RiPPs	Polymetallic	10.80	China: Guizhou, Jiuan
L180322-P-1-LD180319-0003_HL3GTCCXY_L1_clean_bin.46.permissi	OEZ008529	yes	LMSG_G000005213.1	OES073692	terpene	Terpene	Polymetallic	8.02	China: Guizhou, Jiuan
L180322-P-1-LD180319-0003_HL3GTCCXY_L1_clean_bin.47.orig.fa_	OEZ008529	yes	LMSG_G000011174.1	OES073692	arylpolyene	Others	Polymetallic	5.86	China: Guizhou, Jiuan
L180322-P-1-LD180319-0003_HL3GTCCXY_L1_clean_bin.47.orig.fa_	OEZ008529	yes	LMSG_G000011174.1	OES073692	arylpolyene	Others	Polymetallic	27.13	China: Guizhou, Jiuan
L180322-P-1-LD180319-0003_HL3GTCCXY_L1_clean_bin.47.orig.fa_	OEZ008529	yes	LMSG_G000011174.1	OES073692	bacteriocin	RiPPs	Polymetallic	10.91	China: Guizhou, Jiuan
L180322-P-1-LD180319-0003_HL3GTCCXY_L1_clean_bin.48.orig.fa_	OEZ008529	yes	LMSG_G000008594.1	OES073692	other	Others	Polymetallic	7.28	China: Guizhou, Jiuan
L180322-P-1-LD180319-0003_HL3GTCCXY_L1_clean_bin.5.strict.fa	OEZ008529	yes	LMSG_G000009636.1	OES073692	terpene	Terpene	Polymetallic	21.98	China: Guizhou, Jiuan
L180322-P-1-LD180319-0003_HL3GTCCXY_L1_clean_bin.5.strict.fa	OEZ008529	yes	LMSG_G000009636.1	OES073692	terpene	Terpene	Polymetallic	18.33	China: Guizhou, Jiuan
L180322-P-1-LD180319-0003_HL3GTCCXY_L1_clean_bin.50.orig.fa_	OEZ008529	yes	LMSG_G000011080.1	OES073692	arylpolyene	Others	Polymetallic	23.79	China: Guizhou, Jiuan
L180322-P-1-LD180319-0003_HL3GTCCXY_L1_clean_bin.50.orig.fa_	OEZ008529	yes	LMSG_G000011080.1	OES073692	NRPS-like	NRPS	Polymetallic	8.10	China: Guizhou, Jiuan
L180322-P-1-LD180319-0003_HL3GTCCXY_L1_clean_bin.51.orig.fa_	OEZ008529	yes	LMSG_G000010050.1	OES073692	arylpolyene	Others	Polymetallic	13.17	China: Guizhou, Jiuan
L180322-P-1-LD180319-0003_HL3GTCCXY_L1_clean_bin.51.orig.fa_	OEZ008529	yes	LMSG_G000010050.1	OES073692	ladderane	Others	Polymetallic	28.48	China: Guizhou, Jiuan
L180322-P-1-LD180319-0003_HL3GTCCXY_L1_clean_bin.51.orig.fa_	OEZ008529	yes	LMSG_G000010050.1	OES073692	T3PKS	PKSother	Polymetallic	16.88	China: Guizhou, Jiuan
L180322-P-1-LD180319-0003_HL3GTCCXY_L1_clean_bin.51.orig.fa_	OEZ008529	yes	LMSG_G000010050.1	OES073692	bacteriocin	RiPPs	Polymetallic	7.62	China: Guizhou, Jiuan
L180322-P-1-LD180319-0003_HL3GTCCXY_L1_clean_bin.51.orig.fa_	OEZ008529	yes	LMSG_G000010050.1	OES073692	terpene	Terpene	Polymetallic	13.36	China: Guizhou, Jiuan
L180322-P-1-LD180319-0003_HL3GTCCXY_L1_clean_bin.51.orig.fa_	OEZ008529	yes	LMSG_G000010050.1	OES073692	arylpolyene	Others	Polymetallic	18.40	China: Guizhou, Jiuan
L180322-P-1-LD180319-0003_HL3GTCCXY_L1_clean_bin.51.orig.fa_	OEZ008529	yes	LMSG_G000010050.1	OES073692	NRPS-like.ectoine	Others	Polymetallic	46.27	China: Guizhou, Jiuan
L180322-P-1-LD180319-0003_HL3GTCCXY_L1_clean_bin.54.strict.f	OEZ008529	yes	LMSG_G000010831.1	OES073692	acyl_amino_acids	Others	Polymetallic	32.06	China: Guizhou, Jiuan
L180322-P-1-LD180319-0003_HL3GTCCXY_L1_clean_bin.55.orig.fa_	OEZ008529	yes	LMSG_G000011159.1	OES073692	arylpolyene	Others	Polymetallic	12.85	China: Guizhou, Jiuan
L180322-P-1-LD180319-0003_HL3GTCCXY_L1_clean_bin.55.orig.fa_	OEZ008529	yes	LMSG_G000011159.1	OES073692	bacteriocin	RiPPs	Polymetallic	5.95	China: Guizhou, Jiuan
L180322-P-1-LD180319-0003_HL3GTCCXY_L1_clean_bin.56.strict.f	OEZ008529	yes	LMSG_G000008137.1	OES073692	terpene	Terpene	Polymetallic	10.46	China: Guizhou, Jiuan
L180322-P-1-LD180319-0003_HL3GTCCXY_L1_clean_bin.57.orig.fa_	OEZ008529	yes	LMSG_G000010417.1	OES073692	hserlactone	Others	Polymetallic	19.20	China: Guizhou, Jiuan
L180322-P-1-LD180319-0003_HL3GTCCXY_L1_clean_bin.57.orig.fa_	OEZ008529	yes	LMSG_G000010417.1	OES073692	terpene	Terpene	Polymetallic	25.02	China: Guizhou, Jiuan
L180322-P-1-LD180319-0003_HL3GTCCXY_L1_clean_bin.57.orig.fa_	OEZ008529	yes	LMSG_G000010417.1	OES073692	terpene	Terpene	Polymetallic	16.60	China: Guizhou, Jiuan
L180322-P-1-LD180319-0003_HL3GTCCXY_L1_clean_bin.57.orig.fa_	OEZ008529	yes	LMSG_G000010417.1	OES073692	hserlactone	Others	Polymetallic	10.13	China: Guizhou, Jiuan
L180322-P-1-LD180319-0003_HL3GTCCXY_L1_clean_bin.57.orig.fa_	OEZ008529	yes	LMSG_G000010417.1	OES073692	bacteriocin	RiPPs	Polymetallic	9.46	China: Guizhou, Jiuan
L180322-P-1-LD180319-0003_HL3GTCCXY_L1_clean_bin.58.orig.fa_	OEZ008529	yes	LMSG_G000010651.1	OES073692	NRPS.T1PKS	PKS-NRP_Hybrids	Polymetallic	44.05	China: Guizhou, Jiuan
L180322-P-1-LD180319-0003_HL3GTCCXY_L1_clean_bin.58.orig.fa_	OEZ008529	yes	LMSG_G000010651.1	OES073692	NRPS-like	NRPS	Polymetallic	24.41	China: Guizhou, Jiuan
L180322-P-1-LD180319-0003_HL3GTCCXY_L1_clean_bin.58.orig.fa_	OEZ008529	yes	LMSG_G000010651.1	OES073692	NRPS	NRPS	Polymetallic	29.18	China: Guizhou, Jiuan
L180322-P-1-LD180319-0003_HL3GTCCXY_L1_clean_bin.58.orig.fa_	OEZ008529	yes	LMSG_G000010651.1	OES073692	arylpolyene	Others	Polymetallic	41.29	China: Guizhou, Jiuan
L180322-P-1-LD180319-0003_HL3GTCCXY_L1_clean_bin.59.orig.fa_	OEZ008529	yes	LMSG_G000008652.1	OES073692	other	Others	Polymetallic	16.33	China: Guizhou, Jiuan
L180322-P-1-LD180319-0003_HL3GTCCXY_L1_clean_bin.8.orig.fa_k	OEZ008529	yes	LMSG_G000008085.1	OES073692	T1PKS	PKSI	Polymetallic	8.91	China: Guizhou, Jiuan
L180322-P-1-LD180319-0003_HL3GTCCXY_L1_clean_bin.8.orig.fa_k	OEZ008529	yes	LMSG_G000008085.1	OES073692	hglE-KS	PKSother	Polymetallic	10.29	China: Guizhou, Jiuan
L180322-P-1-LD180319-0003_HL3GTCCXY_L1_clean_bin.9.orig.fa_k	OEZ008529	yes	LMSG_G000010668.1	OES073692	bacteriocin	RiPPs	Polymetallic	7.15	China: Guizhou, Jiuan
L180322-P-1-LD180319-0003_HL3GTCCXY_L1_clean_bin.9.orig.fa_k	OEZ008529	yes	LMSG_G000010668.1	OES073692	T1PKS.NRPS-like	PKS-NRP_Hybrids	Polymetallic	45.06	China: Guizhou, Jiuan
L180322-P-1-LD180319-0003_HL3GTCCXY_L1_clean_bin.9.orig.fa_k	OEZ008529	yes	LMSG_G000010668.1	OES073692	betalactone	Others	Polymetallic	29.31	China: Guizhou, Jiuan
L180322-P-1-LD180319-0003_HL3GTCCXY_L1_clean_bin.9.orig.fa_k	OEZ008529	yes	LMSG_G000010668.1	OES073692	NRPS.T1PKS	PKS-NRP_Hybrids	Polymetallic	53.43	China: Guizhou, Jiuan
L180322-P-1-LD180319-0003_HL3GTCCXY_L1_clean_bin.9.orig.fa_k	OEZ008529	yes	LMSG_G000010668.1	OES073692	bacteriocin	RiPPs	Polymetallic	10.90	China: Guizhou, Jiuan
L180322-P-1-LD180319-0003_HL3GTCCXY_L1_clean_bin.9.orig.fa_k	OEZ008529	yes	LMSG_G000010668.1	OES073692	terpene	Terpene	Polymetallic	15.42	China: Guizhou, Jiuan
L180322-P-1-LD180319-0003_HL3GTCCXY_L1_clean_bin.9.orig.fa_k	OEZ008529	yes	LMSG_G000010668.1	OES073692	betalactone	Others	Polymetallic	23.23	China: Guizhou, Jiuan
L180322-P-2-LD180319-0004_HL3KCCCXY_L1_clean_bin.1.orig.fa_k	OEZ008529	yes	LMSG_G000004446.1	OES073694	terpene	Terpene	Polymetallic	15.11	China: Guizhou, Jiuan
L180322-P-2-LD180319-0004_HL3KCCCXY_L1_clean_bin.1.orig.fa_k	OEZ008529	yes	LMSG_G000004446.1	OES073694	terpene	Terpene	Polymetallic	22.05	China: Guizhou, Jiuan
L180322-P-2-LD180319-0004_HL3KCCCXY_L1_clean_bin.1.orig.fa_k	OEZ008529	yes	LMSG_G000004446.1	OES073694	hserlactone	Others	Polymetallic	16.49	China: Guizhou, Jiuan
L180322-P-2-LD180319-0004_HL3KCCCXY_L1_clean_bin.10.strict.f	OEZ008529	yes	LMSG_G000008160.1	OES073694	terpene	Terpene	Polymetallic	23.44	China: Guizhou, Jiuan

L180322-P-2-LD180319-0004_HL3KCCCXY_L1_clean_bin.11.orig.fa_	OEZ008529	yes	LMSG_G000004443.1	OES073694	other	Others	Polymetallic	40.46	China: Guizhou, Jiuan
L180322-P-2-LD180319-0004_HL3KCCCXY_L1_clean_bin.12.permissi	OEZ008529	yes	LMSG_G000004444.1	OES073694	T1PKS	PKSI	Polymetallic	33.69	China: Guizhou, Jiuan
L180322-P-2-LD180319-0004_HL3KCCCXY_L1_clean_bin.12.permissi	OEZ008529	yes	LMSG_G000004444.1	OES073694	betalactone	Others	Polymetallic	27.13	China: Guizhou, Jiuan
L180322-P-2-LD180319-0004_HL3KCCCXY_L1_clean_bin.13.orig.fa_	OEZ008529	yes	LMSG_G000005215.1	OES073694	T1PKS	PKSI	Polymetallic	24.01	China: Guizhou, Jiuan
L180322-P-2-LD180319-0004_HL3KCCCXY_L1_clean_bin.13.orig.fa_	OEZ008529	yes	LMSG_G000005215.1	OES073694	terpene	Terpene	Polymetallic	20.83	China: Guizhou, Jiuan
L180322-P-2-LD180319-0004_HL3KCCCXY_L1_clean_bin.13.orig.fa_	OEZ008529	yes	LMSG_G000005215.1	OES073694	NRPS	NRPS	Polymetallic	25.37	China: Guizhou, Jiuan
L180322-P-2-LD180319-0004_HL3KCCCXY_L1_clean_bin.13.orig.fa_	OEZ008529	yes	LMSG_G000005215.1	OES073694	betalactone	Others	Polymetallic	19.21	China: Guizhou, Jiuan
L180322-P-2-LD180319-0004_HL3KCCCXY_L1_clean_bin.13.orig.fa_	OEZ008529	yes	LMSG_G000005215.1	OES073694	bacteriocin	RiPPs	Polymetallic	10.92	China: Guizhou, Jiuan
L180322-P-2-LD180319-0004_HL3KCCCXY_L1_clean_bin.13.orig.fa_	OEZ008529	yes	LMSG_G000005215.1	OES073694	arylpolyene	Others	Polymetallic	28.31	China: Guizhou, Jiuan
L180322-P-2-LD180319-0004_HL3KCCCXY_L1_clean_bin.15.orig.fa_	OEZ008529	yes	LMSG_G000005216.1	OES073694	T1PKS.hglE-KS	PKSother	Polymetallic	31.60	China: Guizhou, Jiuan
L180322-P-2-LD180319-0004_HL3KCCCXY_L1_clean_bin.16.orig.fa_	OEZ008529	yes	LMSG_G000011081.1	OES073694	arylpolyene	Others	Polymetallic	24.60	China: Guizhou, Jiuan
L180322-P-2-LD180319-0004_HL3KCCCXY_L1_clean_bin.16.orig.fa_	OEZ008529	yes	LMSG_G000011081.1	OES073694	terpene	Terpene	Polymetallic	17.20	China: Guizhou, Jiuan
L180322-P-2-LD180319-0004_HL3KCCCXY_L1_clean_bin.16.orig.fa_	OEZ008529	yes	LMSG_G000011081.1	OES073694	lassopeptide	RiPPs	Polymetallic	28.42	China: Guizhou, Jiuan
L180322-P-2-LD180319-0004_HL3KCCCXY_L1_clean_bin.16.orig.fa_	OEZ008529	yes	LMSG_G000011081.1	OES073694	NRPS-like	NRPS	Polymetallic	13.90	China: Guizhou, Jiuan
L180322-P-2-LD180319-0004_HL3KCCCXY_L1_clean_bin.17.strict.f	OEZ008529	yes	LMSG_G000004445.1	OES073694	terpene	Terpene	Polymetallic	25.02	China: Guizhou, Jiuan
L180322-P-2-LD180319-0004_HL3KCCCXY_L1_clean_bin.17.strict.f	OEZ008529	yes	LMSG_G000004445.1	OES073694	terpene	Terpene	Polymetallic	20.84	China: Guizhou, Jiuan
L180322-P-2-LD180319-0004_HL3KCCCXY_L1_clean_bin.17.strict.f	OEZ008529	yes	LMSG_G000004445.1	OES073694	bacteriocin	RiPPs	Polymetallic	10.88	China: Guizhou, Jiuan
L180322-P-2-LD180319-0004_HL3KCCCXY_L1_clean_bin.18.orig.fa_	OEZ008529	yes	LMSG_G000005217.1	OES073694	NRPS-like	NRPS	Polymetallic	21.27	China: Guizhou, Jiuan
L180322-P-2-LD180319-0004_HL3KCCCXY_L1_clean_bin.18.orig.fa_	OEZ008529	yes	LMSG_G000005217.1	OES073694	NRPS	NRPS	Polymetallic	24.74	China: Guizhou, Jiuan
L180322-P-2-LD180319-0004_HL3KCCCXY_L1_clean_bin.18.orig.fa_	OEZ008529	yes	LMSG_G000005217.1	OES073694	NRPS-like	NRPS	Polymetallic	24.42	China: Guizhou, Jiuan
L180322-P-2-LD180319-0004_HL3KCCCXY_L1_clean_bin.18.orig.fa_	OEZ008529	yes	LMSG_G000005217.1	OES073694	NRPS.T1PKS	PKS-NRP_Hybrids	Polymetallic	44.08	China: Guizhou, Jiuan
L180322-P-2-LD180319-0004_HL3KCCCXY_L1_clean_bin.18.orig.fa_	OEZ008529	yes	LMSG_G000005217.1	OES073694	arylpolyene	Others	Polymetallic	41.29	China: Guizhou, Jiuan
L180322-P-2-LD180319-0004_HL3KCCCXY_L1_clean_bin.19.strict.f	OEZ008529	yes	LMSG_G000007805.1	OES073694	T3PKS	PKSother	Polymetallic	12.59	China: Guizhou, Jiuan
L180322-P-2-LD180319-0004_HL3KCCCXY_L1_clean_bin.20.orig.fa_	OEZ008529	yes	LMSG_G000010267.1	OES073694	arylpolyene	Others	Polymetallic	28.96	China: Guizhou, Jiuan
L180322-P-2-LD180319-0004_HL3KCCCXY_L1_clean_bin.20.orig.fa_	OEZ008529	no	LMSG_G000010267.1	OES073694	terpene	Terpene	Polymetallic	21.16	China: Guizhou, Jiuan
L180322-P-2-LD180319-0004_HL3KCCCXY_L1_clean_bin.21.orig.fa_	OEZ008529	yes	LMSG_G000006228.1	OES073694	NRPS.T1PKS	PKS-NRP_Hybrids	Polymetallic	57.03	China: Guizhou, Jiuan
L180322-P-2-LD180319-0004_HL3KCCCXY_L1_clean_bin.21.orig.fa_	OEZ008529	yes	LMSG_G000006228.1	OES073694	bacteriocin	RiPPs	Polymetallic	10.90	China: Guizhou, Jiuan
L180322-P-2-LD180319-0004_HL3KCCCXY_L1_clean_bin.21.orig.fa_	OEZ008529	yes	LMSG_G000006228.1	OES073694	NRPS-like	NRPS	Polymetallic	16.30	China: Guizhou, Jiuan
L180322-P-2-LD180319-0004_HL3KCCCXY_L1_clean_bin.21.orig.fa_	OEZ008529	yes	LMSG_G000006228.1	OES073694	betalactone	Others	Polymetallic	29.31	China: Guizhou, Jiuan
L180322-P-2-LD180319-0004_HL3KCCCXY_L1_clean_bin.21.orig.fa_	OEZ008529	yes	LMSG_G000006228.1	OES073694	betalactone	Others	Polymetallic	25.76	China: Guizhou, Jiuan
L180322-P-2-LD180319-0004_HL3KCCCXY_L1_clean_bin.21.orig.fa_	OEZ008529	yes	LMSG_G000006228.1	OES073694	T1PKS	PKSI	Polymetallic	17.40	China: Guizhou, Jiuan
L180322-P-2-LD180319-0004_HL3KCCCXY_L1_clean_bin.21.orig.fa_	OEZ008529	yes	LMSG_G000006228.1	OES073694	terpene	Terpene	Polymetallic	19.74	China: Guizhou, Jiuan
L180322-P-2-LD180319-0004_HL3KCCCXY_L1_clean_bin.23.strict.f	OEZ008529	yes	LMSG_G000008142.1	OES073694	terpene	Terpene	Polymetallic	11.84	China: Guizhou, Jiuan
L180322-P-2-LD180319-0004_HL3KCCCXY_L1_clean_bin.23.strict.f	OEZ008529	yes	LMSG_G000008142.1	OES073694	terpene	Terpene	Polymetallic	10.78	China: Guizhou, Jiuan
L180322-P-2-LD180319-0004_HL3KCCCXY_L1_clean_bin.26.orig.fa_	OEZ008529	yes	LMSG_G000004447.1	OES073694	betalactone	Others	Polymetallic	28.23	China: Guizhou, Jiuan
L180322-P-2-LD180319-0004_HL3KCCCXY_L1_clean_bin.26.orig.fa_	OEZ008529	yes	LMSG_G000004447.1	OES073694	betalactone	Others	Polymetallic	16.72	China: Guizhou, Jiuan
L180322-P-2-LD180319-0004_HL3KCCCXY_L1_clean_bin.29.orig.fa_	OEZ008529	yes	LMSG_G000011175.1	OES073694	bacteriocin	RiPPs	Polymetallic	5.83	China: Guizhou, Jiuan
L180322-P-2-LD180319-0004_HL3KCCCXY_L1_clean_bin.31.orig.fa_	OEZ008529	no	LMSG_G000011012.1	OES073694	bacteriocin	RiPPs	Polymetallic	10.83	China: Guizhou, Jiuan
L180322-P-2-LD180319-0004_HL3KCCCXY_L1_clean_bin.31.orig.fa_	OEZ008529	no	LMSG_G000011012.1	OES073694	NRPS-like	NRPS	Polymetallic	42.98	China: Guizhou, Jiuan
L180322-P-2-LD180319-0004_HL3KCCCXY_L1_clean_bin.31.orig.fa_	OEZ008529	no	LMSG_G000011012.1	OES073694	NRPS	NRPS	Polymetallic	5.44	China: Guizhou, Jiuan
L180322-P-2-LD180319-0004_HL3KCCCXY_L1_clean_bin.31.orig.fa_	OEZ008529	no	LMSG_G000011012.1	OES073694	NRPS	NRPS	Polymetallic	6.21	China: Guizhou, Jiuan
L180322-P-2-LD180319-0004_HL3KCCCXY_L1_clean_bin.31.orig.fa_	OEZ008529	no	LMSG_G000011012.1	OES073694	NRPS	NRPS	Polymetallic	44.23	China: Guizhou, Jiuan
L180322-P-2-LD180319-0004_HL3KCCCXY_L1_clean_bin.31.orig.fa_	OEZ008529	no	LMSG_G000011012.1	OES073694	NRPS	NRPS	Polymetallic	53.04	China: Guizhou, Jiuan
L180322-P-2-LD180319-0004_HL3KCCCXY_L1_clean_bin.31.orig.fa_	OEZ008529	no	LMSG_G000011012.1	OES073694	thiopeptide	RiPPs	Polymetallic	33.00	China: Guizhou, Jiuan
L180322-P-2-LD180319-0004_HL3KCCCXY_L1_clean_bin.31.orig.fa_	OEZ008529	no	LMSG_G000011012.1	OES073694	phenazine	Others	Polymetallic	10.23	China: Guizhou, Jiuan
L180322-P-2-LD180319-0004_HL3KCCCXY_L1_clean_bin.31.orig.fa_	OEZ008529	no	LMSG_G000011012.1	OES073694	phenazine	Others	Polymetallic	10.23	China: Guizhou, Jiuan
L180322-P-2-LD180319-0004_HL3KCCCXY_L1_clean_bin.31.orig.fa_	OEZ008529	no	LMSG_G000011012.1	OES073694	NRPS	NRPS	Polymetallic	49.55	China: Guizhou, Jiuan
L180322-P-2-LD180319-0004_HL3KCCCXY_L1_clean_bin.31.orig.fa_	OEZ008529	no	LMSG_G000011012.1	OES073694	bacteriocin	RiPPs	Polymetallic	10.86	China: Guizhou, Jiuan
L180322-P-2-LD180319-0004_HL3KCCCXY_L1_clean_bin.31.orig.fa_	OEZ008529	no	LMSG_G000011012.1	OES073694	NRPS	NRPS	Polymetallic	42.58	China: Guizhou, Jiuan
L180322-P-2-LD180319-0004_HL3KCCCXY_L1_clean_bin.31.orig.fa_	OEZ008529	no	LMSG_G000011012.1	OES073694	betalactone.NRPS-like	Others	Polymetallic	41.86	China: Guizhou, Jiuan
L180322-P-2-LD180319-0004_HL3KCCCXY_L1_clean_bin.31.orig.fa_	OEZ008529	no	LMSG_G000011012.1	OES073694	hserlactone	Others	Polymetallic	20.61	China: Guizhou, Jiuan
L180322-P-2-LD180319-0004_HL3KCCCXY_L1_clean_bin.31.orig.fa_	OEZ008529	no	LMSG_G000011012.1	OES073694	hserlactone	Others	Polymetallic	20.61	China: Guizhou, Jiuan
L180322-P-2-LD180319-0004_HL3KCCCXY_L1_clean_bin.31.orig.fa_	OEZ008529	no	LMSG_G000011012.1	OES073694	NAGGN	Others	Polymetallic	14.76	China: Guizhou, Jiuan
L180322-P-2-LD180319-0004_HL3KCCCXY_L1_clean_bin.32.strict.f	OEZ008529	yes	LMSG_G000008376.1	OES073694	terpene	Terpene	Polymetallic	8.78	China: Guizhou, Jiuan
L180322-P-2-LD180319-0004_HL3KCCCXY_L1_clean_bin.34.permissi	OEZ008529	yes	LMSG_G000010055.1	OES073694	NRPS-like.ectoine	Others	Polymetallic	29.11	China: Guizhou, Jiuan
L180322-P-2-LD180319-0004_HL3KCCCXY_L1_clean_bin.34.permissi	OEZ008529	yes	LMSG_G000010055.1	OES073694	terpene	Terpene	Polymetallic	19.57	China: Guizhou, Jiuan
L180322-P-2-LD180319-0004_HL3KCCCXY_L1_clean_bin.34.permissi	OEZ008529	yes	LMSG_G000010055.1	OES073694	bacteriocin	RiPPs	Polymetallic	10.75	China: Guizhou, Jiuan
L180322-P-2-LD180319-0004_HL3KCCCXY_L1_clean_bin.34.permissi	OEZ008529	yes	LMSG_G000010055.1	OES073694	arylpolyene	Others	Polymetallic	13.51	China: Guizhou, Jiuan
L180322-P-2-LD180319-0004_HL3KCCCXY_L1_clean_bin.34.permissi	OEZ008529	yes	LMSG_G000010055.1	OES073694	terpene	Terpene	Polymetallic	13.30	China: Guizhou, Jiuan
L180322-P-2-LD180319-0004_HL3KCCCXY_L1_clean_bin.34.permissi	OEZ008529	yes	LMSG_G000010055.1	OES073694	arylpolyene	Others	Polymetallic	13.29	China: Guizhou, Jiuan
L180322-P-2-LD180319-0004_HL3KCCCXY_L1_clean_bin.34.permissi	OEZ008529	yes	LMSG_G000010055.1	OES073694	ladderane	Others	Polymetallic	11.42	China: Guizhou, Jiuan
L180322-P-2-LD180319-0004_HL3KCCCXY_L1_clean_bin.34.permissi	OEZ008529	yes	LMSG_G000010055.1	OES073694	T3PKS	PKSother	Polymetallic	9.14	China: Guizhou, Jiuan
L180322-P-2-LD180319-0004_HL3KCCCXY_L1_clean_bin.35.strict.f	OEZ008529	yes	LMSG_G000005219.1	OES073694	hglE-KS	PKSother	Polymetallic	28.20	China: Guizhou, Jiuan
L180322-P-2-LD180319-0004_HL3KCCCXY_L1_clean_bin.35.strict.f	OEZ008529	yes	LMSG_G000005219.1	OES073694	T3PKS	PKSother	Polymetallic	41.06	China: Guizhou, Jiuan
L180322-P-2-LD180319-0004_HL3KCCCXY_L1_clean_bin.35.strict.f	OEZ008529	yes	LMSG_G000005219.1	OES073694	terpene	Terpene	Polymetallic	10.86	China: Guizhou, Jiuan
L180322-P-2-LD180319-0004_HL3KCCCXY_L1_clean_bin.35.strict.f	OEZ008529	yes	LMSG_G000005219.1	OES073694	terpene	Terpene	Polymetallic	12.28	China: Guizhou, Jiuan
L180322-P-2-LD180319-0004_HL3KCCCXY_L1_clean_bin.36.orig.fa_	OEZ008529	yes	LMSG_G000010760.1	OES073694	bacteriocin	RiPPs	Polymetallic	11.75	China: Guizhou, Jiuan
L180322-P-2-LD180319-0004_HL3KCCCXY_L1_clean_bin.36.orig.fa_	OEZ008529	yes	LMSG_G000010760.1	OES073694	acyl_amino_acids	Others	Polymetallic	41.97	China: Guizhou, Jiuan
L180322-P-2-LD180319-0004_HL3KCCCXY_L1_clean_bin.4.strict.fa	OEZ008529	no	LMSG_G000010832.1	OES073694	acyl_amino_acids	Others	Polymetallic	5.71	China: Guizhou, Jiuan
L180322-P-2-LD180319-0004_HL3KCCCXY_L1_clean_bin.6.orig.fa_k	OEZ008529	yes	LMSG_G000011160.1	OES073694	arylpolyene	Others	Polymetallic	33.85	China: Guizhou, Jiuan
L180322-P-2-LD180319-0004_HL3KCCCXY_L1_clean_bin.6.orig.fa_k	OEZ008529	yes	LMSG_G000011160.1	OES073694	arylpolyene	Others	Polymetallic	9.79	China: Guizhou, Jiuan
L180322-P-2-LD180319-0004_HL3KCCCXY_L1_clean_bin.6.orig.fa_k	OEZ008529	yes	LMSG_G000011160.1	OES073694	bacteriocin	RiPPs	Polymetallic	7.50	China: Guizhou, Jiuan
L180322-P-2-LD180319-0004_HL3KCCCXY_L1_clean_bin.7.orig.fa_k	OEZ008529	yes	LMSG_G000010203.1	OES073694	terpene	Terpene	Polymetallic	21.91	China: Guizhou, Jiuan
L180322-P-2-LD180319-0004_HL3KCCCXY_L1_clean_bin.7.orig.fa_k	OEZ008529	yes	LMSG_G000010203.1	OES073694	arylpolyene	Others	Polymetallic	25.42	China: Guizhou, Jiuan
L180322-P-2-LD180319-0004_HL3KCCCXY_L1_clean_bin.7.orig.fa_k	OEZ008529	yes	LMSG_G000010203.1	OES073694	terpene	Terpene	Polymetallic	21.73	China: Guizhou, Jiuan
L180322-P-2-LD180319-0004_HL3KCCCXY_L1_clean_bin.7.orig.fa_k	OEZ008529	yes	LMSG_G000010203.1	OES073694	hserlactone	Others	Polymetallic	20.62	China: Guizhou, Jiuan
L180322-P-2-LD180319-0004_HL3KCCCXY_L1_clean_bin.8.orig.fa_k	OEZ008529	yes	LMSG_G000009267.1	OES073694	TfuA-related	RiPPs	Polymetallic	19.47	China: Guizhou, Jiuan
L180322-P-2-LD180319-0004_HL3KCCCXY_L1_clean_bin.8.orig.fa_k	OEZ008529	yes	LMSG_G000009267.1	OES073694	LAP	RiPPs	Polymetallic	15.81	China: Guizhou, Jiuan
L180322-P-2-LD180319-0004_HL3KCCCXY_L1_clean_bin.8.orig.fa_k	OEZ008529	yes	LMSG_G000009267.1	OES073694	terpene	Terpene	Polymetallic	20.85	China: Guizhou, Jiuan
L180322-P-2-LD180319-0004_HL3KCCCXY_L1_clean_bin.9.orig.fa_k	OEZ008529	yes	LMSG_G000008609.1	OES073694	other	Others	Polymetallic	7.06	China: Guizhou, Jiuan
L180322-P-2-LD180319-0005_HL3KCCCXY_L1_clean_bin.10.orig.fa_	OEZ008529	yes	LMSG_G000010123.1	OES073749	arylpolyene	Others	Polymetallic	7.53	China: Guizhou, Zhijin
L180322-P-2-LD180319-0005_HL3KCCCXY_L1_clean_bin.10.orig.fa_	OEZ008529	yes	LMSG_G000010123.1	OES073749	terpene	Terpene	Polymetallic	11.63	China: Guizhou, Zhijin
L180322-P-2-LD180319-0005_HL3KCCCXY_L1_clean_bin.10.orig.fa_	OEZ008529	yes	LMSG_G000010123.1	OES073749	terpene	Terpene	Polymetallic	12.39	China: Guizhou, Zhijin
L180322-P-2-LD180319-0005_HL3KCCCXY_L1_clean_bin.12.orig.fa_	OEZ008529	yes	LMSG_G000007874.1	OES073749	T1PKS.NRPS-like	PKS-NRP_Hybrids	Polymetallic	19.00	China: Guizhou, Zhijin
L180322-P-2-LD180319-0005_HL3KCCCXY_L1_clean_bin.12.orig.fa_	OEZ008529	yes	LMSG_G000007874.1	OES073749	terpene	Terpene	Polymetallic	7.62	China: Guizhou, Zhijin
L180322-P-2-LD180319-0005_HL3KCCCXY_L1_clean_bin.12.orig.fa_	OEZ008529	yes	LMSG_G000007874.1	OES073749	bacteriocin	RiPPs	Polymetallic	8.92	China: Guizhou, Zhijin
L180322-P-2-LD180319-0005_HL3KCCCXY_L1_clean_bin.12.orig.fa_	OEZ008529	yes	LMSG_G000007874.1	OES073749	NRPS-like	NRPS	Polymetallic	29.87	China: Guizhou, Zhijin
L180322-P-2-LD180319-0005_HL3KCCCXY_L1_clean_bin.12.orig.fa_	OEZ008529	yes	LMSG_G000007874.1	OES073749	terpene	Terpene	Polymetallic	18.60	China: Guizhou, Zhijin
L180322-P-2-LD180319-0005_HL3KCCCXY_L1_clean_bin.12.orig.fa_	OEZ008529	yes	LMSG_G000007874.1	OES073749	NRPS-like	NRPS	Polymetallic	43.23	China: Guizhou, Zhijin
L180322-P-2-LD180319-0005_HL3KCCCXY_L1_clean_bin.14.orig.fa_	OEZ008529	yes	LMSG_G000010126.1	OES073749	terpene	Terpene	Polymetallic	6.24	China: Guizhou, Zhijin
L180322-P-2-LD180319-0005_HL3KCCCXY_L1_clean_bin.14.orig.fa_	OEZ008529	yes	LMSG_G000010126.1	OES073749	arylpolyene	Others	Polymetallic	15.61	China: Guizhou, Zhijin
L180322-P-2-LD180319-0005_HL3KCCCXY_L1_clean_bin.15.orig.fa_	OEZ008529	yes	LMSG_G000005220.1	OES073749	bacteriocin	RiPPs	Polymetallic	6.83	China: Guizhou, Zhijin
L180322-P-2-LD180319-0005_HL3KCCCXY_L1_clean_bin.15.orig.fa_	OEZ008529	yes	LMSG_G000005220.1	OES073749	ectoine	Others	Polymetallic	10.41	China: Guizhou, Zhijin
L180322-P-2-LD180319-0005_HL3KCCCXY_L1_clean_bin.15.orig.fa_	OEZ008529	yes	LMSG_G000005220.1	OES073749	T3PKS	PKSother	Polymetallic	28.30	China: Guizhou, Zhijin
L180322-P-2-LD180319-0005_HL3KCCCXY_L1_clean_bin.15.orig.fa_	OEZ008529	yes	LMSG_G000005220.1	OES073749	terpene	Terpene	Polymetallic	11.65	China: Guizhou, Zhijin
L180322-P-2-LD180319-0005_HL3KCCCXY_L1_clean_bin.15.orig.fa_	OEZ008529	yes	LMSG_G000005220.1	OES073749	terpene	Terpene	Polymetallic	19.45	China: Guizhou, Zhijin
L180322-P-2-LD180319-0005_HL3KCCCXY_L1_clean_bin.16.orig.fa_	OEZ008529	yes	LMSG_G000008438.1	OES073749	terpene	Terpene	Polymetallic	26.50	China: Guizhou, Zhijin
L180322-P-2-LD180319-0005_HL3KCCCXY_L1_clean_bin.19.orig.fa_	OEZ008529	yes	LMSG_G000010802.1	OES073749	T1PKS	PKSI	Polymetallic	6.44	China: Guizhou, Zhijin
L180322-P-2-LD180319-0005_HL3KCCCXY_L1_clean_bin.19.orig.fa_	OEZ008529	no	LMSG_G000010802.1	OES073749	acyl_amino_acids	Others	Polymetallic	5.09	China: Guizhou, Zhijin
L180322-P-2-LD180319-0005_HL3KCCCXY_L1_clean_bin.2.orig.fa_k	OEZ008529	yes	LMSG_G000010188.1	OES073749	T3PKS	PKSother	Polymetallic	17.15	China: Guizhou, Zhijin
L180322-P-2-LD180319-0005_HL3KCCCXY_L1_clean_bin.20.orig.fa_	OEZ008529	yes	LMSG_G000010080.1	OES073749	hglE-KS	PKSother	Polymetallic	17.49	China: Guizhou, Zhijin
L180322-P-2-LD180319-0005_HL3KCCCXY_L1_clean_bin.20.orig.fa_	OEZ008529	yes	LMSG_G000010080.1	OES073749	terpene	Terpene	Polymetallic	5.85	China: Guizhou, Zhijin
L180322-P-2-LD180319-0005_HL3KCCCXY_L1_clean_bin.20.orig.fa_	OEZ008529	yes	LMSG_G000010080.1	OES073749	acyl_amino_acids	Others	Polymetallic	39.54	China: Guizhou, Zhijin
L180322-P-2-LD180319-0005_HL3KCCCXY_L1_clean_bin.20.orig.fa_	OEZ008529	yes	LMSG_G000010080.1	OES073749	NRPS-like	NRPS	Polymetallic	5.78	China: Guizhou, Zhijin
L180322-P-2-LD180319-0005_HL3KCCCXY_L1_clean_bin.20.orig.fa_	OEZ008529	yes	LMSG_G000010080.1	OES073749	T1PKS	PKSI	Polymetallic	24.59	China: Guizhou, Zhijin
L180322-P-2-LD180319-0005_HL3KCCCXY_L1_clean_bin.20.orig.fa_	OEZ008529	yes	LMSG_G000010080.1	OES073749	T1PKS	PKSI	Polymetallic	6.85	China: Guizhou, Zhijin
L180322-P-2-LD180319-0005_HL3KCCCXY_L1_clean_bin.20.orig.fa_	OEZ008529	yes	LMSG_G000010080.1	OES073749	terpene	Terpene	Polymetallic	11.61	China: Guizhou, Zhijin
L180322-P-2-LD180319-0005_HL3KCCCXY_L1_clean_bin.21.orig.fa_	OEZ008529	yes	LMSG_G000008468.1	OES073749	arylpolyene	Others	Polymetallic	41.18	China: Guizhou, Zhijin
L180322-P-2-LD180319-0005_HL3KCCCXY_L1_clean_bin.21.orig.fa_	OEZ008529	yes	LMSG_G000008468.1	OES073749	betalactone	Others	Polymetallic	25.86	China: Guizhou, Zhijin
L180322-P-2-LD180319-0005_HL3KCCCXY_L1_clean_bin.21.orig.fa_	OEZ008529	yes	LMSG_G000008468.1	OES073749	terpene	Terpene	Polymetallic	25.32	China: Guizhou, Zhijin
L180322-P-2-LD180319-0005_HL3KCCCXY_L1_clean_bin.21.orig.fa_	OEZ008529	yes	LMSG_G000008468.1	OES073749	betalactone	Others	Polymetallic	27.74	China: Guizhou, Zhijin
L180322-P-2-LD180319-0005_HL3KCCCXY_L1_clean_bin.22.orig.fa_	OEZ008529	yes	LMSG_G000004448.1	OES073749	terpene	Terpene	Polymetallic	30.13	China: Guizhou, Zhijin
L180322-P-2-LD180319-0005_HL3KCCCXY_L1_clean_bin.22.orig.fa_	OEZ008529	yes	LMSG_G000004448.1	OES073749	bacteriocin	RiPPs	Polymetallic	10.86	China: Guizhou, Zhijin
L180322-P-2-LD180319-0005_HL3KCCCXY_L1_clean_bin.22.orig.fa_	OEZ008529	yes	LMSG_G000004448.1	OES073749	bacteriocin	RiPPs	Polymetallic	10.86	China: Guizhou, Zhijin
L180322-P-2-LD180319-0005_HL3KCCCXY_L1_clean_bin.22.orig.fa_	OEZ008529	yes	LMSG_G000004448.1	OES073749	terpene	Terpene	Polymetallic	21.07	China: Guizhou, Zhijin
L180322-P-2-LD180319-0005_HL3KCCCXY_L1_clean_bin.23.orig.fa_	OEZ008529	yes	LMSG_G000006273.1	OES073749	other	Others	Polymetallic	40.46	China: Guizhou, Zhijin
L180322-P-2-LD180319-0005_HL3KCCCXY_L1_clean_bin.24.permissi	OEZ008529	yes	LMSG_G000004449.1	OES073749	NRPS-like	NRPS	Polymetallic	24.34	China: Guizhou, Zhijin
L180322-P-2-LD180319-0005_HL3KCCCXY_L1_clean_bin.24.permissi	OEZ008529	yes	LMSG_G000004449.1	OES073749	T3PKS	PKSother	Polymetallic	8.60	China: Guizhou, Zhijin
L180322-P-2-LD180319-0005_HL3KCCCXY_L1_clean_bin.25.orig.fa_	OEZ008529	yes	LMSG_G000010127.1	OES073749	terpene	Terpene	Polymetallic	10.10	China: Guizhou, Zhijin
L180322-P-2-LD180319-0005_HL3KCCCXY_L1_clean_bin.25.orig.fa_	OEZ008529	yes	LMSG_G000010127.1	OES073749	hserlactone	Others	Polymetallic	5.43	China: Guizhou, Zhijin
L180322-P-2-LD180319-0005_HL3KCCCXY_L1_clean_bin.25.orig.fa_	OEZ008529	yes	LMSG_G000010127.1	OES073749	terpene	Terpene	Polymetallic	5.48	China: Guizhou, Zhijin
L180322-P-2-LD180319-0005_HL3KCCCXY_L1_clean_bin.25.orig.fa_	OEZ008529	yes	LMSG_G000010127.1	OES073749	arylpolyene	Others	Polymetallic	20.50	China: Guizhou, Zhijin
L180322-P-2-LD180319-0005_HL3KCCCXY_L1_clean_bin.25.orig.fa_	OEZ008529	yes	LMSG_G000010127.1	OES073749	arylpolyene	Others	Polymetallic	7.64	China: Guizhou, Zhijin
L180322-P-2-LD180319-0005_HL3KCCCXY_L1_clean_bin.25.orig.fa_	OEZ008529	yes	LMSG_G000010127.1	OES073749	bacteriocin	RiPPs	Polymetallic	7.50	China: Guizhou, Zhijin
L180322-P-2-LD180319-0005_HL3KCCCXY_L1_clean_bin.26.orig.fa_	OEZ008529	yes	LMSG_G000010111.1	OES073749	terpene	Terpene	Polymetallic	22.14	China: Guizhou, Zhijin
L180322-P-2-LD180319-0005_HL3KCCCXY_L1_clean_bin.26.orig.fa_	OEZ008529	yes	LMSG_G000010111.1	OES073749	terpene	Terpene	Polymetallic	21.70	China: Guizhou, Zhijin
L180322-P-2-LD180319-0005_HL3KCCCXY_L1_clean_bin.26.orig.fa_	OEZ008529	yes	LMSG_G000010111.1	OES073749	T3PKS	PKSother	Polymetallic	41.08	China: Guizhou, Zhijin
L180322-P-2-LD180319-0005_HL3KCCCXY_L1_clean_bin.26.orig.fa_	OEZ008529	yes	LMSG_G000010111.1	OES073749	hserlactone	Others	Polymetallic	20.64	China: Guizhou, Zhijin
L180322-P-2-LD180319-0005_HL3KCCCXY_L1_clean_bin.26.orig.fa_	OEZ008529	yes	LMSG_G000010111.1	OES073749	terpene	Terpene	Polymetallic	20.61	China: Guizhou, Zhijin
L180322-P-2-LD180319-0005_HL3KCCCXY_L1_clean_bin.29.orig.fa_	OEZ008529	yes	LMSG_G000010967.1	OES073749	T1PKS.hglE-KS	PKSother	Polymetallic	23.68	China: Guizhou, Zhijin
L180322-P-2-LD180319-0005_HL3KCCCXY_L1_clean_bin.3.orig.fa_k	OEZ008529	no	LMSG_G000010268.1	OES073749	terpene	Terpene	Polymetallic	21.47	China: Guizhou, Zhijin
L180322-P-2-LD180319-0005_HL3KCCCXY_L1_clean_bin.3.orig.fa_k	OEZ008529	yes	LMSG_G000010268.1	OES073749	arylpolyene	Others	Polymetallic	41.19	China: Guizhou, Zhijin
L180322-P-2-LD180319-0005_HL3KCCCXY_L1_clean_bin.30.orig.fa_	OEZ008529	yes	LMSG_G000005221.1	OES073749	arylpolyene	Others	Polymetallic	7.48	China: Guizhou, Zhijin
L180322-P-2-LD180319-0005_HL3KCCCXY_L1_clean_bin.30.orig.fa_	OEZ008529	yes	LMSG_G000005221.1	OES073749	betalactone	Others	Polymetallic	11.33	China: Guizhou, Zhijin
L180322-P-2-LD180319-0005_HL3KCCCXY_L1_clean_bin.30.orig.fa_	OEZ008529	yes	LMSG_G000005221.1	OES073749	terpene	Terpene	Polymetallic	11.56	China: Guizhou, Zhijin
L180322-P-2-LD180319-0005_HL3KCCCXY_L1_clean_bin.30.orig.fa_	OEZ008529	yes	LMSG_G000005221.1	OES073749	terpene	Terpene	Polymetallic	6.09	China: Guizhou, Zhijin
L180322-P-2-LD180319-0005_HL3KCCCXY_L1_clean_bin.30.orig.fa_	OEZ008529	yes	LMSG_G000005221.1	OES073749	betalactone	Others	Polymetallic	5.86	China: Guizhou, Zhijin
L180322-P-2-LD180319-0005_HL3KCCCXY_L1_clean_bin.31.orig.fa_	OEZ008529	yes	LMSG_G000010661.1	OES073749	NRPS.T1PKS	PKS-NRP_Hybrids	Polymetallic	10.77	China: Guizhou, Zhijin
L180322-P-2-LD180319-0005_HL3KCCCXY_L1_clean_bin.31.orig.fa_	OEZ008529	yes	LMSG_G000010661.1	OES073749	NRPS-like	NRPS	Polymetallic	5.06	China: Guizhou, Zhijin
L180322-P-2-LD180319-0005_HL3KCCCXY_L1_clean_bin.33.orig.fa_	OEZ008529	yes	LMSG_G000008572.1	OES073749	NRPS-like	NRPS	Polymetallic	26.72	China: Guizhou, Zhijin
L180322-P-2-LD180319-0005_HL3KCCCXY_L1_clean_bin.36.strict.f	OEZ008529	yes	LMSG_G000011104.1	OES073749	CDPS.bacteriocin	Others	Polymetallic	15.77	China: Guizhou, Zhijin
L180322-P-2-LD180319-0005_HL3KCCCXY_L1_clean_bin.36.strict.f	OEZ008529	yes	LMSG_G000011104.1	OES073749	terpene	Terpene	Polymetallic	6.15	China: Guizhou, Zhijin
L180322-P-2-LD180319-0005_HL3KCCCXY_L1_clean_bin.37.orig.fa_	OEZ008529	yes	LMSG_G000010141.1	OES073749	terpene	Terpene	Polymetallic	11.72	China: Guizhou, Zhijin
L180322-P-2-LD180319-0005_HL3KCCCXY_L1_clean_bin.37.orig.fa_	OEZ008529	yes	LMSG_G000010141.1	OES073749	T3PKS	PKSother	Polymetallic	30.09	China: Guizhou, Zhijin
L180322-P-2-LD180319-0005_HL3KCCCXY_L1_clean_bin.37.orig.fa_	OEZ008529	yes	LMSG_G000010141.1	OES073749	terpene	Terpene	Polymetallic	10.18	China: Guizhou, Zhijin
L180322-P-2-LD180319-0005_HL3KCCCXY_L1_clean_bin.38.strict.f	OEZ008529	yes	LMSG_G000008611.1	OES073749	other	Others	Polymetallic	39.30	China: Guizhou, Zhijin
L180322-P-2-LD180319-0005_HL3KCCCXY_L1_clean_bin.38.strict.f	OEZ008529	yes	LMSG_G000008611.1	OES073749	other	Others	Polymetallic	9.34	China: Guizhou, Zhijin
L180322-P-2-LD180319-0005_HL3KCCCXY_L1_clean_bin.39.permissi	OEZ008529	yes	LMSG_G000008980.1	OES073749	betalactone	Others	Polymetallic	21.10	China: Guizhou, Zhijin
L180322-P-2-LD180319-0005_HL3KCCCXY_L1_clean_bin.39.permissi	OEZ008529	yes	LMSG_G000008980.1	OES073749	bacteriocin	RiPPs	Polymetallic	10.90	China: Guizhou, Zhijin
L180322-P-2-LD180319-0005_HL3KCCCXY_L1_clean_bin.39.permissi	OEZ008529	yes	LMSG_G000008980.1	OES073749	T1PKS	PKSI	Polymetallic	11.10	China: Guizhou, Zhijin
L180322-P-2-LD180319-0005_HL3KCCCXY_L1_clean_bin.4.orig.fa_k	OEZ008529	yes	LMSG_G000011024.1	OES073749	resorcinol.arylpolyene	Others	Polymetallic	47.51	China: Guizhou, Zhijin
L180322-P-2-LD180319-0005_HL3KCCCXY_L1_clean_bin.4.orig.fa_k	OEZ008529	yes	LMSG_G000011024.1	OES073749	terpene	Terpene	Polymetallic	6.03	China: Guizhou, Zhijin
L180322-P-2-LD180319-0005_HL3KCCCXY_L1_clean_bin.41.orig.fa_	OEZ008529	yes	LMSG_G000005222.1	OES073749	NRPS-like	NRPS	Polymetallic	36.79	China: Guizhou, Zhijin
L180322-P-2-LD180319-0005_HL3KCCCXY_L1_clean_bin.41.orig.fa_	OEZ008529	yes	LMSG_G000005222.1	OES073749	bacteriocin	RiPPs	Polymetallic	10.81	China: Guizhou, Zhijin
L180322-P-2-LD180319-0005_HL3KCCCXY_L1_clean_bin.41.orig.fa_	OEZ008529	yes	LMSG_G000005222.1	OES073749	terpene	Terpene	Polymetallic	16.28	China: Guizhou, Zhijin
L180322-P-2-LD180319-0005_HL3KCCCXY_L1_clean_bin.43.orig.fa_	OEZ008529	yes	LMSG_G000011318.1	OES073749	terpene	Terpene	Polymetallic	7.49	China: Guizhou, Zhijin
L180322-P-2-LD180319-0005_HL3KCCCXY_L1_clean_bin.43.orig.fa_	OEZ008529	yes	LMSG_G000011318.1	OES073749	terpene	Terpene	Polymetallic	19.95	China: Guizhou, Zhijin
L180322-P-2-LD180319-0005_HL3KCCCXY_L1_clean_bin.43.orig.fa_	OEZ008529	yes	LMSG_G000011318.1	OES073749	terpene	Terpene	Polymetallic	21.09	China: Guizhou, Zhijin

L180322-P-2-LD180319-0005_HL3KCCCXY_L1_clean_bin.43.orig.fa_	OEZ008529	yes	LMSG_G000011318.1	OES073749	ladderane	Others	Polymetallic	41.24	China: Guizhou, Zhijin
L180322-P-2-LD180319-0005_HL3KCCCXY_L1_clean_bin.43.orig.fa_	OEZ008529	yes	LMSG_G000011318.1	OES073749	NRPS	NRPS	Polymetallic	21.70	China: Guizhou, Zhijin
L180322-P-2-LD180319-0005_HL3KCCCXY_L1_clean_bin.45.orig.fa_	OEZ008529	yes	LMSG_G000010770.1	OES073749	bacteriocin	RiPPs	Polymetallic	9.22	China: Guizhou, Zhijin
L180322-P-2-LD180319-0005_HL3KCCCXY_L1_clean_bin.46.orig.fa_	OEZ008529	yes	LMSG_G000007797.1	OES073749	terpene	Terpene	Polymetallic	11.43	China: Guizhou, Zhijin
L180322-P-2-LD180319-0005_HL3KCCCXY_L1_clean_bin.46.orig.fa_	OEZ008529	yes	LMSG_G000007797.1	OES073749	terpene	Terpene	Polymetallic	8.25	China: Guizhou, Zhijin
L180322-P-2-LD180319-0005_HL3KCCCXY_L1_clean_bin.46.orig.fa_	OEZ008529	yes	LMSG_G000007797.1	OES073749	T3PKS	PKSother	Polymetallic	24.34	China: Guizhou, Zhijin
L180322-P-2-LD180319-0005_HL3KCCCXY_L1_clean_bin.48.orig.fa_	OEZ008529	yes	LMSG_G000005223.1	OES073749	other	Others	Polymetallic	40.46	China: Guizhou, Zhijin
L180322-P-2-LD180319-0005_HL3KCCCXY_L1_clean_bin.49.orig.fa_	OEZ008529	yes	LMSG_G000010221.1	OES073749	betalactone	Others	Polymetallic	31.27	China: Guizhou, Zhijin
L180322-P-2-LD180319-0005_HL3KCCCXY_L1_clean_bin.49.orig.fa_	OEZ008529	yes	LMSG_G000010221.1	OES073749	terpene	Terpene	Polymetallic	21.04	China: Guizhou, Zhijin
L180322-P-2-LD180319-0005_HL3KCCCXY_L1_clean_bin.49.orig.fa_	OEZ008529	yes	LMSG_G000010221.1	OES073749	terpene	Terpene	Polymetallic	24.90	China: Guizhou, Zhijin
L180322-P-2-LD180319-0005_HL3KCCCXY_L1_clean_bin.5.orig.fa_k	OEZ008529	yes	LMSG_G000005226.1	OES073749	betalactone.terpene	Others	Polymetallic	55.37	China: Guizhou, Zhijin
L180322-P-2-LD180319-0005_HL3KCCCXY_L1_clean_bin.51.orig.fa_	OEZ008529	yes	LMSG_G000007868.1	OES073749	terpene	Terpene	Polymetallic	6.72	China: Guizhou, Zhijin
L180322-P-2-LD180319-0005_HL3KCCCXY_L1_clean_bin.51.orig.fa_	OEZ008529	yes	LMSG_G000007868.1	OES073749	terpene	Terpene	Polymetallic	5.48	China: Guizhou, Zhijin
L180322-P-2-LD180319-0005_HL3KCCCXY_L1_clean_bin.51.orig.fa_	OEZ008529	yes	LMSG_G000007868.1	OES073749	NRPS-like	NRPS	Polymetallic	8.22	China: Guizhou, Zhijin
L180322-P-2-LD180319-0005_HL3KCCCXY_L1_clean_bin.52.orig.fa_	OEZ008529	yes	LMSG_G000008454.1	OES073749	betalactone	Others	Polymetallic	23.97	China: Guizhou, Zhijin
L180322-P-2-LD180319-0005_HL3KCCCXY_L1_clean_bin.52.orig.fa_	OEZ008529	yes	LMSG_G000008454.1	OES073749	terpene	Terpene	Polymetallic	21.06	China: Guizhou, Zhijin
L180322-P-2-LD180319-0005_HL3KCCCXY_L1_clean_bin.54.orig.fa_	OEZ008529	yes	LMSG_G000005224.1	OES073749	terpene	Terpene	Polymetallic	10.34	China: Guizhou, Zhijin
L180322-P-2-LD180319-0005_HL3KCCCXY_L1_clean_bin.54.orig.fa_	OEZ008529	yes	LMSG_G000005224.1	OES073749	terpene	Terpene	Polymetallic	5.08	China: Guizhou, Zhijin
L180322-P-2-LD180319-0005_HL3KCCCXY_L1_clean_bin.54.orig.fa_	OEZ008529	yes	LMSG_G000005224.1	OES073749	terpene	Terpene	Polymetallic	11.90	China: Guizhou, Zhijin
L180322-P-2-LD180319-0005_HL3KCCCXY_L1_clean_bin.54.orig.fa_	OEZ008529	yes	LMSG_G000005224.1	OES073749	T3PKS	PKSother	Polymetallic	7.85	China: Guizhou, Zhijin
L180322-P-2-LD180319-0005_HL3KCCCXY_L1_clean_bin.54.orig.fa_	OEZ008529	yes	LMSG_G000005224.1	OES073749	arylpolyene	Others	Polymetallic	8.64	China: Guizhou, Zhijin
L180322-P-2-LD180319-0005_HL3KCCCXY_L1_clean_bin.55.orig.fa_	OEZ008529	yes	LMSG_G000005225.1	OES073749	T1PKS	PKSI	Polymetallic	12.18	China: Guizhou, Zhijin
L180322-P-2-LD180319-0005_HL3KCCCXY_L1_clean_bin.55.orig.fa_	OEZ008529	yes	LMSG_G000005225.1	OES073749	terpene	Terpene	Polymetallic	17.34	China: Guizhou, Zhijin
L180322-P-2-LD180319-0005_HL3KCCCXY_L1_clean_bin.55.orig.fa_	OEZ008529	yes	LMSG_G000005225.1	OES073749	terpene	Terpene	Polymetallic	12.00	China: Guizhou, Zhijin
L180322-P-2-LD180319-0005_HL3KCCCXY_L1_clean_bin.55.orig.fa_	OEZ008529	yes	LMSG_G000005225.1	OES073749	acyl_amino_acids	Others	Polymetallic	27.53	China: Guizhou, Zhijin
L180322-P-2-LD180319-0005_HL3KCCCXY_L1_clean_bin.55.orig.fa_	OEZ008529	yes	LMSG_G000005225.1	OES073749	terpene	Terpene	Polymetallic	10.26	China: Guizhou, Zhijin
L180322-P-2-LD180319-0005_HL3KCCCXY_L1_clean_bin.56.strict.f	OEZ008529	yes	LMSG_G000008153.1	OES073749	terpene	Terpene	Polymetallic	16.02	China: Guizhou, Zhijin
L180322-P-2-LD180319-0005_HL3KCCCXY_L1_clean_bin.57.orig.fa_	OEZ008529	yes	LMSG_G000010198.1	OES073749	terpene	Terpene	Polymetallic	17.33	China: Guizhou, Zhijin
L180322-P-2-LD180319-0005_HL3KCCCXY_L1_clean_bin.57.orig.fa_	OEZ008529	yes	LMSG_G000010198.1	OES073749	arylpolyene	Others	Polymetallic	29.49	China: Guizhou, Zhijin
L180322-P-2-LD180319-0005_HL3KCCCXY_L1_clean_bin.57.orig.fa_	OEZ008529	yes	LMSG_G000010198.1	OES073749	terpene	Terpene	Polymetallic	21.91	China: Guizhou, Zhijin
L180322-P-2-LD180319-0005_HL3KCCCXY_L1_clean_bin.57.orig.fa_	OEZ008529	yes	LMSG_G000010198.1	OES073749	betalactone	Others	Polymetallic	11.72	China: Guizhou, Zhijin
L180322-P-2-LD180319-0005_HL3KCCCXY_L1_clean_bin.58.permissi	OEZ008529	yes	LMSG_G000009627.1	OES073749	terpene	Terpene	Polymetallic	23.33	China: Guizhou, Zhijin
L180322-P-2-LD180319-0005_HL3KCCCXY_L1_clean_bin.58.permissi	OEZ008529	yes	LMSG_G000009627.1	OES073749	NRPS-like	NRPS	Polymetallic	42.63	China: Guizhou, Zhijin
L180322-P-2-LD180319-0005_HL3KCCCXY_L1_clean_bin.58.permissi	OEZ008529	yes	LMSG_G000009627.1	OES073749	terpene	Terpene	Polymetallic	22.09	China: Guizhou, Zhijin
L180322-P-2-LD180319-0005_HL3KCCCXY_L1_clean_bin.58.permissi	OEZ008529	yes	LMSG_G000009627.1	OES073749	nucleoside	Others	Polymetallic	20.95	China: Guizhou, Zhijin
L180322-P-2-LD180319-0005_HL3KCCCXY_L1_clean_bin.59.orig.fa_	OEZ008529	yes	LMSG_G000010213.1	OES073749	arylpolyene	Others	Polymetallic	38.75	China: Guizhou, Zhijin
L180322-P-2-LD180319-0005_HL3KCCCXY_L1_clean_bin.6.orig.fa_k	OEZ008529	yes	LMSG_G000008429.1	OES073749	terpene	Terpene	Polymetallic	26.20	China: Guizhou, Zhijin
L180322-P-2-LD180319-0005_HL3KCCCXY_L1_clean_bin.6.orig.fa_k	OEZ008529	yes	LMSG_G000008429.1	OES073749	NRPS.betalactone	Others	Polymetallic	51.60	China: Guizhou, Zhijin
L180322-P-2-LD180319-0005_HL3KCCCXY_L1_clean_bin.6.orig.fa_k	OEZ008529	yes	LMSG_G000008429.1	OES073749	T3PKS	PKSother	Polymetallic	38.51	China: Guizhou, Zhijin
L180322-P-2-LD180319-0005_HL3KCCCXY_L1_clean_bin.6.orig.fa_k	OEZ008529	yes	LMSG_G000008429.1	OES073749	NRPS	NRPS	Polymetallic	29.03	China: Guizhou, Zhijin
L180322-P-2-LD180319-0005_HL3KCCCXY_L1_clean_bin.6.orig.fa_k	OEZ008529	yes	LMSG_G000008429.1	OES073749	other	Others	Polymetallic	39.51	China: Guizhou, Zhijin
L180322-P-2-LD180319-0005_HL3KCCCXY_L1_clean_bin.6.orig.fa_k	OEZ008529	yes	LMSG_G000008429.1	OES073749	terpene	Terpene	Polymetallic	23.47	China: Guizhou, Zhijin
L180322-P-2-LD180319-0005_HL3KCCCXY_L1_clean_bin.61.orig.fa_	OEZ008529	yes	LMSG_G000005227.1	OES073749	other	Others	Polymetallic	26.84	China: Guizhou, Zhijin
L180322-P-2-LD180319-0005_HL3KCCCXY_L1_clean_bin.62.orig.fa_	OEZ008529	yes	LMSG_G000010701.1	OES073749	terpene	Terpene	Polymetallic	9.54	China: Guizhou, Zhijin
L180322-P-2-LD180319-0005_HL3KCCCXY_L1_clean_bin.62.orig.fa_	OEZ008529	yes	LMSG_G000010701.1	OES073749	NRPS	NRPS	Polymetallic	8.46	China: Guizhou, Zhijin
L180322-P-2-LD180319-0005_HL3KCCCXY_L1_clean_bin.62.orig.fa_	OEZ008529	yes	LMSG_G000010701.1	OES073749	bacteriocin	RiPPs	Polymetallic	8.58	China: Guizhou, Zhijin
L180322-P-2-LD180319-0005_HL3KCCCXY_L1_clean_bin.62.orig.fa_	OEZ008529	yes	LMSG_G000010701.1	OES073749	arylpolyene	Others	Polymetallic	10.41	China: Guizhou, Zhijin
L180322-P-2-LD180319-0005_HL3KCCCXY_L1_clean_bin.65.orig.fa_	OEZ008529	yes	LMSG_G000007783.1	OES073749	T3PKS	PKSother	Polymetallic	28.58	China: Guizhou, Zhijin
L180322-P-2-LD180319-0005_HL3KCCCXY_L1_clean_bin.65.orig.fa_	OEZ008529	yes	LMSG_G000007783.1	OES073749	bacteriocin	RiPPs	Polymetallic	10.88	China: Guizhou, Zhijin
L180322-P-2-LD180319-0005_HL3KCCCXY_L1_clean_bin.65.orig.fa_	OEZ008529	yes	LMSG_G000007783.1	OES073749	terpene	Terpene	Polymetallic	30.32	China: Guizhou, Zhijin
L180322-P-2-LD180319-0005_HL3KCCCXY_L1_clean_bin.68.orig.fa_	OEZ008529	yes	LMSG_G000010095.1	OES073749	T1PKS	PKSI	Polymetallic	25.24	China: Guizhou, Zhijin
L180322-P-2-LD180319-0005_HL3KCCCXY_L1_clean_bin.68.orig.fa_	OEZ008529	yes	LMSG_G000010095.1	OES073749	terpene	Terpene	Polymetallic	12.31	China: Guizhou, Zhijin
L180322-P-2-LD180319-0005_HL3KCCCXY_L1_clean_bin.68.orig.fa_	OEZ008529	yes	LMSG_G000010095.1	OES073749	cyanobactin	RiPPs	Polymetallic	10.31	China: Guizhou, Zhijin
L180322-P-2-LD180319-0005_HL3KCCCXY_L1_clean_bin.68.orig.fa_	OEZ008529	yes	LMSG_G000010095.1	OES073749	hglE-KS	PKSother	Polymetallic	10.43	China: Guizhou, Zhijin
L180322-P-2-LD180319-0005_HL3KCCCXY_L1_clean_bin.68.orig.fa_	OEZ008529	yes	LMSG_G000010095.1	OES073749	T3PKS	PKSother	Polymetallic	11.88	China: Guizhou, Zhijin
L180322-P-2-LD180319-0005_HL3KCCCXY_L1_clean_bin.68.orig.fa_	OEZ008529	yes	LMSG_G000010095.1	OES073749	T1PKS.hglE-KS	PKSother	Polymetallic	17.29	China: Guizhou, Zhijin
L180322-P-2-LD180319-0005_HL3KCCCXY_L1_clean_bin.68.orig.fa_	OEZ008529	yes	LMSG_G000010095.1	OES073749	terpene	Terpene	Polymetallic	11.79	China: Guizhou, Zhijin
L180322-P-2-LD180319-0005_HL3KCCCXY_L1_clean_bin.68.orig.fa_	OEZ008529	yes	LMSG_G000010095.1	OES073749	T1PKS	PKSI	Polymetallic	9.23	China: Guizhou, Zhijin
L180322-P-2-LD180319-0005_HL3KCCCXY_L1_clean_bin.7.strict.fa	OEZ008529	yes	LMSG_G000008186.1	OES073749	terpene	Terpene	Polymetallic	20.86	China: Guizhou, Zhijin
L180322-P-2-LD180319-0005_HL3KCCCXY_L1_clean_bin.7.strict.fa	OEZ008529	yes	LMSG_G000008186.1	OES073749	betalactone	Others	Polymetallic	31.32	China: Guizhou, Zhijin
L180322-P-2-LD180319-0005_HL3KCCCXY_L1_clean_bin.8.strict.fa	OEZ008529	yes	LMSG_G000008202.1	OES073749	terpene	Terpene	Polymetallic	20.86	China: Guizhou, Zhijin
L180322-P-2-LD180319-0006_HL3KCCCXY_L1_clean_bin.1.orig.fa_k	OEZ008529	yes	LMSG_G000010189.1	OES073750	betalactone.terpene	Others	Polymetallic	45.70	China: Guizhou, Zhijin
L180322-P-2-LD180319-0006_HL3KCCCXY_L1_clean_bin.10.orig.fa_	OEZ008529	yes	LMSG_G000010702.1	OES073750	NRPS	NRPS	Polymetallic	23.39	China: Guizhou, Zhijin
L180322-P-2-LD180319-0006_HL3KCCCXY_L1_clean_bin.10.orig.fa_	OEZ008529	yes	LMSG_G000010702.1	OES073750	arylpolyene	Others	Polymetallic	41.29	China: Guizhou, Zhijin
L180322-P-2-LD180319-0006_HL3KCCCXY_L1_clean_bin.10.orig.fa_	OEZ008529	yes	LMSG_G000010702.1	OES073750	terpene	Terpene	Polymetallic	15.29	China: Guizhou, Zhijin
L180322-P-2-LD180319-0006_HL3KCCCXY_L1_clean_bin.10.orig.fa_	OEZ008529	yes	LMSG_G000010702.1	OES073750	NRPS.T1PKS	PKS-NRP_Hybrids	Polymetallic	27.00	China: Guizhou, Zhijin
L180322-P-2-LD180319-0006_HL3KCCCXY_L1_clean_bin.10.orig.fa_	OEZ008529	yes	LMSG_G000010702.1	OES073750	betalactone	Others	Polymetallic	15.86	China: Guizhou, Zhijin
L180322-P-2-LD180319-0006_HL3KCCCXY_L1_clean_bin.11.strict.f	OEZ008529	yes	LMSG_G000008182.1	OES073750	terpene	Terpene	Polymetallic	20.86	China: Guizhou, Zhijin
L180322-P-2-LD180319-0006_HL3KCCCXY_L1_clean_bin.11.strict.f	OEZ008529	yes	LMSG_G000008182.1	OES073750	betalactone	Others	Polymetallic	31.32	China: Guizhou, Zhijin
L180322-P-2-LD180319-0006_HL3KCCCXY_L1_clean_bin.12.orig.fa_	OEZ008529	yes	LMSG_G000009983.1	OES073750	NRPS-like	NRPS	Polymetallic	5.90	China: Guizhou, Zhijin
L180322-P-2-LD180319-0006_HL3KCCCXY_L1_clean_bin.12.orig.fa_	OEZ008529	yes	LMSG_G000009983.1	OES073750	terpene	Terpene	Polymetallic	20.53	China: Guizhou, Zhijin
L180322-P-2-LD180319-0006_HL3KCCCXY_L1_clean_bin.12.orig.fa_	OEZ008529	yes	LMSG_G000009983.1	OES073750	T3PKS	PKSother	Polymetallic	9.16	China: Guizhou, Zhijin
L180322-P-2-LD180319-0006_HL3KCCCXY_L1_clean_bin.12.orig.fa_	OEZ008529	yes	LMSG_G000009983.1	OES073750	T3PKS	PKSother	Polymetallic	22.19	China: Guizhou, Zhijin
L180322-P-2-LD180319-0006_HL3KCCCXY_L1_clean_bin.12.orig.fa_	OEZ008529	yes	LMSG_G000009983.1	OES073750	terpene	Terpene	Polymetallic	12.76	China: Guizhou, Zhijin
L180322-P-2-LD180319-0006_HL3KCCCXY_L1_clean_bin.12.orig.fa_	OEZ008529	yes	LMSG_G000009983.1	OES073750	arylpolyene	Others	Polymetallic	21.85	China: Guizhou, Zhijin
L180322-P-2-LD180319-0006_HL3KCCCXY_L1_clean_bin.18.orig.fa_	OEZ008529	yes	LMSG_G000008194.1	OES073750	terpene	Terpene	Polymetallic	20.86	China: Guizhou, Zhijin
L180322-P-2-LD180319-0006_HL3KCCCXY_L1_clean_bin.2.orig.fa_k	OEZ008529	no	LMSG_G000011013.1	OES073750	thiopeptide	RiPPs	Polymetallic	33.00	China: Guizhou, Zhijin
L180322-P-2-LD180319-0006_HL3KCCCXY_L1_clean_bin.2.orig.fa_k	OEZ008529	no	LMSG_G000011013.1	OES073750	NRPS	NRPS	Polymetallic	53.04	China: Guizhou, Zhijin
L180322-P-2-LD180319-0006_HL3KCCCXY_L1_clean_bin.2.orig.fa_k	OEZ008529	no	LMSG_G000011013.1	OES073750	phenazine	Others	Polymetallic	10.23	China: Guizhou, Zhijin
L180322-P-2-LD180319-0006_HL3KCCCXY_L1_clean_bin.2.orig.fa_k	OEZ008529	no	LMSG_G000011013.1	OES073750	phenazine	Others	Polymetallic	10.23	China: Guizhou, Zhijin
L180322-P-2-LD180319-0006_HL3KCCCXY_L1_clean_bin.2.orig.fa_k	OEZ008529	no	LMSG_G000011013.1	OES073750	hserlactone	Others	Polymetallic	20.61	China: Guizhou, Zhijin
L180322-P-2-LD180319-0006_HL3KCCCXY_L1_clean_bin.2.orig.fa_k	OEZ008529	no	LMSG_G000011013.1	OES073750	NAGGN	Others	Polymetallic	14.76	China: Guizhou, Zhijin
L180322-P-2-LD180319-0006_HL3KCCCXY_L1_clean_bin.2.orig.fa_k	OEZ008529	no	LMSG_G000011013.1	OES073750	NRPS	NRPS	Polymetallic	49.55	China: Guizhou, Zhijin
L180322-P-2-LD180319-0006_HL3KCCCXY_L1_clean_bin.2.orig.fa_k	OEZ008529	no	LMSG_G000011013.1	OES073750	bacteriocin	RiPPs	Polymetallic	10.86	China: Guizhou, Zhijin
L180322-P-2-LD180319-0006_HL3KCCCXY_L1_clean_bin.2.orig.fa_k	OEZ008529	no	LMSG_G000011013.1	OES073750	NRPS	NRPS	Polymetallic	44.23	China: Guizhou, Zhijin
L180322-P-2-LD180319-0006_HL3KCCCXY_L1_clean_bin.2.orig.fa_k	OEZ008529	no	LMSG_G000011013.1	OES073750	hserlactone	Others	Polymetallic	20.61	China: Guizhou, Zhijin
L180322-P-2-LD180319-0006_HL3KCCCXY_L1_clean_bin.2.orig.fa_k	OEZ008529	no	LMSG_G000011013.1	OES073750	betalactone.NRPS-like	Others	Polymetallic	41.86	China: Guizhou, Zhijin
L180322-P-2-LD180319-0006_HL3KCCCXY_L1_clean_bin.2.orig.fa_k	OEZ008529	no	LMSG_G000011013.1	OES073750	bacteriocin	RiPPs	Polymetallic	10.83	China: Guizhou, Zhijin
L180322-P-2-LD180319-0006_HL3KCCCXY_L1_clean_bin.2.orig.fa_k	OEZ008529	no	LMSG_G000011013.1	OES073750	NRPS-like	NRPS	Polymetallic	42.98	China: Guizhou, Zhijin
L180322-P-2-LD180319-0006_HL3KCCCXY_L1_clean_bin.2.orig.fa_k	OEZ008529	no	LMSG_G000011013.1	OES073750	NRPS	NRPS	Polymetallic	6.21	China: Guizhou, Zhijin
L180322-P-2-LD180319-0006_HL3KCCCXY_L1_clean_bin.2.orig.fa_k	OEZ008529	no	LMSG_G000011013.1	OES073750	NRPS	NRPS	Polymetallic	5.44	China: Guizhou, Zhijin
L180322-P-2-LD180319-0006_HL3KCCCXY_L1_clean_bin.2.orig.fa_k	OEZ008529	no	LMSG_G000011013.1	OES073750	NRPS	NRPS	Polymetallic	42.58	China: Guizhou, Zhijin
L180322-P-2-LD180319-0006_HL3KCCCXY_L1_clean_bin.20.permissi	OEZ008529	yes	LMSG_G000011319.1	OES073750	terpene	Terpene	Polymetallic	16.40	China: Guizhou, Zhijin
L180322-P-2-LD180319-0006_HL3KCCCXY_L1_clean_bin.20.permissi	OEZ008529	yes	LMSG_G000011319.1	OES073750	terpene	Terpene	Polymetallic	21.95	China: Guizhou, Zhijin
L180322-P-2-LD180319-0006_HL3KCCCXY_L1_clean_bin.20.permissi	OEZ008529	yes	LMSG_G000011319.1	OES073750	terpene	Terpene	Polymetallic	17.83	China: Guizhou, Zhijin
L180322-P-2-LD180319-0006_HL3KCCCXY_L1_clean_bin.20.permissi	OEZ008529	yes	LMSG_G000011319.1	OES073750	NRPS	NRPS	Polymetallic	12.89	China: Guizhou, Zhijin
L180322-P-2-LD180319-0006_HL3KCCCXY_L1_clean_bin.22.orig.fa_	OEZ008529	no	LMSG_G000010178.1	OES073750	ectoine	Others	Polymetallic	6.76	China: Guizhou, Zhijin
L180322-P-2-LD180319-0006_HL3KCCCXY_L1_clean_bin.23.orig.fa_	OEZ008529	yes	LMSG_G000007784.1	OES073750	T3PKS	PKSother	Polymetallic	21.92	China: Guizhou, Zhijin
L180322-P-2-LD180319-0006_HL3KCCCXY_L1_clean_bin.23.orig.fa_	OEZ008529	yes	LMSG_G000007784.1	OES073750	bacteriocin	RiPPs	Polymetallic	10.88	China: Guizhou, Zhijin
L180322-P-2-LD180319-0006_HL3KCCCXY_L1_clean_bin.23.orig.fa_	OEZ008529	yes	LMSG_G000007784.1	OES073750	terpene	Terpene	Polymetallic	27.94	China: Guizhou, Zhijin
L180322-P-2-LD180319-0006_HL3KCCCXY_L1_clean_bin.24.orig.fa_	OEZ008529	yes	LMSG_G000005230.1	OES073750	NRPS-like	NRPS	Polymetallic	9.25	China: Guizhou, Zhijin
L180322-P-2-LD180319-0006_HL3KCCCXY_L1_clean_bin.24.orig.fa_	OEZ008529	yes	LMSG_G000005230.1	OES073750	NRPS.T1PKS	PKS-NRP_Hybrids	Polymetallic	14.72	China: Guizhou, Zhijin
L180322-P-2-LD180319-0006_HL3KCCCXY_L1_clean_bin.24.orig.fa_	OEZ008529	yes	LMSG_G000005230.1	OES073750	NRPS-like	NRPS	Polymetallic	5.56	China: Guizhou, Zhijin
L180322-P-2-LD180319-0006_HL3KCCCXY_L1_clean_bin.24.orig.fa_	OEZ008529	yes	LMSG_G000005230.1	OES073750	terpene	Terpene	Polymetallic	11.18	China: Guizhou, Zhijin
L180322-P-2-LD180319-0006_HL3KCCCXY_L1_clean_bin.24.orig.fa_	OEZ008529	yes	LMSG_G000005230.1	OES073750	arylpolyene	Others	Polymetallic	5.73	China: Guizhou, Zhijin
L180322-P-2-LD180319-0006_HL3KCCCXY_L1_clean_bin.24.orig.fa_	OEZ008529	yes	LMSG_G000005230.1	OES073750	NRPS	NRPS	Polymetallic	9.96	China: Guizhou, Zhijin
L180322-P-2-LD180319-0006_HL3KCCCXY_L1_clean_bin.24.orig.fa_	OEZ008529	yes	LMSG_G000005230.1	OES073750	NRPS	NRPS	Polymetallic	7.27	China: Guizhou, Zhijin
L180322-P-2-LD180319-0006_HL3KCCCXY_L1_clean_bin.25.strict.f	OEZ008529	yes	LMSG_G000008203.1	OES073750	terpene	Terpene	Polymetallic	20.86	China: Guizhou, Zhijin
L180322-P-2-LD180319-0006_HL3KCCCXY_L1_clean_bin.26.orig.fa_	OEZ008529	yes	LMSG_G000010222.1	OES073750	betalactone	Others	Polymetallic	31.27	China: Guizhou, Zhijin
L180322-P-2-LD180319-0006_HL3KCCCXY_L1_clean_bin.26.orig.fa_	OEZ008529	yes	LMSG_G000010222.1	OES073750	terpene	Terpene	Polymetallic	19.85	China: Guizhou, Zhijin
L180322-P-2-LD180319-0006_HL3KCCCXY_L1_clean_bin.26.orig.fa_	OEZ008529	yes	LMSG_G000010222.1	OES073750	terpene	Terpene	Polymetallic	23.34	China: Guizhou, Zhijin
L180322-P-2-LD180319-0006_HL3KCCCXY_L1_clean_bin.28.orig.fa_	OEZ008529	yes	LMSG_G000010112.1	OES073750	hserlactone	Others	Polymetallic	20.67	China: Guizhou, Zhijin
L180322-P-2-LD180319-0006_HL3KCCCXY_L1_clean_bin.28.orig.fa_	OEZ008529	yes	LMSG_G000010112.1	OES073750	terpene	Terpene	Polymetallic	14.60	China: Guizhou, Zhijin
L180322-P-2-LD180319-0006_HL3KCCCXY_L1_clean_bin.28.orig.fa_	OEZ008529	yes	LMSG_G000010112.1	OES073750	terpene	Terpene	Polymetallic	22.14	China: Guizhou, Zhijin
L180322-P-2-LD180319-0006_HL3KCCCXY_L1_clean_bin.28.orig.fa_	OEZ008529	yes	LMSG_G000010112.1	OES073750	terpene	Terpene	Polymetallic	20.63	China: Guizhou, Zhijin
L180322-P-2-LD180319-0006_HL3KCCCXY_L1_clean_bin.28.orig.fa_	OEZ008529	yes	LMSG_G000010112.1	OES073750	T3PKS	PKSother	Polymetallic	41.08	China: Guizhou, Zhijin
L180322-P-2-LD180319-0006_HL3KCCCXY_L1_clean_bin.3.orig.fa_k	OEZ008529	yes	LMSG_G000010142.1	OES073750	terpene	Terpene	Polymetallic	11.72	China: Guizhou, Zhijin
L180322-P-2-LD180319-0006_HL3KCCCXY_L1_clean_bin.3.orig.fa_k	OEZ008529	yes	LMSG_G000010142.1	OES073750	T3PKS	PKSother	Polymetallic	31.31	China: Guizhou, Zhijin
L180322-P-2-LD180319-0006_HL3KCCCXY_L1_clean_bin.3.orig.fa_k	OEZ008529	yes	LMSG_G000010142.1	OES073750	terpene	Terpene	Polymetallic	10.03	China: Guizhou, Zhijin
L180322-P-2-LD180319-0006_HL3KCCCXY_L1_clean_bin.30.orig.fa_	OEZ008529	yes	LMSG_G000010098.1	OES073750	terpene	Terpene	Polymetallic	5.73	China: Guizhou, Zhijin
L180322-P-2-LD180319-0006_HL3KCCCXY_L1_clean_bin.30.orig.fa_	OEZ008529	yes	LMSG_G000010098.1	OES073750	NRPS	NRPS	Polymetallic	5.58	China: Guizhou, Zhijin
L180322-P-2-LD180319-0006_HL3KCCCXY_L1_clean_bin.30.orig.fa_	OEZ008529	yes	LMSG_G000010098.1	OES073750	NRPS	NRPS	Polymetallic	9.13	China: Guizhou, Zhijin
L180322-P-2-LD180319-0006_HL3KCCCXY_L1_clean_bin.30.orig.fa_	OEZ008529	yes	LMSG_G000010098.1	OES073750	T1PKS.hglE-KS	PKSother	Polymetallic	6.89	China: Guizhou, Zhijin
L180322-P-2-LD180319-0006_HL3KCCCXY_L1_clean_bin.31.orig.fa_	OEZ008529	yes	LMSG_G000007792.1	OES073750	terpene	Terpene	Polymetallic	6.35	China: Guizhou, Zhijin
L180322-P-2-LD180319-0006_HL3KCCCXY_L1_clean_bin.31.orig.fa_	OEZ008529	yes	LMSG_G000007792.1	OES073750	T3PKS	PKSother	Polymetallic	7.13	China: Guizhou, Zhijin
L180322-P-2-LD180319-0006_HL3KCCCXY_L1_clean_bin.32.permissi	OEZ008529	yes	LMSG_G000010077.1	OES073750	NRPS	NRPS	Polymetallic	26.57	China: Guizhou, Zhijin
L180322-P-2-LD180319-0006_HL3KCCCXY_L1_clean_bin.32.permissi	OEZ008529	yes	LMSG_G000010077.1	OES073750	terpene	Terpene	Polymetallic	12.59	China: Guizhou, Zhijin
L180322-P-2-LD180319-0006_HL3KCCCXY_L1_clean_bin.32.permissi	OEZ008529	yes	LMSG_G000010077.1	OES073750	terpene	Terpene	Polymetallic	13.25	China: Guizhou, Zhijin
L180322-P-2-LD180319-0006_HL3KCCCXY_L1_clean_bin.32.permissi	OEZ008529	yes	LMSG_G000010077.1	OES073750	hglE-KS	PKSother	Polymetallic	14.68	China: Guizhou, Zhijin
L180322-P-2-LD180319-0006_HL3KCCCXY_L1_clean_bin.32.permissi	OEZ008529	yes	LMSG_G000010077.1	OES073750	terpene	Terpene	Polymetallic	12.96	China: Guizhou, Zhijin
L180322-P-2-LD180319-0006_HL3KCCCXY_L1_clean_bin.32.permissi	OEZ008529	yes	LMSG_G000010077.1	OES073750	T1PKS	PKSI	Polymetallic	6.67	China: Guizhou, Zhijin
L180322-P-2-LD180319-0006_HL3KCCCXY_L1_clean_bin.33.orig.fa_	OEZ008529	yes	LMSG_G000010771.1	OES073750	acyl_amino_acids	Others	Polymetallic	39.00	China: Guizhou, Zhijin
L180322-P-2-LD180319-0006_HL3KCCCXY_L1_clean_bin.33.orig.fa_	OEZ008529	yes	LMSG_G000010771.1	OES073750	bacteriocin	RiPPs	Polymetallic	9.22	China: Guizhou, Zhijin
L180322-P-2-LD180319-0006_HL3KCCCXY_L1_clean_bin.34.orig.fa_	OEZ008529	yes	LMSG_G000008469.1	OES073750	arylpolyene	Others	Polymetallic	41.18	China: Guizhou, Zhijin
L180322-P-2-LD180319-0006_HL3KCCCXY_L1_clean_bin.34.orig.fa_	OEZ008529	yes	LMSG_G000008469.1	OES073750	betalactone	Others	Polymetallic	27.74	China: Guizhou, Zhijin
L180322-P-2-LD180319-0006_HL3KCCCXY_L1_clean_bin.34.orig.fa_	OEZ008529	yes	LMSG_G000008469.1	OES073750	terpene	Terpene	Polymetallic	23.14	China: Guizhou, Zhijin
L180322-P-2-LD180319-0006_HL3KCCCXY_L1_clean_bin.34.orig.fa_	OEZ008529	yes	LMSG_G000008469.1	OES073750	betalactone	Others	Polymetallic	25.86	China: Guizhou, Zhijin
L180322-P-2-LD180319-0006_HL3KCCCXY_L1_clean_bin.35.permissi	OEZ008529	yes	LMSG_G000011328.1	OES073750	NRPS-like	NRPS	Polymetallic	6.21	China: Guizhou, Zhijin
L180322-P-2-LD180319-0006_HL3KCCCXY_L1_clean_bin.36.orig.fa_	OEZ008529	yes	LMSG_G000008612.1	OES073750	other	Others	Polymetallic	12.65	China: Guizhou, Zhijin
L180322-P-2-LD180319-0006_HL3KCCCXY_L1_clean_bin.37.orig.fa_	OEZ008529	yes	LMSG_G000010209.1	OES073750	arylpolyene	Others	Polymetallic	5.30	China: Guizhou, Zhijin
L180322-P-2-LD180319-0006_HL3KCCCXY_L1_clean_bin.37.orig.fa_	OEZ008529	yes	LMSG_G000010209.1	OES073750	terpene	Terpene	Polymetallic	7.37	China: Guizhou, Zhijin
L180322-P-2-LD180319-0006_HL3KCCCXY_L1_clean_bin.37.orig.fa_	OEZ008529	yes	LMSG_G000010209.1	OES073750	hserlactone	Others	Polymetallic	8.37	China: Guizhou, Zhijin
L180322-P-2-LD180319-0006_HL3KCCCXY_L1_clean_bin.38.strict.f	OEZ008529	yes	LMSG_G000008662.1	OES073750	NRPS-like	NRPS	Polymetallic	43.81	China: Guizhou, Zhijin
L180322-P-2-LD180319-0006_HL3KCCCXY_L1_clean_bin.4.orig.fa_k	OEZ008529	yes	LMSG_G000008455.1	OES073750	betalactone	Others	Polymetallic	22.65	China: Guizhou, Zhijin
L180322-P-2-LD180319-0006_HL3KCCCXY_L1_clean_bin.4.orig.fa_k	OEZ008529	yes	LMSG_G000008455.1	OES073750	terpene	Terpene	Polymetallic	26.06	China: Guizhou, Zhijin
L180322-P-2-LD180319-0006_HL3KCCCXY_L1_clean_bin.4.orig.fa_k	OEZ008529	yes	LMSG_G000008455.1	OES073750	terpene	Terpene	Polymetallic	21.03	China: Guizhou, Zhijin
L180322-P-2-LD180319-0006_HL3KCCCXY_L1_clean_bin.4.orig.fa_k	OEZ008529	yes	LMSG_G000008455.1	OES073750	betalactone	Others	Polymetallic	23.97	China: Guizhou, Zhijin
L180322-P-2-LD180319-0006_HL3KCCCXY_L1_clean_bin.40.orig.fa_	OEZ008529	yes	LMSG_G000008459.1	OES073750	betalactone	Others	Polymetallic	23.86	China: Guizhou, Zhijin
L180322-P-2-LD180319-0006_HL3KCCCXY_L1_clean_bin.40.orig.fa_	OEZ008529	yes	LMSG_G000008459.1	OES073750	terpene	Terpene	Polymetallic	26.11	China: Guizhou, Zhijin
L180322-P-2-LD180319-0006_HL3KCCCXY_L1_clean_bin.40.orig.fa_	OEZ008529	yes	LMSG_G000008459.1	OES073750	betalactone	Others	Polymetallic	22.75	China: Guizhou, Zhijin
L180322-P-2-LD180319-0006_HL3KCCCXY_L1_clean_bin.40.orig.fa_	OEZ008529	yes	LMSG_G000008459.1	OES073750	terpene	Terpene	Polymetallic	17.89	China: Guizhou, Zhijin
L180322-P-2-LD180319-0006_HL3KCCCXY_L1_clean_bin.41.orig.fa_	OEZ008529	yes	LMSG_G000010968.1	OES073750	bacteriocin	RiPPs	Polymetallic	8.93	China: Guizhou, Zhijin
L180322-P-2-LD180319-0006_HL3KCCCXY_L1_clean_bin.41.orig.fa_	OEZ008529	yes	LMSG_G000010968.1	OES073750	T1PKS.hglE-KS	PKSother	Polymetallic	25.60	China: Guizhou, Zhijin
L180322-P-2-LD180319-0006_HL3KCCCXY_L1_clean_bin.42.orig.fa_	OEZ008529	yes	LMSG_G000010214.1	OES073750	terpene	Terpene	Polymetallic	21.71	China: Guizhou, Zhijin
L180322-P-2-LD180319-0006_HL3KCCCXY_L1_clean_bin.42.orig.fa_	OEZ008529	yes	LMSG_G000010214.1	OES073750	T3PKS	PKSother	Polymetallic	12.26	China: Guizhou, Zhijin
L180322-P-2-LD180319-0006_HL3KCCCXY_L1_clean_bin.42.orig.fa_	OEZ008529	yes	LMSG_G000010214.1	OES073750	arylpolyene	Others	Polymetallic	38.38	China: Guizhou, Zhijin
L180322-P-2-LD180319-0006_HL3KCCCXY_L1_clean_bin.43.orig.fa_	OEZ008529	yes	LMSG_G000006325.1	OES073750	NRPS	NRPS	Polymetallic	26.67	China: Guizhou, Zhijin
L180322-P-2-LD180319-0006_HL3KCCCXY_L1_clean_bin.43.orig.fa_	OEZ008529	yes	LMSG_G000006325.1	OES073750	bacteriocin	RiPPs	Polymetallic	10.57	China: Guizhou, Zhijin

L180322-P-2-LD180319-0006_HL3KCCCXY_L1_clean_bin.43.orig.fa_	OEZ008529	yes	LMSG_G000006325.1	OES073750	bacteriocin	RiPPs	Polymetallic	7.88	China: Guizhou, Zhijin
L180322-P-2-LD180319-0006_HL3KCCCXY_L1_clean_bin.43.orig.fa_	OEZ008529	yes	LMSG_G000006325.1	OES073750	terpene	Terpene	Polymetallic	13.48	China: Guizhou, Zhijin
L180322-P-2-LD180319-0006_HL3KCCCXY_L1_clean_bin.43.orig.fa_	OEZ008529	yes	LMSG_G000006325.1	OES073750	NRPS-like	NRPS	Polymetallic	26.23	China: Guizhou, Zhijin
L180322-P-2-LD180319-0006_HL3KCCCXY_L1_clean_bin.43.orig.fa_	OEZ008529	yes	LMSG_G000006325.1	OES073750	betalactone	Others	Polymetallic	16.32	China: Guizhou, Zhijin
L180322-P-2-LD180319-0006_HL3KCCCXY_L1_clean_bin.43.orig.fa_	OEZ008529	yes	LMSG_G000006325.1	OES073750	T3PKS	PKSother	Polymetallic	35.47	China: Guizhou, Zhijin
L180322-P-2-LD180319-0006_HL3KCCCXY_L1_clean_bin.43.orig.fa_	OEZ008529	yes	LMSG_G000006325.1	OES073750	CDPS	Others	Polymetallic	9.07	China: Guizhou, Zhijin
L180322-P-2-LD180319-0006_HL3KCCCXY_L1_clean_bin.43.orig.fa_	OEZ008529	yes	LMSG_G000006325.1	OES073750	NRPS	NRPS	Polymetallic	34.83	China: Guizhou, Zhijin
L180322-P-2-LD180319-0006_HL3KCCCXY_L1_clean_bin.43.orig.fa_	OEZ008529	yes	LMSG_G000006325.1	OES073750	CDPS	Others	Polymetallic	14.32	China: Guizhou, Zhijin
L180322-P-2-LD180319-0006_HL3KCCCXY_L1_clean_bin.44.orig.fa_	OEZ008529	yes	LMSG_G000005232.1	OES073750	T1PKS	PKSI	Polymetallic	42.82	China: Guizhou, Zhijin
L180322-P-2-LD180319-0006_HL3KCCCXY_L1_clean_bin.44.orig.fa_	OEZ008529	yes	LMSG_G000005232.1	OES073750	cyanobactin	RiPPs	Polymetallic	10.13	China: Guizhou, Zhijin
L180322-P-2-LD180319-0006_HL3KCCCXY_L1_clean_bin.44.orig.fa_	OEZ008529	yes	LMSG_G000005232.1	OES073750	terpene	Terpene	Polymetallic	11.68	China: Guizhou, Zhijin
L180322-P-2-LD180319-0006_HL3KCCCXY_L1_clean_bin.44.orig.fa_	OEZ008529	yes	LMSG_G000005232.1	OES073750	T3PKS	PKSother	Polymetallic	5.66	China: Guizhou, Zhijin
L180322-P-2-LD180319-0006_HL3KCCCXY_L1_clean_bin.44.orig.fa_	OEZ008529	yes	LMSG_G000005232.1	OES073750	terpene	Terpene	Polymetallic	14.76	China: Guizhou, Zhijin
L180322-P-2-LD180319-0006_HL3KCCCXY_L1_clean_bin.44.orig.fa_	OEZ008529	yes	LMSG_G000005232.1	OES073750	T1PKS.hglE-KS	PKSother	Polymetallic	26.61	China: Guizhou, Zhijin
L180322-P-2-LD180319-0006_HL3KCCCXY_L1_clean_bin.44.orig.fa_	OEZ008529	yes	LMSG_G000005232.1	OES073750	hglE-KS	PKSother	Polymetallic	31.86	China: Guizhou, Zhijin
L180322-P-2-LD180319-0006_HL3KCCCXY_L1_clean_bin.45.orig.fa_	OEZ008529	yes	LMSG_G000004450.1	OES073750	terpene	Terpene	Polymetallic	12.68	China: Guizhou, Zhijin
L180322-P-2-LD180319-0006_HL3KCCCXY_L1_clean_bin.47.strict.f	OEZ008529	yes	LMSG_G000010799.1	OES073750	acyl_amino_acids	Others	Polymetallic	6.76	China: Guizhou, Zhijin
L180322-P-2-LD180319-0006_HL3KCCCXY_L1_clean_bin.5.strict.fa	OEZ008529	yes	LMSG_G000008551.1	OES073750	NRPS-like	NRPS	Polymetallic	16.42	China: Guizhou, Zhijin
L180322-P-2-LD180319-0006_HL3KCCCXY_L1_clean_bin.51.orig.fa_	OEZ008529	yes	LMSG_G000005233.1	OES073750	hglE-KS	PKSother	Polymetallic	8.21	China: Guizhou, Zhijin
L180322-P-2-LD180319-0006_HL3KCCCXY_L1_clean_bin.51.orig.fa_	OEZ008529	yes	LMSG_G000005233.1	OES073750	acyl_amino_acids	Others	Polymetallic	32.43	China: Guizhou, Zhijin
L180322-P-2-LD180319-0006_HL3KCCCXY_L1_clean_bin.51.orig.fa_	OEZ008529	yes	LMSG_G000005233.1	OES073750	terpene	Terpene	Polymetallic	6.92	China: Guizhou, Zhijin
L180322-P-2-LD180319-0006_HL3KCCCXY_L1_clean_bin.51.orig.fa_	OEZ008529	yes	LMSG_G000005233.1	OES073750	terpene	Terpene	Polymetallic	16.48	China: Guizhou, Zhijin
L180322-P-2-LD180319-0006_HL3KCCCXY_L1_clean_bin.51.orig.fa_	OEZ008529	yes	LMSG_G000005233.1	OES073750	terpene	Terpene	Polymetallic	13.19	China: Guizhou, Zhijin
L180322-P-2-LD180319-0006_HL3KCCCXY_L1_clean_bin.51.orig.fa_	OEZ008529	yes	LMSG_G000005233.1	OES073750	T1PKS.hglE-KS	PKSother	Polymetallic	11.96	China: Guizhou, Zhijin
L180322-P-2-LD180319-0006_HL3KCCCXY_L1_clean_bin.52.orig.fa_	OEZ008529	yes	LMSG_G000008151.1	OES073750	terpene	Terpene	Polymetallic	13.73	China: Guizhou, Zhijin
L180322-P-2-LD180319-0006_HL3KCCCXY_L1_clean_bin.53.orig.fa_	OEZ008529	yes	LMSG_G000010613.1	OES073750	terpene	Terpene	Polymetallic	18.25	China: Guizhou, Zhijin
L180322-P-2-LD180319-0006_HL3KCCCXY_L1_clean_bin.53.orig.fa_	OEZ008529	no	LMSG_G000010613.1	OES073750	siderophore	Others	Polymetallic	11.85	China: Guizhou, Zhijin
L180322-P-2-LD180319-0006_HL3KCCCXY_L1_clean_bin.53.orig.fa_	OEZ008529	no	LMSG_G000010613.1	OES073750	ectoine	Others	Polymetallic	10.41	China: Guizhou, Zhijin
L180322-P-2-LD180319-0006_HL3KCCCXY_L1_clean_bin.53.orig.fa_	OEZ008529	yes	LMSG_G000010613.1	OES073750	resorcinol	Others	Polymetallic	26.34	China: Guizhou, Zhijin
L180322-P-2-LD180319-0006_HL3KCCCXY_L1_clean_bin.53.orig.fa_	OEZ008529	no	LMSG_G000010613.1	OES073750	phosphonate	Others	Polymetallic	21.21	China: Guizhou, Zhijin
L180322-P-2-LD180319-0006_HL3KCCCXY_L1_clean_bin.53.orig.fa_	OEZ008529	no	LMSG_G000010613.1	OES073750	NRPS-like	NRPS	Polymetallic	31.24	China: Guizhou, Zhijin
L180322-P-2-LD180319-0006_HL3KCCCXY_L1_clean_bin.53.orig.fa_	OEZ008529	yes	LMSG_G000010613.1	OES073750	betalactone	Others	Polymetallic	13.99	China: Guizhou, Zhijin
L180322-P-2-LD180319-0006_HL3KCCCXY_L1_clean_bin.54.orig.fa_	OEZ008529	yes	LMSG_G000007798.1	OES073750	terpene	Terpene	Polymetallic	10.59	China: Guizhou, Zhijin
L180322-P-2-LD180319-0006_HL3KCCCXY_L1_clean_bin.54.orig.fa_	OEZ008529	yes	LMSG_G000007798.1	OES073750	terpene	Terpene	Polymetallic	5.82	China: Guizhou, Zhijin
L180322-P-2-LD180319-0006_HL3KCCCXY_L1_clean_bin.54.orig.fa_	OEZ008529	yes	LMSG_G000007798.1	OES073750	T3PKS	PKSother	Polymetallic	20.51	China: Guizhou, Zhijin
L180322-P-2-LD180319-0006_HL3KCCCXY_L1_clean_bin.55.orig.fa_	OEZ008529	yes	LMSG_G000010652.1	OES073750	terpene	Terpene	Polymetallic	11.91	China: Guizhou, Zhijin
L180322-P-2-LD180319-0006_HL3KCCCXY_L1_clean_bin.55.orig.fa_	OEZ008529	yes	LMSG_G000010652.1	OES073750	arylpolyene	Others	Polymetallic	16.01	China: Guizhou, Zhijin
L180322-P-2-LD180319-0006_HL3KCCCXY_L1_clean_bin.55.orig.fa_	OEZ008529	yes	LMSG_G000010652.1	OES073750	NRPS	NRPS	Polymetallic	24.89	China: Guizhou, Zhijin
L180322-P-2-LD180319-0006_HL3KCCCXY_L1_clean_bin.55.orig.fa_	OEZ008529	yes	LMSG_G000010652.1	OES073750	NRPS-like	NRPS	Polymetallic	6.00	China: Guizhou, Zhijin
L180322-P-2-LD180319-0006_HL3KCCCXY_L1_clean_bin.55.orig.fa_	OEZ008529	yes	LMSG_G000010652.1	OES073750	NRPS.T1PKS	PKS-NRP_Hybrids	Polymetallic	31.46	China: Guizhou, Zhijin
L180322-P-2-LD180319-0006_HL3KCCCXY_L1_clean_bin.56.orig.fa_	OEZ008529	no	LMSG_G000010269.1	OES073750	terpene	Terpene	Polymetallic	8.10	China: Guizhou, Zhijin
L180322-P-2-LD180319-0006_HL3KCCCXY_L1_clean_bin.56.orig.fa_	OEZ008529	yes	LMSG_G000010269.1	OES073750	arylpolyene	Others	Polymetallic	14.01	China: Guizhou, Zhijin
L180322-P-3-LD180319-0007_HL3KCCCXY_L2_clean_bin.11.orig.fa_	OEZ008529	yes	LMSG_G000010430.1	OES073751	terpene	Terpene	Polymetallic	25.02	China: Guizhou, Zhijin
L180322-P-3-LD180319-0007_HL3KCCCXY_L2_clean_bin.11.orig.fa_	OEZ008529	yes	LMSG_G000010430.1	OES073751	bacteriocin	RiPPs	Polymetallic	9.46	China: Guizhou, Zhijin
L180322-P-3-LD180319-0007_HL3KCCCXY_L2_clean_bin.11.orig.fa_	OEZ008529	yes	LMSG_G000010430.1	OES073751	terpene	Terpene	Polymetallic	8.96	China: Guizhou, Zhijin
L180322-P-3-LD180319-0007_HL3KCCCXY_L2_clean_bin.12.orig.fa_	OEZ008529	yes	LMSG_G000006326.1	OES073751	bacteriocin	RiPPs	Polymetallic	7.88	China: Guizhou, Zhijin
L180322-P-3-LD180319-0007_HL3KCCCXY_L2_clean_bin.12.orig.fa_	OEZ008529	yes	LMSG_G000006326.1	OES073751	T3PKS	PKSother	Polymetallic	35.50	China: Guizhou, Zhijin
L180322-P-3-LD180319-0007_HL3KCCCXY_L2_clean_bin.12.orig.fa_	OEZ008529	yes	LMSG_G000006326.1	OES073751	CDPS	Others	Polymetallic	9.07	China: Guizhou, Zhijin
L180322-P-3-LD180319-0007_HL3KCCCXY_L2_clean_bin.12.orig.fa_	OEZ008529	yes	LMSG_G000006326.1	OES073751	NRPS	NRPS	Polymetallic	14.18	China: Guizhou, Zhijin
L180322-P-3-LD180319-0007_HL3KCCCXY_L2_clean_bin.12.orig.fa_	OEZ008529	yes	LMSG_G000006326.1	OES073751	NRPS-like	NRPS	Polymetallic	26.23	China: Guizhou, Zhijin
L180322-P-3-LD180319-0007_HL3KCCCXY_L2_clean_bin.12.orig.fa_	OEZ008529	yes	LMSG_G000006326.1	OES073751	bacteriocin	RiPPs	Polymetallic	7.18	China: Guizhou, Zhijin
L180322-P-3-LD180319-0007_HL3KCCCXY_L2_clean_bin.12.orig.fa_	OEZ008529	yes	LMSG_G000006326.1	OES073751	NRPS	NRPS	Polymetallic	19.73	China: Guizhou, Zhijin
L180322-P-3-LD180319-0007_HL3KCCCXY_L2_clean_bin.12.orig.fa_	OEZ008529	yes	LMSG_G000006326.1	OES073751	CDPS	Others	Polymetallic	16.11	China: Guizhou, Zhijin
L180322-P-3-LD180319-0007_HL3KCCCXY_L2_clean_bin.14.orig.fa_	OEZ008529	yes	LMSG_G000010969.1	OES073751	T1PKS	PKSI	Polymetallic	19.50	China: Guizhou, Zhijin
L180322-P-3-LD180319-0007_HL3KCCCXY_L2_clean_bin.15.orig.fa_	OEZ008529	yes	LMSG_G000005234.1	OES073751	betalactone	Others	Polymetallic	22.57	China: Guizhou, Zhijin
L180322-P-3-LD180319-0007_HL3KCCCXY_L2_clean_bin.15.orig.fa_	OEZ008529	yes	LMSG_G000005234.1	OES073751	terpene	Terpene	Polymetallic	21.91	China: Guizhou, Zhijin
L180322-P-3-LD180319-0007_HL3KCCCXY_L2_clean_bin.15.orig.fa_	OEZ008529	yes	LMSG_G000005234.1	OES073751	NRPS-like	NRPS	Polymetallic	23.97	China: Guizhou, Zhijin
L180322-P-3-LD180319-0007_HL3KCCCXY_L2_clean_bin.15.orig.fa_	OEZ008529	yes	LMSG_G000005234.1	OES073751	terpene	Terpene	Polymetallic	21.69	China: Guizhou, Zhijin
L180322-P-3-LD180319-0007_HL3KCCCXY_L2_clean_bin.16.orig.fa_	OEZ008529	yes	LMSG_G000009995.1	OES073751	terpene	Terpene	Polymetallic	10.37	China: Guizhou, Zhijin
L180322-P-3-LD180319-0007_HL3KCCCXY_L2_clean_bin.16.orig.fa_	OEZ008529	yes	LMSG_G000009995.1	OES073751	NRPS-like	NRPS	Polymetallic	10.91	China: Guizhou, Zhijin
L180322-P-3-LD180319-0007_HL3KCCCXY_L2_clean_bin.18.orig.fa_	OEZ008529	yes	LMSG_G000004451.1	OES073751	terpene	Terpene	Polymetallic	26.12	China: Guizhou, Zhijin
L180322-P-3-LD180319-0007_HL3KCCCXY_L2_clean_bin.18.orig.fa_	OEZ008529	yes	LMSG_G000004451.1	OES073751	betalactone	Others	Polymetallic	23.97	China: Guizhou, Zhijin
L180322-P-3-LD180319-0007_HL3KCCCXY_L2_clean_bin.18.orig.fa_	OEZ008529	yes	LMSG_G000004451.1	OES073751	terpene	Terpene	Polymetallic	21.06	China: Guizhou, Zhijin
L180322-P-3-LD180319-0007_HL3KCCCXY_L2_clean_bin.2.strict.fa	OEZ008529	yes	LMSG_G000008671.1	OES073751	NRPS-like	NRPS	Polymetallic	16.47	China: Guizhou, Zhijin
L180322-P-3-LD180319-0007_HL3KCCCXY_L2_clean_bin.20.orig.fa_	OEZ008529	yes	LMSG_G000009225.1	OES073751	bacteriocin	RiPPs	Polymetallic	8.36	China: Guizhou, Zhijin
L180322-P-3-LD180319-0007_HL3KCCCXY_L2_clean_bin.20.orig.fa_	OEZ008529	yes	LMSG_G000009225.1	OES073751	T3PKS	PKSother	Polymetallic	15.38	China: Guizhou, Zhijin
L180322-P-3-LD180319-0007_HL3KCCCXY_L2_clean_bin.20.orig.fa_	OEZ008529	yes	LMSG_G000009225.1	OES073751	T1PKS.hglE-KS	PKSother	Polymetallic	5.06	China: Guizhou, Zhijin
L180322-P-3-LD180319-0007_HL3KCCCXY_L2_clean_bin.20.orig.fa_	OEZ008529	yes	LMSG_G000009225.1	OES073751	hglE-KS	PKSother	Polymetallic	16.94	China: Guizhou, Zhijin
L180322-P-3-LD180319-0007_HL3KCCCXY_L2_clean_bin.20.orig.fa_	OEZ008529	yes	LMSG_G000009225.1	OES073751	bacteriocin	RiPPs	Polymetallic	10.82	China: Guizhou, Zhijin
L180322-P-3-LD180319-0007_HL3KCCCXY_L2_clean_bin.20.orig.fa_	OEZ008529	yes	LMSG_G000009225.1	OES073751	terpene	Terpene	Polymetallic	21.72	China: Guizhou, Zhijin
L180322-P-3-LD180319-0007_HL3KCCCXY_L2_clean_bin.20.orig.fa_	OEZ008529	yes	LMSG_G000009225.1	OES073751	terpene	Terpene	Polymetallic	9.59	China: Guizhou, Zhijin
L180322-P-3-LD180319-0007_HL3KCCCXY_L2_clean_bin.20.orig.fa_	OEZ008529	yes	LMSG_G000009225.1	OES073751	hserlactone	Others	Polymetallic	8.90	China: Guizhou, Zhijin
L180322-P-3-LD180319-0007_HL3KCCCXY_L2_clean_bin.25.orig.fa_	OEZ008529	yes	LMSG_G000008356.1	OES073751	terpene	Terpene	Polymetallic	9.46	China: Guizhou, Zhijin
L180322-P-3-LD180319-0007_HL3KCCCXY_L2_clean_bin.25.orig.fa_	OEZ008529	yes	LMSG_G000008356.1	OES073751	bacteriocin	RiPPs	Polymetallic	5.87	China: Guizhou, Zhijin
L180322-P-3-LD180319-0007_HL3KCCCXY_L2_clean_bin.25.orig.fa_	OEZ008529	yes	LMSG_G000008356.1	OES073751	NRPS-like	NRPS	Polymetallic	13.62	China: Guizhou, Zhijin
L180322-P-3-LD180319-0007_HL3KCCCXY_L2_clean_bin.25.orig.fa_	OEZ008529	yes	LMSG_G000008356.1	OES073751	bacteriocin	RiPPs	Polymetallic	5.21	China: Guizhou, Zhijin
L180322-P-3-LD180319-0007_HL3KCCCXY_L2_clean_bin.26.strict.f	OEZ008529	yes	LMSG_G000008149.1	OES073751	terpene	Terpene	Polymetallic	23.44	China: Guizhou, Zhijin
L180322-P-3-LD180319-0007_HL3KCCCXY_L2_clean_bin.27.orig.fa_	OEZ008529	yes	LMSG_G000010323.1	OES073751	terpene	Terpene	Polymetallic	11.41	China: Guizhou, Zhijin
L180322-P-3-LD180319-0007_HL3KCCCXY_L2_clean_bin.27.orig.fa_	OEZ008529	yes	LMSG_G000010323.1	OES073751	terpene	Terpene	Polymetallic	7.27	China: Guizhou, Zhijin
L180322-P-3-LD180319-0007_HL3KCCCXY_L2_clean_bin.27.orig.fa_	OEZ008529	yes	LMSG_G000010323.1	OES073751	bacteriocin	RiPPs	Polymetallic	7.71	China: Guizhou, Zhijin
L180322-P-3-LD180319-0007_HL3KCCCXY_L2_clean_bin.28.orig.fa_	OEZ008529	yes	LMSG_G000008573.1	OES073751	NRPS-like	NRPS	Polymetallic	7.80	China: Guizhou, Zhijin
L180322-P-3-LD180319-0007_HL3KCCCXY_L2_clean_bin.28.orig.fa_	OEZ008529	yes	LMSG_G000008573.1	OES073751	other	Others	Polymetallic	12.15	China: Guizhou, Zhijin
L180322-P-3-LD180319-0007_HL3KCCCXY_L2_clean_bin.29.strict.f	OEZ008529	yes	LMSG_G000006274.1	OES073751	terpene	Terpene	Polymetallic	21.89	China: Guizhou, Zhijin
L180322-P-3-LD180319-0007_HL3KCCCXY_L2_clean_bin.29.strict.f	OEZ008529	yes	LMSG_G000006274.1	OES073751	T3PKS	PKSother	Polymetallic	39.47	China: Guizhou, Zhijin
L180322-P-3-LD180319-0007_HL3KCCCXY_L2_clean_bin.29.strict.f	OEZ008529	yes	LMSG_G000006274.1	OES073751	terpene	Terpene	Polymetallic	20.81	China: Guizhou, Zhijin
L180322-P-3-LD180319-0007_HL3KCCCXY_L2_clean_bin.29.strict.f	OEZ008529	yes	LMSG_G000006274.1	OES073751	LAP.bacteriocin	RiPPs	Polymetallic	23.54	China: Guizhou, Zhijin
L180322-P-3-LD180319-0007_HL3KCCCXY_L2_clean_bin.29.strict.f	OEZ008529	yes	LMSG_G000006274.1	OES073751	bacteriocin	RiPPs	Polymetallic	7.07	China: Guizhou, Zhijin
L180322-P-3-LD180319-0007_HL3KCCCXY_L2_clean_bin.31.orig.fa_	OEZ008529	yes	LMSG_G000005235.1	OES073751	terpene	Terpene	Polymetallic	11.47	China: Guizhou, Zhijin
L180322-P-3-LD180319-0007_HL3KCCCXY_L2_clean_bin.31.orig.fa_	OEZ008529	yes	LMSG_G000005235.1	OES073751	arylpolyene	Others	Polymetallic	37.10	China: Guizhou, Zhijin
L180322-P-3-LD180319-0007_HL3KCCCXY_L2_clean_bin.31.orig.fa_	OEZ008529	no	LMSG_G000005235.1	OES073751	hglE-KS	PKSother	Polymetallic	7.15	China: Guizhou, Zhijin
L180322-P-3-LD180319-0007_HL3KCCCXY_L2_clean_bin.31.orig.fa_	OEZ008529	yes	LMSG_G000005235.1	OES073751	T3PKS	PKSother	Polymetallic	25.93	China: Guizhou, Zhijin
L180322-P-3-LD180319-0007_HL3KCCCXY_L2_clean_bin.31.orig.fa_	OEZ008529	yes	LMSG_G000005235.1	OES073751	NRPS	NRPS	Polymetallic	43.88	China: Guizhou, Zhijin
L180322-P-3-LD180319-0007_HL3KCCCXY_L2_clean_bin.31.orig.fa_	OEZ008529	yes	LMSG_G000005235.1	OES073751	NRPS	NRPS	Polymetallic	10.08	China: Guizhou, Zhijin
L180322-P-3-LD180319-0007_HL3KCCCXY_L2_clean_bin.31.orig.fa_	OEZ008529	yes	LMSG_G000005235.1	OES073751	T1PKS	PKSI	Polymetallic	38.18	China: Guizhou, Zhijin
L180322-P-3-LD180319-0007_HL3KCCCXY_L2_clean_bin.31.orig.fa_	OEZ008529	yes	LMSG_G000005235.1	OES073751	T1PKS	PKSI	Polymetallic	62.56	China: Guizhou, Zhijin
L180322-P-3-LD180319-0007_HL3KCCCXY_L2_clean_bin.31.orig.fa_	OEZ008529	yes	LMSG_G000005235.1	OES073751	terpene	Terpene	Polymetallic	17.37	China: Guizhou, Zhijin
L180322-P-3-LD180319-0007_HL3KCCCXY_L2_clean_bin.31.orig.fa_	OEZ008529	yes	LMSG_G000005235.1	OES073751	T1PKS	PKSI	Polymetallic	33.12	China: Guizhou, Zhijin
L180322-P-3-LD180319-0007_HL3KCCCXY_L2_clean_bin.31.orig.fa_	OEZ008529	no	LMSG_G000005235.1	OES073751	NRPS	NRPS	Polymetallic	5.95	China: Guizhou, Zhijin
L180322-P-3-LD180319-0007_HL3KCCCXY_L2_clean_bin.31.orig.fa_	OEZ008529	yes	LMSG_G000005235.1	OES073751	NRPS	NRPS	Polymetallic	27.95	China: Guizhou, Zhijin
L180322-P-3-LD180319-0007_HL3KCCCXY_L2_clean_bin.31.orig.fa_	OEZ008529	yes	LMSG_G000005235.1	OES073751	NRPS	NRPS	Polymetallic	37.69	China: Guizhou, Zhijin
L180322-P-3-LD180319-0007_HL3KCCCXY_L2_clean_bin.31.orig.fa_	OEZ008529	yes	LMSG_G000005235.1	OES073751	ectoine	Others	Polymetallic	5.89	China: Guizhou, Zhijin
L180322-P-3-LD180319-0007_HL3KCCCXY_L2_clean_bin.31.orig.fa_	OEZ008529	yes	LMSG_G000005235.1	OES073751	terpene	Terpene	Polymetallic	15.52	China: Guizhou, Zhijin
L180322-P-3-LD180319-0007_HL3KCCCXY_L2_clean_bin.31.orig.fa_	OEZ008529	yes	LMSG_G000005235.1	OES073751	T1PKS	PKSI	Polymetallic	19.54	China: Guizhou, Zhijin
L180322-P-3-LD180319-0007_HL3KCCCXY_L2_clean_bin.31.orig.fa_	OEZ008529	yes	LMSG_G000005235.1	OES073751	NRPS	NRPS	Polymetallic	45.45	China: Guizhou, Zhijin
L180322-P-3-LD180319-0007_HL3KCCCXY_L2_clean_bin.31.orig.fa_	OEZ008529	yes	LMSG_G000005235.1	OES073751	butyrolactone	Others	Polymetallic	10.98	China: Guizhou, Zhijin
L180322-P-3-LD180319-0007_HL3KCCCXY_L2_clean_bin.31.orig.fa_	OEZ008529	yes	LMSG_G000005235.1	OES073751	arylpolyene	Others	Polymetallic	27.09	China: Guizhou, Zhijin
L180322-P-3-LD180319-0007_HL3KCCCXY_L2_clean_bin.31.orig.fa_	OEZ008529	yes	LMSG_G000005235.1	OES073751	NRPS	NRPS	Polymetallic	50.92	China: Guizhou, Zhijin
L180322-P-3-LD180319-0007_HL3KCCCXY_L2_clean_bin.31.orig.fa_	OEZ008529	yes	LMSG_G000005235.1	OES073751	T1PKS	PKSI	Polymetallic	29.12	China: Guizhou, Zhijin
L180322-P-3-LD180319-0007_HL3KCCCXY_L2_clean_bin.31.orig.fa_	OEZ008529	yes	LMSG_G000005235.1	OES073751	butyrolactone	Others	Polymetallic	6.76	China: Guizhou, Zhijin
L180322-P-3-LD180319-0007_HL3KCCCXY_L2_clean_bin.31.orig.fa_	OEZ008529	yes	LMSG_G000005235.1	OES073751	T1PKS	PKSI	Polymetallic	24.39	China: Guizhou, Zhijin
L180322-P-3-LD180319-0007_HL3KCCCXY_L2_clean_bin.31.orig.fa_	OEZ008529	no	LMSG_G000005235.1	OES073751	NRPS	NRPS	Polymetallic	17.78	China: Guizhou, Zhijin
L180322-P-3-LD180319-0007_HL3KCCCXY_L2_clean_bin.31.orig.fa_	OEZ008529	yes	LMSG_G000005235.1	OES073751	NRPS	NRPS	Polymetallic	21.18	China: Guizhou, Zhijin
L180322-P-3-LD180319-0007_HL3KCCCXY_L2_clean_bin.31.orig.fa_	OEZ008529	yes	LMSG_G000005235.1	OES073751	PKS-like.T2PKS	PKSother	Polymetallic	35.85	China: Guizhou, Zhijin
L180322-P-3-LD180319-0007_HL3KCCCXY_L2_clean_bin.31.orig.fa_	OEZ008529	yes	LMSG_G000005235.1	OES073751	NRPS-like	NRPS	Polymetallic	28.66	China: Guizhou, Zhijin
L180322-P-3-LD180319-0007_HL3KCCCXY_L2_clean_bin.31.orig.fa_	OEZ008529	yes	LMSG_G000005235.1	OES073751	NRPS	NRPS	Polymetallic	82.76	China: Guizhou, Zhijin
L180322-P-3-LD180319-0007_HL3KCCCXY_L2_clean_bin.31.orig.fa_	OEZ008529	yes	LMSG_G000005235.1	OES073751	terpene	Terpene	Polymetallic	12.88	China: Guizhou, Zhijin
L180322-P-3-LD180319-0007_HL3KCCCXY_L2_clean_bin.31.orig.fa_	OEZ008529	yes	LMSG_G000005235.1	OES073751	NRPS-like	NRPS	Polymetallic	8.64	China: Guizhou, Zhijin
L180322-P-3-LD180319-0007_HL3KCCCXY_L2_clean_bin.31.orig.fa_	OEZ008529	yes	LMSG_G000005235.1	OES073751	NRPS	NRPS	Polymetallic	49.82	China: Guizhou, Zhijin
L180322-P-3-LD180319-0007_HL3KCCCXY_L2_clean_bin.31.orig.fa_	OEZ008529	yes	LMSG_G000005235.1	OES073751	NRPS.siderophore	Others	Polymetallic	47.17	China: Guizhou, Zhijin
L180322-P-3-LD180319-0007_HL3KCCCXY_L2_clean_bin.31.orig.fa_	OEZ008529	yes	LMSG_G000005235.1	OES073751	T1PKS.NRPS-like	PKS-NRP_Hybrids	Polymetallic	36.14	China: Guizhou, Zhijin
L180322-P-3-LD180319-0007_HL3KCCCXY_L2_clean_bin.31.orig.fa_	OEZ008529	yes	LMSG_G000005235.1	OES073751	NRPS	NRPS	Polymetallic	52.64	China: Guizhou, Zhijin
L180322-P-3-LD180319-0007_HL3KCCCXY_L2_clean_bin.31.orig.fa_	OEZ008529	yes	LMSG_G000005235.1	OES073751	terpene.resorcinol	Others	Polymetallic	45.59	China: Guizhou, Zhijin
L180322-P-3-LD180319-0007_HL3KCCCXY_L2_clean_bin.31.orig.fa_	OEZ008529	yes	LMSG_G000005235.1	OES073751	NRPS	NRPS	Polymetallic	68.71	China: Guizhou, Zhijin
L180322-P-3-LD180319-0007_HL3KCCCXY_L2_clean_bin.31.orig.fa_	OEZ008529	yes	LMSG_G000005235.1	OES073751	bacteriocin	RiPPs	Polymetallic	10.80	China: Guizhou, Zhijin
L180322-P-3-LD180319-0007_HL3KCCCXY_L2_clean_bin.31.orig.fa_	OEZ008529	yes	LMSG_G000005235.1	OES073751	furan	Others	Polymetallic	11.66	China: Guizhou, Zhijin
L180322-P-3-LD180319-0007_HL3KCCCXY_L2_clean_bin.31.orig.fa_	OEZ008529	yes	LMSG_G000005235.1	OES073751	T1PKS	PKSI	Polymetallic	45.71	China: Guizhou, Zhijin
L180322-P-3-LD180319-0007_HL3KCCCXY_L2_clean_bin.31.orig.fa_	OEZ008529	yes	LMSG_G000005235.1	OES073751	NRPS.terpene	Others	Polymetallic	23.39	China: Guizhou, Zhijin
L180322-P-3-LD180319-0007_HL3KCCCXY_L2_clean_bin.31.orig.fa_	OEZ008529	yes	LMSG_G000005235.1	OES073751	NRPS.T1PKS	PKS-NRP_Hybrids	Polymetallic	50.12	China: Guizhou, Zhijin
L180322-P-3-LD180319-0007_HL3KCCCXY_L2_clean_bin.33.orig.fa_	OEZ008529	yes	LMSG_G000010772.1	OES073751	bacteriocin	RiPPs	Polymetallic	11.75	China: Guizhou, Zhijin
L180322-P-3-LD180319-0007_HL3KCCCXY_L2_clean_bin.33.orig.fa_	OEZ008529	yes	LMSG_G000010772.1	OES073751	acyl_amino_acids	Others	Polymetallic	33.88	China: Guizhou, Zhijin
L180322-P-3-LD180319-0007_HL3KCCCXY_L2_clean_bin.34.orig.fa_	OEZ008529	yes	LMSG_G000004452.1	OES073751	NRPS-like	NRPS	Polymetallic	43.38	China: Guizhou, Zhijin
L180322-P-3-LD180319-0007_HL3KCCCXY_L2_clean_bin.34.orig.fa_	OEZ008529	yes	LMSG_G000004452.1	OES073751	T1PKS	PKSI	Polymetallic	33.56	China: Guizhou, Zhijin
L180322-P-3-LD180319-0007_HL3KCCCXY_L2_clean_bin.35.orig.fa_	OEZ008529	yes	LMSG_G000005236.1	OES073751	betalactone	Others	Polymetallic	7.25	China: Guizhou, Zhijin
L180322-P-3-LD180319-0007_HL3KCCCXY_L2_clean_bin.35.orig.fa_	OEZ008529	yes	LMSG_G000005236.1	OES073751	bacteriocin	RiPPs	Polymetallic	5.41	China: Guizhou, Zhijin
L180322-P-3-LD180319-0007_HL3KCCCXY_L2_clean_bin.37.orig.fa_	OEZ008529	yes	LMSG_G000010128.1	OES073751	hserlactone	Others	Polymetallic	11.57	China: Guizhou, Zhijin
L180322-P-3-LD180319-0007_HL3KCCCXY_L2_clean_bin.37.orig.fa_	OEZ008529	yes	LMSG_G000010128.1	OES073751	terpene	Terpene	Polymetallic	24.21	China: Guizhou, Zhijin
L180322-P-3-LD180319-0007_HL3KCCCXY_L2_clean_bin.39.orig.fa_	OEZ008529	yes	LMSG_G000008620.1	OES073751	NRPS-like	NRPS	Polymetallic	16.62	China: Guizhou, Zhijin
L180322-P-3-LD180319-0007_HL3KCCCXY_L2_clean_bin.4.orig.fa_k	OEZ008529	yes	LMSG_G000008427.1	OES073751	terpene	Terpene	Polymetallic	23.47	China: Guizhou, Zhijin
L180322-P-3-LD180319-0007_HL3KCCCXY_L2_clean_bin.4.orig.fa_k	OEZ008529	yes	LMSG_G000008427.1	OES073751	NRPS.betalactone	Others	Polymetallic	51.61	China: Guizhou, Zhijin
L180322-P-3-LD180319-0007_HL3KCCCXY_L2_clean_bin.4.orig.fa_k	OEZ008529	yes	LMSG_G000008427.1	OES073751	T3PKS	PKSother	Polymetallic	30.19	China: Guizhou, Zhijin
L180322-P-3-LD180319-0007_HL3KCCCXY_L2_clean_bin.4.orig.fa_k	OEZ008529	yes	LMSG_G000008427.1	OES073751	NRPS	NRPS	Polymetallic	29.01	China: Guizhou, Zhijin
L180322-P-3-LD180319-0007_HL3KCCCXY_L2_clean_bin.4.orig.fa_k	OEZ008529	yes	LMSG_G000008427.1	OES073751	terpene	Terpene	Polymetallic	26.20	China: Guizhou, Zhijin
L180322-P-3-LD180319-0007_HL3KCCCXY_L2_clean_bin.4.orig.fa_k	OEZ008529	yes	LMSG_G000008427.1	OES073751	other	Others	Polymetallic	41.63	China: Guizhou, Zhijin
L180322-P-3-LD180319-0007_HL3KCCCXY_L2_clean_bin.40.strict.f	OEZ008529	yes	LMSG_G000008613.1	OES073751	other	Others	Polymetallic	22.50	China: Guizhou, Zhijin
L180322-P-3-LD180319-0007_HL3KCCCXY_L2_clean_bin.41.orig.fa_	OEZ008529	yes	LMSG_G000008460.1	OES073751	terpene	Terpene	Polymetallic	14.09	China: Guizhou, Zhijin
L180322-P-3-LD180319-0007_HL3KCCCXY_L2_clean_bin.41.orig.fa_	OEZ008529	yes	LMSG_G000008460.1	OES073751	betalactone	Others	Polymetallic	22.75	China: Guizhou, Zhijin
L180322-P-3-LD180319-0007_HL3KCCCXY_L2_clean_bin.42.orig.fa_	OEZ008529	yes	LMSG_G000010210.1	OES073751	terpene	Terpene	Polymetallic	7.29	China: Guizhou, Zhijin
L180322-P-3-LD180319-0007_HL3KCCCXY_L2_clean_bin.42.orig.fa_	OEZ008529	yes	LMSG_G000010210.1	OES073751	terpene	Terpene	Polymetallic	5.16	China: Guizhou, Zhijin
L180322-P-3-LD180319-0007_HL3KCCCXY_L2_clean_bin.42.orig.fa_	OEZ008529	yes	LMSG_G000010210.1	OES073751	terpene	Terpene	Polymetallic	6.83	China: Guizhou, Zhijin
L180322-P-3-LD180319-0007_HL3KCCCXY_L2_clean_bin.43.orig.fa_	OEZ008529	yes	LMSG_G000008663.1	OES073751	NRPS-like	NRPS	Polymetallic	25.51	China: Guizhou, Zhijin
L180322-P-3-LD180319-0007_HL3KCCCXY_L2_clean_bin.44.orig.fa_	OEZ008529	yes	LMSG_G000010223.1	OES073751	terpene	Terpene	Polymetallic	14.13	China: Guizhou, Zhijin
L180322-P-3-LD180319-0007_HL3KCCCXY_L2_clean_bin.44.orig.fa_	OEZ008529	yes	LMSG_G000010223.1	OES073751	betalactone	Others	Polymetallic	31.27	China: Guizhou, Zhijin
L180322-P-3-LD180319-0007_HL3KCCCXY_L2_clean_bin.45.orig.fa_	OEZ008529	yes	LMSG_G000011161.1	OES073751	bacteriocin	RiPPs	Polymetallic	6.61	China: Guizhou, Zhijin
L180322-P-3-LD180319-0007_HL3KCCCXY_L2_clean_bin.45.orig.fa_	OEZ008529	yes	LMSG_G000011161.1	OES073751	arylpolyene	Others	Polymetallic	9.61	China: Guizhou, Zhijin
L180322-P-3-LD180319-0007_HL3KCCCXY_L2_clean_bin.46.orig.fa_	OEZ008529	yes	LMSG_G000008204.1	OES073751	terpene	Terpene	Polymetallic	20.86	China: Guizhou, Zhijin
L180322-P-3-LD180319-0007_HL3KCCCXY_L2_clean_bin.47.orig.fa_	OEZ008529	yes	LMSG_G000010215.1	OES073751	T3PKS	PKSother	Polymetallic	22.10	China: Guizhou, Zhijin
L180322-P-3-LD180319-0007_HL3KCCCXY_L2_clean_bin.47.orig.fa_	OEZ008529	yes	LMSG_G000010215.1	OES073751	hserlactone	Others	Polymetallic	20.65	China: Guizhou, Zhijin
L180322-P-3-LD180319-0007_HL3KCCCXY_L2_clean_bin.47.orig.fa_	OEZ008529	yes	LMSG_G000010215.1	OES073751	arylpolyene	Others	Polymetallic	41.19	China: Guizhou, Zhijin

L180322-P-3-LD180319-0007_HL3KCCCXY_L2_clean_bin.47.orig.fa_	OEZ008529	yes	LMSG_G000010215.1	OES073751	terpene	Terpene	Polymetallic	21.71	China: Guizhou, Zhijin
L180322-P-3-LD180319-0007_HL3KCCCXY_L2_clean_bin.47.orig.fa_	OEZ008529	yes	LMSG_G000010215.1	OES073751	terpene	Terpene	Polymetallic	11.95	China: Guizhou, Zhijin
L180322-P-3-LD180319-0007_HL3KCCCXY_L2_clean_bin.47.orig.fa_	OEZ008529	yes	LMSG_G000010215.1	OES073751	hserlactone	Others	Polymetallic	14.00	China: Guizhou, Zhijin
L180322-P-3-LD180319-0007_HL3KCCCXY_L2_clean_bin.49.orig.fa_	OEZ008529	yes	LMSG_G000010060.1	OES073751	arylpolyene	Others	Polymetallic	6.88	China: Guizhou, Zhijin
L180322-P-3-LD180319-0007_HL3KCCCXY_L2_clean_bin.49.orig.fa_	OEZ008529	yes	LMSG_G000010060.1	OES073751	arylpolyene	Others	Polymetallic	9.21	China: Guizhou, Zhijin
L180322-P-3-LD180319-0007_HL3KCCCXY_L2_clean_bin.49.orig.fa_	OEZ008529	yes	LMSG_G000010060.1	OES073751	terpene	Terpene	Polymetallic	7.82	China: Guizhou, Zhijin
L180322-P-3-LD180319-0007_HL3KCCCXY_L2_clean_bin.5.orig.fa_k	OEZ008529	yes	LMSG_G000005238.1	OES073751	TfuA-related	RiPPs	Polymetallic	7.59	China: Guizhou, Zhijin
L180322-P-3-LD180319-0007_HL3KCCCXY_L2_clean_bin.5.orig.fa_k	OEZ008529	yes	LMSG_G000005238.1	OES073751	terpene	Terpene	Polymetallic	8.83	China: Guizhou, Zhijin
L180322-P-3-LD180319-0007_HL3KCCCXY_L2_clean_bin.5.orig.fa_k	OEZ008529	yes	LMSG_G000005238.1	OES073751	NRPS-like	NRPS	Polymetallic	30.99	China: Guizhou, Zhijin
L180322-P-3-LD180319-0007_HL3KCCCXY_L2_clean_bin.5.orig.fa_k	OEZ008529	yes	LMSG_G000005238.1	OES073751	terpene	Terpene	Polymetallic	21.93	China: Guizhou, Zhijin
L180322-P-3-LD180319-0007_HL3KCCCXY_L2_clean_bin.50.orig.fa_	OEZ008529	yes	LMSG_G000005237.1	OES073751	terpene	Terpene	Polymetallic	13.83	China: Guizhou, Zhijin
L180322-P-3-LD180319-0007_HL3KCCCXY_L2_clean_bin.50.orig.fa_	OEZ008529	yes	LMSG_G000005237.1	OES073751	terpene	Terpene	Polymetallic	12.24	China: Guizhou, Zhijin
L180322-P-3-LD180319-0007_HL3KCCCXY_L2_clean_bin.50.orig.fa_	OEZ008529	yes	LMSG_G000005237.1	OES073751	T3PKS	PKSother	Polymetallic	30.12	China: Guizhou, Zhijin
L180322-P-3-LD180319-0007_HL3KCCCXY_L2_clean_bin.51.orig.fa_	OEZ008529	yes	LMSG_G000008439.1	OES073751	terpene	Terpene	Polymetallic	26.50	China: Guizhou, Zhijin
L180322-P-3-LD180319-0007_HL3KCCCXY_L2_clean_bin.53.orig.fa_	OEZ008529	yes	LMSG_G000010653.1	OES073751	NRPS	NRPS	Polymetallic	24.74	China: Guizhou, Zhijin
L180322-P-3-LD180319-0007_HL3KCCCXY_L2_clean_bin.53.orig.fa_	OEZ008529	yes	LMSG_G000010653.1	OES073751	terpene	Terpene	Polymetallic	12.09	China: Guizhou, Zhijin
L180322-P-3-LD180319-0007_HL3KCCCXY_L2_clean_bin.53.orig.fa_	OEZ008529	yes	LMSG_G000010653.1	OES073751	T1PKS.NRPS-like	PKS-NRP_Hybrids	Polymetallic	17.08	China: Guizhou, Zhijin
L180322-P-3-LD180319-0007_HL3KCCCXY_L2_clean_bin.53.orig.fa_	OEZ008529	yes	LMSG_G000010653.1	OES073751	NRPS	NRPS	Polymetallic	13.25	China: Guizhou, Zhijin
L180322-P-3-LD180319-0007_HL3KCCCXY_L2_clean_bin.53.orig.fa_	OEZ008529	yes	LMSG_G000010653.1	OES073751	arylpolyene	Others	Polymetallic	32.79	China: Guizhou, Zhijin
L180322-P-3-LD180319-0007_HL3KCCCXY_L2_clean_bin.53.orig.fa_	OEZ008529	yes	LMSG_G000010653.1	OES073751	NRPS-like	NRPS	Polymetallic	17.08	China: Guizhou, Zhijin
L180322-P-3-LD180319-0007_HL3KCCCXY_L2_clean_bin.54.orig.fa_	OEZ008529	yes	LMSG_G000010135.1	OES073751	terpene	Terpene	Polymetallic	9.05	China: Guizhou, Zhijin
L180322-P-3-LD180319-0007_HL3KCCCXY_L2_clean_bin.55.strict.f	OEZ008529	yes	LMSG_G000011314.1	OES073751	NRPS	NRPS	Polymetallic	43.81	China: Guizhou, Zhijin
L180322-P-3-LD180319-0007_HL3KCCCXY_L2_clean_bin.55.strict.f	OEZ008529	yes	LMSG_G000011314.1	OES073751	terpene	Terpene	Polymetallic	21.06	China: Guizhou, Zhijin
L180322-P-3-LD180319-0007_HL3KCCCXY_L2_clean_bin.55.strict.f	OEZ008529	yes	LMSG_G000011314.1	OES073751	ladderane	Others	Polymetallic	28.96	China: Guizhou, Zhijin
L180322-P-3-LD180319-0007_HL3KCCCXY_L2_clean_bin.55.strict.f	OEZ008529	yes	LMSG_G000011314.1	OES073751	terpene	Terpene	Polymetallic	19.88	China: Guizhou, Zhijin
L180322-P-3-LD180319-0007_HL3KCCCXY_L2_clean_bin.56.orig.fa_	OEZ008529	yes	LMSG_G000007781.1	OES073751	acyl_amino_acids	Others	Polymetallic	6.33	China: Guizhou, Zhijin
L180322-P-3-LD180319-0007_HL3KCCCXY_L2_clean_bin.56.orig.fa_	OEZ008529	yes	LMSG_G000007781.1	OES073751	bacteriocin	RiPPs	Polymetallic	9.36	China: Guizhou, Zhijin
L180322-P-3-LD180319-0007_HL3KCCCXY_L2_clean_bin.56.orig.fa_	OEZ008529	yes	LMSG_G000007781.1	OES073751	terpene	Terpene	Polymetallic	30.32	China: Guizhou, Zhijin
L180322-P-3-LD180319-0007_HL3KCCCXY_L2_clean_bin.56.orig.fa_	OEZ008529	yes	LMSG_G000007781.1	OES073751	T3PKS	PKSother	Polymetallic	31.23	China: Guizhou, Zhijin
L180322-P-3-LD180319-0007_HL3KCCCXY_L2_clean_bin.56.orig.fa_	OEZ008529	yes	LMSG_G000007781.1	OES073751	hglE-KS	PKSother	Polymetallic	26.30	China: Guizhou, Zhijin
L180322-P-3-LD180319-0007_HL3KCCCXY_L2_clean_bin.6.orig.fa_k	OEZ008529	yes	LMSG_G000007799.1	OES073751	terpene	Terpene	Polymetallic	10.21	China: Guizhou, Zhijin
L180322-P-3-LD180319-0007_HL3KCCCXY_L2_clean_bin.6.orig.fa_k	OEZ008529	yes	LMSG_G000007799.1	OES073751	T3PKS	PKSother	Polymetallic	25.34	China: Guizhou, Zhijin
L180322-P-3-LD180319-0007_HL3KCCCXY_L2_clean_bin.7.orig.fa_k	OEZ008529	yes	LMSG_G000010190.1	OES073751	terpene	Terpene	Polymetallic	27.64	China: Guizhou, Zhijin
L180322-P-3-LD180319-0007_HL3KCCCXY_L2_clean_bin.7.orig.fa_k	OEZ008529	yes	LMSG_G000010190.1	OES073751	betalactone	Others	Polymetallic	18.85	China: Guizhou, Zhijin
L180322-P-3-LD180319-0007_HL3KCCCXY_L2_clean_bin.8.orig.fa_k	OEZ008529	yes	LMSG_G000010270.1	OES073751	arylpolyene	Others	Polymetallic	41.19	China: Guizhou, Zhijin
L180322-P-3-LD180319-0007_HL3KCCCXY_L2_clean_bin.8.orig.fa_k	OEZ008529	no	LMSG_G000010270.1	OES073751	terpene	Terpene	Polymetallic	7.64	China: Guizhou, Zhijin
L180322-P-3-LD180319-0007_HL3KCCCXY_L2_clean_bin.9.orig.fa_k	OEZ008529	yes	LMSG_G000005239.1	OES073751	terpene	Terpene	Polymetallic	20.63	China: Guizhou, Zhijin
L180322-P-3-LD180319-0007_HL3KCCCXY_L2_clean_bin.9.orig.fa_k	OEZ008529	yes	LMSG_G000005239.1	OES073751	hserlactone	Others	Polymetallic	20.64	China: Guizhou, Zhijin
L180322-P-3-LD180319-0007_HL3KCCCXY_L2_clean_bin.9.orig.fa_k	OEZ008529	yes	LMSG_G000005239.1	OES073751	terpene	Terpene	Polymetallic	14.26	China: Guizhou, Zhijin
L180322-P-3-LD180319-0007_HL3KCCCXY_L2_clean_bin.9.orig.fa_k	OEZ008529	yes	LMSG_G000005239.1	OES073751	T3PKS	PKSother	Polymetallic	26.84	China: Guizhou, Zhijin
L180322-P-3-LD180319-0008_HL3KCCCXY_L2_clean_bin.1.orig.fa_k	OEZ008529	yes	LMSG_G000008357.1	OES073752	terpene	Terpene	Polymetallic	7.99	China: Guizhou, Zhijin
L180322-P-3-LD180319-0008_HL3KCCCXY_L2_clean_bin.1.orig.fa_k	OEZ008529	yes	LMSG_G000008357.1	OES073752	NRPS-like	NRPS	Polymetallic	35.66	China: Guizhou, Zhijin
L180322-P-3-LD180319-0008_HL3KCCCXY_L2_clean_bin.1.orig.fa_k	OEZ008529	yes	LMSG_G000008357.1	OES073752	bacteriocin	RiPPs	Polymetallic	5.26	China: Guizhou, Zhijin
L180322-P-3-LD180319-0008_HL3KCCCXY_L2_clean_bin.10.strict.f	OEZ008529	yes	LMSG_G000009985.1	OES073752	ladderane	Others	Polymetallic	41.26	China: Guizhou, Zhijin
L180322-P-3-LD180319-0008_HL3KCCCXY_L2_clean_bin.10.strict.f	OEZ008529	yes	LMSG_G000009985.1	OES073752	NRPS-like.ectoine	Others	Polymetallic	49.50	China: Guizhou, Zhijin
L180322-P-3-LD180319-0008_HL3KCCCXY_L2_clean_bin.10.strict.f	OEZ008529	yes	LMSG_G000009985.1	OES073752	terpene	Terpene	Polymetallic	17.10	China: Guizhou, Zhijin
L180322-P-3-LD180319-0008_HL3KCCCXY_L2_clean_bin.10.strict.f	OEZ008529	yes	LMSG_G000009985.1	OES073752	arylpolyene	Others	Polymetallic	29.08	China: Guizhou, Zhijin
L180322-P-3-LD180319-0008_HL3KCCCXY_L2_clean_bin.10.strict.f	OEZ008529	yes	LMSG_G000009985.1	OES073752	terpene	Terpene	Polymetallic	12.67	China: Guizhou, Zhijin
L180322-P-3-LD180319-0008_HL3KCCCXY_L2_clean_bin.10.strict.f	OEZ008529	yes	LMSG_G000009985.1	OES073752	bacteriocin	RiPPs	Polymetallic	10.87	China: Guizhou, Zhijin
L180322-P-3-LD180319-0008_HL3KCCCXY_L2_clean_bin.10.strict.f	OEZ008529	yes	LMSG_G000009985.1	OES073752	T3PKS	PKSother	Polymetallic	9.33	China: Guizhou, Zhijin
L180322-P-3-LD180319-0008_HL3KCCCXY_L2_clean_bin.11.orig.fa_	OEZ008529	yes	LMSG_G000010191.1	OES073752	betalactone.terpene	Others	Polymetallic	55.37	China: Guizhou, Zhijin
L180322-P-3-LD180319-0008_HL3KCCCXY_L2_clean_bin.12.orig.fa_	OEZ008529	yes	LMSG_G000010773.1	OES073752	bacteriocin	RiPPs	Polymetallic	9.22	China: Guizhou, Zhijin
L180322-P-3-LD180319-0008_HL3KCCCXY_L2_clean_bin.12.orig.fa_	OEZ008529	yes	LMSG_G000010773.1	OES073752	acyl_amino_acids	Others	Polymetallic	33.88	China: Guizhou, Zhijin
L180322-P-3-LD180319-0008_HL3KCCCXY_L2_clean_bin.15.strict.f	OEZ008529	yes	LMSG_G000011315.1	OES073752	ladderane	Others	Polymetallic	28.95	China: Guizhou, Zhijin
L180322-P-3-LD180319-0008_HL3KCCCXY_L2_clean_bin.15.strict.f	OEZ008529	yes	LMSG_G000011315.1	OES073752	NRPS-like	NRPS	Polymetallic	28.63	China: Guizhou, Zhijin
L180322-P-3-LD180319-0008_HL3KCCCXY_L2_clean_bin.15.strict.f	OEZ008529	yes	LMSG_G000011315.1	OES073752	terpene	Terpene	Polymetallic	14.02	China: Guizhou, Zhijin
L180322-P-3-LD180319-0008_HL3KCCCXY_L2_clean_bin.15.strict.f	OEZ008529	yes	LMSG_G000011315.1	OES073752	terpene	Terpene	Polymetallic	20.04	China: Guizhou, Zhijin
L180322-P-3-LD180319-0008_HL3KCCCXY_L2_clean_bin.15.strict.f	OEZ008529	yes	LMSG_G000011315.1	OES073752	terpene	Terpene	Polymetallic	12.25	China: Guizhou, Zhijin
L180322-P-3-LD180319-0008_HL3KCCCXY_L2_clean_bin.16.orig.fa_	OEZ008529	yes	LMSG_G000010136.1	OES073752	acyl_amino_acids	Others	Polymetallic	19.28	China: Guizhou, Zhijin
L180322-P-3-LD180319-0008_HL3KCCCXY_L2_clean_bin.16.orig.fa_	OEZ008529	yes	LMSG_G000010136.1	OES073752	terpene	Terpene	Polymetallic	11.41	China: Guizhou, Zhijin
L180322-P-3-LD180319-0008_HL3KCCCXY_L2_clean_bin.16.orig.fa_	OEZ008529	yes	LMSG_G000010136.1	OES073752	terpene	Terpene	Polymetallic	24.84	China: Guizhou, Zhijin
L180322-P-3-LD180319-0008_HL3KCCCXY_L2_clean_bin.16.orig.fa_	OEZ008529	yes	LMSG_G000010136.1	OES073752	terpene	Terpene	Polymetallic	6.10	China: Guizhou, Zhijin
L180322-P-3-LD180319-0008_HL3KCCCXY_L2_clean_bin.17.orig.fa_	OEZ008529	yes	LMSG_G000008617.1	OES073752	other	Others	Polymetallic	20.31	China: Guizhou, Zhijin
L180322-P-3-LD180319-0008_HL3KCCCXY_L2_clean_bin.2.orig.fa_k	OEZ008529	yes	LMSG_G000010113.1	OES073752	terpene	Terpene	Polymetallic	13.87	China: Guizhou, Zhijin
L180322-P-3-LD180319-0008_HL3KCCCXY_L2_clean_bin.2.orig.fa_k	OEZ008529	yes	LMSG_G000010113.1	OES073752	hserlactone	Others	Polymetallic	20.64	China: Guizhou, Zhijin
L180322-P-3-LD180319-0008_HL3KCCCXY_L2_clean_bin.2.orig.fa_k	OEZ008529	yes	LMSG_G000010113.1	OES073752	T3PKS	PKSother	Polymetallic	41.08	China: Guizhou, Zhijin
L180322-P-3-LD180319-0008_HL3KCCCXY_L2_clean_bin.20.orig.fa_	OEZ008529	yes	LMSG_G000007785.1	OES073752	T3PKS	PKSother	Polymetallic	31.30	China: Guizhou, Zhijin
L180322-P-3-LD180319-0008_HL3KCCCXY_L2_clean_bin.20.orig.fa_	OEZ008529	yes	LMSG_G000007785.1	OES073752	bacteriocin	RiPPs	Polymetallic	10.88	China: Guizhou, Zhijin
L180322-P-3-LD180319-0008_HL3KCCCXY_L2_clean_bin.21.orig.fa_	OEZ008529	yes	LMSG_G000010703.1	OES073752	bacteriocin	RiPPs	Polymetallic	5.27	China: Guizhou, Zhijin
L180322-P-3-LD180319-0008_HL3KCCCXY_L2_clean_bin.21.orig.fa_	OEZ008529	yes	LMSG_G000010703.1	OES073752	arylpolyene	Others	Polymetallic	41.22	China: Guizhou, Zhijin
L180322-P-3-LD180319-0008_HL3KCCCXY_L2_clean_bin.21.orig.fa_	OEZ008529	yes	LMSG_G000010703.1	OES073752	NRPS.T1PKS	PKS-NRP_Hybrids	Polymetallic	21.98	China: Guizhou, Zhijin
L180322-P-3-LD180319-0008_HL3KCCCXY_L2_clean_bin.21.orig.fa_	OEZ008529	yes	LMSG_G000010703.1	OES073752	betalactone	Others	Polymetallic	12.34	China: Guizhou, Zhijin
L180322-P-3-LD180319-0008_HL3KCCCXY_L2_clean_bin.21.orig.fa_	OEZ008529	yes	LMSG_G000010703.1	OES073752	terpene	Terpene	Polymetallic	12.59	China: Guizhou, Zhijin
L180322-P-3-LD180319-0008_HL3KCCCXY_L2_clean_bin.22.orig.fa_	OEZ008529	yes	LMSG_G000008205.1	OES073752	terpene	Terpene	Polymetallic	20.86	China: Guizhou, Zhijin
L180322-P-3-LD180319-0008_HL3KCCCXY_L2_clean_bin.23.orig.fa_	OEZ008529	yes	LMSG_G000008588.1	OES073752	other	Others	Polymetallic	10.80	China: Guizhou, Zhijin
L180322-P-3-LD180319-0008_HL3KCCCXY_L2_clean_bin.23.orig.fa_	OEZ008529	yes	LMSG_G000008588.1	OES073752	NRPS-like	NRPS	Polymetallic	6.84	China: Guizhou, Zhijin
L180322-P-3-LD180319-0008_HL3KCCCXY_L2_clean_bin.23.orig.fa_	OEZ008529	yes	LMSG_G000008588.1	OES073752	other	Others	Polymetallic	13.27	China: Guizhou, Zhijin
L180322-P-3-LD180319-0008_HL3KCCCXY_L2_clean_bin.23.orig.fa_	OEZ008529	yes	LMSG_G000008588.1	OES073752	other	Others	Polymetallic	6.10	China: Guizhou, Zhijin
L180322-P-3-LD180319-0008_HL3KCCCXY_L2_clean_bin.24.orig.fa_	OEZ008529	yes	LMSG_G000010748.1	OES073752	bacteriocin	RiPPs	Polymetallic	5.25	China: Guizhou, Zhijin
L180322-P-3-LD180319-0008_HL3KCCCXY_L2_clean_bin.26.strict.f	OEZ008529	yes	LMSG_G000005240.1	OES073752	NRPS-like	NRPS	Polymetallic	31.10	China: Guizhou, Zhijin
L180322-P-3-LD180319-0008_HL3KCCCXY_L2_clean_bin.27.orig.fa_	OEZ008529	yes	LMSG_G000010220.1	OES073752	T3PKS	PKSother	Polymetallic	5.90	China: Guizhou, Zhijin
L180322-P-3-LD180319-0008_HL3KCCCXY_L2_clean_bin.28.orig.fa_	OEZ008529	yes	LMSG_G000008154.1	OES073752	terpene	Terpene	Polymetallic	17.14	China: Guizhou, Zhijin
L180322-P-3-LD180319-0008_HL3KCCCXY_L2_clean_bin.3.orig.fa_k	OEZ008529	yes	LMSG_G000005242.1	OES073752	other	Others	Polymetallic	38.64	China: Guizhou, Zhijin
L180322-P-3-LD180319-0008_HL3KCCCXY_L2_clean_bin.30.orig.fa_	OEZ008529	yes	LMSG_G000010133.1	OES073752	betalactone	Others	Polymetallic	31.27	China: Guizhou, Zhijin
L180322-P-3-LD180319-0008_HL3KCCCXY_L2_clean_bin.30.orig.fa_	OEZ008529	yes	LMSG_G000010133.1	OES073752	terpene	Terpene	Polymetallic	23.33	China: Guizhou, Zhijin
L180322-P-3-LD180319-0008_HL3KCCCXY_L2_clean_bin.30.orig.fa_	OEZ008529	yes	LMSG_G000010133.1	OES073752	terpene	Terpene	Polymetallic	21.04	China: Guizhou, Zhijin
L180322-P-3-LD180319-0008_HL3KCCCXY_L2_clean_bin.31.orig.fa_	OEZ008529	yes	LMSG_G000010654.1	OES073752	arylpolyene	Others	Polymetallic	25.24	China: Guizhou, Zhijin
L180322-P-3-LD180319-0008_HL3KCCCXY_L2_clean_bin.31.orig.fa_	OEZ008529	yes	LMSG_G000010654.1	OES073752	NRPS	NRPS	Polymetallic	29.25	China: Guizhou, Zhijin
L180322-P-3-LD180319-0008_HL3KCCCXY_L2_clean_bin.31.orig.fa_	OEZ008529	yes	LMSG_G000010654.1	OES073752	NRPS	NRPS	Polymetallic	12.51	China: Guizhou, Zhijin
L180322-P-3-LD180319-0008_HL3KCCCXY_L2_clean_bin.31.orig.fa_	OEZ008529	yes	LMSG_G000010654.1	OES073752	NRPS-like	NRPS	Polymetallic	24.41	China: Guizhou, Zhijin
L180322-P-3-LD180319-0008_HL3KCCCXY_L2_clean_bin.31.orig.fa_	OEZ008529	yes	LMSG_G000010654.1	OES073752	terpene	Terpene	Polymetallic	12.83	China: Guizhou, Zhijin
L180322-P-3-LD180319-0008_HL3KCCCXY_L2_clean_bin.31.orig.fa_	OEZ008529	yes	LMSG_G000010654.1	OES073752	T1PKS.NRPS-like	PKS-NRP_Hybrids	Polymetallic	17.61	China: Guizhou, Zhijin
L180322-P-3-LD180319-0008_HL3KCCCXY_L2_clean_bin.31.orig.fa_	OEZ008529	yes	LMSG_G000010654.1	OES073752	NRPS	NRPS	Polymetallic	28.53	China: Guizhou, Zhijin
L180322-P-3-LD180319-0008_HL3KCCCXY_L2_clean_bin.33.strict.f	OEZ008529	yes	LMSG_G000010059.1	OES073752	arylpolyene	Others	Polymetallic	40.68	China: Guizhou, Zhijin
L180322-P-3-LD180319-0008_HL3KCCCXY_L2_clean_bin.33.strict.f	OEZ008529	yes	LMSG_G000010059.1	OES073752	terpene	Terpene	Polymetallic	21.79	China: Guizhou, Zhijin
L180322-P-3-LD180319-0008_HL3KCCCXY_L2_clean_bin.33.strict.f	OEZ008529	yes	LMSG_G000010059.1	OES073752	arylpolyene	Others	Polymetallic	23.03	China: Guizhou, Zhijin
L180322-P-3-LD180319-0008_HL3KCCCXY_L2_clean_bin.33.strict.f	OEZ008529	yes	LMSG_G000010059.1	OES073752	terpene	Terpene	Polymetallic	21.99	China: Guizhou, Zhijin
L180322-P-3-LD180319-0008_HL3KCCCXY_L2_clean_bin.33.strict.f	OEZ008529	yes	LMSG_G000010059.1	OES073752	NRPS-like	NRPS	Polymetallic	23.64	China: Guizhou, Zhijin
L180322-P-3-LD180319-0008_HL3KCCCXY_L2_clean_bin.33.strict.f	OEZ008529	yes	LMSG_G000010059.1	OES073752	ectoine	Others	Polymetallic	10.40	China: Guizhou, Zhijin
L180322-P-3-LD180319-0008_HL3KCCCXY_L2_clean_bin.33.strict.f	OEZ008529	yes	LMSG_G000010059.1	OES073752	arylpolyene	Others	Polymetallic	14.16	China: Guizhou, Zhijin
L180322-P-3-LD180319-0008_HL3KCCCXY_L2_clean_bin.34.orig.fa_	OEZ008529	yes	LMSG_G000005241.1	OES073752	T1PKS.NRPS-like	PKS-NRP_Hybrids	Polymetallic	30.04	China: Guizhou, Zhijin
L180322-P-3-LD180319-0008_HL3KCCCXY_L2_clean_bin.34.orig.fa_	OEZ008529	yes	LMSG_G000005241.1	OES073752	NRPS-like	NRPS	Polymetallic	24.85	China: Guizhou, Zhijin
L180322-P-3-LD180319-0008_HL3KCCCXY_L2_clean_bin.34.orig.fa_	OEZ008529	yes	LMSG_G000005241.1	OES073752	NRPS-like	NRPS	Polymetallic	42.63	China: Guizhou, Zhijin
L180322-P-3-LD180319-0008_HL3KCCCXY_L2_clean_bin.34.orig.fa_	OEZ008529	yes	LMSG_G000005241.1	OES073752	lassopeptide	RiPPs	Polymetallic	14.38	China: Guizhou, Zhijin
L180322-P-3-LD180319-0008_HL3KCCCXY_L2_clean_bin.34.orig.fa_	OEZ008529	yes	LMSG_G000005241.1	OES073752	bacteriocin	RiPPs	Polymetallic	10.88	China: Guizhou, Zhijin
L180322-P-3-LD180319-0008_HL3KCCCXY_L2_clean_bin.34.orig.fa_	OEZ008529	yes	LMSG_G000005241.1	OES073752	terpene	Terpene	Polymetallic	18.74	China: Guizhou, Zhijin
L180322-P-3-LD180319-0008_HL3KCCCXY_L2_clean_bin.35.orig.fa_	OEZ008529	yes	LMSG_G000006327.1	OES073752	bacteriocin	RiPPs	Polymetallic	7.88	China: Guizhou, Zhijin
L180322-P-3-LD180319-0008_HL3KCCCXY_L2_clean_bin.35.orig.fa_	OEZ008529	yes	LMSG_G000006327.1	OES073752	NRPS	NRPS	Polymetallic	33.83	China: Guizhou, Zhijin
L180322-P-3-LD180319-0008_HL3KCCCXY_L2_clean_bin.35.orig.fa_	OEZ008529	yes	LMSG_G000006327.1	OES073752	bacteriocin	RiPPs	Polymetallic	8.93	China: Guizhou, Zhijin
L180322-P-3-LD180319-0008_HL3KCCCXY_L2_clean_bin.35.orig.fa_	OEZ008529	yes	LMSG_G000006327.1	OES073752	NRPS-like	NRPS	Polymetallic	27.97	China: Guizhou, Zhijin
L180322-P-3-LD180319-0008_HL3KCCCXY_L2_clean_bin.35.orig.fa_	OEZ008529	yes	LMSG_G000006327.1	OES073752	T3PKS	PKSother	Polymetallic	32.40	China: Guizhou, Zhijin
L180322-P-3-LD180319-0008_HL3KCCCXY_L2_clean_bin.35.orig.fa_	OEZ008529	yes	LMSG_G000006327.1	OES073752	CDPS	Others	Polymetallic	9.07	China: Guizhou, Zhijin
L180322-P-3-LD180319-0008_HL3KCCCXY_L2_clean_bin.35.orig.fa_	OEZ008529	yes	LMSG_G000006327.1	OES073752	betalactone	Others	Polymetallic	20.90	China: Guizhou, Zhijin
L180322-P-3-LD180319-0008_HL3KCCCXY_L2_clean_bin.35.orig.fa_	OEZ008529	yes	LMSG_G000006327.1	OES073752	CDPS	Others	Polymetallic	15.59	China: Guizhou, Zhijin
L180322-P-3-LD180319-0008_HL3KCCCXY_L2_clean_bin.35.orig.fa_	OEZ008529	yes	LMSG_G000006327.1	OES073752	bacteriocin	RiPPs	Polymetallic	5.59	China: Guizhou, Zhijin
L180322-P-3-LD180319-0008_HL3KCCCXY_L2_clean_bin.35.orig.fa_	OEZ008529	yes	LMSG_G000006327.1	OES073752	terpene	Terpene	Polymetallic	5.24	China: Guizhou, Zhijin
L180322-P-3-LD180319-0008_HL3KCCCXY_L2_clean_bin.36.orig.fa_	OEZ008529	yes	LMSG_G000010211.1	OES073752	hserlactone	Others	Polymetallic	8.26	China: Guizhou, Zhijin
L180322-P-3-LD180319-0008_HL3KCCCXY_L2_clean_bin.36.orig.fa_	OEZ008529	yes	LMSG_G000010211.1	OES073752	terpene	Terpene	Polymetallic	6.14	China: Guizhou, Zhijin
L180322-P-3-LD180319-0008_HL3KCCCXY_L2_clean_bin.37.strict.f	OEZ008529	yes	LMSG_G000008183.1	OES073752	betalactone	Others	Polymetallic	31.32	China: Guizhou, Zhijin
L180322-P-3-LD180319-0008_HL3KCCCXY_L2_clean_bin.37.strict.f	OEZ008529	yes	LMSG_G000008183.1	OES073752	terpene	Terpene	Polymetallic	20.86	China: Guizhou, Zhijin
L180322-P-3-LD180319-0008_HL3KCCCXY_L2_clean_bin.38.orig.fa_	OEZ008529	yes	LMSG_G000008600.1	OES073752	betalactone	Others	Polymetallic	15.45	China: Guizhou, Zhijin
L180322-P-3-LD180319-0008_HL3KCCCXY_L2_clean_bin.38.orig.fa_	OEZ008529	yes	LMSG_G000008600.1	OES073752	other	Others	Polymetallic	12.75	China: Guizhou, Zhijin
L180322-P-3-LD180319-0008_HL3KCCCXY_L2_clean_bin.39.orig.fa_	OEZ008529	no	LMSG_G000010970.1	OES073752	hglE-KS	PKSother	Polymetallic	8.15	China: Guizhou, Zhijin
L180322-P-3-LD180319-0008_HL3KCCCXY_L2_clean_bin.40.strict.f	OEZ008529	yes	LMSG_G000005243.1	OES073752	terpene	Terpene	Polymetallic	22.08	China: Guizhou, Zhijin
L180322-P-3-LD180319-0008_HL3KCCCXY_L2_clean_bin.40.strict.f	OEZ008529	yes	LMSG_G000005243.1	OES073752	ladderane	Others	Polymetallic	21.82	China: Guizhou, Zhijin
L180322-P-3-LD180319-0008_HL3KCCCXY_L2_clean_bin.40.strict.f	OEZ008529	yes	LMSG_G000005243.1	OES073752	arylpolyene	Others	Polymetallic	30.01	China: Guizhou, Zhijin
L180322-P-3-LD180319-0008_HL3KCCCXY_L2_clean_bin.40.strict.f	OEZ008529	yes	LMSG_G000005243.1	OES073752	T3PKS	PKSother	Polymetallic	22.21	China: Guizhou, Zhijin
L180322-P-3-LD180319-0008_HL3KCCCXY_L2_clean_bin.40.strict.f	OEZ008529	yes	LMSG_G000005243.1	OES073752	arylpolyene	Others	Polymetallic	17.02	China: Guizhou, Zhijin
L180322-P-3-LD180319-0008_HL3KCCCXY_L2_clean_bin.40.strict.f	OEZ008529	yes	LMSG_G000005243.1	OES073752	NRPS-like	NRPS	Polymetallic	16.96	China: Guizhou, Zhijin
L180322-P-3-LD180319-0008_HL3KCCCXY_L2_clean_bin.40.strict.f	OEZ008529	yes	LMSG_G000005243.1	OES073752	terpene	Terpene	Polymetallic	13.01	China: Guizhou, Zhijin
L180322-P-3-LD180319-0008_HL3KCCCXY_L2_clean_bin.41.orig.fa_	OEZ008529	yes	LMSG_G000008672.1	OES073752	NRPS-like	NRPS	Polymetallic	15.62	China: Guizhou, Zhijin
L180322-P-3-LD180319-0008_HL3KCCCXY_L2_clean_bin.42.orig.fa_	OEZ008529	yes	LMSG_G000009996.1	OES073752	terpene	Terpene	Polymetallic	18.99	China: Guizhou, Zhijin
L180322-P-3-LD180319-0008_HL3KCCCXY_L2_clean_bin.42.orig.fa_	OEZ008529	yes	LMSG_G000009996.1	OES073752	ectoine	Others	Polymetallic	6.30	China: Guizhou, Zhijin
L180322-P-3-LD180319-0008_HL3KCCCXY_L2_clean_bin.42.orig.fa_	OEZ008529	yes	LMSG_G000009996.1	OES073752	terpene	Terpene	Polymetallic	16.41	China: Guizhou, Zhijin
L180322-P-3-LD180319-0008_HL3KCCCXY_L2_clean_bin.42.orig.fa_	OEZ008529	yes	LMSG_G000009996.1	OES073752	NRPS-like	NRPS	Polymetallic	43.47	China: Guizhou, Zhijin
L180322-P-3-LD180319-0008_HL3KCCCXY_L2_clean_bin.42.orig.fa_	OEZ008529	yes	LMSG_G000009996.1	OES073752	arylpolyene	Others	Polymetallic	6.75	China: Guizhou, Zhijin
L180322-P-3-LD180319-0008_HL3KCCCXY_L2_clean_bin.44.orig.fa_	OEZ008529	yes	LMSG_G000010320.1	OES073752	bacteriocin	RiPPs	Polymetallic	9.88	China: Guizhou, Zhijin
L180322-P-3-LD180319-0008_HL3KCCCXY_L2_clean_bin.44.orig.fa_	OEZ008529	yes	LMSG_G000010320.1	OES073752	terpene	Terpene	Polymetallic	30.13	China: Guizhou, Zhijin
L180322-P-3-LD180319-0008_HL3KCCCXY_L2_clean_bin.44.orig.fa_	OEZ008529	yes	LMSG_G000010320.1	OES073752	bacteriocin	RiPPs	Polymetallic	10.86	China: Guizhou, Zhijin
L180322-P-3-LD180319-0008_HL3KCCCXY_L2_clean_bin.44.orig.fa_	OEZ008529	yes	LMSG_G000010320.1	OES073752	terpene	Terpene	Polymetallic	21.07	China: Guizhou, Zhijin
L180322-P-3-LD180319-0008_HL3KCCCXY_L2_clean_bin.45.orig.fa_	OEZ008529	yes	LMSG_G000008614.1	OES073752	other	Others	Polymetallic	15.57	China: Guizhou, Zhijin
L180322-P-3-LD180319-0008_HL3KCCCXY_L2_clean_bin.45.orig.fa_	OEZ008529	yes	LMSG_G000008614.1	OES073752	other	Others	Polymetallic	22.47	China: Guizhou, Zhijin
L180322-P-3-LD180319-0008_HL3KCCCXY_L2_clean_bin.47.orig.fa_	OEZ008529	yes	LMSG_G000010207.1	OES073752	arylpolyene	Others	Polymetallic	26.48	China: Guizhou, Zhijin
L180322-P-3-LD180319-0008_HL3KCCCXY_L2_clean_bin.48.orig.fa_	OEZ008529	yes	LMSG_G000008428.1	OES073752	NRPS.betalactone	Others	Polymetallic	51.60	China: Guizhou, Zhijin
L180322-P-3-LD180319-0008_HL3KCCCXY_L2_clean_bin.48.orig.fa_	OEZ008529	yes	LMSG_G000008428.1	OES073752	T3PKS	PKSother	Polymetallic	30.19	China: Guizhou, Zhijin
L180322-P-3-LD180319-0008_HL3KCCCXY_L2_clean_bin.48.orig.fa_	OEZ008529	yes	LMSG_G000008428.1	OES073752	betalactone	Others	Polymetallic	20.10	China: Guizhou, Zhijin
L180322-P-3-LD180319-0008_HL3KCCCXY_L2_clean_bin.48.orig.fa_	OEZ008529	yes	LMSG_G000008428.1	OES073752	terpene	Terpene	Polymetallic	26.20	China: Guizhou, Zhijin
L180322-P-3-LD180319-0008_HL3KCCCXY_L2_clean_bin.48.orig.fa_	OEZ008529	yes	LMSG_G000008428.1	OES073752	terpene	Terpene	Polymetallic	23.47	China: Guizhou, Zhijin
L180322-P-3-LD180319-0008_HL3KCCCXY_L2_clean_bin.48.orig.fa_	OEZ008529	yes	LMSG_G000008428.1	OES073752	NRPS	NRPS	Polymetallic	29.06	China: Guizhou, Zhijin
L180322-P-3-LD180319-0008_HL3KCCCXY_L2_clean_bin.48.orig.fa_	OEZ008529	yes	LMSG_G000008428.1	OES073752	other	Others	Polymetallic	41.63	China: Guizhou, Zhijin
L180322-P-3-LD180319-0008_HL3KCCCXY_L2_clean_bin.49.permissi	OEZ008529	yes	LMSG_G000008983.1	OES073752	bacteriocin	RiPPs	Polymetallic	5.64	China: Guizhou, Zhijin
L180322-P-3-LD180319-0008_HL3KCCCXY_L2_clean_bin.49.permissi	OEZ008529	yes	LMSG_G000008983.1	OES073752	hglE-KS	PKSother	Polymetallic	14.29	China: Guizhou, Zhijin
L180322-P-3-LD180319-0008_HL3KCCCXY_L2_clean_bin.5.orig.fa_k	OEZ008529	yes	LMSG_G000005244.1	OES073752	betalactone	Others	Polymetallic	23.86	China: Guizhou, Zhijin
L180322-P-3-LD180319-0008_HL3KCCCXY_L2_clean_bin.5.orig.fa_k	OEZ008529	yes	LMSG_G000005244.1	OES073752	terpene	Terpene	Polymetallic	16.41	China: Guizhou, Zhijin
L180322-P-3-LD180319-0008_HL3KCCCXY_L2_clean_bin.5.orig.fa_k	OEZ008529	yes	LMSG_G000005244.1	OES073752	betalactone	Others	Polymetallic	22.75	China: Guizhou, Zhijin
L180322-P-3-LD180319-0008_HL3KCCCXY_L2_clean_bin.5.orig.fa_k	OEZ008529	yes	LMSG_G000005244.1	OES073752	terpene	Terpene	Polymetallic	26.11	China: Guizhou, Zhijin
L180322-P-3-LD180319-0008_HL3KCCCXY_L2_clean_bin.51.orig.fa_	OEZ008529	yes	LMSG_G000008440.1	OES073752	terpene	Terpene	Polymetallic	26.50	China: Guizhou, Zhijin
L180322-P-3-LD180319-0008_HL3KCCCXY_L2_clean_bin.7.orig.fa_k	OEZ008529	yes	LMSG_G000010143.1	OES073752	terpene	Terpene	Polymetallic	11.72	China: Guizhou, Zhijin

L180322-P-3-LD180319-0008_HL3KCCCXY_L2_clean_bin.7.orig.fa_k	OEZ008529	yes	LMSG_G000010143.1	OES073752	terpene	Terpene	Polymetallic	13.28	China: Guizhou, Zhijin
L180322-P-3-LD180319-0008_HL3KCCCXY_L2_clean_bin.7.orig.fa_k	OEZ008529	yes	LMSG_G000010143.1	OES073752	terpene	Terpene	Polymetallic	7.50	China: Guizhou, Zhijin
L180322-P-3-LD180319-0008_HL3KCCCXY_L2_clean_bin.7.orig.fa_k	OEZ008529	yes	LMSG_G000010143.1	OES073752	T3PKS	PKSother	Polymetallic	31.31	China: Guizhou, Zhijin
L180322-P-3-LD180319-0008_HL3KCCCXY_L2_clean_bin.8.orig.fa_k	OEZ008529	yes	LMSG_G000005245.1	OES073752	T3PKS	PKSother	Polymetallic	15.43	China: Guizhou, Zhijin
L180322-P-3-LD180319-0008_HL3KCCCXY_L2_clean_bin.8.orig.fa_k	OEZ008529	yes	LMSG_G000005245.1	OES073752	arylpolyene	Others	Polymetallic	41.19	China: Guizhou, Zhijin
L180322-P-3-LD180319-0008_HL3KCCCXY_L2_clean_bin.8.orig.fa_k	OEZ008529	yes	LMSG_G000005245.1	OES073752	hserlactone	Others	Polymetallic	20.65	China: Guizhou, Zhijin
L180322-P-3-LD180319-0008_HL3KCCCXY_L2_clean_bin.8.orig.fa_k	OEZ008529	yes	LMSG_G000005245.1	OES073752	terpene	Terpene	Polymetallic	11.95	China: Guizhou, Zhijin
L180322-P-3-LD180319-0008_HL3KCCCXY_L2_clean_bin.8.orig.fa_k	OEZ008529	yes	LMSG_G000005245.1	OES073752	terpene	Terpene	Polymetallic	14.81	China: Guizhou, Zhijin
L180322-P-3-LD180319-0008_HL3KCCCXY_L2_clean_bin.9.orig.fa_k	OEZ008529	no	LMSG_G000010271.1	OES073752	terpene	Terpene	Polymetallic	21.52	China: Guizhou, Zhijin
L180322-P-3-LD180319-0008_HL3KCCCXY_L2_clean_bin.9.orig.fa_k	OEZ008529	yes	LMSG_G000010271.1	OES073752	arylpolyene	Others	Polymetallic	41.18	China: Guizhou, Zhijin
L180322-P-3-LD180319-0009_HL3KCCCXY_L2_clean_bin.1.orig.fa_k	OEZ008529	yes	LMSG_G000011148.1	OES073753	arylpolyene	Others	Polymetallic	8.46	China: Guizhou, Zhijin
L180322-P-3-LD180319-0009_HL3KCCCXY_L2_clean_bin.10.orig.fa_	OEZ008529	yes	LMSG_G000010114.1	OES073753	T3PKS	PKSother	Polymetallic	27.11	China: Guizhou, Zhijin
L180322-P-3-LD180319-0009_HL3KCCCXY_L2_clean_bin.10.orig.fa_	OEZ008529	yes	LMSG_G000010114.1	OES073753	terpene	Terpene	Polymetallic	20.63	China: Guizhou, Zhijin
L180322-P-3-LD180319-0009_HL3KCCCXY_L2_clean_bin.10.orig.fa_	OEZ008529	yes	LMSG_G000010114.1	OES073753	terpene	Terpene	Polymetallic	21.70	China: Guizhou, Zhijin
L180322-P-3-LD180319-0009_HL3KCCCXY_L2_clean_bin.10.orig.fa_	OEZ008529	yes	LMSG_G000010114.1	OES073753	hserlactone	Others	Polymetallic	20.64	China: Guizhou, Zhijin
L180322-P-3-LD180319-0009_HL3KCCCXY_L2_clean_bin.11.orig.fa_	OEZ008529	yes	LMSG_G000008470.1	OES073753	betalactone	Others	Polymetallic	27.74	China: Guizhou, Zhijin
L180322-P-3-LD180319-0009_HL3KCCCXY_L2_clean_bin.11.orig.fa_	OEZ008529	yes	LMSG_G000008470.1	OES073753	terpene	Terpene	Polymetallic	22.69	China: Guizhou, Zhijin
L180322-P-3-LD180319-0009_HL3KCCCXY_L2_clean_bin.11.orig.fa_	OEZ008529	yes	LMSG_G000008470.1	OES073753	arylpolyene	Others	Polymetallic	41.18	China: Guizhou, Zhijin
L180322-P-3-LD180319-0009_HL3KCCCXY_L2_clean_bin.11.orig.fa_	OEZ008529	yes	LMSG_G000008470.1	OES073753	betalactone	Others	Polymetallic	25.68	China: Guizhou, Zhijin
L180322-P-3-LD180319-0009_HL3KCCCXY_L2_clean_bin.14.orig.fa_	OEZ008529	yes	LMSG_G000010673.1	OES073753	terpene	Terpene	Polymetallic	6.36	China: Guizhou, Zhijin
L180322-P-3-LD180319-0009_HL3KCCCXY_L2_clean_bin.14.orig.fa_	OEZ008529	yes	LMSG_G000010673.1	OES073753	NRPS-like	NRPS	Polymetallic	5.80	China: Guizhou, Zhijin
L180322-P-3-LD180319-0009_HL3KCCCXY_L2_clean_bin.14.orig.fa_	OEZ008529	yes	LMSG_G000010673.1	OES073753	T1PKS	PKSI	Polymetallic	6.93	China: Guizhou, Zhijin
L180322-P-3-LD180319-0009_HL3KCCCXY_L2_clean_bin.14.orig.fa_	OEZ008529	yes	LMSG_G000010673.1	OES073753	CDPS.arylpolyene	Others	Polymetallic	33.42	China: Guizhou, Zhijin
L180322-P-3-LD180319-0009_HL3KCCCXY_L2_clean_bin.14.orig.fa_	OEZ008529	yes	LMSG_G000010673.1	OES073753	bacteriocin	RiPPs	Polymetallic	10.41	China: Guizhou, Zhijin
L180322-P-3-LD180319-0009_HL3KCCCXY_L2_clean_bin.15.orig.fa_	OEZ008529	yes	LMSG_G000007782.1	OES073753	T3PKS	PKSother	Polymetallic	16.75	China: Guizhou, Zhijin
L180322-P-3-LD180319-0009_HL3KCCCXY_L2_clean_bin.15.orig.fa_	OEZ008529	yes	LMSG_G000007782.1	OES073753	T3PKS	PKSother	Polymetallic	24.30	China: Guizhou, Zhijin
L180322-P-3-LD180319-0009_HL3KCCCXY_L2_clean_bin.15.orig.fa_	OEZ008529	yes	LMSG_G000007782.1	OES073753	bacteriocin	RiPPs	Polymetallic	10.88	China: Guizhou, Zhijin
L180322-P-3-LD180319-0009_HL3KCCCXY_L2_clean_bin.15.orig.fa_	OEZ008529	yes	LMSG_G000007782.1	OES073753	terpene	Terpene	Polymetallic	30.32	China: Guizhou, Zhijin
L180322-P-3-LD180319-0009_HL3KCCCXY_L2_clean_bin.15.orig.fa_	OEZ008529	yes	LMSG_G000007782.1	OES073753	hglE-KS	PKSother	Polymetallic	43.71	China: Guizhou, Zhijin
L180322-P-3-LD180319-0009_HL3KCCCXY_L2_clean_bin.16.orig.fa_	OEZ008529	yes	LMSG_G000008569.1	OES073753	NRPS-like	NRPS	Polymetallic	34.47	China: Guizhou, Zhijin
L180322-P-3-LD180319-0009_HL3KCCCXY_L2_clean_bin.16.orig.fa_	OEZ008529	yes	LMSG_G000008569.1	OES073753	other	Others	Polymetallic	26.95	China: Guizhou, Zhijin
L180322-P-3-LD180319-0009_HL3KCCCXY_L2_clean_bin.17.strict.f	OEZ008529	yes	LMSG_G000010655.1	OES073753	arylpolyene	Others	Polymetallic	41.29	China: Guizhou, Zhijin
L180322-P-3-LD180319-0009_HL3KCCCXY_L2_clean_bin.17.strict.f	OEZ008529	yes	LMSG_G000010655.1	OES073753	NRPS	NRPS	Polymetallic	23.17	China: Guizhou, Zhijin
L180322-P-3-LD180319-0009_HL3KCCCXY_L2_clean_bin.17.strict.f	OEZ008529	yes	LMSG_G000010655.1	OES073753	NRPS-like	NRPS	Polymetallic	42.88	China: Guizhou, Zhijin
L180322-P-3-LD180319-0009_HL3KCCCXY_L2_clean_bin.17.strict.f	OEZ008529	yes	LMSG_G000010655.1	OES073753	NRPS.T1PKS	PKS-NRP_Hybrids	Polymetallic	31.86	China: Guizhou, Zhijin
L180322-P-3-LD180319-0009_HL3KCCCXY_L2_clean_bin.17.strict.f	OEZ008529	yes	LMSG_G000010655.1	OES073753	terpene	Terpene	Polymetallic	12.02	China: Guizhou, Zhijin
L180322-P-3-LD180319-0009_HL3KCCCXY_L2_clean_bin.18.orig.fa_	OEZ008529	yes	LMSG_G000010704.1	OES073753	bacteriocin	RiPPs	Polymetallic	6.64	China: Guizhou, Zhijin
L180322-P-3-LD180319-0009_HL3KCCCXY_L2_clean_bin.18.orig.fa_	OEZ008529	yes	LMSG_G000010704.1	OES073753	arylpolyene	Others	Polymetallic	23.79	China: Guizhou, Zhijin
L180322-P-3-LD180319-0009_HL3KCCCXY_L2_clean_bin.18.orig.fa_	OEZ008529	yes	LMSG_G000010704.1	OES073753	terpene	Terpene	Polymetallic	12.71	China: Guizhou, Zhijin
L180322-P-3-LD180319-0009_HL3KCCCXY_L2_clean_bin.18.orig.fa_	OEZ008529	yes	LMSG_G000010704.1	OES073753	NRPS.T1PKS	PKS-NRP_Hybrids	Polymetallic	36.76	China: Guizhou, Zhijin
L180322-P-3-LD180319-0009_HL3KCCCXY_L2_clean_bin.19.orig.fa_	OEZ008529	yes	LMSG_G000004453.1	OES073753	terpene	Terpene	Polymetallic	20.10	China: Guizhou, Zhijin
L180322-P-3-LD180319-0009_HL3KCCCXY_L2_clean_bin.19.orig.fa_	OEZ008529	yes	LMSG_G000004453.1	OES073753	ladderane	Others	Polymetallic	38.37	China: Guizhou, Zhijin
L180322-P-3-LD180319-0009_HL3KCCCXY_L2_clean_bin.19.orig.fa_	OEZ008529	yes	LMSG_G000004453.1	OES073753	terpene	Terpene	Polymetallic	20.81	China: Guizhou, Zhijin
L180322-P-3-LD180319-0009_HL3KCCCXY_L2_clean_bin.2.permissiv	OEZ008529	yes	LMSG_G000010066.1	OES073753	bacteriocin	RiPPs	Polymetallic	6.76	China: Guizhou, Zhijin
L180322-P-3-LD180319-0009_HL3KCCCXY_L2_clean_bin.2.permissiv	OEZ008529	yes	LMSG_G000010066.1	OES073753	terpene	Terpene	Polymetallic	5.99	China: Guizhou, Zhijin
L180322-P-3-LD180319-0009_HL3KCCCXY_L2_clean_bin.20.orig.fa_	OEZ008529	yes	LMSG_G000010309.1	OES073753	terpene	Terpene	Polymetallic	15.13	China: Guizhou, Zhijin
L180322-P-3-LD180319-0009_HL3KCCCXY_L2_clean_bin.20.orig.fa_	OEZ008529	yes	LMSG_G000010309.1	OES073753	hserlactone	Others	Polymetallic	11.54	China: Guizhou, Zhijin
L180322-P-3-LD180319-0009_HL3KCCCXY_L2_clean_bin.20.orig.fa_	OEZ008529	yes	LMSG_G000010309.1	OES073753	terpene	Terpene	Polymetallic	10.29	China: Guizhou, Zhijin
L180322-P-3-LD180319-0009_HL3KCCCXY_L2_clean_bin.20.orig.fa_	OEZ008529	yes	LMSG_G000010309.1	OES073753	T3PKS	PKSother	Polymetallic	6.57	China: Guizhou, Zhijin
L180322-P-3-LD180319-0009_HL3KCCCXY_L2_clean_bin.20.orig.fa_	OEZ008529	yes	LMSG_G000010309.1	OES073753	terpene	Terpene	Polymetallic	11.96	China: Guizhou, Zhijin
L180322-P-3-LD180319-0009_HL3KCCCXY_L2_clean_bin.20.orig.fa_	OEZ008529	yes	LMSG_G000010309.1	OES073753	arylpolyene	Others	Polymetallic	7.11	China: Guizhou, Zhijin
L180322-P-3-LD180319-0009_HL3KCCCXY_L2_clean_bin.21.orig.fa_	OEZ008529	yes	LMSG_G000010977.1	OES073753	T1PKS.hglE-KS	PKSother	Polymetallic	29.59	China: Guizhou, Zhijin
L180322-P-3-LD180319-0009_HL3KCCCXY_L2_clean_bin.23.orig.fa_	OEZ008529	yes	LMSG_G000008456.1	OES073753	betalactone	Others	Polymetallic	22.65	China: Guizhou, Zhijin
L180322-P-3-LD180319-0009_HL3KCCCXY_L2_clean_bin.23.orig.fa_	OEZ008529	yes	LMSG_G000008456.1	OES073753	terpene	Terpene	Polymetallic	16.13	China: Guizhou, Zhijin
L180322-P-3-LD180319-0009_HL3KCCCXY_L2_clean_bin.23.orig.fa_	OEZ008529	yes	LMSG_G000008456.1	OES073753	terpene	Terpene	Polymetallic	21.06	China: Guizhou, Zhijin
L180322-P-3-LD180319-0009_HL3KCCCXY_L2_clean_bin.23.orig.fa_	OEZ008529	yes	LMSG_G000008456.1	OES073753	betalactone	Others	Polymetallic	16.37	China: Guizhou, Zhijin
L180322-P-3-LD180319-0009_HL3KCCCXY_L2_clean_bin.24.orig.fa_	OEZ008529	yes	LMSG_G000008441.1	OES073753	terpene	Terpene	Polymetallic	15.82	China: Guizhou, Zhijin
L180322-P-3-LD180319-0009_HL3KCCCXY_L2_clean_bin.24.orig.fa_	OEZ008529	yes	LMSG_G000008441.1	OES073753	terpene	Terpene	Polymetallic	10.66	China: Guizhou, Zhijin
L180322-P-3-LD180319-0009_HL3KCCCXY_L2_clean_bin.26.strict.f	OEZ008529	yes	LMSG_G000008615.1	OES073753	other	Others	Polymetallic	21.49	China: Guizhou, Zhijin
L180322-P-3-LD180319-0009_HL3KCCCXY_L2_clean_bin.27.orig.fa_	OEZ008529	yes	LMSG_G000010321.1	OES073753	bacteriocin	RiPPs	Polymetallic	10.86	China: Guizhou, Zhijin
L180322-P-3-LD180319-0009_HL3KCCCXY_L2_clean_bin.27.orig.fa_	OEZ008529	yes	LMSG_G000010321.1	OES073753	terpene	Terpene	Polymetallic	15.80	China: Guizhou, Zhijin
L180322-P-3-LD180319-0009_HL3KCCCXY_L2_clean_bin.27.orig.fa_	OEZ008529	yes	LMSG_G000010321.1	OES073753	terpene	Terpene	Polymetallic	30.13	China: Guizhou, Zhijin
L180322-P-3-LD180319-0009_HL3KCCCXY_L2_clean_bin.27.orig.fa_	OEZ008529	yes	LMSG_G000010321.1	OES073753	bacteriocin	RiPPs	Polymetallic	10.86	China: Guizhou, Zhijin
L180322-P-3-LD180319-0009_HL3KCCCXY_L2_clean_bin.28.orig.fa_	OEZ008529	yes	LMSG_G000007800.1	OES073753	terpene	Terpene	Polymetallic	7.18	China: Guizhou, Zhijin
L180322-P-3-LD180319-0009_HL3KCCCXY_L2_clean_bin.28.orig.fa_	OEZ008529	yes	LMSG_G000007800.1	OES073753	T3PKS	PKSother	Polymetallic	23.55	China: Guizhou, Zhijin
L180322-P-3-LD180319-0009_HL3KCCCXY_L2_clean_bin.28.orig.fa_	OEZ008529	yes	LMSG_G000007800.1	OES073753	terpene	Terpene	Polymetallic	13.20	China: Guizhou, Zhijin
L180322-P-3-LD180319-0009_HL3KCCCXY_L2_clean_bin.29.orig.fa_	OEZ008529	yes	LMSG_G000008458.1	OES073753	betalactone	Others	Polymetallic	22.75	China: Guizhou, Zhijin
L180322-P-3-LD180319-0009_HL3KCCCXY_L2_clean_bin.29.orig.fa_	OEZ008529	yes	LMSG_G000008458.1	OES073753	terpene	Terpene	Polymetallic	20.83	China: Guizhou, Zhijin
L180322-P-3-LD180319-0009_HL3KCCCXY_L2_clean_bin.29.orig.fa_	OEZ008529	yes	LMSG_G000008458.1	OES073753	betalactone	Others	Polymetallic	23.86	China: Guizhou, Zhijin
L180322-P-3-LD180319-0009_HL3KCCCXY_L2_clean_bin.29.orig.fa_	OEZ008529	yes	LMSG_G000008458.1	OES073753	terpene	Terpene	Polymetallic	26.11	China: Guizhou, Zhijin
L180322-P-3-LD180319-0009_HL3KCCCXY_L2_clean_bin.3.strict.fa	OEZ008529	yes	LMSG_G000009967.1	OES073753	terpene	Terpene	Polymetallic	18.71	China: Guizhou, Zhijin
L180322-P-3-LD180319-0009_HL3KCCCXY_L2_clean_bin.3.strict.fa	OEZ008529	yes	LMSG_G000009967.1	OES073753	NRPS-like	NRPS	Polymetallic	11.54	China: Guizhou, Zhijin
L180322-P-3-LD180319-0009_HL3KCCCXY_L2_clean_bin.3.strict.fa	OEZ008529	yes	LMSG_G000009967.1	OES073753	arylpolyene	Others	Polymetallic	11.28	China: Guizhou, Zhijin
L180322-P-3-LD180319-0009_HL3KCCCXY_L2_clean_bin.3.strict.fa	OEZ008529	yes	LMSG_G000009967.1	OES073753	arylpolyene	Others	Polymetallic	9.73	China: Guizhou, Zhijin
L180322-P-3-LD180319-0009_HL3KCCCXY_L2_clean_bin.30.orig.fa_	OEZ008529	yes	LMSG_G000010144.1	OES073753	terpene	Terpene	Polymetallic	8.88	China: Guizhou, Zhijin
L180322-P-3-LD180319-0009_HL3KCCCXY_L2_clean_bin.30.orig.fa_	OEZ008529	yes	LMSG_G000010144.1	OES073753	T3PKS	PKSother	Polymetallic	15.55	China: Guizhou, Zhijin
L180322-P-3-LD180319-0009_HL3KCCCXY_L2_clean_bin.30.orig.fa_	OEZ008529	yes	LMSG_G000010144.1	OES073753	terpene	Terpene	Polymetallic	12.13	China: Guizhou, Zhijin
L180322-P-3-LD180319-0009_HL3KCCCXY_L2_clean_bin.30.orig.fa_	OEZ008529	no	LMSG_G000010144.1	OES073753	terpene	Terpene	Polymetallic	5.16	China: Guizhou, Zhijin
L180322-P-3-LD180319-0009_HL3KCCCXY_L2_clean_bin.30.orig.fa_	OEZ008529	yes	LMSG_G000010144.1	OES073753	T3PKS	PKSother	Polymetallic	20.44	China: Guizhou, Zhijin
L180322-P-3-LD180319-0009_HL3KCCCXY_L2_clean_bin.31.permissi	OEZ008529	yes	LMSG_G000008986.1	OES073753	bacteriocin	RiPPs	Polymetallic	9.21	China: Guizhou, Zhijin
L180322-P-3-LD180319-0009_HL3KCCCXY_L2_clean_bin.31.permissi	OEZ008529	yes	LMSG_G000008986.1	OES073753	T1PKS	PKSI	Polymetallic	9.80	China: Guizhou, Zhijin
L180322-P-3-LD180319-0009_HL3KCCCXY_L2_clean_bin.31.permissi	OEZ008529	yes	LMSG_G000008986.1	OES073753	hglE-KS	PKSother	Polymetallic	6.25	China: Guizhou, Zhijin
L180322-P-3-LD180319-0009_HL3KCCCXY_L2_clean_bin.32.orig.fa_	OEZ008529	yes	LMSG_G000008664.1	OES073753	NRPS-like	NRPS	Polymetallic	16.11	China: Guizhou, Zhijin
L180322-P-3-LD180319-0009_HL3KCCCXY_L2_clean_bin.34.orig.fa_	OEZ008529	yes	LMSG_G000008206.1	OES073753	terpene	Terpene	Polymetallic	20.86	China: Guizhou, Zhijin
L180322-P-3-LD180319-0009_HL3KCCCXY_L2_clean_bin.35.strict.f	OEZ008529	yes	LMSG_G000011329.1	OES073753	T3PKS	PKSother	Polymetallic	9.07	China: Guizhou, Zhijin
L180322-P-3-LD180319-0009_HL3KCCCXY_L2_clean_bin.35.strict.f	OEZ008529	yes	LMSG_G000011329.1	OES073753	NRPS-like	NRPS	Polymetallic	7.06	China: Guizhou, Zhijin
L180322-P-3-LD180319-0009_HL3KCCCXY_L2_clean_bin.36.orig.fa_	OEZ008529	yes	LMSG_G000010426.1	OES073753	terpene	Terpene	Polymetallic	15.54	China: Guizhou, Zhijin
L180322-P-3-LD180319-0009_HL3KCCCXY_L2_clean_bin.36.orig.fa_	OEZ008529	yes	LMSG_G000010426.1	OES073753	terpene	Terpene	Polymetallic	25.02	China: Guizhou, Zhijin
L180322-P-3-LD180319-0009_HL3KCCCXY_L2_clean_bin.37.orig.fa_	OEZ008529	yes	LMSG_G000010129.1	OES073753	bacteriocin	RiPPs	Polymetallic	10.88	China: Guizhou, Zhijin
L180322-P-3-LD180319-0009_HL3KCCCXY_L2_clean_bin.37.orig.fa_	OEZ008529	yes	LMSG_G000010129.1	OES073753	terpene	Terpene	Polymetallic	19.52	China: Guizhou, Zhijin
L180322-P-3-LD180319-0009_HL3KCCCXY_L2_clean_bin.37.orig.fa_	OEZ008529	yes	LMSG_G000010129.1	OES073753	hserlactone	Others	Polymetallic	13.78	China: Guizhou, Zhijin
L180322-P-3-LD180319-0009_HL3KCCCXY_L2_clean_bin.37.orig.fa_	OEZ008529	yes	LMSG_G000010129.1	OES073753	terpene	Terpene	Polymetallic	11.41	China: Guizhou, Zhijin
L180322-P-3-LD180319-0009_HL3KCCCXY_L2_clean_bin.37.orig.fa_	OEZ008529	yes	LMSG_G000010129.1	OES073753	arylpolyene	Others	Polymetallic	31.34	China: Guizhou, Zhijin
L180322-P-3-LD180319-0009_HL3KCCCXY_L2_clean_bin.38.orig.fa_	OEZ008529	yes	LMSG_G000010662.1	OES073753	NRPS.T1PKS	PKS-NRP_Hybrids	Polymetallic	9.49	China: Guizhou, Zhijin
L180322-P-3-LD180319-0009_HL3KCCCXY_L2_clean_bin.38.orig.fa_	OEZ008529	yes	LMSG_G000010662.1	OES073753	NRPS	NRPS	Polymetallic	5.12	China: Guizhou, Zhijin
L180322-P-3-LD180319-0009_HL3KCCCXY_L2_clean_bin.38.orig.fa_	OEZ008529	yes	LMSG_G000010662.1	OES073753	bacteriocin	RiPPs	Polymetallic	7.45	China: Guizhou, Zhijin
L180322-P-3-LD180319-0009_HL3KCCCXY_L2_clean_bin.39.orig.fa_	OEZ008529	yes	LMSG_G000010800.1	OES073753	acyl_amino_acids	Others	Polymetallic	11.70	China: Guizhou, Zhijin
L180322-P-3-LD180319-0009_HL3KCCCXY_L2_clean_bin.40.strict.f	OEZ008529	yes	LMSG_G000010042.1	OES073753	T3PKS	PKSother	Polymetallic	41.09	China: Guizhou, Zhijin
L180322-P-3-LD180319-0009_HL3KCCCXY_L2_clean_bin.40.strict.f	OEZ008529	yes	LMSG_G000010042.1	OES073753	terpene	Terpene	Polymetallic	21.81	China: Guizhou, Zhijin
L180322-P-3-LD180319-0009_HL3KCCCXY_L2_clean_bin.40.strict.f	OEZ008529	yes	LMSG_G000010042.1	OES073753	arylpolyene	Others	Polymetallic	34.28	China: Guizhou, Zhijin
L180322-P-3-LD180319-0009_HL3KCCCXY_L2_clean_bin.40.strict.f	OEZ008529	yes	LMSG_G000010042.1	OES073753	ladderane	Others	Polymetallic	22.43	China: Guizhou, Zhijin
L180322-P-3-LD180319-0009_HL3KCCCXY_L2_clean_bin.40.strict.f	OEZ008529	yes	LMSG_G000010042.1	OES073753	terpene	Terpene	Polymetallic	10.77	China: Guizhou, Zhijin
L180322-P-3-LD180319-0009_HL3KCCCXY_L2_clean_bin.41.orig.fa_	OEZ008529	yes	LMSG_G000008565.1	OES073753	other	Others	Polymetallic	18.80	China: Guizhou, Zhijin
L180322-P-3-LD180319-0009_HL3KCCCXY_L2_clean_bin.42.orig.fa_	OEZ008529	yes	LMSG_G000008621.1	OES073753	NRPS-like	NRPS	Polymetallic	10.62	China: Guizhou, Zhijin
L180322-P-3-LD180319-0009_HL3KCCCXY_L2_clean_bin.42.orig.fa_	OEZ008529	yes	LMSG_G000008621.1	OES073753	other	Others	Polymetallic	21.28	China: Guizhou, Zhijin
L180322-P-3-LD180319-0009_HL3KCCCXY_L2_clean_bin.43.orig.fa_	OEZ008529	yes	LMSG_G000010192.1	OES073753	betalactone.terpene	Others	Polymetallic	46.91	China: Guizhou, Zhijin
L180322-P-3-LD180319-0009_HL3KCCCXY_L2_clean_bin.46.orig.fa_	OEZ008529	yes	LMSG_G000008552.1	OES073753	NRPS-like	NRPS	Polymetallic	12.57	China: Guizhou, Zhijin
L180322-P-3-LD180319-0009_HL3KCCCXY_L2_clean_bin.47.orig.fa_	OEZ008529	yes	LMSG_G000006328.1	OES073753	CDPS	Others	Polymetallic	9.00	China: Guizhou, Zhijin
L180322-P-3-LD180319-0009_HL3KCCCXY_L2_clean_bin.47.orig.fa_	OEZ008529	yes	LMSG_G000006328.1	OES073753	NRPS-like	NRPS	Polymetallic	26.23	China: Guizhou, Zhijin
L180322-P-3-LD180319-0009_HL3KCCCXY_L2_clean_bin.47.orig.fa_	OEZ008529	yes	LMSG_G000006328.1	OES073753	betalactone	Others	Polymetallic	20.90	China: Guizhou, Zhijin
L180322-P-3-LD180319-0009_HL3KCCCXY_L2_clean_bin.47.orig.fa_	OEZ008529	yes	LMSG_G000006328.1	OES073753	CDPS	Others	Polymetallic	15.57	China: Guizhou, Zhijin
L180322-P-3-LD180319-0009_HL3KCCCXY_L2_clean_bin.47.orig.fa_	OEZ008529	yes	LMSG_G000006328.1	OES073753	bacteriocin	RiPPs	Polymetallic	7.88	China: Guizhou, Zhijin
L180322-P-3-LD180319-0009_HL3KCCCXY_L2_clean_bin.47.orig.fa_	OEZ008529	yes	LMSG_G000006328.1	OES073753	terpene	Terpene	Polymetallic	5.13	China: Guizhou, Zhijin
L180322-P-3-LD180319-0009_HL3KCCCXY_L2_clean_bin.47.orig.fa_	OEZ008529	yes	LMSG_G000006328.1	OES073753	NRPS	NRPS	Polymetallic	19.76	China: Guizhou, Zhijin
L180322-P-3-LD180319-0009_HL3KCCCXY_L2_clean_bin.47.orig.fa_	OEZ008529	yes	LMSG_G000006328.1	OES073753	T3PKS	PKSother	Polymetallic	35.50	China: Guizhou, Zhijin
L180322-P-3-LD180319-0009_HL3KCCCXY_L2_clean_bin.47.orig.fa_	OEZ008529	yes	LMSG_G000006328.1	OES073753	NRPS	NRPS	Polymetallic	43.12	China: Guizhou, Zhijin
L180322-P-3-LD180319-0009_HL3KCCCXY_L2_clean_bin.47.orig.fa_	OEZ008529	yes	LMSG_G000006328.1	OES073753	bacteriocin	RiPPs	Polymetallic	10.57	China: Guizhou, Zhijin
L180322-P-3-LD180319-0009_HL3KCCCXY_L2_clean_bin.48.strict.f	OEZ008529	yes	LMSG_G000008150.1	OES073753	terpene	Terpene	Polymetallic	15.96	China: Guizhou, Zhijin
L180322-P-3-LD180319-0009_HL3KCCCXY_L2_clean_bin.5.orig.fa_k	OEZ008529	yes	LMSG_G000009986.1	OES073753	bacteriocin	RiPPs	Polymetallic	7.82	China: Guizhou, Zhijin
L180322-P-3-LD180319-0009_HL3KCCCXY_L2_clean_bin.5.orig.fa_k	OEZ008529	yes	LMSG_G000009986.1	OES073753	terpene	Terpene	Polymetallic	5.46	China: Guizhou, Zhijin
L180322-P-3-LD180319-0009_HL3KCCCXY_L2_clean_bin.5.orig.fa_k	OEZ008529	yes	LMSG_G000009986.1	OES073753	T3PKS	PKSother	Polymetallic	5.17	China: Guizhou, Zhijin
L180322-P-3-LD180319-0009_HL3KCCCXY_L2_clean_bin.5.orig.fa_k	OEZ008529	yes	LMSG_G000009986.1	OES073753	ladderane	Others	Polymetallic	20.51	China: Guizhou, Zhijin
L180322-P-3-LD180319-0009_HL3KCCCXY_L2_clean_bin.5.orig.fa_k	OEZ008529	yes	LMSG_G000009986.1	OES073753	arylpolyene	Others	Polymetallic	9.89	China: Guizhou, Zhijin
L180322-P-3-LD180319-0009_HL3KCCCXY_L2_clean_bin.5.orig.fa_k	OEZ008529	yes	LMSG_G000009986.1	OES073753	terpene	Terpene	Polymetallic	12.05	China: Guizhou, Zhijin
L180322-P-3-LD180319-0009_HL3KCCCXY_L2_clean_bin.5.orig.fa_k	OEZ008529	yes	LMSG_G000009986.1	OES073753	arylpolyene	Others	Polymetallic	19.60	China: Guizhou, Zhijin
L180322-P-3-LD180319-0009_HL3KCCCXY_L2_clean_bin.5.orig.fa_k	OEZ008529	yes	LMSG_G000009986.1	OES073753	NRPS-like.ectoine	Others	Polymetallic	33.80	China: Guizhou, Zhijin
L180322-P-3-LD180319-0009_HL3KCCCXY_L2_clean_bin.51.orig.fa_	OEZ008529	yes	LMSG_G000010272.1	OES073753	arylpolyene	Others	Polymetallic	29.52	China: Guizhou, Zhijin
L180322-P-3-LD180319-0009_HL3KCCCXY_L2_clean_bin.51.orig.fa_	OEZ008529	no	LMSG_G000010272.1	OES073753	terpene	Terpene	Polymetallic	12.49	China: Guizhou, Zhijin
L180322-P-3-LD180319-0009_HL3KCCCXY_L2_clean_bin.52.orig.fa_	OEZ008529	yes	LMSG_G000010749.1	OES073753	bacteriocin	RiPPs	Polymetallic	5.42	China: Guizhou, Zhijin
L180322-P-3-LD180319-0009_HL3KCCCXY_L2_clean_bin.53.orig.fa_	OEZ008529	yes	LMSG_G000005248.1	OES073753	terpene	Terpene	Polymetallic	21.04	China: Guizhou, Zhijin
L180322-P-3-LD180319-0009_HL3KCCCXY_L2_clean_bin.53.orig.fa_	OEZ008529	yes	LMSG_G000005248.1	OES073753	betalactone	Others	Polymetallic	31.27	China: Guizhou, Zhijin
L180322-P-3-LD180319-0009_HL3KCCCXY_L2_clean_bin.53.orig.fa_	OEZ008529	yes	LMSG_G000005248.1	OES073753	terpene	Terpene	Polymetallic	23.31	China: Guizhou, Zhijin
L180322-P-3-LD180319-0009_HL3KCCCXY_L2_clean_bin.54.orig.fa_	OEZ008529	yes	LMSG_G000008618.1	OES073753	other	Others	Polymetallic	40.71	China: Guizhou, Zhijin
L180322-P-3-LD180319-0009_HL3KCCCXY_L2_clean_bin.55.orig.fa_	OEZ008529	yes	LMSG_G000008358.1	OES073753	terpene	Terpene	Polymetallic	11.24	China: Guizhou, Zhijin
L180322-P-3-LD180319-0009_HL3KCCCXY_L2_clean_bin.55.orig.fa_	OEZ008529	yes	LMSG_G000008358.1	OES073753	NRPS-like	NRPS	Polymetallic	28.09	China: Guizhou, Zhijin
L180322-P-3-LD180319-0009_HL3KCCCXY_L2_clean_bin.55.orig.fa_	OEZ008529	yes	LMSG_G000008358.1	OES073753	bacteriocin	RiPPs	Polymetallic	7.53	China: Guizhou, Zhijin
L180322-P-3-LD180319-0009_HL3KCCCXY_L2_clean_bin.6.orig.fa_k	OEZ008529	yes	LMSG_G000009997.1	OES073753	terpene	Terpene	Polymetallic	11.98	China: Guizhou, Zhijin
L180322-P-3-LD180319-0009_HL3KCCCXY_L2_clean_bin.6.orig.fa_k	OEZ008529	yes	LMSG_G000009997.1	OES073753	arylpolyene	Others	Polymetallic	24.01	China: Guizhou, Zhijin
L180322-P-3-LD180319-0009_HL3KCCCXY_L2_clean_bin.6.orig.fa_k	OEZ008529	yes	LMSG_G000009997.1	OES073753	NRPS-like	NRPS	Polymetallic	32.21	China: Guizhou, Zhijin
L180322-P-3-LD180319-0009_HL3KCCCXY_L2_clean_bin.6.orig.fa_k	OEZ008529	yes	LMSG_G000009997.1	OES073753	terpene	Terpene	Polymetallic	12.17	China: Guizhou, Zhijin
L180322-P-3-LD180319-0009_HL3KCCCXY_L2_clean_bin.6.orig.fa_k	OEZ008529	yes	LMSG_G000009997.1	OES073753	arylpolyene	Others	Polymetallic	25.21	China: Guizhou, Zhijin
L180322-P-3-LD180319-0009_HL3KCCCXY_L2_clean_bin.6.orig.fa_k	OEZ008529	yes	LMSG_G000009997.1	OES073753	ectoine	Others	Polymetallic	7.27	China: Guizhou, Zhijin
L180322-P-3-LD180319-0009_HL3KCCCXY_L2_clean_bin.7.orig.fa_k	OEZ008529	yes	LMSG_G000004454.1	OES073753	betalactone	Others	Polymetallic	15.88	China: Guizhou, Zhijin
L180322-P-3-LD180319-0009_HL3KCCCXY_L2_clean_bin.7.orig.fa_k	OEZ008529	yes	LMSG_G000004454.1	OES073753	NRPS	NRPS	Polymetallic	29.05	China: Guizhou, Zhijin
L180322-P-3-LD180319-0009_HL3KCCCXY_L2_clean_bin.7.orig.fa_k	OEZ008529	yes	LMSG_G000004454.1	OES073753	NRPS.betalactone	Others	Polymetallic	51.61	China: Guizhou, Zhijin
L180322-P-3-LD180319-0009_HL3KCCCXY_L2_clean_bin.7.orig.fa_k	OEZ008529	yes	LMSG_G000004454.1	OES073753	T3PKS	PKSother	Polymetallic	30.19	China: Guizhou, Zhijin
L180322-P-3-LD180319-0009_HL3KCCCXY_L2_clean_bin.7.orig.fa_k	OEZ008529	yes	LMSG_G000004454.1	OES073753	other	Others	Polymetallic	41.51	China: Guizhou, Zhijin
L180322-P-3-LD180319-0009_HL3KCCCXY_L2_clean_bin.7.orig.fa_k	OEZ008529	yes	LMSG_G000004454.1	OES073753	terpene	Terpene	Polymetallic	26.20	China: Guizhou, Zhijin
L180322-P-3-LD180319-0009_HL3KCCCXY_L2_clean_bin.7.orig.fa_k	OEZ008529	yes	LMSG_G000004454.1	OES073753	terpene	Terpene	Polymetallic	23.47	China: Guizhou, Zhijin
L180322-P-3-LD180319-0009_HL3KCCCXY_L2_clean_bin.9.orig.fa_k	OEZ008529	yes	LMSG_G000010125.1	OES073753	terpene	Terpene	Polymetallic	7.69	China: Guizhou, Zhijin
L180322-P-4-LD180319-0010_HL3KCCCXY_L3_clean_bin.1.orig.fa_k	OEZ008529	yes	LMSG_G000010093.1	OES073754	terpene	Terpene	Polymetallic	6.77	China: Guizhou, Zhijin
L180322-P-4-LD180319-0010_HL3KCCCXY_L3_clean_bin.10.orig.fa_	OEZ008529	yes	LMSG_G000010761.1	OES073754	bacteriocin	RiPPs	Polymetallic	9.22	China: Guizhou, Zhijin
L180322-P-4-LD180319-0010_HL3KCCCXY_L3_clean_bin.10.orig.fa_	OEZ008529	yes	LMSG_G000010761.1	OES073754	acyl_amino_acids	Others	Polymetallic	39.00	China: Guizhou, Zhijin
L180322-P-4-LD180319-0010_HL3KCCCXY_L3_clean_bin.11.orig.fa_	OEZ008529	yes	LMSG_G000011162.1	OES073754	arylpolyene	Others	Polymetallic	17.87	China: Guizhou, Zhijin
L180322-P-4-LD180319-0010_HL3KCCCXY_L3_clean_bin.11.orig.fa_	OEZ008529	yes	LMSG_G000011162.1	OES073754	bacteriocin	RiPPs	Polymetallic	7.62	China: Guizhou, Zhijin
L180322-P-4-LD180319-0010_HL3KCCCXY_L3_clean_bin.13.orig.fa_	OEZ008529	yes	LMSG_G000008152.1	OES073754	terpene	Terpene	Polymetallic	20.86	China: Guizhou, Zhijin
L180322-P-4-LD180319-0010_HL3KCCCXY_L3_clean_bin.15.orig.fa_	OEZ008529	yes	LMSG_G000008359.1	OES073754	terpene	Terpene	Polymetallic	16.24	China: Guizhou, Zhijin
L180322-P-4-LD180319-0010_HL3KCCCXY_L3_clean_bin.15.orig.fa_	OEZ008529	yes	LMSG_G000008359.1	OES073754	NRPS-like	NRPS	Polymetallic	36.88	China: Guizhou, Zhijin

L180322-P-4-LD180319-0010_HL3KCCCXY_L3_clean_bin.15.orig.fa_	OEZ008529	yes	LMSG_G000008359.1	OES073754	bacteriocin	RiPPs	Polymetallic	10.81	China: Guizhou, Zhijin
L180322-P-4-LD180319-0010_HL3KCCCXY_L3_clean_bin.16.orig.fa_	OEZ008529	yes	LMSG_G000010705.1	OES073754	terpene	Terpene	Polymetallic	11.14	China: Guizhou, Zhijin
L180322-P-4-LD180319-0010_HL3KCCCXY_L3_clean_bin.16.orig.fa_	OEZ008529	yes	LMSG_G000010705.1	OES073754	NRPS.T1PKS	PKS-NRP_Hybrids	Polymetallic	5.76	China: Guizhou, Zhijin
L180322-P-4-LD180319-0010_HL3KCCCXY_L3_clean_bin.16.orig.fa_	OEZ008529	yes	LMSG_G000010705.1	OES073754	arylpolyene	Others	Polymetallic	15.49	China: Guizhou, Zhijin
L180322-P-4-LD180319-0010_HL3KCCCXY_L3_clean_bin.16.orig.fa_	OEZ008529	yes	LMSG_G000010705.1	OES073754	NRPS	NRPS	Polymetallic	9.78	China: Guizhou, Zhijin
L180322-P-4-LD180319-0010_HL3KCCCXY_L3_clean_bin.17.orig.fa_	OEZ008529	no	LMSG_G000011022.1	OES073754	NRPS-like	NRPS	Polymetallic	42.98	China: Guizhou, Zhijin
L180322-P-4-LD180319-0010_HL3KCCCXY_L3_clean_bin.17.orig.fa_	OEZ008529	no	LMSG_G000011022.1	OES073754	bacteriocin	RiPPs	Polymetallic	10.83	China: Guizhou, Zhijin
L180322-P-4-LD180319-0010_HL3KCCCXY_L3_clean_bin.17.orig.fa_	OEZ008529	no	LMSG_G000011022.1	OES073754	NRPS	NRPS	Polymetallic	44.23	China: Guizhou, Zhijin
L180322-P-4-LD180319-0010_HL3KCCCXY_L3_clean_bin.17.orig.fa_	OEZ008529	no	LMSG_G000011022.1	OES073754	thiopeptide	RiPPs	Polymetallic	33.00	China: Guizhou, Zhijin
L180322-P-4-LD180319-0010_HL3KCCCXY_L3_clean_bin.17.orig.fa_	OEZ008529	no	LMSG_G000011022.1	OES073754	NRPS	NRPS	Polymetallic	53.04	China: Guizhou, Zhijin
L180322-P-4-LD180319-0010_HL3KCCCXY_L3_clean_bin.17.orig.fa_	OEZ008529	no	LMSG_G000011022.1	OES073754	phenazine	Others	Polymetallic	10.23	China: Guizhou, Zhijin
L180322-P-4-LD180319-0010_HL3KCCCXY_L3_clean_bin.17.orig.fa_	OEZ008529	no	LMSG_G000011022.1	OES073754	phenazine	Others	Polymetallic	10.23	China: Guizhou, Zhijin
L180322-P-4-LD180319-0010_HL3KCCCXY_L3_clean_bin.17.orig.fa_	OEZ008529	no	LMSG_G000011022.1	OES073754	hserlactone	Others	Polymetallic	20.61	China: Guizhou, Zhijin
L180322-P-4-LD180319-0010_HL3KCCCXY_L3_clean_bin.17.orig.fa_	OEZ008529	no	LMSG_G000011022.1	OES073754	NAGGN	Others	Polymetallic	14.76	China: Guizhou, Zhijin
L180322-P-4-LD180319-0010_HL3KCCCXY_L3_clean_bin.17.orig.fa_	OEZ008529	no	LMSG_G000011022.1	OES073754	NRPS	NRPS	Polymetallic	6.21	China: Guizhou, Zhijin
L180322-P-4-LD180319-0010_HL3KCCCXY_L3_clean_bin.17.orig.fa_	OEZ008529	no	LMSG_G000011022.1	OES073754	hserlactone	Others	Polymetallic	20.61	China: Guizhou, Zhijin
L180322-P-4-LD180319-0010_HL3KCCCXY_L3_clean_bin.17.orig.fa_	OEZ008529	no	LMSG_G000011022.1	OES073754	betalactone.NRPS-like	Others	Polymetallic	41.86	China: Guizhou, Zhijin
L180322-P-4-LD180319-0010_HL3KCCCXY_L3_clean_bin.17.orig.fa_	OEZ008529	no	LMSG_G000011022.1	OES073754	NRPS	NRPS	Polymetallic	49.55	China: Guizhou, Zhijin
L180322-P-4-LD180319-0010_HL3KCCCXY_L3_clean_bin.17.orig.fa_	OEZ008529	no	LMSG_G000011022.1	OES073754	bacteriocin	RiPPs	Polymetallic	10.86	China: Guizhou, Zhijin
L180322-P-4-LD180319-0010_HL3KCCCXY_L3_clean_bin.17.orig.fa_	OEZ008529	no	LMSG_G000011022.1	OES073754	NRPS	NRPS	Polymetallic	5.44	China: Guizhou, Zhijin
L180322-P-4-LD180319-0010_HL3KCCCXY_L3_clean_bin.17.orig.fa_	OEZ008529	no	LMSG_G000011022.1	OES073754	NRPS	NRPS	Polymetallic	42.58	China: Guizhou, Zhijin
L180322-P-4-LD180319-0010_HL3KCCCXY_L3_clean_bin.18.orig.fa_	OEZ008529	yes	LMSG_G000010971.1	OES073754	T1PKS.hglE-KS	PKSother	Polymetallic	19.15	China: Guizhou, Zhijin
L180322-P-4-LD180319-0010_HL3KCCCXY_L3_clean_bin.2.strict.fa	OEZ008529	yes	LMSG_G000010313.1	OES073754	terpene	Terpene	Polymetallic	15.33	China: Guizhou, Zhijin
L180322-P-4-LD180319-0010_HL3KCCCXY_L3_clean_bin.2.strict.fa	OEZ008529	yes	LMSG_G000010313.1	OES073754	terpene	Terpene	Polymetallic	12.61	China: Guizhou, Zhijin
L180322-P-4-LD180319-0010_HL3KCCCXY_L3_clean_bin.2.strict.fa	OEZ008529	yes	LMSG_G000010313.1	OES073754	T3PKS	PKSother	Polymetallic	7.80	China: Guizhou, Zhijin
L180322-P-4-LD180319-0010_HL3KCCCXY_L3_clean_bin.20.orig.fa_	OEZ008529	yes	LMSG_G000008574.1	OES073754	other	Others	Polymetallic	11.70	China: Guizhou, Zhijin
L180322-P-4-LD180319-0010_HL3KCCCXY_L3_clean_bin.20.orig.fa_	OEZ008529	yes	LMSG_G000008574.1	OES073754	other	Others	Polymetallic	7.29	China: Guizhou, Zhijin
L180322-P-4-LD180319-0010_HL3KCCCXY_L3_clean_bin.20.orig.fa_	OEZ008529	yes	LMSG_G000008574.1	OES073754	NRPS-like	NRPS	Polymetallic	6.91	China: Guizhou, Zhijin
L180322-P-4-LD180319-0010_HL3KCCCXY_L3_clean_bin.21.orig.fa_	OEZ008529	yes	LMSG_G000010079.1	OES073754	T1PKS	PKSI	Polymetallic	20.24	China: Guizhou, Zhijin
L180322-P-4-LD180319-0010_HL3KCCCXY_L3_clean_bin.21.orig.fa_	OEZ008529	yes	LMSG_G000010079.1	OES073754	terpene	Terpene	Polymetallic	21.79	China: Guizhou, Zhijin
L180322-P-4-LD180319-0010_HL3KCCCXY_L3_clean_bin.21.orig.fa_	OEZ008529	yes	LMSG_G000010079.1	OES073754	acyl_amino_acids	Others	Polymetallic	60.82	China: Guizhou, Zhijin
L180322-P-4-LD180319-0010_HL3KCCCXY_L3_clean_bin.21.orig.fa_	OEZ008529	yes	LMSG_G000010079.1	OES073754	hglE-KS	PKSother	Polymetallic	31.53	China: Guizhou, Zhijin
L180322-P-4-LD180319-0010_HL3KCCCXY_L3_clean_bin.21.orig.fa_	OEZ008529	yes	LMSG_G000010079.1	OES073754	terpene	Terpene	Polymetallic	14.93	China: Guizhou, Zhijin
L180322-P-4-LD180319-0010_HL3KCCCXY_L3_clean_bin.21.orig.fa_	OEZ008529	yes	LMSG_G000010079.1	OES073754	hglE-KS	PKSother	Polymetallic	10.39	China: Guizhou, Zhijin
L180322-P-4-LD180319-0010_HL3KCCCXY_L3_clean_bin.21.orig.fa_	OEZ008529	yes	LMSG_G000010079.1	OES073754	terpene	Terpene	Polymetallic	12.82	China: Guizhou, Zhijin
L180322-P-4-LD180319-0010_HL3KCCCXY_L3_clean_bin.21.orig.fa_	OEZ008529	yes	LMSG_G000010079.1	OES073754	NRPS-like	NRPS	Polymetallic	8.81	China: Guizhou, Zhijin
L180322-P-4-LD180319-0010_HL3KCCCXY_L3_clean_bin.23.orig.fa_	OEZ008529	yes	LMSG_G000008442.1	OES073754	terpene	Terpene	Polymetallic	26.50	China: Guizhou, Zhijin
L180322-P-4-LD180319-0010_HL3KCCCXY_L3_clean_bin.24.strict.f	OEZ008529	yes	LMSG_G000005249.1	OES073754	NRPS	NRPS	Polymetallic	15.00	China: Guizhou, Zhijin
L180322-P-4-LD180319-0010_HL3KCCCXY_L3_clean_bin.24.strict.f	OEZ008529	yes	LMSG_G000005249.1	OES073754	NRPS	NRPS	Polymetallic	7.27	China: Guizhou, Zhijin
L180322-P-4-LD180319-0010_HL3KCCCXY_L3_clean_bin.24.strict.f	OEZ008529	yes	LMSG_G000005249.1	OES073754	NRPS-like	NRPS	Polymetallic	6.28	China: Guizhou, Zhijin
L180322-P-4-LD180319-0010_HL3KCCCXY_L3_clean_bin.25.permissi	OEZ008529	yes	LMSG_G000011330.1	OES073754	NRPS-like	NRPS	Polymetallic	12.07	China: Guizhou, Zhijin
L180322-P-4-LD180319-0010_HL3KCCCXY_L3_clean_bin.25.permissi	OEZ008529	yes	LMSG_G000011330.1	OES073754	T3PKS	PKSother	Polymetallic	8.81	China: Guizhou, Zhijin
L180322-P-4-LD180319-0010_HL3KCCCXY_L3_clean_bin.26.orig.fa_	OEZ008529	yes	LMSG_G000010750.1	OES073754	bacteriocin	RiPPs	Polymetallic	5.62	China: Guizhou, Zhijin
L180322-P-4-LD180319-0010_HL3KCCCXY_L3_clean_bin.27.permissi	OEZ008529	yes	LMSG_G000010942.1	OES073754	T3PKS	PKSother	Polymetallic	13.80	China: Guizhou, Zhijin
L180322-P-4-LD180319-0010_HL3KCCCXY_L3_clean_bin.27.permissi	OEZ008529	yes	LMSG_G000010942.1	OES073754	NRPS	NRPS	Polymetallic	11.91	China: Guizhou, Zhijin
L180322-P-4-LD180319-0010_HL3KCCCXY_L3_clean_bin.27.permissi	OEZ008529	yes	LMSG_G000010942.1	OES073754	NRPS	NRPS	Polymetallic	7.82	China: Guizhou, Zhijin
L180322-P-4-LD180319-0010_HL3KCCCXY_L3_clean_bin.28.permissi	OEZ008529	yes	LMSG_G000008984.1	OES073754	bacteriocin	RiPPs	Polymetallic	5.47	China: Guizhou, Zhijin
L180322-P-4-LD180319-0010_HL3KCCCXY_L3_clean_bin.28.permissi	OEZ008529	yes	LMSG_G000008984.1	OES073754	betalactone	Others	Polymetallic	8.85	China: Guizhou, Zhijin
L180322-P-4-LD180319-0010_HL3KCCCXY_L3_clean_bin.28.permissi	OEZ008529	yes	LMSG_G000008984.1	OES073754	T1PKS	PKSI	Polymetallic	6.07	China: Guizhou, Zhijin
L180322-P-4-LD180319-0010_HL3KCCCXY_L3_clean_bin.29.strict.f	OEZ008529	yes	LMSG_G000008673.1	OES073754	NRPS-like	NRPS	Polymetallic	10.71	China: Guizhou, Zhijin
L180322-P-4-LD180319-0010_HL3KCCCXY_L3_clean_bin.3.orig.fa_k	OEZ008529	yes	LMSG_G000010193.1	OES073754	terpene	Terpene	Polymetallic	31.59	China: Guizhou, Zhijin
L180322-P-4-LD180319-0010_HL3KCCCXY_L3_clean_bin.31.permissi	OEZ008529	yes	LMSG_G000010078.1	OES073754	terpene	Terpene	Polymetallic	10.99	China: Guizhou, Zhijin
L180322-P-4-LD180319-0010_HL3KCCCXY_L3_clean_bin.31.permissi	OEZ008529	yes	LMSG_G000010078.1	OES073754	terpene	Terpene	Polymetallic	8.06	China: Guizhou, Zhijin
L180322-P-4-LD180319-0010_HL3KCCCXY_L3_clean_bin.31.permissi	OEZ008529	yes	LMSG_G000010078.1	OES073754	terpene	Terpene	Polymetallic	5.54	China: Guizhou, Zhijin
L180322-P-4-LD180319-0010_HL3KCCCXY_L3_clean_bin.32.orig.fa_	OEZ008529	yes	LMSG_G000010096.1	OES073754	T3PKS	PKSother	Polymetallic	30.05	China: Guizhou, Zhijin
L180322-P-4-LD180319-0010_HL3KCCCXY_L3_clean_bin.32.orig.fa_	OEZ008529	yes	LMSG_G000010096.1	OES073754	hglE-KS	PKSother	Polymetallic	13.98	China: Guizhou, Zhijin
L180322-P-4-LD180319-0010_HL3KCCCXY_L3_clean_bin.32.orig.fa_	OEZ008529	yes	LMSG_G000010096.1	OES073754	terpene	Terpene	Polymetallic	21.81	China: Guizhou, Zhijin
L180322-P-4-LD180319-0010_HL3KCCCXY_L3_clean_bin.32.orig.fa_	OEZ008529	yes	LMSG_G000010096.1	OES073754	hglE-KS	PKSother	Polymetallic	30.62	China: Guizhou, Zhijin
L180322-P-4-LD180319-0010_HL3KCCCXY_L3_clean_bin.32.orig.fa_	OEZ008529	yes	LMSG_G000010096.1	OES073754	T1PKS.hglE-KS	PKSother	Polymetallic	41.03	China: Guizhou, Zhijin
L180322-P-4-LD180319-0010_HL3KCCCXY_L3_clean_bin.32.orig.fa_	OEZ008529	yes	LMSG_G000010096.1	OES073754	terpene	Terpene	Polymetallic	21.35	China: Guizhou, Zhijin
L180322-P-4-LD180319-0010_HL3KCCCXY_L3_clean_bin.32.orig.fa_	OEZ008529	yes	LMSG_G000010096.1	OES073754	T1PKS	PKSI	Polymetallic	27.36	China: Guizhou, Zhijin
L180322-P-4-LD180319-0010_HL3KCCCXY_L3_clean_bin.33.orig.fa_	OEZ008529	yes	LMSG_G000010115.1	OES073754	terpene	Terpene	Polymetallic	21.70	China: Guizhou, Zhijin
L180322-P-4-LD180319-0010_HL3KCCCXY_L3_clean_bin.33.orig.fa_	OEZ008529	yes	LMSG_G000010115.1	OES073754	T3PKS	PKSother	Polymetallic	41.08	China: Guizhou, Zhijin
L180322-P-4-LD180319-0010_HL3KCCCXY_L3_clean_bin.33.orig.fa_	OEZ008529	yes	LMSG_G000010115.1	OES073754	terpene	Terpene	Polymetallic	20.63	China: Guizhou, Zhijin
L180322-P-4-LD180319-0010_HL3KCCCXY_L3_clean_bin.33.orig.fa_	OEZ008529	yes	LMSG_G000010115.1	OES073754	hserlactone	Others	Polymetallic	20.64	China: Guizhou, Zhijin
L180322-P-4-LD180319-0010_HL3KCCCXY_L3_clean_bin.34.orig.fa_	OEZ008529	yes	LMSG_G000010324.1	OES073754	bacteriocin	RiPPs	Polymetallic	9.27	China: Guizhou, Zhijin
L180322-P-4-LD180319-0010_HL3KCCCXY_L3_clean_bin.34.orig.fa_	OEZ008529	yes	LMSG_G000010324.1	OES073754	bacteriocin	RiPPs	Polymetallic	5.88	China: Guizhou, Zhijin
L180322-P-4-LD180319-0010_HL3KCCCXY_L3_clean_bin.34.orig.fa_	OEZ008529	yes	LMSG_G000010324.1	OES073754	terpene	Terpene	Polymetallic	12.74	China: Guizhou, Zhijin
L180322-P-4-LD180319-0010_HL3KCCCXY_L3_clean_bin.37.orig.fa_	OEZ008529	yes	LMSG_G000010145.1	OES073754	T3PKS	PKSother	Polymetallic	35.72	China: Guizhou, Zhijin
L180322-P-4-LD180319-0010_HL3KCCCXY_L3_clean_bin.37.orig.fa_	OEZ008529	yes	LMSG_G000010145.1	OES073754	terpene	Terpene	Polymetallic	11.72	China: Guizhou, Zhijin
L180322-P-4-LD180319-0010_HL3KCCCXY_L3_clean_bin.37.orig.fa_	OEZ008529	yes	LMSG_G000010145.1	OES073754	terpene	Terpene	Polymetallic	17.53	China: Guizhou, Zhijin
L180322-P-4-LD180319-0010_HL3KCCCXY_L3_clean_bin.37.orig.fa_	OEZ008529	yes	LMSG_G000010145.1	OES073754	terpene	Terpene	Polymetallic	6.75	China: Guizhou, Zhijin
L180322-P-4-LD180319-0010_HL3KCCCXY_L3_clean_bin.38.orig.fa_	OEZ008529	no	LMSG_G000010614.1	OES073754	NRPS-like	NRPS	Polymetallic	43.97	China: Guizhou, Zhijin
L180322-P-4-LD180319-0010_HL3KCCCXY_L3_clean_bin.38.orig.fa_	OEZ008529	yes	LMSG_G000010614.1	OES073754	resorcinol	Others	Polymetallic	41.93	China: Guizhou, Zhijin
L180322-P-4-LD180319-0010_HL3KCCCXY_L3_clean_bin.38.orig.fa_	OEZ008529	no	LMSG_G000010614.1	OES073754	phosphonate	Others	Polymetallic	40.83	China: Guizhou, Zhijin
L180322-P-4-LD180319-0010_HL3KCCCXY_L3_clean_bin.38.orig.fa_	OEZ008529	no	LMSG_G000010614.1	OES073754	ectoine	Others	Polymetallic	10.41	China: Guizhou, Zhijin
L180322-P-4-LD180319-0010_HL3KCCCXY_L3_clean_bin.38.orig.fa_	OEZ008529	yes	LMSG_G000010614.1	OES073754	betalactone	Others	Polymetallic	22.57	China: Guizhou, Zhijin
L180322-P-4-LD180319-0010_HL3KCCCXY_L3_clean_bin.38.orig.fa_	OEZ008529	yes	LMSG_G000010614.1	OES073754	terpene	Terpene	Polymetallic	21.71	China: Guizhou, Zhijin
L180322-P-4-LD180319-0010_HL3KCCCXY_L3_clean_bin.38.orig.fa_	OEZ008529	no	LMSG_G000010614.1	OES073754	siderophore	Others	Polymetallic	11.85	China: Guizhou, Zhijin
L180322-P-4-LD180319-0010_HL3KCCCXY_L3_clean_bin.4.strict.fa	OEZ008529	yes	LMSG_G000010801.1	OES073754	acyl_amino_acids	Others	Polymetallic	19.59	China: Guizhou, Zhijin
L180322-P-4-LD180319-0010_HL3KCCCXY_L3_clean_bin.4.strict.fa	OEZ008529	no	LMSG_G000010801.1	OES073754	acyl_amino_acids	Others	Polymetallic	5.47	China: Guizhou, Zhijin
L180322-P-4-LD180319-0010_HL3KCCCXY_L3_clean_bin.42.orig.fa_	OEZ008529	yes	LMSG_G000007786.1	OES073754	terpene	Terpene	Polymetallic	16.97	China: Guizhou, Zhijin
L180322-P-4-LD180319-0010_HL3KCCCXY_L3_clean_bin.42.orig.fa_	OEZ008529	yes	LMSG_G000007786.1	OES073754	bacteriocin	RiPPs	Polymetallic	6.61	China: Guizhou, Zhijin
L180322-P-4-LD180319-0010_HL3KCCCXY_L3_clean_bin.42.orig.fa_	OEZ008529	yes	LMSG_G000007786.1	OES073754	terpene	Terpene	Polymetallic	13.11	China: Guizhou, Zhijin
L180322-P-4-LD180319-0010_HL3KCCCXY_L3_clean_bin.42.orig.fa_	OEZ008529	yes	LMSG_G000007786.1	OES073754	T3PKS	PKSother	Polymetallic	21.87	China: Guizhou, Zhijin
L180322-P-4-LD180319-0010_HL3KCCCXY_L3_clean_bin.43.orig.fa_	OEZ008529	yes	LMSG_G000008653.1	OES073754	other	Others	Polymetallic	27.14	China: Guizhou, Zhijin
L180322-P-4-LD180319-0010_HL3KCCCXY_L3_clean_bin.44.orig.fa_	OEZ008529	yes	LMSG_G000005250.1	OES073754	betalactone	Others	Polymetallic	17.70	China: Guizhou, Zhijin
L180322-P-4-LD180319-0010_HL3KCCCXY_L3_clean_bin.44.orig.fa_	OEZ008529	yes	LMSG_G000005250.1	OES073754	T3PKS	PKSother	Polymetallic	19.80	China: Guizhou, Zhijin
L180322-P-4-LD180319-0010_HL3KCCCXY_L3_clean_bin.44.orig.fa_	OEZ008529	yes	LMSG_G000005250.1	OES073754	bacteriocin	RiPPs	Polymetallic	9.08	China: Guizhou, Zhijin
L180322-P-4-LD180319-0010_HL3KCCCXY_L3_clean_bin.44.orig.fa_	OEZ008529	yes	LMSG_G000005250.1	OES073754	betalactone	Others	Polymetallic	17.01	China: Guizhou, Zhijin
L180322-P-4-LD180319-0010_HL3KCCCXY_L3_clean_bin.44.orig.fa_	OEZ008529	yes	LMSG_G000005250.1	OES073754	terpene	Terpene	Polymetallic	7.59	China: Guizhou, Zhijin
L180322-P-4-LD180319-0010_HL3KCCCXY_L3_clean_bin.45.permissi	OEZ008529	yes	LMSG_G000005251.1	OES073754	terpene	Terpene	Polymetallic	23.27	China: Guizhou, Zhijin
L180322-P-4-LD180319-0010_HL3KCCCXY_L3_clean_bin.45.permissi	OEZ008529	yes	LMSG_G000005251.1	OES073754	terpene	Terpene	Polymetallic	22.08	China: Guizhou, Zhijin
L180322-P-4-LD180319-0010_HL3KCCCXY_L3_clean_bin.45.permissi	OEZ008529	yes	LMSG_G000005251.1	OES073754	NRPS-like	NRPS	Polymetallic	18.49	China: Guizhou, Zhijin
L180322-P-4-LD180319-0010_HL3KCCCXY_L3_clean_bin.45.permissi	OEZ008529	yes	LMSG_G000005251.1	OES073754	nucleoside	Others	Polymetallic	15.75	China: Guizhou, Zhijin
L180322-P-4-LD180319-0010_HL3KCCCXY_L3_clean_bin.46.orig.fa_	OEZ008529	yes	LMSG_G000010663.1	OES073754	terpene	Terpene	Polymetallic	8.21	China: Guizhou, Zhijin
L180322-P-4-LD180319-0010_HL3KCCCXY_L3_clean_bin.46.orig.fa_	OEZ008529	yes	LMSG_G000010663.1	OES073754	NRPS	NRPS	Polymetallic	23.33	China: Guizhou, Zhijin
L180322-P-4-LD180319-0010_HL3KCCCXY_L3_clean_bin.46.orig.fa_	OEZ008529	yes	LMSG_G000010663.1	OES073754	NRPS-like	NRPS	Polymetallic	5.50	China: Guizhou, Zhijin
L180322-P-4-LD180319-0010_HL3KCCCXY_L3_clean_bin.46.orig.fa_	OEZ008529	yes	LMSG_G000010663.1	OES073754	NRPS-like	NRPS	Polymetallic	15.11	China: Guizhou, Zhijin
L180322-P-4-LD180319-0010_HL3KCCCXY_L3_clean_bin.46.orig.fa_	OEZ008529	yes	LMSG_G000010663.1	OES073754	arylpolyene	Others	Polymetallic	5.95	China: Guizhou, Zhijin
L180322-P-4-LD180319-0010_HL3KCCCXY_L3_clean_bin.47.orig.fa_	OEZ008529	yes	LMSG_G000008461.1	OES073754	terpene	Terpene	Polymetallic	21.06	China: Guizhou, Zhijin
L180322-P-4-LD180319-0010_HL3KCCCXY_L3_clean_bin.47.orig.fa_	OEZ008529	yes	LMSG_G000008461.1	OES073754	terpene	Terpene	Polymetallic	21.06	China: Guizhou, Zhijin
L180322-P-4-LD180319-0010_HL3KCCCXY_L3_clean_bin.49.orig.fa_	OEZ008529	yes	LMSG_G000005252.1	OES073754	ectoine	Others	Polymetallic	6.76	China: Guizhou, Zhijin
L180322-P-4-LD180319-0010_HL3KCCCXY_L3_clean_bin.49.orig.fa_	OEZ008529	yes	LMSG_G000005252.1	OES073754	terpene	Terpene	Polymetallic	6.52	China: Guizhou, Zhijin
L180322-P-4-LD180319-0010_HL3KCCCXY_L3_clean_bin.5.orig.fa_k	OEZ008529	yes	LMSG_G000011051.1	OES073754	resorcinol.arylpolyene	Others	Polymetallic	17.78	China: Guizhou, Zhijin
L180322-P-4-LD180319-0010_HL3KCCCXY_L3_clean_bin.5.orig.fa_k	OEZ008529	yes	LMSG_G000011051.1	OES073754	terpene	Terpene	Polymetallic	20.85	China: Guizhou, Zhijin
L180322-P-4-LD180319-0010_HL3KCCCXY_L3_clean_bin.5.orig.fa_k	OEZ008529	yes	LMSG_G000011051.1	OES073754	NRPS-like	NRPS	Polymetallic	10.95	China: Guizhou, Zhijin
L180322-P-4-LD180319-0010_HL3KCCCXY_L3_clean_bin.5.orig.fa_k	OEZ008529	yes	LMSG_G000011051.1	OES073754	arylpolyene	Others	Polymetallic	9.39	China: Guizhou, Zhijin
L180322-P-4-LD180319-0010_HL3KCCCXY_L3_clean_bin.5.orig.fa_k	OEZ008529	yes	LMSG_G000011051.1	OES073754	bacteriocin	RiPPs	Polymetallic	7.26	China: Guizhou, Zhijin
L180322-P-4-LD180319-0010_HL3KCCCXY_L3_clean_bin.52.orig.fa_	OEZ008529	yes	LMSG_G000005253.1	OES073754	terpene	Terpene	Polymetallic	14.97	China: Guizhou, Zhijin
L180322-P-4-LD180319-0010_HL3KCCCXY_L3_clean_bin.52.orig.fa_	OEZ008529	yes	LMSG_G000005253.1	OES073754	NRPS-like	NRPS	Polymetallic	22.96	China: Guizhou, Zhijin
L180322-P-4-LD180319-0010_HL3KCCCXY_L3_clean_bin.52.orig.fa_	OEZ008529	yes	LMSG_G000005253.1	OES073754	NRPS-like	NRPS	Polymetallic	31.91	China: Guizhou, Zhijin
L180322-P-4-LD180319-0010_HL3KCCCXY_L3_clean_bin.52.orig.fa_	OEZ008529	yes	LMSG_G000005253.1	OES073754	RPS.lassopeptide.hserlacton	Others	Polymetallic	49.89	China: Guizhou, Zhijin
L180322-P-4-LD180319-0010_HL3KCCCXY_L3_clean_bin.52.orig.fa_	OEZ008529	yes	LMSG_G000005253.1	OES073754	lassopeptide	RiPPs	Polymetallic	12.78	China: Guizhou, Zhijin
L180322-P-4-LD180319-0010_HL3KCCCXY_L3_clean_bin.53.orig.fa_	OEZ008529	yes	LMSG_G000005254.1	OES073754	terpene	Terpene	Polymetallic	20.86	China: Guizhou, Zhijin
L180322-P-4-LD180319-0010_HL3KCCCXY_L3_clean_bin.53.orig.fa_	OEZ008529	yes	LMSG_G000005254.1	OES073754	terpene	Terpene	Polymetallic	12.67	China: Guizhou, Zhijin
L180322-P-4-LD180319-0010_HL3KCCCXY_L3_clean_bin.53.orig.fa_	OEZ008529	yes	LMSG_G000005254.1	OES073754	arylpolyene	Others	Polymetallic	25.65	China: Guizhou, Zhijin
L180322-P-4-LD180319-0010_HL3KCCCXY_L3_clean_bin.53.orig.fa_	OEZ008529	yes	LMSG_G000005254.1	OES073754	terpene	Terpene	Polymetallic	16.20	China: Guizhou, Zhijin
L180322-P-4-LD180319-0010_HL3KCCCXY_L3_clean_bin.54.orig.fa_	OEZ008529	yes	LMSG_G000010656.1	OES073754	NRPS	NRPS	Polymetallic	24.74	China: Guizhou, Zhijin
L180322-P-4-LD180319-0010_HL3KCCCXY_L3_clean_bin.54.orig.fa_	OEZ008529	yes	LMSG_G000010656.1	OES073754	T1PKS.NRPS-like	PKS-NRP_Hybrids	Polymetallic	17.61	China: Guizhou, Zhijin
L180322-P-4-LD180319-0010_HL3KCCCXY_L3_clean_bin.54.orig.fa_	OEZ008529	yes	LMSG_G000010656.1	OES073754	NRPS-like	NRPS	Polymetallic	15.13	China: Guizhou, Zhijin
L180322-P-4-LD180319-0010_HL3KCCCXY_L3_clean_bin.54.orig.fa_	OEZ008529	yes	LMSG_G000010656.1	OES073754	NRPS	NRPS	Polymetallic	13.25	China: Guizhou, Zhijin
L180322-P-4-LD180319-0010_HL3KCCCXY_L3_clean_bin.54.orig.fa_	OEZ008529	yes	LMSG_G000010656.1	OES073754	NRPS-like	NRPS	Polymetallic	21.13	China: Guizhou, Zhijin
L180322-P-4-LD180319-0010_HL3KCCCXY_L3_clean_bin.55.orig.fa_	OEZ008529	yes	LMSG_G000010097.1	OES073754	terpene	Terpene	Polymetallic	15.23	China: Guizhou, Zhijin
L180322-P-4-LD180319-0010_HL3KCCCXY_L3_clean_bin.55.orig.fa_	OEZ008529	yes	LMSG_G000010097.1	OES073754	hglE-KS	PKSother	Polymetallic	15.35	China: Guizhou, Zhijin
L180322-P-4-LD180319-0010_HL3KCCCXY_L3_clean_bin.55.orig.fa_	OEZ008529	yes	LMSG_G000010097.1	OES073754	cyanobactin	RiPPs	Polymetallic	6.93	China: Guizhou, Zhijin
L180322-P-4-LD180319-0010_HL3KCCCXY_L3_clean_bin.55.orig.fa_	OEZ008529	yes	LMSG_G000010097.1	OES073754	T1PKS.hglE-KS	PKSother	Polymetallic	34.05	China: Guizhou, Zhijin
L180322-P-4-LD180319-0010_HL3KCCCXY_L3_clean_bin.55.orig.fa_	OEZ008529	yes	LMSG_G000010097.1	OES073754	cyanobactin	RiPPs	Polymetallic	19.94	China: Guizhou, Zhijin
L180322-P-4-LD180319-0010_HL3KCCCXY_L3_clean_bin.55.orig.fa_	OEZ008529	yes	LMSG_G000010097.1	OES073754	T1PKS.hglE-KS	PKSother	Polymetallic	17.31	China: Guizhou, Zhijin
L180322-P-4-LD180319-0010_HL3KCCCXY_L3_clean_bin.55.orig.fa_	OEZ008529	yes	LMSG_G000010097.1	OES073754	T3PKS	PKSother	Polymetallic	31.90	China: Guizhou, Zhijin
L180322-P-4-LD180319-0010_HL3KCCCXY_L3_clean_bin.55.orig.fa_	OEZ008529	yes	LMSG_G000010097.1	OES073754	terpene	Terpene	Polymetallic	10.65	China: Guizhou, Zhijin
L180322-P-4-LD180319-0010_HL3KCCCXY_L3_clean_bin.58.orig.fa_	OEZ008529	yes	LMSG_G000008457.1	OES073754	betalactone	Others	Polymetallic	22.65	China: Guizhou, Zhijin
L180322-P-4-LD180319-0010_HL3KCCCXY_L3_clean_bin.58.orig.fa_	OEZ008529	yes	LMSG_G000008457.1	OES073754	terpene	Terpene	Polymetallic	16.13	China: Guizhou, Zhijin
L180322-P-4-LD180319-0010_HL3KCCCXY_L3_clean_bin.58.orig.fa_	OEZ008529	yes	LMSG_G000008457.1	OES073754	betalactone	Others	Polymetallic	16.97	China: Guizhou, Zhijin
L180322-P-4-LD180319-0010_HL3KCCCXY_L3_clean_bin.59.permissi	OEZ008529	yes	LMSG_G000005255.1	OES073754	bacteriocin	RiPPs	Polymetallic	10.37	China: Guizhou, Zhijin
L180322-P-4-LD180319-0010_HL3KCCCXY_L3_clean_bin.59.permissi	OEZ008529	yes	LMSG_G000005255.1	OES073754	bacteriocin	RiPPs	Polymetallic	7.23	China: Guizhou, Zhijin
L180322-P-4-LD180319-0010_HL3KCCCXY_L3_clean_bin.6.orig.fa_k	OEZ008529	yes	LMSG_G000005258.1	OES073754	NRPS-like	NRPS	Polymetallic	13.89	China: Guizhou, Zhijin
L180322-P-4-LD180319-0010_HL3KCCCXY_L3_clean_bin.6.orig.fa_k	OEZ008529	yes	LMSG_G000005258.1	OES073754	terpene	Terpene	Polymetallic	5.97	China: Guizhou, Zhijin
L180322-P-4-LD180319-0010_HL3KCCCXY_L3_clean_bin.6.orig.fa_k	OEZ008529	yes	LMSG_G000005258.1	OES073754	lassopeptide	RiPPs	Polymetallic	23.95	China: Guizhou, Zhijin
L180322-P-4-LD180319-0010_HL3KCCCXY_L3_clean_bin.60.orig.fa_	OEZ008529	yes	LMSG_G000005256.1	OES073754	bacteriocin	RiPPs	Polymetallic	8.32	China: Guizhou, Zhijin
L180322-P-4-LD180319-0010_HL3KCCCXY_L3_clean_bin.60.orig.fa_	OEZ008529	yes	LMSG_G000005256.1	OES073754	terpene	Terpene	Polymetallic	10.61	China: Guizhou, Zhijin
L180322-P-4-LD180319-0010_HL3KCCCXY_L3_clean_bin.60.orig.fa_	OEZ008529	yes	LMSG_G000005256.1	OES073754	terpene	Terpene	Polymetallic	13.73	China: Guizhou, Zhijin
L180322-P-4-LD180319-0010_HL3KCCCXY_L3_clean_bin.60.orig.fa_	OEZ008529	yes	LMSG_G000005256.1	OES073754	arylpolyene	Others	Polymetallic	19.41	China: Guizhou, Zhijin
L180322-P-4-LD180319-0010_HL3KCCCXY_L3_clean_bin.60.orig.fa_	OEZ008529	yes	LMSG_G000005256.1	OES073754	hserlactone	Others	Polymetallic	14.37	China: Guizhou, Zhijin
L180322-P-4-LD180319-0010_HL3KCCCXY_L3_clean_bin.61.orig.fa_	OEZ008529	yes	LMSG_G000010224.1	OES073754	terpene	Terpene	Polymetallic	20.35	China: Guizhou, Zhijin
L180322-P-4-LD180319-0010_HL3KCCCXY_L3_clean_bin.61.orig.fa_	OEZ008529	yes	LMSG_G000010224.1	OES073754	terpene	Terpene	Polymetallic	20.78	China: Guizhou, Zhijin
L180322-P-4-LD180319-0010_HL3KCCCXY_L3_clean_bin.61.orig.fa_	OEZ008529	yes	LMSG_G000010224.1	OES073754	betalactone	Others	Polymetallic	31.27	China: Guizhou, Zhijin
L180322-P-4-LD180319-0010_HL3KCCCXY_L3_clean_bin.61.orig.fa_	OEZ008529	yes	LMSG_G000010224.1	OES073754	betalactone	Others	Polymetallic	24.08	China: Guizhou, Zhijin
L180322-P-4-LD180319-0010_HL3KCCCXY_L3_clean_bin.62.orig.fa_	OEZ008529	yes	LMSG_G000010273.1	OES073754	arylpolyene	Others	Polymetallic	11.01	China: Guizhou, Zhijin
L180322-P-4-LD180319-0010_HL3KCCCXY_L3_clean_bin.62.orig.fa_	OEZ008529	no	LMSG_G000010273.1	OES073754	terpene	Terpene	Polymetallic	7.24	China: Guizhou, Zhijin
L180322-P-4-LD180319-0010_HL3KCCCXY_L3_clean_bin.62.orig.fa_	OEZ008529	yes	LMSG_G000010273.1	OES073754	terpene	Terpene	Polymetallic	14.84	China: Guizhou, Zhijin
L180322-P-4-LD180319-0010_HL3KCCCXY_L3_clean_bin.65.permissi	OEZ008529	yes	LMSG_G000005257.1	OES073754	hglE-KS	PKSother	Polymetallic	7.18	China: Guizhou, Zhijin
L180322-P-4-LD180319-0010_HL3KCCCXY_L3_clean_bin.7.orig.fa_k	OEZ008529	yes	LMSG_G000008207.1	OES073754	terpene	Terpene	Polymetallic	20.86	China: Guizhou, Zhijin
L180322-P-4-LD180319-0010_HL3KCCCXY_L3_clean_bin.8.orig.fa_k	OEZ008529	yes	LMSG_G000010099.1	OES073754	terpene	Terpene	Polymetallic	11.27	China: Guizhou, Zhijin
L180322-P-4-LD180319-0010_HL3KCCCXY_L3_clean_bin.8.orig.fa_k	OEZ008529	yes	LMSG_G000010099.1	OES073754	T1PKS	PKSI	Polymetallic	7.22	China: Guizhou, Zhijin
L180322-P-4-LD180319-0010_HL3KCCCXY_L3_clean_bin.8.orig.fa_k	OEZ008529	yes	LMSG_G000010099.1	OES073754	NRPS	NRPS	Polymetallic	18.08	China: Guizhou, Zhijin
L180322-P-4-LD180319-0010_HL3KCCCXY_L3_clean_bin.8.orig.fa_k	OEZ008529	yes	LMSG_G000010099.1	OES073754	NRPS-like	NRPS	Polymetallic	6.02	China: Guizhou, Zhijin
L180322-P-4-LD180319-0010_HL3KCCCXY_L3_clean_bin.8.orig.fa_k	OEZ008529	yes	LMSG_G000010099.1	OES073754	T1PKS	PKSI	Polymetallic	5.07	China: Guizhou, Zhijin

L180322-P-4-LD180319-0010_HL3KCCCXY_L3_clean_bin.8.orig.fa_k	OEZ008529	yes	LMSG_G000010099.1	OES073754	T3PKS	PKSother	Polymetallic	8.18	China: Guizhou, Zhijin
L180322-P-4-LD180319-0010_HL3KCCCXY_L3_clean_bin.8.orig.fa_k	OEZ008529	yes	LMSG_G000010099.1	OES073754	NRPS	NRPS	Polymetallic	18.98	China: Guizhou, Zhijin
L180322-P-4-LD180319-0010_HL3KCCCXY_L3_clean_bin.8.orig.fa_k	OEZ008529	yes	LMSG_G000010099.1	OES073754	terpene	Terpene	Polymetallic	8.13	China: Guizhou, Zhijin
L180322-P-4-LD180319-0010_HL3KCCCXY_L3_clean_bin.8.orig.fa_k	OEZ008529	yes	LMSG_G000010099.1	OES073754	hglE-KS	PKSother	Polymetallic	9.30	China: Guizhou, Zhijin
L180322-P-4-LD180319-0010_HL3KCCCXY_L3_clean_bin.8.orig.fa_k	OEZ008529	yes	LMSG_G000010099.1	OES073754	hglE-KS	PKSother	Polymetallic	13.87	China: Guizhou, Zhijin
L180322-P-4-LD180319-0010_HL3KCCCXY_L3_clean_bin.8.orig.fa_k	OEZ008529	yes	LMSG_G000010099.1	OES073754	terpene	Terpene	Polymetallic	9.84	China: Guizhou, Zhijin
L180322-P-4-LD180319-0010_HL3KCCCXY_L3_clean_bin.9.orig.fa_k	OEZ008529	yes	LMSG_G000005259.1	OES073754	terpene	Terpene	Polymetallic	21.87	China: Guizhou, Zhijin
L180322-P-4-LD180319-0010_HL3KCCCXY_L3_clean_bin.9.orig.fa_k	OEZ008529	yes	LMSG_G000005259.1	OES073754	terpene	Terpene	Polymetallic	13.84	China: Guizhou, Zhijin
L180322-P-4-LD180319-0010_HL3KCCCXY_L3_clean_bin.9.orig.fa_k	OEZ008529	yes	LMSG_G000005259.1	OES073754	T3PKS	PKSother	Polymetallic	11.99	China: Guizhou, Zhijin
L180322-P-4-LD180319-0011_HL3KCCCXY_L3_clean_bin.1.orig.fa_k	OEZ008529	yes	LMSG_G000005266.1	OES073665	NRPS-like	NRPS	Antimony	17.18	China: Guizhou, Banpo
L180322-P-4-LD180319-0011_HL3KCCCXY_L3_clean_bin.1.orig.fa_k	OEZ008529	yes	LMSG_G000005266.1	OES073665	ladderane	Others	Antimony	15.06	China: Guizhou, Banpo
L180322-P-4-LD180319-0011_HL3KCCCXY_L3_clean_bin.10.orig.fa_	OEZ008529	yes	LMSG_G000005260.1	OES073665	arylpolyene	Others	Antimony	29.49	China: Guizhou, Banpo
L180322-P-4-LD180319-0011_HL3KCCCXY_L3_clean_bin.10.orig.fa_	OEZ008529	yes	LMSG_G000005260.1	OES073665	T1PKS.NRPS-like	PKS-NRP_Hybrids	Antimony	27.36	China: Guizhou, Banpo
L180322-P-4-LD180319-0011_HL3KCCCXY_L3_clean_bin.10.orig.fa_	OEZ008529	yes	LMSG_G000005260.1	OES073665	NRPS	NRPS	Antimony	23.13	China: Guizhou, Banpo
L180322-P-4-LD180319-0011_HL3KCCCXY_L3_clean_bin.14.permissi	OEZ008529	yes	LMSG_G000005262.1	OES073665	T3PKS	PKSother	Antimony	5.20	China: Guizhou, Banpo
L180322-P-4-LD180319-0011_HL3KCCCXY_L3_clean_bin.16.orig.fa_	OEZ008529	yes	LMSG_G000005264.1	OES073665	acyl_amino_acids	Others	Antimony	27.86	China: Guizhou, Banpo
L180322-P-4-LD180319-0011_HL3KCCCXY_L3_clean_bin.16.orig.fa_	OEZ008529	yes	LMSG_G000005264.1	OES073665	NRPS-like	NRPS	Antimony	13.09	China: Guizhou, Banpo
L180322-P-4-LD180319-0011_HL3KCCCXY_L3_clean_bin.16.orig.fa_	OEZ008529	yes	LMSG_G000005264.1	OES073665	terpene	Terpene	Antimony	21.68	China: Guizhou, Banpo
L180322-P-4-LD180319-0011_HL3KCCCXY_L3_clean_bin.17.orig.fa_	OEZ008529	yes	LMSG_G000005265.1	OES073665	hglE-KS	PKSother	Antimony	25.37	China: Guizhou, Banpo
L180322-P-4-LD180319-0011_HL3KCCCXY_L3_clean_bin.18.orig.fa_	OEZ008529	yes	LMSG_G000008966.1	OES073665	NRPS	NRPS	Antimony	8.02	China: Guizhou, Banpo
L180322-P-4-LD180319-0011_HL3KCCCXY_L3_clean_bin.19.orig.fa_	OEZ008529	yes	LMSG_G000009082.1	OES073665	T1PKS	PKSI	Antimony	8.73	China: Guizhou, Banpo
L180322-P-4-LD180319-0011_HL3KCCCXY_L3_clean_bin.19.orig.fa_	OEZ008529	yes	LMSG_G000009082.1	OES073665	terpene	Terpene	Antimony	12.01	China: Guizhou, Banpo
L180322-P-4-LD180319-0011_HL3KCCCXY_L3_clean_bin.19.orig.fa_	OEZ008529	yes	LMSG_G000009082.1	OES073665	terpene	Terpene	Antimony	9.12	China: Guizhou, Banpo
L180322-P-4-LD180319-0011_HL3KCCCXY_L3_clean_bin.20.orig.fa_	OEZ008529	yes	LMSG_G000009493.1	OES073665	TfuA-related	RiPPs	Antimony	15.88	China: Guizhou, Banpo
L180322-P-4-LD180319-0011_HL3KCCCXY_L3_clean_bin.20.orig.fa_	OEZ008529	yes	LMSG_G000009493.1	OES073665	T1PKS.hglE-KS	PKSother	Antimony	34.43	China: Guizhou, Banpo
L180322-P-4-LD180319-0011_HL3KCCCXY_L3_clean_bin.20.orig.fa_	OEZ008529	yes	LMSG_G000009493.1	OES073665	NRPS-like	NRPS	Antimony	34.54	China: Guizhou, Banpo
L180322-P-4-LD180319-0011_HL3KCCCXY_L3_clean_bin.20.orig.fa_	OEZ008529	yes	LMSG_G000009493.1	OES073665	bacteriocin	RiPPs	Antimony	10.86	China: Guizhou, Banpo
L180322-P-4-LD180319-0011_HL3KCCCXY_L3_clean_bin.20.orig.fa_	OEZ008529	yes	LMSG_G000009493.1	OES073665	T3PKS	PKSother	Antimony	25.75	China: Guizhou, Banpo
L180322-P-4-LD180319-0011_HL3KCCCXY_L3_clean_bin.23.orig.fa_	OEZ008529	yes	LMSG_G000011306.1	OES073665	terpene	Terpene	Antimony	10.65	China: Guizhou, Banpo
L180322-P-4-LD180319-0011_HL3KCCCXY_L3_clean_bin.23.orig.fa_	OEZ008529	yes	LMSG_G000011306.1	OES073665	ladderane	Others	Antimony	11.04	China: Guizhou, Banpo
L180322-P-4-LD180319-0011_HL3KCCCXY_L3_clean_bin.24.orig.fa_	OEZ008529	yes	LMSG_G000008542.1	OES073665	betalactone	Others	Antimony	41.46	China: Guizhou, Banpo
L180322-P-4-LD180319-0011_HL3KCCCXY_L3_clean_bin.24.orig.fa_	OEZ008529	yes	LMSG_G000008542.1	OES073665	betalactone	Others	Antimony	30.61	China: Guizhou, Banpo
L180322-P-4-LD180319-0011_HL3KCCCXY_L3_clean_bin.24.orig.fa_	OEZ008529	yes	LMSG_G000008542.1	OES073665	other	Others	Antimony	32.85	China: Guizhou, Banpo
L180322-P-4-LD180319-0011_HL3KCCCXY_L3_clean_bin.26.orig.fa_	OEZ008529	yes	LMSG_G000005267.1	OES073665	terpene	Terpene	Antimony	18.31	China: Guizhou, Banpo
L180322-P-4-LD180319-0011_HL3KCCCXY_L3_clean_bin.27.orig.fa_	OEZ008529	yes	LMSG_G000005268.1	OES073665	T3PKS.terpene	Others	Antimony	38.04	China: Guizhou, Banpo
L180322-P-4-LD180319-0011_HL3KCCCXY_L3_clean_bin.29.orig.fa_	OEZ008529	yes	LMSG_G000010627.1	OES073665	bacteriocin	RiPPs	Antimony	5.97	China: Guizhou, Banpo
L180322-P-4-LD180319-0011_HL3KCCCXY_L3_clean_bin.29.orig.fa_	OEZ008529	no	LMSG_G000010627.1	OES073665	terpene	Terpene	Antimony	13.00	China: Guizhou, Banpo
L180322-P-4-LD180319-0011_HL3KCCCXY_L3_clean_bin.29.orig.fa_	OEZ008529	yes	LMSG_G000010627.1	OES073665	arylpolyene	Others	Antimony	19.13	China: Guizhou, Banpo
L180322-P-4-LD180319-0011_HL3KCCCXY_L3_clean_bin.29.orig.fa_	OEZ008529	no	LMSG_G000010627.1	OES073665	terpene	Terpene	Antimony	10.18	China: Guizhou, Banpo
L180322-P-4-LD180319-0011_HL3KCCCXY_L3_clean_bin.30.orig.fa_	OEZ008529	yes	LMSG_G000005270.1	OES073665	NRPS	NRPS	Antimony	5.25	China: Guizhou, Banpo
L180322-P-4-LD180319-0011_HL3KCCCXY_L3_clean_bin.30.orig.fa_	OEZ008529	yes	LMSG_G000005270.1	OES073665	hserlactone	Others	Antimony	8.85	China: Guizhou, Banpo
L180322-P-4-LD180319-0011_HL3KCCCXY_L3_clean_bin.31.orig.fa_	OEZ008529	yes	LMSG_G000009506.1	OES073665	betalactone	Others	Antimony	17.38	China: Guizhou, Banpo
L180322-P-4-LD180319-0011_HL3KCCCXY_L3_clean_bin.32.orig.fa_	OEZ008529	yes	LMSG_G000005271.1	OES073665	terpene	Terpene	Antimony	16.47	China: Guizhou, Banpo
L180322-P-4-LD180319-0011_HL3KCCCXY_L3_clean_bin.32.orig.fa_	OEZ008529	yes	LMSG_G000005271.1	OES073665	terpene	Terpene	Antimony	7.66	China: Guizhou, Banpo
L180322-P-4-LD180319-0011_HL3KCCCXY_L3_clean_bin.34.orig.fa_	OEZ008529	yes	LMSG_G000010858.1	OES073665	terpene	Terpene	Antimony	20.99	China: Guizhou, Banpo
L180322-P-4-LD180319-0011_HL3KCCCXY_L3_clean_bin.34.orig.fa_	OEZ008529	yes	LMSG_G000010858.1	OES073665	arylpolyene	Others	Antimony	41.19	China: Guizhou, Banpo
L180322-P-4-LD180319-0011_HL3KCCCXY_L3_clean_bin.35.orig.fa_	OEZ008529	yes	LMSG_G000011185.1	OES073665	arylpolyene	Others	Antimony	15.35	China: Guizhou, Banpo
L180322-P-4-LD180319-0011_HL3KCCCXY_L3_clean_bin.39.orig.fa_	OEZ008529	yes	LMSG_G000007945.1	OES073665	bacteriocin	RiPPs	Antimony	10.85	China: Guizhou, Banpo
L180322-P-4-LD180319-0011_HL3KCCCXY_L3_clean_bin.39.orig.fa_	OEZ008529	yes	LMSG_G000007945.1	OES073665	T3PKS	PKSother	Antimony	6.50	China: Guizhou, Banpo
L180322-P-4-LD180319-0011_HL3KCCCXY_L3_clean_bin.39.orig.fa_	OEZ008529	yes	LMSG_G000007945.1	OES073665	T1PKS.hglE-KS	PKSother	Antimony	33.20	China: Guizhou, Banpo
L180322-P-4-LD180319-0011_HL3KCCCXY_L3_clean_bin.39.orig.fa_	OEZ008529	yes	LMSG_G000007945.1	OES073665	ladderane	Others	Antimony	6.01	China: Guizhou, Banpo
L180322-P-4-LD180319-0011_HL3KCCCXY_L3_clean_bin.39.orig.fa_	OEZ008529	yes	LMSG_G000007945.1	OES073665	terpene	Terpene	Antimony	21.08	China: Guizhou, Banpo
L180322-P-4-LD180319-0011_HL3KCCCXY_L3_clean_bin.39.orig.fa_	OEZ008529	yes	LMSG_G000007945.1	OES073665	arylpolyene	Others	Antimony	23.40	China: Guizhou, Banpo
L180322-P-4-LD180319-0011_HL3KCCCXY_L3_clean_bin.39.orig.fa_	OEZ008529	yes	LMSG_G000007945.1	OES073665	T3PKS	PKSother	Antimony	8.23	China: Guizhou, Banpo
L180322-P-4-LD180319-0011_HL3KCCCXY_L3_clean_bin.40.strict.f	OEZ008529	yes	LMSG_G000005272.1	OES073665	T3PKS	PKSother	Antimony	11.42	China: Guizhou, Banpo
L180322-P-4-LD180319-0011_HL3KCCCXY_L3_clean_bin.42.orig.fa_	OEZ008529	yes	LMSG_G000008575.1	OES073665	other	Others	Antimony	15.15	China: Guizhou, Banpo
L180322-P-4-LD180319-0011_HL3KCCCXY_L3_clean_bin.42.orig.fa_	OEZ008529	yes	LMSG_G000008575.1	OES073665	other	Others	Antimony	14.27	China: Guizhou, Banpo
L180322-P-4-LD180319-0011_HL3KCCCXY_L3_clean_bin.43.orig.fa_	OEZ008529	yes	LMSG_G000005273.1	OES073665	other	Others	Antimony	7.01	China: Guizhou, Banpo
L180322-P-4-LD180319-0011_HL3KCCCXY_L3_clean_bin.43.orig.fa_	OEZ008529	yes	LMSG_G000005273.1	OES073665	other	Others	Antimony	37.61	China: Guizhou, Banpo
L180322-P-4-LD180319-0011_HL3KCCCXY_L3_clean_bin.44.orig.fa_	OEZ008529	yes	LMSG_G000010368.1	OES073665	bacteriocin	RiPPs	Antimony	11.69	China: Guizhou, Banpo
L180322-P-4-LD180319-0011_HL3KCCCXY_L3_clean_bin.44.orig.fa_	OEZ008529	yes	LMSG_G000010368.1	OES073665	terpene	Terpene	Antimony	9.21	China: Guizhou, Banpo
L180322-P-4-LD180319-0011_HL3KCCCXY_L3_clean_bin.44.orig.fa_	OEZ008529	yes	LMSG_G000010368.1	OES073665	terpene	Terpene	Antimony	9.72	China: Guizhou, Banpo
L180322-P-4-LD180319-0011_HL3KCCCXY_L3_clean_bin.44.orig.fa_	OEZ008529	yes	LMSG_G000010368.1	OES073665	bacteriocin	RiPPs	Antimony	10.83	China: Guizhou, Banpo
L180322-P-4-LD180319-0011_HL3KCCCXY_L3_clean_bin.44.orig.fa_	OEZ008529	yes	LMSG_G000010368.1	OES073665	NRPS-like	NRPS	Antimony	9.10	China: Guizhou, Banpo
L180322-P-4-LD180319-0011_HL3KCCCXY_L3_clean_bin.46.orig.fa_	OEZ008529	yes	LMSG_G000005274.1	OES073665	NRPS-like	NRPS	Antimony	9.95	China: Guizhou, Banpo
L180322-P-4-LD180319-0011_HL3KCCCXY_L3_clean_bin.46.orig.fa_	OEZ008529	yes	LMSG_G000005274.1	OES073665	terpene	Terpene	Antimony	14.50	China: Guizhou, Banpo
L180322-P-4-LD180319-0011_HL3KCCCXY_L3_clean_bin.46.orig.fa_	OEZ008529	yes	LMSG_G000005274.1	OES073665	T3PKS	PKSother	Antimony	15.35	China: Guizhou, Banpo
L180322-P-4-LD180319-0011_HL3KCCCXY_L3_clean_bin.47.orig.fa_	OEZ008529	yes	LMSG_G000010355.1	OES073665	terpene	Terpene	Antimony	20.85	China: Guizhou, Banpo
L180322-P-4-LD180319-0011_HL3KCCCXY_L3_clean_bin.47.orig.fa_	OEZ008529	yes	LMSG_G000010355.1	OES073665	NRPS-like	NRPS	Antimony	44.07	China: Guizhou, Banpo
L180322-P-4-LD180319-0011_HL3KCCCXY_L3_clean_bin.47.orig.fa_	OEZ008529	yes	LMSG_G000010355.1	OES073665	bacteriocin	RiPPs	Antimony	9.73	China: Guizhou, Banpo
L180322-P-4-LD180319-0011_HL3KCCCXY_L3_clean_bin.47.orig.fa_	OEZ008529	yes	LMSG_G000010355.1	OES073665	bacteriocin	RiPPs	Antimony	10.86	China: Guizhou, Banpo
L180322-P-4-LD180319-0011_HL3KCCCXY_L3_clean_bin.47.orig.fa_	OEZ008529	yes	LMSG_G000010355.1	OES073665	NRPS	NRPS	Antimony	35.65	China: Guizhou, Banpo
L180322-P-4-LD180319-0011_HL3KCCCXY_L3_clean_bin.47.orig.fa_	OEZ008529	yes	LMSG_G000010355.1	OES073665	terpene	Terpene	Antimony	22.06	China: Guizhou, Banpo
L180322-P-4-LD180319-0011_HL3KCCCXY_L3_clean_bin.47.orig.fa_	OEZ008529	yes	LMSG_G000010355.1	OES073665	terpene	Terpene	Antimony	10.61	China: Guizhou, Banpo
L180322-P-4-LD180319-0011_HL3KCCCXY_L3_clean_bin.49.strict.f	OEZ008529	yes	LMSG_G000009675.1	OES073665	NRPS-like	NRPS	Antimony	5.14	China: Guizhou, Banpo
L180322-P-4-LD180319-0011_HL3KCCCXY_L3_clean_bin.5.orig.fa_k	OEZ008529	yes	LMSG_G000005281.1	OES073665	NRPS	NRPS	Antimony	47.73	China: Guizhou, Banpo
L180322-P-4-LD180319-0011_HL3KCCCXY_L3_clean_bin.50.strict.f	OEZ008529	yes	LMSG_G000005275.1	OES073665	terpene	Terpene	Antimony	13.30	China: Guizhou, Banpo
L180322-P-4-LD180319-0011_HL3KCCCXY_L3_clean_bin.51.orig.fa_	OEZ008529	yes	LMSG_G000005276.1	OES073665	NRPS-like	NRPS	Antimony	19.93	China: Guizhou, Banpo
L180322-P-4-LD180319-0011_HL3KCCCXY_L3_clean_bin.54.orig.fa_	OEZ008529	yes	LMSG_G000005278.1	OES073665	terpene	Terpene	Antimony	5.68	China: Guizhou, Banpo
L180322-P-4-LD180319-0011_HL3KCCCXY_L3_clean_bin.54.orig.fa_	OEZ008529	yes	LMSG_G000005278.1	OES073665	terpene	Terpene	Antimony	5.04	China: Guizhou, Banpo
L180322-P-4-LD180319-0011_HL3KCCCXY_L3_clean_bin.55.orig.fa_	OEZ008529	yes	LMSG_G000010342.1	OES073665	T3PKS	PKSother	Antimony	41.07	China: Guizhou, Banpo
L180322-P-4-LD180319-0011_HL3KCCCXY_L3_clean_bin.55.orig.fa_	OEZ008529	yes	LMSG_G000010342.1	OES073665	terpene	Terpene	Antimony	21.72	China: Guizhou, Banpo
L180322-P-4-LD180319-0011_HL3KCCCXY_L3_clean_bin.55.orig.fa_	OEZ008529	yes	LMSG_G000010342.1	OES073665	bacteriocin	RiPPs	Antimony	10.94	China: Guizhou, Banpo
L180322-P-4-LD180319-0011_HL3KCCCXY_L3_clean_bin.55.orig.fa_	OEZ008529	yes	LMSG_G000010342.1	OES073665	NRPS-like	NRPS	Antimony	44.07	China: Guizhou, Banpo
L180322-P-4-LD180319-0011_HL3KCCCXY_L3_clean_bin.55.orig.fa_	OEZ008529	yes	LMSG_G000010342.1	OES073665	terpene	Terpene	Antimony	21.92	China: Guizhou, Banpo
L180322-P-4-LD180319-0011_HL3KCCCXY_L3_clean_bin.55.orig.fa_	OEZ008529	yes	LMSG_G000010342.1	OES073665	terpene	Terpene	Antimony	17.16	China: Guizhou, Banpo
L180322-P-4-LD180319-0011_HL3KCCCXY_L3_clean_bin.55.orig.fa_	OEZ008529	yes	LMSG_G000010342.1	OES073665	hserlactone	Others	Antimony	20.68	China: Guizhou, Banpo
L180322-P-4-LD180319-0011_HL3KCCCXY_L3_clean_bin.56.orig.fa_	OEZ008529	yes	LMSG_G000010520.1	OES073665	NRPS-like	NRPS	Antimony	19.64	China: Guizhou, Banpo
L180322-P-4-LD180319-0011_HL3KCCCXY_L3_clean_bin.56.orig.fa_	OEZ008529	yes	LMSG_G000010520.1	OES073665	arylpolyene	Others	Antimony	22.76	China: Guizhou, Banpo
L180322-P-4-LD180319-0011_HL3KCCCXY_L3_clean_bin.56.orig.fa_	OEZ008529	yes	LMSG_G000010520.1	OES073665	acyl_amino_acids	Others	Antimony	24.57	China: Guizhou, Banpo
L180322-P-4-LD180319-0011_HL3KCCCXY_L3_clean_bin.57.orig.fa_	OEZ008529	yes	LMSG_G000010916.1	OES073665	terpene	Terpene	Antimony	7.38	China: Guizhou, Banpo
L180322-P-4-LD180319-0011_HL3KCCCXY_L3_clean_bin.57.orig.fa_	OEZ008529	yes	LMSG_G000010916.1	OES073665	terpene	Terpene	Antimony	7.71	China: Guizhou, Banpo
L180322-P-4-LD180319-0011_HL3KCCCXY_L3_clean_bin.57.orig.fa_	OEZ008529	yes	LMSG_G000010916.1	OES073665	arylpolyene	Others	Antimony	8.42	China: Guizhou, Banpo
L180322-P-4-LD180319-0011_HL3KCCCXY_L3_clean_bin.58.orig.fa_	OEZ008529	yes	LMSG_G000005279.1	OES073665	T3PKS	PKSother	Antimony	6.62	China: Guizhou, Banpo
L180322-P-4-LD180319-0011_HL3KCCCXY_L3_clean_bin.58.orig.fa_	OEZ008529	yes	LMSG_G000005279.1	OES073665	acyl_amino_acids	Others	Antimony	7.33	China: Guizhou, Banpo
L180322-P-4-LD180319-0011_HL3KCCCXY_L3_clean_bin.58.orig.fa_	OEZ008529	yes	LMSG_G000005279.1	OES073665	terpene	Terpene	Antimony	7.18	China: Guizhou, Banpo
L180322-P-4-LD180319-0011_HL3KCCCXY_L3_clean_bin.58.orig.fa_	OEZ008529	yes	LMSG_G000005279.1	OES073665	terpene	Terpene	Antimony	11.25	China: Guizhou, Banpo
L180322-P-4-LD180319-0011_HL3KCCCXY_L3_clean_bin.59.orig.fa_	OEZ008529	yes	LMSG_G000005280.1	OES073665	bacteriocin	RiPPs	Antimony	10.89	China: Guizhou, Banpo
L180322-P-4-LD180319-0011_HL3KCCCXY_L3_clean_bin.59.orig.fa_	OEZ008529	yes	LMSG_G000005280.1	OES073665	NRPS-like	NRPS	Antimony	39.73	China: Guizhou, Banpo
L180322-P-4-LD180319-0011_HL3KCCCXY_L3_clean_bin.59.orig.fa_	OEZ008529	yes	LMSG_G000005280.1	OES073665	terpene	Terpene	Antimony	15.04	China: Guizhou, Banpo
L180322-P-4-LD180319-0011_HL3KCCCXY_L3_clean_bin.6.orig.fa_k	OEZ008529	yes	LMSG_G000005285.1	OES073665	terpene	Terpene	Antimony	5.80	China: Guizhou, Banpo
L180322-P-4-LD180319-0011_HL3KCCCXY_L3_clean_bin.6.orig.fa_k	OEZ008529	yes	LMSG_G000005285.1	OES073665	NRPS	NRPS	Antimony	5.28	China: Guizhou, Banpo
L180322-P-4-LD180319-0011_HL3KCCCXY_L3_clean_bin.6.orig.fa_k	OEZ008529	yes	LMSG_G000005285.1	OES073665	transAT-PKS-like	PKSother	Antimony	14.89	China: Guizhou, Banpo
L180322-P-4-LD180319-0011_HL3KCCCXY_L3_clean_bin.6.orig.fa_k	OEZ008529	yes	LMSG_G000005285.1	OES073665	transAT-PKS-like	PKSother	Antimony	7.58	China: Guizhou, Banpo
L180322-P-4-LD180319-0011_HL3KCCCXY_L3_clean_bin.6.orig.fa_k	OEZ008529	yes	LMSG_G000005285.1	OES073665	T1PKS	PKSI	Antimony	18.47	China: Guizhou, Banpo
L180322-P-4-LD180319-0011_HL3KCCCXY_L3_clean_bin.6.orig.fa_k	OEZ008529	yes	LMSG_G000005285.1	OES073665	transAT-PKS-like	PKSother	Antimony	5.41	China: Guizhou, Banpo
L180322-P-4-LD180319-0011_HL3KCCCXY_L3_clean_bin.6.orig.fa_k	OEZ008529	yes	LMSG_G000005285.1	OES073665	NRPS	NRPS	Antimony	6.03	China: Guizhou, Banpo
L180322-P-4-LD180319-0011_HL3KCCCXY_L3_clean_bin.6.orig.fa_k	OEZ008529	yes	LMSG_G000005285.1	OES073665	NRPS-like	NRPS	Antimony	6.53	China: Guizhou, Banpo
L180322-P-4-LD180319-0011_HL3KCCCXY_L3_clean_bin.6.orig.fa_k	OEZ008529	yes	LMSG_G000005285.1	OES073665	transAT-PKS	PKSother	Antimony	15.95	China: Guizhou, Banpo
L180322-P-4-LD180319-0011_HL3KCCCXY_L3_clean_bin.6.orig.fa_k	OEZ008529	yes	LMSG_G000005285.1	OES073665	terpene	Terpene	Antimony	8.63	China: Guizhou, Banpo
L180322-P-4-LD180319-0011_HL3KCCCXY_L3_clean_bin.60.strict.f	OEZ008529	yes	LMSG_G000011299.1	OES073665	bacteriocin	RiPPs	Antimony	10.24	China: Guizhou, Banpo
L180322-P-4-LD180319-0011_HL3KCCCXY_L3_clean_bin.60.strict.f	OEZ008529	yes	LMSG_G000011299.1	OES073665	bacteriocin	RiPPs	Antimony	10.19	China: Guizhou, Banpo
L180322-P-4-LD180319-0011_HL3KCCCXY_L3_clean_bin.61.orig.fa_	OEZ008529	yes	LMSG_G000005282.1	OES073665	NRPS-like	NRPS	Antimony	10.95	China: Guizhou, Banpo
L180322-P-4-LD180319-0011_HL3KCCCXY_L3_clean_bin.61.orig.fa_	OEZ008529	yes	LMSG_G000005282.1	OES073665	ladderane	Others	Antimony	7.65	China: Guizhou, Banpo
L180322-P-4-LD180319-0011_HL3KCCCXY_L3_clean_bin.61.orig.fa_	OEZ008529	yes	LMSG_G000005282.1	OES073665	ladderane	Others	Antimony	16.07	China: Guizhou, Banpo
L180322-P-4-LD180319-0011_HL3KCCCXY_L3_clean_bin.61.orig.fa_	OEZ008529	yes	LMSG_G000005282.1	OES073665	T3PKS	PKSother	Antimony	11.90	China: Guizhou, Banpo
L180322-P-4-LD180319-0011_HL3KCCCXY_L3_clean_bin.61.orig.fa_	OEZ008529	yes	LMSG_G000005282.1	OES073665	terpene	Terpene	Antimony	6.21	China: Guizhou, Banpo
L180322-P-4-LD180319-0011_HL3KCCCXY_L3_clean_bin.61.orig.fa_	OEZ008529	yes	LMSG_G000005282.1	OES073665	terpene	Terpene	Antimony	5.61	China: Guizhou, Banpo
L180322-P-4-LD180319-0011_HL3KCCCXY_L3_clean_bin.63.orig.fa_	OEZ008529	yes	LMSG_G000011143.1	OES073665	NRPS.T1PKS	PKS-NRP_Hybrids	Antimony	32.35	China: Guizhou, Banpo
L180322-P-4-LD180319-0011_HL3KCCCXY_L3_clean_bin.63.orig.fa_	OEZ008529	yes	LMSG_G000011143.1	OES073665	NRPS	NRPS	Antimony	39.31	China: Guizhou, Banpo
L180322-P-4-LD180319-0011_HL3KCCCXY_L3_clean_bin.63.orig.fa_	OEZ008529	yes	LMSG_G000011143.1	OES073665	NRPS-like	NRPS	Antimony	41.00	China: Guizhou, Banpo
L180322-P-4-LD180319-0011_HL3KCCCXY_L3_clean_bin.64.orig.fa_	OEZ008529	yes	LMSG_G000007866.1	OES073665	bacteriocin	RiPPs	Antimony	6.44	China: Guizhou, Banpo
L180322-P-4-LD180319-0011_HL3KCCCXY_L3_clean_bin.64.orig.fa_	OEZ008529	yes	LMSG_G000007866.1	OES073665	terpene	Terpene	Antimony	30.07	China: Guizhou, Banpo
L180322-P-4-LD180319-0011_HL3KCCCXY_L3_clean_bin.64.orig.fa_	OEZ008529	yes	LMSG_G000007866.1	OES073665	NRPS-like	NRPS	Antimony	42.66	China: Guizhou, Banpo
L180322-P-4-LD180319-0011_HL3KCCCXY_L3_clean_bin.64.orig.fa_	OEZ008529	yes	LMSG_G000007866.1	OES073665	T3PKS	PKSother	Antimony	26.21	China: Guizhou, Banpo
L180322-P-4-LD180319-0011_HL3KCCCXY_L3_clean_bin.64.orig.fa_	OEZ008529	yes	LMSG_G000007866.1	OES073665	terpene	Terpene	Antimony	7.31	China: Guizhou, Banpo
L180322-P-4-LD180319-0011_HL3KCCCXY_L3_clean_bin.65.orig.fa_	OEZ008529	yes	LMSG_G000007933.1	OES073665	bacteriocin	RiPPs	Antimony	7.50	China: Guizhou, Banpo
L180322-P-4-LD180319-0011_HL3KCCCXY_L3_clean_bin.65.orig.fa_	OEZ008529	yes	LMSG_G000007933.1	OES073665	terpene	Terpene	Antimony	9.24	China: Guizhou, Banpo
L180322-P-4-LD180319-0011_HL3KCCCXY_L3_clean_bin.66.orig.fa_	OEZ008529	yes	LMSG_G000009857.1	OES073665	NRPS-like	NRPS	Antimony	31.33	China: Guizhou, Banpo
L180322-P-4-LD180319-0011_HL3KCCCXY_L3_clean_bin.68.orig.fa_	OEZ008529	yes	LMSG_G000005284.1	OES073665	T1PKS	PKSI	Antimony	16.25	China: Guizhou, Banpo
L180322-P-4-LD180319-0011_HL3KCCCXY_L3_clean_bin.68.orig.fa_	OEZ008529	yes	LMSG_G000005284.1	OES073665	terpene	Terpene	Antimony	8.36	China: Guizhou, Banpo
L180322-P-4-LD180319-0011_HL3KCCCXY_L3_clean_bin.69.orig.fa_	OEZ008529	yes	LMSG_G000004455.1	OES073665	terpene	Terpene	Antimony	20.95	China: Guizhou, Banpo
L180322-P-4-LD180319-0011_HL3KCCCXY_L3_clean_bin.69.orig.fa_	OEZ008529	yes	LMSG_G000004455.1	OES073665	ladderane	Others	Antimony	41.24	China: Guizhou, Banpo
L180322-P-4-LD180319-0011_HL3KCCCXY_L3_clean_bin.69.orig.fa_	OEZ008529	yes	LMSG_G000004455.1	OES073665	NRPS.transAT-PKS	PKS-NRP_Hybrids	Antimony	73.69	China: Guizhou, Banpo
L180322-P-4-LD180319-0011_HL3KCCCXY_L3_clean_bin.69.orig.fa_	OEZ008529	yes	LMSG_G000004455.1	OES073665	terpene	Terpene	Antimony	25.93	China: Guizhou, Banpo
L180322-P-4-LD180319-0011_HL3KCCCXY_L3_clean_bin.69.orig.fa_	OEZ008529	yes	LMSG_G000004455.1	OES073665	transAT-PKS-like	PKSother	Antimony	28.34	China: Guizhou, Banpo
L180322-P-4-LD180319-0011_HL3KCCCXY_L3_clean_bin.70.orig.fa_	OEZ008529	yes	LMSG_G000004456.1	OES073665	terpene	Terpene	Antimony	9.18	China: Guizhou, Banpo
L180322-P-4-LD180319-0011_HL3KCCCXY_L3_clean_bin.70.orig.fa_	OEZ008529	yes	LMSG_G000004456.1	OES073665	terpene	Terpene	Antimony	13.87	China: Guizhou, Banpo
L180322-P-4-LD180319-0011_HL3KCCCXY_L3_clean_bin.70.orig.fa_	OEZ008529	yes	LMSG_G000004456.1	OES073665	bacteriocin	RiPPs	Antimony	10.81	China: Guizhou, Banpo
L180322-P-4-LD180319-0011_HL3KCCCXY_L3_clean_bin.70.orig.fa_	OEZ008529	yes	LMSG_G000004456.1	OES073665	hglE-KS	PKSother	Antimony	5.98	China: Guizhou, Banpo
L180322-P-4-LD180319-0011_HL3KCCCXY_L3_clean_bin.70.orig.fa_	OEZ008529	yes	LMSG_G000004456.1	OES073665	T3PKS	PKSother	Antimony	6.94	China: Guizhou, Banpo
L180322-P-4-LD180319-0011_HL3KCCCXY_L3_clean_bin.71.orig.fa_	OEZ008529	yes	LMSG_G000010775.1	OES073665	bacteriocin	RiPPs	Antimony	11.75	China: Guizhou, Banpo
L180322-P-4-LD180319-0011_HL3KCCCXY_L3_clean_bin.71.orig.fa_	OEZ008529	yes	LMSG_G000010775.1	OES073665	acyl_amino_acids	Others	Antimony	33.24	China: Guizhou, Banpo
L180322-P-4-LD180319-0011_HL3KCCCXY_L3_clean_bin.73.strict.f	OEZ008529	yes	LMSG_G000008592.1	OES073665	other	Others	Antimony	11.62	China: Guizhou, Banpo
L180322-P-4-LD180319-0011_HL3KCCCXY_L3_clean_bin.73.strict.f	OEZ008529	yes	LMSG_G000008592.1	OES073665	betalactone	Others	Antimony	7.96	China: Guizhou, Banpo
L180322-P-4-LD180319-0011_HL3KCCCXY_L3_clean_bin.8.orig.fa_k	OEZ008529	yes	LMSG_G000005287.1	OES073665	T3PKS	PKSother	Antimony	5.94	China: Guizhou, Banpo
L180322-P-4-LD180319-0011_HL3KCCCXY_L3_clean_bin.9.orig.fa_k	OEZ008529	yes	LMSG_G000009226.1	OES073665	bacteriocin	RiPPs	Antimony	9.64	China: Guizhou, Banpo
L180322-P-4-LD180319-0011_HL3KCCCXY_L3_clean_bin.9.orig.fa_k	OEZ008529	yes	LMSG_G000009226.1	OES073665	hglE-KS	PKSother	Antimony	24.17	China: Guizhou, Banpo
L180322-P-4-LD180319-0011_HL3KCCCXY_L3_clean_bin.9.orig.fa_k	OEZ008529	yes	LMSG_G000009226.1	OES073665	terpene	Terpene	Antimony	21.72	China: Guizhou, Banpo
L180322-P-4-LD180319-0011_HL3KCCCXY_L3_clean_bin.9.orig.fa_k	OEZ008529	yes	LMSG_G000009226.1	OES073665	terpene	Terpene	Antimony	17.43	China: Guizhou, Banpo
L180322-P-4-LD180319-0011_HL3KCCCXY_L3_clean_bin.9.orig.fa_k	OEZ008529	yes	LMSG_G000009226.1	OES073665	T3PKS	PKSother	Antimony	15.78	China: Guizhou, Banpo
L180322-P-4-LD180319-0012_HL3KCCCXY_L3_clean_bin.10.orig.fa_	OEZ008529	yes	LMSG_G000009072.1	OES073666	ladderane	Others	Antimony	15.77	China: Guizhou, Banpo
L180322-P-4-LD180319-0012_HL3KCCCXY_L3_clean_bin.10.orig.fa_	OEZ008529	yes	LMSG_G000009072.1	OES073666	terpene	Terpene	Antimony	22.05	China: Guizhou, Banpo
L180322-P-4-LD180319-0012_HL3KCCCXY_L3_clean_bin.101.permiss	OEZ008529	yes	LMSG_G000008829.1	OES073666	NRPS-like	NRPS	Antimony	42.46	China: Guizhou, Banpo
L180322-P-4-LD180319-0012_HL3KCCCXY_L3_clean_bin.101.permiss	OEZ008529	yes	LMSG_G000008829.1	OES073666	T3PKS	PKSother	Antimony	21.06	China: Guizhou, Banpo
L180322-P-4-LD180319-0012_HL3KCCCXY_L3_clean_bin.101.permiss	OEZ008529	yes	LMSG_G000008829.1	OES073666	terpene	Terpene	Antimony	19.43	China: Guizhou, Banpo
L180322-P-4-LD180319-0012_HL3KCCCXY_L3_clean_bin.102.permiss	OEZ008529	yes	LMSG_G000008769.1	OES073666	NRPS-like	NRPS	Antimony	20.00	China: Guizhou, Banpo
L180322-P-4-LD180319-0012_HL3KCCCXY_L3_clean_bin.102.permiss	OEZ008529	yes	LMSG_G000008769.1	OES073666	terpene	Terpene	Antimony	14.82	China: Guizhou, Banpo

L180322-P-4-LD180319-0012_HL3KCCCXY_L3_clean_bin.102.permiss	OEZ008529	yes	LMSG_G000008769.1	OES073666	terpene	Terpene	Antimony	9.61	China: Guizhou, Banpo
L180322-P-4-LD180319-0012_HL3KCCCXY_L3_clean_bin.103.orig.fa	OEZ008529	yes	LMSG_G000009231.1	OES073666	T3PKS.terpene	Others	Antimony	23.72	China: Guizhou, Banpo
L180322-P-4-LD180319-0012_HL3KCCCXY_L3_clean_bin.105.orig.fa	OEZ008529	yes	LMSG_G000005289.1	OES073666	NRPS-like	NRPS	Antimony	5.83	China: Guizhou, Banpo
L180322-P-4-LD180319-0012_HL3KCCCXY_L3_clean_bin.105.orig.fa	OEZ008529	yes	LMSG_G000005289.1	OES073666	terpene	Terpene	Antimony	11.86	China: Guizhou, Banpo
L180322-P-4-LD180319-0012_HL3KCCCXY_L3_clean_bin.105.orig.fa	OEZ008529	yes	LMSG_G000005289.1	OES073666	betalactone	Others	Antimony	16.43	China: Guizhou, Banpo
L180322-P-4-LD180319-0012_HL3KCCCXY_L3_clean_bin.105.orig.fa	OEZ008529	yes	LMSG_G000005289.1	OES073666	T3PKS	PKSother	Antimony	8.12	China: Guizhou, Banpo
L180322-P-4-LD180319-0012_HL3KCCCXY_L3_clean_bin.105.orig.fa	OEZ008529	yes	LMSG_G000005289.1	OES073666	betalactone	Others	Antimony	14.90	China: Guizhou, Banpo
L180322-P-4-LD180319-0012_HL3KCCCXY_L3_clean_bin.105.orig.fa	OEZ008529	yes	LMSG_G000005289.1	OES073666	NRPS-like	NRPS	Antimony	22.19	China: Guizhou, Banpo
L180322-P-4-LD180319-0012_HL3KCCCXY_L3_clean_bin.106.strict.	OEZ008529	yes	LMSG_G000005290.1	OES073666	TfuA-related	RiPPs	Antimony	13.27	China: Guizhou, Banpo
L180322-P-4-LD180319-0012_HL3KCCCXY_L3_clean_bin.106.strict.	OEZ008529	yes	LMSG_G000005290.1	OES073666	T3PKS	PKSother	Antimony	24.33	China: Guizhou, Banpo
L180322-P-4-LD180319-0012_HL3KCCCXY_L3_clean_bin.108.orig.fa	OEZ008529	yes	LMSG_G000007906.1	OES073666	terpene	Terpene	Antimony	6.33	China: Guizhou, Banpo
L180322-P-4-LD180319-0012_HL3KCCCXY_L3_clean_bin.108.orig.fa	OEZ008529	yes	LMSG_G000007906.1	OES073666	terpene	Terpene	Antimony	11.30	China: Guizhou, Banpo
L180322-P-4-LD180319-0012_HL3KCCCXY_L3_clean_bin.108.orig.fa	OEZ008529	yes	LMSG_G000007906.1	OES073666	NRPS-like	NRPS	Antimony	10.96	China: Guizhou, Banpo
L180322-P-4-LD180319-0012_HL3KCCCXY_L3_clean_bin.109.orig.fa	OEZ008529	yes	LMSG_G000005291.1	OES073666	bacteriocin	RiPPs	Antimony	10.83	China: Guizhou, Banpo
L180322-P-4-LD180319-0012_HL3KCCCXY_L3_clean_bin.109.orig.fa	OEZ008529	yes	LMSG_G000005291.1	OES073666	arylpolyene	Others	Antimony	35.73	China: Guizhou, Banpo
L180322-P-4-LD180319-0012_HL3KCCCXY_L3_clean_bin.109.orig.fa	OEZ008529	yes	LMSG_G000005291.1	OES073666	terpene	Terpene	Antimony	15.61	China: Guizhou, Banpo
L180322-P-4-LD180319-0012_HL3KCCCXY_L3_clean_bin.109.orig.fa	OEZ008529	yes	LMSG_G000005291.1	OES073666	hserlactone	Others	Antimony	20.56	China: Guizhou, Banpo
L180322-P-4-LD180319-0012_HL3KCCCXY_L3_clean_bin.109.orig.fa	OEZ008529	yes	LMSG_G000005291.1	OES073666	terpene	Terpene	Antimony	13.27	China: Guizhou, Banpo
L180322-P-4-LD180319-0012_HL3KCCCXY_L3_clean_bin.11.orig.fa_	OEZ008529	yes	LMSG_G000005292.1	OES073666	betalactone.terpene	Others	Antimony	43.29	China: Guizhou, Banpo
L180322-P-4-LD180319-0012_HL3KCCCXY_L3_clean_bin.12.orig.fa_	OEZ008529	yes	LMSG_G000007862.1	OES073666	NRPS-like	NRPS	Antimony	42.66	China: Guizhou, Banpo
L180322-P-4-LD180319-0012_HL3KCCCXY_L3_clean_bin.12.orig.fa_	OEZ008529	yes	LMSG_G000007862.1	OES073666	T3PKS	PKSother	Antimony	41.06	China: Guizhou, Banpo
L180322-P-4-LD180319-0012_HL3KCCCXY_L3_clean_bin.12.orig.fa_	OEZ008529	yes	LMSG_G000007862.1	OES073666	bacteriocin	RiPPs	Antimony	10.86	China: Guizhou, Banpo
L180322-P-4-LD180319-0012_HL3KCCCXY_L3_clean_bin.12.orig.fa_	OEZ008529	yes	LMSG_G000007862.1	OES073666	terpene	Terpene	Antimony	30.00	China: Guizhou, Banpo
L180322-P-4-LD180319-0012_HL3KCCCXY_L3_clean_bin.13.orig.fa_	OEZ008529	yes	LMSG_G000005293.1	OES073666	terpene	Terpene	Antimony	6.56	China: Guizhou, Banpo
L180322-P-4-LD180319-0012_HL3KCCCXY_L3_clean_bin.13.orig.fa_	OEZ008529	yes	LMSG_G000005293.1	OES073666	betalactone	Others	Antimony	15.94	China: Guizhou, Banpo
L180322-P-4-LD180319-0012_HL3KCCCXY_L3_clean_bin.14.orig.fa_	OEZ008529	yes	LMSG_G000008649.1	OES073666	other	Others	Antimony	37.64	China: Guizhou, Banpo
L180322-P-4-LD180319-0012_HL3KCCCXY_L3_clean_bin.15.orig.fa_	OEZ008529	yes	LMSG_G000011186.1	OES073666	terpene	Terpene	Antimony	6.05	China: Guizhou, Banpo
L180322-P-4-LD180319-0012_HL3KCCCXY_L3_clean_bin.16.orig.fa_	OEZ008529	yes	LMSG_G000010516.1	OES073666	NRPS-like	NRPS	Antimony	10.56	China: Guizhou, Banpo
L180322-P-4-LD180319-0012_HL3KCCCXY_L3_clean_bin.16.orig.fa_	OEZ008529	yes	LMSG_G000010516.1	OES073666	acyl_amino_acids	Others	Antimony	33.49	China: Guizhou, Banpo
L180322-P-4-LD180319-0012_HL3KCCCXY_L3_clean_bin.16.orig.fa_	OEZ008529	yes	LMSG_G000010516.1	OES073666	arylpolyene	Others	Antimony	41.19	China: Guizhou, Banpo
L180322-P-4-LD180319-0012_HL3KCCCXY_L3_clean_bin.17.strict.f	OEZ008529	yes	LMSG_G000006318.1	OES073666	CDPS	Others	Antimony	20.74	China: Guizhou, Banpo
L180322-P-4-LD180319-0012_HL3KCCCXY_L3_clean_bin.17.strict.f	OEZ008529	yes	LMSG_G000006318.1	OES073666	NRPS-like	NRPS	Antimony	44.25	China: Guizhou, Banpo
L180322-P-4-LD180319-0012_HL3KCCCXY_L3_clean_bin.17.strict.f	OEZ008529	yes	LMSG_G000006318.1	OES073666	TfuA-related	RiPPs	Antimony	19.42	China: Guizhou, Banpo
L180322-P-4-LD180319-0012_HL3KCCCXY_L3_clean_bin.18.orig.fa_	OEZ008529	yes	LMSG_G000010862.1	OES073666	terpene	Terpene	Antimony	21.65	China: Guizhou, Banpo
L180322-P-4-LD180319-0012_HL3KCCCXY_L3_clean_bin.18.orig.fa_	OEZ008529	yes	LMSG_G000010862.1	OES073666	betalactone.arylpolyene	Others	Antimony	37.90	China: Guizhou, Banpo
L180322-P-4-LD180319-0012_HL3KCCCXY_L3_clean_bin.2.orig.fa_k	OEZ008529	yes	LMSG_G000005296.1	OES073666	arylpolyene	Others	Antimony	7.73	China: Guizhou, Banpo
L180322-P-4-LD180319-0012_HL3KCCCXY_L3_clean_bin.21.orig.fa_	OEZ008529	yes	LMSG_G000009325.1	OES073666	lanthipeptide	RiPPs	Antimony	9.40	China: Guizhou, Banpo
L180322-P-4-LD180319-0012_HL3KCCCXY_L3_clean_bin.21.orig.fa_	OEZ008529	yes	LMSG_G000009325.1	OES073666	NRPS.transAT-PKS	PKS-NRP_Hybrids	Antimony	47.02	China: Guizhou, Banpo
L180322-P-4-LD180319-0012_HL3KCCCXY_L3_clean_bin.21.orig.fa_	OEZ008529	yes	LMSG_G000009325.1	OES073666	bacteriocin	RiPPs	Antimony	6.30	China: Guizhou, Banpo
L180322-P-4-LD180319-0012_HL3KCCCXY_L3_clean_bin.21.orig.fa_	OEZ008529	yes	LMSG_G000009325.1	OES073666	LAP.thiopeptide	RiPPs	Antimony	10.13	China: Guizhou, Banpo
L180322-P-4-LD180319-0012_HL3KCCCXY_L3_clean_bin.22.orig.fa_	OEZ008529	yes	LMSG_G000009858.1	OES073666	NRPS-like	NRPS	Antimony	31.33	China: Guizhou, Banpo
L180322-P-4-LD180319-0012_HL3KCCCXY_L3_clean_bin.23.orig.fa_	OEZ008529	yes	LMSG_G000010343.1	OES073666	terpene	Terpene	Antimony	20.12	China: Guizhou, Banpo
L180322-P-4-LD180319-0012_HL3KCCCXY_L3_clean_bin.23.orig.fa_	OEZ008529	yes	LMSG_G000010343.1	OES073666	T3PKS	PKSother	Antimony	41.07	China: Guizhou, Banpo
L180322-P-4-LD180319-0012_HL3KCCCXY_L3_clean_bin.23.orig.fa_	OEZ008529	yes	LMSG_G000010343.1	OES073666	NRPS-like	NRPS	Antimony	44.07	China: Guizhou, Banpo
L180322-P-4-LD180319-0012_HL3KCCCXY_L3_clean_bin.23.orig.fa_	OEZ008529	yes	LMSG_G000010343.1	OES073666	terpene	Terpene	Antimony	21.92	China: Guizhou, Banpo
L180322-P-4-LD180319-0012_HL3KCCCXY_L3_clean_bin.23.orig.fa_	OEZ008529	yes	LMSG_G000010343.1	OES073666	terpene	Terpene	Antimony	21.72	China: Guizhou, Banpo
L180322-P-4-LD180319-0012_HL3KCCCXY_L3_clean_bin.25.orig.fa_	OEZ008529	yes	LMSG_G000010326.1	OES073666	T1PKS	PKSI	Antimony	33.71	China: Guizhou, Banpo
L180322-P-4-LD180319-0012_HL3KCCCXY_L3_clean_bin.25.orig.fa_	OEZ008529	yes	LMSG_G000010326.1	OES073666	NRPS-like	NRPS	Antimony	22.78	China: Guizhou, Banpo
L180322-P-4-LD180319-0012_HL3KCCCXY_L3_clean_bin.3.orig.fa_k	OEZ008529	no	LMSG_G000010379.1	OES073666	T3PKS	PKSother	Antimony	5.89	China: Guizhou, Banpo
L180322-P-4-LD180319-0012_HL3KCCCXY_L3_clean_bin.3.orig.fa_k	OEZ008529	yes	LMSG_G000010379.1	OES073666	hserlactone	Others	Antimony	14.40	China: Guizhou, Banpo
L180322-P-4-LD180319-0012_HL3KCCCXY_L3_clean_bin.3.orig.fa_k	OEZ008529	no	LMSG_G000010379.1	OES073666	terpene	Terpene	Antimony	6.22	China: Guizhou, Banpo
L180322-P-4-LD180319-0012_HL3KCCCXY_L3_clean_bin.3.orig.fa_k	OEZ008529	yes	LMSG_G000010379.1	OES073666	NRPS-like	NRPS	Antimony	7.49	China: Guizhou, Banpo
L180322-P-4-LD180319-0012_HL3KCCCXY_L3_clean_bin.3.orig.fa_k	OEZ008529	no	LMSG_G000010379.1	OES073666	terpene	Terpene	Antimony	8.49	China: Guizhou, Banpo
L180322-P-4-LD180319-0012_HL3KCCCXY_L3_clean_bin.3.orig.fa_k	OEZ008529	yes	LMSG_G000010379.1	OES073666	bacteriocin	RiPPs	Antimony	5.98	China: Guizhou, Banpo
L180322-P-4-LD180319-0012_HL3KCCCXY_L3_clean_bin.3.orig.fa_k	OEZ008529	yes	LMSG_G000010379.1	OES073666	terpene	Terpene	Antimony	13.66	China: Guizhou, Banpo
L180322-P-4-LD180319-0012_HL3KCCCXY_L3_clean_bin.30.orig.fa_	OEZ008529	yes	LMSG_G000005297.1	OES073666	terpene	Terpene	Antimony	17.70	China: Guizhou, Banpo
L180322-P-4-LD180319-0012_HL3KCCCXY_L3_clean_bin.30.orig.fa_	OEZ008529	yes	LMSG_G000005297.1	OES073666	betalactone	Others	Antimony	16.38	China: Guizhou, Banpo
L180322-P-4-LD180319-0012_HL3KCCCXY_L3_clean_bin.30.orig.fa_	OEZ008529	yes	LMSG_G000005297.1	OES073666	NRPS-like	NRPS	Antimony	34.58	China: Guizhou, Banpo
L180322-P-4-LD180319-0012_HL3KCCCXY_L3_clean_bin.30.orig.fa_	OEZ008529	yes	LMSG_G000005297.1	OES073666	bacteriocin	RiPPs	Antimony	10.20	China: Guizhou, Banpo
L180322-P-4-LD180319-0012_HL3KCCCXY_L3_clean_bin.30.orig.fa_	OEZ008529	yes	LMSG_G000005297.1	OES073666	terpene	Terpene	Antimony	19.21	China: Guizhou, Banpo
L180322-P-4-LD180319-0012_HL3KCCCXY_L3_clean_bin.30.orig.fa_	OEZ008529	yes	LMSG_G000005297.1	OES073666	arylpolyene	Others	Antimony	42.17	China: Guizhou, Banpo
L180322-P-4-LD180319-0012_HL3KCCCXY_L3_clean_bin.30.orig.fa_	OEZ008529	yes	LMSG_G000005297.1	OES073666	lassopeptide	RiPPs	Antimony	22.49	China: Guizhou, Banpo
L180322-P-4-LD180319-0012_HL3KCCCXY_L3_clean_bin.30.orig.fa_	OEZ008529	yes	LMSG_G000005297.1	OES073666	hserlactone	Others	Antimony	13.95	China: Guizhou, Banpo
L180322-P-4-LD180319-0012_HL3KCCCXY_L3_clean_bin.34.orig.fa_	OEZ008529	yes	LMSG_G000010385.1	OES073666	NRPS	NRPS	Antimony	12.87	China: Guizhou, Banpo
L180322-P-4-LD180319-0012_HL3KCCCXY_L3_clean_bin.34.orig.fa_	OEZ008529	yes	LMSG_G000010385.1	OES073666	terpene	Terpene	Antimony	10.67	China: Guizhou, Banpo
L180322-P-4-LD180319-0012_HL3KCCCXY_L3_clean_bin.34.orig.fa_	OEZ008529	yes	LMSG_G000010385.1	OES073666	NRPS-like	NRPS	Antimony	8.49	China: Guizhou, Banpo
L180322-P-4-LD180319-0012_HL3KCCCXY_L3_clean_bin.35.strict.f	OEZ008529	yes	LMSG_G000005301.1	OES073666	betalactone	Others	Antimony	15.04	China: Guizhou, Banpo
L180322-P-4-LD180319-0012_HL3KCCCXY_L3_clean_bin.36.orig.fa_	OEZ008529	yes	LMSG_G000010467.1	OES073666	NRPS-like	NRPS	Antimony	5.58	China: Guizhou, Banpo
L180322-P-4-LD180319-0012_HL3KCCCXY_L3_clean_bin.36.orig.fa_	OEZ008529	yes	LMSG_G000010467.1	OES073666	terpene	Terpene	Antimony	5.63	China: Guizhou, Banpo
L180322-P-4-LD180319-0012_HL3KCCCXY_L3_clean_bin.37.orig.fa_	OEZ008529	yes	LMSG_G000006275.1	OES073666	bacteriocin	RiPPs	Antimony	10.92	China: Guizhou, Banpo
L180322-P-4-LD180319-0012_HL3KCCCXY_L3_clean_bin.37.orig.fa_	OEZ008529	yes	LMSG_G000006275.1	OES073666	NRPS-like	NRPS	Antimony	14.15	China: Guizhou, Banpo
L180322-P-4-LD180319-0012_HL3KCCCXY_L3_clean_bin.37.orig.fa_	OEZ008529	yes	LMSG_G000006275.1	OES073666	NRPS-like	NRPS	Antimony	16.74	China: Guizhou, Banpo
L180322-P-4-LD180319-0012_HL3KCCCXY_L3_clean_bin.37.orig.fa_	OEZ008529	yes	LMSG_G000006275.1	OES073666	terpene	Terpene	Antimony	10.92	China: Guizhou, Banpo
L180322-P-4-LD180319-0012_HL3KCCCXY_L3_clean_bin.37.orig.fa_	OEZ008529	yes	LMSG_G000006275.1	OES073666	hserlactone	Others	Antimony	14.33	China: Guizhou, Banpo
L180322-P-4-LD180319-0012_HL3KCCCXY_L3_clean_bin.37.orig.fa_	OEZ008529	yes	LMSG_G000006275.1	OES073666	lassopeptide	RiPPs	Antimony	22.58	China: Guizhou, Banpo
L180322-P-4-LD180319-0012_HL3KCCCXY_L3_clean_bin.37.orig.fa_	OEZ008529	yes	LMSG_G000006275.1	OES073666	arylpolyene	Others	Antimony	31.85	China: Guizhou, Banpo
L180322-P-4-LD180319-0012_HL3KCCCXY_L3_clean_bin.38.orig.fa_	OEZ008529	yes	LMSG_G000007790.1	OES073666	T3PKS	PKSother	Antimony	5.50	China: Guizhou, Banpo
L180322-P-4-LD180319-0012_HL3KCCCXY_L3_clean_bin.38.orig.fa_	OEZ008529	yes	LMSG_G000007790.1	OES073666	terpene	Terpene	Antimony	7.51	China: Guizhou, Banpo
L180322-P-4-LD180319-0012_HL3KCCCXY_L3_clean_bin.38.orig.fa_	OEZ008529	yes	LMSG_G000007790.1	OES073666	bacteriocin	RiPPs	Antimony	6.35	China: Guizhou, Banpo
L180322-P-4-LD180319-0012_HL3KCCCXY_L3_clean_bin.40.orig.fa_	OEZ008529	yes	LMSG_G000011089.1	OES073666	bacteriocin	RiPPs	Antimony	9.18	China: Guizhou, Banpo
L180322-P-4-LD180319-0012_HL3KCCCXY_L3_clean_bin.40.orig.fa_	OEZ008529	yes	LMSG_G000011089.1	OES073666	terpene	Terpene	Antimony	23.08	China: Guizhou, Banpo
L180322-P-4-LD180319-0012_HL3KCCCXY_L3_clean_bin.40.orig.fa_	OEZ008529	yes	LMSG_G000011089.1	OES073666	arylpolyene	Others	Antimony	23.65	China: Guizhou, Banpo
L180322-P-4-LD180319-0012_HL3KCCCXY_L3_clean_bin.41.strict.f	OEZ008529	yes	LMSG_G000011333.1	OES073666	NRPS-like	NRPS	Antimony	28.86	China: Guizhou, Banpo
L180322-P-4-LD180319-0012_HL3KCCCXY_L3_clean_bin.41.strict.f	OEZ008529	yes	LMSG_G000011333.1	OES073666	bacteriocin	RiPPs	Antimony	10.84	China: Guizhou, Banpo
L180322-P-4-LD180319-0012_HL3KCCCXY_L3_clean_bin.42.orig.fa_	OEZ008529	yes	LMSG_G000010628.1	OES073666	terpene	Terpene	Antimony	20.85	China: Guizhou, Banpo
L180322-P-4-LD180319-0012_HL3KCCCXY_L3_clean_bin.42.orig.fa_	OEZ008529	no	LMSG_G000010628.1	OES073666	arylpolyene	Others	Antimony	27.96	China: Guizhou, Banpo
L180322-P-4-LD180319-0012_HL3KCCCXY_L3_clean_bin.42.orig.fa_	OEZ008529	yes	LMSG_G000010628.1	OES073666	arylpolyene	Others	Antimony	25.30	China: Guizhou, Banpo
L180322-P-4-LD180319-0012_HL3KCCCXY_L3_clean_bin.42.orig.fa_	OEZ008529	yes	LMSG_G000010628.1	OES073666	bacteriocin	RiPPs	Antimony	10.99	China: Guizhou, Banpo
L180322-P-4-LD180319-0012_HL3KCCCXY_L3_clean_bin.42.orig.fa_	OEZ008529	yes	LMSG_G000010628.1	OES073666	terpene	Terpene	Antimony	23.00	China: Guizhou, Banpo
L180322-P-4-LD180319-0012_HL3KCCCXY_L3_clean_bin.43.permissi	OEZ008529	yes	LMSG_G000008967.1	OES073666	NRPS	NRPS	Antimony	24.08	China: Guizhou, Banpo
L180322-P-4-LD180319-0012_HL3KCCCXY_L3_clean_bin.43.permissi	OEZ008529	yes	LMSG_G000008967.1	OES073666	lanthipeptide	RiPPs	Antimony	14.63	China: Guizhou, Banpo
L180322-P-4-LD180319-0012_HL3KCCCXY_L3_clean_bin.43.permissi	OEZ008529	yes	LMSG_G000008967.1	OES073666	NRPS	NRPS	Antimony	13.94	China: Guizhou, Banpo
L180322-P-4-LD180319-0012_HL3KCCCXY_L3_clean_bin.43.permissi	OEZ008529	yes	LMSG_G000008967.1	OES073666	terpene	Terpene	Antimony	12.29	China: Guizhou, Banpo
L180322-P-4-LD180319-0012_HL3KCCCXY_L3_clean_bin.43.permissi	OEZ008529	yes	LMSG_G000008967.1	OES073666	NRPS	NRPS	Antimony	9.51	China: Guizhou, Banpo
L180322-P-4-LD180319-0012_HL3KCCCXY_L3_clean_bin.44.orig.fa_	OEZ008529	yes	LMSG_G000009494.1	OES073666	TfuA-related	RiPPs	Antimony	15.90	China: Guizhou, Banpo
L180322-P-4-LD180319-0012_HL3KCCCXY_L3_clean_bin.44.orig.fa_	OEZ008529	yes	LMSG_G000009494.1	OES073666	T3PKS	PKSother	Antimony	25.70	China: Guizhou, Banpo
L180322-P-4-LD180319-0012_HL3KCCCXY_L3_clean_bin.44.orig.fa_	OEZ008529	yes	LMSG_G000009494.1	OES073666	bacteriocin	RiPPs	Antimony	10.55	China: Guizhou, Banpo
L180322-P-4-LD180319-0012_HL3KCCCXY_L3_clean_bin.44.orig.fa_	OEZ008529	yes	LMSG_G000009494.1	OES073666	T1PKS.hglE-KS	PKSother	Antimony	34.46	China: Guizhou, Banpo
L180322-P-4-LD180319-0012_HL3KCCCXY_L3_clean_bin.49.orig.fa_	OEZ008529	yes	LMSG_G000010356.1	OES073666	bacteriocin	RiPPs	Antimony	7.93	China: Guizhou, Banpo
L180322-P-4-LD180319-0012_HL3KCCCXY_L3_clean_bin.49.orig.fa_	OEZ008529	yes	LMSG_G000010356.1	OES073666	NRPS-like	NRPS	Antimony	24.86	China: Guizhou, Banpo
L180322-P-4-LD180319-0012_HL3KCCCXY_L3_clean_bin.49.orig.fa_	OEZ008529	yes	LMSG_G000010356.1	OES073666	NRPS-like	NRPS	Antimony	15.72	China: Guizhou, Banpo
L180322-P-4-LD180319-0012_HL3KCCCXY_L3_clean_bin.49.orig.fa_	OEZ008529	yes	LMSG_G000010356.1	OES073666	terpene	Terpene	Antimony	12.76	China: Guizhou, Banpo
L180322-P-4-LD180319-0012_HL3KCCCXY_L3_clean_bin.49.orig.fa_	OEZ008529	yes	LMSG_G000010356.1	OES073666	NRPS	NRPS	Antimony	26.39	China: Guizhou, Banpo
L180322-P-4-LD180319-0012_HL3KCCCXY_L3_clean_bin.49.orig.fa_	OEZ008529	yes	LMSG_G000010356.1	OES073666	terpene	Terpene	Antimony	20.85	China: Guizhou, Banpo
L180322-P-4-LD180319-0012_HL3KCCCXY_L3_clean_bin.49.orig.fa_	OEZ008529	yes	LMSG_G000010356.1	OES073666	NRPS-like	NRPS	Antimony	34.44	China: Guizhou, Banpo
L180322-P-4-LD180319-0012_HL3KCCCXY_L3_clean_bin.49.orig.fa_	OEZ008529	yes	LMSG_G000010356.1	OES073666	terpene	Terpene	Antimony	14.62	China: Guizhou, Banpo
L180322-P-4-LD180319-0012_HL3KCCCXY_L3_clean_bin.49.orig.fa_	OEZ008529	yes	LMSG_G000010356.1	OES073666	bacteriocin	RiPPs	Antimony	9.73	China: Guizhou, Banpo
L180322-P-4-LD180319-0012_HL3KCCCXY_L3_clean_bin.5.orig.fa_k	OEZ008529	yes	LMSG_G000010765.1	OES073666	bacteriocin	RiPPs	Antimony	9.22	China: Guizhou, Banpo
L180322-P-4-LD180319-0012_HL3KCCCXY_L3_clean_bin.5.orig.fa_k	OEZ008529	yes	LMSG_G000010765.1	OES073666	acyl_amino_acids	Others	Antimony	33.68	China: Guizhou, Banpo
L180322-P-4-LD180319-0012_HL3KCCCXY_L3_clean_bin.50.orig.fa_	OEZ008529	no	LMSG_G000010528.1	OES073666	terpene	Terpene	Antimony	11.56	China: Guizhou, Banpo
L180322-P-4-LD180319-0012_HL3KCCCXY_L3_clean_bin.51.strict.f	OEZ008529	yes	LMSG_G000004457.1	OES073666	bacteriocin	RiPPs	Antimony	10.75	China: Guizhou, Banpo
L180322-P-4-LD180319-0012_HL3KCCCXY_L3_clean_bin.52.orig.fa_	OEZ008529	yes	LMSG_G000010924.1	OES073666	thiopeptide	RiPPs	Antimony	23.83	China: Guizhou, Banpo
L180322-P-4-LD180319-0012_HL3KCCCXY_L3_clean_bin.53.orig.fa_	OEZ008529	yes	LMSG_G000010120.1	OES073666	terpene	Terpene	Antimony	13.86	China: Guizhou, Banpo
L180322-P-4-LD180319-0012_HL3KCCCXY_L3_clean_bin.54.orig.fa_	OEZ008529	yes	LMSG_G000005304.1	OES073666	ladderane	Others	Antimony	9.86	China: Guizhou, Banpo
L180322-P-4-LD180319-0012_HL3KCCCXY_L3_clean_bin.60.orig.fa_	OEZ008529	yes	LMSG_G000010706.1	OES073666	bacteriocin	RiPPs	Antimony	7.17	China: Guizhou, Banpo
L180322-P-4-LD180319-0012_HL3KCCCXY_L3_clean_bin.60.orig.fa_	OEZ008529	yes	LMSG_G000010706.1	OES073666	NRPS.T1PKS	PKS-NRP_Hybrids	Antimony	49.97	China: Guizhou, Banpo
L180322-P-4-LD180319-0012_HL3KCCCXY_L3_clean_bin.60.orig.fa_	OEZ008529	yes	LMSG_G000010706.1	OES073666	terpene	Terpene	Antimony	15.29	China: Guizhou, Banpo
L180322-P-4-LD180319-0012_HL3KCCCXY_L3_clean_bin.60.orig.fa_	OEZ008529	yes	LMSG_G000010706.1	OES073666	arylpolyene	Others	Antimony	41.29	China: Guizhou, Banpo
L180322-P-4-LD180319-0012_HL3KCCCXY_L3_clean_bin.60.orig.fa_	OEZ008529	yes	LMSG_G000010706.1	OES073666	betalactone	Others	Antimony	11.83	China: Guizhou, Banpo
L180322-P-4-LD180319-0012_HL3KCCCXY_L3_clean_bin.62.orig.fa_	OEZ008529	yes	LMSG_G000010369.1	OES073666	bacteriocin	RiPPs	Antimony	11.69	China: Guizhou, Banpo
L180322-P-4-LD180319-0012_HL3KCCCXY_L3_clean_bin.62.orig.fa_	OEZ008529	yes	LMSG_G000010369.1	OES073666	terpene	Terpene	Antimony	6.21	China: Guizhou, Banpo
L180322-P-4-LD180319-0012_HL3KCCCXY_L3_clean_bin.62.orig.fa_	OEZ008529	yes	LMSG_G000010369.1	OES073666	terpene	Terpene	Antimony	10.90	China: Guizhou, Banpo
L180322-P-4-LD180319-0012_HL3KCCCXY_L3_clean_bin.62.orig.fa_	OEZ008529	yes	LMSG_G000010369.1	OES073666	NRPS-like	NRPS	Antimony	5.24	China: Guizhou, Banpo
L180322-P-4-LD180319-0012_HL3KCCCXY_L3_clean_bin.62.orig.fa_	OEZ008529	yes	LMSG_G000010369.1	OES073666	bacteriocin	RiPPs	Antimony	9.40	China: Guizhou, Banpo
L180322-P-4-LD180319-0012_HL3KCCCXY_L3_clean_bin.62.orig.fa_	OEZ008529	yes	LMSG_G000010369.1	OES073666	terpene	Terpene	Antimony	15.59	China: Guizhou, Banpo
L180322-P-4-LD180319-0012_HL3KCCCXY_L3_clean_bin.65.orig.fa_	OEZ008529	yes	LMSG_G000005309.1	OES073666	terpene	Terpene	Antimony	7.29	China: Guizhou, Banpo
L180322-P-4-LD180319-0012_HL3KCCCXY_L3_clean_bin.65.orig.fa_	OEZ008529	yes	LMSG_G000005309.1	OES073666	terpene	Terpene	Antimony	19.81	China: Guizhou, Banpo
L180322-P-4-LD180319-0012_HL3KCCCXY_L3_clean_bin.66.orig.fa_	OEZ008529	yes	LMSG_G000004458.1	OES073666	hglE-KS	PKSother	Antimony	23.37	China: Guizhou, Banpo
L180322-P-4-LD180319-0012_HL3KCCCXY_L3_clean_bin.66.orig.fa_	OEZ008529	yes	LMSG_G000004458.1	OES073666	bacteriocin	RiPPs	Antimony	10.85	China: Guizhou, Banpo
L180322-P-4-LD180319-0012_HL3KCCCXY_L3_clean_bin.66.orig.fa_	OEZ008529	yes	LMSG_G000004458.1	OES073666	terpene	Terpene	Antimony	21.72	China: Guizhou, Banpo
L180322-P-4-LD180319-0012_HL3KCCCXY_L3_clean_bin.66.orig.fa_	OEZ008529	yes	LMSG_G000004458.1	OES073666	T3PKS	PKSother	Antimony	15.83	China: Guizhou, Banpo
L180322-P-4-LD180319-0012_HL3KCCCXY_L3_clean_bin.66.orig.fa_	OEZ008529	yes	LMSG_G000004458.1	OES073666	terpene	Terpene	Antimony	22.03	China: Guizhou, Banpo
L180322-P-4-LD180319-0012_HL3KCCCXY_L3_clean_bin.66.orig.fa_	OEZ008529	yes	LMSG_G000004458.1	OES073666	hserlactone	Others	Antimony	5.94	China: Guizhou, Banpo
L180322-P-4-LD180319-0012_HL3KCCCXY_L3_clean_bin.68.orig.fa_	OEZ008529	yes	LMSG_G000010878.1	OES073666	terpene	Terpene	Antimony	10.09	China: Guizhou, Banpo
L180322-P-4-LD180319-0012_HL3KCCCXY_L3_clean_bin.69.orig.fa_	OEZ008529	yes	LMSG_G000008660.1	OES073666	betalactone	Others	Antimony	41.46	China: Guizhou, Banpo
L180322-P-4-LD180319-0012_HL3KCCCXY_L3_clean_bin.69.orig.fa_	OEZ008529	yes	LMSG_G000008660.1	OES073666	other	Others	Antimony	32.85	China: Guizhou, Banpo
L180322-P-4-LD180319-0012_HL3KCCCXY_L3_clean_bin.69.orig.fa_	OEZ008529	yes	LMSG_G000008660.1	OES073666	betalactone	Others	Antimony	30.61	China: Guizhou, Banpo
L180322-P-4-LD180319-0012_HL3KCCCXY_L3_clean_bin.7.orig.fa_k	OEZ008529	yes	LMSG_G000005313.1	OES073666	terpene	Terpene	Antimony	18.85	China: Guizhou, Banpo
L180322-P-4-LD180319-0012_HL3KCCCXY_L3_clean_bin.7.orig.fa_k	OEZ008529	yes	LMSG_G000005313.1	OES073666	bacteriocin	RiPPs	Antimony	10.89	China: Guizhou, Banpo
L180322-P-4-LD180319-0012_HL3KCCCXY_L3_clean_bin.7.orig.fa_k	OEZ008529	yes	LMSG_G000005313.1	OES073666	T3PKS	PKSother	Antimony	24.11	China: Guizhou, Banpo
L180322-P-4-LD180319-0012_HL3KCCCXY_L3_clean_bin.7.orig.fa_k	OEZ008529	yes	LMSG_G000005313.1	OES073666	terpene	Terpene	Antimony	13.97	China: Guizhou, Banpo
L180322-P-4-LD180319-0012_HL3KCCCXY_L3_clean_bin.70.strict.f	OEZ008529	no	LMSG_G000010798.1	OES073666	acyl_amino_acids	Others	Antimony	7.39	China: Guizhou, Banpo
L180322-P-4-LD180319-0012_HL3KCCCXY_L3_clean_bin.71.orig.fa_	OEZ008529	yes	LMSG_G000008667.1	OES073666	NRPS-like	NRPS	Antimony	19.61	China: Guizhou, Banpo
L180322-P-4-LD180319-0012_HL3KCCCXY_L3_clean_bin.72.strict.f	OEZ008529	yes	LMSG_G000008777.1	OES073666	betalactone	Others	Antimony	10.73	China: Guizhou, Banpo
L180322-P-4-LD180319-0012_HL3KCCCXY_L3_clean_bin.72.strict.f	OEZ008529	yes	LMSG_G000008777.1	OES073666	T3PKS	PKSother	Antimony	8.54	China: Guizhou, Banpo
L180322-P-4-LD180319-0012_HL3KCCCXY_L3_clean_bin.72.strict.f	OEZ008529	yes	LMSG_G000008777.1	OES073666	NRPS-like	NRPS	Antimony	6.18	China: Guizhou, Banpo
L180322-P-4-LD180319-0012_HL3KCCCXY_L3_clean_bin.73.orig.fa_	OEZ008529	yes	LMSG_G000005310.1	OES073666	arylpolyene	Others	Antimony	27.61	China: Guizhou, Banpo
L180322-P-4-LD180319-0012_HL3KCCCXY_L3_clean_bin.73.orig.fa_	OEZ008529	yes	LMSG_G000005310.1	OES073666	terpene	Terpene	Antimony	20.99	China: Guizhou, Banpo
L180322-P-4-LD180319-0012_HL3KCCCXY_L3_clean_bin.74.orig.fa_	OEZ008529	yes	LMSG_G000010973.1	OES073666	T1PKS.hglE-KS	PKSother	Antimony	24.04	China: Guizhou, Banpo
L180322-P-4-LD180319-0012_HL3KCCCXY_L3_clean_bin.75.orig.fa_	OEZ008529	yes	LMSG_G000005311.1	OES073666	betalactone	Others	Antimony	12.29	China: Guizhou, Banpo
L180322-P-4-LD180319-0012_HL3KCCCXY_L3_clean_bin.76.orig.fa_	OEZ008529	yes	LMSG_G000006230.1	OES073666	bacteriocin	RiPPs	Antimony	7.48	China: Guizhou, Banpo
L180322-P-4-LD180319-0012_HL3KCCCXY_L3_clean_bin.76.orig.fa_	OEZ008529	yes	LMSG_G000006230.1	OES073666	terpene	Terpene	Antimony	10.74	China: Guizhou, Banpo
L180322-P-4-LD180319-0012_HL3KCCCXY_L3_clean_bin.76.orig.fa_	OEZ008529	yes	LMSG_G000006230.1	OES073666	terpene	Terpene	Antimony	15.43	China: Guizhou, Banpo
L180322-P-4-LD180319-0012_HL3KCCCXY_L3_clean_bin.78.strict.f	OEZ008529	yes	LMSG_G000009068.1	OES073666	NRPS-like	NRPS	Antimony	7.58	China: Guizhou, Banpo
L180322-P-4-LD180319-0012_HL3KCCCXY_L3_clean_bin.78.strict.f	OEZ008529	yes	LMSG_G000009068.1	OES073666	terpene	Terpene	Antimony	6.31	China: Guizhou, Banpo
L180322-P-4-LD180319-0012_HL3KCCCXY_L3_clean_bin.80.strict.f	OEZ008529	yes	LMSG_G000008831.1	OES073666	NRPS-like	NRPS	Antimony	22.72	China: Guizhou, Banpo
L180322-P-4-LD180319-0012_HL3KCCCXY_L3_clean_bin.80.strict.f	OEZ008529	yes	LMSG_G000008831.1	OES073666	lanthipeptide	RiPPs	Antimony	17.03	China: Guizhou, Banpo
L180322-P-4-LD180319-0012_HL3KCCCXY_L3_clean_bin.80.strict.f	OEZ008529	yes	LMSG_G000008831.1	OES073666	terpene	Terpene	Antimony	21.71	China: Guizhou, Banpo

L180322-P-4-LD180319-0012_HL3KCCCXY_L3_clean_bin.81.orig.fa_	OEZ008529	yes	LMSG_G000005314.1	OES073666	NRPS-like	NRPS	Antimony	25.42	China: Guizhou, Banpo
L180322-P-4-LD180319-0012_HL3KCCCXY_L3_clean_bin.81.orig.fa_	OEZ008529	yes	LMSG_G000005314.1	OES073666	arylpolyene	Others	Antimony	10.25	China: Guizhou, Banpo
L180322-P-4-LD180319-0012_HL3KCCCXY_L3_clean_bin.81.orig.fa_	OEZ008529	yes	LMSG_G000005314.1	OES073666	bacteriocin	RiPPs	Antimony	9.60	China: Guizhou, Banpo
L180322-P-4-LD180319-0012_HL3KCCCXY_L3_clean_bin.81.orig.fa_	OEZ008529	yes	LMSG_G000005314.1	OES073666	terpene	Terpene	Antimony	11.08	China: Guizhou, Banpo
L180322-P-4-LD180319-0012_HL3KCCCXY_L3_clean_bin.81.orig.fa_	OEZ008529	no	LMSG_G000005314.1	OES073666	arylpolyene	Others	Antimony	9.58	China: Guizhou, Banpo
L180322-P-4-LD180319-0012_HL3KCCCXY_L3_clean_bin.82.orig.fa_	OEZ008529	yes	LMSG_G000005315.1	OES073666	acyl_amino_acids	Others	Antimony	5.08	China: Guizhou, Banpo
L180322-P-4-LD180319-0012_HL3KCCCXY_L3_clean_bin.83.orig.fa_	OEZ008529	yes	LMSG_G000005316.1	OES073666	terpene	Terpene	Antimony	18.52	China: Guizhou, Banpo
L180322-P-4-LD180319-0012_HL3KCCCXY_L3_clean_bin.84.orig.fa_	OEZ008529	yes	LMSG_G000010964.1	OES073666	hglE-KS	PKSother	Antimony	21.82	China: Guizhou, Banpo
L180322-P-4-LD180319-0012_HL3KCCCXY_L3_clean_bin.84.orig.fa_	OEZ008529	yes	LMSG_G000010964.1	OES073666	NRPS	NRPS	Antimony	39.12	China: Guizhou, Banpo
L180322-P-4-LD180319-0012_HL3KCCCXY_L3_clean_bin.85.strict.f	OEZ008529	yes	LMSG_G000004459.1	OES073666	NRPS-like	NRPS	Antimony	42.75	China: Guizhou, Banpo
L180322-P-4-LD180319-0012_HL3KCCCXY_L3_clean_bin.85.strict.f	OEZ008529	yes	LMSG_G000004459.1	OES073666	NRPS.T1PKS	PKS-NRP_Hybrids	Antimony	32.69	China: Guizhou, Banpo
L180322-P-4-LD180319-0012_HL3KCCCXY_L3_clean_bin.85.strict.f	OEZ008529	yes	LMSG_G000004459.1	OES073666	NRPS	NRPS	Antimony	52.67	China: Guizhou, Banpo
L180322-P-4-LD180319-0012_HL3KCCCXY_L3_clean_bin.86.orig.fa_	OEZ008529	yes	LMSG_G000010686.1	OES073666	NRPS.T1PKS	PKS-NRP_Hybrids	Antimony	30.49	China: Guizhou, Banpo
L180322-P-4-LD180319-0012_HL3KCCCXY_L3_clean_bin.86.orig.fa_	OEZ008529	yes	LMSG_G000010686.1	OES073666	terpene	Terpene	Antimony	20.84	China: Guizhou, Banpo
L180322-P-4-LD180319-0012_HL3KCCCXY_L3_clean_bin.86.orig.fa_	OEZ008529	yes	LMSG_G000010686.1	OES073666	NRPS	NRPS	Antimony	15.30	China: Guizhou, Banpo
L180322-P-4-LD180319-0012_HL3KCCCXY_L3_clean_bin.86.orig.fa_	OEZ008529	yes	LMSG_G000010686.1	OES073666	arylpolyene	Others	Antimony	23.31	China: Guizhou, Banpo
L180322-P-4-LD180319-0012_HL3KCCCXY_L3_clean_bin.86.orig.fa_	OEZ008529	yes	LMSG_G000010686.1	OES073666	bacteriocin	RiPPs	Antimony	7.51	China: Guizhou, Banpo
L180322-P-4-LD180319-0012_HL3KCCCXY_L3_clean_bin.88.strict.f	OEZ008529	yes	LMSG_G000008641.1	OES073666	other	Others	Antimony	16.91	China: Guizhou, Banpo
L180322-P-4-LD180319-0012_HL3KCCCXY_L3_clean_bin.88.strict.f	OEZ008529	yes	LMSG_G000008641.1	OES073666	other	Others	Antimony	8.32	China: Guizhou, Banpo
L180322-P-4-LD180319-0012_HL3KCCCXY_L3_clean_bin.9.orig.fa_k	OEZ008529	no	LMSG_G000005322.1	OES073666	resorcinol.arylpolyene	Others	Antimony	8.74	China: Guizhou, Banpo
L180322-P-4-LD180319-0012_HL3KCCCXY_L3_clean_bin.9.orig.fa_k	OEZ008529	yes	LMSG_G000005322.1	OES073666	terpene	Terpene	Antimony	5.09	China: Guizhou, Banpo
L180322-P-4-LD180319-0012_HL3KCCCXY_L3_clean_bin.9.orig.fa_k	OEZ008529	yes	LMSG_G000005322.1	OES073666	arylpolyene	Others	Antimony	22.21	China: Guizhou, Banpo
L180322-P-4-LD180319-0012_HL3KCCCXY_L3_clean_bin.9.orig.fa_k	OEZ008529	no	LMSG_G000005322.1	OES073666	acyl_amino_acids	Others	Antimony	6.64	China: Guizhou, Banpo
L180322-P-4-LD180319-0012_HL3KCCCXY_L3_clean_bin.9.orig.fa_k	OEZ008529	yes	LMSG_G000005322.1	OES073666	bacteriocin	RiPPs	Antimony	5.21	China: Guizhou, Banpo
L180322-P-4-LD180319-0012_HL3KCCCXY_L3_clean_bin.90.strict.f	OEZ008529	yes	LMSG_G000006276.1	OES073666	lanthipeptide	RiPPs	Antimony	26.33	China: Guizhou, Banpo
L180322-P-4-LD180319-0012_HL3KCCCXY_L3_clean_bin.90.strict.f	OEZ008529	yes	LMSG_G000006276.1	OES073666	NRPS.transAT-PKS	PKS-NRP_Hybrids	Antimony	67.47	China: Guizhou, Banpo
L180322-P-4-LD180319-0012_HL3KCCCXY_L3_clean_bin.90.strict.f	OEZ008529	yes	LMSG_G000006276.1	OES073666	butyrolactone	Others	Antimony	10.81	China: Guizhou, Banpo
L180322-P-4-LD180319-0012_HL3KCCCXY_L3_clean_bin.90.strict.f	OEZ008529	yes	LMSG_G000006276.1	OES073666	bacteriocin	RiPPs	Antimony	12.22	China: Guizhou, Banpo
L180322-P-4-LD180319-0012_HL3KCCCXY_L3_clean_bin.90.strict.f	OEZ008529	yes	LMSG_G000006276.1	OES073666	sactipeptide	RiPPs	Antimony	20.14	China: Guizhou, Banpo
L180322-P-4-LD180319-0012_HL3KCCCXY_L3_clean_bin.92.orig.fa_	OEZ008529	no	LMSG_G000005317.1	OES073666	terpene	Terpene	Antimony	5.51	China: Guizhou, Banpo
L180322-P-4-LD180319-0012_HL3KCCCXY_L3_clean_bin.92.orig.fa_	OEZ008529	yes	LMSG_G000005317.1	OES073666	T1PKS.hglE-KS	PKSother	Antimony	18.73	China: Guizhou, Banpo
L180322-P-4-LD180319-0012_HL3KCCCXY_L3_clean_bin.92.orig.fa_	OEZ008529	yes	LMSG_G000005317.1	OES073666	NRPS-like	NRPS	Antimony	5.30	China: Guizhou, Banpo
L180322-P-4-LD180319-0012_HL3KCCCXY_L3_clean_bin.93.orig.fa_	OEZ008529	yes	LMSG_G000005318.1	OES073666	bacteriocin	RiPPs	Antimony	5.24	China: Guizhou, Banpo
L180322-P-4-LD180319-0012_HL3KCCCXY_L3_clean_bin.93.orig.fa_	OEZ008529	yes	LMSG_G000005318.1	OES073666	bacteriocin	RiPPs	Antimony	10.24	China: Guizhou, Banpo
L180322-P-4-LD180319-0012_HL3KCCCXY_L3_clean_bin.94.orig.fa_	OEZ008529	yes	LMSG_G000008606.1	OES073666	other	Others	Antimony	20.60	China: Guizhou, Banpo
L180322-P-4-LD180319-0012_HL3KCCCXY_L3_clean_bin.95.orig.fa_	OEZ008529	yes	LMSG_G000005319.1	OES073666	T3PKS	PKSother	Antimony	24.19	China: Guizhou, Banpo
L180322-P-4-LD180319-0012_HL3KCCCXY_L3_clean_bin.95.orig.fa_	OEZ008529	yes	LMSG_G000005319.1	OES073666	betalactone	Others	Antimony	27.01	China: Guizhou, Banpo
L180322-P-4-LD180319-0012_HL3KCCCXY_L3_clean_bin.95.orig.fa_	OEZ008529	yes	LMSG_G000005319.1	OES073666	NRPS-like.terpene	Others	Antimony	58.16	China: Guizhou, Banpo
L180322-P-4-LD180319-0012_HL3KCCCXY_L3_clean_bin.95.orig.fa_	OEZ008529	yes	LMSG_G000005319.1	OES073666	bacteriocin	RiPPs	Antimony	10.27	China: Guizhou, Banpo
L180322-P-4-LD180319-0012_HL3KCCCXY_L3_clean_bin.95.orig.fa_	OEZ008529	yes	LMSG_G000005319.1	OES073666	terpene	Terpene	Antimony	16.47	China: Guizhou, Banpo
L180322-P-4-LD180319-0012_HL3KCCCXY_L3_clean_bin.96.orig.fa_	OEZ008529	yes	LMSG_G000005320.1	OES073666	terpene	Terpene	Antimony	20.80	China: Guizhou, Banpo
L180322-P-4-LD180319-0012_HL3KCCCXY_L3_clean_bin.96.orig.fa_	OEZ008529	yes	LMSG_G000005320.1	OES073666	bacteriocin	RiPPs	Antimony	10.93	China: Guizhou, Banpo
L180322-P-4-LD180319-0012_HL3KCCCXY_L3_clean_bin.96.orig.fa_	OEZ008529	yes	LMSG_G000005320.1	OES073666	NRPS	NRPS	Antimony	46.83	China: Guizhou, Banpo
L180322-P-4-LD180319-0012_HL3KCCCXY_L3_clean_bin.96.orig.fa_	OEZ008529	yes	LMSG_G000005320.1	OES073666	NRPS	NRPS	Antimony	48.47	China: Guizhou, Banpo
L180322-P-4-LD180319-0012_HL3KCCCXY_L3_clean_bin.96.orig.fa_	OEZ008529	yes	LMSG_G000005320.1	OES073666	T1PKS.NRPS-like	PKS-NRP_Hybrids	Antimony	43.39	China: Guizhou, Banpo
L180322-P-4-LD180319-0012_HL3KCCCXY_L3_clean_bin.96.orig.fa_	OEZ008529	yes	LMSG_G000005320.1	OES073666	betalactone	Others	Antimony	29.38	China: Guizhou, Banpo
L180322-P-4-LD180319-0012_HL3KCCCXY_L3_clean_bin.97.orig.fa_	OEZ008529	yes	LMSG_G000007942.1	OES073666	T3PKS	PKSother	Antimony	12.20	China: Guizhou, Banpo
L180322-P-4-LD180319-0012_HL3KCCCXY_L3_clean_bin.97.orig.fa_	OEZ008529	yes	LMSG_G000007942.1	OES073666	arylpolyene	Others	Antimony	7.72	China: Guizhou, Banpo
L180322-P-4-LD180319-0012_HL3KCCCXY_L3_clean_bin.97.orig.fa_	OEZ008529	yes	LMSG_G000007942.1	OES073666	hglE-KS	PKSother	Antimony	33.11	China: Guizhou, Banpo
L180322-P-4-LD180319-0012_HL3KCCCXY_L3_clean_bin.97.orig.fa_	OEZ008529	yes	LMSG_G000007942.1	OES073666	ladderane	Others	Antimony	6.31	China: Guizhou, Banpo
L180322-P-4-LD180319-0012_HL3KCCCXY_L3_clean_bin.97.orig.fa_	OEZ008529	yes	LMSG_G000007942.1	OES073666	terpene	Terpene	Antimony	11.49	China: Guizhou, Banpo
L180322-P-4-LD180319-0012_HL3KCCCXY_L3_clean_bin.97.orig.fa_	OEZ008529	yes	LMSG_G000007942.1	OES073666	bacteriocin	RiPPs	Antimony	10.85	China: Guizhou, Banpo
L180322-P-4-LD180319-0012_HL3KCCCXY_L3_clean_bin.99.orig.fa_	OEZ008529	yes	LMSG_G000011307.1	OES073666	terpene	Terpene	Antimony	6.33	China: Guizhou, Banpo
L180322-P-4-LD180319-0012_HL3KCCCXY_L3_clean_bin.99.orig.fa_	OEZ008529	yes	LMSG_G000011307.1	OES073666	betalactone	Others	Antimony	12.03	China: Guizhou, Banpo
L180322-P-4-LD180319-0012_HL3KCCCXY_L3_clean_bin.99.orig.fa_	OEZ008529	yes	LMSG_G000011307.1	OES073666	terpene	Terpene	Antimony	6.72	China: Guizhou, Banpo
L180322-P-4-LD180319-0012_HL3KCCCXY_L3_clean_bin.99.orig.fa_	OEZ008529	yes	LMSG_G000011307.1	OES073666	terpene	Terpene	Antimony	6.46	China: Guizhou, Banpo
L180322-P-5-LD180319-0013_HL3MGCCXY_L1_clean_bin.100.orig.fa	OEZ008529	yes	LMSG_G000005323.1	OES073667	transAT-PKS-like.NRPS-like	PKS-NRP_Hybrids	Antimony	5.65	China: Guizhou, Banpo
L180322-P-5-LD180319-0013_HL3MGCCXY_L1_clean_bin.100.orig.fa	OEZ008529	yes	LMSG_G000005323.1	OES073667	arylpolyene	Others	Antimony	20.39	China: Guizhou, Banpo
L180322-P-5-LD180319-0013_HL3MGCCXY_L1_clean_bin.100.orig.fa	OEZ008529	yes	LMSG_G000005323.1	OES073667	bacteriocin	RiPPs	Antimony	7.90	China: Guizhou, Banpo
L180322-P-5-LD180319-0013_HL3MGCCXY_L1_clean_bin.102.permiss	OEZ008529	yes	LMSG_G000011103.1	OES073667	NRPS	NRPS	Antimony	30.62	China: Guizhou, Banpo
L180322-P-5-LD180319-0013_HL3MGCCXY_L1_clean_bin.102.permiss	OEZ008529	yes	LMSG_G000011103.1	OES073667	terpene	Terpene	Antimony	20.80	China: Guizhou, Banpo
L180322-P-5-LD180319-0013_HL3MGCCXY_L1_clean_bin.102.permiss	OEZ008529	yes	LMSG_G000011103.1	OES073667	bacteriocin	RiPPs	Antimony	6.06	China: Guizhou, Banpo
L180322-P-5-LD180319-0013_HL3MGCCXY_L1_clean_bin.103.orig.fa	OEZ008529	yes	LMSG_G000009495.1	OES073667	NRPS-like	NRPS	Antimony	17.45	China: Guizhou, Banpo
L180322-P-5-LD180319-0013_HL3MGCCXY_L1_clean_bin.103.orig.fa	OEZ008529	yes	LMSG_G000009495.1	OES073667	LAP	RiPPs	Antimony	14.49	China: Guizhou, Banpo
L180322-P-5-LD180319-0013_HL3MGCCXY_L1_clean_bin.103.orig.fa	OEZ008529	yes	LMSG_G000009495.1	OES073667	bacteriocin	RiPPs	Antimony	6.19	China: Guizhou, Banpo
L180322-P-5-LD180319-0013_HL3MGCCXY_L1_clean_bin.103.orig.fa	OEZ008529	yes	LMSG_G000009495.1	OES073667	T3PKS	PKSother	Antimony	5.86	China: Guizhou, Banpo
L180322-P-5-LD180319-0013_HL3MGCCXY_L1_clean_bin.103.orig.fa	OEZ008529	yes	LMSG_G000009495.1	OES073667	hglE-KS	PKSother	Antimony	14.51	China: Guizhou, Banpo
L180322-P-5-LD180319-0013_HL3MGCCXY_L1_clean_bin.105.orig.fa	OEZ008529	yes	LMSG_G000010974.1	OES073667	T1PKS.hglE-KS	PKSother	Antimony	34.68	China: Guizhou, Banpo
L180322-P-5-LD180319-0013_HL3MGCCXY_L1_clean_bin.105.orig.fa	OEZ008529	yes	LMSG_G000010974.1	OES073667	bacteriocin	RiPPs	Antimony	10.85	China: Guizhou, Banpo
L180322-P-5-LD180319-0013_HL3MGCCXY_L1_clean_bin.107.orig.fa	OEZ008529	yes	LMSG_G000004460.1	OES073667	lanthipeptide	RiPPs	Antimony	17.63	China: Guizhou, Banpo
L180322-P-5-LD180319-0013_HL3MGCCXY_L1_clean_bin.109.strict.	OEZ008529	yes	LMSG_G000008548.1	OES073667	betalactone	Others	Antimony	22.73	China: Guizhou, Banpo
L180322-P-5-LD180319-0013_HL3MGCCXY_L1_clean_bin.109.strict.	OEZ008529	yes	LMSG_G000008548.1	OES073667	other	Others	Antimony	18.73	China: Guizhou, Banpo
L180322-P-5-LD180319-0013_HL3MGCCXY_L1_clean_bin.111.orig.fa	OEZ008529	yes	LMSG_G000010517.1	OES073667	arylpolyene	Others	Antimony	25.43	China: Guizhou, Banpo
L180322-P-5-LD180319-0013_HL3MGCCXY_L1_clean_bin.111.orig.fa	OEZ008529	yes	LMSG_G000010517.1	OES073667	NRPS-like	NRPS	Antimony	7.87	China: Guizhou, Banpo
L180322-P-5-LD180319-0013_HL3MGCCXY_L1_clean_bin.111.orig.fa	OEZ008529	yes	LMSG_G000010517.1	OES073667	acyl_amino_acids	Others	Antimony	13.04	China: Guizhou, Banpo
L180322-P-5-LD180319-0013_HL3MGCCXY_L1_clean_bin.116.permiss	OEZ008529	yes	LMSG_G000009753.1	OES073667	thiopeptide.LAP	RiPPs	Antimony	30.38	China: Guizhou, Banpo
L180322-P-5-LD180319-0013_HL3MGCCXY_L1_clean_bin.117.orig.fa	OEZ008529	yes	LMSG_G000010386.1	OES073667	betalactone	Others	Antimony	13.24	China: Guizhou, Banpo
L180322-P-5-LD180319-0013_HL3MGCCXY_L1_clean_bin.117.orig.fa	OEZ008529	yes	LMSG_G000010386.1	OES073667	NRPS.T1PKS	PKS-NRP_Hybrids	Antimony	13.81	China: Guizhou, Banpo
L180322-P-5-LD180319-0013_HL3MGCCXY_L1_clean_bin.117.orig.fa	OEZ008529	yes	LMSG_G000010386.1	OES073667	hserlactone	Others	Antimony	8.20	China: Guizhou, Banpo
L180322-P-5-LD180319-0013_HL3MGCCXY_L1_clean_bin.118.orig.fa	OEZ008529	yes	LMSG_G000011192.1	OES073667	terpene	Terpene	Antimony	8.09	China: Guizhou, Banpo
L180322-P-5-LD180319-0013_HL3MGCCXY_L1_clean_bin.121.orig.fa	OEZ008529	yes	LMSG_G000005326.1	OES073667	bacteriocin	RiPPs	Antimony	10.83	China: Guizhou, Banpo
L180322-P-5-LD180319-0013_HL3MGCCXY_L1_clean_bin.121.orig.fa	OEZ008529	yes	LMSG_G000005326.1	OES073667	NRPS-like	NRPS	Antimony	24.92	China: Guizhou, Banpo
L180322-P-5-LD180319-0013_HL3MGCCXY_L1_clean_bin.121.orig.fa	OEZ008529	yes	LMSG_G000005326.1	OES073667	T3PKS	PKSother	Antimony	22.83	China: Guizhou, Banpo
L180322-P-5-LD180319-0013_HL3MGCCXY_L1_clean_bin.123.orig.fa	OEZ008529	yes	LMSG_G000006277.1	OES073667	betalactone	Others	Antimony	25.71	China: Guizhou, Banpo
L180322-P-5-LD180319-0013_HL3MGCCXY_L1_clean_bin.123.orig.fa	OEZ008529	yes	LMSG_G000006277.1	OES073667	T1PKS.hglE-KS	PKSother	Antimony	28.53	China: Guizhou, Banpo
L180322-P-5-LD180319-0013_HL3MGCCXY_L1_clean_bin.123.orig.fa	OEZ008529	yes	LMSG_G000006277.1	OES073667	T1PKS	PKSI	Antimony	46.80	China: Guizhou, Banpo
L180322-P-5-LD180319-0013_HL3MGCCXY_L1_clean_bin.123.orig.fa	OEZ008529	yes	LMSG_G000006277.1	OES073667	hglE-KS	PKSother	Antimony	26.91	China: Guizhou, Banpo
L180322-P-5-LD180319-0013_HL3MGCCXY_L1_clean_bin.126.orig.fa	OEZ008529	yes	LMSG_G000010859.1	OES073667	terpene	Terpene	Antimony	6.09	China: Guizhou, Banpo
L180322-P-5-LD180319-0013_HL3MGCCXY_L1_clean_bin.127.orig.fa	OEZ008529	yes	LMSG_G000008567.1	OES073667	other	Others	Antimony	5.22	China: Guizhou, Banpo
L180322-P-5-LD180319-0013_HL3MGCCXY_L1_clean_bin.15.orig.fa_	OEZ008529	yes	LMSG_G000009069.1	OES073667	terpene	Terpene	Antimony	22.05	China: Guizhou, Banpo
L180322-P-5-LD180319-0013_HL3MGCCXY_L1_clean_bin.15.orig.fa_	OEZ008529	yes	LMSG_G000009069.1	OES073667	T1PKS.hglE-KS	PKSother	Antimony	29.63	China: Guizhou, Banpo
L180322-P-5-LD180319-0013_HL3MGCCXY_L1_clean_bin.17.orig.fa_	OEZ008529	yes	LMSG_G000008645.1	OES073667	other	Others	Antimony	27.93	China: Guizhou, Banpo
L180322-P-5-LD180319-0013_HL3MGCCXY_L1_clean_bin.19.orig.fa_	OEZ008529	yes	LMSG_G000005327.1	OES073667	hglE-KS	PKSother	Antimony	20.42	China: Guizhou, Banpo
L180322-P-5-LD180319-0013_HL3MGCCXY_L1_clean_bin.19.orig.fa_	OEZ008529	yes	LMSG_G000005327.1	OES073667	terpene	Terpene	Antimony	16.34	China: Guizhou, Banpo
L180322-P-5-LD180319-0013_HL3MGCCXY_L1_clean_bin.19.orig.fa_	OEZ008529	yes	LMSG_G000005327.1	OES073667	hglE-KS	PKSother	Antimony	10.34	China: Guizhou, Banpo
L180322-P-5-LD180319-0013_HL3MGCCXY_L1_clean_bin.19.orig.fa_	OEZ008529	yes	LMSG_G000005327.1	OES073667	hglE-KS	PKSother	Antimony	7.47	China: Guizhou, Banpo
L180322-P-5-LD180319-0013_HL3MGCCXY_L1_clean_bin.19.orig.fa_	OEZ008529	yes	LMSG_G000005327.1	OES073667	terpene	Terpene	Antimony	12.48	China: Guizhou, Banpo
L180322-P-5-LD180319-0013_HL3MGCCXY_L1_clean_bin.19.orig.fa_	OEZ008529	yes	LMSG_G000005327.1	OES073667	NRPS-like	NRPS	Antimony	5.09	China: Guizhou, Banpo
L180322-P-5-LD180319-0013_HL3MGCCXY_L1_clean_bin.19.orig.fa_	OEZ008529	yes	LMSG_G000005327.1	OES073667	acyl_amino_acids	Others	Antimony	9.78	China: Guizhou, Banpo
L180322-P-5-LD180319-0013_HL3MGCCXY_L1_clean_bin.19.orig.fa_	OEZ008529	yes	LMSG_G000005327.1	OES073667	acyl_amino_acids	Others	Antimony	27.97	China: Guizhou, Banpo
L180322-P-5-LD180319-0013_HL3MGCCXY_L1_clean_bin.2.orig.fa_k	OEZ008529	yes	LMSG_G000009232.1	OES073667	terpene	Terpene	Antimony	6.42	China: Guizhou, Banpo
L180322-P-5-LD180319-0013_HL3MGCCXY_L1_clean_bin.20.orig.fa_	OEZ008529	yes	LMSG_G000010468.1	OES073667	terpene	Terpene	Antimony	13.29	China: Guizhou, Banpo
L180322-P-5-LD180319-0013_HL3MGCCXY_L1_clean_bin.20.orig.fa_	OEZ008529	yes	LMSG_G000010468.1	OES073667	arylpolyene	Others	Antimony	6.63	China: Guizhou, Banpo
L180322-P-5-LD180319-0013_HL3MGCCXY_L1_clean_bin.23.orig.fa_	OEZ008529	yes	LMSG_G000010375.1	OES073667	T3PKS	PKSother	Antimony	5.98	China: Guizhou, Banpo
L180322-P-5-LD180319-0013_HL3MGCCXY_L1_clean_bin.23.orig.fa_	OEZ008529	yes	LMSG_G000010375.1	OES073667	hserlactone	Others	Antimony	11.23	China: Guizhou, Banpo
L180322-P-5-LD180319-0013_HL3MGCCXY_L1_clean_bin.23.orig.fa_	OEZ008529	yes	LMSG_G000010375.1	OES073667	terpene	Terpene	Antimony	22.62	China: Guizhou, Banpo
L180322-P-5-LD180319-0013_HL3MGCCXY_L1_clean_bin.23.orig.fa_	OEZ008529	yes	LMSG_G000010375.1	OES073667	hserlactone	Others	Antimony	16.15	China: Guizhou, Banpo
L180322-P-5-LD180319-0013_HL3MGCCXY_L1_clean_bin.23.orig.fa_	OEZ008529	yes	LMSG_G000010375.1	OES073667	NRPS-like	NRPS	Antimony	7.67	China: Guizhou, Banpo
L180322-P-5-LD180319-0013_HL3MGCCXY_L1_clean_bin.23.orig.fa_	OEZ008529	yes	LMSG_G000010375.1	OES073667	bacteriocin	RiPPs	Antimony	6.49	China: Guizhou, Banpo
L180322-P-5-LD180319-0013_HL3MGCCXY_L1_clean_bin.23.orig.fa_	OEZ008529	yes	LMSG_G000010375.1	OES073667	terpene	Terpene	Antimony	13.20	China: Guizhou, Banpo
L180322-P-5-LD180319-0013_HL3MGCCXY_L1_clean_bin.23.orig.fa_	OEZ008529	yes	LMSG_G000010375.1	OES073667	betalactone	Others	Antimony	22.80	China: Guizhou, Banpo
L180322-P-5-LD180319-0013_HL3MGCCXY_L1_clean_bin.23.orig.fa_	OEZ008529	yes	LMSG_G000010375.1	OES073667	LAP	RiPPs	Antimony	15.58	China: Guizhou, Banpo
L180322-P-5-LD180319-0013_HL3MGCCXY_L1_clean_bin.24.permissi	OEZ008529	yes	LMSG_G000010941.1	OES073667	terpene	Terpene	Antimony	19.64	China: Guizhou, Banpo
L180322-P-5-LD180319-0013_HL3MGCCXY_L1_clean_bin.24.permissi	OEZ008529	yes	LMSG_G000010941.1	OES073667	NRPS-like	NRPS	Antimony	22.90	China: Guizhou, Banpo
L180322-P-5-LD180319-0013_HL3MGCCXY_L1_clean_bin.24.permissi	OEZ008529	yes	LMSG_G000010941.1	OES073667	T3PKS.bacteriocin	Others	Antimony	18.07	China: Guizhou, Banpo
L180322-P-5-LD180319-0013_HL3MGCCXY_L1_clean_bin.24.permissi	OEZ008529	yes	LMSG_G000010941.1	OES073667	bacteriocin	RiPPs	Antimony	10.86	China: Guizhou, Banpo
L180322-P-5-LD180319-0013_HL3MGCCXY_L1_clean_bin.24.permissi	OEZ008529	yes	LMSG_G000010941.1	OES073667	terpene	Terpene	Antimony	11.82	China: Guizhou, Banpo
L180322-P-5-LD180319-0013_HL3MGCCXY_L1_clean_bin.24.permissi	OEZ008529	yes	LMSG_G000010941.1	OES073667	NRPS-like	NRPS	Antimony	12.76	China: Guizhou, Banpo
L180322-P-5-LD180319-0013_HL3MGCCXY_L1_clean_bin.24.permissi	OEZ008529	yes	LMSG_G000010941.1	OES073667	transAT-PKS	PKSother	Antimony	12.37	China: Guizhou, Banpo
L180322-P-5-LD180319-0013_HL3MGCCXY_L1_clean_bin.24.permissi	OEZ008529	yes	LMSG_G000010941.1	OES073667	terpene	Terpene	Antimony	9.29	China: Guizhou, Banpo
L180322-P-5-LD180319-0013_HL3MGCCXY_L1_clean_bin.24.permissi	OEZ008529	yes	LMSG_G000010941.1	OES073667	T3PKS	PKSother	Antimony	8.90	China: Guizhou, Banpo
L180322-P-5-LD180319-0013_HL3MGCCXY_L1_clean_bin.24.permissi	OEZ008529	yes	LMSG_G000010941.1	OES073667	T3PKS	PKSother	Antimony	8.24	China: Guizhou, Banpo
L180322-P-5-LD180319-0013_HL3MGCCXY_L1_clean_bin.24.permissi	OEZ008529	yes	LMSG_G000010941.1	OES073667	transAT-PKS-like	PKSother	Antimony	8.01	China: Guizhou, Banpo
L180322-P-5-LD180319-0013_HL3MGCCXY_L1_clean_bin.24.permissi	OEZ008529	yes	LMSG_G000010941.1	OES073667	TfuA-related	RiPPs	Antimony	6.56	China: Guizhou, Banpo
L180322-P-5-LD180319-0013_HL3MGCCXY_L1_clean_bin.24.permissi	OEZ008529	yes	LMSG_G000010941.1	OES073667	transAT-PKS-like	PKSother	Antimony	5.55	China: Guizhou, Banpo
L180322-P-5-LD180319-0013_HL3MGCCXY_L1_clean_bin.25.orig.fa_	OEZ008529	yes	LMSG_G000010879.1	OES073667	terpene	Terpene	Antimony	12.45	China: Guizhou, Banpo
L180322-P-5-LD180319-0013_HL3MGCCXY_L1_clean_bin.25.orig.fa_	OEZ008529	yes	LMSG_G000010879.1	OES073667	acyl_amino_acids	Others	Antimony	5.83	China: Guizhou, Banpo
L180322-P-5-LD180319-0013_HL3MGCCXY_L1_clean_bin.25.orig.fa_	OEZ008529	yes	LMSG_G000010879.1	OES073667	NRPS-like	NRPS	Antimony	23.82	China: Guizhou, Banpo
L180322-P-5-LD180319-0013_HL3MGCCXY_L1_clean_bin.26.orig.fa_	OEZ008529	yes	LMSG_G000004461.1	OES073667	terpene	Terpene	Antimony	21.86	China: Guizhou, Banpo
L180322-P-5-LD180319-0013_HL3MGCCXY_L1_clean_bin.26.orig.fa_	OEZ008529	yes	LMSG_G000004461.1	OES073667	T3PKS	PKSother	Antimony	21.62	China: Guizhou, Banpo
L180322-P-5-LD180319-0013_HL3MGCCXY_L1_clean_bin.27.orig.fa_	OEZ008529	yes	LMSG_G000005328.1	OES073667	NRPS-like	NRPS	Antimony	14.67	China: Guizhou, Banpo
L180322-P-5-LD180319-0013_HL3MGCCXY_L1_clean_bin.27.orig.fa_	OEZ008529	yes	LMSG_G000005328.1	OES073667	terpene	Terpene	Antimony	18.24	China: Guizhou, Banpo
L180322-P-5-LD180319-0013_HL3MGCCXY_L1_clean_bin.27.orig.fa_	OEZ008529	yes	LMSG_G000005328.1	OES073667	lassopeptide	RiPPs	Antimony	16.15	China: Guizhou, Banpo
L180322-P-5-LD180319-0013_HL3MGCCXY_L1_clean_bin.27.orig.fa_	OEZ008529	yes	LMSG_G000005328.1	OES073667	bacteriocin	RiPPs	Antimony	6.33	China: Guizhou, Banpo
L180322-P-5-LD180319-0013_HL3MGCCXY_L1_clean_bin.27.orig.fa_	OEZ008529	yes	LMSG_G000005328.1	OES073667	bacteriocin	RiPPs	Antimony	5.30	China: Guizhou, Banpo
L180322-P-5-LD180319-0013_HL3MGCCXY_L1_clean_bin.27.orig.fa_	OEZ008529	yes	LMSG_G000005328.1	OES073667	arylpolyene	Others	Antimony	6.09	China: Guizhou, Banpo
L180322-P-5-LD180319-0013_HL3MGCCXY_L1_clean_bin.30.orig.fa_	OEZ008529	yes	LMSG_G000010090.1	OES073667	terpene	Terpene	Antimony	7.03	China: Guizhou, Banpo
L180322-P-5-LD180319-0013_HL3MGCCXY_L1_clean_bin.31.orig.fa_	OEZ008529	no	LMSG_G000009513.1	OES073667	betalactone	Others	Antimony	6.82	China: Guizhou, Banpo
L180322-P-5-LD180319-0013_HL3MGCCXY_L1_clean_bin.34.strict.f	OEZ008529	yes	LMSG_G000008770.1	OES073667	terpene	Terpene	Antimony	21.81	China: Guizhou, Banpo
L180322-P-5-LD180319-0013_HL3MGCCXY_L1_clean_bin.34.strict.f	OEZ008529	yes	LMSG_G000008770.1	OES073667	terpene	Terpene	Antimony	16.75	China: Guizhou, Banpo
L180322-P-5-LD180319-0013_HL3MGCCXY_L1_clean_bin.34.strict.f	OEZ008529	yes	LMSG_G000008770.1	OES073667	bacteriocin	RiPPs	Antimony	10.77	China: Guizhou, Banpo
L180322-P-5-LD180319-0013_HL3MGCCXY_L1_clean_bin.34.strict.f	OEZ008529	yes	LMSG_G000008770.1	OES073667	NRPS-like	NRPS	Antimony	5.70	China: Guizhou, Banpo
L180322-P-5-LD180319-0013_HL3MGCCXY_L1_clean_bin.35.orig.fa_	OEZ008529	yes	LMSG_G000005330.1	OES073667	terpene	Terpene	Antimony	7.11	China: Guizhou, Banpo
L180322-P-5-LD180319-0013_HL3MGCCXY_L1_clean_bin.35.orig.fa_	OEZ008529	yes	LMSG_G000005330.1	OES073667	NRPS-like	NRPS	Antimony	17.18	China: Guizhou, Banpo
L180322-P-5-LD180319-0013_HL3MGCCXY_L1_clean_bin.35.orig.fa_	OEZ008529	yes	LMSG_G000005330.1	OES073667	terpene	Terpene	Antimony	21.03	China: Guizhou, Banpo
L180322-P-5-LD180319-0013_HL3MGCCXY_L1_clean_bin.35.orig.fa_	OEZ008529	yes	LMSG_G000005330.1	OES073667	bacteriocin	RiPPs	Antimony	10.87	China: Guizhou, Banpo
L180322-P-5-LD180319-0013_HL3MGCCXY_L1_clean_bin.38.strict.f	OEZ008529	yes	LMSG_G000008833.1	OES073667	terpene	Terpene	Antimony	15.15	China: Guizhou, Banpo
L180322-P-5-LD180319-0013_HL3MGCCXY_L1_clean_bin.38.strict.f	OEZ008529	yes	LMSG_G000008833.1	OES073667	NRPS-like	NRPS	Antimony	30.04	China: Guizhou, Banpo
L180322-P-5-LD180319-0013_HL3MGCCXY_L1_clean_bin.39.orig.fa_	OEZ008529	yes	LMSG_G000010344.1	OES073667	terpene	Terpene	Antimony	15.22	China: Guizhou, Banpo
L180322-P-5-LD180319-0013_HL3MGCCXY_L1_clean_bin.39.orig.fa_	OEZ008529	yes	LMSG_G000010344.1	OES073667	terpene	Terpene	Antimony	21.92	China: Guizhou, Banpo
L180322-P-5-LD180319-0013_HL3MGCCXY_L1_clean_bin.39.orig.fa_	OEZ008529	yes	LMSG_G000010344.1	OES073667	T3PKS	PKSother	Antimony	41.07	China: Guizhou, Banpo
L180322-P-5-LD180319-0013_HL3MGCCXY_L1_clean_bin.39.orig.fa_	OEZ008529	yes	LMSG_G000010344.1	OES073667	NRPS-like	NRPS	Antimony	44.07	China: Guizhou, Banpo
L180322-P-5-LD180319-0013_HL3MGCCXY_L1_clean_bin.39.orig.fa_	OEZ008529	yes	LMSG_G000010344.1	OES073667	terpene	Terpene	Antimony	17.16	China: Guizhou, Banpo
L180322-P-5-LD180319-0013_HL3MGCCXY_L1_clean_bin.39.orig.fa_	OEZ008529	yes	LMSG_G000010344.1	OES073667	bacteriocin	RiPPs	Antimony	10.94	China: Guizhou, Banpo
L180322-P-5-LD180319-0013_HL3MGCCXY_L1_clean_bin.41.orig.fa_	OEZ008529	yes	LMSG_G000009507.1	OES073667	betalactone	Others	Antimony	8.54	China: Guizhou, Banpo
L180322-P-5-LD180319-0013_HL3MGCCXY_L1_clean_bin.43.orig.fa_	OEZ008529	yes	LMSG_G000008605.1	OES073667	other	Others	Antimony	15.53	China: Guizhou, Banpo
L180322-P-5-LD180319-0013_HL3MGCCXY_L1_clean_bin.44.orig.fa_	OEZ008529	yes	LMSG_G000007929.1	OES073667	transAT-PKS-like	PKSother	Antimony	9.57	China: Guizhou, Banpo
L180322-P-5-LD180319-0013_HL3MGCCXY_L1_clean_bin.44.orig.fa_	OEZ008529	yes	LMSG_G000007929.1	OES073667	ladderane	Others	Antimony	31.99	China: Guizhou, Banpo

L180322-P-5-LD180319-0013_HL3MGCCXY_L1_clean_bin.44.orig.fa_	OEZ008529	yes	LMSG_G000007929.1	OES073667	terpene	Terpene	Antimony	20.95	China: Guizhou, Banpo
L180322-P-5-LD180319-0013_HL3MGCCXY_L1_clean_bin.44.orig.fa_	OEZ008529	yes	LMSG_G000007929.1	OES073667	NRPS.transAT-PKS-like	PKS-NRP_Hybrids	Antimony	58.77	China: Guizhou, Banpo
L180322-P-5-LD180319-0013_HL3MGCCXY_L1_clean_bin.44.orig.fa_	OEZ008529	yes	LMSG_G000007929.1	OES073667	terpene	Terpene	Antimony	21.06	China: Guizhou, Banpo
L180322-P-5-LD180319-0013_HL3MGCCXY_L1_clean_bin.44.orig.fa_	OEZ008529	yes	LMSG_G000007929.1	OES073667	NRPS-like.transAT-PKS	PKS-NRP_Hybrids	Antimony	33.61	China: Guizhou, Banpo
L180322-P-5-LD180319-0013_HL3MGCCXY_L1_clean_bin.45.orig.fa_	OEZ008529	yes	LMSG_G000009250.1	OES073667	T3PKS	PKSother	Antimony	41.05	China: Guizhou, Banpo
L180322-P-5-LD180319-0013_HL3MGCCXY_L1_clean_bin.45.orig.fa_	OEZ008529	yes	LMSG_G000009250.1	OES073667	TfuA-related	RiPPs	Antimony	18.02	China: Guizhou, Banpo
L180322-P-5-LD180319-0013_HL3MGCCXY_L1_clean_bin.46.strict.f	OEZ008529	yes	LMSG_G000009676.1	OES073667	terpene	Terpene	Antimony	19.32	China: Guizhou, Banpo
L180322-P-5-LD180319-0013_HL3MGCCXY_L1_clean_bin.46.strict.f	OEZ008529	yes	LMSG_G000009676.1	OES073667	T1PKS	PKSI	Antimony	26.19	China: Guizhou, Banpo
L180322-P-5-LD180319-0013_HL3MGCCXY_L1_clean_bin.46.strict.f	OEZ008529	yes	LMSG_G000009676.1	OES073667	NRPS-like	NRPS	Antimony	8.93	China: Guizhou, Banpo
L180322-P-5-LD180319-0013_HL3MGCCXY_L1_clean_bin.48.orig.fa_	OEZ008529	yes	LMSG_G000005333.1	OES073667	terpene	Terpene	Antimony	19.03	China: Guizhou, Banpo
L180322-P-5-LD180319-0013_HL3MGCCXY_L1_clean_bin.48.orig.fa_	OEZ008529	yes	LMSG_G000005333.1	OES073667	T3PKS	PKSother	Antimony	41.07	China: Guizhou, Banpo
L180322-P-5-LD180319-0013_HL3MGCCXY_L1_clean_bin.52.orig.fa_	OEZ008529	yes	LMSG_G000005334.1	OES073667	terpene	Terpene	Antimony	11.21	China: Guizhou, Banpo
L180322-P-5-LD180319-0013_HL3MGCCXY_L1_clean_bin.54.orig.fa_	OEZ008529	yes	LMSG_G000010776.1	OES073667	acyl_amino_acids	Others	Antimony	33.87	China: Guizhou, Banpo
L180322-P-5-LD180319-0013_HL3MGCCXY_L1_clean_bin.54.orig.fa_	OEZ008529	yes	LMSG_G000010776.1	OES073667	bacteriocin	RiPPs	Antimony	10.19	China: Guizhou, Banpo
L180322-P-5-LD180319-0013_HL3MGCCXY_L1_clean_bin.55.orig.fa_	OEZ008529	yes	LMSG_G000005336.1	OES073667	terpene	Terpene	Antimony	17.62	China: Guizhou, Banpo
L180322-P-5-LD180319-0013_HL3MGCCXY_L1_clean_bin.55.orig.fa_	OEZ008529	yes	LMSG_G000005336.1	OES073667	betalactone	Others	Antimony	25.95	China: Guizhou, Banpo
L180322-P-5-LD180319-0013_HL3MGCCXY_L1_clean_bin.57.orig.fa_	OEZ008529	yes	LMSG_G000009227.1	OES073667	hglE-KS	PKSother	Antimony	9.64	China: Guizhou, Banpo
L180322-P-5-LD180319-0013_HL3MGCCXY_L1_clean_bin.57.orig.fa_	OEZ008529	yes	LMSG_G000009227.1	OES073667	bacteriocin	RiPPs	Antimony	10.85	China: Guizhou, Banpo
L180322-P-5-LD180319-0013_HL3MGCCXY_L1_clean_bin.57.orig.fa_	OEZ008529	yes	LMSG_G000009227.1	OES073667	terpene	Terpene	Antimony	22.03	China: Guizhou, Banpo
L180322-P-5-LD180319-0013_HL3MGCCXY_L1_clean_bin.57.orig.fa_	OEZ008529	yes	LMSG_G000009227.1	OES073667	T3PKS	PKSother	Antimony	15.78	China: Guizhou, Banpo
L180322-P-5-LD180319-0013_HL3MGCCXY_L1_clean_bin.57.orig.fa_	OEZ008529	yes	LMSG_G000009227.1	OES073667	terpene	Terpene	Antimony	15.42	China: Guizhou, Banpo
L180322-P-5-LD180319-0013_HL3MGCCXY_L1_clean_bin.59.strict.f	OEZ008529	yes	LMSG_G000011335.1	OES073667	arylpolyene	Others	Antimony	23.65	China: Guizhou, Banpo
L180322-P-5-LD180319-0013_HL3MGCCXY_L1_clean_bin.59.strict.f	OEZ008529	yes	LMSG_G000011335.1	OES073667	NRPS-like	NRPS	Antimony	10.57	China: Guizhou, Banpo
L180322-P-5-LD180319-0013_HL3MGCCXY_L1_clean_bin.6.orig.fa_k	OEZ008529	yes	LMSG_G000005341.1	OES073667	NRPS-like	NRPS	Antimony	40.35	China: Guizhou, Banpo
L180322-P-5-LD180319-0013_HL3MGCCXY_L1_clean_bin.6.orig.fa_k	OEZ008529	yes	LMSG_G000005341.1	OES073667	other	Others	Antimony	16.13	China: Guizhou, Banpo
L180322-P-5-LD180319-0013_HL3MGCCXY_L1_clean_bin.6.orig.fa_k	OEZ008529	yes	LMSG_G000005341.1	OES073667	other	Others	Antimony	10.07	China: Guizhou, Banpo
L180322-P-5-LD180319-0013_HL3MGCCXY_L1_clean_bin.60.orig.fa_	OEZ008529	yes	LMSG_G000004462.1	OES073667	terpene	Terpene	Antimony	20.87	China: Guizhou, Banpo
L180322-P-5-LD180319-0013_HL3MGCCXY_L1_clean_bin.60.orig.fa_	OEZ008529	yes	LMSG_G000004462.1	OES073667	terpene	Terpene	Antimony	15.15	China: Guizhou, Banpo
L180322-P-5-LD180319-0013_HL3MGCCXY_L1_clean_bin.60.orig.fa_	OEZ008529	yes	LMSG_G000004462.1	OES073667	ladderane	Others	Antimony	25.56	China: Guizhou, Banpo
L180322-P-5-LD180319-0013_HL3MGCCXY_L1_clean_bin.60.orig.fa_	OEZ008529	yes	LMSG_G000004462.1	OES073667	terpene	Terpene	Antimony	22.15	China: Guizhou, Banpo
L180322-P-5-LD180319-0013_HL3MGCCXY_L1_clean_bin.60.orig.fa_	OEZ008529	yes	LMSG_G000004462.1	OES073667	terpene	Terpene	Antimony	21.05	China: Guizhou, Banpo
L180322-P-5-LD180319-0013_HL3MGCCXY_L1_clean_bin.60.orig.fa_	OEZ008529	yes	LMSG_G000004462.1	OES073667	betalactone	Others	Antimony	22.67	China: Guizhou, Banpo
L180322-P-5-LD180319-0013_HL3MGCCXY_L1_clean_bin.62.orig.fa_	OEZ008529	yes	LMSG_G000005338.1	OES073667	terpene	Terpene	Antimony	22.05	China: Guizhou, Banpo
L180322-P-5-LD180319-0013_HL3MGCCXY_L1_clean_bin.62.orig.fa_	OEZ008529	yes	LMSG_G000005338.1	OES073667	NRPS-like	NRPS	Antimony	52.61	China: Guizhou, Banpo
L180322-P-5-LD180319-0013_HL3MGCCXY_L1_clean_bin.62.orig.fa_	OEZ008529	yes	LMSG_G000005338.1	OES073667	lassopeptide	RiPPs	Antimony	31.28	China: Guizhou, Banpo
L180322-P-5-LD180319-0013_HL3MGCCXY_L1_clean_bin.64.orig.fa_	OEZ008529	yes	LMSG_G000005339.1	OES073667	bacteriocin	RiPPs	Antimony	10.71	China: Guizhou, Banpo
L180322-P-5-LD180319-0013_HL3MGCCXY_L1_clean_bin.65.orig.fa_	OEZ008529	yes	LMSG_G000005340.1	OES073667	T3PKS	PKSother	Antimony	5.04	China: Guizhou, Banpo
L180322-P-5-LD180319-0013_HL3MGCCXY_L1_clean_bin.65.orig.fa_	OEZ008529	yes	LMSG_G000005340.1	OES073667	terpene	Terpene	Antimony	10.26	China: Guizhou, Banpo
L180322-P-5-LD180319-0013_HL3MGCCXY_L1_clean_bin.65.orig.fa_	OEZ008529	yes	LMSG_G000005340.1	OES073667	terpene	Terpene	Antimony	11.43	China: Guizhou, Banpo
L180322-P-5-LD180319-0013_HL3MGCCXY_L1_clean_bin.67.orig.fa_	OEZ008529	yes	LMSG_G000006231.1	OES073667	terpene	Terpene	Antimony	22.06	China: Guizhou, Banpo
L180322-P-5-LD180319-0013_HL3MGCCXY_L1_clean_bin.67.orig.fa_	OEZ008529	yes	LMSG_G000006231.1	OES073667	NRPS-like	NRPS	Antimony	42.48	China: Guizhou, Banpo
L180322-P-5-LD180319-0013_HL3MGCCXY_L1_clean_bin.67.orig.fa_	OEZ008529	yes	LMSG_G000006231.1	OES073667	T1PKS.hglE-KS	PKSother	Antimony	53.42	China: Guizhou, Banpo
L180322-P-5-LD180319-0013_HL3MGCCXY_L1_clean_bin.68.orig.fa_	OEZ008529	yes	LMSG_G000010357.1	OES073667	terpene	Terpene	Antimony	14.62	China: Guizhou, Banpo
L180322-P-5-LD180319-0013_HL3MGCCXY_L1_clean_bin.68.orig.fa_	OEZ008529	yes	LMSG_G000010357.1	OES073667	NRPS	NRPS	Antimony	35.65	China: Guizhou, Banpo
L180322-P-5-LD180319-0013_HL3MGCCXY_L1_clean_bin.68.orig.fa_	OEZ008529	yes	LMSG_G000010357.1	OES073667	terpene	Terpene	Antimony	14.03	China: Guizhou, Banpo
L180322-P-5-LD180319-0013_HL3MGCCXY_L1_clean_bin.68.orig.fa_	OEZ008529	yes	LMSG_G000010357.1	OES073667	bacteriocin	RiPPs	Antimony	9.79	China: Guizhou, Banpo
L180322-P-5-LD180319-0013_HL3MGCCXY_L1_clean_bin.68.orig.fa_	OEZ008529	yes	LMSG_G000010357.1	OES073667	bacteriocin	RiPPs	Antimony	7.94	China: Guizhou, Banpo
L180322-P-5-LD180319-0013_HL3MGCCXY_L1_clean_bin.68.orig.fa_	OEZ008529	yes	LMSG_G000010357.1	OES073667	NRPS-like	NRPS	Antimony	44.07	China: Guizhou, Banpo
L180322-P-5-LD180319-0013_HL3MGCCXY_L1_clean_bin.68.orig.fa_	OEZ008529	yes	LMSG_G000010357.1	OES073667	terpene	Terpene	Antimony	13.98	China: Guizhou, Banpo
L180322-P-5-LD180319-0013_HL3MGCCXY_L1_clean_bin.7.permissiv	OEZ008529	yes	LMSG_G000008968.1	OES073667	terpene	Terpene	Antimony	12.52	China: Guizhou, Banpo
L180322-P-5-LD180319-0013_HL3MGCCXY_L1_clean_bin.7.permissiv	OEZ008529	yes	LMSG_G000008968.1	OES073667	NRPS	NRPS	Antimony	11.62	China: Guizhou, Banpo
L180322-P-5-LD180319-0013_HL3MGCCXY_L1_clean_bin.71.strict.f	OEZ008529	yes	LMSG_G000009859.1	OES073667	NRPS-like	NRPS	Antimony	31.63	China: Guizhou, Banpo
L180322-P-5-LD180319-0013_HL3MGCCXY_L1_clean_bin.73.orig.fa_	OEZ008529	yes	LMSG_G000005343.1	OES073667	NRPS-like	NRPS	Antimony	7.63	China: Guizhou, Banpo
L180322-P-5-LD180319-0013_HL3MGCCXY_L1_clean_bin.73.orig.fa_	OEZ008529	yes	LMSG_G000005343.1	OES073667	NRPS-like	NRPS	Antimony	6.71	China: Guizhou, Banpo
L180322-P-5-LD180319-0013_HL3MGCCXY_L1_clean_bin.73.orig.fa_	OEZ008529	yes	LMSG_G000005343.1	OES073667	terpene	Terpene	Antimony	7.90	China: Guizhou, Banpo
L180322-P-5-LD180319-0013_HL3MGCCXY_L1_clean_bin.73.orig.fa_	OEZ008529	yes	LMSG_G000005343.1	OES073667	terpene	Terpene	Antimony	20.30	China: Guizhou, Banpo
L180322-P-5-LD180319-0013_HL3MGCCXY_L1_clean_bin.73.orig.fa_	OEZ008529	yes	LMSG_G000005343.1	OES073667	terpene	Terpene	Antimony	13.51	China: Guizhou, Banpo
L180322-P-5-LD180319-0013_HL3MGCCXY_L1_clean_bin.73.orig.fa_	OEZ008529	yes	LMSG_G000005343.1	OES073667	bacteriocin	RiPPs	Antimony	10.09	China: Guizhou, Banpo
L180322-P-5-LD180319-0013_HL3MGCCXY_L1_clean_bin.73.orig.fa_	OEZ008529	yes	LMSG_G000005343.1	OES073667	bacteriocin	RiPPs	Antimony	10.83	China: Guizhou, Banpo
L180322-P-5-LD180319-0013_HL3MGCCXY_L1_clean_bin.74.orig.fa_	OEZ008529	yes	LMSG_G000005344.1	OES073667	T3PKS	PKSother	Antimony	20.11	China: Guizhou, Banpo
L180322-P-5-LD180319-0013_HL3MGCCXY_L1_clean_bin.74.orig.fa_	OEZ008529	yes	LMSG_G000005344.1	OES073667	terpene	Terpene	Antimony	10.81	China: Guizhou, Banpo
L180322-P-5-LD180319-0013_HL3MGCCXY_L1_clean_bin.74.orig.fa_	OEZ008529	yes	LMSG_G000005344.1	OES073667	terpene	Terpene	Antimony	9.70	China: Guizhou, Banpo
L180322-P-5-LD180319-0013_HL3MGCCXY_L1_clean_bin.74.orig.fa_	OEZ008529	yes	LMSG_G000005344.1	OES073667	betalactone	Others	Antimony	21.65	China: Guizhou, Banpo
L180322-P-5-LD180319-0013_HL3MGCCXY_L1_clean_bin.74.orig.fa_	OEZ008529	yes	LMSG_G000005344.1	OES073667	terpene	Terpene	Antimony	9.34	China: Guizhou, Banpo
L180322-P-5-LD180319-0013_HL3MGCCXY_L1_clean_bin.74.orig.fa_	OEZ008529	yes	LMSG_G000005344.1	OES073667	terpene	Terpene	Antimony	16.08	China: Guizhou, Banpo
L180322-P-5-LD180319-0013_HL3MGCCXY_L1_clean_bin.77.orig.fa_	OEZ008529	yes	LMSG_G000004463.1	OES073667	T3PKS	PKSother	Antimony	41.12	China: Guizhou, Banpo
L180322-P-5-LD180319-0013_HL3MGCCXY_L1_clean_bin.77.orig.fa_	OEZ008529	yes	LMSG_G000004463.1	OES073667	NRPS-like	NRPS	Antimony	37.99	China: Guizhou, Banpo
L180322-P-5-LD180319-0013_HL3MGCCXY_L1_clean_bin.77.orig.fa_	OEZ008529	yes	LMSG_G000004463.1	OES073667	terpene	Terpene	Antimony	11.33	China: Guizhou, Banpo
L180322-P-5-LD180319-0013_HL3MGCCXY_L1_clean_bin.77.orig.fa_	OEZ008529	yes	LMSG_G000004463.1	OES073667	bacteriocin	RiPPs	Antimony	6.40	China: Guizhou, Banpo
L180322-P-5-LD180319-0013_HL3MGCCXY_L1_clean_bin.77.orig.fa_	OEZ008529	yes	LMSG_G000004463.1	OES073667	terpene	Terpene	Antimony	15.12	China: Guizhou, Banpo
L180322-P-5-LD180319-0013_HL3MGCCXY_L1_clean_bin.77.orig.fa_	OEZ008529	yes	LMSG_G000004463.1	OES073667	terpene	Terpene	Antimony	21.96	China: Guizhou, Banpo
L180322-P-5-LD180319-0013_HL3MGCCXY_L1_clean_bin.77.orig.fa_	OEZ008529	yes	LMSG_G000004463.1	OES073667	terpene	Terpene	Antimony	6.20	China: Guizhou, Banpo
L180322-P-5-LD180319-0013_HL3MGCCXY_L1_clean_bin.77.orig.fa_	OEZ008529	yes	LMSG_G000004463.1	OES073667	LAP.bacteriocin	RiPPs	Antimony	16.20	China: Guizhou, Banpo
L180322-P-5-LD180319-0013_HL3MGCCXY_L1_clean_bin.78.orig.fa_	OEZ008529	yes	LMSG_G000011303.1	OES073667	terpene	Terpene	Antimony	7.70	China: Guizhou, Banpo
L180322-P-5-LD180319-0013_HL3MGCCXY_L1_clean_bin.78.orig.fa_	OEZ008529	yes	LMSG_G000011303.1	OES073667	terpene	Terpene	Antimony	12.85	China: Guizhou, Banpo
L180322-P-5-LD180319-0013_HL3MGCCXY_L1_clean_bin.78.orig.fa_	OEZ008529	yes	LMSG_G000011303.1	OES073667	terpene	Terpene	Antimony	6.85	China: Guizhou, Banpo
L180322-P-5-LD180319-0013_HL3MGCCXY_L1_clean_bin.79.orig.fa_	OEZ008529	yes	LMSG_G000010116.1	OES073667	terpene	Terpene	Antimony	21.91	China: Guizhou, Banpo
L180322-P-5-LD180319-0013_HL3MGCCXY_L1_clean_bin.79.orig.fa_	OEZ008529	yes	LMSG_G000010116.1	OES073667	terpene	Terpene	Antimony	21.70	China: Guizhou, Banpo
L180322-P-5-LD180319-0013_HL3MGCCXY_L1_clean_bin.79.orig.fa_	OEZ008529	yes	LMSG_G000010116.1	OES073667	T3PKS	PKSother	Antimony	41.08	China: Guizhou, Banpo
L180322-P-5-LD180319-0013_HL3MGCCXY_L1_clean_bin.8.permissiv	OEZ008529	yes	LMSG_G000009070.1	OES073667	arylpolyene	Others	Antimony	58.59	China: Guizhou, Banpo
L180322-P-5-LD180319-0013_HL3MGCCXY_L1_clean_bin.8.permissiv	OEZ008529	yes	LMSG_G000009070.1	OES073667	terpene	Terpene	Antimony	22.05	China: Guizhou, Banpo
L180322-P-5-LD180319-0013_HL3MGCCXY_L1_clean_bin.8.permissiv	OEZ008529	yes	LMSG_G000009070.1	OES073667	lassopeptide	RiPPs	Antimony	29.97	China: Guizhou, Banpo
L180322-P-5-LD180319-0013_HL3MGCCXY_L1_clean_bin.8.permissiv	OEZ008529	yes	LMSG_G000009070.1	OES073667	T1PKS.hglE-KS	PKSother	Antimony	41.04	China: Guizhou, Banpo
L180322-P-5-LD180319-0013_HL3MGCCXY_L1_clean_bin.8.permissiv	OEZ008529	yes	LMSG_G000009070.1	OES073667	NRPS-like	NRPS	Antimony	24.98	China: Guizhou, Banpo
L180322-P-5-LD180319-0013_HL3MGCCXY_L1_clean_bin.80.orig.fa_	OEZ008529	yes	LMSG_G000007948.1	OES073667	NRPS-like	NRPS	Antimony	9.07	China: Guizhou, Banpo
L180322-P-5-LD180319-0013_HL3MGCCXY_L1_clean_bin.80.orig.fa_	OEZ008529	yes	LMSG_G000007948.1	OES073667	T3PKS	PKSother	Antimony	13.73	China: Guizhou, Banpo
L180322-P-5-LD180319-0013_HL3MGCCXY_L1_clean_bin.80.orig.fa_	OEZ008529	yes	LMSG_G000007948.1	OES073667	terpene	Terpene	Antimony	5.40	China: Guizhou, Banpo
L180322-P-5-LD180319-0013_HL3MGCCXY_L1_clean_bin.80.orig.fa_	OEZ008529	yes	LMSG_G000007948.1	OES073667	terpene	Terpene	Antimony	10.69	China: Guizhou, Banpo
L180322-P-5-LD180319-0013_HL3MGCCXY_L1_clean_bin.83.strict.f	OEZ008529	yes	LMSG_G000009048.1	OES073667	T1PKS	PKSI	Antimony	44.85	China: Guizhou, Banpo
L180322-P-5-LD180319-0013_HL3MGCCXY_L1_clean_bin.83.strict.f	OEZ008529	yes	LMSG_G000009048.1	OES073667	T1PKS	PKSI	Antimony	48.45	China: Guizhou, Banpo
L180322-P-5-LD180319-0013_HL3MGCCXY_L1_clean_bin.83.strict.f	OEZ008529	yes	LMSG_G000009048.1	OES073667	betalactone	Others	Antimony	15.97	China: Guizhou, Banpo
L180322-P-5-LD180319-0013_HL3MGCCXY_L1_clean_bin.84.orig.fa_	OEZ008529	yes	LMSG_G000007907.1	OES073667	terpene	Terpene	Antimony	11.40	China: Guizhou, Banpo
L180322-P-5-LD180319-0013_HL3MGCCXY_L1_clean_bin.84.orig.fa_	OEZ008529	yes	LMSG_G000007907.1	OES073667	terpene	Terpene	Antimony	10.59	China: Guizhou, Banpo
L180322-P-5-LD180319-0013_HL3MGCCXY_L1_clean_bin.84.orig.fa_	OEZ008529	yes	LMSG_G000007907.1	OES073667	NRPS-like	NRPS	Antimony	6.19	China: Guizhou, Banpo
L180322-P-5-LD180319-0013_HL3MGCCXY_L1_clean_bin.85.orig.fa_	OEZ008529	yes	LMSG_G000005345.1	OES073667	terpene	Terpene	Antimony	13.86	China: Guizhou, Banpo
L180322-P-5-LD180319-0013_HL3MGCCXY_L1_clean_bin.85.orig.fa_	OEZ008529	yes	LMSG_G000005345.1	OES073667	lassopeptide	RiPPs	Antimony	16.54	China: Guizhou, Banpo
L180322-P-5-LD180319-0013_HL3MGCCXY_L1_clean_bin.85.orig.fa_	OEZ008529	yes	LMSG_G000005345.1	OES073667	NRPS-like	NRPS	Antimony	6.90	China: Guizhou, Banpo
L180322-P-5-LD180319-0013_HL3MGCCXY_L1_clean_bin.85.orig.fa_	OEZ008529	yes	LMSG_G000005345.1	OES073667	T3PKS	PKSother	Antimony	19.54	China: Guizhou, Banpo
L180322-P-5-LD180319-0013_HL3MGCCXY_L1_clean_bin.85.orig.fa_	OEZ008529	yes	LMSG_G000005345.1	OES073667	bacteriocin	RiPPs	Antimony	10.87	China: Guizhou, Banpo
L180322-P-5-LD180319-0013_HL3MGCCXY_L1_clean_bin.85.orig.fa_	OEZ008529	yes	LMSG_G000005345.1	OES073667	NRPS-like	NRPS	Antimony	26.61	China: Guizhou, Banpo
L180322-P-5-LD180319-0013_HL3MGCCXY_L1_clean_bin.87.orig.fa_	OEZ008529	yes	LMSG_G000010687.1	OES073667	terpene	Terpene	Antimony	20.56	China: Guizhou, Banpo
L180322-P-5-LD180319-0013_HL3MGCCXY_L1_clean_bin.87.orig.fa_	OEZ008529	yes	LMSG_G000010687.1	OES073667	arylpolyene	Others	Antimony	16.80	China: Guizhou, Banpo
L180322-P-5-LD180319-0013_HL3MGCCXY_L1_clean_bin.87.orig.fa_	OEZ008529	yes	LMSG_G000010687.1	OES073667	bacteriocin	RiPPs	Antimony	7.50	China: Guizhou, Banpo
L180322-P-5-LD180319-0013_HL3MGCCXY_L1_clean_bin.87.orig.fa_	OEZ008529	yes	LMSG_G000010687.1	OES073667	T1PKS.NRPS-like	PKS-NRP_Hybrids	Antimony	15.78	China: Guizhou, Banpo
L180322-P-5-LD180319-0013_HL3MGCCXY_L1_clean_bin.88.orig.fa_	OEZ008529	yes	LMSG_G000011187.1	OES073667	arylpolyene	Others	Antimony	9.87	China: Guizhou, Banpo
L180322-P-5-LD180319-0013_HL3MGCCXY_L1_clean_bin.88.orig.fa_	OEZ008529	yes	LMSG_G000011187.1	OES073667	terpene	Terpene	Antimony	20.62	China: Guizhou, Banpo
L180322-P-5-LD180319-0013_HL3MGCCXY_L1_clean_bin.9.strict.fa	OEZ008529	yes	LMSG_G000009230.1	OES073667	terpene	Terpene	Antimony	7.85	China: Guizhou, Banpo
L180322-P-5-LD180319-0013_HL3MGCCXY_L1_clean_bin.9.strict.fa	OEZ008529	yes	LMSG_G000009230.1	OES073667	bacteriocin	RiPPs	Antimony	6.97	China: Guizhou, Banpo
L180322-P-5-LD180319-0013_HL3MGCCXY_L1_clean_bin.90.strict.f	OEZ008529	yes	LMSG_G000005346.1	OES073667	betalactone	Others	Antimony	10.11	China: Guizhou, Banpo
L180322-P-5-LD180319-0013_HL3MGCCXY_L1_clean_bin.92.orig.fa_	OEZ008529	yes	LMSG_G000005347.1	OES073667	terpene	Terpene	Antimony	18.94	China: Guizhou, Banpo
L180322-P-5-LD180319-0013_HL3MGCCXY_L1_clean_bin.92.orig.fa_	OEZ008529	yes	LMSG_G000005347.1	OES073667	NRPS-like	NRPS	Antimony	40.09	China: Guizhou, Banpo
L180322-P-5-LD180319-0013_HL3MGCCXY_L1_clean_bin.92.orig.fa_	OEZ008529	yes	LMSG_G000005347.1	OES073667	NRPS-like	NRPS	Antimony	25.64	China: Guizhou, Banpo
L180322-P-5-LD180319-0013_HL3MGCCXY_L1_clean_bin.94.orig.fa_	OEZ008529	yes	LMSG_G000011149.1	OES073667	terpene	Terpene	Antimony	19.92	China: Guizhou, Banpo
L180322-P-5-LD180319-0013_HL3MGCCXY_L1_clean_bin.94.orig.fa_	OEZ008529	yes	LMSG_G000011149.1	OES073667	arylpolyene	Others	Antimony	18.86	China: Guizhou, Banpo
L180322-P-5-LD180319-0013_HL3MGCCXY_L1_clean_bin.96.orig.fa_	OEZ008529	yes	LMSG_G000008778.1	OES073667	NRPS-like	NRPS	Antimony	43.91	China: Guizhou, Banpo
L180322-P-5-LD180319-0013_HL3MGCCXY_L1_clean_bin.96.orig.fa_	OEZ008529	yes	LMSG_G000008778.1	OES073667	bacteriocin	RiPPs	Antimony	10.24	China: Guizhou, Banpo
L180322-P-5-LD180319-0013_HL3MGCCXY_L1_clean_bin.96.orig.fa_	OEZ008529	yes	LMSG_G000008778.1	OES073667	betalactone	Others	Antimony	23.67	China: Guizhou, Banpo
L180322-P-5-LD180319-0013_HL3MGCCXY_L1_clean_bin.99.orig.fa_	OEZ008529	yes	LMSG_G000004464.1	OES073667	T3PKS	PKSother	Antimony	17.84	China: Guizhou, Banpo
L180322-P-5-LD180319-0013_HL3MGCCXY_L1_clean_bin.99.orig.fa_	OEZ008529	yes	LMSG_G000004464.1	OES073667	T1PKS.hglE-KS	PKSother	Antimony	25.78	China: Guizhou, Banpo
L180322-P-5-LD180319-0013_HL3MGCCXY_L1_clean_bin.99.orig.fa_	OEZ008529	yes	LMSG_G000004464.1	OES073667	bacteriocin	RiPPs	Antimony	6.83	China: Guizhou, Banpo
L180322-P-5-LD180319-0013_HL3MGCCXY_L1_clean_bin.99.orig.fa_	OEZ008529	yes	LMSG_G000004464.1	OES073667	terpene	Terpene	Antimony	19.30	China: Guizhou, Banpo
L180322-P-5-LD180319-0013_HL3MGCCXY_L1_clean_bin.99.orig.fa_	OEZ008529	yes	LMSG_G000004464.1	OES073667	ladderane	Others	Antimony	25.74	China: Guizhou, Banpo
L180322-P-5-LD180319-0013_HL3MGCCXY_L1_clean_bin.99.orig.fa_	OEZ008529	yes	LMSG_G000004464.1	OES073667	arylpolyene	Others	Antimony	23.40	China: Guizhou, Banpo
L180322-P-5-LD180319-0014_HL3MGCCXY_L1_clean_bin.10.strict.f	OEZ008529	yes	LMSG_G000005349.1	OES073668	betalactone	Others	Antimony	13.23	China: Guizhou, Banpo
L180322-P-5-LD180319-0014_HL3MGCCXY_L1_clean_bin.10.strict.f	OEZ008529	yes	LMSG_G000005349.1	OES073668	betalactone	Others	Antimony	8.93	China: Guizhou, Banpo
L180322-P-5-LD180319-0014_HL3MGCCXY_L1_clean_bin.10.strict.f	OEZ008529	yes	LMSG_G000005349.1	OES073668	proteusin.bacteriocin	RiPPs	Antimony	7.39	China: Guizhou, Banpo
L180322-P-5-LD180319-0014_HL3MGCCXY_L1_clean_bin.11.orig.fa_	OEZ008529	yes	LMSG_G000010345.1	OES073668	terpene	Terpene	Antimony	22.00	China: Guizhou, Banpo
L180322-P-5-LD180319-0014_HL3MGCCXY_L1_clean_bin.11.orig.fa_	OEZ008529	yes	LMSG_G000010345.1	OES073668	NRPS-like	NRPS	Antimony	41.50	China: Guizhou, Banpo
L180322-P-5-LD180319-0014_HL3MGCCXY_L1_clean_bin.11.orig.fa_	OEZ008529	yes	LMSG_G000010345.1	OES073668	terpene	Terpene	Antimony	17.16	China: Guizhou, Banpo
L180322-P-5-LD180319-0014_HL3MGCCXY_L1_clean_bin.11.orig.fa_	OEZ008529	yes	LMSG_G000010345.1	OES073668	T3PKS	PKSother	Antimony	41.07	China: Guizhou, Banpo
L180322-P-5-LD180319-0014_HL3MGCCXY_L1_clean_bin.11.orig.fa_	OEZ008529	yes	LMSG_G000010345.1	OES073668	terpene	Terpene	Antimony	21.72	China: Guizhou, Banpo
L180322-P-5-LD180319-0014_HL3MGCCXY_L1_clean_bin.11.orig.fa_	OEZ008529	yes	LMSG_G000010345.1	OES073668	bacteriocin	RiPPs	Antimony	10.94	China: Guizhou, Banpo
L180322-P-5-LD180319-0014_HL3MGCCXY_L1_clean_bin.12.orig.fa_	OEZ008529	yes	LMSG_G000009059.1	OES073668	NRPS-like	NRPS	Antimony	35.36	China: Guizhou, Banpo
L180322-P-5-LD180319-0014_HL3MGCCXY_L1_clean_bin.12.orig.fa_	OEZ008529	yes	LMSG_G000009059.1	OES073668	terpene	Terpene	Antimony	22.05	China: Guizhou, Banpo
L180322-P-5-LD180319-0014_HL3MGCCXY_L1_clean_bin.16.orig.fa_	OEZ008529	yes	LMSG_G000005350.1	OES073668	terpene	Terpene	Antimony	24.91	China: Guizhou, Banpo
L180322-P-5-LD180319-0014_HL3MGCCXY_L1_clean_bin.18.orig.fa_	OEZ008529	yes	LMSG_G000004465.1	OES073668	terpene	Terpene	Antimony	21.48	China: Guizhou, Banpo
L180322-P-5-LD180319-0014_HL3MGCCXY_L1_clean_bin.18.orig.fa_	OEZ008529	yes	LMSG_G000004465.1	OES073668	arylpolyene	Others	Antimony	14.42	China: Guizhou, Banpo
L180322-P-5-LD180319-0014_HL3MGCCXY_L1_clean_bin.18.orig.fa_	OEZ008529	yes	LMSG_G000004465.1	OES073668	terpene	Terpene	Antimony	16.25	China: Guizhou, Banpo
L180322-P-5-LD180319-0014_HL3MGCCXY_L1_clean_bin.18.orig.fa_	OEZ008529	yes	LMSG_G000004465.1	OES073668	bacteriocin	RiPPs	Antimony	9.42	China: Guizhou, Banpo
L180322-P-5-LD180319-0014_HL3MGCCXY_L1_clean_bin.2.orig.fa_k	OEZ008529	no	LMSG_G000005352.1	OES073668	NRPS	NRPS	Antimony	17.76	China: Guizhou, Banpo
L180322-P-5-LD180319-0014_HL3MGCCXY_L1_clean_bin.2.orig.fa_k	OEZ008529	yes	LMSG_G000005352.1	OES073668	T3PKS	PKSother	Antimony	21.23	China: Guizhou, Banpo
L180322-P-5-LD180319-0014_HL3MGCCXY_L1_clean_bin.2.orig.fa_k	OEZ008529	no	LMSG_G000005352.1	OES073668	NRPS	NRPS	Antimony	5.38	China: Guizhou, Banpo
L180322-P-5-LD180319-0014_HL3MGCCXY_L1_clean_bin.2.orig.fa_k	OEZ008529	no	LMSG_G000005352.1	OES073668	NRPS	NRPS	Antimony	11.15	China: Guizhou, Banpo
L180322-P-5-LD180319-0014_HL3MGCCXY_L1_clean_bin.2.orig.fa_k	OEZ008529	yes	LMSG_G000005352.1	OES073668	NRPS	NRPS	Antimony	13.54	China: Guizhou, Banpo
L180322-P-5-LD180319-0014_HL3MGCCXY_L1_clean_bin.2.orig.fa_k	OEZ008529	no	LMSG_G000005352.1	OES073668	NRPS	NRPS	Antimony	6.12	China: Guizhou, Banpo
L180322-P-5-LD180319-0014_HL3MGCCXY_L1_clean_bin.2.orig.fa_k	OEZ008529	yes	LMSG_G000005352.1	OES073668	NRPS-like	NRPS	Antimony	16.00	China: Guizhou, Banpo
L180322-P-5-LD180319-0014_HL3MGCCXY_L1_clean_bin.2.orig.fa_k	OEZ008529	no	LMSG_G000005352.1	OES073668	NRPS	NRPS	Antimony	9.79	China: Guizhou, Banpo
L180322-P-5-LD180319-0014_HL3MGCCXY_L1_clean_bin.2.orig.fa_k	OEZ008529	no	LMSG_G000005352.1	OES073668	terpene	Terpene	Antimony	6.50	China: Guizhou, Banpo
L180322-P-5-LD180319-0014_HL3MGCCXY_L1_clean_bin.2.orig.fa_k	OEZ008529	no	LMSG_G000005352.1	OES073668	NRPS	NRPS	Antimony	17.65	China: Guizhou, Banpo
L180322-P-5-LD180319-0014_HL3MGCCXY_L1_clean_bin.2.orig.fa_k	OEZ008529	no	LMSG_G000005352.1	OES073668	NRPS	NRPS	Antimony	13.73	China: Guizhou, Banpo
L180322-P-5-LD180319-0014_HL3MGCCXY_L1_clean_bin.2.orig.fa_k	OEZ008529	no	LMSG_G000005352.1	OES073668	NRPS	NRPS	Antimony	10.44	China: Guizhou, Banpo
L180322-P-5-LD180319-0014_HL3MGCCXY_L1_clean_bin.2.orig.fa_k	OEZ008529	yes	LMSG_G000005352.1	OES073668	NRPS-like	NRPS	Antimony	10.51	China: Guizhou, Banpo
L180322-P-5-LD180319-0014_HL3MGCCXY_L1_clean_bin.2.orig.fa_k	OEZ008529	no	LMSG_G000005352.1	OES073668	T1PKS	PKSI	Antimony	12.38	China: Guizhou, Banpo
L180322-P-5-LD180319-0014_HL3MGCCXY_L1_clean_bin.2.orig.fa_k	OEZ008529	no	LMSG_G000005352.1	OES073668	NRPS-like	NRPS	Antimony	6.91	China: Guizhou, Banpo
L180322-P-5-LD180319-0014_HL3MGCCXY_L1_clean_bin.2.orig.fa_k	OEZ008529	no	LMSG_G000005352.1	OES073668	betalactone	Others	Antimony	9.74	China: Guizhou, Banpo
L180322-P-5-LD180319-0014_HL3MGCCXY_L1_clean_bin.2.orig.fa_k	OEZ008529	no	LMSG_G000005352.1	OES073668	NRPS	NRPS	Antimony	15.67	China: Guizhou, Banpo
L180322-P-5-LD180319-0014_HL3MGCCXY_L1_clean_bin.2.orig.fa_k	OEZ008529	yes	LMSG_G000005352.1	OES073668	NRPS.T1PKS	PKS-NRP_Hybrids	Antimony	31.65	China: Guizhou, Banpo
L180322-P-5-LD180319-0014_HL3MGCCXY_L1_clean_bin.2.orig.fa_k	OEZ008529	no	LMSG_G000005352.1	OES073668	T1PKS.T3PKS	PKSother	Antimony	19.63	China: Guizhou, Banpo

L180322-P-5-LD180319-0014_HL3MGCCXY_L1_clean_bin.2.orig.fa_k	OEZ008529	no	LMSG_G000005352.1	OES073668	NRPS	NRPS	Antimony	6.95	China: Guizhou, Banpo
L180322-P-5-LD180319-0014_HL3MGCCXY_L1_clean_bin.2.orig.fa_k	OEZ008529	no	LMSG_G000005352.1	OES073668	T1PKS	PKSI	Antimony	7.21	China: Guizhou, Banpo
L180322-P-5-LD180319-0014_HL3MGCCXY_L1_clean_bin.2.orig.fa_k	OEZ008529	no	LMSG_G000005352.1	OES073668	T1PKS	PKSI	Antimony	18.77	China: Guizhou, Banpo
L180322-P-5-LD180319-0014_HL3MGCCXY_L1_clean_bin.22.orig.fa_	OEZ008529	no	LMSG_G000010527.1	OES073668	terpene	Terpene	Antimony	15.22	China: Guizhou, Banpo
L180322-P-5-LD180319-0014_HL3MGCCXY_L1_clean_bin.24.orig.fa_	OEZ008529	yes	LMSG_G000010762.1	OES073668	acyl_amino_acids	Others	Antimony	47.31	China: Guizhou, Banpo
L180322-P-5-LD180319-0014_HL3MGCCXY_L1_clean_bin.26.orig.fa_	OEZ008529	yes	LMSG_G000007949.1	OES073668	terpene	Terpene	Antimony	6.64	China: Guizhou, Banpo
L180322-P-5-LD180319-0014_HL3MGCCXY_L1_clean_bin.26.orig.fa_	OEZ008529	yes	LMSG_G000007949.1	OES073668	T3PKS	PKSother	Antimony	21.03	China: Guizhou, Banpo
L180322-P-5-LD180319-0014_HL3MGCCXY_L1_clean_bin.28.orig.fa_	OEZ008529	yes	LMSG_G000007731.1	OES073668	thiopeptide.LAP	RiPPs	Antimony	28.30	China: Guizhou, Banpo
L180322-P-5-LD180319-0014_HL3MGCCXY_L1_clean_bin.28.orig.fa_	OEZ008529	yes	LMSG_G000007731.1	OES073668	terpene	Terpene	Antimony	16.60	China: Guizhou, Banpo
L180322-P-5-LD180319-0014_HL3MGCCXY_L1_clean_bin.28.orig.fa_	OEZ008529	yes	LMSG_G000007731.1	OES073668	terpene	Terpene	Antimony	22.03	China: Guizhou, Banpo
L180322-P-5-LD180319-0014_HL3MGCCXY_L1_clean_bin.28.orig.fa_	OEZ008529	yes	LMSG_G000007731.1	OES073668	terpene	Terpene	Antimony	13.78	China: Guizhou, Banpo
L180322-P-5-LD180319-0014_HL3MGCCXY_L1_clean_bin.28.orig.fa_	OEZ008529	yes	LMSG_G000007731.1	OES073668	T3PKS	PKSother	Antimony	41.07	China: Guizhou, Banpo
L180322-P-5-LD180319-0014_HL3MGCCXY_L1_clean_bin.3.orig.fa_k	OEZ008529	yes	LMSG_G000005357.1	OES073668	NRPS.T1PKS	PKS-NRP_Hybrids	Antimony	44.18	China: Guizhou, Banpo
L180322-P-5-LD180319-0014_HL3MGCCXY_L1_clean_bin.3.orig.fa_k	OEZ008529	yes	LMSG_G000005357.1	OES073668	NRPS	NRPS	Antimony	28.22	China: Guizhou, Banpo
L180322-P-5-LD180319-0014_HL3MGCCXY_L1_clean_bin.3.orig.fa_k	OEZ008529	no	LMSG_G000005357.1	OES073668	T1PKS.T3PKS	PKSother	Antimony	16.96	China: Guizhou, Banpo
L180322-P-5-LD180319-0014_HL3MGCCXY_L1_clean_bin.3.orig.fa_k	OEZ008529	no	LMSG_G000005357.1	OES073668	T1PKS	PKSI	Antimony	5.99	China: Guizhou, Banpo
L180322-P-5-LD180319-0014_HL3MGCCXY_L1_clean_bin.3.orig.fa_k	OEZ008529	yes	LMSG_G000005357.1	OES073668	NRPS.T1PKS	PKS-NRP_Hybrids	Antimony	20.48	China: Guizhou, Banpo
L180322-P-5-LD180319-0014_HL3MGCCXY_L1_clean_bin.3.orig.fa_k	OEZ008529	yes	LMSG_G000005357.1	OES073668	T1PKS.T3PKS	PKSother	Antimony	25.82	China: Guizhou, Banpo
L180322-P-5-LD180319-0014_HL3MGCCXY_L1_clean_bin.3.orig.fa_k	OEZ008529	yes	LMSG_G000005357.1	OES073668	NRPS	NRPS	Antimony	9.73	China: Guizhou, Banpo
L180322-P-5-LD180319-0014_HL3MGCCXY_L1_clean_bin.3.orig.fa_k	OEZ008529	no	LMSG_G000005357.1	OES073668	T1PKS	PKSI	Antimony	5.91	China: Guizhou, Banpo
L180322-P-5-LD180319-0014_HL3MGCCXY_L1_clean_bin.3.orig.fa_k	OEZ008529	yes	LMSG_G000005357.1	OES073668	NRPS-like	NRPS	Antimony	32.20	China: Guizhou, Banpo
L180322-P-5-LD180319-0014_HL3MGCCXY_L1_clean_bin.3.orig.fa_k	OEZ008529	yes	LMSG_G000005357.1	OES073668	T3PKS	PKSother	Antimony	12.82	China: Guizhou, Banpo
L180322-P-5-LD180319-0014_HL3MGCCXY_L1_clean_bin.3.orig.fa_k	OEZ008529	yes	LMSG_G000005357.1	OES073668	bacteriocin	RiPPs	Antimony	10.80	China: Guizhou, Banpo
L180322-P-5-LD180319-0014_HL3MGCCXY_L1_clean_bin.3.orig.fa_k	OEZ008529	yes	LMSG_G000005357.1	OES073668	NRPS	NRPS	Antimony	23.12	China: Guizhou, Banpo
L180322-P-5-LD180319-0014_HL3MGCCXY_L1_clean_bin.3.orig.fa_k	OEZ008529	yes	LMSG_G000005357.1	OES073668	T1PKS	PKSI	Antimony	24.29	China: Guizhou, Banpo
L180322-P-5-LD180319-0014_HL3MGCCXY_L1_clean_bin.3.orig.fa_k	OEZ008529	no	LMSG_G000005357.1	OES073668	terpene	Terpene	Antimony	9.59	China: Guizhou, Banpo
L180322-P-5-LD180319-0014_HL3MGCCXY_L1_clean_bin.3.orig.fa_k	OEZ008529	no	LMSG_G000005357.1	OES073668	T1PKS.NRPS-like	PKS-NRP_Hybrids	Antimony	6.07	China: Guizhou, Banpo
L180322-P-5-LD180319-0014_HL3MGCCXY_L1_clean_bin.3.orig.fa_k	OEZ008529	yes	LMSG_G000005357.1	OES073668	NRPS-like	NRPS	Antimony	8.17	China: Guizhou, Banpo
L180322-P-5-LD180319-0014_HL3MGCCXY_L1_clean_bin.30.strict.f	OEZ008529	yes	LMSG_G000006329.1	OES073668	CDPS	Others	Antimony	20.71	China: Guizhou, Banpo
L180322-P-5-LD180319-0014_HL3MGCCXY_L1_clean_bin.30.strict.f	OEZ008529	yes	LMSG_G000006329.1	OES073668	terpene	Terpene	Antimony	21.00	China: Guizhou, Banpo
L180322-P-5-LD180319-0014_HL3MGCCXY_L1_clean_bin.30.strict.f	OEZ008529	yes	LMSG_G000006329.1	OES073668	bacteriocin	RiPPs	Antimony	10.24	China: Guizhou, Banpo
L180322-P-5-LD180319-0014_HL3MGCCXY_L1_clean_bin.30.strict.f	OEZ008529	yes	LMSG_G000006329.1	OES073668	terpene	Terpene	Antimony	20.86	China: Guizhou, Banpo
L180322-P-5-LD180319-0014_HL3MGCCXY_L1_clean_bin.30.strict.f	OEZ008529	yes	LMSG_G000006329.1	OES073668	bacteriocin	RiPPs	Antimony	10.86	China: Guizhou, Banpo
L180322-P-5-LD180319-0014_HL3MGCCXY_L1_clean_bin.30.strict.f	OEZ008529	yes	LMSG_G000006329.1	OES073668	NRPS-like	NRPS	Antimony	20.60	China: Guizhou, Banpo
L180322-P-5-LD180319-0014_HL3MGCCXY_L1_clean_bin.30.strict.f	OEZ008529	yes	LMSG_G000006329.1	OES073668	transAT-PKS-like.NRPS-like	PKS-NRP_Hybrids	Antimony	19.14	China: Guizhou, Banpo
L180322-P-5-LD180319-0014_HL3MGCCXY_L1_clean_bin.30.strict.f	OEZ008529	yes	LMSG_G000006329.1	OES073668	TfuA-related	RiPPs	Antimony	14.25	China: Guizhou, Banpo
L180322-P-5-LD180319-0014_HL3MGCCXY_L1_clean_bin.30.strict.f	OEZ008529	yes	LMSG_G000006329.1	OES073668	terpene	Terpene	Antimony	12.70	China: Guizhou, Banpo
L180322-P-5-LD180319-0014_HL3MGCCXY_L1_clean_bin.30.strict.f	OEZ008529	yes	LMSG_G000006329.1	OES073668	transAT-PKS	PKSother	Antimony	12.63	China: Guizhou, Banpo
L180322-P-5-LD180319-0014_HL3MGCCXY_L1_clean_bin.30.strict.f	OEZ008529	yes	LMSG_G000006329.1	OES073668	NRPS-like	NRPS	Antimony	6.90	China: Guizhou, Banpo
L180322-P-5-LD180319-0014_HL3MGCCXY_L1_clean_bin.30.strict.f	OEZ008529	yes	LMSG_G000006329.1	OES073668	T3PKS.bacteriocin	Others	Antimony	6.34	China: Guizhou, Banpo
L180322-P-5-LD180319-0014_HL3MGCCXY_L1_clean_bin.31.orig.fa_	OEZ008529	yes	LMSG_G000010082.1	OES073668	terpene	Terpene	Antimony	5.36	China: Guizhou, Banpo
L180322-P-5-LD180319-0014_HL3MGCCXY_L1_clean_bin.31.orig.fa_	OEZ008529	yes	LMSG_G000010082.1	OES073668	acyl_amino_acids	Others	Antimony	36.50	China: Guizhou, Banpo
L180322-P-5-LD180319-0014_HL3MGCCXY_L1_clean_bin.31.orig.fa_	OEZ008529	yes	LMSG_G000010082.1	OES073668	NRPS-like	NRPS	Antimony	42.86	China: Guizhou, Banpo
L180322-P-5-LD180319-0014_HL3MGCCXY_L1_clean_bin.31.orig.fa_	OEZ008529	yes	LMSG_G000010082.1	OES073668	terpene	Terpene	Antimony	20.69	China: Guizhou, Banpo
L180322-P-5-LD180319-0014_HL3MGCCXY_L1_clean_bin.31.orig.fa_	OEZ008529	yes	LMSG_G000010082.1	OES073668	hglE-KS	PKSother	Antimony	26.83	China: Guizhou, Banpo
L180322-P-5-LD180319-0014_HL3MGCCXY_L1_clean_bin.31.orig.fa_	OEZ008529	yes	LMSG_G000010082.1	OES073668	acyl_amino_acids	Others	Antimony	9.36	China: Guizhou, Banpo
L180322-P-5-LD180319-0014_HL3MGCCXY_L1_clean_bin.31.orig.fa_	OEZ008529	yes	LMSG_G000010082.1	OES073668	terpene	Terpene	Antimony	15.97	China: Guizhou, Banpo
L180322-P-5-LD180319-0014_HL3MGCCXY_L1_clean_bin.31.orig.fa_	OEZ008529	yes	LMSG_G000010082.1	OES073668	hglE-KS	PKSother	Antimony	16.17	China: Guizhou, Banpo
L180322-P-5-LD180319-0014_HL3MGCCXY_L1_clean_bin.32.orig.fa_	OEZ008529	yes	LMSG_G000005353.1	OES073668	betalactone	Others	Antimony	10.35	China: Guizhou, Banpo
L180322-P-5-LD180319-0014_HL3MGCCXY_L1_clean_bin.32.orig.fa_	OEZ008529	yes	LMSG_G000005353.1	OES073668	terpene	Terpene	Antimony	8.33	China: Guizhou, Banpo
L180322-P-5-LD180319-0014_HL3MGCCXY_L1_clean_bin.32.orig.fa_	OEZ008529	yes	LMSG_G000005353.1	OES073668	bacteriocin	RiPPs	Antimony	8.89	China: Guizhou, Banpo
L180322-P-5-LD180319-0014_HL3MGCCXY_L1_clean_bin.32.orig.fa_	OEZ008529	yes	LMSG_G000005353.1	OES073668	NRPS-like	NRPS	Antimony	8.12	China: Guizhou, Banpo
L180322-P-5-LD180319-0014_HL3MGCCXY_L1_clean_bin.33.strict.f	OEZ008529	yes	LMSG_G000010427.1	OES073668	terpene	Terpene	Antimony	25.02	China: Guizhou, Banpo
L180322-P-5-LD180319-0014_HL3MGCCXY_L1_clean_bin.33.strict.f	OEZ008529	yes	LMSG_G000010427.1	OES073668	terpene	Terpene	Antimony	11.42	China: Guizhou, Banpo
L180322-P-5-LD180319-0014_HL3MGCCXY_L1_clean_bin.34.orig.fa_	OEZ008529	yes	LMSG_G000005354.1	OES073668	terpene	Terpene	Antimony	24.86	China: Guizhou, Banpo
L180322-P-5-LD180319-0014_HL3MGCCXY_L1_clean_bin.34.orig.fa_	OEZ008529	yes	LMSG_G000005354.1	OES073668	betalactone	Others	Antimony	27.23	China: Guizhou, Banpo
L180322-P-5-LD180319-0014_HL3MGCCXY_L1_clean_bin.34.orig.fa_	OEZ008529	yes	LMSG_G000005354.1	OES073668	betalactone	Others	Antimony	32.64	China: Guizhou, Banpo
L180322-P-5-LD180319-0014_HL3MGCCXY_L1_clean_bin.35.orig.fa_	OEZ008529	yes	LMSG_G000005355.1	OES073668	NRPS-like	NRPS	Antimony	23.07	China: Guizhou, Banpo
L180322-P-5-LD180319-0014_HL3MGCCXY_L1_clean_bin.36.orig.fa_	OEZ008529	yes	LMSG_G000010975.1	OES073668	bacteriocin	RiPPs	Antimony	6.00	China: Guizhou, Banpo
L180322-P-5-LD180319-0014_HL3MGCCXY_L1_clean_bin.37.orig.fa_	OEZ008529	yes	LMSG_G000010672.1	OES073668	bacteriocin	RiPPs	Antimony	8.70	China: Guizhou, Banpo
L180322-P-5-LD180319-0014_HL3MGCCXY_L1_clean_bin.4.orig.fa_k	OEZ008529	yes	LMSG_G000010358.1	OES073668	bacteriocin	RiPPs	Antimony	10.83	China: Guizhou, Banpo
L180322-P-5-LD180319-0014_HL3MGCCXY_L1_clean_bin.4.orig.fa_k	OEZ008529	yes	LMSG_G000010358.1	OES073668	bacteriocin	RiPPs	Antimony	9.10	China: Guizhou, Banpo
L180322-P-5-LD180319-0014_HL3MGCCXY_L1_clean_bin.4.orig.fa_k	OEZ008529	yes	LMSG_G000010358.1	OES073668	NRPS-like	NRPS	Antimony	15.78	China: Guizhou, Banpo
L180322-P-5-LD180319-0014_HL3MGCCXY_L1_clean_bin.4.orig.fa_k	OEZ008529	yes	LMSG_G000010358.1	OES073668	bacteriocin	RiPPs	Antimony	9.79	China: Guizhou, Banpo
L180322-P-5-LD180319-0014_HL3MGCCXY_L1_clean_bin.4.orig.fa_k	OEZ008529	yes	LMSG_G000010358.1	OES073668	terpene	Terpene	Antimony	12.75	China: Guizhou, Banpo
L180322-P-5-LD180319-0014_HL3MGCCXY_L1_clean_bin.4.orig.fa_k	OEZ008529	yes	LMSG_G000010358.1	OES073668	NRPS	NRPS	Antimony	26.10	China: Guizhou, Banpo
L180322-P-5-LD180319-0014_HL3MGCCXY_L1_clean_bin.4.orig.fa_k	OEZ008529	yes	LMSG_G000010358.1	OES073668	NRPS-like	NRPS	Antimony	44.07	China: Guizhou, Banpo
L180322-P-5-LD180319-0014_HL3MGCCXY_L1_clean_bin.4.orig.fa_k	OEZ008529	yes	LMSG_G000010358.1	OES073668	terpene	Terpene	Antimony	12.66	China: Guizhou, Banpo
L180322-P-5-LD180319-0014_HL3MGCCXY_L1_clean_bin.4.orig.fa_k	OEZ008529	yes	LMSG_G000010358.1	OES073668	terpene	Terpene	Antimony	14.04	China: Guizhou, Banpo
L180322-P-5-LD180319-0014_HL3MGCCXY_L1_clean_bin.41.orig.fa_	OEZ008529	yes	LMSG_G000010688.1	OES073668	terpene	Terpene	Antimony	20.84	China: Guizhou, Banpo
L180322-P-5-LD180319-0014_HL3MGCCXY_L1_clean_bin.41.orig.fa_	OEZ008529	yes	LMSG_G000010688.1	OES073668	betalactone	Others	Antimony	23.40	China: Guizhou, Banpo
L180322-P-5-LD180319-0014_HL3MGCCXY_L1_clean_bin.41.orig.fa_	OEZ008529	yes	LMSG_G000010688.1	OES073668	NRPS-like	NRPS	Antimony	23.58	China: Guizhou, Banpo
L180322-P-5-LD180319-0014_HL3MGCCXY_L1_clean_bin.41.orig.fa_	OEZ008529	yes	LMSG_G000010688.1	OES073668	arylpolyene	Others	Antimony	24.33	China: Guizhou, Banpo
L180322-P-5-LD180319-0014_HL3MGCCXY_L1_clean_bin.41.orig.fa_	OEZ008529	yes	LMSG_G000010688.1	OES073668	NRPS	NRPS	Antimony	26.56	China: Guizhou, Banpo
L180322-P-5-LD180319-0014_HL3MGCCXY_L1_clean_bin.42.orig.fa_	OEZ008529	yes	LMSG_G000010378.1	OES073668	LAP	RiPPs	Antimony	10.46	China: Guizhou, Banpo
L180322-P-5-LD180319-0014_HL3MGCCXY_L1_clean_bin.42.orig.fa_	OEZ008529	no	LMSG_G000010378.1	OES073668	terpene	Terpene	Antimony	7.33	China: Guizhou, Banpo
L180322-P-5-LD180319-0014_HL3MGCCXY_L1_clean_bin.42.orig.fa_	OEZ008529	yes	LMSG_G000010378.1	OES073668	terpene	Terpene	Antimony	11.43	China: Guizhou, Banpo
L180322-P-5-LD180319-0014_HL3MGCCXY_L1_clean_bin.42.orig.fa_	OEZ008529	no	LMSG_G000010378.1	OES073668	hserlactone	Others	Antimony	6.26	China: Guizhou, Banpo
L180322-P-5-LD180319-0014_HL3MGCCXY_L1_clean_bin.42.orig.fa_	OEZ008529	yes	LMSG_G000010378.1	OES073668	T3PKS	PKSother	Antimony	15.43	China: Guizhou, Banpo
L180322-P-5-LD180319-0014_HL3MGCCXY_L1_clean_bin.42.orig.fa_	OEZ008529	yes	LMSG_G000010378.1	OES073668	NRPS-like	NRPS	Antimony	7.90	China: Guizhou, Banpo
L180322-P-5-LD180319-0014_HL3MGCCXY_L1_clean_bin.42.orig.fa_	OEZ008529	no	LMSG_G000010378.1	OES073668	terpene	Terpene	Antimony	12.25	China: Guizhou, Banpo
L180322-P-5-LD180319-0014_HL3MGCCXY_L1_clean_bin.43.orig.fa_	OEZ008529	yes	LMSG_G000009502.1	OES073668	T3PKS	PKSother	Antimony	5.32	China: Guizhou, Banpo
L180322-P-5-LD180319-0014_HL3MGCCXY_L1_clean_bin.43.orig.fa_	OEZ008529	yes	LMSG_G000009502.1	OES073668	betalactone	Others	Antimony	12.59	China: Guizhou, Banpo
L180322-P-5-LD180319-0014_HL3MGCCXY_L1_clean_bin.43.orig.fa_	OEZ008529	yes	LMSG_G000009502.1	OES073668	terpene	Terpene	Antimony	11.86	China: Guizhou, Banpo
L180322-P-5-LD180319-0014_HL3MGCCXY_L1_clean_bin.43.orig.fa_	OEZ008529	yes	LMSG_G000009502.1	OES073668	betalactone	Others	Antimony	9.15	China: Guizhou, Banpo
L180322-P-5-LD180319-0014_HL3MGCCXY_L1_clean_bin.44.orig.fa_	OEZ008529	yes	LMSG_G000009228.1	OES073668	hglE-KS	PKSother	Antimony	15.69	China: Guizhou, Banpo
L180322-P-5-LD180319-0014_HL3MGCCXY_L1_clean_bin.44.orig.fa_	OEZ008529	yes	LMSG_G000009228.1	OES073668	terpene	Terpene	Antimony	21.72	China: Guizhou, Banpo
L180322-P-5-LD180319-0014_HL3MGCCXY_L1_clean_bin.44.orig.fa_	OEZ008529	yes	LMSG_G000009228.1	OES073668	terpene	Terpene	Antimony	22.03	China: Guizhou, Banpo
L180322-P-5-LD180319-0014_HL3MGCCXY_L1_clean_bin.44.orig.fa_	OEZ008529	yes	LMSG_G000009228.1	OES073668	bacteriocin	RiPPs	Antimony	10.85	China: Guizhou, Banpo
L180322-P-5-LD180319-0014_HL3MGCCXY_L1_clean_bin.45.orig.fa_	OEZ008529	yes	LMSG_G000010370.1	OES073668	bacteriocin	RiPPs	Antimony	11.69	China: Guizhou, Banpo
L180322-P-5-LD180319-0014_HL3MGCCXY_L1_clean_bin.45.orig.fa_	OEZ008529	yes	LMSG_G000010370.1	OES073668	NRPS-like	NRPS	Antimony	15.75	China: Guizhou, Banpo
L180322-P-5-LD180319-0014_HL3MGCCXY_L1_clean_bin.45.orig.fa_	OEZ008529	yes	LMSG_G000010370.1	OES073668	bacteriocin	RiPPs	Antimony	8.28	China: Guizhou, Banpo
L180322-P-5-LD180319-0014_HL3MGCCXY_L1_clean_bin.45.orig.fa_	OEZ008529	yes	LMSG_G000010370.1	OES073668	terpene	Terpene	Antimony	5.50	China: Guizhou, Banpo
L180322-P-5-LD180319-0014_HL3MGCCXY_L1_clean_bin.45.orig.fa_	OEZ008529	yes	LMSG_G000010370.1	OES073668	terpene	Terpene	Antimony	12.85	China: Guizhou, Banpo
L180322-P-5-LD180319-0014_HL3MGCCXY_L1_clean_bin.45.orig.fa_	OEZ008529	yes	LMSG_G000010370.1	OES073668	terpene	Terpene	Antimony	10.27	China: Guizhou, Banpo
L180322-P-5-LD180319-0014_HL3MGCCXY_L1_clean_bin.47.orig.fa_	OEZ008529	yes	LMSG_G000005358.1	OES073668	other	Others	Antimony	35.44	China: Guizhou, Banpo
L180322-P-5-LD180319-0014_HL3MGCCXY_L1_clean_bin.47.orig.fa_	OEZ008529	yes	LMSG_G000005358.1	OES073668	other	Others	Antimony	17.53	China: Guizhou, Banpo
L180322-P-5-LD180319-0014_HL3MGCCXY_L1_clean_bin.52.orig.fa_	OEZ008529	yes	LMSG_G000009328.1	OES073668	terpene	Terpene	Antimony	6.15	China: Guizhou, Banpo
L180322-P-5-LD180319-0014_HL3MGCCXY_L1_clean_bin.54.orig.fa_	OEZ008529	yes	LMSG_G000010384.1	OES073668	NRPS.T1PKS	PKS-NRP_Hybrids	Antimony	11.01	China: Guizhou, Banpo
L180322-P-5-LD180319-0014_HL3MGCCXY_L1_clean_bin.56.orig.fa_	OEZ008529	yes	LMSG_G000010091.1	OES073668	NRPS-like	NRPS	Antimony	5.89	China: Guizhou, Banpo
L180322-P-5-LD180319-0014_HL3MGCCXY_L1_clean_bin.56.orig.fa_	OEZ008529	yes	LMSG_G000010091.1	OES073668	terpene	Terpene	Antimony	7.41	China: Guizhou, Banpo
L180322-P-5-LD180319-0014_HL3MGCCXY_L1_clean_bin.58.orig.fa_	OEZ008529	yes	LMSG_G000011336.1	OES073668	NRPS-like	NRPS	Antimony	10.70	China: Guizhou, Banpo
L180322-P-5-LD180319-0014_HL3MGCCXY_L1_clean_bin.58.orig.fa_	OEZ008529	yes	LMSG_G000011336.1	OES073668	arylpolyene	Others	Antimony	34.55	China: Guizhou, Banpo
L180322-P-5-LD180319-0014_HL3MGCCXY_L1_clean_bin.62.orig.fa_	OEZ008529	yes	LMSG_G000009060.1	OES073668	terpene	Terpene	Antimony	22.05	China: Guizhou, Banpo
L180322-P-5-LD180319-0014_HL3MGCCXY_L1_clean_bin.62.orig.fa_	OEZ008529	yes	LMSG_G000009060.1	OES073668	arylpolyene	Others	Antimony	58.59	China: Guizhou, Banpo
L180322-P-5-LD180319-0014_HL3MGCCXY_L1_clean_bin.63.orig.fa_	OEZ008529	yes	LMSG_G000010312.1	OES073668	terpene	Terpene	Antimony	18.31	China: Guizhou, Banpo
L180322-P-5-LD180319-0014_HL3MGCCXY_L1_clean_bin.63.orig.fa_	OEZ008529	yes	LMSG_G000010312.1	OES073668	terpene	Terpene	Antimony	14.38	China: Guizhou, Banpo
L180322-P-5-LD180319-0014_HL3MGCCXY_L1_clean_bin.63.orig.fa_	OEZ008529	yes	LMSG_G000010312.1	OES073668	terpene	Terpene	Antimony	6.20	China: Guizhou, Banpo
L180322-P-5-LD180319-0014_HL3MGCCXY_L1_clean_bin.64.orig.fa_	OEZ008529	yes	LMSG_G000009042.1	OES073668	thiopeptide	RiPPs	Antimony	5.85	China: Guizhou, Banpo
L180322-P-5-LD180319-0014_HL3MGCCXY_L1_clean_bin.67.orig.fa_	OEZ008529	yes	LMSG_G000005361.1	OES073668	terpene	Terpene	Antimony	19.40	China: Guizhou, Banpo
L180322-P-5-LD180319-0014_HL3MGCCXY_L1_clean_bin.68.orig.fa_	OEZ008529	yes	LMSG_G000005362.1	OES073668	terpene	Terpene	Antimony	15.82	China: Guizhou, Banpo
L180322-P-5-LD180319-0014_HL3MGCCXY_L1_clean_bin.68.orig.fa_	OEZ008529	yes	LMSG_G000005362.1	OES073668	acyl_amino_acids	Others	Antimony	5.69	China: Guizhou, Banpo
L180322-P-5-LD180319-0014_HL3MGCCXY_L1_clean_bin.68.orig.fa_	OEZ008529	yes	LMSG_G000005362.1	OES073668	arylpolyene	Others	Antimony	25.03	China: Guizhou, Banpo
L180322-P-5-LD180319-0014_HL3MGCCXY_L1_clean_bin.69.strict.f	OEZ008529	yes	LMSG_G000009251.1	OES073668	T3PKS	PKSother	Antimony	41.05	China: Guizhou, Banpo
L180322-P-5-LD180319-0014_HL3MGCCXY_L1_clean_bin.69.strict.f	OEZ008529	yes	LMSG_G000009251.1	OES073668	TfuA-related	RiPPs	Antimony	21.95	China: Guizhou, Banpo
L180322-P-5-LD180319-0014_HL3MGCCXY_L1_clean_bin.7.orig.fa_k	OEZ008529	yes	LMSG_G000011085.1	OES073668	arylpolyene	Others	Antimony	41.19	China: Guizhou, Banpo
L180322-P-5-LD180319-0014_HL3MGCCXY_L1_clean_bin.7.orig.fa_k	OEZ008529	yes	LMSG_G000011085.1	OES073668	terpene	Terpene	Antimony	17.93	China: Guizhou, Banpo
L180322-P-5-LD180319-0014_HL3MGCCXY_L1_clean_bin.7.orig.fa_k	OEZ008529	yes	LMSG_G000011085.1	OES073668	betalactone	Others	Antimony	40.85	China: Guizhou, Banpo
L180322-P-5-LD180319-0014_HL3MGCCXY_L1_clean_bin.7.orig.fa_k	OEZ008529	yes	LMSG_G000011085.1	OES073668	bacteriocin	RiPPs	Antimony	10.92	China: Guizhou, Banpo
L180322-P-5-LD180319-0014_HL3MGCCXY_L1_clean_bin.7.orig.fa_k	OEZ008529	yes	LMSG_G000011085.1	OES073668	NRPS-like	NRPS	Antimony	26.84	China: Guizhou, Banpo
L180322-P-5-LD180319-0014_HL3MGCCXY_L1_clean_bin.7.orig.fa_k	OEZ008529	yes	LMSG_G000011085.1	OES073668	lassopeptide	RiPPs	Antimony	22.58	China: Guizhou, Banpo
L180322-P-5-LD180319-0014_HL3MGCCXY_L1_clean_bin.70.orig.fa_	OEZ008529	yes	LMSG_G000005364.1	OES073668	T3PKS.NRPS-like	PKS-NRP_Hybrids	Antimony	34.24	China: Guizhou, Banpo
L180322-P-5-LD180319-0014_HL3MGCCXY_L1_clean_bin.70.orig.fa_	OEZ008529	yes	LMSG_G000005364.1	OES073668	terpene	Terpene	Antimony	25.01	China: Guizhou, Banpo
L180322-P-5-LD180319-0014_HL3MGCCXY_L1_clean_bin.70.orig.fa_	OEZ008529	yes	LMSG_G000005364.1	OES073668	bacteriocin	RiPPs	Antimony	7.19	China: Guizhou, Banpo
L180322-P-5-LD180319-0014_HL3MGCCXY_L1_clean_bin.70.orig.fa_	OEZ008529	yes	LMSG_G000005364.1	OES073668	bacteriocin	RiPPs	Antimony	10.87	China: Guizhou, Banpo
L180322-P-5-LD180319-0014_HL3MGCCXY_L1_clean_bin.70.orig.fa_	OEZ008529	yes	LMSG_G000005364.1	OES073668	NRPS-like	NRPS	Antimony	42.48	China: Guizhou, Banpo
L180322-P-5-LD180319-0014_HL3MGCCXY_L1_clean_bin.72.orig.fa_	OEZ008529	yes	LMSG_G000004467.1	OES073668	terpene	Terpene	Antimony	22.05	China: Guizhou, Banpo
L180322-P-5-LD180319-0014_HL3MGCCXY_L1_clean_bin.72.orig.fa_	OEZ008529	yes	LMSG_G000004467.1	OES073668	terpene	Terpene	Antimony	20.87	China: Guizhou, Banpo
L180322-P-5-LD180319-0014_HL3MGCCXY_L1_clean_bin.72.orig.fa_	OEZ008529	yes	LMSG_G000004467.1	OES073668	terpene	Terpene	Antimony	21.01	China: Guizhou, Banpo
L180322-P-5-LD180319-0014_HL3MGCCXY_L1_clean_bin.72.orig.fa_	OEZ008529	yes	LMSG_G000004467.1	OES073668	terpene	Terpene	Antimony	21.82	China: Guizhou, Banpo
L180322-P-5-LD180319-0014_HL3MGCCXY_L1_clean_bin.73.permissi	OEZ008529	yes	LMSG_G000009754.1	OES073668	LAP.thiopeptide	RiPPs	Antimony	30.38	China: Guizhou, Banpo
L180322-P-5-LD180319-0014_HL3MGCCXY_L1_clean_bin.74.orig.fa_	OEZ008529	yes	LMSG_G000009497.1	OES073668	NRPS-like	NRPS	Antimony	14.50	China: Guizhou, Banpo
L180322-P-5-LD180319-0014_HL3MGCCXY_L1_clean_bin.74.orig.fa_	OEZ008529	yes	LMSG_G000009497.1	OES073668	TfuA-related	RiPPs	Antimony	7.48	China: Guizhou, Banpo
L180322-P-5-LD180319-0014_HL3MGCCXY_L1_clean_bin.74.orig.fa_	OEZ008529	yes	LMSG_G000009497.1	OES073668	T3PKS	PKSother	Antimony	5.73	China: Guizhou, Banpo
L180322-P-5-LD180319-0014_HL3MGCCXY_L1_clean_bin.74.orig.fa_	OEZ008529	yes	LMSG_G000009497.1	OES073668	hglE-KS	PKSother	Antimony	15.93	China: Guizhou, Banpo
L180322-P-5-LD180319-0014_HL3MGCCXY_L1_clean_bin.74.orig.fa_	OEZ008529	yes	LMSG_G000009497.1	OES073668	bacteriocin	RiPPs	Antimony	7.32	China: Guizhou, Banpo
L180322-P-5-LD180319-0014_HL3MGCCXY_L1_clean_bin.75.orig.fa_	OEZ008529	yes	LMSG_G000009674.1	OES073668	NRPS-like	NRPS	Antimony	39.55	China: Guizhou, Banpo
L180322-P-5-LD180319-0014_HL3MGCCXY_L1_clean_bin.75.orig.fa_	OEZ008529	yes	LMSG_G000009674.1	OES073668	terpene	Terpene	Antimony	18.71	China: Guizhou, Banpo
L180322-P-5-LD180319-0014_HL3MGCCXY_L1_clean_bin.76.strict.f	OEZ008529	yes	LMSG_G000004468.1	OES073668	T3PKS	PKSother	Antimony	20.20	China: Guizhou, Banpo
L180322-P-5-LD180319-0014_HL3MGCCXY_L1_clean_bin.76.strict.f	OEZ008529	yes	LMSG_G000004468.1	OES073668	lanthipeptide	RiPPs	Antimony	17.02	China: Guizhou, Banpo
L180322-P-5-LD180319-0014_HL3MGCCXY_L1_clean_bin.76.strict.f	OEZ008529	yes	LMSG_G000004468.1	OES073668	terpene	Terpene	Antimony	11.12	China: Guizhou, Banpo
L180322-P-5-LD180319-0014_HL3MGCCXY_L1_clean_bin.76.strict.f	OEZ008529	yes	LMSG_G000004468.1	OES073668	NRPS-like	NRPS	Antimony	14.39	China: Guizhou, Banpo
L180322-P-5-LD180319-0014_HL3MGCCXY_L1_clean_bin.77.orig.fa_	OEZ008529	yes	LMSG_G000008646.1	OES073668	other	Others	Antimony	26.54	China: Guizhou, Banpo
L180322-P-5-LD180319-0014_HL3MGCCXY_L1_clean_bin.78.orig.fa_	OEZ008529	yes	LMSG_G000010101.1	OES073668	bacteriocin	RiPPs	Antimony	6.69	China: Guizhou, Banpo
L180322-P-5-LD180319-0014_HL3MGCCXY_L1_clean_bin.79.orig.fa_	OEZ008529	yes	LMSG_G000010707.1	OES073668	NRPS-like	NRPS	Antimony	13.72	China: Guizhou, Banpo
L180322-P-5-LD180319-0014_HL3MGCCXY_L1_clean_bin.79.orig.fa_	OEZ008529	yes	LMSG_G000010707.1	OES073668	bacteriocin	RiPPs	Antimony	6.20	China: Guizhou, Banpo
L180322-P-5-LD180319-0014_HL3MGCCXY_L1_clean_bin.80.orig.fa_	OEZ008529	yes	LMSG_G000008576.1	OES073668	other	Others	Antimony	40.44	China: Guizhou, Banpo
L180322-P-5-LD180319-0014_HL3MGCCXY_L1_clean_bin.80.orig.fa_	OEZ008529	yes	LMSG_G000008576.1	OES073668	NRPS-like	NRPS	Antimony	33.22	China: Guizhou, Banpo
L180322-P-5-LD180319-0014_HL3MGCCXY_L1_clean_bin.81.orig.fa_	OEZ008529	yes	LMSG_G000010629.1	OES073668	terpene	Terpene	Antimony	20.98	China: Guizhou, Banpo
L180322-P-5-LD180319-0014_HL3MGCCXY_L1_clean_bin.81.orig.fa_	OEZ008529	yes	LMSG_G000010629.1	OES073668	bacteriocin	RiPPs	Antimony	10.99	China: Guizhou, Banpo
L180322-P-5-LD180319-0014_HL3MGCCXY_L1_clean_bin.81.orig.fa_	OEZ008529	yes	LMSG_G000010629.1	OES073668	arylpolyene	Others	Antimony	27.84	China: Guizhou, Banpo
L180322-P-5-LD180319-0014_HL3MGCCXY_L1_clean_bin.81.orig.fa_	OEZ008529	yes	LMSG_G000010629.1	OES073668	terpene	Terpene	Antimony	18.31	China: Guizhou, Banpo
L180322-P-5-LD180319-0014_HL3MGCCXY_L1_clean_bin.82.orig.fa_	OEZ008529	yes	LMSG_G000004469.1	OES073668	NRPS-like	NRPS	Antimony	42.81	China: Guizhou, Banpo
L180322-P-5-LD180319-0014_HL3MGCCXY_L1_clean_bin.82.orig.fa_	OEZ008529	yes	LMSG_G000004469.1	OES073668	bacteriocin	RiPPs	Antimony	10.24	China: Guizhou, Banpo
L180322-P-5-LD180319-0014_HL3MGCCXY_L1_clean_bin.82.orig.fa_	OEZ008529	yes	LMSG_G000004469.1	OES073668	terpene	Terpene	Antimony	29.45	China: Guizhou, Banpo
L180322-P-5-LD180319-0014_HL3MGCCXY_L1_clean_bin.82.orig.fa_	OEZ008529	yes	LMSG_G000004469.1	OES073668	T3PKS	PKSother	Antimony	30.25	China: Guizhou, Banpo
L180322-P-5-LD180319-0014_HL3MGCCXY_L1_clean_bin.82.orig.fa_	OEZ008529	yes	LMSG_G000004469.1	OES073668	NRPS-like	NRPS	Antimony	42.51	China: Guizhou, Banpo
L180322-P-5-LD180319-0014_HL3MGCCXY_L1_clean_bin.83.strict.f	OEZ008529	yes	LMSG_G000009049.1	OES073668	T1PKS	PKSI	Antimony	50.73	China: Guizhou, Banpo

L180322-P-5-LD180319-0014_HL3MGCCXY_L1_clean_bin.83.strict.f	OEZ008529	yes	LMSG_G000009049.1	OES073668	betalactone	Others	Antimony	15.64	China: Guizhou, Banpo
L180322-P-5-LD180319-0014_HL3MGCCXY_L1_clean_bin.83.strict.f	OEZ008529	yes	LMSG_G000009049.1	OES073668	T1PKS	PKSI	Antimony	39.50	China: Guizhou, Banpo
L180322-P-5-LD180319-0014_HL3MGCCXY_L1_clean_bin.84.orig.fa_	OEZ008529	yes	LMSG_G000005365.1	OES073668	terpene	Terpene	Antimony	16.62	China: Guizhou, Banpo
L180322-P-5-LD180319-0014_HL3MGCCXY_L1_clean_bin.84.orig.fa_	OEZ008529	yes	LMSG_G000005365.1	OES073668	T1PKS	PKSI	Antimony	22.60	China: Guizhou, Banpo
L180322-P-5-LD180319-0014_HL3MGCCXY_L1_clean_bin.84.orig.fa_	OEZ008529	yes	LMSG_G000005365.1	OES073668	NRPS.T1PKS	PKS-NRP_Hybrids	Antimony	19.35	China: Guizhou, Banpo
L180322-P-5-LD180319-0014_HL3MGCCXY_L1_clean_bin.84.orig.fa_	OEZ008529	yes	LMSG_G000005365.1	OES073668	NRPS.T1PKS	PKS-NRP_Hybrids	Antimony	54.36	China: Guizhou, Banpo
L180322-P-5-LD180319-0014_HL3MGCCXY_L1_clean_bin.84.orig.fa_	OEZ008529	yes	LMSG_G000005365.1	OES073668	NRPS-like	NRPS	Antimony	9.16	China: Guizhou, Banpo
L180322-P-5-LD180319-0014_HL3MGCCXY_L1_clean_bin.85.orig.fa_	OEZ008529	yes	LMSG_G000007934.1	OES073668	T3PKS	PKSother	Antimony	6.37	China: Guizhou, Banpo
L180322-P-5-LD180319-0014_HL3MGCCXY_L1_clean_bin.86.orig.fa_	OEZ008529	yes	LMSG_G000007930.1	OES073668	terpene	Terpene	Antimony	6.50	China: Guizhou, Banpo
L180322-P-5-LD180319-0014_HL3MGCCXY_L1_clean_bin.86.orig.fa_	OEZ008529	yes	LMSG_G000007930.1	OES073668	transAT-PKS-like	PKSother	Antimony	7.40	China: Guizhou, Banpo
L180322-P-5-LD180319-0014_HL3MGCCXY_L1_clean_bin.86.orig.fa_	OEZ008529	yes	LMSG_G000007930.1	OES073668	NRPS-like	NRPS	Antimony	6.66	China: Guizhou, Banpo
L180322-P-5-LD180319-0014_HL3MGCCXY_L1_clean_bin.86.orig.fa_	OEZ008529	yes	LMSG_G000007930.1	OES073668	terpene	Terpene	Antimony	5.80	China: Guizhou, Banpo
L180322-P-5-LD180319-0014_HL3MGCCXY_L1_clean_bin.86.orig.fa_	OEZ008529	yes	LMSG_G000007930.1	OES073668	terpene	Terpene	Antimony	14.34	China: Guizhou, Banpo
L180322-P-5-LD180319-0014_HL3MGCCXY_L1_clean_bin.86.orig.fa_	OEZ008529	yes	LMSG_G000007930.1	OES073668	ladderane	Others	Antimony	9.75	China: Guizhou, Banpo
L180322-P-5-LD180319-0014_HL3MGCCXY_L1_clean_bin.86.orig.fa_	OEZ008529	yes	LMSG_G000007930.1	OES073668	transAT-PKS-like	PKSother	Antimony	17.41	China: Guizhou, Banpo
L180322-P-5-LD180319-0014_HL3MGCCXY_L1_clean_bin.86.orig.fa_	OEZ008529	yes	LMSG_G000007930.1	OES073668	transAT-PKS-like	PKSother	Antimony	16.14	China: Guizhou, Banpo
L180322-P-5-LD180319-0014_HL3MGCCXY_L1_clean_bin.87.orig.fa_	OEZ008529	yes	LMSG_G000008853.1	OES073668	terpene	Terpene	Antimony	21.86	China: Guizhou, Banpo
L180322-P-5-LD180319-0014_HL3MGCCXY_L1_clean_bin.89.orig.fa_	OEZ008529	yes	LMSG_G000010117.1	OES073668	terpene	Terpene	Antimony	21.91	China: Guizhou, Banpo
L180322-P-5-LD180319-0014_HL3MGCCXY_L1_clean_bin.89.orig.fa_	OEZ008529	yes	LMSG_G000010117.1	OES073668	terpene	Terpene	Antimony	21.70	China: Guizhou, Banpo
L180322-P-5-LD180319-0014_HL3MGCCXY_L1_clean_bin.89.orig.fa_	OEZ008529	yes	LMSG_G000010117.1	OES073668	T3PKS	PKSother	Antimony	41.08	China: Guizhou, Banpo
L180322-P-5-LD180319-0014_HL3MGCCXY_L1_clean_bin.89.orig.fa_	OEZ008529	yes	LMSG_G000010117.1	OES073668	hserlactone	Others	Antimony	10.39	China: Guizhou, Banpo
L180322-P-5-LD180319-0014_HL3MGCCXY_L1_clean_bin.9.orig.fa_k	OEZ008529	yes	LMSG_G000010195.1	OES073668	terpene	Terpene	Antimony	18.66	China: Guizhou, Banpo
L180322-P-5-LD180319-0014_HL3MGCCXY_L1_clean_bin.90.orig.fa_	OEZ008529	yes	LMSG_G000005366.1	OES073668	NRPS-like	NRPS	Antimony	6.77	China: Guizhou, Banpo
L180322-P-5-LD180319-0014_HL3MGCCXY_L1_clean_bin.90.orig.fa_	OEZ008529	yes	LMSG_G000005366.1	OES073668	arylpolyene	Others	Antimony	41.19	China: Guizhou, Banpo
L180322-P-5-LD180319-0014_HL3MGCCXY_L1_clean_bin.90.orig.fa_	OEZ008529	yes	LMSG_G000005366.1	OES073668	acyl_amino_acids	Others	Antimony	35.72	China: Guizhou, Banpo
L180322-P-5-LD180319-0015_HL3MGCCXY_L1_clean_bin.1.orig.fa_k	OEZ008529	yes	LMSG_G000009100.1	OES073669	terpene	Terpene	Antimony	25.01	China: Guizhou, Banpo
L180322-P-5-LD180319-0015_HL3MGCCXY_L1_clean_bin.1.orig.fa_k	OEZ008529	yes	LMSG_G000009100.1	OES073669	bacteriocin	RiPPs	Antimony	9.04	China: Guizhou, Banpo
L180322-P-5-LD180319-0015_HL3MGCCXY_L1_clean_bin.1.orig.fa_k	OEZ008529	yes	LMSG_G000009100.1	OES073669	T3PKS	PKSother	Antimony	33.52	China: Guizhou, Banpo
L180322-P-5-LD180319-0015_HL3MGCCXY_L1_clean_bin.10.strict.f	OEZ008529	yes	LMSG_G000004470.1	OES073669	arylpolyene	Others	Antimony	41.10	China: Guizhou, Banpo
L180322-P-5-LD180319-0015_HL3MGCCXY_L1_clean_bin.10.strict.f	OEZ008529	yes	LMSG_G000004470.1	OES073669	NRPS-like	NRPS	Antimony	10.57	China: Guizhou, Banpo
L180322-P-5-LD180319-0015_HL3MGCCXY_L1_clean_bin.11.orig.fa_	OEZ008529	yes	LMSG_G000005367.1	OES073669	arylpolyene	Others	Antimony	10.83	China: Guizhou, Banpo
L180322-P-5-LD180319-0015_HL3MGCCXY_L1_clean_bin.11.orig.fa_	OEZ008529	no	LMSG_G000005367.1	OES073669	terpene	Terpene	Antimony	13.31	China: Guizhou, Banpo
L180322-P-5-LD180319-0015_HL3MGCCXY_L1_clean_bin.11.orig.fa_	OEZ008529	yes	LMSG_G000005367.1	OES073669	hserlactone	Others	Antimony	9.39	China: Guizhou, Banpo
L180322-P-5-LD180319-0015_HL3MGCCXY_L1_clean_bin.12.orig.fa_	OEZ008529	yes	LMSG_G000010118.1	OES073669	terpene	Terpene	Antimony	21.91	China: Guizhou, Banpo
L180322-P-5-LD180319-0015_HL3MGCCXY_L1_clean_bin.12.orig.fa_	OEZ008529	yes	LMSG_G000010118.1	OES073669	hserlactone	Others	Antimony	15.09	China: Guizhou, Banpo
L180322-P-5-LD180319-0015_HL3MGCCXY_L1_clean_bin.12.orig.fa_	OEZ008529	yes	LMSG_G000010118.1	OES073669	terpene	Terpene	Antimony	21.70	China: Guizhou, Banpo
L180322-P-5-LD180319-0015_HL3MGCCXY_L1_clean_bin.12.orig.fa_	OEZ008529	yes	LMSG_G000010118.1	OES073669	T3PKS	PKSother	Antimony	41.08	China: Guizhou, Banpo
L180322-P-5-LD180319-0015_HL3MGCCXY_L1_clean_bin.13.strict.f	OEZ008529	yes	LMSG_G000009050.1	OES073669	betalactone	Others	Antimony	27.46	China: Guizhou, Banpo
L180322-P-5-LD180319-0015_HL3MGCCXY_L1_clean_bin.13.strict.f	OEZ008529	yes	LMSG_G000009050.1	OES073669	T1PKS	PKSI	Antimony	42.43	China: Guizhou, Banpo
L180322-P-5-LD180319-0015_HL3MGCCXY_L1_clean_bin.13.strict.f	OEZ008529	yes	LMSG_G000009050.1	OES073669	T1PKS	PKSI	Antimony	47.90	China: Guizhou, Banpo
L180322-P-5-LD180319-0015_HL3MGCCXY_L1_clean_bin.14.orig.fa_	OEZ008529	yes	LMSG_G000005368.1	OES073669	terpene	Terpene	Antimony	16.65	China: Guizhou, Banpo
L180322-P-5-LD180319-0015_HL3MGCCXY_L1_clean_bin.14.orig.fa_	OEZ008529	yes	LMSG_G000005368.1	OES073669	hserlactone	Others	Antimony	18.38	China: Guizhou, Banpo
L180322-P-5-LD180319-0015_HL3MGCCXY_L1_clean_bin.14.orig.fa_	OEZ008529	yes	LMSG_G000005368.1	OES073669	NRPS.T1PKS	PKS-NRP_Hybrids	Antimony	52.41	China: Guizhou, Banpo
L180322-P-5-LD180319-0015_HL3MGCCXY_L1_clean_bin.14.orig.fa_	OEZ008529	yes	LMSG_G000005368.1	OES073669	hserlactone	Others	Antimony	20.59	China: Guizhou, Banpo
L180322-P-5-LD180319-0015_HL3MGCCXY_L1_clean_bin.14.orig.fa_	OEZ008529	yes	LMSG_G000005368.1	OES073669	betalactone	Others	Antimony	26.80	China: Guizhou, Banpo
L180322-P-5-LD180319-0015_HL3MGCCXY_L1_clean_bin.18.orig.fa_	OEZ008529	yes	LMSG_G000010976.1	OES073669	T1PKS.hglE-KS	PKSother	Antimony	11.91	China: Guizhou, Banpo
L180322-P-5-LD180319-0015_HL3MGCCXY_L1_clean_bin.2.strict.fa	OEZ008529	yes	LMSG_G000005371.1	OES073669	terpene	Terpene	Antimony	20.80	China: Guizhou, Banpo
L180322-P-5-LD180319-0015_HL3MGCCXY_L1_clean_bin.2.strict.fa	OEZ008529	yes	LMSG_G000005371.1	OES073669	bacteriocin	RiPPs	Antimony	10.89	China: Guizhou, Banpo
L180322-P-5-LD180319-0015_HL3MGCCXY_L1_clean_bin.2.strict.fa	OEZ008529	yes	LMSG_G000005371.1	OES073669	NRPS	NRPS	Antimony	27.38	China: Guizhou, Banpo
L180322-P-5-LD180319-0015_HL3MGCCXY_L1_clean_bin.20.orig.fa_	OEZ008529	yes	LMSG_G000010359.1	OES073669	terpene	Terpene	Antimony	12.66	China: Guizhou, Banpo
L180322-P-5-LD180319-0015_HL3MGCCXY_L1_clean_bin.20.orig.fa_	OEZ008529	yes	LMSG_G000010359.1	OES073669	NRPS-like	NRPS	Antimony	23.62	China: Guizhou, Banpo
L180322-P-5-LD180319-0015_HL3MGCCXY_L1_clean_bin.20.orig.fa_	OEZ008529	yes	LMSG_G000010359.1	OES073669	NRPS-like	NRPS	Antimony	16.48	China: Guizhou, Banpo
L180322-P-5-LD180319-0015_HL3MGCCXY_L1_clean_bin.20.orig.fa_	OEZ008529	yes	LMSG_G000010359.1	OES073669	terpene	Terpene	Antimony	13.35	China: Guizhou, Banpo
L180322-P-5-LD180319-0015_HL3MGCCXY_L1_clean_bin.20.orig.fa_	OEZ008529	yes	LMSG_G000010359.1	OES073669	NRPS-like	NRPS	Antimony	24.09	China: Guizhou, Banpo
L180322-P-5-LD180319-0015_HL3MGCCXY_L1_clean_bin.20.orig.fa_	OEZ008529	yes	LMSG_G000010359.1	OES073669	bacteriocin	RiPPs	Antimony	10.86	China: Guizhou, Banpo
L180322-P-5-LD180319-0015_HL3MGCCXY_L1_clean_bin.20.orig.fa_	OEZ008529	yes	LMSG_G000010359.1	OES073669	NRPS	NRPS	Antimony	35.11	China: Guizhou, Banpo
L180322-P-5-LD180319-0015_HL3MGCCXY_L1_clean_bin.20.orig.fa_	OEZ008529	yes	LMSG_G000010359.1	OES073669	terpene	Terpene	Antimony	12.62	China: Guizhou, Banpo
L180322-P-5-LD180319-0015_HL3MGCCXY_L1_clean_bin.20.orig.fa_	OEZ008529	yes	LMSG_G000010359.1	OES073669	bacteriocin	RiPPs	Antimony	9.62	China: Guizhou, Banpo
L180322-P-5-LD180319-0015_HL3MGCCXY_L1_clean_bin.21.strict.f	OEZ008529	yes	LMSG_G000011193.1	OES073669	terpene	Terpene	Antimony	8.77	China: Guizhou, Banpo
L180322-P-5-LD180319-0015_HL3MGCCXY_L1_clean_bin.25.strict.f	OEZ008529	yes	LMSG_G000005370.1	OES073669	bacteriocin	RiPPs	Antimony	5.60	China: Guizhou, Banpo
L180322-P-5-LD180319-0015_HL3MGCCXY_L1_clean_bin.26.orig.fa_	OEZ008529	yes	LMSG_G000010518.1	OES073669	acyl_amino_acids	Others	Antimony	21.53	China: Guizhou, Banpo
L180322-P-5-LD180319-0015_HL3MGCCXY_L1_clean_bin.26.orig.fa_	OEZ008529	yes	LMSG_G000010518.1	OES073669	NRPS-like	NRPS	Antimony	20.54	China: Guizhou, Banpo
L180322-P-5-LD180319-0015_HL3MGCCXY_L1_clean_bin.26.orig.fa_	OEZ008529	yes	LMSG_G000010518.1	OES073669	arylpolyene	Others	Antimony	40.55	China: Guizhou, Banpo
L180322-P-5-LD180319-0015_HL3MGCCXY_L1_clean_bin.28.orig.fa_	OEZ008529	yes	LMSG_G000007932.1	OES073669	terpene	Terpene	Antimony	12.63	China: Guizhou, Banpo
L180322-P-5-LD180319-0015_HL3MGCCXY_L1_clean_bin.29.orig.fa_	OEZ008529	yes	LMSG_G000010346.1	OES073669	terpene	Terpene	Antimony	21.72	China: Guizhou, Banpo
L180322-P-5-LD180319-0015_HL3MGCCXY_L1_clean_bin.29.orig.fa_	OEZ008529	yes	LMSG_G000010346.1	OES073669	bacteriocin	RiPPs	Antimony	10.94	China: Guizhou, Banpo
L180322-P-5-LD180319-0015_HL3MGCCXY_L1_clean_bin.29.orig.fa_	OEZ008529	yes	LMSG_G000010346.1	OES073669	terpene	Terpene	Antimony	15.19	China: Guizhou, Banpo
L180322-P-5-LD180319-0015_HL3MGCCXY_L1_clean_bin.29.orig.fa_	OEZ008529	yes	LMSG_G000010346.1	OES073669	terpene	Terpene	Antimony	21.92	China: Guizhou, Banpo
L180322-P-5-LD180319-0015_HL3MGCCXY_L1_clean_bin.29.orig.fa_	OEZ008529	yes	LMSG_G000010346.1	OES073669	NRPS-like	NRPS	Antimony	43.10	China: Guizhou, Banpo
L180322-P-5-LD180319-0015_HL3MGCCXY_L1_clean_bin.29.orig.fa_	OEZ008529	yes	LMSG_G000010346.1	OES073669	T3PKS	PKSother	Antimony	26.76	China: Guizhou, Banpo
L180322-P-5-LD180319-0015_HL3MGCCXY_L1_clean_bin.3.orig.fa_k	OEZ008529	yes	LMSG_G000010102.1	OES073669	terpene	Terpene	Antimony	21.27	China: Guizhou, Banpo
L180322-P-5-LD180319-0015_HL3MGCCXY_L1_clean_bin.3.orig.fa_k	OEZ008529	yes	LMSG_G000010102.1	OES073669	bacteriocin	RiPPs	Antimony	7.51	China: Guizhou, Banpo
L180322-P-5-LD180319-0015_HL3MGCCXY_L1_clean_bin.3.orig.fa_k	OEZ008529	yes	LMSG_G000010102.1	OES073669	NRPS-like	NRPS	Antimony	8.14	China: Guizhou, Banpo
L180322-P-5-LD180319-0015_HL3MGCCXY_L1_clean_bin.3.orig.fa_k	OEZ008529	yes	LMSG_G000010102.1	OES073669	T3PKS	PKSother	Antimony	26.58	China: Guizhou, Banpo
L180322-P-5-LD180319-0015_HL3MGCCXY_L1_clean_bin.3.orig.fa_k	OEZ008529	yes	LMSG_G000010102.1	OES073669	terpene	Terpene	Antimony	13.95	China: Guizhou, Banpo
L180322-P-5-LD180319-0015_HL3MGCCXY_L1_clean_bin.3.orig.fa_k	OEZ008529	yes	LMSG_G000010102.1	OES073669	LAP.bacteriocin	RiPPs	Antimony	16.21	China: Guizhou, Banpo
L180322-P-5-LD180319-0015_HL3MGCCXY_L1_clean_bin.3.orig.fa_k	OEZ008529	yes	LMSG_G000010102.1	OES073669	terpene	Terpene	Antimony	14.46	China: Guizhou, Banpo
L180322-P-5-LD180319-0015_HL3MGCCXY_L1_clean_bin.31.orig.fa_	OEZ008529	yes	LMSG_G000006232.1	OES073669	betalactone	Others	Antimony	30.48	China: Guizhou, Banpo
L180322-P-5-LD180319-0015_HL3MGCCXY_L1_clean_bin.31.orig.fa_	OEZ008529	yes	LMSG_G000006232.1	OES073669	other	Others	Antimony	32.85	China: Guizhou, Banpo
L180322-P-5-LD180319-0015_HL3MGCCXY_L1_clean_bin.31.orig.fa_	OEZ008529	yes	LMSG_G000006232.1	OES073669	betalactone	Others	Antimony	22.14	China: Guizhou, Banpo
L180322-P-5-LD180319-0015_HL3MGCCXY_L1_clean_bin.32.orig.fa_	OEZ008529	yes	LMSG_G000006330.1	OES073669	bacteriocin	RiPPs	Antimony	10.86	China: Guizhou, Banpo
L180322-P-5-LD180319-0015_HL3MGCCXY_L1_clean_bin.32.orig.fa_	OEZ008529	yes	LMSG_G000006330.1	OES073669	terpene	Terpene	Antimony	20.86	China: Guizhou, Banpo
L180322-P-5-LD180319-0015_HL3MGCCXY_L1_clean_bin.32.orig.fa_	OEZ008529	yes	LMSG_G000006330.1	OES073669	transAT-PKS-like.NRPS-like	PKS-NRP_Hybrids	Antimony	19.92	China: Guizhou, Banpo
L180322-P-5-LD180319-0015_HL3MGCCXY_L1_clean_bin.32.orig.fa_	OEZ008529	yes	LMSG_G000006330.1	OES073669	transAT-PKS	PKSother	Antimony	8.71	China: Guizhou, Banpo
L180322-P-5-LD180319-0015_HL3MGCCXY_L1_clean_bin.32.orig.fa_	OEZ008529	yes	LMSG_G000006330.1	OES073669	bacteriocin	RiPPs	Antimony	10.24	China: Guizhou, Banpo
L180322-P-5-LD180319-0015_HL3MGCCXY_L1_clean_bin.32.orig.fa_	OEZ008529	yes	LMSG_G000006330.1	OES073669	terpene	Terpene	Antimony	21.00	China: Guizhou, Banpo
L180322-P-5-LD180319-0015_HL3MGCCXY_L1_clean_bin.32.orig.fa_	OEZ008529	yes	LMSG_G000006330.1	OES073669	CDPS	Others	Antimony	20.74	China: Guizhou, Banpo
L180322-P-5-LD180319-0015_HL3MGCCXY_L1_clean_bin.32.orig.fa_	OEZ008529	yes	LMSG_G000006330.1	OES073669	T3PKS.bacteriocin	Others	Antimony	36.27	China: Guizhou, Banpo
L180322-P-5-LD180319-0015_HL3MGCCXY_L1_clean_bin.32.orig.fa_	OEZ008529	yes	LMSG_G000006330.1	OES073669	terpene	Terpene	Antimony	20.86	China: Guizhou, Banpo
L180322-P-5-LD180319-0015_HL3MGCCXY_L1_clean_bin.32.orig.fa_	OEZ008529	yes	LMSG_G000006330.1	OES073669	CDPS	Others	Antimony	20.71	China: Guizhou, Banpo
L180322-P-5-LD180319-0015_HL3MGCCXY_L1_clean_bin.32.orig.fa_	OEZ008529	yes	LMSG_G000006330.1	OES073669	TfuA-related	RiPPs	Antimony	14.64	China: Guizhou, Banpo
L180322-P-5-LD180319-0015_HL3MGCCXY_L1_clean_bin.32.orig.fa_	OEZ008529	yes	LMSG_G000006330.1	OES073669	NRPS-like	NRPS	Antimony	39.52	China: Guizhou, Banpo
L180322-P-5-LD180319-0015_HL3MGCCXY_L1_clean_bin.32.orig.fa_	OEZ008529	yes	LMSG_G000006330.1	OES073669	NRPS-like	NRPS	Antimony	25.55	China: Guizhou, Banpo
L180322-P-5-LD180319-0015_HL3MGCCXY_L1_clean_bin.36.orig.fa_	OEZ008529	yes	LMSG_G000008647.1	OES073669	other	Others	Antimony	17.73	China: Guizhou, Banpo
L180322-P-5-LD180319-0015_HL3MGCCXY_L1_clean_bin.37.orig.fa_	OEZ008529	yes	LMSG_G000009071.1	OES073669	arylpolyene	Others	Antimony	58.59	China: Guizhou, Banpo
L180322-P-5-LD180319-0015_HL3MGCCXY_L1_clean_bin.37.orig.fa_	OEZ008529	yes	LMSG_G000009071.1	OES073669	NRPS-like	NRPS	Antimony	24.65	China: Guizhou, Banpo
L180322-P-5-LD180319-0015_HL3MGCCXY_L1_clean_bin.37.orig.fa_	OEZ008529	yes	LMSG_G000009071.1	OES073669	lassopeptide	RiPPs	Antimony	29.96	China: Guizhou, Banpo
L180322-P-5-LD180319-0015_HL3MGCCXY_L1_clean_bin.37.orig.fa_	OEZ008529	yes	LMSG_G000009071.1	OES073669	terpene	Terpene	Antimony	22.05	China: Guizhou, Banpo
L180322-P-5-LD180319-0015_HL3MGCCXY_L1_clean_bin.38.strict.f	OEZ008529	yes	LMSG_G000008577.1	OES073669	NRPS-like	NRPS	Antimony	42.81	China: Guizhou, Banpo
L180322-P-5-LD180319-0015_HL3MGCCXY_L1_clean_bin.38.strict.f	OEZ008529	yes	LMSG_G000008577.1	OES073669	other	Others	Antimony	26.26	China: Guizhou, Banpo
L180322-P-5-LD180319-0015_HL3MGCCXY_L1_clean_bin.39.strict.f	OEZ008529	yes	LMSG_G000005373.1	OES073669	betalactone	Others	Antimony	22.65	China: Guizhou, Banpo
L180322-P-5-LD180319-0015_HL3MGCCXY_L1_clean_bin.39.strict.f	OEZ008529	yes	LMSG_G000005373.1	OES073669	arylpolyene	Others	Antimony	20.51	China: Guizhou, Banpo
L180322-P-5-LD180319-0015_HL3MGCCXY_L1_clean_bin.39.strict.f	OEZ008529	yes	LMSG_G000005373.1	OES073669	T1PKS	PKSI	Antimony	16.74	China: Guizhou, Banpo
L180322-P-5-LD180319-0015_HL3MGCCXY_L1_clean_bin.4.orig.fa_k	OEZ008529	yes	LMSG_G000010777.1	OES073669	bacteriocin	RiPPs	Antimony	9.22	China: Guizhou, Banpo
L180322-P-5-LD180319-0015_HL3MGCCXY_L1_clean_bin.4.orig.fa_k	OEZ008529	yes	LMSG_G000010777.1	OES073669	acyl_amino_acids	Others	Antimony	33.89	China: Guizhou, Banpo
L180322-P-5-LD180319-0015_HL3MGCCXY_L1_clean_bin.4.orig.fa_k	OEZ008529	yes	LMSG_G000010777.1	OES073669	arylpolyene	Others	Antimony	38.97	China: Guizhou, Banpo
L180322-P-5-LD180319-0015_HL3MGCCXY_L1_clean_bin.45.orig.fa_	OEZ008529	yes	LMSG_G000005374.1	OES073669	NRPS-like	NRPS	Antimony	43.91	China: Guizhou, Banpo
L180322-P-5-LD180319-0015_HL3MGCCXY_L1_clean_bin.45.orig.fa_	OEZ008529	yes	LMSG_G000005374.1	OES073669	betalactone	Others	Antimony	23.67	China: Guizhou, Banpo
L180322-P-5-LD180319-0015_HL3MGCCXY_L1_clean_bin.46.orig.fa_	OEZ008529	yes	LMSG_G000005375.1	OES073669	hserlactone	Others	Antimony	17.33	China: Guizhou, Banpo
L180322-P-5-LD180319-0015_HL3MGCCXY_L1_clean_bin.46.orig.fa_	OEZ008529	yes	LMSG_G000005375.1	OES073669	hserlactone	Others	Antimony	18.94	China: Guizhou, Banpo
L180322-P-5-LD180319-0015_HL3MGCCXY_L1_clean_bin.46.orig.fa_	OEZ008529	yes	LMSG_G000005375.1	OES073669	NRPS.T1PKS	PKS-NRP_Hybrids	Antimony	23.83	China: Guizhou, Banpo
L180322-P-5-LD180319-0015_HL3MGCCXY_L1_clean_bin.46.orig.fa_	OEZ008529	yes	LMSG_G000005375.1	OES073669	bacteriocin	RiPPs	Antimony	10.57	China: Guizhou, Banpo
L180322-P-5-LD180319-0015_HL3MGCCXY_L1_clean_bin.47.orig.fa_	OEZ008529	yes	LMSG_G000008969.1	OES073669	NRPS-like	NRPS	Antimony	5.86	China: Guizhou, Banpo
L180322-P-5-LD180319-0015_HL3MGCCXY_L1_clean_bin.48.orig.fa_	OEZ008529	yes	LMSG_G000004471.1	OES073669	NRPS	NRPS	Antimony	5.55	China: Guizhou, Banpo
L180322-P-5-LD180319-0015_HL3MGCCXY_L1_clean_bin.48.orig.fa_	OEZ008529	yes	LMSG_G000004471.1	OES073669	betalactone	Others	Antimony	12.06	China: Guizhou, Banpo
L180322-P-5-LD180319-0015_HL3MGCCXY_L1_clean_bin.50.orig.fa_	OEZ008529	yes	LMSG_G000009508.1	OES073669	betalactone	Others	Antimony	9.13	China: Guizhou, Banpo
L180322-P-5-LD180319-0015_HL3MGCCXY_L1_clean_bin.51.orig.fa_	OEZ008529	yes	LMSG_G000009057.1	OES073669	NRPS-like	NRPS	Antimony	30.48	China: Guizhou, Banpo
L180322-P-5-LD180319-0015_HL3MGCCXY_L1_clean_bin.51.orig.fa_	OEZ008529	yes	LMSG_G000009057.1	OES073669	lassopeptide	RiPPs	Antimony	31.28	China: Guizhou, Banpo
L180322-P-5-LD180319-0015_HL3MGCCXY_L1_clean_bin.51.orig.fa_	OEZ008529	yes	LMSG_G000009057.1	OES073669	NRPS-like	NRPS	Antimony	22.60	China: Guizhou, Banpo
L180322-P-5-LD180319-0015_HL3MGCCXY_L1_clean_bin.51.orig.fa_	OEZ008529	yes	LMSG_G000009057.1	OES073669	terpene	Terpene	Antimony	22.05	China: Guizhou, Banpo
L180322-P-5-LD180319-0015_HL3MGCCXY_L1_clean_bin.52.orig.fa_	OEZ008529	no	LMSG_G000010871.1	OES073669	terpene	Terpene	Antimony	5.38	China: Guizhou, Banpo
L180322-P-5-LD180319-0015_HL3MGCCXY_L1_clean_bin.52.orig.fa_	OEZ008529	no	LMSG_G000010871.1	OES073669	arylpolyene	Others	Antimony	8.10	China: Guizhou, Banpo
L180322-P-5-LD180319-0015_HL3MGCCXY_L1_clean_bin.52.orig.fa_	OEZ008529	yes	LMSG_G000010871.1	OES073669	terpene	Terpene	Antimony	7.19	China: Guizhou, Banpo
L180322-P-5-LD180319-0015_HL3MGCCXY_L1_clean_bin.52.orig.fa_	OEZ008529	no	LMSG_G000010871.1	OES073669	bacteriocin	RiPPs	Antimony	6.08	China: Guizhou, Banpo
L180322-P-5-LD180319-0015_HL3MGCCXY_L1_clean_bin.53.orig.fa_	OEZ008529	yes	LMSG_G000008581.1	OES073669	other	Others	Antimony	6.63	China: Guizhou, Banpo
L180322-P-5-LD180319-0015_HL3MGCCXY_L1_clean_bin.54.strict.f	OEZ008529	yes	LMSG_G000006331.1	OES073669	NRPS-like	NRPS	Antimony	42.75	China: Guizhou, Banpo
L180322-P-5-LD180319-0015_HL3MGCCXY_L1_clean_bin.54.strict.f	OEZ008529	yes	LMSG_G000006331.1	OES073669	NRPS	NRPS	Antimony	39.34	China: Guizhou, Banpo
L180322-P-5-LD180319-0015_HL3MGCCXY_L1_clean_bin.54.strict.f	OEZ008529	yes	LMSG_G000006331.1	OES073669	NRPS.T1PKS	PKS-NRP_Hybrids	Antimony	18.28	China: Guizhou, Banpo
L180322-P-5-LD180319-0015_HL3MGCCXY_L1_clean_bin.54.strict.f	OEZ008529	yes	LMSG_G000006331.1	OES073669	CDPS	Others	Antimony	7.96	China: Guizhou, Banpo
L180322-P-5-LD180319-0015_HL3MGCCXY_L1_clean_bin.54.strict.f	OEZ008529	yes	LMSG_G000006331.1	OES073669	CDPS	Others	Antimony	5.99	China: Guizhou, Banpo
L180322-P-5-LD180319-0015_HL3MGCCXY_L1_clean_bin.58.orig.fa_	OEZ008529	yes	LMSG_G000005377.1	OES073669	terpene	Terpene	Antimony	5.42	China: Guizhou, Banpo
L180322-P-5-LD180319-0015_HL3MGCCXY_L1_clean_bin.58.orig.fa_	OEZ008529	yes	LMSG_G000005377.1	OES073669	T3PKS	PKSother	Antimony	9.83	China: Guizhou, Banpo
L180322-P-5-LD180319-0015_HL3MGCCXY_L1_clean_bin.60.orig.fa_	OEZ008529	yes	LMSG_G000010088.1	OES073669	hglE-KS	PKSother	Antimony	10.71	China: Guizhou, Banpo
L180322-P-5-LD180319-0015_HL3MGCCXY_L1_clean_bin.60.orig.fa_	OEZ008529	yes	LMSG_G000010088.1	OES073669	hglE-KS	PKSother	Antimony	15.97	China: Guizhou, Banpo
L180322-P-5-LD180319-0015_HL3MGCCXY_L1_clean_bin.60.orig.fa_	OEZ008529	no	LMSG_G000010088.1	OES073669	T1PKS	PKSI	Antimony	6.22	China: Guizhou, Banpo
L180322-P-5-LD180319-0015_HL3MGCCXY_L1_clean_bin.60.orig.fa_	OEZ008529	yes	LMSG_G000010088.1	OES073669	T3PKS	PKSother	Antimony	6.73	China: Guizhou, Banpo
L180322-P-5-LD180319-0015_HL3MGCCXY_L1_clean_bin.60.orig.fa_	OEZ008529	yes	LMSG_G000010088.1	OES073669	T1PKS.hglE-KS	PKSother	Antimony	14.59	China: Guizhou, Banpo
L180322-P-5-LD180319-0015_HL3MGCCXY_L1_clean_bin.60.orig.fa_	OEZ008529	yes	LMSG_G000010088.1	OES073669	terpene	Terpene	Antimony	13.52	China: Guizhou, Banpo
L180322-P-5-LD180319-0015_HL3MGCCXY_L1_clean_bin.61.orig.fa_	OEZ008529	yes	LMSG_G000010376.1	OES073669	bacteriocin	RiPPs	Antimony	6.79	China: Guizhou, Banpo
L180322-P-5-LD180319-0015_HL3MGCCXY_L1_clean_bin.61.orig.fa_	OEZ008529	yes	LMSG_G000010376.1	OES073669	LAP	RiPPs	Antimony	10.57	China: Guizhou, Banpo
L180322-P-5-LD180319-0015_HL3MGCCXY_L1_clean_bin.61.orig.fa_	OEZ008529	yes	LMSG_G000010376.1	OES073669	terpene	Terpene	Antimony	7.13	China: Guizhou, Banpo
L180322-P-5-LD180319-0015_HL3MGCCXY_L1_clean_bin.61.orig.fa_	OEZ008529	yes	LMSG_G000010376.1	OES073669	terpene	Terpene	Antimony	13.45	China: Guizhou, Banpo
L180322-P-5-LD180319-0015_HL3MGCCXY_L1_clean_bin.61.orig.fa_	OEZ008529	yes	LMSG_G000010376.1	OES073669	T3PKS	PKSother	Antimony	11.56	China: Guizhou, Banpo
L180322-P-5-LD180319-0015_HL3MGCCXY_L1_clean_bin.61.orig.fa_	OEZ008529	yes	LMSG_G000010376.1	OES073669	betalactone	Others	Antimony	29.77	China: Guizhou, Banpo
L180322-P-5-LD180319-0015_HL3MGCCXY_L1_clean_bin.61.orig.fa_	OEZ008529	yes	LMSG_G000010376.1	OES073669	betalactone	Others	Antimony	29.62	China: Guizhou, Banpo
L180322-P-5-LD180319-0015_HL3MGCCXY_L1_clean_bin.61.orig.fa_	OEZ008529	yes	LMSG_G000010376.1	OES073669	hserlactone	Others	Antimony	18.93	China: Guizhou, Banpo
L180322-P-5-LD180319-0015_HL3MGCCXY_L1_clean_bin.61.orig.fa_	OEZ008529	yes	LMSG_G000010376.1	OES073669	terpene	Terpene	Antimony	9.69	China: Guizhou, Banpo
L180322-P-5-LD180319-0015_HL3MGCCXY_L1_clean_bin.62.strict.f	OEZ008529	yes	LMSG_G000010813.1	OES073669	acyl_amino_acids	Others	Antimony	8.65	China: Guizhou, Banpo
L180322-P-5-LD180319-0015_HL3MGCCXY_L1_clean_bin.66.orig.fa_	OEZ008529	yes	LMSG_G000010240.1	OES073669	terpene	Terpene	Antimony	6.25	China: Guizhou, Banpo
L180322-P-5-LD180319-0015_HL3MGCCXY_L1_clean_bin.67.strict.f	OEZ008529	yes	LMSG_G000005379.1	OES073669	NRPS	NRPS	Antimony	21.93	China: Guizhou, Banpo
L180322-P-5-LD180319-0015_HL3MGCCXY_L1_clean_bin.67.strict.f	OEZ008529	yes	LMSG_G000005379.1	OES073669	T3PKS	PKSother	Antimony	29.98	China: Guizhou, Banpo
L180322-P-5-LD180319-0015_HL3MGCCXY_L1_clean_bin.67.strict.f	OEZ008529	yes	LMSG_G000005379.1	OES073669	NRPS-like	NRPS	Antimony	24.69	China: Guizhou, Banpo
L180322-P-5-LD180319-0015_HL3MGCCXY_L1_clean_bin.67.strict.f	OEZ008529	yes	LMSG_G000005379.1	OES073669	terpene	Terpene	Antimony	11.18	China: Guizhou, Banpo
L180322-P-5-LD180319-0015_HL3MGCCXY_L1_clean_bin.68.orig.fa_	OEZ008529	yes	LMSG_G000010689.1	OES073669	terpene	Terpene	Antimony	20.80	China: Guizhou, Banpo
L180322-P-5-LD180319-0015_HL3MGCCXY_L1_clean_bin.68.orig.fa_	OEZ008529	yes	LMSG_G000010689.1	OES073669	arylpolyene	Others	Antimony	41.25	China: Guizhou, Banpo
L180322-P-5-LD180319-0015_HL3MGCCXY_L1_clean_bin.69.strict.f	OEZ008529	yes	LMSG_G000005380.1	OES073669	LAP.thiopeptide	RiPPs	Antimony	30.38	China: Guizhou, Banpo
L180322-P-5-LD180319-0015_HL3MGCCXY_L1_clean_bin.71.orig.fa_	OEZ008529	yes	LMSG_G000008832.1	OES073669	terpene	Terpene	Antimony	21.71	China: Guizhou, Banpo
L180322-P-5-LD180319-0015_HL3MGCCXY_L1_clean_bin.71.orig.fa_	OEZ008529	yes	LMSG_G000008832.1	OES073669	NRPS-like	NRPS	Antimony	23.02	China: Guizhou, Banpo

L180322-P-5-LD180319-0015_HL3MGCCXY_L1_clean_bin.71.orig.fa_	OEZ008529	yes	LMSG_G000008832.1	OES073669	lanthipeptide	RiPPs	Antimony	17.37	China: Guizhou, Banpo
L180322-P-5-LD180319-0015_HL3MGCCXY_L1_clean_bin.73.orig.fa_	OEZ008529	yes	LMSG_G000010083.1	OES073669	terpene	Terpene	Antimony	13.89	China: Guizhou, Banpo
L180322-P-5-LD180319-0015_HL3MGCCXY_L1_clean_bin.73.orig.fa_	OEZ008529	yes	LMSG_G000010083.1	OES073669	terpene	Terpene	Antimony	14.08	China: Guizhou, Banpo
L180322-P-5-LD180319-0015_HL3MGCCXY_L1_clean_bin.73.orig.fa_	OEZ008529	yes	LMSG_G000010083.1	OES073669	T1PKS.hglE-KS	PKSother	Antimony	53.29	China: Guizhou, Banpo
L180322-P-5-LD180319-0015_HL3MGCCXY_L1_clean_bin.73.orig.fa_	OEZ008529	yes	LMSG_G000010083.1	OES073669	hglE-KS	PKSother	Antimony	18.32	China: Guizhou, Banpo
L180322-P-5-LD180319-0015_HL3MGCCXY_L1_clean_bin.73.orig.fa_	OEZ008529	yes	LMSG_G000010083.1	OES073669	acyl_amino_acids	Others	Antimony	12.91	China: Guizhou, Banpo
L180322-P-5-LD180319-0015_HL3MGCCXY_L1_clean_bin.73.orig.fa_	OEZ008529	yes	LMSG_G000010083.1	OES073669	NRPS-like	NRPS	Antimony	6.11	China: Guizhou, Banpo
L180322-P-5-LD180319-0015_HL3MGCCXY_L1_clean_bin.73.orig.fa_	OEZ008529	yes	LMSG_G000010083.1	OES073669	acyl_amino_acids	Others	Antimony	10.40	China: Guizhou, Banpo
L180322-P-5-LD180319-0015_HL3MGCCXY_L1_clean_bin.74.orig.fa_	OEZ008529	yes	LMSG_G000005381.1	OES073669	NRPS-like	NRPS	Antimony	7.14	China: Guizhou, Banpo
L180322-P-5-LD180319-0015_HL3MGCCXY_L1_clean_bin.74.orig.fa_	OEZ008529	yes	LMSG_G000005381.1	OES073669	lassopeptide	RiPPs	Antimony	13.97	China: Guizhou, Banpo
L180322-P-5-LD180319-0015_HL3MGCCXY_L1_clean_bin.74.orig.fa_	OEZ008529	yes	LMSG_G000005381.1	OES073669	T3PKS	PKSother	Antimony	9.43	China: Guizhou, Banpo
L180322-P-5-LD180319-0015_HL3MGCCXY_L1_clean_bin.75.orig.fa_	OEZ008529	yes	LMSG_G000010886.1	OES073669	arylpolyene	Others	Antimony	12.97	China: Guizhou, Banpo
L180322-P-5-LD180319-0015_HL3MGCCXY_L1_clean_bin.75.orig.fa_	OEZ008529	yes	LMSG_G000010886.1	OES073669	bacteriocin	RiPPs	Antimony	8.22	China: Guizhou, Banpo
L180322-P-5-LD180319-0015_HL3MGCCXY_L1_clean_bin.76.strict.f	OEZ008529	yes	LMSG_G000009272.1	OES073669	T3PKS	PKSother	Antimony	7.22	China: Guizhou, Banpo
L180322-P-5-LD180319-0015_HL3MGCCXY_L1_clean_bin.76.strict.f	OEZ008529	yes	LMSG_G000009272.1	OES073669	terpene	Terpene	Antimony	5.74	China: Guizhou, Banpo
L180322-P-5-LD180319-0015_HL3MGCCXY_L1_clean_bin.77.orig.fa_	OEZ008529	yes	LMSG_G000008834.1	OES073669	NRPS-like	NRPS	Antimony	9.99	China: Guizhou, Banpo
L180322-P-5-LD180319-0015_HL3MGCCXY_L1_clean_bin.77.orig.fa_	OEZ008529	yes	LMSG_G000008834.1	OES073669	terpene	Terpene	Antimony	11.64	China: Guizhou, Banpo
L180322-P-5-LD180319-0015_HL3MGCCXY_L1_clean_bin.78.orig.fa_	OEZ008529	no	LMSG_G000009498.1	OES073669	hglE-KS	PKSother	Antimony	5.48	China: Guizhou, Banpo
L180322-P-5-LD180319-0015_HL3MGCCXY_L1_clean_bin.78.orig.fa_	OEZ008529	yes	LMSG_G000009498.1	OES073669	bacteriocin	RiPPs	Antimony	6.25	China: Guizhou, Banpo
L180322-P-5-LD180319-0015_HL3MGCCXY_L1_clean_bin.78.orig.fa_	OEZ008529	yes	LMSG_G000009498.1	OES073669	T3PKS	PKSother	Antimony	5.48	China: Guizhou, Banpo
L180322-P-5-LD180319-0015_HL3MGCCXY_L1_clean_bin.79.strict.f	OEZ008529	yes	LMSG_G000004472.1	OES073669	hglE-KS	PKSother	Antimony	48.30	China: Guizhou, Banpo
L180322-P-5-LD180319-0015_HL3MGCCXY_L1_clean_bin.79.strict.f	OEZ008529	yes	LMSG_G000004472.1	OES073669	NRPS-like	NRPS	Antimony	42.87	China: Guizhou, Banpo
L180322-P-5-LD180319-0015_HL3MGCCXY_L1_clean_bin.8.strict.fa	OEZ008529	yes	LMSG_G000005387.1	OES073669	terpene	Terpene	Antimony	19.63	China: Guizhou, Banpo
L180322-P-5-LD180319-0015_HL3MGCCXY_L1_clean_bin.8.strict.fa	OEZ008529	yes	LMSG_G000005387.1	OES073669	bacteriocin	RiPPs	Antimony	10.87	China: Guizhou, Banpo
L180322-P-5-LD180319-0015_HL3MGCCXY_L1_clean_bin.8.strict.fa	OEZ008529	yes	LMSG_G000005387.1	OES073669	terpene	Terpene	Antimony	10.58	China: Guizhou, Banpo
L180322-P-5-LD180319-0015_HL3MGCCXY_L1_clean_bin.8.strict.fa	OEZ008529	yes	LMSG_G000005387.1	OES073669	terpene	Terpene	Antimony	10.03	China: Guizhou, Banpo
L180322-P-5-LD180319-0015_HL3MGCCXY_L1_clean_bin.81.orig.fa_	OEZ008529	yes	LMSG_G000010860.1	OES073669	terpene	Terpene	Antimony	6.82	China: Guizhou, Banpo
L180322-P-5-LD180319-0015_HL3MGCCXY_L1_clean_bin.81.orig.fa_	OEZ008529	yes	LMSG_G000010860.1	OES073669	resorcinol	Others	Antimony	7.04	China: Guizhou, Banpo
L180322-P-5-LD180319-0015_HL3MGCCXY_L1_clean_bin.83.orig.fa_	OEZ008529	yes	LMSG_G000008854.1	OES073669	terpene	Terpene	Antimony	21.86	China: Guizhou, Banpo
L180322-P-5-LD180319-0015_HL3MGCCXY_L1_clean_bin.86.orig.fa_	OEZ008529	yes	LMSG_G000010092.1	OES073669	terpene	Terpene	Antimony	12.81	China: Guizhou, Banpo
L180322-P-5-LD180319-0015_HL3MGCCXY_L1_clean_bin.89.orig.fa_	OEZ008529	yes	LMSG_G000005386.1	OES073669	terpene	Terpene	Antimony	10.64	China: Guizhou, Banpo
L180322-P-5-LD180319-0015_HL3MGCCXY_L1_clean_bin.89.orig.fa_	OEZ008529	yes	LMSG_G000005386.1	OES073669	T3PKS	PKSother	Antimony	8.30	China: Guizhou, Banpo
L180322-P-5-LD180319-0015_HL3MGCCXY_L1_clean_bin.89.orig.fa_	OEZ008529	yes	LMSG_G000005386.1	OES073669	NRPS-like	NRPS	Antimony	9.03	China: Guizhou, Banpo
L180322-P-5-LD180319-0015_HL3MGCCXY_L1_clean_bin.9.orig.fa_k	OEZ008529	yes	LMSG_G000007943.1	OES073669	ladderane	Others	Antimony	6.69	China: Guizhou, Banpo
L180322-P-5-LD180319-0015_HL3MGCCXY_L1_clean_bin.9.orig.fa_k	OEZ008529	yes	LMSG_G000007943.1	OES073669	T3PKS	PKSother	Antimony	16.99	China: Guizhou, Banpo
L180322-P-5-LD180319-0015_HL3MGCCXY_L1_clean_bin.9.orig.fa_k	OEZ008529	yes	LMSG_G000007943.1	OES073669	hglE-KS	PKSother	Antimony	29.04	China: Guizhou, Banpo
L180322-P-5-LD180319-0015_HL3MGCCXY_L1_clean_bin.9.orig.fa_k	OEZ008529	yes	LMSG_G000007943.1	OES073669	terpene	Terpene	Antimony	11.12	China: Guizhou, Banpo
L180322-P-5-LD180319-0015_HL3MGCCXY_L1_clean_bin.9.orig.fa_k	OEZ008529	yes	LMSG_G000007943.1	OES073669	bacteriocin	RiPPs	Antimony	10.93	China: Guizhou, Banpo
L180322-P-5-LD180319-0015_HL3MGCCXY_L1_clean_bin.90.strict.f	OEZ008529	yes	LMSG_G000010089.1	OES073669	bacteriocin	RiPPs	Antimony	10.21	China: Guizhou, Banpo
L180322-P-5-LD180319-0015_HL3MGCCXY_L1_clean_bin.90.strict.f	OEZ008529	yes	LMSG_G000010089.1	OES073669	terpene	Terpene	Antimony	10.52	China: Guizhou, Banpo
L180322-P-5-LD180319-0015_HL3MGCCXY_L1_clean_bin.92.orig.fa_	OEZ008529	yes	LMSG_G000007939.1	OES073669	terpene	Terpene	Antimony	19.41	China: Guizhou, Banpo
L180322-P-5-LD180319-0015_HL3MGCCXY_L1_clean_bin.93.orig.fa_	OEZ008529	yes	LMSG_G000005388.1	OES073669	T1PKS.hglE-KS	PKSother	Antimony	18.18	China: Guizhou, Banpo
L180322-P-5-LD180319-0015_HL3MGCCXY_L1_clean_bin.93.orig.fa_	OEZ008529	yes	LMSG_G000005388.1	OES073669	hglE-KS	PKSother	Antimony	23.42	China: Guizhou, Banpo
L180322-P-5-LD180319-0015_HL3MGCCXY_L1_clean_bin.93.orig.fa_	OEZ008529	yes	LMSG_G000005388.1	OES073669	terpene	Terpene	Antimony	22.05	China: Guizhou, Banpo
L180322-P-5-LD180319-0015_HL3MGCCXY_L1_clean_bin.94.orig.fa_	OEZ008529	yes	LMSG_G000009061.1	OES073669	terpene	Terpene	Antimony	22.06	China: Guizhou, Banpo
L180322-P-5-LD180319-0015_HL3MGCCXY_L1_clean_bin.94.orig.fa_	OEZ008529	yes	LMSG_G000009061.1	OES073669	NRPS-like	NRPS	Antimony	42.48	China: Guizhou, Banpo
L180322-P-5-LD180319-0015_HL3MGCCXY_L1_clean_bin.94.orig.fa_	OEZ008529	yes	LMSG_G000009061.1	OES073669	T1PKS.hglE-KS	PKSother	Antimony	53.42	China: Guizhou, Banpo
L180322-P-5-LD180319-0015_HL3MGCCXY_L1_clean_bin.97.orig.fa_	OEZ008529	no	LMSG_G000010164.1	OES073669	terpene	Terpene	Antimony	7.58	China: Guizhou, Banpo
L180322-P-5-LD180319-0015_HL3MGCCXY_L1_clean_bin.97.orig.fa_	OEZ008529	no	LMSG_G000010164.1	OES073669	terpene	Terpene	Antimony	10.99	China: Guizhou, Banpo
L180322-P-5-LD180319-0015_HL3MGCCXY_L1_clean_bin.97.orig.fa_	OEZ008529	no	LMSG_G000010164.1	OES073669	acyl_amino_acids	Others	Antimony	5.71	China: Guizhou, Banpo
L180322-P-6-LD180319-0016_HL3MGCCXY_L2_clean_bin.10.orig.fa_	OEZ008529	yes	LMSG_G000009229.1	OES073670	hserlactone	Others	Antimony	5.94	China: Guizhou, Banpo
L180322-P-6-LD180319-0016_HL3MGCCXY_L2_clean_bin.10.orig.fa_	OEZ008529	yes	LMSG_G000009229.1	OES073670	hglE-KS	PKSother	Antimony	5.19	China: Guizhou, Banpo
L180322-P-6-LD180319-0016_HL3MGCCXY_L2_clean_bin.10.orig.fa_	OEZ008529	yes	LMSG_G000009229.1	OES073670	hglE-KS	PKSother	Antimony	14.24	China: Guizhou, Banpo
L180322-P-6-LD180319-0016_HL3MGCCXY_L2_clean_bin.10.orig.fa_	OEZ008529	yes	LMSG_G000009229.1	OES073670	terpene	Terpene	Antimony	21.72	China: Guizhou, Banpo
L180322-P-6-LD180319-0016_HL3MGCCXY_L2_clean_bin.10.orig.fa_	OEZ008529	yes	LMSG_G000009229.1	OES073670	bacteriocin	RiPPs	Antimony	10.85	China: Guizhou, Banpo
L180322-P-6-LD180319-0016_HL3MGCCXY_L2_clean_bin.10.orig.fa_	OEZ008529	yes	LMSG_G000009229.1	OES073670	T3PKS	PKSother	Antimony	15.72	China: Guizhou, Banpo
L180322-P-6-LD180319-0016_HL3MGCCXY_L2_clean_bin.10.orig.fa_	OEZ008529	yes	LMSG_G000009229.1	OES073670	terpene	Terpene	Antimony	5.78	China: Guizhou, Banpo
L180322-P-6-LD180319-0016_HL3MGCCXY_L2_clean_bin.100.strict.	OEZ008529	yes	LMSG_G000008543.1	OES073670	betalactone	Others	Antimony	41.46	China: Guizhou, Banpo
L180322-P-6-LD180319-0016_HL3MGCCXY_L2_clean_bin.101.orig.fa	OEZ008529	yes	LMSG_G000010371.1	OES073670	bacteriocin	RiPPs	Antimony	7.04	China: Guizhou, Banpo
L180322-P-6-LD180319-0016_HL3MGCCXY_L2_clean_bin.101.orig.fa	OEZ008529	yes	LMSG_G000010371.1	OES073670	bacteriocin	RiPPs	Antimony	11.81	China: Guizhou, Banpo
L180322-P-6-LD180319-0016_HL3MGCCXY_L2_clean_bin.101.orig.fa	OEZ008529	yes	LMSG_G000010371.1	OES073670	NRPS-like	NRPS	Antimony	6.14	China: Guizhou, Banpo
L180322-P-6-LD180319-0016_HL3MGCCXY_L2_clean_bin.101.orig.fa	OEZ008529	yes	LMSG_G000010371.1	OES073670	terpene	Terpene	Antimony	17.69	China: Guizhou, Banpo
L180322-P-6-LD180319-0016_HL3MGCCXY_L2_clean_bin.101.orig.fa	OEZ008529	yes	LMSG_G000010371.1	OES073670	terpene	Terpene	Antimony	10.15	China: Guizhou, Banpo
L180322-P-6-LD180319-0016_HL3MGCCXY_L2_clean_bin.102.permiss	OEZ008529	yes	LMSG_G000006332.1	OES073670	NRPS-like	NRPS	Antimony	25.24	China: Guizhou, Banpo
L180322-P-6-LD180319-0016_HL3MGCCXY_L2_clean_bin.102.permiss	OEZ008529	yes	LMSG_G000006332.1	OES073670	terpene	Terpene	Antimony	13.51	China: Guizhou, Banpo
L180322-P-6-LD180319-0016_HL3MGCCXY_L2_clean_bin.102.permiss	OEZ008529	yes	LMSG_G000006332.1	OES073670	CDPS	Others	Antimony	8.68	China: Guizhou, Banpo
L180322-P-6-LD180319-0016_HL3MGCCXY_L2_clean_bin.103.orig.fa	OEZ008529	yes	LMSG_G000005389.1	OES073670	T1PKS.hglE-KS	PKSother	Antimony	24.21	China: Guizhou, Banpo
L180322-P-6-LD180319-0016_HL3MGCCXY_L2_clean_bin.103.orig.fa	OEZ008529	yes	LMSG_G000005389.1	OES073670	bacteriocin	RiPPs	Antimony	10.75	China: Guizhou, Banpo
L180322-P-6-LD180319-0016_HL3MGCCXY_L2_clean_bin.104.orig.fa	OEZ008529	yes	LMSG_G000010084.1	OES073670	hglE-KS	PKSother	Antimony	21.44	China: Guizhou, Banpo
L180322-P-6-LD180319-0016_HL3MGCCXY_L2_clean_bin.104.orig.fa	OEZ008529	yes	LMSG_G000010084.1	OES073670	terpene	Terpene	Antimony	12.11	China: Guizhou, Banpo
L180322-P-6-LD180319-0016_HL3MGCCXY_L2_clean_bin.104.orig.fa	OEZ008529	yes	LMSG_G000010084.1	OES073670	acyl_amino_acids	Others	Antimony	33.72	China: Guizhou, Banpo
L180322-P-6-LD180319-0016_HL3MGCCXY_L2_clean_bin.104.orig.fa	OEZ008529	yes	LMSG_G000010084.1	OES073670	acyl_amino_acids	Others	Antimony	8.37	China: Guizhou, Banpo
L180322-P-6-LD180319-0016_HL3MGCCXY_L2_clean_bin.104.orig.fa	OEZ008529	yes	LMSG_G000010084.1	OES073670	NRPS-like	NRPS	Antimony	26.88	China: Guizhou, Banpo
L180322-P-6-LD180319-0016_HL3MGCCXY_L2_clean_bin.104.orig.fa	OEZ008529	yes	LMSG_G000010084.1	OES073670	terpene	Terpene	Antimony	6.71	China: Guizhou, Banpo
L180322-P-6-LD180319-0016_HL3MGCCXY_L2_clean_bin.106.orig.fa	OEZ008529	yes	LMSG_G000005390.1	OES073670	terpene	Terpene	Antimony	10.78	China: Guizhou, Banpo
L180322-P-6-LD180319-0016_HL3MGCCXY_L2_clean_bin.106.orig.fa	OEZ008529	yes	LMSG_G000005390.1	OES073670	NRPS-like	NRPS	Antimony	6.23	China: Guizhou, Banpo
L180322-P-6-LD180319-0016_HL3MGCCXY_L2_clean_bin.11.orig.fa_	OEZ008529	yes	LMSG_G000009098.1	OES073670	bacteriocin	RiPPs	Antimony	6.40	China: Guizhou, Banpo
L180322-P-6-LD180319-0016_HL3MGCCXY_L2_clean_bin.11.orig.fa_	OEZ008529	yes	LMSG_G000009098.1	OES073670	lassopeptide	RiPPs	Antimony	12.03	China: Guizhou, Banpo
L180322-P-6-LD180319-0016_HL3MGCCXY_L2_clean_bin.111.orig.fa	OEZ008529	yes	LMSG_G000008642.1	OES073670	other	Others	Antimony	5.03	China: Guizhou, Banpo
L180322-P-6-LD180319-0016_HL3MGCCXY_L2_clean_bin.112.orig.fa	OEZ008529	yes	LMSG_G000008657.1	OES073670	other	Others	Antimony	10.45	China: Guizhou, Banpo
L180322-P-6-LD180319-0016_HL3MGCCXY_L2_clean_bin.112.orig.fa	OEZ008529	yes	LMSG_G000008657.1	OES073670	other	Others	Antimony	20.49	China: Guizhou, Banpo
L180322-P-6-LD180319-0016_HL3MGCCXY_L2_clean_bin.113.orig.fa	OEZ008529	yes	LMSG_G000008648.1	OES073670	other	Others	Antimony	18.10	China: Guizhou, Banpo
L180322-P-6-LD180319-0016_HL3MGCCXY_L2_clean_bin.114.orig.fa	OEZ008529	yes	LMSG_G000005393.1	OES073670	T3PKS	PKSother	Antimony	19.68	China: Guizhou, Banpo
L180322-P-6-LD180319-0016_HL3MGCCXY_L2_clean_bin.114.orig.fa	OEZ008529	yes	LMSG_G000005393.1	OES073670	terpene	Terpene	Antimony	10.54	China: Guizhou, Banpo
L180322-P-6-LD180319-0016_HL3MGCCXY_L2_clean_bin.12.strict.f	OEZ008529	yes	LMSG_G000009051.1	OES073670	T1PKS	PKSI	Antimony	26.81	China: Guizhou, Banpo
L180322-P-6-LD180319-0016_HL3MGCCXY_L2_clean_bin.12.strict.f	OEZ008529	yes	LMSG_G000009051.1	OES073670	T1PKS	PKSI	Antimony	15.80	China: Guizhou, Banpo
L180322-P-6-LD180319-0016_HL3MGCCXY_L2_clean_bin.12.strict.f	OEZ008529	yes	LMSG_G000009051.1	OES073670	betalactone	Others	Antimony	12.61	China: Guizhou, Banpo
L180322-P-6-LD180319-0016_HL3MGCCXY_L2_clean_bin.13.orig.fa_	OEZ008529	yes	LMSG_G000010763.1	OES073670	acyl_amino_acids	Others	Antimony	34.74	China: Guizhou, Banpo
L180322-P-6-LD180319-0016_HL3MGCCXY_L2_clean_bin.13.orig.fa_	OEZ008529	yes	LMSG_G000010763.1	OES073670	bacteriocin	RiPPs	Antimony	9.22	China: Guizhou, Banpo
L180322-P-6-LD180319-0016_HL3MGCCXY_L2_clean_bin.15.orig.fa_	OEZ008529	yes	LMSG_G000011300.1	OES073670	bacteriocin	RiPPs	Antimony	10.24	China: Guizhou, Banpo
L180322-P-6-LD180319-0016_HL3MGCCXY_L2_clean_bin.15.orig.fa_	OEZ008529	yes	LMSG_G000011300.1	OES073670	bacteriocin	RiPPs	Antimony	10.19	China: Guizhou, Banpo
L180322-P-6-LD180319-0016_HL3MGCCXY_L2_clean_bin.15.orig.fa_	OEZ008529	yes	LMSG_G000011300.1	OES073670	arylpolyene	Others	Antimony	10.27	China: Guizhou, Banpo
L180322-P-6-LD180319-0016_HL3MGCCXY_L2_clean_bin.16.strict.f	OEZ008529	yes	LMSG_G000008830.1	OES073670	T3PKS	PKSother	Antimony	18.64	China: Guizhou, Banpo
L180322-P-6-LD180319-0016_HL3MGCCXY_L2_clean_bin.16.strict.f	OEZ008529	yes	LMSG_G000008830.1	OES073670	terpene	Terpene	Antimony	16.77	China: Guizhou, Banpo
L180322-P-6-LD180319-0016_HL3MGCCXY_L2_clean_bin.16.strict.f	OEZ008529	yes	LMSG_G000008830.1	OES073670	NRPS-like	NRPS	Antimony	13.74	China: Guizhou, Banpo
L180322-P-6-LD180319-0016_HL3MGCCXY_L2_clean_bin.17.strict.f	OEZ008529	yes	LMSG_G000008582.1	OES073670	other	Others	Antimony	22.45	China: Guizhou, Banpo
L180322-P-6-LD180319-0016_HL3MGCCXY_L2_clean_bin.17.strict.f	OEZ008529	yes	LMSG_G000008582.1	OES073670	NRPS-like	NRPS	Antimony	25.74	China: Guizhou, Banpo
L180322-P-6-LD180319-0016_HL3MGCCXY_L2_clean_bin.17.strict.f	OEZ008529	yes	LMSG_G000008582.1	OES073670	other	Others	Antimony	20.76	China: Guizhou, Banpo
L180322-P-6-LD180319-0016_HL3MGCCXY_L2_clean_bin.17.strict.f	OEZ008529	yes	LMSG_G000008582.1	OES073670	other	Others	Antimony	17.08	China: Guizhou, Banpo
L180322-P-6-LD180319-0016_HL3MGCCXY_L2_clean_bin.18.orig.fa_	OEZ008529	yes	LMSG_G000010814.1	OES073670	acyl_amino_acids	Others	Antimony	40.91	China: Guizhou, Banpo
L180322-P-6-LD180319-0016_HL3MGCCXY_L2_clean_bin.19.orig.fa_	OEZ008529	yes	LMSG_G000009058.1	OES073670	terpene	Terpene	Antimony	22.05	China: Guizhou, Banpo
L180322-P-6-LD180319-0016_HL3MGCCXY_L2_clean_bin.19.orig.fa_	OEZ008529	yes	LMSG_G000009058.1	OES073670	NRPS-like	NRPS	Antimony	34.85	China: Guizhou, Banpo
L180322-P-6-LD180319-0016_HL3MGCCXY_L2_clean_bin.19.orig.fa_	OEZ008529	yes	LMSG_G000009058.1	OES073670	lassopeptide	RiPPs	Antimony	25.70	China: Guizhou, Banpo
L180322-P-6-LD180319-0016_HL3MGCCXY_L2_clean_bin.22.orig.fa_	OEZ008529	yes	LMSG_G000005396.1	OES073670	T3PKS	PKSother	Antimony	13.75	China: Guizhou, Banpo
L180322-P-6-LD180319-0016_HL3MGCCXY_L2_clean_bin.22.orig.fa_	OEZ008529	yes	LMSG_G000005396.1	OES073670	NRPS-like	NRPS	Antimony	33.14	China: Guizhou, Banpo
L180322-P-6-LD180319-0016_HL3MGCCXY_L2_clean_bin.23.orig.fa_	OEZ008529	yes	LMSG_G000010103.1	OES073670	T3PKS	PKSother	Antimony	14.35	China: Guizhou, Banpo
L180322-P-6-LD180319-0016_HL3MGCCXY_L2_clean_bin.23.orig.fa_	OEZ008529	yes	LMSG_G000010103.1	OES073670	terpene	Terpene	Antimony	15.31	China: Guizhou, Banpo
L180322-P-6-LD180319-0016_HL3MGCCXY_L2_clean_bin.23.orig.fa_	OEZ008529	yes	LMSG_G000010103.1	OES073670	terpene	Terpene	Antimony	21.40	China: Guizhou, Banpo
L180322-P-6-LD180319-0016_HL3MGCCXY_L2_clean_bin.23.orig.fa_	OEZ008529	yes	LMSG_G000010103.1	OES073670	NRPS-like	NRPS	Antimony	29.51	China: Guizhou, Banpo
L180322-P-6-LD180319-0016_HL3MGCCXY_L2_clean_bin.23.orig.fa_	OEZ008529	yes	LMSG_G000010103.1	OES073670	terpene	Terpene	Antimony	21.96	China: Guizhou, Banpo
L180322-P-6-LD180319-0016_HL3MGCCXY_L2_clean_bin.23.orig.fa_	OEZ008529	yes	LMSG_G000010103.1	OES073670	bacteriocin	RiPPs	Antimony	10.11	China: Guizhou, Banpo
L180322-P-6-LD180319-0016_HL3MGCCXY_L2_clean_bin.23.orig.fa_	OEZ008529	yes	LMSG_G000010103.1	OES073670	LAP.bacteriocin	RiPPs	Antimony	11.55	China: Guizhou, Banpo
L180322-P-6-LD180319-0016_HL3MGCCXY_L2_clean_bin.24.permissi	OEZ008529	yes	LMSG_G000006333.1	OES073670	bacteriocin	RiPPs	Antimony	10.86	China: Guizhou, Banpo
L180322-P-6-LD180319-0016_HL3MGCCXY_L2_clean_bin.24.permissi	OEZ008529	yes	LMSG_G000006333.1	OES073670	NRPS-like	NRPS	Antimony	42.82	China: Guizhou, Banpo
L180322-P-6-LD180319-0016_HL3MGCCXY_L2_clean_bin.24.permissi	OEZ008529	yes	LMSG_G000006333.1	OES073670	bacteriocin	RiPPs	Antimony	10.24	China: Guizhou, Banpo
L180322-P-6-LD180319-0016_HL3MGCCXY_L2_clean_bin.24.permissi	OEZ008529	yes	LMSG_G000006333.1	OES073670	terpene	Terpene	Antimony	20.86	China: Guizhou, Banpo
L180322-P-6-LD180319-0016_HL3MGCCXY_L2_clean_bin.24.permissi	OEZ008529	yes	LMSG_G000006333.1	OES073670	NRPS-like	NRPS	Antimony	44.12	China: Guizhou, Banpo
L180322-P-6-LD180319-0016_HL3MGCCXY_L2_clean_bin.24.permissi	OEZ008529	yes	LMSG_G000006333.1	OES073670	CDPS	Others	Antimony	20.71	China: Guizhou, Banpo
L180322-P-6-LD180319-0016_HL3MGCCXY_L2_clean_bin.24.permissi	OEZ008529	yes	LMSG_G000006333.1	OES073670	terpene	Terpene	Antimony	21.00	China: Guizhou, Banpo
L180322-P-6-LD180319-0016_HL3MGCCXY_L2_clean_bin.24.permissi	OEZ008529	yes	LMSG_G000006333.1	OES073670	terpene	Terpene	Antimony	20.86	China: Guizhou, Banpo
L180322-P-6-LD180319-0016_HL3MGCCXY_L2_clean_bin.24.permissi	OEZ008529	yes	LMSG_G000006333.1	OES073670	T3PKS.bacteriocin	Others	Antimony	36.19	China: Guizhou, Banpo
L180322-P-6-LD180319-0016_HL3MGCCXY_L2_clean_bin.24.permissi	OEZ008529	yes	LMSG_G000006333.1	OES073670	TfuA-related	RiPPs	Antimony	14.51	China: Guizhou, Banpo
L180322-P-6-LD180319-0016_HL3MGCCXY_L2_clean_bin.24.permissi	OEZ008529	yes	LMSG_G000006333.1	OES073670	transAT-PKS-like.NRPS-like	PKS-NRP_Hybrids	Antimony	11.18	China: Guizhou, Banpo
L180322-P-6-LD180319-0016_HL3MGCCXY_L2_clean_bin.24.permissi	OEZ008529	yes	LMSG_G000006333.1	OES073670	transAT-PKS-like	PKSother	Antimony	9.11	China: Guizhou, Banpo
L180322-P-6-LD180319-0016_HL3MGCCXY_L2_clean_bin.24.permissi	OEZ008529	yes	LMSG_G000006333.1	OES073670	transAT-PKS	PKSother	Antimony	8.95	China: Guizhou, Banpo
L180322-P-6-LD180319-0016_HL3MGCCXY_L2_clean_bin.29.strict.f	OEZ008529	yes	LMSG_G000009270.1	OES073670	terpene	Terpene	Antimony	20.83	China: Guizhou, Banpo
L180322-P-6-LD180319-0016_HL3MGCCXY_L2_clean_bin.29.strict.f	OEZ008529	yes	LMSG_G000009270.1	OES073670	T3PKS	PKSother	Antimony	21.45	China: Guizhou, Banpo
L180322-P-6-LD180319-0016_HL3MGCCXY_L2_clean_bin.3.strict.fa	OEZ008529	yes	LMSG_G000005401.1	OES073670	T1PKS.hglE-KS	PKSother	Antimony	45.12	China: Guizhou, Banpo
L180322-P-6-LD180319-0016_HL3MGCCXY_L2_clean_bin.3.strict.fa	OEZ008529	yes	LMSG_G000005401.1	OES073670	arylpolyene	Others	Antimony	58.59	China: Guizhou, Banpo
L180322-P-6-LD180319-0016_HL3MGCCXY_L2_clean_bin.3.strict.fa	OEZ008529	yes	LMSG_G000005401.1	OES073670	terpene	Terpene	Antimony	22.05	China: Guizhou, Banpo
L180322-P-6-LD180319-0016_HL3MGCCXY_L2_clean_bin.3.strict.fa	OEZ008529	yes	LMSG_G000005401.1	OES073670	lassopeptide	RiPPs	Antimony	29.97	China: Guizhou, Banpo
L180322-P-6-LD180319-0016_HL3MGCCXY_L2_clean_bin.3.strict.fa	OEZ008529	yes	LMSG_G000005401.1	OES073670	NRPS-like	NRPS	Antimony	24.98	China: Guizhou, Banpo
L180322-P-6-LD180319-0016_HL3MGCCXY_L2_clean_bin.33.orig.fa_	OEZ008529	no	LMSG_G000010377.1	OES073670	hserlactone	Others	Antimony	11.94	China: Guizhou, Banpo
L180322-P-6-LD180319-0016_HL3MGCCXY_L2_clean_bin.33.orig.fa_	OEZ008529	yes	LMSG_G000010377.1	OES073670	terpene	Terpene	Antimony	18.58	China: Guizhou, Banpo
L180322-P-6-LD180319-0016_HL3MGCCXY_L2_clean_bin.33.orig.fa_	OEZ008529	yes	LMSG_G000010377.1	OES073670	bacteriocin	RiPPs	Antimony	7.44	China: Guizhou, Banpo
L180322-P-6-LD180319-0016_HL3MGCCXY_L2_clean_bin.33.orig.fa_	OEZ008529	yes	LMSG_G000010377.1	OES073670	betalactone	Others	Antimony	22.45	China: Guizhou, Banpo
L180322-P-6-LD180319-0016_HL3MGCCXY_L2_clean_bin.33.orig.fa_	OEZ008529	yes	LMSG_G000010377.1	OES073670	T3PKS.hserlactone	Others	Antimony	16.85	China: Guizhou, Banpo
L180322-P-6-LD180319-0016_HL3MGCCXY_L2_clean_bin.35.orig.fa_	OEZ008529	yes	LMSG_G000010399.1	OES073670	terpene	Terpene	Antimony	14.09	China: Guizhou, Banpo
L180322-P-6-LD180319-0016_HL3MGCCXY_L2_clean_bin.35.orig.fa_	OEZ008529	yes	LMSG_G000010399.1	OES073670	NRPS-like	NRPS	Antimony	8.65	China: Guizhou, Banpo
L180322-P-6-LD180319-0016_HL3MGCCXY_L2_clean_bin.35.orig.fa_	OEZ008529	yes	LMSG_G000010399.1	OES073670	betalactone	Others	Antimony	27.01	China: Guizhou, Banpo
L180322-P-6-LD180319-0016_HL3MGCCXY_L2_clean_bin.35.orig.fa_	OEZ008529	yes	LMSG_G000010399.1	OES073670	bacteriocin	RiPPs	Antimony	10.27	China: Guizhou, Banpo
L180322-P-6-LD180319-0016_HL3MGCCXY_L2_clean_bin.35.orig.fa_	OEZ008529	yes	LMSG_G000010399.1	OES073670	terpene	Terpene	Antimony	11.90	China: Guizhou, Banpo
L180322-P-6-LD180319-0016_HL3MGCCXY_L2_clean_bin.35.orig.fa_	OEZ008529	yes	LMSG_G000010399.1	OES073670	T3PKS	PKSother	Antimony	6.83	China: Guizhou, Banpo
L180322-P-6-LD180319-0016_HL3MGCCXY_L2_clean_bin.35.orig.fa_	OEZ008529	yes	LMSG_G000010399.1	OES073670	NRPS-like	NRPS	Antimony	30.91	China: Guizhou, Banpo
L180322-P-6-LD180319-0016_HL3MGCCXY_L2_clean_bin.36.orig.fa_	OEZ008529	yes	LMSG_G000005399.1	OES073670	T1PKS.hglE-KS	PKSother	Antimony	15.88	China: Guizhou, Banpo
L180322-P-6-LD180319-0016_HL3MGCCXY_L2_clean_bin.36.orig.fa_	OEZ008529	no	LMSG_G000005399.1	OES073670	T1PKS.hglE-KS	PKSother	Antimony	9.56	China: Guizhou, Banpo
L180322-P-6-LD180319-0016_HL3MGCCXY_L2_clean_bin.36.orig.fa_	OEZ008529	yes	LMSG_G000005399.1	OES073670	terpene	Terpene	Antimony	9.41	China: Guizhou, Banpo
L180322-P-6-LD180319-0016_HL3MGCCXY_L2_clean_bin.36.orig.fa_	OEZ008529	yes	LMSG_G000005399.1	OES073670	hglE-KS	PKSother	Antimony	7.81	China: Guizhou, Banpo
L180322-P-6-LD180319-0016_HL3MGCCXY_L2_clean_bin.36.orig.fa_	OEZ008529	yes	LMSG_G000005399.1	OES073670	terpene	Terpene	Antimony	10.45	China: Guizhou, Banpo
L180322-P-6-LD180319-0016_HL3MGCCXY_L2_clean_bin.37.orig.fa_	OEZ008529	yes	LMSG_G000010360.1	OES073670	bacteriocin	RiPPs	Antimony	9.62	China: Guizhou, Banpo
L180322-P-6-LD180319-0016_HL3MGCCXY_L2_clean_bin.37.orig.fa_	OEZ008529	yes	LMSG_G000010360.1	OES073670	terpene	Terpene	Antimony	20.85	China: Guizhou, Banpo
L180322-P-6-LD180319-0016_HL3MGCCXY_L2_clean_bin.37.orig.fa_	OEZ008529	yes	LMSG_G000010360.1	OES073670	NRPS-like	NRPS	Antimony	23.87	China: Guizhou, Banpo
L180322-P-6-LD180319-0016_HL3MGCCXY_L2_clean_bin.37.orig.fa_	OEZ008529	yes	LMSG_G000010360.1	OES073670	NRPS	NRPS	Antimony	36.94	China: Guizhou, Banpo
L180322-P-6-LD180319-0016_HL3MGCCXY_L2_clean_bin.37.orig.fa_	OEZ008529	yes	LMSG_G000010360.1	OES073670	terpene	Terpene	Antimony	14.62	China: Guizhou, Banpo
L180322-P-6-LD180319-0016_HL3MGCCXY_L2_clean_bin.37.orig.fa_	OEZ008529	yes	LMSG_G000010360.1	OES073670	terpene	Terpene	Antimony	14.04	China: Guizhou, Banpo
L180322-P-6-LD180319-0016_HL3MGCCXY_L2_clean_bin.39.strict.f	OEZ008529	yes	LMSG_G000005400.1	OES073670	terpene	Terpene	Antimony	14.16	China: Guizhou, Banpo
L180322-P-6-LD180319-0016_HL3MGCCXY_L2_clean_bin.39.strict.f	OEZ008529	yes	LMSG_G000005400.1	OES073670	terpene	Terpene	Antimony	21.98	China: Guizhou, Banpo
L180322-P-6-LD180319-0016_HL3MGCCXY_L2_clean_bin.39.strict.f	OEZ008529	yes	LMSG_G000005400.1	OES073670	T1PKS	PKSI	Antimony	36.20	China: Guizhou, Banpo

L180322-P-6-LD180319-0016_HL3MGCCXY_L2_clean_bin.4.orig.fa_k	OEZ008529	yes	LMSG_G000010630.1	OES073670	bacteriocin	RiPPs	Antimony	10.99	China: Guizhou, Banpo
L180322-P-6-LD180319-0016_HL3MGCCXY_L2_clean_bin.40.orig.fa_	OEZ008529	yes	LMSG_G000009062.1	OES073670	terpene	Terpene	Antimony	22.06	China: Guizhou, Banpo
L180322-P-6-LD180319-0016_HL3MGCCXY_L2_clean_bin.40.orig.fa_	OEZ008529	yes	LMSG_G000009062.1	OES073670	NRPS-like	NRPS	Antimony	42.48	China: Guizhou, Banpo
L180322-P-6-LD180319-0016_HL3MGCCXY_L2_clean_bin.40.orig.fa_	OEZ008529	yes	LMSG_G000009062.1	OES073670	hglE-KS	PKSother	Antimony	23.61	China: Guizhou, Banpo
L180322-P-6-LD180319-0016_HL3MGCCXY_L2_clean_bin.40.orig.fa_	OEZ008529	yes	LMSG_G000009062.1	OES073670	T1PKS.hglE-KS	PKSother	Antimony	29.85	China: Guizhou, Banpo
L180322-P-6-LD180319-0016_HL3MGCCXY_L2_clean_bin.41.strict.f	OEZ008529	yes	LMSG_G000005402.1	OES073670	terpene	Terpene	Antimony	11.74	China: Guizhou, Banpo
L180322-P-6-LD180319-0016_HL3MGCCXY_L2_clean_bin.41.strict.f	OEZ008529	yes	LMSG_G000005402.1	OES073670	T1PKS.hglE-KS	PKSother	Antimony	5.16	China: Guizhou, Banpo
L180322-P-6-LD180319-0016_HL3MGCCXY_L2_clean_bin.43.permissi	OEZ008529	yes	LMSG_G000008751.1	OES073670	terpene	Terpene	Antimony	8.42	China: Guizhou, Banpo
L180322-P-6-LD180319-0016_HL3MGCCXY_L2_clean_bin.44.strict.f	OEZ008529	yes	LMSG_G000008855.1	OES073670	terpene	Terpene	Antimony	7.07	China: Guizhou, Banpo
L180322-P-6-LD180319-0016_HL3MGCCXY_L2_clean_bin.46.orig.fa_	OEZ008529	yes	LMSG_G000010519.1	OES073670	NRPS-like	NRPS	Antimony	14.39	China: Guizhou, Banpo
L180322-P-6-LD180319-0016_HL3MGCCXY_L2_clean_bin.46.orig.fa_	OEZ008529	yes	LMSG_G000010519.1	OES073670	arylpolyene	Others	Antimony	41.19	China: Guizhou, Banpo
L180322-P-6-LD180319-0016_HL3MGCCXY_L2_clean_bin.46.orig.fa_	OEZ008529	yes	LMSG_G000010519.1	OES073670	acyl_amino_acids	Others	Antimony	26.83	China: Guizhou, Banpo
L180322-P-6-LD180319-0016_HL3MGCCXY_L2_clean_bin.47.strict.f	OEZ008529	yes	LMSG_G000009234.1	OES073670	T3PKS.terpene	Others	Antimony	22.83	China: Guizhou, Banpo
L180322-P-6-LD180319-0016_HL3MGCCXY_L2_clean_bin.47.strict.f	OEZ008529	yes	LMSG_G000009234.1	OES073670	terpene	Terpene	Antimony	5.24	China: Guizhou, Banpo
L180322-P-6-LD180319-0016_HL3MGCCXY_L2_clean_bin.49.orig.fa_	OEZ008529	yes	LMSG_G000011305.1	OES073670	betalactone	Others	Antimony	11.85	China: Guizhou, Banpo
L180322-P-6-LD180319-0016_HL3MGCCXY_L2_clean_bin.49.orig.fa_	OEZ008529	yes	LMSG_G000011305.1	OES073670	ladderane	Others	Antimony	25.56	China: Guizhou, Banpo
L180322-P-6-LD180319-0016_HL3MGCCXY_L2_clean_bin.49.orig.fa_	OEZ008529	yes	LMSG_G000011305.1	OES073670	terpene	Terpene	Antimony	21.02	China: Guizhou, Banpo
L180322-P-6-LD180319-0016_HL3MGCCXY_L2_clean_bin.49.orig.fa_	OEZ008529	yes	LMSG_G000011305.1	OES073670	terpene	Terpene	Antimony	9.23	China: Guizhou, Banpo
L180322-P-6-LD180319-0016_HL3MGCCXY_L2_clean_bin.49.orig.fa_	OEZ008529	yes	LMSG_G000011305.1	OES073670	terpene	Terpene	Antimony	12.57	China: Guizhou, Banpo
L180322-P-6-LD180319-0016_HL3MGCCXY_L2_clean_bin.49.orig.fa_	OEZ008529	yes	LMSG_G000011305.1	OES073670	terpene	Terpene	Antimony	15.43	China: Guizhou, Banpo
L180322-P-6-LD180319-0016_HL3MGCCXY_L2_clean_bin.5.orig.fa_k	OEZ008529	yes	LMSG_G000006233.1	OES073670	terpene	Terpene	Antimony	20.58	China: Guizhou, Banpo
L180322-P-6-LD180319-0016_HL3MGCCXY_L2_clean_bin.5.orig.fa_k	OEZ008529	yes	LMSG_G000006233.1	OES073670	NRPS-like	NRPS	Antimony	28.77	China: Guizhou, Banpo
L180322-P-6-LD180319-0016_HL3MGCCXY_L2_clean_bin.5.orig.fa_k	OEZ008529	yes	LMSG_G000006233.1	OES073670	terpene	Terpene	Antimony	5.47	China: Guizhou, Banpo
L180322-P-6-LD180319-0016_HL3MGCCXY_L2_clean_bin.50.orig.fa_	OEZ008529	yes	LMSG_G000010196.1	OES073670	terpene	Terpene	Antimony	21.90	China: Guizhou, Banpo
L180322-P-6-LD180319-0016_HL3MGCCXY_L2_clean_bin.51.strict.f	OEZ008529	yes	LMSG_G000005404.1	OES073670	bacteriocin	RiPPs	Antimony	7.10	China: Guizhou, Banpo
L180322-P-6-LD180319-0016_HL3MGCCXY_L2_clean_bin.52.orig.fa_	OEZ008529	yes	LMSG_G000010347.1	OES073670	bacteriocin	RiPPs	Antimony	10.94	China: Guizhou, Banpo
L180322-P-6-LD180319-0016_HL3MGCCXY_L2_clean_bin.52.orig.fa_	OEZ008529	yes	LMSG_G000010347.1	OES073670	terpene	Terpene	Antimony	21.72	China: Guizhou, Banpo
L180322-P-6-LD180319-0016_HL3MGCCXY_L2_clean_bin.52.orig.fa_	OEZ008529	yes	LMSG_G000010347.1	OES073670	hserlactone	Others	Antimony	16.11	China: Guizhou, Banpo
L180322-P-6-LD180319-0016_HL3MGCCXY_L2_clean_bin.52.orig.fa_	OEZ008529	yes	LMSG_G000010347.1	OES073670	terpene	Terpene	Antimony	15.19	China: Guizhou, Banpo
L180322-P-6-LD180319-0016_HL3MGCCXY_L2_clean_bin.52.orig.fa_	OEZ008529	yes	LMSG_G000010347.1	OES073670	NRPS-like	NRPS	Antimony	44.07	China: Guizhou, Banpo
L180322-P-6-LD180319-0016_HL3MGCCXY_L2_clean_bin.52.orig.fa_	OEZ008529	yes	LMSG_G000010347.1	OES073670	T3PKS	PKSother	Antimony	41.07	China: Guizhou, Banpo
L180322-P-6-LD180319-0016_HL3MGCCXY_L2_clean_bin.52.orig.fa_	OEZ008529	yes	LMSG_G000010347.1	OES073670	terpene	Terpene	Antimony	21.92	China: Guizhou, Banpo
L180322-P-6-LD180319-0016_HL3MGCCXY_L2_clean_bin.52.orig.fa_	OEZ008529	yes	LMSG_G000010347.1	OES073670	NRPS-like	NRPS	Antimony	15.79	China: Guizhou, Banpo
L180322-P-6-LD180319-0016_HL3MGCCXY_L2_clean_bin.55.strict.f	OEZ008529	yes	LMSG_G000009677.1	OES073670	T1PKS	PKSI	Antimony	15.31	China: Guizhou, Banpo
L180322-P-6-LD180319-0016_HL3MGCCXY_L2_clean_bin.55.strict.f	OEZ008529	yes	LMSG_G000009677.1	OES073670	terpene	Terpene	Antimony	10.54	China: Guizhou, Banpo
L180322-P-6-LD180319-0016_HL3MGCCXY_L2_clean_bin.56.orig.fa_	OEZ008529	yes	LMSG_G000005405.1	OES073670	terpene	Terpene	Antimony	20.86	China: Guizhou, Banpo
L180322-P-6-LD180319-0016_HL3MGCCXY_L2_clean_bin.57.strict.f	OEZ008529	yes	LMSG_G000009755.1	OES073670	thiopeptide.LAP	RiPPs	Antimony	21.82	China: Guizhou, Banpo
L180322-P-6-LD180319-0016_HL3MGCCXY_L2_clean_bin.58.orig.fa_	OEZ008529	yes	LMSG_G000009101.1	OES073670	terpene	Terpene	Antimony	25.01	China: Guizhou, Banpo
L180322-P-6-LD180319-0016_HL3MGCCXY_L2_clean_bin.58.orig.fa_	OEZ008529	yes	LMSG_G000009101.1	OES073670	bacteriocin	RiPPs	Antimony	10.84	China: Guizhou, Banpo
L180322-P-6-LD180319-0016_HL3MGCCXY_L2_clean_bin.58.orig.fa_	OEZ008529	yes	LMSG_G000009101.1	OES073670	T3PKS.NRPS-like	PKS-NRP_Hybrids	Antimony	50.93	China: Guizhou, Banpo
L180322-P-6-LD180319-0016_HL3MGCCXY_L2_clean_bin.58.orig.fa_	OEZ008529	yes	LMSG_G000009101.1	OES073670	NRPS-like	NRPS	Antimony	29.39	China: Guizhou, Banpo
L180322-P-6-LD180319-0016_HL3MGCCXY_L2_clean_bin.58.orig.fa_	OEZ008529	yes	LMSG_G000009101.1	OES073670	lassopeptide	RiPPs	Antimony	14.35	China: Guizhou, Banpo
L180322-P-6-LD180319-0016_HL3MGCCXY_L2_clean_bin.60.orig.fa_	OEZ008529	yes	LMSG_G000010395.1	OES073670	lassopeptide	RiPPs	Antimony	6.31	China: Guizhou, Banpo
L180322-P-6-LD180319-0016_HL3MGCCXY_L2_clean_bin.60.orig.fa_	OEZ008529	yes	LMSG_G000010395.1	OES073670	NRPS-like	NRPS	Antimony	9.08	China: Guizhou, Banpo
L180322-P-6-LD180319-0016_HL3MGCCXY_L2_clean_bin.60.orig.fa_	OEZ008529	yes	LMSG_G000010395.1	OES073670	arylpolyene	Others	Antimony	15.26	China: Guizhou, Banpo
L180322-P-6-LD180319-0016_HL3MGCCXY_L2_clean_bin.60.orig.fa_	OEZ008529	yes	LMSG_G000010395.1	OES073670	bacteriocin	RiPPs	Antimony	5.26	China: Guizhou, Banpo
L180322-P-6-LD180319-0016_HL3MGCCXY_L2_clean_bin.60.orig.fa_	OEZ008529	yes	LMSG_G000010395.1	OES073670	hserlactone	Others	Antimony	12.64	China: Guizhou, Banpo
L180322-P-6-LD180319-0016_HL3MGCCXY_L2_clean_bin.60.orig.fa_	OEZ008529	yes	LMSG_G000010395.1	OES073670	hserlactone	Others	Antimony	12.88	China: Guizhou, Banpo
L180322-P-6-LD180319-0016_HL3MGCCXY_L2_clean_bin.60.orig.fa_	OEZ008529	yes	LMSG_G000010395.1	OES073670	betalactone	Others	Antimony	12.34	China: Guizhou, Banpo
L180322-P-6-LD180319-0016_HL3MGCCXY_L2_clean_bin.61.orig.fa_	OEZ008529	yes	LMSG_G000008578.1	OES073670	other	Others	Antimony	26.10	China: Guizhou, Banpo
L180322-P-6-LD180319-0016_HL3MGCCXY_L2_clean_bin.61.orig.fa_	OEZ008529	yes	LMSG_G000008578.1	OES073670	NRPS-like	NRPS	Antimony	32.82	China: Guizhou, Banpo
L180322-P-6-LD180319-0016_HL3MGCCXY_L2_clean_bin.62.orig.fa_	OEZ008529	yes	LMSG_G000011102.1	OES073670	terpene	Terpene	Antimony	17.72	China: Guizhou, Banpo
L180322-P-6-LD180319-0016_HL3MGCCXY_L2_clean_bin.62.orig.fa_	OEZ008529	yes	LMSG_G000011102.1	OES073670	bacteriocin	RiPPs	Antimony	10.93	China: Guizhou, Banpo
L180322-P-6-LD180319-0016_HL3MGCCXY_L2_clean_bin.62.orig.fa_	OEZ008529	yes	LMSG_G000011102.1	OES073670	betalactone	Others	Antimony	29.38	China: Guizhou, Banpo
L180322-P-6-LD180319-0016_HL3MGCCXY_L2_clean_bin.62.orig.fa_	OEZ008529	yes	LMSG_G000011102.1	OES073670	T1PKS.NRPS-like	PKS-NRP_Hybrids	Antimony	25.05	China: Guizhou, Banpo
L180322-P-6-LD180319-0016_HL3MGCCXY_L2_clean_bin.62.orig.fa_	OEZ008529	yes	LMSG_G000011102.1	OES073670	NRPS	NRPS	Antimony	23.80	China: Guizhou, Banpo
L180322-P-6-LD180319-0016_HL3MGCCXY_L2_clean_bin.62.orig.fa_	OEZ008529	yes	LMSG_G000011102.1	OES073670	NRPS	NRPS	Antimony	46.83	China: Guizhou, Banpo
L180322-P-6-LD180319-0016_HL3MGCCXY_L2_clean_bin.62.orig.fa_	OEZ008529	yes	LMSG_G000011102.1	OES073670	NRPS	NRPS	Antimony	24.67	China: Guizhou, Banpo
L180322-P-6-LD180319-0016_HL3MGCCXY_L2_clean_bin.63.orig.fa_	OEZ008529	yes	LMSG_G000007950.1	OES073670	NRPS-like	NRPS	Antimony	23.23	China: Guizhou, Banpo
L180322-P-6-LD180319-0016_HL3MGCCXY_L2_clean_bin.63.orig.fa_	OEZ008529	yes	LMSG_G000007950.1	OES073670	NRPS-like	NRPS	Antimony	15.35	China: Guizhou, Banpo
L180322-P-6-LD180319-0016_HL3MGCCXY_L2_clean_bin.63.orig.fa_	OEZ008529	yes	LMSG_G000007950.1	OES073670	T3PKS	PKSother	Antimony	8.66	China: Guizhou, Banpo
L180322-P-6-LD180319-0016_HL3MGCCXY_L2_clean_bin.63.orig.fa_	OEZ008529	yes	LMSG_G000007950.1	OES073670	terpene	Terpene	Antimony	10.56	China: Guizhou, Banpo
L180322-P-6-LD180319-0016_HL3MGCCXY_L2_clean_bin.64.orig.fa_	OEZ008529	yes	LMSG_G000010119.1	OES073670	terpene	Terpene	Antimony	21.91	China: Guizhou, Banpo
L180322-P-6-LD180319-0016_HL3MGCCXY_L2_clean_bin.64.orig.fa_	OEZ008529	yes	LMSG_G000010119.1	OES073670	hserlactone	Others	Antimony	15.09	China: Guizhou, Banpo
L180322-P-6-LD180319-0016_HL3MGCCXY_L2_clean_bin.64.orig.fa_	OEZ008529	yes	LMSG_G000010119.1	OES073670	terpene	Terpene	Antimony	19.16	China: Guizhou, Banpo
L180322-P-6-LD180319-0016_HL3MGCCXY_L2_clean_bin.64.orig.fa_	OEZ008529	yes	LMSG_G000010119.1	OES073670	T3PKS	PKSother	Antimony	41.08	China: Guizhou, Banpo
L180322-P-6-LD180319-0016_HL3MGCCXY_L2_clean_bin.64.orig.fa_	OEZ008529	yes	LMSG_G000010119.1	OES073670	terpene	Terpene	Antimony	21.70	China: Guizhou, Banpo
L180322-P-6-LD180319-0016_HL3MGCCXY_L2_clean_bin.66.orig.fa_	OEZ008529	yes	LMSG_G000005406.1	OES073670	lanthipeptide	RiPPs	Antimony	14.99	China: Guizhou, Banpo
L180322-P-6-LD180319-0016_HL3MGCCXY_L2_clean_bin.66.orig.fa_	OEZ008529	yes	LMSG_G000005406.1	OES073670	NRPS.transAT-PKS	PKS-NRP_Hybrids	Antimony	54.77	China: Guizhou, Banpo
L180322-P-6-LD180319-0016_HL3MGCCXY_L2_clean_bin.66.orig.fa_	OEZ008529	yes	LMSG_G000005406.1	OES073670	thiopeptide.LAP	RiPPs	Antimony	17.19	China: Guizhou, Banpo
L180322-P-6-LD180319-0016_HL3MGCCXY_L2_clean_bin.67.orig.fa_	OEZ008529	yes	LMSG_G000009496.1	OES073670	TfuA-related	RiPPs	Antimony	15.92	China: Guizhou, Banpo
L180322-P-6-LD180319-0016_HL3MGCCXY_L2_clean_bin.67.orig.fa_	OEZ008529	yes	LMSG_G000009496.1	OES073670	T3PKS	PKSother	Antimony	25.67	China: Guizhou, Banpo
L180322-P-6-LD180319-0016_HL3MGCCXY_L2_clean_bin.67.orig.fa_	OEZ008529	yes	LMSG_G000009496.1	OES073670	T1PKS.hglE-KS	PKSother	Antimony	34.47	China: Guizhou, Banpo
L180322-P-6-LD180319-0016_HL3MGCCXY_L2_clean_bin.67.orig.fa_	OEZ008529	yes	LMSG_G000009496.1	OES073670	bacteriocin	RiPPs	Antimony	10.52	China: Guizhou, Banpo
L180322-P-6-LD180319-0016_HL3MGCCXY_L2_clean_bin.69.orig.fa_	OEZ008529	yes	LMSG_G000008702.1	OES073670	betalactone	Others	Antimony	11.28	China: Guizhou, Banpo
L180322-P-6-LD180319-0016_HL3MGCCXY_L2_clean_bin.7.strict.fa	OEZ008529	yes	LMSG_G000006334.1	OES073670	terpene	Terpene	Antimony	20.80	China: Guizhou, Banpo
L180322-P-6-LD180319-0016_HL3MGCCXY_L2_clean_bin.7.strict.fa	OEZ008529	yes	LMSG_G000006334.1	OES073670	TfuA-related	RiPPs	Antimony	19.12	China: Guizhou, Banpo
L180322-P-6-LD180319-0016_HL3MGCCXY_L2_clean_bin.7.strict.fa	OEZ008529	yes	LMSG_G000006334.1	OES073670	bacteriocin	RiPPs	Antimony	10.89	China: Guizhou, Banpo
L180322-P-6-LD180319-0016_HL3MGCCXY_L2_clean_bin.7.strict.fa	OEZ008529	yes	LMSG_G000006334.1	OES073670	NRPS	NRPS	Antimony	25.20	China: Guizhou, Banpo
L180322-P-6-LD180319-0016_HL3MGCCXY_L2_clean_bin.7.strict.fa	OEZ008529	yes	LMSG_G000006334.1	OES073670	CDPS	Others	Antimony	13.31	China: Guizhou, Banpo
L180322-P-6-LD180319-0016_HL3MGCCXY_L2_clean_bin.71.orig.fa_	OEZ008529	yes	LMSG_G000011337.1	OES073670	arylpolyene	Others	Antimony	41.10	China: Guizhou, Banpo
L180322-P-6-LD180319-0016_HL3MGCCXY_L2_clean_bin.71.orig.fa_	OEZ008529	yes	LMSG_G000011337.1	OES073670	NRPS-like	NRPS	Antimony	10.70	China: Guizhou, Banpo
L180322-P-6-LD180319-0016_HL3MGCCXY_L2_clean_bin.72.strict.f	OEZ008529	no	LMSG_G000010526.1	OES073670	terpene	Terpene	Antimony	15.73	China: Guizhou, Banpo
L180322-P-6-LD180319-0016_HL3MGCCXY_L2_clean_bin.72.strict.f	OEZ008529	yes	LMSG_G000010526.1	OES073670	hserlactone	Others	Antimony	12.28	China: Guizhou, Banpo
L180322-P-6-LD180319-0016_HL3MGCCXY_L2_clean_bin.73.strict.f	OEZ008529	yes	LMSG_G000007944.1	OES073670	arylpolyene	Others	Antimony	21.94	China: Guizhou, Banpo
L180322-P-6-LD180319-0016_HL3MGCCXY_L2_clean_bin.73.strict.f	OEZ008529	yes	LMSG_G000007944.1	OES073670	bacteriocin	RiPPs	Antimony	6.48	China: Guizhou, Banpo
L180322-P-6-LD180319-0016_HL3MGCCXY_L2_clean_bin.73.strict.f	OEZ008529	yes	LMSG_G000007944.1	OES073670	terpene	Terpene	Antimony	21.08	China: Guizhou, Banpo
L180322-P-6-LD180319-0016_HL3MGCCXY_L2_clean_bin.73.strict.f	OEZ008529	yes	LMSG_G000007944.1	OES073670	hglE-KS	PKSother	Antimony	44.33	China: Guizhou, Banpo
L180322-P-6-LD180319-0016_HL3MGCCXY_L2_clean_bin.73.strict.f	OEZ008529	yes	LMSG_G000007944.1	OES073670	ladderane	Others	Antimony	19.79	China: Guizhou, Banpo
L180322-P-6-LD180319-0016_HL3MGCCXY_L2_clean_bin.73.strict.f	OEZ008529	yes	LMSG_G000007944.1	OES073670	T3PKS	PKSother	Antimony	12.42	China: Guizhou, Banpo
L180322-P-6-LD180319-0016_HL3MGCCXY_L2_clean_bin.74.orig.fa_	OEZ008529	yes	LMSG_G000008772.1	OES073670	terpene	Terpene	Antimony	11.32	China: Guizhou, Banpo
L180322-P-6-LD180319-0016_HL3MGCCXY_L2_clean_bin.74.orig.fa_	OEZ008529	yes	LMSG_G000008772.1	OES073670	NRPS-like	NRPS	Antimony	16.05	China: Guizhou, Banpo
L180322-P-6-LD180319-0016_HL3MGCCXY_L2_clean_bin.74.orig.fa_	OEZ008529	yes	LMSG_G000008772.1	OES073670	bacteriocin	RiPPs	Antimony	5.16	China: Guizhou, Banpo
L180322-P-6-LD180319-0016_HL3MGCCXY_L2_clean_bin.74.orig.fa_	OEZ008529	yes	LMSG_G000008772.1	OES073670	terpene	Terpene	Antimony	6.69	China: Guizhou, Banpo
L180322-P-6-LD180319-0016_HL3MGCCXY_L2_clean_bin.75.orig.fa_	OEZ008529	yes	LMSG_G000005407.1	OES073670	NRPS-like	NRPS	Antimony	8.73	China: Guizhou, Banpo
L180322-P-6-LD180319-0016_HL3MGCCXY_L2_clean_bin.75.orig.fa_	OEZ008529	yes	LMSG_G000005407.1	OES073670	bacteriocin	RiPPs	Antimony	7.51	China: Guizhou, Banpo
L180322-P-6-LD180319-0016_HL3MGCCXY_L2_clean_bin.75.orig.fa_	OEZ008529	yes	LMSG_G000005407.1	OES073670	terpene	Terpene	Antimony	20.84	China: Guizhou, Banpo
L180322-P-6-LD180319-0016_HL3MGCCXY_L2_clean_bin.75.orig.fa_	OEZ008529	yes	LMSG_G000005407.1	OES073670	arylpolyene	Others	Antimony	31.14	China: Guizhou, Banpo
L180322-P-6-LD180319-0016_HL3MGCCXY_L2_clean_bin.76.orig.fa_	OEZ008529	yes	LMSG_G000008779.1	OES073670	bacteriocin	RiPPs	Antimony	8.18	China: Guizhou, Banpo
L180322-P-6-LD180319-0016_HL3MGCCXY_L2_clean_bin.78.orig.fa_	OEZ008529	yes	LMSG_G000007865.1	OES073670	NRPS-like	NRPS	Antimony	42.66	China: Guizhou, Banpo
L180322-P-6-LD180319-0016_HL3MGCCXY_L2_clean_bin.78.orig.fa_	OEZ008529	yes	LMSG_G000007865.1	OES073670	bacteriocin	RiPPs	Antimony	6.11	China: Guizhou, Banpo
L180322-P-6-LD180319-0016_HL3MGCCXY_L2_clean_bin.78.orig.fa_	OEZ008529	yes	LMSG_G000007865.1	OES073670	terpene	Terpene	Antimony	30.07	China: Guizhou, Banpo
L180322-P-6-LD180319-0016_HL3MGCCXY_L2_clean_bin.78.orig.fa_	OEZ008529	yes	LMSG_G000007865.1	OES073670	bacteriocin	RiPPs	Antimony	5.23	China: Guizhou, Banpo
L180322-P-6-LD180319-0016_HL3MGCCXY_L2_clean_bin.78.orig.fa_	OEZ008529	yes	LMSG_G000007865.1	OES073670	T3PKS	PKSother	Antimony	14.36	China: Guizhou, Banpo
L180322-P-6-LD180319-0016_HL3MGCCXY_L2_clean_bin.81.orig.fa_	OEZ008529	yes	LMSG_G000006234.1	OES073670	ladderane	Others	Antimony	15.54	China: Guizhou, Banpo
L180322-P-6-LD180319-0016_HL3MGCCXY_L2_clean_bin.81.orig.fa_	OEZ008529	yes	LMSG_G000006234.1	OES073670	terpene	Terpene	Antimony	19.62	China: Guizhou, Banpo
L180322-P-6-LD180319-0016_HL3MGCCXY_L2_clean_bin.85.permissi	OEZ008529	yes	LMSG_G000008918.1	OES073670	NRPS-like	NRPS	Antimony	26.40	China: Guizhou, Banpo
L180322-P-6-LD180319-0016_HL3MGCCXY_L2_clean_bin.86.strict.f	OEZ008529	yes	LMSG_G000009264.1	OES073670	TfuA-related	RiPPs	Antimony	19.60	China: Guizhou, Banpo
L180322-P-6-LD180319-0016_HL3MGCCXY_L2_clean_bin.86.strict.f	OEZ008529	yes	LMSG_G000009264.1	OES073670	T3PKS	PKSother	Antimony	14.26	China: Guizhou, Banpo
L180322-P-6-LD180319-0016_HL3MGCCXY_L2_clean_bin.87.orig.fa_	OEZ008529	yes	LMSG_G000008650.1	OES073670	other	Others	Antimony	36.22	China: Guizhou, Banpo
L180322-P-6-LD180319-0016_HL3MGCCXY_L2_clean_bin.88.strict.f	OEZ008529	yes	LMSG_G000009252.1	OES073670	T3PKS	PKSother	Antimony	32.81	China: Guizhou, Banpo
L180322-P-6-LD180319-0016_HL3MGCCXY_L2_clean_bin.91.orig.fa_	OEZ008529	yes	LMSG_G000009861.1	OES073670	NRPS-like	NRPS	Antimony	31.33	China: Guizhou, Banpo
L180322-P-6-LD180319-0016_HL3MGCCXY_L2_clean_bin.93.strict.f	OEZ008529	yes	LMSG_G000011144.1	OES073670	NRPS-like	NRPS	Antimony	42.75	China: Guizhou, Banpo
L180322-P-6-LD180319-0016_HL3MGCCXY_L2_clean_bin.93.strict.f	OEZ008529	yes	LMSG_G000011144.1	OES073670	NRPS.T1PKS	PKS-NRP_Hybrids	Antimony	26.43	China: Guizhou, Banpo
L180322-P-6-LD180319-0016_HL3MGCCXY_L2_clean_bin.93.strict.f	OEZ008529	yes	LMSG_G000011144.1	OES073670	NRPS	NRPS	Antimony	25.08	China: Guizhou, Banpo
L180322-P-6-LD180319-0016_HL3MGCCXY_L2_clean_bin.93.strict.f	OEZ008529	yes	LMSG_G000011144.1	OES073670	NRPS	NRPS	Antimony	28.47	China: Guizhou, Banpo
L180322-P-6-LD180319-0016_HL3MGCCXY_L2_clean_bin.96.orig.fa_	OEZ008529	yes	LMSG_G000008835.1	OES073670	terpene	Terpene	Antimony	15.54	China: Guizhou, Banpo
L180322-P-6-LD180319-0016_HL3MGCCXY_L2_clean_bin.96.orig.fa_	OEZ008529	yes	LMSG_G000008835.1	OES073670	NRPS-like	NRPS	Antimony	23.02	China: Guizhou, Banpo
L180322-P-6-LD180319-0016_HL3MGCCXY_L2_clean_bin.99.orig.fa_	OEZ008529	yes	LMSG_G000004473.1	OES073670	NRPS	NRPS	Antimony	19.11	China: Guizhou, Banpo
L180322-P-6-LD180319-0016_HL3MGCCXY_L2_clean_bin.99.orig.fa_	OEZ008529	yes	LMSG_G000004473.1	OES073670	NRPS	NRPS	Antimony	6.12	China: Guizhou, Banpo
L180322-P-6-LD180319-0016_HL3MGCCXY_L2_clean_bin.99.orig.fa_	OEZ008529	yes	LMSG_G000004473.1	OES073670	NRPS	NRPS	Antimony	15.49	China: Guizhou, Banpo
L180322-P-6-LD180319-0016_HL3MGCCXY_L2_clean_bin.99.orig.fa_	OEZ008529	yes	LMSG_G000004473.1	OES073670	terpene	Terpene	Antimony	15.96	China: Guizhou, Banpo
L180322-P-6-LD180319-0016_HL3MGCCXY_L2_clean_bin.99.orig.fa_	OEZ008529	yes	LMSG_G000004473.1	OES073670	lanthipeptide	RiPPs	Antimony	14.75	China: Guizhou, Banpo
L180322-P-6-LD180319-0016_HL3MGCCXY_L2_clean_bin.99.orig.fa_	OEZ008529	yes	LMSG_G000004473.1	OES073670	NRPS	NRPS	Antimony	22.01	China: Guizhou, Banpo
L180322-P-6-LD180320-0020_HL3MGCCXY_L2_clean_bin.1.orig.fa_k	OEZ008529	yes	LMSG_G000007801.1	OES073697	terpene	Terpene	Polymetallic	15.02	China: Guizhou, Longli
L180322-P-6-LD180320-0020_HL3MGCCXY_L2_clean_bin.1.orig.fa_k	OEZ008529	yes	LMSG_G000007801.1	OES073697	terpene	Terpene	Polymetallic	10.59	China: Guizhou, Longli
L180322-P-6-LD180320-0020_HL3MGCCXY_L2_clean_bin.10.orig.fa_	OEZ008529	yes	LMSG_G000010991.1	OES073697	T1PKS.hglE-KS	PKSother	Polymetallic	23.94	China: Guizhou, Longli
L180322-P-6-LD180320-0020_HL3MGCCXY_L2_clean_bin.11.orig.fa_	OEZ008529	yes	LMSG_G000008595.1	OES073697	other	Others	Polymetallic	12.52	China: Guizhou, Longli
L180322-P-6-LD180320-0020_HL3MGCCXY_L2_clean_bin.14.orig.fa_	OEZ008529	yes	LMSG_G000005411.1	OES073697	NRPS-like	NRPS	Polymetallic	17.41	China: Guizhou, Longli
L180322-P-6-LD180320-0020_HL3MGCCXY_L2_clean_bin.14.orig.fa_	OEZ008529	yes	LMSG_G000005411.1	OES073697	other	Others	Polymetallic	20.48	China: Guizhou, Longli
L180322-P-6-LD180320-0020_HL3MGCCXY_L2_clean_bin.15.orig.fa_	OEZ008529	yes	LMSG_G000010696.1	OES073697	NRPS	NRPS	Polymetallic	7.39	China: Guizhou, Longli
L180322-P-6-LD180320-0020_HL3MGCCXY_L2_clean_bin.18.orig.fa_	OEZ008529	no	LMSG_G000010887.1	OES073697	terpene	Terpene	Polymetallic	5.27	China: Guizhou, Longli
L180322-P-6-LD180320-0020_HL3MGCCXY_L2_clean_bin.19.orig.fa_	OEZ008529	yes	LMSG_G000008579.1	OES073697	NRPS-like	NRPS	Polymetallic	42.81	China: Guizhou, Longli
L180322-P-6-LD180320-0020_HL3MGCCXY_L2_clean_bin.2.strict.fa	OEZ008529	yes	LMSG_G000009628.1	OES073697	terpene	Terpene	Polymetallic	14.90	China: Guizhou, Longli
L180322-P-6-LD180320-0020_HL3MGCCXY_L2_clean_bin.2.strict.fa	OEZ008529	yes	LMSG_G000009628.1	OES073697	terpene	Terpene	Polymetallic	22.09	China: Guizhou, Longli
L180322-P-6-LD180320-0020_HL3MGCCXY_L2_clean_bin.20.orig.fa_	OEZ008529	yes	LMSG_G000011163.1	OES073697	arylpolyene	Others	Polymetallic	6.37	China: Guizhou, Longli
L180322-P-6-LD180320-0020_HL3MGCCXY_L2_clean_bin.20.orig.fa_	OEZ008529	yes	LMSG_G000011163.1	OES073697	arylpolyene	Others	Polymetallic	12.86	China: Guizhou, Longli
L180322-P-6-LD180320-0020_HL3MGCCXY_L2_clean_bin.20.orig.fa_	OEZ008529	yes	LMSG_G000011163.1	OES073697	NRPS.T1PKS.hserlactone	Others	Polymetallic	34.95	China: Guizhou, Longli
L180322-P-6-LD180320-0020_HL3MGCCXY_L2_clean_bin.20.orig.fa_	OEZ008529	yes	LMSG_G000011163.1	OES073697	bacteriocin	RiPPs	Polymetallic	5.34	China: Guizhou, Longli
L180322-P-6-LD180320-0020_HL3MGCCXY_L2_clean_bin.21.orig.fa_	OEZ008529	yes	LMSG_G000010327.1	OES073697	T1PKS	PKSI	Polymetallic	34.06	China: Guizhou, Longli
L180322-P-6-LD180320-0020_HL3MGCCXY_L2_clean_bin.21.orig.fa_	OEZ008529	yes	LMSG_G000010327.1	OES073697	NRPS-like	NRPS	Polymetallic	43.38	China: Guizhou, Longli
L180322-P-6-LD180320-0020_HL3MGCCXY_L2_clean_bin.23.orig.fa_	OEZ008529	yes	LMSG_G000010778.1	OES073697	bacteriocin	RiPPs	Polymetallic	11.75	China: Guizhou, Longli
L180322-P-6-LD180320-0020_HL3MGCCXY_L2_clean_bin.24.orig.fa_	OEZ008529	yes	LMSG_G000010657.1	OES073697	arylpolyene	Others	Polymetallic	18.89	China: Guizhou, Longli
L180322-P-6-LD180320-0020_HL3MGCCXY_L2_clean_bin.24.orig.fa_	OEZ008529	yes	LMSG_G000010657.1	OES073697	terpene	Terpene	Polymetallic	15.80	China: Guizhou, Longli
L180322-P-6-LD180320-0020_HL3MGCCXY_L2_clean_bin.24.orig.fa_	OEZ008529	yes	LMSG_G000010657.1	OES073697	NRPS.T1PKS	PKS-NRP_Hybrids	Polymetallic	44.05	China: Guizhou, Longli
L180322-P-6-LD180320-0020_HL3MGCCXY_L2_clean_bin.24.orig.fa_	OEZ008529	yes	LMSG_G000010657.1	OES073697	NRPS-like	NRPS	Polymetallic	20.81	China: Guizhou, Longli
L180322-P-6-LD180320-0020_HL3MGCCXY_L2_clean_bin.27.strict.f	OEZ008529	yes	LMSG_G000011311.1	OES073697	terpene	Terpene	Polymetallic	21.06	China: Guizhou, Longli
L180322-P-6-LD180320-0020_HL3MGCCXY_L2_clean_bin.27.strict.f	OEZ008529	yes	LMSG_G000011311.1	OES073697	ladderane	Others	Polymetallic	41.24	China: Guizhou, Longli
L180322-P-6-LD180320-0020_HL3MGCCXY_L2_clean_bin.27.strict.f	OEZ008529	yes	LMSG_G000011311.1	OES073697	NRPS	NRPS	Polymetallic	43.37	China: Guizhou, Longli
L180322-P-6-LD180320-0020_HL3MGCCXY_L2_clean_bin.27.strict.f	OEZ008529	yes	LMSG_G000011311.1	OES073697	terpene	Terpene	Polymetallic	9.45	China: Guizhou, Longli
L180322-P-6-LD180320-0020_HL3MGCCXY_L2_clean_bin.28.orig.fa_	OEZ008529	yes	LMSG_G000010225.1	OES073697	betalactone	Others	Polymetallic	20.28	China: Guizhou, Longli
L180322-P-6-LD180320-0020_HL3MGCCXY_L2_clean_bin.28.orig.fa_	OEZ008529	yes	LMSG_G000010225.1	OES073697	terpene	Terpene	Polymetallic	14.10	China: Guizhou, Longli
L180322-P-6-LD180320-0020_HL3MGCCXY_L2_clean_bin.28.orig.fa_	OEZ008529	yes	LMSG_G000010225.1	OES073697	terpene	Terpene	Polymetallic	19.19	China: Guizhou, Longli
L180322-P-6-LD180320-0020_HL3MGCCXY_L2_clean_bin.29.orig.fa_	OEZ008529	yes	LMSG_G000010130.1	OES073697	hserlactone	Others	Polymetallic	5.48	China: Guizhou, Longli
L180322-P-6-LD180320-0020_HL3MGCCXY_L2_clean_bin.30.orig.fa_	OEZ008529	yes	LMSG_G000009306.1	OES073697	terpene	Terpene	Polymetallic	19.05	China: Guizhou, Longli
L180322-P-6-LD180320-0020_HL3MGCCXY_L2_clean_bin.31.orig.fa_	OEZ008529	yes	LMSG_G000011177.1	OES073697	arylpolyene	Others	Polymetallic	7.87	China: Guizhou, Longli
L180322-P-6-LD180320-0020_HL3MGCCXY_L2_clean_bin.31.orig.fa_	OEZ008529	yes	LMSG_G000011177.1	OES073697	bacteriocin	RiPPs	Polymetallic	8.97	China: Guizhou, Longli
L180322-P-6-LD180320-0020_HL3MGCCXY_L2_clean_bin.32.permissi	OEZ008529	yes	LMSG_G000010553.1	OES073697	arylpolyene	Others	Polymetallic	41.19	China: Guizhou, Longli

L180322-P-6-LD180320-0020_HL3MGCCXY_L2_clean_bin.32.permissi	OEZ008529	yes	LMSG_G000010553.1	OES073697	terpene	Terpene	Polymetallic	23.00	China: Guizhou, Longli
L180322-P-6-LD180320-0020_HL3MGCCXY_L2_clean_bin.32.permissi	OEZ008529	yes	LMSG_G000010553.1	OES073697	bacteriocin	RiPPs	Polymetallic	9.92	China: Guizhou, Longli
L180322-P-6-LD180320-0020_HL3MGCCXY_L2_clean_bin.34.orig.fa_	OEZ008529	yes	LMSG_G000005412.1	OES073697	NRPS-like	NRPS	Polymetallic	23.83	China: Guizhou, Longli
L180322-P-6-LD180320-0020_HL3MGCCXY_L2_clean_bin.34.orig.fa_	OEZ008529	yes	LMSG_G000005412.1	OES073697	terpene	Terpene	Polymetallic	12.51	China: Guizhou, Longli
L180322-P-6-LD180320-0020_HL3MGCCXY_L2_clean_bin.34.orig.fa_	OEZ008529	yes	LMSG_G000005412.1	OES073697	terpene	Terpene	Polymetallic	13.30	China: Guizhou, Longli
L180322-P-6-LD180320-0020_HL3MGCCXY_L2_clean_bin.34.orig.fa_	OEZ008529	yes	LMSG_G000005412.1	OES073697	T3PKS	PKSother	Polymetallic	13.23	China: Guizhou, Longli
L180322-P-6-LD180320-0020_HL3MGCCXY_L2_clean_bin.35.orig.fa_	OEZ008529	yes	LMSG_G000008161.1	OES073697	terpene	Terpene	Polymetallic	11.88	China: Guizhou, Longli
L180322-P-6-LD180320-0020_HL3MGCCXY_L2_clean_bin.37.orig.fa_	OEZ008529	yes	LMSG_G000010397.1	OES073697	hserlactone	Others	Polymetallic	20.33	China: Guizhou, Longli
L180322-P-6-LD180320-0020_HL3MGCCXY_L2_clean_bin.37.orig.fa_	OEZ008529	yes	LMSG_G000010397.1	OES073697	bacteriocin	RiPPs	Polymetallic	10.89	China: Guizhou, Longli
L180322-P-6-LD180320-0020_HL3MGCCXY_L2_clean_bin.37.orig.fa_	OEZ008529	yes	LMSG_G000010397.1	OES073697	bacteriocin	RiPPs	Polymetallic	5.23	China: Guizhou, Longli
L180322-P-6-LD180320-0020_HL3MGCCXY_L2_clean_bin.37.orig.fa_	OEZ008529	yes	LMSG_G000010397.1	OES073697	lassopeptide	RiPPs	Polymetallic	12.39	China: Guizhou, Longli
L180322-P-6-LD180320-0020_HL3MGCCXY_L2_clean_bin.37.orig.fa_	OEZ008529	yes	LMSG_G000010397.1	OES073697	arylpolyene	Others	Polymetallic	7.67	China: Guizhou, Longli
L180322-P-6-LD180320-0020_HL3MGCCXY_L2_clean_bin.37.orig.fa_	OEZ008529	yes	LMSG_G000010397.1	OES073697	betalactone	Others	Polymetallic	15.03	China: Guizhou, Longli
L180322-P-6-LD180320-0020_HL3MGCCXY_L2_clean_bin.37.orig.fa_	OEZ008529	yes	LMSG_G000010397.1	OES073697	terpene	Terpene	Polymetallic	9.27	China: Guizhou, Longli
L180322-P-6-LD180320-0020_HL3MGCCXY_L2_clean_bin.37.orig.fa_	OEZ008529	yes	LMSG_G000010397.1	OES073697	terpene	Terpene	Polymetallic	7.56	China: Guizhou, Longli
L180322-P-6-LD180320-0020_HL3MGCCXY_L2_clean_bin.37.orig.fa_	OEZ008529	yes	LMSG_G000010397.1	OES073697	terpene	Terpene	Polymetallic	5.45	China: Guizhou, Longli
L180322-P-6-LD180320-0020_HL3MGCCXY_L2_clean_bin.37.orig.fa_	OEZ008529	yes	LMSG_G000010397.1	OES073697	NRPS-like	NRPS	Polymetallic	8.88	China: Guizhou, Longli
L180322-P-6-LD180320-0020_HL3MGCCXY_L2_clean_bin.38.orig.fa_	OEZ008529	yes	LMSG_G000007787.1	OES073697	bacteriocin	RiPPs	Polymetallic	10.90	China: Guizhou, Longli
L180322-P-6-LD180320-0020_HL3MGCCXY_L2_clean_bin.38.orig.fa_	OEZ008529	yes	LMSG_G000007787.1	OES073697	terpene	Terpene	Polymetallic	13.26	China: Guizhou, Longli
L180322-P-6-LD180320-0020_HL3MGCCXY_L2_clean_bin.38.orig.fa_	OEZ008529	yes	LMSG_G000007787.1	OES073697	T3PKS	PKSother	Polymetallic	9.05	China: Guizhou, Longli
L180322-P-6-LD180320-0020_HL3MGCCXY_L2_clean_bin.39.orig.fa_	OEZ008529	yes	LMSG_G000007838.1	OES073697	NRPS-like	NRPS	Polymetallic	39.71	China: Guizhou, Longli
L180322-P-6-LD180320-0020_HL3MGCCXY_L2_clean_bin.39.orig.fa_	OEZ008529	yes	LMSG_G000007838.1	OES073697	terpene	Terpene	Polymetallic	11.39	China: Guizhou, Longli
L180322-P-6-LD180320-0020_HL3MGCCXY_L2_clean_bin.39.orig.fa_	OEZ008529	yes	LMSG_G000007838.1	OES073697	terpene	Terpene	Polymetallic	13.96	China: Guizhou, Longli
L180322-P-6-LD180320-0020_HL3MGCCXY_L2_clean_bin.39.orig.fa_	OEZ008529	yes	LMSG_G000007838.1	OES073697	NRPS-like	NRPS	Polymetallic	27.19	China: Guizhou, Longli
L180322-P-6-LD180320-0020_HL3MGCCXY_L2_clean_bin.39.orig.fa_	OEZ008529	yes	LMSG_G000007838.1	OES073697	terpene	Terpene	Polymetallic	9.52	China: Guizhou, Longli
L180322-P-6-LD180320-0020_HL3MGCCXY_L2_clean_bin.39.orig.fa_	OEZ008529	yes	LMSG_G000007838.1	OES073697	T3PKS	PKSother	Polymetallic	41.06	China: Guizhou, Longli
L180322-P-6-LD180320-0020_HL3MGCCXY_L2_clean_bin.40.orig.fa_	OEZ008529	yes	LMSG_G000010216.1	OES073697	T3PKS	PKSother	Polymetallic	18.15	China: Guizhou, Longli
L180322-P-6-LD180320-0020_HL3MGCCXY_L2_clean_bin.40.orig.fa_	OEZ008529	yes	LMSG_G000010216.1	OES073697	terpene	Terpene	Polymetallic	13.07	China: Guizhou, Longli
L180322-P-6-LD180320-0020_HL3MGCCXY_L2_clean_bin.41.strict.f	OEZ008529	yes	LMSG_G000010415.1	OES073697	terpene	Terpene	Polymetallic	20.85	China: Guizhou, Longli
L180322-P-6-LD180320-0020_HL3MGCCXY_L2_clean_bin.41.strict.f	OEZ008529	yes	LMSG_G000010415.1	OES073697	terpene	Terpene	Polymetallic	25.01	China: Guizhou, Longli
L180322-P-6-LD180320-0020_HL3MGCCXY_L2_clean_bin.41.strict.f	OEZ008529	yes	LMSG_G000010415.1	OES073697	betalactone	Others	Polymetallic	22.52	China: Guizhou, Longli
L180322-P-6-LD180320-0020_HL3MGCCXY_L2_clean_bin.43.orig.fa_	OEZ008529	yes	LMSG_G000011052.1	OES073697	NRPS-like	NRPS	Polymetallic	16.90	China: Guizhou, Longli
L180322-P-6-LD180320-0020_HL3MGCCXY_L2_clean_bin.43.orig.fa_	OEZ008529	yes	LMSG_G000011052.1	OES073697	terpene	Terpene	Polymetallic	11.18	China: Guizhou, Longli
L180322-P-6-LD180320-0020_HL3MGCCXY_L2_clean_bin.45.orig.fa_	OEZ008529	yes	LMSG_G000005413.1	OES073697	bacteriocin	RiPPs	Polymetallic	6.82	China: Guizhou, Longli
L180322-P-6-LD180320-0020_HL3MGCCXY_L2_clean_bin.45.orig.fa_	OEZ008529	yes	LMSG_G000005413.1	OES073697	LAP	RiPPs	Polymetallic	7.26	China: Guizhou, Longli
L180322-P-6-LD180320-0020_HL3MGCCXY_L2_clean_bin.45.orig.fa_	OEZ008529	yes	LMSG_G000005413.1	OES073697	terpene	Terpene	Polymetallic	7.54	China: Guizhou, Longli
L180322-P-6-LD180320-0020_HL3MGCCXY_L2_clean_bin.45.orig.fa_	OEZ008529	yes	LMSG_G000005413.1	OES073697	terpene	Terpene	Polymetallic	5.52	China: Guizhou, Longli
L180322-P-6-LD180320-0020_HL3MGCCXY_L2_clean_bin.47.orig.fa_	OEZ008529	yes	LMSG_G000005415.1	OES073697	terpene	Terpene	Polymetallic	6.45	China: Guizhou, Longli
L180322-P-6-LD180320-0020_HL3MGCCXY_L2_clean_bin.47.orig.fa_	OEZ008529	yes	LMSG_G000005415.1	OES073697	arylpolyene	Others	Polymetallic	5.24	China: Guizhou, Longli
L180322-P-6-LD180320-0020_HL3MGCCXY_L2_clean_bin.47.orig.fa_	OEZ008529	yes	LMSG_G000005415.1	OES073697	terpene	Terpene	Polymetallic	10.70	China: Guizhou, Longli
L180322-P-6-LD180320-0020_HL3MGCCXY_L2_clean_bin.48.strict.f	OEZ008529	yes	LMSG_G000008195.1	OES073697	terpene	Terpene	Polymetallic	20.86	China: Guizhou, Longli
L180322-P-6-LD180320-0020_HL3MGCCXY_L2_clean_bin.49.orig.fa_	OEZ008529	yes	LMSG_G000010218.1	OES073697	terpene	Terpene	Polymetallic	13.25	China: Guizhou, Longli
L180322-P-6-LD180320-0020_HL3MGCCXY_L2_clean_bin.49.orig.fa_	OEZ008529	yes	LMSG_G000010218.1	OES073697	arylpolyene	Others	Polymetallic	5.18	China: Guizhou, Longli
L180322-P-6-LD180320-0020_HL3MGCCXY_L2_clean_bin.49.orig.fa_	OEZ008529	yes	LMSG_G000010218.1	OES073697	bacteriocin	RiPPs	Polymetallic	5.05	China: Guizhou, Longli
L180322-P-6-LD180320-0020_HL3MGCCXY_L2_clean_bin.5.orig.fa_k	OEZ008529	yes	LMSG_G000004474.1	OES073697	terpene	Terpene	Polymetallic	8.04	China: Guizhou, Longli
L180322-P-6-LD180320-0020_HL3MGCCXY_L2_clean_bin.5.orig.fa_k	OEZ008529	yes	LMSG_G000004474.1	OES073697	T3PKS	PKSother	Polymetallic	12.90	China: Guizhou, Longli
L180322-P-6-LD180320-0020_HL3MGCCXY_L2_clean_bin.50.orig.fa_	OEZ008529	no	LMSG_G000011188.1	OES073697	NRPS.T1PKS	PKS-NRP_Hybrids	Polymetallic	11.43	China: Guizhou, Longli
L180322-P-6-LD180320-0020_HL3MGCCXY_L2_clean_bin.50.orig.fa_	OEZ008529	yes	LMSG_G000011188.1	OES073697	arylpolyene	Others	Polymetallic	9.63	China: Guizhou, Longli
L180322-P-6-LD180320-0020_HL3MGCCXY_L2_clean_bin.53.orig.fa_	OEZ008529	yes	LMSG_G000005416.1	OES073697	betalactone	Others	Polymetallic	15.52	China: Guizhou, Longli
L180322-P-6-LD180320-0020_HL3MGCCXY_L2_clean_bin.53.orig.fa_	OEZ008529	yes	LMSG_G000005416.1	OES073697	NRPS	NRPS	Polymetallic	37.17	China: Guizhou, Longli
L180322-P-6-LD180320-0020_HL3MGCCXY_L2_clean_bin.53.orig.fa_	OEZ008529	yes	LMSG_G000005416.1	OES073697	bacteriocin	RiPPs	Polymetallic	8.52	China: Guizhou, Longli
L180322-P-6-LD180320-0020_HL3MGCCXY_L2_clean_bin.56.orig.fa_	OEZ008529	yes	LMSG_G000010807.1	OES073697	acyl_amino_acids	Others	Polymetallic	19.96	China: Guizhou, Longli
L180322-P-6-LD180320-0020_HL3MGCCXY_L2_clean_bin.6.strict.fa	OEZ008529	yes	LMSG_G000010666.1	OES073697	NRPS	NRPS	Polymetallic	17.41	China: Guizhou, Longli
L180322-P-6-LD180320-0020_HL3MGCCXY_L2_clean_bin.60.orig.fa_	OEZ008529	yes	LMSG_G000009992.1	OES073697	ectoine	Others	Polymetallic	10.40	China: Guizhou, Longli
L180322-P-6-LD180320-0020_HL3MGCCXY_L2_clean_bin.60.orig.fa_	OEZ008529	yes	LMSG_G000009992.1	OES073697	arylpolyene	Others	Polymetallic	42.25	China: Guizhou, Longli
L180322-P-6-LD180320-0020_HL3MGCCXY_L2_clean_bin.60.orig.fa_	OEZ008529	yes	LMSG_G000009992.1	OES073697	terpene	Terpene	Polymetallic	21.76	China: Guizhou, Longli
L180322-P-6-LD180320-0020_HL3MGCCXY_L2_clean_bin.60.orig.fa_	OEZ008529	yes	LMSG_G000009992.1	OES073697	terpene	Terpene	Polymetallic	21.84	China: Guizhou, Longli
L180322-P-6-LD180320-0020_HL3MGCCXY_L2_clean_bin.60.orig.fa_	OEZ008529	yes	LMSG_G000009992.1	OES073697	arylpolyene	Others	Polymetallic	21.37	China: Guizhou, Longli
L180322-P-6-LD180320-0020_HL3MGCCXY_L2_clean_bin.60.orig.fa_	OEZ008529	yes	LMSG_G000009992.1	OES073697	NRPS-like	NRPS	Polymetallic	44.04	China: Guizhou, Longli
L180322-P-6-LD180320-0020_HL3MGCCXY_L2_clean_bin.60.orig.fa_	OEZ008529	yes	LMSG_G000009992.1	OES073697	arylpolyene	Others	Polymetallic	33.78	China: Guizhou, Longli
L180322-P-6-LD180320-0020_HL3MGCCXY_L2_clean_bin.61.orig.fa_	OEZ008529	no	LMSG_G000010833.1	OES073697	acyl_amino_acids	Others	Polymetallic	10.87	China: Guizhou, Longli
L180322-P-6-LD180320-0020_HL3MGCCXY_L2_clean_bin.61.orig.fa_	OEZ008529	yes	LMSG_G000010833.1	OES073697	T1PKS.hserlactone	Others	Polymetallic	13.06	China: Guizhou, Longli
L180322-P-6-LD180320-0020_HL3MGCCXY_L2_clean_bin.63.orig.fa_	OEZ008529	no	LMSG_G000010274.1	OES073697	terpene	Terpene	Polymetallic	11.69	China: Guizhou, Longli
L180322-P-6-LD180320-0020_HL3MGCCXY_L2_clean_bin.63.orig.fa_	OEZ008529	yes	LMSG_G000010274.1	OES073697	arylpolyene	Others	Polymetallic	28.30	China: Guizhou, Longli
L180322-P-6-LD180320-0020_HL3MGCCXY_L2_clean_bin.64.orig.fa_	OEZ008529	yes	LMSG_G000010351.1	OES073697	terpene	Terpene	Polymetallic	20.73	China: Guizhou, Longli
L180322-P-6-LD180320-0020_HL3MGCCXY_L2_clean_bin.64.orig.fa_	OEZ008529	yes	LMSG_G000010351.1	OES073697	NRPS-like	NRPS	Polymetallic	30.07	China: Guizhou, Longli
L180322-P-6-LD180320-0020_HL3MGCCXY_L2_clean_bin.64.orig.fa_	OEZ008529	yes	LMSG_G000010351.1	OES073697	bacteriocin	RiPPs	Polymetallic	10.91	China: Guizhou, Longli
L180322-P-6-LD180320-0020_HL3MGCCXY_L2_clean_bin.64.orig.fa_	OEZ008529	yes	LMSG_G000010351.1	OES073697	betalactone	Others	Polymetallic	24.44	China: Guizhou, Longli
L180322-P-6-LD180320-0020_HL3MGCCXY_L2_clean_bin.64.orig.fa_	OEZ008529	yes	LMSG_G000010351.1	OES073697	hserlactone	Others	Polymetallic	20.64	China: Guizhou, Longli
L180322-P-6-LD180320-0020_HL3MGCCXY_L2_clean_bin.65.orig.fa_	OEZ008529	yes	LMSG_G000007780.1	OES073697	bacteriocin	RiPPs	Polymetallic	5.85	China: Guizhou, Longli
L180322-P-6-LD180320-0020_HL3MGCCXY_L2_clean_bin.65.orig.fa_	OEZ008529	yes	LMSG_G000007780.1	OES073697	terpene	Terpene	Polymetallic	21.49	China: Guizhou, Longli
L180322-P-6-LD180320-0020_HL3MGCCXY_L2_clean_bin.65.orig.fa_	OEZ008529	yes	LMSG_G000007780.1	OES073697	T3PKS	PKSother	Polymetallic	25.80	China: Guizhou, Longli
L180322-P-6-LD180320-0020_HL3MGCCXY_L2_clean_bin.66.strict.f	OEZ008529	yes	LMSG_G000005417.1	OES073697	terpene	Terpene	Polymetallic	12.38	China: Guizhou, Longli
L180322-P-6-LD180320-0020_HL3MGCCXY_L2_clean_bin.66.strict.f	OEZ008529	yes	LMSG_G000005417.1	OES073697	terpene	Terpene	Polymetallic	14.30	China: Guizhou, Longli
L180322-P-6-LD180320-0020_HL3MGCCXY_L2_clean_bin.66.strict.f	OEZ008529	yes	LMSG_G000005417.1	OES073697	arylpolyene	Others	Polymetallic	7.15	China: Guizhou, Longli
L180322-P-6-LD180320-0020_HL3MGCCXY_L2_clean_bin.66.strict.f	OEZ008529	yes	LMSG_G000005417.1	OES073697	arylpolyene	Others	Polymetallic	7.01	China: Guizhou, Longli
L180322-P-6-LD180320-0020_HL3MGCCXY_L2_clean_bin.68.strict.f	OEZ008529	yes	LMSG_G000011002.1	OES073697	bacteriocin	RiPPs	Polymetallic	8.40	China: Guizhou, Longli
L180322-P-6-LD180320-0020_HL3MGCCXY_L2_clean_bin.68.strict.f	OEZ008529	yes	LMSG_G000011002.1	OES073697	hglE-KS	PKSother	Polymetallic	10.97	China: Guizhou, Longli
L180322-P-6-LD180320-0020_HL3MGCCXY_L2_clean_bin.68.strict.f	OEZ008529	yes	LMSG_G000011002.1	OES073697	T1PKS	PKSI	Polymetallic	6.90	China: Guizhou, Longli
L180322-P-6-LD180320-0020_HL3MGCCXY_L2_clean_bin.69.orig.fa_	OEZ008529	yes	LMSG_G000010361.1	OES073697	terpene	Terpene	Polymetallic	8.68	China: Guizhou, Longli
L180322-P-6-LD180320-0020_HL3MGCCXY_L2_clean_bin.69.orig.fa_	OEZ008529	yes	LMSG_G000010361.1	OES073697	NRPS-like	NRPS	Polymetallic	11.00	China: Guizhou, Longli
L180322-P-6-LD180320-0020_HL3MGCCXY_L2_clean_bin.69.orig.fa_	OEZ008529	yes	LMSG_G000010361.1	OES073697	terpene	Terpene	Polymetallic	12.00	China: Guizhou, Longli
L180322-P-6-LD180320-0020_HL3MGCCXY_L2_clean_bin.69.orig.fa_	OEZ008529	yes	LMSG_G000010361.1	OES073697	terpene	Terpene	Polymetallic	10.54	China: Guizhou, Longli
L180322-P-6-LD180320-0020_HL3MGCCXY_L2_clean_bin.7.orig.fa_k	OEZ008529	yes	LMSG_G000008378.1	OES073697	terpene	Terpene	Polymetallic	5.33	China: Guizhou, Longli
L180322-P-6-LD180320-0020_HL3MGCCXY_L2_clean_bin.72.orig.fa_	OEZ008529	yes	LMSG_G000010204.1	OES073697	terpene	Terpene	Polymetallic	12.90	China: Guizhou, Longli
L180322-P-6-LD180320-0020_HL3MGCCXY_L2_clean_bin.72.orig.fa_	OEZ008529	yes	LMSG_G000010204.1	OES073697	terpene	Terpene	Polymetallic	21.91	China: Guizhou, Longli
L180322-P-6-LD180320-0020_HL3MGCCXY_L2_clean_bin.72.orig.fa_	OEZ008529	yes	LMSG_G000010204.1	OES073697	arylpolyene	Others	Polymetallic	36.97	China: Guizhou, Longli
L180322-P-6-LD180320-0020_HL3MGCCXY_L2_clean_bin.8.orig.fa_k	OEZ008529	yes	LMSG_G000010105.1	OES073697	terpene	Terpene	Polymetallic	13.44	China: Guizhou, Longli
L180322-P-6-LD180320-0020_HL3MGCCXY_L2_clean_bin.8.orig.fa_k	OEZ008529	yes	LMSG_G000010105.1	OES073697	terpene	Terpene	Polymetallic	5.56	China: Guizhou, Longli
L180322-P-6-LD180320-0020_HL3MGCCXY_L2_clean_bin.8.orig.fa_k	OEZ008529	yes	LMSG_G000010105.1	OES073697	betalactone	Others	Polymetallic	15.03	China: Guizhou, Longli
L180322-P-6-LD180320-0020_HL3MGCCXY_L2_clean_bin.8.orig.fa_k	OEZ008529	yes	LMSG_G000010105.1	OES073697	T1PKS	PKSI	Polymetallic	47.19	China: Guizhou, Longli
L180322-P-6-LD180320-0020_HL3MGCCXY_L2_clean_bin.9.orig.fa_k	OEZ008529	yes	LMSG_G000005420.1	OES073697	arylpolyene	Others	Polymetallic	23.59	China: Guizhou, Longli
L180322-P-6-LD180320-0020_HL3MGCCXY_L2_clean_bin.9.orig.fa_k	OEZ008529	yes	LMSG_G000005420.1	OES073697	T3PKS	PKSother	Polymetallic	31.60	China: Guizhou, Longli
L180322-P-6-LD180320-0020_HL3MGCCXY_L2_clean_bin.9.orig.fa_k	OEZ008529	yes	LMSG_G000005420.1	OES073697	terpene	Terpene	Polymetallic	11.64	China: Guizhou, Longli
L180322-P-6-LD180320-0020_HL3MGCCXY_L2_clean_bin.9.orig.fa_k	OEZ008529	yes	LMSG_G000005420.1	OES073697	terpene	Terpene	Polymetallic	18.49	China: Guizhou, Longli
L180322-P-6-LD180320-0021_HL3MGCCXY_L2_clean_bin.13.orig.fa_	OEZ008529	yes	LMSG_G000010134.1	OES073698	terpene	Terpene	Polymetallic	14.13	China: Guizhou, Longli
L180322-P-6-LD180320-0021_HL3MGCCXY_L2_clean_bin.13.orig.fa_	OEZ008529	yes	LMSG_G000010134.1	OES073698	betalactone	Others	Polymetallic	31.27	China: Guizhou, Longli
L180322-P-6-LD180320-0021_HL3MGCCXY_L2_clean_bin.13.orig.fa_	OEZ008529	yes	LMSG_G000010134.1	OES073698	betalactone	Others	Polymetallic	24.05	China: Guizhou, Longli
L180322-P-6-LD180320-0021_HL3MGCCXY_L2_clean_bin.13.orig.fa_	OEZ008529	yes	LMSG_G000010134.1	OES073698	terpene	Terpene	Polymetallic	23.31	China: Guizhou, Longli
L180322-P-6-LD180320-0021_HL3MGCCXY_L2_clean_bin.14.orig.fa_	OEZ008529	yes	LMSG_G000005421.1	OES073698	bacteriocin	RiPPs	Polymetallic	9.92	China: Guizhou, Longli
L180322-P-6-LD180320-0021_HL3MGCCXY_L2_clean_bin.15.orig.fa_	OEZ008529	no	LMSG_G000010275.1	OES073698	terpene	Terpene	Polymetallic	6.72	China: Guizhou, Longli
L180322-P-6-LD180320-0021_HL3MGCCXY_L2_clean_bin.15.orig.fa_	OEZ008529	no	LMSG_G000010275.1	OES073698	terpene	Terpene	Polymetallic	6.90	China: Guizhou, Longli
L180322-P-6-LD180320-0021_HL3MGCCXY_L2_clean_bin.15.orig.fa_	OEZ008529	yes	LMSG_G000010275.1	OES073698	arylpolyene	Others	Polymetallic	32.04	China: Guizhou, Longli
L180322-P-6-LD180320-0021_HL3MGCCXY_L2_clean_bin.16.orig.fa_	OEZ008529	yes	LMSG_G000011147.1	OES073698	bacteriocin	RiPPs	Polymetallic	10.87	China: Guizhou, Longli
L180322-P-6-LD180320-0021_HL3MGCCXY_L2_clean_bin.16.orig.fa_	OEZ008529	yes	LMSG_G000011147.1	OES073698	arylpolyene	Others	Polymetallic	11.57	China: Guizhou, Longli
L180322-P-6-LD180320-0021_HL3MGCCXY_L2_clean_bin.17.orig.fa_	OEZ008529	yes	LMSG_G000007788.1	OES073698	bacteriocin	RiPPs	Polymetallic	10.90	China: Guizhou, Longli
L180322-P-6-LD180320-0021_HL3MGCCXY_L2_clean_bin.17.orig.fa_	OEZ008529	yes	LMSG_G000007788.1	OES073698	T3PKS	PKSother	Polymetallic	10.44	China: Guizhou, Longli
L180322-P-6-LD180320-0021_HL3MGCCXY_L2_clean_bin.18.orig.fa_	OEZ008529	yes	LMSG_G000010779.1	OES073698	bacteriocin	RiPPs	Polymetallic	7.04	China: Guizhou, Longli
L180322-P-6-LD180320-0021_HL3MGCCXY_L2_clean_bin.18.orig.fa_	OEZ008529	yes	LMSG_G000010779.1	OES073698	acyl_amino_acids	Others	Polymetallic	5.11	China: Guizhou, Longli
L180322-P-6-LD180320-0021_HL3MGCCXY_L2_clean_bin.20.permissi	OEZ008529	yes	LMSG_G000011312.1	OES073698	terpene	Terpene	Polymetallic	21.06	China: Guizhou, Longli
L180322-P-6-LD180320-0021_HL3MGCCXY_L2_clean_bin.20.permissi	OEZ008529	yes	LMSG_G000011312.1	OES073698	ladderane	Others	Polymetallic	40.36	China: Guizhou, Longli
L180322-P-6-LD180320-0021_HL3MGCCXY_L2_clean_bin.20.permissi	OEZ008529	yes	LMSG_G000011312.1	OES073698	NRPS	NRPS	Polymetallic	38.76	China: Guizhou, Longli
L180322-P-6-LD180320-0021_HL3MGCCXY_L2_clean_bin.20.permissi	OEZ008529	yes	LMSG_G000011312.1	OES073698	T3PKS	PKSother	Polymetallic	26.58	China: Guizhou, Longli
L180322-P-6-LD180320-0021_HL3MGCCXY_L2_clean_bin.20.permissi	OEZ008529	yes	LMSG_G000011312.1	OES073698	terpene	Terpene	Polymetallic	15.13	China: Guizhou, Longli
L180322-P-6-LD180320-0021_HL3MGCCXY_L2_clean_bin.20.permissi	OEZ008529	yes	LMSG_G000011312.1	OES073698	terpene	Terpene	Polymetallic	9.49	China: Guizhou, Longli
L180322-P-6-LD180320-0021_HL3MGCCXY_L2_clean_bin.21.orig.fa_	OEZ008529	yes	LMSG_G000011189.1	OES073698	arylpolyene	Others	Polymetallic	5.81	China: Guizhou, Longli
L180322-P-6-LD180320-0021_HL3MGCCXY_L2_clean_bin.22.strict.f	OEZ008529	yes	LMSG_G000009297.1	OES073698	terpene	Terpene	Polymetallic	17.04	China: Guizhou, Longli
L180322-P-6-LD180320-0021_HL3MGCCXY_L2_clean_bin.23.orig.fa_	OEZ008529	yes	LMSG_G000011164.1	OES073698	arylpolyene	Others	Polymetallic	11.43	China: Guizhou, Longli
L180322-P-6-LD180320-0021_HL3MGCCXY_L2_clean_bin.23.orig.fa_	OEZ008529	yes	LMSG_G000011164.1	OES073698	bacteriocin	RiPPs	Polymetallic	6.71	China: Guizhou, Longli
L180322-P-6-LD180320-0021_HL3MGCCXY_L2_clean_bin.23.orig.fa_	OEZ008529	yes	LMSG_G000011164.1	OES073698	arylpolyene	Others	Polymetallic	14.37	China: Guizhou, Longli
L180322-P-6-LD180320-0021_HL3MGCCXY_L2_clean_bin.24.orig.fa_	OEZ008529	yes	LMSG_G000010124.1	OES073698	terpene	Terpene	Polymetallic	15.77	China: Guizhou, Longli
L180322-P-6-LD180320-0021_HL3MGCCXY_L2_clean_bin.24.orig.fa_	OEZ008529	yes	LMSG_G000010124.1	OES073698	bacteriocin	RiPPs	Polymetallic	6.52	China: Guizhou, Longli
L180322-P-6-LD180320-0021_HL3MGCCXY_L2_clean_bin.24.orig.fa_	OEZ008529	yes	LMSG_G000010124.1	OES073698	T3PKS	PKSother	Polymetallic	9.45	China: Guizhou, Longli
L180322-P-6-LD180320-0021_HL3MGCCXY_L2_clean_bin.24.orig.fa_	OEZ008529	yes	LMSG_G000010124.1	OES073698	arylpolyene	Others	Polymetallic	11.47	China: Guizhou, Longli
L180322-P-6-LD180320-0021_HL3MGCCXY_L2_clean_bin.25.orig.fa_	OEZ008529	yes	LMSG_G000010362.1	OES073698	terpene	Terpene	Polymetallic	21.72	China: Guizhou, Longli
L180322-P-6-LD180320-0021_HL3MGCCXY_L2_clean_bin.25.orig.fa_	OEZ008529	yes	LMSG_G000010362.1	OES073698	terpene	Terpene	Polymetallic	15.48	China: Guizhou, Longli
L180322-P-6-LD180320-0021_HL3MGCCXY_L2_clean_bin.25.orig.fa_	OEZ008529	yes	LMSG_G000010362.1	OES073698	NRPS-like	NRPS	Polymetallic	16.19	China: Guizhou, Longli
L180322-P-6-LD180320-0021_HL3MGCCXY_L2_clean_bin.25.orig.fa_	OEZ008529	yes	LMSG_G000010362.1	OES073698	terpene	Terpene	Polymetallic	22.06	China: Guizhou, Longli
L180322-P-6-LD180320-0021_HL3MGCCXY_L2_clean_bin.25.orig.fa_	OEZ008529	yes	LMSG_G000010362.1	OES073698	NRPS-like	NRPS	Polymetallic	44.07	China: Guizhou, Longli
L180322-P-6-LD180320-0021_HL3MGCCXY_L2_clean_bin.25.orig.fa_	OEZ008529	yes	LMSG_G000010362.1	OES073698	bacteriocin	RiPPs	Polymetallic	11.72	China: Guizhou, Longli
L180322-P-6-LD180320-0021_HL3MGCCXY_L2_clean_bin.29.orig.fa_	OEZ008529	yes	LMSG_G000007778.1	OES073698	hglE-KS	PKSother	Polymetallic	30.30	China: Guizhou, Longli
L180322-P-6-LD180320-0021_HL3MGCCXY_L2_clean_bin.29.orig.fa_	OEZ008529	yes	LMSG_G000007778.1	OES073698	T3PKS	PKSother	Polymetallic	20.41	China: Guizhou, Longli
L180322-P-6-LD180320-0021_HL3MGCCXY_L2_clean_bin.29.orig.fa_	OEZ008529	yes	LMSG_G000007778.1	OES073698	bacteriocin	RiPPs	Polymetallic	7.87	China: Guizhou, Longli
L180322-P-6-LD180320-0021_HL3MGCCXY_L2_clean_bin.29.orig.fa_	OEZ008529	yes	LMSG_G000007778.1	OES073698	terpene	Terpene	Polymetallic	21.49	China: Guizhou, Longli
L180322-P-6-LD180320-0021_HL3MGCCXY_L2_clean_bin.3.orig.fa_k	OEZ008529	yes	LMSG_G000008762.1	OES073698	terpene	Terpene	Polymetallic	7.39	China: Guizhou, Longli
L180322-P-6-LD180320-0021_HL3MGCCXY_L2_clean_bin.3.orig.fa_k	OEZ008529	yes	LMSG_G000008762.1	OES073698	terpene	Terpene	Polymetallic	5.96	China: Guizhou, Longli
L180322-P-6-LD180320-0021_HL3MGCCXY_L2_clean_bin.30.orig.fa_	OEZ008529	yes	LMSG_G000004475.1	OES073698	terpene	Terpene	Polymetallic	13.82	China: Guizhou, Longli
L180322-P-6-LD180320-0021_HL3MGCCXY_L2_clean_bin.32.orig.fa_	OEZ008529	yes	LMSG_G000005423.1	OES073698	acyl_amino_acids	Others	Polymetallic	8.00	China: Guizhou, Longli
L180322-P-6-LD180320-0021_HL3MGCCXY_L2_clean_bin.34.orig.fa_	OEZ008529	no	LMSG_G000010944.1	OES073698	NRPS	NRPS	Polymetallic	45.06	China: Guizhou, Longli
L180322-P-6-LD180320-0021_HL3MGCCXY_L2_clean_bin.34.orig.fa_	OEZ008529	no	LMSG_G000010944.1	OES073698	thiopeptide	RiPPs	Polymetallic	18.83	China: Guizhou, Longli
L180322-P-6-LD180320-0021_HL3MGCCXY_L2_clean_bin.34.orig.fa_	OEZ008529	no	LMSG_G000010944.1	OES073698	bacteriocin	RiPPs	Polymetallic	8.60	China: Guizhou, Longli
L180322-P-6-LD180320-0021_HL3MGCCXY_L2_clean_bin.34.orig.fa_	OEZ008529	no	LMSG_G000010944.1	OES073698	NRPS.T1PKS	PKS-NRP_Hybrids	Polymetallic	59.17	China: Guizhou, Longli
L180322-P-6-LD180320-0021_HL3MGCCXY_L2_clean_bin.35.orig.fa_	OEZ008529	yes	LMSG_G000005424.1	OES073698	betalactone	Others	Polymetallic	25.72	China: Guizhou, Longli
L180322-P-6-LD180320-0021_HL3MGCCXY_L2_clean_bin.35.orig.fa_	OEZ008529	yes	LMSG_G000005424.1	OES073698	terpene	Terpene	Polymetallic	20.73	China: Guizhou, Longli
L180322-P-6-LD180320-0021_HL3MGCCXY_L2_clean_bin.35.orig.fa_	OEZ008529	yes	LMSG_G000005424.1	OES073698	bacteriocin	RiPPs	Polymetallic	10.91	China: Guizhou, Longli
L180322-P-6-LD180320-0021_HL3MGCCXY_L2_clean_bin.35.orig.fa_	OEZ008529	yes	LMSG_G000005424.1	OES073698	NRPS-like	NRPS	Polymetallic	10.05	China: Guizhou, Longli
L180322-P-6-LD180320-0021_HL3MGCCXY_L2_clean_bin.35.orig.fa_	OEZ008529	yes	LMSG_G000005424.1	OES073698	hserlactone	Others	Polymetallic	12.36	China: Guizhou, Longli
L180322-P-6-LD180320-0021_HL3MGCCXY_L2_clean_bin.36.orig.fa_	OEZ008529	yes	LMSG_G000010413.1	OES073698	betalactone	Others	Polymetallic	19.04	China: Guizhou, Longli
L180322-P-6-LD180320-0021_HL3MGCCXY_L2_clean_bin.36.orig.fa_	OEZ008529	yes	LMSG_G000010413.1	OES073698	hserlactone	Others	Polymetallic	20.63	China: Guizhou, Longli
L180322-P-6-LD180320-0021_HL3MGCCXY_L2_clean_bin.36.orig.fa_	OEZ008529	yes	LMSG_G000010413.1	OES073698	terpene	Terpene	Polymetallic	20.85	China: Guizhou, Longli
L180322-P-6-LD180320-0021_HL3MGCCXY_L2_clean_bin.36.orig.fa_	OEZ008529	yes	LMSG_G000010413.1	OES073698	terpene	Terpene	Polymetallic	25.01	China: Guizhou, Longli
L180322-P-6-LD180320-0021_HL3MGCCXY_L2_clean_bin.37.orig.fa_	OEZ008529	yes	LMSG_G000009993.1	OES073698	NRPS-like	NRPS	Polymetallic	44.04	China: Guizhou, Longli
L180322-P-6-LD180320-0021_HL3MGCCXY_L2_clean_bin.37.orig.fa_	OEZ008529	yes	LMSG_G000009993.1	OES073698	terpene	Terpene	Polymetallic	21.01	China: Guizhou, Longli
L180322-P-6-LD180320-0021_HL3MGCCXY_L2_clean_bin.37.orig.fa_	OEZ008529	yes	LMSG_G000009993.1	OES073698	arylpolyene	Others	Polymetallic	20.99	China: Guizhou, Longli
L180322-P-6-LD180320-0021_HL3MGCCXY_L2_clean_bin.37.orig.fa_	OEZ008529	yes	LMSG_G000009993.1	OES073698	arylpolyene	Others	Polymetallic	39.08	China: Guizhou, Longli
L180322-P-6-LD180320-0021_HL3MGCCXY_L2_clean_bin.37.orig.fa_	OEZ008529	yes	LMSG_G000009993.1	OES073698	terpene	Terpene	Polymetallic	16.46	China: Guizhou, Longli
L180322-P-6-LD180320-0021_HL3MGCCXY_L2_clean_bin.37.orig.fa_	OEZ008529	yes	LMSG_G000009993.1	OES073698	arylpolyene	Others	Polymetallic	33.78	China: Guizhou, Longli
L180322-P-6-LD180320-0021_HL3MGCCXY_L2_clean_bin.37.orig.fa_	OEZ008529	yes	LMSG_G000009993.1	OES073698	ectoine	Others	Polymetallic	10.40	China: Guizhou, Longli

L180322-P-6-LD180320-0021_HL3MGCCXY_L2_clean_bin.39.orig.fa_	OEZ008529	yes	LMSG_G000010697.1	OES073698	arylpolyene	Others	Polymetallic	8.33	China: Guizhou, Longli
L180322-P-6-LD180320-0021_HL3MGCCXY_L2_clean_bin.39.orig.fa_	OEZ008529	yes	LMSG_G000010697.1	OES073698	NRPS.T1PKS	PKS-NRP_Hybrids	Polymetallic	19.44	China: Guizhou, Longli
L180322-P-6-LD180320-0021_HL3MGCCXY_L2_clean_bin.4.orig.fa_k	OEZ008529	yes	LMSG_G000010332.1	OES073698	T1PKS	PKSI	Polymetallic	34.06	China: Guizhou, Longli
L180322-P-6-LD180320-0021_HL3MGCCXY_L2_clean_bin.4.orig.fa_k	OEZ008529	yes	LMSG_G000010332.1	OES073698	NRPS-like	NRPS	Polymetallic	43.38	China: Guizhou, Longli
L180322-P-6-LD180320-0021_HL3MGCCXY_L2_clean_bin.41.strict.f	OEZ008529	yes	LMSG_G000005425.1	OES073698	terpene	Terpene	Polymetallic	16.59	China: Guizhou, Longli
L180322-P-6-LD180320-0021_HL3MGCCXY_L2_clean_bin.41.strict.f	OEZ008529	yes	LMSG_G000005425.1	OES073698	bacteriocin	RiPPs	Polymetallic	10.83	China: Guizhou, Longli
L180322-P-6-LD180320-0021_HL3MGCCXY_L2_clean_bin.41.strict.f	OEZ008529	yes	LMSG_G000005425.1	OES073698	NRPS-like	NRPS	Polymetallic	24.52	China: Guizhou, Longli
L180322-P-6-LD180320-0021_HL3MGCCXY_L2_clean_bin.41.strict.f	OEZ008529	yes	LMSG_G000005425.1	OES073698	terpene	Terpene	Polymetallic	5.53	China: Guizhou, Longli
L180322-P-6-LD180320-0021_HL3MGCCXY_L2_clean_bin.45.strict.f	OEZ008529	yes	LMSG_G000008196.1	OES073698	terpene	Terpene	Polymetallic	20.86	China: Guizhou, Longli
L180322-P-6-LD180320-0021_HL3MGCCXY_L2_clean_bin.47.orig.fa_	OEZ008529	yes	LMSG_G000007802.1	OES073698	terpene	Terpene	Polymetallic	24.73	China: Guizhou, Longli
L180322-P-6-LD180320-0021_HL3MGCCXY_L2_clean_bin.48.orig.fa_	OEZ008529	no	LMSG_G000006235.1	OES073698	acyl_amino_acids	Others	Polymetallic	10.56	China: Guizhou, Longli
L180322-P-6-LD180320-0021_HL3MGCCXY_L2_clean_bin.5.orig.fa_k	OEZ008529	yes	LMSG_G000010205.1	OES073698	arylpolyene	Others	Polymetallic	41.18	China: Guizhou, Longli
L180322-P-6-LD180320-0021_HL3MGCCXY_L2_clean_bin.5.orig.fa_k	OEZ008529	yes	LMSG_G000010205.1	OES073698	terpene	Terpene	Polymetallic	12.89	China: Guizhou, Longli
L180322-P-6-LD180320-0021_HL3MGCCXY_L2_clean_bin.5.orig.fa_k	OEZ008529	yes	LMSG_G000010205.1	OES073698	terpene	Terpene	Polymetallic	21.91	China: Guizhou, Longli
L180322-P-6-LD180320-0021_HL3MGCCXY_L2_clean_bin.50.orig.fa_	OEZ008529	yes	LMSG_G000008144.1	OES073698	terpene	Terpene	Polymetallic	10.40	China: Guizhou, Longli
L180322-P-6-LD180320-0021_HL3MGCCXY_L2_clean_bin.51.orig.fa_	OEZ008529	yes	LMSG_G000010658.1	OES073698	NRPS	NRPS	Polymetallic	7.88	China: Guizhou, Longli
L180322-P-6-LD180320-0021_HL3MGCCXY_L2_clean_bin.51.orig.fa_	OEZ008529	yes	LMSG_G000010658.1	OES073698	NRPS-like	NRPS	Polymetallic	42.85	China: Guizhou, Longli
L180322-P-6-LD180320-0021_HL3MGCCXY_L2_clean_bin.51.orig.fa_	OEZ008529	yes	LMSG_G000010658.1	OES073698	NRPS	NRPS	Polymetallic	29.79	China: Guizhou, Longli
L180322-P-6-LD180320-0021_HL3MGCCXY_L2_clean_bin.51.orig.fa_	OEZ008529	yes	LMSG_G000010658.1	OES073698	NRPS	NRPS	Polymetallic	15.80	China: Guizhou, Longli
L180322-P-6-LD180320-0021_HL3MGCCXY_L2_clean_bin.51.orig.fa_	OEZ008529	yes	LMSG_G000010658.1	OES073698	terpene	Terpene	Polymetallic	13.11	China: Guizhou, Longli
L180322-P-6-LD180320-0021_HL3MGCCXY_L2_clean_bin.51.orig.fa_	OEZ008529	yes	LMSG_G000010658.1	OES073698	arylpolyene	Others	Polymetallic	41.29	China: Guizhou, Longli
L180322-P-6-LD180320-0021_HL3MGCCXY_L2_clean_bin.52.orig.fa_	OEZ008529	yes	LMSG_G000011088.1	OES073698	terpene	Terpene	Polymetallic	7.37	China: Guizhou, Longli
L180322-P-6-LD180320-0021_HL3MGCCXY_L2_clean_bin.52.orig.fa_	OEZ008529	yes	LMSG_G000011088.1	OES073698	bacteriocin	RiPPs	Polymetallic	5.56	China: Guizhou, Longli
L180322-P-6-LD180320-0021_HL3MGCCXY_L2_clean_bin.52.orig.fa_	OEZ008529	yes	LMSG_G000011088.1	OES073698	NRPS-like	NRPS	Polymetallic	5.11	China: Guizhou, Longli
L180322-P-6-LD180320-0021_HL3MGCCXY_L2_clean_bin.6.orig.fa_k	OEZ008529	yes	LMSG_G000004476.1	OES073698	NRPS-like	NRPS	Polymetallic	42.81	China: Guizhou, Longli
L180322-P-6-LD180320-0021_HL3MGCCXY_L2_clean_bin.7.orig.fa_k	OEZ008529	no	LMSG_G000010805.1	OES073698	acyl_amino_acids	Others	Polymetallic	12.58	China: Guizhou, Longli
L180322-P-7-LD180320-0022_HL3MGCCXY_L3_clean_bin.12.orig.fa_	OEZ008529	no	LMSG_G000010888.1	OES073699	terpene	Terpene	Polymetallic	5.15	China: Guizhou, Longli
L180322-P-7-LD180320-0022_HL3MGCCXY_L3_clean_bin.13.permissi	OEZ008529	yes	LMSG_G000005426.1	OES073699	phosphonate	Others	Polymetallic	6.74	China: Guizhou, Longli
L180322-P-7-LD180320-0022_HL3MGCCXY_L3_clean_bin.14.orig.fa_	OEZ008529	yes	LMSG_G000010834.1	OES073699	T1PKS.hserlactone	Others	Polymetallic	13.63	China: Guizhou, Longli
L180322-P-7-LD180320-0022_HL3MGCCXY_L3_clean_bin.14.orig.fa_	OEZ008529	no	LMSG_G000010834.1	OES073699	acyl_amino_acids	Others	Polymetallic	13.20	China: Guizhou, Longli
L180322-P-7-LD180320-0022_HL3MGCCXY_L3_clean_bin.15.orig.fa_	OEZ008529	yes	LMSG_G000010659.1	OES073699	arylpolyene	Others	Polymetallic	31.43	China: Guizhou, Longli
L180322-P-7-LD180320-0022_HL3MGCCXY_L3_clean_bin.15.orig.fa_	OEZ008529	yes	LMSG_G000010659.1	OES073699	NRPS	NRPS	Polymetallic	25.04	China: Guizhou, Longli
L180322-P-7-LD180320-0022_HL3MGCCXY_L3_clean_bin.15.orig.fa_	OEZ008529	yes	LMSG_G000010659.1	OES073699	NRPS-like	NRPS	Polymetallic	25.28	China: Guizhou, Longli
L180322-P-7-LD180320-0022_HL3MGCCXY_L3_clean_bin.15.orig.fa_	OEZ008529	yes	LMSG_G000010659.1	OES073699	NRPS	NRPS	Polymetallic	10.84	China: Guizhou, Longli
L180322-P-7-LD180320-0022_HL3MGCCXY_L3_clean_bin.15.orig.fa_	OEZ008529	yes	LMSG_G000010659.1	OES073699	NRPS.T1PKS	PKS-NRP_Hybrids	Polymetallic	19.57	China: Guizhou, Longli
L180322-P-7-LD180320-0022_HL3MGCCXY_L3_clean_bin.16.orig.fa_	OEZ008529	yes	LMSG_G000008162.1	OES073699	terpene	Terpene	Polymetallic	11.22	China: Guizhou, Longli
L180322-P-7-LD180320-0022_HL3MGCCXY_L3_clean_bin.17.orig.fa_	OEZ008529	yes	LMSG_G000010206.1	OES073699	terpene	Terpene	Polymetallic	13.50	China: Guizhou, Longli
L180322-P-7-LD180320-0022_HL3MGCCXY_L3_clean_bin.17.orig.fa_	OEZ008529	yes	LMSG_G000010206.1	OES073699	arylpolyene	Others	Polymetallic	41.18	China: Guizhou, Longli
L180322-P-7-LD180320-0022_HL3MGCCXY_L3_clean_bin.17.orig.fa_	OEZ008529	yes	LMSG_G000010206.1	OES073699	terpene	Terpene	Polymetallic	21.91	China: Guizhou, Longli
L180322-P-7-LD180320-0022_HL3MGCCXY_L3_clean_bin.18.orig.fa_	OEZ008529	yes	LMSG_G000006278.1	OES073699	arylpolyene	Others	Polymetallic	9.98	China: Guizhou, Longli
L180322-P-7-LD180320-0022_HL3MGCCXY_L3_clean_bin.2.strict.fa	OEZ008529	yes	LMSG_G000004478.1	OES073699	terpene	Terpene	Polymetallic	20.89	China: Guizhou, Longli
L180322-P-7-LD180320-0022_HL3MGCCXY_L3_clean_bin.20.orig.fa_	OEZ008529	yes	LMSG_G000010352.1	OES073699	betalactone	Others	Polymetallic	11.50	China: Guizhou, Longli
L180322-P-7-LD180320-0022_HL3MGCCXY_L3_clean_bin.20.orig.fa_	OEZ008529	yes	LMSG_G000010352.1	OES073699	terpene	Terpene	Polymetallic	20.73	China: Guizhou, Longli
L180322-P-7-LD180320-0022_HL3MGCCXY_L3_clean_bin.20.orig.fa_	OEZ008529	yes	LMSG_G000010352.1	OES073699	hserlactone	Others	Polymetallic	20.64	China: Guizhou, Longli
L180322-P-7-LD180320-0022_HL3MGCCXY_L3_clean_bin.20.orig.fa_	OEZ008529	yes	LMSG_G000010352.1	OES073699	NRPS-like	NRPS	Polymetallic	37.78	China: Guizhou, Longli
L180322-P-7-LD180320-0022_HL3MGCCXY_L3_clean_bin.20.orig.fa_	OEZ008529	yes	LMSG_G000010352.1	OES073699	bacteriocin	RiPPs	Polymetallic	10.91	China: Guizhou, Longli
L180322-P-7-LD180320-0022_HL3MGCCXY_L3_clean_bin.22.orig.fa_	OEZ008529	yes	LMSG_G000005427.1	OES073699	terpene	Terpene	Polymetallic	16.35	China: Guizhou, Longli
L180322-P-7-LD180320-0022_HL3MGCCXY_L3_clean_bin.23.orig.fa_	OEZ008529	yes	LMSG_G000008780.1	OES073699	NRPS-like	NRPS	Polymetallic	6.83	China: Guizhou, Longli
L180322-P-7-LD180320-0022_HL3MGCCXY_L3_clean_bin.24.orig.fa_	OEZ008529	yes	LMSG_G000007869.1	OES073699	terpene	Terpene	Polymetallic	22.21	China: Guizhou, Longli
L180322-P-7-LD180320-0022_HL3MGCCXY_L3_clean_bin.24.orig.fa_	OEZ008529	yes	LMSG_G000007869.1	OES073699	T3PKS	PKSother	Polymetallic	20.49	China: Guizhou, Longli
L180322-P-7-LD180320-0022_HL3MGCCXY_L3_clean_bin.24.orig.fa_	OEZ008529	yes	LMSG_G000007869.1	OES073699	terpene	Terpene	Polymetallic	6.72	China: Guizhou, Longli
L180322-P-7-LD180320-0022_HL3MGCCXY_L3_clean_bin.26.strict.f	OEZ008529	yes	LMSG_G000005429.1	OES073699	bacteriocin	RiPPs	Polymetallic	10.90	China: Guizhou, Longli
L180322-P-7-LD180320-0022_HL3MGCCXY_L3_clean_bin.26.strict.f	OEZ008529	yes	LMSG_G000005429.1	OES073699	T3PKS	PKSother	Polymetallic	28.29	China: Guizhou, Longli
L180322-P-7-LD180320-0022_HL3MGCCXY_L3_clean_bin.26.strict.f	OEZ008529	yes	LMSG_G000005429.1	OES073699	terpene	Terpene	Polymetallic	16.75	China: Guizhou, Longli
L180322-P-7-LD180320-0022_HL3MGCCXY_L3_clean_bin.27.orig.fa_	OEZ008529	yes	LMSG_G000007839.1	OES073699	terpene	Terpene	Polymetallic	12.60	China: Guizhou, Longli
L180322-P-7-LD180320-0022_HL3MGCCXY_L3_clean_bin.27.orig.fa_	OEZ008529	yes	LMSG_G000007839.1	OES073699	NRPS-like	NRPS	Polymetallic	20.15	China: Guizhou, Longli
L180322-P-7-LD180320-0022_HL3MGCCXY_L3_clean_bin.27.orig.fa_	OEZ008529	yes	LMSG_G000007839.1	OES073699	T3PKS	PKSother	Polymetallic	25.87	China: Guizhou, Longli
L180322-P-7-LD180320-0022_HL3MGCCXY_L3_clean_bin.27.orig.fa_	OEZ008529	yes	LMSG_G000007839.1	OES073699	NRPS-like	NRPS	Polymetallic	16.76	China: Guizhou, Longli
L180322-P-7-LD180320-0022_HL3MGCCXY_L3_clean_bin.27.orig.fa_	OEZ008529	yes	LMSG_G000007839.1	OES073699	terpene	Terpene	Polymetallic	5.24	China: Guizhou, Longli
L180322-P-7-LD180320-0022_HL3MGCCXY_L3_clean_bin.29.orig.fa_	OEZ008529	yes	LMSG_G000010106.1	OES073699	terpene	Terpene	Polymetallic	14.42	China: Guizhou, Longli
L180322-P-7-LD180320-0022_HL3MGCCXY_L3_clean_bin.29.orig.fa_	OEZ008529	yes	LMSG_G000010106.1	OES073699	T1PKS	PKSI	Polymetallic	28.81	China: Guizhou, Longli
L180322-P-7-LD180320-0022_HL3MGCCXY_L3_clean_bin.3.orig.fa_k	OEZ008529	no	LMSG_G000010276.1	OES073699	terpene	Terpene	Polymetallic	21.17	China: Guizhou, Longli
L180322-P-7-LD180320-0022_HL3MGCCXY_L3_clean_bin.3.orig.fa_k	OEZ008529	yes	LMSG_G000010276.1	OES073699	arylpolyene	Others	Polymetallic	41.18	China: Guizhou, Longli
L180322-P-7-LD180320-0022_HL3MGCCXY_L3_clean_bin.30.orig.fa_	OEZ008529	yes	LMSG_G000006236.1	OES073699	terpene	Terpene	Polymetallic	5.12	China: Guizhou, Longli
L180322-P-7-LD180320-0022_HL3MGCCXY_L3_clean_bin.33.orig.fa_	OEZ008529	yes	LMSG_G000010328.1	OES073699	T1PKS	PKSI	Polymetallic	34.10	China: Guizhou, Longli
L180322-P-7-LD180320-0022_HL3MGCCXY_L3_clean_bin.33.orig.fa_	OEZ008529	yes	LMSG_G000010328.1	OES073699	NRPS-like	NRPS	Polymetallic	28.65	China: Guizhou, Longli
L180322-P-7-LD180320-0022_HL3MGCCXY_L3_clean_bin.34.strict.f	OEZ008529	yes	LMSG_G000011004.1	OES073699	thiopeptide	RiPPs	Polymetallic	35.39	China: Guizhou, Longli
L180322-P-7-LD180320-0022_HL3MGCCXY_L3_clean_bin.34.strict.f	OEZ008529	yes	LMSG_G000011004.1	OES073699	bacteriocin	RiPPs	Polymetallic	7.53	China: Guizhou, Longli
L180322-P-7-LD180320-0022_HL3MGCCXY_L3_clean_bin.35.orig.fa_	OEZ008529	yes	LMSG_G000010963.1	OES073699	T1PKS.hglE-KS	PKSother	Polymetallic	51.26	China: Guizhou, Longli
L180322-P-7-LD180320-0022_HL3MGCCXY_L3_clean_bin.38.orig.fa_	OEZ008529	yes	LMSG_G000010664.1	OES073699	NRPS	NRPS	Polymetallic	6.93	China: Guizhou, Longli
L180322-P-7-LD180320-0022_HL3MGCCXY_L3_clean_bin.38.orig.fa_	OEZ008529	yes	LMSG_G000010664.1	OES073699	NRPS.T1PKS	PKS-NRP_Hybrids	Polymetallic	6.57	China: Guizhou, Longli
L180322-P-7-LD180320-0022_HL3MGCCXY_L3_clean_bin.38.orig.fa_	OEZ008529	yes	LMSG_G000010664.1	OES073699	NRPS-like	NRPS	Polymetallic	20.52	China: Guizhou, Longli
L180322-P-7-LD180320-0022_HL3MGCCXY_L3_clean_bin.4.orig.fa_k	OEZ008529	yes	LMSG_G000010219.1	OES073699	terpene	Terpene	Polymetallic	13.20	China: Guizhou, Longli
L180322-P-7-LD180320-0022_HL3MGCCXY_L3_clean_bin.4.orig.fa_k	OEZ008529	yes	LMSG_G000010219.1	OES073699	terpene	Terpene	Polymetallic	6.19	China: Guizhou, Longli
L180322-P-7-LD180320-0022_HL3MGCCXY_L3_clean_bin.40.orig.fa_	OEZ008529	yes	LMSG_G000009994.1	OES073699	terpene	Terpene	Polymetallic	21.81	China: Guizhou, Longli
L180322-P-7-LD180320-0022_HL3MGCCXY_L3_clean_bin.40.orig.fa_	OEZ008529	yes	LMSG_G000009994.1	OES073699	arylpolyene	Others	Polymetallic	33.78	China: Guizhou, Longli
L180322-P-7-LD180320-0022_HL3MGCCXY_L3_clean_bin.40.orig.fa_	OEZ008529	yes	LMSG_G000009994.1	OES073699	arylpolyene	Others	Polymetallic	21.37	China: Guizhou, Longli
L180322-P-7-LD180320-0022_HL3MGCCXY_L3_clean_bin.40.orig.fa_	OEZ008529	yes	LMSG_G000009994.1	OES073699	terpene	Terpene	Polymetallic	21.84	China: Guizhou, Longli
L180322-P-7-LD180320-0022_HL3MGCCXY_L3_clean_bin.40.orig.fa_	OEZ008529	yes	LMSG_G000009994.1	OES073699	arylpolyene	Others	Polymetallic	42.25	China: Guizhou, Longli
L180322-P-7-LD180320-0022_HL3MGCCXY_L3_clean_bin.40.orig.fa_	OEZ008529	yes	LMSG_G000009994.1	OES073699	NRPS-like	NRPS	Polymetallic	44.04	China: Guizhou, Longli
L180322-P-7-LD180320-0022_HL3MGCCXY_L3_clean_bin.40.orig.fa_	OEZ008529	yes	LMSG_G000009994.1	OES073699	ectoine	Others	Polymetallic	10.40	China: Guizhou, Longli
L180322-P-7-LD180320-0022_HL3MGCCXY_L3_clean_bin.41.orig.fa_	OEZ008529	yes	LMSG_G000008763.1	OES073699	terpene	Terpene	Polymetallic	5.10	China: Guizhou, Longli
L180322-P-7-LD180320-0022_HL3MGCCXY_L3_clean_bin.41.orig.fa_	OEZ008529	yes	LMSG_G000008763.1	OES073699	terpene	Terpene	Polymetallic	5.34	China: Guizhou, Longli
L180322-P-7-LD180320-0022_HL3MGCCXY_L3_clean_bin.42.orig.fa_	OEZ008529	yes	LMSG_G000004479.1	OES073699	terpene	Terpene	Polymetallic	21.49	China: Guizhou, Longli
L180322-P-7-LD180320-0022_HL3MGCCXY_L3_clean_bin.42.orig.fa_	OEZ008529	yes	LMSG_G000004479.1	OES073699	hglE-KS	PKSother	Polymetallic	23.53	China: Guizhou, Longli
L180322-P-7-LD180320-0022_HL3MGCCXY_L3_clean_bin.42.orig.fa_	OEZ008529	yes	LMSG_G000004479.1	OES073699	bacteriocin	RiPPs	Polymetallic	10.88	China: Guizhou, Longli
L180322-P-7-LD180320-0022_HL3MGCCXY_L3_clean_bin.42.orig.fa_	OEZ008529	yes	LMSG_G000004479.1	OES073699	T3PKS	PKSother	Polymetallic	25.80	China: Guizhou, Longli
L180322-P-7-LD180320-0022_HL3MGCCXY_L3_clean_bin.44.orig.fa_	OEZ008529	yes	LMSG_G000010311.1	OES073699	terpene	Terpene	Polymetallic	20.79	China: Guizhou, Longli
L180322-P-7-LD180320-0022_HL3MGCCXY_L3_clean_bin.44.orig.fa_	OEZ008529	yes	LMSG_G000010311.1	OES073699	hserlactone	Others	Polymetallic	17.48	China: Guizhou, Longli
L180322-P-7-LD180320-0022_HL3MGCCXY_L3_clean_bin.44.orig.fa_	OEZ008529	yes	LMSG_G000010311.1	OES073699	terpene	Terpene	Polymetallic	21.93	China: Guizhou, Longli
L180322-P-7-LD180320-0022_HL3MGCCXY_L3_clean_bin.44.orig.fa_	OEZ008529	yes	LMSG_G000010311.1	OES073699	terpene	Terpene	Polymetallic	16.96	China: Guizhou, Longli
L180322-P-7-LD180320-0022_HL3MGCCXY_L3_clean_bin.44.orig.fa_	OEZ008529	yes	LMSG_G000010311.1	OES073699	terpene	Terpene	Polymetallic	20.92	China: Guizhou, Longli
L180322-P-7-LD180320-0022_HL3MGCCXY_L3_clean_bin.44.orig.fa_	OEZ008529	yes	LMSG_G000010311.1	OES073699	T3PKS	PKSother	Polymetallic	41.06	China: Guizhou, Longli
L180322-P-7-LD180320-0022_HL3MGCCXY_L3_clean_bin.45.orig.fa_	OEZ008529	yes	LMSG_G000005431.1	OES073699	terpene	Terpene	Polymetallic	22.06	China: Guizhou, Longli
L180322-P-7-LD180320-0022_HL3MGCCXY_L3_clean_bin.45.orig.fa_	OEZ008529	yes	LMSG_G000005431.1	OES073699	NRPS-like	NRPS	Polymetallic	31.91	China: Guizhou, Longli
L180322-P-7-LD180320-0022_HL3MGCCXY_L3_clean_bin.45.orig.fa_	OEZ008529	yes	LMSG_G000005431.1	OES073699	bacteriocin	RiPPs	Polymetallic	10.86	China: Guizhou, Longli
L180322-P-7-LD180320-0022_HL3MGCCXY_L3_clean_bin.45.orig.fa_	OEZ008529	yes	LMSG_G000005431.1	OES073699	terpene	Terpene	Polymetallic	20.31	China: Guizhou, Longli
L180322-P-7-LD180320-0022_HL3MGCCXY_L3_clean_bin.45.orig.fa_	OEZ008529	yes	LMSG_G000005431.1	OES073699	bacteriocin	RiPPs	Polymetallic	11.72	China: Guizhou, Longli
L180322-P-7-LD180320-0022_HL3MGCCXY_L3_clean_bin.45.orig.fa_	OEZ008529	yes	LMSG_G000005431.1	OES073699	NRPS	NRPS	Polymetallic	44.50	China: Guizhou, Longli
L180322-P-7-LD180320-0022_HL3MGCCXY_L3_clean_bin.45.orig.fa_	OEZ008529	yes	LMSG_G000005431.1	OES073699	terpene	Terpene	Polymetallic	13.61	China: Guizhou, Longli
L180322-P-7-LD180320-0022_HL3MGCCXY_L3_clean_bin.47.orig.fa_	OEZ008529	yes	LMSG_G000008767.1	OES073699	terpene	Terpene	Polymetallic	8.46	China: Guizhou, Longli
L180322-P-7-LD180320-0022_HL3MGCCXY_L3_clean_bin.47.orig.fa_	OEZ008529	yes	LMSG_G000008767.1	OES073699	NRPS-like	NRPS	Polymetallic	6.84	China: Guizhou, Longli
L180322-P-7-LD180320-0022_HL3MGCCXY_L3_clean_bin.48.strict.f	OEZ008529	yes	LMSG_G000010414.1	OES073699	terpene	Terpene	Polymetallic	25.01	China: Guizhou, Longli
L180322-P-7-LD180320-0022_HL3MGCCXY_L3_clean_bin.48.strict.f	OEZ008529	yes	LMSG_G000010414.1	OES073699	terpene	Terpene	Polymetallic	20.85	China: Guizhou, Longli
L180322-P-7-LD180320-0022_HL3MGCCXY_L3_clean_bin.48.strict.f	OEZ008529	yes	LMSG_G000010414.1	OES073699	hserlactone	Others	Polymetallic	20.63	China: Guizhou, Longli
L180322-P-7-LD180320-0022_HL3MGCCXY_L3_clean_bin.48.strict.f	OEZ008529	yes	LMSG_G000010414.1	OES073699	betalactone	Others	Polymetallic	16.33	China: Guizhou, Longli
L180322-P-7-LD180320-0022_HL3MGCCXY_L3_clean_bin.48.strict.f	OEZ008529	yes	LMSG_G000010414.1	OES073699	phosphonate	Others	Polymetallic	10.96	China: Guizhou, Longli
L180322-P-7-LD180320-0022_HL3MGCCXY_L3_clean_bin.48.strict.f	OEZ008529	yes	LMSG_G000010414.1	OES073699	bacteriocin	RiPPs	Polymetallic	6.80	China: Guizhou, Longli
L180322-P-7-LD180320-0022_HL3MGCCXY_L3_clean_bin.5.orig.fa_k	OEZ008529	yes	LMSG_G000010226.1	OES073699	betalactone	Others	Polymetallic	31.27	China: Guizhou, Longli
L180322-P-7-LD180320-0022_HL3MGCCXY_L3_clean_bin.5.orig.fa_k	OEZ008529	yes	LMSG_G000010226.1	OES073699	T3PKS	PKSother	Polymetallic	11.79	China: Guizhou, Longli
L180322-P-7-LD180320-0022_HL3MGCCXY_L3_clean_bin.5.orig.fa_k	OEZ008529	yes	LMSG_G000010226.1	OES073699	terpene	Terpene	Polymetallic	21.94	China: Guizhou, Longli
L180322-P-7-LD180320-0022_HL3MGCCXY_L3_clean_bin.5.orig.fa_k	OEZ008529	yes	LMSG_G000010226.1	OES073699	terpene	Terpene	Polymetallic	14.12	China: Guizhou, Longli
L180322-P-7-LD180320-0022_HL3MGCCXY_L3_clean_bin.50.strict.f	OEZ008529	yes	LMSG_G000011313.1	OES073699	terpene	Terpene	Polymetallic	16.86	China: Guizhou, Longli
L180322-P-7-LD180320-0022_HL3MGCCXY_L3_clean_bin.50.strict.f	OEZ008529	yes	LMSG_G000011313.1	OES073699	terpene	Terpene	Polymetallic	21.06	China: Guizhou, Longli
L180322-P-7-LD180320-0022_HL3MGCCXY_L3_clean_bin.50.strict.f	OEZ008529	yes	LMSG_G000011313.1	OES073699	NRPS	NRPS	Polymetallic	42.87	China: Guizhou, Longli
L180322-P-7-LD180320-0022_HL3MGCCXY_L3_clean_bin.50.strict.f	OEZ008529	yes	LMSG_G000011313.1	OES073699	ladderane	Others	Polymetallic	28.71	China: Guizhou, Longli
L180322-P-7-LD180320-0022_HL3MGCCXY_L3_clean_bin.50.strict.f	OEZ008529	yes	LMSG_G000011313.1	OES073699	terpene	Terpene	Polymetallic	9.49	China: Guizhou, Longli
L180322-P-7-LD180320-0022_HL3MGCCXY_L3_clean_bin.51.strict.f	OEZ008529	no	LMSG_G000010806.1	OES073699	acyl_amino_acids	Others	Polymetallic	5.24	China: Guizhou, Longli
L180322-P-7-LD180320-0022_HL3MGCCXY_L3_clean_bin.52.orig.fa_	OEZ008529	yes	LMSG_G000005433.1	OES073699	terpene	Terpene	Polymetallic	5.44	China: Guizhou, Longli
L180322-P-7-LD180320-0022_HL3MGCCXY_L3_clean_bin.53.orig.fa_	OEZ008529	yes	LMSG_G000010780.1	OES073699	bacteriocin	RiPPs	Polymetallic	11.75	China: Guizhou, Longli
L180322-P-7-LD180320-0022_HL3MGCCXY_L3_clean_bin.53.orig.fa_	OEZ008529	yes	LMSG_G000010780.1	OES073699	acyl_amino_acids	Others	Polymetallic	35.48	China: Guizhou, Longli
L180322-P-7-LD180320-0022_HL3MGCCXY_L3_clean_bin.54.orig.fa_	OEZ008529	yes	LMSG_G000008597.1	OES073699	other	Others	Polymetallic	11.19	China: Guizhou, Longli
L180322-P-7-LD180320-0022_HL3MGCCXY_L3_clean_bin.6.orig.fa_k	OEZ008529	yes	LMSG_G000008197.1	OES073699	terpene	Terpene	Polymetallic	20.86	China: Guizhou, Longli
L180322-P-7-LD180320-0022_HL3MGCCXY_L3_clean_bin.7.orig.fa_k	OEZ008529	yes	LMSG_G000010398.1	OES073699	bacteriocin	RiPPs	Polymetallic	10.89	China: Guizhou, Longli
L180322-P-7-LD180320-0022_HL3MGCCXY_L3_clean_bin.7.orig.fa_k	OEZ008529	yes	LMSG_G000010398.1	OES073699	terpene	Terpene	Polymetallic	9.08	China: Guizhou, Longli
L180322-P-7-LD180320-0022_HL3MGCCXY_L3_clean_bin.9.strict.fa	OEZ008529	yes	LMSG_G000010554.1	OES073699	arylpolyene	Others	Polymetallic	41.19	China: Guizhou, Longli
L180322-P-7-LD180320-0022_HL3MGCCXY_L3_clean_bin.9.strict.fa	OEZ008529	yes	LMSG_G000010554.1	OES073699	bacteriocin	RiPPs	Polymetallic	10.84	China: Guizhou, Longli
L180322-P-7-LD180320-0022_HL3MGCCXY_L3_clean_bin.9.strict.fa	OEZ008529	yes	LMSG_G000010554.1	OES073699	terpene	Terpene	Polymetallic	23.03	China: Guizhou, Longli
L180322-P-7-LD180320-0022_HL3MGCCXY_L3_clean_bin.9.strict.fa	OEZ008529	yes	LMSG_G000010554.1	OES073699	hserlactone	Others	Polymetallic	11.66	China: Guizhou, Longli
L180322-P-7-LD180320-0023_HL3MGCCXY_L3_clean_bin.1.orig.fa_k	OEZ008529	yes	LMSG_G000004482.1	OES073729	T1PKS	PKSI	Polymetallic	28.03	China: Guangxi, Wuyi
L180322-P-7-LD180320-0023_HL3MGCCXY_L3_clean_bin.1.orig.fa_k	OEZ008529	yes	LMSG_G000004482.1	OES073729	hglE-KS	PKSother	Polymetallic	25.01	China: Guangxi, Wuyi
L180322-P-7-LD180320-0023_HL3MGCCXY_L3_clean_bin.1.orig.fa_k	OEZ008529	yes	LMSG_G000004482.1	OES073729	NRPS.transAT-PKS-like	PKS-NRP_Hybrids	Polymetallic	49.07	China: Guangxi, Wuyi
L180322-P-7-LD180320-0023_HL3MGCCXY_L3_clean_bin.1.orig.fa_k	OEZ008529	yes	LMSG_G000004482.1	OES073729	T1PKS	PKSI	Polymetallic	42.98	China: Guangxi, Wuyi
L180322-P-7-LD180320-0023_HL3MGCCXY_L3_clean_bin.1.orig.fa_k	OEZ008529	yes	LMSG_G000004482.1	OES073729	T1PKS.hglE-KS	PKSother	Polymetallic	34.87	China: Guangxi, Wuyi
L180322-P-7-LD180320-0023_HL3MGCCXY_L3_clean_bin.1.orig.fa_k	OEZ008529	yes	LMSG_G000004482.1	OES073729	T1PKS.hglE-KS	PKSother	Polymetallic	70.86	China: Guangxi, Wuyi
L180322-P-7-LD180320-0023_HL3MGCCXY_L3_clean_bin.1.orig.fa_k	OEZ008529	yes	LMSG_G000004482.1	OES073729	acyl_amino_acids	Others	Polymetallic	60.40	China: Guangxi, Wuyi
L180322-P-7-LD180320-0023_HL3MGCCXY_L3_clean_bin.1.orig.fa_k	OEZ008529	yes	LMSG_G000004482.1	OES073729	arylpolyene	Others	Polymetallic	38.63	China: Guangxi, Wuyi
L180322-P-7-LD180320-0023_HL3MGCCXY_L3_clean_bin.101.orig.fa	OEZ008529	yes	LMSG_G000008837.1	OES073729	T3PKS	PKSother	Polymetallic	20.76	China: Guangxi, Wuyi
L180322-P-7-LD180320-0023_HL3MGCCXY_L3_clean_bin.102.orig.fa	OEZ008529	yes	LMSG_G000010936.1	OES073729	terpene	Terpene	Polymetallic	16.06	China: Guangxi, Wuyi
L180322-P-7-LD180320-0023_HL3MGCCXY_L3_clean_bin.102.orig.fa	OEZ008529	yes	LMSG_G000010936.1	OES073729	acyl_amino_acids	Others	Polymetallic	45.14	China: Guangxi, Wuyi
L180322-P-7-LD180320-0023_HL3MGCCXY_L3_clean_bin.104.orig.fa	OEZ008529	yes	LMSG_G000008951.1	OES073729	terpene	Terpene	Polymetallic	13.76	China: Guangxi, Wuyi
L180322-P-7-LD180320-0023_HL3MGCCXY_L3_clean_bin.104.orig.fa	OEZ008529	yes	LMSG_G000008951.1	OES073729	bacteriocin	RiPPs	Polymetallic	7.99	China: Guangxi, Wuyi
L180322-P-7-LD180320-0023_HL3MGCCXY_L3_clean_bin.105.orig.fa	OEZ008529	yes	LMSG_G000007903.1	OES073729	terpene	Terpene	Polymetallic	15.02	China: Guangxi, Wuyi
L180322-P-7-LD180320-0023_HL3MGCCXY_L3_clean_bin.105.orig.fa	OEZ008529	yes	LMSG_G000007903.1	OES073729	T3PKS	PKSother	Polymetallic	22.88	China: Guangxi, Wuyi
L180322-P-7-LD180320-0023_HL3MGCCXY_L3_clean_bin.105.orig.fa	OEZ008529	yes	LMSG_G000007903.1	OES073729	NRPS-like	NRPS	Polymetallic	8.22	China: Guangxi, Wuyi
L180322-P-7-LD180320-0023_HL3MGCCXY_L3_clean_bin.105.orig.fa	OEZ008529	yes	LMSG_G000007903.1	OES073729	NRPS-like	NRPS	Polymetallic	31.36	China: Guangxi, Wuyi
L180322-P-7-LD180320-0023_HL3MGCCXY_L3_clean_bin.105.orig.fa	OEZ008529	yes	LMSG_G000007903.1	OES073729	terpene	Terpene	Polymetallic	12.81	China: Guangxi, Wuyi
L180322-P-7-LD180320-0023_HL3MGCCXY_L3_clean_bin.106.orig.fa	OEZ008529	yes	LMSG_G000004480.1	OES073729	terpene	Terpene	Polymetallic	16.58	China: Guangxi, Wuyi
L180322-P-7-LD180320-0023_HL3MGCCXY_L3_clean_bin.106.orig.fa	OEZ008529	yes	LMSG_G000004480.1	OES073729	terpene	Terpene	Polymetallic	21.08	China: Guangxi, Wuyi
L180322-P-7-LD180320-0023_HL3MGCCXY_L3_clean_bin.110.orig.fa	OEZ008529	yes	LMSG_G000004481.1	OES073729	terpene	Terpene	Polymetallic	14.45	China: Guangxi, Wuyi
L180322-P-7-LD180320-0023_HL3MGCCXY_L3_clean_bin.110.orig.fa	OEZ008529	yes	LMSG_G000004481.1	OES073729	arylpolyene	Others	Polymetallic	41.18	China: Guangxi, Wuyi
L180322-P-7-LD180320-0023_HL3MGCCXY_L3_clean_bin.110.orig.fa	OEZ008529	yes	LMSG_G000004481.1	OES073729	betalactone	Others	Polymetallic	20.62	China: Guangxi, Wuyi
L180322-P-7-LD180320-0023_HL3MGCCXY_L3_clean_bin.110.orig.fa	OEZ008529	yes	LMSG_G000004481.1	OES073729	betalactone	Others	Polymetallic	7.21	China: Guangxi, Wuyi
L180322-P-7-LD180320-0023_HL3MGCCXY_L3_clean_bin.114.orig.fa	OEZ008529	yes	LMSG_G000009662.1	OES073729	NRPS-like	NRPS	Polymetallic	42.69	China: Guangxi, Wuyi
L180322-P-7-LD180320-0023_HL3MGCCXY_L3_clean_bin.114.orig.fa	OEZ008529	yes	LMSG_G000009662.1	OES073729	terpene	Terpene	Polymetallic	14.23	China: Guangxi, Wuyi
L180322-P-7-LD180320-0023_HL3MGCCXY_L3_clean_bin.117.orig.fa	OEZ008529	yes	LMSG_G000008602.1	OES073729	betalactone	Others	Polymetallic	23.80	China: Guangxi, Wuyi
L180322-P-7-LD180320-0023_HL3MGCCXY_L3_clean_bin.117.orig.fa	OEZ008529	yes	LMSG_G000008602.1	OES073729	other	Others	Polymetallic	18.41	China: Guangxi, Wuyi
L180322-P-7-LD180320-0023_HL3MGCCXY_L3_clean_bin.118.orig.fa	OEZ008529	yes	LMSG_G000008814.1	OES073729	terpene	Terpene	Polymetallic	13.95	China: Guangxi, Wuyi

L180322-P-7-LD180320-0023_HL3MGCCXY_L3_clean_bin.118.orig.fa	OEZ008529	yes	LMSG_G000008814.1	OES073729	T3PKS	PKSother	Polymetallic	21.09	China: Guangxi, Wuyi
L180322-P-7-LD180320-0023_HL3MGCCXY_L3_clean_bin.13.strict.f	OEZ008529	yes	LMSG_G000011201.1	OES073729	arylpolyene	Others	Polymetallic	28.84	China: Guangxi, Wuyi
L180322-P-7-LD180320-0023_HL3MGCCXY_L3_clean_bin.14.orig.fa_	OEZ008529	yes	LMSG_G000009485.1	OES073729	lassopeptide	RiPPs	Polymetallic	22.61	China: Guangxi, Wuyi
L180322-P-7-LD180320-0023_HL3MGCCXY_L3_clean_bin.14.orig.fa_	OEZ008529	yes	LMSG_G000009485.1	OES073729	terpene	Terpene	Polymetallic	21.06	China: Guangxi, Wuyi
L180322-P-7-LD180320-0023_HL3MGCCXY_L3_clean_bin.16.permissi	OEZ008529	yes	LMSG_G000008784.1	OES073729	bacteriocin	RiPPs	Polymetallic	9.04	China: Guangxi, Wuyi
L180322-P-7-LD180320-0023_HL3MGCCXY_L3_clean_bin.16.permissi	OEZ008529	yes	LMSG_G000008784.1	OES073729	NRPS-like	NRPS	Polymetallic	17.42	China: Guangxi, Wuyi
L180322-P-7-LD180320-0023_HL3MGCCXY_L3_clean_bin.16.permissi	OEZ008529	yes	LMSG_G000008784.1	OES073729	NRPS-like	NRPS	Polymetallic	11.70	China: Guangxi, Wuyi
L180322-P-7-LD180320-0023_HL3MGCCXY_L3_clean_bin.18.strict.f	OEZ008529	yes	LMSG_G000009028.1	OES073729	bacteriocin	RiPPs	Polymetallic	10.79	China: Guangxi, Wuyi
L180322-P-7-LD180320-0023_HL3MGCCXY_L3_clean_bin.18.strict.f	OEZ008529	yes	LMSG_G000009028.1	OES073729	terpene	Terpene	Polymetallic	17.22	China: Guangxi, Wuyi
L180322-P-7-LD180320-0023_HL3MGCCXY_L3_clean_bin.18.strict.f	OEZ008529	yes	LMSG_G000009028.1	OES073729	NRPS-like	NRPS	Polymetallic	11.42	China: Guangxi, Wuyi
L180322-P-7-LD180320-0023_HL3MGCCXY_L3_clean_bin.20.strict.f	OEZ008529	yes	LMSG_G000009052.1	OES073729	T1PKS	PKSI	Polymetallic	45.82	China: Guangxi, Wuyi
L180322-P-7-LD180320-0023_HL3MGCCXY_L3_clean_bin.20.strict.f	OEZ008529	yes	LMSG_G000009052.1	OES073729	T1PKS	PKSI	Polymetallic	11.59	China: Guangxi, Wuyi
L180322-P-7-LD180320-0023_HL3MGCCXY_L3_clean_bin.20.strict.f	OEZ008529	yes	LMSG_G000009052.1	OES073729	betalactone	Others	Polymetallic	5.10	China: Guangxi, Wuyi
L180322-P-7-LD180320-0023_HL3MGCCXY_L3_clean_bin.21.orig.fa_	OEZ008529	yes	LMSG_G000008970.1	OES073729	terpene	Terpene	Polymetallic	11.24	China: Guangxi, Wuyi
L180322-P-7-LD180320-0023_HL3MGCCXY_L3_clean_bin.21.orig.fa_	OEZ008529	yes	LMSG_G000008970.1	OES073729	T3PKS	PKSother	Polymetallic	40.82	China: Guangxi, Wuyi
L180322-P-7-LD180320-0023_HL3MGCCXY_L3_clean_bin.22.strict.f	OEZ008529	yes	LMSG_G000008757.1	OES073729	terpene	Terpene	Polymetallic	6.83	China: Guangxi, Wuyi
L180322-P-7-LD180320-0023_HL3MGCCXY_L3_clean_bin.24.orig.fa_	OEZ008529	yes	LMSG_G000005437.1	OES073729	terpene	Terpene	Polymetallic	19.85	China: Guangxi, Wuyi
L180322-P-7-LD180320-0023_HL3MGCCXY_L3_clean_bin.24.orig.fa_	OEZ008529	yes	LMSG_G000005437.1	OES073729	terpene	Terpene	Polymetallic	20.82	China: Guangxi, Wuyi
L180322-P-7-LD180320-0023_HL3MGCCXY_L3_clean_bin.24.orig.fa_	OEZ008529	yes	LMSG_G000005437.1	OES073729	NRPS-like	NRPS	Polymetallic	30.94	China: Guangxi, Wuyi
L180322-P-7-LD180320-0023_HL3MGCCXY_L3_clean_bin.27.orig.fa_	OEZ008529	yes	LMSG_G000005439.1	OES073729	terpene	Terpene	Polymetallic	24.88	China: Guangxi, Wuyi
L180322-P-7-LD180320-0023_HL3MGCCXY_L3_clean_bin.27.orig.fa_	OEZ008529	yes	LMSG_G000005439.1	OES073729	NRPS-like	NRPS	Polymetallic	21.35	China: Guangxi, Wuyi
L180322-P-7-LD180320-0023_HL3MGCCXY_L3_clean_bin.27.orig.fa_	OEZ008529	yes	LMSG_G000005439.1	OES073729	arylpolyene	Others	Polymetallic	41.19	China: Guangxi, Wuyi
L180322-P-7-LD180320-0023_HL3MGCCXY_L3_clean_bin.29.strict.f	OEZ008529	no	LMSG_G000010536.1	OES073729	terpene	Terpene	Polymetallic	15.12	China: Guangxi, Wuyi
L180322-P-7-LD180320-0023_HL3MGCCXY_L3_clean_bin.3.strict.fa	OEZ008529	yes	LMSG_G000007860.1	OES073729	T3PKS	PKSother	Polymetallic	41.06	China: Guangxi, Wuyi
L180322-P-7-LD180320-0023_HL3MGCCXY_L3_clean_bin.3.strict.fa	OEZ008529	yes	LMSG_G000007860.1	OES073729	terpene	Terpene	Polymetallic	20.93	China: Guangxi, Wuyi
L180322-P-7-LD180320-0023_HL3MGCCXY_L3_clean_bin.3.strict.fa	OEZ008529	yes	LMSG_G000007860.1	OES073729	NRPS-like	NRPS	Polymetallic	24.69	China: Guangxi, Wuyi
L180322-P-7-LD180320-0023_HL3MGCCXY_L3_clean_bin.3.strict.fa	OEZ008529	yes	LMSG_G000007860.1	OES073729	lassopeptide	RiPPs	Polymetallic	12.69	China: Guangxi, Wuyi
L180322-P-7-LD180320-0023_HL3MGCCXY_L3_clean_bin.3.strict.fa	OEZ008529	yes	LMSG_G000007860.1	OES073729	hserlactone	Others	Polymetallic	14.13	China: Guangxi, Wuyi
L180322-P-7-LD180320-0023_HL3MGCCXY_L3_clean_bin.31.strict.f	OEZ008529	yes	LMSG_G000004483.1	OES073729	terpene	Terpene	Polymetallic	20.94	China: Guangxi, Wuyi
L180322-P-7-LD180320-0023_HL3MGCCXY_L3_clean_bin.31.strict.f	OEZ008529	yes	LMSG_G000004483.1	OES073729	terpene	Terpene	Polymetallic	21.05	China: Guangxi, Wuyi
L180322-P-7-LD180320-0023_HL3MGCCXY_L3_clean_bin.32.orig.fa_	OEZ008529	yes	LMSG_G000011316.1	OES073729	NRPS-like	NRPS	Polymetallic	14.45	China: Guangxi, Wuyi
L180322-P-7-LD180320-0023_HL3MGCCXY_L3_clean_bin.32.orig.fa_	OEZ008529	yes	LMSG_G000011316.1	OES073729	ladderane	Others	Polymetallic	15.79	China: Guangxi, Wuyi
L180322-P-7-LD180320-0023_HL3MGCCXY_L3_clean_bin.32.orig.fa_	OEZ008529	yes	LMSG_G000011316.1	OES073729	terpene	Terpene	Polymetallic	13.41	China: Guangxi, Wuyi
L180322-P-7-LD180320-0023_HL3MGCCXY_L3_clean_bin.32.orig.fa_	OEZ008529	yes	LMSG_G000011316.1	OES073729	terpene	Terpene	Polymetallic	17.41	China: Guangxi, Wuyi
L180322-P-7-LD180320-0023_HL3MGCCXY_L3_clean_bin.32.orig.fa_	OEZ008529	yes	LMSG_G000011316.1	OES073729	terpene	Terpene	Polymetallic	20.88	China: Guangxi, Wuyi
L180322-P-7-LD180320-0023_HL3MGCCXY_L3_clean_bin.36.strict.f	OEZ008529	yes	LMSG_G000008856.1	OES073729	terpene	Terpene	Polymetallic	5.63	China: Guangxi, Wuyi
L180322-P-7-LD180320-0023_HL3MGCCXY_L3_clean_bin.38.permissi	OEZ008529	yes	LMSG_G000006814.1	OES073729	terpene	Terpene	Polymetallic	7.16	China: Guangxi, Wuyi
L180322-P-7-LD180320-0023_HL3MGCCXY_L3_clean_bin.4.orig.fa_k	OEZ008529	yes	LMSG_G000005443.1	OES073729	NRPS	NRPS	Polymetallic	10.69	China: Guangxi, Wuyi
L180322-P-7-LD180320-0023_HL3MGCCXY_L3_clean_bin.40.strict.f	OEZ008529	yes	LMSG_G000004484.1	OES073729	arylpolyene	Others	Polymetallic	41.13	China: Guangxi, Wuyi
L180322-P-7-LD180320-0023_HL3MGCCXY_L3_clean_bin.40.strict.f	OEZ008529	yes	LMSG_G000004484.1	OES073729	terpene	Terpene	Polymetallic	21.99	China: Guangxi, Wuyi
L180322-P-7-LD180320-0023_HL3MGCCXY_L3_clean_bin.40.strict.f	OEZ008529	yes	LMSG_G000004484.1	OES073729	arylpolyene	Others	Polymetallic	25.55	China: Guangxi, Wuyi
L180322-P-7-LD180320-0023_HL3MGCCXY_L3_clean_bin.40.strict.f	OEZ008529	yes	LMSG_G000004484.1	OES073729	terpene	Terpene	Polymetallic	21.88	China: Guangxi, Wuyi
L180322-P-7-LD180320-0023_HL3MGCCXY_L3_clean_bin.40.strict.f	OEZ008529	yes	LMSG_G000004484.1	OES073729	NRPS-like	NRPS	Polymetallic	43.99	China: Guangxi, Wuyi
L180322-P-7-LD180320-0023_HL3MGCCXY_L3_clean_bin.40.strict.f	OEZ008529	yes	LMSG_G000004484.1	OES073729	arylpolyene	Others	Polymetallic	14.65	China: Guangxi, Wuyi
L180322-P-7-LD180320-0023_HL3MGCCXY_L3_clean_bin.40.strict.f	OEZ008529	yes	LMSG_G000004484.1	OES073729	bacteriocin	RiPPs	Polymetallic	5.36	China: Guangxi, Wuyi
L180322-P-7-LD180320-0023_HL3MGCCXY_L3_clean_bin.41.orig.fa_	OEZ008529	yes	LMSG_G000005442.1	OES073729	bacteriocin	RiPPs	Polymetallic	8.93	China: Guangxi, Wuyi
L180322-P-7-LD180320-0023_HL3MGCCXY_L3_clean_bin.41.orig.fa_	OEZ008529	yes	LMSG_G000005442.1	OES073729	terpene	Terpene	Polymetallic	15.12	China: Guangxi, Wuyi
L180322-P-7-LD180320-0023_HL3MGCCXY_L3_clean_bin.41.orig.fa_	OEZ008529	yes	LMSG_G000005442.1	OES073729	T3PKS	PKSother	Polymetallic	10.99	China: Guangxi, Wuyi
L180322-P-7-LD180320-0023_HL3MGCCXY_L3_clean_bin.42.orig.fa_	OEZ008529	yes	LMSG_G000009206.1	OES073729	T1PKS	PKSI	Polymetallic	12.11	China: Guangxi, Wuyi
L180322-P-7-LD180320-0023_HL3MGCCXY_L3_clean_bin.43.orig.fa_	OEZ008529	yes	LMSG_G000008861.1	OES073729	T3PKS	PKSother	Polymetallic	40.72	China: Guangxi, Wuyi
L180322-P-7-LD180320-0023_HL3MGCCXY_L3_clean_bin.43.orig.fa_	OEZ008529	yes	LMSG_G000008861.1	OES073729	terpene	Terpene	Polymetallic	21.02	China: Guangxi, Wuyi
L180322-P-7-LD180320-0023_HL3MGCCXY_L3_clean_bin.43.orig.fa_	OEZ008529	yes	LMSG_G000008861.1	OES073729	bacteriocin	RiPPs	Polymetallic	8.36	China: Guangxi, Wuyi
L180322-P-7-LD180320-0023_HL3MGCCXY_L3_clean_bin.46.orig.fa_	OEZ008529	yes	LMSG_G000010690.1	OES073729	terpene	Terpene	Polymetallic	20.84	China: Guangxi, Wuyi
L180322-P-7-LD180320-0023_HL3MGCCXY_L3_clean_bin.46.orig.fa_	OEZ008529	yes	LMSG_G000010690.1	OES073729	arylpolyene	Others	Polymetallic	14.75	China: Guangxi, Wuyi
L180322-P-7-LD180320-0023_HL3MGCCXY_L3_clean_bin.46.orig.fa_	OEZ008529	yes	LMSG_G000010690.1	OES073729	NRPS	NRPS	Polymetallic	6.00	China: Guangxi, Wuyi
L180322-P-7-LD180320-0023_HL3MGCCXY_L3_clean_bin.46.orig.fa_	OEZ008529	yes	LMSG_G000010690.1	OES073729	bacteriocin	RiPPs	Polymetallic	9.13	China: Guangxi, Wuyi
L180322-P-7-LD180320-0023_HL3MGCCXY_L3_clean_bin.46.orig.fa_	OEZ008529	yes	LMSG_G000010690.1	OES073729	T1PKS.NRPS-like	PKS-NRP_Hybrids	Polymetallic	17.30	China: Guangxi, Wuyi
L180322-P-7-LD180320-0023_HL3MGCCXY_L3_clean_bin.46.orig.fa_	OEZ008529	yes	LMSG_G000010690.1	OES073729	NRPS.ladderane	Others	Polymetallic	46.16	China: Guangxi, Wuyi
L180322-P-7-LD180320-0023_HL3MGCCXY_L3_clean_bin.48.orig.fa_	OEZ008529	yes	LMSG_G000009692.1	OES073729	NRPS-like	NRPS	Polymetallic	36.44	China: Guangxi, Wuyi
L180322-P-7-LD180320-0023_HL3MGCCXY_L3_clean_bin.48.orig.fa_	OEZ008529	yes	LMSG_G000009692.1	OES073729	hserlactone	Others	Polymetallic	6.10	China: Guangxi, Wuyi
L180322-P-7-LD180320-0023_HL3MGCCXY_L3_clean_bin.5.orig.fa_k	OEZ008529	yes	LMSG_G000008898.1	OES073729	terpene	Terpene	Polymetallic	14.17	China: Guangxi, Wuyi
L180322-P-7-LD180320-0023_HL3MGCCXY_L3_clean_bin.50.strict.f	OEZ008529	yes	LMSG_G000008867.1	OES073729	terpene	Terpene	Polymetallic	10.54	China: Guangxi, Wuyi
L180322-P-7-LD180320-0023_HL3MGCCXY_L3_clean_bin.51.orig.fa_	OEZ008529	yes	LMSG_G000009032.1	OES073729	hglE-KS	PKSother	Polymetallic	13.48	China: Guangxi, Wuyi
L180322-P-7-LD180320-0023_HL3MGCCXY_L3_clean_bin.51.orig.fa_	OEZ008529	yes	LMSG_G000009032.1	OES073729	hglE-KS	PKSother	Polymetallic	20.69	China: Guangxi, Wuyi
L180322-P-7-LD180320-0023_HL3MGCCXY_L3_clean_bin.51.orig.fa_	OEZ008529	yes	LMSG_G000009032.1	OES073729	thiopeptide	RiPPs	Polymetallic	33.34	China: Guangxi, Wuyi
L180322-P-7-LD180320-0023_HL3MGCCXY_L3_clean_bin.51.orig.fa_	OEZ008529	yes	LMSG_G000009032.1	OES073729	bacteriocin	RiPPs	Polymetallic	9.04	China: Guangxi, Wuyi
L180322-P-7-LD180320-0023_HL3MGCCXY_L3_clean_bin.51.orig.fa_	OEZ008529	yes	LMSG_G000009032.1	OES073729	T1PKS	PKSI	Polymetallic	21.60	China: Guangxi, Wuyi
L180322-P-7-LD180320-0023_HL3MGCCXY_L3_clean_bin.51.orig.fa_	OEZ008529	yes	LMSG_G000009032.1	OES073729	arylpolyene	Others	Polymetallic	8.09	China: Guangxi, Wuyi
L180322-P-7-LD180320-0023_HL3MGCCXY_L3_clean_bin.51.orig.fa_	OEZ008529	yes	LMSG_G000009032.1	OES073729	hglE-KS	PKSother	Polymetallic	36.69	China: Guangxi, Wuyi
L180322-P-7-LD180320-0023_HL3MGCCXY_L3_clean_bin.51.orig.fa_	OEZ008529	yes	LMSG_G000009032.1	OES073729	acyl_amino_acids	Others	Polymetallic	23.27	China: Guangxi, Wuyi
L180322-P-7-LD180320-0023_HL3MGCCXY_L3_clean_bin.54.orig.fa_	OEZ008529	yes	LMSG_G000010617.1	OES073729	arylpolyene	Others	Polymetallic	42.30	China: Guangxi, Wuyi
L180322-P-7-LD180320-0023_HL3MGCCXY_L3_clean_bin.54.orig.fa_	OEZ008529	yes	LMSG_G000010617.1	OES073729	terpene	Terpene	Polymetallic	23.01	China: Guangxi, Wuyi
L180322-P-7-LD180320-0023_HL3MGCCXY_L3_clean_bin.58.orig.fa_	OEZ008529	yes	LMSG_G000008823.1	OES073729	terpene	Terpene	Polymetallic	21.69	China: Guangxi, Wuyi
L180322-P-7-LD180320-0023_HL3MGCCXY_L3_clean_bin.59.permissi	OEZ008529	yes	LMSG_G000006810.1	OES073729	terpene	Terpene	Polymetallic	13.45	China: Guangxi, Wuyi
L180322-P-7-LD180320-0023_HL3MGCCXY_L3_clean_bin.6.orig.fa_k	OEZ008529	yes	LMSG_G000005447.1	OES073729	terpene	Terpene	Polymetallic	14.37	China: Guangxi, Wuyi
L180322-P-7-LD180320-0023_HL3MGCCXY_L3_clean_bin.6.orig.fa_k	OEZ008529	yes	LMSG_G000005447.1	OES073729	NRPS-like	NRPS	Polymetallic	32.02	China: Guangxi, Wuyi
L180322-P-7-LD180320-0023_HL3MGCCXY_L3_clean_bin.61.orig.fa_	OEZ008529	yes	LMSG_G000008893.1	OES073729	terpene	Terpene	Polymetallic	15.01	China: Guangxi, Wuyi
L180322-P-7-LD180320-0023_HL3MGCCXY_L3_clean_bin.61.orig.fa_	OEZ008529	yes	LMSG_G000008893.1	OES073729	terpene	Terpene	Polymetallic	20.98	China: Guangxi, Wuyi
L180322-P-7-LD180320-0023_HL3MGCCXY_L3_clean_bin.62.orig.fa_	OEZ008529	yes	LMSG_G000005444.1	OES073729	T3PKS	PKSother	Polymetallic	8.20	China: Guangxi, Wuyi
L180322-P-7-LD180320-0023_HL3MGCCXY_L3_clean_bin.62.orig.fa_	OEZ008529	yes	LMSG_G000005444.1	OES073729	arylpolyene	Others	Polymetallic	21.45	China: Guangxi, Wuyi
L180322-P-7-LD180320-0023_HL3MGCCXY_L3_clean_bin.62.orig.fa_	OEZ008529	yes	LMSG_G000005444.1	OES073729	T3PKS	PKSother	Polymetallic	8.88	China: Guangxi, Wuyi
L180322-P-7-LD180320-0023_HL3MGCCXY_L3_clean_bin.63.orig.fa_	OEZ008529	yes	LMSG_G000005445.1	OES073729	NRPS	NRPS	Polymetallic	6.06	China: Guangxi, Wuyi
L180322-P-7-LD180320-0023_HL3MGCCXY_L3_clean_bin.64.strict.f	OEZ008529	yes	LMSG_G000008842.1	OES073729	NRPS-like	NRPS	Polymetallic	22.79	China: Guangxi, Wuyi
L180322-P-7-LD180320-0023_HL3MGCCXY_L3_clean_bin.64.strict.f	OEZ008529	yes	LMSG_G000008842.1	OES073729	terpene	Terpene	Polymetallic	13.87	China: Guangxi, Wuyi
L180322-P-7-LD180320-0023_HL3MGCCXY_L3_clean_bin.67.orig.fa_	OEZ008529	yes	LMSG_G000005446.1	OES073729	terpene	Terpene	Polymetallic	21.70	China: Guangxi, Wuyi
L180322-P-7-LD180320-0023_HL3MGCCXY_L3_clean_bin.67.orig.fa_	OEZ008529	yes	LMSG_G000005446.1	OES073729	arylpolyene	Others	Polymetallic	36.49	China: Guangxi, Wuyi
L180322-P-7-LD180320-0023_HL3MGCCXY_L3_clean_bin.67.orig.fa_	OEZ008529	yes	LMSG_G000005446.1	OES073729	acyl_amino_acids	Others	Polymetallic	27.76	China: Guangxi, Wuyi
L180322-P-7-LD180320-0023_HL3MGCCXY_L3_clean_bin.67.orig.fa_	OEZ008529	yes	LMSG_G000005446.1	OES073729	NRPS-like	NRPS	Polymetallic	19.53	China: Guangxi, Wuyi
L180322-P-7-LD180320-0023_HL3MGCCXY_L3_clean_bin.67.orig.fa_	OEZ008529	yes	LMSG_G000005446.1	OES073729	terpene	Terpene	Polymetallic	21.94	China: Guangxi, Wuyi
L180322-P-7-LD180320-0023_HL3MGCCXY_L3_clean_bin.71.orig.fa_	OEZ008529	yes	LMSG_G000005449.1	OES073729	terpene	Terpene	Polymetallic	7.54	China: Guangxi, Wuyi
L180322-P-7-LD180320-0023_HL3MGCCXY_L3_clean_bin.71.orig.fa_	OEZ008529	yes	LMSG_G000005449.1	OES073729	terpene	Terpene	Polymetallic	10.54	China: Guangxi, Wuyi
L180322-P-7-LD180320-0023_HL3MGCCXY_L3_clean_bin.71.orig.fa_	OEZ008529	yes	LMSG_G000005449.1	OES073729	T3PKS	PKSother	Polymetallic	10.34	China: Guangxi, Wuyi
L180322-P-7-LD180320-0023_HL3MGCCXY_L3_clean_bin.71.orig.fa_	OEZ008529	yes	LMSG_G000005449.1	OES073729	NRPS-like.bacteriocin	Others	Polymetallic	26.49	China: Guangxi, Wuyi
L180322-P-7-LD180320-0023_HL3MGCCXY_L3_clean_bin.72.orig.fa_	OEZ008529	yes	LMSG_G000004485.1	OES073729	terpene	Terpene	Polymetallic	5.42	China: Guangxi, Wuyi
L180322-P-7-LD180320-0023_HL3MGCCXY_L3_clean_bin.72.orig.fa_	OEZ008529	yes	LMSG_G000004485.1	OES073729	indole	Others	Polymetallic	10.23	China: Guangxi, Wuyi
L180322-P-7-LD180320-0023_HL3MGCCXY_L3_clean_bin.72.orig.fa_	OEZ008529	yes	LMSG_G000004485.1	OES073729	terpene	Terpene	Polymetallic	12.99	China: Guangxi, Wuyi
L180322-P-7-LD180320-0023_HL3MGCCXY_L3_clean_bin.72.orig.fa_	OEZ008529	yes	LMSG_G000004485.1	OES073729	NRPS-like	NRPS	Polymetallic	13.79	China: Guangxi, Wuyi
L180322-P-7-LD180320-0023_HL3MGCCXY_L3_clean_bin.73.orig.fa_	OEZ008529	yes	LMSG_G000010875.1	OES073729	terpene	Terpene	Polymetallic	10.27	China: Guangxi, Wuyi
L180322-P-7-LD180320-0023_HL3MGCCXY_L3_clean_bin.73.orig.fa_	OEZ008529	yes	LMSG_G000010875.1	OES073729	terpene	Terpene	Polymetallic	20.76	China: Guangxi, Wuyi
L180322-P-7-LD180320-0023_HL3MGCCXY_L3_clean_bin.73.orig.fa_	OEZ008529	yes	LMSG_G000010875.1	OES073729	terpene	Terpene	Polymetallic	7.62	China: Guangxi, Wuyi
L180322-P-7-LD180320-0023_HL3MGCCXY_L3_clean_bin.78.orig.fa_	OEZ008529	yes	LMSG_G000010880.1	OES073729	acyl_amino_acids	Others	Polymetallic	9.89	China: Guangxi, Wuyi
L180322-P-7-LD180320-0023_HL3MGCCXY_L3_clean_bin.78.orig.fa_	OEZ008529	yes	LMSG_G000010880.1	OES073729	bacteriocin	RiPPs	Polymetallic	6.79	China: Guangxi, Wuyi
L180322-P-7-LD180320-0023_HL3MGCCXY_L3_clean_bin.78.orig.fa_	OEZ008529	yes	LMSG_G000010880.1	OES073729	terpene	Terpene	Polymetallic	13.72	China: Guangxi, Wuyi
L180322-P-7-LD180320-0023_HL3MGCCXY_L3_clean_bin.78.orig.fa_	OEZ008529	yes	LMSG_G000010880.1	OES073729	acyl_amino_acids	Others	Polymetallic	5.33	China: Guangxi, Wuyi
L180322-P-7-LD180320-0023_HL3MGCCXY_L3_clean_bin.78.orig.fa_	OEZ008529	yes	LMSG_G000010880.1	OES073729	bacteriocin	RiPPs	Polymetallic	5.40	China: Guangxi, Wuyi
L180322-P-7-LD180320-0023_HL3MGCCXY_L3_clean_bin.78.orig.fa_	OEZ008529	no	LMSG_G000010880.1	OES073729	arylpolyene	Others	Polymetallic	9.75	China: Guangxi, Wuyi
L180322-P-7-LD180320-0023_HL3MGCCXY_L3_clean_bin.79.orig.fa_	OEZ008529	yes	LMSG_G000005450.1	OES073729	bacteriocin	RiPPs	Polymetallic	7.10	China: Guangxi, Wuyi
L180322-P-7-LD180320-0023_HL3MGCCXY_L3_clean_bin.79.orig.fa_	OEZ008529	yes	LMSG_G000005450.1	OES073729	NRPS-like	NRPS	Polymetallic	5.04	China: Guangxi, Wuyi
L180322-P-7-LD180320-0023_HL3MGCCXY_L3_clean_bin.79.orig.fa_	OEZ008529	yes	LMSG_G000005450.1	OES073729	phosphonate	Others	Polymetallic	9.46	China: Guangxi, Wuyi
L180322-P-7-LD180320-0023_HL3MGCCXY_L3_clean_bin.8.orig.fa_k	OEZ008529	yes	LMSG_G000005453.1	OES073729	other	Others	Polymetallic	40.46	China: Guangxi, Wuyi
L180322-P-7-LD180320-0023_HL3MGCCXY_L3_clean_bin.8.orig.fa_k	OEZ008529	yes	LMSG_G000005453.1	OES073729	other	Others	Polymetallic	31.42	China: Guangxi, Wuyi
L180322-P-7-LD180320-0023_HL3MGCCXY_L3_clean_bin.80.orig.fa_	OEZ008529	yes	LMSG_G000009045.1	OES073729	thiopeptide	RiPPs	Polymetallic	7.49	China: Guangxi, Wuyi
L180322-P-7-LD180320-0023_HL3MGCCXY_L3_clean_bin.81.orig.fa_	OEZ008529	no	LMSG_G000010165.1	OES073729	terpene	Terpene	Polymetallic	21.71	China: Guangxi, Wuyi
L180322-P-7-LD180320-0023_HL3MGCCXY_L3_clean_bin.81.orig.fa_	OEZ008529	no	LMSG_G000010165.1	OES073729	terpene	Terpene	Polymetallic	16.06	China: Guangxi, Wuyi
L180322-P-7-LD180320-0023_HL3MGCCXY_L3_clean_bin.81.orig.fa_	OEZ008529	no	LMSG_G000010165.1	OES073729	terpene	Terpene	Polymetallic	16.21	China: Guangxi, Wuyi
L180322-P-7-LD180320-0023_HL3MGCCXY_L3_clean_bin.81.orig.fa_	OEZ008529	no	LMSG_G000010165.1	OES073729	acyl_amino_acids	Others	Polymetallic	5.33	China: Guangxi, Wuyi
L180322-P-7-LD180320-0023_HL3MGCCXY_L3_clean_bin.81.orig.fa_	OEZ008529	no	LMSG_G000010165.1	OES073729	ectoine	Others	Polymetallic	10.40	China: Guangxi, Wuyi
L180322-P-7-LD180320-0023_HL3MGCCXY_L3_clean_bin.84.strict.f	OEZ008529	yes	LMSG_G000006319.1	OES073729	CDPS	Others	Polymetallic	10.33	China: Guangxi, Wuyi
L180322-P-7-LD180320-0023_HL3MGCCXY_L3_clean_bin.85.orig.fa_	OEZ008529	yes	LMSG_G000010366.1	OES073729	bacteriocin	RiPPs	Polymetallic	10.83	China: Guangxi, Wuyi
L180322-P-7-LD180320-0023_HL3MGCCXY_L3_clean_bin.85.orig.fa_	OEZ008529	yes	LMSG_G000010366.1	OES073729	bacteriocin	RiPPs	Polymetallic	11.69	China: Guangxi, Wuyi
L180322-P-7-LD180320-0023_HL3MGCCXY_L3_clean_bin.85.orig.fa_	OEZ008529	yes	LMSG_G000010366.1	OES073729	NRPS-like	NRPS	Polymetallic	30.28	China: Guangxi, Wuyi
L180322-P-7-LD180320-0023_HL3MGCCXY_L3_clean_bin.85.orig.fa_	OEZ008529	yes	LMSG_G000010366.1	OES073729	terpene	Terpene	Polymetallic	17.97	China: Guangxi, Wuyi
L180322-P-7-LD180320-0023_HL3MGCCXY_L3_clean_bin.85.orig.fa_	OEZ008529	yes	LMSG_G000010366.1	OES073729	terpene	Terpene	Polymetallic	20.15	China: Guangxi, Wuyi
L180322-P-7-LD180320-0023_HL3MGCCXY_L3_clean_bin.85.orig.fa_	OEZ008529	yes	LMSG_G000010366.1	OES073729	betalactone	Others	Polymetallic	29.82	China: Guangxi, Wuyi
L180322-P-7-LD180320-0023_HL3MGCCXY_L3_clean_bin.85.orig.fa_	OEZ008529	yes	LMSG_G000010366.1	OES073729	terpene	Terpene	Polymetallic	13.77	China: Guangxi, Wuyi
L180322-P-7-LD180320-0023_HL3MGCCXY_L3_clean_bin.88.orig.fa_	OEZ008529	yes	LMSG_G000010049.1	OES073729	ladderane	Others	Polymetallic	28.48	China: Guangxi, Wuyi
L180322-P-7-LD180320-0023_HL3MGCCXY_L3_clean_bin.88.orig.fa_	OEZ008529	yes	LMSG_G000010049.1	OES073729	arylpolyene	Others	Polymetallic	5.81	China: Guangxi, Wuyi
L180322-P-7-LD180320-0023_HL3MGCCXY_L3_clean_bin.88.orig.fa_	OEZ008529	yes	LMSG_G000010049.1	OES073729	NRPS-like.ectoine	Others	Polymetallic	49.65	China: Guangxi, Wuyi
L180322-P-7-LD180320-0023_HL3MGCCXY_L3_clean_bin.88.orig.fa_	OEZ008529	yes	LMSG_G000010049.1	OES073729	terpene	Terpene	Polymetallic	18.73	China: Guangxi, Wuyi
L180322-P-7-LD180320-0023_HL3MGCCXY_L3_clean_bin.88.orig.fa_	OEZ008529	yes	LMSG_G000010049.1	OES073729	bacteriocin	RiPPs	Polymetallic	7.61	China: Guangxi, Wuyi
L180322-P-7-LD180320-0023_HL3MGCCXY_L3_clean_bin.89.orig.fa_	OEZ008529	yes	LMSG_G000008796.1	OES073729	terpene	Terpene	Polymetallic	9.93	China: Guangxi, Wuyi
L180322-P-7-LD180320-0023_HL3MGCCXY_L3_clean_bin.9.strict.fa	OEZ008529	yes	LMSG_G000010751.1	OES073729	acyl_amino_acids	Others	Polymetallic	60.84	China: Guangxi, Wuyi
L180322-P-7-LD180320-0023_HL3MGCCXY_L3_clean_bin.9.strict.fa	OEZ008529	yes	LMSG_G000010751.1	OES073729	bacteriocin	RiPPs	Polymetallic	11.75	China: Guangxi, Wuyi
L180322-P-7-LD180320-0023_HL3MGCCXY_L3_clean_bin.91.orig.fa_	OEZ008529	yes	LMSG_G000008985.1	OES073729	bacteriocin	RiPPs	Polymetallic	8.92	China: Guangxi, Wuyi
L180322-P-7-LD180320-0023_HL3MGCCXY_L3_clean_bin.91.orig.fa_	OEZ008529	yes	LMSG_G000008985.1	OES073729	T1PKS	PKSI	Polymetallic	6.12	China: Guangxi, Wuyi
L180322-P-7-LD180320-0023_HL3MGCCXY_L3_clean_bin.91.orig.fa_	OEZ008529	yes	LMSG_G000008985.1	OES073729	hglE-KS	PKSother	Polymetallic	7.22	China: Guangxi, Wuyi
L180322-P-7-LD180320-0023_HL3MGCCXY_L3_clean_bin.91.orig.fa_	OEZ008529	yes	LMSG_G000008985.1	OES073729	betalactone	Others	Polymetallic	12.72	China: Guangxi, Wuyi
L180322-P-7-LD180320-0023_HL3MGCCXY_L3_clean_bin.92.orig.fa_	OEZ008529	yes	LMSG_G000009657.1	OES073729	NRPS-like	NRPS	Polymetallic	42.54	China: Guangxi, Wuyi
L180322-P-7-LD180320-0023_HL3MGCCXY_L3_clean_bin.92.orig.fa_	OEZ008529	yes	LMSG_G000009657.1	OES073729	terpene	Terpene	Polymetallic	16.38	China: Guangxi, Wuyi
L180322-P-7-LD180320-0023_HL3MGCCXY_L3_clean_bin.92.orig.fa_	OEZ008529	yes	LMSG_G000009657.1	OES073729	NRPS-like	NRPS	Polymetallic	42.69	China: Guangxi, Wuyi
L180322-P-7-LD180320-0023_HL3MGCCXY_L3_clean_bin.93.orig.fa_	OEZ008529	yes	LMSG_G000011338.1	OES073729	T3PKS	PKSother	Polymetallic	30.34	China: Guangxi, Wuyi
L180322-P-7-LD180320-0023_HL3MGCCXY_L3_clean_bin.93.orig.fa_	OEZ008529	yes	LMSG_G000011338.1	OES073729	terpene	Terpene	Polymetallic	20.95	China: Guangxi, Wuyi
L180322-P-7-LD180320-0023_HL3MGCCXY_L3_clean_bin.94.orig.fa_	OEZ008529	yes	LMSG_G000006279.1	OES073729	betalactone	Others	Polymetallic	19.78	China: Guangxi, Wuyi
L180322-P-7-LD180320-0023_HL3MGCCXY_L3_clean_bin.94.orig.fa_	OEZ008529	yes	LMSG_G000006279.1	OES073729	T3PKS	PKSother	Polymetallic	13.90	China: Guangxi, Wuyi
L180322-P-7-LD180320-0023_HL3MGCCXY_L3_clean_bin.94.orig.fa_	OEZ008529	yes	LMSG_G000006279.1	OES073729	bacteriocin	RiPPs	Polymetallic	8.39	China: Guangxi, Wuyi
L180322-P-7-LD180320-0023_HL3MGCCXY_L3_clean_bin.94.orig.fa_	OEZ008529	yes	LMSG_G000006279.1	OES073729	TfuA-related	RiPPs	Polymetallic	17.79	China: Guangxi, Wuyi
L180322-P-7-LD180320-0023_HL3MGCCXY_L3_clean_bin.95.orig.fa_	OEZ008529	yes	LMSG_G000008828.1	OES073729	terpene	Terpene	Polymetallic	21.69	China: Guangxi, Wuyi
L180322-P-7-LD180320-0024_HL3MGCCXY_L3_clean_bin.1.orig.fa_k	OEZ008529	yes	LMSG_G000005462.1	OES073730	bacteriocin	RiPPs	Polymetallic	6.10	China: Guangxi, Wuyi
L180322-P-7-LD180320-0024_HL3MGCCXY_L3_clean_bin.1.orig.fa_k	OEZ008529	no	LMSG_G000005462.1	OES073730	terpene	Terpene	Polymetallic	11.31	China: Guangxi, Wuyi
L180322-P-7-LD180320-0024_HL3MGCCXY_L3_clean_bin.1.orig.fa_k	OEZ008529	yes	LMSG_G000005462.1	OES073730	terpene	Terpene	Polymetallic	18.26	China: Guangxi, Wuyi
L180322-P-7-LD180320-0024_HL3MGCCXY_L3_clean_bin.1.orig.fa_k	OEZ008529	no	LMSG_G000005462.1	OES073730	terpene	Terpene	Polymetallic	12.99	China: Guangxi, Wuyi
L180322-P-7-LD180320-0024_HL3MGCCXY_L3_clean_bin.1.orig.fa_k	OEZ008529	yes	LMSG_G000005462.1	OES073730	arylpolyene	Others	Polymetallic	31.71	China: Guangxi, Wuyi
L180322-P-7-LD180320-0024_HL3MGCCXY_L3_clean_bin.100.strict.	OEZ008529	yes	LMSG_G000005454.1	OES073730	NRPS.T1PKS	PKS-NRP_Hybrids	Polymetallic	28.55	China: Guangxi, Wuyi
L180322-P-7-LD180320-0024_HL3MGCCXY_L3_clean_bin.100.strict.	OEZ008529	yes	LMSG_G000005454.1	OES073730	NRPS-like	NRPS	Polymetallic	22.00	China: Guangxi, Wuyi
L180322-P-7-LD180320-0024_HL3MGCCXY_L3_clean_bin.100.strict.	OEZ008529	yes	LMSG_G000005454.1	OES073730	NRPS-like	NRPS	Polymetallic	20.43	China: Guangxi, Wuyi
L180322-P-7-LD180320-0024_HL3MGCCXY_L3_clean_bin.100.strict.	OEZ008529	yes	LMSG_G000005454.1	OES073730	resorcinol.arylpolyene	Others	Polymetallic	19.25	China: Guangxi, Wuyi
L180322-P-7-LD180320-0024_HL3MGCCXY_L3_clean_bin.100.strict.	OEZ008529	yes	LMSG_G000005454.1	OES073730	ladderane	Others	Polymetallic	12.62	China: Guangxi, Wuyi
L180322-P-7-LD180320-0024_HL3MGCCXY_L3_clean_bin.101.orig.fa	OEZ008529	yes	LMSG_G000008816.1	OES073730	terpene	Terpene	Polymetallic	13.30	China: Guangxi, Wuyi

L180322-P-7-LD180320-0024_HL3MGCCXY_L3_clean_bin.101.orig.fa	OEZ008529	yes	LMSG_G000008816.1	OES073730	T3PKS	PKSother	Polymetallic	41.08	China: Guangxi, Wuyi
L180322-P-7-LD180320-0024_HL3MGCCXY_L3_clean_bin.105.strict.	OEZ008529	yes	LMSG_G000010876.1	OES073730	NRPS-like	NRPS	Polymetallic	17.10	China: Guangxi, Wuyi
L180322-P-7-LD180320-0024_HL3MGCCXY_L3_clean_bin.105.strict.	OEZ008529	yes	LMSG_G000010876.1	OES073730	terpene	Terpene	Polymetallic	10.75	China: Guangxi, Wuyi
L180322-P-7-LD180320-0024_HL3MGCCXY_L3_clean_bin.105.strict.	OEZ008529	yes	LMSG_G000010876.1	OES073730	terpene	Terpene	Polymetallic	6.23	China: Guangxi, Wuyi
L180322-P-7-LD180320-0024_HL3MGCCXY_L3_clean_bin.106.orig.fa	OEZ008529	yes	LMSG_G000006280.1	OES073730	NRPS-like	NRPS	Polymetallic	5.96	China: Guangxi, Wuyi
L180322-P-7-LD180320-0024_HL3MGCCXY_L3_clean_bin.107.orig.fa	OEZ008529	yes	LMSG_G000007904.1	OES073730	T3PKS	PKSother	Polymetallic	8.45	China: Guangxi, Wuyi
L180322-P-7-LD180320-0024_HL3MGCCXY_L3_clean_bin.107.orig.fa	OEZ008529	yes	LMSG_G000007904.1	OES073730	NRPS-like	NRPS	Polymetallic	6.23	China: Guangxi, Wuyi
L180322-P-7-LD180320-0024_HL3MGCCXY_L3_clean_bin.107.orig.fa	OEZ008529	yes	LMSG_G000007904.1	OES073730	terpene	Terpene	Polymetallic	14.08	China: Guangxi, Wuyi
L180322-P-7-LD180320-0024_HL3MGCCXY_L3_clean_bin.109.strict.	OEZ008529	yes	LMSG_G000008868.1	OES073730	terpene	Terpene	Polymetallic	10.95	China: Guangxi, Wuyi
L180322-P-7-LD180320-0024_HL3MGCCXY_L3_clean_bin.109.strict.	OEZ008529	yes	LMSG_G000008868.1	OES073730	terpene	Terpene	Polymetallic	14.55	China: Guangxi, Wuyi
L180322-P-7-LD180320-0024_HL3MGCCXY_L3_clean_bin.116.orig.fa	OEZ008529	yes	LMSG_G000008836.1	OES073730	T3PKS	PKSother	Polymetallic	26.58	China: Guangxi, Wuyi
L180322-P-7-LD180320-0024_HL3MGCCXY_L3_clean_bin.116.orig.fa	OEZ008529	yes	LMSG_G000008836.1	OES073730	NRPS-like	NRPS	Polymetallic	41.25	China: Guangxi, Wuyi
L180322-P-7-LD180320-0024_HL3MGCCXY_L3_clean_bin.116.orig.fa	OEZ008529	yes	LMSG_G000008836.1	OES073730	terpene	Terpene	Polymetallic	18.48	China: Guangxi, Wuyi
L180322-P-7-LD180320-0024_HL3MGCCXY_L3_clean_bin.119.orig.fa	OEZ008529	yes	LMSG_G000010893.1	OES073730	betalactone	Others	Polymetallic	8.69	China: Guangxi, Wuyi
L180322-P-7-LD180320-0024_HL3MGCCXY_L3_clean_bin.119.orig.fa	OEZ008529	yes	LMSG_G000010893.1	OES073730	betalactone	Others	Polymetallic	13.09	China: Guangxi, Wuyi
L180322-P-7-LD180320-0024_HL3MGCCXY_L3_clean_bin.119.orig.fa	OEZ008529	no	LMSG_G000010893.1	OES073730	terpene	Terpene	Polymetallic	5.44	China: Guangxi, Wuyi
L180322-P-7-LD180320-0024_HL3MGCCXY_L3_clean_bin.120.strict.	OEZ008529	yes	LMSG_G000004487.1	OES073730	T3PKS	PKSother	Polymetallic	34.71	China: Guangxi, Wuyi
L180322-P-7-LD180320-0024_HL3MGCCXY_L3_clean_bin.120.strict.	OEZ008529	yes	LMSG_G000004487.1	OES073730	terpene	Terpene	Polymetallic	20.99	China: Guangxi, Wuyi
L180322-P-7-LD180320-0024_HL3MGCCXY_L3_clean_bin.122.orig.fa	OEZ008529	yes	LMSG_G000005458.1	OES073730	T3PKS	PKSother	Polymetallic	8.34	China: Guangxi, Wuyi
L180322-P-7-LD180320-0024_HL3MGCCXY_L3_clean_bin.128.orig.fa	OEZ008529	yes	LMSG_G000010881.1	OES073730	acyl_amino_acids	Others	Polymetallic	40.84	China: Guangxi, Wuyi
L180322-P-7-LD180320-0024_HL3MGCCXY_L3_clean_bin.128.orig.fa	OEZ008529	yes	LMSG_G000010881.1	OES073730	ladderane.bacteriocin	Others	Polymetallic	36.25	China: Guangxi, Wuyi
L180322-P-7-LD180320-0024_HL3MGCCXY_L3_clean_bin.128.orig.fa	OEZ008529	yes	LMSG_G000010881.1	OES073730	terpene	Terpene	Polymetallic	21.42	China: Guangxi, Wuyi
L180322-P-7-LD180320-0024_HL3MGCCXY_L3_clean_bin.128.orig.fa	OEZ008529	yes	LMSG_G000010881.1	OES073730	bacteriocin	RiPPs	Polymetallic	6.06	China: Guangxi, Wuyi
L180322-P-7-LD180320-0024_HL3MGCCXY_L3_clean_bin.128.orig.fa	OEZ008529	yes	LMSG_G000010881.1	OES073730	bacteriocin	RiPPs	Polymetallic	10.82	China: Guangxi, Wuyi
L180322-P-7-LD180320-0024_HL3MGCCXY_L3_clean_bin.128.orig.fa	OEZ008529	yes	LMSG_G000010881.1	OES073730	acyl_amino_acids	Others	Polymetallic	15.00	China: Guangxi, Wuyi
L180322-P-7-LD180320-0024_HL3MGCCXY_L3_clean_bin.128.orig.fa	OEZ008529	yes	LMSG_G000010881.1	OES073730	arylpolyene	Others	Polymetallic	21.73	China: Guangxi, Wuyi
L180322-P-7-LD180320-0024_HL3MGCCXY_L3_clean_bin.13.orig.fa_	OEZ008529	yes	LMSG_G000008952.1	OES073730	bacteriocin	RiPPs	Polymetallic	10.22	China: Guangxi, Wuyi
L180322-P-7-LD180320-0024_HL3MGCCXY_L3_clean_bin.13.orig.fa_	OEZ008529	yes	LMSG_G000008952.1	OES073730	terpene	Terpene	Polymetallic	8.20	China: Guangxi, Wuyi
L180322-P-7-LD180320-0024_HL3MGCCXY_L3_clean_bin.130.strict.	OEZ008529	yes	LMSG_G000006237.1	OES073730	NRPS-like	NRPS	Polymetallic	31.35	China: Guangxi, Wuyi
L180322-P-7-LD180320-0024_HL3MGCCXY_L3_clean_bin.130.strict.	OEZ008529	yes	LMSG_G000006237.1	OES073730	NRPS-like	NRPS	Polymetallic	22.27	China: Guangxi, Wuyi
L180322-P-7-LD180320-0024_HL3MGCCXY_L3_clean_bin.130.strict.	OEZ008529	yes	LMSG_G000006237.1	OES073730	bacteriocin	RiPPs	Polymetallic	7.35	China: Guangxi, Wuyi
L180322-P-7-LD180320-0024_HL3MGCCXY_L3_clean_bin.134.strict.	OEZ008529	yes	LMSG_G000005460.1	OES073730	NRPS-like	NRPS	Polymetallic	27.57	China: Guangxi, Wuyi
L180322-P-7-LD180320-0024_HL3MGCCXY_L3_clean_bin.134.strict.	OEZ008529	yes	LMSG_G000005460.1	OES073730	terpene	Terpene	Polymetallic	21.19	China: Guangxi, Wuyi
L180322-P-7-LD180320-0024_HL3MGCCXY_L3_clean_bin.135.strict.	OEZ008529	yes	LMSG_G000008801.1	OES073730	terpene	Terpene	Polymetallic	8.97	China: Guangxi, Wuyi
L180322-P-7-LD180320-0024_HL3MGCCXY_L3_clean_bin.135.strict.	OEZ008529	yes	LMSG_G000008801.1	OES073730	T3PKS	PKSother	Polymetallic	8.89	China: Guangxi, Wuyi
L180322-P-7-LD180320-0024_HL3MGCCXY_L3_clean_bin.135.strict.	OEZ008529	yes	LMSG_G000008801.1	OES073730	terpene	Terpene	Polymetallic	5.88	China: Guangxi, Wuyi
L180322-P-7-LD180320-0024_HL3MGCCXY_L3_clean_bin.136.orig.fa	OEZ008529	yes	LMSG_G000009848.1	OES073730	nucleoside	Others	Polymetallic	8.93	China: Guangxi, Wuyi
L180322-P-7-LD180320-0024_HL3MGCCXY_L3_clean_bin.14.orig.fa_	OEZ008529	yes	LMSG_G000010931.1	OES073730	acyl_amino_acids	Others	Polymetallic	47.56	China: Guangxi, Wuyi
L180322-P-7-LD180320-0024_HL3MGCCXY_L3_clean_bin.14.orig.fa_	OEZ008529	yes	LMSG_G000010931.1	OES073730	terpene	Terpene	Polymetallic	6.85	China: Guangxi, Wuyi
L180322-P-7-LD180320-0024_HL3MGCCXY_L3_clean_bin.16.orig.fa_	OEZ008529	yes	LMSG_G000008894.1	OES073730	terpene	Terpene	Polymetallic	19.67	China: Guangxi, Wuyi
L180322-P-7-LD180320-0024_HL3MGCCXY_L3_clean_bin.16.orig.fa_	OEZ008529	yes	LMSG_G000008894.1	OES073730	terpene	Terpene	Polymetallic	15.01	China: Guangxi, Wuyi
L180322-P-7-LD180320-0024_HL3MGCCXY_L3_clean_bin.17.orig.fa_	OEZ008529	yes	LMSG_G000006238.1	OES073730	acyl_amino_acids	Others	Polymetallic	45.14	China: Guangxi, Wuyi
L180322-P-7-LD180320-0024_HL3MGCCXY_L3_clean_bin.17.orig.fa_	OEZ008529	yes	LMSG_G000006238.1	OES073730	terpene	Terpene	Polymetallic	21.78	China: Guangxi, Wuyi
L180322-P-7-LD180320-0024_HL3MGCCXY_L3_clean_bin.21.orig.fa_	OEZ008529	yes	LMSG_G000005463.1	OES073730	T1PKS.PUFA	Others	Polymetallic	9.72	China: Guangxi, Wuyi
L180322-P-7-LD180320-0024_HL3MGCCXY_L3_clean_bin.22.orig.fa_	OEZ008529	yes	LMSG_G000010382.1	OES073730	hserlactone	Others	Polymetallic	9.60	China: Guangxi, Wuyi
L180322-P-7-LD180320-0024_HL3MGCCXY_L3_clean_bin.22.orig.fa_	OEZ008529	yes	LMSG_G000010382.1	OES073730	hserlactone	Others	Polymetallic	9.45	China: Guangxi, Wuyi
L180322-P-7-LD180320-0024_HL3MGCCXY_L3_clean_bin.22.orig.fa_	OEZ008529	yes	LMSG_G000010382.1	OES073730	NRPS	NRPS	Polymetallic	9.07	China: Guangxi, Wuyi
L180322-P-7-LD180320-0024_HL3MGCCXY_L3_clean_bin.23.orig.fa_	OEZ008529	yes	LMSG_G000008822.1	OES073730	terpene	Terpene	Polymetallic	16.92	China: Guangxi, Wuyi
L180322-P-7-LD180320-0024_HL3MGCCXY_L3_clean_bin.24.orig.fa_	OEZ008529	yes	LMSG_G000010691.1	OES073730	terpene	Terpene	Polymetallic	20.84	China: Guangxi, Wuyi
L180322-P-7-LD180320-0024_HL3MGCCXY_L3_clean_bin.24.orig.fa_	OEZ008529	yes	LMSG_G000010691.1	OES073730	arylpolyene	Others	Polymetallic	15.59	China: Guangxi, Wuyi
L180322-P-7-LD180320-0024_HL3MGCCXY_L3_clean_bin.24.orig.fa_	OEZ008529	yes	LMSG_G000010691.1	OES073730	T1PKS.NRPS-like	PKS-NRP_Hybrids	Polymetallic	26.92	China: Guangxi, Wuyi
L180322-P-7-LD180320-0024_HL3MGCCXY_L3_clean_bin.24.orig.fa_	OEZ008529	yes	LMSG_G000010691.1	OES073730	betalactone	Others	Polymetallic	20.39	China: Guangxi, Wuyi
L180322-P-7-LD180320-0024_HL3MGCCXY_L3_clean_bin.24.orig.fa_	OEZ008529	yes	LMSG_G000010691.1	OES073730	bacteriocin	RiPPs	Polymetallic	7.14	China: Guangxi, Wuyi
L180322-P-7-LD180320-0024_HL3MGCCXY_L3_clean_bin.24.orig.fa_	OEZ008529	yes	LMSG_G000010691.1	OES073730	NRPS	NRPS	Polymetallic	7.35	China: Guangxi, Wuyi
L180322-P-7-LD180320-0024_HL3MGCCXY_L3_clean_bin.26.orig.fa_	OEZ008529	yes	LMSG_G000009503.1	OES073730	betalactone	Others	Polymetallic	17.08	China: Guangxi, Wuyi
L180322-P-7-LD180320-0024_HL3MGCCXY_L3_clean_bin.27.orig.fa_	OEZ008529	yes	LMSG_G000005465.1	OES073730	arylpolyene	Others	Polymetallic	20.32	China: Guangxi, Wuyi
L180322-P-7-LD180320-0024_HL3MGCCXY_L3_clean_bin.28.strict.f	OEZ008529	yes	LMSG_G000010754.1	OES073730	acyl_amino_acids	Others	Polymetallic	19.34	China: Guangxi, Wuyi
L180322-P-7-LD180320-0024_HL3MGCCXY_L3_clean_bin.28.strict.f	OEZ008529	yes	LMSG_G000010754.1	OES073730	bacteriocin	RiPPs	Polymetallic	7.71	China: Guangxi, Wuyi
L180322-P-7-LD180320-0024_HL3MGCCXY_L3_clean_bin.29.orig.fa_	OEZ008529	yes	LMSG_G000006335.1	OES073730	CDPS	Others	Polymetallic	14.90	China: Guangxi, Wuyi
L180322-P-7-LD180320-0024_HL3MGCCXY_L3_clean_bin.3.orig.fa_k	OEZ008529	yes	LMSG_G000005469.1	OES073730	NRPS-like	NRPS	Polymetallic	26.55	China: Guangxi, Wuyi
L180322-P-7-LD180320-0024_HL3MGCCXY_L3_clean_bin.30.orig.fa_	OEZ008529	yes	LMSG_G000005466.1	OES073730	terpene	Terpene	Polymetallic	7.43	China: Guangxi, Wuyi
L180322-P-7-LD180320-0024_HL3MGCCXY_L3_clean_bin.30.orig.fa_	OEZ008529	yes	LMSG_G000005466.1	OES073730	terpene	Terpene	Polymetallic	5.22	China: Guangxi, Wuyi
L180322-P-7-LD180320-0024_HL3MGCCXY_L3_clean_bin.31.permissi	OEZ008529	yes	LMSG_G000005467.1	OES073730	terpene	Terpene	Polymetallic	14.83	China: Guangxi, Wuyi
L180322-P-7-LD180320-0024_HL3MGCCXY_L3_clean_bin.31.permissi	OEZ008529	yes	LMSG_G000005467.1	OES073730	NRPS-like	NRPS	Polymetallic	24.72	China: Guangxi, Wuyi
L180322-P-7-LD180320-0024_HL3MGCCXY_L3_clean_bin.31.permissi	OEZ008529	yes	LMSG_G000005467.1	OES073730	terpene	Terpene	Polymetallic	22.15	China: Guangxi, Wuyi
L180322-P-7-LD180320-0024_HL3MGCCXY_L3_clean_bin.31.permissi	OEZ008529	yes	LMSG_G000005467.1	OES073730	acyl_amino_acids	Others	Polymetallic	13.73	China: Guangxi, Wuyi
L180322-P-7-LD180320-0024_HL3MGCCXY_L3_clean_bin.34.orig.fa_	OEZ008529	yes	LMSG_G000009488.1	OES073730	NRPS-like	NRPS	Polymetallic	5.14	China: Guangxi, Wuyi
L180322-P-7-LD180320-0024_HL3MGCCXY_L3_clean_bin.35.orig.fa_	OEZ008529	yes	LMSG_G000009029.1	OES073730	NRPS-like	NRPS	Polymetallic	42.68	China: Guangxi, Wuyi
L180322-P-7-LD180320-0024_HL3MGCCXY_L3_clean_bin.35.orig.fa_	OEZ008529	yes	LMSG_G000009029.1	OES073730	PKS-like	PKSother	Polymetallic	23.91	China: Guangxi, Wuyi
L180322-P-7-LD180320-0024_HL3MGCCXY_L3_clean_bin.35.orig.fa_	OEZ008529	yes	LMSG_G000009029.1	OES073730	bacteriocin	RiPPs	Polymetallic	10.71	China: Guangxi, Wuyi
L180322-P-7-LD180320-0024_HL3MGCCXY_L3_clean_bin.36.orig.fa_	OEZ008529	yes	LMSG_G000010380.1	OES073730	T1PKS	PKSI	Polymetallic	10.37	China: Guangxi, Wuyi
L180322-P-7-LD180320-0024_HL3MGCCXY_L3_clean_bin.36.orig.fa_	OEZ008529	yes	LMSG_G000010380.1	OES073730	betalactone	Others	Polymetallic	11.30	China: Guangxi, Wuyi
L180322-P-7-LD180320-0024_HL3MGCCXY_L3_clean_bin.36.orig.fa_	OEZ008529	yes	LMSG_G000010380.1	OES073730	hglE-KS	PKSother	Polymetallic	7.26	China: Guangxi, Wuyi
L180322-P-7-LD180320-0024_HL3MGCCXY_L3_clean_bin.36.orig.fa_	OEZ008529	yes	LMSG_G000010380.1	OES073730	hserlactone	Others	Polymetallic	19.94	China: Guangxi, Wuyi
L180322-P-7-LD180320-0024_HL3MGCCXY_L3_clean_bin.36.orig.fa_	OEZ008529	yes	LMSG_G000010380.1	OES073730	hserlactone	Others	Polymetallic	5.55	China: Guangxi, Wuyi
L180322-P-7-LD180320-0024_HL3MGCCXY_L3_clean_bin.36.orig.fa_	OEZ008529	yes	LMSG_G000010380.1	OES073730	hserlactone	Others	Polymetallic	13.67	China: Guangxi, Wuyi
L180322-P-7-LD180320-0024_HL3MGCCXY_L3_clean_bin.39.orig.fa_	OEZ008529	yes	LMSG_G000005468.1	OES073730	terpene	Terpene	Polymetallic	13.56	China: Guangxi, Wuyi
L180322-P-7-LD180320-0024_HL3MGCCXY_L3_clean_bin.39.orig.fa_	OEZ008529	yes	LMSG_G000005468.1	OES073730	terpene	Terpene	Polymetallic	11.01	China: Guangxi, Wuyi
L180322-P-7-LD180320-0024_HL3MGCCXY_L3_clean_bin.39.orig.fa_	OEZ008529	yes	LMSG_G000005468.1	OES073730	T1PKS	PKSI	Polymetallic	25.90	China: Guangxi, Wuyi
L180322-P-7-LD180320-0024_HL3MGCCXY_L3_clean_bin.40.orig.fa_	OEZ008529	yes	LMSG_G000009678.1	OES073730	T1PKS	PKSI	Polymetallic	11.96	China: Guangxi, Wuyi
L180322-P-7-LD180320-0024_HL3MGCCXY_L3_clean_bin.44.orig.fa_	OEZ008529	yes	LMSG_G000008919.1	OES073730	terpene	Terpene	Polymetallic	18.80	China: Guangxi, Wuyi
L180322-P-7-LD180320-0024_HL3MGCCXY_L3_clean_bin.44.orig.fa_	OEZ008529	yes	LMSG_G000008919.1	OES073730	terpene	Terpene	Polymetallic	20.92	China: Guangxi, Wuyi
L180322-P-7-LD180320-0024_HL3MGCCXY_L3_clean_bin.45.strict.f	OEZ008529	yes	LMSG_G000009334.1	OES073730	sactipeptide	RiPPs	Polymetallic	12.98	China: Guangxi, Wuyi
L180322-P-7-LD180320-0024_HL3MGCCXY_L3_clean_bin.46.orig.fa_	OEZ008529	yes	LMSG_G000007928.1	OES073730	bacteriocin	RiPPs	Polymetallic	9.42	China: Guangxi, Wuyi
L180322-P-7-LD180320-0024_HL3MGCCXY_L3_clean_bin.48.orig.fa_	OEZ008529	yes	LMSG_G000005470.1	OES073730	terpene	Terpene	Polymetallic	24.23	China: Guangxi, Wuyi
L180322-P-7-LD180320-0024_HL3MGCCXY_L3_clean_bin.48.orig.fa_	OEZ008529	yes	LMSG_G000005470.1	OES073730	betalactone	Others	Polymetallic	30.53	China: Guangxi, Wuyi
L180322-P-7-LD180320-0024_HL3MGCCXY_L3_clean_bin.48.orig.fa_	OEZ008529	yes	LMSG_G000005470.1	OES073730	lassopeptide	RiPPs	Polymetallic	22.68	China: Guangxi, Wuyi
L180322-P-7-LD180320-0024_HL3MGCCXY_L3_clean_bin.50.orig.fa_	OEZ008529	yes	LMSG_G000008865.1	OES073730	bacteriocin	RiPPs	Polymetallic	8.36	China: Guangxi, Wuyi
L180322-P-7-LD180320-0024_HL3MGCCXY_L3_clean_bin.50.orig.fa_	OEZ008529	yes	LMSG_G000008865.1	OES073730	terpene	Terpene	Polymetallic	21.02	China: Guangxi, Wuyi
L180322-P-7-LD180320-0024_HL3MGCCXY_L3_clean_bin.50.orig.fa_	OEZ008529	yes	LMSG_G000008865.1	OES073730	T3PKS	PKSother	Polymetallic	41.01	China: Guangxi, Wuyi
L180322-P-7-LD180320-0024_HL3MGCCXY_L3_clean_bin.54.orig.fa_	OEZ008529	yes	LMSG_G000005471.1	OES073730	terpene	Terpene	Polymetallic	23.01	China: Guangxi, Wuyi
L180322-P-7-LD180320-0024_HL3MGCCXY_L3_clean_bin.54.orig.fa_	OEZ008529	yes	LMSG_G000005471.1	OES073730	terpene	Terpene	Polymetallic	18.61	China: Guangxi, Wuyi
L180322-P-7-LD180320-0024_HL3MGCCXY_L3_clean_bin.54.orig.fa_	OEZ008529	yes	LMSG_G000005471.1	OES073730	arylpolyene	Others	Polymetallic	42.30	China: Guangxi, Wuyi
L180322-P-7-LD180320-0024_HL3MGCCXY_L3_clean_bin.56.orig.fa_	OEZ008529	yes	LMSG_G000008752.1	OES073730	terpene	Terpene	Polymetallic	13.35	China: Guangxi, Wuyi
L180322-P-7-LD180320-0024_HL3MGCCXY_L3_clean_bin.56.orig.fa_	OEZ008529	yes	LMSG_G000008752.1	OES073730	terpene	Terpene	Polymetallic	14.08	China: Guangxi, Wuyi
L180322-P-7-LD180320-0024_HL3MGCCXY_L3_clean_bin.57.orig.fa_	OEZ008529	yes	LMSG_G000008899.1	OES073730	terpene	Terpene	Polymetallic	21.77	China: Guangxi, Wuyi
L180322-P-7-LD180320-0024_HL3MGCCXY_L3_clean_bin.58.orig.fa_	OEZ008529	yes	LMSG_G000008797.1	OES073730	terpene	Terpene	Polymetallic	10.08	China: Guangxi, Wuyi
L180322-P-7-LD180320-0024_HL3MGCCXY_L3_clean_bin.59.orig.fa_	OEZ008529	yes	LMSG_G000006239.1	OES073730	acyl_amino_acids	Others	Polymetallic	29.65	China: Guangxi, Wuyi
L180322-P-7-LD180320-0024_HL3MGCCXY_L3_clean_bin.59.orig.fa_	OEZ008529	yes	LMSG_G000006239.1	OES073730	arylpolyene	Others	Polymetallic	6.03	China: Guangxi, Wuyi
L180322-P-7-LD180320-0024_HL3MGCCXY_L3_clean_bin.59.orig.fa_	OEZ008529	yes	LMSG_G000006239.1	OES073730	T1PKS.hglE-KS	PKSother	Polymetallic	29.87	China: Guangxi, Wuyi
L180322-P-7-LD180320-0024_HL3MGCCXY_L3_clean_bin.59.orig.fa_	OEZ008529	yes	LMSG_G000006239.1	OES073730	thiopeptide	RiPPs	Polymetallic	21.30	China: Guangxi, Wuyi
L180322-P-7-LD180320-0024_HL3MGCCXY_L3_clean_bin.59.orig.fa_	OEZ008529	yes	LMSG_G000006239.1	OES073730	hglE-KS	PKSother	Polymetallic	30.37	China: Guangxi, Wuyi
L180322-P-7-LD180320-0024_HL3MGCCXY_L3_clean_bin.59.orig.fa_	OEZ008529	yes	LMSG_G000006239.1	OES073730	bacteriocin	RiPPs	Polymetallic	8.36	China: Guangxi, Wuyi
L180322-P-7-LD180320-0024_HL3MGCCXY_L3_clean_bin.59.orig.fa_	OEZ008529	yes	LMSG_G000006239.1	OES073730	hglE-KS	PKSother	Polymetallic	36.61	China: Guangxi, Wuyi
L180322-P-7-LD180320-0024_HL3MGCCXY_L3_clean_bin.63.orig.fa_	OEZ008529	yes	LMSG_G000004488.1	OES073730	terpene	Terpene	Polymetallic	21.69	China: Guangxi, Wuyi
L180322-P-7-LD180320-0024_HL3MGCCXY_L3_clean_bin.64.orig.fa_	OEZ008529	yes	LMSG_G000008658.1	OES073730	other	Others	Polymetallic	9.14	China: Guangxi, Wuyi
L180322-P-7-LD180320-0024_HL3MGCCXY_L3_clean_bin.66.orig.fa_	OEZ008529	yes	LMSG_G000008818.1	OES073730	NRPS-like	NRPS	Polymetallic	42.61	China: Guangxi, Wuyi
L180322-P-7-LD180320-0024_HL3MGCCXY_L3_clean_bin.66.orig.fa_	OEZ008529	yes	LMSG_G000008818.1	OES073730	terpene	Terpene	Polymetallic	21.73	China: Guangxi, Wuyi
L180322-P-7-LD180320-0024_HL3MGCCXY_L3_clean_bin.7.orig.fa_k	OEZ008529	yes	LMSG_G000011321.1	OES073730	terpene	Terpene	Polymetallic	18.12	China: Guangxi, Wuyi
L180322-P-7-LD180320-0024_HL3MGCCXY_L3_clean_bin.7.orig.fa_k	OEZ008529	yes	LMSG_G000011321.1	OES073730	terpene	Terpene	Polymetallic	19.69	China: Guangxi, Wuyi
L180322-P-7-LD180320-0024_HL3MGCCXY_L3_clean_bin.7.orig.fa_k	OEZ008529	yes	LMSG_G000011321.1	OES073730	terpene	Terpene	Polymetallic	15.28	China: Guangxi, Wuyi
L180322-P-7-LD180320-0024_HL3MGCCXY_L3_clean_bin.78.orig.fa_	OEZ008529	yes	LMSG_G000009486.1	OES073730	NRPS-like	NRPS	Polymetallic	9.85	China: Guangxi, Wuyi
L180322-P-7-LD180320-0024_HL3MGCCXY_L3_clean_bin.78.orig.fa_	OEZ008529	yes	LMSG_G000009486.1	OES073730	lassopeptide	RiPPs	Polymetallic	22.85	China: Guangxi, Wuyi
L180322-P-7-LD180320-0024_HL3MGCCXY_L3_clean_bin.78.orig.fa_	OEZ008529	yes	LMSG_G000009486.1	OES073730	terpene	Terpene	Polymetallic	21.06	China: Guangxi, Wuyi
L180322-P-7-LD180320-0024_HL3MGCCXY_L3_clean_bin.8.permissiv	OEZ008529	yes	LMSG_G000006809.1	OES073730	terpene	Terpene	Polymetallic	13.43	China: Guangxi, Wuyi
L180322-P-7-LD180320-0024_HL3MGCCXY_L3_clean_bin.85.orig.fa_	OEZ008529	yes	LMSG_G000009064.1	OES073730	NRPS-like	NRPS	Polymetallic	5.51	China: Guangxi, Wuyi
L180322-P-7-LD180320-0024_HL3MGCCXY_L3_clean_bin.86.strict.f	OEZ008529	yes	LMSG_G000005475.1	OES073730	NRPS	NRPS	Polymetallic	6.79	China: Guangxi, Wuyi
L180322-P-7-LD180320-0024_HL3MGCCXY_L3_clean_bin.86.strict.f	OEZ008529	yes	LMSG_G000005475.1	OES073730	terpene	Terpene	Polymetallic	5.92	China: Guangxi, Wuyi
L180322-P-7-LD180320-0024_HL3MGCCXY_L3_clean_bin.88.orig.fa_	OEZ008529	yes	LMSG_G000005476.1	OES073730	bacteriocin	RiPPs	Polymetallic	6.87	China: Guangxi, Wuyi
L180322-P-7-LD180320-0024_HL3MGCCXY_L3_clean_bin.90.orig.fa_	OEZ008529	yes	LMSG_G000009034.1	OES073730	arylpolyene	Others	Polymetallic	16.11	China: Guangxi, Wuyi
L180322-P-7-LD180320-0024_HL3MGCCXY_L3_clean_bin.90.orig.fa_	OEZ008529	yes	LMSG_G000009034.1	OES073730	T1PKS	PKSI	Polymetallic	9.94	China: Guangxi, Wuyi
L180322-P-7-LD180320-0024_HL3MGCCXY_L3_clean_bin.90.orig.fa_	OEZ008529	yes	LMSG_G000009034.1	OES073730	T1PKS	PKSI	Polymetallic	5.26	China: Guangxi, Wuyi
L180322-P-7-LD180320-0024_HL3MGCCXY_L3_clean_bin.90.orig.fa_	OEZ008529	yes	LMSG_G000009034.1	OES073730	NRPS.transAT-PKS-like	PKS-NRP_Hybrids	Polymetallic	17.75	China: Guangxi, Wuyi
L180322-P-7-LD180320-0024_HL3MGCCXY_L3_clean_bin.90.orig.fa_	OEZ008529	yes	LMSG_G000009034.1	OES073730	hglE-KS	PKSother	Polymetallic	22.66	China: Guangxi, Wuyi
L180322-P-7-LD180320-0024_HL3MGCCXY_L3_clean_bin.90.orig.fa_	OEZ008529	yes	LMSG_G000009034.1	OES073730	T1PKS	PKSI	Polymetallic	20.78	China: Guangxi, Wuyi
L180322-P-7-LD180320-0024_HL3MGCCXY_L3_clean_bin.90.orig.fa_	OEZ008529	yes	LMSG_G000009034.1	OES073730	hglE-KS	PKSother	Polymetallic	23.02	China: Guangxi, Wuyi
L180322-P-7-LD180320-0024_HL3MGCCXY_L3_clean_bin.90.orig.fa_	OEZ008529	yes	LMSG_G000009034.1	OES073730	acyl_amino_acids	Others	Polymetallic	51.23	China: Guangxi, Wuyi
L180322-P-7-LD180320-0024_HL3MGCCXY_L3_clean_bin.90.orig.fa_	OEZ008529	yes	LMSG_G000009034.1	OES073730	T1PKS.hglE-KS	PKSother	Polymetallic	20.32	China: Guangxi, Wuyi
L180322-P-7-LD180320-0024_HL3MGCCXY_L3_clean_bin.97.orig.fa_	OEZ008529	yes	LMSG_G000010897.1	OES073730	bacteriocin	RiPPs	Polymetallic	11.73	China: Guangxi, Wuyi
L180322-P-7-LD180320-0024_HL3MGCCXY_L3_clean_bin.97.orig.fa_	OEZ008529	yes	LMSG_G000010897.1	OES073730	arylpolyene	Others	Polymetallic	41.19	China: Guangxi, Wuyi
L180322-P-7-LD180320-0024_HL3MGCCXY_L3_clean_bin.97.orig.fa_	OEZ008529	yes	LMSG_G000010897.1	OES073730	terpene	Terpene	Polymetallic	21.94	China: Guangxi, Wuyi
L180322-P-7-LD180320-0024_HL3MGCCXY_L3_clean_bin.97.orig.fa_	OEZ008529	yes	LMSG_G000010897.1	OES073730	NRPS-like	NRPS	Polymetallic	38.66	China: Guangxi, Wuyi
L180322-P-7-LD180320-0024_HL3MGCCXY_L3_clean_bin.97.orig.fa_	OEZ008529	yes	LMSG_G000010897.1	OES073730	terpene	Terpene	Polymetallic	21.70	China: Guangxi, Wuyi
L180322-P-7-LD180320-0024_HL3MGCCXY_L3_clean_bin.97.orig.fa_	OEZ008529	yes	LMSG_G000010897.1	OES073730	acyl_amino_acids	Others	Polymetallic	60.82	China: Guangxi, Wuyi
L180322-P-7-LD180320-0024_HL3MGCCXY_L3_clean_bin.98.orig.fa_	OEZ008529	yes	LMSG_G000010353.1	OES073730	terpene	Terpene	Polymetallic	11.45	China: Guangxi, Wuyi
L180322-P-7-LD180320-0024_HL3MGCCXY_L3_clean_bin.98.orig.fa_	OEZ008529	yes	LMSG_G000010353.1	OES073730	bacteriocin	RiPPs	Polymetallic	7.29	China: Guangxi, Wuyi
L180322-P-7-LD180320-0024_HL3MGCCXY_L3_clean_bin.98.orig.fa_	OEZ008529	yes	LMSG_G000010353.1	OES073730	terpene	Terpene	Polymetallic	5.71	China: Guangxi, Wuyi
L180322-P-7-LD180320-0024_HL3MGCCXY_L3_clean_bin.98.orig.fa_	OEZ008529	yes	LMSG_G000010353.1	OES073730	terpene	Terpene	Polymetallic	8.30	China: Guangxi, Wuyi
L180322-P-7-LD180320-0024_HL3MGCCXY_L3_clean_bin.98.orig.fa_	OEZ008529	yes	LMSG_G000010353.1	OES073730	T3PKS	PKSother	Polymetallic	17.66	China: Guangxi, Wuyi
L180322-P-8-LD180320-0025_HL3MGCCXY_L4_clean_bin.1.strict.fa	OEZ008529	yes	LMSG_G000008895.1	OES073731	terpene	Terpene	Polymetallic	20.98	China: Guangxi, Wuyi
L180322-P-8-LD180320-0025_HL3MGCCXY_L4_clean_bin.1.strict.fa	OEZ008529	yes	LMSG_G000008895.1	OES073731	terpene	Terpene	Polymetallic	14.07	China: Guangxi, Wuyi
L180322-P-8-LD180320-0025_HL3MGCCXY_L4_clean_bin.100.strict.	OEZ008529	yes	LMSG_G000011202.1	OES073731	arylpolyene	Others	Polymetallic	14.37	China: Guangxi, Wuyi
L180322-P-8-LD180320-0025_HL3MGCCXY_L4_clean_bin.102.orig.fa	OEZ008529	yes	LMSG_G000009487.1	OES073731	lassopeptide	RiPPs	Polymetallic	5.75	China: Guangxi, Wuyi
L180322-P-8-LD180320-0025_HL3MGCCXY_L4_clean_bin.102.orig.fa	OEZ008529	yes	LMSG_G000009487.1	OES073731	terpene	Terpene	Polymetallic	21.06	China: Guangxi, Wuyi
L180322-P-8-LD180320-0025_HL3MGCCXY_L4_clean_bin.102.orig.fa	OEZ008529	yes	LMSG_G000009487.1	OES073731	NRPS-like	NRPS	Polymetallic	24.05	China: Guangxi, Wuyi
L180322-P-8-LD180320-0025_HL3MGCCXY_L4_clean_bin.103.orig.fa	OEZ008529	yes	LMSG_G000009672.1	OES073731	NRPS-like	NRPS	Polymetallic	25.74	China: Guangxi, Wuyi
L180322-P-8-LD180320-0025_HL3MGCCXY_L4_clean_bin.104.orig.fa	OEZ008529	yes	LMSG_G000009504.1	OES073731	arylpolyene	Others	Polymetallic	24.11	China: Guangxi, Wuyi
L180322-P-8-LD180320-0025_HL3MGCCXY_L4_clean_bin.104.orig.fa	OEZ008529	yes	LMSG_G000009504.1	OES073731	T3PKS	PKSother	Polymetallic	6.65	China: Guangxi, Wuyi
L180322-P-8-LD180320-0025_HL3MGCCXY_L4_clean_bin.104.orig.fa	OEZ008529	yes	LMSG_G000009504.1	OES073731	TfuA-related	RiPPs	Polymetallic	5.77	China: Guangxi, Wuyi
L180322-P-8-LD180320-0025_HL3MGCCXY_L4_clean_bin.104.orig.fa	OEZ008529	yes	LMSG_G000009504.1	OES073731	bacteriocin	RiPPs	Polymetallic	6.36	China: Guangxi, Wuyi
L180322-P-8-LD180320-0025_HL3MGCCXY_L4_clean_bin.104.orig.fa	OEZ008529	yes	LMSG_G000009504.1	OES073731	betalactone	Others	Polymetallic	19.72	China: Guangxi, Wuyi
L180322-P-8-LD180320-0025_HL3MGCCXY_L4_clean_bin.105.orig.fa	OEZ008529	no	LMSG_G000009510.1	OES073731	betalactone	Others	Polymetallic	5.92	China: Guangxi, Wuyi
L180322-P-8-LD180320-0025_HL3MGCCXY_L4_clean_bin.108.strict.	OEZ008529	yes	LMSG_G000009077.1	OES073731	arylpolyene	Others	Polymetallic	26.04	China: Guangxi, Wuyi
L180322-P-8-LD180320-0025_HL3MGCCXY_L4_clean_bin.109.orig.fa	OEZ008529	yes	LMSG_G000005481.1	OES073731	terpene	Terpene	Polymetallic	10.61	China: Guangxi, Wuyi
L180322-P-8-LD180320-0025_HL3MGCCXY_L4_clean_bin.109.orig.fa	OEZ008529	yes	LMSG_G000005481.1	OES073731	T3PKS	PKSother	Polymetallic	29.21	China: Guangxi, Wuyi
L180322-P-8-LD180320-0025_HL3MGCCXY_L4_clean_bin.112.orig.fa	OEZ008529	yes	LMSG_G000005482.1	OES073731	NRPS-like	NRPS	Polymetallic	44.06	China: Guangxi, Wuyi
L180322-P-8-LD180320-0025_HL3MGCCXY_L4_clean_bin.112.orig.fa	OEZ008529	yes	LMSG_G000005482.1	OES073731	terpene	Terpene	Polymetallic	20.83	China: Guangxi, Wuyi
L180322-P-8-LD180320-0025_HL3MGCCXY_L4_clean_bin.112.orig.fa	OEZ008529	yes	LMSG_G000005482.1	OES073731	terpene	Terpene	Polymetallic	21.72	China: Guangxi, Wuyi

L180322-P-8-LD180320-0025_HL3MGCCXY_L4_clean_bin.112.orig.fa	OEZ008529	yes	LMSG_G000005482.1	OES073731	terpene	Terpene	Polymetallic	22.05	China: Guangxi, Wuyi
L180322-P-8-LD180320-0025_HL3MGCCXY_L4_clean_bin.112.orig.fa	OEZ008529	yes	LMSG_G000005482.1	OES073731	betalactone	Others	Polymetallic	29.82	China: Guangxi, Wuyi
L180322-P-8-LD180320-0025_HL3MGCCXY_L4_clean_bin.112.orig.fa	OEZ008529	yes	LMSG_G000005482.1	OES073731	NRPS-like.bacteriocin	Others	Polymetallic	42.68	China: Guangxi, Wuyi
L180322-P-8-LD180320-0025_HL3MGCCXY_L4_clean_bin.112.orig.fa	OEZ008529	yes	LMSG_G000005482.1	OES073731	bacteriocin	RiPPs	Polymetallic	11.69	China: Guangxi, Wuyi
L180322-P-8-LD180320-0025_HL3MGCCXY_L4_clean_bin.113.orig.fa	OEZ008529	yes	LMSG_G000008819.1	OES073731	NRPS-like	NRPS	Polymetallic	26.16	China: Guangxi, Wuyi
L180322-P-8-LD180320-0025_HL3MGCCXY_L4_clean_bin.113.orig.fa	OEZ008529	yes	LMSG_G000008819.1	OES073731	terpene	Terpene	Polymetallic	21.73	China: Guangxi, Wuyi
L180322-P-8-LD180320-0025_HL3MGCCXY_L4_clean_bin.114.orig.fa	OEZ008529	yes	LMSG_G000008753.1	OES073731	terpene	Terpene	Polymetallic	10.09	China: Guangxi, Wuyi
L180322-P-8-LD180320-0025_HL3MGCCXY_L4_clean_bin.114.orig.fa	OEZ008529	yes	LMSG_G000008753.1	OES073731	terpene	Terpene	Polymetallic	13.09	China: Guangxi, Wuyi
L180322-P-8-LD180320-0025_HL3MGCCXY_L4_clean_bin.115.orig.fa	OEZ008529	yes	LMSG_G000009698.1	OES073731	ladderane	Others	Polymetallic	17.26	China: Guangxi, Wuyi
L180322-P-8-LD180320-0025_HL3MGCCXY_L4_clean_bin.115.orig.fa	OEZ008529	yes	LMSG_G000009698.1	OES073731	ladderane	Others	Polymetallic	6.85	China: Guangxi, Wuyi
L180322-P-8-LD180320-0025_HL3MGCCXY_L4_clean_bin.115.orig.fa	OEZ008529	yes	LMSG_G000009698.1	OES073731	ladderane	Others	Polymetallic	9.43	China: Guangxi, Wuyi
L180322-P-8-LD180320-0025_HL3MGCCXY_L4_clean_bin.126.orig.fa	OEZ008529	yes	LMSG_G000010894.1	OES073731	betalactone	Others	Polymetallic	7.97	China: Guangxi, Wuyi
L180322-P-8-LD180320-0025_HL3MGCCXY_L4_clean_bin.126.orig.fa	OEZ008529	yes	LMSG_G000010894.1	OES073731	bacteriocin	RiPPs	Polymetallic	8.88	China: Guangxi, Wuyi
L180322-P-8-LD180320-0025_HL3MGCCXY_L4_clean_bin.127.orig.fa	OEZ008529	yes	LMSG_G000008798.1	OES073731	terpene	Terpene	Polymetallic	11.59	China: Guangxi, Wuyi
L180322-P-8-LD180320-0025_HL3MGCCXY_L4_clean_bin.13.orig.fa_	OEZ008529	yes	LMSG_G000004490.1	OES073731	terpene	Terpene	Polymetallic	20.56	China: Guangxi, Wuyi
L180322-P-8-LD180320-0025_HL3MGCCXY_L4_clean_bin.13.orig.fa_	OEZ008529	yes	LMSG_G000004490.1	OES073731	T3PKS	PKSother	Polymetallic	41.06	China: Guangxi, Wuyi
L180322-P-8-LD180320-0025_HL3MGCCXY_L4_clean_bin.13.orig.fa_	OEZ008529	yes	LMSG_G000004490.1	OES073731	NRPS-like	NRPS	Polymetallic	24.08	China: Guangxi, Wuyi
L180322-P-8-LD180320-0025_HL3MGCCXY_L4_clean_bin.13.orig.fa_	OEZ008529	yes	LMSG_G000004490.1	OES073731	lassopeptide	RiPPs	Polymetallic	12.34	China: Guangxi, Wuyi
L180322-P-8-LD180320-0025_HL3MGCCXY_L4_clean_bin.13.orig.fa_	OEZ008529	yes	LMSG_G000004490.1	OES073731	NRPS	NRPS	Polymetallic	45.95	China: Guangxi, Wuyi
L180322-P-8-LD180320-0025_HL3MGCCXY_L4_clean_bin.130.orig.fa	OEZ008529	yes	LMSG_G000008826.1	OES073731	terpene	Terpene	Polymetallic	21.69	China: Guangxi, Wuyi
L180322-P-8-LD180320-0025_HL3MGCCXY_L4_clean_bin.132.strict.	OEZ008529	yes	LMSG_G000009035.1	OES073731	acyl_amino_acids	Others	Polymetallic	35.80	China: Guangxi, Wuyi
L180322-P-8-LD180320-0025_HL3MGCCXY_L4_clean_bin.132.strict.	OEZ008529	yes	LMSG_G000009035.1	OES073731	NRPS.transAT-PKS-like	PKS-NRP_Hybrids	Polymetallic	30.71	China: Guangxi, Wuyi
L180322-P-8-LD180320-0025_HL3MGCCXY_L4_clean_bin.132.strict.	OEZ008529	yes	LMSG_G000009035.1	OES073731	T1PKS.hglE-KS	PKSother	Polymetallic	29.00	China: Guangxi, Wuyi
L180322-P-8-LD180320-0025_HL3MGCCXY_L4_clean_bin.132.strict.	OEZ008529	yes	LMSG_G000009035.1	OES073731	T1PKS	PKSI	Polymetallic	24.97	China: Guangxi, Wuyi
L180322-P-8-LD180320-0025_HL3MGCCXY_L4_clean_bin.132.strict.	OEZ008529	yes	LMSG_G000009035.1	OES073731	T1PKS.hglE-KS	PKSother	Polymetallic	21.36	China: Guangxi, Wuyi
L180322-P-8-LD180320-0025_HL3MGCCXY_L4_clean_bin.132.strict.	OEZ008529	yes	LMSG_G000009035.1	OES073731	arylpolyene	Others	Polymetallic	16.70	China: Guangxi, Wuyi
L180322-P-8-LD180320-0025_HL3MGCCXY_L4_clean_bin.132.strict.	OEZ008529	yes	LMSG_G000009035.1	OES073731	arylpolyene	Others	Polymetallic	14.52	China: Guangxi, Wuyi
L180322-P-8-LD180320-0025_HL3MGCCXY_L4_clean_bin.132.strict.	OEZ008529	yes	LMSG_G000009035.1	OES073731	T1PKS	PKSI	Polymetallic	13.97	China: Guangxi, Wuyi
L180322-P-8-LD180320-0025_HL3MGCCXY_L4_clean_bin.135.orig.fa	OEZ008529	yes	LMSG_G000010389.1	OES073731	betalactone	Others	Polymetallic	16.15	China: Guangxi, Wuyi
L180322-P-8-LD180320-0025_HL3MGCCXY_L4_clean_bin.135.orig.fa	OEZ008529	yes	LMSG_G000010389.1	OES073731	NRPS-like	NRPS	Polymetallic	5.44	China: Guangxi, Wuyi
L180322-P-8-LD180320-0025_HL3MGCCXY_L4_clean_bin.136.strict.	OEZ008529	yes	LMSG_G000009066.1	OES073731	NRPS-like	NRPS	Polymetallic	16.44	China: Guangxi, Wuyi
L180322-P-8-LD180320-0025_HL3MGCCXY_L4_clean_bin.136.strict.	OEZ008529	yes	LMSG_G000009066.1	OES073731	terpene	Terpene	Polymetallic	14.25	China: Guangxi, Wuyi
L180322-P-8-LD180320-0025_HL3MGCCXY_L4_clean_bin.137.strict.	OEZ008529	yes	LMSG_G000009833.1	OES073731	LAP	RiPPs	Polymetallic	22.21	China: Guangxi, Wuyi
L180322-P-8-LD180320-0025_HL3MGCCXY_L4_clean_bin.14.orig.fa_	OEZ008529	yes	LMSG_G000004491.1	OES073731	terpene	Terpene	Polymetallic	27.56	China: Guangxi, Wuyi
L180322-P-8-LD180320-0025_HL3MGCCXY_L4_clean_bin.14.orig.fa_	OEZ008529	yes	LMSG_G000004491.1	OES073731	terpene	Terpene	Polymetallic	11.12	China: Guangxi, Wuyi
L180322-P-8-LD180320-0025_HL3MGCCXY_L4_clean_bin.14.orig.fa_	OEZ008529	yes	LMSG_G000004491.1	OES073731	NRPS-like	NRPS	Polymetallic	9.01	China: Guangxi, Wuyi
L180322-P-8-LD180320-0025_HL3MGCCXY_L4_clean_bin.14.orig.fa_	OEZ008529	yes	LMSG_G000004491.1	OES073731	NRPS-like	NRPS	Polymetallic	42.52	China: Guangxi, Wuyi
L180322-P-8-LD180320-0025_HL3MGCCXY_L4_clean_bin.14.orig.fa_	OEZ008529	yes	LMSG_G000004491.1	OES073731	T3PKS	PKSother	Polymetallic	22.02	China: Guangxi, Wuyi
L180322-P-8-LD180320-0025_HL3MGCCXY_L4_clean_bin.17.orig.fa_	OEZ008529	yes	LMSG_G000010898.1	OES073731	terpene	Terpene	Polymetallic	21.93	China: Guangxi, Wuyi
L180322-P-8-LD180320-0025_HL3MGCCXY_L4_clean_bin.17.orig.fa_	OEZ008529	yes	LMSG_G000010898.1	OES073731	bacteriocin	RiPPs	Polymetallic	11.73	China: Guangxi, Wuyi
L180322-P-8-LD180320-0025_HL3MGCCXY_L4_clean_bin.17.orig.fa_	OEZ008529	yes	LMSG_G000010898.1	OES073731	NRPS-like	NRPS	Polymetallic	38.66	China: Guangxi, Wuyi
L180322-P-8-LD180320-0025_HL3MGCCXY_L4_clean_bin.17.orig.fa_	OEZ008529	yes	LMSG_G000010898.1	OES073731	terpene	Terpene	Polymetallic	21.70	China: Guangxi, Wuyi
L180322-P-8-LD180320-0025_HL3MGCCXY_L4_clean_bin.17.orig.fa_	OEZ008529	yes	LMSG_G000010898.1	OES073731	acyl_amino_acids	Others	Polymetallic	60.82	China: Guangxi, Wuyi
L180322-P-8-LD180320-0025_HL3MGCCXY_L4_clean_bin.17.orig.fa_	OEZ008529	yes	LMSG_G000010898.1	OES073731	arylpolyene	Others	Polymetallic	41.19	China: Guangxi, Wuyi
L180322-P-8-LD180320-0025_HL3MGCCXY_L4_clean_bin.18.orig.fa_	OEZ008529	yes	LMSG_G000009658.1	OES073731	NRPS-like	NRPS	Polymetallic	42.54	China: Guangxi, Wuyi
L180322-P-8-LD180320-0025_HL3MGCCXY_L4_clean_bin.18.orig.fa_	OEZ008529	yes	LMSG_G000009658.1	OES073731	terpene	Terpene	Polymetallic	16.37	China: Guangxi, Wuyi
L180322-P-8-LD180320-0025_HL3MGCCXY_L4_clean_bin.18.orig.fa_	OEZ008529	yes	LMSG_G000009658.1	OES073731	NRPS-like	NRPS	Polymetallic	42.69	China: Guangxi, Wuyi
L180322-P-8-LD180320-0025_HL3MGCCXY_L4_clean_bin.19.orig.fa_	OEZ008529	yes	LMSG_G000008802.1	OES073731	T3PKS	PKSother	Polymetallic	12.07	China: Guangxi, Wuyi
L180322-P-8-LD180320-0025_HL3MGCCXY_L4_clean_bin.19.orig.fa_	OEZ008529	yes	LMSG_G000008802.1	OES073731	terpene	Terpene	Polymetallic	22.74	China: Guangxi, Wuyi
L180322-P-8-LD180320-0025_HL3MGCCXY_L4_clean_bin.20.orig.fa_	OEZ008529	yes	LMSG_G000005488.1	OES073731	terpene	Terpene	Polymetallic	7.70	China: Guangxi, Wuyi
L180322-P-8-LD180320-0025_HL3MGCCXY_L4_clean_bin.22.strict.f	OEZ008529	yes	LMSG_G000008869.1	OES073731	terpene	Terpene	Polymetallic	10.88	China: Guangxi, Wuyi
L180322-P-8-LD180320-0025_HL3MGCCXY_L4_clean_bin.26.strict.f	OEZ008529	yes	LMSG_G000007925.1	OES073731	bacteriocin	RiPPs	Polymetallic	7.30	China: Guangxi, Wuyi
L180322-P-8-LD180320-0025_HL3MGCCXY_L4_clean_bin.27.strict.f	OEZ008529	yes	LMSG_G000008659.1	OES073731	other	Others	Polymetallic	23.73	China: Guangxi, Wuyi
L180322-P-8-LD180320-0025_HL3MGCCXY_L4_clean_bin.27.strict.f	OEZ008529	yes	LMSG_G000008659.1	OES073731	other	Others	Polymetallic	10.43	China: Guangxi, Wuyi
L180322-P-8-LD180320-0025_HL3MGCCXY_L4_clean_bin.28.orig.fa_	OEZ008529	yes	LMSG_G000010937.1	OES073731	acyl_amino_acids	Others	Polymetallic	45.11	China: Guangxi, Wuyi
L180322-P-8-LD180320-0025_HL3MGCCXY_L4_clean_bin.28.orig.fa_	OEZ008529	yes	LMSG_G000010937.1	OES073731	terpene	Terpene	Polymetallic	21.82	China: Guangxi, Wuyi
L180322-P-8-LD180320-0025_HL3MGCCXY_L4_clean_bin.3.orig.fa_k	OEZ008529	yes	LMSG_G000005491.1	OES073731	NRPS-like	NRPS	Polymetallic	42.57	China: Guangxi, Wuyi
L180322-P-8-LD180320-0025_HL3MGCCXY_L4_clean_bin.3.orig.fa_k	OEZ008529	yes	LMSG_G000005491.1	OES073731	NRPS-like	NRPS	Polymetallic	42.63	China: Guangxi, Wuyi
L180322-P-8-LD180320-0025_HL3MGCCXY_L4_clean_bin.3.orig.fa_k	OEZ008529	yes	LMSG_G000005491.1	OES073731	terpene	Terpene	Polymetallic	20.66	China: Guangxi, Wuyi
L180322-P-8-LD180320-0025_HL3MGCCXY_L4_clean_bin.3.orig.fa_k	OEZ008529	yes	LMSG_G000005491.1	OES073731	T3PKS	PKSother	Polymetallic	41.06	China: Guangxi, Wuyi
L180322-P-8-LD180320-0025_HL3MGCCXY_L4_clean_bin.33.permissi	OEZ008529	yes	LMSG_G000009514.1	OES073731	T3PKS	PKSother	Polymetallic	8.88	China: Guangxi, Wuyi
L180322-P-8-LD180320-0025_HL3MGCCXY_L4_clean_bin.35.orig.fa_	OEZ008529	yes	LMSG_G000005490.1	OES073731	terpene	Terpene	Polymetallic	20.79	China: Guangxi, Wuyi
L180322-P-8-LD180320-0025_HL3MGCCXY_L4_clean_bin.35.orig.fa_	OEZ008529	yes	LMSG_G000005490.1	OES073731	terpene	Terpene	Polymetallic	20.83	China: Guangxi, Wuyi
L180322-P-8-LD180320-0025_HL3MGCCXY_L4_clean_bin.35.orig.fa_	OEZ008529	yes	LMSG_G000005490.1	OES073731	NRPS-like	NRPS	Polymetallic	42.76	China: Guangxi, Wuyi
L180322-P-8-LD180320-0025_HL3MGCCXY_L4_clean_bin.38.orig.fa_	OEZ008529	yes	LMSG_G000004492.1	OES073731	T3PKS	PKSother	Polymetallic	34.74	China: Guangxi, Wuyi
L180322-P-8-LD180320-0025_HL3MGCCXY_L4_clean_bin.38.orig.fa_	OEZ008529	yes	LMSG_G000004492.1	OES073731	terpene	Terpene	Polymetallic	12.92	China: Guangxi, Wuyi
L180322-P-8-LD180320-0025_HL3MGCCXY_L4_clean_bin.39.strict.f	OEZ008529	yes	LMSG_G000008781.1	OES073731	NRPS-like	NRPS	Polymetallic	19.98	China: Guangxi, Wuyi
L180322-P-8-LD180320-0025_HL3MGCCXY_L4_clean_bin.39.strict.f	OEZ008529	yes	LMSG_G000008781.1	OES073731	bacteriocin	RiPPs	Polymetallic	8.72	China: Guangxi, Wuyi
L180322-P-8-LD180320-0025_HL3MGCCXY_L4_clean_bin.40.orig.fa_	OEZ008529	yes	LMSG_G000005492.1	OES073731	T1PKS	PKSI	Polymetallic	17.79	China: Guangxi, Wuyi
L180322-P-8-LD180320-0025_HL3MGCCXY_L4_clean_bin.40.orig.fa_	OEZ008529	yes	LMSG_G000005492.1	OES073731	NRPS-like	NRPS	Polymetallic	10.44	China: Guangxi, Wuyi
L180322-P-8-LD180320-0025_HL3MGCCXY_L4_clean_bin.40.orig.fa_	OEZ008529	yes	LMSG_G000005492.1	OES073731	terpene	Terpene	Polymetallic	7.96	China: Guangxi, Wuyi
L180322-P-8-LD180320-0025_HL3MGCCXY_L4_clean_bin.41.strict.f	OEZ008529	yes	LMSG_G000010187.1	OES073731	NRPS-like	NRPS	Polymetallic	23.50	China: Guangxi, Wuyi
L180322-P-8-LD180320-0025_HL3MGCCXY_L4_clean_bin.41.strict.f	OEZ008529	yes	LMSG_G000010187.1	OES073731	arylpolyene	Others	Polymetallic	18.92	China: Guangxi, Wuyi
L180322-P-8-LD180320-0025_HL3MGCCXY_L4_clean_bin.41.strict.f	OEZ008529	yes	LMSG_G000010187.1	OES073731	terpene	Terpene	Polymetallic	18.44	China: Guangxi, Wuyi
L180322-P-8-LD180320-0025_HL3MGCCXY_L4_clean_bin.43.orig.fa_	OEZ008529	yes	LMSG_G000004493.1	OES073731	NRPS-like	NRPS	Polymetallic	27.63	China: Guangxi, Wuyi
L180322-P-8-LD180320-0025_HL3MGCCXY_L4_clean_bin.45.orig.fa_	OEZ008529	yes	LMSG_G000005493.1	OES073731	nucleoside	Others	Polymetallic	12.19	China: Guangxi, Wuyi
L180322-P-8-LD180320-0025_HL3MGCCXY_L4_clean_bin.47.orig.fa_	OEZ008529	yes	LMSG_G000008858.1	OES073731	terpene	Terpene	Polymetallic	14.90	China: Guangxi, Wuyi
L180322-P-8-LD180320-0025_HL3MGCCXY_L4_clean_bin.52.orig.fa_	OEZ008529	yes	LMSG_G000007931.1	OES073731	T3PKS	PKSother	Polymetallic	8.50	China: Guangxi, Wuyi
L180322-P-8-LD180320-0025_HL3MGCCXY_L4_clean_bin.55.orig.fa_	OEZ008529	yes	LMSG_G000008920.1	OES073731	terpene	Terpene	Polymetallic	15.88	China: Guangxi, Wuyi
L180322-P-8-LD180320-0025_HL3MGCCXY_L4_clean_bin.6.orig.fa_k	OEZ008529	yes	LMSG_G000009246.1	OES073731	terpene	Terpene	Polymetallic	7.03	China: Guangxi, Wuyi
L180322-P-8-LD180320-0025_HL3MGCCXY_L4_clean_bin.60.orig.fa_	OEZ008529	yes	LMSG_G000009074.1	OES073731	T3PKS.resorcinol.arylpolyene	Others	Polymetallic	29.94	China: Guangxi, Wuyi
L180322-P-8-LD180320-0025_HL3MGCCXY_L4_clean_bin.60.orig.fa_	OEZ008529	yes	LMSG_G000009074.1	OES073731	arylpolyene	Others	Polymetallic	12.87	China: Guangxi, Wuyi
L180322-P-8-LD180320-0025_HL3MGCCXY_L4_clean_bin.62.strict.f	OEZ008529	yes	LMSG_G000008585.1	OES073731	other	Others	Polymetallic	37.21	China: Guangxi, Wuyi
L180322-P-8-LD180320-0025_HL3MGCCXY_L4_clean_bin.62.strict.f	OEZ008529	yes	LMSG_G000008585.1	OES073731	other	Others	Polymetallic	29.66	China: Guangxi, Wuyi
L180322-P-8-LD180320-0025_HL3MGCCXY_L4_clean_bin.65.orig.fa_	OEZ008529	yes	LMSG_G000010348.1	OES073731	terpene	Terpene	Polymetallic	6.83	China: Guangxi, Wuyi
L180322-P-8-LD180320-0025_HL3MGCCXY_L4_clean_bin.65.orig.fa_	OEZ008529	yes	LMSG_G000010348.1	OES073731	NRPS-like	NRPS	Polymetallic	14.34	China: Guangxi, Wuyi
L180322-P-8-LD180320-0025_HL3MGCCXY_L4_clean_bin.65.orig.fa_	OEZ008529	yes	LMSG_G000010348.1	OES073731	terpene	Terpene	Polymetallic	9.03	China: Guangxi, Wuyi
L180322-P-8-LD180320-0025_HL3MGCCXY_L4_clean_bin.67.orig.fa_	OEZ008529	yes	LMSG_G000006336.1	OES073731	CDPS	Others	Polymetallic	5.98	China: Guangxi, Wuyi
L180322-P-8-LD180320-0025_HL3MGCCXY_L4_clean_bin.68.orig.fa_	OEZ008529	yes	LMSG_G000005495.1	OES073731	T1PKS.hglE-KS	PKSother	Polymetallic	11.06	China: Guangxi, Wuyi
L180322-P-8-LD180320-0025_HL3MGCCXY_L4_clean_bin.68.orig.fa_	OEZ008529	yes	LMSG_G000005495.1	OES073731	terpene	Terpene	Polymetallic	8.02	China: Guangxi, Wuyi
L180322-P-8-LD180320-0025_HL3MGCCXY_L4_clean_bin.68.orig.fa_	OEZ008529	yes	LMSG_G000005495.1	OES073731	hglE-KS	PKSother	Polymetallic	22.73	China: Guangxi, Wuyi
L180322-P-8-LD180320-0025_HL3MGCCXY_L4_clean_bin.68.orig.fa_	OEZ008529	yes	LMSG_G000005495.1	OES073731	lanthipeptide	RiPPs	Polymetallic	7.32	China: Guangxi, Wuyi
L180322-P-8-LD180320-0025_HL3MGCCXY_L4_clean_bin.68.orig.fa_	OEZ008529	yes	LMSG_G000005495.1	OES073731	T3PKS	PKSother	Polymetallic	5.94	China: Guangxi, Wuyi
L180322-P-8-LD180320-0025_HL3MGCCXY_L4_clean_bin.68.orig.fa_	OEZ008529	yes	LMSG_G000005495.1	OES073731	T1PKS	PKSI	Polymetallic	8.92	China: Guangxi, Wuyi
L180322-P-8-LD180320-0025_HL3MGCCXY_L4_clean_bin.69.orig.fa_	OEZ008529	yes	LMSG_G000005496.1	OES073731	arylpolyene	Others	Polymetallic	20.68	China: Guangxi, Wuyi
L180322-P-8-LD180320-0025_HL3MGCCXY_L4_clean_bin.69.orig.fa_	OEZ008529	yes	LMSG_G000005496.1	OES073731	bacteriocin	RiPPs	Polymetallic	9.41	China: Guangxi, Wuyi
L180322-P-8-LD180320-0025_HL3MGCCXY_L4_clean_bin.69.orig.fa_	OEZ008529	yes	LMSG_G000005496.1	OES073731	T1PKS	PKSI	Polymetallic	8.45	China: Guangxi, Wuyi
L180322-P-8-LD180320-0025_HL3MGCCXY_L4_clean_bin.69.orig.fa_	OEZ008529	yes	LMSG_G000005496.1	OES073731	terpene	Terpene	Polymetallic	10.61	China: Guangxi, Wuyi
L180322-P-8-LD180320-0025_HL3MGCCXY_L4_clean_bin.7.strict.fa	OEZ008529	yes	LMSG_G000010752.1	OES073731	arylpolyene	Others	Polymetallic	38.90	China: Guangxi, Wuyi
L180322-P-8-LD180320-0025_HL3MGCCXY_L4_clean_bin.7.strict.fa	OEZ008529	yes	LMSG_G000010752.1	OES073731	acyl_amino_acids	Others	Polymetallic	38.92	China: Guangxi, Wuyi
L180322-P-8-LD180320-0025_HL3MGCCXY_L4_clean_bin.7.strict.fa	OEZ008529	yes	LMSG_G000010752.1	OES073731	bacteriocin	RiPPs	Polymetallic	7.46	China: Guangxi, Wuyi
L180322-P-8-LD180320-0025_HL3MGCCXY_L4_clean_bin.72.strict.f	OEZ008529	yes	LMSG_G000010531.1	OES073731	terpene	Terpene	Polymetallic	12.69	China: Guangxi, Wuyi
L180322-P-8-LD180320-0025_HL3MGCCXY_L4_clean_bin.75.orig.fa_	OEZ008529	no	LMSG_G000010866.1	OES073731	terpene	Terpene	Polymetallic	6.21	China: Guangxi, Wuyi
L180322-P-8-LD180320-0025_HL3MGCCXY_L4_clean_bin.75.orig.fa_	OEZ008529	yes	LMSG_G000010866.1	OES073731	betalactone	Others	Polymetallic	12.44	China: Guangxi, Wuyi
L180322-P-8-LD180320-0025_HL3MGCCXY_L4_clean_bin.75.orig.fa_	OEZ008529	yes	LMSG_G000010866.1	OES073731	NRPS-like	NRPS	Polymetallic	9.78	China: Guangxi, Wuyi
L180322-P-8-LD180320-0025_HL3MGCCXY_L4_clean_bin.75.orig.fa_	OEZ008529	yes	LMSG_G000010866.1	OES073731	arylpolyene	Others	Polymetallic	11.84	China: Guangxi, Wuyi
L180322-P-8-LD180320-0025_HL3MGCCXY_L4_clean_bin.76.orig.fa_	OEZ008529	yes	LMSG_G000005497.1	OES073731	terpene	Terpene	Polymetallic	7.57	China: Guangxi, Wuyi
L180322-P-8-LD180320-0025_HL3MGCCXY_L4_clean_bin.76.orig.fa_	OEZ008529	yes	LMSG_G000005497.1	OES073731	NRPS-like	NRPS	Polymetallic	7.23	China: Guangxi, Wuyi
L180322-P-8-LD180320-0025_HL3MGCCXY_L4_clean_bin.76.orig.fa_	OEZ008529	yes	LMSG_G000005497.1	OES073731	lassopeptide	RiPPs	Polymetallic	13.09	China: Guangxi, Wuyi
L180322-P-8-LD180320-0025_HL3MGCCXY_L4_clean_bin.77.strict.f	OEZ008529	yes	LMSG_G000009053.1	OES073731	T1PKS	PKSI	Polymetallic	49.18	China: Guangxi, Wuyi
L180322-P-8-LD180320-0025_HL3MGCCXY_L4_clean_bin.77.strict.f	OEZ008529	yes	LMSG_G000009053.1	OES073731	T1PKS	PKSI	Polymetallic	47.90	China: Guangxi, Wuyi
L180322-P-8-LD180320-0025_HL3MGCCXY_L4_clean_bin.77.strict.f	OEZ008529	yes	LMSG_G000009053.1	OES073731	betalactone	Others	Polymetallic	27.46	China: Guangxi, Wuyi
L180322-P-8-LD180320-0025_HL3MGCCXY_L4_clean_bin.78.orig.fa_	OEZ008529	yes	LMSG_G000005498.1	OES073731	hserlactone	Others	Polymetallic	20.58	China: Guangxi, Wuyi
L180322-P-8-LD180320-0025_HL3MGCCXY_L4_clean_bin.78.orig.fa_	OEZ008529	yes	LMSG_G000005498.1	OES073731	terpene	Terpene	Polymetallic	15.08	China: Guangxi, Wuyi
L180322-P-8-LD180320-0025_HL3MGCCXY_L4_clean_bin.78.orig.fa_	OEZ008529	yes	LMSG_G000005498.1	OES073731	NRPS-like	NRPS	Polymetallic	37.03	China: Guangxi, Wuyi
L180322-P-8-LD180320-0025_HL3MGCCXY_L4_clean_bin.78.orig.fa_	OEZ008529	yes	LMSG_G000005498.1	OES073731	NRPS-like	NRPS	Polymetallic	27.87	China: Guangxi, Wuyi
L180322-P-8-LD180320-0025_HL3MGCCXY_L4_clean_bin.78.orig.fa_	OEZ008529	yes	LMSG_G000005498.1	OES073731	T3PKS	PKSother	Polymetallic	41.09	China: Guangxi, Wuyi
L180322-P-8-LD180320-0025_HL3MGCCXY_L4_clean_bin.80.orig.fa_	OEZ008529	yes	LMSG_G000010692.1	OES073731	bacteriocin	RiPPs	Polymetallic	7.54	China: Guangxi, Wuyi
L180322-P-8-LD180320-0025_HL3MGCCXY_L4_clean_bin.80.orig.fa_	OEZ008529	yes	LMSG_G000010692.1	OES073731	T1PKS.NRPS-like	PKS-NRP_Hybrids	Polymetallic	9.89	China: Guangxi, Wuyi
L180322-P-8-LD180320-0025_HL3MGCCXY_L4_clean_bin.80.orig.fa_	OEZ008529	yes	LMSG_G000010692.1	OES073731	NRPS	NRPS	Polymetallic	24.97	China: Guangxi, Wuyi
L180322-P-8-LD180320-0025_HL3MGCCXY_L4_clean_bin.80.orig.fa_	OEZ008529	yes	LMSG_G000010692.1	OES073731	arylpolyene	Others	Polymetallic	22.25	China: Guangxi, Wuyi
L180322-P-8-LD180320-0025_HL3MGCCXY_L4_clean_bin.80.orig.fa_	OEZ008529	yes	LMSG_G000010692.1	OES073731	terpene	Terpene	Polymetallic	14.84	China: Guangxi, Wuyi
L180322-P-8-LD180320-0025_HL3MGCCXY_L4_clean_bin.80.orig.fa_	OEZ008529	yes	LMSG_G000010692.1	OES073731	transAT-PKS	PKSother	Polymetallic	9.51	China: Guangxi, Wuyi
L180322-P-8-LD180320-0025_HL3MGCCXY_L4_clean_bin.80.orig.fa_	OEZ008529	yes	LMSG_G000010692.1	OES073731	arylpolyene	Others	Polymetallic	26.99	China: Guangxi, Wuyi
L180322-P-8-LD180320-0025_HL3MGCCXY_L4_clean_bin.81.orig.fa_	OEZ008529	yes	LMSG_G000008897.1	OES073731	terpene	Terpene	Polymetallic	10.64	China: Guangxi, Wuyi
L180322-P-8-LD180320-0025_HL3MGCCXY_L4_clean_bin.81.orig.fa_	OEZ008529	yes	LMSG_G000008897.1	OES073731	terpene	Terpene	Polymetallic	11.14	China: Guangxi, Wuyi
L180322-P-8-LD180320-0025_HL3MGCCXY_L4_clean_bin.85.orig.fa_	OEZ008529	yes	LMSG_G000010131.1	OES073731	terpene	Terpene	Polymetallic	18.14	China: Guangxi, Wuyi
L180322-P-8-LD180320-0025_HL3MGCCXY_L4_clean_bin.85.orig.fa_	OEZ008529	yes	LMSG_G000010131.1	OES073731	NRPS-like	NRPS	Polymetallic	31.01	China: Guangxi, Wuyi
L180322-P-8-LD180320-0025_HL3MGCCXY_L4_clean_bin.85.orig.fa_	OEZ008529	yes	LMSG_G000010131.1	OES073731	terpene	Terpene	Polymetallic	10.63	China: Guangxi, Wuyi
L180322-P-8-LD180320-0025_HL3MGCCXY_L4_clean_bin.87.orig.fa_	OEZ008529	yes	LMSG_G000005500.1	OES073731	terpene	Terpene	Polymetallic	21.68	China: Guangxi, Wuyi
L180322-P-8-LD180320-0025_HL3MGCCXY_L4_clean_bin.87.orig.fa_	OEZ008529	yes	LMSG_G000005500.1	OES073731	T3PKS	PKSother	Polymetallic	21.10	China: Guangxi, Wuyi
L180322-P-8-LD180320-0025_HL3MGCCXY_L4_clean_bin.9.orig.fa_k	OEZ008529	yes	LMSG_G000009694.1	OES073731	NRPS-like	NRPS	Polymetallic	7.67	China: Guangxi, Wuyi
L180322-P-8-LD180320-0025_HL3MGCCXY_L4_clean_bin.9.orig.fa_k	OEZ008529	yes	LMSG_G000009694.1	OES073731	hserlactone	Others	Polymetallic	15.60	China: Guangxi, Wuyi
L180322-P-8-LD180320-0025_HL3MGCCXY_L4_clean_bin.90.strict.f	OEZ008529	yes	LMSG_G000009030.1	OES073731	NRPS-like	NRPS	Polymetallic	28.81	China: Guangxi, Wuyi
L180322-P-8-LD180320-0025_HL3MGCCXY_L4_clean_bin.90.strict.f	OEZ008529	yes	LMSG_G000009030.1	OES073731	bacteriocin	RiPPs	Polymetallic	10.79	China: Guangxi, Wuyi
L180322-P-8-LD180320-0025_HL3MGCCXY_L4_clean_bin.90.strict.f	OEZ008529	yes	LMSG_G000009030.1	OES073731	PKS-like	PKSother	Polymetallic	23.81	China: Guangxi, Wuyi
L180322-P-8-LD180320-0025_HL3MGCCXY_L4_clean_bin.90.strict.f	OEZ008529	yes	LMSG_G000009030.1	OES073731	terpene	Terpene	Polymetallic	11.74	China: Guangxi, Wuyi
L180322-P-8-LD180320-0025_HL3MGCCXY_L4_clean_bin.91.orig.fa_	OEZ008529	yes	LMSG_G000011322.1	OES073731	terpene	Terpene	Polymetallic	13.47	China: Guangxi, Wuyi
L180322-P-8-LD180320-0025_HL3MGCCXY_L4_clean_bin.91.orig.fa_	OEZ008529	yes	LMSG_G000011322.1	OES073731	terpene	Terpene	Polymetallic	19.54	China: Guangxi, Wuyi
L180322-P-8-LD180320-0025_HL3MGCCXY_L4_clean_bin.91.orig.fa_	OEZ008529	yes	LMSG_G000011322.1	OES073731	terpene	Terpene	Polymetallic	13.93	China: Guangxi, Wuyi
L180322-P-8-LD180320-0025_HL3MGCCXY_L4_clean_bin.92.orig.fa_	OEZ008529	yes	LMSG_G000010393.1	OES073731	terpene	Terpene	Polymetallic	11.48	China: Guangxi, Wuyi
L180322-P-8-LD180320-0025_HL3MGCCXY_L4_clean_bin.97.strict.f	OEZ008529	yes	LMSG_G000004494.1	OES073731	terpene	Terpene	Polymetallic	21.04	China: Guangxi, Wuyi
L180322-P-8-LD180320-0026_HL3MGCCXY_L4_clean_bin.102.orig.fa	OEZ008529	yes	LMSG_G000010367.1	OES073732	terpene	Terpene	Polymetallic	20.83	China: Guangxi, Wuyi
L180322-P-8-LD180320-0026_HL3MGCCXY_L4_clean_bin.102.orig.fa	OEZ008529	yes	LMSG_G000010367.1	OES073732	terpene	Terpene	Polymetallic	7.55	China: Guangxi, Wuyi
L180322-P-8-LD180320-0026_HL3MGCCXY_L4_clean_bin.102.orig.fa	OEZ008529	yes	LMSG_G000010367.1	OES073732	bacteriocin	RiPPs	Polymetallic	10.83	China: Guangxi, Wuyi
L180322-P-8-LD180320-0026_HL3MGCCXY_L4_clean_bin.102.orig.fa	OEZ008529	yes	LMSG_G000010367.1	OES073732	NRPS-like	NRPS	Polymetallic	27.18	China: Guangxi, Wuyi
L180322-P-8-LD180320-0026_HL3MGCCXY_L4_clean_bin.102.orig.fa	OEZ008529	yes	LMSG_G000010367.1	OES073732	terpene	Terpene	Polymetallic	21.72	China: Guangxi, Wuyi
L180322-P-8-LD180320-0026_HL3MGCCXY_L4_clean_bin.102.orig.fa	OEZ008529	yes	LMSG_G000010367.1	OES073732	terpene	Terpene	Polymetallic	7.05	China: Guangxi, Wuyi
L180322-P-8-LD180320-0026_HL3MGCCXY_L4_clean_bin.102.orig.fa	OEZ008529	yes	LMSG_G000010367.1	OES073732	bacteriocin	RiPPs	Polymetallic	11.69	China: Guangxi, Wuyi
L180322-P-8-LD180320-0026_HL3MGCCXY_L4_clean_bin.102.orig.fa	OEZ008529	yes	LMSG_G000010367.1	OES073732	betalactone	Others	Polymetallic	29.80	China: Guangxi, Wuyi
L180322-P-8-LD180320-0026_HL3MGCCXY_L4_clean_bin.103.orig.fa	OEZ008529	yes	LMSG_G000005502.1	OES073732	T3PKS	PKSother	Polymetallic	8.56	China: Guangxi, Wuyi
L180322-P-8-LD180320-0026_HL3MGCCXY_L4_clean_bin.103.orig.fa	OEZ008529	yes	LMSG_G000005502.1	OES073732	lanthipeptide	RiPPs	Polymetallic	7.44	China: Guangxi, Wuyi
L180322-P-8-LD180320-0026_HL3MGCCXY_L4_clean_bin.106.permiss	OEZ008529	yes	LMSG_G000006811.1	OES073732	terpene	Terpene	Polymetallic	17.22	China: Guangxi, Wuyi
L180322-P-8-LD180320-0026_HL3MGCCXY_L4_clean_bin.107.orig.fa	OEZ008529	yes	LMSG_G000005504.1	OES073732	other	Others	Polymetallic	24.08	China: Guangxi, Wuyi
L180322-P-8-LD180320-0026_HL3MGCCXY_L4_clean_bin.107.orig.fa	OEZ008529	yes	LMSG_G000005504.1	OES073732	betalactone	Others	Polymetallic	23.27	China: Guangxi, Wuyi
L180322-P-8-LD180320-0026_HL3MGCCXY_L4_clean_bin.107.orig.fa	OEZ008529	yes	LMSG_G000005504.1	OES073732	other	Others	Polymetallic	27.74	China: Guangxi, Wuyi
L180322-P-8-LD180320-0026_HL3MGCCXY_L4_clean_bin.11.orig.fa_	OEZ008529	yes	LMSG_G000008953.1	OES073732	bacteriocin	RiPPs	Polymetallic	10.22	China: Guangxi, Wuyi
L180322-P-8-LD180320-0026_HL3MGCCXY_L4_clean_bin.11.orig.fa_	OEZ008529	yes	LMSG_G000008953.1	OES073732	terpene	Terpene	Polymetallic	13.62	China: Guangxi, Wuyi

L180322-P-8-LD180320-0026_HL3MGCCXY_L4_clean_bin.111.orig.fa	OEZ008529	yes	LMSG_G000010938.1	OES073732	terpene	Terpene	Polymetallic	21.82	China: Guangxi, Wuyi
L180322-P-8-LD180320-0026_HL3MGCCXY_L4_clean_bin.111.orig.fa	OEZ008529	yes	LMSG_G000010938.1	OES073732	acyl_amino_acids	Others	Polymetallic	45.46	China: Guangxi, Wuyi
L180322-P-8-LD180320-0026_HL3MGCCXY_L4_clean_bin.111.orig.fa	OEZ008529	yes	LMSG_G000010938.1	OES073732	T1PKS	PKSI	Polymetallic	31.35	China: Guangxi, Wuyi
L180322-P-8-LD180320-0026_HL3MGCCXY_L4_clean_bin.112.strict.	OEZ008529	yes	LMSG_G000008820.1	OES073732	terpene	Terpene	Polymetallic	12.46	China: Guangxi, Wuyi
L180322-P-8-LD180320-0026_HL3MGCCXY_L4_clean_bin.115.strict.	OEZ008529	yes	LMSG_G000009515.1	OES073732	terpene	Terpene	Polymetallic	5.58	China: Guangxi, Wuyi
L180322-P-8-LD180320-0026_HL3MGCCXY_L4_clean_bin.13.orig.fa_	OEZ008529	yes	LMSG_G000005506.1	OES073732	PKS-like	PKSother	Polymetallic	23.91	China: Guangxi, Wuyi
L180322-P-8-LD180320-0026_HL3MGCCXY_L4_clean_bin.13.orig.fa_	OEZ008529	yes	LMSG_G000005506.1	OES073732	NRPS-like	NRPS	Polymetallic	28.95	China: Guangxi, Wuyi
L180322-P-8-LD180320-0026_HL3MGCCXY_L4_clean_bin.13.orig.fa_	OEZ008529	yes	LMSG_G000005506.1	OES073732	terpene	Terpene	Polymetallic	21.63	China: Guangxi, Wuyi
L180322-P-8-LD180320-0026_HL3MGCCXY_L4_clean_bin.13.orig.fa_	OEZ008529	yes	LMSG_G000005506.1	OES073732	bacteriocin	RiPPs	Polymetallic	10.71	China: Guangxi, Wuyi
L180322-P-8-LD180320-0026_HL3MGCCXY_L4_clean_bin.14.strict.f	OEZ008529	yes	LMSG_G000005507.1	OES073732	terpene	Terpene	Polymetallic	12.45	China: Guangxi, Wuyi
L180322-P-8-LD180320-0026_HL3MGCCXY_L4_clean_bin.15.strict.f	OEZ008529	yes	LMSG_G000005508.1	OES073732	bacteriocin	RiPPs	Polymetallic	8.09	China: Guangxi, Wuyi
L180322-P-8-LD180320-0026_HL3MGCCXY_L4_clean_bin.15.strict.f	OEZ008529	yes	LMSG_G000005508.1	OES073732	terpene	Terpene	Polymetallic	8.25	China: Guangxi, Wuyi
L180322-P-8-LD180320-0026_HL3MGCCXY_L4_clean_bin.18.strict.f	OEZ008529	yes	LMSG_G000008921.1	OES073732	terpene	Terpene	Polymetallic	20.94	China: Guangxi, Wuyi
L180322-P-8-LD180320-0026_HL3MGCCXY_L4_clean_bin.18.strict.f	OEZ008529	yes	LMSG_G000008921.1	OES073732	terpene	Terpene	Polymetallic	20.92	China: Guangxi, Wuyi
L180322-P-8-LD180320-0026_HL3MGCCXY_L4_clean_bin.19.orig.fa_	OEZ008529	yes	LMSG_G000010867.1	OES073732	NRPS-like	NRPS	Polymetallic	33.78	China: Guangxi, Wuyi
L180322-P-8-LD180320-0026_HL3MGCCXY_L4_clean_bin.19.orig.fa_	OEZ008529	yes	LMSG_G000010867.1	OES073732	arylpolyene	Others	Polymetallic	34.50	China: Guangxi, Wuyi
L180322-P-8-LD180320-0026_HL3MGCCXY_L4_clean_bin.19.orig.fa_	OEZ008529	yes	LMSG_G000010867.1	OES073732	terpene	Terpene	Polymetallic	12.21	China: Guangxi, Wuyi
L180322-P-8-LD180320-0026_HL3MGCCXY_L4_clean_bin.2.strict.fa	OEZ008529	yes	LMSG_G000005510.1	OES073732	terpene	Terpene	Polymetallic	12.56	China: Guangxi, Wuyi
L180322-P-8-LD180320-0026_HL3MGCCXY_L4_clean_bin.26.orig.fa_	OEZ008529	yes	LMSG_G000004495.1	OES073732	NRPS-like	NRPS	Polymetallic	42.72	China: Guangxi, Wuyi
L180322-P-8-LD180320-0026_HL3MGCCXY_L4_clean_bin.26.orig.fa_	OEZ008529	yes	LMSG_G000004495.1	OES073732	T3PKS	PKSother	Polymetallic	34.50	China: Guangxi, Wuyi
L180322-P-8-LD180320-0026_HL3MGCCXY_L4_clean_bin.26.orig.fa_	OEZ008529	yes	LMSG_G000004495.1	OES073732	NRPS-like	NRPS	Polymetallic	42.69	China: Guangxi, Wuyi
L180322-P-8-LD180320-0026_HL3MGCCXY_L4_clean_bin.26.orig.fa_	OEZ008529	yes	LMSG_G000004495.1	OES073732	terpene	Terpene	Polymetallic	14.23	China: Guangxi, Wuyi
L180322-P-8-LD180320-0026_HL3MGCCXY_L4_clean_bin.3.orig.fa_k	OEZ008529	yes	LMSG_G000005515.1	OES073732	lassopeptide	RiPPs	Polymetallic	15.94	China: Guangxi, Wuyi
L180322-P-8-LD180320-0026_HL3MGCCXY_L4_clean_bin.3.orig.fa_k	OEZ008529	yes	LMSG_G000005515.1	OES073732	NRPS-like	NRPS	Polymetallic	24.05	China: Guangxi, Wuyi
L180322-P-8-LD180320-0026_HL3MGCCXY_L4_clean_bin.3.orig.fa_k	OEZ008529	yes	LMSG_G000005515.1	OES073732	terpene	Terpene	Polymetallic	21.06	China: Guangxi, Wuyi
L180322-P-8-LD180320-0026_HL3MGCCXY_L4_clean_bin.31.orig.fa_	OEZ008529	yes	LMSG_G000009081.1	OES073732	resorcinol.arylpolyene	Others	Polymetallic	5.35	China: Guangxi, Wuyi
L180322-P-8-LD180320-0026_HL3MGCCXY_L4_clean_bin.32.orig.fa_	OEZ008529	yes	LMSG_G000006241.1	OES073732	terpene	Terpene	Polymetallic	6.45	China: Guangxi, Wuyi
L180322-P-8-LD180320-0026_HL3MGCCXY_L4_clean_bin.33.orig.fa_	OEZ008529	yes	LMSG_G000005511.1	OES073732	terpene	Terpene	Polymetallic	10.46	China: Guangxi, Wuyi
L180322-P-8-LD180320-0026_HL3MGCCXY_L4_clean_bin.34.permissi	OEZ008529	yes	LMSG_G000010755.1	OES073732	arylpolyene	Others	Polymetallic	41.28	China: Guangxi, Wuyi
L180322-P-8-LD180320-0026_HL3MGCCXY_L4_clean_bin.34.permissi	OEZ008529	yes	LMSG_G000010755.1	OES073732	acyl_amino_acids	Others	Polymetallic	6.87	China: Guangxi, Wuyi
L180322-P-8-LD180320-0026_HL3MGCCXY_L4_clean_bin.36.orig.fa_	OEZ008529	yes	LMSG_G000011323.1	OES073732	terpene	Terpene	Polymetallic	18.12	China: Guangxi, Wuyi
L180322-P-8-LD180320-0026_HL3MGCCXY_L4_clean_bin.36.orig.fa_	OEZ008529	yes	LMSG_G000011323.1	OES073732	terpene	Terpene	Polymetallic	13.62	China: Guangxi, Wuyi
L180322-P-8-LD180320-0026_HL3MGCCXY_L4_clean_bin.36.orig.fa_	OEZ008529	yes	LMSG_G000011323.1	OES073732	terpene	Terpene	Polymetallic	20.16	China: Guangxi, Wuyi
L180322-P-8-LD180320-0026_HL3MGCCXY_L4_clean_bin.37.orig.fa_	OEZ008529	yes	LMSG_G000005512.1	OES073732	hserlactone	Others	Polymetallic	14.07	China: Guangxi, Wuyi
L180322-P-8-LD180320-0026_HL3MGCCXY_L4_clean_bin.37.orig.fa_	OEZ008529	yes	LMSG_G000005512.1	OES073732	NRPS	NRPS	Polymetallic	16.33	China: Guangxi, Wuyi
L180322-P-8-LD180320-0026_HL3MGCCXY_L4_clean_bin.37.orig.fa_	OEZ008529	yes	LMSG_G000005512.1	OES073732	bacteriocin	RiPPs	Polymetallic	5.19	China: Guangxi, Wuyi
L180322-P-8-LD180320-0026_HL3MGCCXY_L4_clean_bin.37.orig.fa_	OEZ008529	yes	LMSG_G000005512.1	OES073732	terpene	Terpene	Polymetallic	8.87	China: Guangxi, Wuyi
L180322-P-8-LD180320-0026_HL3MGCCXY_L4_clean_bin.39.orig.fa_	OEZ008529	yes	LMSG_G000005514.1	OES073732	terpene	Terpene	Polymetallic	16.92	China: Guangxi, Wuyi
L180322-P-8-LD180320-0026_HL3MGCCXY_L4_clean_bin.40.orig.fa_	OEZ008529	yes	LMSG_G000004497.1	OES073732	NRPS-like	NRPS	Polymetallic	28.07	China: Guangxi, Wuyi
L180322-P-8-LD180320-0026_HL3MGCCXY_L4_clean_bin.40.orig.fa_	OEZ008529	yes	LMSG_G000004497.1	OES073732	hserlactone	Others	Polymetallic	15.59	China: Guangxi, Wuyi
L180322-P-8-LD180320-0026_HL3MGCCXY_L4_clean_bin.41.orig.fa_	OEZ008529	yes	LMSG_G000005516.1	OES073732	terpene	Terpene	Polymetallic	10.42	China: Guangxi, Wuyi
L180322-P-8-LD180320-0026_HL3MGCCXY_L4_clean_bin.5.orig.fa_k	OEZ008529	yes	LMSG_G000004498.1	OES073732	terpene	Terpene	Polymetallic	21.69	China: Guangxi, Wuyi
L180322-P-8-LD180320-0026_HL3MGCCXY_L4_clean_bin.51.orig.fa_	OEZ008529	yes	LMSG_G000008825.1	OES073732	terpene	Terpene	Polymetallic	21.69	China: Guangxi, Wuyi
L180322-P-8-LD180320-0026_HL3MGCCXY_L4_clean_bin.56.strict.f	OEZ008529	yes	LMSG_G000006337.1	OES073732	CDPS	Others	Polymetallic	18.86	China: Guangxi, Wuyi
L180322-P-8-LD180320-0026_HL3MGCCXY_L4_clean_bin.59.strict.f	OEZ008529	yes	LMSG_G000008782.1	OES073732	NRPS-like	NRPS	Polymetallic	14.24	China: Guangxi, Wuyi
L180322-P-8-LD180320-0026_HL3MGCCXY_L4_clean_bin.59.strict.f	OEZ008529	yes	LMSG_G000008782.1	OES073732	NRPS-like	NRPS	Polymetallic	10.40	China: Guangxi, Wuyi
L180322-P-8-LD180320-0026_HL3MGCCXY_L4_clean_bin.59.strict.f	OEZ008529	yes	LMSG_G000008782.1	OES073732	bacteriocin	RiPPs	Polymetallic	5.71	China: Guangxi, Wuyi
L180322-P-8-LD180320-0026_HL3MGCCXY_L4_clean_bin.60.strict.f	OEZ008529	yes	LMSG_G000004499.1	OES073732	acyl_amino_acids.hserlactone	Others	Polymetallic	44.08	China: Guangxi, Wuyi
L180322-P-8-LD180320-0026_HL3MGCCXY_L4_clean_bin.60.strict.f	OEZ008529	yes	LMSG_G000004499.1	OES073732	acyl_amino_acids	Others	Polymetallic	44.83	China: Guangxi, Wuyi
L180322-P-8-LD180320-0026_HL3MGCCXY_L4_clean_bin.60.strict.f	OEZ008529	yes	LMSG_G000004499.1	OES073732	hglE-KS	PKSother	Polymetallic	21.06	China: Guangxi, Wuyi
L180322-P-8-LD180320-0026_HL3MGCCXY_L4_clean_bin.60.strict.f	OEZ008529	yes	LMSG_G000004499.1	OES073732	hglE-KS	PKSother	Polymetallic	42.24	China: Guangxi, Wuyi
L180322-P-8-LD180320-0026_HL3MGCCXY_L4_clean_bin.60.strict.f	OEZ008529	yes	LMSG_G000004499.1	OES073732	NRPS	NRPS	Polymetallic	25.12	China: Guangxi, Wuyi
L180322-P-8-LD180320-0026_HL3MGCCXY_L4_clean_bin.60.strict.f	OEZ008529	yes	LMSG_G000004499.1	OES073732	T1PKS	PKSI	Polymetallic	15.79	China: Guangxi, Wuyi
L180322-P-8-LD180320-0026_HL3MGCCXY_L4_clean_bin.61.orig.fa_	OEZ008529	yes	LMSG_G000008896.1	OES073732	terpene	Terpene	Polymetallic	15.01	China: Guangxi, Wuyi
L180322-P-8-LD180320-0026_HL3MGCCXY_L4_clean_bin.61.orig.fa_	OEZ008529	yes	LMSG_G000008896.1	OES073732	terpene	Terpene	Polymetallic	20.98	China: Guangxi, Wuyi
L180322-P-8-LD180320-0026_HL3MGCCXY_L4_clean_bin.63.orig.fa_	OEZ008529	yes	LMSG_G000008795.1	OES073732	terpene	Terpene	Polymetallic	12.51	China: Guangxi, Wuyi
L180322-P-8-LD180320-0026_HL3MGCCXY_L4_clean_bin.64.orig.fa_	OEZ008529	yes	LMSG_G000005522.1	OES073732	_amino_acids.terpene.bacteri	Others	Polymetallic	54.00	China: Guangxi, Wuyi
L180322-P-8-LD180320-0026_HL3MGCCXY_L4_clean_bin.64.orig.fa_	OEZ008529	yes	LMSG_G000005522.1	OES073732	thiopeptide	RiPPs	Polymetallic	31.19	China: Guangxi, Wuyi
L180322-P-8-LD180320-0026_HL3MGCCXY_L4_clean_bin.64.orig.fa_	OEZ008529	yes	LMSG_G000005522.1	OES073732	terpene	Terpene	Polymetallic	17.55	China: Guangxi, Wuyi
L180322-P-8-LD180320-0026_HL3MGCCXY_L4_clean_bin.65.orig.fa_	OEZ008529	yes	LMSG_G000009036.1	OES073732	hglE-KS	PKSother	Polymetallic	8.28	China: Guangxi, Wuyi
L180322-P-8-LD180320-0026_HL3MGCCXY_L4_clean_bin.65.orig.fa_	OEZ008529	yes	LMSG_G000009036.1	OES073732	hglE-KS	PKSother	Polymetallic	12.08	China: Guangxi, Wuyi
L180322-P-8-LD180320-0026_HL3MGCCXY_L4_clean_bin.65.orig.fa_	OEZ008529	yes	LMSG_G000009036.1	OES073732	NRPS	NRPS	Polymetallic	9.06	China: Guangxi, Wuyi
L180322-P-8-LD180320-0026_HL3MGCCXY_L4_clean_bin.65.orig.fa_	OEZ008529	yes	LMSG_G000009036.1	OES073732	transAT-PKS-like.NRPS-like	PKS-NRP_Hybrids	Polymetallic	7.47	China: Guangxi, Wuyi
L180322-P-8-LD180320-0026_HL3MGCCXY_L4_clean_bin.65.orig.fa_	OEZ008529	yes	LMSG_G000009036.1	OES073732	acyl_amino_acids	Others	Polymetallic	5.83	China: Guangxi, Wuyi
L180322-P-8-LD180320-0026_HL3MGCCXY_L4_clean_bin.66.orig.fa_	OEZ008529	yes	LMSG_G000004500.1	OES073732	terpene	Terpene	Polymetallic	17.64	China: Guangxi, Wuyi
L180322-P-8-LD180320-0026_HL3MGCCXY_L4_clean_bin.66.orig.fa_	OEZ008529	yes	LMSG_G000004500.1	OES073732	NRPS-like	NRPS	Polymetallic	42.54	China: Guangxi, Wuyi
L180322-P-8-LD180320-0026_HL3MGCCXY_L4_clean_bin.66.orig.fa_	OEZ008529	yes	LMSG_G000004500.1	OES073732	NRPS-like	NRPS	Polymetallic	42.69	China: Guangxi, Wuyi
L180322-P-8-LD180320-0026_HL3MGCCXY_L4_clean_bin.69.orig.fa_	OEZ008529	yes	LMSG_G000005523.1	OES073732	terpene	Terpene	Polymetallic	13.94	China: Guangxi, Wuyi
L180322-P-8-LD180320-0026_HL3MGCCXY_L4_clean_bin.72.orig.fa_	OEZ008529	yes	LMSG_G000008817.1	OES073732	T3PKS	PKSother	Polymetallic	21.09	China: Guangxi, Wuyi
L180322-P-8-LD180320-0026_HL3MGCCXY_L4_clean_bin.72.orig.fa_	OEZ008529	yes	LMSG_G000008817.1	OES073732	terpene	Terpene	Polymetallic	13.82	China: Guangxi, Wuyi
L180322-P-8-LD180320-0026_HL3MGCCXY_L4_clean_bin.75.orig.fa_	OEZ008529	yes	LMSG_G000007861.1	OES073732	NRPS-like	NRPS	Polymetallic	24.08	China: Guangxi, Wuyi
L180322-P-8-LD180320-0026_HL3MGCCXY_L4_clean_bin.8.strict.fa	OEZ008529	yes	LMSG_G000009055.1	OES073732	betalactone	Others	Polymetallic	22.12	China: Guangxi, Wuyi
L180322-P-8-LD180320-0026_HL3MGCCXY_L4_clean_bin.8.strict.fa	OEZ008529	yes	LMSG_G000009055.1	OES073732	T1PKS	PKSI	Polymetallic	20.83	China: Guangxi, Wuyi
L180322-P-8-LD180320-0026_HL3MGCCXY_L4_clean_bin.8.strict.fa	OEZ008529	yes	LMSG_G000009055.1	OES073732	T1PKS	PKSI	Polymetallic	16.60	China: Guangxi, Wuyi
L180322-P-8-LD180320-0026_HL3MGCCXY_L4_clean_bin.80.orig.fa_	OEZ008529	yes	LMSG_G000009033.1	OES073732	hglE-KS	PKSother	Polymetallic	11.74	China: Guangxi, Wuyi
L180322-P-8-LD180320-0026_HL3MGCCXY_L4_clean_bin.80.orig.fa_	OEZ008529	yes	LMSG_G000009033.1	OES073732	hglE-KS	PKSother	Polymetallic	17.00	China: Guangxi, Wuyi
L180322-P-8-LD180320-0026_HL3MGCCXY_L4_clean_bin.80.orig.fa_	OEZ008529	yes	LMSG_G000009033.1	OES073732	acyl_amino_acids	Others	Polymetallic	52.19	China: Guangxi, Wuyi
L180322-P-8-LD180320-0026_HL3MGCCXY_L4_clean_bin.80.orig.fa_	OEZ008529	yes	LMSG_G000009033.1	OES073732	T1PKS.hglE-KS	PKSother	Polymetallic	29.87	China: Guangxi, Wuyi
L180322-P-8-LD180320-0026_HL3MGCCXY_L4_clean_bin.80.orig.fa_	OEZ008529	yes	LMSG_G000009033.1	OES073732	T1PKS	PKSI	Polymetallic	18.00	China: Guangxi, Wuyi
L180322-P-8-LD180320-0026_HL3MGCCXY_L4_clean_bin.80.orig.fa_	OEZ008529	yes	LMSG_G000009033.1	OES073732	hglE-KS	PKSother	Polymetallic	27.24	China: Guangxi, Wuyi
L180322-P-8-LD180320-0026_HL3MGCCXY_L4_clean_bin.80.orig.fa_	OEZ008529	yes	LMSG_G000009033.1	OES073732	bacteriocin	RiPPs	Polymetallic	11.91	China: Guangxi, Wuyi
L180322-P-8-LD180320-0026_HL3MGCCXY_L4_clean_bin.80.orig.fa_	OEZ008529	yes	LMSG_G000009033.1	OES073732	arylpolyene	Others	Polymetallic	7.55	China: Guangxi, Wuyi
L180322-P-8-LD180320-0026_HL3MGCCXY_L4_clean_bin.81.orig.fa_	OEZ008529	yes	LMSG_G000011339.1	OES073732	T3PKS	PKSother	Polymetallic	30.22	China: Guangxi, Wuyi
L180322-P-8-LD180320-0026_HL3MGCCXY_L4_clean_bin.81.orig.fa_	OEZ008529	yes	LMSG_G000011339.1	OES073732	terpene	Terpene	Polymetallic	20.99	China: Guangxi, Wuyi
L180322-P-8-LD180320-0026_HL3MGCCXY_L4_clean_bin.82.strict.f	OEZ008529	yes	LMSG_G000009591.1	OES073732	terpene	Terpene	Polymetallic	18.16	China: Guangxi, Wuyi
L180322-P-8-LD180320-0026_HL3MGCCXY_L4_clean_bin.82.strict.f	OEZ008529	yes	LMSG_G000009591.1	OES073732	NRPS-like	NRPS	Polymetallic	24.76	China: Guangxi, Wuyi
L180322-P-8-LD180320-0026_HL3MGCCXY_L4_clean_bin.82.strict.f	OEZ008529	yes	LMSG_G000009591.1	OES073732	terpene	Terpene	Polymetallic	12.65	China: Guangxi, Wuyi
L180322-P-8-LD180320-0026_HL3MGCCXY_L4_clean_bin.83.strict.f	OEZ008529	yes	LMSG_G000011204.1	OES073732	arylpolyene	Others	Polymetallic	16.27	China: Guangxi, Wuyi
L180322-P-8-LD180320-0026_HL3MGCCXY_L4_clean_bin.85.orig.fa_	OEZ008529	yes	LMSG_G000010895.1	OES073732	lassopeptide	RiPPs	Polymetallic	11.35	China: Guangxi, Wuyi
L180322-P-8-LD180320-0026_HL3MGCCXY_L4_clean_bin.86.orig.fa_	OEZ008529	yes	LMSG_G000008900.1	OES073732	terpene	Terpene	Polymetallic	21.77	China: Guangxi, Wuyi
L180322-P-8-LD180320-0026_HL3MGCCXY_L4_clean_bin.87.orig.fa_	OEZ008529	yes	LMSG_G000010693.1	OES073732	T1PKS.NRPS-like	PKS-NRP_Hybrids	Polymetallic	12.07	China: Guangxi, Wuyi
L180322-P-8-LD180320-0026_HL3MGCCXY_L4_clean_bin.87.orig.fa_	OEZ008529	yes	LMSG_G000010693.1	OES073732	terpene	Terpene	Polymetallic	20.80	China: Guangxi, Wuyi
L180322-P-8-LD180320-0026_HL3MGCCXY_L4_clean_bin.87.orig.fa_	OEZ008529	yes	LMSG_G000010693.1	OES073732	bacteriocin	RiPPs	Polymetallic	10.93	China: Guangxi, Wuyi
L180322-P-8-LD180320-0026_HL3MGCCXY_L4_clean_bin.87.orig.fa_	OEZ008529	yes	LMSG_G000010693.1	OES073732	arylpolyene	Others	Polymetallic	20.14	China: Guangxi, Wuyi
L180322-P-8-LD180320-0026_HL3MGCCXY_L4_clean_bin.87.orig.fa_	OEZ008529	yes	LMSG_G000010693.1	OES073732	NRPS	NRPS	Polymetallic	12.62	China: Guangxi, Wuyi
L180322-P-8-LD180320-0026_HL3MGCCXY_L4_clean_bin.87.orig.fa_	OEZ008529	no	LMSG_G000010693.1	OES073732	NRPS	NRPS	Polymetallic	5.16	China: Guangxi, Wuyi
L180322-P-8-LD180320-0026_HL3MGCCXY_L4_clean_bin.9.orig.fa_k	OEZ008529	yes	LMSG_G000009505.1	OES073732	TfuA-related	RiPPs	Polymetallic	10.25	China: Guangxi, Wuyi
L180322-P-8-LD180320-0026_HL3MGCCXY_L4_clean_bin.9.orig.fa_k	OEZ008529	yes	LMSG_G000009505.1	OES073732	betalactone	Others	Polymetallic	10.88	China: Guangxi, Wuyi
L180322-P-8-LD180320-0026_HL3MGCCXY_L4_clean_bin.90.orig.fa_	OEZ008529	yes	LMSG_G000005525.1	OES073732	terpene	Terpene	Polymetallic	8.20	China: Guangxi, Wuyi
L180322-P-8-LD180320-0026_HL3MGCCXY_L4_clean_bin.90.orig.fa_	OEZ008529	yes	LMSG_G000005525.1	OES073732	terpene	Terpene	Polymetallic	5.02	China: Guangxi, Wuyi
L180322-P-8-LD180320-0026_HL3MGCCXY_L4_clean_bin.91.strict.f	OEZ008529	yes	LMSG_G000006320.1	OES073732	CDPS	Others	Polymetallic	20.64	China: Guangxi, Wuyi
L180322-P-8-LD180320-0026_HL3MGCCXY_L4_clean_bin.98.orig.fa_	OEZ008529	no	LMSG_G000010618.1	OES073732	terpene	Terpene	Polymetallic	16.97	China: Guangxi, Wuyi
L180322-P-8-LD180320-0026_HL3MGCCXY_L4_clean_bin.98.orig.fa_	OEZ008529	yes	LMSG_G000010618.1	OES073732	NRPS	NRPS	Polymetallic	8.57	China: Guangxi, Wuyi
L180322-P-8-LD180320-0026_HL3MGCCXY_L4_clean_bin.98.orig.fa_	OEZ008529	yes	LMSG_G000010618.1	OES073732	bacteriocin	RiPPs	Polymetallic	8.03	China: Guangxi, Wuyi
L180322-P-8-LD180320-0026_HL3MGCCXY_L4_clean_bin.98.orig.fa_	OEZ008529	yes	LMSG_G000010618.1	OES073732	terpene	Terpene	Polymetallic	18.19	China: Guangxi, Wuyi
L180322-P-8-LD180320-0026_HL3MGCCXY_L4_clean_bin.99.orig.fa_	OEZ008529	yes	LMSG_G000010381.1	OES073732	betalactone	Others	Polymetallic	15.75	China: Guangxi, Wuyi
L180322-P-8-LD180320-0026_HL3MGCCXY_L4_clean_bin.99.orig.fa_	OEZ008529	yes	LMSG_G000010381.1	OES073732	T1PKS.hglE-KS	PKSother	Polymetallic	30.74	China: Guangxi, Wuyi
L180322-P-8-LD180320-0026_HL3MGCCXY_L4_clean_bin.99.orig.fa_	OEZ008529	yes	LMSG_G000010381.1	OES073732	hserlactone	Others	Polymetallic	20.75	China: Guangxi, Wuyi
L180322-P-8-LD180320-0026_HL3MGCCXY_L4_clean_bin.99.orig.fa_	OEZ008529	yes	LMSG_G000010381.1	OES073732	bacteriocin	RiPPs	Polymetallic	10.85	China: Guangxi, Wuyi
L180322-P-8-LD180320-0026_HL3MGCCXY_L4_clean_bin.99.orig.fa_	OEZ008529	yes	LMSG_G000010381.1	OES073732	hserlactone	Others	Polymetallic	13.13	China: Guangxi, Wuyi
L180322-P-8-LD180320-0027_HL3MGCCXY_L4_clean_bin.101.orig.fa	OEZ008529	yes	LMSG_G000004503.1	OES073733	terpene	Terpene	Polymetallic	18.62	China: Guangxi, Wuyi
L180322-P-8-LD180320-0027_HL3MGCCXY_L4_clean_bin.101.orig.fa	OEZ008529	yes	LMSG_G000004503.1	OES073733	T3PKS	PKSother	Polymetallic	10.92	China: Guangxi, Wuyi
L180322-P-8-LD180320-0027_HL3MGCCXY_L4_clean_bin.103.orig.fa	OEZ008529	yes	LMSG_G000008827.1	OES073733	terpene	Terpene	Polymetallic	21.69	China: Guangxi, Wuyi
L180322-P-8-LD180320-0027_HL3MGCCXY_L4_clean_bin.104.orig.fa	OEZ008529	yes	LMSG_G000008841.1	OES073733	NRPS-like	NRPS	Polymetallic	8.43	China: Guangxi, Wuyi
L180322-P-8-LD180320-0027_HL3MGCCXY_L4_clean_bin.105.orig.fa	OEZ008529	yes	LMSG_G000010619.1	OES073733	bacteriocin	RiPPs	Polymetallic	8.15	China: Guangxi, Wuyi
L180322-P-8-LD180320-0027_HL3MGCCXY_L4_clean_bin.105.orig.fa	OEZ008529	yes	LMSG_G000010619.1	OES073733	terpene	Terpene	Polymetallic	18.19	China: Guangxi, Wuyi
L180322-P-8-LD180320-0027_HL3MGCCXY_L4_clean_bin.105.orig.fa	OEZ008529	no	LMSG_G000010619.1	OES073733	terpene	Terpene	Polymetallic	12.14	China: Guangxi, Wuyi
L180322-P-8-LD180320-0027_HL3MGCCXY_L4_clean_bin.105.orig.fa	OEZ008529	yes	LMSG_G000010619.1	OES073733	arylpolyene	Others	Polymetallic	42.29	China: Guangxi, Wuyi
L180322-P-8-LD180320-0027_HL3MGCCXY_L4_clean_bin.106.orig.fa	OEZ008529	yes	LMSG_G000005527.1	OES073733	nucleoside	Others	Polymetallic	18.09	China: Guangxi, Wuyi
L180322-P-8-LD180320-0027_HL3MGCCXY_L4_clean_bin.107.orig.fa	OEZ008529	yes	LMSG_G000004504.1	OES073733	terpene	Terpene	Polymetallic	20.98	China: Guangxi, Wuyi
L180322-P-8-LD180320-0027_HL3MGCCXY_L4_clean_bin.107.orig.fa	OEZ008529	yes	LMSG_G000004504.1	OES073733	terpene	Terpene	Polymetallic	15.01	China: Guangxi, Wuyi
L180322-P-8-LD180320-0027_HL3MGCCXY_L4_clean_bin.109.orig.fa	OEZ008529	yes	LMSG_G000005528.1	OES073733	NRPS-like	NRPS	Polymetallic	5.18	China: Guangxi, Wuyi
L180322-P-8-LD180320-0027_HL3MGCCXY_L4_clean_bin.111.orig.fa	OEZ008529	yes	LMSG_G000005529.1	OES073733	terpene	Terpene	Polymetallic	16.75	China: Guangxi, Wuyi
L180322-P-8-LD180320-0027_HL3MGCCXY_L4_clean_bin.111.orig.fa	OEZ008529	yes	LMSG_G000005529.1	OES073733	terpene	Terpene	Polymetallic	20.86	China: Guangxi, Wuyi
L180322-P-8-LD180320-0027_HL3MGCCXY_L4_clean_bin.111.orig.fa	OEZ008529	yes	LMSG_G000005529.1	OES073733	terpene	Terpene	Polymetallic	18.35	China: Guangxi, Wuyi
L180322-P-8-LD180320-0027_HL3MGCCXY_L4_clean_bin.112.orig.fa	OEZ008529	no	LMSG_G000010721.1	OES073733	T1PKS	PKSI	Polymetallic	6.59	China: Guangxi, Wuyi
L180322-P-8-LD180320-0027_HL3MGCCXY_L4_clean_bin.112.orig.fa	OEZ008529	yes	LMSG_G000010721.1	OES073733	arylpolyene	Others	Polymetallic	12.10	China: Guangxi, Wuyi
L180322-P-8-LD180320-0027_HL3MGCCXY_L4_clean_bin.112.orig.fa	OEZ008529	yes	LMSG_G000010721.1	OES073733	NRPS	NRPS	Polymetallic	13.62	China: Guangxi, Wuyi
L180322-P-8-LD180320-0027_HL3MGCCXY_L4_clean_bin.112.orig.fa	OEZ008529	yes	LMSG_G000010721.1	OES073733	bacteriocin	RiPPs	Polymetallic	7.79	China: Guangxi, Wuyi
L180322-P-8-LD180320-0027_HL3MGCCXY_L4_clean_bin.113.strict.	OEZ008529	yes	LMSG_G000008924.1	OES073733	terpene	Terpene	Polymetallic	12.91	China: Guangxi, Wuyi
L180322-P-8-LD180320-0027_HL3MGCCXY_L4_clean_bin.114.orig.fa	OEZ008529	yes	LMSG_G000005530.1	OES073733	terpene	Terpene	Polymetallic	14.13	China: Guangxi, Wuyi
L180322-P-8-LD180320-0027_HL3MGCCXY_L4_clean_bin.119.orig.fa	OEZ008529	yes	LMSG_G000005531.1	OES073733	NRPS-like	NRPS	Polymetallic	21.62	China: Guangxi, Wuyi
L180322-P-8-LD180320-0027_HL3MGCCXY_L4_clean_bin.121.strict.	OEZ008529	yes	LMSG_G000008901.1	OES073733	bacteriocin	RiPPs	Polymetallic	5.24	China: Guangxi, Wuyi
L180322-P-8-LD180320-0027_HL3MGCCXY_L4_clean_bin.123.orig.fa	OEZ008529	yes	LMSG_G000008815.1	OES073733	terpene	Terpene	Polymetallic	13.30	China: Guangxi, Wuyi
L180322-P-8-LD180320-0027_HL3MGCCXY_L4_clean_bin.123.orig.fa	OEZ008529	yes	LMSG_G000008815.1	OES073733	T3PKS	PKSother	Polymetallic	21.09	China: Guangxi, Wuyi
L180322-P-8-LD180320-0027_HL3MGCCXY_L4_clean_bin.124.orig.fa	OEZ008529	yes	LMSG_G000004505.1	OES073733	terpene	Terpene	Polymetallic	21.77	China: Guangxi, Wuyi
L180322-P-8-LD180320-0027_HL3MGCCXY_L4_clean_bin.129.strict.	OEZ008529	yes	LMSG_G000008783.1	OES073733	NRPS-like	NRPS	Polymetallic	25.05	China: Guangxi, Wuyi
L180322-P-8-LD180320-0027_HL3MGCCXY_L4_clean_bin.129.strict.	OEZ008529	yes	LMSG_G000008783.1	OES073733	NRPS-like	NRPS	Polymetallic	13.02	China: Guangxi, Wuyi
L180322-P-8-LD180320-0027_HL3MGCCXY_L4_clean_bin.129.strict.	OEZ008529	yes	LMSG_G000008783.1	OES073733	bacteriocin	RiPPs	Polymetallic	8.64	China: Guangxi, Wuyi
L180322-P-8-LD180320-0027_HL3MGCCXY_L4_clean_bin.13.strict.f	OEZ008529	yes	LMSG_G000005536.1	OES073733	terpene	Terpene	Polymetallic	15.07	China: Guangxi, Wuyi
L180322-P-8-LD180320-0027_HL3MGCCXY_L4_clean_bin.13.strict.f	OEZ008529	yes	LMSG_G000005536.1	OES073733	NRPS-like	NRPS	Polymetallic	5.62	China: Guangxi, Wuyi
L180322-P-8-LD180320-0027_HL3MGCCXY_L4_clean_bin.131.orig.fa	OEZ008529	yes	LMSG_G000009027.1	OES073733	terpene	Terpene	Polymetallic	21.63	China: Guangxi, Wuyi
L180322-P-8-LD180320-0027_HL3MGCCXY_L4_clean_bin.131.orig.fa	OEZ008529	yes	LMSG_G000009027.1	OES073733	NRPS-like	NRPS	Polymetallic	22.39	China: Guangxi, Wuyi
L180322-P-8-LD180320-0027_HL3MGCCXY_L4_clean_bin.131.orig.fa	OEZ008529	yes	LMSG_G000009027.1	OES073733	PKS-like	PKSother	Polymetallic	23.94	China: Guangxi, Wuyi
L180322-P-8-LD180320-0027_HL3MGCCXY_L4_clean_bin.131.orig.fa	OEZ008529	yes	LMSG_G000009027.1	OES073733	thiopeptide	RiPPs	Polymetallic	27.17	China: Guangxi, Wuyi
L180322-P-8-LD180320-0027_HL3MGCCXY_L4_clean_bin.131.orig.fa	OEZ008529	yes	LMSG_G000009027.1	OES073733	NRPS-like	NRPS	Polymetallic	7.96	China: Guangxi, Wuyi
L180322-P-8-LD180320-0027_HL3MGCCXY_L4_clean_bin.131.orig.fa	OEZ008529	yes	LMSG_G000009027.1	OES073733	bacteriocin	RiPPs	Polymetallic	10.71	China: Guangxi, Wuyi
L180322-P-8-LD180320-0027_HL3MGCCXY_L4_clean_bin.133.orig.fa	OEZ008529	yes	LMSG_G000005534.1	OES073733	NRPS-like	NRPS	Polymetallic	9.36	China: Guangxi, Wuyi
L180322-P-8-LD180320-0027_HL3MGCCXY_L4_clean_bin.133.orig.fa	OEZ008529	yes	LMSG_G000005534.1	OES073733	NRPS-like	NRPS	Polymetallic	23.96	China: Guangxi, Wuyi
L180322-P-8-LD180320-0027_HL3MGCCXY_L4_clean_bin.133.orig.fa	OEZ008529	yes	LMSG_G000005534.1	OES073733	NRPS-like	NRPS	Polymetallic	11.49	China: Guangxi, Wuyi
L180322-P-8-LD180320-0027_HL3MGCCXY_L4_clean_bin.134.strict.	OEZ008529	yes	LMSG_G000008871.1	OES073733	terpene	Terpene	Polymetallic	10.93	China: Guangxi, Wuyi
L180322-P-8-LD180320-0027_HL3MGCCXY_L4_clean_bin.135.permiss	OEZ008529	yes	LMSG_G000005535.1	OES073733	sactipeptide	RiPPs	Polymetallic	10.18	China: Guangxi, Wuyi
L180322-P-8-LD180320-0027_HL3MGCCXY_L4_clean_bin.137.orig.fa	OEZ008529	yes	LMSG_G000006283.1	OES073733	terpene	Terpene	Polymetallic	9.47	China: Guangxi, Wuyi
L180322-P-8-LD180320-0027_HL3MGCCXY_L4_clean_bin.137.orig.fa	OEZ008529	yes	LMSG_G000006283.1	OES073733	bacteriocin	RiPPs	Polymetallic	5.27	China: Guangxi, Wuyi
L180322-P-8-LD180320-0027_HL3MGCCXY_L4_clean_bin.137.orig.fa	OEZ008529	yes	LMSG_G000006283.1	OES073733	betalactone	Others	Polymetallic	9.39	China: Guangxi, Wuyi
L180322-P-8-LD180320-0027_HL3MGCCXY_L4_clean_bin.141.orig.fa	OEZ008529	yes	LMSG_G000008821.1	OES073733	terpene	Terpene	Polymetallic	12.84	China: Guangxi, Wuyi
L180322-P-8-LD180320-0027_HL3MGCCXY_L4_clean_bin.144.orig.fa	OEZ008529	yes	LMSG_G000009511.1	OES073733	betalactone	Others	Polymetallic	18.58	China: Guangxi, Wuyi
L180322-P-8-LD180320-0027_HL3MGCCXY_L4_clean_bin.146.orig.fa	OEZ008529	yes	LMSG_G000009501.1	OES073733	TfuA-related	RiPPs	Polymetallic	14.00	China: Guangxi, Wuyi

L180322-P-8-LD180320-0027_HL3MGCCXY_L4_clean_bin.146.orig.fa	OEZ008529	yes	LMSG_G000009501.1	OES073733	betalactone	Others	Polymetallic	19.00	China: Guangxi, Wuyi
L180322-P-8-LD180320-0027_HL3MGCCXY_L4_clean_bin.146.orig.fa	OEZ008529	yes	LMSG_G000009501.1	OES073733	arylpolyene	Others	Polymetallic	21.26	China: Guangxi, Wuyi
L180322-P-8-LD180320-0027_HL3MGCCXY_L4_clean_bin.146.orig.fa	OEZ008529	yes	LMSG_G000009501.1	OES073733	bacteriocin	RiPPs	Polymetallic	10.88	China: Guangxi, Wuyi
L180322-P-8-LD180320-0027_HL3MGCCXY_L4_clean_bin.146.orig.fa	OEZ008529	yes	LMSG_G000009501.1	OES073733	T3PKS	PKSother	Polymetallic	13.82	China: Guangxi, Wuyi
L180322-P-8-LD180320-0027_HL3MGCCXY_L4_clean_bin.15.orig.fa_	OEZ008529	no	LMSG_G000010532.1	OES073733	terpene	Terpene	Polymetallic	10.88	China: Guangxi, Wuyi
L180322-P-8-LD180320-0027_HL3MGCCXY_L4_clean_bin.17.orig.fa_	OEZ008529	yes	LMSG_G000009516.1	OES073733	terpene	Terpene	Polymetallic	5.14	China: Guangxi, Wuyi
L180322-P-8-LD180320-0027_HL3MGCCXY_L4_clean_bin.17.orig.fa_	OEZ008529	yes	LMSG_G000009516.1	OES073733	T3PKS	PKSother	Polymetallic	5.32	China: Guangxi, Wuyi
L180322-P-8-LD180320-0027_HL3MGCCXY_L4_clean_bin.20.permissi	OEZ008529	yes	LMSG_G000011203.1	OES073733	arylpolyene	Others	Polymetallic	8.60	China: Guangxi, Wuyi
L180322-P-8-LD180320-0027_HL3MGCCXY_L4_clean_bin.21.orig.fa_	OEZ008529	yes	LMSG_G000011340.1	OES073733	terpene	Terpene	Polymetallic	20.99	China: Guangxi, Wuyi
L180322-P-8-LD180320-0027_HL3MGCCXY_L4_clean_bin.21.orig.fa_	OEZ008529	yes	LMSG_G000011340.1	OES073733	butyrolactone	Others	Polymetallic	10.78	China: Guangxi, Wuyi
L180322-P-8-LD180320-0027_HL3MGCCXY_L4_clean_bin.21.orig.fa_	OEZ008529	yes	LMSG_G000011340.1	OES073733	T3PKS	PKSother	Polymetallic	31.77	China: Guangxi, Wuyi
L180322-P-8-LD180320-0027_HL3MGCCXY_L4_clean_bin.24.orig.fa_	OEZ008529	yes	LMSG_G000008824.1	OES073733	terpene	Terpene	Polymetallic	21.69	China: Guangxi, Wuyi
L180322-P-8-LD180320-0027_HL3MGCCXY_L4_clean_bin.26.permissi	OEZ008529	yes	LMSG_G000006812.1	OES073733	terpene	Terpene	Polymetallic	16.26	China: Guangxi, Wuyi
L180322-P-8-LD180320-0027_HL3MGCCXY_L4_clean_bin.34.orig.fa_	OEZ008529	yes	LMSG_G000010694.1	OES073733	NRPS	NRPS	Polymetallic	13.90	China: Guangxi, Wuyi
L180322-P-8-LD180320-0027_HL3MGCCXY_L4_clean_bin.34.orig.fa_	OEZ008529	yes	LMSG_G000010694.1	OES073733	arylpolyene	Others	Polymetallic	15.52	China: Guangxi, Wuyi
L180322-P-8-LD180320-0027_HL3MGCCXY_L4_clean_bin.34.orig.fa_	OEZ008529	yes	LMSG_G000010694.1	OES073733	NRPS-like	NRPS	Polymetallic	22.48	China: Guangxi, Wuyi
L180322-P-8-LD180320-0027_HL3MGCCXY_L4_clean_bin.34.orig.fa_	OEZ008529	yes	LMSG_G000010694.1	OES073733	transAT-PKS	PKSother	Polymetallic	9.51	China: Guangxi, Wuyi
L180322-P-8-LD180320-0027_HL3MGCCXY_L4_clean_bin.34.orig.fa_	OEZ008529	yes	LMSG_G000010694.1	OES073733	bacteriocin	RiPPs	Polymetallic	7.68	China: Guangxi, Wuyi
L180322-P-8-LD180320-0027_HL3MGCCXY_L4_clean_bin.35.orig.fa_	OEZ008529	yes	LMSG_G000009671.1	OES073733	NRPS-like	NRPS	Polymetallic	27.63	China: Guangxi, Wuyi
L180322-P-8-LD180320-0027_HL3MGCCXY_L4_clean_bin.39.orig.fa_	OEZ008529	yes	LMSG_G000011092.1	OES073733	bacteriocin	RiPPs	Polymetallic	10.92	China: Guangxi, Wuyi
L180322-P-8-LD180320-0027_HL3MGCCXY_L4_clean_bin.39.orig.fa_	OEZ008529	yes	LMSG_G000011092.1	OES073733	NRPS-like	NRPS	Polymetallic	12.75	China: Guangxi, Wuyi
L180322-P-8-LD180320-0027_HL3MGCCXY_L4_clean_bin.39.orig.fa_	OEZ008529	yes	LMSG_G000011092.1	OES073733	terpene	Terpene	Polymetallic	12.67	China: Guangxi, Wuyi
L180322-P-8-LD180320-0027_HL3MGCCXY_L4_clean_bin.4.orig.fa_k	OEZ008529	yes	LMSG_G000005542.1	OES073733	bacteriocin	RiPPs	Polymetallic	10.82	China: Guangxi, Wuyi
L180322-P-8-LD180320-0027_HL3MGCCXY_L4_clean_bin.4.orig.fa_k	OEZ008529	yes	LMSG_G000005542.1	OES073733	ladderane.bacteriocin	Others	Polymetallic	41.16	China: Guangxi, Wuyi
L180322-P-8-LD180320-0027_HL3MGCCXY_L4_clean_bin.4.orig.fa_k	OEZ008529	yes	LMSG_G000005542.1	OES073733	arylpolyene	Others	Polymetallic	31.31	China: Guangxi, Wuyi
L180322-P-8-LD180320-0027_HL3MGCCXY_L4_clean_bin.4.orig.fa_k	OEZ008529	yes	LMSG_G000005542.1	OES073733	acyl_amino_acids	Others	Polymetallic	34.77	China: Guangxi, Wuyi
L180322-P-8-LD180320-0027_HL3MGCCXY_L4_clean_bin.4.orig.fa_k	OEZ008529	yes	LMSG_G000005542.1	OES073733	acyl_amino_acids	Others	Polymetallic	60.79	China: Guangxi, Wuyi
L180322-P-8-LD180320-0027_HL3MGCCXY_L4_clean_bin.4.orig.fa_k	OEZ008529	yes	LMSG_G000005542.1	OES073733	terpene	Terpene	Polymetallic	21.65	China: Guangxi, Wuyi
L180322-P-8-LD180320-0027_HL3MGCCXY_L4_clean_bin.4.orig.fa_k	OEZ008529	yes	LMSG_G000005542.1	OES073733	bacteriocin	RiPPs	Polymetallic	10.85	China: Guangxi, Wuyi
L180322-P-8-LD180320-0027_HL3MGCCXY_L4_clean_bin.41.strict.f	OEZ008529	yes	LMSG_G000004507.1	OES073733	hserlactone	Others	Polymetallic	20.75	China: Guangxi, Wuyi
L180322-P-8-LD180320-0027_HL3MGCCXY_L4_clean_bin.41.strict.f	OEZ008529	yes	LMSG_G000004507.1	OES073733	betalactone	Others	Polymetallic	27.51	China: Guangxi, Wuyi
L180322-P-8-LD180320-0027_HL3MGCCXY_L4_clean_bin.41.strict.f	OEZ008529	yes	LMSG_G000004507.1	OES073733	NRPS-like	NRPS	Polymetallic	12.58	China: Guangxi, Wuyi
L180322-P-8-LD180320-0027_HL3MGCCXY_L4_clean_bin.44.strict.f	OEZ008529	yes	LMSG_G000010753.1	OES073733	acyl_amino_acids	Others	Polymetallic	24.01	China: Guangxi, Wuyi
L180322-P-8-LD180320-0027_HL3MGCCXY_L4_clean_bin.44.strict.f	OEZ008529	yes	LMSG_G000010753.1	OES073733	arylpolyene	Others	Polymetallic	5.87	China: Guangxi, Wuyi
L180322-P-8-LD180320-0027_HL3MGCCXY_L4_clean_bin.46.strict.f	OEZ008529	yes	LMSG_G000009660.1	OES073733	NRPS-like	NRPS	Polymetallic	19.39	China: Guangxi, Wuyi
L180322-P-8-LD180320-0027_HL3MGCCXY_L4_clean_bin.46.strict.f	OEZ008529	yes	LMSG_G000009660.1	OES073733	terpene	Terpene	Polymetallic	16.23	China: Guangxi, Wuyi
L180322-P-8-LD180320-0027_HL3MGCCXY_L4_clean_bin.46.strict.f	OEZ008529	yes	LMSG_G000009660.1	OES073733	NRPS-like	NRPS	Polymetallic	10.58	China: Guangxi, Wuyi
L180322-P-8-LD180320-0027_HL3MGCCXY_L4_clean_bin.48.orig.fa_	OEZ008529	yes	LMSG_G000009663.1	OES073733	NRPS-like	NRPS	Polymetallic	42.57	China: Guangxi, Wuyi
L180322-P-8-LD180320-0027_HL3MGCCXY_L4_clean_bin.48.orig.fa_	OEZ008529	yes	LMSG_G000009663.1	OES073733	NRPS-like	NRPS	Polymetallic	42.63	China: Guangxi, Wuyi
L180322-P-8-LD180320-0027_HL3MGCCXY_L4_clean_bin.48.orig.fa_	OEZ008529	yes	LMSG_G000009663.1	OES073733	T3PKS	PKSother	Polymetallic	41.06	China: Guangxi, Wuyi
L180322-P-8-LD180320-0027_HL3MGCCXY_L4_clean_bin.48.orig.fa_	OEZ008529	yes	LMSG_G000009663.1	OES073733	terpene	Terpene	Polymetallic	21.99	China: Guangxi, Wuyi
L180322-P-8-LD180320-0027_HL3MGCCXY_L4_clean_bin.50.orig.fa_	OEZ008529	yes	LMSG_G000005543.1	OES073733	bacteriocin	RiPPs	Polymetallic	10.22	China: Guangxi, Wuyi
L180322-P-8-LD180320-0027_HL3MGCCXY_L4_clean_bin.50.orig.fa_	OEZ008529	yes	LMSG_G000005543.1	OES073733	terpene	Terpene	Polymetallic	13.27	China: Guangxi, Wuyi
L180322-P-8-LD180320-0027_HL3MGCCXY_L4_clean_bin.53.orig.fa_	OEZ008529	yes	LMSG_G000004508.1	OES073733	terpene	Terpene	Polymetallic	21.92	China: Guangxi, Wuyi
L180322-P-8-LD180320-0027_HL3MGCCXY_L4_clean_bin.53.orig.fa_	OEZ008529	yes	LMSG_G000004508.1	OES073733	bacteriocin	RiPPs	Polymetallic	10.94	China: Guangxi, Wuyi
L180322-P-8-LD180320-0027_HL3MGCCXY_L4_clean_bin.53.orig.fa_	OEZ008529	yes	LMSG_G000004508.1	OES073733	T3PKS	PKSother	Polymetallic	40.75	China: Guangxi, Wuyi
L180322-P-8-LD180320-0027_HL3MGCCXY_L4_clean_bin.53.orig.fa_	OEZ008529	yes	LMSG_G000004508.1	OES073733	NRPS-like	NRPS	Polymetallic	44.07	China: Guangxi, Wuyi
L180322-P-8-LD180320-0027_HL3MGCCXY_L4_clean_bin.53.orig.fa_	OEZ008529	yes	LMSG_G000004508.1	OES073733	terpene	Terpene	Polymetallic	10.72	China: Guangxi, Wuyi
L180322-P-8-LD180320-0027_HL3MGCCXY_L4_clean_bin.53.orig.fa_	OEZ008529	yes	LMSG_G000004508.1	OES073733	terpene	Terpene	Polymetallic	16.26	China: Guangxi, Wuyi
L180322-P-8-LD180320-0027_HL3MGCCXY_L4_clean_bin.6.orig.fa_k	OEZ008529	yes	LMSG_G000008862.1	OES073733	terpene	Terpene	Polymetallic	21.02	China: Guangxi, Wuyi
L180322-P-8-LD180320-0027_HL3MGCCXY_L4_clean_bin.6.orig.fa_k	OEZ008529	yes	LMSG_G000008862.1	OES073733	bacteriocin	RiPPs	Polymetallic	8.36	China: Guangxi, Wuyi
L180322-P-8-LD180320-0027_HL3MGCCXY_L4_clean_bin.6.orig.fa_k	OEZ008529	yes	LMSG_G000008862.1	OES073733	T3PKS	PKSother	Polymetallic	41.07	China: Guangxi, Wuyi
L180322-P-8-LD180320-0027_HL3MGCCXY_L4_clean_bin.60.strict.f	OEZ008529	yes	LMSG_G000006242.1	OES073733	other	Others	Polymetallic	40.44	China: Guangxi, Wuyi
L180322-P-8-LD180320-0027_HL3MGCCXY_L4_clean_bin.60.strict.f	OEZ008529	yes	LMSG_G000006242.1	OES073733	other	Others	Polymetallic	41.52	China: Guangxi, Wuyi
L180322-P-8-LD180320-0027_HL3MGCCXY_L4_clean_bin.66.orig.fa_	OEZ008529	yes	LMSG_G000008754.1	OES073733	terpene	Terpene	Polymetallic	6.64	China: Guangxi, Wuyi
L180322-P-8-LD180320-0027_HL3MGCCXY_L4_clean_bin.66.orig.fa_	OEZ008529	yes	LMSG_G000008754.1	OES073733	terpene	Terpene	Polymetallic	7.26	China: Guangxi, Wuyi
L180322-P-8-LD180320-0027_HL3MGCCXY_L4_clean_bin.66.orig.fa_	OEZ008529	yes	LMSG_G000008754.1	OES073733	terpene	Terpene	Polymetallic	20.66	China: Guangxi, Wuyi
L180322-P-8-LD180320-0027_HL3MGCCXY_L4_clean_bin.69.orig.fa_	OEZ008529	yes	LMSG_G000009207.1	OES073733	hglE-KS	PKSother	Polymetallic	11.89	China: Guangxi, Wuyi
L180322-P-8-LD180320-0027_HL3MGCCXY_L4_clean_bin.69.orig.fa_	OEZ008529	yes	LMSG_G000009207.1	OES073733	terpene	Terpene	Polymetallic	8.67	China: Guangxi, Wuyi
L180322-P-8-LD180320-0027_HL3MGCCXY_L4_clean_bin.70.orig.fa_	OEZ008529	yes	LMSG_G000005546.1	OES073733	NRPS-like	NRPS	Polymetallic	13.09	China: Guangxi, Wuyi
L180322-P-8-LD180320-0027_HL3MGCCXY_L4_clean_bin.70.orig.fa_	OEZ008529	yes	LMSG_G000005546.1	OES073733	arylpolyene	Others	Polymetallic	8.21	China: Guangxi, Wuyi
L180322-P-8-LD180320-0027_HL3MGCCXY_L4_clean_bin.70.orig.fa_	OEZ008529	yes	LMSG_G000005546.1	OES073733	terpene	Terpene	Polymetallic	12.19	China: Guangxi, Wuyi
L180322-P-8-LD180320-0027_HL3MGCCXY_L4_clean_bin.70.orig.fa_	OEZ008529	yes	LMSG_G000005546.1	OES073733	T3PKS	PKSother	Polymetallic	8.54	China: Guangxi, Wuyi
L180322-P-8-LD180320-0027_HL3MGCCXY_L4_clean_bin.70.orig.fa_	OEZ008529	yes	LMSG_G000005546.1	OES073733	lassopeptide	RiPPs	Polymetallic	13.14	China: Guangxi, Wuyi
L180322-P-8-LD180320-0027_HL3MGCCXY_L4_clean_bin.71.strict.f	OEZ008529	yes	LMSG_G000005547.1	OES073733	terpene	Terpene	Polymetallic	7.27	China: Guangxi, Wuyi
L180322-P-8-LD180320-0027_HL3MGCCXY_L4_clean_bin.75.orig.fa_	OEZ008529	yes	LMSG_G000005548.1	OES073733	bacteriocin	RiPPs	Polymetallic	10.84	China: Guangxi, Wuyi
L180322-P-8-LD180320-0027_HL3MGCCXY_L4_clean_bin.76.orig.fa_	OEZ008529	yes	LMSG_G000007905.1	OES073733	NRPS-like	NRPS	Polymetallic	10.27	China: Guangxi, Wuyi
L180322-P-8-LD180320-0027_HL3MGCCXY_L4_clean_bin.77.strict.f	OEZ008529	yes	LMSG_G000007937.1	OES073733	bacteriocin	RiPPs	Polymetallic	10.85	China: Guangxi, Wuyi
L180322-P-8-LD180320-0027_HL3MGCCXY_L4_clean_bin.77.strict.f	OEZ008529	yes	LMSG_G000007937.1	OES073733	terpene	Terpene	Polymetallic	21.07	China: Guangxi, Wuyi
L180322-P-8-LD180320-0027_HL3MGCCXY_L4_clean_bin.77.strict.f	OEZ008529	yes	LMSG_G000007937.1	OES073733	T3PKS	PKSother	Polymetallic	28.12	China: Guangxi, Wuyi
L180322-P-8-LD180320-0027_HL3MGCCXY_L4_clean_bin.78.orig.fa_	OEZ008529	yes	LMSG_G000008860.1	OES073733	T3PKS	PKSother	Polymetallic	14.34	China: Guangxi, Wuyi
L180322-P-8-LD180320-0027_HL3MGCCXY_L4_clean_bin.78.orig.fa_	OEZ008529	yes	LMSG_G000008860.1	OES073733	terpene	Terpene	Polymetallic	6.02	China: Guangxi, Wuyi
L180322-P-8-LD180320-0027_HL3MGCCXY_L4_clean_bin.81.orig.fa_	OEZ008529	yes	LMSG_G000010939.1	OES073733	acyl_amino_acids	Others	Polymetallic	45.46	China: Guangxi, Wuyi
L180322-P-8-LD180320-0027_HL3MGCCXY_L4_clean_bin.81.orig.fa_	OEZ008529	yes	LMSG_G000010939.1	OES073733	terpene	Terpene	Polymetallic	21.82	China: Guangxi, Wuyi
L180322-P-8-LD180320-0027_HL3MGCCXY_L4_clean_bin.82.permissi	OEZ008529	yes	LMSG_G000006813.1	OES073733	terpene	Terpene	Polymetallic	13.43	China: Guangxi, Wuyi
L180322-P-8-LD180320-0027_HL3MGCCXY_L4_clean_bin.83.orig.fa_	OEZ008529	yes	LMSG_G000005550.1	OES073733	T1PKS.hglE-KS	PKSother	Polymetallic	43.59	China: Guangxi, Wuyi
L180322-P-8-LD180320-0027_HL3MGCCXY_L4_clean_bin.83.orig.fa_	OEZ008529	yes	LMSG_G000005550.1	OES073733	hserlactone	Others	Polymetallic	13.58	China: Guangxi, Wuyi
L180322-P-8-LD180320-0027_HL3MGCCXY_L4_clean_bin.83.orig.fa_	OEZ008529	yes	LMSG_G000005550.1	OES073733	bacteriocin	RiPPs	Polymetallic	7.42	China: Guangxi, Wuyi
L180322-P-8-LD180320-0027_HL3MGCCXY_L4_clean_bin.83.orig.fa_	OEZ008529	yes	LMSG_G000005550.1	OES073733	betalactone	Others	Polymetallic	16.56	China: Guangxi, Wuyi
L180322-P-8-LD180320-0027_HL3MGCCXY_L4_clean_bin.83.orig.fa_	OEZ008529	yes	LMSG_G000005550.1	OES073733	hserlactone	Others	Polymetallic	15.10	China: Guangxi, Wuyi
L180322-P-8-LD180320-0027_HL3MGCCXY_L4_clean_bin.87.strict.f	OEZ008529	yes	LMSG_G000005552.1	OES073733	terpene	Terpene	Polymetallic	6.95	China: Guangxi, Wuyi
L180322-P-8-LD180320-0027_HL3MGCCXY_L4_clean_bin.88.orig.fa_	OEZ008529	yes	LMSG_G000009037.1	OES073733	T1PKS	PKSI	Polymetallic	42.98	China: Guangxi, Wuyi
L180322-P-8-LD180320-0027_HL3MGCCXY_L4_clean_bin.88.orig.fa_	OEZ008529	yes	LMSG_G000009037.1	OES073733	NRPS.transAT-PKS-like	PKS-NRP_Hybrids	Polymetallic	49.07	China: Guangxi, Wuyi
L180322-P-8-LD180320-0027_HL3MGCCXY_L4_clean_bin.88.orig.fa_	OEZ008529	yes	LMSG_G000009037.1	OES073733	hglE-KS	PKSother	Polymetallic	25.01	China: Guangxi, Wuyi
L180322-P-8-LD180320-0027_HL3MGCCXY_L4_clean_bin.88.orig.fa_	OEZ008529	yes	LMSG_G000009037.1	OES073733	T1PKS	PKSI	Polymetallic	27.71	China: Guangxi, Wuyi
L180322-P-8-LD180320-0027_HL3MGCCXY_L4_clean_bin.88.orig.fa_	OEZ008529	yes	LMSG_G000009037.1	OES073733	acyl_amino_acids	Others	Polymetallic	52.14	China: Guangxi, Wuyi
L180322-P-8-LD180320-0027_HL3MGCCXY_L4_clean_bin.88.orig.fa_	OEZ008529	yes	LMSG_G000009037.1	OES073733	T1PKS.hglE-KS	PKSother	Polymetallic	51.27	China: Guangxi, Wuyi
L180322-P-8-LD180320-0027_HL3MGCCXY_L4_clean_bin.88.orig.fa_	OEZ008529	yes	LMSG_G000009037.1	OES073733	T1PKS.hglE-KS	PKSother	Polymetallic	70.86	China: Guangxi, Wuyi
L180322-P-8-LD180320-0027_HL3MGCCXY_L4_clean_bin.88.orig.fa_	OEZ008529	yes	LMSG_G000009037.1	OES073733	arylpolyene	Others	Polymetallic	38.63	China: Guangxi, Wuyi
L180322-P-8-LD180320-0027_HL3MGCCXY_L4_clean_bin.89.orig.fa_	OEZ008529	yes	LMSG_G000005553.1	OES073733	NRPS-like	NRPS	Polymetallic	9.33	China: Guangxi, Wuyi
L180322-P-8-LD180320-0027_HL3MGCCXY_L4_clean_bin.89.orig.fa_	OEZ008529	yes	LMSG_G000005553.1	OES073733	terpene	Terpene	Polymetallic	17.24	China: Guangxi, Wuyi
L180322-P-8-LD180320-0027_HL3MGCCXY_L4_clean_bin.93.orig.fa_	OEZ008529	yes	LMSG_G000005555.1	OES073733	terpene	Terpene	Polymetallic	11.26	China: Guangxi, Wuyi
L180322-P-8-LD180320-0027_HL3MGCCXY_L4_clean_bin.94.strict.f	OEZ008529	yes	LMSG_G000005556.1	OES073733	LAP	RiPPs	Polymetallic	8.91	China: Guangxi, Wuyi
L180322-P-8-LD180320-0027_HL3MGCCXY_L4_clean_bin.95.orig.fa_	OEZ008529	yes	LMSG_G000005557.1	OES073733	ladderane	Others	Polymetallic	7.96	China: Guangxi, Wuyi
L180322-P-8-LD180320-0027_HL3MGCCXY_L4_clean_bin.95.orig.fa_	OEZ008529	yes	LMSG_G000005557.1	OES073733	ladderane	Others	Polymetallic	9.70	China: Guangxi, Wuyi
L180322-P-8-LD180320-0027_HL3MGCCXY_L4_clean_bin.95.orig.fa_	OEZ008529	yes	LMSG_G000005557.1	OES073733	phosphonate	Others	Polymetallic	15.61	China: Guangxi, Wuyi
L180322-P-8-LD180320-0027_HL3MGCCXY_L4_clean_bin.95.orig.fa_	OEZ008529	yes	LMSG_G000005557.1	OES073733	ladderane	Others	Polymetallic	14.94	China: Guangxi, Wuyi
L180322-P-8-LD180320-0027_HL3MGCCXY_L4_clean_bin.99.orig.fa_	OEZ008529	yes	LMSG_G000011090.1	OES073733	arylpolyene	Others	Polymetallic	9.84	China: Guangxi, Wuyi
L180322-P-8-LD180320-0027_HL3MGCCXY_L4_clean_bin.99.orig.fa_	OEZ008529	yes	LMSG_G000011090.1	OES073733	NRPS-like	NRPS	Polymetallic	18.37	China: Guangxi, Wuyi
L180322-P-8-LD180320-0027_HL3MGCCXY_L4_clean_bin.99.orig.fa_	OEZ008529	yes	LMSG_G000011090.1	OES073733	arylpolyene	Others	Polymetallic	22.87	China: Guangxi, Wuyi
L180322-P-8-LD180320-0027_HL3MGCCXY_L4_clean_bin.99.orig.fa_	OEZ008529	yes	LMSG_G000011090.1	OES073733	terpene	Terpene	Polymetallic	5.85	China: Guangxi, Wuyi
L180322-P-9-LD180320-0028_HL3MGCCXY_L5_clean_bin.11.orig.fa_	OEZ008529	yes	LMSG_G000010438.1	OES073677	bacteriocin	RiPPs	Tin-Zinc	6.77	China: Guangxi, Dashan
L180322-P-9-LD180320-0028_HL3MGCCXY_L5_clean_bin.11.orig.fa_	OEZ008529	yes	LMSG_G000010438.1	OES073677	arylpolyene	Others	Tin-Zinc	20.85	China: Guangxi, Dashan
L180322-P-9-LD180320-0028_HL3MGCCXY_L5_clean_bin.11.orig.fa_	OEZ008529	yes	LMSG_G000010438.1	OES073677	resorcinol.arylpolyene	Others	Tin-Zinc	14.69	China: Guangxi, Dashan
L180322-P-9-LD180320-0028_HL3MGCCXY_L5_clean_bin.11.orig.fa_	OEZ008529	yes	LMSG_G000010438.1	OES073677	terpene	Terpene	Tin-Zinc	15.84	China: Guangxi, Dashan
L180322-P-9-LD180320-0028_HL3MGCCXY_L5_clean_bin.12.orig.fa_	OEZ008529	yes	LMSG_G000009195.1	OES073677	terpene	Terpene	Tin-Zinc	6.30	China: Guangxi, Dashan
L180322-P-9-LD180320-0028_HL3MGCCXY_L5_clean_bin.12.orig.fa_	OEZ008529	yes	LMSG_G000009195.1	OES073677	T3PKS	PKSother	Tin-Zinc	6.02	China: Guangxi, Dashan
L180322-P-9-LD180320-0028_HL3MGCCXY_L5_clean_bin.15.orig.fa_	OEZ008529	yes	LMSG_G000008413.1	OES073677	TfuA-related.bacteriocin	RiPPs	Tin-Zinc	22.68	China: Guangxi, Dashan
L180322-P-9-LD180320-0028_HL3MGCCXY_L5_clean_bin.15.orig.fa_	OEZ008529	yes	LMSG_G000008413.1	OES073677	terpene	Terpene	Tin-Zinc	8.21	China: Guangxi, Dashan
L180322-P-9-LD180320-0028_HL3MGCCXY_L5_clean_bin.15.orig.fa_	OEZ008529	yes	LMSG_G000008413.1	OES073677	TfuA-related	RiPPs	Tin-Zinc	12.66	China: Guangxi, Dashan
L180322-P-9-LD180320-0028_HL3MGCCXY_L5_clean_bin.16.orig.fa_	OEZ008529	yes	LMSG_G000010252.1	OES073677	arylpolyene	Others	Tin-Zinc	32.48	China: Guangxi, Dashan
L180322-P-9-LD180320-0028_HL3MGCCXY_L5_clean_bin.16.orig.fa_	OEZ008529	no	LMSG_G000010252.1	OES073677	terpene	Terpene	Tin-Zinc	10.53	China: Guangxi, Dashan
L180322-P-9-LD180320-0028_HL3MGCCXY_L5_clean_bin.16.orig.fa_	OEZ008529	no	LMSG_G000010252.1	OES073677	terpene	Terpene	Tin-Zinc	5.55	China: Guangxi, Dashan
L180322-P-9-LD180320-0028_HL3MGCCXY_L5_clean_bin.19.orig.fa_	OEZ008529	yes	LMSG_G000005558.1	OES073677	terpene	Terpene	Tin-Zinc	13.49	China: Guangxi, Dashan
L180322-P-9-LD180320-0028_HL3MGCCXY_L5_clean_bin.19.orig.fa_	OEZ008529	yes	LMSG_G000005558.1	OES073677	T3PKS	PKSother	Tin-Zinc	24.53	China: Guangxi, Dashan
L180322-P-9-LD180320-0028_HL3MGCCXY_L5_clean_bin.2.orig.fa_k	OEZ008529	yes	LMSG_G000010877.1	OES073677	terpene	Terpene	Tin-Zinc	16.36	China: Guangxi, Dashan
L180322-P-9-LD180320-0028_HL3MGCCXY_L5_clean_bin.2.orig.fa_k	OEZ008529	yes	LMSG_G000010877.1	OES073677	NRPS.bacteriocin	Others	Tin-Zinc	27.01	China: Guangxi, Dashan
L180322-P-9-LD180320-0028_HL3MGCCXY_L5_clean_bin.20.orig.fa_	OEZ008529	yes	LMSG_G000008077.1	OES073677	arylpolyene	Others	Tin-Zinc	14.33	China: Guangxi, Dashan
L180322-P-9-LD180320-0028_HL3MGCCXY_L5_clean_bin.20.orig.fa_	OEZ008529	yes	LMSG_G000008077.1	OES073677	other	Others	Tin-Zinc	8.59	China: Guangxi, Dashan
L180322-P-9-LD180320-0028_HL3MGCCXY_L5_clean_bin.20.orig.fa_	OEZ008529	yes	LMSG_G000008077.1	OES073677	hglE-KS	PKSother	Tin-Zinc	5.68	China: Guangxi, Dashan
L180322-P-9-LD180320-0028_HL3MGCCXY_L5_clean_bin.20.orig.fa_	OEZ008529	yes	LMSG_G000008077.1	OES073677	T1PKS	PKSI	Tin-Zinc	10.96	China: Guangxi, Dashan
L180322-P-9-LD180320-0028_HL3MGCCXY_L5_clean_bin.25.orig.fa_	OEZ008529	yes	LMSG_G000008999.1	OES073677	bacteriocin	RiPPs	Tin-Zinc	7.47	China: Guangxi, Dashan
L180322-P-9-LD180320-0028_HL3MGCCXY_L5_clean_bin.25.orig.fa_	OEZ008529	yes	LMSG_G000008999.1	OES073677	terpene	Terpene	Tin-Zinc	5.79	China: Guangxi, Dashan
L180322-P-9-LD180320-0028_HL3MGCCXY_L5_clean_bin.25.orig.fa_	OEZ008529	yes	LMSG_G000008999.1	OES073677	terpene	Terpene	Tin-Zinc	10.23	China: Guangxi, Dashan
L180322-P-9-LD180320-0028_HL3MGCCXY_L5_clean_bin.26.strict.f	OEZ008529	yes	LMSG_G000007951.1	OES073677	terpene	Terpene	Tin-Zinc	17.11	China: Guangxi, Dashan
L180322-P-9-LD180320-0028_HL3MGCCXY_L5_clean_bin.29.orig.fa_	OEZ008529	yes	LMSG_G000005560.1	OES073677	betalactone	Others	Tin-Zinc	28.45	China: Guangxi, Dashan
L180322-P-9-LD180320-0028_HL3MGCCXY_L5_clean_bin.29.orig.fa_	OEZ008529	yes	LMSG_G000005560.1	OES073677	betalactone	Others	Tin-Zinc	22.56	China: Guangxi, Dashan
L180322-P-9-LD180320-0028_HL3MGCCXY_L5_clean_bin.29.orig.fa_	OEZ008529	yes	LMSG_G000005560.1	OES073677	betalactone	Others	Tin-Zinc	24.04	China: Guangxi, Dashan
L180322-P-9-LD180320-0028_HL3MGCCXY_L5_clean_bin.32.orig.fa_	OEZ008529	yes	LMSG_G000010909.1	OES073677	thiopeptide	RiPPs	Tin-Zinc	7.04	China: Guangxi, Dashan
L180322-P-9-LD180320-0028_HL3MGCCXY_L5_clean_bin.32.orig.fa_	OEZ008529	yes	LMSG_G000010909.1	OES073677	bacteriocin	RiPPs	Tin-Zinc	6.83	China: Guangxi, Dashan
L180322-P-9-LD180320-0028_HL3MGCCXY_L5_clean_bin.33.orig.fa_	OEZ008529	yes	LMSG_G000011055.1	OES073677	resorcinol	Others	Tin-Zinc	10.21	China: Guangxi, Dashan
L180322-P-9-LD180320-0028_HL3MGCCXY_L5_clean_bin.33.orig.fa_	OEZ008529	yes	LMSG_G000011055.1	OES073677	arylpolyene	Others	Tin-Zinc	25.49	China: Guangxi, Dashan
L180322-P-9-LD180320-0028_HL3MGCCXY_L5_clean_bin.35.orig.fa_	OEZ008529	yes	LMSG_G000005561.1	OES073677	terpene	Terpene	Tin-Zinc	21.00	China: Guangxi, Dashan
L180322-P-9-LD180320-0028_HL3MGCCXY_L5_clean_bin.37.strict.f	OEZ008529	yes	LMSG_G000005563.1	OES073677	T1PKS	PKSI	Tin-Zinc	8.64	China: Guangxi, Dashan
L180322-P-9-LD180320-0028_HL3MGCCXY_L5_clean_bin.38.orig.fa_	OEZ008529	yes	LMSG_G000008341.1	OES073677	butyrolactone	Others	Tin-Zinc	11.01	China: Guangxi, Dashan
L180322-P-9-LD180320-0028_HL3MGCCXY_L5_clean_bin.38.orig.fa_	OEZ008529	yes	LMSG_G000008341.1	OES073677	other	Others	Tin-Zinc	14.35	China: Guangxi, Dashan
L180322-P-9-LD180320-0028_HL3MGCCXY_L5_clean_bin.38.orig.fa_	OEZ008529	yes	LMSG_G000008341.1	OES073677	bacteriocin	RiPPs	Tin-Zinc	9.44	China: Guangxi, Dashan
L180322-P-9-LD180320-0028_HL3MGCCXY_L5_clean_bin.38.orig.fa_	OEZ008529	yes	LMSG_G000008341.1	OES073677	T3PKS	PKSother	Tin-Zinc	21.68	China: Guangxi, Dashan
L180322-P-9-LD180320-0028_HL3MGCCXY_L5_clean_bin.38.orig.fa_	OEZ008529	yes	LMSG_G000008341.1	OES073677	terpene	Terpene	Tin-Zinc	19.42	China: Guangxi, Dashan
L180322-P-9-LD180320-0028_HL3MGCCXY_L5_clean_bin.39.orig.fa_	OEZ008529	yes	LMSG_G000005564.1	OES073677	bacteriocin	RiPPs	Tin-Zinc	5.55	China: Guangxi, Dashan
L180322-P-9-LD180320-0028_HL3MGCCXY_L5_clean_bin.39.orig.fa_	OEZ008529	yes	LMSG_G000005564.1	OES073677	T1PKS	PKSI	Tin-Zinc	8.15	China: Guangxi, Dashan
L180322-P-9-LD180320-0028_HL3MGCCXY_L5_clean_bin.39.orig.fa_	OEZ008529	yes	LMSG_G000005564.1	OES073677	terpene	Terpene	Tin-Zinc	5.96	China: Guangxi, Dashan
L180322-P-9-LD180320-0028_HL3MGCCXY_L5_clean_bin.41.orig.fa_	OEZ008529	yes	LMSG_G000009523.1	OES073677	NRPS-like	NRPS	Tin-Zinc	27.86	China: Guangxi, Dashan
L180322-P-9-LD180320-0028_HL3MGCCXY_L5_clean_bin.41.orig.fa_	OEZ008529	yes	LMSG_G000009523.1	OES073677	terpene	Terpene	Tin-Zinc	21.16	China: Guangxi, Dashan
L180322-P-9-LD180320-0028_HL3MGCCXY_L5_clean_bin.41.orig.fa_	OEZ008529	yes	LMSG_G000009523.1	OES073677	arylpolyene	Others	Tin-Zinc	41.19	China: Guangxi, Dashan
L180322-P-9-LD180320-0028_HL3MGCCXY_L5_clean_bin.41.orig.fa_	OEZ008529	yes	LMSG_G000009523.1	OES073677	ectoine	Others	Tin-Zinc	6.58	China: Guangxi, Dashan
L180322-P-9-LD180320-0028_HL3MGCCXY_L5_clean_bin.41.orig.fa_	OEZ008529	yes	LMSG_G000009523.1	OES073677	terpene	Terpene	Tin-Zinc	20.94	China: Guangxi, Dashan
L180322-P-9-LD180320-0028_HL3MGCCXY_L5_clean_bin.43.orig.fa_	OEZ008529	yes	LMSG_G000011179.1	OES073677	arylpolyene	Others	Tin-Zinc	41.23	China: Guangxi, Dashan
L180322-P-9-LD180320-0028_HL3MGCCXY_L5_clean_bin.45.orig.fa_	OEZ008529	yes	LMSG_G000008273.1	OES073677	terpene	Terpene	Tin-Zinc	19.12	China: Guangxi, Dashan
L180322-P-9-LD180320-0028_HL3MGCCXY_L5_clean_bin.46.orig.fa_	OEZ008529	yes	LMSG_G000008532.1	OES073677	terpene	Terpene	Tin-Zinc	7.29	China: Guangxi, Dashan
L180322-P-9-LD180320-0028_HL3MGCCXY_L5_clean_bin.47.orig.fa_	OEZ008529	yes	LMSG_G000009243.1	OES073677	T3PKS.terpene	Others	Tin-Zinc	32.30	China: Guangxi, Dashan
L180322-P-9-LD180320-0028_HL3MGCCXY_L5_clean_bin.48.orig.fa_	OEZ008529	yes	LMSG_G000008109.1	OES073677	terpene	Terpene	Tin-Zinc	13.73	China: Guangxi, Dashan
L180322-P-9-LD180320-0028_HL3MGCCXY_L5_clean_bin.48.orig.fa_	OEZ008529	yes	LMSG_G000008109.1	OES073677	terpene	Terpene	Tin-Zinc	21.02	China: Guangxi, Dashan
L180322-P-9-LD180320-0028_HL3MGCCXY_L5_clean_bin.52.orig.fa_	OEZ008529	yes	LMSG_G000010469.1	OES073677	terpene	Terpene	Tin-Zinc	12.63	China: Guangxi, Dashan
L180322-P-9-LD180320-0028_HL3MGCCXY_L5_clean_bin.53.orig.fa_	OEZ008529	yes	LMSG_G000005566.1	OES073677	NRPS-like	NRPS	Tin-Zinc	6.71	China: Guangxi, Dashan
L180322-P-9-LD180320-0028_HL3MGCCXY_L5_clean_bin.53.orig.fa_	OEZ008529	yes	LMSG_G000005566.1	OES073677	terpene	Terpene	Tin-Zinc	18.75	China: Guangxi, Dashan
L180322-P-9-LD180320-0028_HL3MGCCXY_L5_clean_bin.55.orig.fa_	OEZ008529	no	LMSG_G000010576.1	OES073677	bacteriocin	RiPPs	Tin-Zinc	5.64	China: Guangxi, Dashan
L180322-P-9-LD180320-0028_HL3MGCCXY_L5_clean_bin.56.orig.fa_	OEZ008529	yes	LMSG_G000005567.1	OES073677	terpene	Terpene	Tin-Zinc	22.50	China: Guangxi, Dashan

L180322-P-9-LD180320-0028_HL3MGCCXY_L5_clean_bin.56.orig.fa_	OEZ008529	yes	LMSG_G000005567.1	OES073677	arylpolyene.hserlactone	Others	Tin-Zinc	29.26	China: Guangxi, Dashan
L180322-P-9-LD180320-0028_HL3MGCCXY_L5_clean_bin.58.orig.fa_	OEZ008529	yes	LMSG_G000005568.1	OES073677	bacteriocin	RiPPs	Tin-Zinc	9.33	China: Guangxi, Dashan
L180322-P-9-LD180320-0028_HL3MGCCXY_L5_clean_bin.59.orig.fa_	OEZ008529	yes	LMSG_G000005569.1	OES073677	terpene	Terpene	Tin-Zinc	22.67	China: Guangxi, Dashan
L180322-P-9-LD180320-0028_HL3MGCCXY_L5_clean_bin.6.orig.fa_k	OEZ008529	yes	LMSG_G000008701.1	OES073677	terpene	Terpene	Tin-Zinc	5.74	China: Guangxi, Dashan
L180322-P-9-LD180320-0028_HL3MGCCXY_L5_clean_bin.62.orig.fa_	OEZ008529	yes	LMSG_G000008556.1	OES073677	hglE-KS	PKSother	Tin-Zinc	31.99	China: Guangxi, Dashan
L180322-P-9-LD180320-0028_HL3MGCCXY_L5_clean_bin.65.orig.fa_	OEZ008529	yes	LMSG_G000010926.1	OES073677	terpene	Terpene	Tin-Zinc	21.66	China: Guangxi, Dashan
L180322-P-9-LD180320-0028_HL3MGCCXY_L5_clean_bin.65.orig.fa_	OEZ008529	yes	LMSG_G000010926.1	OES073677	thiopeptide	RiPPs	Tin-Zinc	29.82	China: Guangxi, Dashan
L180322-P-9-LD180320-0028_HL3MGCCXY_L5_clean_bin.65.orig.fa_	OEZ008529	yes	LMSG_G000010926.1	OES073677	arylpolyene	Others	Tin-Zinc	41.19	China: Guangxi, Dashan
L180322-P-9-LD180320-0028_HL3MGCCXY_L5_clean_bin.67.orig.fa_	OEZ008529	yes	LMSG_G000008228.1	OES073677	terpene	Terpene	Tin-Zinc	15.24	China: Guangxi, Dashan
L180322-P-9-LD180320-0028_HL3MGCCXY_L5_clean_bin.68.orig.fa_	OEZ008529	yes	LMSG_G000010514.1	OES073677	arylpolyene	Others	Tin-Zinc	7.01	China: Guangxi, Dashan
L180322-P-9-LD180320-0028_HL3MGCCXY_L5_clean_bin.68.orig.fa_	OEZ008529	yes	LMSG_G000010514.1	OES073677	NRPS-like	NRPS	Tin-Zinc	6.11	China: Guangxi, Dashan
L180322-P-9-LD180320-0028_HL3MGCCXY_L5_clean_bin.68.orig.fa_	OEZ008529	yes	LMSG_G000010514.1	OES073677	acyl_amino_acids	Others	Tin-Zinc	6.48	China: Guangxi, Dashan
L180322-P-9-LD180320-0028_HL3MGCCXY_L5_clean_bin.69.orig.fa_	OEZ008529	yes	LMSG_G000010446.1	OES073677	NRPS-like	NRPS	Tin-Zinc	42.80	China: Guangxi, Dashan
L180322-P-9-LD180320-0028_HL3MGCCXY_L5_clean_bin.69.orig.fa_	OEZ008529	yes	LMSG_G000010446.1	OES073677	bacteriocin	RiPPs	Tin-Zinc	7.90	China: Guangxi, Dashan
L180322-P-9-LD180320-0028_HL3MGCCXY_L5_clean_bin.69.orig.fa_	OEZ008529	yes	LMSG_G000010446.1	OES073677	terpene	Terpene	Tin-Zinc	8.51	China: Guangxi, Dashan
L180322-P-9-LD180320-0028_HL3MGCCXY_L5_clean_bin.7.orig.fa_k	OEZ008529	yes	LMSG_G000011115.1	OES073677	terpene	Terpene	Tin-Zinc	5.72	China: Guangxi, Dashan
L180322-P-9-LD180320-0028_HL3MGCCXY_L5_clean_bin.70.orig.fa_	OEZ008529	yes	LMSG_G000009308.1	OES073677	terpene	Terpene	Tin-Zinc	13.62	China: Guangxi, Dashan
L180322-P-9-LD180320-0028_HL3MGCCXY_L5_clean_bin.70.orig.fa_	OEZ008529	yes	LMSG_G000009308.1	OES073677	bacteriocin	RiPPs	Tin-Zinc	9.99	China: Guangxi, Dashan
L180322-P-9-LD180320-0028_HL3MGCCXY_L5_clean_bin.74.orig.fa_	OEZ008529	yes	LMSG_G000005571.1	OES073677	T1PKS	PKSI	Tin-Zinc	10.81	China: Guangxi, Dashan
L180322-P-9-LD180320-0028_HL3MGCCXY_L5_clean_bin.74.orig.fa_	OEZ008529	yes	LMSG_G000005571.1	OES073677	NRPS-like	NRPS	Tin-Zinc	19.81	China: Guangxi, Dashan
L180322-P-9-LD180320-0028_HL3MGCCXY_L5_clean_bin.74.orig.fa_	OEZ008529	yes	LMSG_G000005571.1	OES073677	arylpolyene	Others	Tin-Zinc	20.79	China: Guangxi, Dashan
L180322-P-9-LD180320-0028_HL3MGCCXY_L5_clean_bin.74.orig.fa_	OEZ008529	yes	LMSG_G000005571.1	OES073677	terpene	Terpene	Tin-Zinc	13.96	China: Guangxi, Dashan
L180322-P-9-LD180320-0028_HL3MGCCXY_L5_clean_bin.8.orig.fa_k	OEZ008529	yes	LMSG_G000008291.1	OES073677	terpene	Terpene	Tin-Zinc	13.73	China: Guangxi, Dashan
L180322-P-9-LD180320-0029_HL3MGCCXY_L5_clean_bin.10.orig.fa_	OEZ008529	yes	LMSG_G000009436.1	OES073678	ectoine	Others	Tin-Zinc	10.40	China: Guangxi, Dashan
L180322-P-9-LD180320-0029_HL3MGCCXY_L5_clean_bin.10.orig.fa_	OEZ008529	yes	LMSG_G000009436.1	OES073678	betalactone	Others	Tin-Zinc	22.78	China: Guangxi, Dashan
L180322-P-9-LD180320-0029_HL3MGCCXY_L5_clean_bin.10.orig.fa_	OEZ008529	yes	LMSG_G000009436.1	OES073678	bacteriocin	RiPPs	Tin-Zinc	7.25	China: Guangxi, Dashan
L180322-P-9-LD180320-0029_HL3MGCCXY_L5_clean_bin.10.orig.fa_	OEZ008529	yes	LMSG_G000009436.1	OES073678	betalactone	Others	Tin-Zinc	30.86	China: Guangxi, Dashan
L180322-P-9-LD180320-0029_HL3MGCCXY_L5_clean_bin.11.orig.fa_	OEZ008529	yes	LMSG_G000010401.1	OES073678	terpene	Terpene	Tin-Zinc	23.22	China: Guangxi, Dashan
L180322-P-9-LD180320-0029_HL3MGCCXY_L5_clean_bin.16.orig.fa_	OEZ008529	yes	LMSG_G000010470.1	OES073678	arylpolyene	Others	Tin-Zinc	32.52	China: Guangxi, Dashan
L180322-P-9-LD180320-0029_HL3MGCCXY_L5_clean_bin.16.orig.fa_	OEZ008529	yes	LMSG_G000010470.1	OES073678	terpene	Terpene	Tin-Zinc	18.27	China: Guangxi, Dashan
L180322-P-9-LD180320-0029_HL3MGCCXY_L5_clean_bin.17.orig.fa_	OEZ008529	yes	LMSG_G000010722.1	OES073678	NRPS	NRPS	Tin-Zinc	6.31	China: Guangxi, Dashan
L180322-P-9-LD180320-0029_HL3MGCCXY_L5_clean_bin.17.orig.fa_	OEZ008529	yes	LMSG_G000010722.1	OES073678	arylpolyene	Others	Tin-Zinc	32.58	China: Guangxi, Dashan
L180322-P-9-LD180320-0029_HL3MGCCXY_L5_clean_bin.17.orig.fa_	OEZ008529	yes	LMSG_G000010722.1	OES073678	terpene	Terpene	Tin-Zinc	14.88	China: Guangxi, Dashan
L180322-P-9-LD180320-0029_HL3MGCCXY_L5_clean_bin.17.orig.fa_	OEZ008529	yes	LMSG_G000010722.1	OES073678	NRPS	NRPS	Tin-Zinc	12.36	China: Guangxi, Dashan
L180322-P-9-LD180320-0029_HL3MGCCXY_L5_clean_bin.17.orig.fa_	OEZ008529	yes	LMSG_G000010722.1	OES073678	NRPS-like	NRPS	Tin-Zinc	13.39	China: Guangxi, Dashan
L180322-P-9-LD180320-0029_HL3MGCCXY_L5_clean_bin.18.strict.f	OEZ008529	yes	LMSG_G000008078.1	OES073678	T1PKS.hglE-KS	PKSother	Tin-Zinc	39.85	China: Guangxi, Dashan
L180322-P-9-LD180320-0029_HL3MGCCXY_L5_clean_bin.18.strict.f	OEZ008529	yes	LMSG_G000008078.1	OES073678	other	Others	Tin-Zinc	14.00	China: Guangxi, Dashan
L180322-P-9-LD180320-0029_HL3MGCCXY_L5_clean_bin.18.strict.f	OEZ008529	yes	LMSG_G000008078.1	OES073678	arylpolyene	Others	Tin-Zinc	5.63	China: Guangxi, Dashan
L180322-P-9-LD180320-0029_HL3MGCCXY_L5_clean_bin.19.strict.f	OEZ008529	yes	LMSG_G000009414.1	OES073678	bacteriocin	RiPPs	Tin-Zinc	10.85	China: Guangxi, Dashan
L180322-P-9-LD180320-0029_HL3MGCCXY_L5_clean_bin.19.strict.f	OEZ008529	yes	LMSG_G000009414.1	OES073678	bacteriocin	RiPPs	Tin-Zinc	10.85	China: Guangxi, Dashan
L180322-P-9-LD180320-0029_HL3MGCCXY_L5_clean_bin.19.strict.f	OEZ008529	yes	LMSG_G000009414.1	OES073678	betalactone	Others	Tin-Zinc	24.03	China: Guangxi, Dashan
L180322-P-9-LD180320-0029_HL3MGCCXY_L5_clean_bin.19.strict.f	OEZ008529	yes	LMSG_G000009414.1	OES073678	NRPS	NRPS	Tin-Zinc	27.12	China: Guangxi, Dashan
L180322-P-9-LD180320-0029_HL3MGCCXY_L5_clean_bin.19.strict.f	OEZ008529	yes	LMSG_G000009414.1	OES073678	ectoine	Others	Tin-Zinc	10.17	China: Guangxi, Dashan
L180322-P-9-LD180320-0029_HL3MGCCXY_L5_clean_bin.2.orig.fa_k	OEZ008529	yes	LMSG_G000009196.1	OES073678	terpene	Terpene	Tin-Zinc	6.22	China: Guangxi, Dashan
L180322-P-9-LD180320-0029_HL3MGCCXY_L5_clean_bin.23.orig.fa_	OEZ008529	yes	LMSG_G000005574.1	OES073678	thiopeptide	RiPPs	Tin-Zinc	5.45	China: Guangxi, Dashan
L180322-P-9-LD180320-0029_HL3MGCCXY_L5_clean_bin.23.orig.fa_	OEZ008529	yes	LMSG_G000005574.1	OES073678	acyl_amino_acids	Others	Tin-Zinc	6.19	China: Guangxi, Dashan
L180322-P-9-LD180320-0029_HL3MGCCXY_L5_clean_bin.23.orig.fa_	OEZ008529	yes	LMSG_G000005574.1	OES073678	bacteriocin	RiPPs	Tin-Zinc	6.09	China: Guangxi, Dashan
L180322-P-9-LD180320-0029_HL3MGCCXY_L5_clean_bin.25.orig.fa_	OEZ008529	yes	LMSG_G000005575.1	OES073678	betalactone	Others	Tin-Zinc	6.07	China: Guangxi, Dashan
L180322-P-9-LD180320-0029_HL3MGCCXY_L5_clean_bin.25.orig.fa_	OEZ008529	yes	LMSG_G000005575.1	OES073678	terpene	Terpene	Tin-Zinc	9.02	China: Guangxi, Dashan
L180322-P-9-LD180320-0029_HL3MGCCXY_L5_clean_bin.26.strict.f	OEZ008529	yes	LMSG_G000004510.1	OES073678	terpene	Terpene	Tin-Zinc	14.78	China: Guangxi, Dashan
L180322-P-9-LD180320-0029_HL3MGCCXY_L5_clean_bin.27.orig.fa_	OEZ008529	yes	LMSG_G000005576.1	OES073678	terpene	Terpene	Tin-Zinc	13.69	China: Guangxi, Dashan
L180322-P-9-LD180320-0029_HL3MGCCXY_L5_clean_bin.27.orig.fa_	OEZ008529	yes	LMSG_G000005576.1	OES073678	bacteriocin	RiPPs	Tin-Zinc	6.10	China: Guangxi, Dashan
L180322-P-9-LD180320-0029_HL3MGCCXY_L5_clean_bin.29.orig.fa_	OEZ008529	no	LMSG_G000010277.1	OES073678	terpene	Terpene	Tin-Zinc	21.54	China: Guangxi, Dashan
L180322-P-9-LD180320-0029_HL3MGCCXY_L5_clean_bin.29.orig.fa_	OEZ008529	yes	LMSG_G000010277.1	OES073678	arylpolyene	Others	Tin-Zinc	32.48	China: Guangxi, Dashan
L180322-P-9-LD180320-0029_HL3MGCCXY_L5_clean_bin.3.orig.fa_k	OEZ008529	yes	LMSG_G000005580.1	OES073678	terpene	Terpene	Tin-Zinc	12.51	China: Guangxi, Dashan
L180322-P-9-LD180320-0029_HL3MGCCXY_L5_clean_bin.31.strict.f	OEZ008529	yes	LMSG_G000007973.1	OES073678	TfuA-related.bacteriocin	RiPPs	Tin-Zinc	8.88	China: Guangxi, Dashan
L180322-P-9-LD180320-0029_HL3MGCCXY_L5_clean_bin.32.orig.fa_	OEZ008529	yes	LMSG_G000010918.1	OES073678	thiopeptide	RiPPs	Tin-Zinc	13.07	China: Guangxi, Dashan
L180322-P-9-LD180320-0029_HL3MGCCXY_L5_clean_bin.32.orig.fa_	OEZ008529	yes	LMSG_G000010918.1	OES073678	terpene	Terpene	Tin-Zinc	21.66	China: Guangxi, Dashan
L180322-P-9-LD180320-0029_HL3MGCCXY_L5_clean_bin.32.orig.fa_	OEZ008529	yes	LMSG_G000010918.1	OES073678	arylpolyene	Others	Tin-Zinc	41.19	China: Guangxi, Dashan
L180322-P-9-LD180320-0029_HL3MGCCXY_L5_clean_bin.35.orig.fa_	OEZ008529	yes	LMSG_G000008533.1	OES073678	terpene	Terpene	Tin-Zinc	14.29	China: Guangxi, Dashan
L180322-P-9-LD180320-0029_HL3MGCCXY_L5_clean_bin.37.orig.fa_	OEZ008529	yes	LMSG_G000005578.1	OES073678	T3PKS	PKSother	Tin-Zinc	5.74	China: Guangxi, Dashan
L180322-P-9-LD180320-0029_HL3MGCCXY_L5_clean_bin.4.orig.fa_k	OEZ008529	yes	LMSG_G000010515.1	OES073678	arylpolyene	Others	Tin-Zinc	7.59	China: Guangxi, Dashan
L180322-P-9-LD180320-0029_HL3MGCCXY_L5_clean_bin.4.orig.fa_k	OEZ008529	yes	LMSG_G000010515.1	OES073678	acyl_amino_acids	Others	Tin-Zinc	6.03	China: Guangxi, Dashan
L180322-P-9-LD180320-0029_HL3MGCCXY_L5_clean_bin.40.orig.fa_	OEZ008529	yes	LMSG_G000011056.1	OES073678	arylpolyene	Others	Tin-Zinc	6.48	China: Guangxi, Dashan
L180322-P-9-LD180320-0029_HL3MGCCXY_L5_clean_bin.46.orig.fa_	OEZ008529	yes	LMSG_G000011116.1	OES073678	terpene	Terpene	Tin-Zinc	9.54	China: Guangxi, Dashan
L180322-P-9-LD180320-0029_HL3MGCCXY_L5_clean_bin.46.orig.fa_	OEZ008529	yes	LMSG_G000011116.1	OES073678	arylpolyene	Others	Tin-Zinc	7.62	China: Guangxi, Dashan
L180322-P-9-LD180320-0029_HL3MGCCXY_L5_clean_bin.49.orig.fa_	OEZ008529	yes	LMSG_G000005581.1	OES073678	terpene	Terpene	Tin-Zinc	23.04	China: Guangxi, Dashan
L180322-P-9-LD180320-0029_HL3MGCCXY_L5_clean_bin.49.orig.fa_	OEZ008529	yes	LMSG_G000005581.1	OES073678	resorcinol.arylpolyene	Others	Tin-Zinc	32.75	China: Guangxi, Dashan
L180322-P-9-LD180320-0029_HL3MGCCXY_L5_clean_bin.49.orig.fa_	OEZ008529	yes	LMSG_G000005581.1	OES073678	arylpolyene.hserlactone	Others	Tin-Zinc	35.94	China: Guangxi, Dashan
L180322-P-9-LD180320-0029_HL3MGCCXY_L5_clean_bin.5.orig.fa_k	OEZ008529	yes	LMSG_G000004512.1	OES073678	terpene	Terpene	Tin-Zinc	16.84	China: Guangxi, Dashan
L180322-P-9-LD180320-0029_HL3MGCCXY_L5_clean_bin.5.orig.fa_k	OEZ008529	yes	LMSG_G000004512.1	OES073678	terpene	Terpene	Tin-Zinc	16.10	China: Guangxi, Dashan
L180322-P-9-LD180320-0029_HL3MGCCXY_L5_clean_bin.5.orig.fa_k	OEZ008529	yes	LMSG_G000004512.1	OES073678	betalactone	Others	Tin-Zinc	21.37	China: Guangxi, Dashan
L180322-P-9-LD180320-0029_HL3MGCCXY_L5_clean_bin.50.strict.f	OEZ008529	yes	LMSG_G000005582.1	OES073678	ectoine	Others	Tin-Zinc	6.33	China: Guangxi, Dashan
L180322-P-9-LD180320-0029_HL3MGCCXY_L5_clean_bin.51.orig.fa_	OEZ008529	yes	LMSG_G000009180.1	OES073678	T3PKS	PKSother	Tin-Zinc	36.89	China: Guangxi, Dashan
L180322-P-9-LD180320-0029_HL3MGCCXY_L5_clean_bin.51.orig.fa_	OEZ008529	yes	LMSG_G000009180.1	OES073678	terpene	Terpene	Tin-Zinc	13.53	China: Guangxi, Dashan
L180322-P-9-LD180320-0029_HL3MGCCXY_L5_clean_bin.52.orig.fa_	OEZ008529	yes	LMSG_G000010447.1	OES073678	NRPS-like	NRPS	Tin-Zinc	39.30	China: Guangxi, Dashan
L180322-P-9-LD180320-0029_HL3MGCCXY_L5_clean_bin.52.orig.fa_	OEZ008529	yes	LMSG_G000010447.1	OES073678	terpene	Terpene	Tin-Zinc	10.45	China: Guangxi, Dashan
L180322-P-9-LD180320-0029_HL3MGCCXY_L5_clean_bin.53.orig.fa_	OEZ008529	yes	LMSG_G000009000.1	OES073678	bacteriocin	RiPPs	Tin-Zinc	10.80	China: Guangxi, Dashan
L180322-P-9-LD180320-0029_HL3MGCCXY_L5_clean_bin.53.orig.fa_	OEZ008529	yes	LMSG_G000009000.1	OES073678	terpene	Terpene	Tin-Zinc	16.00	China: Guangxi, Dashan
L180322-P-9-LD180320-0029_HL3MGCCXY_L5_clean_bin.54.orig.fa_	OEZ008529	yes	LMSG_G000009472.1	OES073678	betalactone	Others	Tin-Zinc	19.73	China: Guangxi, Dashan
L180322-P-9-LD180320-0029_HL3MGCCXY_L5_clean_bin.54.orig.fa_	OEZ008529	yes	LMSG_G000009472.1	OES073678	betalactone	Others	Tin-Zinc	22.56	China: Guangxi, Dashan
L180322-P-9-LD180320-0029_HL3MGCCXY_L5_clean_bin.54.orig.fa_	OEZ008529	yes	LMSG_G000009472.1	OES073678	betalactone	Others	Tin-Zinc	30.19	China: Guangxi, Dashan
L180322-P-9-LD180320-0029_HL3MGCCXY_L5_clean_bin.55.strict.f	OEZ008529	yes	LMSG_G000008288.1	OES073678	terpene	Terpene	Tin-Zinc	19.06	China: Guangxi, Dashan
L180322-P-9-LD180320-0029_HL3MGCCXY_L5_clean_bin.56.orig.fa_	OEZ008529	yes	LMSG_G000009406.1	OES073678	bacteriocin	RiPPs	Tin-Zinc	7.33	China: Guangxi, Dashan
L180322-P-9-LD180320-0029_HL3MGCCXY_L5_clean_bin.6.orig.fa_k	OEZ008529	yes	LMSG_G000005585.1	OES073678	terpene	Terpene	Tin-Zinc	11.77	China: Guangxi, Dashan
L180322-P-9-LD180320-0029_HL3MGCCXY_L5_clean_bin.6.orig.fa_k	OEZ008529	yes	LMSG_G000005585.1	OES073678	thiopeptide	RiPPs	Tin-Zinc	22.95	China: Guangxi, Dashan
L180322-P-9-LD180320-0029_HL3MGCCXY_L5_clean_bin.6.orig.fa_k	OEZ008529	yes	LMSG_G000005585.1	OES073678	arylpolyene	Others	Tin-Zinc	41.20	China: Guangxi, Dashan
L180322-P-9-LD180320-0029_HL3MGCCXY_L5_clean_bin.6.orig.fa_k	OEZ008529	yes	LMSG_G000005585.1	OES073678	bacteriocin	RiPPs	Tin-Zinc	10.92	China: Guangxi, Dashan
L180322-P-9-LD180320-0029_HL3MGCCXY_L5_clean_bin.6.orig.fa_k	OEZ008529	yes	LMSG_G000005585.1	OES073678	acyl_amino_acids	Others	Tin-Zinc	36.07	China: Guangxi, Dashan
L180322-P-9-LD180320-0029_HL3MGCCXY_L5_clean_bin.60.orig.fa_	OEZ008529	yes	LMSG_G000008557.1	OES073678	hglE-KS	PKSother	Tin-Zinc	6.61	China: Guangxi, Dashan
L180322-P-9-LD180320-0029_HL3MGCCXY_L5_clean_bin.60.orig.fa_	OEZ008529	yes	LMSG_G000008557.1	OES073678	hglE-KS	PKSother	Tin-Zinc	25.66	China: Guangxi, Dashan
L180322-P-9-LD180320-0029_HL3MGCCXY_L5_clean_bin.61.orig.fa_	OEZ008529	yes	LMSG_G000009565.1	OES073678	terpene	Terpene	Tin-Zinc	23.40	China: Guangxi, Dashan
L180322-P-9-LD180320-0029_HL3MGCCXY_L5_clean_bin.61.orig.fa_	OEZ008529	yes	LMSG_G000009565.1	OES073678	terpene	Terpene	Tin-Zinc	11.80	China: Guangxi, Dashan
L180322-P-9-LD180320-0029_HL3MGCCXY_L5_clean_bin.61.orig.fa_	OEZ008529	yes	LMSG_G000009565.1	OES073678	acyl_amino_acids	Others	Tin-Zinc	11.84	China: Guangxi, Dashan
L180322-P-9-LD180320-0029_HL3MGCCXY_L5_clean_bin.61.orig.fa_	OEZ008529	yes	LMSG_G000009565.1	OES073678	NRPS-like	NRPS	Tin-Zinc	7.79	China: Guangxi, Dashan
L180322-P-9-LD180320-0029_HL3MGCCXY_L5_clean_bin.63.orig.fa_	OEZ008529	yes	LMSG_G000005584.1	OES073678	betalactone	Others	Tin-Zinc	30.02	China: Guangxi, Dashan
L180322-P-9-LD180320-0029_HL3MGCCXY_L5_clean_bin.63.orig.fa_	OEZ008529	yes	LMSG_G000005584.1	OES073678	terpene	Terpene	Tin-Zinc	23.71	China: Guangxi, Dashan
L180322-P-9-LD180320-0029_HL3MGCCXY_L5_clean_bin.63.orig.fa_	OEZ008529	yes	LMSG_G000005584.1	OES073678	acyl_amino_acids	Others	Tin-Zinc	10.92	China: Guangxi, Dashan
L180322-P-9-LD180320-0029_HL3MGCCXY_L5_clean_bin.64.orig.fa_	OEZ008529	yes	LMSG_G000011065.1	OES073678	NRPS-like	NRPS	Tin-Zinc	42.60	China: Guangxi, Dashan
L180322-P-9-LD180320-0029_HL3MGCCXY_L5_clean_bin.64.orig.fa_	OEZ008529	yes	LMSG_G000011065.1	OES073678	NRPS-like	NRPS	Tin-Zinc	29.06	China: Guangxi, Dashan
L180322-P-9-LD180320-0029_HL3MGCCXY_L5_clean_bin.64.orig.fa_	OEZ008529	yes	LMSG_G000011065.1	OES073678	bacteriocin	RiPPs	Tin-Zinc	5.55	China: Guangxi, Dashan
L180322-P-9-LD180320-0029_HL3MGCCXY_L5_clean_bin.64.orig.fa_	OEZ008529	yes	LMSG_G000011065.1	OES073678	hserlactone	Others	Tin-Zinc	19.06	China: Guangxi, Dashan
L180322-P-9-LD180320-0029_HL3MGCCXY_L5_clean_bin.64.orig.fa_	OEZ008529	yes	LMSG_G000011065.1	OES073678	lassopeptide	RiPPs	Tin-Zinc	19.86	China: Guangxi, Dashan
L180322-P-9-LD180320-0029_HL3MGCCXY_L5_clean_bin.64.orig.fa_	OEZ008529	yes	LMSG_G000011065.1	OES073678	terpene	Terpene	Tin-Zinc	21.21	China: Guangxi, Dashan
L180322-P-9-LD180320-0029_HL3MGCCXY_L5_clean_bin.64.orig.fa_	OEZ008529	yes	LMSG_G000011065.1	OES073678	arylpolyene	Others	Tin-Zinc	22.99	China: Guangxi, Dashan
L180322-P-9-LD180320-0029_HL3MGCCXY_L5_clean_bin.64.orig.fa_	OEZ008529	yes	LMSG_G000011065.1	OES073678	NRPS-like	NRPS	Tin-Zinc	27.79	China: Guangxi, Dashan
L180322-P-9-LD180320-0029_HL3MGCCXY_L5_clean_bin.65.strict.f	OEZ008529	yes	LMSG_G000009237.1	OES073678	T3PKS.terpene	Others	Tin-Zinc	44.88	China: Guangxi, Dashan
L180322-P-9-LD180320-0029_HL3MGCCXY_L5_clean_bin.68.orig.fa_	OEZ008529	yes	LMSG_G000008882.1	OES073678	terpene	Terpene	Tin-Zinc	20.98	China: Guangxi, Dashan
L180322-P-9-LD180320-0029_HL3MGCCXY_L5_clean_bin.68.orig.fa_	OEZ008529	yes	LMSG_G000008882.1	OES073678	T3PKS	PKSother	Tin-Zinc	41.07	China: Guangxi, Dashan
L180322-P-9-LD180320-0029_HL3MGCCXY_L5_clean_bin.68.orig.fa_	OEZ008529	yes	LMSG_G000008882.1	OES073678	arylpolyene	Others	Tin-Zinc	32.35	China: Guangxi, Dashan
L180322-P-9-LD180320-0029_HL3MGCCXY_L5_clean_bin.69.orig.fa_	OEZ008529	yes	LMSG_G000010439.1	OES073678	terpene.arylpolyene	Others	Tin-Zinc	36.28	China: Guangxi, Dashan
L180322-P-9-LD180320-0029_HL3MGCCXY_L5_clean_bin.69.orig.fa_	OEZ008529	yes	LMSG_G000010439.1	OES073678	resorcinol.arylpolyene	Others	Tin-Zinc	30.24	China: Guangxi, Dashan
L180322-P-9-LD180320-0029_HL3MGCCXY_L5_clean_bin.7.orig.fa_k	OEZ008529	yes	LMSG_G000008342.1	OES073678	butyrolactone	Others	Tin-Zinc	11.01	China: Guangxi, Dashan
L180322-P-9-LD180320-0029_HL3MGCCXY_L5_clean_bin.7.orig.fa_k	OEZ008529	yes	LMSG_G000008342.1	OES073678	other	Others	Tin-Zinc	5.74	China: Guangxi, Dashan
L180322-P-9-LD180320-0029_HL3MGCCXY_L5_clean_bin.71.orig.fa_	OEZ008529	yes	LMSG_G000008292.1	OES073678	terpene	Terpene	Tin-Zinc	5.86	China: Guangxi, Dashan
L180322-P-9-LD180320-0029_HL3MGCCXY_L5_clean_bin.73.orig.fa_	OEZ008529	yes	LMSG_G000008479.1	OES073678	hglE-KS	PKSother	Tin-Zinc	6.12	China: Guangxi, Dashan
L180322-P-9-LD180320-0029_HL3MGCCXY_L5_clean_bin.76.orig.fa_	OEZ008529	yes	LMSG_G000005586.1	OES073678	TfuA-related	RiPPs	Tin-Zinc	18.10	China: Guangxi, Dashan
L180322-P-9-LD180320-0029_HL3MGCCXY_L5_clean_bin.76.orig.fa_	OEZ008529	yes	LMSG_G000005586.1	OES073678	terpene	Terpene	Tin-Zinc	7.20	China: Guangxi, Dashan
L180322-P-9-LD180320-0029_HL3MGCCXY_L5_clean_bin.76.orig.fa_	OEZ008529	yes	LMSG_G000005586.1	OES073678	TfuA-related.bacteriocin	RiPPs	Tin-Zinc	22.68	China: Guangxi, Dashan
L180322-P-9-LD180320-0029_HL3MGCCXY_L5_clean_bin.77.orig.fa_	OEZ008529	yes	LMSG_G000010952.1	OES073678	T1PKS	PKSI	Tin-Zinc	12.35	China: Guangxi, Dashan
L180322-P-9-LD180320-0029_HL3MGCCXY_L5_clean_bin.77.orig.fa_	OEZ008529	yes	LMSG_G000010952.1	OES073678	bacteriocin	RiPPs	Tin-Zinc	10.85	China: Guangxi, Dashan
L180322-P-9-LD180320-0029_HL3MGCCXY_L5_clean_bin.77.orig.fa_	OEZ008529	yes	LMSG_G000010952.1	OES073678	arylpolyene	Others	Tin-Zinc	9.83	China: Guangxi, Dashan
L180322-P-9-LD180320-0029_HL3MGCCXY_L5_clean_bin.77.orig.fa_	OEZ008529	yes	LMSG_G000010952.1	OES073678	hserlactone	Others	Tin-Zinc	20.84	China: Guangxi, Dashan
L180322-P-9-LD180320-0029_HL3MGCCXY_L5_clean_bin.77.orig.fa_	OEZ008529	yes	LMSG_G000010952.1	OES073678	terpene	Terpene	Tin-Zinc	8.58	China: Guangxi, Dashan
L180322-P-9-LD180320-0029_HL3MGCCXY_L5_clean_bin.9.orig.fa_k	OEZ008529	yes	LMSG_G000009417.1	OES073678	betalactone	Others	Tin-Zinc	26.62	China: Guangxi, Dashan
L180322-P-9-LD180320-0029_HL3MGCCXY_L5_clean_bin.9.orig.fa_k	OEZ008529	yes	LMSG_G000009417.1	OES073678	bacteriocin	RiPPs	Tin-Zinc	10.23	China: Guangxi, Dashan
L180322-P-9-LD180320-0029_HL3MGCCXY_L5_clean_bin.9.orig.fa_k	OEZ008529	yes	LMSG_G000009417.1	OES073678	bacteriocin	RiPPs	Tin-Zinc	10.58	China: Guangxi, Dashan
L180322-P-9-LD180320-0029_HL3MGCCXY_L5_clean_bin.9.orig.fa_k	OEZ008529	yes	LMSG_G000009417.1	OES073678	betalactone	Others	Tin-Zinc	22.76	China: Guangxi, Dashan
L180322-P-9-LD180320-0029_HL3MGCCXY_L5_clean_bin.9.orig.fa_k	OEZ008529	yes	LMSG_G000009417.1	OES073678	ectoine	Others	Tin-Zinc	8.23	China: Guangxi, Dashan
L180322-P-9-LD180320-0030_HL3MGCCXY_L5_clean_bin.10.orig.fa_	OEZ008529	yes	LMSG_G000007766.1	OES073700	T3PKS	PKSother	Polymetallic	22.88	China: Guangxi, Longshenao
L180322-P-9-LD180320-0030_HL3MGCCXY_L5_clean_bin.10.orig.fa_	OEZ008529	yes	LMSG_G000007766.1	OES073700	NRPS-like	NRPS	Polymetallic	5.58	China: Guangxi, Longshenao
L180322-P-9-LD180320-0030_HL3MGCCXY_L5_clean_bin.10.orig.fa_	OEZ008529	yes	LMSG_G000007766.1	OES073700	terpene	Terpene	Polymetallic	29.77	China: Guangxi, Longshenao
L180322-P-9-LD180320-0030_HL3MGCCXY_L5_clean_bin.13.orig.fa_	OEZ008529	yes	LMSG_G000008229.1	OES073700	terpene	Terpene	Polymetallic	14.59	China: Guangxi, Longshenao
L180322-P-9-LD180320-0030_HL3MGCCXY_L5_clean_bin.14.orig.fa_	OEZ008529	yes	LMSG_G000010448.1	OES073700	bacteriocin	RiPPs	Polymetallic	8.80	China: Guangxi, Longshenao
L180322-P-9-LD180320-0030_HL3MGCCXY_L5_clean_bin.14.orig.fa_	OEZ008529	yes	LMSG_G000010448.1	OES073700	terpene	Terpene	Polymetallic	23.16	China: Guangxi, Longshenao
L180322-P-9-LD180320-0030_HL3MGCCXY_L5_clean_bin.14.orig.fa_	OEZ008529	yes	LMSG_G000010448.1	OES073700	NRPS-like	NRPS	Polymetallic	42.80	China: Guangxi, Longshenao
L180322-P-9-LD180320-0030_HL3MGCCXY_L5_clean_bin.2.strict.fa	OEZ008529	yes	LMSG_G000010839.1	OES073700	bacteriocin	RiPPs	Polymetallic	6.59	China: Guangxi, Longshenao
L180322-P-9-LD180320-0030_HL3MGCCXY_L5_clean_bin.22.orig.fa_	OEZ008529	yes	LMSG_G000008384.1	OES073700	NRPS-like	NRPS	Polymetallic	44.28	China: Guangxi, Longshenao
L180322-P-9-LD180320-0030_HL3MGCCXY_L5_clean_bin.22.orig.fa_	OEZ008529	yes	LMSG_G000008384.1	OES073700	terpene	Terpene	Polymetallic	15.12	China: Guangxi, Longshenao
L180322-P-9-LD180320-0030_HL3MGCCXY_L5_clean_bin.22.orig.fa_	OEZ008529	yes	LMSG_G000008384.1	OES073700	T1PKS	PKSI	Polymetallic	22.44	China: Guangxi, Longshenao
L180322-P-9-LD180320-0030_HL3MGCCXY_L5_clean_bin.23.orig.fa_	OEZ008529	yes	LMSG_G000008627.1	OES073700	NRPS-like	NRPS	Polymetallic	18.93	China: Guangxi, Longshenao
L180322-P-9-LD180320-0030_HL3MGCCXY_L5_clean_bin.23.orig.fa_	OEZ008529	yes	LMSG_G000008627.1	OES073700	other	Others	Polymetallic	40.72	China: Guangxi, Longshenao
L180322-P-9-LD180320-0030_HL3MGCCXY_L5_clean_bin.28.strict.f	OEZ008529	yes	LMSG_G000009218.1	OES073700	terpene	Terpene	Polymetallic	21.74	China: Guangxi, Longshenao
L180322-P-9-LD180320-0030_HL3MGCCXY_L5_clean_bin.28.strict.f	OEZ008529	yes	LMSG_G000009218.1	OES073700	terpene	Terpene	Polymetallic	22.05	China: Guangxi, Longshenao
L180322-P-9-LD180320-0030_HL3MGCCXY_L5_clean_bin.28.strict.f	OEZ008529	yes	LMSG_G000009218.1	OES073700	hserlactone	Others	Polymetallic	15.56	China: Guangxi, Longshenao
L180322-P-9-LD180320-0030_HL3MGCCXY_L5_clean_bin.29.orig.fa_	OEZ008529	yes	LMSG_G000005590.1	OES073700	bacteriocin	RiPPs	Polymetallic	5.49	China: Guangxi, Longshenao
L180322-P-9-LD180320-0030_HL3MGCCXY_L5_clean_bin.29.orig.fa_	OEZ008529	no	LMSG_G000005590.1	OES073700	terpene	Terpene	Polymetallic	5.11	China: Guangxi, Longshenao
L180322-P-9-LD180320-0030_HL3MGCCXY_L5_clean_bin.3.strict.fa	OEZ008529	yes	LMSG_G000010056.1	OES073700	arylpolyene	Others	Polymetallic	20.55	China: Guangxi, Longshenao
L180322-P-9-LD180320-0030_HL3MGCCXY_L5_clean_bin.3.strict.fa	OEZ008529	yes	LMSG_G000010056.1	OES073700	terpene	Terpene	Polymetallic	15.51	China: Guangxi, Longshenao
L180322-P-9-LD180320-0030_HL3MGCCXY_L5_clean_bin.3.strict.fa	OEZ008529	yes	LMSG_G000010056.1	OES073700	T3PKS	PKSother	Polymetallic	12.62	China: Guangxi, Longshenao
L180322-P-9-LD180320-0030_HL3MGCCXY_L5_clean_bin.3.strict.fa	OEZ008529	yes	LMSG_G000010056.1	OES073700	terpene	Terpene	Polymetallic	9.90	China: Guangxi, Longshenao
L180322-P-9-LD180320-0030_HL3MGCCXY_L5_clean_bin.3.strict.fa	OEZ008529	yes	LMSG_G000010056.1	OES073700	bacteriocin	RiPPs	Polymetallic	7.21	China: Guangxi, Longshenao
L180322-P-9-LD180320-0030_HL3MGCCXY_L5_clean_bin.3.strict.fa	OEZ008529	yes	LMSG_G000010056.1	OES073700	NRPS-like	NRPS	Polymetallic	7.48	China: Guangxi, Longshenao
L180322-P-9-LD180320-0030_HL3MGCCXY_L5_clean_bin.3.strict.fa	OEZ008529	yes	LMSG_G000010056.1	OES073700	arylpolyene	Others	Polymetallic	7.44	China: Guangxi, Longshenao
L180322-P-9-LD180320-0030_HL3MGCCXY_L5_clean_bin.30.orig.fa_	OEZ008529	yes	LMSG_G000009158.1	OES073700	terpene	Terpene	Polymetallic	18.69	China: Guangxi, Longshenao
L180322-P-9-LD180320-0030_HL3MGCCXY_L5_clean_bin.30.orig.fa_	OEZ008529	yes	LMSG_G000009158.1	OES073700	T3PKS	PKSother	Polymetallic	13.64	China: Guangxi, Longshenao
L180322-P-9-LD180320-0030_HL3MGCCXY_L5_clean_bin.31.orig.fa_	OEZ008529	no	LMSG_G000005591.1	OES073700	NRPS-like	NRPS	Polymetallic	7.96	China: Guangxi, Longshenao
L180322-P-9-LD180320-0030_HL3MGCCXY_L5_clean_bin.34.orig.fa_	OEZ008529	yes	LMSG_G000007257.1	OES073700	terpene	Terpene	Polymetallic	20.92	China: Guangxi, Longshenao

L180322-P-9-LD180320-0030_HL3MGCCXY_L5_clean_bin.4.orig.fa_k	OEZ008529	no	LMSG_G000010299.1	OES073700	terpene	Terpene	Polymetallic	10.41	China: Guangxi, Longshenao
L180322-P-9-LD180320-0030_HL3MGCCXY_L5_clean_bin.4.orig.fa_k	OEZ008529	no	LMSG_G000010299.1	OES073700	arylpolyene	Others	Polymetallic	16.98	China: Guangxi, Longshenao
L180322-P-9-LD180320-0030_HL3MGCCXY_L5_clean_bin.4.orig.fa_k	OEZ008529	no	LMSG_G000010299.1	OES073700	terpene	Terpene	Polymetallic	10.87	China: Guangxi, Longshenao
L180322-P-9-LD180320-0030_HL3MGCCXY_L5_clean_bin.44.orig.fa_	OEZ008529	yes	LMSG_G000008016.1	OES073700	betalactone	Others	Polymetallic	23.60	China: Guangxi, Longshenao
L180322-P-9-LD180320-0030_HL3MGCCXY_L5_clean_bin.44.orig.fa_	OEZ008529	yes	LMSG_G000008016.1	OES073700	bacteriocin	RiPPs	Polymetallic	5.13	China: Guangxi, Longshenao
L180322-P-9-LD180320-0030_HL3MGCCXY_L5_clean_bin.44.orig.fa_	OEZ008529	yes	LMSG_G000008016.1	OES073700	betalactone	Others	Polymetallic	30.01	China: Guangxi, Longshenao
L180322-P-9-LD180320-0030_HL3MGCCXY_L5_clean_bin.44.orig.fa_	OEZ008529	yes	LMSG_G000008016.1	OES073700	bacteriocin	RiPPs	Polymetallic	10.30	China: Guangxi, Longshenao
L180322-P-9-LD180320-0030_HL3MGCCXY_L5_clean_bin.46.permissi	OEZ008529	yes	LMSG_G000009905.1	OES073700	nucleoside	Others	Polymetallic	14.14	China: Guangxi, Longshenao
L180322-P-9-LD180320-0030_HL3MGCCXY_L5_clean_bin.48.orig.fa_	OEZ008529	yes	LMSG_G000008433.1	OES073700	terpene	Terpene	Polymetallic	26.48	China: Guangxi, Longshenao
L180322-P-9-LD180320-0030_HL3MGCCXY_L5_clean_bin.5.orig.fa_k	OEZ008529	yes	LMSG_G000008513.1	OES073700	LAP	RiPPs	Polymetallic	9.03	China: Guangxi, Longshenao
L180322-P-9-LD180320-0030_HL3MGCCXY_L5_clean_bin.5.orig.fa_k	OEZ008529	yes	LMSG_G000008513.1	OES073700	terpene	Terpene	Polymetallic	26.28	China: Guangxi, Longshenao
L180322-P-9-LD180320-0030_HL3MGCCXY_L5_clean_bin.53.orig.fa_	OEZ008529	yes	LMSG_G000008079.1	OES073700	hglE-KS	PKSother	Polymetallic	5.22	China: Guangxi, Longshenao
L180322-P-9-LD180320-0030_HL3MGCCXY_L5_clean_bin.53.orig.fa_	OEZ008529	yes	LMSG_G000008079.1	OES073700	T1PKS	PKSI	Polymetallic	7.43	China: Guangxi, Longshenao
L180322-P-9-LD180320-0030_HL3MGCCXY_L5_clean_bin.54.strict.f	OEZ008529	yes	LMSG_G000009385.1	OES073700	terpene	Terpene	Polymetallic	16.34	China: Guangxi, Longshenao
L180322-P-9-LD180320-0030_HL3MGCCXY_L5_clean_bin.54.strict.f	OEZ008529	yes	LMSG_G000009385.1	OES073700	bacteriocin	RiPPs	Polymetallic	5.39	China: Guangxi, Longshenao
L180322-P-9-LD180320-0030_HL3MGCCXY_L5_clean_bin.54.strict.f	OEZ008529	yes	LMSG_G000009385.1	OES073700	bacteriocin	RiPPs	Polymetallic	6.29	China: Guangxi, Longshenao
L180322-P-9-LD180320-0030_HL3MGCCXY_L5_clean_bin.59.orig.fa_	OEZ008529	yes	LMSG_G000010014.1	OES073700	terpene	Terpene	Polymetallic	16.14	China: Guangxi, Longshenao
L180322-P-9-LD180320-0030_HL3MGCCXY_L5_clean_bin.59.orig.fa_	OEZ008529	yes	LMSG_G000010014.1	OES073700	ladderane	Others	Polymetallic	6.79	China: Guangxi, Longshenao
L180322-P-9-LD180320-0030_HL3MGCCXY_L5_clean_bin.6.orig.fa_k	OEZ008529	no	LMSG_G000009535.1	OES073700	NRPS-like	NRPS	Polymetallic	42.69	China: Guangxi, Longshenao
L180322-P-9-LD180320-0030_HL3MGCCXY_L5_clean_bin.6.orig.fa_k	OEZ008529	no	LMSG_G000009535.1	OES073700	terpene	Terpene	Polymetallic	23.29	China: Guangxi, Longshenao
L180322-P-9-LD180320-0030_HL3MGCCXY_L5_clean_bin.6.orig.fa_k	OEZ008529	no	LMSG_G000009535.1	OES073700	terpene	Terpene	Polymetallic	22.05	China: Guangxi, Longshenao
L180322-P-9-LD180320-0030_HL3MGCCXY_L5_clean_bin.6.orig.fa_k	OEZ008529	yes	LMSG_G000009535.1	OES073700	acyl_amino_acids	Others	Polymetallic	9.03	China: Guangxi, Longshenao
L180322-P-9-LD180320-0030_HL3MGCCXY_L5_clean_bin.60.orig.fa_	OEZ008529	yes	LMSG_G000008677.1	OES073700	T1PKS	PKSI	Polymetallic	25.29	China: Guangxi, Longshenao
L180322-P-9-LD180320-0030_HL3MGCCXY_L5_clean_bin.60.orig.fa_	OEZ008529	yes	LMSG_G000008677.1	OES073700	betalactone	Others	Polymetallic	30.18	China: Guangxi, Longshenao
L180322-P-9-LD180320-0030_HL3MGCCXY_L5_clean_bin.60.orig.fa_	OEZ008529	yes	LMSG_G000008677.1	OES073700	arylpolyene	Others	Polymetallic	28.14	China: Guangxi, Longshenao
L180322-P-9-LD180320-0030_HL3MGCCXY_L5_clean_bin.60.orig.fa_	OEZ008529	yes	LMSG_G000008677.1	OES073700	terpene	Terpene	Polymetallic	26.44	China: Guangxi, Longshenao
L180322-P-9-LD180320-0030_HL3MGCCXY_L5_clean_bin.63.strict.f	OEZ008529	yes	LMSG_G000008323.1	OES073700	terpene	Terpene	Polymetallic	21.09	China: Guangxi, Longshenao
L180322-P-9-LD180320-0030_HL3MGCCXY_L5_clean_bin.63.strict.f	OEZ008529	yes	LMSG_G000008323.1	OES073700	other	Others	Polymetallic	17.03	China: Guangxi, Longshenao
L180322-P-9-LD180320-0030_HL3MGCCXY_L5_clean_bin.64.orig.fa_	OEZ008529	no	LMSG_G000010278.1	OES073700	terpene	Terpene	Polymetallic	21.16	China: Guangxi, Longshenao
L180322-P-9-LD180320-0030_HL3MGCCXY_L5_clean_bin.64.orig.fa_	OEZ008529	yes	LMSG_G000010278.1	OES073700	arylpolyene	Others	Polymetallic	35.55	China: Guangxi, Longshenao
L180322-P-9-LD180320-0030_HL3MGCCXY_L5_clean_bin.65.orig.fa_	OEZ008529	yes	LMSG_G000008505.1	OES073700	terpene	Terpene	Polymetallic	24.04	China: Guangxi, Longshenao
L180322-P-9-LD180320-0030_HL3MGCCXY_L5_clean_bin.65.orig.fa_	OEZ008529	yes	LMSG_G000008505.1	OES073700	lassopeptide	RiPPs	Polymetallic	23.45	China: Guangxi, Longshenao
L180322-P-9-LD180320-0030_HL3MGCCXY_L5_clean_bin.66.orig.fa_	OEZ008529	yes	LMSG_G000009001.1	OES073700	terpene	Terpene	Polymetallic	20.91	China: Guangxi, Longshenao
L180322-P-9-LD180320-0030_HL3MGCCXY_L5_clean_bin.66.orig.fa_	OEZ008529	yes	LMSG_G000009001.1	OES073700	bacteriocin	RiPPs	Polymetallic	10.80	China: Guangxi, Longshenao
L180322-P-9-LD180320-0030_HL3MGCCXY_L5_clean_bin.68.orig.fa_	OEZ008529	yes	LMSG_G000007740.1	OES073700	terpene	Terpene	Polymetallic	13.03	China: Guangxi, Longshenao
L180322-P-9-LD180320-0030_HL3MGCCXY_L5_clean_bin.68.orig.fa_	OEZ008529	yes	LMSG_G000007740.1	OES073700	terpene	Terpene	Polymetallic	8.09	China: Guangxi, Longshenao
L180322-P-9-LD180320-0030_HL3MGCCXY_L5_clean_bin.69.orig.fa_	OEZ008529	yes	LMSG_G000006505.1	OES073700	LAP.thiopeptide	RiPPs	Polymetallic	41.84	China: Guangxi, Longshenao
L180322-P-9-LD180320-0030_HL3MGCCXY_L5_clean_bin.7.orig.fa_k	OEZ008529	yes	LMSG_G000008744.1	OES073700	betalactone	Others	Polymetallic	10.96	China: Guangxi, Longshenao
L180322-P-9-LD180320-0030_HL3MGCCXY_L5_clean_bin.7.orig.fa_k	OEZ008529	yes	LMSG_G000008744.1	OES073700	other	Others	Polymetallic	22.19	China: Guangxi, Longshenao
L180322-P-9-LD180320-0030_HL3MGCCXY_L5_clean_bin.7.orig.fa_k	OEZ008529	yes	LMSG_G000008744.1	OES073700	NRPS-like	NRPS	Polymetallic	23.81	China: Guangxi, Longshenao
L180322-P-9-LD180320-0030_HL3MGCCXY_L5_clean_bin.70.strict.f	OEZ008529	yes	LMSG_G000007979.1	OES073700	TfuA-related.bacteriocin	RiPPs	Polymetallic	9.81	China: Guangxi, Longshenao
L180322-P-9-LD180320-0030_HL3MGCCXY_L5_clean_bin.72.orig.fa_	OEZ008529	yes	LMSG_G000009346.1	OES073700	bacteriocin	RiPPs	Polymetallic	10.81	China: Guangxi, Longshenao
L180322-P-9-LD180320-0030_HL3MGCCXY_L5_clean_bin.74.orig.fa_	OEZ008529	yes	LMSG_G000008314.1	OES073700	other	Others	Polymetallic	21.60	China: Guangxi, Longshenao
L180322-P-9-LD180320-0030_HL3MGCCXY_L5_clean_bin.74.orig.fa_	OEZ008529	yes	LMSG_G000008314.1	OES073700	terpene	Terpene	Polymetallic	21.10	China: Guangxi, Longshenao
L180322-P-9-LD180320-0030_HL3MGCCXY_L5_clean_bin.74.orig.fa_	OEZ008529	yes	LMSG_G000008314.1	OES073700	NRPS-like	NRPS	Polymetallic	25.39	China: Guangxi, Longshenao
L180322-P-9-LD180320-0030_HL3MGCCXY_L5_clean_bin.74.orig.fa_	OEZ008529	yes	LMSG_G000008314.1	OES073700	terpene	Terpene	Polymetallic	23.61	China: Guangxi, Longshenao
L180322-P-9-LD180320-0030_HL3MGCCXY_L5_clean_bin.76.orig.fa_	OEZ008529	yes	LMSG_G000011133.1	OES073700	terpene	Terpene	Polymetallic	22.73	China: Guangxi, Longshenao
L180322-P-9-LD180320-0030_HL3MGCCXY_L5_clean_bin.76.orig.fa_	OEZ008529	yes	LMSG_G000011133.1	OES073700	arylpolyene	Others	Polymetallic	41.16	China: Guangxi, Longshenao
L180322-P-9-LD180320-0030_HL3MGCCXY_L5_clean_bin.77.orig.fa_	OEZ008529	yes	LMSG_G000008558.1	OES073700	hglE-KS	PKSother	Polymetallic	8.11	China: Guangxi, Longshenao
L180322-P-9-LD180320-0030_HL3MGCCXY_L5_clean_bin.77.orig.fa_	OEZ008529	yes	LMSG_G000008558.1	OES073700	hglE-KS	PKSother	Polymetallic	9.75	China: Guangxi, Longshenao
L180322-P-9-LD180320-0030_HL3MGCCXY_L5_clean_bin.8.orig.fa_k	OEZ008529	yes	LMSG_G000009642.1	OES073700	NRPS-like	NRPS	Polymetallic	42.65	China: Guangxi, Longshenao
L180322-P-9-LD180320-0030_HL3MGCCXY_L5_clean_bin.8.orig.fa_k	OEZ008529	yes	LMSG_G000009642.1	OES073700	terpene	Terpene	Polymetallic	22.03	China: Guangxi, Longshenao
L180322-P-9-LD180320-0030_HL3MGCCXY_L5_clean_bin.8.orig.fa_k	OEZ008529	yes	LMSG_G000009642.1	OES073700	terpene	Terpene	Polymetallic	23.49	China: Guangxi, Longshenao
L180322-P-9-LD180320-0030_HL3MGCCXY_L5_clean_bin.88.orig.fa_	OEZ008529	yes	LMSG_G000007750.1	OES073700	terpene	Terpene	Polymetallic	14.83	China: Guangxi, Longshenao
L180322-P-9-LD180320-0030_HL3MGCCXY_L5_clean_bin.88.orig.fa_	OEZ008529	yes	LMSG_G000007750.1	OES073700	NRPS	NRPS	Polymetallic	7.97	China: Guangxi, Longshenao
L180322-P-9-LD180320-0030_HL3MGCCXY_L5_clean_bin.88.orig.fa_	OEZ008529	yes	LMSG_G000007750.1	OES073700	T3PKS	PKSother	Polymetallic	11.43	China: Guangxi, Longshenao
L180322-P-9-LD180320-0030_HL3MGCCXY_L5_clean_bin.88.orig.fa_	OEZ008529	yes	LMSG_G000007750.1	OES073700	terpene	Terpene	Polymetallic	11.62	China: Guangxi, Longshenao
L180322-P-9-LD180320-0030_HL3MGCCXY_L5_clean_bin.89.orig.fa_	OEZ008529	yes	LMSG_G000006500.1	OES073700	terpene	Terpene	Polymetallic	20.88	China: Guangxi, Longshenao
L180322-P-9-LD180320-0030_HL3MGCCXY_L5_clean_bin.91.orig.fa_	OEZ008529	yes	LMSG_G000005594.1	OES073700	LAP	RiPPs	Polymetallic	19.81	China: Guangxi, Longshenao
L180322-P-9-LD180320-0030_HL3MGCCXY_L5_clean_bin.92.strict.f	OEZ008529	yes	LMSG_G000009437.1	OES073700	betalactone	Others	Polymetallic	22.78	China: Guangxi, Longshenao
L180322-P-9-LD180320-0030_HL3MGCCXY_L5_clean_bin.92.strict.f	OEZ008529	yes	LMSG_G000009437.1	OES073700	bacteriocin	RiPPs	Polymetallic	10.85	China: Guangxi, Longshenao
L180322-P-9-LD180320-0030_HL3MGCCXY_L5_clean_bin.92.strict.f	OEZ008529	yes	LMSG_G000009437.1	OES073700	betalactone	Others	Polymetallic	30.86	China: Guangxi, Longshenao
L180322-P-9-LD180320-0030_HL3MGCCXY_L5_clean_bin.92.strict.f	OEZ008529	yes	LMSG_G000009437.1	OES073700	ectoine	Others	Polymetallic	10.40	China: Guangxi, Longshenao
L180322-P-9-LD180320-0030_HL3MGCCXY_L5_clean_bin.92.strict.f	OEZ008529	yes	LMSG_G000009437.1	OES073700	LAP	RiPPs	Polymetallic	21.92	China: Guangxi, Longshenao
L180322-P-9-LD180320-0030_HL3MGCCXY_L5_clean_bin.94.orig.fa_	OEZ008529	yes	LMSG_G000008343.1	OES073700	T3PKS	PKSother	Polymetallic	41.06	China: Guangxi, Longshenao
L180322-P-9-LD180320-0030_HL3MGCCXY_L5_clean_bin.94.orig.fa_	OEZ008529	yes	LMSG_G000008343.1	OES073700	bacteriocin	RiPPs	Polymetallic	10.80	China: Guangxi, Longshenao
L180322-P-9-LD180320-0030_HL3MGCCXY_L5_clean_bin.97.orig.fa_	OEZ008529	yes	LMSG_G000006439.1	OES073700	nucleoside	Others	Polymetallic	19.07	China: Guangxi, Longshenao
L180322-P-9-LD180320-0030_HL3MGCCXY_L5_clean_bin.98.orig.fa_	OEZ008529	no	LMSG_G000008488.1	OES073700	terpene	Terpene	Polymetallic	5.44	China: Guangxi, Longshenao
L180322-P-9-LD180320-0030_HL3MGCCXY_L5_clean_bin.98.orig.fa_	OEZ008529	yes	LMSG_G000008488.1	OES073700	arylpolyene	Others	Polymetallic	20.96	China: Guangxi, Longshenao
L180322-P-9-LD180320-0030_HL3MGCCXY_L5_clean_bin.98.orig.fa_	OEZ008529	yes	LMSG_G000008488.1	OES073700	terpene	Terpene	Polymetallic	10.75	China: Guangxi, Longshenao
L180322-P-9-LD180320-0030_HL3MGCCXY_L5_clean_bin.98.orig.fa_	OEZ008529	yes	LMSG_G000008488.1	OES073700	lassopeptide	RiPPs	Polymetallic	6.39	China: Guangxi, Longshenao
L180322-P-9-LD180320-0030_HL3MGCCXY_L5_clean_bin.99.permissi	OEZ008529	yes	LMSG_G000008274.1	OES073700	terpene	Terpene	Polymetallic	19.06	China: Guangxi, Longshenao
L180330P-1-LD180327-0003_HL3VYCCXY_L7_clean_bin.1.orig.fa_kl	OEZ008529	yes	LMSG_G000008536.1	OES073715	terpene	Terpene	Copper	12.43	China: Anhui, Tongling
L180330P-1-LD180327-0003_HL3VYCCXY_L7_clean_bin.1.orig.fa_kl	OEZ008529	yes	LMSG_G000008536.1	OES073715	NRPS-like	NRPS	Copper	11.97	China: Anhui, Tongling
L180330P-1-LD180327-0003_HL3VYCCXY_L7_clean_bin.10.orig.fa_k	OEZ008529	yes	LMSG_G000009144.1	OES073715	terpene	Terpene	Copper	13.57	China: Anhui, Tongling
L180330P-1-LD180327-0003_HL3VYCCXY_L7_clean_bin.10.orig.fa_k	OEZ008529	yes	LMSG_G000009144.1	OES073715	T3PKS	PKSother	Copper	41.01	China: Anhui, Tongling
L180330P-1-LD180327-0003_HL3VYCCXY_L7_clean_bin.10.orig.fa_k	OEZ008529	yes	LMSG_G000009144.1	OES073715	terpene	Terpene	Copper	20.71	China: Anhui, Tongling
L180330P-1-LD180327-0003_HL3VYCCXY_L7_clean_bin.16.orig.fa_k	OEZ008529	yes	LMSG_G000009159.1	OES073715	terpene	Terpene	Copper	6.14	China: Anhui, Tongling
L180330P-1-LD180327-0003_HL3VYCCXY_L7_clean_bin.16.orig.fa_k	OEZ008529	yes	LMSG_G000009159.1	OES073715	terpene	Terpene	Copper	7.50	China: Anhui, Tongling
L180330P-1-LD180327-0003_HL3VYCCXY_L7_clean_bin.19.orig.fa_k	OEZ008529	yes	LMSG_G000009584.1	OES073715	terpene	Terpene	Copper	12.80	China: Anhui, Tongling
L180330P-1-LD180327-0003_HL3VYCCXY_L7_clean_bin.19.orig.fa_k	OEZ008529	yes	LMSG_G000009584.1	OES073715	acyl_amino_acids	Others	Copper	13.68	China: Anhui, Tongling
L180330P-1-LD180327-0003_HL3VYCCXY_L7_clean_bin.19.orig.fa_k	OEZ008529	yes	LMSG_G000009584.1	OES073715	acyl_amino_acids	Others	Copper	12.03	China: Anhui, Tongling
L180330P-1-LD180327-0003_HL3VYCCXY_L7_clean_bin.19.orig.fa_k	OEZ008529	yes	LMSG_G000009584.1	OES073715	terpene	Terpene	Copper	21.25	China: Anhui, Tongling
L180330P-1-LD180327-0003_HL3VYCCXY_L7_clean_bin.19.orig.fa_k	OEZ008529	yes	LMSG_G000009584.1	OES073715	NRPS-like	NRPS	Copper	23.73	China: Anhui, Tongling
L180330P-1-LD180327-0003_HL3VYCCXY_L7_clean_bin.20.orig.fa_k	OEZ008529	yes	LMSG_G000008685.1	OES073715	ectoine	Others	Copper	10.42	China: Anhui, Tongling
L180330P-1-LD180327-0003_HL3VYCCXY_L7_clean_bin.20.orig.fa_k	OEZ008529	yes	LMSG_G000008685.1	OES073715	NRPS-like	NRPS	Copper	35.65	China: Anhui, Tongling
L180330P-1-LD180327-0003_HL3VYCCXY_L7_clean_bin.20.orig.fa_k	OEZ008529	yes	LMSG_G000008685.1	OES073715	betalactone	Others	Copper	19.38	China: Anhui, Tongling
L180330P-1-LD180327-0003_HL3VYCCXY_L7_clean_bin.20.orig.fa_k	OEZ008529	yes	LMSG_G000008685.1	OES073715	bacteriocin	RiPPs	Copper	10.75	China: Anhui, Tongling
L180330P-1-LD180327-0003_HL3VYCCXY_L7_clean_bin.20.orig.fa_k	OEZ008529	yes	LMSG_G000008685.1	OES073715	terpene	Terpene	Copper	14.97	China: Anhui, Tongling
L180330P-1-LD180327-0003_HL3VYCCXY_L7_clean_bin.22.orig.fa_k	OEZ008529	yes	LMSG_G000009408.1	OES073715	bacteriocin	RiPPs	Copper	9.34	China: Anhui, Tongling
L180330P-1-LD180327-0003_HL3VYCCXY_L7_clean_bin.25.orig.fa_k	OEZ008529	yes	LMSG_G000009556.1	OES073715	terpene	Terpene	Copper	22.06	China: Anhui, Tongling
L180330P-1-LD180327-0003_HL3VYCCXY_L7_clean_bin.25.orig.fa_k	OEZ008529	yes	LMSG_G000009556.1	OES073715	NRPS-like	NRPS	Copper	42.72	China: Anhui, Tongling
L180330P-1-LD180327-0003_HL3VYCCXY_L7_clean_bin.27.permissiv	OEZ008529	yes	LMSG_G000009470.1	OES073715	bacteriocin	RiPPs	Copper	9.62	China: Anhui, Tongling
L180330P-1-LD180327-0003_HL3VYCCXY_L7_clean_bin.30.orig.fa_k	OEZ008529	yes	LMSG_G000008517.1	OES073715	terpene	Terpene	Copper	26.29	China: Anhui, Tongling
L180330P-1-LD180327-0003_HL3VYCCXY_L7_clean_bin.31.orig.fa_k	OEZ008529	yes	LMSG_G000005597.1	OES073715	betalactone	Others	Copper	22.76	China: Anhui, Tongling
L180330P-1-LD180327-0003_HL3VYCCXY_L7_clean_bin.31.orig.fa_k	OEZ008529	yes	LMSG_G000005597.1	OES073715	bacteriocin	RiPPs	Copper	10.82	China: Anhui, Tongling
L180330P-1-LD180327-0003_HL3VYCCXY_L7_clean_bin.31.orig.fa_k	OEZ008529	yes	LMSG_G000005597.1	OES073715	betalactone	Others	Copper	26.72	China: Anhui, Tongling
L180330P-1-LD180327-0003_HL3VYCCXY_L7_clean_bin.31.orig.fa_k	OEZ008529	yes	LMSG_G000005597.1	OES073715	ectoine	Others	Copper	6.97	China: Anhui, Tongling
L180330P-1-LD180327-0003_HL3VYCCXY_L7_clean_bin.34.orig.fa_k	OEZ008529	yes	LMSG_G000008275.1	OES073715	terpene	Terpene	Copper	19.12	China: Anhui, Tongling
L180330P-1-LD180327-0003_HL3VYCCXY_L7_clean_bin.35.orig.fa_k	OEZ008529	yes	LMSG_G000009376.1	OES073715	ectoine	Others	Copper	7.37	China: Anhui, Tongling
L180330P-1-LD180327-0003_HL3VYCCXY_L7_clean_bin.37.strict.fa	OEZ008529	yes	LMSG_G000008007.1	OES073715	bacteriocin	RiPPs	Copper	7.36	China: Anhui, Tongling
L180330P-1-LD180327-0003_HL3VYCCXY_L7_clean_bin.37.strict.fa	OEZ008529	yes	LMSG_G000008007.1	OES073715	TfuA-related.bacteriocin	RiPPs	Copper	9.04	China: Anhui, Tongling
L180330P-1-LD180327-0003_HL3VYCCXY_L7_clean_bin.40.permissiv	OEZ008529	yes	LMSG_G000009438.1	OES073715	betalactone	Others	Copper	22.78	China: Anhui, Tongling
L180330P-1-LD180327-0003_HL3VYCCXY_L7_clean_bin.40.permissiv	OEZ008529	yes	LMSG_G000009438.1	OES073715	bacteriocin	RiPPs	Copper	10.85	China: Anhui, Tongling
L180330P-1-LD180327-0003_HL3VYCCXY_L7_clean_bin.40.permissiv	OEZ008529	yes	LMSG_G000009438.1	OES073715	betalactone	Others	Copper	30.86	China: Anhui, Tongling
L180330P-1-LD180327-0003_HL3VYCCXY_L7_clean_bin.40.permissiv	OEZ008529	yes	LMSG_G000009438.1	OES073715	ectoine	Others	Copper	9.08	China: Anhui, Tongling
L180330P-1-LD180327-0003_HL3VYCCXY_L7_clean_bin.41.orig.fa_k	OEZ008529	yes	LMSG_G000009611.1	OES073715	terpene	Terpene	Copper	22.08	China: Anhui, Tongling
L180330P-1-LD180327-0003_HL3VYCCXY_L7_clean_bin.41.orig.fa_k	OEZ008529	yes	LMSG_G000009611.1	OES073715	terpene	Terpene	Copper	17.15	China: Anhui, Tongling
L180330P-1-LD180327-0003_HL3VYCCXY_L7_clean_bin.42.strict.fa	OEZ008529	yes	LMSG_G000009897.1	OES073715	terpene	Terpene	Copper	19.12	China: Anhui, Tongling
L180330P-1-LD180327-0003_HL3VYCCXY_L7_clean_bin.46.orig.fa_k	OEZ008529	yes	LMSG_G000008345.1	OES073715	T3PKS	PKSother	Copper	5.01	China: Anhui, Tongling
L180330P-1-LD180327-0003_HL3VYCCXY_L7_clean_bin.46.orig.fa_k	OEZ008529	yes	LMSG_G000008345.1	OES073715	terpene	Terpene	Copper	7.00	China: Anhui, Tongling
L180330P-1-LD180327-0003_HL3VYCCXY_L7_clean_bin.48.orig.fa_k	OEZ008529	no	LMSG_G000010166.1	OES073715	terpene	Terpene	Copper	6.44	China: Anhui, Tongling
L180330P-1-LD180327-0003_HL3VYCCXY_L7_clean_bin.48.orig.fa_k	OEZ008529	no	LMSG_G000010166.1	OES073715	ectoine	Others	Copper	10.40	China: Anhui, Tongling
L180330P-1-LD180327-0003_HL3VYCCXY_L7_clean_bin.48.orig.fa_k	OEZ008529	no	LMSG_G000010166.1	OES073715	terpene	Terpene	Copper	21.71	China: Anhui, Tongling
L180330P-1-LD180327-0003_HL3VYCCXY_L7_clean_bin.48.orig.fa_k	OEZ008529	yes	LMSG_G000010166.1	OES073715	terpene	Terpene	Copper	21.95	China: Anhui, Tongling
L180330P-1-LD180327-0003_HL3VYCCXY_L7_clean_bin.50.orig.fa_k	OEZ008529	yes	LMSG_G000005598.1	OES073715	terpene	Terpene	Copper	20.91	China: Anhui, Tongling
L180330P-1-LD180327-0003_HL3VYCCXY_L7_clean_bin.50.orig.fa_k	OEZ008529	yes	LMSG_G000005598.1	OES073715	bacteriocin	RiPPs	Copper	10.80	China: Anhui, Tongling
L180330P-1-LD180327-0003_HL3VYCCXY_L7_clean_bin.51.permissiv	OEZ008529	yes	LMSG_G000008108.1	OES073715	betalactone	Others	Copper	16.26	China: Anhui, Tongling
L180330P-1-LD180327-0003_HL3VYCCXY_L7_clean_bin.51.permissiv	OEZ008529	yes	LMSG_G000008108.1	OES073715	NRPS-like	NRPS	Copper	7.41	China: Anhui, Tongling
L180330P-1-LD180327-0003_HL3VYCCXY_L7_clean_bin.52.orig.fa_k	OEZ008529	yes	LMSG_G000009592.1	OES073715	terpene	Terpene	Copper	22.15	China: Anhui, Tongling
L180330P-1-LD180327-0003_HL3VYCCXY_L7_clean_bin.52.orig.fa_k	OEZ008529	yes	LMSG_G000009592.1	OES073715	terpene	Terpene	Copper	9.79	China: Anhui, Tongling
L180330P-1-LD180327-0003_HL3VYCCXY_L7_clean_bin.52.orig.fa_k	OEZ008529	yes	LMSG_G000009592.1	OES073715	NRPS-like	NRPS	Copper	12.39	China: Anhui, Tongling
L180330P-1-LD180327-0003_HL3VYCCXY_L7_clean_bin.53.orig.fa_k	OEZ008529	yes	LMSG_G000009549.1	OES073715	NRPS-like	NRPS	Copper	42.72	China: Anhui, Tongling
L180330P-1-LD180327-0003_HL3VYCCXY_L7_clean_bin.53.orig.fa_k	OEZ008529	yes	LMSG_G000009549.1	OES073715	ectoine	Others	Copper	9.43	China: Anhui, Tongling
L180330P-1-LD180327-0003_HL3VYCCXY_L7_clean_bin.53.orig.fa_k	OEZ008529	no	LMSG_G000009549.1	OES073715	terpene	Terpene	Copper	23.38	China: Anhui, Tongling
L180330P-1-LD180327-0003_HL3VYCCXY_L7_clean_bin.53.orig.fa_k	OEZ008529	yes	LMSG_G000009549.1	OES073715	terpene	Terpene	Copper	22.06	China: Anhui, Tongling
L180330P-1-LD180327-0003_HL3VYCCXY_L7_clean_bin.54.orig.fa_k	OEZ008529	yes	LMSG_G000007065.1	OES073715	bacteriocin	RiPPs	Copper	6.01	China: Anhui, Tongling
L180330P-1-LD180327-0003_HL3VYCCXY_L7_clean_bin.55.orig.fa_k	OEZ008529	yes	LMSG_G000009468.1	OES073715	arylpolyene	Others	Copper	11.53	China: Anhui, Tongling
L180330P-1-LD180327-0003_HL3VYCCXY_L7_clean_bin.55.orig.fa_k	OEZ008529	yes	LMSG_G000009468.1	OES073715	bacteriocin	RiPPs	Copper	6.39	China: Anhui, Tongling
L180330P-1-LD180327-0003_HL3VYCCXY_L7_clean_bin.55.orig.fa_k	OEZ008529	yes	LMSG_G000009468.1	OES073715	ectoine	Others	Copper	5.74	China: Anhui, Tongling
L180330P-1-LD180327-0003_HL3VYCCXY_L7_clean_bin.56.orig.fa_k	OEZ008529	yes	LMSG_G000009412.1	OES073715	ectoine	Others	Copper	8.24	China: Anhui, Tongling
L180330P-1-LD180327-0003_HL3VYCCXY_L7_clean_bin.56.orig.fa_k	OEZ008529	yes	LMSG_G000009412.1	OES073715	terpene	Terpene	Copper	10.63	China: Anhui, Tongling
L180330P-1-LD180327-0003_HL3VYCCXY_L7_clean_bin.56.orig.fa_k	OEZ008529	yes	LMSG_G000009412.1	OES073715	terpene	Terpene	Copper	20.01	China: Anhui, Tongling
L180330P-1-LD180327-0003_HL3VYCCXY_L7_clean_bin.57.orig.fa_k	OEZ008529	no	LMSG_G000010494.1	OES073715	arylpolyene	Others	Copper	13.28	China: Anhui, Tongling
L180330P-1-LD180327-0003_HL3VYCCXY_L7_clean_bin.57.orig.fa_k	OEZ008529	yes	LMSG_G000010494.1	OES073715	terpene	Terpene	Copper	9.53	China: Anhui, Tongling
L180330P-1-LD180327-0003_HL3VYCCXY_L7_clean_bin.58.strict.fa	OEZ008529	no	LMSG_G000009570.1	OES073715	NRPS-like	NRPS	Copper	42.72	China: Anhui, Tongling
L180330P-1-LD180327-0003_HL3VYCCXY_L7_clean_bin.58.strict.fa	OEZ008529	no	LMSG_G000009570.1	OES073715	terpene	Terpene	Copper	22.05	China: Anhui, Tongling
L180330P-1-LD180327-0003_HL3VYCCXY_L7_clean_bin.58.strict.fa	OEZ008529	no	LMSG_G000009570.1	OES073715	ectoine	Others	Copper	9.65	China: Anhui, Tongling
L180330P-1-LD180327-0003_HL3VYCCXY_L7_clean_bin.58.strict.fa	OEZ008529	no	LMSG_G000009570.1	OES073715	terpene	Terpene	Copper	16.76	China: Anhui, Tongling
L180330P-1-LD180327-0003_HL3VYCCXY_L7_clean_bin.59.strict.fa	OEZ008529	yes	LMSG_G000009403.1	OES073715	NRPS	NRPS	Copper	46.47	China: Anhui, Tongling
L180330P-1-LD180327-0003_HL3VYCCXY_L7_clean_bin.6.strict.fa_	OEZ008529	yes	LMSG_G000008017.1	OES073715	betalactone	Others	Copper	23.60	China: Anhui, Tongling
L180330P-1-LD180327-0003_HL3VYCCXY_L7_clean_bin.6.strict.fa_	OEZ008529	yes	LMSG_G000008017.1	OES073715	betalactone	Others	Copper	30.01	China: Anhui, Tongling
L180330P-1-LD180327-0003_HL3VYCCXY_L7_clean_bin.6.strict.fa_	OEZ008529	yes	LMSG_G000008017.1	OES073715	bacteriocin	RiPPs	Copper	10.30	China: Anhui, Tongling
L180330P-1-LD180327-0003_HL3VYCCXY_L7_clean_bin.6.strict.fa_	OEZ008529	yes	LMSG_G000008017.1	OES073715	bacteriocin	RiPPs	Copper	5.43	China: Anhui, Tongling
L180330P-1-LD180327-0003_HL3VYCCXY_L7_clean_bin.67.orig.fa_k	OEZ008529	yes	LMSG_G000008489.1	OES073715	terpene	Terpene	Copper	8.27	China: Anhui, Tongling
L180330P-1-LD180327-0003_HL3VYCCXY_L7_clean_bin.8.orig.fa_kl	OEZ008529	yes	LMSG_G000005599.1	OES073715	terpene	Terpene	Copper	6.85	China: Anhui, Tongling
L180330P-1-LD180327-0004_HL3VYCCXY_L7_clean_bin.1.orig.fa_kl	OEZ008529	yes	LMSG_G000008686.1	OES073716	betalactone	Others	Copper	20.73	China: Anhui, Tongling
L180330P-1-LD180327-0004_HL3VYCCXY_L7_clean_bin.1.orig.fa_kl	OEZ008529	yes	LMSG_G000008686.1	OES073716	NRPS-like	NRPS	Copper	5.18	China: Anhui, Tongling
L180330P-1-LD180327-0004_HL3VYCCXY_L7_clean_bin.1.orig.fa_kl	OEZ008529	yes	LMSG_G000008686.1	OES073716	ectoine	Others	Copper	10.42	China: Anhui, Tongling
L180330P-1-LD180327-0004_HL3VYCCXY_L7_clean_bin.1.orig.fa_kl	OEZ008529	yes	LMSG_G000008686.1	OES073716	terpene	Terpene	Copper	17.84	China: Anhui, Tongling
L180330P-1-LD180327-0004_HL3VYCCXY_L7_clean_bin.1.orig.fa_kl	OEZ008529	yes	LMSG_G000008686.1	OES073716	bacteriocin	RiPPs	Copper	10.80	China: Anhui, Tongling
L180330P-1-LD180327-0004_HL3VYCCXY_L7_clean_bin.10.orig.fa_k	OEZ008529	yes	LMSG_G000011074.1	OES073716	NRPS-like	NRPS	Copper	37.59	China: Anhui, Tongling
L180330P-1-LD180327-0004_HL3VYCCXY_L7_clean_bin.10.orig.fa_k	OEZ008529	yes	LMSG_G000011074.1	OES073716	bacteriocin	RiPPs	Copper	10.33	China: Anhui, Tongling
L180330P-1-LD180327-0004_HL3VYCCXY_L7_clean_bin.10.orig.fa_k	OEZ008529	yes	LMSG_G000011074.1	OES073716	lassopeptide.hserlactone	Others	Copper	30.47	China: Anhui, Tongling
L180330P-1-LD180327-0004_HL3VYCCXY_L7_clean_bin.10.orig.fa_k	OEZ008529	yes	LMSG_G000011074.1	OES073716	NRPS-like	NRPS	Copper	22.92	China: Anhui, Tongling
L180330P-1-LD180327-0004_HL3VYCCXY_L7_clean_bin.11.orig.fa_k	OEZ008529	yes	LMSG_G000004514.1	OES073716	ectoine	Others	Copper	10.38	China: Anhui, Tongling
L180330P-1-LD180327-0004_HL3VYCCXY_L7_clean_bin.13.permissiv	OEZ008529	yes	LMSG_G000008068.1	OES073716	T1PKS.hglE-KS	PKSother	Copper	42.72	China: Anhui, Tongling
L180330P-1-LD180327-0004_HL3VYCCXY_L7_clean_bin.13.permissiv	OEZ008529	yes	LMSG_G000008068.1	OES073716	arylpolyene	Others	Copper	13.13	China: Anhui, Tongling

L180330P-1-LD180327-0004_HL3VYCCXY_L7_clean_bin.15.strict.fa	OEZ008529	yes	LMSG_G000006285.1	OES073716	terpene	Terpene	Copper	17.12	China: Anhui, Tongling
L180330P-1-LD180327-0004_HL3VYCCXY_L7_clean_bin.15.strict.fa	OEZ008529	yes	LMSG_G000006285.1	OES073716	terpene	Terpene	Copper	9.02	China: Anhui, Tongling
L180330P-1-LD180327-0004_HL3VYCCXY_L7_clean_bin.2.orig.fa_k1	OEZ008529	yes	LMSG_G000009142.1	OES073716	terpene	Terpene	Copper	19.55	China: Anhui, Tongling
L180330P-1-LD180327-0004_HL3VYCCXY_L7_clean_bin.2.orig.fa_k1	OEZ008529	yes	LMSG_G000009142.1	OES073716	T3PKS	PKSother	Copper	16.08	China: Anhui, Tongling
L180330P-1-LD180327-0004_HL3VYCCXY_L7_clean_bin.2.orig.fa_k1	OEZ008529	yes	LMSG_G000009142.1	OES073716	terpene	Terpene	Copper	20.83	China: Anhui, Tongling
L180330P-1-LD180327-0004_HL3VYCCXY_L7_clean_bin.22.orig.fa_k	OEZ008529	no	LMSG_G000010300.1	OES073716	arylpolyene	Others	Copper	34.39	China: Anhui, Tongling
L180330P-1-LD180327-0004_HL3VYCCXY_L7_clean_bin.22.orig.fa_k	OEZ008529	no	LMSG_G000010300.1	OES073716	terpene	Terpene	Copper	21.34	China: Anhui, Tongling
L180330P-1-LD180327-0004_HL3VYCCXY_L7_clean_bin.23.orig.fa_k	OEZ008529	yes	LMSG_G000009593.1	OES073716	terpene	Terpene	Copper	13.86	China: Anhui, Tongling
L180330P-1-LD180327-0004_HL3VYCCXY_L7_clean_bin.23.orig.fa_k	OEZ008529	yes	LMSG_G000009593.1	OES073716	acyl_amino_acids	Others	Copper	23.11	China: Anhui, Tongling
L180330P-1-LD180327-0004_HL3VYCCXY_L7_clean_bin.23.orig.fa_k	OEZ008529	yes	LMSG_G000009593.1	OES073716	terpene	Terpene	Copper	22.15	China: Anhui, Tongling
L180330P-1-LD180327-0004_HL3VYCCXY_L7_clean_bin.23.orig.fa_k	OEZ008529	yes	LMSG_G000009593.1	OES073716	NRPS-like	NRPS	Copper	13.65	China: Anhui, Tongling
L180330P-1-LD180327-0004_HL3VYCCXY_L7_clean_bin.27.orig.fa_k	OEZ008529	yes	LMSG_G000008535.1	OES073716	terpene	Terpene	Copper	26.21	China: Anhui, Tongling
L180330P-1-LD180327-0004_HL3VYCCXY_L7_clean_bin.27.orig.fa_k	OEZ008529	yes	LMSG_G000008535.1	OES073716	NRPS-like	NRPS	Copper	27.46	China: Anhui, Tongling
L180330P-1-LD180327-0004_HL3VYCCXY_L7_clean_bin.27.orig.fa_k	OEZ008529	yes	LMSG_G000008535.1	OES073716	bacteriocin	RiPPs	Copper	9.30	China: Anhui, Tongling
L180330P-1-LD180327-0004_HL3VYCCXY_L7_clean_bin.28.orig.fa_k	OEZ008529	yes	LMSG_G000011066.1	OES073716	bacteriocin	RiPPs	Copper	10.32	China: Anhui, Tongling
L180330P-1-LD180327-0004_HL3VYCCXY_L7_clean_bin.28.orig.fa_k	OEZ008529	yes	LMSG_G000011066.1	OES073716	hserlactone	Others	Copper	18.51	China: Anhui, Tongling
L180330P-1-LD180327-0004_HL3VYCCXY_L7_clean_bin.28.orig.fa_k	OEZ008529	yes	LMSG_G000011066.1	OES073716	NRPS-like	NRPS	Copper	24.67	China: Anhui, Tongling
L180330P-1-LD180327-0004_HL3VYCCXY_L7_clean_bin.30.orig.fa_k	OEZ008529	yes	LMSG_G000007055.1	OES073716	bacteriocin	RiPPs	Copper	9.32	China: Anhui, Tongling
L180330P-1-LD180327-0004_HL3VYCCXY_L7_clean_bin.35.orig.fa_k	OEZ008529	yes	LMSG_G000009377.1	OES073716	ectoine	Others	Copper	5.69	China: Anhui, Tongling
L180330P-1-LD180327-0004_HL3VYCCXY_L7_clean_bin.35.orig.fa_k	OEZ008529	yes	LMSG_G000009377.1	OES073716	hglE-KS	PKSother	Copper	5.62	China: Anhui, Tongling
L180330P-1-LD180327-0004_HL3VYCCXY_L7_clean_bin.38.orig.fa_k	OEZ008529	yes	LMSG_G000009557.1	OES073716	terpene	Terpene	Copper	22.06	China: Anhui, Tongling
L180330P-1-LD180327-0004_HL3VYCCXY_L7_clean_bin.38.orig.fa_k	OEZ008529	yes	LMSG_G000009557.1	OES073716	NRPS-like	NRPS	Copper	42.72	China: Anhui, Tongling
L180330P-1-LD180327-0004_HL3VYCCXY_L7_clean_bin.43.orig.fa_k	OEZ008529	yes	LMSG_G000007082.1	OES073716	betalactone	Others	Copper	31.07	China: Anhui, Tongling
L180330P-1-LD180327-0004_HL3VYCCXY_L7_clean_bin.44.orig.fa_k	OEZ008529	yes	LMSG_G000008231.1	OES073716	terpene	Terpene	Copper	20.86	China: Anhui, Tongling
L180330P-1-LD180327-0004_HL3VYCCXY_L7_clean_bin.45.strict.fa	OEZ008529	yes	LMSG_G000009418.1	OES073716	bacteriocin	RiPPs	Copper	10.67	China: Anhui, Tongling
L180330P-1-LD180327-0004_HL3VYCCXY_L7_clean_bin.45.strict.fa	OEZ008529	yes	LMSG_G000009418.1	OES073716	betalactone	Others	Copper	26.44	China: Anhui, Tongling
L180330P-1-LD180327-0004_HL3VYCCXY_L7_clean_bin.45.strict.fa	OEZ008529	yes	LMSG_G000009418.1	OES073716	betalactone	Others	Copper	22.76	China: Anhui, Tongling
L180330P-1-LD180327-0004_HL3VYCCXY_L7_clean_bin.45.strict.fa	OEZ008529	yes	LMSG_G000009418.1	OES073716	bacteriocin	RiPPs	Copper	9.59	China: Anhui, Tongling
L180330P-1-LD180327-0004_HL3VYCCXY_L7_clean_bin.46.orig.fa_k	OEZ008529	yes	LMSG_G000009550.1	OES073716	terpene	Terpene	Copper	22.06	China: Anhui, Tongling
L180330P-1-LD180327-0004_HL3VYCCXY_L7_clean_bin.46.orig.fa_k	OEZ008529	no	LMSG_G000009550.1	OES073716	terpene	Terpene	Copper	23.38	China: Anhui, Tongling
L180330P-1-LD180327-0004_HL3VYCCXY_L7_clean_bin.46.orig.fa_k	OEZ008529	yes	LMSG_G000009550.1	OES073716	NRPS-like	NRPS	Copper	42.73	China: Anhui, Tongling
L180330P-1-LD180327-0004_HL3VYCCXY_L7_clean_bin.48.orig.fa_k	OEZ008529	yes	LMSG_G000009469.1	OES073716	ectoine	Others	Copper	10.39	China: Anhui, Tongling
L180330P-1-LD180327-0004_HL3VYCCXY_L7_clean_bin.48.orig.fa_k	OEZ008529	yes	LMSG_G000009469.1	OES073716	arylpolyene	Others	Copper	19.88	China: Anhui, Tongling
L180330P-1-LD180327-0004_HL3VYCCXY_L7_clean_bin.48.orig.fa_k	OEZ008529	yes	LMSG_G000009469.1	OES073716	butyrolactone	Others	Copper	10.81	China: Anhui, Tongling
L180330P-1-LD180327-0004_HL3VYCCXY_L7_clean_bin.48.orig.fa_k	OEZ008529	yes	LMSG_G000009469.1	OES073716	betalactone	Others	Copper	20.95	China: Anhui, Tongling
L180330P-1-LD180327-0004_HL3VYCCXY_L7_clean_bin.48.orig.fa_k	OEZ008529	yes	LMSG_G000009469.1	OES073716	bacteriocin	RiPPs	Copper	7.13	China: Anhui, Tongling
L180330P-1-LD180327-0004_HL3VYCCXY_L7_clean_bin.48.orig.fa_k	OEZ008529	yes	LMSG_G000009469.1	OES073716	lassopeptide	RiPPs	Copper	11.16	China: Anhui, Tongling
L180330P-1-LD180327-0004_HL3VYCCXY_L7_clean_bin.49.orig.fa_k	OEZ008529	yes	LMSG_G000010279.1	OES073716	arylpolyene	Others	Copper	22.85	China: Anhui, Tongling
L180330P-1-LD180327-0004_HL3VYCCXY_L7_clean_bin.49.orig.fa_k	OEZ008529	no	LMSG_G000010279.1	OES073716	terpene	Terpene	Copper	15.36	China: Anhui, Tongling
L180330P-1-LD180327-0004_HL3VYCCXY_L7_clean_bin.50.orig.fa_k	OEZ008529	yes	LMSG_G000006286.1	OES073716	NRPS	NRPS	Copper	23.90	China: Anhui, Tongling
L180330P-1-LD180327-0004_HL3VYCCXY_L7_clean_bin.50.orig.fa_k	OEZ008529	yes	LMSG_G000006286.1	OES073716	terpene	Terpene	Copper	20.06	China: Anhui, Tongling
L180330P-1-LD180327-0004_HL3VYCCXY_L7_clean_bin.50.orig.fa_k	OEZ008529	yes	LMSG_G000006286.1	OES073716	NRPS-like	NRPS	Copper	22.21	China: Anhui, Tongling
L180330P-1-LD180327-0004_HL3VYCCXY_L7_clean_bin.52.orig.fa_k	OEZ008529	yes	LMSG_G000009415.1	OES073716	bacteriocin	RiPPs	Copper	7.26	China: Anhui, Tongling
L180330P-1-LD180327-0004_HL3VYCCXY_L7_clean_bin.52.orig.fa_k	OEZ008529	yes	LMSG_G000009415.1	OES073716	NRPS	NRPS	Copper	18.73	China: Anhui, Tongling
L180330P-1-LD180327-0004_HL3VYCCXY_L7_clean_bin.52.orig.fa_k	OEZ008529	yes	LMSG_G000009415.1	OES073716	ectoine	Others	Copper	5.52	China: Anhui, Tongling
L180330P-1-LD180327-0004_HL3VYCCXY_L7_clean_bin.52.orig.fa_k	OEZ008529	yes	LMSG_G000009415.1	OES073716	LAP	RiPPs	Copper	9.52	China: Anhui, Tongling
L180330P-1-LD180327-0004_HL3VYCCXY_L7_clean_bin.53.orig.fa_k	OEZ008529	no	LMSG_G000004515.1	OES073716	hserlactone	Others	Copper	14.29	China: Anhui, Tongling
L180330P-1-LD180327-0004_HL3VYCCXY_L7_clean_bin.53.orig.fa_k	OEZ008529	no	LMSG_G000004515.1	OES073716	arylpolyene	Others	Copper	20.94	China: Anhui, Tongling
L180330P-1-LD180327-0004_HL3VYCCXY_L7_clean_bin.53.orig.fa_k	OEZ008529	no	LMSG_G000004515.1	OES073716	terpene	Terpene	Copper	14.65	China: Anhui, Tongling
L180330P-1-LD180327-0004_HL3VYCCXY_L7_clean_bin.54.orig.fa_k	OEZ008529	yes	LMSG_G000007240.1	OES073716	betalactone	Others	Copper	15.56	China: Anhui, Tongling
L180330P-1-LD180327-0004_HL3VYCCXY_L7_clean_bin.55.orig.fa_k	OEZ008529	yes	LMSG_G000009476.1	OES073716	other	Others	Copper	30.64	China: Anhui, Tongling
L180330P-1-LD180327-0004_HL3VYCCXY_L7_clean_bin.55.orig.fa_k	OEZ008529	yes	LMSG_G000009476.1	OES073716	ectoine	Others	Copper	10.43	China: Anhui, Tongling
L180330P-1-LD180327-0004_HL3VYCCXY_L7_clean_bin.55.orig.fa_k	OEZ008529	yes	LMSG_G000009476.1	OES073716	terpene	Terpene	Copper	11.12	China: Anhui, Tongling
L180330P-1-LD180327-0004_HL3VYCCXY_L7_clean_bin.56.permissiv	OEZ008529	yes	LMSG_G000008018.1	OES073716	betalactone	Others	Copper	23.60	China: Anhui, Tongling
L180330P-1-LD180327-0004_HL3VYCCXY_L7_clean_bin.56.permissiv	OEZ008529	yes	LMSG_G000008018.1	OES073716	betalactone	Others	Copper	30.02	China: Anhui, Tongling
L180330P-1-LD180327-0004_HL3VYCCXY_L7_clean_bin.56.permissiv	OEZ008529	yes	LMSG_G000008018.1	OES073716	bacteriocin	RiPPs	Copper	10.30	China: Anhui, Tongling
L180330P-1-LD180327-0004_HL3VYCCXY_L7_clean_bin.56.permissiv	OEZ008529	yes	LMSG_G000008018.1	OES073716	bacteriocin	RiPPs	Copper	5.21	China: Anhui, Tongling
L180330P-1-LD180327-0004_HL3VYCCXY_L7_clean_bin.57.strict.fa	OEZ008529	yes	LMSG_G000009439.1	OES073716	bacteriocin	RiPPs	Copper	10.85	China: Anhui, Tongling
L180330P-1-LD180327-0004_HL3VYCCXY_L7_clean_bin.57.strict.fa	OEZ008529	yes	LMSG_G000009439.1	OES073716	betalactone	Others	Copper	30.86	China: Anhui, Tongling
L180330P-1-LD180327-0004_HL3VYCCXY_L7_clean_bin.57.strict.fa	OEZ008529	yes	LMSG_G000009439.1	OES073716	betalactone	Others	Copper	22.80	China: Anhui, Tongling
L180330P-1-LD180327-0004_HL3VYCCXY_L7_clean_bin.57.strict.fa	OEZ008529	yes	LMSG_G000009439.1	OES073716	ectoine	Others	Copper	9.46	China: Anhui, Tongling
L180330P-1-LD180327-0004_HL3VYCCXY_L7_clean_bin.57.strict.fa	OEZ008529	yes	LMSG_G000009439.1	OES073716	ectoine	Others	Copper	10.26	China: Anhui, Tongling
L180330P-1-LD180327-0004_HL3VYCCXY_L7_clean_bin.57.strict.fa	OEZ008529	yes	LMSG_G000009439.1	OES073716	ladderane	Others	Copper	20.29	China: Anhui, Tongling
L180330P-1-LD180327-0004_HL3VYCCXY_L7_clean_bin.59.orig.fa_k	OEZ008529	yes	LMSG_G000009612.1	OES073716	NRPS-like	NRPS	Copper	30.24	China: Anhui, Tongling
L180330P-1-LD180327-0004_HL3VYCCXY_L7_clean_bin.59.orig.fa_k	OEZ008529	yes	LMSG_G000009612.1	OES073716	terpene	Terpene	Copper	22.09	China: Anhui, Tongling
L180330P-1-LD180327-0004_HL3VYCCXY_L7_clean_bin.59.orig.fa_k	OEZ008529	yes	LMSG_G000009612.1	OES073716	terpene	Terpene	Copper	21.39	China: Anhui, Tongling
L180330P-1-LD180327-0004_HL3VYCCXY_L7_clean_bin.60.orig.fa_k	OEZ008529	yes	LMSG_G000005601.1	OES073716	terpene	Terpene	Copper	22.09	China: Anhui, Tongling
L180330P-1-LD180327-0004_HL3VYCCXY_L7_clean_bin.62.orig.fa_k	OEZ008529	yes	LMSG_G000008344.1	OES073716	terpene	Terpene	Copper	20.48	China: Anhui, Tongling
L180330P-1-LD180327-0004_HL3VYCCXY_L7_clean_bin.62.orig.fa_k	OEZ008529	yes	LMSG_G000008344.1	OES073716	T3PKS	PKSother	Copper	29.53	China: Anhui, Tongling
L180330P-1-LD180327-0004_HL3VYCCXY_L7_clean_bin.62.orig.fa_k	OEZ008529	yes	LMSG_G000008344.1	OES073716	bacteriocin	RiPPs	Copper	10.60	China: Anhui, Tongling
L180330P-1-LD180327-0004_HL3VYCCXY_L7_clean_bin.62.orig.fa_k	OEZ008529	yes	LMSG_G000008344.1	OES073716	other	Others	Copper	9.21	China: Anhui, Tongling
L180330P-1-LD180327-0004_HL3VYCCXY_L7_clean_bin.62.orig.fa_k	OEZ008529	yes	LMSG_G000008344.1	OES073716	butyrolactone	Others	Copper	11.01	China: Anhui, Tongling
L180330P-1-LD180327-0004_HL3VYCCXY_L7_clean_bin.63.orig.fa_k	OEZ008529	yes	LMSG_G000008518.1	OES073716	terpene	Terpene	Copper	6.00	China: Anhui, Tongling
L180330P-1-LD180327-0004_HL3VYCCXY_L7_clean_bin.66.orig.fa_k	OEZ008529	yes	LMSG_G000009002.1	OES073716	terpene	Terpene	Copper	20.91	China: Anhui, Tongling
L180330P-1-LD180327-0004_HL3VYCCXY_L7_clean_bin.66.orig.fa_k	OEZ008529	yes	LMSG_G000009002.1	OES073716	bacteriocin	RiPPs	Copper	10.80	China: Anhui, Tongling
L180330P-1-LD180327-0004_HL3VYCCXY_L7_clean_bin.67.strict.fa	OEZ008529	yes	LMSG_G000008005.1	OES073716	TfuA-related.bacteriocin	RiPPs	Copper	5.05	China: Anhui, Tongling
L180330P-1-LD180327-0004_HL3VYCCXY_L7_clean_bin.7.strict.fa_	OEZ008529	yes	LMSG_G000005603.1	OES073716	terpene	Terpene	Copper	18.86	China: Anhui, Tongling
L180330P-1-LD180327-0004_HL3VYCCXY_L7_clean_bin.7.strict.fa_	OEZ008529	yes	LMSG_G000005603.1	OES073716	arylpolyene.hserlactone	Others	Copper	10.16	China: Anhui, Tongling
L180330P-2-LD180327-0005_HL3VYCCXY_L8_clean_bin.1.orig.fa_k1	OEZ008529	yes	LMSG_G000005604.1	OES073717	NRPS-like	NRPS	Copper	37.59	China: Anhui, Tongling
L180330P-2-LD180327-0005_HL3VYCCXY_L8_clean_bin.1.orig.fa_k1	OEZ008529	yes	LMSG_G000005604.1	OES073717	NRPS-like	NRPS	Copper	20.97	China: Anhui, Tongling
L180330P-2-LD180327-0005_HL3VYCCXY_L8_clean_bin.1.orig.fa_k1	OEZ008529	yes	LMSG_G000005604.1	OES073717	bacteriocin	RiPPs	Copper	10.33	China: Anhui, Tongling
L180330P-2-LD180327-0005_HL3VYCCXY_L8_clean_bin.1.orig.fa_k1	OEZ008529	yes	LMSG_G000005604.1	OES073717	NRPS-like	NRPS	Copper	5.36	China: Anhui, Tongling
L180330P-2-LD180327-0005_HL3VYCCXY_L8_clean_bin.1.orig.fa_k1	OEZ008529	yes	LMSG_G000005604.1	OES073717	lassopeptide.hserlactone	Others	Copper	30.53	China: Anhui, Tongling
L180330P-2-LD180327-0005_HL3VYCCXY_L8_clean_bin.1.orig.fa_k1	OEZ008529	yes	LMSG_G000005604.1	OES073717	terpene	Terpene	Copper	10.96	China: Anhui, Tongling
L180330P-2-LD180327-0005_HL3VYCCXY_L8_clean_bin.1.orig.fa_k1	OEZ008529	yes	LMSG_G000005604.1	OES073717	arylpolyene	Others	Copper	38.01	China: Anhui, Tongling
L180330P-2-LD180327-0005_HL3VYCCXY_L8_clean_bin.10.orig.fa_k	OEZ008529	no	LMSG_G000010540.1	OES073717	arylpolyene	Others	Copper	20.94	China: Anhui, Tongling
L180330P-2-LD180327-0005_HL3VYCCXY_L8_clean_bin.10.orig.fa_k	OEZ008529	no	LMSG_G000010540.1	OES073717	terpene	Terpene	Copper	14.65	China: Anhui, Tongling
L180330P-2-LD180327-0005_HL3VYCCXY_L8_clean_bin.10.orig.fa_k	OEZ008529	no	LMSG_G000010540.1	OES073717	hserlactone	Others	Copper	13.71	China: Anhui, Tongling
L180330P-2-LD180327-0005_HL3VYCCXY_L8_clean_bin.11.orig.fa_k	OEZ008529	yes	LMSG_G000009625.1	OES073717	acyl_amino_acids	Others	Copper	20.24	China: Anhui, Tongling
L180330P-2-LD180327-0005_HL3VYCCXY_L8_clean_bin.11.orig.fa_k	OEZ008529	yes	LMSG_G000009625.1	OES073717	terpene	Terpene	Copper	7.11	China: Anhui, Tongling
L180330P-2-LD180327-0005_HL3VYCCXY_L8_clean_bin.11.orig.fa_k	OEZ008529	yes	LMSG_G000009625.1	OES073717	terpene	Terpene	Copper	16.73	China: Anhui, Tongling
L180330P-2-LD180327-0005_HL3VYCCXY_L8_clean_bin.11.orig.fa_k	OEZ008529	yes	LMSG_G000009625.1	OES073717	NRPS-like	NRPS	Copper	24.37	China: Anhui, Tongling
L180330P-2-LD180327-0005_HL3VYCCXY_L8_clean_bin.12.orig.fa_k	OEZ008529	yes	LMSG_G000008338.1	OES073717	terpene	Terpene	Copper	8.67	China: Anhui, Tongling
L180330P-2-LD180327-0005_HL3VYCCXY_L8_clean_bin.12.orig.fa_k	OEZ008529	yes	LMSG_G000008338.1	OES073717	other	Others	Copper	9.03	China: Anhui, Tongling
L180330P-2-LD180327-0005_HL3VYCCXY_L8_clean_bin.12.orig.fa_k	OEZ008529	yes	LMSG_G000008338.1	OES073717	butyrolactone	Others	Copper	6.17	China: Anhui, Tongling
L180330P-2-LD180327-0005_HL3VYCCXY_L8_clean_bin.12.orig.fa_k	OEZ008529	yes	LMSG_G000008338.1	OES073717	terpene	Terpene	Copper	5.97	China: Anhui, Tongling
L180330P-2-LD180327-0005_HL3VYCCXY_L8_clean_bin.12.orig.fa_k	OEZ008529	yes	LMSG_G000008338.1	OES073717	bacteriocin	RiPPs	Copper	6.54	China: Anhui, Tongling
L180330P-2-LD180327-0005_HL3VYCCXY_L8_clean_bin.15.permissiv	OEZ008529	yes	LMSG_G000009416.1	OES073717	bacteriocin	RiPPs	Copper	10.85	China: Anhui, Tongling
L180330P-2-LD180327-0005_HL3VYCCXY_L8_clean_bin.15.permissiv	OEZ008529	yes	LMSG_G000009416.1	OES073717	ectoine	Others	Copper	5.96	China: Anhui, Tongling
L180330P-2-LD180327-0005_HL3VYCCXY_L8_clean_bin.16.orig.fa_k	OEZ008529	yes	LMSG_G000009396.1	OES073717	terpene	Terpene	Copper	21.68	China: Anhui, Tongling
L180330P-2-LD180327-0005_HL3VYCCXY_L8_clean_bin.16.orig.fa_k	OEZ008529	yes	LMSG_G000009396.1	OES073717	NRPS	NRPS	Copper	23.90	China: Anhui, Tongling
L180330P-2-LD180327-0005_HL3VYCCXY_L8_clean_bin.16.orig.fa_k	OEZ008529	yes	LMSG_G000009396.1	OES073717	NRPS-like	NRPS	Copper	6.40	China: Anhui, Tongling
L180330P-2-LD180327-0005_HL3VYCCXY_L8_clean_bin.17.orig.fa_k	OEZ008529	yes	LMSG_G000007066.1	OES073717	bacteriocin	RiPPs	Copper	9.52	China: Anhui, Tongling
L180330P-2-LD180327-0005_HL3VYCCXY_L8_clean_bin.21.orig.fa_k	OEZ008529	yes	LMSG_G000009551.1	OES073717	ectoine	Others	Copper	7.63	China: Anhui, Tongling
L180330P-2-LD180327-0005_HL3VYCCXY_L8_clean_bin.21.orig.fa_k	OEZ008529	yes	LMSG_G000009551.1	OES073717	NRPS-like	NRPS	Copper	42.73	China: Anhui, Tongling
L180330P-2-LD180327-0005_HL3VYCCXY_L8_clean_bin.21.orig.fa_k	OEZ008529	yes	LMSG_G000009551.1	OES073717	terpene	Terpene	Copper	22.06	China: Anhui, Tongling
L180330P-2-LD180327-0005_HL3VYCCXY_L8_clean_bin.22.orig.fa_k	OEZ008529	yes	LMSG_G000009558.1	OES073717	ectoine	Others	Copper	9.43	China: Anhui, Tongling
L180330P-2-LD180327-0005_HL3VYCCXY_L8_clean_bin.22.orig.fa_k	OEZ008529	yes	LMSG_G000009558.1	OES073717	NRPS-like	NRPS	Copper	42.72	China: Anhui, Tongling
L180330P-2-LD180327-0005_HL3VYCCXY_L8_clean_bin.22.orig.fa_k	OEZ008529	yes	LMSG_G000009558.1	OES073717	terpene	Terpene	Copper	22.11	China: Anhui, Tongling
L180330P-2-LD180327-0005_HL3VYCCXY_L8_clean_bin.22.orig.fa_k	OEZ008529	no	LMSG_G000009558.1	OES073717	terpene	Terpene	Copper	23.38	China: Anhui, Tongling
L180330P-2-LD180327-0005_HL3VYCCXY_L8_clean_bin.23.orig.fa_k	OEZ008529	yes	LMSG_G000009440.1	OES073717	bacteriocin	RiPPs	Copper	10.85	China: Anhui, Tongling
L180330P-2-LD180327-0005_HL3VYCCXY_L8_clean_bin.23.orig.fa_k	OEZ008529	yes	LMSG_G000009440.1	OES073717	betalactone	Others	Copper	30.86	China: Anhui, Tongling
L180330P-2-LD180327-0005_HL3VYCCXY_L8_clean_bin.23.orig.fa_k	OEZ008529	yes	LMSG_G000009440.1	OES073717	betalactone	Others	Copper	22.78	China: Anhui, Tongling
L180330P-2-LD180327-0005_HL3VYCCXY_L8_clean_bin.23.orig.fa_k	OEZ008529	yes	LMSG_G000009440.1	OES073717	ectoine	Others	Copper	7.19	China: Anhui, Tongling
L180330P-2-LD180327-0005_HL3VYCCXY_L8_clean_bin.25.orig.fa_k	OEZ008529	yes	LMSG_G000007338.1	OES073717	bacteriocin	RiPPs	Copper	8.60	China: Anhui, Tongling
L180330P-2-LD180327-0005_HL3VYCCXY_L8_clean_bin.26.strict.fa	OEZ008529	yes	LMSG_G000009411.1	OES073717	ectoine	Others	Copper	9.00	China: Anhui, Tongling
L180330P-2-LD180327-0005_HL3VYCCXY_L8_clean_bin.26.strict.fa	OEZ008529	yes	LMSG_G000009411.1	OES073717	terpene	Terpene	Copper	16.13	China: Anhui, Tongling
L180330P-2-LD180327-0005_HL3VYCCXY_L8_clean_bin.26.strict.fa	OEZ008529	yes	LMSG_G000009411.1	OES073717	terpene	Terpene	Copper	9.24	China: Anhui, Tongling
L180330P-2-LD180327-0005_HL3VYCCXY_L8_clean_bin.28.orig.fa_k	OEZ008529	yes	LMSG_G000008019.1	OES073717	betalactone	Others	Copper	30.01	China: Anhui, Tongling
L180330P-2-LD180327-0005_HL3VYCCXY_L8_clean_bin.28.orig.fa_k	OEZ008529	yes	LMSG_G000008019.1	OES073717	betalactone	Others	Copper	23.60	China: Anhui, Tongling
L180330P-2-LD180327-0005_HL3VYCCXY_L8_clean_bin.28.orig.fa_k	OEZ008529	yes	LMSG_G000008019.1	OES073717	bacteriocin	RiPPs	Copper	10.30	China: Anhui, Tongling
L180330P-2-LD180327-0005_HL3VYCCXY_L8_clean_bin.29.orig.fa_k	OEZ008529	yes	LMSG_G000007219.1	OES073717	ectoine	Others	Copper	10.38	China: Anhui, Tongling
L180330P-2-LD180327-0005_HL3VYCCXY_L8_clean_bin.3.permissive	OEZ008529	yes	LMSG_G000009378.1	OES073717	ectoine	Others	Copper	10.39	China: Anhui, Tongling
L180330P-2-LD180327-0005_HL3VYCCXY_L8_clean_bin.3.permissive	OEZ008529	yes	LMSG_G000009378.1	OES073717	terpene	Terpene	Copper	18.51	China: Anhui, Tongling
L180330P-2-LD180327-0005_HL3VYCCXY_L8_clean_bin.3.permissive	OEZ008529	yes	LMSG_G000009378.1	OES073717	hglE-KS	PKSother	Copper	14.74	China: Anhui, Tongling
L180330P-2-LD180327-0005_HL3VYCCXY_L8_clean_bin.3.permissive	OEZ008529	yes	LMSG_G000009378.1	OES073717	ladderane	Others	Copper	5.14	China: Anhui, Tongling
L180330P-2-LD180327-0005_HL3VYCCXY_L8_clean_bin.30.orig.fa_k	OEZ008529	yes	LMSG_G000004516.1	OES073717	other	Others	Copper	10.32	China: Anhui, Tongling
L180330P-2-LD180327-0005_HL3VYCCXY_L8_clean_bin.30.orig.fa_k	OEZ008529	yes	LMSG_G000004516.1	OES073717	arylpolyene	Others	Copper	13.68	China: Anhui, Tongling
L180330P-2-LD180327-0005_HL3VYCCXY_L8_clean_bin.30.orig.fa_k	OEZ008529	yes	LMSG_G000004516.1	OES073717	T1PKS.hglE-KS	PKSother	Copper	26.26	China: Anhui, Tongling
L180330P-2-LD180327-0005_HL3VYCCXY_L8_clean_bin.32.strict.fa	OEZ008529	yes	LMSG_G000005605.1	OES073717	terpene	Terpene	Copper	20.86	China: Anhui, Tongling
L180330P-2-LD180327-0005_HL3VYCCXY_L8_clean_bin.36.orig.fa_k	OEZ008529	yes	LMSG_G000005606.1	OES073717	lassopeptide	RiPPs	Copper	10.94	China: Anhui, Tongling
L180330P-2-LD180327-0005_HL3VYCCXY_L8_clean_bin.36.orig.fa_k	OEZ008529	yes	LMSG_G000005606.1	OES073717	ectoine	Others	Copper	10.39	China: Anhui, Tongling
L180330P-2-LD180327-0005_HL3VYCCXY_L8_clean_bin.36.orig.fa_k	OEZ008529	yes	LMSG_G000005606.1	OES073717	bacteriocin	RiPPs	Copper	6.55	China: Anhui, Tongling
L180330P-2-LD180327-0005_HL3VYCCXY_L8_clean_bin.37.orig.fa_k	OEZ008529	yes	LMSG_G000007241.1	OES073717	betalactone	Others	Copper	15.56	China: Anhui, Tongling
L180330P-2-LD180327-0005_HL3VYCCXY_L8_clean_bin.39.orig.fa_k	OEZ008529	yes	LMSG_G000005607.1	OES073717	ectoine	Others	Copper	5.57	China: Anhui, Tongling
L180330P-2-LD180327-0005_HL3VYCCXY_L8_clean_bin.4.strict.fa_	OEZ008529	yes	LMSG_G000008006.1	OES073717	TfuA-related.bacteriocin	RiPPs	Copper	6.36	China: Anhui, Tongling
L180330P-2-LD180327-0005_HL3VYCCXY_L8_clean_bin.40.orig.fa_k	OEZ008529	yes	LMSG_G000009003.1	OES073717	terpene	Terpene	Copper	20.91	China: Anhui, Tongling
L180330P-2-LD180327-0005_HL3VYCCXY_L8_clean_bin.40.orig.fa_k	OEZ008529	yes	LMSG_G000009003.1	OES073717	bacteriocin	RiPPs	Copper	10.80	China: Anhui, Tongling
L180330P-2-LD180327-0005_HL3VYCCXY_L8_clean_bin.43.strict.fa	OEZ008529	yes	LMSG_G000009143.1	OES073717	terpene	Terpene	Copper	19.02	China: Anhui, Tongling
L180330P-2-LD180327-0005_HL3VYCCXY_L8_clean_bin.43.strict.fa	OEZ008529	yes	LMSG_G000009143.1	OES073717	terpene	Terpene	Copper	19.02	China: Anhui, Tongling
L180330P-2-LD180327-0005_HL3VYCCXY_L8_clean_bin.43.strict.fa	OEZ008529	yes	LMSG_G000009143.1	OES073717	T3PKS	PKSother	Copper	12.43	China: Anhui, Tongling
L180330P-2-LD180327-0005_HL3VYCCXY_L8_clean_bin.46.orig.fa_k	OEZ008529	yes	LMSG_G000009594.1	OES073717	terpene	Terpene	Copper	10.26	China: Anhui, Tongling
L180330P-2-LD180327-0005_HL3VYCCXY_L8_clean_bin.46.orig.fa_k	OEZ008529	yes	LMSG_G000009594.1	OES073717	terpene	Terpene	Copper	22.15	China: Anhui, Tongling
L180330P-2-LD180327-0005_HL3VYCCXY_L8_clean_bin.46.orig.fa_k	OEZ008529	yes	LMSG_G000009594.1	OES073717	NRPS-like	NRPS	Copper	12.42	China: Anhui, Tongling
L180330P-2-LD180327-0005_HL3VYCCXY_L8_clean_bin.47.orig.fa_k	OEZ008529	yes	LMSG_G000007079.1	OES073717	betalactone	Others	Copper	31.07	China: Anhui, Tongling
L180330P-2-LD180327-0005_HL3VYCCXY_L8_clean_bin.48.orig.fa_k	OEZ008529	yes	LMSG_G000011067.1	OES073717	bacteriocin	RiPPs	Copper	9.45	China: Anhui, Tongling
L180330P-2-LD180327-0005_HL3VYCCXY_L8_clean_bin.48.orig.fa_k	OEZ008529	yes	LMSG_G000011067.1	OES073717	NRPS-like	NRPS	Copper	9.22	China: Anhui, Tongling
L180330P-2-LD180327-0005_HL3VYCCXY_L8_clean_bin.48.orig.fa_k	OEZ008529	yes	LMSG_G000011067.1	OES073717	NRPS-like	NRPS	Copper	13.44	China: Anhui, Tongling
L180330P-2-LD180327-0005_HL3VYCCXY_L8_clean_bin.50.orig.fa_k	OEZ008529	yes	LMSG_G000005608.1	OES073717	other	Others	Copper	30.59	China: Anhui, Tongling
L180330P-2-LD180327-0005_HL3VYCCXY_L8_clean_bin.50.orig.fa_k	OEZ008529	yes	LMSG_G000005608.1	OES073717	ectoine	Others	Copper	10.43	China: Anhui, Tongling
L180330P-2-LD180327-0005_HL3VYCCXY_L8_clean_bin.54.strict.fa	OEZ008529	yes	LMSG_G000009419.1	OES073717	bacteriocin	RiPPs	Copper	10.42	China: Anhui, Tongling
L180330P-2-LD180327-0005_HL3VYCCXY_L8_clean_bin.54.strict.fa	OEZ008529	yes	LMSG_G000009419.1	OES073717	betalactone	Others	Copper	26.87	China: Anhui, Tongling
L180330P-2-LD180327-0005_HL3VYCCXY_L8_clean_bin.54.strict.fa	OEZ008529	yes	LMSG_G000009419.1	OES073717	ectoine	Others	Copper	7.30	China: Anhui, Tongling
L180330P-2-LD180327-0005_HL3VYCCXY_L8_clean_bin.55.orig.fa_k	OEZ008529	yes	LMSG_G000006287.1	OES073717	terpene	Terpene	Copper	20.94	China: Anhui, Tongling
L180330P-2-LD180327-0005_HL3VYCCXY_L8_clean_bin.55.orig.fa_k	OEZ008529	yes	LMSG_G000006287.1	OES073717	bacteriocin	RiPPs	Copper	9.44	China: Anhui, Tongling
L180330P-2-LD180327-0005_HL3VYCCXY_L8_clean_bin.7.orig.fa_k1	OEZ008529	no	LMSG_G000010301.1	OES073717	arylpolyene	Others	Copper	34.38	China: Anhui, Tongling
L180330P-2-LD180327-0005_HL3VYCCXY_L8_clean_bin.7.orig.fa_k1	OEZ008529	no	LMSG_G000010301.1	OES073717	terpene	Terpene	Copper	24.88	China: Anhui, Tongling
L180330P-2-LD180327-0006_HL3VYCCXY_L8_clean_bin.1.orig.fa_k1	OEZ008529	yes	LMSG_G000008346.1	OES073718	T3PKS	PKSother	Copper	5.56	China: Anhui, Tongling

L180330P-2-LD180327-0006_HL3VYCCXY_L8_clean_bin.1.orig.fa_kl	OEZ008529	yes	LMSG_G000008346.1	OES073718	bacteriocin	RiPPs	Copper	6.25	China: Anhui, Tongling
L180330P-2-LD180327-0006_HL3VYCCXY_L8_clean_bin.1.orig.fa_kl	OEZ008529	yes	LMSG_G000008346.1	OES073718	terpene	Terpene	Copper	18.12	China: Anhui, Tongling
L180330P-2-LD180327-0006_HL3VYCCXY_L8_clean_bin.100.orig.fa_	OEZ008529	no	LMSG_G000010561.1	OES073718	arylpolyene	Others	Copper	25.69	China: Anhui, Tongling
L180330P-2-LD180327-0006_HL3VYCCXY_L8_clean_bin.100.orig.fa_	OEZ008529	yes	LMSG_G000010561.1	OES073718	bacteriocin	RiPPs	Copper	10.84	China: Anhui, Tongling
L180330P-2-LD180327-0006_HL3VYCCXY_L8_clean_bin.100.orig.fa_	OEZ008529	yes	LMSG_G000010561.1	OES073718	terpene	Terpene	Copper	19.17	China: Anhui, Tongling
L180330P-2-LD180327-0006_HL3VYCCXY_L8_clean_bin.100.orig.fa_	OEZ008529	yes	LMSG_G000010561.1	OES073718	T1PKS	PKSI	Copper	19.70	China: Anhui, Tongling
L180330P-2-LD180327-0006_HL3VYCCXY_L8_clean_bin.101.orig.fa_	OEZ008529	yes	LMSG_G000009160.1	OES073718	T3PKS	PKSother	Copper	16.88	China: Anhui, Tongling
L180330P-2-LD180327-0006_HL3VYCCXY_L8_clean_bin.101.orig.fa_	OEZ008529	yes	LMSG_G000009160.1	OES073718	terpene	Terpene	Copper	19.37	China: Anhui, Tongling
L180330P-2-LD180327-0006_HL3VYCCXY_L8_clean_bin.105.orig.fa_	OEZ008529	yes	LMSG_G000005612.1	OES073718	ectoine	Others	Copper	7.51	China: Anhui, Tongling
L180330P-2-LD180327-0006_HL3VYCCXY_L8_clean_bin.105.orig.fa_	OEZ008529	yes	LMSG_G000005612.1	OES073718	T1PKS	PKSI	Copper	6.24	China: Anhui, Tongling
L180330P-2-LD180327-0006_HL3VYCCXY_L8_clean_bin.105.orig.fa_	OEZ008529	yes	LMSG_G000005612.1	OES073718	terpene	Terpene	Copper	5.70	China: Anhui, Tongling
L180330P-2-LD180327-0006_HL3VYCCXY_L8_clean_bin.105.orig.fa_	OEZ008529	yes	LMSG_G000005612.1	OES073718	arylpolyene	Others	Copper	33.95	China: Anhui, Tongling
L180330P-2-LD180327-0006_HL3VYCCXY_L8_clean_bin.107.permissi	OEZ008529	yes	LMSG_G000008134.1	OES073718	betalactone	Others	Copper	27.96	China: Anhui, Tongling
L180330P-2-LD180327-0006_HL3VYCCXY_L8_clean_bin.107.permissi	OEZ008529	yes	LMSG_G000008134.1	OES073718	terpene	Terpene	Copper	20.50	China: Anhui, Tongling
L180330P-2-LD180327-0006_HL3VYCCXY_L8_clean_bin.11.strict.fa	OEZ008529	yes	LMSG_G000008740.1	OES073718	terpene	Terpene	Copper	19.20	China: Anhui, Tongling
L180330P-2-LD180327-0006_HL3VYCCXY_L8_clean_bin.11.strict.fa	OEZ008529	yes	LMSG_G000008740.1	OES073718	NRPS-like	NRPS	Copper	35.66	China: Anhui, Tongling
L180330P-2-LD180327-0006_HL3VYCCXY_L8_clean_bin.11.strict.fa	OEZ008529	yes	LMSG_G000008740.1	OES073718	other	Others	Copper	38.29	China: Anhui, Tongling
L180330P-2-LD180327-0006_HL3VYCCXY_L8_clean_bin.11.strict.fa	OEZ008529	yes	LMSG_G000008740.1	OES073718	betalactone	Others	Copper	23.39	China: Anhui, Tongling
L180330P-2-LD180327-0006_HL3VYCCXY_L8_clean_bin.110.orig.fa_	OEZ008529	yes	LMSG_G000005613.1	OES073718	T3PKS	PKSother	Copper	32.25	China: Anhui, Tongling
L180330P-2-LD180327-0006_HL3VYCCXY_L8_clean_bin.110.orig.fa_	OEZ008529	yes	LMSG_G000005613.1	OES073718	terpene	Terpene	Copper	7.30	China: Anhui, Tongling
L180330P-2-LD180327-0006_HL3VYCCXY_L8_clean_bin.113.orig.fa_	OEZ008529	yes	LMSG_G000008363.1	OES073718	terpene	Terpene	Copper	20.36	China: Anhui, Tongling
L180330P-2-LD180327-0006_HL3VYCCXY_L8_clean_bin.115.strict.f	OEZ008529	yes	LMSG_G000009898.1	OES073718	terpene	Terpene	Copper	19.06	China: Anhui, Tongling
L180330P-2-LD180327-0006_HL3VYCCXY_L8_clean_bin.117.orig.fa_	OEZ008529	yes	LMSG_G000004519.1	OES073718	betalactone	Others	Copper	21.73	China: Anhui, Tongling
L180330P-2-LD180327-0006_HL3VYCCXY_L8_clean_bin.117.orig.fa_	OEZ008529	yes	LMSG_G000004519.1	OES073718	terpene	Terpene	Copper	23.69	China: Anhui, Tongling
L180330P-2-LD180327-0006_HL3VYCCXY_L8_clean_bin.118.orig.fa_	OEZ008529	yes	LMSG_G000009121.1	OES073718	NRPS-like	NRPS	Copper	26.24	China: Anhui, Tongling
L180330P-2-LD180327-0006_HL3VYCCXY_L8_clean_bin.118.orig.fa_	OEZ008529	yes	LMSG_G000009121.1	OES073718	bacteriocin	RiPPs	Copper	10.87	China: Anhui, Tongling
L180330P-2-LD180327-0006_HL3VYCCXY_L8_clean_bin.118.orig.fa_	OEZ008529	yes	LMSG_G000009121.1	OES073718	terpene	Terpene	Copper	25.09	China: Anhui, Tongling
L180330P-2-LD180327-0006_HL3VYCCXY_L8_clean_bin.119.orig.fa_	OEZ008529	yes	LMSG_G000005615.1	OES073718	other	Others	Copper	11.17	China: Anhui, Tongling
L180330P-2-LD180327-0006_HL3VYCCXY_L8_clean_bin.119.orig.fa_	OEZ008529	yes	LMSG_G000005615.1	OES073718	NRPS-like	NRPS	Copper	20.10	China: Anhui, Tongling
L180330P-2-LD180327-0006_HL3VYCCXY_L8_clean_bin.119.orig.fa_	OEZ008529	yes	LMSG_G000005615.1	OES073718	NRPS-like	NRPS	Copper	5.68	China: Anhui, Tongling
L180330P-2-LD180327-0006_HL3VYCCXY_L8_clean_bin.119.orig.fa_	OEZ008529	yes	LMSG_G000005615.1	OES073718	other	Others	Copper	23.27	China: Anhui, Tongling
L180330P-2-LD180327-0006_HL3VYCCXY_L8_clean_bin.122.orig.fa_	OEZ008529	yes	LMSG_G000009397.1	OES073718	terpene	Terpene	Copper	20.06	China: Anhui, Tongling
L180330P-2-LD180327-0006_HL3VYCCXY_L8_clean_bin.122.orig.fa_	OEZ008529	yes	LMSG_G000009397.1	OES073718	NRPS-like	NRPS	Copper	22.71	China: Anhui, Tongling
L180330P-2-LD180327-0006_HL3VYCCXY_L8_clean_bin.122.orig.fa_	OEZ008529	yes	LMSG_G000009397.1	OES073718	NRPS	NRPS	Copper	23.95	China: Anhui, Tongling
L180330P-2-LD180327-0006_HL3VYCCXY_L8_clean_bin.123.orig.fa_	OEZ008529	yes	LMSG_G000010032.1	OES073718	terpene	Terpene	Copper	14.99	China: Anhui, Tongling
L180330P-2-LD180327-0006_HL3VYCCXY_L8_clean_bin.123.orig.fa_	OEZ008529	yes	LMSG_G000010032.1	OES073718	T3PKS	PKSother	Copper	21.20	China: Anhui, Tongling
L180330P-2-LD180327-0006_HL3VYCCXY_L8_clean_bin.123.orig.fa_	OEZ008529	yes	LMSG_G000010032.1	OES073718	terpene	Terpene	Copper	15.16	China: Anhui, Tongling
L180330P-2-LD180327-0006_HL3VYCCXY_L8_clean_bin.127.strict.f	OEZ008529	yes	LMSG_G000010781.1	OES073718	arylpolyene	Others	Copper	6.50	China: Anhui, Tongling
L180330P-2-LD180327-0006_HL3VYCCXY_L8_clean_bin.127.strict.f	OEZ008529	yes	LMSG_G000010781.1	OES073718	acyl_amino_acids	Others	Copper	5.64	China: Anhui, Tongling
L180330P-2-LD180327-0006_HL3VYCCXY_L8_clean_bin.14.orig.fa_k	OEZ008529	yes	LMSG_G000009932.1	OES073718	hserlactone	Others	Copper	20.78	China: Anhui, Tongling
L180330P-2-LD180327-0006_HL3VYCCXY_L8_clean_bin.18.orig.fa_k	OEZ008529	yes	LMSG_G000009441.1	OES073718	betalactone	Others	Copper	30.86	China: Anhui, Tongling
L180330P-2-LD180327-0006_HL3VYCCXY_L8_clean_bin.18.orig.fa_k	OEZ008529	yes	LMSG_G000009441.1	OES073718	betalactone	Others	Copper	22.80	China: Anhui, Tongling
L180330P-2-LD180327-0006_HL3VYCCXY_L8_clean_bin.18.orig.fa_k	OEZ008529	yes	LMSG_G000009441.1	OES073718	ectoine	Others	Copper	7.98	China: Anhui, Tongling
L180330P-2-LD180327-0006_HL3VYCCXY_L8_clean_bin.18.orig.fa_k	OEZ008529	yes	LMSG_G000009441.1	OES073718	bacteriocin	RiPPs	Copper	10.85	China: Anhui, Tongling
L180330P-2-LD180327-0006_HL3VYCCXY_L8_clean_bin.19.orig.fa_k	OEZ008529	yes	LMSG_G000008506.1	OES073718	terpene	Terpene	Copper	17.35	China: Anhui, Tongling
L180330P-2-LD180327-0006_HL3VYCCXY_L8_clean_bin.2.orig.fa_kl	OEZ008529	yes	LMSG_G000010167.1	OES073718	terpene	Terpene	Copper	21.95	China: Anhui, Tongling
L180330P-2-LD180327-0006_HL3VYCCXY_L8_clean_bin.2.orig.fa_kl	OEZ008529	no	LMSG_G000010167.1	OES073718	ectoine	Others	Copper	5.51	China: Anhui, Tongling
L180330P-2-LD180327-0006_HL3VYCCXY_L8_clean_bin.2.orig.fa_kl	OEZ008529	no	LMSG_G000010167.1	OES073718	terpene	Terpene	Copper	21.71	China: Anhui, Tongling
L180330P-2-LD180327-0006_HL3VYCCXY_L8_clean_bin.2.orig.fa_kl	OEZ008529	yes	LMSG_G000010167.1	OES073718	terpene	Terpene	Copper	8.27	China: Anhui, Tongling
L180330P-2-LD180327-0006_HL3VYCCXY_L8_clean_bin.22.strict.fa	OEZ008529	yes	LMSG_G000004520.1	OES073718	ladderane	Others	Copper	41.26	China: Anhui, Tongling
L180330P-2-LD180327-0006_HL3VYCCXY_L8_clean_bin.22.strict.fa	OEZ008529	yes	LMSG_G000004520.1	OES073718	arylpolyene	Others	Copper	39.38	China: Anhui, Tongling
L180330P-2-LD180327-0006_HL3VYCCXY_L8_clean_bin.22.strict.fa	OEZ008529	yes	LMSG_G000004520.1	OES073718	NRPS-like.ectoine	Others	Copper	49.34	China: Anhui, Tongling
L180330P-2-LD180327-0006_HL3VYCCXY_L8_clean_bin.22.strict.fa	OEZ008529	yes	LMSG_G000004520.1	OES073718	terpene	Terpene	Copper	16.12	China: Anhui, Tongling
L180330P-2-LD180327-0006_HL3VYCCXY_L8_clean_bin.22.strict.fa	OEZ008529	yes	LMSG_G000004520.1	OES073718	bacteriocin	RiPPs	Copper	10.68	China: Anhui, Tongling
L180330P-2-LD180327-0006_HL3VYCCXY_L8_clean_bin.22.strict.fa	OEZ008529	yes	LMSG_G000004520.1	OES073718	terpene	Terpene	Copper	13.00	China: Anhui, Tongling
L180330P-2-LD180327-0006_HL3VYCCXY_L8_clean_bin.22.strict.fa	OEZ008529	yes	LMSG_G000004520.1	OES073718	arylpolyene	Others	Copper	18.13	China: Anhui, Tongling
L180330P-2-LD180327-0006_HL3VYCCXY_L8_clean_bin.22.strict.fa	OEZ008529	yes	LMSG_G000004520.1	OES073718	T3PKS	PKSother	Copper	13.78	China: Anhui, Tongling
L180330P-2-LD180327-0006_HL3VYCCXY_L8_clean_bin.25.orig.fa_k	OEZ008529	yes	LMSG_G000009199.1	OES073718	terpene	Terpene	Copper	11.94	China: Anhui, Tongling
L180330P-2-LD180327-0006_HL3VYCCXY_L8_clean_bin.25.orig.fa_k	OEZ008529	yes	LMSG_G000009199.1	OES073718	terpene	Terpene	Copper	12.27	China: Anhui, Tongling
L180330P-2-LD180327-0006_HL3VYCCXY_L8_clean_bin.25.orig.fa_k	OEZ008529	yes	LMSG_G000009199.1	OES073718	T3PKS	PKSother	Copper	11.59	China: Anhui, Tongling
L180330P-2-LD180327-0006_HL3VYCCXY_L8_clean_bin.26.orig.fa_k	OEZ008529	yes	LMSG_G000005617.1	OES073718	bacteriocin	RiPPs	Copper	9.34	China: Anhui, Tongling
L180330P-2-LD180327-0006_HL3VYCCXY_L8_clean_bin.26.orig.fa_k	OEZ008529	yes	LMSG_G000005617.1	OES073718	TfuA-related	RiPPs	Copper	5.77	China: Anhui, Tongling
L180330P-2-LD180327-0006_HL3VYCCXY_L8_clean_bin.30.orig.fa_k	OEZ008529	yes	LMSG_G000010492.1	OES073718	NRPS-like	NRPS	Copper	25.89	China: Anhui, Tongling
L180330P-2-LD180327-0006_HL3VYCCXY_L8_clean_bin.30.orig.fa_k	OEZ008529	yes	LMSG_G000010492.1	OES073718	arylpolyene.hserlactone	Others	Copper	11.66	China: Anhui, Tongling
L180330P-2-LD180327-0006_HL3VYCCXY_L8_clean_bin.31.orig.fa_k	OEZ008529	yes	LMSG_G000008452.1	OES073718	terpene	Terpene	Copper	7.25	China: Anhui, Tongling
L180330P-2-LD180327-0006_HL3VYCCXY_L8_clean_bin.34.orig.fa_k	OEZ008529	yes	LMSG_G000009145.1	OES073718	terpene	Terpene	Copper	18.03	China: Anhui, Tongling
L180330P-2-LD180327-0006_HL3VYCCXY_L8_clean_bin.34.orig.fa_k	OEZ008529	yes	LMSG_G000009145.1	OES073718	terpene	Terpene	Copper	9.46	China: Anhui, Tongling
L180330P-2-LD180327-0006_HL3VYCCXY_L8_clean_bin.34.orig.fa_k	OEZ008529	yes	LMSG_G000009145.1	OES073718	T3PKS	PKSother	Copper	29.62	China: Anhui, Tongling
L180330P-2-LD180327-0006_HL3VYCCXY_L8_clean_bin.35.orig.fa_k	OEZ008529	yes	LMSG_G000005619.1	OES073718	hglE-KS	PKSother	Copper	16.11	China: Anhui, Tongling
L180330P-2-LD180327-0006_HL3VYCCXY_L8_clean_bin.4.orig.fa_kl	OEZ008529	yes	LMSG_G000005620.1	OES073718	lassopeptide	RiPPs	Copper	12.03	China: Anhui, Tongling
L180330P-2-LD180327-0006_HL3VYCCXY_L8_clean_bin.4.orig.fa_kl	OEZ008529	yes	LMSG_G000005620.1	OES073718	betalactone	Others	Copper	29.55	China: Anhui, Tongling
L180330P-2-LD180327-0006_HL3VYCCXY_L8_clean_bin.4.orig.fa_kl	OEZ008529	yes	LMSG_G000005620.1	OES073718	T1PKS.hglE-KS	PKSother	Copper	19.45	China: Anhui, Tongling
L180330P-2-LD180327-0006_HL3VYCCXY_L8_clean_bin.4.orig.fa_kl	OEZ008529	yes	LMSG_G000005620.1	OES073718	other	Others	Copper	13.92	China: Anhui, Tongling
L180330P-2-LD180327-0006_HL3VYCCXY_L8_clean_bin.41.orig.fa_k	OEZ008529	yes	LMSG_G000007363.1	OES073718	bacteriocin	RiPPs	Copper	11.18	China: Anhui, Tongling
L180330P-2-LD180327-0006_HL3VYCCXY_L8_clean_bin.45.strict.fa	OEZ008529	yes	LMSG_G000004522.1	OES073718	TfuA-related.bacteriocin	RiPPs	Copper	14.79	China: Anhui, Tongling
L180330P-2-LD180327-0006_HL3VYCCXY_L8_clean_bin.46.orig.fa_k	OEZ008529	yes	LMSG_G000007853.1	OES073718	NRPS-like	NRPS	Copper	9.44	China: Anhui, Tongling
L180330P-2-LD180327-0006_HL3VYCCXY_L8_clean_bin.46.orig.fa_k	OEZ008529	yes	LMSG_G000007853.1	OES073718	terpene	Terpene	Copper	18.90	China: Anhui, Tongling
L180330P-2-LD180327-0006_HL3VYCCXY_L8_clean_bin.46.orig.fa_k	OEZ008529	yes	LMSG_G000007853.1	OES073718	terpene	Terpene	Copper	10.91	China: Anhui, Tongling
L180330P-2-LD180327-0006_HL3VYCCXY_L8_clean_bin.46.orig.fa_k	OEZ008529	yes	LMSG_G000007853.1	OES073718	T3PKS	PKSother	Copper	8.25	China: Anhui, Tongling
L180330P-2-LD180327-0006_HL3VYCCXY_L8_clean_bin.46.orig.fa_k	OEZ008529	yes	LMSG_G000007853.1	OES073718	NRPS-like	NRPS	Copper	7.18	China: Anhui, Tongling
L180330P-2-LD180327-0006_HL3VYCCXY_L8_clean_bin.47.orig.fa_k	OEZ008529	yes	LMSG_G000009420.1	OES073718	betalactone	Others	Copper	23.82	China: Anhui, Tongling
L180330P-2-LD180327-0006_HL3VYCCXY_L8_clean_bin.47.orig.fa_k	OEZ008529	yes	LMSG_G000009420.1	OES073718	ectoine	Others	Copper	9.96	China: Anhui, Tongling
L180330P-2-LD180327-0006_HL3VYCCXY_L8_clean_bin.47.orig.fa_k	OEZ008529	yes	LMSG_G000009420.1	OES073718	bacteriocin	RiPPs	Copper	9.17	China: Anhui, Tongling
L180330P-2-LD180327-0006_HL3VYCCXY_L8_clean_bin.47.orig.fa_k	OEZ008529	yes	LMSG_G000009420.1	OES073718	bacteriocin	RiPPs	Copper	5.49	China: Anhui, Tongling
L180330P-2-LD180327-0006_HL3VYCCXY_L8_clean_bin.47.orig.fa_k	OEZ008529	yes	LMSG_G000009420.1	OES073718	betalactone	Others	Copper	22.76	China: Anhui, Tongling
L180330P-2-LD180327-0006_HL3VYCCXY_L8_clean_bin.47.orig.fa_k	OEZ008529	yes	LMSG_G000009420.1	OES073718	bacteriocin	RiPPs	Copper	10.58	China: Anhui, Tongling
L180330P-2-LD180327-0006_HL3VYCCXY_L8_clean_bin.51.orig.fa_k	OEZ008529	yes	LMSG_G000009480.1	OES073718	betalactone	Others	Copper	28.44	China: Anhui, Tongling
L180330P-2-LD180327-0006_HL3VYCCXY_L8_clean_bin.52.orig.fa_k	OEZ008529	yes	LMSG_G000009389.1	OES073718	terpene	Terpene	Copper	13.39	China: Anhui, Tongling
L180330P-2-LD180327-0006_HL3VYCCXY_L8_clean_bin.54.orig.fa_k	OEZ008529	yes	LMSG_G000008402.1	OES073718	terpene	Terpene	Copper	26.09	China: Anhui, Tongling
L180330P-2-LD180327-0006_HL3VYCCXY_L8_clean_bin.55.orig.fa_k	OEZ008529	yes	LMSG_G000008080.1	OES073718	other	Others	Copper	10.28	China: Anhui, Tongling
L180330P-2-LD180327-0006_HL3VYCCXY_L8_clean_bin.55.orig.fa_k	OEZ008529	yes	LMSG_G000008080.1	OES073718	T1PKS.hglE-KS	PKSother	Copper	33.98	China: Anhui, Tongling
L180330P-2-LD180327-0006_HL3VYCCXY_L8_clean_bin.56.orig.fa_k	OEZ008529	yes	LMSG_G000010480.1	OES073718	arylpolyene.hserlactone	Others	Copper	41.23	China: Anhui, Tongling
L180330P-2-LD180327-0006_HL3VYCCXY_L8_clean_bin.56.orig.fa_k	OEZ008529	yes	LMSG_G000010480.1	OES073718	terpene	Terpene	Copper	23.04	China: Anhui, Tongling
L180330P-2-LD180327-0006_HL3VYCCXY_L8_clean_bin.58.orig.fa_k	OEZ008529	yes	LMSG_G000005621.1	OES073718	betalactone	Others	Copper	29.70	China: Anhui, Tongling
L180330P-2-LD180327-0006_HL3VYCCXY_L8_clean_bin.58.orig.fa_k	OEZ008529	yes	LMSG_G000005621.1	OES073718	betalactone	Others	Copper	22.76	China: Anhui, Tongling
L180330P-2-LD180327-0006_HL3VYCCXY_L8_clean_bin.59.orig.fa_k	OEZ008529	yes	LMSG_G000006289.1	OES073718	betalactone	Others	Copper	33.89	China: Anhui, Tongling
L180330P-2-LD180327-0006_HL3VYCCXY_L8_clean_bin.59.orig.fa_k	OEZ008529	yes	LMSG_G000006289.1	OES073718	terpene	Terpene	Copper	16.34	China: Anhui, Tongling
L180330P-2-LD180327-0006_HL3VYCCXY_L8_clean_bin.61.orig.fa_k	OEZ008529	yes	LMSG_G000005622.1	OES073718	terpene	Terpene	Copper	6.52	China: Anhui, Tongling
L180330P-2-LD180327-0006_HL3VYCCXY_L8_clean_bin.61.orig.fa_k	OEZ008529	yes	LMSG_G000005622.1	OES073718	bacteriocin	RiPPs	Copper	6.35	China: Anhui, Tongling
L180330P-2-LD180327-0006_HL3VYCCXY_L8_clean_bin.63.orig.fa_k	OEZ008529	yes	LMSG_G000009364.1	OES073718	betalactone	Others	Copper	32.65	China: Anhui, Tongling
L180330P-2-LD180327-0006_HL3VYCCXY_L8_clean_bin.69.orig.fa_k	OEZ008529	yes	LMSG_G000005624.1	OES073718	terpene	Terpene	Copper	6.65	China: Anhui, Tongling
L180330P-2-LD180327-0006_HL3VYCCXY_L8_clean_bin.69.orig.fa_k	OEZ008529	yes	LMSG_G000005624.1	OES073718	NRPS-like	NRPS	Copper	12.36	China: Anhui, Tongling
L180330P-2-LD180327-0006_HL3VYCCXY_L8_clean_bin.70.orig.fa_k	OEZ008529	yes	LMSG_G000008258.1	OES073718	terpene	Terpene	Copper	19.11	China: Anhui, Tongling
L180330P-2-LD180327-0006_HL3VYCCXY_L8_clean_bin.72.orig.fa_k	OEZ008529	no	LMSG_G000010305.1	OES073718	arylpolyene	Others	Copper	23.35	China: Anhui, Tongling
L180330P-2-LD180327-0006_HL3VYCCXY_L8_clean_bin.72.orig.fa_k	OEZ008529	no	LMSG_G000010305.1	OES073718	terpene	Terpene	Copper	22.56	China: Anhui, Tongling
L180330P-2-LD180327-0006_HL3VYCCXY_L8_clean_bin.74.permissiv	OEZ008529	yes	LMSG_G000008000.1	OES073718	bacteriocin	RiPPs	Copper	10.32	China: Anhui, Tongling
L180330P-2-LD180327-0006_HL3VYCCXY_L8_clean_bin.74.permissiv	OEZ008529	yes	LMSG_G000008000.1	OES073718	TfuA-related	RiPPs	Copper	13.21	China: Anhui, Tongling
L180330P-2-LD180327-0006_HL3VYCCXY_L8_clean_bin.8.orig.fa_kl	OEZ008529	yes	LMSG_G000010281.1	OES073718	arylpolyene	Others	Copper	39.69	China: Anhui, Tongling
L180330P-2-LD180327-0006_HL3VYCCXY_L8_clean_bin.80.strict.fa	OEZ008529	yes	LMSG_G000005626.1	OES073718	terpene	Terpene	Copper	23.68	China: Anhui, Tongling
L180330P-2-LD180327-0006_HL3VYCCXY_L8_clean_bin.81.orig.fa_k	OEZ008529	yes	LMSG_G000006243.1	OES073718	bacteriocin	RiPPs	Copper	10.77	China: Anhui, Tongling
L180330P-2-LD180327-0006_HL3VYCCXY_L8_clean_bin.82.orig.fa_k	OEZ008529	yes	LMSG_G000008490.1	OES073718	terpene	Terpene	Copper	20.48	China: Anhui, Tongling
L180330P-2-LD180327-0006_HL3VYCCXY_L8_clean_bin.84.orig.fa_k	OEZ008529	yes	LMSG_G000009358.1	OES073718	betalactone	Others	Copper	25.56	China: Anhui, Tongling
L180330P-2-LD180327-0006_HL3VYCCXY_L8_clean_bin.85.strict.fa	OEZ008529	yes	LMSG_G000008232.1	OES073718	terpene	Terpene	Copper	18.61	China: Anhui, Tongling
L180330P-2-LD180327-0006_HL3VYCCXY_L8_clean_bin.89.strict.fa	OEZ008529	yes	LMSG_G000010015.1	OES073718	ladderane	Others	Copper	10.71	China: Anhui, Tongling
L180330P-2-LD180327-0006_HL3VYCCXY_L8_clean_bin.89.strict.fa	OEZ008529	yes	LMSG_G000010015.1	OES073718	terpene	Terpene	Copper	7.20	China: Anhui, Tongling
L180330P-2-LD180327-0006_HL3VYCCXY_L8_clean_bin.91.orig.fa_k	OEZ008529	yes	LMSG_G000009004.1	OES073718	terpene	Terpene	Copper	20.91	China: Anhui, Tongling
L180330P-2-LD180327-0006_HL3VYCCXY_L8_clean_bin.91.orig.fa_k	OEZ008529	yes	LMSG_G000009004.1	OES073718	bacteriocin	RiPPs	Copper	10.80	China: Anhui, Tongling
L180330P-2-LD180327-0006_HL3VYCCXY_L8_clean_bin.93.orig.fa_k	OEZ008529	yes	LMSG_G000010243.1	OES073718	terpene	Terpene	Copper	14.81	China: Anhui, Tongling
L180330P-2-LD180327-0006_HL3VYCCXY_L8_clean_bin.93.orig.fa_k	OEZ008529	yes	LMSG_G000010243.1	OES073718	terpene	Terpene	Copper	13.07	China: Anhui, Tongling
L180330P-2-LD180327-0006_HL3VYCCXY_L8_clean_bin.93.orig.fa_k	OEZ008529	yes	LMSG_G000010243.1	OES073718	arylpolyene	Others	Copper	14.59	China: Anhui, Tongling
L180330P-2-LD180327-0006_HL3VYCCXY_L8_clean_bin.93.orig.fa_k	OEZ008529	yes	LMSG_G000010243.1	OES073718	bacteriocin	RiPPs	Copper	7.35	China: Anhui, Tongling
L180330P-2-LD180327-0006_HL3VYCCXY_L8_clean_bin.94.orig.fa_k	OEZ008529	yes	LMSG_G000010230.1	OES073718	hserlactone	Others	Copper	20.72	China: Anhui, Tongling
L180330P-2-LD180327-0006_HL3VYCCXY_L8_clean_bin.94.orig.fa_k	OEZ008529	yes	LMSG_G000010230.1	OES073718	arylpolyene	Others	Copper	23.37	China: Anhui, Tongling
L180330P-2-LD180327-0006_HL3VYCCXY_L8_clean_bin.94.orig.fa_k	OEZ008529	yes	LMSG_G000010230.1	OES073718	terpene	Terpene	Copper	17.50	China: Anhui, Tongling
L180330P-2-LD180327-0006_HL3VYCCXY_L8_clean_bin.98.orig.fa_k	OEZ008529	yes	LMSG_G000008680.1	OES073718	ectoine	Others	Copper	10.40	China: Anhui, Tongling
L180330P-2-LD180327-0006_HL3VYCCXY_L8_clean_bin.98.orig.fa_k	OEZ008529	yes	LMSG_G000008680.1	OES073718	betalactone	Others	Copper	21.97	China: Anhui, Tongling
L180330P-2-LD180327-0006_HL3VYCCXY_L8_clean_bin.98.orig.fa_k	OEZ008529	yes	LMSG_G000008680.1	OES073718	bacteriocin	RiPPs	Copper	10.81	China: Anhui, Tongling
L180330P-2-LD180327-0006_HL3VYCCXY_L8_clean_bin.98.orig.fa_k	OEZ008529	yes	LMSG_G000008680.1	OES073718	betalactone	Others	Copper	25.91	China: Anhui, Tongling
L180330P-3-LD180327-0007_HL3H2CCXY_L1_clean_bin.10.orig.fa_k	OEZ008529	yes	LMSG_G000009161.1	OES073725	terpene	Terpene	Polymetallic	20.58	China: Anhui, Wuhu
L180330P-3-LD180327-0007_HL3H2CCXY_L1_clean_bin.10.orig.fa_k	OEZ008529	yes	LMSG_G000009161.1	OES073725	T3PKS	PKSother	Polymetallic	12.72	China: Anhui, Wuhu
L180330P-3-LD180327-0007_HL3H2CCXY_L1_clean_bin.100.orig.fa_	OEZ008529	yes	LMSG_G000010680.1	OES073725	betalactone	Others	Polymetallic	23.62	China: Anhui, Wuhu
L180330P-3-LD180327-0007_HL3H2CCXY_L1_clean_bin.100.orig.fa_	OEZ008529	yes	LMSG_G000010680.1	OES073725	arylpolyene	Others	Polymetallic	8.32	China: Anhui, Wuhu
L180330P-3-LD180327-0007_HL3H2CCXY_L1_clean_bin.100.orig.fa_	OEZ008529	yes	LMSG_G000010680.1	OES073725	NRPS-like	NRPS	Polymetallic	21.91	China: Anhui, Wuhu
L180330P-3-LD180327-0007_HL3H2CCXY_L1_clean_bin.100.orig.fa_	OEZ008529	no	LMSG_G000010680.1	OES073725	arylpolyene	Others	Polymetallic	8.65	China: Anhui, Wuhu
L180330P-3-LD180327-0007_HL3H2CCXY_L1_clean_bin.101.orig.fa_	OEZ008529	yes	LMSG_G000009595.1	OES073725	NRPS-like	NRPS	Polymetallic	12.39	China: Anhui, Wuhu
L180330P-3-LD180327-0007_HL3H2CCXY_L1_clean_bin.103.orig.fa_	OEZ008529	yes	LMSG_G000011109.1	OES073725	terpene	Terpene	Polymetallic	18.50	China: Anhui, Wuhu
L180330P-3-LD180327-0007_HL3H2CCXY_L1_clean_bin.104.orig.fa_	OEZ008529	yes	LMSG_G000008678.1	OES073725	terpene	Terpene	Polymetallic	24.15	China: Anhui, Wuhu
L180330P-3-LD180327-0007_HL3H2CCXY_L1_clean_bin.104.orig.fa_	OEZ008529	yes	LMSG_G000008678.1	OES073725	T1PKS	PKSI	Polymetallic	16.55	China: Anhui, Wuhu
L180330P-3-LD180327-0007_HL3H2CCXY_L1_clean_bin.104.orig.fa_	OEZ008529	yes	LMSG_G000008678.1	OES073725	T1PKS	PKSI	Polymetallic	15.36	China: Anhui, Wuhu
L180330P-3-LD180327-0007_HL3H2CCXY_L1_clean_bin.106.orig.fa_	OEZ008529	yes	LMSG_G000008181.1	OES073725	terpene	Terpene	Polymetallic	21.08	China: Anhui, Wuhu
L180330P-3-LD180327-0007_HL3H2CCXY_L1_clean_bin.108.orig.fa_	OEZ008529	yes	LMSG_G000005628.1	OES073725	terpene	Terpene	Polymetallic	5.58	China: Anhui, Wuhu
L180330P-3-LD180327-0007_HL3H2CCXY_L1_clean_bin.108.orig.fa_	OEZ008529	yes	LMSG_G000005628.1	OES073725	T3PKS	PKSother	Polymetallic	15.36	China: Anhui, Wuhu
L180330P-3-LD180327-0007_HL3H2CCXY_L1_clean_bin.109.orig.fa_	OEZ008529	yes	LMSG_G000008519.1	OES073725	terpene	Terpene	Polymetallic	25.31	China: Anhui, Wuhu
L180330P-3-LD180327-0007_HL3H2CCXY_L1_clean_bin.112.orig.fa_	OEZ008529	yes	LMSG_G000005629.1	OES073725	betalactone	Others	Polymetallic	23.72	China: Anhui, Wuhu
L180330P-3-LD180327-0007_HL3H2CCXY_L1_clean_bin.116.orig.fa_	OEZ008529	yes	LMSG_G000005630.1	OES073725	T3PKS	PKSother	Polymetallic	5.58	China: Anhui, Wuhu
L180330P-3-LD180327-0007_HL3H2CCXY_L1_clean_bin.116.orig.fa_	OEZ008529	yes	LMSG_G000005630.1	OES073725	terpene	Terpene	Polymetallic	15.13	China: Anhui, Wuhu
L180330P-3-LD180327-0007_HL3H2CCXY_L1_clean_bin.117.orig.fa_	OEZ008529	yes	LMSG_G000010057.1	OES073725	terpene	Terpene	Polymetallic	13.64	China: Anhui, Wuhu
L180330P-3-LD180327-0007_HL3H2CCXY_L1_clean_bin.117.orig.fa_	OEZ008529	yes	LMSG_G000010057.1	OES073725	NRPS-like.ectoine	Others	Polymetallic	39.38	China: Anhui, Wuhu
L180330P-3-LD180327-0007_HL3H2CCXY_L1_clean_bin.117.orig.fa_	OEZ008529	yes	LMSG_G000010057.1	OES073725	bacteriocin	RiPPs	Polymetallic	10.67	China: Anhui, Wuhu
L180330P-3-LD180327-0007_HL3H2CCXY_L1_clean_bin.117.orig.fa_	OEZ008529	yes	LMSG_G000010057.1	OES073725	terpene	Terpene	Polymetallic	5.93	China: Anhui, Wuhu
L180330P-3-LD180327-0007_HL3H2CCXY_L1_clean_bin.117.orig.fa_	OEZ008529	yes	LMSG_G000010057.1	OES073725	ladderane	Others	Polymetallic	28.68	China: Anhui, Wuhu
L180330P-3-LD180327-0007_HL3H2CCXY_L1_clean_bin.119.strict.f	OEZ008529	yes	LMSG_G000005631.1	OES073725	terpene	Terpene	Polymetallic	13.79	China: Anhui, Wuhu
L180330P-3-LD180327-0007_HL3H2CCXY_L1_clean_bin.119.strict.f	OEZ008529	yes	LMSG_G000005631.1	OES073725	arylpolyene	Others	Polymetallic	6.88	China: Anhui, Wuhu
L180330P-3-LD180327-0007_HL3H2CCXY_L1_clean_bin.119.strict.f	OEZ008529	yes	LMSG_G000005631.1	OES073725	terpene	Terpene	Polymetallic	6.67	China: Anhui, Wuhu
L180330P-3-LD180327-0007_HL3H2CCXY_L1_clean_bin.119.strict.f	OEZ008529	yes	LMSG_G000005631.1	OES073725	NRPS-like	NRPS	Polymetallic	6.60	China: Anhui, Wuhu
L180330P-3-LD180327-0007_HL3H2CCXY_L1_clean_bin.119.strict.f	OEZ008529	yes	LMSG_G000005631.1	OES073725	arylpolyene	Others	Polymetallic	6.50	China: Anhui, Wuhu
L180330P-3-LD180327-0007_HL3H2CCXY_L1_clean_bin.120.orig.fa_	OEZ008529	yes	LMSG_G000008020.1	OES073725	betalactone	Others	Polymetallic	23.60	China: Anhui, Wuhu
L180330P-3-LD180327-0007_HL3H2CCXY_L1_clean_bin.120.orig.fa_	OEZ008529	yes	LMSG_G000008020.1	OES073725	betalactone	Others	Polymetallic	5.98	China: Anhui, Wuhu

L180330P-3-LD180327-0007_HL3H2CCXY_L1_clean_bin.120.orig.fa_	OEZ008529	yes	LMSG_G000008020.1	OES073725	bacteriocin	RiPPs	Polymetallic	10.30	China: Anhui, Wuhu
L180330P-3-LD180327-0007_HL3H2CCXY_L1_clean_bin.124.orig.fa_	OEZ008529	yes	LMSG_G000005632.1	OES073725	NRPS-like	NRPS	Polymetallic	26.97	China: Anhui, Wuhu
L180330P-3-LD180327-0007_HL3H2CCXY_L1_clean_bin.124.orig.fa_	OEZ008529	yes	LMSG_G000005632.1	OES073725	acyl_amino_acids	Others	Polymetallic	43.29	China: Anhui, Wuhu
L180330P-3-LD180327-0007_HL3H2CCXY_L1_clean_bin.124.orig.fa_	OEZ008529	yes	LMSG_G000005632.1	OES073725	bacteriocin	RiPPs	Polymetallic	8.26	China: Anhui, Wuhu
L180330P-3-LD180327-0007_HL3H2CCXY_L1_clean_bin.124.orig.fa_	OEZ008529	yes	LMSG_G000005632.1	OES073725	bacteriocin	RiPPs	Polymetallic	10.58	China: Anhui, Wuhu
L180330P-3-LD180327-0007_HL3H2CCXY_L1_clean_bin.125.orig.fa_	OEZ008529	yes	LMSG_G000009481.1	OES073725	TfuA-related	RiPPs	Polymetallic	5.02	China: Anhui, Wuhu
L180330P-3-LD180327-0007_HL3H2CCXY_L1_clean_bin.125.orig.fa_	OEZ008529	yes	LMSG_G000009481.1	OES073725	betalactone	Others	Polymetallic	28.44	China: Anhui, Wuhu
L180330P-3-LD180327-0007_HL3H2CCXY_L1_clean_bin.126.orig.fa_	OEZ008529	yes	LMSG_G000009350.1	OES073725	betalactone	Others	Polymetallic	12.00	China: Anhui, Wuhu
L180330P-3-LD180327-0007_HL3H2CCXY_L1_clean_bin.15.orig.fa_k	OEZ008529	yes	LMSG_G000005633.1	OES073725	betalactone	Others	Polymetallic	25.56	China: Anhui, Wuhu
L180330P-3-LD180327-0007_HL3H2CCXY_L1_clean_bin.17.orig.fa_k	OEZ008529	yes	LMSG_G000009613.1	OES073725	terpene	Terpene	Polymetallic	5.91	China: Anhui, Wuhu
L180330P-3-LD180327-0007_HL3H2CCXY_L1_clean_bin.17.orig.fa_k	OEZ008529	yes	LMSG_G000009613.1	OES073725	terpene	Terpene	Polymetallic	21.99	China: Anhui, Wuhu
L180330P-3-LD180327-0007_HL3H2CCXY_L1_clean_bin.17.orig.fa_k	OEZ008529	yes	LMSG_G000009613.1	OES073725	NRPS-like	NRPS	Polymetallic	29.28	China: Anhui, Wuhu
L180330P-3-LD180327-0007_HL3H2CCXY_L1_clean_bin.19.orig.fa_k	OEZ008529	yes	LMSG_G000009171.1	OES073725	NRPS-like	NRPS	Polymetallic	21.18	China: Anhui, Wuhu
L180330P-3-LD180327-0007_HL3H2CCXY_L1_clean_bin.19.orig.fa_k	OEZ008529	yes	LMSG_G000009171.1	OES073725	terpene	Terpene	Polymetallic	23.70	China: Anhui, Wuhu
L180330P-3-LD180327-0007_HL3H2CCXY_L1_clean_bin.2.orig.fa_k1	OEZ008529	yes	LMSG_G000008032.1	OES073725	thiopeptide	RiPPs	Polymetallic	8.72	China: Anhui, Wuhu
L180330P-3-LD180327-0007_HL3H2CCXY_L1_clean_bin.2.orig.fa_k1	OEZ008529	yes	LMSG_G000008032.1	OES073725	lassopeptide	RiPPs	Polymetallic	9.53	China: Anhui, Wuhu
L180330P-3-LD180327-0007_HL3H2CCXY_L1_clean_bin.25.orig.fa_k	OEZ008529	yes	LMSG_G000009289.1	OES073725	terpene	Terpene	Polymetallic	21.73	China: Anhui, Wuhu
L180330P-3-LD180327-0007_HL3H2CCXY_L1_clean_bin.25.orig.fa_k	OEZ008529	yes	LMSG_G000009289.1	OES073725	terpene	Terpene	Polymetallic	22.06	China: Anhui, Wuhu
L180330P-3-LD180327-0007_HL3H2CCXY_L1_clean_bin.28.orig.fa_k	OEZ008529	no	LMSG_G000010499.1	OES073725	arylpolyene	Others	Polymetallic	6.73	China: Anhui, Wuhu
L180330P-3-LD180327-0007_HL3H2CCXY_L1_clean_bin.28.orig.fa_k	OEZ008529	yes	LMSG_G000010499.1	OES073725	hserlactone	Others	Polymetallic	9.31	China: Anhui, Wuhu
L180330P-3-LD180327-0007_HL3H2CCXY_L1_clean_bin.3.orig.fa_k1	OEZ008529	yes	LMSG_G000005635.1	OES073725	other	Others	Polymetallic	23.63	China: Anhui, Wuhu
L180330P-3-LD180327-0007_HL3H2CCXY_L1_clean_bin.3.orig.fa_k1	OEZ008529	yes	LMSG_G000005635.1	OES073725	other	Others	Polymetallic	11.49	China: Anhui, Wuhu
L180330P-3-LD180327-0007_HL3H2CCXY_L1_clean_bin.3.orig.fa_k1	OEZ008529	yes	LMSG_G000005635.1	OES073725	other	Others	Polymetallic	14.22	China: Anhui, Wuhu
L180330P-3-LD180327-0007_HL3H2CCXY_L1_clean_bin.30.orig.fa_k	OEZ008529	no	LMSG_G000008525.1	OES073725	hglE-KS	PKSother	Polymetallic	6.14	China: Anhui, Wuhu
L180330P-3-LD180327-0007_HL3H2CCXY_L1_clean_bin.32.orig.fa_k	OEZ008529	yes	LMSG_G000008545.1	OES073725	betalactone	Others	Polymetallic	29.53	China: Anhui, Wuhu
L180330P-3-LD180327-0007_HL3H2CCXY_L1_clean_bin.34.orig.fa_k	OEZ008529	yes	LMSG_G000009194.1	OES073725	betalactone	Others	Polymetallic	16.26	China: Anhui, Wuhu
L180330P-3-LD180327-0007_HL3H2CCXY_L1_clean_bin.34.orig.fa_k	OEZ008529	yes	LMSG_G000009194.1	OES073725	terpene	Terpene	Polymetallic	10.90	China: Anhui, Wuhu
L180330P-3-LD180327-0007_HL3H2CCXY_L1_clean_bin.34.orig.fa_k	OEZ008529	yes	LMSG_G000009194.1	OES073725	terpene	Terpene	Polymetallic	11.73	China: Anhui, Wuhu
L180330P-3-LD180327-0007_HL3H2CCXY_L1_clean_bin.35.orig.fa_k	OEZ008529	yes	LMSG_G000009387.1	OES073725	terpene	Terpene	Polymetallic	13.56	China: Anhui, Wuhu
L180330P-3-LD180327-0007_HL3H2CCXY_L1_clean_bin.35.orig.fa_k	OEZ008529	yes	LMSG_G000009387.1	OES073725	bacteriocin	RiPPs	Polymetallic	8.03	China: Anhui, Wuhu
L180330P-3-LD180327-0007_HL3H2CCXY_L1_clean_bin.36.orig.fa_k	OEZ008529	yes	LMSG_G000008443.1	OES073725	terpene	Terpene	Polymetallic	26.50	China: Anhui, Wuhu
L180330P-3-LD180327-0007_HL3H2CCXY_L1_clean_bin.38.orig.fa_k	OEZ008529	yes	LMSG_G000007857.1	OES073725	NRPS-like	NRPS	Polymetallic	27.06	China: Anhui, Wuhu
L180330P-3-LD180327-0007_HL3H2CCXY_L1_clean_bin.38.orig.fa_k	OEZ008529	yes	LMSG_G000007857.1	OES073725	NRPS-like	NRPS	Polymetallic	24.28	China: Anhui, Wuhu
L180330P-3-LD180327-0007_HL3H2CCXY_L1_clean_bin.38.orig.fa_k	OEZ008529	yes	LMSG_G000007857.1	OES073725	T3PKS	PKSother	Polymetallic	22.52	China: Anhui, Wuhu
L180330P-3-LD180327-0007_HL3H2CCXY_L1_clean_bin.38.orig.fa_k	OEZ008529	yes	LMSG_G000007857.1	OES073725	terpene	Terpene	Polymetallic	20.00	China: Anhui, Wuhu
L180330P-3-LD180327-0007_HL3H2CCXY_L1_clean_bin.38.orig.fa_k	OEZ008529	yes	LMSG_G000007857.1	OES073725	terpene	Terpene	Polymetallic	21.94	China: Anhui, Wuhu
L180330P-3-LD180327-0007_HL3H2CCXY_L1_clean_bin.39.strict.fa	OEZ008529	yes	LMSG_G000007745.1	OES073725	terpene	Terpene	Polymetallic	22.50	China: Anhui, Wuhu
L180330P-3-LD180327-0007_HL3H2CCXY_L1_clean_bin.39.strict.fa	OEZ008529	yes	LMSG_G000007745.1	OES073725	bacteriocin	RiPPs	Polymetallic	7.08	China: Anhui, Wuhu
L180330P-3-LD180327-0007_HL3H2CCXY_L1_clean_bin.39.strict.fa	OEZ008529	yes	LMSG_G000007745.1	OES073725	T3PKS	PKSother	Polymetallic	20.41	China: Anhui, Wuhu
L180330P-3-LD180327-0007_HL3H2CCXY_L1_clean_bin.4.orig.fa_k1	OEZ008529	yes	LMSG_G000007898.1	OES073725	NRPS	NRPS	Polymetallic	10.39	China: Anhui, Wuhu
L180330P-3-LD180327-0007_HL3H2CCXY_L1_clean_bin.4.orig.fa_k1	OEZ008529	yes	LMSG_G000007898.1	OES073725	NRPS-like	NRPS	Polymetallic	15.09	China: Anhui, Wuhu
L180330P-3-LD180327-0007_HL3H2CCXY_L1_clean_bin.4.orig.fa_k1	OEZ008529	yes	LMSG_G000007898.1	OES073725	NRPS-like	NRPS	Polymetallic	20.37	China: Anhui, Wuhu
L180330P-3-LD180327-0007_HL3H2CCXY_L1_clean_bin.40.permissiv	OEZ008529	yes	LMSG_G000008333.1	OES073725	terpene	Terpene	Polymetallic	23.10	China: Anhui, Wuhu
L180330P-3-LD180327-0007_HL3H2CCXY_L1_clean_bin.42.orig.fa_k	OEZ008529	yes	LMSG_G000007822.1	OES073725	terpene	Terpene	Polymetallic	29.81	China: Anhui, Wuhu
L180330P-3-LD180327-0007_HL3H2CCXY_L1_clean_bin.42.orig.fa_k	OEZ008529	yes	LMSG_G000007822.1	OES073725	T3PKS	PKSother	Polymetallic	32.47	China: Anhui, Wuhu
L180330P-3-LD180327-0007_HL3H2CCXY_L1_clean_bin.44.orig.fa_k	OEZ008529	yes	LMSG_G000008066.1	OES073725	terpene	Terpene	Polymetallic	10.53	China: Anhui, Wuhu
L180330P-3-LD180327-0007_HL3H2CCXY_L1_clean_bin.5.orig.fa_k1	OEZ008529	yes	LMSG_G000005638.1	OES073725	terpene	Terpene	Polymetallic	10.06	China: Anhui, Wuhu
L180330P-3-LD180327-0007_HL3H2CCXY_L1_clean_bin.54.permissiv	OEZ008529	yes	LMSG_G000008324.1	OES073725	terpene	Terpene	Polymetallic	21.09	China: Anhui, Wuhu
L180330P-3-LD180327-0007_HL3H2CCXY_L1_clean_bin.56.orig.fa_k	OEZ008529	yes	LMSG_G000005637.1	OES073725	betalactone	Others	Polymetallic	17.78	China: Anhui, Wuhu
L180330P-3-LD180327-0007_HL3H2CCXY_L1_clean_bin.56.orig.fa_k	OEZ008529	yes	LMSG_G000005637.1	OES073725	NRPS-like	NRPS	Polymetallic	22.63	China: Anhui, Wuhu
L180330P-3-LD180327-0007_HL3H2CCXY_L1_clean_bin.56.orig.fa_k	OEZ008529	yes	LMSG_G000005637.1	OES073725	other	Others	Polymetallic	11.24	China: Anhui, Wuhu
L180330P-3-LD180327-0007_HL3H2CCXY_L1_clean_bin.56.orig.fa_k	OEZ008529	yes	LMSG_G000005637.1	OES073725	terpene	Terpene	Polymetallic	6.48	China: Anhui, Wuhu
L180330P-3-LD180327-0007_HL3H2CCXY_L1_clean_bin.56.orig.fa_k	OEZ008529	yes	LMSG_G000005637.1	OES073725	terpene	Terpene	Polymetallic	8.38	China: Anhui, Wuhu
L180330P-3-LD180327-0007_HL3H2CCXY_L1_clean_bin.58.orig.fa_k	OEZ008529	yes	LMSG_G000007364.1	OES073725	bacteriocin	RiPPs	Polymetallic	11.97	China: Anhui, Wuhu
L180330P-3-LD180327-0007_HL3H2CCXY_L1_clean_bin.6.orig.fa_k1	OEZ008529	yes	LMSG_G000008397.1	OES073725	terpene	Terpene	Polymetallic	21.91	China: Anhui, Wuhu
L180330P-3-LD180327-0007_HL3H2CCXY_L1_clean_bin.64.orig.fa_k	OEZ008529	yes	LMSG_G000007832.1	OES073725	terpene	Terpene	Polymetallic	22.05	China: Anhui, Wuhu
L180330P-3-LD180327-0007_HL3H2CCXY_L1_clean_bin.64.orig.fa_k	OEZ008529	yes	LMSG_G000007832.1	OES073725	NRPS-like	NRPS	Polymetallic	23.86	China: Anhui, Wuhu
L180330P-3-LD180327-0007_HL3H2CCXY_L1_clean_bin.64.orig.fa_k	OEZ008529	yes	LMSG_G000007832.1	OES073725	bacteriocin	RiPPs	Polymetallic	10.88	China: Anhui, Wuhu
L180330P-3-LD180327-0007_HL3H2CCXY_L1_clean_bin.64.orig.fa_k	OEZ008529	yes	LMSG_G000007832.1	OES073725	terpene	Terpene	Polymetallic	18.94	China: Anhui, Wuhu
L180330P-3-LD180327-0007_HL3H2CCXY_L1_clean_bin.65.orig.fa_k	OEZ008529	yes	LMSG_G000008681.1	OES073725	ectoine	Others	Polymetallic	10.40	China: Anhui, Wuhu
L180330P-3-LD180327-0007_HL3H2CCXY_L1_clean_bin.65.orig.fa_k	OEZ008529	yes	LMSG_G000008681.1	OES073725	betalactone	Others	Polymetallic	25.93	China: Anhui, Wuhu
L180330P-3-LD180327-0007_HL3H2CCXY_L1_clean_bin.65.orig.fa_k	OEZ008529	yes	LMSG_G000008681.1	OES073725	terpene	Terpene	Polymetallic	21.87	China: Anhui, Wuhu
L180330P-3-LD180327-0007_HL3H2CCXY_L1_clean_bin.65.orig.fa_k	OEZ008529	yes	LMSG_G000008681.1	OES073725	bacteriocin	RiPPs	Polymetallic	10.81	China: Anhui, Wuhu
L180330P-3-LD180327-0007_HL3H2CCXY_L1_clean_bin.66.strict.fa	OEZ008529	yes	LMSG_G000008121.1	OES073725	terpene	Terpene	Polymetallic	20.50	China: Anhui, Wuhu
L180330P-3-LD180327-0007_HL3H2CCXY_L1_clean_bin.67.orig.fa_k	OEZ008529	yes	LMSG_G000005639.1	OES073725	indole	Others	Polymetallic	12.11	China: Anhui, Wuhu
L180330P-3-LD180327-0007_HL3H2CCXY_L1_clean_bin.69.permissiv	OEZ008529	yes	LMSG_G000009923.1	OES073725	nucleoside	Others	Polymetallic	13.08	China: Anhui, Wuhu
L180330P-3-LD180327-0007_HL3H2CCXY_L1_clean_bin.7.orig.fa_k1	OEZ008529	no	LMSG_G000010566.1	OES073725	bacteriocin	RiPPs	Polymetallic	5.99	China: Anhui, Wuhu
L180330P-3-LD180327-0007_HL3H2CCXY_L1_clean_bin.7.orig.fa_k1	OEZ008529	no	LMSG_G000010566.1	OES073725	terpene	Terpene	Polymetallic	15.65	China: Anhui, Wuhu
L180330P-3-LD180327-0007_HL3H2CCXY_L1_clean_bin.7.orig.fa_k1	OEZ008529	no	LMSG_G000010566.1	OES073725	T1PKS	PKSI	Polymetallic	12.46	China: Anhui, Wuhu
L180330P-3-LD180327-0007_HL3H2CCXY_L1_clean_bin.71.strict.fa	OEZ008529	yes	LMSG_G000009933.1	OES073725	hserlactone	Others	Polymetallic	20.78	China: Anhui, Wuhu
L180330P-3-LD180327-0007_HL3H2CCXY_L1_clean_bin.74.strict.fa	OEZ008529	yes	LMSG_G000009398.1	OES073725	terpene	Terpene	Polymetallic	21.75	China: Anhui, Wuhu
L180330P-3-LD180327-0007_HL3H2CCXY_L1_clean_bin.74.strict.fa	OEZ008529	yes	LMSG_G000009398.1	OES073725	NRPS-like	NRPS	Polymetallic	22.71	China: Anhui, Wuhu
L180330P-3-LD180327-0007_HL3H2CCXY_L1_clean_bin.74.strict.fa	OEZ008529	yes	LMSG_G000009398.1	OES073725	NRPS	NRPS	Polymetallic	23.84	China: Anhui, Wuhu
L180330P-3-LD180327-0007_HL3H2CCXY_L1_clean_bin.76.orig.fa_k	OEZ008529	yes	LMSG_G000008339.1	OES073725	bacteriocin	RiPPs	Polymetallic	6.25	China: Anhui, Wuhu
L180330P-3-LD180327-0007_HL3H2CCXY_L1_clean_bin.76.orig.fa_k	OEZ008529	yes	LMSG_G000008339.1	OES073725	other	Others	Polymetallic	5.33	China: Anhui, Wuhu
L180330P-3-LD180327-0007_HL3H2CCXY_L1_clean_bin.76.orig.fa_k	OEZ008529	yes	LMSG_G000008339.1	OES073725	terpene	Terpene	Polymetallic	19.00	China: Anhui, Wuhu
L180330P-3-LD180327-0007_HL3H2CCXY_L1_clean_bin.77.orig.fa_k	OEZ008529	yes	LMSG_G000005641.1	OES073725	betalactone	Others	Polymetallic	23.90	China: Anhui, Wuhu
L180330P-3-LD180327-0007_HL3H2CCXY_L1_clean_bin.78.permissiv	OEZ008529	yes	LMSG_G000009934.1	OES073725	hserlactone	Others	Polymetallic	12.85	China: Anhui, Wuhu
L180330P-3-LD180327-0007_HL3H2CCXY_L1_clean_bin.79.orig.fa_k	OEZ008529	yes	LMSG_G000005642.1	OES073725	terpene	Terpene	Polymetallic	14.11	China: Anhui, Wuhu
L180330P-3-LD180327-0007_HL3H2CCXY_L1_clean_bin.79.orig.fa_k	OEZ008529	yes	LMSG_G000005642.1	OES073725	NRPS	NRPS	Polymetallic	7.57	China: Anhui, Wuhu
L180330P-3-LD180327-0007_HL3H2CCXY_L1_clean_bin.8.orig.fa_k1	OEZ008529	no	LMSG_G000010168.1	OES073725	terpene	Terpene	Polymetallic	6.07	China: Anhui, Wuhu
L180330P-3-LD180327-0007_HL3H2CCXY_L1_clean_bin.8.orig.fa_k1	OEZ008529	no	LMSG_G000010168.1	OES073725	terpene	Terpene	Polymetallic	21.86	China: Anhui, Wuhu
L180330P-3-LD180327-0007_HL3H2CCXY_L1_clean_bin.8.orig.fa_k1	OEZ008529	no	LMSG_G000010168.1	OES073725	terpene	Terpene	Polymetallic	21.71	China: Anhui, Wuhu
L180330P-3-LD180327-0007_HL3H2CCXY_L1_clean_bin.8.orig.fa_k1	OEZ008529	no	LMSG_G000010168.1	OES073725	ectoine	Others	Polymetallic	5.44	China: Anhui, Wuhu
L180330P-3-LD180327-0007_HL3H2CCXY_L1_clean_bin.81.orig.fa_k	OEZ008529	yes	LMSG_G000009090.1	OES073725	betalactone	Others	Polymetallic	31.07	China: Anhui, Wuhu
L180330P-3-LD180327-0007_HL3H2CCXY_L1_clean_bin.81.orig.fa_k	OEZ008529	yes	LMSG_G000009090.1	OES073725	betalactone	Others	Polymetallic	20.15	China: Anhui, Wuhu
L180330P-3-LD180327-0007_HL3H2CCXY_L1_clean_bin.81.orig.fa_k	OEZ008529	yes	LMSG_G000009090.1	OES073725	acyl_amino_acids.betalactone	Others	Polymetallic	24.78	China: Anhui, Wuhu
L180330P-3-LD180327-0007_HL3H2CCXY_L1_clean_bin.81.orig.fa_k	OEZ008529	yes	LMSG_G000009090.1	OES073725	betalactone	Others	Polymetallic	27.63	China: Anhui, Wuhu
L180330P-3-LD180327-0007_HL3H2CCXY_L1_clean_bin.81.orig.fa_k	OEZ008529	yes	LMSG_G000009090.1	OES073725	thiopeptide	RiPPs	Polymetallic	31.20	China: Anhui, Wuhu
L180330P-3-LD180327-0007_HL3H2CCXY_L1_clean_bin.81.orig.fa_k	OEZ008529	yes	LMSG_G000009090.1	OES073725	terpene	Terpene	Polymetallic	22.04	China: Anhui, Wuhu
L180330P-3-LD180327-0007_HL3H2CCXY_L1_clean_bin.83.orig.fa_k	OEZ008529	no	LMSG_G000008710.1	OES073725	terpene	Terpene	Polymetallic	7.06	China: Anhui, Wuhu
L180330P-3-LD180327-0007_HL3H2CCXY_L1_clean_bin.83.orig.fa_k	OEZ008529	yes	LMSG_G000008710.1	OES073725	terpene	Terpene	Polymetallic	10.29	China: Anhui, Wuhu
L180330P-3-LD180327-0007_HL3H2CCXY_L1_clean_bin.83.orig.fa_k	OEZ008529	yes	LMSG_G000008710.1	OES073725	bacteriocin	RiPPs	Polymetallic	10.59	China: Anhui, Wuhu
L180330P-3-LD180327-0007_HL3H2CCXY_L1_clean_bin.88.orig.fa_k	OEZ008529	yes	LMSG_G000008360.1	OES073725	bacteriocin	RiPPs	Polymetallic	10.81	China: Anhui, Wuhu
L180330P-3-LD180327-0007_HL3H2CCXY_L1_clean_bin.88.orig.fa_k	OEZ008529	yes	LMSG_G000008360.1	OES073725	terpene	Terpene	Polymetallic	8.18	China: Anhui, Wuhu
L180330P-3-LD180327-0007_HL3H2CCXY_L1_clean_bin.88.orig.fa_k	OEZ008529	yes	LMSG_G000008360.1	OES073725	NRPS-like	NRPS	Polymetallic	15.50	China: Anhui, Wuhu
L180330P-3-LD180327-0007_HL3H2CCXY_L1_clean_bin.89.orig.fa_k	OEZ008529	yes	LMSG_G000004526.1	OES073725	other	Others	Polymetallic	12.00	China: Anhui, Wuhu
L180330P-3-LD180327-0007_HL3H2CCXY_L1_clean_bin.89.orig.fa_k	OEZ008529	yes	LMSG_G000004526.1	OES073725	terpene	Terpene	Polymetallic	15.12	China: Anhui, Wuhu
L180330P-3-LD180327-0007_HL3H2CCXY_L1_clean_bin.9.orig.fa_k1	OEZ008529	yes	LMSG_G000009186.1	OES073725	T3PKS	PKSother	Polymetallic	25.64	China: Anhui, Wuhu
L180330P-3-LD180327-0007_HL3H2CCXY_L1_clean_bin.91.orig.fa_k	OEZ008529	yes	LMSG_G000005643.1	OES073725	terpene	Terpene	Polymetallic	16.35	China: Anhui, Wuhu
L180330P-3-LD180327-0007_HL3H2CCXY_L1_clean_bin.92.orig.fa_k	OEZ008529	yes	LMSG_G000008415.1	OES073725	terpene	Terpene	Polymetallic	15.43	China: Anhui, Wuhu
L180330P-3-LD180327-0007_HL3H2CCXY_L1_clean_bin.92.orig.fa_k	OEZ008529	yes	LMSG_G000008415.1	OES073725	betalactone	Others	Polymetallic	31.04	China: Anhui, Wuhu
L180330P-3-LD180327-0007_HL3H2CCXY_L1_clean_bin.95.orig.fa_k	OEZ008529	yes	LMSG_G000010016.1	OES073725	terpene	Terpene	Polymetallic	21.87	China: Anhui, Wuhu
L180330P-3-LD180327-0007_HL3H2CCXY_L1_clean_bin.97.strict.fa	OEZ008529	yes	LMSG_G000009726.1	OES073725	LAP	RiPPs	Polymetallic	22.02	China: Anhui, Wuhu
L180330P-3-LD180327-0007_HL3H2CCXY_L1_clean_bin.98.orig.fa_k	OEZ008529	yes	LMSG_G000009442.1	OES073725	betalactone	Others	Polymetallic	29.63	China: Anhui, Wuhu
L180330P-3-LD180327-0007_HL3H2CCXY_L1_clean_bin.98.orig.fa_k	OEZ008529	yes	LMSG_G000009442.1	OES073725	ectoine	Others	Polymetallic	10.50	China: Anhui, Wuhu
L180330P-3-LD180327-0007_HL3H2CCXY_L1_clean_bin.98.orig.fa_k	OEZ008529	yes	LMSG_G000009442.1	OES073725	betalactone	Others	Polymetallic	22.80	China: Anhui, Wuhu
L180330P-3-LD180327-0007_HL3H2CCXY_L1_clean_bin.98.orig.fa_k	OEZ008529	yes	LMSG_G000009442.1	OES073725	bacteriocin	RiPPs	Polymetallic	6.76	China: Anhui, Wuhu
L180330P-3-LD180327-0008_HL3H2CCXY_L1_clean_bin.1.orig.fa_k1	OEZ008529	yes	LMSG_G000010581.1	OES073727	terpene	Terpene	Polymetallic	11.60	China: Anhui, Wuhu
L180330P-3-LD180327-0008_HL3H2CCXY_L1_clean_bin.1.orig.fa_k1	OEZ008529	yes	LMSG_G000010581.1	OES073727	bacteriocin	RiPPs	Polymetallic	5.19	China: Anhui, Wuhu
L180330P-3-LD180327-0008_HL3H2CCXY_L1_clean_bin.14.orig.fa_k	OEZ008529	no	LMSG_G000010302.1	OES073727	arylpolyene	Others	Polymetallic	25.68	China: Anhui, Wuhu
L180330P-3-LD180327-0008_HL3H2CCXY_L1_clean_bin.14.orig.fa_k	OEZ008529	no	LMSG_G000010302.1	OES073727	terpene	Terpene	Polymetallic	7.31	China: Anhui, Wuhu
L180330P-3-LD180327-0008_HL3H2CCXY_L1_clean_bin.14.orig.fa_k	OEZ008529	no	LMSG_G000010302.1	OES073727	terpene	Terpene	Polymetallic	11.66	China: Anhui, Wuhu
L180330P-3-LD180327-0008_HL3H2CCXY_L1_clean_bin.18.strict.fa	OEZ008529	yes	LMSG_G000005647.1	OES073727	ectoine	Others	Polymetallic	10.39	China: Anhui, Wuhu
L180330P-3-LD180327-0008_HL3H2CCXY_L1_clean_bin.18.strict.fa	OEZ008529	yes	LMSG_G000005647.1	OES073727	LAP.bacteriocin	RiPPs	Polymetallic	9.23	China: Anhui, Wuhu
L180330P-3-LD180327-0008_HL3H2CCXY_L1_clean_bin.19.strict.fa	OEZ008529	yes	LMSG_G000009443.1	OES073727	bacteriocin	RiPPs	Polymetallic	10.85	China: Anhui, Wuhu
L180330P-3-LD180327-0008_HL3H2CCXY_L1_clean_bin.19.strict.fa	OEZ008529	yes	LMSG_G000009443.1	OES073727	betalactone	Others	Polymetallic	22.78	China: Anhui, Wuhu
L180330P-3-LD180327-0008_HL3H2CCXY_L1_clean_bin.19.strict.fa	OEZ008529	yes	LMSG_G000009443.1	OES073727	betalactone	Others	Polymetallic	30.86	China: Anhui, Wuhu
L180330P-3-LD180327-0008_HL3H2CCXY_L1_clean_bin.19.strict.fa	OEZ008529	yes	LMSG_G000009443.1	OES073727	ectoine	Others	Polymetallic	9.18	China: Anhui, Wuhu
L180330P-3-LD180327-0008_HL3H2CCXY_L1_clean_bin.20.orig.fa_k	OEZ008529	yes	LMSG_G000005648.1	OES073727	bacteriocin	RiPPs	Polymetallic	11.13	China: Anhui, Wuhu
L180330P-3-LD180327-0008_HL3H2CCXY_L1_clean_bin.20.orig.fa_k	OEZ008529	yes	LMSG_G000005648.1	OES073727	NRPS	NRPS	Polymetallic	38.80	China: Anhui, Wuhu
L180330P-3-LD180327-0008_HL3H2CCXY_L1_clean_bin.20.orig.fa_k	OEZ008529	yes	LMSG_G000005648.1	OES073727	NRPS-like	NRPS	Polymetallic	7.98	China: Anhui, Wuhu
L180330P-3-LD180327-0008_HL3H2CCXY_L1_clean_bin.20.orig.fa_k	OEZ008529	no	LMSG_G000005648.1	OES073727	terpene	Terpene	Polymetallic	10.34	China: Anhui, Wuhu
L180330P-3-LD180327-0008_HL3H2CCXY_L1_clean_bin.20.orig.fa_k	OEZ008529	yes	LMSG_G000005648.1	OES073727	hserlactone	Others	Polymetallic	9.36	China: Anhui, Wuhu
L180330P-3-LD180327-0008_HL3H2CCXY_L1_clean_bin.21.orig.fa_k	OEZ008529	yes	LMSG_G000005649.1	OES073727	bacteriocin	RiPPs	Polymetallic	6.04	China: Anhui, Wuhu
L180330P-3-LD180327-0008_HL3H2CCXY_L1_clean_bin.21.orig.fa_k	OEZ008529	yes	LMSG_G000005649.1	OES073727	bacteriocin	RiPPs	Polymetallic	10.84	China: Anhui, Wuhu
L180330P-3-LD180327-0008_HL3H2CCXY_L1_clean_bin.21.orig.fa_k	OEZ008529	yes	LMSG_G000005649.1	OES073727	siderophore	Others	Polymetallic	14.11	China: Anhui, Wuhu
L180330P-3-LD180327-0008_HL3H2CCXY_L1_clean_bin.21.orig.fa_k	OEZ008529	no	LMSG_G000005649.1	OES073727	arylpolyene	Others	Polymetallic	8.50	China: Anhui, Wuhu
L180330P-3-LD180327-0008_HL3H2CCXY_L1_clean_bin.21.orig.fa_k	OEZ008529	yes	LMSG_G000005649.1	OES073727	acyl_amino_acids	Others	Polymetallic	17.60	China: Anhui, Wuhu
L180330P-3-LD180327-0008_HL3H2CCXY_L1_clean_bin.21.orig.fa_k	OEZ008529	no	LMSG_G000005649.1	OES073727	terpene	Terpene	Polymetallic	19.84	China: Anhui, Wuhu
L180330P-3-LD180327-0008_HL3H2CCXY_L1_clean_bin.22.strict.fa	OEZ008529	yes	LMSG_G000008198.1	OES073727	terpene	Terpene	Polymetallic	16.68	China: Anhui, Wuhu
L180330P-3-LD180327-0008_HL3H2CCXY_L1_clean_bin.23.orig.fa_k	OEZ008529	yes	LMSG_G000005650.1	OES073727	NRPS	NRPS	Polymetallic	7.06	China: Anhui, Wuhu
L180330P-3-LD180327-0008_HL3H2CCXY_L1_clean_bin.24.orig.fa_k	OEZ008529	no	LMSG_G000010577.1	OES073727	terpene	Terpene	Polymetallic	15.63	China: Anhui, Wuhu
L180330P-3-LD180327-0008_HL3H2CCXY_L1_clean_bin.24.orig.fa_k	OEZ008529	no	LMSG_G000010577.1	OES073727	bacteriocin	RiPPs	Polymetallic	10.85	China: Anhui, Wuhu
L180330P-3-LD180327-0008_HL3H2CCXY_L1_clean_bin.25.orig.fa_k	OEZ008529	yes	LMSG_G000004527.1	OES073727	T3PKS	PKSother	Polymetallic	29.58	China: Anhui, Wuhu
L180330P-3-LD180327-0008_HL3H2CCXY_L1_clean_bin.25.orig.fa_k	OEZ008529	yes	LMSG_G000004527.1	OES073727	terpene	Terpene	Polymetallic	21.86	China: Anhui, Wuhu
L180330P-3-LD180327-0008_HL3H2CCXY_L1_clean_bin.29.strict.fa	OEZ008529	yes	LMSG_G000006290.1	OES073727	cyanobactin	RiPPs	Polymetallic	18.44	China: Anhui, Wuhu
L180330P-3-LD180327-0008_HL3H2CCXY_L1_clean_bin.29.strict.fa	OEZ008529	yes	LMSG_G000006290.1	OES073727	bacteriocin	RiPPs	Polymetallic	10.85	China: Anhui, Wuhu
L180330P-3-LD180327-0008_HL3H2CCXY_L1_clean_bin.29.strict.fa	OEZ008529	yes	LMSG_G000006290.1	OES073727	betalactone	Others	Polymetallic	14.60	China: Anhui, Wuhu
L180330P-3-LD180327-0008_HL3H2CCXY_L1_clean_bin.30.orig.fa_k	OEZ008529	yes	LMSG_G000009596.1	OES073727	acyl_amino_acids	Others	Polymetallic	9.13	China: Anhui, Wuhu
L180330P-3-LD180327-0008_HL3H2CCXY_L1_clean_bin.30.orig.fa_k	OEZ008529	yes	LMSG_G000009596.1	OES073727	terpene	Terpene	Polymetallic	22.15	China: Anhui, Wuhu
L180330P-3-LD180327-0008_HL3H2CCXY_L1_clean_bin.30.orig.fa_k	OEZ008529	yes	LMSG_G000009596.1	OES073727	terpene	Terpene	Polymetallic	14.01	China: Anhui, Wuhu
L180330P-3-LD180327-0008_HL3H2CCXY_L1_clean_bin.33.orig.fa_k	OEZ008529	yes	LMSG_G000010723.1	OES073727	NRPS	NRPS	Polymetallic	11.74	China: Anhui, Wuhu
L180330P-3-LD180327-0008_HL3H2CCXY_L1_clean_bin.33.orig.fa_k	OEZ008529	yes	LMSG_G000010723.1	OES073727	terpene	Terpene	Polymetallic	19.34	China: Anhui, Wuhu
L180330P-3-LD180327-0008_HL3H2CCXY_L1_clean_bin.33.orig.fa_k	OEZ008529	yes	LMSG_G000010723.1	OES073727	bacteriocin	RiPPs	Polymetallic	10.28	China: Anhui, Wuhu
L180330P-3-LD180327-0008_HL3H2CCXY_L1_clean_bin.33.orig.fa_k	OEZ008529	yes	LMSG_G000010723.1	OES073727	NRPS.T1PKS	PKS-NRP_Hybrids	Polymetallic	20.48	China: Anhui, Wuhu
L180330P-3-LD180327-0008_HL3H2CCXY_L1_clean_bin.33.orig.fa_k	OEZ008529	yes	LMSG_G000010723.1	OES073727	arylpolyene	Others	Polymetallic	32.59	China: Anhui, Wuhu
L180330P-3-LD180327-0008_HL3H2CCXY_L1_clean_bin.34.orig.fa_k	OEZ008529	no	LMSG_G000004529.1	OES073727	terpene	Terpene	Polymetallic	20.52	China: Anhui, Wuhu
L180330P-3-LD180327-0008_HL3H2CCXY_L1_clean_bin.34.orig.fa_k	OEZ008529	no	LMSG_G000004529.1	OES073727	T1PKS	PKSI	Polymetallic	23.83	China: Anhui, Wuhu
L180330P-3-LD180327-0008_HL3H2CCXY_L1_clean_bin.34.orig.fa_k	OEZ008529	yes	LMSG_G000004529.1	OES073727	bacteriocin	RiPPs	Polymetallic	5.49	China: Anhui, Wuhu
L180330P-3-LD180327-0008_HL3H2CCXY_L1_clean_bin.34.orig.fa_k	OEZ008529	yes	LMSG_G000004529.1	OES073727	arylpolyene	Others	Polymetallic	22.70	China: Anhui, Wuhu
L180330P-3-LD180327-0008_HL3H2CCXY_L1_clean_bin.34.orig.fa_k	OEZ008529	no	LMSG_G000004529.1	OES073727	bacteriocin	RiPPs	Polymetallic	5.39	China: Anhui, Wuhu
L180330P-3-LD180327-0008_HL3H2CCXY_L1_clean_bin.34.orig.fa_k	OEZ008529	no	LMSG_G000004529.1	OES073727	bacteriocin	RiPPs	Polymetallic	10.92	China: Anhui, Wuhu
L180330P-3-LD180327-0008_HL3H2CCXY_L1_clean_bin.36.strict.fa	OEZ008529	yes	LMSG_G000008803.1	OES073727	bacteriocin	RiPPs	Polymetallic	10.83	China: Anhui, Wuhu
L180330P-3-LD180327-0008_HL3H2CCXY_L1_clean_bin.36.strict.fa	OEZ008529	yes	LMSG_G000008803.1	OES073727	T3PKS	PKSother	Polymetallic	11.28	China: Anhui, Wuhu
L180330P-3-LD180327-0008_HL3H2CCXY_L1_clean_bin.36.strict.fa	OEZ008529	yes	LMSG_G000008803.1	OES073727	terpene	Terpene	Polymetallic	10.54	China: Anhui, Wuhu
L180330P-3-LD180327-0008_HL3H2CCXY_L1_clean_bin.36.strict.fa	OEZ008529	yes	LMSG_G000008803.1	OES073727	T3PKS	PKSother	Polymetallic	7.03	China: Anhui, Wuhu
L180330P-3-LD180327-0008_HL3H2CCXY_L1_clean_bin.37.orig.fa_k	OEZ008529	yes	LMSG_G000005652.1	OES073727	bacteriocin	RiPPs	Polymetallic	7.38	China: Anhui, Wuhu

L180330P-3-LD180327-0008_HL3H2CCXY_L1_clean_bin.37.orig.fa_k	OEZ008529	yes	LMSG_G000005652.1	OES073727	betalactone	Others	Polymetallic	25.82	China: Anhui, Wuhu
L180330P-3-LD180327-0008_HL3H2CCXY_L1_clean_bin.37.orig.fa_k	OEZ008529	yes	LMSG_G000005652.1	OES073727	NRPS	NRPS	Polymetallic	27.50	China: Anhui, Wuhu
L180330P-3-LD180327-0008_HL3H2CCXY_L1_clean_bin.37.orig.fa_k	OEZ008529	yes	LMSG_G000005652.1	OES073727	bacteriocin	RiPPs	Polymetallic	10.85	China: Anhui, Wuhu
L180330P-3-LD180327-0008_HL3H2CCXY_L1_clean_bin.37.orig.fa_k	OEZ008529	yes	LMSG_G000005652.1	OES073727	betalactone	Others	Polymetallic	22.76	China: Anhui, Wuhu
L180330P-3-LD180327-0008_HL3H2CCXY_L1_clean_bin.37.orig.fa_k	OEZ008529	yes	LMSG_G000005652.1	OES073727	thiopeptide.LAP	RiPPs	Polymetallic	9.03	China: Anhui, Wuhu
L180330P-3-LD180327-0008_HL3H2CCXY_L1_clean_bin.37.orig.fa_k	OEZ008529	yes	LMSG_G000005652.1	OES073727	ectoine	Others	Polymetallic	10.16	China: Anhui, Wuhu
L180330P-3-LD180327-0008_HL3H2CCXY_L1_clean_bin.38.orig.fa_k	OEZ008529	yes	LMSG_G000005653.1	OES073727	ectoine	Others	Polymetallic	10.40	China: Anhui, Wuhu
L180330P-3-LD180327-0008_HL3H2CCXY_L1_clean_bin.39.orig.fa_k	OEZ008529	yes	LMSG_G000005654.1	OES073727	arylpolyene	Others	Polymetallic	36.52	China: Anhui, Wuhu
L180330P-3-LD180327-0008_HL3H2CCXY_L1_clean_bin.39.orig.fa_k	OEZ008529	yes	LMSG_G000005654.1	OES073727	terpene	Terpene	Polymetallic	21.66	China: Anhui, Wuhu
L180330P-3-LD180327-0008_HL3H2CCXY_L1_clean_bin.4.orig.fa_kl	OEZ008529	yes	LMSG_G000010139.1	OES073727	ectoine	Others	Polymetallic	10.40	China: Anhui, Wuhu
L180330P-3-LD180327-0008_HL3H2CCXY_L1_clean_bin.4.orig.fa_kl	OEZ008529	yes	LMSG_G000010139.1	OES073727	terpene	Terpene	Polymetallic	14.29	China: Anhui, Wuhu
L180330P-3-LD180327-0008_HL3H2CCXY_L1_clean_bin.4.orig.fa_kl	OEZ008529	yes	LMSG_G000010139.1	OES073727	T3PKS	PKSother	Polymetallic	12.31	China: Anhui, Wuhu
L180330P-3-LD180327-0008_HL3H2CCXY_L1_clean_bin.40.orig.fa_k	OEZ008529	yes	LMSG_G000007067.1	OES073727	bacteriocin	RiPPs	Polymetallic	5.92	China: Anhui, Wuhu
L180330P-3-LD180327-0008_HL3H2CCXY_L1_clean_bin.41.orig.fa_k	OEZ008529	yes	LMSG_G000010681.1	OES073727	arylpolyene	Others	Polymetallic	25.93	China: Anhui, Wuhu
L180330P-3-LD180327-0008_HL3H2CCXY_L1_clean_bin.41.orig.fa_k	OEZ008529	yes	LMSG_G000010681.1	OES073727	NRPS-like	NRPS	Polymetallic	20.56	China: Anhui, Wuhu
L180330P-3-LD180327-0008_HL3H2CCXY_L1_clean_bin.41.orig.fa_k	OEZ008529	yes	LMSG_G000010681.1	OES073727	betalactone	Others	Polymetallic	24.36	China: Anhui, Wuhu
L180330P-3-LD180327-0008_HL3H2CCXY_L1_clean_bin.42.orig.fa_k	OEZ008529	yes	LMSG_G000005655.1	OES073727	NRPS-like	NRPS	Polymetallic	38.12	China: Anhui, Wuhu
L180330P-3-LD180327-0008_HL3H2CCXY_L1_clean_bin.42.orig.fa_k	OEZ008529	yes	LMSG_G000005655.1	OES073727	terpene	Terpene	Polymetallic	23.08	China: Anhui, Wuhu
L180330P-3-LD180327-0008_HL3H2CCXY_L1_clean_bin.42.orig.fa_k	OEZ008529	yes	LMSG_G000005655.1	OES073727	betalactone	Others	Polymetallic	23.63	China: Anhui, Wuhu
L180330P-3-LD180327-0008_HL3H2CCXY_L1_clean_bin.42.orig.fa_k	OEZ008529	yes	LMSG_G000005655.1	OES073727	bacteriocin	RiPPs	Polymetallic	10.85	China: Anhui, Wuhu
L180330P-3-LD180327-0008_HL3H2CCXY_L1_clean_bin.44.orig.fa_k	OEZ008529	no	LMSG_G000005656.1	OES073727	bacteriocin	RiPPs	Polymetallic	7.09	China: Anhui, Wuhu
L180330P-3-LD180327-0008_HL3H2CCXY_L1_clean_bin.46.orig.fa_k	OEZ008529	yes	LMSG_G000005657.1	OES073727	terpene	Terpene	Polymetallic	16.95	China: Anhui, Wuhu
L180330P-3-LD180327-0008_HL3H2CCXY_L1_clean_bin.46.orig.fa_k	OEZ008529	yes	LMSG_G000005657.1	OES073727	bacteriocin	RiPPs	Polymetallic	9.51	China: Anhui, Wuhu
L180330P-3-LD180327-0008_HL3H2CCXY_L1_clean_bin.47.strict.fa	OEZ008529	yes	LMSG_G000009475.1	OES073727	bacteriocin	RiPPs	Polymetallic	10.86	China: Anhui, Wuhu
L180330P-3-LD180327-0008_HL3H2CCXY_L1_clean_bin.6.orig.fa_kl	OEZ008529	yes	LMSG_G000010500.1	OES073727	terpene	Terpene	Polymetallic	14.13	China: Anhui, Wuhu
L180330P-4-LD180327-0009_HL3H2CCXY_L2_clean_bin.10.orig.fa_k	OEZ008529	yes	LMSG_G000007245.1	OES073728	betalactone	Others	Polymetallic	7.85	China: Anhui, Wuhu
L180330P-4-LD180327-0009_HL3H2CCXY_L2_clean_bin.11.orig.fa_k	OEZ008529	yes	LMSG_G000009614.1	OES073728	terpene	Terpene	Polymetallic	22.09	China: Anhui, Wuhu
L180330P-4-LD180327-0009_HL3H2CCXY_L2_clean_bin.12.orig.fa_k	OEZ008529	yes	LMSG_G000008276.1	OES073728	terpene	Terpene	Polymetallic	19.12	China: Anhui, Wuhu
L180330P-4-LD180327-0009_HL3H2CCXY_L2_clean_bin.14.strict.fa	OEZ008529	yes	LMSG_G000008233.1	OES073728	terpene	Terpene	Polymetallic	20.86	China: Anhui, Wuhu
L180330P-4-LD180327-0009_HL3H2CCXY_L2_clean_bin.17.orig.fa_k	OEZ008529	yes	LMSG_G000009524.1	OES073728	ectoine	Others	Polymetallic	10.40	China: Anhui, Wuhu
L180330P-4-LD180327-0009_HL3H2CCXY_L2_clean_bin.17.orig.fa_k	OEZ008529	yes	LMSG_G000009524.1	OES073728	terpene	Terpene	Polymetallic	23.29	China: Anhui, Wuhu
L180330P-4-LD180327-0009_HL3H2CCXY_L2_clean_bin.17.orig.fa_k	OEZ008529	yes	LMSG_G000009524.1	OES073728	arylpolyene	Others	Polymetallic	41.19	China: Anhui, Wuhu
L180330P-4-LD180327-0009_HL3H2CCXY_L2_clean_bin.17.orig.fa_k	OEZ008529	yes	LMSG_G000009524.1	OES073728	NRPS-like	NRPS	Polymetallic	24.92	China: Anhui, Wuhu
L180330P-4-LD180327-0009_HL3H2CCXY_L2_clean_bin.17.orig.fa_k	OEZ008529	yes	LMSG_G000009524.1	OES073728	terpene	Terpene	Polymetallic	22.05	China: Anhui, Wuhu
L180330P-4-LD180327-0009_HL3H2CCXY_L2_clean_bin.18.orig.fa_k	OEZ008529	yes	LMSG_G000007068.1	OES073728	bacteriocin	RiPPs	Polymetallic	6.49	China: Anhui, Wuhu
L180330P-4-LD180327-0009_HL3H2CCXY_L2_clean_bin.2.orig.fa_kl	OEZ008529	yes	LMSG_G000008514.1	OES073728	terpene	Terpene	Polymetallic	26.29	China: Anhui, Wuhu
L180330P-4-LD180327-0009_HL3H2CCXY_L2_clean_bin.20.orig.fa_k	OEZ008529	no	LMSG_G000010599.1	OES073728	T1PKS	PKSI	Polymetallic	12.97	China: Anhui, Wuhu
L180330P-4-LD180327-0009_HL3H2CCXY_L2_clean_bin.20.orig.fa_k	OEZ008529	no	LMSG_G000010599.1	OES073728	terpene	Terpene	Polymetallic	5.11	China: Anhui, Wuhu
L180330P-4-LD180327-0009_HL3H2CCXY_L2_clean_bin.20.orig.fa_k	OEZ008529	no	LMSG_G000010599.1	OES073728	bacteriocin	RiPPs	Polymetallic	8.50	China: Anhui, Wuhu
L180330P-4-LD180327-0009_HL3H2CCXY_L2_clean_bin.21.orig.fa_k	OEZ008529	no	LMSG_G000010489.1	OES073728	terpene	Terpene	Polymetallic	9.02	China: Anhui, Wuhu
L180330P-4-LD180327-0009_HL3H2CCXY_L2_clean_bin.21.orig.fa_k	OEZ008529	yes	LMSG_G000010489.1	OES073728	arylpolyene.hserlactone	Others	Polymetallic	26.93	China: Anhui, Wuhu
L180330P-4-LD180327-0009_HL3H2CCXY_L2_clean_bin.21.orig.fa_k	OEZ008529	yes	LMSG_G000010489.1	OES073728	NRPS-like	NRPS	Polymetallic	14.16	China: Anhui, Wuhu
L180330P-4-LD180327-0009_HL3H2CCXY_L2_clean_bin.22.orig.fa_k	OEZ008529	yes	LMSG_G000006291.1	OES073728	terpene	Terpene	Polymetallic	20.92	China: Anhui, Wuhu
L180330P-4-LD180327-0009_HL3H2CCXY_L2_clean_bin.23.strict.fa	OEZ008529	yes	LMSG_G000009444.1	OES073728	bacteriocin	RiPPs	Polymetallic	10.85	China: Anhui, Wuhu
L180330P-4-LD180327-0009_HL3H2CCXY_L2_clean_bin.23.strict.fa	OEZ008529	yes	LMSG_G000009444.1	OES073728	betalactone	Others	Polymetallic	30.86	China: Anhui, Wuhu
L180330P-4-LD180327-0009_HL3H2CCXY_L2_clean_bin.23.strict.fa	OEZ008529	yes	LMSG_G000009444.1	OES073728	betalactone	Others	Polymetallic	22.80	China: Anhui, Wuhu
L180330P-4-LD180327-0009_HL3H2CCXY_L2_clean_bin.23.strict.fa	OEZ008529	yes	LMSG_G000009444.1	OES073728	ectoine	Others	Polymetallic	8.50	China: Anhui, Wuhu
L180330P-4-LD180327-0009_HL3H2CCXY_L2_clean_bin.24.orig.fa_k	OEZ008529	no	LMSG_G000005660.1	OES073728	terpene	Terpene	Polymetallic	11.04	China: Anhui, Wuhu
L180330P-4-LD180327-0009_HL3H2CCXY_L2_clean_bin.24.orig.fa_k	OEZ008529	no	LMSG_G000005660.1	OES073728	terpene	Terpene	Polymetallic	7.43	China: Anhui, Wuhu
L180330P-4-LD180327-0009_HL3H2CCXY_L2_clean_bin.24.orig.fa_k	OEZ008529	no	LMSG_G000005660.1	OES073728	arylpolyene	Others	Polymetallic	30.36	China: Anhui, Wuhu
L180330P-4-LD180327-0009_HL3H2CCXY_L2_clean_bin.25.orig.fa_k	OEZ008529	yes	LMSG_G000008689.1	OES073728	bacteriocin	RiPPs	Polymetallic	5.84	China: Anhui, Wuhu
L180330P-4-LD180327-0009_HL3H2CCXY_L2_clean_bin.25.orig.fa_k	OEZ008529	yes	LMSG_G000008689.1	OES073728	NRPS-like	NRPS	Polymetallic	6.96	China: Anhui, Wuhu
L180330P-4-LD180327-0009_HL3H2CCXY_L2_clean_bin.25.orig.fa_k	OEZ008529	yes	LMSG_G000008689.1	OES073728	NRPS-like	NRPS	Polymetallic	5.96	China: Anhui, Wuhu
L180330P-4-LD180327-0009_HL3H2CCXY_L2_clean_bin.26.orig.fa_k	OEZ008529	yes	LMSG_G000005661.1	OES073728	betalactone	Others	Polymetallic	26.79	China: Anhui, Wuhu
L180330P-4-LD180327-0009_HL3H2CCXY_L2_clean_bin.26.orig.fa_k	OEZ008529	yes	LMSG_G000005661.1	OES073728	betalactone	Others	Polymetallic	24.88	China: Anhui, Wuhu
L180330P-4-LD180327-0009_HL3H2CCXY_L2_clean_bin.26.orig.fa_k	OEZ008529	yes	LMSG_G000005661.1	OES073728	terpene	Terpene	Polymetallic	12.12	China: Anhui, Wuhu
L180330P-4-LD180327-0009_HL3H2CCXY_L2_clean_bin.26.orig.fa_k	OEZ008529	yes	LMSG_G000005661.1	OES073728	bacteriocin	RiPPs	Polymetallic	10.46	China: Anhui, Wuhu
L180330P-4-LD180327-0009_HL3H2CCXY_L2_clean_bin.26.orig.fa_k	OEZ008529	yes	LMSG_G000005661.1	OES073728	ectoine	Others	Polymetallic	10.40	China: Anhui, Wuhu
L180330P-4-LD180327-0009_HL3H2CCXY_L2_clean_bin.27.orig.fa_k	OEZ008529	yes	LMSG_G000005662.1	OES073728	hserlactone	Others	Polymetallic	9.88	China: Anhui, Wuhu
L180330P-4-LD180327-0009_HL3H2CCXY_L2_clean_bin.27.orig.fa_k	OEZ008529	no	LMSG_G000005662.1	OES073728	arylpolyene	Others	Polymetallic	13.66	China: Anhui, Wuhu
L180330P-4-LD180327-0009_HL3H2CCXY_L2_clean_bin.27.orig.fa_k	OEZ008529	yes	LMSG_G000005662.1	OES073728	terpene	Terpene	Polymetallic	15.76	China: Anhui, Wuhu
L180330P-4-LD180327-0009_HL3H2CCXY_L2_clean_bin.29.strict.fa	OEZ008529	yes	LMSG_G000004530.1	OES073728	ectoine	Others	Polymetallic	10.39	China: Anhui, Wuhu
L180330P-4-LD180327-0009_HL3H2CCXY_L2_clean_bin.29.strict.fa	OEZ008529	yes	LMSG_G000004530.1	OES073728	terpene	Terpene	Polymetallic	20.48	China: Anhui, Wuhu
L180330P-4-LD180327-0009_HL3H2CCXY_L2_clean_bin.29.strict.fa	OEZ008529	yes	LMSG_G000004530.1	OES073728	hglE-KS	PKSother	Polymetallic	24.05	China: Anhui, Wuhu
L180330P-4-LD180327-0009_HL3H2CCXY_L2_clean_bin.30.orig.fa_k	OEZ008529	no	LMSG_G000010169.1	OES073728	ectoine	Others	Polymetallic	10.40	China: Anhui, Wuhu
L180330P-4-LD180327-0009_HL3H2CCXY_L2_clean_bin.30.orig.fa_k	OEZ008529	no	LMSG_G000010169.1	OES073728	terpene	Terpene	Polymetallic	21.95	China: Anhui, Wuhu
L180330P-4-LD180327-0009_HL3H2CCXY_L2_clean_bin.30.orig.fa_k	OEZ008529	no	LMSG_G000010169.1	OES073728	terpene	Terpene	Polymetallic	15.21	China: Anhui, Wuhu
L180330P-4-LD180327-0009_HL3H2CCXY_L2_clean_bin.30.orig.fa_k	OEZ008529	no	LMSG_G000010169.1	OES073728	acyl_amino_acids	Others	Polymetallic	5.39	China: Anhui, Wuhu
L180330P-4-LD180327-0009_HL3H2CCXY_L2_clean_bin.33.orig.fa_k	OEZ008529	yes	LMSG_G000005664.1	OES073728	NRPS-like	NRPS	Polymetallic	23.48	China: Anhui, Wuhu
L180330P-4-LD180327-0009_HL3H2CCXY_L2_clean_bin.33.orig.fa_k	OEZ008529	yes	LMSG_G000005664.1	OES073728	terpene	Terpene	Polymetallic	23.39	China: Anhui, Wuhu
L180330P-4-LD180327-0009_HL3H2CCXY_L2_clean_bin.33.orig.fa_k	OEZ008529	yes	LMSG_G000005664.1	OES073728	arylpolyene	Others	Polymetallic	24.04	China: Anhui, Wuhu
L180330P-4-LD180327-0009_HL3H2CCXY_L2_clean_bin.33.orig.fa_k	OEZ008529	yes	LMSG_G000005664.1	OES073728	acyl_amino_acids	Others	Polymetallic	16.49	China: Anhui, Wuhu
L180330P-4-LD180327-0009_HL3H2CCXY_L2_clean_bin.33.orig.fa_k	OEZ008529	yes	LMSG_G000005664.1	OES073728	ectoine	Others	Polymetallic	8.97	China: Anhui, Wuhu
L180330P-4-LD180327-0009_HL3H2CCXY_L2_clean_bin.34.strict.fa	OEZ008529	yes	LMSG_G000008325.1	OES073728	terpene	Terpene	Polymetallic	21.09	China: Anhui, Wuhu
L180330P-4-LD180327-0009_HL3H2CCXY_L2_clean_bin.36.orig.fa_k	OEZ008529	yes	LMSG_G000005665.1	OES073728	ectoine	Others	Polymetallic	10.39	China: Anhui, Wuhu
L180330P-4-LD180327-0009_HL3H2CCXY_L2_clean_bin.36.orig.fa_k	OEZ008529	yes	LMSG_G000005665.1	OES073728	hglE-KS	PKSother	Polymetallic	17.35	China: Anhui, Wuhu
L180330P-4-LD180327-0009_HL3H2CCXY_L2_clean_bin.37.orig.fa_k	OEZ008529	yes	LMSG_G000005666.1	OES073728	terpene	Terpene	Polymetallic	16.44	China: Anhui, Wuhu
L180330P-4-LD180327-0009_HL3H2CCXY_L2_clean_bin.37.orig.fa_k	OEZ008529	yes	LMSG_G000005666.1	OES073728	arylpolyene.hserlactone	Others	Polymetallic	6.82	China: Anhui, Wuhu
L180330P-4-LD180327-0009_HL3H2CCXY_L2_clean_bin.4.strict.fa_	OEZ008529	yes	LMSG_G000007768.1	OES073728	terpene	Terpene	Polymetallic	7.69	China: Anhui, Wuhu
L180330P-4-LD180327-0009_HL3H2CCXY_L2_clean_bin.41.orig.fa_k	OEZ008529	yes	LMSG_G000009390.1	OES073728	terpene	Terpene	Polymetallic	7.69	China: Anhui, Wuhu
L180330P-4-LD180327-0009_HL3H2CCXY_L2_clean_bin.6.strict.fa_	OEZ008529	yes	LMSG_G000010589.1	OES073728	bacteriocin	RiPPs	Polymetallic	10.43	China: Anhui, Wuhu
L180330P-4-LD180327-0009_HL3H2CCXY_L2_clean_bin.6.strict.fa_	OEZ008529	yes	LMSG_G000010589.1	OES073728	terpene	Terpene	Polymetallic	12.70	China: Anhui, Wuhu
L180330P-4-LD180327-0009_HL3H2CCXY_L2_clean_bin.6.strict.fa_	OEZ008529	yes	LMSG_G000010589.1	OES073728	bacteriocin	RiPPs	Polymetallic	7.45	China: Anhui, Wuhu
L180330P-4-LD180327-0009_HL3H2CCXY_L2_clean_bin.7.orig.fa_kl	OEZ008529	yes	LMSG_G000008491.1	OES073728	terpene	Terpene	Polymetallic	24.60	China: Anhui, Wuhu
L180330P-4-LD180327-0009_HL3H2CCXY_L2_clean_bin.7.orig.fa_kl	OEZ008529	yes	LMSG_G000008491.1	OES073728	lassopeptide	RiPPs	Polymetallic	7.20	China: Anhui, Wuhu
L180330P-4-LD180327-0009_HL3H2CCXY_L2_clean_bin.9.orig.fa_kl	OEZ008529	yes	LMSG_G000005668.1	OES073728	betalactone	Others	Polymetallic	24.71	China: Anhui, Wuhu
L180330P-4-LD180327-0009_HL3H2CCXY_L2_clean_bin.9.orig.fa_kl	OEZ008529	yes	LMSG_G000005668.1	OES073728	bacteriocin	RiPPs	Polymetallic	10.85	China: Anhui, Wuhu
L180330P-4-LD180327-0009_HL3H2CCXY_L2_clean_bin.9.orig.fa_kl	OEZ008529	yes	LMSG_G000005668.1	OES073728	betalactone	Others	Polymetallic	24.67	China: Anhui, Wuhu
L180330P-4-LD180328-0001_HL3H2CCXY_L2_clean_bin.1.orig.fa_kl	OEZ008529	no	LMSG_G000008412.1	OES073707	terpene	Terpene	Magnetite	6.38	China: Anhui, Maanshan
L180330P-4-LD180328-0001_HL3H2CCXY_L2_clean_bin.10.strict.fa	OEZ008529	yes	LMSG_G000009363.1	OES073707	betalactone	Others	Magnetite	31.59	China: Anhui, Maanshan
L180330P-4-LD180328-0001_HL3H2CCXY_L2_clean_bin.102.strict.f	OEZ008529	yes	LMSG_G000008420.1	OES073707	terpene	Terpene	Magnetite	20.64	China: Anhui, Maanshan
L180330P-4-LD180328-0001_HL3H2CCXY_L2_clean_bin.106.orig.fa_	OEZ008529	no	LMSG_G000010303.1	OES073707	arylpolyene	Others	Magnetite	41.19	China: Anhui, Maanshan
L180330P-4-LD180328-0001_HL3H2CCXY_L2_clean_bin.108.permissi	OEZ008529	yes	LMSG_G000008890.1	OES073707	T3PKS	PKSother	Magnetite	22.12	China: Anhui, Maanshan
L180330P-4-LD180328-0001_HL3H2CCXY_L2_clean_bin.108.permissi	OEZ008529	yes	LMSG_G000008890.1	OES073707	terpene	Terpene	Magnetite	20.25	China: Anhui, Maanshan
L180330P-4-LD180328-0001_HL3H2CCXY_L2_clean_bin.115.orig.fa_	OEZ008529	yes	LMSG_G000008711.1	OES073707	bacteriocin	RiPPs	Magnetite	9.83	China: Anhui, Maanshan
L180330P-4-LD180328-0001_HL3H2CCXY_L2_clean_bin.115.orig.fa_	OEZ008529	yes	LMSG_G000008711.1	OES073707	betalactone	Others	Magnetite	24.19	China: Anhui, Maanshan
L180330P-4-LD180328-0001_HL3H2CCXY_L2_clean_bin.115.orig.fa_	OEZ008529	yes	LMSG_G000008711.1	OES073707	terpene	Terpene	Magnetite	16.44	China: Anhui, Maanshan
L180330P-4-LD180328-0001_HL3H2CCXY_L2_clean_bin.115.orig.fa_	OEZ008529	yes	LMSG_G000008711.1	OES073707	LAP	RiPPs	Magnetite	15.59	China: Anhui, Maanshan
L180330P-4-LD180328-0001_HL3H2CCXY_L2_clean_bin.115.orig.fa_	OEZ008529	yes	LMSG_G000008711.1	OES073707	terpene	Terpene	Magnetite	16.34	China: Anhui, Maanshan
L180330P-4-LD180328-0001_HL3H2CCXY_L2_clean_bin.116.orig.fa_	OEZ008529	yes	LMSG_G000008337.1	OES073707	bacteriocin	RiPPs	Magnetite	5.52	China: Anhui, Maanshan
L180330P-4-LD180328-0001_HL3H2CCXY_L2_clean_bin.116.orig.fa_	OEZ008529	yes	LMSG_G000008337.1	OES073707	terpene	Terpene	Magnetite	19.35	China: Anhui, Maanshan
L180330P-4-LD180328-0001_HL3H2CCXY_L2_clean_bin.116.orig.fa_	OEZ008529	yes	LMSG_G000008337.1	OES073707	other	Others	Magnetite	9.40	China: Anhui, Maanshan
L180330P-4-LD180328-0001_HL3H2CCXY_L2_clean_bin.116.orig.fa_	OEZ008529	yes	LMSG_G000008337.1	OES073707	T3PKS	PKSother	Magnetite	8.83	China: Anhui, Maanshan
L180330P-4-LD180328-0001_HL3H2CCXY_L2_clean_bin.116.orig.fa_	OEZ008529	yes	LMSG_G000008337.1	OES073707	butyrolactone	Others	Magnetite	9.31	China: Anhui, Maanshan
L180330P-4-LD180328-0001_HL3H2CCXY_L2_clean_bin.12.strict.fa	OEZ008529	yes	LMSG_G000009388.1	OES073707	terpene	Terpene	Magnetite	21.75	China: Anhui, Maanshan
L180330P-4-LD180328-0001_HL3H2CCXY_L2_clean_bin.121.orig.fa_	OEZ008529	yes	LMSG_G000009162.1	OES073707	T3PKS	PKSother	Magnetite	16.29	China: Anhui, Maanshan
L180330P-4-LD180328-0001_HL3H2CCXY_L2_clean_bin.123.orig.fa_	OEZ008529	yes	LMSG_G000010501.1	OES073707	terpene	Terpene	Magnetite	11.37	China: Anhui, Maanshan
L180330P-4-LD180328-0001_HL3H2CCXY_L2_clean_bin.123.orig.fa_	OEZ008529	yes	LMSG_G000010501.1	OES073707	arylpolyene.hserlactone	Others	Magnetite	6.88	China: Anhui, Maanshan
L180330P-4-LD180328-0001_HL3H2CCXY_L2_clean_bin.126.strict.f	OEZ008529	yes	LMSG_G000007983.1	OES073707	other	Others	Magnetite	32.69	China: Anhui, Maanshan
L180330P-4-LD180328-0001_HL3H2CCXY_L2_clean_bin.126.strict.f	OEZ008529	yes	LMSG_G000007983.1	OES073707	TfuA-related.bacteriocin	RiPPs	Magnetite	13.27	China: Anhui, Maanshan
L180330P-4-LD180328-0001_HL3H2CCXY_L2_clean_bin.126.strict.f	OEZ008529	yes	LMSG_G000007983.1	OES073707	bacteriocin	RiPPs	Magnetite	5.26	China: Anhui, Maanshan
L180330P-4-LD180328-0001_HL3H2CCXY_L2_clean_bin.127.orig.fa_	OEZ008529	yes	LMSG_G000007833.1	OES073707	terpene	Terpene	Magnetite	22.05	China: Anhui, Maanshan
L180330P-4-LD180328-0001_HL3H2CCXY_L2_clean_bin.127.orig.fa_	OEZ008529	yes	LMSG_G000007833.1	OES073707	T3PKS	PKSother	Magnetite	22.74	China: Anhui, Maanshan
L180330P-4-LD180328-0001_HL3H2CCXY_L2_clean_bin.127.orig.fa_	OEZ008529	yes	LMSG_G000007833.1	OES073707	bacteriocin	RiPPs	Magnetite	10.88	China: Anhui, Maanshan
L180330P-4-LD180328-0001_HL3H2CCXY_L2_clean_bin.127.orig.fa_	OEZ008529	yes	LMSG_G000007833.1	OES073707	terpene	Terpene	Magnetite	18.94	China: Anhui, Maanshan
L180330P-4-LD180328-0001_HL3H2CCXY_L2_clean_bin.129.orig.fa_	OEZ008529	yes	LMSG_G000009197.1	OES073707	terpene	Terpene	Magnetite	19.62	China: Anhui, Maanshan
L180330P-4-LD180328-0001_HL3H2CCXY_L2_clean_bin.129.orig.fa_	OEZ008529	yes	LMSG_G000009197.1	OES073707	T3PKS	PKSother	Magnetite	9.02	China: Anhui, Maanshan
L180330P-4-LD180328-0001_HL3H2CCXY_L2_clean_bin.131.orig.fa_	OEZ008529	yes	LMSG_G000008331.1	OES073707	terpene	Terpene	Magnetite	9.44	China: Anhui, Maanshan
L180330P-4-LD180328-0001_HL3H2CCXY_L2_clean_bin.131.orig.fa_	OEZ008529	yes	LMSG_G000008331.1	OES073707	terpene	Terpene	Magnetite	11.54	China: Anhui, Maanshan
L180330P-4-LD180328-0001_HL3H2CCXY_L2_clean_bin.134.orig.fa_	OEZ008529	yes	LMSG_G000009725.1	OES073707	LAP	RiPPs	Magnetite	11.52	China: Anhui, Maanshan
L180330P-4-LD180328-0001_HL3H2CCXY_L2_clean_bin.139.orig.fa_	OEZ008529	yes	LMSG_G000009643.1	OES073707	terpene	Terpene	Magnetite	21.78	China: Anhui, Maanshan
L180330P-4-LD180328-0001_HL3H2CCXY_L2_clean_bin.139.orig.fa_	OEZ008529	yes	LMSG_G000009643.1	OES073707	terpene	Terpene	Magnetite	9.93	China: Anhui, Maanshan
L180330P-4-LD180328-0001_HL3H2CCXY_L2_clean_bin.14.orig.fa_k	OEZ008529	yes	LMSG_G000010231.1	OES073707	terpene.arylpolyene	Others	Magnetite	42.19	China: Anhui, Maanshan
L180330P-4-LD180328-0001_HL3H2CCXY_L2_clean_bin.14.orig.fa_k	OEZ008529	yes	LMSG_G000010231.1	OES073707	terpene	Terpene	Magnetite	20.70	China: Anhui, Maanshan
L180330P-4-LD180328-0001_HL3H2CCXY_L2_clean_bin.141.orig.fa_	OEZ008529	yes	LMSG_G000006310.1	OES073707	terpene	Terpene	Magnetite	21.08	China: Anhui, Maanshan
L180330P-4-LD180328-0001_HL3H2CCXY_L2_clean_bin.16.orig.fa_k	OEZ008529	yes	LMSG_G000005673.1	OES073707	T3PKS	PKSother	Magnetite	9.39	China: Anhui, Maanshan
L180330P-4-LD180328-0001_HL3H2CCXY_L2_clean_bin.16.orig.fa_k	OEZ008529	yes	LMSG_G000005673.1	OES073707	terpene	Terpene	Magnetite	13.29	China: Anhui, Maanshan
L180330P-4-LD180328-0001_HL3H2CCXY_L2_clean_bin.17.orig.fa_k	OEZ008529	yes	LMSG_G000008379.1	OES073707	terpene	Terpene	Magnetite	22.39	China: Anhui, Maanshan
L180330P-4-LD180328-0001_HL3H2CCXY_L2_clean_bin.18.orig.fa_k	OEZ008529	yes	LMSG_G000005674.1	OES073707	bacteriocin	RiPPs	Magnetite	10.89	China: Anhui, Maanshan
L180330P-4-LD180328-0001_HL3H2CCXY_L2_clean_bin.18.orig.fa_k	OEZ008529	yes	LMSG_G000005674.1	OES073707	ectoine	Others	Magnetite	10.41	China: Anhui, Maanshan
L180330P-4-LD180328-0001_HL3H2CCXY_L2_clean_bin.18.orig.fa_k	OEZ008529	yes	LMSG_G000005674.1	OES073707	hserlactone	Others	Magnetite	20.70	China: Anhui, Maanshan
L180330P-4-LD180328-0001_HL3H2CCXY_L2_clean_bin.18.orig.fa_k	OEZ008529	yes	LMSG_G000005674.1	OES073707	betalactone	Others	Magnetite	30.91	China: Anhui, Maanshan
L180330P-4-LD180328-0001_HL3H2CCXY_L2_clean_bin.19.orig.fa_k	OEZ008529	yes	LMSG_G000006292.1	OES073707	terpene	Terpene	Magnetite	21.97	China: Anhui, Maanshan
L180330P-4-LD180328-0001_HL3H2CCXY_L2_clean_bin.19.orig.fa_k	OEZ008529	yes	LMSG_G000006292.1	OES073707	NRPS	NRPS	Magnetite	18.93	China: Anhui, Maanshan
L180330P-4-LD180328-0001_HL3H2CCXY_L2_clean_bin.19.orig.fa_k	OEZ008529	yes	LMSG_G000006292.1	OES073707	NRPS-like	NRPS	Magnetite	28.46	China: Anhui, Maanshan
L180330P-4-LD180328-0001_HL3H2CCXY_L2_clean_bin.21.orig.fa_k	OEZ008529	yes	LMSG_G000008654.1	OES073707	other	Others	Magnetite	14.00	China: Anhui, Maanshan
L180330P-4-LD180328-0001_HL3H2CCXY_L2_clean_bin.21.orig.fa_k	OEZ008529	yes	LMSG_G000008654.1	OES073707	other	Others	Magnetite	20.61	China: Anhui, Maanshan
L180330P-4-LD180328-0001_HL3H2CCXY_L2_clean_bin.23.orig.fa_k	OEZ008529	yes	LMSG_G000005676.1	OES073707	other	Others	Magnetite	6.78	China: Anhui, Maanshan
L180330P-4-LD180328-0001_HL3H2CCXY_L2_clean_bin.23.orig.fa_k	OEZ008529	yes	LMSG_G000005676.1	OES073707	lassopeptide	RiPPs	Magnetite	5.51	China: Anhui, Maanshan
L180330P-4-LD180328-0001_HL3H2CCXY_L2_clean_bin.23.orig.fa_k	OEZ008529	yes	LMSG_G000005676.1	OES073707	terpene	Terpene	Magnetite	26.31	China: Anhui, Maanshan
L180330P-4-LD180328-0001_HL3H2CCXY_L2_clean_bin.25.orig.fa_k	OEZ008529	yes	LMSG_G000008679.1	OES073707	T1PKS	PKSI	Magnetite	16.91	China: Anhui, Maanshan
L180330P-4-LD180328-0001_HL3H2CCXY_L2_clean_bin.25.orig.fa_k	OEZ008529	yes	LMSG_G000008679.1	OES073707	arylpolyene	Others	Magnetite	32.59	China: Anhui, Maanshan
L180330P-4-LD180328-0001_HL3H2CCXY_L2_clean_bin.25.orig.fa_k	OEZ008529	yes	LMSG_G000008679.1	OES073707	T1PKS	PKSI	Magnetite	6.33	China: Anhui, Maanshan
L180330P-4-LD180328-0001_HL3H2CCXY_L2_clean_bin.26.orig.fa_k	OEZ008529	yes	LMSG_G000008507.1	OES073707	terpene	Terpene	Magnetite	20.49	China: Anhui, Maanshan
L180330P-4-LD180328-0001_HL3H2CCXY_L2_clean_bin.26.orig.fa_k	OEZ008529	yes	LMSG_G000008507.1	OES073707	terpene	Terpene	Magnetite	25.81	China: Anhui, Maanshan
L180330P-4-LD180328-0001_HL3H2CCXY_L2_clean_bin.26.orig.fa_k	OEZ008529	yes	LMSG_G000008507.1	OES073707	lassopeptide	RiPPs	Magnetite	23.45	China: Anhui, Maanshan
L180330P-4-LD180328-0001_HL3H2CCXY_L2_clean_bin.27.strict.fa	OEZ008529	yes	LMSG_G000009445.1	OES073707	ectoine	Others	Magnetite	10.40	China: Anhui, Maanshan
L180330P-4-LD180328-0001_HL3H2CCXY_L2_clean_bin.27.strict.fa	OEZ008529	yes	LMSG_G000009445.1	OES073707	bacteriocin	RiPPs	Magnetite	5.59	China: Anhui, Maanshan
L180330P-4-LD180328-0001_HL3H2CCXY_L2_clean_bin.29.orig.fa_k	OEZ008529	yes	LMSG_G000005677.1	OES073707	hserlactone	Others	Magnetite	20.78	China: Anhui, Maanshan
L180330P-4-LD180328-0001_HL3H2CCXY_L2_clean_bin.3.orig.fa_kl	OEZ008529	yes	LMSG_G000009597.1	OES073707	terpene	Terpene	Magnetite	15.77	China: Anhui, Maanshan
L180330P-4-LD180328-0001_HL3H2CCXY_L2_clean_bin.3.orig.fa_kl	OEZ008529	yes	LMSG_G000009597.1	OES073707	terpene	Terpene	Magnetite	11.87	China: Anhui, Maanshan
L180330P-4-LD180328-0001_HL3H2CCXY_L2_clean_bin.3.orig.fa_kl	OEZ008529	yes	LMSG_G000009597.1	OES073707	NRPS-like	NRPS	Magnetite	13.30	China: Anhui, Maanshan
L180330P-4-LD180328-0001_HL3H2CCXY_L2_clean_bin.30.orig.fa_k	OEZ008529	yes	LMSG_G000008326.1	OES073707	terpene	Terpene	Magnetite	21.09	China: Anhui, Maanshan
L180330P-4-LD180328-0001_HL3H2CCXY_L2_clean_bin.33.strict.fa	OEZ008529	yes	LMSG_G000005679.1	OES073707	LAP.thiopeptide	RiPPs	Magnetite	20.76	China: Anhui, Maanshan
L180330P-4-LD180328-0001_HL3H2CCXY_L2_clean_bin.33.strict.fa	OEZ008529	yes	LMSG_G000005679.1	OES073707	lassopeptide	RiPPs	Magnetite	11.58	China: Anhui, Maanshan
L180330P-4-LD180328-0001_HL3H2CCXY_L2_clean_bin.35.strict.fa	OEZ008529	yes	LMSG_G000008741.1	OES073707	NRPS-like	NRPS	Magnetite	31.30	China: Anhui, Maanshan
L180330P-4-LD180328-0001_HL3H2CCXY_L2_clean_bin.35.strict.fa	OEZ008529	yes	LMSG_G000008741.1	OES073707	terpene	Terpene	Magnetite	19.43	China: Anhui, Maanshan
L180330P-4-LD180328-0001_HL3H2CCXY_L2_clean_bin.35.strict.fa	OEZ008529	yes	LMSG_G000008741.1	OES073707	other	Others	Magnetite	17.59	China: Anhui, Maanshan

L180330P-4-LD180328-0001_HL3H2CCXY_L2_clean_bin.35.strict.fa	OEZ008529	yes	LMSG_G000008741.1	OES073707	betalactone	Others	Magnetite	8.33	China: Anhui, Maanshan
L180330P-4-LD180328-0001_HL3H2CCXY_L2_clean_bin.36.orig.fa_k	OEZ008529	yes	LMSG_G000009006.1	OES073707	terpene	Terpene	Magnetite	20.91	China: Anhui, Maanshan
L180330P-4-LD180328-0001_HL3H2CCXY_L2_clean_bin.36.orig.fa_k	OEZ008529	yes	LMSG_G000009006.1	OES073707	bacteriocin	RiPPs	Magnetite	10.80	China: Anhui, Maanshan
L180330P-4-LD180328-0001_HL3H2CCXY_L2_clean_bin.39.strict.fa	OEZ008529	yes	LMSG_G000009615.1	OES073707	terpene	Terpene	Magnetite	21.99	China: Anhui, Maanshan
L180330P-4-LD180328-0001_HL3H2CCXY_L2_clean_bin.4.strict.fa_	OEZ008529	yes	LMSG_G000010062.1	OES073707	NRPS-like	NRPS	Magnetite	21.17	China: Anhui, Maanshan
L180330P-4-LD180328-0001_HL3H2CCXY_L2_clean_bin.4.strict.fa_	OEZ008529	yes	LMSG_G000010062.1	OES073707	arylpolyene	Others	Magnetite	13.66	China: Anhui, Maanshan
L180330P-4-LD180328-0001_HL3H2CCXY_L2_clean_bin.4.strict.fa_	OEZ008529	yes	LMSG_G000010062.1	OES073707	ectoine	Others	Magnetite	5.12	China: Anhui, Maanshan
L180330P-4-LD180328-0001_HL3H2CCXY_L2_clean_bin.4.strict.fa_	OEZ008529	yes	LMSG_G000010062.1	OES073707	arylpolyene	Others	Magnetite	5.32	China: Anhui, Maanshan
L180330P-4-LD180328-0001_HL3H2CCXY_L2_clean_bin.4.strict.fa_	OEZ008529	yes	LMSG_G000010062.1	OES073707	terpene	Terpene	Magnetite	5.11	China: Anhui, Maanshan
L180330P-4-LD180328-0001_HL3H2CCXY_L2_clean_bin.40.strict.fa	OEZ008529	yes	LMSG_G000008316.1	OES073707	terpene	Terpene	Magnetite	12.53	China: Anhui, Maanshan
L180330P-4-LD180328-0001_HL3H2CCXY_L2_clean_bin.43.orig.fa_k	OEZ008529	yes	LMSG_G000005680.1	OES073707	terpene	Terpene	Magnetite	13.83	China: Anhui, Maanshan
L180330P-4-LD180328-0001_HL3H2CCXY_L2_clean_bin.43.orig.fa_k	OEZ008529	yes	LMSG_G000005680.1	OES073707	NRPS-like	NRPS	Magnetite	7.19	China: Anhui, Maanshan
L180330P-4-LD180328-0001_HL3H2CCXY_L2_clean_bin.43.orig.fa_k	OEZ008529	yes	LMSG_G000005680.1	OES073707	betalactone	Others	Magnetite	9.55	China: Anhui, Maanshan
L180330P-4-LD180328-0001_HL3H2CCXY_L2_clean_bin.43.orig.fa_k	OEZ008529	yes	LMSG_G000005680.1	OES073707	betalactone	Others	Magnetite	17.78	China: Anhui, Maanshan
L180330P-4-LD180328-0001_HL3H2CCXY_L2_clean_bin.44.permissiv	OEZ008529	yes	LMSG_G000008388.1	OES073707	NRPS-like	NRPS	Magnetite	44.19	China: Anhui, Maanshan
L180330P-4-LD180328-0001_HL3H2CCXY_L2_clean_bin.44.permissiv	OEZ008529	yes	LMSG_G000008388.1	OES073707	terpene	Terpene	Magnetite	11.58	China: Anhui, Maanshan
L180330P-4-LD180328-0001_HL3H2CCXY_L2_clean_bin.49.orig.fa_k	OEZ008529	yes	LMSG_G000007899.1	OES073707	NRPS	NRPS	Magnetite	11.01	China: Anhui, Maanshan
L180330P-4-LD180328-0001_HL3H2CCXY_L2_clean_bin.49.orig.fa_k	OEZ008529	yes	LMSG_G000007899.1	OES073707	NRPS-like	NRPS	Magnetite	15.21	China: Anhui, Maanshan
L180330P-4-LD180328-0001_HL3H2CCXY_L2_clean_bin.49.orig.fa_k	OEZ008529	yes	LMSG_G000007899.1	OES073707	NRPS-like	NRPS	Magnetite	7.45	China: Anhui, Maanshan
L180330P-4-LD180328-0001_HL3H2CCXY_L2_clean_bin.5.orig.fa_kl	OEZ008529	yes	LMSG_G000005682.1	OES073707	terpene	Terpene	Magnetite	5.49	China: Anhui, Maanshan
L180330P-4-LD180328-0001_HL3H2CCXY_L2_clean_bin.50.orig.fa_k	OEZ008529	yes	LMSG_G000007823.1	OES073707	T3PKS	PKSother	Magnetite	41.09	China: Anhui, Maanshan
L180330P-4-LD180328-0001_HL3H2CCXY_L2_clean_bin.50.orig.fa_k	OEZ008529	yes	LMSG_G000007823.1	OES073707	terpene	Terpene	Magnetite	29.81	China: Anhui, Maanshan
L180330P-4-LD180328-0001_HL3H2CCXY_L2_clean_bin.52.orig.fa_k	OEZ008529	yes	LMSG_G000010017.1	OES073707	ladderane	Others	Magnetite	26.72	China: Anhui, Maanshan
L180330P-4-LD180328-0001_HL3H2CCXY_L2_clean_bin.52.orig.fa_k	OEZ008529	yes	LMSG_G000010017.1	OES073707	terpene	Terpene	Magnetite	21.87	China: Anhui, Maanshan
L180330P-4-LD180328-0001_HL3H2CCXY_L2_clean_bin.53.strict.fa	OEZ008529	yes	LMSG_G000008885.1	OES073707	terpene	Terpene	Magnetite	20.98	China: Anhui, Maanshan
L180330P-4-LD180328-0001_HL3H2CCXY_L2_clean_bin.53.strict.fa	OEZ008529	yes	LMSG_G000008885.1	OES073707	arylpolyene	Others	Magnetite	34.43	China: Anhui, Maanshan
L180330P-4-LD180328-0001_HL3H2CCXY_L2_clean_bin.53.strict.fa	OEZ008529	yes	LMSG_G000008885.1	OES073707	T3PKS	PKSother	Magnetite	41.07	China: Anhui, Maanshan
L180330P-4-LD180328-0001_HL3H2CCXY_L2_clean_bin.55.strict.fa	OEZ008529	yes	LMSG_G000009184.1	OES073707	T3PKS	PKSother	Magnetite	29.86	China: Anhui, Maanshan
L180330P-4-LD180328-0001_HL3H2CCXY_L2_clean_bin.56.orig.fa_k	OEZ008529	yes	LMSG_G000004532.1	OES073707	terpene	Terpene	Magnetite	26.21	China: Anhui, Maanshan
L180330P-4-LD180328-0001_HL3H2CCXY_L2_clean_bin.57.orig.fa_k	OEZ008529	yes	LMSG_G000008682.1	OES073707	bacteriocin	RiPPs	Magnetite	6.31	China: Anhui, Maanshan
L180330P-4-LD180328-0001_HL3H2CCXY_L2_clean_bin.57.orig.fa_k	OEZ008529	yes	LMSG_G000008682.1	OES073707	terpene	Terpene	Magnetite	21.66	China: Anhui, Maanshan
L180330P-4-LD180328-0001_HL3H2CCXY_L2_clean_bin.57.orig.fa_k	OEZ008529	yes	LMSG_G000008682.1	OES073707	ectoine	Others	Magnetite	11.01	China: Anhui, Maanshan
L180330P-4-LD180328-0001_HL3H2CCXY_L2_clean_bin.58.strict.fa	OEZ008529	yes	LMSG_G000009362.1	OES073707	betalactone	Others	Magnetite	31.59	China: Anhui, Maanshan
L180330P-4-LD180328-0001_HL3H2CCXY_L2_clean_bin.60.orig.fa_k	OEZ008529	yes	LMSG_G000008234.1	OES073707	terpene	Terpene	Magnetite	10.84	China: Anhui, Maanshan
L180330P-4-LD180328-0001_HL3H2CCXY_L2_clean_bin.61.orig.fa_k	OEZ008529	yes	LMSG_G000006293.1	OES073707	terpene	Terpene	Magnetite	21.86	China: Anhui, Maanshan
L180330P-4-LD180328-0001_HL3H2CCXY_L2_clean_bin.61.orig.fa_k	OEZ008529	yes	LMSG_G000006293.1	OES073707	T3PKS	PKSother	Magnetite	22.76	China: Anhui, Maanshan
L180330P-4-LD180328-0001_HL3H2CCXY_L2_clean_bin.61.orig.fa_k	OEZ008529	yes	LMSG_G000006293.1	OES073707	NRPS-like	NRPS	Magnetite	24.18	China: Anhui, Maanshan
L180330P-4-LD180328-0001_HL3H2CCXY_L2_clean_bin.61.orig.fa_k	OEZ008529	yes	LMSG_G000006293.1	OES073707	terpene	Terpene	Magnetite	13.90	China: Anhui, Maanshan
L180330P-4-LD180328-0001_HL3H2CCXY_L2_clean_bin.62.strict.fa	OEZ008529	yes	LMSG_G000008126.1	OES073707	terpene	Terpene	Magnetite	21.29	China: Anhui, Maanshan
L180330P-4-LD180328-0001_HL3H2CCXY_L2_clean_bin.63.orig.fa_k	OEZ008529	yes	LMSG_G000011084.1	OES073707	betalactone	Others	Magnetite	21.49	China: Anhui, Maanshan
L180330P-4-LD180328-0001_HL3H2CCXY_L2_clean_bin.63.orig.fa_k	OEZ008529	yes	LMSG_G000011084.1	OES073707	lassopeptide	RiPPs	Magnetite	12.97	China: Anhui, Maanshan
L180330P-4-LD180328-0001_HL3H2CCXY_L2_clean_bin.63.orig.fa_k	OEZ008529	yes	LMSG_G000011084.1	OES073707	NRPS-like	NRPS	Magnetite	14.72	China: Anhui, Maanshan
L180330P-4-LD180328-0001_HL3H2CCXY_L2_clean_bin.63.orig.fa_k	OEZ008529	yes	LMSG_G000011084.1	OES073707	NRPS-like	NRPS	Magnetite	28.08	China: Anhui, Maanshan
L180330P-4-LD180328-0001_HL3H2CCXY_L2_clean_bin.63.orig.fa_k	OEZ008529	yes	LMSG_G000011084.1	OES073707	terpene	Terpene	Magnetite	23.11	China: Anhui, Maanshan
L180330P-4-LD180328-0001_HL3H2CCXY_L2_clean_bin.70.orig.fa_k	OEZ008529	no	LMSG_G000010170.1	OES073707	terpene	Terpene	Magnetite	16.34	China: Anhui, Maanshan
L180330P-4-LD180328-0001_HL3H2CCXY_L2_clean_bin.70.orig.fa_k	OEZ008529	no	LMSG_G000010170.1	OES073707	ectoine	Others	Magnetite	10.40	China: Anhui, Maanshan
L180330P-4-LD180328-0001_HL3H2CCXY_L2_clean_bin.70.orig.fa_k	OEZ008529	no	LMSG_G000010170.1	OES073707	terpene	Terpene	Magnetite	15.38	China: Anhui, Maanshan
L180330P-4-LD180328-0001_HL3H2CCXY_L2_clean_bin.71.orig.fa_k	OEZ008529	yes	LMSG_G000010471.1	OES073707	terpene	Terpene	Magnetite	23.04	China: Anhui, Maanshan
L180330P-4-LD180328-0001_HL3H2CCXY_L2_clean_bin.71.orig.fa_k	OEZ008529	yes	LMSG_G000010471.1	OES073707	arylpolyene	Others	Magnetite	41.23	China: Anhui, Maanshan
L180330P-4-LD180328-0001_HL3H2CCXY_L2_clean_bin.71.orig.fa_k	OEZ008529	yes	LMSG_G000010471.1	OES073707	bacteriocin	RiPPs	Magnetite	10.54	China: Anhui, Maanshan
L180330P-4-LD180328-0001_HL3H2CCXY_L2_clean_bin.72.orig.fa_k	OEZ008529	yes	LMSG_G000009187.1	OES073707	terpene	Terpene	Magnetite	7.79	China: Anhui, Maanshan
L180330P-4-LD180328-0001_HL3H2CCXY_L2_clean_bin.72.orig.fa_k	OEZ008529	yes	LMSG_G000009187.1	OES073707	terpene	Terpene	Magnetite	8.32	China: Anhui, Maanshan
L180330P-4-LD180328-0001_HL3H2CCXY_L2_clean_bin.73.permissiv	OEZ008529	yes	LMSG_G000008021.1	OES073707	betalactone	Others	Magnetite	23.60	China: Anhui, Maanshan
L180330P-4-LD180328-0001_HL3H2CCXY_L2_clean_bin.73.permissiv	OEZ008529	yes	LMSG_G000008021.1	OES073707	bacteriocin	RiPPs	Magnetite	6.56	China: Anhui, Maanshan
L180330P-4-LD180328-0001_HL3H2CCXY_L2_clean_bin.73.permissiv	OEZ008529	yes	LMSG_G000008021.1	OES073707	betalactone	Others	Magnetite	10.37	China: Anhui, Maanshan
L180330P-4-LD180328-0001_HL3H2CCXY_L2_clean_bin.77.orig.fa_k	OEZ008529	yes	LMSG_G000005684.1	OES073707	betalactone	Others	Magnetite	18.03	China: Anhui, Maanshan
L180330P-4-LD180328-0001_HL3H2CCXY_L2_clean_bin.77.orig.fa_k	OEZ008529	yes	LMSG_G000005684.1	OES073707	TfuA-related	RiPPs	Magnetite	11.19	China: Anhui, Maanshan
L180330P-4-LD180328-0001_HL3H2CCXY_L2_clean_bin.79.permissiv	OEZ008529	yes	LMSG_G000009290.1	OES073707	terpene	Terpene	Magnetite	21.73	China: Anhui, Maanshan
L180330P-4-LD180328-0001_HL3H2CCXY_L2_clean_bin.79.permissiv	OEZ008529	yes	LMSG_G000009290.1	OES073707	terpene	Terpene	Magnetite	22.06	China: Anhui, Maanshan
L180330P-4-LD180328-0001_HL3H2CCXY_L2_clean_bin.8.orig.fa_kl	OEZ008529	yes	LMSG_G000005688.1	OES073707	terpene	Terpene	Magnetite	22.57	China: Anhui, Maanshan
L180330P-4-LD180328-0001_HL3H2CCXY_L2_clean_bin.8.orig.fa_kl	OEZ008529	yes	LMSG_G000005688.1	OES073707	terpene	Terpene	Magnetite	15.32	China: Anhui, Maanshan
L180330P-4-LD180328-0001_HL3H2CCXY_L2_clean_bin.8.orig.fa_kl	OEZ008529	yes	LMSG_G000005688.1	OES073707	terpene	Terpene	Magnetite	20.69	China: Anhui, Maanshan
L180330P-4-LD180328-0001_HL3H2CCXY_L2_clean_bin.8.orig.fa_kl	OEZ008529	yes	LMSG_G000005688.1	OES073707	bacteriocin	RiPPs	Magnetite	10.87	China: Anhui, Maanshan
L180330P-4-LD180328-0001_HL3H2CCXY_L2_clean_bin.8.orig.fa_kl	OEZ008529	yes	LMSG_G000005688.1	OES073707	T1PKS	PKSI	Magnetite	28.74	China: Anhui, Maanshan
L180330P-4-LD180328-0001_HL3H2CCXY_L2_clean_bin.81.orig.fa_k	OEZ008529	yes	LMSG_G000009399.1	OES073707	terpene	Terpene	Magnetite	21.68	China: Anhui, Maanshan
L180330P-4-LD180328-0001_HL3H2CCXY_L2_clean_bin.81.orig.fa_k	OEZ008529	yes	LMSG_G000009399.1	OES073707	NRPS-like	NRPS	Magnetite	22.21	China: Anhui, Maanshan
L180330P-4-LD180328-0001_HL3H2CCXY_L2_clean_bin.81.orig.fa_k	OEZ008529	yes	LMSG_G000009399.1	OES073707	NRPS	NRPS	Magnetite	23.91	China: Anhui, Maanshan
L180330P-4-LD180328-0001_HL3H2CCXY_L2_clean_bin.90.orig.fa_k	OEZ008529	yes	LMSG_G000008277.1	OES073707	terpene	Terpene	Magnetite	19.12	China: Anhui, Maanshan
L180330P-4-LD180328-0001_HL3H2CCXY_L2_clean_bin.93.orig.fa_k	OEZ008529	yes	LMSG_G000009375.1	OES073707	betalactone	Others	Magnetite	23.90	China: Anhui, Maanshan
L180330P-4-LD180328-0001_HL3H2CCXY_L2_clean_bin.96.orig.fa_k	OEZ008529	yes	LMSG_G000008444.1	OES073707	terpene	Terpene	Magnetite	26.50	China: Anhui, Maanshan
L180330P-4-LD180328-0001_HL3H2CCXY_L2_clean_bin.98.orig.fa_k	OEZ008529	yes	LMSG_G000010435.1	OES073707	terpene	Terpene	Magnetite	23.10	China: Anhui, Maanshan
L180330P-4-LD180328-0001_HL3H2CCXY_L2_clean_bin.99.strict.fa	OEZ008529	yes	LMSG_G000004534.1	OES073707	terpene	Terpene	Magnetite	22.04	China: Anhui, Maanshan
L180330P-4-LD180328-0001_HL3H2CCXY_L2_clean_bin.99.strict.fa	OEZ008529	yes	LMSG_G000004534.1	OES073707	betalactone	Others	Magnetite	31.07	China: Anhui, Maanshan
L180330P-4-LD180328-0001_HL3H2CCXY_L2_clean_bin.99.strict.fa	OEZ008529	yes	LMSG_G000004534.1	OES073707	thiopeptide	RiPPs	Magnetite	31.20	China: Anhui, Maanshan
L180330P-4-LD180328-0001_HL3H2CCXY_L2_clean_bin.99.strict.fa	OEZ008529	yes	LMSG_G000004534.1	OES073707	betalactone	Others	Magnetite	27.63	China: Anhui, Maanshan
L180330P-4-LD180328-0001_HL3H2CCXY_L2_clean_bin.99.strict.fa	OEZ008529	yes	LMSG_G000004534.1	OES073707	acyl_amino_acids.betalactone	Others	Magnetite	61.45	China: Anhui, Maanshan
L180330P-4-LD180328-0001_HL3H2CCXY_L2_clean_bin.99.strict.fa	OEZ008529	yes	LMSG_G000004534.1	OES073707	betalactone.bacteriocin	Others	Magnetite	29.69	China: Anhui, Maanshan
L180330P-4-LD180328-0002_HL3H2CCXY_L2_clean_bin.10.permissiv	OEZ008529	yes	LMSG_G000008537.1	OES073714	terpene	Terpene	Copper	8.11	China: Anhui, Tongling
L180330P-4-LD180328-0002_HL3H2CCXY_L2_clean_bin.10.permissiv	OEZ008529	yes	LMSG_G000008537.1	OES073714	terpene	Terpene	Copper	7.93	China: Anhui, Tongling
L180330P-4-LD180328-0002_HL3H2CCXY_L2_clean_bin.16.orig.fa_k	OEZ008529	yes	LMSG_G000008289.1	OES073714	terpene	Terpene	Copper	19.12	China: Anhui, Tongling
L180330P-4-LD180328-0002_HL3H2CCXY_L2_clean_bin.18.strict.fa	OEZ008529	no	LMSG_G000010547.1	OES073714	terpene	Terpene	Copper	23.13	China: Anhui, Tongling
L180330P-4-LD180328-0002_HL3H2CCXY_L2_clean_bin.18.strict.fa	OEZ008529	yes	LMSG_G000010547.1	OES073714	T1PKS	PKSI	Copper	20.51	China: Anhui, Tongling
L180330P-4-LD180328-0002_HL3H2CCXY_L2_clean_bin.18.strict.fa	OEZ008529	no	LMSG_G000010547.1	OES073714	T1PKS	PKSI	Copper	10.81	China: Anhui, Tongling
L180330P-4-LD180328-0002_HL3H2CCXY_L2_clean_bin.2.orig.fa_kl	OEZ008529	yes	LMSG_G000007223.1	OES073714	ectoine	Others	Copper	10.31	China: Anhui, Tongling
L180330P-4-LD180328-0002_HL3H2CCXY_L2_clean_bin.24.orig.fa_k	OEZ008529	yes	LMSG_G000008508.1	OES073714	arylpolyene	Others	Copper	10.51	China: Anhui, Tongling
L180330P-4-LD180328-0002_HL3H2CCXY_L2_clean_bin.24.orig.fa_k	OEZ008529	yes	LMSG_G000008508.1	OES073714	lassopeptide	RiPPs	Copper	23.32	China: Anhui, Tongling
L180330P-4-LD180328-0002_HL3H2CCXY_L2_clean_bin.24.orig.fa_k	OEZ008529	yes	LMSG_G000008508.1	OES073714	terpene	Terpene	Copper	26.16	China: Anhui, Tongling
L180330P-4-LD180328-0002_HL3H2CCXY_L2_clean_bin.25.orig.fa_k	OEZ008529	no	LMSG_G000010171.1	OES073714	ectoine	Others	Copper	10.40	China: Anhui, Tongling
L180330P-4-LD180328-0002_HL3H2CCXY_L2_clean_bin.25.orig.fa_k	OEZ008529	no	LMSG_G000010171.1	OES073714	terpene	Terpene	Copper	11.01	China: Anhui, Tongling
L180330P-4-LD180328-0002_HL3H2CCXY_L2_clean_bin.25.orig.fa_k	OEZ008529	no	LMSG_G000010171.1	OES073714	terpene	Terpene	Copper	21.71	China: Anhui, Tongling
L180330P-4-LD180328-0002_HL3H2CCXY_L2_clean_bin.31.strict.fa	OEZ008529	yes	LMSG_G000008008.1	OES073714	TfuA-related.bacteriocin	RiPPs	Copper	7.36	China: Anhui, Tongling
L180330P-4-LD180328-0002_HL3H2CCXY_L2_clean_bin.32.orig.fa_k	OEZ008529	yes	LMSG_G000010495.1	OES073714	terpene	Terpene	Copper	11.14	China: Anhui, Tongling
L180330P-4-LD180328-0002_HL3H2CCXY_L2_clean_bin.32.orig.fa_k	OEZ008529	yes	LMSG_G000010495.1	OES073714	arylpolyene.hserlactone	Others	Copper	17.38	China: Anhui, Tongling
L180330P-4-LD180328-0002_HL3H2CCXY_L2_clean_bin.34.strict.fa	OEZ008529	yes	LMSG_G000011075.1	OES073714	bacteriocin	RiPPs	Copper	10.33	China: Anhui, Tongling
L180330P-4-LD180328-0002_HL3H2CCXY_L2_clean_bin.34.strict.fa	OEZ008529	yes	LMSG_G000011075.1	OES073714	NRPS-like	NRPS	Copper	28.76	China: Anhui, Tongling
L180330P-4-LD180328-0002_HL3H2CCXY_L2_clean_bin.34.strict.fa	OEZ008529	yes	LMSG_G000011075.1	OES073714	NRPS-like	NRPS	Copper	26.30	China: Anhui, Tongling
L180330P-4-LD180328-0002_HL3H2CCXY_L2_clean_bin.34.strict.fa	OEZ008529	yes	LMSG_G000011075.1	OES073714	NRPS-like	NRPS	Copper	28.61	China: Anhui, Tongling
L180330P-4-LD180328-0002_HL3H2CCXY_L2_clean_bin.34.strict.fa	OEZ008529	yes	LMSG_G000011075.1	OES073714	terpene	Terpene	Copper	12.83	China: Anhui, Tongling
L180330P-4-LD180328-0002_HL3H2CCXY_L2_clean_bin.34.strict.fa	OEZ008529	yes	LMSG_G000011075.1	OES073714	lassopeptide	RiPPs	Copper	7.59	China: Anhui, Tongling
L180330P-4-LD180328-0002_HL3H2CCXY_L2_clean_bin.38.orig.fa_k	OEZ008529	yes	LMSG_G000010486.1	OES073714	terpene	Terpene	Copper	17.86	China: Anhui, Tongling
L180330P-4-LD180328-0002_HL3H2CCXY_L2_clean_bin.38.orig.fa_k	OEZ008529	yes	LMSG_G000010486.1	OES073714	hserlactone	Others	Copper	12.22	China: Anhui, Tongling
L180330P-4-LD180328-0002_HL3H2CCXY_L2_clean_bin.38.orig.fa_k	OEZ008529	no	LMSG_G000010486.1	OES073714	arylpolyene	Others	Copper	11.74	China: Anhui, Tongling
L180330P-4-LD180328-0002_HL3H2CCXY_L2_clean_bin.39.strict.fa	OEZ008529	yes	LMSG_G000005694.1	OES073714	bacteriocin	RiPPs	Copper	14.69	China: Anhui, Tongling
L180330P-4-LD180328-0002_HL3H2CCXY_L2_clean_bin.39.strict.fa	OEZ008529	yes	LMSG_G000005694.1	OES073714	TfuA-related	RiPPs	Copper	12.63	China: Anhui, Tongling
L180330P-4-LD180328-0002_HL3H2CCXY_L2_clean_bin.4.permissive	OEZ008529	yes	LMSG_G000009899.1	OES073714	terpene	Terpene	Copper	20.82	China: Anhui, Tongling
L180330P-4-LD180328-0002_HL3H2CCXY_L2_clean_bin.41.strict.fa	OEZ008529	yes	LMSG_G000009410.1	OES073714	betalactone	Others	Copper	18.63	China: Anhui, Tongling
L180330P-4-LD180328-0002_HL3H2CCXY_L2_clean_bin.41.strict.fa	OEZ008529	yes	LMSG_G000009410.1	OES073714	bacteriocin	RiPPs	Copper	10.31	China: Anhui, Tongling
L180330P-4-LD180328-0002_HL3H2CCXY_L2_clean_bin.48.strict.fa	OEZ008529	yes	LMSG_G000009421.1	OES073714	bacteriocin	RiPPs	Copper	10.42	China: Anhui, Tongling
L180330P-4-LD180328-0002_HL3H2CCXY_L2_clean_bin.48.strict.fa	OEZ008529	yes	LMSG_G000009421.1	OES073714	betalactone	Others	Copper	22.76	China: Anhui, Tongling
L180330P-4-LD180328-0002_HL3H2CCXY_L2_clean_bin.48.strict.fa	OEZ008529	yes	LMSG_G000009421.1	OES073714	betalactone	Others	Copper	30.33	China: Anhui, Tongling
L180330P-4-LD180328-0002_HL3H2CCXY_L2_clean_bin.48.strict.fa	OEZ008529	yes	LMSG_G000009421.1	OES073714	ectoine	Others	Copper	7.30	China: Anhui, Tongling
L180330P-4-LD180328-0002_HL3H2CCXY_L2_clean_bin.49.orig.fa_k	OEZ008529	yes	LMSG_G000007056.1	OES073714	bacteriocin	RiPPs	Copper	9.57	China: Anhui, Tongling
L180330P-4-LD180328-0002_HL3H2CCXY_L2_clean_bin.5.permissive	OEZ008529	yes	LMSG_G000009446.1	OES073714	betalactone	Others	Copper	22.78	China: Anhui, Tongling
L180330P-4-LD180328-0002_HL3H2CCXY_L2_clean_bin.5.permissive	OEZ008529	yes	LMSG_G000009446.1	OES073714	bacteriocin	RiPPs	Copper	10.85	China: Anhui, Tongling
L180330P-4-LD180328-0002_HL3H2CCXY_L2_clean_bin.5.permissive	OEZ008529	yes	LMSG_G000009446.1	OES073714	betalactone	Others	Copper	30.86	China: Anhui, Tongling
L180330P-4-LD180328-0002_HL3H2CCXY_L2_clean_bin.5.permissive	OEZ008529	yes	LMSG_G000009446.1	OES073714	ectoine	Others	Copper	7.74	China: Anhui, Tongling
L180330P-4-LD180328-0002_HL3H2CCXY_L2_clean_bin.50.permissiv	OEZ008529	yes	LMSG_G000008235.1	OES073714	terpene	Terpene	Copper	20.86	China: Anhui, Tongling
L180330P-4-LD180328-0002_HL3H2CCXY_L2_clean_bin.51.orig.fa_k	OEZ008529	yes	LMSG_G000009007.1	OES073714	terpene	Terpene	Copper	20.91	China: Anhui, Tongling
L180330P-4-LD180328-0002_HL3H2CCXY_L2_clean_bin.51.orig.fa_k	OEZ008529	yes	LMSG_G000009007.1	OES073714	bacteriocin	RiPPs	Copper	10.80	China: Anhui, Tongling
L180330P-4-LD180328-0002_HL3H2CCXY_L2_clean_bin.57.strict.fa	OEZ008529	yes	LMSG_G000008022.1	OES073714	betalactone	Others	Copper	23.60	China: Anhui, Tongling
L180330P-4-LD180328-0002_HL3H2CCXY_L2_clean_bin.57.strict.fa	OEZ008529	yes	LMSG_G000008022.1	OES073714	betalactone	Others	Copper	30.01	China: Anhui, Tongling
L180330P-4-LD180328-0002_HL3H2CCXY_L2_clean_bin.57.strict.fa	OEZ008529	yes	LMSG_G000008022.1	OES073714	TfuA-related.bacteriocin	RiPPs	Copper	34.94	China: Anhui, Tongling
L180330P-4-LD180328-0002_HL3H2CCXY_L2_clean_bin.6.orig.fa_kl	OEZ008529	yes	LMSG_G000009598.1	OES073714	terpene	Terpene	Copper	10.36	China: Anhui, Tongling
L180330P-4-LD180328-0002_HL3H2CCXY_L2_clean_bin.6.orig.fa_kl	OEZ008529	yes	LMSG_G000009598.1	OES073714	terpene	Terpene	Copper	22.15	China: Anhui, Tongling
L180330P-4-LD180328-0002_HL3H2CCXY_L2_clean_bin.6.orig.fa_kl	OEZ008529	yes	LMSG_G000009598.1	OES073714	acyl_amino_acids	Others	Copper	5.52	China: Anhui, Tongling
L180330P-4-LD180328-0002_HL3H2CCXY_L2_clean_bin.6.orig.fa_kl	OEZ008529	yes	LMSG_G000009598.1	OES073714	NRPS-like	NRPS	Copper	13.64	China: Anhui, Tongling
L180330P-4-LD180328-0002_HL3H2CCXY_L2_clean_bin.60.permissiv	OEZ008529	yes	LMSG_G000009552.1	OES073714	terpene	Terpene	Copper	22.06	China: Anhui, Tongling
L180330P-4-LD180328-0002_HL3H2CCXY_L2_clean_bin.60.permissiv	OEZ008529	no	LMSG_G000009552.1	OES073714	terpene	Terpene	Copper	23.38	China: Anhui, Tongling
L180330P-4-LD180328-0002_HL3H2CCXY_L2_clean_bin.60.permissiv	OEZ008529	yes	LMSG_G000009552.1	OES073714	NRPS-like	NRPS	Copper	42.73	China: Anhui, Tongling
L180330P-4-LD180328-0002_HL3H2CCXY_L2_clean_bin.60.permissiv	OEZ008529	yes	LMSG_G000009552.1	OES073714	ectoine	Others	Copper	9.72	China: Anhui, Tongling
L180330P-4-LD180328-0002_HL3H2CCXY_L2_clean_bin.7.orig.fa_kl	OEZ008529	yes	LMSG_G000005695.1	OES073714	ectoine	Others	Copper	7.53	China: Anhui, Tongling
L180330P-4-LD180328-0002_HL3H2CCXY_L2_clean_bin.7.orig.fa_kl	OEZ008529	yes	LMSG_G000005695.1	OES073714	NRPS-like	NRPS	Copper	42.72	China: Anhui, Tongling
L180330P-4-LD180328-0002_HL3H2CCXY_L2_clean_bin.7.orig.fa_kl	OEZ008529	yes	LMSG_G000005695.1	OES073714	terpene	Terpene	Copper	22.06	China: Anhui, Tongling
L180330P-4-LD180328-0002_HL3H2CCXY_L2_clean_bin.7.orig.fa_kl	OEZ008529	no	LMSG_G000005695.1	OES073714	terpene	Terpene	Copper	20.97	China: Anhui, Tongling
L180330P-4-LD180328-0002_HL3H2CCXY_L2_clean_bin.9.permissive	OEZ008529	yes	LMSG_G000009413.1	OES073714	terpene	Terpene	Copper	13.51	China: Anhui, Tongling
L180330P-4-LD180328-0002_HL3H2CCXY_L2_clean_bin.9.permissive	OEZ008529	yes	LMSG_G000009413.1	OES073714	terpene	Terpene	Copper	9.92	China: Anhui, Tongling
L180330P-5-LD180328-0003_HL3H2CCXY_L3_clean_bin.1.orig.fa_kl	OEZ008529	no	LMSG_G000010172.1	OES073719	terpene	Terpene	Copper	12.80	China: Anhui, Tongling
L180330P-5-LD180328-0003_HL3H2CCXY_L3_clean_bin.1.orig.fa_kl	OEZ008529	yes	LMSG_G000010172.1	OES073719	T1PKS	PKSI	Copper	20.34	China: Anhui, Tongling
L180330P-5-LD180328-0003_HL3H2CCXY_L3_clean_bin.1.orig.fa_kl	OEZ008529	yes	LMSG_G000010172.1	OES073719	hserlactone	Others	Copper	6.91	China: Anhui, Tongling
L180330P-5-LD180328-0003_HL3H2CCXY_L3_clean_bin.1.orig.fa_kl	OEZ008529	yes	LMSG_G000010172.1	OES073719	acyl_amino_acids	Others	Copper	6.89	China: Anhui, Tongling
L180330P-5-LD180328-0003_HL3H2CCXY_L3_clean_bin.1.orig.fa_kl	OEZ008529	no	LMSG_G000010172.1	OES073719	ectoine	Others	Copper	7.95	China: Anhui, Tongling
L180330P-5-LD180328-0003_HL3H2CCXY_L3_clean_bin.1.orig.fa_kl	OEZ008529	no	LMSG_G000010172.1	OES073719	terpene	Terpene	Copper	12.12	China: Anhui, Tongling
L180330P-5-LD180328-0003_HL3H2CCXY_L3_clean_bin.1.orig.fa_kl	OEZ008529	no	LMSG_G000010172.1	OES073719	terpene	Terpene	Copper	17.67	China: Anhui, Tongling
L180330P-5-LD180328-0003_HL3H2CCXY_L3_clean_bin.10.strict.fa	OEZ008529	yes	LMSG_G000009379.1	OES073719	ectoine	Others	Copper	10.39	China: Anhui, Tongling
L180330P-5-LD180328-0003_HL3H2CCXY_L3_clean_bin.10.strict.fa	OEZ008529	yes	LMSG_G000009379.1	OES073719	hglE-KS	PKSother	Copper	23.19	China: Anhui, Tongling
L180330P-5-LD180328-0003_HL3H2CCXY_L3_clean_bin.10.strict.fa	OEZ008529	yes	LMSG_G000009379.1	OES073719	terpene	Terpene	Copper	18.78	China: Anhui, Tongling
L180330P-5-LD180328-0003_HL3H2CCXY_L3_clean_bin.15.strict.fa	OEZ008529	yes	LMSG_G000009553.1	OES073719	NRPS-like	NRPS	Copper	39.40	China: Anhui, Tongling
L180330P-5-LD180328-0003_HL3H2CCXY_L3_clean_bin.15.strict.fa	OEZ008529	yes	LMSG_G000009553.1	OES073719	ectoine	Others	Copper	10.40	China: Anhui, Tongling
L180330P-5-LD180328-0003_HL3H2CCXY_L3_clean_bin.15.strict.fa	OEZ008529	no	LMSG_G000009553.1	OES073719	terpene	Terpene	Copper	11.39	China: Anhui, Tongling
L180330P-5-LD180328-0003_HL3H2CCXY_L3_clean_bin.15.strict.fa	OEZ008529	no	LMSG_G000009553.1	OES073719	terpene	Terpene	Copper	13.21	China: Anhui, Tongling
L180330P-5-LD180328-0003_HL3H2CCXY_L3_clean_bin.15.strict.fa	OEZ008529	no	LMSG_G000009553.1	OES073719	terpene	Terpene	Copper	5.64	China: Anhui, Tongling
L180330P-5-LD180328-0003_HL3H2CCXY_L3_clean_bin.16.orig.fa_k	OEZ008529	yes	LMSG_G000010563.1	OES073719	arylpolyene	Others	Copper	35.01	China: Anhui, Tongling
L180330P-5-LD180328-0003_HL3H2CCXY_L3_clean_bin.16.orig.fa_k	OEZ008529	no	LMSG_G000010563.1	OES073719	terpene	Terpene	Copper	14.73	China: Anhui, Tongling
L180330P-5-LD180328-0003_HL3H2CCXY_L3_clean_bin.17.orig.fa_k	OEZ008529	yes	LMSG_G000007354.1	OES073719	bacteriocin	RiPPs	Copper	9.64	China: Anhui, Tongling
L180330P-5-LD180328-0003_HL3H2CCXY_L3_clean_bin.2.strict.fa_	OEZ008529	yes	LMSG_G000007341.1	OES073719	bacteriocin	RiPPs	Copper	9.13	China: Anhui, Tongling
L180330P-5-LD180328-0003_HL3H2CCXY_L3_clean_bin.20.orig.fa_k	OEZ008529	yes	LMSG_G000010283.1	OES073719	terpene	Terpene	Copper	24.85	China: Anhui, Tongling
L180330P-5-LD180328-0003_HL3H2CCXY_L3_clean_bin.20.orig.fa_k	OEZ008529	yes	LMSG_G000010283.1	OES073719	arylpolyene	Others	Copper	41.19	China: Anhui, Tongling
L180330P-5-LD180328-0003_HL3H2CCXY_L3_clean_bin.21.orig.fa_k	OEZ008529	yes	LMSG_G000009471.1	OES073719	bacteriocin	RiPPs	Copper	10.60	China: Anhui, Tongling

L180330P-5-LD180328-0003_HL3H2CCXY_L3_clean_bin.24.strict.fa	OEZ008529	yes	LMSG_G000010490.1	OES073719	arylpolyene	Others	Copper	21.85	China: Anhui, Tongling
L180330P-5-LD180328-0003_HL3H2CCXY_L3_clean_bin.25.strict.fa	OEZ008529	yes	LMSG_G000005696.1	OES073719	betalactone	Others	Copper	15.08	China: Anhui, Tongling
L180330P-5-LD180328-0003_HL3H2CCXY_L3_clean_bin.25.strict.fa	OEZ008529	yes	LMSG_G000005696.1	OES073719	bacteriocin	RiPPs	Copper	11.96	China: Anhui, Tongling
L180330P-5-LD180328-0003_HL3H2CCXY_L3_clean_bin.27.orig.fa_k	OEZ008529	yes	LMSG_G000006246.1	OES073719	bacteriocin	RiPPs	Copper	9.40	China: Anhui, Tongling
L180330P-5-LD180328-0003_HL3H2CCXY_L3_clean_bin.27.orig.fa_k	OEZ008529	yes	LMSG_G000006246.1	OES073719	bacteriocin	RiPPs	Copper	9.95	China: Anhui, Tongling
L180330P-5-LD180328-0003_HL3H2CCXY_L3_clean_bin.27.orig.fa_k	OEZ008529	yes	LMSG_G000006246.1	OES073719	terpene	Terpene	Copper	22.98	China: Anhui, Tongling
L180330P-5-LD180328-0003_HL3H2CCXY_L3_clean_bin.29.orig.fa_k	OEZ008529	no	LMSG_G000010304.1	OES073719	terpene	Terpene	Copper	21.49	China: Anhui, Tongling
L180330P-5-LD180328-0003_HL3H2CCXY_L3_clean_bin.29.orig.fa_k	OEZ008529	no	LMSG_G000010304.1	OES073719	arylpolyene	Others	Copper	34.38	China: Anhui, Tongling
L180330P-5-LD180328-0003_HL3H2CCXY_L3_clean_bin.30.strict.fa	OEZ008529	yes	LMSG_G000006294.1	OES073719	ectoine	Others	Copper	8.32	China: Anhui, Tongling
L180330P-5-LD180328-0003_HL3H2CCXY_L3_clean_bin.30.strict.fa	OEZ008529	yes	LMSG_G000006294.1	OES073719	terpene	Terpene	Copper	20.12	China: Anhui, Tongling
L180330P-5-LD180328-0003_HL3H2CCXY_L3_clean_bin.30.strict.fa	OEZ008529	yes	LMSG_G000006294.1	OES073719	terpene	Terpene	Copper	10.22	China: Anhui, Tongling
L180330P-5-LD180328-0003_HL3H2CCXY_L3_clean_bin.31.orig.fa_k	OEZ008529	yes	LMSG_G000007069.1	OES073719	bacteriocin	RiPPs	Copper	11.01	China: Anhui, Tongling
L180330P-5-LD180328-0003_HL3H2CCXY_L3_clean_bin.34.strict.fa	OEZ008529	yes	LMSG_G000009599.1	OES073719	terpene	Terpene	Copper	22.15	China: Anhui, Tongling
L180330P-5-LD180328-0003_HL3H2CCXY_L3_clean_bin.34.strict.fa	OEZ008529	yes	LMSG_G000009599.1	OES073719	NRPS-like	NRPS	Copper	24.82	China: Anhui, Tongling
L180330P-5-LD180328-0003_HL3H2CCXY_L3_clean_bin.34.strict.fa	OEZ008529	yes	LMSG_G000009599.1	OES073719	acyl_amino_acids	Others	Copper	18.92	China: Anhui, Tongling
L180330P-5-LD180328-0003_HL3H2CCXY_L3_clean_bin.34.strict.fa	OEZ008529	yes	LMSG_G000009599.1	OES073719	terpene	Terpene	Copper	10.55	China: Anhui, Tongling
L180330P-5-LD180328-0003_HL3H2CCXY_L3_clean_bin.34.strict.fa	OEZ008529	yes	LMSG_G000009599.1	OES073719	ectoine	Others	Copper	5.56	China: Anhui, Tongling
L180330P-5-LD180328-0003_HL3H2CCXY_L3_clean_bin.4.orig.fa_k1	OEZ008529	yes	LMSG_G000010506.1	OES073719	arylpolyene	Others	Copper	8.13	China: Anhui, Tongling
L180330P-5-LD180328-0003_HL3H2CCXY_L3_clean_bin.4.orig.fa_k1	OEZ008529	yes	LMSG_G000010506.1	OES073719	hserlactone	Others	Copper	7.46	China: Anhui, Tongling
L180330P-5-LD180328-0003_HL3H2CCXY_L3_clean_bin.5.orig.fa_k1	OEZ008529	no	LMSG_G000010578.1	OES073719	bacteriocin	RiPPs	Copper	10.85	China: Anhui, Tongling
L180330P-5-LD180328-0003_HL3H2CCXY_L3_clean_bin.5.orig.fa_k1	OEZ008529	no	LMSG_G000010578.1	OES073719	T1PKS	PKSI	Copper	23.87	China: Anhui, Tongling
L180330P-5-LD180328-0003_HL3H2CCXY_L3_clean_bin.5.orig.fa_k1	OEZ008529	yes	LMSG_G000010578.1	OES073719	terpene	Terpene	Copper	20.82	China: Anhui, Tongling
L180330P-5-LD180328-0003_HL3H2CCXY_L3_clean_bin.6.orig.fa_k1	OEZ008529	yes	LMSG_G000009447.1	OES073719	betalactone	Others	Copper	21.58	China: Anhui, Tongling
L180330P-5-LD180328-0003_HL3H2CCXY_L3_clean_bin.6.orig.fa_k1	OEZ008529	yes	LMSG_G000009447.1	OES073719	ectoine	Others	Copper	8.29	China: Anhui, Tongling
L180330P-5-LD180328-0003_HL3H2CCXY_L3_clean_bin.6.orig.fa_k1	OEZ008529	yes	LMSG_G000009447.1	OES073719	bacteriocin	RiPPs	Copper	10.85	China: Anhui, Tongling
L180330P-5-LD180328-0003_HL3H2CCXY_L3_clean_bin.6.orig.fa_k1	OEZ008529	yes	LMSG_G000009447.1	OES073719	betalactone	Others	Copper	30.86	China: Anhui, Tongling
L180330P-5-LD180328-0003_HL3H2CCXY_L3_clean_bin.9.orig.fa_k1	OEZ008529	yes	LMSG_G000007242.1	OES073719	betalactone	Others	Copper	11.32	China: Anhui, Tongling
L180330P-5-LD180328-0004_HL3H2CCXY_L3_clean_bin.10.orig.fa_k	OEZ008529	yes	LMSG_G000009131.1	OES073682	bacteriocin	RiPPs	Lead-Zinc	10.95	China: Guangdong, Fankou
L180330P-5-LD180328-0004_HL3H2CCXY_L3_clean_bin.10.orig.fa_k	OEZ008529	yes	LMSG_G000009131.1	OES073682	betalactone	Others	Lead-Zinc	27.10	China: Guangdong, Fankou
L180330P-5-LD180328-0004_HL3H2CCXY_L3_clean_bin.10.orig.fa_k	OEZ008529	yes	LMSG_G000009131.1	OES073682	terpene	Terpene	Lead-Zinc	25.21	China: Guangdong, Fankou
L180330P-5-LD180328-0004_HL3H2CCXY_L3_clean_bin.10.orig.fa_k	OEZ008529	yes	LMSG_G000009131.1	OES073682	terpene	Terpene	Lead-Zinc	24.96	China: Guangdong, Fankou
L180330P-5-LD180328-0004_HL3H2CCXY_L3_clean_bin.10.orig.fa_k	OEZ008529	yes	LMSG_G000009131.1	OES073682	NRPS-like	NRPS	Lead-Zinc	37.30	China: Guangdong, Fankou
L180330P-5-LD180328-0004_HL3H2CCXY_L3_clean_bin.10.orig.fa_k	OEZ008529	yes	LMSG_G000009131.1	OES073682	lassopeptide	RiPPs	Lead-Zinc	18.02	China: Guangdong, Fankou
L180330P-5-LD180328-0004_HL3H2CCXY_L3_clean_bin.13.orig.fa_k	OEZ008529	yes	LMSG_G000004537.1	OES073682	betalactone	Others	Lead-Zinc	25.93	China: Guangdong, Fankou
L180330P-5-LD180328-0004_HL3H2CCXY_L3_clean_bin.14.orig.fa_k	OEZ008529	yes	LMSG_G000008101.1	OES073682	TfuA-related.bacteriocin	RiPPs	Lead-Zinc	19.10	China: Guangdong, Fankou
L180330P-5-LD180328-0004_HL3H2CCXY_L3_clean_bin.15.strict.fa	OEZ008529	no	LMSG_G000010539.1	OES073682	terpene	Terpene	Lead-Zinc	19.53	China: Guangdong, Fankou
L180330P-5-LD180328-0004_HL3H2CCXY_L3_clean_bin.17.orig.fa_k	OEZ008529	yes	LMSG_G000010472.1	OES073682	terpene	Terpene	Lead-Zinc	12.69	China: Guangdong, Fankou
L180330P-5-LD180328-0004_HL3H2CCXY_L3_clean_bin.17.orig.fa_k	OEZ008529	yes	LMSG_G000010472.1	OES073682	arylpolyene.hserlactone	Others	Lead-Zinc	12.45	China: Guangdong, Fankou
L180330P-5-LD180328-0004_HL3H2CCXY_L3_clean_bin.17.orig.fa_k	OEZ008529	yes	LMSG_G000010472.1	OES073682	NRPS-like	NRPS	Lead-Zinc	19.65	China: Guangdong, Fankou
L180330P-5-LD180328-0004_HL3H2CCXY_L3_clean_bin.17.orig.fa_k	OEZ008529	no	LMSG_G000010472.1	OES073682	arylpolyene	Others	Lead-Zinc	14.83	China: Guangdong, Fankou
L180330P-5-LD180328-0004_HL3H2CCXY_L3_clean_bin.2.orig.fa_k1	OEZ008529	yes	LMSG_G000008368.1	OES073682	terpene	Terpene	Lead-Zinc	19.14	China: Guangdong, Fankou
L180330P-5-LD180328-0004_HL3H2CCXY_L3_clean_bin.20.strict.fa	OEZ008529	yes	LMSG_G000010033.1	OES073682	T3PKS	PKSother	Lead-Zinc	19.84	China: Guangdong, Fankou
L180330P-5-LD180328-0004_HL3H2CCXY_L3_clean_bin.20.strict.fa	OEZ008529	yes	LMSG_G000010033.1	OES073682	terpene	Terpene	Lead-Zinc	17.69	China: Guangdong, Fankou
L180330P-5-LD180328-0004_HL3H2CCXY_L3_clean_bin.20.strict.fa	OEZ008529	yes	LMSG_G000010033.1	OES073682	terpene	Terpene	Lead-Zinc	11.70	China: Guangdong, Fankou
L180330P-5-LD180328-0004_HL3H2CCXY_L3_clean_bin.20.strict.fa	OEZ008529	yes	LMSG_G000010033.1	OES073682	NRPS-like	NRPS	Lead-Zinc	14.55	China: Guangdong, Fankou
L180330P-5-LD180328-0004_HL3H2CCXY_L3_clean_bin.25.orig.fa_k	OEZ008529	yes	LMSG_G000008060.1	OES073682	terpene	Terpene	Lead-Zinc	19.69	China: Guangdong, Fankou
L180330P-5-LD180328-0004_HL3H2CCXY_L3_clean_bin.25.orig.fa_k	OEZ008529	yes	LMSG_G000008060.1	OES073682	terpene	Terpene	Lead-Zinc	12.72	China: Guangdong, Fankou
L180330P-5-LD180328-0004_HL3H2CCXY_L3_clean_bin.28.strict.fa	OEZ008529	yes	LMSG_G000005705.1	OES073682	lassopeptide	RiPPs	Lead-Zinc	12.12	China: Guangdong, Fankou
L180330P-5-LD180328-0004_HL3H2CCXY_L3_clean_bin.28.strict.fa	OEZ008529	yes	LMSG_G000005705.1	OES073682	T3PKS	PKSother	Lead-Zinc	14.76	China: Guangdong, Fankou
L180330P-5-LD180328-0004_HL3H2CCXY_L3_clean_bin.28.strict.fa	OEZ008529	yes	LMSG_G000005705.1	OES073682	bacteriocin	RiPPs	Lead-Zinc	9.80	China: Guangdong, Fankou
L180330P-5-LD180328-0004_HL3H2CCXY_L3_clean_bin.28.strict.fa	OEZ008529	yes	LMSG_G000005705.1	OES073682	terpene	Terpene	Lead-Zinc	8.73	China: Guangdong, Fankou
L180330P-5-LD180328-0004_HL3H2CCXY_L3_clean_bin.29.orig.fa_k	OEZ008529	yes	LMSG_G000004538.1	OES073682	T3PKS	PKSother	Lead-Zinc	41.05	China: Guangdong, Fankou
L180330P-5-LD180328-0004_HL3H2CCXY_L3_clean_bin.29.orig.fa_k	OEZ008529	yes	LMSG_G000004538.1	OES073682	bacteriocin	RiPPs	Lead-Zinc	10.92	China: Guangdong, Fankou
L180330P-5-LD180328-0004_HL3H2CCXY_L3_clean_bin.29.orig.fa_k	OEZ008529	yes	LMSG_G000004538.1	OES073682	terpene	Terpene	Lead-Zinc	21.93	China: Guangdong, Fankou
L180330P-5-LD180328-0004_HL3H2CCXY_L3_clean_bin.29.orig.fa_k	OEZ008529	yes	LMSG_G000004538.1	OES073682	lassopeptide	RiPPs	Lead-Zinc	14.86	China: Guangdong, Fankou
L180330P-5-LD180328-0004_HL3H2CCXY_L3_clean_bin.29.orig.fa_k	OEZ008529	yes	LMSG_G000004538.1	OES073682	terpene	Terpene	Lead-Zinc	19.20	China: Guangdong, Fankou
L180330P-5-LD180328-0004_HL3H2CCXY_L3_clean_bin.29.orig.fa_k	OEZ008529	yes	LMSG_G000004538.1	OES073682	NRPS-like	NRPS	Lead-Zinc	35.98	China: Guangdong, Fankou
L180330P-5-LD180328-0004_HL3H2CCXY_L3_clean_bin.34.strict.fa	OEZ008529	yes	LMSG_G000009211.1	OES073682	terpene	Terpene	Lead-Zinc	17.47	China: Guangdong, Fankou
L180330P-5-LD180328-0004_HL3H2CCXY_L3_clean_bin.34.strict.fa	OEZ008529	yes	LMSG_G000009211.1	OES073682	terpene	Terpene	Lead-Zinc	21.72	China: Guangdong, Fankou
L180330P-5-LD180328-0004_HL3H2CCXY_L3_clean_bin.34.strict.fa	OEZ008529	yes	LMSG_G000009211.1	OES073682	T3PKS	PKSother	Lead-Zinc	7.06	China: Guangdong, Fankou
L180330P-5-LD180328-0004_HL3H2CCXY_L3_clean_bin.35.strict.fa	OEZ008529	yes	LMSG_G000010001.1	OES073682	terpene	Terpene	Lead-Zinc	22.09	China: Guangdong, Fankou
L180330P-5-LD180328-0004_HL3H2CCXY_L3_clean_bin.35.strict.fa	OEZ008529	yes	LMSG_G000010001.1	OES073682	NRPS-like	NRPS	Lead-Zinc	23.06	China: Guangdong, Fankou
L180330P-5-LD180328-0004_HL3H2CCXY_L3_clean_bin.35.strict.fa	OEZ008529	yes	LMSG_G000010001.1	OES073682	terpene.arylpolyene	Others	Lead-Zinc	11.99	China: Guangdong, Fankou
L180330P-5-LD180328-0004_HL3H2CCXY_L3_clean_bin.37.permissiv	OEZ008529	yes	LMSG_G000005708.1	OES073682	terpene	Terpene	Lead-Zinc	21.81	China: Guangdong, Fankou
L180330P-5-LD180328-0004_HL3H2CCXY_L3_clean_bin.37.permissiv	OEZ008529	yes	LMSG_G000005708.1	OES073682	arylpolyene	Others	Lead-Zinc	34.06	China: Guangdong, Fankou
L180330P-5-LD180328-0004_HL3H2CCXY_L3_clean_bin.37.permissiv	OEZ008529	yes	LMSG_G000005708.1	OES073682	arylpolyene	Others	Lead-Zinc	40.44	China: Guangdong, Fankou
L180330P-5-LD180328-0004_HL3H2CCXY_L3_clean_bin.37.permissiv	OEZ008529	yes	LMSG_G000005708.1	OES073682	ladderane	Others	Lead-Zinc	30.81	China: Guangdong, Fankou
L180330P-5-LD180328-0004_HL3H2CCXY_L3_clean_bin.37.permissiv	OEZ008529	yes	LMSG_G000005708.1	OES073682	terpene	Terpene	Lead-Zinc	14.20	China: Guangdong, Fankou
L180330P-5-LD180328-0004_HL3H2CCXY_L3_clean_bin.38.orig.fa_k	OEZ008529	yes	LMSG_G000007959.1	OES073682	betalactone	Others	Lead-Zinc	14.75	China: Guangdong, Fankou
L180330P-5-LD180328-0004_HL3H2CCXY_L3_clean_bin.38.orig.fa_k	OEZ008529	yes	LMSG_G000007959.1	OES073682	terpene	Terpene	Lead-Zinc	26.80	China: Guangdong, Fankou
L180330P-5-LD180328-0004_HL3H2CCXY_L3_clean_bin.39.orig.fa_k	OEZ008529	yes	LMSG_G000010232.1	OES073682	hserlactone	Others	Lead-Zinc	18.12	China: Guangdong, Fankou
L180330P-5-LD180328-0004_HL3H2CCXY_L3_clean_bin.39.orig.fa_k	OEZ008529	yes	LMSG_G000010232.1	OES073682	terpene.arylpolyene	Others	Lead-Zinc	42.19	China: Guangdong, Fankou
L180330P-5-LD180328-0004_HL3H2CCXY_L3_clean_bin.39.orig.fa_k	OEZ008529	yes	LMSG_G000010232.1	OES073682	terpene	Terpene	Lead-Zinc	20.79	China: Guangdong, Fankou
L180330P-5-LD180328-0004_HL3H2CCXY_L3_clean_bin.4.orig.fa_k1	OEZ008529	yes	LMSG_G000005710.1	OES073682	arylpolyene	Others	Lead-Zinc	22.82	China: Guangdong, Fankou
L180330P-5-LD180328-0004_HL3H2CCXY_L3_clean_bin.4.orig.fa_k1	OEZ008529	yes	LMSG_G000005710.1	OES073682	acyl_amino_acids	Others	Lead-Zinc	33.09	China: Guangdong, Fankou
L180330P-5-LD180328-0004_HL3H2CCXY_L3_clean_bin.4.orig.fa_k1	OEZ008529	yes	LMSG_G000005710.1	OES073682	terpene	Terpene	Lead-Zinc	14.91	China: Guangdong, Fankou
L180330P-5-LD180328-0004_HL3H2CCXY_L3_clean_bin.42.strict.fa	OEZ008529	yes	LMSG_G000007878.1	OES073682	T3PKS.terpene	Others	Lead-Zinc	33.88	China: Guangdong, Fankou
L180330P-5-LD180328-0004_HL3H2CCXY_L3_clean_bin.42.strict.fa	OEZ008529	yes	LMSG_G000007878.1	OES073682	T1PKS.NRPS-like	PKS-NRP_Hybrids	Lead-Zinc	36.49	China: Guangdong, Fankou
L180330P-5-LD180328-0004_HL3H2CCXY_L3_clean_bin.42.strict.fa	OEZ008529	yes	LMSG_G000007878.1	OES073682	terpene	Terpene	Lead-Zinc	15.41	China: Guangdong, Fankou
L180330P-5-LD180328-0004_HL3H2CCXY_L3_clean_bin.47.strict.fa	OEZ008529	yes	LMSG_G000005709.1	OES073682	terpene	Terpene	Lead-Zinc	21.66	China: Guangdong, Fankou
L180330P-5-LD180328-0004_HL3H2CCXY_L3_clean_bin.47.strict.fa	OEZ008529	yes	LMSG_G000005709.1	OES073682	NRPS-like	NRPS	Lead-Zinc	27.72	China: Guangdong, Fankou
L180330P-5-LD180328-0004_HL3H2CCXY_L3_clean_bin.47.strict.fa	OEZ008529	yes	LMSG_G000005709.1	OES073682	hserlactone	Others	Lead-Zinc	13.89	China: Guangdong, Fankou
L180330P-5-LD180328-0004_HL3H2CCXY_L3_clean_bin.49.orig.fa_k	OEZ008529	yes	LMSG_G000004539.1	OES073682	terpene	Terpene	Lead-Zinc	20.82	China: Guangdong, Fankou
L180330P-5-LD180328-0004_HL3H2CCXY_L3_clean_bin.49.orig.fa_k	OEZ008529	yes	LMSG_G000004539.1	OES073682	terpene	Terpene	Lead-Zinc	20.83	China: Guangdong, Fankou
L180330P-5-LD180328-0004_HL3H2CCXY_L3_clean_bin.49.orig.fa_k	OEZ008529	yes	LMSG_G000004539.1	OES073682	arylpolyene	Others	Lead-Zinc	41.22	China: Guangdong, Fankou
L180330P-5-LD180328-0004_HL3H2CCXY_L3_clean_bin.49.orig.fa_k	OEZ008529	yes	LMSG_G000004539.1	OES073682	hserlactone	Others	Lead-Zinc	14.00	China: Guangdong, Fankou
L180330P-5-LD180328-0004_HL3H2CCXY_L3_clean_bin.5.strict.fa_	OEZ008529	yes	LMSG_G000005714.1	OES073682	bacteriocin	RiPPs	Lead-Zinc	5.98	China: Guangdong, Fankou
L180330P-5-LD180328-0004_HL3H2CCXY_L3_clean_bin.5.strict.fa_	OEZ008529	yes	LMSG_G000005714.1	OES073682	terpene	Terpene	Lead-Zinc	22.05	China: Guangdong, Fankou
L180330P-5-LD180328-0004_HL3H2CCXY_L3_clean_bin.5.strict.fa_	OEZ008529	yes	LMSG_G000005714.1	OES073682	terpene	Terpene	Lead-Zinc	16.76	China: Guangdong, Fankou
L180330P-5-LD180328-0004_HL3H2CCXY_L3_clean_bin.50.orig.fa_k	OEZ008529	yes	LMSG_G000009080.1	OES073682	arylpolyene	Others	Lead-Zinc	6.80	China: Guangdong, Fankou
L180330P-5-LD180328-0004_HL3H2CCXY_L3_clean_bin.51.orig.fa_k	OEZ008529	yes	LMSG_G000007340.1	OES073682	bacteriocin	RiPPs	Lead-Zinc	9.20	China: Guangdong, Fankou
L180330P-5-LD180328-0004_HL3H2CCXY_L3_clean_bin.52.orig.fa_k	OEZ008529	yes	LMSG_G000005711.1	OES073682	terpene	Terpene	Lead-Zinc	21.97	China: Guangdong, Fankou
L180330P-5-LD180328-0004_HL3H2CCXY_L3_clean_bin.52.orig.fa_k	OEZ008529	yes	LMSG_G000005711.1	OES073682	terpene	Terpene	Lead-Zinc	20.85	China: Guangdong, Fankou
L180330P-5-LD180328-0004_HL3H2CCXY_L3_clean_bin.52.orig.fa_k	OEZ008529	yes	LMSG_G000005711.1	OES073682	T3PKS	PKSother	Lead-Zinc	41.07	China: Guangdong, Fankou
L180330P-5-LD180328-0004_HL3H2CCXY_L3_clean_bin.52.orig.fa_k	OEZ008529	yes	LMSG_G000005711.1	OES073682	bacteriocin	RiPPs	Lead-Zinc	10.87	China: Guangdong, Fankou
L180330P-5-LD180328-0004_HL3H2CCXY_L3_clean_bin.52.orig.fa_k	OEZ008529	yes	LMSG_G000005711.1	OES073682	TfuA-related.terpene	Others	Lead-Zinc	18.05	China: Guangdong, Fankou
L180330P-5-LD180328-0004_HL3H2CCXY_L3_clean_bin.52.orig.fa_k	OEZ008529	yes	LMSG_G000005711.1	OES073682	NRPS-like	NRPS	Lead-Zinc	42.74	China: Guangdong, Fankou
L180330P-5-LD180328-0004_HL3H2CCXY_L3_clean_bin.52.orig.fa_k	OEZ008529	yes	LMSG_G000005711.1	OES073682	bacteriocin	RiPPs	Lead-Zinc	13.89	China: Guangdong, Fankou
L180330P-5-LD180328-0004_HL3H2CCXY_L3_clean_bin.64.strict.fa	OEZ008529	yes	LMSG_G000005716.1	OES073682	terpene	Terpene	Lead-Zinc	21.73	China: Guangdong, Fankou
L180330P-5-LD180328-0004_HL3H2CCXY_L3_clean_bin.64.strict.fa	OEZ008529	yes	LMSG_G000005716.1	OES073682	terpene	Terpene	Lead-Zinc	22.06	China: Guangdong, Fankou
L180330P-5-LD180328-0004_HL3H2CCXY_L3_clean_bin.66.orig.fa_k	OEZ008529	yes	LMSG_G000009109.1	OES073682	NRPS-like	NRPS	Lead-Zinc	30.04	China: Guangdong, Fankou
L180330P-5-LD180328-0004_HL3H2CCXY_L3_clean_bin.66.orig.fa_k	OEZ008529	yes	LMSG_G000009109.1	OES073682	terpene	Terpene	Lead-Zinc	21.97	China: Guangdong, Fankou
L180330P-5-LD180328-0004_HL3H2CCXY_L3_clean_bin.66.orig.fa_k	OEZ008529	yes	LMSG_G000009109.1	OES073682	T3PKS	PKSother	Lead-Zinc	24.00	China: Guangdong, Fankou
L180330P-5-LD180328-0004_HL3H2CCXY_L3_clean_bin.69.orig.fa_k	OEZ008529	yes	LMSG_G000004540.1	OES073682	terpene	Terpene	Lead-Zinc	20.23	China: Guangdong, Fankou
L180330P-5-LD180328-0004_HL3H2CCXY_L3_clean_bin.69.orig.fa_k	OEZ008529	yes	LMSG_G000004540.1	OES073682	bacteriocin	RiPPs	Lead-Zinc	11.94	China: Guangdong, Fankou
L180330P-5-LD180328-0004_HL3H2CCXY_L3_clean_bin.71.orig.fa_k	OEZ008529	yes	LMSG_G000009616.1	OES073682	terpene	Terpene	Lead-Zinc	21.98	China: Guangdong, Fankou
L180330P-5-LD180328-0004_HL3H2CCXY_L3_clean_bin.71.orig.fa_k	OEZ008529	yes	LMSG_G000009616.1	OES073682	terpene	Terpene	Lead-Zinc	10.41	China: Guangdong, Fankou
L180330P-5-LD180328-0004_HL3H2CCXY_L3_clean_bin.73.orig.fa_k	OEZ008529	yes	LMSG_G000009123.1	OES073682	bacteriocin	RiPPs	Lead-Zinc	10.87	China: Guangdong, Fankou
L180330P-5-LD180328-0004_HL3H2CCXY_L3_clean_bin.73.orig.fa_k	OEZ008529	yes	LMSG_G000009123.1	OES073682	terpene	Terpene	Lead-Zinc	25.07	China: Guangdong, Fankou
L180330P-5-LD180328-0004_HL3H2CCXY_L3_clean_bin.73.orig.fa_k	OEZ008529	yes	LMSG_G000009123.1	OES073682	NRPS-like	NRPS	Lead-Zinc	31.81	China: Guangdong, Fankou
L180330P-5-LD180328-0004_HL3H2CCXY_L3_clean_bin.74.orig.fa_k	OEZ008529	yes	LMSG_G000005718.1	OES073682	hserlactone	Others	Lead-Zinc	7.25	China: Guangdong, Fankou
L180330P-5-LD180328-0004_HL3H2CCXY_L3_clean_bin.74.orig.fa_k	OEZ008529	yes	LMSG_G000005718.1	OES073682	NRPS-like	NRPS	Lead-Zinc	23.95	China: Guangdong, Fankou
L180330P-5-LD180328-0004_HL3H2CCXY_L3_clean_bin.74.orig.fa_k	OEZ008529	yes	LMSG_G000005718.1	OES073682	terpene	Terpene	Lead-Zinc	6.12	China: Guangdong, Fankou
L180330P-5-LD180328-0004_HL3H2CCXY_L3_clean_bin.75.strict.fa	OEZ008529	yes	LMSG_G000009665.1	OES073682	NRPS-like	NRPS	Lead-Zinc	33.66	China: Guangdong, Fankou
L180330P-5-LD180328-0004_HL3H2CCXY_L3_clean_bin.75.strict.fa	OEZ008529	yes	LMSG_G000009665.1	OES073682	nucleoside	Others	Lead-Zinc	17.42	China: Guangdong, Fankou
L180330P-5-LD180328-0004_HL3H2CCXY_L3_clean_bin.75.strict.fa	OEZ008529	yes	LMSG_G000009665.1	OES073682	NRPS-like	NRPS	Lead-Zinc	35.83	China: Guangdong, Fankou
L180330P-5-LD180328-0004_HL3H2CCXY_L3_clean_bin.75.strict.fa	OEZ008529	yes	LMSG_G000009665.1	OES073682	hserlactone	Others	Lead-Zinc	20.66	China: Guangdong, Fankou
L180330P-5-LD180328-0004_HL3H2CCXY_L3_clean_bin.75.strict.fa	OEZ008529	yes	LMSG_G000009665.1	OES073682	terpene	Terpene	Lead-Zinc	22.02	China: Guangdong, Fankou
L180330P-5-LD180328-0004_HL3H2CCXY_L3_clean_bin.77.orig.fa_k	OEZ008529	yes	LMSG_G000004541.1	OES073682	terpene	Terpene	Lead-Zinc	16.39	China: Guangdong, Fankou
L180330P-5-LD180328-0004_HL3H2CCXY_L3_clean_bin.77.orig.fa_k	OEZ008529	yes	LMSG_G000004541.1	OES073682	terpene	Terpene	Lead-Zinc	23.88	China: Guangdong, Fankou
L180330P-5-LD180328-0004_HL3H2CCXY_L3_clean_bin.77.orig.fa_k	OEZ008529	yes	LMSG_G000004541.1	OES073682	NRPS-like	NRPS	Lead-Zinc	27.94	China: Guangdong, Fankou
L180330P-5-LD180328-0004_HL3H2CCXY_L3_clean_bin.77.orig.fa_k	OEZ008529	yes	LMSG_G000004541.1	OES073682	terpene	Terpene	Lead-Zinc	21.88	China: Guangdong, Fankou
L180330P-5-LD180328-0004_HL3H2CCXY_L3_clean_bin.77.orig.fa_k	OEZ008529	yes	LMSG_G000004541.1	OES073682	lassopeptide	RiPPs	Lead-Zinc	21.59	China: Guangdong, Fankou
L180330P-5-LD180328-0004_HL3H2CCXY_L3_clean_bin.79.strict.fa	OEZ008529	yes	LMSG_G000009176.1	OES073682	terpene	Terpene	Lead-Zinc	11.44	China: Guangdong, Fankou
L180330P-5-LD180328-0004_HL3H2CCXY_L3_clean_bin.79.strict.fa	OEZ008529	yes	LMSG_G000009176.1	OES073682	terpene	Terpene	Lead-Zinc	11.67	China: Guangdong, Fankou
L180330P-5-LD180328-0004_HL3H2CCXY_L3_clean_bin.81.orig.fa_k	OEZ008529	yes	LMSG_G000007917.1	OES073682	NRPS-like	NRPS	Lead-Zinc	18.56	China: Guangdong, Fankou
L180330P-5-LD180328-0004_HL3H2CCXY_L3_clean_bin.81.orig.fa_k	OEZ008529	yes	LMSG_G000007917.1	OES073682	T3PKS	PKSother	Lead-Zinc	41.09	China: Guangdong, Fankou
L180330P-5-LD180328-0004_HL3H2CCXY_L3_clean_bin.81.orig.fa_k	OEZ008529	yes	LMSG_G000007917.1	OES073682	terpene	Terpene	Lead-Zinc	29.02	China: Guangdong, Fankou
L180330P-5-LD180328-0004_HL3H2CCXY_L3_clean_bin.81.orig.fa_k	OEZ008529	yes	LMSG_G000007917.1	OES073682	NRPS-like	NRPS	Lead-Zinc	39.38	China: Guangdong, Fankou
L180330P-5-LD180328-0004_HL3H2CCXY_L3_clean_bin.81.orig.fa_k	OEZ008529	yes	LMSG_G000007917.1	OES073682	lanthipeptide	RiPPs	Lead-Zinc	23.26	China: Guangdong, Fankou
L180330P-5-LD180328-0004_HL3H2CCXY_L3_clean_bin.81.orig.fa_k	OEZ008529	yes	LMSG_G000007917.1	OES073682	bacteriocin	RiPPs	Lead-Zinc	12.00	China: Guangdong, Fankou
L180330P-5-LD180328-0004_HL3H2CCXY_L3_clean_bin.81.orig.fa_k	OEZ008529	yes	LMSG_G000007917.1	OES073682	NRPS-like	NRPS	Lead-Zinc	25.59	China: Guangdong, Fankou
L180330P-5-LD180328-0004_HL3H2CCXY_L3_clean_bin.85.orig.fa_k	OEZ008529	yes	LMSG_G000007371.1	OES073682	bacteriocin	RiPPs	Lead-Zinc	11.97	China: Guangdong, Fankou
L180330P-5-LD180328-0004_HL3H2CCXY_L3_clean_bin.86.strict.fa	OEZ008529	yes	LMSG_G000004542.1	OES073682	NRPS-like	NRPS	Lead-Zinc	26.46	China: Guangdong, Fankou
L180330P-5-LD180328-0004_HL3H2CCXY_L3_clean_bin.87.strict.fa	OEZ008529	yes	LMSG_G000007999.1	OES073682	TfuA-related.bacteriocin	RiPPs	Lead-Zinc	15.49	China: Guangdong, Fankou
L180330P-5-LD180328-0004_HL3H2CCXY_L3_clean_bin.9.orig.fa_k1	OEZ008529	yes	LMSG_G000008421.1	OES073682	terpene	Terpene	Lead-Zinc	9.94	China: Guangdong, Fankou
L180330P-5-LD180328-0004_HL3H2CCXY_L3_clean_bin.92.orig.fa_k	OEZ008529	yes	LMSG_G000007033.1	OES073682	bacteriocin	RiPPs	Lead-Zinc	9.45	China: Guangdong, Fankou
L180330P-5-LD180328-0004_HL3H2CCXY_L3_clean_bin.98.orig.fa_k	OEZ008529	yes	LMSG_G000008424.1	OES073682	terpene	Terpene	Lead-Zinc	22.54	China: Guangdong, Fankou
L180330P-5-LD180328-0004_HL3H2CCXY_L3_clean_bin.98.orig.fa_k	OEZ008529	yes	LMSG_G000008424.1	OES073682	bacteriocin	RiPPs	Lead-Zinc	10.80	China: Guangdong, Fankou
L180330P-5-LD180328-0004_HL3H2CCXY_L3_clean_bin.98.orig.fa_k	OEZ008529	yes	LMSG_G000008424.1	OES073682	other	Others	Lead-Zinc	11.89	China: Guangdong, Fankou
L180330P-5-LD180328-0004_HL3H2CCXY_L3_clean_bin.98.orig.fa_k	OEZ008529	yes	LMSG_G000008424.1	OES073682	bacteriocin	RiPPs	Lead-Zinc	10.41	China: Guangdong, Fankou
L180330P-5-LD180328-0005_HL3H2CCXY_L3_clean_bin.1.orig.fa_k1	OEZ008529	yes	LMSG_G000009133.1	OES073683	betalactone	Others	Lead-Zinc	26.18	China: Guangdong, Fankou
L180330P-5-LD180328-0005_HL3H2CCXY_L3_clean_bin.1.orig.fa_k1	OEZ008529	yes	LMSG_G000009133.1	OES073683	bacteriocin	RiPPs	Lead-Zinc	9.11	China: Guangdong, Fankou
L180330P-5-LD180328-0005_HL3H2CCXY_L3_clean_bin.1.orig.fa_k1	OEZ008529	yes	LMSG_G000009133.1	OES073683	terpene	Terpene	Lead-Zinc	25.21	China: Guangdong, Fankou
L180330P-5-LD180328-0005_HL3H2CCXY_L3_clean_bin.1.orig.fa_k1	OEZ008529	yes	LMSG_G000009133.1	OES073683	bacteriocin	RiPPs	Lead-Zinc	10.85	China: Guangdong, Fankou
L180330P-5-LD180328-0005_HL3H2CCXY_L3_clean_bin.1.orig.fa_k1	OEZ008529	yes	LMSG_G000009133.1	OES073683	lassopeptide	RiPPs	Lead-Zinc	22.39	China: Guangdong, Fankou
L180330P-5-LD180328-0005_HL3H2CCXY_L3_clean_bin.1.orig.fa_k1	OEZ008529	yes	LMSG_G000009133.1	OES073683	NRPS-like	NRPS	Lead-Zinc	44.44	China: Guangdong, Fankou
L180330P-5-LD180328-0005_HL3H2CCXY_L3_clean_bin.100.orig.fa_	OEZ008529	yes	LMSG_G000011118.1	OES073683	arylpolyene	Others	Lead-Zinc	41.16	China: Guangdong, Fankou
L180330P-5-LD180328-0005_HL3H2CCXY_L3_clean_bin.102.orig.fa_	OEZ008529	yes	LMSG_G000008509.1	OES073683	arylpolyene	Others	Lead-Zinc	17.03	China: Guangdong, Fankou
L180330P-5-LD180328-0005_HL3H2CCXY_L3_clean_bin.102.orig.fa_	OEZ008529	yes	LMSG_G000008509.1	OES073683	lassopeptide	RiPPs	Lead-Zinc	23.45	China: Guangdong, Fankou
L180330P-5-LD180328-0005_HL3H2CCXY_L3_clean_bin.102.orig.fa_	OEZ008529	yes	LMSG_G000008509.1	OES073683	terpene	Terpene	Lead-Zinc	14.34	China: Guangdong, Fankou
L180330P-5-LD180328-0005_HL3H2CCXY_L3_clean_bin.104.strict.f	OEZ008529	yes	LMSG_G000008301.1	OES073683	terpene	Terpene	Lead-Zinc	11.47	China: Guangdong, Fankou
L180330P-5-LD180328-0005_HL3H2CCXY_L3_clean_bin.105.orig.fa_	OEZ008529	yes	LMSG_G000009127.1	OES073683	NRPS-like	NRPS	Lead-Zinc	31.75	China: Guangdong, Fankou
L180330P-5-LD180328-0005_HL3H2CCXY_L3_clean_bin.105.orig.fa_	OEZ008529	yes	LMSG_G000009127.1	OES073683	bacteriocin	RiPPs	Lead-Zinc	10.87	China: Guangdong, Fankou
L180330P-5-LD180328-0005_HL3H2CCXY_L3_clean_bin.105.orig.fa_	OEZ008529	yes	LMSG_G000009127.1	OES073683	terpene	Terpene	Lead-Zinc	25.09	China: Guangdong, Fankou
L180330P-5-LD180328-0005_HL3H2CCXY_L3_clean_bin.107.orig.fa_	OEZ008529	yes	LMSG_G000005721.1	OES073683	terpene	Terpene	Lead-Zinc	14.09	China: Guangdong, Fankou
L180330P-5-LD180328-0005_HL3H2CCXY_L3_clean_bin.107.orig.fa_	OEZ008529	yes	LMSG_G000005721.1	OES073683	NRPS-like	NRPS	Lead-Zinc	16.64	China: Guangdong, Fankou
L180330P-5-LD180328-0005_HL3H2CCXY_L3_clean_bin.107.orig.fa_	OEZ008529	yes	LMSG_G000005721.1	OES073683	terpene	Terpene	Lead-Zinc	11.77	China: Guangdong, Fankou

L180330P-5-LD180328-0005_HL3H2CCXY_L3_clean_bin.107.orig.fa_	OEZ008529	yes	LMSG_G000005721.1	OES073683	betalactone	Others	Lead-Zinc	25.82	China: Guangdong, Fankou
L180330P-5-LD180328-0005_HL3H2CCXY_L3_clean_bin.107.orig.fa_	OEZ008529	yes	LMSG_G000005721.1	OES073683	NRPS	NRPS	Lead-Zinc	14.77	China: Guangdong, Fankou
L180330P-5-LD180328-0005_HL3H2CCXY_L3_clean_bin.11.orig.fa_k	OEZ008529	yes	LMSG_G000011083.1	OES073683	lassopeptide	RiPPs	Lead-Zinc	22.15	China: Guangdong, Fankou
L180330P-5-LD180328-0005_HL3H2CCXY_L3_clean_bin.11.orig.fa_k	OEZ008529	yes	LMSG_G000011083.1	OES073683	terpene	Terpene	Lead-Zinc	23.11	China: Guangdong, Fankou
L180330P-5-LD180328-0005_HL3H2CCXY_L3_clean_bin.11.orig.fa_k	OEZ008529	yes	LMSG_G000011083.1	OES073683	betalactone	Others	Lead-Zinc	31.12	China: Guangdong, Fankou
L180330P-5-LD180328-0005_HL3H2CCXY_L3_clean_bin.11.orig.fa_k	OEZ008529	yes	LMSG_G000011083.1	OES073683	NRPS-like	NRPS	Lead-Zinc	25.38	China: Guangdong, Fankou
L180330P-5-LD180328-0005_HL3H2CCXY_L3_clean_bin.11.orig.fa_k	OEZ008529	yes	LMSG_G000011083.1	OES073683	arylpolyene	Others	Lead-Zinc	41.19	China: Guangdong, Fankou
L180330P-5-LD180328-0005_HL3H2CCXY_L3_clean_bin.111.orig.fa_	OEZ008529	yes	LMSG_G000005723.1	OES073683	arylpolyene	Others	Lead-Zinc	9.71	China: Guangdong, Fankou
L180330P-5-LD180328-0005_HL3H2CCXY_L3_clean_bin.111.orig.fa_	OEZ008529	yes	LMSG_G000005723.1	OES073683	bacteriocin	RiPPs	Lead-Zinc	8.91	China: Guangdong, Fankou
L180330P-5-LD180328-0005_HL3H2CCXY_L3_clean_bin.111.orig.fa_	OEZ008529	yes	LMSG_G000005723.1	OES073683	terpene	Terpene	Lead-Zinc	6.71	China: Guangdong, Fankou
L180330P-5-LD180328-0005_HL3H2CCXY_L3_clean_bin.112.orig.fa_	OEZ008529	yes	LMSG_G000004543.1	OES073683	T1PKS.NRPS-like	PKS-NRP_Hybrids	Lead-Zinc	37.20	China: Guangdong, Fankou
L180330P-5-LD180328-0005_HL3H2CCXY_L3_clean_bin.112.orig.fa_	OEZ008529	yes	LMSG_G000004543.1	OES073683	NRPS-like	NRPS	Lead-Zinc	42.68	China: Guangdong, Fankou
L180330P-5-LD180328-0005_HL3H2CCXY_L3_clean_bin.112.orig.fa_	OEZ008529	yes	LMSG_G000004543.1	OES073683	T3PKS.terpene	Others	Lead-Zinc	41.06	China: Guangdong, Fankou
L180330P-5-LD180328-0005_HL3H2CCXY_L3_clean_bin.112.orig.fa_	OEZ008529	yes	LMSG_G000004543.1	OES073683	terpene	Terpene	Lead-Zinc	15.29	China: Guangdong, Fankou
L180330P-5-LD180328-0005_HL3H2CCXY_L3_clean_bin.113.strict.f	OEZ008529	yes	LMSG_G000009110.1	OES073683	NRPS-like	NRPS	Lead-Zinc	44.42	China: Guangdong, Fankou
L180330P-5-LD180328-0005_HL3H2CCXY_L3_clean_bin.113.strict.f	OEZ008529	yes	LMSG_G000009110.1	OES073683	terpene	Terpene	Lead-Zinc	24.96	China: Guangdong, Fankou
L180330P-5-LD180328-0005_HL3H2CCXY_L3_clean_bin.113.strict.f	OEZ008529	yes	LMSG_G000009110.1	OES073683	T3PKS	PKSother	Lead-Zinc	39.15	China: Guangdong, Fankou
L180330P-5-LD180328-0005_HL3H2CCXY_L3_clean_bin.113.strict.f	OEZ008529	yes	LMSG_G000009110.1	OES073683	terpene	Terpene	Lead-Zinc	21.97	China: Guangdong, Fankou
L180330P-5-LD180328-0005_HL3H2CCXY_L3_clean_bin.119.orig.fa_	OEZ008529	yes	LMSG_G000008492.1	OES073683	terpene	Terpene	Lead-Zinc	22.66	China: Guangdong, Fankou
L180330P-5-LD180328-0005_HL3H2CCXY_L3_clean_bin.119.orig.fa_	OEZ008529	yes	LMSG_G000008492.1	OES073683	arylpolyene	Others	Lead-Zinc	15.57	China: Guangdong, Fankou
L180330P-5-LD180328-0005_HL3H2CCXY_L3_clean_bin.13.orig.fa_k	OEZ008529	yes	LMSG_G000009008.1	OES073683	terpene	Terpene	Lead-Zinc	20.91	China: Guangdong, Fankou
L180330P-5-LD180328-0005_HL3H2CCXY_L3_clean_bin.13.orig.fa_k	OEZ008529	yes	LMSG_G000009008.1	OES073683	bacteriocin	RiPPs	Lead-Zinc	10.80	China: Guangdong, Fankou
L180330P-5-LD180328-0005_HL3H2CCXY_L3_clean_bin.16.orig.fa_k	OEZ008529	yes	LMSG_G000011124.1	OES073683	arylpolyene	Others	Lead-Zinc	40.64	China: Guangdong, Fankou
L180330P-5-LD180328-0005_HL3H2CCXY_L3_clean_bin.16.orig.fa_k	OEZ008529	yes	LMSG_G000011124.1	OES073683	terpene	Terpene	Lead-Zinc	23.11	China: Guangdong, Fankou
L180330P-5-LD180328-0005_HL3H2CCXY_L3_clean_bin.18.orig.fa_k	OEZ008529	yes	LMSG_G000005726.1	OES073683	NRPS-like	NRPS	Lead-Zinc	23.20	China: Guangdong, Fankou
L180330P-5-LD180328-0005_HL3H2CCXY_L3_clean_bin.18.orig.fa_k	OEZ008529	yes	LMSG_G000005726.1	OES073683	terpene	Terpene	Lead-Zinc	15.38	China: Guangdong, Fankou
L180330P-5-LD180328-0005_HL3H2CCXY_L3_clean_bin.18.orig.fa_k	OEZ008529	yes	LMSG_G000005726.1	OES073683	terpene	Terpene	Lead-Zinc	15.45	China: Guangdong, Fankou
L180330P-5-LD180328-0005_HL3H2CCXY_L3_clean_bin.2.orig.fa_kl	OEZ008529	yes	LMSG_G000010002.1	OES073683	terpene.arylpolyene	Others	Lead-Zinc	18.14	China: Guangdong, Fankou
L180330P-5-LD180328-0005_HL3H2CCXY_L3_clean_bin.2.orig.fa_kl	OEZ008529	yes	LMSG_G000010002.1	OES073683	NRPS-like	NRPS	Lead-Zinc	15.94	China: Guangdong, Fankou
L180330P-5-LD180328-0005_HL3H2CCXY_L3_clean_bin.2.orig.fa_kl	OEZ008529	yes	LMSG_G000010002.1	OES073683	arylpolyene	Others	Lead-Zinc	6.18	China: Guangdong, Fankou
L180330P-5-LD180328-0005_HL3H2CCXY_L3_clean_bin.2.orig.fa_kl	OEZ008529	yes	LMSG_G000010002.1	OES073683	ectoine	Others	Lead-Zinc	7.86	China: Guangdong, Fankou
L180330P-5-LD180328-0005_HL3H2CCXY_L3_clean_bin.2.orig.fa_kl	OEZ008529	yes	LMSG_G000010002.1	OES073683	terpene	Terpene	Lead-Zinc	5.77	China: Guangdong, Fankou
L180330P-5-LD180328-0005_HL3H2CCXY_L3_clean_bin.20.strict.fa	OEZ008529	yes	LMSG_G000004545.1	OES073683	bacteriocin	RiPPs	Lead-Zinc	10.86	China: Guangdong, Fankou
L180330P-5-LD180328-0005_HL3H2CCXY_L3_clean_bin.20.strict.fa	OEZ008529	yes	LMSG_G000004545.1	OES073683	terpene	Terpene	Lead-Zinc	25.00	China: Guangdong, Fankou
L180330P-5-LD180328-0005_HL3H2CCXY_L3_clean_bin.20.strict.fa	OEZ008529	yes	LMSG_G000004545.1	OES073683	T3PKS	PKSother	Lead-Zinc	41.05	China: Guangdong, Fankou
L180330P-5-LD180328-0005_HL3H2CCXY_L3_clean_bin.20.strict.fa	OEZ008529	yes	LMSG_G000004545.1	OES073683	lassopeptide	RiPPs	Lead-Zinc	22.81	China: Guangdong, Fankou
L180330P-5-LD180328-0005_HL3H2CCXY_L3_clean_bin.20.strict.fa	OEZ008529	yes	LMSG_G000004545.1	OES073683	NRPS-like	NRPS	Lead-Zinc	28.83	China: Guangdong, Fankou
L180330P-5-LD180328-0005_HL3H2CCXY_L3_clean_bin.21.orig.fa_k	OEZ008529	yes	LMSG_G000004546.1	OES073683	NRPS-like	NRPS	Lead-Zinc	36.87	China: Guangdong, Fankou
L180330P-5-LD180328-0005_HL3H2CCXY_L3_clean_bin.21.orig.fa_k	OEZ008529	yes	LMSG_G000004546.1	OES073683	NRPS-like	NRPS	Lead-Zinc	42.72	China: Guangdong, Fankou
L180330P-5-LD180328-0005_HL3H2CCXY_L3_clean_bin.21.orig.fa_k	OEZ008529	yes	LMSG_G000004546.1	OES073683	terpene	Terpene	Lead-Zinc	12.60	China: Guangdong, Fankou
L180330P-5-LD180328-0005_HL3H2CCXY_L3_clean_bin.21.orig.fa_k	OEZ008529	yes	LMSG_G000004546.1	OES073683	terpene	Terpene	Lead-Zinc	22.05	China: Guangdong, Fankou
L180330P-5-LD180328-0005_HL3H2CCXY_L3_clean_bin.21.orig.fa_k	OEZ008529	yes	LMSG_G000004546.1	OES073683	T3PKS	PKSother	Lead-Zinc	23.08	China: Guangdong, Fankou
L180330P-5-LD180328-0005_HL3H2CCXY_L3_clean_bin.21.orig.fa_k	OEZ008529	yes	LMSG_G000004546.1	OES073683	terpene	Terpene	Lead-Zinc	11.50	China: Guangdong, Fankou
L180330P-5-LD180328-0005_HL3H2CCXY_L3_clean_bin.22.orig.fa_k	OEZ008529	yes	LMSG_G000005727.1	OES073683	T3PKS	PKSother	Lead-Zinc	41.10	China: Guangdong, Fankou
L180330P-5-LD180328-0005_HL3H2CCXY_L3_clean_bin.22.orig.fa_k	OEZ008529	yes	LMSG_G000005727.1	OES073683	terpene	Terpene	Lead-Zinc	21.87	China: Guangdong, Fankou
L180330P-5-LD180328-0005_HL3H2CCXY_L3_clean_bin.22.orig.fa_k	OEZ008529	yes	LMSG_G000005727.1	OES073683	terpene	Terpene	Lead-Zinc	15.69	China: Guangdong, Fankou
L180330P-5-LD180328-0005_HL3H2CCXY_L3_clean_bin.22.orig.fa_k	OEZ008529	yes	LMSG_G000005727.1	OES073683	bacteriocin	RiPPs	Lead-Zinc	10.94	China: Guangdong, Fankou
L180330P-5-LD180328-0005_HL3H2CCXY_L3_clean_bin.23.orig.fa_k	OEZ008529	no	LMSG_G000010173.1	OES073683	terpene	Terpene	Lead-Zinc	9.98	China: Guangdong, Fankou
L180330P-5-LD180328-0005_HL3H2CCXY_L3_clean_bin.23.orig.fa_k	OEZ008529	no	LMSG_G000010173.1	OES073683	ectoine	Others	Lead-Zinc	5.08	China: Guangdong, Fankou
L180330P-5-LD180328-0005_HL3H2CCXY_L3_clean_bin.23.orig.fa_k	OEZ008529	yes	LMSG_G000010173.1	OES073683	betalactone	Others	Lead-Zinc	10.21	China: Guangdong, Fankou
L180330P-5-LD180328-0005_HL3H2CCXY_L3_clean_bin.24.orig.fa_k	OEZ008529	yes	LMSG_G000005728.1	OES073683	terpene	Terpene	Lead-Zinc	10.04	China: Guangdong, Fankou
L180330P-5-LD180328-0005_HL3H2CCXY_L3_clean_bin.24.orig.fa_k	OEZ008529	yes	LMSG_G000005728.1	OES073683	hglE-KS	PKSother	Lead-Zinc	11.88	China: Guangdong, Fankou
L180330P-5-LD180328-0005_HL3H2CCXY_L3_clean_bin.24.orig.fa_k	OEZ008529	yes	LMSG_G000005728.1	OES073683	terpene	Terpene	Lead-Zinc	5.16	China: Guangdong, Fankou
L180330P-5-LD180328-0005_HL3H2CCXY_L3_clean_bin.24.orig.fa_k	OEZ008529	yes	LMSG_G000005728.1	OES073683	TfuA-related	RiPPs	Lead-Zinc	12.59	China: Guangdong, Fankou
L180330P-5-LD180328-0005_HL3H2CCXY_L3_clean_bin.28.orig.fa_k	OEZ008529	yes	LMSG_G000008717.1	OES073683	NRPS	NRPS	Lead-Zinc	30.38	China: Guangdong, Fankou
L180330P-5-LD180328-0005_HL3H2CCXY_L3_clean_bin.28.orig.fa_k	OEZ008529	yes	LMSG_G000008717.1	OES073683	terpene	Terpene	Lead-Zinc	26.45	China: Guangdong, Fankou
L180330P-5-LD180328-0005_HL3H2CCXY_L3_clean_bin.29.permissiv	OEZ008529	yes	LMSG_G000005729.1	OES073683	betalactone	Others	Lead-Zinc	18.20	China: Guangdong, Fankou
L180330P-5-LD180328-0005_HL3H2CCXY_L3_clean_bin.29.permissiv	OEZ008529	yes	LMSG_G000005729.1	OES073683	terpene	Terpene	Lead-Zinc	5.31	China: Guangdong, Fankou
L180330P-5-LD180328-0005_HL3H2CCXY_L3_clean_bin.3.permissive	OEZ008529	yes	LMSG_G000007960.1	OES073683	betalactone	Others	Lead-Zinc	23.17	China: Guangdong, Fankou
L180330P-5-LD180328-0005_HL3H2CCXY_L3_clean_bin.3.permissive	OEZ008529	yes	LMSG_G000007960.1	OES073683	terpene	Terpene	Lead-Zinc	26.80	China: Guangdong, Fankou
L180330P-5-LD180328-0005_HL3H2CCXY_L3_clean_bin.30.orig.fa_k	OEZ008529	yes	LMSG_G000005730.1	OES073683	terpene	Terpene	Lead-Zinc	5.02	China: Guangdong, Fankou
L180330P-5-LD180328-0005_HL3H2CCXY_L3_clean_bin.38.orig.fa_k	OEZ008529	yes	LMSG_G000010233.1	OES073683	terpene	Terpene	Lead-Zinc	20.70	China: Guangdong, Fankou
L180330P-5-LD180328-0005_HL3H2CCXY_L3_clean_bin.38.orig.fa_k	OEZ008529	yes	LMSG_G000010233.1	OES073683	hserlactone	Others	Lead-Zinc	20.72	China: Guangdong, Fankou
L180330P-5-LD180328-0005_HL3H2CCXY_L3_clean_bin.38.orig.fa_k	OEZ008529	yes	LMSG_G000010233.1	OES073683	bacteriocin	RiPPs	Lead-Zinc	10.50	China: Guangdong, Fankou
L180330P-5-LD180328-0005_HL3H2CCXY_L3_clean_bin.38.orig.fa_k	OEZ008529	yes	LMSG_G000010233.1	OES073683	terpene.arylpolyene	Others	Lead-Zinc	42.19	China: Guangdong, Fankou
L180330P-5-LD180328-0005_HL3H2CCXY_L3_clean_bin.39.orig.fa_k	OEZ008529	yes	LMSG_G000004547.1	OES073683	bacteriocin	RiPPs	Lead-Zinc	10.90	China: Guangdong, Fankou
L180330P-5-LD180328-0005_HL3H2CCXY_L3_clean_bin.39.orig.fa_k	OEZ008529	yes	LMSG_G000004547.1	OES073683	T3PKS	PKSother	Lead-Zinc	29.31	China: Guangdong, Fankou
L180330P-5-LD180328-0005_HL3H2CCXY_L3_clean_bin.39.orig.fa_k	OEZ008529	yes	LMSG_G000004547.1	OES073683	NRPS-like	NRPS	Lead-Zinc	42.48	China: Guangdong, Fankou
L180330P-5-LD180328-0005_HL3H2CCXY_L3_clean_bin.39.orig.fa_k	OEZ008529	yes	LMSG_G000004547.1	OES073683	NRPS-like	NRPS	Lead-Zinc	31.14	China: Guangdong, Fankou
L180330P-5-LD180328-0005_HL3H2CCXY_L3_clean_bin.39.orig.fa_k	OEZ008529	yes	LMSG_G000004547.1	OES073683	terpene	Terpene	Lead-Zinc	18.76	China: Guangdong, Fankou
L180330P-5-LD180328-0005_HL3H2CCXY_L3_clean_bin.4.orig.fa_kl	OEZ008529	yes	LMSG_G000009128.1	OES073683	lassopeptide	RiPPs	Lead-Zinc	14.64	China: Guangdong, Fankou
L180330P-5-LD180328-0005_HL3H2CCXY_L3_clean_bin.4.orig.fa_kl	OEZ008529	yes	LMSG_G000009128.1	OES073683	betalactone	Others	Lead-Zinc	27.10	China: Guangdong, Fankou
L180330P-5-LD180328-0005_HL3H2CCXY_L3_clean_bin.4.orig.fa_kl	OEZ008529	yes	LMSG_G000009128.1	OES073683	T3PKS	PKSother	Lead-Zinc	41.11	China: Guangdong, Fankou
L180330P-5-LD180328-0005_HL3H2CCXY_L3_clean_bin.4.orig.fa_kl	OEZ008529	yes	LMSG_G000009128.1	OES073683	NRPS-like	NRPS	Lead-Zinc	37.30	China: Guangdong, Fankou
L180330P-5-LD180328-0005_HL3H2CCXY_L3_clean_bin.4.orig.fa_kl	OEZ008529	yes	LMSG_G000009128.1	OES073683	terpene	Terpene	Lead-Zinc	25.21	China: Guangdong, Fankou
L180330P-5-LD180328-0005_HL3H2CCXY_L3_clean_bin.4.orig.fa_kl	OEZ008529	yes	LMSG_G000009128.1	OES073683	bacteriocin	RiPPs	Lead-Zinc	10.95	China: Guangdong, Fankou
L180330P-5-LD180328-0005_HL3H2CCXY_L3_clean_bin.40.orig.fa_k	OEZ008529	yes	LMSG_G000004548.1	OES073683	terpene	Terpene	Lead-Zinc	19.65	China: Guangdong, Fankou
L180330P-5-LD180328-0005_HL3H2CCXY_L3_clean_bin.40.orig.fa_k	OEZ008529	yes	LMSG_G000004548.1	OES073683	terpene	Terpene	Lead-Zinc	16.42	China: Guangdong, Fankou
L180330P-5-LD180328-0005_HL3H2CCXY_L3_clean_bin.40.orig.fa_k	OEZ008529	yes	LMSG_G000004548.1	OES073683	arylpolyene	Others	Lead-Zinc	28.06	China: Guangdong, Fankou
L180330P-5-LD180328-0005_HL3H2CCXY_L3_clean_bin.40.orig.fa_k	OEZ008529	yes	LMSG_G000004548.1	OES073683	arylpolyene	Others	Lead-Zinc	23.21	China: Guangdong, Fankou
L180330P-5-LD180328-0005_HL3H2CCXY_L3_clean_bin.42.strict.fa	OEZ008529	yes	LMSG_G000005732.1	OES073683	LAP	RiPPs	Lead-Zinc	5.14	China: Guangdong, Fankou
L180330P-5-LD180328-0005_HL3H2CCXY_L3_clean_bin.43.orig.fa_k	OEZ008529	yes	LMSG_G000005733.1	OES073683	bacteriocin	RiPPs	Lead-Zinc	9.53	China: Guangdong, Fankou
L180330P-5-LD180328-0005_HL3H2CCXY_L3_clean_bin.43.orig.fa_k	OEZ008529	yes	LMSG_G000005733.1	OES073683	betalactone	Others	Lead-Zinc	15.78	China: Guangdong, Fankou
L180330P-5-LD180328-0005_HL3H2CCXY_L3_clean_bin.43.orig.fa_k	OEZ008529	yes	LMSG_G000005733.1	OES073683	T1PKS	PKSI	Lead-Zinc	11.92	China: Guangdong, Fankou
L180330P-5-LD180328-0005_HL3H2CCXY_L3_clean_bin.44.orig.fa_k	OEZ008529	yes	LMSG_G000005734.1	OES073683	TfuA-related.bacteriocin	RiPPs	Lead-Zinc	16.26	China: Guangdong, Fankou
L180330P-5-LD180328-0005_HL3H2CCXY_L3_clean_bin.44.orig.fa_k	OEZ008529	yes	LMSG_G000005734.1	OES073683	hglE-KS	PKSother	Lead-Zinc	12.58	China: Guangdong, Fankou
L180330P-5-LD180328-0005_HL3H2CCXY_L3_clean_bin.44.orig.fa_k	OEZ008529	yes	LMSG_G000005734.1	OES073683	ectoine	Others	Lead-Zinc	8.49	China: Guangdong, Fankou
L180330P-5-LD180328-0005_HL3H2CCXY_L3_clean_bin.44.orig.fa_k	OEZ008529	yes	LMSG_G000005734.1	OES073683	hglE-KS	PKSother	Lead-Zinc	14.85	China: Guangdong, Fankou
L180330P-5-LD180328-0005_HL3H2CCXY_L3_clean_bin.45.orig.fa_k	OEZ008529	yes	LMSG_G000010962.1	OES073683	terpene	Terpene	Lead-Zinc	23.10	China: Guangdong, Fankou
L180330P-5-LD180328-0005_HL3H2CCXY_L3_clean_bin.5.orig.fa_kl	OEZ008529	yes	LMSG_G000006988.1	OES073683	bacteriocin	RiPPs	Lead-Zinc	9.91	China: Guangdong, Fankou
L180330P-5-LD180328-0005_HL3H2CCXY_L3_clean_bin.50.orig.fa_k	OEZ008529	yes	LMSG_G000005736.1	OES073683	T3PKS	PKSother	Lead-Zinc	33.61	China: Guangdong, Fankou
L180330P-5-LD180328-0005_HL3H2CCXY_L3_clean_bin.50.orig.fa_k	OEZ008529	yes	LMSG_G000005736.1	OES073683	terpene	Terpene	Lead-Zinc	23.68	China: Guangdong, Fankou
L180330P-5-LD180328-0005_HL3H2CCXY_L3_clean_bin.51.strict.fa	OEZ008529	yes	LMSG_G000004550.1	OES073683	NRPS-like	NRPS	Lead-Zinc	33.39	China: Guangdong, Fankou
L180330P-5-LD180328-0005_HL3H2CCXY_L3_clean_bin.51.strict.fa	OEZ008529	yes	LMSG_G000004550.1	OES073683	terpene	Terpene	Lead-Zinc	20.83	China: Guangdong, Fankou
L180330P-5-LD180328-0005_HL3H2CCXY_L3_clean_bin.51.strict.fa	OEZ008529	yes	LMSG_G000004550.1	OES073683	T3PKS	PKSother	Lead-Zinc	17.38	China: Guangdong, Fankou
L180330P-5-LD180328-0005_HL3H2CCXY_L3_clean_bin.52.orig.fa_k	OEZ008529	yes	LMSG_G000010018.1	OES073683	terpene	Terpene	Lead-Zinc	9.61	China: Guangdong, Fankou
L180330P-5-LD180328-0005_HL3H2CCXY_L3_clean_bin.52.orig.fa_k	OEZ008529	yes	LMSG_G000010018.1	OES073683	terpene	Terpene	Lead-Zinc	21.87	China: Guangdong, Fankou
L180330P-5-LD180328-0005_HL3H2CCXY_L3_clean_bin.52.orig.fa_k	OEZ008529	yes	LMSG_G000010018.1	OES073683	ladderane	Others	Lead-Zinc	15.41	China: Guangdong, Fankou
L180330P-5-LD180328-0005_HL3H2CCXY_L3_clean_bin.55.orig.fa_k	OEZ008529	yes	LMSG_G000008426.1	OES073683	terpene	Terpene	Lead-Zinc	26.31	China: Guangdong, Fankou
L180330P-5-LD180328-0005_HL3H2CCXY_L3_clean_bin.57.orig.fa_k	OEZ008529	yes	LMSG_G000009147.1	OES073683	terpene	Terpene	Lead-Zinc	14.90	China: Guangdong, Fankou
L180330P-5-LD180328-0005_HL3H2CCXY_L3_clean_bin.57.orig.fa_k	OEZ008529	yes	LMSG_G000009147.1	OES073683	terpene	Terpene	Lead-Zinc	11.13	China: Guangdong, Fankou
L180330P-5-LD180328-0005_HL3H2CCXY_L3_clean_bin.57.orig.fa_k	OEZ008529	yes	LMSG_G000009147.1	OES073683	terpene	Terpene	Lead-Zinc	7.33	China: Guangdong, Fankou
L180330P-5-LD180328-0005_HL3H2CCXY_L3_clean_bin.57.orig.fa_k	OEZ008529	yes	LMSG_G000009147.1	OES073683	T3PKS	PKSother	Lead-Zinc	38.72	China: Guangdong, Fankou
L180330P-5-LD180328-0005_HL3H2CCXY_L3_clean_bin.58.orig.fa_k	OEZ008529	yes	LMSG_G000009291.1	OES073683	terpene	Terpene	Lead-Zinc	5.80	China: Guangdong, Fankou
L180330P-5-LD180328-0005_HL3H2CCXY_L3_clean_bin.58.orig.fa_k	OEZ008529	yes	LMSG_G000009291.1	OES073683	terpene	Terpene	Lead-Zinc	22.06	China: Guangdong, Fankou
L180330P-5-LD180328-0005_HL3H2CCXY_L3_clean_bin.60.orig.fa_k	OEZ008529	yes	LMSG_G000004551.1	OES073683	betalactone	Others	Lead-Zinc	33.19	China: Guangdong, Fankou
L180330P-5-LD180328-0005_HL3H2CCXY_L3_clean_bin.60.orig.fa_k	OEZ008529	yes	LMSG_G000004551.1	OES073683	terpene	Terpene	Lead-Zinc	12.94	China: Guangdong, Fankou
L180330P-5-LD180328-0005_HL3H2CCXY_L3_clean_bin.65.strict.fa	OEZ008529	yes	LMSG_G000005737.1	OES073683	terpene	Terpene	Lead-Zinc	11.31	China: Guangdong, Fankou
L180330P-5-LD180328-0005_HL3H2CCXY_L3_clean_bin.65.strict.fa	OEZ008529	yes	LMSG_G000005737.1	OES073683	terpene	Terpene	Lead-Zinc	18.92	China: Guangdong, Fankou
L180330P-5-LD180328-0005_HL3H2CCXY_L3_clean_bin.7.orig.fa_kl	OEZ008529	yes	LMSG_G000008369.1	OES073683	terpene	Terpene	Lead-Zinc	15.58	China: Guangdong, Fankou
L180330P-5-LD180328-0005_HL3H2CCXY_L3_clean_bin.72.orig.fa_k	OEZ008529	yes	LMSG_G000007918.1	OES073683	bacteriocin	RiPPs	Lead-Zinc	12.00	China: Guangdong, Fankou
L180330P-5-LD180328-0005_HL3H2CCXY_L3_clean_bin.72.orig.fa_k	OEZ008529	yes	LMSG_G000007918.1	OES073683	NRPS-like	NRPS	Lead-Zinc	39.38	China: Guangdong, Fankou
L180330P-5-LD180328-0005_HL3H2CCXY_L3_clean_bin.72.orig.fa_k	OEZ008529	yes	LMSG_G000007918.1	OES073683	lanthipeptide	RiPPs	Lead-Zinc	23.26	China: Guangdong, Fankou
L180330P-5-LD180328-0005_HL3H2CCXY_L3_clean_bin.72.orig.fa_k	OEZ008529	yes	LMSG_G000007918.1	OES073683	T3PKS	PKSother	Lead-Zinc	41.09	China: Guangdong, Fankou
L180330P-5-LD180328-0005_HL3H2CCXY_L3_clean_bin.72.orig.fa_k	OEZ008529	yes	LMSG_G000007918.1	OES073683	NRPS-like	NRPS	Lead-Zinc	29.78	China: Guangdong, Fankou
L180330P-5-LD180328-0005_HL3H2CCXY_L3_clean_bin.72.orig.fa_k	OEZ008529	yes	LMSG_G000007918.1	OES073683	terpene	Terpene	Lead-Zinc	29.02	China: Guangdong, Fankou
L180330P-5-LD180328-0005_HL3H2CCXY_L3_clean_bin.74.orig.fa_k	OEZ008529	yes	LMSG_G000007834.1	OES073683	NRPS-like	NRPS	Lead-Zinc	33.09	China: Guangdong, Fankou
L180330P-5-LD180328-0005_HL3H2CCXY_L3_clean_bin.74.orig.fa_k	OEZ008529	yes	LMSG_G000007834.1	OES073683	bacteriocin	RiPPs	Lead-Zinc	10.32	China: Guangdong, Fankou
L180330P-5-LD180328-0005_HL3H2CCXY_L3_clean_bin.74.orig.fa_k	OEZ008529	yes	LMSG_G000007834.1	OES073683	terpene	Terpene	Lead-Zinc	7.91	China: Guangdong, Fankou
L180330P-5-LD180328-0005_HL3H2CCXY_L3_clean_bin.74.orig.fa_k	OEZ008529	yes	LMSG_G000007834.1	OES073683	T3PKS.terpene	Others	Lead-Zinc	32.84	China: Guangdong, Fankou
L180330P-5-LD180328-0005_HL3H2CCXY_L3_clean_bin.79.strict.fa	OEZ008529	yes	LMSG_G000005740.1	OES073683	NRPS-like	NRPS	Lead-Zinc	29.59	China: Guangdong, Fankou
L180330P-5-LD180328-0005_HL3H2CCXY_L3_clean_bin.79.strict.fa	OEZ008529	yes	LMSG_G000005740.1	OES073683	terpene	Terpene	Lead-Zinc	23.99	China: Guangdong, Fankou
L180330P-5-LD180328-0005_HL3H2CCXY_L3_clean_bin.79.strict.fa	OEZ008529	yes	LMSG_G000005740.1	OES073683	T3PKS	PKSother	Lead-Zinc	6.21	China: Guangdong, Fankou
L180330P-5-LD180328-0005_HL3H2CCXY_L3_clean_bin.8.orig.fa_kl	OEZ008529	yes	LMSG_G000005742.1	OES073683	NRPS-like.terpene	Others	Lead-Zinc	37.40	China: Guangdong, Fankou
L180330P-5-LD180328-0005_HL3H2CCXY_L3_clean_bin.8.orig.fa_kl	OEZ008529	yes	LMSG_G000005742.1	OES073683	T3PKS	PKSother	Lead-Zinc	33.44	China: Guangdong, Fankou
L180330P-5-LD180328-0005_HL3H2CCXY_L3_clean_bin.81.orig.fa_k	OEZ008529	no	LMSG_G000009571.1	OES073683	terpene	Terpene	Lead-Zinc	9.46	China: Guangdong, Fankou
L180330P-5-LD180328-0005_HL3H2CCXY_L3_clean_bin.81.orig.fa_k	OEZ008529	no	LMSG_G000009571.1	OES073683	NRPS-like	NRPS	Lead-Zinc	42.72	China: Guangdong, Fankou
L180330P-5-LD180328-0005_HL3H2CCXY_L3_clean_bin.81.orig.fa_k	OEZ008529	no	LMSG_G000009571.1	OES073683	ectoine	Others	Lead-Zinc	10.40	China: Guangdong, Fankou
L180330P-5-LD180328-0005_HL3H2CCXY_L3_clean_bin.81.orig.fa_k	OEZ008529	no	LMSG_G000009571.1	OES073683	terpene	Terpene	Lead-Zinc	10.65	China: Guangdong, Fankou
L180330P-5-LD180328-0005_HL3H2CCXY_L3_clean_bin.82.strict.fa	OEZ008529	yes	LMSG_G000009987.1	OES073683	arylpolyene	Others	Lead-Zinc	24.65	China: Guangdong, Fankou
L180330P-5-LD180328-0005_HL3H2CCXY_L3_clean_bin.82.strict.fa	OEZ008529	yes	LMSG_G000009987.1	OES073683	terpene	Terpene	Lead-Zinc	12.64	China: Guangdong, Fankou
L180330P-5-LD180328-0005_HL3H2CCXY_L3_clean_bin.82.strict.fa	OEZ008529	yes	LMSG_G000009987.1	OES073683	NRPS-like	NRPS	Lead-Zinc	35.41	China: Guangdong, Fankou
L180330P-5-LD180328-0005_HL3H2CCXY_L3_clean_bin.82.strict.fa	OEZ008529	yes	LMSG_G000009987.1	OES073683	terpene	Terpene	Lead-Zinc	21.88	China: Guangdong, Fankou
L180330P-5-LD180328-0005_HL3H2CCXY_L3_clean_bin.82.strict.fa	OEZ008529	yes	LMSG_G000009987.1	OES073683	ectoine	Others	Lead-Zinc	10.40	China: Guangdong, Fankou
L180330P-5-LD180328-0005_HL3H2CCXY_L3_clean_bin.82.strict.fa	OEZ008529	yes	LMSG_G000009987.1	OES073683	arylpolyene	Others	Lead-Zinc	8.94	China: Guangdong, Fankou
L180330P-5-LD180328-0005_HL3H2CCXY_L3_clean_bin.83.permissiv	OEZ008529	yes	LMSG_G000010038.1	OES073683	arylpolyene	Others	Lead-Zinc	37.28	China: Guangdong, Fankou
L180330P-5-LD180328-0005_HL3H2CCXY_L3_clean_bin.83.permissiv	OEZ008529	yes	LMSG_G000010038.1	OES073683	ladderane	Others	Lead-Zinc	23.62	China: Guangdong, Fankou
L180330P-5-LD180328-0005_HL3H2CCXY_L3_clean_bin.83.permissiv	OEZ008529	yes	LMSG_G000010038.1	OES073683	NRPS-like	NRPS	Lead-Zinc	20.32	China: Guangdong, Fankou
L180330P-5-LD180328-0005_HL3H2CCXY_L3_clean_bin.83.permissiv	OEZ008529	yes	LMSG_G000010038.1	OES073683	terpene	Terpene	Lead-Zinc	12.01	China: Guangdong, Fankou
L180330P-5-LD180328-0005_HL3H2CCXY_L3_clean_bin.83.permissiv	OEZ008529	yes	LMSG_G000010038.1	OES073683	T3PKS	PKSother	Lead-Zinc	12.41	China: Guangdong, Fankou
L180330P-5-LD180328-0005_HL3H2CCXY_L3_clean_bin.83.permissiv	OEZ008529	yes	LMSG_G000010038.1	OES073683	bacteriocin	RiPPs	Lead-Zinc	7.77	China: Guangdong, Fankou
L180330P-5-LD180328-0005_HL3H2CCXY_L3_clean_bin.83.permissiv	OEZ008529	yes	LMSG_G000010038.1	OES073683	terpene	Terpene	Lead-Zinc	9.45	China: Guangdong, Fankou
L180330P-5-LD180328-0005_HL3H2CCXY_L3_clean_bin.83.permissiv	OEZ008529	yes	LMSG_G000010038.1	OES073683	ectoine	Others	Lead-Zinc	8.73	China: Guangdong, Fankou
L180330P-5-LD180328-0005_HL3H2CCXY_L3_clean_bin.84.permissiv	OEZ008529	yes	LMSG_G000007995.1	OES073683	bacteriocin	RiPPs	Lead-Zinc	5.83	China: Guangdong, Fankou
L180330P-5-LD180328-0005_HL3H2CCXY_L3_clean_bin.87.strict.fa	OEZ008529	yes	LMSG_G000004552.1	OES073683	arylpolyene	Others	Lead-Zinc	41.18	China: Guangdong, Fankou
L180330P-5-LD180328-0005_HL3H2CCXY_L3_clean_bin.87.strict.fa	OEZ008529	yes	LMSG_G000004552.1	OES073683	betalactone	Others	Lead-Zinc	27.77	China: Guangdong, Fankou
L180330P-5-LD180328-0005_HL3H2CCXY_L3_clean_bin.87.strict.fa	OEZ008529	yes	LMSG_G000004552.1	OES073683	terpene	Terpene	Lead-Zinc	22.11	China: Guangdong, Fankou
L180330P-5-LD180328-0005_HL3H2CCXY_L3_clean_bin.88.permissiv	OEZ008529	yes	LMSG_G000009959.1	OES073683	NRPS-like	NRPS	Lead-Zinc	30.48	China: Guangdong, Fankou
L180330P-5-LD180328-0005_HL3H2CCXY_L3_clean_bin.93.orig.fa_k	OEZ008529	yes	LMSG_G000008061.1	OES073683	terpene	Terpene	Lead-Zinc	21.39	China: Guangdong, Fankou
L180330P-5-LD180328-0005_HL3H2CCXY_L3_clean_bin.93.orig.fa_k	OEZ008529	yes	LMSG_G000008061.1	OES073683	terpene	Terpene	Lead-Zinc	23.25	China: Guangdong, Fankou
L180330P-5-LD180328-0005_HL3H2CCXY_L3_clean_bin.98.orig.fa_k	OEZ008529	yes	LMSG_G000008083.1	OES073683	arylpolyene	Others	Lead-Zinc	12.12	China: Guangdong, Fankou
L180330P-5-LD180328-0005_HL3H2CCXY_L3_clean_bin.98.orig.fa_k	OEZ008529	yes	LMSG_G000008083.1	OES073683	T1PKS.hglE-KS	PKSother	Lead-Zinc	19.94	China: Guangdong, Fankou
L180330P-6-LD180328-0006_HL3H2CCXY_L4_clean_bin.1.strict.fa_	OEZ008529	yes	LMSG_G000005752.1	OES073684	TfuA-related.bacteriocin	RiPPs	Lead-Zinc	14.13	China: Guangdong, Fankou
L180330P-6-LD180328-0006_HL3H2CCXY_L4_clean_bin.10.orig.fa_k	OEZ008529	yes	LMSG_G000009183.1	OES073684	terpene	Terpene	Lead-Zinc	21.54	China: Guangdong, Fankou
L180330P-6-LD180328-0006_HL3H2CCXY_L4_clean_bin.10.orig.fa_k	OEZ008529	yes	LMSG_G000009183.1	OES073684	T3PKS	PKSother	Lead-Zinc	20.80	China: Guangdong, Fankou
L180330P-6-LD180328-0006_HL3H2CCXY_L4_clean_bin.101.orig.fa_	OEZ008529	yes	LMSG_G000010899.1	OES073684	NRPS-like.terpene	Others	Lead-Zinc	51.87	China: Guangdong, Fankou
L180330P-6-LD180328-0006_HL3H2CCXY_L4_clean_bin.101.orig.fa_	OEZ008529	yes	LMSG_G000010899.1	OES073684	hserlactone	Others	Lead-Zinc	7.20	China: Guangdong, Fankou
L180330P-6-LD180328-0006_HL3H2CCXY_L4_clean_bin.104.orig.fa_	OEZ008529	yes	LMSG_G000010917.1	OES073684	terpene	Terpene	Lead-Zinc	21.66	China: Guangdong, Fankou

L180330P-6-LD180328-0006_HL3H2CCXY_L4_clean_bin.104.orig.fa_	OEZ008529	yes	LMSG_G000010917.1	OES073684	arylpolyene	Others	Lead-Zinc	6.61	China: Guangdong, Fankou
L180330P-6-LD180328-0006_HL3H2CCXY_L4_clean_bin.106.orig.fa_	OEZ008529	yes	LMSG_G000008416.1	OES073684	betalactone	Others	Lead-Zinc	33.95	China: Guangdong, Fankou
L180330P-6-LD180328-0006_HL3H2CCXY_L4_clean_bin.107.orig.fa_	OEZ008529	yes	LMSG_G000007919.1	OES073684	NRPS-like	NRPS	Lead-Zinc	25.56	China: Guangdong, Fankou
L180330P-6-LD180328-0006_HL3H2CCXY_L4_clean_bin.107.orig.fa_	OEZ008529	yes	LMSG_G000007919.1	OES073684	lanthipeptide	RiPPs	Lead-Zinc	13.91	China: Guangdong, Fankou
L180330P-6-LD180328-0006_HL3H2CCXY_L4_clean_bin.107.orig.fa_	OEZ008529	yes	LMSG_G000007919.1	OES073684	T3PKS	PKSother	Lead-Zinc	41.09	China: Guangdong, Fankou
L180330P-6-LD180328-0006_HL3H2CCXY_L4_clean_bin.107.orig.fa_	OEZ008529	yes	LMSG_G000007919.1	OES073684	bacteriocin	RiPPs	Lead-Zinc	12.00	China: Guangdong, Fankou
L180330P-6-LD180328-0006_HL3H2CCXY_L4_clean_bin.107.orig.fa_	OEZ008529	yes	LMSG_G000007919.1	OES073684	terpene	Terpene	Lead-Zinc	29.02	China: Guangdong, Fankou
L180330P-6-LD180328-0006_HL3H2CCXY_L4_clean_bin.107.orig.fa_	OEZ008529	yes	LMSG_G000007919.1	OES073684	NRPS-like	NRPS	Lead-Zinc	18.56	China: Guangdong, Fankou
L180330P-6-LD180328-0006_HL3H2CCXY_L4_clean_bin.107.orig.fa_	OEZ008529	yes	LMSG_G000007919.1	OES073684	NRPS-like	NRPS	Lead-Zinc	39.38	China: Guangdong, Fankou
L180330P-6-LD180328-0006_HL3H2CCXY_L4_clean_bin.112.orig.fa_	OEZ008529	yes	LMSG_G000008465.1	OES073684	arylpolyene	Others	Lead-Zinc	30.09	China: Guangdong, Fankou
L180330P-6-LD180328-0006_HL3H2CCXY_L4_clean_bin.112.orig.fa_	OEZ008529	yes	LMSG_G000008465.1	OES073684	betalactone	Others	Lead-Zinc	15.76	China: Guangdong, Fankou
L180330P-6-LD180328-0006_HL3H2CCXY_L4_clean_bin.112.orig.fa_	OEZ008529	yes	LMSG_G000008465.1	OES073684	betalactone	Others	Lead-Zinc	27.77	China: Guangdong, Fankou
L180330P-6-LD180328-0006_HL3H2CCXY_L4_clean_bin.112.orig.fa_	OEZ008529	yes	LMSG_G000008465.1	OES073684	terpene	Terpene	Lead-Zinc	24.70	China: Guangdong, Fankou
L180330P-6-LD180328-0006_HL3H2CCXY_L4_clean_bin.113.orig.fa_	OEZ008529	yes	LMSG_G000010601.1	OES073684	terpene	Terpene	Lead-Zinc	22.97	China: Guangdong, Fankou
L180330P-6-LD180328-0006_HL3H2CCXY_L4_clean_bin.113.orig.fa_	OEZ008529	no	LMSG_G000010601.1	OES073684	arylpolyene	Others	Lead-Zinc	5.66	China: Guangdong, Fankou
L180330P-6-LD180328-0006_HL3H2CCXY_L4_clean_bin.113.orig.fa_	OEZ008529	yes	LMSG_G000010601.1	OES073684	arylpolyene	Others	Lead-Zinc	35.85	China: Guangdong, Fankou
L180330P-6-LD180328-0006_HL3H2CCXY_L4_clean_bin.114.orig.fa_	OEZ008529	yes	LMSG_G000006247.1	OES073684	arylpolyene	Others	Lead-Zinc	41.16	China: Guangdong, Fankou
L180330P-6-LD180328-0006_HL3H2CCXY_L4_clean_bin.114.orig.fa_	OEZ008529	yes	LMSG_G000006247.1	OES073684	terpene	Terpene	Lead-Zinc	23.11	China: Guangdong, Fankou
L180330P-6-LD180328-0006_HL3H2CCXY_L4_clean_bin.117.orig.fa_	OEZ008529	yes	LMSG_G000011106.1	OES073684	terpene	Terpene	Lead-Zinc	23.10	China: Guangdong, Fankou
L180330P-6-LD180328-0006_HL3H2CCXY_L4_clean_bin.120.orig.fa_	OEZ008529	yes	LMSG_G000008371.1	OES073684	T3PKS	PKSother	Lead-Zinc	5.34	China: Guangdong, Fankou
L180330P-6-LD180328-0006_HL3H2CCXY_L4_clean_bin.121.strict.f	OEZ008529	yes	LMSG_G000007758.1	OES073684	terpene	Terpene	Lead-Zinc	18.51	China: Guangdong, Fankou
L180330P-6-LD180328-0006_HL3H2CCXY_L4_clean_bin.121.strict.f	OEZ008529	yes	LMSG_G000007758.1	OES073684	NRPS-like	NRPS	Lead-Zinc	30.59	China: Guangdong, Fankou
L180330P-6-LD180328-0006_HL3H2CCXY_L4_clean_bin.121.strict.f	OEZ008529	yes	LMSG_G000007758.1	OES073684	T3PKS	PKSother	Lead-Zinc	11.41	China: Guangdong, Fankou
L180330P-6-LD180328-0006_HL3H2CCXY_L4_clean_bin.122.orig.fa_	OEZ008529	yes	LMSG_G000009177.1	OES073684	terpene	Terpene	Lead-Zinc	18.90	China: Guangdong, Fankou
L180330P-6-LD180328-0006_HL3H2CCXY_L4_clean_bin.123.orig.fa_	OEZ008529	yes	LMSG_G000008863.1	OES073684	terpene	Terpene	Lead-Zinc	19.72	China: Guangdong, Fankou
L180330P-6-LD180328-0006_HL3H2CCXY_L4_clean_bin.123.orig.fa_	OEZ008529	yes	LMSG_G000008863.1	OES073684	T3PKS	PKSother	Lead-Zinc	41.01	China: Guangdong, Fankou
L180330P-6-LD180328-0006_HL3H2CCXY_L4_clean_bin.125.orig.fa_	OEZ008529	yes	LMSG_G000005745.1	OES073684	terpene	Terpene	Lead-Zinc	24.96	China: Guangdong, Fankou
L180330P-6-LD180328-0006_HL3H2CCXY_L4_clean_bin.125.orig.fa_	OEZ008529	yes	LMSG_G000005745.1	OES073684	T3PKS	PKSother	Lead-Zinc	29.39	China: Guangdong, Fankou
L180330P-6-LD180328-0006_HL3H2CCXY_L4_clean_bin.125.orig.fa_	OEZ008529	yes	LMSG_G000005745.1	OES073684	terpene	Terpene	Lead-Zinc	21.97	China: Guangdong, Fankou
L180330P-6-LD180328-0006_HL3H2CCXY_L4_clean_bin.125.orig.fa_	OEZ008529	yes	LMSG_G000005745.1	OES073684	NRPS-like	NRPS	Lead-Zinc	27.77	China: Guangdong, Fankou
L180330P-6-LD180328-0006_HL3H2CCXY_L4_clean_bin.129.strict.f	OEZ008529	yes	LMSG_G000008278.1	OES073684	terpene	Terpene	Lead-Zinc	9.53	China: Guangdong, Fankou
L180330P-6-LD180328-0006_HL3H2CCXY_L4_clean_bin.13.orig.fa_k	OEZ008529	yes	LMSG_G000009124.1	OES073684	NRPS-like	NRPS	Lead-Zinc	17.17	China: Guangdong, Fankou
L180330P-6-LD180328-0006_HL3H2CCXY_L4_clean_bin.13.orig.fa_k	OEZ008529	yes	LMSG_G000009124.1	OES073684	terpene	Terpene	Lead-Zinc	25.09	China: Guangdong, Fankou
L180330P-6-LD180328-0006_HL3H2CCXY_L4_clean_bin.13.orig.fa_k	OEZ008529	yes	LMSG_G000009124.1	OES073684	bacteriocin	RiPPs	Lead-Zinc	10.87	China: Guangdong, Fankou
L180330P-6-LD180328-0006_HL3H2CCXY_L4_clean_bin.130.orig.fa_	OEZ008529	yes	LMSG_G000007072.1	OES073684	bacteriocin	RiPPs	Lead-Zinc	6.01	China: Guangdong, Fankou
L180330P-6-LD180328-0006_HL3H2CCXY_L4_clean_bin.131.orig.fa_	OEZ008529	yes	LMSG_G000009129.1	OES073684	terpene	Terpene	Lead-Zinc	25.21	China: Guangdong, Fankou
L180330P-6-LD180328-0006_HL3H2CCXY_L4_clean_bin.131.orig.fa_	OEZ008529	yes	LMSG_G000009129.1	OES073684	betalactone	Others	Lead-Zinc	27.10	China: Guangdong, Fankou
L180330P-6-LD180328-0006_HL3H2CCXY_L4_clean_bin.131.orig.fa_	OEZ008529	yes	LMSG_G000009129.1	OES073684	lassopeptide	RiPPs	Lead-Zinc	17.94	China: Guangdong, Fankou
L180330P-6-LD180328-0006_HL3H2CCXY_L4_clean_bin.131.orig.fa_	OEZ008529	yes	LMSG_G000009129.1	OES073684	NRPS-like	NRPS	Lead-Zinc	37.31	China: Guangdong, Fankou
L180330P-6-LD180328-0006_HL3H2CCXY_L4_clean_bin.131.orig.fa_	OEZ008529	yes	LMSG_G000009129.1	OES073684	bacteriocin	RiPPs	Lead-Zinc	10.95	China: Guangdong, Fankou
L180330P-6-LD180328-0006_HL3H2CCXY_L4_clean_bin.137.orig.fa_	OEZ008529	yes	LMSG_G000010934.1	OES073684	arylpolyene	Others	Lead-Zinc	9.92	China: Guangdong, Fankou
L180330P-6-LD180328-0006_HL3H2CCXY_L4_clean_bin.137.orig.fa_	OEZ008529	yes	LMSG_G000010934.1	OES073684	bacteriocin	RiPPs	Lead-Zinc	10.89	China: Guangdong, Fankou
L180330P-6-LD180328-0006_HL3H2CCXY_L4_clean_bin.137.orig.fa_	OEZ008529	yes	LMSG_G000010934.1	OES073684	terpene	Terpene	Lead-Zinc	20.51	China: Guangdong, Fankou
L180330P-6-LD180328-0006_HL3H2CCXY_L4_clean_bin.137.orig.fa_	OEZ008529	yes	LMSG_G000010934.1	OES073684	acyl_amino_acids	Others	Lead-Zinc	11.14	China: Guangdong, Fankou
L180330P-6-LD180328-0006_HL3H2CCXY_L4_clean_bin.138.orig.fa_	OEZ008529	yes	LMSG_G000009666.1	OES073684	NRPS-like	NRPS	Lead-Zinc	5.69	China: Guangdong, Fankou
L180330P-6-LD180328-0006_HL3H2CCXY_L4_clean_bin.139.orig.fa_	OEZ008529	yes	LMSG_G000008927.1	OES073684	bacteriocin	RiPPs	Lead-Zinc	7.43	China: Guangdong, Fankou
L180330P-6-LD180328-0006_HL3H2CCXY_L4_clean_bin.145.orig.fa_	OEZ008529	yes	LMSG_G000008364.1	OES073684	terpene	Terpene	Lead-Zinc	18.13	China: Guangdong, Fankou
L180330P-6-LD180328-0006_HL3H2CCXY_L4_clean_bin.146.orig.fa_	OEZ008529	yes	LMSG_G000009102.1	OES073684	NRPS-like	NRPS	Lead-Zinc	8.94	China: Guangdong, Fankou
L180330P-6-LD180328-0006_HL3H2CCXY_L4_clean_bin.146.orig.fa_	OEZ008529	yes	LMSG_G000009102.1	OES073684	terpene	Terpene	Lead-Zinc	12.53	China: Guangdong, Fankou
L180330P-6-LD180328-0006_HL3H2CCXY_L4_clean_bin.146.orig.fa_	OEZ008529	yes	LMSG_G000009102.1	OES073684	bacteriocin	RiPPs	Lead-Zinc	7.73	China: Guangdong, Fankou
L180330P-6-LD180328-0006_HL3H2CCXY_L4_clean_bin.146.orig.fa_	OEZ008529	yes	LMSG_G000009102.1	OES073684	terpene	Terpene	Lead-Zinc	12.81	China: Guangdong, Fankou
L180330P-6-LD180328-0006_HL3H2CCXY_L4_clean_bin.146.orig.fa_	OEZ008529	yes	LMSG_G000009102.1	OES073684	T3PKS.NRPS-like	PKS-NRP_Hybrids	Lead-Zinc	29.18	China: Guangdong, Fankou
L180330P-6-LD180328-0006_HL3H2CCXY_L4_clean_bin.150.orig.fa_	OEZ008529	yes	LMSG_G000011119.1	OES073684	arylpolyene	Others	Lead-Zinc	41.16	China: Guangdong, Fankou
L180330P-6-LD180328-0006_HL3H2CCXY_L4_clean_bin.150.orig.fa_	OEZ008529	yes	LMSG_G000011119.1	OES073684	terpene	Terpene	Lead-Zinc	23.11	China: Guangdong, Fankou
L180330P-6-LD180328-0006_HL3H2CCXY_L4_clean_bin.153.orig.fa_	OEZ008529	yes	LMSG_G000008478.1	OES073684	terpene	Terpene	Lead-Zinc	5.72	China: Guangdong, Fankou
L180330P-6-LD180328-0006_HL3H2CCXY_L4_clean_bin.155.orig.fa_	OEZ008529	yes	LMSG_G000010904.1	OES073684	NRPS-like	NRPS	Lead-Zinc	24.26	China: Guangdong, Fankou
L180330P-6-LD180328-0006_HL3H2CCXY_L4_clean_bin.155.orig.fa_	OEZ008529	yes	LMSG_G000010904.1	OES073684	terpene	Terpene	Lead-Zinc	8.21	China: Guangdong, Fankou
L180330P-6-LD180328-0006_HL3H2CCXY_L4_clean_bin.16.orig.fa_k	OEZ008529	yes	LMSG_G000007924.1	OES073684	T3PKS	PKSother	Lead-Zinc	6.28	China: Guangdong, Fankou
L180330P-6-LD180328-0006_HL3H2CCXY_L4_clean_bin.17.orig.fa_k	OEZ008529	yes	LMSG_G000005751.1	OES073684	bacteriocin.arylpolyene	Others	Lead-Zinc	47.12	China: Guangdong, Fankou
L180330P-6-LD180328-0006_HL3H2CCXY_L4_clean_bin.17.orig.fa_k	OEZ008529	yes	LMSG_G000005751.1	OES073684	T1PKS	PKSI	Lead-Zinc	47.52	China: Guangdong, Fankou
L180330P-6-LD180328-0006_HL3H2CCXY_L4_clean_bin.17.orig.fa_k	OEZ008529	yes	LMSG_G000005751.1	OES073684	terpene	Terpene	Lead-Zinc	20.89	China: Guangdong, Fankou
L180330P-6-LD180328-0006_HL3H2CCXY_L4_clean_bin.17.orig.fa_k	OEZ008529	yes	LMSG_G000005751.1	OES073684	hserlactone	Others	Lead-Zinc	20.86	China: Guangdong, Fankou
L180330P-6-LD180328-0006_HL3H2CCXY_L4_clean_bin.19.orig.fa_k	OEZ008529	yes	LMSG_G000004555.1	OES073684	NRPS	NRPS	Lead-Zinc	37.97	China: Guangdong, Fankou
L180330P-6-LD180328-0006_HL3H2CCXY_L4_clean_bin.19.orig.fa_k	OEZ008529	yes	LMSG_G000004555.1	OES073684	terpene	Terpene	Lead-Zinc	26.10	China: Guangdong, Fankou
L180330P-6-LD180328-0006_HL3H2CCXY_L4_clean_bin.19.orig.fa_k	OEZ008529	yes	LMSG_G000004555.1	OES073684	bacteriocin	RiPPs	Lead-Zinc	10.82	China: Guangdong, Fankou
L180330P-6-LD180328-0006_HL3H2CCXY_L4_clean_bin.2.orig.fa_kl	OEZ008529	yes	LMSG_G000008529.1	OES073684	bacteriocin	RiPPs	Lead-Zinc	10.80	China: Guangdong, Fankou
L180330P-6-LD180328-0006_HL3H2CCXY_L4_clean_bin.2.orig.fa_kl	OEZ008529	yes	LMSG_G000008529.1	OES073684	betalactone	Others	Lead-Zinc	29.23	China: Guangdong, Fankou
L180330P-6-LD180328-0006_HL3H2CCXY_L4_clean_bin.2.orig.fa_kl	OEZ008529	yes	LMSG_G000008529.1	OES073684	betalactone	Others	Lead-Zinc	36.04	China: Guangdong, Fankou
L180330P-6-LD180328-0006_HL3H2CCXY_L4_clean_bin.2.orig.fa_kl	OEZ008529	yes	LMSG_G000008529.1	OES073684	NRPS	NRPS	Lead-Zinc	58.54	China: Guangdong, Fankou
L180330P-6-LD180328-0006_HL3H2CCXY_L4_clean_bin.2.orig.fa_kl	OEZ008529	yes	LMSG_G000008529.1	OES073684	NRPS	NRPS	Lead-Zinc	47.54	China: Guangdong, Fankou
L180330P-6-LD180328-0006_HL3H2CCXY_L4_clean_bin.2.orig.fa_kl	OEZ008529	yes	LMSG_G000008529.1	OES073684	T1PKS	PKSI	Lead-Zinc	38.39	China: Guangdong, Fankou
L180330P-6-LD180328-0006_HL3H2CCXY_L4_clean_bin.20.orig.fa_k	OEZ008529	yes	LMSG_G000008718.1	OES073684	NRPS.T1PKS	PKS-NRP_Hybrids	Lead-Zinc	50.52	China: Guangdong, Fankou
L180330P-6-LD180328-0006_HL3H2CCXY_L4_clean_bin.20.orig.fa_k	OEZ008529	yes	LMSG_G000008718.1	OES073684	terpene	Terpene	Lead-Zinc	26.27	China: Guangdong, Fankou
L180330P-6-LD180328-0006_HL3H2CCXY_L4_clean_bin.22.strict.fa	OEZ008529	yes	LMSG_G000005753.1	OES073684	arylpolyene	Others	Lead-Zinc	18.32	China: Guangdong, Fankou
L180330P-6-LD180328-0006_HL3H2CCXY_L4_clean_bin.22.strict.fa	OEZ008529	yes	LMSG_G000005753.1	OES073684	hglE-KS	PKSother	Lead-Zinc	10.48	China: Guangdong, Fankou
L180330P-6-LD180328-0006_HL3H2CCXY_L4_clean_bin.23.orig.fa_k	OEZ008529	yes	LMSG_G000004556.1	OES073684	terpene	Terpene	Lead-Zinc	20.98	China: Guangdong, Fankou
L180330P-6-LD180328-0006_HL3H2CCXY_L4_clean_bin.23.orig.fa_k	OEZ008529	yes	LMSG_G000004556.1	OES073684	bacteriocin	RiPPs	Lead-Zinc	11.44	China: Guangdong, Fankou
L180330P-6-LD180328-0006_HL3H2CCXY_L4_clean_bin.23.orig.fa_k	OEZ008529	yes	LMSG_G000004556.1	OES073684	terpene	Terpene	Lead-Zinc	5.53	China: Guangdong, Fankou
L180330P-6-LD180328-0006_HL3H2CCXY_L4_clean_bin.24.orig.fa_k	OEZ008529	yes	LMSG_G000004557.1	OES073684	terpene	Terpene	Lead-Zinc	21.59	China: Guangdong, Fankou
L180330P-6-LD180328-0006_HL3H2CCXY_L4_clean_bin.27.strict.fa	OEZ008529	yes	LMSG_G000010019.1	OES073684	arylpolyene	Others	Lead-Zinc	41.14	China: Guangdong, Fankou
L180330P-6-LD180328-0006_HL3H2CCXY_L4_clean_bin.27.strict.fa	OEZ008529	yes	LMSG_G000010019.1	OES073684	terpene	Terpene	Lead-Zinc	21.81	China: Guangdong, Fankou
L180330P-6-LD180328-0006_HL3H2CCXY_L4_clean_bin.27.strict.fa	OEZ008529	yes	LMSG_G000010019.1	OES073684	arylpolyene	Others	Lead-Zinc	34.55	China: Guangdong, Fankou
L180330P-6-LD180328-0006_HL3H2CCXY_L4_clean_bin.27.strict.fa	OEZ008529	yes	LMSG_G000010019.1	OES073684	terpene	Terpene	Lead-Zinc	14.09	China: Guangdong, Fankou
L180330P-6-LD180328-0006_HL3H2CCXY_L4_clean_bin.27.strict.fa	OEZ008529	yes	LMSG_G000010019.1	OES073684	ladderane	Others	Lead-Zinc	14.02	China: Guangdong, Fankou
L180330P-6-LD180328-0006_HL3H2CCXY_L4_clean_bin.3.orig.fa_kl	OEZ008529	yes	LMSG_G000009009.1	OES073684	terpene	Terpene	Lead-Zinc	20.91	China: Guangdong, Fankou
L180330P-6-LD180328-0006_HL3H2CCXY_L4_clean_bin.3.orig.fa_kl	OEZ008529	yes	LMSG_G000009009.1	OES073684	bacteriocin	RiPPs	Lead-Zinc	10.80	China: Guangdong, Fankou
L180330P-6-LD180328-0006_HL3H2CCXY_L4_clean_bin.31.strict.fa	OEZ008529	yes	LMSG_G000005754.1	OES073684	NRPS-like	NRPS	Lead-Zinc	44.44	China: Guangdong, Fankou
L180330P-6-LD180328-0006_HL3H2CCXY_L4_clean_bin.31.strict.fa	OEZ008529	yes	LMSG_G000005754.1	OES073684	bacteriocin	RiPPs	Lead-Zinc	10.85	China: Guangdong, Fankou
L180330P-6-LD180328-0006_HL3H2CCXY_L4_clean_bin.31.strict.fa	OEZ008529	yes	LMSG_G000005754.1	OES073684	bacteriocin	RiPPs	Lead-Zinc	10.86	China: Guangdong, Fankou
L180330P-6-LD180328-0006_HL3H2CCXY_L4_clean_bin.31.strict.fa	OEZ008529	yes	LMSG_G000005754.1	OES073684	terpene	Terpene	Lead-Zinc	15.15	China: Guangdong, Fankou
L180330P-6-LD180328-0006_HL3H2CCXY_L4_clean_bin.31.strict.fa	OEZ008529	yes	LMSG_G000005754.1	OES073684	bacteriocin	RiPPs	Lead-Zinc	9.26	China: Guangdong, Fankou
L180330P-6-LD180328-0006_HL3H2CCXY_L4_clean_bin.31.strict.fa	OEZ008529	yes	LMSG_G000005754.1	OES073684	lassopeptide	RiPPs	Lead-Zinc	6.98	China: Guangdong, Fankou
L180330P-6-LD180328-0006_HL3H2CCXY_L4_clean_bin.32.orig.fa_k	OEZ008529	yes	LMSG_G000009686.1	OES073684	NRPS-like	NRPS	Lead-Zinc	15.09	China: Guangdong, Fankou
L180330P-6-LD180328-0006_HL3H2CCXY_L4_clean_bin.33.orig.fa_k	OEZ008529	yes	LMSG_G000008174.1	OES073684	terpene	Terpene	Lead-Zinc	21.08	China: Guangdong, Fankou
L180330P-6-LD180328-0006_HL3H2CCXY_L4_clean_bin.37.orig.fa_k	OEZ008529	yes	LMSG_G000004558.1	OES073684	terpene	Terpene	Lead-Zinc	23.04	China: Guangdong, Fankou
L180330P-6-LD180328-0006_HL3H2CCXY_L4_clean_bin.37.orig.fa_k	OEZ008529	yes	LMSG_G000004558.1	OES073684	arylpolyene	Others	Lead-Zinc	9.79	China: Guangdong, Fankou
L180330P-6-LD180328-0006_HL3H2CCXY_L4_clean_bin.38.strict.fa	OEZ008529	yes	LMSG_G000006338.1	OES073684	CDPS	Others	Lead-Zinc	20.67	China: Guangdong, Fankou
L180330P-6-LD180328-0006_HL3H2CCXY_L4_clean_bin.38.strict.fa	OEZ008529	yes	LMSG_G000006338.1	OES073684	hserlactone	Others	Lead-Zinc	20.50	China: Guangdong, Fankou
L180330P-6-LD180328-0006_HL3H2CCXY_L4_clean_bin.39.orig.fa_k	OEZ008529	yes	LMSG_G000005756.1	OES073684	sactipeptide	RiPPs	Lead-Zinc	11.41	China: Guangdong, Fankou
L180330P-6-LD180328-0006_HL3H2CCXY_L4_clean_bin.40.strict.fa	OEZ008529	yes	LMSG_G000005757.1	OES073684	terpene	Terpene	Lead-Zinc	13.31	China: Guangdong, Fankou
L180330P-6-LD180328-0006_HL3H2CCXY_L4_clean_bin.40.strict.fa	OEZ008529	yes	LMSG_G000005757.1	OES073684	terpene	Terpene	Lead-Zinc	14.94	China: Guangdong, Fankou
L180330P-6-LD180328-0006_HL3H2CCXY_L4_clean_bin.40.strict.fa	OEZ008529	yes	LMSG_G000005757.1	OES073684	ladderane	Others	Lead-Zinc	15.46	China: Guangdong, Fankou
L180330P-6-LD180328-0006_HL3H2CCXY_L4_clean_bin.40.strict.fa	OEZ008529	yes	LMSG_G000005757.1	OES073684	NRPS-like	NRPS	Lead-Zinc	12.46	China: Guangdong, Fankou
L180330P-6-LD180328-0006_HL3H2CCXY_L4_clean_bin.40.strict.fa	OEZ008529	yes	LMSG_G000005757.1	OES073684	arylpolyene	Others	Lead-Zinc	9.94	China: Guangdong, Fankou
L180330P-6-LD180328-0006_HL3H2CCXY_L4_clean_bin.40.strict.fa	OEZ008529	yes	LMSG_G000005757.1	OES073684	bacteriocin	RiPPs	Lead-Zinc	7.27	China: Guangdong, Fankou
L180330P-6-LD180328-0006_HL3H2CCXY_L4_clean_bin.40.strict.fa	OEZ008529	yes	LMSG_G000005757.1	OES073684	T3PKS	PKSother	Lead-Zinc	5.76	China: Guangdong, Fankou
L180330P-6-LD180328-0006_HL3H2CCXY_L4_clean_bin.41.orig.fa_k	OEZ008529	yes	LMSG_G000008628.1	OES073684	other	Others	Lead-Zinc	25.67	China: Guangdong, Fankou
L180330P-6-LD180328-0006_HL3H2CCXY_L4_clean_bin.41.orig.fa_k	OEZ008529	yes	LMSG_G000008628.1	OES073684	NRPS-like	NRPS	Lead-Zinc	16.50	China: Guangdong, Fankou
L180330P-6-LD180328-0006_HL3H2CCXY_L4_clean_bin.43.orig.fa_k	OEZ008529	yes	LMSG_G000009978.1	OES073684	NRPS-like	NRPS	Lead-Zinc	6.44	China: Guangdong, Fankou
L180330P-6-LD180328-0006_HL3H2CCXY_L4_clean_bin.45.orig.fa_k	OEZ008529	yes	LMSG_G000007372.1	OES073684	bacteriocin	RiPPs	Lead-Zinc	11.97	China: Guangdong, Fankou
L180330P-6-LD180328-0006_HL3H2CCXY_L4_clean_bin.46.orig.fa_k	OEZ008529	yes	LMSG_G000008062.1	OES073684	terpene	Terpene	Lead-Zinc	19.33	China: Guangdong, Fankou
L180330P-6-LD180328-0006_HL3H2CCXY_L4_clean_bin.46.orig.fa_k	OEZ008529	yes	LMSG_G000008062.1	OES073684	terpene	Terpene	Lead-Zinc	21.39	China: Guangdong, Fankou
L180330P-6-LD180328-0006_HL3H2CCXY_L4_clean_bin.47.strict.fa	OEZ008529	yes	LMSG_G000010071.1	OES073684	arylpolyene	Others	Lead-Zinc	25.59	China: Guangdong, Fankou
L180330P-6-LD180328-0006_HL3H2CCXY_L4_clean_bin.47.strict.fa	OEZ008529	yes	LMSG_G000010071.1	OES073684	arylpolyene	Others	Lead-Zinc	17.75	China: Guangdong, Fankou
L180330P-6-LD180328-0006_HL3H2CCXY_L4_clean_bin.47.strict.fa	OEZ008529	yes	LMSG_G000010071.1	OES073684	NRPS-like	NRPS	Lead-Zinc	5.87	China: Guangdong, Fankou
L180330P-6-LD180328-0006_HL3H2CCXY_L4_clean_bin.49.orig.fa_k	OEZ008529	yes	LMSG_G000007877.1	OES073684	terpene	Terpene	Lead-Zinc	13.10	China: Guangdong, Fankou
L180330P-6-LD180328-0006_HL3H2CCXY_L4_clean_bin.49.orig.fa_k	OEZ008529	yes	LMSG_G000007877.1	OES073684	T1PKS.NRPS-like	PKS-NRP_Hybrids	Lead-Zinc	31.14	China: Guangdong, Fankou
L180330P-6-LD180328-0006_HL3H2CCXY_L4_clean_bin.49.orig.fa_k	OEZ008529	yes	LMSG_G000007877.1	OES073684	NRPS-like	NRPS	Lead-Zinc	41.13	China: Guangdong, Fankou
L180330P-6-LD180328-0006_HL3H2CCXY_L4_clean_bin.49.orig.fa_k	OEZ008529	yes	LMSG_G000007877.1	OES073684	T3PKS.terpene	Others	Lead-Zinc	28.52	China: Guangdong, Fankou
L180330P-6-LD180328-0006_HL3H2CCXY_L4_clean_bin.5.orig.fa_kl	OEZ008529	yes	LMSG_G000009349.1	OES073684	hglE-KS	PKSother	Lead-Zinc	6.22	China: Guangdong, Fankou
L180330P-6-LD180328-0006_HL3H2CCXY_L4_clean_bin.5.orig.fa_kl	OEZ008529	yes	LMSG_G000009349.1	OES073684	ectoine	Others	Lead-Zinc	8.23	China: Guangdong, Fankou
L180330P-6-LD180328-0006_HL3H2CCXY_L4_clean_bin.52.orig.fa_k	OEZ008529	yes	LMSG_G000005758.1	OES073684	lassopeptide	RiPPs	Lead-Zinc	23.45	China: Guangdong, Fankou
L180330P-6-LD180328-0006_HL3H2CCXY_L4_clean_bin.52.orig.fa_k	OEZ008529	yes	LMSG_G000005758.1	OES073684	terpene	Terpene	Lead-Zinc	17.34	China: Guangdong, Fankou
L180330P-6-LD180328-0006_HL3H2CCXY_L4_clean_bin.53.orig.fa_k	OEZ008529	yes	LMSG_G000008493.1	OES073684	terpene	Terpene	Lead-Zinc	16.34	China: Guangdong, Fankou
L180330P-6-LD180328-0006_HL3H2CCXY_L4_clean_bin.53.orig.fa_k	OEZ008529	yes	LMSG_G000008493.1	OES073684	lassopeptide	RiPPs	Lead-Zinc	8.75	China: Guangdong, Fankou
L180330P-6-LD180328-0006_HL3H2CCXY_L4_clean_bin.53.orig.fa_k	OEZ008529	yes	LMSG_G000008493.1	OES073684	arylpolyene	Others	Lead-Zinc	7.84	China: Guangdong, Fankou
L180330P-6-LD180328-0006_HL3H2CCXY_L4_clean_bin.56.orig.fa_k	OEZ008529	yes	LMSG_G000010234.1	OES073684	terpene.arylpolyene	Others	Lead-Zinc	42.19	China: Guangdong, Fankou
L180330P-6-LD180328-0006_HL3H2CCXY_L4_clean_bin.56.orig.fa_k	OEZ008529	yes	LMSG_G000010234.1	OES073684	terpene	Terpene	Lead-Zinc	20.77	China: Guangdong, Fankou
L180330P-6-LD180328-0006_HL3H2CCXY_L4_clean_bin.56.orig.fa_k	OEZ008529	yes	LMSG_G000010234.1	OES073684	hserlactone	Others	Lead-Zinc	20.72	China: Guangdong, Fankou
L180330P-6-LD180328-0006_HL3H2CCXY_L4_clean_bin.59.orig.fa_k	OEZ008529	yes	LMSG_G000009292.1	OES073684	terpene	Terpene	Lead-Zinc	12.46	China: Guangdong, Fankou
L180330P-6-LD180328-0006_HL3H2CCXY_L4_clean_bin.59.orig.fa_k	OEZ008529	yes	LMSG_G000009292.1	OES073684	terpene	Terpene	Lead-Zinc	22.06	China: Guangdong, Fankou
L180330P-6-LD180328-0006_HL3H2CCXY_L4_clean_bin.77.orig.fa_k	OEZ008529	yes	LMSG_G000005763.1	OES073684	terpene	Terpene	Lead-Zinc	5.67	China: Guangdong, Fankou
L180330P-6-LD180328-0006_HL3H2CCXY_L4_clean_bin.77.orig.fa_k	OEZ008529	yes	LMSG_G000005763.1	OES073684	T3PKS	PKSother	Lead-Zinc	10.45	China: Guangdong, Fankou
L180330P-6-LD180328-0006_HL3H2CCXY_L4_clean_bin.78.permissiv	OEZ008529	yes	LMSG_G000009988.1	OES073684	terpene	Terpene	Lead-Zinc	20.53	China: Guangdong, Fankou
L180330P-6-LD180328-0006_HL3H2CCXY_L4_clean_bin.78.permissiv	OEZ008529	yes	LMSG_G000009988.1	OES073684	terpene	Terpene	Lead-Zinc	21.91	China: Guangdong, Fankou
L180330P-6-LD180328-0006_HL3H2CCXY_L4_clean_bin.78.permissiv	OEZ008529	yes	LMSG_G000009988.1	OES073684	arylpolyene	Others	Lead-Zinc	34.36	China: Guangdong, Fankou
L180330P-6-LD180328-0006_HL3H2CCXY_L4_clean_bin.78.permissiv	OEZ008529	yes	LMSG_G000009988.1	OES073684	ectoine	Others	Lead-Zinc	10.40	China: Guangdong, Fankou
L180330P-6-LD180328-0006_HL3H2CCXY_L4_clean_bin.8.orig.fa_kl	OEZ008529	yes	LMSG_G000010724.1	OES073684	terpene	Terpene	Lead-Zinc	17.39	China: Guangdong, Fankou
L180330P-6-LD180328-0006_HL3H2CCXY_L4_clean_bin.8.orig.fa_kl	OEZ008529	yes	LMSG_G000010724.1	OES073684	bacteriocin	RiPPs	Lead-Zinc	10.93	China: Guangdong, Fankou
L180330P-6-LD180328-0006_HL3H2CCXY_L4_clean_bin.8.orig.fa_kl	OEZ008529	yes	LMSG_G000010724.1	OES073684	NRPS	NRPS	Lead-Zinc	18.15	China: Guangdong, Fankou
L180330P-6-LD180328-0006_HL3H2CCXY_L4_clean_bin.8.orig.fa_kl	OEZ008529	yes	LMSG_G000010724.1	OES073684	NRPS	NRPS	Lead-Zinc	6.79	China: Guangdong, Fankou
L180330P-6-LD180328-0006_HL3H2CCXY_L4_clean_bin.8.orig.fa_kl	OEZ008529	yes	LMSG_G000010724.1	OES073684	NRPS.T1PKS	PKS-NRP_Hybrids	Lead-Zinc	27.39	China: Guangdong, Fankou
L180330P-6-LD180328-0006_HL3H2CCXY_L4_clean_bin.8.orig.fa_kl	OEZ008529	yes	LMSG_G000010724.1	OES073684	arylpolyene	Others	Lead-Zinc	24.70	China: Guangdong, Fankou
L180330P-6-LD180328-0006_HL3H2CCXY_L4_clean_bin.85.orig.fa_k	OEZ008529	yes	LMSG_G000010247.1	OES073684	T1PKS.hserlactone	Others	Lead-Zinc	50.02	China: Guangdong, Fankou
L180330P-6-LD180328-0006_HL3H2CCXY_L4_clean_bin.85.orig.fa_k	OEZ008529	yes	LMSG_G000010247.1	OES073684	arylpolyene	Others	Lead-Zinc	6.11	China: Guangdong, Fankou
L180330P-6-LD180328-0006_HL3H2CCXY_L4_clean_bin.85.orig.fa_k	OEZ008529	yes	LMSG_G000010247.1	OES073684	bacteriocin	RiPPs	Lead-Zinc	7.81	China: Guangdong, Fankou
L180330P-6-LD180328-0006_HL3H2CCXY_L4_clean_bin.85.orig.fa_k	OEZ008529	yes	LMSG_G000010247.1	OES073684	terpene	Terpene	Lead-Zinc	15.49	China: Guangdong, Fankou
L180330P-6-LD180328-0006_HL3H2CCXY_L4_clean_bin.85.orig.fa_k	OEZ008529	yes	LMSG_G000010247.1	OES073684	terpene	Terpene	Lead-Zinc	15.27	China: Guangdong, Fankou
L180330P-6-LD180328-0006_HL3H2CCXY_L4_clean_bin.86.strict.fa	OEZ008529	no	LMSG_G000009572.1	OES073684	NRPS-like	NRPS	Lead-Zinc	42.72	China: Guangdong, Fankou
L180330P-6-LD180328-0006_HL3H2CCXY_L4_clean_bin.86.strict.fa	OEZ008529	no	LMSG_G000009572.1	OES073684	ectoine	Others	Lead-Zinc	5.87	China: Guangdong, Fankou
L180330P-6-LD180328-0006_HL3H2CCXY_L4_clean_bin.86.strict.fa	OEZ008529	no	LMSG_G000009572.1	OES073684	terpene	Terpene	Lead-Zinc	23.38	China: Guangdong, Fankou
L180330P-6-LD180328-0006_HL3H2CCXY_L4_clean_bin.86.strict.fa	OEZ008529	no	LMSG_G000009572.1	OES073684	terpene	Terpene	Lead-Zinc	15.02	China: Guangdong, Fankou
L180330P-6-LD180328-0006_HL3H2CCXY_L4_clean_bin.93.orig.fa_k	OEZ008529	yes	LMSG_G000005765.1	OES073684	terpene	Terpene	Lead-Zinc	6.47	China: Guangdong, Fankou
L180330P-6-LD180328-0006_HL3H2CCXY_L4_clean_bin.93.orig.fa_k	OEZ008529	yes	LMSG_G000005765.1	OES073684	NRPS-like	NRPS	Lead-Zinc	42.46	China: Guangdong, Fankou
L180330P-6-LD180328-0006_HL3H2CCXY_L4_clean_bin.93.orig.fa_k	OEZ008529	yes	LMSG_G000005765.1	OES073684	T3PKS	PKSother	Lead-Zinc	8.72	China: Guangdong, Fankou
L180330P-6-LD180328-0006_HL3H2CCXY_L4_clean_bin.97.orig.fa_k	OEZ008529	yes	LMSG_G000009141.1	OES073684	terpene	Terpene	Lead-Zinc	15.35	China: Guangdong, Fankou
L180330P-6-LD180328-0006_HL3H2CCXY_L4_clean_bin.97.orig.fa_k	OEZ008529	yes	LMSG_G000009141.1	OES073684	NRPS-like	NRPS	Lead-Zinc	33.41	China: Guangdong, Fankou
L180330P-6-LD180328-0006_HL3H2CCXY_L4_clean_bin.97.orig.fa_k	OEZ008529	yes	LMSG_G000009141.1	OES073684	T3PKS	PKSother	Lead-Zinc	10.69	China: Guangdong, Fankou

L180330P-6-LD180328-0006_HL3H2CCXY_L4_clean_bin.97.orig.fa_k	OEZ008529	yes	LMSG_G000009141.1	OES073684	terpene	Terpene	Lead-Zinc	21.56	China: Guangdong, Fankou
L180330P-6-LD180328-0006_HL3H2CCXY_L4_clean_bin.99.strict.fa	OEZ008529	yes	LMSG_G000007961.1	OES073684	betalactone	Others	Lead-Zinc	14.55	China: Guangdong, Fankou
L180330P-6-LD180328-0006_HL3H2CCXY_L4_clean_bin.99.strict.fa	OEZ008529	yes	LMSG_G000007961.1	OES073684	terpene	Terpene	Lead-Zinc	26.80	China: Guangdong, Fankou
L180330P-6-LD180328-0007_HL3H2CCXY_L4_clean_bin.1.orig.fa_k1	OEZ008529	yes	LMSG_G000007759.1	OES073685	T3PKS	PKSother	Lead-Zinc	6.39	China: Guangdong, Fankou
L180330P-6-LD180328-0007_HL3H2CCXY_L4_clean_bin.1.orig.fa_k1	OEZ008529	yes	LMSG_G000007759.1	OES073685	terpene	Terpene	Lead-Zinc	7.04	China: Guangdong, Fankou
L180330P-6-LD180328-0007_HL3H2CCXY_L4_clean_bin.1.orig.fa_k1	OEZ008529	yes	LMSG_G000007759.1	OES073685	bacteriocin	RiPPs	Lead-Zinc	9.49	China: Guangdong, Fankou
L180330P-6-LD180328-0007_HL3H2CCXY_L4_clean_bin.108.orig.fa_	OEZ008529	yes	LMSG_G000009010.1	OES073685	terpene	Terpene	Lead-Zinc	11.97	China: Guangdong, Fankou
L180330P-6-LD180328-0007_HL3H2CCXY_L4_clean_bin.109.orig.fa_	OEZ008529	yes	LMSG_G000005769.1	OES073685	arylpolyene	Others	Lead-Zinc	40.30	China: Guangdong, Fankou
L180330P-6-LD180328-0007_HL3H2CCXY_L4_clean_bin.109.orig.fa_	OEZ008529	yes	LMSG_G000005769.1	OES073685	terpene	Terpene	Lead-Zinc	21.66	China: Guangdong, Fankou
L180330P-6-LD180328-0007_HL3H2CCXY_L4_clean_bin.109.orig.fa_	OEZ008529	yes	LMSG_G000005769.1	OES073685	thiopeptide	RiPPs	Lead-Zinc	23.88	China: Guangdong, Fankou
L180330P-6-LD180328-0007_HL3H2CCXY_L4_clean_bin.111.orig.fa_	OEZ008529	yes	LMSG_G000010857.1	OES073685	terpene	Terpene	Lead-Zinc	20.82	China: Guangdong, Fankou
L180330P-6-LD180328-0007_HL3H2CCXY_L4_clean_bin.111.orig.fa_	OEZ008529	yes	LMSG_G000010857.1	OES073685	terpene	Terpene	Lead-Zinc	20.83	China: Guangdong, Fankou
L180330P-6-LD180328-0007_HL3H2CCXY_L4_clean_bin.111.orig.fa_	OEZ008529	yes	LMSG_G000010857.1	OES073685	hserlactone	Others	Lead-Zinc	20.74	China: Guangdong, Fankou
L180330P-6-LD180328-0007_HL3H2CCXY_L4_clean_bin.111.orig.fa_	OEZ008529	yes	LMSG_G000010857.1	OES073685	arylpolyene	Others	Lead-Zinc	41.22	China: Guangdong, Fankou
L180330P-6-LD180328-0007_HL3H2CCXY_L4_clean_bin.113.orig.fa_	OEZ008529	yes	LMSG_G000007920.1	OES073685	NRPS-like	NRPS	Lead-Zinc	22.07	China: Guangdong, Fankou
L180330P-6-LD180328-0007_HL3H2CCXY_L4_clean_bin.113.orig.fa_	OEZ008529	yes	LMSG_G000007920.1	OES073685	lanthipeptide	RiPPs	Lead-Zinc	23.26	China: Guangdong, Fankou
L180330P-6-LD180328-0007_HL3H2CCXY_L4_clean_bin.113.orig.fa_	OEZ008529	yes	LMSG_G000007920.1	OES073685	terpene	Terpene	Lead-Zinc	29.02	China: Guangdong, Fankou
L180330P-6-LD180328-0007_HL3H2CCXY_L4_clean_bin.113.orig.fa_	OEZ008529	yes	LMSG_G000007920.1	OES073685	NRPS-like	NRPS	Lead-Zinc	25.59	China: Guangdong, Fankou
L180330P-6-LD180328-0007_HL3H2CCXY_L4_clean_bin.113.orig.fa_	OEZ008529	yes	LMSG_G000007920.1	OES073685	NRPS-like	NRPS	Lead-Zinc	18.56	China: Guangdong, Fankou
L180330P-6-LD180328-0007_HL3H2CCXY_L4_clean_bin.113.orig.fa_	OEZ008529	yes	LMSG_G000007920.1	OES073685	T3PKS	PKSother	Lead-Zinc	41.09	China: Guangdong, Fankou
L180330P-6-LD180328-0007_HL3H2CCXY_L4_clean_bin.113.orig.fa_	OEZ008529	yes	LMSG_G000007920.1	OES073685	bacteriocin	RiPPs	Lead-Zinc	12.00	China: Guangdong, Fankou
L180330P-6-LD180328-0007_HL3H2CCXY_L4_clean_bin.114.orig.fa_	OEZ008529	yes	LMSG_G000007073.1	OES073685	bacteriocin	RiPPs	Lead-Zinc	6.18	China: Guangdong, Fankou
L180330P-6-LD180328-0007_HL3H2CCXY_L4_clean_bin.117.orig.fa_	OEZ008529	yes	LMSG_G000009120.1	OES073685	bacteriocin	RiPPs	Lead-Zinc	10.87	China: Guangdong, Fankou
L180330P-6-LD180328-0007_HL3H2CCXY_L4_clean_bin.117.orig.fa_	OEZ008529	yes	LMSG_G000009120.1	OES073685	NRPS-like	NRPS	Lead-Zinc	30.09	China: Guangdong, Fankou
L180330P-6-LD180328-0007_HL3H2CCXY_L4_clean_bin.117.orig.fa_	OEZ008529	yes	LMSG_G000009120.1	OES073685	terpene	Terpene	Lead-Zinc	25.09	China: Guangdong, Fankou
L180330P-6-LD180328-0007_HL3H2CCXY_L4_clean_bin.119.orig.fa_	OEZ008529	yes	LMSG_G000011125.1	OES073685	arylpolyene	Others	Lead-Zinc	40.65	China: Guangdong, Fankou
L180330P-6-LD180328-0007_HL3H2CCXY_L4_clean_bin.119.orig.fa_	OEZ008529	yes	LMSG_G000011125.1	OES073685	terpene	Terpene	Lead-Zinc	23.11	China: Guangdong, Fankou
L180330P-6-LD180328-0007_HL3H2CCXY_L4_clean_bin.12.orig.fa_k	OEZ008529	yes	LMSG_G000005771.1	OES073685	T1PKS.hglE-KS	PKSother	Lead-Zinc	49.97	China: Guangdong, Fankou
L180330P-6-LD180328-0007_HL3H2CCXY_L4_clean_bin.12.orig.fa_k	OEZ008529	yes	LMSG_G000005771.1	OES073685	bacteriocin	RiPPs	Lead-Zinc	10.80	China: Guangdong, Fankou
L180330P-6-LD180328-0007_HL3H2CCXY_L4_clean_bin.12.orig.fa_k	OEZ008529	yes	LMSG_G000005771.1	OES073685	NRPS	NRPS	Lead-Zinc	57.13	China: Guangdong, Fankou
L180330P-6-LD180328-0007_HL3H2CCXY_L4_clean_bin.120.orig.fa_	OEZ008529	yes	LMSG_G000008417.1	OES073685	terpene	Terpene	Lead-Zinc	9.52	China: Guangdong, Fankou
L180330P-6-LD180328-0007_HL3H2CCXY_L4_clean_bin.120.orig.fa_	OEZ008529	yes	LMSG_G000008417.1	OES073685	TfuA-related	RiPPs	Lead-Zinc	18.43	China: Guangdong, Fankou
L180330P-6-LD180328-0007_HL3H2CCXY_L4_clean_bin.121.orig.fa_	OEZ008529	yes	LMSG_G000007373.1	OES073685	bacteriocin	RiPPs	Lead-Zinc	11.97	China: Guangdong, Fankou
L180330P-6-LD180328-0007_HL3H2CCXY_L4_clean_bin.122.strict.f	OEZ008529	yes	LMSG_G000005770.1	OES073685	terpene	Terpene	Lead-Zinc	22.08	China: Guangdong, Fankou
L180330P-6-LD180328-0007_HL3H2CCXY_L4_clean_bin.122.strict.f	OEZ008529	yes	LMSG_G000005770.1	OES073685	terpene	Terpene	Lead-Zinc	17.93	China: Guangdong, Fankou
L180330P-6-LD180328-0007_HL3H2CCXY_L4_clean_bin.123.orig.fa_	OEZ008529	yes	LMSG_G000010235.1	OES073685	terpene	Terpene	Lead-Zinc	20.70	China: Guangdong, Fankou
L180330P-6-LD180328-0007_HL3H2CCXY_L4_clean_bin.123.orig.fa_	OEZ008529	yes	LMSG_G000010235.1	OES073685	hserlactone	Others	Lead-Zinc	20.72	China: Guangdong, Fankou
L180330P-6-LD180328-0007_HL3H2CCXY_L4_clean_bin.123.orig.fa_	OEZ008529	yes	LMSG_G000010235.1	OES073685	terpene.arylpolyene	Others	Lead-Zinc	42.19	China: Guangdong, Fankou
L180330P-6-LD180328-0007_HL3H2CCXY_L4_clean_bin.127.strict.f	OEZ008529	yes	LMSG_G000009212.1	OES073685	T3PKS.terpene	Others	Lead-Zinc	27.69	China: Guangdong, Fankou
L180330P-6-LD180328-0007_HL3H2CCXY_L4_clean_bin.127.strict.f	OEZ008529	yes	LMSG_G000009212.1	OES073685	terpene	Terpene	Lead-Zinc	21.72	China: Guangdong, Fankou
L180330P-6-LD180328-0007_HL3H2CCXY_L4_clean_bin.128.orig.fa_	OEZ008529	yes	LMSG_G000010039.1	OES073685	terpene	Terpene	Lead-Zinc	11.43	China: Guangdong, Fankou
L180330P-6-LD180328-0007_HL3H2CCXY_L4_clean_bin.128.orig.fa_	OEZ008529	yes	LMSG_G000010039.1	OES073685	T3PKS	PKSother	Lead-Zinc	6.91	China: Guangdong, Fankou
L180330P-6-LD180328-0007_HL3H2CCXY_L4_clean_bin.128.orig.fa_	OEZ008529	yes	LMSG_G000010039.1	OES073685	terpene	Terpene	Lead-Zinc	12.28	China: Guangdong, Fankou
L180330P-6-LD180328-0007_HL3H2CCXY_L4_clean_bin.128.orig.fa_	OEZ008529	yes	LMSG_G000010039.1	OES073685	ladderane	Others	Lead-Zinc	10.06	China: Guangdong, Fankou
L180330P-6-LD180328-0007_HL3H2CCXY_L4_clean_bin.128.orig.fa_	OEZ008529	yes	LMSG_G000010039.1	OES073685	terpene	Terpene	Lead-Zinc	6.08	China: Guangdong, Fankou
L180330P-6-LD180328-0007_HL3H2CCXY_L4_clean_bin.128.orig.fa_	OEZ008529	yes	LMSG_G000010039.1	OES073685	arylpolyene	Others	Lead-Zinc	8.00	China: Guangdong, Fankou
L180330P-6-LD180328-0007_HL3H2CCXY_L4_clean_bin.130.strict.f	OEZ008529	yes	LMSG_G000010953.1	OES073685	arylpolyene	Others	Lead-Zinc	5.54	China: Guangdong, Fankou
L180330P-6-LD180328-0007_HL3H2CCXY_L4_clean_bin.130.strict.f	OEZ008529	yes	LMSG_G000010953.1	OES073685	T1PKS	PKSI	Lead-Zinc	5.24	China: Guangdong, Fankou
L180330P-6-LD180328-0007_HL3H2CCXY_L4_clean_bin.15.strict.fa	OEZ008529	yes	LMSG_G000009135.1	OES073685	NRPS-like	NRPS	Lead-Zinc	44.35	China: Guangdong, Fankou
L180330P-6-LD180328-0007_HL3H2CCXY_L4_clean_bin.15.strict.fa	OEZ008529	yes	LMSG_G000009135.1	OES073685	betalactone	Others	Lead-Zinc	26.46	China: Guangdong, Fankou
L180330P-6-LD180328-0007_HL3H2CCXY_L4_clean_bin.15.strict.fa	OEZ008529	yes	LMSG_G000009135.1	OES073685	bacteriocin	RiPPs	Lead-Zinc	10.85	China: Guangdong, Fankou
L180330P-6-LD180328-0007_HL3H2CCXY_L4_clean_bin.15.strict.fa	OEZ008529	yes	LMSG_G000009135.1	OES073685	terpene	Terpene	Lead-Zinc	25.21	China: Guangdong, Fankou
L180330P-6-LD180328-0007_HL3H2CCXY_L4_clean_bin.15.strict.fa	OEZ008529	yes	LMSG_G000009135.1	OES073685	lassopeptide	RiPPs	Lead-Zinc	22.39	China: Guangdong, Fankou
L180330P-6-LD180328-0007_HL3H2CCXY_L4_clean_bin.15.strict.fa	OEZ008529	yes	LMSG_G000009135.1	OES073685	bacteriocin	RiPPs	Lead-Zinc	9.05	China: Guangdong, Fankou
L180330P-6-LD180328-0007_HL3H2CCXY_L4_clean_bin.17.orig.fa_k	OEZ008529	no	LMSG_G000010950.1	OES073685	NRPS	NRPS	Lead-Zinc	16.41	China: Guangdong, Fankou
L180330P-6-LD180328-0007_HL3H2CCXY_L4_clean_bin.17.orig.fa_k	OEZ008529	no	LMSG_G000010950.1	OES073685	NRPS.T1PKS	PKS-NRP_Hybrids	Lead-Zinc	13.74	China: Guangdong, Fankou
L180330P-6-LD180328-0007_HL3H2CCXY_L4_clean_bin.17.orig.fa_k	OEZ008529	no	LMSG_G000010950.1	OES073685	NRPS	NRPS	Lead-Zinc	22.21	China: Guangdong, Fankou
L180330P-6-LD180328-0007_HL3H2CCXY_L4_clean_bin.17.orig.fa_k	OEZ008529	no	LMSG_G000010950.1	OES073685	thiopeptide	RiPPs	Lead-Zinc	18.83	China: Guangdong, Fankou
L180330P-6-LD180328-0007_HL3H2CCXY_L4_clean_bin.17.orig.fa_k	OEZ008529	no	LMSG_G000010950.1	OES073685	bacteriocin	RiPPs	Lead-Zinc	7.38	China: Guangdong, Fankou
L180330P-6-LD180328-0007_HL3H2CCXY_L4_clean_bin.2.strict.fa_	OEZ008529	yes	LMSG_G000007996.1	OES073685	TfuA-related.bacteriocin	RiPPs	Lead-Zinc	13.83	China: Guangdong, Fankou
L180330P-6-LD180328-0007_HL3H2CCXY_L4_clean_bin.23.orig.fa_k	OEZ008529	yes	LMSG_G000004561.1	OES073685	betalactone	Others	Lead-Zinc	27.10	China: Guangdong, Fankou
L180330P-6-LD180328-0007_HL3H2CCXY_L4_clean_bin.23.orig.fa_k	OEZ008529	yes	LMSG_G000004561.1	OES073685	terpene	Terpene	Lead-Zinc	25.21	China: Guangdong, Fankou
L180330P-6-LD180328-0007_HL3H2CCXY_L4_clean_bin.23.orig.fa_k	OEZ008529	yes	LMSG_G000004561.1	OES073685	NRPS-like	NRPS	Lead-Zinc	37.31	China: Guangdong, Fankou
L180330P-6-LD180328-0007_HL3H2CCXY_L4_clean_bin.23.orig.fa_k	OEZ008529	yes	LMSG_G000004561.1	OES073685	lassopeptide	RiPPs	Lead-Zinc	22.43	China: Guangdong, Fankou
L180330P-6-LD180328-0007_HL3H2CCXY_L4_clean_bin.23.orig.fa_k	OEZ008529	yes	LMSG_G000004561.1	OES073685	bacteriocin	RiPPs	Lead-Zinc	10.95	China: Guangdong, Fankou
L180330P-6-LD180328-0007_HL3H2CCXY_L4_clean_bin.25.orig.fa_k	OEZ008529	yes	LMSG_G000006989.1	OES073685	bacteriocin	RiPPs	Lead-Zinc	9.55	China: Guangdong, Fankou
L180330P-6-LD180328-0007_HL3H2CCXY_L4_clean_bin.29.orig.fa_k	OEZ008529	yes	LMSG_G000005775.1	OES073685	arylpolyene	Others	Lead-Zinc	19.43	China: Guangdong, Fankou
L180330P-6-LD180328-0007_HL3H2CCXY_L4_clean_bin.29.orig.fa_k	OEZ008529	yes	LMSG_G000005775.1	OES073685	NRPS-like	NRPS	Lead-Zinc	19.73	China: Guangdong, Fankou
L180330P-6-LD180328-0007_HL3H2CCXY_L4_clean_bin.29.orig.fa_k	OEZ008529	yes	LMSG_G000005775.1	OES073685	ectoine	Others	Lead-Zinc	8.50	China: Guangdong, Fankou
L180330P-6-LD180328-0007_HL3H2CCXY_L4_clean_bin.29.orig.fa_k	OEZ008529	yes	LMSG_G000005775.1	OES073685	terpene.arylpolyene	Others	Lead-Zinc	6.51	China: Guangdong, Fankou
L180330P-6-LD180328-0007_HL3H2CCXY_L4_clean_bin.3.strict.fa_	OEZ008529	yes	LMSG_G000005777.1	OES073685	terpene	Terpene	Lead-Zinc	23.10	China: Guangdong, Fankou
L180330P-6-LD180328-0007_HL3H2CCXY_L4_clean_bin.33.orig.fa_k	OEZ008529	yes	LMSG_G000008063.1	OES073685	terpene	Terpene	Lead-Zinc	19.33	China: Guangdong, Fankou
L180330P-6-LD180328-0007_HL3H2CCXY_L4_clean_bin.33.orig.fa_k	OEZ008529	yes	LMSG_G000008063.1	OES073685	terpene	Terpene	Lead-Zinc	21.39	China: Guangdong, Fankou
L180330P-6-LD180328-0007_HL3H2CCXY_L4_clean_bin.34.permissiv	OEZ008529	yes	LMSG_G000005776.1	OES073685	terpene	Terpene	Lead-Zinc	22.09	China: Guangdong, Fankou
L180330P-6-LD180328-0007_HL3H2CCXY_L4_clean_bin.34.permissiv	OEZ008529	yes	LMSG_G000005776.1	OES073685	terpene	Terpene	Lead-Zinc	14.88	China: Guangdong, Fankou
L180330P-6-LD180328-0007_HL3H2CCXY_L4_clean_bin.40.strict.fa	OEZ008529	yes	LMSG_G000004563.1	OES073685	NRPS-like	NRPS	Lead-Zinc	31.13	China: Guangdong, Fankou
L180330P-6-LD180328-0007_HL3H2CCXY_L4_clean_bin.40.strict.fa	OEZ008529	yes	LMSG_G000004563.1	OES073685	terpene	Terpene	Lead-Zinc	22.12	China: Guangdong, Fankou
L180330P-6-LD180328-0007_HL3H2CCXY_L4_clean_bin.40.strict.fa	OEZ008529	yes	LMSG_G000004563.1	OES073685	nucleoside	Others	Lead-Zinc	21.02	China: Guangdong, Fankou
L180330P-6-LD180328-0007_HL3H2CCXY_L4_clean_bin.40.strict.fa	OEZ008529	yes	LMSG_G000004563.1	OES073685	betalactone	Others	Lead-Zinc	20.49	China: Guangdong, Fankou
L180330P-6-LD180328-0007_HL3H2CCXY_L4_clean_bin.41.orig.fa_k	OEZ008529	yes	LMSG_G000009238.1	OES073685	T3PKS.terpene	Others	Lead-Zinc	46.02	China: Guangdong, Fankou
L180330P-6-LD180328-0007_HL3H2CCXY_L4_clean_bin.42.orig.fa_k	OEZ008529	yes	LMSG_G000009989.1	OES073685	terpene	Terpene	Lead-Zinc	15.83	China: Guangdong, Fankou
L180330P-6-LD180328-0007_HL3H2CCXY_L4_clean_bin.42.orig.fa_k	OEZ008529	yes	LMSG_G000009989.1	OES073685	arylpolyene	Others	Lead-Zinc	15.21	China: Guangdong, Fankou
L180330P-6-LD180328-0007_HL3H2CCXY_L4_clean_bin.42.orig.fa_k	OEZ008529	yes	LMSG_G000009989.1	OES073685	arylpolyene	Others	Lead-Zinc	7.76	China: Guangdong, Fankou
L180330P-6-LD180328-0007_HL3H2CCXY_L4_clean_bin.42.orig.fa_k	OEZ008529	yes	LMSG_G000009989.1	OES073685	arylpolyene	Others	Lead-Zinc	6.68	China: Guangdong, Fankou
L180330P-6-LD180328-0007_HL3H2CCXY_L4_clean_bin.42.orig.fa_k	OEZ008529	yes	LMSG_G000009989.1	OES073685	NRPS-like	NRPS	Lead-Zinc	26.56	China: Guangdong, Fankou
L180330P-6-LD180328-0007_HL3H2CCXY_L4_clean_bin.45.strict.fa	OEZ008529	yes	LMSG_G000009085.1	OES073685	betalactone	Others	Lead-Zinc	31.07	China: Guangdong, Fankou
L180330P-6-LD180328-0007_HL3H2CCXY_L4_clean_bin.45.strict.fa	OEZ008529	yes	LMSG_G000009085.1	OES073685	thiopeptide	RiPPs	Lead-Zinc	31.20	China: Guangdong, Fankou
L180330P-6-LD180328-0007_HL3H2CCXY_L4_clean_bin.45.strict.fa	OEZ008529	yes	LMSG_G000009085.1	OES073685	betalactone	Others	Lead-Zinc	27.73	China: Guangdong, Fankou
L180330P-6-LD180328-0007_HL3H2CCXY_L4_clean_bin.45.strict.fa	OEZ008529	yes	LMSG_G000009085.1	OES073685	acyl_amino_acids.betalactone	Others	Lead-Zinc	55.12	China: Guangdong, Fankou
L180330P-6-LD180328-0007_HL3H2CCXY_L4_clean_bin.45.strict.fa	OEZ008529	yes	LMSG_G000009085.1	OES073685	terpene	Terpene	Lead-Zinc	21.74	China: Guangdong, Fankou
L180330P-6-LD180328-0007_HL3H2CCXY_L4_clean_bin.48.orig.fa_k	OEZ008529	yes	LMSG_G000009276.1	OES073685	bacteriocin	RiPPs	Lead-Zinc	11.92	China: Guangdong, Fankou
L180330P-6-LD180328-0007_HL3H2CCXY_L4_clean_bin.48.orig.fa_k	OEZ008529	yes	LMSG_G000009276.1	OES073685	terpene	Terpene	Lead-Zinc	14.95	China: Guangdong, Fankou
L180330P-6-LD180328-0007_HL3H2CCXY_L4_clean_bin.48.orig.fa_k	OEZ008529	yes	LMSG_G000009276.1	OES073685	terpene	Terpene	Lead-Zinc	21.01	China: Guangdong, Fankou
L180330P-6-LD180328-0007_HL3H2CCXY_L4_clean_bin.52.orig.fa_k	OEZ008529	yes	LMSG_G000008466.1	OES073685	terpene	Terpene	Lead-Zinc	26.06	China: Guangdong, Fankou
L180330P-6-LD180328-0007_HL3H2CCXY_L4_clean_bin.52.orig.fa_k	OEZ008529	yes	LMSG_G000008466.1	OES073685	betalactone	Others	Lead-Zinc	27.77	China: Guangdong, Fankou
L180330P-6-LD180328-0007_HL3H2CCXY_L4_clean_bin.52.orig.fa_k	OEZ008529	yes	LMSG_G000008466.1	OES073685	arylpolyene	Others	Lead-Zinc	30.16	China: Guangdong, Fankou
L180330P-6-LD180328-0007_HL3H2CCXY_L4_clean_bin.55.orig.fa_k	OEZ008529	yes	LMSG_G000010034.1	OES073685	terpene	Terpene	Lead-Zinc	13.73	China: Guangdong, Fankou
L180330P-6-LD180328-0007_HL3H2CCXY_L4_clean_bin.55.orig.fa_k	OEZ008529	yes	LMSG_G000010034.1	OES073685	NRPS-like	NRPS	Lead-Zinc	8.06	China: Guangdong, Fankou
L180330P-6-LD180328-0007_HL3H2CCXY_L4_clean_bin.55.orig.fa_k	OEZ008529	yes	LMSG_G000010034.1	OES073685	T3PKS	PKSother	Lead-Zinc	8.71	China: Guangdong, Fankou
L180330P-6-LD180328-0007_HL3H2CCXY_L4_clean_bin.57.orig.fa_k	OEZ008529	yes	LMSG_G000009293.1	OES073685	terpene	Terpene	Lead-Zinc	21.73	China: Guangdong, Fankou
L180330P-6-LD180328-0007_HL3H2CCXY_L4_clean_bin.57.orig.fa_k	OEZ008529	yes	LMSG_G000009293.1	OES073685	terpene	Terpene	Lead-Zinc	22.06	China: Guangdong, Fankou
L180330P-6-LD180328-0007_HL3H2CCXY_L4_clean_bin.6.orig.fa_k1	OEZ008529	yes	LMSG_G000008720.1	OES073685	terpene	Terpene	Lead-Zinc	21.05	China: Guangdong, Fankou
L180330P-6-LD180328-0007_HL3H2CCXY_L4_clean_bin.61.strict.fa	OEZ008529	yes	LMSG_G000010020.1	OES073685	terpene	Terpene	Lead-Zinc	21.87	China: Guangdong, Fankou
L180330P-6-LD180328-0007_HL3H2CCXY_L4_clean_bin.61.strict.fa	OEZ008529	yes	LMSG_G000010020.1	OES073685	arylpolyene	Others	Lead-Zinc	28.59	China: Guangdong, Fankou
L180330P-6-LD180328-0007_HL3H2CCXY_L4_clean_bin.61.strict.fa	OEZ008529	yes	LMSG_G000010020.1	OES073685	ladderane	Others	Lead-Zinc	30.81	China: Guangdong, Fankou
L180330P-6-LD180328-0007_HL3H2CCXY_L4_clean_bin.61.strict.fa	OEZ008529	yes	LMSG_G000010020.1	OES073685	terpene	Terpene	Lead-Zinc	14.21	China: Guangdong, Fankou
L180330P-6-LD180328-0007_HL3H2CCXY_L4_clean_bin.62.orig.fa_k	OEZ008529	yes	LMSG_G000009667.1	OES073685	nucleoside	Others	Lead-Zinc	6.68	China: Guangdong, Fankou
L180330P-6-LD180328-0007_HL3H2CCXY_L4_clean_bin.62.orig.fa_k	OEZ008529	yes	LMSG_G000009667.1	OES073685	terpene	Terpene	Lead-Zinc	9.65	China: Guangdong, Fankou
L180330P-6-LD180328-0007_HL3H2CCXY_L4_clean_bin.62.orig.fa_k	OEZ008529	yes	LMSG_G000009667.1	OES073685	hserlactone	Others	Lead-Zinc	5.49	China: Guangdong, Fankou
L180330P-6-LD180328-0007_HL3H2CCXY_L4_clean_bin.64.orig.fa_k	OEZ008529	yes	LMSG_G000009111.1	OES073685	terpene	Terpene	Lead-Zinc	24.11	China: Guangdong, Fankou
L180330P-6-LD180328-0007_HL3H2CCXY_L4_clean_bin.64.orig.fa_k	OEZ008529	yes	LMSG_G000009111.1	OES073685	NRPS-like	NRPS	Lead-Zinc	27.13	China: Guangdong, Fankou
L180330P-6-LD180328-0007_HL3H2CCXY_L4_clean_bin.64.orig.fa_k	OEZ008529	yes	LMSG_G000009111.1	OES073685	T3PKS	PKSother	Lead-Zinc	22.38	China: Guangdong, Fankou
L180330P-6-LD180328-0007_HL3H2CCXY_L4_clean_bin.64.orig.fa_k	OEZ008529	yes	LMSG_G000009111.1	OES073685	terpene	Terpene	Lead-Zinc	21.97	China: Guangdong, Fankou
L180330P-6-LD180328-0007_HL3H2CCXY_L4_clean_bin.65.orig.fa_k	OEZ008529	yes	LMSG_G000009148.1	OES073685	terpene	Terpene	Lead-Zinc	10.21	China: Guangdong, Fankou
L180330P-6-LD180328-0007_HL3H2CCXY_L4_clean_bin.65.orig.fa_k	OEZ008529	yes	LMSG_G000009148.1	OES073685	NRPS-like	NRPS	Lead-Zinc	27.67	China: Guangdong, Fankou
L180330P-6-LD180328-0007_HL3H2CCXY_L4_clean_bin.69.orig.fa_k	OEZ008529	yes	LMSG_G000005780.1	OES073685	terpene	Terpene	Lead-Zinc	16.61	China: Guangdong, Fankou
L180330P-6-LD180328-0007_HL3H2CCXY_L4_clean_bin.69.orig.fa_k	OEZ008529	yes	LMSG_G000005780.1	OES073685	terpene	Terpene	Lead-Zinc	10.10	China: Guangdong, Fankou
L180330P-6-LD180328-0007_HL3H2CCXY_L4_clean_bin.69.orig.fa_k	OEZ008529	yes	LMSG_G000005780.1	OES073685	NRPS-like	NRPS	Lead-Zinc	36.02	China: Guangdong, Fankou
L180330P-6-LD180328-0007_HL3H2CCXY_L4_clean_bin.7.strict.fa_	OEZ008529	yes	LMSG_G000010248.1	OES073685	terpene	Terpene	Lead-Zinc	21.11	China: Guangdong, Fankou
L180330P-6-LD180328-0007_HL3H2CCXY_L4_clean_bin.7.strict.fa_	OEZ008529	yes	LMSG_G000010248.1	OES073685	T1PKS	PKSI	Lead-Zinc	27.25	China: Guangdong, Fankou
L180330P-6-LD180328-0007_HL3H2CCXY_L4_clean_bin.7.strict.fa_	OEZ008529	no	LMSG_G000010248.1	OES073685	terpene	Terpene	Lead-Zinc	14.70	China: Guangdong, Fankou
L180330P-6-LD180328-0007_HL3H2CCXY_L4_clean_bin.7.strict.fa_	OEZ008529	yes	LMSG_G000010248.1	OES073685	bacteriocin	RiPPs	Lead-Zinc	10.67	China: Guangdong, Fankou
L180330P-6-LD180328-0007_HL3H2CCXY_L4_clean_bin.7.strict.fa_	OEZ008529	yes	LMSG_G000010248.1	OES073685	arylpolyene	Others	Lead-Zinc	13.29	China: Guangdong, Fankou
L180330P-6-LD180328-0007_HL3H2CCXY_L4_clean_bin.70.strict.fa	OEZ008529	yes	LMSG_G000004564.1	OES073685	betalactone	Others	Lead-Zinc	23.13	China: Guangdong, Fankou
L180330P-6-LD180328-0007_HL3H2CCXY_L4_clean_bin.70.strict.fa	OEZ008529	yes	LMSG_G000004564.1	OES073685	terpene	Terpene	Lead-Zinc	26.80	China: Guangdong, Fankou
L180330P-6-LD180328-0007_HL3H2CCXY_L4_clean_bin.71.orig.fa_k	OEZ008529	yes	LMSG_G000006557.1	OES073685	lanthipeptide	RiPPs	Lead-Zinc	14.58	China: Guangdong, Fankou
L180330P-6-LD180328-0007_HL3H2CCXY_L4_clean_bin.77.orig.fa_k	OEZ008529	yes	LMSG_G000010725.1	OES073685	terpene	Terpene	Lead-Zinc	19.24	China: Guangdong, Fankou
L180330P-6-LD180328-0007_HL3H2CCXY_L4_clean_bin.77.orig.fa_k	OEZ008529	yes	LMSG_G000010725.1	OES073685	NRPS.T1PKS	PKS-NRP_Hybrids	Lead-Zinc	63.51	China: Guangdong, Fankou
L180330P-6-LD180328-0007_HL3H2CCXY_L4_clean_bin.77.orig.fa_k	OEZ008529	yes	LMSG_G000010725.1	OES073685	bacteriocin	RiPPs	Lead-Zinc	10.93	China: Guangdong, Fankou
L180330P-6-LD180328-0007_HL3H2CCXY_L4_clean_bin.77.orig.fa_k	OEZ008529	yes	LMSG_G000010725.1	OES073685	arylpolyene	Others	Lead-Zinc	24.70	China: Guangdong, Fankou
L180330P-6-LD180328-0007_HL3H2CCXY_L4_clean_bin.87.orig.fa_k	OEZ008529	yes	LMSG_G000010459.1	OES073685	terpene	Terpene	Lead-Zinc	14.94	China: Guangdong, Fankou
L180330P-6-LD180328-0007_HL3H2CCXY_L4_clean_bin.87.orig.fa_k	OEZ008529	yes	LMSG_G000010459.1	OES073685	arylpolyene	Others	Lead-Zinc	34.81	China: Guangdong, Fankou
L180330P-6-LD180328-0007_HL3H2CCXY_L4_clean_bin.88.strict.fa	OEZ008529	yes	LMSG_G000010069.1	OES073685	arylpolyene	Others	Lead-Zinc	21.36	China: Guangdong, Fankou
L180330P-6-LD180328-0007_HL3H2CCXY_L4_clean_bin.88.strict.fa	OEZ008529	yes	LMSG_G000010069.1	OES073685	arylpolyene	Others	Lead-Zinc	16.27	China: Guangdong, Fankou
L180330P-6-LD180328-0007_HL3H2CCXY_L4_clean_bin.88.strict.fa	OEZ008529	yes	LMSG_G000010069.1	OES073685	terpene	Terpene	Lead-Zinc	12.00	China: Guangdong, Fankou
L180330P-6-LD180328-0007_HL3H2CCXY_L4_clean_bin.88.strict.fa	OEZ008529	yes	LMSG_G000010069.1	OES073685	terpene	Terpene	Lead-Zinc	14.50	China: Guangdong, Fankou
L180330P-6-LD180328-0007_HL3H2CCXY_L4_clean_bin.88.strict.fa	OEZ008529	yes	LMSG_G000010069.1	OES073685	NRPS-like	NRPS	Lead-Zinc	12.45	China: Guangdong, Fankou
L180330P-6-LD180328-0007_HL3H2CCXY_L4_clean_bin.90.orig.fa_k	OEZ008529	yes	LMSG_G000007875.1	OES073685	T3PKS.terpene	Others	Lead-Zinc	26.69	China: Guangdong, Fankou
L180330P-6-LD180328-0007_HL3H2CCXY_L4_clean_bin.90.orig.fa_k	OEZ008529	yes	LMSG_G000007875.1	OES073685	terpene	Terpene	Lead-Zinc	10.12	China: Guangdong, Fankou
L180330P-6-LD180328-0007_HL3H2CCXY_L4_clean_bin.90.orig.fa_k	OEZ008529	yes	LMSG_G000007875.1	OES073685	terpene	Terpene	Lead-Zinc	10.26	China: Guangdong, Fankou
L180330P-6-LD180328-0007_HL3H2CCXY_L4_clean_bin.90.orig.fa_k	OEZ008529	yes	LMSG_G000007875.1	OES073685	NRPS-like	NRPS	Lead-Zinc	27.67	China: Guangdong, Fankou
L180330P-6-LD180328-0007_HL3H2CCXY_L4_clean_bin.90.orig.fa_k	OEZ008529	yes	LMSG_G000007875.1	OES073685	T1PKS.NRPS-like	PKS-NRP_Hybrids	Lead-Zinc	31.01	China: Guangdong, Fankou
L180330P-6-LD180328-0007_HL3H2CCXY_L4_clean_bin.91.orig.fa_k	OEZ008529	yes	LMSG_G000004566.1	OES073685	arylpolyene	Others	Lead-Zinc	41.23	China: Guangdong, Fankou
L180330P-6-LD180328-0007_HL3H2CCXY_L4_clean_bin.91.orig.fa_k	OEZ008529	yes	LMSG_G000004566.1	OES073685	arylpolyene	Others	Lead-Zinc	28.00	China: Guangdong, Fankou
L180330P-6-LD180328-0007_HL3H2CCXY_L4_clean_bin.99.orig.fa_k	OEZ008529	yes	LMSG_G000009333.1	OES073685	NRPS-like	NRPS	Lead-Zinc	7.92	China: Guangdong, Fankou
L180330P-6-LD180328-0007_HL3H2CCXY_L4_clean_bin.99.orig.fa_k	OEZ008529	yes	LMSG_G000009333.1	OES073685	transAT-PKS-like	PKSother	Lead-Zinc	5.65	China: Guangdong, Fankou
L180330P-6-LD180328-0007_HL3H2CCXY_L4_clean_bin.99.orig.fa_k	OEZ008529	yes	LMSG_G000009333.1	OES073685	betalactone	Others	Lead-Zinc	14.95	China: Guangdong, Fankou
L180330P-6-LD180328-0008_HL3H2CCXY_L4_clean_bin.10.orig.fa_k	OEZ008529	yes	LMSG_G000009391.1	OES073671	NRPS	NRPS	Pyrite-Copper	23.90	China: Guangdong, Daobaoshan
L180330P-6-LD180328-0008_HL3H2CCXY_L4_clean_bin.10.orig.fa_k	OEZ008529	yes	LMSG_G000009391.1	OES073671	terpene	Terpene	Pyrite-Copper	21.75	China: Guangdong, Daobaoshan
L180330P-6-LD180328-0008_HL3H2CCXY_L4_clean_bin.10.orig.fa_k	OEZ008529	yes	LMSG_G000009391.1	OES073671	NRPS-like	NRPS	Pyrite-Copper	22.21	China: Guangdong, Daobaoshan
L180330P-6-LD180328-0008_HL3H2CCXY_L4_clean_bin.103.orig.fa_	OEZ008529	yes	LMSG_G000007871.1	OES073671	T1PKS.NRPS-like	PKS-NRP_Hybrids	Pyrite-Copper	48.06	China: Guangdong, Daobaoshan
L180330P-6-LD180328-0008_HL3H2CCXY_L4_clean_bin.103.orig.fa_	OEZ008529	yes	LMSG_G000007871.1	OES073671	terpene	Terpene	Pyrite-Copper	13.77	China: Guangdong, Daobaoshan
L180330P-6-LD180328-0008_HL3H2CCXY_L4_clean_bin.103.orig.fa_	OEZ008529	yes	LMSG_G000007871.1	OES073671	T3PKS	PKSother	Pyrite-Copper	20.56	China: Guangdong, Daobaoshan
L180330P-6-LD180328-0008_HL3H2CCXY_L4_clean_bin.103.orig.fa_	OEZ008529	yes	LMSG_G000007871.1	OES073671	terpene	Terpene	Pyrite-Copper	22.34	China: Guangdong, Daobaoshan
L180330P-6-LD180328-0008_HL3H2CCXY_L4_clean_bin.105.orig.fa_	OEZ008529	yes	LMSG_G000005783.1	OES073671	other	Others	Pyrite-Copper	40.44	China: Guangdong, Daobaoshan
L180330P-6-LD180328-0008_HL3H2CCXY_L4_clean_bin.106.orig.fa_	OEZ008529	yes	LMSG_G000008690.1	OES073671	terpene	Terpene	Pyrite-Copper	7.42	China: Guangdong, Daobaoshan
L180330P-6-LD180328-0008_HL3H2CCXY_L4_clean_bin.106.orig.fa_	OEZ008529	yes	LMSG_G000008690.1	OES073671	ectoine	Others	Pyrite-Copper	5.58	China: Guangdong, Daobaoshan
L180330P-6-LD180328-0008_HL3H2CCXY_L4_clean_bin.106.orig.fa_	OEZ008529	yes	LMSG_G000008690.1	OES073671	NRPS-like	NRPS	Pyrite-Copper	7.71	China: Guangdong, Daobaoshan

L180330P-6-LD180328-0008_HL3H2CCXY_L4_clean_bin.106.orig.fa_	OEZ008529	yes	LMSG_G000008690.1	OES073671	bacteriocin	RiPPs	Pyrite-Copper	8.34	China: Guangdong, Daobaoshan
L180330P-6-LD180328-0008_HL3H2CCXY_L4_clean_bin.108.orig.fa_	OEZ008529	yes	LMSG_G000009044.1	OES073671	thiopeptide	RiPPs	Pyrite-Copper	7.75	China: Guangdong, Daobaoshan
L180330P-6-LD180328-0008_HL3H2CCXY_L4_clean_bin.109.orig.fa_	OEZ008529	yes	LMSG_G000008635.1	OES073671	other	Others	Pyrite-Copper	40.72	China: Guangdong, Daobaoshan
L180330P-6-LD180328-0008_HL3H2CCXY_L4_clean_bin.112.orig.fa_	OEZ008529	no	LMSG_G000008712.1	OES073671	betalactone	Others	Pyrite-Copper	8.11	China: Guangdong, Daobaoshan
L180330P-6-LD180328-0008_HL3H2CCXY_L4_clean_bin.112.orig.fa_	OEZ008529	yes	LMSG_G000008712.1	OES073671	bacteriocin	RiPPs	Pyrite-Copper	6.44	China: Guangdong, Daobaoshan
L180330P-6-LD180328-0008_HL3H2CCXY_L4_clean_bin.112.orig.fa_	OEZ008529	yes	LMSG_G000008712.1	OES073671	terpene	Terpene	Pyrite-Copper	11.45	China: Guangdong, Daobaoshan
L180330P-6-LD180328-0008_HL3H2CCXY_L4_clean_bin.114.orig.fa_	OEZ008529	yes	LMSG_G000010473.1	OES073671	arylpolyene	Others	Pyrite-Copper	41.23	China: Guangdong, Daobaoshan
L180330P-6-LD180328-0008_HL3H2CCXY_L4_clean_bin.114.orig.fa_	OEZ008529	yes	LMSG_G000010473.1	OES073671	terpene	Terpene	Pyrite-Copper	23.04	China: Guangdong, Daobaoshan
L180330P-6-LD180328-0008_HL3H2CCXY_L4_clean_bin.115.strict.f	OEZ008529	yes	LMSG_G000009091.1	OES073671	betalactone.bacteriocin	Others	Pyrite-Copper	29.69	China: Guangdong, Daobaoshan
L180330P-6-LD180328-0008_HL3H2CCXY_L4_clean_bin.115.strict.f	OEZ008529	yes	LMSG_G000009091.1	OES073671	betalactone	Others	Pyrite-Copper	5.12	China: Guangdong, Daobaoshan
L180330P-6-LD180328-0008_HL3H2CCXY_L4_clean_bin.116.orig.fa_	OEZ008529	yes	LMSG_G000008293.1	OES073671	terpene	Terpene	Pyrite-Copper	5.43	China: Guangdong, Daobaoshan
L180330P-6-LD180328-0008_HL3H2CCXY_L4_clean_bin.117.permissi	OEZ008529	yes	LMSG_G000008389.1	OES073671	terpene	Terpene	Pyrite-Copper	12.86	China: Guangdong, Daobaoshan
L180330P-6-LD180328-0008_HL3H2CCXY_L4_clean_bin.12.orig.fa_k	OEZ008529	yes	LMSG_G000008088.1	OES073671	T1PKS.hglE-KS	PKSother	Pyrite-Copper	24.27	China: Guangdong, Daobaoshan
L180330P-6-LD180328-0008_HL3H2CCXY_L4_clean_bin.124.orig.fa_	OEZ008529	yes	LMSG_G000008176.1	OES073671	terpene	Terpene	Pyrite-Copper	21.06	China: Guangdong, Daobaoshan
L180330P-6-LD180328-0008_HL3H2CCXY_L4_clean_bin.127.orig.fa_	OEZ008529	no	LMSG_G000009536.1	OES073671	NRPS-like	NRPS	Pyrite-Copper	39.19	China: Guangdong, Daobaoshan
L180330P-6-LD180328-0008_HL3H2CCXY_L4_clean_bin.127.orig.fa_	OEZ008529	no	LMSG_G000009536.1	OES073671	terpene	Terpene	Pyrite-Copper	23.29	China: Guangdong, Daobaoshan
L180330P-6-LD180328-0008_HL3H2CCXY_L4_clean_bin.127.orig.fa_	OEZ008529	no	LMSG_G000009536.1	OES073671	terpene	Terpene	Pyrite-Copper	20.70	China: Guangdong, Daobaoshan
L180330P-6-LD180328-0008_HL3H2CCXY_L4_clean_bin.128.orig.fa_	OEZ008529	yes	LMSG_G000011120.1	OES073671	arylpolyene	Others	Pyrite-Copper	41.16	China: Guangdong, Daobaoshan
L180330P-6-LD180328-0008_HL3H2CCXY_L4_clean_bin.128.orig.fa_	OEZ008529	yes	LMSG_G000011120.1	OES073671	lassopeptide	RiPPs	Pyrite-Copper	10.83	China: Guangdong, Daobaoshan
L180330P-6-LD180328-0008_HL3H2CCXY_L4_clean_bin.128.orig.fa_	OEZ008529	yes	LMSG_G000011120.1	OES073671	terpene	Terpene	Pyrite-Copper	23.11	China: Guangdong, Daobaoshan
L180330P-6-LD180328-0008_HL3H2CCXY_L4_clean_bin.13.orig.fa_k	OEZ008529	yes	LMSG_G000008589.1	OES073671	other	Others	Pyrite-Copper	20.69	China: Guangdong, Daobaoshan
L180330P-6-LD180328-0008_HL3H2CCXY_L4_clean_bin.13.orig.fa_k	OEZ008529	yes	LMSG_G000008589.1	OES073671	other	Others	Pyrite-Copper	11.18	China: Guangdong, Daobaoshan
L180330P-6-LD180328-0008_HL3H2CCXY_L4_clean_bin.130.permissi	OEZ008529	yes	LMSG_G000008839.1	OES073671	NRPS-like	NRPS	Pyrite-Copper	5.47	China: Guangdong, Daobaoshan
L180330P-6-LD180328-0008_HL3H2CCXY_L4_clean_bin.132.orig.fa_	OEZ008529	yes	LMSG_G000011180.1	OES073671	arylpolyene	Others	Pyrite-Copper	21.51	China: Guangdong, Daobaoshan
L180330P-6-LD180328-0008_HL3H2CCXY_L4_clean_bin.14.orig.fa_k	OEZ008529	yes	LMSG_G000010437.1	OES073671	terpene	Terpene	Pyrite-Copper	19.73	China: Guangdong, Daobaoshan
L180330P-6-LD180328-0008_HL3H2CCXY_L4_clean_bin.14.orig.fa_k	OEZ008529	yes	LMSG_G000010437.1	OES073671	arylpolyene	Others	Pyrite-Copper	20.58	China: Guangdong, Daobaoshan
L180330P-6-LD180328-0008_HL3H2CCXY_L4_clean_bin.14.orig.fa_k	OEZ008529	yes	LMSG_G000010437.1	OES073671	bacteriocin	RiPPs	Pyrite-Copper	10.88	China: Guangdong, Daobaoshan
L180330P-6-LD180328-0008_HL3H2CCXY_L4_clean_bin.16.strict.fa	OEZ008529	yes	LMSG_G000008033.1	OES073671	LAP.thiopeptide	RiPPs	Pyrite-Copper	21.83	China: Guangdong, Daobaoshan
L180330P-6-LD180328-0008_HL3H2CCXY_L4_clean_bin.17.orig.fa_k	OEZ008529	yes	LMSG_G000009373.1	OES073671	betalactone	Others	Pyrite-Copper	29.85	China: Guangdong, Daobaoshan
L180330P-6-LD180328-0008_HL3H2CCXY_L4_clean_bin.19.permissiv	OEZ008529	yes	LMSG_G000008236.1	OES073671	terpene	Terpene	Pyrite-Copper	14.67	China: Guangdong, Daobaoshan
L180330P-6-LD180328-0008_HL3H2CCXY_L4_clean_bin.19.permissiv	OEZ008529	yes	LMSG_G000008236.1	OES073671	other	Others	Pyrite-Copper	5.31	China: Guangdong, Daobaoshan
L180330P-6-LD180328-0008_HL3H2CCXY_L4_clean_bin.20.orig.fa_k	OEZ008529	yes	LMSG_G000006295.1	OES073671	terpene	Terpene	Pyrite-Copper	15.08	China: Guangdong, Daobaoshan
L180330P-6-LD180328-0008_HL3H2CCXY_L4_clean_bin.22.orig.fa_k	OEZ008529	yes	LMSG_G000005785.1	OES073671	T1PKS	PKSI	Pyrite-Copper	10.51	China: Guangdong, Daobaoshan
L180330P-6-LD180328-0008_HL3H2CCXY_L4_clean_bin.22.orig.fa_k	OEZ008529	yes	LMSG_G000005785.1	OES073671	hglE-KS	PKSother	Pyrite-Copper	6.34	China: Guangdong, Daobaoshan
L180330P-6-LD180328-0008_HL3H2CCXY_L4_clean_bin.23.orig.fa_k	OEZ008529	yes	LMSG_G000009054.1	OES073671	T1PKS	PKSI	Pyrite-Copper	47.90	China: Guangdong, Daobaoshan
L180330P-6-LD180328-0008_HL3H2CCXY_L4_clean_bin.23.orig.fa_k	OEZ008529	yes	LMSG_G000009054.1	OES073671	T1PKS	PKSI	Pyrite-Copper	39.21	China: Guangdong, Daobaoshan
L180330P-6-LD180328-0008_HL3H2CCXY_L4_clean_bin.23.orig.fa_k	OEZ008529	yes	LMSG_G000009054.1	OES073671	betalactone	Others	Pyrite-Copper	20.74	China: Guangdong, Daobaoshan
L180330P-6-LD180328-0008_HL3H2CCXY_L4_clean_bin.24.strict.fa	OEZ008529	yes	LMSG_G000007916.1	OES073671	NRPS-like	NRPS	Pyrite-Copper	8.57	China: Guangdong, Daobaoshan
L180330P-6-LD180328-0008_HL3H2CCXY_L4_clean_bin.24.strict.fa	OEZ008529	yes	LMSG_G000007916.1	OES073671	NRPS-like	NRPS	Pyrite-Copper	7.35	China: Guangdong, Daobaoshan
L180330P-6-LD180328-0008_HL3H2CCXY_L4_clean_bin.26.strict.fa	OEZ008529	yes	LMSG_G000008116.1	OES073671	terpene	Terpene	Pyrite-Copper	20.41	China: Guangdong, Daobaoshan
L180330P-6-LD180328-0008_HL3H2CCXY_L4_clean_bin.27.orig.fa_k	OEZ008529	yes	LMSG_G000009163.1	OES073671	terpene	Terpene	Pyrite-Copper	17.11	China: Guangdong, Daobaoshan
L180330P-6-LD180328-0008_HL3H2CCXY_L4_clean_bin.27.orig.fa_k	OEZ008529	yes	LMSG_G000009163.1	OES073671	T3PKS	PKSother	Pyrite-Copper	16.99	China: Guangdong, Daobaoshan
L180330P-6-LD180328-0008_HL3H2CCXY_L4_clean_bin.29.orig.fa_k	OEZ008529	yes	LMSG_G000004567.1	OES073671	NRPS-like	NRPS	Pyrite-Copper	33.08	China: Guangdong, Daobaoshan
L180330P-6-LD180328-0008_HL3H2CCXY_L4_clean_bin.29.orig.fa_k	OEZ008529	yes	LMSG_G000004567.1	OES073671	arylpolyene	Others	Pyrite-Copper	38.00	China: Guangdong, Daobaoshan
L180330P-6-LD180328-0008_HL3H2CCXY_L4_clean_bin.30.orig.fa_k	OEZ008529	yes	LMSG_G000009056.1	OES073671	T1PKS	PKSI	Pyrite-Copper	5.73	China: Guangdong, Daobaoshan
L180330P-6-LD180328-0008_HL3H2CCXY_L4_clean_bin.31.orig.fa_k	OEZ008529	yes	LMSG_G000010927.1	OES073671	arylpolyene	Others	Pyrite-Copper	7.15	China: Guangdong, Daobaoshan
L180330P-6-LD180328-0008_HL3H2CCXY_L4_clean_bin.31.orig.fa_k	OEZ008529	yes	LMSG_G000010927.1	OES073671	terpene	Terpene	Pyrite-Copper	14.33	China: Guangdong, Daobaoshan
L180330P-6-LD180328-0008_HL3H2CCXY_L4_clean_bin.31.orig.fa_k	OEZ008529	yes	LMSG_G000010927.1	OES073671	thiopeptide	RiPPs	Pyrite-Copper	27.84	China: Guangdong, Daobaoshan
L180330P-6-LD180328-0008_HL3H2CCXY_L4_clean_bin.36.orig.fa_k	OEZ008529	yes	LMSG_G000010449.1	OES073671	NRPS-like	NRPS	Pyrite-Copper	42.80	China: Guangdong, Daobaoshan
L180330P-6-LD180328-0008_HL3H2CCXY_L4_clean_bin.36.orig.fa_k	OEZ008529	yes	LMSG_G000010449.1	OES073671	bacteriocin	RiPPs	Pyrite-Copper	8.11	China: Guangdong, Daobaoshan
L180330P-6-LD180328-0008_HL3H2CCXY_L4_clean_bin.36.orig.fa_k	OEZ008529	yes	LMSG_G000010449.1	OES073671	terpene	Terpene	Pyrite-Copper	18.57	China: Guangdong, Daobaoshan
L180330P-6-LD180328-0008_HL3H2CCXY_L4_clean_bin.39.orig.fa_k	OEZ008529	yes	LMSG_G000010523.1	OES073671	arylpolyene	Others	Pyrite-Copper	15.91	China: Guangdong, Daobaoshan
L180330P-6-LD180328-0008_HL3H2CCXY_L4_clean_bin.39.orig.fa_k	OEZ008529	no	LMSG_G000010523.1	OES073671	terpene	Terpene	Pyrite-Copper	7.95	China: Guangdong, Daobaoshan
L180330P-6-LD180328-0008_HL3H2CCXY_L4_clean_bin.39.orig.fa_k	OEZ008529	no	LMSG_G000010523.1	OES073671	bacteriocin	RiPPs	Pyrite-Copper	6.53	China: Guangdong, Daobaoshan
L180330P-6-LD180328-0008_HL3H2CCXY_L4_clean_bin.40.orig.fa_k	OEZ008529	yes	LMSG_G000009490.1	OES073671	NRPS-like	NRPS	Pyrite-Copper	29.10	China: Guangdong, Daobaoshan
L180330P-6-LD180328-0008_HL3H2CCXY_L4_clean_bin.40.orig.fa_k	OEZ008529	yes	LMSG_G000009490.1	OES073671	T1PKS.hglE-KS	PKSother	Pyrite-Copper	28.64	China: Guangdong, Daobaoshan
L180330P-6-LD180328-0008_HL3H2CCXY_L4_clean_bin.43.strict.fa	OEZ008529	yes	LMSG_G000009965.1	OES073671	arylpolyene	Others	Pyrite-Copper	26.52	China: Guangdong, Daobaoshan
L180330P-6-LD180328-0008_HL3H2CCXY_L4_clean_bin.43.strict.fa	OEZ008529	yes	LMSG_G000009965.1	OES073671	terpene	Terpene	Pyrite-Copper	18.80	China: Guangdong, Daobaoshan
L180330P-6-LD180328-0008_HL3H2CCXY_L4_clean_bin.44.permissiv	OEZ008529	yes	LMSG_G000009669.1	OES073671	T3PKS	PKSother	Pyrite-Copper	12.76	China: Guangdong, Daobaoshan
L180330P-6-LD180328-0008_HL3H2CCXY_L4_clean_bin.44.permissiv	OEZ008529	yes	LMSG_G000009669.1	OES073671	NRPS-like	NRPS	Pyrite-Copper	10.98	China: Guangdong, Daobaoshan
L180330P-6-LD180328-0008_HL3H2CCXY_L4_clean_bin.44.permissiv	OEZ008529	yes	LMSG_G000009669.1	OES073671	terpene	Terpene	Pyrite-Copper	5.78	China: Guangdong, Daobaoshan
L180330P-6-LD180328-0008_HL3H2CCXY_L4_clean_bin.45.strict.fa	OEZ008529	yes	LMSG_G000008053.1	OES073671	NRPS	NRPS	Pyrite-Copper	49.59	China: Guangdong, Daobaoshan
L180330P-6-LD180328-0008_HL3H2CCXY_L4_clean_bin.45.strict.fa	OEZ008529	yes	LMSG_G000008053.1	OES073671	T1PKS	PKSI	Pyrite-Copper	51.72	China: Guangdong, Daobaoshan
L180330P-6-LD180328-0008_HL3H2CCXY_L4_clean_bin.47.permissiv	OEZ008529	yes	LMSG_G000008974.1	OES073671	bacteriocin	RiPPs	Pyrite-Copper	5.22	China: Guangdong, Daobaoshan
L180330P-6-LD180328-0008_HL3H2CCXY_L4_clean_bin.47.permissiv	OEZ008529	yes	LMSG_G000008974.1	OES073671	hglE-KS	PKSother	Pyrite-Copper	11.38	China: Guangdong, Daobaoshan
L180330P-6-LD180328-0008_HL3H2CCXY_L4_clean_bin.48.orig.fa_k	OEZ008529	yes	LMSG_G000009239.1	OES073671	T3PKS.terpene	Others	Pyrite-Copper	46.02	China: Guangdong, Daobaoshan
L180330P-6-LD180328-0008_HL3H2CCXY_L4_clean_bin.53.orig.fa_k	OEZ008529	yes	LMSG_G000008317.1	OES073671	NRPS-like	NRPS	Pyrite-Copper	42.47	China: Guangdong, Daobaoshan
L180330P-6-LD180328-0008_HL3H2CCXY_L4_clean_bin.53.orig.fa_k	OEZ008529	yes	LMSG_G000008317.1	OES073671	terpene	Terpene	Pyrite-Copper	20.88	China: Guangdong, Daobaoshan
L180330P-6-LD180328-0008_HL3H2CCXY_L4_clean_bin.58.orig.fa_k	OEZ008529	yes	LMSG_G000008411.1	OES073671	lassopeptide	RiPPs	Pyrite-Copper	5.19	China: Guangdong, Daobaoshan
L180330P-6-LD180328-0008_HL3H2CCXY_L4_clean_bin.59.orig.fa_k	OEZ008529	yes	LMSG_G000008547.1	OES073671	betalactone	Others	Pyrite-Copper	20.98	China: Guangdong, Daobaoshan
L180330P-6-LD180328-0008_HL3H2CCXY_L4_clean_bin.59.orig.fa_k	OEZ008529	yes	LMSG_G000008547.1	OES073671	other	Others	Pyrite-Copper	11.49	China: Guangdong, Daobaoshan
L180330P-6-LD180328-0008_HL3H2CCXY_L4_clean_bin.6.orig.fa_k1	OEZ008529	yes	LMSG_G000008353.1	OES073671	bacteriocin	RiPPs	Pyrite-Copper	8.83	China: Guangdong, Daobaoshan
L180330P-6-LD180328-0008_HL3H2CCXY_L4_clean_bin.60.orig.fa_k	OEZ008529	yes	LMSG_G000011087.1	OES073671	NRPS-like	NRPS	Pyrite-Copper	27.94	China: Guangdong, Daobaoshan
L180330P-6-LD180328-0008_HL3H2CCXY_L4_clean_bin.60.orig.fa_k	OEZ008529	yes	LMSG_G000011087.1	OES073671	bacteriocin	RiPPs	Pyrite-Copper	10.86	China: Guangdong, Daobaoshan
L180330P-6-LD180328-0008_HL3H2CCXY_L4_clean_bin.63.orig.fa_k	OEZ008529	yes	LMSG_G000008403.1	OES073671	terpene	Terpene	Pyrite-Copper	15.45	China: Guangdong, Daobaoshan
L180330P-6-LD180328-0008_HL3H2CCXY_L4_clean_bin.64.orig.fa_k	OEZ008529	yes	LMSG_G000010782.1	OES073671	acyl_amino_acids	Others	Pyrite-Copper	33.88	China: Guangdong, Daobaoshan
L180330P-6-LD180328-0008_HL3H2CCXY_L4_clean_bin.64.orig.fa_k	OEZ008529	yes	LMSG_G000010782.1	OES073671	arylpolyene	Others	Pyrite-Copper	38.97	China: Guangdong, Daobaoshan
L180330P-6-LD180328-0008_HL3H2CCXY_L4_clean_bin.64.orig.fa_k	OEZ008529	yes	LMSG_G000010782.1	OES073671	bacteriocin	RiPPs	Pyrite-Copper	9.36	China: Guangdong, Daobaoshan
L180330P-6-LD180328-0008_HL3H2CCXY_L4_clean_bin.65.orig.fa_k	OEZ008529	yes	LMSG_G000007824.1	OES073671	terpene	Terpene	Pyrite-Copper	12.67	China: Guangdong, Daobaoshan
L180330P-6-LD180328-0008_HL3H2CCXY_L4_clean_bin.65.orig.fa_k	OEZ008529	yes	LMSG_G000007824.1	OES073671	terpene	Terpene	Pyrite-Copper	5.43	China: Guangdong, Daobaoshan
L180330P-6-LD180328-0008_HL3H2CCXY_L4_clean_bin.65.orig.fa_k	OEZ008529	yes	LMSG_G000007824.1	OES073671	terpene	Terpene	Pyrite-Copper	10.27	China: Guangdong, Daobaoshan
L180330P-6-LD180328-0008_HL3H2CCXY_L4_clean_bin.67.orig.fa_k	OEZ008529	yes	LMSG_G000005790.1	OES073671	terpene	Terpene	Pyrite-Copper	22.00	China: Guangdong, Daobaoshan
L180330P-6-LD180328-0008_HL3H2CCXY_L4_clean_bin.67.orig.fa_k	OEZ008529	yes	LMSG_G000005790.1	OES073671	NRPS-like	NRPS	Pyrite-Copper	10.20	China: Guangdong, Daobaoshan
L180330P-6-LD180328-0008_HL3H2CCXY_L4_clean_bin.68.orig.fa_k	OEZ008529	yes	LMSG_G000011035.1	OES073671	arylpolyene	Others	Pyrite-Copper	8.50	China: Guangdong, Daobaoshan
L180330P-6-LD180328-0008_HL3H2CCXY_L4_clean_bin.69.strict.fa	OEZ008529	yes	LMSG_G000009011.1	OES073671	terpene	Terpene	Pyrite-Copper	7.40	China: Guangdong, Daobaoshan
L180330P-6-LD180328-0008_HL3H2CCXY_L4_clean_bin.7.orig.fa_k1	OEZ008529	yes	LMSG_G000011091.1	OES073671	NRPS-like	NRPS	Pyrite-Copper	16.98	China: Guangdong, Daobaoshan
L180330P-6-LD180328-0008_HL3H2CCXY_L4_clean_bin.7.orig.fa_k1	OEZ008529	yes	LMSG_G000011091.1	OES073671	arylpolyene	Others	Pyrite-Copper	41.21	China: Guangdong, Daobaoshan
L180330P-6-LD180328-0008_HL3H2CCXY_L4_clean_bin.7.orig.fa_k1	OEZ008529	yes	LMSG_G000011091.1	OES073671	terpene	Terpene	Pyrite-Copper	22.41	China: Guangdong, Daobaoshan
L180330P-6-LD180328-0008_HL3H2CCXY_L4_clean_bin.7.orig.fa_k1	OEZ008529	yes	LMSG_G000011091.1	OES073671	bacteriocin	RiPPs	Pyrite-Copper	5.76	China: Guangdong, Daobaoshan
L180330P-6-LD180328-0008_HL3H2CCXY_L4_clean_bin.75.orig.fa_k	OEZ008529	no	LMSG_G000010284.1	OES073671	terpene	Terpene	Pyrite-Copper	21.53	China: Guangdong, Daobaoshan
L180330P-6-LD180328-0008_HL3H2CCXY_L4_clean_bin.75.orig.fa_k	OEZ008529	yes	LMSG_G000010284.1	OES073671	arylpolyene	Others	Pyrite-Copper	29.19	China: Guangdong, Daobaoshan
L180330P-6-LD180328-0008_HL3H2CCXY_L4_clean_bin.77.orig.fa_k	OEZ008529	yes	LMSG_G000009499.1	OES073671	T3PKS	PKSother	Pyrite-Copper	25.65	China: Guangdong, Daobaoshan
L180330P-6-LD180328-0008_HL3H2CCXY_L4_clean_bin.77.orig.fa_k	OEZ008529	yes	LMSG_G000009499.1	OES073671	NRPS-like	NRPS	Pyrite-Copper	34.54	China: Guangdong, Daobaoshan
L180330P-6-LD180328-0008_HL3H2CCXY_L4_clean_bin.77.orig.fa_k	OEZ008529	yes	LMSG_G000009499.1	OES073671	T1PKS.hglE-KS	PKSother	Pyrite-Copper	34.47	China: Guangdong, Daobaoshan
L180330P-6-LD180328-0008_HL3H2CCXY_L4_clean_bin.77.orig.fa_k	OEZ008529	yes	LMSG_G000009499.1	OES073671	TfuA-related	RiPPs	Pyrite-Copper	19.67	China: Guangdong, Daobaoshan
L180330P-6-LD180328-0008_HL3H2CCXY_L4_clean_bin.78.orig.fa_k	OEZ008529	yes	LMSG_G000005792.1	OES073671	terpene	Terpene	Pyrite-Copper	12.12	China: Guangdong, Daobaoshan
L180330P-6-LD180328-0008_HL3H2CCXY_L4_clean_bin.78.orig.fa_k	OEZ008529	yes	LMSG_G000005792.1	OES073671	bacteriocin	RiPPs	Pyrite-Copper	10.86	China: Guangdong, Daobaoshan
L180330P-6-LD180328-0008_HL3H2CCXY_L4_clean_bin.78.orig.fa_k	OEZ008529	yes	LMSG_G000005792.1	OES073671	arylpolyene	Others	Pyrite-Copper	20.08	China: Guangdong, Daobaoshan
L180330P-6-LD180328-0008_HL3H2CCXY_L4_clean_bin.78.orig.fa_k	OEZ008529	yes	LMSG_G000005792.1	OES073671	terpene	Terpene	Pyrite-Copper	14.81	China: Guangdong, Daobaoshan
L180330P-6-LD180328-0008_HL3H2CCXY_L4_clean_bin.78.orig.fa_k	OEZ008529	yes	LMSG_G000005792.1	OES073671	NRPS-like	NRPS	Pyrite-Copper	5.82	China: Guangdong, Daobaoshan
L180330P-6-LD180328-0008_HL3H2CCXY_L4_clean_bin.79.orig.fa_k	OEZ008529	yes	LMSG_G000010440.1	OES073671	bacteriocin	RiPPs	Pyrite-Copper	10.88	China: Guangdong, Daobaoshan
L180330P-6-LD180328-0008_HL3H2CCXY_L4_clean_bin.79.orig.fa_k	OEZ008529	yes	LMSG_G000010440.1	OES073671	terpene.arylpolyene	Others	Pyrite-Copper	44.36	China: Guangdong, Daobaoshan
L180330P-6-LD180328-0008_HL3H2CCXY_L4_clean_bin.8.orig.fa_k1	OEZ008529	yes	LMSG_G000009998.1	OES073671	terpene.arylpolyene	Others	Pyrite-Copper	32.53	China: Guangdong, Daobaoshan
L180330P-6-LD180328-0008_HL3H2CCXY_L4_clean_bin.8.orig.fa_k1	OEZ008529	yes	LMSG_G000009998.1	OES073671	arylpolyene	Others	Pyrite-Copper	8.37	China: Guangdong, Daobaoshan
L180330P-6-LD180328-0008_HL3H2CCXY_L4_clean_bin.8.orig.fa_k1	OEZ008529	yes	LMSG_G000009998.1	OES073671	terpene	Terpene	Pyrite-Copper	18.61	China: Guangdong, Daobaoshan
L180330P-6-LD180328-0008_HL3H2CCXY_L4_clean_bin.8.orig.fa_k1	OEZ008529	yes	LMSG_G000009998.1	OES073671	ectoine	Others	Pyrite-Copper	7.68	China: Guangdong, Daobaoshan
L180330P-6-LD180328-0008_HL3H2CCXY_L4_clean_bin.83.orig.fa_k	OEZ008529	yes	LMSG_G000005794.1	OES073671	betalactone	Others	Pyrite-Copper	19.81	China: Guangdong, Daobaoshan
L180330P-6-LD180328-0008_HL3H2CCXY_L4_clean_bin.84.orig.fa_k	OEZ008529	yes	LMSG_G000009473.1	OES073671	bacteriocin	RiPPs	Pyrite-Copper	6.35	China: Guangdong, Daobaoshan
L180330P-6-LD180328-0008_HL3H2CCXY_L4_clean_bin.84.orig.fa_k	OEZ008529	yes	LMSG_G000009473.1	OES073671	NRPS.butyrolactone	Others	Pyrite-Copper	13.94	China: Guangdong, Daobaoshan
L180330P-6-LD180328-0008_HL3H2CCXY_L4_clean_bin.86.orig.fa_k	OEZ008529	yes	LMSG_G000010512.1	OES073671	arylpolyene	Others	Pyrite-Copper	6.65	China: Guangdong, Daobaoshan
L180330P-6-LD180328-0008_HL3H2CCXY_L4_clean_bin.86.orig.fa_k	OEZ008529	yes	LMSG_G000010512.1	OES073671	NRPS-like	NRPS	Pyrite-Copper	7.95	China: Guangdong, Daobaoshan
L180330P-6-LD180328-0008_HL3H2CCXY_L4_clean_bin.87.strict.fa	OEZ008529	yes	LMSG_G000009617.1	OES073671	terpene	Terpene	Pyrite-Copper	23.28	China: Guangdong, Daobaoshan
L180330P-6-LD180328-0008_HL3H2CCXY_L4_clean_bin.87.strict.fa	OEZ008529	yes	LMSG_G000009617.1	OES073671	terpene	Terpene	Pyrite-Copper	22.09	China: Guangdong, Daobaoshan
L180330P-6-LD180328-0008_HL3H2CCXY_L4_clean_bin.87.strict.fa	OEZ008529	yes	LMSG_G000009617.1	OES073671	NRPS-like	NRPS	Pyrite-Copper	31.61	China: Guangdong, Daobaoshan
L180330P-6-LD180328-0008_HL3H2CCXY_L4_clean_bin.88.orig.fa_k	OEZ008529	yes	LMSG_G000005795.1	OES073671	terpene	Terpene	Pyrite-Copper	21.99	China: Guangdong, Daobaoshan
L180330P-6-LD180328-0008_HL3H2CCXY_L4_clean_bin.88.orig.fa_k	OEZ008529	yes	LMSG_G000005795.1	OES073671	terpene	Terpene	Pyrite-Copper	23.40	China: Guangdong, Daobaoshan
L180330P-6-LD180328-0008_HL3H2CCXY_L4_clean_bin.88.orig.fa_k	OEZ008529	yes	LMSG_G000005795.1	OES073671	NRPS-like	NRPS	Pyrite-Copper	42.76	China: Guangdong, Daobaoshan
L180330P-6-LD180328-0008_HL3H2CCXY_L4_clean_bin.89.permissiv	OEZ008529	yes	LMSG_G000005796.1	OES073671	thiopeptide.LAP	RiPPs	Pyrite-Copper	41.86	China: Guangdong, Daobaoshan
L180330P-6-LD180328-0008_HL3H2CCXY_L4_clean_bin.94.orig.fa_k	OEZ008529	no	LMSG_G000010947.1	OES073671	bacteriocin	RiPPs	Pyrite-Copper	8.37	China: Guangdong, Daobaoshan
L180330P-6-LD180328-0008_HL3H2CCXY_L4_clean_bin.94.orig.fa_k	OEZ008529	no	LMSG_G000010947.1	OES073671	NRPS	NRPS	Pyrite-Copper	52.53	China: Guangdong, Daobaoshan
L180330P-6-LD180328-0008_HL3H2CCXY_L4_clean_bin.94.orig.fa_k	OEZ008529	no	LMSG_G000010947.1	OES073671	NRPS.T1PKS	PKS-NRP_Hybrids	Pyrite-Copper	44.98	China: Guangdong, Daobaoshan
L180330P-6-LD180328-0008_HL3H2CCXY_L4_clean_bin.94.orig.fa_k	OEZ008529	no	LMSG_G000010947.1	OES073671	thiopeptide	RiPPs	Pyrite-Copper	18.83	China: Guangdong, Daobaoshan
L180330P-6-LD180328-0008_HL3H2CCXY_L4_clean_bin.95.orig.fa_k	OEZ008529	yes	LMSG_G000005798.1	OES073671	terpene	Terpene	Pyrite-Copper	22.12	China: Guangdong, Daobaoshan
L180330P-6-LD180328-0008_HL3H2CCXY_L4_clean_bin.95.orig.fa_k	OEZ008529	yes	LMSG_G000005798.1	OES073671	hserlactone	Others	Pyrite-Copper	20.74	China: Guangdong, Daobaoshan
L180330P-6-LD180328-0008_HL3H2CCXY_L4_clean_bin.96.orig.fa_k	OEZ008529	yes	LMSG_G000004569.1	OES073671	arylpolyene	Others	Pyrite-Copper	26.70	China: Guangdong, Daobaoshan
L180330P-6-LD180328-0008_HL3H2CCXY_L4_clean_bin.96.orig.fa_k	OEZ008529	yes	LMSG_G000004569.1	OES073671	terpene	Terpene	Pyrite-Copper	21.88	China: Guangdong, Daobaoshan
L180330P-6-LD180328-0008_HL3H2CCXY_L4_clean_bin.96.orig.fa_k	OEZ008529	yes	LMSG_G000004569.1	OES073671	ladderane	Others	Pyrite-Copper	41.28	China: Guangdong, Daobaoshan
L180330P-6-LD180328-0008_HL3H2CCXY_L4_clean_bin.96.orig.fa_k	OEZ008529	yes	LMSG_G000004569.1	OES073671	terpene	Terpene	Pyrite-Copper	20.39	China: Guangdong, Daobaoshan
L180330P-6-LD180328-0008_HL3H2CCXY_L4_clean_bin.96.orig.fa_k	OEZ008529	yes	LMSG_G000004569.1	OES073671	arylpolyene	Others	Pyrite-Copper	19.24	China: Guangdong, Daobaoshan
L180330P-7-LD180328-0009_HL3H2CCXY_L5_clean_bin.10.orig.fa_k	OEZ008529	yes	LMSG_G000010441.1	OES073672	bacteriocin	RiPPs	Pyrite-Copper	5.27	China: Guangdong, Daobaoshan
L180330P-7-LD180328-0009_HL3H2CCXY_L5_clean_bin.10.orig.fa_k	OEZ008529	yes	LMSG_G000010441.1	OES073672	bacteriocin	RiPPs	Pyrite-Copper	10.41	China: Guangdong, Daobaoshan
L180330P-7-LD180328-0009_HL3H2CCXY_L5_clean_bin.10.orig.fa_k	OEZ008529	yes	LMSG_G000010441.1	OES073672	terpene.arylpolyene	Others	Pyrite-Copper	48.67	China: Guangdong, Daobaoshan
L180330P-7-LD180328-0009_HL3H2CCXY_L5_clean_bin.104.orig.fa_	OEZ008529	yes	LMSG_G000005800.1	OES073672	other	Others	Pyrite-Copper	17.51	China: Guangdong, Daobaoshan
L180330P-7-LD180328-0009_HL3H2CCXY_L5_clean_bin.104.orig.fa_	OEZ008529	yes	LMSG_G000005800.1	OES073672	betalactone	Others	Pyrite-Copper	41.46	China: Guangdong, Daobaoshan
L180330P-7-LD180328-0009_HL3H2CCXY_L5_clean_bin.105.strict.f	OEZ008529	yes	LMSG_G000008040.1	OES073672	LAP.thiopeptide	RiPPs	Pyrite-Copper	19.46	China: Guangdong, Daobaoshan
L180330P-7-LD180328-0009_HL3H2CCXY_L5_clean_bin.106.strict.f	OEZ008529	yes	LMSG_G000009474.1	OES073672	bacteriocin	RiPPs	Pyrite-Copper	10.86	China: Guangdong, Daobaoshan
L180330P-7-LD180328-0009_HL3H2CCXY_L5_clean_bin.11.orig.fa_k	OEZ008529	yes	LMSG_G000005801.1	OES073672	terpene	Terpene	Pyrite-Copper	6.70	China: Guangdong, Daobaoshan
L180330P-7-LD180328-0009_HL3H2CCXY_L5_clean_bin.14.orig.fa_k	OEZ008529	yes	LMSG_G000008237.1	OES073672	terpene	Terpene	Pyrite-Copper	20.57	China: Guangdong, Daobaoshan
L180330P-7-LD180328-0009_HL3H2CCXY_L5_clean_bin.17.orig.fa_k	OEZ008529	yes	LMSG_G000008651.1	OES073672	other	Others	Pyrite-Copper	40.44	China: Guangdong, Daobaoshan
L180330P-7-LD180328-0009_HL3H2CCXY_L5_clean_bin.2.strict.fa_	OEZ008529	yes	LMSG_G000009618.1	OES073672	terpene	Terpene	Pyrite-Copper	13.70	China: Guangdong, Daobaoshan
L180330P-7-LD180328-0009_HL3H2CCXY_L5_clean_bin.2.strict.fa_	OEZ008529	yes	LMSG_G000009618.1	OES073672	terpene	Terpene	Pyrite-Copper	21.89	China: Guangdong, Daobaoshan
L180330P-7-LD180328-0009_HL3H2CCXY_L5_clean_bin.2.strict.fa_	OEZ008529	yes	LMSG_G000009618.1	OES073672	NRPS-like	NRPS	Pyrite-Copper	31.85	China: Guangdong, Daobaoshan
L180330P-7-LD180328-0009_HL3H2CCXY_L5_clean_bin.23.orig.fa_k	OEZ008529	yes	LMSG_G000005804.1	OES073672	NRPS-like	NRPS	Pyrite-Copper	33.02	China: Guangdong, Daobaoshan
L180330P-7-LD180328-0009_HL3H2CCXY_L5_clean_bin.23.orig.fa_k	OEZ008529	yes	LMSG_G000005804.1	OES073672	terpene	Terpene	Pyrite-Copper	20.90	China: Guangdong, Daobaoshan
L180330P-7-LD180328-0009_HL3H2CCXY_L5_clean_bin.30.orig.fa_k	OEZ008529	yes	LMSG_G000009153.1	OES073672	terpene	Terpene	Pyrite-Copper	23.70	China: Guangdong, Daobaoshan
L180330P-7-LD180328-0009_HL3H2CCXY_L5_clean_bin.30.orig.fa_k	OEZ008529	yes	LMSG_G000009153.1	OES073672	T3PKS	PKSother	Pyrite-Copper	13.64	China: Guangdong, Daobaoshan
L180330P-7-LD180328-0009_HL3H2CCXY_L5_clean_bin.31.strict.fa	OEZ008529	no	LMSG_G000009537.1	OES073672	terpene	Terpene	Pyrite-Copper	20.72	China: Guangdong, Daobaoshan
L180330P-7-LD180328-0009_HL3H2CCXY_L5_clean_bin.31.strict.fa	OEZ008529	no	LMSG_G000009537.1	OES073672	NRPS-like	NRPS	Pyrite-Copper	39.30	China: Guangdong, Daobaoshan
L180330P-7-LD180328-0009_HL3H2CCXY_L5_clean_bin.33.orig.fa_k	OEZ008529	yes	LMSG_G000010824.1	OES073672	bacteriocin	RiPPs	Pyrite-Copper	10.26	China: Guangdong, Daobaoshan
L180330P-7-LD180328-0009_HL3H2CCXY_L5_clean_bin.33.orig.fa_k	OEZ008529	yes	LMSG_G000010824.1	OES073672	acyl_amino_acids	Others	Pyrite-Copper	30.77	China: Guangdong, Daobaoshan
L180330P-7-LD180328-0009_HL3H2CCXY_L5_clean_bin.35.orig.fa_k	OEZ008529	no	LMSG_G000010285.1	OES073672	terpene	Terpene	Pyrite-Copper	19.88	China: Guangdong, Daobaoshan
L180330P-7-LD180328-0009_HL3H2CCXY_L5_clean_bin.35.orig.fa_k	OEZ008529	yes	LMSG_G000010285.1	OES073672	arylpolyene	Others	Pyrite-Copper	41.19	China: Guangdong, Daobaoshan
L180330P-7-LD180328-0009_HL3H2CCXY_L5_clean_bin.36.orig.fa_k	OEZ008529	yes	LMSG_G000008713.1	OES073672	terpene	Terpene	Pyrite-Copper	18.95	China: Guangdong, Daobaoshan
L180330P-7-LD180328-0009_HL3H2CCXY_L5_clean_bin.36.orig.fa_k	OEZ008529	yes	LMSG_G000008713.1	OES073672	bacteriocin	RiPPs	Pyrite-Copper	5.94	China: Guangdong, Daobaoshan
L180330P-7-LD180328-0009_HL3H2CCXY_L5_clean_bin.36.orig.fa_k	OEZ008529	yes	LMSG_G000008713.1	OES073672	betalactone	Others	Pyrite-Copper	21.31	China: Guangdong, Daobaoshan
L180330P-7-LD180328-0009_HL3H2CCXY_L5_clean_bin.4.orig.fa_k1	OEZ008529	yes	LMSG_G000008168.1	OES073672	terpene	Terpene	Pyrite-Copper	12.86	China: Guangdong, Daobaoshan
L180330P-7-LD180328-0009_HL3H2CCXY_L5_clean_bin.40.orig.fa_k	OEZ008529	yes	LMSG_G000009980.1	OES073672	NRPS-like	NRPS	Pyrite-Copper	7.19	China: Guangdong, Daobaoshan
L180330P-7-LD180328-0009_HL3H2CCXY_L5_clean_bin.40.orig.fa_k	OEZ008529	yes	LMSG_G000009980.1	OES073672	terpene	Terpene	Pyrite-Copper	17.42	China: Guangdong, Daobaoshan

L180330P-7-LD180328-0009_HL3H2CCXY_L5_clean_bin.40.orig.fa_k	OEZ008529	yes	LMSG_G000009980.1	OES073672	terpene	Terpene	Pyrite-Copper	10.49	China: Guangdong, Daobaoshan
L180330P-7-LD180328-0009_HL3H2CCXY_L5_clean_bin.40.orig.fa_k	OEZ008529	yes	LMSG_G000009980.1	OES073672	terpene	Terpene	Pyrite-Copper	7.71	China: Guangdong, Daobaoshan
L180330P-7-LD180328-0009_HL3H2CCXY_L5_clean_bin.40.orig.fa_k	OEZ008529	yes	LMSG_G000009980.1	OES073672	T3PKS	PKSother	Pyrite-Copper	6.45	China: Guangdong, Daobaoshan
L180330P-7-LD180328-0009_HL3H2CCXY_L5_clean_bin.44.orig.fa_k	OEZ008529	yes	LMSG_G000009351.1	OES073672	betalactone	Others	Pyrite-Copper	23.26	China: Guangdong, Daobaoshan
L180330P-7-LD180328-0009_HL3H2CCXY_L5_clean_bin.48.orig.fa_k	OEZ008529	yes	LMSG_G000010363.1	OES073672	terpene	Terpene	Pyrite-Copper	10.40	China: Guangdong, Daobaoshan
L180330P-7-LD180328-0009_HL3H2CCXY_L5_clean_bin.48.orig.fa_k	OEZ008529	yes	LMSG_G000010363.1	OES073672	NRPS-like	NRPS	Pyrite-Copper	5.73	China: Guangdong, Daobaoshan
L180330P-7-LD180328-0009_HL3H2CCXY_L5_clean_bin.48.orig.fa_k	OEZ008529	yes	LMSG_G000010363.1	OES073672	terpene	Terpene	Pyrite-Copper	16.79	China: Guangdong, Daobaoshan
L180330P-7-LD180328-0009_HL3H2CCXY_L5_clean_bin.48.orig.fa_k	OEZ008529	yes	LMSG_G000010363.1	OES073672	terpene	Terpene	Pyrite-Copper	6.70	China: Guangdong, Daobaoshan
L180330P-7-LD180328-0009_HL3H2CCXY_L5_clean_bin.48.orig.fa_k	OEZ008529	yes	LMSG_G000010363.1	OES073672	bacteriocin	RiPPs	Pyrite-Copper	5.18	China: Guangdong, Daobaoshan
L180330P-7-LD180328-0009_HL3H2CCXY_L5_clean_bin.5.orig.fa_kl	OEZ008529	yes	LMSG_G000010174.1	OES073672	bacteriocin	RiPPs	Pyrite-Copper	10.89	China: Guangdong, Daobaoshan
L180330P-7-LD180328-0009_HL3H2CCXY_L5_clean_bin.5.orig.fa_kl	OEZ008529	no	LMSG_G000010174.1	OES073672	terpene	Terpene	Pyrite-Copper	17.15	China: Guangdong, Daobaoshan
L180330P-7-LD180328-0009_HL3H2CCXY_L5_clean_bin.5.orig.fa_kl	OEZ008529	no	LMSG_G000010174.1	OES073672	terpene	Terpene	Pyrite-Copper	11.26	China: Guangdong, Daobaoshan
L180330P-7-LD180328-0009_HL3H2CCXY_L5_clean_bin.5.orig.fa_kl	OEZ008529	no	LMSG_G000010174.1	OES073672	ectoine	Others	Pyrite-Copper	10.40	China: Guangdong, Daobaoshan
L180330P-7-LD180328-0009_HL3H2CCXY_L5_clean_bin.50.orig.fa_k	OEZ008529	yes	LMSG_G000011121.1	OES073672	arylpolyene	Others	Pyrite-Copper	14.69	China: Guangdong, Daobaoshan
L180330P-7-LD180328-0009_HL3H2CCXY_L5_clean_bin.50.orig.fa_k	OEZ008529	yes	LMSG_G000011121.1	OES073672	terpene	Terpene	Pyrite-Copper	16.35	China: Guangdong, Daobaoshan
L180330P-7-LD180328-0009_HL3H2CCXY_L5_clean_bin.53.orig.fa_k	OEZ008529	yes	LMSG_G000005808.1	OES073672	T3PKS	PKSother	Pyrite-Copper	41.06	China: Guangdong, Daobaoshan
L180330P-7-LD180328-0009_HL3H2CCXY_L5_clean_bin.53.orig.fa_k	OEZ008529	yes	LMSG_G000005808.1	OES073672	terpene	Terpene	Pyrite-Copper	23.79	China: Guangdong, Daobaoshan
L180330P-7-LD180328-0009_HL3H2CCXY_L5_clean_bin.53.orig.fa_k	OEZ008529	yes	LMSG_G000005808.1	OES073672	T1PKS.NRPS-like	PKS-NRP_Hybrids	Pyrite-Copper	39.02	China: Guangdong, Daobaoshan
L180330P-7-LD180328-0009_HL3H2CCXY_L5_clean_bin.55.orig.fa_k	OEZ008529	yes	LMSG_G000006296.1	OES073672	bacteriocin	RiPPs	Pyrite-Copper	10.88	China: Guangdong, Daobaoshan
L180330P-7-LD180328-0009_HL3H2CCXY_L5_clean_bin.55.orig.fa_k	OEZ008529	yes	LMSG_G000006296.1	OES073672	terpene.arylpolyene	Others	Pyrite-Copper	52.38	China: Guangdong, Daobaoshan
L180330P-7-LD180328-0009_HL3H2CCXY_L5_clean_bin.56.permissiv	OEZ008529	yes	LMSG_G000005809.1	OES073672	terpene	Terpene	Pyrite-Copper	6.46	China: Guangdong, Daobaoshan
L180330P-7-LD180328-0009_HL3H2CCXY_L5_clean_bin.58.orig.fa_k	OEZ008529	yes	LMSG_G000005810.1	OES073672	arylpolyene	Others	Pyrite-Copper	6.71	China: Guangdong, Daobaoshan
L180330P-7-LD180328-0009_HL3H2CCXY_L5_clean_bin.58.orig.fa_k	OEZ008529	yes	LMSG_G000005810.1	OES073672	bacteriocin	RiPPs	Pyrite-Copper	7.81	China: Guangdong, Daobaoshan
L180330P-7-LD180328-0009_HL3H2CCXY_L5_clean_bin.6.orig.fa_kl	OEZ008529	yes	LMSG_G000005813.1	OES073672	hserlactone	Others	Pyrite-Copper	20.78	China: Guangdong, Daobaoshan
L180330P-7-LD180328-0009_HL3H2CCXY_L5_clean_bin.6.orig.fa_kl	OEZ008529	yes	LMSG_G000005813.1	OES073672	bacteriocin	RiPPs	Pyrite-Copper	10.89	China: Guangdong, Daobaoshan
L180330P-7-LD180328-0009_HL3H2CCXY_L5_clean_bin.6.orig.fa_kl	OEZ008529	yes	LMSG_G000005813.1	OES073672	terpene	Terpene	Pyrite-Copper	20.43	China: Guangdong, Daobaoshan
L180330P-7-LD180328-0009_HL3H2CCXY_L5_clean_bin.6.orig.fa_kl	OEZ008529	yes	LMSG_G000005813.1	OES073672	NAGGN	Others	Pyrite-Copper	14.68	China: Guangdong, Daobaoshan
L180330P-7-LD180328-0009_HL3H2CCXY_L5_clean_bin.6.orig.fa_kl	OEZ008529	yes	LMSG_G000005813.1	OES073672	T3PKS.terpene	Others	Pyrite-Copper	60.80	China: Guangdong, Daobaoshan
L180330P-7-LD180328-0009_HL3H2CCXY_L5_clean_bin.6.orig.fa_kl	OEZ008529	yes	LMSG_G000005813.1	OES073672	NRPS-like	NRPS	Pyrite-Copper	42.88	China: Guangdong, Daobaoshan
L180330P-7-LD180328-0009_HL3H2CCXY_L5_clean_bin.60.orig.fa_k	OEZ008529	yes	LMSG_G000010451.1	OES073672	resorcinol.arylpolyene	Others	Pyrite-Copper	23.62	China: Guangdong, Daobaoshan
L180330P-7-LD180328-0009_HL3H2CCXY_L5_clean_bin.60.orig.fa_k	OEZ008529	yes	LMSG_G000010451.1	OES073672	arylpolyene	Others	Pyrite-Copper	28.80	China: Guangdong, Daobaoshan
L180330P-7-LD180328-0009_HL3H2CCXY_L5_clean_bin.60.orig.fa_k	OEZ008529	yes	LMSG_G000010451.1	OES073672	terpene	Terpene	Pyrite-Copper	17.25	China: Guangdong, Daobaoshan
L180330P-7-LD180328-0009_HL3H2CCXY_L5_clean_bin.61.orig.fa_k	OEZ008529	yes	LMSG_G000010513.1	OES073672	arylpolyene	Others	Pyrite-Copper	7.86	China: Guangdong, Daobaoshan
L180330P-7-LD180328-0009_HL3H2CCXY_L5_clean_bin.62.strict.fa	OEZ008529	yes	LMSG_G000009564.1	OES073672	terpene	Terpene	Pyrite-Copper	22.06	China: Guangdong, Daobaoshan
L180330P-7-LD180328-0009_HL3H2CCXY_L5_clean_bin.62.strict.fa	OEZ008529	yes	LMSG_G000009564.1	OES073672	terpene	Terpene	Pyrite-Copper	23.40	China: Guangdong, Daobaoshan
L180330P-7-LD180328-0009_HL3H2CCXY_L5_clean_bin.62.strict.fa	OEZ008529	yes	LMSG_G000009564.1	OES073672	NRPS-like	NRPS	Pyrite-Copper	32.96	China: Guangdong, Daobaoshan
L180330P-7-LD180328-0009_HL3H2CCXY_L5_clean_bin.63.orig.fa_k	OEZ008529	yes	LMSG_G000010474.1	OES073672	arylpolyene.hserlactone	Others	Pyrite-Copper	41.23	China: Guangdong, Daobaoshan
L180330P-7-LD180328-0009_HL3H2CCXY_L5_clean_bin.64.orig.fa_k	OEZ008529	yes	LMSG_G000010788.1	OES073672	bacteriocin	RiPPs	Pyrite-Copper	9.36	China: Guangdong, Daobaoshan
L180330P-7-LD180328-0009_HL3H2CCXY_L5_clean_bin.64.orig.fa_k	OEZ008529	yes	LMSG_G000010788.1	OES073672	arylpolyene	Others	Pyrite-Copper	38.97	China: Guangdong, Daobaoshan
L180330P-7-LD180328-0009_HL3H2CCXY_L5_clean_bin.64.orig.fa_k	OEZ008529	yes	LMSG_G000010788.1	OES073672	acyl_amino_acids	Others	Pyrite-Copper	39.23	China: Guangdong, Daobaoshan
L180330P-7-LD180328-0009_HL3H2CCXY_L5_clean_bin.65.strict.fa	OEZ008529	yes	LMSG_G000009182.1	OES073672	terpene	Terpene	Pyrite-Copper	23.68	China: Guangdong, Daobaoshan
L180330P-7-LD180328-0009_HL3H2CCXY_L5_clean_bin.65.strict.fa	OEZ008529	yes	LMSG_G000009182.1	OES073672	T3PKS	PKSother	Pyrite-Copper	21.66	China: Guangdong, Daobaoshan
L180330P-7-LD180328-0009_HL3H2CCXY_L5_clean_bin.66.orig.fa_k	OEZ008529	yes	LMSG_G000005812.1	OES073672	thiopeptide	RiPPs	Pyrite-Copper	34.19	China: Guangdong, Daobaoshan
L180330P-7-LD180328-0009_HL3H2CCXY_L5_clean_bin.66.orig.fa_k	OEZ008529	yes	LMSG_G000005812.1	OES073672	terpene	Terpene	Pyrite-Copper	21.66	China: Guangdong, Daobaoshan
L180330P-7-LD180328-0009_HL3H2CCXY_L5_clean_bin.66.orig.fa_k	OEZ008529	yes	LMSG_G000005812.1	OES073672	arylpolyene	Others	Pyrite-Copper	41.19	China: Guangdong, Daobaoshan
L180330P-7-LD180328-0009_HL3H2CCXY_L5_clean_bin.67.orig.fa_k	OEZ008529	yes	LMSG_G000009392.1	OES073672	bacteriocin	RiPPs	Pyrite-Copper	7.64	China: Guangdong, Daobaoshan
L180330P-7-LD180328-0009_HL3H2CCXY_L5_clean_bin.67.orig.fa_k	OEZ008529	yes	LMSG_G000009392.1	OES073672	terpene	Terpene	Pyrite-Copper	21.68	China: Guangdong, Daobaoshan
L180330P-7-LD180328-0009_HL3H2CCXY_L5_clean_bin.67.orig.fa_k	OEZ008529	yes	LMSG_G000009392.1	OES073672	NRPS-like	NRPS	Pyrite-Copper	22.32	China: Guangdong, Daobaoshan
L180330P-7-LD180328-0009_HL3H2CCXY_L5_clean_bin.67.orig.fa_k	OEZ008529	yes	LMSG_G000009392.1	OES073672	NRPS	NRPS	Pyrite-Copper	18.25	China: Guangdong, Daobaoshan
L180330P-7-LD180328-0009_HL3H2CCXY_L5_clean_bin.69.strict.fa	OEZ008529	yes	LMSG_G000011209.1	OES073672	arylpolyene	Others	Pyrite-Copper	6.01	China: Guangdong, Daobaoshan
L180330P-7-LD180328-0009_HL3H2CCXY_L5_clean_bin.72.permissiv	OEZ008529	yes	LMSG_G000009561.1	OES073672	terpene	Terpene	Pyrite-Copper	22.06	China: Guangdong, Daobaoshan
L180330P-7-LD180328-0009_HL3H2CCXY_L5_clean_bin.72.permissiv	OEZ008529	no	LMSG_G000009561.1	OES073672	terpene	Terpene	Pyrite-Copper	20.95	China: Guangdong, Daobaoshan
L180330P-7-LD180328-0009_HL3H2CCXY_L5_clean_bin.72.permissiv	OEZ008529	no	LMSG_G000009561.1	OES073672	NRPS-like	NRPS	Pyrite-Copper	25.98	China: Guangdong, Daobaoshan
L180330P-7-LD180328-0009_HL3H2CCXY_L5_clean_bin.72.permissiv	OEZ008529	yes	LMSG_G000009561.1	OES073672	ectoine	Others	Pyrite-Copper	10.40	China: Guangdong, Daobaoshan
L180330P-7-LD180328-0009_HL3H2CCXY_L5_clean_bin.72.permissiv	OEZ008529	yes	LMSG_G000009561.1	OES073672	acyl_amino_acids	Others	Pyrite-Copper	24.24	China: Guangdong, Daobaoshan
L180330P-7-LD180328-0009_HL3H2CCXY_L5_clean_bin.72.permissiv	OEZ008529	yes	LMSG_G000009561.1	OES073672	acyl_amino_acids	Others	Pyrite-Copper	6.56	China: Guangdong, Daobaoshan
L180330P-7-LD180328-0009_HL3H2CCXY_L5_clean_bin.73.orig.fa_k	OEZ008529	yes	LMSG_G000008546.1	OES073672	betalactone	Others	Pyrite-Copper	27.91	China: Guangdong, Daobaoshan
L180330P-7-LD180328-0009_HL3H2CCXY_L5_clean_bin.73.orig.fa_k	OEZ008529	yes	LMSG_G000008546.1	OES073672	other	Others	Pyrite-Copper	23.57	China: Guangdong, Daobaoshan
L180330P-7-LD180328-0009_HL3H2CCXY_L5_clean_bin.74.orig.fa_k	OEZ008529	yes	LMSG_G000009244.1	OES073672	T3PKS.terpene	Others	Pyrite-Copper	40.83	China: Guangdong, Daobaoshan
L180330P-7-LD180328-0009_HL3H2CCXY_L5_clean_bin.75.strict.fa	OEZ008529	yes	LMSG_G000004570.1	OES073672	T1PKS.hglE-KS	PKSother	Pyrite-Copper	29.39	China: Guangdong, Daobaoshan
L180330P-7-LD180328-0009_HL3H2CCXY_L5_clean_bin.75.strict.fa	OEZ008529	yes	LMSG_G000004570.1	OES073672	bacteriocin	RiPPs	Pyrite-Copper	5.51	China: Guangdong, Daobaoshan
L180330P-7-LD180328-0009_HL3H2CCXY_L5_clean_bin.76.orig.fa_k	OEZ008529	yes	LMSG_G000006506.1	OES073672	LAP	RiPPs	Pyrite-Copper	24.85	China: Guangdong, Daobaoshan
L180330P-7-LD180328-0009_HL3H2CCXY_L5_clean_bin.78.strict.fa	OEZ008529	yes	LMSG_G000009253.1	OES073672	T3PKS	PKSother	Pyrite-Copper	36.40	China: Guangdong, Daobaoshan
L180330P-7-LD180328-0009_HL3H2CCXY_L5_clean_bin.78.strict.fa	OEZ008529	yes	LMSG_G000009253.1	OES073672	TfuA-related	RiPPs	Pyrite-Copper	21.95	China: Guangdong, Daobaoshan
L180330P-7-LD180328-0009_HL3H2CCXY_L5_clean_bin.79.orig.fa_k	OEZ008529	yes	LMSG_G000005814.1	OES073672	nucleoside	Others	Pyrite-Copper	14.61	China: Guangdong, Daobaoshan
L180330P-7-LD180328-0009_HL3H2CCXY_L5_clean_bin.80.orig.fa_k	OEZ008529	yes	LMSG_G000004571.1	OES073672	NRPS	NRPS	Pyrite-Copper	50.88	China: Guangdong, Daobaoshan
L180330P-7-LD180328-0009_HL3H2CCXY_L5_clean_bin.80.orig.fa_k	OEZ008529	yes	LMSG_G000004571.1	OES073672	T1PKS	PKSI	Pyrite-Copper	51.68	China: Guangdong, Daobaoshan
L180330P-7-LD180328-0009_HL3H2CCXY_L5_clean_bin.81.orig.fa_k	OEZ008529	yes	LMSG_G000009491.1	OES073672	NRPS-like	NRPS	Pyrite-Copper	29.10	China: Guangdong, Daobaoshan
L180330P-7-LD180328-0009_HL3H2CCXY_L5_clean_bin.81.orig.fa_k	OEZ008529	yes	LMSG_G000009491.1	OES073672	T1PKS.hglE-KS	PKSother	Pyrite-Copper	28.45	China: Guangdong, Daobaoshan
L180330P-7-LD180328-0009_HL3H2CCXY_L5_clean_bin.83.permissiv	OEZ008529	yes	LMSG_G000004572.1	OES073672	terpene	Terpene	Pyrite-Copper	19.96	China: Guangdong, Daobaoshan
L180330P-7-LD180328-0009_HL3H2CCXY_L5_clean_bin.83.permissiv	OEZ008529	yes	LMSG_G000004572.1	OES073672	NRPS-like	NRPS	Pyrite-Copper	11.45	China: Guangdong, Daobaoshan
L180330P-7-LD180328-0009_HL3H2CCXY_L5_clean_bin.84.orig.fa_k	OEZ008529	yes	LMSG_G000008177.1	OES073672	terpene	Terpene	Pyrite-Copper	21.08	China: Guangdong, Daobaoshan
L180330P-7-LD180328-0009_HL3H2CCXY_L5_clean_bin.86.orig.fa_k	OEZ008529	yes	LMSG_G000009092.1	OES073672	arylpolyene	Others	Pyrite-Copper	7.94	China: Guangdong, Daobaoshan
L180330P-7-LD180328-0009_HL3H2CCXY_L5_clean_bin.86.orig.fa_k	OEZ008529	yes	LMSG_G000009092.1	OES073672	betalactone	Others	Pyrite-Copper	8.44	China: Guangdong, Daobaoshan
L180330P-7-LD180328-0009_HL3H2CCXY_L5_clean_bin.86.orig.fa_k	OEZ008529	yes	LMSG_G000009092.1	OES073672	betalactone	Others	Pyrite-Copper	9.82	China: Guangdong, Daobaoshan
L180330P-7-LD180328-0009_HL3H2CCXY_L5_clean_bin.88.orig.fa_k	OEZ008529	yes	LMSG_G000007825.1	OES073672	terpene	Terpene	Pyrite-Copper	29.81	China: Guangdong, Daobaoshan
L180330P-7-LD180328-0009_HL3H2CCXY_L5_clean_bin.88.orig.fa_k	OEZ008529	yes	LMSG_G000007825.1	OES073672	T3PKS	PKSother	Pyrite-Copper	41.09	China: Guangdong, Daobaoshan
L180330P-7-LD180328-0009_HL3H2CCXY_L5_clean_bin.91.orig.fa_k	OEZ008529	yes	LMSG_G000010443.1	OES073672	bacteriocin	RiPPs	Pyrite-Copper	8.26	China: Guangdong, Daobaoshan
L180330P-7-LD180328-0009_HL3H2CCXY_L5_clean_bin.91.orig.fa_k	OEZ008529	yes	LMSG_G000010443.1	OES073672	terpene	Terpene	Pyrite-Copper	18.34	China: Guangdong, Daobaoshan
L180330P-7-LD180328-0009_HL3H2CCXY_L5_clean_bin.91.orig.fa_k	OEZ008529	yes	LMSG_G000010443.1	OES073672	lassopeptide	RiPPs	Pyrite-Copper	7.81	China: Guangdong, Daobaoshan
L180330P-7-LD180328-0009_HL3H2CCXY_L5_clean_bin.91.orig.fa_k	OEZ008529	yes	LMSG_G000010443.1	OES073672	NRPS-like	NRPS	Pyrite-Copper	42.80	China: Guangdong, Daobaoshan
L180330P-7-LD180328-0009_HL3H2CCXY_L5_clean_bin.91.orig.fa_k	OEZ008529	yes	LMSG_G000010443.1	OES073672	furan	Others	Pyrite-Copper	9.86	China: Guangdong, Daobaoshan
L180330P-7-LD180328-0009_HL3H2CCXY_L5_clean_bin.91.orig.fa_k	OEZ008529	yes	LMSG_G000010443.1	OES073672	lassopeptide	RiPPs	Pyrite-Copper	9.75	China: Guangdong, Daobaoshan
L180330P-7-LD180328-0009_HL3H2CCXY_L5_clean_bin.92.orig.fa_k	OEZ008529	yes	LMSG_G000010074.1	OES073672	arylpolyene	Others	Pyrite-Copper	26.70	China: Guangdong, Daobaoshan
L180330P-7-LD180328-0009_HL3H2CCXY_L5_clean_bin.92.orig.fa_k	OEZ008529	yes	LMSG_G000010074.1	OES073672	ladderane	Others	Pyrite-Copper	41.28	China: Guangdong, Daobaoshan
L180330P-7-LD180328-0009_HL3H2CCXY_L5_clean_bin.92.orig.fa_k	OEZ008529	yes	LMSG_G000010074.1	OES073672	arylpolyene	Others	Pyrite-Copper	19.06	China: Guangdong, Daobaoshan
L180330P-7-LD180328-0009_HL3H2CCXY_L5_clean_bin.92.orig.fa_k	OEZ008529	yes	LMSG_G000010074.1	OES073672	terpene	Terpene	Pyrite-Copper	20.39	China: Guangdong, Daobaoshan
L180330P-7-LD180328-0009_HL3H2CCXY_L5_clean_bin.92.orig.fa_k	OEZ008529	yes	LMSG_G000010074.1	OES073672	terpene	Terpene	Pyrite-Copper	21.88	China: Guangdong, Daobaoshan
L180330P-7-LD180328-0009_HL3H2CCXY_L5_clean_bin.93.orig.fa_k	OEZ008529	yes	LMSG_G000007955.1	OES073672	lassopeptide	RiPPs	Pyrite-Copper	20.99	China: Guangdong, Daobaoshan
L180330P-7-LD180328-0009_HL3H2CCXY_L5_clean_bin.95.orig.fa_k	OEZ008529	yes	LMSG_G000008636.1	OES073672	NRPS-like	NRPS	Pyrite-Copper	28.06	China: Guangdong, Daobaoshan
L180330P-7-LD180328-0009_HL3H2CCXY_L5_clean_bin.95.orig.fa_k	OEZ008529	yes	LMSG_G000008636.1	OES073672	other	Others	Pyrite-Copper	40.72	China: Guangdong, Daobaoshan
L180330P-7-LD180328-0009_HL3H2CCXY_L5_clean_bin.97.orig.fa_k	OEZ008529	yes	LMSG_G000009500.1	OES073672	T3PKS	PKSother	Pyrite-Copper	5.23	China: Guangdong, Daobaoshan
L180330P-7-LD180328-0009_HL3H2CCXY_L5_clean_bin.97.orig.fa_k	OEZ008529	yes	LMSG_G000009500.1	OES073672	bacteriocin	RiPPs	Pyrite-Copper	6.06	China: Guangdong, Daobaoshan
L180330P-7-LD180328-0009_HL3H2CCXY_L5_clean_bin.98.orig.fa_k	OEZ008529	yes	LMSG_G000010511.1	OES073672	NRPS-like	NRPS	Pyrite-Copper	42.86	China: Guangdong, Daobaoshan
L180330P-7-LD180328-0009_HL3H2CCXY_L5_clean_bin.98.orig.fa_k	OEZ008529	yes	LMSG_G000010511.1	OES073672	arylpolyene	Others	Pyrite-Copper	41.27	China: Guangdong, Daobaoshan
L180330P-7-LD180328-0009_HL3H2CCXY_L5_clean_bin.99.orig.fa_k	OEZ008529	yes	LMSG_G000008113.1	OES073672	terpene	Terpene	Pyrite-Copper	18.93	China: Guangdong, Daobaoshan
L180330P-7-LD180328-0009_HL3H2CCXY_L5_clean_bin.99.orig.fa_k	OEZ008529	yes	LMSG_G000008113.1	OES073672	betalactone	Others	Pyrite-Copper	25.92	China: Guangdong, Daobaoshan
L180330P-7-LD180328-0010_HL3H2CCXY_L5_clean_bin.101.orig.fa_	OEZ008529	yes	LMSG_G000010444.1	OES073673	terpene	Terpene	Pyrite-Copper	8.32	China: Guangdong, Daobaoshan
L180330P-7-LD180328-0010_HL3H2CCXY_L5_clean_bin.101.orig.fa_	OEZ008529	yes	LMSG_G000010444.1	OES073673	NRPS-like	NRPS	Pyrite-Copper	42.80	China: Guangdong, Daobaoshan
L180330P-7-LD180328-0010_HL3H2CCXY_L5_clean_bin.101.orig.fa_	OEZ008529	yes	LMSG_G000010444.1	OES073673	bacteriocin	RiPPs	Pyrite-Copper	10.87	China: Guangdong, Daobaoshan
L180330P-7-LD180328-0010_HL3H2CCXY_L5_clean_bin.103.orig.fa_	OEZ008529	yes	LMSG_G000005818.1	OES073673	NRPS-like	NRPS	Pyrite-Copper	37.75	China: Guangdong, Daobaoshan
L180330P-7-LD180328-0010_HL3H2CCXY_L5_clean_bin.103.orig.fa_	OEZ008529	yes	LMSG_G000005818.1	OES073673	acyl_amino_acids	Others	Pyrite-Copper	38.27	China: Guangdong, Daobaoshan
L180330P-7-LD180328-0010_HL3H2CCXY_L5_clean_bin.104.permissi	OEZ008529	yes	LMSG_G000008296.1	OES073673	terpene	Terpene	Pyrite-Copper	22.28	China: Guangdong, Daobaoshan
L180330P-7-LD180328-0010_HL3H2CCXY_L5_clean_bin.104.permissi	OEZ008529	yes	LMSG_G000008296.1	OES073673	terpene	Terpene	Pyrite-Copper	7.10	China: Guangdong, Daobaoshan
L180330P-7-LD180328-0010_HL3H2CCXY_L5_clean_bin.105.orig.fa_	OEZ008529	yes	LMSG_G000005819.1	OES073673	terpene	Terpene	Pyrite-Copper	7.73	China: Guangdong, Daobaoshan
L180330P-7-LD180328-0010_HL3H2CCXY_L5_clean_bin.106.orig.fa_	OEZ008529	yes	LMSG_G000007855.1	OES073673	terpene	Terpene	Pyrite-Copper	5.76	China: Guangdong, Daobaoshan
L180330P-7-LD180328-0010_HL3H2CCXY_L5_clean_bin.106.orig.fa_	OEZ008529	yes	LMSG_G000007855.1	OES073673	NRPS-like	NRPS	Pyrite-Copper	6.73	China: Guangdong, Daobaoshan
L180330P-7-LD180328-0010_HL3H2CCXY_L5_clean_bin.110.orig.fa_	OEZ008529	yes	LMSG_G000010436.1	OES073673	terpene.arylpolyene	Others	Pyrite-Copper	52.38	China: Guangdong, Daobaoshan
L180330P-7-LD180328-0010_HL3H2CCXY_L5_clean_bin.110.orig.fa_	OEZ008529	yes	LMSG_G000010436.1	OES073673	bacteriocin	RiPPs	Pyrite-Copper	10.88	China: Guangdong, Daobaoshan
L180330P-7-LD180328-0010_HL3H2CCXY_L5_clean_bin.112.orig.fa_	OEZ008529	yes	LMSG_G000010450.1	OES073673	resorcinol.arylpolyene	Others	Pyrite-Copper	23.62	China: Guangdong, Daobaoshan
L180330P-7-LD180328-0010_HL3H2CCXY_L5_clean_bin.112.orig.fa_	OEZ008529	yes	LMSG_G000010450.1	OES073673	terpene	Terpene	Pyrite-Copper	23.03	China: Guangdong, Daobaoshan
L180330P-7-LD180328-0010_HL3H2CCXY_L5_clean_bin.112.orig.fa_	OEZ008529	yes	LMSG_G000010450.1	OES073673	arylpolyene	Others	Pyrite-Copper	35.35	China: Guangdong, Daobaoshan
L180330P-7-LD180328-0010_HL3H2CCXY_L5_clean_bin.113.strict.f	OEZ008529	yes	LMSG_G000005820.1	OES073673	T3PKS	PKSother	Pyrite-Copper	33.14	China: Guangdong, Daobaoshan
L180330P-7-LD180328-0010_HL3H2CCXY_L5_clean_bin.113.strict.f	OEZ008529	yes	LMSG_G000005820.1	OES073673	TfuA-related	RiPPs	Pyrite-Copper	21.95	China: Guangdong, Daobaoshan
L180330P-7-LD180328-0010_HL3H2CCXY_L5_clean_bin.115.orig.fa_	OEZ008529	yes	LMSG_G000008590.1	OES073673	other	Others	Pyrite-Copper	24.44	China: Guangdong, Daobaoshan
L180330P-7-LD180328-0010_HL3H2CCXY_L5_clean_bin.115.orig.fa_	OEZ008529	yes	LMSG_G000008590.1	OES073673	other	Others	Pyrite-Copper	5.94	China: Guangdong, Daobaoshan
L180330P-7-LD180328-0010_HL3H2CCXY_L5_clean_bin.116.orig.fa_	OEZ008529	yes	LMSG_G000008714.1	OES073673	bacteriocin	RiPPs	Pyrite-Copper	6.30	China: Guangdong, Daobaoshan
L180330P-7-LD180328-0010_HL3H2CCXY_L5_clean_bin.116.orig.fa_	OEZ008529	no	LMSG_G000008714.1	OES073673	terpene	Terpene	Pyrite-Copper	7.23	China: Guangdong, Daobaoshan
L180330P-7-LD180328-0010_HL3H2CCXY_L5_clean_bin.116.orig.fa_	OEZ008529	yes	LMSG_G000008714.1	OES073673	terpene	Terpene	Pyrite-Copper	5.32	China: Guangdong, Daobaoshan
L180330P-7-LD180328-0010_HL3H2CCXY_L5_clean_bin.117.orig.fa_	OEZ008529	yes	LMSG_G000008736.1	OES073673	terpene	Terpene	Pyrite-Copper	16.73	China: Guangdong, Daobaoshan
L180330P-7-LD180328-0010_HL3H2CCXY_L5_clean_bin.118.strict.f	OEZ008529	yes	LMSG_G000009646.1	OES073673	terpene	Terpene	Pyrite-Copper	13.16	China: Guangdong, Daobaoshan
L180330P-7-LD180328-0010_HL3H2CCXY_L5_clean_bin.118.strict.f	OEZ008529	yes	LMSG_G000009646.1	OES073673	NRPS-like	NRPS	Pyrite-Copper	18.15	China: Guangdong, Daobaoshan
L180330P-7-LD180328-0010_HL3H2CCXY_L5_clean_bin.118.strict.f	OEZ008529	yes	LMSG_G000009646.1	OES073673	terpene	Terpene	Pyrite-Copper	13.01	China: Guangdong, Daobaoshan
L180330P-7-LD180328-0010_HL3H2CCXY_L5_clean_bin.119.orig.fa_	OEZ008529	yes	LMSG_G000008238.1	OES073673	terpene	Terpene	Pyrite-Copper	20.86	China: Guangdong, Daobaoshan
L180330P-7-LD180328-0010_HL3H2CCXY_L5_clean_bin.12.orig.fa_k	OEZ008529	yes	LMSG_G000004574.1	OES073673	TfuA-related	RiPPs	Pyrite-Copper	19.67	China: Guangdong, Daobaoshan
L180330P-7-LD180328-0010_HL3H2CCXY_L5_clean_bin.12.orig.fa_k	OEZ008529	yes	LMSG_G000004574.1	OES073673	T1PKS.hglE-KS	PKSother	Pyrite-Copper	38.35	China: Guangdong, Daobaoshan
L180330P-7-LD180328-0010_HL3H2CCXY_L5_clean_bin.12.orig.fa_k	OEZ008529	yes	LMSG_G000004574.1	OES073673	NRPS-like	NRPS	Pyrite-Copper	34.53	China: Guangdong, Daobaoshan
L180330P-7-LD180328-0010_HL3H2CCXY_L5_clean_bin.12.orig.fa_k	OEZ008529	yes	LMSG_G000004574.1	OES073673	T3PKS	PKSother	Pyrite-Copper	41.05	China: Guangdong, Daobaoshan
L180330P-7-LD180328-0010_HL3H2CCXY_L5_clean_bin.12.orig.fa_k	OEZ008529	yes	LMSG_G000004574.1	OES073673	bacteriocin	RiPPs	Pyrite-Copper	10.86	China: Guangdong, Daobaoshan
L180330P-7-LD180328-0010_HL3H2CCXY_L5_clean_bin.120.orig.fa_	OEZ008529	yes	LMSG_G000011183.1	OES073673	arylpolyene	Others	Pyrite-Copper	21.25	China: Guangdong, Daobaoshan
L180330P-7-LD180328-0010_HL3H2CCXY_L5_clean_bin.120.orig.fa_	OEZ008529	yes	LMSG_G000011183.1	OES073673	bacteriocin	RiPPs	Pyrite-Copper	10.92	China: Guangdong, Daobaoshan
L180330P-7-LD180328-0010_HL3H2CCXY_L5_clean_bin.120.orig.fa_	OEZ008529	yes	LMSG_G000011183.1	OES073673	arylpolyene	Others	Pyrite-Copper	20.35	China: Guangdong, Daobaoshan
L180330P-7-LD180328-0010_HL3H2CCXY_L5_clean_bin.122.strict.f	OEZ008529	yes	LMSG_G000008117.1	OES073673	terpene	Terpene	Pyrite-Copper	20.50	China: Guangdong, Daobaoshan
L180330P-7-LD180328-0010_HL3H2CCXY_L5_clean_bin.124.orig.fa_	OEZ008529	yes	LMSG_G000004573.1	OES073673	arylpolyene	Others	Pyrite-Copper	41.16	China: Guangdong, Daobaoshan
L180330P-7-LD180328-0010_HL3H2CCXY_L5_clean_bin.124.orig.fa_	OEZ008529	yes	LMSG_G000004573.1	OES073673	terpene	Terpene	Pyrite-Copper	23.11	China: Guangdong, Daobaoshan
L180330P-7-LD180328-0010_HL3H2CCXY_L5_clean_bin.125.orig.fa_	OEZ008529	yes	LMSG_G000009164.1	OES073673	T3PKS	PKSother	Pyrite-Copper	17.09	China: Guangdong, Daobaoshan
L180330P-7-LD180328-0010_HL3H2CCXY_L5_clean_bin.125.orig.fa_	OEZ008529	yes	LMSG_G000009164.1	OES073673	terpene	Terpene	Pyrite-Copper	18.95	China: Guangdong, Daobaoshan
L180330P-7-LD180328-0010_HL3H2CCXY_L5_clean_bin.13.orig.fa_k	OEZ008529	yes	LMSG_G000005823.1	OES073673	NRPS-like	NRPS	Pyrite-Copper	38.66	China: Guangdong, Daobaoshan
L180330P-7-LD180328-0010_HL3H2CCXY_L5_clean_bin.13.orig.fa_k	OEZ008529	yes	LMSG_G000005823.1	OES073673	T3PKS	PKSother	Pyrite-Copper	25.03	China: Guangdong, Daobaoshan
L180330P-7-LD180328-0010_HL3H2CCXY_L5_clean_bin.13.orig.fa_k	OEZ008529	yes	LMSG_G000005823.1	OES073673	NRPS-like	NRPS	Pyrite-Copper	16.04	China: Guangdong, Daobaoshan
L180330P-7-LD180328-0010_HL3H2CCXY_L5_clean_bin.15.permissiv	OEZ008529	yes	LMSG_G000008318.1	OES073673	terpene	Terpene	Pyrite-Copper	21.09	China: Guangdong, Daobaoshan
L180330P-7-LD180328-0010_HL3H2CCXY_L5_clean_bin.15.permissiv	OEZ008529	yes	LMSG_G000008318.1	OES073673	NRPS-like	NRPS	Pyrite-Copper	24.51	China: Guangdong, Daobaoshan
L180330P-7-LD180328-0010_HL3H2CCXY_L5_clean_bin.17.orig.fa_k	OEZ008529	yes	LMSG_G000005824.1	OES073673	bacteriocin	RiPPs	Pyrite-Copper	10.86	China: Guangdong, Daobaoshan
L180330P-7-LD180328-0010_HL3H2CCXY_L5_clean_bin.18.orig.fa_k	OEZ008529	yes	LMSG_G000009999.1	OES073673	arylpolyene	Others	Pyrite-Copper	8.67	China: Guangdong, Daobaoshan
L180330P-7-LD180328-0010_HL3H2CCXY_L5_clean_bin.18.orig.fa_k	OEZ008529	yes	LMSG_G000009999.1	OES073673	ectoine	Others	Pyrite-Copper	5.72	China: Guangdong, Daobaoshan
L180330P-7-LD180328-0010_HL3H2CCXY_L5_clean_bin.18.orig.fa_k	OEZ008529	yes	LMSG_G000009999.1	OES073673	terpene	Terpene	Pyrite-Copper	9.89	China: Guangdong, Daobaoshan
L180330P-7-LD180328-0010_HL3H2CCXY_L5_clean_bin.18.orig.fa_k	OEZ008529	yes	LMSG_G000009999.1	OES073673	NRPS-like	NRPS	Pyrite-Copper	10.87	China: Guangdong, Daobaoshan
L180330P-7-LD180328-0010_HL3H2CCXY_L5_clean_bin.19.orig.fa_k	OEZ008529	yes	LMSG_G000005825.1	OES073673	NRPS-like	NRPS	Pyrite-Copper	12.97	China: Guangdong, Daobaoshan
L180330P-7-LD180328-0010_HL3H2CCXY_L5_clean_bin.19.orig.fa_k	OEZ008529	yes	LMSG_G000005825.1	OES073673	hserlactone	Others	Pyrite-Copper	10.75	China: Guangdong, Daobaoshan
L180330P-7-LD180328-0010_HL3H2CCXY_L5_clean_bin.19.orig.fa_k	OEZ008529	yes	LMSG_G000005825.1	OES073673	terpene	Terpene	Pyrite-Copper	11.07	China: Guangdong, Daobaoshan
L180330P-7-LD180328-0010_HL3H2CCXY_L5_clean_bin.19.orig.fa_k	OEZ008529	yes	LMSG_G000005825.1	OES073673	T1PKS	PKSI	Pyrite-Copper	12.98	China: Guangdong, Daobaoshan
L180330P-7-LD180328-0010_HL3H2CCXY_L5_clean_bin.23.orig.fa_k	OEZ008529	yes	LMSG_G000008404.1	OES073673	terpene	Terpene	Pyrite-Copper	12.62	China: Guangdong, Daobaoshan
L180330P-7-LD180328-0010_HL3H2CCXY_L5_clean_bin.25.strict.fa	OEZ008529	yes	LMSG_G000004576.1	OES073673	T1PKS	PKSI	Pyrite-Copper	38.53	China: Guangdong, Daobaoshan
L180330P-7-LD180328-0010_HL3H2CCXY_L5_clean_bin.25.strict.fa	OEZ008529	yes	LMSG_G000004576.1	OES073673	betalactone	Others	Pyrite-Copper	27.46	China: Guangdong, Daobaoshan
L180330P-7-LD180328-0010_HL3H2CCXY_L5_clean_bin.25.strict.fa	OEZ008529	yes	LMSG_G000004576.1	OES073673	T1PKS	PKSI	Pyrite-Copper	47.90	China: Guangdong, Daobaoshan
L180330P-7-LD180328-0010_HL3H2CCXY_L5_clean_bin.28.orig.fa_k	OEZ008529	yes	LMSG_G000010783.1	OES073673	acyl_amino_acids	Others	Pyrite-Copper	33.87	China: Guangdong, Daobaoshan
L180330P-7-LD180328-0010_HL3H2CCXY_L5_clean_bin.28.orig.fa_k	OEZ008529	yes	LMSG_G000010783.1	OES073673	bacteriocin	RiPPs	Pyrite-Copper	9.36	China: Guangdong, Daobaoshan
L180330P-7-LD180328-0010_HL3H2CCXY_L5_clean_bin.3.strict.fa_	OEZ008529	yes	LMSG_G000008300.1	OES073673	terpene	Terpene	Pyrite-Copper	18.17	China: Guangdong, Daobaoshan

L180330P-7-LD180328-0010_HL3H2CCXY_L5_clean_bin.31.orig.fa_k	OEZ008529	yes	LMSG_G000008950.1	OES073673	indole	Others	Pyrite-Copper	13.76	China: Guangdong, Daobaoshan
L180330P-7-LD180328-0010_HL3H2CCXY_L5_clean_bin.32.orig.fa_k	OEZ008529	yes	LMSG_G000010075.1	OES073673	ladderane	Others	Pyrite-Copper	41.28	China: Guangdong, Daobaoshan
L180330P-7-LD180328-0010_HL3H2CCXY_L5_clean_bin.32.orig.fa_k	OEZ008529	yes	LMSG_G000010075.1	OES073673	arylpolyene	Others	Pyrite-Copper	23.12	China: Guangdong, Daobaoshan
L180330P-7-LD180328-0010_HL3H2CCXY_L5_clean_bin.32.orig.fa_k	OEZ008529	yes	LMSG_G000010075.1	OES073673	terpene	Terpene	Pyrite-Copper	20.39	China: Guangdong, Daobaoshan
L180330P-7-LD180328-0010_HL3H2CCXY_L5_clean_bin.32.orig.fa_k	OEZ008529	yes	LMSG_G000010075.1	OES073673	arylpolyene	Others	Pyrite-Copper	26.71	China: Guangdong, Daobaoshan
L180330P-7-LD180328-0010_HL3H2CCXY_L5_clean_bin.32.orig.fa_k	OEZ008529	yes	LMSG_G000010075.1	OES073673	terpene	Terpene	Pyrite-Copper	21.88	China: Guangdong, Daobaoshan
L180330P-7-LD180328-0010_HL3H2CCXY_L5_clean_bin.34.strict.fa	OEZ008529	yes	LMSG_G000009400.1	OES073673	terpene	Terpene	Pyrite-Copper	21.68	China: Guangdong, Daobaoshan
L180330P-7-LD180328-0010_HL3H2CCXY_L5_clean_bin.34.strict.fa	OEZ008529	yes	LMSG_G000009400.1	OES073673	NRPS-like	NRPS	Pyrite-Copper	22.18	China: Guangdong, Daobaoshan
L180330P-7-LD180328-0010_HL3H2CCXY_L5_clean_bin.34.strict.fa	OEZ008529	yes	LMSG_G000009400.1	OES073673	NRPS	NRPS	Pyrite-Copper	24.29	China: Guangdong, Daobaoshan
L180330P-7-LD180328-0010_HL3H2CCXY_L5_clean_bin.36.orig.fa_k	OEZ008529	yes	LMSG_G000010475.1	OES073673	terpene	Terpene	Pyrite-Copper	23.01	China: Guangdong, Daobaoshan
L180330P-7-LD180328-0010_HL3H2CCXY_L5_clean_bin.37.orig.fa_k	OEZ008529	yes	LMSG_G000004577.1	OES073673	T1PKS	PKSI	Pyrite-Copper	23.39	China: Guangdong, Daobaoshan
L180330P-7-LD180328-0010_HL3H2CCXY_L5_clean_bin.37.orig.fa_k	OEZ008529	yes	LMSG_G000004577.1	OES073673	terpene	Terpene	Pyrite-Copper	22.27	China: Guangdong, Daobaoshan
L180330P-7-LD180328-0010_HL3H2CCXY_L5_clean_bin.37.orig.fa_k	OEZ008529	yes	LMSG_G000004577.1	OES073673	terpene	Terpene	Pyrite-Copper	7.82	China: Guangdong, Daobaoshan
L180330P-7-LD180328-0010_HL3H2CCXY_L5_clean_bin.37.orig.fa_k	OEZ008529	yes	LMSG_G000004577.1	OES073673	LAP.phosphonate	Others	Pyrite-Copper	23.62	China: Guangdong, Daobaoshan
L180330P-7-LD180328-0010_HL3H2CCXY_L5_clean_bin.37.orig.fa_k	OEZ008529	yes	LMSG_G000004577.1	OES073673	NRPS-like	NRPS	Pyrite-Copper	12.51	China: Guangdong, Daobaoshan
L180330P-7-LD180328-0010_HL3H2CCXY_L5_clean_bin.37.orig.fa_k	OEZ008529	yes	LMSG_G000004577.1	OES073673	NRPS	NRPS	Pyrite-Copper	14.74	China: Guangdong, Daobaoshan
L180330P-7-LD180328-0010_HL3H2CCXY_L5_clean_bin.37.orig.fa_k	OEZ008529	yes	LMSG_G000004577.1	OES073673	terpene	Terpene	Pyrite-Copper	10.41	China: Guangdong, Daobaoshan
L180330P-7-LD180328-0010_HL3H2CCXY_L5_clean_bin.37.orig.fa_k	OEZ008529	yes	LMSG_G000004577.1	OES073673	T1PKS	PKSI	Pyrite-Copper	11.88	China: Guangdong, Daobaoshan
L180330P-7-LD180328-0010_HL3H2CCXY_L5_clean_bin.38.orig.fa_k	OEZ008529	yes	LMSG_G000008034.1	OES073673	LAP.thiopeptide	RiPPs	Pyrite-Copper	10.21	China: Guangdong, Daobaoshan
L180330P-7-LD180328-0010_HL3H2CCXY_L5_clean_bin.40.strict.fa	OEZ008529	yes	LMSG_G000006507.1	OES073673	thiopeptide	RiPPs	Pyrite-Copper	24.53	China: Guangdong, Daobaoshan
L180330P-7-LD180328-0010_HL3H2CCXY_L5_clean_bin.41.orig.fa_k	OEZ008529	yes	LMSG_G000010825.1	OES073673	acyl_amino_acids	Others	Pyrite-Copper	45.71	China: Guangdong, Daobaoshan
L180330P-7-LD180328-0010_HL3H2CCXY_L5_clean_bin.44.orig.fa_k	OEZ008529	no	LMSG_G000010604.1	OES073673	terpene	Terpene	Pyrite-Copper	5.36	China: Guangdong, Daobaoshan
L180330P-7-LD180328-0010_HL3H2CCXY_L5_clean_bin.45.orig.fa_k	OEZ008529	yes	LMSG_G000008166.1	OES073673	terpene	Terpene	Pyrite-Copper	6.92	China: Guangdong, Daobaoshan
L180330P-7-LD180328-0010_HL3H2CCXY_L5_clean_bin.46.orig.fa_k	OEZ008529	yes	LMSG_G000005828.1	OES073673	arylpolyene.hserlactone	Others	Pyrite-Copper	28.82	China: Guangdong, Daobaoshan
L180330P-7-LD180328-0010_HL3H2CCXY_L5_clean_bin.46.orig.fa_k	OEZ008529	yes	LMSG_G000005828.1	OES073673	terpene	Terpene	Pyrite-Copper	23.04	China: Guangdong, Daobaoshan
L180330P-7-LD180328-0010_HL3H2CCXY_L5_clean_bin.46.orig.fa_k	OEZ008529	yes	LMSG_G000005828.1	OES073673	resorcinol.arylpolyene	Others	Pyrite-Copper	26.30	China: Guangdong, Daobaoshan
L180330P-7-LD180328-0010_HL3H2CCXY_L5_clean_bin.48.orig.fa_k	OEZ008529	yes	LMSG_G000008453.1	OES073673	terpene	Terpene	Pyrite-Copper	5.37	China: Guangdong, Daobaoshan
L180330P-7-LD180328-0010_HL3H2CCXY_L5_clean_bin.49.orig.fa_k	OEZ008529	yes	LMSG_G000010418.1	OES073673	hserlactone	Others	Pyrite-Copper	14.59	China: Guangdong, Daobaoshan
L180330P-7-LD180328-0010_HL3H2CCXY_L5_clean_bin.49.orig.fa_k	OEZ008529	yes	LMSG_G000010418.1	OES073673	terpene	Terpene	Pyrite-Copper	25.02	China: Guangdong, Daobaoshan
L180330P-7-LD180328-0010_HL3H2CCXY_L5_clean_bin.49.orig.fa_k	OEZ008529	yes	LMSG_G000010418.1	OES073673	bacteriocin	RiPPs	Pyrite-Copper	10.86	China: Guangdong, Daobaoshan
L180330P-7-LD180328-0010_HL3H2CCXY_L5_clean_bin.49.orig.fa_k	OEZ008529	yes	LMSG_G000010418.1	OES073673	terpene	Terpene	Pyrite-Copper	14.40	China: Guangdong, Daobaoshan
L180330P-7-LD180328-0010_HL3H2CCXY_L5_clean_bin.52.strict.fa	OEZ008529	yes	LMSG_G000005829.1	OES073673	NRPS-like	NRPS	Pyrite-Copper	12.84	China: Guangdong, Daobaoshan
L180330P-7-LD180328-0010_HL3H2CCXY_L5_clean_bin.52.strict.fa	OEZ008529	yes	LMSG_G000005829.1	OES073673	terpene	Terpene	Pyrite-Copper	7.86	China: Guangdong, Daobaoshan
L180330P-7-LD180328-0010_HL3H2CCXY_L5_clean_bin.53.orig.fa_k	OEZ008529	yes	LMSG_G000007956.1	OES073673	lassopeptide	RiPPs	Pyrite-Copper	19.55	China: Guangdong, Daobaoshan
L180330P-7-LD180328-0010_HL3H2CCXY_L5_clean_bin.54.orig.fa_k	OEZ008529	yes	LMSG_G000007158.1	OES073673	acyl_amino_acids	Others	Pyrite-Copper	5.79	China: Guangdong, Daobaoshan
L180330P-7-LD180328-0010_HL3H2CCXY_L5_clean_bin.56.orig.fa_k	OEZ008529	yes	LMSG_G000009086.1	OES073673	terpene	Terpene	Pyrite-Copper	6.53	China: Guangdong, Daobaoshan
L180330P-7-LD180328-0010_HL3H2CCXY_L5_clean_bin.56.orig.fa_k	OEZ008529	yes	LMSG_G000009086.1	OES073673	betalactone	Others	Pyrite-Copper	11.49	China: Guangdong, Daobaoshan
L180330P-7-LD180328-0010_HL3H2CCXY_L5_clean_bin.56.orig.fa_k	OEZ008529	yes	LMSG_G000009086.1	OES073673	betalactone	Others	Pyrite-Copper	13.94	China: Guangdong, Daobaoshan
L180330P-7-LD180328-0010_HL3H2CCXY_L5_clean_bin.56.orig.fa_k	OEZ008529	yes	LMSG_G000009086.1	OES073673	thiopeptide	RiPPs	Pyrite-Copper	12.86	China: Guangdong, Daobaoshan
L180330P-7-LD180328-0010_HL3H2CCXY_L5_clean_bin.57.orig.fa_k	OEZ008529	yes	LMSG_G000009352.1	OES073673	betalactone	Others	Pyrite-Copper	19.67	China: Guangdong, Daobaoshan
L180330P-7-LD180328-0010_HL3H2CCXY_L5_clean_bin.6.orig.fa_k1	OEZ008529	yes	LMSG_G000007872.1	OES073673	terpene	Terpene	Pyrite-Copper	23.47	China: Guangdong, Daobaoshan
L180330P-7-LD180328-0010_HL3H2CCXY_L5_clean_bin.6.orig.fa_k1	OEZ008529	yes	LMSG_G000007872.1	OES073673	terpene	Terpene	Pyrite-Copper	10.69	China: Guangdong, Daobaoshan
L180330P-7-LD180328-0010_HL3H2CCXY_L5_clean_bin.60.orig.fa_k	OEZ008529	no	LMSG_G000009562.1	OES073673	terpene	Terpene	Pyrite-Copper	14.89	China: Guangdong, Daobaoshan
L180330P-7-LD180328-0010_HL3H2CCXY_L5_clean_bin.60.orig.fa_k	OEZ008529	no	LMSG_G000009562.1	OES073673	ectoine	Others	Pyrite-Copper	9.30	China: Guangdong, Daobaoshan
L180330P-7-LD180328-0010_HL3H2CCXY_L5_clean_bin.60.orig.fa_k	OEZ008529	no	LMSG_G000009562.1	OES073673	terpene	Terpene	Pyrite-Copper	13.20	China: Guangdong, Daobaoshan
L180330P-7-LD180328-0010_HL3H2CCXY_L5_clean_bin.61.strict.fa	OEZ008529	no	LMSG_G000010980.1	OES073673	T1PKS.hglE-KS	PKSother	Pyrite-Copper	8.41	China: Guangdong, Daobaoshan
L180330P-7-LD180328-0010_HL3H2CCXY_L5_clean_bin.62.orig.fa_k	OEZ008529	yes	LMSG_G000005832.1	OES073673	T3PKS	PKSother	Pyrite-Copper	5.15	China: Guangdong, Daobaoshan
L180330P-7-LD180328-0010_HL3H2CCXY_L5_clean_bin.63.orig.fa_k	OEZ008529	yes	LMSG_G000010928.1	OES073673	terpene	Terpene	Pyrite-Copper	21.61	China: Guangdong, Daobaoshan
L180330P-7-LD180328-0010_HL3H2CCXY_L5_clean_bin.63.orig.fa_k	OEZ008529	yes	LMSG_G000010928.1	OES073673	arylpolyene	Others	Pyrite-Copper	41.19	China: Guangdong, Daobaoshan
L180330P-7-LD180328-0010_HL3H2CCXY_L5_clean_bin.63.orig.fa_k	OEZ008529	yes	LMSG_G000010928.1	OES073673	thiopeptide	RiPPs	Pyrite-Copper	27.50	China: Guangdong, Daobaoshan
L180330P-7-LD180328-0010_HL3H2CCXY_L5_clean_bin.65.orig.fa_k	OEZ008529	yes	LMSG_G000008090.1	OES073673	T1PKS.hglE-KS	PKSother	Pyrite-Copper	33.09	China: Guangdong, Daobaoshan
L180330P-7-LD180328-0010_HL3H2CCXY_L5_clean_bin.66.orig.fa_k	OEZ008529	yes	LMSG_G000010286.1	OES073673	arylpolyene	Others	Pyrite-Copper	40.92	China: Guangdong, Daobaoshan
L180330P-7-LD180328-0010_HL3H2CCXY_L5_clean_bin.66.orig.fa_k	OEZ008529	no	LMSG_G000010286.1	OES073673	terpene	Terpene	Pyrite-Copper	21.20	China: Guangdong, Daobaoshan
L180330P-7-LD180328-0010_HL3H2CCXY_L5_clean_bin.67.orig.fa_k	OEZ008529	yes	LMSG_G000009619.1	OES073673	terpene	Terpene	Pyrite-Copper	21.89	China: Guangdong, Daobaoshan
L180330P-7-LD180328-0010_HL3H2CCXY_L5_clean_bin.67.orig.fa_k	OEZ008529	yes	LMSG_G000009619.1	OES073673	terpene	Terpene	Pyrite-Copper	11.08	China: Guangdong, Daobaoshan
L180330P-7-LD180328-0010_HL3H2CCXY_L5_clean_bin.67.orig.fa_k	OEZ008529	yes	LMSG_G000009619.1	OES073673	NRPS-like	NRPS	Pyrite-Copper	17.33	China: Guangdong, Daobaoshan
L180330P-7-LD180328-0010_HL3H2CCXY_L5_clean_bin.68.orig.fa_k	OEZ008529	yes	LMSG_G000005833.1	OES073673	acyl_amino_acids	Others	Pyrite-Copper	13.39	China: Guangdong, Daobaoshan
L180330P-7-LD180328-0010_HL3H2CCXY_L5_clean_bin.68.orig.fa_k	OEZ008529	yes	LMSG_G000005833.1	OES073673	NRPS-like	NRPS	Pyrite-Copper	15.42	China: Guangdong, Daobaoshan
L180330P-7-LD180328-0010_HL3H2CCXY_L5_clean_bin.68.orig.fa_k	OEZ008529	yes	LMSG_G000005833.1	OES073673	terpene	Terpene	Pyrite-Copper	10.33	China: Guangdong, Daobaoshan
L180330P-7-LD180328-0010_HL3H2CCXY_L5_clean_bin.68.orig.fa_k	OEZ008529	yes	LMSG_G000005833.1	OES073673	terpene	Terpene	Pyrite-Copper	15.42	China: Guangdong, Daobaoshan
L180330P-7-LD180328-0010_HL3H2CCXY_L5_clean_bin.69.orig.fa_k	OEZ008529	yes	LMSG_G000008178.1	OES073673	oligosaccharide	Saccharides	Pyrite-Copper	45.54	China: Guangdong, Daobaoshan
L180330P-7-LD180328-0010_HL3H2CCXY_L5_clean_bin.69.orig.fa_k	OEZ008529	yes	LMSG_G000008178.1	OES073673	betalactone	Others	Pyrite-Copper	14.75	China: Guangdong, Daobaoshan
L180330P-7-LD180328-0010_HL3H2CCXY_L5_clean_bin.69.orig.fa_k	OEZ008529	yes	LMSG_G000008178.1	OES073673	terpene	Terpene	Pyrite-Copper	21.08	China: Guangdong, Daobaoshan
L180330P-7-LD180328-0010_HL3H2CCXY_L5_clean_bin.70.orig.fa_k	OEZ008529	yes	LMSG_G000011181.1	OES073673	arylpolyene	Others	Pyrite-Copper	12.13	China: Guangdong, Daobaoshan
L180330P-7-LD180328-0010_HL3H2CCXY_L5_clean_bin.70.orig.fa_k	OEZ008529	yes	LMSG_G000011181.1	OES073673	terpene	Terpene	Pyrite-Copper	10.67	China: Guangdong, Daobaoshan
L180330P-7-LD180328-0010_HL3H2CCXY_L5_clean_bin.72.orig.fa_k	OEZ008529	yes	LMSG_G000009661.1	OES073673	terpene	Terpene	Pyrite-Copper	22.00	China: Guangdong, Daobaoshan
L180330P-7-LD180328-0010_HL3H2CCXY_L5_clean_bin.72.orig.fa_k	OEZ008529	yes	LMSG_G000009661.1	OES073673	NRPS-like	NRPS	Pyrite-Copper	10.20	China: Guangdong, Daobaoshan
L180330P-7-LD180328-0010_HL3H2CCXY_L5_clean_bin.74.orig.fa_k	OEZ008529	yes	LMSG_G000010708.1	OES073673	terpene	Terpene	Pyrite-Copper	12.76	China: Guangdong, Daobaoshan
L180330P-7-LD180328-0010_HL3H2CCXY_L5_clean_bin.74.orig.fa_k	OEZ008529	yes	LMSG_G000010708.1	OES073673	bacteriocin	RiPPs	Pyrite-Copper	10.28	China: Guangdong, Daobaoshan
L180330P-7-LD180328-0010_HL3H2CCXY_L5_clean_bin.74.orig.fa_k	OEZ008529	yes	LMSG_G000010708.1	OES073673	T1PKS.NRPS-like	PKS-NRP_Hybrids	Pyrite-Copper	11.71	China: Guangdong, Daobaoshan
L180330P-7-LD180328-0010_HL3H2CCXY_L5_clean_bin.74.orig.fa_k	OEZ008529	yes	LMSG_G000010708.1	OES073673	arylpolyene	Others	Pyrite-Copper	33.50	China: Guangdong, Daobaoshan
L180330P-7-LD180328-0010_HL3H2CCXY_L5_clean_bin.74.orig.fa_k	OEZ008529	yes	LMSG_G000010708.1	OES073673	betalactone	Others	Pyrite-Copper	16.01	China: Guangdong, Daobaoshan
L180330P-7-LD180328-0010_HL3H2CCXY_L5_clean_bin.75.orig.fa_k	OEZ008529	yes	LMSG_G000005835.1	OES073673	terpene	Terpene	Pyrite-Copper	6.75	China: Guangdong, Daobaoshan
L180330P-7-LD180328-0010_HL3H2CCXY_L5_clean_bin.75.orig.fa_k	OEZ008529	yes	LMSG_G000005835.1	OES073673	terpene	Terpene	Pyrite-Copper	17.49	China: Guangdong, Daobaoshan
L180330P-7-LD180328-0010_HL3H2CCXY_L5_clean_bin.75.orig.fa_k	OEZ008529	yes	LMSG_G000005835.1	OES073673	terpene	Terpene	Pyrite-Copper	10.95	China: Guangdong, Daobaoshan
L180330P-7-LD180328-0010_HL3H2CCXY_L5_clean_bin.75.orig.fa_k	OEZ008529	yes	LMSG_G000005835.1	OES073673	NRPS-like	NRPS	Pyrite-Copper	12.23	China: Guangdong, Daobaoshan
L180330P-7-LD180328-0010_HL3H2CCXY_L5_clean_bin.76.orig.fa_k	OEZ008529	yes	LMSG_G000006249.1	OES073673	arylpolyene	Others	Pyrite-Copper	21.42	China: Guangdong, Daobaoshan
L180330P-7-LD180328-0010_HL3H2CCXY_L5_clean_bin.76.orig.fa_k	OEZ008529	yes	LMSG_G000006249.1	OES073673	terpene	Terpene	Pyrite-Copper	23.01	China: Guangdong, Daobaoshan
L180330P-7-LD180328-0010_HL3H2CCXY_L5_clean_bin.76.orig.fa_k	OEZ008529	yes	LMSG_G000006249.1	OES073673	bacteriocin	RiPPs	Pyrite-Copper	10.87	China: Guangdong, Daobaoshan
L180330P-7-LD180328-0010_HL3H2CCXY_L5_clean_bin.76.orig.fa_k	OEZ008529	yes	LMSG_G000006249.1	OES073673	NRPS-like	NRPS	Pyrite-Copper	33.46	China: Guangdong, Daobaoshan
L180330P-7-LD180328-0010_HL3H2CCXY_L5_clean_bin.76.orig.fa_k	OEZ008529	yes	LMSG_G000006249.1	OES073673	acyl_amino_acids	Others	Pyrite-Copper	60.76	China: Guangdong, Daobaoshan
L180330P-7-LD180328-0010_HL3H2CCXY_L5_clean_bin.79.orig.fa_k	OEZ008529	yes	LMSG_G000010682.1	OES073673	NRPS-like	NRPS	Pyrite-Copper	32.35	China: Guangdong, Daobaoshan
L180330P-7-LD180328-0010_HL3H2CCXY_L5_clean_bin.79.orig.fa_k	OEZ008529	yes	LMSG_G000010682.1	OES073673	arylpolyene	Others	Pyrite-Copper	9.38	China: Guangdong, Daobaoshan
L180330P-7-LD180328-0010_HL3H2CCXY_L5_clean_bin.79.orig.fa_k	OEZ008529	yes	LMSG_G000010682.1	OES073673	betalactone	Others	Pyrite-Copper	22.29	China: Guangdong, Daobaoshan
L180330P-7-LD180328-0010_HL3H2CCXY_L5_clean_bin.8.orig.fa_k1	OEZ008529	yes	LMSG_G000009512.1	OES073673	betalactone	Others	Pyrite-Copper	8.45	China: Guangdong, Daobaoshan
L180330P-7-LD180328-0010_HL3H2CCXY_L5_clean_bin.8.orig.fa_k1	OEZ008529	yes	LMSG_G000009512.1	OES073673	TfuA-related	RiPPs	Pyrite-Copper	7.23	China: Guangdong, Daobaoshan
L180330P-7-LD180328-0010_HL3H2CCXY_L5_clean_bin.80.orig.fa_k	OEZ008529	yes	LMSG_G000007806.1	OES073673	terpene	Terpene	Pyrite-Copper	29.46	China: Guangdong, Daobaoshan
L180330P-7-LD180328-0010_HL3H2CCXY_L5_clean_bin.80.orig.fa_k	OEZ008529	yes	LMSG_G000007806.1	OES073673	T3PKS	PKSother	Pyrite-Copper	41.09	China: Guangdong, Daobaoshan
L180330P-7-LD180328-0010_HL3H2CCXY_L5_clean_bin.81.strict.fa	OEZ008529	yes	LMSG_G000005836.1	OES073673	terpene	Terpene	Pyrite-Copper	17.23	China: Guangdong, Daobaoshan
L180330P-7-LD180328-0010_HL3H2CCXY_L5_clean_bin.83.orig.fa_k	OEZ008529	yes	LMSG_G000008639.1	OES073673	NRPS-like	NRPS	Pyrite-Copper	12.16	China: Guangdong, Daobaoshan
L180330P-7-LD180328-0010_HL3H2CCXY_L5_clean_bin.83.orig.fa_k	OEZ008529	yes	LMSG_G000008639.1	OES073673	other	Others	Pyrite-Copper	5.43	China: Guangdong, Daobaoshan
L180330P-7-LD180328-0010_HL3H2CCXY_L5_clean_bin.87.orig.fa_k	OEZ008529	yes	LMSG_G000008169.1	OES073673	terpene	Terpene	Pyrite-Copper	6.76	China: Guangdong, Daobaoshan
L180330P-7-LD180328-0010_HL3H2CCXY_L5_clean_bin.9.strict.fa_	OEZ008529	yes	LMSG_G000006441.1	OES073673	terpene	Terpene	Pyrite-Copper	5.39	China: Guangdong, Daobaoshan
L180330P-7-LD180328-0010_HL3H2CCXY_L5_clean_bin.90.orig.fa_k	OEZ008529	yes	LMSG_G000009012.1	OES073673	bacteriocin	RiPPs	Pyrite-Copper	10.62	China: Guangdong, Daobaoshan
L180330P-7-LD180328-0010_HL3H2CCXY_L5_clean_bin.90.orig.fa_k	OEZ008529	yes	LMSG_G000009012.1	OES073673	terpene	Terpene	Pyrite-Copper	20.54	China: Guangdong, Daobaoshan
L180330P-7-LD180328-0010_HL3H2CCXY_L5_clean_bin.92.orig.fa_k	OEZ008529	yes	LMSG_G000009525.1	OES073673	acyl_amino_acids	Others	Pyrite-Copper	6.90	China: Guangdong, Daobaoshan
L180330P-7-LD180328-0010_HL3H2CCXY_L5_clean_bin.92.orig.fa_k	OEZ008529	yes	LMSG_G000009525.1	OES073673	NRPS-like	NRPS	Pyrite-Copper	24.40	China: Guangdong, Daobaoshan
L180330P-7-LD180328-0010_HL3H2CCXY_L5_clean_bin.92.orig.fa_k	OEZ008529	no	LMSG_G000009525.1	OES073673	terpene	Terpene	Pyrite-Copper	13.27	China: Guangdong, Daobaoshan
L180330P-7-LD180328-0010_HL3H2CCXY_L5_clean_bin.92.orig.fa_k	OEZ008529	yes	LMSG_G000009525.1	OES073673	terpene	Terpene	Pyrite-Copper	10.34	China: Guangdong, Daobaoshan
L180330P-7-LD180328-0010_HL3H2CCXY_L5_clean_bin.92.orig.fa_k	OEZ008529	yes	LMSG_G000009525.1	OES073673	ectoine	Others	Pyrite-Copper	10.40	China: Guangdong, Daobaoshan
L180330P-7-LD180328-0010_HL3H2CCXY_L5_clean_bin.92.orig.fa_k	OEZ008529	no	LMSG_G000009525.1	OES073673	terpene	Terpene	Pyrite-Copper	7.76	China: Guangdong, Daobaoshan
L180330P-7-LD180328-0010_HL3H2CCXY_L5_clean_bin.92.orig.fa_k	OEZ008529	yes	LMSG_G000009525.1	OES073673	acyl_amino_acids	Others	Pyrite-Copper	7.11	China: Guangdong, Daobaoshan
L180330P-7-LD180328-0010_HL3H2CCXY_L5_clean_bin.97.orig.fa_k	OEZ008529	yes	LMSG_G000005839.1	OES073673	T1PKS	PKSI	Pyrite-Copper	7.26	China: Guangdong, Daobaoshan
L180330P-7-LD180328-0010_HL3H2CCXY_L5_clean_bin.97.orig.fa_k	OEZ008529	yes	LMSG_G000005839.1	OES073673	betalactone	Others	Pyrite-Copper	10.09	China: Guangdong, Daobaoshan
L180330P-7-LD180328-0010_HL3H2CCXY_L5_clean_bin.97.orig.fa_k	OEZ008529	yes	LMSG_G000005839.1	OES073673	hserlactone	Others	Pyrite-Copper	5.40	China: Guangdong, Daobaoshan
L180330P-7-LD180328-0010_HL3H2CCXY_L5_clean_bin.99.orig.fa_k	OEZ008529	no	LMSG_G000010175.1	OES073673	ectoine	Others	Pyrite-Copper	5.51	China: Guangdong, Daobaoshan
L180330P-7-LD180328-0010_HL3H2CCXY_L5_clean_bin.99.orig.fa_k	OEZ008529	no	LMSG_G000010175.1	OES073673	acyl_amino_acids	Others	Pyrite-Copper	31.91	China: Guangdong, Daobaoshan
L180330P-7-LD180328-0010_HL3H2CCXY_L5_clean_bin.99.orig.fa_k	OEZ008529	no	LMSG_G000010175.1	OES073673	terpene	Terpene	Pyrite-Copper	21.71	China: Guangdong, Daobaoshan
L180330P-7-LD180328-0010_HL3H2CCXY_L5_clean_bin.99.orig.fa_k	OEZ008529	no	LMSG_G000010175.1	OES073673	terpene	Terpene	Pyrite-Copper	12.79	China: Guangdong, Daobaoshan
L180330P-7-LD180328-0011_HL3H2CCXY_L5_clean_bin.100.strict.f	OEZ008529	yes	LMSG_G000010913.1	OES073674	T1PKS	PKSI	Pyrite-Copper	18.30	China: Guangdong, Daobaoshan
L180330P-7-LD180328-0011_HL3H2CCXY_L5_clean_bin.100.strict.f	OEZ008529	yes	LMSG_G000010913.1	OES073674	bacteriocin	RiPPs	Pyrite-Copper	6.67	China: Guangdong, Daobaoshan
L180330P-7-LD180328-0011_HL3H2CCXY_L5_clean_bin.105.strict.f	OEZ008529	yes	LMSG_G000005841.1	OES073674	thiopeptide	RiPPs	Pyrite-Copper	13.67	China: Guangdong, Daobaoshan
L180330P-7-LD180328-0011_HL3H2CCXY_L5_clean_bin.106.permissi	OEZ008529	yes	LMSG_G000010789.1	OES073674	acyl_amino_acids	Others	Pyrite-Copper	39.76	China: Guangdong, Daobaoshan
L180330P-7-LD180328-0011_HL3H2CCXY_L5_clean_bin.106.permissi	OEZ008529	yes	LMSG_G000010789.1	OES073674	bacteriocin	RiPPs	Pyrite-Copper	11.75	China: Guangdong, Daobaoshan
L180330P-7-LD180328-0011_HL3H2CCXY_L5_clean_bin.117.strict.f	OEZ008529	yes	LMSG_G000005844.1	OES073674	terpene	Terpene	Pyrite-Copper	11.01	China: Guangdong, Daobaoshan
L180330P-7-LD180328-0011_HL3H2CCXY_L5_clean_bin.118.strict.f	OEZ008529	yes	LMSG_G000010502.1	OES073674	terpene	Terpene	Pyrite-Copper	23.04	China: Guangdong, Daobaoshan
L180330P-7-LD180328-0011_HL3H2CCXY_L5_clean_bin.118.strict.f	OEZ008529	yes	LMSG_G000010502.1	OES073674	hserlactone	Others	Pyrite-Copper	12.57	China: Guangdong, Daobaoshan
L180330P-7-LD180328-0011_HL3H2CCXY_L5_clean_bin.125.permissi	OEZ008529	yes	LMSG_G000008749.1	OES073674	terpene	Terpene	Pyrite-Copper	6.95	China: Guangdong, Daobaoshan
L180330P-7-LD180328-0011_HL3H2CCXY_L5_clean_bin.125.permissi	OEZ008529	yes	LMSG_G000008749.1	OES073674	terpene	Terpene	Pyrite-Copper	6.59	China: Guangdong, Daobaoshan
L180330P-7-LD180328-0011_HL3H2CCXY_L5_clean_bin.127.strict.f	OEZ008529	yes	LMSG_G000010364.1	OES073674	terpene	Terpene	Pyrite-Copper	16.85	China: Guangdong, Daobaoshan
L180330P-7-LD180328-0011_HL3H2CCXY_L5_clean_bin.127.strict.f	OEZ008529	yes	LMSG_G000010364.1	OES073674	NRPS-like	NRPS	Pyrite-Copper	12.56	China: Guangdong, Daobaoshan
L180330P-7-LD180328-0011_HL3H2CCXY_L5_clean_bin.127.strict.f	OEZ008529	yes	LMSG_G000010364.1	OES073674	terpene	Terpene	Pyrite-Copper	6.85	China: Guangdong, Daobaoshan
L180330P-7-LD180328-0011_HL3H2CCXY_L5_clean_bin.127.strict.f	OEZ008529	yes	LMSG_G000010364.1	OES073674	bacteriocin	RiPPs	Pyrite-Copper	6.15	China: Guangdong, Daobaoshan
L180330P-7-LD180328-0011_HL3H2CCXY_L5_clean_bin.127.strict.f	OEZ008529	yes	LMSG_G000010364.1	OES073674	terpene	Terpene	Pyrite-Copper	5.97	China: Guangdong, Daobaoshan
L180330P-7-LD180328-0011_HL3H2CCXY_L5_clean_bin.129.strict.f	OEZ008529	yes	LMSG_G000009604.1	OES073674	terpene	Terpene	Pyrite-Copper	21.99	China: Guangdong, Daobaoshan
L180330P-7-LD180328-0011_HL3H2CCXY_L5_clean_bin.129.strict.f	OEZ008529	yes	LMSG_G000009604.1	OES073674	NRPS-like	NRPS	Pyrite-Copper	31.11	China: Guangdong, Daobaoshan
L180330P-7-LD180328-0011_HL3H2CCXY_L5_clean_bin.129.strict.f	OEZ008529	yes	LMSG_G000009604.1	OES073674	terpene	Terpene	Pyrite-Copper	20.95	China: Guangdong, Daobaoshan
L180330P-7-LD180328-0011_HL3H2CCXY_L5_clean_bin.137.strict.f	OEZ008529	yes	LMSG_G000009205.1	OES073674	terpene	Terpene	Pyrite-Copper	38.07	China: Guangdong, Daobaoshan
L180330P-7-LD180328-0011_HL3H2CCXY_L5_clean_bin.137.strict.f	OEZ008529	yes	LMSG_G000009205.1	OES073674	T1PKS	PKSI	Pyrite-Copper	48.10	China: Guangdong, Daobaoshan
L180330P-7-LD180328-0011_HL3H2CCXY_L5_clean_bin.138.permissi	OEZ008529	yes	LMSG_G000005846.1	OES073674	NRPS-like	NRPS	Pyrite-Copper	38.75	China: Guangdong, Daobaoshan
L180330P-7-LD180328-0011_HL3H2CCXY_L5_clean_bin.138.permissi	OEZ008529	yes	LMSG_G000005846.1	OES073674	bacteriocin	RiPPs	Pyrite-Copper	11.81	China: Guangdong, Daobaoshan
L180330P-7-LD180328-0011_HL3H2CCXY_L5_clean_bin.14.strict.fa	OEZ008529	yes	LMSG_G000005848.1	OES073674	ladderane	Others	Pyrite-Copper	32.56	China: Guangdong, Daobaoshan
L180330P-7-LD180328-0011_HL3H2CCXY_L5_clean_bin.144.strict.f	OEZ008529	yes	LMSG_G000009679.1	OES073674	NRPS-like	NRPS	Pyrite-Copper	18.92	China: Guangdong, Daobaoshan
L180330P-7-LD180328-0011_HL3H2CCXY_L5_clean_bin.144.strict.f	OEZ008529	yes	LMSG_G000009679.1	OES073674	NRPS-like	NRPS	Pyrite-Copper	16.12	China: Guangdong, Daobaoshan
L180330P-7-LD180328-0011_HL3H2CCXY_L5_clean_bin.144.strict.f	OEZ008529	yes	LMSG_G000009679.1	OES073674	terpene	Terpene	Pyrite-Copper	5.76	China: Guangdong, Daobaoshan
L180330P-7-LD180328-0011_HL3H2CCXY_L5_clean_bin.155.strict.f	OEZ008529	yes	LMSG_G000005851.1	OES073674	LAP	RiPPs	Pyrite-Copper	22.05	China: Guangdong, Daobaoshan
L180330P-7-LD180328-0011_HL3H2CCXY_L5_clean_bin.156.permissi	OEZ008529	yes	LMSG_G000007946.1	OES073674	T1PKS.hglE-KS	PKSother	Pyrite-Copper	51.64	China: Guangdong, Daobaoshan
L180330P-7-LD180328-0011_HL3H2CCXY_L5_clean_bin.156.permissi	OEZ008529	yes	LMSG_G000007946.1	OES073674	ladderane	Others	Pyrite-Copper	29.34	China: Guangdong, Daobaoshan
L180330P-7-LD180328-0011_HL3H2CCXY_L5_clean_bin.156.permissi	OEZ008529	yes	LMSG_G000007946.1	OES073674	bacteriocin	RiPPs	Pyrite-Copper	10.85	China: Guangdong, Daobaoshan
L180330P-7-LD180328-0011_HL3H2CCXY_L5_clean_bin.156.permissi	OEZ008529	yes	LMSG_G000007946.1	OES073674	T3PKS	PKSother	Pyrite-Copper	22.64	China: Guangdong, Daobaoshan
L180330P-7-LD180328-0011_HL3H2CCXY_L5_clean_bin.156.permissi	OEZ008529	yes	LMSG_G000007946.1	OES073674	terpene	Terpene	Pyrite-Copper	13.14	China: Guangdong, Daobaoshan
L180330P-7-LD180328-0011_HL3H2CCXY_L5_clean_bin.16.strict.fa	OEZ008529	yes	LMSG_G000006250.1	OES073674	NRPS-like	NRPS	Pyrite-Copper	19.39	China: Guangdong, Daobaoshan
L180330P-7-LD180328-0011_HL3H2CCXY_L5_clean_bin.16.strict.fa	OEZ008529	yes	LMSG_G000006250.1	OES073674	terpene	Terpene	Pyrite-Copper	6.69	China: Guangdong, Daobaoshan
L180330P-7-LD180328-0011_HL3H2CCXY_L5_clean_bin.161.strict.f	OEZ008529	yes	LMSG_G000008737.1	OES073674	terpene	Terpene	Pyrite-Copper	11.37	China: Guangdong, Daobaoshan
L180330P-7-LD180328-0011_HL3H2CCXY_L5_clean_bin.21.strict.fa	OEZ008529	yes	LMSG_G000005854.1	OES073674	ladderane	Others	Pyrite-Copper	30.05	China: Guangdong, Daobaoshan
L180330P-7-LD180328-0011_HL3H2CCXY_L5_clean_bin.21.strict.fa	OEZ008529	yes	LMSG_G000005854.1	OES073674	terpene	Terpene	Pyrite-Copper	11.07	China: Guangdong, Daobaoshan
L180330P-7-LD180328-0011_HL3H2CCXY_L5_clean_bin.24.strict.fa	OEZ008529	yes	LMSG_G000010521.1	OES073674	arylpolyene	Others	Pyrite-Copper	18.81	China: Guangdong, Daobaoshan
L180330P-7-LD180328-0011_HL3H2CCXY_L5_clean_bin.24.strict.fa	OEZ008529	yes	LMSG_G000010521.1	OES073674	acyl_amino_acids	Others	Pyrite-Copper	10.79	China: Guangdong, Daobaoshan
L180330P-7-LD180328-0011_HL3H2CCXY_L5_clean_bin.35.strict.fa	OEZ008529	no	LMSG_G000010632.1	OES073674	arylpolyene	Others	Pyrite-Copper	6.93	China: Guangdong, Daobaoshan
L180330P-7-LD180328-0011_HL3H2CCXY_L5_clean_bin.36.strict.fa	OEZ008529	yes	LMSG_G000005858.1	OES073674	arylpolyene	Others	Pyrite-Copper	41.23	China: Guangdong, Daobaoshan
L180330P-7-LD180328-0011_HL3H2CCXY_L5_clean_bin.36.strict.fa	OEZ008529	yes	LMSG_G000005858.1	OES073674	arylpolyene	Others	Pyrite-Copper	27.73	China: Guangdong, Daobaoshan
L180330P-7-LD180328-0011_HL3H2CCXY_L5_clean_bin.39.strict.fa	OEZ008529	yes	LMSG_G000005859.1	OES073674	NRPS-like	NRPS	Pyrite-Copper	35.65	China: Guangdong, Daobaoshan
L180330P-7-LD180328-0011_HL3H2CCXY_L5_clean_bin.39.strict.fa	OEZ008529	yes	LMSG_G000005859.1	OES073674	terpene	Terpene	Pyrite-Copper	11.58	China: Guangdong, Daobaoshan
L180330P-7-LD180328-0011_HL3H2CCXY_L5_clean_bin.4.strict.fa_	OEZ008529	yes	LMSG_G000005862.1	OES073674	terpene	Terpene	Pyrite-Copper	6.95	China: Guangdong, Daobaoshan
L180330P-7-LD180328-0011_HL3H2CCXY_L5_clean_bin.41.strict.fa	OEZ008529	yes	LMSG_G000008755.1	OES073674	terpene	Terpene	Pyrite-Copper	22.03	China: Guangdong, Daobaoshan
L180330P-7-LD180328-0011_HL3H2CCXY_L5_clean_bin.41.strict.fa	OEZ008529	yes	LMSG_G000008755.1	OES073674	terpene	Terpene	Pyrite-Copper	17.60	China: Guangdong, Daobaoshan
L180330P-7-LD180328-0011_HL3H2CCXY_L5_clean_bin.43.strict.fa	OEZ008529	yes	LMSG_G000004578.1	OES073674	terpene	Terpene	Pyrite-Copper	20.92	China: Guangdong, Daobaoshan
L180330P-7-LD180328-0011_HL3H2CCXY_L5_clean_bin.46.strict.fa	OEZ008529	yes	LMSG_G000010594.1	OES073674	arylpolyene	Others	Pyrite-Copper	21.80	China: Guangdong, Daobaoshan

L180330P-7-LD180328-0011_HL3H2CCXY_L5_clean_bin.46.strict.fa OEZ008529	yes	LMSG_G000010594.1	OES073674	terpene	Terpene	Pyrite-Copper	18.18	China: Guangdong, Daobaoshan
L180330P-7-LD180328-0011_HL3H2CCXY_L5_clean_bin.5.strict.fa_ OEZ008529	yes	LMSG_G000010100.1	OES073674	terpene	Terpene	Pyrite-Copper	16.51	China: Guangdong, Daobaoshan
L180330P-7-LD180328-0011_HL3H2CCXY_L5_clean_bin.5.strict.fa_ OEZ008529	yes	LMSG_G000010100.1	OES073674	T1PKS	PKSI	Pyrite-Copper	15.93	China: Guangdong, Daobaoshan
L180330P-7-LD180328-0011_HL3H2CCXY_L5_clean_bin.5.strict.fa_ OEZ008529	yes	LMSG_G000010100.1	OES073674	terpene	Terpene	Pyrite-Copper	11.14	China: Guangdong, Daobaoshan
L180330P-7-LD180328-0011_HL3H2CCXY_L5_clean_bin.5.strict.fa_ OEZ008529	yes	LMSG_G000010100.1	OES073674	terpene	Terpene	Pyrite-Copper	12.10	China: Guangdong, Daobaoshan
L180330P-7-LD180328-0011_HL3H2CCXY_L5_clean_bin.5.strict.fa_ OEZ008529	yes	LMSG_G000010100.1	OES073674	phosphonate	Others	Pyrite-Copper	8.02	China: Guangdong, Daobaoshan
L180330P-7-LD180328-0011_HL3H2CCXY_L5_clean_bin.5.strict.fa_ OEZ008529	yes	LMSG_G000010100.1	OES073674	NRPS-like	NRPS	Pyrite-Copper	5.54	China: Guangdong, Daobaoshan
L180330P-7-LD180328-0011_HL3H2CCXY_L5_clean_bin.5.strict.fa_ OEZ008529	yes	LMSG_G000010100.1	OES073674	NRPS	NRPS	Pyrite-Copper	5.10	China: Guangdong, Daobaoshan
L180330P-7-LD180328-0011_HL3H2CCXY_L5_clean_bin.52.strict.fa OEZ008529	yes	LMSG_G000006321.1	OES073674	CDPS	Others	Pyrite-Copper	20.56	China: Guangdong, Daobaoshan
L180330P-7-LD180328-0011_HL3H2CCXY_L5_clean_bin.56.strict.fa OEZ008529	yes	LMSG_G000004579.1	OES073674	T3PKS	PKSother	Pyrite-Copper	41.07	China: Guangdong, Daobaoshan
L180330P-7-LD180328-0011_HL3H2CCXY_L5_clean_bin.56.strict.fa OEZ008529	yes	LMSG_G000004579.1	OES073674	terpene	Terpene	Pyrite-Copper	16.19	China: Guangdong, Daobaoshan
L180330P-7-LD180328-0011_HL3H2CCXY_L5_clean_bin.57.strict.fa OEZ008529	yes	LMSG_G000010555.1	OES073674	arylpolyene	Others	Pyrite-Copper	31.83	China: Guangdong, Daobaoshan
L180330P-7-LD180328-0011_HL3H2CCXY_L5_clean_bin.57.strict.fa OEZ008529	no	LMSG_G000010555.1	OES073674	terpene	Terpene	Pyrite-Copper	14.81	China: Guangdong, Daobaoshan
L180330P-7-LD180328-0011_HL3H2CCXY_L5_clean_bin.61.strict.fa OEZ008529	yes	LMSG_G000009680.1	OES073674	NRPS-like	NRPS	Pyrite-Copper	32.13	China: Guangdong, Daobaoshan
L180330P-7-LD180328-0011_HL3H2CCXY_L5_clean_bin.61.strict.fa OEZ008529	yes	LMSG_G000009680.1	OES073674	betalactone	Others	Pyrite-Copper	30.36	China: Guangdong, Daobaoshan
L180330P-7-LD180328-0011_HL3H2CCXY_L5_clean_bin.61.strict.fa OEZ008529	yes	LMSG_G000009680.1	OES073674	NRPS-like	NRPS	Pyrite-Copper	29.44	China: Guangdong, Daobaoshan
L180330P-7-LD180328-0011_HL3H2CCXY_L5_clean_bin.61.strict.fa OEZ008529	yes	LMSG_G000009680.1	OES073674	terpene	Terpene	Pyrite-Copper	22.01	China: Guangdong, Daobaoshan
L180330P-7-LD180328-0011_HL3H2CCXY_L5_clean_bin.64.strict.fa OEZ008529	yes	LMSG_G000009240.1	OES073674	T3PKS.terpene	Others	Pyrite-Copper	46.02	China: Guangdong, Daobaoshan
L180330P-7-LD180328-0011_HL3H2CCXY_L5_clean_bin.70.strict.fa OEZ008529	yes	LMSG_G000008297.1	OES073674	terpene	Terpene	Pyrite-Copper	10.46	China: Guangdong, Daobaoshan
L180330P-7-LD180328-0011_HL3H2CCXY_L5_clean_bin.71.strict.fa OEZ008529	yes	LMSG_G000005868.1	OES073674	terpene	Terpene	Pyrite-Copper	6.25	China: Guangdong, Daobaoshan
L180330P-7-LD180328-0011_HL3H2CCXY_L5_clean_bin.73.strict.fa OEZ008529	yes	LMSG_G000009125.1	OES073674	terpene	Terpene	Pyrite-Copper	6.32	China: Guangdong, Daobaoshan
L180330P-7-LD180328-0011_HL3H2CCXY_L5_clean_bin.75.strict.fa OEZ008529	yes	LMSG_G000011210.1	OES073674	arylpolyene	Others	Pyrite-Copper	13.55	China: Guangdong, Daobaoshan
L180330P-7-LD180328-0011_HL3H2CCXY_L5_clean_bin.75.strict.fa OEZ008529	yes	LMSG_G000011210.1	OES073674	arylpolyene	Others	Pyrite-Copper	6.12	China: Guangdong, Daobaoshan
L180330P-7-LD180328-0011_HL3H2CCXY_L5_clean_bin.8.strict.fa_ OEZ008529	yes	LMSG_G000005874.1	OES073674	thiopeptide	RiPPs	Pyrite-Copper	24.15	China: Guangdong, Daobaoshan
L180330P-7-LD180328-0011_HL3H2CCXY_L5_clean_bin.82.strict.fa OEZ008529	yes	LMSG_G000010287.1	OES073674	arylpolyene	Others	Pyrite-Copper	19.76	China: Guangdong, Daobaoshan
L180330P-7-LD180328-0011_HL3H2CCXY_L5_clean_bin.82.strict.fa OEZ008529	yes	LMSG_G000010287.1	OES073674	terpene	Terpene	Pyrite-Copper	11.59	China: Guangdong, Daobaoshan
L180330P-7-LD180328-0011_HL3H2CCXY_L5_clean_bin.86.strict.fa OEZ008529	yes	LMSG_G000007911.1	OES073674	bacteriocin	RiPPs	Pyrite-Copper	10.71	China: Guangdong, Daobaoshan
L180404P-1-LD180328-0012_HL57HCCXY_L3_clean_bin.1.orig.fa_k1 OEZ008529	yes	LMSG_G000009248.1	OES073675	terpene	Terpene	Pyrite-Copper	19.86	China: Guangdong, Daobaoshan
L180404P-1-LD180328-0012_HL57HCCXY_L3_clean_bin.1.orig.fa_k1 OEZ008529	yes	LMSG_G000009248.1	OES073675	TfuA-related	RiPPs	Pyrite-Copper	16.24	China: Guangdong, Daobaoshan
L180404P-1-LD180328-0012_HL57HCCXY_L3_clean_bin.100.orig.fa_ OEZ008529	yes	LMSG_G000010726.1	OES073675	terpene	Terpene	Pyrite-Copper	12.98	China: Guangdong, Daobaoshan
L180404P-1-LD180328-0012_HL57HCCXY_L3_clean_bin.100.orig.fa_ OEZ008529	yes	LMSG_G000010726.1	OES073675	NRPS	NRPS	Pyrite-Copper	23.81	China: Guangdong, Daobaoshan
L180404P-1-LD180328-0012_HL57HCCXY_L3_clean_bin.100.orig.fa_ OEZ008529	yes	LMSG_G000010726.1	OES073675	arylpolyene	Others	Pyrite-Copper	24.71	China: Guangdong, Daobaoshan
L180404P-1-LD180328-0012_HL57HCCXY_L3_clean_bin.100.orig.fa_ OEZ008529	yes	LMSG_G000010726.1	OES073675	NRPS.T1PKS	PKS-NRP_Hybrids	Pyrite-Copper	32.09	China: Guangdong, Daobaoshan
L180404P-1-LD180328-0012_HL57HCCXY_L3_clean_bin.102.orig.fa_ OEZ008529	yes	LMSG_G000005876.1	OES073675	NRPS-like	NRPS	Pyrite-Copper	34.54	China: Guangdong, Daobaoshan
L180404P-1-LD180328-0012_HL57HCCXY_L3_clean_bin.103.orig.fa_ OEZ008529	yes	LMSG_G000008955.1	OES073675	betalactone	Others	Pyrite-Copper	25.93	China: Guangdong, Daobaoshan
L180404P-1-LD180328-0012_HL57HCCXY_L3_clean_bin.104.orig.fa_ OEZ008529	yes	LMSG_G000010603.1	OES073675	terpene	Terpene	Pyrite-Copper	22.97	China: Guangdong, Daobaoshan
L180404P-1-LD180328-0012_HL57HCCXY_L3_clean_bin.108.strict.f OEZ008529	yes	LMSG_G000005877.1	OES073675	betalactone	Others	Pyrite-Copper	18.94	China: Guangdong, Daobaoshan
L180404P-1-LD180328-0012_HL57HCCXY_L3_clean_bin.108.strict.f OEZ008529	yes	LMSG_G000005877.1	OES073675	sactipeptide	RiPPs	Pyrite-Copper	15.26	China: Guangdong, Daobaoshan
L180404P-1-LD180328-0012_HL57HCCXY_L3_clean_bin.108.strict.f OEZ008529	yes	LMSG_G000005877.1	OES073675	acyl_amino_acids	Others	Pyrite-Copper	6.71	China: Guangdong, Daobaoshan
L180404P-1-LD180328-0012_HL57HCCXY_L3_clean_bin.109.orig.fa_ OEZ008529	yes	LMSG_G000006251.1	OES073675	T3PKS	PKSother	Pyrite-Copper	6.25	China: Guangdong, Daobaoshan
L180404P-1-LD180328-0012_HL57HCCXY_L3_clean_bin.109.orig.fa_ OEZ008529	yes	LMSG_G000006251.1	OES073675	bacteriocin	RiPPs	Pyrite-Copper	6.41	China: Guangdong, Daobaoshan
L180404P-1-LD180328-0012_HL57HCCXY_L3_clean_bin.109.orig.fa_ OEZ008529	yes	LMSG_G000006251.1	OES073675	lassopeptide	RiPPs	Pyrite-Copper	6.66	China: Guangdong, Daobaoshan
L180404P-1-LD180328-0012_HL57HCCXY_L3_clean_bin.11.orig.fa_k OEZ008529	yes	LMSG_G000011068.1	OES073675	hserlactone	Others	Pyrite-Copper	17.60	China: Guangdong, Daobaoshan
L180404P-1-LD180328-0012_HL57HCCXY_L3_clean_bin.11.orig.fa_k OEZ008529	yes	LMSG_G000011068.1	OES073675	NRPS-like	NRPS	Pyrite-Copper	21.33	China: Guangdong, Daobaoshan
L180404P-1-LD180328-0012_HL57HCCXY_L3_clean_bin.11.orig.fa_k OEZ008529	yes	LMSG_G000011068.1	OES073675	NRPS-like	NRPS	Pyrite-Copper	41.97	China: Guangdong, Daobaoshan
L180404P-1-LD180328-0012_HL57HCCXY_L3_clean_bin.11.orig.fa_k OEZ008529	yes	LMSG_G000011068.1	OES073675	terpene	Terpene	Pyrite-Copper	10.88	China: Guangdong, Daobaoshan
L180404P-1-LD180328-0012_HL57HCCXY_L3_clean_bin.111.orig.fa_ OEZ008529	yes	LMSG_G000008719.1	OES073675	terpene	Terpene	Pyrite-Copper	20.36	China: Guangdong, Daobaoshan
L180404P-1-LD180328-0012_HL57HCCXY_L3_clean_bin.114.orig.fa_ OEZ008529	yes	LMSG_G000004580.1	OES073675	terpene	Terpene	Pyrite-Copper	21.39	China: Guangdong, Daobaoshan
L180404P-1-LD180328-0012_HL57HCCXY_L3_clean_bin.114.orig.fa_ OEZ008529	yes	LMSG_G000004580.1	OES073675	terpene	Terpene	Pyrite-Copper	19.33	China: Guangdong, Daobaoshan
L180404P-1-LD180328-0012_HL57HCCXY_L3_clean_bin.115.orig.fa_ OEZ008529	yes	LMSG_G000005878.1	OES073675	terpene	Terpene	Pyrite-Copper	9.43	China: Guangdong, Daobaoshan
L180404P-1-LD180328-0012_HL57HCCXY_L3_clean_bin.116.strict.f OEZ008529	yes	LMSG_G000010003.1	OES073675	terpene	Terpene	Pyrite-Copper	17.48	China: Guangdong, Daobaoshan
L180404P-1-LD180328-0012_HL57HCCXY_L3_clean_bin.116.strict.f OEZ008529	yes	LMSG_G000010003.1	OES073675	terpene	Terpene	Pyrite-Copper	12.19	China: Guangdong, Daobaoshan
L180404P-1-LD180328-0012_HL57HCCXY_L3_clean_bin.116.strict.f OEZ008529	yes	LMSG_G000010003.1	OES073675	ectoine	Others	Pyrite-Copper	7.78	China: Guangdong, Daobaoshan
L180404P-1-LD180328-0012_HL57HCCXY_L3_clean_bin.116.strict.f OEZ008529	yes	LMSG_G000010003.1	OES073675	NRPS-like	NRPS	Pyrite-Copper	7.43	China: Guangdong, Daobaoshan
L180404P-1-LD180328-0012_HL57HCCXY_L3_clean_bin.116.strict.f OEZ008529	yes	LMSG_G000010003.1	OES073675	arylpolyene	Others	Pyrite-Copper	6.64	China: Guangdong, Daobaoshan
L180404P-1-LD180328-0012_HL57HCCXY_L3_clean_bin.117.permissi OEZ008529	yes	LMSG_G000009633.1	OES073675	terpene	Terpene	Pyrite-Copper	18.36	China: Guangdong, Daobaoshan
L180404P-1-LD180328-0012_HL57HCCXY_L3_clean_bin.117.permissi OEZ008529	yes	LMSG_G000009633.1	OES073675	terpene	Terpene	Pyrite-Copper	12.06	China: Guangdong, Daobaoshan
L180404P-1-LD180328-0012_HL57HCCXY_L3_clean_bin.117.permissi OEZ008529	yes	LMSG_G000009633.1	OES073675	nucleoside	Others	Pyrite-Copper	7.77	China: Guangdong, Daobaoshan
L180404P-1-LD180328-0012_HL57HCCXY_L3_clean_bin.117.permissi OEZ008529	no	LMSG_G000009633.1	OES073675	terpene	Terpene	Pyrite-Copper	6.55	China: Guangdong, Daobaoshan
L180404P-1-LD180328-0012_HL57HCCXY_L3_clean_bin.13.orig.fa_k OEZ008529	yes	LMSG_G000006252.1	OES073675	bacteriocin	RiPPs	Pyrite-Copper	10.84	China: Guangdong, Daobaoshan
L180404P-1-LD180328-0012_HL57HCCXY_L3_clean_bin.13.orig.fa_k OEZ008529	yes	LMSG_G000006252.1	OES073675	sactipeptide	RiPPs	Pyrite-Copper	21.45	China: Guangdong, Daobaoshan
L180404P-1-LD180328-0012_HL57HCCXY_L3_clean_bin.13.orig.fa_k OEZ008529	yes	LMSG_G000006252.1	OES073675	sactipeptide	RiPPs	Pyrite-Copper	20.16	China: Guangdong, Daobaoshan
L180404P-1-LD180328-0012_HL57HCCXY_L3_clean_bin.133.orig.fa_ OEZ008529	yes	LMSG_G000010954.1	OES073675	T1PKS	PKSI	Pyrite-Copper	47.52	China: Guangdong, Daobaoshan
L180404P-1-LD180328-0012_HL57HCCXY_L3_clean_bin.133.orig.fa_ OEZ008529	yes	LMSG_G000010954.1	OES073675	terpene	Terpene	Pyrite-Copper	20.89	China: Guangdong, Daobaoshan
L180404P-1-LD180328-0012_HL57HCCXY_L3_clean_bin.133.orig.fa_ OEZ008529	yes	LMSG_G000010954.1	OES073675	hserlactone	Others	Pyrite-Copper	20.86	China: Guangdong, Daobaoshan
L180404P-1-LD180328-0012_HL57HCCXY_L3_clean_bin.133.orig.fa_ OEZ008529	yes	LMSG_G000010954.1	OES073675	bacteriocin.arylpolyene	Others	Pyrite-Copper	47.12	China: Guangdong, Daobaoshan
L180404P-1-LD180328-0012_HL57HCCXY_L3_clean_bin.14.orig.fa_k OEZ008529	yes	LMSG_G000007375.1	OES073675	bacteriocin	RiPPs	Pyrite-Copper	11.97	China: Guangdong, Daobaoshan
L180404P-1-LD180328-0012_HL57HCCXY_L3_clean_bin.15.strict.fa OEZ008529	yes	LMSG_G000007962.1	OES073675	betalactone	Others	Pyrite-Copper	23.13	China: Guangdong, Daobaoshan
L180404P-1-LD180328-0012_HL57HCCXY_L3_clean_bin.15.strict.fa OEZ008529	yes	LMSG_G000007962.1	OES073675	terpene	Terpene	Pyrite-Copper	26.75	China: Guangdong, Daobaoshan
L180404P-1-LD180328-0012_HL57HCCXY_L3_clean_bin.16.orig.fa_k OEZ008529	yes	LMSG_G000008422.1	OES073675	terpene	Terpene	Pyrite-Copper	8.44	China: Guangdong, Daobaoshan
L180404P-1-LD180328-0012_HL57HCCXY_L3_clean_bin.17.orig.fa_k OEZ008529	yes	LMSG_G000010236.1	OES073675	arylpolyene	Others	Pyrite-Copper	23.75	China: Guangdong, Daobaoshan
L180404P-1-LD180328-0012_HL57HCCXY_L3_clean_bin.17.orig.fa_k OEZ008529	yes	LMSG_G000010236.1	OES073675	hserlactone	Others	Pyrite-Copper	20.72	China: Guangdong, Daobaoshan
L180404P-1-LD180328-0012_HL57HCCXY_L3_clean_bin.17.orig.fa_k OEZ008529	yes	LMSG_G000010236.1	OES073675	terpene	Terpene	Pyrite-Copper	20.70	China: Guangdong, Daobaoshan
L180404P-1-LD180328-0012_HL57HCCXY_L3_clean_bin.17.orig.fa_k OEZ008529	yes	LMSG_G000010236.1	OES073675	terpene	Terpene	Pyrite-Copper	19.58	China: Guangdong, Daobaoshan
L180404P-1-LD180328-0012_HL57HCCXY_L3_clean_bin.18.orig.fa_k OEZ008529	yes	LMSG_G000008539.1	OES073675	terpene	Terpene	Pyrite-Copper	13.16	China: Guangdong, Daobaoshan
L180404P-1-LD180328-0012_HL57HCCXY_L3_clean_bin.22.orig.fa_k OEZ008529	yes	LMSG_G000009348.1	OES073675	sactipeptide	RiPPs	Pyrite-Copper	20.15	China: Guangdong, Daobaoshan
L180404P-1-LD180328-0012_HL57HCCXY_L3_clean_bin.23.orig.fa_k OEZ008529	yes	LMSG_G000008521.1	OES073675	T1PKS	PKSI	Pyrite-Copper	11.04	China: Guangdong, Daobaoshan
L180404P-1-LD180328-0012_HL57HCCXY_L3_clean_bin.28.strict.fa OEZ008529	yes	LMSG_G000005883.1	OES073675	other	Others	Pyrite-Copper	13.01	China: Guangdong, Daobaoshan
L180404P-1-LD180328-0012_HL57HCCXY_L3_clean_bin.28.strict.fa OEZ008529	yes	LMSG_G000005883.1	OES073675	other	Others	Pyrite-Copper	9.54	China: Guangdong, Daobaoshan
L180404P-1-LD180328-0012_HL57HCCXY_L3_clean_bin.3.strict.fa_ OEZ008529	yes	LMSG_G000007910.1	OES073675	terpene	Terpene	Pyrite-Copper	13.44	China: Guangdong, Daobaoshan
L180404P-1-LD180328-0012_HL57HCCXY_L3_clean_bin.3.strict.fa_ OEZ008529	yes	LMSG_G000007910.1	OES073675	NRPS-like	NRPS	Pyrite-Copper	8.24	China: Guangdong, Daobaoshan
L180404P-1-LD180328-0012_HL57HCCXY_L3_clean_bin.31.orig.fa_k OEZ008529	no	LMSG_G000009538.1	OES073675	bacteriocin	RiPPs	Pyrite-Copper	5.62	China: Guangdong, Daobaoshan
L180404P-1-LD180328-0012_HL57HCCXY_L3_clean_bin.32.orig.fa_k OEZ008529	yes	LMSG_G000007921.1	OES073675	bacteriocin	RiPPs	Pyrite-Copper	12.00	China: Guangdong, Daobaoshan
L180404P-1-LD180328-0012_HL57HCCXY_L3_clean_bin.32.orig.fa_k OEZ008529	yes	LMSG_G000007921.1	OES073675	lanthipeptide	RiPPs	Pyrite-Copper	23.26	China: Guangdong, Daobaoshan
L180404P-1-LD180328-0012_HL57HCCXY_L3_clean_bin.32.orig.fa_k OEZ008529	yes	LMSG_G000007921.1	OES073675	NRPS-like	NRPS	Pyrite-Copper	25.59	China: Guangdong, Daobaoshan
L180404P-1-LD180328-0012_HL57HCCXY_L3_clean_bin.32.orig.fa_k OEZ008529	yes	LMSG_G000007921.1	OES073675	NRPS-like	NRPS	Pyrite-Copper	18.56	China: Guangdong, Daobaoshan
L180404P-1-LD180328-0012_HL57HCCXY_L3_clean_bin.32.orig.fa_k OEZ008529	yes	LMSG_G000007921.1	OES073675	terpene	Terpene	Pyrite-Copper	29.02	China: Guangdong, Daobaoshan
L180404P-1-LD180328-0012_HL57HCCXY_L3_clean_bin.32.orig.fa_k OEZ008529	yes	LMSG_G000007921.1	OES073675	T3PKS	PKSother	Pyrite-Copper	41.09	China: Guangdong, Daobaoshan
L180404P-1-LD180328-0012_HL57HCCXY_L3_clean_bin.32.orig.fa_k OEZ008529	yes	LMSG_G000007921.1	OES073675	NRPS-like	NRPS	Pyrite-Copper	39.38	China: Guangdong, Daobaoshan
L180404P-1-LD180328-0012_HL57HCCXY_L3_clean_bin.33.orig.fa_k OEZ008529	yes	LMSG_G000006339.1	OES073675	CDPS	Others	Pyrite-Copper	20.67	China: Guangdong, Daobaoshan
L180404P-1-LD180328-0012_HL57HCCXY_L3_clean_bin.33.orig.fa_k OEZ008529	yes	LMSG_G000006339.1	OES073675	hserlactone	Others	Pyrite-Copper	20.50	China: Guangdong, Daobaoshan
L180404P-1-LD180328-0012_HL57HCCXY_L3_clean_bin.35.orig.fa_k OEZ008529	yes	LMSG_G000010249.1	OES073675	terpene	Terpene	Pyrite-Copper	7.96	China: Guangdong, Daobaoshan
L180404P-1-LD180328-0012_HL57HCCXY_L3_clean_bin.35.orig.fa_k OEZ008529	yes	LMSG_G000010249.1	OES073675	T1PKS	PKSI	Pyrite-Copper	19.84	China: Guangdong, Daobaoshan
L180404P-1-LD180328-0012_HL57HCCXY_L3_clean_bin.35.orig.fa_k OEZ008529	yes	LMSG_G000010249.1	OES073675	terpene	Terpene	Pyrite-Copper	16.02	China: Guangdong, Daobaoshan
L180404P-1-LD180328-0012_HL57HCCXY_L3_clean_bin.35.orig.fa_k OEZ008529	yes	LMSG_G000010249.1	OES073675	bacteriocin	RiPPs	Pyrite-Copper	7.29	China: Guangdong, Daobaoshan
L180404P-1-LD180328-0012_HL57HCCXY_L3_clean_bin.37.strict.fa OEZ008529	yes	LMSG_G000007760.1	OES073675	terpene	Terpene	Pyrite-Copper	10.78	China: Guangdong, Daobaoshan
L180404P-1-LD180328-0012_HL57HCCXY_L3_clean_bin.37.strict.fa OEZ008529	yes	LMSG_G000007760.1	OES073675	NRPS-like	NRPS	Pyrite-Copper	5.07	China: Guangdong, Daobaoshan
L180404P-1-LD180328-0012_HL57HCCXY_L3_clean_bin.43.permissiv OEZ008529	yes	LMSG_G000009969.1	OES073675	terpene	Terpene	Pyrite-Copper	19.51	China: Guangdong, Daobaoshan
L180404P-1-LD180328-0012_HL57HCCXY_L3_clean_bin.43.permissiv OEZ008529	yes	LMSG_G000009969.1	OES073675	terpene	Terpene	Pyrite-Copper	14.42	China: Guangdong, Daobaoshan
L180404P-1-LD180328-0012_HL57HCCXY_L3_clean_bin.43.permissiv OEZ008529	yes	LMSG_G000009969.1	OES073675	ectoine	Others	Pyrite-Copper	7.93	China: Guangdong, Daobaoshan
L180404P-1-LD180328-0012_HL57HCCXY_L3_clean_bin.43.permissiv OEZ008529	yes	LMSG_G000009969.1	OES073675	arylpolyene	Others	Pyrite-Copper	12.68	China: Guangdong, Daobaoshan
L180404P-1-LD180328-0012_HL57HCCXY_L3_clean_bin.43.permissiv OEZ008529	yes	LMSG_G000009969.1	OES073675	arylpolyene	Others	Pyrite-Copper	11.34	China: Guangdong, Daobaoshan
L180404P-1-LD180328-0012_HL57HCCXY_L3_clean_bin.43.permissiv OEZ008529	yes	LMSG_G000009969.1	OES073675	arylpolyene	Others	Pyrite-Copper	6.43	China: Guangdong, Daobaoshan
L180404P-1-LD180328-0012_HL57HCCXY_L3_clean_bin.45.orig.fa_k OEZ008529	yes	LMSG_G000007876.1	OES073675	NRPS-like	NRPS	Pyrite-Copper	16.41	China: Guangdong, Daobaoshan
L180404P-1-LD180328-0012_HL57HCCXY_L3_clean_bin.45.orig.fa_k OEZ008529	yes	LMSG_G000007876.1	OES073675	terpene	Terpene	Pyrite-Copper	14.26	China: Guangdong, Daobaoshan
L180404P-1-LD180328-0012_HL57HCCXY_L3_clean_bin.45.orig.fa_k OEZ008529	yes	LMSG_G000007876.1	OES073675	NRPS-like	NRPS	Pyrite-Copper	24.64	China: Guangdong, Daobaoshan
L180404P-1-LD180328-0012_HL57HCCXY_L3_clean_bin.45.orig.fa_k OEZ008529	yes	LMSG_G000007876.1	OES073675	T1PKS	PKSI	Pyrite-Copper	14.44	China: Guangdong, Daobaoshan
L180404P-1-LD180328-0012_HL57HCCXY_L3_clean_bin.45.orig.fa_k OEZ008529	yes	LMSG_G000007876.1	OES073675	T3PKS.terpene	Others	Pyrite-Copper	16.07	China: Guangdong, Daobaoshan
L180404P-1-LD180328-0012_HL57HCCXY_L3_clean_bin.47.orig.fa_k OEZ008529	yes	LMSG_G000004581.1	OES073675	bacteriocin	RiPPs	Pyrite-Copper	11.98	China: Guangdong, Daobaoshan
L180404P-1-LD180328-0012_HL57HCCXY_L3_clean_bin.47.orig.fa_k OEZ008529	yes	LMSG_G000004581.1	OES073675	terpene	Terpene	Pyrite-Copper	21.02	China: Guangdong, Daobaoshan
L180404P-1-LD180328-0012_HL57HCCXY_L3_clean_bin.47.orig.fa_k OEZ008529	yes	LMSG_G000004581.1	OES073675	T3PKS	PKSother	Pyrite-Copper	41.07	China: Guangdong, Daobaoshan
L180404P-1-LD180328-0012_HL57HCCXY_L3_clean_bin.53.orig.fa_k OEZ008529	yes	LMSG_G000011126.1	OES073675	arylpolyene	Others	Pyrite-Copper	41.16	China: Guangdong, Daobaoshan
L180404P-1-LD180328-0012_HL57HCCXY_L3_clean_bin.56.orig.fa_k OEZ008529	yes	LMSG_G000011107.1	OES073675	terpene	Terpene	Pyrite-Copper	23.10	China: Guangdong, Daobaoshan
L180404P-1-LD180328-0012_HL57HCCXY_L3_clean_bin.58.orig.fa_k OEZ008529	yes	LMSG_G000005887.1	OES073675	arylpolyene	Others	Pyrite-Copper	5.88	China: Guangdong, Daobaoshan
L180404P-1-LD180328-0012_HL57HCCXY_L3_clean_bin.58.orig.fa_k OEZ008529	yes	LMSG_G000005887.1	OES073675	terpene	Terpene	Pyrite-Copper	21.66	China: Guangdong, Daobaoshan
L180404P-1-LD180328-0012_HL57HCCXY_L3_clean_bin.6.orig.fa_k1 OEZ008529	yes	LMSG_G000005890.1	OES073675	terpene	Terpene	Pyrite-Copper	20.84	China: Guangdong, Daobaoshan
L180404P-1-LD180328-0012_HL57HCCXY_L3_clean_bin.6.orig.fa_k1 OEZ008529	yes	LMSG_G000005890.1	OES073675	terpene	Terpene	Pyrite-Copper	17.11	China: Guangdong, Daobaoshan
L180404P-1-LD180328-0012_HL57HCCXY_L3_clean_bin.6.orig.fa_k1 OEZ008529	yes	LMSG_G000005890.1	OES073675	lanthipeptide	RiPPs	Pyrite-Copper	22.67	China: Guangdong, Daobaoshan
L180404P-1-LD180328-0012_HL57HCCXY_L3_clean_bin.6.orig.fa_k1 OEZ008529	yes	LMSG_G000005890.1	OES073675	T1PKS	PKSI	Pyrite-Copper	24.87	China: Guangdong, Daobaoshan
L180404P-1-LD180328-0012_HL57HCCXY_L3_clean_bin.61.orig.fa_k OEZ008529	yes	LMSG_G000008928.1	OES073675	betalactone	Others	Pyrite-Copper	24.57	China: Guangdong, Daobaoshan
L180404P-1-LD180328-0012_HL57HCCXY_L3_clean_bin.61.orig.fa_k OEZ008529	yes	LMSG_G000008928.1	OES073675	bacteriocin	RiPPs	Pyrite-Copper	10.87	China: Guangdong, Daobaoshan
L180404P-1-LD180328-0012_HL57HCCXY_L3_clean_bin.62.orig.fa_k OEZ008529	yes	LMSG_G000010460.1	OES073675	bacteriocin	RiPPs	Pyrite-Copper	10.54	China: Guangdong, Daobaoshan
L180404P-1-LD180328-0012_HL57HCCXY_L3_clean_bin.62.orig.fa_k OEZ008529	yes	LMSG_G000010460.1	OES073675	arylpolyene.hserlactone	Others	Pyrite-Copper	41.23	China: Guangdong, Daobaoshan
L180404P-1-LD180328-0012_HL57HCCXY_L3_clean_bin.62.orig.fa_k OEZ008529	yes	LMSG_G000010460.1	OES073675	terpene	Terpene	Pyrite-Copper	8.72	China: Guangdong, Daobaoshan
L180404P-1-LD180328-0012_HL57HCCXY_L3_clean_bin.62.orig.fa_k OEZ008529	yes	LMSG_G000010460.1	OES073675	terpene	Terpene	Pyrite-Copper	10.55	China: Guangdong, Daobaoshan
L180404P-1-LD180328-0012_HL57HCCXY_L3_clean_bin.63.orig.fa_k OEZ008529	yes	LMSG_G000009130.1	OES073675	lassopeptide	RiPPs	Pyrite-Copper	22.43	China: Guangdong, Daobaoshan
L180404P-1-LD180328-0012_HL57HCCXY_L3_clean_bin.63.orig.fa_k OEZ008529	yes	LMSG_G000009130.1	OES073675	betalactone	Others	Pyrite-Copper	27.10	China: Guangdong, Daobaoshan
L180404P-1-LD180328-0012_HL57HCCXY_L3_clean_bin.63.orig.fa_k OEZ008529	yes	LMSG_G000009130.1	OES073675	terpene	Terpene	Pyrite-Copper	25.21	China: Guangdong, Daobaoshan
L180404P-1-LD180328-0012_HL57HCCXY_L3_clean_bin.63.orig.fa_k OEZ008529	yes	LMSG_G000009130.1	OES073675	bacteriocin	RiPPs	Pyrite-Copper	10.95	China: Guangdong, Daobaoshan
L180404P-1-LD180328-0012_HL57HCCXY_L3_clean_bin.63.orig.fa_k OEZ008529	yes	LMSG_G000009130.1	OES073675	NRPS-like	NRPS	Pyrite-Copper	37.30	China: Guangdong, Daobaoshan
L180404P-1-LD180328-0012_HL57HCCXY_L3_clean_bin.64.orig.fa_k OEZ008529	yes	LMSG_G000008788.1	OES073675	NRPS-like	NRPS	Pyrite-Copper	6.78	China: Guangdong, Daobaoshan
L180404P-1-LD180328-0012_HL57HCCXY_L3_clean_bin.64.orig.fa_k OEZ008529	yes	LMSG_G000008788.1	OES073675	bacteriocin	RiPPs	Pyrite-Copper	5.57	China: Guangdong, Daobaoshan
L180404P-1-LD180328-0012_HL57HCCXY_L3_clean_bin.65.orig.fa_k OEZ008529	yes	LMSG_G000009112.1	OES073675	T3PKS	PKSother	Pyrite-Copper	22.40	China: Guangdong, Daobaoshan
L180404P-1-LD180328-0012_HL57HCCXY_L3_clean_bin.65.orig.fa_k OEZ008529	yes	LMSG_G000009112.1	OES073675	terpene	Terpene	Pyrite-Copper	24.96	China: Guangdong, Daobaoshan
L180404P-1-LD180328-0012_HL57HCCXY_L3_clean_bin.65.orig.fa_k OEZ008529	yes	LMSG_G000009112.1	OES073675	NRPS-like	NRPS	Pyrite-Copper	23.19	China: Guangdong, Daobaoshan
L180404P-1-LD180328-0012_HL57HCCXY_L3_clean_bin.65.orig.fa_k OEZ008529	yes	LMSG_G000009112.1	OES073675	terpene	Terpene	Pyrite-Copper	21.97	China: Guangdong, Daobaoshan
L180404P-1-LD180328-0012_HL57HCCXY_L3_clean_bin.66.strict.fa OEZ008529	yes	LMSG_G000009668.1	OES073675	NRPS-like	NRPS	Pyrite-Copper	28.17	China: Guangdong, Daobaoshan
L180404P-1-LD180328-0012_HL57HCCXY_L3_clean_bin.66.strict.fa OEZ008529	yes	LMSG_G000009668.1	OES073675	terpene	Terpene	Pyrite-Copper	20.35	China: Guangdong, Daobaoshan
L180404P-1-LD180328-0012_HL57HCCXY_L3_clean_bin.66.strict.fa OEZ008529	yes	LMSG_G000009668.1	OES073675	nucleoside	Others	Pyrite-Copper	20.95	China: Guangdong, Daobaoshan
L180404P-1-LD180328-0012_HL57HCCXY_L3_clean_bin.66.strict.fa OEZ008529	yes	LMSG_G000009668.1	OES073675	hserlactone	Others	Pyrite-Copper	14.96	China: Guangdong, Daobaoshan
L180404P-1-LD180328-0012_HL57HCCXY_L3_clean_bin.66.strict.fa OEZ008529	yes	LMSG_G000009668.1	OES073675	NRPS-like	NRPS	Pyrite-Copper	8.10	China: Guangdong, Daobaoshan
L180404P-1-LD180328-0012_HL57HCCXY_L3_clean_bin.71.orig.fa_k OEZ008529	yes	LMSG_G000008838.1	OES073675	terpene	Terpene	Pyrite-Copper	21.71	China: Guangdong, Daobaoshan
L180404P-1-LD180328-0012_HL57HCCXY_L3_clean_bin.71.orig.fa_k OEZ008529	yes	LMSG_G000008838.1	OES073675	NRPS-like	NRPS	Pyrite-Copper	42.46	China: Guangdong, Daobaoshan
L180404P-1-LD180328-0012_HL57HCCXY_L3_clean_bin.71.orig.fa_k OEZ008529	yes	LMSG_G000008838.1	OES073675	T3PKS	PKSother	Pyrite-Copper	25.42	China: Guangdong, Daobaoshan
L180404P-1-LD180328-0012_HL57HCCXY_L3_clean_bin.72.orig.fa_k OEZ008529	yes	LMSG_G000010892.1	OES073675	arylpolyene	Others	Pyrite-Copper	21.09	China: Guangdong, Daobaoshan
L180404P-1-LD180328-0012_HL57HCCXY_L3_clean_bin.72.orig.fa_k OEZ008529	no	LMSG_G000010892.1	OES073675	arylpolyene	Others	Pyrite-Copper	5.42	China: Guangdong, Daobaoshan
L180404P-1-LD180328-0012_HL57HCCXY_L3_clean_bin.72.orig.fa_k OEZ008529	yes	LMSG_G000010892.1	OES073675	terpene	Terpene	Pyrite-Copper	17.27	China: Guangdong, Daobaoshan
L180404P-1-LD180328-0012_HL57HCCXY_L3_clean_bin.72.orig.fa_k OEZ008529	yes	LMSG_G000010892.1	OES073675	acyl_amino_acids	Others	Pyrite-Copper	54.82	China: Guangdong, Daobaoshan
L180404P-1-LD180328-0012_HL57HCCXY_L3_clean_bin.73.orig.fa_k OEZ008529	yes	LMSG_G000009620.1	OES073675	NRPS-like	NRPS	Pyrite-Copper	9.27	China: Guangdong, Daobaoshan
L180404P-1-LD180328-0012_HL57HCCXY_L3_clean_bin.73.orig.fa_k OEZ008529	yes	LMSG_G000009620.1	OES073675	terpene	Terpene	Pyrite-Copper	21.89	China: Guangdong, Daobaoshan
L180404P-1-LD180328-0012_HL57HCCXY_L3_clean_bin.73.orig.fa_k OEZ008529	yes	LMSG_G000009620.1	OES073675	terpene	Terpene	Pyrite-Copper	14.16	China: Guangdong, Daobaoshan
L180404P-1-LD180328-0012_HL57HCCXY_L3_clean_bin.74.orig.fa_k OEZ008529	yes	LMSG_G000008629.1	OES073675	other	Others	Pyrite-Copper	40.72	China: Guangdong, Daobaoshan
L180404P-1-LD180328-0012_HL57HCCXY_L3_clean_bin.74.orig.fa_k OEZ008529	yes	LMSG_G000008629.1	OES073675	NRPS-like	NRPS	Pyrite-Copper	7.86	China: Guangdong, Daobaoshan
L180404P-1-LD180328-0012_HL57HCCXY_L3_clean_bin.76.strict.fa OEZ008529	yes	LMSG_G000009282.1	OES073675	terpene	Terpene	Pyrite-Copper	22.06	China: Guangdong, Daobaoshan
L180404P-1-LD180328-0012_HL57HCCXY_L3_clean_bin.76.strict.fa OEZ008529	yes	LMSG_G000009282.1	OES073675	terpene	Terpene	Pyrite-Copper	21.73	China: Guangdong, Daobaoshan
L180404P-1-LD180328-0012_HL57HCCXY_L3_clean_bin.79.orig.fa_k OEZ008529	yes	LMSG_G000008425.1	OES073675	bacteriocin	RiPPs	Pyrite-Copper	10.80	China: Guangdong, Daobaoshan
L180404P-1-LD180328-0012_HL57HCCXY_L3_clean_bin.79.orig.fa_k OEZ008529	yes	LMSG_G000008425.1	OES073675	other	Others	Pyrite-Copper	34.01	China: Guangdong, Daobaoshan
L180404P-1-LD180328-0012_HL57HCCXY_L3_clean_bin.79.orig.fa_k OEZ008529	yes	LMSG_G000008425.1	OES073675	terpene	Terpene	Pyrite-Copper	26.56	China: Guangdong, Daobaoshan

L180404P-1-LD180328-0012_HL57HCCXY_L3_clean_bin.79.orig.fa_k	OEZ008529	yes	LMSG_G000008425.1	OES073675	bacteriocin	RiPPs	Pyrite-Copper	7.22	China: Guangdong, Daobaoshan
L180404P-1-LD180328-0012_HL57HCCXY_L3_clean_bin.8.orig.fa_k1	OEZ008529	yes	LMSG_G000010905.1	OES073675	hserlactone	Others	Pyrite-Copper	17.79	China: Guangdong, Daobaoshan
L180404P-1-LD180328-0012_HL57HCCXY_L3_clean_bin.8.orig.fa_k1	OEZ008529	yes	LMSG_G000010905.1	OES073675	NRPS-like	NRPS	Pyrite-Copper	35.03	China: Guangdong, Daobaoshan
L180404P-1-LD180328-0012_HL57HCCXY_L3_clean_bin.8.orig.fa_k1	OEZ008529	yes	LMSG_G000010905.1	OES073675	terpene	Terpene	Pyrite-Copper	11.67	China: Guangdong, Daobaoshan
L180404P-1-LD180328-0012_HL57HCCXY_L3_clean_bin.8.orig.fa_k1	OEZ008529	yes	LMSG_G000010905.1	OES073675	acyl_amino_acids	Others	Pyrite-Copper	13.50	China: Guangdong, Daobaoshan
L180404P-1-LD180328-0012_HL57HCCXY_L3_clean_bin.82.strict.fa	OEZ008529	yes	LMSG_G000008849.1	OES073675	terpene	Terpene	Pyrite-Copper	21.30	China: Guangdong, Daobaoshan
L180404P-1-LD180328-0012_HL57HCCXY_L3_clean_bin.92.orig.fa_k	OEZ008529	yes	LMSG_G000010021.1	OES073675	ladderane	Others	Pyrite-Copper	32.51	China: Guangdong, Daobaoshan
L180404P-1-LD180328-0012_HL57HCCXY_L3_clean_bin.92.orig.fa_k	OEZ008529	yes	LMSG_G000010021.1	OES073675	terpene	Terpene	Pyrite-Copper	21.87	China: Guangdong, Daobaoshan
L180404P-1-LD180328-0012_HL57HCCXY_L3_clean_bin.92.orig.fa_k	OEZ008529	yes	LMSG_G000010021.1	OES073675	terpene	Terpene	Pyrite-Copper	7.21	China: Guangdong, Daobaoshan
L180404P-1-LD180328-0012_HL57HCCXY_L3_clean_bin.93.orig.fa_k	OEZ008529	yes	LMSG_G000005894.1	OES073675	NRPS-like	NRPS	Pyrite-Copper	8.40	China: Guangdong, Daobaoshan
L180404P-1-LD180328-0012_HL57HCCXY_L3_clean_bin.95.strict.fa	OEZ008529	yes	LMSG_G000007997.1	OES073675	TfuA-related.bacteriocin	RiPPs	Pyrite-Copper	13.25	China: Guangdong, Daobaoshan
L180404P-1-LD180328-0012_HL57HCCXY_L3_clean_bin.95.strict.fa	OEZ008529	yes	LMSG_G000007997.1	OES073675	bacteriocin	RiPPs	Pyrite-Copper	10.32	China: Guangdong, Daobaoshan
L180404P-1-LD180328-0012_HL57HCCXY_L3_clean_bin.98.orig.fa_k	OEZ008529	yes	LMSG_G000005895.1	OES073675	terpene	Terpene	Pyrite-Copper	21.08	China: Guangdong, Daobaoshan
L180404P-1-LD180328-0014_HL57HCCXY_L3_clean_bin.104.orig.fa_	OEZ008529	yes	LMSG_G000009013.1	OES073686	terpene	Terpene	Lead-Zinc	16.04	China: Guangdong, Fankou
L180404P-1-LD180328-0014_HL57HCCXY_L3_clean_bin.104.orig.fa_	OEZ008529	yes	LMSG_G000009013.1	OES073686	bacteriocin	RiPPs	Lead-Zinc	10.80	China: Guangdong, Fankou
L180404P-1-LD180328-0014_HL57HCCXY_L3_clean_bin.106.orig.fa_	OEZ008529	no	LMSG_G000010948.1	OES073686	thiopeptide	RiPPs	Lead-Zinc	11.60	China: Guangdong, Fankou
L180404P-1-LD180328-0014_HL57HCCXY_L3_clean_bin.106.orig.fa_	OEZ008529	no	LMSG_G000010948.1	OES073686	NRPS	NRPS	Lead-Zinc	23.29	China: Guangdong, Fankou
L180404P-1-LD180328-0014_HL57HCCXY_L3_clean_bin.106.orig.fa_	OEZ008529	no	LMSG_G000010948.1	OES073686	NRPS	NRPS	Lead-Zinc	28.06	China: Guangdong, Fankou
L180404P-1-LD180328-0014_HL57HCCXY_L3_clean_bin.109.orig.fa_	OEZ008529	yes	LMSG_G000010476.1	OES073686	terpene	Terpene	Lead-Zinc	19.54	China: Guangdong, Fankou
L180404P-1-LD180328-0014_HL57HCCXY_L3_clean_bin.109.orig.fa_	OEZ008529	yes	LMSG_G000010476.1	OES073686	terpene	Terpene	Lead-Zinc	23.04	China: Guangdong, Fankou
L180404P-1-LD180328-0014_HL57HCCXY_L3_clean_bin.109.orig.fa_	OEZ008529	yes	LMSG_G000010476.1	OES073686	arylpolyene.hserlactone	Others	Lead-Zinc	26.89	China: Guangdong, Fankou
L180404P-1-LD180328-0014_HL57HCCXY_L3_clean_bin.11.strict.fa	OEZ008529	yes	LMSG_G000009201.1	OES073686	terpene	Terpene	Lead-Zinc	8.00	China: Guangdong, Fankou
L180404P-1-LD180328-0014_HL57HCCXY_L3_clean_bin.110.strict.f	OEZ008529	yes	LMSG_G000009105.1	OES073686	terpene	Terpene	Lead-Zinc	25.00	China: Guangdong, Fankou
L180404P-1-LD180328-0014_HL57HCCXY_L3_clean_bin.110.strict.f	OEZ008529	yes	LMSG_G000009105.1	OES073686	NRPS-like	NRPS	Lead-Zinc	27.41	China: Guangdong, Fankou
L180404P-1-LD180328-0014_HL57HCCXY_L3_clean_bin.110.strict.f	OEZ008529	yes	LMSG_G000009105.1	OES073686	bacteriocin	RiPPs	Lead-Zinc	5.97	China: Guangdong, Fankou
L180404P-1-LD180328-0014_HL57HCCXY_L3_clean_bin.111.orig.fa_	OEZ008529	yes	LMSG_G000004584.1	OES073686	other	Others	Lead-Zinc	40.72	China: Guangdong, Fankou
L180404P-1-LD180328-0014_HL57HCCXY_L3_clean_bin.111.orig.fa_	OEZ008529	yes	LMSG_G000004584.1	OES073686	NRPS-like	NRPS	Lead-Zinc	22.29	China: Guangdong, Fankou
L180404P-1-LD180328-0014_HL57HCCXY_L3_clean_bin.112.orig.fa_	OEZ008529	yes	LMSG_G000009990.1	OES073686	arylpolyene	Others	Lead-Zinc	8.35	China: Guangdong, Fankou
L180404P-1-LD180328-0014_HL57HCCXY_L3_clean_bin.112.orig.fa_	OEZ008529	yes	LMSG_G000009990.1	OES073686	terpene	Terpene	Lead-Zinc	12.97	China: Guangdong, Fankou
L180404P-1-LD180328-0014_HL57HCCXY_L3_clean_bin.112.orig.fa_	OEZ008529	yes	LMSG_G000009990.1	OES073686	arylpolyene	Others	Lead-Zinc	9.04	China: Guangdong, Fankou
L180404P-1-LD180328-0014_HL57HCCXY_L3_clean_bin.119.strict.f	OEZ008529	yes	LMSG_G000004585.1	OES073686	terpene	Terpene	Lead-Zinc	17.85	China: Guangdong, Fankou
L180404P-1-LD180328-0014_HL57HCCXY_L3_clean_bin.119.strict.f	OEZ008529	yes	LMSG_G000004585.1	OES073686	bacteriocin	RiPPs	Lead-Zinc	11.85	China: Guangdong, Fankou
L180404P-1-LD180328-0014_HL57HCCXY_L3_clean_bin.119.strict.f	OEZ008529	yes	LMSG_G000004585.1	OES073686	terpene	Terpene	Lead-Zinc	14.87	China: Guangdong, Fankou
L180404P-1-LD180328-0014_HL57HCCXY_L3_clean_bin.12.orig.fa_k	OEZ008529	yes	LMSG_G000007761.1	OES073686	terpene	Terpene	Lead-Zinc	7.54	China: Guangdong, Fankou
L180404P-1-LD180328-0014_HL57HCCXY_L3_clean_bin.12.orig.fa_k	OEZ008529	yes	LMSG_G000007761.1	OES073686	T3PKS	PKSother	Lead-Zinc	7.67	China: Guangdong, Fankou
L180404P-1-LD180328-0014_HL57HCCXY_L3_clean_bin.12.orig.fa_k	OEZ008529	yes	LMSG_G000007761.1	OES073686	NRPS-like	NRPS	Lead-Zinc	9.75	China: Guangdong, Fankou
L180404P-1-LD180328-0014_HL57HCCXY_L3_clean_bin.120.orig.fa_	OEZ008529	yes	LMSG_G000011059.1	OES073686	NRPS-like	NRPS	Lead-Zinc	22.13	China: Guangdong, Fankou
L180404P-1-LD180328-0014_HL57HCCXY_L3_clean_bin.120.orig.fa_	OEZ008529	yes	LMSG_G000011059.1	OES073686	terpene	Terpene	Lead-Zinc	23.23	China: Guangdong, Fankou
L180404P-1-LD180328-0014_HL57HCCXY_L3_clean_bin.120.orig.fa_	OEZ008529	yes	LMSG_G000011059.1	OES073686	NRPS-like	NRPS	Lead-Zinc	41.96	China: Guangdong, Fankou
L180404P-1-LD180328-0014_HL57HCCXY_L3_clean_bin.120.orig.fa_	OEZ008529	yes	LMSG_G000011059.1	OES073686	bacteriocin	RiPPs	Lead-Zinc	10.83	China: Guangdong, Fankou
L180404P-1-LD180328-0014_HL57HCCXY_L3_clean_bin.121.orig.fa_	OEZ008529	yes	LMSG_G000005896.1	OES073686	bacteriocin	RiPPs	Lead-Zinc	9.70	China: Guangdong, Fankou
L180404P-1-LD180328-0014_HL57HCCXY_L3_clean_bin.121.orig.fa_	OEZ008529	yes	LMSG_G000005896.1	OES073686	terpene	Terpene	Lead-Zinc	21.32	China: Guangdong, Fankou
L180404P-1-LD180328-0014_HL57HCCXY_L3_clean_bin.121.orig.fa_	OEZ008529	yes	LMSG_G000005896.1	OES073686	arylpolyene	Others	Lead-Zinc	18.65	China: Guangdong, Fankou
L180404P-1-LD180328-0014_HL57HCCXY_L3_clean_bin.121.orig.fa_	OEZ008529	yes	LMSG_G000005896.1	OES073686	T1PKS	PKSI	Lead-Zinc	26.24	China: Guangdong, Fankou
L180404P-1-LD180328-0014_HL57HCCXY_L3_clean_bin.121.orig.fa_	OEZ008529	yes	LMSG_G000005896.1	OES073686	hserlactone	Others	Lead-Zinc	11.56	China: Guangdong, Fankou
L180404P-1-LD180328-0014_HL57HCCXY_L3_clean_bin.122.orig.fa_	OEZ008529	yes	LMSG_G000004586.1	OES073686	bacteriocin	RiPPs	Lead-Zinc	10.80	China: Guangdong, Fankou
L180404P-1-LD180328-0014_HL57HCCXY_L3_clean_bin.122.orig.fa_	OEZ008529	yes	LMSG_G000004586.1	OES073686	bacteriocin	RiPPs	Lead-Zinc	7.22	China: Guangdong, Fankou
L180404P-1-LD180328-0014_HL57HCCXY_L3_clean_bin.122.orig.fa_	OEZ008529	yes	LMSG_G000004586.1	OES073686	terpene	Terpene	Lead-Zinc	22.54	China: Guangdong, Fankou
L180404P-1-LD180328-0014_HL57HCCXY_L3_clean_bin.18.orig.fa_k	OEZ008529	yes	LMSG_G000008370.1	OES073686	terpene	Terpene	Lead-Zinc	20.58	China: Guangdong, Fankou
L180404P-1-LD180328-0014_HL57HCCXY_L3_clean_bin.21.orig.fa_k	OEZ008529	yes	LMSG_G000008721.1	OES073686	LAP	RiPPs	Lead-Zinc	16.92	China: Guangdong, Fankou
L180404P-1-LD180328-0014_HL57HCCXY_L3_clean_bin.23.orig.fa_k	OEZ008529	yes	LMSG_G000004587.1	OES073686	terpene	Terpene	Lead-Zinc	29.02	China: Guangdong, Fankou
L180404P-1-LD180328-0014_HL57HCCXY_L3_clean_bin.23.orig.fa_k	OEZ008529	yes	LMSG_G000004587.1	OES073686	NRPS-like	NRPS	Lead-Zinc	29.88	China: Guangdong, Fankou
L180404P-1-LD180328-0014_HL57HCCXY_L3_clean_bin.23.orig.fa_k	OEZ008529	yes	LMSG_G000004587.1	OES073686	NRPS-like	NRPS	Lead-Zinc	39.38	China: Guangdong, Fankou
L180404P-1-LD180328-0014_HL57HCCXY_L3_clean_bin.23.orig.fa_k	OEZ008529	yes	LMSG_G000004587.1	OES073686	lanthipeptide	RiPPs	Lead-Zinc	21.13	China: Guangdong, Fankou
L180404P-1-LD180328-0014_HL57HCCXY_L3_clean_bin.23.orig.fa_k	OEZ008529	yes	LMSG_G000004587.1	OES073686	NRPS-like	NRPS	Lead-Zinc	18.56	China: Guangdong, Fankou
L180404P-1-LD180328-0014_HL57HCCXY_L3_clean_bin.23.orig.fa_k	OEZ008529	yes	LMSG_G000004587.1	OES073686	T3PKS	PKSother	Lead-Zinc	41.09	China: Guangdong, Fankou
L180404P-1-LD180328-0014_HL57HCCXY_L3_clean_bin.23.orig.fa_k	OEZ008529	yes	LMSG_G000004587.1	OES073686	bacteriocin	RiPPs	Lead-Zinc	12.00	China: Guangdong, Fankou
L180404P-1-LD180328-0014_HL57HCCXY_L3_clean_bin.24.orig.fa_k	OEZ008529	yes	LMSG_G000005899.1	OES073686	betalactone	Others	Lead-Zinc	26.84	China: Guangdong, Fankou
L180404P-1-LD180328-0014_HL57HCCXY_L3_clean_bin.24.orig.fa_k	OEZ008529	yes	LMSG_G000005899.1	OES073686	bacteriocin	RiPPs	Lead-Zinc	8.27	China: Guangdong, Fankou
L180404P-1-LD180328-0014_HL57HCCXY_L3_clean_bin.24.orig.fa_k	OEZ008529	yes	LMSG_G000005899.1	OES073686	T1PKS	PKSI	Lead-Zinc	24.61	China: Guangdong, Fankou
L180404P-1-LD180328-0014_HL57HCCXY_L3_clean_bin.24.orig.fa_k	OEZ008529	yes	LMSG_G000005899.1	OES073686	betalactone	Others	Lead-Zinc	22.35	China: Guangdong, Fankou
L180404P-1-LD180328-0014_HL57HCCXY_L3_clean_bin.24.orig.fa_k	OEZ008529	yes	LMSG_G000005899.1	OES073686	terpene	Terpene	Lead-Zinc	15.16	China: Guangdong, Fankou
L180404P-1-LD180328-0014_HL57HCCXY_L3_clean_bin.24.orig.fa_k	OEZ008529	yes	LMSG_G000005899.1	OES073686	betalactone	Others	Lead-Zinc	23.27	China: Guangdong, Fankou
L180404P-1-LD180328-0014_HL57HCCXY_L3_clean_bin.24.orig.fa_k	OEZ008529	yes	LMSG_G000005899.1	OES073686	transAT-PKS-like	PKSother	Lead-Zinc	46.27	China: Guangdong, Fankou
L180404P-1-LD180328-0014_HL57HCCXY_L3_clean_bin.25.orig.fa_k	OEZ008529	yes	LMSG_G000009132.1	OES073686	bacteriocin	RiPPs	Lead-Zinc	10.95	China: Guangdong, Fankou
L180404P-1-LD180328-0014_HL57HCCXY_L3_clean_bin.25.orig.fa_k	OEZ008529	yes	LMSG_G000009132.1	OES073686	terpene	Terpene	Lead-Zinc	25.21	China: Guangdong, Fankou
L180404P-1-LD180328-0014_HL57HCCXY_L3_clean_bin.25.orig.fa_k	OEZ008529	yes	LMSG_G000009132.1	OES073686	lassopeptide	RiPPs	Lead-Zinc	14.70	China: Guangdong, Fankou
L180404P-1-LD180328-0014_HL57HCCXY_L3_clean_bin.25.orig.fa_k	OEZ008529	yes	LMSG_G000009132.1	OES073686	betalactone	Others	Lead-Zinc	27.10	China: Guangdong, Fankou
L180404P-1-LD180328-0014_HL57HCCXY_L3_clean_bin.25.orig.fa_k	OEZ008529	yes	LMSG_G000009132.1	OES073686	NRPS-like	NRPS	Lead-Zinc	37.30	China: Guangdong, Fankou
L180404P-1-LD180328-0014_HL57HCCXY_L3_clean_bin.25.orig.fa_k	OEZ008529	yes	LMSG_G000009132.1	OES073686	T3PKS	PKSother	Lead-Zinc	41.11	China: Guangdong, Fankou
L180404P-1-LD180328-0014_HL57HCCXY_L3_clean_bin.27.strict.fa	OEZ008529	yes	LMSG_G000007963.1	OES073686	betalactone	Others	Lead-Zinc	23.10	China: Guangdong, Fankou
L180404P-1-LD180328-0014_HL57HCCXY_L3_clean_bin.27.strict.fa	OEZ008529	yes	LMSG_G000007963.1	OES073686	terpene	Terpene	Lead-Zinc	26.80	China: Guangdong, Fankou
L180404P-1-LD180328-0014_HL57HCCXY_L3_clean_bin.30.strict.fa	OEZ008529	yes	LMSG_G000004588.1	OES073686	terpene	Terpene	Lead-Zinc	21.74	China: Guangdong, Fankou
L180404P-1-LD180328-0014_HL57HCCXY_L3_clean_bin.33.permissiv	OEZ008529	yes	LMSG_G000010004.1	OES073686	terpene.arylpolyene	Others	Lead-Zinc	41.10	China: Guangdong, Fankou
L180404P-1-LD180328-0014_HL57HCCXY_L3_clean_bin.33.permissiv	OEZ008529	yes	LMSG_G000010004.1	OES073686	ectoine	Others	Lead-Zinc	7.63	China: Guangdong, Fankou
L180404P-1-LD180328-0014_HL57HCCXY_L3_clean_bin.33.permissiv	OEZ008529	yes	LMSG_G000010004.1	OES073686	terpene	Terpene	Lead-Zinc	16.95	China: Guangdong, Fankou
L180404P-1-LD180328-0014_HL57HCCXY_L3_clean_bin.34.orig.fa_k	OEZ008529	yes	LMSG_G000007376.1	OES073686	bacteriocin	RiPPs	Lead-Zinc	11.97	China: Guangdong, Fankou
L180404P-1-LD180328-0014_HL57HCCXY_L3_clean_bin.37.orig.fa_k	OEZ008529	yes	LMSG_G000009294.1	OES073686	terpene	Terpene	Lead-Zinc	22.06	China: Guangdong, Fankou
L180404P-1-LD180328-0014_HL57HCCXY_L3_clean_bin.37.orig.fa_k	OEZ008529	yes	LMSG_G000009294.1	OES073686	terpene	Terpene	Lead-Zinc	12.46	China: Guangdong, Fankou
L180404P-1-LD180328-0014_HL57HCCXY_L3_clean_bin.42.strict.fa	OEZ008529	yes	LMSG_G000005901.1	OES073686	NRPS-like	NRPS	Lead-Zinc	11.69	China: Guangdong, Fankou
L180404P-1-LD180328-0014_HL57HCCXY_L3_clean_bin.44.strict.fa	OEZ008529	yes	LMSG_G000008302.1	OES073686	terpene	Terpene	Lead-Zinc	22.49	China: Guangdong, Fankou
L180404P-1-LD180328-0014_HL57HCCXY_L3_clean_bin.45.strict.fa	OEZ008529	yes	LMSG_G000007998.1	OES073686	bacteriocin	RiPPs	Lead-Zinc	10.32	China: Guangdong, Fankou
L180404P-1-LD180328-0014_HL57HCCXY_L3_clean_bin.45.strict.fa	OEZ008529	yes	LMSG_G000007998.1	OES073686	TfuA-related.bacteriocin	RiPPs	Lead-Zinc	8.09	China: Guangdong, Fankou
L180404P-1-LD180328-0014_HL57HCCXY_L3_clean_bin.48.orig.fa_k	OEZ008529	yes	LMSG_G000007840.1	OES073686	NRPS-like	NRPS	Lead-Zinc	36.84	China: Guangdong, Fankou
L180404P-1-LD180328-0014_HL57HCCXY_L3_clean_bin.48.orig.fa_k	OEZ008529	yes	LMSG_G000007840.1	OES073686	terpene	Terpene	Lead-Zinc	13.82	China: Guangdong, Fankou
L180404P-1-LD180328-0014_HL57HCCXY_L3_clean_bin.48.orig.fa_k	OEZ008529	yes	LMSG_G000007840.1	OES073686	T3PKS	PKSother	Lead-Zinc	25.13	China: Guangdong, Fankou
L180404P-1-LD180328-0014_HL57HCCXY_L3_clean_bin.48.orig.fa_k	OEZ008529	yes	LMSG_G000007840.1	OES073686	terpene	Terpene	Lead-Zinc	12.61	China: Guangdong, Fankou
L180404P-1-LD180328-0014_HL57HCCXY_L3_clean_bin.48.orig.fa_k	OEZ008529	yes	LMSG_G000007840.1	OES073686	NRPS-like	NRPS	Lead-Zinc	39.80	China: Guangdong, Fankou
L180404P-1-LD180328-0014_HL57HCCXY_L3_clean_bin.5.strict.fa_	OEZ008529	yes	LMSG_G000009912.1	OES073686	hserlactone	Others	Lead-Zinc	6.82	China: Guangdong, Fankou
L180404P-1-LD180328-0014_HL57HCCXY_L3_clean_bin.53.orig.fa_k	OEZ008529	yes	LMSG_G000009113.1	OES073686	terpene	Terpene	Lead-Zinc	21.25	China: Guangdong, Fankou
L180404P-1-LD180328-0014_HL57HCCXY_L3_clean_bin.53.orig.fa_k	OEZ008529	yes	LMSG_G000009113.1	OES073686	terpene	Terpene	Lead-Zinc	17.66	China: Guangdong, Fankou
L180404P-1-LD180328-0014_HL57HCCXY_L3_clean_bin.53.orig.fa_k	OEZ008529	yes	LMSG_G000009113.1	OES073686	NRPS-like	NRPS	Lead-Zinc	27.77	China: Guangdong, Fankou
L180404P-1-LD180328-0014_HL57HCCXY_L3_clean_bin.54.orig.fa_k	OEZ008529	yes	LMSG_G000008423.1	OES073686	terpene	Terpene	Lead-Zinc	10.47	China: Guangdong, Fankou
L180404P-1-LD180328-0014_HL57HCCXY_L3_clean_bin.57.permissiv	OEZ008529	yes	LMSG_G000005903.1	OES073686	NRPS-like	NRPS	Lead-Zinc	28.89	China: Guangdong, Fankou
L180404P-1-LD180328-0014_HL57HCCXY_L3_clean_bin.58.strict.fa	OEZ008529	yes	LMSG_G000010070.1	OES073686	arylpolyene	Others	Lead-Zinc	41.28	China: Guangdong, Fankou
L180404P-1-LD180328-0014_HL57HCCXY_L3_clean_bin.58.strict.fa	OEZ008529	yes	LMSG_G000010070.1	OES073686	NRPS-like	NRPS	Lead-Zinc	30.01	China: Guangdong, Fankou
L180404P-1-LD180328-0014_HL57HCCXY_L3_clean_bin.58.strict.fa	OEZ008529	yes	LMSG_G000010070.1	OES073686	arylpolyene	Others	Lead-Zinc	30.15	China: Guangdong, Fankou
L180404P-1-LD180328-0014_HL57HCCXY_L3_clean_bin.58.strict.fa	OEZ008529	yes	LMSG_G000010070.1	OES073686	terpene	Terpene	Lead-Zinc	14.73	China: Guangdong, Fankou
L180404P-1-LD180328-0014_HL57HCCXY_L3_clean_bin.59.orig.fa_k	OEZ008529	yes	LMSG_G000005904.1	OES073686	bacteriocin	RiPPs	Lead-Zinc	6.01	China: Guangdong, Fankou
L180404P-1-LD180328-0014_HL57HCCXY_L3_clean_bin.59.orig.fa_k	OEZ008529	yes	LMSG_G000005904.1	OES073686	terpene	Terpene	Lead-Zinc	14.44	China: Guangdong, Fankou
L180404P-1-LD180328-0014_HL57HCCXY_L3_clean_bin.59.orig.fa_k	OEZ008529	yes	LMSG_G000005904.1	OES073686	T1PKS	PKSI	Lead-Zinc	8.13	China: Guangdong, Fankou
L180404P-1-LD180328-0014_HL57HCCXY_L3_clean_bin.59.orig.fa_k	OEZ008529	yes	LMSG_G000005904.1	OES073686	bacteriocin	RiPPs	Lead-Zinc	5.67	China: Guangdong, Fankou
L180404P-1-LD180328-0014_HL57HCCXY_L3_clean_bin.60.orig.fa_k	OEZ008529	yes	LMSG_G000008467.1	OES073686	betalactone	Others	Lead-Zinc	17.84	China: Guangdong, Fankou
L180404P-1-LD180328-0014_HL57HCCXY_L3_clean_bin.60.orig.fa_k	OEZ008529	yes	LMSG_G000008467.1	OES073686	arylpolyene	Others	Lead-Zinc	41.18	China: Guangdong, Fankou
L180404P-1-LD180328-0014_HL57HCCXY_L3_clean_bin.60.orig.fa_k	OEZ008529	yes	LMSG_G000008467.1	OES073686	betalactone	Others	Lead-Zinc	25.89	China: Guangdong, Fankou
L180404P-1-LD180328-0014_HL57HCCXY_L3_clean_bin.60.orig.fa_k	OEZ008529	yes	LMSG_G000008467.1	OES073686	terpene	Terpene	Lead-Zinc	26.17	China: Guangdong, Fankou
L180404P-1-LD180328-0014_HL57HCCXY_L3_clean_bin.63.orig.fa_k	OEZ008529	yes	LMSG_G000009966.1	OES073686	NRPS-like	NRPS	Lead-Zinc	12.02	China: Guangdong, Fankou
L180404P-1-LD180328-0014_HL57HCCXY_L3_clean_bin.63.orig.fa_k	OEZ008529	yes	LMSG_G000009966.1	OES073686	terpene	Terpene	Lead-Zinc	7.41	China: Guangdong, Fankou
L180404P-1-LD180328-0014_HL57HCCXY_L3_clean_bin.63.orig.fa_k	OEZ008529	yes	LMSG_G000009966.1	OES073686	T3PKS	PKSother	Lead-Zinc	15.52	China: Guangdong, Fankou
L180404P-1-LD180328-0014_HL57HCCXY_L3_clean_bin.63.orig.fa_k	OEZ008529	yes	LMSG_G000009966.1	OES073686	terpene	Terpene	Lead-Zinc	8.74	China: Guangdong, Fankou
L180404P-1-LD180328-0014_HL57HCCXY_L3_clean_bin.65.orig.fa_k	OEZ008529	yes	LMSG_G000005906.1	OES073686	hserlactone	Others	Lead-Zinc	20.72	China: Guangdong, Fankou
L180404P-1-LD180328-0014_HL57HCCXY_L3_clean_bin.65.orig.fa_k	OEZ008529	yes	LMSG_G000005906.1	OES073686	terpene.arylpolyene	Others	Lead-Zinc	42.19	China: Guangdong, Fankou
L180404P-1-LD180328-0014_HL57HCCXY_L3_clean_bin.65.orig.fa_k	OEZ008529	yes	LMSG_G000005906.1	OES073686	terpene	Terpene	Lead-Zinc	20.79	China: Guangdong, Fankou
L180404P-1-LD180328-0014_HL57HCCXY_L3_clean_bin.7.orig.fa_k1	OEZ008529	yes	LMSG_G000009126.1	OES073686	NRPS-like	NRPS	Lead-Zinc	25.68	China: Guangdong, Fankou
L180404P-1-LD180328-0014_HL57HCCXY_L3_clean_bin.7.orig.fa_k1	OEZ008529	yes	LMSG_G000009126.1	OES073686	terpene	Terpene	Lead-Zinc	25.07	China: Guangdong, Fankou
L180404P-1-LD180328-0014_HL57HCCXY_L3_clean_bin.7.orig.fa_k1	OEZ008529	yes	LMSG_G000009126.1	OES073686	bacteriocin	RiPPs	Lead-Zinc	10.87	China: Guangdong, Fankou
L180404P-1-LD180328-0014_HL57HCCXY_L3_clean_bin.71.strict.fa	OEZ008529	yes	LMSG_G000009103.1	OES073686	terpene	Terpene	Lead-Zinc	25.00	China: Guangdong, Fankou
L180404P-1-LD180328-0014_HL57HCCXY_L3_clean_bin.71.strict.fa	OEZ008529	yes	LMSG_G000009103.1	OES073686	T3PKS.NRPS-like	PKS-NRP_Hybrids	Lead-Zinc	68.29	China: Guangdong, Fankou
L180404P-1-LD180328-0014_HL57HCCXY_L3_clean_bin.71.strict.fa	OEZ008529	yes	LMSG_G000009103.1	OES073686	NRPS-like	NRPS	Lead-Zinc	42.48	China: Guangdong, Fankou
L180404P-1-LD180328-0014_HL57HCCXY_L3_clean_bin.71.strict.fa	OEZ008529	yes	LMSG_G000009103.1	OES073686	bacteriocin	RiPPs	Lead-Zinc	10.92	China: Guangdong, Fankou
L180404P-1-LD180328-0014_HL57HCCXY_L3_clean_bin.73.orig.fa_k	OEZ008529	yes	LMSG_G000007856.1	OES073686	NRPS-like	NRPS	Lead-Zinc	5.59	China: Guangdong, Fankou
L180404P-1-LD180328-0014_HL57HCCXY_L3_clean_bin.73.orig.fa_k	OEZ008529	yes	LMSG_G000007856.1	OES073686	terpene	Terpene	Lead-Zinc	9.46	China: Guangdong, Fankou
L180404P-1-LD180328-0014_HL57HCCXY_L3_clean_bin.73.orig.fa_k	OEZ008529	yes	LMSG_G000007856.1	OES073686	T3PKS	PKSother	Lead-Zinc	9.02	China: Guangdong, Fankou
L180404P-1-LD180328-0014_HL57HCCXY_L3_clean_bin.77.orig.fa_k	OEZ008529	yes	LMSG_G000008064.1	OES073686	terpene	Terpene	Lead-Zinc	21.39	China: Guangdong, Fankou
L180404P-1-LD180328-0014_HL57HCCXY_L3_clean_bin.77.orig.fa_k	OEZ008529	yes	LMSG_G000008064.1	OES073686	terpene	Terpene	Lead-Zinc	19.33	China: Guangdong, Fankou
L180404P-1-LD180328-0014_HL57HCCXY_L3_clean_bin.81.strict.fa	OEZ008529	yes	LMSG_G000004589.1	OES073686	T3PKS.terpene	Others	Lead-Zinc	43.45	China: Guangdong, Fankou
L180404P-1-LD180328-0014_HL57HCCXY_L3_clean_bin.81.strict.fa	OEZ008529	yes	LMSG_G000004589.1	OES073686	terpene	Terpene	Lead-Zinc	21.72	China: Guangdong, Fankou
L180404P-1-LD180328-0014_HL57HCCXY_L3_clean_bin.82.orig.fa_k	OEZ008529	yes	LMSG_G000009093.1	OES073686	betalactone	Others	Lead-Zinc	25.94	China: Guangdong, Fankou
L180404P-1-LD180328-0014_HL57HCCXY_L3_clean_bin.82.orig.fa_k	OEZ008529	yes	LMSG_G000009093.1	OES073686	terpene	Terpene	Lead-Zinc	22.04	China: Guangdong, Fankou
L180404P-1-LD180328-0014_HL57HCCXY_L3_clean_bin.82.orig.fa_k	OEZ008529	yes	LMSG_G000009093.1	OES073686	betalactone.bacteriocin	Others	Lead-Zinc	29.69	China: Guangdong, Fankou
L180404P-1-LD180328-0014_HL57HCCXY_L3_clean_bin.82.orig.fa_k	OEZ008529	yes	LMSG_G000009093.1	OES073686	thiopeptide	RiPPs	Lead-Zinc	11.30	China: Guangdong, Fankou
L180404P-1-LD180328-0014_HL57HCCXY_L3_clean_bin.82.orig.fa_k	OEZ008529	yes	LMSG_G000009093.1	OES073686	betalactone	Others	Lead-Zinc	22.43	China: Guangdong, Fankou
L180404P-1-LD180328-0014_HL57HCCXY_L3_clean_bin.87.orig.fa_k	OEZ008529	yes	LMSG_G000009134.1	OES073686	NRPS-like	NRPS	Lead-Zinc	44.44	China: Guangdong, Fankou
L180404P-1-LD180328-0014_HL57HCCXY_L3_clean_bin.87.orig.fa_k	OEZ008529	yes	LMSG_G000009134.1	OES073686	terpene	Terpene	Lead-Zinc	25.21	China: Guangdong, Fankou
L180404P-1-LD180328-0014_HL57HCCXY_L3_clean_bin.87.orig.fa_k	OEZ008529	yes	LMSG_G000009134.1	OES073686	bacteriocin	RiPPs	Lead-Zinc	10.85	China: Guangdong, Fankou
L180404P-1-LD180328-0014_HL57HCCXY_L3_clean_bin.87.orig.fa_k	OEZ008529	yes	LMSG_G000009134.1	OES073686	lassopeptide	RiPPs	Lead-Zinc	22.39	China: Guangdong, Fankou
L180404P-1-LD180328-0014_HL57HCCXY_L3_clean_bin.87.orig.fa_k	OEZ008529	yes	LMSG_G000009134.1	OES073686	betalactone	Others	Lead-Zinc	26.18	China: Guangdong, Fankou
L180404P-1-LD180328-0014_HL57HCCXY_L3_clean_bin.87.orig.fa_k	OEZ008529	yes	LMSG_G000009134.1	OES073686	bacteriocin	RiPPs	Lead-Zinc	9.11	China: Guangdong, Fankou
L180404P-1-LD180328-0014_HL57HCCXY_L3_clean_bin.87.orig.fa_k	OEZ008529	yes	LMSG_G000009134.1	OES073686	bacteriocin	RiPPs	Lead-Zinc	10.86	China: Guangdong, Fankou
L180404P-1-LD180328-0014_HL57HCCXY_L3_clean_bin.88.orig.fa_k	OEZ008529	yes	LMSG_G000005911.1	OES073686	T1PKS.hglE-KS	PKSother	Lead-Zinc	16.62	China: Guangdong, Fankou
L180404P-1-LD180328-0014_HL57HCCXY_L3_clean_bin.88.orig.fa_k	OEZ008529	yes	LMSG_G000005911.1	OES073686	other	Others	Lead-Zinc	22.96	China: Guangdong, Fankou
L180404P-1-LD180328-0014_HL57HCCXY_L3_clean_bin.9.orig.fa_k1	OEZ008529	yes	LMSG_G000008540.1	OES073686	terpene	Terpene	Lead-Zinc	21.60	China: Guangdong, Fankou
L180404P-1-LD180328-0014_HL57HCCXY_L3_clean_bin.93.orig.fa_k	OEZ008529	yes	LMSG_G000010602.1	OES073686	terpene	Terpene	Lead-Zinc	13.89	China: Guangdong, Fankou
L180404P-1-LD180328-0014_HL57HCCXY_L3_clean_bin.94.orig.fa_k	OEZ008529	yes	LMSG_G000010022.1	OES073686	terpene	Terpene	Lead-Zinc	15.06	China: Guangdong, Fankou
L180404P-1-LD180328-0014_HL57HCCXY_L3_clean_bin.94.orig.fa_k	OEZ008529	yes	LMSG_G000010022.1	OES073686	terpene	Terpene	Lead-Zinc	21.87	China: Guangdong, Fankou
L180404P-1-LD180328-0014_HL57HCCXY_L3_clean_bin.95.orig.fa_k	OEZ008529	yes	LMSG_G000010727.1	OES073686	T1PKS.NRPS-like	PKS-NRP_Hybrids	Lead-Zinc	5.67	China: Guangdong, Fankou
L180404P-1-LD180328-0014_HL57HCCXY_L3_clean_bin.95.orig.fa_k	OEZ008529	yes	LMSG_G000010727.1	OES073686	bacteriocin	RiPPs	Lead-Zinc	8.89	China: Guangdong, Fankou
L180404P-1-LD180328-0014_HL57HCCXY_L3_clean_bin.95.orig.fa_k	OEZ008529	yes	LMSG_G000010727.1	OES073686	NRPS	NRPS	Lead-Zinc	9.07	China: Guangdong, Fankou
L180404P-1-LD180328-0014_HL57HCCXY_L3_clean_bin.95.orig.fa_k	OEZ008529	yes	LMSG_G000010727.1	OES073686	NRPS	NRPS	Lead-Zinc	8.34	China: Guangdong, Fankou
L180404P-1-LD180328-0014_HL57HCCXY_L3_clean_bin.95.orig.fa_k	OEZ008529	yes	LMSG_G000010727.1	OES073686	terpene	Terpene	Lead-Zinc	9.57	China: Guangdong, Fankou
L180404P-1-LD180328-0014_HL57HCCXY_L3_clean_bin.96.strict.fa	OEZ008529	yes	LMSG_G000008534.1	OES073686	NRPS	NRPS	Lead-Zinc	46.99	China: Guangdong, Fankou
L180404P-1-LD180328-0014_HL57HCCXY_L3_clean_bin.96.strict.fa	OEZ008529	yes	LMSG_G000008534.1	OES073686	other	Others	Lead-Zinc	33.29	China: Guangdong, Fankou
L180404P-1-LD180328-0014_HL57HCCXY_L3_clean_bin.96.strict.fa	OEZ008529	yes	LMSG_G000008534.1	OES073686	terpene	Terpene	Lead-Zinc	26.22	China: Guangdong, Fankou
L180404P-1-LD180328-0014_HL57HCCXY_L3_clean_bin.96.strict.fa	OEZ008529	yes	LMSG_G000008534.1	OES073686	NRPS-like	NRPS	Lead-Zinc	27.22	China: Guangdong, Fankou
L180404P-1-LD180402-0001_HL57HCCXY_L3_clean_bin.10.strict.fa	OEZ008529	yes	LMSG_G000008145.1	OES073691	terpene	Terpene	Polymetallic	11.70	China: Guizhou, Jiuan
L180404P-1-LD180402-0001_HL57HCCXY_L3_clean_bin.11.orig.fa_k	OEZ008529	yes	LMSG_G000006442.1	OES073691	nucleoside	Others	Polymetallic	15.50	China: Guizhou, Jiuan
L180404P-1-LD180402-0001_HL57HCCXY_L3_clean_bin.15.orig.fa_k	OEZ008529	yes	LMSG_G000010058.1	OES073691	NRPS-like	NRPS	Polymetallic	43.25	China: Guizhou, Jiuan
L180404P-1-LD180402-0001_HL57HCCXY_L3_clean_bin.15.orig.fa_k	OEZ008529	yes	LMSG_G000010058.1	OES073691	terpene	Terpene	Polymetallic	11.49	China: Guizhou, Jiuan

L180404P-1-LD180402-0001_HL57HCCXY_L3_clean_bin.15.orig.fa_k	OEZ008529	yes	LMSG_G000010058.1	OES073691	terpene	Terpene	Polymetallic	12.75	China: Guizhou, Jiuan
L180404P-1-LD180402-0001_HL57HCCXY_L3_clean_bin.15.orig.fa_k	OEZ008529	yes	LMSG_G000010058.1	OES073691	T3PKS	PKSother	Polymetallic	9.57	China: Guizhou, Jiuan
L180404P-1-LD180402-0001_HL57HCCXY_L3_clean_bin.15.orig.fa_k	OEZ008529	yes	LMSG_G000010058.1	OES073691	T3PKS	PKSother	Polymetallic	15.39	China: Guizhou, Jiuan
L180404P-1-LD180402-0001_HL57HCCXY_L3_clean_bin.15.orig.fa_k	OEZ008529	yes	LMSG_G000010058.1	OES073691	ladderane	Others	Polymetallic	14.08	China: Guizhou, Jiuan
L180404P-1-LD180402-0001_HL57HCCXY_L3_clean_bin.15.orig.fa_k	OEZ008529	yes	LMSG_G000010058.1	OES073691	bacteriocin	RiPPs	Polymetallic	10.68	China: Guizhou, Jiuan
L180404P-1-LD180402-0001_HL57HCCXY_L3_clean_bin.15.orig.fa_k	OEZ008529	yes	LMSG_G000010058.1	OES073691	arylpolyene	Others	Polymetallic	15.25	China: Guizhou, Jiuan
L180404P-1-LD180402-0001_HL57HCCXY_L3_clean_bin.16.orig.fa_k	OEZ008529	yes	LMSG_G000011046.1	OES073691	terpene	Terpene	Polymetallic	20.85	China: Guizhou, Jiuan
L180404P-1-LD180402-0001_HL57HCCXY_L3_clean_bin.16.orig.fa_k	OEZ008529	yes	LMSG_G000011046.1	OES073691	arylpolyene	Others	Polymetallic	43.60	China: Guizhou, Jiuan
L180404P-1-LD180402-0001_HL57HCCXY_L3_clean_bin.16.orig.fa_k	OEZ008529	yes	LMSG_G000011046.1	OES073691	resorcinol	Others	Polymetallic	20.96	China: Guizhou, Jiuan
L180404P-1-LD180402-0001_HL57HCCXY_L3_clean_bin.16.orig.fa_k	OEZ008529	yes	LMSG_G000011046.1	OES073691	NRPS-like	NRPS	Polymetallic	9.82	China: Guizhou, Jiuan
L180404P-1-LD180402-0001_HL57HCCXY_L3_clean_bin.16.orig.fa_k	OEZ008529	yes	LMSG_G000011046.1	OES073691	terpene	Terpene	Polymetallic	21.98	China: Guizhou, Jiuan
L180404P-1-LD180402-0001_HL57HCCXY_L3_clean_bin.16.orig.fa_k	OEZ008529	yes	LMSG_G000011046.1	OES073691	bacteriocin	RiPPs	Polymetallic	7.21	China: Guizhou, Jiuan
L180404P-1-LD180402-0001_HL57HCCXY_L3_clean_bin.19.orig.fa_k	OEZ008529	yes	LMSG_G000008163.1	OES073691	terpene	Terpene	Polymetallic	23.38	China: Guizhou, Jiuan
L180404P-1-LD180402-0001_HL57HCCXY_L3_clean_bin.20.orig.fa_k	OEZ008529	yes	LMSG_G000008127.1	OES073691	terpene	Terpene	Polymetallic	20.48	China: Guizhou, Jiuan
L180404P-1-LD180402-0001_HL57HCCXY_L3_clean_bin.22.orig.fa_k	OEZ008529	yes	LMSG_G000010288.1	OES073691	terpene	Terpene	Polymetallic	10.65	China: Guizhou, Jiuan
L180404P-1-LD180402-0001_HL57HCCXY_L3_clean_bin.23.orig.fa_k	OEZ008529	no	LMSG_G000011023.1	OES073691	hserlactone	Others	Polymetallic	12.31	China: Guizhou, Jiuan
L180404P-1-LD180402-0001_HL57HCCXY_L3_clean_bin.23.orig.fa_k	OEZ008529	no	LMSG_G000011023.1	OES073691	NRPS	NRPS	Polymetallic	24.27	China: Guizhou, Jiuan
L180404P-1-LD180402-0001_HL57HCCXY_L3_clean_bin.23.orig.fa_k	OEZ008529	no	LMSG_G000011023.1	OES073691	NAGGN	Others	Polymetallic	6.42	China: Guizhou, Jiuan
L180404P-1-LD180402-0001_HL57HCCXY_L3_clean_bin.23.orig.fa_k	OEZ008529	no	LMSG_G000011023.1	OES073691	bacteriocin	RiPPs	Polymetallic	10.86	China: Guizhou, Jiuan
L180404P-1-LD180402-0001_HL57HCCXY_L3_clean_bin.23.orig.fa_k	OEZ008529	no	LMSG_G000011023.1	OES073691	phenazine	Others	Polymetallic	9.62	China: Guizhou, Jiuan
L180404P-1-LD180402-0001_HL57HCCXY_L3_clean_bin.23.orig.fa_k	OEZ008529	no	LMSG_G000011023.1	OES073691	bacteriocin	RiPPs	Polymetallic	6.42	China: Guizhou, Jiuan
L180404P-1-LD180402-0001_HL57HCCXY_L3_clean_bin.23.orig.fa_k	OEZ008529	no	LMSG_G000011023.1	OES073691	NRPS	NRPS	Polymetallic	6.53	China: Guizhou, Jiuan
L180404P-1-LD180402-0001_HL57HCCXY_L3_clean_bin.23.orig.fa_k	OEZ008529	no	LMSG_G000011023.1	OES073691	hserlactone	Others	Polymetallic	17.03	China: Guizhou, Jiuan
L180404P-1-LD180402-0001_HL57HCCXY_L3_clean_bin.23.orig.fa_k	OEZ008529	no	LMSG_G000011023.1	OES073691	betalactone.NRPS-like	Others	Polymetallic	17.40	China: Guizhou, Jiuan
L180404P-1-LD180402-0001_HL57HCCXY_L3_clean_bin.24.orig.fa_k	OEZ008529	yes	LMSG_G000005912.1	OES073691	terpene	Terpene	Polymetallic	22.04	China: Guizhou, Jiuan
L180404P-1-LD180402-0001_HL57HCCXY_L3_clean_bin.24.orig.fa_k	OEZ008529	yes	LMSG_G000005912.1	OES073691	arylpolyene	Others	Polymetallic	41.10	China: Guizhou, Jiuan
L180404P-1-LD180402-0001_HL57HCCXY_L3_clean_bin.24.orig.fa_k	OEZ008529	yes	LMSG_G000005912.1	OES073691	arylpolyene	Others	Polymetallic	11.89	China: Guizhou, Jiuan
L180404P-1-LD180402-0001_HL57HCCXY_L3_clean_bin.24.orig.fa_k	OEZ008529	yes	LMSG_G000005912.1	OES073691	terpene	Terpene	Polymetallic	21.44	China: Guizhou, Jiuan
L180404P-1-LD180402-0001_HL57HCCXY_L3_clean_bin.24.orig.fa_k	OEZ008529	yes	LMSG_G000005912.1	OES073691	NRPS-like	NRPS	Polymetallic	31.33	China: Guizhou, Jiuan
L180404P-1-LD180402-0001_HL57HCCXY_L3_clean_bin.24.orig.fa_k	OEZ008529	yes	LMSG_G000005912.1	OES073691	ladderane	Others	Polymetallic	6.40	China: Guizhou, Jiuan
L180404P-1-LD180402-0001_HL57HCCXY_L3_clean_bin.26.orig.fa_k	OEZ008529	yes	LMSG_G000008028.1	OES073691	lassopeptide	RiPPs	Polymetallic	18.60	China: Guizhou, Jiuan
L180404P-1-LD180402-0001_HL57HCCXY_L3_clean_bin.4.strict.fa_	OEZ008529	yes	LMSG_G000009222.1	OES073691	hserlactone	Others	Polymetallic	19.74	China: Guizhou, Jiuan
L180404P-1-LD180402-0001_HL57HCCXY_L3_clean_bin.4.strict.fa_	OEZ008529	yes	LMSG_G000009222.1	OES073691	terpene	Terpene	Polymetallic	21.74	China: Guizhou, Jiuan
L180404P-1-LD180402-0001_HL57HCCXY_L3_clean_bin.4.strict.fa_	OEZ008529	yes	LMSG_G000009222.1	OES073691	terpene	Terpene	Polymetallic	12.09	China: Guizhou, Jiuan
L180404P-1-LD180402-0001_HL57HCCXY_L3_clean_bin.6.orig.fa_kl	OEZ008529	yes	LMSG_G000005914.1	OES073691	hglE-KS	PKSother	Polymetallic	9.19	China: Guizhou, Jiuan
L180404P-1-LD180402-0001_HL57HCCXY_L3_clean_bin.7.strict.fa_	OEZ008529	yes	LMSG_G000008978.1	OES073691	T1PKS	PKSI	Polymetallic	20.81	China: Guizhou, Jiuan
L180404P-1-LD180402-0001_HL57HCCXY_L3_clean_bin.7.strict.fa_	OEZ008529	yes	LMSG_G000008978.1	OES073691	hglE-KS	PKSother	Polymetallic	5.96	China: Guizhou, Jiuan
L180404P-1-LD180402-0001_HL57HCCXY_L3_clean_bin.8.orig.fa_kl	OEZ008529	yes	LMSG_G000009255.1	OES073691	terpene	Terpene	Polymetallic	20.85	China: Guizhou, Jiuan
L180404P-1-LD180402-0001_HL57HCCXY_L3_clean_bin.9.orig.fa_kl	OEZ008529	yes	LMSG_G000009973.1	OES073691	terpene	Terpene	Polymetallic	9.65	China: Guizhou, Jiuan
L180404P-1-LD180402-0001_HL57HCCXY_L3_clean_bin.9.orig.fa_kl	OEZ008529	yes	LMSG_G000009973.1	OES073691	arylpolyene	Others	Polymetallic	7.95	China: Guizhou, Jiuan
L180404P-1-LD180402-0001_HL57HCCXY_L3_clean_bin.9.orig.fa_kl	OEZ008529	yes	LMSG_G000009973.1	OES073691	terpene	Terpene	Polymetallic	5.80	China: Guizhou, Jiuan
L180404P-1-LD180402-0001_HL57HCCXY_L3_clean_bin.9.orig.fa_kl	OEZ008529	yes	LMSG_G000009973.1	OES073691	arylpolyene	Others	Polymetallic	5.19	China: Guizhou, Jiuan
L180404P-1-LD180402-0001_HL57HCCXY_L3_clean_bin.9.orig.fa_kl	OEZ008529	yes	LMSG_G000009973.1	OES073691	bacteriocin	RiPPs	Polymetallic	6.64	China: Guizhou, Jiuan
L180404P-2-LD180402-0002_HL57HCCXY_L4_clean_bin.1.strict.fa_	OEZ008529	yes	LMSG_G000010073.1	OES073693	arylpolyene	Others	Polymetallic	41.10	China: Guizhou, Jiuan
L180404P-2-LD180402-0002_HL57HCCXY_L4_clean_bin.1.strict.fa_	OEZ008529	yes	LMSG_G000010073.1	OES073693	terpene	Terpene	Polymetallic	22.04	China: Guizhou, Jiuan
L180404P-2-LD180402-0002_HL57HCCXY_L4_clean_bin.1.strict.fa_	OEZ008529	yes	LMSG_G000010073.1	OES073693	terpene	Terpene	Polymetallic	20.33	China: Guizhou, Jiuan
L180404P-2-LD180402-0002_HL57HCCXY_L4_clean_bin.1.strict.fa_	OEZ008529	yes	LMSG_G000010073.1	OES073693	arylpolyene	Others	Polymetallic	11.76	China: Guizhou, Jiuan
L180404P-2-LD180402-0002_HL57HCCXY_L4_clean_bin.1.strict.fa_	OEZ008529	yes	LMSG_G000010073.1	OES073693	ladderane	Others	Polymetallic	6.29	China: Guizhou, Jiuan
L180404P-2-LD180402-0002_HL57HCCXY_L4_clean_bin.10.orig.fa_k	OEZ008529	yes	LMSG_G000005915.1	OES073693	other	Others	Polymetallic	5.78	China: Guizhou, Jiuan
L180404P-2-LD180402-0002_HL57HCCXY_L4_clean_bin.12.orig.fa_k	OEZ008529	yes	LMSG_G000010764.1	OES073693	bacteriocin	RiPPs	Polymetallic	10.47	China: Guizhou, Jiuan
L180404P-2-LD180402-0002_HL57HCCXY_L4_clean_bin.12.orig.fa_k	OEZ008529	yes	LMSG_G000010764.1	OES073693	acyl_amino_acids	Others	Polymetallic	41.97	China: Guizhou, Jiuan
L180404P-2-LD180402-0002_HL57HCCXY_L4_clean_bin.13.orig.fa_k	OEZ008529	yes	LMSG_G000010289.1	OES073693	arylpolyene	Others	Polymetallic	23.15	China: Guizhou, Jiuan
L180404P-2-LD180402-0002_HL57HCCXY_L4_clean_bin.13.orig.fa_k	OEZ008529	yes	LMSG_G000010289.1	OES073693	terpene	Terpene	Polymetallic	11.95	China: Guizhou, Jiuan
L180404P-2-LD180402-0002_HL57HCCXY_L4_clean_bin.15.orig.fa_k	OEZ008529	yes	LMSG_G000008523.1	OES073693	other	Others	Polymetallic	21.36	China: Guizhou, Jiuan
L180404P-2-LD180402-0002_HL57HCCXY_L4_clean_bin.15.orig.fa_k	OEZ008529	yes	LMSG_G000008523.1	OES073693	ectoine	Others	Polymetallic	10.40	China: Guizhou, Jiuan
L180404P-2-LD180402-0002_HL57HCCXY_L4_clean_bin.16.orig.fa_k	OEZ008529	yes	LMSG_G000008656.1	OES073693	other	Others	Polymetallic	36.24	China: Guizhou, Jiuan
L180404P-2-LD180402-0002_HL57HCCXY_L4_clean_bin.19.orig.fa_k	OEZ008529	yes	LMSG_G000006443.1	OES073693	lanthipeptide	RiPPs	Polymetallic	24.37	China: Guizhou, Jiuan
L180404P-2-LD180402-0002_HL57HCCXY_L4_clean_bin.19.orig.fa_k	OEZ008529	yes	LMSG_G000006443.1	OES073693	nucleoside	Others	Polymetallic	14.58	China: Guizhou, Jiuan
L180404P-2-LD180402-0002_HL57HCCXY_L4_clean_bin.21.orig.fa_k	OEZ008529	yes	LMSG_G000005917.1	OES073693	terpene	Terpene	Polymetallic	15.51	China: Guizhou, Jiuan
L180404P-2-LD180402-0002_HL57HCCXY_L4_clean_bin.21.orig.fa_k	OEZ008529	yes	LMSG_G000005917.1	OES073693	bacteriocin	RiPPs	Polymetallic	10.87	China: Guizhou, Jiuan
L180404P-2-LD180402-0002_HL57HCCXY_L4_clean_bin.21.orig.fa_k	OEZ008529	yes	LMSG_G000005917.1	OES073693	arylpolyene	Others	Polymetallic	21.75	China: Guizhou, Jiuan
L180404P-2-LD180402-0002_HL57HCCXY_L4_clean_bin.21.orig.fa_k	OEZ008529	yes	LMSG_G000005917.1	OES073693	terpene	Terpene	Polymetallic	22.00	China: Guizhou, Jiuan
L180404P-2-LD180402-0002_HL57HCCXY_L4_clean_bin.21.orig.fa_k	OEZ008529	yes	LMSG_G000005917.1	OES073693	arylpolyene	Others	Polymetallic	11.34	China: Guizhou, Jiuan
L180404P-2-LD180402-0002_HL57HCCXY_L4_clean_bin.23.orig.fa_k	OEZ008529	yes	LMSG_G000008165.1	OES073693	terpene	Terpene	Polymetallic	23.44	China: Guizhou, Jiuan
L180404P-2-LD180402-0002_HL57HCCXY_L4_clean_bin.24.orig.fa_k	OEZ008529	yes	LMSG_G000005918.1	OES073693	NRPS-like	NRPS	Polymetallic	38.16	China: Guizhou, Jiuan
L180404P-2-LD180402-0002_HL57HCCXY_L4_clean_bin.24.orig.fa_k	OEZ008529	yes	LMSG_G000005918.1	OES073693	terpene	Terpene	Polymetallic	11.95	China: Guizhou, Jiuan
L180404P-2-LD180402-0002_HL57HCCXY_L4_clean_bin.24.orig.fa_k	OEZ008529	yes	LMSG_G000005918.1	OES073693	terpene	Terpene	Polymetallic	19.70	China: Guizhou, Jiuan
L180404P-2-LD180402-0002_HL57HCCXY_L4_clean_bin.28.orig.fa_k	OEZ008529	yes	LMSG_G000005920.1	OES073693	T1PKS.hglE-KS	PKSother	Polymetallic	8.95	China: Guizhou, Jiuan
L180404P-2-LD180402-0002_HL57HCCXY_L4_clean_bin.28.orig.fa_k	OEZ008529	yes	LMSG_G000005920.1	OES073693	hglE-KS	PKSother	Polymetallic	19.18	China: Guizhou, Jiuan
L180404P-2-LD180402-0002_HL57HCCXY_L4_clean_bin.29.orig.fa_k	OEZ008529	no	LMSG_G000011021.1	OES073693	hserlactone	Others	Polymetallic	20.61	China: Guizhou, Jiuan
L180404P-2-LD180402-0002_HL57HCCXY_L4_clean_bin.29.orig.fa_k	OEZ008529	no	LMSG_G000011021.1	OES073693	NAGGN	Others	Polymetallic	12.53	China: Guizhou, Jiuan
L180404P-2-LD180402-0002_HL57HCCXY_L4_clean_bin.29.orig.fa_k	OEZ008529	no	LMSG_G000011021.1	OES073693	bacteriocin	RiPPs	Polymetallic	10.86	China: Guizhou, Jiuan
L180404P-2-LD180402-0002_HL57HCCXY_L4_clean_bin.29.orig.fa_k	OEZ008529	no	LMSG_G000011021.1	OES073693	thiopeptide	RiPPs	Polymetallic	33.00	China: Guizhou, Jiuan
L180404P-2-LD180402-0002_HL57HCCXY_L4_clean_bin.29.orig.fa_k	OEZ008529	no	LMSG_G000011021.1	OES073693	phenazine	Others	Polymetallic	10.23	China: Guizhou, Jiuan
L180404P-2-LD180402-0002_HL57HCCXY_L4_clean_bin.36.strict.fa	OEZ008529	yes	LMSG_G000011053.1	OES073693	terpene	Terpene	Polymetallic	23.02	China: Guizhou, Jiuan
L180404P-2-LD180402-0002_HL57HCCXY_L4_clean_bin.36.strict.fa	OEZ008529	yes	LMSG_G000011053.1	OES073693	bacteriocin	RiPPs	Polymetallic	7.10	China: Guizhou, Jiuan
L180404P-2-LD180402-0002_HL57HCCXY_L4_clean_bin.36.strict.fa	OEZ008529	yes	LMSG_G000011053.1	OES073693	terpene	Terpene	Polymetallic	20.85	China: Guizhou, Jiuan
L180404P-2-LD180402-0002_HL57HCCXY_L4_clean_bin.36.strict.fa	OEZ008529	yes	LMSG_G000011053.1	OES073693	arylpolyene	Others	Polymetallic	21.51	China: Guizhou, Jiuan
L180404P-2-LD180402-0002_HL57HCCXY_L4_clean_bin.37.orig.fa_k	OEZ008529	yes	LMSG_G000008303.1	OES073693	terpene	Terpene	Polymetallic	23.49	China: Guizhou, Jiuan
L180404P-2-LD180402-0002_HL57HCCXY_L4_clean_bin.41.orig.fa_k	OEZ008529	no	LMSG_G000009539.1	OES073693	NRPS-like	NRPS	Polymetallic	42.69	China: Guizhou, Jiuan
L180404P-2-LD180402-0002_HL57HCCXY_L4_clean_bin.41.orig.fa_k	OEZ008529	no	LMSG_G000009539.1	OES073693	terpene	Terpene	Polymetallic	13.70	China: Guizhou, Jiuan
L180404P-2-LD180402-0002_HL57HCCXY_L4_clean_bin.41.orig.fa_k	OEZ008529	no	LMSG_G000009539.1	OES073693	terpene	Terpene	Polymetallic	22.05	China: Guizhou, Jiuan
L180404P-2-LD180402-0002_HL57HCCXY_L4_clean_bin.41.orig.fa_k	OEZ008529	yes	LMSG_G000009539.1	OES073693	acyl_amino_acids	Others	Polymetallic	32.15	China: Guizhou, Jiuan
L180404P-2-LD180402-0002_HL57HCCXY_L4_clean_bin.43.strict.fa	OEZ008529	yes	LMSG_G000008610.1	OES073693	other	Others	Polymetallic	28.57	China: Guizhou, Jiuan
L180404P-2-LD180402-0002_HL57HCCXY_L4_clean_bin.44.orig.fa_k	OEZ008529	yes	LMSG_G000010051.1	OES073693	bacteriocin	RiPPs	Polymetallic	5.89	China: Guizhou, Jiuan
L180404P-2-LD180402-0002_HL57HCCXY_L4_clean_bin.44.orig.fa_k	OEZ008529	yes	LMSG_G000010051.1	OES073693	arylpolyene	Others	Polymetallic	10.19	China: Guizhou, Jiuan
L180404P-2-LD180402-0002_HL57HCCXY_L4_clean_bin.44.orig.fa_k	OEZ008529	yes	LMSG_G000010051.1	OES073693	arylpolyene	Others	Polymetallic	28.93	China: Guizhou, Jiuan
L180404P-2-LD180402-0002_HL57HCCXY_L4_clean_bin.44.orig.fa_k	OEZ008529	yes	LMSG_G000010051.1	OES073693	terpene	Terpene	Polymetallic	7.31	China: Guizhou, Jiuan
L180404P-2-LD180402-0002_HL57HCCXY_L4_clean_bin.44.orig.fa_k	OEZ008529	yes	LMSG_G000010051.1	OES073693	T3PKS	PKSother	Polymetallic	9.97	China: Guizhou, Jiuan
L180404P-2-LD180402-0002_HL57HCCXY_L4_clean_bin.44.orig.fa_k	OEZ008529	yes	LMSG_G000010051.1	OES073693	ladderane	Others	Polymetallic	41.26	China: Guizhou, Jiuan
L180404P-2-LD180402-0002_HL57HCCXY_L4_clean_bin.44.orig.fa_k	OEZ008529	yes	LMSG_G000010051.1	OES073693	terpene	Terpene	Polymetallic	13.78	China: Guizhou, Jiuan
L180404P-2-LD180402-0002_HL57HCCXY_L4_clean_bin.44.orig.fa_k	OEZ008529	yes	LMSG_G000010051.1	OES073693	ectoine	Others	Polymetallic	10.40	China: Guizhou, Jiuan
L180404P-2-LD180402-0002_HL57HCCXY_L4_clean_bin.46.permissiv	OEZ008529	yes	LMSG_G000008390.1	OES073693	terpene	Terpene	Polymetallic	11.61	China: Guizhou, Jiuan
L180404P-2-LD180402-0002_HL57HCCXY_L4_clean_bin.46.permissiv	OEZ008529	yes	LMSG_G000008390.1	OES073693	NRPS-like	NRPS	Polymetallic	5.67	China: Guizhou, Jiuan
L180404P-2-LD180402-0002_HL57HCCXY_L4_clean_bin.5.orig.fa_kl	OEZ008529	yes	LMSG_G000005925.1	OES073693	terpene	Terpene	Polymetallic	17.67	China: Guizhou, Jiuan
L180404P-2-LD180402-0002_HL57HCCXY_L4_clean_bin.5.orig.fa_kl	OEZ008529	yes	LMSG_G000005925.1	OES073693	T3PKS	PKSother	Polymetallic	12.34	China: Guizhou, Jiuan
L180404P-2-LD180402-0002_HL57HCCXY_L4_clean_bin.5.orig.fa_kl	OEZ008529	yes	LMSG_G000005925.1	OES073693	arylpolyene	Others	Polymetallic	13.70	China: Guizhou, Jiuan
L180404P-2-LD180402-0002_HL57HCCXY_L4_clean_bin.5.orig.fa_kl	OEZ008529	yes	LMSG_G000005925.1	OES073693	terpene	Terpene	Polymetallic	10.87	China: Guizhou, Jiuan
L180404P-2-LD180402-0002_HL57HCCXY_L4_clean_bin.5.orig.fa_kl	OEZ008529	yes	LMSG_G000005925.1	OES073693	ladderane	Others	Polymetallic	18.56	China: Guizhou, Jiuan
L180404P-2-LD180402-0002_HL57HCCXY_L4_clean_bin.5.orig.fa_kl	OEZ008529	yes	LMSG_G000005925.1	OES073693	terpene	Terpene	Polymetallic	11.49	China: Guizhou, Jiuan
L180404P-2-LD180402-0002_HL57HCCXY_L4_clean_bin.5.orig.fa_kl	OEZ008529	yes	LMSG_G000005925.1	OES073693	terpene	Terpene	Polymetallic	10.76	China: Guizhou, Jiuan
L180404P-2-LD180402-0002_HL57HCCXY_L4_clean_bin.50.strict.fa	OEZ008529	yes	LMSG_G000009644.1	OES073693	terpene	Terpene	Polymetallic	18.76	China: Guizhou, Jiuan
L180404P-2-LD180402-0002_HL57HCCXY_L4_clean_bin.51.orig.fa_k	OEZ008529	yes	LMSG_G000008462.1	OES073693	terpene	Terpene	Polymetallic	16.67	China: Guizhou, Jiuan
L180404P-2-LD180402-0002_HL57HCCXY_L4_clean_bin.51.orig.fa_k	OEZ008529	yes	LMSG_G000008462.1	OES073693	bacteriocin	RiPPs	Polymetallic	10.80	China: Guizhou, Jiuan
L180404P-2-LD180402-0002_HL57HCCXY_L4_clean_bin.51.orig.fa_k	OEZ008529	yes	LMSG_G000008462.1	OES073693	betalactone	Others	Polymetallic	13.68	China: Guizhou, Jiuan
L180404P-2-LD180402-0002_HL57HCCXY_L4_clean_bin.51.orig.fa_k	OEZ008529	yes	LMSG_G000008462.1	OES073693	NRPS-like	NRPS	Polymetallic	14.90	China: Guizhou, Jiuan
L180404P-2-LD180402-0002_HL57HCCXY_L4_clean_bin.52.orig.fa_k	OEZ008529	yes	LMSG_G000010428.1	OES073693	bacteriocin	RiPPs	Polymetallic	9.46	China: Guizhou, Jiuan
L180404P-2-LD180402-0002_HL57HCCXY_L4_clean_bin.52.orig.fa_k	OEZ008529	yes	LMSG_G000010428.1	OES073693	terpene	Terpene	Polymetallic	25.02	China: Guizhou, Jiuan
L180404P-2-LD180402-0002_HL57HCCXY_L4_clean_bin.52.orig.fa_k	OEZ008529	yes	LMSG_G000010428.1	OES073693	hserlactone	Others	Polymetallic	16.67	China: Guizhou, Jiuan
L180404P-2-LD180402-0002_HL57HCCXY_L4_clean_bin.52.orig.fa_k	OEZ008529	yes	LMSG_G000010428.1	OES073693	terpene	Terpene	Polymetallic	12.45	China: Guizhou, Jiuan
L180404P-2-LD180402-0002_HL57HCCXY_L4_clean_bin.7.orig.fa_kl	OEZ008529	yes	LMSG_G000005926.1	OES073693	LAP	RiPPs	Polymetallic	11.70	China: Guizhou, Jiuan
L180404P-2-LD180402-0002_HL57HCCXY_L4_clean_bin.9.orig.fa_kl	OEZ008529	yes	LMSG_G000008279.1	OES073693	terpene	Terpene	Polymetallic	19.12	China: Guizhou, Jiuan
L180404P-2-LD180402-0003_HL57HCCXY_L4_clean_bin.11.orig.fa_k	OEZ008529	no	LMSG_G000010579.1	OES073724	bacteriocin	RiPPs	Polymetallic	10.85	China: Anhui, Wuhu
L180404P-2-LD180402-0003_HL57HCCXY_L4_clean_bin.11.orig.fa_k	OEZ008529	no	LMSG_G000010579.1	OES073724	T1PKS	PKSI	Polymetallic	23.87	China: Anhui, Wuhu
L180404P-2-LD180402-0003_HL57HCCXY_L4_clean_bin.11.orig.fa_k	OEZ008529	yes	LMSG_G000010579.1	OES073724	terpene	Terpene	Polymetallic	20.83	China: Anhui, Wuhu
L180404P-2-LD180402-0003_HL57HCCXY_L4_clean_bin.11.orig.fa_k	OEZ008529	no	LMSG_G000010579.1	OES073724	bacteriocin	RiPPs	Polymetallic	7.13	China: Anhui, Wuhu
L180404P-2-LD180402-0003_HL57HCCXY_L4_clean_bin.11.orig.fa_k	OEZ008529	yes	LMSG_G000010579.1	OES073724	bacteriocin.hserlactone	Others	Polymetallic	11.71	China: Anhui, Wuhu
L180404P-2-LD180402-0003_HL57HCCXY_L4_clean_bin.13.orig.fa_k	OEZ008529	yes	LMSG_G000010503.1	OES073724	hserlactone	Others	Polymetallic	8.35	China: Anhui, Wuhu
L180404P-2-LD180402-0003_HL57HCCXY_L4_clean_bin.13.orig.fa_k	OEZ008529	yes	LMSG_G000010503.1	OES073724	terpene	Terpene	Polymetallic	13.14	China: Anhui, Wuhu
L180404P-2-LD180402-0003_HL57HCCXY_L4_clean_bin.2.strict.fa_	OEZ008529	yes	LMSG_G000009448.1	OES073724	betalactone	Others	Polymetallic	22.78	China: Anhui, Wuhu
L180404P-2-LD180402-0003_HL57HCCXY_L4_clean_bin.2.strict.fa_	OEZ008529	yes	LMSG_G000009448.1	OES073724	betalactone	Others	Polymetallic	30.86	China: Anhui, Wuhu
L180404P-2-LD180402-0003_HL57HCCXY_L4_clean_bin.2.strict.fa_	OEZ008529	yes	LMSG_G000009448.1	OES073724	bacteriocin	RiPPs	Polymetallic	10.85	China: Anhui, Wuhu
L180404P-2-LD180402-0003_HL57HCCXY_L4_clean_bin.2.strict.fa_	OEZ008529	yes	LMSG_G000009448.1	OES073724	ectoine	Others	Polymetallic	9.57	China: Anhui, Wuhu
L180404P-2-LD180402-0003_HL57HCCXY_L4_clean_bin.3.orig.fa_kl	OEZ008529	yes	LMSG_G000009423.1	OES073724	bacteriocin	RiPPs	Polymetallic	7.50	China: Anhui, Wuhu
L180404P-2-LD180402-0003_HL57HCCXY_L4_clean_bin.3.orig.fa_kl	OEZ008529	yes	LMSG_G000009423.1	OES073724	cyanobactin	RiPPs	Polymetallic	5.95	China: Anhui, Wuhu
L180404P-2-LD180402-0003_HL57HCCXY_L4_clean_bin.4.orig.fa_kl	OEZ008529	yes	LMSG_G000010491.1	OES073724	terpene	Terpene	Polymetallic	17.41	China: Anhui, Wuhu
L180404P-2-LD180402-0003_HL57HCCXY_L4_clean_bin.5.strict.fa_	OEZ008529	yes	LMSG_G000008199.1	OES073724	terpene	Terpene	Polymetallic	20.86	China: Anhui, Wuhu
L180404P-2-LD180402-0003_HL57HCCXY_L4_clean_bin.6.orig.fa_kl	OEZ008529	yes	LMSG_G000007070.1	OES073724	bacteriocin	RiPPs	Polymetallic	11.01	China: Anhui, Wuhu
L180404P-2-LD180402-0003_HL57HCCXY_L4_clean_bin.8.strict.fa_	OEZ008529	no	LMSG_G000011007.1	OES073724	hserlactone	Others	Polymetallic	11.14	China: Anhui, Wuhu
L180404P-2-LD180402-0003_HL57HCCXY_L4_clean_bin.8.strict.fa_	OEZ008529	no	LMSG_G000011007.1	OES073724	betalactone	Others	Polymetallic	19.34	China: Anhui, Wuhu
L180404P-2-LD180402-0003_HL57HCCXY_L4_clean_bin.8.strict.fa_	OEZ008529	no	LMSG_G000011007.1	OES073724	NRPS	NRPS	Polymetallic	17.91	China: Anhui, Wuhu
L180404P-2-LD180402-0003_HL57HCCXY_L4_clean_bin.8.strict.fa_	OEZ008529	no	LMSG_G000011007.1	OES073724	siderophore	Others	Polymetallic	12.58	China: Anhui, Wuhu
L180404P-2-LD180402-0003_HL57HCCXY_L4_clean_bin.8.strict.fa_	OEZ008529	no	LMSG_G000011007.1	OES073724	NRPS	NRPS	Polymetallic	7.34	China: Anhui, Wuhu
L180404P-2-LD180402-0003_HL57HCCXY_L4_clean_bin.8.strict.fa_	OEZ008529	no	LMSG_G000011007.1	OES073724	NRPS-like	NRPS	Polymetallic	5.88	China: Anhui, Wuhu
L180404P-2-LD180402-0004_HL57HCCXY_L4_clean_bin.10.orig.fa_k	OEZ008529	yes	LMSG_G000005927.1	OES073755	NRPS-like	NRPS	Copper	42.69	China: Fujian, Zijin
L180404P-2-LD180402-0004_HL57HCCXY_L4_clean_bin.10.orig.fa_k	OEZ008529	yes	LMSG_G000005927.1	OES073755	T3PKS	PKSother	Copper	37.98	China: Fujian, Zijin
L180404P-2-LD180402-0004_HL57HCCXY_L4_clean_bin.11.orig.fa_k	OEZ008529	yes	LMSG_G000011317.1	OES073755	terpene	Terpene	Copper	21.13	China: Fujian, Zijin
L180404P-2-LD180402-0004_HL57HCCXY_L4_clean_bin.11.orig.fa_k	OEZ008529	yes	LMSG_G000011317.1	OES073755	terpene	Terpene	Copper	22.52	China: Fujian, Zijin
L180404P-2-LD180402-0004_HL57HCCXY_L4_clean_bin.11.orig.fa_k	OEZ008529	yes	LMSG_G000011317.1	OES073755	terpene	Terpene	Copper	11.84	China: Fujian, Zijin
L180404P-2-LD180402-0004_HL57HCCXY_L4_clean_bin.11.orig.fa_k	OEZ008529	yes	LMSG_G000011317.1	OES073755	NRPS	NRPS	Copper	24.30	China: Fujian, Zijin
L180404P-2-LD180402-0004_HL57HCCXY_L4_clean_bin.13.orig.fa_k	OEZ008529	yes	LMSG_G000011327.1	OES073755	NRPS-like	NRPS	Copper	24.41	China: Fujian, Zijin
L180404P-2-LD180402-0004_HL57HCCXY_L4_clean_bin.13.orig.fa_k	OEZ008529	yes	LMSG_G000011327.1	OES073755	bacteriocin	RiPPs	Copper	7.14	China: Fujian, Zijin
L180404P-2-LD180402-0004_HL57HCCXY_L4_clean_bin.13.orig.fa_k	OEZ008529	yes	LMSG_G000011327.1	OES073755	T3PKS	PKSother	Copper	15.44	China: Fujian, Zijin
L180404P-2-LD180402-0004_HL57HCCXY_L4_clean_bin.15.orig.fa_k	OEZ008529	yes	LMSG_G000008147.1	OES073755	terpene	Terpene	Copper	5.87	China: Fujian, Zijin
L180404P-2-LD180402-0004_HL57HCCXY_L4_clean_bin.19.orig.fa_k	OEZ008529	yes	LMSG_G000010943.1	OES073755	NRPS-like	NRPS	Copper	25.00	China: Fujian, Zijin
L180404P-2-LD180402-0004_HL57HCCXY_L4_clean_bin.19.orig.fa_k	OEZ008529	yes	LMSG_G000010943.1	OES073755	T3PKS	PKSother	Copper	9.44	China: Fujian, Zijin
L180404P-2-LD180402-0004_HL57HCCXY_L4_clean_bin.19.orig.fa_k	OEZ008529	yes	LMSG_G000010943.1	OES073755	lassopeptide	RiPPs	Copper	23.95	China: Fujian, Zijin
L180404P-2-LD180402-0004_HL57HCCXY_L4_clean_bin.19.orig.fa_k	OEZ008529	yes	LMSG_G000010943.1	OES073755	NRPS	NRPS	Copper	32.30	China: Fujian, Zijin
L180404P-2-LD180402-0004_HL57HCCXY_L4_clean_bin.19.orig.fa_k	OEZ008529	yes	LMSG_G000010943.1	OES073755	NRPS-like	NRPS	Copper	7.47	China: Fujian, Zijin
L180404P-2-LD180402-0004_HL57HCCXY_L4_clean_bin.19.orig.fa_k	OEZ008529	yes	LMSG_G000010943.1	OES073755	butyrolactone.NRPS-like	Others	Copper	17.85	China: Fujian, Zijin
L180404P-2-LD180402-0004_HL57HCCXY_L4_clean_bin.19.orig.fa_k	OEZ008529	yes	LMSG_G000010943.1	OES073755	NRPS	NRPS	Copper	14.90	China: Fujian, Zijin
L180404P-2-LD180402-0004_HL57HCCXY_L4_clean_bin.19.orig.fa_k	OEZ008529	yes	LMSG_G000010943.1	OES073755	NRPS.T1PKS	PKS-NRP_Hybrids	Copper	22.76	China: Fujian, Zijin
L180404P-2-LD180402-0004_HL57HCCXY_L4_clean_bin.2.orig.fa_kl	OEZ008529	yes	LMSG_G000010660.1	OES073755	NRPS	NRPS	Copper	46.34	China: Fujian, Zijin
L180404P-2-LD180402-0004_HL57HCCXY_L4_clean_bin.23.orig.fa_k	OEZ008529	yes	LMSG_G000011105.1	OES073755	terpene	Terpene	Copper	14.77	China: Fujian, Zijin
L180404P-2-LD180402-0004_HL57HCCXY_L4_clean_bin.23.orig.fa_k	OEZ008529	yes	LMSG_G000011105.1	OES073755	NRPS	NRPS	Copper	32.48	China: Fujian, Zijin
L180404P-2-LD180402-0004_HL57HCCXY_L4_clean_bin.23.orig.fa_k	OEZ008529	yes	LMSG_G000011105.1	OES073755	CDPS.bacteriocin	Others	Copper	20.38	China: Fujian, Zijin
L180404P-2-LD180402-0004_HL57HCCXY_L4_clean_bin.23.orig.fa_k	OEZ008529	yes	LMSG_G000011105.1	OES073755	TfuA-related	RiPPs	Copper	12.20	China: Fujian, Zijin
L180404P-2-LD180402-0004_HL57HCCXY_L4_clean_bin.24.orig.fa_k	OEZ008529	yes	LMSG_G000010784.1	OES073755	bacteriocin	RiPPs	Copper	9.22	China: Fujian, Zijin

L180404P-2-LD180402-0004_HL57HCCXY_L4_clean_bin.24.orig.fa_k	OEZ008529	yes	LMSG_G000010784.1	OES073755	acyl_amino_acids	Others	Copper	33.26	China: Fujian, Zijin
L180404P-2-LD180402-0004_HL57HCCXY_L4_clean_bin.25.permissiv	OEZ008529	yes	LMSG_G000009629.1	OES073755	terpene	Terpene	Copper	22.09	China: Fujian, Zijin
L180404P-2-LD180402-0004_HL57HCCXY_L4_clean_bin.26.orig.fa_k	OEZ008529	yes	LMSG_G000008208.1	OES073755	terpene	Terpene	Copper	20.86	China: Fujian, Zijin
L180404P-2-LD180402-0004_HL57HCCXY_L4_clean_bin.28.orig.fa_k	OEZ008529	yes	LMSG_G000010940.1	OES073755	bacteriocin	RiPPs	Copper	8.86	China: Fujian, Zijin
L180404P-2-LD180402-0004_HL57HCCXY_L4_clean_bin.28.orig.fa_k	OEZ008529	yes	LMSG_G000010940.1	OES073755	NRPS-like	NRPS	Copper	18.18	China: Fujian, Zijin
L180404P-2-LD180402-0004_HL57HCCXY_L4_clean_bin.28.orig.fa_k	OEZ008529	yes	LMSG_G000010940.1	OES073755	NRPS	NRPS	Copper	34.16	China: Fujian, Zijin
L180404P-2-LD180402-0004_HL57HCCXY_L4_clean_bin.28.orig.fa_k	OEZ008529	yes	LMSG_G000010940.1	OES073755	T3PKS	PKSother	Copper	19.63	China: Fujian, Zijin
L180404P-2-LD180402-0004_HL57HCCXY_L4_clean_bin.28.orig.fa_k	OEZ008529	yes	LMSG_G000010940.1	OES073755	NRPS	NRPS	Copper	14.95	China: Fujian, Zijin
L180404P-2-LD180402-0004_HL57HCCXY_L4_clean_bin.28.orig.fa_k	OEZ008529	yes	LMSG_G000010940.1	OES073755	bacteriocin	RiPPs	Copper	7.88	China: Fujian, Zijin
L180404P-2-LD180402-0004_HL57HCCXY_L4_clean_bin.28.orig.fa_k	OEZ008529	yes	LMSG_G000010940.1	OES073755	betalactone	Others	Copper	18.17	China: Fujian, Zijin
L180404P-2-LD180402-0004_HL57HCCXY_L4_clean_bin.29.orig.fa_k	OEZ008529	yes	LMSG_G000010081.1	OES073755	terpene	Terpene	Copper	9.02	China: Fujian, Zijin
L180404P-2-LD180402-0004_HL57HCCXY_L4_clean_bin.3.orig.fa_kl	OEZ008529	yes	LMSG_G000009968.1	OES073755	terpene	Terpene	Copper	13.32	China: Fujian, Zijin
L180404P-2-LD180402-0004_HL57HCCXY_L4_clean_bin.3.orig.fa_kl	OEZ008529	yes	LMSG_G000009968.1	OES073755	NRPS-like	NRPS	Copper	27.55	China: Fujian, Zijin
L180404P-2-LD180402-0004_HL57HCCXY_L4_clean_bin.3.orig.fa_kl	OEZ008529	yes	LMSG_G000009968.1	OES073755	arylpolyene	Others	Copper	11.91	China: Fujian, Zijin
L180404P-2-LD180402-0004_HL57HCCXY_L4_clean_bin.3.orig.fa_kl	OEZ008529	yes	LMSG_G000009968.1	OES073755	arylpolyene	Others	Copper	22.80	China: Fujian, Zijin
L180404P-2-LD180402-0004_HL57HCCXY_L4_clean_bin.3.orig.fa_kl	OEZ008529	yes	LMSG_G000009968.1	OES073755	terpene	Terpene	Copper	15.30	China: Fujian, Zijin
L180404P-2-LD180402-0004_HL57HCCXY_L4_clean_bin.3.orig.fa_kl	OEZ008529	yes	LMSG_G000009968.1	OES073755	arylpolyene	Others	Copper	12.81	China: Fujian, Zijin
L180404P-2-LD180402-0004_HL57HCCXY_L4_clean_bin.30.orig.fa_k	OEZ008529	yes	LMSG_G000008430.1	OES073755	NRPS.betalactone	Others	Copper	32.43	China: Fujian, Zijin
L180404P-2-LD180402-0004_HL57HCCXY_L4_clean_bin.30.orig.fa_k	OEZ008529	yes	LMSG_G000008430.1	OES073755	NRPS	NRPS	Copper	46.92	China: Fujian, Zijin
L180404P-2-LD180402-0004_HL57HCCXY_L4_clean_bin.30.orig.fa_k	OEZ008529	yes	LMSG_G000008430.1	OES073755	terpene	Terpene	Copper	16.76	China: Fujian, Zijin
L180404P-2-LD180402-0004_HL57HCCXY_L4_clean_bin.6.orig.fa_kl	OEZ008529	yes	LMSG_G000006253.1	OES073755	T1PKS.hglE-KS	PKSother	Copper	39.09	China: Fujian, Zijin
L180404P-2-LD180402-0004_HL57HCCXY_L4_clean_bin.6.orig.fa_kl	OEZ008529	yes	LMSG_G000006253.1	OES073755	betalactone	Others	Copper	24.88	China: Fujian, Zijin
L180404P-2-LD180402-0004_HL57HCCXY_L4_clean_bin.6.orig.fa_kl	OEZ008529	yes	LMSG_G000006253.1	OES073755	bacteriocin	RiPPs	Copper	10.90	China: Fujian, Zijin
L180404P-2-LD180402-0004_HL57HCCXY_L4_clean_bin.8.orig.fa_kl	OEZ008529	yes	LMSG_G000008155.1	OES073755	terpene	Terpene	Copper	11.70	China: Fujian, Zijin
L180404P-2-LD180402-0004_HL57HCCXY_L4_clean_bin.9.orig.fa_kl	OEZ008529	yes	LMSG_G000010146.1	OES073755	terpene	Terpene	Copper	11.72	China: Fujian, Zijin
L180404P-2-LD180402-0004_HL57HCCXY_L4_clean_bin.9.orig.fa_kl	OEZ008529	yes	LMSG_G000010146.1	OES073755	T3PKS	PKSother	Copper	31.31	China: Fujian, Zijin
L180404P-2-LD180402-0004_HL57HCCXY_L4_clean_bin.9.orig.fa_kl	OEZ008529	yes	LMSG_G000010146.1	OES073755	terpene	Terpene	Copper	10.25	China: Fujian, Zijin
L180404P-3-LD180402-0006_HL57HCCXY_L5_clean_bin.1.strict.fa_	OEZ008529	yes	LMSG_G000009094.1	OES073706	betalactone	Others	Magnetite	27.63	China: Anhui, Maanshan
L180404P-3-LD180402-0006_HL57HCCXY_L5_clean_bin.1.strict.fa_	OEZ008529	yes	LMSG_G000009094.1	OES073706	betalactone	Others	Magnetite	31.07	China: Anhui, Maanshan
L180404P-3-LD180402-0006_HL57HCCXY_L5_clean_bin.1.strict.fa_	OEZ008529	yes	LMSG_G000009094.1	OES073706	betalactone.bacteriocin	Others	Magnetite	29.69	China: Anhui, Maanshan
L180404P-3-LD180402-0006_HL57HCCXY_L5_clean_bin.1.strict.fa_	OEZ008529	yes	LMSG_G000009094.1	OES073706	betalactone	Others	Magnetite	25.97	China: Anhui, Maanshan
L180404P-3-LD180402-0006_HL57HCCXY_L5_clean_bin.1.strict.fa_	OEZ008529	yes	LMSG_G000009094.1	OES073706	betalactone	Others	Magnetite	19.92	China: Anhui, Maanshan
L180404P-3-LD180402-0006_HL57HCCXY_L5_clean_bin.12.orig.fa_k	OEZ008529	yes	LMSG_G000009982.1	OES073706	terpene	Terpene	Magnetite	13.33	China: Anhui, Maanshan
L180404P-3-LD180402-0006_HL57HCCXY_L5_clean_bin.12.orig.fa_k	OEZ008529	yes	LMSG_G000009982.1	OES073706	ectoine	Others	Magnetite	7.55	China: Anhui, Maanshan
L180404P-3-LD180402-0006_HL57HCCXY_L5_clean_bin.12.orig.fa_k	OEZ008529	yes	LMSG_G000009982.1	OES073706	terpene	Terpene	Magnetite	11.73	China: Anhui, Maanshan
L180404P-3-LD180402-0006_HL57HCCXY_L5_clean_bin.13.orig.fa_k	OEZ008529	yes	LMSG_G000008023.1	OES073706	betalactone	Others	Magnetite	12.72	China: Anhui, Maanshan
L180404P-3-LD180402-0006_HL57HCCXY_L5_clean_bin.15.orig.fa_k	OEZ008529	yes	LMSG_G000005930.1	OES073706	nucleoside	Others	Magnetite	13.06	China: Anhui, Maanshan
L180404P-3-LD180402-0006_HL57HCCXY_L5_clean_bin.20.strict.fa	OEZ008529	yes	LMSG_G000007985.1	OES073706	bacteriocin	RiPPs	Magnetite	10.32	China: Anhui, Maanshan
L180404P-3-LD180402-0006_HL57HCCXY_L5_clean_bin.20.strict.fa	OEZ008529	yes	LMSG_G000007985.1	OES073706	TfuA-related.bacteriocin	RiPPs	Magnetite	13.15	China: Anhui, Maanshan
L180404P-3-LD180402-0006_HL57HCCXY_L5_clean_bin.26.orig.fa_k	OEZ008529	yes	LMSG_G000008035.1	OES073706	lassopeptide	RiPPs	Magnetite	13.85	China: Anhui, Maanshan
L180404P-3-LD180402-0006_HL57HCCXY_L5_clean_bin.26.orig.fa_k	OEZ008529	yes	LMSG_G000008035.1	OES073706	LAP.thiopeptide	RiPPs	Magnetite	21.23	China: Anhui, Maanshan
L180404P-3-LD180402-0006_HL57HCCXY_L5_clean_bin.27.strict.fa	OEZ008529	yes	LMSG_G000008280.1	OES073706	terpene	Terpene	Magnetite	19.33	China: Anhui, Maanshan
L180404P-3-LD180402-0006_HL57HCCXY_L5_clean_bin.29.strict.fa	OEZ008529	yes	LMSG_G000008129.1	OES073706	terpene	Terpene	Magnetite	20.48	China: Anhui, Maanshan
L180404P-3-LD180402-0006_HL57HCCXY_L5_clean_bin.30.strict.fa	OEZ008529	yes	LMSG_G000009900.1	OES073706	terpene	Terpene	Magnetite	10.63	China: Anhui, Maanshan
L180404P-3-LD180402-0006_HL57HCCXY_L5_clean_bin.34.orig.fa_k	OEZ008529	yes	LMSG_G000010567.1	OES073706	terpene	Terpene	Magnetite	22.97	China: Anhui, Maanshan
L180404P-3-LD180402-0006_HL57HCCXY_L5_clean_bin.34.orig.fa_k	OEZ008529	yes	LMSG_G000010567.1	OES073706	hserlactone	Others	Magnetite	20.44	China: Anhui, Maanshan
L180404P-3-LD180402-0006_HL57HCCXY_L5_clean_bin.36.orig.fa_k	OEZ008529	yes	LMSG_G000009283.1	OES073706	terpene	Terpene	Magnetite	13.82	China: Anhui, Maanshan
L180404P-3-LD180402-0006_HL57HCCXY_L5_clean_bin.36.orig.fa_k	OEZ008529	yes	LMSG_G000009283.1	OES073706	terpene	Terpene	Magnetite	22.06	China: Anhui, Maanshan
L180404P-3-LD180402-0006_HL57HCCXY_L5_clean_bin.36.orig.fa_k	OEZ008529	yes	LMSG_G000009283.1	OES073706	bacteriocin	RiPPs	Magnetite	6.48	China: Anhui, Maanshan
L180404P-3-LD180402-0006_HL57HCCXY_L5_clean_bin.38.orig.fa_k	OEZ008529	yes	LMSG_G000005931.1	OES073706	acyl_amino_acids	Others	Magnetite	34.51	China: Anhui, Maanshan
L180404P-3-LD180402-0006_HL57HCCXY_L5_clean_bin.38.orig.fa_k	OEZ008529	yes	LMSG_G000005931.1	OES073706	NRPS-like	NRPS	Magnetite	25.85	China: Anhui, Maanshan
L180404P-3-LD180402-0006_HL57HCCXY_L5_clean_bin.38.orig.fa_k	OEZ008529	yes	LMSG_G000005931.1	OES073706	bacteriocin	RiPPs	Magnetite	13.46	China: Anhui, Maanshan
L180404P-3-LD180402-0006_HL57HCCXY_L5_clean_bin.44.orig.fa_k	OEZ008529	yes	LMSG_G000010795.1	OES073706	acyl_amino_acids	Others	Magnetite	43.29	China: Anhui, Maanshan
L180404P-3-LD180402-0006_HL57HCCXY_L5_clean_bin.44.orig.fa_k	OEZ008529	yes	LMSG_G000010795.1	OES073706	acyl_amino_acids	Others	Magnetite	15.67	China: Anhui, Maanshan
L180404P-3-LD180402-0006_HL57HCCXY_L5_clean_bin.44.orig.fa_k	OEZ008529	yes	LMSG_G000010795.1	OES073706	bacteriocin	RiPPs	Magnetite	10.58	China: Anhui, Maanshan
L180404P-3-LD180402-0006_HL57HCCXY_L5_clean_bin.45.permissiv	OEZ008529	yes	LMSG_G000008391.1	OES073706	terpene	Terpene	Magnetite	13.29	China: Anhui, Maanshan
L180404P-3-LD180402-0006_HL57HCCXY_L5_clean_bin.45.permissiv	OEZ008529	yes	LMSG_G000008391.1	OES073706	NRPS-like	NRPS	Magnetite	11.81	China: Anhui, Maanshan
L180404P-3-LD180402-0006_HL57HCCXY_L5_clean_bin.48.permissiv	OEZ008529	yes	LMSG_G000008330.1	OES073706	terpene	Terpene	Magnetite	21.09	China: Anhui, Maanshan
L180404P-3-LD180402-0006_HL57HCCXY_L5_clean_bin.7.orig.fa_kl	OEZ008529	yes	LMSG_G000005932.1	OES073706	terpene	Terpene	Magnetite	23.38	China: Anhui, Maanshan
L180404P-3-LD180402-0006_HL57HCCXY_L5_clean_bin.8.orig.fa_kl	OEZ008529	yes	LMSG_G000010023.1	OES073706	terpene	Terpene	Magnetite	10.05	China: Anhui, Maanshan
L180404P-3-LD180402-0006_HL57HCCXY_L5_clean_bin.8.orig.fa_kl	OEZ008529	yes	LMSG_G000010023.1	OES073706	terpene	Terpene	Magnetite	5.82	China: Anhui, Maanshan
L180404P-3-LD180402-0006_HL57HCCXY_L5_clean_bin.8.orig.fa_kl	OEZ008529	yes	LMSG_G000010023.1	OES073706	arylpolyene	Others	Magnetite	17.73	China: Anhui, Maanshan
L180404P-3-LD180402-0007_HL57HCCXY_L5_clean_bin.1.strict.fa_	OEZ008529	yes	LMSG_G000005934.1	OES073708	LAP	RiPPs	Magnetite	21.63	China: Anhui, Maanshan
L180404P-3-LD180402-0007_HL57HCCXY_L5_clean_bin.13.orig.fa_k	OEZ008529	yes	LMSG_G000010035.1	OES073708	NRPS-like	NRPS	Magnetite	8.36	China: Anhui, Maanshan
L180404P-3-LD180402-0007_HL57HCCXY_L5_clean_bin.13.orig.fa_k	OEZ008529	yes	LMSG_G000010035.1	OES073708	terpene	Terpene	Magnetite	7.66	China: Anhui, Maanshan
L180404P-3-LD180402-0007_HL57HCCXY_L5_clean_bin.14.strict.fa	OEZ008529	yes	LMSG_G000007986.1	OES073708	bacteriocin	RiPPs	Magnetite	10.32	China: Anhui, Maanshan
L180404P-3-LD180402-0007_HL57HCCXY_L5_clean_bin.14.strict.fa	OEZ008529	yes	LMSG_G000007986.1	OES073708	TfuA-related.bacteriocin	RiPPs	Magnetite	13.42	China: Anhui, Maanshan
L180404P-3-LD180402-0007_HL57HCCXY_L5_clean_bin.16.orig.fa_k	OEZ008529	yes	LMSG_G000009975.1	OES073708	ladderane	Others	Magnetite	17.12	China: Anhui, Maanshan
L180404P-3-LD180402-0007_HL57HCCXY_L5_clean_bin.16.orig.fa_k	OEZ008529	yes	LMSG_G000009975.1	OES073708	terpene	Terpene	Magnetite	15.00	China: Anhui, Maanshan
L180404P-3-LD180402-0007_HL57HCCXY_L5_clean_bin.16.orig.fa_k	OEZ008529	yes	LMSG_G000009975.1	OES073708	NRPS-like	NRPS	Magnetite	15.58	China: Anhui, Maanshan
L180404P-3-LD180402-0007_HL57HCCXY_L5_clean_bin.16.orig.fa_k	OEZ008529	yes	LMSG_G000009975.1	OES073708	arylpolyene	Others	Magnetite	15.50	China: Anhui, Maanshan
L180404P-3-LD180402-0007_HL57HCCXY_L5_clean_bin.16.orig.fa_k	OEZ008529	yes	LMSG_G000009975.1	OES073708	bacteriocin	RiPPs	Magnetite	10.88	China: Anhui, Maanshan
L180404P-3-LD180402-0007_HL57HCCXY_L5_clean_bin.16.orig.fa_k	OEZ008529	yes	LMSG_G000009975.1	OES073708	arylpolyene	Others	Magnetite	5.39	China: Anhui, Maanshan
L180404P-3-LD180402-0007_HL57HCCXY_L5_clean_bin.18.orig.fa_k	OEZ008529	yes	LMSG_G000007842.1	OES073708	NRPS-like	NRPS	Magnetite	34.06	China: Anhui, Maanshan
L180404P-3-LD180402-0007_HL57HCCXY_L5_clean_bin.18.orig.fa_k	OEZ008529	yes	LMSG_G000007842.1	OES073708	terpene	Terpene	Magnetite	13.80	China: Anhui, Maanshan
L180404P-3-LD180402-0007_HL57HCCXY_L5_clean_bin.18.orig.fa_k	OEZ008529	yes	LMSG_G000007842.1	OES073708	terpene	Terpene	Magnetite	12.61	China: Anhui, Maanshan
L180404P-3-LD180402-0007_HL57HCCXY_L5_clean_bin.18.orig.fa_k	OEZ008529	yes	LMSG_G000007842.1	OES073708	NRPS-like	NRPS	Magnetite	23.84	China: Anhui, Maanshan
L180404P-3-LD180402-0007_HL57HCCXY_L5_clean_bin.18.orig.fa_k	OEZ008529	yes	LMSG_G000007842.1	OES073708	T3PKS	PKSother	Magnetite	25.07	China: Anhui, Maanshan
L180404P-3-LD180402-0007_HL57HCCXY_L5_clean_bin.19.permissiv	OEZ008529	yes	LMSG_G000005933.1	OES073708	terpene	Terpene	Magnetite	21.77	China: Anhui, Maanshan
L180404P-3-LD180402-0007_HL57HCCXY_L5_clean_bin.19.permissiv	OEZ008529	yes	LMSG_G000005933.1	OES073708	terpene	Terpene	Magnetite	21.41	China: Anhui, Maanshan
L180404P-3-LD180402-0007_HL57HCCXY_L5_clean_bin.19.permissiv	OEZ008529	yes	LMSG_G000005933.1	OES073708	terpene	Terpene	Magnetite	13.28	China: Anhui, Maanshan
L180404P-3-LD180402-0007_HL57HCCXY_L5_clean_bin.24.orig.fa_k	OEZ008529	yes	LMSG_G000008036.1	OES073708	LAP.thiopeptide	RiPPs	Magnetite	21.27	China: Anhui, Maanshan
L180404P-3-LD180402-0007_HL57HCCXY_L5_clean_bin.24.orig.fa_k	OEZ008529	yes	LMSG_G000008036.1	OES073708	lassopeptide	RiPPs	Magnetite	11.73	China: Anhui, Maanshan
L180404P-3-LD180402-0007_HL57HCCXY_L5_clean_bin.28.strict.fa	OEZ008529	yes	LMSG_G000008281.1	OES073708	terpene	Terpene	Magnetite	20.86	China: Anhui, Maanshan
L180404P-3-LD180402-0007_HL57HCCXY_L5_clean_bin.3.strict.fa_	OEZ008529	yes	LMSG_G000008130.1	OES073708	terpene	Terpene	Magnetite	20.48	China: Anhui, Maanshan
L180404P-3-LD180402-0007_HL57HCCXY_L5_clean_bin.31.orig.fa_k	OEZ008529	yes	LMSG_G000007859.1	OES073708	terpene	Terpene	Magnetite	19.97	China: Anhui, Maanshan
L180404P-3-LD180402-0007_HL57HCCXY_L5_clean_bin.31.orig.fa_k	OEZ008529	yes	LMSG_G000007859.1	OES073708	T3PKS	PKSother	Magnetite	8.66	China: Anhui, Maanshan
L180404P-3-LD180402-0007_HL57HCCXY_L5_clean_bin.31.orig.fa_k	OEZ008529	yes	LMSG_G000007859.1	OES073708	terpene	Terpene	Magnetite	21.86	China: Anhui, Maanshan
L180404P-3-LD180402-0007_HL57HCCXY_L5_clean_bin.31.orig.fa_k	OEZ008529	yes	LMSG_G000007859.1	OES073708	NRPS-like	NRPS	Magnetite	24.18	China: Anhui, Maanshan
L180404P-3-LD180402-0007_HL57HCCXY_L5_clean_bin.31.orig.fa_k	OEZ008529	yes	LMSG_G000007859.1	OES073708	NRPS-like	NRPS	Magnetite	16.15	China: Anhui, Maanshan
L180404P-3-LD180402-0007_HL57HCCXY_L5_clean_bin.33.orig.fa_k	OEZ008529	yes	LMSG_G000009621.1	OES073708	terpene	Terpene	Magnetite	22.09	China: Anhui, Maanshan
L180404P-3-LD180402-0007_HL57HCCXY_L5_clean_bin.35.strict.fa	OEZ008529	yes	LMSG_G000010477.1	OES073708	terpene	Terpene	Magnetite	20.83	China: Anhui, Maanshan
L180404P-3-LD180402-0007_HL57HCCXY_L5_clean_bin.35.strict.fa	OEZ008529	yes	LMSG_G000010477.1	OES073708	NRPS-like	NRPS	Magnetite	19.33	China: Anhui, Maanshan
L180404P-3-LD180402-0007_HL57HCCXY_L5_clean_bin.35.strict.fa	OEZ008529	yes	LMSG_G000010477.1	OES073708	arylpolyene.hserlactone	Others	Magnetite	13.27	China: Anhui, Maanshan
L180404P-3-LD180402-0007_HL57HCCXY_L5_clean_bin.38.orig.fa_k	OEZ008529	yes	LMSG_G000005936.1	OES073708	ladderane	Others	Magnetite	6.98	China: Anhui, Maanshan
L180404P-3-LD180402-0007_HL57HCCXY_L5_clean_bin.38.orig.fa_k	OEZ008529	yes	LMSG_G000005936.1	OES073708	ectoine	Others	Magnetite	10.40	China: Anhui, Maanshan
L180404P-3-LD180402-0007_HL57HCCXY_L5_clean_bin.38.orig.fa_k	OEZ008529	yes	LMSG_G000005936.1	OES073708	arylpolyene	Others	Magnetite	10.34	China: Anhui, Maanshan
L180404P-3-LD180402-0007_HL57HCCXY_L5_clean_bin.38.orig.fa_k	OEZ008529	yes	LMSG_G000005936.1	OES073708	terpene	Terpene	Magnetite	20.23	China: Anhui, Maanshan
L180404P-3-LD180402-0007_HL57HCCXY_L5_clean_bin.38.orig.fa_k	OEZ008529	yes	LMSG_G000005936.1	OES073708	arylpolyene	Others	Magnetite	20.80	China: Anhui, Maanshan
L180404P-3-LD180402-0007_HL57HCCXY_L5_clean_bin.38.orig.fa_k	OEZ008529	yes	LMSG_G000005936.1	OES073708	terpene	Terpene	Magnetite	18.50	China: Anhui, Maanshan
L180404P-3-LD180402-0007_HL57HCCXY_L5_clean_bin.38.orig.fa_k	OEZ008529	yes	LMSG_G000005936.1	OES073708	arylpolyene	Others	Magnetite	15.32	China: Anhui, Maanshan
L180404P-3-LD180402-0007_HL57HCCXY_L5_clean_bin.4.orig.fa_kl	OEZ008529	yes	LMSG_G000009015.1	OES073708	bacteriocin	RiPPs	Magnetite	5.77	China: Anhui, Maanshan
L180404P-3-LD180402-0007_HL57HCCXY_L5_clean_bin.4.orig.fa_kl	OEZ008529	yes	LMSG_G000009015.1	OES073708	terpene	Terpene	Magnetite	9.39	China: Anhui, Maanshan
L180404P-3-LD180402-0007_HL57HCCXY_L5_clean_bin.40.orig.fa_k	OEZ008529	yes	LMSG_G000007057.1	OES073708	bacteriocin	RiPPs	Magnetite	11.01	China: Anhui, Maanshan
L180404P-3-LD180402-0007_HL57HCCXY_L5_clean_bin.40.orig.fa_k	OEZ008529	yes	LMSG_G000007057.1	OES073708	bacteriocin	RiPPs	Magnetite	6.37	China: Anhui, Maanshan
L180404P-3-LD180402-0007_HL57HCCXY_L5_clean_bin.5.orig.fa_kl	OEZ008529	yes	LMSG_G000006671.1	OES073708	nucleoside	Others	Magnetite	20.60	China: Anhui, Maanshan
L180404P-3-LD180402-0007_HL57HCCXY_L5_clean_bin.51.orig.fa_k	OEZ008529	yes	LMSG_G000009974.1	OES073708	NRPS-like	NRPS	Magnetite	17.37	China: Anhui, Maanshan
L180404P-3-LD180402-0007_HL57HCCXY_L5_clean_bin.51.orig.fa_k	OEZ008529	yes	LMSG_G000009974.1	OES073708	arylpolyene	Others	Magnetite	7.24	China: Anhui, Maanshan
L180404P-3-LD180402-0007_HL57HCCXY_L5_clean_bin.51.orig.fa_k	OEZ008529	yes	LMSG_G000009974.1	OES073708	terpene	Terpene	Magnetite	15.10	China: Anhui, Maanshan
L180404P-3-LD180402-0007_HL57HCCXY_L5_clean_bin.51.orig.fa_k	OEZ008529	yes	LMSG_G000009974.1	OES073708	arylpolyene	Others	Magnetite	25.51	China: Anhui, Maanshan
L180404P-3-LD180402-0007_HL57HCCXY_L5_clean_bin.51.orig.fa_k	OEZ008529	yes	LMSG_G000009974.1	OES073708	ectoine	Others	Magnetite	10.40	China: Anhui, Maanshan
L180404P-3-LD180402-0007_HL57HCCXY_L5_clean_bin.52.strict.fa	OEZ008529	yes	LMSG_G000009095.1	OES073708	betalactone	Others	Magnetite	31.07	China: Anhui, Maanshan
L180404P-3-LD180402-0007_HL57HCCXY_L5_clean_bin.52.strict.fa	OEZ008529	yes	LMSG_G000009095.1	OES073708	terpene	Terpene	Magnetite	22.04	China: Anhui, Maanshan
L180404P-3-LD180402-0007_HL57HCCXY_L5_clean_bin.52.strict.fa	OEZ008529	yes	LMSG_G000009095.1	OES073708	betalactone	Others	Magnetite	27.63	China: Anhui, Maanshan
L180404P-3-LD180402-0007_HL57HCCXY_L5_clean_bin.52.strict.fa	OEZ008529	yes	LMSG_G000009095.1	OES073708	thiopeptide	RiPPs	Magnetite	31.20	China: Anhui, Maanshan
L180404P-3-LD180402-0007_HL57HCCXY_L5_clean_bin.52.strict.fa	OEZ008529	yes	LMSG_G000009095.1	OES073708	betalactone.bacteriocin	Others	Magnetite	29.69	China: Anhui, Maanshan
L180404P-3-LD180402-0007_HL57HCCXY_L5_clean_bin.52.strict.fa	OEZ008529	yes	LMSG_G000009095.1	OES073708	acyl_amino_acids.betalactone	Others	Magnetite	31.54	China: Anhui, Maanshan
L180404P-3-LD180402-0007_HL57HCCXY_L5_clean_bin.53.permissiv	OEZ008529	yes	LMSG_G000009970.1	OES073708	arylpolyene	Others	Magnetite	23.13	China: Anhui, Maanshan
L180404P-3-LD180402-0007_HL57HCCXY_L5_clean_bin.53.permissiv	OEZ008529	yes	LMSG_G000009970.1	OES073708	ectoine	Others	Magnetite	6.46	China: Anhui, Maanshan
L180404P-3-LD180402-0007_HL57HCCXY_L5_clean_bin.53.permissiv	OEZ008529	yes	LMSG_G000009970.1	OES073708	NRPS-like	NRPS	Magnetite	15.94	China: Anhui, Maanshan
L180404P-3-LD180402-0007_HL57HCCXY_L5_clean_bin.53.permissiv	OEZ008529	yes	LMSG_G000009970.1	OES073708	arylpolyene	Others	Magnetite	12.36	China: Anhui, Maanshan
L180404P-3-LD180402-0007_HL57HCCXY_L5_clean_bin.53.permissiv	OEZ008529	yes	LMSG_G000009970.1	OES073708	terpene	Terpene	Magnetite	7.67	China: Anhui, Maanshan
L180404P-3-LD180402-0007_HL57HCCXY_L5_clean_bin.59.orig.fa_k	OEZ008529	yes	LMSG_G000009284.1	OES073708	terpene	Terpene	Magnetite	22.06	China: Anhui, Maanshan
L180404P-3-LD180402-0007_HL57HCCXY_L5_clean_bin.59.orig.fa_k	OEZ008529	yes	LMSG_G000009284.1	OES073708	terpene	Terpene	Magnetite	21.73	China: Anhui, Maanshan
L180404P-3-LD180402-0007_HL57HCCXY_L5_clean_bin.6.orig.fa_kl	OEZ008529	yes	LMSG_G000010024.1	OES073708	terpene	Terpene	Magnetite	7.02	China: Anhui, Maanshan
L180404P-3-LD180402-0007_HL57HCCXY_L5_clean_bin.65.orig.fa_k	OEZ008529	yes	LMSG_G000010063.1	OES073708	terpene	Terpene	Magnetite	18.24	China: Anhui, Maanshan
L180404P-3-LD180402-0007_HL57HCCXY_L5_clean_bin.65.orig.fa_k	OEZ008529	yes	LMSG_G000010063.1	OES073708	ladderane	Others	Magnetite	11.08	China: Anhui, Maanshan
L180404P-3-LD180402-0007_HL57HCCXY_L5_clean_bin.65.orig.fa_k	OEZ008529	yes	LMSG_G000010063.1	OES073708	ectoine	Others	Magnetite	7.50	China: Anhui, Maanshan
L180404P-3-LD180402-0007_HL57HCCXY_L5_clean_bin.71.permissiv	OEZ008529	yes	LMSG_G000008976.1	OES073708	hglE-KS	PKSother	Magnetite	6.50	China: Anhui, Maanshan
L180404P-3-LD180402-0007_HL57HCCXY_L5_clean_bin.9.orig.fa_kl	OEZ008529	yes	LMSG_G000007757.1	OES073708	NRPS.T1PKS	PKS-NRP_Hybrids	Magnetite	34.96	China: Anhui, Maanshan
L180404P-3-LD180402-0007_HL57HCCXY_L5_clean_bin.9.orig.fa_kl	OEZ008529	yes	LMSG_G000007757.1	OES073708	terpene	Terpene	Magnetite	26.06	China: Anhui, Maanshan
L180404P-3-LD180402-0007_HL57HCCXY_L5_clean_bin.9.orig.fa_kl	OEZ008529	yes	LMSG_G000007757.1	OES073708	T1PKS	PKSI	Magnetite	15.06	China: Anhui, Maanshan
L180404P-3-LD180402-0008_HL57HCCXY_L5_clean_bin.11.orig.fa_k	OEZ008529	yes	LMSG_G000009295.1	OES073709	terpene	Terpene	Magnetite	15.26	China: Anhui, Maanshan
L180404P-3-LD180402-0008_HL57HCCXY_L5_clean_bin.11.orig.fa_k	OEZ008529	yes	LMSG_G000009295.1	OES073709	terpene	Terpene	Magnetite	22.06	China: Anhui, Maanshan
L180404P-3-LD180402-0008_HL57HCCXY_L5_clean_bin.16.orig.fa_k	OEZ008529	yes	LMSG_G000009979.1	OES073709	terpene	Terpene	Magnetite	7.30	China: Anhui, Maanshan
L180404P-3-LD180402-0008_HL57HCCXY_L5_clean_bin.17.orig.fa_k	OEZ008529	yes	LMSG_G000009188.1	OES073709	T3PKS	PKSother	Magnetite	7.00	China: Anhui, Maanshan
L180404P-3-LD180402-0008_HL57HCCXY_L5_clean_bin.22.orig.fa_k	OEZ008529	yes	LMSG_G000007987.1	OES073709	bacteriocin	RiPPs	Magnetite	10.14	China: Anhui, Maanshan
L180404P-3-LD180402-0008_HL57HCCXY_L5_clean_bin.23.orig.fa_k	OEZ008529	yes	LMSG_G000009210.1	OES073709	terpene	Terpene	Magnetite	8.04	China: Anhui, Maanshan
L180404P-3-LD180402-0008_HL57HCCXY_L5_clean_bin.23.orig.fa_k	OEZ008529	yes	LMSG_G000009210.1	OES073709	terpene	Terpene	Magnetite	9.27	China: Anhui, Maanshan
L180404P-3-LD180402-0008_HL57HCCXY_L5_clean_bin.33.orig.fa_k	OEZ008529	yes	LMSG_G000010796.1	OES073709	bacteriocin	RiPPs	Magnetite	8.26	China: Anhui, Maanshan
L180404P-3-LD180402-0008_HL57HCCXY_L5_clean_bin.33.orig.fa_k	OEZ008529	yes	LMSG_G000010796.1	OES073709	acyl_amino_acids	Others	Magnetite	23.00	China: Anhui, Maanshan
L180404P-3-LD180402-0008_HL57HCCXY_L5_clean_bin.33.orig.fa_k	OEZ008529	yes	LMSG_G000010796.1	OES073709	NRPS-like	NRPS	Magnetite	30.09	China: Anhui, Maanshan
L180404P-3-LD180402-0008_HL57HCCXY_L5_clean_bin.34.orig.fa_k	OEZ008529	yes	LMSG_G000009645.1	OES073709	terpene	Terpene	Magnetite	10.97	China: Anhui, Maanshan
L180404P-3-LD180402-0008_HL57HCCXY_L5_clean_bin.34.orig.fa_k	OEZ008529	yes	LMSG_G000009645.1	OES073709	acyl_amino_acids	Others	Magnetite	19.54	China: Anhui, Maanshan
L180404P-3-LD180402-0008_HL57HCCXY_L5_clean_bin.37.orig.fa_k	OEZ008529	yes	LMSG_G000009087.1	OES073709	betalactone	Others	Magnetite	31.07	China: Anhui, Maanshan
L180404P-3-LD180402-0008_HL57HCCXY_L5_clean_bin.37.orig.fa_k	OEZ008529	yes	LMSG_G000009087.1	OES073709	betalactone	Others	Magnetite	20.68	China: Anhui, Maanshan
L180404P-3-LD180402-0008_HL57HCCXY_L5_clean_bin.37.orig.fa_k	OEZ008529	yes	LMSG_G000009087.1	OES073709	acyl_amino_acids	Others	Magnetite	9.69	China: Anhui, Maanshan
L180404P-3-LD180402-0008_HL57HCCXY_L5_clean_bin.37.orig.fa_k	OEZ008529	yes	LMSG_G000009087.1	OES073709	terpene	Terpene	Magnetite	22.04	China: Anhui, Maanshan
L180404P-3-LD180402-0008_HL57HCCXY_L5_clean_bin.37.orig.fa_k	OEZ008529	yes	LMSG_G000009087.1	OES073709	betalactone	Others	Magnetite	27.63	China: Anhui, Maanshan
L180404P-3-LD180402-0008_HL57HCCXY_L5_clean_bin.37.orig.fa_k	OEZ008529	yes	LMSG_G000009087.1	OES073709	betalactone.bacteriocin	Others	Magnetite	29.69	China: Anhui, Maanshan
L180404P-3-LD180402-0008_HL57HCCXY_L5_clean_bin.38.orig.fa_k	OEZ008529	yes	LMSG_G000008282.1	OES073709	terpene	Terpene	Magnetite	20.86	China: Anhui, Maanshan
L180404P-3-LD180402-0008_HL57HCCXY_L5_clean_bin.39.orig.fa_k	OEZ008529	yes	LMSG_G000010064.1	OES073709	ladderane	Others	Magnetite	12.36	China: Anhui, Maanshan
L180404P-3-LD180402-0008_HL57HCCXY_L5_clean_bin.39.orig.fa_k	OEZ008529	yes	LMSG_G000010064.1	OES073709	terpene	Terpene	Magnetite	21.78	China: Anhui, Maanshan
L180404P-3-LD180402-0008_HL57HCCXY_L5_clean_bin.39.orig.fa_k	OEZ008529	yes	LMSG_G000010064.1	OES073709	bacteriocin	RiPPs	Magnetite	6.35	China: Anhui, Maanshan
L180404P-3-LD180402-0008_HL57HCCXY_L5_clean_bin.39.orig.fa_k	OEZ008529	yes	LMSG_G000010064.1	OES073709	terpene	Terpene	Magnetite	5.45	China: Anhui, Maanshan
L180404P-3-LD180402-0008_HL57HCCXY_L5_clean_bin.39.orig.fa_k	OEZ008529	yes	LMSG_G000010064.1	OES073709	arylpolyene	Others	Magnetite	14.86	China: Anhui, Maanshan
L180404P-3-LD180402-0008_HL57HCCXY_L5_clean_bin.39.orig.fa_k	OEZ008529	yes	LMSG_G000010064.1	OES073709	ectoine	Others	Magnetite	6.09	China: Anhui, Maanshan
L180404P-3-LD180402-0008_HL57HCCXY_L5_clean_bin.4.permissive	OEZ008529	yes	LMSG_G000008393.1	OES073709	NRPS-like	NRPS	Magnetite	22.40	China: Anhui, Maanshan

L180404P-3-LD180402-0008_HL57HCCXY_L5_clean_bin.4.permissive	OEZ008529	yes	LMSG_G000008393.1	OES073709	terpene	Terpene	Magnetite	8.11	China: Anhui, Maanshan
L180404P-3-LD180402-0008_HL57HCCXY_L5_clean_bin.4.permissive	OEZ008529	yes	LMSG_G000008393.1	OES073709	terpene	Terpene	Magnetite	6.09	China: Anhui, Maanshan
L180404P-3-LD180402-0008_HL57HCCXY_L5_clean_bin.41.orig.fa_k	OEZ008529	yes	LMSG_G000009165.1	OES073709	T3PKS	PKSother	Magnetite	7.41	China: Anhui, Maanshan
L180404P-3-LD180402-0008_HL57HCCXY_L5_clean_bin.42.strict.fa	OEZ008529	yes	LMSG_G000008037.1	OES073709	thiopeptide.LAP	RiPPs	Magnetite	11.71	China: Anhui, Maanshan
L180404P-3-LD180402-0008_HL57HCCXY_L5_clean_bin.42.strict.fa	OEZ008529	yes	LMSG_G000008037.1	OES073709	lassopeptide	RiPPs	Magnetite	8.68	China: Anhui, Maanshan
L180404P-3-LD180402-0008_HL57HCCXY_L5_clean_bin.49.orig.fa_k	OEZ008529	yes	LMSG_G000008131.1	OES073709	terpene	Terpene	Magnetite	20.49	China: Anhui, Maanshan
L180404P-3-LD180402-0008_HL57HCCXY_L5_clean_bin.50.orig.fa_k	OEZ008529	yes	LMSG_G000007841.1	OES073709	terpene	Terpene	Magnetite	15.15	China: Anhui, Maanshan
L180404P-3-LD180402-0008_HL57HCCXY_L5_clean_bin.50.orig.fa_k	OEZ008529	yes	LMSG_G000007841.1	OES073709	NRPS-like	NRPS	Magnetite	15.46	China: Anhui, Maanshan
L180404P-3-LD180402-0008_HL57HCCXY_L5_clean_bin.50.orig.fa_k	OEZ008529	yes	LMSG_G000007841.1	OES073709	NRPS-like	NRPS	Magnetite	23.97	China: Anhui, Maanshan
L180404P-3-LD180402-0008_HL57HCCXY_L5_clean_bin.50.orig.fa_k	OEZ008529	yes	LMSG_G000007841.1	OES073709	T3PKS	PKSother	Magnetite	25.11	China: Anhui, Maanshan
L180404P-3-LD180402-0008_HL57HCCXY_L5_clean_bin.50.orig.fa_k	OEZ008529	yes	LMSG_G000007841.1	OES073709	terpene	Terpene	Magnetite	13.80	China: Anhui, Maanshan
L180404P-3-LD180402-0008_HL57HCCXY_L5_clean_bin.8.orig.fa_k1	OEZ008529	yes	LMSG_G000010478.1	OES073709	terpene	Terpene	Magnetite	17.89	China: Anhui, Maanshan
L180404P-3-LD180402-0008_HL57HCCXY_L5_clean_bin.8.orig.fa_k1	OEZ008529	yes	LMSG_G000010478.1	OES073709	arylpolyene.hserlactone	Others	Magnetite	41.20	China: Anhui, Maanshan
L180404P-4-LD180402-0009_HL57HCCXY_L6_clean_bin.1.strict.fa_	OEZ008529	yes	LMSG_G000009449.1	OES073726	betalactone	Others	Polymetallic	30.86	China: Anhui, Wuhu
L180404P-4-LD180402-0009_HL57HCCXY_L6_clean_bin.1.strict.fa_	OEZ008529	yes	LMSG_G000009449.1	OES073726	bacteriocin	RiPPs	Polymetallic	10.85	China: Anhui, Wuhu
L180404P-4-LD180402-0009_HL57HCCXY_L6_clean_bin.1.strict.fa_	OEZ008529	yes	LMSG_G000009449.1	OES073726	betalactone	Others	Polymetallic	22.80	China: Anhui, Wuhu
L180404P-4-LD180402-0009_HL57HCCXY_L6_clean_bin.1.strict.fa_	OEZ008529	yes	LMSG_G000009449.1	OES073726	ectoine	Others	Polymetallic	10.40	China: Anhui, Wuhu
L180404P-4-LD180402-0009_HL57HCCXY_L6_clean_bin.6.orig.fa_k1	OEZ008529	no	LMSG_G000006254.1	OES073726	T1PKS	PKSI	Polymetallic	23.87	China: Anhui, Wuhu
L180404P-4-LD180402-0009_HL57HCCXY_L6_clean_bin.6.orig.fa_k1	OEZ008529	no	LMSG_G000006254.1	OES073726	bacteriocin	RiPPs	Polymetallic	8.63	China: Anhui, Wuhu
L180404P-4-LD180402-0009_HL57HCCXY_L6_clean_bin.6.orig.fa_k1	OEZ008529	yes	LMSG_G000006254.1	OES073726	bacteriocin	RiPPs	Polymetallic	10.62	China: Anhui, Wuhu
L180404P-4-LD180402-0009_HL57HCCXY_L6_clean_bin.6.orig.fa_k1	OEZ008529	yes	LMSG_G000006254.1	OES073726	bacteriocin	RiPPs	Polymetallic	7.66	China: Anhui, Wuhu
L180404P-4-LD180402-0009_HL57HCCXY_L6_clean_bin.6.orig.fa_k1	OEZ008529	yes	LMSG_G000006254.1	OES073726	terpene	Terpene	Polymetallic	22.21	China: Anhui, Wuhu
L180404P-4-LD180402-0009_HL57HCCXY_L6_clean_bin.6.orig.fa_k1	OEZ008529	no	LMSG_G000006254.1	OES073726	bacteriocin	RiPPs	Polymetallic	10.85	China: Anhui, Wuhu
L180404P-4-LD180402-0009_HL57HCCXY_L6_clean_bin.9.permissive	OEZ008529	yes	LMSG_G000008200.1	OES073726	terpene	Terpene	Polymetallic	5.31	China: Anhui, Wuhu
L180404P-4-LD180402-0010_HL57HCCXY_L6_clean_bin.11.permissiv	OEZ008529	yes	LMSG_G000008327.1	OES073720	terpene	Terpene	Copper	15.41	China: Anhui, Tongling
L180404P-4-LD180402-0010_HL57HCCXY_L6_clean_bin.12.orig.fa_k	OEZ008529	yes	LMSG_G000004590.1	OES073720	ectoine	Others	Copper	6.69	China: Anhui, Tongling
L180404P-4-LD180402-0010_HL57HCCXY_L6_clean_bin.12.orig.fa_k	OEZ008529	yes	LMSG_G000004590.1	OES073720	LAP	RiPPs	Copper	23.80	China: Anhui, Tongling
L180404P-4-LD180402-0010_HL57HCCXY_L6_clean_bin.12.orig.fa_k	OEZ008529	yes	LMSG_G000004590.1	OES073720	terpene	Terpene	Copper	21.99	China: Anhui, Tongling
L180404P-4-LD180402-0010_HL57HCCXY_L6_clean_bin.12.orig.fa_k	OEZ008529	yes	LMSG_G000004590.1	OES073720	T3PKS	PKSother	Copper	18.63	China: Anhui, Tongling
L180404P-4-LD180402-0010_HL57HCCXY_L6_clean_bin.12.orig.fa_k	OEZ008529	yes	LMSG_G000004590.1	OES073720	betalactone	Others	Copper	22.91	China: Anhui, Tongling
L180404P-4-LD180402-0010_HL57HCCXY_L6_clean_bin.12.orig.fa_k	OEZ008529	yes	LMSG_G000004590.1	OES073720	other	Others	Copper	41.28	China: Anhui, Tongling
L180404P-4-LD180402-0010_HL57HCCXY_L6_clean_bin.12.orig.fa_k	OEZ008529	yes	LMSG_G000004590.1	OES073720	bacteriocin	RiPPs	Copper	10.80	China: Anhui, Tongling
L180404P-4-LD180402-0010_HL57HCCXY_L6_clean_bin.12.orig.fa_k	OEZ008529	yes	LMSG_G000004590.1	OES073720	terpene	Terpene	Copper	20.88	China: Anhui, Tongling
L180404P-4-LD180402-0010_HL57HCCXY_L6_clean_bin.2.orig.fa_k1	OEZ008529	yes	LMSG_G000007059.1	OES073720	bacteriocin	RiPPs	Copper	6.57	China: Anhui, Tongling
L180404P-4-LD180402-0010_HL57HCCXY_L6_clean_bin.6.strict.fa_	OEZ008529	yes	LMSG_G000009450.1	OES073720	bacteriocin	RiPPs	Copper	10.85	China: Anhui, Tongling
L180404P-4-LD180402-0010_HL57HCCXY_L6_clean_bin.6.strict.fa_	OEZ008529	yes	LMSG_G000009450.1	OES073720	betalactone	Others	Copper	22.80	China: Anhui, Tongling
L180404P-4-LD180402-0010_HL57HCCXY_L6_clean_bin.6.strict.fa_	OEZ008529	yes	LMSG_G000009450.1	OES073720	betalactone	Others	Copper	30.86	China: Anhui, Tongling
L180404P-4-LD180402-0010_HL57HCCXY_L6_clean_bin.6.strict.fa_	OEZ008529	yes	LMSG_G000009450.1	OES073720	ectoine	Others	Copper	8.58	China: Anhui, Tongling
L180404P-4-LD180402-0011_HL57HCCXY_L6_clean_bin.1.orig.fa_k1	OEZ008529	yes	LMSG_G000008239.1	OES073721	terpene	Terpene	Copper	18.68	China: Anhui, Tongling
L180404P-4-LD180402-0011_HL57HCCXY_L6_clean_bin.10.orig.fa_k	OEZ008529	yes	LMSG_G000007116.1	OES073721	terpene	Terpene	Copper	20.94	China: Anhui, Tongling
L180404P-4-LD180402-0011_HL57HCCXY_L6_clean_bin.12.strict.fa	OEZ008529	yes	LMSG_G000008319.1	OES073721	terpene	Terpene	Copper	12.45	China: Anhui, Tongling
L180404P-4-LD180402-0011_HL57HCCXY_L6_clean_bin.15.permissiv	OEZ008529	yes	LMSG_G000005945.1	OES073721	bacteriocin	RiPPs	Copper	10.86	China: Anhui, Tongling
L180404P-4-LD180402-0011_HL57HCCXY_L6_clean_bin.15.permissiv	OEZ008529	yes	LMSG_G000005945.1	OES073721	T3PKS	PKSother	Copper	29.09	China: Anhui, Tongling
L180404P-4-LD180402-0011_HL57HCCXY_L6_clean_bin.18.orig.fa_k	OEZ008529	yes	LMSG_G000004591.1	OES073721	betalactone	Others	Copper	11.32	China: Anhui, Tongling
L180404P-4-LD180402-0011_HL57HCCXY_L6_clean_bin.19.permissiv	OEZ008529	yes	LMSG_G000007980.1	OES073721	bacteriocin	RiPPs	Copper	5.34	China: Anhui, Tongling
L180404P-4-LD180402-0011_HL57HCCXY_L6_clean_bin.23.orig.fa_k	OEZ008529	no	LMSG_G000010559.1	OES073721	arylpolyene	Others	Copper	32.69	China: Anhui, Tongling
L180404P-4-LD180402-0011_HL57HCCXY_L6_clean_bin.23.orig.fa_k	OEZ008529	yes	LMSG_G000010559.1	OES073721	bacteriocin	RiPPs	Copper	10.80	China: Anhui, Tongling
L180404P-4-LD180402-0011_HL57HCCXY_L6_clean_bin.23.orig.fa_k	OEZ008529	yes	LMSG_G000010559.1	OES073721	bacteriocin	RiPPs	Copper	5.35	China: Anhui, Tongling
L180404P-4-LD180402-0011_HL57HCCXY_L6_clean_bin.23.orig.fa_k	OEZ008529	no	LMSG_G000010559.1	OES073721	terpene	Terpene	Copper	13.40	China: Anhui, Tongling
L180404P-4-LD180402-0011_HL57HCCXY_L6_clean_bin.23.orig.fa_k	OEZ008529	yes	LMSG_G000010559.1	OES073721	T1PKS	PKSI	Copper	19.51	China: Anhui, Tongling
L180404P-4-LD180402-0011_HL57HCCXY_L6_clean_bin.24.permissiv	OEZ008529	yes	LMSG_G000009380.1	OES073721	terpene	Terpene	Copper	11.70	China: Anhui, Tongling
L180404P-4-LD180402-0011_HL57HCCXY_L6_clean_bin.24.permissiv	OEZ008529	yes	LMSG_G000009380.1	OES073721	ectoine	Others	Copper	7.49	China: Anhui, Tongling
L180404P-4-LD180402-0011_HL57HCCXY_L6_clean_bin.28.orig.fa_k	OEZ008529	yes	LMSG_G000008283.1	OES073721	terpene	Terpene	Copper	19.12	China: Anhui, Tongling
L180404P-4-LD180402-0011_HL57HCCXY_L6_clean_bin.4.orig.fa_k1	OEZ008529	yes	LMSG_G000007377.1	OES073721	bacteriocin	RiPPs	Copper	11.18	China: Anhui, Tongling
L180404P-4-LD180402-0011_HL57HCCXY_L6_clean_bin.6.orig.fa_k1	OEZ008529	yes	LMSG_G000007060.1	OES073721	bacteriocin	RiPPs	Copper	9.71	China: Anhui, Tongling
L180404P-4-LD180402-0011_HL57HCCXY_L6_clean_bin.8.strict.fa_	OEZ008529	yes	LMSG_G000005946.1	OES073721	betalactone	Others	Copper	22.78	China: Anhui, Tongling
L180404P-4-LD180402-0011_HL57HCCXY_L6_clean_bin.8.strict.fa_	OEZ008529	yes	LMSG_G000005946.1	OES073721	betalactone	Others	Copper	30.86	China: Anhui, Tongling
L180404P-4-LD180402-0011_HL57HCCXY_L6_clean_bin.8.strict.fa_	OEZ008529	yes	LMSG_G000005946.1	OES073721	bacteriocin	RiPPs	Copper	10.85	China: Anhui, Tongling
L180404P-4-LD180402-0011_HL57HCCXY_L6_clean_bin.8.strict.fa_	OEZ008529	yes	LMSG_G000005946.1	OES073721	ectoine	Others	Copper	10.05	China: Anhui, Tongling
L180404P-4-LD180402-0011_HL57HCCXY_L6_clean_bin.9.orig.fa_k1	OEZ008529	yes	LMSG_G000007769.1	OES073721	T3PKS	PKSother	Copper	38.97	China: Anhui, Tongling
L180404P-4-LD180402-0011_HL57HCCXY_L6_clean_bin.9.orig.fa_k1	OEZ008529	yes	LMSG_G000007769.1	OES073721	terpene	Terpene	Copper	24.69	China: Anhui, Tongling
L180404P-5-LD180402-0005_HL57HCCXY_L4_L6_L7_clean_R_bin.1.st	OEZ008529	yes	LMSG_G000008038.1	OES073705	lassopeptide	RiPPs	Magnetite	11.68	China: Anhui, Maanshan
L180404P-5-LD180402-0005_HL57HCCXY_L4_L6_L7_clean_R_bin.1.st	OEZ008529	yes	LMSG_G000008038.1	OES073705	thiopeptide	RiPPs	Magnetite	7.48	China: Anhui, Maanshan
L180404P-5-LD180402-0005_HL57HCCXY_L4_L6_L7_clean_R_bin.13.s	OEZ008529	yes	LMSG_G000008886.1	OES073705	T3PKS	PKSother	Magnetite	24.89	China: Anhui, Maanshan
L180404P-5-LD180402-0005_HL57HCCXY_L4_L6_L7_clean_R_bin.13.s	OEZ008529	yes	LMSG_G000008886.1	OES073705	terpene	Terpene	Magnetite	19.81	China: Anhui, Maanshan
L180404P-5-LD180402-0005_HL57HCCXY_L4_L6_L7_clean_R_bin.13.s	OEZ008529	yes	LMSG_G000008886.1	OES073705	arylpolyene	Others	Magnetite	23.28	China: Anhui, Maanshan
L180404P-5-LD180402-0005_HL57HCCXY_L4_L6_L7_clean_R_bin.15.s	OEZ008529	yes	LMSG_G000009285.1	OES073705	terpene	Terpene	Magnetite	22.06	China: Anhui, Maanshan
L180404P-5-LD180402-0005_HL57HCCXY_L4_L6_L7_clean_R_bin.15.s	OEZ008529	yes	LMSG_G000009285.1	OES073705	terpene	Terpene	Magnetite	21.73	China: Anhui, Maanshan
L180404P-5-LD180402-0005_HL57HCCXY_L4_L6_L7_clean_R_bin.16.o	OEZ008529	yes	LMSG_G000007988.1	OES073705	bacteriocin	RiPPs	Magnetite	7.91	China: Anhui, Maanshan
L180404P-5-LD180402-0005_HL57HCCXY_L4_L6_L7_clean_R_bin.17.s	OEZ008529	yes	LMSG_G000009096.1	OES073705	betalactone.bacteriocin	Others	Magnetite	29.69	China: Anhui, Maanshan
L180404P-5-LD180402-0005_HL57HCCXY_L4_L6_L7_clean_R_bin.17.s	OEZ008529	yes	LMSG_G000009096.1	OES073705	thiopeptide	RiPPs	Magnetite	31.20	China: Anhui, Maanshan
L180404P-5-LD180402-0005_HL57HCCXY_L4_L6_L7_clean_R_bin.17.s	OEZ008529	yes	LMSG_G000009096.1	OES073705	betalactone	Others	Magnetite	20.84	China: Anhui, Maanshan
L180404P-5-LD180402-0005_HL57HCCXY_L4_L6_L7_clean_R_bin.17.s	OEZ008529	yes	LMSG_G000009096.1	OES073705	betalactone	Others	Magnetite	20.96	China: Anhui, Maanshan
L180404P-5-LD180402-0005_HL57HCCXY_L4_L6_L7_clean_R_bin.17.s	OEZ008529	yes	LMSG_G000009096.1	OES073705	betalactone	Others	Magnetite	21.06	China: Anhui, Maanshan
L180404P-5-LD180402-0005_HL57HCCXY_L4_L6_L7_clean_R_bin.17.s	OEZ008529	yes	LMSG_G000009096.1	OES073705	acyl_amino_acids	Others	Magnetite	6.49	China: Anhui, Maanshan
L180404P-5-LD180402-0005_HL57HCCXY_L4_L6_L7_clean_R_bin.2.st	OEZ008529	yes	LMSG_G000008132.1	OES073705	terpene	Terpene	Magnetite	20.48	China: Anhui, Maanshan
L180404P-5-LD180402-0005_HL57HCCXY_L4_L6_L7_clean_R_bin.20.s	OEZ008529	yes	LMSG_G000010568.1	OES073705	bacteriocin	RiPPs	Magnetite	10.95	China: Anhui, Maanshan
L180404P-5-LD180402-0005_HL57HCCXY_L4_L6_L7_clean_R_bin.20.s	OEZ008529	yes	LMSG_G000010568.1	OES073705	arylpolyene	Others	Magnetite	22.79	China: Anhui, Maanshan
L180404P-5-LD180402-0005_HL57HCCXY_L4_L6_L7_clean_R_bin.24.p	OEZ008529	yes	LMSG_G000011110.1	OES073705	terpene	Terpene	Magnetite	19.20	China: Anhui, Maanshan
L180404P-5-LD180402-0005_HL57HCCXY_L4_L6_L7_clean_R_bin.4.or	OEZ008529	yes	LMSG_G000008243.1	OES073705	terpene	Terpene	Magnetite	7.33	China: Anhui, Maanshan
L180404P-5-LD180402-0012_HL57HCCXY_L7_clean_bin.13.orig.fa_k	OEZ008529	yes	LMSG_G000007247.1	OES073722	betalactone	Others	Copper	11.99	China: Anhui, Tongling
L180404P-5-LD180402-0012_HL57HCCXY_L7_clean_bin.14.orig.fa_k	OEZ008529	yes	LMSG_G000007770.1	OES073722	T3PKS	PKSother	Copper	41.02	China: Anhui, Tongling
L180404P-5-LD180402-0012_HL57HCCXY_L7_clean_bin.14.orig.fa_k	OEZ008529	yes	LMSG_G000007770.1	OES073722	terpene	Terpene	Copper	24.65	China: Anhui, Tongling
L180404P-5-LD180402-0012_HL57HCCXY_L7_clean_bin.15.strict.fa	OEZ008529	yes	LMSG_G000009451.1	OES073722	bacteriocin	RiPPs	Copper	10.85	China: Anhui, Tongling
L180404P-5-LD180402-0012_HL57HCCXY_L7_clean_bin.15.strict.fa	OEZ008529	yes	LMSG_G000009451.1	OES073722	betalactone	Others	Copper	25.25	China: Anhui, Tongling
L180404P-5-LD180402-0012_HL57HCCXY_L7_clean_bin.15.strict.fa	OEZ008529	yes	LMSG_G000009451.1	OES073722	betalactone	Others	Copper	22.80	China: Anhui, Tongling
L180404P-5-LD180402-0012_HL57HCCXY_L7_clean_bin.15.strict.fa	OEZ008529	yes	LMSG_G000009451.1	OES073722	ectoine	Others	Copper	10.40	China: Anhui, Tongling
L180404P-5-LD180402-0012_HL57HCCXY_L7_clean_bin.16.orig.fa_k	OEZ008529	yes	LMSG_G000008284.1	OES073722	terpene	Terpene	Copper	20.86	China: Anhui, Tongling
L180404P-5-LD180402-0012_HL57HCCXY_L7_clean_bin.8.orig.fa_k1	OEZ008529	yes	LMSG_G000008328.1	OES073722	NRPS.hserlactone	Others	Copper	11.53	China: Anhui, Tongling
L180404P-5-LD180402-0012_HL57HCCXY_L7_clean_bin.8.orig.fa_k1	OEZ008529	yes	LMSG_G000008328.1	OES073722	terpene	Terpene	Copper	21.09	China: Anhui, Tongling
L180404P-5-LD180402-0012_HL57HCCXY_L7_clean_bin.9.orig.fa_k1	OEZ008529	yes	LMSG_G000010529.1	OES073722	bacteriocin	RiPPs	Copper	10.84	China: Anhui, Tongling
L180404P-5-LD180402-0012_HL57HCCXY_L7_clean_bin.9.orig.fa_k1	OEZ008529	yes	LMSG_G000010529.1	OES073722	T1PKS	PKSI	Copper	20.17	China: Anhui, Tongling
L180404P-5-LD180402-0012_HL57HCCXY_L7_clean_bin.9.orig.fa_k1	OEZ008529	yes	LMSG_G000010529.1	OES073722	arylpolyene	Others	Copper	40.67	China: Anhui, Tongling
L180404P-5-LD180402-0012_HL57HCCXY_L7_clean_bin.9.orig.fa_k1	OEZ008529	yes	LMSG_G000010529.1	OES073722	T1PKS	PKSI	Copper	47.31	China: Anhui, Tongling
L180404P-5-LD180402-0012_HL57HCCXY_L7_clean_bin.9.orig.fa_k1	OEZ008529	yes	LMSG_G000010529.1	OES073722	terpene	Terpene	Copper	22.97	China: Anhui, Tongling
L180404P-5-LD180402-0013_HL57HCCXY_L7_clean_bin.12.orig.fa_k	OEZ008529	yes	LMSG_G000008329.1	OES073723	terpene	Terpene	Copper	21.09	China: Anhui, Tongling
L180404P-5-LD180402-0013_HL57HCCXY_L7_clean_bin.13.orig.fa_k	OEZ008529	yes	LMSG_G000011325.1	OES073723	bacteriocin	RiPPs	Copper	7.68	China: Anhui, Tongling
L180404P-5-LD180402-0013_HL57HCCXY_L7_clean_bin.13.orig.fa_k	OEZ008529	yes	LMSG_G000011325.1	OES073723	T3PKS	PKSother	Copper	11.31	China: Anhui, Tongling
L180404P-5-LD180402-0013_HL57HCCXY_L7_clean_bin.4.orig.fa_k1	OEZ008529	yes	LMSG_G000008285.1	OES073723	terpene	Terpene	Copper	19.12	China: Anhui, Tongling
L180404P-5-LD180402-0013_HL57HCCXY_L7_clean_bin.5.orig.fa_k1	OEZ008529	yes	LMSG_G000007061.1	OES073723	bacteriocin	RiPPs	Copper	6.19	China: Anhui, Tongling
L180404P-5-LD180402-0013_HL57HCCXY_L7_clean_bin.6.orig.fa_k1	OEZ008529	yes	LMSG_G000009452.1	OES073723	betalactone	Others	Copper	30.48	China: Anhui, Tongling
L180404P-5-LD180402-0013_HL57HCCXY_L7_clean_bin.6.orig.fa_k1	OEZ008529	yes	LMSG_G000009452.1	OES073723	betalactone	Others	Copper	22.77	China: Anhui, Tongling
L180404P-5-LD180402-0013_HL57HCCXY_L7_clean_bin.6.orig.fa_k1	OEZ008529	yes	LMSG_G000009452.1	OES073723	ectoine	Others	Copper	10.40	China: Anhui, Tongling
L180404P-5-LD180402-0013_HL57HCCXY_L7_clean_bin.6.orig.fa_k1	OEZ008529	yes	LMSG_G000009452.1	OES073723	bacteriocin	RiPPs	Copper	10.85	China: Anhui, Tongling
L180404P-5-LD180402-0013_HL57HCCXY_L7_clean_bin.9.orig.fa_k1	OEZ008529	yes	LMSG_G000007117.1	OES073723	terpene	Terpene	Copper	20.94	China: Anhui, Tongling
L180404P-5-LD180402-0014_HL57HCCXY_L7_clean_bin.11.orig.fa_k	OEZ008529	yes	LMSG_G000007941.1	OES073676	arylpolyene	Others	Pyrite-Copper	9.95	China: Guangdong, Daobaoshan
L180404P-5-LD180402-0014_HL57HCCXY_L7_clean_bin.11.orig.fa_k	OEZ008529	yes	LMSG_G000007941.1	OES073676	resorcinol	Others	Pyrite-Copper	22.76	China: Guangdong, Daobaoshan
L180404P-5-LD180402-0014_HL57HCCXY_L7_clean_bin.16.orig.fa_k	OEZ008529	yes	LMSG_G000005948.1	OES073676	arylpolyene	Others	Pyrite-Copper	33.81	China: Guangdong, Daobaoshan
L180404P-5-LD180402-0014_HL57HCCXY_L7_clean_bin.16.orig.fa_k	OEZ008529	yes	LMSG_G000005948.1	OES073676	terpene	Terpene	Pyrite-Copper	21.70	China: Guangdong, Daobaoshan
L180404P-5-LD180402-0014_HL57HCCXY_L7_clean_bin.18.strict.fa	OEZ008529	yes	LMSG_G000008201.1	OES073676	terpene	Terpene	Pyrite-Copper	14.62	China: Guangdong, Daobaoshan
L180404P-5-LD180402-0014_HL57HCCXY_L7_clean_bin.2.orig.fa_k1	OEZ008529	yes	LMSG_G000010595.1	OES073676	terpene	Terpene	Pyrite-Copper	23.02	China: Guangdong, Daobaoshan
L180404P-5-LD180402-0014_HL57HCCXY_L7_clean_bin.20.orig.fa_k	OEZ008529	yes	LMSG_G000008240.1	OES073676	terpene	Terpene	Pyrite-Copper	6.51	China: Guangdong, Daobaoshan
L180404P-5-LD180402-0014_HL57HCCXY_L7_clean_bin.22.orig.fa_k	OEZ008529	yes	LMSG_G000005950.1	OES073676	terpene	Terpene	Pyrite-Copper	9.01	China: Guangdong, Daobaoshan
L180404P-5-LD180402-0014_HL57HCCXY_L7_clean_bin.25.strict.fa	OEZ008529	yes	LMSG_G000009697.1	OES073676	NRPS-like	NRPS	Pyrite-Copper	26.20	China: Guangdong, Daobaoshan
L180404P-5-LD180402-0014_HL57HCCXY_L7_clean_bin.3.orig.fa_k1	OEZ008529	yes	LMSG_G000005955.1	OES073676	terpene	Terpene	Pyrite-Copper	21.74	China: Guangdong, Daobaoshan
L180404P-5-LD180402-0014_HL57HCCXY_L7_clean_bin.32.orig.fa_k	OEZ008529	yes	LMSG_G000008756.1	OES073676	terpene	Terpene	Pyrite-Copper	15.74	China: Guangdong, Daobaoshan
L180404P-5-LD180402-0014_HL57HCCXY_L7_clean_bin.32.orig.fa_k	OEZ008529	yes	LMSG_G000008756.1	OES073676	terpene	Terpene	Pyrite-Copper	22.00	China: Guangdong, Daobaoshan
L180404P-5-LD180402-0014_HL57HCCXY_L7_clean_bin.33.strict.fa	OEZ008529	yes	LMSG_G000005953.1	OES073676	terpene	Terpene	Pyrite-Copper	22.01	China: Guangdong, Daobaoshan
L180404P-5-LD180402-0014_HL57HCCXY_L7_clean_bin.33.strict.fa	OEZ008529	yes	LMSG_G000005953.1	OES073676	NRPS-like	NRPS	Pyrite-Copper	23.25	China: Guangdong, Daobaoshan
L180404P-5-LD180402-0014_HL57HCCXY_L7_clean_bin.33.strict.fa	OEZ008529	yes	LMSG_G000005953.1	OES073676	NRPS-like	NRPS	Pyrite-Copper	19.67	China: Guangdong, Daobaoshan
L180404P-5-LD180402-0014_HL57HCCXY_L7_clean_bin.34.orig.fa_k	OEZ008529	yes	LMSG_G000005954.1	OES073676	terpene	Terpene	Pyrite-Copper	10.14	China: Guangdong, Daobaoshan
L180404P-5-LD180402-0014_HL57HCCXY_L7_clean_bin.34.orig.fa_k	OEZ008529	yes	LMSG_G000005954.1	OES073676	lanthipeptide	RiPPs	Pyrite-Copper	13.96	China: Guangdong, Daobaoshan
L180404P-5-LD180402-0014_HL57HCCXY_L7_clean_bin.35.orig.fa_k	OEZ008529	yes	LMSG_G000006509.1	OES073676	thiopeptide	RiPPs	Pyrite-Copper	35.98	China: Guangdong, Daobaoshan
L180404P-5-LD180402-0014_HL57HCCXY_L7_clean_bin.4.orig.fa_k1	OEZ008529	yes	LMSG_G000008891.1	OES073676	terpene	Terpene	Pyrite-Copper	21.01	China: Guangdong, Daobaoshan
L180404P-5-LD180402-0014_HL57HCCXY_L7_clean_bin.4.orig.fa_k1	OEZ008529	yes	LMSG_G000008891.1	OES073676	T3PKS	PKSother	Pyrite-Copper	26.57	China: Guangdong, Daobaoshan
L180404P-5-LD180402-0014_HL57HCCXY_L7_clean_bin.40.orig.fa_k	OEZ008529	yes	LMSG_G000010026.1	OES073676	NRPS-like	NRPS	Pyrite-Copper	7.19	China: Guangdong, Daobaoshan
L180404P-5-LD180402-0014_HL57HCCXY_L7_clean_bin.40.orig.fa_k	OEZ008529	yes	LMSG_G000010026.1	OES073676	terpene	Terpene	Pyrite-Copper	9.48	China: Guangdong, Daobaoshan
L180404P-5-LD180402-0014_HL57HCCXY_L7_clean_bin.40.orig.fa_k	OEZ008529	yes	LMSG_G000010026.1	OES073676	terpene	Terpene	Pyrite-Copper	17.80	China: Guangdong, Daobaoshan
L180404P-5-LD180402-0014_HL57HCCXY_L7_clean_bin.40.orig.fa_k	OEZ008529	yes	LMSG_G000010026.1	OES073676	T3PKS	PKSother	Pyrite-Copper	25.40	China: Guangdong, Daobaoshan
L180404P-5-LD180402-0014_HL57HCCXY_L7_clean_bin.42.orig.fa_k	OEZ008529	yes	LMSG_G000010785.1	OES073676	acyl_amino_acids	Others	Pyrite-Copper	33.89	China: Guangdong, Daobaoshan
L180404P-5-LD180402-0014_HL57HCCXY_L7_clean_bin.42.orig.fa_k	OEZ008529	yes	LMSG_G000010785.1	OES073676	arylpolyene	Others	Pyrite-Copper	8.60	China: Guangdong, Daobaoshan
L180404P-5-LD180402-0014_HL57HCCXY_L7_clean_bin.49.orig.fa_k	OEZ008529	yes	LMSG_G000008790.1	OES073676	NRPS-like	NRPS	Pyrite-Copper	6.25	China: Guangdong, Daobaoshan
L180404P-5-LD180402-0014_HL57HCCXY_L7_clean_bin.49.orig.fa_k	OEZ008529	yes	LMSG_G000008790.1	OES073676	bacteriocin	RiPPs	Pyrite-Copper	9.48	China: Guangdong, Daobaoshan
L180404P-5-LD180402-0014_HL57HCCXY_L7_clean_bin.49.orig.fa_k	OEZ008529	yes	LMSG_G000008790.1	OES073676	NRPS-like	NRPS	Pyrite-Copper	6.89	China: Guangdong, Daobaoshan
L180404P-5-LD180402-0014_HL57HCCXY_L7_clean_bin.5.strict.fa_	OEZ008529	yes	LMSG_G000004592.1	OES073676	NRPS-like	NRPS	Pyrite-Copper	32.14	China: Guangdong, Daobaoshan
L180404P-5-LD180402-0014_HL57HCCXY_L7_clean_bin.5.strict.fa_	OEZ008529	yes	LMSG_G000004592.1	OES073676	NRPS-like	NRPS	Pyrite-Copper	42.63	China: Guangdong, Daobaoshan
L180404P-5-LD180402-0014_HL57HCCXY_L7_clean_bin.5.strict.fa_	OEZ008529	yes	LMSG_G000004592.1	OES073676	betalactone	Others	Pyrite-Copper	30.36	China: Guangdong, Daobaoshan
L180404P-5-LD180402-0014_HL57HCCXY_L7_clean_bin.5.strict.fa_	OEZ008529	yes	LMSG_G000004592.1	OES073676	terpene	Terpene	Pyrite-Copper	22.01	China: Guangdong, Daobaoshan
L180404P-5-LD180402-0014_HL57HCCXY_L7_clean_bin.52.orig.fa_k	OEZ008529	yes	LMSG_G000009043.1	OES073676	thiopeptide	RiPPs	Pyrite-Copper	5.37	China: Guangdong, Daobaoshan
L180404P-5-LD180402-0014_HL57HCCXY_L7_clean_bin.54.strict.fa	OEZ008529	yes	LMSG_G000008039.1	OES073676	LAP.thiopeptide	RiPPs	Pyrite-Copper	19.97	China: Guangdong, Daobaoshan
L180404P-5-LD180402-0014_HL57HCCXY_L7_clean_bin.54.strict.fa	OEZ008529	yes	LMSG_G000008039.1	OES073676	lassopeptide	RiPPs	Pyrite-Copper	11.84	China: Guangdong, Daobaoshan
L180404P-5-LD180402-0014_HL57HCCXY_L7_clean_bin.55.permissiv	OEZ008529	yes	LMSG_G000009296.1	OES073676	terpene	Terpene	Pyrite-Copper	21.73	China: Guangdong, Daobaoshan
L180404P-5-LD180402-0014_HL57HCCXY_L7_clean_bin.55.permissiv	OEZ008529	yes	LMSG_G000009296.1	OES073676	terpene	Terpene	Pyrite-Copper	22.06	China: Guangdong, Daobaoshan
L180404P-5-LD180402-0014_HL57HCCXY_L7_clean_bin.56.orig.fa_k	OEZ008529	yes	LMSG_G000008133.1	OES073676	terpene	Terpene	Pyrite-Copper	20.48	China: Guangdong, Daobaoshan
L180404P-5-LD180402-0014_HL57HCCXY_L7_clean_bin.57.strict.fa	OEZ008529	yes	LMSG_G000005959.1	OES073676	LAP	RiPPs	Pyrite-Copper	10.75	China: Guangdong, Daobaoshan
L180404P-5-LD180402-0014_HL57HCCXY_L7_clean_bin.60.orig.fa_k	OEZ008529	yes	LMSG_G000010429.1	OES073676	NRPS	NRPS	Pyrite-Copper	30.53	China: Guangdong, Daobaoshan
L180404P-5-LD180402-0014_HL57HCCXY_L7_clean_bin.60.orig.fa_k	OEZ008529	yes	LMSG_G000010429.1	OES073676	hserlactone	Others	Pyrite-Copper	13.03	China: Guangdong, Daobaoshan
L180404P-5-LD180402-0014_HL57HCCXY_L7_clean_bin.60.orig.fa_k	OEZ008529	yes	LMSG_G000010429.1	OES073676	bacteriocin	RiPPs	Pyrite-Copper	9.46	China: Guangdong, Daobaoshan
L180404P-5-LD180402-0014_HL57HCCXY_L7_clean_bin.60.orig.fa_k	OEZ008529	yes	LMSG_G000010429.1	OES073676	terpene	Terpene	Pyrite-Copper	25.02	China: Guangdong, Daobaoshan
L180404P-5-LD180402-0014_HL57HCCXY_L7_clean_bin.61.permissiv	OEZ008529	yes	LMSG_G000007990.1	OES073676	bacteriocin	RiPPs	Pyrite-Copper	9.20	China: Guangdong, Daobaoshan
L180404P-5-LD180402-0014_HL57HCCXY_L7_clean_bin.61.permissiv	OEZ008529	yes	LMSG_G000007990.1	OES073676	TfuA-related.bacteriocin	RiPPs	Pyrite-Copper	6.75	China: Guangdong, Daobaoshan
L180404P-5-LD180402-0014_HL57HCCXY_L7_clean_bin.62.strict.fa	OEZ008529	yes	LMSG_G000005961.1	OES073676	bacteriocin	RiPPs	Pyrite-Copper	10.83	China: Guangdong, Daobaoshan
L180404P-5-LD180402-0014_HL57HCCXY_L7_clean_bin.63.orig.fa_k	OEZ008529	no	LMSG_G000009540.1	OES073676	NRPS-like	NRPS	Pyrite-Copper	39.14	China: Guangdong, Daobaoshan
L180404P-5-LD180402-0014_HL57HCCXY_L7_clean_bin.63.orig.fa_k	OEZ008529	no	LMSG_G000009540.1	OES073676	terpene	Terpene	Pyrite-Copper	22.05	China: Guangdong, Daobaoshan
L180404P-5-LD180402-0014_HL57HCCXY_L7_clean_bin.64.orig.fa_k	OEZ008529	yes	LMSG_G000005962.1	OES073676	ladderane	Others	Pyrite-Copper	41.16	China: Guangdong, Daobaoshan
L180404P-5-LD180402-0014_HL57HCCXY_L7_clean_bin.7.orig.fa_k1	OEZ008529	yes	LMSG_G000007826.1	OES073676	terpene	Terpene	Pyrite-Copper	29.81	China: Guangdong, Daobaoshan
L180404P-5-LD180402-0014_HL57HCCXY_L7_clean_bin.7.orig.fa_k1	OEZ008529	yes	LMSG_G000007826.1	OES073676	T3PKS	PKSother	Pyrite-Copper	32.41	China: Guangdong, Daobaoshan
L180404P-5-LD180402-0014_HL57HCCXY_L7_clean_bin.8.strict.fa_	OEZ008529	yes	LMSG_G000005965.1	OES073676	betalactone	Others	Pyrite-Copper	17.72	China: Guangdong, Daobaoshan

L180404P-5-LD180402-0014_HL57HCCXY_L7_clean_bin.8.strict.fa_	OEZ008529	yes	LMSG_G000005965.1	OES073676	betalactone	Others	Pyrite-Copper	28.41	China: Guangdong, Daobaoshan
L180404P-5-LD180402-0014_HL57HCCXY_L7_clean_bin.8.strict.fa_	OEZ008529	yes	LMSG_G000005965.1	OES073676	terpene	Terpene	Pyrite-Copper	20.10	China: Guangdong, Daobaoshan
L180404P-5-LD180402-0014_HL57HCCXY_L7_clean_bin.9.orig.fa_k1	OEZ008529	yes	LMSG_G000005966.1	OES073676	T3PKS	PKSother	Pyrite-Copper	14.38	China: Guangdong, Daobaoshan
L180404P-5-LD180402-0014_HL57HCCXY_L7_clean_bin.9.orig.fa_k1	OEZ008529	yes	LMSG_G000005966.1	OES073676	terpene	Terpene	Pyrite-Copper	15.33	China: Guangdong, Daobaoshan
SHC_1601_R_bin.1.orig.fa_k141_3772.region001.fasta	OEZ008529	yes	LMSG_G000007197.1	OES074477	bacteriocin	RiPPs	Pyrite	6.03	China: Guangdong, Guangzhou
SHC_1601_R_bin.3.orig.fa_k141_9455.region001.fasta	OEZ008529	yes	LMSG_G000007224.1	OES074477	ectoine	Others	Pyrite	8.66	China: Guangdong, Guangzhou
SHC_1601_R_bin.4.orig.fa_k141_51481.region001.fasta	OEZ008529	yes	LMSG_G000004595.1	OES074477	betalactone	Others	Pyrite	28.72	China: Guangdong, Guangzhou
SHC_1601_R_bin.5.orig.fa_k141_1330.region001.fasta	OEZ008529	no	LMSG_G000010541.1	OES074477	hserlactone	Others	Pyrite	20.55	China: Guangdong, Guangzhou
SHC_1601_R_bin.5.orig.fa_k141_34152.region001.fasta	OEZ008529	yes	LMSG_G000010541.1	OES074477	terpene	Terpene	Pyrite	23.13	China: Guangdong, Guangzhou
SHC_1601_R_bin.5.orig.fa_k141_39919.region001.fasta	OEZ008529	yes	LMSG_G000010541.1	OES074477	arylpolyene	Others	Pyrite	41.19	China: Guangdong, Guangzhou
SHC_1601_R_bin.6.orig.fa_k141_15052.region001.fasta	OEZ008529	no	LMSG_G000009554.1	OES074477	terpene	Terpene	Pyrite	23.38	China: Guangdong, Guangzhou
SHC_1601_R_bin.6.orig.fa_k141_18659.region001.fasta	OEZ008529	no	LMSG_G000009554.1	OES074477	terpene	Terpene	Pyrite	12.86	China: Guangdong, Guangzhou
SHC_1601_R_bin.6.orig.fa_k141_45722.region001.fasta	OEZ008529	yes	LMSG_G000009554.1	OES074477	ectoine	Others	Pyrite	6.71	China: Guangdong, Guangzhou
SHC_1601_R_bin.6.orig.fa_k141_49968.region001.fasta	OEZ008529	yes	LMSG_G000009554.1	OES074477	NRPS-like	NRPS	Pyrite	31.37	China: Guangdong, Guangzhou
SHC_1601_R_bin.8.orig.fa_k141_11857.region001.fasta	OEZ008529	no	LMSG_G000010524.1	OES074477	terpene	Terpene	Pyrite	8.53	China: Guangdong, Guangzhou
SHC_1601_R_bin.8.orig.fa_k141_40824.region001.fasta	OEZ008529	yes	LMSG_G000010524.1	OES074477	arylpolyene	Others	Pyrite	5.23	China: Guangdong, Guangzhou
SHC_1606_R_bin.11.orig.fa_k141_15914.region001.fasta	OEZ008529	yes	LMSG_G000005967.1	OES074478	bacteriocin	RiPPs	Pyrite	8.78	China: Guangdong, Guangzhou
SHC_1606_R_bin.11.orig.fa_k141_45296.region001.fasta	OEZ008529	yes	LMSG_G000005967.1	OES074478	terpene	Terpene	Pyrite	21.98	China: Guangdong, Guangzhou
SHC_1606_R_bin.11.orig.fa_k141_54505.region001.fasta	OEZ008529	yes	LMSG_G000005967.1	OES074478	arylpolyene	Others	Pyrite	13.26	China: Guangdong, Guangzhou
SHC_1606_R_bin.2.orig.fa_k141_46682.region001.fasta	OEZ008529	yes	LMSG_G000007080.1	OES074478	betalactone	Others	Pyrite	29.69	China: Guangdong, Guangzhou
SHC_1606_R_bin.9.permissive.fa_c00004_NODE_4_...region001.fa	OEZ008529	yes	LMSG_G000010542.1	OES074478	terpene	Terpene	Pyrite	23.13	China: Guangdong, Guangzhou
SHC_1606_R_bin.9.permissive.fa_c00005_NODE_5_...region001.fa	OEZ008529	yes	LMSG_G000010542.1	OES074478	arylpolyene	Others	Pyrite	41.19	China: Guangdong, Guangzhou
SHC_1606_R_bin.9.permissive.fa_c00017_NODE_17...region001.fa	OEZ008529	no	LMSG_G000010542.1	OES074478	hserlactone	Others	Pyrite	20.55	China: Guangdong, Guangzhou
SHC_160711_S7_L001_R_bin.1.orig.fa_k141_15170.region001.fast	OEZ008529	no	LMSG_G000010543.1	OES074479	terpene	Terpene	Pyrite	16.37	China: Guangdong, Guangzhou
SHC_160711_S7_L001_R_bin.1.orig.fa_k141_45420.region001.fast	OEZ008529	yes	LMSG_G000010543.1	OES074479	bacteriocin	RiPPs	Pyrite	8.64	China: Guangdong, Guangzhou
SHC_160711_S7_L001_R_bin.1.orig.fa_k141_62290.region001.fast	OEZ008529	yes	LMSG_G000010543.1	OES074479	arylpolyene	Others	Pyrite	41.19	China: Guangdong, Guangzhou
SHC_160711_S7_L001_R_bin.10.orig.fa_k141_10889.region001.fas	OEZ008529	yes	LMSG_G000007225.1	OES074479	ectoine	Others	Pyrite	9.55	China: Guangdong, Guangzhou
SHC_160711_S7_L001_R_bin.11.strict.fa_c00013_NODE_13...regio	OEZ008529	yes	LMSG_G000009422.1	OES074479	betalactone	Others	Pyrite	24.74	China: Guangdong, Guangzhou
SHC_160711_S7_L001_R_bin.11.strict.fa_c00016_NODE_16...regio	OEZ008529	yes	LMSG_G000009422.1	OES074479	betalactone	Others	Pyrite	22.76	China: Guangdong, Guangzhou
SHC_160711_S7_L001_R_bin.11.strict.fa_c00034_NODE_34...regio	OEZ008529	yes	LMSG_G000009422.1	OES074479	ectoine	Others	Pyrite	10.40	China: Guangdong, Guangzhou
SHC_160711_S7_L001_R_bin.11.strict.fa_c00081_NODE_81...regio	OEZ008529	yes	LMSG_G000009422.1	OES074479	bacteriocin	RiPPs	Pyrite	10.64	China: Guangdong, Guangzhou
SHC_160711_S7_L001_R_bin.11.strict.fa_c00115_NODE_11...regio	OEZ008529	yes	LMSG_G000009422.1	OES074479	bacteriocin	RiPPs	Pyrite	9.09	China: Guangdong, Guangzhou
SHC_160711_S7_L001_R_bin.2.permissive.fa_c00012_NODE_12...re	OEZ008529	yes	LMSG_G000009601.1	OES074479	terpene	Terpene	Pyrite	22.15	China: Guangdong, Guangzhou
SHC_160711_S7_L001_R_bin.2.permissive.fa_c00027_NODE_27...re	OEZ008529	yes	LMSG_G000009601.1	OES074479	NRPS-like	NRPS	Pyrite	24.92	China: Guangdong, Guangzhou
SHC_160711_S7_L001_R_bin.2.permissive.fa_c00047_NODE_47...re	OEZ008529	yes	LMSG_G000009601.1	OES074479	acyl_amino_acids	Others	Pyrite	16.16	China: Guangdong, Guangzhou
SHC_160711_S7_L001_R_bin.2.permissive.fa_c00050_NODE_50...re	OEZ008529	yes	LMSG_G000009601.1	OES074479	terpene	Terpene	Pyrite	14.11	China: Guangdong, Guangzhou
SHC_160711_S7_L001_R_bin.2.permissive.fa_c00066_NODE_66...re	OEZ008529	yes	LMSG_G000009601.1	OES074479	ectoine	Others	Pyrite	6.44	China: Guangdong, Guangzhou
SHC_160711_S7_L001_R_bin.3.orig.fa_k141_14168.region001.fast	OEZ008529	no	LMSG_G000005969.1	OES074479	terpene	Terpene	Pyrite	23.38	China: Guangdong, Guangzhou
SHC_160711_S7_L001_R_bin.3.orig.fa_k141_22113.region001.fast	OEZ008529	yes	LMSG_G000005969.1	OES074479	terpene	Terpene	Pyrite	22.06	China: Guangdong, Guangzhou
SHC_160711_S7_L001_R_bin.3.orig.fa_k141_47536.region001.fast	OEZ008529	yes	LMSG_G000005969.1	OES074479	NRPS.hserlactone	Others	Pyrite	27.69	China: Guangdong, Guangzhou
SHC_160711_S7_L001_R_bin.3.orig.fa_k141_505.region001.fasta	OEZ008529	yes	LMSG_G000005969.1	OES074479	ectoine	Others	Pyrite	10.40	China: Guangdong, Guangzhou
SHC_160711_S7_L001_R_bin.3.orig.fa_k141_63540.region001.fast	OEZ008529	yes	LMSG_G000005969.1	OES074479	NRPS-like	NRPS	Pyrite	42.73	China: Guangdong, Guangzhou
SHC_160711_S7_L001_R_bin.4.strict.fa_c00003_NODE_3_...region	OEZ008529	yes	LMSG_G000005970.1	OES074479	acyl_amino_acids	Others	Pyrite	69.04	China: Guangdong, Guangzhou
SHC_160711_S7_L001_R_bin.4.strict.fa_c00005_NODE_5_...region	OEZ008529	yes	LMSG_G000005970.1	OES074479	terpene	Terpene	Pyrite	23.32	China: Guangdong, Guangzhou
SHC_160711_S7_L001_R_bin.4.strict.fa_c00006_NODE_6_...region	OEZ008529	yes	LMSG_G000005970.1	OES074479	NRPS-like	NRPS	Pyrite	42.66	China: Guangdong, Guangzhou
SHC_160711_S7_L001_R_bin.4.strict.fa_c00026_NODE_26...region	OEZ008529	yes	LMSG_G000005970.1	OES074479	terpene	Terpene	Pyrite	21.03	China: Guangdong, Guangzhou
SHC_160711_S7_L001_R_bin.4.strict.fa_c00034_NODE_34...region	OEZ008529	yes	LMSG_G000005970.1	OES074479	nucleoside	Others	Pyrite	20.94	China: Guangdong, Guangzhou
SHC_160711_S7_L001_R_bin.5.orig.fa_k141_54635.region001.fast	OEZ008529	yes	LMSG_G000008241.1	OES074479	terpene	Terpene	Pyrite	6.98	China: Guangdong, Guangzhou
SHC_160711_S7_L001_R_bin.8.orig.fa_k141_1011.region001.fasta	OEZ008529	yes	LMSG_G000009559.1	OES074479	terpene	Terpene	Pyrite	11.43	China: Guangdong, Guangzhou
SHC_160711_S7_L001_R_bin.8.orig.fa_k141_34531.region001.fast	OEZ008529	yes	LMSG_G000009559.1	OES074479	ectoine	Others	Pyrite	6.71	China: Guangdong, Guangzhou
SHC_160711_S7_L001_R_bin.8.orig.fa_k141_4619.region001.fasta	OEZ008529	yes	LMSG_G000009559.1	OES074479	NRPS-like	NRPS	Pyrite	20.55	China: Guangdong, Guangzhou
SHC1608-300-2_S2_S8_L001_R_bin.4.orig.fa_k141_29220.region00	OEZ008529	yes	LMSG_G000009555.1	OES074480	terpene	Terpene	Pyrite	22.06	China: Guangdong, Guangzhou
SHC1608-300-2_S2_S8_L001_R_bin.4.orig.fa_k141_31435.region00	OEZ008529	no	LMSG_G000009555.1	OES074480	terpene	Terpene	Pyrite	23.38	China: Guangdong, Guangzhou
SHC1608-300-2_S2_S8_L001_R_bin.4.orig.fa_k141_31903.region00	OEZ008529	yes	LMSG_G000009555.1	OES074480	NRPS-like	NRPS	Pyrite	42.72	China: Guangdong, Guangzhou
SHC1608-300-2_S2_S8_L001_R_bin.4.orig.fa_k141_5987.region001	OEZ008529	yes	LMSG_G000009555.1	OES074480	NRPS.hserlactone	Others	Pyrite	27.63	China: Guangdong, Guangzhou
SHC1608-300-2_S2_S8_L001_R_bin.4.orig.fa_k141_8383.region001	OEZ008529	yes	LMSG_G000009555.1	OES074480	ectoine	Others	Pyrite	9.12	China: Guangdong, Guangzhou
SHC1608-300-2_S2_S8_L001_R_bin.8.orig.fa_k141_1373.region001	OEZ008529	yes	LMSG_G000009602.1	OES074480	terpene	Terpene	Pyrite	22.15	China: Guangdong, Guangzhou
SHC1608-300-2_S2_S8_L001_R_bin.8.orig.fa_k141_505.region001.	OEZ008529	yes	LMSG_G000009602.1	OES074480	NRPS-like	NRPS	Pyrite	42.62	China: Guangdong, Guangzhou
SHC1608-300-2_S2_S8_L001_R_bin.8.orig.fa_k141_67445.region00	OEZ008529	yes	LMSG_G000009602.1	OES074480	acyl_amino_acids	Others	Pyrite	5.70	China: Guangdong, Guangzhou
SHC1608-300-2_S2_S8_L001_R_bin.8.orig.fa_k141_8906.region001	OEZ008529	yes	LMSG_G000009602.1	OES074480	ectoine	Others	Pyrite	7.49	China: Guangdong, Guangzhou
SHC1608-300-2_S2_S8_L001_R_bin.8.orig.fa_k141_9848.region001	OEZ008529	yes	LMSG_G000009602.1	OES074480	terpene	Terpene	Pyrite	14.13	China: Guangdong, Guangzhou
SRR10333320_bin.1.orig.fa_k141_1021128.region001.fasta	OEZ008529	yes	LMSG_G000005974.1	SRS5562336	acyl_amino_acids	Others	Coal	6.91	China: Guizhou, Fuquan
SRR10333320_bin.1.orig.fa_k141_549596.region001.fasta	OEZ008529	yes	LMSG_G000005974.1	SRS5562336	NRPS-like	NRPS	Coal	6.49	China: Guizhou, Fuquan
SRR10333320_bin.1.orig.fa_k141_549596_0.region001.fasta	OEZ008529	yes	LMSG_G000005974.1	SRS5562336	NRPS-like	NRPS	Coal	6.49	China: Guizhou, Fuquan
SRR10333320_bin.3.strict.fa_c00006_NODE_6_...region001.fasta	OEZ008529	yes	LMSG_G000005975.1	SRS5562336	bacteriocin	RiPPs	Coal	10.89	China: Guizhou, Fuquan
SRR10333320_bin.3.strict.fa_c00007_NODE_7_...region001.fasta	OEZ008529	yes	LMSG_G000005975.1	SRS5562336	terpene	Terpene	Coal	24.94	China: Guizhou, Fuquan
SRR10333320_bin.3.strict.fa_c00066_NODE_66...region001.fasta	OEZ008529	yes	LMSG_G000005975.1	SRS5562336	terpene	Terpene	Coal	14.74	China: Guizhou, Fuquan
SRR10333320_bin.3.strict.fa_c00073_NODE_73...region001.fasta	OEZ008529	yes	LMSG_G000005975.1	SRS5562336	lassopeptide.lanthipeptide	RiPPs	Coal	18.08	China: Guizhou, Fuquan
SRR10333320_bin.3.strict.fa_c00094_NODE_94...region001.fasta	OEZ008529	yes	LMSG_G000005975.1	SRS5562336	T3PKS	PKSother	Coal	14.27	China: Guizhou, Fuquan
SRR10333320_bin.3.strict.fa_c00178_NODE_17...region001.fasta	OEZ008529	yes	LMSG_G000005975.1	SRS5562336	NRPS-like	NRPS	Coal	7.02	China: Guizhou, Fuquan
SRR10333320_bin.4.orig.fa_k141_754036.region001.fasta	OEZ008529	yes	LMSG_G000007936.1	SRS5562336	terpene	Terpene	Coal	25.62	China: Guizhou, Fuquan
SRR10333320_bin.4.orig.fa_k141_8401.region001.fasta	OEZ008529	yes	LMSG_G000007936.1	SRS5562336	T3PKS	PKSother	Coal	18.14	China: Guizhou, Fuquan
SRR10333320_bin.4.orig.fa_k141_964913.region001.fasta	OEZ008529	yes	LMSG_G000007936.1	SRS5562336	bacteriocin	RiPPs	Coal	9.92	China: Guizhou, Fuquan
SRR10333321_bin.2.orig.fa_k141_1149707.region001.fasta	OEZ008529	yes	LMSG_G000005976.1	SRS5562335	TfuA-related	RiPPs	Coal	9.20	China: Guizhou, Fuquan
SRR10333321_bin.2.orig.fa_k141_780531.region001.fasta	OEZ008529	yes	LMSG_G000005976.1	SRS5562335	NRPS-like	NRPS	Coal	7.60	China: Guizhou, Fuquan
SRR10333321_bin.4.orig.fa_k141_18418.region001.fasta	OEZ008529	yes	LMSG_G000004596.1	SRS5562335	terpene	Terpene	Coal	25.66	China: Guizhou, Fuquan
SRR10333321_bin.4.orig.fa_k141_373778.region001.fasta	OEZ008529	yes	LMSG_G000004596.1	SRS5562335	bacteriocin	RiPPs	Coal	10.91	China: Guizhou, Fuquan
SRR10333321_bin.4.orig.fa_k141_497254.region001.fasta	OEZ008529	yes	LMSG_G000004596.1	SRS5562335	T3PKS	PKSother	Coal	31.95	China: Guizhou, Fuquan
SRR10333321_bin.5.orig.fa_k141_256157.region001.fasta	OEZ008529	yes	LMSG_G000005978.1	SRS5562335	indole	Others	Coal	17.40	China: Guizhou, Fuquan
SRR10333321_bin.5.orig.fa_k141_273517.region001.fasta	OEZ008529	yes	LMSG_G000005978.1	SRS5562335	terpene	Terpene	Coal	10.57	China: Guizhou, Fuquan
SRR10333321_bin.5.orig.fa_k141_486141.region001.fasta	OEZ008529	yes	LMSG_G000005978.1	SRS5562335	bacteriocin	RiPPs	Coal	10.89	China: Guizhou, Fuquan
SRR10333321_bin.5.orig.fa_k141_52097.region001.fasta	OEZ008529	yes	LMSG_G000005978.1	SRS5562335	NRPS-like	NRPS	Coal	22.68	China: Guizhou, Fuquan
SRR10333321_bin.6.strict.fa_c00006_NODE_6_...region001.fasta	OEZ008529	yes	LMSG_G000005979.1	SRS5562335	ladderane.arylpolyene	Others	Coal	23.81	China: Guizhou, Fuquan
SRR10333321_bin.6.strict.fa_c00063_NODE_63...region001.fasta	OEZ008529	yes	LMSG_G000005979.1	SRS5562335	lanthipeptide	RiPPs	Coal	10.69	China: Guizhou, Fuquan
SRR10333321_bin.6.strict.fa_c00075_NODE_75...region001.fasta	OEZ008529	yes	LMSG_G000005979.1	SRS5562335	NRPS	NRPS	Coal	10.29	China: Guizhou, Fuquan
SRR10333321_bin.6.strict.fa_c00115_NODE_11...region001.fasta	OEZ008529	yes	LMSG_G000005979.1	SRS5562335	lanthipeptide	RiPPs	Coal	7.99	China: Guizhou, Fuquan
SRR10333321_bin.6.strict.fa_c00118_NODE_11...region001.fasta	OEZ008529	yes	LMSG_G000005979.1	SRS5562335	NRPS	NRPS	Coal	7.91	China: Guizhou, Fuquan
SRR10333321_bin.6.strict.fa_c00135_NODE_13...region001.fasta	OEZ008529	yes	LMSG_G000005979.1	SRS5562335	bacteriocin	RiPPs	Coal	7.37	China: Guizhou, Fuquan
SRR10333321_bin.6.strict.fa_c00151_NODE_15...region001.fasta	OEZ008529	yes	LMSG_G000005979.1	SRS5562335	terpene	Terpene	Coal	6.98	China: Guizhou, Fuquan
SRR10333321_bin.6.strict.fa_c00226_NODE_22...region001.fasta	OEZ008529	yes	LMSG_G000005979.1	SRS5562335	NRPS	NRPS	Coal	5.25	China: Guizhou, Fuquan
SRR10333322_bin.2.orig.fa_k141_425969.region001.fasta	OEZ008529	yes	LMSG_G000004597.1	SRS5562334	NRPS-like	NRPS	Coal	33.60	China: Guizhou, Fuquan
SRR10333322_bin.2.orig.fa_k141_754251.region001.fasta	OEZ008529	yes	LMSG_G000004597.1	SRS5562334	T3PKS	PKSother	Coal	26.48	China: Guizhou, Fuquan
SRR10333322_bin.5.orig.fa_k141_170381.region001.fasta	OEZ008529	yes	LMSG_G000005982.1	SRS5562334	LAP	RiPPs	Coal	14.95	China: Guizhou, Fuquan
SRR10333322_bin.5.orig.fa_k141_438985.region001.fasta	OEZ008529	yes	LMSG_G000005982.1	SRS5562334	terpene	Terpene	Coal	20.87	China: Guizhou, Fuquan
SRR10333322_bin.5.orig.fa_k141_558989.region001.fasta	OEZ008529	yes	LMSG_G000005982.1	SRS5562334	T3PKS	PKSother	Coal	38.34	China: Guizhou, Fuquan
SRR10333322_bin.5.orig.fa_k141_606418.region001.fasta	OEZ008529	yes	LMSG_G000005982.1	SRS5562334	terpene	Terpene	Coal	25.01	China: Guizhou, Fuquan
SRR10333322_bin.5.orig.fa_k141_769223.region001.fasta	OEZ008529	yes	LMSG_G000005982.1	SRS5562334	TfuA-related	RiPPs	Coal	22.00	China: Guizhou, Fuquan
SRR10333322_bin.6.permissive.fa_c00007_NODE_7_...region001.f	OEZ008529	yes	LMSG_G000005983.1	SRS5562334	NRPS	NRPS	Coal	11.31	China: Guizhou, Fuquan
SRR10333322_bin.6.permissive.fa_c00058_NODE_58...region001.f	OEZ008529	yes	LMSG_G000005983.1	SRS5562334	NRPS	NRPS	Coal	7.21	China: Guizhou, Fuquan
SRR10333322_bin.6.permissive.fa_c00070_NODE_70...region001.f	OEZ008529	yes	LMSG_G000005983.1	SRS5562334	NRPS	NRPS	Coal	6.73	China: Guizhou, Fuquan
SRR10333322_bin.6.permissive.fa_c00089_NODE_89...region001.f	OEZ008529	yes	LMSG_G000005983.1	SRS5562334	NRPS	NRPS	Coal	6.39	China: Guizhou, Fuquan
SRR10333322_bin.6.permissive.fa_c00112_NODE_11...region001.f	OEZ008529	yes	LMSG_G000005983.1	SRS5562334	NRPS	NRPS	Coal	5.84	China: Guizhou, Fuquan
SRR10333322_bin.6.permissive.fa_c00113_NODE_11...region001.f	OEZ008529	yes	LMSG_G000005983.1	SRS5562334	NRPS-like	NRPS	Coal	5.83	China: Guizhou, Fuquan
SRR10333322_bin.6.permissive.fa_c00136_NODE_13...region001.f	OEZ008529	yes	LMSG_G000005983.1	SRS5562334	NRPS	NRPS	Coal	5.41	China: Guizhou, Fuquan
SRR10333322_bin.6.permissive.fa_c00156_NODE_15...region001.f	OEZ008529	yes	LMSG_G000005983.1	SRS5562334	NRPS	NRPS	Coal	5.18	China: Guizhou, Fuquan
SRR10333322_bin.7.orig.fa_k141_305465.region001.fasta	OEZ008529	yes	LMSG_G000005984.1	SRS5562334	NRPS.T1PKS	PKS-NRP_Hybrids	Coal	52.27	China: Guizhou, Fuquan
SRR10333322_bin.7.orig.fa_k141_311953.region001.fasta	OEZ008529	yes	LMSG_G000005984.1	SRS5562334	bacteriocin	RiPPs	Coal	8.84	China: Guizhou, Fuquan
SRR10333322_bin.7.orig.fa_k141_679665.region001.fasta	OEZ008529	yes	LMSG_G000005984.1	SRS5562334	arylpolyene	Others	Coal	40.56	China: Guizhou, Fuquan
SRR10333322_bin.9.orig.fa_k141_329075.region001.fasta	OEZ008529	yes	LMSG_G000007922.1	SRS5562334	NRPS-like	NRPS	Coal	8.22	China: Guizhou, Fuquan
SRR10333322_bin.9.orig.fa_k141_474361.region001.fasta	OEZ008529	yes	LMSG_G000007922.1	SRS5562334	T3PKS	PKSother	Coal	5.02	China: Guizhou, Fuquan
SRR10333322_bin.9.orig.fa_k141_589943.region001.fasta	OEZ008529	yes	LMSG_G000007922.1	SRS5562334	terpene	Terpene	Coal	7.35	China: Guizhou, Fuquan
SRR10333322_bin.9.orig.fa_k141_884106.region001.fasta	OEZ008529	yes	LMSG_G000007922.1	SRS5562334	NRPS-like	NRPS	Coal	8.80	China: Guizhou, Fuquan
SRR10333323_bin.1.orig.fa_k141_121977.region001.fasta	OEZ008529	yes	LMSG_G000004599.1	SRS5562333	T3PKS	PKSother	Coal	41.05	China: Guizhou, Fuquan
SRR10333323_bin.1.orig.fa_k141_766800.region001.fasta	OEZ008529	yes	LMSG_G000004599.1	SRS5562333	terpene	Terpene	Coal	29.00	China: Guizhou, Fuquan
SRR10333323_bin.1.orig.fa_k141_833779.region001.fasta	OEZ008529	yes	LMSG_G000004599.1	SRS5562333	NRPS-like	NRPS	Coal	22.09	China: Guizhou, Fuquan
SRR10333323_bin.1.orig.fa_k141_872176.region001.fasta	OEZ008529	yes	LMSG_G000004599.1	SRS5562333	lassopeptide	RiPPs	Coal	31.47	China: Guizhou, Fuquan
SRR10333323_bin.2.orig.fa_k141_152281.region001.fasta	OEZ008529	yes	LMSG_G000011184.1	SRS5562333	bacteriocin	RiPPs	Coal	7.83	China: Guizhou, Fuquan
SRR10333323_bin.2.orig.fa_k141_201484.region001.fasta	OEZ008529	no	LMSG_G000011184.1	SRS5562333	NRPS.T1PKS	PKS-NRP_Hybrids	Coal	11.07	China: Guizhou, Fuquan
SRR10333323_bin.2.orig.fa_k141_248406.region001.fasta	OEZ008529	yes	LMSG_G000011184.1	SRS5562333	NRPS-like	NRPS	Coal	13.24	China: Guizhou, Fuquan
SRR10333323_bin.2.orig.fa_k141_267093.region001.fasta	OEZ008529	yes	LMSG_G000011184.1	SRS5562333	arylpolyene	Others	Coal	24.79	China: Guizhou, Fuquan
SRR10333323_bin.3.orig.fa_k141_1027466.region001.fasta	OEZ008529	yes	LMSG_G000005985.1	SRS5562333	terpene	Terpene	Coal	11.44	China: Guizhou, Fuquan
SRR10333323_bin.3.orig.fa_k141_928913.region001.fasta	OEZ008529	yes	LMSG_G000005985.1	SRS5562333	NRPS-like	NRPS	Coal	7.23	China: Guizhou, Fuquan
SRR10333323_bin.4.strict.fa_c00003_NODE_3_...region001.fasta	OEZ008529	yes	LMSG_G000005986.1	SRS5562333	terpene	Terpene	Coal	13.17	China: Guizhou, Fuquan
SRR10333323_bin.4.strict.fa_c00106_NODE_10...region001.fasta	OEZ008529	yes	LMSG_G000005986.1	SRS5562333	NRPS	NRPS	Coal	5.56	China: Guizhou, Fuquan
SRR10333324_bin.1.strict.fa_c00002_NODE_2_...region001.fasta	OEZ008529	yes	LMSG_G000005988.1	SRS5562332	NRPS.T1PKS	PKS-NRP_Hybrids	Coal	54.38	China: Guizhou, Fuquan
SRR10333324_bin.1.strict.fa_c00003_NODE_3_...region001.fasta	OEZ008529	yes	LMSG_G000005988.1	SRS5562332	NRPS.T1PKS	PKS-NRP_Hybrids	Coal	66.87	China: Guizhou, Fuquan
SRR10333324_bin.1.strict.fa_c00006_NODE_6_...region001.fasta	OEZ008529	yes	LMSG_G000005988.1	SRS5562332	T3PKS.bacteriocin	Others	Coal	32.55	China: Guizhou, Fuquan
SRR10333324_bin.1.strict.fa_c00008_NODE_8_...region001.fasta	OEZ008529	yes	LMSG_G000005988.1	SRS5562332	T1PKS	PKSI	Coal	43.82	China: Guizhou, Fuquan
SRR10333324_bin.1.strict.fa_c00016_NODE_16...region001.fasta	OEZ008529	yes	LMSG_G000005988.1	SRS5562332	bacteriocin	RiPPs	Coal	10.85	China: Guizhou, Fuquan
SRR10333324_bin.1.strict.fa_c00025_NODE_25...region001.fasta	OEZ008529	yes	LMSG_G000005988.1	SRS5562332	lanthipeptide	RiPPs	Coal	13.77	China: Guizhou, Fuquan
SRR10333324_bin.1.strict.fa_c00026_NODE_26...region001.fasta	OEZ008529	yes	LMSG_G000005988.1	SRS5562332	NRPS-like	NRPS	Coal	39.60	China: Guizhou, Fuquan
SRR10333324_bin.1.strict.fa_c00028_NODE_28...region001.fasta	OEZ008529	yes	LMSG_G000005988.1	SRS5562332	NRPS	NRPS	Coal	47.69	China: Guizhou, Fuquan
SRR10333324_bin.1.strict.fa_c00035_NODE_35...region001.fasta	OEZ008529	yes	LMSG_G000005988.1	SRS5562332	terpene	Terpene	Coal	22.27	China: Guizhou, Fuquan
SRR10333324_bin.1.strict.fa_c00038_NODE_38...region001.fasta	OEZ008529	yes	LMSG_G000005988.1	SRS5562332	NRPS	NRPS	Coal	38.37	China: Guizhou, Fuquan
SRR10333324_bin.1.strict.fa_c00064_NODE_64...region001.fasta	OEZ008529	yes	LMSG_G000005988.1	SRS5562332	bacteriocin	RiPPs	Coal	10.87	China: Guizhou, Fuquan
SRR10333324_bin.1.strict.fa_c00067_NODE_67...region001.fasta	OEZ008529	yes	LMSG_G000005988.1	SRS5562332	NRPS.lanthipeptide	Others	Coal	25.03	China: Guizhou, Fuquan
SRR10333324_bin.2.orig.fa_k141_115263.region001.fasta	OEZ008529	yes	LMSG_G000004600.1	SRS5562332	bacteriocin	RiPPs	Coal	10.85	China: Guizhou, Fuquan
SRR10333324_bin.2.orig.fa_k141_177651.region001.fasta	OEZ008529	yes	LMSG_G000004600.1	SRS5562332	NRPS.T1PKS	PKS-NRP_Hybrids	Coal	51.49	China: Guizhou, Fuquan
SRR10333324_bin.2.orig.fa_k141_177651.region002.fasta	OEZ008529	yes	LMSG_G000004600.1	SRS5562332	hserlactone	Others	Coal	10.85	China: Guizhou, Fuquan
SRR10333324_bin.2.orig.fa_k141_451080.region001.fasta	OEZ008529	yes	LMSG_G000004600.1	SRS5562332	arylpolyene	Others	Coal	41.26	China: Guizhou, Fuquan
SRR10333324_bin.3.strict.fa_c00012_NODE_12...region001.fasta	OEZ008529	yes	LMSG_G000006299.1	SRS5562332	T3PKS.terpene	Others	Coal	27.22	China: Guizhou, Fuquan
SRR10333324_bin.3.strict.fa_c00150_NODE_15...region001.fasta	OEZ008529	yes	LMSG_G000006299.1	SRS5562332	terpene	Terpene	Coal	9.43	China: Guizhou, Fuquan
SRR10333324_bin.6.orig.fa_k141_498989.region001.fasta	OEZ008529	yes	LMSG_G000005989.1	SRS5562332	lassopeptide	RiPPs	Coal	13.37	China: Guizhou, Fuquan
SRR10333325_bin.1.orig.fa_k141_1064294.region001.fasta	OEZ008529	yes	LMSG_G000005994.1	SRS5562331	terpene	Terpene	Coal	6.23	China: Guizhou, Fuquan
SRR10333325_bin.1.orig.fa_k141_1166988.region001.fasta	OEZ008529	yes	LMSG_G000005994.1	SRS5562331	hserlactone	Others	Coal	8.98	China: Guizhou, Fuquan
SRR10333325_bin.1.orig.fa_k141_260353.region001.fasta	OEZ008529	yes	LMSG_G000005994.1	SRS5562331	NRPS-like	NRPS	Coal	11.15	China: Guizhou, Fuquan
SRR10333325_bin.1.orig.fa_k141_264167.region001.fasta	OEZ008529	yes	LMSG_G000005994.1	SRS5562331	NRPS-like	NRPS	Coal	13.05	China: Guizhou, Fuquan
SRR10333325_bin.1.orig.fa_k141_780195.region001.fasta	OEZ008529	yes	LMSG_G000005994.1	SRS5562331	lassopeptide	RiPPs	Coal	15.51	China: Guizhou, Fuquan
SRR10333325_bin.1.orig.fa_k141_867557.region001.fasta	OEZ008529	yes	LMSG_G000005994.1	SRS5562331	betalactone	Others	Coal	24.40	China: Guizhou, Fuquan
SRR10333325_bin.1.orig.fa_k141_998953.region001.fasta	OEZ008529	yes	LMSG_G000005994.1	SRS5562331	arylpolyene	Others	Coal	22.26	China: Guizhou, Fuquan
SRR10333325_bin.10.orig.fa_k141_102329.region001.fasta	OEZ008529	yes	LMSG_G000008570.1	SRS5562331	NRPS-like	NRPS	Coal	24.94	China: Guizhou, Fuquan
SRR10333325_bin.10.orig.fa_k141_991760.region001.fasta	OEZ008529	yes	LMSG_G000008570.1	SRS5562331	other	Others	Coal	13.96	China: Guizhou, Fuquan
SRR10333325_bin.12.orig.fa_k141_1053471.region001.fasta	OEZ008529	yes	LMSG_G000005990.1	SRS5562331	terpene	Terpene	Coal	8.32	China: Guizhou, Fuquan
SRR10333325_bin.12.orig.fa_k141_297099.region001.fasta	OEZ008529	yes	LMSG_G000005990.1	SRS5562331	T3PKS	PKSother	Coal	20.53	China: Guizhou, Fuquan
SRR10333325_bin.13.strict.fa_c00001_NODE_1_...region001.fast	OEZ008529	yes	LMSG_G000007746.1	SRS5562331	terpene	Terpene	Coal	17.60	China: Guizhou, Fuquan
SRR10333325_bin.13.strict.fa_c00021_NODE_21...region001.fast	OEZ008529	yes	LMSG_G000007746.1	SRS5562331	bacteriocin	RiPPs	Coal	8.93	China: Guizhou, Fuquan
SRR10333325_bin.13.strict.fa_c00105_NODE_10...region001.fast	OEZ008529	yes	LMSG_G000007746.1	SRS5562331	T3PKS	PKSother	Coal	6.90	China: Guizhou, Fuquan
SRR10333325_bin.14.orig.fa_k141_201961.region001.fasta	OEZ008529	yes	LMSG_G000005991.1	SRS5562331	terpene	Terpene	Coal	15.34	China: Guizhou, Fuquan

SRR10333325_bin.14.orig.fa_k141_913171.region001.fasta	OEZ008529	yes	LMSG_G000005991.1	SRS5562331	bacteriocin	RiPPs	Coal	9.91	China: Guizhou, Fuquan
SRR10333325_bin.15.orig.fa_k141_1157376.region001.fasta	OEZ008529	yes	LMSG_G000011048.1	SRS5562331	resorcinol.arylpolyene	Others	Coal	32.07	China: Guizhou, Fuquan
SRR10333325_bin.15.orig.fa_k141_130989.region001.fasta	OEZ008529	yes	LMSG_G000011048.1	SRS5562331	NRPS-like	NRPS	Coal	18.46	China: Guizhou, Fuquan
SRR10333325_bin.15.orig.fa_k141_289805.region001.fasta	OEZ008529	yes	LMSG_G000011048.1	SRS5562331	terpene	Terpene	Coal	19.00	China: Guizhou, Fuquan
SRR10333325_bin.15.orig.fa_k141_291810.region001.fasta	OEZ008529	yes	LMSG_G000011048.1	SRS5562331	arylpolyene	Others	Coal	41.79	China: Guizhou, Fuquan
SRR10333325_bin.15.orig.fa_k141_30242.region001.fasta	OEZ008529	yes	LMSG_G000011048.1	SRS5562331	terpene	Terpene	Coal	20.85	China: Guizhou, Fuquan
SRR10333325_bin.16.orig.fa_k141_202256.region001.fasta	OEZ008529	yes	LMSG_G000005992.1	SRS5562331	TfuA-related	RiPPs	Coal	13.09	China: Guizhou, Fuquan
SRR10333325_bin.16.orig.fa_k141_385052.region001.fasta	OEZ008529	yes	LMSG_G000005992.1	SRS5562331	T3PKS	PKSother	Coal	28.11	China: Guizhou, Fuquan
SRR10333325_bin.19.orig.fa_k141_379165.region001.fasta	OEZ008529	yes	LMSG_G000005993.1	SRS5562331	bacteriocin	RiPPs	Coal	8.68	China: Guizhou, Fuquan
SRR10333325_bin.19.orig.fa_k141_754733.region001.fasta	OEZ008529	yes	LMSG_G000005993.1	SRS5562331	lassopeptide	RiPPs	Coal	11.16	China: Guizhou, Fuquan
SRR10333325_bin.20.orig.fa_k141_205457.region001.fasta	OEZ008529	yes	LMSG_G000004601.1	SRS5562331	terpene	Terpene	Coal	20.84	China: Guizhou, Fuquan
SRR10333325_bin.20.orig.fa_k141_367450.region001.fasta	OEZ008529	yes	LMSG_G000004601.1	SRS5562331	resorcinol.arylpolyene	Others	Coal	49.15	China: Guizhou, Fuquan
SRR10333325_bin.20.orig.fa_k141_536488.region001.fasta	OEZ008529	yes	LMSG_G000004601.1	SRS5562331	bacteriocin	RiPPs	Coal	6.17	China: Guizhou, Fuquan
SRR10333325_bin.20.orig.fa_k141_697946.region001.fasta	OEZ008529	yes	LMSG_G000004601.1	SRS5562331	betalactone	Others	Coal	32.83	China: Guizhou, Fuquan
SRR10333325_bin.23.orig.fa_k141_104257.region001.fasta	OEZ008529	yes	LMSG_G000008715.1	SRS5562331	NRPS	NRPS	Coal	7.63	China: Guizhou, Fuquan
SRR10333325_bin.23.orig.fa_k141_1077618.region001.fasta	OEZ008529	yes	LMSG_G000008715.1	SRS5562331	NRPS.nucleoside	Others	Coal	38.54	China: Guizhou, Fuquan
SRR10333325_bin.23.orig.fa_k141_1118486.region001.fasta	OEZ008529	yes	LMSG_G000008715.1	SRS5562331	NRPS	NRPS	Coal	20.87	China: Guizhou, Fuquan
SRR10333325_bin.23.orig.fa_k141_115639.region001.fasta	OEZ008529	yes	LMSG_G000008715.1	SRS5562331	terpene	Terpene	Coal	17.82	China: Guizhou, Fuquan
SRR10333325_bin.23.orig.fa_k141_180502.region001.fasta	OEZ008529	no	LMSG_G000008715.1	SRS5562331	NRPS	NRPS	Coal	6.36	China: Guizhou, Fuquan
SRR10333325_bin.23.orig.fa_k141_18347.region001.fasta	OEZ008529	yes	LMSG_G000008715.1	SRS5562331	NRPS	NRPS	Coal	13.28	China: Guizhou, Fuquan
SRR10333325_bin.23.orig.fa_k141_195643.region001.fasta	OEZ008529	yes	LMSG_G000008715.1	SRS5562331	NRPS	NRPS	Coal	15.57	China: Guizhou, Fuquan
SRR10333325_bin.23.orig.fa_k141_254245.region001.fasta	OEZ008529	yes	LMSG_G000008715.1	SRS5562331	NRPS-like	NRPS	Coal	24.20	China: Guizhou, Fuquan
SRR10333325_bin.23.orig.fa_k141_371274.region001.fasta	OEZ008529	yes	LMSG_G000008715.1	SRS5562331	lanthipeptide.bacteriocin	RiPPs	Coal	25.90	China: Guizhou, Fuquan
SRR10333325_bin.23.orig.fa_k141_434046.region001.fasta	OEZ008529	yes	LMSG_G000008715.1	SRS5562331	NRPS	NRPS	Coal	11.34	China: Guizhou, Fuquan
SRR10333325_bin.23.orig.fa_k141_456021.region001.fasta	OEZ008529	yes	LMSG_G000008715.1	SRS5562331	resorcinol	Others	Coal	41.76	China: Guizhou, Fuquan
SRR10333325_bin.23.orig.fa_k141_468854.region001.fasta	OEZ008529	yes	LMSG_G000008715.1	SRS5562331	NRPS	NRPS	Coal	15.07	China: Guizhou, Fuquan
SRR10333325_bin.23.orig.fa_k141_599907.region001.fasta	OEZ008529	yes	LMSG_G000008715.1	SRS5562331	NRPS-like.terpene	Others	Coal	24.92	China: Guizhou, Fuquan
SRR10333325_bin.23.orig.fa_k141_605721.region001.fasta	OEZ008529	no	LMSG_G000008715.1	SRS5562331	NRPS	NRPS	Coal	5.06	China: Guizhou, Fuquan
SRR10333325_bin.23.orig.fa_k141_659500.region001.fasta	OEZ008529	yes	LMSG_G000008715.1	SRS5562331	bacteriocin	RiPPs	Coal	10.83	China: Guizhou, Fuquan
SRR10333325_bin.23.orig.fa_k141_756275.region001.fasta	OEZ008529	yes	LMSG_G000008715.1	SRS5562331	NRPS	NRPS	Coal	15.84	China: Guizhou, Fuquan
SRR10333325_bin.23.orig.fa_k141_834200.region001.fasta	OEZ008529	yes	LMSG_G000008715.1	SRS5562331	NRPS.bacteriocin	Others	Coal	51.95	China: Guizhou, Fuquan
SRR10333325_bin.23.orig.fa_k141_91906.region001.fasta	OEZ008529	no	LMSG_G000008715.1	SRS5562331	NRPS	NRPS	Coal	5.57	China: Guizhou, Fuquan
SRR10333325_bin.23.orig.fa_k141_932614.region001.fasta	OEZ008529	yes	LMSG_G000008715.1	SRS5562331	NRPS	NRPS	Coal	69.03	China: Guizhou, Fuquan
SRR10333325_bin.23.orig.fa_k141_955128.region001.fasta	OEZ008529	yes	LMSG_G000008715.1	SRS5562331	NRPS	NRPS	Coal	11.71	China: Guizhou, Fuquan
SRR10333325_bin.25.orig.fa_k141_1177690.region001.fasta	OEZ008529	yes	LMSG_G000005996.1	SRS5562331	terpene	Terpene	Coal	18.58	China: Guizhou, Fuquan
SRR10333325_bin.25.orig.fa_k141_229480.region001.fasta	OEZ008529	yes	LMSG_G000005996.1	SRS5562331	T3PKS	PKSother	Coal	27.27	China: Guizhou, Fuquan
SRR10333325_bin.25.orig.fa_k141_272166.region001.fasta	OEZ008529	yes	LMSG_G000005996.1	SRS5562331	bacteriocin	RiPPs	Coal	5.28	China: Guizhou, Fuquan
SRR10333325_bin.25.orig.fa_k141_509702.region001.fasta	OEZ008529	yes	LMSG_G000005996.1	SRS5562331	NRPS-like	NRPS	Coal	20.78	China: Guizhou, Fuquan
SRR10333325_bin.25.orig.fa_k141_804436.region001.fasta	OEZ008529	yes	LMSG_G000005996.1	SRS5562331	T3PKS	PKSother	Coal	41.07	China: Guizhou, Fuquan
SRR10333325_bin.26.orig.fa_k141_1121965.region001.fasta	OEZ008529	yes	LMSG_G000004602.1	SRS5562331	betalactone	Others	Coal	26.68	China: Guizhou, Fuquan
SRR10333325_bin.26.orig.fa_k141_340568.region001.fasta	OEZ008529	yes	LMSG_G000004602.1	SRS5562331	betalactone	Others	Coal	24.15	China: Guizhou, Fuquan
SRR10333325_bin.27.orig.fa_k141_298657.region001.fasta	OEZ008529	yes	LMSG_G000011172.1	SRS5562331	arylpolyene	Others	Coal	12.61	China: Guizhou, Fuquan
SRR10333325_bin.27.orig.fa_k141_389863.region001.fasta	OEZ008529	yes	LMSG_G000011172.1	SRS5562331	bacteriocin	RiPPs	Coal	5.64	China: Guizhou, Fuquan
SRR10333325_bin.28.strict.fa_c00011_NODE_11...region001.fast	OEZ008529	yes	LMSG_G000007870.1	SRS5562331	terpene	Terpene	Coal	21.68	China: Guizhou, Fuquan
SRR10333325_bin.28.strict.fa_c00040_NODE_40...region001.fast	OEZ008529	yes	LMSG_G000007870.1	SRS5562331	NRPS-like	NRPS	Coal	36.66	China: Guizhou, Fuquan
SRR10333325_bin.28.strict.fa_c00051_NODE_51...region001.fast	OEZ008529	yes	LMSG_G000007870.1	SRS5562331	T3PKS	PKSother	Coal	20.88	China: Guizhou, Fuquan
SRR10333325_bin.28.strict.fa_c00067_NODE_67...region001.fast	OEZ008529	yes	LMSG_G000007870.1	SRS5562331	T1PKS	PKSI	Coal	19.18	China: Guizhou, Fuquan
SRR10333325_bin.30.orig.fa_k141_1021583.region001.fasta	OEZ008529	yes	LMSG_G000005997.1	SRS5562331	bacteriocin	RiPPs	Coal	10.87	China: Guizhou, Fuquan
SRR10333325_bin.30.orig.fa_k141_1105181.region001.fasta	OEZ008529	yes	LMSG_G000005997.1	SRS5562331	T3PKS	PKSother	Coal	26.56	China: Guizhou, Fuquan
SRR10333325_bin.30.orig.fa_k141_1108408.region001.fasta	OEZ008529	yes	LMSG_G000005997.1	SRS5562331	terpene	Terpene	Coal	17.80	China: Guizhou, Fuquan
SRR10333325_bin.30.orig.fa_k141_1129575.region001.fasta	OEZ008529	yes	LMSG_G000005997.1	SRS5562331	NRPS-like	NRPS	Coal	21.58	China: Guizhou, Fuquan
SRR10333325_bin.30.orig.fa_k141_180696.region001.fasta	OEZ008529	yes	LMSG_G000005997.1	SRS5562331	terpene	Terpene	Coal	19.97	China: Guizhou, Fuquan
SRR10333325_bin.30.orig.fa_k141_712653.region001.fasta	OEZ008529	yes	LMSG_G000005997.1	SRS5562331	T3PKS	PKSother	Coal	36.66	China: Guizhou, Fuquan
SRR10333325_bin.30.orig.fa_k141_722101.region001.fasta	OEZ008529	yes	LMSG_G000005997.1	SRS5562331	bacteriocin	RiPPs	Coal	10.85	China: Guizhou, Fuquan
SRR10333325_bin.30.orig.fa_k141_864138.region001.fasta	OEZ008529	yes	LMSG_G000005997.1	SRS5562331	arylpolyene	Others	Coal	41.23	China: Guizhou, Fuquan
SRR10333325_bin.31.orig.fa_k141_265777.region001.fasta	OEZ008529	yes	LMSG_G000005998.1	SRS5562331	bacteriocin	RiPPs	Coal	6.84	China: Guizhou, Fuquan
SRR10333325_bin.31.orig.fa_k141_647268.region001.fasta	OEZ008529	yes	LMSG_G000005998.1	SRS5562331	NRPS-like	NRPS	Coal	32.63	China: Guizhou, Fuquan
SRR10333325_bin.31.orig.fa_k141_859791.region001.fasta	OEZ008529	yes	LMSG_G000005998.1	SRS5562331	terpene	Terpene	Coal	20.85	China: Guizhou, Fuquan
SRR10333325_bin.33.orig.fa_k141_1118382.region001.fasta	OEZ008529	yes	LMSG_G000009256.1	SRS5562331	TfuA-related	RiPPs	Coal	6.71	China: Guizhou, Fuquan
SRR10333325_bin.34.orig.fa_k141_17127.region001.fasta	OEZ008529	yes	LMSG_G000009173.1	SRS5562331	T3PKS	PKSother	Coal	22.01	China: Guizhou, Fuquan
SRR10333325_bin.34.orig.fa_k141_3391.region001.fasta	OEZ008529	yes	LMSG_G000009173.1	SRS5562331	terpene	Terpene	Coal	15.93	China: Guizhou, Fuquan
SRR10333325_bin.38.orig.fa_k141_1040445.region001.fasta	OEZ008529	yes	LMSG_G000006002.1	SRS5562331	terpene	Terpene	Coal	9.09	China: Guizhou, Fuquan
SRR10333325_bin.38.orig.fa_k141_687828.region001.fasta	OEZ008529	yes	LMSG_G000006002.1	SRS5562331	T3PKS	PKSother	Coal	40.95	China: Guizhou, Fuquan
SRR10333325_bin.39.orig.fa_k141_1101428.region001.fasta	OEZ008529	yes	LMSG_G000004603.1	SRS5562331	NRPS-like	NRPS	Coal	16.63	China: Guizhou, Fuquan
SRR10333325_bin.39.orig.fa_k141_1124663.region001.fasta	OEZ008529	yes	LMSG_G000004603.1	SRS5562331	lassopeptide.terpene	Others	Coal	35.42	China: Guizhou, Fuquan
SRR10333325_bin.39.orig.fa_k141_644675.region001.fasta	OEZ008529	yes	LMSG_G000004603.1	SRS5562331	T1PKS	PKSI	Coal	13.27	China: Guizhou, Fuquan
SRR10333325_bin.4.strict.fa_c00005_NODE_5_...region001.fasta	OEZ008529	yes	LMSG_G000004606.1	SRS5562331	terpene	Terpene	Coal	23.35	China: Guizhou, Fuquan
SRR10333325_bin.4.strict.fa_c00006_NODE_6_...region001.fasta	OEZ008529	yes	LMSG_G000004606.1	SRS5562331	terpene	Terpene	Coal	22.13	China: Guizhou, Fuquan
SRR10333325_bin.4.strict.fa_c00007_NODE_7_...region001.fasta	OEZ008529	yes	LMSG_G000004606.1	SRS5562331	NRPS-like	NRPS	Coal	42.73	China: Guizhou, Fuquan
SRR10333325_bin.4.strict.fa_c00019_NODE_19...region001.fasta	OEZ008529	yes	LMSG_G000004606.1	SRS5562331	arylpolyene	Others	Coal	18.70	China: Guizhou, Fuquan
SRR10333325_bin.42.strict.fa_c00003_NODE_3_...region001.fast	OEZ008529	yes	LMSG_G000009166.1	SRS5562331	terpene	Terpene	Coal	20.72	China: Guizhou, Fuquan
SRR10333325_bin.42.strict.fa_c00004_NODE_4_...region001.fast	OEZ008529	yes	LMSG_G000009166.1	SRS5562331	terpene	Terpene	Coal	23.71	China: Guizhou, Fuquan
SRR10333325_bin.42.strict.fa_c00006_NODE_6_...region001.fast	OEZ008529	yes	LMSG_G000009166.1	SRS5562331	betalactone	Others	Coal	32.75	China: Guizhou, Fuquan
SRR10333325_bin.42.strict.fa_c00038_NODE_38...region001.fast	OEZ008529	yes	LMSG_G000009166.1	SRS5562331	T3PKS	PKSother	Coal	25.05	China: Guizhou, Fuquan
SRR10333325_bin.43.strict.fa_c00005_NODE_5_...region001.fast	OEZ008529	yes	LMSG_G000004605.1	SRS5562331	NRPS-like	NRPS	Coal	42.57	China: Guizhou, Fuquan
SRR10333325_bin.43.strict.fa_c00006_NODE_6_...region001.fast	OEZ008529	yes	LMSG_G000004605.1	SRS5562331	NRPS	NRPS	Coal	45.75	China: Guizhou, Fuquan
SRR10333325_bin.5.orig.fa_k141_1170627.region001.fasta	OEZ008529	yes	LMSG_G000006003.1	SRS5562331	terpene	Terpene	Coal	12.46	China: Guizhou, Fuquan
SRR10333325_bin.5.orig.fa_k141_155641.region001.fasta	OEZ008529	yes	LMSG_G000006003.1	SRS5562331	NRPS-like	NRPS	Coal	27.07	China: Guizhou, Fuquan
SRR10333325_bin.5.orig.fa_k141_996567.region001.fasta	OEZ008529	yes	LMSG_G000006003.1	SRS5562331	NRPS-like	NRPS	Coal	42.86	China: Guizhou, Fuquan
SRR10333325_bin.6.orig.fa_k141_259017.region001.fasta	OEZ008529	yes	LMSG_G000004607.1	SRS5562331	terpene	Terpene	Coal	23.82	China: Guizhou, Fuquan
SRR10333325_bin.6.orig.fa_k141_691531.region001.fasta	OEZ008529	yes	LMSG_G000004607.1	SRS5562331	betalactone	Others	Coal	28.88	China: Guizhou, Fuquan
SRR10333325_bin.6.orig.fa_k141_700116.region001.fasta	OEZ008529	yes	LMSG_G000004607.1	SRS5562331	T3PKS	PKSother	Coal	41.72	China: Guizhou, Fuquan
SRR10333325_bin.6.orig.fa_k141_931779.region001.fasta	OEZ008529	yes	LMSG_G000004607.1	SRS5562331	bacteriocin	RiPPs	Coal	5.91	China: Guizhou, Fuquan
SRR10333326_bin.10.orig.fa_k141_350221.region001.fasta	OEZ008529	yes	LMSG_G000011025.1	SRS5562330	resorcinol.arylpolyene	Others	Coal	8.05	China: Guizhou, Fuquan
SRR10333326_bin.12.orig.fa_k141_193235.region001.fasta	OEZ008529	yes	LMSG_G000004608.1	SRS5562330	T3PKS	PKSother	Coal	9.80	China: Guizhou, Fuquan
SRR10333326_bin.12.orig.fa_k141_561805.region001.fasta	OEZ008529	yes	LMSG_G000004608.1	SRS5562330	terpene	Terpene	Coal	20.39	China: Guizhou, Fuquan
SRR10333326_bin.13.orig.fa_k141_399474.region001.fasta	OEZ008529	yes	LMSG_G000011095.1	SRS5562330	bacteriocin	RiPPs	Coal	10.89	China: Guizhou, Fuquan
SRR10333326_bin.15.strict.fa_c00010_NODE_10...region001.fast	OEZ008529	yes	LMSG_G000009273.1	SRS5562330	T3PKS	PKSother	Coal	40.89	China: Guizhou, Fuquan
SRR10333326_bin.15.strict.fa_c00031_NODE_31...region001.fast	OEZ008529	yes	LMSG_G000009273.1	SRS5562330	TfuA-related	RiPPs	Coal	17.82	China: Guizhou, Fuquan
SRR10333326_bin.16.strict.fa_c00024_NODE_24...region001.fast	OEZ008529	yes	LMSG_G000004609.1	SRS5562330	NRPS	NRPS	Coal	34.14	China: Guizhou, Fuquan
SRR10333326_bin.17.orig.fa_k141_114284.region001.fasta	OEZ008529	yes	LMSG_G000006007.1	SRS5562330	betalactone	Others	Coal	6.50	China: Guizhou, Fuquan
SRR10333326_bin.17.orig.fa_k141_757559.region001.fasta	OEZ008529	yes	LMSG_G000006007.1	SRS5562330	betalactone	Others	Coal	7.52	China: Guizhou, Fuquan
SRR10333326_bin.20.permissive.fa_c00135_NODE_13...region001.	OEZ008529	yes	LMSG_G000006008.1	SRS5562330	terpene	Terpene	Coal	5.62	China: Guizhou, Fuquan
SRR10333326_bin.21.strict.fa_c00002_NODE_2_...region001.fast	OEZ008529	yes	LMSG_G000004611.1	SRS5562330	terpene	Terpene	Coal	23.40	China: Guizhou, Fuquan
SRR10333326_bin.21.strict.fa_c00005_NODE_5_...region001.fast	OEZ008529	yes	LMSG_G000004611.1	SRS5562330	NRPS	NRPS	Coal	43.27	China: Guizhou, Fuquan
SRR10333326_bin.21.strict.fa_c00016_NODE_16...region001.fast	OEZ008529	yes	LMSG_G000004611.1	SRS5562330	T3PKS	PKSother	Coal	30.30	China: Guizhou, Fuquan
SRR10333326_bin.21.strict.fa_c00023_NODE_23...region001.fast	OEZ008529	yes	LMSG_G000004611.1	SRS5562330	betalactone	Others	Coal	25.89	China: Guizhou, Fuquan
SRR10333326_bin.21.strict.fa_c00045_NODE_45...region001.fast	OEZ008529	yes	LMSG_G000004611.1	SRS5562330	terpene	Terpene	Coal	15.62	China: Guizhou, Fuquan
SRR10333326_bin.21.strict.fa_c00068_NODE_68...region001.fast	OEZ008529	yes	LMSG_G000004611.1	SRS5562330	butyrolactone	Others	Coal	7.26	China: Guizhou, Fuquan
SRR10333326_bin.22.orig.fa_k141_312720.region001.fasta	OEZ008529	yes	LMSG_G000006009.1	SRS5562330	bacteriocin	RiPPs	Coal	6.58	China: Guizhou, Fuquan
SRR10333326_bin.22.orig.fa_k141_82863.region001.fasta	OEZ008529	yes	LMSG_G000006009.1	SRS5562330	arylpolyene	Others	Coal	31.34	China: Guizhou, Fuquan
SRR10333326_bin.23.orig.fa_k141_238432.region001.fasta	OEZ008529	yes	LMSG_G000006010.1	SRS5562330	terpene	Terpene	Coal	20.84	China: Guizhou, Fuquan
SRR10333326_bin.23.orig.fa_k141_804809.region001.fasta	OEZ008529	yes	LMSG_G000006010.1	SRS5562330	TfuA-related	RiPPs	Coal	24.52	China: Guizhou, Fuquan
SRR10333326_bin.23.orig.fa_k141_95273.region001.fasta	OEZ008529	yes	LMSG_G000006010.1	SRS5562330	LAP	RiPPs	Coal	22.24	China: Guizhou, Fuquan
SRR10333326_bin.26.permissive.fa_c00050_NODE_50...region001.	OEZ008529	yes	LMSG_G000006011.1	SRS5562330	terpene	Terpene	Coal	5.58	China: Guizhou, Fuquan
SRR10333326_bin.6.orig.fa_k141_582577.region001.fasta	OEZ008529	yes	LMSG_G000009260.1	SRS5562330	terpene	Terpene	Coal	8.37	China: Guizhou, Fuquan
SRR10333326_bin.6.orig.fa_k141_742229.region001.fasta	OEZ008529	yes	LMSG_G000009260.1	SRS5562330	T3PKS	PKSother	Coal	5.45	China: Guizhou, Fuquan
SRR10333326_bin.6.orig.fa_k141_929342.region001.fasta	OEZ008529	yes	LMSG_G000009260.1	SRS5562330	terpene	Terpene	Coal	8.97	China: Guizhou, Fuquan
SRR10333326_bin.7.orig.fa_k141_456535.region001.fasta	OEZ008529	yes	LMSG_G000008380.1	SRS5562330	terpene	Terpene	Coal	5.89	China: Guizhou, Fuquan
SRR10333326_bin.9.orig.fa_k141_612245.region001.fasta	OEZ008529	yes	LMSG_G000006012.1	SRS5562330	terpene	Terpene	Coal	6.31	China: Guizhou, Fuquan
SRR10333327_bin.1.orig.fa_k141_620732.region001.fasta	OEZ008529	yes	LMSG_G000009190.1	SRS5562329	terpene	Terpene	Coal	12.53	China: Guizhou, Fuquan
SRR10333327_bin.10.orig.fa_k141_244787.region001.fasta	OEZ008529	yes	LMSG_G000006013.1	SRS5562329	lassopeptide	RiPPs	Coal	5.12	China: Guizhou, Fuquan
SRR10333327_bin.10.orig.fa_k141_637332.region001.fasta	OEZ008529	yes	LMSG_G000006013.1	SRS5562329	NRPS	NRPS	Coal	32.93	China: Guizhou, Fuquan
SRR10333327_bin.10.orig.fa_k141_801223.region001.fasta	OEZ008529	yes	LMSG_G000006013.1	SRS5562329	terpene	Terpene	Coal	22.36	China: Guizhou, Fuquan
SRR10333327_bin.11.orig.fa_k141_479519.region001.fasta	OEZ008529	yes	LMSG_G000008733.1	SRS5562329	terpene	Terpene	Coal	7.32	China: Guizhou, Fuquan
SRR10333327_bin.11.orig.fa_k141_881241.region001.fasta	OEZ008529	yes	LMSG_G000008733.1	SRS5562329	other	Others	Coal	6.76	China: Guizhou, Fuquan
SRR10333327_bin.13.orig.fa_k141_423534.region001.fasta	OEZ008529	yes	LMSG_G000009174.1	SRS5562329	terpene	Terpene	Coal	15.67	China: Guizhou, Fuquan
SRR10333327_bin.13.orig.fa_k141_685024.region001.fasta	OEZ008529	yes	LMSG_G000009174.1	SRS5562329	T3PKS	PKSother	Coal	9.57	China: Guizhou, Fuquan
SRR10333327_bin.15.strict.fa_c00002_NODE_2_...region001.fast	OEZ008529	yes	LMSG_G000009167.1	SRS5562329	terpene	Terpene	Coal	23.71	China: Guizhou, Fuquan
SRR10333327_bin.15.strict.fa_c00007_NODE_7_...region001.fast	OEZ008529	yes	LMSG_G000009167.1	SRS5562329	terpene	Terpene	Coal	20.50	China: Guizhou, Fuquan
SRR10333327_bin.15.strict.fa_c00012_NODE_12...region001.fast	OEZ008529	yes	LMSG_G000009167.1	SRS5562329	betalactone	Others	Coal	29.91	China: Guizhou, Fuquan
SRR10333327_bin.15.strict.fa_c00021_NODE_21...region001.fast	OEZ008529	yes	LMSG_G000009167.1	SRS5562329	T3PKS	PKSother	Coal	29.85	China: Guizhou, Fuquan
SRR10333327_bin.16.orig.fa_k141_413336.region001.fasta	OEZ008529	yes	LMSG_G000006014.1	SRS5562329	NRPS-like	NRPS	Coal	5.67	China: Guizhou, Fuquan
SRR10333327_bin.16.orig.fa_k141_644481.region001.fasta	OEZ008529	yes	LMSG_G000006014.1	SRS5562329	terpene	Terpene	Coal	5.71	China: Guizhou, Fuquan
SRR10333327_bin.16.orig.fa_k141_770256.region001.fasta	OEZ008529	yes	LMSG_G000006014.1	SRS5562329	terpene	Terpene	Coal	5.98	China: Guizhou, Fuquan
SRR10333327_bin.17.orig.fa_k141_139067.region001.fasta	OEZ008529	yes	LMSG_G000011165.1	SRS5562329	terpene	Terpene	Coal	13.22	China: Guizhou, Fuquan
SRR10333327_bin.17.orig.fa_k141_20655.region001.fasta	OEZ008529	yes	LMSG_G000011165.1	SRS5562329	lanthipeptide	RiPPs	Coal	22.64	China: Guizhou, Fuquan
SRR10333327_bin.17.orig.fa_k141_229301.region001.fasta	OEZ008529	yes	LMSG_G000011165.1	SRS5562329	arylpolyene	Others	Coal	30.03	China: Guizhou, Fuquan
SRR10333327_bin.17.orig.fa_k141_563505.region001.fasta	OEZ008529	yes	LMSG_G000011165.1	SRS5562329	bacteriocin	RiPPs	Coal	10.98	China: Guizhou, Fuquan
SRR10333327_bin.17.orig.fa_k141_860267.region001.fasta	OEZ008529	yes	LMSG_G000011165.1	SRS5562329	arylpolyene	Others	Coal	8.41	China: Guizhou, Fuquan
SRR10333327_bin.18.orig.fa_k141_857518.region001.fasta	OEZ008529	yes	LMSG_G000008723.1	SRS5562329	terpene	Terpene	Coal	16.89	China: Guizhou, Fuquan
SRR10333327_bin.2.permissive.fa_c00007_NODE_7_...region001.f	OEZ008529	yes	LMSG_G000006019.1	SRS5562329	NRPS.T1PKS	PKS-NRP_Hybrids	Coal	28.79	China: Guizhou, Fuquan
SRR10333327_bin.2.permissive.fa_c00049_NODE_49...region001.f	OEZ008529	yes	LMSG_G000006019.1	SRS5562329	terpene	Terpene	Coal	24.54	China: Guizhou, Fuquan
SRR10333327_bin.2.permissive.fa_c00124_NODE_12...region001.f	OEZ008529	yes	LMSG_G000006019.1	SRS5562329	NRPS-like	NRPS	Coal	10.23	China: Guizhou, Fuquan
SRR10333327_bin.2.permissive.fa_c00132_NODE_13...region001.f	OEZ008529	yes	LMSG_G000006019.1	SRS5562329	NRPS	NRPS	Coal	9.28	China: Guizhou, Fuquan
SRR10333327_bin.2.permissive.fa_c00141_NODE_14...region001.f	OEZ008529	yes	LMSG_G000006019.1	SRS5562329	T3PKS	PKSother	Coal	8.57	China: Guizhou, Fuquan
SRR10333327_bin.20.orig.fa_k141_442005.region001.fasta	OEZ008529	yes	LMSG_G000006015.1	SRS5562329	terpene	Terpene	Coal	12.68	China: Guizhou, Fuquan
SRR10333327_bin.21.orig.fa_k141_174960.region001.fasta	OEZ008529	yes	LMSG_G000006016.1	SRS5562329	NRPS-like	NRPS	Coal	8.69	China: Guizhou, Fuquan
SRR10333327_bin.21.orig.fa_k141_718583.region001.fasta	OEZ008529	yes	LMSG_G000006016.1	SRS5562329	NRPS	NRPS	Coal	37.79	China: Guizhou, Fuquan
SRR10333327_bin.21.orig.fa_k141_742069.region001.fasta	OEZ008529	yes	LMSG_G000006016.1	SRS5562329	bacteriocin	RiPPs	Coal	6.75	China: Guizhou, Fuquan
SRR10333327_bin.21.orig.fa_k141_792019.region001.fasta	OEZ008529	yes	LMSG_G000006016.1	SRS5562329	terpene	Terpene	Coal	16.94	China: Guizhou, Fuquan
SRR10333327_bin.23.orig.fa_k141_735106.region001.fasta	OEZ008529	yes	LMSG_G000008562.1	SRS5562329	terpene	Terpene	Coal	12.17	China: Guizhou, Fuquan
SRR10333327_bin.23.orig.fa_k141_930731.region001.fasta	OEZ008529	yes	LMSG_G000008562.1	SRS5562329	NRPS-like	NRPS	Coal	38.44	China: Guizhou, Fuquan
SRR10333327_bin.23.orig.fa_k141_930731.region002.fasta	OEZ008529	yes	LMSG_G000008562.1	SRS5562329	bacteriocin	RiPPs	Coal	10.81	China: Guizhou, Fuquan
SRR10333327_bin.24.orig.fa_k141_431038.region001.fasta	OEZ008529	yes	LMSG_G000006017.1	SRS5562329	bacteriocin	RiPPs	Coal	10.81	China: Guizhou, Fuquan
SRR10333327_bin.24.orig.fa_k141_740398.region001.fasta	OEZ008529	yes	LMSG_G000006017.1	SRS5562329	betalactone	Others	Coal	32.24	China: Guizhou, Fuquan
SRR10333327_bin.24.orig.fa_k141_926657.region001.fasta	OEZ008529	yes	LMSG_G000006017.1	SRS5562329	other	Others	Coal	30.59	China: Guizhou, Fuquan
SRR10333327_bin.26.orig.fa_k141_253191.region001.fasta	OEZ008529	yes	LMSG_G000004613.1	SRS5562329	T3PKS	PKSother	Coal	5.30	China: Guizhou, Fuquan
SRR10333327_bin.26.orig.fa_k141_395967.region001.fasta	OEZ008529	yes	LMSG_G000004613.1	SRS5562329	terpene	Terpene	Coal	14.61	China: Guizhou, Fuquan
SRR10333327_bin.27.orig.fa_k141_256153.region001.fasta	OEZ008529	yes	LMSG_G000006018.1	SRS5562329	terpene	Terpene	Coal	10.90	China: Guizhou, Fuquan
SRR10333327_bin.27.orig.fa_k141_365112.region001.fasta	OEZ008529	yes	LMSG_G000006018.1	SRS5562329	NRPS	NRPS	Coal	11.93	China: Guizhou, Fuquan
SRR10333327_bin.27.orig.fa_k141_803090.region001.fasta	OEZ008529	yes	LMSG_G000006018.1	SRS5562329	bacteriocin	RiPPs	Coal	6.37	China: Guizhou, Fuquan
SRR10333327_bin.27.orig.fa_k141_829925.region001.fasta	OEZ008529	yes	LMSG_G000006018.1	SRS5562329	terpene	Terpene	Coal	10.12	China: Guizhou, Fuquan
SRR10333327_bin.27.orig.fa_k141_905923.region001.fasta	OEZ008529	yes	LMSG_G000006018.1	SRS5562329	other	Others	Coal	26.93	China: Guizhou, Fuquan
SRR10333327_bin.28.orig.fa_k141_268818.region001.fasta	OEZ008529	yes	LMSG_G000010314.1	SRS5562329	bacteriocin	RiPPs	Coal	7.26	China: Guizhou, Fuquan
SRR10333327_bin.28.orig.fa_k141_298645.region001.fasta	OEZ008529	yes	LMSG_G000010314.1	SRS5562329	NRPS-like	NRPS	Coal	9.29	China: Guizhou, Fuquan
SRR10333327_bin.28.orig.fa_k141_532035.region001.fasta	OEZ008529	yes	LMSG_G000010314.1	SRS5562329	T3PKS	PKSother	Coal	14.97	China: Guizhou, Fuquan
SRR10333327_bin.28.orig.fa_k141_535657.region001.fasta	OEZ008529	yes	LMSG_G000010314.1	SRS5562329	terpene	Terpene	Coal	8.18	China: Guizhou, Fuquan

SRR10333327_bin.28.orig.fa_k141_794494.region001.fasta	OEZ008529	yes	LMSG_G000010314.1	SRS5562329	arylpolyene	Others	Coal	5.29	China: Guizhou, Fuquan
SRR10333327_bin.33.orig.fa_k141_177397.region001.fasta	OEZ008529	yes	LMSG_G000011173.1	SRS5562329	arylpolyene	Others	Coal	5.60	China: Guizhou, Fuquan
SRR10333327_bin.33.orig.fa_k141_290823.region001.fasta	OEZ008529	yes	LMSG_G000011173.1	SRS5562329	NRPS.T1PKS	PKS-NRP_Hybrids	Coal	44.33	China: Guizhou, Fuquan
SRR10333327_bin.34.orig.fa_k141_899194.region001.fasta	OEZ008529	yes	LMSG_G000008373.1	SRS5562329	terpene	Terpene	Coal	9.25	China: Guizhou, Fuquan
SRR10333327_bin.35.orig.fa_k141_754860.region001.fasta	OEZ008529	yes	LMSG_G000009482.1	SRS5562329	T1PKS.hglE-KS	PKSother	Coal	8.91	China: Guizhou, Fuquan
SRR10333327_bin.37.orig.fa_k141_122567.region001.fasta	OEZ008529	yes	LMSG_G000011047.1	SRS5562329	terpene	Terpene	Coal	23.09	China: Guizhou, Fuquan
SRR10333327_bin.37.orig.fa_k141_198154.region001.fasta	OEZ008529	yes	LMSG_G000011047.1	SRS5562329	arylpolyene	Others	Coal	42.13	China: Guizhou, Fuquan
SRR10333327_bin.37.orig.fa_k141_721833.region001.fasta	OEZ008529	yes	LMSG_G000011047.1	SRS5562329	bacteriocin	RiPPs	Coal	6.91	China: Guizhou, Fuquan
SRR10333327_bin.37.orig.fa_k141_796681.region001.fasta	OEZ008529	yes	LMSG_G000011047.1	SRS5562329	resorcinol.arylpolyene	Others	Coal	19.98	China: Guizhou, Fuquan
SRR10333327_bin.37.orig.fa_k141_853475.region001.fasta	OEZ008529	yes	LMSG_G000011047.1	SRS5562329	NRPS-like	NRPS	Coal	23.51	China: Guizhou, Fuquan
SRR10333327_bin.37.orig.fa_k141_887490.region001.fasta	OEZ008529	yes	LMSG_G000011047.1	SRS5562329	terpene	Terpene	Coal	20.85	China: Guizhou, Fuquan
SRR10333327_bin.38.orig.fa_k141_165667.region001.fasta	OEZ008529	yes	LMSG_G000006301.1	SRS5562329	bacteriocin	RiPPs	Coal	10.80	China: Guizhou, Fuquan
SRR10333327_bin.38.orig.fa_k141_556688.region001.fasta	OEZ008529	yes	LMSG_G000006301.1	SRS5562329	ladderane	Others	Coal	30.96	China: Guizhou, Fuquan
SRR10333327_bin.38.orig.fa_k141_659474.region001.fasta	OEZ008529	yes	LMSG_G000006301.1	SRS5562329	terpene	Terpene	Coal	20.11	China: Guizhou, Fuquan
SRR10333327_bin.41.orig.fa_k141_645243.region001.fasta	OEZ008529	yes	LMSG_G000006020.1	SRS5562329	NRPS	NRPS	Coal	24.18	China: Guizhou, Fuquan
SRR10333327_bin.41.orig.fa_k141_849283.region001.fasta	OEZ008529	yes	LMSG_G000006020.1	SRS5562329	terpene	Terpene	Coal	19.96	China: Guizhou, Fuquan
SRR10333327_bin.42.orig.fa_k141_355695.region001.fasta	OEZ008529	yes	LMSG_G000004618.1	SRS5562329	arylpolyene	Others	Coal	21.65	China: Guizhou, Fuquan
SRR10333327_bin.42.orig.fa_k141_723096.region001.fasta	OEZ008529	yes	LMSG_G000004618.1	SRS5562329	bacteriocin	RiPPs	Coal	8.11	China: Guizhou, Fuquan
SRR10333327_bin.42.orig.fa_k141_908419.region001.fasta	OEZ008529	yes	LMSG_G000004618.1	SRS5562329	betalactone	Others	Coal	11.67	China: Guizhou, Fuquan
SRR10333327_bin.43.strict.fa_c00014_NODE_14...region001.fast	OEZ008529	yes	LMSG_G000004619.1	SRS5562329	terpene	Terpene	Coal	14.56	China: Guizhou, Fuquan
SRR10333327_bin.43.strict.fa_c00042_NODE_42...region001.fast	OEZ008529	yes	LMSG_G000004619.1	SRS5562329	arylpolyene	Others	Coal	9.33	China: Guizhou, Fuquan
SRR10333327_bin.44.orig.fa_k141_21570.region001.fasta	OEZ008529	yes	LMSG_G000004620.1	SRS5562329	NRPS	NRPS	Coal	29.86	China: Guizhou, Fuquan
SRR10333327_bin.44.orig.fa_k141_721643.region001.fasta	OEZ008529	yes	LMSG_G000004620.1	SRS5562329	NRPS-like	NRPS	Coal	37.92	China: Guizhou, Fuquan
SRR10333327_bin.44.orig.fa_k141_728148.region001.fasta	OEZ008529	yes	LMSG_G000004620.1	SRS5562329	NRPS-like	NRPS	Coal	30.17	China: Guizhou, Fuquan
SRR10333327_bin.8.orig.fa_k141_130747.region001.fasta	OEZ008529	yes	LMSG_G000004621.1	SRS5562329	NRPS	NRPS	Coal	26.91	China: Guizhou, Fuquan
SRR10333327_bin.8.orig.fa_k141_27878.region001.fasta	OEZ008529	yes	LMSG_G000004621.1	SRS5562329	NRPS.nucleoside	Others	Coal	56.99	China: Guizhou, Fuquan
SRR10333327_bin.8.orig.fa_k141_291838.region001.fasta	OEZ008529	yes	LMSG_G000004621.1	SRS5562329	NRPS.lassopeptide	Others	Coal	23.05	China: Guizhou, Fuquan
SRR10333327_bin.8.orig.fa_k141_366220.region001.fasta	OEZ008529	yes	LMSG_G000004621.1	SRS5562329	terpene	Terpene	Coal	20.96	China: Guizhou, Fuquan
SRR10333327_bin.8.orig.fa_k141_410405.region001.fasta	OEZ008529	yes	LMSG_G000004621.1	SRS5562329	bacteriocin	RiPPs	Coal	10.83	China: Guizhou, Fuquan
SRR10333327_bin.8.orig.fa_k141_415782.region001.fasta	OEZ008529	yes	LMSG_G000004621.1	SRS5562329	NRPS	NRPS	Coal	9.39	China: Guizhou, Fuquan
SRR10333327_bin.8.orig.fa_k141_452465.region001.fasta	OEZ008529	yes	LMSG_G000004621.1	SRS5562329	bacteriocin	RiPPs	Coal	8.98	China: Guizhou, Fuquan
SRR10333327_bin.8.orig.fa_k141_482059.region001.fasta	OEZ008529	yes	LMSG_G000004621.1	SRS5562329	NRPS	NRPS	Coal	11.55	China: Guizhou, Fuquan
SRR10333327_bin.8.orig.fa_k141_482093.region001.fasta	OEZ008529	no	LMSG_G000004621.1	SRS5562329	NRPS	NRPS	Coal	5.57	China: Guizhou, Fuquan
SRR10333327_bin.8.orig.fa_k141_642953.region001.fasta	OEZ008529	yes	LMSG_G000004621.1	SRS5562329	NRPS	NRPS	Coal	12.73	China: Guizhou, Fuquan
SRR10333327_bin.8.orig.fa_k141_653176.region001.fasta	OEZ008529	yes	LMSG_G000004621.1	SRS5562329	lanthipeptide	RiPPs	Coal	15.33	China: Guizhou, Fuquan
SRR10333327_bin.8.orig.fa_k141_686089.region001.fasta	OEZ008529	yes	LMSG_G000004621.1	SRS5562329	lanthipeptide	RiPPs	Coal	16.81	China: Guizhou, Fuquan
SRR10333327_bin.8.orig.fa_k141_753065.region001.fasta	OEZ008529	yes	LMSG_G000004621.1	SRS5562329	NRPS	NRPS	Coal	15.07	China: Guizhou, Fuquan
SRR10333327_bin.8.orig.fa_k141_863411.region001.fasta	OEZ008529	yes	LMSG_G000004621.1	SRS5562329	NRPS	NRPS	Coal	20.88	China: Guizhou, Fuquan
SRR10333327_bin.8.orig.fa_k141_867259.region001.fasta	OEZ008529	yes	LMSG_G000004621.1	SRS5562329	NRPS	NRPS	Coal	69.03	China: Guizhou, Fuquan
SRR10333327_bin.8.orig.fa_k141_881398.region001.fasta	OEZ008529	yes	LMSG_G000004621.1	SRS5562329	NRPS-like	NRPS	Coal	24.20	China: Guizhou, Fuquan
SRR10333327_bin.8.orig.fa_k141_906111.region001.fasta	OEZ008529	yes	LMSG_G000004621.1	SRS5562329	resorcinol	Others	Coal	41.76	China: Guizhou, Fuquan
SRR10333327_bin.8.orig.fa_k141_999890.region001.fasta	OEZ008529	yes	LMSG_G000004621.1	SRS5562329	terpene	Terpene	Coal	17.48	China: Guizhou, Fuquan
SRR10333327_bin.8.orig.fa_k141_999890.region002.fasta	OEZ008529	yes	LMSG_G000004621.1	SRS5562329	NRPS.bacteriocin	Others	Coal	63.69	China: Guizhou, Fuquan
SRR10333328_bin.10.strict.fa_c00002_NODE_2_...region001.fast	OEZ008529	yes	LMSG_G000009652.1	SRS5562328	terpene	Terpene	Coal	22.09	China: Guizhou, Fuquan
SRR10333328_bin.10.strict.fa_c00012_NODE_12...region001.fast	OEZ008529	yes	LMSG_G000009652.1	SRS5562328	terpene	Terpene	Coal	14.73	China: Guizhou, Fuquan
SRR10333328_bin.10.strict.fa_c00015_NODE_15...region001.fast	OEZ008529	yes	LMSG_G000009652.1	SRS5562328	NRPS-like	NRPS	Coal	32.35	China: Guizhou, Fuquan
SRR10333328_bin.11.orig.fa_k141_286218.region001.fasta	OEZ008529	yes	LMSG_G000008374.1	SRS5562328	terpene	Terpene	Coal	8.41	China: Guizhou, Fuquan
SRR10333328_bin.14.orig.fa_k141_307967.region001.fasta	OEZ008529	yes	LMSG_G000008722.1	SRS5562328	terpene	Terpene	Coal	10.58	China: Guizhou, Fuquan
SRR10333328_bin.14.orig.fa_k141_459472.region001.fasta	OEZ008529	yes	LMSG_G000008722.1	SRS5562328	NRPS	NRPS	Coal	8.16	China: Guizhou, Fuquan
SRR10333328_bin.14.orig.fa_k141_463093.region001.fasta	OEZ008529	yes	LMSG_G000008722.1	SRS5562328	NRPS	NRPS	Coal	9.62	China: Guizhou, Fuquan
SRR10333328_bin.17.orig.fa_k141_121417.region001.fasta	OEZ008529	yes	LMSG_G000011166.1	SRS5562328	terpene	Terpene	Coal	13.22	China: Guizhou, Fuquan
SRR10333328_bin.17.orig.fa_k141_215025.region001.fasta	OEZ008529	yes	LMSG_G000011166.1	SRS5562328	bacteriocin	RiPPs	Coal	6.63	China: Guizhou, Fuquan
SRR10333328_bin.17.orig.fa_k141_261010.region001.fasta	OEZ008529	yes	LMSG_G000011166.1	SRS5562328	arylpolyene	Others	Coal	16.39	China: Guizhou, Fuquan
SRR10333328_bin.17.orig.fa_k141_529142.region001.fasta	OEZ008529	yes	LMSG_G000011166.1	SRS5562328	arylpolyene	Others	Coal	9.70	China: Guizhou, Fuquan
SRR10333328_bin.2.orig.fa_k141_109099.region001.fasta	OEZ008529	yes	LMSG_G000008716.1	SRS5562328	NRPS	NRPS	Coal	28.55	China: Guizhou, Fuquan
SRR10333328_bin.2.orig.fa_k141_146827.region001.fasta	OEZ008529	yes	LMSG_G000008716.1	SRS5562328	NRPS	NRPS	Coal	15.24	China: Guizhou, Fuquan
SRR10333328_bin.2.orig.fa_k141_238043.region001.fasta	OEZ008529	yes	LMSG_G000008716.1	SRS5562328	NRPS	NRPS	Coal	10.10	China: Guizhou, Fuquan
SRR10333328_bin.2.orig.fa_k141_24097.region001.fasta	OEZ008529	yes	LMSG_G000008716.1	SRS5562328	terpene	Terpene	Coal	15.43	China: Guizhou, Fuquan
SRR10333328_bin.2.orig.fa_k141_26029.region001.fasta	OEZ008529	yes	LMSG_G000008716.1	SRS5562328	NRPS	NRPS	Coal	6.41	China: Guizhou, Fuquan
SRR10333328_bin.2.orig.fa_k141_292438.region001.fasta	OEZ008529	yes	LMSG_G000008716.1	SRS5562328	bacteriocin.lanthipeptide	RiPPs	Coal	25.90	China: Guizhou, Fuquan
SRR10333328_bin.2.orig.fa_k141_29506.region001.fasta	OEZ008529	yes	LMSG_G000008716.1	SRS5562328	NRPS	NRPS	Coal	6.60	China: Guizhou, Fuquan
SRR10333328_bin.2.orig.fa_k141_310368.region001.fasta	OEZ008529	yes	LMSG_G000008716.1	SRS5562328	NRPS	NRPS	Coal	11.55	China: Guizhou, Fuquan
SRR10333328_bin.2.orig.fa_k141_356962.region001.fasta	OEZ008529	no	LMSG_G000008716.1	SRS5562328	NRPS	NRPS	Coal	7.40	China: Guizhou, Fuquan
SRR10333328_bin.2.orig.fa_k141_360157.region001.fasta	OEZ008529	yes	LMSG_G000008716.1	SRS5562328	NRPS-like	NRPS	Coal	12.56	China: Guizhou, Fuquan
SRR10333328_bin.2.orig.fa_k141_436029.region001.fasta	OEZ008529	yes	LMSG_G000008716.1	SRS5562328	NRPS	NRPS	Coal	11.14	China: Guizhou, Fuquan
SRR10333328_bin.2.orig.fa_k141_441288.region001.fasta	OEZ008529	yes	LMSG_G000008716.1	SRS5562328	bacteriocin	RiPPs	Coal	5.90	China: Guizhou, Fuquan
SRR10333328_bin.2.orig.fa_k141_449847.region001.fasta	OEZ008529	yes	LMSG_G000008716.1	SRS5562328	resorcinol	Others	Coal	16.74	China: Guizhou, Fuquan
SRR10333328_bin.2.orig.fa_k141_450063.region001.fasta	OEZ008529	yes	LMSG_G000008716.1	SRS5562328	bacteriocin	RiPPs	Coal	6.17	China: Guizhou, Fuquan
SRR10333328_bin.2.orig.fa_k141_523953.region001.fasta	OEZ008529	yes	LMSG_G000008716.1	SRS5562328	NRPS	NRPS	Coal	7.46	China: Guizhou, Fuquan
SRR10333328_bin.2.orig.fa_k141_545783.region001.fasta	OEZ008529	yes	LMSG_G000008716.1	SRS5562328	terpene	Terpene	Coal	14.52	China: Guizhou, Fuquan
SRR10333328_bin.22.orig.fa_k141_148670.region001.fasta	OEZ008529	yes	LMSG_G000010315.1	SRS5562328	bacteriocin	RiPPs	Coal	6.02	China: Guizhou, Fuquan
SRR10333328_bin.22.orig.fa_k141_216057.region001.fasta	OEZ008529	yes	LMSG_G000010315.1	SRS5562328	arylpolyene	Others	Coal	7.45	China: Guizhou, Fuquan
SRR10333328_bin.22.orig.fa_k141_3363.region001.fasta	OEZ008529	yes	LMSG_G000010315.1	SRS5562328	bacteriocin	RiPPs	Coal	10.85	China: Guizhou, Fuquan
SRR10333328_bin.22.orig.fa_k141_383029.region001.fasta	OEZ008529	yes	LMSG_G000010315.1	SRS5562328	T3PKS	PKSother	Coal	8.51	China: Guizhou, Fuquan
SRR10333328_bin.22.orig.fa_k141_460690.region001.fasta	OEZ008529	yes	LMSG_G000010315.1	SRS5562328	terpene	Terpene	Coal	8.94	China: Guizhou, Fuquan
SRR10333328_bin.22.orig.fa_k141_54989.region001.fasta	OEZ008529	yes	LMSG_G000010315.1	SRS5562328	T3PKS	PKSother	Coal	5.21	China: Guizhou, Fuquan
SRR10333328_bin.26.strict.fa_c00021_NODE_21...region001.fast	OEZ008529	yes	LMSG_G000006025.1	SRS5562328	NRPS-like	NRPS	Coal	14.20	China: Guizhou, Fuquan
SRR10333328_bin.26.strict.fa_c00041_NODE_41...region001.fast	OEZ008529	yes	LMSG_G000006025.1	SRS5562328	other	Others	Coal	11.67	China: Guizhou, Fuquan
SRR10333328_bin.26.strict.fa_c00058_NODE_58...region001.fast	OEZ008529	yes	LMSG_G000006025.1	SRS5562328	terpene	Terpene	Coal	10.34	China: Guizhou, Fuquan
SRR10333328_bin.26.strict.fa_c00151_NODE_15...region001.fast	OEZ008529	yes	LMSG_G000006025.1	SRS5562328	butyrolactone	Others	Coal	5.42	China: Guizhou, Fuquan
SRR10333328_bin.26.strict.fa_c00189_NODE_18...region001.fast	OEZ008529	yes	LMSG_G000006025.1	SRS5562328	terpene	Terpene	Coal	5.44	China: Guizhou, Fuquan
SRR10333328_bin.28.orig.fa_k141_493743.region001.fasta	OEZ008529	yes	LMSG_G000009192.1	SRS5562328	T3PKS	PKSother	Coal	11.45	China: Guizhou, Fuquan
SRR10333328_bin.28.orig.fa_k141_552032.region001.fasta	OEZ008529	yes	LMSG_G000009192.1	SRS5562328	terpene	Terpene	Coal	18.94	China: Guizhou, Fuquan
SRR10333328_bin.29.orig.fa_k141_18398.region001.fasta	OEZ008529	yes	LMSG_G000006026.1	SRS5562328	terpene	Terpene	Coal	10.20	China: Guizhou, Fuquan
SRR10333328_bin.29.orig.fa_k141_388318.region001.fasta	OEZ008529	yes	LMSG_G000006026.1	SRS5562328	terpene	Terpene	Coal	5.10	China: Guizhou, Fuquan
SRR10333328_bin.31.orig.fa_k141_354981.region001.fasta	OEZ008529	yes	LMSG_G000009630.1	SRS5562328	terpene	Terpene	Coal	6.58	China: Guizhou, Fuquan
SRR10333328_bin.31.orig.fa_k141_365665.region001.fasta	OEZ008529	yes	LMSG_G000009630.1	SRS5562328	terpene	Terpene	Coal	9.64	China: Guizhou, Fuquan
SRR10333328_bin.31.orig.fa_k141_442940.region001.fasta	OEZ008529	yes	LMSG_G000009630.1	SRS5562328	nucleoside	Others	Coal	6.43	China: Guizhou, Fuquan
SRR10333328_bin.31.orig.fa_k141_504793.region001.fasta	OEZ008529	yes	LMSG_G000009630.1	SRS5562328	NRPS-like	NRPS	Coal	9.42	China: Guizhou, Fuquan
SRR10333328_bin.32.orig.fa_k141_329315.region001.fasta	OEZ008529	yes	LMSG_G000006027.1	SRS5562328	terpene	Terpene	Coal	20.85	China: Guizhou, Fuquan
SRR10333328_bin.32.orig.fa_k141_344072.region001.fasta	OEZ008529	yes	LMSG_G000006027.1	SRS5562328	bacteriocin	RiPPs	Coal	6.83	China: Guizhou, Fuquan
SRR10333328_bin.32.orig.fa_k141_460898.region001.fasta	OEZ008529	yes	LMSG_G000006027.1	SRS5562328	NRPS-like	NRPS	Coal	10.00	China: Guizhou, Fuquan
SRR10333328_bin.32.orig.fa_k141_504230.region001.fasta	OEZ008529	yes	LMSG_G000006027.1	SRS5562328	arylpolyene	Others	Coal	27.36	China: Guizhou, Fuquan
SRR10333328_bin.32.orig.fa_k141_523809.region001.fasta	OEZ008529	yes	LMSG_G000006027.1	SRS5562328	terpene	Terpene	Coal	23.09	China: Guizhou, Fuquan
SRR10333328_bin.32.orig.fa_k141_559193.region001.fasta	OEZ008529	yes	LMSG_G000006027.1	SRS5562328	resorcinol.arylpolyene	Others	Coal	20.08	China: Guizhou, Fuquan
SRR10333328_bin.41.strict.fa_c00002_NODE_2_...region001.fast	OEZ008529	yes	LMSG_G000010040.1	SRS5562328	T3PKS	PKSother	Coal	41.07	China: Guizhou, Fuquan
SRR10333328_bin.41.strict.fa_c00022_NODE_22...region001.fast	OEZ008529	yes	LMSG_G000010040.1	SRS5562328	terpene	Terpene	Coal	21.84	China: Guizhou, Fuquan
SRR10333328_bin.41.strict.fa_c00032_NODE_32...region001.fast	OEZ008529	yes	LMSG_G000010040.1	SRS5562328	ladderane	Others	Coal	29.84	China: Guizhou, Fuquan
SRR10333328_bin.41.strict.fa_c00034_NODE_34...region001.fast	OEZ008529	yes	LMSG_G000010040.1	SRS5562328	arylpolyene	Others	Coal	29.15	China: Guizhou, Fuquan
SRR10333328_bin.41.strict.fa_c00087_NODE_87...region001.fast	OEZ008529	yes	LMSG_G000010040.1	SRS5562328	bacteriocin	RiPPs	Coal	6.70	China: Guizhou, Fuquan
SRR10333328_bin.41.strict.fa_c00151_NODE_15...region001.fast	OEZ008529	yes	LMSG_G000010040.1	SRS5562328	NRPS-like	NRPS	Coal	10.76	China: Guizhou, Fuquan
SRR10333328_bin.41.strict.fa_c00220_NODE_22...region001.fast	OEZ008529	yes	LMSG_G000010040.1	SRS5562328	terpene	Terpene	Coal	6.53	China: Guizhou, Fuquan
SRR10333328_bin.42.orig.fa_k141_149913.region001.fasta	OEZ008529	yes	LMSG_G000006028.1	SRS5562328	terpene	Terpene	Coal	17.09	China: Guizhou, Fuquan
SRR10333328_bin.42.orig.fa_k141_151398.region001.fasta	OEZ008529	yes	LMSG_G000006028.1	SRS5562328	T1PKS.hglE-KS	PKSother	Coal	5.75	China: Guizhou, Fuquan
SRR10333328_bin.42.orig.fa_k141_415202.region001.fasta	OEZ008529	yes	LMSG_G000006028.1	SRS5562328	hglE-KS	PKSother	Coal	20.56	China: Guizhou, Fuquan
SRR10333328_bin.43.orig.fa_k141_101196.region001.fasta	OEZ008529	yes	LMSG_G000006029.1	SRS5562328	terpene	Terpene	Coal	5.14	China: Guizhou, Fuquan
SRR10333328_bin.46.orig.fa_k141_142554.region001.fasta	OEZ008529	yes	LMSG_G000008561.1	SRS5562328	betalactone	Others	Coal	27.80	China: Guizhou, Fuquan
SRR10333328_bin.46.orig.fa_k141_147370.region001.fasta	OEZ008529	yes	LMSG_G000008561.1	SRS5562328	betalactone	Others	Coal	24.94	China: Guizhou, Fuquan
SRR10333328_bin.46.orig.fa_k141_163080.region001.fasta	OEZ008529	yes	LMSG_G000008561.1	SRS5562328	arylpolyene	Others	Coal	31.86	China: Guizhou, Fuquan
SRR10333328_bin.46.orig.fa_k141_332424.region001.fasta	OEZ008529	yes	LMSG_G000008561.1	SRS5562328	NRPS	NRPS	Coal	29.19	China: Guizhou, Fuquan
SRR10333328_bin.47.orig.fa_k141_108601.region001.fasta	OEZ008529	yes	LMSG_G000004622.1	SRS5562328	terpene	Terpene	Coal	20.35	China: Guizhou, Fuquan
SRR10333328_bin.47.orig.fa_k141_429373.region001.fasta	OEZ008529	yes	LMSG_G000004622.1	SRS5562328	betalactone	Others	Coal	32.75	China: Guizhou, Fuquan
SRR10333328_bin.47.orig.fa_k141_448286.region001.fasta	OEZ008529	yes	LMSG_G000004622.1	SRS5562328	terpene	Terpene	Coal	23.71	China: Guizhou, Fuquan
SRR10333328_bin.47.orig.fa_k141_469697.region001.fasta	OEZ008529	yes	LMSG_G000004622.1	SRS5562328	T3PKS	PKSother	Coal	23.13	China: Guizhou, Fuquan
SRR10333328_bin.48.orig.fa_k141_314064.region001.fasta	OEZ008529	yes	LMSG_G000008563.1	SRS5562328	terpene	Terpene	Coal	6.99	China: Guizhou, Fuquan
SRR10333328_bin.48.orig.fa_k141_467138.region001.fasta	OEZ008529	yes	LMSG_G000008563.1	SRS5562328	NRPS	NRPS	Coal	25.28	China: Guizhou, Fuquan
SRR10333328_bin.48.orig.fa_k141_93528.region001.fasta	OEZ008529	yes	LMSG_G000008563.1	SRS5562328	other	Others	Coal	23.02	China: Guizhou, Fuquan
SRR10333328_bin.48.orig.fa_k141_96143.region001.fasta	OEZ008529	yes	LMSG_G000008563.1	SRS5562328	bacteriocin	RiPPs	Coal	6.43	China: Guizhou, Fuquan
SRR10333328_bin.50.strict.fa_c00002_NODE_2_...region001.fast	OEZ008529	yes	LMSG_G000007895.1	SRS5562328	NRPS	NRPS	Coal	45.75	China: Guizhou, Fuquan
SRR10333328_bin.50.strict.fa_c00007_NODE_7_...region001.fast	OEZ008529	yes	LMSG_G000007895.1	SRS5562328	NRPS-like	NRPS	Coal	42.63	China: Guizhou, Fuquan
SRR10333328_bin.50.strict.fa_c00008_NODE_8_...region001.fast	OEZ008529	yes	LMSG_G000007895.1	SRS5562328	NRPS-like	NRPS	Coal	42.63	China: Guizhou, Fuquan
SRR10333328_bin.9.strict.fa_c00011_NODE_11...region001.fasta	OEZ008529	yes	LMSG_G000009542.1	SRS5562328	terpene	Terpene	Coal	11.92	China: Guizhou, Fuquan
[truncated: 150,692 more chars]
