# Supplementary material for: Mining of novel secondary metabolite biosynthetic gene clusters from acid mine drainage
Source: Sci Data. 2022 Dec 9;9:760. doi: 10.1038/s41597-022-01866-6 (PMC9734747; doi:10.1038/s41597-022-01866-6)
Supplement: Supplementary file 5 — Supplementary Table 4 [file 41597_2022_1866_MOESM5_ESM.pdf]

Supplementary Table 4. The smBGC distribution in 1,316 dRep clusters

Genome ID	dRep cluster ID	Retained after dRep	Number of NRPS	Number of Others	Number of PKS I	Number of PKS-NRP_Hybrids	Number of PKSother	Number of RiPPs	Number of saccharides	Number of terpene
LMSG_G000005313.1	1000_1	yes					1	1		2
LMSG_G000007731.1	1000_1	no					1	1		3
LMSG_G000006154.1	1001_0	yes					1	1		1
LMSG_G000005209.1	1002_0	yes	1				1	1		2
LMSG_G000004716.1	1003_0	yes	2		1	1		4		2
LMSG_G000004976.1	1004_0	yes					1			
LMSG_G000004410.1	1007_0	yes	1							
LMSG_G000009857.1	1008_1	no	1							
LMSG_G000009858.1	1008_1	no	1							
LMSG_G000009859.1	1008_1	no	1							
LMSG_G000009861.1	1008_1	no	1							
LMSG_G000005674.1	1009_0	yes		3				1		
LMSG_G000004429.1	1010_0	yes								1
LMSG_G000004971.1	1012_0	yes		1						
LMSG_G000011303.1	1014_1	no								3
LMSG_G000004467.1	1014_1	yes								4
LMSG_G000011308.1	1015_1	no		1						4
LMSG_G000011306.1	1015_1	no		1						1
LMSG_G000011307.1	1015_1	no		1						3
LMSG_G000004462.1	1015_1	yes		2						4
LMSG_G000011305.1	1015_1	no		2						4
LMSG_G000008989.1	1016_1	no						1		1
LMSG_G000008990.1	1016_1	no						1		1
LMSG_G000008991.1	1016_1	no						1		1
LMSG_G000008988.1	1016_1	no						1		1
LMSG_G000008992.1	1016_1	no						1		1
LMSG_G000008993.1	1016_1	no						1		1
LMSG_G000008994.1	1016_1	no						1		1
LMSG_G000008995.1	1016_1	no						1		1
LMSG_G000008996.1	1016_1	no						1		
LMSG_G000008998.1	1016_1	no						1		1
LMSG_G000008999.1	1016_1	no						1		2
LMSG_G000009000.1	1016_1	no						1		1
LMSG_G000009001.1	1016_1	no						1		1
LMSG_G000005598.1	1016_1	yes						1		1
LMSG_G000009002.1	1016_1	no						1		1
LMSG_G000009003.1	1016_1	no						1		1
LMSG_G000009004.1	1016_1	no						1		1
LMSG_G000009006.1	1016_1	no						1		1
LMSG_G000009007.1	1016_1	no						1		1
LMSG_G000009008.1	1016_1	no						1		1
LMSG_G000009009.1	1016_1	no						1		1
LMSG_G000009010.1	1016_1	no								1
LMSG_G000009011.1	1016_1	no								1
LMSG_G000009012.1	1016_1	no						1		1
LMSG_G000009013.1	1016_1	no						1		1
LMSG_G000009015.1	1016_1	no						1		1
LMSG_G000009340.1	1017_1	no						1		
LMSG_G000004914.1	1017_1	yes						1		
LMSG_G000009343.1	1017_1	no						1		
LMSG_G000009344.1	1017_1	no						1		
LMSG_G000009338.1	1017_1	no						1		
LMSG_G000009345.1	1017_1	no						1		
LMSG_G000009346.1	1017_1	no						1		
LMSG_G000011304.1	1018_1	no	2		1		2			1
LMSG_G000005285.1	1018_1	yes	3		1		4			2
LMSG_G000006191.1	1019_0	yes		1						
LMSG_G000004344.1	1020_1	yes						2		2
LMSG_G000009230.1	1020_1	no						1		1
LMSG_G000009225.1	1022_1	no		1			3	2		2
LMSG_G000009226.1	1022_1	no					2	1		2
LMSG_G000004458.1	1022_1	yes		1			2	1		2
LMSG_G000009227.1	1022_1	no					2	1		2
LMSG_G000009228.1	1022_1	no					1	1		2
LMSG_G000009229.1	1022_1	no		1			3	1		2
LMSG_G000005137.1	1023_0	yes					2	1		2
LMSG_G000004456.1	1024_0	yes					2	1		2
LMSG_G000006152.1	1025_0	yes								1
LMSG_G000004674.1	1026_0	yes	1	2	1		2	3		2

LMSG_G000004771.1	1028_1	yes	1	1				1	1
LMSG_G000007828.1	1028_1	no	1				1	1	2
LMSG_G000007829.1	1028_1	no	1				1	2	2
LMSG_G000007830.1	1028_1	no					1		1
LMSG_G000007831.1	1028_1	no					1	2	2
LMSG_G000007832.1	1028_1	no	1					1	2
LMSG_G000007833.1	1028_1	no					1	1	2
LMSG_G000007834.1	1028_1	no	1	1				1	1
LMSG_G000004950.1	1029_0	yes		2					
LMSG_G000004347.1	1030_1	yes	1						
LMSG_G000004881.1	1031_0	yes	1						
LMSG_G000005986.1	1032_0	yes	1						1
LMSG_G000007897.1	1033_1	no	3						
LMSG_G000007900.1	1033_1	no	3						
LMSG_G000007898.1	1033_1	no	3						
LMSG_G000007899.1	1033_1	no	3						
LMSG_G000004620.1	1033_1	yes	3						
LMSG_G000007895.1	1033_1	no	3						
LMSG_G000007896.1	1033_1	no	3						
LMSG_G000004605.1	1033_2	yes	2						
LMSG_G000005197.1	1035_0	yes						1	
LMSG_G000005727.1	1036_1	yes					1	1	2
LMSG_G000005259.1	1037_1	yes					1		2
LMSG_G000005284.1	1038_0	yes			1				1
LMSG_G000010088.1	1041_1	no			1		4		1
LMSG_G000005399.1	1041_1	yes					3		2
LMSG_G000010089.1	1042_1	no						1	1
LMSG_G000005402.1	1042_1	yes					1		1
LMSG_G000010094.1	1043_1	no	1						2
LMSG_G000010093.1	1043_1	no							1
LMSG_G000010090.1	1043_1	no							1
LMSG_G000010091.1	1043_1	no	1						1
LMSG_G000010092.1	1043_1	no							1
LMSG_G000006233.1	1043_1	yes	1						2
LMSG_G000010095.1	1044_1	no			2		3	1	2
LMSG_G000005232.1	1044_1	yes			1		3	1	2
LMSG_G000010096.1	1044_1	no			1		4		2
LMSG_G000004671.1	1045_1	yes			1		3	1	2
LMSG_G000010097.1	1045_1	no					4	2	2
LMSG_G000004669.1	1046_0	yes	2			1	3	3	2
LMSG_G000004936.1	1047_0	yes			1		2	3	
LMSG_G000004714.1	1048_1	yes	3	1			2		2
LMSG_G000010098.1	1048_1	no	2				1		1
LMSG_G000010099.1	1048_1	no	3		2		3		3
LMSG_G000004745.1	1049_1	yes	3			1	3	2	2
LMSG_G000005445.1	1049_2	yes	1						
LMSG_G000004352.1	1050_0	yes	4	1	2	1	1	3	2
LMSG_G000004685.1	1051_0	yes			2		2	1	2
LMSG_G000006325.1	1052_1	yes	3	3			1	2	1
LMSG_G000006326.1	1052_1	no	3	2			1	2	
LMSG_G000006327.1	1052_1	no	2	3			1	3	1
LMSG_G000006328.1	1052_1	no	3	3			1	2	1
LMSG_G000010942.1	1052_1	no	2				1		
LMSG_G000010940.1	1052_1	no	3	1			1	2	
LMSG_G000005138.1	1053_0	yes				1			2
LMSG_G000010412.1	1054_1	no	1	1					2
LMSG_G000004437.1	1054_1	yes		2					2
LMSG_G000010415.1	1054_1	no		1					2
LMSG_G000010413.1	1054_1	no		2					2
LMSG_G000010414.1	1054_1	no		3				1	2
LMSG_G000010416.1	1055_1	no						2	2
LMSG_G000010419.1	1055_1	no							2
LMSG_G000010422.1	1055_1	no		1					2
LMSG_G000010423.1	1055_1	no							1
LMSG_G000010420.1	1055_1	no							2
LMSG_G000010424.1	1055_1	no	1	1					2
LMSG_G000010425.1	1055_1	no		1					
LMSG_G000010421.1	1055_1	no		2				1	2
LMSG_G000010411.1	1055_1	no		1				1	2
LMSG_G000010417.1	1055_1	no		2				1	2
LMSG_G000004445.1	1055_1	yes						1	2
LMSG_G000010430.1	1055_1	no						1	2
LMSG_G000010426.1	1055_1	no							2
LMSG_G000010427.1	1055_1	no							2
LMSG_G000010418.1	1055_1	no		1				1	2
LMSG_G000010428.1	1055_1	no		1				1	2
LMSG_G000010429.1	1055_1	no	1	1				1	1

LMSG_G000004634.1	1058_0	yes		5	1	1	
LMSG_G000004367.1	1060_1	yes		1			2
LMSG_G000011301.1	1060_1	no		1			2
LMSG_G000008898.1	1062_1	no					1
LMSG_G000008899.1	1062_1	no					1
LMSG_G000008897.1	1062_1	no					2
LMSG_G000008900.1	1062_1	no					1
LMSG_G000004505.1	1062_1	yes					1
LMSG_G000004609.1	1063_1	yes	1				
LMSG_G000006033.1	1064_0	yes	1				
LMSG_G000009973.1	1065_1	no		2		1	2
LMSG_G000005917.1	1065_1	yes		2		1	2
LMSG_G000010349.1	1066_1	no	1	2		1	1
LMSG_G000010350.1	1066_1	no	1	2		1	1
LMSG_G000010351.1	1066_1	no	1	2		1	1
LMSG_G000005424.1	1066_1	yes	1	2		1	1
LMSG_G000010352.1	1066_1	no	1	2		1	1
LMSG_G000006148.1	1067_0	yes	1				
LMSG_G000011338.1	1068_1	no			1		1
LMSG_G000004487.1	1068_1	yes			1		1
LMSG_G000011339.1	1068_1	no			1		1
LMSG_G000011340.1	1068_1	no		1	1		1
LMSG_G000009074.1	1070_1	no		2			
LMSG_G000005120.1	1071_1	yes		1			
LMSG_G000009077.1	1071_1	no		1			
LMSG_G000009080.1	1071_1	no		1			
LMSG_G000005102.1	1072_0	yes		2	1		
LMSG_G000009698.1	1073_1	no		3			
LMSG_G000005557.1	1073_1	yes		4			
LMSG_G000009336.1	1074_1	no		2			
LMSG_G000004841.1	1075_0	yes				1	
LMSG_G000004900.1	1076_1	yes	1	2		1	1
LMSG_G000011058.1	1076_1	no	1			1	
LMSG_G000011059.1	1076_1	no	2			1	1
LMSG_G000011060.1	1076_1	no	1	1			1
LMSG_G000011061.1	1077_1	no	3			1	1
LMSG_G000006266.1	1077_1	yes	3	2		2	1
LMSG_G000011062.1	1077_1	no	5	2		2	1
LMSG_G000011063.1	1077_1	no	2	1		1	1
LMSG_G000011064.1	1077_1	no	3	1			1
LMSG_G000011070.1	1077_1	no	2	1		1	
LMSG_G000011071.1	1077_1	no	2	3		2	1
LMSG_G000011065.1	1077_1	no	3	2		2	1
LMSG_G000011066.1	1077_1	no	1	1		1	
LMSG_G000011067.1	1077_1	no	2			1	
LMSG_G000011068.1	1077_1	no	2	1			1
LMSG_G000011069.1	1077_1	no	1	2		3	1
LMSG_G000011072.1	1078_1	no	1	1		2	1
LMSG_G000011073.1	1078_1	no	2	1		1	1
LMSG_G000011074.1	1078_1	no	2	1		1	
LMSG_G000005604.1	1078_1	yes	3	2		1	1
LMSG_G000011075.1	1078_1	no	3			2	1
LMSG_G000006124.1	1078_2	yes		2			1
LMSG_G000006111.1	1079_0	yes	2	2		2	1
LMSG_G000004743.1	1080_0	yes		1			1
LMSG_G000005192.1	1081_0	yes		2		3	1
LMSG_G000011076.1	1082_1	no	1	1		1	1
LMSG_G000011077.1	1082_1	no	2	1		2	1
LMSG_G000011078.1	1082_1	no	1	1			
LMSG_G000004386.1	1082_1	yes	1	1		2	1
LMSG_G000011079.1	1082_1	no	1			1	2
LMSG_G000011080.1	1082_1	no	1	1			
LMSG_G000011081.1	1082_1	no	1	1		1	1
LMSG_G000005328.1	1082_2	yes	1	1		3	1
LMSG_G000004826.1	1083_0	yes	1	1		2	1
LMSG_G000011082.1	1084_1	no	2	2		1	1
LMSG_G000005018.1	1084_1	yes	2	2		1	1
LMSG_G000005020.1	1085_0	yes	1	3		2	1
LMSG_G000004848.1	1086_1	yes	2	1			
LMSG_G000004958.1	1086_2	yes	1	2			1
LMSG_G000004803.1	1087_0	yes		1		2	1
LMSG_G000004775.1	1088_1	yes	2	1			1
LMSG_G000011084.1	1088_1	no	2	1		1	1
LMSG_G000011083.1	1088_1	no	1	2		1	1
LMSG_G000006275.1	1089_1	yes	2	2		2	1
LMSG_G000011085.1	1089_1	no	1	2		2	1
LMSG_G000004957.1	1089_2	yes	1	1		1	1

LMSG_G000004673.1	1090_0	yes	1	1				2	1
LMSG_G000005253.1	1091_0	yes	2	1				1	1
LMSG_G000005994.1	1092_0	yes	2	3				1	1
LMSG_G000006003.1	1093_1	yes	2						1
LMSG_G000011095.1	1093_1	no						1	
LMSG_G000005978.1	1094_0	yes	1	1				1	1
LMSG_G000005007.1	1095_1	yes		2		1			
LMSG_G000004682.1	1097_0	yes						2	
LMSG_G000009335.1	1099_1	no		3					
LMSG_G000004405.1	1099_1	yes		3					
LMSG_G000004818.1	1100_0	yes		1				1	
LMSG_G000009376.1	1104_1	no		1					
LMSG_G000009377.1	1104_1	no		1			1		
LMSG_G000009378.1	1104_1	no		2			1		1
LMSG_G000004530.1	1104_1	yes		1			1		1
LMSG_G000009379.1	1104_1	no		1			1		1
LMSG_G000009380.1	1104_1	no		1					1
LMSG_G000005653.1	1105_1	yes		1					
LMSG_G000004766.1	1106_1	yes	1					1	
LMSG_G000009459.1	1106_1	no						2	
LMSG_G000004768.1	1107_1	yes						1	
LMSG_G000009460.1	1107_1	no						1	
LMSG_G000009461.1	1107_1	no						2	
LMSG_G000004764.1	1108_1	yes						1	
LMSG_G000009466.1	1108_1	no						1	
LMSG_G000009462.1	1108_1	no						2	
LMSG_G000009463.1	1108_1	no	1					1	
LMSG_G000009464.1	1108_1	no	1					1	
LMSG_G000004355.1	1109_1	yes		2					1
LMSG_G000009382.1	1109_1	no		1				1	1
LMSG_G000009381.1	1109_1	no						1	1
LMSG_G000009383.1	1109_1	no							1
LMSG_G000009384.1	1109_1	no						1	1
LMSG_G000009385.1	1109_1	no						2	1
LMSG_G000006114.1	1109_2	yes	1	1				1	1
LMSG_G000006123.1	1110_0	yes						1	1
LMSG_G000005665.1	1112_0	yes		1			1		
LMSG_G000005458.1	1113_1	yes					1		
LMSG_G000009514.1	1113_1	no					1		
LMSG_G000009515.1	1113_1	no							1
LMSG_G000009516.1	1113_1	no					1		1
LMSG_G000011149.1	1114_1	no		1					1
LMSG_G000005361.1	1114_1	yes							1
LMSG_G000005571.1	1115_1	yes	1	1	1				1
LMSG_G000010952.1	1115_1	no		2	1			1	1
LMSG_G000006055.1	1115_2	yes		3					1
LMSG_G000010959.1	1115_2	no		3				1	1
LMSG_G000011024.1	1116_1	no		1					1
LMSG_G000004601.1	1116_1	yes		2				1	1
LMSG_G000011025.1	1116_1	no		1					
LMSG_G000006046.1	1116_2	yes		1				1	1
LMSG_G000011027.1	1117_1	no		2				1	
LMSG_G000011026.1	1117_1	no		2				1	1
LMSG_G000004892.1	1117_1	yes		2				1	1
LMSG_G000011028.1	1117_1	no		1				1	
LMSG_G000011029.1	1117_1	no		2				1	2
LMSG_G000011030.1	1117_1	no		1					1
LMSG_G000011031.1	1117_1	no		2				1	
LMSG_G000011032.1	1117_1	no		2				1	2
LMSG_G000011033.1	1117_1	no		2				1	1
LMSG_G000011034.1	1117_1	no	1	2				1	2
LMSG_G000011035.1	1117_1	no		1					
LMSG_G000011037.1	1117_2	no	1	3				1	2
LMSG_G000011038.1	1117_2	no	1	2				1	1
LMSG_G000011039.1	1117_2	no	1	2				1	2
LMSG_G000011040.1	1117_2	no	1	3				1	2
LMSG_G000011041.1	1117_2	no	1	1				1	2
LMSG_G000011042.1	1117_2	no	1	1				1	1
LMSG_G000011043.1	1117_2	no	1	2				1	1
LMSG_G000011049.1	1117_2	no	1	2				1	1
LMSG_G000011044.1	1117_2	no	1	2				1	2
LMSG_G000011045.1	1117_2	no	1	2				1	2
LMSG_G000011050.1	1117_2	no	1	2				1	2
LMSG_G000011051.1	1117_2	no	1	2				1	1
LMSG_G000011052.1	1117_2	no	1						1
LMSG_G000011055.1	1117_2	no		2					
LMSG_G000011056.1	1117_2	no		1					

LMSG_G000011046.1	1117_2	no	1	2		1	2
LMSG_G000011053.1	1117_2	no		1		1	2
LMSG_G000011048.1	1117_2	no	1	2			2
LMSG_G000011047.1	1117_2	no	1	2		1	2
LMSG_G000006027.1	1117_2	yes	1	2		1	2
LMSG_G000011054.1	1117_2	no	1	2		1	1
LMSG_G000011086.1	1118_1	no		1			1
LMSG_G000005129.1	1118_1	yes		2			
LMSG_G000011087.1	1118_1	no	1			1	
LMSG_G000011089.1	1119_1	no		1		1	1
LMSG_G000011088.1	1119_1	no	1			1	1
LMSG_G000006278.1	1119_1	yes		1			
LMSG_G000011090.1	1119_1	no	1	2			1
LMSG_G000011091.1	1119_1	no	1	1		1	1
LMSG_G000004710.1	1119_2	yes	2	1		1	1
LMSG_G000011092.1	1119_2	no	1			1	1
LMSG_G000011094.1	1120_1	no		1			
LMSG_G000006145.1	1120_1	yes	1	1			1
LMSG_G000004343.1	1121_0	yes	1			1	2
LMSG_G000005330.1	1122_0	yes	1			1	2
LMSG_G000004650.1	1123_1	yes			1		2
LMSG_G000009274.1	1123_1	no			1		2
LMSG_G000009275.1	1123_1	no			1		2
LMSG_G000006002.1	1124_0	yes			1		1
LMSG_G000005268.1	1125_1	yes		1			
LMSG_G000009231.1	1125_1	no		1			
LMSG_G000009232.1	1125_1	no					1
LMSG_G000009234.1	1125_1	no		1			1
LMSG_G000004810.1	1125_2	yes		1			
LMSG_G000009241.1	1125_2	no			1		2
LMSG_G000009242.1	1125_2	no			1		1
LMSG_G000009235.1	1125_2	no		1			
LMSG_G000009236.1	1125_2	no		1			
LMSG_G000009246.1	1125_2	no					1
LMSG_G000009243.1	1125_2	no		1			
LMSG_G000009237.1	1125_2	no		1			
LMSG_G000009238.1	1125_2	no		1			
LMSG_G000009239.1	1125_2	no		1			
LMSG_G000009244.1	1125_2	no		1			
LMSG_G000009240.1	1125_2	no		1			
LMSG_G000004866.1	1126_0	yes			1		
LMSG_G000004597.1	1127_0	yes	1		1		
LMSG_G000005132.1	1128_1	yes				1	
LMSG_G000009248.1	1128_1	no				1	1
LMSG_G000009249.1	1128_2	no				1	
LMSG_G000005031.1	1128_2	yes					1
LMSG_G000009262.1	1128_2	no				1	1
LMSG_G000004982.1	1128_3	yes				1	1
LMSG_G000009263.1	1129_1	no				1	
LMSG_G000009250.1	1129_1	no			1	1	
LMSG_G000009251.1	1129_1	no			1	1	
LMSG_G000009252.1	1129_1	no			1		
LMSG_G000009253.1	1129_1	no			1	1	
LMSG_G000005820.1	1129_1	yes			1	1	
LMSG_G000005290.1	1129_2	yes			1	1	
LMSG_G000009264.1	1129_2	no			1	1	
LMSG_G000009266.1	1130_1	no				2	1
LMSG_G000009254.1	1130_1	no				1	1
LMSG_G000004441.1	1130_1	yes				1	1
LMSG_G000009267.1	1130_1	no				2	1
LMSG_G000009255.1	1130_1	no					1
LMSG_G000009256.1	1130_2	no				1	
LMSG_G000006008.1	1130_2	yes					1
LMSG_G000009269.1	1131_2	no				1	1
LMSG_G000009259.1	1132_1	no			1	1	
LMSG_G000009258.1	1132_1	no				1	
LMSG_G000004402.1	1132_1	yes				1	
LMSG_G000004681.1	1133_0	yes			1	1	
LMSG_G000005481.1	1134_1	yes			1		1
LMSG_G000008860.1	1134_1	no			1		1
LMSG_G000005832.1	1135_0	yes			1		
LMSG_G000004720.1	1137_0	yes					1
LMSG_G000009414.1	1138_1	no	1	2		2	
LMSG_G000009415.1	1138_1	no	1	1		2	
LMSG_G000009416.1	1138_1	no		1		1	
LMSG_G000005652.1	1138_1	yes	1	3		3	
LMSG_G000009417.1	1139_1	no		3		2	

LMSG_G000005597.1	1139_1	yes		3		1	
LMSG_G000009418.1	1139_1	no		2		2	
LMSG_G000009419.1	1139_1	no		2		1	
LMSG_G000009420.1	1139_1	no		3		3	
LMSG_G000009421.1	1139_1	no		3		1	
LMSG_G000009422.1	1139_1	no		3		2	
LMSG_G000009427.1	1140_1	no		1		1	
LMSG_G000009428.1	1140_1	no		1		1	
LMSG_G000009429.1	1140_1	no		3		1	
LMSG_G000009425.1	1140_1	no		3		1	
LMSG_G000009430.1	1140_1	no		3		1	
LMSG_G000009431.1	1140_1	no		3		1	
LMSG_G000009432.1	1140_1	no		3		2	
LMSG_G000009433.1	1140_1	no		3		1	
LMSG_G000009434.1	1140_1	no		3		1	
LMSG_G000009435.1	1140_1	no		3		1	
LMSG_G000009436.1	1140_1	no		3		1	
LMSG_G000009437.1	1140_1	no		3		2	
LMSG_G000009438.1	1140_1	no		3		1	
LMSG_G000009439.1	1140_1	no		5		1	
LMSG_G000009440.1	1140_1	no		3		1	
LMSG_G000009441.1	1140_1	no		3		1	
LMSG_G000009442.1	1140_1	no		3		1	
LMSG_G000009443.1	1140_1	no		3		1	
LMSG_G000009444.1	1140_1	no		3		1	
LMSG_G000009445.1	1140_1	no		1		1	
LMSG_G000009446.1	1140_1	no		3		1	
LMSG_G000009447.1	1140_1	no		3		1	
LMSG_G000009448.1	1140_1	no		3		1	
LMSG_G000009449.1	1140_1	no		3		1	
LMSG_G000009450.1	1140_1	no		3		1	
LMSG_G000005946.1	1140_1	yes		3		1	
LMSG_G000009451.1	1140_1	no		3		1	
LMSG_G000009452.1	1140_1	no		3		1	
LMSG_G000009470.1	1141_1	no				1	
LMSG_G000009471.1	1141_1	no				1	
LMSG_G000009468.1	1143_1	no		2		1	
LMSG_G000009469.1	1143_1	no		4		2	
LMSG_G000005606.1	1143_1	yes		1		2	
LMSG_G000005668.1	1144_0	yes		2		1	
LMSG_G000005560.1	1145_1	yes		3			
LMSG_G000009472.1	1145_1	no		3			
LMSG_G000009475.1	1146_1	no				1	
LMSG_G000009473.1	1146_1	no		1		1	
LMSG_G000009474.1	1146_1	no				1	
LMSG_G000005824.1	1146_1	yes				1	
LMSG_G000004418.1	1147_1	yes				1	1
LMSG_G000009389.1	1147_1	no					1
LMSG_G000009387.1	1147_1	no				1	1
LMSG_G000009390.1	1147_1	no					1
LMSG_G000009388.1	1147_1	no					1
LMSG_G000009393.1	1148_1	no	2			1	1
LMSG_G000009394.1	1148_1	no	2				1
LMSG_G000009401.1	1148_1	no	1				1
LMSG_G000009395.1	1148_1	no	1			1	1
LMSG_G000009403.1	1148_1	no	1				
LMSG_G000006286.1	1148_1	yes	2				1
LMSG_G000009396.1	1148_1	no	2				1
LMSG_G000009397.1	1148_1	no	2				1
LMSG_G000009398.1	1148_1	no	2				1
LMSG_G000009399.1	1148_1	no	2				1
LMSG_G000009391.1	1148_1	no	2				1
LMSG_G000009392.1	1148_1	no	2			1	1
LMSG_G000009400.1	1148_1	no	2				1
LMSG_G000011309.1	1149_1	no	1	1			3
LMSG_G000011310.1	1149_1	no	1	1			3
LMSG_G000011318.1	1149_1	no	1	1			3
LMSG_G000011319.1	1149_1	no	1				3
LMSG_G000011314.1	1149_1	no	1	1			2
LMSG_G000011315.1	1149_1	no	1	1			3
LMSG_G000004453.1	1149_1	yes		1			2
LMSG_G000011311.1	1149_1	no	1	1			2
LMSG_G000011312.1	1149_1	no	1	1	1		3
LMSG_G000011313.1	1149_1	no	1	1			3
LMSG_G000011316.1	1149_1	no	1	1			3
LMSG_G000011317.1	1149_1	no	1				3
LMSG_G000006308.1	1149_2	yes	1	1			2

LMSG_G000005568.1	1151_1	yes						1	
LMSG_G000009406.1	1151_1	no						1	
LMSG_G000009408.1	1151_2	no						1	
LMSG_G000009410.1	1151_2	no		1				1	
LMSG_G000005696.1	1151_2	yes		1				1	
LMSG_G000004384.1	1152_0	yes							1
LMSG_G000009277.1	1155_1	no							1
LMSG_G000009276.1	1155_1	no						1	2
LMSG_G000004585.1	1155_1	yes						1	2
LMSG_G000009286.1	1156_1	no							2
LMSG_G000009287.1	1156_1	no							2
LMSG_G000009278.1	1156_1	no							2
LMSG_G000009279.1	1156_1	no						1	2
LMSG_G000009280.1	1156_1	no							2
LMSG_G000009281.1	1156_1	no							2
LMSG_G000009289.1	1156_1	no							2
LMSG_G000009290.1	1156_1	no							2
LMSG_G000005716.1	1156_1	yes							2
LMSG_G000009291.1	1156_1	no							2
LMSG_G000009292.1	1156_1	no							2
LMSG_G000009293.1	1156_1	no							2
LMSG_G000009282.1	1156_1	no							2
LMSG_G000009294.1	1156_1	no							2
LMSG_G000009283.1	1156_1	no						1	2
LMSG_G000009284.1	1156_1	no							2
LMSG_G000009295.1	1156_1	no							2
LMSG_G000009285.1	1156_1	no							2
LMSG_G000009296.1	1156_1	no							2
LMSG_G000004659.1	1158_0	yes	1						
LMSG_G000005297.1	1159_1	yes	1	3				2	2
LMSG_G000010395.1	1159_1	no	1	4				2	
LMSG_G000010396.1	1159_2	no		1					1
LMSG_G000010397.1	1159_2	no	1	3				3	3
LMSG_G000010398.1	1159_2	no						1	1
LMSG_G000006173.1	1159_2	yes	1	3				2	
LMSG_G000005319.1	1160_1	yes		2			1	1	1
LMSG_G000010399.1	1160_1	no	2	1			1	1	2
LMSG_G000004399.1	1161_0	yes	1	4			1	1	2
LMSG_G000004730.1	1162_0	yes						3	
LMSG_G000005309.1	1163_0	yes							2
LMSG_G000004455.1	1164_1	yes		1		1	1		2
LMSG_G000007929.1	1164_1	no		1		2	1		2
LMSG_G000007930.1	1164_1	no	1	1			3		3
LMSG_G000006290.1	1165_1	yes		1				2	
LMSG_G000009423.1	1165_1	no						2	
LMSG_G000009424.1	1165_1	no		1				1	
LMSG_G000006285.1	1166_1	yes							2
LMSG_G000009411.1	1166_1	no		1					2
LMSG_G000009412.1	1167_1	no		1					2
LMSG_G000009413.1	1167_1	no							2
LMSG_G000006294.1	1167_1	yes		1					2
LMSG_G000005621.1	1168_0	yes		2					
LMSG_G000006204.1	1169_0	yes	2	1			1		
LMSG_G000010958.1	1170_1	no		3	1			1	1
LMSG_G000010956.1	1170_1	no		3	1			1	1
LMSG_G000010957.1	1170_1	no		3	1			1	1
LMSG_G000006256.1	1170_1	yes		3	1				1
LMSG_G000005751.1	1171_1	yes		2	1				1
LMSG_G000010953.1	1171_1	no		1	1				
LMSG_G000010954.1	1171_1	no		2	1				1
LMSG_G000010955.1	1171_1	no		1					
LMSG_G000006070.1	1172_0	yes						1	1
LMSG_G000004696.1	1173_0	yes	1						1
LMSG_G000006218.1	1174_0	yes		1					1
LMSG_G000009208.1	1175_1	no					1		3
LMSG_G000009209.1	1175_1	no					1		2
LMSG_G000005085.1	1175_1	yes					1		2
LMSG_G000009210.1	1175_1	no							2
LMSG_G000009211.1	1176_1	no					1		2
LMSG_G000009212.1	1176_1	no		1					1
LMSG_G000004589.1	1176_1	yes		1					1
LMSG_G000005809.1	1177_0	yes							1
LMSG_G000009203.1	1178_1	no					1	1	
LMSG_G000009204.1	1178_1	no							1
LMSG_G000005495.1	1178_1	yes			1		3	1	1
LMSG_G000005121.1	1179_0	yes	1		1		1	1	1
LMSG_G000005066.1	1180_1	yes			1				2

LMSG_G000009205.1	1180_1	no			1			1
LMSG_G000005890.1	1181_0	yes			1		1	2
LMSG_G000005125.1	1182_0	yes				1		1
LMSG_G000009206.1	1183_1	no			1			
LMSG_G000005468.1	1183_1	yes			1			2
LMSG_G000009207.1	1183_1	no				1		1
LMSG_G000005119.1	1184_0	yes			1	1	1	2
LMSG_G000004724.1	1185_1	yes			1			
LMSG_G000010964.1	1185_1	no	1			1		
LMSG_G000010963.1	1185_1	no				1		
LMSG_G000010978.1	1186_1	no				1		
LMSG_G000010967.1	1186_1	no				1		
LMSG_G000010968.1	1186_1	no				1	1	
LMSG_G000010969.1	1186_1	no			1			
LMSG_G000010970.1	1186_1	no				1		
LMSG_G000010977.1	1186_1	no				1		
LMSG_G000010971.1	1186_1	no				1		
LMSG_G000010973.1	1186_1	no				1		
LMSG_G000010974.1	1186_1	no				1	1	
LMSG_G000010975.1	1186_1	no					1	
LMSG_G000010976.1	1186_1	no				1		
LMSG_G000005389.1	1186_1	yes				1	1	
LMSG_G000010979.1	1187_1	no			1			
LMSG_G000010984.1	1187_1	no			1			
LMSG_G000010985.1	1187_1	no				1		
LMSG_G000010986.1	1187_1	no				1		
LMSG_G000005216.1	1187_1	yes				1		
LMSG_G000010991.1	1187_1	no				1		
LMSG_G000010980.1	1187_1	no				1		
LMSG_G000010962.1	1189_1	no						1
LMSG_G000011106.1	1189_1	no						1
LMSG_G000005777.1	1189_1	yes						1
LMSG_G000011107.1	1189_1	no						1
LMSG_G000010432.1	1189_2	no						1
LMSG_G000010434.1	1189_2	no						1
LMSG_G000011109.1	1189_2	no						1
LMSG_G000010435.1	1189_2	no						1
LMSG_G000011110.1	1189_2	no						1
LMSG_G000004889.1	1190_0	yes						1
LMSG_G000004833.1	1193_1	yes					1	
LMSG_G000010999.1	1193_1	no					1	
LMSG_G000004707.1	1194_0	yes			1	1		
LMSG_G000004995.1	1195_0	yes	1				2	
LMSG_G000005000.1	1196_0	yes	1				1	2
LMSG_G000004428.1	1197_0	yes	1			1		1
LMSG_G000010507.1	1198_1	no		1				
LMSG_G000006144.1	1198_1	yes	1	1				
LMSG_G000010508.1	1199_1	no	1	2				
LMSG_G000010509.1	1199_1	no	1	1				
LMSG_G000005038.1	1199_1	yes		2				
LMSG_G000005818.1	1199_2	yes	1	1				
LMSG_G000007951.1	1_1	no						1
LMSG_G000004510.1	1_1	yes						1
LMSG_G000004567.1	1200_1	yes	1	1				
LMSG_G000010511.1	1200_1	no	1	1				
LMSG_G000010520.1	1201_1	no	1	2				
LMSG_G000010516.1	1201_1	no	1	2				
LMSG_G000010517.1	1201_1	no	1	2				
LMSG_G000005366.1	1201_1	yes	1	2				
LMSG_G000010518.1	1201_1	no	1	2				
LMSG_G000010519.1	1201_1	no	1	2				
LMSG_G000010514.1	1201_1	no	1	2				
LMSG_G000010515.1	1201_1	no		2				
LMSG_G000010512.1	1201_1	no	1	1				
LMSG_G000010513.1	1201_1	no		1				
LMSG_G000010521.1	1201_1	no		2				
LMSG_G000006249.1	1202_0	yes	1	2			1	1
LMSG_G000004641.1	1203_1	yes		1		1		1
LMSG_G000005444.1	1204_1	yes		1		2		
LMSG_G000007931.1	1204_1	no				1		
LMSG_G000007933.1	1204_2	no					1	1
LMSG_G000007934.1	1204_2	no				1		
LMSG_G000007932.1	1204_2	no						1
LMSG_G000006077.1	1205_1	yes		2			2	1
LMSG_G000010318.1	1205_1	no		1			2	1
LMSG_G000007948.1	1206_1	no	1			1		2
LMSG_G000007949.1	1206_1	no				1		1

LMSG_G000005386.1	1206_1	yes	1			1		1
LMSG_G000007950.1	1206_1	no	2			1		1
LMSG_G000007917.1	1207_1	no	3			1	2	1
LMSG_G000007918.1	1207_1	no	2			1	2	1
LMSG_G000007919.1	1207_1	no	3			1	2	1
LMSG_G000007920.1	1207_1	no	3			1	2	1
LMSG_G000007921.1	1207_1	no	3			1	2	1
LMSG_G000004587.1	1207_1	yes	3			1	2	1
LMSG_G000005682.1	1208_1	yes						1
LMSG_G000004644.1	1209_0	yes		2			1	1
LMSG_G000010872.1	1216_1	no		1			1	2
LMSG_G000010873.1	1216_1	no	1	2			1	2
LMSG_G000005077.1	1216_1	yes		3			2	2
LMSG_G000005780.1	1218_1	yes	1					2
LMSG_G000010875.1	1218_2	no						3
LMSG_G000010876.1	1218_2	no	1					2
LMSG_G000005490.1	1218_2	yes	1					2
LMSG_G000010877.1	1218_2	no		1				1
LMSG_G000005111.1	1218_3	yes	1	1				3
LMSG_G000005016.1	1219_0	yes	1	1			1	1
LMSG_G000004834.1	1220_0	yes		1				2
LMSG_G000010868.1	1221_1	no		1				1
LMSG_G000010869.1	1221_1	no					1	1
LMSG_G000004817.1	1222_1	yes		1			2	2
LMSG_G000010607.1	1222_1	no		1			1	2
LMSG_G000005291.1	1222_2	yes		2			1	2
LMSG_G000010909.1	1223_1	no					2	
LMSG_G000005574.1	1223_1	yes		1			2	
LMSG_G000005585.1	1224_0	yes		2			2	1
LMSG_G000006150.1	1225_0	yes					1	1
LMSG_G000010910.1	1226_1	no		1	1		1	1
LMSG_G000010911.1	1226_1	no		1	1		1	1
LMSG_G000006313.1	1226_1	yes		1	1		1	1
LMSG_G000010912.1	1226_1	no		1	1		1	1
LMSG_G000010915.1	1227_1	no		1	1		1	1
LMSG_G000005067.1	1227_1	yes		1	1		1	1
LMSG_G000010913.1	1227_1	no			1		1	
LMSG_G000010914.1	1227_1	no		1	1		1	1
LMSG_G000010916.1	1228_1	no		1				2
LMSG_G000010917.1	1228_1	no		1				1
LMSG_G000005887.1	1228_1	yes		1				1
LMSG_G000005769.1	1228_2	yes		1			1	1
LMSG_G000010870.1	1229_1	no					1	1
LMSG_G000005012.1	1229_1	yes		1			1	1
LMSG_G000004839.1	1230_1	yes					2	1
LMSG_G000010871.1	1230_1	no		1			1	2
LMSG_G000006172.1	1230_2	yes		1			2	1
LMSG_G000006156.1	1231_0	yes		1			2	
LMSG_G000006169.1	1232_0	yes					1	1
LMSG_G000010855.1	1233_1	no					1	1
LMSG_G000006059.1	1233_1	yes					1	1
LMSG_G000010856.1	1233_1	no					1	1
LMSG_G000005280.1	1234_0	yes	1				1	1
LMSG_G000004539.1	1235_1	yes		2				2
LMSG_G000010857.1	1235_1	no		2				2
LMSG_G000010858.1	1236_1	no		1				1
LMSG_G000005310.1	1236_1	yes		1				1
LMSG_G000010859.1	1236_1	no						1
LMSG_G000010860.1	1236_1	no		1				1
LMSG_G000010861.1	1236_1	no		1				1
LMSG_G000005281.1	1237_0	yes	1					
LMSG_G000006153.1	1238_0	yes		2				1
LMSG_G000004788.1	1239_0	yes						1
LMSG_G000010896.1	1240_1	no		1				2
LMSG_G000005446.1	1240_1	yes	1	2				2
LMSG_G000010897.1	1240_1	no	1	2			1	2
LMSG_G000010898.1	1240_1	no	1	2			1	2
LMSG_G000005833.1	1240_2	yes	1	1				2
LMSG_G000010906.1	1241_1	no						1
LMSG_G000010907.1	1241_1	no						1
LMSG_G000010908.1	1241_1	no						1
LMSG_G000006226.1	1241_1	yes						1
LMSG_G000010900.1	1242_1	no	1	2				1
LMSG_G000010901.1	1242_1	no	1	2				1
LMSG_G000005063.1	1242_1	yes	1	2				1
LMSG_G000010902.1	1242_1	no	1	2				1
LMSG_G000010904.1	1242_1	no	1					1

LMSG_G000010905.1	1242_1	no	1	2			1
LMSG_G000010608.1	1243_1	no		2			1
LMSG_G000010609.1	1243_1	no		2			1
LMSG_G000005100.1	1243_1	yes		3			1
LMSG_G000005103.1	1244_0	yes		1			1
LMSG_G000004643.1	1245_0	yes	2	1		2	2
LMSG_G000006193.1	1246_0	yes	1				1
LMSG_G000010920.1	1247_1	no		1		1	1
LMSG_G000010921.1	1247_1	no		1		1	1
LMSG_G000010922.1	1247_1	no		1		1	1
LMSG_G000010923.1	1247_1	no		1			1
LMSG_G000010919.1	1247_1	no		1		1	1
LMSG_G000010924.1	1247_1	no				1	
LMSG_G000010926.1	1247_1	no		1		1	1
LMSG_G000010918.1	1247_1	no		1		1	1
LMSG_G000010927.1	1247_1	no		1		1	1
LMSG_G000005812.1	1247_1	yes		1		1	1
LMSG_G000010928.1	1247_1	no		1		1	1
LMSG_G000010933.1	1248_1	no				1	
LMSG_G000005022.1	1248_1	yes				1	1
LMSG_G000010932.1	1248_1	no					1
LMSG_G000005108.1	1248_2	yes				1	1
LMSG_G000006072.1	1249_0	yes		2		2	1
LMSG_G000005718.1	1250_1	yes	1	1			1
LMSG_G000010899.1	1250_1	no		2			
LMSG_G000005709.1	1250_2	yes	1	1			1
LMSG_G000005497.1	1251_1	yes	1			1	1
LMSG_G000010895.1	1251_1	no				1	
LMSG_G000006139.1	1252_0	yes	1				
LMSG_G000004981.1	1253_0	yes		1			
LMSG_G000011145.1	1254_1	no		1		1	
LMSG_G000011146.1	1254_1	no				1	
LMSG_G000011148.1	1254_1	no		1			
LMSG_G000011147.1	1254_1	no		1		1	
LMSG_G000006009.1	1254_1	yes		1		1	
LMSG_G000011150.1	1255_1	no		1		2	1
LMSG_G000011151.1	1255_1	no		2		1	1
LMSG_G000011155.1	1255_1	no		2	1	1	1
LMSG_G000011156.1	1255_1	no		2		1	
LMSG_G000005087.1	1255_1	yes		2		1	1
LMSG_G000011152.1	1255_1	no		1			1
LMSG_G000011153.1	1255_1	no		2			1
LMSG_G000011154.1	1255_1	no		1		1	1
LMSG_G000011157.1	1255_1	no		1			
LMSG_G000011158.1	1255_1	no		1		1	1
LMSG_G000011159.1	1255_1	no		1		1	
LMSG_G000011160.1	1255_1	no		2		1	
LMSG_G000011161.1	1255_1	no		1		1	
LMSG_G000011162.1	1255_1	no		1		1	
LMSG_G000011163.1	1255_1	no		3		1	
LMSG_G000011164.1	1255_1	no		2		1	
LMSG_G000011165.1	1255_1	no		2		2	1
LMSG_G000011166.1	1255_1	no		2		1	1
LMSG_G000011167.1	1255_1	no	1	3		2	
LMSG_G000011182.1	1256_1	no		1		1	
LMSG_G000011183.1	1256_1	no		2		1	
LMSG_G000006304.1	1256_1	yes		1		1	
LMSG_G000011171.1	1257_1	no		1			
LMSG_G000011169.1	1257_1	no		1			
LMSG_G000011168.1	1257_1	no		1			
LMSG_G000011170.1	1257_1	no		1			
LMSG_G000011176.1	1257_1	no		1			
LMSG_G000011174.1	1257_1	no		2		1	
LMSG_G000011175.1	1257_1	no				1	
LMSG_G000011177.1	1257_1	no		1		1	
LMSG_G000011172.1	1257_1	no		1		1	
LMSG_G000011173.1	1257_1	no		1	1		
LMSG_G000004656.1	1257_1	yes		1		1	
LMSG_G000004998.1	1258_0	yes		1		1	
LMSG_G000005984.1	1259_1	yes		1	1	1	
LMSG_G000011184.1	1259_1	no	1	1	1	1	
LMSG_G000011185.1	1260_1	no		1			
LMSG_G000011186.1	1260_1	no					1
LMSG_G000011187.1	1260_1	no		1			1
LMSG_G000005405.1	1260_1	yes					1
LMSG_G000011188.1	1260_1	no		1	1		
LMSG_G000011189.1	1260_1	no		1			

LMSG_G000006056.1	1261_0	yes		1		1		
LMSG_G000004742.1	1262_0	yes		3			1	1
LMSG_G000005339.1	1263_0	yes					1	
LMSG_G000004902.1	1264_0	yes		1				
LMSG_G000005656.1	1265_0	yes					1	
LMSG_G000004600.1	1266_0	yes		2		1	1	
LMSG_G000005095.1	1267_0	yes				1		
LMSG_G000010617.1	1268_1	no		1				1
LMSG_G000005471.1	1268_1	yes		1				2
LMSG_G000005462.1	1269_1	yes		1			1	3
LMSG_G000010618.1	1269_1	no	1				1	2
LMSG_G000010619.1	1269_1	no		1			1	2
LMSG_G000010621.1	1269_2	no		1			1	2
LMSG_G000010622.1	1269_2	no		2			1	1
LMSG_G000006267.1	1269_2	yes		2			1	2
LMSG_G000010624.1	1269_2	no		2			1	2
LMSG_G000010625.1	1269_2	no		1			1	1
LMSG_G000010623.1	1269_2	no		1			1	2
LMSG_G000010620.1	1269_2	no		2			1	1
LMSG_G000010631.1	1269_2	no		1			1	2
LMSG_G000010627.1	1269_2	no		1			1	2
LMSG_G000010628.1	1269_2	no		2			1	2
LMSG_G000010629.1	1269_2	no		1			1	2
LMSG_G000010630.1	1269_2	no					1	
LMSG_G000010632.1	1269_2	no		1				
LMSG_G000004529.1	1269_3	yes		1	1		3	1
LMSG_G000004824.1	1270_1	yes		2			2	2
LMSG_G000010929.1	1270_1	no		2			2	2
LMSG_G000010930.1	1270_1	no		2			2	2
LMSG_G000010931.1	1270_1	no		1				1
LMSG_G000004813.1	1271_0	yes		1			1	1
LMSG_G000006557.1	127_1	no					1	
LMSG_G000005370.1	1272_0	yes					1	
LMSG_G000005032.1	1273_1	yes		2			1	1
LMSG_G000010934.1	1273_1	no		2			1	1
LMSG_G000005127.1	1274_0	yes		2				
LMSG_G000005649.1	1275_0	yes		3			2	1
LMSG_G000005654.1	1276_0	yes		1				1
LMSG_G000010935.1	1277_1	no		2			1	
LMSG_G000006088.1	1277_1	yes		2			2	
LMSG_G000006090.1	1278_0	yes		1	1		3	1
LMSG_G000006063.1	1279_1	yes		1			1	1
LMSG_G000010638.1	1279_1	no		1			1	1
LMSG_G000010637.1	1280_1	no				1	1	3
LMSG_G000006058.1	1280_1	yes				1	1	1
LMSG_G000004899.1	1281_0	yes	3	3		1	1	
LMSG_G000005931.1	1282_0	yes	1	1			1	
LMSG_G000004631.1	1283_1	yes			1		1	1
LMSG_G000010616.1	1283_1	no					1	1
LMSG_G000006263.1	1284_0	yes		1				1
LMSG_G000010633.1	1286_1	no		1				1
LMSG_G000005021.1	1286_1	yes					1	1
LMSG_G000010645.1	1287_1	no					1	1
LMSG_G000005094.1	1287_1	yes					1	1
LMSG_G000010647.1	1287_1	no					1	1
LMSG_G000005648.1	1288_0	yes	2	1			1	1
LMSG_G000006190.1	1289_0	yes		1			1	1
LMSG_G000005179.1	1290_0	yes				1		
LMSG_G000005974.1	1291_0	yes	2	1				
LMSG_G000004901.1	1292_0	yes		3			1	1
LMSG_G000010648.1	1293_1	no	3	2	1			1
LMSG_G000010649.1	1293_1	no	3	1		2		
LMSG_G000010650.1	1293_1	no	2	1		1		1
LMSG_G000010651.1	1293_1	no	2	1		1		
LMSG_G000005217.1	1293_1	yes	3	1		1		
LMSG_G000010652.1	1293_1	no	2	1		1		1
LMSG_G000010653.1	1293_1	no	3	1		1		1
LMSG_G000010654.1	1293_1	no	4	1		1		1
LMSG_G000010655.1	1293_1	no	2	1		1		1
LMSG_G000010656.1	1293_1	no	4			1		
LMSG_G000010657.1	1293_1	no	1	1		1		1
LMSG_G000010658.1	1293_1	no	4	1				1
LMSG_G000010659.1	1293_1	no	3	1		1		
LMSG_G000010660.1	1293_1	no	1					
LMSG_G000010665.1	1294_1	no		1				
LMSG_G000010661.1	1294_1	no	1			1		
LMSG_G000005230.1	1294_1	yes	4	1		1		1

LMSG_G000010662.1	1294_1	no	1			1		1	
LMSG_G000010663.1	1294_1	no	3	1					1
LMSG_G000010666.1	1294_1	no	1						
LMSG_G000010664.1	1294_1	no	2			1			
LMSG_G000010669.1	1295_1	no		2	1	1		1	
LMSG_G000010668.1	1295_1	no		2		2		2	1
LMSG_G000006228.1	1295_1	yes	1	2	1	1		1	1
LMSG_G000005029.1	1296_1	yes	1	1	1			1	1
LMSG_G000005236.1	1296_2	yes		1				1	
LMSG_G000010670.1	1297_1	no	1	2				1	1
LMSG_G000010671.1	1297_1	no	1	2				1	1
LMSG_G000010672.1	1297_1	no						1	
LMSG_G000005416.1	1297_1	yes	1	1				1	
LMSG_G000005075.1	1297_2	yes	1	1				1	1
LMSG_G000010673.1	1298_1	no	1	1	1			1	1
LMSG_G000005314.1	1298_1	yes	1	2				1	1
LMSG_G000010675.1	1298_2	no	1	1					1
LMSG_G000004821.1	1298_2	yes		2		1		1	1
LMSG_G000010674.1	1298_2	no		2		1		1	1
LMSG_G000004683.1	1298_3	yes		1			1	1	1
LMSG_G000010676.1	1299_1	no	2	1				1	1
LMSG_G000005003.1	1299_1	yes	2	1					
LMSG_G000010677.1	1299_2	no	1	2					
LMSG_G000010678.1	1299_2	no	1	3					
LMSG_G000010679.1	1299_2	no	2	2					1
LMSG_G000004906.1	1299_2	yes	2	3					1
LMSG_G000010684.1	1299_2	no	1	3					1
LMSG_G000010680.1	1299_2	no	1	3					
LMSG_G000010681.1	1299_2	no	1	2					
LMSG_G000010682.1	1299_2	no	1	2					
LMSG_G000004827.1	1300_0	yes	1	2				1	1
LMSG_G000005874.1	130_0	yes						1	
LMSG_G000010685.1	1301_1	no	1	1				1	1
LMSG_G000010686.1	1301_1	no	1	1		1		1	1
LMSG_G000010687.1	1301_1	no		1		1		1	1
LMSG_G000010688.1	1301_1	no	2	2					1
LMSG_G000010689.1	1301_1	no		1					1
LMSG_G000005407.1	1301_1	yes	1	1				1	1
LMSG_G000010690.1	1301_1	no	1	2		1		1	1
LMSG_G000010691.1	1301_1	no	1	2		1		1	1
LMSG_G000010692.1	1301_1	no	1	2		1	1	1	1
LMSG_G000010693.1	1301_1	no	2	1		1		1	1
LMSG_G000010694.1	1301_1	no	2	1			1	1	
LMSG_G000005178.1	1302_1	yes	1	1					1
LMSG_G000010695.1	1302_1	no	1	2				1	
LMSG_G000010696.1	1302_1	no	1						
LMSG_G000010697.1	1302_1	no		1		1			
LMSG_G000005496.1	1302_2	yes		1	1			1	1
LMSG_G000010698.1	1303_1	no						1	1
LMSG_G000010699.1	1303_1	no		3		1		1	1
LMSG_G000010700.1	1303_1	no	1			1			1
LMSG_G000010709.1	1303_1	no	1	1		1		1	1
LMSG_G000005215.1	1303_1	yes	1	2	1			1	1
LMSG_G000010701.1	1303_1	no	1	1				1	1
LMSG_G000010702.1	1303_1	no	1	2		1			1
LMSG_G000010703.1	1303_1	no		2		1		1	1
LMSG_G000010704.1	1303_1	no		1		1		1	1
LMSG_G000010705.1	1303_1	no	1	1		1			1
LMSG_G000010706.1	1303_1	no		2		1		1	1
LMSG_G000010707.1	1303_1	no	1					1	
LMSG_G000010708.1	1303_1	no		2		1		1	1
LMSG_G000005433.1	1304_0	yes							1
LMSG_G000010714.1	1305_1	no						1	1
LMSG_G000010715.1	1305_1	no	1	1		1			1
LMSG_G000010716.1	1305_1	no	2	1				1	1
LMSG_G000004919.1	1305_1	yes	2	3		1		1	1
LMSG_G000010717.1	1305_1	no				1			
LMSG_G000010718.1	1305_1	no	1	1		1			1
LMSG_G000010719.1	1305_1	no	3	1	1			1	1
LMSG_G000010720.1	1305_1	no	3	2		1		1	1
LMSG_G000010721.1	1305_1	no	1	1	1			1	
LMSG_G000010722.1	1305_1	no	3	1					1
LMSG_G000010723.1	1305_1	no	1	1		1		1	1
LMSG_G000010724.1	1305_1	no	2	1		1		1	1
LMSG_G000010725.1	1305_1	no		1		1		1	1
LMSG_G000010726.1	1305_1	no	1	1		1			1
LMSG_G000010727.1	1305_1	no	2			1		1	1

LMSG_G000010728.1	1305_1	no	1	1		1	1	
LMSG_G000010729.1	1305_1	no		1				1
LMSG_G000010711.1	1306_1	no	2	1				1
LMSG_G000004630.1	1306_1	yes	1	1		1		1
LMSG_G000010712.1	1306_1	no	1	1		1		1
LMSG_G000005260.1	1307_0	yes	1	1		1		
LMSG_G000005006.1	1308_0	yes	1	1	1	1		
LMSG_G000005264.1	1309_1	yes	1	1				1
LMSG_G000010878.1	1309_1	no						1
LMSG_G000010879.1	1309_1	no	1	1				1
LMSG_G000005985.1	1310_0	yes	1					1
LMSG_G000010893.1	1311_1	no		2				1
LMSG_G000010894.1	1311_1	no		1			1	
LMSG_G000006283.1	1311_1	yes		1			1	1
LMSG_G000006007.1	1312_0	yes		2				
LMSG_G000006048.1	1313_0	yes	1	3			1	1
LMSG_G000005322.1	1314_1	yes		3			1	1
LMSG_G000010886.1	1314_1	no		1			1	
LMSG_G000004819.1	1315_0	yes		4				
LMSG_G000010889.1	1316_1	no		2				1
LMSG_G000010890.1	1316_1	no		2				1
LMSG_G000010891.1	1316_1	no		2				1
LMSG_G000010887.1	1316_1	no						1
LMSG_G000010888.1	1316_1	no						1
LMSG_G000005710.1	1316_1	yes		2				1
LMSG_G000010892.1	1316_1	no		3				1
LMSG_G000005276.1	1317_0	yes	1					
LMSG_G000006181.1	1318_0	yes	1				2	
LMSG_G000004816.1	1319_0	yes	1	1			1	1
LMSG_G000006071.1	1320_0	yes					1	1
LMSG_G000006170.1	1321_0	yes	1	1			1	1
LMSG_G000010880.1	1322_1	no		3			2	1
LMSG_G000010881.1	1322_1	no		4			2	1
LMSG_G000005542.1	1322_1	yes		4			2	1
LMSG_G000010882.1	1323_1	no						1
LMSG_G000010883.1	1323_1	no					1	
LMSG_G000005476.1	1323_1	yes					1	
LMSG_G000010884.1	1323_1	no		1			1	1
LMSG_G000006178.1	1324_0	yes	1	1			2	
LMSG_G000005323.1	1325_0	yes		1		1	1	
LMSG_G000005423.1	1327_1	yes		1				
LMSG_G000005421.1	1328_1	yes					1	
LMSG_G000010737.1	1328_2	no					1	
LMSG_G000010734.1	1328_2	no					1	
LMSG_G000006305.1	1328_2	yes		1			1	
LMSG_G000010736.1	1328_2	no					1	
LMSG_G000010733.1	1328_2	no					1	
LMSG_G000005182.1	1330_0	yes		1				
LMSG_G000010744.1	1332_1	no					1	
LMSG_G000010748.1	1332_1	no					1	
LMSG_G000010749.1	1332_1	no					1	
LMSG_G000010750.1	1332_1	no					1	
LMSG_G000005104.1	1333_0	yes					1	
LMSG_G000006137.1	1334_0	yes					1	
LMSG_G000004353.1	1335_1	yes		2			1	
LMSG_G000010756.1	1335_1	no		1			1	
LMSG_G000010786.1	1335_1	no		2				
LMSG_G000010757.1	1335_1	no		1			1	
LMSG_G000010766.1	1335_1	no		1			1	
LMSG_G000010767.1	1335_1	no		1				
LMSG_G000010787.1	1335_1	no					1	
LMSG_G000010768.1	1335_1	no		1			1	
LMSG_G000010758.1	1335_1	no		1			1	
LMSG_G000010759.1	1335_1	no		1			1	1
LMSG_G000010769.1	1335_1	no					1	
LMSG_G000010760.1	1335_1	no		1			1	
LMSG_G000010770.1	1335_1	no					1	
LMSG_G000010771.1	1335_1	no		1			1	
LMSG_G000010772.1	1335_1	no		1			1	
LMSG_G000010773.1	1335_1	no		1			1	
LMSG_G000010761.1	1335_1	no		1			1	
LMSG_G000010775.1	1335_1	no		1			1	
LMSG_G000010765.1	1335_1	no		1			1	
LMSG_G000010776.1	1335_1	no		1			1	
LMSG_G000010762.1	1335_1	no		1				
LMSG_G000010777.1	1335_1	no		2			1	
LMSG_G000010763.1	1335_1	no		1			1	

LMSG_G000010778.1	1335_1	no					1	
LMSG_G000010779.1	1335_1	no		1			1	
LMSG_G000010780.1	1335_1	no		1			1	
LMSG_G000010751.1	1335_1	no		1			1	
LMSG_G000010754.1	1335_1	no		1			1	
LMSG_G000010752.1	1335_1	no		2			1	
LMSG_G000010755.1	1335_1	no		2				
LMSG_G000010753.1	1335_1	no		2				
LMSG_G000010781.1	1335_1	no		2				
LMSG_G000010782.1	1335_1	no		2			1	
LMSG_G000010788.1	1335_1	no		2			1	
LMSG_G000010783.1	1335_1	no		1			1	
LMSG_G000010789.1	1335_1	no		1			1	
LMSG_G000010764.1	1335_1	no		1			1	
LMSG_G000010784.1	1335_1	no		1			1	
LMSG_G000010785.1	1335_1	no		2				
LMSG_G000010811.1	1336_1	no		1				
LMSG_G000010808.1	1336_1	no		1				
LMSG_G000010809.1	1336_1	no	1	1				
LMSG_G000010810.1	1336_1	no		1				
LMSG_G000010792.1	1336_1	no	1	1			1	
LMSG_G000010793.1	1336_1	no	1	1				
LMSG_G000010794.1	1336_1	no		1				
LMSG_G000010790.1	1336_1	no	1				1	
LMSG_G000010804.1	1336_1	no		2				
LMSG_G000010802.1	1336_1	no		1	1			
LMSG_G000010799.1	1336_1	no		1				
LMSG_G000010800.1	1336_1	no		1				
LMSG_G000010801.1	1336_1	no		2				
LMSG_G000010798.1	1336_1	no		1				
LMSG_G000010807.1	1336_1	no		1				
LMSG_G000010805.1	1336_1	no		1				
LMSG_G000010806.1	1336_1	no		1				
LMSG_G000005632.1	1336_1	yes	1	1			2	
LMSG_G000010795.1	1336_1	no		2			1	
LMSG_G000010796.1	1336_1	no	1	1			1	
LMSG_G000010797.1	1336_1	no	1	1				
LMSG_G000010815.1	1337_1	no		2				
LMSG_G000010816.1	1337_1	no		2				
LMSG_G000010817.1	1337_1	no		1				
LMSG_G000010818.1	1337_1	no		1				
LMSG_G000010819.1	1337_1	no		1				
LMSG_G000004907.1	1337_1	yes		1				
LMSG_G000010820.1	1337_1	no		1				
LMSG_G000010821.1	1337_1	no		2				
LMSG_G000010822.1	1337_1	no		1				
LMSG_G000010823.1	1337_1	no		1				
LMSG_G000010813.1	1337_1	no		1				
LMSG_G000010814.1	1337_1	no		1				
LMSG_G000010824.1	1337_1	no		1			1	
LMSG_G000010825.1	1337_1	no		1				
LMSG_G000010826.1	1337_1	no		1			1	
LMSG_G000006166.1	1337_2	yes		1				
LMSG_G000010827.1	1337_2	no			1			
LMSG_G000010829.1	1337_3	no		2				
LMSG_G000010828.1	1337_3	no		2				
LMSG_G000010830.1	1337_3	no		1				
LMSG_G000010831.1	1337_3	no		1				
LMSG_G000010832.1	1337_3	no		1				
LMSG_G000010833.1	1337_3	no		2				
LMSG_G000006235.1	1337_3	yes		1				
LMSG_G000010834.1	1337_3	no		2				
LMSG_G000006168.1	1338_0	yes		3				
LMSG_G000010835.1	1339_1	no					1	
LMSG_G000004752.1	1339_1	yes					2	
LMSG_G000006231.1	1341_1	yes	1			1		1
LMSG_G000009060.1	1341_1	no		1				1
LMSG_G000009061.1	1341_1	no	1			1		1
LMSG_G000009062.1	1341_1	no	1			2		1
LMSG_G000004370.1	1342_1	yes	1	1		1		1
LMSG_G000009063.1	1342_1	no	1	1		2		
LMSG_G000009068.1	1343_1	no	1					1
LMSG_G000009064.1	1343_1	no	1					
LMSG_G000009066.1	1343_1	no	1					1
LMSG_G000005536.1	1343_1	yes	1					1
LMSG_G000009069.1	1344_1	no				1		1
LMSG_G000005388.1	1344_1	yes				2		1

LMSG_G000009070.1	1345_1	no	1	1			1	1		1
LMSG_G000009071.1	1345_1	no	1	1				1		1
LMSG_G000005401.1	1345_1	yes	1	1			1	1		1
LMSG_G000004984.1	1346_0	yes								1
LMSG_G000005279.1	1347_0	yes		1			1			2
LMSG_G000007923.1	1348_1	no						1		
LMSG_G000007925.1	1348_1	no						1		
LMSG_G000007924.1	1348_1	no					1			
LMSG_G000006251.1	1348_1	yes					1	2		
LMSG_G000007927.1	1349_1	no						1		
LMSG_G000005404.1	1349_1	yes						1		
LMSG_G000007928.1	1350_1	no						1		
LMSG_G000005548.1	1350_1	yes						1		
LMSG_G000004975.1	1350_2	yes						1		
LMSG_G000006207.1	1351_0	yes						1		
LMSG_G000004831.1	1352_1	yes		4				1		
LMSG_G000009041.1	1354_1	no	1	1				2		
LMSG_G000005465.1	1354_1	yes		1						
LMSG_G000005114.1	1355_0	yes						1		
LMSG_G000009680.1	1357_1	no	2	1						1
LMSG_G000004592.1	1357_1	yes	2	1						1
LMSG_G000009692.1	1358_1	no	1	1						
LMSG_G000009694.1	1358_1	no	1	1						
LMSG_G000004497.1	1358_1	yes	1	1						
LMSG_G000004649.1	1359_1	yes	1	1			1			
LMSG_G000009695.1	1359_1	no		1						
LMSG_G000009696.1	1359_1	no	1	1						
LMSG_G000005266.1	1359_2	yes	1	1						
LMSG_G000009697.1	1359_2	no	1							
LMSG_G000004711.1	1360_0	yes	1	1						2
LMSG_G000005819.1	1362_0	yes								1
LMSG_G000009028.1	1363_1	no	1					1		1
LMSG_G000009029.1	1363_1	no	1				1	1		
LMSG_G000009030.1	1363_1	no	1				1	1		1
LMSG_G000005506.1	1363_1	yes	1				1	1		1
LMSG_G000009027.1	1363_1	no	2				1	2		1
LMSG_G000005460.1	1364_0	yes	1							1
LMSG_G000004424.1	1366_0	yes		1				2		
LMSG_G000004461.1	1369_1	yes					1			1
LMSG_G000008853.1	1369_1	no								1
LMSG_G000008854.1	1369_1	no								1
LMSG_G000008855.1	1369_1	no								1
LMSG_G000005954.1	1370_0	yes						1		1
LMSG_G000004625.1	1371_0	yes		2			1			1
LMSG_G000005289.1	1372_1	yes	2	2			1			1
LMSG_G000009502.1	1372_1	no		2			1			1
LMSG_G000006279.1	1373_1	yes		1			1	2		
LMSG_G000009503.1	1373_1	no		1						
LMSG_G000009504.1	1373_1	no		2			1	2		
LMSG_G000009505.1	1373_1	no		1				1		
LMSG_G000009501.1	1373_1	no		2			1	2		
LMSG_G000005287.1	1373_2	yes					1			
LMSG_G000009506.1	1374_1	no		1						
LMSG_G000009507.1	1374_1	no		1						
LMSG_G000009508.1	1374_1	no		1						
LMSG_G000009510.1	1374_1	no		1						
LMSG_G000009511.1	1374_1	no		1						
LMSG_G000009512.1	1374_1	no		1				1		
LMSG_G000009513.1	1375_1	no		1						
LMSG_G000004471.1	1375_1	yes	1	1						
LMSG_G000004426.1	1376_0	yes		1			2	3		
LMSG_G000005443.1	1377_0	yes	1							
LMSG_G000004850.1	1378_1	yes	1				1			
LMSG_G000009490.1	1378_1	no	1				1			
LMSG_G000009491.1	1378_1	no	1				1			
LMSG_G000009492.1	1379_1	no	1				2	3		
LMSG_G000009493.1	1379_1	no	1				2	2		
LMSG_G000009494.1	1379_1	no					2	2		
LMSG_G000009495.1	1379_1	no	1				2	2		
LMSG_G000009497.1	1379_1	no	1				2	2		
LMSG_G000009498.1	1379_1	no					2	1		
LMSG_G000009496.1	1379_1	no					2	2		
LMSG_G000009499.1	1379_1	no	1				2	1		
LMSG_G000009500.1	1379_1	no					1	1		
LMSG_G000004574.1	1379_1	yes	1				2	2		
LMSG_G000004701.1	1380_0	yes	1	1						1
LMSG_G000009032.1	1381_1	no		2	1		3	2		

LMSG_G000006239.1	1381_1	yes		2			3	2	
LMSG_G000009033.1	1381_1	no		2	1		4	1	
LMSG_G000004482.1	1382_1	yes		2	2	1	3		
LMSG_G000009034.1	1382_1	no		2	3	1	3		
LMSG_G000009035.1	1382_1	no		3	2	1	2		
LMSG_G000009036.1	1382_1	no	1	1		1	2		
LMSG_G000009037.1	1382_1	no		2	2	1	3		
LMSG_G000009042.1	1383_1	no						1	
LMSG_G000009045.1	1383_2	no						1	
LMSG_G000009044.1	1383_2	no						1	
LMSG_G000005841.1	1383_2	yes						1	
LMSG_G000009043.1	1383_2	no						1	
LMSG_G000004378.1	1384_0	yes							1
LMSG_G000009072.1	1385_1	no		1					1
LMSG_G000006234.1	1385_1	yes		1					1
LMSG_G000005304.1	1386_0	yes		1					
LMSG_G000006264.1	1387_0	yes					1		1
LMSG_G000005338.1	1388_1	yes	1					1	1
LMSG_G000009059.1	1388_1	no	1						1
LMSG_G000009057.1	1388_1	no	2					1	1
LMSG_G000009058.1	1388_1	no	1					1	1
LMSG_G000005024.1	1389_1	yes	1	1					1
LMSG_G000005118.1	1389_2	yes	1	1					1
LMSG_G000005023.1	1390_0	yes	1						1
LMSG_G000004822.1	1391_0	yes		2			1		
LMSG_G000005317.1	1392_0	yes	1				1		1
LMSG_G000007938.1	1394_1	no					1	2	1
LMSG_G000007939.1	1394_1	no							1
LMSG_G000005442.1	1394_1	yes					1	1	1
LMSG_G000007937.1	1394_1	no					1	1	1
LMSG_G000005854.1	1395_1	yes		1					1
LMSG_G000007941.1	1395_1	no		2					
LMSG_G000007945.1	1395_2	no		2			3	1	1
LMSG_G000007942.1	1395_2	no		2			2	1	1
LMSG_G000004464.1	1395_2	yes		2			2	1	1
LMSG_G000007943.1	1395_2	no		1			2	1	1
LMSG_G000007944.1	1395_2	no		2			2	1	1
LMSG_G000007946.1	1395_2	no		1			2	1	1
LMSG_G000010387.1	1396_1	no		1			1		2
LMSG_G000010388.1	1396_1	no		2			1		2
LMSG_G000005449.1	1396_1	yes		1			1		2
LMSG_G000010389.1	1396_1	no	1	1					
LMSG_G000010390.1	1397_1	no	1	1		1		2	2
LMSG_G000005014.1	1397_1	yes	1	1		1		2	2
LMSG_G000010391.1	1397_1	no		1		1		2	2
LMSG_G000010392.1	1397_1	no	1	1		2		2	2
LMSG_G000010393.1	1397_1	no							1
LMSG_G000005019.1	1398_1	yes	1	4			1	1	2
LMSG_G000010394.1	1398_1	no	1	5			1	1	2
LMSG_G000004830.1	1399_0	yes		2				1	
LMSG_G000007908.1	1400_1	no	3						2
LMSG_G000005015.1	1400_1	yes	4		1				2
LMSG_G000004670.1	1401_0	yes	1			1		1	3
LMSG_G000010610.1	1402_1	no	1	4					1
LMSG_G000010611.1	1402_1	no	1	5					1
LMSG_G000005211.1	1402_1	yes	1	5					1
LMSG_G000010613.1	1402_1	no	1	5					1
LMSG_G000010614.1	1402_1	no	1	5					1
LMSG_G000004734.1	1403_0	yes		2					
LMSG_G000011014.1	1404_1	no	5	6				3	
LMSG_G000011015.1	1404_1	no	6	6				3	
LMSG_G000011016.1	1404_1	no	7	3				1	
LMSG_G000004923.1	1404_1	yes	6	6				3	
LMSG_G000011017.1	1404_1	no	6	6				3	
LMSG_G000011008.1	1404_1	no	6	6				3	
LMSG_G000011018.1	1404_1	no	5	6				3	
LMSG_G000011019.1	1404_1	no	11	3				3	
LMSG_G000011009.1	1404_1	no	6	6				3	
LMSG_G000011010.1	1404_1	no	7	6				3	
LMSG_G000011020.1	1404_1	no	7	6				3	
LMSG_G000011011.1	1404_1	no	7	6				3	
LMSG_G000011012.1	1404_1	no	7	6				3	
LMSG_G000011013.1	1404_1	no	7	6				3	
LMSG_G000011022.1	1404_1	no	7	6				3	
LMSG_G000011023.1	1404_1	no	2	5				2	
LMSG_G000011021.1	1404_1	no		3				2	
LMSG_G000005655.1	1405_0	yes	1	1				1	1

LMSG_G000004689.1	1406_0	yes	1						
LMSG_G000010317.1	1408_1	no		1					1
LMSG_G000005220.1	1408_1	yes		1			1	1	2
LMSG_G000006079.1	1409_1	yes		1				1	1
LMSG_G000010319.1	1409_1	no							1
LMSG_G000004638.1	1410_0	yes		1				1	
LMSG_G000005466.1	1411_1	yes							2
LMSG_G000008924.1	1411_1	no							1
LMSG_G000010408.1	1413_1	no	1					1	1
LMSG_G000010409.1	1413_1	no	2						1
LMSG_G000006064.1	1413_1	yes				1		1	1
LMSG_G000010410.1	1413_1	no				1		1	1
LMSG_G000006069.1	1414_0	yes							1
LMSG_G000004639.1	1415_1	yes		2			1		2
LMSG_G000010406.1	1415_1	no		1			1		1
LMSG_G000006084.1	1416_0	yes						1	1
LMSG_G000010404.1	1417_1	no		1				1	1
LMSG_G000005130.1	1417_1	yes							1
LMSG_G000010405.1	1418_1	no							1
LMSG_G000005096.1	1418_1	yes		1					1
LMSG_G000004414.1	1420_0	yes					1	1	2
LMSG_G000006073.1	1421_0	yes		1	1		1	1	
LMSG_G000010403.1	1422_1	no		1			1		1
LMSG_G000004635.1	1422_1	yes		1			1		1
LMSG_G000010402.1	1422_1	no		1					
LMSG_G000006094.1	1423_0	yes							1
LMSG_G000005470.1	1424_0	yes		1				1	1
LMSG_G000006076.1	1425_0	yes		1					1
LMSG_G000006092.1	1426_0	yes		1	1			1	1
LMSG_G000005569.1	1427_1	yes							1
LMSG_G000010401.1	1427_1	no							1
LMSG_G000010400.1	1428_1	no							1
LMSG_G000004640.1	1428_1	yes					1		1
LMSG_G000006083.1	1429_0	yes					1	1	1
LMSG_G000004741.1	1430_1	yes		1	1				1
LMSG_G000010105.1	1430_1	no		1	1				2
LMSG_G000010106.1	1430_1	no			1				1
LMSG_G000005578.1	1431_1	yes					1		
LMSG_G000005763.1	1432_0	yes					1		1
LMSG_G000010380.1	1433_1	no		4	1		1		
LMSG_G000010381.1	1433_1	no		3			1	1	
LMSG_G000005550.1	1433_1	yes		3			1	1	
LMSG_G000010382.1	1434_1	no	1	2					
LMSG_G000005512.1	1434_1	yes	1	1				1	1
LMSG_G000004840.1	1435_1	yes	3	1				1	1
LMSG_G000010383.1	1435_1	no	1	2		1		1	1
LMSG_G000010384.1	1435_2	no				1			
LMSG_G000005375.1	1435_2	yes		2		1		1	
LMSG_G000005270.1	1436_0	yes	1	1					
LMSG_G000010385.1	1437_1	no	2						1
LMSG_G000010386.1	1437_1	no		2		1			
LMSG_G000005368.1	1437_1	yes		3		1			1
LMSG_G000006082.1	1438_0	yes		2				1	
LMSG_G000006093.1	1439_0	yes						1	1
LMSG_G000005413.1	1440_0	yes						2	2
LMSG_G000005564.1	1441_0	yes			1			1	1
LMSG_G000005293.1	1442_0	yes		1					1
LMSG_G000010322.1	1444_1	no						1	2
LMSG_G000004448.1	1444_1	yes						2	2
LMSG_G000010323.1	1444_1	no						1	2
LMSG_G000010320.1	1444_1	no						2	2
LMSG_G000010321.1	1444_1	no						2	2
LMSG_G000010324.1	1444_1	no						2	1
LMSG_G000004661.1	1445_0	yes						1	2
LMSG_G000010107.1	1446_1	no	1	3				1	2
LMSG_G000010108.1	1446_1	no		1				2	3
LMSG_G000005047.1	1446_1	yes	1	2				1	2
LMSG_G000010109.1	1446_1	no	1	2				2	1
LMSG_G000010110.1	1446_1	no	1					2	1
LMSG_G000005976.1	1447_0	yes	1					1	
LMSG_G000005546.1	1450_0	yes	1	1			1	1	1
LMSG_G000005975.1	1451_0	yes	1				1	2	2
LMSG_G000004703.1	1452_0	yes	1						2
LMSG_G000005390.1	1453_0	yes	1						1
LMSG_G000004874.1	1454_0	yes		2				1	2
LMSG_G000005463.1	1455_0	yes		1					
LMSG_G000010340.1	1456_1	no		1	1	1	1	3	

LMSG_G000006255.1	1456_1	yes		1	1	1	1	3	
LMSG_G000010341.1	1456_1	no		1	1	1	1	3	
LMSG_G000010374.1	1457_1	no	1	1				3	1
LMSG_G000006095.1	1457_1	yes	1	1				3	1
LMSG_G000006057.1	1458_1	yes	1	1		1		1	2
LMSG_G000010373.1	1458_1	no	1			1		1	2
LMSG_G000006080.1	1458_2	yes	1			1		1	2
LMSG_G000006089.1	1459_0	yes	1						2
LMSG_G000004847.1	1460_0	yes	1		1				
LMSG_G000010342.1	1461_1	no	1	1			1	1	3
LMSG_G000010343.1	1461_1	no	1				1		3
LMSG_G000010344.1	1461_1	no	1				1	1	3
LMSG_G000010345.1	1461_1	no	1				1	1	3
LMSG_G000010346.1	1461_1	no	1				1	1	3
LMSG_G000010347.1	1461_1	no	2	1			1	1	3
LMSG_G000010353.1	1461_1	no					1	1	3
LMSG_G000010348.1	1461_1	no	1						2
LMSG_G000004508.1	1461_1	yes	1				1	1	3
LMSG_G000006432.1	146_1	no							1
LMSG_G000006433.1	146_1	no							1
LMSG_G000010354.1	1462_1	no	3					2	3
LMSG_G000010355.1	1462_1	no	2					2	3
LMSG_G000010356.1	1462_1	no	4					2	3
LMSG_G000010357.1	1462_1	no	2					2	3
LMSG_G000010358.1	1462_1	no	3					3	3
LMSG_G000010359.1	1462_1	no	4					2	3
LMSG_G000010360.1	1462_1	no	2					1	3
LMSG_G000010361.1	1462_1	no	1						3
LMSG_G000010362.1	1462_1	no	2					1	3
LMSG_G000005431.1	1462_1	yes	2					2	3
LMSG_G000010363.1	1462_1	no	1					1	3
LMSG_G000010364.1	1462_1	no	1					1	3
LMSG_G000010365.1	1463_1	no	1					1	
LMSG_G000010366.1	1463_1	no	1	1				2	3
LMSG_G000005482.1	1463_1	yes	1	2				1	3
LMSG_G000010367.1	1463_1	no	1	1				2	4
LMSG_G000010368.1	1463_2	no	1					2	2
LMSG_G000010369.1	1463_2	no	1					2	3
LMSG_G000005343.1	1463_2	yes	2					2	3
LMSG_G000010370.1	1463_2	no	1					2	3
LMSG_G000010371.1	1463_2	no	1					2	2
LMSG_G000011321.1	1464_1	no							3
LMSG_G000011322.1	1464_1	no							3
LMSG_G000011323.1	1464_1	no							3
LMSG_G000005529.1	1464_1	yes							3
LMSG_G000004624.1	1465_0	yes	2	1		2	1	2	1
LMSG_G000010138.1	1466_1	no	1						2
LMSG_G000010137.1	1466_1	no		2					
LMSG_G000005037.1	1466_1	yes	1	2					2
LMSG_G000004773.1	1466_2	yes	1	1					2
LMSG_G000005918.1	1467_0	yes	1						2
LMSG_G000010217.1	1468_1	no		3			1		2
LMSG_G000004642.1	1468_1	yes		1			1		1
LMSG_G000010184.1	1469_1	no							1
LMSG_G000010185.1	1469_1	no					1	1	3
LMSG_G000006306.1	1469_1	yes		1			1	1	2
LMSG_G000010186.1	1469_1	no					1		2
LMSG_G000006099.1	1470_0	yes					1		3
LMSG_G000004375.1	1471_0	yes		1					1
LMSG_G000006446.1	147_1	no		1					
LMSG_G000006435.1	147_1	no		1					
LMSG_G000006438.1	147_1	no		1					1
LMSG_G000006439.1	147_1	no		1					
LMSG_G000005814.1	147_1	yes		1					
LMSG_G000006441.1	147_1	no							1
LMSG_G000006442.1	147_1	no		1					
LMSG_G000006443.1	147_1	no		1				1	
LMSG_G000005189.1	1472_0	yes	1	2	1		1	3	1
LMSG_G000006098.1	1473_1	yes			1		1		3
LMSG_G000010179.1	1473_1	no		1	1		1		3
LMSG_G000010149.1	1474_1	no		2					1
LMSG_G000004763.1	1474_1	yes		1					3
LMSG_G000010150.1	1474_1	no		1					2
LMSG_G000010151.1	1474_1	no		2					3
LMSG_G000010153.1	1474_1	no		3					3
LMSG_G000010154.1	1474_1	no		1					
LMSG_G000010155.1	1474_1	no		2					3

LMSG_G000010156.1	1474_1	no		1				3
LMSG_G000010157.1	1474_1	no		1				3
LMSG_G000010158.1	1474_1	no		1				3
LMSG_G000010159.1	1474_1	no		2				1
LMSG_G000010160.1	1474_1	no		2				2
LMSG_G000010161.1	1474_1	no		2				2
LMSG_G000010162.1	1474_1	no		2				
LMSG_G000010163.1	1474_1	no		2				2
LMSG_G000010178.1	1474_1	no		1				
LMSG_G000010164.1	1474_1	no		1				2
LMSG_G000010165.1	1474_1	no		2				3
LMSG_G000010166.1	1474_1	no		1				3
LMSG_G000010167.1	1474_1	no		1				3
LMSG_G000010168.1	1474_1	no		1				3
LMSG_G000010169.1	1474_1	no		2				2
LMSG_G000010170.1	1474_1	no		1				2
LMSG_G000010171.1	1474_1	no		1				2
LMSG_G000010172.1	1474_1	no		3	1			3
LMSG_G000010173.1	1474_1	no		2				1
LMSG_G000010174.1	1474_1	no		1			1	2
LMSG_G000010175.1	1474_1	no		2				2
LMSG_G000010177.1	1474_1	no		2				2
LMSG_G000004811.1	1475_1	yes		1				2
LMSG_G000010139.1	1475_1	no		1		1		1
LMSG_G000006100.1	1475_2	yes	1	1		1		3
LMSG_G000010181.1	1475_2	no		2		1		2
LMSG_G000010180.1	1475_2	no	1	1	1	1		3
LMSG_G000010182.1	1476_1	no		1		1		3
LMSG_G000006104.1	1476_1	yes				1		3
LMSG_G000010183.1	1476_1	no				1		3
LMSG_G000010140.1	1477_1	no						2
LMSG_G000010147.1	1477_1	no				1		1
LMSG_G000010148.1	1477_1	no				1		1
LMSG_G000010141.1	1477_1	no				1		2
LMSG_G000010142.1	1477_1	no				1		2
LMSG_G000005237.1	1477_1	yes				1		2
LMSG_G000010143.1	1477_1	no				1		3
LMSG_G000010144.1	1477_1	no				2		3
LMSG_G000010145.1	1477_1	no				1		3
LMSG_G000010146.1	1477_1	no				1		2
LMSG_G000004718.1	1478_0	yes		1				3
LMSG_G000006101.1	1479_0	yes		1				2
LMSG_G000005439.1	1480_1	yes	1	1				1
LMSG_G000010187.1	1480_1	no	1	1				1
LMSG_G000004761.1	1481_1	yes						1
LMSG_G000010188.1	1481_1	no				1		
LMSG_G000005226.1	1482_1	yes		1				
LMSG_G000010189.1	1482_1	no		1				
LMSG_G000010190.1	1482_1	no		1				1
LMSG_G000010191.1	1482_1	no		1				
LMSG_G000010192.1	1482_1	no		1				
LMSG_G000010193.1	1482_1	no						1
LMSG_G000005292.1	1482_2	yes		1				
LMSG_G000010195.1	1482_2	no						1
LMSG_G000010196.1	1482_2	no						1
LMSG_G000010197.1	1483_1	no		1		1		2
LMSG_G000005420.1	1483_1	yes		1		1		2
LMSG_G000010198.1	1484_1	no		2				2
LMSG_G000005234.1	1484_1	yes	1	1				2
LMSG_G000010199.1	1485_1	no		1				2
LMSG_G000010200.1	1485_1	no		1				2
LMSG_G000005199.1	1485_1	yes		1				2
LMSG_G000010201.1	1485_1	no		1				2
LMSG_G000010202.1	1485_1	no		1				2
LMSG_G000010203.1	1485_1	no		2				2
LMSG_G000010207.1	1485_1	no		1				
LMSG_G000010204.1	1485_1	no		1				2
LMSG_G000010205.1	1485_1	no		1				2
LMSG_G000010206.1	1485_1	no		1				2
LMSG_G000006105.1	1486_0	yes						2
LMSG_G000005353.1	1487_1	yes	1	1			1	1
LMSG_G000004723.1	1488_0	yes		1			1	1
LMSG_G000010209.1	1489_1	no		2				1
LMSG_G000010210.1	1489_1	no						3
LMSG_G000010211.1	1489_1	no		1				1
LMSG_G000005252.1	1489_1	yes		1				1
LMSG_G000010213.1	1490_1	no		1				

LMSG_G000010214.1	1490_1	no		1		1		1
LMSG_G000010215.1	1490_1	no		3		1		2
LMSG_G000005245.1	1490_1	yes		2		1		2
LMSG_G000010216.1	1490_1	no				1		1
LMSG_G000006035.1	149_0	yes					1	
LMSG_G000005354.1	1491_0	yes		2				1
LMSG_G000010111.1	1492_1	no		1		1		3
LMSG_G000010112.1	1492_1	no		1		1		3
LMSG_G000005239.1	1492_1	yes		1		1		2
LMSG_G000010113.1	1492_1	no		1		1		1
LMSG_G000010114.1	1492_1	no		1		1		2
LMSG_G000010115.1	1492_1	no		1		1		2
LMSG_G000010116.1	1492_1	no				1		2
LMSG_G000010117.1	1492_1	no		1		1		2
LMSG_G000010118.1	1492_1	no		1		1		2
LMSG_G000010119.1	1492_1	no		1		1		3
LMSG_G000004698.1	1493_0	yes				1		3
LMSG_G000005997.1	1495_1	yes	1	1		2	2	2
LMSG_G000010314.1	1495_1	no	1	1		1	1	1
LMSG_G000010315.1	1495_1	no		1		2	2	1
LMSG_G000010316.1	1495_1	no				1	1	1
LMSG_G000004420.1	1496_0	yes		1		1	5	1
LMSG_G000005711.1	1497_0	yes	1	1		1	2	2
LMSG_G000005813.1	1498_0	yes	1	3			1	1
LMSG_G000010227.1	1499_1	no		2				1
LMSG_G000010237.1	1499_1	no		2				2
LMSG_G000010228.1	1499_1	no		2				1
LMSG_G000010229.1	1499_1	no		2				1
LMSG_G000010238.1	1499_1	no		2				3
LMSG_G000010230.1	1499_1	no		2				1
LMSG_G000010231.1	1499_1	no		1				1
LMSG_G000010232.1	1499_1	no		2				1
LMSG_G000010233.1	1499_1	no		2			1	1
LMSG_G000010234.1	1499_1	no		2				1
LMSG_G000010235.1	1499_1	no		2				1
LMSG_G000010236.1	1499_1	no		2				2
LMSG_G000005906.1	1499_1	yes		2				1
LMSG_G000004765.1	1500_1	yes		1			1	
LMSG_G000010241.1	1500_1	no		1				2
LMSG_G000010242.1	1500_1	no		1			1	
LMSG_G000010240.1	1500_1	no						1
LMSG_G000010243.1	1500_1	no		1			1	2
LMSG_G000010244.1	1501_1	no		2			1	2
LMSG_G000010245.1	1501_1	no		2			1	1
LMSG_G000010246.1	1501_1	no		2			1	2
LMSG_G000010247.1	1501_1	no		2			1	2
LMSG_G000010248.1	1501_1	no		1	1		1	2
LMSG_G000010249.1	1501_1	no			1		1	2
LMSG_G000005896.1	1501_1	yes		2	1		1	1
LMSG_G000010290.1	1501_2	no		1				1
LMSG_G000010291.1	1501_2	no		1				1
LMSG_G000010293.1	1501_2	no		1				2
LMSG_G000010294.1	1501_2	no		1				2
LMSG_G000010306.1	1501_2	no		1				1
LMSG_G000010295.1	1501_2	no		1				1
LMSG_G000010296.1	1501_2	no		1				1
LMSG_G000010297.1	1501_2	no		1				2
LMSG_G000010298.1	1501_2	no						1
LMSG_G000010299.1	1501_2	no		1				2
LMSG_G000010300.1	1501_2	no		1				1
LMSG_G000010301.1	1501_2	no		1				1
LMSG_G000010305.1	1501_2	no		1				1
LMSG_G000010302.1	1501_2	no		1				2
LMSG_G000005660.1	1501_2	yes		1				2
LMSG_G000010303.1	1501_2	no		1				
LMSG_G000010304.1	1501_2	no		1				1
LMSG_G000010253.1	1502_1	no		1				
LMSG_G000010254.1	1502_1	no		2				1
LMSG_G000010255.1	1502_1	no		1				1
LMSG_G000010256.1	1502_1	no		1				1
LMSG_G000010257.1	1502_1	no		1				1
LMSG_G000004954.1	1502_1	yes		1				1
LMSG_G000010258.1	1502_1	no		1				1
LMSG_G000010259.1	1502_1	no		1				1
LMSG_G000010250.1	1502_1	no		1				1
LMSG_G000010251.1	1502_1	no		2				1
LMSG_G000010260.1	1502_1	no		1				1

LMSG_G000010261.1	1502_1	no		1					1
LMSG_G000010262.1	1502_1	no		1					1
LMSG_G000010263.1	1502_1	no		1					1
LMSG_G000010264.1	1502_1	no		1					1
LMSG_G000010265.1	1502_1	no		1					1
LMSG_G000010266.1	1502_1	no		1					1
LMSG_G000010267.1	1502_1	no		1					1
LMSG_G000010268.1	1502_1	no		1					1
LMSG_G000010269.1	1502_1	no		1					1
LMSG_G000010270.1	1502_1	no		1					1
LMSG_G000010271.1	1502_1	no		1					1
LMSG_G000010272.1	1502_1	no		1					1
LMSG_G000010273.1	1502_1	no		1					2
LMSG_G000010274.1	1502_1	no		1					1
LMSG_G000010275.1	1502_1	no		1					2
LMSG_G000010276.1	1502_1	no		1					1
LMSG_G000010252.1	1502_1	no		1					2
LMSG_G000010277.1	1502_1	no		1					1
LMSG_G000010278.1	1502_1	no		1					1
LMSG_G000010279.1	1502_1	no		1					1
LMSG_G000010281.1	1502_1	no		1					
LMSG_G000010283.1	1502_1	no		1					1
LMSG_G000010284.1	1502_1	no		1					1
LMSG_G000010285.1	1502_1	no		1					1
LMSG_G000010286.1	1502_1	no		1					1
LMSG_G000010287.1	1502_1	no		1					1
LMSG_G000010288.1	1502_1	no							1
LMSG_G000010289.1	1502_1	no		1					1
LMSG_G000005009.1	1502_2	yes		1					
LMSG_G000005267.1	1503_1	yes							1
LMSG_G000010120.1	1503_1	no							1
LMSG_G000004366.1	1504_1	yes						1	1
LMSG_G000010121.1	1504_1	no		1				1	1
LMSG_G000010122.1	1504_1	no						1	1
LMSG_G000005991.1	1504_2	yes						1	1
LMSG_G000005072.1	1505_1	yes	1	1		1		1	3
LMSG_G000010307.1	1505_1	no						1	1
LMSG_G000006026.1	1506_1	yes							2
LMSG_G000005723.1	1506_2	yes		1				1	1
LMSG_G000010310.1	1507_1	no		1			1		3
LMSG_G000005224.1	1507_1	yes		1			1		3
LMSG_G000010309.1	1507_1	no		2			1		3
LMSG_G000005221.1	1508_0	yes		3					2
LMSG_G000010123.1	1509_1	no		1					2
LMSG_G000005254.1	1509_1	yes		1					3
LMSG_G000005185.1	1510_1	yes		1					2
LMSG_G000010126.1	1510_1	no		1					1
LMSG_G000010220.1	1510_1	no					1		
LMSG_G000010125.1	1510_1	no							1
LMSG_G000010218.1	1510_1	no		1				1	1
LMSG_G000010124.1	1510_1	no		1			1	1	1
LMSG_G000010219.1	1510_1	no							2
LMSG_G000010127.1	1511_1	no		3				1	2
LMSG_G000010128.1	1511_1	no		1					1
LMSG_G000010129.1	1511_1	no		2				1	2
LMSG_G000005256.1	1511_1	yes		2				1	2
LMSG_G000010130.1	1511_1	no		1					
LMSG_G000004748.1	1511_2	yes		1				1	1
LMSG_G000005062.1	1511_3	yes		3				1	2
LMSG_G000005437.1	1512_1	yes	1						2
LMSG_G000010131.1	1512_1	no	1						2
LMSG_G000010132.1	1513_1	no		1					2
LMSG_G000010221.1	1513_1	no		1					2
LMSG_G000010222.1	1513_1	no		1					2
LMSG_G000010223.1	1513_1	no		1					1
LMSG_G000010133.1	1513_1	no		1					2
LMSG_G000005248.1	1513_1	yes		1					2
LMSG_G000010224.1	1513_1	no		2					2
LMSG_G000010225.1	1513_1	no		1					2
LMSG_G000010134.1	1513_1	no		2					2
LMSG_G000010226.1	1513_1	no		1			1		2
LMSG_G000005225.1	1514_1	yes		1	1				3
LMSG_G000010135.1	1514_1	no							1
LMSG_G000010136.1	1514_1	no		1					3
LMSG_G000005688.1	1515_0	yes			1			1	3
LMSG_G000005238.1	1516_0	yes	1					1	2
LMSG_G000006125.1	1517_0	yes		2	1		1		5

LMSG_G000006151.1	1518_0	yes						1	3
LMSG_G000007903.1	1519_1	no	2				1		2
LMSG_G000007904.1	1519_1	no	1				1		1
LMSG_G000004491.1	1519_1	yes	2				1		2
LMSG_G000007905.1	1519_1	no	1						
LMSG_G000006828.1	15_1	no						1	
LMSG_G000006830.1	15_1	no						1	
LMSG_G000007906.1	1520_1	no	1						2
LMSG_G000007907.1	1520_1	no	1						2
LMSG_G000005393.1	1520_1	yes					1		1
LMSG_G000004690.1	1520_2	yes	4				1	1	2
LMSG_G000007858.1	1521_1	no					1		1
LMSG_G000007857.1	1521_1	no	2				1		2
LMSG_G000006293.1	1521_1	yes	1				1		2
LMSG_G000007859.1	1521_1	no	2				1		2
LMSG_G000004538.1	1522_0	yes	1				1	2	2
LMSG_G000005340.1	1523_1	yes					1		2
LMSG_G000010431.1	1523_1	no					1		1
LMSG_G000005296.1	1524_0	yes		1					
LMSG_G000004658.1	1525_0	yes						1	
LMSG_G000005184.1	1526_1	yes		1			1		4
LMSG_G000010311.1	1526_1	no		1			1		4
LMSG_G000005344.1	1527_1	yes		1			1		4
LMSG_G000010312.1	1527_1	no							3
LMSG_G000005196.1	1528_1	yes					1		3
LMSG_G000010313.1	1528_1	no					1		2
LMSG_G000006103.1	1529_0	yes	1		1			1	2
LMSG_G000004888.1	1530_1	yes	1			1		3	
LMSG_G000010945.1	1530_1	no	1			1		2	
LMSG_G000010949.1	1530_1	no	1			1		3	
LMSG_G000010944.1	1530_1	no	1			1		2	
LMSG_G000010950.1	1530_1	no	2			1		2	
LMSG_G000010947.1	1530_1	no	1			1		2	
LMSG_G000010948.1	1530_1	no	2					1	
LMSG_G000004939.1	1531_0	yes	1					1	
LMSG_G000010862.1	1532_1	no		1					1
LMSG_G000005362.1	1532_1	yes		2					1
LMSG_G000006210.1	1533_1	yes	1	2					1
LMSG_G000010863.1	1533_1	no	1	2					1
LMSG_G000010864.1	1533_1	no		3					1
LMSG_G000010866.1	1533_1	no	1	2					1
LMSG_G000010867.1	1533_1	no	1	1					1
LMSG_G000011179.1	1534_1	no		1					
LMSG_G000011180.1	1534_1	no		1					
LMSG_G000005810.1	1534_1	yes		1				1	
LMSG_G000011181.1	1534_1	no		1					1
LMSG_G000004712.1	1535_1	yes	1	1			1	2	1
LMSG_G000010379.1	1535_1	no	1	1			1	1	3
LMSG_G000010375.1	1535_1	no	1	3			1	2	2
LMSG_G000010378.1	1535_1	no	1	1			1	1	3
LMSG_G000010376.1	1535_1	no		3			1	2	3
LMSG_G000010377.1	1535_1	no		3				1	1
LMSG_G000005982.1	1536_0	yes					1	2	2
LMSG_G000010437.1	1537_1	no		1				1	1
LMSG_G000006296.1	1537_1	yes		1				1	
LMSG_G000010436.1	1537_1	no		1				1	
LMSG_G000006262.1	1538_1	yes		2				1	
LMSG_G000010438.1	1538_1	no		2				1	1
LMSG_G000010439.1	1538_1	no		2					
LMSG_G000006261.1	1539_1	yes		2				1	1
LMSG_G000010440.1	1539_1	no		1				1	
LMSG_G000010441.1	1539_1	no		1				2	
LMSG_G000004341.1	1540_1	yes	1	2					1
LMSG_G000010451.1	1540_1	no		2					1
LMSG_G000010450.1	1540_1	no		2					1
LMSG_G000005567.1	1540_2	yes		1					1
LMSG_G000005666.1	1541_0	yes		1					1
LMSG_G000004928.1	1542_0	yes							1
LMSG_G000005828.1	1543_0	yes		2					1
LMSG_G000010452.1	1544_1	no		2					
LMSG_G000004920.1	1544_1	yes	1	1					1
LMSG_G000010453.1	1545_1	no		2					1
LMSG_G000005581.1	1545_1	yes		2					1
LMSG_G000010454.1	1546_1	no	1	1					1
LMSG_G000004891.1	1546_1	yes		1					1
LMSG_G000010455.1	1546_1	no		1					1
LMSG_G000010456.1	1546_1	no		1					

LMSG_G000010457.1	1546_1	no	1	1				1
LMSG_G000010463.1	1547_1	no		1				1
LMSG_G000010464.1	1547_1	no	1	1				1
LMSG_G000010479.1	1547_1	no						1
LMSG_G000010461.1	1547_1	no		1				1
LMSG_G000010462.1	1547_1	no		2				
LMSG_G000010482.1	1547_1	no		2			1	1
LMSG_G000010465.1	1547_1	no		1				1
LMSG_G000010481.1	1547_1	no		2				
LMSG_G000010458.1	1547_1	no		1				1
LMSG_G000010466.1	1547_1	no		1				1
LMSG_G000010467.1	1547_1	no	1					1
LMSG_G000010468.1	1547_1	no		1				1
LMSG_G000010469.1	1547_1	no						1
LMSG_G000010470.1	1547_1	no		1				1
LMSG_G000010480.1	1547_1	no		1				1
LMSG_G000010471.1	1547_1	no		1			1	1
LMSG_G000010472.1	1547_1	no	1	2				1
LMSG_G000004558.1	1547_1	yes		1				1
LMSG_G000010459.1	1547_1	no		1				1
LMSG_G000010473.1	1547_1	no		1				1
LMSG_G000010474.1	1547_1	no		1				
LMSG_G000010475.1	1547_1	no						1
LMSG_G000010460.1	1547_1	no		1			1	2
LMSG_G000010476.1	1547_1	no		1				2
LMSG_G000010477.1	1547_1	no	1	1				1
LMSG_G000010478.1	1547_1	no		1				1
LMSG_G000004801.1	1548_1	yes		1				1
LMSG_G000010488.1	1548_1	no		2				1
LMSG_G000010483.1	1548_1	no		1				1
LMSG_G000010484.1	1548_1	no		2				1
LMSG_G000010487.1	1548_1	no		2				
LMSG_G000010485.1	1548_1	no		2				1
LMSG_G000010486.1	1548_1	no		2				1
LMSG_G000005603.1	1548_2	yes		1				1
LMSG_G000010493.1	1549_1	no						1
LMSG_G000010492.1	1549_1	no	1	1				
LMSG_G000005657.1	1549_1	yes					1	1
LMSG_G000010489.1	1549_1	no	1	1				1
LMSG_G000010490.1	1549_1	no		1				
LMSG_G000010491.1	1549_1	no						1
LMSG_G000010496.1	1549_2	no		2				1
LMSG_G000010504.1	1549_2	no		1				
LMSG_G000010498.1	1549_2	no		1				
LMSG_G000010505.1	1549_2	no		1				1
LMSG_G000010494.1	1549_2	no		1				1
LMSG_G000010499.1	1549_2	no		2				
LMSG_G000010500.1	1549_2	no						1
LMSG_G000005662.1	1549_2	yes		2				1
LMSG_G000010501.1	1549_2	no		1				1
LMSG_G000010495.1	1549_2	no		1				1
LMSG_G000010506.1	1549_2	no		2				
LMSG_G000010502.1	1549_2	no		1				1
LMSG_G000010503.1	1549_2	no		1				1
LMSG_G000004770.1	1550_0	yes		3				1
LMSG_G000004931.1	1551_0	yes		2			1	1
LMSG_G000005381.1	1552_1	yes	1			1	1	
LMSG_G000009098.1	1552_1	no					2	
LMSG_G000009099.1	1553_1	no					1	1
LMSG_G000005083.1	1553_1	yes	1					1
LMSG_G000005345.1	1554_1	yes	2			1	2	1
LMSG_G000009100.1	1554_1	no				1	1	1
LMSG_G000009101.1	1554_1	no	1		1		2	1
LMSG_G000009104.1	1555_1	no	2			1	2	1
LMSG_G000004547.1	1555_1	yes	2			1	1	1
LMSG_G000009102.1	1555_1	no	1		1		1	2
LMSG_G000009103.1	1555_1	no	1		1		1	1
LMSG_G000005364.1	1556_0	yes	1		1		2	1
LMSG_G000006049.1	1557_0	yes	1				2	1
LMSG_G000009106.1	1558_1	no	1			1	2	1
LMSG_G000004545.1	1558_1	yes	1			1	2	1
LMSG_G000009105.1	1558_1	no	1				1	1
LMSG_G000004804.1	1559_1	yes	1				1	1
LMSG_G000009107.1	1559_1	no	1				1	
LMSG_G000009108.1	1560_1	no	1			1		2
LMSG_G000009109.1	1560_1	no	1			1		1
LMSG_G000009110.1	1560_1	no	1			1		2

LMSG_G000005745.1	1560_1	yes	1				1		2
LMSG_G000009111.1	1560_1	no	1				1		2
LMSG_G000009112.1	1560_1	no	1				1		2
LMSG_G000009113.1	1560_1	no	1						2
LMSG_G000004797.1	1561_0	yes	1					1	1
LMSG_G000004949.1	1562_0	yes	1				1	1	2
LMSG_G000005705.1	1563_0	yes					1	2	1
LMSG_G000008842.1	1564_1	no	1						1
LMSG_G000006280.1	1564_1	yes	1						
LMSG_G000008841.1	1564_1	no	1						
LMSG_G000010936.1	1565_1	no		1					1
LMSG_G000006238.1	1565_1	yes		1					1
LMSG_G000010937.1	1565_1	no		1					1
LMSG_G000010938.1	1565_1	no		1	1				1
LMSG_G000010939.1	1565_1	no		1					1
LMSG_G000009114.1	1566_1	no	1					1	1
LMSG_G000009122.1	1566_1	no						1	1
LMSG_G000009115.1	1566_1	no	1					1	1
LMSG_G000009116.1	1566_1	no	1					1	1
LMSG_G000009117.1	1566_1	no	1					1	1
LMSG_G000005051.1	1566_1	yes	1					1	1
LMSG_G000009118.1	1566_1	no	1					1	1
LMSG_G000009119.1	1566_1	no	1					1	1
LMSG_G000009121.1	1566_1	no	1					1	1
LMSG_G000009123.1	1566_1	no	1					1	1
LMSG_G000009127.1	1566_1	no	1					1	1
LMSG_G000009124.1	1566_1	no	1					1	1
LMSG_G000009120.1	1566_1	no	1					1	1
LMSG_G000009125.1	1566_1	no							1
LMSG_G000009126.1	1566_1	no	1					1	1
LMSG_G000009131.1	1567_1	no	1	1				2	2
LMSG_G000009128.1	1567_1	no	1	1			1	2	1
LMSG_G000009129.1	1567_1	no	1	1				2	1
LMSG_G000004561.1	1567_1	yes	1	1				2	1
LMSG_G000009130.1	1567_1	no	1	1				2	1
LMSG_G000009132.1	1567_1	no	1	1			1	2	1
LMSG_G000009133.1	1568_1	no	1	1				3	1
LMSG_G000005754.1	1568_1	yes	1					4	1
LMSG_G000009135.1	1568_1	no	1	1				3	1
LMSG_G000009134.1	1568_1	no	1	1				4	1
LMSG_G000009991.1	1569_1	no	1	4					2
LMSG_G000004436.1	1569_1	yes	1	4					2
LMSG_G000009995.1	1569_1	no	1						1
LMSG_G000009996.1	1569_1	no	1	2					2
LMSG_G000009997.1	1569_1	no	1	3					2
LMSG_G000009992.1	1569_1	no	1	4					2
LMSG_G000009993.1	1569_1	no	1	4					2
LMSG_G000009994.1	1569_1	no	1	4					2
LMSG_G000009974.1	1569_1	no	1	3					1
LMSG_G000004351.1	1570_1	yes	1	3				1	2
LMSG_G000009975.1	1570_1	no	1	3				1	1
LMSG_G000009976.1	1571_1	no	1	2				1	1
LMSG_G000005415.1	1571_1	yes		1					2
LMSG_G000007733.1	1572_1	no	1						2
LMSG_G000006146.1	1572_1	yes	2				1		2
LMSG_G000004666.1	1573_1	yes	1					1	1
LMSG_G000005979.1	1574_0	yes	3	1				3	1
LMSG_G000005988.1	1575_0	yes	3	2	1	2		3	1
LMSG_G000006155.1	1576_1	yes	1		1			1	1
LMSG_G000010710.1	1576_1	no	1					1	1
LMSG_G000009485.1	1578_1	no						1	1
LMSG_G000009486.1	1578_1	no	1					1	1
LMSG_G000009487.1	1578_1	no	1					1	1
LMSG_G000005515.1	1578_1	yes	1					1	1
LMSG_G000009488.1	1579_1	no	1						
LMSG_G000005553.1	1579_1	yes	1						1
LMSG_G000005989.1	1581_0	yes						1	
LMSG_G000004514.1	158_1	yes		1					
LMSG_G000007219.1	158_1	no		1					
LMSG_G000007223.1	158_1	no		1					
LMSG_G000007224.1	158_1	no		1					
LMSG_G000007225.1	158_1	no		1					
LMSG_G000005320.1	1584_1	yes	2	1		1		1	1
LMSG_G000011102.1	1584_1	no	3	1		1		1	1
LMSG_G000006318.1	1585_1	yes	1	1				1	
LMSG_G000006332.1	1585_1	no	1	1					1
LMSG_G000011104.1	1586_1	no		1					1

LMSG_G000011103.1	1586_1	no	1					1	1
LMSG_G000005371.1	1586_1	yes	1					1	1
LMSG_G000006334.1	1586_1	no	1	1				2	1
LMSG_G000011105.1	1586_1	no	1	1				1	1
LMSG_G000005258.1	1587_1	yes	1					1	1
LMSG_G000010943.1	1587_1	no	4	1		1	1	1	
LMSG_G000006323.1	1588_1	yes	2	3		1	1	3	3
LMSG_G000010941.1	1588_1	no	2	1			5	2	3
LMSG_G000006329.1	1588_1	no	2	2		1	1	3	3
LMSG_G000006330.1	1588_1	no	2	3		1	1	3	3
LMSG_G000006333.1	1588_1	no	2	2		1	2	3	3
LMSG_G000005346.1	1589_1	yes		1					
LMSG_G000004744.1	1590_0	yes	5					1	
LMSG_G000005249.1	1591_0	yes	3						
LMSG_G000005607.1	159_1	yes		1					
LMSG_G000010960.1	1592_1	no	1				1		
LMSG_G000006311.1	1592_1	yes	1				1	1	
LMSG_G000004837.1	1593_0	yes	8	3				1	
LMSG_G000004760.1	1594_0	yes					1		
LMSG_G000004988.1	1595_0	yes		4					
LMSG_G000005829.1	1597_0	yes	1						1
LMSG_G000005379.1	1599_0	yes	2				1		1
LMSG_G000005082.1	1600_0	yes		1				1	1
LMSG_G000004499.1	1601_0	yes	1	2	1		2		
LMSG_G000007230.1	160_1	no		2					
LMSG_G000006206.1	1603_0	yes		1			1		1
LMSG_G000004715.1	1604_0	yes		1			1		1
LMSG_G000005502.1	1606_0	yes					1	1	
LMSG_G000005945.1	1607_1	yes					1	1	
LMSG_G000011325.1	1607_1	no					1	1	
LMSG_G000004688.1	1608_0	yes	1				1	2	
LMSG_G000006085.1	1609_0	yes					1		
LMSG_G000007257.1	161_1	no							1
LMSG_G000005068.1	1612_1	yes	1						
LMSG_G000011326.1	1612_1	no	1						
LMSG_G000006257.1	1614_0	yes	1						
LMSG_G000004785.1	1615_0	yes		1					
LMSG_G000004657.1	1616_0	yes						1	
LMSG_G000004449.1	1617_1	yes	1				1		
LMSG_G000011328.1	1617_1	no	1						
LMSG_G000011329.1	1617_1	no	1				1		
LMSG_G000011330.1	1617_1	no	1				1		
LMSG_G000011327.1	1617_1	no	1				1	1	
LMSG_G000005927.1	1619_0	yes	1				1		
LMSG_G000004686.1	1622_1	yes	2	1				2	
LMSG_G000011331.1	1622_1	no	1						
LMSG_G000005450.1	1623_0	yes	1	1				1	
LMSG_G000004749.1	1624_1	yes	1					1	
LMSG_G000011333.1	1624_1	no	1					1	
LMSG_G000011335.1	1625_1	no	1	1					
LMSG_G000011336.1	1625_1	no	1	1					
LMSG_G000004470.1	1625_1	yes	1	1					
LMSG_G000011337.1	1625_1	no	1	1					
LMSG_G000004695.1	1626_0	yes	1					3	
LMSG_G000004708.1	1627_0	yes						1	
LMSG_G000005966.1	1632_0	yes					1		1
LMSG_G000009048.1	1636_1	no		1	2				
LMSG_G000009049.1	1636_1	no		1	2				
LMSG_G000009050.1	1636_1	no		1	2				
LMSG_G000009051.1	1636_1	no		1	2				
LMSG_G000009052.1	1636_1	no		1	2				
LMSG_G000009053.1	1636_1	no		1	2				
LMSG_G000009055.1	1636_1	no		1	2				
LMSG_G000009054.1	1636_1	no		1	2				
LMSG_G000004576.1	1636_1	yes		1	2				
LMSG_G000009056.1	1637_1	no			1				
LMSG_G000005839.1	1637_1	yes		2	1				
LMSG_G000008864.1	1638_1	no					1		1
LMSG_G000008861.1	1638_1	no					1	1	1
LMSG_G000008865.1	1638_1	no					1	1	1
LMSG_G000008862.1	1638_1	no					1	1	1
LMSG_G000008863.1	1638_1	no					1		1
LMSG_G000004581.1	1638_1	yes					1	1	1
LMSG_G000008872.1	1639_1	no							2
LMSG_G000008867.1	1639_1	no							1
LMSG_G000008868.1	1639_1	no							2
LMSG_G000008869.1	1639_1	no							1

LMSG_G000008871.1	1639_1	no						1
LMSG_G000004556.1	1639_1	yes					1	2
LMSG_G000004362.1	1640_1	yes				1		1
LMSG_G000008876.1	1640_1	no						1
LMSG_G000008878.1	1640_1	no				1		1
LMSG_G000006295.1	1641_0	yes						1
LMSG_G000007243.1	164_1	no		1				
LMSG_G000007244.1	164_1	no		1				
LMSG_G000007240.1	164_1	no		1				
LMSG_G000007241.1	164_1	no		1				
LMSG_G000007245.1	164_1	no		1				
LMSG_G000007242.1	164_1	no		1				
LMSG_G000004591.1	164_1	yes		1				
LMSG_G000007247.1	164_1	no		1				
LMSG_G000006188.1	1642_0	yes			1			
LMSG_G000004357.1	1643_1	yes		1		1		1
LMSG_G000008879.1	1643_1	no		1		1		1
LMSG_G000008883.1	1643_1	no		1		1		1
LMSG_G000008884.1	1643_1	no				1		
LMSG_G000008880.1	1643_1	no		1		1		1
LMSG_G000008881.1	1643_1	no	1	1		1		1
LMSG_G000008882.1	1643_1	no		1		1		1
LMSG_G000008885.1	1643_1	no		1		1		1
LMSG_G000008886.1	1643_1	no		1		1		1
LMSG_G000004865.1	1644_1	yes				1		2
LMSG_G000008866.1	1644_1	no				1		2
LMSG_G000008889.1	1644_1	no				1		
LMSG_G000008890.1	1644_1	no				1		1
LMSG_G000004579.1	1645_1	yes				1		1
LMSG_G000008891.1	1645_1	no				1		1
LMSG_G000006011.1	1646_0	yes						1
LMSG_G000004606.1	1647_1	yes	1	1				2
LMSG_G000009542.1	1647_1	no	1	1				2
LMSG_G000009544.1	1647_1	no	1					2
LMSG_G000008892.1	1648_1	no						2
LMSG_G000008893.1	1648_1	no						2
LMSG_G000008894.1	1648_1	no						2
LMSG_G000008895.1	1648_1	no						2
LMSG_G000008896.1	1648_1	no						2
LMSG_G000004504.1	1648_1	yes						2
LMSG_G000004964.1	1649_0	yes		1				
LMSG_G000005262.1	1652_0	yes				1		
LMSG_G000004713.1	1656_0	yes					1	
LMSG_G000006241.1	1658_1	yes						1
LMSG_G000005523.1	1659_0	yes						1
LMSG_G000005447.1	1660_1	yes	1					1
LMSG_G000008818.1	1660_1	no	1					1
LMSG_G000008819.1	1660_1	no	1					1
LMSG_G000008820.1	1660_1	no						1
LMSG_G000008821.1	1660_1	no						1
LMSG_G000008822.1	1661_1	no						1
LMSG_G000005514.1	1661_1	yes						1
LMSG_G000007082.1	166_1	no		1				
LMSG_G000007079.1	166_1	no		1				
LMSG_G000004595.1	166_1	yes		1				
LMSG_G000007080.1	166_1	no		1				
LMSG_G000008814.1	1662_1	no				1		1
LMSG_G000008816.1	1662_1	no				1		1
LMSG_G000005500.1	1662_1	yes				1		1
LMSG_G000008817.1	1662_1	no				1		1
LMSG_G000008815.1	1662_1	no				1		1
LMSG_G000008823.1	1663_1	no						1
LMSG_G000004488.1	1663_1	yes						1
LMSG_G000008825.1	1663_1	no						1
LMSG_G000008824.1	1663_1	no						1
LMSG_G000008828.1	1665_1	no						1
LMSG_G000008826.1	1665_1	no						1
LMSG_G000004498.1	1665_1	yes						1
LMSG_G000008827.1	1665_1	no						1
LMSG_G000005274.1	1666_1	yes	1			1		1
LMSG_G000008829.1	1666_1	no	1			1		1
LMSG_G000008830.1	1666_1	no	1			1		1
LMSG_G000008831.1	1668_1	no	1				1	1
LMSG_G000004468.1	1668_1	yes	1			1	1	1
LMSG_G000008832.1	1668_1	no	1				1	1
LMSG_G000008833.1	1669_1	no	1					1
LMSG_G000008834.1	1669_1	no	1					1

LMSG_G000008835.1	1669_1	no	1					1
LMSG_G000008837.1	1669_1	no				1		
LMSG_G000008836.1	1669_1	no	1			1		1
LMSG_G000005765.1	1669_1	yes	1			1		1
LMSG_G000008839.1	1669_1	no	1					
LMSG_G000008838.1	1669_1	no	1			1		1
LMSG_G000005511.1	1670_0	yes						1
LMSG_G000005530.1	1671_0	yes						1
LMSG_G000004557.1	1673_1	yes						1
LMSG_G000008849.1	1673_1	no						1
LMSG_G000005488.1	1674_2	yes						1
LMSG_G000005525.1	1675_0	yes						2
LMSG_G000006319.1	1683_1	yes		1				
LMSG_G000006337.1	1683_1	no		1				
LMSG_G000006209.1	1685_0	yes				1		1
LMSG_G000006321.1	1690_0	yes		1				
LMSG_G000007197.1	169_1	no					1	
LMSG_G000007196.1	169_1	no						1
LMSG_G000007195.1	169_1	no						1
LMSG_G000005134.1	1694_0	yes				1		
LMSG_G000004660.1	170_0	yes						1
LMSG_G000009312.1	1703_1	no					2	
LMSG_G000005054.1	1703_1	yes					1	
LMSG_G000004832.1	1706_1	yes	1					1
LMSG_G000010605.1	1706_1	no	1					1
LMSG_G000005026.1	1717_0	yes	1	1			1	
LMSG_G000005961.1	1718_0	yes					1	
LMSG_G000005140.1	1727_0	yes		2				1
LMSG_G000008916.1	1731_1	no					1	
LMSG_G000006066.1	1731_1	yes					1	
LMSG_G000008915.1	1731_1	no					1	
LMSG_G000005053.1	1733_0	yes		1				
LMSG_G000011216.1	1748_3	no		1				
LMSG_G000004397.1	1750_0	yes		3		1		1
LMSG_G000006047.1	1751_0	yes		1				
LMSG_G000007362.1	175_1	no					1	
LMSG_G000004396.1	175_1	yes					1	
LMSG_G000007367.1	175_1	no					1	
LMSG_G000007368.1	175_1	no					1	
LMSG_G000007365.1	175_1	no					1	
LMSG_G000007363.1	175_1	no					1	
LMSG_G000007364.1	175_1	no					1	
LMSG_G000007371.1	175_1	no					1	
LMSG_G000007372.1	175_1	no					1	
LMSG_G000007373.1	175_1	no					1	
LMSG_G000007375.1	175_1	no					1	
LMSG_G000007376.1	175_1	no					1	
LMSG_G000007377.1	175_1	no					1	
LMSG_G000005547.1	1755_0	yes						1
LMSG_G000005962.1	1756_0	yes		1				
LMSG_G000009848.1	1761_1	no		1				
LMSG_G000005493.1	1761_1	yes		1				
LMSG_G000005131.1	1767_0	yes		1			1	
LMSG_G000006322.1	1774_0	yes		1				
LMSG_G000006335.1	1786_1	no		1				
LMSG_G000006336.1	1786_1	no		1				
LMSG_G000006320.1	1786_1	yes		1				
LMSG_G000005959.1	1791_0	yes					1	
LMSG_G000006291.1	179_1	yes						1
LMSG_G000007116.1	179_1	no						1
LMSG_G000007117.1	179_1	no						1
LMSG_G000005527.1	1797_1	yes		1				
LMSG_G000009771.1	1803_1	no					1	
LMSG_G000009770.1	1803_1	no					1	
LMSG_G000004994.1	18_0	yes		1				1
LMSG_G000009775.1	1815_1	no					1	
LMSG_G000006159.1	1815_1	yes					1	
LMSG_G000005135.1	1818_1	yes		1				
LMSG_G000006338.1	1818_1	no		2				
LMSG_G000006339.1	1818_1	no		2				
LMSG_G000007158.1	182_1	no		1				
LMSG_G000009833.1	1827_1	no					1	
LMSG_G000005556.1	1827_1	yes					1	
LMSG_G000005531.1	1829_0	yes	1					
LMSG_G000004700.1	1837_1	yes	1			1		3
LMSG_G000010077.1	1837_1	no	1		1	1		3
LMSG_G000010078.1	1837_1	no						3

LMSG_G000007064.1	186_1	no						1	
LMSG_G000007051.1	186_1	no						1	
LMSG_G000007052.1	186_1	no						1	
LMSG_G000006215.1	186_1	yes						1	
LMSG_G000007049.1	186_1	no						1	
LMSG_G000007053.1	186_1	no						1	
LMSG_G000007050.1	186_1	no						1	
LMSG_G000007054.1	186_1	no						1	
LMSG_G000007065.1	186_1	no						1	
LMSG_G000007055.1	186_1	no						1	
LMSG_G000007066.1	186_1	no						1	
LMSG_G000007067.1	186_1	no						1	
LMSG_G000007068.1	186_1	no						1	
LMSG_G000007056.1	186_1	no						1	
LMSG_G000007069.1	186_1	no						1	
LMSG_G000007070.1	186_1	no						1	
LMSG_G000007057.1	186_1	no						2	
LMSG_G000007059.1	186_1	no						1	
LMSG_G000007060.1	186_1	no						1	
LMSG_G000007061.1	186_1	no						1	
LMSG_G000007062.1	186_1	no						1	
LMSG_G000007063.1	186_1	no						1	
LMSG_G000004883.1	187_1	yes						1	
LMSG_G000007072.1	187_1	no						1	
LMSG_G000007073.1	187_1	no						1	
LMSG_G000007334.1	198_1	no						1	
LMSG_G000007335.1	198_1	no						1	
LMSG_G000007336.1	198_1	no						1	
LMSG_G000004916.1	198_1	yes						1	
LMSG_G000007338.1	198_1	no						1	
LMSG_G000007341.1	198_1	no						1	
LMSG_G000007340.1	198_1	no						1	
LMSG_G000007354.1	198_2	no						1	
LMSG_G000007345.1	198_2	no						1	
LMSG_G000007350.1	198_2	no						1	
LMSG_G000005033.1	199_0	yes						2	
LMSG_G000008774.1	214_1	no						2	1
LMSG_G000006988.1	215_1	no						1	
LMSG_G000006989.1	215_1	no						1	
LMSG_G000007033.1	220_1	no						1	
LMSG_G000006857.1	22_1	no						1	
LMSG_G000006859.1	22_1	no						1	
LMSG_G000004783.1	225_0	yes					1		
LMSG_G000006180.1	226_0	yes		1				1	
LMSG_G000004828.1	227_0	yes	1						
LMSG_G000006274.1	228_0	yes					1	2	2
LMSG_G000009325.1	230_1	no				1		3	
LMSG_G000005406.1	230_1	yes				1		2	
LMSG_G000006276.1	231_0	yes		1		1		3	
LMSG_G000004483.1	232_1	yes							2
LMSG_G000008919.1	232_1	no							2
LMSG_G000008920.1	232_1	no							1
LMSG_G000008921.1	232_1	no							2
LMSG_G000004578.1	233_0	yes							1
LMSG_G000009959.1	234_1	no	1						
LMSG_G000005903.1	234_1	yes	1						
LMSG_G000006269.1	236_0	yes		1					
LMSG_G000004812.1	244_1	yes					1		
LMSG_G000008917.1	244_1	no					1		
LMSG_G000005507.1	245_0	yes							1
LMSG_G000005983.1	246_0	yes	8						
LMSG_G000006810.1	247_1	no							1
LMSG_G000006809.1	247_1	no							1
LMSG_G000004494.1	247_1	yes							1
LMSG_G000006811.1	247_1	no							1
LMSG_G000006812.1	247_1	no							1
LMSG_G000006814.1	248_1	no							1
LMSG_G000005510.1	248_1	yes							1
LMSG_G000006813.1	248_1	no							1
LMSG_G000004350.1	250_0	yes	1	1	1			1	1
LMSG_G000004652.1	251_0	yes						1	
LMSG_G000004457.1	252_0	yes						1	
LMSG_G000009334.1	253_1	no						1	
LMSG_G000005535.1	253_1	yes						1	
LMSG_G000005180.1	254_1	yes	2		1		2		1
LMSG_G000006037.1	254_2	yes		1					
LMSG_G000004786.1	255_0	yes		2				1	

LMSG_G000004825.1	256_0	yes		2				1	
LMSG_G000004416.1	257_1	yes	2	4					
LMSG_G000011007.1	257_1	no	3	3					
LMSG_G000004382.1	258_0	yes		2				1	
LMSG_G000011100.1	259_1	no					1		
LMSG_G000005123.1	259_1	yes					1		
LMSG_G000011096.1	259_1	no					1		
LMSG_G000011097.1	259_1	no					1		
LMSG_G000011099.1	259_1	no					1		
LMSG_G000010951.1	262_1	no		3				1	
LMSG_G000004787.1	262_1	yes		1			1	1	
LMSG_G000004965.1	263_0	yes		1					
LMSG_G000006187.1	264_0	yes	1	1					
LMSG_G000004736.1	265_0	yes		1					
LMSG_G000004636.1	266_0	yes					1		2
LMSG_G000004963.1	267_0	yes						1	
LMSG_G000004395.1	268_0	yes		1					
LMSG_G000006065.1	269_1	yes						1	
LMSG_G000008910.1	269_1	no						1	
LMSG_G000008911.1	270_1	no		1					
LMSG_G000004792.1	270_1	yes		1					
LMSG_G000008796.1	272_1	no							1
LMSG_G000008797.1	272_1	no							1
LMSG_G000008798.1	272_1	no							1
LMSG_G000008795.1	272_1	no							1
LMSG_G000008800.1	272_1	no							1
LMSG_G000006307.1	272_1	yes							1
LMSG_G000005377.1	274_1	yes					1		1
LMSG_G000008801.1	275_1	no					1		2
LMSG_G000008802.1	275_1	no					1		1
LMSG_G000004503.1	275_1	yes					1		1
LMSG_G000006211.1	276_1	yes					1	1	1
LMSG_G000008804.1	276_1	no					1	1	1
LMSG_G000008803.1	276_1	no					2	1	1
LMSG_G000008806.1	276_1	no					1	1	1
LMSG_G000008805.1	276_1	no						1	1
LMSG_G000006202.1	277_0	yes					1		1
LMSG_G000005334.1	282_1	yes							1
LMSG_G000008810.1	284_1	no		1			1		
LMSG_G000006060.1	284_1	yes		1			1		
LMSG_G000004633.1	284_2	yes		1			1	1	2
LMSG_G000010854.1	286_1	no						1	
LMSG_G000010852.1	286_1	no						1	
LMSG_G000010839.1	287_1	no						1	
LMSG_G000006303.1	288_1	yes						2	
LMSG_G000010850.1	288_1	no						2	
LMSG_G000006324.1	290_1	yes		1					
LMSG_G000004398.1	291_0	yes		2					1
LMSG_G000011143.1	292_1	no	2			1			
LMSG_G000004459.1	292_1	yes	2			1			
LMSG_G000006331.1	292_1	no	2	2		1			
LMSG_G000011144.1	292_1	no	3			1			
LMSG_G000007922.1	293_1	no	2				1		1
LMSG_G000004599.1	293_1	yes	1				1	1	1
LMSG_G000004472.1	294_1	yes	1				1		
LMSG_G000008918.1	294_1	no	1						
LMSG_G000005508.1	298_1	yes						1	1
LMSG_G000008901.1	298_1	no						1	
LMSG_G000005333.1	299_1	yes					1		1
LMSG_G000009272.1	299_1	no					1		1
LMSG_G000009270.1	299_1	no					1		1
LMSG_G000009753.1	301_1	no						1	
LMSG_G000009754.1	301_1	no						1	
LMSG_G000005380.1	301_1	yes						1	
LMSG_G000009755.1	301_1	no						1	
LMSG_G000004337.1	302_0	yes						2	
LMSG_G000005255.1	304_0	yes						2	
LMSG_G000005846.1	305_0	yes	1					1	
LMSG_G000010634.1	306_1	no						1	1
LMSG_G000006061.1	306_1	yes						1	
LMSG_G000010635.1	306_1	no						1	
LMSG_G000010644.1	307_1	no				1			1
LMSG_G000004790.1	307_1	yes			1	1			1
LMSG_G000010640.1	308_1	no			1	2			
LMSG_G000010641.1	308_1	no	1		1	2			
LMSG_G000010642.1	308_1	no	1		1				
LMSG_G000006068.1	308_1	yes	1		1	2			1

LMSG_G000008760.1	309_1	no							2
LMSG_G000005190.1	309_1	yes							2
LMSG_G000008762.1	309_1	no							2
LMSG_G000008763.1	309_1	no							2
LMSG_G000008764.1	310_1	no						1	2
LMSG_G000008765.1	310_1	no	1						2
LMSG_G000005425.1	310_1	yes	1					1	2
LMSG_G000008767.1	310_1	no	1						1
LMSG_G000004735.1	311_1	yes	1					1	2
LMSG_G000008768.1	311_1	no	1					1	2
LMSG_G000008773.1	312_1	no	1					1	2
LMSG_G000008769.1	312_1	no	1						2
LMSG_G000008770.1	312_1	no	1					1	2
LMSG_G000005387.1	312_1	yes						1	3
LMSG_G000008772.1	312_1	no	1					1	2
LMSG_G000004346.1	313_0	yes							1
LMSG_G000004338.1	315_1	yes	3					1	1
LMSG_G000008965.1	315_1	no	3					1	1
LMSG_G000008966.1	316_1	no	1						
LMSG_G000008967.1	316_1	no	3					1	1
LMSG_G000008968.1	316_1	no	1						1
LMSG_G000008969.1	316_1	no	1						
LMSG_G000004473.1	316_1	yes	4					1	1
LMSG_G000005272.1	317_1	yes					1		
LMSG_G000004376.1	318_1	yes							2
LMSG_G000008757.1	318_1	no							1
LMSG_G000008752.1	318_1	no							2
LMSG_G000008753.1	318_1	no							2
LMSG_G000008754.1	318_1	no							3
LMSG_G000008755.1	318_1	no							2
LMSG_G000008756.1	318_1	no							2
LMSG_G000004680.1	3_1	yes							3
LMSG_G000008751.1	3_1	no							1
LMSG_G000008749.1	3_1	no							2
LMSG_G000006243.1	320_0	yes						1	
LMSG_G000008972.1	322_1	no			1				
LMSG_G000008974.1	322_1	no					1	1	
LMSG_G000004570.1	322_1	yes					1	1	
LMSG_G000008976.1	322_1	no					1		
LMSG_G000005193.1	323_1	yes			1			1	
LMSG_G000011002.1	323_1	no			1		1	1	
LMSG_G000011004.1	323_1	no						2	
LMSG_G000008978.1	324_1	no			1		1		
LMSG_G000005920.1	324_1	yes					2		
LMSG_G000008979.1	324_2	no					1	1	
LMSG_G000008980.1	324_2	no		1	1			1	
LMSG_G000008983.1	324_2	no					1	1	
LMSG_G000008986.1	324_2	no			1		1	1	
LMSG_G000008984.1	324_2	no		1	1			1	
LMSG_G000008985.1	324_2	no		1	1		1	1	
LMSG_G000006253.1	324_2	yes		1			1	1	
LMSG_G000005326.1	325_0	yes	1				1	1	
LMSG_G000004349.1	326_1	yes	1				1	1	
LMSG_G000005933.1	3_2	yes							3
LMSG_G000006075.1	335_0	yes						1	
LMSG_G000006186.1	338_0	yes							1
LMSG_G000010961.1	34_1	no	8	2		1	1	2	1
LMSG_G000005013.1	34_1	yes	2	1			1	1	1
LMSG_G000005282.1	346_0	yes	1	2			1		2
LMSG_G000005528.1	347_0	yes	1						
LMSG_G000008780.1	350_1	no	1						
LMSG_G000006317.1	35_0	yes	2	1		1	1		
LMSG_G000004740.1	352_0	yes	1				1		
LMSG_G000009894.1	354_1	no							1
LMSG_G000009892.1	354_1	no							1
LMSG_G000009893.1	354_1	no							1
LMSG_G000009895.1	354_1	no							1
LMSG_G000009915.1	354_1	no							1
LMSG_G000005174.1	354_1	yes							1
LMSG_G000009897.1	354_1	no							1
LMSG_G000009898.1	354_1	no							1
LMSG_G000009899.1	354_1	no							1
LMSG_G000009900.1	354_1	no							1
LMSG_G000006129.1	355_0	yes							1
LMSG_G000007954.1	357_1	no	1					1	
LMSG_G000007957.1	357_1	no	1						
LMSG_G000004389.1	357_1	yes	1					1	

LMSG_G000007955.1	357_1	no				1	
LMSG_G000007956.1	357_1	no				1	
LMSG_G000009923.1	365_1	no		1			
LMSG_G000005930.1	365_1	yes		1			
LMSG_G000009932.1	368_1	no		1			
LMSG_G000009933.1	368_1	no		1			
LMSG_G000005677.1	368_1	yes		1			
LMSG_G000009934.1	370_1	no		1			
LMSG_G000009901.1	373_1	no		1			
LMSG_G000009903.1	373_1	no		1			
LMSG_G000009905.1	373_1	no		1			
LMSG_G000009912.1	373_1	no		1			
LMSG_G000004789.1	390_0	yes				1	
LMSG_G000005934.1	401_0	yes				1	
LMSG_G000008775.1	402_1	no				1	
LMSG_G000004460.1	402_1	yes				1	
LMSG_G000005732.1	404_1	yes				1	
LMSG_G000009723.1	405_1	no				1	
LMSG_G000009711.1	405_1	no				1	
LMSG_G000005594.1	405_1	yes				1	
LMSG_G000009725.1	405_1	no				1	
LMSG_G000009726.1	406_1	no				1	
LMSG_G000004726.1	416_0	yes					2
LMSG_G000004336.1	418_0	yes				1	1
LMSG_G000004588.1	421_0	yes					1
LMSG_G000004632.1	425_1	yes				2	
LMSG_G000010104.1	425_1	no				2	
LMSG_G000010000.1	427_1	no		2			2
LMSG_G000009977.1	427_1	no		1			2
LMSG_G000010001.1	427_1	no	1	1			1
LMSG_G000010002.1	427_1	no	1	3			1
LMSG_G000009978.1	427_1	no	1				
LMSG_G000005775.1	427_1	yes	1	3			
LMSG_G000009998.1	427_1	no		3			1
LMSG_G000009999.1	427_1	no	1	2			1
LMSG_G000010003.1	427_1	no	1	2			2
LMSG_G000010004.1	427_1	no		2			1
LMSG_G000005041.1	427_2	yes	1	2			1
LMSG_G000010006.1	428_1	no		2			1
LMSG_G000010007.1	428_1	no		1			1
LMSG_G000010008.1	428_1	no		1			
LMSG_G000010009.1	428_1	no					1
LMSG_G000010010.1	428_1	no		2			2
LMSG_G000010011.1	428_1	no		2			2
LMSG_G000010012.1	428_1	no		1			1
LMSG_G000010013.1	428_1	no		1			1
LMSG_G000010014.1	428_1	no		1			1
LMSG_G000010015.1	428_1	no		1			1
LMSG_G000010016.1	428_1	no					1
LMSG_G000010017.1	428_1	no		1			1
LMSG_G000005708.1	428_1	yes		3			2
LMSG_G000010018.1	428_1	no		1			2
LMSG_G000010019.1	428_1	no		3			2
LMSG_G000010020.1	428_1	no		2			2
LMSG_G000010021.1	428_1	no		1			2
LMSG_G000010022.1	428_1	no					2
LMSG_G000010023.1	428_1	no		1			2
LMSG_G000010024.1	428_1	no					1
LMSG_G000009979.1	428_1	no					1
LMSG_G000009965.1	428_2	no		1			1
LMSG_G000005868.1	428_2	yes					1
LMSG_G000010027.1	429_1	no	1				2
LMSG_G000010028.1	429_1	no	1		1		1
LMSG_G000010025.1	429_1	no	1		1		2
LMSG_G000010029.1	429_1	no	1		1		2
LMSG_G000010030.1	429_1	no	1		1		2
LMSG_G000004912.1	429_1	yes	1		1		2
LMSG_G000010031.1	429_1	no					2
LMSG_G000010032.1	429_1	no			1		2
LMSG_G000010033.1	429_1	no	1		1		2
LMSG_G000010034.1	429_1	no	1		1		1
LMSG_G000009980.1	429_1	no	1		1		3
LMSG_G000009966.1	429_1	no	1		1		2
LMSG_G000009982.1	429_1	no		1			2
LMSG_G000010035.1	429_1	no	1				1
LMSG_G000010026.1	429_1	no	1		1		2
LMSG_G000010036.1	430_1	no	1	2	2		1

LMSG_G000010037.1	430_1	no		2	1		2
LMSG_G000010038.1	430_1	no	1	3	1	1	2
LMSG_G000005757.1	430_1	yes	1	2	1	1	2
LMSG_G000010039.1	430_1	no		2	1		3
LMSG_G000010040.1	430_1	no	1	2	1	1	2
LMSG_G000005208.1	431_0	yes	1	1	1		2
LMSG_G000005925.1	432_0	yes		2	1		4
LMSG_G000010041.1	433_1	no		2			1
LMSG_G000005069.1	433_1	yes		3	1		3
LMSG_G000009983.1	434_1	no	1	1	2		2
LMSG_G000005243.1	434_1	yes	1	3	1		2
LMSG_G000010042.1	434_1	no		2	1		2
LMSG_G000010043.1	435_1	no		4	1	1	2
LMSG_G000009984.1	435_1	no	1	1			
LMSG_G000010053.1	435_1	no	1	2	1	1	1
LMSG_G000010044.1	435_1	no		4	1	1	2
LMSG_G000010045.1	435_1	no	1	4	1	1	2
LMSG_G000010046.1	435_1	no		3	1	1	2
LMSG_G000010047.1	435_1	no		4	1	1	2
LMSG_G000010054.1	435_1	no		4		1	2
LMSG_G000010048.1	435_1	no		3	1	1	1
LMSG_G000010050.1	435_1	no		4	1	1	1
LMSG_G000010055.1	435_1	no		4	1	1	2
LMSG_G000009985.1	435_1	no		3	1	1	2
LMSG_G000009986.1	435_1	no		4	1	1	2
LMSG_G000010049.1	435_1	no		3		1	1
LMSG_G000010056.1	435_1	no	1	2	1	1	2
LMSG_G000004520.1	435_1	yes		4	1	1	2
LMSG_G000010057.1	435_1	no		2		1	2
LMSG_G000010058.1	435_1	no	1	2	2	1	2
LMSG_G000010051.1	435_1	no		4	1	1	2
LMSG_G000010060.1	436_1	no		2			1
LMSG_G000010059.1	436_1	no	1	4			2
LMSG_G000009967.1	436_1	no	1	2			1
LMSG_G000004484.1	436_1	yes	1	3		1	2
LMSG_G000009968.1	436_1	no	1	3			2
LMSG_G000004390.1	437_1	yes		3			2
LMSG_G000010061.1	437_1	no	1	2			1
LMSG_G000009987.1	437_1	no	1	3			2
LMSG_G000009988.1	437_1	no		2			2
LMSG_G000009989.1	437_1	no	1	3			1
LMSG_G000009969.1	437_1	no		4			2
LMSG_G000009990.1	437_1	no		2			1
LMSG_G000005631.1	437_2	yes	1	2			2
LMSG_G000010062.1	437_2	no	1	3			1
LMSG_G000009970.1	437_2	no	1	3			1
LMSG_G000009971.1	438_1	no		1			
LMSG_G000010068.1	438_1	no					1
LMSG_G000010066.1	438_1	no				1	1
LMSG_G000004465.1	438_1	yes		1		1	2
LMSG_G000010063.1	438_1	no		2			1
LMSG_G000010064.1	438_1	no		3		1	2
LMSG_G000010072.1	439_1	no		2			2
LMSG_G000004548.1	439_1	yes		2			2
LMSG_G000010071.1	439_1	no	1	2			
LMSG_G000010069.1	439_1	no	1	2			2
LMSG_G000010070.1	439_1	no	1	2			1
LMSG_G000005912.1	440_1	yes	1	3			2
LMSG_G000010073.1	440_1	no		3			2
LMSG_G000010076.1	441_1	no		1			1
LMSG_G000004569.1	441_1	yes		3			2
LMSG_G000010074.1	441_1	no		3			2
LMSG_G000010075.1	441_1	no		3			2
LMSG_G000005936.1	441_2	yes		5			2
LMSG_G000005257.1	442_0	yes			1		
LMSG_G000005107.1	443_1	yes		1		1	
LMSG_G000008927.1	443_1	no				1	
LMSG_G000008928.1	443_1	no		1		1	
LMSG_G000005475.1	444_1	yes	1				1
LMSG_G000009972.1	446_1	no		1			
LMSG_G000005417.1	446_1	yes		2			2
LMSG_G000005092.1	447_0	yes	1				1
LMSG_G000005878.1	448_0	yes					1
LMSG_G000006138.1	449_1	yes	3	1			1
LMSG_G000008925.1	449_1	no	6	1			1
LMSG_G000008926.1	449_1	no	2				
LMSG_G000004335.1	450_0	yes	1		1		2

LMSG_G000005350.1	451_0	yes						1
LMSG_G000007958.1	452_1	no		1				1
LMSG_G000007964.1	452_1	no						1
LMSG_G000007965.1	452_1	no		1				1
LMSG_G000007966.1	452_1	no						1
LMSG_G000007967.1	452_1	no						1
LMSG_G000007959.1	452_1	no		1				1
LMSG_G000007960.1	452_1	no		1				1
LMSG_G000007961.1	452_1	no		1				1
LMSG_G000004564.1	452_1	yes		1				1
LMSG_G000007962.1	452_1	no		1				1
LMSG_G000007963.1	452_1	no		1				1
LMSG_G000004522.1	453_1	yes					1	
LMSG_G000007993.1	454_1	no					1	
LMSG_G000007994.1	454_1	no					1	
LMSG_G000008001.1	454_1	no					1	
LMSG_G000008002.1	454_1	no					1	
LMSG_G000008003.1	454_1	no					1	
LMSG_G000008004.1	454_1	no					1	
LMSG_G000008009.1	454_1	no					2	
LMSG_G000007991.1	454_1	no					2	
LMSG_G000007989.1	454_1	no					2	
LMSG_G000007981.1	454_1	no					2	
LMSG_G000007982.1	454_1	no					1	
LMSG_G000007974.1	454_1	no					1	
LMSG_G000007992.1	454_1	no					2	
LMSG_G000007975.1	454_1	no					2	
LMSG_G000007976.1	454_1	no					1	
LMSG_G000007977.1	454_1	no					1	
LMSG_G000007978.1	454_1	no					1	
LMSG_G000007973.1	454_1	no					1	
LMSG_G000007979.1	454_1	no					1	
LMSG_G000008007.1	454_1	no					2	
LMSG_G000008005.1	454_1	no					1	
LMSG_G000008006.1	454_1	no					1	
LMSG_G000008000.1	454_1	no					2	
LMSG_G000007983.1	454_1	no		1			2	
LMSG_G000008008.1	454_1	no					1	
LMSG_G000007999.1	454_1	no					1	
LMSG_G000007995.1	454_1	no					1	
LMSG_G000005752.1	454_1	yes					1	
LMSG_G000007996.1	454_1	no					1	
LMSG_G000007997.1	454_1	no					2	
LMSG_G000007998.1	454_1	no					2	
LMSG_G000007985.1	454_1	no					2	
LMSG_G000007986.1	454_1	no					2	
LMSG_G000007987.1	454_1	no					1	
LMSG_G000007980.1	454_1	no					1	
LMSG_G000007988.1	454_1	no					1	
LMSG_G000007990.1	454_1	no					2	
LMSG_G000005694.1	454_2	yes					2	
LMSG_G000008010.1	456_1	no		2			1	
LMSG_G000008011.1	456_1	no		2			3	
LMSG_G000008012.1	456_1	no		1			2	
LMSG_G000008013.1	456_1	no		2			1	
LMSG_G000008014.1	456_1	no		2			2	
LMSG_G000008015.1	456_1	no		2			1	
LMSG_G000005173.1	456_1	yes		2			2	
LMSG_G000008016.1	456_1	no		2			2	
LMSG_G000008017.1	456_1	no		2			2	
LMSG_G000008018.1	456_1	no		2			2	
LMSG_G000008019.1	456_1	no		2			1	
LMSG_G000008020.1	456_1	no		2			1	
LMSG_G000008021.1	456_1	no		2			1	
LMSG_G000008022.1	456_1	no		2			1	
LMSG_G000008023.1	456_1	no		1				
LMSG_G000004779.1	456_2	yes		2				
LMSG_G000006042.1	458_0	yes				1		1
LMSG_G000008971.1	462_1	no	1					
LMSG_G000005894.1	462_1	yes	1					
LMSG_G000005454.1	463_1	yes	2	2	1			
LMSG_G000009081.1	463_1	no		1				
LMSG_G000009308.1	464_1	no					1	1
LMSG_G000005576.1	464_1	yes					1	1
LMSG_G000006265.1	466_0	yes	2					
LMSG_G000005349.1	467_0	yes		2			1	
LMSG_G000009333.1	468_1	no	1	1		1		

LMSG_G000005899.1	468_1	yes		3	1	1	1	1
LMSG_G000006230.1	469_0	yes					1	2
LMSG_G000005862.1	470_0	yes						1
LMSG_G000009298.1	471_1	no		2				
LMSG_G000004403.1	471_1	yes		2				
LMSG_G000004444.1	472_1	yes		1	1			
LMSG_G000006227.1	472_2	yes		2				
LMSG_G000005315.1	473_1	yes		1				
LMSG_G000009300.1	474_1	no		1				
LMSG_G000005426.1	474_1	yes		1				
LMSG_G000009328.1	475_1	no						1
LMSG_G000009331.1	476_1	no		2			1	
LMSG_G000006224.1	476_1	yes		2				
LMSG_G000005877.1	477_0	yes		2			1	
LMSG_G000009302.1	478_1	no		2				
LMSG_G000009301.1	478_1	no		2				
LMSG_G000004447.1	478_1	yes		2				
LMSG_G000005005.1	478_2	yes		2				
LMSG_G000005965.1	479_0	yes		2				1
LMSG_G000005106.1	480_1	yes		2				
LMSG_G000005756.1	482_1	yes					1	
LMSG_G000009348.1	482_1	no					1	
LMSG_G000009305.1	486_1	no						1
LMSG_G000009307.1	486_1	no						1
LMSG_G000009306.1	486_1	no						1
LMSG_G000009297.1	486_1	no						1
LMSG_G000004478.1	486_1	yes						1
LMSG_G000005213.1	486_2	yes						1
LMSG_G000005204.1	487_0	yes		1				
LMSG_G000004527.1	488_0	yes				1		1
LMSG_G000009310.1	489_1	no						1
LMSG_G000005650.1	489_1	yes	1					
LMSG_G000005647.1	490_0	yes		1			1	
LMSG_G000005582.1	491_0	yes		1				
LMSG_G000004784.1	493_1	yes	1				1	
LMSG_G000008748.1	493_1	no					1	
LMSG_G000004684.1	494_0	yes		1		1		3
LMSG_G000004577.1	496_1	yes	2	1	2			3
LMSG_G000010100.1	496_1	no	2	1	1			3
LMSG_G000004401.1	497_0	yes		2		1	3	
LMSG_G000004463.1	498_1	yes	1			1	2	4
LMSG_G000010101.1	498_1	no					1	
LMSG_G000010102.1	498_1	no	1			1	2	3
LMSG_G000010103.1	498_1	no	1			1	2	3
LMSG_G000004469.1	499_0	yes	2			1	1	1
LMSG_G000006497.1	50_1	no						1
LMSG_G000006498.1	50_1	no						1
LMSG_G000006499.1	50_1	no						1
LMSG_G000006500.1	50_1	no						1
LMSG_G000006201.1	50_1	yes						1
LMSG_G000009082.1	502_1	no			1			2
LMSG_G000005400.1	502_1	yes			1			2
LMSG_G000009097.1	503_1	no		4				
LMSG_G000009088.1	503_1	no		3			1	1
LMSG_G000009083.1	503_1	no		5			1	1
LMSG_G000009084.1	503_1	no		2				1
LMSG_G000009089.1	503_1	no		2			1	
LMSG_G000009090.1	503_1	no		4			1	1
LMSG_G000004534.1	503_1	yes		4			1	1
LMSG_G000009085.1	503_1	no		3			1	1
LMSG_G000009091.1	503_1	no		2				
LMSG_G000009092.1	503_1	no		3				
LMSG_G000009086.1	503_1	no		2			1	1
LMSG_G000009093.1	503_1	no		3			1	1
LMSG_G000009094.1	503_1	no		5				
LMSG_G000009095.1	503_1	no		4			1	1
LMSG_G000009087.1	503_1	no		5				1
LMSG_G000009096.1	503_1	no		5			1	
LMSG_G000005522.1	504_0	yes		1			1	1
LMSG_G000005336.1	505_0	yes		1				1
LMSG_G000008333.1	506_1	no						1
LMSG_G000008331.1	506_1	no						2
LMSG_G000005932.1	506_1	yes						1
LMSG_G000004345.1	507_0	yes	1					2
LMSG_G000005552.1	509_1	yes						1
LMSG_G000005926.1	5_0	yes					1	
LMSG_G000005275.1	512_1	yes						1

LMSG_G000011192.1	512_1	no							1
LMSG_G000011193.1	512_1	no							1
LMSG_G000005848.1	513_1	yes		1					
LMSG_G000004894.1	515_1	yes		1				1	2
LMSG_G000009214.1	515_1	no		1				1	2
LMSG_G000009215.1	516_1	no					1	1	2
LMSG_G000005714.1	516_1	yes						1	2
LMSG_G000009219.1	516_2	no					1		
LMSG_G000009216.1	516_2	no		1					2
LMSG_G000009217.1	516_2	no		1					2
LMSG_G000004435.1	516_2	yes		1					2
LMSG_G000009218.1	516_2	no		1					2
LMSG_G000009220.1	517_1	no		1					2
LMSG_G000009221.1	517_1	no		1					2
LMSG_G000009224.1	517_1	no							1
LMSG_G000004446.1	517_1	yes		1					2
LMSG_G000009222.1	517_1	no		1					2
LMSG_G000004887.1	518_1	yes		1					2
LMSG_G000009223.1	518_1	no		1					2
LMSG_G000005955.1	519_0	yes							1
LMSG_G000008970.1	520_1	no					1		1
LMSG_G000004492.1	520_1	yes					1		1
LMSG_G000006502.1	52_1	no						1	
LMSG_G000006504.1	52_1	no						1	
LMSG_G000006505.1	52_1	no						1	
LMSG_G000005796.1	52_1	yes						1	
LMSG_G000006506.1	52_1	no						1	
LMSG_G000006507.1	52_1	no						1	
LMSG_G000006509.1	52_1	no						1	
LMSG_G000005798.1	523_1	yes		1					1
LMSG_G000005836.1	523_2	yes							1
LMSG_G000005844.1	525_0	yes							1
LMSG_G000008856.1	526_1	no							1
LMSG_G000008858.1	526_1	no							1
LMSG_G000005516.1	526_1	yes							1
LMSG_G000005914.1	527_0	yes					1		
LMSG_G000010080.1	528_1	no	1	1	2		1		2
LMSG_G000005233.1	528_1	yes		1			2		3
LMSG_G000010079.1	528_1	no	1	1	1		2		3
LMSG_G000010081.1	528_1	no							1
LMSG_G000010085.1	528_2	no	1	1			2		3
LMSG_G000005327.1	528_2	yes	1	2			3		2
LMSG_G000010082.1	528_2	no	1	2			2		3
LMSG_G000010083.1	528_2	no	1	2			2		2
LMSG_G000010084.1	528_2	no	1	2			1		2
LMSG_G000010329.1	530_1	no	1						
LMSG_G000010330.1	530_1	no	1						
LMSG_G000010331.1	530_1	no			1				
LMSG_G000010325.1	530_1	no	1		1				
LMSG_G000004452.1	530_1	yes	1		1				
LMSG_G000010326.1	530_1	no	1		1				
LMSG_G000010327.1	530_1	no	1		1				
LMSG_G000010332.1	530_1	no	1		1				
LMSG_G000010328.1	530_1	no	1		1				
LMSG_G000006067.1	534_1	yes				2	1		1
LMSG_G000008813.1	534_1	no	1			2	1		1
LMSG_G000008777.1	535_1	no	1	1			1		
LMSG_G000008778.1	535_1	no	1	1				1	
LMSG_G000005374.1	535_1	yes	1	1					
LMSG_G000008779.1	535_1	no						1	
LMSG_G000008785.1	536_1	no	2	3				1	
LMSG_G000008789.1	536_1	no	1					1	
LMSG_G000008786.1	536_1	no	2					1	
LMSG_G000008784.1	536_1	no	2					1	
LMSG_G000006237.1	536_1	yes	2					1	
LMSG_G000008781.1	536_1	no	1					1	
LMSG_G000008782.1	536_1	no	2					1	
LMSG_G000008783.1	536_1	no	2					1	
LMSG_G000008788.1	536_1	no	1					1	
LMSG_G000008790.1	536_1	no	2					1	
LMSG_G000005027.1	537_0	yes		1					1
LMSG_G000004722.1	538_0	yes		1					2
LMSG_G000011113.1	571_1	no		1					1
LMSG_G000011114.1	571_1	no		1					
LMSG_G000011115.1	571_1	no							1
LMSG_G000011116.1	571_1	no		1					1
LMSG_G000011118.1	571_1	no		1					

LMSG_G000011119.1	571_1	no		1			1
LMSG_G000011120.1	571_1	no		1		1	1
LMSG_G000011121.1	571_1	no		1			1
LMSG_G000004573.1	571_1	yes		1			1
LMSG_G000011122.1	572_1	no		1			1
LMSG_G000011123.1	572_1	no		1			1
LMSG_G000011124.1	572_1	no		1			1
LMSG_G000006247.1	572_1	yes		1			1
LMSG_G000011125.1	572_1	no		1			1
LMSG_G000011126.1	572_1	no		1			
LMSG_G000004374.1	573_0	yes		1			1
LMSG_G000011127.1	574_1	no		1			1
LMSG_G000011129.1	574_1	no		1			1
LMSG_G000011130.1	574_1	no		1			1
LMSG_G000011131.1	574_1	no		1			1
LMSG_G000011132.1	574_1	no					1
LMSG_G000011128.1	574_1	no					1
LMSG_G000004434.1	574_1	yes		1			1
LMSG_G000011133.1	574_1	no		1			1
LMSG_G000004619.1	575_1	yes		1			1
LMSG_G000005534.1	578_0	yes	3				
LMSG_G000006097.1	579_0	yes		1	1		
LMSG_G000006096.1	581_0	yes		1		1	
LMSG_G000004637.1	583_1	yes		4		1	1
LMSG_G000010372.1	583_1	no		3		1	1
LMSG_G000005017.1	584_0	yes	1	1			2
LMSG_G000004358.1	595_1	yes				1	
LMSG_G000006258.1	596_0	yes	1				
LMSG_G000008029.1	599_1	no				2	
LMSG_G000008024.1	599_1	no				1	
LMSG_G000008026.1	599_1	no				3	
LMSG_G000008032.1	599_1	no				2	
LMSG_G000005679.1	599_1	yes				2	
LMSG_G000008033.1	599_1	no				1	
LMSG_G000008040.1	599_1	no				1	
LMSG_G000008034.1	599_1	no				1	
LMSG_G000008028.1	599_1	no				1	
LMSG_G000008035.1	599_1	no				2	
LMSG_G000008036.1	599_1	no				2	
LMSG_G000008037.1	599_1	no				2	
LMSG_G000008038.1	599_1	no				2	
LMSG_G000008039.1	599_1	no				2	
LMSG_G000008122.1	600_1	no					1
LMSG_G000004354.1	600_1	yes					1
LMSG_G000008135.1	600_1	no					1
LMSG_G000008118.1	600_1	no					1
LMSG_G000008114.1	600_1	no		1			1
LMSG_G000008115.1	600_1	no					1
LMSG_G000008136.1	600_1	no					1
LMSG_G000008119.1	600_1	no					1
LMSG_G000008123.1	600_1	no					2
LMSG_G000008124.1	600_1	no					1
LMSG_G000008134.1	600_1	no		1			1
LMSG_G000008121.1	600_1	no					1
LMSG_G000008126.1	600_1	no					1
LMSG_G000008116.1	600_1	no					1
LMSG_G000008113.1	600_1	no		1			1
LMSG_G000008117.1	600_1	no					1
LMSG_G000008127.1	600_1	no					1
LMSG_G000008129.1	600_1	no					1
LMSG_G000008130.1	600_1	no					1
LMSG_G000008131.1	600_1	no					1
LMSG_G000008132.1	600_1	no					1
LMSG_G000008133.1	600_1	no					1
LMSG_G000008120.1	600_1	no					1
LMSG_G000008138.1	601_1	no					1
LMSG_G000008139.1	601_1	no					1
LMSG_G000008140.1	601_1	no					2
LMSG_G000008141.1	601_1	no					1
LMSG_G000008137.1	601_1	no					1
LMSG_G000008142.1	601_1	no					2
LMSG_G000008144.1	601_1	no					1
LMSG_G000006236.1	601_1	yes					1
LMSG_G000008145.1	601_1	no					1
LMSG_G000008147.1	601_1	no					1
LMSG_G000005152.1	602_1	yes	1			1	1
LMSG_G000010442.1	602_1	no	1			1	1

LMSG_G000010445.1	602_1	no	1		1	1
LMSG_G000010446.1	602_1	no	1		1	1
LMSG_G000010447.1	602_1	no	1			1
LMSG_G000010448.1	602_1	no	1		1	1
LMSG_G000010449.1	602_1	no	1		1	1
LMSG_G000010443.1	602_1	no	1	1	3	1
LMSG_G000010444.1	602_1	no	1		1	1
LMSG_G000008156.1	603_1	no				1
LMSG_G000008157.1	603_1	no				1
LMSG_G000008158.1	603_1	no				1
LMSG_G000008164.1	603_1	no				1
LMSG_G000008159.1	603_1	no				1
LMSG_G000008160.1	603_1	no				1
LMSG_G000008153.1	603_1	no				1
LMSG_G000008151.1	603_1	no				1
LMSG_G000008149.1	603_1	no				1
LMSG_G000008154.1	603_1	no				1
LMSG_G000008150.1	603_1	no				1
LMSG_G000008152.1	603_1	no				1
LMSG_G000008161.1	603_1	no				1
LMSG_G000004475.1	603_1	yes				1
LMSG_G000008162.1	603_1	no				1
LMSG_G000008166.1	603_1	no				1
LMSG_G000008163.1	603_1	no				1
LMSG_G000008165.1	603_1	no				1
LMSG_G000008155.1	603_1	no				1
LMSG_G000006214.1	604_1	yes				1
LMSG_G000008213.1	604_1	no				1
LMSG_G000004381.1	605_1	yes				1
LMSG_G000008214.1	605_1	no				1
LMSG_G000008215.1	605_1	no				1
LMSG_G000008216.1	605_1	no				1
LMSG_G000004648.1	605_2	yes				1
LMSG_G000008217.1	606_1	no				1
LMSG_G000008218.1	606_1	no				1
LMSG_G000008219.1	606_1	no				1
LMSG_G000008220.1	606_1	no				1
LMSG_G000008221.1	606_1	no				1
LMSG_G000008222.1	606_1	no				1
LMSG_G000008223.1	606_1	no				2
LMSG_G000008224.1	606_1	no				1
LMSG_G000008225.1	606_1	no				1
LMSG_G000008226.1	606_1	no				1
LMSG_G000008227.1	606_1	no				1
LMSG_G000008228.1	606_1	no				1
LMSG_G000008229.1	606_1	no				1
LMSG_G000008231.1	606_1	no				1
LMSG_G000005605.1	606_1	yes				1
LMSG_G000008232.1	606_1	no				1
LMSG_G000008233.1	606_1	no				1
LMSG_G000008234.1	606_1	no				1
LMSG_G000008235.1	606_1	no				1
LMSG_G000008236.1	606_1	no		1		1
LMSG_G000008237.1	606_1	no				1
LMSG_G000008238.1	606_1	no				1
LMSG_G000008239.1	606_1	no				1
LMSG_G000008240.1	606_1	no				1
LMSG_G000008241.1	606_1	no				1
LMSG_G000008242.1	606_1	no				1
LMSG_G000008259.1	607_1	no				1
LMSG_G000008260.1	607_1	no				1
LMSG_G000008255.1	607_1	no				1
LMSG_G000008261.1	607_1	no				1
LMSG_G000008256.1	607_1	no				1
LMSG_G000008262.1	607_1	no				1
LMSG_G000008263.1	607_1	no				1
LMSG_G000008264.1	607_1	no				1
LMSG_G000008287.1	607_1	no				1
LMSG_G000008265.1	607_1	no				1
LMSG_G000008257.1	607_1	no				1
LMSG_G000008266.1	607_1	no				1
LMSG_G000008267.1	607_1	no				1
LMSG_G000008268.1	607_1	no				1
LMSG_G000008269.1	607_1	no				1
LMSG_G000008270.1	607_1	no				1
LMSG_G000008271.1	607_1	no				1
LMSG_G000008273.1	607_1	no				1

LMSG_G000008288.1	607_1	no					1
LMSG_G000008274.1	607_1	no					1
LMSG_G000008275.1	607_1	no					1
LMSG_G000008258.1	607_1	no					1
LMSG_G000005638.1	607_1	yes					1
LMSG_G000008276.1	607_1	no					1
LMSG_G000008277.1	607_1	no					1
LMSG_G000008289.1	607_1	no					1
LMSG_G000008278.1	607_1	no					1
LMSG_G000008279.1	607_1	no					1
LMSG_G000008280.1	607_1	no					1
LMSG_G000008281.1	607_1	no					1
LMSG_G000008282.1	607_1	no					1
LMSG_G000008283.1	607_1	no					1
LMSG_G000008243.1	607_1	no					1
LMSG_G000008284.1	607_1	no					1
LMSG_G000008285.1	607_1	no					1
LMSG_G000008286.1	607_1	no					1
LMSG_G000006118.1	608_0	yes					1
LMSG_G000008306.1	609_1	no	1				1
LMSG_G000008304.1	609_1	no	1				1
LMSG_G000008305.1	609_1	no	1	1			1
LMSG_G000005035.1	609_1	yes	1	1			1
LMSG_G000008307.1	609_1	no	1				
LMSG_G000004987.1	610_1	yes		1			1
LMSG_G000008310.1	610_2	no					1
LMSG_G000005156.1	611_1	yes	1	2			2
LMSG_G000008313.1	611_1	no	1	2			2
LMSG_G000008311.1	611_1	no	1	1			2
LMSG_G000008312.1	611_1	no	1	1			2
LMSG_G000008314.1	611_1	no	1	1			2
LMSG_G000008316.1	612_1	no					1
LMSG_G000005729.1	612_1	yes		1			1
LMSG_G000008317.1	613_1	no	1				1
LMSG_G000005804.1	613_1	yes	1				1
LMSG_G000008318.1	613_1	no	1				1
LMSG_G000005915.1	613_2	yes		1			
LMSG_G000008320.1	616_1	no		1			1
LMSG_G000008321.1	616_1	no					1
LMSG_G000005168.1	616_1	yes		1			1
LMSG_G000008322.1	616_1	no		1			1
LMSG_G000008323.1	616_1	no		1			1
LMSG_G000008324.1	616_1	no					1
LMSG_G000008325.1	616_1	no					1
LMSG_G000008326.1	616_1	no					1
LMSG_G000008330.1	616_1	no					1
LMSG_G000008327.1	616_1	no					1
LMSG_G000008319.1	616_1	no					1
LMSG_G000008328.1	616_1	no		1			1
LMSG_G000008329.1	616_1	no					1
LMSG_G000005563.1	617_1	yes			1		
LMSG_G000008479.1	617_1	no				1	
LMSG_G000005301.1	618_1	yes		1			
LMSG_G000006277.1	619_0	yes		1	1	2	
LMSG_G000004550.1	621_1	yes	1			1	1
LMSG_G000009141.1	621_1	no	1			1	2
LMSG_G000006126.1	622_0	yes	1				
LMSG_G000004377.1	623_1	yes				1	2
LMSG_G000009146.1	623_1	no					1
LMSG_G000009144.1	623_1	no				1	2
LMSG_G000009142.1	623_1	no				1	2
LMSG_G000009143.1	623_1	no				1	2
LMSG_G000009145.1	623_1	no				1	2
LMSG_G000009147.1	623_1	no				1	3
LMSG_G000009148.1	623_1	no	1				1
LMSG_G000009154.1	624_1	no				1	
LMSG_G000009155.1	624_1	no				1	1
LMSG_G000009156.1	624_1	no				1	1
LMSG_G000004431.1	624_1	yes				1	1
LMSG_G000009151.1	624_1	no				1	1
LMSG_G000009152.1	624_1	no				1	1
LMSG_G000009157.1	624_1	no				1	1
LMSG_G000009158.1	624_1	no				1	1
LMSG_G000009159.1	624_1	no					2
LMSG_G000009160.1	624_1	no				1	1
LMSG_G000009161.1	624_1	no				1	1
LMSG_G000009162.1	624_1	no				1	

LMSG_G000009163.1	624_1	no			1		1
LMSG_G000009153.1	624_1	no			1		1
LMSG_G000009164.1	624_1	no			1		1
LMSG_G000009165.1	624_1	no			1		
LMSG_G000004953.1	625_0	yes			1		
LMSG_G000005584.1	626_0	yes		2			1
LMSG_G000009166.1	627_1	no		1	1		2
LMSG_G000009167.1	627_1	no		1	1		2
LMSG_G000004622.1	627_1	yes		1	1		2
LMSG_G000009168.1	627_1	no			1		2
LMSG_G000009170.1	628_1	no					1
LMSG_G000009171.1	628_1	no	1				1
LMSG_G000004647.1	628_1	yes					2
LMSG_G000009173.1	629_1	no			1		1
LMSG_G000004608.1	629_1	yes			1		1
LMSG_G000009174.1	629_1	no			1		1
LMSG_G000009172.1	629_1	no			1		1
LMSG_G000009175.1	630_1	no					1
LMSG_G000005626.1	630_1	yes					1
LMSG_G000009178.1	630_2	no					2
LMSG_G000009176.1	630_2	no					2
LMSG_G000005737.1	630_2	yes					2
LMSG_G000009177.1	630_2	no					1
LMSG_G000009179.1	631_1	no					1
LMSG_G000004474.1	631_1	yes			1		1
LMSG_G000009181.1	631_2	no			1		1
LMSG_G000005558.1	631_2	yes			1		1
LMSG_G000009180.1	631_2	no			1		1
LMSG_G000009182.1	631_2	no			1		1
LMSG_G000009186.1	632_1	no			1		
LMSG_G000009184.1	632_1	no			1		
LMSG_G000005736.1	632_1	yes			1		1
LMSG_G000009183.1	632_1	no			1		1
LMSG_G000009185.1	632_1	no					1
LMSG_G000005055.1	632_2	yes					1
LMSG_G000009189.1	632_2	no					1
LMSG_G000009187.1	632_2	no					2
LMSG_G000009188.1	632_2	no			1		
LMSG_G000009190.1	632_2	no					1
LMSG_G000004334.1	633_1	yes			1		2
LMSG_G000009260.1	633_1	no			1		2
LMSG_G000004759.1	634_0	yes		1		1	
LMSG_G000009195.1	636_1	no			1		1
LMSG_G000009196.1	636_1	no					1
LMSG_G000009199.1	636_1	no			1		2
LMSG_G000005628.1	636_1	yes			1		1
LMSG_G000009197.1	636_1	no			1		1
LMSG_G000009201.1	636_1	no					1
LMSG_G000005992.1	637_1	yes			1	1	
LMSG_G000009273.1	637_1	no			1	1	
LMSG_G000004665.1	638_0	yes				1	
LMSG_G000006010.1	639_0	yes				2	1
LMSG_G000004678.1	641_0	yes			1	1	2
LMSG_G000005619.1	642_0	yes			1		
LMSG_G000006299.1	643_0	yes		1			1
LMSG_G000009476.1	644_1	no		2			1
LMSG_G000005608.1	644_1	yes		2			
LMSG_G000005617.1	645_0	yes				2	
LMSG_G000009478.1	646_1	no		1			
LMSG_G000009479.1	646_1	no		1			
LMSG_G000009480.1	646_1	no		1			
LMSG_G000009481.1	646_1	no		1		1	
LMSG_G000005684.1	646_1	yes		1		1	
LMSG_G000005622.1	647_0	yes				1	1
LMSG_G000005734.1	649_1	yes		1	2	1	
LMSG_G000009349.1	649_1	no		1	1		
LMSG_G000005128.1	651_1	yes		1			
LMSG_G000009350.1	651_1	no		1			
LMSG_G000009351.1	651_1	no		1			
LMSG_G000009352.1	651_1	no		1			
LMSG_G000006220.1	653_1	yes		1			
LMSG_G000006219.1	654_0	yes		1	1		
LMSG_G000009355.1	655_1	no		2			
LMSG_G000005001.1	655_1	yes		2			
LMSG_G000004967.1	656_0	yes		1			
LMSG_G000004849.1	657_0	yes			1		
LMSG_G000009356.1	659_1	no	1	1			

LMSG_G000009359.1	659_1	no		1						
LMSG_G000009360.1	659_1	no		1						
LMSG_G000009357.1	659_1	no		1						
LMSG_G000009358.1	659_1	no		1						
LMSG_G000005633.1	659_1	yes		1						
LMSG_G000009362.1	659_1	no		1						
LMSG_G000005629.1	660_1	yes		1						
LMSG_G000009363.1	660_1	no		1						
LMSG_G000009365.1	661_1	no		1						
LMSG_G000009374.1	661_1	no		1						
LMSG_G000009367.1	661_1	no		1						
LMSG_G000009364.1	661_1	no		1						
LMSG_G000005641.1	661_1	yes		1						
LMSG_G000009375.1	661_1	no		1						
LMSG_G000009373.1	661_1	no		1						
LMSG_G000005373.1	662_0	yes		2	1					
LMSG_G000005093.1	664_0	yes		2			1	1		
LMSG_G000005235.1	666_0	yes	15	9	7	2	3	1		4
LMSG_G000005639.1	670_1	yes		1						
LMSG_G000008950.1	670_1	no		1						
LMSG_G000005794.1	671_0	yes		1						
LMSG_G000008951.1	672_1	no						1		1
LMSG_G000008952.1	672_1	no						1		1
LMSG_G000008953.1	672_1	no						1		1
LMSG_G000005543.1	672_1	yes						1		1
LMSG_G000004537.1	673_1	yes		1						
LMSG_G000008955.1	673_1	no		1						
LMSG_G000004425.1	676_0	yes						1		
LMSG_G000008054.1	686_1	no			1					
LMSG_G000008055.1	686_1	no			1					
LMSG_G000008053.1	686_1	no	1		1					
LMSG_G000004571.1	686_1	yes	1		1					
LMSG_G000005785.1	687_0	yes			1		1			
LMSG_G000008291.1	688_1	no								1
LMSG_G000008292.1	688_1	no								1
LMSG_G000008293.1	688_1	no								1
LMSG_G000008294.1	689_1	no								1
LMSG_G000004905.1	689_1	yes								1
LMSG_G000008295.1	689_1	no								1
LMSG_G000008296.1	689_1	no								2
LMSG_G000008297.1	689_1	no								1
LMSG_G000008298.1	689_1	no								1
LMSG_G000004893.1	690_1	yes								1
LMSG_G000008299.1	690_1	no								1
LMSG_G000008300.1	690_1	no								1
LMSG_G000008171.1	691_1	no								1
LMSG_G000005561.1	691_1	yes								1
LMSG_G000008168.1	691_1	no								1
LMSG_G000008169.1	691_1	no								1
LMSG_G000008173.1	692_1	no								1
LMSG_G000008172.1	692_1	no								1
LMSG_G000008174.1	692_1	no								1
LMSG_G000008176.1	692_1	no								1
LMSG_G000008177.1	692_1	no								1
LMSG_G000008178.1	692_1	no		1					1	1
LMSG_G000005895.1	692_1	yes								1
LMSG_G000008179.1	692_2	no		1						1
LMSG_G000008181.1	692_2	no								1
LMSG_G000006310.1	692_2	yes								1
LMSG_G000004480.1	693_0	yes								2
LMSG_G000004767.1	694_1	yes								1
LMSG_G000008301.1	694_1	no								1
LMSG_G000008302.1	694_1	no								1
LMSG_G000008303.1	694_1	no								1
LMSG_G000008184.1	695_1	no		1						1
LMSG_G000008185.1	695_1	no		1						1
LMSG_G000008187.1	695_1	no		1						1
LMSG_G000006222.1	695_1	yes		1						1
LMSG_G000008186.1	695_1	no		1						1
LMSG_G000008182.1	695_1	no		1						1
LMSG_G000008183.1	695_1	no		1						1
LMSG_G000008189.1	696_1	no								1
LMSG_G000008188.1	696_1	no		1						1
LMSG_G000008191.1	696_1	no								1
LMSG_G000005183.1	696_1	yes								1
LMSG_G000008192.1	696_1	no								1
LMSG_G000008194.1	696_1	no								1

LMSG_G000008195.1	696_1	no					1
LMSG_G000008196.1	696_1	no					1
LMSG_G000008197.1	696_1	no					1
LMSG_G000008198.1	696_1	no					1
LMSG_G000008199.1	696_1	no					1
LMSG_G000008200.1	696_1	no					1
LMSG_G000008201.1	696_1	no					1
LMSG_G000008202.1	697_1	no					1
LMSG_G000008203.1	697_1	no					1
LMSG_G000008204.1	697_1	no					1
LMSG_G000008205.1	697_1	no					1
LMSG_G000008206.1	697_1	no					1
LMSG_G000008207.1	697_1	no					1
LMSG_G000008208.1	697_1	no					1
LMSG_G000008209.1	697_1	no					1
LMSG_G000008210.1	697_1	no		1			1
LMSG_G000008211.1	697_1	no					1
LMSG_G000004654.1	697_1	yes					1
LMSG_G000008212.1	697_1	no					1
LMSG_G000008424.1	698_1	no		1		2	1
LMSG_G000008425.1	698_1	no		1		2	1
LMSG_G000004586.1	698_1	yes				2	1
LMSG_G000005676.1	699_1	yes		1		1	1
LMSG_G000008426.1	699_1	no					1
LMSG_G000008429.1	701_1	no	1	2	1		2
LMSG_G000008427.1	701_1	no	1	2	1		2
LMSG_G000008428.1	701_1	no	1	3	1		2
LMSG_G000004454.1	701_1	yes	1	3	1		2
LMSG_G000008430.1	701_1	no	1	1			1
LMSG_G000008431.1	702_1	no					1
LMSG_G000008434.1	702_1	no					1
LMSG_G000008432.1	702_1	no					1
LMSG_G000004433.1	702_1	yes					1
LMSG_G000008433.1	702_1	no					1
LMSG_G000008446.1	703_1	no					1
LMSG_G000008435.1	703_1	no					1
LMSG_G000008447.1	703_1	no					1
LMSG_G000008448.1	703_1	no				1	1
LMSG_G000008449.1	703_1	no					1
LMSG_G000008436.1	703_1	no					1
LMSG_G000008437.1	703_1	no					1
LMSG_G000008438.1	703_1	no					1
LMSG_G000004450.1	703_1	yes					1
LMSG_G000008439.1	703_1	no					1
LMSG_G000008440.1	703_1	no					1
LMSG_G000008441.1	703_1	no					2
LMSG_G000008442.1	703_1	no					1
LMSG_G000008452.1	703_1	no					1
LMSG_G000008443.1	703_1	no					1
LMSG_G000008444.1	703_1	no					1
LMSG_G000008453.1	703_1	no					1
LMSG_G000005112.1	704_0	yes				2	
LMSG_G000006252.1	705_0	yes				3	
LMSG_G000004590.1	706_0	yes		3	1	2	2
LMSG_G000004387.1	707_0	yes	1				1
LMSG_G000008057.1	708_1	no				1	1
LMSG_G000008058.1	708_1	no					2
LMSG_G000008059.1	708_1	no					2
LMSG_G000008065.1	708_1	no					1
LMSG_G000008066.1	708_1	no					1
LMSG_G000008060.1	708_1	no					2
LMSG_G000008061.1	708_1	no					2
LMSG_G000008062.1	708_1	no					2
LMSG_G000008063.1	708_1	no					2
LMSG_G000004580.1	708_1	yes					2
LMSG_G000008064.1	708_1	no					2
LMSG_G000004618.1	709_0	yes		2		1	
LMSG_G000006044.1	710_0	yes		1		2	
LMSG_G000009482.1	711_1	no			1		
LMSG_G000006028.1	711_1	yes			2		1
LMSG_G000009483.1	711_1	no					1
LMSG_G000005728.1	712_0	yes			1	1	2
LMSG_G000004613.1	713_1	yes			1		1
LMSG_G000009192.1	713_1	no			1		1
LMSG_G000009191.1	713_1	no					1
LMSG_G000006015.1	714_0	yes					1
LMSG_G000007936.1	715_1	no			1	1	1

LMSG_G000004596.1	715_1	yes				1	1	1
LMSG_G000008405.1	717_1	no						1
LMSG_G000008395.1	717_1	no						1
LMSG_G000008399.1	717_1	no						1
LMSG_G000008400.1	717_1	no						1
LMSG_G000005149.1	717_1	yes						1
LMSG_G000008401.1	717_1	no						1
LMSG_G000008396.1	717_1	no						1
LMSG_G000008402.1	717_1	no						1
LMSG_G000008397.1	717_1	no						1
LMSG_G000008403.1	717_1	no						1
LMSG_G000008404.1	717_1	no						1
LMSG_G000006029.1	718_0	yes						1
LMSG_G000004602.1	719_1	yes		2				
LMSG_G000004519.1	720_1	yes		1				1
LMSG_G000009194.1	720_1	no		1				2
LMSG_G000008454.1	721_1	no		1				1
LMSG_G000008455.1	721_1	no		2				2
LMSG_G000004451.1	721_1	yes		1				2
LMSG_G000008456.1	721_1	no		2				2
LMSG_G000008457.1	721_1	no		2				1
LMSG_G000008459.1	722_1	no		2				2
LMSG_G000008460.1	722_1	no		1				1
LMSG_G000005244.1	722_1	yes		2				2
LMSG_G000008458.1	722_1	no		2				2
LMSG_G000008461.1	722_1	no						2
LMSG_G000008463.1	723_1	no		1				1
LMSG_G000008464.1	723_1	no	1	1			1	1
LMSG_G000004439.1	723_1	yes		1			1	1
LMSG_G000008462.1	723_1	no	1	1			1	1
LMSG_G000004552.1	724_1	yes		2				1
LMSG_G000008465.1	724_1	no		3				1
LMSG_G000008466.1	724_1	no		2				1
LMSG_G000008467.1	724_1	no		3				1
LMSG_G000008468.1	725_1	no		3				1
LMSG_G000008469.1	725_1	no		3				1
LMSG_G000008470.1	725_1	no		3				1
LMSG_G000004481.1	725_1	yes		3				1
LMSG_G000005680.1	725_2	yes	1	2				1
LMSG_G000005148.1	726_1	yes		1	2			1
LMSG_G000008674.1	726_1	no		2	2		1	1
LMSG_G000008675.1	726_1	no		1	1			1
LMSG_G000008676.1	726_1	no		1	2			1
LMSG_G000008677.1	726_1	no		2	1			1
LMSG_G000008678.1	726_1	no			2			1
LMSG_G000008679.1	726_1	no		1	2			
LMSG_G000004918.1	726_2	yes		1	2		1	1
LMSG_G000005612.1	727_0	yes		2	1			1
LMSG_G000008680.1	728_1	no		3			1	
LMSG_G000008681.1	728_1	no		2			1	1
LMSG_G000005661.1	728_1	yes		3			1	1
LMSG_G000008682.1	728_1	no		1			1	1
LMSG_G000008683.1	729_1	no	1	2			1	1
LMSG_G000008687.1	729_1	no	1	2			1	1
LMSG_G000004383.1	729_1	yes	1	2			1	1
LMSG_G000008688.1	729_1	no	1	1			1	1
LMSG_G000008684.1	729_1	no	1	2			1	1
LMSG_G000008685.1	729_1	no	1	2			1	1
LMSG_G000008686.1	729_1	no	1	2			1	1
LMSG_G000008689.1	729_1	no	2				1	
LMSG_G000008690.1	729_1	no	1	1			1	1
LMSG_G000005721.1	730_1	yes	2	1				2
LMSG_G000006020.1	730_2	yes	1					1
LMSG_G000005998.1	731_1	yes	1				1	1
LMSG_G000008562.1	731_1	no	1				1	1
LMSG_G000006671.1	73_1	no		1				
LMSG_G000006018.1	732_1	yes	1	1			1	2
LMSG_G000008563.1	732_1	no	1	1			1	1
LMSG_G000008561.1	733_1	no	1	3				
LMSG_G000006032.1	733_1	yes	1	3				
LMSG_G000005993.1	734_0	yes					2	
LMSG_G000008481.1	735_1	no					1	1
LMSG_G000008482.1	735_1	no		1				1
LMSG_G000004999.1	735_1	yes		1			1	1
LMSG_G000008483.1	735_1	no					1	1
LMSG_G000008484.1	735_1	no					1	1
LMSG_G000008485.1	735_1	no					1	1

LMSG_G000008486.1	735_1	no	1				1	1
LMSG_G000008487.1	735_1	no		1			1	
LMSG_G000008480.1	735_1	no						1
LMSG_G000008488.1	735_1	no		1			1	2
LMSG_G000008489.1	735_1	no						1
LMSG_G000008490.1	735_1	no						1
LMSG_G000008491.1	735_1	no					1	1
LMSG_G000008492.1	735_1	no		1				1
LMSG_G000008493.1	735_1	no		1			1	1
LMSG_G000008499.1	736_1	no					1	1
LMSG_G000008500.1	736_1	no					1	1
LMSG_G000008501.1	736_1	no					2	1
LMSG_G000008502.1	736_1	no		1			1	1
LMSG_G000008496.1	736_1	no					2	1
LMSG_G000008497.1	736_1	no					1	1
LMSG_G000008495.1	736_1	no		1			1	1
LMSG_G000008510.1	736_1	no					1	1
LMSG_G000008498.1	736_1	no					1	1
LMSG_G000008503.1	736_1	no					1	1
LMSG_G000008504.1	736_1	no					1	1
LMSG_G000008505.1	736_1	no					1	1
LMSG_G000008506.1	736_1	no						1
LMSG_G000008507.1	736_1	no					1	2
LMSG_G000008508.1	736_1	no		1			1	1
LMSG_G000008509.1	736_1	no		1			1	1
LMSG_G000005758.1	736_1	yes					1	1
LMSG_G000008511.1	736_1	no		1			1	1
LMSG_G000008515.1	737_1	no					1	1
LMSG_G000008512.1	737_1	no						1
LMSG_G000004432.1	737_1	yes					1	1
LMSG_G000008516.1	737_1	no						1
LMSG_G000008513.1	737_1	no					1	1
LMSG_G000008517.1	737_1	no						1
LMSG_G000008518.1	737_1	no						1
LMSG_G000008519.1	737_1	no						1
LMSG_G000008514.1	737_1	no						1
LMSG_G000006128.1	738_0	yes						1
LMSG_G000008521.1	739_1	no			1			
LMSG_G000005911.1	739_1	yes		1		1		
LMSG_G000008524.1	740_1	no				1	1	
LMSG_G000008526.1	740_1	no		1			1	
LMSG_G000008522.1	740_1	no				1	1	
LMSG_G000005620.1	740_1	yes		2		1	1	
LMSG_G000008525.1	740_1	no				1		
LMSG_G000008523.1	740_1	no		2				
LMSG_G000008407.1	741_1	no	1					
LMSG_G000008406.1	741_1	no	1					
LMSG_G000006271.1	741_1	yes	1					1
LMSG_G000004802.1	742_1	yes						1
LMSG_G000008408.1	742_1	no						1
LMSG_G000008409.1	743_1	no		1				1
LMSG_G000008410.1	743_1	no		1				1
LMSG_G000006289.1	743_1	yes		1				1
LMSG_G000008411.1	743_1	no					1	
LMSG_G000005643.1	743_2	yes						1
LMSG_G000008412.1	743_2	no						1
LMSG_G000008414.1	744_1	no					2	1
LMSG_G000008413.1	744_1	no					2	1
LMSG_G000005586.1	744_1	yes					2	1
LMSG_G000008418.1	745_1	no		1			1	1
LMSG_G000008415.1	745_1	no		1				1
LMSG_G000008420.1	745_1	no						1
LMSG_G000008421.1	745_1	no						1
LMSG_G000004551.1	745_1	yes		1				1
LMSG_G000008416.1	745_1	no		1				
LMSG_G000008417.1	745_1	no					1	1
LMSG_G000008422.1	745_1	no						1
LMSG_G000008423.1	745_1	no						1
LMSG_G000005730.1	748_0	yes						1
LMSG_G000004871.1	749_1	yes		1				1
LMSG_G000008472.1	749_1	no						1
LMSG_G000008473.1	749_2	no					1	1
LMSG_G000008474.1	749_2	no				1	2	1
LMSG_G000005590.1	749_2	yes					1	1
LMSG_G000008475.1	750_1	no		1				1
LMSG_G000008476.1	750_1	no		1				3
LMSG_G000008477.1	750_1	no		1				1

LMSG_G000006315.1	750_1	yes		1		1		2
LMSG_G000008478.1	750_1	no						1
LMSG_G000005630.1	751_0	yes				1		1
LMSG_G000005599.1	752_0	yes						1
LMSG_G000006273.1	753_1	yes		1				
LMSG_G000008565.1	753_1	no		1				
LMSG_G000008347.1	754_1	no					1	2
LMSG_G000008348.1	754_1	no		1		1	1	2
LMSG_G000008349.1	754_1	no		2			1	1
LMSG_G000008350.1	754_1	no		1		1	1	1
LMSG_G000008351.1	754_1	no		1		1	1	1
LMSG_G000004412.1	754_1	yes		1		1	1	
LMSG_G000008352.1	754_1	no		1		1	1	2
LMSG_G000008334.1	754_1	no		1		1	1	1
LMSG_G000008335.1	754_1	no				1	1	
LMSG_G000008340.1	754_1	no				1	1	
LMSG_G000008336.1	754_1	no		1		1	1	1
LMSG_G000008341.1	754_1	no		2		1	1	1
LMSG_G000008342.1	754_1	no		2				
LMSG_G000008343.1	754_1	no				1	1	
LMSG_G000008345.1	754_1	no				1		1
LMSG_G000008344.1	754_1	no		2		1	1	1
LMSG_G000008338.1	754_1	no		2			1	2
LMSG_G000008346.1	754_1	no				1	1	1
LMSG_G000008339.1	754_1	no		1			1	1
LMSG_G000008337.1	754_1	no		2		1	1	1
LMSG_G000008353.1	754_1	no					1	
LMSG_G000004408.1	755_0	yes		2		1	1	1
LMSG_G000005733.1	756_1	yes		1	1		1	
LMSG_G000008529.1	756_1	no	2	2	1		1	
LMSG_G000005771.1	757_0	yes	1			1	1	
LMSG_G000006016.1	758_1	yes	2				1	1
LMSG_G000008530.1	758_1	no	1					1
LMSG_G000008531.1	759_1	no						2
LMSG_G000008532.1	759_1	no						1
LMSG_G000008533.1	759_1	no						1
LMSG_G000004532.1	759_1	yes						1
LMSG_G000006292.1	760_1	yes	2					1
LMSG_G000008534.1	760_1	no	2	1				1
LMSG_G000005624.1	761_0	yes	1					1
LMSG_G000008536.1	762_1	no	1					1
LMSG_G000008535.1	762_1	no	1				1	1
LMSG_G000006287.1	762_1	yes					1	1
LMSG_G000008537.1	762_1	no						2
LMSG_G000004903.1	763_1	yes					1	1
LMSG_G000008541.1	763_1	no					1	1
LMSG_G000008539.1	763_1	no						1
LMSG_G000008540.1	763_1	no						1
LMSG_G000004886.1	763_2	yes	1				1	2
LMSG_G000004555.1	764_0	yes	1				1	1
LMSG_G000005177.1	765_0	yes	1	3			1	1
LMSG_G000006013.1	766_0	yes	1				1	1
LMSG_G000005203.1	767_1	yes		1				
LMSG_G000008355.1	767_1	no	1					
LMSG_G000005222.1	768_1	yes	1				1	1
LMSG_G000008356.1	768_1	no	1				2	1
LMSG_G000008357.1	768_1	no	1				1	1
LMSG_G000008358.1	768_1	no	1				1	1
LMSG_G000008359.1	768_1	no	1				1	1
LMSG_G000008360.1	768_1	no	1				1	1
LMSG_G000008362.1	770_1	no						1
LMSG_G000004391.1	770_1	yes						1
LMSG_G000008366.1	770_1	no						1
LMSG_G000008367.1	770_1	no						1
LMSG_G000008363.1	770_1	no						1
LMSG_G000008368.1	770_1	no						1
LMSG_G000008369.1	770_1	no						1
LMSG_G000008364.1	770_1	no						1
LMSG_G000008370.1	770_1	no						1
LMSG_G000004869.1	771_0	yes						1
LMSG_G000005990.1	772_0	yes				1		1
LMSG_G000006014.1	773_0	yes	1					2
LMSG_G000005613.1	774_1	yes				1		1
LMSG_G000008371.1	774_1	no				1		
LMSG_G000008372.1	775_1	no						1
LMSG_G000004442.1	775_1	yes						1
LMSG_G000008376.1	775_1	no						1

LMSG_G000008378.1	775_1	no				1
LMSG_G000008373.1	775_1	no				1
LMSG_G000008374.1	775_1	no				1
LMSG_G000008375.1	775_1	no				1
LMSG_G000004526.1	776_1	yes		1		1
LMSG_G000008379.1	776_1	no				1
LMSG_G000008380.1	778_1	no				1
LMSG_G000004687.1	779_0	yes				1
LMSG_G000004411.1	780_0	yes	1	2		1
LMSG_G000008249.1	782_1	no		1		
LMSG_G000008250.1	782_1	no		1		
LMSG_G000004896.1	782_1	yes		1		
LMSG_G000008254.1	782_1	no		1		
LMSG_G000008251.1	782_1	no		1		
LMSG_G000008253.1	782_1	no		1		
LMSG_G000005851.1	784_0	yes			1	
LMSG_G000008567.1	785_1	no		1		
LMSG_G000008568.1	788_1	no	1	1		
LMSG_G000008571.1	788_1	no	1			
LMSG_G000008580.1	788_1	no	1	1		
LMSG_G000008572.1	788_1	no	1			
LMSG_G000008573.1	788_1	no	1	1		
LMSG_G000008569.1	788_1	no	1	1		
LMSG_G000008574.1	788_1	no	1	2		
LMSG_G000008575.1	788_1	no		2		
LMSG_G000008576.1	788_1	no	1	1		
LMSG_G000008577.1	788_1	no	1	1		
LMSG_G000008578.1	788_1	no	1	1		
LMSG_G000008579.1	788_1	no	1			
LMSG_G000004476.1	788_1	yes	1			
LMSG_G000008570.1	788_1	no	1	1		
LMSG_G000005341.1	789_1	yes	1	2		
LMSG_G000008581.1	789_1	no		1		
LMSG_G000008582.1	789_1	no	1	3		
LMSG_G000004363.1	790_1	yes		2		
LMSG_G000008584.1	790_1	no		2		
LMSG_G000008583.1	790_1	no		2		
LMSG_G000004340.1	790_2	yes		2		
LMSG_G000008586.1	791_1	no		1		
LMSG_G000008585.1	791_1	no		2		
LMSG_G000006242.1	791_1	yes		2		
LMSG_G000008587.1	792_1	no		2		
LMSG_G000004944.1	792_1	yes		2		
LMSG_G000008588.1	792_1	no	1	3		
LMSG_G000008592.1	792_1	no		2		
LMSG_G000008589.1	792_1	no		2		
LMSG_G000008590.1	792_1	no		2		
LMSG_G000005883.1	792_2	yes		2		
LMSG_G000005186.1	793_1	yes		1		
LMSG_G000008593.1	793_1	no		1		
LMSG_G000008594.1	793_1	no		1		
LMSG_G000008600.1	793_1	no		2		
LMSG_G000008595.1	793_1	no		1		
LMSG_G000008597.1	793_1	no		1		
LMSG_G000008603.1	794_1	no		2		
LMSG_G000008604.1	794_1	no		2		
LMSG_G000008606.1	794_1	no		1		
LMSG_G000008605.1	794_1	no		1		
LMSG_G000008602.1	794_1	no		2		
LMSG_G000005504.1	794_1	yes		3		
LMSG_G000004860.1	795_0	yes		1		
LMSG_G000008616.1	796_1	no		1		
LMSG_G000008607.1	796_1	no		1		
LMSG_G000008608.1	796_1	no		3		
LMSG_G000005205.1	796_1	yes		2		
LMSG_G000008609.1	796_1	no		1		
LMSG_G000008611.1	796_1	no		2		
LMSG_G000008612.1	796_1	no		1		
LMSG_G000008613.1	796_1	no		1		
LMSG_G000008614.1	796_1	no		2		
LMSG_G000008615.1	796_1	no		1		
LMSG_G000008610.1	796_1	no		1		
LMSG_G000004754.1	797_0	yes		1		
LMSG_G000005227.1	798_1	yes		1		
LMSG_G000008617.1	798_1	no		1		
LMSG_G000008618.1	798_1	no		1		
LMSG_G000008619.1	799_1	no	1	1		

LMSG_G000005411.1	799_1	yes	1	1		
LMSG_G000008623.1	800_1	no		1		
LMSG_G000008620.1	800_1	no	1			
LMSG_G000005242.1	800_1	yes		1		
LMSG_G000008621.1	800_1	no	1	1		
LMSG_G000008630.1	801_1	no	1	1		
LMSG_G000008631.1	801_1	no	1	1		
LMSG_G000008637.1	801_1	no		1		
LMSG_G000008632.1	801_1	no	1	1		
LMSG_G000008633.1	801_1	no	1	1		
LMSG_G000008624.1	801_1	no		1		
LMSG_G000008625.1	801_1	no	1	1		
LMSG_G000008626.1	801_1	no	1	1		
LMSG_G000008634.1	801_1	no	1	1		
LMSG_G000008627.1	801_1	no	1	1		
LMSG_G000008628.1	801_1	no	1	1		
LMSG_G000008635.1	801_1	no		1		
LMSG_G000008636.1	801_1	no	1	1		
LMSG_G000008629.1	801_1	no	1	1		
LMSG_G000004584.1	801_1	yes	1	1		
LMSG_G000008640.1	802_1	no		1		
LMSG_G000008638.1	802_1	no		1		
LMSG_G000005615.1	802_1	yes	2	2		
LMSG_G000008639.1	802_1	no	1	1		
LMSG_G000004968.1	803_0	yes	1	1		
LMSG_G000005273.1	804_1	yes		2		
LMSG_G000008641.1	804_1	no		2		
LMSG_G000008642.1	804_1	no		1		
LMSG_G000008645.1	805_1	no		1		
LMSG_G000008646.1	805_1	no		1		
LMSG_G000008647.1	805_1	no		1		
LMSG_G000008648.1	805_1	no		1		
LMSG_G000008649.1	806_1	no		1		
LMSG_G000008650.1	806_1	no		1		
LMSG_G000005783.1	806_1	yes		1		
LMSG_G000008651.1	806_1	no		1		
LMSG_G000004339.1	806_2	yes		3		
LMSG_G000008652.1	807_1	no		1		
LMSG_G000005223.1	807_2	yes		1		
LMSG_G000008653.1	807_2	no		1		
LMSG_G000006109.1	807_3	yes		2		
LMSG_G000005635.1	808_1	yes		3		
LMSG_G000008654.1	808_1	no		2		
LMSG_G000004443.1	808_2	yes		1		
LMSG_G000008656.1	808_2	no		1		
LMSG_G000005358.1	808_3	yes		2		
LMSG_G000008657.1	808_3	no		2		
LMSG_G000005453.1	808_4	yes		2		
LMSG_G000008658.1	808_4	no		1		
LMSG_G000008659.1	808_4	no		2		
LMSG_G000008542.1	809_1	no		3		
LMSG_G000008660.1	809_1	no		3		
LMSG_G000006232.1	809_1	yes		3		
LMSG_G000008543.1	809_1	no		1		
LMSG_G000008544.1	810_1	no		2		
LMSG_G000004388.1	810_1	yes		2		
LMSG_G000008545.1	810_1	no		1		
LMSG_G000008546.1	810_1	no		2		
LMSG_G000008548.1	810_2	no		2		
LMSG_G000008547.1	810_2	no		2		
LMSG_G000005800.1	810_2	yes		2		
LMSG_G000004372.1	811_0	yes		2		
LMSG_G000008661.1	812_1	no	1			
LMSG_G000004653.1	812_1	yes	1			
LMSG_G000008662.1	813_1	no	1			
LMSG_G000008663.1	813_1	no	1			
LMSG_G000005240.1	813_1	yes	1			
LMSG_G000008664.1	813_1	no	1			
LMSG_G000008666.1	814_1	no	1			
LMSG_G000008669.1	814_1	no	1			
LMSG_G000008551.1	814_1	no	1			
LMSG_G000008671.1	814_1	no	1			
LMSG_G000008672.1	814_1	no	1			
LMSG_G000008552.1	814_1	no	1			
LMSG_G000008673.1	814_1	no	1			
LMSG_G000008667.1	814_1	no	1			
LMSG_G000008381.1	815_1	no	1		1	1

LMSG_G000004430.1	815_1	yes	1		1			1
LMSG_G000008382.1	815_1	no	1		1			1
LMSG_G000008383.1	815_1	no	1		1			1
LMSG_G000008385.1	815_1	no			1			1
LMSG_G000008384.1	815_1	no	1		1			1
LMSG_G000008386.1	816_1	no	1					1
LMSG_G000008388.1	816_1	no	1					1
LMSG_G000008389.1	816_1	no						1
LMSG_G000004572.1	816_1	yes	1					1
LMSG_G000008390.1	816_1	no	1					1
LMSG_G000008391.1	816_1	no	1					1
LMSG_G000008393.1	816_1	no	1					2
LMSG_G000008069.1	817_1	no		1		1		
LMSG_G000008070.1	817_1	no		1		1		
LMSG_G000008071.1	817_1	no		2		1		
LMSG_G000008072.1	817_1	no		2		1		
LMSG_G000008073.1	817_1	no		1		1		
LMSG_G000008074.1	817_1	no		2		1		
LMSG_G000008075.1	817_1	no				1		
LMSG_G000008076.1	817_1	no				1		
LMSG_G000008077.1	817_1	no		2	1	1		
LMSG_G000008078.1	817_1	no		2		1		
LMSG_G000008079.1	817_1	no			1	1		
LMSG_G000008068.1	817_1	no		1		1		
LMSG_G000004516.1	817_1	yes		2		1		
LMSG_G000008080.1	817_1	no		1		1		
LMSG_G000008081.1	818_1	no		1		1		
LMSG_G000008082.1	818_1	no		1		1		
LMSG_G000008083.1	818_1	no		1		1		
LMSG_G000005753.1	818_1	yes		1		1		
LMSG_G000008089.1	819_1	no				1		
LMSG_G000005201.1	819_1	yes				1		
LMSG_G000008086.1	819_1	no				1		
LMSG_G000008085.1	819_1	no			1	1		
LMSG_G000008088.1	819_1	no				1		
LMSG_G000008090.1	819_1	no				1		
LMSG_G000008101.1	820_1	no					1	
LMSG_G000005065.1	821_1	yes	1	1				
LMSG_G000008105.1	821_1	no		1				
LMSG_G000008106.1	821_1	no	1	1				
LMSG_G000008104.1	821_1	no	1	2				
LMSG_G000008108.1	821_1	no	1	1				
LMSG_G000008109.1	823_1	no						2
LMSG_G000004512.1	823_1	yes		1				2
LMSG_G000004851.1	824_1	yes						1
LMSG_G000008559.1	825_1	no				1		
LMSG_G000008553.1	825_1	no				1		
LMSG_G000008554.1	825_1	no				1		
LMSG_G000005166.1	825_1	yes				1		
LMSG_G000008555.1	825_1	no				1		
LMSG_G000008556.1	825_1	no				1		
LMSG_G000008557.1	825_1	no				2		
LMSG_G000008558.1	825_1	no				2		
LMSG_G000006017.1	826_0	yes		2			1	
LMSG_G000004915.1	827_0	yes		1				
LMSG_G000008733.1	828_1	no		1				1
LMSG_G000006025.1	828_1	yes	1	2				2
LMSG_G000004607.1	829_0	yes		1		1	1	1
LMSG_G000004611.1	830_0	yes	1	2		1		2
LMSG_G000004541.1	831_0	yes	1				1	3
LMSG_G000005064.1	832_0	yes	1	1				1
LMSG_G000004603.1	833_0	yes	1	1	1			
LMSG_G000004845.1	834_0	yes	1					
LMSG_G000004485.1	835_0	yes	1	1				2
LMSG_G000008734.1	836_1	no						1
LMSG_G000008735.1	836_1	no						1
LMSG_G000008738.1	836_1	no						1
LMSG_G000005801.1	836_1	yes						1
LMSG_G000008736.1	836_1	no						1
LMSG_G000008737.1	836_1	no						1
LMSG_G000008739.1	837_1	no	1					1
LMSG_G000005164.1	837_1	yes	1					1
LMSG_G000008742.1	837_2	no	1					1
LMSG_G000008743.1	837_2	no	1					1
LMSG_G000008744.1	837_2	no	1	2				
LMSG_G000008740.1	837_2	no	1	2				1
LMSG_G000005637.1	837_2	yes	1	2				2

LMSG_G000008741.1	837_2	no	1	2					1
LMSG_G000004772.1	838_0	yes		2					1
LMSG_G000004719.1	839_0	yes	1						1
LMSG_G000004440.1	840_0	yes	1	1					
LMSG_G000004972.1	846_0	yes	2	1	1		1		1
LMSG_G000004979.1	847_0	yes	1	3		1	1		1
LMSG_G000005355.1	849_0	yes	1						
LMSG_G000004400.1	850_0	yes		2			1		
LMSG_G000004974.1	852_0	yes	1				1		1
LMSG_G000006081.1	854_0	yes					1		1
LMSG_G000004983.1	855_0	yes		1					1
LMSG_G000005396.1	857_0	yes	1				1		
LMSG_G000004985.1	858_0	yes		1					
LMSG_G000005591.1	859_0	yes	1						
LMSG_G000004961.1	860_0	yes	1	5			2	2	2
LMSG_G000004973.1	861_0	yes	1	5			1		2
LMSG_G000004991.1	862_0	yes	3	4	2		1	3	2
LMSG_G000005352.1	863_0	yes	14	1	3	1	2		1
LMSG_G000005357.1	864_0	yes	5		3	3	3	1	1
LMSG_G000004959.1	865_0	yes			1			1	2
LMSG_G000004394.1	866_0	yes	4	4	2		1		6
LMSG_G000004993.1	867_0	yes		2			2	1	
LMSG_G000005575.1	868_0	yes		1					1
LMSG_G000008708.1	869_1	no		1				1	2
LMSG_G000008705.1	869_1	no		1				2	
LMSG_G000008704.1	869_1	no						2	2
LMSG_G000004955.1	869_1	yes		1				1	1
LMSG_G000008709.1	869_1	no		1				2	1
LMSG_G000008707.1	869_1	no		1				3	1
LMSG_G000008710.1	869_1	no						1	2
LMSG_G000008711.1	869_1	no		1				2	2
LMSG_G000008712.1	869_1	no		1				1	1
LMSG_G000008713.1	869_1	no		1				1	1
LMSG_G000008714.1	869_1	no						1	2
LMSG_G000005904.1	869_2	yes			1			2	1
LMSG_G000008715.1	870_1	no	13	4				2	1
LMSG_G000004621.1	870_1	yes	9	4				4	2
LMSG_G000008716.1	870_1	no	10	1				3	2
LMSG_G000004540.1	871_1	yes						1	1
LMSG_G000008717.1	871_1	no	1						1
LMSG_G000008718.1	871_1	no				1			1
LMSG_G000008720.1	871_1	no							1
LMSG_G000008719.1	871_1	no							1
LMSG_G000008721.1	871_1	no						1	
LMSG_G000008723.1	871_2	no							1
LMSG_G000008722.1	871_2	no	2						1
LMSG_G000006039.1	871_2	yes							1
LMSG_G000006301.1	872_1	yes		1				1	1
LMSG_G000008724.1	872_1	no		1				1	1
LMSG_G000005250.1	873_1	yes		2			1	1	1
LMSG_G000006036.1	874_0	yes	2	3		4	3	2	1
LMSG_G000004702.1	875_0	yes	1						
LMSG_G000004962.1	878_0	yes							1
LMSG_G000008701.1	879_1	no							1
LMSG_G000005580.1	879_1	yes							1
LMSG_G000005311.1	880_1	yes		1					
LMSG_G000008702.1	880_1	no		1					
LMSG_G000005271.1	881_1	yes							2
LMSG_G000005278.1	881_2	yes							2
LMSG_G000004913.1	882_0	yes	1	1				1	
LMSG_G000005265.1	883_0	yes					1		
LMSG_G000004769.1	884_1	yes							1
LMSG_G000007741.1	884_1	no					1		1
LMSG_G000007742.1	884_1	no					1		1
LMSG_G000007743.1	884_1	no					1	1	2
LMSG_G000007744.1	884_1	no					1	1	2
LMSG_G000007736.1	884_1	no							1
LMSG_G000007737.1	884_1	no							1
LMSG_G000007738.1	884_1	no							1
LMSG_G000007739.1	884_1	no							1
LMSG_G000007740.1	884_1	no							2
LMSG_G000006116.1	884_2	yes					1		1
LMSG_G000007745.1	885_1	no					1	1	1
LMSG_G000005673.1	885_1	yes					1		1
LMSG_G000007746.1	886_1	no					1	1	1
LMSG_G000006012.1	886_1	yes							1
LMSG_G000005214.1	888_0	yes					1	1	1

LMSG_G000007747.1	889_1	no	1						1
LMSG_G000007749.1	889_1	no	1	1			1		3
LMSG_G000007748.1	889_1	no	1						1
LMSG_G000007750.1	889_1	no	1				1		2
LMSG_G000005642.1	889_1	yes	1						1
LMSG_G000004969.1	890_0	yes	3			1	1		1
LMSG_G000007751.1	891_1	no		1	3	1			2
LMSG_G000007752.1	891_1	no	1	1	2	1			1
LMSG_G000007753.1	891_1	no	2	2		1		1	1
LMSG_G000007754.1	891_1	no	2	1		1			1
LMSG_G000007755.1	891_1	no		1	1		1		
LMSG_G000007756.1	891_1	no				2			1
LMSG_G000005210.1	891_1	yes		1	1	2			1
LMSG_G000007757.1	891_1	no			1	1			1
LMSG_G000005740.1	892_1	yes	1				1		1
LMSG_G000007758.1	892_1	no	1				1		1
LMSG_G000007759.1	892_1	no					1	1	1
LMSG_G000007760.1	892_1	no	1						1
LMSG_G000007761.1	892_2	no	1				1		1
LMSG_G000006040.1	892_2	yes	1						1
LMSG_G000005365.1	893_0	yes	1		1	2			1
LMSG_G000007767.1	894_1	no	1				1		1
LMSG_G000004921.1	894_1	yes	2				1		1
LMSG_G000007771.1	894_1	no	1				1		1
LMSG_G000007773.1	894_1	no					1		1
LMSG_G000007775.1	894_1	no					1		1
LMSG_G000007772.1	894_1	no	1				1		1
LMSG_G000007774.1	894_1	no	1				1		1
LMSG_G000007762.1	894_1	no	1				1		1
LMSG_G000007763.1	894_1	no					1		1
LMSG_G000007764.1	894_1	no	1				1		1
LMSG_G000007765.1	894_1	no	1				1		2
LMSG_G000007766.1	894_1	no	1				1		1
LMSG_G000007768.1	894_1	no							1
LMSG_G000007769.1	894_1	no					1		1
LMSG_G000007770.1	894_1	no					1		1
LMSG_G000006019.1	895_0	yes	2			1	1		1
LMSG_G000005151.1	896_0	yes	1				1		
LMSG_G000005412.1	897_0	yes	1				1		2
LMSG_G000005147.1	898_0	yes					1		
LMSG_G000005996.1	899_0	yes	1				2	1	1
LMSG_G000007835.1	900_1	no	2				1		3
LMSG_G000007836.1	900_1	no		1			1		2
LMSG_G000007837.1	900_1	no	1						2
LMSG_G000005206.1	900_1	yes	2	1			1		2
LMSG_G000007838.1	900_1	no	2				1		3
LMSG_G000007839.1	900_1	no	2				1		2
LMSG_G000007847.1	901_1	no	1						
LMSG_G000007844.1	901_1	no					1		
LMSG_G000007845.1	901_1	no	1				1		2
LMSG_G000007848.1	901_1	no	1						1
LMSG_G000004546.1	901_1	yes	2				1		3
LMSG_G000007840.1	901_1	no	2				1		2
LMSG_G000007842.1	901_1	no	2				1		2
LMSG_G000007841.1	901_1	no	2				1		2
LMSG_G000007846.1	901_1	no	2				1		2
LMSG_G000007850.1	902_1	no	1	1			1		1
LMSG_G000007851.1	902_1	no	1				1		2
LMSG_G000007849.1	902_1	no		1			1		1
LMSG_G000007852.1	902_1	no	2				1		2
LMSG_G000005074.1	902_1	yes	2				1		2
LMSG_G000007853.1	902_1	no	2				1		2
LMSG_G000005742.1	902_2	yes		1			1		
LMSG_G000004645.1	903_0	yes	1				1		2
LMSG_G000004728.1	904_0	yes	1	1					1
LMSG_G000004739.1	905_0	yes		1			1		
LMSG_G000004663.1	906_0	yes						1	1
LMSG_G000004667.1	907_0	yes	1					1	2
LMSG_G000004932.1	908_0	yes	1						1
LMSG_G000007860.1	909_1	no	1	1			1	1	1
LMSG_G000004490.1	909_1	yes	2				1	1	1
LMSG_G000007861.1	909_1	no	1						
LMSG_G000007863.1	910_1	no					1	1	1
LMSG_G000004751.1	910_1	yes	1				1	1	1
LMSG_G000007866.1	910_1	no	1				1	1	2
LMSG_G000007862.1	910_1	no	1				1	1	1
LMSG_G000007865.1	910_1	no	1				1	2	1

LMSG_G000007873.1	911_1	no	1				1		1
LMSG_G000007867.1	911_1	no	1				1		1
LMSG_G000007868.1	911_1	no	1						2
LMSG_G000007869.1	911_1	no					1		2
LMSG_G000007871.1	911_1	no				1	1		2
LMSG_G000005808.1	911_1	yes				1	1		1
LMSG_G000007872.1	911_1	no							2
LMSG_G000007870.1	911_1	no	1		1		1		1
LMSG_G000005316.1	912_0	yes							1
LMSG_G000007874.1	913_1	no	2			1		1	2
LMSG_G000005241.1	913_1	yes	2			1		2	1
LMSG_G000004757.1	914_1	yes	1			1	1		2
LMSG_G000005212.1	914_2	yes	1			1			2
LMSG_G000004934.1	915_0	yes	2		1	1	1		1
LMSG_G000007777.1	916_1	no					2	1	1
LMSG_G000007779.1	916_1	no					1	1	2
LMSG_G000007783.1	916_1	no					1	1	1
LMSG_G000007784.1	916_1	no					1	1	1
LMSG_G000007781.1	916_1	no		1			2	1	1
LMSG_G000007785.1	916_1	no					1	1	
LMSG_G000007782.1	916_1	no					3	1	1
LMSG_G000007786.1	916_1	no					1	1	2
LMSG_G000007780.1	916_1	no					1	1	1
LMSG_G000007778.1	916_1	no					2	1	1
LMSG_G000004479.1	916_1	yes					2	1	1
LMSG_G000007789.1	917_1	no					1	1	1
LMSG_G000007791.1	917_1	no					1	1	
LMSG_G000007792.1	917_1	no					1		1
LMSG_G000007790.1	917_1	no					1	1	1
LMSG_G000007787.1	917_1	no					1	1	1
LMSG_G000007788.1	917_1	no					1	1	
LMSG_G000005429.1	917_1	yes					1	1	1
LMSG_G000004746.1	918_0	yes					1	1	1
LMSG_G000007793.1	919_1	no							1
LMSG_G000007794.1	919_1	no					1		2
LMSG_G000007795.1	919_1	no					1		1
LMSG_G000007796.1	919_1	no					1		1
LMSG_G000007797.1	919_1	no					1		2
LMSG_G000007798.1	919_1	no					1		2
LMSG_G000007799.1	919_1	no					1		1
LMSG_G000007800.1	919_1	no					1		2
LMSG_G000007801.1	919_1	no							2
LMSG_G000007802.1	919_1	no							1
LMSG_G000005427.1	919_1	yes							1
LMSG_G000007803.1	920_1	no					2		1
LMSG_G000007804.1	920_1	no					2		1
LMSG_G000005219.1	920_1	yes					2		2
LMSG_G000006260.1	921_0	yes					1	1	1
LMSG_G000006272.1	922_1	yes					1		1
LMSG_G000007805.1	922_1	no					1		
LMSG_G000004368.1	923_1	yes					1	1	1
LMSG_G000007807.1	923_1	no					1		
LMSG_G000007808.1	923_1	no			1		1		1
LMSG_G000007809.1	923_1	no					1		1
LMSG_G000007810.1	923_1	no					1		1
LMSG_G000007811.1	923_1	no					1	1	1
LMSG_G000007812.1	923_1	no					1		2
LMSG_G000007813.1	923_1	no					1		
LMSG_G000007814.1	923_1	no					1		
LMSG_G000007815.1	923_1	no					1		1
LMSG_G000007816.1	923_1	no					1		1
LMSG_G000007817.1	923_1	no					1		1
LMSG_G000007818.1	923_1	no					1	1	1
LMSG_G000007819.1	923_1	no					1		1
LMSG_G000007820.1	923_1	no					1		1
LMSG_G000007821.1	923_1	no					1		1
LMSG_G000007822.1	923_1	no					1		1
LMSG_G000007823.1	923_1	no					1		1
LMSG_G000007824.1	923_1	no							3
LMSG_G000007825.1	923_1	no					1		1
LMSG_G000007806.1	923_1	no					1		1
LMSG_G000007826.1	923_1	no					1		1
LMSG_G000007827.1	923_1	no					1	1	1
LMSG_G000004664.1	924_0	yes	3				1		1
LMSG_G000005071.1	925_0	yes	1						2
LMSG_G000005089.1	926_1	yes	1						
LMSG_G000007855.1	926_1	no	1						1

LMSG_G000007856.1	926_1	no	1				1		1
LMSG_G000007878.1	927_1	no		1		1			1
LMSG_G000004543.1	927_1	yes	1	1		1			1
LMSG_G000007877.1	927_1	no	1	1		1			1
LMSG_G000007875.1	927_1	no	1	1		1			2
LMSG_G000007876.1	927_1	no	2	1	1				1
LMSG_G000005792.1	928_0	yes	1	1				1	2
LMSG_G000004493.1	929_1	yes	1						
LMSG_G000009671.1	929_1	no	1						
LMSG_G000005469.1	930_1	yes	1						
LMSG_G000009672.1	930_1	no	1						
LMSG_G000005347.1	931_1	yes	2						1
LMSG_G000009674.1	931_1	no	1						1
LMSG_G000009675.1	932_1	no	1						
LMSG_G000009676.1	932_1	no	1		1				1
LMSG_G000009677.1	932_1	no			1				1
LMSG_G000009678.1	932_1	no			1				
LMSG_G000005492.1	932_1	yes	1		1				1
LMSG_G000009679.1	932_2	no	2						1
LMSG_G000005953.1	932_2	yes	2						1
LMSG_G000004423.1	932_3	yes	1		1				1
LMSG_G000006270.1	933_1	yes	7	3		1	8	2	2
LMSG_G000009311.1	933_1	no	4	1		1	9	1	1
LMSG_G000004563.1	936_1	yes	1	2					1
LMSG_G000006225.1	937_0	yes	1				1		1
LMSG_G000011299.1	938_1	no						2	
LMSG_G000005318.1	938_1	yes						2	
LMSG_G000011300.1	938_1	no		1				2	
LMSG_G000004542.1	939_1	yes	1						
LMSG_G000009687.1	940_1	no	1						
LMSG_G000009690.1	940_1	no	1						
LMSG_G000009688.1	940_1	no	1						
LMSG_G000009689.1	940_1	no	1						
LMSG_G000009686.1	940_1	no	1						
LMSG_G000005876.1	940_1	yes	1						
LMSG_G000004829.1	942_0	yes	1						
LMSG_G000004507.1	943_0	yes	1	2					
LMSG_G000004970.1	944_0	yes		1					
LMSG_G000009657.1	945_1	no	2						1
LMSG_G000009658.1	945_1	no	2						1
LMSG_G000004500.1	945_1	yes	2						1
LMSG_G000009660.1	945_1	no	2						1
LMSG_G000006250.1	945_2	yes	1						1
LMSG_G000005790.1	946_1	yes	1						1
LMSG_G000009661.1	946_1	no	1						1
LMSG_G000009662.1	947_1	no	1						1
LMSG_G000004495.1	947_1	yes	2				1		1
LMSG_G000005498.1	948_0	yes	2	1			1		1
LMSG_G000005491.1	949_1	yes	2				1		1
LMSG_G000009663.1	949_1	no	2				1		1
LMSG_G000009665.1	950_1	no	2	2					1
LMSG_G000009666.1	950_1	no	1						
LMSG_G000009667.1	950_1	no		2					1
LMSG_G000009669.1	950_1	no	1				1		1
LMSG_G000005823.1	950_1	yes	2				1		
LMSG_G000009668.1	950_1	no	2	2					1
LMSG_G000005825.1	951_0	yes	1	1	1				1
LMSG_G000009517.1	952_1	no	1	3					2
LMSG_G000009518.1	952_1	no	1	3					2
LMSG_G000009519.1	952_1	no	1	3					2
LMSG_G000009520.1	952_1	no	1	3					2
LMSG_G000009521.1	952_1	no		1					1
LMSG_G000009522.1	952_1	no	1	2					2
LMSG_G000005034.1	952_1	yes	1	3					2
LMSG_G000009523.1	952_1	no	1	2					2
LMSG_G000009524.1	952_1	no	1	2					2
LMSG_G000009525.1	952_1	no	1	3					3
LMSG_G000009526.1	952_1	no	1	1					2
LMSG_G000009527.1	953_1	no	1						2
LMSG_G000009528.1	953_1	no	1	1					2
LMSG_G000009529.1	953_1	no	1	1					2
LMSG_G000009530.1	953_1	no							2
LMSG_G000009531.1	953_1	no							2
LMSG_G000006216.1	953_1	yes	1						2
LMSG_G000009532.1	953_1	no							1
LMSG_G000009533.1	953_1	no							1
LMSG_G000009534.1	953_1	no		1					2

LMSG_G000009535.1	953_1	no	1	1			2
LMSG_G000009536.1	953_1	no	1				2
LMSG_G000009537.1	953_1	no	1				1
LMSG_G000009538.1	953_1	no				1	
LMSG_G000009539.1	953_1	no	1	1			2
LMSG_G000009540.1	953_1	no	1				1
LMSG_G000009541.1	953_1	no	1				
LMSG_G000005191.1	954_0	yes	1				2
LMSG_G000010596.1	955_1	no				1	
LMSG_G000010597.1	955_1	no				1	
LMSG_G000010598.1	955_1	no					1
LMSG_G000010599.1	955_1	no			1	1	1
LMSG_G000006197.1	955_1	yes				1	1
LMSG_G000009584.1	956_1	no	1	2			2
LMSG_G000005601.1	956_1	yes					1
LMSG_G000009586.1	957_1	no					1
LMSG_G000009585.1	957_1	no		1			1
LMSG_G000009587.1	957_1	no	1				2
LMSG_G000009588.1	957_1	no	1	1			2
LMSG_G000009589.1	957_1	no		2			2
LMSG_G000009590.1	957_1	no	1	1			2
LMSG_G000005467.1	957_1	yes	1	1			2
LMSG_G000009591.1	957_1	no	1				2
LMSG_G000009592.1	957_1	no	1				2
LMSG_G000009593.1	957_1	no	1	1			2
LMSG_G000009594.1	957_1	no	1				2
LMSG_G000009595.1	957_1	no	1				
LMSG_G000009596.1	957_1	no		1			2
LMSG_G000009597.1	957_1	no	1				2
LMSG_G000009598.1	957_1	no	1	1			2
LMSG_G000009599.1	957_1	no	1	2			2
LMSG_G000009601.1	957_1	no	1	2			2
LMSG_G000009602.1	957_1	no	1	2			2
LMSG_G000009603.1	957_1	no	1	1			2
LMSG_G000009624.1	958_1	no	1	3			1
LMSG_G000009605.1	958_1	no	1				2
LMSG_G000009606.1	958_1	no					2
LMSG_G000009607.1	958_1	no	1				2
LMSG_G000009608.1	958_1	no	1				1
LMSG_G000009609.1	958_1	no	1				2
LMSG_G000009610.1	958_1	no	1				2
LMSG_G000009611.1	958_1	no					2
LMSG_G000009612.1	958_1	no	1				2
LMSG_G000009613.1	958_1	no	1				2
LMSG_G000009614.1	958_1	no					1
LMSG_G000009615.1	958_1	no					1
LMSG_G000009616.1	958_1	no					2
LMSG_G000005770.1	958_1	yes					2
LMSG_G000009617.1	958_1	no	1				2
LMSG_G000009618.1	958_1	no	1				2
LMSG_G000009619.1	958_1	no	1				2
LMSG_G000009604.1	958_1	no	1				2
LMSG_G000009620.1	958_1	no	1				2
LMSG_G000009621.1	958_1	no					1
LMSG_G000009622.1	958_1	no	1				2
LMSG_G000004780.1	959_1	yes	1	2			2
LMSG_G000009625.1	959_1	no	1	1			2
LMSG_G000009632.1	960_1	no	1	1			2
LMSG_G000009626.1	960_1	no	1	1			3
LMSG_G000009627.1	960_1	no	1	1			2
LMSG_G000005251.1	960_1	yes	1	1			2
LMSG_G000009628.1	960_1	no					2
LMSG_G000009629.1	960_1	no					1
LMSG_G000009630.1	960_1	no	1	1			2
LMSG_G000009631.1	960_1	no	1				2
LMSG_G000009635.1	960_2	no	1	3			2
LMSG_G000005776.1	960_2	yes					2
LMSG_G000009633.1	960_2	no		1			3
LMSG_G000009634.1	960_2	no					1
LMSG_G000005726.1	961_0	yes	1				2
LMSG_G000006113.1	962_0	yes	1				1
LMSG_G000006787.1	96_2	no		1			
LMSG_G000005181.1	963_1	yes					1
LMSG_G000009636.1	963_1	no					2
LMSG_G000009637.1	964_1	no	1				2
LMSG_G000009638.1	964_1	no	1				2
LMSG_G000005159.1	964_1	yes					2

LMSG_G000009640.1	964_1	no					2
LMSG_G000009641.1	964_1	no	1				2
LMSG_G000009642.1	964_1	no	1				2
LMSG_G000009643.1	964_1	no					2
LMSG_G000009646.1	964_1	no	1				2
LMSG_G000009644.1	964_1	no					1
LMSG_G000009645.1	964_1	no		1			1
LMSG_G000004956.1	965_1	yes	1	2			2
LMSG_G000009653.1	965_1	no	1	2			2
LMSG_G000009655.1	965_1	no					1
LMSG_G000009647.1	965_2	no	1	1			2
LMSG_G000009648.1	965_2	no		1			2
LMSG_G000009649.1	965_2	no					2
LMSG_G000009651.1	965_2	no	1				3
LMSG_G000005970.1	965_2	yes	1	2			2
LMSG_G000009650.1	965_2	no		1			2
LMSG_G000009567.1	966_1	no	1	1			2
LMSG_G000009568.1	966_1	no	1	3			1
LMSG_G000006205.1	966_1	yes	1	1			2
LMSG_G000009569.1	966_2	no	1				1
LMSG_G000009570.1	966_2	no	1	1			2
LMSG_G000009571.1	966_2	no	1	1			2
LMSG_G000009572.1	966_2	no	1	1			2
LMSG_G000009573.1	966_2	no	1	1			2
LMSG_G000009574.1	966_2	no	1	1			2
LMSG_G000009575.1	966_2	no	1	1			2
LMSG_G000009580.1	966_2	no	1	1			2
LMSG_G000009576.1	966_2	no	1	1			2
LMSG_G000009581.1	966_2	no	1	1			2
LMSG_G000009577.1	966_2	no	1	1			2
LMSG_G000009578.1	966_2	no		1			2
LMSG_G000009579.1	966_2	no	1	1			2
LMSG_G000004627.1	966_2	yes	1	1			2
LMSG_G000009582.1	966_2	no	1				2
LMSG_G000009583.1	966_2	no	1	1			2
LMSG_G000009545.1	967_1	no	1	1			2
LMSG_G000009546.1	967_1	no	1	2			2
LMSG_G000009547.1	967_1	no	1	2			2
LMSG_G000009548.1	967_1	no	1	1			2
LMSG_G000009549.1	967_1	no	1	1			2
LMSG_G000009550.1	967_1	no	1				2
LMSG_G000009551.1	967_1	no	1	1			1
LMSG_G000009552.1	967_1	no	1	1			2
LMSG_G000009553.1	967_1	no	1	1			3
LMSG_G000009554.1	967_1	no	1	1			2
LMSG_G000005969.1	967_1	yes	1	2			2
LMSG_G000009555.1	967_1	no	1	2			2
LMSG_G000009563.1	968_1	no					1
LMSG_G000009556.1	968_1	no	1				1
LMSG_G000009557.1	968_1	no	1				1
LMSG_G000009558.1	968_1	no	1	1			2
LMSG_G000005695.1	968_1	yes	1	1			2
LMSG_G000009561.1	968_1	no	1	3			2
LMSG_G000009562.1	968_1	no		1			2
LMSG_G000009559.1	968_1	no	1	1			1
LMSG_G000005664.1	969_0	yes	1	3			1
LMSG_G000009652.1	970_1	no	1				2
LMSG_G000006034.1	970_1	yes	1				2
LMSG_G000005795.1	971_1	yes	1				2
LMSG_G000009564.1	971_1	no	1				2
LMSG_G000009566.1	972_1	no	1				2
LMSG_G000005566.1	972_1	yes	1				1
LMSG_G000009565.1	972_1	no	1	1			2
LMSG_G000010587.1	973_1	no				2	1
LMSG_G000010585.1	973_1	no		1			2
LMSG_G000010586.1	973_1	no					1
LMSG_G000010588.1	973_1	no					1
LMSG_G000010583.1	973_1	no					1
LMSG_G000010584.1	973_1	no				1	1
LMSG_G000010581.1	973_1	no				1	1
LMSG_G000010589.1	973_1	no				2	1
LMSG_G000006246.1	973_1	yes				2	1
LMSG_G000010591.1	974_1	no		1			1
LMSG_G000004379.1	974_1	yes		1	1		1
LMSG_G000010592.1	974_1	no		1			1
LMSG_G000010593.1	974_1	no		1			1
LMSG_G000010594.1	974_1	no		1			1

LMSG_G000010595.1	974_1	no				1
LMSG_G000010590.1	974_1	no				1
LMSG_G000005948.1	975_0	yes	1			1
LMSG_G000010575.1	976_1	no			1	
LMSG_G000010576.1	976_1	no			1	
LMSG_G000010577.1	976_1	no			1	1
LMSG_G000010578.1	976_1	no		1	1	1
LMSG_G000010579.1	976_1	no	1	1	2	1
LMSG_G000006254.1	976_1	yes		1	4	1
LMSG_G000010600.1	977_1	no				1
LMSG_G000010601.1	977_1	no	2			1
LMSG_G000010604.1	977_1	no				1
LMSG_G000010603.1	977_1	no				1
LMSG_G000010602.1	977_1	no				1
LMSG_G000010522.1	978_1	no	2		1	1
LMSG_G000010525.1	978_1	no				1
LMSG_G000010523.1	978_1	no	1		1	1
LMSG_G000010524.1	978_1	no	1			1
LMSG_G000005967.1	978_1	yes	1		1	1
LMSG_G000010528.1	979_1	no				1
LMSG_G000010527.1	979_1	no				1
LMSG_G000005367.1	979_1	yes	2			1
LMSG_G000010526.1	979_1	no	1			1
LMSG_G000010550.1	979_2	no	3		1	1
LMSG_G000010548.1	979_2	no	1	1		1
LMSG_G000010544.1	979_2	no	2			1
LMSG_G000010545.1	979_2	no	3	1		
LMSG_G000010546.1	979_2	no	2	1		
LMSG_G000010549.1	979_2	no	1			
LMSG_G000004515.1	979_2	yes	2			1
LMSG_G000010540.1	979_2	no	2			1
LMSG_G000010547.1	979_2	no		2		1
LMSG_G000010539.1	979_2	no				1
LMSG_G000010541.1	979_2	no	2			1
LMSG_G000010542.1	979_2	no	2			1
LMSG_G000010543.1	979_2	no	1		1	1
LMSG_G000010556.1	980_1	no	1		1	1
LMSG_G000010564.1	980_1	no	1	1		1
LMSG_G000010558.1	980_1	no	1			1
LMSG_G000010574.1	980_1	no			1	2
LMSG_G000010569.1	980_1	no	1		1	1
LMSG_G000010570.1	980_1	no	1		1	
LMSG_G000010571.1	980_1	no	1		1	
LMSG_G000010572.1	980_1	no			2	1
LMSG_G000010573.1	980_1	no	1		1	
LMSG_G000010557.1	980_1	no	1			1
LMSG_G000010552.1	980_1	no	1		1	
LMSG_G000010551.1	980_1	no	3		1	1
LMSG_G000010553.1	980_1	no	1		1	1
LMSG_G000010554.1	980_1	no	2		1	1
LMSG_G000010561.1	980_1	no	1	1	1	1
LMSG_G000010566.1	980_1	no		1	1	1
LMSG_G000010563.1	980_1	no	1			1
LMSG_G000010555.1	980_1	no	1			1
LMSG_G000010567.1	980_1	no	1			1
LMSG_G000010559.1	980_1	no	1	1	2	1
LMSG_G000010568.1	980_1	no	1		1	
LMSG_G000010529.1	980_1	no	1	2	1	1
LMSG_G000010560.1	980_1	no	2	1	1	1
LMSG_G000006167.1	980_1	yes	1		1	1
LMSG_G000010562.1	980_1	no	1	1		1
LMSG_G000010565.1	980_1	no	1			1
LMSG_G000005187.1	980_2	yes	1		3	1
LMSG_G000010533.1	981_1	no				1
LMSG_G000010534.1	981_1	no				1
LMSG_G000010535.1	981_1	no		1		1
LMSG_G000010530.1	981_1	no				1
LMSG_G000010536.1	981_1	no				1
LMSG_G000010531.1	981_1	no				1
LMSG_G000010532.1	981_1	no				1
LMSG_G000004762.1	982_1	yes	1		3	1
LMSG_G000010537.1	982_1	no			3	1
LMSG_G000010538.1	982_1	no				1
LMSG_G000006142.1	983_1	yes	1			
LMSG_G000011206.1	984_1	no	1			
LMSG_G000011201.1	984_1	no	1			
LMSG_G000011202.1	984_1	no	1			

LMSG_G000011204.1	984_1	no		1			
LMSG_G000011203.1	984_1	no		1			
LMSG_G000011209.1	984_1	no		1			
LMSG_G000011210.1	984_1	no		2			
LMSG_G000004875.1	985_0	yes		1			
LMSG_G000005858.1	987_0	yes		2			
LMSG_G000004566.1	988_0	yes		2			
LMSG_G000005555.1	989_0	yes					1
LMSG_G000005950.1	990_0	yes					1
LMSG_G000006141.1	991_0	yes	1		1		
LMSG_G000007916.1	992_1	no	2				
LMSG_G000005835.1	992_1	yes	1				3
LMSG_G000006147.1	993_0	yes	2				
LMSG_G000004806.1	994_1	yes	1		1	1	2
LMSG_G000007915.1	994_1	no			1		1
LMSG_G000007910.1	995_1	no	1				1
LMSG_G000005901.1	995_1	yes	1				
LMSG_G000004809.1	996_1	yes				1	1
LMSG_G000007911.1	996_1	no				1	
LMSG_G000004794.1	997_1	yes	2				1
LMSG_G000007909.1	997_1	no	2				1
LMSG_G000007912.1	998_1	no	1			1	1
LMSG_G000004392.1	998_1	yes	1			1	1
LMSG_G000007913.1	998_1	no	1			1	1
LMSG_G000007914.1	998_1	no	1			1	1
LMSG_G000005039.1	998_2	yes					1
LMSG_G000005859.1	999_0	yes	1				1
